
JOURNAL INFORMATION
==============================
NLM Title Abbreviation: Insights Imaging
Journal ID: Insights Imaging
NLM Journal ID: 101532453

Insights into Imaging

EISSN: 1869-4101
Publisher: Springer

ARTICLE INFORMATION
==============================
PMCID: PMC13323440
DOI: 10.1186/s13244-026-02345-x
Article ID: 2345
Article version: 1
Subjects: Meeting Abstracts

ECR 2026 Book of Abstracts

Electronic publication date: 2026 Jul 1
Publication date: 2026 Jul
Volume: 17
Issue: Suppl 1
Issue sponsor: Publication of this supplement was funded by the European Congress of Radiology.
Electronic Location ID: 175
Copyright: © The Author(s) 2026
Copyright year: 2026
License: Open Access This article is licensed under a Creative Commons Attribution 4.0 International License, which permits use, sharing, adaptation, distribution and reproduction in any medium or format, as long as you give appropriate credit to the original author(s) and the source, provide a link to the Creative Commons licence, and indicate if changes were made. The images or other third party material in this article are included in the article's Creative Commons licence, unless indicated otherwise in a credit line to the material. If material is not included in the article's Creative Commons licence and your intended use is not permitted by statutory regulation or exceeds the permitted use, you will need to obtain permission directly from the copyright holder. To view a copy of this licence, visit http://creativecommons.org/licenses/by/4.0/.
License URL: https://creativecommons.org/licenses/by/4.0/

Conference name: European Congress of Radiology 2026
Conference location: Vienna, Austria
Conference date: 4-8 March 2026
issue-copyright-statement: © European Society of Radiology (ESR) 2026

==============================

### ECR 2026

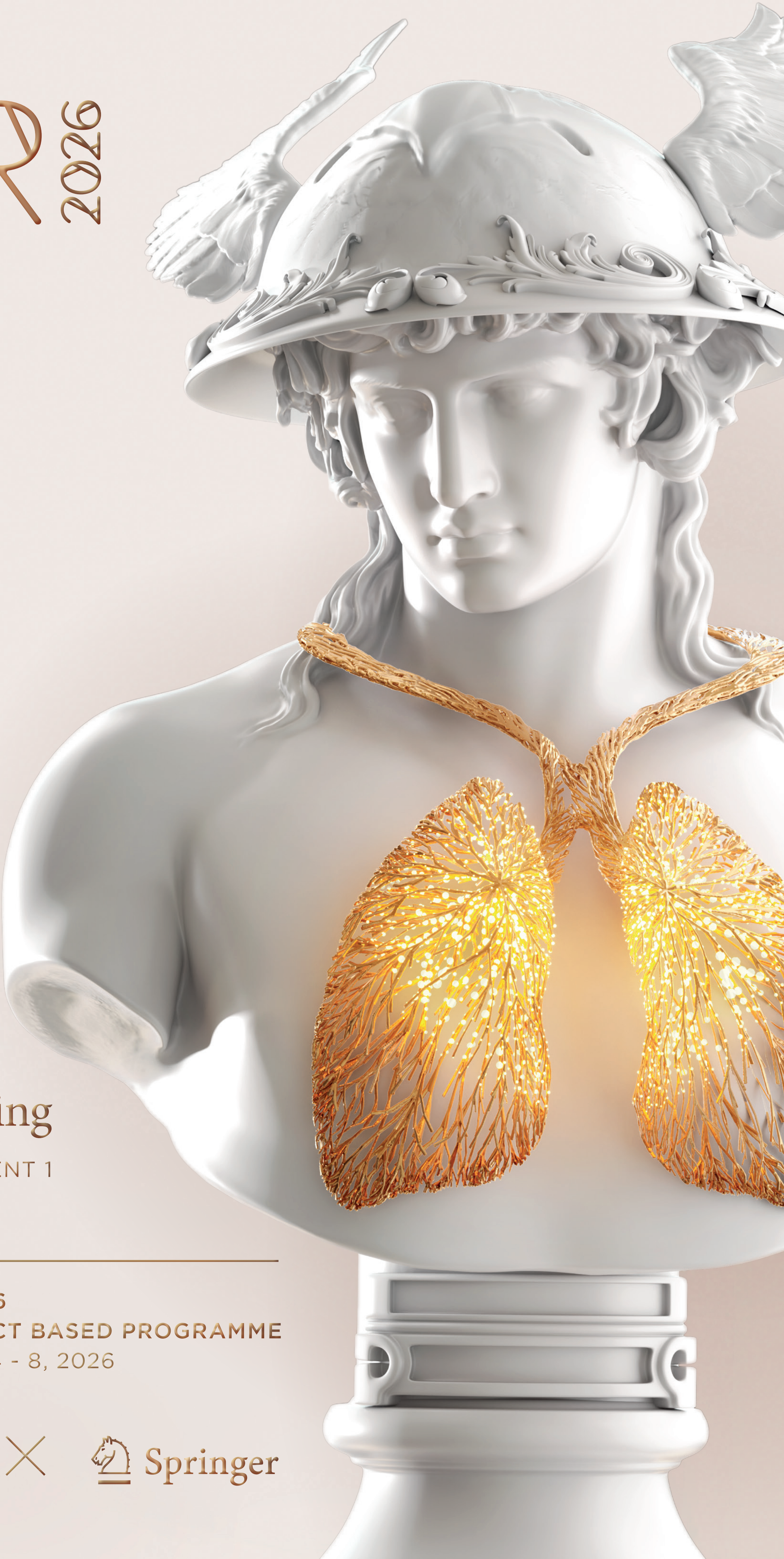

Insights into Imaging

VOLUME 17 | SUPPLEMENT 1

**CONTENTS**

3 - 410 **ECR 2026**
**ABSTRACT BASED PROGRAMME**
MARCH 4 - 8, 2026

ESR EUROPEAN SOCIETY OF RADIOLOGY × Springer

**Disclaimer**

The ECR 2026 Book of Abstracts is published by the European Society of Radiology (ESR) and summarises the presentations accepted to be held at the European Congress of Radiology 2026 (Vienna, Austria, March 4 - March 8, 2026).

Abstracts were submitted by the authors warranting that good scientific practice, copyrights and data privacy regulations have been observed and relevant conflicts of interest declared.

Abstracts reflect the authors' opinions and knowledge. The ESR does not give any warranty about the accuracy or completeness of medical procedures, diagnostic procedures or treatments contained in the material included in this publication. The views and opinions presented in ECR abstracts and presentations, including scientific, educational and professional matters, do not necessarily reflect the views and opinions of the ESR.

In no event will the ESR be liable for any direct or indirect, special, incidental, consequential, punitive or exemplary damages arising from the use of these abstracts.

The Book of Abstracts and all of its component elements are for general educational purposes for health care professionals only and must not take the place of professional medical advice. Those seeking medical advice should always consult their physician or other medical professional.

In preparing this publication, every effort has been made to provide the most current, accurate, and clearly expressed information possible. Nevertheless, inadvertent errors in information can occur. The ESR is not responsible for typographical errors, accuracy, completeness or timeliness of the information contained in this publication.

The ECR 2026 Book of Abstracts is a supplement to Insights into Imaging (1869-4101) and published under Creative Commons Attribution 4.0 International License.

Insights Imaging (2026) Vol 17 (Suppl 1)
https://doi.org/10.1186/s13244-026-02345-x
Published: 01 July 2026

### ECR 2026
### Abstract-based Programme

Research Presentation Sessions (RPS)

**Wednesday, March 4 .................... 3**
**Thursday, March 5......................106**
**Friday, March 6 ..........................191**
**Saturday, March 7 ......................277**
**Sunday, March 8........................366**

### Wednesday, March 4

**08:00-09:30 Research Stage 5**

### Research Presentation Session: Abdominal and Gastrointestinal

### RPS 101

### Frontiers in colorectal cancer imaging: from diagnosis to precision therapy

**Moderator**
Monique Maas; Amsterdam/NL
*(moniquemaas@live.nl)*

### Gut decisions based on the liver: A radiomics approach to boost colorectal cancer screening

*A. Hinterberger*[1], J. Bohn[1], J. Betge[2], S. Grosu[3], S. O. Schönberg[2], D. Nörenberg[2], J. N. Kather[4], F. Grawe[1], K. Maier-Hein[1]; [1]Heidelberg/DE, [2]Mannheim/DE, [3]Munich/DE, [4]Dresden/DE
*(hinterbergera@me.com)*

**Purpose:** Noninvasive colorectal cancer (CRC) screening represents a key opportunity to improve colonoscopy participation rates and reduce CRC mortality. This study explores the potential of the gut-liver-axis for predicting colorectal neoplasia through liver-derived radiomic features extracted from routine CT images.
**Methods or Background:** In this retrospective study, we analyzed data of 1,997 patients who underwent colonoscopy and abdominal CT. Patients either had no colorectal neoplasia (n=1,189) or colorectal neoplasia (ntotal=808; adenomas n=423, CRC n=385). Radiomic features were extracted from 3D liver segmentations using the Radiomics Processing ToolKit (RPTK), which performed feature extraction, filtering, and classification. The dataset was split into training (n=1,397) and test (n=600) cohorts. Five machine learning models were trained with 5-fold cross-validation on the 20 most informative features and ensembles.
**Results or Findings:** The radiomics-based XGBoost model achieved best validation performance with a test AUROC of 0.810 [95% CI: 0.767–0.837], clearly outperforming the best clinical-only model (test AUROC: 0.457 [95% CI: 0.411–0.506]). After Youden index-based threshold optimization, the final model reached a test sensitivity of 74.1% and specificity of 72.3% for predicting the presence of colorectal neoplasia. Subclassification between colorectal cancer and adenoma showed lower accuracy (test AUROC: 0.674 [95% CI: 0.606–0.741]).
**Conclusion:** Our findings establish proof-of-concept that liver-derived radiomics from routine abdominal CT can predict colorectal neoplasia. Beyond offering a pragmatic, widely accessible adjunct to CRC screening, this approach highlights the gut–liver axis as a novel biomarker source and sparks new mechanistic hypotheses for future translational research.
**Limitations:** This single-center, retrospective study was intended as a proof-of-concept to demonstrate technical feasibility. Validation in larger, prospective, and multicenter cohorts will be essential to confirm generalizability and clinical utility.
**Funding for this study:** No funding or grant support was received for this study.
**Has your study been approved by an ethics committee?** Yes
**Ethics committee - additional information:** Ethikkommission II (2023-887-AF 11)
**Author Disclosures:**
Jakob Nikolas Kather: Shareholder: StratifAI, Synagen, Tremont AI, and Ignition Labs Grant Recipient: GSK, AstraZeneca, Bayer, Daiichi Sankyo, Eisai, Janssen, Merck, MSD, BMS, Roche, Pfizer, and Fresenius Consultant: Panakeia, AstraZeneca, MultiplexDx, Mindpeak, Owkin, DoMore Diagnostics, and Bioptimus.
Stefan Oswald Schönberg: Nothing to disclose
Freba Grawe: Nothing to disclose
Sergio Grosu: Nothing to disclose
Jonas Bohn: Nothing to disclose
Dominik Nörenberg: Nothing to disclose
Anna Hinterberger: Nothing to disclose
Klaus Maier-Hein: Nothing to disclose
Johannes Betge: Nothing to disclose

### Computed tomography based texture analysis and Node-RADS in colon cancer

*J. Leonhardi*, A-K. Höhn, M. Mehdorn, T. Denecke, H-J. Meyer; Leipzig/DE
*(jakob.leonhardi@medizin.uni-leipzig.de)*

**Purpose:** Texture analysis provides quantitative imaging markers and might improve tumor tissue characterization. Node-RADS classification is a classification system to improve lymph node description in oncological imaging. The present analysis investigated the diagnostic benefit of both methods to categorize and stage lymph nodes in patients with colon cancer.
**Methods or Background:** For Node-RADS analysis, 108 patients (n = 49 females, 45.3%, age 70.08 ± 14.34 years) were included. All patients were surgically resected and lymph nodes were histopathological analyzed. Lymph nodes were scored in accordance to the Node-RADS classification by two experienced radiologists. 85 patients were eligible for texture analysis of the lymph nodes (n = 39 females, 45.9%). Interreader variability was assessed with Cohen's kappa analysis, discrimination analysis was performed with Mann-Whitney-U test and diagnostic accuracy was assessed with ROC curve analysis.
**Results or Findings:** Out of 279 extracted CT texture features, 7 parameters independently showed statistically significant differences between lymph node metastasis to negative lymphnodes. A multivariate model based on n = 7 independent texture parameters reached an AUC of 0.79 (95% CI: 0.69-0.89) with sensitivity of 0.77 and specificity of 0.70. Node-RADS scores showed statistically significant differences between N-/N + stage (N- mean 1.89 ± 1.09 score versus N+ 2.93 ± 1.62 score, p = 0.001). ROC curve analysis showed an AUC of 0.68 for Node-RADS-based lymph node discrimination, with an optimal sensitivity of 0.62 and specificity of 0.71.
**Conclusion:** Texture analysis can improve the diagnostic accuracy for nodal CT staging in patients with colon cancer. Node-RADS scores showed only limited diagnostic accuracy in colon cancer with high interreader variability. This should question clinical translation.
**Limitations:** This was a retrospective study design with possible known inherent bias. Further validation studies are needed to confirm present results.
**Funding for this study:** No Funding was received for this study.
**Has your study been approved by an ethics committee?** Yes
**Ethics committee - additional information:** This retrospective, observational study involving human participants was performed in accordance with the ethical standards of the institutional and/or national research committee and with the 1964 Helsinki Declaration and its later amendments or comparable ethical standards. This retrospective study was approved by our institutional ethics review board (Ethics Committee of the University of Leipzig EK: 243-14-14072014), and the requirement for informed consent of patients was waived.
**Author Disclosures:**
Timm Denecke: Nothing to disclose
Matthias Mehdorn: Nothing to disclose
Anne-Kathrin Höhn: Nothing to disclose
Hans-Jonas Meyer: Nothing to disclose
Jakob Leonhardi: Nothing to disclose

### Feasibility of 40-keV Virtual Monoenergetic Images for Evaluating Extramural Vascular Invasion in Rectal Cancer: Comparison with Rectal MRI as Reference Standard

*M. Asano*, Y. Noda, N. Kawai, T. Suto, T. Kaga, S. Omata, T. Miyoshi, H. Kato, M. Matsuo; Gifu/JP
*(masa.asa0612@gmail.com)*

**Purpose:** To evaluate the feasibility of 40-keV virtual monoenergetic images (VMIs) derived from dual-energy CT for assessing extramural vascular invasion (EMVI) in patients with rectal cancer.
**Methods or Background:** Fifty-six patients with rectal cancer underwent both contrast-enhanced dual-energy CT and rectal MRI were retrospectively analyzed. The 40-keV VMIs at the portal venous phase were reconstructed for all patients. Two radiologists evaluated the EMVI scores on 40-keV VMIs. Another radiologist interpreted rectal MRI and created the reference standard. The diagnostic performance for detecting EMVI (EMVI scores of 3 and 4) in 40-keV VMIs was compared with rectal MRI findings as a reference standard using McNemar test.
**Results or Findings:** The sensitivity and specificity for detecting EMVI in 40-keV VMIs were 85.7% and 88.6%, respectively. When comparing with rectal MRI, the 40-keV VMIs showed no statistical difference in the sensitivity (P =.25) and specificity (P =.13). The κ value was 0.42 in 40-keV VMIs, indicating moderate interobserver agreement.
**Conclusion:** When compared with rectal MRI, the 40-keV VMIs had no statistical difference in the sensitivity and specificity for diagnosing EMVI in patients with rectal cancer.
**Limitations:** Small sample size, evaluated only EMVI of rectal cancer, not compared with the pathological results, and only used rapid-kilovoltage-switching DECT.
**Funding for this study:** This study received no funding.
**Has your study been approved by an ethics committee?** Yes

**Ethics committee - additional information:** Our institutional review board approved this retrospective study and waived written informed consent due to the retrospective nature of the study.
**Author Disclosures:**
Tetsuro Kaga: Nothing to disclose
Toshiharu Miyoshi: Nothing to disclose
Taketo Suto: Nothing to disclose
Masashi Asano: Nothing to disclose
Yoshifumi Noda: Nothing to disclose
Masayuki Matsuo: Nothing to disclose
Nobuyuki Kawai: Nothing to disclose
Shingo Omata: Nothing to disclose
Hiroki Kato: Nothing to disclose

**Reliability of MRI in Detecting Lymph Node Involvement in Rectal Cancer**
N. Gündüz, *M. Gezgin*, B. Akgun, H. Seneldir, B. Atalay, O. Alimoglu, G. Erdem; Istanbul/TR
*(me.egezgin@hotmail.com)*

**Purpose:** Rectal cancer affects more than 700,000 people annually worldwide and accounts for 3.2% of cancer-related deaths.Regional lymph node(LN) status is a critical determinant in TNM staging, treatment planning, and prognosis.Magnetic resonance imaging(MRI) is the preferred modality for local staging, yet its diagnostic reliability for LN involvement remains debated. This study aimed to evaluate the diagnostic performance and reliability of MRI in assessing LN metastasis in rectal cancer.
**Methods or Background:** This retrospective study included patients with rectal cancer who underwent preoperative pelvic MRI and subsequent surgical resection between 2020 and 2023. Patients without either imaging or pathology data were excluded. Two abdominal radiologists(1–10 years of experience) independently reviewed MRIs using the 2016 ESGAR criteria.LN morphology(short-axis size, round shape, irregular border, heterogeneity) was assessed. Nodes ≥9 mm, 5–9 mm with ≥2 malignant features, or <5 mm with ≥3 features were classified as malignant; mucinous nodes were also considered malignant. In patients receiving neoadjuvant therapy, nodes >5 mm were classified malignant. MRI findings were compared with histopathology. Interobserver agreement was analyzed with kappa statistics.
**Results or Findings:** Of 68 patients, 42 were eligible (mean age 57.6 years; 24 male, 18 female).Histopathology confirmed LN metastases in 16 cases, all detected by MRI(sensitivity 100%). Of 26 LN-negative cases, MRI correctly identified 15 but overcalled 11, yielding specificity 57.7%. Overall diagnostic accuracy was 73.8%, with positive predictive value 59.3% and negative predictive value 100%. Interobserver agreement was excellent (κ=0.94).
**Conclusion:** MRI demonstrated high sensitivity but relatively low specificity in detecting LN involvement in rectal cancer, consistent with prior literature. Awareness of potential false positives is essential, and refinement of morphologic malignancy criteria may improve specificity.
**Limitations:** Single-center design, retrospective analysis, modest sample size, and inability to stratify histopathological LN subtypes may limit generalizability.
**Funding for this study:** No funding
**Has your study been approved by an ethics committee?** Yes
**Ethics committee - additional information:** Approved by Istanbul Medeniyet University Ethics Committee, 2023/0292
**Author Disclosures:**
Merve Gezgin: Nothing to disclose
Burcin Akgun: Nothing to disclose
Gülnur Erdem: Nothing to disclose
Nesrin Gündüz: Nothing to disclose
Hatice Seneldir: Nothing to disclose
Orhan Alimoglu: Nothing to disclose
Başak Atalay: Nothing to disclose

**Postinduction Response Evaluation in Total Neoadjuvant Therapy of Rectal Cancer: Toward Response-guided Management Strategy**
*Y. Shen*[1], T. Jiang[1], X. Gong[2], W. Meng[1], Z. Wang[1]; [1]Chengdu/CN, [2]Cheng du/CN
*(hi_moke@163.com)*

**Purpose:** Total neoadjuvant therapy (TNT) has improved the pathological complete response (pCR) rate for locally advanced rectal cancer (LARC). The relationship between tumor response after the induction chemotherapy (INCT) and pCR has not been decided. We aimed to evaluate the association between the post-induction (PI) tumor response after INCT and the ultimate treatment response in LARCs undergoing TNT.
**Methods or Background:** From 2018 to 2022, consecutive patients diagnosed with LARC who received TNT before radical surgery and had MRI examinations at both PI and pre-operation (PO) were included. 360 LARC patients were enrolled for initial assessment, with 224 patients included in the final analysis. The primary outcome was the pCR rate. MR-TRG and tumor longitudinal length reduction rate (TLLR) were used to evaluate the tumor response to INCT at PI. The combination of MR-TRG and DWI was used to determine the presence of MRI complete response (mriCR) at PO. A univariate logistic regression model was used to establish associations between PI response and PO response, PI response and pathological response.
**Results or Findings:** The current study included 224 patients (median [IQR] age, 56 [49-65] years; 136 [60.7%] male). Compared with patients who did not achieve partial response (PR) at PI, patients who achieved PR at PI have higher PO mriCR rate (OR 3.788, 95%CI 1.740-8.202, p<0.001) and higher final pCR rate (OR 4.412, 95%CI 1.881-10.347, p<0.001).
**Conclusion:** Tumor response to INCT is related to the final pathological response in LARC patients who underwent TNT. Good responders to INCT have higher odds of reaching a mriCR or pCR, supporting mid-term tumor response evaluation after INCT in a response-guided treatment strategy.
**Limitations:** Survival outcomes were not included in the current study because the data were still unavailable.
**Funding for this study:** This work was supported by the Postdoctoral Fellowship Program of CPSF (No. GZB20250525), the National Natural Science Foundation of China (82103541, 82203474, 82203394, 82103918), Department of Science and Technology of Sichuan Province (No. 2022YFS0162, 2021YFS0025, 2022YFS0209), Sichuan Province Natural Science Foundation Project (2022NSFSC0840, 2023NSFSC1834), 1·3·5 Project for Disciplines of Excellence-Clinical Research Incubation Project, and West China Hospital, Sichuan University (No. 22HXFH001, 2019HXFH031).
**Has your study been approved by an ethics committee?** Yes
**Ethics committee - additional information:** The study received institutional review board approval and written informed consent was obtained from all participants.
**Author Disclosures:**
Xiaoling Gong: Nothing to disclose
Tianxiang Jiang: Nothing to disclose
Wenjian Meng: Nothing to disclose
Yu Shen: Nothing to disclose
Ziqiang Wang: Nothing to disclose

**Offering behavioural economics messages or sigmoidoscopy or CT-colonography to increase participation of non-respondents in population-based colorectal cancer screening: a randomized controlled trial**
G. Gorini[1], *L. Sali*[1], B. Mallardi[1], C. Campanino[1], E. Betti[1], F. Battisti[1], S. Stoffel[2], P. Mantellini[1]; [1]Florence/IT, [2]Basel/CH
*(lapo.sali@yahoo.it)*

**Purpose:** To evaluate if offering behavioural economics messages or alternative tests improve participation of non-respondents in immunochemical faecal test (FIT) based colorectal cancer (CRC) screening.
**Methods or Background:** 20,225 non-respondents to FIT (aged 50-70 years) in a CRC screening program were randomized into six groups and invited by mail: 1) to FIT with standard letter (SL); 2) to FIT with SL plus feedback message (F); 3) to FIT with SL plus social norm message (MN); 4) to FIT with SL and combination of F+MN; 5) to sigmoidoscopy ; 6) to CT colonography. The primary outcome measure was the participation rate within 90 days from invitation. The trial was registered: ISRCTN11841256.
**Results or Findings:** Overall 1,106 non-respondents to FIT partecipated. Screening participation rates were 5.7% in SL, 7.4% in F , 6.7% in MN, 6.6% in F+MN, 2.0% in sigmoidoscopy, 4.1% in CT colonography groups. F group invitees were more likely to participate as compared to SL control group (aOR=1.32; 95%CI: 1.10-1.57), to sigmoidoscopy group (aOR=0.32; 95%CI: 0.24–0.42) and to CT colonography group (aOR=0.67, 95%CI:0.52–0.86).
**Conclusion:** Behavioural economics interventions can increase participation in FIT-based CRC screening programs more than offering different screening tests. In non-respondents to FIT CT colonography recorded a two-fold participation to screening as compared to sigmoidscopy.
**Limitations:** Detection rates of colorectal neoplasia were not evaluated in presente study.
**Funding for this study:** Tuscany Region (CUP F18D20000120002, 2018)
**Has your study been approved by an ethics committee?** Yes
**Ethics committee - additional information:** Comitato Etico Regione Toscana- Area Vasta Centro (CEAVC): ID: 16875_spe, approved 19/01/21
**Author Disclosures:**
Chiara Campanino: Nothing to disclose
Sandro Stoffel: Nothing to disclose
Elisa Betti: Nothing to disclose
Paola Mantellini: Nothing to disclose
Giuseppe Gorini: Nothing to disclose
Francesca Battisti: Nothing to disclose
Beatrice Mallardi: Nothing to disclose
Lapo Sali: Nothing to disclose

**Diagnostic Accuracy of Hounsfield Units in Virtual Non-Contrast (VNC) vs. True Non-Contrast (TNC) for Differentiating Hepatic Cysts and Colorectal Cancer Liver Metastases**

*M. Kabiri*, M. Nejati, C. Booz, I. Yel, K. Eichler, T. Vogl; Frankfurt am Main/DE
*(moh3nkabir@gmail.com)*

**Purpose:** The differentiation of hepatic cysts and colorectal cancer liver metastases (CRLM) is critical for accurate diagnosis and treatment planning. This study evaluates the diagnostic accuracy of Hounsfield Units (HU) in Virtual Non-Contrast (VNC) imaging compared to True Non-Contrast (TNC) imaging for distinguishing these lesions.
**Methods or Background:** A retrospective analysis was conducted on 120 patients who underwent Dual-Energy Computed Tomography (DECT) scans between January 2020 and December 2022. Patients with confirmed hepatic cysts or CRLM were included. HU values were measured in regions of interest (ROIs) within the lesions. Statistical analyses included paired t-tests and ROC curve analysis to evaluate diagnostic performance.
**Results or Findings:** The TNC method achieved an AUC of 0.864 (sensitivity: 70.51%, specificity: 82.26%), while the VNC method achieved an AUC of 0.861 (sensitivity: 66.88%, specificity: 84.62%). No significant difference was observed between the two methods ($p = 0.850$). VNC imaging demonstrated slightly higher mean HU values for both cysts and metastases compared to TNC imaging.
**Conclusion:** VNC imaging offers comparable diagnostic accuracy to TNC imaging for differentiating hepatic cysts and CRLM, with the added benefit of reduced radiation exposure. These findings support the integration of VNC imaging into routine clinical practice.
**Limitations:** The limitations of this study are minimal and unlikely to impact the validity of the findings.
**Funding for this study:** The authors declare that they have no known competing financial interests or personal relationships that could have appeared to influence the work reported in this paper.
**Has your study been approved by an ethics committee?** Yes
**Ethics committee - additional information:** The institutional ethic review board approved the present retrospective single-center study with a waiver for written informed consent.
**Author Disclosures:**
Thomas Vogl: Nothing to disclose
Ibrahim Yel: Nothing to disclose
Christian Booz: Nothing to disclose
Katrin Eichler: Nothing to disclose
Mohsen Kabiri: Nothing to disclose
Maral Nejati: Nothing to disclose

**08:00 - 09:30** **Research Stage 6**

### Research Presentation Session: Artificial Intelligence and Imaging Informatics

### RPS 105

### Exploring the AI-radiomics interface in lung cancer

**Moderator**
Harry J. De Koning; Rotterdam/NL
*(h.dekoning@erasmusmc.nl)*
**Author Disclosures:**
Harry J. De Koning: Grant Recipient: 4-IN-THE-LUNG-RUN trial+LAPIN, Speaker: Astra Zeneca

**Evaluation of the influence of human factors towards a new AI-CADe/x system for lung cancer risk stratification in the radiology workflow**

C. Santos[1], M. Santos[1], J. H. R. Cairns[2], A. Johnstone[2], M. Darby[2], C. Arteta[1], *A. Scarsbrook*[2]; [1]Oxford/UK, [2]Leeds/UK
*(a.scarsbrook@nhs.net)*

**Purpose:** Integration of AI-based computer aided detection (CADe) and computer aided diagnosis (CADx) tools could help improve and standardize CT reporting and increase efficiency. However, clinical benefit may in part depend on user's attitudes towards AI technologies. The effect of human factors on a new AI-CADe/x tool for assisting CT-based lung nodule risk stratification was evaluated.
**Methods or Background:** A Multiple Reader Multiple Case (MRMC) study assessed the effect of an AI-CADe/x tool on readers estimates of lung cancer likelihood from CT scans. This facilitated pulmonary nodule detection, segmentation, and lung cancer risk prediction. Twelve radiologists evaluated 240 cases (95 cancers), with and without AI-assistance. Technology acceptance was modelled using a 5-point Likert Scale survey assessing Perceived Ease of Use (PEO) and Usefulness (PU), attitudes towards AI and trust. These were correlated with mean effect size (ES), determined via the Dorfman-Berbaum-Metz method (z-test used for difference between ES; 95% confidence intervals reported).
**Results or Findings:** The AI tool increased user reporting confidence, median survey score (interquartile range) was 4 (4-4.25), being highly rated for PEO (4 (4-5)) and PU (4 (4-5)). Participants expressed greater trust towards CADe (4 (3-4)) than CADx (3 (2-4)) components. ES was affected by levels of trust, automation, aversion and confirmation biases. Participants without tendency for confirmation bias (n=5), i.e. who considered the AI suggestion even when it challenged their initial assessment, showed a significantly higher ES from AI-assistance ($p=0.028$) than those exhibiting bias, with an ES of 6.25% [-1.95, 8.35] versus 3.02% [2.02, 6.31], respectively.
**Conclusion:** Attitudes towards AI influence radiologist interaction with AI-based CADe/CADx tools. Positive attitudes and greater trust resulted in improved benefits from AI assistance for lung cancer risk-stratification.
**Limitations:** Preliminary technology acceptance modelling across twelve radiologists.
**Funding for this study:** The study was jointly funded by the National Institute for Health and Care Research (NIHR) and the Office for Life Sciences (OLS) under project ID NIHR207547.
**Has your study been approved by an ethics committee?** Not applicable
**Author Disclosures:**
Carlos Arteta: Employee: Optellum Ltd
James Henry Robert Cairns: Nothing to disclose
Mauro Santos: Employee: Optellum Ltd
Annette Johnstone: Nothing to disclose
Andrew Scarsbrook: Grant Recipient: National Institute of Health and Research
Catarina Santos: Employee: Optellum Ltd
Michael Darby: Nothing to disclose

**Commercially available AI products for CT-based lung cancer screening: capabilities, clinical evidence, and alignment with international screening frameworks**

*N. Antonissen*[1], S. S. Schalekamp[1], H. Hahn[2], K. G. Van Leeuwen[3], C. Jacobs[1]; [1]Nijmegen/NL, [2]Bremen/DE, [3]Utrecht/NL
*(noa.antonissen@radboudumc.nl)*

**Purpose:** To evaluate certified capabilities of CE-marked AI products for lung nodule analysis in CT lung cancer screening, assess their alignment with international screening frameworks, and analyze their supporting peer-reviewed evidence.
**Methods or Background:** Six core clinical tasks (nodule detection, classification, measurement, growth assessment, malignancy risk estimation, structured management) were defined by analyzing four major screening frameworks: the Lung CT Screening Reporting and Data System (Lung-RADS) version 2022, British Thoracic Society (BTS) guidelines, European Union Position Statement (EUPS) recommendations, and European Society of Thoracic Imaging (ESTI) nodule management recommendations. CE-marked AI products were identified through the Health AI Register. Vendors confirmed certified capabilities using a standardized questionnaire; public documentation supplemented non-responders. Scientific evidence was evaluated using a six-level efficacy framework and assessed for study characteristics.
**Results or Findings:** In total, 16 products from 16 vendors were included, and 10 vendors completed the questionnaire. Regarding capabilities, 14 products detect and measure solid and subsolid nodules, 12 support growth assessment, and none support endobronchial or cystic lesion evaluation. For risk estimation, 9 products provide malignancy-risk outputs: 5 use the PanCan model and 4 provide proprietary AI-based scores. Six products had no peer-reviewed publications. The remaining 10 products were supported by 60 studies, of which 7% were prospective and 45% vendor-independent, with external testing including multicenter (40%), multinational (7%), and screening cohort (40%) datasets. Overall, evidence was concentrated at lower efficacy levels: 70% assessed standalone diagnostic accuracy, 25% examined effects on diagnostic decision-making, and none reported patient or societal outcomes.
**Conclusion:** CE-marked AI solutions fulfill core functions for nodule assessment but lack certified capabilities for endobronchial and cystic lesions and are supported by limited prospective, higher-level clinical evidence.
**Limitations:** Product capability assessment relied on publicly available and vendor-reported data.
**Funding for this study:** This project is co-funded by the SOLACE project, funded under the EU4Health Programme 2021–2027 under grant agreement no. 101101187.
**Has your study been approved by an ethics committee?** Not applicable

**Author Disclosures:**
Horst Hahn: Other: HH is affiliated with Fraunhofer MEVIS as Managing Director
Steven S. Schalekamp: Nothing to disclose
Kicky Gerhilde Van Leeuwen: Other: KvL is co-owner of Romion Health and Health AI Register and has received speaker fees from Siemens Healthineers and Bayer Pharmaceuticals.
Noa Antonissen: Nothing to disclose
Colin Jacobs: Other: CJ receives research grants and royalties to host institution from MeVis Medical Solutions, public-private research grant with Philips Medical Systems, and public-private research grant with Siemens Healthineers. CJ received speaker fees from Canon Medical Systems and Johnson & Johnson.

**Diagnostic performance of Harrison.ai CT chest algorithm for detection of lung nodules using the DUKE Lung Cancer Screening Dataset**

*M. Ryan*[1], M. Steele[1], G. Sanderson[1], J. Tang[2]; [1]Sydney/AU, [2]Victoria/AU
*(melissa.ryan@annalise.ai)*

**Purpose:** To evaluate the performance of an artificial intelligence (AI) algorithm for pulmonary nodule detection on low-dose CT chest scans to determine the clinical utility of AI-based nodule detection as a decision support tool in lung cancer screening.
**Methods or Background:** Low-dose CT chest scans (n=1,060), available at https://doi.org/10.5281/zenodo.10782890, as part of the DUKE lung cancer screening dataset were evaluated using the Harrison.ai CT chest device. The dataset represents a cohort of screening CT scans from a high-volume contemporary lung cancer screening center with associated Lung-RADS scores, and where applicable, histologic type and stage of future lung cancer. All images were analysed by the device, which automatically identifies cases with identified nodules, as well as localisation of each nodule instance within an image series. Patient-level and instance-level sensitivity were evaluated. Stratified performance based on gender and malignancy was also performed.
**Results or Findings:** Preliminary results of 1,037 cases that were successfully processed demonstrated patient-level sensitivity of 91% and instance-level sensitivity of 62%, with an average of 1.12 false positive instances per case. False positive rate may be in part due to AI detection of nodules below the datasets minimum size threshold (4 mm).
**Conclusion:** The Harrison.ai CT chest device demonstrated excellent diagnostic performance at the case level which supports the potential role for AI in enhancing efficiency and reliability in lung cancer screening programs using low-dose CT.
**Limitations:** The retrospective design and reliance on a single institutional dataset limit generalisability. The algorithm's performance may vary with differing scanner protocols, populations, and nodule characteristics not fully represented in the DUKE dataset.
**Funding for this study:** No funding was received for this study
**Has your study been approved by an ethics committee?** Not applicable
**Author Disclosures:**
Megan Steele: Employee: Harrison.ai
Jennifer Tang: Employee: Harrison.ai
Georgina Sanderson: Employee: Harrison.ai
Melissa Ryan: Employee: Harrison.ai

**AI-Assistance Improving Lung Nodule Risk Assessment in Lung Cancer Screening: Results from RELIVE, A Multi-Reader Multi-Case Study to Evaluate the Performance of an end-to-end AI/ML-based CADe/CADx**

*C. M. Voyton*, P-H. Siot, B. Renoust, B. Huet, V. Bourdes; Valbonne/FR
*(charles.voyton@mediantechnologies.com)*

**Purpose:** Evaluation of LDCT exams for lung cancer screening (LCS) is time intensive, and nodule risk stratification is challenging and prone to false positives. This multi-reader multi-case (MRMC) study evaluated the improvement in radiological review and nodule risk assessment with the support of an end-to-end AI/ML-based CADe/CADx.
**Methods or Background:** In this paired split plot (PSP) design MRMC, 480 retrospective LCS patients (160 cancer, 320 non-cancer) and 16 US board-certified radiologists were split into 4 balanced blocks of 120 patients and 4 radiologists respectively; each case within each block was read twice (with and without the assistance of the AI/ML-based CADe/CADx) with at least 28 days of washout in between. Readers were asked to locate, measure and assign a 1-100 risk score per nodule as well as assign a recommended patient management.
**Results or Findings:** AUROC (AUC) and specificity (sp) with the support of the AI (AUC: 0.843, sp: 0.700) significantly increased ($p < 0.05$) versus without (AUC: 0.828, sp: 0.648) demonstrating LCS report improved diagnostic performance. Less experienced radiologists (2-6 years of experience) showed the largest increase in AUC of 4.77% ($p = 0.0308$). The inter-reader agreement was also significantly improved ($p<0.0001$) for readers using the device with an increase in intraclass correlation coefficient (ICC) from 0.707 to 0.830.
**Conclusion:** The AI system enhanced radiologists' diagnostic accuracy for pulmonary nodule malignancy risk assessment in a LCS cohort. These findings support its potential role as a key decision-support tool in clinical practice, especially for early career clinicians.
**Limitations:** Retrospective study on an enriched cohort that does not fully reflect real-world cancer and nodule characteristic prevalences.
**Funding for this study:** This study was funded by Median Technologies On Behalf of the REALITY/RELIVE Investigators: (participating centers and investigators: Baptist Cancer Center, Memphis, TN, USA – Raymond Osarogiagbon; Clínica Universidad de Navarra, Madrid, Spain – Luis Seijo, Gorka Bastarrika; Penn Medicine, Philadelphia, PA, USA – Anil Vachani; Hospital Universitario Fundación Jiménez Díaz, Madrid, Spain – Carolina Gotera; The University of Texas MD Anderson Cancer Center, Houston, TX, USA – Edwin Ostrin, Jennifer Dennison).
**Has your study been approved by an ethics committee?** Yes
**Ethics committee - additional information:** clinicaltrials.gov number: NCT06751576
**Author Disclosures:**
Benjamin Renoust: Author: Median Technologies
Charles Michael Voyton: Author: Median Technologies
Benoit Huet: Author: Median Technologies
Valerie Bourdes: Author: Median Technologies
Pierre-Henri Siot: Author: Median Technologies

**A 'Grounded Theory' exploration of stakeholders' opinions of Radiology AI for Prioritised Imaging and Diagnosis of lung cancer (the 'RAPID-LC' study)**

*C. Rainey*[1], A. Gill[2], S. L. Mcfadden[2]; [1]Cork/IE, [2]Belfast/UK
*(clare.rainey@ucc.ie)*

**Purpose:** Lung cancer is a leading cause of death globally and projected to reach 3·55 million deaths by 2050. Currently many patients in Northen Ireland (NI) are identified at advanced stages leading to a poor survival rate. AI has been proposed to assist clinicians, however the perceptions of the clinician and patient need to be considered . This will ensure that AI is used optimally and designed with the end-user in mind.
**Methods or Background:** A grounded theory methodology was adopted. 1:1 interviews were conducted with members of the public (patients) and clinicians (two nurses, an oncologist, a medical physicist, a radiographer and an anesthetist) currently working in the lung cancer pathway in NI. Data were transcribed verbatim and Braun and Clarkes thematic analysis used.
**Results or Findings:** Seven themes and eight subthemes emerged from the data. Patients trusted the clinicians' scrutiny of the AI system whilst the clinician required robust ongoing validation and education. The public/patient participants are unconcerned about the cost implications of AI. Both groups were positive about using AI to streamline care/reduce waiting times. They were more cautious in its use for symptomatic presentations. Whilst results are optimistic, various concerns exist around (i) trust, (ii) communication, (iii) validation (iv) infrastructure. These remain central to discussions around AI adoption in NI and internationally.
**Conclusion:** Clinicians require robust and transparent validation, while the public are happy to trust the clinician. Neither group feels that autonomous AI is acceptable currently. Both groups feel strongly that AI is needed to support healthcare systems. Education and co-design have been proposed to facilitate adoption.
**Limitations:** This study was conducted with participants from NI, where the lung cancer pathway may differ slightly from the rest of Europe.
**Funding for this study:** SBRI Research grant
**Has your study been approved by an ethics committee?** Yes
**Ethics committee - additional information:** UU Nursing and Health Research Filter Committee
**Author Disclosures:**
Clare Rainey: Grant Recipient: This study was funded by an SBRI grant
Sonyia Lorraine Mcfadden: Grant Recipient: This study was funded by an SBRI grant
Avneet Gill: Grant Recipient: This study was funded by an SBRI grant

**A preliminary study: Homology-based detection of lung cancer lesion in CT images**

*K. Doi*, H. Numasaki, Y. Anetai, M. Imai, Y. Natsume-Kitatani; Osaka/JP
*(doi@nibn.go.jp)*

**Purpose:** We proposed the homology-based feature (HF) map to pulmonary quantify fibrotic lesions in CT images (Doi, et al. IJCARS 2025). However, lung cancer lesions reduce this quantitatively performance. This study addressed this issue by detecting lung cancer lesions prior to the quantification of pulmonary fibrosis through the optimizing the HF map calculation.
**Methods or Background:** We utilized publicly available forty CT images and the corresponding radiotherapy structure datasets from the cancer image archive. These datasets include the coordinate information of lung cancer lesions defined by radiologists, so the coordinate information was utilized as a ground truth. HF maps were obtained from the collected CT images, and predicted lung cancer lesions were generated by the threshold processing for HF maps. This method can be regulated by hyperparameters such as the tile size, tile shift size, range of CT value for analyses, and threshold value of HF map. Here, these hyperparameters were optimized using the DICE coefficient as an objective function, which can indicate similarity between a predicted lung cancer lesion and a ground truth.
**Results or Findings:** The DICE coefficient has achieved 0.93 by optimized hyperparameters as followings: the tile size, 16 pixels; tile shift size, 2; range of CT value for analyses; -800 to 100; and threshold value of HF map, 85% of the maximum value of HF maps. The range of CT values differs from that of the quantification of fibrosis, which might be caused by differences in those CT values.
**Conclusion:** We have demonstrated the potential of the HF map to detect lung cancer lesions in CT images.
**Limitations:** This methodology will be optimized for each lung cancer type related to the CT value. Moreover, more deeply validation of this study using validation datasets is required.
**Funding for this study:** This work has been supported by JSPS KAKENHI [grant number 24K23909] and the Japan Science and Technology Agency COI-NEXT [grant JPMJPF2018].
**Has your study been approved by an ethics committee?** Not applicable
**Author Disclosures:**
Masato Imai: Nothing to disclose
Kentaro Doi: Nothing to disclose
Yayoi Natsume-Kitatani: Nothing to disclose
Hodaka Numasaki: Nothing to disclose
Yusuke Anetai: Nothing to disclose

### In-vivo classification between lung adenocarcinoma and squamous cell lung cancer through late fusion of a clinical model and an MRI radiomic model

J-E. Al Khoury[1], C. Nioche[1], M. Lacroix[2], M. Hamadouche[3], C. Suchel[3], P-Y. Brillet[3], *F. Frouin*[1]; [1]Orsay/FR, [2]Paris/FR, [3]Bobigny/FR
*(frederique.frouin@inserm.fr)*

**Purpose:** Improving a classification model between lung adenocarcinoma (ADK) from squamous cell lung cancer (SQc) through the integration of clinical and MRI data.
**Methods or Background:** Clinical and MRI radiomic logistic regression models were developed from a training/validation set of 80 patients with advanced lung cancer (59 ADK and 21 SQc). A late fusion model combining the probabilities of the two models was defined. The three models were tested on 50 patients (30 ADK and 20 SQc). Clinical data were recorded including age, weight, BMI, smoking status, and TNM staging. Following brain MRI to discover possible brain metastases, a contrast-enhanced T1-weighted (CET1w) chest MRI was performed. After preprocessing of CET1w MRI, including intensity normalization and spatial resampling (1 mm3 isotropic voxels), the lung tumor was delineated in 3D. Radiomic features including radial intensity measures were extracted using LIFEx software (version 25.9.4). Model training was based on a forward selection of features that maximises balanced accuracy.
**Results or Findings:** Only one parameter (age) was selected for the clinical model. Only one RIM feature (mean gradient calculated between the mean intensities of successive layers within the tumor) was selected for the radiomic model. Based on a five-fold cross-validation framework, the balanced accuracy was 0.62 for clinical, 0.61 for radiomic, and 0.68 for late fusion models in the validation phase. When applied to the test set, the balanced accuracies were 0.62 for clinical, 0.62 for radiomic, and 0.67 for late fusion models.
**Conclusion:** The late fusion of the clinical model and the MRI radiomic model shows improved results for the classification task (ADK compared to SQc) when compared to each individual model. All three models were robust and showed stable performance in the test set.
**Limitations:** No limitations were identified
**Funding for this study:** Technical and logistic help from GE HealthCare
**Has your study been approved by an ethics committee?** Yes
**Ethics committee - additional information:** IRB protocol 32-2016, study number: 2016-A00813-48
**Author Disclosures:**
Macil Hamadouche: Nothing to disclose
Frédérique Frouin: Nothing to disclose
Pierre-Yves Brillet: Nothing to disclose
Christophe Nioche: Nothing to disclose
Chloé Suchel: Nothing to disclose
Joseph-Elias Al Khoury: Nothing to disclose
Maxime Lacroix: Nothing to disclose

### Benchmarking lung tumour segmentation models: stratified performance of deep learning models across tumour sizes and cancer stages

*A. Cerrato Nieto*, E. Scholten, S. S. Schalekamp, M. Prokop, C. Jacobs; Nijmegen/NL
*(alonso.cerratonieto@radboudumc.nl)*

**Purpose:** Accurate lung tumour segmentation in CT scans is crucial for staging, radiotherapy planning, and treatment monitoring. Published deep learning models for lung tumour segmentation show varying consistency across tumour stages and may fail in complex tumours. This study compared five publicly available models with an in-house model trained to be robust to variation in tumour sizes and cancer stages.
**Methods or Background:** A dataset of 588 CT scans from lung cancer patients (2006-2020) was retrospectively collected and annotated at Radboud University Medical Center. A deep learning model was trained using the nnU-Net architecture on subsets of Radboud patients (n=505), the NSCLC-Radiomics dataset (n=362) and the Medical Segmentation Decathlon dataset (n=56). Our model was compared with five publicly available models, including the Universal Lesion Segmentation baseline model, the Medical Segmentation Decathlon lung model, DuneAI, TotalSegmentator and nnInteractive. Segmentation accuracy was assessed using volumetric and boundary metrics, including stratified analyses by tumour size (≤30 mm, >30–50 mm, >50–70 mm, >70 mm) on our internal test dataset (n=83).
**Results or Findings:** Our proposed model performed equal to or superior to the best public models regarding volumetric Dice scores (median ≥0.87), showing an increase ranging from 0.01 to 0.28, depending on the model and the tumour size group. The model demonstrated substantially improved consistency with interquartile ranges ≤ 0.10 for all tumour sizes. It also achieved higher surface Dice together with lower Hausdorff distance, indicating improved tumour border accuracy. Performance remained superior in clinically demanding cases with cavities, local invasion, and large masses.
**Conclusion:** Our model improves performance and robustness over prior models across tumour sizes, including challenging cases. It represents a promising step towards automated evaluation of lung tumours.
**Limitations:** Independent validation in larger multicentre datasets is required.
**Funding for this study:** Public private consortium with funding from NWO, Dutch Ministry of Economic Affairs, and MeVis Medical Solutions, Bremen, Germany.
**Has your study been approved by an ethics committee?** Yes
**Ethics committee - additional information:** The institutional review board waived the need for informed consent because of the retrospective design and data pseudonymization.
**Author Disclosures:**
Steven S. Schalekamp: Nothing to disclose
Mathias Prokop: Research/Grant Support: Canon Medical Systems Speaker: Canon Medical Systems (Speaker fee to institution)
Ernst Scholten: Nothing to disclose
Alonso Cerrato Nieto: Research/Grant Support: Dutch Science Foundation (NWO), Dutch Ministry of Economic Affairs, and MeVis Medical Solutions AG, Bremen, Germany.
Colin Jacobs: Research/Grant Support: MeVis Medical Solutions AG, Bremen, Germany and Philips Medical System, Best, Netherlands; Other: Royalties to institution from MeVis Medical Solutions AG, Bremen, Germany

### Benchmarking of Artificial Intelligence and Radiologists for Indeterminate Lung Nodule Malignancy Risk Estimation on Screening CT: Results of the LUNA25 Challenge

D. Peeters[1], *B. Obreja*[1], N. Antonissen[1], Z. Saghir[2], U. Pastorino[3], G. De Bock[4], R. Vliegenthart[4], M. Prokop[1], C. Jacobs[1]; [1]Nijmegen/NL, [2]Hellerup/DK, [3]Milan/IT, [4]Groningen/NL
*(bogdan.obreja@radboudumc.nl)*

**Purpose:** Accurate risk classification of indeterminate (5-15mm) lung nodules can reduce unnecessary follow-up in lung cancer screening. AI may assist in risk classification, however, benchmarking studies are limited. Here, we present the results of the LUNA25 challenge, a public competition that evaluates AI and radiologist performance for malignancy risk estimation of indeterminate nodules at screening CT.
**Methods or Background:** LUNA25 consists of an AI study and a reader study. For AI development, participants had access to a public dataset of 4069 CT scans from the National Lung Cancer Screening Trial (NLST), with 555 malignant and 5608 benign nodules. AI evaluation was performed on an external test set with 156 malignant and 312 benign indeterminate solid and part-solid nodules from baseline scans of the Danish (DLCST), Dutch-Belgian (NELSON), and Italian (MILD) lung cancer screening trials. For the reader study, radiologists assessed 300 nodules from the test set, assigning each a malignancy risk score (0–100) and management recommendation (low, intermediate, or high-risk). Performance was compared using area under the ROC curve (AUC), sensitivity, and specificity.

**Results or Findings:** On the subset of 300 nodules, the top-performing AI system showed a statistically superior AUC of 0.78 (95% CI :0.73-0.84, p<0.001) in comparison to the average AUC of 75 readers with an AUC of 0.69 (95% CI :0.64-0.74). At the ≥ indeterminate risk threshold, the AI correctly classified 12% more malignant cases at matched specificity, and 20% fewer false-positives at matched sensitivity.
**Conclusion:** The top-performing AI system demonstrated statistically significant superior performance compared to the average radiologist in estimating malignancy risk for indeterminate lung nodules detected on screening CT, highlighting its potential use as a decision-support tool.
**Limitations:** LUNA25 only benchmarks AI's stand-alone performance and does not address workflow integration or radiologist-AI interaction.
**Funding for this study:** This work was supported by a public-private research projectwith funding from the Dutch Research Council (NWO), the Dutch Ministry of Economic Affairs, and MeVis Medical Solutions (Bremen, Germany), as well as by a public-private project with funding from the Dutch Cancer Society (KWF Kankerbestrijding, project number 9037) and Siemens Healthineers, as well a project with funding from the Dutch Cancer Society (KWF Kankerbestrijding, project number 14113).
**Has your study been approved by an ethics committee?** Yes
**Ethics committee - additional information:** Ethical approval for the training set was granted with the NLST trial receiving institutional review board approval at all 33 centers participating in the trial. In addition, informed consent was provided by all participants involved in the trial. Access to this dataset was granted through the National Cancer Institute's Cancer Data Access System (CDAS) under project number NLST-74, NLST-111, NLST-164 and NLST-267. Ethical approvals for the testing set were obtained from the Ethics Committee of Copenhagen County (DLCST), the institutional review board of Fondazione IRCCS Istituto Nazionale Tumori di Milano (MILD), and the Dutch Minister of Health with support from the Dutch Health Council (NELSON), along with authorization from the Ethical Boards of participating centres.
**Author Disclosures:**
Geertruida De Bock: Nothing to disclose
Dre Peeters: Other: The host institution of D.P. receives research grants and royalties from MeVis Medical Solutions, Bremen, Germany.
Mathias Prokop: Other: The host institution of M.P. receives research grants and royalties from MeVis Medical Solutions, Bremen, Germany. M.P. receives grants from Canon Medical Systems, and Siemens Healthineers.
Zaigham Saghir: Nothing to disclose
Rozemarijn Vliegenthart: Nothing to disclose
Ugo Pastorino: Nothing to disclose
Noa Antonissen: Other: The host institution of N.A. receives research grants and royalties from MeVis Medical Solutions, Bremen, Germany.
Bogdan Obreja: Other: The host institution of B.O. receives research grants and royalties from MeVis Medical Solutions, Bremen, Germany.
Colin Jacobs: Other: The host institution of C.J. receives research grants and royalties from MeVis Medical Solutions, Bremen, Germany. C.J. is a collaborator in a public-private research project where Radboudumc collaborates with Philips Medical Systems (Best, the Netherlands).

### Can traditional radiomics help in ultra-low dose early lung cancer diagnosis?

*A. Mrukwa*[1], M. Socha[1], J. Polanska[1], R. Dziadziuszko[2]; [1]Gliwice/PL, [2]Gdańsk/PL
*(anna.mrukwa@polsl.pl)*

**Purpose:** Lung cancer is the deadliest cancer in the world due to the usually late diagnosis. If diagnosed early on, during the screening programs, many patients could be saved. Nodules in LDCT scans are miniscule and hard to spot, so it is important to create a system to assist with malignancy assessment while retaining method explainability. Radiomic features were designed for diagnosis in advanced stages, thus may not be suitable for this case.
**Methods or Background:** Nodules were extracted from Pilot Pomeranian Lung Cancer Screening Program using MiMSeg on lungs without bronchovascular bundles. Only changes meeting radiomics calculation size requirements are considered, disqualifying 49 of them. 363 malignant nodules were used and 1054 benign. Pyradiomics radiomic features and additionally introduced sphericity and spikularity are evaluated with Kruskal-Wallis test and post hoc analysis using Dunn's test.
**Results or Findings:** Effect size between groups is medium at most, whereas post hoc analysis showed that the most different group is calcification, with the suspicious changes not distinct from other groups. Thus, binary multilayer perceptron trained on the features achieved balanced accuracy of only 54.9% and 53.3% for medium effect features. For multiclass classification, balanced accuracy is 22.8% for all features, 25.7% for selected. This improvement comes from calcification (over half correctly classified) and is tradeoff for others (accuracy for suspicious dropping from 0.189 to 0.057).
**Conclusion:** Traditional methods do not perform well with LDCT and do not provide a reliable method for early lung cancer diagnosis. Additionally, the smallest nodules are not suited for this analysis type. Thus, introduction of Deep Learning methods seems to give the biggest promise.
**Limitations:** No limitations were identified.
**Funding for this study:** No funding was received for this study.
**Has your study been approved by an ethics committee?** Not applicable
**Author Disclosures:**
Anna Mrukwa: Nothing to disclose
Rafal Dziadziuszko: Nothing to disclose
Marek Socha: Nothing to disclose
Joanna Polanska: Nothing to disclose

### Evaluating research quality of lung cancer CT radiomics using RQS 1.0 and METRICS

*M. Balbi*[1], V. Giannini[1], N. C. Culasso[2], A. Defeudis[3], A. U. Cavallo[4], A. Stanzione[5], A. Ponsiglione[5], R. Cuocolo[6], A. Veltri[1]; [1]Turin/IT, [2]Orbassano/IT, [3]Candiolo/IT, [4]Rome/IT, [5]Naples/IT, [6]Baronissi/IT

**Purpose:** To assess the methodological quality of lung cancer CT radiomics studies using the Radiomic Quality Score (RQS) 1.0 and the METhodological RadiomICs Score (METRICS).
**Methods or Background:** Lung cancer CT radiomics studies published up to December 31, 2024, were scored by 6 human readers of different expertise using RQS 1.0 and METRICS. Median scores were computed, and differences across clinical aim, publication year, and patient number were assessed using the Kruskal-Wallis test. In cases of significant differences, pairwise Mann–Whitney U tests were performed to evaluate group-wise comparisons.
**Results or Findings:** A total of 834 studies were included. Median METRICS and RQS 1.0 percentage scores were 0.63 (IQR, 0.51–0.72) and 0.36 (IQR, 0.52–0.71), respectively. The main methodological limitations included the lack of external validation or any validation strategy, the unavailability of open data, and the retrospective, single-center study design. Median METRICS scores of studies published in 2024 (0.67) and 2023 (0.69) were significantly higher than those published before 2023 (≤0.65; all p < 0.04). Similarly, median RQS 1.0 scores of studies published in 2024 (0.42) were significantly higher than those previously published (≤0.39; all p < 0.01). Studies including >100 patients showed significantly higher METRICS and RQS 1.0 scores than those with ≤100 patients (all p < 0.001). Differences across clinical aims were observed, with patterns varying between METRICS and RQS 1.0.
**Conclusion:** Research quality in lung cancer CT radiomics was rated as good by METRICS while RQS 1.0 yielded comparatively lower scores. Both tools highlighted an improvement in quality over time and higher scores in studies involving >100 patients.
**Limitations:** -Lack of reproducibility analyses. -Lack of evaluation according to RQS 2.0 (published after data collection and analysis).
**Funding for this study:** No funding.
**Has your study been approved by an ethics committee?** Not applicable
**Author Disclosures:**
Valentina Giannini: Nothing to disclose
Andrea Veltri: Nothing to disclose
Noemi Cristina Culasso: Nothing to disclose
Andrea Ponsiglione: Other: The Author was part of the METRICS team
Arnaldo Stanzione: Other: The Author was part of the METRICS team
Maurizio Balbi: Nothing to disclose
Renato Cuocolo: Other: The Author was part of the METRICS team
Armando Ugo Cavallo: Other: The Author was part of the METRICS team
Arianna Defeudis: Nothing to disclose

### Comparative analysis of multiple AI software packages on detection performance of the reference nodule for lung cancer screening application

*X. Ouyang*[1], K. Togka[2], D. Han[2], I. Schuldink[2], B. Jiang[1], C. Van Der Aalst[1], H. J. De Koning[1], M. Oudkerk[2]; [1]Rotterdam/NL, [2]Groningen/NL
*(x.ouyang@erasmusmc.nl)*

**Purpose:** Over the past five years, artificial intelligence(AI) for pulmonary nodule detection on Computed Tomography(CT) has rapidly advanced, and multiple commercial AI software packages are now available. However, independent validation—particularly at the reference nodule level—remains limited. This study compares four CE-certified commercial AI software packages for reference nodule detection on low-dose CT, aiming to provide scientific evidence for their application in lung cancer screening(LCS).
**Methods or Background:** A total of 560 baseline LDCT scans were consecutively selected from three Dutch 4-IN-THE-LUNG-RUN(4ITLR) centers following the trial's protocol. Expert radiologists established consensus reference standards. Four CE-certified(three FDA-cleared) commercial AI packages(A–D) were evaluated using an internal automatic platform.

Finally, 554 participants were divided into two cohorts per the NELSON 2.0-European Position Statement by reference standard: positive(77 with the largest nodule ≥100 mm³) and negative(477 with the largest nodule <100 mm³ or no nodules). AI detection in the positive cohort was classified as "Correct positive" or "Negative misclassification." Subgroup analyses were performed by nodule volume, pulmonary-lobe location, and attachment. Logistic regression was applied to examine their effect on AI detection.
**Results or Findings:** Correct positive rates were: Software A: 60/77(77.9%), B: 71/77(92.2%), and C: 59/77(76.6%), D: 66/77(85.7%). Software A showed increasing detection with nodule volume, while D showed negative coefficients. Both C and D each missed 2/6 masses. All packages performed better for right-lung nodules and non-attached nodules, with significant inter-software differences ($p<0.05$). Logistic regression confirmed higher detectability for right-sided and non-attached nodules (log-odds>0).
**Conclusion:** Despite high reported performance in the literature, commercial AI packages show variable detection in a low-dose CT lung cancer screening dataset. Reference nodule detectability is influenced by pulmonary location, nodule volume, and attachment, highlighting the need for independent, comprehensive validation before and during LCS application.
**Limitations:** Not applicable
**Funding for this study:** Not applicable
**Has your study been approved by an ethics committee?** Not applicable
**Author Disclosures:**
Matthijs Oudkerk: Nothing to disclose
Beibei Jiang: Nothing to disclose
Harry J. De Koning: Nothing to disclose
Katerina Togka: Nothing to disclose
Daiwei Han: Nothing to disclose
Xiaotong Ouyang: Nothing to disclose
Ilona Schuldink: Nothing to disclose
Carlijn Van Der Aalst: Nothing to disclose

**Automated CT Quantification of Interstitial Lung Abnormalities and Prognostic Associations in Lung Cancer Screening**
*P. Vasudev*, M. Azimbagirad, B. Selvarajah, A. Bhamani, S. Aslani, D. Alexander, SUMMIT consortium, S. Janes, J. Jacob; London/UK
*(pardeep.vasudev.19@ucl.ac.uk)*

**Purpose:** Interstitial lung abnormalities (ILA) are increasingly recognised in lung cancer screening cohorts and may indicate early fibrotic change. Their prognostic importance has been shown, but large-scale reproducible assessment remains challenging. Automated CT-based quantification offers a scalable solution. The aim was to determine whether automated measures are associated with mortality and whether prognostic performance improves with expert radiologist ILA diagnosis.
**Methods or Background:** This was a retrospective analysis of a multi-centre prospective lung cancer screening cohort. Low-dose chest CT scans from 4,411 participants were included, with ILA status determined by expert radiologist assessment: 3,622 had no ILA and 789 had ILA (17.9% of participants). An automated pipeline segmented the lungs, airways, and vessels, and quantified abnormal parenchymal density by normalising CT intensities and applying mean- and standard deviation-based thresholds to generate density maps. Multivariable Cox proportional hazards regression assessed associations between parenchymal density metrics, expert radiologist ILA diagnosis, and mortality, adjusting for age, sex, smoking history, and forced vital capacity (FVC). Hazard ratios (HR) with 95% confidence intervals (CI) and p-values were reported.
**Results or Findings:** Abnormal high-density lung volume was independently associated with increased mortality (HR 1.03, 95% CI 1.01–1.04, $p=2.04\times10^{-3}$). Prognostic associations strengthened when expert radiologist ILA diagnosis was incorporated (HR 1.03, 95% CI 1.01–1.04, $p=9.72\times10^{-9}$). Significant covariates included age (HR 1.08, 95% CI 1.06–1.11, $p=8.18\times10^{-15}$), sex (HR 0.60, 95% CI 0.45–0.79, $p=2.21\times10^{-4}$), and FVC (HR 0.97, 95% CI 0.97–0.98, $p=1.42\times10^{-10}$). Smoking history was not statistically significant.
**Conclusion:** Automated CT quantification of parenchymal density abnormalities was associated with mortality in lung cancer screening participants. Prognostic associations were stronger when expert radiologist ILA diagnosis was combined with automated measures, supporting their use as objective biomarkers for further validation.
**Limitations:** No limitations were identified.
**Funding for this study:** No funding was received for this study.
**Has your study been approved by an ethics committee?** Yes
**Ethics committee - additional information:** Ethical approval for the lung cancer screening study was obtained from a NHS Research Ethics committee (17/LO/2004) and the NHS Health Research Authority's Confidentiality Advisory Group (18/CAG/0054).
**Author Disclosures:**
Sam Janes: Nothing to disclose
Shahab Aslani: Nothing to disclose
Amyn Bhamani: Nothing to disclose
Daniel Alexander: Nothing to disclose
Brintha Selvarajah: Nothing to disclose
Pardeep Vasudev: Nothing to disclose
Joseph Jacob: Nothing to disclose
Mehran Azimbagirad: Nothing to disclose
Summit Consortium: Nothing to disclose

**08:00 - 09:30** **Research Stage 1**

### Research Presentation Session: Head and Neck

### RPS 108

### Integration of advanced imaging techniques and AI in the head and neck

**Moderator**
Lorenzo Ugga; Naples/IT
*(lorenzo.ugga@gmail.com)*

**Image Quality Improvement in Head and Neck Angiography based on Dual-energy CT and Deep Learning**
*H. Zhang*, Y. Meng; Xuzhou/CN
*(314390107@qq.com)*

**Purpose:** Compare image quality of image reconstructed using DLIR and IR algorithms for head and neck dual-energy CT angiography (DECTA).
**Methods or Background:** This prospective study comprised patients with head and neck DECTA. Images reconstructed by four algorithms (120-kVp-like with ASIR-V40%,50 keV with ASIR-V40%,50 keV with DLIR-M,50 keV with DLIR-H) were compared. CT attenuation, image noise, SNR, CNR, edge-rise distance (ERD) and edge-rise slope (ERS) were calculated. Subjective image quality scores were evaluated.
**Results or Findings:** CT attenuation of vessels in 120kVp-like images were lower than 3 sets of 50 keV images with significant difference. In 50 keV images, both sternocleidomastoid muscle and white matter had a minimum noise in DLIR-H, and a maximum in ASIR-V40% group with significant difference. SNR and CNR in 50 keV images of all vessels had the same results: highest in DLIR-H group and lowest in ASIR-V40% group with significant differences. The mean value of ERD showed no significant difference among four groups. While 120kVp-like images had the lowest ERS, which showed statistically significant difference with the other groups. In terms of overall image quality, sharpness, and artifacts, the scores of DLIR-M and DLIR-H at 50 keV were not statistically different, and were higher than ASIR-V40% at 50 keV images, and higher than ASIR-V40% at 120 kVp-like. The scores of DLIR-H at 50 keV were highest in terms of noise and average scores.
**Conclusion:** DLIR is a potential solution for DECTA reconstruction since it can greatly reduce image noise, improving image quality of head and neck DECTA at 50 keV.
**Limitations:** First, our population was relatively small. Secondly, our study only compared 40%ASIR-V and DLIR, without including higher levels of ASIR-V for comparison. Finally, patients with BMI out of the normal range were excluded.
**Funding for this study:** We acknowledge financial support from the Jiangsu Traditional Chinese Medicine Science and Technology Development Plan Project (MS2021100), and the Key Research and Development Program of Xuzhou Science and Technology Bureau (KC20159).
**Has your study been approved by an ethics committee?** Yes
**Ethics committee - additional information:** Ethical approval was obtained from the Affiliated Hospital of Xuzhou Medical University, (approval number XYFY2024-KL456-01)
**Author Disclosures:**
Yankai Meng: Author: No remunerated Author: No remunerated
He Zhang: Author: No remunerated

**Beyond the T: Volumetric MRI Predicts Lymphatic Spread in HNSCC**
*D. Giardino*, A-I. Nica, P. Thoenissen, T. J. Vogl, I. Yel, R. Sader, C. Booz; Frankfurt/DE

**Purpose:** To investigate the relationship between primary tumor volume, the spatial distribution of cervical lymphnode metastases, and TNM staging in patients with oral and pharyngeal squamous cell carcinoma (HNSCC) using high-resolution MRI.
**Methods or Background:** This retrospective analysis evaluated 116 predominantly male (62.9%) patients (age 67.7 ± 11.5 years) with histologically confirmed HNSCC who underwent surgical resection with neck dissection or definitive chemoradiation. MRI-based volumetry and center-to-center distance measurements between primary tumor and cervical lymphnode metastases were performed using 3D postprocessing software.
**Results or Findings:** Tumor-to-lymphnode distances ranged from 11.7 to 117.3 mm (mean: 47.6 ± 19.4 mm). Tumor volume varied from 0.5 to 87.2 $cm^3$ (median: 13.3 ± 13.2 $cm^3$), and was higher in male patients. Significant correlation was observed between tumor volume and lymphnode distance and pT-stage ($p<0.0001$). The correlation between tumor volume and the distance to metastatic lymphnodes was stronger (Spearman's $r=0.4541$, $p<0.0001$) than the correlation between the pT-stage (TNM classification) and nodal distance (Spearman's $r=0.2682$, $p=0.0036$).
**Conclusion:** MRI-based assessment revealed that tumor volume is a stronger predictor of the spatial extent of lymph node metastases than T-stage alone. Larger tumors were associated with greater distances to metastatic lymph nodes. These findings highlight the value of quantitative 3D MRI-based volume and distance analyses in potentially improving surgical and radiotherapeutic planning.
**Limitations:** Limitations of the study include its retrospective nature.
**Funding for this study:** No funding was obtained for this study.
**Has your study been approved by an ethics committee?** Yes
**Ethics committee - additional information:** This study received approval from the Ethics Committee.
**Author Disclosures:**
Christian Booz: Speaker: I have received speaking fees from Siemens Healthineers.
Ibrahim Yel: Nothing to disclose
Andreea-Ioana Nica: Nothing to disclose
Thomas J. Vogl: Nothing to disclose
Phillip Thoenissen: Nothing to disclose
Robert Sader: Nothing to disclose
Davide Giardino: Nothing to disclose

**Surveillance Imaging in Head & Neck Cancer: Node-RADS Validation in the Treated Neck**
*A. H. Shah*, S. Sen, A. Gehani, A. Chandra, P. Ghosh, S. Mukhopadhyay, A. Chatterjee, J. Khoda, A. Patra; Kolkata/IN
*(shahakshat1788@gmail.com)*

**Purpose:** Node-RADS was originally designed for staging untreated lymph nodes. Its performance in the post-treatment neck, where fibrosis and necrosis mimic recurrence, is unknown. We confirmed Node-RADS in treated head and neck cancer patients, with subsite-specific analysis across CT and MRI cohorts.
**Methods or Background:** We retrospectively reviewed 602 patients with head and neck squamous cell carcinoma treated between 2012 and 2024, yielding 1,098 post-treatment nodes. Imaging modality was decided by subsite: oral cavity and larynx underwent CT, while oropharynx and nasopharynx were assessed with MRI (no dual-modality cases). Two head and neck radiologists independently assigned Node-RADS categories (1-5). Reference standards included histopathology, MDT consensus, or ≥12-month imaging follow-up. Diagnostic performance and predictive values were calculated overall and by subsite; inter-observer agreement was assessed (κ).
**Results or Findings:** Node-RADS categories correlated strongly with recurrence risk ($p<0.001$). At a cutoff of Node-RADS ≥4, sensitivity was 81% and specificity 86%, with overall accuracy 84%. NPV exceeded 92% for Node-RADS 1-2, while Node-RADS 5 achieved PPV 89%. Subsite analysis showed comparable performance across CT-evaluated subsites (oral cavity, larynx) and MRI-evaluated subsites (oropharynx, nasopharynx). Interobserver agreement was substantial ($\kappa = 0.74$), aligning with prior untreated Node-RADS studies (κ ~0.6–0.7). Clinical concordance was high: 71% of Node-RADS 4–5 prompted MDT-directed biopsy or salvage, while most Node-RADS 1–2 remained under surveillance.
**Conclusion:** This is the first validation of Node-RADS in the treated neck. Structured nodal reporting retains strong predictive value post-therapy, with high reproducibility and clear MDT alignment. Node-RADS offers a practical lexicon for post-treatment surveillance, bridging radiology and clinical decision-making.
**Limitations:** Single-center retrospective design
**Funding for this study:** None
**Has your study been approved by an ethics committee?** Not applicable
**Author Disclosures:**
Aditi Chandra: Nothing to disclose
Anisha Gehani: Nothing to disclose
Sumit Mukhopadhyay: Nothing to disclose
Argha Chatterjee: Nothing to disclose
Saugata Sen: Nothing to disclose
Akshat Hitesh Shah: Nothing to disclose
Anurima Patra: Nothing to disclose
Priya Ghosh: Nothing to disclose
Jeevitesh Khoda: Nothing to disclose

**Intraoperative and Histopathological Evaluation of Paragangliomas Following Preoperative Embolization: Comparing Outcomes of Particles, Onyx, and Glubran**
*H. Steinberg*[1], N. van Landeghem[1], D. Van Landeghem[1], B. M. Schaarschmidt[1], M. Forsting[1], I. Wanke[2], C. Deuschl[1], Y. Li [1]; [1]Essen/DE, [2]Zürich/DE

**Purpose:** Head and neck paragangliomas are rare, hypervascular tumors for which preoperative embolization is often required to mitigate intraoperative blood loss. This study compares intraoperative and histopathological outcomes of paragangliomas following preoperative embolization using particles, Onyx, or Glubran.
**Methods or Background:** Retrospective analysis of patients undergoing preoperative embolization for paragangliomas at a tertiary care center from 2010 to 2024. Outcomes included post-embolization angiographic devascularization, intraoperative bleeding, histopathological necrosis, inflammatory response, and resection margins.
**Results or Findings:** Twenty-two patients (15 female; mean age 52.6 years) were included. The mean interval between embolization and surgery was 5.1 days. Liquid embolic agents (Onyx: 54.5%, Glubran: 22.7%) were used in 77.3% of cases, with particles in the rest. Liquid embolics demonstrated superior tumor devascularization (median 90% vs. 50%, $p=0.05$) and necrosis (20% vs. 0%, $p=0.039$) compared to particles. However, no significant differences were observed in intraoperative blood loss (hemoglobin loss 1.4 g/dL for liquid embolics vs. 0.5 g/dL, $p=0.676$), procedure duration (134 vs. 96 minutes, $p=0.085$), or peritumoral inflammation. Complete resection (R0) was achieved in 81.25% of liquid embolic cases versus 100% with particles. Onyx and Glubran showed comparable efficacy.
**Conclusion:** Liquid embolic agents (Onyx/Glubran) achieved greater tumor devascularization and necrosis than particles, though surgical outcomes (blood loss, complete resection rates) were comparable. Agent selection may depend on procedural goals, balancing angiographic efficacy against surgical feasibility.
**Limitations:** The limitations of this study are its retrospective and single-center study design.
**Funding for this study:** No funding was received for this study.
**Has your study been approved by an ethics committee?** Yes
**Ethics committee - additional information:** The study was approved by the ethics committee of the University of Duisburg-Essen.
**Author Disclosures:**
Michael Forsting: Nothing to disclose
Yan Li: Nothing to disclose
Benedikt Michael Schaarschmidt: Nothing to disclose
Cornelius Deuschl: Nothing to disclose
Natalie Van Landeghem: Nothing to disclose
Daniel Van Landeghem: Nothing to disclose
Isabel Wanke: Nothing to disclose
Hannah Steinberg: Nothing to disclose

**The Prognostic Value of Intra- and Peri-tumoral Habitat Analysis in Nasopharyngeal Carcinoma: Combining IVIM and ASL**
*F. Yang*, M. Lin, H. Zhang; Beijing/CN
*(y731582237@163.com)*

**Purpose:** To evaluate the value of intravoxel incoherent motion (IVIM) and arterial spin labeling (ASL)-based habitat analysis of intra- and peri-tumoral regions for predicting overall survival (OS) and progression-free survival (PFS) in NPC.
**Methods or Background:** 106 patients were prospectively included. Primary tumors were delineated on T2-weighted imaging, and three peritumoral regions (3, 5, and 10 mm) were automatically dilated and manually corrected according to the clinical tumor volume outlining criteria. The pure diffusion coefficient (D) and mean kurtosis (MK) maps of IVIM, and blood flow (BF) map of ASL were used for habitat analysis. Volume fraction and histogram parameters (Mean, Kurtosis and Skewness) were extracted for each subregion. Univariate and multivariate Cox analyses were used to construct intra- and peri-tumoral, Clinic, and combined models, which were assessed by the C-index. Nomogram, calibration curves, and Kaplan-Meier curves were also plotted.

**Results or Findings:** The intra-tumoral subregion 4 was characterized by high D and low BF and MK value, representing the radiotherapy-resistant region and patients with treatment failure had higher proportion of subregion 4. The Intra-score independently predicted OS (hazard radio [HR]: 1.023, P = 0.002) and PFS (HR: 1.028, P = 0.001). Combined clinical factors with intra- and peri-tumoral habitat features showed the highest performance (C-index: 0.780 [OS] and 0.721 [PFS]) and outperformed the Clinic model (C-index: 0.691 [OS] and 0.666 [PFS], P ≤ 0.03). The post-hoc subgroup analysis confirmed robustness.
**Conclusion:** Peri-tumoral regions provide valuable prognostic information. Combining IVIM-ASL-based habitat analysis offers a non-invasive approach to predicting treatment outcomes.
**Limitations:** Small sample size and lack of histopathological confirmation information were the limitations.
**Funding for this study:** None
**Has your study been approved by an ethics committee?** Yes
**Ethics committee - additional information:** Chinese Academy of Medical Science
**Author Disclosures:**
Fan Yang: Nothing to disclose
Meng Lin: Nothing to disclose
Hongmei Zhang: Nothing to disclose

**Deep learning-enhanced MRI assessment of the optic nerves: A focus on AiCE denoising**

A. Sajust De Bergues De Escalup, A. Lecler, *E. O'Shaughnessy*; Paris/FR

**Purpose:** Orbital MRI is essential for assessing optic nerve disorders but is limited by motion artifacts, low signal-to-noise ratio (SNR), and susceptibility effects. Deep learning–based reconstruction improves image quality and shortens acquisition time. Canon’s Advanced intelligent Clear-IQ Engine (AiCE) reduces noise while preserving detail. This study evaluated AiCE’s impact on image quality and diagnostic confidence in coronal T2- and post-contrast T1-weighted orbital MRI.
**Methods or Background:** This retrospective single-center study included 72 orbital MRI exams on a 3T MRI. Coronal T2-WI (n=71) and post-contrast T1-WI (n=25) were reconstructed with and without AiCE. Two blinded radiologists separately reviewed series, assessing optic nerve hyperintensity, atrophy, and qualitative features (optic nerve sharpness, brain sharpness, overall quality) on 5-point scales. Motion artifacts were rated separately. SNR and contrast-to-noise ratio (CNR) were measured using. Wilcoxon and McNemar tests with Bonferroni correction were used. Agreement was assessed with Cohen’s κ and ICC.
**Results or Findings:** In coronal T2-WI, mean SNR rose from 25.94 ± 16.94 (non-AiCE) to 49.04 ± 26.01 (AiCE), and CNR from 14.29 ± 15.87 to 25.57 ± 22.48 (p < 0.001). In post-contrast T1-WI, SNR and CNR were unchanged (p = 1; p = 0.72). AiCE significantly improved brain and optic nerve sharpness and overall quality (p ≤ 0.001). Detection of optic nerve hyperintensity and atrophy was unchanged. Inter-reader κ for diagnostic features ranged 0.795–0.966; intra-reader κ for diagnostic features was 0.87–1; weighted κ for qualitative metrics was 0.209–0.803. ICCs ranged 0.234–0.624.
**Conclusion:** AiCE improved orbital MRI quality, especially in coronal T2-WI, without compromising diagnostic feature detection. These gains support its use for confident optic nerve assessment in routine practice.
**Limitations:** Quantitative gains were variable, with higher intra- than inter-reader agreement. Further studies should optimize its use across centers.
**Funding for this study:** No funding was provided for this study.
**Has your study been approved by an ethics committee?** Not applicable
**Author Disclosures:**
Aurore Sajust De Bergues De Escalup: Nothing to disclose
Emma O'Shaughnessy: Nothing to disclose
Augustin Lecler: Speaker: Received speaker honoraria from Canon, Biogen, and Bayer for participation in symposia.

**ADC-Based Radiomics for Cholesteatoma Diagnosis: Comparison with Conventional Imaging**

*R. O. Önder*[1], T. Bekçi[1], A. Tosun[2]; [1]Giresun/TR, [2]Trabzon/TR
*(orkunonder535@gmail.com)*

**Purpose:** To evaluate the diagnostic performance of conventional radiological findings (CT and DWI) and radiomic features derived from ADC maps in the diagnosis of cholesteatoma, and to assess the added value of their artificial intelligence-based combination.
**Methods or Background:** In this retrospective study, 51 patients with suspected cholesteatoma (January 2020–August 2024) were analyzed. Non-contrast temporal bone CT and DWI were assessed, and radiomic features were extracted from ADC maps. Reproducibility was tested with intraclass correlation coefficients (ICC ≥0.75). Machine learning (ML) models, including a three-layer artificial neural network, were trained using radiology, radiomics, and combined datasets. An independent validation group (n=15) was used to confirm generalizability.
**Results or Findings:** Of 51 lesions, 23 (45.1%) were histopathologically confirmed as cholesteatoma. Cholesteatomatous lesions had higher CT HU values (p=0.004), more frequent diffusion restriction (p<0.001), and lower ADC values (p<0.001). Radiomic analysis identified 20 significant features. The combined ML model achieved the best performance (accuracy 98.0%, AUC 0.982), which remained robust in the validation cohort (accuracy 93.3%, AUC 0.951).
**Conclusion:** Artificial intelligence and ADC radiomics provide complementary diagnostic value to conventional radiology in the detection of cholesteatoma. The artificial intelligence-assisted integration of radiomics and radiology achieves superior performance, thereby supporting its potential role in non-invasive diagnosis and the clinical decision-making process.
**Limitations:** Single-center, retrospective design with a limited sample size; external validation is required.
**Funding for this study:** No funding was received for this study.
**Has your study been approved by an ethics committee?** Yes
**Ethics committee - additional information:** This retrospective study was approved by the Scientific Research Ethics Committee of Giresun Training and Research Hospital on 4 September 2024 (decision no. 17).
**Author Disclosures:**
Ramazan Orkun Önder: Nothing to disclose
Tümay Bekçi: Nothing to disclose
Alptekin Tosun: Nothing to disclose

**Prediction of Ki-67 status in tongue squamous cell carcinoma using histogram features of spectral images derived from dual-layer spectral-detector CT**

*H. K. Zhang*[1], F. Chen[1], Y. Liao[2], X. Liu[2]; [1]Haikou/CN, [2]Guangzhou/CN
*(xiaomin.liu@philips.com)*

**Purpose:** The Ki-67 status is often correlated with the clinical course of cancer. This study aimed to investigate the feasibility of predicting Ki-67 status in TSCC using histogram features of spectral images acquired with dual-layer spectral-detector CT(DLCT).
**Methods or Background:** A retrospective cohort of 69 patients with TSCC (50 Ki-67-positive and 19 Ki-67-negative, with a threshold of 20%) who underwent surgery with preoperative DLCT scans was collected. Conventional images and spectral images, including Virtual Monoenergetic Images (VMIs) at 40KeV, 70KeV, 100KeV, and 130KeV, iodine density maps, effective atomic number maps, virtual non-contrast (VNC) images, were generated based on venous-phase CT data. Tumor regions of interest (ROIs) were manually delineated on the VMI 40KeV images and then copied and pasted onto other images. A total of 12 histogram parameters were extracted from each image. Feature selection was performed using Spearman correlation analysis (threshold = 0.9) and backward stepwise regression. Logistic regression was used to construct predictive models. Model performance was assessed with receiver operating characteristic (ROC) analysis, and compared with the DeLong's test.
**Results or Findings:** In the discrimination of Ki-67 status based on individual features, the energy from VNC obtained the highest area under the curve (AUC) of 0.727 (95% CI: 0.578-0.876), followed by the energy from VIMs 70keV with an AUC of 0.719 (0.571-0.867). The model combining valuable features from all spectral images achieved the highest AUC of 0.863(0.765-0.962), which was significantly outperformed than the model corporating valuable features from conventional images with an AUC of 0.663(0.505-0.822), and a P<0.01.
**Conclusion:** The histogram features of spectral images exhibit promising performance in preoperatively predicting Ki-67 status in patients with TSCC.
**Limitations:** Retrospective, single-center study with a small sample size
**Funding for this study:** Not applicable
**Has your study been approved by an ethics committee?** Yes
**Ethics committee - additional information:** The Ethics Committee granted a waiver of informed consent for this retrospective study.
**Author Disclosures:**
Xiaomin Liu: Nothing to disclose
Hang Kai Zhang: Nothing to disclose
Feng Chen: Nothing to disclose
Yuting Liao: Nothing to disclose

**Deep learning-based body composition analysis: Multiple independent prognostic biomarkers from routine CT in head and neck cancer**

E. Frodl, L. Golla, L. Gantner, M. Harth, J. Dietz, T. Vogl, P. Thoenissen, *A. M. Bucher*; Frankfurt/DE
*(bucher@med.uni-frankfurt.de)*

**Purpose:** To identify independent body composition predictors of overall survival in head and neck squamous cell carcinoma patients using automated deep learning analysis of routine baseline CT at L3 and abdominal cavity levels.

**Methods or Background:** This retrospective study analysed 181 HNSCC patients (mean age 63.2±12.4 years; 50.8% female) using deep learning-based automated body composition analysis from baseline venous-phase contrast-enhanced CT (91.7%, 5mm slice thickness). The algorithm quantified tissue volumes and densities including intermuscular adipose tissue infiltration (IMAT), subcutaneous adipose tissue (SAT), muscle, and bone. Additionally, muscle-to-bone ratio (MBR) and IMAT/TAT ratio were calculated. Cox regression with multivariate adjustment for age, gender, T-stage, N-stage, and UICC stage identified independent predictors.
**Results or Findings:** Patients presented with advanced disease (UICC IVA: 31.5%, III: 22.6%, I: 21.6%) with 41.4% nodal involvement. Multiple body composition metrics showed independent prognostic value in multivariate analysis. IMAT/TAT ratios remained significant predictors at both abdominal cavity (volume: HR=1.490, p=0.0008, 95%CI: 1.180-1.882; attenuation: HR=1.465, p=0.0015, 95%CI: 1.158-1.854) and L3 levels (volume: HR=1.458, p=0.0011, 95%CI: 1.163-1.827; attenuation: HR=1.457, p=0.0007, 95%CI: 1.171-1.812). SAT attenuation independently predicted survival at both levels (abdominal: HR=1.469, p=0.0017, 95%CI: 1.156-1.867; L3: HR=1.486, p=0.0005, 95%CI: 1.189-1.857). Uniquely, muscle-bone volume ratio at abdominal cavity showed protective effects (HR=0.741, p=0.0477, 95%CI: 0.550-0.997), unavailable at single-slice L3. All metrics maintained significance alongside UICC stage (HR=2.435, p=0.0015) in multivariate models.
**Conclusion:** Deep learning-based body composition analysis identifies multiple independent predictors of overall survival in HNSCC. IMAT infiltration and SAT attenuation maintain prognostic value across both anatomical levels. The protective muscle-bone volume ratio, unique to abdominal cavity assessment, supports volumetric analysis. These automatically derived biomarkers enhance risk stratification beyond traditional staging without additional radiation exposure.
**Limitations:** The limitations of the study are the single-centre retrospective design and pending external validation.
**Funding for this study:** Funding was provided by the German Federal Ministry of Education and Research through the RACOON project (reference number 01KX2021).
**Has your study been approved by an ethics committee?** Yes
**Ethics committee - additional information:** The study was approved by University Medicine Frankfurt (UCT-9-2023).
**Author Disclosures:**
Eric Frodl: Nothing to disclose
Julia Dietz: Nothing to disclose
Lisa Golla: Nothing to disclose
Thomas Vogl: Nothing to disclose
Lorna Gantner: Nothing to disclose
Phillip Thoenissen: Nothing to disclose
Andreas Michael Bucher: Nothing to disclose
Maurice Harth: Nothing to disclose

**Time-Saving Potential of Deep Learning-Based Reconstructions in Head and Neck MRI: A Comparative Study**
*F. Albisinni*, C. Carbone, M. Ravanelli, D. Farina, G. Corciulo; Brescia/IT
*(f.albisinni@unibs.it)*

**Purpose:** This study aimed to evaluate the time-saving potential of deep learning (DL)-based reconstructions in T2-weighted head and neck MRI, while assessing their impact on image quality.
**Methods or Background:** Fifty-two patients underwent three TSE T2 sequences: (A) standard acquisition without DL (3 signal averages, 2'35"), (B) DL with intermediate strength (2 averages, 1'25"), and (C) DL with maximal strength (1 average, 43"). Images were blindly assessed at two anatomical levels (nasopharynx and oral cavity) by three radiologists of varying experience. Image quality was rated on a 3-point Likert scale across four categories: overall quality, artifacts, edge sharpness, and noise. Readers also attempted to identify the sequence type. Inter-rater agreement and image quality comparisons were analyzed statistically.
**Results or Findings:** A total of 636 images were evaluated. Acquisition time was reduced by 45% in sequence B and by over 70% in sequence C compared to standard imaging. Despite the substantial time savings, DL-based sequences maintained comparable image quality: overall quality scores for sequences A, B, and C were 2.63, 2.52, and 2.52, respectively (p = 0.055). Differences in artifacts and noise were not statistically significant. A minor reduction in edge sharpness was observed between sequences A and B (p = 0.02). Inter-rater agreement remained low, and the ability to identify the sequence type was limited (44% accuracy).
**Conclusion:** Deep learning-based reconstructions enable significant reductions in scan time—up to 70%—in head and neck MRI without compromising diagnostic image quality. These findings highlight DL's potential to support faster, more efficient imaging protocols in clinical practice.
**Limitations:** This study is limited by low inter-rater agreement, evaluation restricted to T2-weighted images at two anatomical levels, and the absence of diagnostic accuracy analysis.
**Funding for this study:** This study received no external funding.
**Has your study been approved by an ethics committee?** Not applicable
**Author Disclosures:**
Giulia Corciulo: Nothing to disclose
Davide Farina: Nothing to disclose
Flavia Albisinni: Nothing to disclose
Marco Ravanelli: Nothing to disclose
Carmela Carbone: Nothing to disclose

**08:00 - 09:30** **Research Stage 2**

### Research Presentation Session: Musculoskeletal

### RPS 110

### What is new in sarcopenia and body composition

**Moderator**
Christos Loupatatzis; Männedorf/CH
*(c.loupatatzis@spitalmaennedorf.ch)*

**Sex-Specific Hip and Thigh Muscle Composition in GLP-1RA Users: Automated MRI Phenotyping in the UK Biobank**
*M. Thanaj*, B. Whitcher, H. Raza, M. Niglas, C. Bell-Bradford, E. L. Thomas, D. Amiras, J. D. Bell; London/UK
*(m.thanaj@westminster.ac.uk)*

**Purpose:** Glucagon-like peptide-1 receptor agonists (GLP-1RA) are recommended for treatment of type 2 diabetes (T2D) and obesity, but concerns regarding sarcopenia are reported. We developed automated methods to quantify volume and fat infiltration of 20 hip and thigh muscles, evaluating sex- and GLP-1RA–specific differences.
**Methods or Background:** UK Biobank participants prescribed with GLP-1RA before the baseline imaging visit (N = 67; mean duration 5.9 ± 2.9 years, range 0.1-12) were matched by sex, age (±1.5 years), BMI (±1.5 kg/m²), Townsend index (±1.5), T2D, hypertension, cardiovascular disease, muscle disorders, sedentary lifestyle (>10.6 h/day), and cholesterol medication with non-GLP-1RA controls. Sensitivity analyses included dynapenia (handgrip <16 kg women, <27 kg men) and falls (>1). Twenty hip and thigh muscles were segmented from T1w Dixon MRI scans using a deep learning model with MONAI (Medical Open Network for AI) Swin-UNETR architecture. Median fat fraction per muscle group was calculated. Paired t-tests were performed with false discovery rate (FDR) correction.
**Results or Findings:** In men (N=60), GLP-1RA use showed a higher pectineus fat fraction (mean diff: 0.013, FDR=0.043) and rectus femoris fat fraction (0.018, FDR=0.038), while adductor longus (-42 mL, FDR=0.038), gluteus medius (-67 mL, FDR=0.038), and rectus femoris (-79 mL, FDR=$4\times10^{-4}$) volumes were lower, showing no significant differences in women (N=28). Including dynapenia and falls (44M/20F) eliminated differences in muscle volume and fat fraction.
**Conclusion:** Our scalable deep learning pipeline shows GLP-1RA–related adductor longus, rectus femoris, and gluteus medius volume reduction and pectineus and rectus femoris fat fraction increase in men, with no differences in women or when including dynapenia and falls. These findings suggest possible sex-specific GLP-1RA effects that require validation in larger cohorts.
**Limitations:** Small sample size, limited longitudinal data, heterogeneity in GLP-1 exposure, and medication type may restrict interpretation
**Funding for this study:** No funding was recieved
**Has your study been approved by an ethics committee?** Yes
**Ethics committee - additional information:** Fully anonymised images and participant metadata were obtained through UK Biobank Access Application number 23889. The UK Biobank has approval from the North West Multi-Centre Research Ethics Committee (REC reference: 11/NW/0382), and obtained written informed consent from all participants before the study. All methods were performed in accordance with the relevant guidelines and regulations as presented by the appropriate authorities, including the Declaration of Helsinki.

**Author Disclosures:**
Brandon Whitcher: Shareholder: Hyperfine Shareholder: Beam Therapeutics Shareholder: Regeneron
Dimitri Amiras: Shareholder: MedicaliSight Ltd
E. Louise Thomas: Nothing to disclose
Marili Niglas: Nothing to disclose
Hamzah Raza: Nothing to disclose
Jimmy D. Bell: Nothing to disclose
Camilo Bell-Bradford: Nothing to disclose
Marjola Thanaj: Nothing to disclose

**Decreased Chest CT-Derived Lumbar Skeletal Muscle Mass Associated with Adverse Outcomes in Sepsis Patients: A Multicenter Retrospective**

*L. Fu*, X-G. Peng; Nanjing/CN
*(t17861522268@163.com)*

**Purpose:** While the lumbar skeletal muscle index (SMI) predicts outcomes in sepsis, the significance of its dynamic changes remains unclear. This study evaluated the association between longitudinal changes in CT-derived body composition parameters and clinical outcomes in sepsis.
**Methods or Background:** We retrospectively included sepsis patients from three tertiary centers. Two non-contrast chest CT scans at the L1 level were analyzed: a baseline scan within 48 hours of diagnosis and a follow-up scan ≥5 days later. Parameters included SMI, skeletal muscle density (SMD), subcutaneous and visceral fat area (SFA, VFA) and density (SFD, VFD). X-tile determined the optimal ΔSMI cutoff for mortality. Patients were classified into high and low skeletal muscle wasting (SMW) groups. Survival was analyzed using Kaplan–Meier and log-rank tests. Cox regression identified mortality-associated factors.
**Results or Findings:** A total of 561 patients (median age, 69 years; interquartile range, 58–79 years; 378 males) were analyzed. The optimal ΔSMI cutoff for defining high SMW was > 4.04 cm²/m². Patients in the high SMW group had significantly higher 28-day, 3-month, and 1-year mortality Among 561 patients (median age 69; 378 male), the optimal ΔSMI cutoff for high SMW was > 4.04 cm²/m². The high SMW group had significantly higher 28-day (56.5% vs 3.0%), 3-month (82.4% vs 6.8%), and 1-year mortality (88.6% vs 17.4%) (all P<0.001). No other Δparameters correlated with mortality. Multivariate Cox analysis identified ΔSMI, age, Charlson Index, mechanical ventilation, APACHE II, and creatinine as independent predictors. ΔSMI was the strongest predictor (HR: 1.165 per 1 cm²/m² loss; 95% CI: 1.133–1.198; P<0.001).
**Conclusion:** Longitudinal loss of lumbar muscle mass, quantified by serial CT, independently predicts mortality in sepsis. Monitoring muscle dynamics could enhance risk stratification and guide targeted interventions to improve outcomes.
**Limitations:** This study requires prospective validation.
**Funding for this study:** Longitudinal loss of lumbar muscle mass, quantified by serial CT, independently predicts mortality in sepsis. Monitoring muscle dynamics could enhance risk stratification and guide targeted interventions to improve outcomes.
**Has your study been approved by an ethics committee?** Yes
**Ethics committee - additional information:** 2024ZDSYLL060-p01
**Author Disclosures:**
Lin Fu: Nothing to disclose
Xin-Gui Peng: Nothing to disclose

**The Body Beneath: CT Body Composition and Its Impact on Transplant Success**

*G. Boscaro*, M. Visoná, S. Silvestrin, D. Trevisani, A. Michielin, E. Faccioli, A. Dell'Amore, R. Stramare, C. Giraudo; Padova/IT
*(giulio.boscaro@gmail.com)*

**Purpose:** To assess the role of CT-based body composition in bilateral lung transplant candidates (BLTc).
**Methods or Background:** We included BLTc referring to our tertiary center who performed at least one HRCT <18 months before the transplant. The paravertebral muscle and subcutaneous tissue were segmented at the level of the 12th thoracic vertebra to extract density (Hu) and area (cm2); the skeletal muscle index (SMI) was then computed. According to Moon et al, the following SMI thresholds, 7.13cm2/m2 for women and 8.67cm2/m2 for men, were used to define sarcopenia. Using the Spearman correlation coefficient, the relationship between muscle strength, hand-grip test, sarcopenia, muscle density, and area was explored. By the ANOVA for repeated measures any change in body composition over three time points (before the transplant, ≤36 months after the transplant, and ≤48 months after the transplant) was investigated. The logistic regression analysis was used to evaluate if demographics, spirometry metrics, and sarcopenia before the transplant predicted the survival.
**Results or Findings:** Sixty-two BLTc were included (24 female; average age 53±13 years; 25 died). Twenty-three (37.1%) BLTc were affected by sarcopenia before the transplant. The ANOVA demonstrated a significant decrease of muscle area at first follow-up after BLT (p<0.001). An inverse, significant but moderate correlation emerged between sarcopenia and muscle strength as well as grip test (r=-.310,p=0.014 and r=-.409,p<0.001, respectively) and a positive moderate correlation occurred between muscle area and muscle strength and grip test (r=445,p<0.001 and r=.342,p=0.007). The multivariate logistic regression showed that sarcopenia predicts mortality (p=0.014, 95%CI for sarcopenia 1.523–45.429).
**Conclusion:** More than a third of BLTc are affected by sarcopenia which may have an impact on the success of the transplant.
**Limitations:** Single center study.
**Funding for this study:** No.
**Has your study been approved by an ethics committee?** Yes
**Ethics committee - additional information:** local IRB
**Author Disclosures:**
Andrea Dell'Amore: Nothing to disclose
Stefano Silvestrin: Nothing to disclose
Eleonora Faccioli: Nothing to disclose
Roberto Stramare: Nothing to disclose
Anna Michielin: Nothing to disclose
Martina Visoná: Nothing to disclose
Giulio Boscaro: Nothing to disclose
Doriana Trevisani: Nothing to disclose
Chiara Giraudo: Nothing to disclose

**Simplifying CT-Based Muscle Quality Assessment Using Local Muscle Surrogates: Validation in a Large, Heterogeneous Imaging Cohort**

J. Gotta, *L. D. Grünewald*, V. Koch, S. Mahmoudi, T. Vogl; Frankfurt am Main/DE

**Purpose:** Advanced CT-derived body composition metrics are physiologically relevant but computationally demanding. This study aimed to evaluate whether attenuation and volume measurements from individual muscle groups, specifically autochthonous, gluteal, and iliopsoas muscles, can serve as surrogates for established metrics of muscle quality and quantity, including skeletal muscle radiodensity (SMRA), myosteatosis, skeletal muscle area (SMA), and muscle volume.
**Methods or Background:** In this retrospective analysis of 25,252 abdominal CT scans, attenuation and volume metrics were extracted for the iliopsoas, autochthonous, and gluteal muscles. Generalized additive models (GAMs) were fitted to assess their association with reference myosteatosis metrics, including L3-SMRA, volumetric muscle radiodensity, intramuscular fat proportion, L3-SMA, and volumetric muscle percentage. All predictors were modeled as continuous spline terms, and model performance was evaluated using pseudo $R^2$ (deviance explained). Laterality was excluded. Relationships were visually assessed for linearity.
**Results or Findings:** Attenuation of the autochthonous muscles was the strongest surrogate for L3-SMRA ($R^2$ = 0.88), followed by gluteus medius ($R^2$ = 0.73), gluteus maximus ($R^2$=0.70), and iliopsoas ($R^2$ = 0.69). For volumetric abdominal myosteatosis, gluteus maximus HU achieved the highest explained variance ($R^2$ =0.59), followed by autochthonous ($R^2$=0.57), gluteus medius ($R^2$=0.54), and iliopsoas ($R^2$=0.43). Normalized L3-SMA was best predicted by iliopsoas muscle volume ($R^2$=0.69), followed by autochthonous ($R^2$ = 0.64), gluteus medius ($R^2$=0.60), and gluteus maximus ($R^2$ = 0.49). Associations with volumetric muscle percentage were consistently weaker across all compartments ($R^2$≤0.35). All results were statistically significant (p < 0.001).
**Conclusion:** Attenuation metrics of the autochthonous and gluteal muscles are strong linear surrogates for SMRA and myosteatosis, while iliopsoas muscle volume most accurately predicts L3-SMA. These findings suggest that selective muscle-based surrogates can approximate advanced body composition metrics with lower computational cost than full segmentation.
**Limitations:** Retrospective study
**Funding for this study:** No funding was received.
**Has your study been approved by an ethics committee?** Yes
**Ethics committee - additional information:** Under waiver.
**Author Disclosures:**
Thomas Vogl: Nothing to disclose
Vitali Koch: Nothing to disclose
Scherwin Mahmoudi: Nothing to disclose
Leon David Grünewald: Nothing to disclose
Jennifer Gotta: Nothing to disclose

**Preoperative Visceral Adipose Accumulation and Skeletal Muscle Density Reduction Predict Adverse Outcomes in Kidney Transplantation: A Prospective Cohort Study**

*Y. Zhang*, J. Tang, Z. Li; Chengdu/CN
*(zzyy_0514@126.com)*

**Purpose:** This study aimed to use computed tomography (CT) to assess muscle reduction and adipose deposition and examined the predictive values of those indicators on clinical outcomes after kidney transplantation.
**Methods or Background:** Abnormal body composition has been widely observed in kidney transplant recipients. However, how muscle reduction and adipose deposition affect the prognosis after kidney transplantation is not fully understood, especially in Chinese population. This prospective study examined a consecutive series of patients who underwent their first kidney transplant at our medical center between June 1, 2020 and June 30, 2023. Within 30 days before surgery, skeletal muscle mass index (SMI), skeletal muscle density (SMD), visceral adipose area index (VAI) and subcutaneous adipose area index (SAI) were evaluated using abdominal computed tomography. Multivariable logistic regression and cox regression were used to explore associations between each index and prognosis in terms of infection, delayed graft function and rejection after kidney transplantation.
**Results or Findings:** The final analysis included 455 patients (147 women, mean age 34.5 ± 10.18 years), who were followed up for a median of 47.67 (IQR, 34.23 – 51.72) months. In the multivariable Cox regression model, each one standard deviation increase in SMD was associated with a 20% lower risk of urinary tract infections (HR, 0.80; 95% CI, 0.69–0.95; p = 0.008), indicating that patients with lower SMD were at significantly higher risk. Elevated VAI independently predicted lung infections (HR, 1.25, 95 CI%, 1.03-1.52, p = 0.022). Patients with high VAI increased delayed graft function risk 1.44-fold (HR 1.44, 95%CI 1.04-1.98, p = 0.028).
**Conclusion:** Visceral adipose accumulation and skeletal muscle density reduction before kidney transplantation are independently associated with greater risk of poor prognosis on clinical outcomes after kidney transplantation.
**Limitations:** This was a single-center study.
**Funding for this study:** None.
**Has your study been approved by an ethics committee?** Yes
**Ethics committee - additional information:** the Ethics Committee of West China Hospital
**Author Disclosures:**
Jing Tang: Nothing to disclose
Yu Zhang: Nothing to disclose
Zhenli Li: Nothing to disclose

**Sarcopenia in patients undergoing vertebroplasty and kyphoplasty: diagnostic definitions and impact on clinical outcomes. Systematic review and meta-analysis**

*M. Basile*, M. Zanardo, S. Gitto, D. Albano, L. M. Sconfienza; Milano/IT

**Purpose:** This systematic review and meta-analysis evaluated the impact of sarcopenia on clinical outcomes after percutaneous vertebroplasty (PVP) and kyphoplasty (PKP) in patients with osteoporotic vertebral compression fractures (OVCFs), and examined the heterogeneity in sarcopenia definitions across studies.
**Methods or Background:** A comprehensive literature search was conducted in PubMed, Embase, and Scopus through June 2025. Eligible studies assessed sarcopenia in OVCF patients treated with PVP or PKP. Extracted data included diagnostic modalities (CT, MRI, DEXA, BIA), anatomical landmarks, muscle indices (skeletal muscle index [SMI], psoas muscle index, psoas-to-lumbar vertebral index, appendicular muscle index), functional parameters (handgrip strength, gait speed), clinical outcomes (refracture, residual low back pain [RBP]), and statistical measures.
**Results or Findings:** Eighteen studies (3,531 patients; 1,357 sarcopenic) were included. CT was the most frequently employed diagnostic tool (72%), followed by MRI (27%), DEXA (11%), and BIA (0.2%). The most common reference levels were L3 (28%) and T12 (28%). SMI was reported in 7 studies (38%), with cut-off values ranging from 29–30.6 $cm^2/m^2$ in males and 36–42.6 $cm^2/m^2$ in females. Six studies (33%) assessed functional parameters, while only three (17%) fully adhered to EWGSOP2 criteria. Pooled analysis demonstrated that sarcopenic patients had a markedly higher risk of refracture (HR 3.61; 95% CI 2.49–5.23; p<0.05) and an increased likelihood of RBP (OR 2.78; 95% CI 1.50-5.00).
**Conclusion:** Sarcopenia significantly worsens outcomes after PVP and PKP in OVCF patients. Adoption of a standardized diagnostic framework—integrating CT-based muscle mass quantification (particularly SMI at T12), muscle strength (handgrip), and physical performance (gait speed)—is recommended to enhance risk stratification. Tailored management strategies for sarcopenic patients may improve postoperative outcomes.
**Limitations:** Heterogeneity in sarcopenia definitions, the predominance of retrospective study designs, and limited generalizability underscore the need for further prospective, standardized, multicenter studies.
**Funding for this study:** Not applicable
**Has your study been approved by an ethics committee?** Not applicable
**Author Disclosures:**
Salvatore Gitto: Nothing to disclose
Luca Maria Sconfienza: Nothing to disclose
Moreno Zanardo: Nothing to disclose
Mariachiara Basile: Nothing to disclose
Domenico Albano: Nothing to disclose

**Sarcopenia in patients with colorectal carcinoma undergoing chemotherapy**

*D. Vogele*, S. Waibel, K. Mueller, T. Ettrich, M. J. Beer; Ulm/DE
*(daniel.vogele@uniklinik-ulm.de)*

**Purpose:** Sarcopenia is associated with poor oncologic outcomes, increased risk of falls, hospitalization, and reduced quality of life. However, diagnostic approaches remain heterogeneous. This study evaluated a simple radiological method for sarcopenia detection in colorectal cancer patients using routine staging CT scans, and assessed its prognostic relevance. The role of radiomic features combined with machine learning for diagnosing sarcopenia and predicting tumor progression was also analyzed.
**Methods or Background:** CT scans of 57 rectal and 46 sigmoid carcinoma patients undergoing chemotherapy were retrospectively reviewed. At the third lumbar vertebra, muscle areas of the psoas major, quadratus lumborum, erector spinae, and rectus abdominis were measured. The Psoas Muscle Index (PMI) was calculated as psoas muscle area/$height^2$. Sarcopenia was defined using Bahat et al. cut-offs. Tumor response was assessed by RECIST at diagnosis (T1) and after 9 months (T3). Sex and tumor entity differences were evaluated. Radiomic features of skeletal muscles were extracted, and feature selection performed by lasso regression to train a neural network.
**Results or Findings:** Psoas muscle assessment yielded high imaging quality in 97.7% of cases; unilateral and bilateral measures were equivalent (p=0.064). Low PMI showed a non-significant trend toward predicting progressive disease (PD) in univariate regression (OR=0.79; p=0.07), but not in multivariate analysis (OR=1; p>0.5). No association was observed between PMI and PD-free survival. Machine learning achieved 0.71±0.08 accuracy (AUC=0.79±0.07) for sarcopenia detection, but only 0.63±0.04 (AUC=0.66±0.06) for PD prediction.
**Conclusion:** The psoas muscle is a simple, reproducible CT marker for sarcopenia in colorectal cancer. Although PMI was not an independent predictor of PD, a protective trend was observed. Machine learning showed promise for sarcopenia detection but limited value for predicting progression. Larger prospective studies are warranted.
**Limitations:** Small cohort (n=103), retrospective design, single measurement timepoint
**Funding for this study:** None
**Has your study been approved by an ethics committee?** Yes
**Ethics committee - additional information:** The study was approved by the local ethics committee (no. 219/20).
**Author Disclosures:**
Thomas Ettrich: Nothing to disclose
Sebastian Waibel: Nothing to disclose
Daniel Vogele: Nothing to disclose
Konstantin Mueller: Nothing to disclose
Meinrad Johannes Beer: Nothing to disclose

**Associations of gestational diabetes mellitus and reduced skeletal muscle quality in premenopausal women assessed using texture analysis of quantitative magnetic resonance imaging**

*Y. Stohldreier*[1], M. Dieckmeyer[2], O. Dietrich[1], S. Schläger[1], J. Seißler[1], U. Ferrari[1], N. Hesse[1], A. Gersing[1]; [1]Munich/DE, [2]Bern/CH
*(yannick.stohldreier@med.uni-muenchen.de)*

**Purpose:** To evaluate associations between metabolic status and proton density fat fraction (PDFF) and second-order texture features of vertebral bone marrow (VBM) and autochthonous (AM) and psoas muscles on magnetic resonance imaging (MRI) in premenopausal women with and without recent gestational diabetes mellitus (GDM).
**Methods or Background:** Thirty-six premenopausal women (GDM, n=19; healthy controls (HC), n=17; mean age, 36.3±3.9 years) underwent MRI at 11.0±2.4 months postpartum. Multivariable logistic regression models adjusting for age and BMI were used to assess associations between GDM and metabolic-syndrome parameters (triglycerides, HDL, waist circumference, fasting plasma glucose and blood pressure) and differences in muscle and bone marrow quality, measured using texture analysis of PDFF maps.
**Results or Findings:** PDFF values of the thoracic (PDFF T9-12) and lumbar (PDFF L1-4) VBM were significantly higher in the GDM group compared to HC (PDFF T9-12 41.6±12.3% vs. 33.9±7.6%, p=0.03; PDFF L1-4 46.7±12.4% vs. 39.5±8.1%, p<0.05), whereas AM and psoas showed no significant PDFF difference (p>0.05). After adjustment for age and BMI, PDFF of VBM was significantly associated with GDM status (PDFF T9-12 p=0.02; PDFF L1-4

p=0.03). In addition, several muscle second-order texture features also remained associated with GDM status (dissimilarity AM (OR 4.30 [95% CI 1.61, 16.78], p=0.01), homogeneity AM (OR 0.26 [95% CI 0.07, 0.63], p=0.01), dissimilarity psoas (OR 3.98 [95% CI 1.57, 14.25], p=0.01) and homogeneity psoas (OR 0.29 [95% CI 0.09, 0.71], p=0.01)), indicating a reduced muscle quality in GDM patients.
**Conclusion:** Women with recent GDM exhibit more heterogenous paraspinal skeletal muscle tissue after adjusting for age and BMI, suggesting a reduced muscle quality in patients with GDM compared to healthy controls, which indicates that muscle PDFF may be a useful biomarker for muscle health in patients with metabolic impairment.
**Limitations:** Retrospective monocentric study.
**Funding for this study:** The author(s) declare financial support was received for the research, authorship, and/or publication of this article. This work was funded by the Munich Clinician Scientist Program (MCSP) of the University of Munich (LMU; grant number ACS-10), LMU Klinikum, the German Center for Diabetes Research (DZD), and the Helmholtz Zentrum München.
**Has your study been approved by an ethics committee?** Yes
**Ethics committee - additional information:** The study was approved by the local institutional review board (Ethics Commission of the Medical Faculty, Ludwig-Maximilians Universität München) and all study participants provided written informed consent prior to their participation in the study, which was conducted in accordance with the declaration of Helsinki.
**Author Disclosures:**
Olaf Dietrich: Nothing to disclose
Jochen Seißler: Nothing to disclose
Sarah Schläger: Nothing to disclose
Yannick Stohldreier: Nothing to disclose
Alexandra Sophia Gersing: Nothing to disclose
Michael Dieckmeyer: Nothing to disclose
Nina Hesse: Nothing to disclose
Uta Ferrari: Nothing to disclose

**Using vision transformer to classify sarcopenia severity on two-dimensional magnetic resonance images of the foot in patients with diabetic foot disease**
*M. Ahmad*, D. Amiras, J. Shalhoub, A. Davies, A. G. Rockall; London/UK
*(manalahmad.ma@gmail.com)*

**Purpose:** Vision transformer (ViT) is a recent development in the world of deep learning models (AI) and is an alternative to existing convolutional neural networks. ViTs aim to classify, detect and segment images. Diabetic foot disease (DFD) is a complex disease and is associated with lower limb amputation. Magnetic resonance imaging (MRI) is commonly used in patients with DFD. Our aim was to explore the use of deep learning models to identify predictors on MRI for limb loss in patients with DFD by using sarcoepenia as a potential surrogate marker by stratifying the extent of sarcopenia on MRI images of the foot.
**Methods or Background:** Two-dimensional images of the foot at the base of the 1st metatarsal were classified as having mild, moderate or severe sarcopenia. A subset of 50 images were also graded by a musculoskeletal radiologist to establish the inter-rater reliability. 824 images were annotated. Following data pre-processing and data augmentation, 1740 images were available for the deep learning models which were split into a 70:20:10 ratio for training:validation:testing. A ViT model was applied to classify the images as mild, moderate or severe.
**Results or Findings:** The inter-rater reliability was 0.827 [95% CI 0.726-0.928; p-value <0.001]. ViT had an accuracy of 78.7% with an F1 score of 79.9% in classifying sarcopenia severity on two-dimensional MRI pictures. The model had a high precision (81.5%) and recall (78.7%). The confidence threshold could be set to 51% without any deterioration in the model's performance.
**Conclusion:** ViT is a useful deep learning model in classifying the severity of sarcopenia in patients with diabetic foot disease.
**Limitations:** Further external validation is required to test the robustness of the model on different datasets.
**Funding for this study:** Imperial College Healthcare NHS Trust radiology pump priming fund.
**Has your study been approved by an ethics committee?** Yes
**Ethics committee - additional information:** Ethics approval received from Health Research Authority UK
**Author Disclosures:**
Dimitri Amiras: Nothing to disclose
Alun Davies: Nothing to disclose
Manal Ahmad: Nothing to disclose
Joseph Shalhoub: Nothing to disclose
Andrea Grace Rockall: Board Member: ESR

**CT-based Body Adipose Mass Quantification and Correlation with MRI-based Meniscal Tears**
*H. Abbasguliyev*[1], A. Yalçın[2], M. S. Ateş Çetin[2], F. Alper[2]; [1]Muğla/TR, [2]Erzurum/TR
*(hasanabbasguliyev@gmail.com)*

**Purpose:** This study aimed to quantify intra-abdominal visceral adipose tissue (VAT) using CT and assess its relationship with BMI. Additionally, we evaluated the association between meniscal damage on knee MRI and both BMI and intra-abdominal fat, while also testing the reproducibility of automated fat quantification.
**Methods or Background:** From January 2010 to December 2023, patients who underwent abdominal CT and also had knee MRI within one year were retrospectively identified. BMI values were classified into four groups (underweight, normal, overweight, obese). Meniscal signal changes on T2WI were graded in five stages (1, 2a, 2b, 2c, 3). Subcutaneous adipose tissue (SCAT) and VAT volumes were calculated using 3D Slicer software. Correlations between BMI, SCAT, VAT, and meniscal damage were analyzed ($p < 0.05$).
**Results or Findings:** Forty-one patients were included (21 men, 20 women; mean age 30 ± 9 years; mean BMI 25.9 ± 4.6). Mean SCAT volume was 260.31 ± 145.74 and VAT volume 315.74 ± 226.96. VAT showed a negative correlation with BMI (r = –0.3, p = 0.01), while SCAT did not (r = 0.2, p = 0.8). Medial meniscus damage correlated strongly with BMI (r = 0.7, p = 0.01), but not with SCAT. Lateral meniscus damage showed no significant association with BMI, SCAT, or VAT (p = 0.6, 0.8, 0.8, respectively).
**Conclusion:** This study demonstrates a novel link between VAT, BMI, and medial meniscus damage. Automated VAT quantification shows promise as a reproducible index, warranting larger studies to clarify its potential as a complement to BMI.
**Limitations:** The retrospective design required exclusion of patients with motion artifacts on knee MRI or abdominal CT, as well as those failing to meet at least one inclusion criterion.
**Funding for this study:** N/A
**Has your study been approved by an ethics committee?** Yes
**Ethics committee - additional information:** Atatuk University Non-Interventional Clinical Research Ethics Committee Decision No: B.30.2.ATA.0.01.00/306
**Author Disclosures:**
Ahmet Yalçın: Nothing to disclose
Hasan Abbasguliyev: Nothing to disclose
Melek Sevde Ateş Çetin: Nothing to disclose
Fatih Alper: Nothing to disclose

**Reliability of ultrasound measurements of upper arm muscle thickness in relaxed and maximally contracted states, and their relationship to strength**
*M. Pavošev*[1], D. Wieman[2], J. Menzinga[2], C. Chimbunde[3], F. Ernst[4], L. Sardinha[5], G. D. J. Colombo[6], C. D' Helft[7]; [1]Ljubljana/SI, [2]Groningen/NL, [3]Bloemfontein/ZA, [4]Cormondrèche/CH, [5]Lisbon/PT, [6]Milan/IT, [7]Dublin/IE
*(pavosevmichel@gmail.com)*

**Purpose:** Ultrasound (US) assessment of muscle thickness might be a safe and accessible method for the evaluation of sarcopenia. US can reliably measure muscle thickness during relaxation, but its functional meaning is limited. Therefore, this study investigates whether US-measured upper arm muscle thickness during contraction, incorporating neuromuscular activation, can also be assessed reliably, and how it is correlated to strength across physical activity levels and arm dominance.
**Methods or Background:** Twenty-four subjects completed a digital survey capturing demographics, sporting habits and arm dominance. US and maximal isometric strength measurements were taken respectively by the SonoScape Doppler E2 and MicroFET2 dynamometer, in Fowler's position with the arm stabilized in ~135° elbow flexion. Reliability was assessed using the intraclass correlation coefficient (ICC). Correlations were computed for the relaxed and contracted state between muscle thickness and strength for the entire study population, and subgroups based on physical activity level and arm dominance.
**Results or Findings:** ICC values indicated good to excellent intra-observer (>0.837) and inter-observer (>0.781) reliability in both relaxed and contracted states. Significant correlations (p<0.001) between muscle thickness and strength were observed: rs=0.737 (relaxed) and rs =0.748 (contracted). In the low-to-moderate activity group (n=15), correlations were respectively rs =0.829 and rs =0.850. No significant correlations were found in the high activity group (n=9). For arm dominance (n=21), significant correlations (p<0.001) were found for both dominant and non-dominant arms: rs =0.748 and rs =0.700 (relaxed), rs =0.670 and rs =0.778 (contracted).
**Conclusion:** Upper arm muscle thickness can be reliably measured using US in both relaxed and contracted states, and shows strong correlation with strength.

**Limitations:** Population size is limited and age is unevenly distributed, consisting mainly of young adults.
**Funding for this study:** No funding was received for this study.
**Has your study been approved by an ethics committee?** Yes
**Ethics committee - additional information:** Joint approval of the University of Ljubljana and UCD
**Author Disclosures:**
Michel Pavošev: Nothing to disclose
Jacob Menzinga: Nothing to disclose
Desi Wieman: Nothing to disclose
Laura Sardinha: Nothing to disclose
Chantel Chimbunde: Nothing to disclose
Félicia Ernst: Nothing to disclose
Catherine D' Helft: Nothing to disclose
Giorgio Diego Junior Colombo: Nothing to disclose

**Automated muscle compartment volume assessment: a deep learning approach on whole-body Dixon MRI**
*C. J. M. L. Edeline*, P. Margain, P. Omoumi; Lausanne/CH
*(charlesedeline@gmail.com)*

**Purpose:** Assessing muscle volume is crucial for understanding how training, aging, immobilization, and disease affect muscle health. Whole-body Dixon MRI enables robust tissue characterization, but manual segmentation for volume estimation is time-consuming. This study developed and evaluated a deep learning model for automated volume estimation of nine thigh and leg muscle compartments, assessing accuracy versus manual labels and test - retest repeatability.
**Methods or Background:** Nine compartments were analyzed: posterior, anterior, and medial thigh; sartorius; gluteal muscles; and superficial posterior, deep posterior, anterior tibial, and lateral tibial leg compartments. Whole-body Dixon MRI (1.95×1.95×4.00 mm; ~5 min) from 21 asymptomatic participants (12 males, 9 females; 44.2 ± 16.9 y; BMI 22.3 ± 2.5 kg/m²) from the Lausanne Knee Study were used. Manual segmentations were created on four sequences (fat-sat, water-sat, in-phase, out-of-phase). A 3D U-Net trained on in-phase images with five-fold cross-validation was evaluated for accuracy (MAE, Pearson r) and scan–rescan repeatability; test–retest metrics (ICC and coefficient of variation [CV]) were computed on n = 9 scans.
**Results or Findings:** High accuracy was observed, with mean absolute errors (MAE) of 4.4–30.4 cm³ (1.1–6.3% of mean volume). Pearson correlations exceeded 0.95 for most compartments (p < 0.001). Test–retest repeatability (n = 9 scans) was excellent: ICC = 0.98–0.99 and CV = 0.33–1.75% across the nine compartments.
**Conclusion:** A 3D U-Net can accurately and repeatably quantify muscle compartment volumes on whole-body Dixon MRI. The combination of high accuracy and excellent reliability supports application in large-scale cohorts and longitudinal or clinical studies, pending external validation.
**Limitations:** Limitations include data derived from a single center and a single protocol, as well as the use of an asymptomatic cohort. Therefore, the model should be validated in participants with relevant conditions, such as sarcopenia.
**Funding for this study:** This work was funded by the Swiss National Science Foundation, Switzerland (SNSF Grant #CRSII5_177155)
**Has your study been approved by an ethics committee?** Yes
**Ethics committee - additional information:** The study was approved by the Cantonal Ethics Committee for Research on Human Beings (CER-VD).
**Author Disclosures:**
Charles Jean Marie Louis Edeline: Nothing to disclose
Paul Margain: Nothing to disclose
Patrick Omoumi: Nothing to disclose

**Assessment of Sarcopenia and Body Composition Using Low-Dose CT from PET-CT**
F. Abubacker Sulaiman, *A. A. Basheer Ahmed*; Chennai/IN
*(dr.ashrafbasheer@gmail.com)*

**Purpose:** To evaluate the feasibility of assessing sarcopenia and body composition using low-dose CT (LDCT) data obtained from routine ^18F-FDG PET/CT scans and to correlate these measurements with clinical parameters and treatment outcomes in oncologic patients.
**Methods or Background:** A prospective study was conducted on 70 patients who underwent whole-body ^18F-FDG PET/CT for staging or response assessment. Cross-sectional muscle and fat areas were analyzed at the third lumbar vertebral (L3) level using LDCT images reconstructed from PET/CT datasets. Skeletal muscle index (SMI), visceral fat area (VFA), and subcutaneous fat area (SFA) were quantified using semi-automated segmentation software. Sarcopenia was defined using established sex-specific SMI cutoffs. Associations between SMI, BMI, serum albumin, and treatment response were statistically analyzed using Pearson's correlation and regression models.
**Results or Findings:** Sarcopenia was identified in 32 patients (45.7%), predominantly among older and low-BMI individuals. Mean SMI values showed strong correlation with serum albumin levels (r = 0.72, p < 0.001) and moderate correlation with treatment response on follow-up PET/CT (r = 0.64, p < 0.01). Patients with sarcopenia exhibited higher incidence of adverse events and poorer metabolic response. LDCT-based measurements were reproducible, with excellent interobserver agreement (ICC = 0.92). Radiation dose from LDCT did not exceed 2.5 mSv per scan.
**Conclusion:** Low-dose CT from routine PET/CT provides a reliable, opportunistic tool for assessing sarcopenia and body composition without additional imaging or radiation exposure, adding valuable prognostic information in oncology.
**Limitations:** Single-center design, limited sample size, and absence of longitudinal survival analysis. Larger multicentric studies are warranted for validation.
**Funding for this study:** No external funding was received for this study.
**Has your study been approved by an ethics committee?** Yes
**Ethics committee - additional information:** Institutional ethical committee approval was obtained.
**Author Disclosures:**
Farook Abubacker Sulaiman: Nothing to disclose
Ashraf Ahmed Basheer Ahmed: Nothing to disclose

**08:00 - 09:30** **Research Stage 3**

### Research Presentation Session: Neuro

### RPS 111

### Glymphatic system, venous and small vessel disease: small lesions, big impact

**Moderator**
Milos A. Lucic; Sremska Kamenica/RS
*(milos.a.lucic@gmail.com)*

**Evaluation of the Ocular Glymphatic System and Optic Nerve in Humans Using Magnetic Resonance Imaging**
*M. Can*, A. Karaosmanoğlu, R. Göçmen, N. P. Acar Özen, M. A. Tuncer, G. F. Yavaş, I. Koç, A. I. Colpak Işıkay, E. Gümeler; Ankara/TR
*(95canmert@gmail.com)*

**Purpose:** We aimed to determine whether an ocular glymphatic pathway exists on magnetic resonance imaging (MRI) and how it relates to ocular findings.
**Methods or Background:** Twelve healthy volunteers (age 24–52) with 23 normal fundoscopic exams were prospectively enrolled. MRI was performed with 3 T MR scanner (GE SignaTM Architect) with 48 channel head coil. Heavily T2-weighted 3D Fluid attenuated inversion recovery (FLAIR) sequence (TR/TE/TI: 9000/520/2250 ms) was used to detect subtle signal intensity (SI) changes. Three subjects were scanned at baseline and at 4, 8, 12, 18, and 24 hours following IV gadobutrol (1.0 mmol/mL) to evaluate contrast clearance from the optic nerve (ON) sheath. Based on these results, the remaining subjects were scanned at 0, 4, and 18 hours (figure 1). Subtracted images were generated (baseline vs. post-injection) using SPM25 after normalization and coregistration. SI volume in the ON sheath was semi-automatically measured. We measured enhanced volume from subtracted image of baseline from 4th hour and residual volume from subtracted image of baseline from 18th hour. We calculated clearance volume and ratio of clearance with this information (figure 2). We performed Spearman's correlation analysis between age, intraocular pressure (IOP), retinal nerve fiber layer (RFNL) thickness and these measurements.
**Results or Findings:** Correlation analysis revealed a significant negative correlation between IOP and clearance volume (r=-.575, p=0.004) and clearance ratio (r=-.549, p=0.007). No correlation was found with age or RNFL thickness.
**Conclusion:** Our findings support the presence of a bidirectional ocular glymphatic system in humans, modulated by IOP and the intraocular–intracranial pressure gradient. While existing literature contains limited evidence for a similar pathway in humans, our preliminary findings provide the first obvious demonstration of this correlation in a human cohort.
**Limitations:** Intracranial pressure, concentration of contrast media not available
**Funding for this study:** Funded by Hacettepe University Scientific Research Projects Coordination Unit
**Has your study been approved by an ethics committee?** Yes

**Ethics committee - additional information:** Hacettepe University Clinical Research Ethics Committee
**Author Disclosures:**
Mert Can: Nothing to disclose
Ekim Gümeler: Nothing to disclose
Ayça Karaosmanoğlu: Nothing to disclose
Irem Koç: Nothing to disclose
Rahşan Göçmen: Nothing to disclose
Ayşe Ilksen Colpak Işıkay: Nothing to disclose
Güliz Fatma Yavaş: Nothing to disclose
Nazire Pınar Acar Özen: Nothing to disclose
Meryem Aslı Tuncer: Nothing to disclose

**Enlarged choroid plexus drives longitudinal hippocampal atrophy**

*K. Yuba*[1], K. Watanabe[1], N. Kuriyama[2], E. Ozaki[1], D. Matsui[1], Y. Marunaka[1], K. Akazawa[1], K. Hayakawa[1], K. Yamada[1]; [1]Kyoto/JP, [2]Shizuoka/JP
*(ken1@koto.kpu-m.ac.jp)*

**Purpose:** The glymphatic system is a brain-wide clearance pathway for metabolic waste. Although dysfunction of this system has been implicated in neurodegenerative disorders, its long-term impact on brain structure remains unclear. The choroid plexus (CP) is thought to play a critical role in glymphatic function. Enlargement of CP has been reported in Alzheimer's disease and mild cognitive impairment, suggesting a link with CP dysfunction. We hypothesized that CP dysfunction is associated with subsequent hippocampal atrophy, given the hippocampus is a key structure for memory and spatial cognition. This study aimed to investigate whether baseline CP volume predicts longitudinal hippocampal volume change.
**Methods or Background:** We analyzed 154 community residents (median age, 67 years; 64% male) from a brain health check-up program who underwent 1.5-T MRI at baseline (2012-2013) and follow-up (2018-2019), after excluding those scoring <27 on the Mini-Mental State Examination (MMSE) to restrict the sample to cognitively normal individuals. Three-dimensional T1-weighted images were processed using the Sequence Adaptive Multimodal SEGmentation tool implemented in FreeSurfer (v7.3.2). Linear regression models were applied to assess the association between baseline CP volume and hippocampal volume loss over six years, adjusting for age, sex, intracranial volume, and MMSE.
**Results or Findings:** Baseline CP volume was a significant predictor of hippocampal volume loss over the subsequent six years ($\beta = -0.05$, $p < 0.01$) after adjustment for covariates, with CP showing a significant increase and hippocampal a significant decrease during this period.
**Conclusion:** In cognitively normal older adults, larger CP volume at baseline was linked to subsequent hippocampal atrophy over six years. These findings suggest that impairment in the glymphatic system may contribute to accelerated hippocampal atrophy.
**Limitations:** CP volume increase may reflect other factors such as inflammation and immunity, warranting further study.
**Funding for this study:** No funding.
**Has your study been approved by an ethics committee?** Yes
**Ethics committee - additional information:** This study was approved by the Ethical Review Board of the Kyoto Prefectural University of Medicine (approval number: G-144), and written informed consent was obtained from all participants.
**Author Disclosures:**
Daisuke Matsui: Nothing to disclose
Kentaro Yuba: Nothing to disclose
Kentaro Akazawa: Nothing to disclose
Nagato Kuriyama: Nothing to disclose
Yoshinori Marunaka: Nothing to disclose
Etsuko Ozaki: Nothing to disclose
Kei Yamada: Nothing to disclose
Katsumi Hayakawa: Nothing to disclose
Keita Watanabe: Nothing to disclose

**White matter hyperintensity-associated iron overload links glymphatic system dysfunction to cognitive impairment in cerebral small vessel disease**

*Y. Qiu*, Y. Hu, Q. Fu, W. Hu, Y. Wang, Q. Xu, Y. Dai, Y. Sun, Y. Zhou; Shanghai/CN
*(jacobqiu1@163.com)*

**Purpose:** Glymphatic dysfunction is implicated in cognitive impairment in cerebral small vessel disease (CSVD). Iron overload within white matter hyperintensity (WMH), indicating metabolic abnormalities, may be a key mechanism underneath. This study aimed to investigate the relationships between WMH iron burden, glymphatic function, and cognition in CSVD.
**Methods or Background:** A total of 102 patients with CSVD and 29 matched healthy controls (HCs) were enrolled. WMH iron burden was quantified using the sub-voxel approach iterAtive magnetic suscePtibility sources sepARaTion Quantitative Susceptibility Mapping (APART-QSM), and glymphatic function was assessed with the Diffusion Tensor Image Analysis aLong the Perivascular Space (DTI-ALPS) index. Correlation and mediation analyses were performed.
**Results or Findings:** CSVD patients exhibited higher WMH iron burden, lower DTI-ALPS index, and poorer cognitive performance than HCs. Elevated WMH iron burden was associated with deficits in attention-executive (att-exe), memory, and visual-spatial domains, whereas reduced DTI-ALPS index correlated with impaired att-exe and memory function. More importantly, WMH iron burden fully mediated the link between DTI-ALPS index and both att-exe and memory function in CSVD patients.
**Conclusion:** These findings non-invasively identify WMH iron overload, a probable representative of microglial activation, as a critical mediator between glymphatic dysfunction and cognitive decline in CSVD, prompting a potential therapeutic target.
**Limitations:** The limitations of the study are as follows. First, the DTI-ALPS index is an indirect measure of glymphatic flow and does not quantify clearance efficiency directly; future multimodal imaging could provide a more holistic assessment. Second, in some subjects, regions of interest for DTI-ALPS were shifted to avoid WMH, which may introduce bias by excluding areas of potential glymphatic dysfunction. Third, future studies using habitat analysis and longitudinal designs are needed to clarify the spatiotemporal dynamics of iron deposition.
**Funding for this study:** Funding was provided by the National Natural Science Foundation of China [grant number 82171885, 82302142], Eastern Talent Plan Leading Project [grant number LJ2023127], the Shanghai Science and Technology Committee Project [Explorer Project Funding, grant number 24TS1414800], the Leading Talent Program of Shanghai Municipal Health Commission [grant number 2022LJ023], the Technology Standardized Management and Promotion Projects of Shanghai Shenkang Hospital Development Center [grant number SHDC22023022], National Natural Science Foundation Promotion Project of Ren Ji Hospital [grant number RJTJ25-MS-110], the Shanghai YRD Foundation for Innovation in Health Industry [grant number 2025-YRDFHI-086], and Medical Engineering Cross Research Foundation of Shanghai Jiao Tong University [grant number YG2022QN035, YG2022QN037].
**Has your study been approved by an ethics committee?** Yes
**Ethics committee - additional information:** This study was approved by the Ethics Committee of Ren Ji Hospital, School of Medicine, Shanghai Jiao Tong University (reference number KY2021-179-B).
**Author Disclosures:**
Yuanzheng Wang: Nothing to disclose
Yage Qiu: Nothing to disclose
Qingyang Fu: Nothing to disclose
Yongming Dai: Nothing to disclose
Yan Zhou: Nothing to disclose
Qun Xu: Nothing to disclose
Wentao Hu: Nothing to disclose
Yawen Sun: Nothing to disclose
Ying Hu: Nothing to disclose

**The association of Choroid Plexus volume with Clinical Status of Parkinson's Disease**

B. Atalay, *O. Cetin*, M. Gezgin, M. B. Doğan, H. Yıldız, F. B. Ozdilek; Istanbul/TR

**Purpose:** The choroid plexus, which regulates cerebrospinal fluid dynamics and contributes to the glymphatic clearance system, has been implicated in neurodegenerative processes. This study aimed to investigate the association between the choroid plexus volume (CPV), and clinical status of Parkinson's disease (PD).
**Methods or Background:** We retrospectively analyzed 57 patients with PD, and 45 controls who underwent brain MRI. CPV was manually segmented using ITK-SNAP on T1-weighted FSPGR images and normalized to intracranial volüme (ICV), which was calculated using volBrain software. Clinical parameters included Mini Mental State Examination (MMSE), Unified Parkinson's Disease Rating Scale (UPDRS), Hoehn and Yahr (HY) scale, disease duration, and levodopa equivalent daily dose (LEDD). Group comparisons were conducted using Mann–Whitney U, and chi-square tests where appropriate. Correlation analyses were performed using Spearman's test.
**Results or Findings:** Mean CPV was 27.6±4.2 cm3 in patients and 28.5±7.2 cm3 in controls ($p = 0.68$). Within the Parkinson's cohort, CPV and CPV/ICV showed significant negative correlations with MMSE ($r = –0.38$, $p = 0.01$; $r = –0.45$, $p = 0.01$, respectively). Conversely, positive correlations were observed with UPDRS-III ($r = 0.30$, $p = 0.03$; $r = 0.27$, $p = 0.04$, respectively) and HY

Wednesday stage (r = 0.32, p = 0.02; r = 0.33, p = 0.02, respectively). CPV was also negatively correlated with LEDD (r = –0.31, p = 0.03). Furthermore, CPV was significantly higher in patients with dementia (2.18 ± 0.65) compared to those without dementia (1.53 ± 0.70; p = 0.01).
**Conclusion:** CPV correlates with cognitive and motor symptoms severity in PD, highlighting its potential as an imaging biomarker of disease progression.
**Limitations:** The retrospective design, use of manual segmentation, and absence of longitudinal data limit causal interpretation.
**Funding for this study:** Not applicable
**Has your study been approved by an ethics committee?** Not applicable
**Author Disclosures:**
Merve Gezgin: Nothing to disclose
Omer Cetin: Nothing to disclose
Fatma Betül Ozdilek: Nothing to disclose
Başak Atalay: Nothing to disclose
Hüseyin Yıldız: Nothing to disclose
Mahmut Bilal Doğan: Nothing to disclose

**Ultra–High–In-Plane–Resolution 2D T2-Weighted Imaging at 5T Compared to 3T: Visualization of Enlarged Perivascular Spaces**
*J. Lu*[1], X. Zhang[1], Y. Wang[2], R. Tang[2], B. Zhang[1]; [1]Nanjing/CN, [2]Shanghai/CN
*(b101230039@gmail.com)*

**Purpose:** To compare PVS visualization and quantification between 3T and 5T ultra–high in-plane resolution 2D T2-weighted imaging, and to examine associations with glymphatic-related diffusion and volumetric metrics, as well as behavioral measures.
**Methods or Background:** From September 2025 to May 2025, 36 healthy adults (mean age ± SD, 26.6 ± 3.3 years; 12 men) underwent same-day 3T and 5T brain MRI, including 2D T2-weighted imaging, 3D T1-weighted imaging, and multi-shell diffusion prospectively. PVS were segmented from Enhanced PVS Contrast images, and five morphological metrics (volume, volume fraction [VF], diameter, solidity, length) were extracted for whole brain, basal ganglia, and hemispheric regions. Associations with free water fraction (FWF), ALPS index, choroid plexus volume (CPV), and clinical scales (HAMA, BMI, PSQI) were evaluated. Paired t-tests and interaction models were applied.
**Results or Findings:** Median qualitative PVS visualization scores were higher at 5T versus 3T (5.0 vs 3.0, P<.001). Quantitatively, 5T imaging yielded increases of 49.5% in PVS volume, 37.2% in VF, 5.6% in diameter, 14.5% in solidity, and 14.5% in length (all P<.001). 5T-derived PVS metrics showed stronger associations with FWF, ALPS index, and CPV ($R^2$ up to 0.41) than 3T ($R^2 < .01$), with significant field strength interactions (P = .032). Only 5T detected significant correlations between PVS morphology and behavioral measures, including HAMA, BMI, and PSQI.
**Conclusion:** 5T MRI substantially improves PVS visualization and quantification compared with 3T, enabling stronger detection of structural–functional coupling and behavioral associations relevant to glymphatic function.
**Limitations:** This study is limited by its focus on healthy young adults, the limited availability of 5T MRI systems, and its cross-sectional design, which precludes causal inference. These factors may restrict generalizability and immediate clinical translation.
**Funding for this study:** None
**Has your study been approved by an ethics committee?** Yes
**Ethics committee - additional information:** the institutional review board of Nanjing Drum Tower Hospital
**Author Disclosures:**
Bing Zhang: Nothing to disclose
Jiaming Lu: Nothing to disclose
Ran Tang: Nothing to disclose
Xin Zhang: Nothing to disclose
Yichen Wang: Nothing to disclose

**Alterations in Cortical Structural Networks and Imaging–Transcriptomic Mechanisms in Cerebral Small Vessel Disease Patients with Mild Cognitive Impairment**
*M. Xu*, H. Mao, J. Yang, M. Li, Q. Gao, Y. Shi, L. Ma, F. Wang, X. Fang; Wuxi/CN

**Purpose:** To investigate alterations in cortical individualized structural networks and their molecular basis in patients with cerebral small vessel disease and mild cognitive impairment (CSVD-MCI), using a multi-modal imaging–transcriptomic approach, and to compare the identified molecular signatures with peripheral blood transcriptomic profiles.
**Methods or Background:** T1-weighted images were processed with FreeSurfer to extract five cortical morphological features and construct individualized structural networks. Regional group differences were assessed, followed by spatial transcriptomic analysis using Allen Human Brain Atlas data and partial least squares (PLS) regression. Significant genes were identified by bootstrap and FDR correction. Functional enrichment and cross-system comparisons were performed with peripheral blood RNA-seq.
**Results or Findings:** In this prospective study, 245 CSVD patients (152 NCI, 93 MCI) underwent neuropsychological testing and MRI. Compared with the NCI group, MCI patients showed significantly lower whole-brain mean MIND values (p = 0.002) and regional reductions across 90 cortical regions, mainly in the frontal, parietal, temporal, insular, and cingulate cortices. Network analyses showed pronounced decreases in default mode and dorsal attention networks (p < 0.005). PLS1 explained the strongest spatial covariance (ρ = 0.397, p_spin < 0.001), identifying 860 positively and 594 negatively weighted genes. Enrichment analyses indicated positive weights for ion transport and synaptic signaling, and negative weights for protein translation and MAPK regulation. Overlapping pathways were observed with peripheral blood profiles.
**Conclusion:** CSVD-MCI involves widespread cortical network reorganization with distinct transcriptional signatures, partially mirrored in peripheral blood, supporting their potential as molecular markers of cognitive impairment. These findings highlight a novel cross-system molecular link, offering opportunities for early risk stratification and targeted interventions.
**Limitations:** Cross-sectional design and reliance on postmortem transcriptomic data may limit causal inference.
**Funding for this study:** Medical Expert Team Program of Wuxi Taihu Talent Plan (THRC-TD-YXYXK-2021),Wuxi Medical Innovation Team Program (CXTD2021002)
**Has your study been approved by an ethics committee?** Yes
**Ethics committee - additional information:** The Affliated Wuxi People's Hospital of Nanjing Medical University
**Author Disclosures:**
Lin Ma: Nothing to disclose
Mingyu Li: Nothing to disclose
Xiangming Fang: Nothing to disclose
Min Xu: Nothing to disclose
Feng Wang: Nothing to disclose
Yachen Shi: Nothing to disclose
Qianqian Gao: Nothing to disclose
Jiayi Yang: Nothing to disclose
Haixia Mao: Nothing to disclose

**Baseline QSM Metrics as Predictors of Cognitive Decline in Cerebral Small Vessel Disease**
*Y. Hao*[1], X. Fan[1], E. Li[2], H. You[1], Y. Zhu[1], F. Feng[1]; [1]Beijing/CN, [2]Shanghai/CN
*(haoweiweiweier@outlook.com)*

**Purpose:** Cerebral small vessel disease (CSVD) is associated with microstructural changes in subcortical gray matter that relate to cognitive performance, with potential regional heterogeneity across subregions. This study investigates the predictive value of baseline quantitative susceptibility mapping (QSM) metrics for cognitive function in patients with CSVD
**Methods or Background:** From September 2022 to June 2025, a prospective cohort of 248 participants [90 with mild CSVD vs 108 with severe CSVD vs 50 healthy control (HC)] were recruited. All participants had cognitive examination including Montreal Cognitive Assessment (MoCA) and Mini-Mental State Examination (MMSE) and MRI including multiecho gradient echo (mGRE) sequence. QSM images were segmented into 32 subcortical gray matter regions, with baseline QSM metrics (mean, median, interquartile range [IQR], and maximum susceptibility) analyzed. Propensity score matching adjusted for baseline confounders. Cognitive function were evaluated using Cox regression models, and risk stratification was performed based on thresholds identified through receiver operating characteristic (ROC) analysis
**Results or Findings:** After adjusting for potential confounders, the susceptibility values in right posterior caudate (β = 4.675, P = 0.003) and the right posterior hippocampus (β = 1.516, P = 0.025) were positively associated with CSVD severity, and those in the globus pallidus, putamen and caudate nucleus were negatively associated with cognitive function. Baseline QSM metrics, particularly the mean susceptibility (QSMmean) (AUC=0.833) and the median susceptibility (QSMmedian) (AUC=0.750) demonstrated moderate-to-substantial predictive performance for cognitive decline. Patients with severe CSVD showed a 5.2-fold increased risk of cognitive impairment compared to those with mild CSVD
**Conclusion:** QSM metrics, serving as a complementary imaging biomarker, demonstrated significant predictive value for cognitive function. Validation in larger, independent cohorts is warranted

**Limitations:** Susceptibility values are affected by noniron-related molecules, especially myelin, which are consistently found in structures like the thalamus
**Funding for this study:** This study was supported by the National Natural Science Foundation of China (grants 82371946 and 82401557), the Chinese Academy of Medical Sciences Innovation Fund for Medical Sciences (grant 2021-I2M-1-025), and the National Postdoctoral Researcher Funding Program (No. GZC20240138)
**Has your study been approved by an ethics committee?** Yes
**Ethics committee - additional information:** This study was approved by the Ethics Committee of Peking Union Medical College Hospital (approval number: 1-23PJ2044)
**Author Disclosures:**
Feng Feng: Nothing to disclose
Yuwei Hao: Nothing to disclose
Hui You: Nothing to disclose
Xiaoyuan Fan: Nothing to disclose
Yicheng Zhu: Nothing to disclose
Enhui Li: Nothing to disclose

**Causal Structural Covariance Network Revealing Atrophy Progression In Cerebral Small Vessel Disease Continuum**
*R. Mu*[1], P. Yang[1], W. Zheng[1], X. Qin[1], J. Lv[1], B. Huang[1], X. Liu[2], L. Cheng[1], X. Zhu[1]; [1]Guilin/CN, [2]Guangzhou/CN
*(ronghuamu@outlook.com)*

**Purpose:** Structural covariance networks (SCNs) provide an important framework for understanding large-scale brain network damage across the continuum of cerebral small vessel disease (CSVD). However, it remains unclear how progressive cross-regional structural injury drives SCN disruption.
**Methods or Background:** From May 2020 to November 2021, 500 community participants were enrolled, including patients with vascular dementia, vascular mild cognitive impairment, CSVD with preserved cognition, and cognitively normal elderly controls. A multimodal CSVD cognitive impairment database was established, integrating neuroimaging (3D T1-weighted MRI), neuropsychological assessments, and clinical data. T1-weighted images were automatically preprocessed using a standardized SPM12 pipeline. Morphology-based independent component analysis was applied to extract gray matter SCNs, enabling network-level characterization of CSVD-related structural alterations and whole-brain atrophy patterns. Causal structural covariance networks (CaSCNs) were further constructed with a sliding time-window approach to identify the temporal causal relationships between key driving nodes and progressive atrophy. Finally, functional decoding was used to map structural alterations to behavioral domains and to explore their associations with cognitive impairment.
**Results or Findings:** Compared with normal aging, SCNs exhibited progressive disruption in CSVD with preserved cognition, vascular mild cognitive impairment, and vascular dementia. Dynamic CaSCN analysis based on composite cognitive scores identified hippocampus and frontal lobes as key causal hubs, with their atrophy significantly predicting subsequent atrophy in other regions. Functional decoding confirmed specific associations between network-level damage originating from these regions and impairments in executive function, attention, and memory.
**Conclusion:** This study systematically delineated the spatial distribution and interregional associations of brain atrophy across the CSVD continuum. Hippocampus and frontal lobes emerged as central origins of atrophy, whose degeneration predicted widespread secondary atrophy. These findings highlight the causal and temporal dynamics of whole-brain atrophy and provide novel network-level insights into CSVD progression.
**Limitations:** None
**Funding for this study:** None
**Has your study been approved by an ethics committee?** Yes
**Ethics committee - additional information:** None
**Author Disclosures:**
Luqi Cheng: Nothing to disclose
Peng Yang: Nothing to disclose
Xiaomin Liu: Nothing to disclose
Wei Zheng: Nothing to disclose
Bingqin Huang: Nothing to disclose
Ronghua Mu: Nothing to disclose
Xiaoyan Qin: Nothing to disclose
Xiqi Zhu: Nothing to disclose
Jian Lv: Nothing to disclose

**Combining plaques and cerebral small vessel diseases imaging characteristics for ischemic stroke prediction in intracranial atherosclerotic disease**
*J. Zhang*, J. Miao; Shanghai/CN

**Purpose:** The aim of this study was to investigate the predictive value of intracranial plaques and cerebral small vessel diseases (CSVDs) imaging characteristics for ischemic stroke, further elucidated the impact of coexisted large and small intracranial vessel diseases on stroke events.
**Methods or Background:** Patients who underwent contrast-enhanced higher–resolution vessel wall MRI (HRVW-MRI) with intracranial atherosclerotic disease were recruited from January 2021 to April 2024. Plaque features like intraplaque hemorrhage (IPH), enhancement grade, maximum wall thickness (Max WT), lumen area (LA) were assessed in HRVW-MRI. We used total CSVDs score based on 4 CSVDs imaging markers, including lacunes, white matter hyperintensities (WMLs), cerebral microbleeds (CBMs), and enlarged perivascular spaces (EPVSs), and the overall CSVDs burden score was 0–4. Binary logistic regression analysis and receiver operating characteristic (ROC) curve were used for data analysis.
**Results or Findings:** Of the total 237 patients (the median age was 63, 154 males), 163 patients occurred stroke. Binary logistic regression revealed that patients with greater Max WT, larger LA, more CBMs and EPVSs, higher enhancement grade, total CSVDs and WMLs score were more likely occurred stroke (all $p < 0.05$). The combination of these variables achieved a higher area under the curve (0.85, 95% CI 0.80-0.96, p＜0.000) than that of the plaques (0.77, 95% CI 0.71-0.89, p＜0.000) and CSVDs (0.79, 95% CI 0.72-0.87, p＜0.000) imaging characteristics alone.
**Conclusion:** Plaque features combined with CSVDs imaging makers provides incremental value for stroke prediction, which is helpful for the clinical management of high-risk patients.
**Limitations:** Firstly, the sample size was small. Secondly, the present study was restricted to patients with anterior circulation infarct. Thirdly, this was a retrospective study with bias.
**Funding for this study:** Shanghai Explorer Program (Grant no. 24TS1410900)
**Has your study been approved by an ethics committee?** Not applicable
**Author Disclosures:**
Jun Zhang: Nothing to disclose
Jiali Miao: Nothing to disclose

**Arterial spin labeling perfusion in accidental carbon monoxide intoxication: a longitudinal study**
*T. Torgan*, W. Norhøy, C. M. Page, F. Fridtjof Heyerdahl, A. Bjørnerud, L. Tjelta Westlye, P. W. Sowa; Oslo/NO
*(torstein.torgan@gmail.com)*

**Purpose:** To investigate longitudinal changes in gray matter (GM) cerebral blood flow (CBF) following accidental carbon monoxide (CO) poisoning, using arterial spin labeling (ASL).
**Methods or Background:** Survivors of CO intoxication may experience persistent or recurrent neurological symptoms, even when conventional MRI findings are minimal. ASL perfusion imaging may detect subtle changes. Twenty-six young adults (average 24.6 years old) with varying degrees of CO poisoning were admitted to the hospital and recruited in this longitudinal study. MRI was done at four timepoints (TP), after average of 5.1 days (TP1), 2.1 months (TP2), 7.6 months (TP3), and 18.2 months (TP4). ASL data were processed using ExploreASL and compared with HbCO levels. CBF was assessed globally, in subcortical regions, and across vascular-territorial cortical areas. Linear and mixed effects regression were used to evaluate the association between HbCO levels and CBF at each TP, and across all TPs.
**Results or Findings:** There was decrease of CBF in TP1 in Putamen, and negative correlation between CBF and HbCO levels at TP2 in all but one evaluated brain regions (CBF falling 0.34 ml/100g/min per 1 % increase HbCO for global grey matter, p=0.031). The mixed-effects analysis showed association between HbCO levels and CBF across all TPs in most areas, with the strongest effect in Putamen (CBF falling 0.63ml/100g/min per 1 % increase in HbCO, p=0.001), as well as effect of TP4 for CBF reduction in thalamus (p=0.049).
**Conclusion:** GM ASL perfusion is reduced in CO poisoning after 2.1 months (earliest and most strongly in putamen), indicating usefulness of ASL for evaluating neurotoxicity. There is a dose-dependent association between HbCO levels and reduced perfusion. Perfusion in thalamus appears to be affected also at a later timepoint.
**Limitations:** Small study, variations in CO exposure, new technique
**Funding for this study:** Funded by Oslo University Hospital
**Has your study been approved by an ethics committee?** Yes

**Ethics committee - additional information:** REK sør-øst C
**Author Disclosures:**
Atle Bjørnerud: Nothing to disclose
Lars Tjelta Westlye: Nothing to disclose
Christian Magnus Page: Nothing to disclose
Fridtjof Fridtjof Heyerdahl: Nothing to disclose
Piotr Wladyslaw Sowa: Nothing to disclose
Torstein Torgan: Nothing to disclose
Wibeke Norhøy: Nothing to disclose

**The multimodal imaging features and temporal evolution of SLE in patients with MELAS: A multicenter cohort study**
Q. Yu, R. Wang, C. Sun, D. Geng, J. Lin, *S. Piao*, Y. Li; Shanghai/CN

**Purpose:** Mitochondrial encephalomyopathy with lactic acidosis and stroke-like episodes (MELAS) is a rare maternally inherited disease. As a recurrent paroxysmal clinical event, the heterogeneity underlying the progression of stroke-like episode (SLE) remains unclear.
**Methods or Background:** In this retrospective, multicenter, observational cohort study, we sought to confirm the clinical, genetics, radiological and temporal evolution characteristics of SLEs. Brain atrophy was assessed using Global Cortical Atrophy (GCA) and quantitative brain volumetric measures(QBVM). Both methods were employed to explore brain atrophy changes over time among these patients.
**Results or Findings:** Between August 2012 and December 2024, we identified 171 MELAS patients who developed SLEs. Based on the age of first SLE, these patients were divided into two groups, juvenile group and adult group. Hearing loss and diabetes mellitus were significantly more frequent in the adult group ($p<0.05$). Nausea/vomiting was significantly more frequent in the juvenile group ($p=0.017$). SLEs were significantly more prone to involve the frontal lobe in the juvenile group ($p=0.002$). Stroke-like lesion (SLL) areas were significantly larger in the juvenile group ($p=0.005$). Furthermore, a total of 67 patients with available follow-up imaging data were assessed for GCA and QBVM. MELAS patients showed rapidly annual increasing scores according to the GCA (median, IQR; 2.55, [1.48,5.14]), and significant ventricular enlargement based on QBVM (median annual change rate, IQR; 0.05, [-0.01, 0.14]). In the longitudinal assessment of GCA , an exponential-like change was observed. By approximately two years of follow-up, the progression appeared to stabilize, entering a phase where changes were markedly less pronounced.
**Conclusion:** Our findings highlight the clinical, radiological and temporal evolution characteristics of SLEs between both groups. We demonstrated that MELAS shows a rapid brain atrophy progression within a 2-year interval and follows different natural courses.
**Limitations:** Not applicable.
**Funding for this study:** This work was supported by Science and Technology Commission of Shanghai Municipality (22TS1400900, 22ZR1409500, 23S31904100, 24SF1904200, 24SF1904201); and Huashan Hospital Foundation (Basic Research Youth Cultivation Program) (2024JC018).
**Has your study been approved by an ethics committee?** Yes
**Ethics committee - additional information:** The studies involving humans were approved by the Institutional Review Board of Huashan hospital.
**Author Disclosures:**
Sirong Piao: Nothing to disclose
Daoying Geng: Nothing to disclose
Yuxin Li: Nothing to disclose
Qingyun Yu: Nothing to disclose
Chong Sun: Nothing to disclose
Rong Wang: Nothing to disclose
Jie Lin: Nothing to disclose

**08:00 - 09:30 Research Stage 4**

### Research Presentation Session: Oncologic Imaging

### RPS 116

### Innovations in AI applications for oncology

**Moderator**
Boj Friedrich Hoppe; Munich/DE

**Foundation Model Based Lesion Tracking For The Longitudinal Follow-Up of Solid Tumors on CT and MRI**
*L. Machado*, A. Prat, H. Philippe, T. Danielou, K. Le Floch, M. Ronot, D. Tordjman, P. Manceron, P. Hérent; Paris/FR
*(dr.machado.leo@gmail.com)*

**Purpose:** RECIST 1.1 remains the reference for longitudinal assessment of solid tumors but is slow, labor-intensive, and prone to inter-reader variability. Most AI tools are unimodal, organ-specific, and restricted to single timepoints, limiting clinical use. We present a pan-tumor, CT/MRI lesion-tracking pipeline for semi-automated longitudinal follow-up using foundation models.
**Methods or Background:** To achieve lesion tracking, we used features from Curia, a multimodal foundation model (Dancette et al., 2025, arXiv). From the baseline lesion, Curia enables the generation of a bounding-box on the follow-up exam, localizing the follow-up lesion. This bounding-box is then processed by the segmentation model Oncopilot-v2 to obtain the follow-up mask. Oncopilot-v2 is a fine-tuning of Raps3D (Danielou et al., 2025, arXiv) on 16,035 lesions (e.g., liver, lung, brain, lymph nodes) in CT and MRI, following the same procedure as Oncopilot (Machado et al., 2025, NPJ Precision Oncology). Evaluation used a multicenter dataset of 238 patients with 391 baseline target lesions (mean 1.64/patient; CT/MRI ratio 1.15; mean follow-up 290 days). Longest diameter and short axis were computed on baseline and follow-up masks, and RECIST classification derived from the sum of largest diameters (SLD).
**Results or Findings:** Median DICE was 0.80. CT lesion tracking was superior to MRI (0.82 vs 0.78). Median absolute error on SLD was 3.2 mm (8.5% of SLD). RECIST classification accuracy reached 71% for PR/SD/PD and 79% for PD vs non-PD.
**Conclusion:** This pipeline enables longitudinal propagation of target-lesion masks on CT and MRI, advancing toward a semi-automatic RECIST workflow with potential productivity gains and reduced variability.
**Limitations:** Full RECIST requires integration of non-target lesions. Complete-response cases were absent. The lesion tracking accuracy could be improved. Radiologist oversight remains essential to validate and refine segmentation. Prospective studies are needed to confirm clinical utility.
**Funding for this study:** Not applicable
**Has your study been approved by an ethics committee?** Not applicable
**Author Disclosures:**
Pierre Manceron: Founder: Raidium
Maxime Ronot: Nothing to disclose
Korentin Le Floch: Employee: Raidium
Léo Machado: Employee: Raidium
Hélène Philippe: Employee: Raidium
Paul Hérent: Founder: Raidium
Théo Danielou: Employee: Raidium
Amaury Prat: Employee: Raidium
Daniel Tordjman: Employee: Raidium

**Deep-Learning (DL) image reconstruction and Chemical Shift Correction (CSC) algorithm boosts the performance of Zero Echo Time (ZTE) sequence to detect lytic myeloma lesions**
*D. Lepot*[1], C. Chabot[1], G. Duchêne[1], S. Mandava[2], M. Fung[3], J. Poujol[4], N. Michoux[1], P. Triqueneaux[1], F. Lecouvet[1]; [1]Brussels/BE, [2]Atlanta, GA/US, [3]New York, NY/US, [4]Buc/FR
*(darius.lepot@gmail.com)*

**Purpose:** The mineral bone has traditionally been inaccessible to MRI. Pseudo-CT MRI sequences now allow visualization of the bone mineral structure for applications like oncology. However these pseudo-CT sequences still have limitations. We compared the accuracy of three pseudo-CT MRI sequences (native Zero-Echo-Time (ZTE), Deep Learning (DL)-Chemical Shift

Wednesday

Correction (CSC) reconstructed ZTE (ZTE-DL), and gradient-echo Black-Bone (BB)) in detecting osteolytic multiple myeloma (MM) lesions, using CT as reference.
**Methods or Background:** Newly diagnosed MM patients underwent ZTE and BB sequences of the lumbar spine, pelvis, and proximal femurs within a 3T whole-body MRI study (Signa Premier, GE HealthCare) (prospective trial: NCT05381077). ZTE-DL was obtained by reconstructing ZTE raw data using an algorithm combining AIR™ Recon DL (ARDL) and CSC. All patients also underwent 18FDG-PET/CT including an optimized CT within a week. Ten bone regions and two scores (lesion presence/absence, lesion number) were evaluated by three readers. Repeatability and reproducibility (Gwet's AC1/AC2), differences in lesion number, and accuracy (Acc) were assessed by sequence, region, and reader.
**Results or Findings:** Ten participants were included. Repeatability was moderate for ZTE (AC1≥0.45), good for ZTE-DL and BB (AC1≥0.60) and very good for CT (AC1≥0.80). Reproducibility was fair for ZTE and BB (AC2≥0.20), good for ZTE-DL (AC2≥0.60) and very good for CT (AC2≥0.80). AccZTE-DL ranged from 80 to 93% with an increase in accuracy ranging from +23% to +32% compared to AccZTE, and equal to +19% compared to AccBB. ZTE-DL detected more lesions than ZTE/BB (+30%/+25%, respectively).
**Conclusion:** DL and CSC reconstruction improves repeatability, reproducibility, and accuracy of ZTE sequences for detecting MM lesions. ZTE-DL detects more lesions than ZTE/BB.
**Limitations:** The limitation of the study were a mononcentric/ small cohort and a study only covering the lumbar spine, pelvis, proximal femurs.
**Funding for this study:** Frederic E. Lecouvet's research works are funded by the Peterbroeck Research Professorship fund and Allard-Janssen Fund for Cancer Research, Belgian nonprofit organizations. Caroline Chabot is a PhD student supported by the Fondation Saint Luc, a Belgian non-profit organization.
**Has your study been approved by an ethics committee?** Yes
**Ethics committee - additional information:** Nct05381077
**Author Disclosures:**
Caroline Chabot: Grant Recipient: Fondation Saint Luc, a Belgian non-profit organization
Gaëtan Duchêne: Nothing to disclose
Nicolas Michoux: Nothing to disclose
Perrine Triqueneaux: Nothing to disclose
Darius Lepot: Nothing to disclose
Julie Poujol: Employee: GE HealthCare
Sagar Mandava: Employee: GE HealthCare
Maggie Fung: Employee: GE HealthCare
Frédéric Lecouvet: Research/Grant Support: Peterbroeck Research Professorship fund and Allard-Janssen Fund for Cancer Research, Belgian nonprofit organizations

**AI-enhanced prostate MRI: unveiling unreported prostate incidental findings through routine screening for prostate cancer**
*D. Skwierawska*[1], S. Heidarikahkesh[1], D. Bounias[2], R. J. Jóźwiak[3], D. Hadler[1], M. Bachl[1], M. Uder[1], F. B. Laun[1], S. Bickelhaupt[1]; [1]Erlangen/DE, [2]Heidelberg/DE, [3]Warsaw/PL
*(dominika.skwierawska@gmail.com)*

**Purpose:** To evaluate the feasibility of automated detection and segmentation of incidental findings in prostate MRI.
**Methods or Background:** This IRB-approved, retrospective study included n=425 prostate MRI examinations (1.5T and 3.0T), comprising n=306 internal cases from our institution and n=119 external cases from three independent datasets. Manual segmentations were performed for sigmoid diverticulosis (SD), perirectal lymph nodes (PLN), urinary bladder diverticula (UBD), bladder wall thickenings (BWT), inguinal hernias (IH), degenerative changes of the hip (DC), synovial cysts (SC) and hydrocele testis (HT) on T2-weighted images for n=265 internal cases (n=520 ROIs). An nnU-Net model was trained on n=213 of these cases, and the remaining independent n=52 cases were used for the quantitative evaluation of model performance. Further, n =160 additional examinations (n=41 internal, n=119 from three independent external datasets) were evaluated by a radiologist.
**Results or Findings:** Segmentation performance varied between the incidental findings. Quantitatively, the highest mean Dice scores were achieved for SD (0.80 ± 0.14), HT (0.76 ± 0.20), and DC (0.70 ± 0.07). Radiologist evaluation across four datasets (one internal and three external) demonstrated accuracies of 0.98/0.77/0.93/0.93 for PLN, 0.98/0.87/0.85/0.88 for SD, 0.93/0.90/0.80/0.88 for IH, and 0.93/0.85/0.76/0.80 for DC, with accuracies for most other incidental findings also exceeding 0.85.
**Conclusion:** Our method can automatically detect and segment incidental findings in prostate MRI across four independent datasets, demonstrating potential to enhance the efficiency and consistency of reporting, supporting further research with larger, more diverse datasets including additional annotations and targets.
**Limitations:** The dataset was curated to maximise all targeted findings, and despite reflecting real-world prevalence, it remained imbalanced. Rarer and more subjective findings, such as mild bladder wall thickenings or degenerative changes of the hip, were challenging to annotate due to subtle appearances and patient-specific variations.
**Funding for this study:** Funding from the Bavarian Academic Center for Central, Eastern, and Southeastern Europe (BAYHOST) Scholarship, and from the German Research Foundation (DFG; project 500397400) is gratefully acknowledged.
**Has your study been approved by an ethics committee?** Yes
**Ethics committee - additional information:** Name of the ethics committee: Ethics committee of the Friedrich-Alexander-Universität Erlangen – Nürnberg, Medizinische Fakultät Approval Code: 25-67-Br Approval Date: 25.03.2025
**Author Disclosures:**
Dimitrios Bounias: Nothing to disclose
Sebastian Bickelhaupt: Nothing to disclose
Dominika Skwierawska: Nothing to disclose
Frederik Bernd Laun: Nothing to disclose
Rafał Janusz Jóźwiak: Nothing to disclose
Michael Uder: Nothing to disclose
Dominique Hadler: Nothing to disclose
Maximilian Bachl: Nothing to disclose
Shirin Heidarikahkesh: Nothing to disclose

**Direct breast cancer segmentation in k-space from dynamic contrast-enhanced MRI using a novel deep learning model**
*L. T. Rotkopf*[1], M. Rempe[2], C. Strack[2], H-P. Schlemmer[1], J. Kleesiek[2]; [1]Heidelberg/DE, [2]Essen/DE

**Purpose:** Medical image segmentation models predominantly operate in the image domain and require fully reconstructed images as input. We investigate a novel deep learning model that directly predicts the k-space representation of breast cancer segmentations from DCE-MRI data and compare its performance to a state-of-the-art image-domain framework.
**Methods or Background:** The DUKE subset cohort of the publicly available MAMA-MIA breast MRI dataset was selected for this study, consisting of 291 examinations in patients with confirmed breast cancer. For our proposed method, axial k-space slices were generated from the subtraction DCE-MRI volumes using Fourier transforms. A custom 2D U-Net was trained via 5-fold cross-validation to predict the complex-valued k-space tumor mask directly from subtraction k-space. This was compared against a standard 3D nnU-Net baseline trained on reconstructed 3D volumes. The pooled slice-level Dice similarity coefficient for the foreground tumor class was used as primary evaluation metric.
**Results or Findings:** The proposed k-space segmentation model achieved a mean Dice score of 0.69 ± 0.02 across the five cross-validation folds. The 3D nnU-Net baseline in the image domain achieved a mean Dice score of 0.62 ± 0.26. These results show direct k-space prediction can achieve performance comparable to established image-domain methods.
**Conclusion:** Our results demonstrate the potential of performing tumor segmentation directly in the k-space MRI domain, with performance levels competitive to established image-domain baselines. Operating in k-space is a novel approach that could open new avenues for developing integrated reconstruction and segmentation pipelines, and may offer advantages in accelerated, undersampled MRI acquisitions.
**Limitations:** This study was conducted retrospectively on a single-center cohort, which may limit the generalizability of the findings.
**Funding for this study:** This study was supported by the Jöster Foundation.
**Has your study been approved by an ethics committee?** Not applicable
**Author Disclosures:**
Jens Kleesiek: Nothing to disclose
Lukas Thomas Rotkopf: Nothing to disclose
Moritz Rempe: Nothing to disclose
Christian Strack: Nothing to disclose
Heinz-Peter Schlemmer: Nothing to disclose

**Evaluating medical AI image segmentation models using augmentation**
*M. Neitzel*, M. Sayed, B. Wichtlhuber, E. Frodl, J. Dietz, T. Vogl, P. Reschke, A. M. Bucher; Frankfurt/DE
*(ma.neitzel@med.uni-frankfurt.de)*

**Purpose:** Unreliable AI segmentations can compromise clinical decisions, so we developed and validated Seg-Eval, a model-agnostic, ground-truth-free pipeline that estimates segmentation quality from uncertainty maps of ROI masks on clinical CT.
**Methods or Background:** We conducted a retrospective multi-cohort study (GastricBCA n=161, PDAC Surgical n=362, PDAC Palliative n=309; total 832 CTs). For each scan, 10 random augmentations (rigid, elastic, or combined)

were generated and segmented with TotalSegmentator into 117 ROIs. Masks were mapped back to native space, yielding 8,320 inverse-segmentation masks (ISMs), with the native TotalSegmentator masks on original scans serving as reference. We computed ROI-wise DICE (ISMs vs. native masks), ASSD, coefficient of variation of DICE, augmentation loss, uncertainty (1 − modal agreement), and Pearson correlations; a subset of ROIs (n=90) underwent reader scoring and correction to link uncertainty with DICE improvement.
**Results or Findings:** Across all cohorts, we observed consistent inverse associations between segmentation quality and variability. DICE correlated strongly and negatively with uncertainty (Gastric r=−0.76; Surgical r=−0.59; Palliative r=−0.57; all p<0.001), indicating that lower performance was accompanied by higher predictive uncertainty. Similarly, higher augmentation loss was linked to increased uncertainty (r=0.70, 0.60, 0.42; all p<0.001), while higher DICE scores were consistently associated with lower variability (r=−0.93, −0.90, −0.91; all p<0.001). Pooling data across cohorts revealed a strong negative correlation between average uncertainty and DICE (Pearson r=−0.69, p<0.001), underlining robustness across subgroups. Reader analysis confirmed that higher-uncertainty ROIs yielded larger DICE gains after correction (r=−0.86, p<0.001).
**Conclusion:** Seg-Eval provides ground-truth-free, model-agnostic QC by flagging uncertain ROIs and passing reliable results, reducing workload and error propagation while supporting standardised, vendor-neutral integration into clinical workflows.
**Limitations:** The limitation of the study is that only one segmentation engine was tested.
**Funding for this study:** Funding was provided by the German Federal Ministry of Education and Research through the RACOON project (reference number 01KX2021).
**Has your study been approved by an ethics committee?** Yes
**Ethics committee - additional information:** Ethics approval was obtained by University Medicine Frankfurt (Reference 274/18)
**Author Disclosures:**
Eric Frodl: Nothing to disclose
Julia Dietz: Nothing to disclose
Thomas Vogl: Nothing to disclose
Matthias Neitzel: Nothing to disclose
Mattin Sayed: Nothing to disclose
Philipp Reschke: Nothing to disclose
Benedikt Wichtlhuber: Nothing to disclose
Andreas Michael Bucher: Nothing to disclose

### Automated response assessment from free-text radiology reports in metastatic melanoma using privacy-preserving Large Language Models

*J. C. Holzschuh*, F. Wäscher, F. Winneknecht, C. H. Ziener, J. Hassel, H-P. Schlemmer, L. T. Rotkopf; Heidelberg/DE

**Purpose:** Response evaluation in oncology trials relies on manual annotations and therefore limits trial sizes. Because free-text radiology reports are routinely generated, we hypothesize that large language models (LLMs) can automatically convert them into accurate, structured assessments. However, reliance on cloud-based or closed models raises privacy concerns. We therefore evaluate local, on-premises LLMs to accurately extract RECIST response categories from radiology reports.
**Methods or Background:** In this retrospective study we included 63 patients from clinical trials evaluating immunotherapy for metastatic melanoma. Two state-of-the-art LLMs (MicrosoftPhi-4-14B and DeepSeekR1-32B) were prompted to predict the RECIST category at the first follow-up imaging from the unstructured baseline and follow-up clinical radiology reports. Ground-truth targets were validated clinical RECIST assessments. Performance was measured using multiclass accuracy and sensitivity and specificity for each response category.
**Results or Findings:** Overall multiclass accuracies for RECIST 1.1 prediction at the first follow-up were 0.56 and 0.64 for Microsoft Phi-4 and DeepSeek-R1, respectively. Class-wise accuracies were 0.55 vs 0.40 for PR, 0.00 vs 0.33 for CR, 0.30 vs 0.55 for SD, and 0.90 vs 1.00 for PD. For predicting PD, Phi-4 and DeepSeek-R1 achieved sensitivities of 0.90 and 1.00, respectively, and specificities of 0.65 and 0.70. No significant differences between models were found (all p>0.05).
**Conclusion:** On-premises, privacy-preserving LLMs demonstrate promising capabilities in extracting RECIST response categories from free-text radiology reports, especially for differentiating progressive from non-progressive disease. This automated approach potentially reduces the burden of manual evaluation in future clinical trials.
**Limitations:** Limited include the relatively small sample size, single-institution data, and focus on metastatic melanoma.
**Funding for this study:** No funding.
**Has your study been approved by an ethics committee?** Yes
**Ethics committee - additional information:** Heidelberg
**Author Disclosures:**
Fabian Wäscher: Nothing to disclose
Christian Herbert Ziener: Nothing to disclose
Lukas Thomas Rotkopf: Nothing to disclose
Julius C Holzschuh: Nothing to disclose
Jessica Hassel: Nothing to disclose
Felix Winneknecht: Nothing to disclose
Heinz-Peter Schlemmer: Nothing to disclose

### Multimodal Volumetric Body Composition Analysis with AI: Agreement Between Whole-Body MRI and PET/CT

*J. C. Holzschuh*[1], L. Rotkopf[1], F. Bauer[2], C. Sachpekidis[1], A. Dimitrakopoulou-Strauss[1], H. Goldschmidt[1], M-S. Raab[1], H-P. Schlemmer[1], M. Wennmann[1]; [1]Heidelberg/DE, [2]Cologne/DE

**Purpose:** Imaging-derived body composition metrics are increasingly recognized as prognostic biomarkers in oncology. Advances in deep learning now allow automated segmentation of skeletal muscle and adipose tissue, enabling large-scale and potentially routine clinical application. However, the reproducibility of these measures across imaging modalities, particularly between computed tomography (CT) and magnetic resonance imaging (MRI), remains uncertain.
**Methods or Background:** We analysed same-day whole-body PET/CT and T1-weighted MRI scans from 42 patients from the prospective GMMG-HD7- and GMMG-HD8-/DSMM-XIX-study. A fully automated deep learning pipeline based on the TotalSegmentator Framework was created to segment skeletal muscle, visceral and subcutaneous adipose tissue to derive volume measurements. Volumetric agreement between CT- and MRI-based metrics was assessed using pearson correlation coefficient (r), intraclass correlation coefficients (ICC), and Bland-Altman analyses.
**Results or Findings:** CT- and MRI-derived body composition measures demonstrated strong correlations (r > 0.97) and high reproducibility for volumetric metrics (ICC > 0.95) across the analyzed compartments, although systematic modality-specific volumetric differences were observed. Compared with CT, MRI yielded lower estimates of adipose tissue and skeletal muscle volumes as Bland-Altman analysis demonstrated a mean bias of -353 cm3 (95% LOA: -2576 to 1869) for subcutaneous adipose tissue, -211 cm3 (-774 to 351) for visceral abdominal adipose tissue, and -2653 cm3 (-4854 to -452) for skeletal muscle.
**Conclusion:** Automated deep learning-based body composition analysis is feasible and reproducible across CT and MRI, although systematic modality-specific biases were identified. While these are small relative to the overall variance, they should be accounted for in research and clinical implementation. These findings contribute towards using multimodal, imaging-derived body composition biomarkers, potentially guiding future personalized cancer care.
**Limitations:** While the standardized single-scanner T1-weighted MRI protocol ensures consistency, future studies are required to validate generalizability across scanners, sequences, and larger cohorts.
**Funding for this study:** No Funding
**Has your study been approved by an ethics committee?** Yes
**Ethics committee - additional information:** Heidelberg
**Author Disclosures:**
Christos Sachpekidis: Nothing to disclose
Hartmut Goldschmidt: Nothing to disclose
Fabian Bauer: Nothing to disclose
Antonia Dimitrakopoulou-Strauss: Nothing to disclose
Julius C Holzschuh: Nothing to disclose
Lukas Rotkopf: Nothing to disclose
Marc-Steffen Raab: Nothing to disclose
Markus Wennmann: Nothing to disclose
Heinz-Peter Schlemmer: Nothing to disclose

### Evaluating the AI and Radiologist scoring for PIRADS in Prostate cancer diagnosis

*R. Hermawan*[1], T. Budianto[1], S. J. S. Gardezi[2], D. Kumar[3], A. Gandhamal[3], G. Marcel[1]; [1]Jakarta/ID, [2]Sharjah/AE, [3]Delhi/IN
*(dr_riohermawan@yahoo.com)*

**Purpose:** Prostate cancer is one of the most frequently diagnosed cancers in men worldwide, representing about 30% of the male cancer burden. Early diagnosis and treatment is key for long-term and progression-free survival in prostate cancer[1]. Artificial intelligence (AI) techniques leveraging deep learning (DL), are increasingly applied in prostate cancer detection to enhance accuracy and lower costs. This study aims to leverage DL for automatic detection , diagnosis and classification of prostate cancer.

**Methods or Background:** This retrospective study included 40 patients (mean age: 62 years) diagnosed with prostate cancer at Dharmais Cancer Hospital, Indonesia. PIRADS scores were computed using a deep learning–based assessment application (United Imaging Intelligence®). tumor diameter, volume along with right–left (RL), superior–inferior (SI), and anterior–posterior (AP) diameters, were computed. The AI system automatically classified detected prostate tumors into PIRADS categories based on the obtained scores.
**Results or Findings:** A strong correlation was found between radiologist- and AI-derived prostate volumes ($r = 0.976$, $p = 2.47 \times 10^{-23}$). Over 40% of cases were classified as PIRADS 5, 22.5% as PIRADS 4, with the remainder in lower categories. Moreover the category wise PIRADS analysis showed highest agreement for PIRADS 5 (62.5%), moderate for PIRADS 3 (42.9%), minimal for PIRADS 4 (11.1%), and no matches for PIRADS 1–2 . Overall the agreement was more reliable in higher categories and minimal in lower categories.
**Conclusion:** The study underscores the of AI-powered DL application for prostate cancer detection and PIRADS classification. The results exhibit a strong correlation with radiologist-derived prostate volumes and achieved higher agreement in advanced PIRADS categories, highlighting the utility of AI in supporting radiologists.
**Limitations:** However, more diverse cohort, along with multi-radiologist evaluations, are required to improve reliability across all PIRADS categories and establish broader clinical usage.
**Funding for this study:** None
**Has your study been approved by an ethics committee?** Not applicable
**Author Disclosures:**
Syed Jamal Safdar Gardezi: Nothing to disclose
Taufik Budianto: Nothing to disclose
Gamael Marcel: Nothing to disclose
Akash Gandhamal: Nothing to disclose
Dileep Kumar: Nothing to disclose
Rio Hermawan: Nothing to disclose

**Spatial Mapping of Tumor Response and Mesorectal Involvement on Pretreatment Contrast-Enhanced CT in Locally Advanced Rectal Cancer**
H. Gan, *J. Li*, Z. Li, Z. Zhou; Wuhan/CN

**Purpose:** To explore spatiotemporal features from large-scale multi-center contrast-enhanced computed tomography (CECT) data to construct quantitative spatiotemporal models for tumor response in locally advanced rectal cancer(LARC) patients to neoadjuvant chemoradiotherapy(NCRT).
**Methods or Background:** This study retrospectively collected CECT and clinical information of 337 pathologically confirmed LARC patients from three centers. Two radiologists performed the annotation of tumors and the mesorectum on dual-phase CECT. The cohort was randomly partitioned into training (70%), validation (20%), and test sets (10%). Radiomic extraction yielded 1,223 features per phase. Pathologic response to CRT was assessed histopathologically and graded using the AJCC tumor regression grade (TRG) system (0–3). Treating TRG as a four-class outcome, we evaluated the performance of the radiomics-based multiclass classifier using a one-vs-rest scheme with macro-averaged ROC AUC. We trained thirteen models to predict tumor, mesorectal involvement. Two input schemes were evaluated: single-phase arterial (A) or venous (V) data and a dual-phase V–A difference design, which leverages multiphasic information for improved detectability. Bayesian optimization was used for hyperparameter tuning.
**Results or Findings:** Among the total population, TRG0 accounts for 9.7%, TRG1 for 13.3%, TRG2 for 41.5%, and TRG3 for 35.5%. For tumor model, arterial phase achieves the highest AUC, at 0.7844; for mesorectum model, the V-A difference performs best, with an AUC of 0.7527. Across Phases A, V, and V-A difference, the tumor model consistently demonstrates better performance than mesorectum , with respective ΔAUC of 0.057, 0.0384, and 0.0097.
**Conclusion:** The tumour-mesorectum model can effectively explain the spatiotemporal changes of tumors in LARC after NCRT by integrating features across multiple phases and characteristics of different spatial regions. It provides a quantitative tool for evaluating tumor response and mesorectal involvement, and addressing poor efficacy prediction accuracy.
**Limitations:** Retrospective study.
**Funding for this study:** None
**Has your study been approved by an ethics committee?** Yes
**Ethics committee - additional information:** The IRB of Tongji Hospital, Tongji Medical College, Huazhong University of Science and Technology approved this work( TJ-IRB202401065).
**Author Disclosures:**
Zhen Li: Nothing to disclose
Ziling Zhou: Nothing to disclose
Jiali Li: Nothing to disclose
Huiyu Gan: Nothing to disclose

**Investigating longitudinal radiomic features variation on MRI to predict tumour upgrading of prostate cancer during active surveillance**
*L. V. Sciacqua*, B. Dionisi Ferrera, E. Gioscio, C. Marenghi, F. Badenchini, T. Rancati, N. Nicolai, A. Casale, A. Messina; Milan/IT
*(luvisci97@gmail.com)*

**Purpose:** We investigated whether changes in whole-prostate MRI-derived radiomic-features RFs during active-surveillance are associated with histopathological upgrading at repeat biopsy. Specifically, we tested whether increasing time from diagnosis allows aggressive tumor foci to become more detectable on mp-MRI (predictive delta-RFs). We also examined whether these RFs provide signals at diagnostic MRI and whether baseline radiomics adds predictive value to clinical models based on PSA density and number of positive biopsy cores. Frequent ISUP upgrades outside MRI-visible lesions support whole-prostate radiomics.
**Methods or Background:** We studied 147 consecutive patients (60 with upgrading) from a prospective single-center active-surveillance cohort, monitored with mpMRI (diagnosis, years 1, 2, 4) and targeted biopsies for PIRADS >2 lesions (years 1, 4). Candidate delta-RFs (between diagnosis and last-available mp-MRI) were identified using multiple-ANOVA, and logistic models were built from MANOVA-selected RFs at diagnosis. RFs (T1, T2, ADC maps) were extracted from diagnostic and last-available MRI using a standardized pipeline to calculate absolute baseline RFs and delta-RFs. Associations of delta-RFs with upgrading were tested with multiple-ANOVA, and logistic models were developed using baseline RFs.
**Results or Findings:** The number of positive cores (OR=1.8, 1 core vs >1) and PSA density (OR=1.6 per 0.05 ng/ml/cc increase) were linked to upgrading (AUC=0.68). The MANOVA model included six delta-RFs: four T2, one T1, one ADC. ADC (morph_vol_dens_aabb) explained most variance. Baseline morph_vol_dens_aabb was associated with decreased upgrading risk (OR=0.90, AUC=0.65). Adding morph_vol_dens_aabb to the clinical-pathological model improved AUC (=0.73).
**Conclusion:** Prostate radiomics aspects related to the coherence and compactness of ADC maps predict upgrading, indicating prostates with globally organized diffusion are less likely to harbor undetected higher-grade foci.
**Limitations:** The limitations of the study are the need for independent external datasets to ensure robustness and further analyses of lesion-level RFs.
**Funding for this study:** No funding was received for this study.
**Has your study been approved by an ethics committee?** Not applicable
**Author Disclosures:**
Lucilla Violetta Sciacqua: Nothing to disclose
Tiziana Rancati: Nothing to disclose
Eliana Gioscio: Nothing to disclose
Benedetta Dionisi Ferrera: Nothing to disclose
Antonella Messina: Nothing to disclose
Fabio Badenchini: Nothing to disclose
Cristina Marenghi: Nothing to disclose
Nicola Nicolai: Nothing to disclose
Alessandra Casale: Nothing to disclose

**Deep learning radiomics models for the identification of HER2 expression using super-resolution ultrasound images**
*J. Zhuang*, C. Yuefan, Y. Xie, Q. Ye; Fuzhou/CN

**Purpose:** The human epidermal growth factor receptor 2 (HER2) expression status varies across different histological types and staging stages of bladder cancer, making it crucial for prognosis assessment. This study investigates the potential of deep learning radiomics models using ultrasound images reconstructed through super-resolution for HER2 status identification.
**Methods or Background:** A total of 113 patients with confirmed bladder urothelial carcinoma were retrospectively analyzed and divided into the train and test sets in a ratio of eitht to two. A super-resolution (SR) reconstruction technique based on a generative adversarial network (GAN) was applied to increase the spatial resolution of ultrasound images by two and four times. Radiomic features were extracted from the regions of interest (ROIs). The deep learning features were extracted based on ResNet101, and the principal component analysis (PCA) was used for dimensionality reduction. Least absolute shrinkage and selection operator (LASSO) regression was conducted to further screen the radiomic features, deep learning features, and clinical features. Finally, the screened features were involved for machine learning (ML) modeling.
**Results or Findings:** Four models were developed, including logistic regression (LR), support vector machine (SVM), k-nearest neighbor (KNN), and light gradient boosting machine (LightGBM). Overall, the combined models based on ultrasound images with four times resolution improvement showed the best performance. Among them, the SVM achieved the highest AUC of 0.994 and 0.929 in the train and test sets, respectively. The decision curve analysis (DCA) suggested its potential for assisting clinical decision-making.

Wednesday

**Conclusion:** The super-resolution (SR) sampling helps to improve the performance of ultrasound-based prediction models. A machine learning model combining clinical features, radiomics features, and deep learning features shows potential for HER2 detection in urothelial carcinoma of the bladder.
**Limitations:** No limitations were identified.
**Funding for this study:** Funding was provided by Medical Foundation of Fujian Health Commission of China (no. CX23A010)
**Has your study been approved by an ethics committee?** Yes
**Ethics committee - additional information:** The ethics committee notification can be found under the number 2021KY013 of the Ethics Committee of Fujian Medical University Union Hospital
**Author Disclosures:**
Chen Yuefan: Nothing to disclose
Jiajing Zhuang: Nothing to disclose
Qin Ye: Nothing to disclose
Yihan Xie: Nothing to disclose

**10:00 - 11:00** **Research Stage 2**

### Research Presentation Session: Breast

### RPS 202

### Beyond the basics: the new era of breast ultrasound

**Moderator**
Jessica Muscat; Msida/MT
*(jessica2860@gmail.com)*

### Observer Agreement in Sonographic Glandular Tissue Component Classification and Breast Composition Assessment on Ultrasound: A Multi-Institutional Study

*T. Uematsu*, Japan Association of Breast and Thyroid Sonology; Shizuoka/JP
*(t.uematsu@scchr.jp)*

**Purpose:** The sonographic glandular tissue component (GTC) has emerged as a potential imaging biomarker for stratifying breast cancer risk in women with dense breasts. Anticipating its inclusion in the upcoming BI-RADS 6th edition, this study aimed to assess the reliability and clinical utility of GTC classification and sonographic breast composition through a multi-institutional observer performance study.
**Methods or Background:** Fifteen readers (9 physicians, 6 sonographers) from 12 institutions independently assessed 100 paired ultrasound images twice, 1 month apart. GTC was qualitatively classified into four categories by fibroglandular tissue proportion (minimal, mild, moderate, marked); minimal/mild were grouped as low GTC and moderate/marked as high GTC. Breast composition was categorized as dense (high GTC), nondense (fat lobules visible), or intermediate, standardized by an FGT-to-FAT (subcutaneous and retromammary fat) ratio cutoff of 1. Inter- and intra-observer agreement was evaluated using κ statistics.
**Results or Findings:** Inter-observer agreement for GTC classifications was substantial for both the four-category (κ = 0.66 and 0.73 for the first and second readings, respectively) and dichotomized (κ = 0.61 and 0.64) schemes. Agreement for sonographic breast composition was similarly substantial (κ = 0.65 and 0.67). Intra-observer agreement was also substantial for GTC (four-category κ = 0.78; two-category κ = 0.75) and for breast composition (κ = 0.77).
**Conclusion:** Both inter- and intra-observer agreement were substantial, indicating that qualitative assessment of GTC and sonographic breast composition is reproducible and reliable. These findings support the integration of GTC into routine clinical practice and research as a practical tool for risk stratification in women with dense breasts.
**Limitations:** First, its retrospective design may have introduced selection bias. Second, evaluations were based on static images, which do not fully capture the dynamic information available in real-time ultrasound.
**Funding for this study:** None
**Has your study been approved by an ethics committee?** Yes
**Ethics committee - additional information:** This multi-institutional study was approved by the Institutional Review Board of our hospital (Approval No. T2024-11-2024-10-3).
**Author Disclosures:**
Ultrasound Glandular Tissue Component (Gtc) Research Committee Japan Association Of Breast And Thyroid Sonology: Nothing to disclose
Takayoshi Uematsu: Nothing to disclose

### Non-mass lesions on breast ultrasound: decoding predictors of malignancy through clinico-pathological correlation

*E. Jain*, R. Bansal, Z. Khan, B. Aggarwal; New Delhi/IN
*(eetajain06@gmail.com)*

**Purpose:** Non-mass lesions (NMLs) are frequently encountered in breast imaging but are not formally defined in the 5th edition of the BI-RADS ultrasound lexicon. This study aimed to characterise their ultrasound features, evaluate corresponding findings on mammography and MRI when available, and identify predictors of malignancy through clinical and pathological correlation.
**Methods or Background:** This prospective single-centre study included 120 women with non-mass lesions (NMLs) detected on ultrasound between December 2024 and August 2025. Ultrasound served as the index test, with features assessed according to the Japan Association of Breast and Thyroid Sonology (JABTS) criteria, classifying NMLs as ductal abnormalities, hypoechoic areas, architectural distortion, microcysts, or echogenic foci without a hypoechoic area. Histopathology of ultrasound-guided biopsies was the reference standard, with malignant lesions further characterised by immunohistochemistry (ER, PR, HER2, Ki-67). Benign and malignant NMLs were compared for demographics, clinical presentation, and imaging features. Logistic regression was used to identify independent predictors, and receiver operating characteristic (ROC) analysis compared the diagnostic accuracy of BI-RADS with the predictive model.
**Results or Findings:** Of 120 NMLs, 62.5% were malignant and 37.5% benign. Independent predictors of malignancy were posterior shadowing ($p < 0.001$), echogenic foci ($p = 0.008$), and architectural distortion ($p = 0.006$), while hypoechoic areas were highly sensitive but non-specific. Malignancy rates increased progressively across BI-RADS categories, with BI-RADS ≥4B yielding a sensitivity of 75%, specificity of 80%, and accuracy of 77%. The regression-based predictive model demonstrated superior diagnostic performance (AUC 0.87) compared with BI-RADS alone (AUC 0.78).
**Conclusion:** Posterior shadowing, echogenic foci, and architectural distortion are key predictors of malignancy in NMLs. As these lesions remain undefined in the BI-RADS lexicon, this study underscores the need for standardised descriptors and integrated imaging to improve diagnostic accuracy.
**Limitations:** Not applicable.
**Funding for this study:** No funding was provided for this study.
**Has your study been approved by an ethics committee?** Yes
**Ethics committee - additional information:** The ethics committee UID number is 27731.
**Author Disclosures:**
Zaara Khan: Nothing to disclose
Eeta Jain: Nothing to disclose
Richa Bansal: Nothing to disclose
Bharat Aggarwal: Nothing to disclose

### Preliminary Results Comparing Diagnostic Accuracy of 12G vs 14G CNB in Non-Mass Breast Lesions

*Z. H. Temiz*, M. A. Nazli; Istanbul/TR
*(zisantemiz1@hotmail.com)*

**Purpose:** Non-mass breast lesions present diagnostic difficulties due to their subtle imaging features and varied pathology. Although 14-gauge (14G) core needle biopsy (CNB) is standard, 12-gauge (12G) devices may offer better tissue yield. This study aims to compare the diagnostic performance of 12G and 14G CNB in non-mass breast lesions, focusing on tissue yield, sample adequacy, radiology–pathology concordance, upgrade rates of high-risk lesions (B3).
**Methods or Background:** This retrospective study analyzed 106 non-mass breast lesions (12G: 38; 14G: 68) detected by mammography, ultrasound, or MRI. Biopsies were ultrasound and histopathology was categorized as benign, high-risk, or malignant. Evaluated outcomes included radiology–pathology concordance, calcification, upgrade after surgery/re-biopsy, and management. Statistical analysis used Fisher's exact test with ORs and 95% CI; $p<0.05$ was significant.
**Results or Findings:** Radiology–pathology discordance was lower in the 12G group (10.5%) compared to the 14G group (20.6%) (OR=0.45, p=0.23). Calcification sampling was significantly higher with 12G (39.5%) versus 14G (17.6%) (p=0.02). Upgrade rates were similar: 12G 13.2% vs 14G 10.3% (OR=1.33, p=0.75). Among calcification-positive lesions, discordance was lower in the 12G group (13.3%) than in the 14G group (33.3%) (OR=0.31, p=0.36), and upgrade rates were also lower (20.0% vs 41.7%; OR=0.35, p=0.40).

**Conclusion:** The 12G core needle biopsy demonstrated a higher success rate in sampling calcifications and showed a trend toward lower radiology–pathology discordance compared to 14G device, although the difference was not statistically significant. While upgrade rates remained similar between the two groups, these preliminary findings indicate that the 12G needle may provide more reliable sampling, particularly in calcification-rich non-mass breast lesions.
**Limitations:** This study is limited by its retrospective single-center design, small sample size, and incomplete follow-up, which may affect the accuracy of upgrade rates. Additionally, needle selection bias could have influenced group distribution.
**Funding for this study:** This study received no funding.
**Has your study been approved by an ethics committee?** Yes
**Ethics committee - additional information:** Ethics approval was obtained from the IRB of Başakşehir Çam and Sakura City Hospital.
**Author Disclosures:**
Mehmet Ali Nazli: Nothing to disclose
Zişan Hayriye Yücel: Nothing to disclose

**The number of biopsies taken and the pathological accuracy rate of breast diseases**
*A. K. K. Soydan*, M. Demir; Istanbul/TR
*(ahmet_kursat97@hotmail.com)*

**Purpose:** To evaluate the cumulative diagnostic adequacy of sequential 14-gauge tru-cut biopsy samples in breast lesions visible on ultrasonography and determine the optimal number of core samples required for diagnostic accuracy.
**Methods or Background:** Prospective study conducted June-December 2024 including 86 patients with 86 breast lesions (BI-RADS 3, 4, or 5). Three sequential core biopsy samples were obtained from each lesion under US guidance using 14-G automated biopsy needle. Each sample was sent separately for pathological evaluation. Cumulative diagnostic adequacy was assessed for one, two, and three samples.
**Results or Findings:** Cumulative diagnostic adequacy: 84.9% (73/86) with first core, 94.2% (81/86) with two cores, 98.8% (85/86) with three cores. All malignant lesions (n=15) were diagnosed with first two samples (100%). Diagnostic adequacy increase was significant from first to second sample ($p<0.05$) but not from second to third ($p>0.05$). First core diagnostic rate was higher for solid lesions (87.3%) versus cystic lesions (50.0%) ($p=0.049$).
**Conclusion:** Three core samples in US-guided tru-cut biopsy provide very high diagnostic adequacy (98.8%). Two high-quality core samples may be sufficient for diagnosing malignant lesions, suggesting biopsy protocols could be optimized to be less invasive without compromising oncologic safety.
**Limitations:** Single-center study limiting generalizability. Small sample size of malignant lesions (n=15). Single pathologist evaluation precluding inter-observer reliability assessment. Benign lesion reference standard included follow-up rather than surgical excision in all cases.
**Funding for this study:** No Not specified
**Has your study been approved by an ethics committee?** Yes
**Ethics committee - additional information:** Study approved by Clinical Research Ethics Committee of Ümraniye Training and Research Hospital. Conducted according to Declaration of Helsinki principles. Written informed consent obtained from all participants.
**Author Disclosures:**
Ahmet Kürşat Kursat Soydan: Nothing to disclose
Mustafa Demir: Nothing to disclose

**Comparison of Shear wave elastography (SWE) characteristics according to receptor status in breast malignancies**
*U. Chouksey*, P. B. Thakkar, A. Katdare, P. Haria, N. Shetty, S. Kulkarni; Mumbai/IN
*(drunnatichouksey@gmail.com)*

**Purpose:** Evaluation of qualitative and quantitative characteristics of Shear wave elastography (SWE) among malignant breast lesions according to their receptor status.
**Methods or Background:** This study was a subset analysis of a larger prospective observational study including 468 lesions in 404 patients. Among these, hormonal and molecular receptor statuses were available for 388 malignant cases. These lesions were broadly categorized into three groups, i.e. first group being receptor positive, that is either ER or PR positive, the second group being HER – 2 enriched and the third group being triple negative. The mean values along with measures of distribution for Emax and Emean was calculated for each category. Qualitative characteristics were also evaluated, like presence of perilesional stiff rim. These lesions were accordingly differently classified based on the qualitative internal stiffness and perilesional stiffness into four categories: perilesional stiff rim present and qualitatively soft, perilesional stiff rim present and qualitatively hard, perilesional stiff rim absent and qualitatively soft, and perilesional stiff rim absent and qualitatively hard.
**Results or Findings:** Majority of the lesions with triple negative receptor status showed a perilesional stiff ring and were internally soft. While 90 percent of the lesions in the HER-2 subgroup demonstrated a perilesional stiff ring (including both hard and soft lesions). No such trend was observed in the receptor-positive subgroup.
**Conclusion:** 1. A significant difference was found among the values of Emean among the three classifications of molecular and hormonal subtypes but not among Emax values.2. There was significant association of the perilesional ring present, qualitative soft subset with both TNBC and HER-2 enriched subset. There was no significant association of any of the four categories with the Receptor positive subgroup.
**Limitations:** Evaluation of qualitative parameters suffered from observer bias.
**Funding for this study:** None
**Has your study been approved by an ethics committee?** Yes
**Ethics committee - additional information:** This was a subset analysis of a prospective observational study, approved by the institutional ethics committee.
**Author Disclosures:**
Aparna Katdare: Nothing to disclose
Suyash Kulkarni: Nothing to disclose
Purvi Haria: Nothing to disclose
Nitin Shetty: Nothing to disclose
Palak Bhavesh Thakkar: Nothing to disclose
Unnati Chouksey: Nothing to disclose

**New Ultrasound Techniques for Breast Lesion Characterization: Prospective Comparison of BI-RADS, Histology, CAD "Live BreastAssist", Strain Elastography, Shear Wave, and MV Flow**
*M. Renda*, C. Catalano, G. Bonito, C. Solito, F. Raponi, V. Dolcetti, G. Maglia, G. Lo Conte, V. Cantisani; Rome/IT
*(maurizio.renda@uniroma1.it)*

**Purpose:** The purpose of our study was to compare the diagnostic performance of the new ultrasound techniques in the differentiation between benign and malignant breast lesions, in accordance to the BI-RADS classification, evaluating the agreement between the software “Live Breast Assist”, human investigator, and the diagnostic accuracy of Elastography performed with Strain Ratio (SR), 2D-Shear Wave (STE) Point Shear Wave (STQ), and advanced Doppler technique MV-Flow. Inter-operator variability will also be analyzed.
**Methods or Background:** 92 patients have been enrolled, with a total of 110 focal breast lesions evaluated by an experienced breast imaging radiologist who assigned BI-RADS categories. All patients were analyzed using Strain Elastography (SE), Shear Wave Elastography (STE),using B-mode ultrasound, color Doppler, MV-FLOW, semi-automatic “Live Breast Assist” software analysis. All patients underwent biopsy, with histopathology serving as the gold standard.
**Results or Findings:** A total of 110 breast lesions were analyzed (14% benign, 7.6% B3, 78.4% malignant), with fibroadenomas and fibrocystic mastopathy being the most common benign findings, and infiltrating ductal carcinoma the predominant malignancy. The Live Breast Assist software demonstrated excellent agreement with the operator in assessing size, margins, orientation, and echostructure. Concordance with histological BI-RADS classification was high, while operator–histology agreement was good. Elastography identified a Strain Ratio cut-off of 1.89, with strain elastography outperforming 2D SWE and pSWE. The MV FLOW software, which provides advantages over conventional color Doppler, has demonstrated that higher measured values in breast lesions are correlated with the histopathological grade of lesion severity
**Conclusion:** New advanced ultrasound techniques show promising results for differentiating breast lesions, potentially reducing the need for biopsies, and serving as a support tool in discriminating a suspicious lesion.
**Limitations:** The limitations of this study include the small sample size, which, however, can be readily increased.
**Funding for this study:** No
**Has your study been approved by an ethics committee?** Not applicable
**Author Disclosures:**
Maurizio Renda: Author: Università di roma la sapienza Author: rome sapienza university
Gianluca Maglia: Nothing to disclose
Giacomo Bonito: Nothing to disclose
Gianmarco Lo Conte: Nothing to disclose
Vincenzo Dolcetti: Nothing to disclose
Carmen Solito: Nothing to disclose
Flavia Raponi: Nothing to disclose
Vito Cantisani: Nothing to disclose
Carlo Catalano: Nothing to disclose

**A Novel Deep Learning Radiomics Nomogram Integrating B-mode Ultrasound and Contrast-enhanced Ultrasound for Preoperative Prediction of Lymphovascular Invasion in Invasive Breast Cancer**

*Z. L. Wang*; Beijing/CN
*(wzllg@sina.com)*

**Purpose:** To develop a deep learning radiomics nomogram (DLRN) that integrated B-mode ultrasound (BMUS) and contrast-enhanced ultrasound (CEUS) images for preoperative lymphovascular invasion (LVI) prediction in invasive breast cancer (IBC).
**Methods or Background:** 981 patients with IBC from three hospitals were retrospectively enrolled. Of 834 patients recruited from Hospital I, 688 were designated as the training cohort and 146 as the internal test cohort, whereas 147 patients from Hospitals II and III were assigned to constitute the external test cohort. Deep learning and handcrafted radiomics features of BMUS and CEUS images were extracted from breast cancer to construct a deep learning radiomics (DLR) signature. The DLRN was developed by integrating the DLR signature and independent clinicopathological parameters. The performance of the DLRN was evaluated with respect to discrimination, calibration, and clinical benefit.
**Results or Findings:** The DLRN exhibited good performance in predicting LVI, with areas under the receiver operating characteristic curves (AUCs) of 0.885 (95% confidence interval [CI], 0.858-0.912), 0.914 (95% CI, 0.868-0.960) and 0.914 (95% CI, 0.867-0.960) in the training, internal test, and external test cohorts, respectively. The DLRN exhibited good stability and clinical practicability, as demonstrated by the calibration curve and decision curve analysis. In addition, the DLRN outperformed the traditional clinical model and the DLR signature for LVI prediction in the internal and external test cohorts (all $P < 0.05$).
**Conclusion:** The DLRN exhibited good performance in predicting LVI, representing a non-invasive approach to preoperatively determining LVI status in IBC.
**Limitations:** This study only included patients with unifocal IBC due to the difficulty in identifying the lesion causing ALN metastasis in multifocal and multicentric lesions. The DLRN was developed based on static breast CEUS images rather than CEUS videos due to the retrospective study.
**Funding for this study:** This study has received funding by National Key Research and Development Program of China (2023YFC2414203), and National Natural Science Foundation of China (No. 82371972).
**Has your study been approved by an ethics committee?** Yes
**Ethics committee - additional information:** This study was authorized by the institutional review board of the Hospital and adhered to the Declaration of Helsinki (S2024-801-01).
**Author Disclosures:**
Zhi Li Wang: Nothing to disclose

**Energy sustainability of breast ultrasound: Comparison between automated and handheld ultrasound systems**

*J. T. Lee*[1], B. K. Seo[1], J. Y. Lee[1], M. S. Bae[1], Y. Moon[1], K. R. Cho[2], O. Woo[2], S. E. Song[2], S-Y. Kim[2]; [1]Ansan/KR, [2]Seoul/KR
*(jeoungtaek@gmail.com)*

**Purpose:** Breast cancer is the most common female malignancy worldwide, and ultrasound (US) is widely used for diagnosis and screening. While its clinical utility is well established, the environmental impact has rarely been assessed. This study compared the energy consumption of automated breast ultrasound (ABUS) and handheld ultrasound (HHUS) systems and explored strategies for optimized ultrasound management.
**Methods or Background:** In this prospective study, one ABUS and two HHUS systems (HHUS-1 and HHUS-2) from different vendors were evaluated using a power meter (1-Hz sampling). A total of 240 patients without prior breast surgery underwent B-mode US (80 ABUS, 80 HHUS-1, 80 HHUS-2). For HHUS, four breast radiologists (16–26 years of experience) each performed 20 examinations per system. We measured average power (kW) during active, idle, and off modes, scan time (min), energy use (kWh), and carbon emissions (kg$CO_2$e). Group comparisons were performed using the Mann-Whitney U test.
**Results or Findings:** Compared with HHUS-1, ABUS required 28%, 35%, and 100% less average power in active, idle, and off modes, respectively, and 24% shorter scan time, resulting in a 46% reduction in energy consumption and carbon emissions (all $p < 0.05$). Compared with HHUS-2, ABUS showed similar active-mode power but 26% and 100% reductions in idle and off modes and 18% shorter scan time. These changes yielded a 7% overall reduction in energy and emissions; the decreases in idle, off, and scan time were statistically significant ($p < 0.05$). Reducing idle-mode duration further lowered energy consumption by 12–16%.
**Conclusion:** ABUS demonstrated lower power demand, energy consumption, and carbon emissions compared with HHUS. Optimizing ultrasound workflows, particularly minimizing idle time, may improve environmental sustainability while maintaining clinical utility.
**Limitations:** Not applicable
**Funding for this study:** National Research Foundation of Korea funded by the Korea government (RS-2024-00347290)
**Has your study been approved by an ethics committee?** Yes
**Ethics committee - additional information:** Not applicable because no patient data are involved
**Author Disclosures:**
Min Sun Bae: Nothing to disclose
Okhee Woo: Nothing to disclose
Soo-Yeon Kim: Nothing to disclose
Bo Kyoung Seo: Nothing to disclose
Sung Eun Song: Nothing to disclose
Yangin Moon: Nothing to disclose
Ji Young Lee: Nothing to disclose
Jeong Taek Lee: Nothing to disclose
Kyu Ran Cho: Nothing to disclose

**10:00 - 11:00** **Research Stage 4**

### Research Presentation Session: Artificial Intelligence and Imaging Informatics

### RPS 205

### Improving diagnosis and prognosis through AI in CNS diseases

**Moderator**
Armando Ugo Cavallo; Rome/IT

**Assessment of the stability of intracranial aneurysms using a deep learning model based on computed tomography angiography**

*L. Zeng*; Chongqing/CN
*(825140736@qq.com)*

**Purpose:** The aim of this study was to construct a deep learning model (DLM) to identify unstable aneurysms on computed tomography angiography (CTA) images.
**Methods or Background:** The clinical data of 1041 patients with 1227 aneurysms were retrospectively analyzed from August 2011 to May 2021. Patients with aneurysms were divided into unstable (ruptured, evolving and symptomatic aneurysms) and stable (fortuitous, nonevolving and asymptomatic aneurysms) groups and randomly divided into training (833 patients with 991 aneurysms) and internal validation (208 patients with 236 aneurysms) sets. One hundred and ninety-seven patients with 229 aneurysms from another hospital were included in the external validation set. Six models based on a convolutional neural network (CNN) or logistic regression were constructed on the basis of clinical, morphological and deep learning (DL) features. The area under the curve (AUC), accuracy, sensitivity and specifcity were calculated to evaluate the discriminating ability of the models.
**Results or Findings:** The AUCs of Models A (clinical), B (morphological) and C (DL features from the CTA image) in the external validation set were 0.5706, 0.9665 and 0.8453, respectively. The AUCs of Model D (clinical and DL features), Model E (clinical and morphological features) and Model F (clinical, morphological and DL features) in the external validation set were 0.8395, 0.9597 and 0.9696, respectively.
**Conclusion:** The CNN-based DLM, which integrates clinical, morphological and DL features, outperforms other models in predicting IA stability. The DLM has the potential to assess IA stability and support clinical decision-making.
**Limitations:** The maximum slices of aneurysm images were used to construct 2D models, and some important factors that may affect aneurysm stability may have been missed, possibly leading to bias. More advanced DL algorithms are needed.
**Funding for this study:** This study was supported by the Science and Technology Commission of Chongqing City, China (CSTB2023NSCQ-MSX0668), the Joint Project of Science and Health of Chongqing City, China (2023MSXM022) and the Science and Technology Research Program of Chongqing Municipal Education Commission (KJQN202200407).
**Has your study been approved by an ethics committee?** Yes
**Ethics committee - additional information:** The local ethics committee (Banan Hospital, 2021015; Xinqiao Hospital, 202248201) approved this retrospective study and agreed to the informed consent waiver of patients.
**Author Disclosures:**
Lu Zeng: Nothing to disclose

**Glioblastoma Response Prediction and Tumor and Organ-at-Risk Segmentation with Radiomics and Deep Learning**

*A. Mora Rubio*[1], C. Bravo Vergara[1], M. Beser-Robles[1], G. Ribas[1], P. Garcia Verdu[1], I. Popp[2], A. L. Grosu[2], L. Marti-Bonmati[1], M. Carles Fariña[1]; [1]Valencia/ES, [2]Freiburg/DE

**Purpose:** The current standard treatment for Glioblastoma Multiforme (GBM) involves radiation therapy (RT) and requires manual tumour segmentation, which is labour-intensive and susceptible to inter-observer variability. Additionally, given the high recurrence rate of GBM patients, accurate response prediction methods can help to improve patient prognosis stratification and optimize treatment plans. The aim of this study is to develop and evaluate automatic segmentation methods based on deep learning and assess the ability of mathematical models employing clinical and radiomics features (RF) to improve the accuracy of response prediction.
**Methods or Background:** The study included 253 patients with primary/recurrent GBM, prospectively (185) and a retrospectively recruited in 13 institutions. The open-source cohort BraTS2021 of primary glioma patients was also used. The nnU-Net open-source framework was used to develop models for Enhancing Tumour, Peritumoral Edema, RT Planning Target Volume and six Organs-at-Risk, based on segmentation performed by neuroradiologist and radiation oncologist. For response prediction in recurrent GBM of the prospective cohort, the Cox Proportional Hazard and Logistic Regression models for overall survival, time to progression, and early recurrence prediction, were applied.
**Results or Findings:** The segmentation models achieved good to excellent performance, with average DSC scores in the test set of 0.79 (0.69-0.98). Clinical and MR-RF showed significant discrimination between patients with early and late progression on validation and test sets ($p < 0.05$ in Kaplan-Meier curves). Wavelet transform RF and clinical features like age, methylation status, and tumour localisation were notably significant predictors.
**Conclusion:** The good performance of the automatic segmentation models supports their use in clinical workflows to simplify procedures, reduce time investment, and increase robustness. Radiomics analysis suggested that the MR-RF model has potential for predicting time to progression.
**Limitations:** Ongoing work is about the FET-PET complementary information.
**Funding for this study:** This work was supported by the MATTO-GBM Project under the European TRANSCAN-3 ERA-NET 2022 Program, funded by ISCIII (AC23_1/00012), FAECC (TRANSCAN2022-784-104), and the European Union through the Next Generation EU Funds. The funders had no role in study design, data collection and analysis, decision to publish, or preparation of the manuscript.
**Has your study been approved by an ethics committee?** Yes
**Ethics committee - additional information:** All patients gave written informed consent according to institutional and federal guidelines. All study protocols were approved by the corresponding ethics committee.
**Author Disclosures:**
Anca Ligia Grosu: Nothing to disclose
Carolina Bravo Vergara: Nothing to disclose
Luis Marti-Bonmati: Nothing to disclose
Ilinca Popp: Nothing to disclose
Maria Beser-Robles: Nothing to disclose
Gloria Ribas: Nothing to disclose
Alejandro Mora Rubio: Nothing to disclose
Montserrat Carles Fariña: Nothing to disclose
Pablo Garcia Verdu: Nothing to disclose

**Diagnostic accuracy of a commercial AI tool for intracranial large- and medium-vessel occlusion detection in a multicenter emergency-CTA cohort**

*H. Andersson*, B. Hansen, J. Wassélius; Lund/SE
*(henrik.x.andersson@skane.se)*

**Purpose:** To evaluate the diagnostic accuracy of an AI tool (AIDOC VO), the first commercial AI tool designed to detect both large (LVO) and medium vessel occlusions (MeVO) on head-and-neck CT angiography (CTA), in a region-wide multicenter emergency setting.
**Methods or Background:** Prospective diagnostic-accuracy study of consecutive emergency CTAs from 3 031 adults (mean age 67 years; 51 % women), acquired 1 March - 8 July 2024 across a ten hospitals healthcare region. AI analysed each scan; the routine radiology report served as comparator. Examinations flagged positive or doubtful by either test underwent rereading by interventional neuroradiologists to establish the reference standard. Sensitivity, specificity, predictive value, and accuracy were calculated for the primary VO analysis, with prespecified sub-analyses for LVO and MeVO. Paired differences were tested with McNemar's test.
**Results or Findings:** Of 3 031 CTAs, 2 804 (92.5 %) yielded valid AI output, among which VO was identified in 224 (8 %) examinations. VO sensitivity/specificity were 81.7%/99.6% for AI versus 81.2%/99.3% for the clinical radiology report ($p = 0.91$/$p = 0.12$). LVO sensitivity was 92.8% versus 87.0% ($p=0.42$); MeVO 76.1% versus 79.2% ($p=0.55$). Paired overall accuracy showed no significant differences (VO $p=0.38$; LVO $p=0.06$; MeVO $p=0.76$). AI identified VO in 42 examinations missed by radiologists (18.8% enhanced detection; 15 per 1,000) and generated 11 false alerts (3.9 per 1,000).
**Conclusion:** Stand-alone AI matched radiologist performance, with a similar number of enhanced detections and few false alerts, supporting complementary use in emergency stroke workflows.
**Limitations:** About 7.5% of CTAs failed quality control, only positive/doubtful cases were reread so some false negatives may have been missed, AI heat-map localization was not verified, and effects of AI assistance on readers were not assessed.
**Funding for this study:** Funding was provided by the Crafoord Foundation, VINNOVA, and SUS Stiftelser & Fonder; sponsors had no role in the study.
**Has your study been approved by an ethics committee?** Yes
**Ethics committee - additional information:** The Swedish Ethical Review Authority approved the study (#2023-00387-01) and waived informed consent.
**Author Disclosures:**
Johan Wassélius: Nothing to disclose
Henrik Andersson: Nothing to disclose
Björn Hansen: Nothing to disclose

**Development of predictive models to identify the intracranial aneurysm responsible for subarachnoid hemorrhage in patients with multiple saccular aneurysms**

*G. Wang*; Chongqing/CN
*(wgxlove1234@163.com)*

**Purpose:** To develop and test machine learning (ML) models using CTA to identify the intracranial aneurysm (IA) responsible for subarachnoid hemorrhage (SAH) accurately in patients with multiple saccular IAs and to determine whether these models outperform traditional predictive markers.
**Methods or Background:** 207 SAH patients with 460 IAs from four hospitals were included and randomly divided into training (80%) and internal validation (20%) sets. Additionally, an external validation set comprising 65 patients with 147 IAs from other four hospitals was used. The predictive models were developed using ML methods that integrated the morphological features of IAs (e.g., size and shape) to identify the responsible IA. These models were then compared with traditional predictive markers that relies on hemorrhage patterns and the maximum IA size.
**Results or Findings:** The areas under the curves (AUCs) for the hemorrhage patterns and the maximum IA size were 0.496–0.505, 0.502–0.523, and 0.488–0.498 in the training, internal validation, and external validation sets, respectively. Among the 13 ML models, the best-performing models were the Gaussian process, logistic regression, and quadratic discriminant analysis models, with AUCs of 0.912, 0.894, and 0.890, respectively, for the training set; 0.869, 0.872, and 0.853, respectively, for the internal validation set; and 0.898, 0.892, and 0.897, respectively, for the external validation set. DeLong tests revealed no significant differences among these models, but all the models outperformed traditional predictive markers ($P<0.001$).
**Conclusion:** ML models that integrate multiple morphological features can predict the IA responsible for SAH accurately in patients with multiple IAs. These models outperform traditional predictive markers in identifying the responsible IA, thereby facilitating prompt and effective treatment.
**Limitations:** The results may not be applicable for predicting the rupture risk of unruptured IAs or for patients with a single IA.
**Funding for this study:** This study was supported by the Science and Technology Commission of Chongqing City, China (CSTB2023NSCQ-MSX0668) and the Science and Technology Research Program of Chongqing Municipal Education Commission (KJQN202200407).
**Has your study been approved by an ethics committee?** Yes
**Ethics committee - additional information:** This multicenter study received approval from the ethics committees of our hospitals. Since this was a retrospective study, informed consent from patients was not needed.
**Author Disclosures:**
Guangxian Wang: Speaker: No Author: No

**Diffusion-based deep learning model for automated detection of focal cortical dysplasia using multi-modality PET-MRI**

*M. Alshammari*[1], M. Yakubu[1], E. Guedj[2], N. Girard[2], J. Cardoso[1], A. Hammers[1]; [1]London/UK, [2]Marseille/FR
*(maha.alshammari@kcl.ac.uk)*

**Purpose:** Co-registration of FDG PET and MRI has been shown to improve detectability of epileptogenic lesions in focal cortical dysplasia (FCD). In previous work, we generated pseudo-normal PET using a diffusion model, which achieved high sensitivity but resulted in an average of 22 false positives per case. Here, we integrate multi-modality data (co-registered PET and MRI) for the generation of pseudo-normal PET.

**Methods or Background:** A weakly-supervised 2D-UNet-based diffusion model with dual input channels (co-registered PET and MRI) was trained on 280 slices from 35 healthy controls, with synthetic FCD lesions in 140 slices (50%). The model was trained using 5-fold cross-validation, yielding 5 independent models. Each test case was processed through all 5 cross-validated models at 2 different noise levels with 5 samples each, generating 50 pseudo-normal reconstructions. Deviations between original and pseudo-normal images were quantified as voxel-wise robust Z-scores, enhanced using Probabilistic Threshold-Free Cluster Enhancement (pTFCE), and thresholded at FWER-corrected value. Performance was evaluated on 10 independent synthetically lesioned test cases.
**Results or Findings:** The multi-modality model achieved 80% sensitivity (8/10 lesions detected) with 2.0±2.72 false-positive detections per case. This represents an 11-fold improvement in specificity compared to our previous PET-only approach while maintaining comparable detection sensitivity.
**Conclusion:** The multi-modality diffusion model successfully detects FCD lesions with dramatically improved specificity. The reduction from 22 to 2 false positives per case demonstrates significant clinical potential for automated epileptogenic zone localisation in presurgical epilepsy evaluation.
**Limitations:** Validation was performed on synthetically lesioned cases only. Further evaluation with clinical FCD cases and larger diverse datasets is required to establish robust real-world performance.
**Funding for this study:** Funding for this study: The School of Biomedical Engineering and Imaging Sciences is supported by the Wellcome EPSRC Centre for Medical Engineering at King's College London (WT 203148/Z/16/Z) and the Department of Health via the National Institute for Health Research (NIHR) comprehensive Biomedical Research Centre award to Guy's & St Thomas' NHS Foundation Trust in partnership with King's College London and King's College Hospital NHS Foundation Trust. MA is supported by the Saudi Arabia Cultural Bureau in London under the Saudi scholarship program.
**Has your study been approved by an ethics committee?** Not applicable
**Author Disclosures:**
Jorge Cardoso: Nothing to disclose
Alexander Hammers: Nothing to disclose
Eric Guedj: Nothing to disclose
Mubaraq Yakubu: Nothing to disclose
Nadine Girard: Nothing to disclose
Maha Alshammari: Nothing to disclose

### Unmasking Bias in BrainAGE: The Role of Scanner Manufacturer, Field Strength and Sequence Parameters

V. L. Ivan[1], M. Vach[1], J. Caspers[1], D. Weiß[1], *C. B. Fink*[1], D. M. Hedderich[2], C. Rubbert[1]; [1]Düsseldorf/DE, [2]Munich/DE
*(bijanfink92@gmail.com)*

**Purpose:** Brain Age Gap Estimation (BrainAGE) has emerged as a promising biomarker of brain health and disease. Yet, systematic bias may arise not only from study design but also from technical factors such as scanner manufacturer, field strength, or sequence parameters. This study aimed to quantify their influence on BrainAGE in healthy subjects.
**Methods or Background:** We included 2,414 cognitively normal participants from four population-based studies (ADNI, HCPA, OASIS3, PPMI). BrainAGE was computed using a standardized pipeline (CAT12/SPM12 preprocessing, PCA, Gaussian process regression) and a model trained on 2,953 independent controls. Differences in BrainAGE were assessed across cohorts, scanner manufacturers, field strengths (1.5T vs 3T), and T1-sequence acceleration using Welch's t-tests or ANOVA with Tukey post-hoc tests. Mean absolute error (MAE) was also calculated.
**Results or Findings:** Mean BrainAGE differed significantly between cohorts (ADNI: –5.9±5.5 years, HCPA: –4.1±6.2, OASIS3: –4.8±5.4, PPMI: –3.0±5.8; p<0.0001). Field strength had a strong impact: 1.5T scans yielded smaller BrainAGE (–2.2±4.8 years, MAE ~4.2) compared to 3T scans (–5.1±5.6, MAE ~6.7; p<0.05 across cohorts). Scanner manufacturer also mattered: in ADNI, Philips scanners produced a significantly smaller BrainAGE (–4.9±5.1, MAE 6.0) compared to GE (–6.3±6.2, MAE 7.3; p=0.008), while in PPMI Siemens differed from GE (p=0.008). By contrast, accelerated vs. unaccelerated 3T T1 acquisitions showed no significant effect (p=0.47).
**Conclusion:** Scanner manufacturer and field strength systematically bias BrainAGE in healthy cohorts, while sequence acceleration does not. These findings highlight that BrainAGE is not scanner-independent and emphasize caution when comparing results across studies or pooling multi-cohort data.
**Limitations:** The retrospective use of heterogeneous cohort data with only limited scanner parameter detail may confound effects of acquisition protocols with cohort-specific factors.
**Funding for this study:** None
**Has your study been approved by an ethics committee?** Yes
**Ethics committee - additional information:** Positive ethical approval
**Author Disclosures:**
Marius Vach: Nothing to disclose
Julian Caspers: Nothing to disclose
Christian Rubbert: Nothing to disclose
Daniel Weiß: Nothing to disclose
Vivien Lorena Ivan: Nothing to disclose
Christian Bijan Fink: Nothing to disclose
Dennis M. Hedderich: Nothing to disclose

### Synergistic A.I.: Unique Approach of Dual Algorithm Integration Transforms Trauma Imaging Workflow

*C. M. Toxopeus*, D. Duyndam, M. Gorzeman, A. Driessen; Amsterdam/NL
*(cmtoxopeus@gmail.com)*

**Purpose:** Increasing CT scan demand, particularly brain and cervical spine trauma imaging, places significant pressure on emergency departments (ED). OLVG Hospital began evaluating algorithms from AIDOC for these scans in 2021. Previous studies in our hospital demonstrated that AIDOC implementation significantly reduced trauma patient throughput times for CT brain in context of trauma. Since these scans are often combined with CT cervical spine, we investigated the potential for further ED throughput time reduction through a cervical spine fracture algorithm. Algorithm accuracy was thoroughly tested before release to emergency physicians.
**Methods or Background:** We analyzed 2,564 cases (June 2022 - June 2023) to assess algorithm accuracy. Training modules were developed for emergency physicians. Given lower sensitivity for cervical fractures in severe arthrosis, algorithm use was restricted to patients <65 years, excluding high-energy trauma or neurological deficits. Emergency physicians could consult radiologists for excluded categories. During pilot testing (December 2023 - April 2024), emergency physicians used the cervical algorithm during shifts.
**Results or Findings:** The accuracy study yielded a positive predictive value >50% and negative predictive value >99.45% and deemed safe within the context of our Level II trauma center. Emergency physician evaluation of cervical spine algorithm use was consistently positive, leading to definitive integration into our collaborative ED workflow.
**Conclusion:** This study demonstrates that combined application of AIDOC algorithms for CT brain and cervical spine in a Level II trauma center, together with emergency physician training: 1) is safe, 2) results in significant workload reduction for radiologists during shifts, 3) increases efficiency of patient flow in the ED, and 4) has a positive effect on collaboration between radiologists and clinicians.
**Limitations:** This study was conducted at a Level II trauma center, which may limit generalizability to other trauma centers.
**Funding for this study:** Innovation Fund of OLVG Hospital.
**Has your study been approved by an ethics committee?** Not applicable
**Author Disclosures:**
Debbie Duyndam: Nothing to disclose
Carolien Margot Toxopeus: Nothing to disclose
Annet Driessen: Nothing to disclose
Michiel Gorzeman: Nothing to disclose

**10:00 - 11:00** **Research Stage 5**

### Research Presentation Session: Genitourinary

### RPS 207

### Scrotal and penile imaging

**Moderator**
Mustafa Seçil; İzmir/TR
*(mustafa.secil@deu.edu.tr)*

### Structured Reporting and Contrast-Enhanced Ultrasound in Testicular Tumor Assessment: A Retrospective Study to Enhance Diagnostic Clarity and Clinical Decision-Making

*M. L. Schnitzer*, F. Herr, J. Rübenthaler; Munich/DE

**Purpose:** Structured reporting (SR) offers standardized radiological documentation, enhancing clarity and reproducibility. However, its role in contrast-enhanced ultrasound (CEUS) for testicular tumors remains underexplored. This study evaluates SR's impact on diagnostic accuracy, clinical decision-making, and workflow efficiency compared to free-text reporting (FTR).

**Methods or Background:** In this retrospective, single-center study, 65 male patients with suspected testicular tumors underwent CEUS at LMU University Hospital. Reports were initially documented as FTRs by an experienced radiologist and later converted into SRs using Smart Reporting software. Four board-certified urologists independently assessed both formats using a structured questionnaire. Completeness, readability, trust, and impact on clinical decision-making were evaluated. Statistical analysis included McNemar's test and the Wilcoxon signed-rank test, with $\alpha = 0.05$.
**Results or Findings:** SRs significantly improved readability (97.3% vs. 10.0%, $p < 0.001$) and information extraction (98.8% vs. 91.9%, $p < 0.001$). However, completeness (56.9% vs. 60.8%, $p = 0.427$) and clinical decision support (85.7% vs. 84.9%, $p = 0.152$) were comparable. Trust in SRs was lower than in FTRs (4.92 vs. 5.22, $p < 0.001$), likely due to missing diagnostic parameters and retrospective SR generation.
**Conclusion:** SR enhances standardization and workflow efficiency but does not yet outperform FTRs in completeness or clinical decision-making. Interdisciplinary collaboration in template development and the integration of classification systems could improve SR's diagnostic value. Future prospective, multicenter studies should assess real-time SR implementation and its potential impact on diagnostic accuracy and patient management.
**Limitations:** This study's limitations include its retrospective, single-center design and small sample size, which may limit generalizability. The SRs were generated retrospectively from FTRs, potentially affecting trust and completeness. Additionally, missing diagnostic parameters and lack of real-time SR implementation could have influenced clinical decision-making outcomes.
**Funding for this study:** No funding.
**Has your study been approved by an ethics committee?** Yes
**Ethics committee - additional information:** LMU Munich Ethics Board
**Author Disclosures:**
Moritz Ludwig Schnitzer: Nothing to disclose
Johannes Rübenthaler: Nothing to disclose
Felix Herr: Nothing to disclose

**Pharmaco-penile Doppler ultrasound in erectile dysfunction: experience from a Malagasy tertiary centre**
R. Franquine, *R. Ny Ako*, G. E. P. Andrianah, L. H. N. O. N. Rajaonarison, R. Hasina Dina, A. Ahmad; Antananarivo/MG
*(nktsmb@gmail.com)*

**Purpose:** To describe the indications, findings, and diagnostic contribution of pharmaco-penile Doppler ultrasound in patients with erectile dysfunction in a Malagasy university hospital.
**Methods or Background:** This descriptive cross-sectional study was carried out at the imaging centre of Joseph Ravoahangy Andrianavalona University Hospital from 04.2022 to 12.2024. All patients who underwent pharmaco-penile Doppler ultrasound were included. Data collected from reports included demographics, clinical history, indications, drugs used (alprostadil), and haemodynamic parameters over time.
**Results or Findings:** Forty patients were included (mean age 50.3 years; range 22–78). Hypertension and diabetes were common comorbidities. The main indication was erectile dysfunction, with differentiation of vascular versus psychogenic causes. A vascular origin was found in 67.5% of cases, with arterial insufficiency in 42.5% (mean peak systolic velocity 14.3 ± 4.7 cm/s), venous leak in 20% (mean end-diastolic velocity 11.3 ± 0.7 cm/s), and mixed pattern in 5%. Psychogenic dysfunction was suspected in 32.5%. Pathological findings varied with age, with vascular causes predominating after 50 years. The technique was well tolerated and provided key data for therapeutic orientation.
**Conclusion:** Pharmaco-penile Doppler ultrasound is a minimally invasive and highly informative tool for the evaluation of erectile dysfunction. In our Malagasy cohort, vascular aetiologies were predominant, particularly arterial insufficiency and venous leak. This first structured local series highlights the central role of penile Doppler in differentiating vascular from psychogenic dysfunction and guiding multidisciplinary management. It also demonstrates that advanced functional ultrasound is feasible in resource-limited settings, with findings of international clinical relevance.
**Limitations:** The limitations of the study are its small sample size, retrospective design, and single-centre setting.
**Funding for this study:** No funding was received for this study.
**Has your study been approved by an ethics committee?** Not applicable
**Author Disclosures:**
Randrianantenaina Franquine: Nothing to disclose
Lova Hasina Ny Ony Narindra Rajaonarison: Nothing to disclose
Ranoharison Hasina Dina: Nothing to disclose
Ratsimbasoa Ny Ako: Nothing to disclose
Gabrielle Emmylou Prisca Andrianah: Nothing to disclose
Ahmad Ahmad: Nothing to disclose

**Broken or Bruised? The Role of MRI in suspected Penile fracture**
*J. Bjergfelt*, P. Peter Krysfeldt Hansen, R. Mirón Mombiela; Herlev/DK
*(johannes@bjergfelt.dk)*

**Purpose:** Penile fracture is a rare but acute urological condition that is most often diagnosed clinically. In cases with classic findings such as swelling, discoloration, an audible "snap," and sudden loss of erection, urgent surgery is recommended without further imaging. However, in cases with an unclear clinical presentation, diagnostic uncertainty may arise, and MRI can be considered a valuable diagnostic tool. The purpose of the study was to investigate the role of MRI in patients with suspected penile fractures.
**Methods or Background:** We conducted a retrospective case series of 16 patients referred for MRI with suspected penile fracture between March 2024 and July 2025. Data were collected from the electronic medical record and PACS. Clinical history, physical findings, MRI results, treatment decisions, complications, surgical outcomes, and time intervals were analyzed.
**Results or Findings:** MRI confirmed penile fracture in 7 of 16 patients. Only 4 underwent surgery. The remaining 3 were managed conservatively: 2 due to proximal fractures and 1 due to a complex history with previous trauma. The remaining 9 patients got an alternative diagnosis and avoided unnecessary surgery. No major short-term complications were observed. All MRI's were performed within 12 hours.
**Conclusion:** MRI is a cost-effective tool in the diagnostic work-up of patients with clinically uncertain penile fractures. The modality may contribute to a more precise treatment decision, particularly in cases with proximal lesions where surgery may carry a higher risk of complications. In this series, MRI did not delay treatment and avoided unnecessary surgery.
**Limitations:** This study was limited by its retrospective single-center design, the small number of patients, and the absence of a control group of patients who went straight to surgery.
**Funding for this study:** None
**Has your study been approved by an ethics committee?** Not applicable
**Author Disclosures:**
Peter Peter Krysfeldt Hansen: Nothing to disclose
Johannes Bjergfelt: Nothing to disclose
Rebeca Mirón Mombiela: Nothing to disclose

**Radiomics-based detection of Germ Cell Neoplasia In Situ using volumetric ADC and FA histogram features: a retrospective study**
*M. V. Christodoulou*, O. Pappa, L. G. Astrakas, E. Lampri, A. Paliouras, E. Douvli, N. Sofikitis, M. I. Argyropoulou, A. C. Tsili; Ioannina/GR
*(maria.veatriki@gmail.com)*

**Purpose:** Germ Cell Neoplasia In Situ (GCNIS) is the precursor of most testicular germ cell tumors (TGCTs). The aim of this study was to evaluate whether first-order radiomics features derived from volumetric diffusion tensor imaging (DTI) metrics—specifically apparent diffusion coefficient (ADC) and fractional anisotropy (FA) histogram parameters—can detect GCNIS.
**Methods or Background:** This study included 15 men with TGCTs and 10 controls who underwent scrotal MRI, including DTI. Volumetric ADC and FA histogram metrics were calculated for the following tissues: group 1, TGCT; group 2: testicular parenchyma adjacent to tumor, histologically positive for GCNIS; and group 3, normal testis. Non-parametric statistics assessed group differences in ADC and FA histogram parameters. Pearson's correlation analysis was followed by ordinal regression analysis to identify key predictive metrics.
**Results or Findings:** Widespread distributional differences ($p < 0.05$) were observed for many ADC and FA variables, with both TGCTs and GCNIS showing significant divergence from normal testes. Among the ADC statistics, the 10th percentile and skewness ($p = 0.042$), range ($p = 0.023$), interquartile range ($p = 0.021$), total energy ($p = 0.033$), entropy and kurtosis ($p = 0.027$) proved the most significant predictors for tissue classification. FA_energy ($p = 0.039$) was the most significant fingerprint of carcinogenesis among the FA metrics. These parameters correctly characterized 88.8% of TGCTs, 87.5% of GCNIS tissues and 100% of normal testes.
**Conclusion:** Radiomics features derived from volumetric ADC and FA histogram analysis may serve as valuable non-invasive biomarkers for the early detection of GCNIS and provide insights into testicular tumorigenesis.
**Limitations:** Single-center, retrospective design with a small sample size; manual, consensus-based segmentation; lack of independent validation cohort; analysis restricted to first-order radiomics features; and, lack of histological confirmation in controls.
**Funding for this study:** No funding was received for this study.
**Has your study been approved by an ethics committee?** Yes
**Ethics committee - additional information:** The study was approved by the Institutional Review Board and Local Ethics Committee.

**Author Disclosures:**
Athanasios Paliouras: Nothing to disclose
Loukas G Astrakas: Nothing to disclose
Ourania Pappa: Nothing to disclose
Erriketi Douvli: Nothing to disclose
Maria Veatriki Christodoulou: Nothing to disclose
Evangeli Lampri: Nothing to disclose
Maria I. Argyropoulou: Nothing to disclose
Nikolaos Sofikitis: Nothing to disclose
Athina C. Tsili: Nothing to disclose

**Ultrasound evaluation of inguino-scrotal pathologies: experience from a Malagasy university hospital**
C. Randrianandrasana, *R. Ny Ako*, L. H. N. O. N. Rajaonarison, G. E. P. Andrianah, R. Hasina Dina, A. Ahmad; Antananarivo/MG
*(nktsmb@gmail.com)*

**Purpose:** To describe the spectrum of inguino-scrotal pathologies on ultrasound, with emphasis on urgent entities such as complicated hernia and necrotic testicular torsion, and to determine their prevalence in a hospital-based Malagasy cohort.
**Methods or Background:** This descriptive cross-sectional study was carried out at the radiology department of CHUJRA Antananarivo from 18.04.2025 to 07.07.2025. All patients undergoing inguino-scrotal ultrasound were included. Demographic data, indications, and sonographic findings were analysed, focusing on prevalence, age distribution, and complications of hernia and torsion.
**Results or Findings:** Inguino-scrotal ultrasound represented 4.7% of examinations during the study period. The most affected age group was 0–5 years (27.2%). The predominant pathology was inguinal hernia (19.4% of cases). Complications were frequent: 35% of hernias were complicated, and 40% of testicular torsions showed necrosis. Other findings included hydrocoele, varicocele, epididymo-orchitis, and rare tumours. Ultrasound was decisive in differentiating benign from urgent conditions and in guiding immediate management.
**Conclusion:** Ultrasound is the modality of choice for inguino-scrotal diseases due to its accessibility, non-invasiveness, and high diagnostic value. In our Malagasy cohort, inguinal hernia and testicular torsion were the leading causes, with a significant proportion of complications, reflecting delayed consultation and limited resources. This study underlines the pivotal role of ultrasound in early diagnosis and triage of inguino-scrotal emergencies. As one of the first structured series from Madagascar, it highlights the feasibility of generating clinically impactful imaging data in resource-limited settings, making it of international relevance.
**Limitations:** The limitations of the study are its monocentric design and short study period.
**Funding for this study:** No funding was received for this study.
**Has your study been approved by an ethics committee?** Not applicable
**Author Disclosures:**
Lova Hasina Ny Ony Narindra Rajaonarison: Nothing to disclose
Ranoharison Hasina Dina: Nothing to disclose
Ratsimbasoa Ny Ako: Nothing to disclose
Gabrielle Emmylou Prisca Andrianah: Nothing to disclose
Ahmad Ahmad: Nothing to disclose
Claudio Randrianandrasana: Nothing to disclose

**10:00 - 11:00 Research Stage 1**

### Research Presentation Session: Neuro

### RPS 211

### Modern imaging in stroke

**Moderator**
Milos A. Lucic; Sremska Kamenica/RS
*(milos.a.lucic@gmail.com)*

**The prognostic value of baseline CT-perfusion parameters in ischemic stroke patients treated with endovascular thrombectomy in MRPREDICTS prediction model**
*C. Khan*, B. Roozenbeek, P. J. van Doormaal, A. Van Der Lugt, D. Bos, CONTRAST consortium; Rotterdam/NL
*(c.khan@erasmusmc.nl)*

**Purpose:** Endovascular thrombectomy (EVT) has reduced disability in ischemic stroke patients, but significant outcome variability persists despite recanalisation. It is unknown whether CT-perfusion can improve functional outcome prediction. This study investigates the prognostic value of baseline CT-perfusion parameters for functional independence in acute ischemic stroke patients treated with thrombectomy in MR PREDICTS.
**Methods or Background:** Ischemic patients treated with EVT from MR CLEAN NOIV, MR CLEAN MED, and MR CLEAN LATE, and with available baseline CT-perfusion were included. Post-processing was performed using Siemens syngo.via.VB80C to retrieve penumbra (cerebral blood flow<27ml/100g/min) and ischemic core volumes (cerebral blood volume (CBV)<1.2ml/100ml), hypoperfusion intensity ratio (brain tissue volume Tmax>10s / brain tissue volume Tmax>6s) and CBV-index (average CBV in Tmax>6s within ischemic hemisphere / average CBV in Tmax≤4s tissues). Primary outcome was functional independence defined as modified Rankin Scale (mRS) 0-2 versus 3-6 at 90 days. Secondary outcome was functional outcome using ordinal mRS (0-6). The prediction model MR PREDICTS was used as benchmark and CT-perfusion parameters were added and compared to the benchmark using likelihood ratio tests. Bootstrapping was used for internal validation. Discrimination was reported using (ordinal) c-statistic.
**Results or Findings:** A total of 354 patients were included. Ischemic core significantly improved the benchmark model (p<0.05) and increased the c-statistic for functional independence (0.81, 95% CI 0.77–0.86 versus 0.80, 95% CI 0.76–0.84) and ordinal functional outcome (0.73, 95% CI 0.70–0.76 versus 0.72, 95% CI 0.69–0.75). CBV-index yielded similar results to ischemic core.
**Conclusion:** Baseline CT-perfusion may slightly improve the MR PREDICTS model for functional independence and outcome in ischemic stroke patients treated with EVT. Future studies should investigate whether CT-perfusion can improve treatment effect prediction for clinical decision making.
**Limitations:** This study lacks external validation and CT-perfusion post-processing is vendor specific.
**Funding for this study:** The MR CLEAN NOIV, MR CLEAN MED, and MR CLEAN LATE trials were supported by the Collaboration for New Treatments of Acute Stroke (CONTRAST) consortium, which is supported by the Netherlands Cardiovascular Research Initiative, an initiative of the Dutch Heart Foundation (CVON2015-01: CONTRAST); the Brain Foundation Netherlands (HA2015.01.06); the Ministry of Economic Affairs by means of the PPP Allowance made available by Top Sector Life Sciences and Health to stimulate public–private partnerships (LSHM17016); and unrestricted funding by Stryker, Medtronic, and Cerenovus. CLEOPATRA study led by Amsterdam UMC, the Netherlands, has contributed to the collection of CTP data and is sponsored by Zorgevaluatie Leading the Change.
**Has your study been approved by an ethics committee?** Yes
**Ethics committee - additional information:** The MR CLEAN NOIV, MR CLEAN MED, and MR CLEAN LATE were approved by the Medical and Ethical Review Committee in the Netherlands as part of the original trial protocols. All patient records and images were anonymised before analysis, and written informed consent was obtained from all patients or their legal representatives.
**Author Disclosures:**
Aad Van Der Lugt: Nothing to disclose
Ching Khan: Nothing to disclose
Pieter Jan Van Doormaal: Nothing to disclose
CONTRAST consortium: Nothing to disclose
Bob Roozenbeek: Nothing to disclose
Daniel Bos: Nothing to disclose

**Predictive Value of Dual-Energy CT Parameters for Hemorrhagic Transformation After Endovascular Therapy in Acute Ischemic Stroke**
*Y. h. Y ou*, Y. Xiong, W. Zhu; Wuhan/CN
*(1442823824@qq.com)*

**Purpose:** This study aims to evaluate the predictive value of dual-energy CT (DECT) parameters compared to CT perfusion (CTP) for hemorrhagic transformation (HT) after endovascular therapy (EVT) in acute ischemic stroke (AIS) patients, and to analyze their correlations with clinical parameters.
**Methods or Background:** We included 112 AIS patients who underwent scanning on Revolution CT Power and received EVT. Based on post-treatment HT occurrence, patients were divided into HT (n=25; age 42-89; male=14) and non-HT groups (n=87; age 31-87; male=54). DECT parameters [effective atomic number (Effective-Z), iodine concentration (Iodine Water)], CTP parameters [cerebral blood flow<30% volume (CBF<30%), time to maximum>6s volume (Tmax>6s)], and clinical data were collected. Univariate and multivariate binary logistic regression were used to identify independent predictors and construct prediction models. Predictive performance was evaluated using receiver operating characteristic (ROC) curve analysis.
**Results or Findings:** Significant intergroup differences (P<0.05) were observed in Effective-Z, Iodine Water, CBF<30% volume, Tmax>6s volume, age, diabetes history, stroke history, prior endovascular intervention/surgery, modified Rankin Scale (mRS) score, and Activities of Daily Living (ADL) score. Multivariate analysis identified Effective-Z, age, diabetes history, and mRS

score as independent predictors of HT. ROC analysis showed comparable predictive efficacy between DECT parameters (Effective-Z, AUC=0.67; Iodine Water, AUC=0.66) and CTP parameters (CBF<30% volume, AUC=0.71; Tmax>6s volume, AUC=0.65). The combined model integrating DECT parameters with clinical independent predictors demonstrated optimal predictive performance (AUC=0.80). DECT parameters significantly correlated with both CTP parameters and neurological function scores in Spearman analysis.
**Conclusion:** DECT parameters demonstrate predictive capability for HT post-EVT in AIS patients comparable to CTP. The combined model incorporating DECT parameters and clinical factors provides the highest predictive value, serving as an effective tool for hemorrhage risk assessment.
**Limitations:** Not applicable.
**Funding for this study:** No funding was provided for this study.
**Has your study been approved by an ethics committee?** Not applicable
**Author Disclosures:**
Ying Xiong: Nothing to disclose
Ya Hui Y Ou: Nothing to disclose
Wenzhen Zhu: Nothing to disclose

**A comparative study of cerebral arteries' morphological and haemodynamic features between post-stroke patients and age-matched non-stroke adults as assessed by advanced ultrasonography techniques**
*S. T. Gunda*, J. H-Y. Yip, V. T-K. Ng, Z. Chen, X. Han, J. Qu, X. Chen, M. Y-C. Pang, M. Ying; Kowloon/HK
*(stgunda@polyu.edu.hk)*

**Purpose:** Stroke poses a serious public health burden due to high morbidity and mortality worldwide. Ultrasonography has advanced over recent years, and emerging techniques such as three-dimensional(3D) and arterial stiffness analysis may provide new insights on stroke risk biomarkers, critical for preventative and therapeutic management. This study compared cerebral arteries' morphological and haemodynamic features between post-stroke patients and non-stroke (controls) as assessed by novel ultrasonography techniques.
**Methods or Background:** A cross-sectional study involving post-stroke patients and age-matched (controls) aged 50 yrs and above was conducted. Novel ultrasonography applications, including auto CIMT, 3D, and arterial stiffness analysis assessed extracranial cerebral arteries' morphology and hemodynamic features across the two groups, whereas transcranial color-coded Doppler ultrasound assessed intracranial hemodynamics.
**Results or Findings:** A total of 124 participants (post-stroke, n=57 and controls, n=67) were enrolled. The carotid beta-stiffness index (CAS β), elastic modulus (CAS kPa), and pulse wave velocity (CAS PWV) were significantly higher in post-stroke patients compared to controls (15.8±26.7 vs. 9.3±7.7, 208.9±333 kPa vs. 123.7±112 kPa, and 7.8±3.9 m/s vs. 6.5±2.2 m/s, all (p<0.05)), respectively. Conversely, carotid compliance and distensibility coefficient were higher in non-stroke (all p<0.05). However, no between group difference in 3D lumen volume stenosis was observed suggesting stenosis-related hemodynamic failure was unlikely the primary stroke mechanism in this population. Furthermore, post-stroke patients showed reduced cerebral blood flow (all DCCA parameters, ICA EDV, and MCA MFV, all p<0.05).
**Conclusion:** Our study observed significant differences in cerebral arteries' morphological and haemodynamic features, with post-stroke patients exhibiting higher arterial stiffness and reduced cerebral blood flow compared to non-stroke individuals. Reference values for 3D ultrasound-based and arterial stiffness features are provided.
**Limitations:** No limitations identified.
**Funding for this study:** This research project was supported by a research studentship grant (R006) and a research project fund (P0035203) of The Hong Kong Polytechnic University, Hung Hom, Kowloon, Hong Kong SAR, China.
**Has your study been approved by an ethics committee?** Yes
**Ethics committee - additional information:** The study was approved by the Institutional Review Board (or Ethics Committee) of The Hong Kong Polytechnic University (HSEARS20220714001)
**Author Disclosures:**
Veronica Tsam-Kit Ng: Nothing to disclose
Marco Yiu-Chung Pang: Nothing to disclose
Simon Takadiyi Gunda: Nothing to disclose
Ziman Chen: Nothing to disclose
Xiangyan Chen: Nothing to disclose
Jerica Hiu-Yui Yip: Nothing to disclose
Jingguo Qu: Nothing to disclose
Michael Ying: Nothing to disclose
Xinyang Han: Nothing to disclose

**Application of Extended Angio-CT from the Diaphragm in the Stroke Code: Diagnostic Value and Clinical Impact**
*S. De Gracia Nájera*, E. Saureu Rufach, C. Miró Ballesté, T. Barrera Yudego, J. A. Rojas Cristancho, G. Mauri Capdevilla, F. Purroy García, R. Mitjana Penella; Lleida/ES

**Purpose:** Given the low detection rate of cardioembolic sources with transthoracic echocardiography (1%) and the limited availability of transesophageal echocardiography, alternative non-invasive approaches are required for the etiological diagnosis of ischemic stroke, such as extended CT angiography (CTA) without cardiac synchronization performed during the hyperacute phase of stroke.The main objective of this study is to evaluate whether extended CTA provides diagnostic-quality images and enables detection of cardiovascular pathology as a possible stroke etiology. Secondary objectives include evaluating incidental pulmonary or mediastinal findings with potential therapeutic implications for patient management.
**Methods or Background:** A descriptive, prospective study was conducted including a cohort of 249 patients assessed within the stroke code protocol from January 2024 to September 2025, using extended CTA from the diaphragms upward without cardiac synchronization.
**Results or Findings:** Out of 249 studies, 223 (89.6%) were considered of moderate or good image quality. A total of 149 patients were diagnosed with ischemic stroke (59.8%). Among this group, 58 were women (38.9%) and 91 were men (61.1%), with an average age of 71 years. In 15 patients (10%), cardiovascular pathology was identified as a potential stroke etiology: 2 with intracardiac thrombus (1.3%), 3 with aortic dissection (2%), 3 with ulcerated plaques in the ascending aorta (2%), and 7 with irregular atherosclerosis in the aortic arch (4.7%). Additionally, concomitant pathologies were observed in 17 patients (11.4%), including pulmonary thromboembolism, neoplastic disease, and infections.
**Conclusion:** The inclusion of extended CTA in the stroke code protocol may contribute to identifying cardiovascular pathology potentially involved in the etiology of stroke, as well as detecting concomitant conditions that may impact therapeutic management.
**Limitations:** Not applicable.
**Funding for this study:** No
**Has your study been approved by an ethics committee?** Not applicable
**Author Disclosures:**
Carla Miró Ballesté: Nothing to disclose
Francisco Purroy García: Nothing to disclose
Teresa Barrera Yudego: Nothing to disclose
Eugènia Saureu Rufach: Nothing to disclose
Gerard Mauri Capdevilla: Nothing to disclose
Raquel Mitjana Penella: Nothing to disclose
Jhonatan Andrés Rojas Cristancho: Nothing to disclose
Sara De Gracia Nájera: Nothing to disclose

**Beyond ASPECTS: multimodal CT with perfusion and collateral scoring improves prediction of stroke outcome**
*T. B. Plojović*, D. Janjic, D. Vasin, F. Crnovršanin, N. Spasojević, S. M. Lekic; Belgrade/RS
*(tarik95@hotmail.rs)*

**Purpose:** To evaluate the prognostic value of CT perfusion (CTP) parameters in predicting functional outcome after acute ischemic stroke, to compare their performance with the Alberta Stroke Program Early CT Score (ASPECTS) on non-contrast CT (NCCT), and to assess whether collateral scoring on CT angiography (CTA) provides additional predictive value.
**Methods or Background:** This retrospective study included 122 consecutive patients with acute ischemic stroke admitted to the Emergency Center between January 2023 and December 2024. All patients underwent NCCT, CTA, and CTP as part of the acute stroke protocol. ASPECTS was calculated on NCCT. CTP maps were analyzed for standard thresholds of core (CBF <30%) and penumbra (Tmax >6s). Collateral status was graded on CTA using a semi-quantitative collateral score. Functional outcome was assessed at 90 days using the modified Rankin Scale (mRS), dichotomized into favorable (mRS 0–2) and unfavorable outcome (mRS 3–6).
**Results or Findings:** NCCT ASPECTS correlated significantly with outcome (p<0.05) but showed limited discriminatory ability (AUC 0.68). CTP-derived parameters, particularly core volume (CBF <30%) and penumbra volume (Tmax >6s), demonstrated stronger correlation with unfavorable outcome (p<0.001) and higher predictive accuracy (AUC 0.82). The addition of CTA collateral score further improved the prognostic model (combined AUC 0.89), outperforming both ASPECTS and CTP alone.
**Conclusion:** CT perfusion provides superior prognostic value compared with NCCT ASPECTS in predicting functional outcome after acute ischemic stroke. Incorporating CTA collateral assessment further enhances predictive accuracy, underscoring the importance of a multimodal CT approach.

**Limitations:** Limitations of the study include its retrospective, single-center design, potential software-related variability in perfusion analysis, and the lack of long-term outcome data beyond 90 days
**Funding for this study:** No funding for this study.
**Has your study been approved by an ethics committee?** Yes
**Ethics committee - additional information:** This study was conducted in accordance with the Declaration of Helsinki and approved by the Institutional Ethics Committee.
**Author Disclosures:**
Snezana Milika Lekic: Nothing to disclose
Doroteja Janjic: Nothing to disclose
Faris Crnovršanin: Nothing to disclose
Dragan Vasin: Nothing to disclose
Tarik Binasa Plojović: Nothing to disclose
Natalija Spasojević: Nothing to disclose

**Code stroke in patients on anticoagulants: Added value of embolic source detection by hyperacute cardiac CT**
*P. Gilabert Núñez*, H. Cuellar Calabria, M. De Dios, T. Alejandro, A. Roque, A. Calderwood, C. Meza, F. Rizzo, J. Pagola; Barcelona/ES
*(pablo.gilabert@vallhebron.cat)*

**Purpose:** To determine the rate of positive CT results for stroke (CTs+) in patients with a history of atrial fibrillation under a regimen of direct-acting oral anticoagulants (DOAC-AF). To detect patients with thrombus in the heart or ascending aorta (T+) on a hyperacute cardiac CT in a venous phase. To find out whether thrombus and left atrial size (LAs) has added value in this clinical presentation.
**Methods or Background:** DOAC-AF patients admitted to the emergency department with a suspicion of stroke underwent multimodal code stroke CT followed by venous-phase cardiac CT (n=75, 55% men, 81±9 years). T+ and LAs were correlated with NIHSS (National Institutes of Health Stroke Scale) at admission and discharge and with CTs+, defined as arterial occlusion or ischemia.
**Results or Findings:** Rates of CTs+ and T+ were 56% and 13%, respectively. Ninety per cent of T+ were CTs+, in contrast to 51% of T- (p=0.02), with sensitivity, specificity, positive and negative predictive values of 21%, 97%, 90% and 49%, respectively. Patients with a severely dilated LAs≥33.2 cm² had rates of S+ and E+ of 90% and 52%, respectively (p=0.002 for comparison with LAs<33.2 cm²). T+ patients presented with higher NIHSS at baseline (16.5 versus 6;p=0.02). None was discharged with NIHSS-0, compared to 39% of T- (p=0.02).
**Conclusion:** A modified code stroke CT protocol including a venous-phase cardiac CT allowed for the detection of cardiac thrombi in 13% of patients with a history of medically treated atrial fibrillation. This group showed more severely dilated left atria, presented with higher NIHSS scores at admission and suffered more sequelae than patients without source lesions.
**Limitations:** The limitatios of this study is that most patients did not undergo transesophageal ultrasound owing to poor clinical condition.
**Funding for this study:** Funding was provided by European Regional Development Fund 24-00333.
**Has your study been approved by an ethics committee?** Yes
**Ethics committee - additional information:** CEIm EOM(AG)060/2024(6343)
**Author Disclosures:**
Federica Rizzo: Nothing to disclose
Hug Cuellar Calabria: Nothing to disclose
Pablo Gilabert Núñez: Nothing to disclose
Jorge Pagola: Nothing to disclose
Ana Calderwood: Nothing to disclose
Albert Roque: Nothing to disclose
Marta De Dios: Nothing to disclose
Claudia Meza: Nothing to disclose
Tomasello Alejandro: Nothing to disclose

**Endovascular Thrombectomy for Subterminus Internal Carotid Artery (ICA-I) Occlusions – analysis of 3730 patients**
*E. Hall*, B. Hansen, T. Ullberg, J. Wasselius; Lund/SE
*(emma.hall@med.lu.se)*

**Purpose:** Subterminus intracranial internal carotid artery (ICA-I) occlusion is an underrecognized stroke subtype which may delay access to endovascular thrombectomy (EVT). This study aimed to: 1) characterise clinical presentation; and 2) assess radiological reporting accuracy in EVT-treated patients with ICA-I occlusions
**Methods or Background:** EVT-treated patients with ICA-I, ICA terminus (ICA-T), and middle cerebral artery M1 occlusions were identified from a nationwide registry (2016–2022). Outcomes included NIHSS, ASPECT, recanalization success, and 90-day mRS. In a single-center cohort (2016–2025), stroke severity and perfusion deficits were analyzed in relation to anterior and posterior circulation variants, and accuracy of baseline radiology reports was compared with ICA-T and M1 occlusion controls.
**Results or Findings:** Of 3730 EVT patients, 356 (9.5%) had ICA-I, 657 (17.6%) ICA-T, and 2717 (72.8%) M1 occlusions. Median preoperative NIHSS was 17 (variance 34) for ICA-I, 19 (variance 25) for ICA-T, and 16 (variance 37) for M1 (p<0.001 between groups). While recanalization rates and 24-hour NIHSS were similar, patients with M1 occlusions had better 90-day outcomes than those with ICA-I or ICA-T occlusions. Low and high ASPECT were more common for ICA-I occlusions while the intermediate ASPECT values were more common in ICA-T and M1 occlusions (p<0.001). In ICA-I cases, anterior circulation variants had a greater impact on stroke severity, compared to posterior circulation variants. Only 29% of ICA-I occlusions were correctly identified in initial radiology reports, compared with 56% for ICA-T and 100% for M1 occlusions.
**Conclusion:** ICA-I occlusions present with high variability and are often underrecognized on baseline imaging. Greater awareness among radiologists and clinicians is needed to improve diagnostic accuracy and treatment pathways.
**Limitations:** There is a potential risk of underreporting of ICA-I occlusions due to mild or atypical symptoms. In Riksstroke, functional outcome is self-reported.
**Funding for this study:** This study was funded by grants from Teggerstiftelsen to EH, and regional ALF grants to EH, TU and JW, the Crafoord Foundation to JW, VINNOVA to JW, and by SUS Stiftelser & Fonder to JW and BMH. None of the funding bodies had any involvement in the planning of methodology, data retrieval or analysis in the study.
**Has your study been approved by an ethics committee?** Yes
**Ethics committee - additional information:** The Swedish Ethical Review Authority approved the study and waived individual informed consent (#2024-03485-01).
**Author Disclosures:**
Teresa Ullberg: Nothing to disclose
Johan Wasselius: Speaker: Medtronic Speaker: Siemens Healtineers
Emma Hall: Nothing to disclose
Björn Hansen: Nothing to disclose

**10:00 - 11:00 Research Stage 6**

### Research Presentation Session: Vascular

### RPS 215

### Advancing the boundaries of vascular ultrasound

**Moderator**
Lorenzo E. Derchi; Genoa/IT

**Association between Ultrasonographic Morphological Features of CP-RADS Category 2 Carotid Plaques and the Risk of Ischemic Stroke Events**
J. Yiwen, J. Wang, M. Chen, *Y. Yang*; Shanghai/CN

**Purpose:** Objective: To investigate the independent predictive value of plaque characteristics based on conventional ultrasound and contrast-enhanced ultrasound (CEUS)—specifically a composite model integrating intraplaque punctate calcification and contrast enhancement—for new-onset cerebral infarction in patients with CP-RADS category 2 carotid plaques, and to develop and validate a nomogram prediction model incorporating clinical and imaging features.
**Methods or Background:** Methods: This retrospective study included 744 patients with CP-RADS category 2 carotid plaques who underwent carotid ultrasound and CEUS. Following follow-up, 404 patients were randomized 7:3 into training (n=283) and validation (n=121) sets. Independent predictors of new-onset cerebral infarction were identified through univariate and multivariate logistic regression, and used to develop a nomogram. Model performance was assessed by ROC analysis, AUC, sensitivity, and specificity.

**Results or Findings:** The plaque characteristics (the composite model of intraplaque punctate calcification and contrast enhancemen) is an independent predictor of new-onset cerebral infarction in CP-RADS Category 2 carotid plaques. Our nomogram demonstrates accurate and stable prediction, offering a reliable tool for early risk stratification and individualized intervention.
**Conclusion:** Conclusion: Plaque characteristics (the composite model of intraplaque punctate calcification and contrast enhancement) serve as an independent predictor of new-onset cerebral infarction in patients with CP-RADS Category 2 carotid plaques. The nomogram model developed in this study exhibits good predictive accuracy and stability, providing an intuitive and reliable quantitative tool for early identification of high-risk populations and implementation of individualized interventions.
**Limitations:** This single-center retrospective study has inherent limitations. The single-institution sample limits generalizability, and the retrospective design may introduce systematic bias. To minimize bias, we exclusively enrolled patients with recent cerebral infarction (within 6 months) to clearly define cerebrovascular events. Future multi-center studies with extended follow-up are needed to validate the model's predictive performance and clinical utility.
**Funding for this study:** No
**Has your study been approved by an ethics committee?** Yes
**Ethics committee - additional information:** This study strictly adheres to the ethical principles of the Declaration of Helsinki (2013 revision) and has been approved by the Ethics Committee of Shanghai Tongren Hospital
**Author Disclosures:**
Jingyi Wang: Nothing to disclose
Jin Yiwen: Nothing to disclose
Yuxin Yang: Nothing to disclose
Man Chen: Nothing to disclose

**From Gut to Vessel Wall: TMAO and Carotid Ultrasound in Pediatric Obesity**
*A. Borlea*, D. I. Stoian, S. Mihuta, A. Bena, C. Paul; Timisoara/RO

**Purpose:** PURPOSE. To investigate the relationship between serum trimethylamine N-oxide (TMAO), a gut-derived metabolite, and carotid intima–media thickness (CIMT) as an ultrasound marker of subclinical atherosclerosis in children with obesity.
**Methods or Background:** METHODS. We enrolled 70 children aged 4–18 years: 50 obese (BMI >95th percentile, waist circumference >90th percentile) and 20 normal-weight controls. Clinical evaluation included BMI, waist-to-height ratio, Tanner stage, and signs of insulin resistance. Serum TMAO was measured by liquid chromatography–mass spectrometry, alongside fasting glucose, insulin, lipid profile, transaminases, creatinine, uric acid, and vitamin D. CIMT was assessed bilaterally with high-resolution ultrasound. Arterial stiffness parameters (pulse wave velocity [PWV], augmentation index, and central and peripheral blood pressures) were recorded for comparison.
**Results or Findings:** RESULTS. Obese children had significantly higher TMAO levels (median 289.5 vs 218.7 µg/L, $p=0.02$) and CIMT (0.56 ± 0.07 vs 0.49 ± 0.05 mm, $p<0.001$) compared to controls. Serum TMAO correlated positively with CIMT ($\rho=0.41$, $p<0.001$), PWV ($\rho=0.36$, $p=0.004$), and systolic blood pressure ($\rho=0.33$, $p=0.007$). In multivariable regression, TMAO remained an independent predictor of CIMT ($\beta=0.37$, $p<0.001$), even after adjusting for BMI, waist measures, age, and puberty stage. Children with insulin resistance had higher CIMT and PWV than their non-insulin-resistant peers, but TMAO levels did not differ significantly between subgroups. Logistic regression showed that higher TMAO was associated with increased odds of acanthosis nigricans ($p=0.03$).
**Conclusion:** CONCLUSION. Serum TMAO is elevated in pediatric obesity and independently predicts carotid intima–media thickness, reinforcing the role of ultrasound as the key imaging modality to detect early vascular injury in this population.
**Limitations:** Limitations: cross-sectional design; limited control sample; absence of dietary/microbiome profiling
**Funding for this study:** No funding
**Has your study been approved by an ethics committee?** Yes
**Ethics committee - additional information:** The Ethics Committee for Scientific Research of the “Pius Brinzeu” Emergency County Hospital has approved the study (No. 349/15.11.2022)
**Author Disclosures:**
Corina Paul: Nothing to disclose
Dana I. Stoian: Nothing to disclose
Andreea Bena: Nothing to disclose
Andreea Borlea: Nothing to disclose
Simina Mihuta: Nothing to disclose

**Value of Carotid-Plaque Viscoelastic Ultrasound for Predicting Ischemic Cerebrovascular Risk in Patients with Mild (<50 %) Carotid Stenosis**
H. Jin, H. Yunqian, *Q. Chen*, Y. Yang, M. Chen; Shanghai/CN

**Purpose:** To determine whether ultrasound-derived viscoelastic properties of carotid plaques can identify individuals with mild (<50 %) carotid stenosis who are at heightened risk of an ischemic cerebrovascular event.
**Methods or Background:** Consecutive patients with mild carotid stenosis examined between April 2023 and June 2024 were enrolled. Ninety-seven had sustained an ischemic cerebrovascular event within the preceding 6 months; 592 had remained event-free. Gray-scale and viscoelastic ultrasound were used to quantify plaque elasticity and viscosity. Logistic regression was employed to assess the association between viscoelastic parameters and incident events while adjusting for clinical covariates. ROC analysis evaluated the predictive performance of these parameters.
**Results or Findings:** Plaques in the event group exhibited significantly lower mean elastic modulus (P-Emean) and mean viscous modulus (P-Vmean) than those in the asymptomatic group. Both P-Emean and P-Vmean emerged as independent predictors of events. Combined viscoelastic models yielded excellent discrimination, with the elastic-component model achieving an AUC of 0.871.
**Conclusion:** In patients with mild carotid stenosis, plaque biomechanics are closely linked to cerebrovascular events. Ultrasound assessment of plaque viscoelasticity can improve risk stratification and lays the groundwork for precision management of this population.
**Limitations:** Histological validation of the viscoelastic parameters was not performed.
**Funding for this study:** This research was supported by Shanghai Tongren Hospital, with project numbers TRYJ2022LC09, tx2023xk18, and TK2017r04
**Has your study been approved by an ethics committee?** Yes
**Ethics committee - additional information:** This study was approved by the ethics committee of our hospital.
**Author Disclosures:**
Haowei Jin: Nothing to disclose
Huang Yunqian: Nothing to disclose
Qiao Chen: Nothing to disclose
Yuxin Yang: Nothing to disclose
Man Chen: Nothing to disclose

**Analysis of ultrasound characteristics of plaques in patients with mild carotid stenosis and embolic stroke of unknown cause (ESUS)**
M. Kader, L. Zhang, H. Yunqian, M. Chen, *Y. Yang*, Q. Chen; Shanghai/CN

**Purpose:** This study aimed to investigate the ultrasonic characteristics of plaques in patients with mild carotid stenosis (stenosis <50%) complicated with embolic stroke of unknown cause (ESUS), in order to provide new ideas and basis for the etiological diagnosis and individualized treatment of ESUS.
**Methods or Background:** This retrospective study analyzed carotid plaque characteristics in 106 stroke patients admitted from May to December 2019 and divided into three groups: Embolic Stroke of Undetermined Source (ESUS, n=23), Extracranial Large Artery Atherosclerosis (LAA, n=28), and a non-stroke control group (n=55), using ultrasound and contrast-enhanced ultrasound. The echo intensity, surface morphology and perfusion pattern of carotid plaques and other related ultrasound parameters were compared between different groups. The binary logistic regression model, ROC curve and multivariate analysis were used to evaluate the diagnostic efficacy of ultrasound parameters and clinical factors.
**Results or Findings:** Both LAA and ESUS groups exhibited significantly higher frequencies of microbubble perfusion and "inside-to-outside" perfusion patterns compared to controls. Key plaque features including hypoechoic areas, irregular morphology, and incomplete fibrous cap showed significant intergroup differences ($P < 0.05$). The combination of plaque thickness <3.4 mm and absence of "inside-to-outside" perfusion pattern achieved an AUC of 0.802 for ESUS diagnosis with 80.4% accuracy.
**Conclusion:** These findings suggest that integrated assessment of plaque thickness and CEUS perfusion patterns enhances stroke subtype differentiation and prognostic risk assessment, providing valuable guidance for individualized diagnosis and treatment strategies.
**Limitations:** The single-center design with small sample size (n=106) affects generalizability, ultrasound assessments carry inherent subjectivity, comprehensive evaluation of embolic sources requires additional imaging modalities, and insufficient follow-up duration prevents long-term outcome assessment.
**Funding for this study:** Not applicable
**Has your study been approved by an ethics committee?** Not applicable

**Author Disclosures:**
Man Chen: Nothing to disclose
Huang Yunqian: Nothing to disclose
Qiao Chen: Nothing to disclose
Yuxin Yang: Nothing to disclose
Lixia Zhang: Nothing to disclose
Maraba Kader: Nothing to disclose

**Diagnostic Performance of Microvascular Ultrasound for Endoleak Detection after EVAR: Comparison with CEUS and CTA**
*M. Czeczelewski*, A. Drelich-Zbroja, M. A. Kuczyńska; Lublin/PL
*(marcin.czeczelewski@gmail.com)*

**Purpose:** Patients following endovascular aneurysm repair (EVAR) require long-term imaging surveillance to detect late complications such as endoleaks. CTA remains the gold standard for follow-up but exposes patients to ionizing radiation and iodinated contrast agents. Microvascular ultrasound (MVUS) is a novel, contrast-free technique capable of visualizing slow microvascular flow. This study aimed to compare the diagnostic accuracy of MVUS with CEUS and CTA in detecting endoleaks after EVAR.
**Methods or Background:** A prospective cross-sectional study was conducted in 73 consecutive patients (60 males, 13 females) who underwent EVAR. Within one week post-procedure, each participant underwent ultrasound assessment including both MVUS and CEUS. CEUS was performed after intravenous administration of 1.6 mL of SonoVue (Bracco, Italy). Within one month, all patients underwent triple-phase CTA, which served as the reference standard. Endoleaks were classified according to the Mayo Clinic classification.
**Results or Findings:** Among the 73 patients, the stent graft types included Zenith Flex (19; 26%), Zenith t-Branch (27; 37%), Gore Excluder (17; 23%), and Medtronic Endurant II (10; 14%). Ultrasound prior to discharge revealed 42 endoleaks, including 33 detected by both MVUS and CEUS, and 9 detected only by MVUS. One month later, CTA confirmed 28 of the 33 endoleaks initially detected by CEUS and MVUS, while 3 had resolved and 3 patients were lost to follow-up. Of the 9 endoleaks detected only by MVUS, one was confirmed by CTA. These MVUS-only endoleaks were characterized by fine, linear flow signals perpendicular to the transducer.
**Conclusion:** MVUS demonstrates high sensitivity for detecting endoleaks following EVAR.
**Limitations:** This single-center study had a limited sample size and short follow-up duration. CTA was performed up to one month after ultrasound, which limits its reliability as a reference standard.
**Funding for this study:** ESR Research Seed Grants 2024
**Has your study been approved by an ethics committee?** Yes
**Ethics committee - additional information:** Medical University of Lublin Bioethics committee KE-0254/57/03/2024
**Author Disclosures:**
Marcin Czeczelewski: Nothing to disclose
Maryla Anna Kuczyńska: Nothing to disclose
Anna Drelich-Zbroja: Nothing to disclose

**Morphological and functional vascular imaging markers of diabetic atherosclerosis**
*D. J. Petrovic*; Belgrade/RS
*(dusanpetrovic736@gmail.com)*

**Purpose:** The objective of the survey was to establish the correlation between carotid intima-media thickness (CIMT) and type 2 diabetes mellitus (T2DM), as well as to assess the hemodynamic parameters and their relationship to T2DM.
**Methods or Background:** 186 participants between the ages of 42 and 84 with proven T2DM (112 men and 74 women; mean age ± SD, 63.13 ± 6.96) were retrospectively examined (prospective design or retrospective analysis of a prospective data set) by the carotid ultrasound Doppler examination measuring carotid morpho-functional features. Subjects were followed for 26 months from the inclusion in the study (regular control examinations and phone contact). Data were recorded on new cases of mortality (CV mortality) and adverse CV events (myocardial infarction - MI, cardiac insufficiency, surgical revascularization - coronary or stroke, etc.).
**Results or Findings:** In this study, the value of CIMT varies between 0.56 and 1.41mm (mean CIMT=1.05 ± 0.12mm) while functional results were (RI=0.81 ± 7; PI =1.16±0.185; PSV=132.5 ± 16.65; EDV=28.33 ± 9.04; carotid artery diameter = 6.5 ± 0.92mm). After 26 months of time frame follow-up, 24 vascular events were noted (MI, TIA, stroke, and coronary angioplasty).
**Conclusion:** The evaluation of morphological (CIMT) and functional (RI, PSV, EDV) parameters is a reliable indicator and barometer for assessing the degree of diabetic vascular remodeling during atherosclerosis progression.
**Limitations:** Relatively limited sample size, retrospective nature.
**Funding for this study:** No fundings
**Has your study been approved by an ethics committee?** Not applicable
**Author Disclosures:**
Dusan Jordan Petrovic: Nothing to disclose

**10:00 - 11:00** **Research Stage 3**

### Research Presentation Session: Professional Challenges: Shaping our Professional Future

### RPS 230
### Building a sustainable future for radiology

**Moderator**
Elisabetta Giannotti; Cambridge/UK

**The Role of Radiology in Promoting Environmental Sustainability: A Policy Perspective**
*J. Lydia*, F. Abubacker Sulaiman, R. Praveenkumar, M. Nivitha, D. Dharshini, A. Raashid Ibrahim; Chennai/IN
*(merlinlydia99@gmail.com)*

**Purpose:** Evaluate the environmental impact of radiology services. Identify policy gaps in promoting sustainable practices in radiology. Assess awareness and actions related to sustainability among radiology professionals. Demonstrate practical departmental initiatives toward sustainable radiology practices.
**Methods or Background:** Literature review of global and regional studies on sustainable radiology practices. Analysis of existing healthcare policies related to sustainability. Survey of radiology professionals to assess awareness of sustainable development and adoption of environmentally friendly practices. Documentation and evaluation of actions implemented in our radiology department, including energy-efficient protocols, waste reduction, and recycling initiatives aimed at future sustainability.
**Results or Findings:** Survey results revealed moderate awareness among radiology professionals, with varying adoption of sustainable practices. Departmental initiatives led to measurable reductions in energy use and waste. Existing policies are broad and lack modality-specific guidance for radiology.
**Conclusion:** Radiology can play a key role in healthcare sustainability through awareness, practical initiatives, and policy integration. Structured support at the policy level is essential to standardize and incentivize environmentally responsible practices.
**Limitations:** None
**Funding for this study:** No funding was provided
**Has your study been approved by an ethics committee?** Not applicable
**Author Disclosures:**
Rathinamoorthy Praveenkumar: Nothing to disclose
Deva Dharshini: Nothing to disclose
Mohanam Nivitha: Nothing to disclose
Johnbosco Lydia: Nothing to disclose
Farook Abubacker Sulaiman: Nothing to disclose
Abubacker Raashid Ibrahim: Nothing to disclose

**Safe and Sustainable Contrast Media Use: Balancing Patient and Environmental Impact**
A. Cue Castro, *A. Camacho Palomino*; Mexico City/MX

**Purpose:** To evaluate current evidence on the biological and environmental effects of iodinated and gadolinium-based contrast media (CM) and to propose justification and dose-optimization strategies that ensure diagnostic quality while minimizing risks to patients and the environment.
**Methods or Background:** A comprehensive literature review (PubMed, ESUR, ACR, WHO, 2015–2025) analyzed data on nephrotoxicity, gadolinium deposition, hypersensitivity, and environmental persistence of CM. International guidelines were reviewed to identify best practices in patient risk assessment, indication justification, and imaging protocol optimization.
**Results or Findings:** Evidence confirms that repeated CM exposure may induce renal and systemic effects in susceptible patients and that gadolinium and iodine residues persist in wastewater, impacting aquatic ecosystems. Personalized dosing (weight- and renal function–based), use of macrocyclic gadolinium chelates and low- or iso-osmolar iodinated agents, and optimized CT/MRI protocols reduce administered doses without compromising diagnostic value. Standardized justification checklists decrease unnecessary contrast use by up to 30–40%.
**Conclusion:** Rational and justified use of contrast media is essential to balance diagnostic benefit with patient and environmental safety. Radiology departments should integrate contrast monitoring systems, dose optimization protocols, and education programs promoting responsible, evidence-based use of iodinated and gadolinium agents. Professional education should be prioritized to ensure responsible imaging practice.
**Limitations:** Data heterogeneity and variability in study design limit direct comparison across contrast agents and imaging modalities. Environmental data remain regionally restricted and require broader, multicenter monitoring.
**Funding for this study:** Own
**Has your study been approved by an ethics committee?** Not applicable
**Author Disclosures:**
Araceli Cue Castro: Employee: Bracco, medical affairs and techinovigilance &farmacovigilance -LATAM Hub
Alejandro Camacho Palomino: Nothing to disclose

**Reducing Idle CT Scanner Energy Consumption Between Examinations: Operational Feasibility and Impact of a Rapid-Reactivation Power Save Mode**
*P. Hehenkamp*[1], M. Obmann[1], S. Kamber[1], D. Kraft[2], M. Loser[2], E. M. Merkle[1], T. Heye[1], J. Vosshenrich[1]; [1]Basel/CH, [2]Forchheim/DE
*(paul.hehenkamp@usb.ch)*

**Purpose:** To assess the efficiency and operational feasibility of a novel CT power save mode designed to reduce inter-examination energy consumption without affecting clinical workflows.
**Methods or Background:** CT scanners consume substantial energy during idle periods. While powering down systems has been shown to provide substantial energy, cost, and carbon emission benefits, this is infeasible for short inter-examination intervals. To address this current limitation, one CT scanner (SOMATOM X.ceed; Siemens Healthineers) was equipped with a newly developed power save mode. The scanner's power draw was prospectively measured over a 28-week period (Nov 2024–Jun 2025). Data was segmented into active, idle and power save states. Energy and emission savings were calculated, and usability and impact on workflow were assessed via a survey of CT technologists.
**Results or Findings:** Across 124 workdays, the power save mode reduced power draw by 26.8% compared with idle (1.6 ± 0.1 kW vs. 2.1 ± 0.1 kW), yielding a 15.6% reduction in nonproductive and 7.2% reduction in total operational energy use. Nonproductive time accounted for 66.1% of scanner on-hours with the power save mode being active during 58.1% of this time. 19 CT technologists responded to the structured survey. Awareness of the mode's functionality was high (84%), and 79% of the technologists had manually activated the power save mode at least once. All technologists reported the activation process as "very easy." Importantly, all technologists (100%) denied any technical issues or workflow disruptions.
**Conclusion:** A novel rapid-reactivation CT power save mode substantially reduces nonproductive energy consumption during inter-examination idle periods without disrupting clinical workflows, representing a practical step toward a more sustainable operation of CT.
**Limitations:** Only one specific CT scanner model was investigated. Relative and absolute savings may vary by vendor, model, and practice setting.
**Funding for this study:** No funding was received for this study
**Has your study been approved by an ethics committee?** Not applicable
**Author Disclosures:**
Tobias Heye: Research/Grant Support: Siemens Healthineers
Markus Obmann: Nothing to disclose
Doreen Kraft: Employee: Siemens Healthineers
Elmar M. Merkle: Research/Grant Support: Siemens Healthineers
Paul Hehenkamp: Nothing to disclose
Michael Loser: Employee: Siemens Healthineers
Jan Vosshenrich: Nothing to disclose
Sven Kamber: Nothing to disclose

**Energy Consumption Analysis of Six MRI Generations: Implications for Sustainable Imaging**
*N. Maalouf*, F. K. E. Mankertz, J. Berger, F. Raab, J. Herrmann, K. Nikolaou, T. Küstner, S. Afat; Tübingen/DE

**Purpose:** The purpose of this study is to compare the energy consumption of six Siemens MRI systems and assess the implications for sustainability and cost efficiency.
**Methods or Background:** Aera, AvantoFit, and Sola (1.5 T) and PrismaFit, Vida, and VidaFit (3 T) scanners were evaluated under routine clinical conditions (6.2024–3.2025). Scan data were sent to the manufacturer for centralized analysis of energy consumption (kWh), power output (kW), and time-normalized power (kW/min). Deviations from the fleet mean were calculated across sequence types, with scan duration used to adjust for time-dependent variability. Scanner model and field strength (1.5 T vs 3 T) were analyzed as determinants of energy efficiency. Performance differences were visualized with deviation plots and radar charts. Statistical analysis used confidence intervals.
**Results or Findings:** Energy use differed markedly across scanners. VidaFit showed the highest deviations (+66% normalized power, +57% energy) and greatest consumption, while AvantoFit and Vida consumed least (−24% to −31% below mean). Radar analysis confirmed VidaFit as the main outlier, with other models clustering near the fleet average.
**Conclusion:** Energy use differs across Siemens MRI generations despite similar clinical operation, reflecting hardware and gradient design. Selecting lower-consumption scanners for routine exams can cut costs and reduce carbon footprint. Integrating energy-efficiency metrics into MRI procurement supports sustainable radiology without affecting diagnostic quality.
**Limitations:** A single-center study may limit generalizability, although the trends remained consistent over several months of monitoring.
**Funding for this study:** No funding was received.
**Has your study been approved by an ethics committee?** Yes
**Ethics committee - additional information:** IRB approval was obtained.
**Author Disclosures:**
Judith Herrmann: Nothing to disclose
Konstantin Nikolaou: Nothing to disclose
Florian Raab: Nothing to disclose
Saif Afat: Nothing to disclose
Josephine Berger: Nothing to disclose
Fiona Karola Elisabeth Mankertz: Nothing to disclose
Nour Maalouf: Nothing to disclose
Thomas Küstner: Nothing to disclose

**Greening with AI-MRI: Smart, Short, and Sustainable**
*F. Mariotti*, A. Borgheresi, A. Agostini, L. Reversi, M. Valenti, A. Giovagnoni; Ancona/IT
*(dottfrancescomariotti@gmail.com)*

**Purpose:** Magnetic Resonance Imaging (MRI) has high energy demands, raising sustainability concerns in abdominal applications. With growing demand for liver MRI, there is a need to balance diagnostic value with environmental impact. Abbreviated protocols (AMRI) and AI-based acceleration are promising strategies to reduce scan time and electricity use. This study quantifies potential energy and $CO_2$ savings in upper abdominal MRI while assessing trade-offs between energy neutrality and patient throughput.
**Methods or Background:** Five literature-based scenarios were modeled: (A) standard multiphasic protocol with contrast (20–30 min, ~20 kWh/exam); (B) abbreviated non-contrast (7–9 min); (C) abbreviated contrast-enhanced (10–12 min); (D) standard with AI-based acceleration (30% scan time reduction); and (E) abbreviated with AI acceleration. Assumptions included an active phase representing 40–60% of total energy, baseline active consumption of 8–12 kWh, and Italian electricity carbon intensity of 0.233 kg $CO_2$e/kWh (2024). Per-exam savings, annual throughput, and energy neutrality thresholds—the maximum exams within a 114,000 kWh/year budget—were then calculated.
**Results or Findings:** Compared with the standard protocol, abbreviated non-contrast saved 41–51% of active energy (0.96–1.70 kg $CO_2$e), abbreviated contrast 70–72% (1.35–2.24), AI ~30% (0.56–0.84), and abbreviated with AI 59–87% (1.23–2.40). With a 114,000 kWh/year budget, standard protocols allowed ~5,700 exams, abbreviated non-contrast ~7,972, abbreviated contrast ~9,268, AI ~6,705, and abbreviated with AI ~9,819. Depending on the protocol, between 25% and 72% more liver MRI exams could be performed annually without exceeding the energy budget.
**Conclusion:** Abbreviated and AI-accelerated liver MRI protocols reduce active energy use by up to 87% and allow thousands of additional exams annually within the same energy budget. Despite trade-offs between diagnostic completeness and efficiency, these strategies support greener radiology, improved productivity, and progress toward sustainable healthcare
**Limitations:** Theoretical study based on literature-derived data
**Funding for this study:** Not applicable
**Has your study been approved by an ethics committee?** Not applicable
**Author Disclosures:**
Luca Reversi: Nothing to disclose
Marco Valenti: Nothing to disclose
Andrea Agostini: Nothing to disclose
Francesco Mariotti: Nothing to disclose
Alessandra Borgheresi: Nothing to disclose
Andrea Giovagnoni: Nothing to disclose

**Carbon Footprint Comparison: CT vs MRI vs Ultrasound**
*F. Abubacker Sulaiman*; Chennai/IN
*(fasulaiman@gmail.com)*

**Purpose:** To quantitatively compare the carbon footprint and energy consumption of computed tomography (CT), magnetic resonance imaging (MRI), and ultrasound examinations, and to identify potential strategies for sustainable imaging practice.
**Methods or Background:** A prospective observational study was conducted over six months in a tertiary care imaging center. A total of 300 diagnostic procedures were analyzed—100 each of CT, MRI, and ultrasound. Energy consumption was measured using integrated power meters on imaging systems and converted to carbon dioxide equivalents ($CO_2e$) based on local emission factors. Ancillary energy use (HVAC, lighting, computing) was proportionally attributed per examination. Patient throughput and scan duration were factored to calculate per-scan carbon footprint. Data were statistically analyzed using ANOVA and correlation with machine age and utilization.
**Results or Findings:** Average carbon footprint per examination was CT: 9.2 kg $CO_2e$, MRI: 17.1 kg $CO_2e$, and Ultrasound: 0.8 kg $CO_2e$. MRI demonstrated the highest footprint due to long acquisition times and continuous cooling system requirements, whereas ultrasound showed minimal emissions. Optimization of scan protocols and standby power management reduced CT and MRI footprints by approximately 22% and 18%, respectively. Equipment utilization efficiency showed strong inverse correlation with per-scan carbon footprint ($r = –0.78$, $p < 0.001$).
**Conclusion:** Among cross-sectional imaging modalities, ultrasound remains the most sustainable, while MRI contributes the highest carbon emissions. Implementing energy-efficient hardware, optimized scheduling, and eco-mode settings can significantly mitigate environmental impact in radiology departments.
**Limitations:** Single-center design, exclusion of contrast media production impact, and regional energy variability may limit generalizability.
**Funding for this study:** No external funding was received for this study.
**Has your study been approved by an ethics committee?** Yes
**Ethics committee - additional information:** Institutional ethical committee approval was obtained.
**Author Disclosures:**
Farook Abubacker Sulaiman: Nothing to disclose

**Human-Centric AI Adoption in Swiss Radiology: A Comprehensive Radiologist Sentiment Assessment Methodology for Sustainable Technology Integration**
*S. Morozov*, N. Heracleous, B. Rizk, C. Thouly, B. Dufour, O. Novarina; Sion/CH
*(dr.morozov.sergey@gmail.com)*

**Purpose:** To develop and validate a systematic methodology for evaluating radiologist AI adoption patterns, innovation readiness, and user experience across a multi-site radiology network, providing actionable insights for sustainable AI integration in clinical practice.
**Methods or Background:** Building on four years of AI implementation since 2021 with 10 AI solutions processing 100,000 exams annually, a comprehensive mixed-methods survey was conducted among 58 radiologists across 20 sites of 3R Swiss Imaging Network in June 2025, achieving 91.4% response rate. The methodology combined quantitative metrics including Net Promoter Score (NPS) analysis for 12 AI solutions, sentiment scoring (1-5 scale), and usage frequency assessment. A composite Innovation Index was calculated incorporating AI adoption rate, usage intensity, technology relevance, and innovation sentiment. Qualitative thematic analysis categorized improvement suggestions. Statistical analysis included Shapiro-Wilk normality testing and Spearman correlations to identify predictors of AI acceptance.
**Results or Findings:** The group achieved a General Innovation Index of 73/100, positioning it in the "Early Adopter" phase among European radiology networks. AI adoption rate reached 91.4/100 with 53/58 radiologists using AI tools. Usage intensity scored 70.3/100 with 66% reporting frequent AI use. NPS varied dramatically across solutions (range: -55.3 to +86.7). Top performers included X-ray Bone Age estimation (NPS +86.7), Trauma and Orthopedic diagnostics (NPS +65.2). Critical improvement themes emerged: speed/latency (48% of concerns), advanced workflow integration (19%), and reliability issues (11%). Strong correlations were found between trust, quality perception, and clinical confidence ($r=0.66-0.68$).
**Conclusion:** This human-centric assessment framework successfully identified innovation profiles, user segments, and systematic barriers to AI adoption. The methodology enables evidence-based change management, targeted interventions, and sustainable innovation strategies in radiology practices.
**Limitations:** Longitudinal validation needed to confirm predictive value of innovation metrics.
**Funding for this study:** Institution funded.
**Has your study been approved by an ethics committee?** Not applicable
**Author Disclosures:**
Cyril Thouly: Shareholder: SMA - 3R Holding
Sergey Morozov: Research/Grant Support: GEHC, Screenpoint, Lunit, Bracco, Guerbet
Benoît Dufour: Shareholder: SMA - 3R Holding
Natalie Heracleous: Nothing to disclose
Benoît Rizk: Shareholder: SMA - 3R Holding
Octave Novarina: Nothing to disclose

**Patient Acceptance of Sustainable Practices at a Holistic Women Imaging Clinic in Egypt: WAFI-GREEN Pilot**
*H. S. Gewefel*[1], M. Amin[2], S. Khodair[2], M. Zaher[1], A. Mahmoud[2];
[1]6th of October City/EG, [2]Cairo/EG
*(hsg276@mail.harvard.edu)*

**Purpose:** To assess feasibility and patient acceptance of pragmatic sustainability measures (“green radiology”) in the Women And Fetal Imaging (WAFI) private imaging center in Giza, Egypt.
**Methods or Background:** Single-center cross-sectional survey of adult women attending mammography and or breast ultrasound at WAFI, the women age was 52.9 ± 13. An online, self-administered questionnaire containing 19 questions was completed by 82 women, capturing their acceptance across a range of domains; (1) energy-conscious scheduling (night/morning slots), (2) digital intake and electronic reports, (3) Heating, Ventilation, and Air Conditioning (HVAC) optimization with preserved comfort, (4) travel consolidation (combining services in one visit), and (5) reassurance about lower-dose X-ray protocols. Survey items were rated on a five-point Likert scale (1 = strongly disagree, 5 = strongly agree). Responses of “agree” or “strongly agree” were classified as indicating acceptance. Data were analyzed using descriptive statistics and reported as proportions (n/N).
**Results or Findings:** Eighty-two respondents completed the survey, revealing strong acceptance of green radiology practices across multiple domains. Key findings included high interest in clustered/morning appointments (80%) and a willingness to combine services in one visit (82.9%). Most participants preferred digital intake and reports (79%), with 65.7% finding them easy to use, though a small minority (14.6%) still wanted a situational paper option. Patient comfort and reassurance were also highly rated, with 89% satisfied with HVAC optimization and 86.6% confident in the safety of lower-dose X-ray protocols. Overall care satisfaction was exceptionally high at 97.6%.
**Conclusion:** Conclusion Women in a real-world breast imaging unit were highly receptive to sustainability measures. This pilot showed that targeted educational strategies improve women's knowledge and engagement with sustainable radiology practices.
**Limitations:** This study was performed at a single centre and included a relatively small number of participants.
**Funding for this study:** Acknowledgements: This work was granted funding by The European Institute for Biomedical Imaging Research (EIBIR) and The European Society of Radiology (ESR).
**Has your study been approved by an ethics committee?** Not applicable
**Author Disclosures:**
Hanan Soliman Gewefel: Consultant: Reviewing Grant Recipient: Implementation Grant Recipient: Analysis Grant Recipient: Research
Sara Khodair: Owner: Data Collection Research/Grant Support: Data collection Research/Grant Support: Data Collection Consultant: Audit
Malak Zaher: Employee: Project manager Employee: Project Manager
Ahmed Mahmoud: Nothing to disclose
Manar Amin: Nothing to disclose

**11:30 - 12:30** **Research Stage 2**

### Research Presentation Session: Cardiac

### RPS 303

### Coronary CT angiography, plaque burden and risk stratification: coronary plaque composition, adipose tissue and functional insights

**Moderator**
Iva Žuža; Rijeka/ Kostrena/HR
*(iva.zuza276@gmail.com)*

**Volumetry of coronary plaque components on CT coronary angiography: Predictor of MACE?**
*E. Ekkelenkamp*[1], S. Dasselaar[2], J. Van Dijk[1], B. Vendel[1], J. Van Dalen[1], R. Vliegenthart[3]; [1]Zwolle/NL, [2]Enschede/NL, [3]Groningen/NL

**Purpose:** Computed tomography coronary angiography (CTCA) is a valuable tool to detect coronary plaques. Plaque composition may improve risk stratification. Our aim was to establish the value of coronary plaque composition quantification in addition to standard risk factors and CAD-RADS classification to predict occurrence of MACE.
**Methods or Background:** We identified 1700 patients who underwent CTCA between 2019 and 2021 for whom 2y follow-up was available. The 63 patients who experienced a MACE including all-cause death within 2 years were 1:1 propensity-score-matched to 63 patients without MACE based on CAD-RADS and standard risk factors. In all 126 matched patients we measured plaque volumes using HU-based thresholds for non-calcified (≤ 30HU), fibrotic (31-350HU) and calcified plaques (>350HU) with commercial software (Advantage Workstation 3.2, GE Healthcare). Volume measurements were categorized into tertiles of low, middle or high plaque volume, for each plaque type. We compared plaque-volume tertiles in terms of MACE-occurrence using a Cox proportional hazards model, with lowest tertile as reference category.
**Results or Findings:** For the non-MACE and MACE groups, the median volumes of total (508 vs. 527mm3, p=0.36), non-calcified (51 vs. 58mm3, p=0.14), fibrotic (418 vs. 438mm3, p=0.32) and calcified plaque (44 vs. 38mm3, p=0.25) did not differ. Hazard ratios of total and non-calcified plaque tertiles for MACE ranged from 1.11 (p=0.77) to 1.66 (p=0.25), respectively. HR of the middle tertile for fibrotic plaque was 2.93 (p=0.04), and for the highest tertile HR 1.37 (p=0.73). Middle and high calcified plaque tertiles had HRs of 0.59 (p=0.12) and 0.60 (p=0.16), respectively.
**Conclusion:** We did not find evidence for benefit of CTCA-based volume measurements of coronary plaque components to identify patients at risk of MACE.
**Limitations:** Relatively small sample due to matched design based on MACE patient cohort.
**Funding for this study:** Not applicable.
**Has your study been approved by an ethics committee?** Not applicable
**Author Disclosures:**
Jorn Van Dalen: Research/Grant Support: GE Healthcare
Joris Van Dijk: Research/Grant Support: GE Healthcare
Eline Ekkelenkamp: Research/Grant Support: GE Healthcare
Rozemarijn Vliegenthart: Nothing to disclose
Brian Vendel: Research/Grant Support: GE Healthcare
Simon Dasselaar: Nothing to disclose

**Association of Socioeconomic Status with Coronary Artery Plaque and MACE: Insights from the DISCHARGE Trial**
*A-C. Stahl*[1], K. Schulze[1], R. Bockelmann[1], F. Biavati[1], P. Maurovich-Horvat[2], K. F. Kofoed[3], J. Dodd[4], M. Dewey[1]; [1]Berlin/DE, [2]Budapest/HU, [3]Copenhagen/DK, [4]Dublin/IL
*(ann-christine.stahl@charite.de)*

**Purpose:** To assess the association between socioeconomic status (SES), measured by income, and coronary artery plaque on computed tomography (CT) as well as major adverse cardiovascular events (MACE), defined as cardiovascular death, nonfatal myocardial infarction, or nonfatal stroke in both randomisation groups of the Diagnostic Imaging Strategies for Patients With Stable Chest Pain and Intermediate Risk of Coronary Artery Disease (DISCHARGE) trial.
**Methods or Background:** This is a post-hoc analysis of the multicentre European DISCHARGE trial. Patients ≥30 years with stable chest pain referred for invasive coronary angiography (ICA) were randomised to ICA or CT. SES was determined by monthly net income, adjusted for country-specific price levels and normalised to the national median in the year of randomisation. Patients were categorised into low (≤1) and high (>1) SES groups. Cox survival analysis was used to assess associations between SES and MACE while a general linear model was used for associations with plaque volumes.
**Results or Findings:** Of 3561 patients, SES data were available for 2624 (1380 low SES). CT-based quantitative plaque analysis was performed in 1232 patients. After adjustment for age, sex, and cardiovascular risk factors, there was no significant difference in MACE between the low and high SES group (hazard ratio: 1.08, p=0.76). Coronary plaque volume was numerically higher in the low SES group, but differences were not statistically significant for total plaque volume (TPV, p=0.48), non-calcified plaque volume (p=0.66), or calcified plaque volume (p=0.36). SES remained not independently associated with TPV after adjustment (p=0.78).
**Conclusion:** SES was not associated with MACE or plaque volume suggesting that clinical risk factors outweigh socioeconomic influences in stable chest pain patients.
**Limitations:** SES was based on self-reported income, which may introduce misclassification. Therefore, residual confounding cannot be excluded.
**Funding for this study:** Funded by the European Union Seventh Framework Program and others; DISCHARGE ClinicalTrials.gov number: NCT02400229.
**Has your study been approved by an ethics committee?** Yes
**Ethics committee - additional information:** The study was approved by ethics committee at Charité – Universitätsmedizin Berlin (EA1/294/13) as the coordinating centre, by the German Federal Office for Radiation Protection, and by local or national ethics committees.
**Author Disclosures:**
Kenrick Schulze: Nothing to disclose
Jonathan Dodd: Grant Recipient: Dodd has received grant support from the Irish Lung Foundation, the St. Vincent'sHospital Group Foundation, University College Dublin and the FP7 Program of the European Commission for the randomized multicenter DISCHARGE trial (603266-2, HEALTH-2012.2.4.-2) Author: author in the Stat-Dx book Series Diagnostic Imaging – Cardiovascular and the textbook CT and MRI in Cardiology, Elsevier Other: Associate Editor of Radiology, Respirology and the Quarterly Journal of Medicine Board Member: Editorial Board member of Radiology Cardiothoracic Imaging Speaker: Dodd has received speaker fees from boehringer ingelheim.
Klaus F. Kofoed: Nothing to disclose
Pál Maurovich-Horvat: Nothing to disclose
Robin Bockelmann: Nothing to disclose
Marc Dewey: Other: Editor: Cardiac CT (Springer Nature) Other: Hands-on cardiac CT courses (www.ct-kurs.de) Grant Recipient: Grants: EU (EC-GA 603266 in HEALTH.2013.2.4.2-2) DFG (DE 1361/14-1, DE 1361/18-1, BIOQIC GRK 2260/1, Radiomics DE 1361/19-1 [428222922] and 20-1 [428223139] in SPP 2177/1), GUIDE-IT (DE 1361/24-1), Berlin University Alliance (GC_SC_PC 27), G-BA (01NVF23002), Berlin Institute of Health (Digital Health Accelerator) Other: Institutional research agreements: Siemens, General Electric, Philips, Canon Patent Holder: Patent on fractal analysis of perfusion imaging (jointly with Florian Michallek, EPO 2022 EP3350773A1, and USPTO 2021 10,991,109, approved)
Ann-Christine Stahl: Nothing to disclose
Federico Biavati: Nothing to disclose

**Epicardial Adipose Tissue CT Attenuation in Kawasaki Disease and Association with Coronary Artery Aneurysms**
*U. Anser*, S. Azhe, L. Wen; Chengdu/CN
*(usama.anser2017@gmail.com)*

**Purpose:** To quantify epicardial adipose tissue (EAT) CT attenuation in children with Kawasaki disease (KD) and evaluate its associations with demographic and clinical factors, and the risk of adverse cardiovascular outcomes, aiming to establish EAT as a potential imaging biomarker of coronary risk.
**Methods or Background:** This retrospective study included 100 KD patients (mean age 8.2 ± 3.9 years) and 35 controls (mean age 8.4 ± 2.8 years) who underwent non-contrast cardiac CT between 2018 and 2024. EAT density was segmented manually (–190 to –30 HU) from the pulmonary bifurcation to the diaphragm. CT scans were performed in both acute and chronic stages of KD, reflecting the heterogeneous timing of imaging in clinical practice. Group comparisons were performed with the Mann–Whitney U test, correlations with Spearman's rho, and predictors of EAT density with linear regression. Statistical significance was set at p<0.05.
**Results or Findings:** EAT CT attenuation was significantly lower in KD patients (–83.9 ± 2.7 HU) compared with controls (–86.3 ± 2.8 HU)(p<0.001). Within KD, lower EAT CT attenuation was associated with thrombosis (p=0.04) and calcification (p=0.03). A moderate positive correlation was observed between EAT CT attenuation and coronary Z-score (ρ=0.53, p<0.001). In regression analysis, both Z-score (β=0.27, 95% CI: 0.17–0.38, p<0.001) and age (β=0.15, 95% CI: 0.01–0.30, p=0.035) independently predicted higher EAT CT attenuation, whereas sex and BMI were not significant. The multivariable model explained 29% of the variance in EAT density ($R^2$=0.293).
**Conclusion:** These findings suggest that EAT CT attenuation may serve as a potential imaging marker for coronary risk in KD, although larger studies are needed for validation.
**Limitations:** None
**Funding for this study:** Supported by the National Natural Science Foundation of China.
**Has your study been approved by an ethics committee?** Yes
**Ethics committee - additional information:** This study was approved by the Institutional Ethics Committee of West China Second University Hospital, Sichuan University. Given the retrospective design and anonymized use of clinical and imaging data, the requirement for informed consent was waived. All procedures were conducted in accordance with the Declaration of Helsinki (2013 revision).
**Author Disclosures:**
Lingyi Wen: Nothing to disclose
Shiganmo Azhe: Nothing to disclose
Usama Anser: Nothing to disclose

**CT angiography analysis of the characteristics of epicardium and pericoronary adipose tissue before coronary artery plaque formation in patients with type 2 diabetes**
X. Chen[1], Z. Li[1], X. Wan[1], T. Xu[1], *X. Liu*[2], Y. Liao[2], Y. Xu[2]; [1]Dongguan/CN, [2]Guangzhou/CN
*(xiaomin.liu@philips.com)*

**Purpose:** To investigate pre-plaque epicardial adipose tissue (EAT), pericoronary fat attenuation index (FAI), and CT-derived fractional flow reserve (CT-FFR) on coronary computed tomography angiography (CCTA) as biomarkers for early cardiovascular risk in type 2 diabetes mellitus (T2DM) patients.
**Methods or Background:** We retrospectively collected 268 patients(from two hospitals, January 2022 – December 2024) with suspected coronary heart disease, normal coronary arteries on CCTA, newly diagnosed prediabetes(Pre-DM, n=132) or T2DM (n = 136) without prior treatment, and 142 healthy controls with normal CCTA and glucose profiles. Differences in EAT, FAI, and CT-FFR among the three groups were compared. Continuous variables were analyzed via Kruskal-Wallis H test with Bonferroni pairwise comparisons, and categorical variables via chi-square test.
**Results or Findings:** Kruskal-Wallis H test revealed significant intergroup differences in multiple parameters (EATV, EATV/BMI, FAI-Max, FAI-Mean, FAI-LAD, FAI-LCX; all p<0.05). Post-hoc analyses showed that most parameters significantly differed between the healthy and T2DM groups (EATV, EATV/BMI, FAI-Max, FAI-Mean, FAI-LAD, FAI-LCX; all p<0.05), while only FAI-Max significantly differed between healthy and Pre-DM groups (P=0.025). EATV was the sole parameter differing between Pre-DM and T2DM groups (P=0.03). CT-FFR, including all individual branches and the mean value, did not differ significantly among groups (all P>0.05).
**Conclusion:** In patients with early T2DM patients (including Pre-DM) but no plaques or functional myocardial ischemia (CT-FFR > 0.8), EATV and FAI (especially FAI-Max) were significantly higher than in healthy individuals. FAI-Max changed significantly at the Pre-DM stage, and EATV further increased with T2DM progression. CCTA-based Imaging features (such as EATV and FAI-Max) are expected to become non-invasive imaging biomarkers for assessing early cardiovascular risk in T2DM patients, providing important information for early clinical intervention.
**Limitations:** Single-center study with a smal l cohort
**Funding for this study:** None
**Has your study been approved by an ethics committee?** Yes
**Ethics committee - additional information:** The Ethics Committee granted a waiver of informed consent for this retrospective study.
**Author Disclosures:**
Zhaoyong Li: Nothing to disclose
Xiaomin Liu: Nothing to disclose
Xinyu Wan: Nothing to disclose
Tuanxin Xu: Nothing to disclose
Xudong Chen: Nothing to disclose
Yongzhou Xu: Nothing to disclose
Yuting Liao: Nothing to disclose

**Association between allergic inflammatory diseases and computerized tomography-derived coronary inflammation and atherosclerotic disease extent and phenotype: the ALLERGIC-CAD study**
*C. Martini*, F. L. Gurgoglione, E. Ridolo, A. A. Palumbo, G. Cicala, F. Nicoletta, C. Baldari, M. De Filippo, G. Niccoli; Parma/IT
*(chiara.martini@unipr.it)*

**Purpose:** To evaluate whether patients with suspected chronic coronary syndromes (CCS) and allergic inflammatory diseases show differences in coronary CT angiography (CCTA)-derived atherosclerotic markers — including plaque extent, vulnerable phenotype, and pericoronary fat attenuation index (pFAI) — compared to non-allergic patients.
**Methods or Background:** Allergic inflammatory diseases are linked to higher risks of myocardial infarction and major adverse cardiovascular events. CCTA detects vulnerable plaques and inflammation markers, such as pFAI, enhancing risk stratification. This study analyzed patients with suspected CCS undergoing clinically indicated CCTA at Parma University Hospital, stratified by allergic history. Plaque burden, vulnerable phenotype (≥2 high-risk features: positive remodeling, low-attenuation plaque, napkin-ring sign, spotty calcification), and pFAI were assessed at patient and lesion levels.
**Results or Findings:** A total of 289 patients (58 allergic, 20.1%) and 953 lesions were analyzed. CAD prevalence, extent, and severity were similar between groups. Allergic patients showed higher pFAI values (RCA: -65.72 vs -69.36, p=.012; LAD: -64.64 vs -68.81, p=.022; LCX: -61.36 vs -63.05, p=.024) and more vulnerable plaques (53 vs 186, p<.001), including more LAPs (51 vs 127, p<.001), spotty calcifications (31 vs 116, p=.015), and napkin-ring signs (27 vs 67, p<.001). Diabetes (OR 1.143, p<.001), allergy (OR 1.122, p=.003), and pFAI-RCA (OR 1.004, p=.014) predicted vulnerable plaque phenotype.
**Conclusion:** In suspected CCS, CAD prevalence and severity were similar regardless of allergy, but allergic patients showed greater pericoronary inflammation and more vulnerable plaques.
**Limitations:** - Single-center design - Partial inclusion of allergic diseases - Incomplete allergy characterization - Lack of systemic inflammatory markers - Exclusion of acute/prior CVD patients - No clinical follow-up
**Funding for this study:** Nothing to declare
**Has your study been approved by an ethics committee?** Yes
**Ethics committee - additional information:** Protocol number of approval by the Ethics Committee of the University Hospital of Parma (AOUPR): 150/2023/OSS/AOUPR SIRER ID 5860
**Author Disclosures:**
Filippo Luca Gurgoglione: Nothing to disclose
Anselmo Alessandro Palumbo: Nothing to disclose
Gloria Cicala: Nothing to disclose
Massimo De Filippo: Nothing to disclose
Erminia Ridolo: Nothing to disclose
Francesca Nicoletta: Nothing to disclose
Giampaolo Niccoli: Nothing to disclose
Chiara Martini: Nothing to disclose
Chiara Baldari: Nothing to disclose

**Hemodynamic force derived from cardiac MRI outperforms strain imaging in identifying heart failure with preserved ejection fraction among patients with acute myocarditis**
*Y. Hou*, H. Zhou, L. Weihua; Changsha/CN

**Purpose:** To investigate the diagnostic potential of left ventricular (LV) hemodynamic force (HDF) parameters to differentiate between acute myocarditis patients with heart failure with preserved ejection fraction (HFpEF) and those without heart failure (non-HF), while systematically comparing their performance against conventional LV strain parameters.
**Methods or Background:** We retrospectively analysis included 226 consecutive patients with acute myocarditis stratified by heart failure status: non-heart failure (non-HF) group and HF group (with preserved ejection fraction defined as LVEF≥50%). HDF parameters were calculated (I) as the root mean square of the apex-base directions during systole [HDF(Sys)] and diastole [HDF(Dia)]; (II) as the force ratios of the lateral-septal/apex-base directions during systole [R-Sys)], and diastole [R-(Dia)]. Additionally, LV global longitudinal strain (GLS), global circumferential strain (GCS) and global radial strain (GRS) were measured using a CMR feature tracking technique.
**Results or Findings:** Among 226 myocarditis patients, 53 had HF [HFpEF: 20 (8.8%); HFmrEF/HFrEF: 33 (14.6%)]. Compared to the non-HF group, HFpEF patients exhibited: I. Reduced LVEF (53.55±4.80 vs. 57.78±4.68, P=0.011); II. Impaired left ventricular strain parameters (LVGLS: −20.23±4.02 vs. −22.73±3.77, P=0.020); III. Increased R-Sys (19.49±7.17 vs. 12.90±3.89, P<0.001). R-Sys (AUC 0.836, cutoff 14.40%) distinguished HFpEF with 85.0% sensitivity and 76.3% specificity, outperforming LVEF (AUC 0.758, cutoff 51.98%) and GLS (AUC 0.675, cutoff 21.48%). R-Sys's discriminative power was significantly better than GLS (AUC 0.836 vs 0.675, p=0.043).

**Conclusion:** The LV hemodynamic force parameter (R-Sys) showed greater diagnostic accuracy than myocardial strain parameter (GLS) for detecting HFpEF in acute myocarditis patients.
**Limitations:** This study is a retrospective study with potential selection bias.
**Funding for this study:** This study was supported by the National Natural Science Foundation of China (82071984, 82071894, and 91959117), the Science and Technology Innovation Program of Hunan Province (2020RC4007), and The Scientific Research Program of FuRong Laboratory 2024PT5110.
**Has your study been approved by an ethics committee?** Yes
**Ethics committee - additional information:** Medical Ethics Review Committee of Xiangya Hospital Central South University (2025061065)
**Author Disclosures:**
Yangzhen Hou: Nothing to disclose
Liao Weihua: Nothing to disclose
Hui Zhou: Nothing to disclose

**Coronary sinus: added value to estimate mitral regurgitation severity**
G. Cabrelle, A. Lupi, G. Ruperti, F. Mastro, M. Scordari, *T. Melis*, C. Zanon, G. Gerosa, A. Pepe; Padua/IT

**Purpose:** To evaluate whether coronary sinus (CS) size measured by CMR can discriminate between moderate and severe mitral regurgitation (MR).
**Methods or Background:** Retrospective, single-centre study from Padua University Hospital including patients referred for CMR from July 2024 to June 2025, affected by moderate-severe MR. Exclusion criteria: significant non-mitral valvular disease, history of cardiac surgery, major congenital heart disease, persistent left superior vena cava and ICDs. Cine SSFP sequences (width 6 mm, gap 0) sagittal to mitral anulus were analysed. CS maximum/minimum sagittal caliper and area were measured within 1 cm from CS ostium, together with indexed atrial and ventricular volumes, ejection fraction, mitral regurgitant volume and fraction, global longitudinal and circumferential strain.
**Results or Findings:** Thirty-four patients (66 ± 11 years, 17F). Moderate MR: 21 patients; severe MR: 13 patients. Ten patients underwent surgery. CS area showed a significant positive association with right indexed atrial volume ($\beta$ = 3.15, p = 0.004). In multiple regression analysis, CS area was the strongest independent predictor of MR severity ($\beta$ = 0.95, p < 0.0001), with adjusted $R^2$ = 0.75 for the model (p = 0.0002). A CS maximum caliper threshold of 14 mm and area of 0.97 $cm^2$ provided the best discriminatory performance.
**Conclusion:** CS maximum caliper and area emerge as predictors of MR severity and may serve as complementary parameters to improve diagnostic accuracy and pre-surgical stratification by CMR in patients with MR.
**Limitations:** Single center study Sparse population
**Funding for this study:** None
**Has your study been approved by an ethics committee?** Not applicable
**Author Disclosures:**
Amalia Lupi: Nothing to disclose
Alessia Pepe: Nothing to disclose
Giulio Cabrelle: Nothing to disclose
Chiara Zanon: Nothing to disclose
Gino Gerosa: Nothing to disclose
Giovanni Ruperti: Nothing to disclose
Florinda Mastro: Nothing to disclose
Matteo Scordari: Nothing to disclose
Thomas Melis: Nothing to disclose

**11:30 - 12:30** **Research Stage 6**

### Research Presentation Session: Chest Hot Topic with Keynote Lecture

### RPS 304

### Hot Topic: AI-driven lung cancer screening

**Moderator**
Guillaume Chassagnon; Paris/FR
**Author Disclosures:**
Guillaume Chassagnon: Consultant: Gleamer, Insmed, Speaker: Chiesi, Boehringer Ingelheim

**Keynote Lecture**
Justus Erasmus Roos; Lucerne/CH
*(justus.roos@luks.ch)*

**Assessing Low-Dose CT Image Quality in ESTI's Lung Cancer Screening Protocol: Insights into Patient Size Influence**
*D. C. Rotzinger*, C. Pozzessere, G. Fahrni, T. Saliba, C. Chevallier, Y. Siddiki, V. Vitzthum, C. Von Garnier, L. Gallego Manzano; Lausanne/CH
*(david.rotzinger@chuv.ch)*

**Purpose:** To qualitatively and quantitatively evaluate the European Society of Thoracic Imaging (ESTI) lung cancer screening (LCS) low-dose CT protocol's image quality of the at three weight-based CTDIvol levels, and to assess the effect of participant size on image quality.
**Methods or Background:** ASiR-V80 lung-kernel reconstructions of 173 LCS participants (69 females, mean age 62.9 years) were retrospectively analyzed, and size metrics (weight, thoracic perimeter) obtained. Three readers independently rated diagnostic quality, noise and sharpness on a 5-point Likert scale. Mean, median, standard deviation, categorical percentage, and inter-reader agreement were calculated. Dose-normalized contrast-to-noise ratio (CNR/CTDIvol) was evaluated as a function of size by linear regression.
**Results or Findings:** Mean weight and thoracic perimeter were 78kg (43-180) and 107cm (81-142.6). Median CTDIvol was 0.40mGy (0.40–0.51, n=5), 0.85mGy (0.60–2.95, n=96), and 1.69mGy (0.86–3.06, n=72). Reader scores (mean/median/SD) were 4.02/4/0.73 for quality, 3.95/4/0.73 for noise, and 4.06/4/0.72 for sharpness. Quality was rated good/excellent in 77.8% of cases, moderate in 20.4%, and poor/non-diagnostic in 1.7%. Noise and sharpness were rated minimal/excellent in 67.6% and 78% of cases, respectively. Inter-reader agreement was 59.0% for quality, 47.4% for noise, and 58.4% for sharpness. CNR/CTDIvol decreased with increasing weight and perimeter ($p \leq 0.001$), with perimeter explaining more variance in heavier patients ($R^2$ 0.432 vs 0.168 in >80kg; 0.250 vs 0.291 in 50–80kg). At the conservative 25th-percentile, log-CTDI models suggested dose tiers of ~0.4mGy at <63kg/<30cm, 0.9mGy at 87kg/34cm, and 1.6mGy at 98kg/36.3cm.
**Conclusion:** The ESTI LCS protocol provides high-quality images with potential for further dose reduction. Thoracic perimeter appears more robust than weight in balancing image quality and dose, particularly in heavier patients.
**Limitations:** Retrospective, single-center design; small <50kg subgroup sample size; single reconstruction algorithm
**Funding for this study:** No external funding
**Has your study been approved by an ethics committee?** Yes
**Ethics committee - additional information:** Ethics Committee of the Canton de Vaud - CER-VD
**Author Disclosures:**
Chiara Pozzessere: Nothing to disclose
Veronika Vitzthum: Nothing to disclose
Christophe Von Garnier: Nothing to disclose
David Christian Rotzinger: Nothing to disclose
Lucia Gallego Manzano: Nothing to disclose
Yasmina Siddiki: Nothing to disclose
Thomas Saliba: Nothing to disclose
Guillaume Fahrni: Nothing to disclose
Christine Chevallier: Nothing to disclose

**Training and Education in Lung Cancer Screening: Preliminary Results from a European Survey**

*R. Mura*[1], A. Kerpel-Fronius[2], T. G. Blum[3], J. Chorostowska-Wynimko[4], P. Zolda[1], H. Prosch[1]; [1]Vienna/AT, [2]Budapest/HU, [3]Berlin/DE, [4]Warsaw/PL

**Purpose:** To explore the current status of lung cancer screening (LCS) training practices, identify existing gaps, and further define key competencies to be included in LCS educational curricula.
**Methods or Background:** As part of the European SOLACE project, a structured cross-sectional survey, consisting of 11 closed- and open-ended items, was designed and administered to a panel of European LCS experts. Participants were contacted via email and invited to individual Zoom interviews (May-August 2025), each lasting approximately 30 minutes. Descriptive statistics were used for data analysis.
**Results or Findings:** Seventeen LCS experts were interviewed (response rate 85%, 17/20) from 11 European countries, including 10 radiologists, 4 pulmonologists, 2 thoracic surgeons, and 1 project manager. Reported training activities ranged from established programs (4/11 countries, mandatory in 2/4), to learning initiatives (4/11) and/or planned programs (4/11). Radiologists (100%), pulmonologists (71%), and thoracic surgeons (67%) were identified as the main target groups for training. On a 6-point Likert scale (0= not important, 5= extremely important), experts indicated limited awareness of training needs as the most relevant gap (mean score 3.1±2.2). Key educational areas to be strengthened included management of incidental findings (4.2±0.6), collaborative (4.1±1.2) and communicative skills (3.8±1.5). Among essential competencies for professionals involved in LCS, the top-rated were familiarity with guideline-based nodule management (4.7±0.5), proficiency in managing of incidental findings (4.5±0.6), and basic knowledge of AI-tools (4.5±0.8).
**Conclusion:** The findings reveal a highly heterogeneous LCS training landscape across Europe, further challenged by limited awareness of training needs, and highlight the importance of developing shared European curricula. Priorities include technical competencies as well as collaborative and communication skills.
**Limitations:** Not applicable.
**Funding for this study:** The SOLACE project is co-funded under the EU4Health Programme 2021–2027 under grant agreement no. 101101187. Funded by the European Union. Views and opinions expressed are however those of the author(s) only and do not necessarily reflect those of the European Union or HaDEA. Neither the European Union nor the granting authority can be held responsible for them.
**Has your study been approved by an ethics committee?** Not applicable
**Author Disclosures:**
Joanna Chorostowska-Wynimko: Nothing to disclose
Anna Kerpel-Fronius: Nothing to disclose
Pamela Zolda: Nothing to disclose
Helmut Prosch: Speaker: AstraZeneca, Boehringer Ingelheim, Janssen, MSD, Sanofi
Torsten Gerriet Blum: Nothing to disclose
Rebecca Mura: Nothing to disclose

**Artificial Intelligence–Supported Early Detection of Lung Cancer from Chest X-Ray in Routine Clinical Practice: Real-World, Multicenter Study Across Czech Hospitals**

K. Kvaková[1], *D. Kvak*[2], J. Dandár[2], M. Stastny[2], J. Olejko[3], J. Kultan[3], J. Gerold[4], A. Kotoucova[5], C. Vojtek[6], P. Struna[7], E. Dvorackova[8], J. F. Smetana[8]; [1]Brno/CZ, [2]Prague/CZ, [3]Olomouc/CZ, [4]Sumperk/CZ, [5]Benešov/CZ, [6]Nový Jičín/CZ, [7]Slaný/CZ, [8]Kladno/CZ
*(daniel.kvak@carebot.com)*

**Purpose:** Although AI systems for chest X-ray (CXR) interpretation may enable earlier detection of thoracic malignancy, large-scale real-world evidence remains limited. We present results of a joint Carebot–Bristol Myers Squibb program integrating AI decision-support into routine workflows to evaluate feasibility, flagged-case yield, and downstream actions for suspected malignancy (primary lung cancer or pulmonary metastases).
**Methods or Background:** All CXRs across nine Czech hospitals (regional and tertiary; two pneumo-oncology centers) from Jan 1–Jun 30, 2025 were automatically analyzed with commercial-stage AI software (Carebot AI CXR; Carebot s.r.o., Czechia). The AI flagged abnormalities; a joint panel of hospital and interim Carebot radiologists classified exams as suspicious for thoracic malignancy. Suspicious cases entered fast-track diagnostics. Primary outcomes: (i) proportion flagged as suspicious, (ii) patients referred for diagnostic work-up, and (iii) confirmed malignancies.
**Results or Findings:** Among 96 459 CXRs, the algorithm identified abnormalities in 16 030 exams (16.6%). Multidisciplinary review classified 837 CXRs (0.87%) as suspicious. Follow-up status was available in 561/837 (67.0%): 211 (25.2% of all suspicious; 37.6% of those with status) were referred for immediate diagnostic work-up, 350 (41.8% of all; 62.4% of recorded) were cleared without further work-up, and 276 (33.0%) remain under evaluation. Among the 211 investigated, 54 previously undiagnosed thoracic cancers were detected, 70 confirmed known malignancies or pulmonary metastases, 38 await confirmation of nodule origin (biopsy or PET/CT), 20 entered long-term radiologic surveillance, and 29 were recalled owing to missing clinical data.
**Conclusion:** In this single-country, real-world, multicenter analysis of routine CXR exams during H1-2025, the Carebot–BMS AI triage workflow enabled early identification of thoracic malignancies within routine care while triaging <1% of exams for expedited review. Ongoing follow-up will further characterize the scalability of this AI-supported workflow.
**Limitations:** This observational study lacks a contemporaneous control.
**Funding for this study:** Carebot s.r.o. and Bristol Myers Squibb supported software integration and coordination; no public grant funding was received.
**Has your study been approved by an ethics committee?** Not applicable
**Author Disclosures:**
Alena Kotoucova: Nothing to disclose
Jiri Gerold: Nothing to disclose
Pavel Struna: Nothing to disclose
Juraj Kultan: Nothing to disclose
Jakub Dandár: Employee: Carebot s.r.o.
Eliska Dvorackova: Nothing to disclose
Karolína Kvaková: Nothing to disclose
Daniel Kvak: Board Member: Carebot s.r.o.
Cyril Vojtek: Nothing to disclose
Marek Stastny: Employee: Bristol Myers Squibb
Jakub Olejko: Nothing to disclose
Jan František Smetana: Nothing to disclose

**The potential role of Digital Tomosynthesis in improving the efficiency of Lung Cancer Screening at reduced radiation doses, costs and radiologists burden**

*N. Shabshin*[1], Y. Kimmel[2], M. Armoni[3], Y. Schiffenbauer[2], A. Grubstein[3], L. Roshkovan[1], A. Iannessi[4], A. F. Nitu[5], E. Atar[3]; [1]Philadelphia, Afula/IL, [2]Petah Tikva/IL, [3]Petach Tikva/IL, [4]Nice/FR, [5]Bucharest/RO
*(nogah.shabshin@gmail.com)*

**Purpose:** Lung Cancer Screening (LCS) programs are expanding worldwide. However, barriers including high costs, radiologist shortages, and radiation concerns remain. Within LCS populations, 84% present Lung-RADS 1,2, requiring 12-month follow-up, while scores 0,3,4 require closer monitoring. Chest Digital Tomosynthesis (DTS) offers lower radiation, faster reading, and reduced costs compared to CT. Prior studies found DTS may have a potential role in LCS. This study aims to evaluate whether cold-cathode DTS is comparable to CT when classifying patients to 2 groups: A:12-month follow-up (Lung-RADS 1,2) vs. B closer follow-up/workup (Lung-RADS 0,3,4).
**Methods or Background:** A cohort of 38 patients with a diagnostic chest CT underwent a supine DTS (Nanox.ARC). Three radiologists reviewed the DTS and CTs blindly and independently maintaining a one-month gap between modalities. Patients were classified into groups A and B. The classification was compared between DTS and CT. Analyses included negative and positive predictive value (NPV, PPV), inter/intra-reader agreement, and concordance between DTS and CT.
**Results or Findings:** On both CT and DTS, 25 patients were categorized as group B and 13 as group A. None of the patients migrated from one group to the other when compared to CT and therefore DTS and CT showed no difference in follow-up recommendations. NPVs ranged 72.7–90.0% and PPVs 87.5–92.0%. Inter-reader agreement for DTS was comparable to CT (91.4%, 82.9%, 88.6% vs. 88.6%, 82.9%, 88.6%). Inter- and intra-reader agreement were strong, with kappa values >0.65.
**Conclusion:** Preliminary results suggest DTS can classify patients to those who can continue with 12-months follow-up (Lung-RADS 1–2) and others that require closer follow-up/workup. DTS can potentially reduce the use of CT and thus reduce radiation, costs, and reading time in LCS..
**Limitations:** small population of clinical patients, not LCS patients
**Funding for this study:** Study was funded by Nano-x Imaging
**Has your study been approved by an ethics committee?** Yes
**Ethics committee - additional information:** IRB approvals were granted
**Author Disclosures:**
Eli Atar: Nothing to disclose
Mor Armoni: Nothing to disclose
Leonid Roshkovan: Nothing to disclose
Andra- Florina Nitu: Nothing to disclose
Yael Schiffenbauer: Employee: Nano-x imaging
Nogah Shabshin: Consultant: Nano-x imaging
Ahuva Grubstein: Nothing to disclose
Antoine Iannessi: Nothing to disclose
Yotam Kimmel: Employee: Nano-x imaging

**Italung2-CCM lung cancer screening pilot: quantitative LDCT-analysis of emphysema and coronary visual score in a baseline cohort of 663 patients as potential risk-based prognostic stratification**

L. Gozzi, *I. Cecchi*, D. Cozzi, E. Cavigli, G. Picozzi, G. Gorini, A. Bindi, C. Moroni, V. Miele; Firenze/IT
*(ilaria.cecchi@unifi.it)*

**Purpose:** To investigate the association between emphysema, assessed both visually and quantitatively, socio-demographic, smoking, clinical variables, and radiological signs of lung cancer (LC) and coronary artery calcifications (CAC), in a population of 663 patients enrolled in the Tuscan Italung2-CCM lung cancer screening (LCS) programme.
**Methods or Background:** We included 663 patients aged 55–75 years, with ≥30 pack-years smoking history. Baseline low-dose CT (LDCT) was evaluated both visually for emphysema/CAC and quantitatively using Pulmo 3D Syngo.Via software (LV, MLD, LAV, P15).
**Results or Findings:** Among the participants, 96.1% had negative/downgraded LDCT findings, while 26 people (4%) were classified as Lung-RADS 4B/4X; 16 of them were confirmed lung cancers. Emphysema was visually detected in 43% of participants and was significantly more frequent in men (OR=1.60), heavy smokers ≥ 40 pack-years (OR=1.96), and LC patients (OR=8.76). Quantitative emphysema values correlated with male sex, age and former smoker status. CAC was present in 67% of scans, more common in men, older participants, and smokers, but showed no independent association with LC and emphysema. Visual scoring detected more emphysema cases (288) compared to software-based quantification (171), likely due to the masking effect of smoking-induced bronchiolitis on density-based measurements.
**Conclusion:** This study confirms emphysema as an independent predictor of LC, while CAC reflects shared cardiovascular risks without independent LC association. Visual evaluation outperforms quantitative assessment in detecting emphysema, especially in current smokers, highlighting the need to integrate standardised evaluation of emphysema, CAC, and smoking history in LCS lung programmes.
**Limitations:** The limitations of the study are inter-scanner variability across centres, potentially affecting CT measures. Moreover, focusing our analysis purely on subjects enrolled in the ITALUNG2-CCM cohort limits the generalisability of its findings. Future studies should harmonize protocols and expand recruitment for broader external validity.
**Funding for this study:** No funding was received for this study.
**Has your study been approved by an ethics committee?** Not applicable
**Author Disclosures:**
Alessandra Bindi: Nothing to disclose
Vittorio Miele: Nothing to disclose
Ilaria Cecchi: Nothing to disclose
Giulia Picozzi: Nothing to disclose
Luca Gozzi: Nothing to disclose
Edoardo Cavigli: Nothing to disclose
Giuseppe Gorini: Nothing to disclose
Chiara Moroni: Nothing to disclose
Diletta Cozzi: Nothing to disclose

**Deep learning to predict mortality risk from lung cancer screening CT in heavy smokers**

*J. Jahn*, R. T. Schirrmeister, F. Bamberg, J. Weiß; Freiburg im Breisgau/DE
*(johannes.jahn94@gmx.de)*

**Purpose:** Altered body composition (BC), such as reduced muscle mass or excess adipose tissue, is an independent predictor of cardiovascular and cancer-related mortality. It remains unclear whether deep learning can estimate mortality risk directly from chest CT slices. Here, we developed a deep learning model (CTchest-risk) to estimate mortality risk from lung screening chest CTs in heavy smokers.
**Methods or Background:** Data from the CT arm of the National Lung Screening Trial (NLST) were used. CTchest-risk was developed in two steps: (1) segmentation of skeletal muscle (SM), subcutaneous adipose tissue (SAT), and intramuscular adipose tissue (IMAT); (2) training of a second model using segmentation masks combined with five axial slices centered at the thoracic vertebra T5 to predict mortality. The training dataset included 14,208 participants (39,304 individual scans). Independent testing was performed on 8,971 individuals not used for development. Primary endpoint was 6-year mortality. CTchest-risk was categorized into three groups (<15%; 15–85% [reference]; >85%). Survival was assessed with Kaplan–Meier analysis and Cox regression adjusted for age, sex, race, BMI, smoking status, diabetes, hypertension, and history of stroke or heart disease. Model performance was evaluated with Harrell's C-index and nested likelihood-ratio tests (LRT).
**Results or Findings:** In the test cohort (mean age 61.3±5 years; 41.0% female), 462 deaths (5.1%) occurred over a median follow-up of 6.5 years. Kaplan–Meier estimates showed a graded association between CTchest-risk groups (log-rank p<0.0001). High-risk individuals (>85%) had 38% higher adjusted mortality compared with the reference group (aHR 1.38, 95% CI 1.11–1.71, p=0.004). In nested model comparison, adding CTchest-risk to clinical factors improved prognostic performance (C-index 0.725 vs. 0.731; LRT p<0.001).
**Conclusion:** CTchest-risk identifies high-risk individuals from lung screening chest CT and predicts mortality beyond traditional risk factors in heavy smokers.
**Limitations:** No external validation set.
**Funding for this study:** No funding was received for this study.
**Has your study been approved by an ethics committee?** Yes
**Ethics committee - additional information:** All NLST-participants provided written informed consent for the original trial and secondary use of the data was approved by the local IRB.
**Author Disclosures:**
Jakob Weiß: Nothing to disclose
Robin Tibor Schirrmeister: Nothing to disclose
Fabian Bamberg: Nothing to disclose
Johannes Jahn: Nothing to disclose

**Development and validation of deep learning model for pulmonary nodule detection**

*R. C. Yarol*, S. Gezer, E. Unel, D. Unay, O. Dicle; Izmir/TR
*(raifyarol@gmail.com)*

**Purpose:** This study aimed to develop a deep learning based decision support system to assist radiologists in the detection, follow-up, and diagnostic evaluation of nodules on thorax CT images.
**Methods or Background:** Patients who underwent thorax CT and had at least one pulmonary nodule between January 2022 and December 2023 at one institution were retrospectively screened. A dataset consisting of 2,152 nodules from 205 patients was compiled. In addition to nodule annotations, the location (intrapulmonary, subpleural, perivascular) and density (solid, semisolid, ground-glass) of each nodule were recorded for analysis. The dataset was divided into training and testing subsets. A 2D U-Net-based model was employed for lung and lobar segmentation. Vessel structures were labeled using a thresholding method. A 3D U-Net model was utilized for nodule detection and segmentation. The model's performance on the test set was evaluated using sensitivity, specificity, and F1-score. Causes of false positives and false negatives were retrospectively analyzed.
**Results or Findings:** The developed model achieved a sensitivity of 0.979, specificity of 0.774, and F1-score of 0.864. The models sensitivity for nodules larger than 6 mm was 92.6%, precision was 74.3%, and F1- score was 0.824. For nodules smaller than 6 mm, the sensitivity was 98.8%, precision was 77.9%, and F1-score was 0.871. The most common cause of false positives (48.8%) was found to be subpleural fibrotic strands. In the analysis of false negatives, there was a statistically significant association between nodule density and false-negative predictions by the model (p<0.001). No statistically significant relationship was found between nodule location and false-negative results.
**Conclusion:** Our deep learning model demonstrated high accuracy in pulmonary nodule detection, highlighting its potential to support radiologists, though further multicenter validation is warranted.
**Limitations:** Retrospective nature
**Funding for this study:** This study is funded by TUBITAK and X- Focus AI
**Has your study been approved by an ethics committee?** Yes
**Ethics committee - additional information:** Approved by Ethics Committee (6779-GOA)
**Author Disclosures:**
Sinem Gezer: Consultant: Consultant in the project developing the model
Oğuz Dicle: Consultant: Consultant in the project developing the model
Emre Unel: Employee: Employee of the company that developed the model
Raif Can Yarol: Nothing to disclose
Devrim Unay: Consultant: Consultant in the project developing the model

**11:30 - 12:30** **Research Stage 4**

### Research Presentation Session: Head and Neck

### RPS 308

### Thyroid and parathyroid imaging

**Moderator**
Andrew Sinclair McQueen; Newcastle Upon Tyne/UK
*(andrew.mcqueen1@nhs.net)*

### Ultrasonographic features of ACR TIRADS 3 and 4 thyroid nodules predicting malignant potential

*J. Bosschee*; Eindhoven/NL

**Purpose:** To identify ultrasonographic features in ACR TIRADS 3 and 4 thyroid nodules to predict malignant potential.
**Methods or Background:** Thyroid carcinoma incidence in the Netherlands has increased from 372 new cases in 2000 to 904 in 2023, primarily due to population aging and improved imaging techniques. Fortunately the overall prognosis is relatively favorable with a 30 year survival rate of 95%. The inevitable accompanying increase of thyroid ultrasound and ultrasound guided fine needle aspirations (FNA) may result in overdiagnosis and unnecessary patient burden. The revised Dutch thyroid cancer guidelines aim to reduce this overdiagnosis with clinical and radiological recommendations. However, recommendations regarding ACR TIRADS 3 and 4 nodules remain ambiguous, with clinical management relying on expert consensus. Thyroid ultrasound and FNA results of patients presented to the Catharina hospital in Eindhoven (Netherlands) from 2019-2022 were retrospectively analyzed. All pathological specimens were assigned a Besthesda score and a pathological diagnosis. Predictive ultrasonographic features regarding malignant potential were investigated.
**Results or Findings:** A total of 341 FNA were analyzed of which 101 (30%) were TIRADS 3 nodules and 124 (36%) were TIRADS 4 nodules. Pathology results were available for 96 TIRADS 3 and 116 TIRADS 4 nodules. A total of 29 (30%) TIRADS 3 noduli scored a Bethesda score 3 or higher and five (5%) turned out to be malignant. 55 (47%) TIRADS 4 noduli scored a Bethesda 3 or higher and 17 (15%) were malignant.
**Conclusion:** This study will further investigate the predictive ultrasonographic features of TIRADS 3 and 4 nodules that turn out to be thyroid carcinoma. Our goal is to aid radiologists in deciding on ultrasound follow-up and/ or FNA in order to prevent overdiagnosis and consequently unnecessary patient burden and health care costs.
**Limitations:** Retrospective study.
**Funding for this study:** No funding was received for this study.
**Has your study been approved by an ethics committee?** Not applicable
**Author Disclosures:**
Joris Bosschee: Nothing to disclose

### Stiffness Matters: Elastography Enhances Detection of Thyroid Cancer Across Histologic Subtypes

*D. I. Stoian*, M. Latia, *A. Borlea*; Timisoara/RO

**Purpose:** PURPOSE To evaluate whether elastography improves diagnostic performance beyond TIRADS in papillary (PTC), follicular (FTC), and medullary (MTC) thyroid cancers, particularly where ultrasound-based stratification underperforms.
**Methods or Background:** METHODS We retrospectively analyzed 452 thyroid cancers over 10 years. Subgroups included 29 PTC with 65 benign controls, 32 FTC with 65 Bethesda IV controls, and 20 MTC without controls. All nodules underwent B-mode ultrasound, TIRADS categorization, and strain (SE) and/or shear wave elastography (SWE). Stiffness was integrated as an additional risk factor and compared with TIRADS alone.
**Results or Findings:** RESULTS In PTC, 31 nodules were intermediate- and 16 high-risk by TIRADS. Elastography upgraded 8 nodules using SWE and 14 using SE. Diagnostic accuracy improved, with AUC increasing from 0.764 (B-mode) to 0.886 (TIRADS+SWE) and 0.861 (TIRADS+SE). In FTC, only 13/32 nodules (40.6%) were identified as high-risk by TIRADS, versus 27/32 (84.4%) when stiffness was added. In MTC, TIRADS flagged ~60% as high-risk; on SE, 75% scored ES 3–4 (mean ES 3.2), and SWE with a 30.5 kPa cut-off correctly identified 87.5% of malignancies. DISCUSSION Elastography significantly enhances TIRADS across histologies. Across subtypes, most nodules were stiff, contradicting the traditional view that FTC and MTC are “soft” cancers. While B-mode alone often misses FTC and MTC, stiffness integration markedly improves cancer detection and supports surgical decision-making.
**Conclusion:** CONCLUSION Stiffness is not a stand-alone criterion but consistently augments TIRADS. Elastography should be incorporated into routine assessment of intermediate and high-risk thyroid nodules.
**Limitations:** LIMITATIONS Retrospective design, sample sizes, monocentric study.
**Funding for this study:** No funding
**Has your study been approved by an ethics committee?** Not applicable
**Ethics committee - additional information:** The studies involving humans were approved by The Ethics Committee of the Victor Babes University of Medicine and Pharmacy. The studies were conducted in accordance with the local legislation and institutional requirements. The participants provided their written informed consent to participate in this study.
**Author Disclosures:**
Monica Latia: Nothing to disclose
Dana I Stoian: Nothing to disclose
Andreea Borlea: Nothing to disclose

Wednesday

### Predictive Value of Arterial Enhancement Fraction Derived from Dual-Layer Spectral Computed Tomography for Thyroid Microcarcinoma

*J. Yu*[1], Y. Chen[1], Z. Song[1], X. Zhang[2], D. Zhang[1], X. Liu[3]; [1]Chongqing/CN, [2]Chengdu/CN, [3]Guangzhou/CN
*(xiaomin.liu@philips.com)*

**Purpose:** To evaluate the predictive value of dual-layer spectral computed tomography (DSCT)-derived arterial enhancement fraction (AEF) in diagnosing thyroid microcarcinomas.
**Methods or Background:** A total of 321 pathologically confirmed thyroid micronodules (131 benign, 190 malignant) underwent preoperative DSCT. Quantitative parameters, including normalized iodine concentration (NIC), normalized effective atomic number (NZeff), and the slope of the spectral Hounsfield unit curve (λHU(40–100)), were assessed. Both single-energy CT-derived AEF (AEFS) and DSCT-derived AEF (AEFD) were calculated. Conventional features like microcalcifications were also analyzed. Correlation between AEFD and AEFS was determined using Spearman’s correlation coefficient. Diagnostic performance was evaluated by calculating the area under the curve (AUC) using receiver operating characteristic (ROC) analysis.
**Results or Findings:** Malignant nodules showed significantly lower AEFD and AEFS than benign nodules ($p < 0.001$). Arterial phase spectral parameters differed significantly between groups ($p < 0.001$), unlike venous phase parameters. Multivariate analysis identified λHU(40–100) as an independent predictor of malignancy (OR: 0.600, $p = 0.002$). AEFD and AEFS were strongly correlated ($r = 0.710$, $p < 0.001$). AEFD demonstrated superior diagnostic performance (AUC: 0.794; sensitivity: 70.5%; specificity: 81.7%; accuracy: 75.1%) compared to AEFS, APλHU(40–100), and calcification. Clinically, using AEFD reduced the unnecessary biopsy rate to 18.3%, preventing 107 procedures.
**Conclusion:** AEFD correlates strongly with AEFS and shows favorable diagnostic efficacy for thyroid micronodules, significantly reducing unnecessary biopsies and optimizing patient management.
**Limitations:** Study limitations include its retrospective, single-center design and the need to validate AEFD for other histological subtypes. Future work should automate ROI placement and integrate molecular biomarkers.
**Funding for this study:** The study was funded by the Post-Doctoral Science Foundation of Chongqing, China (No. CSTB2022NSCQ-BHX0737), the Medical Research Program of the Combination of Chongqing National Health Commission and Chongqing Science and Technology Bureau, China (No. 2024MSXM094), and the Medical Research Youth Program, a joint initiative of the Chongqing National Health Commission and the Chongqing Science and Technology Bureau, China (No. 2024QNXM047).
**Has your study been approved by an ethics committee?** Yes
**Ethics committee - additional information:** his retrospective study was approved by the Medical Ethics Committee of Chongqing General Hospital with a waiver of informed consent.
**Author Disclosures:**
Xiaomin Liu: Nothing to disclose
Yuwei Chen: Nothing to disclose
Jiayi Yu: Nothing to disclose
Xiaodi Zhang: Nothing to disclose
Zuhua Song: Nothing to disclose
Dan Zhang: Nothing to disclose

**Ultrasound Detection and Mapping of Suspicious Cervical Lymph Nodes in Patients with PTC: How We Do It?!**

*T. Charkhalashvili*; Kutaisi/GE
*(tea_charxalashvili@yahoo.com)*

**Purpose:** According to Global Cancer Observatory (GLOBOCAN 2024), thyroid cancer is among 10 most frequent cancers worldwide. It's most common subtype - PTC spreads to the regional lymph nodes in 20-50% of cases. Accurate preoperative ultrasound (US) evaluation is crucial, as the detection and precise localisation of metastatic nodes directly influence the extent of surgical dissection and better patient outcome. Purpose of this abstract is to highlighting diagnostic criteria, scanning technique and practical tips for mapping LNs and easy communication with surgeons.
**Methods or Background:** Patients, previously diagnosed with PTC by Thyroid FNA, are scanned with high frequency ultrasound transducer (12-15MHz) prior to surgery in supine position, extending head cranially, with pillow under shoulders, for maximal identity whit surgical position. All the cervical levels are scanned step-by-step on right and left sides. Nodes are assessed for suspicious features: rounded morphology, absence of fatty hilum, microcalcifications, cystic changes and atypical vascularity. LNs are labeled on the neck with marker, as well as on the schematic template for patient history.
**Results or Findings:** Systematic scanning prevented missed nodes, while structured reporting improved preoperative planning. In our series of standardised Ultrasound mapping of cervical lymph nodes we have high detection rates(80-92%) of regional metastatic LN, and better communications with colleagues.
**Conclusion:** A standardised systematic ultrasound protocol for cervical lymph node assessment in PTC increases detection rate, accurate cevical level mapping and effective communication with surgeons. Adoption of such protocols improves preoperative planning and contributes to optimised patient outcomes.
**Limitations:** Small or deeply located nodes may be difficult to visualise, especially in obese patients or those with short necks. Reactive or inflammatory LNs may mimic metastases, lowering specificity, also, early metastases or micrometastases are not be detectable by US.
**Funding for this study:** N/A
**Has your study been approved by an ethics committee?** Not applicable
**Author Disclosures:**
Tea Charkhalashvili: Nothing to disclose

**Thyroid Metastases from Non-Thyroid Malignancies: Systematic Review and Radiological Features**

*C. Peter*, T. Denecke, H-J. Meyer; Leipzig/DE
*(christian.peter1@web.de)*

**Purpose:** To systematically review clinical and radiological characteristics of thyroid metastases from non-thyroid primary tumors.
**Methods or Background:** Metastases to the thyroid gland from non-thyroid malignancies are rare but clinically relevant. Reported frequencies vary. Prior reviews mainly addressed clinical and histopathological aspects, while analyses of radiological features remain limited. We conducted a systematic PubMed search (2010–2025) according to PRISMA guidelines. Eligible studies included case reports, case series and observational studies on histologically confirmed thyroid metastases from non-thyroid primaries with radiological data (ultrasound, CT, or MRI). Studies on primary thyroid malignancies or non-human data were excluded. Extracted data included demographics, primary tumor, interval between primary diagnosis and thyroid metastasis, clinical presentation, and imaging findings
**Results or Findings:** A total of 215 studies, including 1,006 patients, with mean age of 61.7 years were identified. The most common primary tumors metastasizing to the thyroid are lung cancer, breast cancer and renal cell cancer. Most metastasis are identified metachronous after the initial tumor diagnosis. Most cases present as a painless neck mass and as a unilateral manifestation. On ultrasound, most lesions were hypoechogenic with a blurred border. On CT, metastases were predominantly hypodense. On MRI, lesions were typically intermediate on T2-weighted images and iso- to hyperintense on T1-weighted images. A misdiagnosis is common in up to 31% of cases.
**Conclusion:** Metastases to the thyroid gland are rare but should be considered in the differential diagnosis of thyroid lesions, especially in patients with a history of malignancy. This systematic review provides an overview of clinical and radiological features, which may aid in diagnosis and differentiation from primary thyroid tumors.
**Limitations:** Heterogeneity of included studies, small case numbers of case reports with potential publication bias, incomplete radiological data, restricting generalizability to broader clinical practice
**Funding for this study:** None
**Has your study been approved by an ethics committee?** Not applicable
**Author Disclosures:**
Timm Denecke: Nothing to disclose
Hans-Jonas Meyer: Nothing to disclose
Christian Peter: Nothing to disclose

**Diagnostic Accuracy of ACR-TIRADS Versus S-Detect in Thyroid Nodule Characterization: Comparison with FNAC as the Gold Standard**

P. Pacini, M. Renda, C. Catalano, *V. Dolcetti*, C. Solito, F. Raponi, G. Bonito, G. Maglia, V. Cantisani; Rome/IT

**Purpose:** To compare the diagnostic performance of ACR-TIRADS, assessed by an experienced radiologist, with that of the S-Detect system in the evaluation of thyroid nodules, and to validate both methods against the cytological gold standard, fine-needle aspiration cytology (FNAC).
**Methods or Background:** This prospective study included 120 patients (35–75 years) with thyroid nodules or lesions detected clinically or on routine neck ultrasound at Umberto I Hospital, Rome. Thyroid nodules are a common finding, with a prevalence exceeding 50% in women and in individuals >60 years. While FNAC remains the diagnostic gold standard, it carries significant financial costs for healthcare systems and psychological stress for patients. Recent efforts have focused on improving ultrasound-based diagnostic accuracy to reduce unnecessary FNAC procedures.
**Results or Findings:** ACR-TIRADS demonstrated a sensitivity of >97% and specificity of 92%. S-Detect achieved a sensitivity of 94% and specificity of 90%. When compared with FNAC, ACR-TIRADS showed a sensitivity of 94% and S-Detect 91%, confirming strong diagnostic performance for both tools.
**Conclusion:** ACR-TIRADS demonstrated excellent agreement with FNAC and remains a reliable tool in thyroid nodule characterization. S-Detect also showed high diagnostic accuracy, supporting its role as a complementary, cost-effective aid in clinical practice.
**Limitations:** A sample size that needs to be expanded
**Funding for this study:** No
**Has your study been approved by an ethics committee?** Yes
**Ethics committee - additional information:** Roma sapienza universit?
**Author Disclosures:**
Maurizio Renda: Nothing to disclose
Patrizia Pacini: Nothing to disclose
Gianluca Maglia: Nothing to disclose
Giacomo Bonito: Nothing to disclose
Vincenzo Dolcetti: Nothing to disclose
Carmen Solito: Nothing to disclose
Flavia Raponi: Nothing to disclose
Vito Cantisani: Nothing to disclose
Carlo Catalano: Nothing to disclose

**Quantitative CEUS Assessment of Thyroid Nodules**

*A. Borlea*, S. Bunceanu, D. I. Stoian; Timisoara/RO
*(andreea.brl3@gmail.com)*

**Purpose:** To evaluate the diagnostic performance of quantitative contrast-enhanced ultrasound (CEUS) parameters in differentiating benign from malignant thyroid nodules.
**Methods or Background:** Thirty thyroid nodules with histopathologic confirmation (15 benign, 15 malignant: 12 papillary, 2 follicular, 1 medullary) underwent CEUS. Quantitative time–intensity curve (TIC) analysis included: Arrival Time (AT), Rise Time (RT), Time to Peak (TTP), Fall Time or Wash-out Time (FT), Mean Transit Time (mTT), Peak Intensity (PKI), Area Under Curve (AUC), Wash-in AUC (WiAUC), Wash-out AUC (WoAUC), Ascending Slope (AS), Descending Slope (DS), Slope Ratio (SR), and mean TIC.
**Results or Findings:** Malignant nodules showed significantly shorter AT (12.3 ± 3.5 vs. 16.7 ± 4.2 s, p=0.01), faster RT (7.1 ± 2.2 vs. 9.8 ± 2.6 s, p=0.02), and earlier TTP (19.4 ± 4.6 vs. 26.5 ± 5.3 s, p<0.01). FT was prolonged (31.2 ± 8.4 vs. 22.5 ± 6.7 s, p=0.02), yielding longer mTT (38.3 ± 9.2 vs. 32.3 ± 7.1 s, p=0.04). PKI was higher in malignant nodules (28.6 ± 5.7 vs. 21.4 ± 4.9 a.u., p=0.01). Perfusion integrals were increased: AUC 1520 vs. 980 a.u.*s (p=0.01), WiAUC 650 vs. 410 (p=0.02), WoAUC 870 vs. 570 (p=0.03). Slopes were steeper in malignancy: AS 2.3 vs. 1.6 a.u./s (p=0.03), DS 2.1 vs. 1.3 (p=0.04), with higher SR (0.91 vs. 0.78). ROC analysis showed AUC 0.86 for TTP and 0.88 for PKI; combining PKI+TTP achieved 0.92 sensitivity and 0.88 specificity.
**Conclusion:** Malignant thyroid nodules present rapid wash-in, higher peak enhancement, and delayed wash-out. Quantitative CEUS parameters, particularly TTP and PKI, significantly improve diagnostic accuracy and may complement TIRADS and elastography in risk stratification.
**Limitations:** Small, single-center cohort; larger studies needed for threshold validation.
**Funding for this study:** No funding
**Has your study been approved by an ethics committee?** Yes
**Ethics committee - additional information:** UMFT Victor Babes Ethics Committee
**Author Disclosures:**
Dana I. Stoian: Nothing to disclose
Andreea Borlea: Nothing to disclose
Stefania Bunceanu: Nothing to disclose

**Analysis of Contrast Enhancement Patterns and Diagnostic Performance of 4D CT in the Detection of Abnormal Parahtyroid Glands in Patient with Primary Hyperparathyroidism**

*F. Zengin*, M. M. Geres, A. Gemalmaz, A. Özel; Istanbul/TR

**Purpose:** This study aimed to evaluate the diagnostic performance of 4D-CT in patients with PHPT, focusing on preoperative localization of abnormal parathyroid glands, comparing enhancement patterns with those described in the literature, and determining overall diagnostic efficacy.
**Methods or Background:** This retrospective study included 215 parathyroid lesions from 194 PHPT patients who underwent 4D-CT and surgery. An experienced radiologist, blinded to surgical and pathological findings, assessed the scans for lesion detection, size, polar vessel presence, and enhancement characteristics. HU values of lesions and normal thyroid tissue were measured in pre-contrast, arterial, and venous phases.
**Results or Findings:** 4D-CT localized 90.7% of lesions. Sensitivity, PPV, and accuracy were higher in single-gland cases (94%, 97.5%, 92%) than multiglandular ones (82.3%, 87.5%, 73.7%). Most lesions showed Bahl Type B enhancement (74.4%), but all mislocalizations occurred in this group, suggesting limited specificity. Parathyroid lesions had lower pre-contrast HU than thyroid tissue; however, the <58 HU threshold was only supportive (AUC ≈ 0.72). Arterial enhancement rose by 321.9%, and venous washout reached 42.0%. Absolute (61.3%) and relative (46.5%) washout ratios were strong differentiators. Larger lesions (>11 mm) were associated with polar vessels (p < 0.001). Lesion size correlated with arterial HU, washout, PTH, and calcium. Ectopic lesions (4.2%) had fewer polar vessels (p = 0.010). The study proposes a novel pattern-based approach incorporating arterial HU ratio and washout rates, suggesting their combination may improve preoperative localization.
**Conclusion:** 4D-CT is highly effective for preoperative localization in PHPT. Dynamic contrast parameters, particularly arterial enhancement and washout, improve accuracy beyond classical classifications and may further benefit from radiomics integration.
**Limitations:** The single-center design and the limited number of rare enhancement patterns, non-localized lesions, and multiglandular cases underscore the need for further multicenter studies to validate these findings.
**Funding for this study:** This research received no specific grant from any funding agency.
**Has your study been approved by an ethics committee?** Yes
**Ethics committee - additional information:** University of Health Sciences Şişli Hamidiye Etfal Training and Research Hospital Clinical Research Ethics Committee-4802
**Author Disclosures:**
Feyza Zengin: Nothing to disclose
Ali Gemalmaz: Nothing to disclose
Alper Özel: Nothing to disclose
Mehmet Mert Geres: Nothing to disclose

**Comparison of 4D-CT imaging findings and biochemical parameters in parathyroid adenomas: The role of polar vessel presence**

*B. G. Ürkmez*, A. Gürsoy Çoruh, M. Kul, S. N. Yılmazer, M. K. Gökcan, Ç. Uzun; Ankara/TR
*(toyga118@gmail.com)*

**Purpose:** To investigate the association between radiological findings of parathyroid adenomas (PA) on 4 dimensional computed tomography (4D-CT) and biochemical parameters, as well as the impact of polar vessel presence on these relationship.
**Methods or Background:** This retrospective study included patients who underwent 4D-CT imaging for parathyroid adenoma between 2015 and 2025, with the diagnosis confirmed histopathologically. PV presence was evaluated in consensus by two observers. Densities of PA were measured in pre-contrast, arterial, and venous phases using the largest region of interest excluding cystic/calcified areas. Volume was calculated as 0.523 × long axis × short axis × craniocaudal length. Enhancement indices ('wash-in', 'wash-out', and 'retained') were calculated. Serum parathyroid hormone (PTH), calcium (Ca), and creatinine levels were recorded. Correlations between imaging and biochemical findings were analyzed; Differences according to PV presence were assessed using the t-test/Mann–Whitney U test.
**Results or Findings:** A total of 84 patients (mean age: 56.42 ± 15.04 years) were included, comprising 45 PV-positive (PV+) and 39 PV-negative (PV−). PV+ patients had higher PTH (p=0.044), higher arterial phase density (p=0.020), and significantly greater wash-in and wash-out values (p=0.010 and p=0.033, respectively). PA volume correlated positively with PTH (r=0.300, p=0.006) and Ca (r=0.432, p=0.014). Pre-contrast density correlated negatively with PTH (r=−0.248, p=0.023). No other significant correlations between densities and biochemical parameters were observed.
**Conclusion:** The presence of a polar vessel is associated with higher arterial enhancement, greater wash-in/wash-out, and elevated PTH levels, supporting its diagnostic role in 4D-CT evaluation of PA. Larger adenoma volume correlates with higher biochemical activity, suggesting a contribution to hypercalcemia through increased PTH secretion. PV assessment should be considered an important imaging finding in the diagnostic work-up.
**Limitations:** Retrospective design, moderate sample size, and lack of fine-needle aspiration PTH measurements.
**Funding for this study:** No funding was provided for this study.
**Has your study been approved by an ethics committee?** Not applicable
**Author Disclosures:**
Melahat Kul: Nothing to disclose
Sezer Nil Yılmazer: Nothing to disclose
Ayşegül Gürsoy Çoruh: Nothing to disclose
Büşra Gözde Ürkmez: Nothing to disclose
Mustafa Kürşat Gökcan: Nothing to disclose
Çağlar Uzun: Nothing to disclose

**11:30 - 12:30** **Research Stage 5**

### Research Presentation Session: Interventional Radiology

### RPS 309

### More than needles and wires in the world of interventional radiology

**Moderator**
Thomas Karl Helmberger; Munich/DE
*(thomas.helmberger@muenchen-klinik.de)*

**Real-Time Digital Documentation in Interventional Radiology: Modular Tools for Efficiency and Quality**

*M. Schönfeld*, E. Ulrich, J. Kottlors, T. Schömig, K. R. Laukamp; Köln/DE
*(michael.schoenfeld@gmx.de)*

**Purpose:** Structured documentation of interventional radiology procedures is critical for quality assurance, billing, and research. In routine practice documentation is often retrospective, time-consuming, and error-prone. We developed a modular digital solution that enables structured, real-time documentation directly during the procedure.
**Methods or Background:** The tools cover the full spectrum of interventional radiology, including neurovascular (mechanical thrombectomy, carotid stenting, vasospasm treatment, aneurysm, subdural hematoma embolization), oncological/embolization (fibroid, prostate, tumor embolization, TACE, SIRT, BBA, bleeding), percutaneous (biopsy, drainage, cryoablation, microwave ablation), and vascular access procedures (PICC, port, TIPS). All modules run on sterile-clickable touchscreens in the angiography suite, capturing timestamps, techniques, materials, and findings in real time. From these inputs the system automatically generates: (1) a standardized editable report, (2) procedure codes, (3) structured quality assurance data, and (4) prepared registry entries. Data from the first 95 consecutive procedures documented with the system were analyzed and compared with 100 procedures performed before implementation.
**Results or Findings:** In the first 95 procedures, the mean time for report creation decreased from 12.2 ± 6.1 minutes before implementation to 1.3 ± 0.5 minutes, with reports now available immediately after the procedure instead of with an average delay of more than 48 hours. The time required for procedure coding was reduced from 11.4 ± 3.2 minutes, including frequent corrections, to 1.2 ± 0.4 minutes. The duration of quality assurance documentation decreased from 8.5 ± 2.8 minutes to 2.1 ± 1.3 minutes. Registry entries also became significantly faster, dropping from 8.2 ± 3.6 minutes to 2.0 ± 1.2 minutes, while completeness increased from less than 20% to more than 90%.
**Conclusion:** Real-time digital documentation across the spectrum of interventional radiology is feasible and leads to substantial gains in efficiency, accuracy, and quality assurance.
**Limitations:** None
**Funding for this study:** None
**Has your study been approved by an ethics committee?** Not applicable
**Author Disclosures:**
Kai Roman Laukamp: Nothing to disclose
Elias Ulrich: Nothing to disclose
Michael Schönfeld: Owner: Michael Schönfeld is the developer of the described tools. No financial conflict of interest exists.
Jonathan Kottlors: Nothing to disclose
Thomas Schömig: Nothing to disclose

**Unveiling the immunomodulatory effects of transarterial chemoembolisation: insights into local T-cell infiltration in hepatocellular carcinoma**

*L. Heidemann*, R. Schmidt, E. Y. Yilmaz, Y. He, J. Böhme, K. Harb, D. Müller, B. Gebauer, L. J. Savic; Berlin/DE
*(luisa.heidemann@charite.de)*

**Purpose:** To investigate local immunomodulatory effects of conventional transarterial chemoembolisation (cTACE) in hepatocellular carcinoma (HCC).
**Methods or Background:** This single-centre prospective study included HCC patients undergoing interstitial brachytherapy in afterloading technique (iBT) only or cTACE with consecutive iBT (10.2020-08.2023). IRB approval and informed consent were obtained. Biopsies were acquired from untreated HCC before iBT (iBT group) or 24 hours post-cTACE and before iBT (cTACE/iBT group). Based on histopathology and peri-interventional CT images, biopsies were graded as intra- and peritumoural. Immunohistochemistry staining was performed for CD3+, CD4+, CD8+, PD-1+ T cells, and Ki67. On digitized samples, T cell populations were quantified as a percentage of all counted cells using QuPath software. Cell counts were correlated with response according to RECIST version 1.1 assessed on MRI eight weeks post-treatment. Statistics included Shapiro-Wilk and Mann-Whitney U tests.
**Results or Findings:** This study cohort comprised 82 patients (66 male), with a mean age of 69.6 ± 9.9 years. CD3+ and CD4+ T-cell infiltration was higher in the cTACE/iBT group (n=39) compared to the iBT group (n=43; p=0.043 and p=0.03, respectively). CD3+ (p=0.02), CD4+ (p=0.01), and CD8+ T cell counts (p=0.01) were higher in the peritumoural compartment than intratumourally. In the cTACE/iBT group, responders (n=11) showed higher median CD3+ (p=0.49), CD4+ (p=0.19), and CD8+ T-cell counts (p=0.07) compared to non-responders (n=25).
**Conclusion:** This study demonstrates early effects of cTACE on T-cell infiltration into the tumour microenvironment, contributing to a greater understanding of peripheral lymphocyte depletion and migration following cTACE.
**Limitations:** Firstly, longitudinal effects post-cTACE are not captured. Secondly, findings of a single biopsy may not be representative of the entire tumour. Lastly, outcome was measured after iBT or cTACE/iBT, while biopsies were obtained before iBT from untreated tumours or post-cTACE.
**Funding for this study:** Guerbet
**Has your study been approved by an ethics committee?** Yes
**Ethics committee - additional information:** Institutional review board approval (Charité – Universitätsmedizin Berlin, EA2/091/19) and written informed consent were obtained from all patients.
**Author Disclosures:**
Kudor Harb: Nothing to disclose
Bernhard Gebauer: Nothing to disclose
Emine Yaren Yilmaz: Nothing to disclose
Yubei He: Nothing to disclose
Dominik Müller: Nothing to disclose
Luisa Heidemann: Nothing to disclose
Robin Schmidt: Nothing to disclose
Lynn Jeanette Savic: Nothing to disclose
Judith Böhme: Nothing to disclose

**An immune-active peptide-based shear-thinning soraphinib-loading hydrogel embolic agent in VX2 kidney tumor**

*S. Zhang*, Z. Wang, Z. Jin; Beijing/CN
*(839035620@qq.com)*

**Purpose:** This study developed an innovative immune-active peptide-based shear-thinning hydrogel loaded with sorafenib (AbSNP-CIR) as a multifunctional transcatheter arterial embolization (TAE) agent for treating VX2 kidney tumors, evaluating its therapeutic efficacy, immunomodulatory effects, and biocompatibility.
**Methods or Background:** We formulated a CIR host defense peptide hydrogel incorporating albumin-bound sorafenib nanoparticles (AbSNP). The material's shear-thinning properties, injectability, and embolic characteristics were validated in vitro. Twenty-four New Zealand white rabbits with VX2 kidney tumors were randomized into four TAE groups: AbSNP-CIR, CIR (drug-free), Lipiodol®, and control (angiography only). Tumor progression was monitored via contrast-enhanced CT at days 0, 7, and 14. Comprehensive blood analyses assessed systemic toxicity. Histopathological examination of major organs and tumors was performed at endpoint. Single-cell RNA sequencing analyzed immune microenvironment alterations between CIR and Lipiodol® groups. Survival benefits were evaluated using Kaplan-Meier analysis with Log-rank tests.
**Results or Findings:** Both CIR and AbSNP-CIR hydrogels demonstrated excellent injectability and effective vessel occlusion, inducing significant tumor necrosis. The AbSNP-CIR group exhibited superior tumor growth suppression. scRNA-seq revealed CIR-based hydrogels reversed immunosuppression by significantly downregulating LGALS3 and upregulating CD83 compared to Lipiodol®. Survival analysis demonstrated AbSNP-CIR significantly prolonged median survival (40.5 days) versus control (23 days, p=0.028), CIR (26.5 days, p=0.035), and Lipiodol® (27.5 days, p=0.049). Blood parameters showed no significant systemic toxicity.
**Conclusion:** The AbSNP-CIR hydrogel represents an advanced embolic strategy that integrates mechanical occlusion, localized sorafenib delivery, and active immunomodulation, significantly enhancing antitumor efficacy and survival in kidney tumor models.
**Limitations:** Small sample size and single animal model require validation in larger studies.
**Funding for this study:** This study has received funding from the National Natural Science Foundation of China (Nos. 22232006, Biomolecular Condensates: Phase Separation Modulation and Functionalization)
**Has your study been approved by an ethics committee?** Yes
**Ethics committee - additional information:** Approval number: XHDW-2023-017
**Author Disclosures:**
Zhiwei Wang: Nothing to disclose
Zhengyu Jin: Nothing to disclose
Shenbo Zhang: Nothing to disclose

**Personalized Dosimetry Enhances Hepatocellular Carcinoma Response in Yttrium-90 Resin Microsphere Radioembolization**

*F. Calabrese*[1], T. T. Makoto[2], E. Di Gaeta[1], A. Casadei-Gardini Andrea[1], C. Canevari[1], L. Strigari[2], F. Ratti[1], C. Mosconi[2], F. De Cobelli[1]; [1]Milan/IT, [2]Bologna/IT
*(francescacalabrese18@gmail.com)*

**Purpose:** The aim of the study is to determine a tumor mean absorbed dose (Dmean) able to predict the therapeutic response to Yttrium-90 (Y-90) resin-microsphere transarterial radioembolization (TARE) in hepatocellular carcinoma (HCC), and evaluate its efficacy and safety profile.
**Methods or Background:** A retrospective analysis was conducted on HCC patients treated with TARE at two institutions between January 2020 and May 2024. Clinical, radiological and procedural data were collected. Outcomes included objective response rate (ORR) for target lesions (complete or partial), complete response (CR), overall response, time-to-local progression (TLP) and time-to-progression (TTP), assessed on contrast-enhanced CT. Receiver operating characteristic (ROC) analysis identified the optimal Dmean for achieving ORR and CR. ORR comparisons used Fisher's exact test, survival was analyzed using Kaplan-Meier curves, and Cox regression was used for univariable and multivariable analysis.
**Results or Findings:** Seventy-six lesions from 64 patients (mean age 71.3 ± 9.6; 54 male) were analyzed, with an average tumor size of 54.8 ± 31.5 mm. CR at 3 months was obtained in 41 lesions. Mean TLP and overall survival (OS) were 26.7 ± 2.5 and 36.2 ± 2.9 months, respectively. The calculated Dmean to obtain ORR was 296.74 Gy (specificity 100, PPV 100%). Patients receiving doses < 296.74 Gy experienced shorter TLP (p = 0.001) and a higher progression risk (HR 6.9; p = 0.009). No increase in complications was observed at Dmean >296.74 Gy. A Dmean > 435.11 Gy was associated to CR. OS was negatively influenced by BCLC stage C (HR 6.0; p = 0.003) and portal vein invasion (HR 3.5; p = 0.04).
**Conclusion:** A tumor Dmean of 296.74 Gy stands out as a powerful predictor of treatment success, delivering strong therapeutic results without compromising patient safety.
**Limitations:** Retrospective study
**Funding for this study:** Na
**Has your study been approved by an ethics committee?** Yes
**Ethics committee - additional information:** 64/Int/2021
**Author Disclosures:**
Taninokuchi Tomassoni Makoto: Nothing to disclose
Francesco De Cobelli: Nothing to disclose
Andrea Casadei-Gardini Andrea: Nothing to disclose
Cristina Mosconi: Nothing to disclose
Carla Canevari: Nothing to disclose
Francesca Calabrese: Nothing to disclose
Ettore Di Gaeta: Nothing to disclose
Lidia Strigari: Nothing to disclose
Francesca Ratti: Nothing to disclose

**Dual-Energy CT Assessment of TIPS Patency: Diagnostic Accuracy, Confidence, and Quantitative Iodine Analysis**
*M. Oberparleiter*, J. Vosshenrich, H-C. Breit, A. C. Seifert, P. J. Kaiser, P. Hehenkamp, D. Boll, C. J. Zech, M. Obmann; Basel/CH

**Purpose:** To compare diagnostic performance, confidence, and reading time between conventional and dual-energy CT (DECT) protocols in evaluating TIPS patency and assess the value of DECT-derived quantitative iodine measurements.
**Methods or Background:** This retrospective single-centre study included 57 patients with TIPS who underwent portal-venous DECT between 11/2014 and 07/2024. For each case, a conventional CT dataset and a DECT dataset (additional iodine maps and virtual non-contrast images [VNC]) were created. Two abdominal radiologists and three residents assessed TIPS patency and rated diagnostic confidence (5-point Likert scale). Sensitivity and specificity were compared using McNemar’s test; confidence and reading time with the Wilcoxon signed-rank test. Iodine concentrations were measured in the inferior vena cava, portal vein, and TIPS using three circular ROIs each. Diagnostic performance of absolute iodine values and iodine ratios (TIPS/portal vein, TIPS/inferior vena cava) was assessed with ROC analysis. Thresholds were defined via Youden index.
**Results or Findings:** TIPS occlusion was present in 14 patients. Sensitivity was similar for conventional CT and DECT (93% vs. 97%, p=.219), while specificity increased from 93% to 98% with DECT (p<.01). Diagnostic confidence improved (median 4 [IQR 3–4] vs. 4 [IQR 4–5], p<.01), and reading time decreased (109 s vs. 98 s, p<.001). Mean iodine concentrations (mg/ml) were: IVC 3.99, portal vein 4.65, open TIPS 4.49, occluded TIPS 0.67. ROC-AUCs were 0.998 (absolute iodine), 1.000 (TIPS/portal vein), and 0.995 (TIPS/IVC). Optimal thresholds were 1.40 mg/ml, 0.262, and 0.263, yielding sensitivities/specificities of 95%/100%, 100%/100%, and 98%/100%, respectively.
**Conclusion:** Adding iodine maps and VNC images to DECT improves diagnostic specificity, confidence, and efficiency in assessing TIPS patency. Quantitative iodine measurements allow for accurate, objective evaluation and may be useful in inconclusive cases.
**Limitations:** This was a single-centre retrospective study with a limited sample size.
**Funding for this study:** This study did not receive any external funding.
**Has your study been approved by an ethics committee?** Yes
**Ethics committee - additional information:** The need for informed consent was waived.
**Author Disclosures:**
Markus Obmann: Nothing to disclose
Hanns-Christian Breit: Nothing to disclose
Philippe Julien Kaiser: Nothing to disclose
Christoph Johannes Zech: Nothing to disclose
Daniel Boll: Nothing to disclose
Paul Hehenkamp: Nothing to disclose
Jan Vosshenrich: Nothing to disclose
Alina Carolin Seifert: Nothing to disclose
Moritz Oberparleiter: Nothing to disclose

**Construction of a Postoperative Infection Risk Prediction Model for Microwave Ablation of Hepatic Malignant Tumors and Rational Application Strategy for Prophylactic Antibiotics**
*Z. FU*, Y. Liu, E. Xu; Guangdong/CN

**Purpose:** This study aimed to identify independent risk factors for post-MWA infections and develop a risk stratification system to guide prophylactic antibiotic use.
**Methods or Background:** In this retrospective study of 359 patients who underwent MWA for liver malignancies (2019-2025), we collected comprehensive perioperative data including baseline characteristics, preoperative indicators, imaging features, procedural parameters, and infection-related outcomes. Risk factors were identified through univariate and multivariate analyses, and a risk stratification system was established (low-risk: <5%; intermediate-risk: 5%-15%; high-risk: >15%).
**Results or Findings:** Post-procedural infections occurred in 46 patients (12.8%). Multivariate analysis identified three independent risk factors: history of biliary tract procedures (OR=5.79, 95% CI: 1.53-21.97), presence of five or more tumors (OR=3.69, 95% CI: 1.17-11.63), and larger tumor size (OR=1.03 per mm, 95% CI: 1.00-1.05). The risk stratification model demonstrated good discrimination (AUC=0.82), with infection rates showing significant gradient distribution across risk groups (1.4% vs. 7.8% vs. 34.8%, P<0.001).
**Conclusion:** Three independent risk factors for post-MWA infections were identified. The risk prediction model may guide rational prophylactic antibiotic use in clinical practice.
**Limitations:** The single-center design and sample size may limit generalizability, requiring external validation.
**Funding for this study:** 2025 Futian District Health System Research Project Approval
**Has your study been approved by an ethics committee?** Yes
**Ethics committee - additional information:** Ethics Committee of the Eighth Affiliated Hospital of Sun Yat-sen University
**Author Disclosures:**
Ying Liu: Nothing to disclose
Zexin Fu: Nothing to disclose
Erjiao Xu: Nothing to disclose

**11:30 - 12:30** **Research Stage 3**

### Research Presentation Session: Paediatric

### RPS 312

### Congenital and acquired paediatric cardiothoracic radiology

**Moderator**
Jana Polakova Mistinova; Bratislava/SK

**Imaging and evaluation of cervicothoracic lymphatic drainage pathways in single ventricle patients with Fontan circulation using the mDixon steady state MR angiography**
*P. Langguth*, M. Huhndorf, H. Seoudy, O. Jansen, A. Uebing, I. Voges; Kiel/DE

**Purpose:** Patients with Fontan circulation after single ventricle palliation are at increased risk of pathological alterations in the cervicothoracic lymphatic drainage system, including chylothorax and plastic bronchitis. Visualization and classification of these lymphatic pathways may provide prognostic information and help guide clinical management. This study aimed to demonstrate that a 3D mDixon steady-state MR-angiography sequence, commonly used for cardiovascular assessment, can simultaneously visualize lymphatic structures with high diagnostic quality.
**Methods or Background:** We retrospectively analyzed MR examinations of 88 pediatric and young adult Fontan patients. The 3D mDixon steady-state MR-angiography was assessed for image quality and diagnostic yield regarding cervicothoracic lymphatic pathways. Ventricular volumes, ejection fraction, and mass were quantified from cine imaging. Lymphatic pathways were classified according to an established four-tier system (Types 1–4).
**Results or Findings:** Image quality of the lymphatic pathways was rated “very good” or “good” in over 90% of cases. Six patients presented with lymphatic complications; five (83.3%) showed higher-grade pathways (Type 3 or 4). Among 88 patients, the distribution was: 31.8% Type 1, 18.2% Type 2, 39.8% Type 3, and 9.1% Type 4. No significant associations were observed between lymphatic type and single ventricle morphology, age at Fontan completion, protein levels, NT-proBNP, or central venous pressure. Likewise, no correlation was found between lymphatic type and ventricular parameters (volumes, ejection fraction, mass).
**Conclusion:** 3D mDixon steady-state MR-angiography enables high-quality, simultaneous visualization of cardiovascular and cervicothoracic lymphatic anatomy in Fontan patients without additional imaging sequences. Patients with lymphatic complications tended to present with higher lymphatic types, suggesting a role for this technique in risk stratification and follow-up.
**Limitations:** This was a retrospective study. T2-weighted lymphangiography, the current reference standard for lymphatic imaging, was not systematically performed for comparison. Abdominal lymphatic abnormalities were not evaluated.
**Funding for this study:** No fundings
**Has your study been approved by an ethics committee?** Yes
**Ethics committee - additional information:** Christian-Albrechts University
**Author Disclosures:**
Anselm Uebing: Nothing to disclose
Inga Voges: Nothing to disclose
Patrick Langguth: Nothing to disclose
Olav Jansen: Nothing to disclose
Monika Huhndorf: Nothing to disclose
Hatim Seoudy: Nothing to disclose

**Late Gadolinium Enhancement in Kawasaki Disease: its Distribution, Association with Coronary Artery Lesions and Myocardial Strain**
S. Azhe, Z. Zhou, Y-K. Guo, L-Y. Wen, *X. Deng*; Chengdu/CN

**Purpose:** To investigate the distribution patterns of late gadolinium enhancement (LGE) in the chronic phase of Kawasaki disease (KD) and its relationship with coronary artery lesions and myocardial strain.
**Methods or Background:** In this retrospective, single-center study, 185 KD patients who underwent cardiac magnetic resonance (CMR) during the chronic phase were enrolled. They were divided into an LGE-positive group (n=25) and an LGE-negative group (n=160). CMR image analysis was performed using commercial software.
**Results or Findings:** Left ventricular (LV) LGE was present in 25 (13.5%) patients, involving 15.07 ± 0.12% of the LV mass. The LGE distribution was as follows: longitudinally, the basal (68%) and mid-ventricular (68%) segments were most frequently affected; circumferentially, the lateral (68%) and anterior (44%) walls were predominant; radially, subendocardial (24%), transmural (32%), and subepicardial (32%) patterns were observed. Coronary artery stenosis (OR: 5.48) and a history of thrombosis (OR: 3.87) were identified as independent risk factors for LGE (all P < 0.05). The LGE location matched the perfusion territory of stenotic or thrombotic coronary arteries in 19 patients, while 6 LGE-positive patients had no coronary artery lesions. Myocardial strain (global and segmental) was significantly impaired in the LGE+ group across all directions (longitudinal, radial, circumferential) compared to the LGE- group (all p < 0.05).
**Conclusion:** In KD patients, LGE is primarily located in the basal and mid lateral walls of the left ventricle. Coronary artery stenosis and thrombosis history are independent risk factors for LGE. The presence of LGE is associated with subclinical left ventricular dysfunction.
**Limitations:** This was a single-center, single-vendor, observational study; there was potential for selection bias, and the patient population may not fully represent the global population with KD.
**Funding for this study:** This study is supported by the National Natural Science Foundation of China (82102020, 82471970) and the Special Project for the Central Government to Guide the Development of Local Science and Technology in Sichuan Province(2023ZYD0100).
**Has your study been approved by an ethics committee?** Yes
**Ethics committee - additional information:** This study protocol was approved by the Institutional Review Board at our University Hospital (No. 2023095)
**Author Disclosures:**
Zhongqin Zhou: Author: The authors have no potential conflicts of interest to disclose
Ying-Kun Guo: Author: The authors have no potential conflicts of interest to disclose
Shiganmo Azhe: Speaker: The authors have no potential conflicts of interest to disclose
Ling-Yi Wen: Author: The authors have no potential conflicts of interest to disclose
Xinrui Deng: Nothing to disclose

**Differentiating genetic and viral pediatric dilated cardiomyopathy: a pilot study**
*T. Banaz*, F. Köse, M. F. Kazanbaş, B. Tütüncüoğlu, M. Kadıoğlu, S. Ozkök; Istanbul/TR
*(tubabanaz59@gmail.com)*

**Purpose:** Dilated cardiomyopathy (DCM) is a major cause of heart failure and transplantation in children. While genetic testing can aid diagnosis, it is often costly, slow, and inconclusive. Viral myocarditis is a common cause of pediatric DCM, but distinguishing it from genetic forms is difficult due to overlapping clinical and imaging features. This study aims to investigate whether magnetic resonance imaging (MRI) signal characteristics of lymphoid organs can non-invasively distinguish between viral and genetic DCM.
**Methods or Background:** This retrospective study included 131 pediatric DCM patients (<18 years) treated between January 2022 and December 2024. Thirty-one patients with confirmed genetic (n=13) or viral (n=18) etiology were analyzed. Exclusion criteria were congenital heart disease, autoimmune disease, malignancy, or missing data. Diagnoses were based on molecular/genetic testing or pathogen detection. Myocarditis diagnoses were based on clinical, laboratory, ECG, echocardiographic, cardiac MRI findings. No biopsies were performed. Patients underwent cardiac MRI with T1&T2 mapping. Quantitative values were extracted from the myocardium, spleen, and thymus. Median and interquartile ranges were calculated, intergroup comparisons were performed using independent t-tests.
**Results or Findings:** No significant differences were observed in conventional cardiac MRI parameters, including biventricular volume indices, ejection fractions, native T1&T2 mapping, or myocardial contrast enhancement percentage (p>0.05). Native T1 values were significantly higher in the genetic group in the thymus (p = 0.033) and in the spleen (p = 0.049). Splenic T2 mapping values were higher in the genetic group (p = 0.048).
**Conclusion:** Conventional MRI may not clearly differentiate genetic and viral DCM. Genetic DCM is linked to prolonged inflammation, with elevated T1 values in thymus and spleen indicating fibrosis or ongoing inflammation, and T2 values in spleen indicating subclinical edema.
**Limitations:** Retrospective design, small sample size, age/immune/technical variability in lymphoid MRI signals
**Funding for this study:** No funding was received for this study.
**Has your study been approved by an ethics committee?** Yes
**Ethics committee - additional information:** This study was approved by the Ethics Committee of Istanbul Medipol University, Istanbul/Turkey.
**Author Disclosures:**
Serçin Ozkök: Nothing to disclose
Berk Tütüncüoğlu: Nothing to disclose
Fadime Köse: Nothing to disclose
Muhammed Faruk Kazanbaş: Nothing to disclose
Tuba Banaz: Nothing to disclose
Mehmet Kadıoğlu: Nothing to disclose

Wednesday

**Generalised Arterial Calcifications of Infancy (GACI)- the criticality of Time and the importance of early diagnostics**
*H. Witowicz*, W. Grajkowska, E. Jurkiewicz; Warsaw/PL
*(helena.witowicz@wp.pl)*

**Purpose:** The purpose of our study was to assess very rare genetic disease - Generalised Arterial Calcifications of Infants (GACI) and to find possible differences in management with other rare condition - idiopatic artery calcifications from our Institure.
**Methods or Background:** We retrospectively assessed four new patients by genetic background, clinical symptoms, diagnostic process and treatment responce. Every patient had CT. For the purpose of our study paper review was performed. From 1899 were published aproximatelly 256 cases of GACI.
**Results or Findings:** In the first case the patient was admitted to the hospital at the right time- in the first day after birth, which gave doctors enough time to make correct diagnosis(CT reveled typical arterial calcifications, ENPP1 mutation) and the right treatment (bisphosphonates)was started with almost total regression of the calcifications in 3 months. In the second case the circumstances of addmission was different-the infant was redirected to our Institute too late,three weeks after birth. In this case CT revealed advanced large and middle-size artierial calcifications. No gene analisis were performed. No bisphosphonate treatment was inclueded. Patient died of sudden cardiac arrest.The right diagnosis (GACI) was made postmortem. This case shoud be an important lesson how fast calcifications could develop without treatment and how crucial is early diagnosis with the the right treatment. In the third and fourth cases of idiopathic artery calcifications no genetic alterations(typical for GACI) were revealed. Calcifications receded by itself without bisphosphonates treatment.
**Conclusion:** Our study higlights the importance of early diagnostic of GACI and early start of the treatment with bisphosphonates. Time is crucial in this type of diseases. Every day/week of waiting could worsening the disease significantly with plenty of possible complications. That why time is the most valuable resource.
**Limitations:** N/A
**Funding for this study:** N/A
**Has your study been approved by an ethics committee?** Not applicable
**Author Disclosures:**
Helena Witowicz: Nothing to disclose
Wiesława Grajkowska: Nothing to disclose
Elżbieta Jurkiewicz: Nothing to disclose

**Synergistic and Interpretable AI for Predicting Paediatric RMPP: A Multi-modal Fusion of Clinical and CT Data**
*L. Deng*, Z. Jia; Yulin/CN
*(denglin3819@sina.com)*

**Purpose:** Delayed diagnosis of paediatric refractory Mycoplasma pneumoniae pneumonia (RMPP) leads to severe sequelae, as current single-modality predictors lack accuracy. We aimed to develop and validate a synergistic, multi-modal AI framework, integrating heterogeneous clinical data with Swin Transformer-based CT analysis, for high-fidelity and clinically trustworthy early risk stratification

**Methods or Background:** This retrospective study of 466 children with MPP fused four data pillars: clinical/laboratory data, quantified radiologist knowledge, automated objective CT biomarkers, and deep sub-visual features from a Swin Transformer. An XGBoost model integrated these features, with SHAP analysis ensuring decision transparency. The framework was validated on an independent test set.
**Results or Findings:** On an independent test set, the fusion model showed excellent discrimination (AUC: 0.92; 95% CI: 0.88-0.96) with high sensitivity (0.85) and specificity (0.88), enabling robust identification of high-risk patients. Ablation studies confirmed the multi-modal approach significantly outperformed single-modality baselines. SHAP analysis revealed a synergy between known clinical factors (e.g., LDH, CRP) and novel, influential sub-visual CT patterns from the Swin Transformer, confirming the data fusion's synergistic gain.
**Conclusion:** This study's core contribution is quantitatively confirming a significant synergistic effect among four heterogeneous data dimensions via ablation study, establishing the necessity of multi-modal fusion for RMPP prediction. The proposed explainability framework transforms opaque model decision-making into a transparent, auditable evidence pathway, providing a reliable basis for clinical decisions. This work offers an empirical foundation for a safer, more precise human-machine collaborative model, aiming to improve outcomes through timely intervention.
**Limitations:** Not applicable
**Funding for this study:** Yulin City Youth Talent Support Program
**Has your study been approved by an ethics committee?** Yes
**Ethics committee - additional information:** Yulin Hospital,The First Affiliated Hospital of Xi'an Jiaotong University:2025048
**Author Disclosures:**
Zhe Jia: Nothing to disclose
Lin Deng: Nothing to disclose

**MRI in Fetal and Neonatal Circulation**
D. Velan, F. Abubacker Sulaiman, R. Praveenkumar, J. Lydia, M. Nivitha, *K. Saravanan*; Chennai/IN

**Purpose:** To explore the feasibility and diagnostic potential of 4D Flow MRI in evaluating dynamic hemodynamics of fetal and neonatal circulation, including intracardiac flow patterns, shunt physiology, and cerebroplacental perfusion. The study aims to establish normative flow parameters and identify early markers of circulatory compromise in high-risk pregnancies.
**Methods or Background:** Conventional Doppler ultrasound provides limited two-dimensional assessment of blood flow velocity and direction, lacking volumetric and temporal resolution. 4D Flow MRI, with its time-resolved three-dimensional velocity encoding, enables comprehensive quantification of flow vectors, wall shear stress, and vorticity throughout the cardiac cycle. In this prospective study, 40 fetuses (28–38 weeks) and 20 neonates underwent 4D Flow MRI using a prototype highly accelerated phase-contrast sequence with retrospective gating. Post-processing included vessel segmentation, streamline visualization, and quantitative analysis of flow through the foramen ovale, ductus arteriosus, and umbilical vessels.
**Results or Findings:** 4D Flow MRI successfully delineated intracardiac and extracardiac flow pathways in 95% of cases. Quantitative analysis revealed consistent flow patterns correlating with gestational age. In growth-restricted fetuses, altered shunt flow ratios and reduced cerebral perfusion indices were observed. Neonatal 4D Flow data demonstrated transitional circulatory changes post-delivery, validating physiologic closure of fetal shunts
**Conclusion:** 4D Flow MRI provides unparalleled insights into fetal and neonatal hemodynamics, overcoming limitations of Doppler ultrasound. It holds transformative potential for early detection of circulatory dysfunction, particularly in placental insufficiency and congenital heart disease.
**Limitations:** Motion artifacts, fetal heart rate variability, and prolonged acquisition times currently restrict routine clinical application. Absence of real-time gating and small sample size limit statistical generalizability. Further optimization of faster, motion-robust sequences and standardized fetal cardiac gating are required for broader clinical translation.
**Funding for this study:** None
**Has your study been approved by an ethics committee?** Not applicable
**Author Disclosures:**
Rathinamoorthy Praveenkumar: Nothing to disclose
K. Saravanan: Nothing to disclose
Mohanam Nivitha: Nothing to disclose
Johnbosco Lydia: Nothing to disclose
Dhanush Velan: Nothing to disclose
Farook Abubacker Sulaiman: Nothing to disclose

**Cardiac MRI Evaluation of Foetal Ductus Arteriosus in Right Aortic Arch**
*S-Z. Dong*; Shanghai/CN
*(dongsuzhen@126.com)*

**Purpose:** To show the types of ductus arteriosus (DA) position and course, and further measure the diameters of DA in foetal different branching patterns of the right aortic arch (RAA) using prenatal cardiac magnetic resonance
**Methods or Background:** 88 foetuses with RAA were analysed retrospectively. RAA mirror-image branching with left anterior DA (n=16), RAA with aberrant left subclavian artery (ALSCA) and left posterior DA (n=50), RAA mirror-image branching with left retroesophageal DA (n=9), RAA mirror-image branching with right DA (n=11), RAA with ALSCA and bilateral DA (n=1) and RAA mirror-image branching with bilateral DA (n=1). Two radiologists measured the diameters of DA respectively, and then statistically analysed the data of the DA diameters obtained from all foetuses. Statistical comparisons between the z score of DA diameter were analysed using the non-parametric Mann-Whitney U test.
**Results or Findings:** Of the 88 foetuses, the mean z score of the DA diameter with 16 foetuses with RAA mirror-image branching with left anterior DA was significantly less than that of the 11 cases with RAA with right DA ($P < 0.05$); the DA of 50 cases with RAA with ALSCA were larger than that of 9 cases with RAA with left retroesophageal DA ($P < 0.05$). Eleven neonates (12.5%) had respiratory symptoms, nine with RAA with ALSCA and left posterior DA, one with RAA and left anterior DA, and one with RAA and left retroesophageal DA.
**Conclusion:** Foetal CMR can describe the position and course of DA, and further measure the diameters of DA in different branching patterns of RAA.
**Limitations:** All cases were followed up only during the neonatal period, therefore, only a few cases have clinical symptoms in this study.
**Funding for this study:** National Natural Science Foundation of China (81971582, 81571628), Shanghai Pujiang Programme (2019PJD030)
**Has your study been approved by an ethics committee?** Yes
**Ethics committee - additional information:** This study was authorized by the ethics commission of our hospital.
**Author Disclosures:**
Su-Zhen Dong: Nothing to disclose

**The actual use of fetal gated cardiovascular magnetic resonance imaging: a worldwide survey**
*G. Gourincour*[1], H. Boeth[2], F. Kording[2], D. Grevent[3], R. Chaoui[4], L. J. Salomon[3]; [1]Marseille/FR, [2]Hamburg/DE, [3]Paris/FR, [4]Berlin/DE
*(guillaume.gorincour@gmail.com)*

**Purpose:** To study the clinical and research use of fetal smart-sync cardiovascular magnetic resonance imaging (F-ss-CV-MRI) worldwide
**Methods or Background:** Extensive survey sent to 70 centers equipped with the device. Questions concerned project-leading specialty, country, setting, quantitative and qualitative user analysis, degree of satisfaction and remaining needs
**Results or Findings:** 30 centers answered (43 %). In 19 (63 %) team was lead by radiologists, in 8 (27 %) by cardiologists, and in 3 (10 %) by researchers. Thirteen centers were in the US (43 %), 4 (13 %) in Germany, 3 (10 %) in France, 2 (6.7 %) in the Netherlands, Sweden and the UK, and 1 (3.3 %) in Italy, Switzerland and Israel. Setting was academic in 23 (77 %), hospital-based in 16 (54 %) and private in 2 (6.7 %). Centers have been users for a mean of 21.6 months (Min 1, max 48), with a mean number of exams of 33.2 (Min 2, Max 200). Twenty-eight centers perform MRI between 30 and 35 weeks' gestation (WG), 8 after 35 WG and 4 before 30 WG. In 19 centers it is performed for clinics and research, in 6 only for clinics and in 6 only research. Twenty-four centers (80 %) rate as high/very high the improvement and would recommend it. Clinical situations when it would be recommended were mostly unclear complex congenital heart disease with need for improved delineation
**Conclusion:** F-ss-CV-MRI is a promising technique which needs shorter sequences, better consistency and wider diffusion
**Limitations:** Image quality for 10 centers (33 %), convincing their referrals for 8 (27 %), and MR accessibility for 8 (27). Fourteen centers (47 %) consider that it has not yet an impact on diagnosis but 24 (80 %) believe it will have
**Funding for this study:** None
**Has your study been approved by an ethics committee?** Not applicable
**Author Disclosures:**
Fabian Kording: CEO: Northh Medical
Rabih Chaoui: Nothing to disclose
Laurent J. Salomon: Nothing to disclose
David Grevent: Nothing to disclose
Guillaume Gourincour: Nothing to disclose
Heide Boeth: Employee: Northh Medical

**11:30 - 12:30** **Room G1**

### Research Presentation Session: Radiographers Hot Topic with Keynote Lecture

### RPS 314

### Hot Topic: AI-augmented practice

**Moderators**
Clare Rainey; Cork/IE
*(clare.rainey@ucc.ie)*
Ramprabananth Sivanandan; Asker/NO
*(sivram@vestreviken.no)*

**Keynote Lecture**
Jonathan Loui Portelli; Msida/MT
*(jonathan.portelli@um.edu.mt)*

### Artificial intelligence-assisted interpretation of lung cancer on chest CT using Google AI Studio

*Z. D. Aljneibi*, S. Almenhali, L. J. O. C. L. Lança; Abudhabi/AE
*(zainab_alj5@hotmail.com)*

**Purpose:** This study explored the diagnostic performance and interpretative behaviour of an artificial intelligence (AI)-enhanced model for detecting lung cancer on chest computed tomography (CT). Particular focus was given to the qualitative consistency of the AI-generated reports.
**Methods or Background:** An exploratory analysis was conducted using the publicly available IQ-OTH/NCCD dataset, comprising 110 CT cases (55 normal, 15 benign, 40 malignant). A pre-trained convolutional neural network in Google AI Studio was fine-tuned on a subset of images and applied to independent test cases. Quantitative outcomes included diagnostic accuracy, sensitivity, specificity, and area under the receiver operating characteristic curve (AUC). Qualitative analysis evaluated report structure, terminology use, and error patterns.
**Results or Findings:** The AI model achieved an overall accuracy of 75.5% (sensitivity 74.5%, specificity 76.4%). Malignant cases were identified with high discriminative performance (AUC = 0.902; 95 % CI: 0.830–0.964), while benign cases proved more difficult to classify (AUC = 0.615; 95 % CI: 0.477–0.754). Qualitative analysis showed consistent use of radiological terminology, with structured descriptions resembling human reporting. In normal cases, terms such as “clear lung fields” confirmed accurate recognition, though occasional references to “ground-glass opacities” reflected oversensitivity to minor changes. Benign cases were described with bilateral opacities and fibrotic changes, but overlapping terminology sometimes led to misclassification. Malignant cases were strongly aligned with features such as “mass” and “lesion.” These interpretative tendencies highlight effective feature recognition but limited specificity in non-malignant contexts.
**Conclusion:** Google AI Studio showed potential in detecting malignant disease and produced structured reports with consistent terminology. However, its interpretative oversensitivity and limited specificity highlight the need for human oversight and further refinement to reduce misclassification in benign and normal presentations.
**Limitations:** This study's retrospective design and reliance on a public dataset may limit generalizability.
**Funding for this study:** Not applicable
**Has your study been approved by an ethics committee?** Not applicable
**Author Disclosures:**
Shayaa Almenhali: Nothing to disclose
Zainab Dawood Aljneibi: Nothing to disclose
Luis J. O. C. Luis Lança: Nothing to disclose

### AI-enabled environmental sustainability in radiology: a systematic review

*N. Stogiannos*[1], K. Konstantinidis[2], S. Kalari[3], S. C. Shelmerdine[1], S. Ursprung[4], T. Küstner[4], K. Nikolaou[4], A. G. Rockall[5], C. Malamateniou[1]; [1]London/UK, [2]Kifisia/GR, [3]Athens/GR, [4]Tübingen/DE, [5]Godalming/UK

**Purpose:** To explore the potential role of AI-powered applications in enhancing environmental sustainability in radiology.
**Methods or Background:** Searches were performed across eight different electronic bibliographic databases: Google Scholar, PubMed, Wiley Online library, Embase, Web of Science platform, the Cochrane library, CINAHL Ultimate, and the MedRxiv database for preprint work, for the publication years 2020-2025. Specific keywords were combined with Boolean operators and filters to optimise searches. Duplicate removal, screening of titles and abstracts, as well as full-text evaluation were done using the Rayyan.ai platform. The CASP checklists were used to perform risk of bias assessment. Extracted data were analysed using a narrative synthesis approach.
**Results or Findings:** Out of 87120 initial sources of evidence, 18 articles were finally included in this systematic review. AI was identified as an important factor in reducing energy consumption in radiology, both by minimising equipment idle time and by accelerating MRI acquisition, automating planning, and simplifying workflows. In addition, AI could facilitate low-field imaging or significantly reduce contrast agent dosage, by generating virtually enhanced images, or restoring signal with minimal contrast agent waste. AI could contribute to sustainability in imaging by reducing unnecessary examinations through clinical decision support, optimising workforce and patient scheduling, and assisting image analysis to minimise workstation use which together lower energy consumption and resource use.
**Conclusion:** This systematic review demonstrates that AI-powered applications hold promise with regard to environmental sustainability in radiology, helping to optimise image acquisition, image analysis, contrast administration, and workflow efficiency. Balance must be struck between energy-intensive AI usage and AI-enhanced energy saving, while any costs and energy demands associated with training AI models and data storage should be always considered.
**Limitations:** n/a
**Funding for this study:** n/a
**Has your study been approved by an ethics committee?** Not applicable
**Author Disclosures:**
Konstantin Nikolaou: Nothing to disclose
Selmina Kalari: Nothing to disclose
Nikolaos Stogiannos: Nothing to disclose
Susan Cheng Shelmerdine: Nothing to disclose
Andrea Grace Rockall: Nothing to disclose
Stephan Ursprung: Nothing to disclose
Kleanthis Konstantinidis: Nothing to disclose
Christina Malamateniou: Nothing to disclose
Thomas Küstner: Nothing to disclose

### AI-Based Cardiac MRI for Tissue Characterization in Acute Myocardial Infarction: a reproducibility study

*V. Tambè*, D. Capra, B. Loscocco, C. Casamassima, M. Memmedova, F. Secchi; Milan/IT
*(valentina.tambe@gmail.com)*

**Purpose:** This study investigated the impact of AI-based image denoising and super-resolution in cardiac magnetic resonance (CMR) imaging in patients with acute myocardial infarction who underwent coronary revascularization.
**Methods or Background:** Myocardial mass, edema, fibrosis and no-reflow were segmented by an expert reader using a semi-automatic software, in both native and AI-enhanced images. The analysis focused on Short Tau Inversion Recovery (STIR) and Late Gadolinium Enhancement (LGE) sequences.
**Results or Findings:** Twenty-seven patients were included, with no-reflow present in 13. AI-derived measurements demonstrated good agreement with native imaging. For edema percentage, the bias was 3% with limits of agreement (LoA) from –24.3% to +30.4%; for LGE percentage, the bias was 1% with LoA from –17.6% to +19.6%. AI reconstructions tended to underestimate myocardial mass: in T1 sequences, the bias was –9.1 g (LoA –26.2 to +8.0 g), and in T2 sequences, –5.3 g (LoA –49.9 to +39.3 g). Similarly, no-reflow mass and volume were underestimated, with biases of –6.5 g (LoA –16.4 to +3.8) and –6.2 ml (LoA –15.6 to +3.3), respectively. In both native and AI-enhanced datasets, significant negative correlations were observed between LVEF and LGE% (native: $\rho = -0.616$, $p<0.001$; AI: $\rho = -0.551$, $p=0.003$) as well as between LVEF and no-reflow mass (native: $\rho = -0.652$, $p=0.016$; AI $\rho = -0.57$, $p = 0.04$), whereas edema showed a moderate negative correlation (native: $\rho = -0.501$, $p=0.008$; AI: $\rho = -0.402$, $p=0.038$).
**Conclusion:** A negligible bias was observed between native and AI-derived measurements. Although the limits of agreement were wide, AI-enhanced CMR consistently correlated with systolic function. These findings support the use of AI-based denoising and super-resolution in CMR without significantly affecting myocardial tissue quantification.
**Limitations:** The limited sample size may have affected the limits of agreement.
**Funding for this study:** None
**Has your study been approved by an ethics committee?** Yes

**Ethics committee - additional information:** IRCCS MULTIMEDICA - ethics committee
**Author Disclosures:**
Chiara Casamassima: Nothing to disclose
Francesco Secchi: Nothing to disclose
Valentina Tambè: Nothing to disclose
Beatrice Loscocco: Nothing to disclose
Malaka Memmedova: Nothing to disclose
Davide Capra: Nothing to disclose

**AI-Assisted Onboarding for Radiographers: A Qualitative Evaluation of the Pair Chat Assistant**

*C. Tan*, L. A. J. Chong; Singapore/SG
*(celine.tan.y.y@sgh.com.sg)*

**Purpose:** Onboarding for newly recruited radiographers often relies on didactic lectures and online learning to introduce protocols and practice. While effective, this limits engagement, flexibility, and self-directed learning. Pair Chat is an in-house AI large language model platform that enables staff to build personalised AI bots. A customised bot was created within the Radiography Department to provide interactive, on-demand support for new radiographers during onboarding. This study evaluated the perceived effectiveness of AI-assisted onboarding using the Pair Chat assistant compared with the conventional methods of learning.
**Methods or Background:** A qualitative exploratory design was adopted. Eight radiographers who had used the Pair Chat assistant for three months participated in a focus group discussion. Data were transcribed verbatim and analysed through a hybrid content–thematic approach. Domains explored included learning effectiveness, confidence, engagement, efficiency, and improvement suggestions.
**Results or Findings:** Three themes emerged. First, accessibility barriers: reliance on work desktops limited spontaneous use, highlighting the need for mobile access. Second, a trust hierarchy: although valued for rapid, non-judgemental clarification, participants continued to prioritise colleagues and institutional references. This conditional trust reinforced the perception of the AI as a supplementary rather than replacement tool. Third, complementarity: while the assistant offered detailed, searchable content, the absence of graphics and experiential depth reduced its utility compared with lectures.
**Conclusion:** AI-assisted onboarding can enhance flexibility and learner autonomy, but its effectiveness depends on accessibility and integration with mentorship. A blended model may provide the most sustainable approach.
**Limitations:** This pilot study is limited by its small sample size, single-site context, and short evaluation period. Findings relied on self-reported perceptions, with potential bias from participant self-selection and group dynamics, and may not be generalisable. Results should therefore be interpreted as preliminary insights to inform larger-scale studies.
**Funding for this study:** Not Applicable.
**Has your study been approved by an ethics committee?** Not applicable
**Author Disclosures:**
Letty Ai Jia Chong: Nothing to disclose
Celine Tan: Nothing to disclose

**AI failures during AI implementation into clinical practice**

*N. Stogiannos*[1], R. Cuocolo[2], A. D'Antonoli[3], D. Pinto Dos Santos[4], H. Harvey[1], M. Huisman[5], B. Koçak[6], M. Klontzas[7], C. Malamateniou[1]; [1]London/UK, [2]Naples/IT, [3]Basel/CH, [4]Mainz/DE, [5]Nijmegen/NL, [6]Instanbul/TR, [7]Heraklion/GR

**Purpose:** To map out different potential causes of AI failures, that could potentially impede AI implementation, lead to poor patient outcomes, increase financial costs, and add burden to clinical workflows. Potential solutions to mitigate these errors are also presented.
**Methods or Background:** A diverse group of AI experts in medical imaging, including radiologists, radiographers, computer scientists, and technical physicians, hand-searched all available literature to identify studies related to AI failures in radiology, as well as potential solutions. All eligible papers were then analysed by three group members and assigned to specific categories.
**Results or Findings:** Three distinct categories of AI failures were identified: a) errors related to AI models (algorithmic bias, lack of diverse and inclusive datasets, poor internal/external testing, failures due to unseen real-world data, suboptimal post-market surveillance, and data safety during AI model decommissioning), b) infrastructure-related failures (hardware/software issues, poor integration into PACS/RIS, network deficiencies), and c) human factors (human-AI interaction, automation bias, resistance to change, publication mishaps, annotation/interpretation errors, ergonomics). Adequate AI training and literacy, continuous monitoring of AI tools, standardized reporting of AI studies, multidisciplinary collaboration, effective leadership, and funding were suggested as potential solutions to the above failures.
**Conclusion:** AI models can fail at any stage through their lifecycle. Infrastructure issues and human related factors may present an important cause of AI failures.
**Limitations:** This is not an exhaustive list of AI failures in radiology as it is challenging to identify AI failures in a culture that mostly celebrates success. Also, identification is challenging due to publication bias and the evolving nature of the field.
**Funding for this study:** N/A
**Has your study been approved by an ethics committee?** Not applicable
**Author Disclosures:**
Michail Klontzas: Nothing to disclose
Burak Koçak: Nothing to disclose
Hugh Harvey: Nothing to disclose
Akıncı D'Antonoli: Nothing to disclose
Merel Huisman: Nothing to disclose
Nikolaos Stogiannos: Nothing to disclose
Renato Cuocolo: Nothing to disclose
Daniel Pinto Dos Santos: Nothing to disclose
Christina Malamateniou: Nothing to disclose

**Collaborative development of an ML-based CT protocol recommender**

A. Li[1], *B. Dufour*[2], S. Morozov[2], N. Heracleous[2], B. Rizk[2], C. Thouly[2]; [1]Milwaukee/US, [2]Sion/CH
*(benoit.dufour@groupe3r.ch)*

**Purpose:** To collaboratively develop and evaluate a lightweight, data-driven machine learning model for recommending CT Body protocols, and to analyze the effect of procedure-based filtering on performance under data-scarce conditions.
**Methods or Background:** Efficient CT protocol selection often requires synthesis of multiple data sources and coordination among staff, creating variability and delay; AI-augmented decision support may streamline this step in the radiology workflow. A multicenter French-language dataset of 42,286 adult CT Body studies across 27 protocols was assembled; to emulate scarcity, training was limited to 5,000 studies with stratified partitioning of the remainder into validation (8%) and test (20%). Inputs were imaging order and free-text clinical indication; features were derived via a statistical text extraction and used as inputs to a decision tree-based model, XGBoost. Given the class imbalance, micro F1 was the primary metric.
**Results or Findings:** Procedure–protocol analysis showed certain procedures mapped to multiple protocols, mirroring local workflow. Baseline model (all procedures) achieved micro F1 58%. After filtering one high-volume procedure (43% of exams), micro F1 increased to 67%. Aggressive filtering (two procedures totaling 60% of exams) reached micro F1 80%. In the baseline model, high-confidence predictions (≥0.80 probability) covered 33% of the test set with 91% accuracy, predominantly for well-defined protocols (e.g., spine). Confusion analysis indicated strong performance in 18/27 protocols, with most errors among protocols with overlapping anatomic coverage.
**Conclusion:** A simple decision tree–based model, coupled with procedure filtering, achieved the target 80% micro F1 on a CT Body dataset under constrained training size, suggesting AI-augmented protocoling can reliably handle a substantial subset of studies while flagging ambiguous cases for expert review.
**Limitations:** Generalizability to other sites, languages, and clinical domains requires validation; comparisons with macro-weighted metrics may further characterize class-specific behavior.
**Funding for this study:** Research grant from GEHC
**Has your study been approved by an ethics committee?** Yes
**Ethics committee - additional information:** Approved by CER-VD
**Author Disclosures:**
Cyril Thouly: Shareholder: SMA - 3R Holding
Sergey Morozov: Nothing to disclose
Benoît Dufour: Shareholder: SMA - 3R Holding
Natalie Heracleous: Nothing to disclose
Benoît Rizk: Shareholder: SMA - 3R Holding
Allison Li: Employee: GEHC

**Discussion**

11:30 - 12:30 **Research Stage 1**

### Research Presentation Session: Oncologic Imaging Hot Topic with Keynote Lecture

### RPS 316

### Hot Topic: metastasis detection and oligometastasis

**Moderator**
Antoine Iannessi; Nice/FR
*(antoineiannessi@gmail.com)*
**Author Disclosures:**
Antoine Iannessi: Employee: Mediantechnologies, Founder: Therapixel

**Keynote Lecture**
Gaurang Pradipkumar Raval; Rajkot/IN
*(drgaurang.raval@gmail.com)*

**The use of oligometastatic disease in routine radiology practice: a real world data analysis**
J. Wu[1], A. De Araujo[2], S. Khozin[3], *M. J. Willemink*[1]; [1]Palo Alto, CA/US, [2]São Paulo/BR, [3]Cary, NC/US
*(martin@segmed.ai)*

**Purpose:** Oligometastatic disease (OMD) refers to a state of metastatic cancer, characterized by a limited number of metastatic lesions. Local treatments such as ablative therapy could potentially be curative to OMD. OMD-diagnosis is based exclusively on imaging findings. OMD challenges the older binary view of cancer as either localized or metastatic. Given the importance of imaging findings for diagnosing OMD, we explored the use of OMD in real-world radiological practice.
**Methods or Background:** We evaluated clinical radiology-reports from USA-located healthcare systems. Healthcare providers ranged from imaging centers, small- and medium-sized hospitals, to academic and oncology-focused providers. A real-world imaging data platform was used to search, filter, and select radiology reports (Segmed).
**Results or Findings:** A total of 33,747,977 reports of 11 radiology modalities (including X-ray, MRI, CT, and PET) from healthcare providers in 40 states were included in the search. OMD was mentioned in 164 radiology reports from 109 different patients. Clinicians provided OMD-information in the indication and history in 148 radiology reports of 95 patients, while radiologists mentioned OMD in 18 reports of 17 patients. OMD was mentioned in 20 different primary tumor-types, most commonly breast (n=39 reports of 22 patients), lung (n=33 reports of 26 patients), and prostate cancer (n=16 reports of 13 patients). Modalities with OMD-mention included CT (n=58), X-ray Angiography (n=44), PET/CT (n=35), MRI (n=26), and X-ray (n=1). The first mention of OMD was in 2017 (n=2 reports), which increased to n=33 in 2024.
**Conclusion:** In this large scale observational study, we found that despite the importance of imaging, the term OMD is rarely used in routine radiology practice in the USA. These results suggest the need to improve the definition and guidelines around OMD.
**Limitations:** Retrospective study with only US data, no EU data.
**Funding for this study:** None
**Has your study been approved by an ethics committee?** Not applicable
**Author Disclosures:**
Sean Khozin: Nothing to disclose
Aline De Araujo: Employee: Segmed, Inc.
Martin J. Willemink: Founder: Segmed, Inc.
Jie Wu: Founder: Segmed, Inc.

**Optimizing Risk Stratification: Diagnostic Performance and Clinical Utility of Node-RADS for Predicting Lymph Node Metastasis in Cervical Cancer**
*J. Chen*, Z. Yuan, Y. Li, Y-L. He, Z. Jin, H-D. Xue; Beijing/CN
*(15860859313@163.com)*

**Purpose:** To develop and validate the first nomogram that integrates Node-RADS with clinical parameters for predicting lymph node metastasis (LNM) in cervical cancer. We further aim to evaluate the clinical utility of this integrated model across various scenarios, particularly in fertility-sparing candidates and cases with indeterminate lymph nodes.
**Methods or Background:** This retrospective study included 465 cervical cancer patients. Two radiologists independently evaluated lymph nodes using Node-RADS on preoperative MRI. Diagnostic metrics (sensitivity, specificity, etc.) were calculated. A combined model incorporating Node-RADS and clinicopathological predictors was developed and presented as a nomogram. Diagnostic performance was evaluated via ROC analysis and decision curve analysis. Subgroup analyses were performed in fertility-sparing candidates (including age <45 years or tumor size ≤2 cm), lymph nodes with subcentimeter metastasis (short-axis diameter <1 cm), and those assigned a Node-RADS score of 3.
**Results or Findings:** Node-RADS score 3 provided optimal diagnostic balance (sensitivity 68.7%, specificity 88.2%, NPV 92.8%), while scores 4–5 showed high specificity (≥99%). The combined model outperformed Node-RADS alone (AUC 0.872 vs. 0.827), with good calibration (p = 0.44) and a greater net benefit at threshold probabilities of 10–40%. The nomogram enabled individualized risk estimation. Subgroup analysis confirmed strong performance in fertility-sparing candidates (AUC 0.884 for age<45; 0.851 for tumor ≤2 cm), identified patients with subcentimeter LNM（AUC 0.877）, and improved stratification of Node-RADS 3 nodes (AUC 0.838; LR+ 2.61).
**Conclusion:** Node-RADS is a robust independent predictor for preoperative lymph node metastasis assessment in cervical cancer. Node-RADS-integrated nomogram represents a powerful and clinically useful tool for the individualized preoperative prediction of LNM in cervical cancer, potentially enhancing decision-making for personalized treatment strategies.
**Limitations:** The generalizability of our findings may be limited by the study's retrospective design and single-center patient cohort.
**Funding for this study:** The CAMS Innovation Fund for Medical Sciences (grant no. 2023-I2M-C&T-B-020), National High-Level Hospital Clinical Research Funding (grant no. 2025-PUMCH-C-029).
**Has your study been approved by an ethics committee?** Not applicable
**Author Disclosures:**
Yong-Lan He: Nothing to disclose
Hua-Dan Xue: Nothing to disclose
Jing Chen: Nothing to disclose
Zhilin Yuan: Nothing to disclose
Zhengyu Jin: Nothing to disclose
Yuan Li: Nothing to disclose

**CT-based Node-RADS for regional lymph node metastasis in pancreatic ductal adenocarcinoma: diagnostic performance and prognostic impact**
*X-P. Meng*; Wuxi/CN
*(mengxp0711@163.com)*

**Purpose:** To evaluate the diagnostic performance and prognostic value of the Node Reporting and Data System 1.0 (Node-RADS) for regional lymph node (LN) metastasis in pancreatic ductal adenocarcinoma (PDAC) using preoperative CT.
**Methods or Background:** This single-center retrospective study included patients with PDAC who underwent contrast-enhanced CT followed by surgical resection between January 2017 and July 2023. Each regional LN station was assessed using Node-RADS, in which nodal size and morphologic features were integrated into a five-point scoring system (1–5, from very low to very high suspicion), with histopathology serving as the reference standard. Diagnostic performance, including sensitivity and specificity, was evaluate at the patient, LN group, and station levels. Cox regression analyses were conducted to evaluate the prognostic impact of Node-RADS after adjusting for clinicopathological factors.
**Results or Findings:** A total of 216 patients (mean age ± standard deviation, 61.2 ± 10.0 years; 138 males) were included, with LN metastases detected in 154 of 216 patients (71.3%). A Node-RADS score of 3 or greater yielded the optimal cutoff for predicting lymph node metastasis, with sensitivity and specificity of 77.3% (119/154) and 83.9% (52/62) at the patient level, 74.8% (104/139) and 81.8% (63/77) for Group 1, and 77.3% (34/44) and 82.6% (142/172) for Group 2. Across regional nodal stations, Node-RADS achieved a sensitivity of 60.0–85.7% and a specificity of 79.5–98.8%. Higher Node-RADS categories were independently associated with worse overall survival (hazard ratio [HR], 1.34; 95% CI: 1.15–1.56; p = 0.012) and disease-free survival (HR, 1.45; 95% CI: 1.17–1.92; p = 0.007).
**Conclusion:** Node-RADS provides robust diagnostic accuracy for LN metastasis in PDAC and may serve as an effective preoperative prognostic predictor.
**Limitations:** Retrospective; Single-center study
**Funding for this study:** the foundation of the Wuxi Municipal Bureau on Science and Technology (Y20232002)
**Has your study been approved by an ethics committee?** Yes
**Ethics committee - additional information:** This single-center retrospective study was approved by the Ethics Review Committee of the Affiliated Wuxi People's Hospital of Nanjing Medical University, and the requirement for informed consent was waived due to the retrospective design.
**Author Disclosures:**
Xiang-Pan Meng: Nothing to disclose

**Improving CT detection of subcentimeter liver metastases: AI-augmented contrast boosting enhances sensitivity and conspicuity in choroidal melanoma**

*J. Berger*, F. K. E. Mankertz, N. Maalouf, A. S. Brendlin, J. Herrmann, K. Nikolaou, S. Afat; Tübingen/DE
*(josephine.berger@med.uni-tuebingen.de)*

**Purpose:** To evaluate whether AI-based contrast boosting of single-phase CT improves the detection and visual conspicuity of subcentimetre liver metastases from choroidal melanoma, using liver MRI as the reference standard.
**Methods or Background:** This retrospective single-centre study included 68 patients (mean age, 69.6 ± 11.2 years; 46% men) with histologically confirmed choroidal melanoma and suspected liver metastases, enrolled between January 2019 and July 2022. All underwent portal venous phase contrast-enhanced whole-body CT and dedicated liver MRI. CT data were post-processed using a commercial AI-based contrast-boosting algorithm (ClariACE, ClariPi, Seoul, South Korea). MRI-identified metastases served as the reference standard and were stratified by size (<5 mm, 5-10 mm, >10 mm). Two radiologists independently assessed visibility on standard and AI-boosted CT, recording binary detection and conspicuity on a five-point Likert scale. Detection rates were compared using McNemar's test, conspicuity using the Wilcoxon signed-rank test, and inter-reader agreement using Cohen's kappa and intraclass correlation coefficient.
**Results or Findings:** A total of 104 liver metastases were identified on MRI, including 58 <5 mm. For sub-5 mm lesions, AI-boosted CT significantly improved sensitivity from 57% to 81% ($p < 0.001$). A modest, non-significant increase was observed for 5-10 mm lesions (82% to 89%, $p = 0.09$), while detection of >10 mm lesions remained 100%. Median conspicuity scores for sub-5 mm lesions increased from 3.0 to 4.3 ($p < 0.001$). Inter-reader agreement was excellent for detection ($\kappa = 0.84$) and conspicuity (ICC = 0.87). Increased conspicuity correlated with improved detection of small lesions.
**Conclusion:** AI-based contrast enhancement significantly improves sensitivity and conspicuity for detecting subcentimetre liver metastases, especially those <5 mm, without additional patient risk and may facilitate earlier detection, staging, and treatment planning.
**Limitations:** Retrospective, single-centre design and modest sample size may limit generalisability.
**Funding for this study:** None.
**Has your study been approved by an ethics committee?** Yes
**Ethics committee - additional information:** Local IRB board approval was obtained.
**Author Disclosures:**
Judith Herrmann: Nothing to disclose
Konstantin Nikolaou: Nothing to disclose
Andreas Stefan Brendlin: Nothing to disclose
Saif Afat: Nothing to disclose
Josephine Berger: Nothing to disclose
Fiona Karola Elisabeth Mankertz: Nothing to disclose
Nour Maalouf: Nothing to disclose

**Small Liver Metastases from Uveal Melanoma: detection on MRI comparing Gd-EOB-DTPA hepatobiliary phase with DWI sequences and dynamic phase**

*C. Pontiggia*, V. Magni, M. Vaiani; Milan/IT

**Purpose:** To compare Gd-EOB-DTPA hepatobiliary phase and DWI sequences with dynamic phase on contrast-enhanced MRI for detection of liver metastases from uveal melanoma, as assessed by two readers with different levels of expertise.
**Methods or Background:** This retrospective study included patients with liver metastases from uveal melanoma who underwent surgery between 2017 and 2025. Preoperative liver-focused MRI scans were independently reviewed by two readers with different expertise. Two imaging protocols were evaluated: T1/T2-weighted with dynamic sequences, and T1/T2-weighted with DWI/ADC and hepatobiliary phase sequences. Histopathological results from surgery and follow-up MRI for non-resected lesions were retrieved. Lesion counts were compared using the Wilcoxon signed-rank test. Inter-reader agreement was assessed with the ICC.
**Results or Findings:** 16 patients were included. Senior reader detected 68 lesions with DWI/hepatobiliary phase sequences and 25 with dynamic contrast-enhanced sequences, while junior reader detected 45 and 33 lesions, respectively. Of the 68 lesions detected by senior reader on DWI/hepatobiliary sequences, 60 had histopathological confirmation, seven were confirmed at follow-up MRI, and one was a false positive (positive predictive value of 98.5%). In 9/16 patients (56.3%), MRI detected all surgically proven lesions, whereas in 7/16 patients surgery revealed additional lesions not visible on MRI. Senior reader identified significantly more lesions with DWI/hepatobiliary than with dynamic sequences (median 3.5 versus 1.0, $p = 0.0014$). Junior reader also detected significantly more lesions with DWI/hepatobiliary than with dynamic sequences (median 2.0 versus 1.0, $p = 0.017$). Inter-reader agreement was moderate (ICC of 0.66 for dynamic and 0.64 for DWI/hepatobiliary sequences).
**Conclusion:** DWI/hepatobiliary MRI sequences improved lesion detection compared with dynamic sequences, particularly for the experienced reader; furthermore, they showed a high positive predictive value, with complete concordance to surgery in over half of patients.
**Limitations:** Small sample size.
**Funding for this study:** No funding was received for this study.
**Has your study been approved by an ethics committee?** Not applicable
**Author Disclosures:**
Chiara Pontiggia: Nothing to disclose
Veronica Magni: Nothing to disclose
Marta Vaiani: Nothing to disclose

**Prognostic factors for metastatic patients: Numeric tumor fraction in liquid biopsy, tumor volume and anthropometry in CT scans**

*A. Choucair*[1], J. Dupont[2], G. H. Jardali[1], L. Dawi[1], L. Lawrance[3], S. Ammari[2], C. Massard[2], A. Italiano[4], N. Lassau[2]; [1]Paris/FR, [2]Villejuif/FR, [3]Villepinte/FR, [4]Bordeaux/FR
*(jules.dupont@gustaveroussy.fr)*

**Purpose:** Total tumor volume (TTV) on CT scans, circulating tumor DNA (CTDNA) from liquid biopsy (LB), known as tumor fraction (TF), as well as 3Danthropometric measurements, are indicators for predicting overall survival (OS) in metastatic patients.
**Methods or Background:** Patients with a baseline CT scan and LB (STING, NCT04932525, Gustave Roussy) were included from January 2021 to December 2023 in this study. CT scans were annonymized and manually annotated in 2D by radiologists, with a 3D TTV estimated (Tumor volume = 2/3 xMesh surface area x Minor axis). TF was reported as a percentage. The Anthropometer3DNet software provided measurements for muscle, superficial, and deep fat masses; combined with TTV and with Cox model coefficients to generate a CT scan risk-score. Patients were grouped based on TF (≥0%) and divided into two subgroups according to the CT scan risk-score. OS was the primary endpoint, and the analyses were carried out using Kaplan-Meier estimators and log-rankstatistics.
**Results or Findings:** Among 720 patients with 38,283 annotated metastases, the median OS was 13.1 months, with a median TF and TTV of 3.3% and 91.6 $cm^3$. A total of 213 patients had TF=0% despite measurable tumor burden (median TTV 35.3 $cm^3$), and their OS was shorter (10.2 months) compared to TF>0% (25.5 months). Within the TF=0% group, patients with a low CT-scan risk score lived longer (35.4 months) than those with a higher score (19.4 months). In the TF>0% group, OS was 13.2 months for low CT-scan risk-score but dropped to 7.5 months for those with TTV above the median.
**Conclusion:** TTV and anthropometric measurements, when combined with TF offer a more precise prediction of OS.
**Limitations:** This is a retrospective single-center study, relying on 2D TTV estimation with a heterogeneity in the cohort.
**Funding for this study:** No fundings.
**Has your study been approved by an ethics committee?** Not applicable
**Author Disclosures:**
Lama Dawi: Nothing to disclose
Littisha Lawrance: Nothing to disclose
Christophe Massard: Nothing to disclose
Antoine Italiano: Nothing to disclose
Samy Ammari: Nothing to disclose
Nathalie Lassau: Nothing to disclose
Aurelie Choucair: Nothing to disclose
Ghina Hilal Jardali: Nothing to disclose
Jules Dupont: Nothing to disclose

**13:00 - 14:30 Research Stage 1**

### Research Presentation Session: Cardiac

### RPS 403

### Inflammation, immunometabolism and cardiac imaging

**Moderator**
Iacopo Carbone; Rome/IT
*(iacopo.carbone@uniroma1.it)*

**The role of cardiac computed tomography and echocardiography in diagnosis of infective endocarditis**

*A. Č. Petkovic*, N. Menkovic, O. Petrovic, I. Bilbija, D. Stanisavljevic, S. Putnik, B. Ivanovic, R. Maksimović; Belgrade/RS
*(ancipetkovic@gmail.com)*

**Purpose:** Infective endocarditis (IE) is rare disease with high mortality rate and rising incidence, requiring timely and precise diagnosis in order to choose appropriate therapy. Imaging of morphologic lesions is integrative part of diagnosis. Artifacts and patient's habitus make echocardiography difficult to visualize advanced-form IE. Cardiac computed tomography (CCT) constantly shows an additive diagnostic value due to high spatial resolution of cardiac anatomy. Joint application of both diagnostic tests improves overall sensitivity and specificity in diagnosing IE.
**Methods or Background:** Diagnostic study of 83 patients who satisfied category of definite and possible IE, based on modified Duke's criteria 2015, was conducted at University Clinical Center of Serbia, between May 2013 and April 2023. Patients underwent transthoracic echocardiography (TTE), transesophageal echocardiography (TEE), and CCT. We analyzed valvular and paravalvular IE lesions in three imaging methods and compared them to surgical or autopsy findings. We calculated sensitivity, specificity, diagnostic accuracy, and positive and negative predictive value of imaging tests individually and jointly used.
**Results or Findings:** We examined 78 patients and analyzed 85 valves (70 native, 13 prosthetic, and 2 corrected due to Ozaki procedure, central shunt and 4 pacemaker leads). As single test, sensitivity and specificity of CCT, TTE, and TEE for valvular lesions were 91.6/20%, 65.5/57.9%, and 60/84%, and paravalvular lesions were 100/0%, 46/10.5%, and 14.7/100%. When combined together, sensitivity and specificity for valvular lesions rose to 96.6/0% and paravalvular lesions to 100/0%. We also analyzed the diagnostic performance for each test in single and mutual application, per specific IE lesion.
**Conclusion:** CCT in comparison to TTE and TEE shows better diagnostic performance in detection of valvular and paravalvular lesions. In joint application, there is a statistically significant difference in performance compared to their single use.
**Limitations:** Not applicable.
**Funding for this study:** No funding was provided for this study.
**Has your study been approved by an ethics committee?** Yes
**Ethics committee - additional information:** Ethics Committee of the School of Medicine of the University of Belgrade (No: 17/IV-26, 11 April 2023, Belgrade, Serbia)
**Author Disclosures:**
Ilija Bilbija: Nothing to disclose
Dejana Stanisavljevic: Nothing to disclose
Ana Čedomir Petkovic: Nothing to disclose
Ružica Maksimović: Nothing to disclose
Svetozar Putnik: Nothing to disclose
Olga Petrovic: Nothing to disclose
Nemanja Menkovic: Nothing to disclose
Branislava Ivanovic: Nothing to disclose

**Pericoronary Adipose Tissue Attenuation in Patients with Spontaneous Coronary Artery Dissection According to Emotional or Physical Triggers: Insights from the INSIGHT-SCAD Study**

*C. Martini*, M. Covani, F. L. Gurgoglione, M. Della Bella, G. Cicala, L. Vignali, M. De Filippo, G. Niccoli, E. Solinas; Parma/IT
*(chiara.martini@unipr.it)*

**Purpose:** To evaluate whether pericoronary adipose tissue (PCAT) attenuation, a marker of vascular inflammation, differs according to the type of trigger (emotional vs. physical) in patients with spontaneous coronary artery dissection (SCAD), and to explore the potential link between emotional stress, vascular inflammation, and SCAD pathophysiology.
**Methods or Background:** Inflammation may play a pivotal role in predisposing vascular fragility to spontaneous coronary artery dissection (SCAD). PCAT attenuation, derived from coronary computed tomography angiography (CCTA), is a recognized imaging biomarker of coronary inflammation. This analysis, part of the INSIDE-SCAD study, included patients with SCAD who underwent CCTA within 24 hours of the index event and had a clearly identifiable trigger. Patients were stratified by trigger type (emotional vs. physical). PCAT attenuation was measured in culprit and non-culprit vessels.
**Results or Findings:** Twenty-five SCAD patients were included (mean age 55±11 years; 80% female). Emotional triggers were reported in 17 patients (68%), and physical triggers in 8 (32%). Obesity was more prevalent in patients with physical triggers (62.5% vs. 5.9%, p=0.010), while Type 2 dissections were more frequent in the emotional trigger group (64.7% vs. 25.0%, p=0.040). Patients with emotional triggers exhibited significantly higher PCAT attenuation compared to those with physical triggers in the culprit vessel (−61.6 vs. −70.9 HU, p=0.019), LAD (−59.7 vs. −70.0 HU, p=0.023), and RCA (−66.7 vs. −75.4 HU, p=0.005).
**Conclusion:** Patients with SCAD showed elevated PCAT attenuation, particularly in cases associated with emotional triggers. These findings suggest that vascular inflammation may represent a predisposing factor for SCAD and that autonomic dysregulation may contribute to this inflammatory state.
**Limitations:** - Small sample size - Observational sub-study design - Selection bias (trigger-defined inclusion) - Absence of a control group - Lack of follow-up and outcome data
**Funding for this study:** Nothing to disclose
**Has your study been approved by an ethics committee?** Yes
**Ethics committee - additional information:** Protocol number of approval by the Ethics Committee of the University Hospital of Parma (AOUPR): 605/2023/OSS/AOUPR SIRER ID 6728
**Author Disclosures:**
Filippo Luca Gurgoglione: Nothing to disclose
Gloria Cicala: Nothing to disclose
Emilia Solinas: Nothing to disclose
Massimo De Filippo: Nothing to disclose
Matteo Della Bella: Nothing to disclose
Giampaolo Niccoli: Nothing to disclose
Luigi Vignali: Nothing to disclose
Chiara Martini: Nothing to disclose
Marco Covani: Nothing to disclose

**External Validation and CMR Optimization of the 2025 ESC Myocarditis Guideline Risk Stratification Strategy**

*Y. Wang*, M. Lu; Beijing/CN
*(wangyining97@126.com)*

**Purpose:** To externally validate the myocarditis risk stratification proposed in the 2025 European Society of Cardiology (ESC) guideline. A further aim was to determine whether cardiovascular magnetic resonance (CMR) parameters, specifically replacing late gadolinium enhancement (LGE) segment count with quantitative LGE extent and adding extracellular volume (ECV), could improve prognostic discrimination. The feasibility of a CMR-only risk stratification approach was also evaluated.
**Methods or Background:** We retrospectively included 308 patients with CMR-diagnosed acute myocarditis. We compared: (a) the original ESC 2025 three-tier model; (b) Model 1, substituting LGE extent for LGE segment count (threshold ≥9.5%, ROC-derived); (c) Model 2, adding ECV (threshold ≥37.5%) to refine low/intermediate risk; and (d) Model 3, a CMR-only three-tier scheme using ESC-defined LVEF categories together with dichotomized LGE extent and ECV. Risk discrimination was assessed by Kaplan–Meier analysis and Cox regression.
**Results or Findings:** A total of 55 major adverse cardiac events (17.9%) were observed during a median follow-up of 3.0 years. In the original ESC 2025 model, high-risk patients were effectively identified (HR 7.26, p=0.006), but intermediate vs. low risk was not significant (HR 1.79, p=0.467). In Model 1, both intermediate (HR 4.53, p=0.046) and high-risk groups (HR 16.93, p<0.001) were significantly different from low risk. In Model 2, prognostic separation further improved (intermediate HR 6.55, p=0.013; high HR 20.83, p<0.001). In Model 3 (CMR-only), risk stratification remained robust (intermediate HR 3.92, p=0.002; high HR 14.11, p<0.001). Model 2 achieved the highest C-index (0.787).
**Conclusion:** While the ESC 2025 scheme reliably identifies high-risk patients with myocarditis, quantitative LGE extent and ECV provide meaningful incremental value for distinguishing low and intermediate risk. A CMR-only stratification is feasible and shows robust prognostic separation.
**Limitations:** Single-center retrospective design; potential variability in CMR acquisition and analysis.
**Funding for this study:** This work was supported by The Construction Research Project of the Key Laboratory (Cultivation) of Chinese Academy of Medical Sciences (2019PT310025); National Natural Science Foundation of China (82471973); Noncommunicable Chronic Diseases-National Science and Technology Major Project (2023ZD0504502); Chinese Academy of Medical

Sciences Innovation Fund for Medical Sciences (CIFMS, 2021-I2M-1-063); Clinical and Translational Fund of Chinese Academy of Medical Sciences (2019XK320063); Youth Key Program of High-level Hospital Clinical Research (2022-GSP-QZ-5).
**Has your study been approved by an ethics committee?** Yes
**Ethics committee - additional information:** The study was approved by the ethics committee of our Hospital (no. 2022–1770)
**Author Disclosures:**
Minjie Lu: Nothing to disclose
Yining Wang: Nothing to disclose

**Evaluation of the Diagnostic Performance of Iodine Maps Derived from a Dual-Layer CT for the Diagnosis of Pericarditis**

*L. R. M. Lanzafame*, D. Masi, G. Ascenti, S. Mazziotti, T. D'Angelo; Messina/IT
*(ludovicalanzafame@gmail.com)*

**Purpose:** To evaluate the diagnostic performance of iodine maps generated from dual-layer spectral CT in detecting pericarditis.
**Methods or Background:** Patients with and without pericardial effusion who underwent coronary computed tomography angiography (CCTA) between February 2023 and December 2024 using a dual-layer spectral CT scanner were retrospectively enrolled. Regions of interest (ROIs) were positioned on the pericardial layers in the iodine maps to quantify iodine concentration (mg/mL). Furthermore, pericardial thickness was measured in both spectral and conventional reconstructions. Pericardial thickness was assessed in both spectral and conventional reconstructions. The diagnostic accuracy of iodine concentration and pericardial thickness in identifying pericarditis was assessed using the European Society of Cardiology (ESC) clinical criteria as reference standard.
**Results or Findings:** The study included 105 patients. The median iodine concentration in patients with pericarditis was 1.79 mg/mL [IQR:1.11–2.24], significantly higher than in negative patients (0.55 mg/mL [IQR:0.42–0.66]; p<0.0001). On conventional reconstructions pericardial thickness was 3.5 mm [IQR:2.7–4.2] in positive patients and 1.2 mm [IQR:1.15–1.3] in negative patients (p<0.0001). On iodine maps, corresponding values were 2.7 mm [IQR: 2.3–3.7] and 1.2 mm [IQR:1.1–1.3] (p<0.0001). Iodine maps achieved an AUC of 0.99 (95%CI:0.94–0.99), with 93.9% sensitivity (95%CI:79.8–99.3) and 95.8% (95%CI: 88.3–99.1) specificity, for a threshold of iodine concentration >0.82 mg/mL. For a pericardial thickness >1.6 mm, iodine maps showed an AUC of 1.00 (95%CI:0.97–1.00), with 100% sensitivity (95%CI:89.4–100) and 100% specificity (95%CI:95.0–100). Conventional reconstructions demonstrated an AUC of 1.00 (95%CI:0.95–1.00), with 100% sensitivity (95%CI:89.4–100) and 100% specificity (95%CI:90.5–100) in case of pericardial thickness >1.8 mm.
**Conclusion:** Spectral iodine maps demonstrated excellent diagnostic accuracy in detecting pericarditis based on iodine concentration and pericardial thickness.
**Limitations:** Retrospective, single-center design with modest sample size, exclusive use of dual-layer spectral CT.
**Funding for this study:** This research received no external funding.
**Has your study been approved by an ethics committee?** Yes
**Ethics committee - additional information:** CT-OPTIMUM (Protocol No. 79-23)
**Author Disclosures:**
Giorgio Ascenti: Speaker: Philips Speaker: Bracco
Danilo Masi: Nothing to disclose
Silvio Mazziotti: Nothing to disclose
Ludovica Rosa Maria Lanzafame: Nothing to disclose
Tommaso D'Angelo: Speaker: Bracco Speaker: Philips

**Improved Reclassification of In-Stent Restenosis Using Ultra-High-Resolution Photon-Counting Detector-CT: An Intra-Individual Comparative Study**

*C. Lisi*[1], F. Catapano[1], S. Figliozzi[1], L. S. Politi[1], A. Laghi[1], M. Francone[2]; [1]Milan/IT, [2]Rome/IT

**Purpose:** To compare ultra-high-resolution photon-counting CT (UHR-PCD-CT) with energy-integrating detector CT (EID-CT) and standard-resolution PCD-CT for intra-individual evaluation of coronary stent patency.
**Methods or Background:** This prospective single-center study enrolled 51 patients (mean age, 64.5 ± 9.2 years; 16 women) with 67 coronary stents between November 2024 and April 2025. Patients were randomized to EID-CT plus coronary CT perfusion (n=26; 31 stents) or EID-CT plus UHR-PCD-CT (n=25; 36 stents). Image quality, diagnostic confidence, blooming index, and lumen diameters were compared among EID-CT, standard-resolution (SR) PCD-CT, and UHR-PCD-CT. In-stent restenosis (ISR) was defined as ≥50% stenosis and/or inducible ischemia. Radiation exposure was recorded. Statistical analyses included chi-square, t-test, Mann–Whitney U, and Cohen's K.
**Results or Findings:** Subjective image quality rated “excellent” was higher with UHR-PCD-CT (88.9%) vs SR-PCD-CT (45.2%, p<0.001) and EID-CT (22.6%, p<0.001). Internal lumen diameter was significantly larger with UHR-PCD-CT (2.65 ± 0.30 mm) vs SR-PCD-CT (2.40 ± 0.32 mm, p<0.01) and EID-CT (2.13 ± 0.36 mm, p<0.0001). Blooming artifacts decreased from 55.3% (EID-CT) and 42.8% (SR-PCD-CT) to 29.5% with UHR-PCD-CT (p<0.0001). Good/excellent diagnostic confidence (Likert 3–4) was achieved in 94.4% of UHR-PCD-CT stents vs 61.1% (SR-PCD-CT, p<0.001) and 41.9% (EID-CT, p<0.001). All stents <3 mm were assessable with UHR-PCD-CT vs 65% with EID-CT. Radiation dose of UHR-PCD-CT (12.0 ± 2.8 mSv) was comparable to EID-CT plus CTP (11.5 ± 2.5 mSv; p=0.42).
**Conclusion:** UHR-PCD-CT markedly improves lumen visualization, reduces blooming artifacts, and increases diagnostic confidence in coronary stent evaluation, particularly for stents <3 mm, with radiation exposure comparable to combined EID-CT and CT perfusion.
**Limitations:** Single-center design and modest sample size. Lack of systematic invasive coronary angiography validation and of long-term clinical outcomes.
**Funding for this study:** No funding, but this work was funded by the National Plan for NRRP Complementary Investments (PNC, established with the decree-law 6 May 2021, n. 59, converted by law n. 101 of 2021) in the call for the funding of research initiatives for technologies and innovative trajectories in the health and care sectors (Directorial Decree n. 931 of 06-06-2022) - project n. PNC0000003 - AdvaNced Technologies for Human-centrEd Medicine (project acronym: ANTHEM). This work reflects only the authors' views and opinions, neither the Ministry for University and Research nor the European Commission can be considered responsible for them.
**Has your study been approved by an ethics committee?** Not applicable
**Author Disclosures:**
Costanza Lisi: Nothing to disclose
Letterio Salvatore Politi: Nothing to disclose
Federica Catapano: Nothing to disclose
Andrea Laghi: Nothing to disclose
Marco Francone: Nothing to disclose
Stefano Figliozzi: Nothing to disclose

**Noncalcified Coronary Plaque Burden Mediates the Association of Hepatic Steatosis With Major Adverse Cardiovascular Events: Insights from the PROMISE Trial**

*J. M. Brendel*[1], T. Mayrhofer[1], N. Kerkovits[1], S. Ersözlü[1], M. Kolossvary[2], M. T. Lu[1], M. Ferencik[3], P. Douglas[4], B. Foldyna[1]; [1]Boston, MA/US, [2]Budapest/HU, [3]Portland/US, [4]Durham/US
*(jan.brendel@med.uni-tuebingen.de)*

**Purpose:** To investigate whether HS is related to plaque volume, plaque burden, and MACE, and whether coronary plaque composition mediates the relationship between HS and MACE.
**Methods or Background:** Hepatic steatosis (HS) has been linked to major adverse cardiovascular events (MACE) independently of other cardiovascular risk factors and the extent of coronary artery disease. However, the association between HS, advanced plaque measures, and MACE remains unclear. A central core laboratory analyzed PROMISE participants randomized to the CT arm. HS was assessed on non-contrast CT using standard hepatic and splenic attenuation methods. Coronary CT angiography was used to quantify total, calcified, noncalcified, and low-attenuation plaque volume and burden (% vessel volume). Multivariable regression and mediation analyses assessed relationships between HS, plaque components, and MACE (death, myocardial infarction, unstable angina hospitalization; median follow-up 25 months, IQR: 18–33).
**Results or Findings:** Among 3,637 patients (60.6±8.2 years, 51.4% female), 25.5% had HS and were slightly younger, more often male, had more cardiovascular risk factors, and a higher MACE rate (4.1% vs. 2.5%), all p<0.05. After adjustment for clinical risk factors, HS was associated exclusively with greater noncalcified plaque burden (NCPB, β 1.25%; 95%CI 0.02–2.49; p=0.047). HS conferred increased MACE risk independent of atherosclerotic cardiovascular disease risk score, obesity, obstructive stenosis, and NCPB (aHR 1.69; 95%CI, 1.12–2.54; p=0.012). NCPB accounted for 11% of the association between HS and MACE.
**Conclusion:** HS is linked to greater NCPB and to MACE, independent of clinical risk factors and advanced CT plaque measures. HS should be considered when stratifying cardiovascular risk and may inform medical therapy.
**Limitations:** Median follow-up of just over 2 years limits the ability to assess long-term outcomes.
**Funding for this study:** This study was supported by NIH/NHLBI grants #1R01HL098236, #1R01HL098237, #1R01HL098305, #1R01HL170877-01, #1R01HL146145-01A1. The content is solely the responsibility of the authors and does not necessarily represent the official views of the National Institutes of Health.
**Has your study been approved by an ethics committee?** Yes

**Ethics committee - additional information:** All participants provided written informed consent, and the trial was approved by local or central institutional review boards (ClinicalTrials.gov NCT01174550)
**Author Disclosures:**
Jan Michael Brendel: Research/Grant Support: Deutsche Forschungsgemeinschaft (DFG, German Research Foundation) #540505270
Marton Kolossvary: Consultant: Elucid Bioimaging Inc.
Nóra Kerkovits: Nothing to disclose
Sara Ersözlü: Other: Alnylam Pharmaceuticals Speaker: Amicus Therapeutics
Pamela Douglas: Nothing to disclose
Thomas Mayrhofer: Nothing to disclose
Borek Foldyna: Grant Recipient: National Heart, Lung, and Blood Institute Consultant: Cleerly
Maros Ferencik: Consultant: Cleerly
Michael T. Lu: Grant Recipient: American Heart Association Grant Recipient: Medicine, National Heart, Lung, and Blood Institute

**Diagnostic performance of coronary CT angiography to diagnose acute spontaneous coronary artery dissection**
*L. J. Moser*[1], T. T. Demmert[1], K. Klambauer[1], V. Mergen[1], B. Stähli[2], R. Manka[1], M. Eberhard[1], C. Templin[3], H. Alkadhi[1]; [1]Zürich/CH, [2]Zurich/CH, [3]Greifswald/DE

**Purpose:** The purpose of this study was to evaluate the performance of coronary CTA for the diagnosis of acute SCAD.
**Methods or Background:** This prospective single-center study included 52 patients with SCAD confirmed by invasive coronary angiography (ICA) and 70 patients with chest pain but without SCAD (control group), who all underwent coronary CTA. Two independent readers evaluated coronary CTA for SCAD, including vessel abnormalities and myocardial hypodensity. Accuracy, sensitivity, and specificity with 95% confidence intervals (CI) were calculated using ICA as the reference standard. Coronary CTA imaging features of SCAD were correlated with diagnostic confidence using multivariable regression analysis. Interreader agreement was determined by Cohen's Kappa.
**Results or Findings:** Patient-level sensitivity of coronary CTA to diagnose SCAD ranged from 52% (CI:38%-66%)-58% (CI:43%-71%), with a specificity of 97% (CI:90%-100%) and accuracy of 78% (CI:69%-85%) and 80% (CI:72%-87%) for both readers (Kappa=0.891). Sensitivity and accuracy were higher for proximal (sensitivity 67% (CI:38%-88%) and 73% (CI:45%-92%); accuracy 93% (CI:87%-97%) and 94% (CI:89%-98%)) than for distal SCAD (sensitivity 40% (CI:26%-56%) and 49% (CI:34%-64%); accuracy 76% (CI:68%-83%) and 80% (CI:71%-86%)), while specificity was high irrespective of location (97%; CI:91%-99%, resp.100%). Additional myocardial hypodensity increased readers' confidence (beta coefficient -0.38; p=0.11 for reader 1, and beta coefficient of -0.90; p=0.004 for reader 2) and sensitivity (71% (CI:57%-83%) and 65% (CI:51%-78%), respectively), while reducing specificity (both 91%, CI:82%-97%).
**Conclusion:** Coronary CTA has a poor sensitivity for the diagnosis of SCAD, while specificity is high irrespective of SCAD location. Patients with coronary CTA positive for SCAD might be deferred from ICA after careful consideration and when conservative management is intended.
**Limitations:** The limitations of the study are as follows: Single center study. Different generations of CT scanners. Not all patients in the control cohort underwent ICA.
**Funding for this study:** No funding was received for this study.
**Has your study been approved by an ethics committee?** Yes
**Ethics committee - additional information:** Approval by local ethics committee.
**Author Disclosures:**
Barbara Stähli: Nothing to disclose
Christian Templin: Nothing to disclose
Tristan Thorben Demmert: Nothing to disclose
Victor Mergen: Nothing to disclose
Matthias Eberhard: Nothing to disclose
Lukas Jakob Moser: Nothing to disclose
Robert Manka: Nothing to disclose
Konstantin Klambauer: Nothing to disclose
Hatem Alkadhi: Nothing to disclose

**CMR-derived Left Atrial Strain Provides Incremental Prognostic Value in Kawasaki Disease**
Z. Zhou, *L-Y. Wen*, S. Azhe, L. Hu, Y. Zhu; Chengdu/CN
*(lizzievane@126.com)*

**Purpose:** To evaluate the role of CMR-derived Left Atrial (LA) strain in prognostic assessment in patients with Kawasaki disease (KD) and to investigate their incremental value beyond coronary artery lesions and late gadolinium enhancement (LGE).
**Methods or Background:** The prospective study analyzed 214 KD patients (median age, 5.5 years [IQR, 3.4-7.7 years]; 142 male) who under CMR examination. Clinical composite endpoint events included cardiac death, decreased left ventricular systolic function, cardiac cerebral ischemic syndrome, syncope, readmission for treatment due to chest pain, and malignant arrhythmias. Cox survival analysis was used to assess the association between variables and time to the end points. C index and chi-square value were used to determine the incremental value of LA strain.
**Results or Findings:** During a median follow-up of 32.1 months (IQR, 20.2–46.3 months), 33 of 214 (15.4%) patients experienced clinical composite endpoint events. Multivariable Cox regression analysis of Model 1 (including coronary thrombosis and LGE positive), revealed that both soronary thrombosis (HR: 3.007; 95% CI: 1.114–8.121; P = 0.03) and LGE positive (HR: 4.104; 95% CI: 1.471–11.453; P = 0.007) were independent predictors for the clinical composite endpoint. Multivariate Cox regression of Model 2 (including Model 1 and εe), showed that εe was independent predictors of the clinical composite endpoint events (HR: 0.972; 95%CI: 0.945–0.999, P=0.042). Compared with Model 1, Model 2 showed a significantly higher chi-square value (57.36 vs. 52.46, P<0.001) and C index (0.73 vs. 0.66, P<0.001).
**Conclusion:** εe was the independent predictor of clinical composite endpoint events. Moreover, εe provides incremental value to coronary thrombosis and LGE positive in predicting clinical composite endpoint events.
**Limitations:** The primary limitation of this study is its small single-center sample size.
**Funding for this study:** This work was supported by the National Natural Science Foundation of China (82471970), Sichuan Science and Technology Program (2024YFFK0258).
**Has your study been approved by an ethics committee?** Yes
**Ethics committee - additional information:** The ethics was approved by the Ethics committee of West China Second University Hospital.
**Author Disclosures:**
Zhongqin Zhou: Nothing to disclose
Lei Hu: Nothing to disclose
Ling-Yi Wen: Nothing to disclose
Shiganmo Azhe: Nothing to disclose
Yaoji Zhu: Nothing to disclose

**Extracardiac Thoracoabdominal Atherosclerotic Plaque Burden in Heart Transplant Candidates is not Associated with Cardiovascular Standard Modifiable Risk Factors**
*T. R. Readford*[1], M. Ugander[2], P. Kench[1], G. Figtree[1], J. Nadel[1], N. Giannotti[1]; [1]Sydney/AU, [2]Solna/SE
*(nicola.giannotti@sydney.edu.au)*

**Purpose:** Contemporary strategies for detection and management of atherosclerosis focus on controlling Standard Modifiable Risk Factors (SMuRFs) including diabetes, hypertension, hyperlipidaemia, and smoking. However, it is unclear if extracardiac thoracoabdominal atherosclerotic plaque burden is associated with SMuRFs. The purpose of this study was to compare extracardiac thoracoabdominal atherosclerotic plaque burden by computed tomography angiography (CTA) between patients with and without SMuRFs among heart transplant candidates with ischaemic or non-ischaemic cardiomyopathy (ICM, NICM).
**Methods or Background:** This study was a retrospective, single-centre observational study. Heart transplant candidates with ICM or NICM undergoing thoracoabdominal CTA were matched for age and sex. Patients were classified as those with SMuRFs or SMuRF-less based on medical records. Extracardiac thoracoabdominal non-calcified and calcified plaque was classified as present or absent across 80 arterial segments per patient.
**Results or Findings:** Among included patients (n=167, median [interquartile range] age 58 [53-63] years, 16% female, 51% NICM), 40 patients (24%) were SMuRF-less (ICM: 16/82 (20%), NICM: 24/85 (28%), age 56 [50-67] years). Overall, out of 13,360 arterial segments, 1,746 (13%) were affected by atherosclerotic plaque (9 [4-15] segments per patient). Compared to NICM, ICM had higher total plaque burden (11 [7-18] vs 6 [3-11] segments per patient, p<0.001). However, SMuRF-less patients showed no difference in non-calcified, calcified, and total plaque burden compared to patients with SMuRFs among all patients (ICM+NICM) (p>0.17 for all), and within the ICM and NICM groups, respectively (p>0.30 for all).
**Conclusion:** The burden of extracardiac thoracoabdominal atherosclerotic plaque does not differ among heart transplant candidates that are SMuRF-less or those with SMuRFs, regardless of underlying ICM or NICM. Prevalence of SMuRFs is not an effective marker to determine the need to screen for extracardiac atherosclerotic plaque among heart transplant candidates.
**Limitations:** Retrospective observational design.
**Funding for this study:** No funding was received for this study.
**Has your study been approved by an ethics committee?** Yes
**Ethics committee - additional information:** This study was approved by the local human subject research ethics committee with a retrospective waiver of individual informed consent.

Author Disclosures:
Peter Kench: Nothing to disclose
Martin Ugander: Nothing to disclose
Nicola Giannotti: Nothing to disclose
Thomas Ramsay Readford: Nothing to disclose
Gemma Figtree: Nothing to disclose
James Nadel: Nothing to disclose

**13:00 - 14:30** **Research Stage 4**

### Research Presentation Session: Chest

### RPS 404

### Photon counting CT: advances in chest imaging

**Moderator**
Pierluigi Ciet; Rotterdam/NL
*(p.ciet@erasmusmc.nl)*

### Photon-Counting CT at Radiography Equivalent Dose: Impact on Quantitative Lung Parameters

*J. Kroschke*[1], B. J. Kerber[1], O. Von Stackelberg[2], F. Ensle[1], L. Jungblut[1], M. O. Wielpütz[3], T. Frauenfelder[1], H-U. Kauczor[4], O. Weinheimer[4]; [1]Zurich/CH, [2]Mannheim/DE, [3]Greifswald/DE, [4]Heidelberg/DE
*(jonas.kroschke@usz.ch)*

**Purpose:** Photon-counting computed tomography (PCCT) enables dose reductions to levels comparable to chest radiography, which is of clinical interest for applications such as lung cancer screening. However, quantitative CT (QCT) metrics may be altered at ultra-low dose (ULD). This study evaluated the impact of dose reduction on lung QCT.
**Methods or Background:** In 101 patients (mean age 61.0±12.5 years), non-contrast low-dose (LD-CT, CTDIvol: ~0.64±0.15 mGy) and radiography-comparable ULD-CT (~0.11±0.03 mGy) scans were acquired in the same session on PCCT, with LD-CT as reference. Using YACTA software fully automated QCT analysis was performed on images reconstructed with a soft kernel (Br36u, strength level 3) for lung parenchyma and a hard kernel (Bl64u, strength level 3) for airways. Two expert readers assessed image quality, emphysema, and airway wall features.
**Results or Findings:** ULD-CT showed a 66.5% increase in noise for Br36u and 63.9% for Bl64u, reflected in lower visual image quality ($p<0.001$, $\kappa=0.59$). Automated airway segmentation decreased (−41.4, $p<0.001$), consistent with fewer visible generations ($p<0.001$, ICC=0.81). Wall percentage remained stable across dose levels, with no significant differences in visually assessed wall thickening (R1 p=0.89, R2 p=0.20). At ULD, mean lung density (MLD) was reduced 13.5 HU ($p<0.001$; $r^2=0.93$), leading to a lower 15th percentile (Perc15: −20.2 HU, $p<0.001$; $r^2=0.93$). Applying a sponge model correction for volume differences decreased bias (MLD: −12.5 HU; Perc15: −19.9 HU) and improved correlations ($r^2=0.97$–$0.98$). Visually, emphysema extent was consistently underestimated at ULD ($p<0.001$, $\kappa=0.92$).
**Conclusion:** PCCT at radiography-comparable dose alters QCT metrics, particularly lung density and emphysema quantification. While sponge model correction mitigates these effects, both automated and visual emphysema assessments remain biased, highlighting limitations of the conventional −950 HU cut-off at ULD.
**Limitations:** Single-center, single-vendor study with limited patient numbers. No HR-CT as reference standard.
**Funding for this study:** No external funding was provided for this study.
**Has your study been approved by an ethics committee?** Yes
**Ethics committee - additional information:** Approval for this study was granted by the local ethics committee (KEK-ZH-NR. 2022-D0008)
**Author Disclosures:**
Mark O. Wielpütz: Nothing to disclose
Thomas Frauenfelder: Nothing to disclose
Oliver Weinheimer: Nothing to disclose
Hans-Ulrich Kauczor: Nothing to disclose
Oyunbileg Von Stackelberg: Nothing to disclose
Falko Ensle: Nothing to disclose
Lisa Jungblut: Nothing to disclose
Bjarne Jonas Kerber: Nothing to disclose
Jonas Kroschke: Nothing to disclose

### Quantitative Assessment of Idiopathic Inflammatory Myopathy-Associated Interstitial Lung Disease Using Photon-Counting CT: Comparison of Standard-Dose and Tin-Filtered Low-Dose Protocols

*Z. Zhu*[1], K. Xu[1], L. Nicodeme[2], Q. Wang[1], L. Song[1], Z. Jin[1]; [1]Beijing/CN, [2]Vienna/AT

**Purpose:** The accurate quantification of interstitial lung disease (ILD) in idiopathic inflammatory myopathies (IIM) is critical for prognosis but is limited by the radiation dose constraints of conventional CT. This study aimed to evaluate the clinical utility of photon-counting CT (PCCT)-derived quantitative parameters for ILD severity assessment in IIM, comparing standard-dose (SD) and tin-filtered low-dose (LD) protocols.
**Methods or Background:** In this prospective single-center study, outpatients with IIM-ILD underwent consecutive SD (120 kVp) and LD (Sn100 kVp) PCCT scans between September 2023 and November 2024. Images were reconstructed with 1.0-mm slice thickness. Quantitative ILD features were extracted using an EU-MDR–approved deep learning software. Fibrosis extent was defined as the sum of reticulation, honeycombing, and bronchiectasis. Visual scoring was performed for severity classification. Comparisons were analyzed using Bland–Altman and receiver operating characteristic analyses; correlation tests were assessed with Spearman's rank correlation coefficients.
**Results or Findings:** A total of 48 adults with IIM-ILD (40 females; mean age, 53.88 ± 11.13 years) were included. LD scans achieved a substantial dose reduction (CTDVol, 0.77 vs 5.40 mGy) while maintaining high agreement with SD (net differences <1% for all parameters). Low-dose protocols slightly underestimated fibrotic changes, with a mean net difference of -0.64% (95% LOA: −1.83% to 0.54%), whereas they overestimated the extent of ground-glass opacity, with a mean net difference of 0.98% (95% LOA: −1.27% to 3.24%). The reticulation was strongly correlated with FVC% ($\rho_{max}$ = –0.615, P<0.001) and DLCO% ($\rho_{max}$ = –0.726, P<0.001). The total ILD burden strongly distinguished limited from extensive disease (AUCs, 0.876 to 0.881).
**Conclusion:** LD protocols markedly reduce radiation exposure without compromising accuracy. PCCT provides a reliable and reproducible quantitative assessment of ILD severity in IIM and has the potential to improve longitudinal monitoring in IIM-ILD.
**Limitations:** None.
**Funding for this study:** No funding was provided for this study.
**Has your study been approved by an ethics committee?** Yes
**Ethics committee - additional information:** This study was approved by the Ethics Committee of Peking Union Medical College Hospital, and all participants provided written informed consent (I-23PJ1459).
**Author Disclosures:**
Zhenchen Zhu: Nothing to disclose
Kepei Xu: Nothing to disclose
Lan Song: Nothing to disclose
Zhengyu Jin: Nothing to disclose
Qian Wang: Nothing to disclose
Luc Nicodeme: Equipment Support Recipient: The quantitative ILD analysis in this study was performed using software tools from contextflow. Luc Nicodeme is an employee of contextflow. However, he was not involved in the study design, data interpretation, or the writing of the abstract.

### Photon-Counting CT-based Lung Ventilation and Perfusion Metrics: Reference Ranges and Regional Variability in 103 Healthy Subjects

J. Dittrich[1], C. Van den Berg[1], S. Scharm[1], C. M. Schaefer-Prokop[2], S. Dettmer[1], F. Wacker[1], G. H. Pöhler[1], *H-O. Shin*[1]; [1]Hannover/DE, [2]Amersfoort/NL
*(shin.hoen-oh@mh-hannover.de)*

**Purpose:** To establish normative reference ranges for photon-counting CT (PCCT)-derived pulmonary ventilation and perfused blood volume (PBV) in healthy adults, using pulmonary function tests (PFTs) as the clinical reference standard.
**Methods or Background:** In this prospective, single-centre study (December 2022 - April 2024), 103 healthy adults underwent spirometry-guided inspiratory and expiratory contrast-enhanced PCCT in supine position. Automated lobar segmentation was performed using TotalSegmentator. CT-derived total lung capacity (CT-TLC), residual volume (CT-RV), and vital capacity (CT-VC) were compared with corresponding PFT metrics. Ventilation (%) was quantified as the relative air-volume change between inspiration and expiration from nonlinearly registered virtual non-contrast images, normalised to inspiratory lung volume. PBV was calculated from inspiratory datasets, normalised to left atrial iodine attenuation, and corrected for lung inflation by fixing mean parenchymal attenuation at -850 Hounsfield Units. Reference ranges were expressed as mean ± standard deviation and 5th-95th percentiles. Associations with sex, age, lung region, and gravitational orientation were statistically analysed.

**Results or Findings:** The final cohort included 91 participants (mean age, 53±12 years; 49 men). CT-derived lung volumes were lower than corresponding PFT values but strongly correlated (CT-TLC r=0.89; CT-RV r=0.80; CT-VC r=0.82). Mean ventilation was 59.5 ± 8.5% (42.1%-72.5%), normalised mean PBV was 12.4 ± 2.5% (8.8%-16.8%). PBV was higher in women than men (P=0.03), and both ventilation and PBV declined with age (P<0.001). Regionally, ventilation was higher in lower versus upper lobes (P=0.003), whereas PBV was higher in upper versus lower lobes (P=0.01). Both parameters exhibited a dorsal>ventral gradient (P<0.001).
**Conclusion:** This study defines normative reference ranges for PCCT-derived lung ventilation and PBV in healthy adults. Both parameters are influenced by demographic and regional factors and demonstrate characteristic gravitational gradients.
**Limitations:** Single-centre design; no assessment of scan-rescan repeatability.
**Funding for this study:** Part of the study was funded by DigiStrucMed (Else-Kröner-Foundation).
**Has your study been approved by an ethics committee?** Yes
**Ethics committee - additional information:** This study was approved by the German Federal Office for Radiation Protection (BfS) and by the local ethics committee of Medical School Hannover (number 10077_BO_S_2021 StrSchVO).
**Author Disclosures:**
Cornelia M. Schaefer-Prokop: Nothing to disclose
Charlotte Van Den Berg: Nothing to disclose
Sabine Dettmer: Nothing to disclose
Hoen-Oh Shin: Nothing to disclose
Sarah Scharm: Nothing to disclose
Gesa Helen Pöhler: Nothing to disclose
Julian Dittrich: Nothing to disclose
Frank Wacker: Nothing to disclose

**Ultra-high-resolution photon-counting CT in the assessment of interstitial lung disease in systemic sclerosis**
*F-j. Feretz*, Y. Gaillandre, A. Hutt, S. Khung, P. Felloni, B. Longere, C. V. Gkizas, J-B. Faivre, F. A. Pontana; Lille/FR
*(fjferetz@hotmail.fr)*

**Purpose:** To compare the detection and characterization of interstitial lung disease (ILD) elementary lesions in systemic sclerosis using ultra-high-resolution (UHR) photon-counting detector CT (PCCT) versus high-resolution CT (HRCT) from a third-generation dual-source energy-integrating detector CT.
**Methods or Background:** This retrospective study included 22 patients with systemic sclerosis and ILD (15 women; mean age: 62.8 years), each undergoing both HRCT and PCCT-UHR for follow-up with clinical and functional stability between scans. HRCT parameters: collimation 2×96×0.6 mm; pitch 2; Sn150kV; 150 mAs with automatic modulation; 1 mm slices; $512^2$ matrix; Bl57 kernel; iterative reconstruction (ADMIRE level 4). PCCT-UHR parameters: collimation 2×120×0.2 mm; pitch 2; Sn100kV or Sn140kV depending on body habitus; automatic tube current modulation (IQ level 36); 0.2 mm slices; $1024^2$ matrix; Bl60 kernel; QIR level 4. Image analysis included tracheal noise measurement and qualitative scoring (0-3 scale) of ground-glass opacities, intralobular reticulations, bronchiectasis, bronchiolectasis, and honeycombing on 1 mm slices.
**Results or Findings:** The mean interval between scans was 33 ± 25 months. Despite higher image noise with PCCT-UHR (67.14 ± 13.18 HU vs 33.63 ± 5.81 HU; P < 0.0001), overall image quality was subjectively superior (P = 0.0348). PCCT-UHR yielded significantly improved visualization of ground-glass opacities (P = 0.0003), intralobular reticulations (P = 0.0045), bronchiectasis (P = 0.0001), and bronchiolectasis (P = 0.0002), with no significant difference for honeycombing (P = 0.125). Radiation dose was significantly lower with PCCT-UHR (CTDIvol: 2.35 ± 0.82 mGy; DLP: 77.64 ± 25.38 mGy·cm) compared to HRCT (CTDIvol: 3.35 ± 1.01 mGy; DLP: 116.77 ± 33.45 mGy·cm; P = 0.0008).
**Conclusion:** Ultra-high-resolution PCCT enables more accurate and lower-dose detection of early fibrotic changes in systemic sclerosis-related ILD, potentially improving disease monitoring and therapeutic decision-making.
**Limitations:** Monocentric retrospective study
**Funding for this study:** None
**Has your study been approved by an ethics committee?** Yes
**Ethics committee - additional information:** Waiver of informed consent
**Author Disclosures:**
Yann Gaillandre: Nothing to disclose
Jean-Baptiste Faivre: Nothing to disclose
Benjamin Longere: Nothing to disclose
Paul Felloni: Nothing to disclose
Christos Vasileiou Gkizas: Nothing to disclose
Suonita Khung: Nothing to disclose
François-Joseph Feretz: Nothing to disclose
François Ascagne Pontana: Nothing to disclose
Antoine Hutt: Nothing to disclose

**Low-dose photon-counting CT for IM-ILD: Preserving detection of key pulmonary abnormalities with reduced radiation**
*K. Xu*, Z. Zhu, L. Song; Beijing/CN
*(xkepei@163.com)*

**Purpose:** To evaluate whether low-dose photon-counting CT (PCD-CT) can preserve the detection of key pulmonary abnormalities related to interstitial lung disease (ILD) while reducing radiation exposure, and to explore the incremental value of thinner-slice low-dose images.
**Methods or Background:** We prospectively enrolled patients with inflammatory myopathy–associated ILD patients(IM-ILD) who underwent same-day standard-dose (SD) PCD-CT and low-dose (LD) PCD-CT between October 2023 and November 2024. Four image series—SD-1.0 mm and LD-1.0 mm/0.4 mm/0.2 mm—were assessed for subjective image quality, noise, and visualization of normal structures using a 5-point Likert scale. Subsequently, two thoracic radiologists further evaluated the presence and confidence of ILD-related findings, including ground-glass opacity with reticulation (GG+R), ground-glass opacity with traction bronchiectasis (GG+B), and honeycombing.
**Results or Findings:** Fifty patients were included (mean age 53 ± 11 years; 9 males). The mean effective dose was significantly lower for LD vs SD (0.32 ± 0.07 mSv vs 2.25 ± 0.56 mSv; p < 0.001). Although SD showed better results than LD in overall image quality and image noise (p < 0.05), detection rates for GG+R, GG+B, consolidation, and honeycombing showed no significant differences across the four series, except for GG+B between SD-1 mm and LD-0.2 mm. Reader confidence was also comparable, except for lower confidence in GG+B on LD-0.2 mm. Thinner LD-0.4 mm images provided incremental detection in a small number of cases of GG+B missed on 1 mm images, whereas LD-0.2 mm showed no added benefit.
**Conclusion:** Low-dose PCD-CT substantially reduced radiation exposure while still providing good capability in detecting ILD-related pulmonary abnormalities compared with standard-dose PCD-CT, with thinner slices offering additional diagnostic value.
**Limitations:** The sample size was relatively small.
**Funding for this study:** This study has received funding from the National Natural Science Foundation of China (NSFC no. 82171934) and the National High-Level Hospital Clinical Research Funding (2022-PUMCH-B-069).
**Has your study been approved by an ethics committee?** Yes
**Ethics committee - additional information:** The Institutional Review Board of our hospital approved this prospective study [I-23PJ1459].
**Author Disclosures:**
Zhenchen Zhu: Nothing to disclose
Kepei Xu: Nothing to disclose
Lan Song: Nothing to disclose

**Systemic Sclerosis-Associated Interstitial Lung Disease: A Comparison of Automated Lung Texture Analysis in Photon-Counting-Detector and conventional Energy-Integrating- Detector CT**
*J. Happe*[1], C. Bruni[2], L. Jungblut[1], N. Landini[3], C. Blüthgen[1], A. R. Larici[3], T. Frauenfelder[1], O. Distler[2], J. Kroschke[1]; [1]Zürich/CH, [2]Zurich/CH, [3]Rome/IT
*(jasmin.happe@web.de)*

**Purpose:** To investigate whether the superior image quality of photon-counting detector CT (PCD-CT) facilitates the application of automated lung texture analysis (LTA) for detecting interstitial lung disease (ILD) in systemic sclerosis (SSc) compared to conventional energy-integrating detector CT (EID-CT).
**Methods or Background:** 186 patients with systemic sclerosis (SSc), who had undergone clinically indicated EID-CT or PCD-CT, were included. Matching was performed based on comparable demographical and rheumatological profiles to ensure comparability. Visual ILD assessment by three expert radiologists served as the reference standard. Image quality was assessed both subjectively using Likert-scales and objectively by measuring signal-to-noise ratios (SNR). Quantitative ILD features and disease extent were extracted using LTA based on the CALIPER algorithm (Imbio). Diagnostic accuracy was evaluated through ROC-AUC analysis.
**Results or Findings:** LTA-based ILD-assessment showed higher AUC for PCD-CT than EID-CT in detecting ILD (PCD-CT AUC: 0.846 vs. EID-CT AUC: 0.772) and specific ILD features, including groundglass opacities, reticulation, and honeycombing. However, EID-CT showed higher AUCs than PCD-CT in detecting extensive ILD (>20% lung involvement; AUC = 0.978 vs. 0.842). Despite reduced radiation exposure, PCD-CT still delivered comparable SNR alongside improved image quality rating scores.
**Conclusion:** LTA demonstrated acceptable to excellent AUC values for ILD detection on both, PCD-CT and EID-CT scans, with PCD-CT-based LTA consistently achieving superior AUCs in detecting individual ILD features in systemic sclerosis. Notably, LTA models trained on EIDCT data performed robustly when applied to PCD-CT images. Accordingly, the improved image quality and reduced radiation dose of PCD-CT support its potential for longitudinal ILD monitoring using LTA. Nonetheless, further multicenter validation is necessary to confirm these findings.

**Limitations:** Post-hoc-analysis with the use of visual assessment by experienced thoracic radiologists as the reference standard for LTA-derived parameters, due to the lack of pathological confirmation.
**Funding for this study:** No funding was provided for this study.
**Has your study been approved by an ethics committee?** Yes
**Ethics committee - additional information:** The ethics committee notification can be found under the number BASEC 2018-02165.
**Author Disclosures:**
Nicholas Landini: Nothing to disclose
Thomas Frauenfelder: Speaker: speaker fees from Bayer, Bracco, AstraZeneca, and Boehringer Ingelheim
Anna Rita Larici: Nothing to disclose
Christian Blüthgen: Research/Grant Support: research support from the Promedica Foundation, Chur, Switzerland
Oliver Distler: Consultant: 4P-Pharma, Abbvie, Acepodia, Aera, AnaMar, Anaveon, Argenx, AstraZeneca, Boehringer Ingelheim, BMS, Calluna, Cantargia AB, CSL Behring, EMD Serono, Galderma, Galapagos, Gossamer, Hemetron, Innovaderm, Janssen, Lilly, MSD Merck, Nkarta, Novartis, Oorja Bio, Orion, Pilan, Prometheus, Quell, Redxpharma, Scleroderma Research Foundation, Sumitomo, Topadur, UCB and Umlaut bio Founder: Co-founder of CITUS AG Speaker: 4P-Pharma, Abbvie, Acepodia, Aera, AnaMar, Anaveon, Argenx, AstraZeneca, Boehringer Ingelheim, BMS, Calluna, Cantargia AB, CSL Behring, EMD Serono, Galderma, Galapagos, Gossamer, Hemetron, Innovaderm, Janssen, Lilly, MSD Merck, Nkarta, Novartis, Oorja Bio, Orion, Pilan, Prometheus, Quell, Redxpharma, Scleroderma Research Foundation, Sumitomo, Topadur, UCB and Umlaut bio.
Jasmin Happe: Nothing to disclose
Lisa Jungblut: Nothing to disclose
Jonas Kroschke: Nothing to disclose
Cosimo Bruni: Nothing to disclose

**Photon-Counting Detector CT vs. Energy-Integrating Detector CT for Pulmonary Nodule Diagnosis: A Comparative Evaluation with Radiologist and Automated Detection Software**
*M. Zhang*, B. Kun, L. Zhifei, H. K. Zhang, J. Shen, K. Liang; Shanghai/CN
*(zgzm1985@163.com)*

**Purpose:** To compare the image quality and diagnostic performance of photon-counting detector CT (PCCT) with energy-integrating detector CT (EID-CT) for pulmonary nodule diagnosis by using both radiologist assessment and computer-assisted diagnosis (CAD).
**Methods or Background:** Thirty-four patients were retrospectively included (EID-CT, n=17; PCCT, n=17). All chest CTs were acquired at 120 kVp with automatic tube current modulation. Both EID-CT and PCCT images were reconstructed using hybrid iterative reconstruction (HIR) with 512×512 and 1024×1024 matrices. A radiologist recorded radiation dose and evaluated image quality using a five-point scale (1=non-diagnostic, 5=excellent). Solid (SN), ground-glass (GGN), and part-solid (PSN) nodules were independently identified by both a radiologist and CAD. Nodule detection performance was evaluated across the four image groups, using a blinded senior radiologist's assessment of all available reconstructions served as the reference standard.
**Results or Findings:** There were no significant differences in baseline characteristics (gender, age, BMI) or nodule distribution between the groups (P>0.05). The effective radiation dose with PCCT was 70.2% lower than with EID-CT (2.94±0.49 vs. 9.85±1.13mSv; p<0.05). PCCT (HIR, 1024×1024) achieved the highest subjective image quality, significantly outperforming the best-performing EID-CT at the same matrix size (4.82±0.39 vs. 3.88 ± 0.49, p<0.05). For all three nodule types, PCCT outperformed the best-performing EID-CT (HIR, 1024×1024) for both the radiologist and CAD. Radiologist detection rates on PCCT were 88.1%(37/42), 92.3%(12/13), and 100%(2/2) for SN, GGN, and PSN, respectively; CAD on PCCT achieved 100.0% across all subtypes, versus 75.4%(43/57), 77.8%(14/18), and 50.0%(1/2) for the radiologist and 64.9%(37/57), 100.0%(18/18), and 50.0%(1/2) for CAD on EID-CT.
**Conclusion:** PCCT provides higher spatial resolution and lower radiation dose, as confirmed by both radiologist evaluation and automated detection software, supporting its clinical value for pulmonary nodule detection.
**Limitations:** Small sample size; limited number of part-solid nodules.
**Funding for this study:** N/A
**Has your study been approved by an ethics committee?** Yes
**Ethics committee - additional information:** The study protocol was approved by the Institutional Review Board.
**Author Disclosures:**
Li Zhifei: Nothing to disclose
Bai Kun: Nothing to disclose
Jianjun Shen: Nothing to disclose
Kaiyi Liang: Nothing to disclose
Hao Kang Zhang: Nothing to disclose
Ming Zhang: Nothing to disclose

**Comparative lung perfusion analysis in chronic thromboembolic pulmonary hypertension with V/Q-SPECT and photon-counting CT**
*M. Moeskes*[1], T. Derlin[2], A. M. Hunkemöller[1], C. M. Schaefer-Prokop[3], J. Eckstein[1], B. C. Meyer[1], J. Vogel-Claussen[1], F. Wacker[1], H-o. Shin[1]; [1]Hannover/DE, [2]Bad Oeynhausen/DE, [3]Amersfoort/NL
*(moeskes.matthias@mh-hannover.de)*

**Purpose:** The purpose of this study was to compare quantitative lung perfusion assessment using photon-counting computed tomography (PCCT) with ventilation/perfusion single-photon emission computed tomography (V/Q-SPECT), the current reference standard, in patients with suspected or confirmed chronic thromboembolic pulmonary hypertension (CTEPH).
**Methods or Background:** This retrospective, single-centre study included twenty-three patients (13 men, ten women; mean age 67.9 ± 10.7 years) who underwent both PCCT and V/Q-SPECT between 01.10.2021 and 31.12.2024. Lobar and whole-lung perfusion were quantified using PCCT-derived perfused blood volume maps and V/Q-SPECT perfusion images. Lung and lobe segmentation was performed with an artificial intelligence-based tool. Perfusion values were normalised (95% confidence interval z-score scaling). Agreement between modalities was analysed with Pearson correlation and Bland–Altman analysis, and perfusion defect volumes were compared across thresholds.
**Results or Findings:** Whole-lung mean perfusion correlated strongly between PCCT and V/Q-SPECT (r = 0.72, p < 0.05). Lobar correlations ranged from r = 0.62 in the middle lobe to r = 0.85 in the left lower lobe. PCCT yielded slightly higher mean perfusion values (0.50 ± 0.04) compared to V/Q-SPECT (0.49 ± 0.09). Bland–Altman analysis showed a bias of +0.015 with limits of agreement from –0.13 to +0.16. Perfusion defect volume correlation was moderate, with whole-lung r = 0.60 and lobar r = 0.49–0.77. Image quality was affected by venous contrast artefacts in PCCT and respiratory misregistration in V/Q-SPECT.
**Conclusion:** PCCT-based perfusion imaging demonstrates high concordance with V/Q-SPECT, supporting its potential as a comprehensive single-modality approach for functional and anatomical evaluation of CTEPH.
**Limitations:** The limitations of the study are its single-centre, retrospective design and the relatively small sample size.
**Funding for this study:** No funding was received for this study.
**Has your study been approved by an ethics committee?** Yes
**Ethics committee - additional information:** All individuals provided written informed consent, and the requirement for ethics approval was waived by the institutional review board (No. 10724_BO_K_2023).
**Author Disclosures:**
Matthias Moeskes: Nothing to disclose
Cornelia M. Schaefer-Prokop: Nothing to disclose
Thorsten Derlin: Nothing to disclose
Jens Vogel-Claussen: Nothing to disclose
Bernhard C. Meyer: Nothing to disclose
Hoen-Oh Shin: Nothing to disclose
Jan Eckstein: Nothing to disclose
Anna M. Hunkemöller: Nothing to disclose
Frank Wacker: Nothing to disclose

**Normalized iodine concentrations derived from photon counting computed tomography: Correlation of histopathological features of lung cancer subtypes**
*M-J. Sähn*, E. Topal, J. Ottemöller, R. I. Danebrock, J. Borggrefe, A. S. Surov; Minden/DE
*(marwin.saehn@web.de)*

**Purpose:** Photon counting computed tomography (PCCT) is a novel imaging modality able to measures iodine concentration within tumors. However, correlations between iodine concentration and histopathological features are not well established in lung cancer. This study aims to adress the issue.
**Methods or Background:** NSCLC (adenocarcinoma, squamous cell carcinoma) cases were evaluated using standardized protocols for measuring normalized iodine concentration (NIC) in PCCT and correlated it with Ki67 proliferation rate, number of tumor cells per unit area („cell count"), tumor proportion score (TPS), combined positive score (CPS), and immune cell (IC) score. SCLC NIC was correlated with Ki67 proliferation rate. All NICs were tested for subtype differences via Wilcoxon test. Interobserver reliability was assessed using intraclass correlation coefficients (ICCs).
**Results or Findings:** A total of 92 cases (NSCLC n=58, SCLC n=34) were included. The ICCs showed excellent inter-observer consistency for iodine concentration measurements in both NSCLC and small-cell lung cancer (SCLC). Correlation analysis revealed no significant association between iodine concentration and Ki67 proliferation rate, TPS, CPS, IC score or cell count in most tumor subtypes. However, a weak, statistically significant positive correlation was found with TPS in adenocarcinoma (0.21, p=0.007). No significant difference in iodine concentration between lung cancer subtypes was observed.

**Conclusion:** Our study demonstrates excellent interobserver consistency for measuring iodine concentration using PCCT in NSCLC and SCLC. While limited correlation with histopathological features was observed, the results suggest that iodine concentration may be useful to predict TPS in adenocarcinoma.
**Limitations:** Retrospective study.
**Funding for this study:** None.
**Has your study been approved by an ethics committee?** Yes
**Ethics committee - additional information:** Ruhr-University Bochum (2021–827)
**Author Disclosures:**
Alexey Surov: Nothing to disclose
Emirhan Topal: Nothing to disclose
Marwin-Jonathan Sähn: Nothing to disclose
Jonas Ottemöller: Nothing to disclose
Raihanatou Ina Danebrock: Nothing to disclose
Jan Borggrefe: Nothing to disclose

**Clinical-Radiomics Nomogram Based on Dual-Energy CT for Differentiating Benign and Malignant Solid Pulmonary Nodules with External Validation**
Y. Y. Wang, H. Wu, *Y. Wang*, X. Yu; Shanghai/CN

**Purpose:** To investigate the added value of multiparametric dual energy CT (DECT) radiomics integrated with clinical-radiological features for noninvasive differentiation of benign and malignant solid pulmonary nodules (SPNs), with emphasis on external validation and diagnostic performance in sub-centimeter nodules.
**Methods or Background:** This retrospective study enrolled 441 patients with pathologically confirmed SPNs who underwent preoperative DECT ( IQon CT and CT7500, Philips Healthcare), divided into training (n=252), internal test (n=112), and external test (n=77) cohorts. Radiomics features were extracted from conventional and virtual monoenergetic images (40 and 70 keV), and material decomposition images (including iodine density (ID), Z-effective atomic number (Zeff), electron density (ED) maps) in arterial (AP) and venous phases (VP). Logistic regression constructed radiomics models, and combined clinical-radiomics model was visualized as a nomogram. Subgroup analysis was performed by nodule size (≤10 mm vs >10 mm), and diagnostic accuracy was compared with that of two radiologists.
**Results or Findings:** A total of 441 SPNs (112 benign, 329 malignant) were included. The optimal radiomics model, comprising features from ID in VP and Zeff in both AP and VP, achieved area under the curve (AUC) of 0.835, 0.804, and 0.772 in the training, internal, and external cohorts, respectively. The combined model (age, lobulation, and 10 radiomic features) outperformed the clinical-radiological model in all cohorts (AUC: 0.889 vs 0.816; 0.865 vs 0.795; 0.823 vs 0.742; $p<0.05$). It maintained strong performance for ≤10 mm and >10 mm nodules, with AUC of 0.875 and 0.888 (internal test) and 1.000 and 0.794 (external test).
**Conclusion:** A DECT-based multiparametric radiomics model integrated with clinical radiological features enables accurate and noninvasive differentiation between benign and malignant SPNs, including sub-centimeter nodules.
**Limitations:** Retrospective design may introduce selection bias.
**Funding for this study:** National Key Research and Development Program of China (No. 2024YFF0728600)
**Has your study been approved by an ethics committee?** Yes
**Ethics committee - additional information:** Approved by the institutional review board of Renji Hospital affiliated to Shanghai Jiao Tong University (approval number: LY2025-036-B) and Sun Yat-sen University Cancer Center (approval number: B2025-189-01).
**Author Disclosures:**
Yu Wang: Nothing to disclose
Xiao Yu: Nothing to disclose
Yan Yan Wang: Nothing to disclose
Huawei Wu: Nothing to disclose

**The Power of Photon-Counting CT in Pulmonary Sarcoidosis**
*L. Piscopo*[1], G. Rea[2], A. G. Fois[1], D. Turilli[1], G. De Paula[1], L. M. Fattacciu[1], E. Solinas[1], S. Masala[1], M. Scaglione[1]; [1]Sassari/IT, [2]Napoli/IT
*(leandra.piscopo@gmail.com)*

**Purpose:** Sarcoidosis is a multisystem granulomatous disease, and the lung is the most commonly affected organ. The aim of this prospective study was to assess the morphological performance of Photon-counting CT (PCCT), with dual reconstruction kernel (HR and soft tissue kernels) in patients with pulmonary sarcoidosis, focusing on its diagnostic accuracy, radiation dose reduction, ultra-high-resolution (UHR) and spectral capabilities.
**Methods or Background:** Forty consecutive patients with pulmonary sarcoidosis underwent PCCT at our institution. Two chest radiologists blinded the imaging reports and functional data. Detailed evaluation of the granulomatous alterations with HRCT imaging (nodular, ground glass opacity, consolidative reticular, fibrosing) was the object of the radiological evaluation with the further aim of identifying elements of sub-millimetric detail (intercepted excellently by the PCCT) that could increase the diagnostic power. Radiation dose indices (CTDIvol, DLP, effective dose) were compared with standard energy-integrating detector CT (EID-CT).
**Results or Findings:** PCCT with its iterative algorithm reconstruction and UHR protocols (0.4 mm, with select reconstructions at 0.2 mm), provided a sharper depiction of parenchymal microstructures, interlobular septa, nodules, fibrotic changes, and pleural abnormalities. Compared to EID-CT, PCCT significantly enhanced the diagnostic accuracy with great potential in predictive value, while the radiation dose analysis demonstrated a 30–50% reduction in CTDIvol and effective dose.
**Conclusion:** PCCT provides significant advantages in granulomatous disorders, particularly in pulmonary sarcoidosis. Its UHR morphological detail, using 0.25 mm isotropic voxels, facilitates clear visualization of tiny micronodules near 0.5 mm and subtle early fibrotic reticulations in order to reduce the radiant load, expand spectral/quantitative potential, and improve its diagnostic accuracy and positive predictive value. Therefore, PCCT could improve the staging, therapy monitoring, and future integration into precision imaging pathways for granulomatosis lung diseases.
**Limitations:** Lack of standardization for new imaging method technology.
**Funding for this study:** This research received no external funding
**Has your study been approved by an ethics committee?** Yes
**Ethics committee - additional information:** The study was conducted according to the guidelines of the Declaration of Helsinki. The local Institutional review board approved the study. The Ethical Committee’s approval was obtained (2022030).
**Author Disclosures:**
Davide Turilli: Nothing to disclose
Liala Mirella Fattacciu: Nothing to disclose
Gaetano Rea: Nothing to disclose
Mariano Scaglione: Nothing to disclose
Giovanni De Paula: Nothing to disclose
Salvatore Masala: Nothing to disclose
Leandra Piscopo: Nothing to disclose
Alessandro Giuseppe Fois: Nothing to disclose
Emma Solinas: Nothing to disclose

**High-Resolution Ultra-Low-Dose Photon-Counting CT in Lung Nodule Assessment: Application Value and Comparative Analysis with Standard-Dose EID-CT**
*X. Tong*[1], J. Sun[1], J. Zhou[1], Y. Li[2], M. Jiang[3], L. Jie[1], B. Li[3], X. Liang[2]; [1]Chengdu/CN, [2]ShangHai/CN, [3]ShenYang/CN
*(tongxy1226@163.com)*

**Purpose:** To analyze the image quality and diagnostic performance of high-resolution ultra-low-dose photon-counting CT (PCCT) in evaluating lung nodules with varying sizes and densities, and perform a comparative analysis with standard-dose energy-integrating detector CT (EID-CT). The ultimate goal is to verify the superiority of high-resolution ultra-low-dose PCCT in lung nodule imaging and its potential for clinical application.
**Methods or Background:** Fifteen spherical nodules, covering five diameters (3 mm, 5 mm, 8 mm, 10 mm, 12 mm) and three density levels (100 HU, -630 HU, -800 HU), were randomly embedded in a commercial lung phantom and scanned. High-resolution low-dose scanning was performed using PCCT (NeuViz P10); while standard-dose scanning was conducted with EID-CT (NeuViz Epoch+). Lung nodules were automatically detected using CAD software and subsequently evaluated in terms of contrast-to-noise ratio (CNR). Differences among scanning protocols were assessed using repeated measures ANOVA.
**Results or Findings:** The effective dose of PCCT was 0.524 mSv, compared with 2.425 mSv for EID-CT, corresponding to an approximately 78.4% reduction in radiation dose. For data reconstructed at 512 matrix size, PCCT showed higher CNR than EID-CT at both 512 ($t = 4.812$, $p = 0.001$) and 1024 ($t = 4.743$, $p = 0.001$) matrix sizes. Similarly, for data reconstructed at 1024 matrix size, PCCT showed higher CNR than EID-CT at both 512 ($t = 3.691$, $p = 0.006$) and 1024 ($t = 3.166$, $p = 0.013$) matrix sizes.
**Conclusion:** Despite approach 80% decrease in radiation dose, PCCT remains superior to large-matrix EID-CT when evaluating lung nodules and overall image quality, This finding strongly indicates that PCCT holds great potential as a reliable substitute for standard-dose EID-CT in applications like lung nodule assessment.
**Limitations:** These findings require further validation across diverse parameter settings and in vivo.

Wednesday

**Funding for this study:** No funding was received for this study.
**Has your study been approved by an ethics committee?** Not applicable
**Author Disclosures:**
Bingsheng Li: Employee: Neusoft Medical Systems
Jie Zhou: Employee: Neusoft Medical Systems
Xiaoyu Tong: Nothing to disclose
Xiaoyun Liang: Employee: Neusoft Medical Systems
Liu Jie: Employee: Neusoft Medical Systems
Yi Li: Employee: Neusoft Medical Systems
Jiayu Sun: Nothing to disclose
Mingkuan Jiang: Employee: Neusoft Medical Systems

**13:00 - 14:30** **Research Stage 3**

### Research Presentation Session: Hybrid, Molecular and Translational Imaging

### RPS 406

### Moving forward in clinical molecular oncologic imaging

**Moderator**
Désirée Deandreis; Villejuif/FR
*(desiree.deandreis@gustaveroussy.fr)*
**Author Disclosures:**
Désirée Deandreis: Advisory Board: ICPO, Consultant: Novartis, GE Healthcare, TELIX, OTD, Spectrum Dynamics, ITM, Immedica, Investigator: Novartis, Research Grant/Support: Spectrum Dynamics, Speaker: Spectrum Dynamics, Novartis, Telix

### Multicentre Analysis of Focal Bone Uptake on [18F]PSMA-1007 PET/CT in Prostate Cancer – Comparative Effectiveness of PSMA-RADS v2.0 vs bone uptake metastatic probability (BUMP) score

*J. H. R. Cairns*[1], S. J. Arain[1], A. Challapalli[2], A. Bahl[2], T. Ali[3], J. Sage[4], R. Frood[1], G. Petrides[3], A. Scarsbrook[1]; [1]Leeds/UK, [2]Bristol/UK, [3]Newcastle/UK, [4]Oxford/UK
*(james.cairns2@nhs.net)*

**Purpose:** Prostate-specific membrane antigen (PSMA) PET/CT is widely used in prostate cancer management. Indeterminate bone lesions (IBLs) remain a diagnostic challenge when using [18F]PSMA-1007. The study compared the bone uptake metastatic probability (BUMP) score - a composite model combining imaging and clinical parameters with PSMA Reporting and Data System (PSMA-RADS) Version 2.0 for predicting skeletal metastases in IBLs.
**Methods or Background:** [18F]PSMA-1007 PET/CT scans from three tertiary cancer centres were retrospectively analysed. Bone lesions with focal PSMA uptake were evaluated. For each lesion SUVmax, CT-derived mean Hounsfield unit and androgen deprivation therapy status were recorded to calculate the BUMP score. Lesional PSMA-RADS scores were determined by experienced radiologists. Ground truth was established using follow-up imaging, prostate specific antigen (PSA) kinetics and multidisciplinary consensus. Receiver operating characteristic (ROC) analysis was used to assess diagnostic performance of both methods.
**Results or Findings:** In total 136 patients (median age 70.5) with 427 bone lesions were analysed; 58.8% (n=251) of lesions were metastatic. Median SUVmax for malignant lesions was 7.55 (Interquartile range (IQR) 10.3) versus 3.53 (IQR 1.51) for benign lesions. BUMP achieved an area under the ROC curve (AUROC) of 0.81 (95% confidence interval (CI): 0.78 - 0.85) with an optimal threshold at 0.125 yielding an F1 score of 0.77. PSMA-RADS 2 outperformed BUMP with higher sensitivity and specificity (98% and 96%, versus 74% and 75% respectively). In equivocal PSMA-RADS 3 lesions (n=44), BUMP had reduced discriminative power (AUROC 0.61, 95%: CI 0.40-0.79).
**Conclusion:** BUMP performed well in predicting metastases but underperformed compared to expert PSMA-RADS assessment. In equivocal lesions BUMP offered limited value, highlighting importance of expert interpretation.
**Limitations:** Retrospective design, limited subgroup power and reliance on clinical rather than histopathological ground truth.
**Funding for this study:** No specific funding was obtained for this study.
**Has your study been approved by an ethics committee?** Not applicable
**Author Disclosures:**
James Henry Robert Cairns: Nothing to disclose
Amit Bahl: Nothing to disclose
George Petrides: Nothing to disclose
Andrew Scarsbrook: Nothing to disclose
Russell Frood: Nothing to disclose
Amarnath Challapalli: Nothing to disclose
Tamir Ali: Nothing to disclose
John Sage: Employee: Mirada
Salman Javed Arain: Nothing to disclose

### Personalized SUVmean–Based Semiautomatic PSMA PET/CT Segmentation Predicting 12–Month Progression in mCRPC Patients Undergoing [177Lu]Lu–PSMA–617 Therapy

*W. Jutidamrongphan*[1], J. Hu[1], J. Wang[1], Y. Chen[1], Y. Rakvongthai[2], R. Seifert[1], K. Shi[1], A. Rominger[1], A. Afshar-Oromieh[1]; [1]Bern/CH, [2]Bangkok/TH
*(petit_222@hotmail.com)*

**Purpose:** Appropriate patient selection is crucial for effective [177Lu]Lu–prostate–specific membrane antigen (PSMA) radioligand therapy (RLT) in metastatic castration–resistant prostate cancer (mCRPC). While quantitative tumor burden assessment from PSMA PET/CT is prognostically valuable, its accuracy depends on segmentation methodology. This study compares fixed SUV 4 thresholding (SUV_4) with personalized SUVmean–based segmentation (SUV_MEAN) for predicting 12–month disease progression.
**Methods or Background:** This single–center study retrospectively analyzed 51 mCRPC patients treated with [177Lu]Lu–PSMA–617. Pre–treatment PSMA PET/CT was performed with [68Ga]Ga–PSMA–11 (n=6) or [18F]F–PSMA–1007 (n=45). Whole–body molecular tumor volume (MTV), total lesion uptake (TLU), and total lesion quotient (TLQ) were derived using both segmentation methods. Progression–free survival (PFS) was assessed with Cox regression; 12–month progression was evaluated using LASSO–penalized multivariable logistic regression.
**Results or Findings:** Mean age was 70.6 years (range 54–89); median PSA 264 ng/mL (range 1.6–6946); median treatment cycles 4 (range 1–11). At 12 months, 27 patients (53%) progressed. Both high MTV_4 and MTV_MEAN were associated with shorter PFS (HR 2.50 [95% CI 1.30–4.80], p=0.006), as were high TLU_4 and TLU_MEAN (HR 2.02 [1.06–3.85], p=0.032). For 12–month progression prediction, the SUV_MEAN–based model (MTV_MEAN, treatment cycles, baseline PSA) achieved superior performance with AUC 0.923 (95% CI 0.849–0.996) and cross–validated AUC 0.842 (0.691–0.993), with MTV_MEAN as the only significant PET–derived predictor. No SUV_4–derived metrics achieved significance in the final model.
**Conclusion:** Personalized SUV_MEAN segmentation provides superior prognostic performance compared to fixed SUV_4 thresholding for predicting early progression after [177Lu]Lu–PSMA–617 RLT. By incorporating patient–specific tumor uptake patterns, SUV_MEAN generates more clinically representative and reproducible tumor burden metrics. Validation in larger multicenter cohorts could improve patient selection and provide a standardized foundation for AI–driven prognostic tools.
**Limitations:** Not applicable
**Funding for this study:** No funding was provided for this study
**Has your study been approved by an ethics committee?** Yes
**Ethics committee - additional information:** The ethics committee approval can be found under the number 2023–01877
**Author Disclosures:**
Yothin Rakvongthai: Nothing to disclose
Jiahui Wang: Nothing to disclose
Kuangyu Shi: Nothing to disclose
Yizhou Chen: Nothing to disclose
Jiaxi Hu: Nothing to disclose
Warissara Jutidamrongphan: Nothing to disclose
Ali Afshar-Oromieh: Nothing to disclose
Axel Rominger: Nothing to disclose
Robert Seifert: Nothing to disclose

### FDG PET/CT Biomarkers Beyond Deauville: Added Value of Dmax and MTV in Predicting Outcome in Lymphoma

*Y. Dauytova*, Z. J. Zholdybay, Z. Zhakenova, Z. M. Amankulov, A. Dyo, L. Aitzhanova, M. S. Telkhazhaeyeva; Almaty/KZ
*(juldiz.dauytova@mail.ru)*

**Purpose:** To assess the added prognostic value of MTV and Dmax beyond the Deauville score in predicting progression-free survival (PFS) in lymphoma patients
**Methods or Background:** 18F-FDG PET/CT is indispensable in the management of lymphoma. While the Deauville 5-point scale remains the cornerstone for response assessment, it often fails to capture the full biological heterogeneity of disease. Novel quantitative biomarkers such as metabolic tumor volume (MTV) and the maximum distance between the two most metabolically active lesions (Dmax) may provide complementary insights into tumor burden and dissemination. We retrospectively evaluated 120 patients with newly diagnosed Hodgkin and non-Hodgkin lymphoma who underwent baseline and interim ^18F-FDG PET/CT. MTV was derived from semi-automated segmentation, and Dmax was calculated as the Euclidean distance between the most distant FDG-avid lesions. Patients were stratified into high vs. low MTV and Dmax groups. Kaplan–Meier and multivariate Cox analyses were performed with PFS as the primary endpoint.
**Results or Findings:** Patients with high baseline Dmax (>30 cm) and high MTV had significantly inferior outcomes. The 2-year PFS was 82% in the low-Dmax group versus 48% in the high-Dmax group ($p < 0.01$), and 80% versus 50% for low vs. high MTV ($p < 0.01$). On multivariate analysis, both Dmax and MTV remained independent predictors of PFS beyond interim PET response. The combined model (Deauville + MTV + Dmax) achieved superior risk stratification, identifying a subgroup with poor prognosis despite negative interim PET findings.
**Conclusion:** MTV and Dmax extend the prognostic power of FDG PET/CT beyond the Deauville scale. Their integration into routine reporting can refine risk stratification and support personalized treatment strategies in lymphoma.
**Limitations:** N/a
**Funding for this study:** No funding was received for this work
**Has your study been approved by an ethics committee?** Not applicable
**Author Disclosures:**
Zhandos Mukhtaruly Amankulov: Nothing to disclose
Andrei Dyo: Nothing to disclose
Zhanar Zhakenova: Nothing to disclose
Meruyert Seitbekovna Telkhazhaeyeva: Nothing to disclose
Yulduzkhan Dauytova: Nothing to disclose
Zhamilya J. Zholdybay: Nothing to disclose
Laura Aitzhanova: Nothing to disclose

### Longitudinal integrated diagnostics in GEP-NET patients undergoing PRRT

F. Herr, *C. A. Dascalescu*, R. Ebner, M. L. Schnitzer, J. Ricke, M. Heimer, M. Brendel, R. Werner, C. C. Cyran; Munich/DE
*(christian.dascalescu@med.uni-muenchen.de)*

**Purpose:** To evaluate the prognostic value of integrated clinical and laboratory biomarkers for longitudinal overall survival (OS) prediction in patients with gastroenteropancreatic neuroendocrine tumors (GEP-NETs) undergoing [177Lu]Lu-DOTA-TATE peptide receptor radionuclide therapy (PRRT).
**Methods or Background:** In this retrospective single-center study, 178 patients with histologically confirmed, well-differentiated (Grade 1–2) GEP-NETs treated with PRRT between 2012 and 2023 were included. Clinical and laboratory parameters (BMI, CRP, albumin, leukocytes, erythrocytes, hemoglobin, platelets, NSE, CgA) were collected at baseline, follow-up 1 (FU1; after 2 cycles), and follow-up 2 (FU2; after 4 cycles). Optimal cut-offs were determined using maximally selected rank statistics. Univariate and multivariate Cox regression analyses were performed; model performance was assessed by Harrell's concordance index (C-index) and Akaike Information Criterion (AIC). Kaplan–Meier analysis with log-rank testing was applied.
**Results or Findings:** Median OS across the cohort was 70.1 months after a median follow-up of 60.8 months. At FU1, albumin ≤ 4.1 g/dL and CgA > 299 ng/mL were independent predictors of shorter OS. At FU2, albumin ≤ 4.5 g/dL, NSE > 29.1 ng/mL, and CgA > 179 ng/mL retained independent prognostic value. Models combining these biomarkers showed superior prognostic accuracy (C-index up to 0.77) compared with single-parameter approaches. Kaplan–Meier analyses confirmed significant separation between high- and low-risk groups.
**Conclusion:** Longitudinal assessment of albumin, NSE, and CgA provides independent and complementary prognostic information in PRRT-treated GEP-NET patients. Integration of these biomarkers into multivariable models improves OS prediction beyond baseline or single-marker assessment, supporting their role in personalized follow-up and treatment planning.
**Limitations:** Retrospective, single-center design; external validation in multicenter cohorts is required.
**Funding for this study:** No
**Has your study been approved by an ethics committee?** Yes
**Ethics committee - additional information:** This retrospective single-center study was approved by the local ethics committee (approval number 19–027). Due to the retrospective nature, informed consent was waived.
**Author Disclosures:**
Moritz Ludwig Schnitzer: Nothing to disclose
Rudolf Werner: Nothing to disclose
Clemens C. Cyran: Nothing to disclose
Ricarda Ebner: Nothing to disclose
Maurice Heimer: Nothing to disclose
Felix Herr: Nothing to disclose
Matthias Brendel: Nothing to disclose
Christian Alexander Dascalescu: Nothing to disclose
Jens Ricke: Nothing to disclose

### Value of pre-operative 18F-FDG-PET/CT in restaging NSCLC patients after neoadjuvant immunochemotherapy

*D. Kifjak*, M. J. Hochmair, A. Korajac, S. Pochepnia, R-I. Milos, A. Hoda, K. Sinn, L. Beer, H. Prosch; Vienna/AT

**Purpose:** To evaluate the clinical impact of pre-operative 18F-FDG-PET/CT in non-small cell lung cancer (NSCLC) patients who received neoadjuvant immunochemotherapy.
**Methods or Background:** This retrospective analysis included 68 potentially operable NSCLC patients (39 female, 29 male) who were treated with neoadjuvant immunochemotherapy and underwent subsequent pre-operative 18F-FDG-PET/CT for surgical planning. Histological subtypes included 42 adenocarcinomas and 26 squamous cell carcinomas. Pre-operative 18F-FDG-PET/CT scans after neoadjuvant immunochemotherapy were assessed for new metastatic disease and their influence on treatment decisions. Patient treatment decisions, including surgical resection and reasons for non-operative management, were reviewed.
**Results or Findings:** Of the 68 patients, 62 underwent surgical resection. Six patients did not undergo surgery. Among these, one patient was found to have new osseous metastases in the pelvis on pre-operative 18F-FDG-PET/CT, that were only detected on the metabolic PET component, which excluded surgery and therefore altered the treatment decision. Five patients did not undergo surgery for reasons unrelated to 18F-FDG-PET/CT findings: one declined surgery, one was excluded by multidisciplinary tumor board decision due to a large primary tumor, one was referred for radiotherapy because of N3 disease, and in two patients surgical resection was technically not feasible. Thus, pre-operative 18F-FDG-PET/CT changed the treatment strategy in only one patient (1.5%).
**Conclusion:** While the current National Comprehensice Cancer Network (NCCN) guidelines recommend contrast-enhanced CT with optional 18F-FDG-PET/CT for restaging prior to surgery, our results suggest that pre-operative 18F-FDG-PET/CT may not be warranted, raising an ethical dilemma regarding addional costs, radiation exposure and patient burden given its minimal impact on treatment decision-making.
**Limitations:** This was a retrospective analysis of a relatively small cohort size.
**Funding for this study:** The financial support by the Austrian Federal Ministry for Digital and Economic Affairs, the National Foundation for Research, Technology and Development and the Christian Doppler Research Association is gratefully acknowledged.
**Has your study been approved by an ethics committee?** Yes
**Ethics committee - additional information:** Approved by the institutional ethics committee (EC No: 1521/2015).
**Author Disclosures:**
Katharina Sinn: Nothing to disclose
Maximilian Johannes Hochmair: Nothing to disclose
Helmut Prosch: Speaker: Boehringer Ingelheim, Astra Zeneca, MSD, Sanofi und Jansen
Lucian Beer: Nothing to disclose
Aida Korajac: Nothing to disclose
Daria Kifjak: Nothing to disclose
Ruxandra-Iulia Milos: Nothing to disclose
Alireza Hoda: Nothing to disclose
Svitlana Pochepnia: Nothing to disclose

### Functional Imaging Modalities in Multiple Myeloma: Comparative Diagnostic Performance of Whole-Body MRI, FDG and FCH PET/CT

*G. Kaya*[1], O. Kodaz[2], A. E. Yıldız[2], S. Akın[2], P. Ozgen Kıratlı[2]; [1]Yozgat/TR, [2]Ankara/TR
*(kayagursan@gmail.com)*

**Purpose:** Recent ESMO-EANM guidelines recognize $^{18}$F-FDG-PET/CT and whole-body diffusion-weighted MRI (WB-MRI) as key imaging modalities in multiple myeloma (MM), while $^{18}$F-Fluorocholine-(FCH)-PET/CT has emerged as a potentially more sensitive alternative. However, no prior study has directly compared all three modalities in the same cohort. This study aimed to evaluate the diagnostic yield of FCH PET/CT, FDG PET/CT, and WB-MRI in newly diagnosed MM patients and correlate imaging findings with laboratory and histopathological markers.
**Methods or Background:** This retrospective approved-study included 7 adults with newly diagnosed MM (median age: 63). All underwent WB-MRI, FDG-PET/CT, and FCH-PET/CT within four weeks. PET scans were evaluated using IMPeTUs criteria; WB-MRI was scored semi-quantitatively. Lesion- and patient-based analyses were performed. SUVmax and ADC were recorded for three target lesions per patient.
**Results or Findings:** FCH-PET/CT and WB-MRI detected disease in all patients(100%), outperforming FDG-PET/CT (positive in 6/7). FCH identified significantly more lesions than FDG or WB-MRI ($p = 0.005$), especially in diffuse marrow involvement. FDG showed better performance in cases with low CD138 expression. Imaging patterns varied markedly, even in similar disease phenotypes. SUVmax and ADC values showed weak correlation ($r < 0.4$). FDG Deauville scores correlated strongly with urinary kappa light chains, while FCH

scores correlated with focal WB-MRI lesion counts and lower ADC values. Interestingly, higher $\beta_2$-microglobulin levels were inversely associated with FCH scores. WB-MRI findings correlated with urinary lambda light chains and protein levels in patients with focal lesions. These findings underscore FC-PET/CT's translational potential as a sensitive and widely accessible tool in MM staging.
**Conclusion:** FCH-PET/CT provided the most extensive disease detection, particularly in diffuse marrow infiltration. The complementary nature of FCH, FDG, and WB-MRI imaging suggests value in combined multimodality approaches.
**Limitations:** Retrospective nature, small cohort.
**Funding for this study:** This research received no specific grant from any funding agency in the public, commercial, or not-for-profit sectors.
**Has your study been approved by an ethics committee?** Yes
**Ethics committee - additional information:** Hacettepe Üniversitesi Sağlık Bilimleri Araştırma Etik Kurulu; Hacettepe University Institutional Review Board for Health Sciences Research; SBA25/604
**Author Disclosures:**
Pınar Ozgen Kıratlı: Nothing to disclose
Oguz Kodaz: Nothing to disclose
Gürsan Kaya: Nothing to disclose
Serkan Akın: Nothing to disclose
Adalet Elçin Yıldız: Nothing to disclose

**Evaluation of standardized uptake value (SUV) and apparent diffusion coefficient (ADC) as prognostic imaging parameters for primary Ewing Sarcoma in hybrid 18F-FDG-PET/MRI**
*M. A. Drews*, A. Milosevic, S. Zensen, M. Opitz, L. Umutlu, W. Fendler, K. Herrmann, U. Dirksen, B. M. Schaarschmidt; Essen/DE
*(marcel.a.drews@gmail.com)*

**Purpose:** Ewing sarcoma (EwS) is the second most prevalent malignant bone tumour among paediatric and juvenile patients. Due to its highly malignant character, an accurate diagnosis is crucial to determine most suitable treatment and to identify patients with unfavourable courses as early as possible. In this context, hybrid 18F-FDG-PET/MRI combines high-contrast morphological information with functional diffusion imaging and metabolic data which could deliver valuable prognostic information for treatment response and survival prediction. Therefore, we evaluated correlations between ADC and SUV as established parameters for tumour cellularity and metabolic activity and Salzer-Kuntschik scale(SKS) for treatment response as well as overall survival(OS) in this rare entity.
**Methods or Background:** In a monocentric retrospective study, 39 patients with histopathologically proven EwS who underwent 18F-FDG PET/MRI before therapy start were evaluated(14female, median age 16yrs [IQR:11-23]). A polygonal region of interest was drawn encompassing the entire primary tumour mass on fused PET/MR images to determine SUVmax and SUVmean and copied onto the ADC maps to quantify ADCmean and ADCmin. Dependencies between these parameters and SKS were analysed using Spearman's correlation coefficients and Hazard ratio was used for OS.
**Results or Findings:** A strong inverse correlation between SUVmax and ADCmean($\rho$=-0.709,$p<0.001$) and SUVmean and ADCmean($\rho$=-0.689,$p<0.001$) was observed. Furthermore, moderate correlations between SKS and SUVmax($\rho$=0.502,p=0.009), SUVmean($\rho$=0.389,p=0.049) and ADCmean($\rho$=-0.425,p=0.03) were seen as well as correlations between OS and SUVmax(HR:5.324,95%-CI 1.065-26.615,p=0.042) and SUVmean(HR: 5.558,95%-CI 1.114-27.733,p=0.037).
**Conclusion:** Our study demonstrates a strong correlation between metabolic activity(SUV) and tumour cellularity(ADC) in EwS. SUVmax and SUVmean can further be used as prognostic markers for neoadjuvant treatment response and OS, while ADCmean also correlates inversely with treatment response but does not deliver complementary information.
**Limitations:** Limitations lie in the retrospective design and small patient number, but which is high for this rare entity.
**Funding for this study:** Marcel Drews received funding by the Junior Clinical Scientist Program at the University of Essen Medical School to carry out this project.
**Has your study been approved by an ethics committee?** Yes
**Ethics committee - additional information:** Approval by the local ethics committee was obtained and the need for informed consent was waived due to the retrospective nature of this study (application number: 24-11729-BO; approval date: 23.02.2024).
**Author Disclosures:**
Ken Herrmann: Nothing to disclose
Wolfgang Fendler: Nothing to disclose
Marcel Alexander Drews: Nothing to disclose
Benedikt Michael Schaarschmidt: Nothing to disclose
Uta Dirksen: Nothing to disclose
Sebastian Zensen: Nothing to disclose
Lale Umutlu: Nothing to disclose
Aleksandar Milosevic: Nothing to disclose
Marcel Opitz: Nothing to disclose

**13:00 - 14:30 Research Stage 6**

### Research Presentation Session: Genitourinary

### RPS 407
### Bladder cancer and ureteral cancer imaging

**Moderator**
Emanuele Messina; Rome/IT
*(emanuele.messina@uniroma1.it)*

**An artificial intelligence framework based on contrast-enhanced CT for predicting postoperative recurrence risk in non-muscle-invasive bladder cancer: a multicenter study**
*J. Wu*, J. Li, J. Han, R. Liu; Inner Mongolia Autonomous Region/CN
*(2539663885@qq.com)*

**Purpose:** To determine whether the deep learning model based on automatic segmentation of CT images can provide a robust prediction of postoperative recurrence for non-muscle-invasive bladder cancer (NMIBC).
**Methods or Background:** Preoperative CT scans were retrospectively acquired in patients with surgically proven NMIBC at multiple centers from January 2015 to May 2023. Follow-up was extended until May 2025, and cases were categorized as recurrent or nonrecurrent based on postoperative outcomes. The proposed framework comprised four modules, including a 3D tumor segmentation model by 3D-UNet, a deep learning feature extraction module by Swin Transformer, a clinical-radiological feature screening module, and a fully-connected classification module that combines features from different sources. The predictive performance of the model was evaluated based on discrimination, calibration, and clinical usefulness.
**Results or Findings:** The training data set was comprised of 310 patients from three centers, and 115 and 72 patients were included in the two external test data sets. The average Dice coefficient of the 3D-UNet automatic segmentation network in the test sets was 0.88 and 0.83. Subsequently, the model's ability to predict the risk of postoperative recurrence was evaluated. The integrated model exhibited excellent performance with AUCs of 0.92 in the training set, 0.85 and 0.88 in the external validation set, along with satisfactory calibration across all cohorts ($P>0.05$). Furthermore, the integrated model significantly outperformed both the clinical-radiological model and deep learning model ($P<0.05$).
**Conclusion:** The integrated model based on clinical features and deep learning features focusing on NMIBC patients undergoing the transurethral resection of bladder tumor(TURBT), accurately identifies cases at high-risk of postoperative recurrence, which outperformed the clinical-radiological feature model.
**Limitations:** This is a retrospective study and we only included NMIBC with a pathological diagnosis after TURBT, with some selection bias.
**Funding for this study:** This work was supported in part by the Key Scientific Research and Development Achievement Transformation Programme of the Department of Science and Technology of the Inner Mongolia Autonomous Region (2025YFSH0050); and partially supported by the Public Hospital Research Joint Fund Programme of Inner Mongolia Medical University (2023GLLH0086, 2024GLLH0013).
**Has your study been approved by an ethics committee?** Yes
**Ethics committee - additional information:** This study was approved by the Institutional Review Board, and written informed consent was obtained.
**Author Disclosures:**
Rui Liu: Nothing to disclose
Junlin Li: Nothing to disclose
Jiayue Han: Nothing to disclose
Jialu Wu: Nothing to disclose

**Utility of nacVI-RADS for Predicting Pathological Complete Response After Chemoradiotherapy in Muscle-Invasive Bladder Cancer**

*K. Fukui*[1], M. Kobayashi[1], S. Yoshida[1], Y. Arita[2], K. Yamagiwa[1], J. Tsuchiya[1], Y. Fujii[1], U. Tateishi[1]; [1]Tokyo/JP, [2]San Francisco, CA/US

**Purpose:** Accurate post-treatment imaging evaluation remains a key challenge in muscle-invasive bladder cancer (MIBC), particularly for assessing the effect of chemoradiotherapy (CRT) in the context of bladder-sparing strategies. To pathologically validate CRT response, analysis of a patient cohort who underwent partial or radical cystectomy after CRT is required. This study evaluated the effectiveness of nacVI-RADS on multiparametric MRI for predicting pathological complete response (pCR) after CRT and explored its potential role in standardized post-treatment assessment to guide bladder-preserving management.
**Methods or Background:** In this single-center retrospective study, 73 patients with non-metastatic MIBC who underwent CRT (40 Gy) followed by cystectomy and had multiparametric MRI both pre-TURBT and post-CRT were included. Two board-certified radiologists with genitourinary subspecialty experience (9 and >10 years), blinded to pathology, independently assigned nacVI-RADS on post-CRT MRI. Diagnostic performance for predicting pCR was assessed using a prespecified threshold of nacVI-RADS <3; inter-reader agreement was quantified with Cohen's κ.
**Results or Findings:** Median age was 70 years; 30% were female. Clinical stages were T2 55%, T3 43%, and T4 3%. Radical and partial cystectomy were performed in 37% and 63%, respectively. pCR occurred in 67% (49/73). Patients with nacVI-RADS <3 had significantly higher pCR rates than those with ≥3 (reader 1: 81% vs 42%, p=0.002; reader 2: 77% vs 46%, p=0.021). For predicting pCR, sensitivity/specificity of nacVI-RADS <3 were 78%/63% (reader 1) and 80%/50% (reader 2). Inter-reader agreement was almost perfect (κ=0.84).
**Conclusion:** To our knowledge, this is the first study to validate nacVI-RADS as a reproducible imaging biomarker for pathological response assessment after CRT. These results support its potential role in standardized post-treatment MRI evaluation and in guiding bladder-preserving strategies in MIBC.
**Limitations:** Retrospective single-center design with a modest cohort and a fixed CRT dose regimen.
**Funding for this study:** No funding was received for this study.
**Has your study been approved by an ethics committee?** Not applicable
**Author Disclosures:**
Ukihide Tateishi: Nothing to disclose
Kenichi Fukui: Nothing to disclose
Soichiro Yoshida: Nothing to disclose
Ken Yamagiwa: Nothing to disclose
Junichi Tsuchiya: Nothing to disclose
Yuki Arita: Nothing to disclose
Masaki Kobayashi: Nothing to disclose
Yasuhisa Fujii: Nothing to disclose

**Optimizing Multi-b-Value DWI Quantitative Parameters for Assessing Detrusor Muscle Invasion and Histological Grade in Bladder Tumors**

Y. Wu, X. Zhu, *Y. Shang*, C. Liu, Y. Tong, L. Ai, Z. Xiaolong; Kunming/CN
*(mri2507@126.com)*

**Purpose:** Given bladder tumors' high incidence, malignancy, and invasiveness, traditional VI-RADS scoring, based on morphology, inadequately captures tumor microstructure. This study innovatively evaluates MRI multi-b-value DWI parameters' predictive power for muscular invasion and histological grading in bladder urothelial carcinoma, exploring their synergy with VI-RADS.
**Methods or Background:** Prospectively, 160 patients with suspected bladder masses underwent conventional MRI and multi-b-value DWI (11 b-values, 0-2000 s/mm²). Among them, 106 pathologically confirmed cases (age 68±11 years, 84.9% male) were analyzed. Using iCareSpinx software, entire lesion volumes were delineated to compute 15 diffusion parameters from six non-Gaussian models (CTRW, DKI, FROC, IVIM, SEM, SE). Statistical tests compared parameter differences between groups with varying muscular invasion and pathological grades. Binary logistic regression constructed combined diagnostic models, and ROC curves with AUC values evaluated diagnostic performance.
**Results or Findings:** Significant differences were observed in CTRW-ADC, DKI-ADC, IVIM-D slow, SEM-ADC, SM-ADC, SM-Theta, and monoADC for discriminating muscular invasion and pathological grading (all P < 0.05). Individually, these parameters showed moderate AUCs (0.704-0.779 for invasion, 0.629-0.712 for grading). VI-RADS alone had AUCs of 0.774 (95% CI: 0.681-0.867) for invasion and 0.720 (95% CI: 0.621-0.818) for grading. Combining parameters with VI-RADS improved AUCs (e.g., 0.870 for invasion with four parameters, outperforming traditional monoADC-VI-RADS combination's 0.845; 0.849 for grading, outperforming 0.765).
**Conclusion:** MRI multi-b-value DWI quantitative parameters offer clinical value in assessing bladder urothelial tumor invasiveness and grading. While their individual efficacy is limited, their combination with VI-RADS significantly enhances diagnostic performance, providing a more reliable tool.
**Limitations:** Firstly, the single-center study has a relatively small sample size. Secondly, not delineating smaller or unclear-boundary lesions may omit some. However, volume-based delineation minimizes this bias. Additionally, post-processing times for some models are lengthy, necessitating further optimization to enhance clinical practicality.
**Funding for this study:** Yunnan Provincial Education Department Project: Research on Predicting Pathological Grading of Bladder Cancer Based on MRI Radiomics Model (2024J03480) General Project of Yunnan Provincial Science and Technology Department: Comprehensive Study on Assessing Bladder Cancer Aggressiveness, Prognosis, and Therapeutic Efficacy Using MRI-Based Deep Learning Models (202401AY070001-337)
**Has your study been approved by an ethics committee?** Yes
**Ethics committee - additional information:** None
**Author Disclosures:**
Zhou Xiaolong: Nothing to disclose
Canli Liu: Nothing to disclose
Yuyun Tong: Nothing to disclose
Xiaofang Zhu: Nothing to disclose
Liping Ai: Nothing to disclose
Yangying Wu: Nothing to disclose
Yunyun Shang: Nothing to disclose

**VI-RADS quality score: reproducibility validation study**

*L. De Giacomo*, A. Dehghanpour, A. Borrelli, G. Bincoletto, M. Pecoraro, V. Panebianco; Rome/IT
*(luca.degiacomo@uniroma1.it)*

**Purpose:** To retrospectively validate the reproducibility of the recently proposed Vesical Imaging Quality Score (VI-RADS QS) for multiparametric magnetic resonance imaging (mpMRI) of the bladder, assessing inter-reader agreement across radiologists with different levels of expertise.
**Methods or Background:** One hundred consecutive bladder mpMRI examinations acquired at 1.5T and 3T scanners were retrospectively retrieved and anonymized. Three radiologists with varying experience in genitourinary imaging (expert, intermediate, beginner) independently scored each examination according to the VI-RADS QS, which stratifies image quality as inadequate, adequate, or optimal per sequence (T2-weighted, diffusion-weighted, and dynamic contrast-enhanced) and overall. Inter- and intra-reader agreement were measured using Gwet's AC1 statistics.
**Results or Findings:** Preliminary analyses are expected to show moderate to substantial inter-reader agreement for the overall VI-RADS QS (anticipated Gwet's AC1 ≈ 0.50–0.70), with higher reproducibility among expert readers compared to beginners. Per-sequence agreement is projected to be higher for DWI and DCE compared to T2-weighted imaging, reflecting the key role of these sequences in bladder cancer staging.
**Conclusion:** This retrospective study on 150 bladder mpMRI examinations will provide the first validation of the VI-RADS Quality Score, demonstrating its reproducibility across readers of varying expertise. Adoption of the VI-RADS QS may facilitate protocol optimization, harmonize reporting across institutions, and strengthen the reliability of bladder MRI in multidisciplinary decision-making.
**Limitations:** Small sample size; single center retrospective study
**Funding for this study:** None
**Has your study been approved by an ethics committee?** Not applicable
**Author Disclosures:**
Gaia Bincoletto: Nothing to disclose
Valeria Panebianco: Nothing to disclose
Antonella Borrelli: Nothing to disclose
Ailin Dehghanpour: Nothing to disclose
Luca De Giacomo: Nothing to disclose
Martina Pecoraro: Nothing to disclose

**Added value of synthetic MRI in refining VI-RADS for predicting muscle-invasive bladder cancer**

*Z. Zhao*, X-G. Peng; Nanjing/CN
*(zhaozihui0216@163.com)*

**Purpose:** To evaluate the diagnostic value of synthetic MRI (SyMRI)-derived T1 and T2 values in predicting muscle-invasive bladder cancer (MIBC) and assess the feasibility of integrating them into biparametric MRI VI-RADS (bpVI-RADS).
**Methods or Background:** This prospective, dual-center study included 36 patients with histologically confirmed urothelial carcinoma who underwent standardized bladder MRI. Two radiologists independently assigned bpVI-RADS scores based on T2WI and DWI, blinded to pathological. Discrepancies were resolved by another senior radiologist. Tumor volumes were manually delineated on SyMRI-derived maps to extract T1 and T2 values. Two modified models (bpVI-RADS+T1 and bpVI-RADS+T2) were developed by adjusting bpVI-RADS scores based on predefined T1 or T2 thresholds. Diagnostic performance was compared among original and modified scoring systems using ROC analysis.
**Results or Findings:** Thirty-six patients (mean age: 71.25 ± 10.27 years, 22.2% MIBC) were enrolled in this study. MIBC lesions showed significantly lower T1 and T2 values than non–muscle-invasive bladder cancer (NMIBC). Optimal cutoff values for differentiating MIBC from NMIBC were 1370 msec for T1 (sensitivity: 87.5%, specificity: 89.3%) and 113 msec for T2 (sensitivity: 87.5%, specificity: 100%). Among all scoring methods, bpVI-RADS+T2 achieved the highest diagnostic performance at the ≥3 cutoff, with higher specificity (89.3% vs. 67.9%, p=0.031), accuracy (91.7% vs. 75.0%, p=0.031), and AUC (0.993 vs. 0.900, p=0.048) compared to bpVI-RADS. The bpVI-RADS+T1 showed non-significant AUC improvement (0.973 vs. 0.900, P = 0.144).
**Conclusion:** Incorporating SyMRI-derived T2 values into the bpVI-RADS framework significantly enhances diagnostic performance for predicting MIBC.
**Limitations:** First, the modest sample size and limited MIBC cases may reduce statistical power and generalizability. Second, inter-center variations in MRI equipment and software could have introduced bias despite high reader consistency. Finally, the modified thresholds were derived from this cohort without external validation, warranting cautious clinical application.
**Funding for this study:** This study has received funding by National Natural Science Foundation of China (82272064), Jiangsu Provincial Science and Technique Program (BK20221461), Zhongda Hospital Affiliated to Southeast University, Jiangsu Province High-Level Hospital Pairing Assistance Construction (zdlyg08), Postgraduate Research & Practice Innovation Program of Jiangsu Province (KYCX23_0323 and KYCX22_0297).
**Has your study been approved by an ethics committee?** Yes
**Ethics committee - additional information:** Approval No. 2024ZDSYLL061-P01
**Author Disclosures:**
Zihui Zhao: Nothing to disclose
Xin-Gui Peng: Nothing to disclose

### Optimizing VI-RADS: Tumor Contact Length for Precise Bladder Cancer Staging

*D. Gupta*, A. Bhandari, N. Gupta; Jaipur/IN
*(drdivyanshrad@gmail.com)*

**Purpose:** To investigate VI-RADS diagnostic performance on multiparametric MRI for predicting detrusor muscle invasion in bladder cancer, with histopathology as gold standard, and evaluate tumor contact length (TCL) as a quantitative biomarker for equivocal cases.
**Methods or Background:** This prospective cross-sectional study enrolled 70 patients with suspected bladder masses undergoing 3.0T Mp-MRI prior to transurethral resection (June 2023-July 2024). Inclusion criteria: gross hematuria and untreated bladder cancer. Exclusion criteria: prior treatment, MRI contraindications, or elevated creatinine. Imaging protocol included T2-weighted imaging, diffusion-weighted imaging with ADC mapping, and dynamic contrast-enhanced sequences. VI-RADS scoring used standardized algorithms with DWI as dominant sequence. TCL was measured as maximum curvilinear tumor-bladder wall interface on T2-weighted images. Statistical analysis included ROC analysis, Youden index optimization, and multivariate logistic regression.
**Results or Findings:** The cohort comprised 44 patients (62.9%) with non-muscle-invasive bladder cancer (NMIBC) and 26 patients (37.1%) with muscle-invasive bladder cancer (MIBC). VI-RADS (≥3 threshold) achieved 96.2% sensitivity, 84.7% specificity, 89.7% accuracy, and 95.5% negative predictive value. Diffusion-weighted imaging showed superior individual sequence performance (92.5% sensitivity, 88.1% specificity). TCL (≥3.2cm threshold) demonstrated 96.2% sensitivity, 94.6% specificity, and 98.6% accuracy. Within challenging VI-RADS 2-3 intermediate cases (n=36), TCL integration achieved 89% sensitivity, 95% specificity, and 94% accuracy. Multivariate analysis confirmed VI-RADS ≥3 (OR 4.2, p<0.05) and TCL (OR 8.7, p<0.01) as independent predictors.
**Conclusion:** VI-RADS provides highly accurate bladder cancer staging. TCL integration transforms equivocal intermediate-risk lesions into definitive clinical decisions, fundamentally improving patient management using standard imaging protocols.
**Limitations:** single-center design, moderate sample size, predominance of urothelial carcinoma, and dependence on TURBT reference standard with inherent sampling variability.
**Funding for this study:** The authors received no specific funding for this work
**Has your study been approved by an ethics committee?** Yes
**Ethics committee - additional information:** Ethics approval obtained from SMS Medical college Ethics Committee. Written informed consent obtained from all participants prior to enrollment."
**Author Disclosures:**
Anu Bhandari: Nothing to disclose
Naman Gupta: Nothing to disclose
Divyansh Gupta: Nothing to disclose

### Dual-layer detector spectral CT parameters for differentiating low-grade and high-grade bladder urothelial cancer and predicting muscular invasion

*J. Lv*[1], R. X. Mu[1], X. Li[1], W. Zheng[1], P. Yang[1], X. Liu[2], X. Qin[1], X. Zhu[3]; [1]Guilin/CN, [2]Guangzhou/CN, [3]Baise/CN
*(lvjnxs@163.com)*

**Purpose:** To evaluate the diagnostic efficacy of dual-layer detector spectral CT (DLCT) in distinguishing low-grade (LG) from high-grade (HG) bladder urothelial cancer and in predicting muscular invasion (MI).
**Methods or Background:** A total of 116 patients (74 HG, 42 LG) who underwent preoperative DLCT scan were retrospectively included. Quantitative parameters included iodine density (ID) and extracellular volume (ECV) fractions in the arterial (AECV), venous (VECV), and equilibrium (EECV) phases were calculated. ECV was calculated as (1–hematocrit) × (lesion iodine concentration / blood pool iodine concentration). Univariate and multivariate regression analysis were performed to construct the combined diagnostic mode for predicting bladder cancer grading, as well as MI versus non muscular invasion (NMI) within HG and LG tumors, respectively. ROC analysis was used to assess diagnostic performance.
**Results or Findings:** V-ID, AECV, VECV, and EECV significantly differentiated HG from LG tumors, with AUCs of 0.801, 0.727, 0.770, and 0.833, respectively. Tumor calcification was identified as a risk factor, achieving an AUC of 0.638. The combined diagnostic model significantly improved the discriminative performance for HG versus LG bladder cancer, yielding an AUC of 0.891 (sensitivity 0.905, specificity 0.833). For predicting MI within HG tumors, heterogeneous muscular enhancement (HME) at VMI 40 keV, VECV and EECV served as predictors, with AUCs of 0.733, 0.692, and 0.714, respectively. The combined model for MI prediction achieved an AUC of 0.788 (sensitivity=0.800, specificity=0.708) within HG tumors. No spectral parameters demonstrated statistically significant difference in predicting MI within the LG subgroup.
**Conclusion:** DLCT-derived parameters, particularly ECV, allow reliable differentiation between low- and high-grade bladder urothelial carcinoma and provide valuable prediction of muscular invasion in HG tumors.
**Limitations:** None
**Funding for this study:** None
**Has your study been approved by an ethics committee?** Yes
**Ethics committee - additional information:** None
**Author Disclosures:**
Xiaomin Liu: Nothing to disclose
Wei Zheng: Nothing to disclose
Ronghua Xiqi Mu: Nothing to disclose
Peng Yang: Nothing to disclose
Xiaoyan Qin: Nothing to disclose
Xiqi Zhu: Nothing to disclose
Xin Li: Nothing to disclose
Jian Lv: Nothing to disclose

### INcidence of malignant ureteric obStruction In patienTs with non-organ confined abdominopElvic malignancy (INSITE MUO) a multi-centre observational study

*O. Llewellyn*[1], J. Blackmur[1], J. Aning[2], N. Shaida[3], M. Bagkeris[4], T. Barrett[3], A. Laird[1], BAUS Oncology, UNITE Collaborative[1]; [1]Edinburgh/UK, [2]Bristol/UK, [3]Cambridge/UK, [4]London/UK
*(oliver.llewellyn@nhs.scot)*

**Purpose:** Interventional radiologists and Urologists commonly encounter malignant ureteric obstruction (MUO). Capturing this patient population is challenging due to the pan-cancer aetiology. The aim of this study was to estimate the incidence of MUO in advanced abdominopelvic cancer and provide groundwork for further prospective work.
**Methods or Background:** National multicentre observational research study using national Interventional Radiology and Urology stakeholder networks (UNITE & BAUS). 24 sites contributed HRA approved, RCR funded study. Study teams contained at least one post-FRCR Trainee/Consultant. Cases of advanced cancer involving the abdomen and cases of MUO were identified by trainees filtering reports. To do this, a standardised list of CT examination protocols involving the abdomen/pelvis over a 4 week study period was acquired at each site. Pseudonymised data was collected centrally from sites using the REDCap platform.
**Results or Findings:** 6781 abdominopelvic malignancy cases were identified. 2972 cancers were classified as advanced. 1387 cases missing TNM data were excluded from this interim analysis. The incidence of MUO in advanced cancer was 5.2% and the prevalence of was 9.8%. Survival analysis (Kaplan-Meier) indicated a protective influence of intervention (nephrostomy or stent) on overall survival in MUO in advanced cancer, p = 0.049. Intra-abdominal involvement from cancers originating outside the abdomen represented the largest proportion of the advanced intra-abdominal cancers (22.7%). The most common cause for MUO was prostate cancer (18.1%).

**Conclusion:** The incidence of MUO in patients with advanced abdominopelvic cancer was 5.2% and the prevalence of was 9.8%. Intervention (nephrostomy or ureteric stent) in MUO in advanced cancer appears to have a protective effect on overall survival.
**Limitations:** Observational study methodology
**Funding for this study:** Royal college of radiologists Kodak fellowship grant
**Has your study been approved by an ethics committee?** Not applicable
**Author Disclosures:**
Nadeem Shaida: Nothing to disclose
Manolis Bagkeris: Nothing to disclose
James Blackmur: Nothing to disclose
Oliver Llewellyn: Nothing to disclose
Tristan Barrett: Nothing to disclose
Unite Collaborative: Nothing to disclose
Jon Aning: Nothing to disclose
Baus Oncology: Nothing to disclose
Alexander Laird: Nothing to disclose

**Preliminary evaluation of 5T MRI for bladder imaging: feasibility and image quality**
*Z. Zhao*, X-G. Peng; Nanjing/CN
*(zhaozihui0216@163.com)*

**Purpose:** To evaluate the feasibility and image quality of ultra-high-field 5T MRI for bladder imaging.
**Methods or Background:** This prospective study included 6 healthy volunteers undergoing 5T MRI and 7 bladder cancer patients undergoing 3T MRI between June and July 2025, with one patient scanned at both field strengths, yielding 14 bladder MRI datasets. Imaging sequences included T1-weighted, T2-weighted, and diffusion-weighted imaging (DWI). All participants drank 300–500 mL of water 40 minutes before scanning or voided 1–2 hours beforehand, with intravenous spasmolytic administered 15 minutes prior. Three experienced radiologists independently evaluated T2WI and DWI sequences using a 5-point Likert scale for bladder distension, artifacts, tumor–bladder wall interface clarity (or bladder wall clarity in volunteers), and overall image quality (a weighted composite score of the above metrics, on a 5-point scale). 5T images were compared with 3T images.
**Results or Findings:** Among 5T scans, 6/7 (85.7%) achieved optimal bladder distension, 1/7 (14.3%) showed notable artifacts, and all 7 (100%) exhibited clear bladder wall or tumor–bladder wall interface details. Notably, two volunteers prepared via waiting 1–2 hours after voiding showed almost no artifacts. Compared with 3T MRI, 5T MRI demonstrated marginally superior overall image quality (4.4±0.6 vs. 3.7±0.7, p=0.065), with better bladder distension, clearer bladder wall details, and fewer artifacts, although differences did not reach statistical significance (p > 0.05). Visual assessment in one patient scanned on both field strengths confirmed better image quality at 5T.
**Conclusion:** Ultra-high-field 5T MRI shows excellent feasibility for bladder imaging, with trends toward improved anatomic delineation and artifact reduction over 3T.
**Limitations:** The study is limited by a small, unevenly distributed sample, and most results were not statistically significant. Larger prospective studies are needed to confirm the clinical value of 5T bladder MRI.
**Funding for this study:** This study has received funding by National Natural Science Foundation of China (82272064), Jiangsu Provincial Science and Technique Program (BK20221461), Zhongda Hospital Affiliated to Southeast University, Jiangsu Province High-Level Hospital Pairing Assistance Construction (zdlyg08), Postgraduate Research & Practice Innovation Program of Jiangsu Province (KYCX23_0323 and KYCX22_0297).
**Has your study been approved by an ethics committee?** Yes
**Ethics committee - additional information:** Approval No. 2024ZDSYLL061-P01
**Author Disclosures:**
Zihui Zhao: Nothing to disclose
Xin-Gui Peng: Nothing to disclose

**MRI-based Intratumoral Heterogeneity Quantification Accessing Early Neoadjuvant Treatment Response in Bladder Cancer**
*L. Kong*, Y. Qin, H. Wang; Guangzhou/CN
*(konglm@mail2.sysu.edu.cn)*

**Purpose:** To develop a quantitative measure of Intratumoral Heterogeneity (ITH) on pretreatment MRI scans and test its performance for predicting pathologic complete response (pCR) after neoadjuvant treatment (NAT) in bladder cancer (BCa)
**Methods or Background:** Pretreatment MRI scans, including contrast-enhanced (CE) and non-contrast-enhanced (NE) T1-weighted images (WI), were retrospectively acquired in patients with BCa who received NAT followed by surgery from April 2020 and August 2024. Tumor regions on MRI scans were clustered and integrated with global pixel distribution patterns to calculate ITH scores. Multivariable logistic regression analysis was used to identify variables associated with pCR, and significant variables, including clinicopathologic variables and ITH index, were combined into a predictive model for which performance was assessed using the area under the receiver operating characteristic curve (AUC).
**Results or Findings:** Among the 74 patients, the median age was 64 years (IQR: 57, 71); with 61 (82%) being male. Forty patients (54%) achieved pCR. The combined model showed good performance for predicting pCR to NAT in the training data set (AUC, 0.976 ) and test data sets (AUC, 0.875).
**Conclusion:** ITH analysis on pre-therapeutic CE- and NE-MRI demonstrated promising predictive ability for NAT responsiveness prior to treatment initiation.
**Limitations:** All patients in this study were recruited from single center, which need a further verification of the generalizability of the findings. Second, in this study, the use of manual tumor delineation was time consuming and also could affect the stability and repeatability of radiomic features. In future, it is imperative to employ an automated tumor segmentation method.
**Funding for this study:** This study received funding from the following sources: National Natural Science Foundation of China (Nos. 82372075, 82371911), Natural Science Outstanding Youth Fund Project of Guangdong province, China (No. 2024B1515020061)
**Has your study been approved by an ethics committee?** Yes
**Ethics committee - additional information:** the Institutional Review Board of the First Affiliated Hospital
**Author Disclosures:**
Lingmin Kong: Nothing to disclose
Yanjin Qin: Nothing to disclose
Huanjun Wang: Nothing to disclose

**The role of multiparametric MRI and VIRADS in the preoperative differentiation of Ta and T1 bladder cancer**
*M. N. Tasdemir*, U. Eryürük, S. Aslan; Giresun/TR
*(mervetsdmr@gmail.com)*

**Purpose:** To assess multiparametric MRI's utility, using VI-RADS and quantitative metrics – ADC, Tumor Contact Lenght (TCL) – , for distinguishing Ta from T1 stage non-muscle-invasive bladder cancer (NMIBC).
**Methods or Background:** This retrospective study enrolled patients with histopathologically confirmed NMIBC (2021–2025) who underwent mpMRI. Index tumors were evaluated using mp-VIRADS, and ADC values and TCL were measured. ROC analysis determined optimal cut-offs for ADC and TCL. The Mann-Whitney U and chi-square tests compared continuous variables and VI-RADS associations, respectively.
**Results or Findings:** Among 157 patients (83 Ta, 74 T1), T1 tumors exhibited significantly lower mean tumor ADC (1136 vs. 1326 x$10^{-3}$ mm²/s) and mean normalized ADC (0.47 vs. 0.55), and higher mean TCL (30.4 vs. 14.0 mm, all p<0.001). ROC analysis for Ta-T1 discrimination yielded AUCs of 0.72 for tumor ADC (cut-off: 1216, sensitivity 79%, specificity 74%), 0.72 for normalized ADC (cut-off: 0.51, sensitivity 79%, specificity 68%), and 0.75 for TCL (cut-off: 17 mm, sensitivity 66%, specificity 79%), identifying TCL as the strongest discriminator. T1 stage tumors were significantly more frequent with higher mpVIRADS scores, with a prevalence of 22.2% (6/27) for score 1, 46.7% (42/90) for score 2, 52.4% (11/21) for score 3, 66.7% (6/9) for score 4, and 90.0% (9/10) for score 5 (p < 0.001).
**Conclusion:** MpMRI effectively differentiates Ta from T1 NMIBC. Both VI-RADS system and quantitative assessments are valuable, with TCL being the most potent discriminator. These findings underscore mpMRI's pivotal role in preoperative planning.
**Limitations:** This was a retrospective study.
**Funding for this study:** None.
**Has your study been approved by an ethics committee?** Yes
**Ethics committee - additional information:** The study was approved by the local ethics commitee.
**Author Disclosures:**
Uluhan Eryürük: Nothing to disclose
Merve Nur Tasdemir: Nothing to disclose
Serdar Aslan: Nothing to disclose

**13:00 - 14:30** **Research Stage 5**

### Research Presentation Session: Neuro

### RPS 411

### Neuroanatomy and epilepsy imaging

**Moderator**
Nuria Bargallo I Alabart; Barcelona/ES
*(bargallo@clinic.cat)*

### Sub-voxel quantitative susceptibility mapping for assessing whole-brain magnetic susceptibility in temporal lobe epilepsy: A preliminary study

*Z. Huang*, Y. Li, N. Zhu, L. Mazu, J. Chu; Guangzhou/CN
*(huangzh236@mail2.sysu.edu.cn)*

**Purpose:** This study aims to use the recently proposed sub-voxel QSM, namely APART-QSM, to simultaneously quantify the paramagnetic and diamagnetic susceptibility sources in temporal lobe epilepsy (TLE).
**Methods or Background:** In this retrospective analysis, a total of 52 healthy subjects (HCs) and 68 patients with TLE were recruited. According to the ILAE 2020 definition, the patients were divided into 30 non-refractory, 31 refractory TLE and 7 patients were lost to follow-up. APART-QSM could distinguish the paramagnetic susceptibility map (Xpara) and diamagnetic susceptibility map (Xdia).
**Results or Findings:** In the voxel-wise analysis, compared with HCs, TLE patients exhibited deceased Xpara and increased Xdia in a large scale of brain regions. Remarkably, refractory TLE demonstrated significantly decreased Xpara and increased Xdia in both thalamus relative to non-refractory TLE. Decreased Xdia was found in the left supramarginal gyrus in refractory TLE patients compared to non-refractory cohorts (all FDR-q < 0.05 & cluster extent > 100 voxels). No significant differences were observed in disease duration between patient groups. The log-transformed seizure frequency is higher in refractory TLE than that in non-refractory patients (ln [seizure frequency] 0.31 ± 0.34 vs 0.80 ± 0.67, P = 0.001). In the patient cohorts, the disease duration was negatively correlated with Xpara in the left thalamus (r = -0.284, P = 0.026), right thalamus (r = -0.367, P = 0.004) and positive with Xdia in the right thalamus (r = 0.395, P = 0.002). The log-transformed seizure frequency is negatively correlated with Xdia in the left supramarginal gyrus (r = -0.258, P = 0.045).
**Conclusion:** The sub-voxel QSM offers significant insights into the magnetic susceptibility perturbation of the whole brain in TLE patients.
**Limitations:** This work is lacking of pathological evidence.
**Funding for this study:** None.
**Has your study been approved by an ethics committee?** Yes
**Ethics committee - additional information:** The institutional ethical committee of the First Affiliated Hospital of Sun Yat-Sen University.
**Author Disclosures:**
Zihuan Huang: Nothing to disclose
Yizhen Li: Nothing to disclose
Jianping Chu: Nothing to disclose
Nengjin Zhu: Nothing to disclose
Liwei Mazu: Nothing to disclose

### Cortical Structural Parameters and Cognitive Performance in Juvenile Myoclonic Epilepsy: A Surface-Based Morphometry Study

*O. A. Kargin*, R. Ozun Kargin, A. Ceyhan Dirican, H. D. Atakli; Istanbul/TR
*(aykankargin@gmail.com)*

**Purpose:** Juvenile myoclonic epilepsy (JME) is the most prevalent idiopathic generalized epilepsy syndrome, with a typical onset at adolescence. Beyond seizures, JME has increasingly been recognized as a condition involving widespread cognitive dysfunction, particularly affecting memory, executive functions, and attention. This study aimed to investigate cortical structural alterations in JME using surface-based morphometry and to explore their relationship with cognitive performance, with the goal of identifying potential imaging biomarkers.
**Methods or Background:** Fifty-five patients with JME and 65 healthy controls matched for age, sex, and educational status underwent high-resolution 3D T1-weighted brain MRI. Cortical thickness, sulcal depth, gyrification index, and fractal dimension were extracted using the CAT12/SPM pipeline. Cognitive functions were assessed with a comprehensive neuropsychological battery covering multiple domains including memory, verbal fluency, attention, executive functions, and psychomotor speed. Vertex-wise analyses were conducted using threshold-free cluster enhancement (TFCE). All models were adjusted for age, educational status, depression/anxiety scores, and Euler number.
**Results or Findings:** Compared to controls, JME patients exhibited significant cortical thinning and increased gyrification, most prominently in perirolandic and medial prefrontal cortices, extending to frontal, parietal, and occipital regions (pFWE<0.001). These effects remained robust after controlling for global mean cortical thickness. Correlation analyses demonstrated moderate positive associations between cortical thickness in frontal regions and cognitive performance, particularly in memory, verbal fluency, and executive domains (pFWE<0.05, r≤0.57).
**Conclusion:** Our findings highlight region-specific cortical alterations in JME that extend beyond global effects, linking frontal structural abnormalities to clinically relevant cognitive deficits. These results support the role of surface-based morphometric parameters as promising neuroimaging biomarkers of cognitive impairment in JME.
**Limitations:** This is a cross-sectional study without longitudinal follow-up.
**Funding for this study:** No funding was received for this study.
**Has your study been approved by an ethics committee?** Yes
**Ethics committee - additional information:** The study was approved by the ethics committee of University of Health Sciences, Bakirkoy Prof. Dr. Mazhar Osman Training and Research Hospital for Psychiatric, Neurologic, and Neurosurgical Diseases.
**Author Disclosures:**
Hayrunnisa Dilek Atakli: Nothing to disclose
Rabia Ozun Kargin: Nothing to disclose
Osman Aykan Kargin: Nothing to disclose
Ayten Ceyhan Dirican: Nothing to disclose

### From Static to Dynamic: Multiscale Connectivity Features of Resting-State Brain Network Reconfiguration in SeLECTS

*X. Chen*, L. Song, A. J. Zhang, X. Yang, L. Jiang; Zunyi City/CN
*(cxu2025@163.com)*

**Purpose:** To reveal network-level functional reconfiguration in children with self-limited epilepsy with centrotemporal spikes (SeLECTS) by integrating static functional connectivity (sFNC), dynamic functional connectivity (dFNC), and hidden Markov model (HMM) analysis.
**Methods or Background:** Sixty-one children with SeLECTS and sixty-nine healthy controls (HCs) underwent resting-state fMRI. Independent component analysis identified 34 components, 15 of which were matched to six canonical networks. sFNC was computed from full time series. dFNC was assessed using sliding-window and k-means clustering. And the HMM was applied to resolve dynamic brain activation states.
**Results or Findings:** The sFNC analysis showed significantly reduced connectivity within the DAN (IC_31–IC_32 and IC_32–IC_33) in SeLECTS compared to HCs. The dFNC analysis identified four recurrent states. In state 1 (IC_32–IC_33) and state 3 (IC_31–IC_32), both reflecting intra-DAN connectivity, SeLECTS showed significantly reduced connectivity compared to HCs. No group differences were found in states 2 and 4. State 2 was characterized by more balanced connectivity and showed fewer fractional windows and shorter mean dwell time in SeLECTS. In contrast, States 1 and 3 represented two extreme connectivity patterns, respectively reflecting low integration and over-integration. The HMM analysis also identified four dynamic states, including distinct extremes of brain network activation—one with markedly increased activation and another with globally reduced activation. Additionally, SeLECTS exhibited prolonged dwell time in a state characterized by suppressed DAN activity.
**Conclusion:** This study indicates that persistent disruption of DAN connectivity may underlie resting-state network abnormalities in SeLECTS. Imbalanced connectivity states revealed by dFNC and HMM from distinct dynamic perspectives further support network reorganization in SeLECTS.. This static-to-dynamic integrated analysis offers new perspectives on its neuropathological mechanisms.
**Limitations:** Limitations include cross-sectional design, lack of EEG-fMRI, and uncontrolled medication effects potentially confounding connectivity findings.
**Funding for this study:** This work was supported by the National Natural Science Foundation of China (Grants No. 82160328), Natural Science Foundation of Guizhou Province (Project No. Qiankehejichu-ZK [2021] yiban 479, Qiankehejichu-ZK [2022] yiban 582), Scientific and Technological Innovation Talent Team for Functional Imaging and Artificial Intelligence Applications in Guizhou Province (Project: QKHRC- CXD(2025)047).
**Has your study been approved by an ethics committee?** Not applicable
**Author Disclosures:**
Xiyao Yang: Nothing to disclose
An Jie Zhang: Nothing to disclose
Linfeng Song: Nothing to disclose
Lin Jiang: Nothing to disclose
Xu Chen: Nothing to disclose

**Retrospective Evaluation of Hippocampal Morphology in Temporal Lobe Epilepsy: Toward Interpretable and Automated Detection of Sclerosis**

*T. M. C. Nguyen*; Hanoi/VN
*(thuminhchou@gmail.com)*

**Purpose:** Hippocampal sclerosis (HS) is the most frequent pathological finding in temporal lobe epilepsy (TLE) but may be subtle or missed on conventional MRI, limiting pre-surgical decision-making. HipUnfold, an automated surface-based morphometry tool, has shown promise for HS detection and lateralisation. However, prior work focused mainly on volumetry and asymmetry indices. We extended this approach using unsupervised clustering of shape features and compare imaging-derived clusters with histopathological severity.
**Methods or Background:** We retrospectively included 30 unilateral HS patients who underwent surgical resection and 30 age- and sex-matched controls. Preoperative T1-weighted MRI was processed using HipUnfold to reconstruct hippocampal surfaces and extract structural features (volume, thickness, gyrification, curvature). Morphological features were compared across sclerotic, contralateral, and control hippocampi. Principal component analysis (PCA) was applied to shape-normalized features, followed by unsupervised k-mean clustering.
**Results or Findings:** HS patients showed significant reductions in hippocampal volume (−24.6%, $p < 0.001$) with decreased gyrification (−25.6%, $p < 0,0001$) and increased mean curvature (+9.55%, $p < 0.05$). PCA explained 84.4% of variance and identified three clusters: Cluster 1 (n = 17) with severe atrophy and curvature change, and Cluster 2 (n = 29) with milder alterations and Cluster 3 (n=10) with preserved morphology, likely corresponding to non-sclerotic hippocampi. Cluster membership corresponded closely with histopathological grading of sclerosis, providing greater diagnostic granularity than volumetry or asymmetry alone.
**Conclusion:** Though HipUnfold is validated for HS detection, our study demonstrates that unsupervised clustering of surface features captures HS phenotypes aligned with histopathological sclerotic severity, offering added interpretability for presurgical planning in TLE
**Limitations:** This pilot study had a modest sample size, which limits statistical power and cluster stability. Most surgical cases showed clear HS on imaging, introducing selection bias.. Larger prospective studies with broader anatomical coverage are needed for validation.
**Funding for this study:** This study received no external funding.
**Has your study been approved by an ethics committee?** Yes
**Ethics committee - additional information:** The study were approved by the ethics committee of the Hanoi Medical University (no 4339/QĐ-ĐHYHN)
**Author Disclosures:**
Thu Minh Chau Nguyen: Nothing to disclose

**Correlation of morphological features of the arcuate and uncinate fasciculi to language lateralization: a combined fMRI and DTI study**

*J. Yoo*, R. Stepponat, F. Fischmeister, G. Kasprian; Vienna/AT
*(n11909299@students.meduniwien.ac.at)*

**Purpose:** Understanding the organization of language functions is one of the most rigorously researched neuroscientific topics, as it carries significant importance in clinical settings, such as preoperative planning in neurosurgery, and disease comprehension in neurological disorders. In our combined DTI tractography and fMRI study, we aim to determine whether experts who work closely with the above-mentioned imaging methods can identify the correct language lateralization solely with DTI tractography in TLE patients as well as healthy controls.
**Methods or Background:** We performed a questionnaire study with experts who routinely work with DTI tractography as well as fMRI in patients. The experts were shown randomized, partially mirrored images of DTI tractographies of the arcuate and uncinate fasciculi and then attempted to determine the language lateralization based on the images. The results were further validated by calculating the inter-rater variability. Furthermore, fMRI- and DTI-laterality indices (LI) were correlated in order to validate our data.
**Results or Findings:** The exploratory preliminary results show a correlation of the arcuate fascicle structure to the fMRI LI as proven by previous studies. Furthermore, certain visual qualities of the arcuate fascicle such as a dense, highly organized fiber structure spanning between the temporal and frontal lobe showed to be a visual marker for language lateralization to the same side.
**Conclusion:** The preliminary results suggest that an evaluation by experts of language lateralization solely by DTI tractography is possible. This finding can help clinicians determine language lateralization or increase their confidence in identifying the dominant hemisphere when fMRI is unavailable or the patient is not able to perform fMRI.
**Limitations:** Due to the distribution of language laterality by fMRI, atypically lateralized patients are under-represented and further investigation with a larger pool of atypically lateralized patients may be needed.
**Funding for this study:** Medical University of Vienna
**Has your study been approved by an ethics committee?** Yes
**Ethics committee - additional information:** The study has been performed under amendment of ethics application 1141/2023
**Author Disclosures:**
Florian Fischmeister: Nothing to disclose
Radheshyam Stepponat: Nothing to disclose
Jiyeon Yoo: Nothing to disclose
Gregor Kasprian: Nothing to disclose

**Stability of spontaneous brain activity and functional connectivity in Subcortical gray matter using test–retest ultrahigh field 7-Tesla resting-state functional magnetic resonance imaging**

*H. M. H. Sbaihat*, B. Bellenberg, A. K. Roenneke, B. Krieger, D. Müller, C. Lukas; Bochum/DE
*(hasansbaihat@gmail.com)*

**Purpose:** Reliable biomarkers are needed in psychiatry and neurology to improve diagnosis and treatment. While the reliability of resting-state (RS) fMRI has been studied at standard field strengths, it remains underexplored at ultrahigh field (UHF) strengths such as 7 Tesla. This study evaluated the stability of spontaneous brain activity (RSA) and functional connectivity in subcortical gray matter (SCGM), regions central to cognition, emotion, and memory. We assessed RSA with the amplitude of low-frequency fluctuations (ALFF), local connectivity with regional homogeneity (ReHo), and global connectivity with degree centrality (DC).
**Methods or Background:** Sixteen healthy participants (mean age = 25.3 ± 2.0 years) were selected from a publicly available dataset (Gorgolewski et al., 2015). fMRI and T1-weighted images were acquired on a Siemens MAGNETOM 7T scanner in two sessions one week apart. Preprocessing was performed, and then ALFF, ReHo, and DC were computed in native space for each session. Test–retest reliability was assessed using Lin's concordance correlation coefficient across SCGM regions, including the amygdala, basal ganglia, caudate, hippocampus, insula, pallidum, putamen, and thalamus.
**Results or Findings:** Our results indicate moderate-to-strong stability across all selected subcortical gray matter regions, except for the RSA in pallidum. The amygdala, insular cortex, and thalamus demonstrated strong reliability across ALFF, DC, and ReHo. The basal ganglia and caudate were moderately stable for ALFF and ReHo but strong for DC. The hippocampus and putamen showed moderate stability across all measures, while the pallidum exhibited weak ALFF stability but moderate DC and ReHo.
**Conclusion:** RS measures of SCGM exhibit generally moderate-to-strong stability. These findings support the potential of ultrahigh-field 7T fMRI for reliably assessing spontaneous brain activity and connectivity in subcortical regions, providing a foundation for future biomarker development in psychiatry and neurological disorders.
**Limitations:** Not applicable.
**Funding for this study:** No funding was provided for this study.
**Has your study been approved by an ethics committee?** Yes
**Ethics committee - additional information:** publicly available dataset (Gorgolewski and colleagues, 2015)
**Author Disclosures:**
Britta Krieger: Nothing to disclose
Barbara Bellenberg: Nothing to disclose
Hasan M. H. Sbaihat: Nothing to disclose
Anna Katharina Roenneke: Nothing to disclose
Carsten Lukas: Nothing to disclose
Dajana Müller: Nothing to disclose

**Fetal MRI of suspected cortical malformations: predicting epileptogenic outcomes**

*S. Chambers*, S. Glatter, L. Krepler, T. Dorittke, J. Binder, M. Weber, D. Prayer, G. Kasprian; Vienna/AT
*(stefanie.chambers@meduniwien.ac.at)*

**Purpose:** Predictions of epileptogenicity are often biased by inferring risk retrospectively from symptom onset. Fetal MRI allows identification of malformations of cortical development (MCD) before clinical manifestation. We present a preliminary analysis of the epileptogenicity of suspected MCD in prenatal MRI, validated through postnatal follow-up. Our aim is to stratify epilepsy risk by MCD subtype and location, providing less biased estimates to support the transition from reactive to preemptive, disease-modifying interventions.
**Methods or Background:** We retrospectively reviewed fetal MRIs performed at the Medical University of Vienna (2005–2024) with suspected MCD. Scans included T2-weighted sequences in three planes at 1.5/3T, with ≥1 year postnatal follow-up. Two neuroradiologists independently assessed primary MCD features (e.g., schizencephaly, polymicrogyria, dysplasia) and lesion location. Clinical outcomes included epilepsy and/or neurodevelopmental delay. Group comparisons used chi-squared/Fisher's exact tests; stepwise logistic regression assessed multivariable effects.

**Results or Findings:** Forty-four cases (18 female) were included with mean follow-up of 6.3 years. Sixteen patients (36.4%) developed epilepsy and 19 (43.2%) neurodevelopmental delay. Microlissencephaly and dysgyria were strongly associated with epilepsy (both p=0.013), with frontal lobe involvement being the strongest predictor (OR=13, p<0.001), remaining significant in multivariable regression. Combining location to pathology, schizencephaly also reached significance (p=0.013), whereas periventricular nodular heterotopia and hippocampal malrotation showed nonsignificant associations.
**Conclusion:** Our exploratory, preliminary analysis highlights the epileptogenicity of microlissencephaly, dysgyria, and frontal lobe involvement, while schizencephaly and polymicrogyria may be less predictive in a univariate analysis than assumed. Our findings support the questionable epileptogenicity of periventricular nodular heterotopia and suggest possible two-hit mechanisms.
**Limitations:** The limitations are the small sample size and consequent difficulty of disentangling co-occurring features, a multi-center study is currently ongoing. Furthermore, our assessment is limited to early-onset epilepsy due to the mean follow-up period of 6 years.
**Funding for this study:** No funding was received for this study.
**Has your study been approved by an ethics committee?** Yes
**Ethics committee - additional information:** The IRB number is EK 2167/2023
**Author Disclosures:**
Tim Dorittke: Nothing to disclose
Sarah Glatter: Nothing to disclose
Julia Binder: Nothing to disclose
Stefanie Chambers: Nothing to disclose
Daniela Prayer: Nothing to disclose
Lara Krepler: Nothing to disclose
Gregor Kasprian: Nothing to disclose
Michael Weber: Nothing to disclose

### What you see is not always what you get - MRI-Based Ganglionic Eminence Volumetry Challenges Subjective Assessment in CNS Anomalies

*M. Stuempflen*, P. Kienast, V. Schmidbauer, M. Weber, J. Tischer, T. Dorittke, J. Binder, D. Prayer, G. Kasprian; Vienna/AT

**Purpose:** Failure of fetal interneuron migration arising from the ganglionic eminence (GE) may lead to a wide spectrum of neuropsychiatric and neurodevelopmental disorders. Early detection of anomalies may improve the MRI phenotyping of neurodevelopmental diseases. This atlas-based fetal MRI study aimed to quantitatively assess development of and alterations in GE volume in fetuses with previously diagnosed abnormal GE volumes based on subjective assessment only.
**Methods or Background:** This retrospective study investigated 17 fetuses (20 fetal MRIs, mean gestational age 26.3 weeks, SD 3.3) with subjectively enlarged ganglionic eminence with concurrent structural central nervous system anomalies based on assessment of experienced fetal neuroimaging experts. Three-dimensional volumetry was performed based on super-resolution fetal MRI and compared to age-matched neurotypical controls (94 fetuses, 100 MRIs, mean gestational age 27.2 weeks, SD 3.6).
**Results or Findings:** Only 25% were found to include patients with GE hyperplasia, while 60% were found to show normal and 15% smaller GE volumes than healthy references. Most patients (80%) were found to have increased total brain volume - mostly due to high ventriculomegaly (75%). Brain parenchyma volume was enlarged in only 20%. No correlation was found between GE volumes and volumes of ten other substructures of the fetal head.
**Conclusion:** The study highlights the unreliability of expert visual assessment of GE size, despite excellent examination conditions and emphasizes the necessity of three-dimensional volumetry. Specifically in patients with structural CNS anomalies quantitative fetal neuroimaging will serve as an emerging tool for deep phenotyping, which identifies abnormalities within the GE prior to the structural emergence of cortical malformations.
**Limitations:** The findings of this study should be confirmed in larger patients cohorts and histological studies, which were unavailable due to the in vivo nature of this study.
**Funding for this study:** None.
**Has your study been approved by an ethics committee?** Yes
**Ethics committee - additional information:** Approval of ethics committee obtained (number EK 1585/2021).
**Author Disclosures:**
Victor Schmidbauer: Nothing to disclose
Tim Dorittke: Nothing to disclose
Marlene Stuempflen: Nothing to disclose
Julia Binder: Nothing to disclose
Johannes Tischer: Nothing to disclose
Daniela Prayer: Nothing to disclose
Patric Kienast: Nothing to disclose
Gregor Kasprian: Speaker: Alexion, BRACCO, Lilly
Michael Weber: Nothing to disclose

**13:00 - 14:30** **Room G1**

### Research Presentation Session: Radiographers

### RPS 414

### Educating radiographers for enhanced patient care

**Moderators**
Laura Jurša; Rače/SI
Huadan Xue; Beijing/CN
*(bjdanna95@163.com)*

### Evaluating the use of complementary therapies amongst patients undergoing radiotherapy in Malta

*K. Borg Grima*, A. Xuereb; Msida/MT
*(karen.borg-grima@um.edu.mt)*

**Purpose:** Complementary therapies (CTs) are increasingly recognised for their potential to improve quality of life in oncology care. However, evidence regarding their awareness, utilisation, and acceptance among Maltese cancer patients remains limited. This study aimed to assess patient awareness, explore demographic influences, and evaluate willingness to use CTs within the state oncology hospital.
**Methods or Background:** A cross-sectional, non-experimental, prospective research was conducted with patients undergoing radical radiotherapy. A self-designed questionnaire, incorporating both quantitative and limited qualitative components, was distributed to 140 eligible participants. Ninety-one completed responses were analysed (65% response rate). Data was processed using IBM SPSS® version 27. Chi-square, Kruskal–Wallis, and Friedman tests were applied to evaluate associations between demographics and CT use, with statistical significance set at p<0.05.
**Results or Findings:** Over half of the participants (57.8% females; 42.2% males) reported prior use of CTs. Younger patients (76.9% aged 18–47 years) and those with tertiary education (82.6%) were most likely to use CTs. Awareness of at least one CT was reported by 88% of respondents, with massage (68.1%), yoga (58.2%), dietary supplements (46.2%), and meditation (46.2%) being the most commonly cited. Willingness to use CTs if offered freely within the hospital was expressed by 95.6% of patients. The Friedman test indicated the strongest agreement that a wider range of CTs should be available in Malta (mean 4.27), and that more patient education is required before using such additional therapies.
**Conclusion:** The findings highlight strong awareness, favourable attitudes, and clear demand for CT integration within oncology services in Malta. Younger and better-educated patients were most likely to utilise CTs. Implementation may improve holistic cancer care and patient wellbeing, while promoting patient education on additional cancer therapies.
**Limitations:** Study limitations included a reduced participant sample size, limiting generalisability.
**Funding for this study:** No funding was used for this study
**Has your study been approved by an ethics committee?** Yes
**Ethics committee - additional information:** The study was approved by the University Research Ethics Committee (reference number: 9094_15062021_).
**Author Disclosures:**
Karen Borg Grima: Nothing to disclose
Amy Xuereb: Nothing to disclose

### Knowledge and Perceptions of MRI Safety: A Cross-Sectional Survey Among Healthcare Professionals and the General Population

M. Signorini[1], *M. Garziera*[2], D. Cerchiaro[2], S. Fontani[1], B. Bragagnolo[2], G. Iannucci[2], V. Iurilli[2]; [1]Rovigo/IT, [2]Vicenza/IT
*(matteogarziera@gmail.com)*

**Purpose:** To evaluate the level of knowledge and perceptions regarding MRI safety among healthcare professionals and the general population, identifying well-understood topics and critical misconceptions, in order to inform targeted educational strategies.
**Methods or Background:** A cross-sectional online survey was developed (Google Forms) and distributed between June–August 2025 via hospital networks and social media. The questionnaire comprised demographic items, a self-assessment of knowledge (5-point Likert scale), 13 core safety questions and 6 advanced safety questions. Respondents were categorized as: radiologists, radiographers (TSRM), non-radiologist physicians, other

Wednesday healthcare workers, students, and general population. Data were analyzed with $\chi^2$ tests, Kruskal–Wallis, Spearman correlation, and logistic regression.
**Results or Findings:** A total of 524 valid responses were collected: 30% general population and 70% healthcare professionals. Overall, healthcare workers achieved significantly higher accuracy than the general population across all 13 core questions ($p<0.001$, large effect sizes). Radiologists obtained the highest mean accuracy (72.4%), followed by radiographers (66.8%) and non-radiologist physicians (61.2%), while other healthcare workers (54.1%) and students (58.7%) scored lower. Critical knowledge gaps emerged across all groups regarding removal of contact lenses (<30% correct) and recognition of common transient effects (≤13%). Logistic regression confirmed radiologists were significantly more likely to answer correctly on advanced items such as ventriculo-peritoneal shunts (OR 2.85, $p<0.001$) and long-term effects (OR 2.12, $p=0.005$). Self-perceived knowledge correlated with actual accuracy among professionals ($\rho=0.14$, $p=0.009$), but not in the general population.
**Conclusion:** Substantial differences exist between professionals and laypersons, and across professional subgroups, in MRI safety knowledge. While radiologists demonstrate the highest competence, recurrent misconceptions persist in all categories, underscoring the need for targeted educational initiatives.
**Limitations:** Convenience sampling via online distribution may introduce selection bias; self-reported professional categories were not externally verified.
**Funding for this study:** None
**Has your study been approved by an ethics committee?** Not applicable
**Author Disclosures:**
Matteo Garziera: Nothing to disclose
Giuseppe Iannucci: Author: I have nothing to disclose
Manuel Signorini: Nothing to disclose
Vincenzo Iurilli: Nothing to disclose
Silvia Fontani: Nothing to disclose
Davide Cerchiaro: Author: I have nothing to disclose
Barbara Bragagnolo: Nothing to disclose

**Structured SimZones in CT education: enhancing clinical reasoning with immersive simulation**
E. Stefani[1], M. Centenaro[1], *R. P. P. Almeida*[2]; [1]Padova/IT, [2]Esch-sur-Alzette/LU
*(rui.pereiradealmeida@uni.lu)*

**Purpose:** Traditional CT education often emphasizes technical competence, with limited opportunities to practice decision-making and clinical reasoning. The SimZones framework (Roussin & Weinstock, 2017) provides a structured pathway of increasing complexity for simulation-based learning. Its integration with immersive high-fidelity technologies may enhance diagnostic judgment and bridge the gap between classroom and clinical environments. To evaluate the effectiveness of a structured CT simulation program based on the SimZones model in strengthening clinical reasoning and operational confidence among undergraduate radiography students.
**Methods or Background:** Four immersive simulation sessions focusing on CT modules were delivered to 24 second- and third-year students. The program was designed within SimZones 2 and 3 and included pre-briefing, guided scenarios, and reflective debriefing. Effectiveness was assessed using the validated Italian version of the Simulation Effectiveness Tool–Modified (SET-M). Data analysis included descriptive statistics of 19 items and correlation across cognitive, technical, and decision-making domains.
**Results or Findings:** Overall SET-M scores were high (mean 2.4/3). Decision-making achieved a mean of 2.41, while debriefing received the highest rating (2.55). A strong correlation ($r = 0.75$) was observed between pre-briefing quality and the ability to manage complex scenarios. Moderate correlations ($r = 0.68$) were found between technical knowledge, patient safety, and decision-making autonomy. Qualitative feedback highlighted increased situational clinical reasoning, confidence, and awareness of professional roles.
**Conclusion:** Structured CT simulation based on the SimZones framework, combined with immersive technologies, effectively enhances clinical reasoning and confidence in radiography students. This approach strengthens the educational impact of simulation, bridging academic learning with clinical practice, and offers a sustainable, replicable model for next-generation radiography education.
**Limitations:** None
**Funding for this study:** None
**Has your study been approved by an ethics committee?** Not applicable
**Author Disclosures:**
Michela Centenaro: Nothing to disclose
Rui Pedro Pereira Almeida: Nothing to disclose
Eleonora Stefani: Nothing to disclose

**Personalised CT/MRI Placements: Tailored Clinical Training**
*A. Devetti*, S. Da Dalt; Udine/IT
*(angiedevetti@hotmail.it)*

**Purpose:** This project details the implementation and evaluation of a personalised, advanced clinical placement model in CT and MRI for undergraduate radiographers. The overarching objective is to deepen and specialise students' practical proficiency in both modalities whilst fostering reflective self-assessment and self-efficacy.
**Methods or Background:** The advanced placement, structured as a mixed-modality block following two standard, dedicated 6-week continuous blocks in CT and MRI, is collaboratively designed with each student upon demonstrating high foundational competence and autonomy. This phase shifts emphasis from core principles to refining advanced skills in areas of high clinical relevance or student interest (3T prostate MRI, DECT urography, cardiac imaging). Learning objectives are deliberately flexible, adapting to the host clinical site's available caseload. Students who achieved lower prior performance primarily focus on consolidation of core competencies.
**Results or Findings:** The preliminary introduction of this personalised model suggests a marked increase in student engagement and the acquisition of highly targeted, specialised technical skills. The individualised learning pathways allowed high-performing students to effectively manage and contribute to advanced procedures, exceeding standard undergraduate placement outcomes. Conversely, the tailored objectives successfully remediated skill deficits in lower-performing cohorts.
**Conclusion:** The personalised, mixed-modality placement represents a sophisticated pedagogical evolution in radiographer training. By linking specific learning objectives to prior performance and individual inclination, this approach ensures maximal clinical exposure relevance and promotes the development of highly capable and motivated practitioners.
**Limitations:** The model's efficacy, tested on a small cohort of 16 students, was inherently restricted by clinical site casemix and operational flow, potentially limiting exposure to every pre-agreed objective. To counteract inherent self-report bias in the final reflective assessment, the students' individual reports were robustly cross-referenced with the summative evaluation provided by experienced placement mentors.
**Funding for this study:** There was no funding for this study.
**Has your study been approved by an ethics committee?** Not applicable
**Author Disclosures:**
Stefano Da Dalt: Nothing to disclose
Angie Devetti: Nothing to disclose

**Radiographers' Role and Practice in Suspected Cases of Child Physical Abuse in Malta**
*E. Buttigieg*, J. L. Portelli; Msida/MT
*(emabuttigieg@gmail.com)*

**Purpose:** Radiographers are frequently among the first healthcare professionals to encounter patients presenting with signs or injuries that may be indicative of physical abuse. The purpose of this study was to examine the role and current clinical practices of radiographers in Malta in the detection and management of Suspected Child Physical Abuse (SCPA).
**Methods or Background:** From a target population of 44 radiographers working in the A&E department of the public general hospital and/or public health centres in Malta, 36 (81.88%) completed an online questionnaire developed for this study.
**Results or Findings:** Most radiographers (66.7%) had encountered SCPA, with 47.2% reporting occasional cases. All recognised physical and behavioural indicators, however 83.4% reported limited familiarity with radiological signs of SCPA, indicating potential training gaps. While 61.1% identified the detection and reporting of SCPA as part of their professional duties, only 41.7% were aware of a legal obligation to report, and 63.9% lacked knowledge of departmental protocols. Although 77.8% stated they would report suspected cases, confidence varied: 77.8% felt competent in producing high-quality images for legal proceedings, whereas 55.6% reported only moderate competence in providing court testimony. Training gaps were notable, with half indicating they had never received formal training and 66.7% unaware of available training opportunities. Nevertheless, 83.3% valued specialised training in SCPA, with 94.4% supporting mandatory periodic training programmes.
**Conclusion:** Radiographers play a pivotal role in safeguarding children, but variability in knowledge, awareness, and radiographic interpretation, may impede timely intervention. While most acknowledged their responsibilities and demonstrated understanding of SCPA, gaps in knowledge and familiarity with procedural frameworks were evident. Structured education, targeted training, and clear protocols are needed to strengthen radiographers' contribution to multidisciplinary child protection.
**Limitations:** As a single-centre, cross-sectional study, the findings may not be generalisable.

**Funding for this study:** None
**Has your study been approved by an ethics committee?** Yes
**Ethics committee - additional information:** Formal ethical approval was obtained from the University Research Ethics Committee (UREC) and the Faculty Research Ethics Committee (FREC) under reference number FHS-2024-00359.
**Author Disclosures:**
Emma Buttigieg: Nothing to disclose
Jonathan Loui Portelli: Nothing to disclose

**AIMIROE - Artificial Intelligence in Medical Imaging and Radiation Oncology (MIRO) Education: A European Survey**

*H. G. Erenstein*[1], R. G. L. Decoster[2], J. Menzinga[1], N. Stogiannos[3], C. Malamateniou[3]; [1]Groningen, Gr/NL, [2]Brussels/BE, [3]London/UK
*(h.erenstein@pl.hanze.nl)*

**Purpose:** AI is reshaping Medical Imaging Radiation Oncology (MIRO), but limited awareness and education hinder adoption. This EuSoMII-led study, in collaboration with EFRS, identifies EU-based AI programs for MIRO and aims to centralize them on an accessible platform to support ethical use, compliance, and professional growth.
**Methods or Background:** A Qualtrics-based electronic survey was developed and piloted with six MIRO-professionals to collect data on European AI course characteristics (e.g. format and delivery, content, and audience target audience). Ethical approval was granted by KU Leuven (Ref: G-2024082214). The survey was disseminated via snowball sampling through social media, and mailing lists of EuSoMII and EFRS between September 16, 2024, and January 16, 2025.
**Results or Findings:** This study identified 30 MIRO-related AI courses across Europe. Most (55.2%) were offered by universities, with industry and professional bodies contributing 20.7% and 17.2%. Courses targeted radiographers, medical physicists, and radiologists (40–60%), mainly at EQF7 level (42.9%). Most were standalone (76.7%), online (53.3%), and free (43.3%), with nearly half lasting under a week. English was the primary language (76.7%), and 80% were openly accessible.
**Conclusion:** Short AI courses for MIRO professionals across Europe offer accessible entry points but often lack depth and hands-on experience. Expanding formats, multilingual access, and global inclusivity, especially in low-resource settings, are essential for meaningful, practical, and equitable AI integration.
**Limitations:** The study used self-reported data and snowball sampling, which may have introduced selection bias and limited course coverage. Conducting the survey in English may have excluded non-English responses and overlooked multilingual offerings. Course details were based on publicly available information, and rapid changes in AI education may affect the relevance of findings.
**Funding for this study:** No external funding was received for this study.
**Has your study been approved by an ethics committee?** Yes
**Ethics committee - additional information:** Ethical approval was granted by KU Leuven (Ref: G-2024082214)
**Author Disclosures:**
Robin Germain Lucien Decoster: Nothing to disclose
Hendrik Garbrand Erenstein: Nothing to disclose
Jacob Menzinga: Nothing to disclose
Nikolaos Stogiannos: Nothing to disclose
Christina Malamateniou: Nothing to disclose

**Transformative e-learning to enhance radiographers' care of older adults: outcomes from a multi-module educational course**

*A. Er*, L. H. Sng; Singapore/SG
*(amandaertw@gmail.com)*

**Purpose:** With a rapidly ageing population, radiographers are increasingly caring for older adults who require unique physical and cognitive considerations. Yet, radiography curricula often provide limited training in this area, potentially compromising patient-centred care. This study evaluated the effectiveness of a transformative e-learning course in enhancing qualified radiographers' knowledge, communication strategies, and reflective practices when caring for older adult patients.
**Methods or Background:** A total of 189 radiographers from a Singapore healthcare cluster completed a two-hour, four-module self-directed e-learning course. The programme was grounded in transformative learning theory, encouraging participants to challenge assumptions and engage in reflective practice. Knowledge was assessed through pre- and post-course multiple-choice questions, and learning effectiveness was analysed using paired t-tests. Additionally, open-ended reflective responses were subjected to thematic analysis to capture qualitative insights into changes in practice.
**Results or Findings:** Knowledge scores improved significantly, rising from a mean of 76.8% pre-course to 96.0% post-course ($p<0.001$). Thematic analysis of reflective responses revealed four dominant themes: patient communication and rapport building, compassionate care approaches, workplace challenges and adaptations, and professional growth and self-reflection. Together, these findings suggest both cognitive and attitudinal benefits of the intervention.
**Conclusion:** A short, theory-informed e-learning course produced significant improvements in radiographers' knowledge and reflective practice when caring for older adults. While participants highlighted the value of communication and compassion, tensions between best practice and workplace time constraints remain an ongoing barrier to implementation.
**Limitations:** Voluntary participation introduced self-selection bias, limiting generalisability of findings.
**Funding for this study:** Funding was provided by the SingHealth Duke-NUS Radiological Sciences Clinical Programme (Project No.: 10/FY2023/P1/03-A18).
**Has your study been approved by an ethics committee?** Not applicable
**Author Disclosures:**
Amanda Er: Nothing to disclose
Li Hoon Sng: Nothing to disclose

**Person-centred care education in practice: students' and academics' evaluation of a postgraduate radiography module**

R. Van de Venter[1], *E. Skelton*[2], B. Potts[2], C. J. C. Parish[2], N. Shiner[3], E. Hyde[2], J. St John-Matthews[2], B. Ohene-Botwe[2], C. Malamateniou[2]; [1]Port Elizabeth/ZA, [2]London/UK, [3]Keele/UK
*(emily.skelton.2@city.ac.uk)*

**Purpose:** To evaluate a new postgraduate educational module delivered by a UK-based University designed to empower radiographers with the knowledge, skills, and confidence to embed person-centred approaches to care provision within the clinical practices.
**Methods or Background:** A qualitative, online survey consisting of six open-ended questions was developed to capture perspectives from students enrolled on the module, and teaching faculty delivering the module. Participants were also asked about what improvements could be made to future iterations of the module. An inductive, open, and descriptive coding approach was utilised, informed by a social constructivist epistemology and relativist ontology. Responses were received from sixteen participants (n=10 enrolled students, n=6 teaching faculty).
**Results or Findings:** Four themes were developed from the qualitative analysis: 1) Stimulating a culture of person-centred care: strengths of the module; 2) Module aspects that limited relatability and learning; 3) The ideal person-centred care module: suggestions for improvement, and 4) Becoming champions of person-centred care: reflection on the module impact. Collectively, these themes represented how participants felt empowered to apply theoretical knowledge acquired during the module to implement person-centred care approaches within their own radiography practices, and support colleagues within their clinical departments.
**Conclusion:** Focused educational modules may be an effective strategy to help develop a culture of person-centred care in radiography environments. The findings of this evaluation will inform future iterations of the module, and may be used to develop other educational interventions focussed on person-centred care in different clinical contexts.
**Limitations:** Self-selection of participants may incur bias in the findings by over- or under-representing specific perspectives. The small sample size and focus on a unique educational module may limit generalisability of the findings outside of this study.
**Funding for this study:** None.
**Has your study been approved by an ethics committee?** Not applicable
**Author Disclosures:**
Riaan Van De Venter: Nothing to disclose
Emily Skelton: Nothing to disclose
Connor James Claude Parish: Nothing to disclose
Naomi Shiner: Nothing to disclose
Benard Ohene-Botwe: Nothing to disclose
Emma Hyde: Nothing to disclose
Janice St John-Matthews: Nothing to disclose
Ben Potts: Nothing to disclose
Christina Malamateniou: Nothing to disclose

**A focus group study of student experiences of an Interprofessional Education Workshop (IPE) for learning about dementia together**

J. Keane, *N. Moore*, M. F. Mcentee, C. Rainey, L. McLaughlin, T. O'Sullivan, J. O'Neill, P. C. Murphy, A. England; Cork/IE

**Purpose:** Dementia encompasses a group of disorders characterised by progressive cognitive decline that significantly impairs daily functioning, most notably memory and problem-solving abilities. Inter-professional education (IPE) involves learners from multiple disciplines engaging collaboratively to enhance understanding of roles and approaches to care. This qualitative focus group study aimed to evaluate the effectiveness of an IPE workshop in improving communication and role recognition in the management of dementia.

**Methods or Background:** A half-day workshop on dementia care was delivered to healthcare students from eleven disciplines (medicine, nursing, dentistry, physiotherapy, radiography, radiation therapy, audiology, speech and language therapy, pharmacy, occupational therapy, and paramedicine). The workshop employed a multimodal approach, integrating lectures, small-group discussions, and case-based learning. Post-workshop, a number of participants engaged in inter-professional online focus groups, and the data were analysed using Braun and Clarke's thematic analysis framework.
**Results or Findings:** Two focus groups comprising eleven participants from five healthcare disciplines were conducted in November 2024, six weeks following the workshop. Thematic analysis identified four overarching themes: (1) awareness of disciplinary roles in dementia care, (2) the importance of teamwork and interprofessional communication, (3) the influence of patient advocacy and awareness, and (4) communication skills for supporting people with dementia.
**Conclusion:** This workshop was regarded as highly beneficial, offering valuable inter-professional learning opportunities and highlighting the importance of patient advocacy in educational contexts. While only minor adjustments in timing and broader disciplinary inclusion were suggested, participants reported improved understanding of role boundaries, communication challenges, and care for people with dementia
**Limitations:** Not all disciplines were represented in the focus groups.
**Funding for this study:** None
**Has your study been approved by an ethics committee?** Yes
**Ethics committee - additional information:** Social Research Ethics committee (SREC) UCC approval was granted for this study.
**Author Disclosures:**
John O'Neill: Nothing to disclose
John Keane: Nothing to disclose
Mark F. Mcentee: Nothing to disclose
Niamh Moore: Nothing to disclose
Clare Rainey: Nothing to disclose
Laura Mclaughlin: Nothing to disclose
Andrew England: Nothing to disclose
Peter C. Murphy: Nothing to disclose
Trish O'Sullivan: Nothing to disclose

**Global Classrooms: Virtual Exchange Strengthens Students' Ethical and Patient Safety Competence**
*B. T. Andersson*[1], J. Gardling[1], H. Muller[2], I-K. Sebelego[2]; [1]Lund/SE, [2]Bloemfontein/ZA
*(bodil-t.andersson@med.lu.se)*

**Purpose:** 1. Understand the Global Implementation of Virtual Classes in Ethics and Patient Safety. 2. Identify the Advantages of Virtual Learning in Ethics and Patient Safety Education . 3. Explore Effective Strategies for Managing Virtual Classes in Ethics and Patient Safety.
**Methods or Background:** Radiographers play a pivotal role in upholding patient safety and ethical standards in medical imaging. To foster global dialogue on these critical issues, a university in South Africa and a university in south of Sweden, conducted a series of collaborative virtual classes in 2024. Students engaged in scenario-based discussions on "patient safety" and "ethics of care from a global perspective". This study evaluates radiography students' perceptions of these international educational virtual collaborations. Following the virtual sessions, first-year (n = 88) and second-year (n = 80) radiography students completed an online survey. The instrument consisted of nine Likert-scale questions (five-point scale) that assessed technical performance, participation, engagement, and learning outcomes. Two open-ended questions gathered qualitative feedback on experiences and suggestions for improvement.
**Results or Findings:** Over 80% of participants reported actively contributing opinions during the classes. Quantitative results showed that more than 80% of students agreed or strongly agreed with all nine positive survey statements. Qualitatively, the analysis revealed a strong alignment of ethical values between the two international student cohorts. Key suggestions for improvement included allocating more time for peer interaction and using smaller breakout groups to facilitate deeper discussions.
**Conclusion:** Virtual international collaborations are an effective tool for promoting global knowledge exchange in radiography education. It significantly strengthen students' capacity to integrate best practices in patient safety and ethics into their professional development.
**Limitations:** Reduced Interpersonal Interaction and limited opportunities for face-to-face debate, emotional expression, and observing professional behavior in practice.
**Funding for this study:** None
**Has your study been approved by an ethics committee?** Not applicable
**Author Disclosures:**
Bodil T. Andersson: Nothing to disclose
Henra Muller: Nothing to disclose
Ida-Keshia Sebelego: Nothing to disclose
Jenny Gardling: Nothing to disclose

**Radiographers as knowledge translators**
*M. De Summa*, S. Spinosa; Roma/IT

**Purpose:** To investigate radiographers' role as knowledge translators in Italian hospitals by mapping their activities bridging technological innovation and clinical practice, identifying factors that facilitate or impede research integration and evaluating training programmes that improve competence and confidence in adopting emerging technologies.
**Methods or Background:** Radiology evolves rapidly due to technological advances, yet a gap persists between theory and clinical practice. Radiographers often make decisions based on experience rather than evidence, and knowledge translation in radiology remains nascent. A multicentre mixed-methods study will combine a questionnaire survey (~300 participants), semi-structured interviews with radiographers and trainers and ethnographic observation of intra-hospital training. Quantitative data will be analysed descriptively and by regression; qualitative data will undergo thematic analysis.
**Results or Findings:** The study expects to identify three radiographer profiles: expert facilitators engaged in communities of practice; clinical pragmatists with strong practical skills but limited evidence use; and digital learners interested in AI but lacking experience. Organisational support, protected training time and mentoring are anticipated facilitators, whereas resource constraints and absence of formal updating pathways may emerge as barriers. Combined training interventions (webinars, mentorship and simulations) should improve confidence and knowledge translation.
**Conclusion:** Radiographers act as vital knowledge translators, ensuring innovations are adopted safely and effectively. Developing knowledge translation skills within intra-hospital training can reduce the theory–practice gap, foster evidence-based practice and encourage integration of AI. Communities of practice, mentoring and interdisciplinary collaboration reinforce the radiographer's identity as a mediator between technology and patient care and support sustainable service delivery overall.
**Limitations:** The study relies on self-reported data and observations from a limited number of hospitals; findings may not generalise to all settings. As this is a prospective design, actual outcomes may differ from expectations.
**Funding for this study:** None
**Has your study been approved by an ethics committee?** Not applicable
**Author Disclosures:**
Marco De Summa: Nothing to disclose
Sandro Spinosa: Nothing to disclose

**From preparation to perception in rectal MRI: Improving patient experiences with self-administered micro-enemas**
*L. Molenaar*, M. A. van der Lubbe, M. De Haan, K. P. Chupetlovska, R. Beets-Tan, D. M. J. Lambregts; Amsterdam/NL
*(l.molenaar@nki.nl)*

**Purpose:** A micro-enema is known to improve image quality on rectal MRI (particularly DWI) by reducing gas-induced artefacts. However, little is known about the patient burden of this preparatory step. This study aimed to assess and improve patient experience when self-administrating a micro-enema before rectal MRI.
**Methods or Background:** At our institution, patients are routinely instructed to self-administer a micro-enema before rectal MRI. During an initial 4-month period (Period A), patients completed a questionnaire assessing discomfort (none, mild, severe) and provided feedback with suggestions for improvement. Based on results of this questionnaire, corrective actions were taken. The questionnaire was repeated in a subsequent period (Period B) to assess the impact of these improvements. Two board-certified radiologists analyzed the presence of DWI artefacts (none/non-significant vs. clinically significant) to confirm the micro-enema's effect on image quality.
**Results or Findings:** 83 patients completed the questionnaire (27 in period A, 56 in period B). In period A, 33% of patients reported mild discomfort, versus 21% in period B. The majority of patients reported no discomfort (67% in period A, 77% in period B). Main causes of discomfort were suboptimal toilet facilities (15%), fear of incorrect administration (11%), and concerns about leakage during MRI (7%). Many patients, especially in period A, did not understand the micro-enema's purpose. After improving facilities and patient information, complaints about toilets ceased, and anxiety about administration dropped to 5%. Image quality was high, with 96% of cases showing no clinically significant artefacts.

**Conclusion:** This study shows that a self-administered micro-enema is generally well-tolerated by patients and leads to good image quality. Clear instructions, patient support, and adequate facilities enable this step to be successfully integrated into routine protocols.
**Limitations:** Relatively small an unequally sized patient cohorts.
**Funding for this study:** None.
**Has your study been approved by an ethics committee?** Not applicable
**Author Disclosures:**
Lyanne Molenaar: Nothing to disclose
Margriet De Haan: Nothing to disclose
Regina G.H. Beets-Tan: Nothing to disclose
Doenja Marina Johanna Lambregts: Nothing to disclose
Megan Anna Van Der Lubbe: Nothing to disclose
Kalina Plamenova Chupetlovska: Nothing to disclose

**Radiographers' awareness and patients' needs on risk-benefit communication in nuclear medicine: A Cross-Sectional Study**
K. Borg Grima, *N. Agius*; Msida/MT
*(nicoleagius02@gmail.com)*

**Purpose:** Limited research exploring radiographers' communication practices in NM was found, despite European regulations requiring radiation risk disclosure to patients. This study evaluated radiographers' awareness and practices in risk-benefit communication, alongside patient expectations and needs, within nuclear medicine (NM). The focus was on cardiac stress scans and routine 18F-FDG PET/CT examinations performed in Malta, where a single NM centre services the national population.
**Methods or Background:** A quantitative, prospective, cross-sectional design was employed, complemented by qualitative elements. Two anonymous questionnaires were distributed: one to radiographers (n= 9) and one to patients (n=57). The radiographers' survey assessed demographics, training, knowledge of radiation risks, and communication practices, while the patients' survey examined understanding, clarity of information provided, and unmet informational needs. Data were analysed using SPSS v29, chi-squared tests for categorical associations and content analysis for open-ended responses.
**Results or Findings:** Discrepancies were identified between radiographers' awareness and knowledge, and patients' stated needs. While 96.5% of patients found the language used to be clear, only 10.5% fully understood that benefits outweighed risks. All radiographers expressed interest in further training, with 55% highlighting that structured education would improve their confidence levels in communication. Barriers to information giving included limited time, fear of alarming patients, lack of standardised materials and training. Patients frequently requested more information on radiation exposure to themselves, relatives, and the general public.
**Conclusion:** Despite radiographers' intention to communicate risk-benefit information, knowledge gaps and inconsistencies persist within the local scenario resulting in patient misunderstandings. Standardised communication protocols, visual aids, plain-language explanations and continuous professional development are needed to ensure accurate, patient-centred risk-benefit discussions in NM and PET/CT.
**Limitations:** Overall there was a low response rate to the patients' questionnaire, therefore results cannot be generalised to the entire target population.
**Funding for this study:** No funding was received for this study.
**Has your study been approved by an ethics committee?** Yes
**Ethics committee - additional information:** The study was approved by the university of Malta research ethics committee (reference number: FHS – 2024 – 00417).
**Author Disclosures:**
Karen Borg Grima: Nothing to disclose
Nicole Agius: Nothing to disclose

**Impact of flow rate on Patient comfort, extravasation incidence and allergic-like reactions during administration of iodinated contrast media at room temperature: CATCHY III Trial**
*L. Stammen*, C. R. Jeukens, T. Flohr, J. E. Wildberger, L. Brandts, B. Martens; Maastricht/NL
*(lion.stammen@mumc.nl)*

**Purpose:** The randomized CATCHY I trial and the subsequent large follow-up CATCHY II found no benefit of pre-warming iodinated contrast media (CM) over room-temperature administration regarding patient comfort, extravasation, or allergic-like reactions. However, as both used relatively low injection flow rates, applicability to higher flow rates remains uncertain. This study examines patient comfort, extravasation, and allergic like reactions when administering CM at room temperature with high flow rates.
**Methods or Background:** This retrospective study included consecutive outpatients undergoing Coronary CT Angiography (May-October 2025); from August onward, a new CM protocol applied higher flow rates. CM injections (300mgI/ml, room temperature) were performed via an 18-gauge needle (GFR=103ml/min), using weight-adapted protocols. Post-scan, patients rated comfort (5-point Likert scale, excellent-very poor), pain (no/mild, moderate, severe), and any side feelings (yes/no). Technicians reported extravasations or allergic like reactions. Low-flow (2-4 ml/s) and high-flow (7-10 ml/s) groups were compared using the Mann-Whitney U test or the Fisher's exact test.
**Results or Findings:** Preliminary results indicated no significant differences between the low flow rate group (n=15) and the high flow rate group (n=14) regarding comfort (p=.949) and pain score (p=.683). Side feelings showed no significant differences, accept for heat sensation, where 92.9% of the participants in the high flow rate group reported to feel a heat sensation, compared to only 46.7% in the low flow rate group (p=.014).
**Conclusion:** These preliminary findings indicate that using higher flow rates when administering CM at room-temperature does not increase the incidence of extravasation or allergic-like reactions, and has no significant impact on comfort or pain scores, although patients are more likely to experience a heat sensation.
**Limitations:** Single-center study and small sample size due to preliminary results.
**Funding for this study:** This study received no funding
**Has your study been approved by an ethics committee?** Not applicable
**Author Disclosures:**
Thomas Flohr: Nothing to disclose
Lion Stammen: Nothing to disclose
Joachim E. Wildberger: Speaker: speakers bureau from Siemens and Bayer Research/Grant Support: institutional research grants from Adora/Oldelft, Anaconda, Bard, Bayer, Bentley, Boston, Brainlab, GE, Inari Medical, Johnson & Johnson, Merit Medical, Nico-Lab, Philips, Sectra, Siemens and Stryker.
Bibi Martens: Speaker: speakers bureau from Bayer
Cécile Rlpn Jeukens: Nothing to disclose
Lloyd Brandts: Nothing to disclose

**13:00 - 14:30** **Research Stage 2**

### Research Presentation Session: Vascular

### RPS 415
### Technical innovations in vascular imaging

**Moderator**
Patrick Krumm; Tübingen/DE
**Author Disclosures:**
Patrick Krumm: Research Grant/Support: Bayer Healthcare, Siemens Healthineers

**In Vitro and In Vivo Stability Assessment of the Investigational, Macrocyclic Gadolinium-Based Contrast Agent Gadoquatrane**
*S. Gruendemann*, T. Frenzel, J. Lohrke, J. Boyken, G. Jost, M. Berger, H-F. Ulbrich, H. Pietsch; Berlin/DE
*(stephan.gruendemann@bayer.com)*

**Purpose:** Gadoquatrane is a tetrameric extracellular macrocyclic gadolinium-based contrast agent (GBCA) with a T1 relaxivity of 11.8 L/(mmol Gd*s) at 1.41 T in human plasma, which has been submitted for regulatory approval in several countries. This study evaluated its stability in comparison with approved macrocyclic GBCAs in vitro and in vivo.
**Methods or Background:** All assays were conducted at equimolar Gd concentrations. Dissociation kinetics were measured for gadoquatrane, gadoteridol, gadobutrol, gadoterate, and gadopiclenol at pH 1.2 and 37°C using a complexometric assay. Stability in human plasma at pH 7.4 and 37°C was analyzed by ion exchange chromatography with ICP-MS. Rats received a single injection of gadoquatrane, gadobutrol, or gadopiclenol (0.6 mmol Gd/kg; human equivalent 0.1 mmol Gd/kg), and Gd distribution in bone was quantified by laser ablation ICP-MS one week later.
**Results or Findings:** At pH 1.2, dissociation half-lives were 28.6 days (gadoquatrane), 14.2 days (gadopiclenol), 2.7 days (gadoterate), 14.1 h (gadobutrol), and 2.2 h (gadoteridol). After 15 days in plasma (pH 7.4), no detectable Gd release was observed for gadoquatrane or gadoterate, while gadobutrol, gadoteridol, and gadopiclenol released 0.12%, 0.20%, and 0.20%, respectively. In rats, bone marrow Gd levels were similar across compounds (2.3-3.0 nmol/g). In epiphysis, concentrations were 1.2 nmol/g for gadoquatrane and gadobutrol, and 2.2 nmol/g for gadopiclenol. In diaphysis, levels were 0.5, 1.0, and 2.7 nmol/g, respectively. Elemental imaging showed lowest Gd in mineralized bone for gadoquatrane (<1 nmol/g) compared to gadobutrol and gadopiclenol.
**Conclusion:** Gadoquatrane demonstrated the highest kinetic inertness under acidic conditions and no measurable Gd release under physiological conditions in plasma. Its high stability was supported by very low Gd concentrations in mineralized bone in rats.
**Limitations:** These preclinical data do not allow direct conclusions regarding safety in humans.
**Funding for this study:** None
**Has your study been approved by an ethics committee?** Not applicable
**Author Disclosures:**
Hannes-Friedrich Ulbrich: Employee: Bayer AG
Markus Berger: Employee: Bayer AG
Gregor Jost: Employee: Bayer AG
Thomas Frenzel: Employee: Bayer AG
Jessica Lohrke: Employee: Bayer AG
Stephan Gruendemann: Employee: Bayer AG
Janina Boyken: Employee: Bayer AG
Hubertus Pietsch: Employee: Bayer AG

**One-year follow-up of gadolinium presence and distribution in rat bones: Evaluation of the novel, high-relaxivity contrast agent gadoquatrane compared to gadopiclenol and gadobutrol**
G. Jost, J. Boyken, J. Lohrke, S. Gruendemann, *H. Pietsch*; Berlin/DE
*(hubertus.pietsch@bayer.com)*

**Purpose:** There is ongoing scientific research on the presence of gadolinium (Gd) in bones after administration of Gd-based contrast agents (GBCAs). Gadoquatrane is a novel, macrocyclic GBCA with significantly higher r1-relaxivity, enabling MRI at reduced Gd dose. Gadoquatrane is in clinical development and was recently investigated in phase III studies at a dose of 0.04 mmol Gd/kg. Our study evaluates the presence of Gd in rat femurs compared to standard doses of gadopiclenol and gadobutrol.
**Methods or Background:** Rats (Wistar-Han, n=40 per GBCA) received a single intravenous injection of gadoquatrane (0.24 mmolGd/kg), gadopiclenol (0.3 mmolGd/kg) or gadobutrol (0.6 mmolGd/kg), representing human doses of 0.04, 0.05 and 0.1 mmol Gd/kg, respectively. After a treatment-free period of 1,4,22 and 52 weeks the Gd concentrations of the dissected femur (n=10 rats) were measured by inductively coupled plasma mass spectrometry (ICP-MS) and laser-ablation ICP-MS imaging (LA-ICP-MS).
**Results or Findings:** The Gd concentrations in bone epiphysis and diaphysis were comparable after administration of gadobutrol and gadopiclenol, whereas 2-to 6-fold lower concentrations were observed after injection of gadoquatrane. All GBCAs showed a limited Gd wash-out over the one-year follow up. In bone diaphysis the Gd concentration (geometric mean in nmol/g) after 1 and 52 weeks was 0.2±1.4 and 0.2±1.3 (gadoquatrane), 1.0±1.9 and 0.6±1.9 (gadopiclenol), 0.6±1.8 and 0.5±1.2 (gadobutrol). LA-ICP-MS imaging revealed different Gd distribution patterns. A local accumulation of Gd in the cortical bone was present after injection of gadopiclenol, which was barely visible after injection of gadobutrol. No local accumulation was detected for gadoquatrane.
**Conclusion:** The presence and distribution of Gd in the rat femur after a single injection of human equivalent clinical Gd doses differ among the three GBCAs. For gadoquatrane considerably lower Gd concentrations without local accumulations were detected.
**Limitations:** Preclinical study
**Funding for this study:** None
**Has your study been approved by an ethics committee?** Not applicable
**Author Disclosures:**
Gregor Jost: Employee: Bayer AG
Jessica Lohrke: Employee: Bayer AG
Stephan Gruendemann: Employee: Bayer AG
Janina Boyken: Employee: Bayer AG
Hubertus Pietsch: Employee: Bayer AG

**First imaging results of a non-metallic aortic stent graft for MRI**
*F. Wegner*[1], M. R. Meyer[2], V. Gesché[3], P. Borm[4], A. Dell[1], G. Männel[1], D. Wendt[1], T. Friedrich[1], M. M. Sieren[1]; [1]Lübeck/DE, [2]Duisburg/DE, [3]Aachen/DE, [4]Düsseldorf/DE
*(franz.wegner@uksh.de)*

**Purpose:** Commercially available endovascular stents are typically manufactured from metals. Due to their ferromagnetic properties, these devices can cause significant imaging artefacts, potentially limiting follow-up assessments with MRI and posing challenges for future MRI-guided EVAR procedures. The aim of this study was to develop a non-metallic aortic stent graft and evaluate its imaging characteristics in MRI.
**Methods or Background:** The stent struts were printed in a Z-shaped configuration using a custom-built 3D-printer with PEEK polymer filaments. The printer employed a rotational principle to apply the polymer onto a tubular graft textile. The stent graft was placed via a tube of 20 mm in diameter in a 3D-printed aortic phantom (material: Vero/Agilus, Shore 70A). The phantom was filled with gadolinium-based contrast agent (dilution 1:200) and subsequently imaged using a clinical MRI-scanner. To evaluate potential stent artefacts, the SNRs inside the stent and outside the stent were calculated. The stent strut thickness displayed on MR-images was measured at six locations and averaged.
**Results or Findings:** The rotational printing process enabled a stable connection between the stent struts and the textile. The resulting stent measured 30 mm in outer diameter and 100 mm in length. The strut thickness was measured by a caliper to be 1.6 mm. After the placement in the phantom, the stent fully expanded and showed no macroscopic loss of integrity. The stent struts were clearly delineable in MRI with a measured strut thickness of 1.7 ± 0.1 mm. The SNR inside the stent was 86.4 and outside 88.1, indicating no relevant stent-induced artefacts.
**Conclusion:** A non-metallic stent graft can be successfully manufactured using a rotational 3D-printing process with PEEK polymer, enabling artefact-free stent imaging in MRI.
**Limitations:** Only static measurements have been performed.
**Funding for this study:** Federal Ministry of Education and Research (BMBF), grant number 13GW0608F
**Has your study been approved by an ethics committee?** Not applicable
**Author Disclosures:**
Franz Wegner: Nothing to disclose
Marius R. Meyer: Nothing to disclose
Thomas Friedrich: Nothing to disclose
Dennis Wendt: Nothing to disclose
Valentine Gesché: CEO: Founder of the company Peragraft
Paul Borm: Founder: Co-Founder of Nano4Imaging
Malte Maria Sieren: Nothing to disclose
Georg Männel: Nothing to disclose
Annika Dell: Nothing to disclose

**Development of a degradable stent marking technology for Magnetic Particle Imaging**
*P. Elfers*[1], K. Lüdtke-Buzug[2], A. Malhotra[2], J. Ackers[2], J. C. Engster[2], D. Melenberg[2], M. Ahlborg[2], T. Friedrich[2], F. Wegner[1]; [1]Lübeck/DE, [2]Luebeck/DE

**Purpose:** Magnetic Particle Imaging (MPI) is a preclinical imaging modality on the verge to first human studies. It is based on the real-time visualisation of magnetic nanoparticles (MNPs). MPI is particularly well suited for cardiovascular imaging and enables artefact-free imaging of stent lumina. To visualise stents during MPI-guided interventions and to prevent interferences during follow-up examinations, a bioresorbable coating is required that degrades after a defined period. The aim of this work was to develop a coating based on polylactic acid (PLA) and MNPs to make stents temporarily visible in MPI.
**Methods or Background:** Glass capillaries were coated with a PLA-MNP-mixture by dip-coating. The degradation of the coating was investigated in a static water bath experiment at 37 °C. For this purpose, each sample was stored individually in a glass vial filled with isotonic saline solution. Ten different exposure durations (one hour to 28 days) were investigated and the samples were removed for analysis at the respective time. Before and after the water bath, the samples were dried, weighed, their MPI signal was measured, and their morphology was examined by using micro-CT.
**Results or Findings:** A continuous mass reduction of the markers was observed after exposure to the water bath (approx. 6 % after one hour, approx. 90 % after 28 days). Micro-CT showed only a slight volume decrease, whereas cavities formed inside the markers with increasing exposure time. A detectable MPI signal was present throughout the entire observation period.
**Conclusion:** PLA seems to be a suitable polymer for degradable stent markers. The observed properties provide a promising basis for future applications of MPI in cardiovascular and periinterventional imaging.
**Limitations:** The samples were investigated under static conditions only. Flow experiments are required to transfer the results.
**Funding for this study:** This work was supported in part by the Clinician Scientist Program of the University of Lübeck under Grant CS10-2021. The Fraunhofer IMTE and this work are supported by the EU, the State Schleswig-Holstein, Germany, and by Internal Programs (Grants 12420002/LPWE1.1.1/1536, 12524009/LPW21L/2.2/262 and 139–600251).
**Has your study been approved by an ethics committee?** Not applicable
**Author Disclosures:**
Franz Wegner: Nothing to disclose
Johann Christopher Engster: Nothing to disclose
Justin Ackers: Nothing to disclose
David Melenberg: Nothing to disclose
Thomas Friedrich: Nothing to disclose
Kerstin Lüdtke-Buzug: Nothing to disclose
Ankit Malhotra: Nothing to disclose
Patrick Elfers: Nothing to disclose
Mandy Ahlborg: Nothing to disclose

**Valve stent crimper enables faster reloading compared to the traditional tourniquet technique - Bench-top testing of a novel approach for PMEG reloading**

*P. Osztrogonacz*, J. Csőre, A. Szentiványi, Á. Bérczi, A. Hüttl, C. Csobay-Novák; Budapest/HU

**Purpose:** During physician-modified endograft (PMEG) preparation, reloading the stent graft into its delivery sheath is a time-sensitive step. Traditionally, this is performed using the tourniquet (T) technique. At our quaternary aortic referral center, valve stent crimper (C) has emerged as a potential alternative, offering a controlled and possibly more reproducible method. Our study aimed to compare the C and T techniques in terms of reloading time.
**Methods or Background:** A Medtronic Valiant 38x200mm thoracic stent graft was reloaded a total of 18 times in a bench-top setting using either the T-technique (n=9) or the C-technique (n=9). Reloading times were recorded in minutes. Statistical analysis was performed using the Mann–Whitney U test to compare reloading times between groups. Results are reported in median (interquartile ranges (IQR)).
**Results or Findings:** The C-group demonstrated significantly faster reloading times, with a median time of 3.98 minutes (3.27–5.23), compared to 11.48 minutes (8.82–17.35) in the T-group (p = .001). The C group also exhibited narrower variability, suggesting improved reproducibility.
**Conclusion:** The crimper-technique offers a faster and more consistent method for thoracic stent graft reloading when compared to the traditional tourniquet technique. Its use may improve procedural efficiency and reproducibility in complex endovascular aortic aneurysm repair.
**Limitations:** Bench-top design, single stent graft type assessed, sample size may not detect rare occurrences of infolding / device failure
**Funding for this study:** None.
**Has your study been approved by an ethics committee?** Not applicable
**Author Disclosures:**
Peter Osztrogonacz: Nothing to disclose
Ákos Bérczi: Nothing to disclose
András Szentiványi: Nothing to disclose
Artúr Hüttl: Nothing to disclose
Csaba Csobay-Novák: Speaker: CCN is an international and domestic trainer, proctor, speaker for Medtronic, Cook Medical, Gore Medical and Terumo Aortic.
Judit Csőre: Nothing to disclose

**Development and validation of a pulmonary vessel segmentation model based on deep learning for detecting chronic thromboembolic pulmonary hypertension at CT pulmonary angiography**

*Y. Lin*, S. Xie; Beijing/CN
*(18811150617@163.com)*

**Purpose:** To develop, externally test, and evaluate clinical acceptability of a deep learning (DL) segmentation model of pulmonary vessels for detecting chronic thromboembolic pulmonary hypertension (CTEPH) at CT pulmonary angiography (CTPA).
**Methods or Background:** A multicenter, retrospective study was conducted involving 621 consecutive patients who underwent right heart catheterization (RHC), including individuals with confirmed CTEPH, CTED, and normal controls. Among them, 503 patients were assigned to the model development and internal test cohort, and the remaining 118 formed the external validation cohort. Using CTPA, the pulmonary vasculature was reconstructed via a DL method to separate arteries from veins and compute vascular volumes based on cross-sectional area (CSA) and tortuosity for each branch. Statistically significant features were selected to develop the segmentation model. Diagnostic performance was evaluated using AUC, and the correlation between predicted and actual mean pulmonary arterial pressure (mPAP) was assessed across two institutions.
**Results or Findings:** The CTEPH cohort in the training set showed significantly larger total vessel volume, large vessel volume, total arterial volume, and greater arterial and venous tortuosity compared to both the CTED and control cohorts (all $P < 0.001$). Seventeen features were selected to develop a segmentation model, which demonstrated excellent diagnostic performance for identifying CTEPH using RHC results as the gold standard. The model achieved high AUC values (0.96–0.99) in both the training and internal test sets, and maintained strong accuracy in external validation. Additionally, predicted mPAP showed good agreement with real mPAP across two institutions ($R^2 = 0.91$ and 0.77, respectively).
**Conclusion:** Automated quantification methods using CT imaging can provide a imaging marker for identification of CTEPH and CTED.
**Limitations:** The findings are limited by a small sample size and multiple comparisons, which increased the risk of false-positive results.
**Funding for this study:** Beijing Natural Science Foundation
**Has your study been approved by an ethics committee?** Yes
**Ethics committee - additional information:** This project was approved by the Ethics Committee of China-Japan Friendship Hospital
**Author Disclosures:**
Sheng Xie: Nothing to disclose
Yue Lin: Nothing to disclose

**Non-Contrast QISS MRI Outperforms Angiography in Detecting Lower-Limb Arterial Patency**

*J. Csőre*[1], A. Crichton[2], E. Pomozi[2], M. Drake[2], P. Haddad[2], J. Lamichhane[2], A. B. Lumsden[2], T. L. Roy[2]; [1]Budapest/HU, [2]Houston, TX/US

**Purpose:** Chronic limb-threatening ischemia (CLTI) is often linked to chronic kidney disease (CKD), making vascular imaging in this population challenging. Digital subtraction angiography (DSA) remains the diagnostic reference standard but is invasive and requires iodinated contrast. Quiescent Interval Single Shot (QISS) MRI is a newer non-contrast-enhanced method that mitigates these risks. This study aimed to determine whether QISS MRI identifies a greater number of patent arterial segments than DSA, and whether this advantage is most pronounced in patients with advanced CKD (stages 4/5).
**Methods or Background:** Patients with CLTI who underwent both QISS MRI and DSA were included. Popliteal and infrapopliteal arteries were classified as patent or occluded across three sub-segments (e.g., AT1=proximal, AT2=mid, AT3=distal). Two blinded reviewers independently evaluated all images. Participants were stratified by GFR into <30, 30-59, ≥60 mL/min/1.73 m² groups. Primary outcome was the proportion of patent arterial segments identified by QISS compared to DSA across these GFR categories. Secondary outcomes included TASC and infrapopliteal GLASS score changes between modalities.
**Results or Findings:** Among 57 patients (752 vessel segments), QISS MRI demonstrated a significantly higher proportion of patent segments compared to DSA (67.2% vs 57.3%, $p<0.001$). Differences were significant in the <30 and ≥60 GFR groups, but not in the 30-59 group. TASC and GLASS scores were significantly downgraded using QISS MRI (mean TASC 2.2 vs 2.3, $p=0.049$; GLASS 2.6 vs 2.2, $p<0.001$).
**Conclusion:** QISS MRI identifies more patent vessel segments than DSA, especially in patients with advanced renal dysfunction. As earlier studies using older non-contrast MR techniques have shown comparable outcomes in bypasses to arteries visible only on MRI but not on DSA after three years, these results further support incorporating QISS MRI into routine limb-salvage assessment protocols.
**Limitations:** Single-center design, modest sample size
**Funding for this study:** Jerold B. Katz Academy of Translational Science under project number 15790002 (recipient's name: Trisha Roy), the American Heart Association Transformational Award under project ID 17590004 (recipient's name: Trisha Roy), and the National Institutes of Health Research Project grant (R01) under award number R01HL174587 (recipient's name: Trisha Roy).
**Has your study been approved by an ethics committee?** Yes
**Ethics committee - additional information:** Study ID 15790002
**Author Disclosures:**
Madeline Drake: Nothing to disclose
Alexander Crichton: Nothing to disclose
Enikő Pomozi: Nothing to disclose
Janak Lamichhane: Nothing to disclose
Paul Haddad: Nothing to disclose
Judit Csőre: Nothing to disclose
Alan B. Lumsden: Nothing to disclose
Trisha L. Roy: Founder: Magellan Biomedical Inc. Research/Grant Support: Baylis Medical Technologies, Boston Scientific, Light Matter Interaction

**15:00 - 16:00** **Research Stage 4**

### Research Presentation Session: Abdominal and Gastrointestinal

### RPS 501

### Interventional oncology and treatment assessment in liver cancer

**Moderator**
Christoph Johannes Zech; Basel/CH
*(christoph.zech@usb.ch)*

### MR Radiomics to Predict Recurrence Location in Nonviable Hepatocellular Carcinoma After Transarterial Chemoembolization

*S-H. Zhang*[1], Y. Song[2], Y-C. Wang[1]; [1]Jiangsu/CN, [2]Shanghai/CN
*(shuhangzhang@outlook.com)*

**Purpose:** To develop a predictive model that integrates radiomics features from contrast-enhanced MRI with conventional radiologic features to identify early recurrence locations in nonviable hepatocellular carcinoma (HCC) after transarterial chemoembolization (TACE).
**Methods or Background:** This multicenter retrospective study included HCC patients treated with TACE whose contrast-enhanced MRI at 1–2 months post-treatment showed LR-TR nonviable lesions ≥1.0 cm with at least one-year follow-up. A 1-cm peritumoral ring was divided into eight sectors for radiomics feature extraction to build a radiomics model. A fusion model combined these features with two radiologic characteristics (non-smooth margin and peritumoral hyperintensity on T2- or diffusion-weighted images). Model performance was evaluated using ROC curves, and differences were assessed by the DeLong test.
**Results or Findings:** A total of 616 sectors from 77 lesions were included in the study, including 96 (15.6%) sectors with recurrence within one year of follow-up. The radiomics model achieved AUC of 0.738 and 0.718 in the training and test cohorts. The radiologic model, constructed using non-smooth margin and peritumoral hyperintensity on T2-weighted or diffusion-weighted images, achieved AUC of 0.751 and 0.744 in the training and test cohorts, respectively. The fusion model combining three radiomics features and two radiologic features achieved AUC of 0.794 and 0.765 in the training and test cohorts, respectively. The DeLong test demonstrated a statistically significant difference between the fusion model and the radiologic model in the training cohort (P = 0.026).
**Conclusion:** The fusion model combining selected radiomics features and conventional radiologic features demonstrated excellent performance in predicting the site of recurrence in LR-TR nonviable HCC lesions following TACE.
**Limitations:** Its retrospective design may introduce selection bias, and the limited sample size raises concerns about potential overfitting and restricted generalizability, highlighting the need for prospective validation.
**Funding for this study:** This study has received funding by National Natural Science Foundation of China (NSFC, No. 82271978).
**Has your study been approved by an ethics committee?** Yes
**Ethics committee - additional information:** This multicenter, retrospective study was reviewed and approved by Institutional Ethics Committee for clinical research of the hospital, approval number [2024ZDSYLL149-P01], with a waiver for informed consent.
**Author Disclosures:**
Yuan-Cheng Wang: Nothing to disclose
Yang Song: Nothing to disclose
Shu-Hang Zhang: Nothing to disclose

### Prognostic Prediction Models Incorporating Longitudinal MRI Features in Hepatocellular Carcinoma Patients after Transarterial Chemoembolization

B. Li, *S-H. Zhang*, Y-C. Wang; Nanjing/CN
*(shuhangzhang@outlook.com)*

**Purpose:** Current prognostic prediction models of hepatocellular carcinoma (HCC) who treated with transarterial chemoembolization (TACE) include only the preoperative imaging and clinical features, with their available repeated postoperative MRI features measurements underutilized. HCC prognostic prediction models were constructed in this study to clarify whether and to what extent the inclusion of postoperative longitudinal measurements of longest diameter of active lesions, number of tumors, longest diameter of target lesions and Apparent Diffusion Coefficient (ADC) value of target lesions can improve the model performance and perform a dynamic prediction.
**Methods or Background:** The training cohort study included 56 HCC patients who underwent TACE, with preoperative measurement and three or more measurements within 24 months after TACE, respectively. Prediction models to predict HCC untreatable tumor-aggressive progression (macrovascular invasion, extrahepatic spread, >50% liver involvement) were constructed with clinical variables, by incorporating preoperative quantitative MRI imaging features , as well as their postoperative longitudinal measurements.
**Results or Findings:** The prediction model with postoperative longitudinal measurements of longest diameter of active lesions and number of tumors within 24 months after TACE outperformed the model including preoperative imaging and clinical features only, with the better area under the receiver operating characteristic curves (AUCs: 0.859 vs 0.797), (AUCs: 0.923 vs 0.815) and (AUCs: 0.824 vs 0.786) at 12, 24, and 36 months after TACE. The proposed longitudinal prediction model can provide a personalized dynamic prediction for a new patient, with estimated survival probability updated when a new measurement is collected during 24 months after TACE.
**Conclusion:** Prediction models including longitudinal measurements of longest diameter of active lesions and number of tumors have improved accuracy in predicting the prognosis of HCC patients.
**Limitations:** The proposed prediction models were developed solely on a single-institution cohort without external validation.
**Funding for this study:** This study has received funding by National Natural Science Foundation of China (NSFC, No. 82271978, 92359304, 82330060)
**Has your study been approved by an ethics committee?** Not applicable
**Author Disclosures:**
Yuan-Cheng Wang: Nothing to disclose
Binrong Li: Nothing to disclose
Shu-Hang Zhang: Nothing to disclose

### HCC nodules in cirrhotic patients: prognostic value of iodine concentration density derived from dual energy CT iodine maps

*P. A. Bonaffini*, P. Majoro, A. Celestino, A. Barbaro, C. Gargiulo, P. Marra, S. Sironi; Monza/IT
*(pa.bonaffini@gmail.com)*

**Purpose:** To assess the prognostic value of iodine concentration differences measured on iodine maps derived by dual energy CT (DECT) in hepatocellular carcinoma (HCC) in cirrhosis, comparing untreated nodules (disease progression/stability) and those with recurrence after loco-regional therapies.
**Methods or Background:** DECT of suspected HCC (LI-RADS 3-5) were retrospectively reviewed (March 2022-September 2023; minimum 3 months follow-up) . Lesions were divided in untreated (group 1) and undergoing loco-regional treatments (group 2). Progression was defined as ≥20% size increase (mRECIST). Iodine maps were generated in the hepatic arterial and portal venous phases. In each phase iodine concentration density (ICD, mg/ml) was measured by ROI placed in homogeneously enhancing portion of the target nodule (aICDnodule, vICDnodule) and in the non-nodular parenchyma (aICDliver, vICDliver). Lesion-to-normal liver ICD ratio (aLNR, vLNR) to quantify nodule enhancement behavior and ratio of LNR during the arterial to the venous phase (ravLNR) were calculated and compared.
**Results or Findings:** Twenty-two patients (mean age 69.5 years) with 41 lesions (19 LI-RADS 3; 6 LI-RADS 4; 16 LI-RADS 5) were included in group 1. In lesions demonstrating progression (14/41) baseline ICD values were significantly lower compared to stable nodules: aICDnodule 26.0 (IQR 20.5-30.5) vs 33 (IQR 27-40), vICDnodule 22 (IQR 19-25) vs 25.5 (IQR 27-40), respectively. Twenty-two patients (mean age 70.3 years) with 31 lesions (8 LI-RADS 3; 2 LI-RADS 4; 21 LI-RADS 5) were included in group 2. The comparison of the median iodine concentrations (aICD, vICD, aLNR, vLNR, ravLNR) at baseline between lesions relapsing after treatment and those without recurrence showed no significant differences.
**Conclusion:** Quantification of iodine concentration on DECT may support the prognostic stratification of suspected HCC in cirrhotic patients, helping to predict the risk of progression in non-treated lesions.
**Limitations:** Retrospective Small sample size
**Funding for this study:** Not applicable
**Has your study been approved by an ethics committee?** Not applicable
**Author Disclosures:**
Antonio Celestino: Nothing to disclose
Polycarpe Majoro: Nothing to disclose
Paolo Marra: Nothing to disclose
Pietro Andrea Bonaffini: Nothing to disclose
Alessandro Barbaro: Nothing to disclose
Carlotta Gargiulo: Nothing to disclose
Sandro Sironi: Nothing to disclose

**Early Intrazonal Recurrence in HCC Treated with Ablation Therapies - Focus on CEUS**

*P. Buscemi*[1], M. D'Amico[1], M. G. Vallone[1], F. Meloni[2]; [1]Palermo/IT, [2]Milan/IT
*(palbusc@gmail.com)*

**Purpose:** Hepatocellular carcinoma (HCC) is the most common primary liver tumor, with an increasing global incidence. Early recurrence detection post-ablation improves retreatment outcomes and survival. This study evaluates the role of CEUS in identifying early CC recurrence through the characterization of patent arteries observed 24 hours post-ablation via CECT and follow-up imaging.
**Methods or Background:** Ninety-eight patients (66 men, 32 women) with 121 HCCs treated with RFA (62) or MWA (59) between January 2016 and December 2019 were analyzed. Post-treatment imaging included CT and CEUS at 24 hours and CT/MRI with CEUS every four months. Peri-ablative vessels (5-10 mm from the periphery) were identified in 51/121 lesions (42.1%), while intranecrotic vessels were found in 28/51 lesions (54.9%).
**Results or Findings:** Intralesional vessels increased in caliber and length in 75% of cases, leading to the development of enhancement with wash-in and wash-out consistent with intrazonal recurrence. CEUS detected intralesional vascular recurrence earlier than CT/MRI, with only 2/21 cases (9.5%) visible on CT/MRI. A significant correlation was found between local recurrence and perilesional vessels ($p<0.01$), as well as between periablation and intralesional vessels ($p<0.01$), highlighting a microenvironment favorable to neo-angiogenesis. Perilesional vascularization was significantly associated with RFA ($p<0.01$).
**Conclusion:** Persistent vascularization near the ablation site may promote intra- and perizonal recurrence by sustaining residual tumor cells. CEUS demonstrated superior sensitivity in detecting early intralesional recurrence compared to CT/MRI, supporting its role in post-ablation surveillance.
**Limitations:** Single-center retrospective design
**Funding for this study:** This study received no funding
**Has your study been approved by an ethics committee?** Not applicable
**Author Disclosures:**
Mario D'Amico: Nothing to disclose
Paolo Buscemi: Nothing to disclose
Franca Meloni: Nothing to disclose
Mario Giuseppe Vallone: Nothing to disclose

**LI-RADS CT/MRI Nonradiation Treatment Response Assessment Version 2024: Patterns of Recurrence after Surgical Resection**

*S-H. Chiu*[1], D. Delitto[2], L. S. Yoon[2], A. Kamaya[2], J. Tse[2]; [1]Taipei/TW, [2]Palo alto, CA/US
*(doc62207@gmail.com)*

**Purpose:** The LI-RADS CT/MRI Nonradiation Treatment Response Algorithm (v2024) extends its application to assessing the surgical margin after hepatocellular carcinoma (HCC) resection. This study aims to apply v2024 to surgically-resected HCCs, determine rates of local recurrence defined by v2024, and evaluate the timing of recurrence during postoperative surveillance.
**Methods or Background:** This retrospective study included patients with surgically-resected HCC and postoperative liver CT/MRI for ≥6 months. v2024 and LI-RADS CT/MRI Diagnosis Version 2018 (v2018) were applied to the surgical margin. Two abdominal radiologists assessed for local recurrence (LR-TR Viable meeting ≥LR-4 by v2018 at diagnosis or follow-up), intrahepatic recurrence away from the margin (new lesion meeting ≥LR-4), and extrahepatic disease. Discrepancies were resolved by a third radiologist.
**Results or Findings:** Two hundred patients (142 men and 58 women; median age 67 years) were followed for a median of 30.5 months [IQR 13.7-60.1]. LR-TR Viable at the surgical margin occurred in 57 patients (28.5%) after a median of 5.1 months. At diagnosis, 26 LR-TR Viable observations (45.6%) already met v2018 criteria for ≥LR-4, while 31 (54.4%) did not; of these, 30 progressed to ≥LR-4 after a median of 3.2 months, and 1 resolved with no residual HCC at transplantation. Intrahepatic recurrence away from the margin developed in 77 patients (38.5%) after a median of 10.9 months, and extrahepatic recurrence in 37 patients (18.5%) after 5.4 months. Inter-reader agreement for LR-TR category was excellent ($\kappa=0.93$), and its positive predictive value for local recurrence was 98.2%.
**Conclusion:** In about half of cases, v2024 detects local recurrence at the surgical margin roughly 3 months before HCC criteria are met, enabling earlier diagnosis of local recurrence.
**Limitations:** Single-center, retrospective study, and relied on v2018 rather than histologic confirmation as the reference standard for recurrence.
**Funding for this study:** None
**Has your study been approved by an ethics committee?** Yes
**Ethics committee - additional information:** Stanford University IRB eProtocol 77441
**Author Disclosures:**
Daniel Delitto: Nothing to disclose
Luke S Yoon: Nothing to disclose
Sung-Hua Chiu: Nothing to disclose
Aya Kamaya: Nothing to disclose
Justin Tse: Consultant: Justin R Tse is or was recently a consultant for Ascelia Pharma, Intuitive Surgical, Inc., and AbSolutions Med, Inc. Grant Recipient: Justin R Tse receives or recently received grant funding from GE HealthCare and Bayer AG.

**Quantitative CT Perfusion and Radiomics Reveal Complementary Markers of Treatment Response in HCC Patients Undergoing TACE**

*J. N. Nonninger*[1], J. Slavicek[1], N. Fezoulidis[1], K. Hergan[2], S. Zandieh[1]; [1]Vienna/AT, [2]Salzburg/AT

**Purpose:** Hepatocellular carcinoma (HCC), the most common primary liver cancer, is often treated with transarterial chemoembolization (TACE). Standard criteria (RECIST, mRECIST) may miss early changes, underestimating therapeutic effects. Quantitative imaging may improve early assessment and guide therapy. This study analyzed CT perfusion and radiomic features of HCC before and after TACE and their association with tumor burden.
**Methods or Background:** In this retrospective, single-center study, 32 patients with histologically confirmed HCC underwent CT perfusion and radiomic analysis prior to and following TACE. Multiple quantitative perfusion parameters (arterial flow, perfusion flow, perfusion index) and radiomic features were extracted. Statistical comparisons were performed using the Wilcoxon signed-rank test and Spearman’s correlation.
**Results or Findings:** Arterial flow significantly decreased from a median of 56.5 to 47.7 mL/100 mL/min after TACE ($p = 0.009$), while nonsignificant increases in perfusion flow (from 101.3 to 107.8 mL/100 mL/min, $p = 0.44$) and decreases in the perfusion index (from 38.6% to 35.7%, $p = 0.25$) were observed. Perfusion flow was strongly and positively correlated with tumor size ($\rho = 0.94$, $p < 0.001$). Five radiomic texture features especially ShortRunHighGrayLevelEmphasis ($\Delta = +2.11$, $p = 0.0001$) and LargeAreaHighGrayLevelEmphasis ($\Delta = +75{,}706$, $p = 0.0006$)—changed significantly after treatment. These changes were more pronounced in tumors ≥50 mm.
**Conclusion:** CT perfusion and radiomics offer complementary insights into the treatment response of patients with HCC. While perfusion parameters reflect macroscopic vascular changes and are correlated with tumor burden, radiomic features can indicate microstructural changes after TACE. This combined imaging approach may improve early therapeutic assessment and support precision oncology strategies.
**Limitations:** Despite its strengths, the study has limitations, including its retrospective design and potential observer bias from manual segmentation, which was mitigated by consensus reading.
**Funding for this study:** No funds were used in this study.
**Has your study been approved by an ethics committee?** Yes
**Ethics committee - additional information:** Ethics Committee of the City of Vienna
**Author Disclosures:**
Shahin Zandieh: Nothing to disclose
Klaus Hergan: Nothing to disclose
Jakob Slavicek: Nothing to disclose
Julian Niklas Nonninger: Nothing to disclose
Nicolas Fezoulidis: Nothing to disclose

**15:00 - 16:00** **Research Stage 2**

### Research Presentation Session: Cardiac

### RPS 503

### Imaging in structural heart disease

**Moderator**
Birgitta K Velthuis; Utrecht/NL
**Author Disclosures:**
Birgitta K Velthuis: Research Grant/Support: Philips Healthcare, Speaker: Philips Healthcare

### Prevalence of mitral annular disjunction (MAD), mitral valve prolapse (MVP), and curling in a healthy population: assessment by cardiac MRI and correlation with left ventricular myocardial T1 mapping

*P. Franceschi*[1], F. Calo'[2], V. Russo[1], F. Niro[1], F. Buia[1], D. Attinà[1], C. Sportoletti[1], E. Rasciti[1], L. Lovato[1]; [1]Bologna/IT, [2]Brindisi/IT

**Purpose:** To assess the prevalence of mitral annular disjunction (MAD), mitral valve prolapse (MVP), and curling in healthy individuals using cardiac magnetic resonance (CMR), and to evaluate correlations between MAD length and native myocardial T1 mapping values.
**Methods or Background:** We prospectively enrolled 141 healthy volunteers (mean age 39 years; 57% female) without cardiovascular risk factors. CMR at 3T included ECG-triggered cine balanced steady-state free precession acquisitions in 2-, 3-, and 4-chamber long-axis views. Native T1 mapping was performed in three short-axis slices. Global and segmental values were extracted.
**Results or Findings:** MAD was detected in 63 of 140 subjects (45%), mostly in the inferior segment, with lengths between 1–12 mm. MVP was identified in 65 of 137 subjects (47%), predominantly monoleaflet (39%). MVP was more prevalent in subjects with MAD (56%), with significantly longer disjunction in bileaflet prolapse compared with monoleaflet or absent MVP (5±3 mm vs. 1±2 mm; p<0.001). Curling was observed in 3 of 140 cases (2%); two of these also presented with MAD and MVP. MAD length was greater in individuals with curling (7±2 mm vs. 1±2 mm; p<0.001). Native T1 showed no significant differences between participants with or without MAD, MVP, or curling.
**Conclusion:** MAD is a frequent finding in healthy individuals, predominantly at the inferior annulus. Native T1 values remained normal across groups, suggesting preserved myocardial tissue. While isolated MAD seems benign, longer disjunction associated with MVP and curling indicates structural remodeling of the atrioventricular junction and may represent an arrhythmogenic substrate. CMR enables simultaneous morphological and tissue characterization, supporting its role in early identification of at-risk phenotypes.
**Limitations:** The limitation of the study is the lack of correlation with echocardiographic, ECG and fibrosis data (contrast agent was not administered in healthy volunteers).
**Funding for this study:** No funding was received for this study.
**Has your study been approved by an ethics committee?** Yes
**Ethics committee - additional information:** The study was approved by the Ethics Committee of Area Vasta Emilia Centro (CE-AVEC) (reference number 864/2022/Sper/AOUBo).
**Author Disclosures:**
Fabio Niro: Nothing to disclose
Camilla Sportoletti: Nothing to disclose
Francesco Buia: Nothing to disclose
Francesca Calo': Nothing to disclose
Luigi Lovato: Nothing to disclose
Vincenzo Russo: Nothing to disclose
Edoardo Rasciti: Nothing to disclose
Domenico Attinà: Nothing to disclose
Paola Franceschi: Nothing to disclose

### CMR-Based Prediction of Arrhythmic Risk in Mitral Valve Prolapse

*Y. B. Uluman*, E. İsmailov, F. Köse, M. Kadıoğlu, B. Tütüncüoğlu, F. Alpaslan, T. Banaz, K. B. Bekaroğlu, S. Baş Özkök; Istanbul/TR
*(yusufuluman@icloud.com)*

**Purpose:** To investigate the association between cardiac arrhythmias and cardiac magnetic resonance (CMR) findings in mitral valve prolapse (MVP).
**Methods or Background:** MVP is usually benign, but some patients develop malignant arrhythmias and sudden cardiac death; CMR may help identify structural risk markers. We retrospectively analyzed 27 MVP patients underwent 1.5 Tesla CMR with cine, T1/T2 mapping, and late-gadolinium-enhancement (LGE). Assessed parameters included ventricular volume/function, prolapse height, valve thickening, mitral annular disjunction, and LGE extent/localization. Prolapse height was defined relative to the atrioventricular junction, and measurements were performed using automated segmentation with manual correction. ECG/Holter were used to assessed arrhythmia. Continuous variables were presented as mean±SD and compared with the Mann–Whitney U test; categorical variables with the chi-square test. Correlations were examined using Pearson or Spearman coefficients, and multivariate logistic regression identified independent arrhythmia predictors. Significance was set at p<0.05.
**Results or Findings:** Mean prolapse height was 9.3±3.0 mm, and valve thickening was present in 29.6% of patients. MAD was identified in 66.7% (true/pseudo ratio: 11/8). Arrhythmia occurred in 44.4%, while biventricular systolic function was preserved. LGE was observed in 81.5% of patients, mostly limited (<5% of myocardial volume in 86.4%), involving the inferolateral wall in 29.6% and papillary muscles in 18.5%. Documented arrhythmias includes ventricular extrasystole (n=5), atrial fibrillation (n=2), and sustained ventricular tachycardia (n=5); no sudden death occurred. LGE extent and inferolateral wall involvement were not associated with arrhythmia, whereas papillary muscle fibrosis showed a significant correlation (OR=31.1,p=0.02,AUC=0.89).
**Conclusion:** This study demonstrated that papillary muscle fibrosis is a strong independent predictor of arrhythmia in patients with mitral valve prolapse. The findings suggest that papillary involvement, rather than global myocardial fibrosis, may represent the key structural substrate for arrhythmogenesis in this population.
**Limitations:** Relatively small, single-centre sample size.
**Funding for this study:** N/A
**Has your study been approved by an ethics committee?** Yes
**Ethics committee - additional information:** Number: E-96317027-514.10-251444224 Subject: KAEK/26.06.2024.70
**Author Disclosures:**
Serçin Baş Özkök: Nothing to disclose
Berk Tütüncüoğlu: Nothing to disclose
Fatih Alpaslan: Nothing to disclose
Fadime Köse: Nothing to disclose
Kemal Burak Bekaroğlu: Nothing to disclose
Eitibar Ismailov: Nothing to disclose
Yusuf Bahtiyar Uluman: Nothing to disclose
Tuba Banaz: Nothing to disclose
Mehmet Kadıoğlu: Nothing to disclose

### Diagnostic Accuracy of High Pitch Spectral Imaging Photon-Counting CT for the Assessment of Coronary Artery Disease in Single Scan Pre-Transcatheter Aortic Valve Workup

*C. Booz*[1], S. Waldeck[2], L. S. Alizadeh[1], I. Yel[1], T. Vogl[1], A-I. Nica[1], V. Koch[1], L. D. Grünewald[1], D. Overhoff[2]; [1]Frankfurt/DE, [2]Koblenz/DE
*(boozchristian@gmail.com)*

**Purpose:** To evaluate the diagnostic accuracy of high pitch coronary CT angiography (CCTA) spectral datasets derived from a single CTA for the assessment of coronary artery disease (CAD) in transcatheter aortic valve implantation (TAVI) imaging using invasive coronary angiography (ICA) as reference standard.
**Methods or Background:** Patients with severe aortic stenosis who underwent photon-counting CT TAVI pre-procedural planning between April 2022 and February 2023 were retrospectively selected. The spectral CCTA datasets were used for the evaluation of objective and subjective image quality. Additionally, the diagnostic accuracy was assessed by determining, sensibility, specificity, positive predictive value (PPV) and negative predictive value (NPV) using ICA as reference standard both on a per-vessel and a per-segment basis. Furthermore, patients were divided in two groups according to calcium score values to define the impact of calcium burden on the diagnostic performance.
**Results or Findings:** The final study population included 93 patients (mean age, 80±8.4 years; 58 men). The mean overall signal-to-noise ratio was 8.7±2.5 while the contrast-to-noise ratio set on mean values of 9.6±2.5. Image quality median value was 1 [1-2] for the subjective assessment. The diagnostic accuracy for the detection of CAD resulted in a sensitivity of 90.3% (95%CI: 82.9-95.2), specificity of 79.9% (95%CI: 74.5-84.6), PPV of 64.1% (95%CI: 58.2-69.7), NPV of 95.4% (95%CI: 92-97.4). On a per-segment basis sensitivity was 86.4% (95%CI: 79.1-91.9), specificity was 87.6% (95%CI: 85-90), PPV was 56% (95%CI: 50.7-61.1) and NPV was 97.3% (95%CI: 95.8-98.2).

**Conclusion:** Spectral datasets based on high pitch photon-counting single scan CCTA allow for high diagnostic accuracy in the evaluation of CAD in high-risk patients.
**Limitations:** Limitations of the study include its retrospective nature.
**Funding for this study:** No funding was obtained for this study.
**Has your study been approved by an ethics committee?** Yes
**Ethics committee - additional information:** This study received approval from our Ethics Committee.
**Author Disclosures:**
Christian Booz: Speaker: I have received speaking fees from Siemens Healthineers.
Thomas Vogl: Nothing to disclose
Vitali Koch: Nothing to disclose
Ibrahim Yel: Nothing to disclose
Daniel Overhoff: Nothing to disclose
Andreea-Ioana Nica: Nothing to disclose
Stephan Waldeck: Nothing to disclose
Leona Soraja Alizadeh: Nothing to disclose
Leon David Grünewald: Nothing to disclose

**Total body CT-derived opportunistic parameters for predicting TAVI outcomes: a data-driven approach**
*M. Liberotti*[1], A. Colombo[1], V. Dubreuil[2], C. Gnasso[1], D. Vignale[1], A. Palmisano[1], A. Esposito[1]; [1]Milan/IT, [2]London/UK
*(ma.liberotti@gmail.com)*

**Purpose:** Transcatheter aortic valve implantation (TAVI) is widely adopted treatment for severe aortic stenosis (AS) in high-risk patients. Accurate pre-TAVI risk stratification is crucial to predict post-procedural outcomes, and planning CT could provide opportunistic biomarkers that objectively capture patient frailty and comorbidities. This study aims to automatically extract opportunistic parameters from TAVI-planning CT to create a data-driven model to predict 1-year outcomes.
**Methods or Background:** In this single-center retrospective observational study, TAVI-planning CTs were automatically segmented using a pre-trained open-source nnUNet model. An automated pipeline extracted volume ($mm^3$) and density metrics (HU) from over 120 anatomical structures per patient. From 1330 initial features, stable variables were identified via 5-fold cross-validated Random Survival Forest, filtered with univariate Cox, refined by Cox-LASSO and backward elimination. A multivariable Cox model predicting 1-year mortality was built and its performance assessed using the mean concordance index (C-index).
**Results or Findings:** The study included 458 severe AS patients undergoing TAVI (F/M=234/224; median age 82, IQR: 78-85). At 12-month follow-up post-TAVI, 70 deaths and 24 hospitalizations for heart failure occurred. Following multistage feature selection, five variables informed the final model with four independent event predictors: left atrial volume (HR 1.41s, p=0.03), HU standard deviation in left upper lung lobe (HR 1.30, p<0.005) and brachiocephalic arterial trunk (HR 1.20, p=0.01) and 95th percentile HU in intervertebral discs (HR 1.27, p<0.005). The multivariable Cox model, adjusted for demographic and clinical variables, achieved a mean C-index of 0.72 (± 0.06), demonstrating strong prognostic performance.
**Conclusion:** This study confirms the potential of total body CT-derived parameters to predict post TAVI outcomes for severe AS patients, by integrating multiple features into a unified predictive model.
**Limitations:** Retrospective and single-center design may introduce potential bias. The model was not externally validated.
**Funding for this study:** This study was partially granted by: European Union - Next Generation EU, Mission 4 Component 1, CUP D53D23 021100001”, Bando PRIN PNRR 2022 (P2022JBKN2), Italian Ministry of University and Research (MUR).
**Has your study been approved by an ethics committee?** Yes
**Ethics committee - additional information:** This monocentric observational study was approved by the institutional review board (CT-based myocardial characterization study: CTMyoC 112/INT/2019 ). The study is a retrospective analysis of prospectively collected data and was conducted according to the Declaration of Helsinki. Written informed consent was obtained for all participants.
**Author Disclosures:**
Davide Vignale: Nothing to disclose
Antonio Esposito: Nothing to disclose
Marta Liberotti: Nothing to disclose
Anna Palmisano: Nothing to disclose
Alberto Colombo: Nothing to disclose
Chiara Gnasso: Nothing to disclose
Vanessa Dubreuil: Nothing to disclose

**Photon-Counting Detector CT versus Cardiac Magnetic Resonance for ECV Quantification: Impact of Reconstruction Phase and Myocardial Layer Analysis**
*S. N. Li*, X. Tong, W. Tian, N. Yang, B. Zhu, J. Nan, G. Wang, Z. Chen; Lanzhou, China/CN
*(13571236891@163.com)*

**Purpose:** To assess the agreement in myocardial extracellular volume fraction(ECV) quantification between photon-counting detector CT (PCD-CT) and cardiovascular magnetic resonance (CMR) in patients with cardiovascular disease, and to identify the optimal PCD-CT reconstruction phase and myocardial layer for measurement.
**Methods or Background:** This retrospective study included 11 cardiovascular disease patients who underwent both PCD-CT(with late iodine enhancement) and CMR. ECV derived from CMR served as the reference standard, calculated via a standardized formula. PCD-CT-based ECV was measured at both systolic and diastolic reconstruction phases, with regions of interest placed in the endocardial, mid-myocardial, and epicardial layers, as well as their average.
**Results or Findings:** A significant correlation was found between PCD-CT-systolic-epicardial-ECV and CMR-ECV(r = 0.835, 95% CI: 0.471–0.956, p = 0.001), and a strong correlation between PCD-CT-diastolic-endocardial-ECV and CMR-ECV (r = 0.807, 95% CI: 0.402–0.948, p = 0.003). Overall correlation was higher in systole than diastole, with PCD-CT-systolic-epicardial-ECV demonstrating the strongest correlation across all phases and layers. The mean difference between PCD-CT-systolic-epicardial-ECV and CMR-ECV was –1.856% (95% LoA: –7.068 to 3.355).
**Conclusion:** PCD-CT-derived ECV measurements show strong agreement with CMR,particularly when acquired during the systolic phase at the epicardial layer, supporting its potential as a clinically viable alternative for myocardial interstitial fibrosis.
**Limitations:** Small sample size.
**Funding for this study:** This study was supported by Gansu Province Health Industry Research Project (GSWSKY2023-05), Gansu Province Science and Technology Plan Project (24JRRA310).
**Has your study been approved by an ethics committee?** Yes
**Ethics committee - additional information:** The First Hospital of Lanzhou University
**Author Disclosures:**
Shu Nan Li: Nothing to disclose
Gang Wang: Nothing to disclose
Na Yang: Nothing to disclose
Wei Tian: Nothing to disclose
Bobin Zhu: Nothing to disclose
Zixian Chen: Nothing to disclose
Jiang Nan: Nothing to disclose
Xi Tong: Nothing to disclose

**Inferolateral LGE: additional clinical significance of mitral annular disjunction (MAD) in arrhythmogenesis compared with non-MAD disease**
*V. Vespucci*, D. Boccetti, S. Ciambellini, F. Carusi, C. Confalone, S. Di Terlizzi, P. Palumbo, E. Di Cesare; L'Aquila/IT
*(vespucci.vanessa@hotmail.com)*

**Purpose:** Mitral annular disjunction (MAD) is recognized as a potentially arrhythmogenic substrate, yet its clinical impact on disease progression and outcomes remains uncertain. Cardiac magnetic resonance (CMR) with late gadolinium enhancement (LGE) may help refine individual risk stratification in these patients. The objective of this study was to investigate the prognostic role of CMR findings in patients with MAD compared with individuals presenting a similar non-ischemic LGE phenotype but without MAD.
**Methods or Background:** In this retrospective-prospective study, 82 patients were enrolled and classified into three groups: MAD+/LGE- (n=26), MAD+/LGE+ (n=21), and MAD-/LGE+ (n=35, control group). The primary endpoint was the occurrence of major adverse cardiac events (MACE), defined as sudden cardiac death (SCD), uncontrolled ventricular arrhythmias (NCVA), catheter ablation or implantation of an implantable cardioverter defibrillator (ICD) or pacemaker (PMK).
**Results or Findings:** During a mean follow-up of 1.8 ± 1.1 years, 22 patients (32%) experienced MACE, with a comparable incidence across the three groups. Arrhythmic burden did not differ significantly among groups. Neither MAD presence/absence, MAD severity (MADmax, p=0.175), nor LGE extent (p=0.387) predicted adverse outcomes. However, MAD-/LGE+ patients showed lower GLS values, consistent with subclinical systolic dysfunction and a higher short- to mid-term MACE risk compared with MAD+/LGE+ patients (OR 4.463, 95% CI 1.506–13.230; p=0.007).
**Conclusion:** MAD should be regarded as a potential risk factor for arrhythmias and adverse cardiac events. Nonetheless, its prognostic weight appears lower than that of non-MAD disease with a comparable LGE phenotype.

**Limitations:** Study limitations include the small sample size, the retrospective–prospective design, and the absence of T1 mapping. As reported in previous studies, T1 mapping and extracellular volume (ECV) assessment could provide further insights into interstitial fibrosis and risk stratification in MAD.
**Funding for this study:** No fund
**Has your study been approved by an ethics committee?** Not applicable
**Author Disclosures:**
Carlo Confalone: Nothing to disclose
Vanessa Vespucci: Nothing to disclose
Sandro Di Terlizzi: Nothing to disclose
Danilo Boccetti: Nothing to disclose
Pierpaolo Palumbo: Nothing to disclose
Ernesto Di Cesare: Nothing to disclose
Francesca Carusi: Nothing to disclose
Silvia Ciambellini: Nothing to disclose

**Right Atrial and Right Ventricular Strain in Dilated Cardiomyopathy vs. Healthy Population Controls: A Comparative CMR Analysis from Two Large Population Cohorts**
*T. Geyer*, P. Franke, B. J. Wintersperger, R. Lorbeer; Munich/DE
*(thomas.geyer@med.uni-muenchen.de)*

**Purpose:** Cardiovascular magnetic resonance (CMR) strain imaging is increasingly applied for functional characterization of dilated cardiomyopathy (DCM). While left-sided strain has been extensively described, large-scale reference data for right atrial and ventricular strain in healthy populations remain limited, complicating clinical interpretation. Our objective was to establish normal RA and RV strain values in cardiovascular (CV) disease–free individuals and compare them with patients with DCM from a population-based registry using harmonized imaging and post-processing protocols.
**Methods or Background:** We studied 162 patients with non-ischemic DCM from the German Centre for Cardiovascular Research (DZHK) nationwide multicenter databank with standardized clinical and imaging protocols. Normal reference data were derived from 400 CV disease–free participants of the population-based KORA (Cooperative Health Research in Augsburg) MRI sub-study. All underwent 1.5T or 3T CMR. RA reservoir strain (RA-RS), RA longitudinal strain (RA-LS), RV radial strain (RV-RS), RV longitudinal strain (RV-LS), and RV circumferential strain (RV-CS) were quantified using feature tracking (cvi42, Circle CVI). Group differences were assessed by multivariable linear regression adjusted for age and sex.
**Results or Findings:** In controls, mean values were RA-RS 50.5%, RA-LS 20.0%, RV-RS 24.9%, RV-LS 21.6%, and RV-CS 14.3%. Patients with DCM showed significant reductions in all parameters (all $p<0.001$): RA-RS 40.8% (−9.7), RA-LS 15.4% (−4.6), RV-RS 16.1% (−8.8), RV-LS 17.8% (−3.8), and RV-CS 9.2% (−5.1). The largest adjusted differences were observed for RA-RS (β=−9.7%; 95%CI −13.5 to −4.7) and RV-RS (β=−8.8%; 95%CI −10.3 to −6.2).
**Conclusion:** This study provides robust, population-based reference values for right heart strain and demonstrates the utility of CMR-derived strain for detecting early dysfunction in DCM. Such reference values may improve diagnostic precision and support clinical decision-making.
**Limitations:** Limitations include the cross-sectional design and lack of longitudinal outcome data, which may restrict prognostic interpretation.
**Funding for this study:** None.
**Has your study been approved by an ethics committee?** Yes
**Ethics committee - additional information:** The study was approved by the ethics committee of the Bavarian Chamber of Physicians and the ethics committee of the Ludwig-Maximilians-University Munich and complies with the Declaration of Helsinki.
**Author Disclosures:**
Philipp Franke: Nothing to disclose
Roberto Lorbeer: Nothing to disclose
Thomas Geyer: Nothing to disclose
Bernd J. Wintersperger: Nothing to disclose

**Cardiac magnetic resonance evaluation of Mavacamten therapy in patients with severe hypertrophic obstructive cardiomyopathy: preliminary results**
*M. Dodaro*, L. Conia, G. Marchionni, M. Alfarano, C. Chimenti, C. Catalano, N. Galea; Rome/IT
*(martina.dodaro@uniroma1.it)*

**Purpose:** Hypertrophic obstructive cardiomyopathy (HOCM), characterized by left ventricular (LV) hypertrophy and systolic anterior motion (SAM) of the mitral valve, remains a clinically and therapeutically challenging disease. Mavacamten, a selective cardiac myosin inhibitor, has shown clinical and functional benefits, but its structural effects assessed by cardiac magnetic resonance (CMR) imaging are not fully defined. This study explored structural and functional changes after Mavacamten therapy using advanced CMR techniques.
**Methods or Background:** Twelve patients with severe HOCM and SAM were prospectively enrolled and received Mavacamten for at least 17 weeks. CMR was performed at 3T, before and after therapy, including cine balanced steady-state free precession, pre- and post-contrast Modified Look-Locker Inversion Recovery, and T1-weighted inversion recovery imaging for late gadolinium enhancement (LGE). Measurements included ventricular wall thickness, LV and right ventricle (RV) volumes, LGE, global/segmental native T1 and extracellular volume fraction (ECV). Statistical analysis was performed with paired t-tests, with significance set at $p\leq0.05$.
**Results or Findings:** After an average follow-up of 31±5.6 weeks, 9 of 12 patients demonstrated resolution of LV outflow tract obstruction and disappearance of SAM. Significant decreases in wall thickness were observed in septal and lateral basal-ventricular segments (p=0.04 and p=0.03), as well as in anterior and septal mid-ventricular walls (p=0.05 and p=0.03). RV anterior and inferior wall thickness also declined (p=0.004 and p=0.02). Significant reductions in indexed LV mass (p=0.004), RV stroke volume (p=0.004) and RV ejection fraction (p=0.01) were observed, although RV values ramained normal. No significant changes were seen in native T1, ECV, or LGE areas.
**Conclusion:** These preliminary data suggest that Mavacamten could induce early favorable reverse remodeling in patients with severe HOCM. Larger cohorts are required to better define myocardial tissue effects and remodeling.
**Limitations:** Monocentric study; small population; short-time follow-up
**Funding for this study:** None
**Has your study been approved by an ethics committee?** Not applicable
**Author Disclosures:**
Martina Dodaro: Nothing to disclose
Cristina Chimenti: Nothing to disclose
Giulia Marchionni: Nothing to disclose
Maria Alfarano: Nothing to disclose
Luca Conia: Nothing to disclose
Nicola Galea: Nothing to disclose
Carlo Catalano: Nothing to disclose

**15:00 - 16:00** **Research Stage 5**

### Research Presentation Session: Artificial Intelligence and Imaging Informatics

### RPS 505

### The segmentation evolution continues: innovative solutions and applications for today's quantitative imaging

**Moderator**
Andreas Stefan Brendlin; Tübingen/DE

**A Hospital-Integrated Human-in-the-Loop Platform for Reproducible Musculoskeletal Segmentation and AI Deployment**
*J. Garcia-Henao*, N. Bünger, B. Herzog, S. Caprara; Zurich/CH
*(john.garciahenao@balgrist.ch)*

**Purpose:** To evaluate the Medical Imaging Research Orchestration (MIRO) platform for integrating human-in-the-loop (HITL) annotation and AI-assisted segmentation within hospital infrastructure, with a focus on improving reproducibility and collaboration in musculoskeletal imaging.
**Methods or Background:** The development of accurate AI-assisted segmentation models in orthopaedics requires standardized datasets, clinical validation, and reproducible workflows. The MIRO platform was developed within the MedTwins Agil.IT Trusted Research Environment (TRE) to connect hospital PACS systems with secure research storage and high-performance computing resources. This study used the Spine Segmentation Dataset, which includes 37 cadaveric CT scans acquired at the Balgrist Research Center and the Swiss Center for Musculoskeletal Imaging. The dataset was designed to be accessible to the scientific community and serves as a benchmark for reproducible spine segmentation. Three segmentation models, TotalSegmentator, MedSAM2, and SegmentAnyBone, were evaluated using the MIRO platform. Radiologists and surgeons accessed the images and segmentations through the MIRO web interface, enabling direct visualization, refinement, and comparison. Segmentation accuracy was measured using Dice similarity, 95th percentile Hausdorff distance, and surface Dice coefficients.

**Results or Findings:** TotalSegmentator provided the most consistent delineation of vertebrae. MedSAM2 achieved high performance with guided prompts, and SegmentAnyBone produced detailed segmentation in complex regions. Integration within MIRO allowed efficient dataset management, traceability of annotations, and reproducible comparison across annotators and models.
**Conclusion:** The MIRO platform enables secure, collaborative, and reproducible segmentation research for musculoskeletal imaging within hospital environments.
**Limitations:** Preliminary findings are based on early-stage evaluations; large-scale multi-institutional validation is ongoing.
**Funding for this study:** This study was supported by the Digitalization Initiative of the Zurich Higher Education Institutions (DIZH) under the project MedTwins Agil.IT (2024–2026).
**Has your study been approved by an ethics committee?** Not applicable
**Author Disclosures:**
John Garcia-Henao: Nothing to disclose
Sebastiano Caprara: Nothing to disclose
Nicholas Bünger: Nothing to disclose
Benedikt Herzog: Nothing to disclose

**Zero-shot performance of promptable medical images segmentation model**
*A. Jaiswal*, F. Meyer, L. Oberlinkels, R-U. Müller, N. Große Hokamp, L. Caldeira, T. Persigehl; Cologne/DE

**Purpose:** Robust segmentation of structures of interest such as organs, tumors is crucial for clinical applications including diagnosis, treatment planning, and disease monitoring. In this work, we performed evaluation of two public medical image segmentation models.
**Methods or Background:** We collected two datasets (DS1, DS2) from University Hospital Cologne. DS1 includes 102 3D MR-scans of 50 autosomal dominant polycystic kidney disease patients. DS2 consists of 40 MR-scans from 40 prostate cancer patients. We evaluated the performance of TotalSegmentator MRI (TS)[1] and prompt based model nnInteractive (NI)[2] for polycystic kidney segmentation in DS1 and prostate segmentation in DS2. NI was evaluated using a single interaction via a randomly inflated 2D bounding box around each kidney and around the prostate. Dice scores and Wilcoxon Signed-Rank Test were used to compare the performance.
**Results or Findings:** For kidney segmentation, TS resulted in dice score of 0.32±0.28 (0.03, 0.90). NI outperformed with a dice of 0.78±0.17 (0.09, 0.97)(p<.001). Polycystic kidneys are deformed due to multiple cysts and have different appearance compared to normal kidneys. Though nnInteractive was not trained on polycystic kidney data, it captured previously unseen objects well even with single interaction. The prostate region often did not have clear boundaries, likely confusing the NI model resulting in dice of 0.80±0.14 (0.31, 0.92). For prostate segmentation, TS outperformed NI with a dice of 0.85±0.03 (0.74, 0.91)(p<.001).
**Conclusion:** NI generalizes well on the new tasks and allows generating high quality segmentations with one or few interactions.
**Limitations:** In future, we will test NI on different datasets and with multiple interactions including different prompts. References: [1] D'Antonoli et. al., "Totalsegmentator mri: Robust sequence-independent segmentation of multiple anatomic structures in mri." Radiology 2025. [2] Isensee et. al., "nninteractive: Redefining 3d promptable segmentation." arXiv preprint 2025.
**Funding for this study:** This work has been supported by RACOON „NUM 2.0“(FKZ: 01KX2121).
**Has your study been approved by an ethics committee?** Yes
**Ethics committee - additional information:** The approval numbers are 15-323-retro, 23-1193-retro.
**Author Disclosures:**
Nils Große Hokamp: Nothing to disclose
Liliana Caldeira: Nothing to disclose
Astha Jaiswal: Nothing to disclose
Roman-Ulrich Müller: Nothing to disclose
Laura Oberlinkels: Nothing to disclose
Thorsten Persigehl: Nothing to disclose
Franziska Meyer: Nothing to disclose

**Federated Learning for Brain Tumor Segmentation: Privacy-Preserving Multi-Institutional AI Model Development**
*E. Abdullayev*; Baku/AZ
*(elshan.ae@gmail.com)*

**Purpose:** To develop and validate a federated learning approach for automated brain tumor segmentation in MRI that maintains data privacy while leveraging multi-institutional datasets for improved model performance.
**Methods or Background:** This retrospective multi-center study implemented a federated learning framework across five institutions using 3,420 brain MRI scans (T2-weighted, FLAIR, T1-Gd) from patients with glioblastoma, meningioma, and metastatic lesions collected between 2019 and 2024. A 3D U-Net architecture was trained using a federated averaging algorithm, where each institution trained locally without sharing raw data. Ground truth segmentations were established by consensus of two neuroradiologists. Model performance was compared against centralized learning and single-institution models using Dice similarity coefficient (DSC), Hausdorff distance (HD95), and sensitivity/specificity metrics. Statistical significance was assessed using Wilcoxon signed-rank tests.
**Results or Findings:** The federated learning model achieved a mean DSC of 0.763 ± 0.124 for the whole tumor, 0.698 ± 0.156 for the tumor core, and 0.612 ± 0.189 for the enhancing tumor regions. Compared to single-institution models, federated learning showed moderate improvement in DSC (0.763 vs 0.721, p=0.023) and HD95 (8.4mm vs 11.2mm, p=0.041). Performance was slightly lower than centralized learning (DSC: 0.763 vs 0.791, p=0.031) but maintained complete data privacy. The model showed variable performance across different institutions (DSC range: 0.698-0.812), with challenges in generalizing different MRI protocols. Training convergence required 15% more iterations compared to centralized approaches due to data heterogeneity.
**Conclusion:** Federated learning enables the development of brain tumor segmentation models while preserving patient data privacy, though with some performance trade-offs compared to centralized approaches. Despite challenges with data heterogeneity across institutions, this method shows promise for collaborative AI development in medical imaging.
**Limitations:** Single time-point analysis, heterogeneous scanner types, absence of an external validation cohort, and limited tumor subtype representation.
**Funding for this study:** No funding received for this study.
**Has your study been approved by an ethics committee?** Not applicable
**Author Disclosures:**
Elshan Abdullayev: Nothing to disclose

**Multimodal learning for automated segmentation and preoperative risk stratification of endometrial cancer via multi-sequence MRI**
*X. Nie*[1], G. Liu[2], Y. Dong[2], X. Wang[1]; [1]Hong Kong/HK, [2]Shenyang/CN
*(xpnie@surgery.cuhk.edu.hk)*

**Purpose:** Risk stratification in endometrial cancer (EC) is essential for treatment planning, but is currently determined by postoperative pathology. This study aims to develop and validate a multimodal learning model for non-invasive, preoperative risk prediction in EC patients using multi-sequence MRI, thereby enabling more individualized surgical and adjuvant treatment strategies.
**Methods or Background:** This multicenter retrospective study included 623 paired axial contrast-enhanced T1-weighted (CE-T1W) and fat-suppressed T2-weighted (FS-T2W) MRI scans obtained from EC patients across three sources. A two-stage multimodal learning framework was designed: in Stage I, the model was pretrained for 3D tumor segmentation and radiomics feature extraction; in Stage II, the model was further finetuned to integrate traditional radiomics features and multiscale deep learning image features for risk stratification into low-, intermediate-, and high-risk groups according to the ESGO/ESMO/ESP 2020 guidelines. Experiments on multicenter cohorts evaluated tumor segmentation and risk stratification with standard metrics.
**Results or Findings:** The proposed AI model outperformed the widely used nnUNet in 3D tumor segmentation, achieving Dice scores of 0.753 ± 0.149 on CE-T1W MRI and 0.764 ± 0.129 on FS-T2W MRI in the internal validation cohort, and maintained high accuracy in two external validation cohorts. For preoperative risk stratification, the model achieved an AUC of 0.801 (95% CI: 0.712-0.880) in the internal validation cohort, with AUCs of 0.852, 0.746, and 0.799 for the high-, intermediate-, and low-risk groups, respectively. Moreover, the performance observed in external validation cohorts (AUCs of 0.740 and 0.724) demonstrated the model's robust generalizability for risk stratification.
**Conclusion:** Our results highlight the model's potential to support preoperative risk stratification in EC patients, particularly by accurately identifying high-risk groups. This may aid preoperative clinical decision-making and ultimately improve patient outcomes.
**Limitations:** Not applicable
**Funding for this study:** Not applicable
**Has your study been approved by an ethics committee?** Yes
**Ethics committee - additional information:** The ethics committee notification can be found under the number XJS202506107.
**Author Disclosures:**
Yue Dong: Nothing to disclose
Guanyu Liu: Nothing to disclose
Xiuping Nie: Nothing to disclose
Xin Wang: Nothing to disclose

**Deep Learning-Based MRI Segmentation of the Uterus for Early Adenomyosis Detection Across Menstruation and Ovulation**

*C. Tappermann*[1], M. S. May[2], L. Siegler[2], T. Rüttinger[2], M. Fenske[2], M. B. Bauer[2], L. Kratzsch[2], S. Arndt[2], B. Lassen-Schmidt[1]; [1]Bremen/DE, [2]Erlangen/DE

**Purpose:** The RACOON FADEN project investigates MRI-based uterine biomarkers for early adenomyosis detection, a gynaecological condition in which endometrial tissue grows into the muscular wall of the uterus, often associated with uterine enlargement, pelvic pain, and infertility. This research includes volumetric segmentation of the myometrium (MM), junctional zone (JZ), and endometrium (EM) during menstruation and ovulation. Within the project, tailored deep learning models are trained to partially automate this process. Their performance should be assessed for both phases.
**Methods or Background:** The test dataset includes 16 females from six German university hospitals. Reference segmentations were created by medical students and reviewed by radiologists using a CuraMate workflow based on predefined guidelines. MM, JZ, and EM were segmented up to the cervical junction on T2-weighted short-axis uterine images using motion-insensitive, multi-shot TSE BLADE sequences. Models were trained iteratively in three rounds on additional training data, incorporating more data each time (32/98/122 samples, comprising both menstruation and ovulation phases).
**Results or Findings:** Model performance was measured with the Dice Similarity Coefficient (DSC), resulting in DSC 0.71 (MM), 0.68 (JZ), 0.63 (EM) during menstruation and DSC 0.66 (MM), 0.65 (JZ), 0.74 (EM) during ovulation for model 1. Model 2 achieved DSC 0.76 (MM), 0.74 (JZ), 0.75 (EM) during menstruation and DSC 0.74 (MM), 0.77 (JZ), 0.87 (EM) during ovulation. Model 3 reached DSC 0.73 (MM), 0.70 (JZ), 0.75 (EM) during menstruation and DSC 0.72 (MM), 0.73 (JZ), 0.84 (EM) during ovulation. No significant differences were observed between paired DSC values for menstruation and ovulation (Wilcoxon signed-rank test; all $p > 0.1$).
**Conclusion:** Our deep learning models reliably segment MM, JZ, and EM on T2-weighted MRI during both phases, with no significant performance differences, supporting automated assessment of early adenomyosis.
**Limitations:** No Limitations.
**Funding for this study:** Funding was provided by the Bundesministerium für Bildung und Forschung via Netzwerk Universitätsmedizin (NUM 2.0, FKZ: 01KX2121).
**Has your study been approved by an ethics committee?** Yes
**Ethics committee - additional information:** The RACOON FADEN study was approved by the ethics committee of all participating university hospitals. The study protocol complies with the declaration of Helsinki.
**Author Disclosures:**
Marlene Bettina Bauer: Nothing to disclose
Lieselotte Kratzsch: Nothing to disclose
Sebastian Arndt: Nothing to disclose
Lisa Siegler: Nothing to disclose
Matthias Stefan May: Nothing to disclose
Bianca Lassen-Schmidt: Nothing to disclose
Chiara Tappermann: Nothing to disclose
Theresa Rüttinger: Nothing to disclose
Martin Fenske: Nothing to disclose

**MRIvals - TotalSegmentator vs MRSegmentator. Validation of major organs segmentation in abdominal scans. A comparative study**

*G. Lappas*[1], A. Afentouli[1], N. Patlakas[1], P. Giannikopoulos[1], M. Triantafyllou[2], G. I. Kalaitzakis[2], M. Klontzas[2], K. Petropoulos[1]; [1]Athens/GR, [2]Heraklion/GR
*(glappas@iknowhealth.com)*

**Purpose:** This study aims to validate and compare the open-source TotalSegmentator 3mm (TS3MR) and MRSegmentator (MRS) models for the segmentation of major abdominal organs in Magnetic Resonance (MR) scans.
**Methods or Background:** The models' segmentation capability was quantified using Dice Similarity Coefficient (DSC) and Normalized Surface Distance (NSD) across four heterogeneous datasets (N≈1600 individuals & 100 scanners). Exploratory Data Analysis, including basic statistics, was performed to investigate the suitability of the datasets for benchmarking. Additionally, explainable AI (i.e., Grad-CAM maps) was utilized focusing on disentangling the black box of the model decision-making. Clinical rating of the predicted segmentations' quality was conducted by one assistant professor of radiology, and one senior radiology resident from Greek hospital scans (N=10 patients).
**Results or Findings:** Overall, despite the high data variability, projected in normalized volume (0.44±0.21) and intensity (0.36±0.22), both MR models can accurately segment most abdominal organs, e.g., average DSC > 90%. MRS was more accurate across all organs compared to TS3MR as DSC scores were boosted from 4% to 28% depending on sequence, pathology and anatomical region. Poorer and less robust performance was found for the gallbladder, and pancreas with an average DSC ≈ 63% for both models. Grad-CAM maps revealed the models' inconsistencies. The clinical evaluation findings showed high-quality and clinical acceptable segmentations for both models while fine-grained details were generated by MRS.
**Conclusion:** Both TotalSegmentator 3mm and MRSegmentator models can accurately segment most of the major abdominal organs in MR scans while the latter is more accurate across all organs and modalities.
**Limitations:** The available MR data presents lower variability (for scanning protocols and demographics) for kidneys, spleen and gallbladder. Segmentation of lower abdomen organs such as prostate is not supported by MRSegmentator even though routine examined.
**Funding for this study:** None
**Has your study been approved by an ethics committee?** Not applicable
**Author Disclosures:**
Michail Klontzas: Nothing to disclose
Konstantinos Petropoulos: Board Member: CTO and member of BoD at IKnowHealth S.A.
Aikaterini Afentouli: Employee: AI/ML engineer at IKnowHealth S.A.
Nektarios Patlakas: Employee: AI/ML engineer at IKnowHealth S.A.
Georgios Ioannis Kalaitzakis: Nothing to disclose
Petros Giannikopoulos: Employee: Software engineer at IKnowHealth S.A.
Georgios Lappas: Employee: AI/ML engineer at IKnowHealth S.A.
Matthaios Triantafyllou: Nothing to disclose

**Deep Learning (DL)-Based Detection and Segmentation of Acute Pulmonary Thromboembolism (PTE) in Computed Tomography Pulmonary Angiography (CTPA): A Clinical Validation Study**

M. A. Kamar[1], M. M. Baris[2], E. Konukoglu[3], *E. Cinar*[2], N. Cakir[2], T. Yonka[2], A. Ozgen Alpaydin[2], N. S. Gezer[2]; [1]İzmir/TR, [2]Izmir/TR, [3]Zurich/CH
*(cinarekin98@gmail.com)*

**Purpose:** To clinically validate a DL model for fully automated detection and segmentation of acute PTE on CTPA, and to assess the impact of imaging artifacts and contrast enhancement quality on model performance.
**Methods or Background:** A total of 530 CTPA examinations (157 PTE-positive, 373 PTE-negative) were retrospectively evaluated. Emboli were manually segmented slice-by-slice by experienced radiologist to establish the ground truth. Each CTPA scan is classified by the DL model as positive or negative; then voxel-level segmentation of emboli across all slices in a fully automated end-to-end manner is performed for positive scans. The model's performance in embolus detection was evaluated. Subgroup analysis classified emboli from the main pulmonary artery to lobar branches as central, and segmental to subsegmental emboli as peripheral. False positive(FP) and false negative(FN) cases were analyzed for image artifacts and pulmonary artery attenuation (HU). Embolus volume analysis was also performed.
**Results or Findings:** The AI model achieved overall sensitivity of 91.7%, specificity of 92.0%, precision of 82.8%, and accuracy of 91.9%. In peripheral emboli cases (n=57), the model showed 84.2% sensitivity. In central emboli (n=100), sensitivity reached 96.0%. Among 13 FN cases, 10 were associated with technical limitations including low contrast density in the main pulmonary artery (<250HU), streak artifacts, motion artifacts, and low tube current. Similarly, 22 of 30 FP cases were linked to similar artifacts, particularly vena cava streaks and suboptimal contrast timing. Only 3 FN and 8 FP cases occurred without any identifiable artifact.
**Conclusion:** The DL model demonstrated high performance especially for centrally located emboli. Image artifacts and low pulmonary artery contrast enhancement significantly contributed to false results, highlighting the importance of standardized acquisition protocols for reliable AI-assisted diagnosis.
**Limitations:** The model's volumetric segmentation errors were primarily in small-volume thrombi.
**Funding for this study:** None
**Has your study been approved by an ethics committee?** Yes
**Ethics committee - additional information:** This study was approved by the Non-Interventional Research Ethics Committee of Dokuz Eylül University Hospital
**Author Disclosures:**
Ender Konukoglu: Consultant: Provided scientific consultancy for the TUBITAK-supported R&D project in which the product was developed
Ekin Cinar: Nothing to disclose
Mustafa Mahmut Baris: Nothing to disclose
Tayfun Yonka: Employee: Machine learning engineer of the company who contributed to the development of pulmonary embolism detection model
Nurullah Cakir: Nothing to disclose
Naciye Sinem Gezer: Consultant: Provided scientific consultancy for the TUBITAK-supported R&D project in which the product was developed
Aylin Ozgen Alpaydin: Nothing to disclose
Mehmet Akif Kamar: Nothing to disclose

**Autosegmentation of Tumor-Bearing Bladders on CT: A Comparison of U-Net Variants and an Ensemble Model**

*L. Chen*[1], E. Guo[1], Z. Wu[2], Z. Jin[1], G. Zhang[1], H. Xue[1], H. Sun[1]; [1]Beijing/CN, [2]Fushun/CN
*(li.cherish@outlook.com)*

**Purpose:** To evaluate and compare the performance of 2D U-Net, 3D U-Net, Dual Swin Transformer U-Net (DS-TransUNet), and an Ensemble Segmentation Model (ESM) for automated segmentation of tumor-bearing bladders on CT, a critical task in computer-aided diagnosis of bladder cancer (BCa).
**Methods or Background:** This retrospective study included 435 BCa patients (397 internal, 38 external) with manual segmentation as ground truth. Models were trained and validated through five-fold cross-validation within the nnU-Net framework. The ESM was constructed by aggregating predictions from three individual models using majority voting. Performance metrics included the Dice similarity coefficient (DSC), average surface distance, and 95th-percentile Hausdorff distance, with subgroup analyses by gender and muscle invasion status (muscle-invasive BCa / non-muscle-invasive BCa).
**Results or Findings:** The ESM achieved the highest DSC in both internal (0.980±0.024) and external (0.986±0.014) cohorts, significantly outperforming DS-TransUNet ($P < 0.001$) and 3D U-Net in internal validation ($P = 0.047$), and again outperformed DS-TransUNet ($P < 0.001$) and showed a marginally significant advantage over 2D U-Net ($P = 0.045$) in external validation. 3D U-Net and 2D U-Net also showed strong performance, particularly in external validation, while the DS-TransUNet model had the worst relative segmentation efficiency. Subgroup analyses confirmed stable performance of ESM across gender and tumor invasion categories.
**Conclusion:** The 3D U-Net excels in segmentation accuracy, while the 2D U-Net is efficient and consistent. DS-TransUNet, though promising, needs refinement for robustness. The ESM model, integrating advantages from multiple U-Net-based architectures, demonstrated optimal accuracy and robustness, highlighting its potential clinical utility.
**Limitations:** First, the relatively small external validation cohort potentially limits broader applicability. Additionally, cases with significant imaging artifacts were excluded, potentially affecting real-world generalizability.
**Funding for this study:** This work was supported by Peking Union Medical College Hospital Talent Cultivation Program (Category D) [grant number UHB11588]; National High- Level Hospital Clinical Research Funding [grant numbers, 2022-PUMCH-A-035, 2022-PUMCH-B-069, 2022-PUMCH-A-033, and 2022-PUMCH-B-068]; the Beijing Municipal Natural Science Foundation [grant number L232133] and the CAMS Innovation Fund for Medical Sciences (CIFMS) [grant number 2024-I2M-C&T-C-004].
**Has your study been approved by an ethics committee?** Yes
**Ethics committee - additional information:** The approval of the Institutional Review Board of the affiliated hospital of Peking Union Medical College has been obtained.
**Author Disclosures:**
Erjia Guo: Nothing to disclose
Zhe Wu: Nothing to disclose
Gumuyang Zhang: Nothing to disclose
Hao Sun: Nothing to disclose
Li Chen: Nothing to disclose
Zhengyu Jin: Nothing to disclose
Huadan Xue: Nothing to disclose

**15:00 - 16:00 Research Stage 3**

### Research Presentation Session: Head and Neck

### RPS 508

### New developments in imaging of the oral cavity and the oropharynx

**Moderator**
Carlos Buitrago Tellez; Solothurn/CH
*(carlos.buitrago@spital.so.ch)*

**Oral Cavity Lesion Mimicker: How prevalent is the Mylohyoid Boutonnière on MRI?**

*A. R. J. Péporté*[1], J. Kostova[1], F. B. Schön[2], G. Andreisek[2], L. Diem[3], F. Wagner[4]; [1]Frauenfeld/CH, [2]Münsterlingen/CH, [3]Lucerne/CH, [4]Aarau/CH
*(peporte_anne@hotmail.com)*

**Purpose:** The mylohyoid muscle is commonly considered a continuous muscular sling, but it is frequently discontinuous, forming mylohyoid boutonnières (MHB) that can contain salivary tissue, fat, blood vessels, or lymph nodes. The MR prevalence remains largely unexplored. This study assesses the prevalence, age- dependence, anatomical distribution, and content of MHBs on MRI.
**Methods or Background:** A retrospective review of MRI scans of the head and neck from 294 patients between 2016 and 2020 was conducted. MR images were analyzed for the presence, location, and contents of MHBs. Inter-rater agreement from the three independent readers with different levels of experience and statistical analysis were performed to assess consistency across readers.
**Results or Findings:** MHBs were identified in 50.7% of individuals, with bilateral deficiencies occurring in 45.6% of cases. Most defects were located in the anterior (45.6%) and middle (51.0%) thirds of the mylohyoid muscle. The herniated content consisted predominantly of salivary tissue (69.3%), followed by fat (15.9%), and blood vessels (14.2%). No significant association was found between MHB prevalence and age. There was a high level of inter-rater agreement among all three raters regarding the presence, side, location, and content of the MHB, with no statistically significant discrepancies observed across the assessed parameters.
**Conclusion:** A MHB is a true and common anatomical variant, with a prevalence of 50.7% in this study, and most defects are bilateral. This knowledge aids in preventing diagnostic errors when evaluating oral cavity lesions, minimizing the need for unnecessary invasive procedures.
**Limitations:** This retrospective study, while involving a heterogeneous referral population and excluding about 20% of cases due to MRI artifacts, benefits from a large sample size, multi-reader interpretation, and provides valuable non-invasive anatomical assessment complementary to cadaveric data.
**Funding for this study:** No funding was received.
**Has your study been approved by an ethics committee?** Yes
**Ethics committee - additional information:** Institutional board approval was obtained (Req-2024-01600; EKOS 24/237) from the Eastern Swiss Ethics Committee.
**Author Disclosures:**
Joana Kostova: Nothing to disclose
Franca Wagner: Nothing to disclose
Anne Renée Juliette Péporté: Nothing to disclose
Gustav Andreisek: Nothing to disclose
Fabian Bernhard Schön: Nothing to disclose
Lara Diem: Nothing to disclose

**Gingival ultrasonography in evaluation of post-operative healing in post collagen tissue graft for gingival recession - A novel cohort study in a tertiary care center and teaching hospital**

*R. B. Venkatasubramanian*, R. Sivakumar, V. Sai, R. Ramachandran, B. Dev, R. Rangasami, M. Lokesh, H. Sivakumar; Chennai/IN
*(vrajeshwarbalaji@gmail.com)*

**Purpose:** To set gingival ultrasonography protocols as a screening tool to evaluate post operative healing in collage tissue graft patients for gingival recession.
**Methods or Background:** Gingival recession is a common condition when gingival margin moves proximally beyond cemento-enamel junction. It can increase risk of dental carries and have cosmetic issues. Root coverage procedures like autologous collagen tissue graft are a common surgery for gingival recession. A cohort of 15 patients of gingival recession planned for autologous collagen tissue graft, aged 30-50, were sonographically assessed pre-operatively, 1 and 2 weeks post-op for healing assessment. Anterior gingival thickness and vascularity(spectral Doppler) were assessed pre-op. Surgical site echogenicity, neovascularisation status(Power and spectral Doppler) and anterior gingival thickness were assessed on post-op follow up. Protocols were set to assess anterior gingival thickness from anterior gingival margin to interdentine papilla at incisor plane, low PRF for Doppler evaluation.
**Results or Findings:** All 15 patients(100%) had decreased mean anterior gingival thickness - 1.3+/-0.3 mm, demostrating high resistance flow (RI>0.7) in Doppler. Post surgery, mean anterior gingival thickness was - 1.6+/-0.4 mm in week 1 and 1.5+/-0.2 mm in week 2. 14 out of 15 patients (93.3%) showed increased relative echogenicity on both follow up scans signifying inflammation. One patient had ~0.5 cc persistent collection in surgical site. Post operative Doppler showed low resistance flow (RI<0.6) with decreased PSV in all cases signifying inflammation/healing.
**Conclusion:** Post operative intraoral/gingival ultrasonography is an effective tool to assess inflammation, post-operative changes, abscess formation and neovascularisation in post autologous collagen tissue graft, in adjunct to clinical and visual assessment. Presence of hypoechoic areas, collections, high resistance vascular flow and decreased gingival thickness are markers of poor prognosis.
**Limitations:** Limited sample size can be expanded to cover other dental surgeries.
**Funding for this study:** Self funded.
**Has your study been approved by an ethics committee?** Yes
**Ethics committee - additional information:** Part of MD thesis dissertation.
**Author Disclosures:**
Rajalakshmi Sivakumar: Nothing to disclose
Venkata Sai: Nothing to disclose
Bhawna Dev: Nothing to disclose
Hariram Sivakumar: Nothing to disclose
Rajoo Ramachandran: Nothing to disclose
Murali Lokesh: Nothing to disclose
Rajeshwar Balaji Venkatasubramanian: Nothing to disclose
Rajeswaran Rangasami: Nothing to disclose

### Interobserver agreement of Neck Imaging Reporting and Data System (NI-RADS) in MRI-based surveillance of oropharyngeal squamous cell carcinoma

*M. Fusina*, M. Parillo, M. Neri, M. Antolini, D. Vo, F. De Rosa, C. C. Quattrocchi; Trento/IT
*(marianna.fusina@unitn.it)*

**Purpose:** To investigate the level of agreement among radiologists with varying levels of expertise when interpreting MRI scans using NI-RADS system for monitoring oropharyngeal squamous cell carcinoma (OPSCC) patients.
**Methods or Background:** An observational retrospective study was conducted to analyze follow-up MRIs in patients treated for OPSCC. NI-RADS scores were assigned by five radiologists (two residents in radiology, one general radiologist and two head and neck experts). Agreement was evaluated using percentage of agreement (POA) and kappa values (κ) for NI-RADS final score and for each MRI specific feature of the NI-RADS (lymph nodes, primary tumor size, primary site signal on T2-weighted, contrast-enhanced and diffusion-weighted images). Concordance was assessed separately for the first follow-up MRI and subsequent follow-up scans.
**Results or Findings:** A total of 91 patients were evaluated, and each reader analyzed 218 MRI. Overall inter-reader agreement was moderate for NI-RADS score (κ = 0.53, POA = 89%) and for individual MRI features (κ = 0.42–0.52, POA = 84%–93%). Agreement was notably lower between the expert radiologist and the two radiology residents during the first follow-up MRI, particularly for assessing contrast-enhancement at the primary site (κ = 0.25–0.36, POA = 77%–90%) and lymph nodes (κ = 0.38–0.41, POA = 72%–88%).
**Conclusion:** MRI NI-RADS in the surveillance of OPSCC patients shows moderate agreement among readers with varying expertise, especially in the evaluation of the first post-treatment scans. Routine use of NI-RADS in clinical practice and educational programs could improve uniformity in imaging assessments.
**Limitations:** We included examinations from different MRI scanners within a considerable timeframe. In a limited number of instances, DWI sequences were missing because standardized head and neck imaging protocols were not in place during earlier examinations.
**Funding for this study:** The authors declare that no funds, grants, or other support were received during the preparation of this manuscript.
**Has your study been approved by an ethics committee?** Yes
**Ethics committee - additional information:** The retrospective study was approved by the institutional review board and conducted in accordance with the principles of the Declaration of Helsinki. Informed consent to participate in the study was waived due to the retrospective-observational study design.
**Author Disclosures:**
Flora De Rosa: Nothing to disclose
Marinella Neri: Nothing to disclose
Monica Antolini: Nothing to disclose
Daniele Vo: Nothing to disclose
Carlo Cosimo Quattrocchi: Nothing to disclose
Marianna Fusina: Nothing to disclose
Marco Parillo: Nothing to disclose

### Refining Clinical T-Staging of Oral Tongue Squamous Cell Carcinoma with Structured MRI Reporting to Guide Neoadjuvant Chemoimmunotherapy

*J. Pan*, X. Hui, L-Z. Liu, W. Huang; Guangzhou/CN
*(panjie@sysucc.org.cn)*

**Purpose:** Current T-staging for oral tongue squamous cell carcinoma (OTSCC) primarily relies on postoperative pathology, which offers suboptimal preoperative risk stratification, limiting precision in the neoadjuvant therapy era. This study aimed to optimize the clinical T-staging for OTSCC using a structured MRI reporting system and evaluate its value in guiding patient selection for neoadjuvant chemoimmunotherapy (NACI).
**Methods or Background:** In this retrospective study, we analyzed patients with pathologically confirmed OTSCC who underwent pretreatment MRI between January 2010 and January 2025. Based on a structured MRI report, patients were stratified into four risk groups: "Very-low-risk" (no structural invasion), "Low-risk" (hyoglossus/styloglossus invasion), "Medium-risk" (Mild structural invasion: eg., lingual artery, sulcus terminalis, sublingual gland), and "High-risk" (Severe structural invasion: eg., mylohyoid, retromolar trigone, oropharynx). Network and survival analyses were employed to assess prognostic differences. The survival benefit of NACI was specifically evaluated in the high-risk group defined by the new staging system.
**Results or Findings:** Among 1202 enrolled patients, the optimized T-staging system showed significantly superior risk stratification for 5-year overall survival (OS) (48.1%, 74.8%, 85.9%, 95.1% for the four groups, P <0.001) versus the current T-staging (86.9%, 77.8%, 70.4%, 53.1%, P>0.05). Crucially, high-risk patients receiving NACI had markedly better 5-year OS than those who did not (84.2% vs. 45.9%, p<0.05). The revised T-staging system showed better stratification in OS (C-index: 0.712 vs. 0.652, p < 0.05).
**Conclusion:** A structured MRI-based T-staging system provides superior prognostic stratification and effectively identifies high-risk OTSCC patients who derive significant survival benefit from NACI, facilitating preoperative personalized treatment planning.
**Limitations:** This was a retrospective, single-center study. Additionally, there is inherent subjectivity in interpreting structural invasion, although excellent inter-observer agreement was confirmed (kappa > 0.82 for all evaluated structures).
**Funding for this study:** The National Natural Science Foundation of China (No. 82171906) and the Guangdong Basic and Applied Basic Research Foundation (2025A1515011590, 2024A1515140147).
**Has your study been approved by an ethics committee?** Yes
**Ethics committee - additional information:** This retrospective study was conducted in accordance with the ethical guidelines outlined in the 1964 Declaration of Helsinki and approved by the ethics committee (approval number: B2019-047-Y02; date of approval: November 07, 2022).
**Author Disclosures:**
Li-Zhi Liu: Nothing to disclose
Wenjie Huang: Nothing to disclose
Xie Hui: Nothing to disclose
Jie Pan: Nothing to disclose

### High-Resolution Intraoral Ultrasound for Depth of Invasion Assessment in Early Tongue Squamous Cell Carcinoma: Technical Refinements and Validation

*M. P. Belfiore*, M. Balzano, M. Montella, G. Colella, D. Setola, D. Di Stasio; Naples/IT
*(mariapaola.belfiore@unicampania.it)*

**Purpose:** To validate the accuracy and reproducibility of high-resolution intraoral ultrasound (ioUS) in measuring depth of invasion (DOI) in early-stage oral squamous cell carcinoma (OSCC) of the tongue and to address common methodological inconsistencies in current imaging practices.
**Methods or Background:** Thirty-five patients (21 males, 14 females; mean age 64.7 ± 10.3 years) with histologically confirmed OSCC of the tongue (dorsal, lateral, or ventral surfaces) and clinical tumor size ≤2 cm (T1–T2) were prospectively enrolled. ioUS imaging was performed using an 18 MHz linear probe in a non-contact technique. For each patient, three high-quality images were selected based on bilateral visualization of intact epithelial margins.

A horizontal DOI reference line was defined at the epithelium-connective tissue interface, and DOI was measured perpendicularly to the deepest hypoechoic area. Images with acoustic shadowing were excluded. DOI values were normalized to correct for tissue shrinkage during histopathological processing. Measurements were performed using ImageJ software. Statistical analysis included Spearman's correlation, intraclass correlation coefficient (ICC), and Mann-Whitney U test.
**Results or Findings:** ioUS demonstrated a strong correlation with pathological DOI (Spearman's $\rho = 0.93$; $p < 0.001$), and all lesions were correctly staged as T1 or T2, with 100% concordance with pathological staging. Normalized DOI data showed excellent reproducibility (ICC >0.90). No significant differences were found between ioUS and pathological DOI measurements ($p > 0.05$).
**Conclusion:** High-resolution ioUS provides accurate and reproducible measurements of DOI in early tongue OSCC, with staging performance equivalent to histopathological analysis. Technical refinements, including standardized image acquisition and definition of reference lines, are critical for clinical reliability.
**Limitations:** No limitations were identified.
**Funding for this study:** No funding was received for this study.
**Has your study been approved by an ethics committee?** Not applicable
**Author Disclosures:**
Maria Paola Belfiore: Nothing to disclose
Donato Setola: Nothing to disclose
Dario Di Stasio: Nothing to disclose
Giuseppe Colella: Nothing to disclose
Marco Montella: Nothing to disclose
Marialuisa Balzano: Nothing to disclose

**Preoperative prediction of perineural invasion in tongue squamous cell carcinoma with spectral detector CT-derived histogram parameters and clinical features**
*H. K. Zhang*[1], F. Chen[1], Y. Liao[2], X. Liu[2]; [1]Haikou/CN, [2]Guangzhou/CN
*(xiaomin.liu@philips.com)*

**Purpose:** This study aims to investigate the feasibility of preoperative prediction of perineural invasion (PNI) status in tongue squamous cell carcinoma (TSCC) using dual-layer spectral-detector CT(DLCT) histogram features and clinical features.
**Methods or Background:** We retrospectively analyzed 85 patients with TSCC, comprising 30 PNI-positive and 55 PNI-negative cases. All patients underwent preoperative contrast-enhanced DLCT, and spectral images (including virtual mono-energetic images at 40KeV, 70KeV, 100KeV, 130KeV, iodine density, effective atomic number, and virtual non-contrast images) were reconstructed from venous phase data. A single radiologist manually delineated the tumor region of interest (ROI) on 40-keV virtual mono-energetic images, which was propagated to other spectral images to extract seven histogram features from each image. Clinical and histogram features were compared using appropriate statistical tests (t-test, Wilcoxon, or chi-square). Feature selection involved Spearman correlation filtering (threshold = 0.9) followed by backward stepwise regression. Three predictive models—clinical, spectral-histogram, and a combined model—were developed using logistic regression. Model performance was evaluated and compared via receiver operating characteristic (ROC) analysis and DeLong's test.
**Results or Findings:** Pathological T-stage differed significantly between PNI-positive and negative groups ($P<0.05$). Most spectral histogram features also showed significant intergroup differences (all $P<0.05$). The clinical, spectral-histogram, and combined models achieved AUCs of 0.751 (0.653–0.849), 0.760 (0.655–0.865), and 0.828 (0.736–0.920), respectively. The spectral-histogram and combined models both significantly outperformed the clinical model ($P<0.05$).
**Conclusion:** The combined model integrating DLCT-derived spectral histogram features with clinical data demonstrates promising efficacy for the preoperative prediction of perineural invasion in tongue squamous cell carcinoma.
**Limitations:** Retrospective, single-center study with a small sample size
**Funding for this study:** Not applicable
**Has your study been approved by an ethics committee?** Yes
**Ethics committee - additional information:** The Ethics Committee granted a waiver of informed consent for this retrospective study.
**Author Disclosures:**
Xiaomin Liu: Nothing to disclose
Hang Kai Zhang: Nothing to disclose
Feng Chen: Nothing to disclose
Yuting Liao: Nothing to disclose

**Prevalence and Distribution of Anterior Disc Displacement of the Temporomandibular Joint: A Retrospective MRI Study in a Large German Cohort**
*M. L. Schnitzer*, F. Herr, L. Göbel, J. Rübenthaler; Munich/DE

**Purpose:** Craniomandibular disorders (CMD) are common musculoskeletal conditions, often linked to anterior disc displacement (ADD) of the temporomandibular joint (TMJ). MRI is the diagnostic standard of reference. This study assessed prevalence and demographic patterns of ADD in a large patient cohort.
**Methods or Background:** We retrospectively analyzed 512 TMJ MRI examinations (2017–2022, LMU Munich). ADD was graded: 1 (10–11 o'clock), 2 (9–10 o'clock), 3 (6–9 o'clock). Borderline cases were assigned to the higher grade.
**Results or Findings:** The cohort included 382 females and 130 males (mean age 40.9 ± 18.7 years). ADD was present in 63.5%: Grade 1, 20.3%; Grade 2, 10.7%; Grade 3, 32.4%. No displacement was found in 36.5%. Distribution showed polarization (Grade 0 vs. Grade 3). Bilateral symmetry occurred in 58% ($r = 0.50$, $p < 0.000005$). Females were more frequently affected by higher-grade ADD ($p < 0.001$). Younger patients showed more severe grades. A pandemic-related shift was observed, with female predominance in 2020 and male rebound in 2021.
**Conclusion:** ADD is frequent and shows polarized severity. Female predominance, younger age association, and pandemic-related shifts underline its multifactorial nature. MRI provides essential diagnostic and epidemiological insights.
**Limitations:** This retrospective, single-center study may reflect referral bias and is not fully representative of the general population. The cross-sectional design precludes causal inference between ADD severity, age, sex, and pandemic-related trends. MRI interpretation was based on categorical grading, which may oversimplify complex disc dynamics. Variability between 1.5T and 3.0T scanners, as well as interobserver differences, could influence diagnostic consistency. Clinical correlation with symptoms and treatment outcomes was beyond the study's scope, limiting assessment of functional relevance.
**Funding for this study:** No Third Party funding for this study
**Has your study been approved by an ethics committee?** Yes
**Ethics committee - additional information:** Approved by local ethics committee
**Author Disclosures:**
Moritz Ludwig Schnitzer: Nothing to disclose
Lilly Göbel: Nothing to disclose
Johannes Rübenthaler: Nothing to disclose
Felix Herr: Nothing to disclose

**Dental radiology at a glance**
*M. Smoljan*; Zagreb/HR
*(miasmoljan@gmail.com)*

**Purpose:** The purpose of this presentation is to review the most common pathologies of the maxillofacial region, as well as their imaging characteristics on different imaging modalities, and their differentials.
**Methods or Background:** Dental radiology is still a relatively unfamiliar part of radiology for many radiologists, even though the maxillofacial region is seen on many neuroradiological examinations, like brain and cervical spine scans, and included in most head & neck scans. Therefore, radiologists come across different odontogenic and non-odontogenic lesions of the jaws and other pathological lesions of this area in their everyday practice.
**Results or Findings:** Different odontogenic and non-odontogenic lesions of maxillofacial region can be seen on X-rays, CT scans and MRIs. When analyzing brain, head, neck or cervical spine images, radiologists should pay attention to maxillofacial structures as well. Whether the indication for the radiological examination is to diagnose an already suspected dental/maxillofacial pathology or the pathology found is an incidental finding in the examination performed for other indications, the radiologists should be familiar with the nomenclature, the lesions typical for this region, and their differentials.
**Conclusion:** Odontogenic and non-odontogenic lesions of the jaws, as well as other maxillofacial pathologies, account for a substantial number of pathological changes visible on different radiological modalities. Adding them to the radiological reports may assist the clinicians solve the clinical inquiry and help the patient maintain their oral health.
**Limitations:** No limitations were identified.
**Funding for this study:** No funding was received.
**Has your study been approved by an ethics committee?** Not applicable
**Author Disclosures:**
Mia Smoljan: Nothing to disclose

**15:00 - 16:00** **Research Stage 1**

### Research Presentation Session: Physics in Medical Imaging

### RPS 513

### Unlocking the power of photon-counting CT

**Moderator**
Louise Bowden; Dublin/IE
*(louise.bowden@gmail.com)*

### Small Pixel Effect and Tin Effect in Energy-Integrating and in Photon-Counting CT

J. Wucherpfennig[1], L. T. Rotkopf[1], H-P. Schlemmer[1], M. Frölich[2], S. O. Schönberg[2], S. Sawall[1], *M. Kachelrieß*[1]; [1]Heidelberg/DE, [2]Mannheim/DE
*(marc.kachelriess@dkfz.de)*

**Purpose:** To evaluate the separate and combined influence of the small pixel effect (SPE) and tin prefiltration in terms of signal-to-noise ratio at unit dose (SNRD) in energy-integrating CT (EICT) and photon-counting CT (PCCT).
**Methods or Background:** SPE refers to higher SNRD for scans with small detector pixels versus larger pixels at the same MTF. Tin effect refers to the selective removal of low-energy photons before reaching the patient. Fully exploiting the tin effect requires adjusting tube voltages, which was not possible in our setting with the same tube voltages and spectra on both scanners. Both effects improve the SNRD. To quantify, images of semi-anthropomorphic abdomen phantoms of sizes 20×30 cm (S), 25×35 cm (M), 30×40 cm (L), and 35×45 cm (XL) were acquired with an EICT (Somatom Definition Flash, Siemens Healthineers) and a PCCT (Naeotom Alpha.Peak, Siemens Healthineers) at 140 kV and 140 kV Sn. PCCT scans were done in standard and ultra-high-resolution mode to exploit the SPE. All reconstructions within each scanner were performed with the same MTF.
**Results or Findings:** SPE allowed for 36% (S), 30% (M) and 6% (L) dose reduction. Tin effect in PCCT was observed only in the XL phantom, with 25% dose reduction. The unobserved tin effect in smaller phantoms and the reduced SPE in larger phantoms are presumably due to a proprietary raw-data-dependent filter implemented in the PCCT. Tin effect in EICT was consistent across all phantom sizes with a dose reduction of 20% (S), 29% (M) and 30% (L).
**Conclusion:** EICT benefits from the tin effect. PCCT allows to exploit the small pixel effect. Making use of the expected combined effect in PCCT, however, seems to require an adaptation of the vendor-proprietory rawdata filter.
**Limitations:** Findings are based on phantoms.
**Funding for this study:** None.
**Has your study been approved by an ethics committee?** Not applicable
**Author Disclosures:**
Stefan Oswald Schönberg: Nothing to disclose
Matthias Frölich: Nothing to disclose
Lukas Thomas Rotkopf: Nothing to disclose
Jöken Wucherpfennig: Nothing to disclose
Heinz-Peter Schlemmer: Nothing to disclose
Marc Kachelrieß: Nothing to disclose
Stefan Sawall: Nothing to disclose

### Effects of Pulse Pileup Correction on the Signal and Noise Characteristics in Clinical Photon-Counting CT

*X. Liu*, F. Dong, K. Li; Houston/US
*(xliu@mdanderson.org)*

**Purpose:** In the NAEOTOM Alpha photon-counting detector CT (PCD-CT), the mean CT number of a given tissue has shown good consistency across varying dose levels. This observation has been interpreted as evidence that pulse pileups are negligible in Alpha. However, such stability in CT numbers can also result from effective pileup correction. The aim of this study was to evaluate the impact of pileup correction on the signal and noise characteristics of the Alpha PCD-CT.
**Methods or Background:** Images were acquired using an Alpha scanner at four tube potentials and twelve tube current settings. The vendor's pulse pileup correction was automatically applied during reconstruction. Experimental measurements included image variance, noise power spectrum (NPS), MTF, and mean HU. The dependence of each metric on tube current and dose level was analyzed.
**Results or Findings:** The MTF, the shape of the NPS, and the mean HU showed no significant variation with tube current or dose. However, the image variance deviated notably from the classical inverse relationship with tube current (variance ∝ 1/mA). Specifically, the observed variance was higher than predicted by this relationship, with the deviation becoming more pronounced at higher mA levels. At tube currents above 300 mA at 120 kV, corresponding to a flux of 1E8 counts-per-second/pixel, the variance even increased with mA. This counterintuitive behavior can only be explained by the presence of a pileup correction mechanism.
**Conclusion:** Pulse pileup correction was automatically applied in the Alpha PCD-CT imaging chain. This correction linearizes the mean detector counts with respect to tube current but results in elevated image noise. The correction appears to be applied on a pixel-by-pixel basis, as it does not affect spatial resolution or alter the noise texture.
**Limitations:** The study is based only on phantoms.
**Funding for this study:** None
**Has your study been approved by an ethics committee?** Not applicable
**Author Disclosures:**
Frank Dong: Nothing to disclose
Xinming Liu: Nothing to disclose
Ke Li: Consultant: Pulmera Inc.

### Dose and noise texture effects on lung nodule volumetry in photon counting CT

E. Pimenta[1], G. Boiset[1], G. Negro[1], M. Bettencourt[1], A. Tomal[2], L. J. Oostveen[3], M. Caballo[4], *I. Sechopoulos*[3], P. R. Costa[1]; [1]São Paulo/BR, [2]Campinas/BR, [3]Nijmegen/NL, [4]'s-Hertogenbosch/NL
*(ioannis.sechopoulos@radboudumc.nl)*

**Purpose:** To evaluate the effect of noise texture at different dose levels on volumetric accuracy and precision of synthetic solid nodules (SNs) and ground-glass opacities (GGOs) imaged with photon counting CT (PCCT).
**Methods or Background:** A 3D-printed SN and a GGO were inserted into an anthropomorphic lung phantom. Ground-truth volumes were quantified using a µCT (U-SPECT6CThr, MILabs). The phantom was scanned five times with repositioning in a prototype PCCT system (Canon Medical Systems) at four CTDIvol levels (0.3, 0.5, 0.8 and 1.1 mGy). Images were reconstructed using hybrid iterative reconstruction (AIDR 3D/FC54 kernel). Volumes were segmented (3DSlicer, v5.6.2) by readers blinded to the imaging parameters. Accuracy was assessed using median relative error and Bland-Altman plots. Precision was evaluated using the coefficient of variation (CV, IQR-based). Noise power spectra (NPS) were calculated from four ROIs around each nodule. Associations between volumetric performance, NPS features (area, downslope $\sigma$, peak frequency fpeak, and average frequency fave) and CTDIvol were evaluated using ordinary least squares and quantile regression (QReg, $\tau=0.5$).
**Results or Findings:** SN volumetry showed strong dose-dependence for accuracy (QReg $R^2=0.91$; CTDIvol $\beta=0.93$, $p=0.034$), whereas GGO accuracy modeling was weaker ($R^2=0.46$). In contrast, GGO precision was dose-dependent ($R^2=0.57$; $\beta=7.71$, $p=0.022$). For SN, precision strongly correlated with NPS features (QReg $R^2=0.78$), including fpeak ($\beta=-154.8$, $p<0.001$), fave ($\beta=170.5$, $p<0.001$), and $\sigma$ ($\beta=-151.6$, $p<0.001$).
**Conclusion:** For the SN, accuracy was dose-driven while precision was associated with noise texture. For the GGO, precision was predominantly dose-dependent. These findings highlight the importance of tailored protocol optimization in PCCT.
**Limitations:** A limited number of nodules and a single reconstruction method/kernel combination, which restrict generalizability.
**Funding for this study:** The authors would like to acknowledge financial support of Fundação de Amparo à Pesquisa do Estado de São Paulo (FAPESP) (grants 2018/059820, 2021/14688-0, 2022/114570 and 2023/03945-8), Conselho Nacional de Desenvolvimento Científico e Tecnológico (CNPq) (grants 141335/2021-1, 131691/2021-0, 138533/2022-9, 302986/2023-5 and 311657/20214).
**Has your study been approved by an ethics committee?** Not applicable
**Author Disclosures:**
Paulo Roberto Costa: Nothing to disclose
Marco Caballo: Employee: Marco Caballo is employed as a scientist by Canon Medical Systems Europe. His contribution pertained providing technical and scientific expertise, and he had no influence or control in any data or result generated in this study.
Luuk J. Oostveen: Nothing to disclose
Gisell Boiset: Nothing to disclose
Elsa Pimenta: Nothing to disclose
Miguel Bettencourt: Nothing to disclose
Alessandra Tomal: Nothing to disclose
Ioannis Sechopoulos: Research/Grant Support: Research grant support from Canon Medical
Giovanna Negro: Nothing to disclose

**Patient-specific resolution measurements in low-dose chest CT: adapting mesh-based algorithms for high noise images**
*K. Torfs*, K. Merken, D. Petrov, H. Bosmans; Leuven/BE
*(kwinten.torfs@uzleuven.be)*

**Purpose:** Mesh-based resolution algorithms quantify sharpness on patient images via modulation transfer functions (MTF) taken along the patient air-skin interface, but often fail for noisy chest images (e.g. sharp kernels/low dose). We introduce a high-noise-adapted mesh algorithm (AM), with altered edge-profile conditioning, and compare it to the current mesh algorithm (CM) and a phantom-based circular-edge method (CE), considered gold standard.
**Methods or Background:** High-contrast circular phantom images were simulated with resolution and noise texture characteristics derived from analytical ground-truth MTFs. Ideal coefficients for the analytical model were fitted to the CE MTF of nine Siemens SOMATOM Edge kernels. Ten repeated stacks were simulated per combination of: • Kernel (Br32–Br60, Qr32–Qr60, Bl57) • Simulated phantom tilting (0°-10° in 2° increments) • Noise magnitude (0-200HU in 25-HU steps) • Clothing (present/absent) Percentage error (PE) was compared across CE, AM and CM.
**Results or Findings:** CE performance was largely noise-independent, but sensitive to high tilting (>6°: PE=60±40%). For tilting <6° (PE=2.5±1.9%), CE was more accurate (p<0.001) than both AM/CM, while AM outperformed (p<0.001) for tilting >6°. Mesh-based algorithms were tilt-resistant but noise-sensitive: • Low noise (0-50 HU): PE of AM lower than CM (p<0.001: 3±3% vs. 5±3%) • Medium noise (75-125 HU): PE of AM lower than CM (p<0.001: 8±5% vs. 40±30%) • High noise (150-200 HU): PE of AM lower than CM (p<0.001: 17±9% vs. 70±30%) Similar tendencies were observed with clothing present, only at slightly higher PE, and CM outperformed AM (p<0.05) for noises 0-25 HU.
**Conclusion:** For patient-based resolution measurements, tested on non-circular objects, AM reduces error significantly versus CM in most scenarios, limiting PE to maximally 10% for noise up to 125 HU. CE remains optimal for circular phantoms, used in quality control, given precise positioning.
**Limitations:** None
**Funding for this study:** Funding was provided by Kom op Tegen Kanker (G0B1922N) in light of a doctoral grant
**Has your study been approved by an ethics committee?** Yes
**Ethics committee - additional information:** Approved under internal reference number S68527
**Author Disclosures:**
Kwinten Torfs: Nothing to disclose
Hilde Bosmans: Nothing to disclose
Karen Merken: Nothing to disclose
Dimitar Petrov: Nothing to disclose

**CT Image Denoising with Noise Augmentation**
G. Kristof, R. Von Stritzky, E. Eulig, *M. Kachelrieß*; Heidelberg/DE
*(marc.kachelriess@dkfz.de)*

**Purpose:** To improve AI-based CT image denoising, aiming at more naturally looking output images with less hallucinations, by providing noise covariance information to denoising neural networks.
**Methods or Background:** CT image noise is correlated between neighboring voxels. The correlation strongly depends on the patient's global attenuation properties. We provide this information to deep denoising neural networks by simulating several additional noise-only images as additional network input. Up to ten of these noise only images and the low-dose image, which is the one to be denoised, are now input into a denoising network. We tested this noise-augmented deep denoising (NADD) for the CNN10, ResNet, and WGAN-VGG denoising networks. Our training, validation and test dataset consists of approximately 50,000 clinical CT images. For each network, we compared the NADD version with its original version.
**Results or Findings:** The NADD versions of the networks performed significantly better than the original versions. The structural similarity index measure (SSIM) improved from 0.937 to 0.956 for CNN10, from 0.942 to 0.954 for ResNet and from 0.917 to 0.940 for WGAN-VGG. In particular, a significant qualitative improvement of the images is observed and far less hallucinations are apparent with NADD.
**Conclusion:** Using additional noise-only realizations as network input significantly improves the denoising capability of denoising networks. A downside is the requirement of additional image reconstructions corresponding to the number of additional noise realizations used. NADD may help to lower the effective dose to and therefore cancer risk of the patient.
**Limitations:** Our study only considers 2D, image-by-image, denoising. Future work should extend the denoising networks to true 3D networks that can denoise a whole volume in a 3D fashion.
**Funding for this study:** This study was supported in parts by the German Federal Ministry for the Environment, Nature Conservation, Nuclear Safety and Consumer Protection (BMUV) under grant 67KI2036B, and by the Helmholtz International Graduate School for Cancer Research, Heidelberg, Germany.
**Has your study been approved by an ethics committee?** Not applicable
**Author Disclosures:**
Gernot Kristof: Nothing to disclose
Roman Von Stritzky: Nothing to disclose
Marc Kachelrieß: Nothing to disclose
Elias Eulig: Nothing to disclose

**CT-DImQ_SSIM: GUI-based platform for evaluating Structural Similarity Index Measure (SSIM) for CT image quality assessment: AI vs iterative reconstruction phantom study**
*A. Kazhybekova*, J. Cooke, M. Kelly, I. Hernandez-Giron; Dublin/IE
*(ainur.kazhybekova@ucdconnect.ie)*

**Purpose:** To present an open-access CT image quality evaluation platform for automated comparison of reconstruction algorithms (AI/DLR vs iterative), based on structural similarity index (SSIM) and histogram analyses.
**Methods or Background:** CT-DImQ_SSIM is a Python-based GUI (PySide6, scikit-image library) that evaluates SSIM (Wang2004) between two images/stacks, with user-selected SSIM parameters and input window level/width mimicking different visualization conditions. SSIM local maps are automatically created globally and in selected VOIs and saved (TIFF), and average SSIM-values calculated. As an application the Kyoto Kagaku lung paediatric phantom with 3D-printed nodules was scanned (GE-Revolution, standard-thorax, clinical iterative reconstruction, ASIR50-lung and DLR, TrueFidelityHigh). SSIM analysis was performed in selected VOIs (nodules, vessels, rib, vertebra, heart).
**Results or Findings:** High similarity between both reconstructions (82% pixels, 0.8<SSIM<1), supports the global anatomical structure DLR preservation observed. Smoother edges and reduced apparent enhancement is reflected in low local SSIM-values, especially in high contrast transitions (boundaries, nodules and bones edges). The latter showed the lowest SSIM edge values with up to 10% of pixels SSIM<0.5. Histograms showed a narrower distribution (more uniformity) for DLR (FWHM, nodule={IR:160HU, DLR:30 HU}, rib={IR:245HU, DLR:50HU}, vessel={IR:160HU, TFH:30HU}, heart {IR:120HU, DLR:25HU}).
**Conclusion:** CT-DImQ_SSIM is an open-access tool for fast and automated SSIM CT image analysis, for imaging protocol and reconstruction algorithms comparison. SSIM maps are a useful visual and quantitative tool to identify potential areas in the image where CT reconstructions/settings may differ, with potential use to identify DLR hallucinations or significant CT-value changes. In an anthropomorphic thorax phantom study, DLR preserved image structural information overall, whereas in edges, especially in high contrast regions it showed low similarity compared to the standard iterative reconstruction.
**Limitations:** The phantom lacks lung parenchyma which can introduce a bias in comparison with patients reconstructed with same algorithms.
**Funding for this study:** Ainur Kazhybekova holds an UCD Ad astra PhD Scholarship.
**Has your study been approved by an ethics committee?** Not applicable
**Author Disclosures:**
Michael Kelly: Nothing to disclose
Jennie Cooke: Nothing to disclose
Ainur Kazhybekova: Nothing to disclose
Irene Hernandez-Giron: Nothing to disclose

**Ultra-high and high-resolution color K-edge imaging using Spectral Photon-Counting CT for ytterbium and gadolinium imaging: a phantom study**
*R. Coulibaly*[1], A. Robert[1], E. Thomas[1], C. A. Hernandez-Fajardo[1], A. J. Gutwinska[1], M. N. Antonuccio[2], P. Ravipati[1], J. Greffier[3], S. A. Si-Mohamed[1]; [1]Villeurbanne/FR, [2]Suresnes/FR, [3]Nîmes/FR
*(ramata.coulibaly@creatis.insa-lyon.fr)*

**Purpose:** To assess the impact of ultra-high resolution (Detailed2) and high-resolution (HRB) reconstruction kernels on K-edge imaging of ytterbium and gadolinium using spectral photon-counting CT (SPCCT).
**Methods or Background:** A custom-made cylindrical phantom containing five fillable holes was used. Two configurations were tested: configuration1 contained holes with Gd (0.5, 1, 2, and 10mg/mL) and configuration2 contained holes with Yb at the same concentrations. For each configuration, nine helical acquisitions were performed on the phantom at 120kVp and 75mAs. Conventional images, as well as color K-edge images of Yb (61keV) and Gd (50.2keV), were reconstructed using the HRB and Detailed2 kernels. A three-basis (photoelectric/Compton/K-edge) projection-based material decomposition was used to obtain the K-edge images. Image quality was evaluated in terms of noise power spectrum (NPS), and task-based transfer function (TTF).
**Results or Findings:** In conventional images, the Detailed2 kernel resulted in higher noise (34.87UH vs 61.57UH configuration1; 34.90UH vs 61.55UH configuration2) and shifted the NPS peak to higher spatial frequencies (0.30mm$^{-1}$ vs 0.80mm$^{-1}$ configuration1; 0.33mm$^{-1}$vs 0.80mm$^{-1}$ configuration2) compared with HRB. In K-edge images, both kernels showed similar noise (0.07mg/mL gadolinium; 0.08mg/mL ytterbium) with stable $f_{peak}$ values

(0.33mm⁻¹ gadolinium; 0.35 vs 0.32mm⁻¹ ytterbium). In conventional images, Detailed2 provided higher spatial resolution. As concentration increased from 0.5 to 10mg/mL in configuration2, $f_{50}$ ranged 0.242–0.586mm⁻¹ with Detailed2 and 0.247–0.459mm⁻¹ with HRB. In configuration1, Detailed2 ranged 0.330–0.545mm⁻¹, HRB 0.295–0.448mm⁻¹. In K-edge images, HRB performed better at low concentrations. At 0.5mg/mL, $f_{50}$ was 0.239 vs 0.231mm⁻¹ for ytterbium and 0.280 vs 0.250mm⁻¹ for gadolinium. At 10mg/mL, Detailed2 outperformed HRB: for ytterbium, 0.732 vs 0.535mm⁻¹; for gadolinium, 0.626 vs 0.451mm⁻¹.
**Conclusion:** For K-edge imaging, noise was similar, but Detailed2 gave higher resolution at high concentrations, while HRB was slightly better at low concentrations.
**Limitations:** Phantom study.
**Funding for this study:** This work was supported by the ERC starting Grant KOLOR SPCCT Imaging (N°101118079).
**Has your study been approved by an ethics committee?** Not applicable
**Author Disclosures:**
Antoine Robert: Nothing to disclose
Priusha Ravipati: Nothing to disclose
Eloïse Thomas: Nothing to disclose
Salim Aymeric Si-Mohamed: Nothing to disclose
Christian Alejandro Hernandez-Fajardo: Nothing to disclose
Agnieszka Joanna Gutwinska: Nothing to disclose
Joel Greffier: Nothing to disclose
Ramata Coulibaly: Nothing to disclose
Maria Nicole Antonuccio: Nothing to disclose

**Liver Fat and Iron Quantification with Spectral Localizer Radiographs from Photon-Counting Detector CT**
*A. Tognella*[1], T. Flohr[2], J. M. Fröhlich[3], T. Nowak[2], M. A. Fischer[3], H. Alkadhi[3], S. Stocker[3]; [1]Zürich/CH, [2]Forchheim/DE, [3]Zurich/CH

**Purpose:** Liver fat quantification on CT is often confounded by hepatic iron deposition and the use of iodinated contrast agents. This phantom study aimed to assess the feasibility and accuracy of quantifying liver fat content (LFC) in the presence of iron using pre-contrast spectral localizer radiographs (SLR) from a photon-counting detector CT (PCD-CT).
**Methods or Background:** Sixteen phantoms were created using mixtures of liver tissue, fat, and iron to simulate four levels of LFC (0%, 10%, 30%, 50%) and four levels of liver iron concentration (LIC: 0, 1.5, 3, and 6 mg/mL). All phantoms were scanned on a PCD-CT using three tube current settings (10 mA, 50 mA, 300 mA), acquiring SLRs. Material decomposition of high- and low-energy bin data yielded water and hydroxyapatite (HA) maps. HA-values were analyzed as a function of LFC and LIC, and water-values were correlated with corresponding HA-values.
**Results or Findings:** Increasing LFC resulted in a linear decrease in HA-values, consistent across LIC levels (slopes ranging from -0.0016 to -0.0023, r = 0.997 – 1.0). Conversely, increasing LIC led to a linear increase in HA values, independent of LFC (slopes ranging from 0.0147 to 0.017, r = 0.978 – 1.0). When combined with water values in a two-dimensional material space, these consistent linear trends enabled accurate estimation of LFC regardless of LIC. Findings were reproducible across all tube current settings.
**Conclusion:** This phantom study demonstrates that SLRs from PCD-CT enable accurate estimation of liver fat content, even in the presence of iron deposition. If validated in vivo, this technique may facilitate opportunistic screening for hepatic steatosis and iron overload in clinical practice solely using SLRs.
**Limitations:** Proof-of-principle study with phantom setup offers simplified conditions. No evaluation of patient data. Limited to a specific PCD-CT.
**Funding for this study:** None
**Has your study been approved by an ethics committee?** Not applicable
**Author Disclosures:**
Thomas Flohr: Employee: Former employee from Siemens Healthineers
Andrin Tognella: Nothing to disclose
Johannes Malte Fröhlich: Nothing to disclose
Soleen Stocker: Research/Grant Support: Institutional research grants from Bayer, Canon, Guerbet, and Siemens
Tristan Nowak: Employee: Current employee from Siemens Healthineers
Michael Alexander Fischer: Nothing to disclose
Hatem Alkadhi: Research/Grant Support: Institutional research grants from Bayer, Canon, Guerbet, and Siemens

**Detecting Iodine in a Semi-Anthropomorphic Liver Phantom – Evaluating the Performance of a 4th Generation Prototype Deep-Silicon Photon Counting CT system**
*L. G. Pedersen*[1], A. Schulz[1], A. Dybwad[1], D. Crotty[2], H. Linder[3], J. Levy[4], K. Jensen[1]; [1]Oslo/NO, [2]Cork/IE, [3]Stockholm/SE, [4]Greenwich, NY/US
*(ligped@ous-hf.no)*

**Purpose:** Smaller metastatic liver lesions can be difficult to detect on conventional energy-integrating (EID) CT. The improved spatial and spectral resolution of Photon Counting CT (PCCT) is expected to improve iodine contrast detection in liver imaging.
**Methods or Background:** A semi-anthropomorphic liver phantom (PhantomLab) was scanned using GSI on a Revolution Apex EID CT and subsequently on a 4th-generation prototype silicon-based PCCT system (both GE HealthCare). The phantom contains both homogeneous inserts without Iodine and Iodine inserts of varying concentration (1-3mg/mL) to mimic metastatic liver lesions of diameter 3-15mm. Similar CTDIvol levels (5-25mGy) were used to image on both systems with other technique parameters prescribed as similar as possible. Using prototype image reconstruction algorithms, Virtual Monoenergetic Images (VMI) from 40-70keV and Iodine and Water material maps were reconstructed. Voxel-based Hounsfield Unit (HU), image noise (measured as standard deviation (SD)), and Contrast-to-Noise (CNR) were quantified. A two-alternative forced choice reader study was performed to visually detect iodine inserts.
**Results or Findings:** For the 15mm lesion, PCCT VMI reconstructed using the prototype image reconstruction process demonstrate increased CNR as CTDIvol increases and keV reduces. At 70keV, the CNR of the 15mm lesion in PCCT increased from 1.5-3.3 with increasing CTDIvol. At equal CTDIvol (10mGy), PCCT images (8.9-12 HU) showed a significant decrease in background image noise range relative to EID images (11.6-35.1 HU). While PCCT image noise is reduced, the iodine HU stays consistent across CTDIvol, resulting in up to 60% increased CNR for all reconstructed VMIs on the PCCT relative to EID.
**Conclusion:** Reader study participants observed the 3 mm lesion in 90% of cases in PCCT images obtained between 15 and 20 mGy.
**Limitations:** Only phantom study, results should be confirmed in a patient study.
**Funding for this study:** None.
**Has your study been approved by an ethics committee?** Not applicable
**Author Disclosures:**
Kristin Jensen: Nothing to disclose
Josh Levy: Nothing to disclose
Dominic Crotty: Nothing to disclose
Anselm Schulz: Nothing to disclose
Line Gaard Pedersen: Nothing to disclose
Anniken Dybwad: Nothing to disclose
Hugo Linder: Nothing to disclose

**15:00 - 16:00 Research Stage 6**

### Research Presentation Session: Emergency Imaging

### RPS 517

### New approaches in trauma imaging in peace and war times

**Moderator**
Elizabeth Dick; London/UK
*(elizabethdick2010@gmail.com)*
**Author Disclosures:**
Elizabeth Dick: Speaker: Everlight, Other: hosted by Everlight Nov 2024 in Sydney

**Advancing Emergency Radiology: The Critical Role of Imaging Near Catastrophic Events**
U. Pidvalna, *D. Beshley*; Lviv/UA
*(dbeshley@ukr.net)*

**Purpose:** 1. Understand the role of radiological modalities (X-ray, ultrasound, CT) in triage and evacuation of casualties. 2. Identify imaging findings of combat-related injuries across the head, chest, abdomen, pelvis, and extremities. 3. Recognize key challenges in applying diagnostic imaging in mobile military hospitals.
**Methods or Background:** Military trauma has evolved with the advancement of modern warfare, creating new challenges in managing battlefield injuries. Timely diagnosis and decision-making are crucial in the resource-limited environment of a mobile military hospital. Radiological tools, including X-ray, ultrasound, and CT, are indispensable for frontline medical teams, enhancing decision-making during patient evacuation and trauma care.

**Results or Findings:** Imaging in mobile military hospitals reveals a broad spectrum of traumatic injuries, particularly in the head, chest, abdomen, pelvis, and extremities. Key findings include: Head injuries: Brain trauma and fractures. Chest injuries: Pneumothorax, hemothorax, heart and vessel injuries. Abdominal injuries: Hemoperitoneum, solid organ trauma. Pelvic and extremity injuries: Amputations and fractures. X-ray and ultrasound provide rapid initial assessments, while CT is employed for complex injuries. This integration enables faster diagnosis and informed decision-making, ultimately improving patient outcomes.
**Conclusion:** Radiology significantly enhances trauma care in mobile military hospitals, enabling swift diagnosis and triage. While logistical challenges remain, including limited resources, diagnostic imaging has proven vital in improving survival rates and optimizing patient care on the battlefield. The integration of imaging technologies in mobile settings continues to play a key role in modern military medicine.
**Limitations:** Single center, potential biases in imaging interpretation due to varying expertise levels, constraints in radiological equipment, and resources in a combat setting.
**Funding for this study:** No
**Has your study been approved by an ethics committee?** Not applicable
**Author Disclosures:**
Uliana Pidvalna: Nothing to disclose
Dmytro Beshley: Nothing to disclose

**MRI safety recommendations in patients with unknown metallic fragments after war-related injuries: a pragmatic workflow**
*M. Khurtsylava*; Kyiv/UA
*(khurtsylavaa@gmail.com)*

**Purpose:** To present practical considerations and a step-by-step workflow for MRI safety in patients with unknown metallic fragments, a patient group that has become increasingly common due to war-related injuries.
**Methods or Background:** Patients with shrapnel wounds or metallic foreign bodies of undocumented origin are frequently referred for MRI. The exact composition of fragments is often unknown, while clinical questions usually concern anatomical regions distant from the fragments (e.g. brain MRI in a patient with fragments in the leg). Literature review and analysis of local clinical experience were used to design a pragmatic workflow for risk assessment and decision-making.
**Results or Findings:** Metallic fragments may include steel, copper/bronze, lead, aluminium, or tungsten. Risks range from ferromagnetic displacement (steel), RF-induced heating, to severe image artefacts. Proposed workflow: 1. Focused history and inspection for scars. 2. X-ray/CT for localisation and density assessment. 3. Identification of critical sites (orbit, spinal canal, vessels, heart). 4. Risk–benefit evaluation: MRI performed only if clinical necessity outweighs potential risks. 5. Technical precautions: low-SAR protocols, local transmit/receive coils, patient monitoring, clear documentation. When fragments are remote from the scanned region, mechanical risks are low but heating remains possible; each case requires individual assessment.
**Conclusion:** Patients with unknown metallic fragments represent a growing challenge in MRI practice. A structured workflow helps improve safety and avoid unnecessary refusals of clinically important examinations.
**Limitations:** This workflow is based on literature and local practice. Prospective multicentre validation is required.
**Funding for this study:** No funding was received for this study.
**Has your study been approved by an ethics committee?** Not applicable
**Author Disclosures:**
Mariia Khurtsylava: Nothing to disclose

**Comparative CT findings in polytrauma: differences between living patients and post-mortem CT**
*M. Palermo*, R. A. Trapani, M. Pane, F. Tiralongo, S. Palmucci, P. V. Foti, C. Pomara, A. Basile; Catania/IT
*(monica.palermo91@gmail.com)*

**Purpose:** To compare CT findings in polytrauma patients due to traffict accident undergoing emergency CT scan with those observed in deceased patients examined with post-mortem CT (PMCT), focusing on the most frequent fatal injuries reported in the literature and our experience.
**Methods or Background:** We performed a retrospective evaluation of findings in patients who underwent emergency whole-body CT (WBCT) in our emergency department due to traffic accidents and of findings on PMCT of trauma victims performed in our Institution. We also performed a literature review on main findings in this setting of patients and on more common fatal injuries detectable with PMCT.
**Results or Findings:** In living patients, WBCT typically demonstrated limb fractures, intracranial hemorrhages, thoracic and abdominal bleeding, and solid organ injuries, often supporting timely therapeutic decisions. In deceased trauma victims, in accordance with Schmitt-Sody et al. , the predominant fatal injuries on PMCT were: • Severe thoracic injuries (~65%), including massive hemothorax, pulmonary lacerations, and major vascular ruptures, especially aortic rupture. Severe cranio-cerebral trauma (~47%), such as extensive intracranial hemorrhage or destruction of brain parenchyma. Massive intra-abdominal hemorrhage (e.g., liver or splenic rupture), less frequent but still relevant.
**Conclusion:** CT in living polytrauma patients reveals a broad spectrum of injuries, many of which are potentially treatable if promptly identified. PMCT, on the other hand, consistently highlights the lesions that are most often fatal—severe thoracic hemorrhage, aortic rupture, extensive cranial trauma, and massive abdominal bleeding. Awareness of these patterns from PMCT can help radiologists maintain a higher index of suspicion during emergency reporting, facilitating early recognition of critical injuries and potentially life-threatening complications, ultimately improving patient management in the acute setting.
**Limitations:** Retrospective study, small number of cases
**Funding for this study:** No fundings received.
**Has your study been approved by an ethics committee?** Not applicable
**Author Disclosures:**
Riccardo Alvise Trapani: Nothing to disclose
Monica Palermo: Nothing to disclose
Pietro Valerio Foti: Nothing to disclose
Cristoforo Pomara: Nothing to disclose
Mario Pane: Nothing to disclose
Antonio Basile: Nothing to disclose
Stefano Palmucci: Nothing to disclose
Francesco Tiralongo: Nothing to disclose

**Diffusion-Weighted Imaging in the Early MRI Follow-up of Abdominal Solid Organ Injuries: Diagnostic Value and Protocol Optimization**
*F. Iacobellis*, C. Camillo, M. Di Serafino, L. Barbuto, R. Ronza, D. Grimaldi, C. Rinaldo, F. Verde, S. Giovine; Naples/IT
*(iacobellisf@gmail.com)*

**Purpose:** To assess the diagnostic value of diffusion-weighted imaging (DWI) in the early MRI follow-up of abdominal solid organ injuries in polytraumatized patients, comparing its performance with conventional and contrast-enhanced sequences, and to define an optimized protocol tailored to trauma-related findings.
**Methods or Background:** MRI studies performed between 2018 and 2022 for early follow-up of high-energy abdominal trauma were retrospectively reviewed. Each protocol included T1- and T2-weighted sequences (with and without fat suppression), DWI, and 3D T1-weighted fat-saturated sequences before and after intravenous (IV) gadolinium-based contrast administration. Three radiologists in consensus evaluated the contribution of each sequence to lesion detection, characterization, and follow-up assessment.
**Results or Findings:** Forty-six MRI examinations were analyzed. Conventional T1W, T2W, and T2W fat-sat sequences provided essential morphological and evolutionary information about parenchymal lesions and hemoperitoneum. DWI sequences improved lesion conspicuity compared to unenhanced sequences in 50% of cases, were equivalent in 18.7%, and inferior in 14.5%. Compared to post-contrast sequences, DWI showed equivalent diagnostic performance in 54.1%, superior in 27.1%, and inferior in 18.7% of cases. Contrast-enhanced sequences remained essential in confirming contained vascular injuries (8.6%) and urinary leakage.
**Conclusion:** DWI provides substantial diagnostic value in the early follow-up of abdominal parenchymal injuries, frequently matching or exceeding the information obtained from contrast-enhanced imaging. Incorporating DWI as a core component of trauma MRI protocols may reduce dependence on contrast agents, shorten scan times, and improve diagnostic safety in patients with renal impairment or critical conditions.
**Limitations:** Retrospective design of the study, limited sample size, heterogeneity of trauma severity and timing of follow-up MRI. No quantitative analysis of ADC values was performed.
**Funding for this study:** None
**Has your study been approved by an ethics committee?** Not applicable
**Author Disclosures:**
Dario Grimaldi: Nothing to disclose
Francesca Iacobellis: Nothing to disclose
Costanza Camillo: Nothing to disclose
Sabrina Giovine: Nothing to disclose
Francesco Verde: Nothing to disclose
Roberto Ronza: Nothing to disclose
Marco Di Serafino: Nothing to disclose
Luigi Barbuto: Nothing to disclose
Chiara Rinaldo: Nothing to disclose

**Prognostic relevance of contrast extravasation in patients undergoing endovascular embolization of acute bleeding**

*H-J. Meyer*, S. Ebel, T. Denecke, M. Struck; Leipzig/DE
*(jonas90.meyer@web.de)*

**Purpose:** : Contrast extravasation on imaging studies is a clinical surrogate for bleeding severity. However, the prognostic relevance of this imaging sign needs to be evaluated. The aim of this study was to analyze the impact of contrast extravasation defined by computed tomography (CT) and angiography on massive transfusion and 30-day mortality in patients with acute bleeding undergoing transarterial embolization (TAE).
**Methods or Background:** A mixed cohort of patients with acute bleeding requiring treatment with TAE between 2018 and 2022 was retrospectively evaluated. All patients underwent triphasic CT to localize the source of bleeding and to calculate extravasation volumes in the arterial and portal venous phases. The bleeding rate k was calculated from the CT images.
**Results or Findings:** A total of 128 patients (79 male, 61.7%) with a mean age of 67.4 years (range 21-95 years) and an all-cause 30-day mortality rate of 34.4% were included in the present analysis. A moderate positive correlation was identified between transfused packed red blood cell units and bleeding rate k (r=0.33, p<0.001). In multivariable logistic regression analysis, bleeding rate k was identified as an independent prognostic factor for massive transfusion (OR 25.77, 95% CI 1.35-493.61, p=0.031, area under the receiver operating characteristic curve (AUROC) of the model: 0.847) and 30-day mortality (OR 25.04, 95% CI 2.29-273.42, p=0.008, AUROC of the model: 0.781).
**Conclusion:** CT-defined bleeding rate k is a prognostic factor for massive transfusion and 30-day mortality in patients with acute bleeding undergoing TAE and may be superior to the volume of contrast extravasation volume alone. Further studies are needed to confirm this finding.
**Limitations:** The study has to address the limitation of a retrospective single center, and the heterogenous nature of the patient cohort.
**Funding for this study:** None
**Has your study been approved by an ethics committee?** Not applicable
**Author Disclosures:**
Timm Denecke: Nothing to disclose
Manuel Struck: Nothing to disclose
Sebastian Ebel: Nothing to disclose
Hans-Jonas Meyer: Nothing to disclose

**Quadruple-Rule-Out CT Angiography: Dual-Energy CT for Comprehensive Evaluation of Acute Chest Pain**

*M. E. Çevik*[1], E. Zengin[2], K. B. Memiş[3]; [1]Kırıkkale/TR, [2]Ankara/TR, [3]Erzincan/TR
*(drmehmetemircevik@gmail.com)*

**Purpose:** Computerized tomography (CT) has been increasingly utilized in the differential diagnosis of acute chest pain. Combining the triple rule out CT angiography (TRO-CT) approach with dual-energy CT (DECT) can enhance the diagnostic capability by identifying myocardial perfusion deficiencies. This combination can yield a quadruple-rule-out computed tomography angiography (QRO-CT) technique. The aim of this study is to determine the efficacy of the QRO-CT.
**Methods or Background:** Intraluminal diseases and abnormalities in the main coronary arteries and branches were investigated. The myocardial dark spots on the color-coded iodine map were identified as perfusion deficiencies. Pulmonary arteries and aorta were also evaluated.
**Results or Findings:** The study population consisted of 211 patients. The sensitivity, specificity, and positive and negative predictive values of QRO-CT for pulmonary embolism were 93.5%, 100%, 100%, and 95.3%, respectively. For obstructive coronary artery disease, the values were 96.1%, 93.4%, 89.2%, and 97.7%, respectively. For myocarditis, the values were 69.2%, 100%, 100%, and 93.6%, respectively.
**Conclusion:** the QRO-CT method may successfully evaluate myocardial perfusion deficits, hence expanding the differential diagnosis capabilities of the standard TRO-CT method for myocarditis. It can provide useful information on myocardial perfusion, which may influence the choice to perform invasive catheterization in cases of coronary artery obstruction.
**Limitations:** Main limitations are the single-center design, moderate sample size, incomplete troponin data in some patients, and the fact that not all patients had MRI correlation.
**Funding for this study:** No external funding was received.
**Has your study been approved by an ethics committee?** Yes
**Ethics committee - additional information:** Our study was approved by the ethics committee. The ethics committee approval number is as follows: 14.12.2021-EBYU-KAEK-21/12-18.
**Author Disclosures:**
Mehmet Emir Çevik: Nothing to disclose
Kemal Buğra Memiş: Nothing to disclose
Ece Zengin: Nothing to disclose

**Pelvic Radiographs in Polytrauma: Diagnostic Accuracy and Interobserver Agreement. A Retrospective CT Correlation Study**

*N. Al Balushi*, S. Raniga, A. K. Mittal, N. M. Fazaldad; Muscat/OM
*(xnoor.b@gmail.com)*

**Purpose:** Pelvic fractures in polytrauma patients are a critical concern due to their association with vascular injuries, haemorrhage and shock. While CT is the gold standard for evaluation of bony and articular injuries, pelvic radiographs remain the primary screening imaging modality in many trauma protocols. This study aims to assess the diagnostic accuracy of anteroposterior pelvic radiographs for detecting pelvic fractures using CT as reference and to evaluate interobserver agreement among radiologists.
**Methods or Background:** In this retrospective, cross sectional study approved by the institutional ethics board, adult patients presenting with acute pelvic trauma between 2016-2022 who underwent both pelvic radiography and CT were included. Two staff radiologists independently reviewed the pelvic radiographs, blinded to CT results. Diagnostic accuracy was assessed using CT as the reference standard, calculating sensitivity, specificity, positive predictive value (PPV) and negative predictive value (NPV). Interobserver agreement was analysed using Fleiss multirater kappa statistics. Findings were compared with those reported in existing literature.
**Results or Findings:** Total of 99 patients were included (mean age 33.2±13.1 years, 77.5% male). Acetabular fractures were the most frequently missed, with 53% of cases falsely negative and 1.2% false positives. Sacral fractures had a 50% miss rate on radiographs. The overall interobserver agreement was substantial (kappa= 0.788), with highest agreement seen in iliac, pubic and proximal femur fractures and lowest in acetabular and sacral fractures.
**Conclusion:** Anteroposterior pelvic radiographs have limited sensitivity for detecting acetabular and posterior ring fractures. In our cohort, all unstable fractures were detected on radiographs, supporting their continued use as effective initial screening tools in polytrauma.
**Limitations:** The study is constrained by limited sample size and evolving image quality. Radiographic interpretation may have been impacted by patient positioning and obscuring bowel gases, particularly in subtle fractures.
**Funding for this study:** None
**Has your study been approved by an ethics committee?** Yes
**Ethics committee - additional information:** Approved
**Author Disclosures:**
Noor Muhammad Fazaldad: Nothing to disclose
Alok Kumar Mittal: Nothing to disclose
Noor Al Balushi: Nothing to disclose
Sameer Raniga: Nothing to disclose

**Morel-Lavallée Lesions in Emergency Radiology – How Often Are They Missed?**

*M. Mitrašinović*, D-S. Petkovic, A. Popovic, S. Milosevic, D. Vasin; Belgrade/RS
*(marijamitrasinovic7@gmail.com)*

**Purpose:** Morel-Lavallée lesions (MLLs) are rare soft tissue injuries occurring in trauma setting, characterized by degloving of subcutaneous tissue from muscular fascia, creating a potential space filled with blood and lymph. They are often overlooked leading to inadequate treatment, complications and recurrence. This study aimed to evaluate the diagnostic role of CT in detecting MLLs and to increase awareness of their recognition on CT, which is usually the first-line imaging tool in polytrauma patients.
**Methods or Background:** We conducted a retrospective review of trauma patients between 2022 and 2025 based on two criteria: emergency admission for polytrauma and description of a subcutaneous hematoma on CT involving the upper limb, gluteal region, knee, elbow, scapular region, or abdominal wall. These CT scans were then reassessed for misdiagnosed lesions. Diagnostic CT criteria included: (1) acute, hyperdense collections between subcutaneous fat and fascia; and (2) chronic-appearing, encapsulated collections with fluid–fluid or gas levels, fat lobules, or septations. Patient clinical records were analyzed for confirmation.
**Results or Findings:** Of 110 trauma patients, 35 (31.8%) showed CT imaging features consistent with MLLs. Clinical confirmation was obtained in 14 patients (12.7% of the cohort; 40% of CT-suspected lesions). The CT-suspected group comprised 22 men (62.9%) and 13 women (37.1%). Most lesions occurred in the gluteal/femoral region (71.4%), followed by scapular (14.3%), knee (8.6%), and elbow (5.7%). CT demonstrated a positive predictive value (PPV) of 40.0% (95% CI: 24.6–57.7%), with a 60% false-positive rate.

**Conclusion:** CT can raise suspicion for MLLs but does not provide sufficient specificity for definitive diagnosis. While CT is valuable in the acute setting, further evaluation with MRI and clinical examination is essential. Improved awareness of CT features among radiologists may enhance recognition, ensure timely follow-up, and guide management.
**Limitations:** No
**Funding for this study:** No
**Has your study been approved by an ethics committee?** Not applicable
**Author Disclosures:**
Stefan Milosevic: Nothing to disclose
Aneta Popovic: Nothing to disclose
Marija Mitrašinović: Nothing to disclose
Dragan Vasin: Nothing to disclose
Dunja-Simona Petkovic: Nothing to disclose

**To assess whether brief structural reporting system can reduce the report turn-around time**
*Y-C. Wong*; New Taipei City/TW
*(ycwong@cgmh.org.tw)*

**Purpose:** Whole-body CT for trauma is a rapid and safe imaging modality. But with hundreds of images per trauma patient, the workload has burned out radiologists and the pressure on timeliness of reporting is increasing. Experimental studies have demonstrated that structured reporting systems can reduce report turn-around time by approximately 25% to 50% (the denominator is about 25 minutes). The purpose of this study is to assess whether brief structural reporting system can reduce the report turn-around time.
**Methods or Background:** From January 2024 to June 2025, we retrospectively reviewed whole-body CT of trauma patients. We assessed whether brief structured reporting system that includes five key questions can further reduce report turn-around time. The five questions included (1) intracranial midline shift, (2) tension pneumothorax, (3) cardiac tamponade or aortic injury, (4) abdominal contrast medium extravasation and (5) unstable spine or pelvic fracture.
**Results or Findings:** Among 62 whole-body CT reports, there were 35 males and 27 females, mean age was 46.1 ± 19.1 years, ISS was 25.7 ± 12.6, (min 4 - max 50), report turn-around time was 3.97 ± 1.85 minutes, (min 1 - max 7 minutes). There were 12 intracranial midline shifts, 7 tension pneumothorax, 2 cardiac tamponade or aortic injury, 9 abdominal contrast medium extravasation and 13 unstable spine or pelvic fracture.
**Conclusion:** The brief structural reporting system could report the whole-body CT, and it took a mean of 3.97 minutes to accomplish.
**Limitations:** We do not disclose the reliability among different readers. We also do not disclose the impact of the reports on the therapeutic planning of trauma surgeons. Lastly, this initial study is carried out in a single center and the inclusion number is small.
**Funding for this study:** Chang Gung Medical Foundation Research
**Has your study been approved by an ethics committee?** Yes
**Ethics committee - additional information:** Chang Gung Medical Foundation Ethics Committee
**Author Disclosures:**
Yon-Cheong Wong: Nothing to disclose

**16:30 - 17:30 Research Stage 3**

### Research Presentation Session: Abdominal and Gastrointestinal

### RPS 601

### Chronic liver disease and portal hypertension

**Moderator**
Henri-Marcel Hoogewoud; Corminboeuf/CH

**Role of 2D shear wave elastography in screening for portal hypertension and varices in children with chronic liver disease**
*D. Chaurasia*, R. Batra, R. Dixit; New Delhi/IN
*(deeksha.chaurasia@gmail.com)*

**Purpose:** The AASLD adopts Baveno VI/VII consensus and recommends specific cut-offs for non-invasive assessment of portal hypertension (PH) and varices using elastography in adults; however, there are no established guidelines for the pediatric population due to limited high-quality data. This study aimed to evaluate the utility of hepatic stiffness (HS) and splenic stiffness (SS) values in predicting PH and assessing the risk of varices in children with chronic liver disease (CLD) using 2D shear wave elastography (2D-SWE).
**Methods or Background:** This cross-sectional analytical study included 64 participants under 18 years of age, comprising 34 children with CLD (26 children with PH and 11 children with varices) and 30 age-matched healthy controls. Ultrasound of the liver and spleen was performed using a curvilinear (C1-6) probe on a GE LOGIQ S8 system in a fasting state, and ten 2D-SWE measurements (in m/s) were obtained. Mean values were calculated and considered reliable if interquartile range to median ratio was ≤15%. HS and SS values were analyzed, and ROC curves were drawn to predict PH. The splenic-to-hepatic stiffness (S/L) ratio was calculated to differentiate patients with varices.
**Results or Findings:** HS and SS values were significantly ($p < 0.05$) higher in children with PH, and the S/L ratio was significantly ($p < 0.05$) elevated in those with varices as compared to those without.
**Conclusion:** SS can serve as a non-invasive surrogate marker for PH in children. The S/L ratio demonstrated superior performance over HS or SS values alone and may aid both in screening and in identifying children at increased risk of varices, allowing for tailored monitoring and timely intervention.
**Limitations:** Small sample size and no validated pediatric cut-offs. Larger, multicenter studies are needed to confirm the clinical utility in pediatric risk stratification.
**Funding for this study:** The study was institutionally supported by the Department of Radiology, Maulana Azad Medical College.
**Has your study been approved by an ethics committee?** Yes
**Ethics committee - additional information:** The study was approved by the Institutional Ethics Committee of Maulana Azad Medical College, New Delhi.
**Author Disclosures:**
Deeksha Chaurasia: Nothing to disclose
Rashmi Dixit: Nothing to disclose
Radhika Batra: Nothing to disclose

**The performance of CT-based radiomic models of Liver and Spleen in predicting Clinically Significant Portal Hypertension**
*A. M. Morariu-Barb*, M. A. Socaciu, B. Boca, H. Stefanescu, B. Procopet, M. Lupsor-Platon; Cluj-Napoca/RO

**Purpose:** Clinically significant portal hypertension(CSPH) is the main determinant of complications in chronic liver disease(CLD). The gold standard is hepatic venous pressure gradient (HVPG)(>10 mmHg), but this method is invasive and not widely available. Standard CT offers little value beyond morphological assessment, but CT radiomics may represent a more accurate alternative.This study investigated whether CT-based radiomics can predict CSPH, comparing segmentation strategies (2Dvs.3D) and regions of interest (liver,spleen or both).

**Methods or Background:** We retrospectively analyzed 52 patients with biopsy-proven CLD who underwent HVPG and contrast-enhanced CT within three months. Nine healthy controls were included based on FibroScan F0 values and absence of signs of portal hypertension. 2D and 3D segmentations of liver and spleen were performed in portal-venous phase using Slicer software. Radiomic features were extracted and neural networks were trained to classify CSPH. Six models were developed: 2Dliver, 2Dspleen, 2Dliver+spleen, 3Dliver, 3Dspleen and 3Dliver+spleen. Performance was evaluated using area under the receiver operating characteristic curve(AUROC) and accuracy.
**Results or Findings:** CSPH was present in 26 patients(42.6%).The combined 3D liver and spleen model achieved the highest performance with an AUROC of 0.899 and accuracy of 66.7%. The 3Dspleen and 3Dliver models followed, with AUROCs of 0.811 and 0.816 and accuracies of 81.4% and 68%.Among the 2D models, the combined liver and spleen model performed best (AUROC 0.825,accuracy 53.3%), followed by the 2Dspleen model (AUROC 0.757,accuracy 66.7%) and the 2Dliver model (AUROC 0.703,accuracy 52.9%).
**Conclusion:** CT-based radiomic analysis of liver and spleen predicted CSPH with good performance.3D segmentations and combined liver-spleen models outperformed 2D and single-organ models. These findings support CT radiomics as a promising non-invasive tool for CSPH detection and prognostic stratification, extending the utility of routine CT.
**Limitations:** Single-center study Small size cohort Lack of external validation
**Funding for this study:** None
**Has your study been approved by an ethics committee?** Not applicable
**Author Disclosures:**
Horia Stefanescu: Nothing to disclose
Monica Lupsor-Platon: Nothing to disclose
Andreea Mihaela Morariu-Barb: Nothing to disclose
Bianca Boca: Nothing to disclose
Mihai Adrian Socaciu: Nothing to disclose
Bogdan Procopet: Nothing to disclose

**Fontan-Associated liver disease: radiological findings and prevalence of hepatocellular carcinoma in an adult patient cohort**
*S. Rivera Rojas*, S. Morón Hodge, N. Saturio Galán, P. Castillo Grau, M. Cuadros Martínez, M. E. Velilla Aparicio, M. Cabero, E. Rodríguez, C. Martín Hervás; Madrid/ES
*(santiriverarojas@gmail.com)*

**Purpose:** The Fontan procedure is a surgical palliation for patients with a univentricular heart. Hemodynamic changes after the procedure lead to hepatic fibrosis and increase the risk of hepatocellular carcinoma (HCC), highlighting the need for multidisciplinary follow-up with a key role for radiology. This study aims to describe the radiological findings associated with Fontan-associated liver disease (FALD) and HCC, and to document the time to onset of these findings.
**Methods or Background:** Retrospective cohort study of 87 patients at a referral hospital with abdominal imaging available in the electronic medical record (ultrasound, MRI, and/or CT). All imaging studies were reviewed. We recorded the interval from the Fontan procedure to the first imaging study, the time to diagnosis of FALD, their frequency, and the prevalence of HCC based on radiological and histological criteria.
**Results or Findings:** Among the 87 patients, 59.8% were male. During follow-up 92.0% of patients were diagnosed with FALD, with a mean age of 29 years (SD 8.5). Median interval from Fontan procedure to the first imaging study was 17.5 years (IQR 12). FALD-related findings were observed on the initial imaging study in 77.0%. Radiological features of FALD included heterogeneous liver parenchyma (67.4%), irregular hepatic margins (61.2%), caudate lobe hypertrophy (55.3%), splenomegaly (52.3%), suprahepatic vein dilation (33.7%), and ascites (16.3%). Nodules were detected in 43.0% of patients. Nodules suggestive of HCC developed in 6 patients (6.9%), with histological confirmation in 3 ( 3.4%).
**Conclusion:** Radiological findings associated with FALD are common from the first imaging study in patients post-Fontan. Although HCC is infrequent, it can develop in a subset, emphasizing the importance of standardized long-term imaging surveillance for early detection and management.
**Limitations:** This was a retrospective cohort study.
**Funding for this study:** None.
**Has your study been approved by an ethics committee?** Not applicable
**Author Disclosures:**
Nuria Saturio Galán: Nothing to disclose
Carmen Martín Hervás: Nothing to disclose
Santiago Rivera Rojas: Nothing to disclose
Sara Morón Hodge: Nothing to disclose
Miguel Cabero: Nothing to disclose
Marta Cuadros Martínez: Nothing to disclose
Elena Rodríguez: Nothing to disclose
Maria Elena Velilla Aparicio: Nothing to disclose
Pilar Castillo Grau: Nothing to disclose

**Assessment of Liver Functional Reserve in Cirrhosis Using Dual-Energy CT–Derived Extracellular Volume Fraction**
*S. Hong*, J. E. Kim; Jinju/KR
*(iamgoodbaba@hanmail.net)*

**Purpose:** This study assessed whether dual-energy CT (DECT)–derived liver extracellular volume fraction (fECV) can reliably evaluate liver functional reserve in cirrhotic patients, as determined by Child–Pugh classification (CP class), compared with other non-invasive markers.
**Methods or Background:** This retrospective study included 258 patients with liver cirrhosis. fECV score was measured from iodine maps derived from equilibrium-phase images at 100/140 Sn kVp. The diagnostic performance of fECV and other non-invasive markers (aspartate transaminase-to-platelet ratio index [APRI], Fibrosis-4 [FIB-4], Model for End-Stage Liver Disease [MELD]) for differentiating CP classes was assessed by comparing the area under the receiver operating characteristic curve (AUC) using DeLong's test with Holm correction for multiple comparisons.
**Results or Findings:** The fECV score was positively correlated with CP class ($r = 0.53$, $p < 0.01$). For differentiating CP class A from B, the AUCs were similar among fECV (0.84), APRI (0.83; $p = 1$), and FIB-4 (0.83; $p = 1$), whereas MELD demonstrated a significantly higher AUC (0.94; $p < 0.05$). For differentiating CP classes B and C, the fECV score demonstrated a significantly higher AUC (0.78), compared with FIB-4 (0.50, $p = 0.04$) and APRI (0.49, $p = 0.04$), whereas no significant difference was observed between fECV and MELD (0.92, $p = 0.12$).
**Conclusion:** Dual energy CT-derived fECV score is a useful non-invasive marker for assessing liver functional reserve based on the CP class.
**Limitations:** First, this study was a retrospective study conducted at a single institution. Second, iodine quantification results may vary depending on the type of DECT system. Third, there was a relatively small number of patients with Child–Pugh class C (n = 13) compared with classes A (n = 207) and B (n = 38).
**Funding for this study:** No funding was received for this study.
**Has your study been approved by an ethics committee?** Yes
**Ethics committee - additional information:** This retrospective study was approved by Gyeongsang National University Hospital review board (IRB No. 2025-05-005).
**Author Disclosures:**
Ji Eun Kim: Nothing to disclose
Seokjin Hong: Nothing to disclose

**Clinical application of ultrasound localization microscopy in cirrhosis**
*J. Jin*[1], C. Huang[2], Y. Ma[1], W. Zhang[1], Y. Tan[1], S. Chen[2], J. Ren[1]; [1]Guangzhou/CN, [2]Rochester, MN/US
*(jinjy3@mail.sysu.edu.cn)*

**Purpose:** Liver microcirculatory dysfunction plays a crucial role in the development and progression of cirrhosis. There is an urgent clinical need for effective methods to assess hepatic microcirculation. This study explores the feasibility of in vivo ultrasound localization microscopy (ULM) imaging and quantitative analysis in humans. We aim to qualitatively and quantitatively investigate the similarities and differences in hepatic microcirculation between healthy volunteers and patients with cirrhosis using ULM.
**Methods or Background:** This study consists of two parts. Part I involves in vivo comparative imaging of normal volunteers using Color Doppler (CD), ultra-micro angiography (UMA), and ULM to validate the clinical feasibility of ULM by assessing image resolution. Part II compares microcirculatory characteristics between normal and cirrhotic livers based on ULM-derived images and quantitative parameters.
**Results or Findings:** Compared with CD and UMA, ULM demonstrates superior capability in detecting subcapsular hepatic microcirculation. Among the quantitative parameters based on total blood flow, vessel density (VD), velocity (V), perfusion index (PI), distance metric (DM), sum of angles metric (SOAM), number of vessel branches per unit area (NOBPA) and number of branch points per unit area (NOBPPA) were higher in cirrhotic group than in control group. Overall, higher density and perfusion, higher tortuosity of microvessels were revealed in cirrhotic groups.
**Conclusion:** ULM enables in vivo, non-invasive, visual, and quantitative assessment of hepatic microcirculation, holding promise as a pivotal tool for early diagnosis of cirrhosis and monitoring therapeutic efficacy.
**Limitations:** This study did not conduct a longitudinal comparison of microvascular imaging and quantitative microcirculation parameters across different stages of liver fibrosis. We aim to include patients with varying degrees of liver fibrosis in order to better characterize the overall progression of microcirculatory changes throughout the development of liver fibrosis.
**Funding for this study:** National Natural Science Foundation of China (No. 82202192) Basic and Applied Basic Research Foundation of Guangdong Province (No. 2021A1515110591) Guangzhou Municipal Science and Technology Project (No. SL2024A04J01248)
**Has your study been approved by an ethics committee?** Yes

Wednesday

**Ethics committee - additional information:** This study was approved by the Institutional Review Board (IRB) of the Third Affiliated Hospital of Sun Yat-Sen University.
**Author Disclosures:**
Yuxue Tan: Nothing to disclose
Yanping Ma: Nothing to disclose
Jie Ren: Nothing to disclose
Wei Zhang: Nothing to disclose
Chengwu Huang: Nothing to disclose
Jieyang Jin: Nothing to disclose
Shigao Chen: Nothing to disclose

**A Dual-layer Spectral Nomogram Integrating Structural, Functional, and morphological Parameters for Risk Stratification of Esophageal Varices in Cirrhosis**

*J. Li*[1], J. Lei[1], B. Wang[1], X. Zhang[2]; [1]Lanzhou/CN, [2]Xi An/CN
*(296066255@qq.com)*

**Purpose:** To develop and validate a noninvasive dual-layer spectral computed tomography (DSCT)-based nomogram model integrating quantitative multiparameters and clinical indicators to optimize risk assessment and hemorrhage prediction of esophageal varices (EVs) in patients with cirrhosis.
**Methods or Background:** This retrospective study enrolled 284 cirrhotic patients undergoing DSCT , divided into training (n=194) and validation (n=90) cohorts. Patients were stratified according to the presence of high-risk esophageal varices (HREV), red color signs (RC signs), and esophageal variceal bleeding (EVB), with endoscopy as reference. Quantitative parameters were analyzed, including extracellular volume fraction (ECV) and iodine washout rate (IWR) of liver and spleen, and esophageal variceal diameter (EVD). Multivariable logistic regression identified predictors for nomogram construction. Model performance was evaluated via AUC, calibration curves, decision curve analysis (DCA), and predictive metrics [(sensitivity, specificity, positive/negative predictive value (PPV/NPV) ]in both cohorts.
**Results or Findings:** EVD, ECVliver, ECVspleen, and IWRliver differed significantly across subgroups stratified by HREV, RC(+), and EVB status (P < 0.05). Multivariate analysis identified EVD, ECVspleen, and IWRliver as independent predictors of HREV; EVD, ECVspleen, IWRliver, and total bilirubin for RC sign; and EVD, ECVspleen, aspartate aminotransferase, and red blood counts for EVB (all P < 0.05). The nomogram demonstrated high predictive accuracy for HREV (AUC: 0.963/0.968), RC signs (0.898/0.854), and EVB (0.847/0.785) in training/validation cohorts. DCA confirmed clinical utility, with net benefit across low-to-medium risk thresholds (0.0-0.6). At EVD thresholds of 5 mm and 3 mm, the model achieved specificity/PPV of 97.1-100.0%/97.7-100.0% and sensitivity/NPV of 94.4-94.2%/88.7-91.2% for HREV in the training and validation cohorts, respectively.
**Conclusion:** This DSCT-based nomogram synergizes structural (ECVspleen), functional (IWRliver), and morphological (EVD) biomarkers to noninvasively stratify risks of HREV, RC signs, and EVB in cirrhosis.
**Limitations:** Lack external validation.
**Funding for this study:** None
**Has your study been approved by an ethics committee?** Not applicable
**Author Disclosures:**
Xiaoyue Zhang: Nothing to disclose
Junqiang Lei: Nothing to disclose
Baoyuan Wang: Nothing to disclose
Jinkui Li: Nothing to disclose

**Diffusion-based virtual elastography (DvE) at 3T for liver fibrosis quantification in cirrhosis during HCC screening: a prospective single-centre study**

*F. Delavari*, C. Dromain, L. Haefliger, L. Fernandes, M. Fraga Christinet, I. Jelescu, N. Vietti Violi; Lausanne/CH

**Purpose:** Non-invasive quantification of liver fibrosis in cirrhosis guides prognosis, surveillance, and therapy. Biopsy is invasive and sampling-limited; vibration-controlled transient elastography (VCTE) samples a small right-lobe volume and may be affected by obesity/inflammation; MR elastography requires dedicated hardware. The purpose of this work is to implement and evaluate a 3T Diffusion-based virtual elastography (DvE) workflow that converts two-point shifted ADC into voxel-wise stiffness (μ_diff, kPa) for quantifying liver fibrosis, and to test its association withVCTE in adults with cirrhosis undergoing HCC screening; secondary aims were to quantify intrahepatic heterogeneity and assess repeatability in 6 months.
**Methods or Background:** Prospective, single-centre study with MRI and VCTE at each visit. DWI: ss-EPI, b=200/1500 s/mm². μ_diff was computed from sADC using a calibrated linear model. Processing included liver masking with vessel exclusion, variability filtering, a right-lobe region of interest (ROI) mirroring VCTE practice, and a whole-liver heterogeneity index (HI=coefficient of variation of μ_diff). Associations were tested with Spearman's ρ; repeatability with two-way random-effects, absolute-agreement inter-class correlation (ICC).
**Results or Findings:** Eighty-two MRI–VCTE pairs were analysed. Right-lobe μ_diff correlated with VCTE stiffness (ρ=0.34, p=0.034). In 24 rescans, right-lobe μ_diff showed moderate repeatability (ICC=0.58). Whole-liver μ_diff showed a weaker, non-significant association (ρ=0.18, p=0.09). HI>20% classified 26.8% of livers as heterogeneous; excluding these strengthened the whole-liver association to significance (ρ=0.27, p=0.043; n=60), indicating spatial variability as a confounder when comparing local vs global stiffness.
**Conclusion:** DvE provides simple MRI-integrated, whole-liver stiffness maps for fibrosis quantification in cirrhosis screening. Observing the association withVCTE, which samples a right-lobe ROI, we propose intrahepatic heterogeneity can drive discordance. DvE may complement VCTE as for non-invasive whole-liver fibrosis assessment within routine HCC surveillance.
**Limitations:** Empirical calibration out of the dataset; diffusion artefacts and SNR/motion, limited sample size.
**Funding for this study:** SNSF - Project funding in biology and medicine (division III) 2021 October - 207944
**Has your study been approved by an ethics committee?** Yes
**Ethics committee - additional information:** Prospective uniccentric study with local ethics approval (CER-VD 2022-D0116)
**Author Disclosures:**
Montserrat Fraga Christinet: Nothing to disclose
Laura Haefliger: Nothing to disclose
Farnaz Delavari: Nothing to disclose
Ileana Jelescu: Nothing to disclose
Lucia Fernandes: Nothing to disclose
Naïk Vietti Violi: Nothing to disclose
Clarisse Dromain: Nothing to disclose

**AI-automated liver volumetry and MRI data extraction for characterisation of patients with chronic liver disease**

*D. Catucci*[1], L. Hulbert[1], L. Zbinden[1], M. P. Brönnimann[1], V. Obmann[1], A. Berzigotti[1], A. Christe[1], R. Sznitman[1], A. T. Huber[2]; [1]Bern/CH, [2]Lucerne/CH

**Purpose:** To analyse whether artificial intelligence (AI)-automated volumetry of liver structures and data extraction from liver T1 mapping can differentiate stages of chronic liver disease (CLD) and detect clinically significant portal hypertension (CSPH).
**Methods or Background:** In this retrospective study, 159 patients underwent liver MRI including T1 maps before (T1 long) and 20 minutes after Gd-EOB-DTPA-administration (T1 short) and native T1 Dixon-sequence for volumetry between 09/2018 and 05/2019. CLD-patients were grouped based on liver biopsy into early CLD (F0-F2; n=34), advanced CLD (F3-F4; n=27) and advanced CLD with CSPH (F3-F4 with presence of portosystemic collaterals, splenomegaly >12 cm with thrombocytopenia <100 G/L or ascites; n=53). 45 patients without CLD served as a negative control group. Total liver volume (TLV), hepatic veins volume (HVV), liver segmental volumes I-VIII, T1 long and T1 short were automatically measured using an in-house developed liver-segmentation-algorithm. A T1 reduction rate (ΔT1) was calculated as (T1 long – T1 short)/T1 long. In addition, a functional-right-lobe-volume-ratio (FRLVR) was calculated as (volumes segments IV-VIII/TLV)*ΔT1 and a hepatic-veins-ratio (HVR) was calculated as (HVV/TLV)*1000. Parameters were compared between groups using the Kruskal-Wallis-test. Univariate logistic regression analysis was used to assess the discriminatory power of parameters for group-differentiation.
**Results or Findings:** All parameters differed significantly between the groups (p<0.05). TLV initially increased with CLD-severity and decreased at advanced CLD-stages with CSPH. In contrast, ΔT1, FRLVR and HVR continuously decreased with increasing CLD-severity. HVR and FRLVR showed strong discriminatory power for CLD (odds-ratio OR 0.71 and 0.88), advanced CLD (OR 0.72 and 0.86) and CSPH (OR 0.66 and 0.87), all with p<0.001.
**Conclusion:** Our findings highlight the potential of integrating AI-automated liver segmentation for MRI data extraction and volumetry into clinical workflows to improve management of CLD patients.
**Limitations:** Retrospective study design.
**Funding for this study:** This study received funding from the Swiss National Science Foundation (grant number 10003604).
**Has your study been approved by an ethics committee?** Yes
**Ethics committee - additional information:** Cantonal ethics comittee of Bern, Switzerland.
**Author Disclosures:**
Lukas Zbinden: Nothing to disclose
Raphael Sznitman: Nothing to disclose
Damiano Catucci: Nothing to disclose
Verena Obmann: Nothing to disclose
Leona Hulbert: Nothing to disclose
Michael P. Brönnimann: Nothing to disclose
Andreas Christe: Nothing to disclose
Adrian Thomas Huber: Nothing to disclose
Annalisa Berzigotti: Nothing to disclose

**16:30 - 17:30** **Research Stage 2**

### Research Presentation Session: Breast

### RPS 602

### Advances and emerging trends in breast MRI

**Moderator**
Pascal A.T. Baltzer; Vienna/AT

**High Spatiotemporal Resolution in Breast DCE-MRI: Evaluation of a Novel 4D Stack-of-Stars Technique**

*N. Mesropyan*[1], C. Katemann[2], A. Isaak[1], J. A. Luetkens[1]; [1]Bonn/DE, [2]Hamburg/DE
*(m20narine@gmail.com)*

**Purpose:** To clinically implement and evaluate a 4D diamond-shaped pseudo–golden angle stack-of-stars (SoS) acquisition with k-space weighted image contrast (KWIC) reconstruction for breast dynamic contrast-enhanced MRI (4D-DCE), assessing image quality, diagnostic confidence, and BI-RADS agreement across conventional and ultrafast protocols.
**Methods or Background:** This retrospective study included female patients who underwent breast MRI at 3T using the 4D-DCE sequence. Three protocol types were generated: (1) conventional (4 × 60 s), (2) ultrafast (20× 3s), and (3) combined (ultrafast followed by 3 × 60 s). Two readers independently or in consensus rated image quality (overall quality, artifacts, sharpness, lesion conspicuity, and morphology) and diagnostic confidence using a 5-point Likert scale. BI-RADS scores were compared to the final reference standard (histology or ≥2-year imaging follow-up). Agreement was assessed using Cohen's kappa and ICC.
**Results or Findings:** A total of 167 patients (mean age: 59 ± 11 years) were included. Despite high temporal resolution of the ultrafast 4D-DCE, image quality was good to excellent and was comparable to the standard-resolution post-contrast T1 mDixon sequence (e.g., overall quality: 4.8±0.4 vs. 4.8±0.3, P=.99).The combined 4D-DCE protocol yielded the highest diagnostic confidence by BI-RADS assignment in both readers, with the most pronounced improvement observed in patients with high background parenchymal enhancement (e.g., reader 1: 3.2 ± 0.6 [conventional] vs. 4.2 ±0.4[ultrafast] vs. 4.9 ± 0.3[combined],P<.001). BI-RADS agreement with the final reference standard was good to excellent across all DCE protocols, with the highest agreement achieved using the combined 4D-DCE(e.g., reader 1: κ=0.89, 95% CI:0.84–0.95).
**Conclusion:** The proposed 4D-DCE technique enables robust breast DCE-MRI with high spatial and temporal resolution. Combining ultrafast and conventional acquisitions within a single protocol improves diagnostic confidence and BI-RADS agreement.
**Limitations:** Intraindividual study comparing different 4D-DCE approaches within the same examination
**Funding for this study:** None
**Has your study been approved by an ethics committee?** Yes
**Ethics committee - additional information:** Number 2025-317-BO, Ethic committee of the University Hospital Bonn
**Author Disclosures:**
Julian Alexander Luetkens: Nothing to disclose
Christoph Katemann: Other: Supported the development and adaptation of the proposed MRI sequence Other: Co-author
Narine Mesropyan: Nothing to disclose
Alexander Isaak: Nothing to disclose

**Prognostic value of kinetic parameters in pre-treatment ultrafast dynamic contrast-enhanced MRI of invasive breast cancers**

*L. Y. Chan*, P. L. Lam, D. Fenn, W. P. Cheung, L. W. Lo, W. S. Mak, K. M. L. Wong, E. P. Y. Fung; Hong Kong/HK

**Purpose:** Ultrafast dynamic contrast-enhanced MRI (UF-DCE MRI) is a novel sequence which captures contrast wash-in in breast lesions in the very early post-contrast period with high spatial and temporal resolution, allowing analysis of an early wash-in kinetic curve. This study aims to evaluate the correlation between kinetic parameters in UF-DCE MRI and histopathological characteristics of invasive breast cancers.
**Methods or Background:** This is a retrospective study performed in an acute general hospital. 49 consecutive female adult patients (median age, 54.2 years; range, 30.0-85.4 years) with 53 pathologically proven invasive breast cancers who underwent pretreatment MRI breast imaging were included from June 2023 to June 2025. The ultrafast MRI protocol consisted of a pre-contrast and 12 T1-weighted post-contrast high-temporal-resolution images in the first minute using TWIST-VIBE technique. Kinetic parameters of index breast cancers were obtained from ultrafast sequences, including time-to-enhancement (TTE), maximum slope (MS) and arterial-venous interval (AVI). TTE, MS and AVI among breast cancers with different histological grade (Modified Bloom-Richardson grade), ER/PR/HER2 positivity and tumour subtype were compared using the Mann-Whitney U test and Kruskal-Wallis test as appropriate.
**Results or Findings:** High histologic grade cancers had larger MS compared to low to intermediate grade cancers (high grade=14.9%/s vs low to intermediate grade=11.4%/s, p=0.049). ER-negative cancers showed shorter TTE compared to ER-positive cancers (ER-negative=4.5s vs ER-positive=9.0s, p=0.037). HER2-positive cancers showed shorter TTE than HER2-negative cancers (HER2-positive=4.5s vs HER2-negative=9.0s, p=0.032).
**Conclusion:** Ultrafast MRI–derived kinetic parameters TTE and MS were associated with histopathologic characteristics in invasive breast cancers. UF-DCE demonstrated potential to identify more aggressive breast cancers and provide prognostic value to guide breast cancer management.
**Limitations:** The limitations of the study are single-centre retrospective study with limited sample size.
**Funding for this study:** No funding was provided for this study.
**Has your study been approved by an ethics committee?** Yes
**Ethics committee - additional information:** This study was approved by the Central Institutional Review Board (CIRB) of the Hospital Authority (HA) in Hong Kong Special Administrative Region (reference number: CIRB-2025-249-5). The requirement for informed consent was waived by CIRB.
**Author Disclosures:**
Wai Pan Cheung: Nothing to disclose
Ka Man Lily Wong: Nothing to disclose
Eliza Po Yan Fung: Nothing to disclose
Wing Sze Mak: Nothing to disclose
Lok Yee Chan: Nothing to disclose
Lai Wan Lo: Nothing to disclose
Pak Lun Lam: Nothing to disclose
Dickson Fenn: Nothing to disclose

**Predictive model for tumor-infiltrating lymphocyte levels in breast cancer using multi-phase DCE-MRI radiomics and semantic features**

*W. Yang*, X-G. Peng; Nanjing/CN
*(ywj001019@163.com)*

**Purpose:** This study aims to develop predictive models for tumor-infiltrating lymphocyte (TIL) levels in breast cancer patients using multi-phase dynamic contrast-enhanced MRI (DCE-MRI) radiomics and semantic features from tumor and peritumoral regions.
**Methods or Background:** A total of 298 pathologically confirmed BC patients were stratified into low (<10%, n=102) and high (≥10%, n=196) TIL groups. Tumor and peritumoral volumes were manually delineated across six DCE-MRI phases using 3D-Slicer, with radiomics features extracted via FAE software. Ten machine learning algorithms were evaluated using five-fold cross-validation. Traditional imaging features were selected through univariate and multivariate logistic regression. Model performance was assessed by the area under the receiver operating characteristic curve (AUC), and statistical comparisons were performed with DeLong's test.
**Results or Findings:** 1,688 radiomics features were extracted from tumor and peritumoral VOIs. Among intratumoral models, DCE_Phase 5 (DCE_P5) showed the best performance (training AUC = 0.835; validation AUC = 0.714), and DCE_Phase 3 (DCE_P3) was optimal for peritumoral models (training AUC = 0.793; validation AUC = 0.703). The combined model using DCE_P5 and DCE_P3 achieved AUCs of 0.878 (training) and 0.772 (validation). In semantic feature analysis, axillary lymph node status (P = 0.017) and fibroglandular tissue content (P = 0.019) were significant predictors. The combined model using DCE_P5 tumor and semantic features achieved AUCs of 0.853 (training) and 0.706 (validation), while the DCE_P3 and semantic feature model reached AUCs of 0.792 and 0.705. The final integrated model achieved the highest performance (AUC_train = 0.881; AUC_valid = 0.773).
**Conclusion:** This study presents a noninvasive, clinically applicable model combining multi-phase DCE-MRI and semantic features to predict TIL levels in BC, aiding in immunotherapy decision-making.
**Limitations:** The retrospective study with a limited sample size may affect generalizability. External validation in larger cohorts is needed.

**Funding for this study:** This study has received funding by National Natural Science Foundation of China (82272064), Jiangsu Provincial Science and Technique Program (BK20221461), Zhongda Hospital Affiliated to Southeast University, Jiangsu Province High-Level Hospital Pairing Assistance Construction (zdlyg08), Postgraduate Research & Practice Innovation Program of Jiangsu Province (KYCX23_0323, and KYCX22_0297).
**Has your study been approved by an ethics committee?** Yes
**Ethics committee - additional information:** This study was approved by the Ethics Committee of the Affiliated Zhongda Hospital of Southeast University (Approval Number: 2024ZDSYLL369-P01). All participants provided informed consent, and the study was conducted in accordance with the ethical principles outlined in the Declaration of Helsinki.
**Author Disclosures:**
Wenjun Yang: Nothing to disclose
Xin-Gui Peng: Nothing to disclose

**From Research to Clinic: First Prospective Evaluation of Volumetric Deep-Learning Super-Resolution in 3D T2-Weighted Breast MRI**
*N. Mesropyan*[1], O. Weber[2], A. Isaak[1], H. Peeters[3], C. Katemann[2], J. A. Luetkens[1]; [1]Bonn/DE, [2]Hamburg/DE, [3]Best/NL

**Purpose:** To evaluate, a fully volumetric deep-learning(DL) super-resolution research prototype combining compressed sensing with denoising and resolution upscaling for 3D T2-weighted (T2w) breast MRI, and to quantify its impact on acquisition time, image quality, and BI-RADS agreement.
**Methods or Background:** This prospective single-center study was conducted on a 1.5-T MRI. Two Cartesian 3D T2w acquisitions with low and normal resolution were acquired and reconstructed with the DL framework to yield T2DL and T2NR+DL. Acquisition time, quantitative image quality (apparent signal-to-noise (aSNR), apparent contrast-to-noise (aCNR)) and qualitative scores (5-point Likert scale: overall quality, sharpness, conspicuity, morphology, artifacts) were obtained in randomized, blinded reading sessions. BI-RADS agreement between different protocol types with the final score were assessed. Kruskal–Wallis one-way analysis of variance followed by Dunn's post hoc test, intraclass correlation coefficients (ICCs), and Cohen k test were used for statistical analysis.
**Results or Findings:** 64 women (mean age 55±13 years; range 28–84) were investigated. Acquisition time decreased by 41% for T2DL vs T2NR (126 vs 215 seconds). Quantitative quality improved with DL (e.g., for aSNR: 28.5±7.3 [T2NR] vs 36.6±10.9 [T2DL] vs 45.7±10.3 [T2NR+DL]; for aCNR: 21.8±5.6 [T2NR] vs 25.9±8.6 [T2DL] vs 35.3±9.1 [T2NR+DL]; all P<.001). Qualitative image quality was highest for T2NR+DL (for reader 2: overall quality: for T2NR+DL 5 [IQR 5–5] vs 4 [4-5] for T2 DL vs. 4 [3–4] for T2NR; P<.001). Inter-reader agreement for image quality assessment was good to excellent, ICCs rages 0.79–0.96 (CI:0.65-0.98). BI-RADS agreement was strong across all 3D DL protocols, Cohen k ranges 0.98–0.99 (CI: 0.98-0.99).
**Conclusion:** This volumetric DL super-resolution approach enables faster 3D T2w breast MRI while enhancing image quality. Alternatively, at unchanged acquisition time, it can further improve the quality of normal-resolution images.
**Limitations:** Research prototype was used in this study.
**Funding for this study:** None
**Has your study been approved by an ethics committee?** Yes
**Ethics committee - additional information:** Approved by the ethic committee of the University Hospital Bonn.
**Author Disclosures:**
Julian Alexander Luetkens: Nothing to disclose
Christoph Katemann: Other: Supported the development and adaptation of the proposed MRI sequence Other: Co-author
Narine Mesropyan: Nothing to disclose
Alexander Isaak: Nothing to disclose
Oliver Weber: Other: Supported the development of the sequence Other: Co-author
Hans Peeters: Other: Co-author Other: Supported the development of the sequence

**Breast ductal carcinoma in situ: MRI predictors of HER2 positivity and molecular characteristics**
*A. Zeynalova*, G. Esen, A. Dolu, M. Akin, N. Önder Denizoğlu, F. Tokat, C. Uras; Istanbul/TR
*(dr.amaliyazeynalova@yahoo.com)*

**Purpose:** To evaluate the association between molecular subtypes and magnetic resonance imaging (MRI) findings in ductal carcinoma in situ (DCIS), with a focus on identifying MRI features characteristic of the HER2-positive subtype.
**Methods or Background:** This retrospective observational study included 55 female patients who underwent preoperative breast MRI and had pure or microinvasive DCIS confirmed by surgical pathology between 2010 and 2024. Lesion size, lesion type, nipple extention, morphology and kinetic features were evaluated by two experienced breast radiologists in consensus. Age, clinical presentation, lesion location, tumour grade, necrosis, tumour size, surgical margin status, axillary lymph node involvement, receptor status were recorded. Receptor status was re-evaluated by a pathologist if initially unavailable.
**Results or Findings:** HER2 positivity was detected in 43.6% of cases. HER2-positive DCIS showed a larger mean MRI lesion size (44.7 mm, p=0.004) and more frequent nipple extension (p=0.032). ER negativity was higher in HER2-positive cases (p=0.003), and high nuclear grade was more common (p=0.052). Receiver operating characteristic (ROC) analysis identified a 22 mm lesion size threshold for predicting HER2 positivity (AUC=0.740). MRI lesion size, nipple extension, ER negativity and high nuclear grade were independent predictors of HER2 positivity, while no other imaging or pathological variables showed significant differences.
**Conclusion:** Recent studies have shown that HER-2 positivity may be associated with a worse prognosis and more frequent progression to invasive disease in DCIS. There is very limited data in literature about MRI features of different molecular subtypes of DCIS. Our results show that MRI features, particularly large lesion size and nipple extension are associated with HER2 positivity in DCIS and may aid molecular subtype prediction and surgical planning.
**Limitations:** The limitations of the study are its retrospective single-centre design and the small sample size.
**Funding for this study:** No funding was provided for this study.
**Has your study been approved by an ethics committee?** Yes
**Ethics committee - additional information:** The ethics committee notification can be found under the number 2023-21/704.
**Author Disclosures:**
Alev Dolu: Nothing to disclose
Meliha Akin: Nothing to disclose
Nurper Önder Denizoğlu: Nothing to disclose
Cihan Uras: Nothing to disclose
Gul Esen: Nothing to disclose
Amalya Zeynalova: Nothing to disclose
Fatma Tokat: Nothing to disclose

**Beyond the Breast: Radiomics of Axillary Nodes on Routine Breast MRI as a Biomarker of Nodal Metastasis**
*A. H. Shah*, A. Chandra, S. Sen, P. Ghosh; Kolkata/IN
*(shahakshat1788@gmail.com)*

**Purpose:** Axillary nodal status is critical in breast cancer staging, yet MRI assessment relies on subjective criteria such as cortical thickening or hilum loss, which are only moderately accurate. We evaluated whether radiomics of axillary nodes visible on routine breast MRI can provide a reproducible, objective predictor of nodal metastasis.
**Methods or Background:** We retrospectively analyzed 212 women with biopsy-proven breast cancer who underwent preoperative breast MRI (2013–2024) and subsequent nodal sampling or dissection. The most conspicuous axillary node per patient was segmented on post-contrast T1- and T2-weighted sequences. PyRadiomics extracted first-order, shape, and texture features. Machine-learning models were trained to classify nodal status and compared against conventional MRI descriptors (short-axis >10 mm, cortical thickness >3 mm, hilum loss).
**Results or Findings:** Of 212 patients, 118 had metastatic and 94 benign nodes. Radiomics features reflecting shape irregularity, enhancement heterogeneity, and cortical entropy were most predictive. The radiomics model achieved an AUC of 0.87, outperforming conventional MRI criteria (AUC 0.71, p<0.01). Sensitivity and specificity were 82% and 80%, respectively. Radiomics also improved prediction of extranodal extension (AUC 0.79 vs 0.62). Interobserver reproducibility was excellent for radiomics scores, compared with only moderate agreement for visual assessment (κ=0.46).
**Conclusion:** Radiomics of axillary nodes on standard breast MRI provides a reproducible, quantitative biomarker of nodal metastasis, clearly outperforming conventional visual descriptors. This is the largest dedicated study to date demonstrating that data already embedded in routine breast MRI can be unlocked to refine nodal staging, with direct implications for surgical and systemic treatment planning.
**Limitations:** Single-center retrospective design; segmentation restricted to one node per patient; no external validation.
**Funding for this study:** None
**Has your study been approved by an ethics committee?** Not applicable
**Author Disclosures:**
Aditi Chandra: Nothing to disclose
Saugata Sen: Nothing to disclose
Akshat Hitesh Shah: Nothing to disclose
Priya Ghosh: Nothing to disclose

**16:30 - 17:30** **Research Stage 4**

### Research Presentation Session: Artificial Intelligence and Imaging Informatics

### RPS 605

### Does AI really help? Real-world performance data in fracture diagnostics

**Moderator**
Patrick Mc Laughlin; Cork/IE
*(mclaughlin.paddy@gmail.com)*
**Author Disclosures:**
Patrick Mc Laughlin: Other: Clinical Safety Officer Change Healthcare 2020-2023. Chief Medical Officer Change Healthcare 2017-2020.

### Retrospective validation of three artificial intelligence -based fracture detection systems using local data

*J. H. J. Ketola*, S. Inkinen, T. Mäkelä, K. Pohto, M. Kortesniemi, S. Syväranta; Helsinki/FI

**Purpose:** Artificial intelligence (AI)-based fracture detection systems are being implemented worldwide to accelerate diagnostic workflows and improve accuracy. In this study, we evaluated three commercial AI fracture detection systems retrospectively using a diverse local trauma X-ray dataset. The objective was to evaluate the performance of each system and to assess their respective strengths and limitations.
**Methods or Background:** A local dataset comprising 1,891 trauma X-ray images (675 adults, 1,216 paediatric) spanning various anatomical regions was processed using three different AI systems. AI results were compared to primary radiologist reports, calculating accuracy, sensitivity, and specificity for each solution. An experienced radiologist verified any discrepancies, and fractures identified by AI but not included in the original reports were classified as additional AI-findings.
**Results or Findings:** For adults, accuracies ranged from 0.94 to 0.96, sensitivities from 0.89 to 0.93, and specificities from 0.96 to 0.97. In paediatric cases, accuracies ranged from 0.96 to 0.97, sensitivities from 0.89 to 0.95, and specificities from 0.96 to 0.98. AI-enhanced detection rates for adults ranged from 4.9% to 5.9%, and for paediatrics from 1.1% to 2.1%. Performance varied by anatomical area, bone, and fracture type.
**Conclusion:** All three solutions showed similar performance, differing only by a few percentage points. Depending on the clinical use-case, prioritizing either higher sensitivity or specificity may be preferable. Our findings underscore the importance of validating AI fracture detection systems with local data to reveal their unique advantages and limitations.
**Limitations:** Testing was performed using fixed operating points. More comprehensive evaluation based on receiver-operating characteristics was not feasible due to unavailable raw output probabilities. A detailed cost-benefit assessment would require a longer, prospective testing period.
**Funding for this study:** No external funding
**Has your study been approved by an ethics committee?** Not applicable
**Author Disclosures:**
Suvi Syväranta: Nothing to disclose
Mika Kortesniemi: Nothing to disclose
Satu Inkinen: Nothing to disclose
Teemu Mäkelä: Nothing to disclose
Kati Pohto: Nothing to disclose
Juuso Heikki Jalmari Ketola: Nothing to disclose

### Role of artificial intelligence in assisting non-MSK specialist radiologists with tibial plateau fracture detection

*A. I. Hernáiz Ferrer*, E. M. Preda, D. Meccariello, J. Bosio, L. Carone, C. Bortolotto, L. Preda; Pavia/IT

**Purpose:** We evaluated the diagnostic performance of two AI software programs, BoneView (AI-1) and RBfracture (AI-2), in assisting two non-specialist radiologists (NSR-1 and NSR-2) with the detection of tibial plateau fractures on conventional knee X-rays. These fractures are often challenging to identify due to their subtle appearance.
**Methods or Background:** In this retrospective monocentric study we analyzed 673 radiographs from 324 patients with knee trauma. All patients included in the study underwent a knee CT scan after the X-ray, which served as the gold standard. Diagnostic performance was assessed using sensitivity, specificity and area under the curve (AUC).
**Results or Findings:** The average patient age was 62 years, and 52.5% were female. CT scans confirmed tibial plateau fractures in 145 patients (44.8%) with AO/OTA B2 fractures being the most common type (35 patients, 24.1%). When evaluating X-rays, the AI tools performed similarly to NSRs (AUC: AI-1=0.88, AI-2=0.86, NSR1=0.86, NSR2=0.78). AI significantly improved the diagnostic performance of NSR-2 when combined with AI-1 and AI-2 (AUC=0.85 and 0.86; p=0.0001 and p=0.001, respectively), and of NSR-1 when combined with AI-2 (AUC=0.90; p=0.018). The overall performance of both AI systems for detecting other fracture types visible on radiographs (femur, patella, fibula) was also evaluated. AI-1 achieved an AUC of 0.842 and AI-2 an AUC of 0.798 (p = 0.017). Since AI-2 also detects lipohemarthrosis, we assessed its performance, finding a sensitivity of 61.7%, specificity of 98.5%, and an AUC of 0.801.
**Conclusion:** Both AI tools can assist non-specialist radiologists in detecting tibial plateau fractures on standard X-rays. They can also detect other fractures, with AI-2 showing particular promise in improving specificity through the detection of lipohemarthrosis.
**Limitations:** This study has some limitations, including its retrospective design. The results presented are preliminary.
**Funding for this study:** This study received no financial support and was conducted using free trial versions of the AI software.
**Has your study been approved by an ethics committee?** Yes
**Ethics committee - additional information:** The study was approved by the IRB Comitato Etico Territoriale Lombardia 6.
**Author Disclosures:**
Emma Maria Preda: Nothing to disclose
Jacopo Bosio: Nothing to disclose
Ana Isabel Hernáiz Ferrer: Nothing to disclose
Luisa Carone: Nothing to disclose
Lorenzo Preda: Nothing to disclose
Davide Meccariello: Nothing to disclose
Chandra Bortolotto: Nothing to disclose

### Budget-Impact Analysis of AI-Supported Fracture Detection: A Multi-Center Study in Moravian–Silesian Hospitals

M. Rehor[1], J. Orság[2], *D. Kvak*[2]; [1]Jindřichův Hradec/CZ, [2]Prague/CZ
*(daniel.kvak@carebot.com)*

**Purpose:** We quantified the one-year budget impact and return on investment (ROI) of deploying an AI tool for fracture detection in emergency musculoskeletal radiography across five Moravian–Silesian hospitals.
**Methods or Background:** We built a decision-tree comparing standard care (radiologist-only; sensitivity 82.4%, specificity 95.7%) with an AI-assisted pathway in which all 339,828 annual MSK X-rays were pre-read by commercial software (Carebot AI Bones; €1 per scan; sensitivity 92.1%, specificity 89.7%). AI-positive studies proceeded to radiologist confirmation, whereas AI-negative studies were cleared. First-diagnosis fracture prevalence was set at 6% (20,390). Downstream costs comprised an emergency revisit per false negative (€150) plus a one-day admission for 25% of false negatives (€194), and outpatient referrals for 10% of false positives (€20). Radiologist time savings were valued at one minute per avoided read at €27/hour. Litigation costs were analysed separately and excluded from the primary model.
**Results or Findings:** Relative to standard care, AI assistance reduced false negatives by 55% (3,587 to 1,614) and increased false positives by 140% (13,735 to 32,891). False-negative costs fell from €711,954 to €320,379 (saving €391,575). False-positive referrals increased from €27,470 to €65,782 (increment €38,312). Avoiding 288,161 reads saved 4,803 radiologist hours, valued at €129,681. After AI fees of €339,828, net regional savings were €143,116 (≈€28,623 per hospital). The benefit–cost ratio was 1.42, corresponding to an ROI of 42%. At €0.75 and €0.50 per scan, ROI rose to 90% and 184%, respectively.
**Conclusion:** In high-throughput emergency workflows, the AI triage model can halve missed fractures, lower downstream care costs, and deliver a positive ROI while focusing human review on AI-positive studies.
**Limitations:** Assumptions include negative-clearing without routine spot checks, static test performance, tariff-based unit costs, and single-region generalisability; prospective, real-time validation is required.
**Funding for this study:** Carebot s.r.o. supported software integration and modelling; no public grant funding was received.
**Has your study been approved by an ethics committee?** Not applicable
**Author Disclosures:**
Jiří Orság: Nothing to disclose
Marek Rehor: Nothing to disclose
Daniel Kvak: Nothing to disclose

**Comparison of deep learning object detection models for fracture diagnosis in X-ray images**

*C. Jeon*, J. Park; Seoul/KR
*(violet1252@naver.com)*

**Purpose:** Automated fracture detection via deep learning-based object detection can enhance diagnostic efficiency from radiographs. While one-stage models offer real-time processing, two-stage models provide higher precision. This study compares YOLOv8, RetinaNet, and Faster R-CNN on the FracAtlas X-ray dataset to determine the model offering the optimal trade-off between accuracy and practical applicability for clinical use.
**Methods or Background:** Object detection models were trained on the FracAtlas dataset (4,083 radiographs, 922 annotated fractures) using Python 3.9.0 with Detectron2 and Ultralytics frameworks. Model performance was assessed via confusion matrix-based indicators (Precision, Recall, F1-score), mean Average Precision at IoU 0.50 (mAP@0.50), and inference speed (frames per second, FPS).
**Results or Findings:** Faster R-CNN achieved the highest diagnostic accuracy with mAP@0.50 = 0.82, Precision = 0.92, Recall = 0.75, and F1-score = 0.82, but had a limited inference speed of 5 FPS. YOLOv8 demonstrated real-time performance with 45 FPS but lower accuracy (mAP@0.50 = 0.62, Recall = 0.57), particularly struggling with subtle fracture detection. RetinaNet produced intermediate results, yielding mAP@0.50 = 0.67 and 10 FPS. The superior feature extraction of Faster R-CNN underscores the clinical benefit of two-stage approaches when diagnostic accuracy is critical.
**Conclusion:** Faster R-CNN delivers the highest fracture detection accuracy, making it well-suited for clinical use, though its low FPS restricts real-time deployment. One-stage models excel in speed but fall short for complex diagnostic demands. Future research should explore lightweight optimisation of Faster R-CNN and larger datasets to enhance generalizability. Two-stage models remain valuable for high-precision medical image analysis.
**Limitations:** This study was limited to a single FracAtlas X-ray image dataset and an imbalanced fractured-to-non-fractured ratio (1:4.6), which may affect model performance.
**Funding for this study:** No funding was received for this study.
**Has your study been approved by an ethics committee?** Not applicable
**Author Disclosures:**
Jongwoon Park: Nothing to disclose
Changmin Jeon: Nothing to disclose

**Systematic Assessment of the Medical Utility of Radiology Artificial Intelligence in Fracture Detection (SAMURAI-Fracture): A multi-centre prospective cluster randomised cross-over trial**

*J. Vaz*[1], S. Ather[1], M. J. Lundemann[2], T. Bentabol Munoz[1], S. A. Beer[1], A. Espinosa[1], K. Nash[1], A. Novak[1]; [1]Oxford/UK, [2]København K/DK
*(james.vaz@ouh.nhs.uk)*

**Purpose:** To evaluate whether implementation of an AI-assisted fracture detection tool reduces unnecessary healthcare contacts arising from misdiagnosed fractures in emergency departments (EDs) and minor injuries units (MIUs).
**Methods or Background:** A multicentre, prospective, cluster randomised cross-over trial is being conducted across four NHS Trusts, encompassing level 1 EDs and MIUs. All patients aged ≥2 years undergoing X-ray for suspected fracture are eligible, with exclusions for skeletal surveys for non-accidental injury and skull, facial, dental, and cervical spine radiographs. Approximately 45,000 patients are expected to be recruited during a six-month period (October 2025–March 2026). An MHRA-approved AI tool is integrated into PACS at each site. Randomisation determines whether sites commence with AI active or inactive, alternating monthly between “on” and “off” periods. During “on” periods, clinicians access AI-annotated images as adjunctive decision support; during “off” periods, standard practice applies. The primary outcome is the proportion of unnecessary NHS contacts, defined as re-attendances or referrals resulting from false negatives and false positives. Secondary outcomes include diagnostic accuracy compared with radiology reports, subgroup analyses by anatomical region and demographics, length of stay in ED, and patient/clinician experiences captured via questionnaires.
**Results or Findings:** Results will compare unnecessary NHS contacts, diagnostic accuracy, and service outcomes between AI “on” and “off” periods.
**Conclusion:** This study will provide the first large-scale prospective evidence on the clinical, service, and health-economic impact of AI fracture detection in real-world NHS settings.
**Limitations:** As a pragmatic cluster trial, differences in staffing, patient mix, or workflow between periods may influence results.
**Funding for this study:** Small Business Research Initiative (SBRI) Healthcare Urgent and Emergency Care Grant
**Has your study been approved by an ethics committee?** Yes
**Ethics committee - additional information:** UK Research Ethics Committee Approval Obtained (IRAS 357391)
**Author Disclosures:**
Teresa Bentabol Munoz: Nothing to disclose
James Vaz: Grant Recipient: NIHR Oxford BRC
Michael Juncker Lundemann: Employee: Radiobotics, Denmark
Katrina Nash: Nothing to disclose
Abdala Espinosa: Nothing to disclose
Alex Novak: Nothing to disclose
Sarim Ather: Board Member: RAIQC Ltd
Sally Anne Beer: Nothing to disclose

**Multi-center Post-Implementation Monitoring of an AI Fracture Detection algorithm on Trauma Patients: Workflow Redesign, Safety Signals and Governance**

R. Sivanandan[1], *J. Vardal*[2], L. Tveiten[2], K. G. Brurberg[2], B. A. Graff[2]; [1]Asker/NO, [2]Drammen/NO
*(jvardal@gmail.com)*

**Purpose:** To evaluate the performance, workflow impact, and patient safety of a CE-marked AI application for fracture detection on x-rays with history of trauma following deployment across two hospitals within a regional healthcare trust.
**Methods or Background:** A retrospective, multi-center post-implementation study was conducted over two months at Site-A (n=1284) and Site-B (n=1177) hospitals. AI analyzed trauma-related X-rays, with radiologist reports serving as ground truth. Patient data included demographics, X-ray region, AI/radiologist findings, fracture types, additional observations, patient disposition, and follow-up imaging. Workflow was redesigned to allow patient discharge based on AI results obtained within two minutes approx., while radiologist reports were issued later the same or next day, enabling faster clinical decision-making with post-hoc expert validation.
**Results or Findings:** Fracture prevalence among the study population (2-99 years) was 34–37% (≈450 – 480 per site). AI and radiologist results showed high overall concordance across both hospitals 86 – 89%. However, 2.4 % (23-35 per site) cases were missed by AI; in 0.9% (11-12 per site) of cases, patients were prematurely discharged based on AI results. Four cases with fractures were recalled for treatment later, while the rest 0.8% (19 cases) had conservative treatment with follow-up scan following radiologists’ report. Workflow redesign reduced discharge time substantially compared with historical baseline, with consistent patterns across hospitals.
**Conclusion:** Multi-center monitoring confirmed consistent AI performance and notable workflow acceleration, while also identifying safety concerns such as missed fractures—underscoring the critical need for radiologist. These findings emphasize the importance of structured post-deployment governance to improve operational efficiency and human oversight involvement in AI-integrated patient care.
**Limitations:** Limitations include the restricted one-month observation periods at each site and absence of long-term or continous monitoring of patient outcome data, which could be addressed in future.
**Funding for this study:** Not funded
**Has your study been approved by an ethics committee?** Yes
**Ethics committee - additional information:** Ref-ID 23/07690-3, from the data protection officer in Vestre Viken Hospital Trust (HT)
**Author Disclosures:**
Jonas Vardal: Nothing to disclose
Kjetil Gundro Brurberg: Nothing to disclose
Line Tveiten: Nothing to disclose
Ramprabananth Sivanandan: Nothing to disclose
Bjørn Anton Graff: Nothing to disclose

**Comparative evaluation of commercial AI algorithms for fracture detection on musculoskeletal radiographs**

*J. Vaz*, K. Nash, A. Novak, C. Mihaiu, N. Salik, S. Ather; Oxford/UK
*(james.vaz@ouh.nhs.uk)*

**Purpose:** To conduct a comparative evaluation of commercial AI tools designed to detect fractures on musculoskeletal radiographs, assessing diagnostic performance across vendors.
**Methods or Background:** Six commercial vendors were invited of which three participated in this retrospective evaluation. A dataset of 500 anonymised radiographs was collected from Oxford University Hospitals and enriched to ~50% fracture prevalence to ensure anatomical and pathological diversity. Each case's ground truth was labelled through independent review by two musculoskeletal radiologists (>10 years’ experience), with arbitration from a third where required. All images were processed in batch by vendors. Vendors had up to 72 hours to return results.

**Results or Findings:** All participating vendors returned results within the 72-hour period. One vendor processed all images within their intended use case; whilst two vendors did not return results for between 16-18 cases. Accuracy ranged from 80.4% (95% CI: 76.4-83.8) to 87.2% (83.9-89.8). Sensitivity ranged from 70.4% (63.5-76.5) to 89.7% (84.8-93.2) and specificity from 73.9% (68.5-78.7) to 87.7% (83.1-91.2). McNemar's test revealed significant differences in accuracy, sensitivity, and specificity across tools (p <0.001), with some tools excelling in sensitivity and others in specificity.
**Conclusion:** Multiple commercial AI tools demonstrated high overall accuracy in detecting fractures on radiographs. However, there was notable variation in diagnostic performance between vendors. Sensitivity and specificity showed the greatest divergence, two tools prioritised either specificity or sensitivity, and one offered a more balanced performance profile. These differences may influence clinical outcomes and should be carefully considered when selecting AI tools for deployment.
**Limitations:** Commercial vendors were required to adapt their algorithms to a binary output (‘fracture present’ vs. ‘fracture absent’), whereas in routine use they may also provide a third category (‘possible fracture’). This constraint may have influenced the reported sensitivity and specificity.
**Funding for this study:** Innovate UK
**Has your study been approved by an ethics committee?** Yes
**Ethics committee - additional information:** This study was conducted in accordance with the Declaration of Helsinki. Ethics approval was granted for this study under Simulation Training in the Emergency Department clinical trials identifier NCT05427838.
**Author Disclosures:**
James Vaz: Grant Recipient: NIHR Oxford BRC
Cosmin Mihaiu: Employee: RAIQC Ltd
Katrina Nash: Nothing to disclose
Alex Novak: Nothing to disclose
Sarim Ather: Shareholder: RAIQC Ltd
Nabeeha Salik: Employee: RAIQC Ltd

**AI-Enhanced Fracture Detection in the ED: A Real-World Assessment of Diagnostic Accuracy and Imaging Utilisation**
*M. Godson Treacy*, S. Doherty, O. O'Brien, F. Desmond, F. Husson, P. J. Macmahon; Dublin/IE
*(mgodsontreacy@gmail.com)*

**Purpose:** Accurate and timely diagnosis of pelvic, hip, and spinal fractures in the Emergency Department (ED) is critical to reducing morbidity and mortality. Gleamer BoneView, an AI-based fracture detection tool, was implemented in our hospital in 2024. This study aims to evaluate the impact that the use of Gleamer BoneView, had on fracture detection in a real-world ED setting. We hypothesise that AI assistance would improve detection rates without increasing downstream imaging.
**Methods or Background:** We retrospectively analysed cases of adult ED patients undergoing pelvic/hip or spinal radiographs for suspected fracture from June–August 2024 (AI-assisted reporting) and for the same patient cohort in June-August 2023 (radiologist-only reported). Fracture detection was determined by the final radiologist's report. Detection rates were compared using chi-squared tests (p<0.05). CT and MRI utilisation, along with AI diagnostic performance (sensitivity, specificity, PPV, NPV), were also assessed.
**Results or Findings:** Pelvic/hip fracture detection increased from 12.7% (49/386) in 2023 to 19.2% (70/365) in 2024 (p=0.02). Spinal fracture detection increased from 14.7% (67/457) in 2023 to 21.3% (64/301) in 2024 (p=0.03). Downstream CT use remained stable for both pelvic/hip (11.7% vs. 10.4%) and spinal radiographs (7.8% vs. 7.6%). Downstream MRI use for indeterminate spinal radiographs decreased from 5.3% in 2023 to 3.3% in 2024. AI diagnostic performance was as follows: Spine radiographs: Sensitivity 90%, Specificity 93%, PPV 79%, NPV 97%. Pelvis/hip radiographs: Sensitivity 85%, Specificity 92%, PPV 71%, NPV 96%.
**Conclusion:** AI-assisted radiograph interpretation significantly improved fracture detection rates in the ED, without increased CT usage and with reduced MRI demand. These results support the integration of AI tools into acute imaging pathways to enhance diagnostic accuracy and streamline patient management.
**Limitations:** Short timeframe/small patient cohort. Single Centre Study.
**Funding for this study:** N/A
**Has your study been approved by an ethics committee?** Yes
**Ethics committee - additional information:** Hospital ethics committee.
**Author Disclosures:**
Fiona Desmond: Nothing to disclose
Oliver O'Brien: Nothing to disclose
Peter Joseph Macmahon: Nothing to disclose
Francois Husson: Nothing to disclose
Sadhbh Doherty: Nothing to disclose
Molly Godson Treacy: Nothing to disclose

**16:30 - 17:30** **Research Stage 1**

### Research Presentation Session: Interventional Radiology Hot Topic with Keynote Lecture

### RPS 609
### Hot Topic: AI-driven image interventions

**Moderator**
Andreas H. Mahnken; Bochum/DE

**Keynote Lecture**
Okan Gürkan; Istanbul/TR
*(drokan@gmail.com)*

**Artificial Intelligence in Pulmonary Nodule Biopsy: Comparing Predictive Strategies with Interventional Radiologists**
D. G. Castiglione, *L. Musmeci*, G. Failla, F. Libra, D. Falsaperla, F. Vacirca, F. Tiralongo, S. Palmucci, A. Basile; Catania/IT
*(lorenz.musmec@gmail.com)*

**Purpose:** This study aimed to evaluate the capability of two artificial intelligence (AI) models to predict optimal biopsy strategies for pulmonary nodules and to compare their decision-making process with approaches adopted by interventional radiologists.
**Methods or Background:** We retrospectively reviewed 49 lung biopsy procedures performed at our institution between November 2024 and February 2025. Clinical and imaging data, including lesion size, pleural distance, and complication rates, were collected. Lung nodules were manually delineated on CT scans and submitted in three orthogonal planes to two AI systems. Each AI was asked to simulate key procedural decisions: imaging guidance modality (CT vs US), patient positioning, and needle trajectory. AI outputs were compared with actual biopsy strategies retrieved from RIS/PACS. Subgroup analysis was performed according to operator experience (>10 years vs <10 years). Inter-reader agreement was measured with Cohen’s kappa.
**Results or Findings:** Overall AI–human agreement was poor (κ = 0.01–0.20). Fair agreement emerged between AI-2 and operators regarding guidance modality and entry point (κ = 0.21–0.40). Moderate agreement was observed when comparing AI-2 with radiologists with >10 years of experience, particularly for selecting CT vs US guidance and trajectory planning (κ = 0.41–0.60). AI-1 displayed weaker overall performance, with minimal alignment to clinical practice.
**Conclusion:** Current AI tools show limited reliability in replicating expert interventional radiologists’ decision-making in lung biopsy planning. While modest concordance was observed with more experienced operators, both systems lack sufficient robustness for autonomous use. AI may serve as an adjunctive decision-support tool but cannot yet substitute for clinical expertise. Further refinement with larger datasets and advanced training is warranted.
**Limitations:** N/A
**Funding for this study:** N/A
**Has your study been approved by an ethics committee?** Yes
**Ethics committee - additional information:** N/A
**Author Disclosures:**
Giovanni Failla: Nothing to disclose
Daniele Falsaperla: Nothing to disclose
Davide Giuseppe Castiglione: Nothing to disclose
Federica Libra: Nothing to disclose
Antonio Basile: Nothing to disclose
Francesco Vacirca: Nothing to disclose
Stefano Palmucci: Nothing to disclose
Lorenzo Musmeci: Nothing to disclose
Francesco Tiralongo: Nothing to disclose

**Smartphone Augmented Reality Guidance for Needle Intervention in Phantom and In Vivo**
*L. Saccenti*, N. Varble, J. Karanian, W. F. Pritchard, M. Li, B. J. Wood; Bethesda, MD/US

**Purpose:** To evaluate in a phantom and in vivo the accuracy of needle insertions using a smartphone augmented reality (AR) application with integrated needle guide and deep learning-based body tracking registration.

**Methods or Background:** A smartphone AR application was developed (Unity, Vuforia) with a surface tracking tool based on deep learning, to automatically overlay and scroll CT imaging data on a body, without fiducials or tracking hardware. A smartphone cover with an integrated needle guide (Civco) was designed and 3D printed, permitting use in percutaneous interventions. The target and entry point were selected through the smartphone application. Needle insertion along the planned path was performed with smartphone sensor feedback. Accuracy of needle insertions was assessed in a phantom (Sun Nuclear) (N=30; 5 physicians), and in vivo (N=14 needle insertions, 3 swine) under Institutional Animal Care and Use Committee approval. Accuracy (tip-to-target-center distance) and angular error were evaluated on post-insertion CT (iQon, Philips).
**Results or Findings:** In phantom, median accuracy was 4.3mm [IQR 2.2-6.7mm], and median angular error was 2.9° [1.5-4.7°]. In vivo, a total of 14 needle insertions (kidney N=5, liver N=4, and tight muscle N=5) were performed. Median accuracy was 8.8mm (IQR 5.5-11.5mm), and median angular error was 3.8° (IQR 2.4-5.7°).
**Conclusion:** A smartphone augmented reality application enabled automatic overlay of CT with body surface tracking and demonstrated accuracy of 4.3mm in phantom and 8.8mm in vivo for needle insertions. This smartphone AR application has a simple workflow without pre-procedural CT with fiducials or bulky hardware and does not require internal segmentation.
**Limitations:** Larger studies will include more operators to confirm those results
**Funding for this study:** Intramural Research grant from the National Institutes of Health
**Has your study been approved by an ethics committee?** Yes
**Ethics committee - additional information:** Institutional Animal Care and Use Committee approval for in vivo study
**Author Disclosures:**
Laetitia institutes of Saccenti: Research/Grant Support: Clinical Translational Fellowship Program of the NIH Clinical Center and the Intramural Research Program of the National Institute of Biomedical Imaging and Bioengineering.
Nicole Varble: Employee: Philips
John Karanian: Nothing to disclose
Brad J. Wood: Nothing to disclose
Ming Li: Nothing to disclose
William F Pritchard: Nothing to disclose

### Delta habitat analysis and 2.5D deep learning for predicting complete response of colorectal cancer lung metastases treated by radiofrequency ablation

*H. Huang*, W. Li, W. Li; Shanghai/CN

**Purpose:** To develop and validate a multi-omics integrated prediction model for complete response (CR) of colorectal cancer (CRC) lung metastases treated by radiofrequency ablation by combining radiomics, pathomics, and clinical features using delta habitat analysis and 2.5D deep learning.
**Methods or Background:** We analyzed pre- and post-treatment CT images of 249 CRC lung metastases from two centers between August 2016 and June 2022. Delta habitat subregions were generated via K-means clustering of local radiomic features. A 2.5D deep learning approach extracted spatial features from the largest cross-sectional tumor slices, while whole-slide pathology images were processed using weakly supervised multi-instance learning. Feature selection involved the minimum redundancy maximum relevance (mRMR) and least absolute shrinkage and selection operator (LASSO) regression. Models were built using support vector machine (SVM), random forest, and other classifiers, and integrated via multi-omics fusion. Performance was evaluated using receiver operating characteristic (ROC) analysis, calibration curves, and decision curve analysis (DCA).
**Results or Findings:** A total of 150 lung metastases (60%) achieved CR. The Delta Habitat signature achieved an AUC of 0.818 in the validation cohort, outperforming traditional delta radiomics (AUC = 0.741) and pre-habitat models (AUC = 0.740). The 2.5D multi-instance learning and pathomics signatures showed AUCs of 0.711 and 0.780, respectively. The combined multi-omics model achieved the highest AUC of 0.872, with improved calibration and net benefit in DCA.
**Conclusion:** The integrated multi-omics model significantly enhances CR prediction accuracy by leveraging complementary information from radiomic, pathomic, and clinical data, demonstrating strong potential for supporting clinical decision-making.
**Limitations:** Larger external validation data is required to confirm generalizability. Computational complexity and dependency on high-quality imaging may limit clinical deployment.
**Funding for this study:** 1. National Key R&D Program of China (Grant No.2023YFC2411404); 2. Shanghai Anticancer Association EYAS PROJECT (SACA-CY23B03)
**Has your study been approved by an ethics committee?** Yes
**Ethics committee - additional information:** Ethics Committee of Fudan University, Shanghai Cancer Center (Approval Number: 1612167–18).
**Author Disclosures:**
Wentao Li: Nothing to disclose
Wenchang Li: Nothing to disclose
Haozhe Huang: Nothing to disclose

### Novel Acousto-Optic Sensor Stylet for Needle Tip Tracking, Needle Tip Imaging, and Tissue Characterization Compared to Capabilities of Electromagnetic Tracking

*L. Saccenti*, O. Pena, L. Hazen, D. Singh, A. Gallagher, A. Mikhail, W. F. Pritchard, J. Karanian, M. Li; Bethesda, MD/US

**Purpose:** To assess the accuracy of a novel acousto-optic sensor for needle tip localization within ultrasound(US) imaging compared to electromagnetic(EM) tracking in phantoms. The ability to localize tumor and reconstruct US view from the needle tip was also evaluated.
**Methods or Background:** An acousto-optic 50µm sensor was prototyped and integrated into a 20G stylet. The sensor was interfaced with US and a visualization software (DeepSight Technology). It provided 3D location of the needle tip relative to US plane and an internal US view from the needle tip. A phantom with target tumors (Sun Nuclear) was punctured using the acousto-optic sensor and using a commercial EM tracking system (PercuNav, Philips). The EM tracking system was composed of a US transducer sensor, a needle holder sensor and an EM field generator. Needle insertions were completed when the tracking system indicated the needle tip was located at the center of the target. Accuracies (distances from needle tip to target center) on CT imaging (iQon, Philips) were compared (paired t-test, Rstudio v2024).
**Results or Findings:** Tumors (5-13cm depth) were targeted using the acousto-optic tracking system (N=10) and then the EM tracking system (N=10). The mean accuracy was 2.85mm (SD 1.32mm) for the acousto-optic tracking system and 3.57mm (SD 1.18mm; P=0.19) for the EM tracking system. Using the acousto-optic system, US imaging from needle tip was feasible for each insertion, with sufficient resolution to allow characterization of target-specific echogenicity around the needle tip. The EM tracking system did not allow internal imaging or tissue characterization capabilities.
**Conclusion:** This novel acousto-optic sensor could successfully localize the needle tip in 3 dimensions with accuracy similar to EM tracking in phantom, while enabling internal US imaging from the needle tip.
**Limitations:** Phantom study
**Funding for this study:** NIH intramural grant
**Has your study been approved by an ethics committee?** Not applicable
**Author Disclosures:**
Deven Singh: Nothing to disclose
Ashley Gallagher: Nothing to disclose
Laetitia institutes of Saccenti: Research/Grant Support: Clinical Translational Fellowship Program of the NIH Clinical Center and the Intramural Research Program of the National Institute of Biomedical Imaging and Bioengineering.
Lindsey Hazen: Nothing to disclose
John Karanian: Nothing to disclose
Olivia Pena: Nothing to disclose
Andrew Mikhail: Nothing to disclose
Ming Li: Nothing to disclose
William F Pritchard: Nothing to disclose

### Robotic in-bore MRI-guided prostate biopsy: experience in PI-RADS 4 and 5 lesions at a public university hospital

*S. De la Chica Bolaños*, J. Salazar, D. C. Gimenez, A. Soldevila, F. A. Armas Terry, S. Guillén Rodriguez, N. De La Torre Rubio, R. Castañeda Argaiz, M. Figols Gorina; Manresa/ES

**Purpose:** To describe our experience with robotic in-bore MRI-guided prostate biopsy with the Soteria® system in PI-RADS 4 and 5 lesions within a fast-track diagnostic pathway at a public university hospital, and to analyse effectiveness and safety.
**Methods or Background:** A single-centre retrospective study (01.08.2024–31.07.2025) was conducted at Hospital Sant Joan de Déu – Fundació Althaia, Manresa, Spain, in patients referred from a fast-track diagnostic pathway and undergoing in-bore MRI-guided prostate biopsy with the Soteria® system for PI-RADS 4 or 5 lesions. Clinical data, MRI lesion characteristics (size, location), and histopathological outcomes were analysed. Complications (rectal bleeding, haematuria, fever, emergency visits) were recorded.
**Results or Findings:** 72 cases were analysed. Mean age was 71 ± 8.1 years and median PSA 8.8 ng/mL (IQR 5.7–14.6). The malignancy rate was 61.1% (95% CI: 48.9–72.4). Of these, 47 were PI-RADS 4 and 24 PI-RADS 5. Malignancy was more frequent in PI-RADS 5 than in PI-RADS 4 (87.5% vs 48.9%; p=0.0015). Most tumours were in the peripheral zone (68.2%), followed by transitional (27.3%) and central (2.3%). Histologically, PI-RADS 5 lesions showed a higher proportion of ISUP grade ≥4 compared with PI-RADS 4 lesions (47.6%; p=0.033). Concordance between biopsy and surgical specimen ISUP was substantial (Weighted Kappa=0.77, 95% CI: 0.49–1.00) in the five patients with surgery. Complications were infrequent, reported in three of 54

patients with follow-up (5.6%): one mild rectal bleeding, two febrile episodes, and one emergency visit.
**Conclusion:** Robotic in-bore MRI-guided prostate biopsy is safe, effective, and achieves high cancer detection. PI-RADS 5 correlated with malignancy and higher ISUP. Substantial concordance was observed between biopsy and surgical ISUP.
**Limitations:** The small number of surgical cases for ISUP comparison.
**Funding for this study:** No funding was received for this study.
**Has your study been approved by an ethics committee?** Not applicable
**Author Disclosures:**
Noelia De La Torre Rubio: Nothing to disclose
Mariona Figols Gorina: Nothing to disclose
Roberto Castañeda Argaiz: Nothing to disclose
Sergio De La Chica Bolaños: Nothing to disclose
Felix Adrian Armas Terry: Nothing to disclose
Juan Salazar: Nothing to disclose
David Coll Gimenez: Nothing to disclose
Sergio Guillén Rodriguez: Nothing to disclose
Anna Soldevila: Nothing to disclose

**Performance of AI in predicting HCC recurrence after thermal ablation: a systematic review**
*A. Posa*, P. Barbieri, M. Lippi, G. D'Aniello, E. V. Andreani, R. Iezzi; Rome/IT
*(alessandro.posa@gmail.com)*

**Purpose:** To evaluate the effectiveness of AI-driven predictive models in predicting HCC recurrence.
**Methods or Background:** Recurrence prediction of hepatocellular carcinoma (HCC) after thermal ablation represents a challenge that can impact patients' quality of life. Artificial Intelligence (AI)-based radiomics models, applied to various imaging modalities, can improve recurrence prediction, therefore guiding therapeutic decisions. A systematic literature search in PubMed and Scopus has been performed. A total of 17 studies were selected to be included in this systematic review. All studies included response prediction evaluation with AI models for patients who underwent thermal ablation for HCC. Deep learning and machine learning algorithms were confronted to evaluate the predictive performance and accuracy using metrics such as the area under the curve (AUC) and concordance index (C-index).
**Results or Findings:** The developed models demonstrated high accuracy in predicting local progression and recurrence, allowing a solid risk stratification. In particular, the integration of imaging data and clinical-laboratory variables optimized treatment selection, highlighting the superior ability of imaging models to predict therapeutic outcomes compared to clinical parameters alone. Furthermore, radiomic analysis of follow-up imaging enabled highly accurate detection of ablation site recurrence.
**Conclusion:** AI-driven predictive models based on multimodal radiomic analyses integrated with clinical data represent promising tools for predicting tumour recurrence after thermal ablation in HCC patients.
**Limitations:** Mostly retrospective and monocentric studies. Different reported outcome measures.
**Funding for this study:** None.
**Has your study been approved by an ethics committee?** Not applicable
**Author Disclosures:**
Pierluigi Barbieri: Nothing to disclose
Roberto Iezzi: Nothing to disclose
Edoardo Vincenzo Andreani: Nothing to disclose
Giovanni D'Aniello: Nothing to disclose
Alessandro Posa: Nothing to disclose
Marcello Lippi: Nothing to disclose

**16:30 - 17:30 Research Stage 6**

### Research Presentation Session: Musculoskeletal

### RPS 610

### Imaging in action: musculoskeletal interventions

**Moderator**
Eugen Divjak; Zagreb/HR
*(edivjak@gmail.com)*

**Platelet-rich plasma versus corticosteroid–hyaluronic acid treatment for patients with knee osteoarthritis: a retrospective comparative study**
*M. Adinolfi Falcone*, L. Rinvenuto, F. Arrigoni, L. Zugaro, A. Barile, E. Di Cesare; L'Aquila/IT
*(marica.adi@outlook.it)*

**Purpose:** Platelet-rich plasma (PRP) and hyaluronic acid (HA) are established intra-articular therapies. This study aimed to compare the short- and long-term efficacy of PRP with a corticosteroid–HA combination in patients with knee osteoarthritis.
**Methods or Background:** A retrospective single-center study was conducted on 100 patients under 60 years of age (mean age 51) with knee osteoarthritis classified according to the International Cartilage Repair Society (ICRS). Patients were divided into two groups: Group A received three intra-articular PRP injections at 2-week intervals; Group B received one intra-articular corticosteroid injection followed by two HA injections at the same intervals. Symptom severity was assessed using the Visual Analog Scale (VAS) at baseline and at 1, 3, 6, and 12 months. Statistical analysis was performed, with $p<0.05$ considered significant.
**Results or Findings:** Baseline VAS scores were comparable between groups. At 1 month, corticosteroid–HA showed greater early symptom relief (VAS decrease: 4.30 ± 2.31, $p<0.001$). At 3 months, no significant difference was observed ($p=0.860$). PRP demonstrated slower onset but superior long-term efficacy at 6 and 12 months (VAS decrease: 5.56 ± 1.50, $p<0.001$). Within the corticosteroid–HA group, subgroup analysis by ICRS grade demonstrated more durable effects in grades I–II, whereas grades III–IV showed improvement limited to the short-term.
**Conclusion:** PRP injections provided significantly greater long-term symptom relief compared with corticosteroid–HA. While PRP showed slower onset, its efficacy was sustained up to 12 months. Corticosteroid–HA offered more transient benefits, with duration influenced by baseline ICRS grade. These findings support PRP as a more effective long-term treatment option for knee osteoarthritis.
**Limitations:** No limitations were identified.
**Funding for this study:** No funding was received for this study.
**Has your study been approved by an ethics committee?** Not applicable
**Author Disclosures:**
Francesco Arrigoni: Nothing to disclose
Luigi Rinvenuto: Nothing to disclose
Ernesto Di Cesare: Nothing to disclose
Marica Adinolfi Falcone: Nothing to disclose
Antonio Barile: Nothing to disclose
Luigi Zugaro: Nothing to disclose

**Ultrasound guided intramuscular administration and MRI monitoring of non-viral gene editing delivery technology for intramuscular treatment of Duchenne muscular dystrophy**
*O. Fındık Şener*, F. Sage, E. H. Niks, S. S. Rauh, M. C. De Ruiter, M. A. Engelse, N. Geijsen, M. C. Burgmans, H. E. Kan; Leiden/NL
*(o.findik_sener@lumc.nl)*

**Purpose:** Duchenne muscular dystrophy is characterized by progressive muscle wasting. Current gene-editing trials using systemic delivery face challenges in systemic side effects and tissue exposure. Intramuscular dosing may increase tissue concentrations and limit side effects, but requires an optimal injection technique. We aim to develop ultrasound-guided intramuscular delivery and MRI monitoring of fluid distribution in a preclinical setting.

**Methods or Background:** Dose-escalation and repeatability were assessed in two sets of four porcine extensor digitorum brevis muscles in situ. Four volumes (2.5-10ml of an ink and iTOP (induced transduction by osmocytosis and propanebetaine) solution (dilution 1/10) were injected using a 20G Chiba needle under ultrasound guidance. The entire muscle was targeted longitudinally from four directions. Muscle volume and ink distribution (%stained vs total) were quantified on 2cm cross-sections. Repeatability was assessed with the intraclass correlation-coefficient (ICC). In four other porcine hindlimbs, fluid distribution was quantified using 3T MRI, pre and post-injection scans (pixel-intensity threshold method on the TSE Dixon water-only image (TE=94ms)). 2.5 and 5ml injections are completed so far, with 7.6 and 10ml expected to be completed by March 2026.
**Results or Findings:** Muscle volumes were 26.9±4.1cm3. The injection to volume ratio was 0.1; 0.2; 0.3 and 0.3 (2.5ml to 10ml). In the dose-escalation experiment, higher injection volumes showed increased fluid distribution (ranging from 54.8% for 2.5ml to 99.4% for 10ml). Repeatability was excellent with an ICC of 0.99. Post-injection MR images showed a clear increase in signal intensity, with up to 65% elevated pixels.
**Conclusion:** Ultrasound-guided four longitudinal intramuscular injections provided up to 99.4% fluid distribution and excellent consistency between the measurements. MRI enabled in-situ monitoring of fluid distribution, suggesting that this method can also be used in-vivo.
**Limitations:** The limitation of the study is having non-living models.
**Funding for this study:** Funding was provided by the Novo Nordisk Foundation Center for Stem Cell Medicine (reNEW), (Grant number NNF21CC0073729)
**Has your study been approved by an ethics committee?** Not applicable
**Author Disclosures:**
Ozge Fındık Şener: Nothing to disclose
Erik H. Niks: Nothing to disclose
Marten A. Engelse: Nothing to disclose
Hermien E. Kan: Research/Grant Support: H.E. Kan reports research support from Philips Healthcare during the conduct of the study, and trial support from ImagingDMD-UF outside the submitted work; all reimbursements for H.E. Kan were received by the LUMC, and no personal financial benefits were received
Marco C. De Ruiter: Nothing to disclose
Mark C. Burgmans: Nothing to disclose
Fanny Sage: Nothing to disclose
Niels Geijsen: Nothing to disclose
Susi S. Rauh: Nothing to disclose

**Ultrasound-guided platelet-rich plasma and corticosteroid injection for subacromial-subdeltoid bursitis: a prospective comparative study**
*G. Kalidonis*, V. Nikovia, E. A. Ezerska, K. Demetriou, M. Klontzas, M. Raissaki, A. Karantanas, E. E. Vassalou; Heraklion/GR
*(grg.kalidonis@gmail.com)*

**Purpose:** To compare the efficacy of ultrasound-guided subacromial-subdeltoid (SASD) bursa platelet-rich plasma (PRP) and corticosteroid injections in patients with SASD bursitis over 1-year and to assess the predictive value of several factors related to outcome.
**Methods or Background:** From January 2021 to November 2023, consecutive patients with SASD bursitis secondary to shoulder impingement syndrome (SIS) were prospectively enrolled. Patients with rotator cuff tendinosis/tears were excluded. Participants were randomly assigned into two groups, (i)PRP-group receiving a single, ultrasound-guided SASD bursa injection with 10mL of autologous PRP (n=41) and (ii)corticosteroid-group, receiving a 10mL mixture of triamcinolone acetonide, lidocaine and saline (n-42). Clinical outcomes were assessed using the Constant-Murley Score (CMS) at baseline, 1-week, 3-months, 6-months, and 1-year post-treatment. Statistical analyses were performed using the Mann-Whitney and Hosmer-Lemeshow tests. A multivariable logistic regression model was developed to ascertain the effect of six factors on CMS. P<.05 denoted significance.
**Results or Findings:** The PRP-group included 26-females/15-males (mean age, 43.4 years) and the corticosteroid-group 24-females/18-males (mean age, 42.3 years). Baseline CMS showed no significant difference (p=.11). In both groups CMS improved significantly at all time-points compared to baseline (p<.05). At 6-months no significant difference was observed (p=.076); the corticosteroid-arm showed improved outcome at all other time-points (p<.05). Treatment method and maximum bursa width were significant predictors for achieving patient acceptable symptom state at 3-months while patient age and baseline CMS were significant at 1-year.
**Conclusion:** Both corticosteroids and PRP appeared effective for SASD bursitis, with results sustained up to 1-year. PRP achieved comparable effect in the mid-term. Corticosteroids showed superior outcome at other time-points. These findings support PRP as an alternative therapeutic option for SIS-related SASD bursitis.
**Limitations:** CMS, although widely accepted, presents certain drawbacks as a clinical tool for evaluating functional capacity.
**Funding for this study:** None
**Has your study been approved by an ethics committee?** Not applicable
**Author Disclosures:**
Michail Klontzas: Nothing to disclose
Georgios Kalidonis: Nothing to disclose
Karolos Demetriou: Nothing to disclose
Evangelia E. Vassalou: Nothing to disclose
Eleni Anna Ezerska: Nothing to disclose
Maria Raissaki: Nothing to disclose
Vasiliki Nikovia: Nothing to disclose
Apostolos Karantanas: Nothing to disclose

**Ultrasound-guided triple-target "sandwich" technique for flare-ups of chronic Achilles tendinopathy: preliminary experience**
*G. Reverchon*, A. Bazzocchi, F. Ponti, G. Bilancia, D. Romeo, P. Spinnato; Bologna/IT
*(giorgio.reverchon@gmail.com)*

**Purpose:** Flare-ups of chronic Achilles tendinopathy (AT) represent a therapeutic challenge. Conservative options may fail, while surgery is often too invasive. We present our initial experience with a novel ultrasound-guided interventional procedure – the triple-target "sandwich" technique – designed to manage AT flare-ups in a minimally invasive manner.
**Methods or Background:** Between January and June, 7 procedures were performed on 5 patients with chronic AT flare-ups (2 women, 3 men; mean age 50 years). Under ultrasound guidance, with previous subcutanous and peritendinous tissue local anestesia, injections were performed at three levels: (1) intratendinous tissue with hyperosmolar glucose, (2) pre-Achilles bursa with anesthetic and triamcinolone, and (3) retro-Achilles bursa with anesthetic and water-soluble corticosteroid. Prolotherapy works by injecting an hyperosmolar glucose solution that mildly irritates the tissue, stimulating the body's natural healing response. This helps the body produce new collagen, making the tendons stronger and reducing the pain.Patients were subsequently referred for rehabilitation. Pain scores (NRS) and range of motion (complete restoration, subtotal, partial and unchanged) were assessed at baseline, 1 week, 3 months, and 6 months.
**Results or Findings:** Mean pain scores significantly decreased from 8.8 at baseline to 1.5 at 1 week, 0.8 at 3 months, and 3.1 at 6 months (p=0.002). At 3-month follow-up, 42% of patients achieved complete recovery of motion, 42% subtotal recovery, and 14% partial recovery. At 6 months, complete recovery was maintained in 28%, subtotal in 28%, and partial in 42%. No complications or adverse events were reported.
**Conclusion:** The ultrasound-guided triple-target "sandwich" technique appears to be a safe, well-tolerated, and cost-effective procedure for the management of AT flare-ups. Despite the limitations of a small sample and short follow-up, these preliminary findings suggest promising clinical potential.
**Limitations:** Small patient cohort and limited follow-up.
**Funding for this study:** No funding was provided.
**Has your study been approved by an ethics committee?** Yes
**Ethics committee - additional information:** local IRB approval obtained.
**Author Disclosures:**
Alberto Bazzocchi: Nothing to disclose
Federico Ponti: Nothing to disclose
Gabriele Bilancia: Nothing to disclose
Giorgio Reverchon: Nothing to disclose
Domenico Romeo: Nothing to disclose
Paolo Spinnato: Nothing to disclose

**CT-Guided Facet Joint Cyst Rupture: Clinical Outcomes of a Minimally Invasive Technique**
*S. Mandal*[1], D. Dalili[2]; [1]London/UK, [2]Oxford/UK

**Purpose:** To assess outcomes of CT-guided facet joint cyst rupture over five years, focusing on surgical avoidance and the need for subsequent operative management.
**Methods or Background:** Facet joint cysts, a sequela of degenerative spinal disease, may produce nerve compression and pain. While surgical excision remains standard, CT-guided cyst rupture offers a minimally invasive alternative, with reported surgical avoidance rates of 55–66%. We retrospectively reviewed all patients undergoing CT-guided cyst rupture at Epsom Hospital (2020–2025). Follow-up data were derived from spinal surgeon clinic records. Of 35 cases identified, 14 were excluded (no cyst at procedure, spontaneous resolution, incomplete follow-up, or technical failure), leaving 21 patients for analysis.
**Results or Findings:** The cohort comprised 10 men (47%) and 11 women (53%), mean age 68 years. Most cysts were at L4/L5 (76%). Ten patients (48%) underwent an indirect approach; 11 (52%) received combined indirect and direct techniques. Eight patients (38%) required surgery (completed or scheduled), with a mean interval of 85 days post-rupture. Thirteen patients (62%) avoided surgery; of these, five (24%) required additional interventions (epidural or Racz catheter), while eight (38%) required no further treatment and derived sustained benefit.

**Conclusion:** CT-guided facet joint cyst rupture is a safe, effective, and minimally invasive treatment, with 62% avoiding surgery and over a third achieving durable relief without further intervention. These outcomes align with published data, supporting its consideration as a first-line option, particularly for patients at elevated surgical risk or preferring less invasive management.
**Limitations:** Small sample size, heterogenous follow-up, and retrospective design. We are still collecting data for a larger study to assess validation, generalisability and reproducibility.
**Funding for this study:** Not applicable
**Has your study been approved by an ethics committee?** Not applicable
**Author Disclosures:**
Danoob Dalili: Nothing to disclose
Sumona Mandal: Nothing to disclose

**Image-Guided Management of Chronic Post-TKA Knee Pain: Genicular Artery Embolization and Genicular Nerve Ablation**
*R. Marcello*, G. Marcello, M. Cuccarelli; Roma/IT
*(robermarcello@gmail.com)*

**Purpose:** To evaluate the clinical indications, technical approaches, and outcomes of two interventional radiology techniques—genicular artery embolization (GAE) and genicular nerve ablation (GNA)—in the management of chronic knee pain following total knee arthroplasty (TKA).
**Methods or Background:** We conducted a retrospective cohort analysis of patients with chronic (>3 months) post-TKA knee pain unresponsive to conservative treatment. Patients underwent either GAE, GNA, or a sequential combined approach. • GAE: performed under fluoroscopic guidance and microcatheter 1.7 Fr. superselective embolization of hyperemic genicular branches. Vascular approach was antegrade percutaneous US-guided common femoral artery access in most cases whereas controlateral cross over access was indicated in obese patients or in case of others anatomical limitations. • GNA: performed using radiofrequency ablation of the superomedial, superolateral, and inferomedial genicular nerves after a diagnostic nerve block. A dedicated RF generator was applied. Pain relief was assessed via VAS score at baseline and during follow-up (1, 3, and 6 months), along with functional improvement (WOMAC) and complication rates.
**Results or Findings:** • Total patients: 13 (GAE n=8 GNA n=2; combined n=3) • Mean pain reduction (VAS at 3 months): o GAE: –3.5 points o GNA: –4.1 points o Combined: –5.2 points • Functional improvement (WOMAC): significant in all groups (p<0.01), particularly in the combined group. • Complications: no major complications observed; minor side effects included transient skin discoloration (GAE) and local paresthesia (GNA). • Technical success: 100% in all procedures.
**Conclusion:** GAE and GNA are safe and effective image-guided procedures for chronic pain management after TKA, each addressing different pain mechanisms (inflammatory vs. neuropathic). Combined approaches may offer enhanced pain relief in selected cases. These minimally invasive techniques represent promising alternatives to revision surgery.
**Limitations:** Any
**Funding for this study:** Any
**Has your study been approved by an ethics committee?** Not applicable
**Author Disclosures:**
Gianmarco Marcello: Nothing to disclose
Roberto Marcello: Nothing to disclose
Martina Cuccarelli: Nothing to disclose

**Botulinum toxin for the belly: beyond aesthetics in abdominal wall hernia repair**
*A. M. Delgado Brito*, J. D. Aquerreta, I. Soriano, M. Elorz Carlón, M. Jiménez Vázquez, D. A. Zambrano, M. R. López De La Torre Carretero, A. Paternain Nuin; Pamplona/ES

**Purpose:** To evaluate the safety and feasibility of ultrasound-guided botulinum toxin type A (BTX-A) injection into the lateral abdominal wall as a preoperative adjunct for abdominal wall hernia repair, with attention to anatomy, technique, and outcomes.
**Methods or Background:** Consecutive case series of 23 patients. Abdominal wall anatomy was reviewed to guide infiltration of the lateral abdominal muscles (external oblique, internal oblique, transversus abdominis), which contribute to retraction and tension of the hernia defect. Under ultrasound guidance, 3–5 injection sites per side were used, tailored to abdominal wall dimensions. A total dose of 500 IU was diluted and evenly distributed across target muscles. The median interval from injection to surgery was 29 days. Patients were followed for hernia recurrence and procedure-related adverse events.
**Results or Findings:** Mean age 61.4 ± 12.3 years; mean body mass index (BMI) 28.9 ± 3.6 kg/m². Most patients (22/23; 95.7%) had at least one risk factor (smoking, diabetes, chronic obstructive pulmonary disease (COPD), or overweight). The procedure was well tolerated, with no major BTX-A–related complications. After a median follow-up of 881 days, no hernia recurrences were observed, including in two patients operated <48 hours after injection.
**Conclusion:** Ultrasound-guided BTX-A injection of the lateral abdominal wall is a safe, minimally invasive, and reproducible preoperative technique. By inducing temporary muscle relaxation, it facilitates fascial closure and optimizes repair. In this series, no medium-term recurrences were observed despite multiple comorbidities, supporting further comparative studies.
**Limitations:** The limitations of the study are its small sample size and the absence of a control group. Subsequent studies should enroll larger cohorts and incorporate a suitable control arm to improve robustness and generalizability.
**Funding for this study:** No funding was received for this study.
**Has your study been approved by an ethics committee?** Not applicable
**Author Disclosures:**
Jesús Dámaso Aquerreta: Nothing to disclose
Ignacio Soriano: Nothing to disclose
Manuel Rafael López De La Torre Carretero: Nothing to disclose
Adolfo Manuel Delgado Brito: Nothing to disclose
Daniel Alfonso Zambrano: Nothing to disclose
Mariana Elorz Carlón: Nothing to disclose
Alberto Paternain Nuin: Nothing to disclose
Marcos Jiménez Vázquez: Nothing to disclose

**16:30 - 17:30** **Research Stage 5**

### Research Presentation Session: Physics in Medical Imaging

### RPS 613

### Modern strategies, risk, and safety management in medical imaging

**Moderator**
Annalisa Trianni; Trento/IT
*(annalisa.trianni@asuit.tn.it)*

**Automated CT quality assurance analysis and monitoring framework for CT with open-source code - initial international experiences within IAEA project**
*J. I. Peltonen*[1], A-M. Hoyos-Garcia[2], D. Y. Nersissian[3], T. Li Kuo[4], I. Fitton[5], V. Tsapaki[6], O. Ciraj Bjelac[6], M. Kortesniemi[1]; [1]Helsinki/FI, Mexico City/MX, [3]São Paulo/BR, [4]Kuala Lumpur/MY, [5]Paris/FR, [6]Vienna/AT
*(juha.peltonen@hus.fi)*

**Purpose:** To test previously created automated quality assurance (QA) framework for computed tomography (CT) as a multi-center international pilot use with various different CT models as part of the International Atomic Energy Agency (IAEA) Coordinated Research Project (CRP) entitled “Advanced Tools for Quality and Dosimetry of Digital Imaging in Radiology” (E24025).
**Methods or Background:** The QA framework was built on free open-source code components and libraries to facilitate future development and for freely sharing knowledge within global medical physics and radiation protection community. The framework core components included results database, in-house built database communication interface, interactive user interface, data processing backend and a DICOM server. The QA framework requires only the standard CT calibration (vendor specific) phantom with homogenous section included in any commercial CT system installation. The QA analysis provides all essential technical image quality features (e.g. CT numbers and their variation, contrast, noise, resolution) including also model-observer based values (detectability index).
**Results or Findings:** The pilot installation was deployed in 6 countries with 12 CT systems in total representing various scanner generations and platforms, including conventional multi-slice scanners and new photon counting detector-based CT. The results demonstrated excellent applicability with different systems and environments.

**Conclusion:** Large scale CT QA framework built on free open-source code has been successfully tested on multi-center international environment within IAEA pilot project. The framework can be prepared for large scale global use.
**Limitations:** This research outlines early findings from the system's broad implementation.
**Funding for this study:** No dedicated funding was provided for this study.
**Has your study been approved by an ethics committee?** Not applicable
**Author Disclosures:**
Ana-Mariel Hoyos-Garcia: Nothing to disclose
Olivera Ciraj Bjelac: Nothing to disclose
Juha Ilmari Peltonen: Nothing to disclose
Tan Li Kuo: Nothing to disclose
Virginia Tsapaki: Nothing to disclose
Isabelle Fitton: Nothing to disclose
Mika Kortesniemi: Nothing to disclose
Denise Yanikian Nersissian: Nothing to disclose

### How to quality-assure new CT devices using a dose management system – analysis of scan parameter use, radiation exposure and technique

A. Romanyukha[1], L. Garajová[2], *N. Fitousi*[1], A. M. Sprinkart[2]; [1]Leuven/BE, [2]Bonn/DE
*(niki.fitousi@qaelum.com)*

**Purpose:** New scanner technology comes with promise of lower exposure and improved workflow. The purpose of this study was to QA a new spectral CT device by evaluating the difference in standard scan parameters, exposure and technique between old and new scanners of the same vendor.
**Methods or Background:** 6 months of data was exported from the dose management system including study compositions, scan parameters (kVp, pitch, collimation, exposure time per rotation, scanlength), and indicators of exposure (CTDIvol, SSDEWED) and technique (positioning offsets, lengths of scans outside localizer borders) for the same exams on iCT 256 (old) and spectral CT 7500 (new) scanners (Philips Healthcare): chest-abdomen with contrast (n=295 and n=694, respectively), chest with contrast (n=106 and n=220, respectively) and chest without contrast (n=252 and n=393, respectively). All data was collected from radiation dose structured reports.
**Results or Findings:** Study compositions, kVp and collimation were identical, while pitch was 1.8 times higher and exposure time per rotation was 0.06 s lower on the spectral CT 7500 for all protocols. Median scanlength was 1-6% higher on the new scanner for standard exams, likely attributed to patient differences. CTDIvol and SSDEWED were 50% lower for both chest protocols on the new scanner, and 14% and 22% lower, respectively, for the chest-abdomen protocol on the new scanner. Higher ranges of CTDIvol per patient WED were observed on the new scanner. Vertical and horizontal positioning offsets improved by 47-86% and 57-89%, respectively, on the new scanner. Scanning outside localizer borders was uncommon on both scanners and reduced further on the new scanner.
**Conclusion:** Differences in scan parameters were found, and spectral CT 7500 demonstrated reduced dose and improved technique. Investigation into scan settings and their impact on dose will be performed.
**Limitations:** None
**Funding for this study:** None
**Has your study been approved by an ethics committee?** Not applicable
**Author Disclosures:**
Alois M. Sprinkart: Nothing to disclose
Laura Garajová: Nothing to disclose
Anna Romanyukha: Employee: Qaelum NV
Niki Fitousi: Employee: Qaelum NV

### Development of a Practical Phantom for Quality Assurance in Lung Cancer Screening CT

*S. Sawall*, L. T. Rotkopf, T. D. Do, H-U. Kauczor, C. H. Ziener, H-P. Schlemmer, M. Kachelrieß; Heidelberg/DE
*(stefan.sawall@dkfz.de)*

**Purpose:** To design and evaluate a cost-efficient, semi-anthropomorphic phantom for quality assurance of CT lung cancer screening protocols, with emphasis on accessibility and applicability in resource-limited settings.
**Methods or Background:** The phantom was built from readily available materials for task-based image quality assessment at clinically relevant intersection lengths and dose levels. Candidate materials including ABS, PMMA, polycarbonate, polyethylene, and polyurethane (PU) foams were tested for machinability, attenuation, and water equivalence. A semi-anthropomorphic phantom with thoracic dimensions of 20 × 30 cm approximating adults was constructed. It includes features for the assessment of task transfer function, slice sensitivity profile, noise power spectrum, image noise, and lesion detectability. Cylindrical inserts and nodules of varying sizes and contrasts were embedded to mimic solid and ground-glass nodules. Imaging experiments were performed on an energy-integrating CT (Somatom Force) and a photon-counting CT (Naeotom Alpha.Peak) at multiple tube voltages and prefilters. Furthermore, a calibration approach is introduced in which CT values are modeled as a function of PU density or alternative materials, ensuring consistent evaluation despite local material variability.
**Results or Findings:** ABS and PU foams proved most suitable for simulating tissue and low-contrast lesions. ABS served as the phantom body and solid nodules, while PU foams of different densities represented lung parenchyma and ground-glass nodules. These materials provided water-equivalent attenuation, stable CT values across scanners and voltages, and realistic intersection lengths. The phantom enabled robust image quality assessment.
**Conclusion:** Our low-cost, reproducible phantom for CT lung cancer screening QA can be manufactured on site with simple tools, supporting broad use in clinical practice. To support adoption, design files and specifications will be made available.
**Limitations:** Manual fabrication may reduce structural accuracy, although CNC machining can improve precision where available.
**Funding for this study:** N/A
**Has your study been approved by an ethics committee?** Not applicable
**Author Disclosures:**
Thuy Duong Do: Nothing to disclose
Christian Herbert Ziener: Nothing to disclose
Hans-Ulrich Kauczor: Nothing to disclose
Lukas Thomas Rotkopf: Nothing to disclose
Heinz-Peter Schlemmer: Nothing to disclose
Marc Kachelrieß: Nothing to disclose
Stefan Sawall: Nothing to disclose

### Beyond One Size Fits All: Personalized Dosimetry in CT and Fluoroscopy Using NCICT and NCIRF

*C. Lee*; Rockville/US
*(choonsik.lee@nih.gov)*

**Purpose:** Traditional dosimetry methods in diagnostic imaging often rely on reference patient models, overlooking the diversity of age, sex, body size, and pregnancy status in real-world practice. This “one-size-fits-all” approach limits accuracy and underestimates variability in radiation dose. Recent advances with the National Cancer Institute dosimetry systems for Computed Tomography (NCICT) and Radiography and Fluoroscopy (NCIRF) move beyond population averages to provide personalized dose estimates.
**Methods or Background:** NCICT and NCIRF integrate Monte Carlo–derived dose libraries with advanced computational human phantoms that span pediatric through adult anatomies, both sexes, varying body habitus, and more recently pregnant patients. Organ dose estimates are individualized by merging scanner- or procedure-specific parameters with anatomically matched phantoms. NCIRF further models spatial dose distributions to estimate local peak skin dose—critical for patients undergoing complex, high-dose interventions.
**Results or Findings:** By accounting for patient-specific characteristics, individualized organ dose estimates can differ several-fold from reference values, particularly in pediatric, obese, or pregnant cohorts. NCIRF’s peak skin dose calculations enable proactive management of deterministic effects. Both systems are designed for seamless integration with dose reports, enabling high-throughput monitoring in clinical practice and retrospective dose reconstruction in research cohorts. Large-scale applications have demonstrated their utility for personalized radiation protection, patient counseling, and epidemiological studies.
**Conclusion:** NCICT and NCIRF represent a paradigm shift in diagnostic imaging dosimetry—moving beyond “one size fits all” to precision dose assessment. By leveraging the most advanced computational human phantoms and real-world imaging data, these systems provide powerful tools for patient-specific monitoring and long-term risk evaluation in both CT and fluoroscopy.
**Limitations:** Although NCICT and NCIRF employ individualized computational phantoms to improve accuracy, residual anatomical discrepancies from actual patients remain and may introduce uncertainty in organ and skin dose estimates.
**Funding for this study:** This research was supported by the Intramural Research Program of the National Institutes of Health (NIH). The contributions of the NIH author(s) were made as part of their official duties as NIH federal employees, are in compliance with agency policy requirements, and are considered Works of the United States Government. However, the findings and conclusions presented in this paper are those of the author(s) and do not necessarily reflect the views of the NIH or the U.S. Department of Health and Human Services.
**Has your study been approved by an ethics committee?** Not applicable
**Author Disclosures:**
Choonsik Lee: Nothing to disclose

**X-ray imaging dosimeters: target uncertainty for revealing errors**
*P. Toroi*[1], I. Stojanović[2], M. Živanović[2], A. Ciccotelli[3], N. Kiiskinen[4], M. Borowski[5]; [1]Vantaa/FI, [2]Vinča, Belgrade/RS, [3]Rome/IT, [4]Helsinki/FI, [5]Braunschweig/DE
*(paula.toroi@stuk.fi)*

**Purpose:** Measurement results should always be reported together with the estimated uncertainties. Measurement uncertainties must be sufficiently low to achieve the accuracy requested for clinical measurements. For air kerma measurements the target uncertainty is well established e.g., in IAEA TRS-457. However, for the other quality control (QC) parameters such as tube voltage, half-value layer, and irradiation time, the target uncertainty has not yet been clearly defined [Komatina I, et al. 2025 Physica Medica 136 105055].
**Methods or Background:** The dosimeter performance and associated uncertainties were analyzed within a European project using data from surveys, publications and experimental measurements. The achievable uncertainties were compared with established targets, and their clinical relevance was evaluated.
**Results or Findings:** For air kerma measurements, a target uncertainty of 5% (k = 2) can be reliably achieved with proper calibration. In contrast, for the other QC parameters the situation is less clear. IEC standards provide some acceptance criteria. For example, tube voltage should be within 8% in general radiology and 5% in mammography. To verify compliance with such limits, the measurement uncertainty must be considerably lower than the acceptance threshold. For the other QC parameters, an uncertainty of 2% is often set as a goal, although this is challenging to achieve. While reproducibility can be excellent (uncertainty <1%), absolute accuracy strongly depends on proper calibration. If a device is used under conditions different from those of its calibration, systematic errors exceeding 5% may occur.
**Conclusion:** Dosimeters used in X-ray imaging enable measurement of a wide range of QC parameters. However, for quantities other than air kerma, the associated uncertainties are still poorly known. This study clarifies achievable uncertainty levels and supports a more informed evaluation of whether observed measurement deviations are significant.
**Limitations:** Not applicable.
**Funding for this study:** This work was funded by the project 22NRM01 TraMeXI which has received funding from the European Partnership on Metrology, cofinanced from the European Union's Horizon Europe Research and Innovation Programme and by the Participating States. Funded by the European Union.
**Has your study been approved by an ethics committee?** Not applicable
**Author Disclosures:**
Niko Kiiskinen: Research/Grant Support: Funding through 22NRM01 TraMeXI research project
Paula Toroi: Research/Grant Support: Funding through 22NRM01 TraMeXI research project
Markus Borowski: Research/Grant Support: Funding through 22NRM01 TraMeXI research project
Alessia Ciccotelli: Research/Grant Support: Funding through 22NRM01 TraMeXI research project
Miloš Živanović: Research/Grant Support: Funding through 22NRM01 TraMeXI research project
Ivana Stojanović: Research/Grant Support: Funding through 22NRM01 TraMeXI research project

**Estimation of radiation risks from PET/CT examinations: what is the contribution of CT exposure versus FDG administration?**
*V. Mari*, N. Ntoufas, S. Koukouraki, K. Perisinakis; Iraklion/GR
*(vereniki1999@yahoo.gr)*

**Purpose:** To estimate the contribution of CT exposure and FDG administration to the life attributable radiogenic risk (LAR) of cancer associated with PET/CT examinations and compare the total LAR to the nominal life intrinsic risk (LIR) of cancer.
**Methods or Background:** The radiation dose to radiosensitive organs was calculated in a series of 24 patients subjected to PET/CT. Organ radiation doses from the administered FDG activity were determined using organ dose per activity factors. To estimate CT exposure-related organ doses, a Monte Carlo simulation software package was used that simulates the actual patient CT exposure on a patient-specific mathematical phantom generating dose images in 1-1 correspondence to original CT images. The FDG-related, CT-related, cumulative organ doses and published organ-specific radiogenic cancer risk factors were employed to estimate corresponding LAR estimates. The mean total LAR was compared to recently published LIR of cancer.
**Results or Findings:** The FDG-related organ doses ranged from 1.3 mGy (skin) to 27.9 mGy (bladder). The CT exposure-related organ doses ranged from 7.2 mGy (uterus) to 18.1 mGy (thyroid). The CT-related and FDG-related LAR was estimated to be 3.9 x10-4 and 2.0 x10-4 for males and 10.4 x10-4 and 3.4 x10-4 for females, respectively. The total LAR from a PET/CT examination was found to increase the risk for carcinogenesis to a cancer-free individual subjected to PET/CT by only 1.0015 and 1.0042 for male and female individuals, respectively.
**Conclusion:** The main contributor to the radiogenic risk of cancer from PET/CT examinations is CT, contributing by 78% and 65% to the total LAR of female and male patients, respectively. The increase of the nominal risk of cancer induction following a PET/CT examination with a modern system is < 0.42% and may be considered marginal.
**Limitations:** Cohort size.
**Funding for this study:** None.
**Has your study been approved by an ethics committee?** Not applicable
**Author Disclosures:**
Konstantinos Perisinakis: Nothing to disclose
Sophia Koukouraki: Nothing to disclose
Vereniki Mari: Nothing to disclose
Nick Ntoufas: Nothing to disclose

**The current status of reporting to accidental or unintended radiation exposure for patients and workers in Lithuania**
*B. Gricienė*, I. Andriulevičiūtė, K. Skovorodko, L. Krynke, M. Jeršova, V. Statkus, A. Samuilis; Vilnius/LT
*(birute.griciene@santa.lt)*

**Purpose:** The aim of this study is to provide an overview of the Lithuanian legal framework for the registration of accidental and unintended radiation exposures and to present national statistics on patient and occupational exposure cases exceeding investigation levels.
**Methods or Background:** Across Europe, the requirements for recording and reporting unplanned or emergency exposure situations vary considerably, reflecting differences in national radiation protection strategies and regulatory approaches. In Lithuania, these requirements established through national hygiene norms, which provide the legal basis for mandatory reporting. In addition, hospitals are required to implement local orders that regulate the reporting and analysis of unintended events. Patient cases in which radiation exposure during interventional radiology procedures leads to observable skin effects, except when associated with complex procedures, must be reported to the competent authority. For occupational exposures, hospitals are obliged to establish local investigation levels. These serve as threshold values that trigger further analysis, corrective actions, and preventive measures. In practice, this requires the systematic registration of unintended events, the application of optimization strategies, and the use of structured procedures for case analysis.
**Results or Findings:** Statistics on unintended events in diagnostic radiology reported by Lithuanian hospitals from 2020 to 2025 were analyzed. The majority of events were related to human error or equipment malfunction. Occupational exposure cases exceeding investigation levels were analysed, with most associated with demanding workloads and complex fluoroscopy guided interventional procedures.
**Conclusion:** Our findings highlight the importance and challenges of implementing a clear legal framework for the systematic registration of accidental and unintended radiation exposure cases. National statistics confirm that the application of error system registration and investigation levels provides a practical and effective tool for harmonizing case analysis, supporting optimization, ensuring consistent radiation protection standards.
**Limitations:** Not applicable.
**Funding for this study:** No funding was provided for this study
**Has your study been approved by an ethics committee?** Not applicable
**Author Disclosures:**
Artūras Samuilis: Nothing to disclose
Inga Andriulevičiūtė: Nothing to disclose
Marija Jeršova: Nothing to disclose
Kirill Skovorodko: Nothing to disclose
Vaidas Statkus: Nothing to disclose
Birutė Gricienė: Nothing to disclose
Leonid Krynke: Nothing to disclose

**From risk to reliability: advancing patient safety in imaging and radiotherapy**
*A. Papachristodoulou*[1], C. Paraskevopoulou[2], C. Domingos Filho[3], D. Baltazar[4], A. Roncacci[5]; [1]Thessaloniki/GR, [2]Athens/GR, [3]Barcelona/ES, [4]Amadora/PT, [5]The Hague/NL
*(nasia.papachristodoulou@affidea.com)*

**Purpose:** To evaluate trends in clinical incidents related to patient identification and procedure matching across a European diagnostic and radiotherapy network, and to assess the impact of targeted safety interventions.
**Methods or Background:** Clinical incident (CI) reports were collected from 286 diagnostic imaging and radiotherapy centres across 15 European countries. Incidents included repeated examinations, unintended radiation exposures, wrong procedure, wrong patient, wrong side and wrong site imaged or treated. Data was analyzed from January 2023 to August 2025. An action plan was introduced in response to peaks observed in March 2023 and April

2024. Safety interventions included extensive staff training, safety campaigns, systematic review of incidents, and deployment of digital identification tools.
**Results or Findings:** In 2023, 36 incidents were reported in January, with reductions to 10–18 per month from May onward, after the first intervention. The overall burden was highest in 2023 compared with subsequent years. In 2024, incident counts peaked at 21 (March) but stabilized at lower averages (8–13). Data from 2025 (Q1–Q3) showed further improvement, with monthly counts between 2–13, consistently below 2023–2024 levels. High-risk categories such as wrong patient and wrong site incidents declined significantly, while repeated examinations remained the most frequent category. The downward trend correlates with the roll-out of the action plan and harmonized safety protocols across countries.
**Conclusion:** A structured patient safety program, triggered by peak periods and supported by training, campaigns, and clinical review, produced sustained reductions in errors. Embedding these actions into clinical governance frameworks advances value-based healthcare by reducing harm and optimizing processes and resources. Continuous analysis of incidents is essential to identify trends and enable proactive mitigating actions that further strengthen safety culture across imaging networks.
**Limitations:** N/A
**Funding for this study:** N/A
**Has your study been approved by an ethics committee?** Not applicable
**Author Disclosures:**
Alessandro Roncacci: Nothing to disclose
Athanasia Papachristodoulou: Nothing to disclose
Chrysa Paraskevopoulou: Nothing to disclose
Clodoaldo Domingos Filho: Nothing to disclose
David Baltazar: Nothing to disclose

**Realization of Actionable Ultrasound Quality Issues in a Multi-Site Radiology Unit**
*J. H. J. Ketola*, S. Inkinen; Helsinki/FI

**Purpose:** Diagnostic ultrasound (US) transducers are prone to mechanical damage due to wearing or impact. Many defects can be identified by evaluating in-air reverberation patterns. Defective transducers can be either repaired or replaced. This study aimed to evaluate the outcomes associated with our technical quality control (QC) program for US transducers in our unit covering over 80 US devices with ca. 300 transducers.
**Methods or Background:** In January 2024, our hospital district implemented a digital quality control platform. Each diagnostic transducer is tested monthly by taking in-air reverberation images, which are available on the platform. If a transducer is found defective, medical engineering staff are notified to arrange repair or replacement.
**Results or Findings:** By October 2025, a total of 187 transducer defects had been identified by medical physicists or local radiographers/sonographers. Crystal damage was confirmed in 53 transducers through phantom measurements. Of these, 20 transducers were repaired and 33 were replaced — one with an existing unused probe, seven under warranty claims, and 25 through new purchases. Electrical safety assessments detected excess leakage current in three transducers, while one was found to have a short circuit. Faulty connectors and beamformers accounted for eight defects. Following repairs, five transducers exhibited recurring issues, prompting compensation claims. Additionally, one repaired transducer demonstrated reduced penetration depth in phantom testing and was subsequently replaced.
**Conclusion:** Our technical quality control program effectively detects transducer defects. Significant cost savings can be achieved by utilizing warranty claims for replacement and opting to transducer repairs rather than acquiring new transducers. However, our recent findings emphasize importance of QC for repaired transducers as they may present recurrent or novel issues, potentially invalidating financial benefits.
**Limitations:** No limitations were identified.
**Funding for this study:** No external funding.
**Has your study been approved by an ethics committee?** Not applicable
**Author Disclosures:**
Satu Inkinen: Nothing to disclose
Juuso Heikki Jalmari Ketola: Nothing to disclose

### Thursday, March 5

**08:00-09:30** **Research Stage 3**

### Research Presentation Session: Abdominal and Gastrointestinal

### RPS 701

### Quantitative imaging and biomarkers

**Moderator**
Ekaterina Khristenko; Frankfurt am Main/DE
*(katkhristenko@gmail.com)*

**Imaging Biomarkers for Prognostication in Primary Sclerosing Cholangitis: A Radiomics Approach**
*C. Maino*, P. N. Franco, C. R. G. L. O. M. Talei Franzesi, E. De Bernardi, L. Cristoferi, D. Ippolito; Monza/IT
*(mainocesare@gmail.com)*

**Purpose:** Risk assessment in primary sclerosing cholangitis (PSC) by magnetic resonance imaging (MRI) relies on semi-quantitative analysis, which can result in interpretation variability. Radiomics may offer a quantitative approach for risk stratification. This study aims to explore and validate MRI-derived radiomic features to identify high-risk PSC patients.
**Methods or Background:** In this prospective study (January 2019–December 2022), consecutive PSC patients undergoing routine gadoxetate disodium-enhanced MRI were recruited. Using PyRadiomics, whole liver parenchyma features were extracted from five MRI sequences according to the Image Biomarker Standardisation Initiative (IBSI). Patients were categorised into risk groups based on the Mayo risk score (MRS) and liver stiffness measurement (LSM). Features associated with high-risk patients were selected and validated in an independent cohort. A survival analysis was conducted in the combined cohort to assess the prognostic value of the radiomic features for clinical events.
**Results or Findings:** One hundred and two PSC patients were enrolled in this study. Five radiomics features were associated with high risk in the training cohort. In the validation setting, GLRLM-Run Entropy in the fat-saturation T2 weighted imaging (FS-T2W) sequence was the only significant feature, with an odds ratio of 3.90 (CI 1.46–10.42, p = 0.007) for MRS and 2.97 (CI 1.33–6.66, p = 0.008) for LSM. Its prognostic potential on clinical outcome was confirmed by Cox regression analysis in the combined cohort (hazard ratio per 0.1 increase = 1.480, CI 1.226–1.786), showing excellent predictive performance (C-index = 0.857).
**Conclusion:** GLRLM-Run Entropy in FS-T2W is a novel radiomics-based biomarker for risk stratification in PSC patients. It is quantitative, standardised, easy to compute and cost-free, positioning it as a potential key innovation in PSC radiology-based biomarkers
**Limitations:** Retrospective Single institution
**Funding for this study:** None/not used
**Has your study been approved by an ethics committee?** Yes
**Ethics committee - additional information:** Approved
**Author Disclosures:**
Laura Cristoferi: Nothing to disclose
Cesare Maino: Nothing to disclose
Cammillo Roberto Giovanni Leopoldo Oreste Massimiliano Talei Franzesi: Nothing to disclose
Elisabetta De Bernardi: Nothing to disclose
Paolo Niccolò Franco: Nothing to disclose
Davide Ippolito: Nothing to disclose

**Stability of Hepatic Radiomics Features Across Monoenergetic Reconstructions Using Portal Venous Phase as Benchmark**
*L. Sannikova*, S. Gusarova, I. Gruzdev, N. Karelskaya, V. Shirokov, V. Aznaurov, A. Ustalov, S. A. Shmeleva, E. V. Kondratyev; Moscow/RU
*(ls89613503160@yandex.ru)*

**Purpose:** To investigate how reproducible liver radiomics features are when derived from monoenergetic (MonoE) CT reconstructions, using portal venous phase imaging as the reference standard with a consistent 3D-ROI approach.
**Methods or Background:** The study included 64 abdominal CT examinations. A spherical VOI was positioned within a homogeneous segment of liver parenchyma devoid of vessels. This VOI, initially delineated in the portal venous phase, was automatically propagated across MonoE reconstructions (40, 55, 70, and 200 keV). Radiomic features were extracted and normalized via z-score transformation. Reproducibility was quantified using concordance correlation coefficient, intra-class correlation coefficient, and paired statistical tests with FDR adjustment.
**Results or Findings:** Reconstructions at 40 and 55 keV showed the closest alignment with portal imaging, yielding CCC values above 0.90 for most features, with ~45% qualifying as reproducible. The 70 keV level demonstrated intermediate reproducibility, especially limited for texture-based features, with only ~30% classified as reproducible. The 200 keV reconstructions performed poorly, with a high proportion of features below CCC 0.70, and evidence of systematic bias. Across all reconstructions, shape and first-order features remained the most robust. Similarity analyses and PCA revealed clustering of portal with MonoE40 and MonoE55, while MonoE200 consistently diverged from other groups.
**Conclusion:** Virtual monoenergetic reconstructions at 40 and 55 keV demonstrate the highest reproducibility of hepatic radiomics features and can be regarded as reliable surrogates for portal venous phase imaging. Importantly, these findings indicate that spectral CT data can be integrated alongside conventional radiomics models. Substantial energy shifts (e.g., high-keV reconstructions) generate significant alterations in feature behavior. Such investigations may not only refine technical reproducibility but also reveal hidden imaging phenotypes or disease-specific characteristics that remain undetectable at standard energy levels.
**Limitations:** CT-scanner from only one vendor (Philips) was used.
**Funding for this study:** Nothing to disclosure
**Has your study been approved by an ethics committee?** Yes
**Ethics committee - additional information:** This study was approved by the local ethics committee of the A. V. Vishnevsky National Medical Research Center of Surgery.
**Author Disclosures:**
Evgeny V Kondratyev: Nothing to disclose
Natalia Karelskaya: Nothing to disclose
Svetlana Gusarova: Nothing to disclose
Sofiia Antonovna Shmeleva: Nothing to disclose
Andrey Ustalov: Nothing to disclose
Vladimir Aznaurov: Nothing to disclose
Ivan Gruzdev: Nothing to disclose
Vadim Shirokov: Nothing to disclose
Lyudmila Sannikova: Nothing to disclose

**CT Radiomics for Preoperative Prediction of Vessels Encapsulating Tumor Clusters in Hepatocellular Carcinoma**
L. Jia[1], *S. N. Li*[1], Y. Li[2], J. Li[1], J. Lei[1]; [1]Lanzhou/CN, [2]Beijing/CN

**Purpose:** Vessels encapsulating tumor clusters (VETC) represent a distinct histologic vascular pattern strongly associated with metastatic spread in hepatocellular carcinoma (HCC). This study aimed to develop and validate a CT-based radiomics model for the preoperative prediction of VETC positivity using a dual-center design.
**Methods or Background:** In this retrospective study, we included patients with pathologically confirmed HCC who underwent contrast-enhanced CT between January 2019 and January 2023. The training cohort consisted of 142 patients from Center 1, and an independent external validation cohort included 61 patients from Center 2. Radiomic features were extracted from arterial and portal venous phase images. A radiomic signature was developed using a K-nearest neighbor classifier and compared to clinical-only and combined clinical-radiomic models. Performance was assessed using AUC, calibration curves, and decision curve analysis (DCA).
**Results or Findings:** The radiomics model significantly outperformed the clinical model, with an AUC of 0.894 (95% CI: 0.863–0.916) in the training set and 0.85 (95% CI: 0.78–0.92) in the external validation set. There was no significant difference between the radiomics and combined clinical-radiomics models (AUC: 0.922 vs. 0.894; p = 0.182). DCA demonstrated robust clinical utility of the radiomics model.
**Conclusion:** The proposed CT radiomics model noninvasively predicts VETC pattern in HCC with strong generalizability across institutions. This tool shows promise for identifying aggressive HCC subtypes preoperatively, potentially supporting personalized treatment strategies.
**Limitations:** None
**Funding for this study:** None
**Has your study been approved by an ethics committee?** Yes
**Ethics committee - additional information:** This retrospective study was approved by the Institutional Review Board (LDYYLL-2024-398)
**Author Disclosures:**
Shu Nan Li: Speaker: RPS 503, Photon-Counting Detector CT versus Cardiac Magnetic Resonance for ECV Quantification: Impact of Reconstruction Phase and Myocardial Layer, Analysis Session title: Imaging in structural heart disease Session number: Day: March 4, 2026 Time: 15:00-16:00
Lulu Jia: Nothing to disclose
Junqiang Lei: Nothing to disclose
Jinkui Li: Nothing to disclose
Yang Li: Nothing to disclose

**CT-Based Hepatic Vascular Biomarkers for Preoperative Prediction of 2-Year Recurrence-Free Survival in Hepatocellular Carcinoma**
*J. Cui*, Y. Lei, C. Ma, Y. Xie, Z. Luo; Jiangmen Central Hospital/CN
*(1004018713@qq.com)*

**Purpose:** To develop and validate a machine learning (ML) model using preoperative CT-derived hepatic vascular features for predicting 2-year recurrence-free survival (RFS) in hepatocellular carcinoma (HCC) patients after hepatectomy.
**Methods or Background:** This multicenter retrospective study enrolled 361 patients from two centers, allocated into training (n=181), internal validation (n=120), and external test (n=60) cohorts. From preoperative arterial phase and portal venous phase CT, we extracted 3D vascular features including volumes of the hepatic artery, portal vein, and hepatic vein, their vessel-to-liver volume ratios, and 3D fractal dimension as a measure of vascular complexity. We built three ML models to predict 2-year recurrence risk: a vascular model, a clinical model, and an integrated model combining both. Model performance was evaluated using the area under the receiver operating characteristic curve (AUC). Kaplan-Meier and log-rank tests assessed RFS differences between model-stratified risk groups.
**Results or Findings:** The integrated model demonstrated superior predictive performance for 2-year RFS, achieving the highest AUC values of 0.8057 in the internal validation set and 0.7736 in the external test set. It significantly outperformed the vascular model (AUCs: 0.7572 and 0.6900) and the clinical model (AUCs: 0.5848 and 0.6146). Furthermore, patients stratified as high-risk by the integrated model exhibited significantly worse actual RFS outcomes than those in the low-risk group (all $P < 0.05$).
**Conclusion:** Our study validates that the combined ML model, integrating 3D vascular features and clinical factors, serves as an effective non-invasive tool for predicting the risk of recurrence within 2 years after hepatectomy in HCC patients. Its superior performance over single-source models underscores the synergistic value of vascular complexity for improved prognostic stratification.
**Limitations:** This study is limited by its retrospective nature and requires larger, prospective, multi-center cohorts for further validation to ensure generalizability.
**Funding for this study:** No funding was received for this study
**Has your study been approved by an ethics committee?** Not applicable
**Author Disclosures:**
Yunxuan Xie: Nothing to disclose
Jin Cui: Nothing to disclose
Yan Lei: Nothing to disclose
Zhixin Luo: Nothing to disclose
Changyi Ma: Nothing to disclose

**Pharmacokinetic modeling of MRI-based liver function for risk assessment in primary sclerosing cholangitis: a prospective pilot study**
*W. C. Bartholomä*, S. Cai, C. Simonsson, M. Karlsson, S. Kechagias, M. Woisetschlager, N. Dahlström, P. Lundberg; Linköping/SE

**Purpose:** Primary sclerosing cholangitis (PSC) is a rare fibroinflammatory liver disease with a highly variable course, ranging from indolent to rapidly progressive cases leading to cirrhosis, liver failure, or hepatobiliary malignancies. Accurate identification of patients at risk for poor outcomes remains challenging. MRI-based approaches such as DiStrict, Anali, and relative enhancement show promise but are limited by operator dependency or static measurements. Pharmacokinetic modeling offers a dynamic, quantitative alternative for risk assessment in PSC.
**Methods or Background:** A prospective cohort of 26 PSC patients underwent up to four annual MRI exams with a total clinical follow-up of 7.5 years. Clinical endpoints included liver transplantation, decompensated cirrhosis, and cholangiocarcinoma. The pharmacokinetic model's performance was compared to MELD, the Amsterdam-Oxford Model (AOM), Relative Liver Enhancement (RE) and the Anali Score. Correlation and ROC analyses with laboratory values and established risk scores were performed. A segmental approach was also explored.
**Results or Findings:** The pharmacokinetic model (Globalki ) showed significant correlations with MELD (r = -0.429, p = 0.029), AOM (r = -0.557, p = 0.003), and endpoint events (r = -0.605, p = 0.001). ROC analysis indicated that Globalki (AUC 0.943) outperformed the Anali Scores (AUC 0.800 – 0.829) in identifying patients at risk for an endpoint event and was comparable to MELD (AUC 0.857) and AOM (AUC 0.900). The model showed no significant correlation with laboratory values, suggesting its potential as an independent biomarker.
**Conclusion:** The pharmacokinetic model based on hepatobiliary contrast uptake in MRI provides relevant independent prognostic information in PSC, comparable to established models such as AOM or MELD.
**Limitations:** Small sample size and limited number of endpoint events. Mayo Risk score could not be calculated for the patient cohort as a complement to the Amsterdam-Oxford Model.
**Funding for this study:** RFoU medel för doktorander (Regional Research and Development Funding for PhD students, provided by the Region of Östergötland (Östergötland Medical County). Additional funding from Vinnova (Sweden's Innovation Agency), and the Swedish Research Council (Engineering and Natural Sciences (VR/NT)).
**Has your study been approved by an ethics committee?** Yes
**Ethics committee - additional information:** Approved by Institutional Review board, Dnr 2011/8-24-21/2, 2018/1111-32, and 2018/1494-31/3
**Author Disclosures:**
Nils Dahlström: Nothing to disclose
Peter Lundberg: Nothing to disclose
Christian Simonsson: Nothing to disclose
Shan Cai: Nothing to disclose
Wolf Claus Bartholomä: Nothing to disclose
Mischa Woisetschlager: Nothing to disclose
Stergios Kechagias: Nothing to disclose
Markus Karlsson: Nothing to disclose

**Portal-to-Hepatic Vein Transit Time in Autoimmune Liver Diseases: A Pilot Study of CEUS Utility and Correlation with Histology**
*A. Fedak*[1], T. J. Popiela[2], M. Zwolińska-Wcisło[2], K. Mirowski[2], J. Jamroś[2]; [1]Kraków/PL, [2]Krakow/PL
*(andrzejfedak@gmail.com)*

**Purpose:** Background: Contrast-enhanced ultrasound (CEUS) is widely used for focal liver lesions, but its role in autoimmune liver diseases (AILD: autoimmune hepatitis [AIH], primary biliary cholangitis [PBC], and overlap) is unclear. Core biopsy is the gold standard but invasive. Non-invasive tools, including shear-wave elastography (SWE) and biochemical indices (e.g., FIB-4), are increasingly applied yet may not fully reflect microvascular changes. In AILD, lobular pathology alters resistance and promotes shunts. CEUS, by measuring portal-to-hepatic vein transit time (PV/HV TT), may capture these alterations.
**Methods or Background:** Methods: We prospectively studied 25 patients (22–66 years, 68% female) with histologically confirmed AIH (n=19), PBC (n=4), or overlap (n=2). All underwent core liver biopsy as the reference standard. Exclusion criteria were decompensated cirrhosis and coagulopathy. Patients undergoing CEUS for unrelated indications served as controls. The protocol included laboratory testing, FIB-4, Doppler ultrasound, SWE (Metavir), and CEUS with parametric imaging. Correlations with histology were analyzed using Spearman's rank.
**Results or Findings:** Results: Advanced fibrosis by FIB-4 was found in 48% of patients; SWE indicated significant fibrosis (Metavir ≥F3) in 62%. A threshold of PV/HV TT <3.5 s emerged as the most accurate discriminator of lobular injury, yielding 92% sensitivity versus 72% for laboratory tests (p=0.18). In controls, mean PV/HV TT was 7.8 s. No patient showed concordant negativity across CEUS and laboratory testing. PV/HV TT negatively correlated with FIB-4 (ρ=–0.42, p=0.035). All imaging and laboratory indices correlated significantly with biopsy.
**Conclusion:** Conclusion: This pilot study shows that CEUS sensitively detects microvascular alterations in AILD and complements SWE and biochemical indices. With further validation, CEUS may reduce reliance on biopsy and improve patient monitoring
**Limitations:** As a pilot investigation the limitation of the study is small amount of participants.
**Funding for this study:** Jagiellonian University grant No 1072.6120.268.2022
**Has your study been approved by an ethics committee?** Yes
**Ethics committee - additional information:** Obrazowanie ultrasonograficzne unaczynienia wątroby z wykorzystaniem środków kontrastujących w chorobach przebiegających z włóknieniem : niealkoholowa choroba stłuszczeniowa wątroby (NAFLD) autoimmunologiczne zapalenie wątroby (AIH), pierwotne zapalenie dróg żółciowych (PBC)" 1072.6120.268.2022, Acceptance of Bioethic Commitee of Jagiellonian University Medical College
**Author Disclosures:**
Tadeusz J. Popiela: Nothing to disclose
Krystian Mirowski: Nothing to disclose
Małgorzata Zwolińska-Wcisło: Nothing to disclose
Andrzej Fedak: Nothing to disclose
Jan Jamroś: Nothing to disclose

**Volumetric body composition analysis outperforms L3-derived metrics for survival prediction in cholangiocarcinoma**
J. Gotta, *L. D. Grünewald*, V. Koch, S. Mahmoudi, T. Vogl; Frankfurt/DE

**Purpose:** Single-slice analysis at the third lumbar vertebra (L3) is widely used in oncologic imaging to assess body composition. However, three-dimensional volumetric analysis may better reflect tissue distribution. This study compared the prognostic performance of volumetric and L3-derived body composition metrics in patients with cholangiocarcinoma.

**Methods or Background:** In this retrospective study, 147 patients with histologically confirmed intrahepatic (n = 65), perihilar (n = 46), or distal (n = 36) cholangiocarcinoma (47 women, 100 men; median age 67.0 years, IQR 58.0–73.0) underwent native-phase CT. Body composition was analysed using nnU-Net-based segmentation with manual quality control. Volumetric parameters included muscle percentage, myosteatosis percentage, visceral adipose tissue (VAT) percentage, and total adipose tissue (TAT) percentage. L3-derived metrics comprised skeletal muscle area (SMA_L3), muscle radiation attenuation (SMRA_L3), intramuscular fat (IMAT_L3), VAT_L3, and VAT HU_L3. Prognostic value was assessed using Kaplan–Meier and Cox proportional hazards models.
**Results or Findings:** Volumetric muscle percentage and myosteatosis percentage were the strongest survival predictors (log-rank = 17.43 and 17.67; HR = 0.93 and 1.06; both p < 0.001), followed by VAT and TAT percentages (log-rank = 12.26 and 8.20; both p = 0.01). These volumetric metrics showed strong statistical significance and clear survival stratification. Among L3-derived metrics, IMAT_L3 (HR = 1.09; p = 0.002), SMRA_L3 (HR = 0.97; p =0.005), VAT HU_L3 (HR = 0.98; p =0.004), and SMA_L3 (HR = 0.99; p = 0.02) were associated with survival but with lower log-rank values and less consistent group separation. VAT_L3 and SAT_L3 were not significant.
**Conclusion:** Volumetric CT-derived body composition metrics showed stronger and more consistent associations with survival than corresponding L3-based measures. Despite representing similar compartments, volumetric analysis may better capture the heterogeneity and extent of tissue distribution, improving prognostic accuracy in cholangiocarcinoma.
**Limitations:** Retrospective Study
**Funding for this study:** No funding was received for this study.
**Has your study been approved by an ethics committee?** Yes
**Ethics committee - additional information:** Waiver due to retrospective nature
**Author Disclosures:**
Thomas Vogl: Nothing to disclose
Vitali Koch: Nothing to disclose
Scherwin Mahmoudi: Nothing to disclose
Leon David Grünewald: Nothing to disclose
Jennifer Gotta: Nothing to disclose

**Noninvasive liver biomechanical mapping identifies a pre-fibrotic niche and early conditions favoring HCC development**
V. Koch[1], L. D. Grünewald[1], J. Gotta[1], S. Mahmoudi[1], R. Hammerstingl[1], K. Eichler[1], O. Darwish[2], T. Vogl[1], *R. Sinkus*[2]; [1]Frankfurt/DE, [2]Paris/FR
*(ralph.sinkus@inserm.fr)*

**Purpose:** The rise in metabolic syndrome is driving diffuse liver disease and HCC, often without advanced fibrosis. Identifying at-risk individuals in the pre-fibrotic stage is challenging. This study developed and validated a 3D MRE–based biomechanical imaging framework for early liver disease detection and characterization of HCC-related alterations in non-fibrotic livers.
**Methods or Background:** In this prospective study, 3D MRE was consecutively performed in 193 participants (mean age, 56 years; 83 women) with suspected or confirmed liver disease, including 73 biopsy-confirmed cases (38%) and 26 HCCs (14%). 20 healthy volunteers served as controls. The complex shear modulus was decomposed into classical stiffness (|G*|), shear wave attenuation (α), and phase angle (Y), yielding a two-dimensional "biomechanical fingerprint" of liver tissue. Biomechanical parameters were correlated with histopathology, blood biomarkers, and clinical characteristics.
**Results or Findings:** We identified three biomechanical regions: healthy tissue, a pre-fibrotic niche with inflammation but near-normal stiffness, and fibrotic/cirrhotic tissue. In the pre-fibrotic niche, α was markedly reduced despite preserved stiffness, accompanied by decreased E-cadherin and early α-smooth muscle actin upregulation. An α threshold <50 $m^{-1}$ was linked to higher HCC risk, even without fibrosis, and the biomechanical continuum mirrored gradual changes in clinical and molecular biomarkers (FIB-4, ASAT, PDFF, platelet count).
**Conclusion:** Our findings show that 3D MRE allows organ-specific, noninvasive mapping of liver disease and identifies a biomechanical state permissive to HCC before fibrosis. This approach extends elastography beyond fibrosis staging and enables early, individualized HCC risk stratification.
**Limitations:** This study has limitations, including predominance of fatty liver disease, reliance on biopsy with inherent variability, reduced biopsy numbers due to MRI eligibility, and its observational, hypothesis-generating design. Technical constraints (single frequency, single vendor, research transducer) and reliance on presumed F0 in most healthy volunteers may limit reproducibility.
**Funding for this study:** This work was supported by the German Doktor Robert Pfleger Foundation, the Wilhelm Vaillant Foundation, the French Integrated Cancer Research Center "SiRIC InsiTu" (INCa-DGOS-INSERM-ITMO Cancer_18008), and the French National Research Agency RHU Operandi (ANR-21-RHUS-0012).
**Has your study been approved by an ethics committee?** Yes
**Ethics committee - additional information:** The institutional ethics review board approved this prospective, cross-sectional, single-center study that complies with the Declaration of Helsinki.
**Author Disclosures:**
Omar Darwish: Nothing to disclose
Thomas Vogl: Nothing to disclose
Vitali Koch: Board Member: Editorial Board Member Radiology In Training
Scherwin Mahmoudi: Nothing to disclose
Renate Hammerstingl: Nothing to disclose
Ralph Sinkus: Nothing to disclose
Leon David Grünewald: Nothing to disclose
Katrin Eichler: Nothing to disclose
Jennifer Gotta: Nothing to disclose

**Feasibility of Photon-counting Detector CT-derived Extracellular Volume in Liver Fibrosis Evaluation: A Head-to-head Correlation with MRE**
*L. Huimin*, X. Wu, Z. Xu, F. Yan; Shanghai/CN
*(lhm12362@rjh.com.cn)*

**Purpose:** Dual-energy CT-derived hepatic extracellular volume (ECV) has shown promise in liver fibrosis quantification but is limited by the higher radiation dose of dual-energy acquisitions and the need for specific scan protocols. Photon-counting detector CT (PCCT), representing a new generation of multienergy CT, offers intrinsic spectral images and improved dose efficiency. This study aimed to investigate the feasibility of photon-counting CT-based ECV for liver fibrosis evaluation, using MR elastography (MRE) as the reference standard.
**Methods or Background:** Between June 2024 and July 2025, 53 participants were prospectively recruited to undergo multiphase abdominal PCCT and MRE. Delayed-phase PCCT images were acquired after the portal venous phase for ECV quantification. Correlation between ECV and MRE-derived liver stiffness measurements (LSMs) was assessed in a head-to-head comparison, and stratification performance for different fibrosis stages was evaluated with receiver operating characteristic curve analysis.
**Results or Findings:** According to established MRE thresholds (3.50 kPa, 4.30 kPa, 6.50 kPa) for liver fibrosis staging, 11 participants had F1, 12 had F2, 20 had F3, and 10 had cirrhosis. ECV correlated strongly with LSM (Spearman ρ = 0.903 [95% CI: 0.834, 0.944], P < .0001). For fibrosis stratification, ECV demonstrated excellent diagnostic performance for ≥ F2, ≥ F3, and F4, with areas under the receiver operating characteristic curve of 0.96 (0.91-1.00), 0.93 (0.86-0.99), and 0.97 (0.94-1.00), respectively. The corresponding cutoff values were 23.35%, 24.15%, and 27.28%.
**Conclusion:** These findings demonstrated excellent correlation between ECV and LSM, supporting PCCT-derived ECV as a feasible alternative for noninvasive liver fibrosis quantification.
**Limitations:** This study was limited by its relatively small sample size from a single center, which may restrict the generalizability of the findings. Larger multicenter studies are warranted to validate the performance of PCCT-derived ECV in liver fibrosis evaluation.
**Funding for this study:** No funding was provided for this study.
**Has your study been approved by an ethics committee?** Yes
**Ethics committee - additional information:** The ethics committee notification can be found under the number KY2024-187.
**Author Disclosures:**
Xinyu Wu: Nothing to disclose
Zhihan Xu: Employee: Siemens Healthineers CT Collaboration
Fuhua Yan: Nothing to disclose
Lin Huimin: Nothing to disclose

**08:00 - 09:30** **Research Stage 4**

### Research Presentation Session: Cardiac

### RPS 703

### CMR for myocardial tissue characterisation and function: myocardial fibrosis, mapping and hemodynamic forces in CMR

**Moderator**
Violeta Vassileva Groudeva; Sofia/BG
*(violetagroudeva@gmail.com)*

**CMR-derived haemodynamic forces predict adverse LV remodelling after STEMI: a multicentre cohort study**
M. Hu[1], X. Yue[1], J. Qi[1], Y. Chen[2], T. Li[3], K. He[1], *S. Gui*[4]; [1]BeiJing/CN, [2]Shanghai/CN, [3]Beijing/CN, [4]Wuhan/CN
*(shen.gui@philips.com)*

**Purpose:** To determine whether early cardiac MRI–derived haemodynamic force (HDF) parameters predict adverse left-ventricular remodelling (ALVR) after primary PCI for STEMI, and to assess their incremental prognostic value beyond conventional markers.
**Methods or Background:** In this retrospective multicentre cohort, 147 STEMI patients (92 internal, 55 external) underwent CMR within 14 days post-PCI and at 5 months. HDFs were computed non-invasively from cine sequences (Medis). ALVR was defined as a ≥15% increase in LV end-systolic volume. Group differences were analysed and multivariable logistic regression identified independent predictors. Incremental value of systolic HDF metrics over traditional predictors was assessed using likelihood-ratio tests, net reclassification improvement (NRI) and integrated discrimination improvement (IDI).
**Results or Findings:** Independent predictors of ALVR were peak troponin-T (OR 1.079, 95% CI 1.004–1.160; p=0.038), infarct percentage (OR 1.139, 95% CI 1.039–1.249; p=0.026), systolic lateral HDF RMS (OR 0.370, 95% CI 0.138–0.991; p=0.048) and systolic HDF impulse (OR 0.661, 95% CI 0.462–0.946; p=0.024). Adding systolic HDF parameters significantly improved model performance across base models, AUC increased from 0.704 to 0.778 in the derivation cohort and from 0.655 to 0.807 in the validation cohort (NRI 0.714 and 1.012; IDI 0.122 and 0.284; likelihood-ratio p=0.007 and 0.009), indicating meaningful reclassification over conventional predictors.
**Conclusion:** Systolic HDF metrics—particularly lateral HDF RMS and impulse—are independent predictors of ALVR after STEMI and provide incremental prognostic value beyond established markers, supporting their use for post-infarction risk stratification and clinical decision-making.
**Limitations:** The retrospective nature of the study introduces a potential for selection bias. Therefore, larger prospective studies are needed to validate HDF prediction models and their therapeutic guidance.
**Funding for this study:** This work was supported in part by the National Key R&D Program of China under Grant 2024YFF0505704.
**Has your study been approved by an ethics committee?** Yes
**Ethics committee - additional information:** This retrospective multicentre study complied with the Declaration of Helsinki; institutional approvals/consent waivers were obtained where required.
**Author Disclosures:**
Xiuzheng Yue: Nothing to disclose
Shen Gui: Nothing to disclose
Kunlun He: Nothing to disclose
Miao Hu: Nothing to disclose
Tao Li: Nothing to disclose
Yinyin Chen: Nothing to disclose
Jing Qi: Nothing to disclose

**Hemodynamic force derived from cardiac MRI reveal subclinical left ventricular dysfunction in patients with acute myocarditis**
*Y. Hou*, H. Zhou, L. Weihua; Changsha/CN

**Purpose:** To investigate the potential role of left ventricular (LV) hemodynamic force (HDF) for detecting cardiac dysfunction in acute myocarditis patients.
**Methods or Background:** We retrospectively enrolled 173 consecutive acute myocarditis patients without heart failure (non-HF) along with 64 age- and sex-matched healthy controls. HDF parameters were calculated (I) as the root mean square during systole [HDF(Sys)] and diastole [HDF(Dia)]; (II) as the mean forces during distinct phases—systolic thrust [HDF(A)], systole-diastole transition [HDF(B)], diastolic deceleration [HDF(C)], and late diastolic filling [HDF(D)]. Additionally, LV global longitudinal strain (GLS), global circumferential strain (GCS) and global radial strain (GRS) were measured using a CMR feature tracking technique.
**Results or Findings:** Compared with controls, non-HF patients exhibited impaired diastolic HDF parameters, including reduced HDF(Dia) (13.27±5.07 vs 16.00±6.40, p=0.007), decreased mean force in phase B [HDF(B)] (11.09±3.75 vs 12.92±4.51, p=0.026), phase C [HDF(C)] (12.61±5.94 vs 16.07±6.82, p=0.001), and elevated mean force in phase D [HDF(D)] (2.82±2.19 vs 1.25±1.60, p<0.001); while systolic HDF parameters, including HDF(Sys) (24.19±4.57 vs 24.25±5.43, p=0.314) and HDF(A) (22.15±4.93 vs 23.02±6.18, p=0.885) showed no statistically significant variations. There was also no significant difference in LV ejection fraction (EF) or strain between healthy controls and non-HF patients (p =0.099 for LVEF, p = 0.934 for GLS, p = 0.608 for GCS, and p = 0.155 for GRS).
**Conclusion:** In contrast to healthy controls, non-HF acute myocarditis patients already showed early LV HDF parameters impairment, while maintaining normal LVEF and strain measurements.
**Limitations:** This study is a retrospective study with potential selection bias.
**Funding for this study:** This study was supported by the National Natural Science Foundation of China (82071984, 82071894, and 91959117), the Science and Technology Innovation Program of Hunan Province (2020RC4007), and The Scientific Research Program of FuRong Laboratory 2024PT5110.
**Has your study been approved by an ethics committee?** Yes
**Ethics committee - additional information:** Medical Ethics Review Committee of Xiangya Hospital Central South University (No. 2025061065)
**Author Disclosures:**
Yangzhen Hou: Nothing to disclose
Liao Weihua: Nothing to disclose
Hui Zhou: Nothing to disclose

**High temporal resolution MR phase contrast imaging for evaluation of the isovolumic relaxation time**
*C. Reiter*, G. Reiter, A. Schmidt, E. Kolesnik, D. Scherr, M. Fuchsjäger, U. Reiter; Graz/AT
*(clemens.reiter@medunigraz.at)*

**Purpose:** To evaluate the utility of a high temporal resolution 2D phase-contrast (PC) MRI sequence for the assessment of the isovolumic relaxation time (IVRT) - a key parameter in evaluation of left ventricular diastolic dysfunction - compared to a standard 2D PC sequence using echocardiography as reference.
**Methods or Background:** Twenty-five healthy subjects (13 females; age 60 ± 9 years) were prospectively recruited for cardiac MRI at 3T and echocardiography. Echocardiographic evaluation of the IVRT (IVRT-Echo) was performed according to current guidelines. A retrospective ECG-gated high temporal resolution cine in-plane 2D PC measurement (measured temporal resolution = 18 ms interpolated to 70 frames per cardiac cycle) was acquired in the 3-chamber view under free breathing. The IVRT was evaluated as interval between the end of aortic flow and onset of transmitral inflow from this measurement (IVRT-highTR). To study the impact of temporal resolution, the IVRT was also evaluated from a standard transmitral 2D PC measurement (measured temporal resolution = 45 ms interpolated to 30 frames per cardiac cycle; IVRT-standard). Results were analyzed using paired t-tests and correlation analysis.
**Results or Findings:** Cardiac intervals did not differ between modalities and measurements (899 ± 127 ms, 880 ± 127 ms and 889 ± 123 ms for echocardiography, IVRT-highTR and IVRT-standard, respectively; p, 0.267-0.674). Echocardiographic IVRT (79 ± 16 ms) showed no significant bias compared to neither IVRT-highTR (77 ± 15 ms, p = 0.054) nor IVRT-standard (75 ± 17 ms, p = 0.101). A stronger correlation was observed between IVRT-highTR (0.944, p<0.001) and echocardiography than IVRT-standard (0.745, p<0.001).
**Conclusion:** High temporal resolution 2D PC MRI allows precise assessment of the IVRT compared to echocardiography as reference, outperforming a standard temporal resolution transmitral 2D flow measurement.
**Limitations:** Small sample size.
**Funding for this study:** OeNB Anniversary Fund 17934
**Has your study been approved by an ethics committee?** Yes
**Ethics committee - additional information:** Local ethic number: 24-126 ex 11/12

**Author Disclosures:**
Ursula Reiter: Nothing to disclose
Gert Reiter: Employee: Siemens Healthcare Diagnostics GmbH
Michael Fuchsjäger: Nothing to disclose
Ewald Kolesnik: Nothing to disclose
Daniel Scherr: Nothing to disclose
Clemens Reiter: Nothing to disclose
Albrecht Schmidt: Nothing to disclose

**The role of myocardial strain assessment using cardiac magnetic resonance imaging in patients with non-compacted left ventricular myocardium: determination of subclinical contractility disorders**
*D. Filatova*, E. Mershina, E. Gagarina, R. Myasnikov, O. Kulikova, A. Meshkov, A. Kiseleva, V. E. Sinitsyn; Moscow/RU
*(dariafilatova.msu@mail.ru)*

**Purpose:** Non-compaction of left ventricular myocardium (NCLVM) is a morphological variant characterized by myocardial two-layer structure. Patients often develop contractile dysfunction, leading to heart failure. However, increased trabeculation is observed in other diseases, such as dilated cardiomyopathy (DCM), making diagnosis challenging when LV ejection fraction (LVEF) is preserved and LV dilation is minimal. The study purpose: to determine the role of myocardial strain, assessed by cardiac magnetic resonance imaging (CMR), in identifying subclinical contractile dysfunction in patients with NCLVM and preserved LVEF.
**Methods or Background:** Study enrolled patients with genetically confirmed NCLVM and preserved LVEF, patients with DCM, and individuals without cardiovascular disease (control group). All participants underwent CMR with intravenous contrast administration. Myocardial strain analysis was performed using cvi42 software.
**Results or Findings:** The study included 112 participants: 16 patients with NCLVM and preserved LVEF (mean age 37.8±13.8 years;9 men), 51 control subjects with no pathological findings on CMR (37.7±13 years;31 men), and 45 patients with DCM (45.6±14.8 years;29 men). Significant reduction in global and segmental strain was observed in DCM. Patients with NCLVM demonstrated reduced global strain, decreased longitudinal and circumferential strain (LS, CS) in mid and apical segments (10,12-14 and 6,7,10-12,14, respectively). Radial strain (RS) was reduced in most segments (1,4-16) compared to control group ($p<0.05$). ROC analysis revealed that global LS, RS and CS provided the optimal AUC values for differentiating NCLVM from DCM. The optimal AUC values were obtained for global LS, RS and CS, as well as for LS in segment 15, RS in segment 5, and CS in segment 9.
**Conclusion:** Myocardial strain assessment is valuable diagnostic tool for detecting contractile dysfunction in patients with NCLVM and preserved LVEF. It is more sensitive to functional impairment than LVEF measurement alone.
**Limitations:** N/A
**Funding for this study:** N/A
**Has your study been approved by an ethics committee?** Yes
**Ethics committee - additional information:** The study was approved by local ethic commitee of Medical Research and Educational Institute of Lomonosov Moscow State University.
**Author Disclosures:**
Evgeniia Gagarina: Nothing to disclose
Valentin E. Sinitsyn: Nothing to disclose
Anna Kiseleva: Nothing to disclose
Aleksei Meshkov: Nothing to disclose
Olga Kulikova: Nothing to disclose
Elena Mershina: Nothing to disclose
Daria Filatova: Nothing to disclose
Roman Myasnikov: Nothing to disclose

**Genotype–Phenotype and Imaging Correlations in Pediatric Cardiomyopathies**
*B. Tütüncüoğlu*, F. Alpaslan, M. F. Kazanbaş, E. İsmailov, T. Y. Kılıç, T. Banaz, F. Köse, M. Kadıoğlu, S. Ozkök; Istanbul/TR
*(berk.tutun97@gmail.com)*

**Purpose:** To investigate genotype–phenotype relationships and cardiac magnetic resonance (CMR) correlates in pediatric patients with cardiomyopathy, focusing on fibrosis and ventricular dysfunction.
**Methods or Background:** This retrospective cohort included 32 pediatric patients diagnosed with cardiomyopathy and followed at our center. Clinical data, genetic testing and CMR results were analyzed. Patients were classified as dilated cardiomyopathy (DCM), hypertrophic cardiomyopathy (HCM) or other subtypes including restrictive and noncompaction. Genetic testing was performed in 25 patients, identifying pathogenic variants. Imaging evaluation emphasized late gadolinium enhancement (LGE) as a marker of myocardial fibrosis and ventricular functional parameters.
**Results or Findings:** The cohort comprised 15 patients with DCM (46.9%), 13 with HCM (40.6%) and 4 with other subtypes (12.5%). Pathogenic variants were detected in 14 of 25 tested patients (56%). In HCM, genotype-positive patients demonstrated greater wall thickness (mean 19.5 mm vs. 17 mm) and a higher fibrosis prevalence. LGE was present in 87.5% of genotype-positive versus 20% of genotype-negative cases. In DCM, TTN splice variants were predominant genetic etiology, consistent with their known familial role. All DCM patients with LGE exhibited severely reduced systolic function (LVEF <30%), underscoring the combined prognostic impact of fibrosis and contractile impairment. Notably, in HCM, hypertrophy severity did not directly correlate with fibrosis burden, highlighting the limitation of wall thickness alone as a risk marker.
**Conclusion:** Integration of genetic and imaging data provides a comprehensive framework for assessing pediatric cardiomyopathies. Pathogenic variants were associated with more severe phenotypes and higher fibrosis prevalence, as quantified by CMR. Fibrosis in HCM represents an independent risk marker beyond hypertrophy, while in DCM, scar formation consistently paralleled advanced dysfunction. Combined genetic testing and CMR enhance risk stratification and guide personalized management in this vulnerable population.
**Limitations:** Small sample size and retrospective design
**Funding for this study:** Not receive any financial support
**Has your study been approved by an ethics committee?** Not applicable
**Author Disclosures:**
Serçin Ozkök: Nothing to disclose
Berk Tütüncüoğlu: Nothing to disclose
Fatih Alpaslan: Nothing to disclose
Taha Yiğid Kılıç: Nothing to disclose
Fadime Köse: Nothing to disclose
Muhammed Faruk Kazanbaş: Nothing to disclose
Eitibar Ismailov: Nothing to disclose
Tuba Banaz: Nothing to disclose
Mehmet Kadıoğlu: Nothing to disclose

**Association between Fine Particulate Air Pollution Exposure and Subclinical Myocardial Fibrosis in Structurally Normal Hearts: a CMR-based study**
*E. Locatelli*, S. Figliozzi, F. Catapano, A. Laghi, M. Francone; Milan/IT
*(locatellielena99@gmail.com)*

**Purpose:** To investigate the potential association between long-term exposure to airborne particulate matter (PM2.5 and PM10) and early cardiac changes with cardiac magnetic resonance (CMR) mapping in individuals with structurally normal hearts, to elucidate the link between air pollution and myocardial remodeling.
**Methods or Background:** The impact of air pollution on early myocardial remodeling in individuals without overt cardiovascular disease remains incompletely understood. CMR mapping provides sensitive markers of myocardial fibrosis and inflammation that may reveal subclinical injury. We retrospectively studied 231 patients undergoing CMR with structurally normal hearts, preserved function, and no LGE. PM2.5 and PM10 exposure was derived from the nearest European Environment Agency monitoring stations. CMR parameters included left ventricular end-diastolic volume indexed, left ventricular ejection fraction, native T1, and T2 mapping. Associations with pollutant exposure were tested with multivariable linear and logistic regression adjusted for demographic, clinical, and socioeconomic factors.
**Results or Findings:** Higher PM2.5 exposure was associated with higher native T1 values ( $p = 0.020$); no associations were found with other CMR parameters or with PM10 exposure. Patients with high PM2.5 exposure had significantly higher T1 compared with those with low exposure. PM2.5 was associated with pathological T1, with male sex and diabetes being other independent associates.
**Conclusion:** In individuals with structurally normal hearts, chronic PM2.5 exposure was independently associated with higher native T1 values and pathological T1, suggesting early diffuse myocardial fibrosis.
**Limitations:** Its retrospective, single-center design and relatively small sample size; secondly, exposure assessment was based on residential proximity to air quality monitoring stations, which may not represent individual variability in exposure; finally, the association with PM2.5 was statistically significant but the absolute effect size was modest and most T1 values remained within the normal range.
**Funding for this study:** None
**Has your study been approved by an ethics committee?** Yes
**Ethics committee - additional information:** The study protocol was conducted in accordance with the principles of the Declaration of Helsinki and was approved by the Institutional Review Board's Ethics Committee.

**Author Disclosures:**
Federica Catapano: Author: Humanitas Research Hospital
Andrea Laghi: Author: Humanitas Research Hospital
Marco Francone: Author: Humanitas Research Hospital
Stefano Figliozzi: Author: Humanitas Research Hospital
Elena Locatelli: Nothing to disclose

**Prognostic Implications of CMR-Derived Infarct Burden for Therapeutic Strategies in Chronic Multivessel Coronary Artery Disease**
*Z. Tian*[1], T. Liu[2], M. Lu[1]; [1]Beijing/CN, [2]Shenyang/CN

**Purpose:** This study aimed to assess whether the extent of late gadolinium enhancement (LGE), quantified as myocardial infarction percentage (MI%), influences long-term outcomes under different therapeutic strategies in patients with chronic multivessel coronary artery disease (MVD) without prior revascularization.
**Methods or Background:** A multicenter retrospective cohort of 715 patients with chronic MVD who underwent contrast-enhanced cardiac magnetic resonance was analyzed. Patients were stratified according to LGE-derived MI% and subsequently grouped by therapeutic management with optimal medical therapy (OMT), percutaneous coronary intervention (PCI), or coronary artery bypass grafting (CABG). The primary endpoint was cardiac death. Secondary endpoints were all-cause mortality and major adverse cardiac and cerebrovascular events (MACCE). Kaplan–Meier and multivariable Cox regression analyses were performed to evaluate associations between treatment strategies, infarct burden, and outcomes.
**Results or Findings:** During a median follow-up of 6.7 years, patients with higher MI% exhibited significantly increased risks of cardiac death, all-cause mortality, and MACCE. Within this subgroup, both PCI and CABG were associated with lower event rates compared with OMT ($P < 0.001$ for all). Moreover, PCI was associated with a more favorable prognosis than CABG, reflected by lower rates of cardiac death and MACCE. In patients with lower MI%, no significant differences in outcomes were observed across treatment strategies.
**Conclusion:** In patients with chronic MVD without prior revascularization, CMR-derived infarct burden identifies a high-risk population in whom revascularization, particularly PCI, provides superior long-term survival compared with medical therapy or CABG. These findings emphasize the importance of integrating myocardial tissue characterization into contemporary Heart Team decision-making to refine therapeutic selection.
**Limitations:** This study is limited by its retrospective design and the possibility of residual confounding despite multivariable adjustment.
**Funding for this study:** This study was supported by National Natural Science Foundation of China (grant number 82471973); Beijing Natural Science Foundation (grant number 7242110); National High Level Hospital Clinical Research Funding (grant number 2022-GSP-QZ-5).
**Has your study been approved by an ethics committee?** Yes
**Ethics committee - additional information:** Ethics Committee of Fuwai Hospital, CAMS&PUMC 2022-1770
**Author Disclosures:**
Minjie Lu: Nothing to disclose
Zhaoxin Tian: Nothing to disclose
Ting Liu: Nothing to disclose

**A CMR-Guided Stepwise Approach to Diagnose Arrhythmogenic Cardiomyopathy with Left Ventricular Involvement: Insights from Explanted Hearts**
*H. Zhang*, M. Lu; Peking/CN
*(zhy_dreamer@163.com)*

**Purpose:** This study aimed to analyze the clinical and imaging features of end-stage arrhythmogenic cardiomyopathy (ACM) with left ventricular (LV) involvement, and distinguish it from dilated cardiomyopathy (DCM).
**Methods or Background:** We retrospectively studied heart transplantation (HTx) recipients with pathology-confirmed ACM (n=29) or DCM (n=36). All patients underwent comprehensive clinical, imaging and pathological evaluation. Cardiac magnetic resonance (CMR) protocols included cine, late gadolinium enhancement (LGE), and T1 mapping sequences. Explanted hearts received gross and histologic evaluations.
**Results or Findings:** The ACM cohort had earlier symptom onset (30.5 years) and longer diagnostic delay (27.8 months vs. DCM 0.1 months, p=0.006). The misdiagnosis rate of ACM before HTx was 27.6%, predominantly misclassified as DCM. Qualitative and quantitative CMR analyses revealed distinct differences between ACM and DCM in morphological, functional, and tissue-specific parameters. Specifically, ACM patients exhibited more frequent right ventricular (RV) motion abnormalities, greater RV dilation (185.8 mL/m² vs. 101.4 mL/m², p<0.001) and larger LV-LGE extent (31.3% ± 12.4% vs. 18.8% ± 14.5%, p<0.001). These hallmark variables were selected to construct a three-step diagnostic model, which achieved an exceptional performance for ACM diagnosis (area under the curve 0.98, sensitivity 100%). Pathologic analysis confirmed severe RV fibrofatty replacement in ACM.
**Conclusion:** A CMR-guided stepwise model combining qualitative, quantitative, and tissue characterization parameters effectively differentiates ACM with LV involvement from DCM, offering high diagnostic accuracy and sensitivity. This approach addresses diagnostic challenges in end-stage heart failure, enabling precise diagnosis in transplant candidates and optimizing treatment strategies.
**Limitations:** The retrospective nature of data and specimen collection precluded genotype-phenotype and precise radiological-pathological correlation analysis.
**Funding for this study:** National Natural Science Foundation of China (grant no. 82471973)
**Has your study been approved by an ethics committee?** Yes
**Ethics committee - additional information:** This study was approved by the local Institutional Ethics Committee (No. 2022-1770).
**Author Disclosures:**
Minjie Lu: Nothing to disclose
Huaying Zhang: Nothing to disclose

**Right Atrial Strain as a Novel Marker for High-Risk Phenotypes in Dilated Cardiomyopathy: Insights from a Multicenter Prospective Cohort**
*T. Geyer*, P. Franke, R. Lorbeer, B. J. Wintersperger; Munich/DE
*(thomas.geyer@med.uni-muenchen.de)*

**Purpose:** Right atrial (RA) dysfunction is an important prognostic factor in heart failure, but its significance in dilated cardiomyopathy (DCM) is often overlooked. Although CMR-derived RA strain has become a sensitive indicator of right heart impairment, its connection with common DCM comorbidities remains unclear. We aimed to explore the relationship between RA strain and major cardiovascular comorbidities to improve risk assessment in DCM.
**Methods or Background:** Datasets of 162 patients with non-ischemic DCM, all undergoing standardized 1.5T/3T CMR from the prospective German Centre for Cardiovascular Research (DZHK) nationwide multicenter databank, were analyzed. RA and right ventricular (RV) strain—reservoir (RS), longitudinal (LS), and circumferential (CS)—were quantified using feature tracking (cvi42, Circle CVI). Multivariable linear regression models, adjusted for age and sex, examined associations between strain parameters and predefined cardiovascular comorbidities, including heart failure, valvular disease, and atrial fibrillation (AF).
**Results or Findings:** Lower RA-RS was a consistent and significant finding in patients with comorbidities that increase right heart load. Specifically, RA-RS was independently associated with heart failure ($\beta=-14.96\%$; 95% CI: −25.42 to −4.49), valvular disease ($\beta=-13.68\%$; 95% CI: −24.65 to −2.72), AF ($\beta=-10.16\%$; 95% CI: −18.95 to −1.38), and combined valvular disease + AF ($\beta=-11.38\%$; 95% CI: −19.36 to −3.39), all p<0.05. Associations between RV strain and comorbidities were less consistent. Notably, RV-RS was unexpectedly higher in coronary artery disease (CAD), a finding that warrants further study ($\beta=6.14\%$; 95% CI: 0.35 to 11.93; p<0.05).
**Conclusion:** In this large, prospectively phenotyped DCM cohort, RA reservoir strain is a robust, non-invasive CMR marker consistently reduced in patients with comorbidities that impose additional right heart load. These findings support RA strain as a useful parameter for identifying higher-risk patients with DCM.
**Limitations:** Limitations include the cross-sectional design and lack of outcome data.
**Funding for this study:** None.
**Has your study been approved by an ethics committee?** Yes
**Ethics committee - additional information:** The study was approved by the ethics committee of the Bavarian Chamber of Physicians and the ethics committee of the Ludwig-Maximilians-University Munich and complies with the Declaration of Helsinki.
**Author Disclosures:**
Philipp Franke: Nothing to disclose
Roberto Lorbeer: Nothing to disclose
Thomas Geyer: Nothing to disclose
Bernd J. Wintersperger: Nothing to disclose

**Impact of Physical Inactivity on Subclinical Left Ventricular Function: A Population-Based CMR Strain Analysis**
P. Franke, *T. Geyer*, B. J. Wintersperger, R. Lorbeer; Munich/DE
*(thomas.geyer@med.uni-muenchen.de)*

**Purpose:** The association of physical inactivity with subclinical alteration of cardiac structure and function is not wellcharacterized. We investigated the impact of physical inactivity on left ventricular (LV) strain assessed byfeature-tracking cardiac MRI in a population-based cohort free of overt cardiovascular disease (CVD).

**Methods or Background:** We analyzed 360 participants (156 women; 39–73 years) from the MRI sub-study of the community-basedKORA (Cooperative Health Research in the Region of Augsburg) cohort. Physical activity was assessed atbaseline (Exam 1), 7-year follow-up (Exam 2), and 14-year follow-up (Exam 3). CMR with cine imagingand feature-tracking strain analysis was performed at Exam 3. Global longitudinal, circumferential, andradial strain were quantified. Associations of current and past inactivity with strain values were evaluatedin cross-sectional and longitudinal models adjusted for demographics and risk factors.
**Results or Findings:** Current physical inactivity (Exam 3) was associated with reduced longitudinal strain (β=-1.09%; 95%CI-2.02; -0.15; p=0.023), radial strain (β=-2.64%; 95%CI -4.79; -0.49; p=0.016) and circumferential strain(β=-0.90%; 95%CI -1.60; -0.20; p=0.012). Physical inactivity at baseline (Exam 1) also predicted lowerstrain values at Exam 3. Repeated reports of inactivity across two or more examinations were associatedwith progressively lower radial (β −2.09%; 95%CI −3.71 to −0.47; p=0.012) and circumferential strain (β−0.69%; 95%CI −1.22 to −0.16; p=0.011). Associations were more pronounced in men than in women.
**Conclusion:** Current and long-term physical inactivity were linked to impaired LV strain in asymptomatic adults withoutcardiovascular disease, with more pronounced effects in men. CMR strain thus provides sensitive markersof subclinical LV dysfunction associated with lifestyle factors.
**Limitations:** Limitations include MRI data acquisition only at one time point in a subsample, questionnaire-based assessment of physical activity, and restriction to a European ancestry cohort.
**Funding for this study:** None.
**Has your study been approved by an ethics committee?** Yes
**Ethics committee - additional information:** The study was approved by the ethics committee of the Bavarian Chamber of Physicians, Munich (Exam 1: EC No. 99186 and 05004, Exams 2 and 3: EC No. 06068). The MRI examination protocol was approved by the ethics committee of the Ludwig Maximilian University Hospital, Munich.
**Author Disclosures:**
Philipp Franke: Nothing to disclose
Roberto Lorbeer: Nothing to disclose
Thomas Geyer: Nothing to disclose
Bernd J. Wintersperger: Nothing to disclose

**CMR-Derived Mechanics parameters and Tissue Characterization: Prognostic Role in Hypertrophic Cardiomyopathy**
*F. Carusi*, V. Vespucci, S. Ciambellini, P. Palumbo, E. Di Cesare; L'Aquila/IT

**Purpose:** The aim of the study is to investigate the prognostic relevance of CMR-derived ventricular and atrial strain and strain rate, and tissue composition via late gadolinium enhancement (LGE), in patients with HCM.
**Methods or Background:** This retrospective observational study included 56 patients. Functional assessment included bi-atrial and bi-ventricular volumetric indices, along with myocardial strain and strain rate analysis (global longitudinal [GLS], circumferential [GCS], and radial strain [GRS], and peak early diastolic strain rate [PEDSR]). Also atrial reservoir, conduit and booster pump strain and SR were analyzed. Myocardial fibrosis was quantified using LGE. Clinical follow-up was conducted via clinical interviews. Binary logistic regression was used to identify predictors of adverse events.
**Results or Findings:** In univariate analysis, left ventricular ejection fraction (LVEF), right and left atrial volumes, extent of LGE, GLS, GCS, GRS, PEDSR and strain reservoir were all significantly associated with adverse clinical outcomes. However, in multivariate analysis, only PEDSR remained an independent predictor of adverse events (p = 0.037; OR < 0.001, 95% CI: 0.000–0.22). LVEF (p = 0.08) and %LGE (p = 0.074) demonstrated a trend toward statistical significance. Among different model including conventional functional marker (model I)(AUC = 0.82), %LGE (model II = model I + %LGE) (AUC = 0.83), strain value (model III = model II + all strain)(AUC = 0.84) and PEDSR (model IV = model III + PEDSR), best C-index was reached only whit PEDSR evaluation (AUC = 0.92).
**Conclusion:** In patients with HCM, CMR-derived parameters of ventricular mechanics and myocardial fibrosis provide significant prognostic value. Among them, impaired diastolic function, as indicated by reduced PEDSR, emerges as a strong and independent predictor of adverse clinical outcomes, underscoring the importance of advanced functional assessment for effective risk stratification.
**Limitations:** Small sample size.
**Funding for this study:** This research did not receive any specific grant from funding agencies in the public, commercial, or not-for-profit sectors.
**Has your study been approved by an ethics committee?** Not applicable
**Author Disclosures:**
Vanessa Vespucci: Nothing to disclose
Pierpaolo Palumbo: Nothing to disclose
Ernesto Di Cesare: Nothing to disclose
Francesca Carusi: Nothing to disclose
Silvia Ciambellini: Nothing to disclose

**Normal variations of myocardial T1, T2 and T2* values at 1.5 T cardiac MRI in sex-matched healthy volunteers**
*F. Cadour*[1], J. Caudron[2], A. Gillibert[3], S. Normant[3], J-N. Dacher[3]; [1]Toronto, ON/CA, [2]Le Havre/FR, [3]Rouen/FR
*(farah.cadour@hotmail.fr)*

**Purpose:** The purpose of this study was to determine the normal variations of myocardial T1, T2, and T2* relaxation times on cardiac MRI obtained at 1.5 T in healthy, sex-balanced volunteers aged between 18 and 69 years.
**Methods or Background:** A total of 172 healthy volunteers were recruited prospectively. They were further divided into seven sex-balanced age groups (18–19 years, 20–24 years, 25–29 years, 30–39 years, 40–49 years, 50–59 years, and 60–69 years). T1, T2, and T2* mapping were acquired in a single short-axis slice at the mid-level of the left ventricle. Global T1, T2, and T2* values were the mean of all segments. Comparisons between females and males were performed in each age group using independent samples t-test or Wilcoxon rank sum test, as appropriate. Multivariable linear effects models were used to analyze the effect of heart rate, body mass index, left ventricular mass, age, and sex on T1, T2, and T2* values.
**Results or Findings:** There were 83 males and 89 females, with a mean age of 37.3 ± 15.6 years. Females had greater T1 values (980.9 ± 26.2 ms) compared to males (949.7 ± 18.3 ms) (P < 0.001). T1 values decreased with age (P < 0.001), with smaller sex-related differences in older participants. Male sex and age were independently associated with lower values of T1 mapping. Age in females was independently associated with lower T1, T2, and T2* values.
**Conclusion:** T1, T2, and T2* values are influenced by age and sex, emphasizing the need to read and calibrate MRI values with respect to patient characteristics to avoid misdiagnosis.
**Limitations:** The limitations of the study are T1 values evaluated only in mid-ventricle, absence of extreme age recruitment, absence of extracellular volume performance and single vendor evaluation.
**Funding for this study:** No funding was received for this study.
**Has your study been approved by an ethics committee?** Yes
**Ethics committee - additional information:** The study was approved by institutional ethic committee, and study was registered under NCT01712581.
**Author Disclosures:**
Farah Cadour: Nothing to disclose
Sébastien Normant: Nothing to disclose
Jean-Nicolas Dacher: Nothing to disclose
André Gillibert: Nothing to disclose
Jérôme Caudron: Nothing to disclose

**08:00 - 09:30** **Research Stage 2**

### Research Presentation Session: Chest

### RPS 704

### Latest developments in interstitial lung disease (ILD) imaging

**Moderator**
Axel Vanrossomme; Gembloux/BE

**Interstitial lung abnormalities in oncologic patients with drug-induced pneumonitis: prevalence, radiological and clinical correlations**
*R. H. Hayoz*, N. Mansouri, C. Bongard, L. Righi, M. Obeid, C. Dromain, D. C. Rotzinger, C. Pozzessere; Lausanne/CH
*(roschan.hayoz@outlook.com)*

**Purpose:** Interstitial lung abnormalities (ILA) have been associated with increased all-cause mortality. In oncologic patients, the relationship between pre-existing ILA and drug-induced pneumonitis (DIP) remains poorly understood. This study aimed to assess the prevalence of pre-existing ILA in patients who developed DIP, investigate their influence on DIP severity and radiological pattern, and evaluate ILA progression after DIP.
**Methods or Background:** We retrospectively analyzed CTs of cancer patients with confirmed DIP between 2020 and 2025. Two blinded readers assessed the presence and type of ILA at baseline CT, DIP pattern at the time of pneumonitis, and ILA progression on subsequent scans (≥30days post-DIP). Categorical variables were reported as percentages. Statistical analysis included percentages for categorical variables, Cohen's Kappa for inter-reader ILA agreement, and Spearman's rank correlation for ILA-DIP severity association.

**Results or Findings:** ILA were identified in 21/88 patients (24%; 6 females; median age 70 years, range 59–82). The predominant ILA pattern was subpleural non-fibrotic (81%, n=17). Inter-reader agreement was substantial (κ=0.655). ILA was mentioned in only one baseline radiological report. Organizing pneumonia was the most frequent DIP pattern for both non-ILA and ILA subgroups (50% and 62%, respectively). Grade ≥2 pneumonitis was recorded in 44 (50%) non-ILA and in 12 (57%) ILA patients. A weak, non-significant, positive correlation emerged between ILA and DIP severity (rho=0.174, p=0.126). Following DIP, ILA progression was detected in 11 patients (52%).
**Conclusion:** ILA are relatively common in oncologic patients but remain substantially underreported. Although their correlation with DIP severity was weak and not statistically significant, ILA progression occurred in over half of cases post-DIP, suggesting a potential impact on long-term pulmonary outcomes and patient management.
**Limitations:** retrospective design, single-center, relatively small ILA sample size, mixed cancer types and oncologic treatments
**Funding for this study:** None
**Has your study been approved by an ethics committee?** Yes
**Ethics committee - additional information:** Comité d'éthique de la recherche du canton de Vaud. CER-VD 2022-01536
**Author Disclosures:**
Michel Obeid: Nothing to disclose
Lorenzo Righi: Nothing to disclose
Chiara Pozzessere: Nothing to disclose
Roschan Hélène Hayoz: Nothing to disclose
Cédric Bongard: Nothing to disclose
David Christian Rotzinger: Nothing to disclose
Nahal Mansouri: Nothing to disclose
Clarisse Dromain: Nothing to disclose

**Detecting Interstitial Lung Disease and Identifying Extensive Disease in Systemic Sclerosis: Cut-Offs for CT Lung Texture Analysis**
*N. Landini*[1], L. Jungblut[2], C. Strappa[1], C. Blüthgen[2], A. R. Larici[1], M. Matucci-Cerinic[3], T. Frauenfelder[2], O. Distler[2], C. Bruni[2]; [1]Rome/IT, [2]Zürich/CH, [3]Milan/IT
*(nikolandini@hotmail.it)*

**Purpose:** To identify and validate optimal thresholds of ILD extent quantified through Lung Texture Analysis (LTA) to detect the presence of ILD and extensive involvement in SSc patients.
**Methods or Background:** SSc patients visiting the Rheumatology Departments of University Hospital Zurich and the Careggi University Hospital Florence were identified for enrollment. Technically suitable images were analyzed through LTA™ (Imbio), quantifying the percentage of lung volume occupied by ILD. Two radiologists independently reviewed the CT scans to identify ILD and extensive disease, the latter defined as >20% of lung parenchyma involved, with disagreements resolved by consensus or third reviewer. Patients were randomly split 2:1 into derivation and validation cohorts. Receiver operating characteristic (ROC) curves with area under the curve (AUC) were computed to identify the optimal LTA ILD extent threshold for detecting ILD and extensive disease. Cox regression analysis was performed to determine the impact of ILD and extensive disease on mortality, adjusted for confounders.
**Results or Findings:** A total of 664 were eligible for the study. Visual analysis identified ILD in 313 (47%) cases, of whom 103 (33%) extensive ILD. In the derivation cohort (433 patients, 206 ILD, 38% extensive), ROC analysis identified the optimal ILD extent threshold for ILD detection at 1% (AUC 0.83), and at 7% for extensive ILD (AUC 0.84). In the validation cohort (231 patients, 104 ILD, 25% extensive), these thresholds achieved 78% sensitivity/71% specificity for ILD presence, and 81% sensitivity/70% specificity for extensive ILD. Over a median 5 years follow-up, 84 (13%) patients died. Both ILD presence and extensive ILD independently predicted mortality, with comparable results by visual and LTA™ assessments.
**Conclusion:** Our results lay the foundation for expanding the use of post-processing analysis in SSc-ILD towards automated diagnosis and stratification.
**Limitations:** Retrospective study.
**Funding for this study:** None
**Has your study been approved by an ethics committee?** Yes
**Ethics committee - additional information:** Ethic Committee Canton Zurich PB Nr 2018-0216 Comitato Etico Toscana Area Vasta Centro protocol 12300/oss and 15220/oss
**Author Disclosures:**
Nicholas Landini: Nothing to disclose
Cecilia Strappa: Nothing to disclose
Thomas Frauenfelder: Nothing to disclose
Anna Rita Larici: Nothing to disclose
Christian Blüthgen: Nothing to disclose
Oliver Distler: Nothing to disclose
Marco Matucci-Cerinic: Nothing to disclose
Lisa Jungblut: Nothing to disclose
Cosimo Bruni: Nothing to disclose

**Quantitative HRCT in Interstitial Lung Disease Secondary to Systemic Sclerosis: Evaluation of Disease Progression and Therapeutic Response**
*V. Uggenti*[1], M. Di Battista[1], A. Della Rossa[1], L. Tavanti[1], A. De Liperi[1], B. Bartholmai[2], M. Mosca[1], E. Neri[1], C. Romei[1]; [1]Pisa/IT, [2]Rochester, MN/US
*(v.uggenti@studenti.unipi.it)*

**Purpose:** This study aimed to assess, using CALIPER-derived quantitative parameters, the longitudinal course of interstitial lung disease secondary to systemic sclerosis (SSc-ILD), a complex condition characterized by alternating inflammatory exacerbations and progressive fibrotic changes.
**Methods or Background:** 50 SSc-ILD patients were included in the study (36 females; 14 males), with a mean age at baseline HRCT of 57.1 ± 13.0 years and a mean disease duration of 60.9 ± 81.5 months. Among them, 43 (86%) were receiving immunosuppressive therapy, and 7 (14%) were receiving antifibrotic treatment. All patients underwent at least two follow-up HRCT scans. All HRCTs were processed using CALIPER, which performs lung texture analysis, providing percentages of honeycombing (HC), reticulations (RET), ground glass (GG), vascular-related structures (VRS), and normal lung. ILD% was defined as the sum of GG%, RET%, and HC%.
**Results or Findings:** Pulmonary arterial hypertension (PAH) was associated with higher ILD (p=0.006) and VRS (p<0.001) values. Among patients receiving immunosuppressive therapy, RET+HC showed a progressive increase over time. Conversely, GG displayed a distinct trend: in patients with baseline GG% <20%, follow-up oscillations suggested higher responsiveness to treatment; in contrast, patients with extensive baseline involvement (>20%) exhibited a progressive increase in GG. ILD% was an independent predictor of pulmonary function: higher ILD% was associated with reduced FVC (β = –0.50, SE = 0.12, p<0.001) and DLCO (β = –0.77, SE = 0.11, p<0.001).
**Conclusion:** Higher ILD% and VRS% scores correlate with PAH. The subanalysis of individual interstitial alterations suggests that GG% oscillations reflect responsiveness to immunosuppressive therapy. The inverse correlation between ILD% and both FVC and DLCO supports the prognostic value of quantitative parameters. Quantitative HRCT may support tailored therapeutic management in SSc-ILD.
**Limitations:** Single-center, retrospective design, with potential selection bias. Imbalanced treatment groups. Heterogeneous follow-up.
**Funding for this study:** None.
**Has your study been approved by an ethics committee?** Yes
**Ethics committee - additional information:** Interstiziopatia polmonare in corso di malattie autoimmuni sistemiche: correlazione del fenotipo polmonare con caratteristiche cliniche e sierologiche, uno studio osservazionale (AISILD); Azienda Ospedaliero-Universitaria Pisana, Via Roma 67, Pisa; UO Reumatologia AOUP; Protocollo versione 2 del 06/04/2024.
**Author Disclosures:**
Vincenzo Uggenti: Nothing to disclose
Emanuele Neri: Nothing to disclose
Chiara Romei: Nothing to disclose
Brian Bartholmai: Nothing to disclose
Marco Di Battista: Nothing to disclose
Marta Mosca: Nothing to disclose
Laura Tavanti: Nothing to disclose
Alessandra Della Rossa: Nothing to disclose
Annalisa De Liperi: Nothing to disclose

**Assessment of the Relationship Between CT-Derived Lung Metrics and Pulmonary Function Tests in Interstitial Lung Disease**
E. Temel, B. S. Akhan, H. Erturk, M. e. Sahin, *E. A. Tan Kaynar*; Ankara/TR
*(eceaylintan@gmail.com)*

**Purpose:** Interstitial lung diseases (ILDs) are characterized by inflammation and fibrosis leading to impaired lung function. High-resolution computed tomography (HRCT) allows quantitative assessment of lung structure, while pulmonary function tests (PFTs) evaluate function. The relationship between these measures over time remains underexplored. This study aims to investigate correlations between changes in CT-derived lung volumetric and densitometric parameters and alterations in PFT results in ILD patients.

**Methods or Background:** This retrospective cohort study included 142 ILD patients who underwent HRCT and PFT at two distinct time points. Lung volume and density metrics—total lung volume (TLV), total lung density (TLD), fibrotic parenchymal volume (FPV), and fibrotic percentage—were quantified. Changes (∆) were manually calculated and correlated with ∆FEV1, ∆FVC, and ∆FEV1/FVC ratio using Pearson's correlation.
**Results or Findings:** ∆TLV correlated positively with ∆FEV1 ($r = 0.192$, $p < 0.05$) and ∆FVC ($r = 0.228$, $p < 0.05$). ∆TLD correlated negatively with ∆FEV1 ($r = −0.249$, $p < 0.05$). Both ∆FPV and fibrotic percentage inversely correlated with ∆FEV1 ($r = −0.272$ and −0.330, respectively, $p < 0.05$) and ∆FVC ($r = −0.188$ and −0.293, respectively, $p < 0.05$). Subgroup analysis showed significant correlations mainly in nonspecific interstitial pneumonia (NSIP) patients.
**Conclusion:** Changes in CT-derived volumetric and densitometric lung metrics correlate with pulmonary function changes in ILD, especially NSIP, supporting combined imaging and functional assessment for disease monitoring.
**Limitations:** This study has several limitations. First, its retrospective design may introduce selection bias and limits control over confounding variables. Second, the sample size, although sufficient for overall analysis, may be insufficient to detect subtle differences within some ILD subtypes, especially less common ones.Lastly, the study was conducted at a single center, which may limit the generalizability of the findings to broader populations.
**Funding for this study:** No funding was received for this study
**Has your study been approved by an ethics committee?** Not applicable
**Ethics committee - additional information:** Ethical approval of the study was obtained from the Ankara Atatürk Sanatorium Training and Research Hospital Clinical Research Ethics Committee (2024-BCEK/278, date: 14.05.2025).
**Author Disclosures:**
Bilge Sezin Akhan: Nothing to disclose
Mustafa Engın Sahin: Nothing to disclose
Ece Aylin Tan Kaynar: Nothing to disclose
Hakan Erturk: Nothing to disclose
Esra Temel: Nothing to disclose

### Dynamic concordance of subpleural regional volume in upper lung fields with global lung volume in chronic interstitial pneumonia: association with prognosis and upper lung subpleural irregular lines

*Y. Nagatani*, H. Nakagawa, Y. Tsunoda, R. Uemura, K. Fukunaga, Y. Nakano, Y. Watanabe; Otsu/JP
*(yatsushi@belle.shiga-med.ac.jp)*

**Purpose:** To investigate the relationship among dynamic concordance in upper lung subpleural normal-appearing lung parenchyma (SNAL) assessed on dynamic ventilation computed tomography (DVCT), the presence of subpleural irregular lines (SIL)reflecting pathological UIP pattern, clinical symptoms, and long-term prognosis in chronic interstitial pneumonia (IP).
**Methods or Background:** Fifty-four chronic IP patients underwent DVCT and pulmonary function tests (PFTs) in a single visit. Spherical volumes of interest (VOI) with a diameter of 10 mm were set at peak-inspiration in 8 subpleural SNAL, in ventral and dorsal mid sagittal plane. Automatic trucking functions obtained variable VOIs in 13-16 frame images (0.35 seconds/frame). Dynamic concordance of regional volume for SNAL with global lung volume (GLV) was assessed by cross-correlation coefficients in the volume (CCCv). The ratio of honeycomb volume to GLV (%HV) was measured on peak-inspiratory CT. In addition to the mean CCCv, PFT parameters and %HV were compared between survival and non-survival groups, between weak (modified MRC 0 or 1) and strong dyspnea groups (modified MRC 2 to 4), and between regions with SIL and those without using Mann-Whitney U test.
**Results or Findings:** CCCv for regions with SIL (0.61±0.50), was lower compared with those without (0.83±0.25)($p<0.0001$). No difference in CCCv was found between weak and strong dyspnea groups. The mean CCCv (0.66±0.31) as well as %FVC, and % FEV1, in the non-survival group was lower than that in the survival group (0.81±0.15)($p=0.009$). In addition, %HV in the non-survival groups (3.68±2.52) was higher compared with the survival group (2.17±2.15) ($p=0.005$).
**Conclusion:** DVCT demonstrated that dynamic discordance of the upper lung SNAL with global lung field was associated with subpleural irregular lines and long-term poor prognosis for chronic IP patients.
**Limitations:** The number of included patients was not so large.
**Funding for this study:** Grants-in-Aid for Scientific Research by Ministry of Education, Culture, Sports, Science and Technology of Japan (17K10361)
**Has your study been approved by an ethics committee?** Yes
**Ethics committee - additional information:** IRB of Shiga University of Medical Science
**Author Disclosures:**
Yoko Tsunoda: Nothing to disclose
Ryo Uemura: Nothing to disclose
Yasutaka Nakano: Nothing to disclose
Yukihiro Nagatani: Nothing to disclose
Kentaro Fukunaga: Nothing to disclose
Yoshiyuki Watanabe: Nothing to disclose
Hiroaki Nakagawa: Nothing to disclose

### Quantitative CT Analysis in Interstitial Pneumonia with Autoimmune Features: Diagnostic and Prognostic Insights from a Retrospective Cohort Study

*M. Puseljic*, A. J. Schlemmer, I. Vlasicek, A-K. Kaufmann-Bühler, F. Moazedi-Fürst, M. Fuchsjäger, E. Talakic; Graz/AT

**Purpose:** The position of interstitial pneumonia with autoimmune features (IPAF) within the interstitial lung disease (ILD) spectrum remains unclear, with limited data on the role of quantitative CT (QCT). This study aims to evaluate threshold-based QCT for distinguishing IPAF from other ILD subtypes and to assess the prognostic value of specific QCT features.
**Methods or Background:** In this retrospective single-center study, 227 patients (mean age, 63.6 ± 12.8 years) with CTD-ILD (n = 123), IPAF (n = 54), or IPF (n = 50) diagnosed between January 2005 and October 2024 were included. QCT assessed ground-glass opacity (GGO), consolidation, emphysema, affected lung, and the GGO-to-consolidation ratio. A general linear model was used for group comparison of QCT features; progression-free survival (PFS) was analysed with Kaplan–Meier and Cox regression to identify QCT-based risk factors for the whole study sample and for each subgroup.
**Results or Findings:** Lung consolidation was significantly higher in IPAF than in CTD-ILD ($p = 0.046$), while CTD-ILD showed higher GGO-to-consolidation ratios than IPAF ($p < 0.001$) and IPF ($p = 0.009$). IPAF had shorter PFS than CTD-ILD but longer than IPF. Higher GGO-to-consolidation ratios (HR, 0.87; 95% CI: 0.79–0.97; $p = 0.011$) and higher emphysema percentage (HR, 0.96; 95% CI: 0.93–0.99; $p = 0.011$) were associated with reduced risk of progression, whereas the usual interstitial pneumonia pattern was linked to higher risk (HR, 1.70; 95% CI: 1.07–2.71; $p = 0.024$). The GGO-to-consolidation ratio was associated with lower PFS only in the CTD-ILD group.
**Conclusion:** QCT demonstrated significant differences in imaging features and prognostic implications when comparing IPAF with other ILD subtypes.
**Limitations:** CT scans were performed on different scanners, and there was an imbalance between subgroup sample sizes.
**Funding for this study:** No fundings recieved.
**Has your study been approved by an ethics committee?** Yes
**Ethics committee - additional information:** Ethics Committee Medical University of Graz (36-202 ex 23/24)
**Author Disclosures:**
Ann-Katrin Kaufmann-Bühler: Nothing to disclose
Michael Fuchsjäger: Nothing to disclose
Marijan Puseljic: Nothing to disclose
Igor Vlasicek: Nothing to disclose
Anselm Johannes Schlemmer: Nothing to disclose
Emina Talakic: Nothing to disclose
Florentine Moazedi-Fürst: Nothing to disclose

### Respiratory discordance of global bronchial volume against global lung volume measured on dynamic ventilation computed tomography: association with prognosis of chronic interstitial pneumonia patients

*Y. Nagatani*, H. Nakagawa, R. Uemura, Y. Tsunoda, K. Fukunaga, Y. Nakano, Y. Watanabe; Otsu/JP
*(yatsushi@belle.shiga-med.ac.jp)*

**Purpose:** To investigate the association of dynamic concordance between global lung volume (GLV) and global bronchial volume (GBV) measured on dynamic ventilation computed tomography (DVCT) with prognosis
**Methods or Background:** Sixty-seven chronic interstitial pneumonia patients, including 22 idiopathic pulmonary fibrosis (IPF), underwent DVCT. GLV and GBV were measured in each of 13 to 17 frame images (0.35 seconds/frame), and their change ratio (GLVcr and GBVcr) were calculated. Cross-correlation coefficients between GLV and GBV (CCCv), and between GLVcr and GBVcr (CCCvcr) were assessed. Percentage of honeycomb volume to GLV (%HV) was measured as pulmonary fibrosis extent on peak-inspiratory CT. Mortality and the latest survival data were recorded. Following two analyses were performed in IPF, non-IPF as well as the total population: 1) CCCv, CCCvcr and %HV were compared between alive and non-alive groups. 2) Using cutoff values determined based on the area under the receiver operating characteristic curve, mortality difference for the 3 parameters were assessed by log-rank test under Kaplan-Meier curve.

**Results or Findings:** In addition to value difference between the 2 groups, patients with %HV of 2.05 or more had lower survival rates compared to those without (median survival time, 1756 vs 1933 days; p=0.002) for non-IPF as well as total. In contrast, CCCvcr for non-alive group (0.584±0.254) was lower than alive group (0.814±0.092) for IPF as well as total (p=0.008). Moreover, patients with CCCvcr of 0.795 or less had lower survival rates compared to those without (median survival time, 932 vs 1701 days; p=0.034) for IPF as well as non-IPF and total.
**Conclusion:** Respiratory discordance of GBVcr with GLVcr assessed on DVCT as well as %HV could be a useful indicator of prognosis especially for IPF.
**Limitations:** The number of included patients was not so large.
**Funding for this study:** Grants-in-Aid for Scientific Research by Ministry of Education, Culture, Sports, Science and Technology of Japan (17K10361)
**Has your study been approved by an ethics committee?** Yes
**Ethics committee - additional information:** IRB of Shiga University of Medical Science
**Author Disclosures:**
Yoko Tsunoda: Nothing to disclose
Ryo Uemura: Nothing to disclose
Yasutaka Nakano: Nothing to disclose
Yukihiro Nagatani: Nothing to disclose
Kentaro Fukunaga: Nothing to disclose
Yoshiyuki Watanabe: Nothing to disclose
Hiroaki Nakagawa: Nothing to disclose

### Three CT Visual Scores versus Two Lung Texture Analyses in Systemic Sclerosis-Related Interstitial Lung Disease: Associations with Pulmonary Function Tests

*M. E. Diana*[1], G. Pellegrino[2], D. Mohammad Reza Beigi[1], M. Orlandi[3], C. Bruni[4], V. Riccieri[1], M. Matucci-Cerinic[2], V. Panebianco[1], N. Landini[1]; [1]Rome/IT, [2]Milan/IT, [3]Modena/IT, [4]Florence/IT

**Purpose:** To compare the relationships of three CT Visual Scores (VSs) of lung alterations and two Lung Texture Analyses (LTAs) with pulmonary function tests in Systemic Sclerosis-Interstitial Lung Disease (SSc-ILD), identifying which method best reflects functional decline.
**Methods or Background:** Patients followed by two Rheumatology Units were evaluated. Chest CTs with Forced Vital Capacity (FVC%) and Lung Carbon Monoxide Diffusing Capacity (DLCO%) assessed within one month were included. Two radiologists performed three visual scores in consensus: Visual1 (involvement assessed at five levels), Visual2 (six levels), and Visual3 (six zones, whole lung evaluation). LTAs were performed with two software (Aview and IMBIO). A linear regression including clinical covariates (age, sex, smoking habits, antibody subset, pulmonary hypertension) was applied. The model fit was assessed using adjusted R2 ($p<0.05$). The agreement between the best VS and LTA was also evaluated using Bland–Altman analyses.
**Results or Findings:** 55 patients were included (44 women, median age 59 years). The median ILD extent ranged 17.0%-20% with VSs, and 6%-9.7% with LTAs. Linear regression with FVC% yielded $R^2$ values ranging from 0.267 to 0.371 for VSs, and from 0.223 to 0.478 for LTAs ($p<0.01$). Aview provided the best fit, followed by Visual3. Linear regression with DLCO% produced $R^2$ values ranging from 0.143 to 0.283 for VSs and from 0.223 to 0.230 for LTAs ($p<0.05$). Visual3 provided the best fit, followed by Aview. As ILD extent increased, the difference between Aview and Visual3 tended to increase (mean difference -13%, slope -0.213).
**Conclusion:** Aview and Visual3 assessments provided the best relationship with FVC% and DLCO%, respectively. The difference in ILD extent between the two methods may not be negligible at higher extents, where Aview tends to underestimate ILD compared to Visual3.
**Limitations:** Retrospective study, patients number
**Funding for this study:** None.
**Has your study been approved by an ethics committee?** Yes
**Ethics committee - additional information:** Rif. 7284 Prot. 0681/2023 Comitato Etico Territoriale Lazio Area 1 Careggi, Florence, 27299/2019, code 15220/oss CESC Treviso-Belluno, 641/CECEAV
**Author Disclosures:**
Davide Mohammad Reza Beigi: Nothing to disclose
Valeria Panebianco: Nothing to disclose
Nicholas Landini: Nothing to disclose
Valeria Riccieri: Nothing to disclose
Marco Matucci-Cerinic: Nothing to disclose
Greta Pellegrino: Nothing to disclose
Martina Orlandi: Nothing to disclose
Marco Emanuele Diana: Nothing to disclose
Cosimo Bruni: Nothing to disclose

### Interstitial Lung Abnormalities (ILA) in the Post-COVID19 Era: A call for radiologists awarness

*Z. Chahbi*, S. Wakrim; Agadir/MA
*(zchahbi@gmail.com)*

**Purpose:** Interstitial lung abnormalities (ILA) are incidental radiological findings involving at least 5% of the lung parenchyma in asymptomatic individuals. The COVID-19 pandemic has raised concerns about post-viral lung damage contributing to ILA and its progression to pulmonary fibrosis. This study aims to assess the prevalence and characteristics of ILA in a post-COVID-19 population and explore potential associations with fibrotic changes.
**Methods or Background:** A retrospective analysis was conducted on 343 chest CT scans of patients over 40 years old with no prior diagnosis of interstitial lung disease (ILD) from January 2023 to January 2024. ILAs were classified into three subtypes based on Fleischner Society criteria: non-subpleural ILA, subpleural non-fibrosing ILA, and subpleural fibrosing ILA. CT scans were evaluated for key radiological features, including ground-glass opacities, reticulations, septal thickening, traction bronchiectasis, cysts, honeycombing, and architectural distortion. Statistical analysis was performed using IBM SPSS Statistics.
**Results or Findings:** The overall prevalence of ILA was 18%, with subpleural fibrosing ILA and subpleural non-fibrosing ILA each accounting for 8%, and non-subpleural ILA for 2%. A female predominance (58%) was observed, with a mean patient age of 59 years. Compared to pre-pandemic literature, which reported a 7% prevalence in general populations, our findings suggest an increase in ILA prevalence, particularly in fibrosing subtypes.
**Conclusion:** The study highlights a potentially higher detection rate of fibrosing ILAs in the post-COVID era, raising concerns about long-term lung remodeling and progression to ILD. The increased proportion of fibrosing ILA warrants further longitudinal research to assess its evolution and the need for modified follow-up strategies. Radiologists play a crucial role in early identification and risk stratification to prevent irreversible lung damage.
**Limitations:** None
**Funding for this study:** None
**Has your study been approved by an ethics committee?** Yes
**Ethics committee - additional information:** Approved
**Author Disclosures:**
Zakaria Chahbi: Nothing to disclose
Soukaina Wakrim: Nothing to disclose

### Deep Learning-Based Airway Segmentation in Systemic Lupus Erythematosus Patients with Interstitial Lung Disease (SLE-ILD): A High-Resolution CT Analysis

*S. Piao*, B. Li, L. Zhao, R. Zhao, J. Wang, R. Xiao, Y. Ming, W. Song; Beijing/CN

**Purpose:** To characterize lobar and segmental airway volume differences between systemic lupus erythematosus (SLE) patients with interstitial lung disease (ILD) and those without ILD (non-ILD) using a deep learning-based approach on non-contrast chest high-resolution CT (HRCT).
**Methods or Background:** A retrospective analysis was conducted on 106 SLE patients (27 SLE-ILD, 79 SLE-non-ILD) who underwent HRCT. A customized deep learning framework based on the U-Net architecture was developed to automatically segment airway structures at the lobar and segmental levels via HRCT. Volumetric measurements of lung lobes and segments derived from the segmentations were statistically compared between the two groups using two-sample t-tests (significance threshold: $p < 0.05$).
**Results or Findings:** At lobar level, significant airway volume enlargement in SLE-ILD patients was observed in the right upper lobe (p=0.009) and left upper lobe (p=0.039) compared to SLE-non-ILD. At the segmental level, significant differences were found in segments including R1 (p=0.016), R3 ($p<0.001$), and L3 (p=0.038), with the most marked changes in the upper lung zones, while lower zones showed non-significant trends.
**Conclusion:** Our study demonstrates that an automated deep learning-based approach can effectively quantify airway volumes on HRCT scans and reveal significant, region-specific airway dilation in patients with SLE-ILD compared to those without ILD. The pattern of involvement, predominantly affecting the upper lobes and specific segments, highlights a distinct topographic phenotype of SLE-ILD and implicates airway structural alterations as a potential biomarker for disease presence. This AI-powered quantitative imaging biomarker holds promise for enhancing the early detection and monitoring of ILD in the SLE population, ultimately contributing to more personalized patient management.
**Limitations:** It was a retrospective and single-center study, and the generalizability of our findings needs validation in larger, multi-ethnic and longitudinal cohorts.
**Funding for this study:** Peking Union Medical College Hospital Research Funding for Postdoc (kyfyjj202412); China Postdoctoral Science Foundation(2025M772188 )

**Has your study been approved by an ethics committee?** Yes
**Ethics committee - additional information:** This study was approved by the Institutional Review Board of Peking Union Medical College Hospital .
**Author Disclosures:**
Sirong Piao: Nothing to disclose
Wei Song: Nothing to disclose
Ran Xiao: Nothing to disclose
Bing Li: Nothing to disclose
Ruijie Zhao: Nothing to disclose
Jiaru Wang: Nothing to disclose
Ying Ming: Nothing to disclose
Longfei Zhao: Nothing to disclose

**Longitudinal CT Progression, Quantitative CT Parameters, and Prognostic Implications in Anti-synthetase Antibody Syndrome Associated Interstitial Lung Disease**

*J. Wang*, R. Zhao, R. Xiao, S. Piao, R. Zhao, Y. Ming, X. Sui, W. Song; Beijing/CN

**Purpose:** To characterize longitudinal chest CT changes in patients with antisynthetase antibody syndrome–associated interstitial lung disease (ASS-ILD), evaluate their relationship with pulmonary function, and assess the prognostic value of quantitative CT parameters.
**Methods or Background:** This retrospective study included 60 newly diagnosed ASS-ILD patients (April 2014–December 2022) with positive anti-synthetase antibodies and ≥2 chest CT scans. Clinical, serological, pulmonary function test, and CT data were analyzed. CT abnormalities and patterns were assessed visually and quantified using semi-quantitative CT scores and automated quantitative CT software. Patients were stratified by clinical outcomes (regression, stability, deterioration) and baseline disease severity.
**Results or Findings:** Median follow-up was 25 months with 404 CT scans reviewed. Ground-glass opacities, linear opacities, and consolidations predominated at baseline. Over time, traction bronchiectasis significantly progressed ($p<0.001$), while consolidation, pleural thickening, and OP patterns declined. Fibrotic patterns increased, particularly in anti-PL-12 (100%), anti-Jo-1 (77.4%), and anti-EJ (77.8%) subgroups. Baseline traction bronchiectasis was more frequent in stability/deterioration groups, whereas OP pattern and consolidation favored regression. CT scores and quantitative CT parameters (MLD, HAV%) correlated strongly with FVC%, $FEV_1$%, TLC, and VC, and moderately with DLco%. The more advanced group exhibited higher CT scores, MLD values and HAV% and lower LAV%.
**Conclusion:** Longitudinal CT assessment in ASS-ILD reveals progression to fibrotic patterns, with strong correlations between quantitative CT parameters and pulmonary function test. Both CT scores and quantitative CT parameters provide prognostic insights and may guide individualized management.
**Limitations:** First, it was a single-center, retrospective study with relatively small subgroup sizes, which may limit the generalizability of our findings. Second, the follow-up period was relatively short, preventing evaluation of the association between imaging scores and patient survival outcomes.
**Funding for this study:** National Natural Science Foundation of China (No. 82173623)
**Has your study been approved by an ethics committee?** Not applicable
**Author Disclosures:**
Sirong Piao: Nothing to disclose
Wei Song: Nothing to disclose
Ran Xiao: Nothing to disclose
Ruijie Zhao: Nothing to disclose
Jiaru Wang: Nothing to disclose
Xin Sui: Nothing to disclose
Rui Zhao: Nothing to disclose
Ying Ming: Nothing to disclose

**Deep Learning-Based Segmentation of Pulmonary Vasculature in Systemic Lupus Erythematosus Patients with Interstitial Lung Disease (SLE-ILD): A High-Resolution CT Analysis**

*S. Piao*, B. Li, L. Zhao, Y. Ming, R. Zhao, J. Wang, R. Xiao, R. Zhao, W. Song; Beijing/CN

**Purpose:** To investigate the structural differences in pulmonary arteries and veins at lobar and segmental levels between systemic lupus erythematosus (SLE) patients with interstitial lung disease (ILD) and those without ILD (non-ILD) using deep learning-driven chest high-resolution CT (HRCT) analysis.
**Methods or Background:** A retrospective cohort of 106 SLE patients (27 SLE-ILD, 79 SLE-non-ILD) underwent chest HRCT. A deep learning algorithm (U-Net architecture) was trained to segment pulmonary arteries and veins at lobar and segmental levels via non-contrast chest HRCT, and vascular volumes were calculated. Group-wise comparisons were performed using two sample t-test, with significance set at $p<0.05$.
**Results or Findings:** Significant differences in pulmonary vasculature were observed between SLE-ILD and SLE-non-ILD groups. At lobar Level, SLE-ILD patients demonstrated significantly larger arterial volumes in the right upper lobe ($p<0.001$) and left upper lobe ($p=0.002$) compared to SLE-non-ILD. SLE-ILD patients exhibited increased venous volumes in the right upper lobe ($p=0.001$) and reduced volumes in the left lower lobe ($p=0.030$). At segmental Level, SLE-ILD patients showed higher arterial volumes in segments R1 ($p=0.010$), R3 ( $p<0.001$), R7 ($p=0.010$), and L3 ($p=0.001$), but lower volumes in L9 ($p=0.005$) and L10 ($p=0.019$). SLE-ILD patients had elevated venous volumes in segments R1 ($p=0.001$), R2 ($p=0.003$), R3 ($p=0.002$), L1-2 ($p=0.017$), and L3 ( $p=0.019$), but reduced volumes in L9 ($p=0.003$) and L10 ($p=0.004$).
**Conclusion:** Our study demonstrates a characteristic pattern of upper lobe arterial and venous enlargement coupled with lower lobe venous reduction in SLE-ILD, suggesting a complex interplay between SLE-associated vasculopathy and interstitial changes. AI-derived vascular metrics hold promise for improving the early diagnosis, risk stratification, and monitoring of SLE-ILD.
**Limitations:** It was a retrospective and single-center study, and the generalizability of our findings needs validation in larger, multi-ethnic and longitudinal cohorts.
**Funding for this study:** Peking Union Medical College Hospital Research Funding for Postdoc (kyfyjj202412); China Postdoctoral Science Foundation(2025M772188 )
**Has your study been approved by an ethics committee?** Yes
**Ethics committee - additional information:** This study was approved by the Institutional Review Board of Peking Union Medical College Hospital .
**Author Disclosures:**
Sirong Piao: Nothing to disclose
Wei Song: Nothing to disclose
Ran Xiao: Nothing to disclose
Bing Li: Nothing to disclose
Ruijie Zhao: Nothing to disclose
Rui Zhao: Nothing to disclose
Jiaru Wang: Nothing to disclose
Ying Ming: Nothing to disclose
Longfei Zhao: Nothing to disclose

**08:00 - 09:30** **Research Stage 5**

### Research Presentation Session: Artificial Intelligence and Imaging Informatics

### RPS 705

### Foundation models: building blocks or building hype?

**Moderator**
Christian Hinge; Copenhagen/DK
*(christian.hinge@regionh.dk)*
**Author Disclosures:**
Christian Hinge: Research Grant/Support: My PhD salary is supported by a grant from Siemens Healthineers to Rigshospitalet, Copenhagen

**Monitoring Black-Box AI Tools for Radiology with a Local Foundation Model**

*C. Gonzalez*, Z. Fang, H. S. Na, D. Larson, A. Chaudhari; Palo Alto, CA/US
*(camgonza@stanford.edu)*

**Purpose:** The offering of AI tools for radiology is expanding rapidly, yet their performance on local data is often unclear. Manually annotating site-specific studies or training local models for each use case is unfeasible. We show how a single vision-language model trained on routinely collected scans and radiology reports can estimate the confidence of commercial AI tools.
**Methods or Background:** We trained a vision–language model on 4,648 in-house non-contrast head CT studies (median patient age 69.4 years, 43.7% female) and associated radiology reports. We extracted zero-shot predictions for intracranial hemorrhage, midline shift, mass effect, and ischemic stroke by formulating textual prompts and calculating the cosine similarity between positive and negative prompts in the latent space. We utilized those predictions and density-based uncertainty quantification to calibrate three black-box AI models, including an FDA-cleared tool.

**Results or Findings:** The vendor model reached a sensitivity of 0.64 and specificity of 0.83 on the in-house test set. Distance distributions between false negative and true negative black-box predictions differed significantly across all splits ($p < 0.001$; Mann-Whitney U), showing that misclassifications can be identified from image embeddings. Selecting different confidence thresholds on validation data increased sensitivity to 0.75 and 0.81, with only moderate rises in false positives.
**Conclusion:** A single vision-language model trained on routinely collected data can help evaluate the usability of black-box AI tools both before and after deployment. By providing scalable oversight across multiple products and clinical tasks with minimal overhead, the proposed framework supports safer integration of AI into radiology practice.
**Limitations:** Our current results are limited to data from a single hospital. We plan to make the proposed framework openly available and extend validation to additional clinics in the future.
**Funding for this study:** No funding
**Has your study been approved by an ethics committee?** Not applicable
**Author Disclosures:**
David Larson: Shareholder: Bunker Hill Health
Zhongnan Fang: Nothing to disclose
Akshay Chaudhari: Consultant: Patient Square Capital, Chondrometrics GmbH, and Elucid Bioimaging Shareholder: Cognita Imaging, Subtle Medical, LVIS Corp, and Brain Key Founder: Cognita Imaging Research/Grant Support: GE Healthcare, Philips, Microsoft, Amazon, Google, NVIDIA, and Stability.
Hye Sun Na: Nothing to disclose
Camila Gonzalez: Nothing to disclose

**Enhancing Ultrasound Image Analysis: Domain Adaptation of Vision-Language Models with Adapters**
*J. Qu*, X. Han, T. Xiao, J. Qin, A. D. King, W. C. W. Chu, J. Cai, M. Ying; Hong Kong/HK
*(jingguo.qu@connect.polyu.hk)*

**Purpose:** To develop and evaluate domain adaptation methods for vision-language foundation models (VLMs) to enhance medical ultrasound (US) image analysis. This study addresses the performance limitations of existing VLMs caused by the domain shift from natural to medical images, aiming to improve automated segmentation and classification of lesions in US scans.
**Methods or Background:** We adapted a pre-trained CLIP model using parameter-efficient fine-tuning. Specifically, we integrated a novel multi-cognitive visual adapter (Mona) into the vision transformer backbone of a frozen CLIP model. For downstream tasks, we designed lightweight segmentation and classification heads incorporating feature map up-sampling and adaptive average pooling to handle variations in lesion size and reduce computational overhead. The framework was evaluated on six public and in-house US datasets (including one external testing set) for lymph nodes, breast, thyroid, and prostate.
**Results or Findings:** For segmentation, our adapted CLIP model with the Mona adapter without fine-tuning, outperformed all available models and achieved 0.831 in Dice score on the interal lymph node dataset. For classification, supervised fine-tuning was necessary, as zero-shot accuracy was near random chance. The fine-tuned model achieved up to 0.738 accuracy and 0.850 AUC on an external lymph node test set. Fine-tuning on small datasets led to performance degradation in segmentation, indicating catastrophic forgetting.
**Conclusion:** Our study demonstrated that adapting large-scale VLMs pre-trained on natural images is a highly effective strategy for ultrasound image segmentation. Our proposed method demonstrates superior segmentation capabilities. However, robust classification and overcoming the negative effects of fine-tuning on small datasets remain key challenges for future work.
**Limitations:** Key limitations include poor zero-shot classification, requiring supervised data for diagnosis, and catastrophic forgetting, where fine-tuning on small datasets degrades robust pre-trained features, especially for segmentation.
**Funding for this study:** This work was supported by General Research Funds of the Research Grant Council of Hong Kong (Reference no. 15102222 and 15102524).
**Has your study been approved by an ethics committee?** Not applicable
**Author Disclosures:**
Ann Dorothy King: Nothing to disclose
Jing Cai: Nothing to disclose
Jingguo Qu: Nothing to disclose
Tonghuan Xiao: Nothing to disclose
Jing Qin: Nothing to disclose
Michael Ying: Nothing to disclose
Xinyang Han: Nothing to disclose
Winnie C. W. Chu: Nothing to disclose

**ThighSeg: Foundation Model Adaptation for Thigh Composition Analysis in Multi-Sequence MRI**
*S. Huang*, G. Hu, Q. Wang, D. Zhang, Z. Jin, H. Xue; Beijing/CN

**Purpose:** To develop and validate a universal deep learning framework, based on the adaptation of a large-scale medical vision foundation model, for automated thigh composition analysis across diverse MRI sequences.
**Methods or Background:** Multisequence MRI data (e.g., T1, T2, STIR, and Dixon sequences) from five public datasets and two local datasets were annotated for sartorius, quadriceps, adductor muscles, gracilis, hamstrings, femur, and subcutaneous tissue. Training set included 498 sequences (222 participants) from TotalSegmentatorMRI, UFATS, HuashanMyo, and local cohort 1; internal testing used 109 sequences (55 participants); two external test sets comprised 54 sequences (27 participants, Folkhälsan) and 154 sequences (19 participants, MyoSegmenTUM) respectively. A 2D nnUNet model fine-tuned from MedDINOv3 for 100 epochs was applied to segment thigh components. Segmentation and measurements were evaluated using Dice similarity coefficient (DSC) and intraclass correlation coefficient (ICC). Six-week resistance-training effects were evaluated in 12 participants from local cohort 1 using paired t-tests; age-related changes were assessed in 1017 participants from local cohort 2 using Pearson correlation.
**Results or Findings:** The model demonstrated robust segmentation performance on internal (DSC: 0.889-0.956) and external test sets (DSC: 0.806-0.938 for Folkhälsan, 0.834-0.900 for MyoSegmenTUM). Automated measurements of muscle volume and muscle fat fraction showed strong agreement with reference values in both healthy volunteers (ICC: 0.931-0.995) and patients with neuromuscular diseases (ICC: 0.905-0.988). The model detected significant resistance-exercise-induced increases in the volume of the sartorius, quadriceps, and hamstrings ($P < 0.01$). Muscle volumes correlated negatively with age in the local population cohort, especially for the quadriceps femoris ($r = -0.546$ in females, -0.450 in males).
**Conclusion:** ThighSeg is an automated and robust tool for thigh composition analysis.
**Limitations:** The limitations of the study include the retrospective design and the insufficient assessment of generalization ability to unseen sequences.
**Funding for this study:** No funding was received for this study.
**Has your study been approved by an ethics committee?** Yes
**Ethics committee - additional information:** The study was approved by the Ethics Committee of Peking Union Medical College Hospital.
**Author Disclosures:**
Ge Hu: Nothing to disclose
Daming Zhang: Nothing to disclose
Qin Wang: Nothing to disclose
Zhengyu Jin: Nothing to disclose
Huadan Xue: Nothing to disclose
Shengqian Huang: Nothing to disclose

**Distinguishing ILA from Atelectasis on Radiographs Using a Foundation Model with Multi-Institutional Validation**
*T. Schnitzler*[1], A. P. Gehret[1], H. Zaytoun[1], A. Nowroozi[2], M. Bondarenko[2], J. H. Sohn[2]; [1]Aarau/CH, [2]San Francisco, CA/US
*(tician.schnitzler@ksa.ch)*

**Purpose:** To evaluate the performance of a radiology vision foundation model (RAD-DINO) combined with a lightweight multi-layer perceptron (MLP) classifier for distinguishing interstitial lung abnormalities (ILA) from atelectasis on chest radiographs, validated externally across institutional datasets.
**Methods or Background:** A classification pipeline was developed using RAD-DINO as a frozen feature extractor with an appended MLP classifier. Training utilized a curated dataset comprising posteroanterior (PA) chest radiographs of confirmed ILA and atelectasis cases, verified through same-day CT imaging. The internal dataset included 542 ILA and 1,167 atelectasis cases. The external validation cohort consisted of 85 ILA and 100 atelectasis cases. Data were split into training, validation, and internal test sets using an 80:10:10 ratio. Given the limited number of ILA cases, the RAD-DINO backbone remained frozen to mitigate overfitting. Random sampling ensured balanced representation of atelectasis cases during training epochs. The model was trained for 10 epochs using cross-entropy loss and the Adam optimizer. Performance metrics included accuracy, precision, recall, F1 score, and ROC-AUC, assessed on internal and external test datasets.
**Results or Findings:** The final model demonstrated robust performance with an accuracy of 79.8% on internal validation and 81.1% externally. ROC-AUC scores were 0.865 (internal validation) and 0.890 (external test). The frozen RAD-DINO effectively provided generalizable features, while random sampling enhanced training stability and performance consistency across diverse datasets.
**Conclusion:** Radiological vision foundation models, combined with a lightweight MLP classifier, effectively distinguish ILA from atelectasis on chest radiographs. Freezing the RAD-DINO backbone facilitated robust transfer learning with limited labeled data, maintaining high generalizability and diagnostic accuracy across multiple institutions.

**Limitations:** No Limitations.
**Funding for this study:** Swiss Society of Radiology Gottfried & Julia Bangerter-Rhyner Foundation
**Has your study been approved by an ethics committee?** Yes
**Ethics committee - additional information:** Institutional Review Board Nr:17-22317.
**Author Disclosures:**
Masha Bondarenko: Nothing to disclose
Adrienne Patrizia Gehret: Nothing to disclose
Tician Schnitzler: Grant Recipient: 2024 RSNA Research Fellow Grant Grant Recipient: Swiss Society of Radiology Grant Recipient: Gottfried&Julia Bangerter-Rhyner Foundation Grant Recipient: ForMe Grant Switzerland Consultant: bray-Z AG, Zurich
Hasan Zaytoun: Nothing to disclose
Jae Ho Sohn: Speaker: Siemens Healthineers
Ali Nowroozi: Nothing to disclose

**How Many Samples to Label for an Application given a Chest X-Ray Foundation Model?**
N. Nechaev, D. Umerenkov, V. Gombolevskiy, E. Przhezdzetskaya, *A. Khardin*, D. Dylov; Moscow/RU
*(khardintherapevt@gmail.com)*

**Purpose:** Estimating how many labeled cases are needed to meet a clinical performance target is essential for planning cost-effective model development. We investigate whether power-law fits to early learning curves can predict the training size required to reach an ROC-AUC threshold for chest X-ray (CXR) pathology classifiers built on top of foundation models.
**Methods or Background:** We constructed pathology-specific binary CXR datasets from MIMIC-CXR using RadGraph-derived labels normalized into 20 distinct classes. For each pathology, we formed train/val/test splits and sampled training subsets, adding negatives at a 1:5 ratio. We evaluated feature-based transfer learning with four encoders: RadDINO-MAIRA-2, XrayCLIP, XraySigLIP, and a ResNet-50 baseline. We then fit a power-law to the observed learning curves and estimated the number of positive cases needed to reach ROC-AUC 0.90—using fits built from limited early points.
**Results or Findings:** Foundation models substantially reduced labeled data needs versus ResNet-50. Across several pathologies, XraySigLIP/XrayCLIP achieved strong ROC-AUC with n@90 in the tens to low hundreds, while ResNet-50 often required orders of magnitude more data. For example, n@90 dropped from thousands or millions with ResNet-50 to double-digit counts with XraySigLIP. Crucially, fits using ≤50 positive cases provided reliable extrapolations of the eventual plateau. Early-slope magnitude correlated with final ROC-AUC across model–pathology pairs, supporting its use as a planning signal.
**Conclusion:** A simple protocol—train on small, incremented subsets; fit a power law; extrapolate to a target ROC-AUC—enables practical sample-size estimation for CXR pathologies with foundation-model features. In many cases, ~50–100 positive cases suffice to predict (and often achieve) clinically competitive performance, guiding annotation budgets and deployment timelines.
**Limitations:** Results are derived from one public dataset with RadGraph-based labels and frozen encoders; prospective, multi-center validation, end-to-end finetuning, and alternative targets (e.g., F1, sensitivity at fixed specificity) warrant study.
**Funding for this study:** No external funding was received; the work was conducted as part of the authors' institutional duties.
**Has your study been approved by an ethics committee?** Not applicable
**Author Disclosures:**
Victor Gombolevskiy: Nothing to disclose
Dmitry Dylov: Nothing to disclose
Anton Khardin: Nothing to disclose
Evgenia Przhezdzetskaya: Nothing to disclose
Nikolay Nechaev: Nothing to disclose
Dmitry Umerenkov: Nothing to disclose

**Automated monitoring framework for foundation model-generated draft reporting: Example use case in chest radiography**
*L. Brink*[1], A. Burade[2], N. Bhatia[2], K. Schmidt[1], M. K. Kalra[2], S. Mercaldo[2], L. Coombs[1], B. C. Bizzo[2]; [1]Reston, VA/US, [2]Boston, MA/US
*(lbrink@acr.org)*

**Purpose:** To develop and test a framework for automated performance monitoring of draft reports from foundation models using chest radiographs (CXR) as a use case example.
**Methods or Background:** Our retrospective, single-site study used 147 adult patients' CXR reports with radiologist-annotated findings (625 positive findings in total) to evaluate large language models (LLMs) for automated extraction of 233 predefined findings (regardless of criticality, significance, and severity) from each report. The best prompting strategy was then applied to AI draft reports generated with a commercial visual language model (VLM, Harrison.ai) from additional 121 CXRs. Radiologist reports served as the reference. Performance metrics with 95% confidence intervals (CIs) were calculated per finding and summarized at the case level and by finding criticality.
**Results or Findings:** Both one-shot and zero-shot prompting with Claude-3.5 achieved high accuracy (99.2%, CI:99.1–99.3) and specificity (99.6%, CI:99.6–99.7) for extracting reported findings, with minor sensitivity differences (one-shot: 77.8%, CI:74.4–80.9; zero-shot: 74.9%, CI:71.4–78.2). Using the selected extraction approach, the VLM achieved high accuracy (98.2%, CI:98.0–98.3) but substantially lower sensitivity (39.2%, CI:34.6–44.0) and F1 score (0.38, CI:0.34–0.43), indicating lower performance in finding-positive reports and high performance in normal reports.
**Conclusion:** Our framework can help automate monitoring of VLM-derived radiology report drafts. Since the findings extraction methods were highly accurate, the framework exposed the VLM's limited ability to capture true findings despite the high overall accuracy. We expect to scale the framework for additional LLMs and VLMs with temporal performance monitoring.
**Limitations:** Small sample size, single-site data, single-country LLMs, and the lack of stratified PA/portable CXR analyses limit the generalizability evaluation of our approach.
**Funding for this study:** No funding was received for this study.
**Has your study been approved by an ethics committee?** Yes
**Ethics committee - additional information:** IRB detials 2020P001792
**Author Disclosures:**
Kendall Schmidt: Nothing to disclose
Laura Coombs: Nothing to disclose
Bernardo Canedo Bizzo: Nothing to disclose
Laura Brink: Nothing to disclose
Mannudeep K. Kalra: Nothing to disclose
Sarah Mercaldo: Nothing to disclose
Anushree Burade: Nothing to disclose
Neil Bhatia: Nothing to disclose

**Diagnostic Accuracy of Pretrained Medical Foundation Models on Knee X-Rays**
H. Moon, *H. Ko*, Y. Kim, D. Lee, H. D. Chae, C. M. Park; Seoul/KR
*(lucasko1994@snu.ac.kr)*

**Purpose:** To investigate whether general medical foundation models can effectively adapt to downstream imaging tasks beyond their original training domains. Specifically, we evaluate their diagnostic accuracy in knee radiograph analysis—including Kellgren–Lawrence grading, effusion detection, and fracture identification—and compare them with a knee-specialised expert model trained on domain-specific data to assess the added value of task-focused adaptation.
**Methods or Background:** We retrospectively analysed 110,734 knee radiographic studies (2003–2023) from a tertiary hospital, reserving the most recent two years (10,248 studies) as a temporally separated test set. Labels for Kellgren–Lawrence grade (0–4), effusion, and fractures (acute and periprosthetic) were extracted from structured radiology reports using a rule-based approach. Three models with identical architectures were evaluated: MedSigLIP, a general medical foundation model pretrained via Google Med-Gemma; a knee-specialised model further trained with our images and reports; and a randomly initialised baseline. Performance was assessed using accuracy, F1-score, and AUC on both frontal views and multi-view inputs (frontal, lateral, skyline).
**Results or Findings:** The knee-specialised model consistently outperformed both MedSigLIP and the randomly initialised baseline. For effusion (all views), accuracy/F1/AUC were 87.7/87.3/93.4 for the knee-specialised model versus 82.2/82.2/91.8 for MedSigLIP and 80.7/79.8/89.1 for random ($p<.001$ for knee-specialised vs both). For fracture, values were 77.1/78.1/83.2 (knee-specialised) vs 68.9/67.3/72.8 (MedSigLIP) and 70.9/68.6/76.4 (random) ($p<.001$). For KL grading, accuracies were 56.5%, 54.1%, and 51.1%, respectively, with the knee-specialised model showing modest but significant gain over MedSigLIP ($p<.05$). Multi-view inputs improved effusion and fracture detection, while frontal views remained superior for KL grading.
**Conclusion:** General medical foundation models improved performance over models without pretraining, supporting their role in enhancing downstream imaging tasks. Additional task-specific adaptation further boosted diagnostic accuracy, underscoring the complementary value of both general pretraining and domain-specialised refinement.
**Limitations:** Findings are restricted to internal validation.
**Funding for this study:** This study was supported by a grant from the Korea Health Industry Development Institute (KHIDI)
**Has your study been approved by an ethics committee?** Yes
**Ethics committee - additional information:** IRB approval obtained (Seoul University Hospital, No. 2405-070-1536).

**Author Disclosures:**
Chang Min Park: Nothing to disclose
Dongheon Lee: Nothing to disclose
Henry Moon: Nothing to disclose
Yoorim Kim: Nothing to disclose
Hanbin Ko: Nothing to disclose
Hee Dong Chae: Nothing to disclose

**A Foundation Model Framework for Multi-View MRI Classification of Extramural Vascular Invasion and Mesorectal Fascia Invasion in Rectal Cancer**

*Y. Zhang*[1], S. A. Mali[1], H. C. Woodruff[1], S. Amirrajab[1], E. I. Crespo[2], A. Jimenez-Pastor[2], L. Marti-Bonmati[2], Z. Salahuddin[1], P. Lambin[1]; [1]Maastricht/NL, [2]Valencia/ES
*(yumeng.zhang@maastrichtuniversity.nl)*

**Purpose:** Accurate MRI-based identification of extramural vascular invasion (EVI) and mesorectal fascia invasion (MFI) is crucial for risk-stratified rectal cancer treatment. However, subjective visual assessment and inter-institutional variability limit diagnostic consistency. Therefore, this study aims to develop and externally evaluate a multi-center, foundation-model-driven framework that automatically classifies EVI and MFI on axial and sagittal T2-weighted MRI.
**Methods or Background:** 331 pre-treatment rectal-cancer MRI scans from three European hospitals (La Fe University and Polytechnic Hospital, Unidade Local de Saúde Hospital, and Centre Hospitalier Universitaire d'Angers) were retrospectively analyzed. A self-supervised frequency-domain harmonization pipeline was used to reduce scanner variability. Three classifiers—SeResNet, the universal biomedical pretrained transformer (UMedPT) with a multilayer perceptron (MLP) head, and a logistic-regression variant using frozen UMedPT features (UMedPT_LR)—were trained (n=265) and tested (n=66). Gradient-weighted class activation mapping (Grad-CAM) visualized model predictions.
**Results or Findings:** UMedPT_LR achieved superior EVI classification using fused axial and sagittal features (area under the receiver operating characteristic curve, AUC = 0.82). Optimal MFI detection occurred with UMedPT using axial harmonized images (AUC = 0.77); these results outperform the challenge winners. Frequency-domain harmonization enhanced MFI performance, with variable effects on EVI. Multi-view fusion, which combined axial and sagittal features, consistently improved EVI classification. Conventional convolutional neural networks (CNNs) underperformed, especially in F1 score and balanced accuracy. Grad-CAM demonstrated appropriate model attention on peritumoral regions (EVI) and mesorectal fascia margins (MFI).
**Conclusion:** The proposed foundation-model-driven framework leveraging frequency-domain harmonization and multi-view feature fusion achieves state-of-the-art performance in automated MRI classification of EVI and MFI, demonstrating excellent generalizability across multiple centers.
**Limitations:** Limitations include modest sample size, no center-specific analyses, and limited validation. Larger multi-institutional cohorts, advanced imaging, and in silico trials are needed to improve generalizability and clinical translation.
**Funding for this study:** Authors acknowledge financial support from ERC advanced grant (ERC-ADG-2015 n° 694812 - Hypoximmuno),, ERC-2020-PoC: 957565-AUTO.DISTINCT. Authors also acknowledge financial support from the European Union's Horizon research and innovation programme under grant agreement: CHAIMELEON n° 952172 (main contributor), ImmunoSABR n° 733008, EuCanImage n° 952103, TRANSCAN Joint Transnational Call 2016 (JTC2016 CLEARLY n° UM 2017-8295), IMI-OPTIMA n° 101034347, AIDAVA (HORIZON-HLTH-2021-TOOL-06) n°101057062, REALM (HORIZON-HLTH-2022-TOOL-11) n° 101095435, RADIOVAL (HORIZON-HLTH-2021-DISEASE-04-04) n°101057699 and EUCAIM (DIGITAL-2022-CLOUD-AI-02) n°101100633. This study was also supported by the China Scholarship Council grant (202208110055).
**Has your study been approved by an ethics committee?** Yes
**Ethics committee - additional information:** The study was approved by the Ethics Committees of all participating centers: La Fe University and Polytechnic Hospital (Valencia, Spain), Unidade Local de Saúde Hospital (Portugal), and Centre Hospitalier Universitaire d'Angers (France).
**Author Disclosures:**
Luis Marti-Bonmati: Nothing to disclose
Eduardo Ibor Crespo: Nothing to disclose
Ana Jimenez-Pastor: Nothing to disclose
Zohaib Salahuddin: Nothing to disclose
Yumeng Zhang: Nothing to disclose
Henry Christian Woodruff: Nothing to disclose
Sina Amirrajab: Nothing to disclose
Shruti Atul Mali: Nothing to disclose
Philippe Lambin: Nothing to disclose

**Best Practices for CT Foundation Model Embeddings: Ablation Studies and Cancer Immunotherapy Outcome Prediction**

*C. Mendoza-Moreno*, D. Navarro-Garcia, C. Zatse, A. Marcos Morales, O. Llorian-Salvador, R. Perez Lopez; Barcelona/ES
*(cristinamendoza@vhio.net)*

**Purpose:** Foundation models (FMs) offer powerful representations of medical imaging data, with the potential to improve performance in downstream tasks. Yet, it is unclear how their performance is affected by common machine learning (ML) pipeline choices. This study evaluates how design choices in data handling and model construction influence the predictive power of FM-derived features, benchmarked against hand-crafted radiomics.
**Methods or Background:** We analyzed the pre-treatment CT scans from 593 immunotherapy-treated cancer patients. Using a 10-iteration, 5-fold nested cross-validation framework, we conducted ablation studies to compare FM embeddings with hand-crafted radiomics across three endpoints: (a) clinical benefit, (b) lesion growth, and (c) lesion location. We examined the influence of five critical ML pipeline variables on predictive performance (AUROC): (1) class imbalance correction, (2) sample size variation, (3) feature normalization, (4) feature selection optimization, and (5) classifier–feature selector combinations.
**Results or Findings:** FMs consistently outperformed radiomics, particularly FMCIB embeddings (AUROC up to 0.66 vs. 0.59 for radiomics) for clinical benefit prediction . Standard preprocessing, such as feature normalization, improved performance when using hand-crafted radiomics but had no impact for FM embeddings. Performance did not uniformly degrade with reduced sample size, suggesting robustness to limited data in some tasks. Optimal feature number and classifier–selector combinations varied by endpoint. No universal "best" pipeline existed, and performance was highly context-dependent.
**Conclusion:** FM embeddings demonstrate superior performance compared to hand-crafted radiomics but require tailored pipelines. Moreover, both the prediction endpoint and the choice of FM embeddings significantly influence performance, underscoring the need for FM-specific workflows rather than one-size-fits-all solutions.
**Limitations:** This was a single-center study with limited external validation. Binary outcome definitions (e.g., clinical benefit, tumor growth) may oversimplify treatment response, and FM-specific architectural differences were not fully disentangled.
**Funding for this study:** Not applicable.
**Has your study been approved by an ethics committee?** Yes
**Ethics committee - additional information:** This retrospective study was approved by the institutional review board (PR(AG)70/2018). Informed consent was obtained from all patients participating in the clinical trials. The requirement for additional consent for computational image analysis was waived.
**Author Disclosures:**
Adrià Marcos Morales: Nothing to disclose
Oscar Llorian-Salvador: Nothing to disclose
Raquel Perez Lopez: Nothing to disclose
Christina Zatse: Nothing to disclose
Cristina Mendoza-Moreno: Nothing to disclose
Daniel Navarro-Garcia: Nothing to disclose

**Non-invasive Prediction of MGMT Promoter Methylation in Glioblastoma from Routine MRI Using Foundation Models**

*Z. Salahuddin*, C. Cortenraede, S. Amirrajab, H. C. Woodruff, P. Lambin; Maastricht/NL

**Purpose:** MGMT promoter methylation informs prognosis and treatment response in glioblastoma (GBM), but it is typically assessed invasively. We evaluated whether open-source foundation models can non-invasively predict MGMT methylation status from preoperative MRI, and explored model interpretability and fairness.
**Methods or Background:** We used multi-sequence MRI (T1, cT1, T2, FLAIR) from the UCSF PDGM and UPenn GBM datasets, restricted to WHO grade 4 GBM. After standardized tumor-centric preprocessing, we obtained 637 patients (70% training, 10% validation, 20% testing) for MGMT methylation status prediction. We benchmarked supervised, self-supervised, and multi-task foundation models (FMCIB, UMedPT, BrainIAC, Models Genesis, Med3D) under three training regimes: (1) frozen extractor + logistic regression, (2) frozen extractor + MLP, (3) full fine-tuning. Input fusion strategies (early/late) and augmentation ablations were tested. The primary metric was ROC-AUC.
**Results or Findings:** For MGMT status prediction, Models Genesis fine-tuned on FLAIR achieved a ROC-AUC of 0.73, exceeding previously reported MRI-only benchmarks (AUC 0.63). Logistic regression trained on UMedPT embeddings achieved an AUC of 0.69. Kaplan–Meier curves stratified by predicted MGMT labels showed separation comparable to curves stratified by true MGMT labels on the held-out test set. Counterfactual explanations indicated that the model primarily attends to peritumoral tissue rather than the enhancing core. MGMT prediction performance varied by age: AUCs were 0.74 (Q1: 17–54), 0.79 (Q2: 54–63), 0.61 (Q3: 63–71), and 0.48 (Q4: 71–94). Gender performance was balanced (Male: 0.675, Female: 0.658).
**Conclusion:** Foundation models enable improved, non-invasive MGMT methylation prediction from routine MRI in a clinically meaningful way, with interpretability indicating reliance on peritumoral context. While gender fairness is well maintained, age-related performance disparities warrant mitigation.
**Limitations:** Further work is needed to explore semantic and clinical feature fusion. Prospective validation and domain adaptation on unseen external datasets are required.
**Funding for this study:** Authors acknowledge financial support from ERC advanced grant (ERC-ADG-2015 n° 694812 - Hypoximmuno), ERC-2020-PoC: 957565-AUTO.DISTINCT. Authors also acknowledge financial support from the European Union's Horizon research and innovation programme under grant agreement: CHAIMELEON n° 952172 (main contributor), ImmunoSABR n° 733008, EuCanImage n° 952103, TRANSCAN Joint Transnational Call 2016 (JTC2016 CLEARLY n° UM 2017-8295), IMI-OPTIMA n° 101034347, AIDAVA (HORIZON-HLTH-2021-TOOL-06) n°101057062, REALM (HORIZON-HLTH-2022-TOOL-11) n° 101095435, RADIOVAL (HORIZON-HLTH-2021-DISEASE-04-04) n°101057699 and EUCAIM (DIGITAL-2022-CLOUD-AI-02) n°101100633.
**Has your study been approved by an ethics committee?** Not applicable
**Author Disclosures:**
Zohaib Salahuddin: Nothing to disclose
Henry Christian Woodruff: Shareholder: H.C.W. owns minority shares in Radiomics SA.
Cédric Cortenraede: Nothing to disclose
Sina Amirrajab: Nothing to disclose
Philippe Lambin: Patent Holder: P.L. is a co-inventor on two issued patents with royalties on radiomics (PCT/NL2014/050248 and PCT/NL2014/050728) licensed to Radiomics SA, one issued patent on mtDNA (PCT/EP2014/059089) licensed to ptTheragnostic/DNAmito, one granted patent on LSRT (PCT/P126537PC00, US patent no. 12,102,842) licensed to Varian, one issued patent on prodrugs (WO2019EP64112) without royalties, one non-issued, non-licensed patent on deep・learning・radiomics (N2024889) Research/Grant Support: P.L. has received grants and sponsored research agreements from Convert Pharmaceuticals SA, LivingMed Biotech srl and Radiomics SA; has received presenter fees and/or reimbursement of travel costs or consultancy fees (all in cash or in kind) from AstraZeneca, BHV srl and Roche. Shareholder: P.L. holds or has held minority shares in Bactam srl, Convert Pharmaceuticals SA, Comunicare SA, LivingMed Biotech srl and Radiomics SA.

### A Vision Language Foundation Model for MRI Harmonization Achieves High Accuracy in Modality and Anatomy Classification

D. Wang[1], T. C. Arnold[2], A. Shankaranarayanan[1], *G. Zaharchuk*[1];
[1]Menlo Park/US, [2]Philadelphia, PA/US
*(gregz@stanford.edu)*

**Purpose:** Magnetic Resonance Imaging (MRI) is widely used in both clinical and research settings. However, there is no standard system for naming or categorizing MRI sequences. As a result, MRI sequences often vary in appearance due to differences in imaging protocols, scanner vendors, and institutional practices. The goal of this study is to create an efficient streamlined method to standarize the MRI sequences.
**Methods or Background:** A vision-language model was designed with BERT and 3D ResNet-18 for text/image feature extraction (36 meta tags and 32 slices). Six transformer layers with two classification heads were then applied for modality and anatomy classification. An expert rule-based method performs final verification by checking metadata fields for sequence types better identified by rules, including MRA, SSFP, and ADC/DWI. We minimize reliance on the original Series Description tag, which is often inconsistent and potentially misleading.
**Results or Findings:** Anatomies include Neuro (Brain, Neck, CSPINE, TSPINE, LSPINE) and MSK (Wrist, Hip, Elbow, Knee). Modalities include T1, T1c, T2, T2-FLAIR, DWI, MRA, T2*, SWI, SSFP, ASL, ADC, CAL, and LOC, covering nearly all common MRI sequence types. Neuro achieved 97.38% modality and 92.02% anatomy accuracy, while MSK achieved 96.7% modality and 99.5% anatomy accuracy on a comprehensive test dataset comprising 1,501 cases. Our next step is to improve Neuro anatomy accuracy.
**Conclusion:** The proposed method enables MRI metadata standardization, with high accuracy in MRI sequence classification. This improves the reliability and efficiency of MRI data organization, retrieval, and downstream analysis.
**Limitations:** Model inference is 8–10 s per series on the app side; future work will also aim to improve inference speed.
**Funding for this study:** None
**Has your study been approved by an ethics committee?** Not applicable
**Author Disclosures:**
Greg Zaharchuk: Nothing to disclose
Thomas Campbell Arnold: Nothing to disclose
Ajit Shankaranarayanan: Nothing to disclose
Dayang Wang: Nothing to disclose

### Explainable Anatomy-Guided AI for Prostate MRI: Foundation Models and In Silico Clinical Trials for Virtual Biopsy-based Risk Assessment

*Z. Salahuddin*[1], D. Khan[1], Y. Zhang[1], S. Kuang[1], S. A. Mali[1], H. C. Woodruff[1], S. Amirrajab[1], R. Cavill[1], E. I. Crespo[2], A. Jimenez-Pastor[2], A. Galiana-Bordera[2], P. Jimenez[2], L. Marti-Bonmati[2], P. Lambin[1];
[1]Maastricht/NL, [2]Valencia/ES

**Purpose:** To develop and validate a fully automated, anatomically guided deep-learning pipeline that combines foundation models with counterfactual explainability for prostate-cancer (PCa) risk stratification on routine magnetic-resonance imaging (MRI).
**Methods or Background:** The pipeline includes an nnU-Net module that segments the prostate and its zones on axial T2-weighted MRI, a classification module that fine-tunes the UMedPT Swin-Transformer on 3D patches with optional gland or zonal priors and clinical variables, and a VAE-GAN framework that generates counterfactuals to highlight image regions driving model decisions. Development used 1,500 PI-CAI cases for segmentation and 617 multicentre biparametric MRI exams with clinical data from the CHAIMELEON challenge for classification (70% training, 10% validation, 20% testing). Clinical utility was tested in a paired multicentre in-silico trial where 20 clinicians interpreted a 125-case test set with and without AI support after a 60-day washout.
**Results or Findings:** The incorporation of gland priors boosted the foundation model's Area Under the Curve (AUC) from 0.69 to 0.72, and a three-scale ensemble (patch sizes 160–224) obtained the best test performance (AUC = 0.79), surpassing the 2024 CHAIMELEON challenge winners. Counterfactual heat-maps consistently highlighted lesion-containing regions within the segmented gland, providing intuitive, voxel-level explanations of risk predictions. In the prospective in silico trial, AI assistance increased mean diagnostic accuracy from 0.72 to 0.77 and Cohen's κ from 0.43 to 0.53, while cutting average review time per case from 5.3 min to 3.1 min (≈ 40 % gain).
**Conclusion:** Anatomy-aware foundation models enriched with gland priors and counterfactual explanations deliver accurate, transparent and time-saving PCa risk stratification on standard MRI, supporting their integration as virtual biopsies in clinical workflows.
**Limitations:** Domain adaptation and prospective trials are needed to confirm robustness in real-world settings.
**Funding for this study:** Authors acknowledge financial support from ERC advanced grant (ERC-ADG-2015 n° 694812 - Hypoximmuno), ERC-2020-PoC: 957565-AUTO.DISTINCT. Authors also acknowledge financial support from the European Union's Horizon research and innovation programme under grant agreement: CHAIMELEON n° 952172 (main contributor), ImmunoSABR n° 733008, EuCanImage n° 952103, TRANSCAN Joint Transnational Call 2016 (JTC2016 CLEARLY n° UM 2017-8295), IMI-OPTIMA n° 101034347, AIDAVA (HORIZON-HLTH-2021-TOOL-06) n°101057062, REALM (HORIZON-HLTH-2022-TOOL-11) n° 101095435, RADIOVAL (HORIZON-HLTH-2021-DISEASE-04-04) n°101057699 and EUCAIM (DIGITAL-2022-CLOUD-AI-02) n°101100633.
**Has your study been approved by an ethics committee?** Yes
**Ethics committee - additional information:** All datasets were retrospective, and de-identified. No additional institutional review board approval was required.
**Author Disclosures:**
Sheng Kuang: Nothing to disclose
Luis Marti-Bonmati: Nothing to disclose
Sina Amirrajab: Nothing to disclose
Eduardo Ibor Crespo: Nothing to disclose
Paula Jimenez: Nothing to disclose
Ana Jimenez-Pastor: Nothing to disclose
Zohaib Salahuddin: Nothing to disclose
Yumeng Zhang: Nothing to disclose
Henry Christian Woodruff: Shareholder: H.C.W. owns minority shares in Radiomics SA.
Rachel Cavill: Nothing to disclose
Adrian Galiana-Bordera: Nothing to disclose
Shruti Atul Mali: Nothing to disclose
Danial Khan: Nothing to disclose
Philippe Lambin: Research/Grant Support: P.L. has received grants and sponsored research agreements from Convert Pharmaceuticals SA, LivingMed Biotech srl and Radiomics SA; has received presenter fees and/or reimbursement of travel costs or consultancy fees (all in cash or in kind) from AstraZeneca, BHV srl and Roche. Shareholder: P.L. holds or has held minority shares in Bactam srl, Convert Pharmaceuticals SA, Comunicare SA, LivingMed

Biotech srl and Radiomics SA Patent Holder: P.L. is a co-inventor on two issued patents with royalties on radiomics (PCT/NL2014/050248 and PCT/NL2014/050728) licensed to Radiomics SA, one issued patent on mtDNA (PCT/EP2014/059089) licensed to ptTheragnostic/DNAmito, one granted patent on LSRT (PCT/P126537PC00, US patent no. 12,102,842) licensed to Varian, one issued patent on prodrugs (WO2019EP64112) without royalties, one non-issued, non-licensed patent on deeplearningradiomics (N2024889).

**Are the foundation models the answer for radiology AI: Evaluating deep learning and foundation model embeddings for binary classification of pediatric foreign body aspiration on chest radiographs**

*I. Ö. Koska*, I. Genişol; Izmir/TR
*(ozgurkoska@icloud.com)*

**Purpose:** Foreign body aspiration (FBA) in children is a common emergency, yet its early detection on chest radiographs is challenging. We aimed to develop a binary classifier to distinguish FBA from chronic cough patients using chest X-rays and to evaluate the performance of both conventional transfer learning and foundation model embeddings in this context
**Methods or Background:** A dataset of 251 pediatric chest X-rays was used, including 51 FBA cases and 200 chronic cough cases. A MobileNetV2 model was trained using transfer learning with weighted binary cross-entropy and focal loss to handle class imbalance. Additionally, embeddings were extracted from DINOv2 and TorchXRay foundation models and used to train classical machine learning classifiers: XGBoost, LightGBM, and Support Vector Machine (SVM). Models were evaluated using accuracy, F1-score, and area under the ROC curve (AUC).
**Results or Findings:** The best-performing model was the MobileNetV2 transfer learning approach, which utilized class-weighted binary cross-entropy loss and the ADAM optimizer, achieving an accuracy of 0.82, a weighted average F1-score of 0.84, and an AUC of 0.91. Among foundation models, the best performance was achieved by DinoV2 embeddings, achieving 0.79 accuracy, 0.79 F1-score, and AUC 0.80 Models built on foundation model embeddings demonstrated inferior performance, highlighting the limitation of directly applying large self-supervised models to radiology tasks with limited datasets.
**Conclusion:** In this small, imbalanced pediatric chest X-ray dataset, transfer learning with a lightweight CNN (MobileNetV2) outperformed models built on foundation model embeddings. This suggests that foundation models, while promising, may not yet provide a universal solution for radiology AI, particularly in specialized or small-scale clinical datasets.
**Limitations:** A small dataset and single-center design were the main limitations of this study.
**Funding for this study:** None
**Has your study been approved by an ethics committee?** Yes
**Ethics committee - additional information:** Behçet Uz Children's Hospital (İzmir) Local Ethical Committee Approval
**Author Disclosures:**
Ilker Özgür Koska: Nothing to disclose
Incinur Genişol: Nothing to disclose

**Building a Multicenter Radiology Foundation Model with Privacy-Preserving Swarm Learning: The ODELIA Consortium Initiative**

*J. Zhu*, O. Lester Saldanha; Heidelberg/DE

**Purpose:** To develop a multicenter radiology foundation model for breast MRI screening using privacy-preserving swarm learning (SL) within the ODELIA consortium, demonstrating feasibility, performance, and clinical relevance across European institutions.
**Methods or Background:** Swarm learning enables decentralized training of AI models without centralizing sensitive data, addressing privacy and regulatory barriers. The ODELIA consortium connects >8 academic hospitals and research institutes across Europe, pooling an estimated >20,000 breast MRI examinations from heterogeneous scanners and protocols. We implemented an open-source SL framework (Mediswarm) built with Python, NVFlare, Docker, and cross-platform-based coordination. Foundation model pretraining was performed on public imaging datasets, followed by weakly supervised fine-tuning with case-level diagnostic labels. Data never leaves local sites; secure aggregation, differential privacy, and active learning loops ensure robustness and efficiency.
**Results or Findings:** The swarm-trained foundation model achieved robust performance across institutions. For breast cancer detection, sensitivity reached 94% and specificity 92%, surpassing locally trained models (average sensitivity 88%, specificity 85%). Cross-institutional validation confirmed superior generalizability, with consistent AUROC >0.93 across diverse MRI protocols. Communication overhead was reduced by ~30% and computational load by ~25% compared with centralized training. Training remained stable under simulated node failures, and active learning improved minority-class performance, yielding a 7% gain in detecting small (<15 mm) tumors.
**Conclusion:** This work represents the first multinational SL implementation in radiology, delivering a scalable, open-source framework for collaborative foundation model training. ODELIA demonstrates that radiology foundation models can achieve robust, generalizable, and privacy-preserving performance in breast MRI, with potential for direct clinical translation and future expansion to additional oncologic imaging modalities.
**Limitations:** Preliminary results are based on ongoing training; full evaluation on all partner datasets and prospective clinical validation are pending.
**Funding for this study:** Funded by the European Union Horizon programme (Grant HORIZON-HLTH-2021-CARE-05-02, ODELIA project)
**Has your study been approved by an ethics committee?** Yes
**Ethics committee - additional information:** This study has been approved by the local ethics committees of all participating ODELIA consortium institutions. Imaging data are anonymised and processed in compliance with GDPR and national regulations. No raw patient data are exchanged between sites; all model training is performed via privacy-preserving swarm learning.
**Author Disclosures:**
Oliver Lester Saldanha: Nothing to disclose
Jiefu Zhu: Nothing to disclose

**08:00 - 09:30** **Research Stage 1**

### Research Presentation Session: Paediatric

### RPS 712

### Paediatric abdominal radiology, new developments in ultrasound, MRI and CT

**Moderator**
Stéphanie Franchi-Abella; Le Kremlin-Bicêtre/FR
*(stephanie.franchi@aphp.fr)*

**Quantitative contrast-enhanced ultrasound parameters correlation in the acute phase of paediatric inflammatory bowel disease: a comparative analysis with magnetic resonance imaging and colonoscopy**

*D. Pelaez Sanchez*, L. Vara de Andres, A. M. Lancharro Zapata, C. Marin Rodriguez, M. Sánchez Pérez, A. Aguado Del Hoyo, M. d. M. Tolin Hernandi, C. Miranda Cid, C. Sanchez Sanchez; Madrid/ES
*(daniel.pelaezsanchez@gmail.com)*

**Purpose:** Contrast-enhanced intestinal ultrasound (CEUS) is a less invasive and more cost-effective imaging modality compared to magnetic resonance enterography (MRE) and colonoscopy for assessing the lower gastrointestinal tract and related complications in Crohn's disease. Its use in paediatric settings is gaining interest due to its safety profile and real-time functional imaging capabilities.
**Methods or Background:** This study included 48 CEUS measurements performed in paediatric patients during the acute phase of inflammatory bowel disease. Quantitative CEUS parameters - including area under the curve (AUC), time to peak (TTP), and peak intensity (PI) - were correlated with findings from MRE and colonoscopy, as well as with faecal calprotectin levels, to evaluate their diagnostic utility against parameters in subacute and chronic phases - with no active inflamation - in order to establish a clincally useful threshold.
**Results or Findings:** The CEUS parameters showed a strong correlation with faecal calprotectin levels and with imaging and endoscopic findings consistent with active inflammation. Increases in AUC, TTP, and PI were directly proportional to the degree of inflammatory activity identified through MRE and colonoscopy.
**Conclusion:** Quantitative parameters obtained from CEUS appear to reliably reflect the presence and severity of active inflammation in paediatric Crohn's disease. These findings support the potential role of CEUS as a non-invasive, reproducible, and effective tool for diagnosis and follow-up in this patient population.
**Limitations:** The limitations of the study are the small sample size which may limit the statistical power and generalisability of the findings, the retrospective and observational analysis, which may introduce potential biases and limit causal inferences and the single-hospital focus, which may restrict the applicability of the results to broader or more diverse paediatric populations..
**Funding for this study:** No.
**Has your study been approved by an ethics committee?** Not applicable

**Author Disclosures:**
Cesar Sanchez Sanchez: Nothing to disclose
Daniel Pelaez Sanchez: Nothing to disclose
Loreto Vara De Andres: Nothing to disclose
Alejandra Aguado Del Hoyo: Nothing to disclose
María Sánchez Pérez: Nothing to disclose
Carlos Marin Rodriguez: Nothing to disclose
Angel Miguel Lancharro Zapata: Nothing to disclose
Carmen Miranda Cid: Nothing to disclose
Maria Del Mar Tolin Hernandi: Nothing to disclose

**Ultrasound-derived fat fraction for identifying pediatric metabolic dysfunction-associated steatotic liver disease: a prospective study**
*Y. Dong*, Y. Huang, Y. Qiu, Y. Wang, J. Chen, L. Wei, R. Cheng; Shanghai/CN
*(dr_mimi@163.com)*

**Purpose:** To evaluate the diagnostic performance of ultrasound-derived fat fraction (UDFF) in diagnosing hepatic steatosis in pediatric patients with metabolic dysfunction-associated steatotic liver disease (MASLD).
**Methods or Background:** In this prospective study, pediatric patients diagnosed with MASLD were enrolled. All patients underwent UDFF measurements (six acquisitions) in V/VIII hepatic segments, using the Acuson Sequoia ultrasound system (Siemens Healthineers, USA) with a DAX probe (1.0 – 3.5 MHz) before magnetic resonance imaging proton density fat fraction (MRI-PDFF) measurement within one week. Bland-Altman analysis and Spearman correlation coefficient were performed. Area under the receiver operating characteristic curves (AUCs) was performed to assess the diagnostic performance of UDFF measurements in hepatic steatosis. Intraclass correlation coefficients (ICCs) were calculated to estimate the consistency of UDFF measurements.
**Results or Findings:** From May 2024 to December 2024, 27 patients (median age, 12 years; median body mass index, 28.4 kg/m2) were included. The median UDFF value was 13.0 % (IQR: 6.0 – 21.5). UDFF and MRI-PDFF had a mean bias of 3.4 %, with a 95 % LOA ranging from -17.3 % to 24.0 %. UDFF values showed a positive correlation with MRI-PDFF (r = 0.69). Taking MRI-PDFF ≥ 5 %, ≥ 15 %, and ≥ 25 % as the reference standard for diagnosing hepatic steatosis grade ≥ S1, ≥ S2, and S3, the AUCs of UDFF were 0.80, 0.84, and 0.82, with the cutoff values of 9.5 %, 10.5 %, and 12.8 %, respectively. The ICC of UDFF measurements was 0.92 (95 % CI: 0.86 – 0.96).
**Conclusion:** UDFF measurement demonstrates good diagnostic performance in diagnosing and grading hepatic steatosis in pediatric MASLD patients.
**Limitations:** The sample size is limited because it includes pediatric patients with metabolic dysfunction-associated steatotic liver disease from one center.
**Funding for this study:** This project was supported by the Sino-German Mobility Program of NSFC and DFG (Grant No. M-0504) and the National Natural Science Foundation of China (Grant No. 82071942).
**Has your study been approved by an ethics committee?** Yes
**Ethics committee - additional information:** Ethics Committee of Xinhua Hospital Affiliated to Shanghai Jiao Tong University School of Medicine (Approval Number: XHEC-C-2022-121-2)
**Author Disclosures:**
Yunlin Huang: Nothing to disclose
Rui Cheng: Nothing to disclose
Yi Dong: Nothing to disclose
Yijie Qiu: Nothing to disclose
Juan Chen: Nothing to disclose
Ying Wang: Nothing to disclose
Li Wei: Nothing to disclose

**Normal values for shear wave elastography, shear wave dispersion and attenuation imaging in healthy paediatric livers**
*M. Zellner*, M. S. Schmidt, F. Huber, C. Paverd, A. Martin, S. Micic, V. Spyropoulou, C. J. J. Kellenberger; Zürich/CH

**Purpose:** The burden of liver disorders in children, including metabolic dysfunction-associated steatotic liver disease, underscores the need for non-invasive diagnostic tools. Ultrasound modalities namely Shear wave elastography (SWE), shear wave dispersion (SWD), and attenuation imaging (ATI) are potential alternatives to invasive methods. However, robust paediatric reference data for these techniques remain scarce.
**Methods or Background:** This retrospective study aimed to define normative values for SWE, SWD, and ATI in a healthy paediatric population and explore how these parameters are influenced by age, sex, body mass index (BMI), and fasting state. From a larger cohort of 734 children, 264 (135 girls; median age 11.5 years) with no evidence of liver pathology were included. All participants underwent liver ultrasound with a standardized protocol, acquiring five ATI and ten SWE/SWD measurements per child. Only high-quality acquisitions were analyzed.
**Results or Findings:** Median values were: ATI 0.54 dB/cm/MHz (IQR: 0.50–0.58), SWE 1.24 m/s (IQR: 1.14–1.33), and SWD 11.70 (m/s)/kHz (IQR: 10.84–12.13). ATI and SWD were inversely associated with age (p = 0.0003 and p = 0.0048), and SWD showed a significant negative correlation with BMI z-score (p < 0.0001) and was lower in females (p = 0.0013). SWE demonstrated a weak positive relationship with depth of measurement (p = 0.0261). Fasting duration did not significantly affect any parameter.
**Conclusion:** These findings establish paediatric reference ranges for SWE, SWD, and ATI, highlighting the influence of age and sex—especially on SWD and ATI and emphasizing the importance of contextual interpretation in clinical practice.
**Limitations:** This study has several limitations. Its retrospective design introduces potential for selection and information bias. Despite careful screening, undetected minor illnesses may have subtly affected liver viscoelasticity. Finally, the predominantly white study population may limit generalizability to more diverse ethnic groups.
**Funding for this study:** The authors disclose that the retrospective data analysis was supported by funding from Canon Medical Systems, received by the first author
**Has your study been approved by an ethics committee?** Yes
**Ethics committee - additional information:** Documentation of written informed consent from parents or legal guardians was reviewed retrospectively prior to data analysis. Clinical data and laboratory data were extracted from digital patient records. The study was approved by the local ethics committee
**Author Disclosures:**
Florian Huber: Nothing to disclose
Catherine Paverd: Nothing to disclose
Michael Zellner: Research/Grant Support: Canon Medical Systems
Vasiliki Spyropoulou: Nothing to disclose
Alexander Martin: Nothing to disclose
Christian J. J. Kellenberger: Nothing to disclose
Srdjan Micic: Nothing to disclose
Magdalena Sophie Schmidt: Nothing to disclose

**Diagnostic yield of post-mortem Micro-CT for abdominal and pelvic anomalies**
*I. C. Simcock*[1], A. Lamouroux[2], S. C. Shelmerdine[1], C. Hutchinson[1], N. Sebire[1], O. Arthurs[1]; [1]London/UK, [2]Montpellier/FR
*(ian.simcock@gosh.nhs.uk)*

**Purpose:** To demonstrate the yield of abdominal and pelvic diagnoses at less-invasive autopsy following a post-mortem fetal micro-CT investigation for early gestation pregnancy loss.
**Methods or Background:** Micro-CT provides high-resolution imaging for early gestation fetuses (usually below 20 weeks, or <300 g bodyweight), typically following a miscarriage or termination of pregnancy, allowing parents a less invasive autopsy investigation. We retrospectively analysed micro-CT diagnoses made on an unselected population of over 1200 early gestation fetuses between 2017 and 2024.
**Results or Findings:** We made 277 individual abdominal/pelvic diagnoses on Micro-CT, in 196/1200 patients (16.3%). 100/277 (36.1%) were solely abdominal, 61/277 (22.0%) solely pelvic, 27/277 (9.7%) were both, and 96/277 (34.7%) were external anomalies. We demonstrate the spectrum of disorders detected using Micro-CT: the commonest were abdominal-pelvic wall defects 84/277 (30.3%) and renal anomalies 77/277 (27.8%). The commonest renal anomalies were horseshoe kidneys 16/77 (20.8%), and cystic kidneys 13/77 (16.9%). Imaging was diagnostic in almost all cases except for 17/1200 (1.4%) non-diagnostic scans due to severe maceration.
**Conclusion:** We present the spectrum of abdominal/pelvic anomalies identified using post-mortem fetal Micro-CT. This information provides less-invasive autopsy for parents and clinical audit. The findings may be used to inform the likelihood of fetal abnormalities in subsequent pregnancies.
**Limitations:** Single centre data from a large specialist centre. Not all parents consented to invasive autopsy, so not all diagnoses could be histologically confirmed.
**Funding for this study:** Funding for this work was received from the the National Institute for Health and Care Research - UK (ICS, SCS, OJA) and the Mobility funding grant - Montpellier and Nimes University Hospital, France (AL). The views expressed are those of the author(s) and not necessarily those of the NIHR or the Department of Health and Social Care.
**Has your study been approved by an ethics committee?** Yes
**Ethics committee - additional information:** Ethics approval (17/WS/0089).
**Author Disclosures:**
Ian C. Simcock: Nothing to disclose
Neil Sebire: Nothing to disclose
Audrey Lamouroux: Nothing to disclose
Ciaran Hutchinson: Nothing to disclose
Susan Cheng Shelmerdine: Nothing to disclose
Owen Arthurs: Nothing to disclose

**Ultra Micro Angiography for Evaluating Activity of Crohn’s Disease in Pediatric Patients：: A Prospective Study**

*Y. Wang*, Y. Dong, H. Feng, L. Wei, R. Cheng, Y. Huang, Y. Wang; Shanghai/CN

**Purpose:** To investigate the value of Ultra Micro Angiography (UMA) imaging in evaluating the inflammation activity of pediatric Crohn's disease (CD) patients.
**Methods or Background:** The Pediatric Crohn’s Disease Activity Index (PCDAI) was regarded as the reference standard to evaluate the activity of CD. Before treatment, pediatric CD patients underwent B mode ultrasound (BMUS) and recorded the parameters. Color Doppler Flow Imaging (CDFI) and UMA were used to evaluate the blood flow in the most thickened diseased bowel wall using Limberg scores. The diagnostic performance of UMA and CDFI in assessing the inflammatory activity of pediatric CD was compared.
**Results or Findings:** A total of 54 pediatric CD patients were included and divided into three groups: inactive, mild and moderate-to-severe CD. When assessing bowel wall blood flow using CDFI, there was no statistically significant difference in bowel wall blood flow signals between the mild CD and the inactive CD ($P > 0.05$). There was statistically significant difference in blood flow signals between the mild and moderate-to-severe CD groups ($P < 0.05$). When assessing bowel wall blood flow using UMA, there were statistically significant differences in blood flow signals among all three groups (all $P < 0.05$). ROC analysis revealed that diagnostic performance of the UMA-BMUS model was significantly better than that of the CDFI-BMUS model (AUC 0.911 vs. 0.819; sensitivity 86.9 % vs. 72.2 %; specificity 94.5 % vs. 91.7 %; $P < 0.05$).
**Conclusion:** By sensitively depicting the low-velocity and tiny blood flow signals of the inflamed bowel wall, UMA is an effective noninvasive imaging method to make semi-quantitative evaluation of the activity of pediatric CD.
**Limitations:** The assessment of pediatric CD activity was based on semi-quantitative evaluation. Further multi-center studies are still necessary to verify our results.
**Funding for this study:** National Natural Science Foundation of China (Grant No. 82071942)
**Has your study been approved by an ethics committee?** Yes
**Ethics committee - additional information:** The university hospital’s ethics committee : ID No. XHEC-C-2024-038-2
**Author Disclosures:**
Yunlin Huang: Nothing to disclose
Rui Cheng: Nothing to disclose
Ying Wang: Nothing to disclose
Haixia Feng: Nothing to disclose
Ying Wang: Author: first authorship
Li Wei: Nothing to disclose
Yi Dong: Author: Correspondence

**Optimizing MRI-PDFF prediction in pediatric based on AC and BSC considering respiratory effect**

*S. Song*, J. Kim, M-J. Lee, K. H. Han, Y. Kwon, H. Koh, Y. W. Kim, E. J. Lee, H. Yoon; Seoul/KR
*(ct3b07@yuhs.ac)*

**Purpose:** To evaluate correlation between MR-proton density fat fraction (PDFF) and ultrasound-based parameters (attenuation coefficient [AC] and backscatter coefficient [BSC]) in pediatric patients, assess respiratory influence, and derive the optimal predictive model under various breathing conditions.
**Methods or Background:** This prospective study included consecutive pediatric patients with suspected fatty liver disease who underwent abdominal ultrasound and MRI with PDFF at baseline. AC and BSC were each acquired ten times during free breathing (FB) and breath holding (BH). Pearson correlation and linear regression assessed associations of AC and BSC with MR-PDFF. Multiple regression incorporating AC and BSC evaluated respiratory interactions. Additionally, 12 novel predictive models were constructed under FB and BH, with performance compared using adjusted $R^2$ and root mean square error (RMSE).
**Results or Findings:** Finally 44 patients (mean age 14.1 years) were included. AC and BSC showed significant correlations with MR-PDFF regardless of breathing condition (all $p < 0.001$). Multiple regression models during BH demonstrated better performance compared with FB (adjusted $R^2$, 0.64 vs 0.58; RMSE, 7.5 vs 8.0). There was no significant interaction effects of AC and BSC on MR-PDFF with breathing condition. Among 12 predictive models, the ordinary least squares (OLS) model performed best under BH (RMSE 7.14), while the support vector machine (SVM) model performed best under FB (RMSE 7.66).
**Conclusion:** In conclusion, AC and BSC are significantly correlated with MR-PDFF in pediatric patients under both breathing conditions. Predictive performance is slightly higher with BH, but the difference is minimal. The SVM model under FB still provided clinically acceptable accuracy, may be helpful for children with limited respiratory cooperation.
**Limitations:** Limitations in our study include small sample size, single-center design, relatively older cohort, and lack of external validation.
**Funding for this study:** None.
**Has your study been approved by an ethics committee?** Yes
**Ethics committee - additional information:** This study was approved by our hospital’s institutional review board. Written informed consent was obtained from all participants.
**Author Disclosures:**
Yong Woo Kim: Nothing to disclose
Hong Koh: Nothing to disclose
Yonghan Kwon: Nothing to disclose
Haesung Yoon: Nothing to disclose
Mi-Jung Lee: Nothing to disclose
Jisoo Kim: Nothing to disclose
Seunghyun Song: Nothing to disclose
Kyung Hwa Han: Nothing to disclose
Eun Joo Lee: Nothing to disclose

**Up-to-date paediatric sonographic reference values and percentile curves for abdominal organ dimensions in a large European cohort**

*E. Höhne*, S. Fischer, T. Vogl, V. Koch, S. Mahmoudi, I. Yel, K. Eichler, L. D. Grünewald; Frankfurt/DE

**Purpose:** Accurate assessment of liver, spleen, and kidney size is essential in pediatric diagnostics, where ultrasound provides a safe, accessible, and non-invasive tool. This study aimed to establish contemporary, population-based reference values for abdominal organ dimensions in children and adolescents in order to reflect current growth patterns and support reliable clinical decision-making.
**Methods or Background:** A total of 27,696 abdominal ultrasound examinations performed in children and adolescents (0–18 years) between 2000 and 2025 at a large tertiary referral center in Germany were retrospectively evaluated. Organ dimensions were measured by experienced pediatric radiologists using standardized protocols. Age-related percentile curves (P3–P97) were established with Generalized Additive Models for Location, Scale, and Shape (GAMLSS) to account for non-linear growth dynamics.
**Results or Findings:** Organ-specific datasets included 12,254 livers, 12,703 spleens, 16,254 right kidneys, and 16,516 left kidneys. All organs exhibited characteristic non-linear growth trajectories. No significant sex-related differences were identified for spleen ($p = 0.195$), liver ($p = 0.517$), right kidney ($p = 0.712$), or left kidney ($p = 0.797$), supporting the use of unified reference values. The left kidney consistently exceeded the right in size ($p < 0.0001$), with a mean difference of 2–5 mm.
**Conclusion:** This study provides updated, population-specific percentile curves for liver, spleen, and kidney dimensions, derived from one of the largest pediatric ultrasound datasets in Central Europe. These normative values offer a robust reference framework to support the differentiation between physiological growth variation and pathological organ enlargement in children and adolescents.
**Limitations:** The retrospective, single-center design and the absence of anthropometric parameters such as height or weight may limit the generalizability of the presented age-based reference values.
**Funding for this study:** No funding was obtained for this study.
**Has your study been approved by an ethics committee?** Yes
**Ethics committee - additional information:** This retrospective cohort study was approved by the institutional review board (approval #2024-1854_1) with waiver of informed consent.
**Author Disclosures:**
Thomas Vogl: Nothing to disclose
Vitali Koch: Nothing to disclose
Scherwin Mahmoudi: Nothing to disclose
Ibrahim Yel: Nothing to disclose
Sebastian Fischer: Nothing to disclose
Leon David Grünewald: Nothing to disclose
Katrin Eichler: Nothing to disclose
Elena Höhne: Nothing to disclose

**Portal vein stenosis after paediatric liver transplantation: ultrasound criteria predictive of stenosis and risk factors for recurrence after balloon angioplasty**

*A. Martin-Champetier*, B. Binselim, E. Dammann, B. Roquelaure, A. Fabre, O. Boillot, A. Dabadie, P. Petit; Marseille/FR
*(ant.champetier@gmail.com)*

**Purpose:** Portal vein stenosis is a common complication after paediatric liver transplantation. The value of ultrasound compared with portal angiography in detecting stenosis and its recurrence after balloon angioplasty has been little studied.

**Methods or Background:** Children who underwent liver transplantation and subsequently received portal angioplasty in our centre were retrospectively included. Demographic, clinical, biological, and ultrasound data were collected and correlated with the presence of angiographic portal vein stenosis. In cases of recurrence after balloon dilatation, the previously collected data as well as angiographic findings were analysed.
**Results or Findings:** Twenty-one children (median age 42 months) underwent 43 portographies. Angiographic stenosis was confirmed in 37 procedures (86.0%), and recurrence occurred after 22 procedures (56.4%). In multivariate analysis, no criterion was associated with the presence of stenosis or recurrence. ROC curves suggested thresholds associated with angiographically proven stenosis (AUC > 0.5) for the following criteria: "B-mode measurement of stenosis", "increase in trans-anastomotic velocity compared with postoperative ultrasound", and "trans-anastomotic pressure gradient". The ROC curve also suggested that the criterion "balloon size" was associated with recurrence (AUC > 0.5).
**Conclusion:** Our study did not identify predictive criteria for stenosis or recurrence in multivariate analysis. Further studies are required to reduce the number of portal angiographies and improve the management of these stenoses.
**Limitations:** This retrospective design resulted in missing data for certain variables, such as splenic elastography. Although this represents the largest cohort reported in the paediatric literature, the relatively small sample size may have limited the statistical power of the analysis.
**Funding for this study:** No external funding was received for the conduct of this study.
**Has your study been approved by an ethics committee?** Yes
**Ethics committee - additional information:** This study received approval from our institution's health data commission and is registered under file number ZJCRST.
**Author Disclosures:**
Olivier Boillot: Nothing to disclose
Alexia Dabadie: Nothing to disclose
Antoine Martin-Champetier: Nothing to disclose
Badr Binselim: Nothing to disclose
Alexandre Fabre: Nothing to disclose
Bertrand Roquelaure: Nothing to disclose
Elena Dammann: Nothing to disclose
Philippe Petit: Nothing to disclose

### Updated sonographic reference values and percentile curves for thyroid volume in children and adolescents (0–18 years) from a large European cohort

*E. Höhne*, S. Fischer, T. Vogl, S. Mahmoudi, P. Reschke, J. Gotta, V. Koch, L. D. Grünewald; Frankfurt/DE

**Purpose:** Reference values for thyroid volume are essential for diagnosing goiter and interpreting pediatric thyroid imaging, yet comprehensive data across the full pediatric age range remain scarce. This study aimed to establish updated sonographic reference values and percentile curves in children and adolescents (0–18 years) from a large European cohort.
**Methods or Background:** This retrospective single-centre cohort study included pediatric thyroid ultrasound examinations performed between 2000 and 2025 at a tertiary referral hospital. Thyroid lobe volumes were calculated from standardized measurements using the formula length × width × depth × 0.5, excluding the isthmus. In total, 4,965 left and 4,948 right lobes fulfilled quality criteria and were included in the analysis. Age-dependent percentile curves (P3–P97) were modeled using GAMLSS.
**Results or Findings:** Thyroid volume (Tvol) increased steadily from birth to late adolescence in both sexes. In boys, mean Tvol rose from 0.71 ml at birth to 8.09 ml at 18 years, and in girls mean Tvol increased from 0.53 ml to 7.46 ml. Peak annual growth occurred earlier in girls (11–12 years, +0.99 ml) than in boys (13–14 years, +0.73 ml), but overall mean Tvol did not differ significantly between sexes ($p = 0.28$). Across all ages, the right lobe was consistently larger than the left ($p < 0.0001$).
**Conclusion:** This large single-centre study provides updated sonographic reference values and percentile curves for thyroid volume across the entire pediatric age range. The results confirm established growth patterns, demonstrate consistent right–left asymmetry, and highlight population-specific deviations from WHO standards, supporting accurate clinical assessment in children and adolescents.
**Limitations:** Limitations include the retrospective single-centre design; validation in independent cohorts is warranted.
**Funding for this study:** No external funding received.
**Has your study been approved by an ethics committee?** Yes
**Ethics committee - additional information:** This retrospective cohort study received approval from the institutional review board (approval number 2024-1854_1), with informed consent waived.
**Author Disclosures:**
Thomas Vogl: Nothing to disclose
Vitali Koch: Nothing to disclose
Scherwin Mahmoudi: Nothing to disclose
Philipp Reschke: Nothing to disclose
Sebastian Fischer: Nothing to disclose
Leon David Grünewald: Nothing to disclose
Jennifer Gotta: Nothing to disclose
Elena Höhne: Nothing to disclose

### Quantitative Analysis of Placental Perfusion in Twin Pregnancies Using Arterial Spin Labeling: A New Imaging Tool for Assessing Asymmetry and Hypoperfusion

*X. Zhang*[1], L. Ji[1], R. Tang[2], M. Zhao[1]; [1]Nanjing/CN, [2]Shanghai/CN

**Purpose:** To evaluate the feasibility of using arterial spin labeling (ASL) to quantitatively assess placental perfusion in twin pregnancies and compare the results with singleton pregnancies.
**Methods or Background:** ASL imaging was performed on twin and singleton pregnancies to measure placental blood flow (BF). Gaussian mixture modeling was applied to partition twin placentas into regions corresponding to each fetus. Asymmetry index (AI) and hypoperfusion fraction (HF) were calculated for each fetus and compared between groups.
**Results or Findings:** Singleton placentas exhibited a mean AI close to 0%, with HF around 10% and BF_mean ranging from 150–400 ml/100g/min. Twin pregnancies showed a wider AI distribution (median -30%, range -120% to +90%) and higher HF in Twin A (11%) compared to Twin B (7%). The automated partitioning algorithm demonstrated stability in identifying perfusion regions.
**Conclusion:** ASL provides a non-invasive method to quantitatively assess placental perfusion in twin pregnancies, revealing significant perfusion asymmetry. This technique offers potential for early identification of at-risk fetuses and may complement existing imaging modalities in clinical practice.
**Limitations:** This study is a small-sample exploratory attempt, which may limit the generalizability of the findings. ASL acquisition was restricted to a few thick slices, potentially missing parts of the placenta. In twin pregnancies, placental vascular anastomoses make perfusion partitioning challenging, and our clustering-based segmentation may not fully match anatomical territories.
**Funding for this study:** Maternal and Child Health Hospital High-level Construction Project(GZL2529)
**Has your study been approved by an ethics committee?** Yes
**Ethics committee - additional information:** It was approved by the institutional ethics committee of The First Affiliated Hospital of Nanjing Medical University.
**Author Disclosures:**
Ran Tang: Nothing to disclose
Liangyu Ji: Nothing to disclose
Xuan Zhang: Nothing to disclose
Meng Zhao: Nothing to disclose

**08:00 - 09:30** **Research Stage 6**

### Research Presentation Session: Oncologic Imaging

### RPS 716

### Novel developments in neuro-oncology

**Moderator**
Irina Trofimenko; Lisbon/PT
*(itrof-01@yandex.ru)*

**Tissue discrimination in brain metastases using neurite orientation dispersion and density imaging MRI**
*M. Bauer*, S. Mangesius, M. Wagner, J. Kerschbaumer, D. Pinggera, J. Mangesius, A. E. Grams, E. R. Gizewski, C. Birkl; Innsbruck/AT

**Purpose:** Brain metastases exhibit complex microstructural heterogeneity, challenging accurate tissue characterisation. This study aimed to investigate whether the MRI model Neurite Orientation Dispersion and Density Imaging (NODDI) can differentiate tissue types across entire regions in brain metastases patients.
**Methods or Background:** This single-centre, prospective study included nine patients with 39 confirmed brain metastases, restricted to lesions of sufficient size for clinically relevant differential diagnosis. Head MRI scans were performed at 3 T using a multi-shell diffusion protocol. NODDI parameter maps were calculated for Orientation Dispersion Index (ODI), Neurite Density Index (NDI) and Free Water Fraction (FWF) using the Watson model. Whole-tissue regions representing oedema, necrosis, neoplasm, white matter, deep grey matter and cortical grey matter were segmented automatically in 3D Slicer using the MONAI deep learning pipeline on standard sequences. Univariate analyses of pairwise tissue differences employed the Wilcoxon signed-rank and paired Student's t-test. Multivariate analyses used Hotelling's T2, combining all three NODDI parameters to evaluate their joint discrimination power.
**Results or Findings:** Univariate analyses revealed multiple statistically significant differences in NODDI parameters, with the single non-significant result for all parameters when comparing neoplasm and cortex (ODI: $p = .55$; NDI: $p = .41$; FWF: $p = .17$). FWF and ODI showed significant differences between pathological and normal appearing regions ($p < .02$), suggesting their value as markers of microstructural alteration. Multivariate analyses demonstrated significant discriminations across all tissue comparisons ($p < .04$), except between cortex and necrosis ($p = .06$).
**Conclusion:** NODDI parameters differentiate necrosis, oedema and neoplasm in patients with clinically relevant brain metastases. Incorporating multivariate analyses offers the combined evaluation of NODDI parameters, providing complementary information.
**Limitations:** The used NODDI model may underestimate complex fibre dispersions in tumours. Automatic segmentation was not validated by experts.
**Funding for this study:** Funding was provided by SNSF Postdoc.Mobility Fellowship PM221964 to Melanie Bauer.
**Has your study been approved by an ethics committee?** Yes
**Ethics committee - additional information:** The study was approved by institutional review board and written informed consent was obtained from all participants.
**Author Disclosures:**
Melanie Bauer: Nothing to disclose
Daniel Pinggera: Nothing to disclose
Elke Ruth Gizewski: Nothing to disclose
Astrid E. Grams: Nothing to disclose
Michaela Wagner: Nothing to disclose
Christoph Birkl: Nothing to disclose
Julian Mangesius: Nothing to disclose
Johannes Kerschbaumer: Nothing to disclose
Stephanie Mangesius: Nothing to disclose

**Differential effects of IDH genotypes on corticospinal tract integrity and neurological functions in glioma patients: a study using neurite orientation dispersion and density imaging**
*K. Deng*, Y. Xue, R. Jiang; Fuzhou/CN
*(315986323@qq.com)*

**Purpose:** To explore the relationship among isocitrate dehydrogenase (IDH) genotypes of glioma,the diffusion MRI features of corticospinal tract (CST) and the neurologic assessment in neuro-oncology (NANO) score.
**Methods or Background:** 65 patients with gliomas adjacent to CST and 32 healthy controls underwent structural and diffusion MRI.Diffusion metrics, including DTI and NODDI, were calculated along the CST.Neurologic function was assessed using NANO scale.NANO score and subscores were compared between IDH wild-type and IDH mutant.The CST features were analysed between affected and healthy side for glioma patients and between left and right side for healthy controls.The relative CST features were evaluated among the three groups.Spearman rank correlation analysis was used to evaluate the correlation between the relative CST features and NANO score.
**Results or Findings:** Results showed significantly higher NANO scores, indicating worse function, in IDH wild-type patients compared to IDH mutant patients. Significant differences were found in motor subscores (gait, strength, ataxia, facial strength).CST microstructure on the affected side was significantly altered in both groups, with more extensive changes in IDH wild-type gliomas.Significant differences in relative CST metrics (ICVF, MD, RD) were found between IDH genotypes.There were differences in the relative CST features between the control group and different IDH genotypes.Furthermore, AD and MD correlated positively with NANO score, while ICVF and ODI correlated negatively.
**Conclusion:** Neurological impairment, particularly motor function,is more severe in IDH wild-type gliomas,likely due to greater CST microstructural injury. NODDI provides sensitive microstructural detection, confirming that CST integrity is better preserved in IDH mutant gliomas.
**Limitations:** There was no direct correlation between diffusion-sampled data and surgical findings. Therefore, it could not be ascertained whether the glioma truly infiltrated the CST. However, the decrease in motor function may indirectly reflect the injury of CST.
**Funding for this study:** This work was supported by Fujian Provincial Natural ScienceFoundation of China (Grant number: 2025J01800), Fujian provincial health technology project (Grant number: 2021QNA013), the Education and Research Program for Middle-aged and Young Teachers in Fujian Province (Grant number: JAT210108) .
**Has your study been approved by an ethics committee?** Yes
**Ethics committee - additional information:** This study was approved by the Institutional Review Board of Fujian Medical University Union Hospital (2020KY0132), and written informed consent was obtained from all the participants.
**Author Disclosures:**
Kaiji Deng: Nothing to disclose
Yunjing Xue: Nothing to disclose
Rifeng Jiang: Nothing to disclose

**Enhancing brain metastasis detection in CT imaging: the impact of Maximum Intensity Projection (MIP) among radiologists with different levels of expertise**
*G. Pentassuglia*[1], M. Martucci[2], M. L. Angeli[2], F. Lisi[1], L. Ruscelli[3], A. Infante[2], S. Gaudino[1]; [1]Rome/IT, [2]Roma/IT, [3]Bergamo/IT
*(gianni.penta98@gmail.com)*

**Purpose:** Brain metastases are the most common intracranial tumors in adults and are critical for oncologic staging and treatment. Although MRI is the gold standard, contrast-enhanced head CT is frequently performed during whole-body oncologic follow-up and often interpreted by general radiologists, risking underdiagnosis. Maximum Intensity Projection (MIP) is a CT post-processing technique widely used in thoracic and vascular imaging but underutilized in neuroimaging. This study evaluated whether MIP can improve CT detection of brain metastases, particularly for less-experienced readers.
**Methods or Background:** This retrospective single-center study included 100 oncologic patients who underwent brain MRI within 30 days of contrast-enhanced whole-body CT. MRI served as the reference standard. Two radiologists—a general radiologist (R1) and a neuroradiologist (R2)—reviewed CT scans in two sessions: standard axial CT and CT with MIP reconstructions. The number of detected lesions, lesion size, and reporting time were recorded.
**Results or Findings:** MRI identified significantly more metastases than CT (mean ± SD: MRI = 12.4 ± 33.6; CT without MIP: R1 = 3.5 ± 5.9, R2 = 4.9 ± 10.0; CT with MIP: R1 = 3.8 ± 6.0, R2 = 4.9 ± 9.6; $p < 0.001$). MIP modestly improved detection for R1, while R2's results remained stable. R1's performance with MIP approximated R2's with standard CT, reducing inter-reader variability. MIP also shortened report times (−1 min for R1; −2 min for R2). In oligometastatic patients (<5 lesions), CT_MIP aligned more closely with MRI, improving reliability of findings relevant for treatment planning.
**Conclusion:** Although MRI remains superior, MIP-enhanced CT increases lesion detection and reduces report times, particularly for general radiologists. Incorporating MIP into routine CT protocols may improve diagnostic accuracy and efficiency in oncologic follow-up, especially in non-specialist or resource-limited settings.
**Limitations:** No limitations were identified.
**Funding for this study:** No funding was received for this study.
**Has your study been approved by an ethics committee?** Not applicable
**Ethics committee - additional information:** The study is retrospective.

**Author Disclosures:**
Amato Infante: Nothing to disclose
Luigi Ruscelli: Nothing to disclose
Simona Gaudino: Nothing to disclose
Francesca Lisi: Nothing to disclose
Maria Luigia Angeli: Nothing to disclose
Matia Martucci: Nothing to disclose
Giovanni Pentassuglia: Nothing to disclose

**Decoding Glioma Phenotypes: Quantitative Paramagnetic and Diamagnetic Susceptibility Mapping Using APART-QSM**

*L. Mazu*[1], S. Zeng[1], W. Zhou[2], S. He[1], N. Zhu[1], J. Wu[1], Z. Yang[1], H. Wei[2], J. Chu[1]; [1]GuangZhou/CN, [2]Shanghai/CN
*(mazlw@mail2.sysu.edu.cn)*

**Purpose:** This study aimed to (1) assess the value of quantitative paramagnetic( χpara) and diamagnetic( χdia) susceptibility in predicting glioma grading, IDH status, and prognosis; and (2)verify their correlations with intratumoral iron and tumor-associated macrophages (TAMs).
**Methods or Background:** We retrospectively enrolled 81 patients with diffuse gliomas (44 men, mean age 50 years) who underwent MRI including QSM. χpara and χdia were derived using a novel APART-QSM technique. Histological analysis included iron quantification (Perls stain) and multiplex immunofluorescence for TAM markers (CD68, CD86, CD163). Diagnostic models for tumor grade and IDH status were developed. Survival analysis and Spearman correlation with pathological indicators were performed.
**Results or Findings:** The diagnostic models for IDH status and tumor grade achieved AUCs of 0.81 (95%CI 0.71-0.90) and 0.87 (95% CI 0.78-0.97), respectively. The results showed that χdia was an independent predictor for IDH-mutant status (p=0.016.OR=0.12), while χpara was significantly associated with high-grade glioma (p=0.01.OR=8.16).Calibration curves and decision curve analyses confirmed good predictive performance and clinical utility. Kaplan-Meier analysis showed significant overall survival differences stratified by χpara/χdia. Both χpara and χdia positively correlated with iron concentration (r=0.32, r=0.29; p<0.05). Negative correlations were observed between χpara/QSM and $CD163^{+}$ macrophage infiltration (r=-0.27, r=-0.25; p<0.05). Additionally, there was no significant correlation among the other variables.
**Conclusion:** χpara and χdia serve as valuable imaging biomarkers for predicting molecular subtype, grading, and prognosis in gliomas. Their correlations with iron deposition were confirmed, supporting their biological relevance. These findings may aid in non-invasive glioma characterization and personalized treatment planning.
**Limitations:** Not applicable.
**Funding for this study:** This study was funded by National Natural Science Foundation of China (NSFC 82172015)
**Has your study been approved by an ethics committee?** Yes
**Ethics committee - additional information:** This retrospective study received ethical approval of the Clinical Research and Laboratory Animal Ethics Committee, The First Affiliated Hospital of Sun Yat-sen University (number: [2024]763).
**Author Disclosures:**
Hongjiang Wei: Nothing to disclose
Siyuan He: Nothing to disclose
Wenyu Zhou: Nothing to disclose
Jianping Chu: Nothing to disclose
Nengjin Zhu: Nothing to disclose
Liwei Mazu: Nothing to disclose
Jiajing Wu: Nothing to disclose
Zhiyun Yang: Nothing to disclose
Shanmei Zeng: Nothing to disclose

**Diagnosis and Management of Radiation-Induced Gliomas in Children: An Interdisciplinary Perspective**

*N. Plakhotina*, D. Drozdovskaya, A. V. Smirnova, D. I. Kuplevatskaya, K. Boiko, A. Brusser; Saint Petersburg/RU
*(plahotinadezhda@gmail.com)*

**Purpose:** To define the imaging features and outcomes of pediatric radiation-induced brain tumors (RIBT) using a multicenter cohort. The study aimed to apply diagnostic criteria, ensure lesion-field concordance, utilize advanced imaging (MRI, amino-acid PET), and establish realistic follow-up expectations.
**Methods or Background:** We reviewed 14 young patients treated between 2019 and 2025 (average age, 5.4 years). To confirm these were radiation-induced tumors, we checked: did the tumor appear where radiation was given before, was it at least 4 years after radiation, was it a different tumor type than the original disease, and was there no family cancer history. Image analysis assessed field geometry, contrast enhancement, diffusion-weighted imaging, and radiopharmaceutical uptake on amino acid PET.
**Results or Findings:** All tumors were high-grade gliomas located within the previous radiation fields, emerging after a median latency of 7 years. MRI showed infiltrative tumors with heterogeneous contrast enhancement. Tumors were located in the cerebral hemispheres (54%), cerebellum (33%), and brainstem (13%). Differential diagnoses included neuroleukemia relapse and other encephalopathies. Amino acid PET confirmed high radiopharmaceutical uptake in the tumor foci, aiding visualization. Outcomes were poor: 7 patients died, median overall survival was 13.8 months, and progression-free survival was 8 months. Progression was predominantly local (~90%); extraneural spread was rare (0,5%). In a separate sub-cohort receiving repeat proton irradiation (n=8), median overall survival was 19 months.
**Conclusion:** In children, RIBT typically manifest as high-grade gliomas within irradiated areas after a long latency. Diagnosis relies on correlating radiation therapy history with modified criteria, supported by multiparametric MRI and amino acid PET. Despite modern treatments, the prognosis remains uncertain, necessitating careful, lifelong neuro-oncological monitoring for survivors of cranial irradiation. Imaging is critical for guiding biopsy timing and therapy selection.
**Limitations:** N/A
**Funding for this study:** N/A
**Has your study been approved by an ethics committee?** Not applicable
**Author Disclosures:**
Konstantin Boiko: Nothing to disclose
Nadezhda Plakhotina: Nothing to disclose
Alina Vyacheslavovna Smirnova: Nothing to disclose
Darya Drozdovskaya: Nothing to disclose
Anastasia Brusser: Nothing to disclose
Daria Igorevna Kuplevatskaya: Nothing to disclose

**MR Imaging correlation of survival in patients with high-grade gliomas**

*S. Namkhainyam*, D. Khashbat, T. Dashjamts, A. Bat-Arildii, E. Ochirjav; Ulaanbaatar/MN
*(sharwaa.01122@gmail.com)*

**Purpose:** To evaluate the survival of patients diagnosed with malignant gliomas and to identify MRI imaging features associated with prognosis.
**Methods or Background:** The study was conducted between 2018 and 2022 by sampling 125 clients who were diagnosed with malignant glioma brain tumor by undergoing MRI scan. Retrospective cohort analysis was done selecting 34 cases according to the inclusion and exclusion criteria.
**Results or Findings:** Total of 34 cases, 28 (60.5%) were male and 6 (29.5%) were female, with a median age of 50.67 ± 19.43 years. The median survival time after diagnosis was 15.7 months. For patients diagnosed with WHO Grade III malignant gliomas, the median survival time was 35.9 ± 10.1 months, while for those with Grade IV malignant gliomas, it was 12.2 ± 8.2 months. The overall survival rates among the study participants were 92% at 1month, 82% at 3months, 67% at 6months, 61% at 9months, 60% at 1 year, 12% at 3 years, and 0% at 5 years. According to the comparison of survival rate with MRI imaging features were significantly associated with prognosis: high tumor square (P=0.011), multiple hemorrhagic components (P=0.047), high edema index (P=0.007), necrosis (P=0.0001), thickness of contrast enhancement (P=0.003) and midline shift (P=0.019).
**Conclusion:** The average survival time following diagnosis of high grade glioma was 15.7 months. For patients with WHO grade III gliomas, the mean survival time was 35.9 ± 10.1 months, whereas for grade IV gliomas 12.2 ± 8.2 months. Patients with WHO grade IV gliomas had significantly shorter survival than those with grade III gliomas. MRI features such as tumor volume, peritumoral edema index, necrosis, hemorrhagic content, and mass effect were found to be negative prognostic factors for survival.
**Limitations:** Our study was conducted onrelatively small number ofcases.
**Funding for this study:** This research did not receive any specific grant from funding agencies in the public, commercial, or not-for-profit sectors. ID:2024/3-06
**Has your study been approved by an ethics committee?** Yes
**Ethics committee - additional information:** This study was approved by ethics Committee pf the Mongolian National University of Medical Sciences and National Pathology center of Ulaanbaatar Mongolia
**Author Disclosures:**
Ariuntungalag Bat-Arildii: Author: co-author
Tuvshinjargal Dashjamts: Author: co-author
Enkhee Ochirjav: Author: co-author
Delgerdalai Khashbat: Author: Corresponding author
Sharavjamts Namkhainyam: Author: Author

**QSM-derived histogram and intratumoral susceptibility features for noninvasive Glioma tumor grading**

*V. Shahmaei*[1], F. Sodaei[2]; [1]Tehran/IR, [2]Odense/DK
*(fsodaei@health.sdu.dk)*

**Purpose:** Accurate preoperative grading of gliomas is crucial for treatment planning and prognosis. Conventional MRI lacks specificity, whereas quantitative susceptibility mapping (QSM) sensitively detects magnetic susceptibility changes from iron, calcification, and microhemorrhage. Intratumoral susceptibility signals (ITSS), reflecting microvascular proliferation and hemorrhage, are closely associated with tumor angiogenesis and aggressiveness, offering a promising noninvasive biomarker for glioma grading.
**Methods or Background:** A retrospective analysis was conducted on 40 pediatric patients with histopathologically confirmed gliomas, categorized into low-grade (WHO grade II) and high-grade (WHO grade III–IV) groups. Multi-echo T2* gradient-echo MRI data were processed using the Morphology Enabled Dipole Inversion (MEDI) QSM pipeline. Tumor regions were delineated, and both histogram-derived susceptibility metrics (mean, percentiles, skewness, kurtosis) and ITSS counts were quantified. Statistical comparisons between groups were performed using independent-samples t-tests, and diagnostic performance was assessed with receiver operating characteristic (ROC) analysis.
**Results or Findings:** High-grade gliomas exhibited significantly higher mean susceptibility values and greater histogram heterogeneity (90th percentile, kurtosis; $p < 0.05$), as well as markedly increased ITSS counts, compared with low-grade tumors. ITSS alone achieved an area under the ROC curve (AUC) of 0.87 (sensitivity 82%, specificity 83%), while histogram-derived susceptibility metrics yielded an AUC of 0.88 (sensitivity 85%, specificity 80%). The combined model, integrating both histogram features and ITSS, demonstrated the highest performance, with an AUC of 0.92, sensitivity of 90%, and specificity of 85%.
**Conclusion:** Histogram features derived from QSM, together with ITSS quantification, provide robust, noninvasive biomarkers for glioma grading. In this study, incorporating ITSS improved the differentiation of glioma grades, highlighting the potential of QSM as a valuable adjunct to conventional MRI for preoperative tumor evaluation.
**Limitations:** The limitations of the study are the retrospective design and small sample size, which may limit generalizability.
**Funding for this study:** No funding was received for this study.
**Has your study been approved by an ethics committee?** Yes
**Ethics committee - additional information:** The study was approved by the ethics committee of Mahak Hospital.
**Author Disclosures:**
Forough Sodaei: Nothing to disclose
Vahid Shahmaei: Nothing to disclose

**Comparative Analysis of 5.0T versus 3.0T Susceptibility-Weighted Imaging in Glioma: A Pair-Matched Study**

*Y. Zhao*, N. Mei, Z. Ruan, T. Xu, Y. Lu, B. Yin; Shanghai/CN
*(zhaoyj92918@163.com)*

**Purpose:** To evaluate the feasibility of 5T SWI imaging in the diagnosis of gliomas, we conducted a comparative analysis with 3T imaging to assess image quality and determine the clinical value of 5T SWI in the preoperative evaluation of gliomas.
**Methods or Background:** Between 2024 and 2025, the glioma patients underwent 5T MRI scans. Each patient was individually matched with a control subject selected from the 3T MRI cohort. Radiologist scoring of image quality using a Likert scale, SNR, CNR, contrast ratio, and ITSS assessment were obtained. Paired Wilcoxon signed rank tests were used to compare characteristics derived at 3 T and 5T . The correlation between ITSS scores and molecular pathology was analyzed.
**Results or Findings:** A total of 27 participants were included undergoing 5T MRI. Each case was individually matched with one control subject selected from the 3.0T MRI cohort. Median image quality scores were 4.0 at 3 T and5.0 at 5T . SNR and CNR at 5 T was higher than at 3T. The degree of ITSS in 5.0 T cohort was significantly higher than that in 3T cohort . In the 5T cohort, a statistically significant association was observed between ITSS and WHO grades . The ROC curve analysis indicated that 1.5 was the best cutoff value to predict pathological grade. It shows that gliomas with a Ki-67 ≥ 10% exhibit a significantly higher ITSS than those with a Ki-67 < 10% .
**Conclusion:** SWI at 5T was found to be feasible and provided visualization of distal small vessel branches. And the image quality of 5T enables more precise ITSS delineation and demonstrating correlation with tumor proliferative activity
**Limitations:** The limitations of this study include its single-center design and small sample size, requiring future validation
**Funding for this study:** Not applicable
**Has your study been approved by an ethics committee?** Not applicable
**Author Disclosures:**
Bo Yin: Nothing to disclose
Yiping Lu: Nothing to disclose
Zhouying Ruan: Nothing to disclose
Yajing Zhao: Nothing to disclose
Nan Mei: Nothing to disclose
Tianqi Xu: Nothing to disclose

**Tertiary Lymphoid Structures in Glioblastoma: Association with Multiparametric MRI Imaging Phenotypic Features and Patient Survival**

*Q. Zhou*, J. Zhou; Lanzhou/CN
*(408027919@qq.com)*

**Purpose:** Tertiary lymphoid structures (TLSs) are associated with improved survival in patients with various malignancies. However, their prognostic value and correlation with imaging features in glioblastoma (GBM) remain unclear. This study aimed to examine the relationship between TLSs and survival outcomes, and their association with imaging characteristics in patients with GBM.
**Methods or Background:** A retrospective analysis was conducted on 190 newly diagnosed patients with GBM. TLS-positive (TLSs+) and TLS-negative (TLSs-) groups were classified via hematoxylin-eosin staining to identify lymphocyte aggregates. Multiplex immunofluorescence staining (for CD20, CD3, CD21, and CD23) assessed TLS maturity, categorizing them into mature (mTLSs) and immature (imTLSs) subgroups. Survival was analyzed using the Kaplan-Meier method, while logistic regression developed TLS predictive models. Clinicopathological and Visually Accessible Rembrandt Images (VASARI) imaging features were compared between groups.
**Results or Findings:** Among 190 cases, 85 (44.73% positivity rate) were TLSs+, including 47 imTLSs and 38 mTLSs. Significant differences in overall survival were observed between TLSs+TLSs- groups and mTLSs/imTLSs groups (all $p < 0.05$). For VASARI features, the TLSs+/TLSs- groups differed significantly in F2, F8, F17, F22 ($p < 0.05$), while the mTLSs/imTLSs groups differed in F6, F16, F18 ($p < 0.05$); these features were independent predictors of TLS presence and maturity.
**Conclusion:** Conclusion: TLSs exist at different maturation stages in GBM and serve as favorable prognostic biomarkers. TLS presence and maturity are significantly correlated with patients' overall survival and specific VASARI features. Preoperative prediction of TLSs via imaging may support risk stratification and individualized treatment for patients with GBM.
**Limitations:** First, this was a single-center, retrospective investigation. Second, the patient cohort size was relatively small, which may have limited the robustness of the findings.
**Funding for this study:** This study is supported by grants from the National Natural Science Foundation of China (grant number: 82371914), the Cuiying Science and Technology Innovation Program of Lanzhou University Second Hospital (grant number:CY2023-YB-A03),the Science and Technology Program of Lanzhou City(grant number:2024-9-122), and the Science and Technology Program of Gansu Province(grant number: 24JRRA376).
**Has your study been approved by an ethics committee?** Yes
**Ethics committee - additional information:** This study was approved by the Medical Ethics Committee of our institution (Ethics Approval No. 2025A-364), and the research protocol adhered to the principles of the Declaration of Helsinki.
**Author Disclosures:**
Junlin Zhou: Nothing to disclose
Qing Zhou: Speaker: Department of Radiology, Lanzhou University Second Hospital ; E-mail address: 408027919@qq.com

**Contribution of Advanced MRI Biomarkers in the Characterization of Brain Lesions After Stereotactic Radiotherapy: Radionecrosis Versus Progression**

*J. Hadchiti*[1], S. Haffiane[1], S. Ganjoo[2], M. El-Aichi[1], H. J. Howell[2], G. Garcia[1], N. Lassau[1], C. S. Balleyguier[1], S. Ammari[1]; [1]Villejuif/FR, [2]New York, NY/US
*(joyahadchiti@hotmail.com)*

**Purpose:** Evaluate the predictive performance of a new set of imaging biomarkers related to microvasculature, blood flow, and tumor oxygen use in distinguishing tumor recurrence from radiation necrosis compared to the commonly used cerebral blood volume (CBV) biomarker
**Methods or Background:** 119 symptomatic patients with metastatic brain lesions post-radiotherapy were enrolled . 205 total lesions were analyzed across this sample. The diagnosis was tumor recurrence (n= 71 in test cohort and n = 7 in validation cohort) and radiation necrosis (n = 111 in test cohort and n = 16 in validation cohort) using expert-consensus derived from pathology and long-term follow-up as a reference standard. We studied 9 radiologist-reported imaging biomarkers (corrected CBV, non-corrected CBV, Delay, CTH, CTH Max, OEF, rLeakage, rCMRO2, COV). We performed univariate analyses to compare the area under the receiver operating characteristic curve (AUC)

Thursday for the ratio values across all biomarkers in the test and validation cohorts. We additionally used a Random Forest model to identify the optimal combination of biomarkers with the highest predictive performance.
**Results or Findings:** In the test cohort, Delay_ratio, CTH_ratio, CTH Max_ratio , OEF_ratio , rCMRO2_ratio , and COV_ratio all significantly outperformed the predictive performance of CBV_corrected_ratio and CBV_noncorrected_ratio. The Random Forest algorithm demonstrated that the optimal model for differentiating between TR and RN was comprised of five biomarkers: CBV_corrected_ratio, CBV_noncorrected_ratio, Delay_ratio, OEF_ratio, and COV_ratio with Delay_ratio having the highest feature importance and CBV_noncorrected_ratio having the lowest.
**Conclusion:** The novel imaging biomarkers investigated in this study demonstrate promising predictive performance in distinguishing between tumor recurrence and radionecrosis that warrants prospective validation
**Limitations:** Variation in patient numbers across different primary tumor types Single institution study
**Funding for this study:** No funding
**Has your study been approved by an ethics committee?** Yes
**Ethics committee - additional information:** Local ethic comimittee of Gustave Roussy Institute
**Author Disclosures:**
Gabriel Garcia: Nothing to disclose
Shonik Ganjoo: Nothing to disclose
Samia Haffiane: Nothing to disclose
Mohammed El-Aichi: Nothing to disclose
Samy Ammari: Nothing to disclose
Nathalie Lassau: Nothing to disclose
Joya Hadchiti: Nothing to disclose
Corinne S. Balleyguier: Nothing to disclose
Harrison J. Howell: Nothing to disclose

**A Study on the Predictive Value of a Multi-Parameter Model Combining Diffusion and Perfusion MRI Based on Histogram Analysis for MGMT Status and Prognosis in Glioblastoma**
*F. Yu*, W. Chen, P. Chen, J. F. Chen; Chongqing, China/CN
*(18381507977@163.com)*

**Purpose:** Methylation of the $O^6$-methylguanine-DNA methyltransferase (MGMT) promoter is a key molecular marker for guiding treatment strategies and predicting prognosis in glioblastoma. This study investigated the value of diffusion tensor imaging (DTI) and arterial spin labeling (ASL) histogram parameters for non-invasively predicting MGMT status, and explored the mediating role in how MGMT status influences patient survival outcomes.
**Methods or Background:** We retrospectively analyzed DTI and ASL data from 349 glioblastoma patients, extracting histogram parameters including fractional anisotropy (FA), mean diffusivity (MD), and cerebral blood flow (CBF). Intergroup comparisons were performed based on MGMT status. Predictive performance for MGMT status was evaluated using ROC curves, calibration curves and the Hosmer-Lemeshow goodness-of-fit test. For survival assessment, Kaplan-Meier and Cox regression analyses evaluated associations between imaging features, molecular markers, and overall survival (OS). Bootstrap mediation analysis assessed whether imaging parameters mediated the relationship between MGMT status and survival.
**Results or Findings:** The DTI model (AUC = 0.842) was significantly superior to the ASL model (AUC = 0.619) in predicting MGMT status. Kaplan-Meier analysis confirmed that MGMT promoter methylation was a significant protective prognostic factor (Log-rank P = 0.041). However, Cox proportional hazards regression analysis showed that while imaging parameters were not independent OS prognostic factors, mediation analysis identified FA 10th and MD skewness as complete mediators in the association between MGMT status and OS.
**Conclusion:** DTI histogram parameters are effective non-invasive biomarkers for predicting MGMT status. Specifically, FA 10th and MD skewness serve as complete mediators, clarifying the key mechanism by which unmethylated MGMT drives poor prognosis through inducing microstructural disruption and increased tissue heterogeneity in tumors. This provides important imaging insights for understanding glioblastoma biology and may suggest new targets for future targeted treatment strategies.
**Limitations:** Not applicable
**Funding for this study:** Not applicable
**Has your study been approved by an ethics committee?** Yes
**Ethics committee - additional information:** The study was approved by the Ethics Committee of the First Affiliated Hospital of the Army Medical University (KY2024109).
**Author Disclosures:**
Jia Fei Chen: Nothing to disclose
Pinzhen Chen: Nothing to disclose
Wei Chen: Nothing to disclose
Fengwei Yu: Nothing to disclose

**Mapping the MRI Patterns of Spinal Metastases: Compartment Distribution and Primary Tumor Insights from 12405 Patients**
*A. K. Nawaz*, S. Ahmed, A. Iqbal, K. Siddique; Lahore/PK
*(dr.arubanawaz@gmail.com)*

**Purpose:** Spinal metastasis are the most common malignant lesions of spine and major cause of neurological morbidity. Large-scale data on lesion distribution across spinal compartments and their primary tumor origins are limited. This study analyzes MRI patterns of spinal metastasis and summarizes primary tumor types in a large cohort.
**Methods or Background:** We retrospectively reviewed MRI reports of 12,405 patients with biopsy-proven or clinically confirmed spinal metastasis from 2020-2025. Lesions were categorized into vertebral body, paravertebral soft tissues, epidural, intradural–extramedullary and intramedullary compartments. Primary tumor types and presence of cord compression were recorded. Counts and percentages were calculated.
**Results or Findings:** Compartment distribution: Epidural 10,918 [88%], vertebral body 5262 [42%], paravertebral 397 [3%], intradural–extramedullary 124 [1%], intramedullary 50 [less than 1%]. Primary tumors: Lung 4962 [40%], breast 3722 [30%], prostate 1737 [14%], renal 744 [6%], thyroid 248 [2%], gastrointestinal [496 [4%], melanoma 124 [1%], sarcoma to 48 [2%], lymphoma 124 [1%]. Epidural lesions were most frequent. Rare intradural and extramedullary metastasis were observed, particularly among melanoma, renal and lung cancers. Compartment based evaluation improved distinction from mimics and provided a structured framework for reporting in multidisciplinary planning.
**Conclusion:** In this large cohort, spinal metastasis demonstrate distinct MRI patterns across anatomical compartments. Systemic compartment based assessment enhances diagnostic accuracy, facilitates early detection of cord compression and informs multidisciplinary management. Rarer intramedullary lesions, though uncommon, required high vigilance. Key points: 1. MRI is essential for comprehensive assessment of spinal metastasis. 2. Compartment analysis improves differentiation from mimics. 3. Primary tumor data provides context without implying strict compartment correlations. 4. Rarer intramedullary lesions demand careful attention.
**Limitations:** None
**Funding for this study:** None
**Has your study been approved by an ethics committee?** Yes
**Ethics committee - additional information:** This retrospective anonymized review was conducted in accordance with our institutional IRB guidelines. No additional patient consent was required.
**Author Disclosures:**
Aruba Nawaz Khattak: Nothing to disclose
Kashif Siddique: Nothing to disclose
Adila Iqbal: Nothing to disclose
Shahroze Ahmed: Nothing to disclose

**Microstructure mapping with time-dependent diffusion MRI differentiates primary central nervous system lymphoma from glioblastoma**
W. Jun, L. Jue, *S. Gui*, J. Wang; Wuhan/CN
*(shen.gui@philips.com)*

**Purpose:** This study aimed to evaluate the diagnostic utility of time-dependent diffusion MRI (td-dMRI)-derived microstructural parameters in differentiating PCNSL from GBM, and to correlate these parameters with histopathologic findings.
**Methods or Background:** This retrospective study included 32 GBM and 19 PCNSL patients who underwent 3.0-T MRI with oscillating gradient spin-echo (OGSE) and pulsed gradient spin-echo (PGSE) sequences. Microstructural parameters (intracellular volume fraction (Vin), cell diameter, cellularity, extracellular diffusivity (Dex)) were compared between them. The area under the receiver operating characteristic curve (AUC) was used to evaluate the diagnostic performance of these indices. Histopathologic validation was performed by correlating td-dMRI parameters with hematoxylin-eosin (H&E) stained sections.
**Results or Findings:** In enhancing tumor regions, PCNSL showed significantly lower cell diameter and Dex, but higher Vin and cellularity than GBM (all $p < 0.001$). Vin demonstrated the highest diagnostic accuracy (AUC = 0.901, sensitivity = 0.737, specificity = 0.906). No significant differences were found in peritumoral regions. Vin correlated strongly with histopathologic nuclear volume fraction ($r = 0.76$, $p < 0.001$).
**Conclusion:** Td-dMRI-derived microstructural parameters, particularly Vin, effectively differentiate PCNSL from GBM, offering a noninvasive approach to improve preoperative diagnosis.
**Limitations:** First, due to the small sample size, the model may be overfitted, so combined diagnostic performance was not evaluated. Larger sample sizes are necessary to verify this model in the future. Second, ROIs were manually defined, automated tumor segmentation methods may reduce the inter-operator variability and improve the accuracy. Third, histopathologic slides were obtained by sampling a limited area of the tumor, whereas td-dMRI-based microstructural parameters were derived from the entire tumor region, leading to a lack of precise matching. Finally, we didn't combine td-dMRI parameters with other MRI methods, which may further improve the diagnostic performance of a single indicator.
**Funding for this study:** This work was supported by the National Natural Science Foundation of China (82202230, 82371945).
**Has your study been approved by an ethics committee?** Yes
**Ethics committee - additional information:** Institutional Review Board approval was obtained.
**Author Disclosures:**
Wu Jun: Nothing to disclose
Shen Gui: Nothing to disclose
Lu Jue: Nothing to disclose
Jing Wang: Nothing to disclose

**10:00 - 11:00** **Research Stage 2**

### Research Presentation Session: Abdominal and Gastrointestinal

### RPS 801

### Advances in inflammatory bowel disease (IBD) imaging

**Moderator**
Jaap Stoker; Amsterdam/NL
**Author Disclosures:**
Jaap Stoker: Grant Recipient: Crohn's & Colitis Foundation Litwin IBD Pioneers grant

### MRI in Perianal Fistulizing Crohn's Disease: Inter-Reader Agreement of MAGNIFI-CD and 3D Volume Measurements

*K. Johnson*[1], I. De Kock[2], K. J. H. Bengtsson[3], B. Janssens[1], A. Menys[4], G. Bislenghi[1], C. W. P. Greer[4], G. Bhatnagar[4], B. Verstockt[1]; [1]Leuven/BE, [2]Ghent/BE, [3]Lund/SE, [4]London/UK
*(kristin.johnson@med.lu.se)*

**Purpose:** The Magnetic Resonance Novel Index for Fistula Imaging in Crohn's Disease (MAGNIFI-CD) and 3D volumetry are emerging metrics for quantifying and visualizing perianal Crohn's disease (pCD). The purpose of the study was to assess inter-reader agreement for both metrics in different pCD TOpClass categories among radiologists with varying levels of expertise in inflammatory bowel disease (IBD).
**Methods or Background:** Fifty pelvic MRIs from 50 unique pCD patients were retrospectively and randomly selected from a single-center IBD database: 25 represented minimal disease (suitable for repair; TOpClass 1–2a), while 25 represented disease requiring symptom control interventions/proctectomy (TOpClass 2b–3). Six radiologists independently assessed each MRI using a commercial MAGNIFI-CD module (Entrolytics by Motilent), blinded to clinical data. Three were IBD experts (>50 fistula MRI reads/year), three were non-IBD experts (<10/year). MAGNIFI-CD (0–25) was scored, and contrast-enhancing fistula volumes were manually segmented on T1 post-contrast axial images. Inter-reader agreement was analyzed through intraclass correlation coefficient (ICC).
**Results or Findings:** Inter-reader agreement for MAGNIFI-CD was ICC = 0.747 (95% CI: 0.640–0.835), among experts 0.786 (0.681–0.865) and non-experts 0.780 (0.610–0.877). Agreement for 3D volume was 0.887 (0.830–0.929), among experts 0.872 (0.792–0.923) and non-experts 0.893 (0.796–0.942). In TOpClass 1–2a, agreement for MAGNIFI-CD was 0.706 (0.561–0.834) and volume 0.537 (0.367–0.715). In TOpClass 2b–3, agreement was 0.664 (0.482–0.816) for MAGNIFI-CD and 0.885 (0.800–0.943) for volume.
**Conclusion:** MAGNIFI-CD and 3D fistula volume measurements showed substantial to almost perfect inter-reader agreement with comparable performance between experts and non-experts, underscoring their reliability for research and clinical use. Agreement for volume was, however, only moderate in mild disease (TOpClass 1–2a), indicating greater assessment difficulty in this subgroup.
**Limitations:** Single-center, small sub-groups.
**Funding for this study:** Swedish governmental funding of clinical research ALF, Maggie Stephens foundation, Gastroenterological Research Fund Sweden, the Royal Physiographic Society of Lund, Medical Society in Lund, Olle Olsson foundation, Nils-Magnus and Irma Ohlsson Foundation, Swedish Society of Radiology
**Has your study been approved by an ethics committee?** Yes
**Ethics committee - additional information:** Local ethics committee approval (S69002)
**Author Disclosures:**
Gauraang Bhatnagar: Board Member: IBD UK board member for BSGAR Consultant: Motilent, Alimentiv Other: IBUS trainer: Workshop 1 and Workshop 2 Shareholder: Motilent, Fetch Health Patent Holder: P295276.US.02, SYSTEM TO CHARACTERIZE TOPOLOGY AND MORPHOLOGY OF FISTULAE FROM MEDICAL IMAGING DATA
Kristin Johnson: Consultant: Spago Nanomedical Speaker: Pfizer, Siemens Healthineers Shareholder: Sectra
Isabelle De Kock: Board Member: ESGAR Consultant: Takeda, Motilent and Alimentiv
Alex Menys: CEO: Motilent
Johan Bengtsson: Consultant: Bayer
Bram Verstockt: Grant Recipient: AbbVie, Biora Therapeutics, Celltrion, Landos, Pfizer, Sanofi, Sossei Heptares/Nxera and Takeda Speaker: Abbvie, Agomab, Alfasigma, Biogen, Bristol Myers Squibb, Celltrion, Eli Lily, Falk, Ferring, Galapagos, Materia Prima, Johnson and Johnson, Pfizer, Sandoz, Takeda, Tillots Pharma, Truvion and Viatris Shareholder: Vagustim and Thethis Pharma Consultant: Abbvie, Alfasigma, Alimentiv, Anaptys Bio, Applied Strategic, Astrazeneca, Atheneum, BenevolentAI, Biora Therapeutics, Boxer Capital, Bristol Myers Squibb, Domain Therapeutics, Eli Lily, Galapagos, Guidepont, Landos, Merck, Mirador Therapeutics, Mylan, Nxera, Inotrem, Ipsos, Johnson and Johnson, Pfizer, Sandoz, Sanofi, Santa Ana Bio, Sapphire Therapeutics, Sosei Heptares, Takeda, Tillots Pharma and Viatris.
Birgitt Janssens: Nothing to disclose
Christian William Paul Greer: Consultant: Motilent
Gabriele Bislenghi: Nothing to disclose

### T2 Mapping as a quantitative biomarker for assessing intestinal inflammation in Crohn's disease: preliminary evidence

*P. Minelli*, A. Colarieti, B. Mattio, C. Garlisi, A. Carriero; Novara/IT
*(pierlucaminelli@gmail.com)*

**Purpose:** To investigate the diagnostic performance of T2 mapping for the detection and quantification of bowel wall oedema in patients with Crohn's disease and to assess its correlation with fecal calprotectin levels as a biochemical marker of intestinal inflammation.
**Methods or Background:** This retrospective, single-center study included 18 patients with histologically confirmed Crohn's disease who underwent magnetic resonance enterography at 3T. T2-mapping sequences were acquired using a single-breath-hold axial gradient-echo protocol. Regions of interest (ROIs) were delineated on thickened bowel segments previously identified on standard T2-weighted images. Statistical analyses included Kolmogorov–Smirnov testing, paired Student's t-tests, and Pearson correlation analyses.
**Results or Findings:** Quantitative evaluation on non-contrast T2-weighted images revealed pathological mural thickening (>5 mm) in 15 of 18 patients, with a mean thickness of 9.5 ± 2.5 mm. T2 relaxation times followed a normal distribution in both normal ($p = 0.21$) and thickened ($p = 0.11$) bowel segments. Mean T2 values were significantly higher in thickened walls compared to normal segments (105.6 ± 4.3 ms vs. 51.8 ± 9.4 ms; $p < 10^{-6}$). No significant correlation was found between T2 values and fecal calprotectin levels.
**Conclusion:** T2-mapping provides a quantitative alternative for evaluating intestinal inflammation in patients with contraindications to contrast agents, serving as a dependable tool for disease monitoring during follow-up.
**Limitations:** This study is limited by its monocentric, retrospective design and the relatively small patient cohort. Furthermore, as T2 mapping is a parametric sequence originally developed for cardiac magnetic resonance, its application to bowel imaging requires further validation in larger, multicenter studies.
**Funding for this study:** No funding was received for this study.
**Has your study been approved by an ethics committee?** Yes
**Ethics committee - additional information:** Approved by the local institutional ethics committee of AOU Maggiore della Carità, Novara, Italy (approval number: CE 090/2025).
**Author Disclosures:**
Anna Colarieti: Nothing to disclose
Pierluca Minelli: Nothing to disclose
Beatrice Mattio: Nothing to disclose
Alessandro Carriero: Nothing to disclose
Christian Garlisi: Nothing to disclose

**Classification of different type of radiological errors done by radiology residents in reporting MR Perianal fistulography**

*K. A. Bhagwat*; Harapanahalli/IN
*(bhagwatkishan@gmail.com)*

**Purpose:** Learn different types of radiological reporting errors in MR Perianal Fistulography as per Kim and Mansfield classification
**Methods or Background:** MR Fistulography reporting needs adequate knowlegde of MR anatomy , acqusition , history , clinical examination to correctly report the study. However preliminary reports done by residents will have errors. 40 MRI Fistulography studies were marked on PACS , anonymized and were provided to 15 residents of radiology in third year of training to report. Structured reporting format were provided and a line diagram were drawn for coronal and axial plane by residents. Two consultant radiologists reviewed the reports done by residents as well as the reports done by consultants and tabulated the erros done by residents as compared to final reports. The consultants were blinded to reduce bias.
**Results or Findings:** All the reports done by 15 residents for 40 MR fistulograms( Total of 600 reports ) and report in PACS done by Consultants were reviewed for errors. The errors were reviewed classified as per Kim and Mansfield classification ; into twelve types of errors. Type 4 error, underreading was most common error found among the reports. Example : Tract from the second cutaneous opening was not identified. Type 10 error, satisfaction of search , was the next common error . The secondary tract was being missed , once primary fistula tract is obvious.
**Conclusion:** Human error in interpretation of radiological images does occur for various reasons.. Understanding the errors done by residnets in training in reporting the MR fistulography will help in training them better.
**Limitations:** Since the residents were reporting the past studies done, they may not be in real time situation of doing a preliminary reporting or final report by the consultant.
**Funding for this study:** None.
**Has your study been approved by an ethics committee?** Not applicable
**Author Disclosures:**
Kishan Ashok Bhagwat: Nothing to disclose

**Diagnostic Accuracy of Non-Contrast MRI Sequences Versus Contrast-Enhanced Imaging in Inflammatory Bowel Disease: Is Contrast Administration Still Necessary?**

*R. A. Pugliesi*, M. Triscari Barberi, G. Roccella, R. Cannella, A. Mavaro, G. Lo Re; Palermo/IT

**Purpose:** To compare the diagnostic performance of non–contrast MRI sequences—balanced turbo/fast field echo (BTFE/BFFE), T2-weighted spectral attenuated inversion recovery (T2 SPAIR), and diffusion-weighted imaging (DWI)—with contrast-enhanced imaging for evaluating small-bowel disease activity in inflammatory bowel disease (IBD).
**Methods or Background:** This retrospective study included 207 adult patients who underwent magnetic resonance enterography: 145 with IBD and 62 without. Imaging features were recorded, and diagnostic performance of MRI sequences was assessed using the area under the curve (AUC), sensitivity, and specificity. Pairwise comparisons were performed with the DeLong test.
**Results or Findings:** IBD patients showed significantly higher prevalence of intestinal wall thickening (91.0% vs. 14.5%), ulcerations (34.5% vs. 0%), pseudopolyps (29.7% vs. 0%), vasa recta engorgement (66.9% vs. 1.6%), hypomobility (50.3% vs. 6.5%), adipose hypertrophy (68.3% vs. 3.2%), mesenteric lymphadenopathy (55.9% vs. 9.7%), and fistulas (14.5% vs. 1.6%) (all $p < 0.01$). Contrast-enhanced imaging achieved the highest diagnostic accuracy (AUC 0.860; sensitivity 80.0%; specificity 91.9%). Among non-contrast sequences, DWI (AUC 0.811; sensitivity 70.3%; specificity 91.9%) and BTFE/BFFE (AUC 0.800; sensitivity 64.8%; specificity 95.2%) performed significantly better than T2 SPAIR (AUC 0.670; sensitivity 46.9%; specificity 87.1%). Contrast-enhanced imaging was superior to all non-contrast sequences ($p \leq 0.036$), while DWI and BTFE/BFFE showed no significant difference ($p = 0.701$).
**Conclusion:** Contrast-enhanced MRI demonstrated the highest diagnostic accuracy and should remain the reference standard in settings requiring maximal diagnostic confidence. However, DWI and BTFE/BFFE sequences showed high specificity, indicating their potential as reliable alternatives when contrast use is contraindicated or in follow-up scenarios requiring repeated imaging.
**Limitations:** Limitations include the retrospective design, potential selection bias
**Funding for this study:** n/a
**Has your study been approved by an ethics committee?** Yes
**Ethics committee - additional information:** study submitted and approved by the University of Palermo ethics committee
**Author Disclosures:**
Rosa Alba Pugliesi: Nothing to disclose
Giovanni Roccella: Nothing to disclose
Anna Mavaro: Nothing to disclose
Roberto Cannella: Nothing to disclose
Marika Triscari Barberi: Nothing to disclose
Giuseppe Lo Re: Nothing to disclose

**Relationship between MRI quantified ileo-anal J-Pouch motility, pouch inflammation and patient bowel frequency and symptom load**

*C. Dennis*, W. Weston, W. Blad, I. Naim, A. Menys, S. Taylor; London/UK
*(chloe.dennis@ucl.ac.uk)*

**Purpose:** Patients with ileo-anal pouches often develop intrusive increased bowel frequency. GLP-1 agonists reduce frequency possibly inhibiting motility. Using motility MRI (mMRI), we investigated the relationship between pouch motility, endoscopic inflammation and symptoms
**Methods or Background:** 30 ileoanal pouch patients (mean age 44 years, 9 female) and 10 controls (mean age 44, 5 male: 5 with non-colonic Crohns disease, 5 with ulcerative colitis) underwent MR enterography including a breath-hold balanced steady-state gradient echo motility sequence processed using GIQuant (Motilent) A radiologist placed regions of interest around the wall of the pouch and rectum (for controls), deriving a motility metric (expressed in arbitrary units (AU). Pouchoscopy was dichotomized into inflammation vs. no inflammation. Daily patient bowel frequency was dichotomized into =>10 and <10 , and symptoms into “highly 'symptomatic' vs 'coping. Motility was compared using Mann Whitney statistic.
**Results or Findings:** Five patients were excluded due to suboptimal pouch visualisation. Mean pouch motility was higher than rectal motility 157AU (25 to 391) vs. 59AU (23 to 104), $p = 0.002$. Patients with endoscopic inflammation (n=11) had lower motility than those without (119AU vs 185AU, $P = 0.05$). Motility was higher in those with =>10 bowel movements (n=13) compared to <10 (205AU vs. 116AU, $P = 0.007$. Pouch motility was correlated to bowel movement frequency (Rho = 0.46, $p = 0.01$), but not significantly different between highly symptomatic patients (n=9) vs. those coping (183AU vs. 132AU) $P = 0.1$.
**Conclusion:** mMRI quantified pouch motility is associated with bowel frequency and inflammation providing mechanistic insights for new therapeutic agents
**Limitations:** A retrospective study with a small sample size.
**Funding for this study:** n/a
**Has your study been approved by an ethics committee?** Yes
**Ethics committee - additional information:** Data sharing ethics
**Author Disclosures:**
Chloe Dennis: Nothing to disclose
Stuart Taylor: Shareholder: Motilent
Alex Menys: CEO: Motilent
Iyad Naim: Employee: Motilent
William Weston: Nothing to disclose
William Blad: Nothing to disclose

**10:00 - 11:00** **Research Stage 3**

### Research Presentation Session: Chest

### RPS 804

### Current topics in lung cancer imaging

**Moderator**
Peter Beddy; Dublin/IE
*(peterbeddy@gmail.com)*

**Thoracic Body Composition Across Age and Smoking Status in a Lung Cancer Screening Cohort: Insights from the NELSON Study**

*Y. Xin*[1], S. Z. Erick[1], M. A. Heuvelmans[1], G. De Bock[1], R. Vliegenthart[1], B. Edwin[2], M. Van Tuinen[1], F. Mohamed Hoesein[2]; [1]Groningen/NL, [2]Utrecht/NL
*(yexinme@gmail.com)*

**Purpose:** CT-based body composition measures improve prediction of mortality in oncology, but reference values are lacking. We evaluated age- and smoking-related variation in body composition in a lung cancer screening cohort.

**Methods or Background:** We used artificial intelligence-based automated analysis of body composition measures on baseline low-dose chest CT from male participants in the NELSON lung cancer screening trial. Skeletal muscle area (SMA) and subcutaneous adipose tissue area (SAT) were quantified at T5, T8, and T10 levels. The mean values of these levels were used to derive a single SMA and SAT metric per participant; the fat-to-muscle ratio (FMR) was calculated. Age in 5-year groups and smoking status were analyzed in relation to body composition measures. Smoking pack-years was included as covariate in regression analyses.
**Results or Findings:** We included 4,435 men with mean age 59.4 (SD=5.6) years and mean smoking pack-years 42.2 (SD=29.7). Current smokers (55.0%%) had significantly lower SAT (372 vs. 441 cm², p<0.001), SMA (501 vs. 507 cm², p<0.001) and FMR (0.74 vs. 0.87, p<0.001), compared with former smokers(45.0%). Across 5-year age groups, SMA declined from 515 cm² in the 50–54 years group to 472 cm² in the ≥70 years group (p<0.001), while SAT increased from 376 to 443 cm² (p<0.001) and FMR from 0.70 to 0.90 (p<0.001); associations remained significant after adjusting for smoking status and pack-years.
**Conclusion:** In men undergoing lung cancer screening, higher age was associated with muscle loss and fat gain, while current smoking was associated with lower muscle and fat compared to former smoking. Reference values of chest CT-based body composition measures may help risk stratification in screening.
**Limitations:** Our further analyses should confirm the relationship of body composition markers to outcomes in lung cancer screening setting.
**Funding for this study:** Funding was provided by the Dutch Cancer Society and Siemens Healthineers. The first author is supported by funding from the Affiliated Hospital of Southwest Medical University, Luzhou, Sichuan, China.
**Has your study been approved by an ethics committee?** Yes
**Ethics committee - additional information:** Health Council, WBO Committee. Population Screening Act: CT screening on lung cancer. The Hague: Health Council of the Netherlands, 2000.
**Author Disclosures:**
Marjolein A. Heuvelmans: Nothing to disclose
Geertruida De Bock: Nothing to disclose
Marcel Van Tuinen: Nothing to disclose
Rozemarijn Vliegenthart: Nothing to disclose
Bennink Edwin: Nothing to disclose
Ye Xin: Nothing to disclose
Suazo Zepeda Erick: Nothing to disclose
Firdaus Mohamed Hoesein: Nothing to disclose

**Airspace enlargement with fibrosis on CT is a strong predictor of mortality after lung cancer surgery**
*G. Chassagnon*, A. Lupo, M. Royer, M. Alifano, N. Roche, M-P. Revel; Paris/FR

**Purpose:** Preoperative risk stratification before lung cancer surgery mainly relies on clinical indicators. This study aimed to determine whether chest computed tomography (CT)-derived parameters could provide additional prognostic value in identifying patients at increased risk of peri-operative mortality.
**Methods or Background:** We retrospectively analyzed data from 720 patients who underwent lung cancer resection at our tertiary center in 2018-2019. Clinical, functional and imaging data were analyzed, including for the latter, coronary artery calcium score, pulmonary artery and aorta diameters, presence of emphysema, interstitial lung abnormalities, or airspace enlargement with fibrosis (AEF). Multivariable logistic and Cox regression models were applied to identify independent predictors of 30-day and 90-day mortality, as well as overall survival.
**Results or Findings:** AEF on CT (Odds Ratio (OR) 10.87, p < 0.001) was a significant predictor of 30-day mortality, while AEF (OR 7.04, p<0.001), as well as a higher ECOG status (OR 1.98, p=0.029) and a lower $FEV_1$ (OR 0.97, p=0.023) were associated with increased 90-day mortality. AEF was present in 8.2% of patients and was observed in 45.5% of patients who died within 30 days postoperatively. AEF was a nearly significant predictor of long-term survival in the multivariate Cox regression (HR = 1.57, 95% CI: 0.99–2.47, p = 0.046).
**Conclusion:** CT can provide valuable prognostic information in patients undergoing lung cancer surgery. AEF on CT is a major independent prognostic marker, especially for perioperative mortality. Our results support recognizing AEF as a distinct radiological entity that should be systematically assessed.
**Limitations:** Firstly, AEF was defined radiologically rather than histologically. Histological confirmation of AEF was unavailable because the affected lung areas were not systematically sampled and analysed.. Secondly, it was a single-centre retrospective study, which may limit the generalizability of the findings.
**Funding for this study:** This study received no funding This study received non funding
**Has your study been approved by an ethics committee?** Yes
**Ethics committee - additional information:** The study protocol was approved by the local Institutional Review Board (CLEP Decision N°: AAA-2025-10005), which waived the need for patient consent.
**Author Disclosures:**
Guillaume Chassagnon: Other: Beneficiary of AI solutions from Gleamer, Nevis, Deephealth, Coreline
Marco Alifano: Nothing to disclose
Nicolas Roche: Nothing to disclose
Marie Royer: Nothing to disclose
Audrey Lupo: Nothing to disclose
Marie-Pierre Revel: Other: Beneficiary of AI solutions from Gleamer, DeepHealth, Nevis, Coreline

**CT acquisition protocols in lung cancer screening: Insights from an international survey disseminated beyond the SOLACE consortium**
*M. F. G. Konrad*[1], E. Nischwitz[1], J. Chorostowska-Wynimko[2], J. Vogel-Claussen[3], J. Moes-Sosnowska[2], M. Adamek[4], A. Kerpel-Fronius[5], H. Prosch[6], H-U. Kauczor[1]; [1]Heidelberg/DE, [2]Warsaw/PL, [3]Hannover/DE, [4]Gdańsk/PL, [5]Budapest/HU, [6]Vienna/AT
*(mathis.konrad@med.uni-heidelberg.de)*

**Purpose:** Assessing the current status of applied CT image acquisition protocols in lung cancer screening (LCS) worldwide, focusing on technical factors linked to radiation exposure.
**Methods or Background:** The survey was expanded from an internal SOLACE version to include broader dissemination, collecting data from personnel responsible for the definition of CT protocols at LCS centres worldwide. Data were collected through a baseline survey between June 2024 and September 2025 to represent the most current status.
**Results or Findings:** Survey responses were received from 71 screening centres from 29 countries (19 Europe, 10 other continents). Institutional factors influencing CT protocols encompass the responsibility of establishment and modification of protocols by personnel (radiologists, radiographers, medical physicists, manufacturer personnel). In 33% of the institutions the protocol was established by a multiprofessional team. CT protocols were mostly modifiable (85%). Technical questions were partially answered. In 88% of the centres (43 of 49) automatic exposure control was implemented. Reconstructed slice thickness ranged 0.625-1.5 mm; 1.0 mm dominating with 64%. Increment ranged 0.5-1.25 mm, where 0.625 mm, 0.7 mm, and 1.0 mm were distributed more evenly with 18%, 25%, and 30%, respectively. Screening-specific software was used at 87% of sites; mainly for nodule detection, volumetry, and calculation of volume doubling time (32, 31, and 25 centres, respectively). Reconstruction algorithm types included filtered-back projection (4), iterative reconstruction with statistical modeling (30), and iterative reconstruction with deep learning support (7).
**Conclusion:** LCS imaging often reaches the technical limits of currently operated devices. Multiprofessional establishment of CT protocols is an area for improvement. Variations in reconstruction algorithm types warrant further research regarding their influence on volumetry calculations. Protocol optimization is essential to balance radiation exposure reduction and diagnostic quality.
**Limitations:** The survey likely reflects centres with research interests.
**Funding for this study:** This project is co-funded under the EU4Health Programme 2021–2027 under grant agreement no. 101101187
**Has your study been approved by an ethics committee?** Not applicable
**Author Disclosures:**
Joanna Chorostowska-Wynimko: Nothing to disclose
Anna Kerpel-Fronius: Nothing to disclose
Joanna Moes-Sosnowska: Nothing to disclose
Helmut Prosch: Research/Grant Support: Boehringer Ingelheim, AstraZeneca, Siemens Healthineers and the Christian Doppler Research Association, EU Commission (EU4Health, Horizon Europe Health) Speaker: AstraZeneca, BMS, Boehringer Ingelheim, Bracco, Daiichi Sankyo, Janssen, MSD, Novartis, Roche, Sanofi, Siemens Healthineers, Takeda.
Hans-Ulrich Kauczor: Nothing to disclose
Jens Vogel-Claussen: Nothing to disclose
Emily Nischwitz: Nothing to disclose
Mathis Franz Georg Konrad: Other: This contribution has been prepared on behalf of the SOLACE consortium
Mariusz Adamek: Nothing to disclose

**Rethinking RECIST in the era of neoadjuvant treatment for lung cancer**
*F. Palmeri*[1], A. Del Gaudio[2], M. Zerunian[2], F. Di Gregorio[2], A. Ferraris[1], D. Caruso[2], M. Francone[2], A. Laghi[3]; [1]Torino/IT, [2]Rome/IT, [3]Pieve Emanuele/IT
*(federica.palmeri@uniroma1.it)*

**Purpose:** To assess the accuracy of RECIST 1.1 in evaluating treatment response after neoadjuvant chemotherapy, with or without immunotherapy, in patients with stage IIIA–IIIB non-small cell lung cancer (NSCLC) undergoing surgical resection, and to highlight its limitations in reflecting true pathological outcomes.
**Methods or Background:** In a prospective pilot study, 33 patients with stage IIIA–IIIB NSCLC received neoadjuvant chemotherapy prior to surgery; 11 also received pembrolizumab. All patients underwent baseline and post-treatment contrast-enhanced CT scans, evaluated using RECIST 1.1 or iRECIST by two radiologists in consensus. Surgical specimens were analyzed histopathologically to determine complete or partial response. Radiological and pathological responses were then compared.
**Results or Findings:** Of the 33 patients, 12 achieved complete pathological response (pCR). None of these patients were classified as complete responders by RECIST 1.1 on post-treatment imaging. The remaining 21 patients showed partial pathological response, with residual viable tumor ranging from 10% to 80%. Only 9 of these (43%) met RECIST criteria for partial response, while the others were categorized as stable disease despite substantial histological regression. This discordance was more pronounced in patients treated with combined chemo-immunotherapy, suggesting that lesion size alone may fail to capture treatment-induced changes such as immune-related remodeling or necrosis.
**Conclusion:** RECIST 1.1 appears insufficiently sensitive to capture complete or substantial tumor response after neoadjuvant therapy in stage IIIA–IIIB NSCLC. The discrepancy is most evident with immunotherapy, which may induce changes not reflected by lesion size. As a result, radiological assessments often underestimate true pathological response. In locally advanced NSCLC, alternative or complementary imaging biomarkers are needed to better predict pathological outcomes and guide clinical decisions, particularly when chemotherapy is combined with immunotherapy.
**Limitations:** The limitations are the small sample size and single-center design
**Funding for this study:** No funding was received for this study.
**Has your study been approved by an ethics committee?** Yes
**Ethics committee - additional information:** Written informed consent was acquired for all patients and Istitutional Review Board approval was obtained.
**Author Disclosures:**
Damiano Caruso: Nothing to disclose
Marta Zerunian: Nothing to disclose
Federica Palmeri: Nothing to disclose
Francesca Di Gregorio: Nothing to disclose
Andrea Ferraris: Nothing to disclose
Andrea Laghi: Nothing to disclose
Antonella Del Gaudio: Nothing to disclose
Marco Francone: Nothing to disclose

**Beyond biopsy: predicting PD-L1 status in non-small cell lung cancer through CT radiomics**
*F. Palmeri*[1], V. Solimene[2], A. Del Gaudio[2], M. Zerunian[2], F. Di Gregorio[2], A. Ferraris[1], D. Caruso[2], M. Francone[2], A. Laghi[3]; [1]Torino/IT, [2]Roma/IT, [3]Pieve Emanuele/IT
*(federica.palmeri@uniroma1.it)*

**Purpose:** To develop and validate machine learning (ML) models based on radiomic features extracted from CT imaging to differentiate between high (PD-L1 ≥50%) and low (PD-L1 <50%) expression in non-small cell lung cancer (NSCLC). The aim was to explore whether imaging biomarkers could provide a noninvasive alternative to histological profiling for guiding immunotherapy decisions.
**Methods or Background:** This retrospective study included 210 patients with histologically confirmed NSCLC and pre-treatment contrast-enhanced chest CT. Among them, 46 (21.9%) showed high PD-L1 expression, while 164 (78.1%) showed low expression. Tumors were manually segmented, and radiomic features were extracted following Image Biomarker Standardization Initiative guidelines. Five ML models were built for binary classification ("PD-L1 ≥50%" vs. "PD-L1 <50%"), using Random Forest, Support Vector Machine, K-Nearest Neighbors, Multi-Layer Perceptron, and Logistic Regression classifiers. Histological PD-L1 served as the reference standard. Statistical significance was set at $P < .05$.
**Results or Findings:** The Multi-Layer Perceptron model achieved the best performance, with a ROC-AUC of 0.61 (95% CI: 0.57–0.66, $P < .005$) and an accuracy of 64% (95% CI: 60–68%). Sensitivity reached 70% (95% CI: 65–75%) and PPV 82% (95% CI: 80–84%), while specificity and NPV were lower at 44% (95% CI: 36–52%) and 29% (95% CI: 24–35%), respectively. The F1 score was 75% (95% CI: 71–78%).
**Conclusion:** Radiomic analysis of pre-treatment CT images using a Multi-Layer Perceptron classifier showed potential in distinguishing PD-L1 expression levels in NSCLC, with good sensitivity, PPV, and overall F1 score. However, the model's limited specificity and NPV suggest it may be more effective in identifying patients likely to express high PD-L1, rather than excluding them. Radiomic-based ML models could aid noninvasive prediction of PD-L1, supporting immunotherapy selection when biopsy is inconclusive or risky.
**Limitations:** The limitations are the retrospective single-center design.
**Funding for this study:** No funding was received for this study.
**Has your study been approved by an ethics committee?** Yes
**Ethics committee - additional information:** Written informed consent was acquired for all patients and Istitutional Review Board approval was obtained.
**Author Disclosures:**
Damiano Caruso: Nothing to disclose
Marta Zerunian: Nothing to disclose
Federica Palmeri: Nothing to disclose
Francesca Di Gregorio: Nothing to disclose
Andrea Ferraris: Nothing to disclose
Andrea Laghi: Nothing to disclose
Antonella Del Gaudio: Nothing to disclose
Marco Francone: Nothing to disclose
Vincenzo Solimene: Nothing to disclose

**10:00 - 11:00** **Research Stage 4**

### Research Presentation Session: Artificial Intelligence and Imaging Informatics

### RPS 805

### Automating diagnosis and pattern recognition: AI performance in chest radiography and lung disease

**Moderator**
Joe Barnett; St Albans/UK
**Author Disclosures:**
Joe Barnett: Consultant: Heart Lung Health LTD, part owned subsidiary of DeepHealth LTD

**Artificial intelligence solution in B-lines detection on lung ultrasound**
*M. Stevik*[1], M. Malík[1], A. Dzian[1], F. Babic[2], Š. Vetešková[1], M. Bundzel[2], J. Magyar[2], K. Zelenak[1]; [1]Martin/SK, [2]Košice/SK

**Purpose:** The main limitation of LUS is its high operator dependency. This has led to significant interest in developing artificial intelligence (AI) approaches for the interpretation of LUS imaging. The primary aim of this study was to evaluate the accuracy of a trained AI model in B-lines detection in the LUS movies using a novel designed hybrid solution that combines the convolutional neural network (CNN) and analytical approach. The secondary aim was to evaluate the accuracy of a radiology resident beginner in LUS in B-lines detection and to evaluate the educational potential of AI in LUS.
**Methods or Background:** In this single-center prospective study, a machine learning based software, the LUS AI solution, was used for automated detection and marking of B-lines in the LUS footages. 75 consecutive patients were enrolled, total of 300 LUS videos. The LUS videos were reviewed and evaluated for the presence of the B-line by two radiologists expert and one radiology resident. Then radiology resident was allowed to revise the initial conclusion regarding B-lines presence.
**Results or Findings:** Accuracy, sensitivity, specificity, positive and negative predictive values of artificial inelligence in B-line detection were 0.85, 0.9, 0.832, 0.661 and 0.958 respectively. The resident's values were 0.69, 0.575, 0.732, 0.438 and 0.958 respectively. The resident's values after correction based on artificial intelligence results were 0.823, 0.912, 0.791, 0.613 and 0.961 respectively.
**Conclusion:** Artificial intelligence solution showed higher accuracy in B – lines detection. It could play role in residents' education.
**Limitations:** There was overall a relatively small number of patients recruited. Our presented AI program results still show some limitations, which is due to the limited amount of data and inability to use some data augmentation methods to address this issue.

**Funding for this study:** This research is funded by the Slovak Research and Development Agency, grant number APVV 20-24-0454.
**Has your study been approved by an ethics committee?** Yes
**Ethics committee - additional information:** Approval was granted by the Ethics Committee of Jessenius Faculty of Medicine in Martin (No. EK 44/2021). Date of approval: 29 June 2021.
**Author Disclosures:**
Marek Bundzel: Nothing to disclose
Frantisek Babic: Nothing to disclose
Štefánia Vetešková: Nothing to disclose
Jan Magyar: Nothing to disclose
Kamil Zelenak: Nothing to disclose
Martin Stevik: Nothing to disclose
Anton Dzian: Nothing to disclose
Marek Malík: Nothing to disclose

**Design of a CT-Based Deep Learning Model to Predict the Metastatic Potential of Sub-centimetric Pulmonary Nodules in Oncology Patients**
N. E. Özen[1], O. Yeniceri[1], *F. Dinç*[1], S. Yılmaz[2], N. G. Narin[2]; [1]Mugla/TR, [2]Muğla/TR
*(fundadi@yahoo.com)*

**Purpose:** Accurate determination of the malignant potential of pulmonary nodules smaller than 1 cm in oncology patients remains a diagnostic challenge. The smaller the nodule, the greater the uncertainty in interpretation, which can lead to staging inaccuracies and delays. This study aimed to investigate the utility of deep learning methods in predicting the malignancy of sub-centimetric pulmonary nodules detected on lung CT scans at the time of initial diagnosis.
**Methods or Background:** This study represents a preliminary report of a project that we have just started. A total of 933 nodules were analyzed, comprising 443 retrospectively confirmed benign nodules from patients without known malignancy and 490 malignant nodules that demonstrated interval growth and were reported as metastatic. Malignant nodules originated from primary tumors of the rectum, colon, renal cell carcinoma, prostate, uterus, cervix, ovary, larynx, and breast. Of the total dataset, 653 nodules were allocated to the training set, 139 to the validation set, and 141 to the test set. Model development was based on the ResNet-50 architecture with transfer learning.
**Results or Findings:** Using a dataset of 933 nodules, the model achieved a best validation accuracy of 91.3% and a test accuracy of 83.69%. The area under the ROC curve (AUC) was 0.896, demonstrating strong discriminative performance.
**Conclusion:** Findings from this preliminary study suggest that deep learning–based approaches may provide valuable support in the staging process at the time of diagnosis, particularly in oncology patients presenting with sub-centimetric pulmonary nodules on CT scans in the presence of a known primary tumor elsewhere.
**Limitations:** This paper is a preliminary report of a deep learning study using a relatively small dataset. A larger dataset is in preparation.
**Funding for this study:** This study is currently being conducted without funding. However, an application has been submitted to TÜBİTAK 1001 for funding.
**Has your study been approved by an ethics committee?** Not applicable
**Author Disclosures:**
Nida Gökçe Narin: Nothing to disclose
Nizamettin Emre Özen: Nothing to disclose
Funda Dinç: Nothing to disclose
Onder Yeniceri: Nothing to disclose
Selim Yılmaz: Nothing to disclose

**Predicting rapid progression and prognosis of idiopathic inflammatory myopathies-associated interstitial lung disease using AI-based quantitative CT analysis of pulmonary vessel-related structures**
*Y. Qiang*, H. Wang, M. Liu, H. Dai; Beijing/CN
*(qiangyuhui199841@163.com)*

**Purpose:** Pulmonary vessel-related structure (PVRS) abnormalities in idiopathic inflammatory myopathies-related interstitial lung disease (IIM-ILD) remains poorly understood. This two-center study investigated PVRS parameters on HRCT as predictors of rapid progression and prognosis in IIM-ILD.
**Methods or Background:** 578 IIM-ILD patients (412 females, median age 53) came from the prospective ILD cohort of two centers. AI-based quantification of baseline HRCT assessed PVRS and interstitial lesions. An independent external cohort of 64 IIM-ILD patients (43 females, median age 54) from the second center was used to validate the generalizability of PVRS in predicting IIM-ILD progression.
**Results or Findings:** In the first center, 249 patients with rapidly progressive ILD (RP-ILD) exhibited significantly higher mean pulmonary vascular diameter (mPVD) ($P<0.05$) at shorter vascular-pleural distances, increased PVRS volume, and greater standard deviation of pulmonary vascular diameter (sdPVD) ($P<0.001$) compared to non-RP-ILD. Age (HR: 1.03, 95% CI: 1.01-1.06), ground glass opacity (GGO) percentage (HR: 1.04, 95% CI: 1.02-1.06), and sdPVD at 6mm and 18mm from the pleura were identified as independent risk factors for poor prognosis in anti-synthetase syndrome (ASS) patients (concordance index = 0.819). In contrast, age (HR: 1.06, 95% CI: 1.02-1.11), mPVD at 6mm from the pleura, and lactic dehydrogenase were independent risk factors for poor prognosis in anti-MDA5-positive dermatomyositis (MDA5+ DM) patients (concordance index = 0.835). Validation in the second center using the multivariate Cox regression model from the internal training cohort revealed predictive C-indices of 0.841 (ASS) and 0.814 (MDA5+ DM) in the external cohort.
**Conclusion:** Baseline PVRS parameters on HRCT serve as prognostic indicators for rapid progression and adverse prognosis in IIM-ILD.
**Limitations:** The quantitative PVRS could not differentiate between arterial and venous vessels.
**Funding for this study:** National Key Technologies R & D Program Precision Medicine Research, and the National Natural Science Foundation of China.
**Has your study been approved by an ethics committee?** Yes
**Ethics committee - additional information:** China-Japan friendship hospital
**Author Disclosures:**
Min Liu: Nothing to disclose
Hongyi Wang: Nothing to disclose
Yuhui Qiang: Nothing to disclose
Huaping Dai: Nothing to disclose

**Per-pixel Bone Attenuation Contribution Map Generation using Machine Learning for Chest Radiographs**
*T. Dorosti*, L. Kaster, M. Lochschmidt, J. B. Thalhammer, S. Peterhansl, F. Schaff, F. Pfeiffer, D. Pfeiffer; Munich/DE
*(tina.dorosti@tum.de)*

**Purpose:** We aim to generate attenuation contribution masks for bone structures present in real and synthetic frontal chest radiographs (CXR) on a pixel level using machine learning. Such bone attenuation contribution (BAC) maps will allow for a personalized, per-pixel correction of beam hardening artifacts in novel imaging modalities such as X-ray dark-field (DF) imaging.
**Methods or Background:** A total of 5959 chest CT scans were retrieved from two publicly available datasets of the Luna16 (n=656) and the RSNA PE challenge (n=5303). Additionally, CXRs from 72 subjects (33 healthy: 20 men, mean age[range]=62.4[34, 80]; 39 with COPD: 25 men, mean age[range]=69.0[47, 91]) were retrospectively selected (10.2018-12.2019) from our in-house dataset. All CT scans and their corresponding 3D binary bone segmentations were forward projected using a simulated X-ray spectrum to generate synthetic CXRs and relative bone thickness projections referred to as BAC maps, respectively. A U-Net model was trained and tested on synthetic radiographs from the public datasets. Model performance was assessed quantitatively for the public synthetic data with the mean absolute percentage error (MAPE), Pearson correlation, and two-sided Student t distribution. For the real in-house CXRs, data was assessed qualitatively, as no reference BAC data is available for real radiographs.
**Results or Findings:** The predicted BAC maps showed low error rates and strong correlations with the reference. Specifically, for the Luna16 test set (n=131), an MAPE=18.1% and a correlation of 0.81 ($P<0.001$) were achieved. For the RSNA PE test data (n=1060), an MAPE=12.5% and a correlation of 0.91 ($P<0.001$) were obtained.
**Conclusion:** The U-Net successfully generated per-pixel BAC maps for synthetic and real CXRs, demonstrating potential for applications in DF image processing.
**Limitations:** The sample of real radiographs was restricted to healthy and COPD subjects from a single medical center.
**Funding for this study:** We acknowledge financial support through the European Research Council (ERC Synergy Grant SmartX, SyG 101167328), and the Free State of Bavaria under the Excellence Strategy of the Federal Government and the States, as well as by the Technical University of Munich – Institute for Advanced Study.
**Has your study been approved by an ethics committee?** Yes

**Ethics committee - additional information:** All data was analyzed retrospectively and anonymously. The study was approved by the ethical review committee and was conducted in accordance with the regulations of our institution (approval code: 87/18 S, Institutional Review Board of the Faculty of Medicine, Technical University of Munich, Germany).
**Author Disclosures:**
Tina Dorosti: Nothing to disclose
Johannes Benedikt Thalhammer: Nothing to disclose
Sebastian Peterhansl: Nothing to disclose
Lennard Kaster: Nothing to disclose
Franz Pfeiffer: Nothing to disclose
Maximilian Lochschmidt: Nothing to disclose
Florian Schaff: Nothing to disclose
Daniela Pfeiffer: Nothing to disclose

**Comparative evaluation of an open source versus commercial artificial intelligence solution for detection of acute lung pathologies on chest radiographs**
*L. Y. T. Chan*, S. Y. Yee, Y. J. Toh, P. Yogendra; Singapore/SG

**Purpose:** To evaluate the accuracy of an open-source solution (CheXNeXt) and a commercial product (Rayscape CXR) for chest radiograph interpretation compared with board-certified radiologists.
**Methods or Background:** Chest radiographs (CXRs) are among the most frequently requested first-line investigations for suspected cardiopulmonary pathology. Rising imaging volumes and radiologist shortages have driven interest in Artificial Intelligence (AI) solutions, which have potential to triage urgent cases for expedited reporting and serve as diagnostic adjuncts to enhance efficiency and accuracy. This single-institution retrospective study analysed 1003 emergency department CXRs performed after hours. Radiology reports served as the reference standard, whereby outcomes were extracted directly from report documentation. Two AI models (CheXNeXt and Rayscape CXR) independently analysed CXRs for pneumonia, pleural effusion, pneumothorax, and pulmonary oedema. Model performance was evaluated using sensitivity, specificity, and Gwet's AC1, with agreement strength interpreted according to Landis and Koch. McNemar's test assessed statistical differences between models.
**Results or Findings:** Rayscape CXR demonstrated higher overall sensitivity (97.4% vs. 91.7%), while CheXNeXt showed higher specificity (71.5% vs. 40.0%). For effusion, pneumothorax, and oedema, both models achieved almost perfect agreement with the reference standard, with Rayscape CXR (0.902–0.997) outperforming CheXNeXt (0.812–0.890). Conversely, CheXNeXt outperformed Rayscape CXR in pneumonia detection (0.829 vs. 0.765). McNemar's tests revealed significant differences in error patterns across all pathologies ($p < 0.001$).
**Conclusion:** Rayscape CXR had greater agreement for effusion, pneumothorax and oedema while CheXNeXt demonstrated greater agreement for pneumonia. Overall, Rayscape CXR demonstrated higher sensitivity and CheXNeXt showed higher specificity. These complementary strengths suggest context-specific deployment strategies: sensitivity-optimised models may aid triage of urgent cases, while specificity-focused models may provide confirmatory support.
**Limitations:** The study was limited by differing training definitions for pathologies used by each AI algorithm, potentially influencing comparative performance.
**Funding for this study:** No funding was received for this study.
**Has your study been approved by an ethics committee?** Not applicable
**Author Disclosures:**
Praveen Yogendra: Nothing to disclose
Ying Jie Toh: Nothing to disclose
Li Yi Tammy Chan: Nothing to disclose
Sze Ying Yee: Nothing to disclose

**Repeated evaluation of AI for lung nodule detection in chest radiographs: version-to-version evaluation in a multicentre study**
*M. Besouw*, M. De Rooij, M. J. Rutten, B. Van Ginneken, S. S. Schalekamp; Nijmegen/NL
*(marlie.besouw@radboudumc.nl)*

**Purpose:** Artificial intelligence (AI) for lung nodule detection in chest radiographs is increasingly implemented, yet reproducible methods to evaluate new and updated AI product versions are lacking. Our Project AIR framework was established to benchmark CE-certified AI tools, and most recently performed in 2023. In this follow-up study, updated versions of AI products for lung nodule detection were assessed.
**Methods or Background:** Sixteen vendors were invited to participate in a new round for the evaluation of lung nodule detection in chest radiographs. Up to now, four commercial AI products have been evaluated. Performance was tested on the same hidden multicentre dataset of 386 scans. The primary outcome was the area under the receiver operating characteristic curve (AUC). Statistical comparison between versions was performed, and all results were benchmarked against the average performance of radiologists.
**Results or Findings:** Three of four products showed an increase in AUC (+0.07 to +0.09). The average AUC of these products significantly increased from 0.84 (95% CI 0.79–0.87) to 0.89 (95% CI 0.86–0.92) ($p<0.05$). For the other system, identical case-level outputs resulted in an unchanged AUC of 0.88. Across the evaluated products, AUCs in the current analysis ranged from 0.87 to 0.91. Three of the four products performed significantly better than the average radiologist, with an AUC of 0.81 (95% CI 0.77–0.85).
**Conclusion:** Our project AIR framework enables repeated testing of new and updated AI products. For three out of four AI products, performance in lung nodule detection improved compared to 2023. All currently evaluated AI products performed at or above the average radiologist benchmark
**Limitations:** Two vendor submissions were still being processed at the time of analysis, and not all products provided updated versions. Results are preliminary and limited to a single standardised dataset.
**Funding for this study:** This study was supported by the Netherlands Organisation for Scientific Research (NWO), project no. OSF23.1.018.
**Has your study been approved by an ethics committee?** Not applicable
**Author Disclosures:**
Steven S Schalekamp: Nothing to disclose
Marlie Besouw: Nothing to disclose
Bram Van Ginneken: Founder: Plain Medical
Matthieu J.C.M. Rutten: Nothing to disclose
Maarten De Rooij: Nothing to disclose

**Agreement and Diagnostic Accuracy of an FDA- and CE-Cleared AI Solution Versus Junior Radiologist for Chest Radiographs**
*N. Sadaf*, S. K. Marupaka, M. M. Sameer, R. P. Babu; Hyderabad/IN
*(sadafnahdiya@gmail.com)*

**Purpose:** To compare the diagnostic performance of an FDA- and CE-cleared computer-aided detection (CADe) solution with a junior radiologist (JR) for chest radiograph (CXR) interpretation, using a senior radiologist (SR) as reference
**Methods or Background:** In this retrospective, single-center study, 906 CXRs from patients aged ≥12 years (mean 47.7 years; M/F: 455/451) were analyzed. Each CXR was independently reviewed by: (a) an FDA- and CE-cleared CADe solution (DeepTek.ai), (b) a JR (<5 years' experience), and (c) an SR (10 years' experience). Diagnostic performance metrics (sensitivity, specificity, PPV, NPV, accuracy) were calculated using SR as reference. Reader agreement was assessed via Cohen's κ. Institutional Review Board approval was obtained with a waiver of consent.
**Results or Findings:** AI demonstrated superior sensitivity for detecting suspicious CXRs (65% vs 53% for JR; $p < 0.001$), corresponding to 60 additional suspicious cases detected compared with JR. Agreement with SR in suspicious cases was also higher for AI (64.6% vs 53.4%). JR exhibited higher specificity for non-suspicious cases (90.3% vs 78.1%; $p < 0.001$). Overall accuracy was slightly higher for AI (70% vs 68%), with moderate overall concordance (κ=0.41 vs 0.40).
**Conclusion:** The AI solution outperformed a junior radiologist in detecting suspicious findings and showed strong concordance with an experienced radiologist, highlighting its potential as a reliable second reader in CXR interpretation. While JR excelled in non-suspicious-case specificity, AI's superior detection of suspicious cases underscores its value in supporting early diagnosis and clinical decision-making.
**Limitations:** This study is limited by the use of a single senior radiologist as the reference standard, which could introduce bias, and by its retrospective, single-centre design, which may restrict the generalizability of the findings.
**Funding for this study:** This research received no external funding
**Has your study been approved by an ethics committee?** Not applicable
**Author Disclosures:**
Masthangari Mohammad Sameer: Nothing to disclose
Sravan Kumar Marupaka: Nothing to disclose
Nahdiya Sadaf: Nothing to disclose
Ravindra Prakash Babu: Nothing to disclose

**10:00 - 11:00** **Research Stage 6**

### Research Presentation Session: Interventional Radiology

### RPS 809

### From tissue to therapy: innovations in biopsy and ablation

**Moderator**
Alban Denys; Lausanne/CH
*(alban.denys@chuv.ch)*

### Technical Success, Diagnostic Yield, and Safety of Image-Guided Renal Biopsies in a Multicenter Cohort

*R. J. Ocker-Serger*[1], M. Opitz[1], B. M. Schaarschmidt[1], L. V. Klüner[1], M. A. Drews[1], J. Haubold[1], J. Nadjiri[2], S. Zensen[1]; [1]Essen/DE, [2]Munich/DE

**Purpose:** To assess the technical success, diagnostic yield, and complication profile of image-guided percutaneous renal biopsies in a real-world, multicenter setting.
**Methods or Background:** This retrospective registry analysis included 5,235 renal biopsies performed between 2018 and 2024 at 176 centers in Germany, Austria, and Switzerland. Technical success was defined as confirmed needle placement within the target lesion. Diagnostic yield referred to histological adequacy for clinical decision-making. Complications were classified according to the Society of Interventional Radiology (SIR) grading system.
**Results or Findings:** Technical success was achieved in 98.4% (5,150/5,235) of interventions, and diagnostic yield was 94.9% (3,943/4,154). Outpatient procedures showed slightly higher technical success (99.3%, 267/269) than inpatient procedures (98.3%, 4,883/4,966; p = 0.241), with comparable diagnostic yield and safety profiles. The overall complication rate was 5.0% (264/5,235), with major complications in 0.7% (39/5,235). Bleeding was the most frequent complication, including venous (33.0%), parenchymal (30.3%), and arterial (17.1%) events. Coagulation abnormalities were significantly associated with higher complication rates.
**Conclusion:** Image-guided percutaneous renal biopsies are effective and safe across a large multicenter cohort. The results support their use in outpatient settings, provided coagulation status is optimized and adequate tissue sampling is achieved
**Limitations:** The main limitations of this registry-based study are potential selection bias, reliance on site-reported data with incomplete documentation, and the absence of a central histopathological review.
**Funding for this study:** No funding for this study.
**Has your study been approved by an ethics committee?** Yes
**Ethics committee - additional information:** Local ethic committee University Duisburg-Essen 22-10865-BO
**Author Disclosures:**
Laura Valentina Klüner: Nothing to disclose
Marcel Alexander Drews: Nothing to disclose
Benedikt Michael Schaarschmidt: Grant Recipient: not related to this paper
Johannes Haubold: Nothing to disclose
Sebastian Zensen: Nothing to disclose
Raya Juliane Ocker-Serger: Nothing to disclose
Marcel Opitz: Nothing to disclose
Jonathan Nadjiri: Nothing to disclose

### Can systematic biopsy be avoided? clinical impact of saturation target biopsy in clinically significative prostate cancer (csPCa) detection

*S. Inturri*[1], E. Raimondi[1], M. Tilli[1], M. Dugo[1], F. Ermili[1], M. Montesi[1], R. Bisi[2], M. Bandi[1], R. Rizzati[1]; [1]Lagosanto/IT, [2]Ferrara/IT
*(sebinturri95@gmail.com)*

**Purpose:** To evaluate whether saturation target biopsy (sTB) alone can replace the combined approach of sTB plus systematic biopsy (SB) for csPCa detection in patients with PI-RADS ≥3 lesions on multiparametric MRI (mpMRI).
**Methods or Background:** We retrospectively analyzed 390 MRI–US fusion biopsy patients with single prostatic lesion PI-RADS ≥3 at mpMRI. Patients were divided into three groups according to PI-RADS score: n=77 with PI-RADS 3 (19.8%), n=186 with PI-RADS 4 (47.6%), and n=127 with PI-RADS 5 (32.6%). For each group, histology from target (T), regional saturation target (RST), and contralateral systematic biopsy (CSB) was compared. Patients were then classified as negative, T and/or RST ≥ to CSB, or T and/or RST < to CSB.
**Results or Findings:** In the PI-RADS 3 group, histology was negative in 52 patients (67.5%), positive with T and/or RST ≥ to CSB in 18 patients (23.4%), and positive with T and/or RST < to CSB in 7 patients (9.1%). In the PI-RADS 4 group, histology was negative in 20 patients (10.7%), positive with T and/or RST ≥ to CSB in 161 patients (86.6%), and positive with T and/or RST < to CSB in 5 patients (2.7%). In the PI-RADS 5 group, histology was negative in 4 patients (3.1%), positive with T and/or RST ≥ to CSB in 120 patients (94.5%), and positive with T and/or RST < to CSB in 3 patients (2.4%). Only 7 out of 390 patients (1.8%) were positive exclusively at CSB. Among these, 5 (71.5%) had Gleason score 3+3 (ISUP 1), while 2 (28.5%) had Gleason score 3+4 (ISUP 2).
**Conclusion:** SB in addition to sTB provided no significant additional diagnostic value for csPCa in patients with PI-RADS ≥3 lesions on mpMRI.
**Limitations:** Not applicable
**Funding for this study:** No funding was provided for this study
**Has your study been approved by an ethics committee?** Not applicable
**Author Disclosures:**
Riccardo Bisi: Nothing to disclose
Edoardo Raimondi: Nothing to disclose
Massimo Tilli: Nothing to disclose
Martina Bandi: Nothing to disclose
Marco Dugo: Nothing to disclose
Roberto Rizzati: Nothing to disclose
Marco Montesi: Nothing to disclose
Sebastiano Inturri: Nothing to disclose
Francesca Ermili: Nothing to disclose

### Reduction of incidence of pneumothorax in CT-guided lung biopsies by saline infiltration during coaxial needle extraction

*G. Viviano*, A. Adebanjo, M. De Filippo; Parma/IT
*(giulia.viviano@unipr.it)*

**Purpose:** This study evaluates whether saline infiltration during coaxial needle extraction reduces the incidence of pneumothorax (PTX) in CT-guided lung biopsies, potentially enhancing procedural safety.
**Methods or Background:** A retrospective analysis of 200 consecutive CT-guided core needle biopsies (18G) was conducted using our hospital's Radiology Department database. All procedures were performed between February 2022 and December 2024 by a single operator with 20 years of experience. Patients were divided into two groups: Group A (100 patients), receiving 5–10 ml of 0.9% saline solution during coaxial needle extraction, and Group B (100 patients), where no saline was infiltrated. Descriptive and inferential statistical analyses assessed the relationship between saline infusion and PTX incidence.
**Results or Findings:** PTX occurred in 24% of Group B patients versus 7% in Group A (p < 0.001). Multivariate analysis confirmed saline infusion as a significant protective factor, even in high-risk patients (e.g., those with severe emphysema or in lateral decubitus). The odds ratio (OR = 3.9) indicates that the absence of saline increases PTX risk nearly fourfold.
**Conclusion:** Saline infiltration during coaxial needle removal significantly reduces PTX incidence in CT-guided lung biopsies, offering a simple, effective strategy to enhance patient safety.
**Limitations:** None
**Funding for this study:** None
**Has your study been approved by an ethics committee?** Yes
**Ethics committee - additional information:** Università di Parma
**Author Disclosures:**
Adenike Adebanjo: Nothing to disclose
Massimo De Filippo: Nothing to disclose
Giulia Viviano: Nothing to disclose

### The new era of non-invasive interventional oncology with histotripsy - early results and experience on hepatic tumours from Asia

*N. R. Mahboobani*, P. Chan, K. H. Lee, C. Tsai, J. F. Griffith; Hong Kong/HK
*(neerajmahboobani@cuhk.edu.hk)*

**Purpose:** Histotripsy uses high intensity pulsed ultrasound waves to mechanically destroy targeted tissue whilst sparing surrounding tissues and structures. We report results and experience from one of the first sites in Asia using histotripsy to treat focal liver lesions.
**Methods or Background:** 19 patients (12 males, 7 females) with mean age of 70.5 years had 22 focal liver lesions treated with histotripsy between April and September 2025. All treatment procedures were done under general anaesthesia. Clinical parameters, laboratory test results and imaging were reviewed.
**Results or Findings:** 15 hepatocellular carcinomas and 7 liver metastases were treated. Mean size of the lesions was 2.2 cm (range 0.9 to 4.0 cm). 21 lesions were successfully treated as evident by liquefaction on post-treatment day 1 MRI, with technical success rate 95.4%. One small lesion in segment I

showed no treatment related changes on post-treatment day 1 MRI. One patient developed transient thrombosis in right portal vein branch which subsequently resolved on day 7. Minor complications included transient derangement of liver function (15 patients), mild ascites (8), mild abdominal wall oedema (5), and mild right pleural effusion (4). All minor complications subsequently resolved. 17 patients have had post-treatment 1 month MRI, out of which 8 have also had post-treatment 3 month MRI. These show progressive shrinkage of the liquefied treatment volume with no evidence of recurrence.
**Conclusion:** Histotripsy is an effective non-invasive treatment for focal liver lesions which can be clearly visualised on ultrasound. It is suitable for lesions which are in close proximity to structures such as the biliary tract or vessels. Patient and lesion selection, patient positioning and ventilation strategies during general anaesthesia to reduce respiratory excursion of the liver are important factors for successful treatment.
**Limitations:** No limitations were identified.
**Funding for this study:** No funding was received for this study.
**Has your study been approved by an ethics committee?** Yes
**Ethics committee - additional information:** The study was approved by The Chinese University of Hong Kong Medical Centre (CUHKMC CREC-202504)
**Author Disclosures:**
James F. Griffith: Nothing to disclose
Patricia Chan: Nothing to disclose
Chris Tsai: Nothing to disclose
Kam Ho Lee: Nothing to disclose
Neeraj Ramesh Mahboobani: Nothing to disclose

**Ultra long-term renal function post image-guided ablation and partial nephrectomy for stage 1 renal cell carcinomas**
H. Whittaker, V. Chan, *R. Miller*, J. Cartledge, J. Zhong, O. Abdel-Hadi, J. Smith, J. Lenton, T. M. Wah; Leeds/UK
*(rhys.miller5@nhs.net)*

**Purpose:** Long-term renal function post image-guided ablation (IGA) and partial nephrectomy (PN) is underreported. This study aims to outline the ultra-long term renal function of IGA and PN patients.
**Methods or Background:** This is a retrospective study of localised RCC (T1) patients undergoing image-guided cryoablation, radiofrequency ablation (RFA) or laparoscopic PN from 2004-2015 at our institution. Cox regression and log-rank analysis were used for survival analysis of long-term renal function, alongside t-test, chi-squared tests.
**Results or Findings:** A total of 295 patients undergoing image-guided cryoablation (104), image-guided RFA (99), and laparoscopic PN (92) were included. Median follow-up was 136 months (IQR 76). IGA patients were older (mean difference 13.8 years, p<0.001) and more comorbid (mean difference in Charlson index 1.47, p<0.001). Pre-treatment CKD is significantly more prevalent in patients undergoing IGA (29.1%) than PN (10.9%) (p=0.001). At 10-years follow-up, eGFR decreased 16.6% in IGA vs 8.4% in PN (p=0.12). New CKD (Grade 3 or above) developed in 28.3% of IGA patients vs 15.2% of PN patients (p=0.027) at follow-up. However, on a multivariable cox regression model, when taking account into age and Charlson comorbidity index, new-CKD free survival is similar between IGA and PN (HR 0.72, 95% CI 0.34-1.52, p=0.390). Six patients underwent renal dialysis, all IGA patients due to pre-treatment CKD. CKD-specific deaths were similar (HR 0.26, 95%CI 0.03-2.2, p=0.218).
**Conclusion:** Despite an older, co-morbid population pre-treatment, IGA achieved similar ultra-long-term renal function to PN.
**Limitations:** However, this study is limited by selection bias, and long-term results from randomised trials are warranted.
**Funding for this study:** No funding.
**Has your study been approved by an ethics committee?** Not applicable
**Author Disclosures:**
Vinson Chan: Nothing to disclose
Hannah Whittaker: Nothing to disclose
Jim Zhong: Nothing to disclose
Tze Min Wah: Nothing to disclose
Jon Cartledge: Nothing to disclose
Rhys Miller: Nothing to disclose
James Lenton: Nothing to disclose
Jonathan Smith: Nothing to disclose
Omar Abdel-Hadi: Nothing to disclose

**Differential Outcomes of Cryoablation for Pulmonary Metastases: Local Efficacy and Systemic Effects on Target vs. Non-Target Lesions**
*G. Liang*, S. Huang, W. Xiao, S. Gong; Hangzhou/CN
*(lgc_zju@163.com)*

**Purpose:** To evaluate the safety and efficacy of cryoablation for pulmonary metastases and investigate its differential impact on targeted versus non-targeted lesions.
**Methods or Background:** This retrospective study included 74 patients (39 male, mean age 61.7±8.3 years) with 102 pathologically proven pulmonary metastases treated with cryoablation between July 2020 and September 2024. Demographics, lesion characteristics, procedural details, and complications were recorded. Patients underwent follow-up CT scans at 3, 6, and 12 months. The primary outcome was the progression status of the targeted lesion at one year. Secondary outcomes included the progression of non-targeted lesions at each interval and overall survival. Changes in lesion size (length/short-diameter) were evaluated.
**Results or Findings:** The most common primary cancer was colorectal cancer (72%). The procedure was technically successful in all cases. Complication rates were low, with 24% of patients experiencing minor events (e.g., pneumothorax, hemoptysis); only two cases (2%) required chest tube drainage for moderate pneumothorax. For targeted lesions, larger lesion size (length and short-diameter) and a shorter distance from the ablation margin significantly predicted local progression (all p<0.001). For non-targeted lesions, the location of the ablated metastasis was a significant predictor of progression. Treating subpleural lesions resulted in significantly better control of non-targeted metastases at 3, 6, and 12 months compared to intrapulmonary lesions (Odds Ratios: 0.33, 0.33, 0.38; all p<0.05).
**Conclusion:** Cryoablation is a safe and effective treatment for pulmonary metastases. Outcomes for targeted lesions are influenced by their size and the achieved ablation margin. Notably, ablating subpleural metastases is associated with superior subsequent control of non-targeted lesions, suggesting a potential systemic benefit.
**Limitations:** The retrospective nature of this study.
**Funding for this study:** This study has received funding by National Natural Science Foundation of China (NSFC, No. 82330060 and 82370738).
**Has your study been approved by an ethics committee?** Yes
**Ethics committee - additional information:** This study was approved by Clinical Research Ethics Committee of the First Affiliated Hospital of Zhejiang University School of Medicine (IIT20240466A)
**Author Disclosures:**
Guangcai Liang: Nothing to disclose
Shan Huang: Nothing to disclose
Wenbo Xiao: Nothing to disclose
Shaolin Gong: Nothing to disclose

**10:00 - 11:00** **Research Stage 1**

### Research Presentation Session: Musculoskeletal Hot Topic with Keynote Lecture

### RPS 810

### Hot Topic: musculoskeletal imaging meets sustainability

**Moderator**
Chiara Giraudo; Padua/IT
*(chiara.giraudo@unipd.it)*

**Keynote Lecture**
Emma Rowbotham; Leeds/UK

**Real-World Implementation of a Deep Learning–Based Reconstruction Algorithm in Musculoskeletal MRI: Impact on Workflow, Image Quality, and Sustainability**
*J. Mendonca*, J. D. A. Batista Araújo Filho, A. G. Ormond Filho, T. R. Giacometti, A. Motoyama Caiado, B. A. Rocha, N. Y. Hashimoto, M. Nico, D. Bahia; São Paulo/BR
*(angela.caiado@grupofleury.com.br)*

**Purpose:** To describe our institutional experience deploying a deep learning reconstruction (DLR) algorithm for musculoskeletal MRI in an outpatient setting, assessing its impact on MRI acquisition time, image quality, and collaborative workflow adaptation.
**Methods or Background:** This retrospective study evaluated MSK MRI exams performed at a large private radiology network in Brazil. Following vendor-guided scanner upgrades, a k-space-based DLR algorithm (AIR™ Recon DL) was integrated into native reconstruction pipelines across multiple systems. Workflow adjustments and protocol standardization were co-developed in collaboration with the vendor and internal radiologist leads. We compared MRI studies from 12 months before (Group 1) and after (Group 2) DLR implementation. Acquisition time was automatically calculated per exam. A subset of cases was randomly selected for blinded qualitative image review using a 5-point Likert scale. Estimated energy use and $CO_2$e emissions were calculated using scanner-specific consumption metrics and regional emission factors.
**Results or Findings:** A total of 22,165 MSK MRI scans were analyzed: 10,492 in Group 1 and 11,673 in Group 2. Median scan duration decreased by 53% post-implementation, with the most substantial reductions in shoulder (62%), wrist (59%), knee (52%), spine (38%) and hip (33%) scans. Workflow optimization reduced scanner idle time, improved patient throughput, and lowered anxiety- and motion-related interruptions. Radiologist image quality assessments remained stable, with occasional feedback on altered image texture. Annual energy savings exceeded 2.3 MWh per scanner, avoiding over 1 metric ton of $CO_2$e emissions.
**Conclusion:** This real-world implementation of AI-driven DLR demonstrated tangible workflow optimization, improved patient experience, and meaningful environmental benefits. Ongoing collaboration among vendors, radiologists, and technologists was essential for successful adoption, underscoring the importance of clinician–industry partnerships in ensuring safe and efficient AI deployment.
**Limitations:** Retrospective study with limited image quality sampling and no cost-effectiveness analysis.
**Funding for this study:** None
**Has your study been approved by an ethics committee?** Yes
**Ethics committee - additional information:** Approved in 05-14-2025
**Author Disclosures:**
Tiago Rangon Giacometti: Nothing to disclose
Alipio Gomes Ormond Filho: Nothing to disclose
Julio Mendonca: Nothing to disclose
Angela Motoyama Caiado: Nothing to disclose
Marcelo Nico: Nothing to disclose
José De Arimatéia Batista Araújo Filho: Nothing to disclose
Noé Yoitiro Hashimoto: Nothing to disclose
Daniella Bahia: Nothing to disclose
Bruno Aragao Rocha: Nothing to disclose

### 3D isotropic FastView MRI Localizer allows reliable torsion measurements of the lower limb

*F. Herr*, N. Hohmann, C. A. Dascalescu, V. M. Schäfer, H. Gildein, B. Hoppe, J. Ricke, J. Arnholdt, P. Reidler; Munich/DE
*(felix.herr@med.uni-muenchen.de)*

**Purpose:** To evaluate whether a 3D isotropic MRI localizer (FastView) can reliably and efficiently assess femoral and tibial torsion compared to a dedicated MRI protocol, offering a faster alternative to a conventional dedicated MRI protocol.
**Methods or Background:** In this retrospective single-center study, 60 lower limbs from 30 patients (mean age 27.1 ± 11.5 years; 19 female, 11 male) were examined using FastView and a dedicated MRI protocol. FastView imaged the entire lower limb in 17.4 seconds (voxel size 5×5×5 mm$^3$), whereas the standard protocol required nearly 7 minutes. Two independent readers measured femoral and tibial torsion angles. Agreement between methods was evaluated using intraclass correlation coefficients (ICCs), Bland–Altman plots, and Pearson's $R^2$.
**Results or Findings:** Across 60 limbs, no significant differences were observed in all measured angles between FastView and the dedicated protocol ($p > 0.05$). Femoral torsion showed excellent inter-modality agreement (ICC: 0.91–0.96), with mean differences ranging from –2.2 ± 3.4° (p = 0.001) to –3.7 ± 4.4° ($p < 0.001$). Tibial torsion also showed excellent agreement (ICC: 0.91–0.94), with mean differences from –1.2 ± 4.6° (p = 0.16) to –2.1 ± 4.6° (p = 0.02). Inter-reader agreement was consistently high for both modalities (ICC: 0.95–0.99). Bland–Altman analyses and Pearson's $R^2$ (range: 0.84–0.94) confirmed strong correlation and reliability.
**Conclusion:** FastView MRI localizer showed excellent agreement with the dedicated torsion protocol for femoral and tibial measurements. By reducing scan time and avoiding radiation, FastView promotes a more sustainable approach and may replace conventional multi-sequence protocols in practice.
**Limitations:** This retrospective single-center study has limited generalizability, and the sample size was relatively small. Although scan acquisition with FastView is reduced to ~17 seconds, preparation time remains similar to standard protocols.
**Funding for this study:** No funding was received for this study.
**Has your study been approved by an ethics committee?** Yes
**Ethics committee - additional information:** This study was approved by the institutional review board (approval number: 23-0020; date of approval: 09 March 2023).
**Author Disclosures:**
Hannah Gildein: Nothing to disclose
Jörg Arnholdt: Nothing to disclose
Boj Hoppe: Nothing to disclose
Verena Martina Schäfer: Nothing to disclose
Natascha Hohmann: Nothing to disclose
Felix Herr: Nothing to disclose
Paul Reidler: Nothing to disclose
Christian Alexander Dascalescu: Nothing to disclose
Jens Ricke: Nothing to disclose

### 3D MRI- Based Virtual Radiographs Of The Pelvis For Assessment Of Acetabular Coverage And Version

J. Roshardt[1], S. Steppacher[1], M. K. Meier[1], S. Sommer[2], R. Sutter[2], *F. Schmaranzer*[2]; [1]Bern/CH, [2]Zürich/CH
*(fschmaranzer@gmail.com)*

**Purpose:** Hip deformities require precise diagnosis before joint-preserving surgery, but standard radiographs and CT scans involve ionizing radiation, raising concerns about cumulative exposure, particularly in younger patients. MRI offers detailed, radiation-free imaging of bone morphology and soft tissues. Advances in deep learning enable automatic segmentation and MRI-based 3D pelvic modeling, allowing virtual radiographic-like projections. This study determined if standard radiographic parameters (lateral center-edge angle [LCEA], retroversion index [RI], and acetabular index [AI]) can be accurately measured on MRI-based projections compared to conventional anteroposterior (AP) pelvic radiographs.
**Methods or Background:** Fifty patients (100 hips, mean age 28 years) with symptomatic hip deformities underwent evaluation with standard AP pelvic radiographs and direct hip MR arthrography (including T1-w VIBE DIXON). Osseous pelvis was segmented using a validated 3D nnU-Net, cortical bone contrast augmentation and radiograph-like projections were generated using a cone beam projection algorithm. Arthrography and non-contrast sides were analyzed separately. Parameters were measured on both imaging modalities and compared using t-tests and Pearson's correlation.
**Results or Findings:** The LCEA showed no significant difference between conventional and MRI-based projections, with absolute mean differences of 0.2±1.2° (arthrography, p=0.71) and 0.6±1.7° (non-contrast, p=0.29). Similarly, AI showed no significant difference (0.6±1.5° arthrography, p=0.20); 1.±2.1° (non-contrast, p=0.075). For RI, absolute mean differences were 0.5±4% (arthrography, p=0.80) and 2.±5.3% (non-contrast, p=0.22), indicating no significant difference. Moderate to strong correlations were found between MRI-based and conventional radiographs (LCEA: r=0.84/0.77, AI: r=0.75/0.66, RI: r=0.60/0.66 for arthrography/non-contrast; all p<0.001).
**Conclusion:** MRI-based radiographic-like projections, whether with or without contrast agents, provide measurements comparable to AP pelvic radiographs for key hip morphology parameters. This approach eliminates radiation exposure while maintaining diagnostic accuracy, offering significant advantages for young patients requiring repeated imaging for hip deformity assessment and surgical planning.
**Limitations:** No external validation
**Funding for this study:** Funding received by the Swiss National Science Foundation (porject no. 205091)
**Has your study been approved by an ethics committee?** Yes
**Ethics committee - additional information:** Ethics committee of the University of Bern
**Author Disclosures:**
Jose Roshardt: Nothing to disclose
Stefan Sommer: Employee: Siemens Healthineers
Florian Schmaranzer: Grant Recipient: Swiss National Science Foundation
Simon Steppacher: Grant Recipient: Swiss National Science Foundation
Malin Kristin Meier: Nothing to disclose
Reto Sutter: Nothing to disclose

### Do All Scaphoid Fractures Need a CT Scan? Insights from a European Dual-Centre Study to Guide Clinical Decision-Making

*M. Avanesov*[1], C. Zickert[2], M. Stojkova[3], G. F. Peter[1], J. Yamamura[1], H. Ragab[1]; [1]Hamburg/DE, [2]Lüneburg/DE, [3]Duisburg/DE
*(maxim.avanesov@gmx.de)*

**Purpose:** This dual-centre study analysed the additional value of CT imaging besides conventional radiography (x-ray) on the diagnosis and outcome of acute scaphoid fractures at two university medical centres in Europe.
**Methods or Background:** Two hundred and twelve consecutive patients (103 patients from Hamburg, Germany (mean age 38 ±19 years, 82 males) and 109 patients from Skopje, North Macedonia (mean age 38 ±18 years, 86 males)) who were diagnosed with scaphoid fractures were included in the European dual-centre study. All patients underwent conventional radiography in three views and all fractures were staged according to the dichotomic Herbert classification (Herbert A: stable fracture; Herbert B: unstable fracture). In Hamburg, all patients underwent additional CT imaging, whereas in Skopje only patients with suspected instability (Herbert Type B) underwent additional CT imaging. Demographics, treatment strategies, as well as clinical and functional outcomes were retrospectively compared between the two centres.
**Results or Findings:** All patients with a scaphoid fracture in Hamburg underwent additional CT imaging, whereas only 23 out of 109 patients (21 %) in Skopje had a complementary CT scan (p < .001). Fracture type distribution and stability were comparable between centres. While 42 out of 103 patients (41%) underwent surgical treatment in Hamburg, only 13 out of 109 patients (12%) were operated in Skopje (p < .001). However, there were no statistically significant differences in complication rates between the two sides (p = .489).
**Conclusion:** This dual-centre study suggests that using additional CT imaging only for unstable acute scaphoid fractures does not lead to worse clinical or functional outcomes or an increased rate of complications.
**Limitations:** Retrospective study design Limited follow-up period MRI imaging was not investigated
**Funding for this study:** No external funding sources.
**Has your study been approved by an ethics committee?** Yes
**Ethics committee - additional information:** Ethical approval was provided by the local institutional ethics committee. The requirement for written informed consent was waived due to the retrospective nature of the study. The study was performed in accordance with the ethical standards as laid down in the 1964 Declaration of Helsinki and its later amendments.
**Author Disclosures:**
Maxim Avanesov: Nothing to disclose
Carolina Zickert: Nothing to disclose
Jin Yamamura: Nothing to disclose
Gregor Fritz Peter: Nothing to disclose
Marija Stojkova: Nothing to disclose
Haissam Ragab: Nothing to disclose

### Deep Learning Accelerated MRI in Muscle Injuries of Professional Soccer Athletes: Enhancing Efficiency and Sustainability

*S. Mahmoudi*, K. Eichler, L. D. Grünewald, T. J. Vogl, I. Yel; Frankfurt/DE
*(scherwin.mahmoudi@gmail.com)*

**Purpose:** The purpose of this study was to investigate the impact of deep learning-accelerated MRI on acquisition time and image quality in muscle injuries of professional soccer athletes.
**Methods or Background:** 28 professional male soccer athletes (mean age 25.4±3.4) who underwent 3-Tesla-MRI of the lower limb due to suspicion of muscle injuries were included. Standard sequences were acquired consisting of PDw imaging in axial, paracoronal and parasagittal plane, as well as T1-weighted imaging in axial plane. Additionally, PDw imaging sequences were acquired using the deep learning algorithm (PDDL). Subjective evaluation was independently performed by three blinded radiologists. 5-point Likert scales were utilized to subjectively assess diagnostic confidence, image quality, and lesion sharpness. Objective analysis encompassed time efficiency and quantitative imaging parameters, including signal-to-noise-ratio (SNR) and contrast-to-noise-ratio (CNR), as well as operational costs including electricity and personnel.
**Results or Findings:** MRI revealed structural injuries in 21 of 28 athletes (75%). Subjective evaluations of PDDL sequences revealed excellent diagnostic confidence (median 5, interquartile range (IQR): 5-5), image quality (median 5, IQR: 5-5), and lesion sharpness (median 5, IQR: 4.5-5). In 91.7 % of cases, PDDL sequences were favored over conventional PD imaging. Objective image analysis of PDDL revealed significant higher SNR (115.0±9.1) and CNR values (80.5±12.8) when compared to conventional PD sequences (81.3±5.9 and 54.3±5.4, respectively)(p<0.05). Acquisition times could be reduced by an average of 39.7% using PDDL sequences.
**Conclusion:** Our findings suggest that deep learning MRI of muscle injuries among professional athletes allow a reduction in acquisition time while additionally improving image quality. The integration of these sequences may enhance operational efficiency and reduce ongoing costs, promoting a more sustainable and value-based approach in radiology.
**Limitations:** The study population is relatively small, which is attributable to the specific subcohort examined.
**Funding for this study:** No funding
**Has your study been approved by an ethics committee?** Yes
**Ethics committee - additional information:** The study was approved by the ethics committee of the University Hospital Frankfurt
**Author Disclosures:**
Scherwin Mahmoudi: Nothing to disclose
Ibrahim Yel: Speaker: Siemens
Thomas J. Vogl: Nothing to disclose
Leon David Grünewald: Nothing to disclose
Katrin Eichler: Nothing to disclose

### Buddy Imaging in MSK: Adding Value to Routine Scans for a Greener Future

*C. Zhu*[1], I. d. Mata[2], C. Zhu[3], E. Santana[2]; [1]Barcelos/PT, [2]Lisboa/PT, [3]Lisbon/PT

**Purpose:** Healthcare sustainability requires imaging strategies that maximize diagnostic yield while minimizing resource use. Musculoskeletal (MSK) findings are frequently visible on routine scans obtained for other indications, such as abdominal CT, pelvic MRI, or vascular imaging. Leveraging these "incidental windows" provides clinically relevant information without additional examinations, contributing to more efficient and environmentally responsible practice. The aim of this presentation is to demonstrate how MSK findings can be opportunistically assessed on routine non-MSK scans, to discuss their role in the early detection of conditions such as osteoporosis or sarcopenia, and to illustrate how this approach contributes to sustainable imaging by reducing redundant examinations while enhancing patient care.
**Methods or Background:** A literature review and pictorial synthesis were performed, focusing on CT and MRI examinations where bone and soft-tissue structures are included but not primarily targeted. Examples include vertebral bone mineral density from abdominal CT, femoral muscle bulk in pelvic MRI, and vascular CT revealing both arterial calcifications and adjacent bone health.
**Results or Findings:** Analysis of routine scans demonstrates that clinically relevant MSK information can often be extracted without additional imaging. Vertebral Hounsfield units derived from abdominal CT correlate with osteoporosis risk, muscle cross-sectional area on pelvic MRI reflects sarcopenia, and joint or tendon morphology may be partially assessed on pelvic or vascular studies. Incorporating this opportunistic evaluation into structured reporting allows early recognition of disease, reduces the demand for dedicated MSK examinations, and contributes to a more sustainable imaging workflow.
**Conclusion:** "Buddy imaging" transforms routine scans into multipurpose tools, aligning clinical value with sustainability goals. Recognizing MSK features beyond the primary indication supports earlier intervention, avoids duplicate examinations, and exemplifies a greener radiology practice.
**Limitations:** No limitations were identified.
**Funding for this study:** No funding was received for this study.
**Has your study been approved by an ethics committee?** Not applicable
**Author Disclosures:**
Chenyi Zhu: Nothing to disclose
Inês Da Mata: Nothing to disclose
Eduardo Santana: Nothing to disclose
Caixia Zhu: Nothing to disclose

### A Unified Model for Mutlianatomy Musculoskeletal Image Synthesis in MRI

*T. C. Arnold*[1], L. Wang[1], A. Shankaranarayanan[1], L. N. Tanenbaum[2]; [1]Menlo Park, CA/US, [2]Riverside, CT/US

**Purpose:** Musculoskeletal MR protocols include multiple sequences with different acquisition planes (axial, sagittal, coronal) and contrasts (T1w, T2w, T2w fat-sat, PD, PD fat-sat). The exact combination of sequences varies widely across institutions. Previously, we demonstrated the ability to synthesize sequences in knee protocols, thus enabling faster or more comprehensive exams. However, that algorithm's utility is limited to knee exams. In this study, we present a unified model capable of synthesizing sequences across a wide range of musculoskeletal anatomies.
**Methods or Background:** We retrospectively analyzed 554 musculoskeletal MR exams to identify image synthesis sets containing a target image, a sequence-type match, and an acquisition-plane match. We held out 55 subjects for testing, which spanned 6 target sequences, 8 musculoskeletal regions, and 39 target/sequence-type/acquisition-plane combinations. The remaining subjects were used to train a patch-based neural network. Model performance was quantitative assessed using peak signal-to-noise ratio (PSNR) and structural similarity index (SSIM).
**Results or Findings:** Across all synthesis combinations, average PSNR was 23.6±3.3 dB and SSIM 0.66±0.15. Averaging across target sequence, quantitative values ranged from PSNR=[22.5-25.7 dB] and SSIM=[0.59-0.80] with Ax PD FS having the highest (25.7±5.0 dB, 0.80±0.12) followed by Ax T2 FS (24.8±0.2 dB, 0.77±0.01). Across anatomies, values ranged from PSNR=[20.1-27.8 dB] and SSIM=[0.59-0.81] with elbow and ankle showing the best performance and wrist having the worst performance.
**Conclusion:** We provide a preliminary demonstration that a single model can synthesize a range of sequences from various musculoskeletal anatomies. This offers a flexible and generalizable approach that can match site-specific musculoskeletal protocols and adapt to protocol changes.
**Limitations:** Future studies should include a qualitative reader study by radiologists.
**Funding for this study:** Funding provided by Subtle Medical.
**Has your study been approved by an ethics committee?** Not applicable

**Author Disclosures:**
Long Wang: Employee: Subtle Medical
Lawrence Neil Tanenbaum: Consultant: Subtle Medical
Thomas Campbell Arnold: Employee: Subtle Medical
Ajit Shankaranarayanan: Employee: Subtle Medical

**10:00 - 11:00 Research Stage 5**

### Research Presentation Session: Neuro

### RPS 811

### Multiple sclerosis: advanced MRI biomarkers and imaging innovations

**Moderator**
Alejandro Rovira Cañellas; Barcelona/ES
*(alex.rovira.idi@gencat.cat)*
**Author Disclosures:**
Alejandro Rovira Cañellas: Advisory Board: Novartis, Sanofi-Genzyme, Roche, and Bayer; Share Holder: Tensor Medical, Speaker: Bayer, Sanofi-Genzyme, Merck, Teva Pharmaceutical Industries Ltd, Novartis, Roche, BMS, Neuroaxpharm and Biogen

**Optic Nerve Lesion Volume, White Matter Hyperintensities, and Brain Volumetrics in Multiple Sclerosis: A Multi-Sequence MRI-Based Analysis**
*A. Korbecki*, T. Konopczyński, O. Hawro, A. Blachucik, K. Winiarczyk, K. Litwinowicz, M. Sobański, J. Bladowska, A. Zimny; Wroclaw/PL
*(adrian.korbecki.md@gmail.com)*

**Purpose:** To examine the association between optic nerve lesion volume (ONLV) on double inversion recovery (DIR) MRI and other imaging biomarkers of disease burden in multiple sclerosis (MS), including white matter hyperintensities (WMHs), T1-weighted hypointensities, and brain volumetrics. The study explores whether ONLV reflects a more severe neurodegenerative profile and may serve as a marker of disease severity.
**Methods or Background:** In this cross-sectional study, 212 MS patients underwent 3T MRI including 3D T1-weighted, FLAIR, and DIR sequences. Optic nerve lesions were manually segmented on DIR and quantified volumetrically. Patients were categorized by optic nerve involvement: none (n = 59), unilateral (n = 60), or bilateral (n = 93). WMHs were segmented and anatomically classified using an AI-based tool. T1W hypointensities and brain volumetrics were extracted using FreeSurfer and a machine-learning algorithm applied to 3D T1W and FLAIR images. Models were adjusted for intracranial volume.
**Results or Findings:** ONLV positively correlated with periventricular ($r = 0.365$, $p < 0.001$), deep ($r = 0.165$, $p = 0.005$), and juxtacortical ($r = 0.163$, $p = 0.007$) WMHs. Bilateral involvement was associated with higher WMH burden, increased T1W hypointensities ($\beta = 10.91$, $p < 0.001$), and greater white-matter atrophy ($\beta = -107.02$, $p = 0.016$), particularly along visual pathways. Periventricular WMHs also correlated with global cortical and subcortical gray-matter loss.
**Conclusion:** ONLV is associated with greater lesion load and neurodegeneration in MS, supporting its potential as a biomarker of disease severity. Longitudinal studies are needed to confirm prognostic value.
**Limitations:** Cross-sectional design limits assessment of longitudinal progression; future studies should track ONLV evolution and its radiological/clinical impact. Manual segmentation is prone to variability; AI-based tools could improve accuracy and reproducibility. Integrating comprehensive clinical data and more uniform cohorts would strengthen future investigations.
**Funding for this study:** No funding was provided for this study.
**Has your study been approved by an ethics committee?** Yes
**Ethics committee - additional information:** The study was conducted according to the guidelines of the Declaration of Helsinki and approved by the Institutional Review Board of the Wroclaw Medical University Ethics Committee for conducting research involving humans.
**Author Disclosures:**
Anna Zimny: Nothing to disclose
Krzysztof Winiarczyk: Nothing to disclose
Oktawian Hawro: Nothing to disclose
Tomasz Konopczyński: Nothing to disclose
Aleksandra Blachucik: Nothing to disclose
Adrian Korbecki: Nothing to disclose
Michał Sobański: Nothing to disclose
Kamil Litwinowicz: Nothing to disclose
Joanna Bladowska: Nothing to disclose

**Analysis of Spinal Cord MRI in Multiple Sclerosis: Implications for Monitoring Disease Progression**
*H. Larassati*[1], S. M. Sceppacuercia[2], A. H. M. E. Hammam[1], O. Sarwani[1], W. Brownlee[1], C. Auger[2], A. Rovira Cañellas[2], T. A. Yousry[1]; [1]London/UK, [2]Barcelona/ES

**Purpose:** To evaluate the benefits of spinal cord MRI in multiple sclerosis (MS) by assessing lesion dynamics over time, their relationship with brain lesions, and disability progression.
**Methods or Background:** This retrospective, multicentre longitudinal study included MS patients from London (UK) and Barcelona (Spain) who underwent brain and spinal cord MRI at baseline and follow-up. Clinical data included relapse history, Expanded Disability Status Scale (EDSS), and disease-modifying therapy (DMT) use. MRI reports were extracted from PACS, with independent reads by two neuroradiologists to assess inter- and intra-observer reliability. Regression models evaluated associations with disability.
**Results or Findings:** 127 patients (75.6% females, 24.4% males) were included, mean age of 43 years old, median follow-up interval at 19 months (11–29 months), 56.8% were on DMT at follow up. 82.7% of patients had spinal cord lesions at baseline, and 15.1% developed at least one lesion at follow-up. New spinal cord lesions were most frequent at 11–12 months (28.6%) and less common at later follow-ups (8–22%). New spinal cord lesions were more common in the relapsing MS, and was significantly associated with EDSS worsening ($p=0.036$). In progressive MS, new lesion incidence was lower and showed weaker correlation with disability progression. Spinal-only activity was present in 7.9% patients at baseline and occurred in additional cases at follow-up. Inter- and intra-rater agreement for lesion burden and new lesion detection was moderate to high ($\kappa=0.60–0.83$; ICC=0.56–0.94).
**Conclusion:** New spinal cord lesions predict disability worsening and reveal spinal-only activity missed on brain MRI. New spinal lesion accrual was relatively low and the benefit of spinal MRI may vary by patient phenotype and timing. A selective, phenotype- and timing-based approach to spinal cord imaging may optimise its use in MS monitoring.
**Limitations:** Small sample size, limited follow-up period
**Funding for this study:** None
**Has your study been approved by an ethics committee?** Yes
**Ethics committee - additional information:** University College London Hospitals NHS Foundation Trust
**Author Disclosures:**
Sofia Maria Sceppacuercia: Nothing to disclose
Cristina Auger: Nothing to disclose
Tarek A. Yousry: Nothing to disclose
Hana Larassati: Nothing to disclose
Wallace Brownlee: Nothing to disclose
Alejandro Rovira Cañellas: Speaker: Speaker honoraria from Bayer, Sanofi-Genzyme, Merck-Serono, Teva Pharmaceutical Industries Ltd, Novartis, Roche, Bristol-Myers and Biogen Research/Grant Support: Fondo de Investigación en Salud (PI19/00950 and PI22/01589) and Instituto de Salud Carlos III, Spain Advisory Board: Novartis, Sanofi-Genzyme, Synthetic MR, TensorMedical, Roche, and Biogen Other: CMO and co-founder of Tensor Medical.
Ahmed Hassan Mohamed Elzeki Hammam: Nothing to disclose
Omran Sarwani: Nothing to disclose

**Paramagnetic Rim Lesions: Potential Biomarkers of Disease Activity and Prognosis in Multiple Sclerosis**
B. Atalay, *M. Yamer*, M. B. Doğan, I. Aydın Cantürk; Istanbul/TR
*(mizginyamer1@gmail.com)*

**Purpose:** To investigate the association of paramagnetic rim lesion subtypes—complete, incomplete, patch-like, which serve as biomarkers of chronic active lesions in multiple sclerosis—with the Expanded Disability Status Scale (EDSS) and multiple sclerosis subtypes.

**Methods or Background:** This retrospective study included patients with a confirmed diagnosis of MS who underwent brain MRI. PRLs were evaluated by count, type (complete rim, incomplete rim, or patch-like) and location using phase images from SWI sequences. The presence of active lesions was also noted. Statistical analyses examined associations between PRL characteristics and clinical parameters using non-parametric tests and Spearman correlations.
**Results or Findings:** Thirty-two patients (75% female; mean age 46.1 ± 15.7 years) were included. PRLs were present in 62.5% of patients, with 28.1% complete and 12.5% incomplete rims, and the remainder were patch-like. PRL count, type, and location did not differ significantly across MS subtypes (p = 0.93, 0.21, and 0.08, respectively). Patients with active lesions exhibited a significantly higher number of PRLs compared to those without (p = 0.02). However, PRL location and type were not associated with the presence of active lesions (p = 0.07 and p = 0.15). No significant correlations were observed between PRL count and either EDSS score or disease duration (p = 0.83 and p = 0.87). Likewise, EDSS score and disease duration did not significantly differ between patients with and without PRLs (p = 0.23 and p = 0.14, respectively).
**Conclusion:** PRLs are common in patients with MS and are associated with the presence of active lesions, but not with MS subtype, EDSS score, or disease duration. Their role in clinical prognosis should be clarified in larger, prospective studies.
**Limitations:** The limitations of our study include its retrospective design, the relatively small patient sample size.
**Funding for this study:** No funding for this study.
**Has your study been approved by an ethics committee?** Yes
**Ethics committee - additional information:** Our study was approved by an ethics committee.
**Author Disclosures:**
Mizgin Yamer: Nothing to disclose
Ilknur Aydın Cantürk: Nothing to disclose
Başak Atalay: Nothing to disclose
Mahmut Bilal Doğan: Nothing to disclose

**Effect of Lesion Filling on Brain Age Estimation in Multiple Sclerosis**
*S. Hannoun*, G. Fayad, N. El-Ayoubi, S. Khoury; Beirut/LB
*(sh156@aub.edu.lb)*

**Purpose:** To investigate whether lesion filling improves the accuracy and interpretability of brain age estimation in multiple sclerosis (MS), and to assess its associations with clinical disability and structural MRI markers.
**Methods or Background:** We retrospectively analyzed 571 relapsing-remitting MS patients. Brain age was estimated using the BrainAgeR pipeline on both non-lesion-filled and lesion-filled T1-weighted images. Bias correction was applied to adjust for age-related prediction bias, and the Brain Age Gap (BAG) was computed as the difference between corrected predicted age and chronological age. Agreement between approaches was assessed using Bland–Altman analysis, Pearson correlation, and intraclass correlation coefficient (ICC). Associations of BAG with disability measures (EDSS, 9HPT, SDMT, 25FWT) and volumetric MRI metrics (global and subcortical volumes) were evaluated using multivariable regression with Bonferroni correction.
**Results or Findings:** Lesion-filled and non-lesion-filled brain age estimates showed excellent agreement (r=0.97, ICC=0.962), with a mean difference of 1.23 years. The mean absolute error was slightly lower for lesion-filled predictions (8.12 vs 9.40 years). Both BAG measures were significantly associated with EDSS, 9HPT, and SDMT (p<0.001), but not with 25FWT. Lesion-filled BAG demonstrated stronger associations with gray matter, thalamic, and hippocampal volumes, with higher explained variance compared to non-lesion-filled BAG. These associations remained significant after multiple comparison correction.
**Conclusion:** Brain age estimation is robust to lesion effects in MS, with lesion filling offering modest improvements in alignment with structural imaging markers but limited impact on clinical correlations. Lesion correction should be considered when precise structural interpretability is required.
**Limitations:** This cross-sectional, single-center study included only relapsing-remitting patients without healthy controls. Scanner variability and potential circularity between lesion-filled volumetrics and brain age may have influenced associations. Longitudinal, multi-center studies are warranted.
**Funding for this study:** None
**Has your study been approved by an ethics committee?** Yes
**Ethics committee - additional information:** This study was approved by the American University of Beirut Biomedical Institutional Review Board (IRB), which explicitly waived the requirement for written informed consent due to the retrospective nature of the study and minimal risk to participants.
**Author Disclosures:**
Samia Khoury: Nothing to disclose
Salem Hannoun: Nothing to disclose
Nabil El-Ayoubi: Nothing to disclose
Grace Fayad: Nothing to disclose

**Deep Learning Algorithm Boosts Contrast Signal and Lesion Visualization in Multiple Sclerosis Patients: A Multi-Reader Clinical Performance Study**
*S. Pasumarthi Venkata*[1], C. Arnold[1], P. Gulaka[1], S. Colombo Serra[2], G. Erb[3], G. D'Anna[4], A. Shankaranarayanan[1], G. Zaharchuk[5]; [1]Menlo Park, CA/US, [2]Colleretto Giacosa/IT, [3]Konstanz/DE, [4]Milan/IT, [5]Stanford, CA/US
*(sonia.colombo@bracco.com)*

**Purpose:** In the treatment of Multiple Sclerosis (MS), contrast-enhanced (CE) images are crucial in differentiating between new and chronic lesions. Recently, an FDA-cleared contrast boosting (CB) deep learning algorithm was developed that boosted contrast signals present in T1w standard contrast-enhanced (SC) images, improving lesion visualization without increasing dosage. In this work, we evaluate the clinical performance of the CB algorithm on a public dataset consisting of T1w pre and CE images from MS patients.
**Methods or Background:** From the Open MS Dataset, T1w-pre and SC images from 30 patients (23 Females; 39±10) were used in this study. Contrast boosted (CB) images were generated from T1w-pre and SC using the CB algorithm. Three board-certified radiologists were asked to score the SC and CB images for lesion contrast enhancement, border delineation and internal morphology on a 4-point Likert scale. Readers also scored on any False Lesions (FL) found on CB images and their impact on diagnosis. Contrast-to-noise ratio (CNR), lesion-to-brain ratio (LBR) and contrast-enhancement-percentage (CEP) were computed on SC and CB images.
**Results or Findings:** The CB images were rated higher (p<0.05) than SC images for enhancement (3.66±0.56 vs 2.25±0.84), delineation (3.62±0.71 vs 2.25±0.84) and morphology (3.41±0.77 vs 2.12±0.89). CB images had higher (p<0.01) CNR, LBR and CEP. The readers found a few enhancing lesions that were almost missed on SC but were clearly visualized on CB. 12% of cases had FLs that could potentially impact diagnosis, but could be ruled out using T2-FLAIR.
**Conclusion:** We showed that the CB algorithm has superior clinical performance on MS lesions when compared to SC images. The CB algorithm clearly visualized a few lesions that were almost undetectable from the SC images.
**Limitations:** This study is limited to a small number of cases.
**Funding for this study:** n/a
**Has your study been approved by an ethics committee?** Not applicable
**Author Disclosures:**
Srivathsa Pasumarthi Venkata: Employee: Subtle Medical Inc
Greg Zaharchuk: Nothing to disclose
Gunter Erb: Employee: Bracco Imaging Deutschland GmbH
Sonia Colombo Serra: Employee: Bracco Imaging SpA
Praveen Gulaka: Employee: Subtle Medical Inc
Thomas Campbell Arnold: Employee: Subtle Medical Inc
Ajit Shankaranarayanan: Employee: Subtle Medical Inc
Gennaro D'Anna: Nothing to disclose

**Diagnostic values of IVIM parametric maps in predicting disabilities for relapsing-remitting multiple sclerosis patients**
*O. Alomair*[1], S. A. Alghamdi[1], A. Abujamea[1], M. S. Alshuhri[2], S. Aljarallah[1], N. Alkhawajah[1], H. Al-Mubarak[3], Y. Alashban[1], N. Kurniawan[4]; [1]Riyadh/SA, [2]Al Kharj/SA, [3]Glasgow/UK, [4]Brisbane QLD/AU
*(oalomir@ksu.edu.sa)*

**Purpose:** In this abstract, we achieved three aims previously published in three papers. First, evaluate intravoxel incoherent motion (IVIM) diffusion and perfusion MRI metrics for various types of MS lesions, including enhanced, non-enhanced, and black hole lesions. Second, investigate the correlation and predictive values of the IVIM diffusion and perfusion MRI metrics with disability status. Third, utilise radiomics features to evaluate the predictive value of IVIM diffusion parameters in relation to disability severity.
**Methods or Background:** This cross-sectional study retrospectively analysed quantitative IVIM parameters and MRI data from 197 MS patients. Multiple linear regression was applied to identify independent predictors of EDSS score. Machine learning (ML) techniques, such as XGB, Random Forest, and ANN, were employed to explore the relationships between radiomic IVIM and clinical variables.
**Results or Findings:** In this abstract, we presented the results previously published in three papers. First, ADC, D, and D* values for MS black hole lesions were significantly higher (p < 0.0001) than those for other MS lesions. Second, in the multivariate regression analysis, only the number of MS lesions and relapses emerged as independent predictors of EDSS score (p-value < 0.001). Third, for disability prediction, IVIM-D and D* radiomics strongly correlated with EDSS: Random Forest achieved 89% accuracy (AUC = 0.90), while CNN achieved 90% accuracy (AUC = 0.95).
**Conclusion:** These three published studies demonstrate the utility of IVIM parameters in detecting microstructural alterations associated with MS impairment. Machine learning analyses of IVIM metrics provided independent predictors of functional impairment and disability in MS. It validated our results.

**Limitations:** This study has several limitations, which include a single time point study, and it was limited to analysis of MS lesions without considering normal-appearing white or grey matter.
**Funding for this study:** This research was funded by the King Salman Center for Disability Research through Research Group no. KSRG-2024-197. The presented work based on three published paper; Investigating the Role of Intravoxel Incoherent Motion Diffusion-Weighted Imaging in Evaluating Multiple Sclerosis Lesions, The Utility of Intravoxel Incoherent Motion Metrics in Assessing Disability in Relapsing–Remitting Multiple Sclerosis and IVIM-DWI-Based Radiomics for Lesion Phenotyping and Clinical Status Prediction in Relapsing–Remitting Multiple Sclerosis.
**Has your study been approved by an ethics committee?** Yes
**Ethics committee - additional information:** This study was conducted in accordance with the Declaration of Helsinki and approved by the Local Ethics Committee from King Saud University, Medical City (No. E-23-7517; approval date—22 January 2023; date of renewal of ethical certificate—30 June 2025).
**Author Disclosures:**
Sami A Alghamdi: Nothing to disclose
Nuha Alkhawajah: Nothing to disclose
Yazeed Alashban: Nothing to disclose
Abdullah Abujamea: Nothing to disclose
Salman Aljarallah: Nothing to disclose
Haitham Al-Mubarak: Nothing to disclose
Nyoman Kurniawan: Nothing to disclose
Othman Alomair: Nothing to disclose
Mohammed Shenan Alshuhri: Nothing to disclose

**Generalizable DIR-like Image Synthesis Across Multisite MRI Data for Improved Multiple Sclerosis Lesion Assessment**
L. Wang, C. Arnold, Z. Zhou, L. Xiang, A. Shankaranarayanan, S. Bash, L. N. Tanenbaum, *S. Pasumarthi Venkata*; Menlo Park, CA/US
*(nuromri@gmail.com)*

**Purpose:** Double inversion recovery(DIR) MRI provides superior gray–white matter differentiation and lesion visibility in multiple sclerosis(MS), but is rarely acquired in routine practice due to long scan times. We repurposed a synthesis model originally developed for STIR and applied it to multiple brain MS datasets to evaluate its cross-domain generalization. Despite being trained in a different anatomical setting, the model demonstrates strong performance in generating DIR-like (Syn-DIR) images, with consistent improvements across multisite MS datasets.
**Methods or Background:** The model was directly applied to T1-weighted and T2-FLAIR brain scans to generate DIR-like images without additional training. Two external datasets were analyzed: open_ms_data(30 subjects) and MSLesSeg(75 subjects). Evaluation included structural fidelity (SSIM and Dice scores on brain volume analysis), tissue contrast (gray-to-white-matter ratio and lesion-to-white-matter ratio), multisite consistency analysis, and longitudinal stability analysis.
**Results or Findings:** FLAIR and SynDIR showed high structural fidelity across sites (mean SSIM: $0.8719 \pm 0.0217$), with Dice scores >0.85 for all major brain structures. SynDIR images exhibited significantly improved tissue and lesion contrast compared with FLAIR. Specifically,gray-to-white-matter ratio increased from $1.2688 \pm 0.0617$ to $1.6134 \pm 0.1946$, and lesion-to-white-matter ratio increased from $1.4035 \pm 0.1309$ to $1.9335 \pm 0.3833$ ($p < 0.001$). The t-test on the ratio of gray-to-white-matter ratio to lesion-to-white-matter between the two cohorts showed no significant difference, indicating consistent performance across sites. In addition, the Wilcoxon signed-rank test showed no significant difference between time points ($p = 0.1478$).
**Conclusion:** A spine-trained synthesis model generalized effectively to brain MS cohorts, generating DIR-like images that enhanced lesion visibility and showed consistent performance across sites and timepoints. This repurposed approach enables advanced contrasts for MS studies without acquiring the DIR series.
**Limitations:** In future work, these methods should undergo qualitative review by radiologists.
**Funding for this study:** n/a
**Has your study been approved by an ethics committee?** Not applicable
**Author Disclosures:**
Srivathsa Pasumarthi Venkata: Employee: Subtle Medical
Suzie Bash: Nothing to disclose
Long Wang: Nothing to disclose
Lei Xiang: Nothing to disclose
Lawrence Neil Tanenbaum: Nothing to disclose
Thomas Campbell Arnold: Nothing to disclose
Ajit Shankaranarayanan: Nothing to disclose
Zechen Zhou: Nothing to disclose

**The Role of AI in Multiple Sclerosis Follow-up: Improving Accuracy and Reducing Reporting Times**
*E. Masiello*, A. Diena, G. M. Agazzi, A. Falini, N. E. Anzalone; Milan/IT
*(e.masiello@studenti.unisr.it)*

**Purpose:** In multiple sclerosis (MS) MRI is essential for diagnosis and follow-up. Detecting new or enlarging lesions can be challenging and reporting is time-consuming. This study aimed to evaluate the role of an AI platform in lesion detection compared with neuroradiologists of different experience levels, while also assessing its impact on reading time.
**Methods or Background:** This retrospective observational study included 59 adult patients with relapsing–remitting MS who underwent two brain MRI examinations with standardized 3D-FLAIR sequences at baseline and follow-up. Two neuroradiologists with >10 years and 3 years of experience independently reviewed anonymized scans for new or enlarging lesions, blinded to AI output and each other. The AI software (TensorMedical, Spain) automatically quantified new and slowly expanding lesions by co-registering longitudinal scans. Consensus between the two radiologists was considered the ground truth. Diagnostic accuracy, Cohen’s kappa agreement, and reading times with and without AI support were analyzed.
**Results or Findings:** The cohort included 40 females and 19 males (mean age=43.2 years). The average time interval between baseline and follow-up MRI was 14.2 months (range 2.3–51.1). The mean difference in lesion count compared to ground truth was $0.322 \pm 0.797$ for the senior reader (N.A), $0.373 \pm 1.410$ for the junior reader (A.D), and $-0.763 \pm 4.440$ for AI. Overall differences across groups were statistically significant (ANOVA, $p=0.0409$), but post-hoc comparisons didn't reach significance. Agreement with ground truth was almost perfect for the senior reader ($\kappa=0.898$), substantial for the junior reader ($\kappa=0.695$), and substantial for AI ($\kappa=0.797$). AI assistance reduced median reading time from 5.5 to 2.0 minutes
**Conclusion:** AI performance was comparable to that of an experienced neuroradiologist and superior to a junior reader. Integration of AI reduced reading time and enhance consistency, particularly for less experienced neuroradiologists.
**Limitations:** Retrospective, single-center, limited sample size.
**Funding for this study:** No funding.
**Has your study been approved by an ethics committee?** Not applicable
**Author Disclosures:**
Andrea Falini: Nothing to disclose
Nicoletta Emanuela Anzalone: Nothing to disclose
Edoardo Masiello: Nothing to disclose
Alberto Diena: Nothing to disclose
Giorgio Maria Agazzi: Nothing to disclose

**13:00 - 14:00** **Research Stage 4**

### Research Presentation Session: Abdominal and Gastrointestinal

### RPS 901

### Advances in small bowel imaging: from ischaemia to perforation

**Moderator**
Pierre-Alexandre Aloïs Poletti; Geneva/CH
*(pierre-alexandre.poletti@hug.ch)*

**Arterial occlusive acute mesenteric ischaemia: prevalence and prognosis of small bowel wall hypoenhancement on CT**
S. Thavarajah[1], H. Garnier[1], S. Vilain[1], M. Ronot[1], *L. Garzelli*[2]; [1]Clichy/FR, [2]Paris/FR
*(lorenzo.garzelli@aphp.fr)*

**Purpose:** To evaluate the prevalence, imaging features, and prognostic significance of bowel hypoenhancement on CT in arterial occlusive acute mesenteric ischaemia (AOAMI).
**Methods or Background:** Retrospective, observational, single-centre study of 220 patients referred to our intestinal stroke unit between 2006 and 2023. All patients with AOAMI and available portal-venous phase abdominal CT were reviewed. Clinical, biological, treatment, and outcome data were retrieved from a prospective database. The primary objective was prevalence and imaging description of bowel wall hypoenhancement; the secondary objective was its association with bowel resection-free survival and other prognostic factors.

**Results or Findings:** Among 220 patients (median age 68 years; 55% men), bowel hypoenhancement was present in 127 (57%), predominantly in the ileum (95%). It was was more frequent in embolic than atherosclerotic occlusion (61% vs 38%) and was associated with decreased venous outflow in 58%. Compared with patients without bowel hypoenhancement, these patients had higher lactate (2.8 vs 1.7 mmol/L, p<0.001), more frequent leukocytosis >15G/L (63% vs 40%, p=0.001), and a greater prevalence of portomesenteric gas (14% vs 1%, p<0.001). Resection was more frequent (72% vs 31%, p<0.001) with longer segments removed (154 vs 85 cm, p=0.007), and patients more often required home parenteral nutrition (17% vs 5%, p=0.018). Resection-free survival was significantly lower in patients with hypoenhancement (log-rank p<0.001).
**Conclusion:** Bowel hypoenhancement on CT is frequent in AOAMI and constitutes a marker of disease severity. Its early recognition may help identify high-risk patients, and anticipate adverse outcomes.
**Limitations:** Limitations include retrospective, single-centre design, possible selection bias, and reduced reproducibility as hypoenhancement was assessed by a single reader.
**Funding for this study:** None
**Has your study been approved by an ethics committee?** Not applicable
**Author Disclosures:**
Sarah Thavarajah: Nothing to disclose
Maxime Ronot: Nothing to disclose
Hippolyte Garnier: Nothing to disclose
Lorenzo Garzelli: Nothing to disclose
Sarah Vilain: Nothing to disclose

**Clinical value of the distribution and type of superior mesenteric lesions in acute occlusive arterial mesenteric ischaemia**
S. Vilain[1], H. Garnier[1], M. Dioguardi Burgio[1], J. Gregory[1], M. Ronot[1], *L. Garzelli*[2]; [1]Clichy/FR, [2]Paris/FR
*(lorenzo.garzelli@aphp.fr)*

**Purpose:** The prognostic impact of superior mesenteric artery (SMA) lesion type and distribution on management and outcomes in acute occlusive arterial mesenteric ischaemia (AOAMI) remains uncertain.
**Methods or Background:** Retrospective single-centre study including patients treated for AOAMI between 2016 and 2024 in our intestinal stroke centre unit. Patients with incomplete SMA occlusion were excluded. SMA lesions were classified according to Tual et al. as proximal (S1), median (S2), or distal (S3), and graded as stenosis, occlusion with downstream patency, or occlusion without downstream patency. Lesion burden was defined as the number of occluded segments. Outcomes included revascularisation strategy, gastrointestinal morbidity (short bowel syndrome [SBS], home parenteral nutrition [HPN]), and mortality.
**Results or Findings:** One hundred forty-two patients were included (mean age 67 years, 60% men); 68% had embolic and 30% atherothrombotic AOAMI. Median lactate was 2.5 mmol/L; Revascularisation was attempted in 86%, and 85 patients required bowel resection. Overall mortality was 39%. At follow-up, 18% were alive without gastrointestinal sequelae, 22% had SBS, and 14% required HPN. The dominant lesions were most frequently S2 (48%), followed by S1 (37%) and S3 (15%); mean length was 51 mm, with 36% >50 mm. Occlusion without downstream patency occurred in 47%, and 23% involved >2 territories without patency. Lesion distribution influenced endovascular strategy: stenting mainly for S1 with downstream patency, thrombolysis for S3, and thrombectomy for S2–S3. Lesion burden correlated with gastrointestinal morbidity but not mortality. Mortality was significantly associated with inferior mesenteric artery occlusion and splanchnic arterial calcifications (p=0.038 and <0.01).
**Conclusion:** SMA lesion type and distribution determine endovascular strategy in AOAMI, while lesion burden predicts gastrointestinal morbidity.
**Limitations:** Retrospective design and potential selection bias
**Funding for this study:** None
**Has your study been approved by an ethics committee?** Not applicable
**Author Disclosures:**
Jules Gregory: Nothing to disclose
Marco Dioguardi Burgio: Nothing to disclose
Maxime Ronot: Nothing to disclose
Hippolyte Garnier: Nothing to disclose
Lorenzo Garzelli: Nothing to disclose
Sarah Vilain: Nothing to disclose

**A Morbidity-Mortality Scale to Assess Outcome in Occlusive Arterial Acute Mesenteric Ischaemia**
H. Garnier[1], S. Thavarajah[1], S. Vilain[1], M. Ronot[1], *L. Garzelli*[2]; [1]Clichy/FR, [2]Paris/FR
*(lorenzo.garzelli@aphp.fr)*

**Purpose:** Outcomes of arterial occlusive acute mesenteric ischaemia (AOAMI) are usually reported as short-term survival, which underestimates long-term morbidity. We aimed to propose a morbidity-mortality scale to provide a more comprehensive outcome measure for clinical trials in patients with AOAMI.
**Methods or Background:** Retrospective, single-centre study including patients admitted for AOAMI (2016–2023). Clinical, biological, imaging, treatment and outcomes data were analysed. A four-level morbidity-mortality scale was developed according to post-AMI status: 0 = bowel preservation, 1 = bowel resection, 2 = intestinal failure and 3 = death. The primary outcome was the distribution of the scale at 1, 3, 6 or 12 months.
**Results or Findings:** A total of 256 patients were included (median age: 68; 44% female). Overall, 149 (58%) underwent bowel resection and 73 (29%) developed short bowel syndrome during follow-up. Thirty-five patients (14%) required long-term parenteral nutrition, including 16 (6%) with >4 infusions per week. At last follow-up, one-third of patients (33%) had a digestive stoma. Scale distribution was as follows: scale 0 (bowel preservation) stable at 32%, 30%, 30% 30% at 1, 3, 6 and 12 months ; scale 1 (resection) stable at 24%, 24%, 25% 24% at 1, 3, 6 and 12 months ; scale 2 (intestinal failure) decreased from 25% to 15% from 1 to 12 months ; scale 3 (death) increased from 19% to 31% from 1 to 12 months.
**Conclusion:** This new morbidity-mortality scale captures both survival and functional outcomes, providing a comprehensive measure of disease burden in AOAMI. It could serve as a novel endpoint in future therapeutic trials.
**Limitations:** Limitations include the retrospective, single-centre design, which may entail missing data, selection bias, and limited generalisability.
**Funding for this study:** None
**Has your study been approved by an ethics committee?** Not applicable
**Author Disclosures:**
Sarah Thavarajah: Nothing to disclose
Maxime Ronot: Nothing to disclose
Hippolyte Garnier: Nothing to disclose
Lorenzo Garzelli: Nothing to disclose
Sarah Vilain: Nothing to disclose

**Morphometric Analysis of Normal Small Bowel on CT: Variations by Age and Segment in More Than 500 patients**
J. André[1], T. Pesce[2], C. Iosif[2], M. Ronot[3], M. Zappa[1], *L. Garzelli*[3]; [1]Cayenne/GF, [2]Fort de France/FR, [3]Paris/FR
*(lorenzo.garzelli@aphp.fr)*

**Purpose:** Normal CT dimensions of the small bowel are not established, although calibre and wall thickness are routinely measured in ischaemic, inflammatory, and obstructive diseases. This study aimed to define normative values in adults without intestinal abnormalities.
**Methods or Background:** In this prospective bicentric study, adults from French Guiana and Martinique (french overseas territories) undergoing portal venous phase abdominal CT between May and October 2023 were screened. Patients with abdominal abnormalities or previous surgery were excluded. Calibre and wall thickness were measured at six arterial landmarks (proximal, mid, and distal jejunum and ileum). Associations with age, sex, and geographic origin were analysed. Inter- and intra-observer reproducibility was assessed in 10% of cases using intraclass correlation coefficients (ICCs).
**Results or Findings:** Among 2,336 screened patients, 502 were included (mean age 52 years; 56% women; French Guiana n=249; Martinique n=253). Mean jejunal calibre and thickness were 24.6 mm (± 2.0) and 2.6 mm (± 0.4), compared with 19.8 mm (± 3.0) and 1.3 (± 0.2) mm in the ileum. Jejunal values were significantly greater than ileal ones (p<0.01). Calibre increased with age, whereas thickness decreased (both p<0.01; r=0.70 and r=−0.64). Neither sex (calibre p=0.36; thickness p=0.63) nor geographic origin (calibre p=0.42; thickness p=0.54) significantly influenced measurements. Reproducibility was substantial, with ICCs of 0.78 (intra) and 0.73 (inter) for calibre, and 0.70 (intra) and 0.72 (inter) for wall thickness.
**Conclusion:** This large bicentric study provides reference CT values for small bowel calibre and wall thickness by segment, showing a progressive reduction from proximal jejunum to distal ileum with variations across age. These benchmarks may enhance standardisation for clinical diagnosis and research.
**Limitations:** Limitations are underrepresentation of non-American populations, absence of anthropometric data, and potential bias from trauma cases.
**Funding for this study:** None

**Has your study been approved by an ethics committee?** Not applicable
**Author Disclosures:**
Jimmy André: Nothing to disclose
Christina Iosif: Nothing to disclose
Thibaud Pesce: Nothing to disclose
Maxime Ronot: Nothing to disclose
Magaly Zappa: Nothing to disclose
Lorenzo Garzelli: Nothing to disclose

**Who needs MRI in suspected endometriosis? A retrospective observational diagnostic study of added value beyond TVUS**

*G. Shaden*, N. Lev-Cohain; Jerusalem/IL
*(gshaden95@gmail.com)*

**Purpose:** Magnetic resonance (MR) imaging is considered a second-line technique after transvaginal ultrasonography (TVUS) in suspected endometriosis. Updated MRI indications have recently been defined by the ESUR consensus. This study aimed to evaluate, in our institutional cohort, which patients benefit from MRI beyond TVUS, and to assess adherence to ESUR indications in routine practice.
**Methods or Background:** We retrospectively reviewed 103 women with suspected or confirmed endometriosis who underwent both TVUS and pelvic MRI between 2020 and 2025 at a tertiary endometriosis centre. All TVUS were performed by a single expert gynaecological sonographer, and all MRIs interpreted by the same senior radiologist specialising in endometriosis imaging. Findings were compared across compartments (anterior, middle, posterior, and extra-pelvic). Interpretations were categorised as: (1) normal TVUS with abnormal MRI; or (2) abnormal TVUS, with assessment of MRI's incremental diagnostic contribution.
**Results or Findings:** Among 23% of patients (24/103) with normal TVUS, MRI revealed deep endometriosis in 58% (14/24), confirming its added value in symptomatic women with negative ultrasound. When both modalities demonstrated endometriosis, MRI consistently contributed further diagnostic information. The clearest difference was in bowel assessment: MRI detected bowel involvement in 55% (28/51) of patients missed by TVUS, and in 29% (15/51) of those with TVUS-suspected bowel disease, MRI more accurately defined depth and extent of infiltration.
**Conclusion:** MRI is essential in symptomatic patients with negative TVUS, in line with ESUR consensus. In addition, when TVUS suggests deep endometriotic plaques—particularly in locations where ultrasound is less sensitive—MRI should be performed, as it provides superior delineation of disease extent and involvement.
**Limitations:** Retrospective single-centre design. Variable TVUS–MRI intervals (median 35 days; 25% >6 months) may have influenced discrepancies.
**Funding for this study:** None.
**Has your study been approved by an ethics committee?** Not applicable
**Author Disclosures:**
Ghrayeb Shaden: Nothing to disclose
Naama Lev-Cohain: Nothing to disclose

**A study on CT-based diagnosis of gastrointestinal perforation sites based on the anatomical relationships between the gastrointestinal tract and the peritoneum**

*X. Liu*, R. Chen; Zhuhai, Guangdong/CN
*(liuxiujuan2005@126.com)*

**Purpose:** The selection of surgical approach and incision for gastrointestinal perforation depends on factors such as the location and etiology of the perforation. Accurate preoperative localization and etiological assessment are critical for optimal treatment planning. Currently, computed tomography (CT) is the unique preoperative method for localization; however, its accuracy is limited and it's time-consuming. Existing studies focus on specific imaging signs and adopt the mechanical anatomical grouping based on the sequence of gastrointestinal tract, neglecting the hydrodynamic characteristics of the peritoneal compartments. This study intends to group based on gastrointestinal-peritoneal relationships to evaluate the distribution pattern of free gas and characteristic CT signs of perforation in each group, in order to improve the preoperative localization accuracy and shorten the diagnostic time.
**Methods or Background:** In a retrospective study, 250 patients with surgically confirmed gastrointestinal perforations were classified into three anatomical groups: intraperitoneal, interperitoneal, and extraperitoneal. CT studies were reviewed using tailored window settings and multiplanar reconstructions to assess the distribution of free gas and specific imaging signs. Comparative statistical analysis was performed.
**Results or Findings:** Free gas distribution demonstrated strong anatomical dependence: intraperitoneal perforations primarily involved the supracolic compartment; interperitoneal perforations localized to the mesenteric root space; and extraperitoneal perforations were characterized by retroperitoneal gas. The most prevalent ancillary signs were ascites, tiny intra-abdominal gas bubbles, and fat stranding.
**Conclusion:** Leveraging anatomical and radiopathological correlations, this study proposes a novel diagnostic pathway: "pneumoperitoneum distribution → anatomical grouping → targeted lesion identification." This systematic approach facilitates efficient and accurate perforation localization by utilizing gas distribution patterns to prioritize anatomical segments. A stepwise evaluation sequence—beginning with the retroperitoneum, followed by the mesenteric sinuses, and finally the supracolic space—is recommended, augmented by attention to key CT signs and optimized windowing.
**Limitations:** No
**Funding for this study:** The Clinical Research Promotion Project of Zhuhai People's Hospital(Grant No. 2023LCTS-03)
**Has your study been approved by an ethics committee?** Yes
**Ethics committee - additional information:** No
**Author Disclosures:**
Riling Chen: Author: Responsible for the completion of experiments, thesis writing and revision
Xiujuan Liu: Speaker: speaking Owner: writing

**Gastrografin in the management of adhesional small bowel obstruction: scanned too soon?**

*U. Dutta*, N. Chantima, I. Miglior, J. Chmelo, J. Brown, A. Phillips; Newcastle upon Tyne/UK
*(utkarsh.dutta2@nhs.net)*

**Purpose:** To audit gastrografin use and timing of follow-up abdominal X-rays (AXRs) in adhesive small bowel obstruction (ASBO) management at a tertiary-care centre.
**Methods or Background:** The 2017 Bologna guidelines recommend water-soluble contrast in the conservative management of ASBO. Consensus favours obtaining a follow-up AXR at least 6 hours post-gastrografin administration to allow adequate bowel transit time. We conducted a retrospective audit over a 6-month period of all acute surgical admissions with uncomplicated ASBO confirmed on computed tomography (CT). We examined gastrografin prescription, administration timing, and follow-up AXR intervals. Following educational interventions (departmental posters and teaching sessions), we re-audited practice between April-August 2025.
**Results or Findings:** Of 74 ASBO patients, 57 received gastrografin with follow-up AXRs. Nine patients (18%) were scanned <6 hours post-administration, with mean prescription-to-AXR time of 6.6 ± 2.0 hours and administration-to-AXR time of 4.9 ± 1.0 hours. Successful transit to colon was seen in approximately two-third of cases. Ten patients underwent multiple AXRs; eight received repeat gastrografin. Root-cause analysis revealed that in 48/57 cases (84%), follow-up AXRs were requested at prescription rather than administration, failing to account for the mean 1.7 ± 1.0 hour prescription-administration delay. Re-audit demonstrated significant improvements better adherence to current recommendations: only 3/45 patients (7%) scanned <6 hours, with mean AXR timing improved to 7.0 ± 1.5 hours.
**Conclusion:** Enhanced communication between clinicians, nurses, and radiographers optimizes AXR timing in ASBO management.
**Limitations:** The paucity of national guidelines and indeed a local protocol on the use of gastrografin is a key limitation towards sustained improvement.
**Funding for this study:** Not applicable.
**Has your study been approved by an ethics committee?** Not applicable
**Author Disclosures:**
Joshua Brown: Nothing to disclose
Utkarsh Dutta: Nothing to disclose
Jakub Chmelo: Nothing to disclose
Alexander Phillips: Nothing to disclose
Natawadee Chantima: Nothing to disclose
Isabel Miglior: Nothing to disclose

**13:00 - 14:00** **Research Stage 1**

### Research Presentation Session: Cardiac Hot Topic with Keynote Lecture

### RPS 903

### Hot Topic: low-field MRI

**Moderator**
Matthias Gutberlet; Leipzig/DE
*(matthias.gutberlet@helios-kliniken.de)*

**Keynote Lecture**
Tilman Stephan Emrich; Mainz/DE
*(tilman.emrich@gmail.com)*

### Association Between Myocardial Lipomatous Metaplasia and Sudden Cardiac Death in Patients with Prior Myocardial Infarction

*K. Aisikaier*; Beijing/CN

**Purpose:** This study aims to noninvasively assess the prevalence of LM using cardiac magnetic resonance (CMR) and explore its association with sudden cardiac death (SCD) in patients with prior myocardial infarction (PMI).
**Methods or Background:** In this single-center retrospective study, 896 patients with PMI were included. PMI was confirmed by CMR evidence of subendocardial or transmural late gadolinium enhancement (LGE) in a coronary artery-specific distribution. LM was confirmed using frequency-based sequences (chemical shift or fat-water separation sequence) or T1 contrast-based sequences (short T1 inversion recovery [STIR] or pre-contrast T1-weighted sequence). The primary endpoint was a composite of SCD, aborted SCD, or appropriate implantable cardioverter-defibrillator (ICD) therapy.
**Results or Findings:** LM was detected in 127 (14.2%) patients. These patients had larger infarcts (23.4% vs. 19.9%, $p < 0.001$) and higher SCD event rates (11.8% vs. 6.1%, $p = 0.035$). Over 64 months median follow-up, 62 (6.9%) reached the endpoint. LM independently predicted SCD (adjusted HR: 2.28; 95% CI: 1.27–4.11; $p = 0.006$) after adjusting for after adjusting for age, sex, LVEF≤35%, gray zone, LGE extent, and end-diastolic volume index (EDVi). LM improved SCD risk prediction beyond LVEF≤35% (C-statistic: 0.58 to 0.62; log-likelihood: -398 to -394, $p = 0.007$) , especially in patients with LVEF >35% or LGE≤24.4%(optimal cutoff 24.4%; AUC=0.751, 95% CI 0.697–0.806) .
**Conclusion:** In patients with PMI, the presence of LM was independently associated with SCD risk, demonstrating incremental prognostic value beyond conventional risk markers, including LVEF. Furthermore, LM maintained significant predictive value in patients with preserved LVEF (>35%), suggesting its particular utility for risk stratification in the current guideline-defined lower-risk population.
**Limitations:** As a large retrospective cohort study conducted at a tertiary referral center, this investigation carries an inherent risk of referral bias.
**Funding for this study:** This study is supported by the National Key R&D Program of China (Nos. 2021YFF0501400 and 2021YFF0501404) and Key Project of National Natural Science Foundation of China (No. 82430066).
**Has your study been approved by an ethics committee?** Not applicable
**Author Disclosures:**
Kaisaierjiang Aisikaier: Nothing to disclose

### Nonlinear Associations Between Left Ventricular End-Diastolic Diameter and Myocardial Strain Parameters in Metabolic Syndrome: A Cardiac Magnetic Resonance Study

*J-K. Li*, Y. Zhigang, R. Shi, W-R. Li; Chengdu/CN
*(leejke_33@163.com)*

**Purpose:** To investigate the nonlinear associations between left ventricular end-diastolic diameter (LVEDD) and left ventricular strain parameters derived from cardiac magnetic resonance (CMR) in patients with metabolic syndrome (Mets), and to determine the relative impact of metabolic factors including diabetes mellitus (DM), hypertension (HTN), and body mass index (BMI) on strain behavior.
**Methods or Background:** We retrospectively analyzed cine CMR data from August 2019 to November 2021 from patients with Mets. Global myocardial strain parameters—LV radial peak strain (LVPRS), circumferential peak strain (LVPCS), longitudinal peak strain (LVPLS), and early diastolic radial strain rate (LV0PDSR-L0)—were quantified. Nonlinear polynomial regression models were constructed to examine associations between LVEDD (short-axis view) and strain parameters, adjusting for sex, age, DM, HTN, and BMI.
**Results or Findings:** A total of 105 patients with Mets (51.64 ± 12.49 years, 70.5% male) were recruited. LVEDD exhibited a significant nonlinear association with all strain parameters, including LVPRS ($R^2 = 0.507$, $p<0.001$), U-shaped trend; LVPCS ($R^2 = 0.491$, $p<0.001$), inverted U-shaped; LVPLS ($R^2 = 0.315$, $p=0.017$), mildly inverted U-shaped; LV0PDSR-L0 ($R^2 = 0.204$, $p=0.058$), weak U-shaped trend. After adjusting for sex, age, DM, HTN, and BMI in Mets patients, LVEDD and its quadratic term were the only two significant predictors in LVPRS and LVPCS models($p<0.05$). Among the covariates, only sex showed a significant independent association with LVPLS($p=0.033$).
**Conclusion:** In patients with Mets, LVEDD is nonlinearly associated with myocardial strain parameters, reflecting complex remodeling dynamics. CMR-derived LV strain analysis may serve as a sensitive marker for subclinical myocardial dysfunction beyond traditional metabolic risk factors and early treatments could be given.
**Limitations:** Firstly, this is a retrospective single-center study with a limited sample size. Secondly, the causality and temporal sequence relationship between LVEDD and strain parameters cannot to be comfirmed.
**Funding for this study:** None.
**Has your study been approved by an ethics committee?** Yes
**Ethics committee - additional information:** This study was approved by the Ethics Committee of West China Hospital of Sichuan University. Written informed consent was obtained. This study complied with the Declaration of Helsinki.
**Author Disclosures:**
Rui Shi: Nothing to disclose
Jia-Ke Li: Nothing to disclose
Wen-Rong Li: Nothing to disclose
Yang Zhigang: Nothing to disclose

### Application of Four-dimensional flow cardiac magnetic resonance in risk stratification of connective tissue disease-associated pulmonary arterial hypertension

*N. Xu*, Y. Xu; Jiangsu Province/CN
*(xun729781983@163.com)*

**Purpose:** To explore the value of four-dimensional flow cardiovascular magnetic resonance (4D Flow CMR) in connective tissue disease-associated pulmonary arterial hypertension (CTD-PAH) risk stratification by comparing its diagnostic efficacy and parameter consistency with two-dimensional phase-contrast magnetic resonance imaging (2D PC-MRI).
**Methods or Background:** Eighty-five CTD-PAH patients were retrospectively enrolled and categorized into two groups per 2022 ESC/ERS guidelines: low/intermediate-low risk (Group 1, n=67) and intermediate-high/high risk groups (Group 2, n=18). All underwent 2D PC-MRI and 4D Flow CMR. Right ventricular (RV) function parameters and pulmonary artery [main (MPA), left (LPA), right (RPA)] hemodynamic parameters were extracted. Inter-group comparisons, logistic regression, ROC analysis (DeLong test), and consistency (intraclass correlation coefficient, ICC) analyses were performed.
**Results or Findings:** Compared with Group1, Group2 showed increased RV end-systolic volume index (RVESVI), RV end-diastolic volume index (RVEDVI), RV myocardial mass index (RVMMI) and decreased RV ejection fraction (RVEF) (all $P<0.05$). 2D PC-MRI revealed lower relative area change (RAC), peak velocity (PV) and mean velocity (MV) of MPA (all $P<0.01$). 4D Flow CMR revealed prolonged MPA vortex duration (24% vs 12%, $P<0.01$), and lower PV/MV in MPA/LPA/RPA (all $P<0.05$). Multivariate Logistic analysis showed MPA PV and vortex duration from 4D Flow CMR and MPA PV from 2D PC-MRI were independent factors for distinguishing medium-high/high-risk patients. The consistency of MPA PV between the two techniques was good (ICC=0.765), while that of MPA MV was moderate (ICC=0.562).
**Conclusion:** 4D-derived MPA PV and vortex duration are independent predictors for CTD-PAH risk stratification, the combined model based on 4D parameters has superior diagnostic efficacy over 2D PC-MRI, improving CTD-PAH risk stratification accuracy.
**Limitations:** Not applicable
**Funding for this study:** Not applicable
**Has your study been approved by an ethics committee?** Not applicable
**Author Disclosures:**
Yi Xu: Nothing to disclose
Nan Xu: Nothing to disclose

**Evaluation of the effect of contrast agent use on cardiac functions in cine MRI**

M. F. Kazanbaş, B. Tütüncüoğlu, T. Banaz, M. Kadıoğlu, F. Köse, *T. Y. Kılıç*, N. Okumuş, A. Arslan, S. Ozkök; Istanbul/TR
*(taha.yigit.kilic@gmail.com)*

**Purpose:** This study aimed to evaluate the effect of contrast administration on volumetric and functional parameters of the left ventricle(LV) measured by cardiac magnetic resonance cine imaging, with particular emphasis on paired comparison and Bland–Altman agreement analysis.
**Methods or Background:** Cardiac cine MRI is a reference method for quantifying LV volumes and ejection fraction (EF). However, the influence of contrast administration on volumetric assessment remains debated. In this retrospective analysis, 42 patients undergoing both pre- and post-contrast cine underwent 1.5 Tesla CMR were included. LV end-diastolic volume index(EDVI), end-systolic volume index(ESVI), stroke volume index (SVI), ejection fraction(EF), and heart rate(BPM) were measured. ECG/Holter were used to assessed arrhythmia. Results were expressed as median(25th–75th percentile) and compared using paired analysis with Bland–Altman evaluation. Significance was set at $p<0.05$.
**Results or Findings:** Pre-contrast cine measurements demonstrated a median LV-EDVI of 72.5 mL/m2, LV-ESVI 27.5 mL/m2, LV-SVI 41.0 mL/m2, LV-EF 60.5%, and heart rate 86.5 bpm(68.0–101.8). Post-contrast, LV-EDVI was 69.0 mL/m2, LV-ESVI 36.5 mL/m2, LV-SVI 33.5 mL/m2, LV-EF 52.5%, and heart rate 87.5 bpm(76.8–111.3). Compared with pre-contrast, post-contrast cine demonstrated a significant reduction in LV-EF($\Delta$–10.4 ± 9.6%, $p<0.01$) and LV-SVI ($\Delta$–9.0 ± 12.3 mL/m2, p=0.02), while changes in LV-EDVI and LV-ESVI were not significant. Heart rate significantly increased ($\Delta$ +12.8±21.3, p=0.04). Bland–Altman analysis confirmed systematically lower post-contrast values, with a mean bias of –10.4%(LoA –35.2 to +14.5) for LV-EF and –9.0 mL/m2(LoA –56.9 to +38.9) for LV-SVI, indicating wide inter-individual variability.
**Conclusion:** Contrast administration may lead to systematic underestimation of EF and SVI on cine MRI, whereas EDVI and ESVI remain stable. Awareness of this effect is important for consistent longitudinal follow-up and clinical decision-making.
**Limitations:** The small sample size of only 42 patients restricts the generalizability of the findings.
**Funding for this study:** No funding was received for this study.
**Has your study been approved by an ethics committee?** Yes
**Ethics committee - additional information:** An application was made to the ethics committee of Başakşehir Çam and Sakura City Hospital.
**Author Disclosures:**
Serçin Ozkök: Nothing to disclose
Berk Tütüncüoğlu: Nothing to disclose
Taha Yiğid Kılıç: Nothing to disclose
Fadime Köse: Nothing to disclose
Ahmet Arslan: Nothing to disclose
Muhammed Faruk Kazanbaş: Nothing to disclose
Nurgül Okumuş: Nothing to disclose
Tuba Banaz: Nothing to disclose
Mehmet Kadıoğlu: Nothing to disclose

**Cardiac Magnetic Resonance Assessment - Functional and Tissue Characteristics in Antiphospholipid Syndrome**

*K. Xu*, W. Liu, Y. Xu; Nan Jing/CN
*(xuke090919@163.com)*

**Purpose:** To evaluate the value of multiparametric cardiac magnetic resonance (CMR) in assessing myocardial involvement in patients with antiphospholipid syndrome (APS).
**Methods or Background:** This retrospective study enrolled 30 APS patients and 30 healthy controls who underwent CMR between January 2022 and June 2025. CMR assessment encompassed biventricular structural and functional parameters, and myocardial tissue characterization parameters. Subgroup analyses were performed based on secondary APS (SAPS) or primary APS (PAPS), and positive or negative late gadolinium enhancement (LGE). Spearman correlation analysis was used to explore the correlation between CMR parameters and laboratory indicators.
**Results or Findings:** Compared with the control group,APS group showed reduced biventricular ejection fractions and increased left ventricular end systolic volume index (LVESVI), native T1, T2 values and extracellular volume (ECV) (all $P < 0.01$). Subgroup analysis revealed that right ventricular end diastolic volume index (RVEDVI), stroke volume index (SVI), cardiac index (CI), native T1, and ECV (32.55 ± 4.45% vs. 27.94 ± 1.86%, P = 0.020) in SAPS group were significantly higher than those in PAPS group (all $P < 0.05$). In addition, LGE in SAPS group was more frequently localized in the left ventricular anterior wall, inferior wall, and interventricular septum, and subendocardial involvement was more common. The LGE-positive group exhibited lower left ventricular ejection fraction (LVEF) and higher left ventricular end systolic volume index (LVESVI) and mass index (MI) (all $P < 0.05$). LGE burden positively correlated with logNT-proBNP (r = 0.493, p = 0.006).
**Conclusion:** CMR reveals biventricular dysfunction and myocardial fibrosis in APS. SAPS patients demonstrated more severe involvement, characterized by elevated LGE burden and distinct subendocardial patterns. The correlation between LGE and NT-proBNP indicates that CMR has important value in risk stratification and prognostic assessment in APS.
**Limitations:** Not applicable
**Funding for this study:** Not applicable
**Has your study been approved by an ethics committee?** Not applicable
**Author Disclosures:**
Wangyan Liu: Nothing to disclose
Ke Xu: Nothing to disclose
Yi Xu: Nothing to disclose

**An AI-Based Hybrid CNN–SVM and U-Net Platform for Automated Classification, Scar Quantification, and 3D Visualization in Cardiac LGE MRI**

*C. Y. Chen*, F. Y. Yang, S-J. Chen, W-J. Lee, Y-S. Huang, Y-F. Tsai, P-Y. Chiang, Y. W. Guo, S. Y. Huang; Taipei/TW
*(hersheychen39@gmail.com)*

**Purpose:** To develop and validate an AI platform integrating Hybrid CNN–SVM classification, U-Net segmentation, and 3D scar visualization for automated myocardial scar analysis in LGE cardiac MRI, aiming to reduce manual annotation workload and improve diagnostic consistency.
**Methods or Background:** Ninety LGE MRI datasets were analyzed. A convolutional neural network (CNN) with four convolutional layers was trained for feature extraction, followed by a support vector machine (SVM) classifier with five-fold cross-validation to distinguish ischemic from non-ischemic cardiomyopathy. Scar segmentation was performed using a U-Net and compared with manual annotations (2SD, 3SD, 5SD, FWHM). Performance metrics included accuracy, AUC, Dice coefficient, and concordance with manual references. Grad-CAM was applied for interpretability.
**Results or Findings:** The Hybrid CNN–SVM achieved an accuracy of 0.851 and an AUC of 0.920. U-Net segmentation demonstrated strong concordance with manual contours (Dice = 0.801 ± 0.021) while substantially reducing annotation workload. Automated analysis markedly shortened labeling time compared with manual delineation and improved reproducibility across observers. In addition, the platform generated 3D scar visualizations, providing intuitive assessment of scar burden and transmural extent.
**Conclusion:** This AI-based platform demonstrates robust performance for automated classification and scar segmentation in LGE MRI. By improving workflow efficiency, reducing inter-observer variability, and integrating 3D visualization, it offers strong potential for adoption into routine radiology practice for standardized CMR interpretation.
**Limitations:** While the platform demonstrated robust performance, limitations include the relatively small, single-center dataset and occasional segmentation errors in blood pool regions. Future studies with larger, multi-center cohorts are warranted.
**Funding for this study:** This research was funded by National Science and Technology Council of Taiwan (no. MOST 114-2314-B-031-001)
**Has your study been approved by an ethics committee?** Yes
**Ethics committee - additional information:** The study was approved by the Research Ethics Committee of the National Taiwan University Hospital (Reference number: 202509157RINE).
**Author Disclosures:**
Feng Yi Yang: Nothing to disclose
Wen-Jeng Lee: Nothing to disclose
Yu-Sen Huang: Nothing to disclose
Yi-Fei Tsai: Nothing to disclose
Sheng Yao Huang: Nothing to disclose
Yu Wei Guo: Nothing to disclose
Pin-Yi Chiang: Nothing to disclose
Shyh-Jye Chen: Nothing to disclose
Chin-Yu Chen: Nothing to disclose

**13:00 - 14:00 Research Stage 2**

### Research Presentation Session: Artificial Intelligence and Imaging Informatics

### RPS 905

### Typing your question instead of googling it: how chatbots are changing radiology practice

**Moderator**
Nina Kottler; Carlsbad/US
*(nina.kottler@radpartners.com)*
**Author Disclosures:**
Nina Kottler: Employee: Radiology Partners and Mosaic Clinical Technologies, Owner: I own some of the practices at Radiology Partners, Share Holder: I am a partner and hence share holder at Radiology Partners; Radiology Partners holds equity with aidoc and Rad AI and owns Cognita.ai.

**Promoting Sustainable Breast Imaging and Interventional Practices with an AI-Based Chatbot**
*G. Della Pepa*, G. Irmici, C. De Berardinis, E. D'Ascoli, L. Corradini, G. Rossini, C. Depretto, G. P. Scaperrotta; Milan/IT

**Purpose:** To develop and evaluate an educational chatbot powered by a low-footprint Large Language Model (LLM), aimed at increasing awareness and knowledge of sustainable clinical practices in breast imaging among radiology professionals.
**Methods or Background:** GreenBreastBot was developed as a Custom GPT using GPT-3.5, a pre-trained LLM, ensuring negligible energy consumption per interaction. The chatbot was populated with bilingual (Italian/English) structured content derived from the ESR Green Radiology position paper, WHO climate-health recommendations, and internal Breast Unit guidelines. The tool adapts its explanations based on user expertise and delivers microlearning units, interactive quizzes, flashcards, and clinical scenarios. A four-week pilot was conducted in a tertiary Breast Unit. Participants included radiologists, residents, and radiographers. Outcomes included usage frequency, satisfaction (5-point Likert scale), and self-reported awareness before and after interaction.
**Results or Findings:** Twenty-nine professionals participated: 12 consultants, 11 residents, and 6 radiographers. Users completed an average of 3.6 chatbot sessions per week. Overall satisfaction was high (mean 4.5/5); 91% found the chatbot useful or very useful. Post-intervention, 67% of participants reported improved awareness of sustainable imaging practices, with greatest gains in understanding paperless consent workflows, appropriateness in follow-up imaging, and environmentally conscious interventional preparation. No significant technical barriers were reported.
**Conclusion:** GreenBreastBot demonstrates that a pre-trained, low-energy LLM chatbot can effectively deliver eco-education in breast imaging. Its integration of institutional and international guidelines enables scalable, impactful, and environmentally coherent training for radiology teams.
**Limitations:** The main limitations are the monocentric design and the absence of objective performance measures.
**Funding for this study:** None
**Has your study been approved by an ethics committee?** Not applicable
**Author Disclosures:**
Gianmarco Della Pepa: Nothing to disclose
Elisa D'Ascoli: Nothing to disclose
Gabriele Rossini: Nothing to disclose
Gianfranco Paride Scaperrotta: Nothing to disclose
Catherine Depretto: Nothing to disclose
Lisa Corradini: Nothing to disclose
Claudia De Berardinis: Nothing to disclose
Giovanni Irmici: Nothing to disclose

**Chatgpt for structured reporting in ct brain: resident experience**
*K. S. Chhabra*[1], D. B. Dahiphale[2], S. S. Sarda[2]; [1]Jalandhar/IN, [2]Aurangabad/IN
*(dr.karanschhabra@gmail.com)*

**Purpose:** To assess the feasibility and utility of ChatGPT in generating structured CT brain reports, evaluate its impact on reporting time and accuracy, and document resident perspectives in a tertiary care setting.
**Methods or Background:** This pilot study included 20 radiology residents who reported 200 CT brain examinations between January and April 2025. Each case was reported twice: once using conventional free-text reporting and once with ChatGPT-assisted structured reporting. Residents used a standardised prompt library covering common CT brain findings such as haemorrhage, infarct, mass effect, hydrocephalus, and extra-axial collections. Metrics assessed included reporting time, completeness (based on a 10-point checklist), inter-observer consistency, and resident satisfaction. Accuracy was validated against consultant-reviewed reference reports.
**Results or Findings:** ChatGPT-assisted structured reporting reduced mean reporting time from 14.2 minutes to 9.1 minutes (36% reduction). Completeness scores improved significantly (mean 9.3/10 vs 7.8/10, $p<0.01$), with better coverage of critical elements such as haemorrhage location, mass effect, and ventricular status. Inter-observer agreement improved, particularly for standardised terminology. Accuracy compared with consultant reports was maintained, with no significant increase in errors. Resident feedback highlighted improved clarity and confidence, though some noted occasional generic or redundant phrasing requiring manual refinement.
**Conclusion:** ChatGPT shows promise as a practical tool for structured reporting in CT brain studies, enhancing efficiency, completeness, and inter-observer consistency without compromising accuracy. While human oversight remains essential, integration of AI-driven structured templates can support radiology training and streamline reporting in high-volume settings.
**Limitations:** Single-centre pilot design.
**Funding for this study:** No funding was received for this study.
**Has your study been approved by an ethics committee?** Not applicable
**Author Disclosures:**
Devidas Baburao Dahiphale: Nothing to disclose
Sanket Sanjay Sarda: Nothing to disclose
Karanvir Singh Chhabra: Nothing to disclose

**Achieving Truly Informed Consent? A Prospective Controlled Trial Using Retrieval-Augmented Generation Before CT Examinations**
*F. Busch*, T. Lemke, S. Ziegelmayer, M. Graf, A. W. Marka, P. Prucker, M. R. Makowski, K. K. Bressem, L. C. Adams; Munich/DE
*(felix.busch@tum.de)*

**Purpose:** This prospective comparative study aimed to investigate the feasibility, usability, and effectiveness of a Retrieval-Augmented Generation (RAG)-powered Patient Information Assistant (PIA) chatbot for pre-CT information counseling, compared to standard physician-led consultation and informed consent procedures.
**Methods or Background:** Eighty-six patients scheduled for CT imaging (November-December 2024) were randomly assigned to either the PIA group (n=43), receiving pre-CT information via a RAG-powered chatbot, or the control group (n=43), receiving standard doctor-led consultation. Patient satisfaction, information clarity, comprehension, and concerns were assessed using six ten-point Likert-scale questions. Consultation duration was recorded, and patients in the PIA group indicated their preferred mode of future counseling. Two radiologists independently evaluated each PIA session based on five criteria: overall quality, scientific and clinical evidence, clinical usefulness and relevance, consistency, and up-to-dateness.
**Results or Findings:** Both groups reported similarly high ratings for information clarity (PIA: $8.64 \pm 1.69$; control: $8.86 \pm 1.28$; $p=0.82$) and overall comprehension (PIA: $8.81 \pm 1.40$; control: $8.93 \pm 1.61$; $p=0.35$). Physician-led consultations more effectively alleviated patient concerns ($8.30 \pm 2.63$ vs. $6.46 \pm 3.29$; $p=0.003$). Patients in the PIA group required significantly shorter subsequent consultation times (median: 120 s [IQR: 100-140] vs. 195 s [IQR: 170-220]; $p=0.04$). Radiologists rated PIA chats favorably across all evaluated categories.
**Conclusion:** A RAG-powered PIA chatbot can effectively deliver pre-CT information while reducing physician consultation time. Although patient satisfaction and comprehension were comparable to standard consultations, physician-led interactions remained superior in addressing patient concerns. These findings highlight the potential of AI-based chatbot solutions to streamline patient counseling for imaging procedures. At the same time, physician engagement remains crucial for addressing patient worries, suggesting a complementary role for both approaches in clinical practice.
**Limitations:** Single-center, sample size, self-reported outcomes.
**Funding for this study:** None.
**Has your study been approved by an ethics committee?** Yes
**Ethics committee - additional information:** Technical University of Munich (2024–469-S-KK)

**Author Disclosures:**
Tristan Lemke: Nothing to disclose
Philipp Prucker: Nothing to disclose
Keno K. Bressem: Nothing to disclose
Markus Graf: Nothing to disclose
Marcus R. Makowski: Nothing to disclose
Lisa C. Adams: Nothing to disclose
Alexander Wolfgang Marka: Nothing to disclose
Sebastian Ziegelmayer: Nothing to disclose
Felix Busch: Nothing to disclose

**AI-Supported MR Safety Assessment of Implanted Devices: First Clinical Evaluation**
*H. Kreutzer*, D. Rashid, D. Truhn, S. Nebelung; Aachen/DE
*(hannakreutzer22@gmail.com)*

**Purpose:** MR safety checks for patients with implanted devices are time-consuming and error-prone. Clinicians must identify the exact device model, retrieve the manufacturer's handbook, and extract applicable scanning conditions. We developed an AI-agent that streamlines device-specific MR eligibility assessment using manufacturer documentation and scientific literature.
**Methods or Background:** The agent is built in LangGraph with a router node classifying user queries. Device-specific queries are directed to a retrieval-augmented generation (RAG) pipeline that utilizes manufacturer handbooks. General MR safety queries are handled by a separate RAG pipeline that utilizes peer-reviewed scientific literature. A central GPT-4.1 node composes the final output. A web-based interface (chatbot-like) allows free-text queries or image uploads of implant ID-cards, which are analysed with GPT-4.1 Vision. The interface displays both the reasoning steps and the retrieved handbook/literature pages for transparency. Evaluation was performed using consecutive patients with cardiac devices from our hospital. An MR-physicist documented the final safety decision (scan eligibility and protocol parameters), which served as the reference standard.
**Results or Findings:** The agent's recommendation was correct in 15/19 cases. In the remaining four cases, the system flagged missing documentation, thereby avoiding unsupported recommendations. Importantly, no incorrect recommendations were made. Correct guideline source pages were displayed in 13 of the 15 correct cases.
**Conclusion:** An AI-agent grounded in manufacturer guidance can reliably answer MR safety questions. Early testing demonstrates promising accuracy and interpretability, with transparent display of reasoning and sources. If scaled beyond cardiac devices and expanded into comprehensive device databases, such agents have the potential to fundamentally transform MR safety practice by accelerating workflows, reducing errors, and setting new standards for patient safety in radiology.
**Limitations:** Some device manuals were unavailable. Evaluation was restricted to cardiac devices. Use of GPT-4.1 requires anonymised data.
**Funding for this study:** This research is supported by the Deutsche Forschungsgemeinschaft - DFG (701010997, 517243167, 515639690) , the German Federal Ministry of Research, Technology and Space (Transform Liver - 031L0312C, DECIPHER-M, 01KD2420B) and the European Union Research and Innovation Programme (ODELIA - GA 101057091, SAGMA – GA 101222556).
**Has your study been approved by an ethics committee?** Not applicable
**Author Disclosures:**
Daniel Rashid: Nothing to disclose
Daniel Truhn: Nothing to disclose
Hanna Kreutzer: Nothing to disclose
Sven Nebelung: Nothing to disclose

**Energy Usage of Large Language Models and Segmentation Models in Radiology**
*M. Segeroth*, S. Yang, J. Wasserthal, J. Cyriac, T. Heye, E. M. Merkle, M. Bach, J. Vosshenrich; Basel/CH
*(martin.segeroth@gmail.com)*

**Purpose:** Neural networks, in particular large language models (LLMs), are increasingly valuable tools that support human tasks rather than simply automating them. However, their use requires substantial amounts of energy. In clinical practice, justified privacy concerns favor the evaluation of open-source models, which allows for assessing their energy consumption.
**Methods or Background:** Within our institutional healthcare network, we deployed privateGPT and Ollama as the primary platforms for LLM utilization, and Nora for image analysis. The models were hosted on a server equipped with eight NVIDIA A100 GPUs (80 GB each). For LLM experiments, we tested Llama3-70B, and for medical image segmentation, we used TotalSegmentator. Task scheduling was managed with Slurm 23.11.4, while energy consumption was monitored using nvidia-smi 550.163.01 and turbostat 2023.11.07. Additional overall server-level measurements were performed.
**Results or Findings:** The server's eight GPUs allow a maximum power of 400 W each, yet during our tests total peak power consumption reached 4235 W, with more than 1000 W attributable to non-GPU components. Idle consumption was 63 W per GPU and 1150 W for the full server. A single LLM request consumed 5.94 Wh (95% CI: 5.87–5.98 Wh), with GPU utilization at 86.39% (CI: 86.39–86.39%). TotalSegmentator training for MRI segmentations required 8389.14 Wh (CI: 8193.84–8730.37 Wh), with GPU utilization at 78.93% (CI: 78.93–78.93%). Inference with TotalSegmentator consumed 0.96 Wh (CI: 0.96–0.97 Wh) per case for tissue types, and complete MRI segmentation required 1.47 Wh (CI: 1.47–1.48 Wh).
**Conclusion:** Neural networks in clinical deployment consume a noticeable amount of energy, with individual tasks requiring 1–6 Wh, several times more than a typical Google search (~0.2 Wh). Nonetheless, their ability to augment clinical performance and support decision-making can justify the additional energy expenditure.
**Limitations:** Additional models and hardware are under evaluation.
**Funding for this study:** None
**Has your study been approved by an ethics committee?** Not applicable
**Author Disclosures:**
Tobias Heye: Nothing to disclose
Michael Bach: Nothing to disclose
Jacob Wasserthal: Nothing to disclose
Joshy Cyriac: Nothing to disclose
Martin Segeroth: Nothing to disclose
Elmar M. Merkle: Nothing to disclose
Jan Vosshenrich: Nothing to disclose
Shan Yang: Nothing to disclose

**13:00 - 14:00** **Research Stage 6**

### Research Presentation Session: Hybrid, Molecular and Translational Imaging Hot Topic with Keynote Lecture

### RPS 906

### Hot Topic: novel radiopharmaceuticals for imaging targets and therapy

**Moderator**
Clemens Mingels; Sacramento/US
*(clemens.mingels@insel.ch)*

**Keynote Lecture**
Sikandar Mohd Shaikh; Hyderabad/IN
*(idrsikandar@gmail.com)*

**Non-Invasive Monitoring of BRAF-MEK Inhibitor Therapy in a Human Melanoma Mouse Model Using [18F]-ML-10 PET/CT**
*J. Stock*, A. C. Klose, F. Herr, M. J. Antons, R. Werner, J. Ricke, M. Heimer, P. M. Kazmierczak, C. C. Cyran; Munich/DE

**Purpose:** To evaluate whether the PET tracer [18F]-ML-10 enables early, non-invasive in vivo assessment of therapy-induced apoptotic changes in BRAF-mutant malignant melanoma under combined BRAF/MEK inhibitor treatment.
**Methods or Background:** A human melanoma xenograft model (A375) in 15 immunodeficient mice was used to evaluate [18F]-ML-10 PET/CT for monitoring BRAF-MEK inhibitor therapy (treatment: n = 7; control: n = 8). PET/CT scans were performed at baseline and day 7. Quantitative analysis assessed target-to-background ratios (TBR-BP, TBR-M). Histological validation included cleaved caspase-3 immunohistochemistry, TUNEL assay, and CD31 staining for microvessel density.
**Results or Findings:** [18F]-ML-10 PET/CT demonstrated a marked increase in [18F]-ML-10 uptake in the treatment group between baseline and follow-up but no significant change in the control group. Quantitative PET analysis revealed significantly higher TBR-BP (23.48 ± 20.61 vs. 5.81 ± 3.63; $p = 0.015$), and TBR-M values (42.04 ± 42.25 vs. 11.36 ± 9.54; $p = 0.031$) in the treatment group at follow-up. Histologically, no significant differences in apoptotic cell counts were observed between groups with cleaved caspase-3 (239.4 ± 102.7 vs. 270.4 ± 101.4 cells/HPF; $p = 0.33$) or TUNEL-staining (83.3

Thursday

± 34.0 vs. 64.5 ± 21.9; p = 0.29), however mean values showed a similar trend to PET results. CD31-immunohistochemistry demonstrated a significantly lower microvessel density in the treatment group (30.7 ± 10.2 vs. 43.6 ± 14.7; p = 0.041), consistent with the anti-angiogenic effects of BRAF-MEK inhibition.
**Conclusion:** [18F]-ML-10 PET/CT may enable non-invasive detection of therapy-associated changes suggestive of increased apoptosis in melanoma xenografts under BRAF-MEK inhibitor therapy. The findings support the potential of [18F]-ML-10 PET/CT for early therapy monitoring in targeted cancer treatment.
**Limitations:** Small sample size and the restriction to a single imaging time point may have limited the detection of transient apoptotic effects.
**Funding for this study:** Not applicable.
**Has your study been approved by an ethics committee?** Yes
**Ethics committee - additional information:** All animal experiments were performed in accordance with the guidelines for the use of living animals in scientific studies and the animal study was officially approved.
**Author Disclosures:**
Philipp M Kazmierczak: Nothing to disclose
Rudolf Werner: Nothing to disclose
Clemens C. Cyran: Nothing to disclose
Maurice Heimer: Nothing to disclose
Felix Herr: Nothing to disclose
Jonathan Stock: Nothing to disclose
Ann Christin Klose: Nothing to disclose
Melissa J. Antons: Nothing to disclose
Jens Ricke: Nothing to disclose

**Imaging the Breakdown of Tumor-Promoting Desmoplasia via Adoptive Transfer of Fibroblast Activation Protein-Directed CAR T Cells**
*Y. Sun*, Q. Sang, L. Yize, Z. Xiao; Nanjing/CN

**Purpose:** The therapeutic potential of chimeric antigen receptor (CAR) T-cell therapy in solid malignancies is frequently limited by inadequate infiltration, suboptimal localization, and functional exhaustion of T cells within the tumor microenvironment (TME). Previous studies have shown that targeting tumor stroma with fibroblast activation protein (FAP)-specific CAR T cells can promote T-cell penetration and suppress tumor growth in pancreatic ductal adenocarcinoma (PDAC). However, the temporal dynamics of FAP-CAR T-cell migration and their interactions with stromal and extracellular matrix (ECM) components remain poorly defined. The objective of this study was to characterize these processes to guide optimization of CAR T-cell performance in solid tumors.
**Methods or Background:** Multiplex immunostaining combined with intravital two-photon microscopy of PDAC tumor explants was used to assess FAP-targeted CAR T-cell behavior at defined time points. Analyses focused on localization, activation status, and migration patterns in relation to stromal and ECM structures.
**Results or Findings:** Within 1–3 days post-transfer, FAP-CAR T cells accumulated predominantly in stromal regions encasing tumor nests but were largely excluded from the nests themselves. In these stromal zones, CAR T cells displayed activation and migrated through areas of loosely organized collagen, whereas movement was severely hindered in dense ECM. At tumor margins, aligned collagen fibers guided T-cell migration but restricted entry into tumor nests. By day 5, depletion of immunosuppressive stromal cells and disruption of peritumoral fibrillar collagen were observed, enabling penetration of CAR T cells into tumor nests. Co-treatment with hyaluronidase further enhanced infiltration.
**Conclusion:** These findings indicate that stromal remodeling, achieved by depletion of FAP⁺ stromal cells and degradation of ECM, facilitates CAR T-cell accumulation and functional engagement within the desmoplastic architecture of PDAC.
**Limitations:** No limitations
**Funding for this study:** This work was supported by the National Natural Science Foundation of China （24HAA00816，82572294，32500802 and 3290002501C2 from Z.X.）
**Has your study been approved by an ethics committee?** Yes
**Ethics committee - additional information:** All animal experiments were approved by the Institutional Animal Care and Use Committee (IACUC) of the Southeast University.
**Author Disclosures:**
Qinghua Sang: Nothing to disclose
Li Yize: Nothing to disclose
Zebin Xiao: Nothing to disclose
Yumeng Sun: Nothing to disclose

**The Role of [$^{68}$Ga]Ga-FAPi -2286PET/CT in the Evaluation of Treatment Response in Gastrointestinal System Malignancies**
N. Alan Selcuk, *G. Beydagi*, K. Akcay, A. Görmez, T. Toklu, L. Kabasakal; Istanbul/TR
*(gamzebeydagi@gmail.com)*

**Purpose:** This study aimed to investigate the potential efficacy of [$^{68}$Ga]Ga-FAPi PET/CT in evaluating treatment response in patients with gastrointestinal system malignancies.
**Methods or Background:** Patients with gastrointestinal malignancies who underwent [$^{68}$Ga]Ga-FAPi PET/CT for treatment response assessment were included. The maximum standardized uptake values (SUVmax), total tumor volumes, and total lesion FAP expression values, tumor marker levels were recorded. Imaging responses were assessed separately according to PERCIST criteria on [$^{68}$Ga]Ga-FAPi PET/CT and RECIST criteria on CT. Survival outcomes were analyzed using Kaplan–Meier method, and their association with treatment response was assessed using Log-Rank (Mantel–Cox) test.
**Results or Findings:** A total of 100 [$^{68}$Ga]Ga-FAPi PET/CT scans were performed in 50 patients, both before and after treatment. Among the included patients, 70% had gastric cancer, 14% had colon cancer, 8% had pancreatic cancer, 4% had appendiceal cancer, 2% had hepatocellular carcinoma, and 2% had cholangiocellular carcinoma. A strong correlation was found between tumor volumes and total lesion FAP expression and the corresponding serum tumor markers (AUC = 0.875). There was a statistically significant and near-perfect concordance between [$^{68}$Ga]Ga-FAPi PET PERCIST and CT RECIST criteria (Kappa = 0.833, p < 0.05). Moderate agreement was found for primary tumors (Kappa = 0.526) and bone metastases (Kappa = 0.657), while excellent agreement was observed for lymph node (Kappa = 1.0), peritoneal (Kappa = 0.815), and visceral metastases (Kappa = 1.0). Treatment response assessed by [$^{68}$Ga]Ga-FAPi PET/CT was predictive of overall survival (p=0.02). Median overall survival was 7.6 months in patients with progressive disease, 19.8 months in those with stable disease and not reached in patients showing partial or complete response, as defined by PERCIST criteria.
**Conclusion:** [$^{68}$Ga]Ga-FAPi PET/CT appears to be an effective tool for assessing treatment response and monitoring gastrointestinal malignancies.
**Limitations:** N/A
**Funding for this study:** N/A
**Has your study been approved by an ethics committee?** Yes
**Ethics committee - additional information:** Ethics Committee of Yeditepe University (Date: 02.03.2020 / No: 1576).
**Author Disclosures:**
Levent Kabasakal: Nothing to disclose
Turkay Toklu: Nothing to disclose
Kaan Akcay: Nothing to disclose
Gamze Beydagi: Nothing to disclose
Ayşegül Görmez: Nothing to disclose
Nalan Alan Selcuk: Nothing to disclose

**First clinical experience of 68Ga-FAPI PET/CT in Kazakhstan**
Z. J. Zholdybay, *M. Gabdullina*, Z. Zhakenova, G. Alisherov, Z. M. Amankulov; Almaty/KZ
*(kydyralievamadina28@gmail.com)*

**Purpose:** We aimed to investigate the use of 68Ga-FAPI PET/CT in patients with various oncological diseases by analyzing the pathological uptake in tumors as well as physiological uptake of the tracer.
**Methods or Background:** Inclusion criteria included: 1) age ≥ 18 years; 2) confirmed diagnosis or clinical suspicion of a primary solid malignancy; 3) referral for cancer staging or evaluation of suspected recurrence; 4) written informed consent. Exclusion criteria included: 1) pregnancy or lactation; 2) lymphoma or melanoma; 3) known hypersensitivity or adverse reaction to the 68Ga-FAPI; 4) refusal to undergo biopsy or surgery; 5) renal impairment.
**Results or Findings:** Between January 10, 2024, and February 28, 2025, a total of 199 cancer patients aged 24–80 years (mean age 52.6 ± 15.4 years) were included in the study. The majority of patients (64%) were female. Indications for 68Ga-FAPI PET/CT included evaluation of primary solid tumors arising in the breast, hepatobiliary system, gastrointestinal tract, lung, head and neck region, ovary, and pancreas; a smaller subset of patients underwent imaging for less common malignancies such as choroidal tumors, histiocytic disorders, seminoma, renal neoplasms and others. 68Ga-FAPI-04 demonstrated minimal physiological uptake in normal brain regions and intestinal loops, with comparatively low activity in the liver and oropharynx. Uptake was also low in the thyroid gland, salivary glands, lung parenchyma, esophagus, myocardium, and glandular breast tissue, thereby enhancing lesion contrast in these organs.
**Conclusion:** In conclusion, 68Ga-FAPI-04 appears to be a promising radiotracer for the molecular imaging of most malignant neoplasms.

**Limitations:** First, biopsies could not be performed for all detected lesions, limiting histopathological confirmation. Second, the study was conducted in a single center. Finally, there was no direct comparison with 18F-FDG PET/CT.
**Funding for this study:** Committee of Science of the Ministry of Science and Higher Education of the Republic of Kazakhstan (Grant No.AP19679719)
**Has your study been approved by an ethics committee?** Yes
**Ethics committee - additional information:** This prospective study was approved by the Local Ethics Committee of the Asfendiyarov Kazakh National Medical University (27 September 2023, protocol number 7 (143)).
**Author Disclosures:**
Zhandos Mukhtaruly Amankulov: Nothing to disclose
Zhanar Zhakenova: Nothing to disclose
Madina Gabdullina: Nothing to disclose
Galymzhan Alisherov: Nothing to disclose
Zhamilya J. Zholdybay: Nothing to disclose

### Stability, Biodistribution, and Patient Dosimetry of [177Lu]Lu-FAP-2286 Radionuclide Therapy

K. Akcay, T. Toklu, *G. Beydagi*, L. Kabasakal, B. Caner, N. Alan Selcuk; Istanbul/TR
*(gamzebeydagi@gmail.com)*

**Purpose:** Fibroblast activation protein (FAP) is an attractive target for tumor imaging and therapy. While $^{68}$Ga-labeled FAPi-04 and FAPi-46 are widely used diagnostically, their brief retention limits therapeutic use. FAP-2286 has emerged as a candidate for radionuclide therapy. This study evaluated the in-vitro/in-vivo stability, biodistribution, and organ/tumor dosimetry of [$^{177}$Lu]Lu-FAP-2286.
**Methods or Background:** Seven treatments were administered to five patients (mean age 61.0±15.3 years; F/M: 2/3) with positive uptake on [$^{68}$Ga]Ga-FAPi-46 or [$^{68}$Ga]Ga-FAP-2286. The mean administered activity was 215±14.7 mCi. Whole-body anterior/posterior planar scans and SPECT/CT were obtained at 4, 24, 72, and 120 h post-infusion with corrections for attenuation, scatter, and detector/collimator response. Blood samples up to 120 h supported red marrow dosimetry and in-vivo stability assessment. Whole-body ROIs and VOIs for kidneys, liver, and lesions were segmented. Time-activity curves were fitted with mono- or bi-exponentials in MATLAB R2013a to derive cumulated activities. Absorbed doses were estimated using IDAC-Dose 2.1.
**Results or Findings:** Two patients had pancreatic adenocarcinoma, one neuroendocrine carcinoma, and two soft-tissue sarcoma. In-vitro stability was at least 41 h; in-vivo blood stability persisted ≥24 h. Mean absorbed doses were 0.50±0.19 Gy/GBq (kidneys), 0.030±0.014 Gy/GBq (liver), and 0.054±0.014 Gy/GBq (red marrow). The mean tumor dose was 5.97±3.85 Gy (range 1.10–18.52 Gy).
**Conclusion:** No adverse events occurred, and all patients tolerated therapy well. At the administered activities, renal and marrow toxicity thresholds were not exceeded. Bone marrow doses were comparable to the only published series, whereas renal doses were lower. Because prior data reported doses only for bone metastases, direct comparison with the soft-tissue tumor doses presented here is not possible. Overall, [$^{177}$Lu]Lu-FAP-2286 shows favorable stability, dosimetry, and safety, supporting its potential as a therapeutic option across multiple malignancies.
**Limitations:** N/A
**Funding for this study:** N/A
**Has your study been approved by an ethics committee?** Yes
**Ethics committee - additional information:** Ethics Committee of Yeditepe University
**Author Disclosures:**
Levent Kabasakal: Nothing to disclose
Turkay Toklu: Nothing to disclose
Biray Caner: Nothing to disclose
Kaan Akcay: Nothing to disclose
Gamze Beydagi: Nothing to disclose
Nalan Alan Selcuk: Nothing to disclose

### A glypican-3 targeted diagnostic radiopharmaceutical for hepatocellular carcinoma molecular imaging

*H. Guo*, X. Ma; Changsha, Hunan/CN
*(guohonghui2021@163.com)*

**Purpose:** Glypican-3 (GPC3) is upregulated in hepatocellular carcinoma (HCC), is implicated in tumor progression, and correlates with poor prognosis. These properties make it promising for both targeted immunotherapy and early detection via PET imaging. Here, we developed a novel cyclic peptide molecular probe via Phage Display Biopanning and validated the imaging ability in HCC xenografts and explored its application for HCC patients.
**Methods or Background:** The novel novel cyclic peptide molecular probe, namely ZSQT-3B010, was labeled with Gallium-68 (68Ga) to obtain [68Ga]Ga-ZSQT-3B010. Cell uptake assays, small-animal PET imaging, and biodistribution studies were performed to evaluate its targeting ability. Pharmacokinetics studies were confirmed in healthy volunteers (HV, n=6). In the first-in-human study, 17 patients (10 HCC and 7 Non-HCC patients) underwent [68Ga]Ga-ZSQT-3B010 PET/MR scan. Radiotracer uptake in tumors and normal tissues was quantified, and tumor-to-liver ratios (TLR) were calculated.
**Results or Findings:** [68Ga]Ga-ZSQT-3B010 was synthesized with high radiochemical purity and exhibited specific uptake and efficient internalization in GPC3-positive cells. [68Ga]Ga-ZSQT-3B010 effectively visualized HCC tumors, reaching a peak SUV uptake of 5.09 at 4h p.i., 3.40 and 50.00 fold higher than in GPC3 moderate and low expression tumors. The [68Ga]Ga-ZSQT-3B010 demonstrated favorable pharmacokinetics and a low radiation dose in HV. In preliminary clinical studies, no adverse events was observed. Totally 29 lesions were detected and the mean SUVmax was 15.80±3.14 at 60 min p.i. and 19.16±4.26 at 120min p.i., with a minimum detectable lesion size ≤1 cm. The TLR is 4.53-to-28.67. Further, a positive correction between GPC3 expression and SUVmax (R=0.84, P<0.05). Notably, the stomach uptake is very low compared to other reported targeted GPC3 agents.
**Conclusion:** The [68Ga]Ga-ZSQT-3B010 has high GPC3 binding affinity and specificity for HCC detection, and may further benifit GPC3 targeting therapy.
**Limitations:** No limitations were identified.
**Funding for this study:** Funding was provided by the Project of Research on New Targeted Peptide Radioactive Drugs for Tumor (A-202406-026-CD).
**Has your study been approved by an ethics committee?** Yes
**Ethics committee - additional information:** The study was approved by the Ethics Committee of the Second Xiangya Hospital of Central South University (Num：LYF20250186).
**Author Disclosures:**
Xiaowei Ma: Nothing to disclose
Honghui Guo: Nothing to disclose

### Optimal scan time for [18F]SiTATE PET/CT imaging of patients with neuroendocrine tumors

*R. Ebner*, F. Herr, K. Klimek, J. Ricke, C. C. Cyran, R. Werner, G. Sheikh; Munich/DE

**Purpose:** Somatostatin receptor (SSR)-targeting tracer [$^{18}$F]SiTATE is gaining momentum in clinical use as a promising alternative to commonly used gallium-68-labelled SSR-targeting compounds, demonstrating excellent image quality and clinical utility. Currently, imaging is performed 90 minutes after tracer injection, complicating integration into a multi-tracer PET environment, where most tracers are imaged 60 minutes after tracer injection. We therefore investigated whether comparable image quality and quantification can be achieved at earlier time points more compatible with clinical routine.
**Methods or Background:** Eight patients with histologically confirmed metastasized GEP-NET received [$^{18}$F]SiTATE-PET/CT with repeated scans 60, 90, and 120 minutes post-injection. SUVmax, SUVpeak and SUVmean were measured in visceral organs and 49 SSR-positive lesions (lymph node, liver, bone), and tumor-to-background ratios were calculated. Statistical comparison was performed across time points. Scans were visually assessed for noticeable differences by four readers of varying experience.
**Results or Findings:** No significant differences in tumor-to-background ratios were observed across timepoints for liver (p=0.6613), lymph nodes (p=0.5413), bone (p=0.8478), or overall lesion assessment (p=0.7248). Post hoc multiple comparisons using tukey's test revealed no significant pairwise differences (p=0.5103–0.999). Readers did not report qualitative visual difference between the time points, all lesions could be clearly identified against the surrounding background.
**Conclusion:** The results demonstrate consistent tracer uptake over time, with minimal variation in tumor-to-background contrast across metastatic sites. Imaging at 60 minutes post-injection provides equivalent lesion detectability and quantitative contrast compared to 90 and 120 minutes. These findings highlight the clinical utility of early [$^{18}$F]SiTATE imaging for NET patients, facilitating workflow and improving patient comfort without compromising diagnostic accuracy.
**Limitations:** The main limitation of our study is the small sample size and its single-center design, which may limit generalizability of the findings.
**Funding for this study:** None.
**Has your study been approved by an ethics committee?** Yes
**Ethics committee - additional information:** The data analysis received approval from the institutional ethics board of LMU Munich.
**Author Disclosures:**
Rudolf Werner: Nothing to disclose
Konrad Klimek: Nothing to disclose
Clemens C. Cyran: Nothing to disclose
Ricarda Ebner: Nothing to disclose
Gabriel Sheikh: Nothing to disclose
Felix Herr: Nothing to disclose
Jens Ricke: Nothing to disclose

**18F-FES-PET/MRI for Axillary Staging in ER+ Breast Cancer: Diagnostic Accuracy and Optimal SUVmax Cutoff**

*T. Spiegel*[1], D. A. Resch[1], S. Rasul[1], O. Lafcı[1], N. Pötsch[1], P. Clauser[1], P. A. Baltzer[1], K. Pinker-Domenig[2], T. H. Helbich[1]; [1]Vienna/AT, [2]New York, NY/US
*(thomas.spiegel@meduniwien.ac.at)*

**Purpose:** 18F-Fluoroestradiol (18F-FES) is a novel radiotracer, and little is known about its role in axillary lymph node (LN) staging in estrogen receptor (ER)-positive breast cancer (BC) on PET/MRI. This study aims to evaluate the diagnostic performance of 18F-FES-PET/MRI for axillary LN staging in ER+ BC and to define optimal thresholds.
**Methods or Background:** This retrospective analysis of prospectively acquired single-center study data includes 37 female patients with 38 ER+ BCs, who underwent 18F-FES-PET/MRI using a Biograph mMR system (Siemens, Germany). Semiquantitative PET parameters including SUVmax of axillary LNs were assessed by placing a volume of interest (syngo.via, Siemens) on the most suspicious LN identified on MRI. Histopathology from biopsy or surgery served as the reference standard. Statistical analysis included Shapiro-Wilk test, t-test, and ROC analysis with AUC and Youden's index; p<0.05 was considered significant.
**Results or Findings:** Histopathology confirmed 21 metastatic and 17 benign LNs. Metastatic LNs showed higher uptake (mean SUVmax 2.60, 95% CI 1.91-3.29) than benign LNs (0.98, 95% CI 0.81-1.16; p<0.001), with an AUC of 0.852. An SUVmax cutoff of ≥1.56 yielded a specificity of 100% (17/17), sensitivity of 66.7% (14/21), PPV of 100% (14/14), NPV of 70.8% (17/24), and accuracy of 81.6% (31/38). Lower thresholds improved sensitivity but markedly reduced specificity. Subgroup analysis showed a trend toward higher uptake of metastatic LNs in invasive lobular carcinomas (ILC, n=5, mean SUVmax 3.61, 95% CI 1.33-5.89) compared to invasive ductal carcinomas (IDC, n=16, mean SUVmax 2.29, 95% CI 1.59-2.98; p=0.087).
**Conclusion:** 18F-FES-PET/MRI allows reliable LN characterization in ER+ BC. An SUVmax cutoff of ≥1.56 offers excellent specificity and overall accuracy, with higher uptake trends in lobular compared to ductal carcinoma.
**Limitations:** Retrospective, single-center design, and limited sample size.
**Funding for this study:** OeNB Anniversary Fund AB18207ONB
**Has your study been approved by an ethics committee?** Yes
**Ethics committee - additional information:** EK Nr. 510/2009
**Author Disclosures:**
Pascal A.T. Baltzer: Nothing to disclose
Nina Pötsch: Nothing to disclose
Daphne Ariane Resch: Nothing to disclose
Thomas Spiegel: Nothing to disclose
Katja Pinker-Domenig: Nothing to disclose
Thomas H. Helbich: Nothing to disclose
Oğuz Lafcı: Nothing to disclose
Sazan Rasul: Nothing to disclose
Paola Clauser: Nothing to disclose

**13:00 - 14:00** **Research Stage 3**

### Research Presentation Session: Neuro

### RPS 911

### Skull-based essentials: from the sellar region to the cerebellopontine angle

**Moderator**
Joanna Bladowska; Wrocław/PL
*(joanna.bladowska@pwr.edu.pl)*

**Multiparametric MRI biomarkers predicting atypical pituitary adenomas: Correlation with surgical complexity and histopathologic markers of aggressive behavior**

S. Nalubolu, *R. Hiregouja Eranna*; Bengaluru/IN
*(drroja95@gmail.com)*

**Purpose:** While most pituitary adenomas are indolent, a subset exhibit atypical morphology characterized by increased firmness, invasiveness, and proliferative histopathology. These features contribute to surgical complexity and early postoperative recurrence. Preoperative identification of such atypical adenomas using quantitative MRI biomarkers—particularly diffusion, T2 signal, and enhancement pattern—may aid surgical planning and risk stratification.
**Methods or Background:** A retrospective review was conducted on 128 surgically resected pituitary adenomas (2020–2023). Preoperative 3 T MRI (T1, T2, DWI/ADC, dynamic contrast-enhanced sequences) and CT were assessed for cavernous sinus invasion (Knosp grade), dural contact, margin irregularity, T2 signal, cystic/necrotic change, and osseous invasion. Mean ADC values (b = 0, 1000 s/mm²) were measured from tumour ROIs. Atypicality was defined by intraoperative firmness and/or proliferative indices (Ki-67 > 3%, p53 positivity). Interobserver agreement (κ) and ROC analyses were performed. Follow-up MRI and hormonal data (6–18 months) assessed persistence or progression.
**Results or Findings:** Atypical lesions had a substantially lower mean ADC than typical ones ($0.72 \pm 0.09$ vs. $0.91 \pm 0.11 \times 10^{-3}$ mm²/s; $p < 0.001$).T2 hypointensity occurred in 63% versus 31% (p = 0.002), correlating with intraoperative firmness (κ = 0.81).Cavernous sinus invasion (sensitivity 85%, specificity 82%) and irregular margins (74%, 68%) were strong predictors. The combined model with T2 hypointensity + invasion and ADC < $0.80 \times 10^{-3}$ mm²/s had an AUC of 0.91. Early progression was seen in 17% of atypical versus 4% of typical adenomas (p = 0.03).
**Conclusion:** Atypical pituitary adenomas exhibit a reproducible MRI signature—low ADC, T2 hypointensity, and invasive margins—reflecting fibrous, highly cellular tissue. These multiparametric MRI biomarkers predict surgical difficulty and early recurrence, supporting their integration into preoperative evaluation and biologically guided management.
**Limitations:** Single-center limitation.
**Funding for this study:** No funding was provided for this study.
**Has your study been approved by an ethics committee?** Not applicable
**Author Disclosures:**
Shivakanth Nalubolu: Nothing to disclose
Roja Hiregouja Eranna: Nothing to disclose

**Identifying Pituitary Microadenomas Using Dynamic Enhancement Patterns: A Descriptive Study**

*D. A. Zambrano*, A. M. Delgado Brito, C. D. Solano, M. R. López De La Torre Carretero, J. M. Rodríguez Ortega, M. Calvo Imirizaldu; Pamplona/ES
*(dazambrano@unav.es)*

**Purpose:** Describe and quantify dynamic perfusion-curve patterns in pituitary lesions (mainly microadenomas) and compare them with normal pituitaries.
**Methods or Background:** Pituitary microadenomas (<10 mm) are benign, often asymptomatic tumors with low clinical risk, typically identified through MRI and hormonal evaluation. However, their small size and subtle enhancement can lead to diagnostic uncertainty. Dynamic contrast-enhanced (DCE) MRI curve patterns (types I–III) have been proposed as diagnostic aids. This retrospective descriptive study included 30 patients (20 with pituitary microadenomas and 10 controls) evaluated between January 2022 and December 2024. Imaging was performed on 3T and 1.5T scanners using 3D T1-weighted DCE sequences, followed by motion correction and temporal signal-intensity curve analysis. Regions of interest (ROIs) were placed in the lesion and contralateral pituitary tissue (or bilaterally in controls). Perfusion curves were classified as type I, II, or III for descriptive comparison between microadenomas and normal pituitaries.
**Results or Findings:** In this study of 30 patients (20 with microadenomas, 10 controls), 95% of microadenomas exhibited a type II DCE curve, whereas all controls showed normal physiological curves. These results indicate a strong association between microadenomas and type II perfusion patterns, suggesting that DCE-curve analysis may support visual diagnosis in uncertain cases. However, due to the retrospective design and small sample size, findings are exploratory. Further research is needed to standardize metrics, evaluate reproducibility, and validate diagnostic performance before adopting type II curves as a formal criterion.
**Conclusion:** Most pituitary microadenomas exhibited a type IIb DCE pattern, suggesting that perfusion-curve analysis may aid in their detection. Further studies are needed to validate these findings.
**Limitations:** Retrospective design and small sample size render findings exploratory. Standardization of quantitative metrics, interobserver reproducibility testing, and validation in larger prospective studies are required before clinical adoption.
**Funding for this study:** The authors did not receive any funding for this study
**Has your study been approved by an ethics committee?** Not applicable
**Author Disclosures:**
José Marlon Rodríguez Ortega: Nothing to disclose
Manuel Rafael López De La Torre Carretero: Nothing to disclose
Adolfo Manuel Delgado Brito: Nothing to disclose
Marta Calvo Imirizaldu: Nothing to disclose
Daniel Alfonso Zambrano: Nothing to disclose
Carlos Delgado Solano: Nothing to disclose

**Craniopharyngioma magnetic resonance imaging, progression, gender, age and histologic aspects: a retrospective cohort**

*M. O. Sousa*, R. Silva; São Luís/BR
*(marciarad2012@gmail.com)*

**Purpose:** Evaluate magnetic resonance imaging (MRI) craniopharyngioma diagnosis patients, correlating them with histological subtypes, age and gender. Furthermore, observe differential diagnosis, and the disease evolution based on MRI aspect.
**Methods or Background:** Retrospective cohort analysis of 111 MRIs from 34 craniopharyngioma diagnosis patients performed between 2018 and 2025, comparing histological diagnosis, age, gender and progression. Descriptive statistical analysis, Kruskal-Wallis test and Fisher's exact test were performed using Stata software.
**Results or Findings:** Most females patients ranging from 2 to 71 years old, median of 13, interquartile range (IQR) = 8-24 years old. Twenty-nine were craniopharyngioma confirmed, two pituitary macroadenoma, two suprasellar pilocytic astrocytoma, and one Rathke's pouch cyst. Adamatinomatous craniopharyngiomas (86,67%), papillary (12,33%). Kruskal-Wallis test being statistically significant for the association between the histological type and age group (P = 0,27) but not for progression (P = 0,53). Fisher's exact test demonstrated no association between progression and histological type (P = 1,0).
**Conclusion:** In this study sample, we observed a predominance of adamatinomatous subtype craniopharyngiomas in children and young adults, with a small percentage of elderly individuals affected by the papillary subtype, predominantly female. These lesions presented challenging imaging diagnoses and was confused with macroadenomas, Rathke's pouch cysts, and pilocytic astrocytomas. Although most presented reduction, many progressed despite treatment, and some died, characterizing the aggressive aspect of the tumor.
**Limitations:** The study was limited by the small sample size given the rarity of the disease. Other limitations were the high number of missing information on histological subtype and the lack of follow-up in some cases.
**Funding for this study:** No funding
**Has your study been approved by an ethics committee?** Not applicable
**Author Disclosures:**
Marcia Oliveira Sousa: Nothing to disclose
Rodrigo Silva: Nothing to disclose

**Facial nerve characterisation in CPA tumors: from tractography to multimodal predictive models**

A. Mangili[1], A. Arrigoni[1], G. Pezzetti[2], B. Frigeni[2], R. Bivona[2], S. Capelli[1], G. Danesi[2], A. Caroli[1], *S. Gerevini*[2]; [1]Ranica/IT, [2]Bergamo/IT
*(sgerevini@asst-pg23.it)*

**Purpose:** Cerebellopontine angle (CPA) tumors are often benign but can compress adjacent structures, including the facial nerve (FN). Surgical resection is standard treatment but carries a risk of iatrogenic injury and facial palsy. This study investigates the role of anatomical and diffusion-weighted MRI (DW-MRI) in presurgical planning, focusing on FN reconstruction. Radiomic features and diffusion tensor imaging (DTI) biomarkers were extracted and integrated with clinical variables to train machine learning models predicting FN integrity, postoperative outcomes, and long-term function.
**Methods or Background:** Forty-seven CPA patients who underwent preoperative MRI and surgery were analyzed. MRI protocols included DW-MRI AP (b0 and 1500; 50 directions; voxel size 2×2×2 mm), b0 PA, post-contrast volumetric T1-w, and volumetric T2-w scans. A custom pipeline, implemented in Python with MRtrix3Tissue and FSL, corrected noise and artifacts, segmented lesions, and performed a SS3T-CSD-based tractography using the iFOD2 algorithm with anatomically guided seeding.
**Results or Findings:** The FN was successfully reconstructed in all patients. The tumor-affected side showed higher fractional anisotropy (FA) and lower mean diffusivity (MD) than the contralateral side. Diffusion metrics correlated with conventional prognostic markers, including blink reflex and Koos grade. Compound Muscle Action Potential (CMAP) correlated inversely with diffusion values and FN tract length. Postoperative and 1-year House-Brackmann (HB) grades were significantly associated with lesion volume, FN length, and CMAP. Machine learning models achieved accuracies of 0.76 for predicting FN adherence, 0.82 for postoperative HB, and 0.90 for 12-month HB outcomes.
**Conclusion:** Diffusion tractography enhances CPA presurgical planning. Combined radiomic, clinical, and neurophysiological data improve prediction of FN integrity, short-term function, and long-term outcomes after resection.
**Limitations:** The study is limited by its single-center design, modest sample size, and lack of external validation.
**Funding for this study:** The study didn’t receive any funding.
**Has your study been approved by an ethics committee?** Yes
**Ethics committee - additional information:** Protocol title: Facial nerve Assessment in patients with Cerebellopontine angle tumours: a quantitative Evaluation from pre-surgical brain MRI Scan (FACES)
**Author Disclosures:**
Serena Capelli: Nothing to disclose
Andrea Mangili: Nothing to disclose
Giulio Pezzetti: Nothing to disclose
Rachele Bivona: Nothing to disclose
Barbara Frigeni: Nothing to disclose
Alberto Arrigoni: Nothing to disclose
Anna Caroli: Nothing to disclose
Giovanni Danesi: Nothing to disclose
Simonetta Gerevini: Nothing to disclose

**Predictive Assessment of Pituitary Neuroendocrine Tumor Recurrence Using MRI Fractal Analysis and Radiomics**

*C. Chen*, J. Zhou; LanZhou/CN

**Purpose:** Although most pituitary neuroendocrine tumors (PitNETs) are benign with good postoperative outcomes, they frequently recur despite multidisciplinary treatment. Early prediction of PitNET recurrence is clinically vital. This study aims to explore the value of preoperative assessment of PitNET recurrence based on MRI fractal analysis and radiomic features.
**Methods or Background:** A retrospective study included 123 PitNET patients who underwent MRI, divided into recurrence (n=50) and non-recurrence (n=73) groups based on follow-up. Clinical, pathological, and conventional MRI data were collected. Fractal dimension was calculated from sagittal CE-T1WI using ImageJ. Radiomic features were extracted from whole-tumor images on axial T2, sagittal CE-T1WI, and coronal CE-T1WI sequences via Darwin platform. Group differences in fractal parameters, radiomic features, clinical/MRI/pathological traits were analyzed to construct an optimal predictive model.
**Results or Findings:** Statistically significant differences in fractal dimension were observed between the two groups ($P < 0.001$). Among clinical features, preoperative ACTH levels, tumor texture, extent of tumor resection, presence of tumor apoplexy, and CSF leakage all differed significantly (all $P < 0.05$). For conventional MRI signs, statistically significant differences were found in maximum tumor diameter/height, tumor hemorrhage, carotid artery encasement, and cavernous sinus invasion (all $P < 0.05$). Pathologically, P53 positivity showed a significant difference ($P = 0.03$). The SVM model based on radiomic features outperformed others. The SVM model constructed using statistically significant fused features achieved the highest performance, with AUC values of 0.951 in the test set and 0.778 in the validation set .
**Conclusion:** Combining MRI fractal analysis, radiomics, and clinical/pathological data enhances clinical decision-making to improve treatment and patient quality of life.
**Limitations:** This is a retrospective study. Multi-dimensional parameters such as molecular omics and genomics will be integrated to refine the model.
**Funding for this study:** National Natural Science Foundation Project (82371914)
**Has your study been approved by an ethics committee?** Not applicable
**Author Disclosures:**
Junlin Zhou: Author: corresponding author
Chunhui Chen: Author: the first author

**13:00 - 14:00** **Research Stage 5**

### Research Presentation Session: Oncologic Imaging

### RPS 916

### The evolving landscape of hybrid imaging for oncology

**Moderator**
Johannes Rübenthaler; Munich/DE

### 18F-FES-PET/MRI in Breast Cancer: Distinguishing ER Status and Subtype Uptake Patterns

*T. Spiegel*[1], D. A. Resch[1], S. Rasul[1], O. Lafcı[1], N. Pötsch[1], P. Clauser[1], P. A. Baltzer[1], K. Pinker-Domenig[2], T. H. Helbich[1]; [1]Vienna/AT, [2]New York, NY/US
*(thomas.spiegel@meduniwien.ac.at)*

**Purpose:** 18F-Fluoroestradiol (18F-FES) is a novel radiotracer primarily used for staging ER-positive breast cancer (BC). However, understanding its uptake characteristics in both benign and malignant breast lesions is essential. This study evaluates 18F-FES-PET/MRI uptake patterns across receptor status, molecular BC subtypes, and benign breast lesions.
**Methods or Background:** This retrospective analysis of prospectively acquired data includes 41 women with 48 breast lesions (42 malignant, 6 benign) who underwent 18F-FES-PET/MRI. All patients underwent simultaneous 18F-FES-PET/MRI performed using a Biograph mMR system (Siemens, Germany). Quantitative PET parameters, including SUVmax, and lesion size were assessed. Histopathology in malignant tumors and histopathology or follow-up >2 years in benign lesions was the reference standard. Mann-Whitney U test and ROC analyses were performed; $p<0.05$ was considered significant.
**Results or Findings:** Among 42 BCs, 39 were ER-positive (8 Luminal A, 31 Luminal B) and 3 triple-negative breast cancers (TNBC). ER-positive BCs had statistically significantly higher SUVmax (median 2.40, IQR 1.31-3.59) than ER-negatives (SUVmax <1.00, p=0.014). 6 ER-positive BCs with lesion sizes <9 mm failed to show any uptake. AUC for SUVmax in lesions ≥10 mm to differentiate between ER-positive and ER-negative BCs was 1.00 and when including all lesions AUC 0.902. SUVmax tended to be higher in Luminal B than Luminal A (p=0.173), both exceeding TNBC (p<0.001 and p=0.036). Benign lesions (n=6) showed SUVmax values between 0.72-1.57, statistically significantly lower than in BCs (p<0.001).
**Conclusion:** 18F-FES uptake is predominantly observed in ER-positive breast cancers and reliably distinguishes ER-positive from ER-negative tumors ≥10 mm. However, overlap exists among molecular subtypes, particularly Luminal A and B, and benign lesions may also show variable uptake.
**Limitations:** Retrospective design and small sample sizes in certain subgroups.
**Funding for this study:** OeNB Anniversary Fund AB18207ONB
**Has your study been approved by an ethics committee?** Yes
**Ethics committee - additional information:** EK Nr. 510/2009
**Author Disclosures:**
Pascal A.T. Baltzer: Nothing to disclose
Nina Pötsch: Nothing to disclose
Daphne Ariane Resch: Nothing to disclose
Thomas Spiegel: Nothing to disclose
Katja Pinker-Domenig: Nothing to disclose
Thomas H. Helbich: Nothing to disclose
Oğuz Lafcı: Nothing to disclose
Sazan Rasul: Nothing to disclose
Paola Clauser: Nothing to disclose

### Comparison of PET/CT with 18F-FDG and 68Ga-FAPI in the detection of liver tumors

*E. Davydova*, M. A. Komarova, V. E. Sinitsyn, A. Martinovich, O. Ryzhova; Moscow/RU

**Purpose:** To compare the diagnostic accuracy of FAPI PET/CT and FDG PET/CT in patients with liver tumor.
**Methods or Background:** 15 patients (mean age 57,5 y.o, 7male/8 female) diagnosed with liver cancer who underwent FAPI and FDG PET/CT between 7 days were included in the study. 4pts were examined to detect progression of the underlying disease (had the previous treatment). The all liver lesions had morphological verification (11pts had cholangiocarcinoma G1-3, 2pts. - hepatocellular carcinoma, 1- intraductal papillary tumor of the bile duct and 1pts - calcifying nested stromal-epithelial tumor). Uptake intensity(SUVmax) and target-to-background ratio(TBR) of the primary liver tumor and lymph nodes metastasis were compared between two imaging modalities. Sensitivity(Se), specificity(Sp), positive predictive value(PPV), negative predictive value(NPV) and accuracy(Ac) were calculated for each modality on a per-region basis.
**Results or Findings:** 14 pts were found primary tumor and 3 pts of them had metastases in regional lymph nodes or lung. There was a better contrast with 68Ga-FAPI, with SUVmax of the liver tumors of 14,9 [8,94-23,48] compared with SUVmax of 7,81 [3,55-10,21] of liver tumors with 18F-FDG (p=0,02). The TBR of primary tumor were higher in 68Ga-FAPI compared with 18F-FDG - 10,56[4,15-16,46] vs. 2,5[1,17-3,67] respectively, p=0,0014. For detection of primary liver tumor and metastasis on region based level Se, Sp, PPV, NPV and Ac of Ga-68 FAPI PET/CT were higher than F18-FDG PET/CT (97,3%, 71,43 %, 90,00%, 90,91% and 90,20% vs. 78,38%, 57,14%, 82,86%,50,00% and 72,55 %, respectively).
**Conclusion:** FAPI PET-CT is a very promising tool for evaluation of patients with liver tumor because more accurate than FDG PET-CT. High level of FAPI uptake in the primary hepatic lesions is indicate the potential of FAP-targeted radionuclide therapy.
**Limitations:** No
**Funding for this study:** Without funding
**Has your study been approved by an ethics committee?** Not applicable
**Author Disclosures:**
Olga Ryzhova: Nothing to disclose
Maria Aleksandrovna Komarova: Nothing to disclose
Valentin Евгеньевич Sinitsyn: Nothing to disclose
Ekaterina Davydova: Nothing to disclose
Alexander Martinovich: Nothing to disclose

### Toward Structured Axillary Nodal Staging in Breast Cancer: Prospective Correlation of the Proposed Axillary Node Reporting and Data System (AN-RADS) with PET-CT and Histopathology

*N. A. Umretiya*, V. Mahajan, R. Arafath; Chennai/IN
*(nidhiumarethiya@gmail.com)*

**Purpose:** To evaluate the diagnostic performance of the proposed Axillary Node Reporting and Data System (AN-RADS) ultrasound classification for axillary nodes in breast cancer staging and to correlate AN-RADS categories with PET-CT and histopathology.
**Methods or Background:** This prospective observational study enrolled 15 patients with biopsy-proven breast carcinoma undergoing baseline staging between 1st August and 1st September 2025. All patients underwent axillary ultrasound with AN-RADS scoring (categories 1–5) and 18F-FDG PET-CT. PET positivity was defined as SUVmax ≥2.5. Histopathology (SLNB, core, or excision biopsy) or FNAC was available in 13/15 patients; 2 were excluded from diagnostic accuracy calculations due to lack of biopsy. AN-RADS ≥4 was considered ultrasound-positive. Statistical analysis included malignancy rates per category, diagnostic accuracy metrics with exact 95% confidence intervals, and Cohen's κ for concordance.
**Results or Findings:** Median patient age was 54 years (range: 42–68). AN-RADS distribution was: category 2 in 5 patients (33.3%), category 3 in 1 (6.7%), category 4 in 3 (20.0%), and category 5 in 6 (40.0%). Among 13 evaluable cases, 8/13 (61.5%; 95% CI 31.6–86.1) demonstrated metastatic nodes. Malignancy rates were 0% in AN-RADS 2 and 100% in AN-RADS 4–5. With AN-RADS ≥4 as threshold, ultrasound achieved sensitivity, specificity, PPV, NPV, and accuracy of 100%. PET-CT using SUVmax ≥2.5 showed identical diagnostic performance. Ultrasound, PET-CT, and histopathology showed perfect concordance (κ = 1.0).
**Conclusion:** The proposed AN-RADS system demonstrates a clear malignancy gradient and excellent correlation with PET-CT and histopathology, suggesting its potential as a structured, reliable framework for axillary nodal staging in breast cancer.
**Limitations:** Small sample size, short study period, and selection bias limit generalizability. The perfect performance observed is likely influenced by the restricted dataset.
**Funding for this study:** No external funding was received.
**Has your study been approved by an ethics committee?** Not applicable
**Author Disclosures:**
Nidhi Atulbhai Umretiya: Nothing to disclose
Vandana Mahajan: Nothing to disclose
Rasheed Arafath: Nothing to disclose

**Diagnostic performance of whole-body MRI (wb-MRI) and 68Ga-PSMA PET/CT for staging and re-staging of high-risk prostate cancer (PCa)**
*R. Regazzo*, A. Cattabriga, C. Gaudiano, A. Bardelli, L. Bianchi, A. Farolfi, S. Fanti, R. Schiavina, C. Mosconi; Bologna/IT

**Purpose:** PSMA-PET is the gold standard for staging and restaging high-risk prostate cancer (PCa), while whole-body MRI (wb-MRI) may represent a radiation-free alternative despite limited evidence. This prospective single-center study aimed to assess the diagnostic non-inferiority of wb-MRI compared to PSMA-PET for metastatic evaluation in high-risk patients at primary treatment and in those with biochemical recurrence (BCR).
**Methods or Background:** We enrolled 89 PCa patients: 47 high/very high-risk men undergoing radical prostatectomy with extended pelvic lymph node dissection (RARP + ePLND, Group 1) and 42 men with PSA persistence or BCR after surgery (Group 2). All patients underwent PSMA-PET and wb-MRI within 30 days. Reference standards were histopathology in Group 1 and clinical/imaging follow-up in Group 2. Sensitivity, specificity, PPV, NPV, accuracy, and AUC from ROC analyses were compared between modalities.
**Results or Findings:** In Group 1, pathological N1 disease was found in 42%. PSMA-PET vs wb-MRI for nodal staging showed sensitivity 53% vs 41%, specificity 90% vs 87%, and AUC 0.71 vs 0.64, with no significant differences. In Group 2, 21% had PSA persistence and 79% BCR (median time 28 months, median PSA 0.3 ng/mL). PSMA-PET vs wb-MRI detected local recurrence in 17% vs 29%, nodal disease in 12% vs 19%, and distant metastases in 33% vs 19%. Diagnostic performance was comparable (sensitivity 54% vs 46%, specificity 86% vs 89%, AUC 0.80 vs 0.78).
**Conclusion:** Wb-MRI demonstrates diagnostic performance comparable to PSMA-PET for staging and restaging high-risk PCa and may represent a valid alternative in selected patients.
**Limitations:** Preliminary single-center results; limited sample size; heterogeneous follow-up in the BCR group.
**Funding for this study:** No funding was received for this study.
**Has your study been approved by an ethics committee?** Not applicable
**Author Disclosures:**
Andrea Bardelli: Nothing to disclose
Riccardo Schiavina: Nothing to disclose
Lorenzo Bianchi: Nothing to disclose
Andrea Farolfi: Nothing to disclose
Stefano Fanti: Nothing to disclose
Cristina Mosconi: Nothing to disclose
Arrigo Cattabriga: Nothing to disclose
Riccardo Regazzo: Nothing to disclose
Caterina Gaudiano: Nothing to disclose

**Predictive Value of Baseline Metabolic Tumor Volume (MTV) and Total Lesion Glycolysis (TLG) in Oncologic PET/CT**
*F. Abubacker Sulaiman*[1], A. Asokan[2], S. Sundaraiya[1]; [1]Chennai/IN, [2]Coimbatore/IN
*(fasulaiman@gmail.com)*

**Purpose:** To assess the prognostic and predictive value of baseline metabolic tumor volume (MTV) and total lesion glycolysis (TLG) derived from ^18F-FDG PET/CT in patients with solid malignancies, and to correlate these volumetric biomarkers with treatment response and progression-free survival (PFS).
**Methods or Background:** A prospective study was performed on 75 patients with biopsy-proven malignancies, including gynecologic, gastrointestinal, and thoracic cancers, who underwent baseline ^18F-FDG PET/CT prior to therapy. Quantitative parameters—SUVmax, SUVmean, MTV, and TLG—were obtained using semi-automated segmentation (SUV threshold ≥41% of SUVmax). Treatment response was assessed using PERCIST criteria at 3 and 6 months. Patients were followed up for 12 months to evaluate PFS. Statistical analysis included Kaplan–Meier survival curves, Cox regression, and Pearson's correlation.
**Results or Findings:** Baseline MTV and TLG values demonstrated strong predictive correlation with treatment outcomes and PFS. Patients with high MTV (>120 $cm^3$) and TLG (>850 g·$cm^3$) showed significantly lower response rates and shorter PFS (median 6.4 months) compared to those with low MTV/TLG (median 14.8 months; $p < 0.001$). While SUVmax alone failed to predict long-term outcome, combined volumetric parameters (MTV + TLG) achieved the highest prognostic accuracy (AUC = 0.91). Multivariate analysis confirmed MTV and TLG as independent predictors of disease progression.
**Conclusion:** Baseline metabolic tumor volume and total lesion glycolysis are robust quantitative biomarkers for predicting treatment response and survival, outperforming conventional SUV metrics in oncologic PET/CT.
**Limitations:** Single-center design, heterogeneous tumor population, and short follow-up duration limit generalizability. Multicentric studies with standardized segmentation protocols are warranted.
**Funding for this study:** No external funding was received for this study.
**Has your study been approved by an ethics committee?** Yes
**Ethics committee - additional information:** Institutional ethical committee approval was obtained.
**Author Disclosures:**
Sumati Sundaraiya: Nothing to disclose
Amritha Asokan: Nothing to disclose
Farook Abubacker Sulaiman: Nothing to disclose

**Correlation of FDG-PETCT derived Metabolic Tumor Volume (MTV) with DWI MRI for the assessment of colon cancer liver metastases**
*N. C. Balci*, G. Iuppa, A. K. Ansari, C. Hajj, A. Khan; Abu Dhabi/AE
*(ncbalci@gmail.com)*

**Purpose:** FDG-PETCT derived MTV is determinant parameter in oncologic assessment in colon cancer liver metastases. This study aims to identify if DWI can provide correlative results.
**Methods or Background:** FDG PETCT and DWI MRI of 50 patients with colon cancer liver metastases were retrospectively enrolled in the study, PETCT derived MTV was measured using automated software . DWI MRI was acquired in the scanner with highest b value of 800 mm2/sec and synthetic higher b value images were created in workstation with the values of 1000 mm2/sec, 1500 mm2/sec and 2000 mm2/sec. For each patient, the total number of liver lesions, total MTV and total lesion volumes on DWI MRI were calculated both on PETCT and on four different b value images. Correlation of total liver lesions was made with Pearson's r test. Correlation of MTV with DWI was made using paired t-test.
**Results or Findings:** Average number lesions for each patient were: 3.04 for PETCT, 3.67 for b= 800 mm2/sec (Pearson r=0.97), 3.45 for b=1000 mm2/sec (Pearson r=0.98), 3.07 for b=1500 mm2/sec (Pearson r= 0.99) and 2.97 for b=2000 mm2/sec (p Pearson r=0.98). The average total MTV was 56.25 cc, average total tumor volumes on DWI were 59.98 cc for b=800 mm2/sec, $p < 0.001$ (significantly higher than PET-CT); 58.41 cc for b=1000 mm2/sec, $p < 0.001$ (significantly higher than PET-CT); 56.41 cc for b=1500 mm2/sec, $p = 0.021$ (least significant difference); and 54.22cc for b=2000 mm2/sec, $p < 0.001$ (significantly lower than PET-CT).
**Conclusion:** The result of this study reveals significant correlation of number of lesions and total tumor volume compared to PETCT lesion count and MTV when b value of 1500 is assessed on DWI.
**Limitations:** Histopathology correlation of all metastases missing.
**Funding for this study:** Imaging Institute Cleveland Clinic
**Has your study been approved by an ethics committee?** Yes
**Ethics committee - additional information:** Research Ethics Committee Cleveland Clinic Abu Dhabi
**Author Disclosures:**
Amanullah Khan: Nothing to disclose
Ayham Khan Ansari: Nothing to disclose
Carla Hajj: Nothing to disclose
Giuseppe Iuppa: Nothing to disclose
Numan Cem Balci: Nothing to disclose

**Prospective assessment of baseline staging and treatment response in newly diagnosed multiple myeloma with combined 18F-FDG PET/CT and Whole-Body Diffusion-Weighted MRI**
*A. Cattabriga*, M. Talarico, C. Nanni, R. Regazzo, G. Pertile, S. Brocchi, S. Fanti, E. Zamagni, C. Mosconi; Bologna/IT
*(arrigo.cattabriga@unibo.it)*

**Purpose:** The aim of this study was to compare the diagnostic performance of PET/CT and WBMRI in the diagnosis and staging of smoldering multiple myeloma (SMM) and newly diagnosed multiple myeloma (NDMM), to correlate both imaging modalities with markers of disease burden and prognostic features and to validate in clinical practice the imaging response criteria IMPeTUs and MY-RADS.
**Methods or Background:** 205 patients (74 SMM, 131 NDMM) were prospectively enrolled between October 2022 and April 2025. All underwent baseline assessment with WBMRI and FDG-PET/CT. In NDMM, baseline-positive imaging was repeated before maintenance in transplant-eligible patients and after one year of treatment in transplant-ineligible patients. Deauville/IMPeTUs and MY-RADS criteria were applied to define focal lesions (FLs), diffuse disease and treatment response. Imaging findings were correlated with biochemical parameters and prognostic scores (ISS/R-ISS).
**Results or Findings:** FLs and paraskeletal disease were detected more frequently by WBMRI vs PET/CT ($p<0.05$). Both modalities showed concordance in detecting extramedullary disease ($\kappa=1$). WBMRI-detected FLs significantly correlated with worse prognosis by ISS/R-ISS. Diffuse disease was detected more frequently by PET/CT ($p<0.05$). However, WBMRI-defined diffuse disease correlated with multiple clinical parameters (hemoglobin <10 g/dL, bone marrow plasma cells, paraprotein, urinary protein), whereas PET-defined diffuse disease did not. In SMM, detection of FLs and diffuse disease was similar across techniques. In the preliminary analysis of the first 45 reassessed patients, WBMRI and PET/CT showed good concordance in response assessment ($\kappa=0.69$).

**Conclusion:** WBMRI was superior to PET/CT in detecting FLs and paraskeletal disease and correlated better with disease burden and prognosis. Both modalities showed concordance for extramedullary disease and response assessment.
**Limitations:** Main limitations are the small sample size in some subgroups (extramedullary disease, post-treatment reassessment). These cohorts are expected to expand; updated data will be presented.
**Funding for this study:** This study received no external funding. All expenses were covered by our institution's research funding
**Has your study been approved by an ethics committee?** Yes
**Ethics committee - additional information:** The institutional ethics board of our institution (IRCCS Policlinico di Sant'Orsola, Bologna) approved this study prior to patient enrollment
**Author Disclosures:**
Elena Zamagni: Nothing to disclose
Stefano Brocchi: Nothing to disclose
Marco Talarico: Nothing to disclose
Cristina Nanni: Nothing to disclose
Stefano Fanti: Nothing to disclose
Cristina Mosconi: Nothing to disclose
Arrigo Cattabriga: Nothing to disclose
Riccardo Regazzo: Nothing to disclose
Giacomo Pertile: Nothing to disclose

**Can the PET PSMA Primary Score offer similar performance to the PIRADS V2.1 from Multiparametric Magnetic Resonance to detect suspicious lesions of prostate cancer to guide prostate biopsy ?**

*M. Garcia Fontes*, M. Rodriguez Parodi, L. Valuntas, E. Otero, L. Mouro, V. Gigirey, L. Servente, G. Dos Santos; Montevideo/UY
*(margarita.garciafontes@cudim.org)*

**Purpose:** The detection of suspicious lesions of prostate cancer using Multiparametric Magnetic Resonance(mpMRI) is challenging because of the many pitfalls that often lead to unnecessary biopsies. The aim of the present study is to compare the concordance of the PIRADS Scale V 2.1 of mpMRI with the PRIMARY SCORE of the PSMA PET CT in the detection of suspicious lesions of clinically significative prostate cancer to determine if it can add sensitivity or specificity in order to guide more effective biopsies.
**Methods or Background:** Fifty patients aged between 48 and 85 years were included. They underwent mpMRI using 3T equipment, fusion biopsy using MRI and ultrasound, and PSMA PET/CT for initial staging. The number of PIRADS lesions detected by mpMRI were compared with the uptake areas using the PRIMARY PSMA Score and both methods were correlated with the pathological anatomy findings.
**Results or Findings:** A statistically significant correlation was found between PIRADS 4 and 5 lesions on mpMRI and SCORE 3 and 4 lesions on PSMA PET for Gleason 7 and higher grades found in the pahtologic anatomy. In one patient with a hip prosthesis, the PET PSMA Primary Score allowed the detection of suspicious lesions that were very doubtful due to artifacts in the mpMRI.
**Conclusion:** The analysis of multiparametric prostate MRI using the PIRADS V2.1 and the PSMA PET Primary Score has similar sensitivity for detecting suspicious lesions for prostate cancer to guide prostate biopsy. The PET PSMA Primary Score has slightly higher specificity than PIRADS V2.1 of mpMRI for clinically significant cancers. Both methods could be use together to detect suspicious lesions of prostate cancer increasing sensitivity. In patients with contraindications for mpMRI, PSMA PET SCORE seems to be a useful tool.
**Limitations:** Not aplicable
**Funding for this study:** No funding was provided for this study
**Has your study been approved by an ethics committee?** Yes
**Ethics committee - additional information:** Yes
**Author Disclosures:**
Gerardo Dos Santos: Nothing to disclose
Laura Valuntas: Nothing to disclose
Elena Otero: Nothing to disclose
Martin Rodriguez Parodi: Nothing to disclose
Veronica Gigirey: Nothing to disclose
Margarita Garcia Fontes: Nothing to disclose
Laura Mouro: Nothing to disclose
Liliana Servente: Nothing to disclose

**14:30 - 16:00 Research Stage 2**

### Research Presentation Session: Breast

### RPS 1002

### Breast cancer screening: technology, tools, and trends

**Moderator**
Sophia Zackrisson; Malmö/SE
*(sophia.zackrisson@med.lu.se)*
**Author Disclosures:**
Sophia Zackrisson: Advisory Board: Siemens and Bayer Advisory boards during 202, Board Member: EUSOBI and Swedish Society of Radiology, Founder: Z Imagination AB, consultancy firm in radiology, teaching and research, Grant Recipient: The Swedish Cancer Society, The Swedish Breast Cancer Association, Governmental funding for clinical research, Wallenberg Foundation, The Swedish Research Council, EU, Patent Holder: Patent approved, US (application no PCT/EP2014/057372)

**Evaluation of recall rates in the Irish National Breast Screening Programme: Insights from two million screening mammograms**

*S. Murphy*, T. Mooney, N. Phelan, A. Smith, A. Connors, A. Larke, S. Mcnally, P. Fitzpatrick, M. Mullooly; Dublin/IE
*(sophieacmurphy@gmail.com)*

**Purpose:** To examine recall patterns and characteristics within the population-based breast screening programme in Ireland.
**Methods or Background:** Breast cancer screening aims to reduce breast cancer mortality and morbidity through early detection and treatment. Recall rate is a key performance indicator of population-based breast screening, representing the proportion of women recalled for further evaluation. Guidance on acceptable recall rates vary internationally. An anonymous aggregate retrospective study of 2,031,995 mammography screening examination results, was conducted between 2000 and 2019. Descriptive patterns of recall rates and characteristics were examined and stratified by prevalent and incident examinations. Differences across the time-periods (2000-2008, 2009-2017 and 2018-2019) were assessed using Chi-square tests.
**Results or Findings:** Recall rate for screening examinations conducted during the full study period was 4.05% (n=82,338/2,031,995). Across three time-periods examined, recall rates among the prevalent screening examination group, increased, from 5.5% to 8.0% to 10.0% and within the incident group from 2.3%->2.8%->3.0%. Recalls due to calcifications and asymmetry increased over the time periods, most notably within the prevalent examinations where recalls due to calcification increased from 6.0/1,00->9.0/1,000->13.4/1,000(p<0.001),whilst recalls due to asymmetry increased from 17.1/1,000->31.3/1,000->41.0/1,000(p<0.001). Overall, among both prevalent and incident screening examinations, an increase in the cancer detection rate (CDR) was observed(p=0.005 and p<0.001 respectively). However, the overall positive predictive value(PPV) remained relatively stable.
**Conclusion:** This study highlights the upward trajectory of recall within Ireland's national breast screening service. The findings highlight the need for discussions among a diverse range of stakeholders, including national and international screening networks, to determine the optimal recall rate to ensure the benefits of screening are maximised and all potential harms are minimised.
**Limitations:** Lack of information regarding detailed characteristics of the cancers detected and information regarding breast density.
**Funding for this study:** No
**Has your study been approved by an ethics committee?** Yes
**Ethics committee - additional information:** As this study utilises existing anonymised data, formal ethical approval was not required in accordance with local ethics review board guidelines.
**Author Disclosures:**
Sorcha Mcnally: Nothing to disclose
Maeve Mullooly: Nothing to disclose
Alan Smith: Nothing to disclose
Patricia Fitzpatrick: Nothing to disclose
Alissa Connors: Nothing to disclose
Niall Phelan: Nothing to disclose
Therese Mooney: Nothing to disclose
Aideen Larke: Nothing to disclose
Sophie Murphy: Nothing to disclose

**Recall Rate and Cancer Detection Rate of the first round of Digital Breast Tomosynthesis screening in a Dutch population-based trial**

*L. Kregting*, L. Pennings, M. J. A. Smid-Geirnaerdt, A. Bluekens, J. Gommers, S. D. Verboom, I. Sechopoulos, M. Broeders; Nijmegen/NL
*(lindy.kregting@radboudumc.nl)*

**Purpose:** To evaluate the short-term screening performance of Digital Breast Tomosynthesis (DBT) in the first round of the DBT with Advanced Reading Methods (STREAM) prospective screening trial being performed in the Dutch Breast Cancer Screening Programme.
**Methods or Background:** The Dutch screening programme includes biennial four-view digital mammography (DM) for women 50 to 75 years old. All examinations are independently double-read by screening radiologists with consensus reading or arbitration in case of disagreement. STREAM trial participants underwent the first of two rounds of four-view DBT screening instead of DM between July 2023 and May 2024. A contemporaneous control group received regular DM screening. Current analyses include first round results for recall rate (RR) , stratified by Breast Imaging Reporting and Data System (BI-RADS) category 0, 4, or 5, and cancer detection rate (CDR) .
**Results or Findings:** Out of 44,682 invited screenees, 18,186 participated in STREAM (41%). Among them, 488 were recalled (RR: 26.8 per 1,000), of whom 199 (1.1%) were assessed as BI-RADS 0, 257 (1.4%) as BI-RADS 4, and 32 (0.2%) as BI-RADS 5. Of the recalled women, 166 women were diagnosed with screen-detected breast cancer (CDR: 9.1 per 1,000). The control group consisted of 95,052 participants of DM screening. Among them, 2,252 were recalled (RR: 23.7 per 1,000), of whom 661 were diagnosed with breast cancer (CDR: 7.0 per 1,000). This results in an increase in RR of 2.9 per 1,000 (p=0.012) and an increase in CDR of 2.1 per 1,000 (p=0.002) for DBT screening compared to DM.
**Conclusion:** First round results suggest a modest increase in RR and an increase in CDR for DBT screening compared to DM in the Dutch breast cancer screening programme.
**Limitations:** Interval cancer and second round data not available yet.
**Funding for this study:** This study is funded by ZonMW (grantnr.:5550402130002) and KWF (grantnr.:13710).
**Has your study been approved by an ethics committee?** Yes
**Ethics committee - additional information:** Trial approved by the Dutch Minister of Health, Welfare and Sport in view of the Population Screening Act (WBO). (registrationnr.:3574709-1044141-PG).
**Author Disclosures:**
Mireille Broeders: Grant Recipient: Screenpoint Medical, Sectra Benelux, Hologic, Volpara Solutions, Lunit, iCAD Speaker: Hologic, Siemens Healthcare.
Sarah Delaja Verboom: Research/Grant Support: Screenpoint Medical
Jessie Gommers: Nothing to disclose
Adriana Bluekens: Nothing to disclose
Lindy Kregting: Nothing to disclose
Ioannis Sechopoulos: Research/Grant Support: Siemens Healthcare, Canon Medical Systems, Screenpoint Medical, Sectra Benelux, Volpara Healthcare, Lunit Advisory Board: Koning Corp Speaker: Canon Medical.
Maartje Julma Alwine Smid-Geirnaerdt: Nothing to disclose
Lian Pennings: Nothing to disclose

**Missed cancers at mammography: histological and biological characterisation in the P.I.N.K. study**

*L. A. Incardona*[1], M. Franchini[2], S. Molinaro[2], S. Pieroni[2], J. Nori[3], G. P. Scaperrotta[4], A. Nicolucci[3], E. Cassano[5], E. Montrucchio[1]; [1]La Spezia/IT, [2]Pisa/IT, [3]Florence/IT, [4]Milano/IT, [5]Milan/IT
*(incardonaludovica@gmail.com)*

**Purpose:** Mammography (MX) is the cornerstone of breast cancer screening but has limited sensitivity in specific subgroups. The P.I.N.K. Study was designed to evaluate the role of multimodality imaging in breast cancer diagnosis. This sub-analysis aimed to characterise cancers missed by MX and detected only by additional imaging.
**Methods or Background:** The P.I.N.K. database included 29,360 women undergoing 60,270 integrated diagnostic exams between 2017 and 2025. Among 1,246 surgically confirmed breast cancers, 233 (18.7%) were not visible on MX and were diagnosed exclusively by ultrasound (US), digital breast tomosynthesis (DBT), or magnetic resonance imaging (MRI). Breast density, histology and biological subtype were analysed.
**Results or Findings:** Missed cancers accounted for 233 of 1,246 cases (18.7%). Most were invasive (80%, 186/233), multifocal in nearly one third (32%, 74/233), and predominantly found in dense breasts (75%, 175/233). Younger women under fifty years were affected (40%). Histologically, invasive lobular carcinoma represented about 20% (47/233), while invasive ductal carcinoma accounted for the majority. Regarding biological profile, Luminal A was the most frequent subtype (55%, 128/233), followed by Luminal B (25%, 58/233), with HER2-positive (12%, 28/233) and triple-negative tumours (8%, 19/233) less common. US detected 142 of 233 missed cancers (60.9%), DBT 56 (24.0%), and MRI 35 (15.0%). These findings indicate that missed cancers are clinically significant and not indolent.
**Conclusion:** In this large multicentre cohort, almost one in five breast cancers were missed by MX but detected through integrated imaging. Their profile (dense breasts, younger women, invasive histology, and distinct biological features) highlights the clinical relevance of multimodality approaches. P.I.N.K. shows that the value of integrated imaging is both quantitative and qualitative, preventing missed cancers from becoming interval cancers and supporting personalised diagnostic strategies.
**Limitations:** This was a retrospective analysis of observational data.
**Funding for this study:** No funding was received for this study.
**Has your study been approved by an ethics committee?** Not applicable
**Author Disclosures:**
Alberto Nicolucci: Nothing to disclose
Sabrina Molinaro: Nothing to disclose
Jacopo Nori: Nothing to disclose
Edgardo Montrucchio: Nothing to disclose
Stefania Pieroni: Nothing to disclose
Gianfranco Paride Scaperrotta: Nothing to disclose
Michela Franchini: Nothing to disclose
Ludovica Anna Incardona: Nothing to disclose
Enrico Cassano: Nothing to disclose

**Supplemental MRI screening for women with extremely dense breasts: results of three screening rounds of the DENSE trial**

*S. V. Grinsven*[1], E. Monninkhof[1], R. Mann[2], W. B. Veldhuis[1], C. Van Gils[1], The Dense Trial Study Group[1]; [1]Utrecht/NL, [2]Nijmegen/NL
*(s.e.l.vangrinsven-2@umcutrecht.nl)*

**Purpose:** To study the effect of supplemental MRI screening for women with extremely dense breasts on advanced breast cancers.
**Methods or Background:** Dutch women with extremely dense breasts and a negative mammogram were pre-randomized to either the MRI-invitation (n=8,061) or control group (n=32,312, standard mammography). In the first (prevalent) round, supplemental MRI led to higher cancer detection and fewer interval cancers. The rate of advanced breast cancers in subsequent (incident) screening rounds serves as a further important measure to assess the impact on health outcomes. Since many women randomized to MRI-invitation did not participate, intention-to-treat analyses dilute the true effect. Therefore, our main analysis is a per-protocol approach, comparing advanced breast cancer (TNM stage II+) rates, adjusted for age and socioeconomic status using inverse probability weighting. Rate differences (RD) were calculated with 95% confidence intervals.
**Results or Findings:** As expected, in the first round, the advanced breast cancer rate per 1000 women was similar between MRI participants and controls (RD: 0.8 [95% CI: -0.6, 2.2]). In the second round, the rate was lower in the MRI group but not yet statistically significant (RD: 1.4 [95% CI: -0.3, 3.2]). By the third round, the rate in the MRI group was significantly lower than in the control group (RD: 2.6 [95% CI: 0.9, 4.3]).
**Conclusion:** From the second round, MRI participants had lower advanced cancer rates, reaching significance in the third round. These findings show that the health benefits of MRI screening likely extend beyond lowering interval cancers.
**Limitations:** The primary outcome used a per-protocol and not an intention-to-treat approach. As mortality could not be studied due to limited sample size and follow-up, advanced breast cancer was used as a surrogate. These results will inform mortality modelling.
**Funding for this study:** Supported by the University Medical Center Utrecht (project number, UMCU DENSE), the Netherlands Organization for Health Research and Development (project numbers, ZonMW-200320002-UMCU and ZonMW Preventie 50-53125-98-014), the Dutch Cancer Society (project numbers, DCS-UU-2009-4348, UU-2014-6859, and UU-2014-7151), the Dutch Pink Ribbon–A Sister's Hope (project number, Pink Ribbon-10074), Bayer Pharmaceuticals (project number, BSP-DENSE), and Stichting Kankerpreventie Midden-West. For research purposes, Volpara Health Technologies provided Volpara Imaging Software, version 1.5, for installation on servers in the screening units.
**Has your study been approved by an ethics committee?** Yes
**Ethics committee - additional information:** The Dutch Minister of Health, Welfare and Sport, who was advised by the Health Council of the Netherlands (2011/2019 WBO, The Hague, The Netherlands), approved the DENSE trial on November 11, 2011.
**Author Disclosures:**
Carla Van Gils: Nothing to disclose
Evelyn Monninkhof: Nothing to disclose
Wouter B. Veldhuis: Nothing to disclose
The Dense Trial Study Group: Nothing to disclose
Sophie Van Grinsven: Nothing to disclose
Ritse Mann: Nothing to disclose

**From Screening to Clinical Diagnosis: Can AI Match Expert Eyes in Mammography?**

*M. R. López De La Torre Carretero*[1], D. A. Zambrano[1], A. M. Delgado Brito[1], C. D. Solano[1], A. Elizalde[1], L. J. Pina Insausti[1], P. Martinez Miravete[2]; [1]Pamplona/ES, [2]Zaragoza/ES
*(mltorrec@unav.es)*

**Purpose:** To evaluate the performance of an artificial intelligence (AI) tool in classifying mammograms from opportunistic screening versus clinical populations when compared with expert radiologist interpretation.
**Methods or Background:** This prospective multicenter study included 2,574 patients who underwent digital mammography, tomosynthesis, and targeted ultrasound when indicated. Patients were stratified into opportunistic screening (76.7%) or clinical setting (symptomatic, oncologic follow-up...) groups. Mammograms were analyzed by a commercially available AI tool, which categorized studies as "normal" or "suspicious". Radiologists also classified studies as "normal"/"suspicious", blinded to AI. The reference standard was histopathology (when available) or the final radiologist assessment after ultrasound. Performance was compared using chi-square and McNemar tests, both globally and across patient subgroups.
**Results or Findings:** Mean age was 56.4 years (55.4 in screening, 59.7 in clinical). The AI classified 38% of cases as suspicious (31.1% in screening and 58% in clinical patients; $p < 0.0001$). Meanwhile, radiologists classified 20% of cases as suspicious (22% in screening vs 13% in clinical; $p < 0.0001$). In the global analysis, AI sensitivity was 87.9%, with limited specificity (63.9%), with 64.4% accuracy. Radiologists showed 91.3% sensitivity, with significantly higher specificity (86.3%), overall precision (86.5%), and agreement with the reference standard (kappa = 0.20 vs. 0.06 for AI). Stratified analysis showed that AI performed better in screening (sensitivity 94.3%, specificity 70.1%) than in clinical settings (sensitivity 84.6%, specificity 43.4%). In clinical cases, radiologists achieved 98% sensitivity and 81.2% specificity (kappa = 0.27), outperforming AI across all measures. All comparisons between AI and radiologists were statistically significant ($p < 0.0001$).
**Conclusion:** AI demonstrated robust performance in screening settings. However, its limited specificity and lower accuracy in clinical scenarios highlight the need for substantial improvements before it can match expert radiologist diagnosis.
**Limitations:** Retrospective
**Funding for this study:** None
**Has your study been approved by an ethics committee?** Yes
**Ethics committee - additional information:** Research Ethics Committee - Clínica Universidad de Navarra
**Author Disclosures:**
Paula Martinez Miravete: Nothing to disclose
Arlette Elizalde: Nothing to disclose
Manuel Rafael López De La Torre Carretero: Nothing to disclose
Adolfo Manuel Delgado Brito: Nothing to disclose
Daniel Alfonso Zambrano: Nothing to disclose
Carlos Delgado Solano: Nothing to disclose
Luis Javier Pina Insausti: Nothing to disclose

**Screen-detected high-risk lesions of the breast: adherence to surveillance and probability of breast cancer development**

*E. L. Van Der Veer*[1], A. Bluekens[1], A. M. P. Coolen-Janssen[1], W. Vreuls[2], A. Voogd[3], L. Duijm[2]; [1]Tilburg/NL, [2]Nijmegen/NL, [3]Maastricht/NL
*(e.l.vanderveer93@gmail.com)*

**Purpose:** The prevalence of breast lesions of uncertain malignant potential, commonly referred to as high-risk lesions (HRLs), detected at screening mammography is increasing, which stresses the importance of the recently published guidelines on their management. In this study, we present the incidence, upgrade rate, adherence, and follow-up outcomes in a screened population.
**Methods or Background:** A total of 17,809 recalled women, who took part in the Dutch screening program between January 2009 and July 2019, were included in this retrospective analysis of a prospectively obtained database. A HRL was identified in 537 recalled women and their incidence rate, diagnostic work-up and follow-up, and any upgrade to (pre)malignancy after surgical excision and during follow-up were examined.
**Results or Findings:** The incidence rate of HRLs was 0.87 per 1,000 screens and 3.02 per 100 recalls. The majority of HRLs consisted of papillary lesions (32.4%) and atypical ductal hyperplasia (ADH) (21.6%). Surgical excision was performed for 254 (47.3%) of the 537 HRLs, resulting in 59 (23.2%) HRLs being upgraded to (pre)malignancy. Invasive tumors were mainly found in papillary lesions with atypia (n=9/17) and ADH (n=6/17). Of the women diagnosed with an HRL, 60.7% had an indication for radiologic follow-up. Of these women, 79.1% actually underwent follow-up with 43.0% undergoing 5-year surveillance. During follow-up, 28 (10.9%) of the women with an HRL developed a (pre)malignancy.
**Conclusion:** Of women with screen-detected HRLs, 16.2% developed breast cancer, either after surgical excision or during follow-up. Less than half of the women completed the 5-year surveillance, highlighting the importance of continued attention to obtain maximal follow-up adherence.
**Limitations:** ADH diagnosis varies between observers due to its similarity to low-grade DCIS, with biopsy type and sample size affecting outcomes. Some subgroups were too small for comparison with guidelines.
**Funding for this study:** This research did not receive any funding.
**Has your study been approved by an ethics committee?** Not applicable
**Author Disclosures:**
Willem Vreuls: Nothing to disclose
Angela M P Coolen-Janssen: Nothing to disclose
Lucien Duijm: Nothing to disclose
Adriana Bluekens: Nothing to disclose
Eline Leontine Van Der Veer: Nothing to disclose
Adri Voogd: Nothing to disclose

**Factors that impact upon mammographic interpretation: differences between Radiography Advanced Practitioners and Radiologists caution against inter-cohort generalisations**

N. F. Clerkin[1], C. Ski[1], *P. Brennan*[2], R. M. Strudwick[3]; [1]Belfast/UK, [2]Sydney/AU, [3]Ipswich/UK

**Purpose:** Radiography Advanced Practitioners (RAPs) have interpreted mammograms in the United Kingdom since 1995 (1). Evidence on factors influencing RAP diagnostic performance remains limited. This study aimed to identify reader and image-based factors affecting RAP performance in mammography interpretation.
**Methods or Background:** The research comprised of three components. First, a systematic literature review, identifying 38 studies on performance variation in mammography. Second, experimental work which involved 18 UK based RAPs interpreting 60 cloud hosted mammograms with known truth; results were analysed against reader characteristics. Third, a comparative observer study focussed on factors that impact performance, RAPs (n=18) and radiologists (n=24) interpreted the same cases. Difficulty indices were calculated for all cases using an established methodology and causal agents influencing performance were explored.
**Results or Findings:** The review highlighted a paucity of RAP specific performance data. A range of findings included, higher ROC values with: less compared with more than 10 years' experience (p=0.004); more compared with less than 100 cases read per week (p=0.036); consistent rather than sporadic use of prior mammograms (p=0.0231); recent rather than distant eye testing (p=0.021). Specificity was lower amongst RAPs who stated their emotional mindset impacted their reading (88) compared to those who did not (66.5) (p=0.034). Strong correlations were found between RAPs and radiologists for cases with specific levels of difficulty (cancer: r = 0.83; normal: r = 0.73), uniquely to RAPs, soft tissue cancers compared with calcifications as well as cases without prior images were more challenging.
**Conclusion:** For the first time, factors that impact performance of RAPs have been identified. Whilst some factors are common for radiologists and RAPs, some are unique, suggesting that extending findings to both must be done with caution.
**Limitations:** Limitations included questionnaire responses and gender representation.
**Funding for this study:** None
**Has your study been approved by an ethics committee?** Yes
**Ethics committee - additional information:** Ethical approval was gained from the University of Suffolk Post Graduate Research Committee on the 14th of January 2022 (RETH(P)21/006) . NHS Ethical considerations were achieved through the NHS Health Research Authority (HRA). The HRA confirmed no Research Ethics Committee review was required. Ethical exemption was also provided by the University of Sydney to include radiologist performance data in the study.
**Author Disclosures:**
Noelle Frances Clerkin: Nothing to disclose
Patrick Brennan: Nothing to disclose
Ruth Mary Strudwick: Nothing to disclose
Chantal Ski: Nothing to disclose

**Participation of the transgender population in breast and prostate cancer screening**

*T. Jezek*[1], A. Beck-Toelly[1], N. Miłecki[1], N. Ailec[1], N. Pötsch[1], A. D'Angelo[2], P. A. Baltzer[1], T. H. Helbich[1], P. Clauser[1]; [1]Vienna/AT, [2]Rome/IT
*(n11817305@students.meduniwien.ac.at)*

**Purpose:** The objective of the study was to assess the participation of the transgender (TG) population in breast and prostate cancer screening programs.
**Methods or Background:** This retrospective, monocentric study was approved from the local ethics committee. All transgender patients attending the dedicated local clinic were included. Data on breast imaging examination and prostate specific antigen (PSA) controls were retrieved by three investigators from the local information system. Exclusion criteria were lack of data in the system, no hormonal therapy. Data retrieved were type and duration of the hormonal therapy, family history for breast and prostate cancer, personal history of breast or prostate cancer and previous PSA and mammography exams. Descriptive statistics was used to present the data.
**Results or Findings:** To date, 750 TG individuals have been evaluated (mean age 34.7, standard deviation SD 16.9; 389 TG male and 361 TG female). 68 had a positive family history for breast or ovarian cancer (9.1%), and 16 (2.1%) for prostate cancer. Only one individual with a personal history of breast cancer was identified (TG male). Of the 209 individuals above 40 years old (mean age 53.6, SD 9.5), only 13 (one TG male, 12 TG female) participated in mammography screening (6.2%). Among the 126 TG females, 50 underwent a PSA test (39.7%). No breast or prostate cancers were diagnosed during or after the transition.
**Conclusion:** Our findings show a very low participation in breast cancer screening among the TG population, even in individuals with a positive family history. Participation in prostate cancer testing was higher, most likely because it is conducted through a simple blood test. This indicates a widespread lack of awareness regarding available screening programs and their relevance.
**Limitations:** Retrospective study involving many patients with incomplete data.
**Funding for this study:** No funding was received for this study.
**Has your study been approved by an ethics committee?** Yes
**Ethics committee - additional information:** The study was approved by the local ethics committee (1183/2023).
**Author Disclosures:**
Teresa Jezek: Nothing to disclose
Pascal A.T. Baltzer: Nothing to disclose
Nina Pötsch: Nothing to disclose
Thomas H. Helbich: Nothing to disclose
Andrea Beck-Toelly: Nothing to disclose
Nick Ailec: Nothing to disclose
Nastassja Miłecki: Nothing to disclose
Paola Clauser: Grant Recipient: Siemens Healthineers
Anna D'Angelo: Nothing to disclose

**Radiofrequency-Based Imaging for Breast Screening: Interim Analysis Results from MammoScreen Clinical Trial**

*G. Tiberi*[1], D. Álvarez Sánchez-Bayuela[2], N. Ghavami[1], R. Loretoni[1], T. Frauenfelder[3], C. Alves[4], M. Álvarez-Benito[5], M. Calabrese[6], A. S. Tagliafico[6], J. Lubinski[7], C. Romero Castellano[2]; [1]Perugia/IT, [2]Toledo/ES, [3]Zürich/CH, [4]Lisbon/PT, [5]Córdoba/ES, [6]Genoa/IT, [7]Szczecin/PL
*(gianluigi@ubt-tech.com)*

**Purpose:** To present interim results of the study "A Clinical Investigation to Evaluate Microwave Imaging via MammoWave in a Population-based Screening Program for Early Breast Cancer (BC) Detection" (ClinicalTrials.gov ID:NCT06291896), activated within HORIZON-MISS-2021-CANCER-02-01 scheme.
**Methods or Background:** Women undergoing routine screening mammograms were invited to join the study. After providing informed consent, participants underwent microwave breast imaging using MammoWave. MammoWave's output was automatically labelled by an artificial intelligence (AI) model (based on hierarchical machine-learning approach), classifying each breast as "NSF" (no-suspicious findings, no lesion or lesion with low suspicion) or "WSF" (with-suspicious findings, indicating presence of a suspicious lesion). MammoWave's results are compared to a reference standard, defined as the outcome of the conventional breast examination pathway—including histological confirmation—with a two-year follow-up. Reference standard will be classified as 'positive' when BC is confirmed by histology, and 'negative' otherwise. Primary outcomes are sensitivity/specificity of MammoWave's AI model in BC detection.
**Results or Findings:** We report findings from the first 3,000 volunteers enrolled at 9 hospitals across 5 European countries. The interim analysis was performed on 5,896 breasts (12 'positive' cases), from 2,967 subjects included in full analysis set population (mean age 57.2 years±7.5 [SD]). This interim analysis allowed us to evaluate AI model's performance, with 42% sensitivity [95%CI:13.8-69.6] and 75% specificity [95%CI:74.1-76.3]. We proceeded to update AI model by fusing information of hierarchical machine-learning approach and statistical classifiers. Updated AI model allowed us to (retrospectively) reach: 67% sensitivity [95%CI:29–99] (80% in dense breasts [95%CI:45–99]); 81% specificity 95%CI:79.2-83.2] (80% in dense breasts [95%CI:77–83]).
**Conclusion:** MammoWave's AI model demonstrates promise in breast cancer screening, particularly in terms of specificity; sensitivity requires further optimization. Final results of this trial on 10,000 volunteers will provide more insights.
**Limitations:** Non-randomized design; small cancer sample size.
**Funding for this study:** This work was supported by the funding received by the MammoScreen project, co-funded by the European Union's Horizon research and innovation framework programme, Grant agreement 101097079.
**Has your study been approved by an ethics committee?** Yes
**Ethics committee - additional information:** This study was approved by the Research Ethics Committee of the Liguria Region (CET), Italy, on 13 November 2023 (approval number: CET - Liguria: 524/2023 - DB id 13399), the Research Ethics Committee of Complejo Hospitalario de Toledo (CEIC), Spain, on 29 November 2023 (approval number: CEIC - 1094), the National Ethics Committee for Clinical Research (CEIC), Portugal on 12 January 2024 (approval number: CEIC - 2311KC814), the Bioethical Committee of Pomeranian Medical University in Szczecin, Poland (approval number: KB-006/23/2024) on 13 March 2024, and the Zurich Cantonal Ethics Commission, Swiss (BASEC 2023-D0101) on 7 June 2024.
**Author Disclosures:**
Celeste Alves: Nothing to disclose
Marina Álvarez-Benito: Nothing to disclose
Thomas Frauenfelder: Nothing to disclose
Daniel Álvarez Sánchez-Bayuela: Nothing to disclose
Alberto Stefano Tagliafico: Nothing to disclose
Jan Lubinski: Nothing to disclose
Cristina Romero Castellano: Nothing to disclose
Gianluigi Tiberi: Founder: Gianluigi Tiberi is co-founder of UBT - Umbria Bioengineering Technologies.
Riccardo Loretoni: Nothing to disclose
Navid Ghavami: Founder: Navid Ghavami is co-founder of UBT - Umbria Bioengineering Technologies.
Massimo Calabrese: Nothing to disclose

**Breast Arterial Calcifications on Mammography as a Surrogate Marker for Coronary Artery Disease in Women Aged 40–50 Years: A TriNetX Database Study**

*V. Batra*, J. Harvey; Rochester, NY/US
*(vivekbatramd@gmail.com)*

**Purpose:** Breast arterial calcifications (BAC) on mammography are frequently underreported. Growing evidence suggests that BAC correlate with coronary artery disease (CAD) and may serve as a surrogate marker of cardiovascular risk in women undergoing routine breast cancer screening. In women without a known history of cardiovascular disease, consistent recognition and reporting of BAC could facilitate early risk stratification and preventive intervention.
**Methods or Background:** Using the TriNetX database, we conducted a retrospective analysis of women aged 40–50 years who underwent screening mammography, identified through ICD-10 codes. We quantified the total number of screening exams, the proportion with reported calcifications, and the subset of patients with documented CAD. We also examined the potential underestimation of BAC prevalence due to their frequent classification as BI-RADS 1 (negative) rather than BI-RADS 2 (benign findings).
**Results or Findings:** Across 109 U.S. healthcare organizations, 6,783,908 screening mammograms were performed in women aged 40–50 years. Of these, 2,396,182 examinations were reported as abnormal or inconclusive. A total of 6,070,184 women in this age group had a diagnosis of CAD. The intersection of women with both BAC and CAD included only 4,420 patients—substantially lower than expected—suggesting significant underreporting likely related to BI-RADS coding variability.
**Conclusion:** Coronary artery disease remains the leading cause of death among women in the United States. The inconsistent reporting of vascular calcifications on mammography may obscure an important opportunity for early cardiovascular risk detection. Standardizing the reporting of BAC as BI-RADS 2 could enhance identification of subclinical atherosclerosis and support earlier, targeted cardiovascular prevention in women without known heart disease.
**Limitations:** This study is limited by its retrospective design and reliance on ICD-10 coding within the TriNetX database, which may lead to underreporting or misclassification of vascular calcifications and coronary artery disease.
**Funding for this study:** None
**Has your study been approved by an ethics committee?** Not applicable
**Author Disclosures:**
Vivek Batra: Nothing to disclose
Jennifer Harvey: Nothing to disclose

**AI-Enabled Detection of Breast Arterial Calcifications in Mammography: Diagnostic Performance and Impact of Image Quality in a Large Real-World Screening Population**

*J. Sänger*[1], J. Happe[1], A. Dudle[2], C. Ruppert[2], A. Weber[3], A. Ciritsis[2], T. Frauenfelder[1], A. Boss[3]; [1]Zürich/CH, [2]Zurich/CH, [3]Wetzikon/CH
*(jonathan.saenger@usz.ch)*

**Purpose:** To determine the prevalence of breast arterial calcifications (BAC) in a large cohort of opportunistic mammography breast cancer screening using artificial intelligence (AI) and to highlight both their potential diagnostic overlap with grouped microcalcifications and their role as an opportunistic biomarker for cardiovascular risk assessment.

**Methods or Background:** This retrospective study included 4692 women who underwent a total of 6,061 mammographic examinations with a total of 24,519 mammographic images between 2011 and 2017 at a tertiary centre. A YOLO-based object detection algorithm was trained on standard mammographic projections (CC and MLO) to detect and differentiate BAC and non-vascular calcifications, assigning BI-RADS categories to the latter.
**Results or Findings:** BAC was found with AI-based analysis in 652 of 4,692 women (13.9%, 95% CI 12.9–14.9). Among 6,061 individual examinations, BAC were present in 800 (13.2%, 95% CI 12.4–14.1). Considering all 24,519 mammographic images, BAC was detected in 1,644 images (6.7%, 95% CI 6.4–7.0). At the time of the first mammogram, BAC-positive woman had a mean age of 66.6 ± 10.9 years, whereas those without BAC had a mean age of 56.3 ± 11.3 years ($p < 0.001$).
**Conclusion:** Artificial intelligence applied to mammography revealed that one in seven women has BAC. Early BAC can be challenging to distinguish from suspicious grouped microcalcifications, and systematic AI-based screening during routine breast imaging may aid differentiation. Moreover, BAC recognition is clinically relevant as it correlates with CAC and cardiovascular risk. Embedding automated BAC detection into established breast cancer screening programmes may therefore provide a low-cost, scalable opportunity for opportunistic cardiovascular risk assessment, with the potential to enable earlier identification of women at risk and contribute to risk reduction.
**Limitations:** The limitations of the study are the retrospective design and the single-centre setting.
**Funding for this study:** Funding was provided by the Swiss Cancer League (KFS-5524-02-2022) and the Clinical Research Priority Program “Artificial Intelligence in oncological Imaging” of the University of Zurich.
**Has your study been approved by an ethics committee?** Yes
**Ethics committee - additional information:** The study was approved on 6 July 2021 by the Institutional Review Board (No. 2021-01095).
**Author Disclosures:**
Andreas Boss: Shareholder: b-rayZ
Carlotta Ruppert: Employee: b-rayZ
Thomas Frauenfelder: Nothing to disclose
Anna Weber: Nothing to disclose
Jasmin Happe: Nothing to disclose
Alexander Ciritsis: Employee: b-rayZ
Alice Dudle: Employee: b-rayZ
Jonathan Sänger: Nothing to disclose

**14:30 - 16:00 Research Stage 6**

### Research Presentation Session: Cardiac

### RPS 1003

### Ischemia and myocardial infarction

**Moderator**
Konstantin Nikolaou; Tübingen/DE
**Author Disclosures:**
Konstantin Nikolaou: Research Grant/Support: Siemens Healthineers, Bayer Healthcare

### Left Ventricular Ejection Fraction Partially Mediates the Relationship Between Myocardial Replacement Fibrosis and Sudden Cardiac Death Post Myocardial Infarction

*K. Aisikaier*, P. Zhou; Beijing/CN

**Purpose:** To evaluate the potential mediating role of left ventricular ejection fraction (LVEF) in the relationship between replacement fibrosis (assessed by late gadolinium enhancement (LGE)) and sudden cardiac death (SCD) in patients post-myocardial infarction (MI), and also to assess this mediation effect in subgroups based on LVEF≤35% and>35% according to implantable cardioverter-defibrillator (ICD) selection criterion.
**Methods or Background:** A retrospective analysis was conducted on 917 post-MI patients (mean age: 56.3±11.0 years, 88.8% male) who underwent cardiac MR from January 2017 to August 2021. The endpoint for SCDs included SCD, aborted SCD, and appropriate ICD discharges. The association of LGE with LVEF was quantified using linear regression models. The associations of LGE and LVEF with SCDs were evaluated using competition risk models. Mediation analysis was then used to decompose the total effect of LGE on SCDs into direct and indirect (mediated through LVEF) effects using accelerated failure time models.
**Results or Findings:** Over a median follow-up of 63.3 (IQR, 43.6 to 76.6) months, 65 patients (7.1%) experienced SCDs. LGE was significantly associated with lower LVEF ($\beta$=-0.35, $p<0.001$). Both LGE and LVEF independently predicted SCDs (sHR=1.06, $p<0.001$; sHR=0.95, $p=0.03$, respectively). Mediation analysis showed that LVEF accounted for 19.7% of the total effect of LGE on SCDs ($p<0.001$). This mediation effect was 40.4% in patients with LVEF>35% ($p=0.02$), while no mediation was observed in patients with LVEF≤35% ($p=0.08$).
**Conclusion:** LVEF partially mediated the effect of LGE on the SCD, accounting for less than one-fifth of the total effect. LVEF alone inadequately captured the whole SCD risk, irrespective of whether LVEF is greater than 35% or 35% or less.
**Limitations:** Due to the retrospective nature of the study, the interval between the last MI and CMR varied, which may introduce selection bias.
**Funding for this study:** This study is supported by the National Key R&D Program of China (Nos. 2021YFF0501400 and 2021YFF0501404), the Key Project of National Natural Science Foundation of China (No. 82430066), and the China International Medical Foundation (No. Z-2014-07-2101).
**Has your study been approved by an ethics committee?** Yes
**Ethics committee - additional information:** The study adhered to the principles outlined in the Declaration of Helsinki and received approval from the Institutional Review Board (IRB) and the hospital’s clinical audit department.
**Author Disclosures:**
Pengyu Zhou: Nothing to disclose
Kaisaierjiang Aisikaier: Nothing to disclose

### Cardiac Spectral PCCT Radiomics and Clinical Data for Optimizing Cath Lab Triage in ACS

*U. Schmid*, A. S. Brendlin; Tübingen/DE

**Purpose:** Many ACS patients undergo cardiac CT and same-day catheterization, yet only a subset require stenting, leading to possible resource overuse. Photon-counting detector CT (PCCT) provides unique spectral data. We evaluated five classification approaches: clinical data alone (C), radiomics from mixed (M) or spectral high/low energy bins (HL), and early fusion of clinical with mixed (CM) or spectral (CHL) data. We hypothesized PCCT’s inherent informational gain could enhance resource stratification.
**Methods or Background:** We retrospectively analyzed PCCT cardiac scans from 96 patients undergoing same-day catheterization (15 stented). Clinical data and radiomics from automated AHA myocardial segments (Syngo.via) entered an identical ML pipeline: SMOTETomek, StandardScaler, sequential feature selection (univariate, XGBoost importance, ElasticNet, correlation filtering, RFECV), and an XGBoost classifier with BayesSearchCV tuning. Performance was assessed on a held-out test set via Matthews Correlation Coefficient (MCC), with Bayesian (posterior distributions, BFs, HDIs) and frequentist (bootstrap, adj. p) comparisons.
**Results or Findings:** Clinical data (C) alone was limited (MCC: 0.427, HDI [0.321–0.530]). Radiomics-only spectral (HL: MCC 0.726, HDI [0.617–0.826]) outperformed mixed (M: MCC 0.513, HDI [0.393–0.626]), confirmed by frequentist ($P<0.001$) and Bayesian analyses ($BF_{10}$=2.56). Fused models performed best: CHL (MCC 0.966, HDI [0.929–0.996]) and CM (MCC 0.954, HDI [0.911–0.991]). Bayesian comparisons showed CHL superior to C, M, and HL ($BF_{10}>100$) but not decisively over CM ($\Delta$=0.012, HDI [−0.049–0.069]; $BF_{10}$=0.04, $P$=0.737).
**Conclusion:** Clinical data alone is insufficient. Spectral radiomics outperform mixed, and fusion (CM, CHL) markedly improves classification. Fusing clinical with spectral radiomics shows highest accuracy, supporting optimized post-CT stratification and cath lab triage.
**Limitations:** Retrospective single-center design, small cohort, and vendor-specific PCCT/AID implementation. Radiomics extraction and ML pipeline choices may limit generalizability. Clinical endpoints were surrogates without outcome validation. Findings require external validation across centers, scanners, algorithms, and larger populations.
**Funding for this study:** None
**Has your study been approved by an ethics committee?** Yes
**Ethics committee - additional information:** University Hospital Tuebingen
**Author Disclosures:**
Andreas Stefan Brendlin: Nothing to disclose
Ulrich Schmid: Nothing to disclose

### Diagnostic performance of a coronary CT angiography-based Deep Learning Model for the prediction of vessel-specific ischemia

*B. Peters*[1], R. Symons[2], A. Nchimi Longang[3], O. Ghekiere[1]; [1]Schilde/BE, [2]Olv-Waver/BE, [3]Strassen/LU

**Purpose:** Intracoronary pressure measurement is the standard of care for assessing vessel specific ischemia of coronary stenosis. New techniques using artificial intelligence emerge to compute Fractional Flow Reserve(FFR) based on coronary computed tomography angiography(CCTA) images(FFRAI). We assess a new FFRAI Deep Learning Model for the prediction of vessel specific ischemia compared to two validated invasive physiological measurements.
**Methods or Background:** We retrospectively selected 322 vessels from 275 patients at two different centers who underwent CCTA and intracoronary catheter measurements with FFR or instantaneous wave-free ratio(iFR) within three months. Two radiologist at each center generated curvilinear reformats of each coronary artery for FFRAI≤0.80 prediction. Sensitivity, specificity, positive predictive value(PPV), negative predictive value(NPV) and diagnostic accuracy of FFRAI were calculated using FFR≤0.80(n=224) and iFR≤0.89(n=238) as standards of reference. Radiologists' concordance was assessed with Cohen's κ. A multinomial logistic regression model including all FFRAI false-positive and false-negative was built to evaluate the influence of patient- and CCTA-related parameters on the diagnostic value of FFRAI.
**Results or Findings:** Sensitivity, specificity, PPV, NPV, and diagnostic accuracy of FFRAI in predicting FFR≤0.80 and iFR≤0.89 were 85%(58/68) and 91%(78/86), 82%(128/156) and 78%(119/152), 67%(58/86) and 70%(78/111), 93%(128/138) and 94%(119/127), and 83%(186/224) and 83%(197/238), respectively. Concordance between senior and junior radiologists' supervision was substantial(κ=0.725,P<0.001). Calcium score of the stenotic vessel has a significant detrimental effect (p<0.001;OR,1.002;95%CI1.001–1.003) on the diagnostic value of FFRAI.
**Conclusion:** FFRAI has a high diagnostic performance and reliability for the diagnosis of vessel specific coronary ischemia compared to two intracoronary catheter assessments. Missed FFRAI diagnoses were associated with coronary calcifications.
**Limitations:** Retrospective design. CT-FFRAI relies on manually adjusted cMPR images, introducing susceptibility to user,. CT-FFRAI uses a dichotomous outcome with a threshold of 0.80, which overlooks clinical information pertaining to lesions in the grey-zone.
**Funding for this study:** No funding
**Has your study been approved by an ethics committee?** Yes
**Ethics committee - additional information:** Ethical approval in 2 centers: Center 1: 2022/130, 2022; Center 2: 13/06/2023
**Author Disclosures:**
Olivier Ghekiere: Nothing to disclose
Rolf Symons: Nothing to disclose
Benjamin Peters: Nothing to disclose
Alain Nchimi Longang: Nothing to disclose

### Patterns in Use of Diagnostic Imaging and Initiation of Medical and Revascularisation Therapies for Patients with Suspected Chronic Coronary Syndrome in the Contemporary Era

T. Kido[1], H. Taniguchi[2], *M. Takeichi*[2], H. Fujii[3], T. Fujioka[2], N. Fujikawa[2], T. Yamada[2], S. Okami[2], S. Kohsaka[3]; [1]Toon, Ehime/JP, [2]Osaka/JP, [3]Tokyo/JP
*(naoto.fujikawa@bayer.com)*

**Purpose:** Purpose: For patients with suspected chronic coronary syndrome (CCS), selecting appropriate imaging examinations is crucial for accurate diagnosis and improved outcomes. Advancements in CCTA technology, such as higher resolution and lower radiation doses, have led to new guideline recommendations and changes in reimbursement. However, comprehensive analyses of patient trajectories and practice shifts in the contemporary era have been scarcely studied. The JOURNEY study aims to elucidate these patterns of imaging, medication, and revascularization in real-world settings.
**Methods or Background:** Methods: The JOURNEY study is a retrospective observational study utilising Japanese hospital administrative database provided by Medical Data Vision Co., Ltd. Patients aged ≥40 years who underwent their first imaging examination (i.e., CCTA, SPECT or CAG) for suspected CCS between 01 March 2015 and 31 January 2023 were included. Patients were followed from the date of their first imaging examination to Day 90. Multivariate logistic regression analysis was performed to examine factors associated with the use of CCTA.
**Results or Findings:** Results: A total of 123,050 patients with suspected CCS were included. The mean age was 70.0 (SD 10.7) years, and 56.2% were male. CCTA was the first imaging examination for 61.2% of patients, followed by SPECT (19.7%) and CAG (19.1%). Among the patients, 15.7% required the second imaging examination. By Day 90, 20.7% initiated antiplatelet therapy, 17.4% were confirmed to have CCS, and 9.0% received revascularization therapy. Recently enrolled patients underwent CCTA more often than those enrolled earlier (index year 2021-2023 versus 2015-2017, adjusted OR: 1.48 [95% CI: 1.43-1.53]).
**Conclusion:** Conclusion: CCTA was the most commonly performed initial imaging examination for suspected CCS, with increased usage in recent years.
**Limitations:** Limitations: The MDV data, collected for reimbursement rather than research, may introduce biases in data accuracy and representativeness.
**Funding for this study:** Funding was provided by Bayer AG (Leverkusen, Germany).
**Has your study been approved by an ethics committee?** Yes
**Ethics committee - additional information:** The study was approved by the independent ethics committee of the specified nonprofit organization, MINS (reference number: 240221).
**Author Disclosures:**
Hironori Taniguchi: Employee: Bayer Yakuhin, Ltd.
Shun Kohsaka: Consultant: Bayer Yakuhin, Ltd. Speaker: Bayer Yakuhin, Ltd.
Teruhito Kido: Consultant: Bayer Yakuhin, Ltd. Speaker: Bayer Yakuhin, Ltd.
Naoto Fujikawa: Employee: Bayer Yakuhin, Ltd.
Hiroyuki Fujii: Employee: Ark Medical Solutions Inc.
Suguru Okami: Employee: Bayer Yakuhin, Ltd.
Taisuke Fujioka: Employee: Bayer Yakuhin, Ltd.
Makiko Takeichi: Employee: Bayer Yakuhin, Ltd.
Takashi Yamada: Employee: Bayer Yakuhin, Ltd.

### Introducing a new score to quantify the distribution of coronary artery calcification based on coronary CT angiography

*M. Di Giovanni*, L. Száraz, S. Beke, K. Nagy, N. Nagy, H. Balogh, S. Veres, J. Simon, P. Maurovich-Horvat; Budapest/HU
*(markdigiovanni28@gmail.com)*

**Purpose:** The coronary artery calcium score (CACS) is an independent predictor of mortality. However, CACS does not provide information on the spatial distribution of calcified plaques. The aim of the present study is to determine the predictive value of the coronary calcium entropy score proposed by our research group taking into account the distribution of calcified plaques regarding the need for further testing after coronary CT angiography (CCTA).
**Methods or Background:** We retrospectively included consecutive patients who underwent CCTA for suspected coronary artery disease (CAD). We used multivariate logistic regression to fit three diagnostic models. Model 1 included clinical data (age, gender, type of angina, heart rate, and rhythm), which was augmented by CACS (Model 2) and CACS and calcium entropy score (Model 3). Chi-squared test was used to compare the models.
**Results or Findings:** After exclusion, data from 325 patients was analyzed (35.1% female, 63.7±10.6 years); 55 patients (16.8%) were recommended for further testing. For the clinical parameters (Model 1), presence of arrhythmia significantly increased the likelihood of further investigation. For Model 2, a CACS greater than 100 was a significant predictor (101-400: p=0.029, 401-1000: p=0.02, >1000: p<0.001). When supplemented with the calcium entropy score (Model 3), even a moderately high entropy score (>33th pc) was found to be a significant predictor (34th-66th percentile: p=0.034, >67th percentile: p=0.003). Overall, Model 3 significantly outperformed Model 2 (p<0.001).
**Conclusion:** The coronary calcium entropy score was found to be a significant predictor of the need for further testing following CCTA beyond clinical data and CACS. Based on our results, the formula developed for the distribution of calcified plaques may have a potential role in the investigation of CAD.
**Limitations:** This was a single-center study with a retrospective study design.
**Funding for this study:** M.D.G. received a Research Excellence Grant from Semmelweis University (EFOP-3.6.3-VEKOP-16-2017-00009) and was supported by the New National Excellence Program of the Ministry of Innovation and Technology of Hungary (ÚNKP-23-2-III-SE-43).
**Has your study been approved by an ethics committee?** Yes
**Ethics committee - additional information:** The study was approved by the Scientific and Research Ethics Committee of the Hungarian Medical Research Council (IV/665-3/2022/EKU) and was carried out in accordance with the tenets of the Declaration of Helsinki.
**Author Disclosures:**
Lili Száraz: Nothing to disclose
Pál Maurovich-Horvat: Nothing to disclose
Norbert Nagy: Nothing to disclose
Márk Di Giovanni: Nothing to disclose
Judit Simon: Nothing to disclose
Hanna Balogh: Nothing to disclose
Samuel Beke: Nothing to disclose
Kristóf Nagy: Nothing to disclose
Szilárd Veres: Nothing to disclose

### Assessment of Left Ventricular Function with Reduced Phase Cardiac CT Reconstructions: Preliminary Results from the CT-STEMI Study

*M. Moretti*[1], F. Troise[2], A. Maiorana[1], L. Blasi[1], D. Verna[1], T. D'Angelo[3], M. Olivieri[4], R. Faletti[1], M. Gatti[1]; [1]Torino/IT, [2]Bari/IT, [3]Messina/IT, [4]Chieti/IT
*(marta.moretti@unito.it)*

**Purpose:** To evaluate the agreement between cardiac computed tomography (CCT) and cardiac magnetic resonance (CMR) in assessing left ventricular (LV) function in post-STEMI patients, and to investigate the impact of CCT acquisitions on restricted R–R intervals, with a view toward potential prospective protocols.
**Methods or Background:** Forty-eight patients (mean age 62 ± 11 years, 87.5% male) were prospectively enrolled in the multicenter CT-STEMI study (NCT05941585, November 2023 – October 2024). Patients were randomized to undergo either CCT or CMR at 6.8 ± 1.8 days after STEMI, followed by the complementary exam within 5.3 ± 2.8 days. LVEDV, LVESV, and LVEF were measured using CVI42 software. The inclusion of true systolic and diastolic

Thursday phases was assessed across 40–80%, 30–90%, and 30–100% R–R ranges, along with their corresponding functional estimates.
**Results or Findings:** Full-cycle CCT demonstrated excellent concordance with CMR (LVEF: 46 ± 11% vs 45 ± 10.9%, p=0.40). The 40–80% interval included 26/48 systolic and 2/48 diastolic phases, with a mean LVEF underestimation of –8.7% (p<0.0001). The 30–90% interval captured all systolic but only 5/48 diastolic phases, leading to an underestimation of –3.4% (p<0.0001). The 30–100% range consistently included both systolic and diastolic phases in all patients (48/48), providing functional measurements comparable to CMR.
**Conclusion:** CCT shows high agreement with CMR in the evaluation of LV function. However, a 40–80% acquisition window significantly underestimates LVEF. When precise functional assessment is required, extending the reconstruction range to 30–100% allows comprehensive and reliable analysis.
**Limitations:** These results should be interpreted in light of study limitations, including the small sample size, preliminary nature of the data, variable imaging timing after STEMI, reliance on a single software platform, and lack of outcome or radiation analysis.
**Funding for this study:** This research was supported by a grant from the Italian Ministry of Health under the “Ricerca Finalizzata 2021 – Giovani Ricercatori” program, project number GR-2021-12372092. The funding was allocated to the project titled “Cardiac Computed Tomography for Comprehensive Risk Stratification of Arrhythmic, Atherothrombotic, and Heart Failure Events Following Reperfused ST-Segment Elevation Myocardial Infarction”. The funder had no role in the design, data collection, analysis, or interpretation of this study
**Has your study been approved by an ethics committee?** Yes
**Ethics committee - additional information:** No additional information
**Author Disclosures:**
Marco Gatti: Nothing to disclose
Ludovica Blasi: Nothing to disclose
Riccardo Faletti: Nothing to disclose
Antonio Maiorana: Nothing to disclose
Marta Moretti: Nothing to disclose
Tommaso D'Angelo: Nothing to disclose
Marzia Olivieri: Nothing to disclose
Daniele Verna: Nothing to disclose
Francesca Troise: Nothing to disclose

### Machine learning of Coronary Wall Radiomics Reveals Hidden CAD Phenotypes on CCTA

*F. Pisu*[1], D. Vignale[1], D. Serra[1], A. Palmisano[1], L. Saba[2], A. Esposito[1]; [1]Milan/IT, [2]Cagliari/IT
*(fra.pisu1@gmail.com)*

**Purpose:** Perivascular imaging seeks to quantify inflammation, fibrosis, and remodeling linked to coronary artery disease (CAD). Fat-related biomarkers (e.g., mean attenuation of perivascular adipose tissue) are promising, yet their sensitivity to scanner, protocol and body habitus variations limits reproducibility. Coronary wall (CW) radiomics may reveal more reproducible and subtler remodeling beyond mean attenuation. We tested whether unsupervised machine learning (ML) of CW radiomics can separate obstructive from non-obstructive CAD (ObCAD/NObCAD) and generalize across cohorts.
**Methods or Background:** We analyzed 40 CCTA cases (public ASOCA dataset: 20 patients with CAD, median stenosis 30% [IQR 25–70], 20 controls). CW radiomics were extracted from the proximal 5 cm of the LAD, RCA, and LCx and harmonized for site/scanners differences. We clustered patients using two principal components from the seven most informative features. Alignment with ObCAD status (≥50% stenosis) and Agatston coronary calcium (CAC) was assessed. Generalizability was tested by projecting 49 institutional patients (38 CAD patients ≤70% stenosis, CCTA for suspected CAD) into the learned cluster space.
**Results or Findings:** Two radiomic phenotypes separated CAD status with high rule-out performance (specificity 94%, NPV 91%). In the external cohort, 34/49 (69%) mapped to the NObCAD-like and 15/49 (31%) to the ObCAD-like phenotype (specificity 78%, NPV 85%). Misclassified cases had borderline stenosis (50–65%), older age (median 75y) and had more CV risk factors. CAC did not differ between phenotypes (median CAC 0[0–130] vs 18[0–95], p=0.76), supporting remodeling beyond calcium burden.
**Conclusion:** Machine learning of CW radiomics yields data-driven coronary phenotypes that separate obstructive from non-obstructive CAD and generalize across cohorts, indicating a feasible pathway to subclinical risk stratification beyond stenosis.
**Limitations:** Small, retrospective cohorts with limited clinical data and no adjudicated outcomes; obstructive CAD defined by stenosis only; residual site/scanner effects despite harmonization.
**Funding for this study:** The project PNRR-MAD-2022-12376633 is funded by the European Union– Next Generation EU – PNRR M6C2 - Investimento 2.1 Valorizzazione e potenziamento della ricerca biomedica del SSN
**Has your study been approved by an ethics committee?** Yes
**Ethics committee - additional information:** All study methods received approval from the ethics committee of IRCCS San Raffaele Scientific Institute (study registration number CE 158/INT/2022) and adhered to relevant guidelines.
**Author Disclosures:**
Davide Vignale: Nothing to disclose
Davide Serra: Nothing to disclose
Antonio Esposito: Nothing to disclose
Anna Palmisano: Nothing to disclose
Francesco Pisu: Nothing to disclose
Luca Saba: Nothing to disclose

**14:30 - 16:00** **Research Stage 3**

### Research Presentation Session: Genitourinary

### RPS 1007
### Imaging of benign female pelvic disorders

**Moderator**
Rahel Kubik-Huch; Baden/CH
*(kubik@hispeed.ch)*

### Added Value of T2 Fat-Saturated MRI Sequences in detection of Nerve Involvement in Lateral Deep Pelvic Infiltrating Endometriosis

*S. Viganò*, S. Annibali, C. Cazzella, P. A. Bonaffini, P. Marra, S. Sironi; Bergamo/IT
*(s.vigano36@campus.unimib.it)*

**Purpose:** To assess the added diagnostic value of T2-weighted fat-saturated (T2-FS) MRI sequences for the detection of nerve involvement in lateral deep infiltrating endometriosis (DIE), in comparison to the standard MRI protocol.
**Methods or Background:** Patients who underwent a pelvic MRI for clinical and/ or ultrasound lateral DIE suspicion were retrospectively enrolled (February 2021- April 2023). Two radiologists, with 10 and 2 years of experience respectively, independently assessed the presence of lateral DIE and of nerve involvement, first evaluating the standard MRI protocol and then with the addition of T2-FS sequences. The findings of the senior reader were considered the reference standard. Interobserver agreement was assessed with Cohen’s kappa.
**Results or Findings:** Fifty-one patients (mean age 34 years) were included. The senior radiologist detected nerve involvement in 13/51 patients (25,5%) with the standard protocol, increasing to 17/51 patients (33,3%) at re-evaluation with the addition of T2-FS sequences. Nerve structures most frequently involved were the sacral plexus, particularly the pudendal nerve. Interobserver agreement increased from poor with the standard protocol to moderate with T2-FS sequences. The additional T2-FS sequences particularly enhanced the junior radiologist’s ability to identify indirect sign of nerve involvement, improving diagnostic confidence.
**Conclusion:** T2-FS MRI sequences may provide added value in the evaluation of nerve involvement in lateral DIE. Although radiological experience remains crucial, their inclusion in pelvic MRI protocols may support less experienced radiologists and should be considered when nerve involvement is suspected.
**Limitations:** The limited sample size. Lack of surgical correlation.
**Funding for this study:** None.
**Has your study been approved by an ethics committee?** Not applicable
**Author Disclosures:**
Simona Annibali: Nothing to disclose
Paolo Marra: Nothing to disclose
Pietro Andrea Bonaffini: Nothing to disclose
Caterina Cazzella: Nothing to disclose
Sandro Sironi: Nothing to disclose
Sara Viganò: Nothing to disclose

**The Diagnostic Value of High-Resolution 3D T2-Weighted MRI in Preoperative Evaluation and Classification of Herlyn-Werner-Wunderlich Syndrome: A Prospective Study**

*Y. Qi*, Y. Zhang, X. Gao, P. Zhang, Y. He, S. Wang, H. Xue, L. Zhu; Beijing/CN
*(qiyafei19910603@163.com)*

**Purpose:** HWWS is a rare triad of didelphys uterus, oblique vaginal septum and ipsilateral renal agenesis. Accurate preoperative diagnosis and classification are crucial for surgical planning, This study aims to investigate the diagnostic value of high-resolution three-dimensional T2-weighted MRI in the preoperative evaluation and classification of HWWS.
**Methods or Background:** The study prospectively enrolled 70 patients clinically diagnosed with HWWS between 2020 and 2024. All patients underwent pelvic MRI examinations, including high-resolution 3D T2WI (1 mm slice thickness) and conventional T2 TSE sequences, supplemented by large-FOV T2 coronal imaging for urinary system evaluation and full-spine X-ray examinations for skeletal assessment. Two radiologists blinded to clinical data recorded uterine morphology, obstruction level and communications, and related complications. Consistency analysis was performed between imaging findings and surgical results in 52 surgical patients.
**Results or Findings:** The results showed the following uterine morphological classifications: didelphys uterus (54 cases, 77.1%), complete septate uterus (14 cases, 20%), and bicornuate uterus (2 cases, 2.9%). Among surgical patients, 31 cases had vaginal-level obstruction (18 Type I, 11 Type II, and 2 Type III), while 21 cases had cervical-level obstruction (19 Type IV and 2 special Type III+IV). 3D-T2 detected 7/11 vaginal-septum fistulas that TSE missed; both sequences showed 2 cervical communications. Associated anomalies including ipsilateral renal agenesis 69 (99%), ovarian endometriosis 16 (type IV predominance), hematosalpinx 16, scoliosis 26 (37%), mild scoliosis 29 (41%).
**Conclusion:** Sub-millimeter 3D-T2 MRI significantly outperforms TSE in identifying vaginal communications and precisely localizing HWWS obstruction, enabling confident surgical planning. Routine adoption of high-resolution 3D protocols plus renal/spinal imaging is recommended.
**Limitations:** Despite 3D MRI's clear benefit for detecting cervix–septum fistulas, collapsed vaginal walls occasionally obscured the tract; with consent, we will trial gel distension to sharpen vaginal anatomy.
**Funding for this study:** National High Level Hospital Clinical Research Funding（2022-PUMCH-B-069）
**Has your study been approved by an ethics committee?** Yes
**Ethics committee - additional information:** This study was approved by the PUMC Ethics Committee (No. K24C2950).
**Author Disclosures:**
Shu Wang: Nothing to disclose
Xin Gao: Nothing to disclose
Lan Zhu: Nothing to disclose
Yiwei Zhang: Nothing to disclose
Huadan Xue: Nothing to disclose
Yonglan He: Nothing to disclose
Yafei Qi: Nothing to disclose
Peng Zhang: Nothing to disclose

**Mri based #enzian classification as a preoperative guide in deep infiltrating endometriosis - a comparative study with intraoperative findings**

*P. Dhakar*, A. Bose, V. Mishra, P. Sukhani, J. Chowdhary; Jaipur/IN
*(parkexports04@gmail.com)*

**Purpose:** Evaluate the efficacy of the ENZIAN score by comparing magnetic resonance imaging (MRI) results with intraoperative findings in cases of deep infiltrating endometriosis
**Methods or Background:** This retrospective study examined 50 patients with endometriomas and suspected deep infiltrating endometriosis of age ranging from 25 to 52 years (mean age 38.5 years), who underwent surgery between October 1, 2025 and September 31, 2024, at our institution (n = 50). The study compared preoperative MRI findings with intraoperative results using the #ENZIAN score and assessed the sensitivity , specificity , positive predictive value and negative predictive value of this approach.
**Results or Findings:** The diagnostic accuracy of MRI in detecting various endometriotic lesions by application of #ENZIAN Score was evaluated, with results confirmed through surgical findings. Sensitivity and negative predictive values of MRI confirmed by surgery were 98% and 92.6% (endometriomas), 95.2% and 91.7% (lesions in the vaginal/rectovaginal space), 78.4% and 56% (uterosacral ligaments), 91.4% and 89.7% (rectum/sigmoid colon), 57.1% and 94.1% (myometrium), 85.7% and 98.3% (bladder), and 73.3% and 92.2% (intestine), respectively
**Conclusion:** The #ENZIAN score serves as a detailed anatomical map that closely aligns with surgical findings, thereby facilitating more effective surgical planning. It demonstrates a strong correlation between MRI and intraoperative results, enhancing diagnostic efficacy, and serves as a valuable communication tool between radiologists and gynecologists.
**Limitations:** Despite its high sensitivity and NPV, MRI has limitations in detecting certain forms of DIE, particularly those involving the uterosacral ligaments and myometrium. Additionally, MRI's ability to accurately assess the severity of lesions in each compartment may be influenced by the operator's experience, the quality of the imaging, and the resolution of the MRI sequences used.
**Funding for this study:** None
**Has your study been approved by an ethics committee?** Not applicable
**Author Disclosures:**
Anindita Bose: Nothing to disclose
Vineet Mishra: Nothing to disclose
Jai Chowdhary: Nothing to disclose
Paresh Sukhani: Nothing to disclose
Palak Dhakar: Nothing to disclose

**The clinical usefulness of the preoperative #Enzian score for estimating operative time in endometriosis surgery**

*H. M. M. Smid*, O. d. f. Henneman; Den Haag/NL
*(hannah-smid@hotmail.com)*

**Purpose:** Analysing clinical usefulness of preoperative #Enzian score as predictor for operative time planning in endometriosis surgery.
**Methods or Background:** Retrospective study in which 369 patients with deep infiltrating endometriosis had a procedural time planning in a multidisciplinary meeting. #Enzian score was used to assess endometriosis in abdominal compartments with preoperative transvaginal ultrasound (TVUS) and MRI. Procedural time was estimated (EPT) after discussing patient history, symptoms, physical examination and #Enzian-scored imaging. Difference between EPT and actual operating time (AOT) was calculated. Accurate EPT was defined as AOT within 30 minutes of EPT. Sensitivity and specificity of dichotomised imaging #Enzian scores were calculated for individual compartments. Correlating and non-correlating MRI and surgical #Enzian scores were analysed per compartment, in relation to three outcomes: AOT >30 minutes shorter than EPT, accurate AOT (+/- <30 minutes of EPT) and AOT >30 minutes longer than EPT.
**Results or Findings:** Sensitivity and specificity of pre-operative imaging differs between compartments and is lower in most compartments compared to literature. In total, 39.3% of operations were shorter than accurate EPT, 48.2% were within accurate EPT and 12.5% were longer than accurate EPT. There was no significant difference in AOT distribution in individual compartments comparing correlating and non-correlating #Enzian scores.
**Conclusion:** There is no correlation between mismatch in pre-/peroperative #Enzian scores in individual compartments and non-accurate EPT. As the EPT is set in a multidisciplinary meeting, personal experience of surgeon, patient history and physical examination play a large role in assessing EPT, next to the imaging results. The complexity of accurate EPT cannot only be defined in one (preoperative) classification.
**Limitations:** Retrospective study
**Funding for this study:** No funding for this study
**Has your study been approved by an ethics committee?** Yes
**Ethics committee - additional information:** Local ethics committee
**Author Disclosures:**
Hannah Maartje Maria Smid: Nothing to disclose
Onno Dirk Fransiscus Henneman: Nothing to disclose

**Transvaginal Ultrasound versus MRI for Assessment of Lower Uterine Segment Cesarean Scars: A Prospective Comparative Study**

*R. Praveenkumar*, F. Abubacker Sulaiman, J. Lydia, D. Velan; Chennai/IN
*(fasulaiman@gmail.com)*

**Purpose:** To compare the accuracy of transvaginal ultrasound (USG) and magnetic resonance imaging (MRI) in measuring lower uterine segment (LUS) scar thickness and detecting defective or thinned cesarean scars, and to assess their predictive value for uterine rupture or dehiscence in subsequent pregnancies.
**Methods or Background:** In this prospective study, 60 women with a history of one previous cesarean section and planned repeat delivery were enrolled. LUS scar thickness was measured using transvaginal USG and 1.5T MRI (T2-weighted sequences). Presence of scar defects such as niches or thinning was documented. Imaging findings were correlated with intraoperative observations at the time of repeat cesarean delivery. Statistical analysis included paired t-tests for scar thickness comparison and sensitivity/specificity calculation for defect detection.d

**Results or Findings:** Mean scar thickness measured by USG was 3.8 ± 1.1 mm, while MRI measured 4.1 ± 1.2 mm (p = 0.04). MRI detected scar defects in 22/60 patients, whereas USG detected defects in 14/60. MRI demonstrated higher sensitivity (88%) and specificity (92%) for detecting scar defects compared to USG (sensitivity 64%, specificity 85%). All cases of intraoperative dehiscence (n = 4) were correctly identified preoperatively by MRI, whereas USG identified 2/4.
**Conclusion:** MRI provides superior detection of defective or thinned cesarean scars and offers more reliable prediction of uterine dehiscence than USG. Transvaginal USG remains useful for routine scar thickness assessment, but MRI may be recommended for high-risk patients to guide clinical management.
**Limitations:** Single-center study with a small sample size; operator dependency of USG; MRI limited to 1.5T; findings may not generalize to women planning VBAC
**Funding for this study:** Not applicable
**Has your study been approved by an ethics committee?** Yes
**Ethics committee - additional information:** Institutional Ethics Committee approval obtained; informed consent taken from all participants
**Author Disclosures:**
Rathinamoorthy Praveenkumar: Nothing to disclose
Johnbosco Lydia: Nothing to disclose
Dhanush Velan: Nothing to disclose
Farook Abubacker Sulaiman: Nothing to disclose

**Ultrasound-Guided Sclerotherapy with Lauromacrogol for Ovarian Endometriomas: A Prospective Study of Efficacy, Safety, and Ovarian Reserve**
*Y. Luo*, M. Zhang, Z. Huang, X. Zhang; Guangzhou/CN
*(15920908796@163.com)*

**Purpose:** To prospectively assess the efficacy, safety, and impact on ovarian reserve of ultrasound-guided lauromacrogol sclerotherapy (UGLS) for ovarian endometriomas (OE).
**Methods or Background:** This prospective study enrolled patients undergoing UGLS between April 2024 and April 2025. Preoperative assessment comprised cyst maximum diameter, cyst volume, serum cancer antigen 125 (CA-125), serum anti-Müllerian hormone (AMH), and visual analogue scale (VAS) pain scores. The UGLS procedure involved cyst aspiration, irrigation, and instillation of 1% lauromacrogol. Follow-up assessments at 1, 3, and 6 months evaluated cyst reduction rate, relevant biomarker levels, and pain scores.
**Results or Findings:** This study enrolled 78 patients (106 lesions) undergoing UGLS. The mean age of the participants was 30.17 ± 3.75 years. At baseline, the mean VAS scores were 4.66 ± 2.82 for dysmenorrhea and the mean volume was 105.06 ± 96.65 ml. After the operative, the technical success rate was 97.81%. Significant reductions in cyst volume was observed at 6 months (P<0.001), with volume reduction rates of 81.51 ± 15.65%(1 month), 90.63 ± 12.02%(3 months), and 93.21 ± 9.51% (6 months), and the mean cyst volume was 18.53 ± 74.24 ml at 6 months. CA-125 levels decreased postoperatively, while no significant change in AMH levels was observed (P＞0.05). The incidence of mild complications was 5.13%, which were relieved after observation.
**Conclusion:** UGLS is an effective, minimally invasive treatment for OE that successfully preserves ovarian reserve, and demonstrating promise for patients with diminished ovarian reserve.
**Limitations:** This article is a single arm, single center study
**Funding for this study:** supported by“the Third Affiliated Hospital of Sun Yat-sen University, Clinical Research Program (QHJH202303)
**Has your study been approved by an ethics committee?** Yes
**Ethics committee - additional information:** the Third Affiliated Hospital of Sun Yat-sen University (RG2024-021-01)
**Author Disclosures:**
Yingxin Luo: Author: No conflict of interest Author: No conflict of interest
Xinling Zhang: Founder: No conflict of interest Founder: No conflict of interest
Zeping Huang: Author: No conflict of interest Author: No conflict of interest
Man Zhang: Author: No conflict of interest Author: No conflict of interest

**Concordance and Diagnostic Accuracy of Repeat Placental MRI for the Assessment of Placental Accreta Spectrum Disorder**
*Y. H. Song*, A. Chua, S. Tan, D. Richmond, E. E. Alibrahim, J. Keene, C. Shadbolt, S-F. Oon; Melbourne/AU
*(yoonho.song@outlook.com)*

**Purpose:** While placental MRI is well-established as a useful diagnostic tool for the evaluation of placenta accreta spectrum disorder (PASD), the optimal gestational age for assessment and diagnostic value of repeat MRI remains uncertain. The aim of this study was to retrospectively assess the utility of routine repeat placental MRIs performed at a tertiary Specialist Women's Imaging referral centre. We evaluated inter-study concordance and diagnostic accuracy of MRI performed at 28- and 32-weeks gestation at diagnosing PASD, using placental pathology as the reference standard.
**Methods or Background:** We retrospectively reviewed all placental MRI studies performed from 2015-2025, identifying patients who underwent two placental MRIs for PASD assessment beyond 24-weeks gestation. Diagnostic accuracy was assessed by comparing dichotomised MRI findings with placental pathology at delivery. Sensitivity and specificity with 95% confidence intervals (CIs) were calculated for the initial and repeat studies. Inter-study concordance was assessed using Cohen’s kappa coefficient with 95% CIs for disease severity (normal, accreta/increta, or percreta) and diagnosis of PASD.
**Results or Findings:** Between 2015 and 2025, 61 women had two placental MRI studies beyond 24-weeks gestation for PASD assessment. For diagnosis of PASD, the initial scan (median gestation 29-weeks) demonstrated 97.4% sensitivity (95% CI 86.5–99.5) and 60.0% specificity (95% CI 31.3–83.2). The repeat scan (median gestation 31-weeks) demonstrated 100.0% sensitivity (95% CI 90.8–100.0) and 50.0% specificity (95% CI 23.7–76.7). Inter-study concordance was substantial for disease severity (κ = 0.72, 95% CI 0.58–0.87), PASD diagnosis (κ = 0.77, 95% CI 0.59–0.96), and placenta percreta diagnosis (κ = 0.77, 95% CI 0.63–0.95).
**Conclusion:** There is comparable accuracy and substantial concordance between initial and repeat placental MRI studies performed beyond 24-weeks gestation. This suggests limited diagnostic benefit in routinely repeating MRI for the diagnostic evaluation of PASD, but larger studies would be required to confirm this.
**Limitations:** The study results are limited by a small sample size but could contribute to future meta-analyses.
**Funding for this study:** No funding was provided for this study.
**Has your study been approved by an ethics committee?** Yes
**Ethics committee - additional information:** The study was approved by the Royal Women’s Hospital Ethics Committee (Reference QA/111988/RWHV-2024-455133).
**Author Disclosures:**
Ekaterina Ekaterina Alibrahim: Nothing to disclose
Sheng-Fei Oon: Nothing to disclose
Clair Shadbolt: Nothing to disclose
Yoonho Song: Nothing to disclose
Samantha Tan: Nothing to disclose
Jacqui Keene: Nothing to disclose
Danielle Richmond: Nothing to disclose
Alexander Chua: Nothing to disclose

**Comparing Intraplacental Vascular Impairment and Microperfusion in patients with hypertensive disorders of Pregnancy using IntraVoxel Incoherent Motion MRI of the placenta**
*E. A. Kirillova*, I. Mashchenko, P. Kozlova, N. Osipova, V. Markina, I. Zazerskaya, G. Trufanov; St. Petersburg/RU
*(kirillovaelizaveta30@gmail.com)*

**Purpose:** Hypertensive disorders of pregnancy (HDPs) are major causes of maternal morbidity worldwide. A hallmark of these conditions is impaired placental perfusion. Intravoxel incoherent motion (IVIM) MRI enables non-invasive quantification of placental microcirculation and may serve as a useful biomarker of placental disorders in this cohort. The study aimed at comparing IVIM parameters in whole placenta and maternal/foetal compartments in patients with or without HPDs.
**Methods or Background:** 16 women aged 26-43 years underwent placental IVIM-MRI, including ten patients in the HDP group and six patients in the control group. Mean gestational age at the time of 3T-MRI was 31.6 weeks (95% CI: 29.4–33.8) in the HDP group and 30.5 weeks (95% CI: 27.6–33.3) in the control group. A multi-b-value DWI protocol (0–800 s/mm²) was applied. Group differences and subgroup trends were analysed descriptively. Subgroups included patients with severe preeclampsia (PE) ± chronic hypertension (CH), moderate PE ± CH, and isolated CH.
**Results or Findings:** Whole-placenta perfusion fraction (f) value reflecting villous vascularity was reduced in patients with HDPs (19.2%, 95% CI 15.2–23.1) vs controls (24.8%, 95% CI 16.0–33.7). Maternal f (18.6% vs 24.0%) and foetal f (19.2% vs 25.8%) values showed similar decreases. The diffusion coefficient (D and D*) parameters indicating water diffusion in the tissue and the microvascular flow, respectively, also tended to be lower in the HDP group. The subgroup analysis revealed the most pronounced reductions of all IVIM parameters in severe PE ± CH vs controls.
**Conclusion:** IVIM parameters of the placenta showed consistent reductions in patients with HDPs compared to the control group, reflecting impaired placental microperfusion in this cohort.
**Limitations:** The limitation of the study was a small sample size.
**Funding for this study:** No funding was received for this study
**Has your study been approved by an ethics committee?** Yes
**Ethics committee - additional information:** All participants were informed about scientific purposes of this study and gave their informed consents.

**Author Disclosures:**
Elizaveta Aleksandrovna Kirillova: Nothing to disclose
Irina Mashchenko: Nothing to disclose
Irina Zazerskaya: Nothing to disclose
Polina Kozlova: Nothing to disclose
Valentina Markina: Nothing to disclose
Natalia Osipova: Nothing to disclose
Gennadiy Trufanov: Nothing to disclose

**Therapeutic Decision-Making in Patients with Bowel Endometriosis: The Clinical Value of MRI**
*R. V. Ninkova*, F. Curti, S. Riccardi, C. Cutonilli, M. Gennarini, F. Frezza, C. Amoroso, C. Catalano, L. Manganaro; Rome/IT
*(roberta.ninkova@gmail.com)*

**Purpose:** To evaluate the role of magnetic resonance imaging (MRI) in guiding treatment choice between medical and surgical management in patients with bowel endometriosis, based on detailed analysis of lesion morphology and anatomical involvement.
**Methods or Background:** A retrospective study was conducted on 215 patients with bowel endometriosis who underwent MRI between 2017 and March 2024, with at least one year of follow-up. Among them, 135 received surgical treatment (alone or combined with medical therapy), while 80 underwent exclusive medical treatment. MRI evaluation focused on lesion location (low, mid, or upper rectum; rectosigmoid junction; extrapelvic sites), number of nodules, longitudinal extension, thickness, and circumferential involvement. Clinical symptoms were recorded using the NRS scale, comparing pre- and post-treatment scores. The occurrence of bowel obstruction and post-surgical complications were also documented.
**Results or Findings:** The upper rectum was the most frequently affected site (33%), followed by the rectosigmoid junction (22%) and mid-rectum (20%). Bowel obstruction occurred in 5.1% of cases, exclusively in lesions ≥10 cm from the anal verge with ≥4/8 circumferential involvement. Surgical treatment achieved a significantly greater reduction in symptom severity in patients with lesions >14 mm and ≥4/8 circumferential extension (mean NRS reduction = 4.3 ± 1.2 vs. 2.1 ± 1.0; $p < 0.001$). In smaller or less extensive lesions, outcomes were comparable between medical and surgical therapy.Postoperative complications occurred in 65.2% of surgical cases, most commonly bladder incontinence (26.7%).
**Conclusion:** MRI proved essential for precise lesion characterization and for guiding personalized therapeutic decision-making based on features associated with symptom severity.
**Limitations:** This study is limited by its retrospective, single-center design and the absence of randomization, which may introduce selection bias. The follow-up duration and subjective assessment of symptoms using the NRS scale could also affect result consistency.
**Funding for this study:** None
**Has your study been approved by an ethics committee?** Yes
**Ethics committee - additional information:** This study was performed according to the principles of the Declaration of Helsinki
**Author Disclosures:**
Marco Gennarini: Nothing to disclose
Claudia Cutonilli: Nothing to disclose
Sandrine Riccardi: Nothing to disclose
Roberta Valerieva Ninkova: Nothing to disclose
Federica Curti: Nothing to disclose
Flaminia Frezza: Nothing to disclose
Chiara Amoroso: Nothing to disclose
Carlo Catalano: Nothing to disclose
Lucia Manganaro: Nothing to disclose

**Post-abortion Complications: How Not to Be Misled !**
*M. Bouhlali*, P. Verpillat; Lille/FR
*(mohamed.bouhlali.fr@gmail.com)*

**Purpose:** To understand the clinico-biological principles of post-abortion follow-up. To recognize simple trophoblastic retention on imaging. To detail the main differential diagnoses of trophoblastic retention. To differentiate trophoblastic retention from acquired arteriovenous fistula.
**Methods or Background:** Pelvic ultrasound and/or MRI evaluation was performed at Lille University Hospital, in patients after induced abortion, in cases of metrorrhagia or abnormal decline of β-hCG levels.
**Results or Findings:** Trophoblastic retention should be suspected in cases of metrorrhagia or plateauing β-hCG levels persisting more than 3 weeks after abortion. An empty uterine cavity with a thin endometrium and negative β-hCG excludes trophoblastic retention. Trophoblastic retention appears as heterogeneous echogenic intrauterine material, respecting the endometrium–myometrium interface, with vascularization on color Doppler, peak systolic velocities generally not exceeding 40 cm/s. Major role of color Doppler ultrasound in differential diagnoses: Intrauterine clot: strictly intracavitary echogenic mass, avascular on color Doppler, with negative β-hCG. Arteriovenous fistula: endo-myometrial lesion with marked hypervascularization on color Doppler, peak velocities exceeding 1 m/s. Ectopic pregnancy : empty uterine cavity, thickened gravid endometrium, β-hCG > 1500, with or without direct visualization of an extrauterine gestational sac MRI may reveal signs of an arteriovenous fistula on dynamic contrast-enhanced sequences, such as early venous return in the gonadal vein and helps differentiate it from a refluxing gonadal vein Molar pregnancy or gestational trophoblastic tumor (GTT) should be suspected based on clinico-biological criteria (very high β-hCG levels) and imaging features.
**Conclusion:** Preserved endometrium–myometrium interface with PSV < 40 cm/s → trophoblastic retention. Myometrial invasion with PSV > 1 m/s → acquired AVF/AVM, with early venous return on dynamic MRI. Empty uterine cavity + β-hCG > 1500 → ectopic pregnancy until proven otherwise. Very high β-hCG → molar pregnancy or GTT until proven otherwise.
**Limitations:** Comorbidity
**Funding for this study:** Lille university Hospital
**Has your study been approved by an ethics committee?** Not applicable
**Author Disclosures:**
Mohamed Bouhlali: Nothing to disclose
Pauline Verpillat: Nothing to disclose

**Associations Between Pelvic Fat Distribution and Severity of Deep Infiltrating Endometriosis: A Retrospective MRI-based Analysis**
*A-K. Kaufmann-Bühler*[1], M. Kopetzky[1], M. Puseljic[1], I. Vlasicek[1], B. Catalano[2], M. Fuchsjäger[1], M. Wölfler[1], W. Schöll[1], E. Talakic[1]; [1]Graz/AT, [2]Rome/IT
*(ann-katrin.kaufmann-buehler@uniklinikum.kages.at)*

**Purpose:** To investigate whether the distribution of pelvic fat compartments, such as subcutaneous fat (SCF), intramuscular fat (IMF), and pelvic visceral fat (PVF), is associated with the severity of deep endometriosis (DE), based on pelvic MRI volumetry.
**Methods or Background:** In this retrospective single-center study, 71 women (mean age 33.4 ± 6.5 years) with surgically and/or histologically confirmed DE underwent standardized pelvic MRI between 2021-2023. Volumes of SCF, IMF, PVF, and gluteus maximus muscle (MV), were segmented semi-automatically using 3D Slicer software. Disease severity was classified according to the #Enzian system. Spearman's correlation and non-parametric group comparisons were used to assess associations between body composition and disease severity. Multivariate linear regression models served to identify independent predictors of disease severity.
**Results or Findings:** PV correlated negatively with severity in compartment B (uterosacral ligaments/pelvic wall; $r = −0.28$, $p = 0.008$). IMF correlated positively with extragenital disease (compartment F; $r = 0.26$, $p = 0.015$) and with the overall #Enzian score ($r = 0.21$, $p = 0.047$. No significant associations were found for SCF or MV. Linear regression models identified IMF($\beta = 4.87$, $p = 0.023$) and IMF/MV ratio ($\beta = 31.6$, $p = 0.023$) as independent predictors of #Enzian score, with a stronger normalized effect of IMF relative to MV.
**Conclusion:** Correlating with compartment-specific and overall disease severity, MRI-based pelvic fat and muscle volumes may serve as a novel non-invasive biomarker of disease burden in DE. These findings support the hypothesis that pelvic fat distribution reflects underlying pathophysiological mechanisms and could support non-invasive staging strategies. Prospective validation and integration with hormonal and clinical markers are warranted.
**Limitations:** Limitations of this study include its retrospective and cross-sectional design, precluding causal inference.
**Funding for this study:** None
**Has your study been approved by an ethics committee?** Yes
**Ethics committee - additional information:** Ethics Committee of the Medical University of Graz (Reference number: EK 429 Ex: 22/23).
**Author Disclosures:**
Ann-Katrin Kaufmann-Bühler: Nothing to disclose
Bianca Catalano: Nothing to disclose
Michael Fuchsjäger: Nothing to disclose
Monika Wölfler: Nothing to disclose
Marijan Puseljic: Nothing to disclose
Michaela Kopetzky: Nothing to disclose
Igor Vlasicek: Nothing to disclose
Emina Talakic: Nothing to disclose
Wolfgang Schöll: Nothing to disclose

**Intraprocedural assessment of treatment efficacy using intravoxel incoherent motion (IVIM)-analysis during magnetic resonance-guided focused ultrasound (MRgFUS) treatment of uterine fibroids**

*L. Knorren*[1], J. D. J. Slotman[1], I. M. Nijholt[1], J. Schutte[1], J. Huirne[2], C. M. Tax[3], L. W. Bartels[3], M. F. Boomsma[1]; [1]Zwolle/NL, [2]Amsterdam/NL, [3]Utrecht/NL
*(e.r.knorren@gmail.com)*

**Purpose:** Reliable intraprocedural assessment of treatment efficacy during magnetic resonance–guided focused ultrasound (MRgFUS) for uterine fibroids may optimize outcomes and reduce procedure time. The non-perfused volume ratio (NPVr) is conventionally calculated post-treatment using contrast-enhanced T1-weighted imaging (CET1w). However, contrast administration precludes further ablation. In this explorative pilot study, the potential of intravoxel incoherent motion (IVIM)-derived perfusion fraction (f)- and apparent diffusion coefficient (D)-maps for real-time MRgFUS treatment monitoring was evaluated.
**Methods or Background:** Women undergoing MRgFUS treatment between September 2024 and July 2025 at our institution were consecutively enrolled. DWI was obtained at baseline, at 45-minute intervals during ablation and after final sonication. CET1w was subsequently performed and used to calculate NPVr from manual 3D segmentations of the total fibroid volume and non-perfused volume. IVIM-maps were generated post-procedurally using non-linear least-squares fitting. Explorative visual analysis was performed by two experienced researchers, with CET1w as reference for the final IVIM-maps.
**Results or Findings:** Fourteen women were included with a median of five DWI-scans per patient (range 3-6). Mean NPVr was 43,7±25,9% (range: 4,4-97,6%). In thirteen cases, progressive hypointense regions on f-maps and hyperintense regions on D-maps were observed intraprocedurally. In one patient, a global hypointense pattern on the f-map appeared 45 minutes after the start of treatment and persisted, corresponding to complete ablation (NPVr 97,6%). Absent or minimal changes on IVIM were associated with low NPVr (<10%, n=2). In eleven cases partial changes of the fibroid on IVIM-maps were observed, visually corresponding with intermediate ablation outcomes on CET1w (NPVr 12,3–66,7%).
**Conclusion:** Intraprocedural visual changes of fibroid tissue on f- and D-maps shows potential as a tool for visualizing intraprocedural progression of MRgFUS treatment. Clinical applicability will be evaluated in a future study.
**Limitations:** Small sample size
**Funding for this study:** None
**Has your study been approved by an ethics committee?** Yes
**Ethics committee - additional information:** (MEC number: 200723, protocol ID: NL74716.075.20)
**Author Disclosures:**
Judith Huirne: Nothing to disclose
Jorik Derk Jan Slotman: Nothing to disclose
Loes Knorren: Nothing to disclose
Chantal M.W. Tax: Nothing to disclose
Lambertus W. Bartels: Nothing to disclose
Joke Schutte: Nothing to disclose
Martijn Franklin Boomsma: Nothing to disclose
Ingrid M. Nijholt: Nothing to disclose

**14:30 - 16:00** **Research Stage 1**

### Research Presentation Session: Interventional Radiology

### RPS 1009

### Emerging trends in neuro and head and neck interventional radiology

**Moderator**
Malgorzata Szczerbo-Trojanowska; Lublin/PL
*(mst.trojanowska@gmail.com)*

**Validation of a Web-Based Device Compatibility Program for Neurointervention**

*J. H. Choi*, B. Kwon, Y. Song, D. H. Lee; Seoul/KR
*(heatpro1@naver.com)*

**Purpose:** Neurointerventional procedures often require simultaneous use of multiple devices, necessitating compatibility checks. Because device specifications are reported in mixed units (e.g., catheter ID in inches, OD in French, wire OD in inches), direct calculation is difficult. In addition, when more than two devices are used in a guiding catheter, simply summing outer diameters can be misleading. This study evaluated the usefulness of a self-developed, web-based compatibility checking program.
**Methods or Background:** A device database was built using specifications of neurointerventional devices in use since 2016. Spatial compatibility was assessed with circle geometry, and length compatibility incorporated catheter hub length and additional device placement. Clinical experience with specific combinations was also integrated. Program performance was tested using cases from 2020 to 2024 in which compatibility checks were required. Results were compared with actual clinical use or in-vitro testing to determine sensitivity, specificity, accuracy, and predictive values. Three users also measured the time required for compatibility evaluation using the program versus manual calculation.
**Results or Findings:** A total of 355 compatibility checks were analyzed: 126 with one insert device, 173 with two, and 56 with three. The program showed sensitivity of 94.6%, specificity of 86.4%, accuracy of 91.4%, positive predictive value of 91.5%, and negative predictive value of 91.2%. Evaluation time with the program was consistently shorter than with manual calculation across all users.
**Conclusion:** This web-based compatibility program provides rapid access to device specifications and supports optimal device selection by combining mathematical modeling with clinical data. Broader use across centers and accumulation of additional clinical experience are expected to enhance its reliability and clinical value.
**Limitations:** Although the database was constructed based on specifications provided by the manufacturers, the acceptable tolerance ranges of the devices were not specified.
**Funding for this study:** None
**Has your study been approved by an ethics committee?** Yes
**Ethics committee - additional information:** This retrospective study was approved by the Institutional Review Board of our institution (approval number: No. 2025-1217)
**Author Disclosures:**
Joon Ho Choi: Nothing to disclose
Boseong Kwon: Nothing to disclose
Yunsun Song: Nothing to disclose
Deok Hee Lee: Nothing to disclose

**Risk of Clopidogrel Resistance in Endovascular Aneurysm Stenting**

*M. Stanton*; Brisbane/AU

**Purpose:** Thromboembolic complications remain a significant risk in endovascular aneurysm stenting, with clopidogrel resistance implicated as a major contributing factor. This study aims to evaluate thromboembolic outcomes in clopidogrel-resistant patients undergoing endovascular aneurysm stenting and to assess whether switching to ticagrelor mitigates these risks.
**Methods or Background:** This single-centre retrospective cohort study included 228 patients treated between 2020 and 2024. All patients underwent VerifyNow P2Y12 testing prior to the procedure. Clopidogrel non-responders (<40% inhibition) were switched to ticagrelor. The primary endpoint was thromboembolic complication, defined as radiological or clinical infarct, aiming to capture all patients with thromboembolic complication, whether symptomatic or not. Secondary analyses evaluated aneurysm characteristics and associated risk factors.
**Results or Findings:** Of the 228 patients, a significant 122 patients (54%) were found to be non-responders and switched to ticagrelor. No statistically significant differences were observed in rates of clinical infarct (13% vs 11%, p=0.7) between clopidogrel responders and ticagrelor-treated non-responders. Evidence of radiological infarct was included to incorporate any asymptomatic patients and also was found to have no difference between groups (19% vs 12%, p=0.2) . Larger aneurysm size correlated with both higher number of radiological (p=0.011) and clinical infarcts (p=0.002). Diabetes and advanced age were associated with multiple aneurysms. Younger patients with prior subarachnoid haemorrhage were more likely to undergo stent-assisted coiling.
**Conclusion:** Preoperative platelet function testing and switching clopidogrel non-responders to ticagrelor resulted in comparable thromboembolic complication rates to clopidogrel responders. These findings support the role of routine platelet function testing and individualized antiplatelet management in endovascular aneurysm treatment.
**Limitations:** Limitations for this study are that it's a single centre, retrospective study. Although powered adequately, some subgroup analyses may still be underpowered.
**Funding for this study:** No funding was received for this study
**Has your study been approved by an ethics committee?** Not applicable
**Author Disclosures:**
Mitchell Stanton: Nothing to disclose

**Small versus Large Woven EndoBridge Devices for Intracranial Aneurysms: Results from the WorldWideWEB Multicenter Study**

*F. Dugar*[1], T. D. L. Nguyen-Kim[1], A. Dmytriw[2], D. Coluccia[1], M-N. Psychogios[3], P. Sporns[1]; [1]Zurich/CH, [2]Boston, MA/US, [3]Basel/CH
*(franja.dugar95@gmail.com)*

**Purpose:** Smaller Woven EndoBridge (WEB) devices (≤ 4.5 mm) for the treatment of intracranial aneurysms are technically challenging to deploy, and data on their safety and efficacy remain scarce. This study compared functional, anatomic, and safety outcomes of small WEBs with those of large (> 4.5 mm) and very large (> 7.5 mm) devices.
**Methods or Background:** The WorldWideWEB consortium is a retrospective, multicenter collaboration across 30 international institutions, including adults with intracranial aneurysms treated with WEB. Patients were stratified into small (≤ 4.5 mm) and large (> 4.5 mm) groups; a subanalysis compared small and very large (> 7.5 mm) devices. The primary outcome was retreatment rate. Secondary outcomes comprised mRS, anatomic outcomes periprocedurally and at last follow-up (FU), and safety events, including intracranial hemorrhage (ICH) and thromboembolic complications (TECs).
**Results or Findings:** Among 1473 patients, 229 (15.5%) received a small WEB. Baseline characteristics were similar. Retreatment was less frequent (4.3% vs 8.8%, p = 0.037) and complete occlusion higher with small WEBs (periprocedurally: 57.1% vs 36.6%, p < 0.001; last FU: 76.2% vs 58.5%, p < 0.001). Median mRS at last FU was comparable (1 [1–2] vs 1 [1–2], p = 0.88), as were safety events (ICH 2.6% vs 0.9%, p = 0.102; TECs 3.1% vs 3.9%, p = 0.686). Very large WEBs had higher retreatment (16.1%, p < 0.001) and lower complete occlusion (periprocedurally: 32.2%, p < 0.001; last FU: 50.5%, p < 0.001), with comparable safety outcomes (ICH 0.9%, p = 0.266; TECs 4.5%, p = 0.512).
**Conclusion:** Small WEBs showed superior anatomic outcomes and lower retreatment rates compared with large devices, without significant differences in functional or safety outcomes.
**Limitations:** Absence of core laboratory adjudication for anatomic outcomes and retrospective design.
**Funding for this study:** No funding was provided for this study.
**Has your study been approved by an ethics committee?** Not applicable
**Author Disclosures:**
Adam Dmytriw: Nothing to disclose
Peter Sporns: Nothing to disclose
Marios-Nikos Psychogios: Nothing to disclose
Daniel Coluccia: Nothing to disclose
Thi Dan Linh Nguyen-Kim: Nothing to disclose
Franja Dugar: Nothing to disclose

**A Comparison of Mechanical Thrombectomy Outcomes between M2 occlusion and M1 occlusion**

N. Çetinkaya, *F. Zengin*, U. Erdem, E. Çamurcuoğlu; Istanbul/TR
*(feyzengin@gmail.com)*

**Purpose:** Mechanical thrombectomy has been shown to be effective for large vessel occlusions in the anterior circulation, but its safety and success in treating distal vasculature remain unclear. This study evaluates outcomes of M2 versus M1 occlusions in acute ischemic stroke.
**Methods or Background:** A retrospective analysis of prospectively collected data was performed on patients with similar NIHSS scores who underwent stent retriever-based mechanical thrombectomy for isolated M1 or M2 MCA occlusions. Between March 2022 and May 2024, 198 patients were treated: 134 (67.7%) with M1 and 64 (32.3%) with M2 occlusions. Primary outcomes included recanalization success (TICI 2b/3), periprocedural complications, hemorrhage, 90-day mRS scores, and mortality.
**Results or Findings:** Mechanical thrombectomy is a safe, effective treatment for MCA occlusions in both M1 and M2 segments. Recanalization rates were higher in M1 (88%) than M2 (70.3%). Procedure time averaged 91.2 minutes for M1 and 104.6 for M2. Fewer attempts were required in M2 cases (1.57 vs. 2.4). Anticoagulant use occurred in 44.2% of M2 and 40.1% of M1 cases. M2 patients were slightly older (73.0 vs. 71.8 years). Despite lower recanalization, M2 outcomes and complication rates, including hemorrhage and mortality, were comparable. NIHSS scores at admission did not differ significantly (p = 0.296). Aspiration showed high efficacy in both groups.
**Conclusion:** The findings indicate that. M2 thrombectomy is safe, with many patients achieving good outcomes. Angiographic and clinical results were similar to M1. Endovascular treatment of severe M2 cases does not increase procedural risk or bleeding. Poor outcomes were linked to older age and prior anticoagulant use. Therefore, stent-retriever thrombectomy should be considered for severe acute M2 occlusion.
**Limitations:** This study was limited by its single-center design and lack of outcome assessment beyond 90 days.
**Funding for this study:** This research received no specific grant from any funding agency.
**Has your study been approved by an ethics committee?** Yes
**Ethics committee - additional information:** University of Health Sciences Şişli Hamidiye Etfal Training and Research Hospital Clinical Research Ethics Committee-4430
**Author Disclosures:**
Feyza Zengin: Nothing to disclose
Umut Erdem: Nothing to disclose
Eyüp Çamurcuoğlu: Nothing to disclose
Nazim Çetinkaya: Nothing to disclose

**Neurovascular Stent-Assisted Coil Embolization for Wide-Neck Visceral Artery Aneurysms: The NEVAR Technique**

*C. Sallemi*, C. Viola, F. Bodini, M. Pegorer, R. Bellosta; Brescia/IT
*(claudiosallemi@hotmail.it)*

**Purpose:** To describe a novel endovascular approach using low-profile neurovascular stents for coil embolization of wide-neck visceral artery aneurysms (VAAs), with focus on a dual-stent “kissing” configuration for bifurcation aneurysms and tortuous anatomy.
**Methods or Background:** Thirteen patients (12 women, 1 man; mean age 57 years) with wide-neck VAAs were treated between 2020 and 2025 using intracranial remodeling stents (Solitaire AB, Medtronic; Neuroform Atlas, Stryker) adapted for visceral circulation. Aneurysm locations included splenic (n=7), renal (n=3), hepatic (n=1), and pancreaticoduodenal (n=2) arteries. A dual-stent “kissing” configuration was used in eight anatomically complex cases (bifurcation aneurysms n=6, severe tortuosity n=2). All patients received dual antiplatelet therapy with aspirin and clopidogrel (Duoplavin) starting 5 days before the procedure, continued for 1 month postoperatively, followed by aspirin monotherapy for 6 months. Technical success, periprocedural events, and contrast-enhanced CT follow-up at 6, 12, and 24 months were assessed.
**Results or Findings:** Technical success was achieved in all cases (100%). Mean procedural time was 95 minutes, with radiation exposure consistent with comparable endovascular procedures. Dual-stent cases involved larger aneurysms (mean 22 mm) and required more coils (mean 7.8) than single-stent cases (mean 19.8 mm; 3.2 coils). No periprocedural complications or delayed ischemic events occurred. Median hospital stay was 2 days. Follow-up CT at 6, 12, and 24 months confirmed durable aneurysm exclusion, preserved parent artery flow, and maintained stent patency in all patients.
**Conclusion:** The NEVAR technique is a safe and effective solution for wide-neck visceral aneurysms. The dual-stent “kissing” configuration provides stable scaffolding and precise coil delivery while maintaining branch perfusion. This approach may represent a paradigm shift for treating complex visceral aneurysms previously requiring open surgery or parent vessel sacrifice.
**Limitations:** Single-center, retrospective design with a small cohort; long-term validation is warranted.
**Funding for this study:** No external funding was received for this study
**Has your study been approved by an ethics committee?** Yes
**Ethics committee - additional information:** This study was approved by the Territorial Ethics Committee Lombardia 6 (Pavia, Italy), in the session held on July 29, 2025 (Protocol No. NEVAR, Prot. 0049213/25).
**Author Disclosures:**
Costanza Viola: Nothing to disclose
Claudio Sallemi: Nothing to disclose
Matteo Pegorer: Nothing to disclose
Flavio Bodini: Nothing to disclose
Raffaello Bellosta: Nothing to disclose

**Primary Coiling Revisited: Safety and Efficacy for Ruptured Intracranial Aneurysms**

*F. Ozden*, İ. Özer, U. Mustak, O. Taydaş, M. Ozdemir, E. Arık, V. Taşçi, Ö. F. Topaloğlu, M. H. Öztürk; Sakarya/TR
*(furkanozden02@gmail.com)*

**Purpose:** To evaluate the current role and effectiveness of primary coiling in managing acute aneurysmal subarachnoid hemorrhage (aSAH).
**Methods or Background:** We conducted a retrospective single-center study involving all patients who were admitted with aSAH and treated with primary coiling between January 2019 and March 2025. The information extracted included patient demographics, such as age and sex, as well as specific details about the aneurysm, including its location, size, and morphology. The immediate angiographic outcomes were assessed based on the grade of aneurysm occlusion. Our primary focus was on two key endpoints: the rebleeding rate following the procedure and the overall mortality rate one year post-treatment.
**Results or Findings:** A total of 127 patients (mean age 57.0 ± 12.6 years; 76 female, 65%) who underwent primary coiling for aSAH were included in this study. The most common location for the aneurysms was the internal carotid artery (ICA) at 35.5%. The mean size of the aneurysms was 9.0 ± 5.6 mm. Immediate post-procedural angiography showed complete aneurysm occlusion (Raymond-Roy Class 1) in 35 patients (27.6%). Partial occlusion (Raymond-Roy Class 2 or 3) was observed in the remaining 92 patients (72.4%). During the 1-year follow-up period, the overall mortality rate was 20.5% (26 of 127 patients). Rebleeding from the treated aneurysm occurred in 2 patients (1.6%).
**Conclusion:** Primary coiling is a safe and effective method for managing aSAH. Its minimally invasive nature makes it one of the preferred treatments for aneurysms.
**Limitations:** Firstly, the retrospective design of this study introduces inherent selection bias and depends on the accuracy of medical records. Secondly, the lack of a direct comparative group, such as surgical clipping, prevents us from making definitive conclusions about the superiority of the treatment.
**Funding for this study:** This study did not receive funding from any external sources.
**Has your study been approved by an ethics committee?** Yes
**Ethics committee - additional information:** Sakarya University/E-43012747-050.04-463752/07.03.2025
**Author Disclosures:**
Ismail Özer: Nothing to disclose
Ömer Faruk Topaloğlu: Nothing to disclose
Erbil Arık: Nothing to disclose
Mustafa Ozdemir: Nothing to disclose
Onur Taydaş: Nothing to disclose
Furkan Ozden: Nothing to disclose
Mehmet Halil Öztürk: Nothing to disclose
Umit Mustak: Nothing to disclose
Volkan Taşçi: Nothing to disclose

**Effect of Copper Filtration on Radiation Dose in Cerebral 3D Rotational Angiography During Endovascular Aneurysm Treatment**
*N. Schmitt*[1], A. Berting[2], C. Herweh[1], T. Hilgenfeld[1], F. Preisner[1], L. Wucherpfennig[1], M. Bendszus[1], D. F. Vollherbst[1], M. Möhlenbruch[1]; [1]Heidelberg/DE, [2]Forchheim/DE

**Purpose:** Cerebral angiography remains the gold standard for diagnosing and endovascular management of cerebral aneurysms. Three-dimensional rotational angiography (3D-RA) provides superior ana-tomical detail compared to conventional 2D imaging; however, it is associated with relatively high radiation exposure, raising concerns regarding patient safety. This study aimed to evaluate the potential of copper (Cu) filtration to reduce radiation dose in 3D-RA.
**Methods or Background:** Forty subsequent patients undergoing endovascular treatment of unruptured cerebral aneu-rysms were included. All underwent 3D-RA using the ARTIS icono angiography system (Siemens Healthineers). In 20 patients, standard hardware with a 0.8mm aluminum (Al) filter was applied; in the subsequent 20, the Al filter was replaced by a 0.1mm Cu filter. Image quality was assessed quantitatively via contrast-to-noise ratio (CNR) and qualitatively using a five-point scale. Radiati-on dose was evaluated using entrance-skin dose (mGy) and dose-area product (Gy·cm²).
**Results or Findings:** Image quality was preserved in both groups with no differences in CNR (CNR±SD, Al: 20.72±1.82 vs. Cu: 20.66±1.54; p=0.93) or qualitative scoring (score±SD, Al: 4.55 ± 0.54 vs. Cu: 4.63±0.46; p=0.75). Total radiation dose was lower with Cu filtration (e.g., mGy±SD, Al: 110.63±10.75 vs. Cu: 68.70±6.03; Gy·cm²±SD, Al: 6.26±1.57 vs. Cu: 3.35±0.6733, p<0.001 respectively), corre-sponding to a dose reduction of 38% (entrance-skin dose) and 46% (dose-area product). These findings highlight Cu filtration as an effective and easily implementable strategy for dose opti-mization in 3D-RA. Given its compatibility with existing imaging protocols and its capacity to reduce patient exposure without compromising image quality, this approach is especially valu-able in young patients or in patients requiring repeated imaging.
**Conclusion:** Copper filtration substantially reduces radiation dose during 3D-RA while preserving image qua-lity, supporting its adoption as a practical improvement for patient safety in clinical practice.
**Limitations:** Retrospective. Single-center study.
**Funding for this study:** This study was supported by research funding from Siemens Healthineers. The authors declare no other conflicts of interest.
**Has your study been approved by an ethics committee?** Yes
**Ethics committee - additional information:** Approval for data collection was obtained from the local ethics committee and written informed consent was provided by all participants.
**Author Disclosures:**
Lena Wucherpfennig: Nothing to disclose
Fabian Preisner: Nothing to disclose
Tim Hilgenfeld: Nothing to disclose
Christian Herweh: Nothing to disclose
Martin Bendszus: Nothing to disclose
Andreas Berting: Employee: Siemens Healthineers
Dominik F. Vollherbst: Nothing to disclose
Markus Möhlenbruch: Research/Grant Support: This study was supported by research funding from Siemens Healthineers.
Niclas Schmitt: Research/Grant Support: This study was supported by research funding from Siemens Healthineers.

**Management of Intracranial Dural AV Fistulas: Endovascular Approach and Outcome Analysis**
*M. Szmygin*, T. Roman, P. Szmygin, M. Zbroja-Putowska, M. Czeczelewski, R. Rola; Lublin/PL
*(mszmygin@gmail.com)*

**Purpose:** Intracranial dural arteriovenous fistulas (DAVFs) are rare vascular malformations characterized by abnormal connections between meningeal arteries and dural venous sinuses or cortical veins. They might result from trauma, surgery, or infection. Clinical presentation varies widely, ranging from benign symptoms to life-threatening intracranial hemorrhage. This study aims to present our center's experience with the endovascular management of DAVFs, with emphasis on technical aspects of the procedures and associated clinical outcomes.
**Methods or Background:** We retrospectively reviewed all patients with intracranial DAVFs Cognard grade IIb-V treated via endovascular embolization at our institution in last 10 years. DAVFs characteristics, demographics as well as procedura and clinical data were collected and evaluated. Complications and mortality rate was calculated.
**Results or Findings:** In total 48 patients (19 female, mean age of 53 years) were included. Most common presenting symptoms include tinnitus, headaches and neurological deficits. In majority of cases, embolizations were carried out via arterial access and treatment decisions were based by fistula angioarchitecture and venous drainage patterns. All fistulas were treated with liquid embolic (glue and ethylene vinyl alcohol copolymers). Targeted vessels included middle meningeal artery, occipital artery and meningohypophyseal trunk. Obliteration of >90% of fistula was observed in 71% of cases. Satisfactory clinical outcome (defined as >75% symptoms alleviation) was achieved in 79%. Procedural complications occurred in 2 cases. One patient died due to intracranial hemorrhage caused by embolization.
**Conclusion:** Endovascular embolization is a safe and effective first-line therapy for intracranial DAVFs. Careful evaluation of fistula angioarchitecture, especially venous drainage and individualized procedural planning are essential for optimal clinical outcome.
**Limitations:** Relatively small sample size, monocentric and retrospective character of the study as well as lack of control arm with patients treated with different methods.
**Funding for this study:** None
**Has your study been approved by an ethics committee?** Yes
**Ethics committee - additional information:** Medical University of Lublin
**Author Disclosures:**
Tomasz Roman: Nothing to disclose
Maciej Szmygin: Nothing to disclose
Monika Zbroja-Putowska: Nothing to disclose
Radosław Rola: Nothing to disclose
Marcin Czeczelewski: Nothing to disclose
Paweł Szmygin: Nothing to disclose

**Reperfusion Therapy in Anterior Cerebral Artery Occlusions: Insights from a Multicenter Cohort**
*M. Szmygin*[1], P. Luchowski[1], M. Zbroja-Putowska[1], M. Czeczelewski[1], T. J. Popiela[2]; [1]Lublin/PL, [2]Krakow/PL
*(mszmygin@gmail.com)*

**Purpose:** While mechanical thrombectomy (MT) is an established treatment for acute ischemic stroke due to large vessel occlusion, its role in anterior cerebral artery (ACA) occlusions remains uncertain due to their rarity and conflicting clinical outcomes. This study aims to evaluate outcomes of MT in patients with primary or secondary ACA occlusions across two tertiary stroke centers in Poland.
**Methods or Background:** This retrospective, multicenter study included patients treated with MT for ACA occlusions between 2016 and 2024. Inclusion criteria included: ACA occlusion (segments A1–A4), NIHSS ≥6, age ≥18, and pre-stroke mRS ≤3. Clinical and procedural parameters, reperfusion success, complications and functional outcomes at 90 days were analyzed.
**Results or Findings:** Of the 134 patients (median age 74; 57% female), 63% had primary ACA occlusions. Risk factors included hypertension (78%), coronary artery disease (67%), and atrial fibrillation (51%). The average baseline NIHSS was 18. Intravenous thrombolysis was administered in 58% of cases. Successful reperfusion (mTICI 2B-3) was achieved in 86% of cases, with a first-pass effect in 40%. Symptomatic intracranial hemorrhage (sICH) occurred in 5% of patients . At 90 days, favorable functional outcomes (mRS 0–2) was observed in 41 patients (31%) and overall mortality rate was 37%. Higher NIHSS scores at admission and the presence of sICH were identified as independent predictors of 90-day mortality.
**Conclusion:** MT for ACA occlusions demonstrates high rates of successful recanalization and acceptable safety profiles. However, the moderate rate of favorable outcomes and relatively high mortality rate underscore the need for further prospective studies to define its efficacy compared to best medical therapy in this patient subgroup.

**Limitations:** Retrospective design of the study, absence of a control group treated with intravenous rt-PA alone and high percentage of aspiration used as a primary technique.
**Funding for this study:** None
**Has your study been approved by an ethics committee?** Yes
**Ethics committee - additional information:** The ethics committee of Medical University of Lublin (KE-0254/285/2019)
**Author Disclosures:**
Maciej Szmygin: Nothing to disclose
Monika Zbroja-Putowska: Nothing to disclose
Tadeusz J. Popiela: Nothing to disclose
Marcin Czeczelewski: Nothing to disclose
Piotr Luchowski: Nothing to disclose

**Analysis of malignant thyroid nodules that do not meet all recommended criteria for fine-needle aspiration according to the six existing international TIRADS guidelines**
*A. Görmez*, B. Uzunoğlu, T. Orhan, O. M. Topcuoğlu; Istanbul/TR
*(gundogdu.aysegul@gmail.com)*

**Purpose:** The American College of Radiology Thyroid Imaging and Reporting Data System (ACR-TIRADS), Kwak-TIRADS, Korean TIRADS (K-TIRADS), European TIRADS (EU-TIRADS), American Thyroid Association (ATA), and China (C-TIRADS) provide recommendations for fine needle aspiration biopsy (FNAB) based on ultrasound findings and size in thyroid nodules. However, some malignant nodules do not meet the biopsy criteria and are missed. This study aimed to evaluate the sonographic features and clinical outcomes of malignant thyroid nodules that do not meet the biopsy criteria of the six existing international guidelines.
**Methods or Background:** Ultrasound images of pathologically proven malignant thyroid nodules between January 2007-2025 were retrospectively evaluated by two radiologists with 15 years of experience in thyroid ultrasonography and two radiology assistants. Nodules were classified according to ACR-TIRADS, Kwak-TIRADS, K-TIRADS, EU-TIRADS, ATA, and C-TIRADS. Categorical variables were expressed as percentages, and continuous variables as mean ± standard deviation. Chi-square and Mann–Whitney U tests were used for intergroup comparisons.
**Results or Findings:** A total of 277 malignant nodules with a mean diameter of 14.8±10.6 mm (range 3–74 mm) were included. Pathological confirmation was obtained by FNAB in 123 nodules and by surgery in 154 nodules. A total of 129 nodules were classified as ACR-TIRADS 5, and 56.6% of these were <10 mm. Similarly, 218 nodules were classified as EU-TIRADS 5, and 50.4% of these were <10 mm. Overall, 110 malignant nodules (39.7%) were ≤10 mm. Only 6 nodules were in TR3 category, while the remaining 104 nodules (94.5%) were in TR4 or TR5 category. However, due to the size criterion, biopsy was not recommended.
**Conclusion:** The current thresholds for performing and following up on FNAB are debatable. Larger study populations may determine the optimal size threshold for thyroid nodule management.
**Limitations:** No limitations were identified.
**Funding for this study:** No funding was received for this study.
**Has your study been approved by an ethics committee?** Yes
**Ethics committee - additional information:** The study was approved by Yeditepe University Ethics Committee (202308Y0442).
**Author Disclosures:**
Tolga Orhan: Nothing to disclose
Betül Uzunoğlu: Nothing to disclose
Ayşegül Görmez: Nothing to disclose
Osman Melih Topcuoğlu: Nothing to disclose

**Expanding the Role of Pre-Operative Transarterial Embolization of Thyroid Arteries in Hyperthyroidism Patients: A Proposal for Thyroid Specimen Weighting over 120 grams**
*T. C. Yeh*, C-K. Wang, W-C. Lin; Kaoshiung City/TW
*(ytc182@gmail.com)*

**Purpose:** According to a previous study by Kosho Yamanouchi et al., thyroids that weigh more than 200 grams present as a predictive factor for excessive bleeding during surgery for Graves' disease. We would like to propose that preoperative transarterial embolization (pre-op TAE) can effectively improve the intraoperative and postoperative outcomes in hyperthyroidism patients undergoing thyroidectomy, especially those with a thyroid weight of more than 120 grams.
**Methods or Background:** This is a single-center, retrospective study in which patients with clinically diagnosed overt hyperthyroidism that eventually underwent thyroidectomy were selected. We divided the patients into two subgroups, patients that underwent direct surgical resection and patients that underwent pre-operative embolization of thyroid arteries.
**Results or Findings:** A total of 55 patients were enrolled, including 36 in the resection-only group and 19 in the pre-op TAE group. The mean intraoperative blood loss in the pre-op TAE group was significantly reduced (99.5 ml) compared with the resection-only group (210.7 ml; p= 0.041) with a p-value of . Similarly, the mean postoperative hospital stay was shorter in the pre-op TAE group (1.18 days vs. 2.15 days; p<0.001). Postoperative pain, evaluated by the numerical rating scale, was also less severe in the pre-op TAE group (scores of 1–2) compared with the resection-only group (scores of 2–3; p<0.001). All differences reached statistical significance. Further stratification by thyroid specimen weight supported the hypothesis that patients with glands weighing more than 120 grams particularly benefited from pre-op TAE.
**Conclusion:** Pre-operative thyroid TAE can reduce the amount of intraoperative blood loss, shorten the length of hospital stay and lessen the degree of wound pain compared to those that underwent thyroidectomy directly.
**Limitations:** Small sample size. Potential bias in pain evaluation.
**Funding for this study:** This study received no specific grant from any funding agency in the public, commercial, or not-for-profit sectors.
**Has your study been approved by an ethics committee?** Not applicable
**Author Disclosures:**
Cheng-Kang Wang: Nothing to disclose
Wei-Che Lin: Nothing to disclose
Tzu Chi Yeh: Nothing to disclose

**Predicting therapeutic response to thermal ablation for benign thyroid nodules using a nomogram: A multicenter retrospective investigation**
*X. Shan*; Shanghai/CN
*(shanxuexiawx@163.com)*

**Purpose:** To develop and validate a nomogram based on clinical and ultrasound (US) parameters for preoperative prediction of the complete absorption rate following thermal ablation (TA) in benign thyroid nodules (BTNs).
**Methods or Background:** This multicenter retrospective study included 372 patients with 395 BTNs treated with TA from May 2018 to September 2021. Complete absorption was defined as a volume reduction rate (VRR) of 100% at 12 months. Predictors were identified by logistic regression with LASSO selection and incorporated into a nomogram. Model performance was assessed with receiver operating characteristic analysis, calibration, decision curve analysis, and external validation.
**Results or Findings:** Of the 395 nodules included, 87 (22.0%) nodules achieved complete absorption at the 12-month follow-up after TA. Predictive factors included in the nomogram are location between nodule and dangerous triangle area (OR, 0.36 [95% confidence interval [CI]: 0.15, 0.84]; P =0.018), morphology (OR, 0.18 [95% CI: 0.05, 0.65]; P =0.009), vascularity (OR, 0.32 [95% CI: 0.11, 0.92]; P <0.034), volume (OR, 0.08 [95% CI: 0.01, 0.69]; P =0.021), enhancement mode (OR, 0.25 [95% CI: 0.11, 0.59]; P =0.001). This prognostic nomogram showed great calibration and discrimination with an AUC of 0.818 (95% CI, 0.759–0.878) and 0.710 (95% CI, 0.597–0.823) in the training and external validating sets, respectively.
**Conclusion:** A clinical nomogram was successfully established to preoperatively and noninvasively predict the complete absorption rate for nodules undergoing TA. The model might guide physicians in evaluating the efficacy of TA and to facilitate developing individualized treatment and prognostic plans.
**Limitations:** The number of cases is not large enough.
**Funding for this study:** None
**Has your study been approved by an ethics committee?** Not applicable
**Author Disclosures:**
Xuexia Shan: Nothing to disclose

**CT- and Fluoroscopy-Guided Transfacet Fixation Under Local Anesthesia: A Minimally Invasive Alternative to Lumbar Fusion after Laminectomy**
*K. Desalos*, N. Amoretti; Nice/FR
*(kevindesalos@gmail.com)*

**Purpose:** To evaluate the feasibility, safety, and clinical outcomes of percutaneous transfacet screw fixation under local anesthesia as a minimally invasive alternative for post-laminectomy instability.
**Methods or Background:** Prospective monocentric study (2022–2025) including 30 patients with lumbar segmental instability after laminectomy. All underwent CT- and fluoroscopy-guided percutaneous transfacet fixation under local or regional anesthesia. Pain (VAS) and quality of life (EQ-5D) were assessed preoperatively and at 1, 6, and 12 months and 2 years follow-up.
**Results or Findings:** Technical success rate: 100%. Significant pain reduction (mean VAS −3 points, p<0.05) and improved EQ-5D (median reduction of 2 points) were observed at 6 and 12 months. No major adverse events were reported. No cases required conversion to open fusion. Screws could be safely repositioned or removed if needed

**Conclusion:** CT- and fluoroscopy-guided transfacet fixation under local anesthesia is safe, feasible, and effective. It provides durable pain relief and improved quality of life, while avoiding general anesthesia, conventional surgery, and prolonged hospitalization.
**Limitations:** Single-center, limited sample size, and relatively short follow-up for some patients. Larger multicenter trials are required to validate long-term outcomes
**Funding for this study:** No funding was received for this study.
**Has your study been approved by an ethics committee?** Not applicable
**Author Disclosures:**
Kevin Desalos: Nothing to disclose
Nicolas Amoretti: Nothing to disclose

**14:30 - 16:00** **Research Stage 4**

### Research Presentation Session: Professional Challenges: Shaping our Professional Future

### RPS 1030

### Imaging wisely: balancing protection, practice, and performance

**Moderator**
Christian J. Johannes Herold; Vienna/AT
**Author Disclosures:**
Christian J. Johannes Herold: Advisory Board: Contextflow, Roclub, CEO: Medical University of Vienna International GmbH, Share Holder: various companies

### Repeat Imaging Trends over a Decade: Entering a New Era of Imaging Surveillance

*J. Sarnecki*, M. T. Meyer, T. Heye; Basel/CH
*(jedrzej.sarnecki@gmail.com)*

**Purpose:** To investigate trends in per-patient imaging utilization and follow-up intervals over the last decade.
**Methods or Background:** Trends in imaging utilization per patient and the time intervals between examinations were analyzed for 332’008 adult patients who underwent 1’660’034 studies (CR, US, CT, MRI, PET-CT) between 2014-2024 at a tertiary referral hospital. Patients were categorized into two age groups: <65yrs. and ≥65yrs.
**Results or Findings:** Over the last decade, examinations per patient increased by 12% for inpatients (1.66 to 1.86) versus 2.7% for outpatients (1.30 to 1.35). The main driver of change was CT imaging, with 12.0% (<65yrs.) and 12.7% (>65yrs.) increase among inpatients (MRI: -4% and +1.6%, respectively). The strongest increase was in CTs of abdomen/pelvis (+14%), chest (+12.5%), and chest-abdomen-pelvis (+8.9%). Exemplarily, the number of patients with 5 examinations rose by 31.9% (7’183 to 9’477), those with 15 by 112.4% (1’875 to 3’983), with 30 examinations by 341.5% (342 to 1’510). In 2014 the most frequent repeat-imaging modality was CR; in 2024, CR, US, CT were similar, with CT most frequent in the older group. Median time between CT examinations decreased by 29.1%, from 70 days in 2014 to 49.7 days in 2024 (25th percentile: 11.8 to 7 days). Among inpatients, the reduction was greater at 55.4% (36 to 16 days) versus 7.7% among outpatients (91 to 84 days). Repeat CT examinations increased across all follow-up intervals: within 7 days (+53.3%), within 30 days (+46.9%), within 90 days (+52.6%), and >90 days (+47%).
**Conclusion:** The results show an increase in imaging utilization per patient and a reduction in the time between examinations, underscoring the growing role of radiology in patient care. The most significant increase is seen in CT imaging during inpatient stays.
**Limitations:** Retrospective, single-centre study.
**Funding for this study:** None
**Has your study been approved by an ethics committee?** Not applicable
**Author Disclosures:**
Tobias Heye: Nothing to disclose
Jed Sarnecki: Nothing to disclose
Manfred Tobias Meyer: Nothing to disclose

### Variability in CT Referral Data Completeness: Insights from the EU-JUST-CT Project

C. Singer[1], M. Saban[2], L. Bergovoy-Yellin[1], B. Brkljačić[3], A. Karoussou-Schreiner[4], *J. Sosna*[5]; [1]Ramat Gan/IL, [2]Tel Aviv/IL, [3]Zagreb/HR, [4]Luxembourg/LU, [5]Jerusalem/IL
*(jacobs@hadassah.org.il)*

**Purpose:** To evaluate the quality and completeness of CT referrals using audited data from the EU-JUST-CT project across seven European countries: Belgium, Denmark, Estonia, Finland, Greece, Hungary, and Slovenia (n=6,734 referrals).
**Methods or Background:** Each referral was evaluated using seven binary indicators: Clinical data sufficient; Examination specified; Referrer specialty; Prior exams; Patient status (in-/outpatient); Patient gender and age. We computed a Completeness Score (sum of all seven indicators) and a Critical Score based on four essential items (clinical data, exam type, gender, and age). We examined the distribution of both scores by country and calculated the proportion of fully complete referrals (Completeness Score = 7) and fully critical-complete referrals (Critical Score = 4).
**Results or Findings:** The median Completeness Score was 7 in Denmark; 6 in Belgium, Greece, Hungary, and Slovenia; 5 in Estonia; and 4 in Finland. Denmark had the highest mean Completeness Score (6.6), while Finland had the lowest (4.3). The proportion of fully complete referrals ranged from 60.6% (Denmark) to 0.3% (Finland). For the Critical Score, all countries had a median of 4, but inter-country variation in the proportion of referrals with full critical data was observed: (67-98%). The mean Critical Score was highest for Belgium (3.98) and lowest for Slovenia (3.64).
**Conclusion:** Incomplete information on CT referrals was prevalent, with differences observed between countries. The results presented raise the need for unified protocols and standards, possibly anchored in the national legislation, which dictates what minimal information is required in a CT referral in order to be approved.
**Limitations:** Our dataset, while large and geographically diverse within Europe, may not generalize to other regions with different healthcare structures, referral styles, or disease prevalence. Additionally, this study did not assess the clinical consequences of incomplete referrals.
**Funding for this study:** No
**Has your study been approved by an ethics committee?** Yes
**Ethics committee - additional information:** local research ethics committees or IRBs at each of the 125 participating imaging facilities across the seven countries. At each site, the IRB either approved the study or waived the requirement for informed consent due to the retrospective nature and de-identification of the data.
**Author Disclosures:**
Boris Brkljačić: Nothing to disclose
Mor Saban: Nothing to disclose
Jacob Sosna: Nothing to disclose
Lucia Bergovoy-Yellin: Nothing to disclose
Alexandra Karoussou-Schreiner: Nothing to disclose
Clara Singer: Nothing to disclose

### Referring Physicians' Perspectives on Radiology Appointment Management: A Cross-sectional Survey of General Practitioners and Hospital Physicians

*P. Reschke*, K. Eichler, T. Vogl, A. M. Bucher; Frankfurt/DE
*(philipp.reschke@outlook.de)*

**Purpose:** Radiology appointment delays compromise healthcare efficiency and patient satisfaction, yet referring physicians’ perspectives on scheduling challenges remain underexplored. This study investigates these perspectives and identifies targeted strategies for improvement, comparing general practitioners (GPs) and hospital physicians.
**Methods or Background:** We conducted a cross-sectional survey across Germany from June 2023 to June 2024, recruiting 220 actively practicing physicians via systematic random sampling. This included 79 GPs, who regularly refer outpatients to hospital-based radiology departments, and 141 hospital physicians, who coordinate imaging for inpatients. An anonymous online questionnaire assessed the perceived impact of imaging delays and coordination difficulties (7-point Likert scales), preferred improvement strategies (multiple choice), and prioritization of quality metrics (weighted ranking).
**Results or Findings:** GPs had significantly more clinical experience than hospital physicians (20 ± 14 vs. 7 ± 9 years; $p < 0.001$), with no gender differences ($p = 0.67$). GPs reported a greater impact of imaging delays on patient care (4.39 ± 1.50 vs. 4.02 ± 1.49; $p = 0.02$) and more coordination challenges (4.55 ± 1.42 vs. 4.30 ± 1.50; $p < 0.05$). Hospital physicians preferred real-time workflow tracking (34.8% vs. 9.1%; $p < 0.001$) and automated reminders (20.2% vs. 6.1%; $p = 0.007$), while GPs favored centralized scheduling (33.3% vs. 19.1%; $p = 0.03$) and urgent case prioritization (42.4% vs. 23.6%; $p = 0.008$). Short-term appointment availability was the top priority across both groups (24.9%; $\chi^2(4) = 107.2$, $p < 0.001$).

**Conclusion:** Radiology scheduling delays remain a key barrier to timely patient care. The findings support the implementation of referrer-specific appointment management strategies in order to optimize imaging coordination.
**Limitations:** Our analysis focused exclusively on referring physicians' perspectives, excluding viewpoints from radiologists, administrative staff, and patients.
**Funding for this study:** None
**Has your study been approved by an ethics committee?** Not applicable
**Author Disclosures:**
Thomas Vogl: Nothing to disclose
Philipp Reschke: Nothing to disclose
Katrin Eichler: Nothing to disclose
Andreas Michael Bucher: Nothing to disclose

**Exploring Care of People Who Identify as Transgender or Non-binary [TGNB] Accessing Radiology/Radiotherapy Services in the UK and the Republic of Ireland**
*T. Miller*[1], N. Cook[1], J. Daly Lynn[1], L. McLaughlin[2], S. L. Mcfadden[1]; [1]Derry/Londonderry/UK, [2]Cork/IE
*(miller-t4@ulster.ac.uk)*

**Purpose:** It is a legal requirement to verify biological sex and pregnancy status prior to exposing people to the dangers of ionising radiation. Sensitivities that exist when ascertaining pregnancy status can result in ethically challenging scenarios for healthcare workers (HCWs) and leave TGNB people uncomfortable with their care.
**Methods or Background:** Two validated surveys were conducted: one assessing the radiology/radiotherapy experiences of TGNB people and the other examining HCWs attitudes towards TGNB identities. The surveys were disseminated via professional conferences, social media, and voluntary support organisations. Data collection will be completed on 31st December 2025. SPSS version 28 is being used to conduct descriptive and inferential statistics.
**Results or Findings:** To date 76 HCWs and 53 TGNB participants have responded to the surveys. Preliminary results report 37% (n=28) of HCWs had experienced transphobic behaviours from colleagues. While 20% (n=15) disagreed or strongly disagreed that knowing the gender identity of the person in their care was important. Of the TGNB respondents 51% (n=27) had not experienced affirming interactions with staff. Meanwhile 62% (n=33) had not been asked about their chosen name and 66% (n=35) had not been asked about their preferred pronouns. Additionally, 25% (n=13) of TGNB respondents did not understand the significance of pregnancy status questions.
**Conclusion:** Further education is needed for both sets of participants TGNB communities must be educated on the rationale for pregnancy status questions and HCWs must be provided with the tools to uphold legal obligations while maintaining the dignity of TGNB identities.
**Limitations:** This is a very sensitive topic which may hinder patients from participating. Hence, resulting in a limited sample size. The data is representative of the UK and Ireland population only and may not be fully reflective of the international opinion.
**Funding for this study:** Department for Education funding is enabling completion of the PhD
**Has your study been approved by an ethics committee?** Yes
**Ethics committee - additional information:** Ethical permission for this study has been granted by Ulster University's Nursing and Health Research Ethics Filter Committee on 15th March 2025.
**Author Disclosures:**
Tracy Miller: Nothing to disclose
Jean Daly Lynn: Nothing to disclose
Laura Mclaughlin: Nothing to disclose
Sonyia Lorraine Mcfadden: Nothing to disclose
Neal Cook: Nothing to disclose

**Interventions to reduce MRI access time – a systematic review on the existing scientific evidence**
*C. Debats*, M. Van Deursen - Luijten, C. Adang, F. Van Kasteren, N. Visschers, E. Peters, F. M. Zijta, J. E. Wildberger, T. Van Nijnatten; Maastricht/NL
*(charles.debats@mumc.nl)*

**Purpose:** Growing demand for advanced imaging techniques like MRI has resulted in long waiting lists and delayed patient diagnosis by exceeding facility capacity, with consequently prolonged MRI access time. This study defines MRI access time as the time-interval between requesting an MR exam and the performance of the MR exam. The aim of this systematic review was to provide an overview of the available evidence on interventions to reduce MRI access time.
**Methods or Background:** Pubmed, Embase, Cochrane and Web of Science databases were searched up to September 1st 2025. Two reviewers independently screened all studies and performed data extraction. Eligible studies were original investigations reporting on MRI access times and provided results before and after the proposed intervention to reduce access time. Reviews, meta-analyses, conference papers, editorials, technical reports and case reports were excluded.
**Results or Findings:** The literature search yielded 3875 records, of which 29 records were fully reviewed, and 10 met inclusion criteria. Five studies evaluated simulated interventions, three studies reported implemented interventions. Reported interventions can be subdivided in three categories: matching capacity and demand (e.g., increasing MRI capacity); waiting list segmentation (e.g., grouping patients with similar characteristics to reduce coil-changing time); and operational redesigns of the MRI scheduling process (e.g., implementing a decentralized integrated online booking system). Sample sizes ranged from 264 until 172,000 patients. Reported effectiveness of interventions varied widely: most successful solution combined multiple interventions, achieving a reduction in MRI access time of 73.3 days. Multiple intervention studies did not show any measurable improvement to reduce MRI access time.
**Conclusion:** Current interventions to reduce MRI access time have shown limited success, with insufficient evidence supporting the impact of specified interventions, necessitating urgent research on novel interventions to be performed.
**Limitations:** N/a
**Funding for this study:** N/a
**Has your study been approved by an ethics committee?** Not applicable
**Author Disclosures:**
Esther Peters: Nothing to disclose
Charles Debats: Nothing to disclose
Flip Van Kasteren: Nothing to disclose
Thiemo Van Nijnatten: Advisory Board: GE Healthcare, Bayer, Screenpoint Medical Speaker: GE Healthcare, Bayer, Hologic Inc Research/Grant Support: GE Healthcare, Bayer
Chris Adang: Nothing to disclose
Joachim E. Wildberger: Nothing to disclose
Marieke Van Deursen - Luijten: Nothing to disclose
Nol Visschers: Nothing to disclose
Frank Marinus Zijta: Nothing to disclose

**Radiation exposure during CT: Patients´ knowledge, perception and request for information**
*N. Seiß*, M. Mohamed, F. Yavuz, F. Biavati, M. Rief, T. A. Auer, M. Dewey, A-C. Stahl; Berlin/DE
*(nathalie.seiss@charite.de)*

**Purpose:** To assess patients' existing knowledge and their desired level of information about radiation exposure during CT.
**Methods or Background:** This single-centre survey was conducted at a large European university hospital. Patients (≥ 18 years) capable of giving informed consent and scheduled for a clinically indicated CT were eligible for inclusion. A 17-question survey covering demographic data, radiation exposure of different imaging examinations, and patients' desired level of information was used to assess patients´ existing knowledge and requests for information about radiation exposure. Analysis was done by calculating frequencies of given answers. As not all patients answered all questions, the denominators differ. Chi-Square and Mann-Whitney U tests were performed to determine differences between genders.
**Results or Findings:** 285 questionnaires were collected with recruitment still ongoing. The median age of the patients was 62 years (IQR: 53-73), with 42% (118/280) being female. The majority, 81% (171/211) correctly stated that CT is an examination with radiation exposure, with no significant difference between female and male (85% vs. 77%, p=0.187). Interestingly, also 35% (74/211) selected MRI as an examination involving radiation exposure. Patients estimated that the radiation exposure of a thoracic CT would only be two times higher (IQR: 1-4) than that of a thoracic X-ray in two planes, again without significant gender differences (p=0.794). Overall, 71% (147/206) felt to be sufficiently to very well informed about the radiation exposure of CT and 36% (78/219) requested to receive further information.
**Conclusion:** While most patients are aware that CT involves radiation exposure, they tend to underestimate this exposure compared to X-rays. Most of the patients felt adequately informed with a relevant number of patients still wishing for further information.
**Limitations:** This is a single-centre study limiting the generalisability of the results.
**Funding for this study:** No funding was received for this study.
**Has your study been approved by an ethics committee?** Yes
**Ethics committee - additional information:** The study was approved by the local ethics committee at Charité - Universitätsmedizin Berlin (EA4/273/21).

**Author Disclosures:**
Nathalie Seiß: Nothing to disclose
Matthias Rief: Other: Institutional master research agreements exist with Siemens, General Electric, Philips, and Canon. The terms of these arrangements are managed by the legal department of Charité – Universitätsmedizin Berlin. Grant Recipient: Rief received grant support from German Research Foundation (DFG).
Mahmoud Mohamed: Nothing to disclose
Ferhat Yavuz: Nothing to disclose
Marc Dewey: Other: M.D. is European Society of Radiology (ESR) Publications Chair (2022-2025); the opinions expressed in this presentation are the author's own and do not represent the view of ESR. Other: Editor: Cardiac CT (Springer Nature). Other: Hands-on cardiac CT courses (www.ct-kurs.de) Other: Patent on fractal analysis of perfusion imaging (jointly with Florian Michallek, EPO 2022 EP3350773A1, and USPTO 2021 10,991,109, approved) Grant Recipient: Grants: EU (EC-GA 603266 in HEALTH.2013.2.4.2-2) DFG (DE 1361/14-1, DE 1361/18-1, BIOQIC GRK 2260/1, Radiomics DE 1361/19-1 [428222922] and 20-1 [428223139] in SPP 2177/1), GUIDE-IT (DE 1361/24-1), Berlin University Alliance (GC_SC_PC 27), G-BA (01NVF23002), Berlin Institute of Health (Digital Health Accelerator). Other: Institutional research agreements: Siemens, General Electric, Philips, Canon.
Ann-Christine Stahl: Nothing to disclose
Timo Alexander Auer: Nothing to disclose
Federico Biavati: Nothing to disclose

**Do doctors routinely working with ionising radiation have adequate knowledge of radiation safety and risk?**
*H. Said*; Chelmsford/UK
*(hadil.said@hotmail.co.uk)*

**Purpose:** To explore physician knowledge regarding radiation doses, the different modalities used, and important safety precautions. To determine the follow up research necessary to improve safety.
**Methods or Background:** Radiology is a growing specialty, and ionising radiation is increasingly being used by physicians across specialties. It is essential to understand basic radiation safety and take necessary precautions. This review synthesises the evidence on whether doctors have adequate knowledge of radiation safety and risk. A systematic review was performed, following PRISMA (Preferred Reporting Items for Systematic Reviews and Meta-Analyses) guidelines. Boolean operators were used to search Embase and PubMed databases. Inclusion criteria: English language articles within the last 10 years. Exclusion criteria: case reports, editorials, case presentations, animal studies, mammography articles. All relevant papers involved surveys; therefore a thematic analysis was performed.
**Results or Findings:** The initial search gave 52 results, after applying inclusion and exclusion criteria this reduced to 25 results. 11 articles were relevant to the research question. Doctors' knowledge of radiation safety and risk was inadequate across specialties, and geographical locations. Safety measures (e.g. providing and wearing appropriate personal protective equipment) were either ignored, or inadequate, despite clear legislation within this area. Seniority did not translate to better knowledge or safety practices.
**Conclusion:** The evidence demonstrates that doctors have inadequate knowledge of radiation safety. The provision of radiation safety training for physicians working with ionising radiation is inadequate. Standards and guidelines should follow national/international guidelines, rather than local policy.
**Limitations:** This review used Braun & Clarke's reflexive thematic analysis, in which it is good practice to enlist a single coder. This means that there is one consistent approach throughout the review, but it lacks other reviewers and perspectives to corroborate paper selection and agree on the analysis.
**Funding for this study:** No funding was received for this study. Ethical approval was not required as this was an systematic review using publicly available data.
**Has your study been approved by an ethics committee?** Not applicable
**Author Disclosures:**
Hadil Said: Nothing to disclose

**Health Hazards Among Radiology Professionals: A Descriptive Survey**
*J. Lydia*, F. Abubacker Sulaiman, R. Praveenkumar, M. Nivitha, D. Dharshini, D. Velan, A. Raashid Ibrahim, A. Selvam; Chennai/IN
*(merlinlydia99@gmail.com)*

**Purpose:** Radiology professionals are exposed to various health hazards due to the nature of their work. Occupational risks include radiation, chemical exposure, infections, and musculoskeletal strain, while non-occupational risks stem from sedentary work, screen exposure, shift duties, and stress.
**Methods or Background:** A structured Google Forms survey was conducted among radiologists, radiographers, and nurses to assess occupational and non-occupational hazards. Responses were analyzed, and the prevalence of each risk factor was expressed as a percentage. Preventive measures were also evaluated.
**Results or Findings:** The most common occupational hazards were radiation exposure (78%), musculoskeletal strain (65%), chemical/contrast exposure (42%), biological hazards (38%), and work-related stress (55%). Non-occupational risks included sedentary lifestyle (70%), eye strain (62%), sleep disturbances (48%), and lifestyle-related stress (50%). Key preventive strategies included personal protective equipment, ergonomic adjustments, vaccination, optimized shift schedules, and lifestyle modifications
**Conclusion:** Radiology professionals face multiple health hazards. Awareness and implementation of safety protocols, ergonomic practices, and healthy lifestyle measures are essential to reduce risks and ensure long-term professional well-being.
**Limitations:** The study relied on self-reported data from a limited sample, which may introduce bias and limit generalizability. Objective measurements of exposure and long-term effects were not included.
**Funding for this study:** No funding was provided
**Has your study been approved by an ethics committee?** Not applicable
**Author Disclosures:**
Rathinamoorthy Praveenkumar: Nothing to disclose
Deva Dharshini: Nothing to disclose
Mohanam Nivitha: Nothing to disclose
Johnbosco Lydia: Nothing to disclose
Dhanush Velan: Nothing to disclose
Farook Abubacker Sulaiman: Nothing to disclose
Abubacker Raashid Ibrahim: Nothing to disclose
Atchaya Selvam: Nothing to disclose

**Virtopsy with Photon-Counting CT: New Perspectives in Forensic Post-Mortem Imaging**
*E. Solinas*, L. Piscopo, G. De Paula, D. Turilli, M. Scaglione, S. A. Masala; Sassari/IT
*(solinas.emma@gmail.com)*

**Purpose:** Virtopsy, or virtual autopsy, is an established method in post-mortem investigations, offering a non-invasive alternative to conventional autopsy. The recent introduction of photon-counting CT (PCCT) provides new perspectives in forensic medicine, thanks to its ability to directly convert incident photons into electrical charges, thereby reducing electronic noise and improving spatial resolution and offering spectral imaging, improving tissue and material characterization.
**Methods or Background:** We performed 15 post-mortem examinations using PCCT, comparing image quality and diagnostic performance with conventional CT, focusing on fracture detection, identification of foreign bodies, and differentiation of metallic materials. Particular attention was given to firearm-related deaths, where metallic artifacts often compromise conventional CT.
**Results or Findings:** PCCT provided high-definition images even at low energies, enabling detailed assessment of skeletal structures and soft tissues without contrast medium. Metallic artifact reduction allowed reliable discrimination between different bullets' type, significantly enhancing firearm injury evaluation. Improved visualization of trauma patterns and organ damage further support the diagnostic value. Compared to conventional CT, PCCT demonstrated superior accuracy, reproducibility, and artifact reduction.
**Conclusion:** Virtopsy with PCCT is a rapid, reliable, and body-preserving imaging method with clear advantages over conventional CT. Our preliminary series of 15 cases highlights its potential to improve forensic investigations and forensic anthropology, with promising applications in both forensic and academic contexts.
**Limitations:** No limitations were identified.
**Funding for this study:** No funding was received for this study.
**Has your study been approved by an ethics committee?** Not applicable
**Author Disclosures:**
Davide Turilli: Nothing to disclose
Mariano Scaglione: Nothing to disclose
Giovanni De Paula: Nothing to disclose
Salvatore Masala: Nothing to disclose
Leandra Piscopo: Nothing to disclose
Emma Solinas: Nothing to disclose

**Radiological Imaging and Image-Guided Procedures in Palliative Inpatients: A Retrospective Review at a Tertiary Centre**
*R. Cusack*, E. Jones, J. Drought, C. Murphy, S. O'Neill, R. Kernan, N. Sheehy, N. O'Leary; Dublin/IE
*(rcusack@tcd.ie)*

**Purpose:** Radiology plays a key role in diagnosis, monitoring, and intervention for advanced disease, yet its value at the end of life is debated. Prior studies have shown high imaging utilisation with limited impact on management[1,2]. This study evaluated the utilisation and clinical value of radiological imaging and image-guided procedures in palliative inpatients, aiming to identify opportunities to optimise practice in end-of-life care.
**Methods or Background:** We retrospectively reviewed inpatients who died in St James's Hospital between January and December 2023. Patients were identified using the hospital's RIP Excel Report, and imaging data were extracted from electronic records. Radiological investigations in the last four weeks of life were included: plain radiography, CT, MRI, ultrasound, nuclear medicine, and image-guided procedures (ultrasound- and interventional radiology–guided). Frequency, modality, timing, and interval between last imaging and death were recorded. Imaging was classed as high-value if it established a new diagnosis that altered management or informed prognosis; otherwise, it was low-value.
**Results or Findings:** A total of 260 patients were included. The median number of investigations was 5. Plain radiography (median 3) and CT (median 1) were most common, while MRI (n=32), ultrasound (n=41), and nuclear medicine (n=10) were less frequent. Forty-two image-guided procedures were performed. The median interval between last imaging and death was 4 days. Of imaging within three days of death, 44 (25%) were high-value and 131 (75%) low-value.
**Conclusion:** Imaging and procedures are common in the last month of life, often close to death, but most were of limited clinical value from a radiological perspective. Greater collaboration between radiology and palliative care may help reduce low-yield imaging.
**Limitations:** This study is limited by its retrospective, single-centre design and reliance on clinical documentation.
**Funding for this study:** No external funding was received.
**Has your study been approved by an ethics committee?** Yes
**Ethics committee - additional information:** Ethical approval was granted by the St James's Hospital/Tallaght University Hospital Joint Research Ethics Committee.
**Author Disclosures:**
Ronan Kernan: Nothing to disclose
Niall Sheehy: Nothing to disclose
Norma O'Leary: Nothing to disclose
Eva Jones: Nothing to disclose
John Drought: Nothing to disclose
Caitriona Murphy: Nothing to disclose
Ruth Cusack: Nothing to disclose
Susan O'Neill: Nothing to disclose

**Radiology in Crisis: Preparing for Catastrophes, Conflict Zones, and Military Actions**
*U. Pidvalna*, D. Beshley; Lviv/UA
*(upidvalna@gmail.com)*

**Purpose:** Radiologists play an important, but often underrecognized, role in managing the medical consequences of crises: from natural disasters and mass-casualty events to armed conflicts and war zones. As global instability rises, the need for a structured and resilient radiology response becomes paramount.
**Methods or Background:** This abstract addresses the practical, technical, and ethical challenges faced by radiologists working under extreme conditions. It is not limited to power outages, air raid sirens, infrastructure damage, limited internet access, triage, and scarce resources, but also to moral and ethical issues.
**Results or Findings:** Drawing on experiences from war zones, this discussion provides a realistic, system-based overview of radiological practice in unstable environments. How to maintain imaging services during power outages? Do we need to adapt workflows without PACS/RIS systems? Should we ensure communication in the absence of digital networks? Managing triage imaging in high-pressure scenarios? Emphasis on the mental and physical strain placed on radiology teams, as well as the need for international cooperation, modular protocols, and mobile radiology units.
**Conclusion:** Sharing pragmatic strategies and organizational insights equips radiologists with the tools and mindset necessary to prepare for and function effectively during crises, wherever they may arise.
**Limitations:** Limitations include the variability of crisis settings, the lack of standardized protocols, and the reliance on personal experiences.
**Funding for this study:** No funding.
**Has your study been approved by an ethics committee?** Not applicable
**Author Disclosures:**
Uliana Pidvalna: Nothing to disclose
Dmytro Beshley: Nothing to disclose

**16:30 - 18:00** **Research Stage 1**

### Research Presentation Session: Abdominal and Gastrointestinal Hot Topic with Keynote Lecture

### RPS 1101
### Hot Topic: prognostication

**Moderator**
Felix Hahn; Mainz/DE

**Keynote Lecture**
Susanna Lee; Boston/US
*(slee0@mgh.harvard.edu)*

**Integrated clinical and contrast-enhanced CT parameters for predicting therapeutic response in colorectal cancer liver metastasis treated with chemotherapy and bevacizumab**
*L. Yuan*, J. Zhou; Lanzhou/CN
*(yuanl075@163.com)*

**Purpose:** To investigate the predictive value of clinical and pre- and post-treatment CT parameters for the efficacy of bevacizumab in patients with colorectal liver metastasis(CRLM) .
**Methods or Background:** This retrospective study included 290 patients with CRLM and 455 liver metastases treated with bevacizumab in our hospital . The morphological features of CRLM after four treatment cycles were assessed using CT images. CT values at baseline and after four treatment cycles in different phases (PS, AP, VP, and DP), and density change values (ΔCT values) before and after treatment were calculated. According to RECIST 1.1, CRLM after 12 treatment cycles was categorized into responsive and non-responsive groups. Differences between the two groups were searched for using the T-test, Mann−Whitney U, or chi-square test, and diagnostic performances of the different variables were evaluated using the receiver operating characteristic (ROC) curve.
**Results or Findings:** RAS mutant-type CRLM was more prevalent in the responsive group than in the wild-type group, with a significant difference ($P<0.001$). CRLM with a lobulated shape and heterogeneous texture after four treatment cycles showed a poorer treatment response than those with a round shape and homogeneous texture, with a significant difference ($P<0.05$). Additionally, CT values of AP-pre, VP-pre, DP-pre, VP-post, and ΔAP were higher in the responsive group than in the non-responsive group. The combined use of clinical and contrast-enhanced CT parameters demonstrated better efficacy (AUC >0.7) than single parameters. Parameters such as RAS, texture-post, and AP-pre combined showed higher predictive efficacy.
**Conclusion:** The combination of clinical parameters and contrast-enhanced CT parameters before and after treatment can effectively predict the early therapeutic response in patients with CRLM.
**Limitations:** It is single-center, small-sample, and retrospective nature may have affected the robustness of the results.
**Funding for this study:** This work was supported by grants of the National Natural Science Foundation of China (No. 82371914) and the Cuiying Scientific and Technological Innovation Program of Lanzhou University Second Hospital (CY2021-ZD-01).
**Has your study been approved by an ethics committee?** Yes
**Ethics committee - additional information:** The study was approved by the institutional ethical review board (2023A-379).
**Author Disclosures:**
Junlin Zhou: Nothing to disclose
Long Yuan: Nothing to disclose

**The Impact of Sarcopenia on the Risk of Gallbladder Perforation in Acute Cholecystitis: A Retrospective Analysis**
*M. V. Gökay*, K. Kadirli, F. S. Bayramoğlu, H. Mutlu; Istanbul/TR
*(m.vasfigokay@gmail.com)*

**Purpose:** To evaluate the association and prognostic value of CT-derived body composition parameters with gallbladder perforation in patients with acute cholecystitis.

**Methods or Background:** We retrospectively analyzed 80 surgically treated patients with acute cholecystitis (Jan 2018–Dec 2024). Gallbladder perforation was intraoperatively confirmed in 21 cases (26.2%), while 59 non-perforated patients, matched by age, sex, and comorbidity, served as controls. Preoperative non-contrast abdominal CTs were assessed at the L3 level. Skeletal muscles (psoas, paraspinal, abdominal wall) and visceral/subcutaneous fat were segmented semi-automatically using ImageJ v1.54m. Thresholds: –29 to +150 HU for muscle, –190 to –30 HU for fat. All areas were normalized to vertebral surface area. Measurements were repeated twice by two readers. Comorbidities were grouped as none, 1, 2, or ≥3. Collected variables included demographics, CRP, leukocytes, and time intervals (symptom–CT, symptom–admission, admission–surgery). Univariate tests identified predictors of perforation. Significant variables were entered into multivariate logistic regression (backward LR). ROC assessed diagnostic performance, and VIF tested collinearity. Variables with <20% missingness were handled via listwise deletion, leading to minor sample variations.
**Results or Findings:** Univariate analysis showed significantly higher IMAT/vertebra ratio in the perforation group (p=0.050). Multivariate analysis identified IMAT/vertebra ratio (OR: 4.63; 95% CI: 1.41–15.15; p=0.011) and leukocyte count (OR: 1.14; 95% CI: 1.02–1.29; p=0.026) as independent predictors. Symptom-to-CT interval showed borderline association (OR: 1.14; 95% CI: 1.00–1.29; p=0.054). Interobserver agreement was excellent (ICC = 0.89).
**Conclusion:** Our findings show that the IMAT/vertebra ratio is an independent predictor of gallbladder perforation in acute cholecystitis, reflecting inflammatory burden and reduced physiological reserve. Validation in larger multicenter cohorts is needed to confirm its prognostic value and support integration into routine preoperative assessment.
**Limitations:** Single-center, retrospective design and small sample size.
**Funding for this study:** None.
**Has your study been approved by an ethics committee?** Yes
**Ethics committee - additional information:** The study was approved by Koşuyolu High Specialization Training and Research Hospital Ethics Committee for Scientific Research (Approval No: 2025/08/1129)
**Author Disclosures:**
Kenan Kadirli: Nothing to disclose
Fatma Sibel Bayramoğlu: Nothing to disclose
Hakan Mutlu: Nothing to disclose
Muhammet Vasfi Gökay: Nothing to disclose

**Prediction of Microsatellite Instability in Colorectal Cancer Using Two Internally Validated Radiomic Models**
*A. Galluzzo*, L. Scalzone, C. Mugnaini, G. Danti, V. Miele; Firenze/IT
*(antonio.galluzzo@unifi.it)*

**Purpose:** To develop two different radiomic models (RMs) based on preoperative portal phase computed tomography (PP CT) to predict microsatellite instability (MSI) in patients with colorectal cancer (CRC) before surgery.
**Methods or Background:** PP CT scans of 115 CC patients were segmented using 3DSlicer (v5.6.1). Model I included images from three different scanners (GE, Siemens, Philips), while Model II used only one scanner (GE). For Model I, 80 patients were used for training and 35 for internal validation; for Model II, 46 and 24 patients were used, respectively. Data on sex, age, tumor location, and MSI genomic status were collected. Significant radiomic features (RFs) were identified using the t-test or Mann–Whitney test (p<0.05), and the most robust RFs were selected using the LASSO regression method. Both RMs were internally validated.
**Results or Findings:** Model I, based on 2 RFs and 1 clinical features (LOCATION) achieved an AUC of 0.76 (95% CI: 0.65–0.87) in the training cohort and 0.74 (95% CI: 0.56–0.92) in the validation cohort. Model II, based on 3 RFs, achieved an AUC of 0.85 (95% CI: 0.73–0.96) in the training cohort and 0.72 (95% CI: 0.50–0.94) in the validation cohort.
**Conclusion:** Both RMs performed well in distinguishing MSI from non-MSI tumors, potentially reducing the need for invasive histology and improving treatment timing. Despite a higher AUC, Model II showed overfitting compared to Model I, which included two RFs and one clinical feature (LOCATION). Developing models on larger, more diverse datasets is preferable to improve generalizability and limit overfitting.
**Limitations:** This study is limited by its retrospective design, small sample size, lack of external validation, and absence of follow-up. Future studies should use larger, prospective cohorts. Standardized imaging and multicentre trials are key for clinical implementation of radiomics.
**Funding for this study:** No funding was received for this study
**Has your study been approved by an ethics committee?** Yes
**Ethics committee - additional information:** The study was conducted in accordance with the Declaration of Helsinki, and approved by the Ethics Committee of Careggi University Hospital (protocol code 13261_OSS).

**Author Disclosures:**
Vittorio Miele: Nothing to disclose
Ginevra Danti: Nothing to disclose
Ludovica Scalzone: Nothing to disclose
Antonio Galluzzo: Nothing to disclose
Camilla Mugnaini: Nothing to disclose

**Opportunistic CT-derived Assessment of Spleen volume Predicts Outcome in Patients undergoing MitraClip**
*B. Salam*, M. Weber, A. M. Sprinkart, S. Nowak, M. Theis, A. Isaak, J. A. Luetkens, J. Vogelhuber, D. Kütting; Bonn/DE

**Purpose:** The spleen is an essential component of the immune system and closely associated with cardiac function (cardio-splenic axis). In this context, spleen volume (SV) emerges as a potential new prognostic indicator for cardiovascular risk. This study aimed to investigate the prognostic value of SV measured from routine preinterventional CT in patients undergoing MitraClip for treatment of recurrent mitral regurgitation.
**Methods or Background:** Consecutive patients undergoing MitraClip between February 2011 and November 2022 at the Heart Center Bonn were retrospectively evaluated. SV was determined from pre-interventional CT scans using a dedicated deep learning segmentation model for body composition analysis (TotalSegmentor).
**Results or Findings:** A total of 214 patients (mean age: 77.6±8.2 years, mean EuroSCORE II: 5.0±3.6%) were investigated. 1-year survivors had a significantly lower SV compared to non-survivors (0.19±0.12 cm3 vs. 0.35±0.35 cm3, P<0.001). According to their SV, patients were dichotomized by the median value and defined to have low (<0.182 cm3) and high SV (≥0.182 cm3), respectively. Following MitraClip, high SV was related to acute kidney injury (53.3% vs. 71.0%, P=0.020) as well as increased 30-day (2.8% vs. 12.1%, P=0.009), 1-year (10.3% vs. 27.1%, P=0.001), 2-year (15.0% vs. 33.6%; P=0.001), and 3-year mortality (15.9% vs. 34.6%; P=0.002). On multivariate Cox regression analysis, SV (Hazard Ratio 8.92 [95% Confidence Interval: 2.67-29.90]; P<0.001), as well as NT-proBNP (HR 1.00 [95% CI: 1.00-1.00]; P=0.015) were identified as independent predictors of 1-year mortality.
**Conclusion:** Our results indicate CT-derived SV as a promising new imaging biomarker, which provides additional information for risk stratification in MitraClip patients. Future studies should explore the clinical value of SV compared with other established risk stratification tools and the prognostic role of SV for other cardiovascular and oncologic diseases.
**Limitations:** Single-center retrospective design limits generalizability.
**Funding for this study:** N/A
**Has your study been approved by an ethics committee?** Not applicable
**Author Disclosures:**
Julian Alexander Luetkens: Nothing to disclose
Alexander Isaak: Nothing to disclose
Johanna Vogelhuber: Nothing to disclose
Alois M. Sprinkart: Nothing to disclose
Marcel Weber: Nothing to disclose
Sebastian Nowak: Nothing to disclose
Maike Theis: Nothing to disclose
Daniel Kütting: Nothing to disclose
Babak Salam: Nothing to disclose

**Tumor mutation burden drives survival outcomes in pancreatic ductal adenocarcinoma and enables noninvasive prediction via spectral CT**
*S. Shi*, J. Liu, Y. Luo, S-T. Feng, W. Deng; Guangzhou/CN

**Purpose:** To investigate the prognostic value of tumor mutation burden (TMB) in pancreatic ductal adenocarcinoma (PDAC) and the predictive utility of dual-layer spectral CT (DLCT) for TMB assessment.
**Methods or Background:** This retrospective study included patients with histologically confirmed PDAC who underwent DLCT from June 2019 to December 2023. The association between TMB and progression-free survival (PFS) was analyzed using survival analysis and the optimal cutoff was calculated to group patients. The Kaplan-Meier survival curves were used to compare the PFS between both groups. Independent TMB predictors were identified through univariate and LASSO regression. Predictive performance was assessed via ROC and precision-recall (PR) curve analyses.
**Results or Findings:** Among 75 enrolled patients (mean age 60.4 ± 11.2 years, 41 male), 52 received combination immunotherapy. Median TMB was 2.13 mutations per megabase (mut/Mb; interquartile range: 1.00–4.26). An integer TMB cutoff of 5 mut/Mb (optimized from 5.03 for clinical practicality) stratified patients into distinct prognostic groups, with low-TMB cases demonstrating inferior PFS (median PFS: 5 vs. 7 months, p=0.02); this trend persisted in the immunotherapy subgroup (4 vs. 7 months, p=0.02). Normalized iodine concentration in the pancreatic phase ($nIC_a$) emerged as the sole independent TMB predictor (area under the curve [AUC] of ROC=0.901, cutoff=0.089; accuracy=0.893, sensitivity=0.818, specificity=0.906), surpassing normalized conventional CT attenuation values in the pancreatic phase ($nCT_a$,

AUC=0.834), pancreatic tumor infiltration (AUC=0.679), and a combined model (nCTa+PTI, AUC=0.864). PR curves confirmed $nIC_a$'s superior efficacy. Patients stratified by $nIC_a$-predicted TMB status exhibited significant PFS differences (7 vs. 5 months, p=0.04).
**Conclusion:** Low TMB is a negative prognostic biomarker in PDAC, associated with shorter PFS. DLCT-derived $nIC_a$ enables accurate, noninvasive TMB prediction, supporting its potential role in therapeutic stratification.
**Limitations:** The single-center retrospective design with a limited sample size ， a single vendor platform.
**Funding for this study:** This study has received funding by National Natural Science Foundation of China (82472096) and Natural Science Foundation of Guangdong Province (2024A1515011968) to Yanji Luo.
**Has your study been approved by an ethics committee?** Yes
**Ethics committee - additional information:** The study design was approved by the ethics review board of our hospital and informed consent was waived owing to the retrospective nature of the study.
**Author Disclosures:**
Shi-Ting Feng: Nothing to disclose
Jiawei Liu: Nothing to disclose
Yanji Luo: Nothing to disclose
Siya Shi: Nothing to disclose
Weiwei Deng: Nothing to disclose

### AI-driven longitudinal body composition analysis from routine CT imaging for predicting survival in pancreatic ductal adenocarcinoma

*F. Herr*, M. Vornhülz, C. A. Dascalescu, A. E. Sint, J. Ricke, M. Ingrisch, L. K. Shiyam Sundar, J. Mayerle, S. Grosu; Munich/DE
*(felix.herr@med.uni-muenchen.de)*

**Purpose:** To evaluate the prognostic relevance of longitudinal changes in body composition derived from routine CT scans in patients with pancreatic ductal adenocarcinoma (PDAC) using fully automated image analysis.
**Methods or Background:** In this retrospective single-center study, 55 patients with histologically confirmed PDAC from the prospective METAPAC trial were included. All had at least one venous-phase abdominal CT within 90 days from baseline. Fully automated segmentation was used to quantify volumetric indices for visceral fat (VF), subcutaneous fat (SF), muscle (M), and compute ratios (VF/SF, SF/M). Relative changes from baseline were calculated. Optimal cutoffs were determined using maximally selected rank statistics. Associations with overall survival (OS) were analyzed via Cox regression and evaluated by Harrell's C-index and AIC.
**Results or Findings:** A total of 88 CT scans from 55 patients (mean age 67 ± 11 years; 28 male) were analyzed. An increase in subcutaneous fat index (SFI) (HR: 5.17; C-index: 0.60; p = .028) and a decrease in VF/SF ratio (HR: 2.70; C-index: 0.67; p = .014) were significantly associated with reduced OS. Both metrics remained prognostic in multivariable analysis (SFI: HR: 4.67; p = .0398; VF/SF: HR: 2.75; p = .0199; C-index: 0.73). Absolute baseline or follow-up values were not predictive. Kaplan–Meier analysis confirmed shorter OS in patients with SFI > 0.77 (median OS 319 days vs. not reached; p = .015) and VF/SF ≤ 0.97 (median OS 131 vs. 809 days; p = .011).
**Conclusion:** In PDAC patients, dynamic fat distribution changes, particularly rising SFI and falling VF/SF ratios, were associated with poorer survival. Automated body composition analysis from routine CT may serve as a non-invasive imaging biomarker for early risk stratification.
**Limitations:** Key limitations of our study include the small sample size and retrospective, single-center design.
**Funding for this study:** No funding was received for this study.
**Has your study been approved by an ethics committee?** Yes
**Ethics committee - additional information:** This single-center retrospective study was approved by the local ethics committee (approval number 19-027).
**Author Disclosures:**
Alena Elisabeth Sint: Nothing to disclose
Julia Mayerle: Nothing to disclose
Sergio Grosu: Nothing to disclose
Marlies Vornhülz: Nothing to disclose
Michael Ingrisch: Nothing to disclose
Felix Herr: Nothing to disclose
Lalith Kumar Shiyam Sundar: Nothing to disclose
Christian Alexander Dascalescu: Nothing to disclose
Jens Ricke: Nothing to disclose

### Automated Abdominal CT Biomarkers Predict 5 and 10 year Mortality in a Large, Multi-Hospital Cohort

*A. Suri*[1], M. A. B. C. Rockenbach[2], B. C. Bizzo[2], J. Garrett[3], D. C. Elton[2], P. J. Pickhardt[3]; [1]Los Angeles, CA/US, [2]Boston, MA/US, [3]Madison, WI/US

**Purpose:** To investigate whether abdominal CT biomarkers explored in prior literature remain predictive of 5 and 10 year all-cause mortality in a novel, large cohort.
**Methods or Background:** 180,935 CT patients (with and without contrast, imaging the abdomen, one scan per patient, mean avg 56.6±17.4, 55.7% female) from our multi-hospital institution were selected for usage in this study. Automatically extractable imaging biomarkers (derived from muscle attenuation, bone mineral density, visceral & subcutaneous adipose tissue volume, and abdominal aorta Agatston score at the L3 vertebral body) were calculated on all scans. Date of death of each patient was also gathered. 80% of scans (144,748) were used to train/validate 14 different algorithms to predict 5 or 10 year all-cause mortality. The best algorithm on the training set (based on AUC over 5-fold cross-validation) was evaluated on the hold out test set.
**Results or Findings:** The best performing algorithm for 5-year mortality prediction was XGBoost which achieved an AUC of 0.84 on the hold out test set. The algorithm did not vary significantly in AUC for age ≤65=0.82 vs >65=0.85. The best performing algorithm for 10-year mortality prediction was Light Gradient Boosting Machines (AUC=0.79) with minimal variation according to age (AUC≤65=0.77 vs AUC>65=0.81). For both 5 and 10 year mortality prediction, the most important values relied on visceral adipose tissue attenuation followed by visceral:subcutaneous adipose tissue volume ratio. The least important feature was abdominal aorta Agatston score.
**Conclusion:** Automated abdominal CT biomarkers are strong predictors of all-cause mortality even in large, multi-hospital settings. These imaging biomarkers remain predictive regardless of clinical demographics such as age.
**Limitations:** Follow up data on patients is limited by their interaction with our institution. As a result, information on the death of patients may be incomplete.
**Funding for this study:** No funding sources to declare.
**Has your study been approved by an ethics committee?** Not applicable
**Author Disclosures:**
Perry J. Pickhardt: Nothing to disclose
Bernardo Canedo Bizzo: Nothing to disclose
Daniel C. Elton: Nothing to disclose
Marcio Aloisio Bezerra Cavalcanti Rockenbach: Nothing to disclose
John Garrett: Nothing to disclose
Abhinav Suri: Nothing to disclose

### Signal Intensity–Derived Apparent Extracellular Volume: Can It Predict Liver Fibrosis?

A. Buz, *K. N. Kılıçarslan*; Bolu/TR
*(kubranurkilicarslan@gmail.com)*

**Purpose:** This study aims to evaluate the feasibility of signal intensity–based apparent extracellular volume derived from dynamic liver MRI as a noninvasive and accessible quantitative method to improve the diagnostic reliability of liver fibrosis assessment.
**Methods or Background:** A total of 61 adult patients with chronic liver parenchymal disease who underwent contrast-enhanced dynamic liver MRI between July 2021 and August 2025 were included in the study. In the dynamic MRI, signal intensity measurements of the aorta and liver parenchyma from both lobes were obtained in the pre-contrast and late hepatic phases LAVA series. Patients were divided into three groups based on their FIB-4 index to explore the correlation with liver ECV. All data analyses were performed using R version 4.4.1.
**Results or Findings:** A total of 61 participants were included in the study, comprising 34 males and 27 females. The median age was 65 years (IQR: 12; range: 26–82 years). The median ECV was 0.291 (IQR: 0.104) in the low-risk group (n = 6), 0.316 (IQR: 0.129) in the intermediate-risk group (n = 19), and 0.283 (IQR: 0.101) in the high-risk group (n = 36). Overall, no clear trend of increasing or decreasing ECV with higher FIB-4 value was observed.
**Conclusion:** Signal intensity–derived liver ECV is a feasible, noninvasive measure, but it did not consistently correlate with FIB-4–based fibrosis risk in this cohort. Incorporating T1 mapping may improve its accuracy, and further studies are needed to determine its predictive value for liver fibrosis.
**Limitations:** Single center-retrospective study design.
**Funding for this study:** N/A
**Has your study been approved by an ethics committee?** Yes
**Ethics committee - additional information:** The study was approved by Bolu Abant İzzet Baysal University Non-Interventional Clinical Research Ethics Committee.
**Author Disclosures:**
Kübra Nur Kılıçarslan: Nothing to disclose
Ayşenur Buz: Nothing to disclose

**16:30 - 18:00** **Research Stage 2**

### Research Presentation Session: Breast

### RPS 1102

### Revolutionising breast imaging with artificial intelligence

**Moderator**
Fredrik Strand; Stockholm/SE
*(fredrik.strand@ki.se)*
**Author Disclosures:**
Fredrik Strand: Consultant: Voio Inc, Founder: ClearScanAI AB, Grant Recipient: Various Swedish government and non-profit grants, Owner: Minority share in Voio Inc and ClearScanAI AB

**The effect of AI on retrospectively visible interval cancers in mammography screening – results from the randomised controlled MASAI trial**
*V. Hernström*, H. Sartor, O. Hagberg, K. Lang; Lund/SE
*(vhernstrom@gmail.com)*

**Purpose:** To investigate whether the distribution of interval cancer classification groups differ with or without the use of artificial intelligence (AI) in mammography screening.
**Methods or Background:** The MASAI trial is a randomised, controlled, non-inferiority, screening study comparing AI-supported mammography screening with standard double reading. In the intervention group, AI was used for triage to single or double reading and for detection support. Participants with interval cancers were retrospectively reviewed by a panel of breast radiologists and classified as true negative, showing minimal signs or missed at screening. The distribution of classification groups was compared between the intervention and control groups, and in relation to AI risk scores. Differences in distribution were assessed with a Chi-2 test.
**Results or Findings:** There were fewer interval cancers showing minimal signs (14 [17%] vs 26 [28%]) and a similar number of missed (9 [11%] vs 10 [11%]) and true negatives (59 [72%] vs 57 [61%]), in the intervention group compared to the control group. The distribution was however not statistically significantly different (p=0.22). Of the retrospectively visible interval cancers (missed or minimal signs) in the intervention group, 78% (18/23) had intermediate or high AI risk scores and 65% (15/23) were correctly localised by AI.
**Conclusion:** The use of AI in mammography screening yielded fewer interval cancers with minimal signs at screening compared with standard double reading, indicating its ability to aid in detecting subtle malignancies. A further reduction of interval cancer may be achievable since a substantial proportion of the retrospectively visible interval cancers were assigned elevated risk scores and were correctly localised by AI.
**Limitations:** Single-institution trial, single AI-system, informed review of classification groups
**Funding for this study:** Swedish Cancer Society, Confederation of Regional Cancer Centres, and governmental funding for clinical research.
**Has your study been approved by an ethics committee?** Yes
**Ethics committee - additional information:** Swedish ethical review authority, number NCT04838756.
**Author Disclosures:**
Veronica Hernström: Nothing to disclose
Kristina Lang: Nothing to disclose
Hanna Sartor: Nothing to disclose
Oskar Hagberg: Nothing to disclose

**Influence of AI-informed Disease Prevalence on Radiologist Performance: Insights from the Mammography Screening with Artificial Intelligence trial (MASAI)**
*J. Gommers*[1], C. Abbey[2], O. Hagberg[3], K. Lang[3]; [1]Nijmegen/NL, [2]Santa Barbara, CA/US, [3]Malmö/SE
*(jessie.gommers@radboudumc.nl)*

**Purpose:** To investigate how knowledge of AI-informed disease prevalence influenced radiologists' screening performance by comparing outcomes across AI risk scores when reading with and without AI.
**Methods or Background:** This study used data from the prospective MASAI trial, designed to compare AI-supported mammography screening, where AI triaged examinations to single (AI scores 1-9) or double (AI score 10) reading and was used as decision support, with standard double reading without AI. Recall rates (RR), cancer detection rates (CDR), and false positive rates (FPR) were compared between the AI-supported group (intervention) and double reading group (control) using Fisher's exact tests with results stratified by AI risk scores. Single-reader assessments in the control group were approximated for examinations with AI scores 1-9.
**Results or Findings:** In total, 105,087 women were included in this study, of which 52,576 in the control group and 52,511 in the intervention group. For examinations classified as low risk (AI score 1–7), AI-supported reading led to a reduction in RR (0.50% vs 0.61%, P=.043) and FPR (0.49% vs 0.59%, P=.049 ), without affecting CDR (0.17‰ vs 0.22‰, P=.804), compared to standard reading. For intermediate-risk examinations (AI scores 8–9), AI-supported reading resulted in increased RR (2.29% vs 1.58%, P<.001) and FPR (1.90% vs 1.30%, P=.003), with no change in CDR (3.94‰ vs 2.75‰, P=.224). For high-suspicion examinations (AI score 10), AI-supported reading increased RR (14.41% vs 9.44%, P<.001), FPR (6.69% vs 3.40%, P<.001), and CDR (77.23‰ vs 60.34‰, P=.004).
**Conclusion:** Knowledge about AI-informed prevalence affects radiologists differently across AI risk categories, enhancing cancer detection in high-risk examinations and reducing false positives in low-risk examinations.
**Limitations:** Interval cancer data not yet included.
**Funding for this study:** Swedish Cancer Society, Confederation of Regional Cancer Centres, and Swedish governmental funding for clinical research.
**Has your study been approved by an ethics committee?** Yes
**Ethics committee - additional information:** The Swedish Ethical Review Authority approved the study (2020-04936, 2023-026848-02) and waived the need for obtaining written informed consent.
**Author Disclosures:**
Kristina Lang: Founder: N23 Health
Jessie Gommers: Nothing to disclose
Craig Abbey: Consultant: Canon Medical Advisory Board: Izotropic Corp.
Oskar Hagberg: Nothing to disclose

**Artificial Intelligence in Mammography Screening in Norway (AIMS Norway): a randomized controlled trial**
Å. S. Holen, M. Larsen, *S. Hofvind*; Oslo/NO
*(solveig.hofvind@kreftregisteret.no)*

**Purpose:** The randomized controlled trial Artificial Intelligence in Mammography Screening in Norway (AIMS Norway) evaluates the performance of artificial intelligence (AI) combined with one or two radiologists, compared to standard independent double reading in BreastScreen Norway. The objective is to demonstrate that AI-assisted screening is non-inferior to the current standard in detecting breast cancer.
**Methods or Background:** This randomized, controlled, non-inferiority, parallel-group, single-blind trial is recruiting participants through written consent at the time of mammography screening. Participants are randomized into either a study or control group. In the study group, examinations are analyzed using Transpara® v2.1 (ScreenPoint Medical) and triaged by AI score, indicating risk of malignancy. Exams with low scores (1–7) are single-read, while those with intermediate to high scores (8–10) undergo independent double reading (standard of care). Radiologists are blinded to both the AI results and whether the exam is single- or double-read during primary reading; AI scores and AI-generated annotations are only available during consensus meetings. The control group follows standard double reading.
**Results or Findings:** Recruitment for the trial began in November 2024 in Western Norway and in September 2025 in the Central region. Recruitment in Northern Norway is planned to start by the end of 2025. The trial aims to enroll 140,000 women. As of October 2025, 79% of women attending the screening program in participating regions have consented participation in the study.
**Conclusion:** The AIMS Norway randomized controlled trial has begun recruiting participants in two health regions, with a 79% acceptance rate. Recruitment will continue until the target number of participants is reached to demonstrate non-inferiority in screen-detected cancer rates.
**Limitations:** None
**Funding for this study:** Norwegian Cancer Society (Pink Ribbon, #214931) and the Western (#F-12858-D11417), Central (#2024-36740) and Northern (#HNF1723-24) Norway Regional Health Authorities.
**Has your study been approved by an ethics committee?** Yes
**Ethics committee - additional information:** Regional Ethical Committee in South East Norway.
**Author Disclosures:**
Åsne Sørlien Holen: Nothing to disclose
Solveig Hofvind: Nothing to disclose
Marthe Larsen: Nothing to disclose

**AI-only for assessing breast cancer screening mammograms – the evolution with AI as an independent reader**

*K. E. Dembrower*[1], F. Pilblad[1], M. Arango Lievano[2]; [1]Stockholm/SE, [2]Montpellier/FR
*(karin.dembrower@capiostgoran.se)*

**Purpose:** Since June 2023 an AI-algorithm replaced one human reader in a population based screening program for breast cancer, redefining the standard of care for this center as AI and one radiologist instead of two radiolgists. This study aims at assessing the safety of an AI only pathway for highly confidence normal examinations.
**Methods or Background:** If either AI or the human reader flagged an examination, it was referred to the consensus discussion where two breast radiologists finally decide to declare the woman as healthy or recall her for further work-up. The AI-algorithm generated an abnormality score (AS) from zero to one hundred where zero is no abnormality detected and one hundred is most likely cancer. Images were generated from equipment by one manufacturer. We reported which cancer cases were flagged by a human reader, by AI and if the examination was flagged because of clinical symptoms (and/or/not AI-positive). The ground truth was pathologically proven breast cancer. We analyzed the AS of all examinations to determine a safety threshold where no SDC was missed by AI.
**Results or Findings:** All screening mammograms between 15/01/2025 and 31/08/2025 were assessed independently by AI and one human reader. In total 25 172 women were screened. And total of 158 cancer cases were diagnosed with a score between 3.95 and 99.2. The lowest AI-score for a cancer case was set 3,95. If we set the safety threshold at AS=2, the proportion triaged images is 17,2% (4 330 examinations).
**Conclusion:** These results demonstrate that assessment of low score screening examinations (AI score below two) with AI only is safe for almost 20% of the screening examinations, further decreasing radiolgists' workload.
**Limitations:** Not applicable
**Funding for this study:** None
**Has your study been approved by an ethics committee?** Not applicable
**Author Disclosures:**
Margarita Arango Lievano: Nothing to disclose
Karin Elisabeth Dembrower: Nothing to disclose
Frida Pilblad: Nothing to disclose

**Artificial intelligence reveals early mammographic signs of breast cancers diagnosed at subsequent screening**

*E. Di Gaetano*, C. M. Weiss, R. Cerniato, E. Cattarin, G. Soppelsa, I. Vinci, G. Morana; Treviso/IT
*(eleonora.digaetano@aulss2.veneto.it)*

**Purpose:** Artificial intelligence (AI) generates continuous risk scores (RS) and identifies regions of interest (ROI) on screening mammograms (SM). We evaluated whether baseline RS and ROI in an AI-supported screening (AISS) could predict breast cancers (BC) diagnosed at the subsequent round, and whether results were consistent across BI-RADS breast density (BD) and lesion types.
**Methods or Background:** We retrospectively analysed 321 women diagnosed with BC at the subsequent AISS round (November 2023–July 2025), performed on average after 777 (median 768;range 691–1,192) days following a negative SM. Side-specific RS at baseline and subsequent round were compared using the Wilcoxon signed-rank test. ROI were deemed concordant if they overlapped the tumour in at least one projection. McNemar's test assessed within-subject ROI changes, and conditional odds ratios (OR) with 95% confidence intervals (CI) were calculated. Analyses were stratified by BD and lesion type.
**Results or Findings:** Baseline RS was significantly higher in breasts that developed BC than in cancer-free sides (16.9vs10.1;p<0.001), and increased further at the subsequent round (47.6vs10.2;p<0.001). Concordant ROI were present at baseline in 111/321 BCs (34.6%). Mean baseline RS was similar for opacities (15.9), microcalcifications (15.8), and distortions (19.6); combined lesions had higher values (20.4). Baseline ROI concordance was found in 31.3% (50/160) of opacities, 28.8% (21/73) of microcalcifications, 40.0% (18/45) of distortions, and 51.2% (22/43) of combined lesions. When grouped by BD, concordant ROI were more frequent in BD C–D (46/122;37.7%) than in A–B (65/199;32.7%).
**Conclusion:** AI assigned higher RS to the breast that later developed cancer and localized one third of BCs at baseline with concordant ROI, especially in combined lesions. These findings highlight the potential of AI to anticipate BC detection in population-based screening.
**Limitations:** No limitations
**Funding for this study:** No Funding for this study
**Has your study been approved by an ethics committee?** Not applicable
**Author Disclosures:**
Giorgia Soppelsa: Nothing to disclose
Eleonora Di Gaetano: Nothing to disclose
Elisa Cattarin: Nothing to disclose
Giovanni Morana: Nothing to disclose
Ivana Vinci: Nothing to disclose
Claudia Maria Weiss: Nothing to disclose
Roberta Cerniato: Nothing to disclose

**AI risk score for a new versus old version of a CE-marked AI model for breast cancer detection**

M. Larsen, Å. S. Holen, N. Moshina, M. B. Bergan, *S. Hofvind*; Oslo/NO
*(solveig.hofvind@kreftregisteret.no)*

**Purpose:** To explore screening examinations with and without breast cancer with the same screening mammograms, but with higher AI risk score in the new versus old version of an AI model.
**Methods or Background:** This retrospective cohort study used data from 117,709 screening examinations performed in BreastScreen Norway 2009-2018. The same screening mammograms were processed by two versions of the commercially available AI model, Transpara (version 1.7 and 2.1, ScreenPoint Medical). We used the categorical AI risk score assessment, AI score 1-10. Scores between 1-7 were defined as low risk, 8-9 as intermediate risk, and 10 as high risk of malignancy. Changes in risk scores below refers to the low, intermediate and high-risk groups.
**Results or Findings:** A total of 5.3% (6272/117,709) of all screening examinations had higher AI score in version 2.1 as in 1.7. Among the screen-detected cancer cases, 7.9% (58/737) had higher AI score in the new versus old version, while 85.6% (631/737) had stable high AI risk score. We found 1.5% (11/737) to have a lower (10 -> 1-9) and 5.0% (37/737) to have stable low or stable intermediate score (1-9 -> 1-9). Among the interval cancers, 11.5% (23/200) had higher score in the new versus old version.
**Conclusion:** An updated version of the AI model resulted in a higher detection of cancers in the high AI risk score group. However, the total number of examinations in the high-risk group did also increase, which resulted in a higher proportion of high AI score, but no cancer detected.
**Limitations:** Exploring location of AI markings and comparison with true cancer location were not included in this study.
**Funding for this study:** The Norwegian Cancer Society and the Pink Ribbon Campaign have supported performance of the study.
**Has your study been approved by an ethics committee?** Yes
**Ethics committee - additional information:** Approved by the Regional Ethical Committee in South Eastern Norway
**Author Disclosures:**
Nataliia Moshina: Nothing to disclose
Marie Burns Bergan: Nothing to disclose
Åsne Sørlien Holen: Nothing to disclose
Solveig Hofvind: Nothing to disclose
Marthe Larsen: Nothing to disclose

**Empirically determined effect of dataset size and enrichment on threshold selection of a commercial mammography AI algorithm**

*N. Payne*[1], J. Rothwell[1], R. Black[1], S. Hickman[2], F. Kilburn-Toppin[1], B. Kasmai[3], A. Juette[3], F. J. Gilbert[1]; [1]Cambridge/UK, [2]London/UK, [3]Norwich/UK
*(np520@cam.ac.uk)*

**Purpose:** To empirically show the varying precision and accuracy of selecting AI operating thresholds based on dataset size and composition in prospective use.
**Methods or Background:** 22,608 full-field digital consecutive mammograms from a single site and vendor (including 952 screen detected cancers (SDC) and 471 interval cancers (IC)) were scored by a commercial AI tool (Lunit INSIGHT-MMGv1.1.7) to establish 'true' performance. Nine data subsets were defined by degree of cancer enrichment (representative [8/1000 SDC, 4/1000 IC], mildly enriched [20/1000 SDC, 10/1000 IC], and heavily enriched [100/1000 SDC, 50/1000 IC]) and size (small [n=250], medium [n=1000], large [n=5000]). Each subset was used to measure area under the receiver operating characteristic curve (AUC) and to set thresholds at 90% specificity, 70% sensitivity, and 5% recall which were then applied to the full data to find their 'actual' performance. Subsampling was repeated 1000 times.
**Results or Findings:** 'True' AUC was 0.899. Using larger and enriched subsets increased the likelihood of an 'accurate' measure of AUC. Enrichment was beneficial for threshold setting based on sensitivity, however, even using large heavily enriched subsets, the 'actual' sensitivity ranged by >5% points. There is a detrimental impact of enrichment when threshold setting based on recall rate, as oversampling of cancer cases leads to a reduced recall rate when applied to the population. Mean recall rate of thresholds set using the representative, mildly-, and heavily enriched subsets were ~5%, ~4% and ~0.5% respectively.

**Conclusion:** When selecting a dataset to evaluate AI tools and set operating thresholds, larger datasets are beneficial provided they are relevant to the metric used. Enrichment allows smaller datasets to better assess AUC and sensitivity, however, it is vital to use representative datasets when assessing recall rates.
**Limitations:** Single site and single vendor.
**Funding for this study:** Funding was provided by the Future Dreams Breast Cancer charity, the National Institute for Health and Care Research (NIHR) Cambridge Biomedical Research Centre (NIHR203312) and the Cancer Research UK early detection program grant (C543/A26884). The views expressed are those of the authors and not necessarily those of the NIHR or the Department of Health and Social Care.
**Has your study been approved by an ethics committee?** Yes
**Ethics committee - additional information:** This study used the Cambridge Cohort Mammography East Anglia Digital Imaging Archive (‘CC-MEDIA’) dataset, which has ethical approval in which informed consent is waived (Health Research Authority Research Ethics Committee 25/LO/0220, Health Research Authority Confidentiality Advisory Group 20/CAG/0009, Public Health England Research Advisory Committee BSPRAC_090).
**Author Disclosures:**
Fiona J. Gilbert: Research/Grant Support: ScreenPoint Research/Grant Support: Bayer Healthcare Research/Grant Support: Lunit Research/Grant Support: Therapixel Research/Grant Support: iCAD Research/Grant Support: Vara Research/Grant Support: GE Healthcare
Arne Juette: Nothing to disclose
Fleur Kilburn-Toppin: Nothing to disclose
Sarah Hickman: Nothing to disclose
Nicholas Payne: Research/Grant Support: Therapixel Research/Grant Support: Lunit Research/Grant Support: iCAD Research/Grant Support: GE Healthcare Research/Grant Support: Bayer Healthcare Research/Grant Support: Vara Research/Grant Support: ScreenPoint
Joshua Rothwell: Research/Grant Support: ScreenPoint Research/Grant Support: iCAD Research/Grant Support: Lunit Research/Grant Support: Therapixel Research/Grant Support: Vara
Richard Black: Nothing to disclose
Bahman Kasmai: Nothing to disclose

### A Fully Automated Deep Learning Framework for Lesion Segmentation and Survival Prediction in Breast Cancer Patients

K. Wang[1], S. Wang[1], S. Huang[2], J. Xie[3], *S. Dong*[2], M. Xu[1], R. Zhang[1];
[1]Hangzhou/CN, [2]Beijing/CN, [3]Shanghai/CN
*(mail_dong33@163.com)*

**Purpose:** To develop and validate a fully automated deep learning framework (FA-SurvNet) that integrates lesion segmentation and survival prediction for breast cancer patients using preoperative MRI.
**Methods or Background:** In this retrospective study, 573 female breast cancer patients from two medical centers who underwent preoperative MRI were enrolled. We developed the FA-SurvNet model, which integrates an nnU-Net for automated tumor segmentation with a CNN-Cox regression network for survival analysis. The segmentation performance was evaluated using the Dice Similarity Coefficient (DSC), Positive Predictive Value (PPV), and Sensitivity (SEN). The prognostic performance of FA-SurvNet was compared against two baseline models: a clinical Traditional Chinese Medicine (TCM) model and a radiomics model, both based on conventional Cox regression. Model evaluation included Harrell's concordance index (C-index), time-dependent area under the curve (AUC), and decision curve analysis (DCA) for net clinical benefit.
**Results or Findings:** The nnU-Net segmentation model achieved high performance in the training (DSC: 0.85, PPV: 0.85, SEN: 0.88) and testing cohorts (DSC: 0.84, PPV: 0.87, SEN: 0.84). The FA-SurvNet model demonstrated excellent predictive ability for recurrence risk, with C-indexes of 0.88 (training) and 0.84 (testing). It outperformed the radiomics model (training C-index: 0.84; testing C-index: 0.75) and performed comparably to the clinical TCM model (training C-index: 0.89; testing C-index: 0.88). The time-dependent AUCs of FA-SurvNet for predicting 3- and 5-year recurrence-free survival (RFS) were 0.89 and 0.90 in the training cohort, and 0.84 and 0.87 in the testing cohort, respectively.
**Conclusion:** The FA-SurvNet framework successfully automates the entire prognostic pipeline from lesion segmentation to survival risk estimation, offering a powerful and efficient tool for personalizing breast cancer management.
**Limitations:** None
**Funding for this study:** None
**Has your study been approved by an ethics committee?** Yes
**Ethics committee - additional information:** None
**Author Disclosures:**
Shushan Dong: Nothing to disclose
Sicong Huang: Nothing to disclose
Jingyu Xie: Nothing to disclose
Shiwei Wang: Nothing to disclose
Kaiting Wang: Nothing to disclose
Ruixin Zhang: Nothing to disclose
Maosheng Xu: Nothing to disclose

### Artificial intelligence–assisted risk stratification of atypical breast lesions: correlation of pathology, imaging features and Lunit abnormality score

*R. B. Souza*, L. F. Chala, G. G. N. Mello, T. C. d. M. Tucunduva, M. Siepcich, B. A. Rocha, I. Oliveria, M. P. F. Ananias, V. L. N. Aguillar; São Paulo/BR
*(rafael.boava@unifesp.br)*

**Purpose:** Atypical breast lesions (B3), including atypical ductal hyperplasia (ADH), lobular neoplasia (LN), and flat epithelial atypia (FEA), diagnosed by stereotactic vacuum-assisted biopsy (VAB) of suspicious calcifications carry variable risk of underestimation and upgrade to ductal carcinoma in situ (DCIS) or invasive carcinoma. This study evaluated upgrade rates of B3 lesions, predictive factors, and the role of Lunit INSIGHT MMG abnormality score as an AI-based biomarker.
**Methods or Background:** This retrospective study included 271 women (Jan 2020–Dec 2024) with VAB-diagnosed B3; 157 underwent surgery and 114 clinical-radiological follow-up. Analyzed variables included age, personal/family history, calcification morphology and distribution, residual calcifications, and pathological subtype. Lunit scores were retrieved for 100 cases. Analyses included chi-square, Fisher’s exact, Mann-Whitney U, and ROC.
**Results or Findings:** Overall upgrade rate was 16.6% (26/157): 73% DCIS, 27% invasive carcinoma. Pathology was the strongest predictor. Borderline ADH demonstrated the highest upgrade risk (77.8%, $p<0.001$), followed by ADH (21.9%, $p=0.012$), FEA (13%), and LN (10%). No difference was observed between isolated and multiple atypias ($p=0.801$), both lower than borderline ADH. Pleomorphic linear/branching calcifications were significantly associated with malignancy ($p=0.007$). Residual calcifications showed a non-significant trend toward higher risk (22% vs. 11.4%). Clinical variables were not predictive. Lunit scores were higher in upgraded vs non-upgraded cases (mean 36.2 vs 23.2; median 32.2 vs 11.9), though not statistically significant ($p=0.149$). ROC analysis yielded an AUC of 0.615, with optimal cut-off of approximately 29 (sensitivity 62.5%, specificity 67.9%).
**Conclusion:** Borderline ADH is the strongest predictor of upgrade, followed by suspicious calcification morphology. Although not statistically significant, higher Lunit scores trended toward upgraded lesions and moderate discriminatory performance. AI-assisted mammographic analysis may complement pathology and imaging in risk models, supporting individualized management of atypical calcifications.
**Limitations:** No limitations were identified.
**Funding for this study:** No funding was received for this study.
**Has your study been approved by an ethics committee?** Yes
**Ethics committee - additional information:** This study was approved by the Research Ethics Committee of Grupo Fleury.
**Author Disclosures:**
Tatiana Cardoso De Mello Tucunduva: Employee: Fleury Medicina e Saúde
Giselle Guedes Netto Mello: Employee: Fleury Medicina e Saúde
Luciano Fernandes Chala: Employee: Fleury Medicina e Saúde
Rafael Boava Souza: Nothing to disclose
Mariana Pinheiro Ferri Ananias: Employee: Fleury Medicina e Saúde
Vera Lúcia Nunes Aguillar: Employee: Fleury Medicina e Saúde
Bruno Aragao Rocha: Employee: Fleury Medicina e Saúde
Monica Siepcich: Employee: Fleury Medicina e Saúde
Igor Oliveria: Employee: Fleury Medicina e Saúde

### Real versus Virtual Contrast Enhancement for AI-Based Breast MRI Classification

*T-T. Nguyen*, S. Heidarikahkesh, H. Schreiter, L. Brock, L. A. Kapsner, A. Liebert, A. Maier, M. Uder, S. Bickelhaupt; Erlangen/DE
*(tri-thien.nguyen@fau.de)*

**Purpose:** Breast MRI offers high sensitivity and specificity; however, widespread use remains limited. Contrast-free protocols and AI tools might improve accessibility, for example in screening. This study evaluated the diagnostic performance of an AI model trained on early contrast-enhanced subtraction (T1sub) images for breast cancer classification, and tested its application on both real contrast-enhanced T1sub and virtually generated contrast-enhanced (T1virtual) images from non-contrast sequences.

**Methods or Background:** This IRB-approved retrospective study included 1,712 women undergoing routine multiparametric 3T breast MRI, some with multiple exams. Images were split by breast side, yielding 3,685 samples, including 488 test cases. A Medical Slice Transformer was trained on T1sub images to classify BI-RADS 1–3 versus 4–6. Model performance was evaluated on both T1sub and T1virtual test images. Statistics used McNemar's test for accuracy and DeLong's test for AUC; specificity at 90%, 95%, and 97.5% sensitivity was reported. Attention map analysis assessed whether the model focused on clinically relevant regions, using a three-point Likert scale (good, moderate, poor) rated by a board-certified radiologist.
**Results or Findings:** The model achieved higher performance on T1sub compared with T1virtual images (AUC 0.80±0.03 vs 0.74±0.03, p=0.035; accuracy 0.85 vs 0.81, p=0.0056). At sensitivity thresholds of 90%, 95%, and 97.5%, specificity was 0.33/0.21/0.12 on T1sub and 0.21/0.13/0.09 on T1virtual. Attention maps showed similar lesion focus, with most cases rated 'Good' (55–60%) or 'Moderate' (30–35%).
**Conclusion:** An AI model trained on contrast-enhanced T1sub achieved 4% higher accuracy on T1sub than T1virtual, a statistically significant difference. While performance was lower on virtual contrast, the approach remains promising and warrants further validation to expand accessibility of AI-assisted breast cancer detection, particularly in screening scenarios.
**Limitations:** Single-center retrospective study; model trained exclusively on contrast-enhanced data and applied without re-training to virtual images.
**Funding for this study:** This project is partially funded by the Bavarian Ministry of Economic Affairs, Regional Development and Energy
**Has your study been approved by an ethics committee?** Yes
**Ethics committee - additional information:** This study was approved by the Institutional Review Board of the Friedrich-Alexander-Universität Erlangen-Nürnberg (Ethik-Kommission) with waived informed consent (approval number 23-281-Br) due to its retrospective nature and use of pseudonymized data.
**Author Disclosures:**
Sebastian Bickelhaupt: Nothing to disclose
Lorenz A. Kapsner: Nothing to disclose
Tri-Thien Nguyen: Nothing to disclose
Andreas Maier: Nothing to disclose
Michael Uder: Nothing to disclose
Hannes Schreiter: Nothing to disclose
Luise Brock: Nothing to disclose
Shirin Heidarikahkesh: Nothing to disclose
Andrzej Liebert: Nothing to disclose

### Can artificial intelligence detect additional cancers on screening digital breast tomosynthesis?

*V. Dahlblom*, K. Johnson, M. Dustler, A. Tingberg, S. Zackrisson; Malmö/SE
*(victor@dahlblom.nu)*

**Purpose:** Several studies have indicated that AI systems can detect additional cancers on screening digital mammography (DM) examinations compared with unaided radiologists, but it is less studied if this also applies for digital breast tomosynthesis (DBT). We want to investigate if a DBT AI system can detect cancers that were not detected in screening.
**Methods or Background:** The study is based on the Malmö Breast Tomosynthesis Screening Trial, where women were screened with double-read DBT once. Following screenings used DM only. DBT examinations from 14368 women were analysed with ScreenPoint Transpara 2.1, which gives a score between 1 and 10, where 10 means the highest cancer suspicion. The AI scores from the DBT screening examinations of women with screening-detected cancers in the two upcoming screening rounds (1.5 or 2 years interval depending on age) – or diagnosed with interval cancers, were compared with scores from women without breast cancer.
**Results or Findings:** The mean AI score at screening DBT was clearly higher for women with cancer diagnosed in the interval until (mean 6.1) or at the first following screening round (mean 5.9), compared to women without cancer diagnosed during the two subsequent screening rounds (mean 3.3). During the first interval and screening round, 23% (6/22) of the interval cancers and 26% (15/57) of the screening-detected cancers had score 10 at DBT screening, compared to 3.8% (527/13986) among women without cancer. For cancers in the second following screening round, the DBT examination had score 10 in 11% (3/27) of interval cancers and 10% (6/56) of screening-detected cancers.
**Conclusion:** AI analysis of DBT screening images could potentially detect a substantial amount of additional cancers compared to radiologist double reading of DBT.
**Limitations:** Single-centre, single-vendor, single-view DBT at one screening occasion.
**Funding for this study:** Skåne University Hospital
**Has your study been approved by an ethics committee?** Yes
**Ethics committee - additional information:** Covered by the ethical approval for the MBTST (official records number: 2009/770)
**Author Disclosures:**
Victor Dahlblom: Nothing to disclose
Kristin Johnson: Consultant: Spago Nanomedicals Speaker: Pfizer, Siemens Healthineers
Anders Tingberg: Research/Grant Support: Siemens Healthineers
Magnus Dustler: Nothing to disclose
Sophia Zackrisson: Equipment Support Recipient: ScreenPoint Medical Speaker: Siemens Healthineers

### Effect of an Annotation-Free AI System for Simultaneous Detection and Diagnosis on Breast Ultrasound: Multireader, Multicase Study Across Diverse Healthcare Professionals

*H. Park*[1], K. H. Han[2], H-W. Kim[3], W. H. Kim[4], J. Kim[4], J. H. Yoon[1];
[1]Seoul, Republic of Korea/KR, [2]Seoul/KR, [3]iksan/KR, [4]Daegu/KR
*(immint3@yuhs.ac)*

**Purpose:** To evaluate how a deep learning-based artificial intelligence(AI) system that provides autonomous lesion detection and simultaneous differential diagnosis for breast ultrasound(US) affects the diagnostic performance of readers with diverse professional backgrounds.
**Methods or Background:** This study included 1,000 US images (500 cancer, 250 benign, 250 negative). Fifteen readers of various medical profession were recruited: 6 radiologists, 3 breast surgeons, 3 physicians, and 3 radiographers. Image interpretation was conducted in two sessions; session 1: without AI, and after 2-weeks washout, session 2: with AI. Reader performances were evaluated and compared between the two sessions.
**Results or Findings:** Overall reader-averaged area under the localization receiver operating characteristic curve(LROC) significantly increased in session 2 vs. session 1 [0.910 (95% CI: 0.888, 0.931) vs 0.864 (95% CI: 0.831, 0.898)] (P=0.002). LROC of standalone AI was 0.909 (95% CI: 0.889, 0.930). Of subgroups according to profession, radiographers showed significantly improved LROC in session 2 vs. session 1, 0.922 vs 0.868, (P=0.032). Average sensitivity and accuracy were significantly improved for all 15 readers in session 2 vs. session 1; 95.0% vs. 85.3% and 85.7% vs. 82.3%, respectively (all P<0.001). Physicians (82.3% vs. 75.7%) and radiographers (81.7% vs. 76.7%) demonstrated significant decrease of specificity in session 2 vs. session 1 (all P<0.001), respectively. Radiologists without fellowship training or <8 years of experience, showed significant improvement in sensitivity and accuracy but decreased specificity in session 2 (all P<0.05), respectively.
**Conclusion:** Using AI for breast US interpretation significantly enhanced the overall diagnostic performances in readers of diverse healthcare professionals. AI application may result in different consequences across readers of different levels of expertise, that should be considered in clinical application.
**Limitations:** Cancer enriched population was used for reader study.
**Funding for this study:** This study was supported by the Korea Medical Device Development Fund grant funded by the Korea government (the Ministry of Science and ICT, the Ministry of Trade, Industry and Energy, the Ministry of Health & Welfare, the Ministry of Food and Drug Safety) (Project Number: 1711197554, RS-2023-00227526).
**Has your study been approved by an ethics committee?** Yes
**Ethics committee - additional information:** This study has been approved by the institutional review board (IRB) of three institutions (Severance Hospital, IRB No. 1-2023-0073, Wonkwang University Hospital IRB No. 2023-07-017-002, Kyungpook National University Chilgok Hospital IRB No. KNUCH 2023-11-029), with a waiver for informed consent.
**Author Disclosures:**
Jung Hyun Yoon: Nothing to disclose
Won Hwa Kim: CEO: Authors W.H.K. and J.K. are the CEOs of BeamWorks Inc., which provided the equipment for this study.
Jaeil Kim: CEO: Authors W.H.K. and J.K. are the CEOs of BeamWorks Inc., which provided the equipment for this study.
Hasang Park: Nothing to disclose
Kyung Hwa Han: Nothing to disclose
Hye-Won Kim: Nothing to disclose

### Application of an artificial intelligence-assisted diagnostic system for breast ultrasound: a prospective study

*Z. L. Wang*; Beijing/CN
*(jinzhiying0810@163.com)*

**Purpose:** Accurate diagnosis of breast cancer is of great importance to improve the prognosis of patients. AI-assisted diagnostic system for breast ultrasound is gradually being applied in the identification of benign and malignant breast lesions. This study aimed to evaluate the diagnostic performance and optimal application of AI-assisted ultrasonography for breast lesions in clinical setting.

**Methods or Background:** A total of 501 consecutive patients with 679 breast lesions were prospectively included in the study. Junior and senior radiologists were asked to interpret images of lesions with and without AI assistance, respectively. Three application modes of AI were employed: AI alone, adjusted BI-RADS, and second reading mode. The diagnostic performances of these application modes were analyzed and compared.
**Results or Findings:** The AUC of junior radiologists increased from 0.879 to 0.921 in BI-RADSsecond reading, which was higher than that in BI-RADSadjusted (0.901), similar to that in AI alone (0.924), and lower than that obtained by senior radiologists (0.950). Using BI-RADS category 4A as the threshold, the sensitivity of junior radiologists was found to increase from 0.83 to 0.92 (P<0.001). Furthermore, the specificity increased from 0.79 to 0.85, which was higher than those of AI alone and BI-RADSadjusted (P<0.001). The unnecessary biopsy rate decreased by 14.70% (P=0.01). For senior radiologists, the sensitivity increased from 0.91 to 0.96 (P=0.01). Similar results were observed in the subgroup analysis of lesions ≤2 cm. For lesions >2 cm, only the specificity of junior radiologists increased from 0.39 to 0.52 (P=0.03).
**Conclusion:** AI-assisted ultrasound is useful for the diagnosis of breast lesions, particularly for junior radiologists and lesions ≤2 cm. The use of the second reading mode can achieve excellent diagnostic performance.
**Limitations:** The sample size of breast lesions >2 cm was relatively small.
**Funding for this study:** This work was supported by the National Natural Science Foundation of China (No. 82071925), the Military Health Project (No. 22BJZ23), and the Equipment Comprehensive Research Project (No. LB20211A010011).
**Has your study been approved by an ethics committee?** Yes
**Ethics committee - additional information:** This prospective study was approved by the Ethics Committee of the Chinese PLA General Hospital (No. S2021-683-01), and informed consent was obtained from all patients.
**Author Disclosures:**
Xiaomeng Zou: Nothing to disclose
Zhi-Ying Jin: Nothing to disclose
Zhi Li Wang: Nothing to disclose

**16:30 - 18:00** **Research Stage 4**

### Research Presentation Session: Artificial Intelligence and Imaging Informatics

### RPS 1105

### AI-radiomics and clinical data convergence across the breast cancer continuum

**Moderator**
Annie Ng; London/UK
*(annie.ng@deephealth.com)*
**Author Disclosures:**
Annie Ng: Employee: DeepHealth, Share Holder: DeepHealth

### Same studies, different scores: how RQS, RQS2, and METRICS shape radiomics quality assessment

*M. Bobowicz*, M. Kosno, K. P. Brzozowski, E. Szurowska; Gdańsk/PL
*(bobowicz@gmail.com)*

**Purpose:** Radiomics has emerged as a powerful method for extracting quantitative insights from medical imaging, with the potential to enhance diagnostic and prognostic precision. We aimed to critically evaluate RQS, RQS2, and METRICS tools to assess their robustness, reliability, and practical utility, thereby guiding reproducible and high-quality radiomics research.
**Methods or Background:** A comprehensive search of PubMed, Embase, Scopus, Web of Science, and IEEE Xplore databases identified studies employing predicting pathological complete response (pCR) in breast cancer patients receiving neoadjuvant therapy based on MRI radiomics. The study quality was assessed using RQS, RQS2 and METRICS. We compared metrics across seven overarching categories: study design and protocol, imaging protocol quality, image preparation and processing, segmentation and ROI definition, feature extraction and selection, model building and validation, and reporting, transparency, and open science. Inter-reader agreement was evaluated with Cohen's κ, and overall score reliability was determined using ICC.
**Results or Findings:** RQS and RQS2 emphasise clinical aspects, while METRICS provides more holistic perspective. The result of this approach is reflected in the higher median score achieved by METRICS compared to RQS and RQS2. The correlation between total scores was weak to moderate. The RQS vs. RQS2 analysis yielded a result of $\rho = 0.312$ ($p \approx 0.068$). Similarly, the RQS vs. METRICS analysis produced a result of $\rho = 0.180$ ($p \approx 0.302$). Finally, the RQS2 vs. METRICS comparison yielded a result of $\rho = 0.412$ ($p = 0.014$).
**Conclusion:** The METRICS tool is the most equitable choice, as each supercategory addresses multiple facets of the issue. Each research problem has unique characteristics, and the effectiveness of RQS, RQS2, or METRICS may differ. Therefore, when evaluating a model's quality, at least two forms should be used.
**Limitations:** None
**Funding for this study:** This project has received funding from the Digital Europe Programme under grant agreement No. 101100633 (EUCAIM); the European Union's Horizon Europe and Horizon 2020 research and innovation programme under grant agreement No. 101057699 (RadioVal) and No. 952103 (EuCanImage)
**Has your study been approved by an ethics committee?** Not applicable
**Author Disclosures:**
Maciej Bobowicz: Nothing to disclose
Michał Kosno: Nothing to disclose
Edyta Szurowska: Nothing to disclose
Krystian Paweł Brzozowski: Nothing to disclose

### Diagnostic Performance of Virtual vs. True Non-Contrast Dual-Energy CT for Differentiating Hepatic Cysts from Liver Metastases in Breast Cancer

A. Gökduman, I. Yel, P. Reschke, *J. Gotta*, S. Mahmoudi, V. Koch, M. Dimitrova, T. Vogl, C. Booz; Frankfurt/DE

**Purpose:** To assess the diagnostic performance and Hounsfield unit (HU) correlation between virtual non-contrast (VNC) and true non-contrast (TNC) dual-energy CT (DECT) for differentiating hepatic cysts from liver metastases in patients with breast cancer.
**Methods or Background:** This retrospective study included 668 liver lesions (334 cysts, 334 metastases) in patients with histologically confirmed breast cancer who underwent DECT between January 2020 and December 2022. HU values were measured in lesion-specific regions of interest (ROIs) from both TNC and VNC images. Receiver operating characteristic (ROC) analysis was performed to identify optimal cut-off values using the Youden index. Diagnostic performance was assessed using AUC, sensitivity, specificity, positive predictive value (PPV), negative predictive value (NPV), and overall accuracy. Correlation between TNC and VNC HU values was analyzed. The reference standard for lesion classification was either contrast-enhanced MRI or image-guided biopsy.
**Results or Findings:** TNC achieved an area under the curve (AUC) of 0.869, while VNC demonstrated a comparable AUC of 0.866; both results were statistically significant ($p < 0.001$). TNC showed a sensitivity of 87.7%, specificity of 63.5%, and accuracy of 75.6%. VNC yielded a sensitivity of 85.0%, specificity of 61.4%, and accuracy of 73.2%. A strong positive correlation was observed between TNC and VNC HU values ($r = 0.977$; $p < 0.001$). In this cohort, omission of the true non-contrast phase and exclusive use of VNC would have resulted in a documented CTDI reduction of approximately 40–50%.
**Conclusion:** VNC imaging demonstrated diagnostic performance nearly equivalent to that of TNC, with minimal clinically relevant differences. Given the strong HU correlation, replacing TNC with VNC could significantly reduce radiation exposure in oncological liver staging, streamline imaging workflows, and maintain high diagnostic performance.
**Limitations:** Minor attenuation discrepancies could influence lesion classification in borderline cases.
**Funding for this study:** No funding.
**Has your study been approved by an ethics committee?** Yes
**Ethics committee - additional information:** The local ethics committee approved the study protocol.
**Author Disclosures:**
Christian Booz: Nothing to disclose
Ibrahim Yel: Nothing to disclose
Mirela Dimitrova: Nothing to disclose
Thomas Vogl: Nothing to disclose
Vitali Koch: Nothing to disclose
Scherwin Mahmoudi: Nothing to disclose
Philipp Reschke: Nothing to disclose
Aynur Gökduman: Nothing to disclose
Jennifer Gotta: Nothing to disclose

**Implementation of AI in a National Breast Cancer Screening System**
*O. Tervonen*; Oulu/FI
*(osmo.tervonen@oulu.fi)*

**Purpose:** The purpose of this study was to study which mandatory steps should be achieved to implement AI as a part of a national breast cancer screening program.
**Methods or Background:** The study consisted of following steps: 1. Finding of an AI software ready be implemented; 2. Health care service provider commitment; 3. Compliance with Hospital IT system 4. Compliance with hospital data security quidelines; 5. Rediness of the ministry for legislation change of the national screening process; 6. National Radiology Society participation; 7. Defining the new screening process with the health care professionals; 8. Compliance with the national and EU GDPR regulation
**Results or Findings:** Seven out of eight required steps were achieved. We defined an impementation process, where AI process is running parallely with the present well defined two radiologist screening process, and after experience, define the new national screening process. Multiple AI software were considered applicaple and the one with an independent reader process model was selected. The health care provider was committed to co-work based on proven short and long-term benefits. The national legislator indicated readiness in starting legislation change based on the results. However, as a part of GDPR regulation, the National Office of Data Protection Ombudsman, which is the highest national authority, made a statement that all the screening data has to be removed from the archives of the AI vendor after the process, which was impossible to comply by the vendor.
**Conclusion:** The current GDPR regulation and how it is implemented nationally does not allow to proceed in taking AI as a part of a national screening program
**Limitations:** The data used for the evaluetion was based on review of the literature and assement by the board members of the study,.
**Funding for this study:** This study has not received external funding.
**Has your study been approved by an ethics committee?** Yes
**Ethics committee - additional information:** The ethicch committee of the hospital has apptoved the study.
**Author Disclosures:**
Osmo Tervonen: Nothing to disclose

**Automated Classification of Malignant versus Benign Breast Lesions on DCE-MRI: A Comparative Study of 3D Deep Learning Architectures and Input Preprocessing Strategies**
*M. A. Kukal*, M. Gitmez, T. Orhan, B. Uzunoğlu, A. Hamamci, F. Celebi; Istanbul/TR
*(mustafaardakukal@icloud.com)*

**Purpose:** To systematically evaluate different deep learning architectures (DL) and input preprocessing strategies for automated differentiation of malignant from benign breast lesions on dynamic contrast-enhanced MRI (DCE-MRI).
**Methods or Background:** This retrospective study analyzed breast DCE-MRI examinations acquired on 1.5T and 3T scanners. Post-contrast second phase dynamic T1-weighted sequences were processed using three input strategies: full image volumes, segmentation masks, and lesion-centered patches (96×96×64 voxels). Data were partitioned into training (80%) and independent test (20%) sets, with patient-stratified 5-fold cross-validation used for model selection. Five 3D convolutional neural networks were evaluated: ResNet3D-18, R(2+1)D-18, MC3-18, DenseNet3D-121, and X3D-S. All architectures were adapted for single-channel volumetric input with binary classification output. Class imbalance was addressed through stratified sampling, focal loss, and data augmentation. Performance was assessed using precision, recall, F1-score, area under the precision-recall curve (AUC-PR), and accuracy.
**Results or Findings:** The cohort included 163 patients (54 benign, 109 malignant lesions) with median age 44 years (IQR: 40-51) and median lesion size 12 mm (IQR: 8-20). All classifications were validated against histopathological standards. Patch-based ResNet3D-18 demonstrated the most ballanced and robust performance on the independent test set: precision 0.85, recall 0.81, F1-score 0.83, PR-AUC 0.83, and accuracy 0.80. Patch-based preprocessing consistently yielded the most robust performance across all architectures.
**Conclusion:** Patch-based input preprocessing demonstrated superior performance compared to full volumes and segmentation masks for automated breast lesion classification on DCE-MRI. The achieved diagnostic accuracy meets clinically acceptable standards. While improved performance is expected with larger datasets, current findings support clinical feasibility. Multi-institutional validation studies are warranted to confirm generalizability.
**Limitations:** Not Applicable
**Funding for this study:** No funding was provided for this study.
**Has your study been approved by an ethics committee?** Yes
**Ethics committee - additional information:** Yeditepe University, IRB #E.83321821-805.02.03-460
**Author Disclosures:**
Tolga Orhan: Nothing to disclose
Mustafa Gitmez: Nothing to disclose
Filiz Celebi: Nothing to disclose
Betül Uzunoğlu: Nothing to disclose
Mustafa Arda Kukal: Nothing to disclose
Andaç Hamamci: Nothing to disclose

**A Physics-Informed Neural Network for Robust Pharmacokinetic Parameter Estimation from Ultrafast Breast MRI Time-Intensity Profiles with Varying Temporal Resolution**
*G. Ertaş*, F. Celebi, D. Yildirim; Istanbul/TR
*(gokhan.ertas@yeditepe.edu.tr)*

**Purpose:** Ultrafast dynamic contrast-enhanced magnetic resonance imaging (DCE-MRI) achieves high temporal resolution for breast cancer assessment but yields sparsely sampled time-intensity profiles (TIPs). This study develops and validates a physics-informed neural network (PINN) that simultaneously reconstructs continuous TIPs and estimates pharmacokinetic (PK) parameters from sparse ultrafast DCE-MRI data, comparing its accuracy with conventional curve-fitting methods.
**Methods or Background:** The PINN comprised four fully connected layers with hyperbolic tangent activations, trained to minimize a composite loss combining data-fidelity (mean-squared error) and physics-informed terms that penalize residuals of the simplified Tofts ordinary differential equation. Ktrans and Vp were optimized jointly with network weights. Conventional nonlinear least squares (NLLS) fitting using Levenberg-Marquardt algorithm provided comparison. Both methods were evaluated on synthetic datasets with known ground-truth values: benign lesions (median Ktrans=0.013, range 0.0026–0.17; Vp=0.0022, range 0.00025–0.017) and malignant lesions (Ktrans=0.073, range 0.013–0.14;Vp=0.021, range 0.002–0.099) across temporal resolutions from 5-13 seconds at 1-second intervals. Performance was quantified using adjusted R2 (adjR2) for goodness-of-fit and median percentage absolute error (MdPAE) for parameter accuracy.
**Results or Findings:** Across 1000 synthetic sparse TIPs, PINN generated smooth continuous reconstructions with excellent agreement (adjR2=0.993) and low parameter errors (MdPAE: Ktrans=0.5%, Vp=1.4%). NLLS achieved superior curve fitting (adjR2=0.999) but substantially higher parameter errors (MdPAE: Ktrans=3.1%, Vp=24.7%). PINN consistently delivered more accurate PK estimates than NLLS, with greatest improvement for Vp estimation.
**Conclusion:** Integrating tracer-kinetic equations within neural networks enables robust TIP reconstruction and more accurate PK parameter estimates from sparse ultrafast DCE-MRI, enhancing quantitative biomarker mapping reliability in breast MRI.
**Limitations:** Not applicable.
**Funding for this study:** No funding was provided for this study.
**Has your study been approved by an ethics committee?** Not applicable
**Author Disclosures:**
Gökhan Ertaş: Nothing to disclose
Düzgün Yildirim: Nothing to disclose
Filiz Celebi: Nothing to disclose

**Can large language models replace traditional textbooks in breast radiology education for medical students?**
*U. Yildiz*, O. Adiguzel Karaoysal, A. Karadayi Buyukozsoy, N. Voyvoda; Istanbul/TR
*(umitcanyldz21@gmail.com)*

**Purpose:** This study aimed to systematically assess the content validity and educational adequacy of lecture notes on breast imaging for medical students, produced by five prominent Large Language Models (LLMs), in comparison to a standard radiology textbook.
**Methods or Background:** Five large language models (LLMs)—GPT-4o, Claude 4 Sonnet, Gemini 2.5 Pro, DeepSeek V3, and Grok 3—were tasked with generating lecture notes on breast imaging. A rubric consisting of 14 themes and 43 subthemes was constructed based on a reference textbook. Three radiologists, each with distinct professional backgrounds, independently evaluated the outputs of these models for accuracy, scope, clarity, and educational value using the Content Validity Index (CVI) metrics.
**Results or Findings:** None of the LLMs achieved the recommended CVI thresholds, which are indicative of high content validity. The most effective model addressed only 34.9% of the subthemes and 78.6% of the themes, whereas the other models demonstrated inferior performance. Notably, there was significant inter-rater variability among the expert evaluators, underscoring subjective differences in assessment.
**Conclusion:** Current general-purpose LLMs are insufficient as standalone resources for specialised medical education purposes. While they can address some topics, performance variability necessitates domain-specific fine-tuning, advanced prompting strategies, and robust evaluation frameworks. They are best used as supplementary tools within structured, instructor-led learning environments.
**Limitations:** The limitations of the study are its use of a single, simple prompt that may not reflect the models' full potential and an evaluation that lacked student input. The study was also limited by its focus on a single topic, its restriction to the English language, and the uncontrolled design variations among the LLMs.
**Funding for this study:** No funding was received for this study.
**Has your study been approved by an ethics committee?** Yes
**Ethics committee - additional information:** The ethics committee notification can be found under the number UID 2025/0l0.99/l8/3.
**Author Disclosures:**
Nuray Voyvoda: Nothing to disclose
Ozge Adiguzel Karaoysal: Nothing to disclose
Umitcan Yildiz: Nothing to disclose
Aysegul Karadayi Buyukozsoy: Nothing to disclose

**Performance evaluation of mammography AI detection software on symptomatic breast cohort**
*N. Sharma*[1], M. M. M. McMahon[1], M. Fletcher[1], D. Manuel[1], S. Rajan[1], A. Kshirsagar[2], F. Eskandari[1], J. Simpson[1]; [1]Leeds/UK, [2]Santa Clara, CA/US
*(nisha.sharma2@nhs.net)*

**Purpose:** Several studies have reported standalone performance of artificial intelligence (AI) software in the screening population. The purpose of this study is to assess the performance of commercially available AI software in symptomatic population.
**Methods or Background:** This prospective study included a total of 308 subjects (79 with dense breasts and 229 with fatty breasts) imaged at diagnostic clinic from August 2024 to March 2025. All women with clinically suspected cancer had bilateral DBT and those recalled for a mammographic abnormality on 2D mammogram had synthetic 2D and 3D imaging of the affected breast only. The cohort contained 87 malignant cases confirmed by biopsy, 46 benign cases confirmed by biopsy and 175 cases deemed benign after follow-up imaging. Results of Genius AI® Detection 2.0 were available for each subject and were reviewed by a breast imaging expert to determine TP, TN, FP and FN by AI for each patient based on location of AI marks and the pathological outcome of the subject. Sensitivity, specificity, PPV and NPV for AI were calculated as well as ROC analysis was performed.
**Results or Findings:** Sensitivity of AI software to detect biopsy proven cancers was 93.1% (81/87) with a 95% CI of 85.0-97.2%. Specificity of AI software to correctly identify benign cases was 52.9% (117/221) with a 95% CI of 46.2%-59.7%. Positive predictive value of AI for this cohort was 43.8% (81/185) with a 95% CI of 36.6%-51.3%. Negative predictive value of AI for this cohort was 95.1% (117/123) with a 95% CI of 89.2%-98.0%. AI software model achieved an area under ROC curve (AUC) of 0.89 with a 95% CI of 0.85 to 0.93.
**Conclusion:** The AI detection software demonstrated high sensitivity and high specificity in symptomatic population.
**Limitations:** Single centre study
**Funding for this study:** None
**Has your study been approved by an ethics committee?** Not applicable
**Author Disclosures:**
Julie Simpson: Nothing to disclose
Nisha Sharma: Nothing to disclose
Dilani Manuel: Nothing to disclose
Ferdows Eskandari: Nothing to disclose
Ashwini Kshirsagar: Nothing to disclose
Maggie Fletcher: Nothing to disclose
Michelle Michelle Mcmahon Mcmahon: Nothing to disclose
Sabrina Rajan: Nothing to disclose

**How Fair Are We? A Systematic Review of Bias in MRI Radiomics Models for Predicting Breast Cancer Treatment Response**
*M. Bobowicz*[1], D. A. Kessler[2], M. Kosno[1], S. Joshi[2], K. Marias[3], O. Diaz[2], K. Lekadir[2]; [1]Gdańsk/PL, [2]Barcelona/ES, [3]Heraklion/GR
*(bobowicz@gmail.com)*

**Purpose:** Radiomics is a widely employed technique for predicting pathological complete response (pCR) to neoadjuvant therapy in breast cancer. Despite its popularity, several problems remain related to fairness, generalizability, and methodological quality. In this review, a thorough analysis was conducted, thereby exposing the potential sources of bias and limitations in the existing studies.
**Methods or Background:** A comprehensive search was conducted in major bibliographic databases, including PubMed, Embase, Scopus, Web of Science, and IEEE Xplore, to identify studies that utilised MRI-based radiomics for predicting pCR in breast cancer patients undergoing neoadjuvant therapy. The methodological quality of the included studies was assessed using the METhodological RadiomICs Score (METRICS), while a custom framework adapted from the QUADAS-2 tool was used to determine fairness. Additionally, a GUI-based sample size calculator was developed, integrating the events per variable (EPV) rule and the Cox-Snell $R^2$ approach to evaluate the adequacy of study power.
**Results or Findings:** A total of 35 studies met the inclusion criteria and were thus deemed eligible for further analysis. The majority of these studies (80%) were retrospective and based on single-centre cohorts, with limited demographic detail and infrequent external validation (14%). A mere five studies (14%) assessed the model's performance across demographic or clinical subgroups. The sample sizes of the studies were often small, and 75% of the studies were underpowered based on established EPV and $R^2$ criteria.
**Conclusion:** This analysis enables us to provide practical recommendations for enhancing the reliability, generalizability, and equity of radiomics-based models for predicting pCR and potentially beyond. This is achieved by underscoring standard practices and pinpointing potential sources of bias, along with developing sample size calculators based on dual methodologies: the EPV rule and the Cox-Snell $R^2$ approach.
**Limitations:** Not specified in the abstract
**Funding for this study:** Digital Europe Programme grant No. 101100633 (EUCAIM); Horizon Europe and Horizon 2020 grant No. 101057699 (RadioVal) and No. 952103 (EuCanImage)
**Has your study been approved by an ethics committee?** Not applicable
**Author Disclosures:**
Oliver Diaz: Nothing to disclose
Kostas Marias: Nothing to disclose
Karim Lekadir: Nothing to disclose
Maciej Bobowicz: Nothing to disclose
Michał Kosno: Nothing to disclose
Smriti Joshi: Nothing to disclose
Dimitri Alexander Kessler: Nothing to disclose

**Is Digital Breast Tomosynthesis (DBT)-based Artificial Intelligence (AI) Better than 2D Mammography-based AI?**
*G. J. W. Partridge*[1], H. Jupp[1], J. James[1], M. Michell[2], Y. Chen[1]; [1]Nottingham/UK, [2]London/UK
*(george.partridge2@nottingham.ac.uk)*

**Purpose:** Digital Breast Tomosynthesis (DBT) has been shown to improve sensitivity and specificity for cancer detection compared with 2D digital mammography (2DDM) when interpreted by human readers. It is therefore expected that AI trained on DBT would also outperform AI based on 2DDM. In this study, we directly compare the performance of DBT-AI and 2DDM-AI using a dataset of paired DBT+2DDM cases from the UK DBT screening PROSPECTS Trial.
**Methods or Background:** In the UK, the multi-centre PROSPECTS randomised trial has been conducted to investigate the cost-effectiveness of replacing 2DDM with DBT in screening. Between January 2019-October 2022, 14,479 women were randomised into the intervention arm of PROSPECTS and screened with 2-view combo DBT+2DDM. Malignant cases are pathology proven and normal cases will have a negative 3-year follow-up screening episode. A DBT- and a 2DDM-based AI algorithm from the same commercial AI vendor (Lunit) will analyse the DBT and 2DDM images from this cohort, respectively. AI case scores between modalities will be compared using linear correlation and Bland-Altman plots, and ROC analysis will compare overall model performance.
**Results or Findings:** The eligible cases from the PROSPECTS dataset are currently being analysed by the AI algorithms. Data will be presented at ECR if accepted.
**Conclusion:** As the popularity of DBT based screening grows, it’s important to know how the performance of DBT-based cancer detection AI systems compare to 2DDM-based counterparts. If DBT-based AI performance is superior to 2DDM, this may also support wider adoption of DBT in screening.
**Limitations:** Cases from the PROSPECTS Trial are from one vendor (Hologic) which may limit the generalisability of the findings. Relatively small size of evaluation test-set; low cancer prevalence (but screening setting).
**Funding for this study:** Lunit
**Has your study been approved by an ethics committee?** Not applicable
**Author Disclosures:**
Harry Jupp: Nothing to disclose
Michael Michell: Nothing to disclose
George J W Partridge: Nothing to disclose
Jonathan James: Nothing to disclose
Yan Chen: Grant Recipient: Funding provided by Lunit

**Development of an Integrated Radiopathomics Signature for Oncotype DX Prediction in Breast Cancer: a pilot study**
*V. Giannini*, G. Nicoletti, A. Defeudis, E. Battista, I. Castellano, M. Durando; Torino/IT
*(valentina.giannini@unito.it)*

**Purpose:** To evaluate the feasibility of developing an Artificial Intelligence (AI)-based integrated biomarker to predict Oncotype DX using magnetic resonance imaging(MRI) and H&E-fixed histopathological Whole Slide Images(WSI).
**Methods or Background:** A total of 120 patients (94 from the Duke-Breast-Cancer-MRI dataset and 26 from an internal cohort) were used for the radiomics signature, while 55 were included in the pathomics analysis; 19 were common to both. In the pathomics pipeline, H&E WSI were divided into 224×224pixel patches, and color normalized with the Macenko method. First-order and texture features were extracted with PyRadiomics and aggregated at patient level using descriptive statistics. Redundant features (Pearson correlation≥0.8) were removed. The most relevant were retained (correlation>0.3 with the output) and used to train multiple classifiers with leave-one-out cross-validation (CV). For radiomics, images were Z-score standardized, tumors manually segmented, and first-order, texture, and shape features extracted. Feature selection was performed with mRMR algorithm, and classifiers were trained with stratified CV on the Duke dataset and tested on the internal cohort.
**Results or Findings:** In pathomics, two features were retained, with ElasticNet yielding the best performance. In radiomics, four features were selected, with the Decision Tree performing best. On the 19 patients common to both datasets, each model reached 74% accuracy. The pathomics model misclassified five patients (two as low risk, three as high risk), while the radiomics model misclassified five (one as low risk, four as high risk). When combined into a radiopathomics signature, accuracy increased to 84%, with three misclassifications (one as low risk, two as high risk).
**Conclusion:** This pilot study supports the feasibility of a radiopathomics signature for Oncotype DX prediction.
**Limitations:** The small sample size and monocentric design of the pathomics cohort limit generalizability, highlighting the need for larger, multicentric validation.
**Funding for this study:** Not Applicable
**Has your study been approved by an ethics committee?** Not applicable
**Author Disclosures:**
Valentina Giannini: Nothing to disclose
Manuela Durando: Nothing to disclose
Giulia Nicoletti: Nothing to disclose
Isabella Castellano: Nothing to disclose
Eleonora Battista: Nothing to disclose
Arianna Defeudis: Nothing to disclose

**Artificial intelligence scores as a biomarker for ductal carcinoma in situ upgrade risk**
*M. Bahl*, A. Kniss, H. Kim, K-S. Kim, S. Do, L. Lamb; Boston, MA/US
*(mbahl1@mgh.harvard.edu)*

**Purpose:** Artificial intelligence (AI) is increasingly used for mammography to enhance breast cancer detection. The purpose of this study was to evaluate whether AI scores for screening-detected ductal carcinoma in situ (DCIS) are associated with upgrade to invasive carcinoma at surgery.
**Methods or Background:** This retrospective study included consecutive screening digital breast tomosynthesis (DBT) examinations performed between 2014 and 2021 at a single academic institution. All cases were radiologist-initiated screening recalls with biopsy-confirmed pure DCIS. A commercially available, FDA-cleared AI algorithm (Genius AI® Detection 2.0; Hologic, Inc.) assigned a score (0-100) to each examination; scores ≥22 were classified as positive per vendor recommendation. Upgrade status to microinvasive (≤1 mm) or invasive (>1 mm) carcinoma was determined from surgical pathology reports. Associations between AI scores and upgrade risk were evaluated using standard statistical tests, with $p < 0.05$ considered significant.
**Results or Findings:** The study cohort included 344 women (mean age 62 ± 12 years) with biopsy-proven DCIS. Positive AI scores (≥22) were observed in 94.8% (326/344) of cases. At surgery, 15.1% (52/344) were upgraded to invasive carcinoma, including 25.0% (13/52) with microinvasive disease. Upgraded cases had a significantly higher mean AI score compared with nonupgraded cases (78 vs. 60, $p < 0.001$). No significant difference in AI scores was observed between upgrades to invasive carcinoma versus microinvasive disease (80 vs. 69, $p = 0.11$).
**Conclusion:** Higher AI scores on screening DBT were associated with DCIS cases upgraded to invasive carcinoma. These findings suggest that AI-derived scores may serve as a potential biomarker of biologically aggressive DCIS and could inform risk stratification and management strategies.
**Limitations:** The limitations of the study are single-center, retrospective design and use of one vendor/algorithm with a vendor-defined threshold, which may limit generalizability.
**Funding for this study:** Funding was provided by Hologic, Inc. The authors, none of whom are employees of Hologic, maintained full control over the data and the submitted information.
**Has your study been approved by an ethics committee?** Yes
**Ethics committee - additional information:** This retrospective Health Insurance Portability and Accountability Act (HIPAA)-compliant study was granted an exemption from the requirement for written informed consent by the institutional review board at the Massachusetts General Hospital (Protocol #: 2023P003130).
**Author Disclosures:**
Kyung-Su Kim: Nothing to disclose
Ariel Kniss: Nothing to disclose
Leslie Lamb: Nothing to disclose
Manisha Bahl: Research/Grant Support: Hologic, Inc. Consultant: Hologic, Inc.
Synho Do: Nothing to disclose
Hyunji Kim: Nothing to disclose

**Influence of Mammographic Positioning Quality, BI-RADS Density, and Mammography Image Type on the Discriminatory Performance of an AI Short-Term Breast Cancer Risk Model**
*G. Spear*[1], M. Abdolell[2], L. R. Margolies[3], K. Yao[4]; [1]Park Ridge, IL/US, [2]Halifax, NS/CA, [3]New York, NY/US, [4]Evanston, IL/US
*(ggspear21@gmail.com)*

**Purpose:** To evaluate how mammographic positioning quality, breast density, and image type influence the discriminatory performance of an image-based AI model for short-term breast cancer risk, with implications for deployment in clinical practice.
**Methods or Background:** A retrospective cohort study of 59,352 screening mammograms (1,822 cancers; 57,530 controls) acquired between May 2017 and April 2023 was performed at an urban health system. Eligible exams comprised standard four-view sets without implants; controls required a negative follow-up ≥1 year after the index exam. AI algorithms categorized positioning quality using a 12-level PGMI scale, breast density using ACR BI-RADS 5th edition categories (A–D), and Hologic image type (FFDM, C-view synthetic 2D, Intelligent 2D). Short-term risk predictions were generated with the Mirai model and evaluated by 2-year area under the ROC curve (AUC). Stratified analyses compared performance across positioning, density, and image type strata; $p<0.05$ considered statistically significant.
**Results or Findings:** Mirai overall 2-year model performance was AUC=0.73 but varied significantly across strata. Exams rated P+/P achieved higher discrimination (AUC=0.80) than those rated P–/G+/G/G–/M+/M/M–/I+/I/I– (AUC=0.72), p=0.02. BI-RADS A mammograms achieved higher discrimination (AUC=0.76) than denser categories B–D (AUC=0.72), p=0.05. FFDM images achieved higher accuracy (AUC=0.80) relative to synthetic 2D formats (AUC=0.72), $p<0.001$. Positioning quality, breast density, and image type exerted independent and measurable effects on model performance.
**Conclusion:** Suboptimal positioning, higher breast density, and synthetic 2D images reduce the discriminatory performance of an AI-based short-term breast cancer risk model. Ensuring optimal positioning quality, accounting for density, and recognizing modality type are critical for robust AI-driven risk assessment and successful clinical implementation.
**Limitations:** Single health system retrospective design; potential residual confounding. Future work will involve external validation across multiple vendors and sites to confirm generalizability and assess clinical utility.
**Funding for this study:** NorthShore University HealthSystem
**Has your study been approved by an ethics committee?** Yes
**Ethics committee - additional information:** Approved by the NorthShore University HealthSystem Institutional Review Board (EH22-163)
**Author Disclosures:**
Laurie R. Margolies: Advisory Board: Sectra, Screenpoint Medical, GE Healthcare, Siemens Heathineers
Katharine Yao: Nothing to disclose
Georgia Spear: Nothing to disclose
Mohamed Abdolell: CEO: Densitas Inc.

**16:30 - 18:00** **Research Stage 3**

### Research Presentation Session: Genitourinary

### RPS 1107

### Prostate cancer MR imaging: today and tomorrow

**Moderator**
Raphaele Marie Renard Penna; Paris/FR
*(raphaele.renardpenna@psl.aphp.fr)*

**Biparametric and multiparametric MRI for prostate cancer detection (Gleason grade group 1, ≥2 and ≥3) – an analysis of the PRIME trial**
*A. Ng*, Prime Trial Group; London/UK

**Purpose:** Multiparametric MRI (mpMRI) ± prostate biopsy is internationally recommended for diagnosing clinically significant prostate cancer (csPCa). However, resource limitations limit global adoption. The PRIME trial (NCT04571840) aimed to evaluate whether biparametric MRI (bpMRI) without intravenous contrast, was non-inferior to mpMRI in detecting csPCa.
**Methods or Background:** In a prospective, multicentre, within-patient, non-inferiority trial, biopsy naïve men with suspected PCa underwent mpMRI. Radiologists first reported bpMRI (T2-weighted and diffusion weighted imaging) blinded to the dynamic contrast enhanced (DCE) sequences. After unblinding to DCE, radiologists reported the full mpMRI. Men with bpMRI or mpMRI suggesting PCa underwent MRI-targeted ± systematic biopsy. Men whose MRI were not suggestive of PCa, but PSA density ≥0.15 ng/mL/mL received systematic biopsy. The primary outcome was the proportion of men diagnosed with csPCa (Gleason grade group (GGG) ≥2). Secondary outcomes included the proportion of men diagnosed with clinically insignificant PCa (ciPCa) (GGG 1), and csPCa (GGG ≥3).
**Results or Findings:** 490 patients included in the primary outcome analysis were recruited from 22 centres across 12 countries. bpMRI detected csPCa (GGG ≥2) in 143/490 (29.2%) compared to 145/490 (29.6%) on mpMRI (adjusted difference 0.4%; 95% CI -1.2 to 0.4; p=0.5). Results were consistent on a lesion-level analysis. bpMRI detected ciPCa (GGG 1) in 45/490 (9.2%) compared to 47/490 (9.6%) on mpMRI (adjusted difference 0.4%; 95% CI -1.2 to 0.4). bpMRI detected csPCa (GGG ≥3) in 73/490 (14.9%) compared to 75/490 (15.3%) on mpMRI (adjusted difference 0.4%; 95% CI -1.2 to 0.4).
**Conclusion:** In men with suspected prostate cancer, bpMRI with or without targeted biopsy was non-inferior to mpMRI in the detection of GGG 1, ≥2 or ≥3 prostate cancer.
**Limitations:** Radiologists were generally very experienced and scans were of good quality.
**Funding for this study:** The PRIME trial is supported by The John Black Charitable Foundation, Prostate Cancer UK, the European Association of Urology Research Foundation and Wolfgang.Dieckmann Foundation.
**Has your study been approved by an ethics committee?** Yes
**Ethics committee - additional information:** The UK National REC (West Midlands Black Country Research Ethics Committee, Nottingham) gave favourable approval for PRIME protocol V.2.0 on 28 June 2021 (ref: 21/WM/0091)
**Author Disclosures:**
Alexander Ng: Nothing to disclose
Prime Trial Group: Nothing to disclose

**Modelling apparent diffusion coefficient (ADC) variability in multicentre prostate MRI to identify significant acquisition parameters and clinical factors**
S. Yu[1], J. Almeida[2], B. Whitcher[1], S. J. Withey[1], M. Blackledge[1], D-M. Koh[1], N. Papanikolaou[2], *J. Winfield*[1]; [1]London/UK, [2]Porto/PT
*(jessica.winfield@rmh.nhs.uk)*

**Purpose:** Apparent diffusion coefficient (ADC) estimates from diffusion-weighted MRI (DWI) have shown good repeatability and ability to identify clinically-significant prostate cancer. Despite these findings, ADC values are not used quantitatively in clinical practice due to the lack of defined thresholds for interpretation and variation in ADC estimates between different imaging protocols. The impact of imaging protocol variations and inter-scanner differences on ADC estimates has not been quantified in large international datasets. This study aims to model ADC values of the prostate, including variation with tumour grade, in a large multicentre dataset of DWI data to identify the imaging protocol and inter-scanner differences that affect ADC estimates.
**Methods or Background:** Mean ADC estimates of the prostate gland of 6413 patients from the ProCAncer-I repository including tumour grade and imaging metadata are used in the analysis. The whole prostate glands were segmented using methods developed in the ProCAncer-I project. A list of potentially significant imaging protocol parameters were compiled, based on literature review. Clinical data, including age, pathological Gleason Grade, and radiological PI-RADS scores were also collected.
**Results or Findings:** Random forest and Lasso regression identified significant factors using repeated 5-fold cross validation, with echo time, field of view, pixel spacing and PI-RADS=5 found consistently to be the most important factors correlating with ADC values. Lasso coefficients indicate that ADC value will change by -6.78, +98.58(10-6 mm2/s) per ms and mm increase in TE and pixel spacing, and +56.87, -34.84(10-6 mm2/s) for small-field-of-view scans and PI-RADS category 5, independently.
**Conclusion:** Using a real-world multi-centre prostate MRI dataset has allowed us to identify significant MR parameters and clinical factors that affect ADC estimates, which can inform on future multi-centre collaborations and protocol standardisation.
**Limitations:** N/A
**Funding for this study:** N/A
**Has your study been approved by an ethics committee?** Not applicable
**Author Disclosures:**
Jose Almeida: Nothing to disclose
Brandon Whitcher: Nothing to disclose
Dow-Mu Koh: Nothing to disclose
Matthew Blackledge: Nothing to disclose
Jessica Winfield: Nothing to disclose
Nikolaos Papanikolaou: Nothing to disclose
Sheng Yu: Nothing to disclose
Samuel Joseph Withey: Nothing to disclose

**Improving Prostate MRI Quality Assessment Across Diverse Evaluators and Institutional Practice Through a PI-QUAL Lecture**
*L. Xu*[1], F. Giganti[2], C. M. Moore[2], H. Sun[3], X. Wang[1], L. Shi[1]; [1]Hangzhou/CN, [2]London/UK, [3]Beijing/CN
*(xu2016lili@163.com)*

**Purpose:** To evaluate the impact of a Prostate Imaging Quality (PI-QUAL) v2 lecture on accuracy and inter-reader agreement of prostate MRI image quality assessment among radiologists and technologists from diverse professional backgrounds and institutions, and to evaluate its role in institutional-level quality improvement through a quality control (QC) process.
**Methods or Background:** Ten participants (five radiologists and five technologists) from five different hospitals independently evaluated 15 prostate MRIs before and after a standardized PI-QUAL lecture. Reference scores were established by two experienced radiologists. Assessment accuracy was evaluated by the area under the ROC curve (AUC). Inter-reader agreement was measured using percent agreement (PA) and Gwet's AC1. Subgroup analyses were conducted based on participant characteristics. For the quality assurance and QC (QA/QC), each center submitted 15 prostate MRIs before and after the lecture. Image quality was evaluated centrally to determine institutional improvement.
**Results or Findings:** The overall AUC improved from 0.629 to 0.842 ($p < 0.001$), with subgroup improvements across all participant types (pre: 0.500–0.688; post: 0.806–0.896). Inter-reader agreement improved from PA 57.6% to 72.6% and Gwet's AC1 from 0.214 to 0.466. Through the QA/QC process, three centers initially failed to meet PI-QUAL v2 technical criteria. After feedback, image quality markedly improved, with all post-intervention scans achieving PI-QUAL v2 scores >1.
**Conclusion:** A structured PI-QUAL v2 lecture significantly improved both the accuracy and inter-reader agreement of prostate MRI image quality assessments among diverse evaluators. Incorporating QA/QC further enhanced institutional image quality. PI-QUAL v2 training is a feasible strategy for standardizing prostate MRI quality.
**Limitations:** First, the number of cases evaluated was limited. Second, no hands-on workshop was conducted.
**Funding for this study:** Not available.
**Has your study been approved by an ethics committee?** Yes
**Ethics committee - additional information:** This study was approved by the Institutional Review Board (IRB No. IRB-2025-683 (IIT)).
**Author Disclosures:**
Hao Sun: Nothing to disclose
Lili Xu: Nothing to disclose
Caroline M Moore: Nothing to disclose
Lei Shi: Nothing to disclose
Xu Wang: Nothing to disclose
Francesco Giganti: Nothing to disclose

**mpMRI Surveillance After Focal Therapy for Localized Prostate Cancer: A Comparative Analysis of TARGET and PI-FAB Scores**

G. Hang[1], R. Z. Lee[1], A. L. C. Y. Lai[1], N. Lath[1], J. Velaga[2], K. J. Tay[1], N. T. Ngo[1], *Y. M. Law*[1]; [1]Singapore/SG, [2]Brunswick East/AU
*(law.yan.mee@singhealth.com.sg)*

**Purpose:** The growing adoption of focal therapy (FT) for localized prostate cancer (PCa) emphasizes the need for standardized MRI-based frameworks to detect recurrent clinically significant prostate cancer (csPCa). The Transatlantic Recommendations for Prostate Gland Evaluation with MRI after Focal Therapy (TARGET) and Prostate Imaging after Focal Ablation (PI-FAB) systems have recently been proposed to guide post-FT multiparametric MRI (mpMRI) assessment. This study compared the diagnostic performance and inter-reader agreement of TARGET and PI-FAB.
**Methods or Background:** Patients who underwent FT for localized csPCa were recruited as part of a prospective phase II trial and an extended cohort. All patients underwent mpMRI and prostate biopsy. Two genitourinary radiologists evaluated post-FT mpMRI independently using TARGET and PI-FAB. Diagnostic metrics (sensitivity, specificity, positive predictive value [PPV], negative predictive value [NPV], accuracy) were analyzed. Inter-reader agreement was assessed using quadratic weighted Cohen's kappa (κ).
**Results or Findings:** Seventy-nine patients with 139 ablated sites were included. For both TARGET and PI-FAB, radiologist 1 achieved the same sensitivity 100%, specificity of 82.6%, PPV of 62%, NPV of 100%, and an accuracy of 74.8%. Radiologist 2 achieved a sensitivity of 72.4% and 78.5%, a specificity of 65.4% and 64.8%, a PPV of 52.2% and 43.1%, an NPV 90% and 92.3%, and an accuracy of 66.9 % and 67.6% in TARGET and PI-FAB, respectively. Inter-reader agreement was moderate (κ=0.59 for TARGET, κ=0.60 for PI-FAB)
**Conclusion:** TARGET and PI-FAB demonstrated comparable diagnostic performance in detecting in-field recurrence of csPCa following FT. Despite different reader experience, moderate agreement was achieved, supporting their role in standardized post-FT mpMRI evaluation.
**Limitations:** This study was conducted at a single center with a modest sample size, which limits generalizability. Only two radiologists participated, restricting assessment of variability across broader expertise levels.
**Funding for this study:** Dr KJ Tay received funding support from National Medical Research Council, Singapore. Grant number - NCT04138914.
**Has your study been approved by an ethics committee?** Yes
**Ethics committee - additional information:** Singapore General Hospital IRB
**Author Disclosures:**
Anna Lois, Chooi Yan Lai: Nothing to disclose
Guanqi Hang: Nothing to disclose
Kae Jack Tay: Nothing to disclose
Narayan Lath: Nothing to disclose
Rebekah Zhuyi Lee: Nothing to disclose
Jyothirmayi Velaga: Nothing to disclose
Nye Thane Ngo: Nothing to disclose
Yan Mee Law: Nothing to disclose

**Visualizing Invasive Cribriform Architecture and Intraductal Carcinoma in Prostate Cancer via Time-Dependent Diffusion MRI**

*Y. Chen*, H. Wang, Y. Guo; Guangzhou/CN
*(chenyling59@mail.sysu.edu.cn)*

**Purpose:** To investigate the feasibility of time-dependent diffusion MRI-based microstructural mapping for noninvasively characterizing the intraductal carcinoma (IDC) and invasive cribriform (Cr) histologic patterns in prostate cancer (PCa).
**Methods or Background:** This retrospective study enrolled men with pathologically confirmed PCa based on radical prostatectomy specimens between March 2023 and March 2025. Time-dependent diffusion MRI was performed using pulsed and oscillating gradient diffusion MRI sequences. The microstructural parameters, including cell diameter(d), extracellular diffusivity(Dex), intracellular fraction (fin), and cellularity were estimated with a two-compartment model. Apparent diffusion coefficient (ADC) maps were generated for each diffusion frequency (0 Hz, 17 Hz, 33 Hz). These parameters were compared between PCa with and without Cr/IDC patterns. Their diagnostic performance in detecting Cr/IDC architecture were assessed.
**Results or Findings:** A total of 81 participants were enrolled, of which 69.5% (57/81) PCa exhibited Cr/IDC patterns. Among all measurements derived from time-dependent diffusion MRI, fin and cellularity were significantly higher in the Cr/IDC-positive group compared to the negative group ($P < 0.001$ and $P = 0.002$, respectively), while ADC0Hz and ADC17Hz values were significantly lower ($P = 0.015$ and $P = 0.029$, respectively). No significant differences were observed in Dex, d and ADC33Hz (P = 0.151, 0.689, and 0.055, respectively). For detecting Cr/IDC morphology, fin yielded the highest area under the receiver operating characteristic curve (AUC) of 0.758 (95% CI: 0.639–0.877). Cellularity, ADC0Hz and ADC17Hz showed AUCs of 0.732 (95% CI: 0.605–0.858), 0.671 (95% CI: 0.545–0.798), and 0.654 (95% CI: 0.519–0.790), respectively.
**Conclusion:** Microstructural mapping via time-dependent diffusion MRI demonstrates strong potential for noninvasively characterizing histologic subtypes of PCa, showing high efficacy in identifying cribriform architecture and intraductal carcinoma.
**Limitations:** Sensitivity analyses adjusted for Gleason grade were not performed due to the small cohort.
**Funding for this study:** National Natural Science Foundation of China (82372075, 82371911, 82402415); Natural Science Outstanding Youth Fund Project of Guangdong Province (2024B1515020061); Youth Medical Innovation and Practice Research Program of Guangzhou (2023QNYXYB009)
**Has your study been approved by an ethics committee?** Yes
**Ethics committee - additional information:** The study was approved by the institutional Medical Research Ethics Committee of the First Affiliated Hospital, Sun Yat-sen University.
**Author Disclosures:**
Yanling Chen: Nothing to disclose
Huanjun Wang: Nothing to disclose
Yan Guo: Nothing to disclose

**Pre-treatment MRI as an Independent Prognostic Marker for Biochemical Recurrence, Metastasis, and Prostate Cancer Mortality After Radical Prostatectomy: Systematic Review and Meta-analysis**

*G. Agrotis*, S. Ursprung, P. Van Leeuwen, H. Van der Poel, U. van der Heide, I. G. Schoots; Amsterdam/NL
*(g.agrotis@hotmail.com)*

**Purpose:** To systematically review and meta-analyze the prognostic value of pre-treatment MRI for oncological outcomes in men undergoing radical prostatectomy.
**Methods or Background:** A systematic literature search of MEDLINE, Embase, and Scopus was performed from inception through March 2025. Eligible studies evaluated pre-treatment MRI in men undergoing radical prostatectomy and reported long-term oncological outcomes, including biochemical recurrence, metastatic failure, overall survival, or cancer-specific mortality. Two reviewers independently extracted data and assessed study quality using the Quality in Prognostic Studies tool. Random-effects meta-analysis was performed to pool hazard ratios (HRs). The primary outcome was biochemical recurrence. Secondary outcomes included metastatic failure and prostate cancer–specific mortality.
**Results or Findings:** Forty-four studies (27,453 patients) were included. MRI-detected extracapsular extension (mT3a disease) (HR 2.16 [95% CI, 1.85–2.52]) and seminal vesicle invasion (mT3b disease) (HR 3.32 [2.68–4.12]) were strong independent predictors of biochemical recurrence. PI-RADS score (HR 2.22 [1.86–2.64]) and tumor apparent diffusion coefficient values (HR 2.39 [1.65–3.47]) also showed prognostic value. Additional MRI features associated with recurrence included tumor diameter (HR 1.04 [1.01–1.07]), tumor volume (HR 1.56 [1.20–2.02]), peripheral zone tumor location (HR 3.64 [1.71–7.72]), and MRI-detected positive lymph nodes (HR 2.33 [1.78–3.04]). For metastatic failure, MRI-detected extracapsular extension (HR 4.11 [2.24–7.571]), seminal vesicle invasion (HR 8.36 [2.21–31.6]), and tumor diameter (HR 1.04 [1.02–1.06]) were independent predictors. MRI-detected extracapsular extension was also associated with prostate cancer–specific mortality (HR 7.86 [4.51–13.7]).
**Conclusion:** Pre-treatment MRI provides independent prognostic value for biochemical recurrence, metastatic failure, and cancer-specific mortality in men undergoing radical prostatectomy. Integration of specific MRI-derived predictors into risk models may improve preoperative stratification, guide treatment planning, and inform post-treatment management.
**Limitations:** retrospective, heterogeneity of MRI protocols and variability of readers
**Funding for this study:** No funding to disclose
**Has your study been approved by an ethics committee?** Not applicable
**Author Disclosures:**
Henk Van Der Poel: Nothing to disclose
Pim Van Leeuwen: Nothing to disclose
Georgios Agrotis: Nothing to disclose
Uulke Van Der Heide: Nothing to disclose
Ivo Gerardus Schoots: Nothing to disclose
Stephan Ursprung: Nothing to disclose

**Value of additional systematic cores during MRI-guided targeted prostate biopsy in prostate cancer screening in young men – results from the PROBASE trial**

*M. Boschheidgen*[1], R. Al-Monajjed[1], A. Kilaviciute[2], J. P. Radtke[1], H-P. Schlemmer[2], G. Antoch[1], P. Albers[1], L. Schimmöller[3]; [1]Düsseldorf/DE, [2]Heidelberg/DE, [3]Herne/DE
*(matthias.boschheidgen@med.uni-duesseldorf.de)*

**Purpose:** While MRI-guided targeted biopsy (TB) is becoming an integral part for early prostate cancer (PCa) detection, its role in screening of younger men remains unclear. Here we analyse the additional value of systematic biopsy (SB) to improve detection of clinically significant PCa (csPCa).
**Methods or Background:** 525 men aged 45–54 years with confirmed PSA ≥3.0 ng/ml underwent mpMRI followed by combined TB and SB between February 2014 and August 2023 within a multicenter, prospective screening trial in Germany. Software-based MRI/ultrasound fusion 2-core TB combined with SB was performed transrectally or transperineally. Primary objective was to analyse differences in csPC detection rates between SB versus TB in correlation to MRI. Secondary objectives were overall PCa detection and ISUP grade group (GG) distribution by SB and/or TB.
**Results or Findings:** PCa was detected in 209 men (39%). 148 were csPCa (71%; ISUP GG ≥2). SB missed 24 cases of csPCa (16%) and TB missed 49 (33%). SB detected 25 more low-risk PCa than TB (n=51 vs. n=26). In 64% of the cases in which SB detected a higher ISUP GG (n=89; including ISUP GG 1) the positive cores were located within the MRI-detected lesions. 5 ISUP GG ≥3 PCa were not identified on MRI.
**Conclusion:** A relevant proportion of csPCa was missed by 2-core TB although they were correctly identified on MRI, suggesting limitations in targeting accuracy and/or fusion technique. Thus, SB or at least perilesional sampling should become the standard biopsy technique in PCa detection in younger men until targeting can be further optimized.
**Limitations:** Limitations are the lack of centralized MRI review before biopsy, variability in biopsy technique, retrospective subgroup analysis, and short follow-up.
**Funding for this study:** Deutsche Krebshilfe
**Has your study been approved by an ethics committee?** Yes
**Ethics committee - additional information:** Ethikkommission Düsseldorf
**Author Disclosures:**
Jan Philipp Radtke: Nothing to disclose
Peter Albers: Nothing to disclose
Rouvier Al-Monajjed: Nothing to disclose
Matthias Boschheidgen: Nothing to disclose
Lars Schimmöller: Nothing to disclose
Agne Kilaviciute: Nothing to disclose
Heinz-Peter Schlemmer: Nothing to disclose
Gerald Antoch: Nothing to disclose

**Improving Prostate Cancer Detection with Targeted Biopsy: Focus on DWI-definition of Lesion-Core and Penumbra**

*L. Laschena*, F. Mezzapesa, L. De Giacomo, S. Novelli, E. Messina, V. Panebianco; Rome/IT
*(ludovica.laschena@uniroma1.it)*

**Purpose:** To explore the potential role of DWI in defining the "lesion-core" in the prostate peripheral zone and to assess whether targeting biopsies specifically on the DWI-based "lesion-core" can improve the diagnostic performance in detecting csPCa.
**Methods or Background:** Prospective,single-center study; patients with clinical suspicion of PCa underwent mpMRI according to PI-RADS v2.1 recommendations and those with a positive scan (PI-RADS 5, 4 and PI-RADS 3 with PSA-density ≥0.15) were directed to a transrectal-fusion-targeted-biopsy (TBx). Only peripheral zone lesions with a mismatch in terms of size or extent between DWI and T2WI were considered. During pre-biopsy lesion segmentation performed by an experienced uro-radiologist, the "lesion-core" was indicated as the most restricting area on DWI, while the "penumbra" as the exceeding area visible on T2WI.During the procedure cores from each region were collected and separately stored. Statistical analyses included McNemar's,Wilcoxon, and ROC.
**Results or Findings:** 111 patients were enrolled;152 lesions identified.Overall, 12 (10.8%) had no evidence of prostate cancer, 18 (16.2%) had ciPCa, and 81 (72.8%) were diagnosed with csPCa.When stratifying by biopsy site, significant differences emerged between "lesion-core" and "penumbra".Detection of csPCa was higher in the lesion-core (107-70.3%) compared to the penumbra (79-52.0%), with a significant difference (p=0.01).Similarly, Gleason scores were higher in the lesion-core than in the penumbra.ROC analysis confirmed the superior diagnostic performance of lesion-core biopsies.The AUC for csPCa detection was 0.86 for lesion-core targeting compared to 0.66 for penumbra targeting, with the difference reaching statistical significance (p=0.031).
**Conclusion:** DWI-based lesion-core targeting provides higher diagnostic yield for csPCa compared to penumbra sampling. This approach refines biopsy accuracy, reduces overdiagnosis of indolent disease, and may improve patient stratification. DWI should therefore be considered the dominant sequence to guide targeted biopsy in peripheral zone lesions.
**Limitations:** Single-center study, sample-size
**Funding for this study:** None
**Has your study been approved by an ethics committee?** Yes
**Ethics committee - additional information:** N/A
**Author Disclosures:**
Luca De Giacomo: Nothing to disclose
Valeria Panebianco: Nothing to disclose
Emanuele Messina: Nothing to disclose
Simone Novelli: Nothing to disclose
Ludovica Laschena: Nothing to disclose
Francesca Mezzapesa: Nothing to disclose

**Quantitative Assessment of Inter-Observer Variability in PI-RADS Evaluation of Transition Zone Lesions: Impact of Reader Expertise on Diagnostic Performance and Cost-Effectiveness**

F. Spoto[1], E. Demozzi[1], G. Sala[1], C. Cicciò[1], L. Monterubbiano[1], N. Cardobi[2], G. Cacciatori[2], M. D'Onofrio[2], *G. Foti*[1]; [1]Negrar/IT, [2]Verona/IT
*(gfoti81@yahoo.it)*

**Purpose:** To quantify the impact of reader expertise on diagnostic accuracy for transition zone lesions using histopathological validation, and evaluate associated clinical and economic implications.
**Methods or Background:** This retrospective single-center study analyzed 315 consecutive patients with PI-RADS ≥3 transition zone lesions who underwent multiparametric MRI followed by MRI-guided in-bore biopsy between January 2018 and December 2024. Cases underwent blinded re-interpretation by either community radiologists (n=164) or expert readers at specialized center (n=151). Assessment included diagnostic accuracy metrics, PI-RADS category-specific performance, inter-observer agreement, and multivariable logistic regression. Economic impact was assessed through cost analysis and incremental cost-effectiveness ratios.
**Results or Findings:** Expert readers achieved superior diagnostic accuracy compared to community radiologists (47.0% versus 35.4%, p<0.001), representing 11.6% absolute improvement. Positive predictive value for clinically significant cancer improved 15.2% (43.3% versus 28.1%, p<0.001). For PI-RADS 4 lesions (56.8% of cohort), specialized centers achieved 76.9% cancer detection versus 42.6% for non-specialized centers (p<0.001), nearly doubling detection while reducing false positives by 60%. In multivariable analysis, PI-RADS score remained the strongest predictor (OR 3.06, p<0.001), while expertise effect was attenuated after adjustment (OR 1.12, p=0.68). Inter-observer agreement between experts (κ=0.603) exceeded agreement with community readers (κ=0.291, p=0.002). Economic analysis revealed €627 cost reduction per cancer detected through expert interpretation.
**Conclusion:** Reader expertise substantially impacts diagnostic accuracy for transition zone lesions, particularly for PI-RADS 4 lesions where specialized centers nearly double cancer detection rates while reducing false positives by 60%.
**Limitations:** Study limitations include retrospective design, potential selection bias, and lack of external validation. Economic analysis was limited to direct medical costs from a healthcare system perspective, without considering indirect costs or long-term treatment expenses beyond initial diagnosis.
**Funding for this study:** This study received no external funding.
**Has your study been approved by an ethics committee?** Not applicable
**Author Disclosures:**
Giuseppe Sala: Nothing to disclose
Nicolò Cardobi: Nothing to disclose
Giada Cacciatori: Nothing to disclose
Leonardo Monterubbiano: Nothing to disclose
Mirko D'Onofrio: Nothing to disclose
Giovanni Foti: Nothing to disclose
Flavio Spoto: Nothing to disclose
Emanuele Demozzi: Nothing to disclose
Carmelo Cicciò: Nothing to disclose

**Diagnostic accuracy and reliability of MR based Node-RADS for prostate cancer**

*R. C. Yarol*, C. Sinirsiz, M. S. Ozer, I. B. Akin, O. Bozkurt, V. Sen, E. B. Tuna, K. Yorukoglu, C. Altay; Izmir/TR
*(raifyarol@gmail.com)*

**Purpose:** The aim of this study is to investigate the ability of the Node-RADS in predicting lymph node metastasis of prostate cancer.
**Methods or Background:** The Node-RADS score was provided a standized assessment of lymph nodes with scores ranging from 1 to 5, accounting size and configuration criteria. In thıs study, 102 patients with prostate cancer who have undergone radical prostatectomy and lymph node dissection from 2021 to 2025 were retrospectively evaluated for detection of lymph nodes and scored according to Node-RADS system. Node-RADS evaluations were performed by two radiologists based on pre-operative MR examinations. Diagnostic accuracy was assessed with receiver-operating characteristics (ROC) curve analysis, sensitivity, specificity, positive predictive value (PPV), and negative predictive value (NPV). Interreader agreement was calculated using Cohen's kappa.
**Results or Findings:** Overall, 102 patients with a mean age of 67.26 ± 6.20 years were included. Lymph node metastasis was histopathologically proven 12 among 102 patients (11,8%). One patient with a score of 1 (1/80) and one patient with a score of 2 (1/8) had a positive node. Considering a Node RADS 4–5 positive and a Node RADS 1–2 negative, PPV was 85.7% and the NPV was 97.7%. Node-RADS achieved a sensitivity of 75% and a specificity of 98,8%. ROC curve analysis for lymph node discrimination showed an AUC of 0.897.
**Conclusion:** Node-RADS is a reliable and effective tool for predicting nodal metastasis in prostate cancer. Pre-operative routine use of it may enhance radiological staging and support treatment decisions.
**Limitations:** Retrospective nature and limited number of patients
**Funding for this study:** No funding
**Has your study been approved by an ethics committee?** Yes
**Ethics committee - additional information:** This study is approved by ethics committee.
**Author Disclosures:**
Canan Altay: Nothing to disclose
Kutsal Yorukoglu: Nothing to disclose
Can Sinirsiz: Nothing to disclose
Ozan Bozkurt: Nothing to disclose
Isil Başara Akin: Nothing to disclose
Volkan Sen: Nothing to disclose
Raif Can Yarol: Nothing to disclose
Emine Burcin Tuna: Nothing to disclose
Muhammed Selçuk Ozer: Nothing to disclose

**Is It Safe to Omit Contrast in Prostate Cancer Patients on Active Surveillance?**
A. Dehghanpour, M. Bicchetti, A. Borrelli, S. Novelli, E. Messina, V. Panebianco, *L. Laschena*; Rome/IT
*(ludovica.laschena@uniroma1.it)*

**Purpose:** Active surveillance (AS) is the recommended strategy for men with low-risk prostate cancer. The Prostate Cancer Radiological Estimation of Change in Sequential Evaluation (PRECISE) criteria were designed to standardise the reporting of tumour progression on serial MRI, and PRECISE v2 has recently refined definitions and incorporated minimum quality standards. Multiple studies have demonstrated the non-inferiority of biparametric MRI (bpMRI) compared to multiparametric MRI (mpMRI) for the detection of csPCa. The aim of this study was to evaluate whether contrast administration can also be safely omitted in patients undergoing AS.
**Methods or Background:** We retrospectively analysed a prospectively maintained single-centre AS cohort of 185 men with clinically insignificant prostate cancer and ≥3 years of follow-up. 120 underwent repeat biopsy; the remainder were clinically and radiologically stable. Two expert radiologists independently reviewed all MRI examinations and assigned PRECISE v2 scores once using mpMRI and once using bpMRI. Image quality was scored with PI-QUAL v2. The reference standard was ISUP group grade on biopsy or stable follow-up. Diagnostic performance was assessed with ROC analysis for a PRECISE cut-off ≥4.
**Results or Findings:** For mpMRI, PRECISE ≥4 predicted ISUP upgrading with sensitivity 70%, specificity 75%, PPV 58%, NPV 84%, and accuracy 72% (AUC 0.77). In scans with PI-QUAL 3, particularly DWI score 4, bpMRI yielded comparable performance: sensitivity 66%, specificity 73%, PPV 55%, NPV 82%, accuracy 70% (AUC 0.75). When PI-QUAL was <3, bpMRI showed inferior performance with mpMRI significantly outperforming bpMRI ($p<0.05$).
**Conclusion:** PRECISE scoring system is an accurate tool for predicting disease progression in men on AS, both with mpMRI and bpMRI when image quality is optimal. However, in suboptimal scans mpMRI clearly outperforms bpMRI, and it is therefore not yet safe to omit contrast administration.
**Limitations:** Retrospective study.
**Funding for this study:** None
**Has your study been approved by an ethics committee?** Yes
**Ethics committee - additional information:** Institutional Review Board and the Ethics Committee
**Author Disclosures:**
Marco Bicchetti: Nothing to disclose
Valeria Panebianco: Nothing to disclose
Antonella Borrelli: Nothing to disclose
Emanuele Messina: Nothing to disclose
Ailin Dehghanpour: Nothing to disclose
Simone Novelli: Nothing to disclose
Ludovica Laschena: Nothing to disclose

**Prostate Cancer: Value of volume-adjusted PSA density in recurrence assessment after HIFU**
*C. Elsner*, A-M. Pausch, T. Rabadi, N. Rupp, D. Eberli, A. M. Hötker; Zürich/CH

**Purpose:** Optimal follow-up and biopsy stratification after high-intensity focused ultrasound (HIFU) ablation of localized prostate cancer are challenging, as PSA levels are often unreliable due to inflammation and variable residual tissue. This study aimed to evaluate the diagnostic performance of the Prostate Imaging after Focal Ablation (PI-FAB) score on multiparametric MRI in combination with volume-adjusted PSA density (vaPSA-D, derived from volumetrically assessed residual vital prostate tissue) in recurrence detection.
**Methods or Background:** This IRB-approved, retrospective single-center study included 119 men treated with HIFU for localized prostate cancer. Follow-up mpMRI and prostate biopsies were performed at 6, 12, and 36 months. PI-FAB scores and vaPSA-D were assessed independently. Diagnostic performance was analyzed using histopathology from biopsy as the reference standard.
**Results or Findings:** The PI-FAB score demonstrated good diagnostic performance, particularly at 36 months (AUC 0.92). VaPSA-D likewise showed solid performance, with steadily increasing accuracy over time (AUC 0.68, 0.81, 0.84 at 6, 12, and 36 months; overall 0.78), outperforming PI-FAB at 12 months post-HIFU. Integrating vaPSA-D into PI-FAB subgroups significantly enhanced recurrence assessment: PI-FAB 1: sensitivity/specificity 75%/78% (threshold 0.14 ng/ml); PI-FAB ≥2: sensitivity/specificity 67%/85% (threshold: 0.18 ng/ml); PI-FAB 3: sensitivity/specificity 69%/90% (threshold: 0.18 ng/ml).
**Conclusion:** Both PI-FAB and vaPSA-D perform well in the post-HIFU setting, each with distinct strengths: PI-FAB shows excellent performance at late follow-up, while vaPSA-D ensures high sensitivity and reliable recurrence exclusion across all time points. Their combined application provides the most accurate assessment of in-field recurrence after HIFU, supporting a complementary rather than exclusive use of these tools to potentially reduce unnecessary biopsies.
**Limitations:** Limitations include the retrospective design, single-center setting, and an overall low recurrence prevalence.
**Funding for this study:** No funding for this study.
**Has your study been approved by an ethics committee?** Yes
**Ethics committee - additional information:** Cantonal ethics committee Zurich
**Author Disclosures:**
Niels Rupp: Nothing to disclose
Daniel Eberli: Nothing to disclose
Antonia-Maria Pausch: Nothing to disclose
Toni Rabadi: Nothing to disclose
Clara Elsner: Nothing to disclose
Andreas M. Hötker: Nothing to disclose

**Evaluation of Diagnostic Performance and Inter-reader agreement of the Transatlantic Recommendations for Prostate Gland Evaluation with Magnetic Resonance Imaging After Focal Therapy (TAREGT)**
G. Hang[1], R. Z. Lee[1], A. L. C. Y. Lai[1], N. Lath[1], J. Velaga[2], K. J. Tay[1], N. T. Ngo[1], *Y. M. Law*[1]; [1]Singapore/SG, [2]Brunswick East/AU
*(law.yan.mee@singhealth.com.sg)*

**Purpose:** Architectural distortion after focal therapy (FT) presents challenges for post-treatment surveillance using multiparametric magnetic resonance imaging (mpMRI). To address this, the Transatlantic Recommendations for Prostate Gland Evaluation with Magnetic Resonance Imaging After Focal Therapy (TAREGT) scoring system—a 5-point scale—was recently developed to evaluate residual cancer or in-field recurrence at ablation sites. This study aimed to assess the diagnostic performance and inter-rater reliability of the TARGET system in post-FT mpMRI.
**Methods or Background:** Patients were enrolled through a phase II clinical trial and an extended prospective cohort. Eligible participants had localized clinically significant prostate cancer (csPCa) and underwent cryotherapy or irreversible electroporation (IRE) between October 2019 and January 2024. All underwent post-FT mpMRI and subsequent prostate biopsy. Two genitourinary radiologists with 11 and 5 years of experience independently assessed mpMRI using TARGET. Sensitivity, specificity, positive predictive value (PPV), negative predictive value (NPV), and accuracy were calculated. Inter-rater reliability was measured with quadratic weighted Cohen's kappa (κ).
**Results or Findings:** Seventy-nine patients with 139 treated sites were analyzed. Radiologist 1 achieved sensitivity 100 %, specificity 82.6 %, PPV 62 %, NPV 100 %, and accuracy 74.8 %. Radiologist 2 achieved sensitivity 72.4 %, specificity 65.4 %, PPV 52.2 %, NPV 90 %, and accuracy 66.9 %. Cohen's κ was 0.59, indicating moderate agreement.
**Conclusion:** The TARGET scoring system demonstrated high diagnostic accuracy in detecting in-field recurrence after FT. Despite moderate inter-reader agreement, diagnostic performance was higher for the more experienced radiologist, highlighting the influence of expertise. These findings support TARGET as a valid framework for standardized post-FT mpMRI surveillance.
**Limitations:** This study was conducted at a single center with a modest sample size, which may limit generalizability. Only two radiologists participated, restricting assessment of variability across broader expertise levels.
**Funding for this study:** Dr KJ Tay received funding support from National Medical Research Council, Singapore. Grant number - NCT04138914.
**Has your study been approved by an ethics committee?** Yes
**Ethics committee - additional information:** The study has been approved by Singapore General Hospital IRB
**Author Disclosures:**
Anna Lois, Chooi Yan Lai: Nothing to disclose
Guanqi Hang: Nothing to disclose
Kae Jack Tay: Nothing to disclose
Narayan Lath: Nothing to disclose
Rebekah Zhuyi Lee: Nothing to disclose
Jyothirmayi Velaga: Nothing to disclose
Nye Thane Ngo: Nothing to disclose
Yan Mee Law: Nothing to disclose

**16:30 - 18:00 Research Stage 5**

### Research Presentation Session: Neuro

### RPS 1111

### Neuro-oncology and spine disorders: cutting-edge MRI approaches

**Moderator**
Lukas Haider; London/UK

### When Sulcal Enhancement is Not Leptomeningeal Metastasis: Diagnostic Value of T1–T2FLAIR Matching for Differentiating Benign Contrast Leakage

L. Joo, J. B. Lee, Y. Yim, *N. H. Kim*; Seoul/KR
*(knhpaul0823@gmail.com)*

**Purpose:** The purpose of this study is to characterize ISE observed in patients undergoing brain MRI for the evaluation of brain metastasis
**Methods or Background:** This retrospective study included patients who underwent enhanced brain MRI between November, 2023 and September, 2024 for the purpose of evaluating brain metastasis. All patients underwent CE-T2FLAIR, CE-3D MPRAGE, and CE-3D SPACE imaging. The obtained contrast-enhanced T2FLAIR images were evaluated by two independent radiologists to identify patients with sulcal enhancement, and in case of disagreement, a consensus conclusion was reached. The patients were then divided into true leptomeningeal metastasis and ISE based on imaging and clinical findings, and the incidence of ISE was investigated, and logistic regression analysis was performed to identify variables with statistically significant associations with risk factors such as age, microbleed (3 categories; 0, 1-4, 5≤), and previous brain radiation therapy (RT) history.
**Results or Findings:** A total of 387 patients who suspected malignancy underwent brain MRI, and 50 were excluded, resulting in 337 brain MRIs included in this analysis. Among these, 72 patients showed sulcal enhancement on CE-T2FLAIR. Of these, 11 were diagnosed as true leptomeningeal metastasis, and 61 were classified as ISE, yielding an incidence of 18.1% (61/337). Logistic regression identified age, microbleed, and previous RT history as significant risk factors. Multivariate logistic regression results showed that age, microbleed, and RT history were independent risk factors to predict ISE on CE-T2FLAIR, regardless of gender. (Odds ratio; age: 1.1, 1.0 ~ 1.1; microbleeds: 1-4: 2.9, 1.5 ~ 5.8; ≥5: 7.3, 2.6 ~ 21.0; RT: 7.7, 2.1 ~ 28.0)
**Conclusion:** The incidence of ILE performed to evaluate brain metastasis was 18.1% . Significant independent risk factors for detecting ILE include age, microbleed, and RT history.
**Limitations:** Not applicable
**Funding for this study:** The study was supported by grant from the Central Medical Service (CMS) Co., Ltd. Research Fund.
**Has your study been approved by an ethics committee?** Yes
**Ethics committee - additional information:** Chungang University Hospital 2501-016-19560
**Author Disclosures:**
Jung Bin Lee: Author: Manuscript writing
Nam Hoon Kim: Speaker: Presentation
Younghee Yim: Research/Grant Support: Project administration
Leehi Joo: Consultant: Validation

### Metabolic, Microvascular, and Macrovascular DSC-PWI-Derived Metrics for Presurgical Differentiation of Glioblastoma and Solitary Brain Metastasis Across Different Acquisition Protocols

*C. García*[1], I. Martinez-Zalacain[2], P. Naval-Baudin[2], D. Choque[2], A. M. Viveros Castaño[2], M. Cos Domingo[2], C. Majós[2], A. Pons Escoda[2]; [1]Murcia/ES, [2]Hospitalet de Llobregat/ES
*(clemente292@gmail.com)*

**Purpose:** The aim of this study is to quantify the diagnostic performance of a multiparametric, bicompartmental perfusion analysis, and to establish technical-scenario-specific thresholds and classifiers for the presurgical differentiation between glioblastomas and metastases.
**Methods or Background:** Patients: Retrospective cohort with 101 glioblastomas IDH-wt (52 males, median age 61) and 52 metastases (29 males, median age 63). DSC-Perfusion: Double-acquisition protocol: 1st without pre-bolus, flip angle 75°; 2nd with pre-bolus (using prior contrast), flip angle 60°. Segmentations: Semi-automatic and 3D. Two tumor compartments: enhancing tumor and peritumoral T2-FLAIR abnormality (edema). Masks co-registered to DSC space. Perfusion metrics: Commercial AIF-based Bayesian vascular model. Voxel-wise and normalized to normal-appearing white matter. Macrovascular metrics: rCBV (relative-cerebral-blood-volume), rCBF (relative-cerebral-blood-flow), MTT (mean-transit-time). Microvascular: CTH (capillary-transit-time-heterogeneity), TTD (delay), COV (coefficient-of-variance). Metabolic: OEF (oxygen-extraction-fraction), rCMRO2 (relative-cerebral-metabolic-rate-of-oxygen). Data analysis: Computation of best single-metric/single-statistic performance per protocol and tumor compartment. Combination of metrics using logistic regressions on three-metric sets that maximized discrimination for each acquisition scenario and compartment, as well as bicompartmental models.
**Results or Findings:** Single-metric performance: The highest AUC was 0.78 for peritumoral rCBFmax in the second protocol. Remaining best single-metric AUCs: protocol-1/enhancing CTHp25 = 0.73; protocol-1/edema rCBFmax = 0.77; protocol-2/enhancing MTTmin = 0.74. Trivariable classifiers: Scenario-specific peak AUCs were protocol-2/enhancing 0.79 (MTTmin + TTDmean + rCMRO2mean); protocol-2/edema 0.79 (CTHmin + OEFmin + rCBFmax). Within protocols, bicompartmental models outperformed single-compartment models—protocol-1 0.82 (vs 0.75); protocol-2 0.80 (vs 0.79). All models ranged 0.75–0.83. In exploratory subanalysis, both single metrics and classifiers remained stable in lab-simulated heterogeneous settings, with AUCs 0.73–0.84.
**Conclusion:** Multiparametric bicompartmental perfusion analysis using innovative commercial software demonstrates excellent discriminatory power and robustness across technical settings. Strengthening biological plausibility, classifiers combining vascular and metabolic information from distinct tumor habitats achieved the highest performance.
**Limitations:** Single-centre, retrospective design.
**Funding for this study:** No funding was received for this study.
**Has your study been approved by an ethics committee?** Yes
**Ethics committee - additional information:** The study was approved by the Research Ethics Committee of Hospital Universitari de Bellvitge.
**Author Disclosures:**
Carlos Majós: Nothing to disclose
Pablo Naval-Baudin: Nothing to disclose
Albert Pons Escoda: Nothing to disclose
Ignacio Martinez-Zalacain: Nothing to disclose
Mónica Cos Domingo: Nothing to disclose
Ady Mildred Viveros Castaño: Nothing to disclose
Clemente García: Nothing to disclose
Diego Choque: Nothing to disclose

### EGFR Mutation Subtypes Influence MRI Patterns and Survival Outcomes in Leptomeningeal Metastasis from Lung Adenocarcinoma with Intraventricular Chemotherapy via Ommaya Reservoir

*Y. Jia*, M. Yuan; Nanjing/CN
*(zhenyijia2480@126.com)*

**Purpose:** Leptomeningeal metastasis (LM) is a devastating complication of advanced lung adenocarcinoma (LUAD), with high prevalence in EGFR-mutant patients. Imaging and prognostic differences among EGFR subtypes remain poorly understood, especially in those receiving intraventricular chemotherapy via Ommaya reservoir. This study investigated MRI features and survival outcomes across EGFR mutation subtypes in this context.

**Methods or Background:** In this multicenter retrospective study, LUAD patients with cerebrospinal fluid (CSF)-confirmed LM (November 2021–June 2024) underwent contrast-enhanced brain MRI and CSF-based next-generation sequencing (NGS). Patients were classified as EGFR exon 21 L858R (EGFR 21), exon 19 deletion (EGFR 19), or non-classic/other mutations (NCOM). Imaging features were compared using chi-square tests, and intracranial progression-free survival (iPFS) and overall survival (OS) were analyzed with Kaplan–Meier and Cox regression models.
**Results or Findings:** A total of 110 patients (mean age 55.6 ± 10.2 years) were included. MRI-negative LM was more frequent in EGFR 19 (65.5%) versus EGFR 21 (34.9%) and NCOM (34.2%) ($p = 0.01$). EGFR 21 was associated with localized disease (<4 lobes: 85.7%) compared with NCOM (≥4 lobes: 45.5%) ($p = 0.01$). Median iPFS and OS were longer in EGFR 21 (12.0 and 18.0 months) than in NCOM (6.5 and 10.2 months). EGFR 19 conferred no survival benefit. EGFR subtype independently predicted iPFS ($p = 0.03$), but not OS ($p = 0.12$).
**Conclusion:** EGFR mutation subtypes are linked to distinct MRI features and survival outcomes in LUAD patients with LM treated via Ommaya reservoir, providing insights for diagnosis, risk stratification, and treatment planning.
**Limitations:** This study is limited by modest sample size and lack of CSF biomarker correlation. The high proportion of MRI-negative LM in EGFR 19 underscores the need for multimodal imaging and molecular integration in future multicenter studies.
**Funding for this study:** None
**Has your study been approved by an ethics committee?** Yes
**Ethics committee - additional information:** he First Affiliated Hospital of Nanjing Medical University Ethics Review Board
**Author Disclosures:**
Mei Yuan: Nothing to disclose
Yizhen Jia: Nothing to disclose

**Differentiating Radiation-Induced Brain Injury from Tumor Recurrence in Brain Metastases after Radiotherapy: A Deep Learning Model Integrating MRI and Clinical Data**
Y. Liang, Z. Chen, *M. Ge*, Y. Wang; Beijing/CN
*(2234823639@qq.com)*

**Purpose:** Differentiating radiation-Induced brain Injury (RIBI) from tumor recurrence (TR) in brain metastases after stereotactic radiosurgery (SRS) remains a significant clinical challenge due to their similar appearance on conventional MRI. As management strategies differ substantially, this study aims to develop a deep learning model integrating multiparametric MRI with clinical data for accurate discrimination.
**Methods or Background:** This retrospective study enrolled 82 patients with brain metastases treated with SRS between January 2016 and December 2024. Diagnosis was confirmed by pathology or ≥6 months of follow-up. Based on post-radiation evolution, lesions were classified as RIBI (49 lesions) if showing initial growth followed by spontaneous regression or stability >6 months, or as TR (43 lesions) if demonstrating continuous progression. Multiparametric MRI sequences (T1WI, contrast-enhanced T1WI, T2WI) were analyzed, with all lesions manually delineated by radiologists. A deep learning model was developed to fuse imaging features with embedded clinical data (including patient demographics, tumor characteristics, and treatment parameters) for end-to-end classification. Model performance was evaluated via cross-validation using accuracy, sensitivity, specificity, and AUC.
**Results or Findings:** The integrated model demonstrated high performance, achieving an accuracy of 91.30%, sensitivity of 84.21%, specificity of 97.14%, and AUC of 0.92. Compared to the MRI-only model (accuracy 89.13%, AUC 0.885), clinical data integration improved accuracy by 2% and AUC by 0.035, validating the added value of multi-modal fusion.
**Conclusion:** The deep learning model integrating MRI and clinical data shows significant potential for accurately differentiating RIBI from TR in post-SRS brain metastases, providing a high-precision, non-invasive tool for clinical decision-making that may help avoid unnecessary invasive procedures.
**Limitations:** Not applicable
**Funding for this study:** Not applicable
**Has your study been approved by an ethics committee?** Not applicable
**Author Disclosures:**
Zelong Chen: Nothing to disclose
Menglin Ge: Nothing to disclose
Yuhan Liang: Nothing to disclose
Yulin Wang: Nothing to disclose

**Percentage signal recovery and relative cerebral blood volume: a dual-parameter strategy to differentiate post-stereotactic radiosurgery tumour progression from radiation necrosis in brain metastases**
V. Sawlani[1], *N. Mei*[2], R. Flintham[1], S. Meade[1], H. Benghiat[1], S. Nagaraju[1], U. Pohl[1], P. Sanghera[1], V. Wykes[1]; [1]Birmingham/UK, [2]Shanghai/CN

**Purpose:** Stereotactic radiosurgery (SRS) is widely used for brain metastases, but differentiating tumour progression from radiation necrosis on conventional MRI remains difficult. Percentage signal recovery (PSR), derived from dynamic susceptibility contrast (DSC) perfusion MRI, reflects signal recovery post-contrast and offers insights into capillary permeability. This study aimed to evaluate PSR and relative cerebral blood volume (rCBV) and assess their combined diagnostic value in post-SRS brain metastases.
**Methods or Background:** Patients with enlarging post-SRS brain metastases and diagnostic uncertainty were retrospectively included. PSR and rCBV were extracted from DSC-MRI and normalized to contralateral white matter. The dataset was split into training and validation cohorts using stratified sampling. Logistic regression with 5-fold cross-validation and bootstrap validation was used. Diagnostic performance was assessed by ROC analysis.
**Results or Findings:** Sixty-one patients (62 lesions; 26 progression, 36 necrosis) were included. Progression showed higher rCBV (2.84 vs. 0.76) and lower PSR (95% vs. 176%) (both $p < 0.001$). Both were significant in univariate analysis; PSR remained independently predictive ($p = 0.04$) in multivariate analysis. PSR outperformed rCBV in ROC analysis (AUC = 0.960 vs. 0.898); the combined model improved accuracy (95.9%) without loss of sensitivity or specificity. Bootstrap-derived thresholds were 108% (PSR) and 1.96 (rCBV). A nomogram was developed for individualized risk estimation.
**Conclusion:** PSR and rCBV provide complementary diagnostic information for post-SRS lesion assessment. PSR offers added value without additional scanning, and integration of both parameters enhances diagnostic confidence. Routine inclusion of PSR and rCBV in post-SRS imaging protocols is recommended.
**Limitations:** This retrospective single-centre study may be subject to selection bias and limited generalizability. Using maximum rCBV and PSR values likely improved sensitivity for detecting focal tumour recurrence but may introduce variability in heterogeneous lesions; prospective multicentre validation is warranted.
**Funding for this study:** None
**Has your study been approved by an ethics committee?** Yes
**Ethics committee - additional information:** Institutional Review Board of Queen Elizabeth Hospital Birmingham
**Author Disclosures:**
Helen Benghiat: Nothing to disclose
Sara Meade: Nothing to disclose
Santhosh Nagaraju: Nothing to disclose
Ute Pohl: Nothing to disclose
Victoria Wykes: Nothing to disclose
Nan Mei: Nothing to disclose
Paul Sanghera: Nothing to disclose
Vijay Sawlani: Nothing to disclose
Robert Flintham: Nothing to disclose

**PSR: an accurate marker in differentiating between radionecrosis and progression of brain metastases treated with stereotactic radiosurgery**
*Z. H. Chen Zhou*, A. Hilario Barrio, E. Salvador Alvarez, A. Cardenas, J. Romero Coronado, C. Lechuga Vázquez, A. Martínez De Aragón Calvo, R. D´Ambrosi, A. Ramos Gonzalez; Madrid/ES
*(zhaohuichenzhou@gmail.com)*

**Purpose:** Stereotactic radiosurgery (SRS) is the treatment of choice for local control of isolated brain metastases. However, after SRS, it is common for metastases to show increased enhancement, making it difficult to distinguish between tumour progression and treatment-related effects. DSC perfusion is useful for evaluating haemodynamic changes, with rCBV being the most extensively studied parameter. Nevertheless, the percentage signal recovery (PSR), which is an indicator of blood–brain barrier integrity, has been assessed less frequently. This study aimed to compare the diagnostic performance of rCBV and PSR.
**Methods or Background:** A retrospective analysis was performed on patients with brain metastases who were treated with SRS between January 2020 and December 2024. The patients showed increased post-treatment enhancement and had available DSC perfusion. The final diagnosis of progression or radionecrosis was established by either surgical resection or clinical–radiological follow-up. Images were processed to obtain rCBV and PSR values. Statistical tests (Mann–Whitney U and Student's t-test) were applied to compare the two groups.

**Results or Findings:** A total of 79 treated brain metastases that subsequently grew were included in the analysis (45 cases of radionecrosis and 34 cases of progression). rCBV was significantly higher in cases of tumour progression (p < 0.001), while PSR was significantly lower (p < 0.001). Using a cut-off value of 1.7, rCBV achieved a sensitivity of 91.2%, a specificity of 60%, and an area under the curve (AUC) of 0.87. For PSR, the cutoff value was 70%. Sensitivity was 94.1%, specificity was 97.8%, and the AUC was 0.99.
**Conclusion:** Although traditionally overlooked, PSR is more accurate than rCBV at differentiating tumour progression from radionecrosis after radiosurgery. This establishes PSR as a highly useful diagnostic tool in clinical practice.
**Limitations:** Retrospectiva and single center study.
**Funding for this study:** No funding.
**Has your study been approved by an ethics committee?** Yes
**Ethics committee - additional information:** The hospital's local ethics committee affirms that it complies with ethical standards and good clinical practice.
**Author Disclosures:**
Amaya Hilario Barrio: Nothing to disclose
Carmen Lechuga Vázquez: Nothing to disclose
Elena Salvador Alvarez: Nothing to disclose
Ana Martínez De Aragón Calvo: Nothing to disclose
Agustin Cardenas: Nothing to disclose
Ana Ramos Gonzalez: Nothing to disclose
Rafael D´Ambrosi: Nothing to disclose
Juan Romero Coronado: Nothing to disclose
Zhao Hui Chen Zhou: Nothing to disclose

**Prediction of incidental asymptomatic meningioma at high risk of tumor growth using a multiparametric MRI-based machine learning approach**
*N. Mei*[1], Y. Chen[2], V. Sawlani[3], X. Li[1], J. Cui[1], Z. Zheng[4], D. Wang[1], Y. Lu[1], B. Yin[1]; [1]Shanghai/CN, [2]Jiangsu/CN, [3]Birmingham/UK, [4]Shandong/CN

**Purpose:** Tumor growth imposes a considerable psychological impact on patients with incidental asymptomatic meningiomas. This study aimed to identify clinical, semantic, and multiparametric MRI features associated with growth potential and to develop a machine learning model for risk prediction, thereby informing personalized surveillance and management strategies.
**Methods or Background:** This retrospective multi-center study enrolled adult patients with incidental asymptomatic meningiomas confirmed by routine MRI. Tumors were manually segmented on CE-T1WI images. Radiomics features were extracted from CE-T1WI, T2-FLAIR, and ADC images and selected using correlation and Cox regression analyses. A random survival forest model was developed with five-fold cross-validation to predict tumor growth. Model performance was assessed by C-index and time-dependent ROC curves. Risk stratification was evaluated using Kaplan-Meier analysis.
**Results or Findings:** 421 patients with incidental asymptomatic meningiomas from Institution A were randomly split into training, validation, and testing sets, with an independent external validation set comprising 39 patients from Institutions B and C. Eleven significant predictors were incorporated into a random survival forest model, which demonstrated strong performance with C-indices of 0.928, 0.874, 0.872, 0.860 in the training, validation, testing, and external validation sets, respectively. The model achieved consistently high time-dependent AUCs (> 0.80) at 1-, 2-, 3-, and 5-year follow-up, and stratified patients into significantly low and high growth-risk groups on Kaplan-Meier analysis.
**Conclusion:** Our MRI-based machine learning model reliably predicts growth risk in incidental asymptomatic meningiomas, enabling personalized surveillance. This may improve clinical decision-making and reduce unnecessary interventions and, importantly, patient anxiety.
**Limitations:** Patient inclusion was based on meningiomas identified at initial MRI, which may have led to rare misclassification of solitary fibrous tumor; however, follow-up likely minimized this risk. Only reproducible, interpretable radiomics features were included to reduce redundancy and overfitting.
**Funding for this study:** This work is sponsored by the Explorers Program of Shanghai (Grant no. 24TS1410800) and the National Natural Science Foundation of China (82281966).
**Has your study been approved by an ethics committee?** Yes
**Ethics committee - additional information:** Institutional Review Board of Huashan Hospital
**Author Disclosures:**
Bo Yin: Nothing to disclose
Yiping Lu: Nothing to disclose
Yao Chen: Nothing to disclose
Dongdong Wang: Nothing to disclose
Xuanxuan Li: Nothing to disclose
Jin Cui: Nothing to disclose
Nan Mei: Nothing to disclose
Vijay Sawlani: Nothing to disclose
Zhisong Zheng: Nothing to disclose

**Imaging Perspectives on Angiomatous Meningioma and Solitary Fibrous Tumor: Diagnostic Challenges and Differentiation**
*H-H. Chen*, F-C. Chang; New Taipei City/TW
*(drchenhsiaohsuan@gmail.com)*

**Purpose:** Intracranial solitary fibrous tumors(SFT) are rare hypervascular dural based tumor, which showed overlapping imaging appearance with angiomatous meningioma(AM), another hypervascular dural based tumor. This study aim to differentiate the two different tumor on a routine MRI sequence.
**Methods or Background:** We retrospectively reviewed the pathology database at VGHTPE from January 2015 to September 2025, and included 20 cases of SFT and AM respectively. Preoperative MRI features was analyzed. Group comparisons were performed using chi-square or t-tests.
**Results or Findings:** SFT typically demonstrated T1 and T2 isointensity, while AM more often showed T1 hypointensity and T2 hyperintensity. Both tumors were hypervascular with marked enhancement and no significant restricted diffusion overall. Although AM more frequently exhibited mild restricted diffusion in peripheral region. The presence of peripheral restricted diffusion showed a sensitivity of 50%, specificity of 85%, and overall accuracy of 67.5% in identifying AM.
**Conclusion:** AM and SFT may exhibit overlapping imaging features but differ markedly in biological behavior and prognosis. Accurate differentiation is essential for guiding appropriate therapeutic strategies. Peripheral restricted diffusion appears to be a promising imaging marker for distinguishing AM from SFT.
**Limitations:** Limitations include small sample size and retrospective single-center design. Possible bias due to manual drawing and visual positioning of the ROI.
**Funding for this study:** Not applicable
**Has your study been approved by an ethics committee?** Not applicable
**Author Disclosures:**
Hsiao-Hsuan Chen: Nothing to disclose
Feng-Chi Chang: Nothing to disclose

**Correlation of Elastography Stiffness with ADC and Conventional MRI Sequences in Meningiomas**
*T. Orhan*, M. Oral, Z. Firat, G. Ekinci; Istanbul/TR
*(tolgaorhan_95@hotmail.com)*

**Purpose:** To investigate the relationship between magnetic resonance (MR) elastography-derived stiffness values and diffusion (ADC) as well as conventional MR signal intensity ratios (SIR) in meningiomas
**Methods or Background:** We retrospectively analyzed 40 patients with histologically proven meningiomas who underwent preoperative MRI, including elastography. Elastography stiffness scores were compared with ADC values and SIRs from T1, T2, and FLAIR sequences.
**Results or Findings:** Elastography scores showed a significant negative correlation with ADC values (p<0,01), indicating that stiffer tumors tend to have lower ADC. In contrast, no significant associations were found between elastography and SIR values on T1, T2, or FLAIR sequences (p > 0.05 for all). A secondary finding was noted: in the subgroup of patients with elevated T2 SIR (>1) but reduced FLAIR SIR (<1), elastography scores were higher (median = 4) compared to other patients (median = 3). This difference was statistically significant (p<0.05).
**Conclusion:** Preoperative elastography stiffness is inversely related to ADC in meningiomas, suggesting complementary roles of these imaging biomarkers. While conventional SIR values alone did not correlate with stiffness, the observation of high elastography scores in tumors with a “T2/FLAIR mismatch” may represent a promising secondary marker. Further studies with larger cohorts are warranted to validate this novel finding.
**Limitations:** Lack of direct correlation between the elastography stiffness values and a gold standard Retrospective study Small sample size Single centred study
**Funding for this study:** None
**Has your study been approved by an ethics committee?** Yes
**Ethics committee - additional information:** The institutional review board approved this retrospective study (Yeditepe University, IRB #E.83321821-805.02.03-624, 18.04.2025).
**Author Disclosures:**
Gazanfer Ekinci: Nothing to disclose
Zeynep Firat: Nothing to disclose
Müge Oral: Nothing to disclose
Tolga Orhan: Nothing to disclose

**Association Between Facet Joint Orientation, Facet Tropism, and Degenerative Lumbar Spondylolisthesis**
*B. Akgun*, E. Gülay, B. Baysal; Istanbul/TR
*(burcin.akgun@hotmail.com)*

**Purpose:** Spondylolisthesis is defined as the anterior displacement of one vertebra relative to the caudal vertebra.This study aimed to evaluate the relationship between facet tropism and disc and facet joint degeneration in degenerative lumbar spondylolisthesis and to identify potential radiographic risk factors by comparison with healthy controls.
**Methods or Background:** Seventy patients with DLS at L3/4, L4/5, and L5/S1 and seventy controls were included.Lumbar MR images were independently assessed by two radiology resident.Intervertebral disc degeneration was graded using the Pfirrmann system, while facet joint degeneration was evaluated with Weishaupt and Fujiwara scores.Right and left facet angles and tropism were recorded.The reference plane was defined as a line through the disc midline and base of the spinous process;the facet line connected the anteromedial and posteromedial borders of the superior articular facet.Facet angle was calculated between the reference plane and facet line. Tropism was defined as an asymmetry ≥8°. Inter-observer agreement was assessed with Cohen's Kappa; correlations were analyzed using Spearman's test.
**Results or Findings:** Among patients,65.5% were male and 34.5% female. DLS involvement was 43.7% at L4-5, 40.8% at L5-S1, and 15.5% at L3-4.Pfirrmann scores at L5-S1 were significantly higher in DLS than controls($p<0.05$), while L3-4 and L4-5 showed no difference.Weishaupt and Fujiwara scores at L4-5 were significantly higher in DLS($p<0.05$); other levels showed no difference.Facet tropism prevalence did not differ significantly. Right-sided facet angle negatively correlated with Weishaupt and Fujiwara scores($p<0.05$); no significant left-sided correlation was observed.At L3-4 and L5-S1, tropism was associated with higher degeneration scores($p<0.01$).
**Conclusion:** Facet joint degeneration, along with facet morphology, may play important roles in DLS development.These findings suggest that facet morphology and tropism may be linked to degeneration and could serve as potential radiographic risk factors in DLS.
**Limitations:** No limitations were identified.
**Funding for this study:** No funding was provided for this study.
**Has your study been approved by an ethics committee?** Yes
**Ethics committee - additional information:** Istanbul Medeniyet University non-interventional health research ethics committee
**Author Disclosures:**
Burcin Akgun: Nothing to disclose
Begumhan Baysal: Nothing to disclose
Ertuğrul Gülay: Nothing to disclose

**Intraoperative MRI for Resection of Intramedullary Spinal Cord Tumors: A Case Series of 15 Patients**
*K-C. Lin*, Y. Huang; Taipei City/TW
*(kerrapple@gmail.com)*

**Purpose:** Intraoperative MRI (ioMR) is an underutilized examination for facilitating resection of intramedullary spinal cord tumors. In the current case series, the authors present their technical and interpretation-related experiences with ioMR.
**Methods or Background:** A total of 15 patients who underwent ioMR for intramedullary spinal cord tumor resection from April to December 2024 were retrospectively included in this single-center series. Unique intraoperative imaging findings were evaluated (intraoperative contrast leakage [IOCL], blood product in the surgical cavity, residual high T2 signal in the spinal cord, and suspicious nodular enhancement), and ioMR imaging quality was assessed on a Likert scale. Technical details were recorded for ioMR sequences.
**Results or Findings:** The average scanning time was 58.9 ± 8.7 minutes. Residual tumors were identified in eight patients (53.3%), and two patients (13.3%) underwent additional resection. IOCL was the most prevalent imaging finding, observed in 73.3%–80.0% of patients, with good interobserver agreement. The median imaging quality was acceptable (Likert scale = 3), as assessed by two reviewers, with thoracic spine images exhibiting the worst quality among all segments.
**Conclusion:** Total resection can be achieved in patients with intramedullary spinal cord tumors under the assistance of ioMR. Radiologists should note unique imaging findings that have been previously identified, with particular attention given to IOCL, to correctly interpret intraoperative spine MRI.
**Limitations:** First, the relatively small number of patients with heterogeneous tumor pathology and surgical goals may render our experiences non-generalizable experience to all patients undergoing intramedullary tumor resection. Second, the average ioMR scan time was approximately 1 hour, and this time increased with patient transfer. Whether the benefits of ioMR for surgical outcomes justify the longer operating time and greater resources expended should be examined in further studies.
**Funding for this study:** Nil
**Has your study been approved by an ethics committee?** Not applicable
**Author Disclosures:**
Yucheng Huang: Nothing to disclose
Keng-Chian Lin: Nothing to disclose

**CoMPaSS-NMD: Unlocking genotype-phenotype associations in neuromuscular disorders on MRI with machine learning**
*L. Piorecki*[1], M. Socha[1], J. Verdú-Díaz[2], J. Diaz-Manera[2], V. Straub[2], R. Tupler[3], J. Polanska[1]; [1]Poland/PL, [2]Newcastle/UK, [3]Modena/IT
*(lukasz.piorecki@polsl.pl)*

**Purpose:** Neuromuscular disorders (NMDs) are genetically heterogeneous and often display subtle, visually indistinct MRI patterns. Automated stratification and classification may facilitate diagnosis, enable patient grouping, and enhance understanding of genotype–phenotype associations in NMD.
**Methods or Background:** The original dataset consisted of 1,351 T1-weighted MRI scans of the lower limbs from 616 patients with 9 different genetically confirmed neuromuscular disorders, collected within the MYO-GUIDE project between 2023 and 2025. The dataset was cleaned, and the MRI scans were preprocessed by removing skin, subcutaneous tissue and osseous structures. The thigh region in the MR volumes was found by referencing the femur. After cleaning, the data were partitioned into training, validation, and hold-out subsets, containing 139, 47, and 51 scans, respectively, with multiple timepoints of the same patient assigned exclusively to one subset. Three feature extraction approaches were investigated: two based on radiomic descriptors and one employing a convolutional autoencoder. Feature dimensionality was reduced using Boruta and CellBRF selection algorithms. Classifier optimization was performed using four-fold cross-validation with a grid search across Logistic Regression (LR), Decision Tree (DT), Random Forest (RF), and Support Vector Machine (SVM) models, as well as their parameters, for each feature selection method. The best classifier was chosen based on the weighted AUC.
**Results or Findings:** For radiomic descriptors, Boruta outperformed CellBRF, whereas CellBRF performed better for autoencoder-derived features with higher dimensionality. The highest classification metrics were achieved for radiomics per pie slice feature space, reduced by Boruta and LR as the classifier. Achieving a weighted AUC metric of 0.87 and 0.90 for the validation and holdout subsets, respectively.
**Conclusion:** This approach suggests the potential of MRI-based automation to support differential diagnosis in neuromuscular disorders and contribute to the development of imaging biomarkers.
**Limitations:** Not applicable.
**Funding for this study:** This work was financed by CoMPaSS-NMD, Computational Models for new Patients Stratification Strategies of Neuromuscular Disorders, HORIZON RIA, Tools and Technologies for Healthy Society, ID: GAP-101080874
**Has your study been approved by an ethics committee?** Not applicable
**Author Disclosures:**
José Verdú-Díaz: Nothing to disclose
Marek Socha: Nothing to disclose
Lukasz Piorecki: Nothing to disclose
Joanna Polanska: Nothing to disclose
Rossella Tupler: Nothing to disclose
Volker Straub: Nothing to disclose
Jordi Diaz-Manera: Nothing to disclose

### Friday, March 6

**08:00-09:00** **Research Stage 1**

### Research Presentation Session: Abdominal and Gastrointestinal

### RPS 1201

### Technical advances in abdominal imaging

**Moderator**
Sergio Grosu; Munich/DE

**Enhancing Image Quality and Diagnostic Confidence through AI-Based Spectral Reconstruction in Abdominal Imaging**

*L. Hieronymi*[1], J. Lueckel[1], S. Skornitzke[2], N. Große Hokamp[1], D. Maintz[1]; [1]Cologne/DE, [2]Hamburg/DE

**Purpose:** To evaluate a prototype deep learning–based spectral reconstruction algorithm (SAI, Philips) for spectral detector dual-energy CT (sdDECT) in abdominal imaging. The algorithm aims to reduce image noise while preserving texture in both conventional and virtual monoenergetic images (VMI), addressing known limitations of low-keV VMI.
**Methods or Background:** This retrospective study included 67 patients undergoing contrast-enhanced abdominal sdDECT. Conventional images were reconstructed with hybrid-iterative (HI-R) and spectral algorithm (SAI-R). VMI (40–200keV) were generated accordingly (HI-VMI, SAI-VMI). Quantitative analysis involved 22 ROIs in the liver, pancreas, spleen, kidneys, psoas muscle, and fat. Signal-to-noise and contrast-to-noise ratios (SNR/CNR) were calculated. Two blinded radiologists compared randomized, patient-matched images (HI-R/VMI vs. SAI-R/VMI, 40–70keV) across liver, pancreas, and kidney, rating image quality, noise, texture, lesion conspicuity, and diagnostic confidence using a two-alternative forced choice design. Statistical analysis was performed with ANOVA and Tukey's post hoc test.
**Results or Findings:** Attenuation was higher in low-keV VMI (40–60keV) compared to conventional reconstructions ($p \leq 0.05$). Noise increased with decreasing keV, with SAI-VMI showing lower noise than HI-VMI at all levels (e.g., muscle at 40keV: 14.39±4.88HU vs. 16.15±4.90HU; $p \leq 0.05$). Importantly, SAI-VMI demonstrated lower noise at low-keV compared to HI-VMI at higher keV, highlighting superior noise performance at lower energy levels (e.g., liver: SAI at 40keV 12.17±3.71HU vs. HI at 60keV 13.10±3.34HU; $p \leq 0.05$). SNR and CNR were significantly higher with SAI-R and SAI-VMI, particularly at low-keV (e.g. liver CNR at 40 keV: SAI-VMI 8.18±2.67 vs. HI-VMI 7.15±2.41; $p \leq 0.05$). Reader preference strongly favored SAI-R/VMI (averaged: HI-R 0.83%±1.31% vs. SAI-R 31.67%±4.97%; $p \leq 0.05$). SAI-VMI at 40keV was preferred over all other reconstructions (97.92%±2.36%, $p \leq 0.05$).
**Conclusion:** AI-based spectral reconstruction improves abdominal sdDECT by reducing noise and enhancing SNR/CNR, while further increasing diagnostic benefits of VMI.
**Limitations:** The study is limited by its retrospective design.
**Funding for this study:** This work was funded by Philips Healthcare. The funding source had no involvement in study design, collection or interpretation of data.
**Has your study been approved by an ethics committee?** Yes
**Ethics committee - additional information:** This study is IRB-approved.
**Author Disclosures:**
Nils Große Hokamp: Nothing to disclose
David Maintz: Nothing to disclose
Lina Hieronymi: Nothing to disclose
Julia Lueckel: Nothing to disclose
Stephan Skornitzke: Employee: Philips GmbH Market DACH

**Improved visualization of pancreas and tumor boundaries using high-frequency kernels with deep-learning image reconstruction at high-strength level**

*N. Kawai*, Y. Noda, T. Kaga, K. Matsuoka, S. Omata, Y. Takai, M. Asano, H. Kato, M. Matsuo; Gifu/JP

**Purpose:** The combination of high-frequency reconstruction kernels and deep-learning image reconstruction (DLIR) may enhance structure edges while maintaining image quality in abdominal CT. This study aimed to investigate the feasibility of combining high-frequency reconstruction kernels and DLIR at high-strength level (DLIR-H) for improving visualization of the pancreas and tumor boundaries on pancreatic protocol CT.
**Methods or Background:** This retrospective study included 30 patients (median age, 75 years; 16 women; median body mass index, 22 kg/m²) who underwent pancreatic protocol CT for assessing pancreatic tumors from January 2024 to July 2024. Four image sets were reconstructed using DLIR-H in combination with either standard, bone, bone-plus, or lung kernels. Edge sharpness between the pancreas and retroperitoneal fat tissue (pancreas-to-fat) and between the pancreas and pancreatic ductal adenocarcinoma (pancreas-to-PDAC) was quantitatively assessed using edge rise slope (ERS) measurements. Two radiologists qualitatively examined the sharpness of the pancreas, tumor boundary, and overall image quality.
**Results or Findings:** Pancreas-to-fat ERS was greater in lung kernel images than in standard and bone kernel images (P = 0.001). Pancreas-to-PDAC ERS was greater in lung kernel images than in other kernel images (P < 0.001). Sharpness of the pancreas and tumor boundaries was better in lung kernel images than in other kernel images (P < 0.001 for both). Overall image quality in lung kernel images was comparable to the standard and superior to the bone kernel (P < 0.001).
**Conclusion:** The combination of lung kernel and DLIR-H in pancreatic protocol CT improves both quantitative and qualitative sharpness of the pancreas and tumor boundaries while maintaining the overall image quality.
**Limitations:** Limitations include small sample size, lower body mass index than Western populations, no diagnostic performance assessment, and restriction to one vendor and DLIR-H.
**Funding for this study:** No funding was received for this study.
**Has your study been approved by an ethics committee?** Yes
**Ethics committee - additional information:** Medical Research Ethics Review Committee, Gifu University Graduate School of Medicine
**Author Disclosures:**
Tetsuro Kaga: Nothing to disclose
Yukiko Takai: Nothing to disclose
Masashi Asano: Nothing to disclose
Yoshifumi Noda: Nothing to disclose
Masayuki Matsuo: Nothing to disclose
Nobuyuki Kawai: Nothing to disclose
Kota Matsuoka: Nothing to disclose
Shingo Omata: Nothing to disclose
Hiroki Kato: Nothing to disclose

**Assessing Technical Performance of 3T Spin-Echo EPI Liver MR Elastography: Insights from a Large Tertiary Cohort**

*A. Y. Yitik*, R. Ambros, S. Ba-Ssalamah, S. Pötter-Lang, N. Bastati-Huber, A. Kristic, V. Vetchy, U. I. Attenberger, A. Ba-Ssalamah; Vienna/AT
*(ahmetyasinyitik@gmail.com)*

**Purpose:** To evaluate determinants of technical failure of 3T liver MR elastography (MRE) using a 2D spin-echo EPI sequence in a large tertiary cohort.
**Methods or Background:** This retrospective study included 2,731 consecutive patients (mean age 55 ± 6 years; 52% male) who underwent 3T MRE between January 2020 and December 2023. Exams were performed with a 2D spin-echo EPI sequence. Two radiologists independently analyzed MRE and anatomic images. Liver iron concentration (LIC) and proton density fat fraction (PDFF) were measured on a 3D multi-echo GRE sequence. Technical failure was defined as the absence of pixels with >95% confidence index and/or lack of visible shear waves. Logistic regression was used to identify predictors of failure.
**Results or Findings:** The overall failure rate was 8% (205/2,731). On univariate analysis, higher body mass index (BMI), liver iron deposition, massive ascites, T1-mapping values, MELD, APRI, and ALBI scores were significantly associated with failure (all p < 0.05). Age, sex, PDFF, and FIB-4 were not. Multivariable analysis identified BMI, liver iron deposition, and massive ascites as independent predictors. $R2^*$ demonstrated fair accuracy for predicting failure (AUC = 0.783, 95% CI: 0.694–0.872, p < 0.001) with an optimal threshold of ≥171 $s^{-1}$ at 3T.
**Conclusion:** The technical failure rate of 3T MRE using 2D spin-echo EPI was 8%. Massive ascites, iron overload, and elevated BMI were independent predictors of unsuccessful exams. Identifying these risk factors may guide patient selection and optimize MRE performance in clinical practice.
**Limitations:** This single-center retrospective study using 3T 2D SE-EPI MRE is subject to design and selection bias.
**Funding for this study:** None
**Has your study been approved by an ethics committee?** Yes
**Ethics committee - additional information:** Ethics approval number: 1759/2023

**Author Disclosures:**
Antonia Kristic: Nothing to disclose
Sarah Pötter-Lang: Speaker: Bayer
Raphael Ambros: Nothing to disclose
Sami Ba-Ssalamah: Nothing to disclose
Ahmed Ba-Ssalamah: Consultant: Bayer Speaker: Bayer, Siemens and Sanofi
Ahmet Yasin Yitik: Nothing to disclose
Ulrike I. Attenberger: Speaker: Siemens Healthineers Consultant: Bureau, Bayer
Nina Bastati-Huber: Nothing to disclose
Veronika Vetchy: Nothing to disclose

**Utility of slice selection gradient reversal and multi-shot echo planar imaging in rectal DWI at 3 tesla**

*N. Kawai*[1], Y. Noda[1], T. Kaga[1], Y. Ueda[2], M. Yoneyama[2], M. Asano[1], S. Ido[1], K. Kajita[1], M. Matsuo[1]; [1]Gifu/JP, [2]Tokyo/JP

**Purpose:** This study aimed to evaluate whether slice selection gradient reversal (SSGR) and multi-shot EPI (ms-EPI) could improve image quality compared with conventional single-shot EPI (ss-EPI) with spectral attenuated inversion recovery (SPAIR) in rectal DWI.
**Methods or Background:** This prospective study included 24 participants (median age, 70 years; 16 men) who underwent rectal MRI at 3T between March and August 2025. DWI was acquired using three sequences: ss-EPI with SPAIR, ss-EPI with SSGR, and ms-EPI with SSGR (two shots). Acquisition time was equalized by adjusting the number of signal averages (NSA; 8 for ss-EPI and 4 for ms-EPI). Two radiologists qualitatively assessed sharpness, distortion, artifact, lesion conspicuity, and overall image quality using a four-point Likert scale. Quantitative analysis included signal-to-noise ratio (SNR), signal intensity ratio (SIR), and apparent diffusion coefficient (ADC) value for rectal cancer.
**Results or Findings:** Rectal cancer was confirmed in 18 participants (75%). Sharpness, lesion conspicuity, and overall image quality were significantly better in the order of ms-EPI with SSGR, ss-EPI with SSGR, and ss-EPI with SPAIR images ($P < 0.001$ for all). Distortion and artifact were significantly reduced in ms-EPI with SSGR than in the other two images ($P < 0.001$ for both). SNR was higher in ss-EPI with SSGR than the other two images ($P < 0.001$). SIR was significantly higher in the order of ms-EPI with SSGR, ss-EPI with SSGR, and ss-EPI with SPAIR ($P < 0.001$). No significant differences were observed in ADC value ($P = 0.84$).
**Conclusion:** SSGR improved image quality compared with SPAIR. With equal acquisition times, ms-EPI showed lower SNR but was superior in reducing distortion and artifacts.
**Limitations:** Small sample size, use of a single-vendor 3T system, and adjustment of NSA to equalize scan time.
**Funding for this study:** No funding was received for this study.
**Has your study been approved by an ethics committee?** Yes
**Ethics committee - additional information:** Medical Research Ethics Review Committee, Gifu University Graduate School of Medicine
**Author Disclosures:**
Masami Yoneyama: Nothing to disclose
Tetsuro Kaga: Nothing to disclose
Yu Ueda: Nothing to disclose
Kimihiro Kajita: Nothing to disclose
Satoshi Ido: Nothing to disclose
Masashi Asano: Nothing to disclose
Yoshifumi Noda: Nothing to disclose
Masayuki Matsuo: Nothing to disclose
Nobuyuki Kawai: Nothing to disclose

**Contrast economy without diagnostic compromise: hospital-scale savings from a simple 1.0 mL/kg, low-kVp abdominal CT protocol**

J. T. d. S. d. Castro, H. d. J. Ferreira, D. C. Novais da Silva, *D. Yamada*, S. San Juan Dertkigil, F. Reis; Campinas - Sao Paulo/BR
*(danielay@unicamp.br)*

**Purpose:** Reducing iodine dose while maintaining diagnostic enhancement is crucial for patient safety, cost control, and environmental responsibility. The purpose was to quantify the per-patient and cohort-level contrast/iodine savings achieved after implementing a simple weight-based dosing table (1.0 mL/kg) aligned with low tube potential (80–100 kVp), compared with a conventional fixed-dose protocol.
**Methods or Background:** This was a single-center, real-world cohort study (pre/post implementation) analyzing 562 consecutive multiphasic abdominal CTs. Groups were: Conventional (pre-implementation, fixed dose) and three weight tiers in the post-implementation phase: G1 (≤90 kg @ 80 kVp), G2 (~91–120 kg @ 100 kVp), and G3 (≥120 kg, exploratory). A counterfactual baseline contrast volume was estimated for each post-implementation patient based on the Conventional group's median mL/kg for that weight band. Per-patient saving was calculated as baseline minus observed volume. Total cohort saving (L) and enhancement results (from Poster 1) were the primary outcomes.
**Results or Findings:** The new protocol delivered meaningful contrast savings across all groups: G1 (≤90 kg, n=232): Achieved the highest per-patient savings (mean 24.9 mL; median 27.2 mL). G2 (~91–120 kg, n=38): Showed modest but clear savings (mean 5.6 mL; median 6.4 mL). Total Savings: The cohort yielded a total saving of 7.40 L (G1) and 0.27 L (G2) within the dataset. Crucially, these substantial volume reductions co-occurred with higher proportions of exams meeting target portal enhancement (110–130 HU) and IRHR≥50 HU, indicating no diagnostic compromise.
**Conclusion:** A pragmatic 1.0 mL/kg weight-based dosing table integrated with 80–100 kVp delivers significant iodine/contrast savings at scale while successfully sustaining or improving diagnostic enhancement targets. This approach supports cost stewardship and environmental responsibility alongside demonstrable quality gains.
**Limitations:** Single-center, pre/post implementation cohort study, limiting the generalizability of specific savings figures to other institutions or protocols.
**Funding for this study:** Not applicable.
**Has your study been approved by an ethics committee?** Yes
**Ethics committee - additional information:** The work has been submitted to the university's Research Ethics Committee (CEP) and is currently under review for evaluation and final opinion.
**Author Disclosures:**
Hortência De Jesus Ferreira: Nothing to disclose
Fabiano Reis: Nothing to disclose
José Thiago De Souza De Castro: Nothing to disclose
Danielle Cristina Novais Da Silva: Nothing to disclose
Sergio San Juan Dertkigil: Nothing to disclose
Daniel Akio Yamada: Nothing to disclose

**Impact of monoenergetic imaging with second-generation dual-layer spectral CT on abdominal contrast enhancement and iodine dose reduction**

*A. Ciprani*, T. Polidori, G. Tremamunno, D. De Santis, F. Virgili, F. Pacelli, M. Zerunian, D. Caruso, M. Francone; Roma/IT
*(asiaciprani@gmail.com)*

**Purpose:** To evaluate and compare the effect on quantitative image quality of two different contrast medium (CM) injection protocols using conventional and virtual monoenergetic imaging (VMI) at 50keV.
**Methods or Background:** In this retrospective study, Group A (n=30) underwent abdominal CT with 700mgI/kg of CM based on lean body weight (LBW) using conventional images, while Group B (n=30) received 500mgI/kg of CM using spectral derived images at 50keV. Signal-to-noise ratio (SNR) and contrast-to-noise ratio (CNR) were measured in the liver, spleen, kidneys, pancreas, aorta, and portal vein during arterial and portal phases. Comparisons were performed with the Wilcoxon signed-rank test and Bonferroni correction, and effect sizes (Cohen’s d) were calculated.
**Results or Findings:** Despite the lower iodine dose, Group B showed higher image quality. In the arterial phase, pancreatic SNR increased from 25.4 to 40.7 (+60%) and aortic SNR from 18.2 to 46.0 (+153%, $p<0.001$). Pancreatic CNR increased from 15.1 to 24.2 (+60%) and aortic CNR from 20.5 to 52.1 (+154%, $p<0.001$). In the portal venous phase, liver SNR increased from 28.6 to 48.0 (+68%) and portal vein SNR from 22.4 to 38.5 (+72%), with CNR showing the same trend (all $p<0.001$). Although spectral reconstructions had slightly higher noise (8.3 vs. 7.5 HU, $p<0.05$), the overall increase in SNR and CNR was substantial. Effect size analysis confirmed these differences, with Cohen’s d >0.8 for most structures and >1.2 for aortic and hepatic measurements.
**Conclusion:** VMI at 50keV showed higher SNR and CNR than conventional reconstructions, despite lower iodine dose. Improvements were consistent in both arterial and portal phases, indicating that low-keV VMI enables iodine reduction without compromising image quality, with potential benefits for safety and diagnostic confidence.
**Limitations:** Single-center study with small sample size and no clinical impact assessment.
**Funding for this study:** None
**Has your study been approved by an ethics committee?** Not applicable
**Author Disclosures:**
Damiano Caruso: Nothing to disclose
Fiammetta Pacelli: Nothing to disclose
Federico Virgili: Nothing to disclose
Marta Zerunian: Nothing to disclose
Giuseppe Tremamunno: Nothing to disclose
Domenico De Santis: Nothing to disclose
Asia Ciprani: Nothing to disclose
Tiziano Polidori: Nothing to disclose
Marco Francone: Nothing to disclose

**Utility of 40 keV Virtual Monoenergetic Reconstructions in Preoperative Staging of Pancreatic Ductal Adenocarcinoma**

*N. É. Balogh*, B. K. Budai, A. I. Vigh, E. Tóth, Á. Szücs, A. Szijártó, P. Maurovich Horvat, I. Dudás; Budapest/HU

**Purpose:** We aimed to evaluate whether 40 keV virtual monoenergetic image (VMI) reconstructions from photon-counting detector CT (PCD-CT) offer improved diagnostic utility over standard 70 keV in preoperative assessment of pancreatic ductal adenocarcinoma (PDAC).
**Methods or Background:** Three-phase contrast-enhanced PCD-CT scans (arterial, portal, and venous) were analyzed in 50 patients with histologically confirmed PDAC. Lesion diameters, image quality, and diagnostic confidence (1–5 scale) were assessed at both 40 and 70 keV. Morphologic and staging features were compared; moreover, one radiologist rated whether 40 keV reconstructions aided interpretation.
**Results or Findings:** Lesion diameters did not differ significantly between 40 and 70 keV, though isolated cases showed discrepancies up to 18 mm. Image quality and diagnostic confidence were significantly higher at 40 keV across all phases (p < 0.001). Morphologic and staging features were highly consistent, with T-stage altered in only three patients. Subjective assessment indicated that 40 keV reconstructions were helpful in 86% of cases. The portal venous phase was perceived as the most beneficial, with 40 keV aiding interpretation in 37/50 (74%) patients, followed by the venous phase in 36/50 (72%) and the arterial phase in 21/50 (42%). In 16/50 (32%) patients, the reconstructions were helpful across all three phases, and in an additional 19/50 (38%) patients, in two phases.
**Conclusion:** 40 keV VMI reconstructions significantly improve subjective image quality and diagnostic confidence in PDAC imaging, without compromising objective lesion measurements or staging interpretation. Occasional large differences in tumor size and consistently positive subjective feedback support the clinical utility of 40 keV, particularly in the portal venous phase, highlighting its value in preoperative staging and surgical planning.
**Limitations:** This was a single-center study with a retrospective study design.
**Funding for this study:** No funding was received for this study.
**Has your study been approved by an ethics committee?** Yes
**Ethics committee - additional information:** The study was approved by the institutional ethics committee.
**Author Disclosures:**
Attila Szijártó: Nothing to disclose
Ákos Szücs: Nothing to disclose
Ibolyka Dudás: Nothing to disclose
András István Vigh: Nothing to disclose
Enikő Tóth: Nothing to disclose
Pál Maurovich Horvat: Nothing to disclose
Nóra Éva Balogh: Nothing to disclose
Bettina Katalin Budai: Nothing to disclose

**Automated CT-Based Liver Volumetry for Donor Weight Estimation: A Retrospective Study of 103 Cases**

*F. Minonne*, A. P. Micheletti, F. Giorgino, G. De Santis, S. Tibaldi, M. Moretti, R. Aroasio, M. Gatti, R. Faletti; Turin/IT
*(francescominonne97@gmail.com)*

**Purpose:** Accurate preoperative estimation of donor liver weight is a critical step in optimizing organ allocation and surgical planning for liver transplantation. This study evaluated the feasibility of automated computed tomography (CT)–based volumetric analysis to predict liver weight and its correlation with clinical parameters.
**Methods or Background:** We conducted a retrospective, single-center observational study at the Città della Salute e della Scienza di Torino, including 103 deceased donors between 2014 and 2025 (age range: 13–84 years). Each subject underwent abdominal CT close to organ procurement for orthotopic liver transplantation (OLT). Donors were stratified into two groups according to imaging protocol: non-contrast CT and contrast-enhanced CT. Each group was further split into a training cohort, used to refine weight estimation algorithms, and a validation cohort, designed to test predictive accuracy.
**Results or Findings:** Liver segmentation was performed using the open-source software TotalSegmentator. Mean processing time was under two minutes per scan. Across both imaging groups, predicted liver weight closely approximated actual graft weight at explant, with a mean absolute error of ±0.14 kg, corresponding to about 9% of the true value. This degree of precision was consistent between non-contrast and contrast-enhanced acquisitions.
**Conclusion:** These findings support the feasibility of fully automated, convolutional neural network–based approaches for liver volumetry in transplantation. The proposed workflow demonstrated speed, reproducibility, and satisfactory accuracy. By reducing the time and variability associated with manual volumetric assessments, this method could improve efficiency in clinical practice and enhance the reliability of preoperative planning in liver transplantation.
**Limitations:** No
**Funding for this study:** No
**Has your study been approved by an ethics committee?** Yes
**Ethics committee - additional information:** No Additional Information
**Author Disclosures:**
Anna Pia Micheletti: Nothing to disclose
Francesco Minonne: Nothing to disclose
Marco Gatti: Nothing to disclose
Fabio Giorgino: Nothing to disclose
Riccardo Faletti: Nothing to disclose
Marta Moretti: Nothing to disclose
Giovanni De Santis: Nothing to disclose
Stefano Tibaldi: Nothing to disclose
Roberta Aroasio: Nothing to disclose

**When Experts Disagree: Using AI to optimize splenic volumetry**

*E. Petrash*, K. Poliakov, M. Basova, M. Dugova, R. Gareeva, A. Zakharov, V. Gombolevskiy; Moscow/RU
*(cherkun.ekaterina@gmail.com)*

**Purpose:** Splenic volumetry is important for splenomegaly detection. The aim is to assess agreement and robustness of spleen volume estimation methods on CT. Manual linear measurements used for volume estimation may be slice- and reader-dependent, whereas AI segmentation is expected to provide reproducible, protocol-independent volumes once the software version is fixed.
**Methods or Background:** Experienced radiologists independently measured craniocaudal length (L), maximal diameter (D), and thickness (T) on abdominal CT and computed volumes via the formula V =30+0.58× L×D×T. The same scans were processed by a CNN-based tool Spleen-IRA which automatically extracted dimensions and volumes. Algorithm-generated masks were validated by two radiologists with more than 12 years experience, disagreements were reviewed to ensure quality. Verified AI measurements were designated as reference for splenic volume.
**Results or Findings:** Statistical tests demonstrated significant differences between readers and between reader-derived and AI-derived spleen volumes ($p < 10^{-7}$), underscoring the variability of manual measurements compared to the reproducibility of AI-based volumetry.
**Conclusion:** Manual spleen volumetry is time-consuming and inter-reader variable. Moreover, the observed expert disagreement suggests that the linear-measurement methodology itself is imperfect (landmarking and slice-selection dependent). AI segmentation delivers reader-independent spleen volumes without geometric approximations. With fixed inference settings, AI outputs are consistent on identical inputs. We therefore recommend AI segmentation as the reference method, and using the curated, quality-controlled mask dataset to audit legacy formulae and develop protocol-specific alternatives for settings where AI cannot be deployed.
**Limitations:** None
**Funding for this study:** IRA labs
**Has your study been approved by an ethics committee?** Not applicable
**Author Disclosures:**
Victor Gombolevskiy: Employee: IRA labs
Kirill Poliakov: Employee: IRA labs
Regina Gareeva: Employee: IRA labs
Alexey Zakharov: Employee: IRA labs
Mariya Dugova: Employee: IRA labs
Ekaterina Petrash: Employee: IRA labs
Maria Basova: Employee: IRA labs

08:00 - 09:00 Research Stage 4

### Research Presentation Session: Artificial Intelligence and Imaging Informatics

### RPS 1205

### Training tomorrow's radiologists and improving our clinical practice: the LLM-powered revolution

**Moderator**
Lukas Lambert; Prague/CZ
*(lambert.lukas@gmail.com)*
**Author Disclosures:**
Lukas Lambert: Advisory Board: Eli Lilly s.r.o.

**Structured data extraction from non-English brain MRI reports improves with few-shot prompting of an open-weight large language model**
*K. Mouheb*[1], A. Pomp[1], A. Manenti[2], H. Seelaar[1], F. Mattace-Raso[1], M. W. Vernooij[1], F. Wolters[1], S. Klein[1], E. Bron[1]; [1]Rotterdam/NL, [2]Toulouse/FR
*(k.mouheb@erasmusmc.nl)*

**Purpose:** Automatic data extraction from free-text radiology reports enables large-scale research, but current open-weight large language models (LLMs) may underperform because they lack domain knowledge. We evaluate whether few-shot prompting with annotated examples improves extraction from non-English neuroradiology reports.
**Methods or Background:** We analyzed 947 Dutch free-text brain MRI reports (2016-2021) from a memory clinic. Trained medical students annotated 24 variables. We used the open-weight LLM LLaMA 3.1 to extract these variables. In zero-shot prompting, the model received only task instructions. In few-shot prompting, we provided three annotated examples selected using three strategies: random, fixed, and structural similarity-based selection. Performance was evaluated using overall accuracy, balanced accuracy for categorical, accuracy for numerical, and text similarity for free-text fields, averaged across 10 random train-test splits.
**Results or Findings:** The overall accuracy of LLaMA 3.1 obtained with few-shot prompting with similarity-based selection (89%, [CI: 87–90%]) was significantly higher than that obtained with zero-shot prompting (81%, [81–82%]). Similar improvements were seen with other selection strategies (random: 84%, [82–86%]; fixed: 86% [83–90%]). Notably, few-shot prompting with similarity-based selection showed significantly higher accuracies in extracting microbleed counts (92% [90-93%]) and infarct counts (81% [77-85%]) compared to zero-shot prompting (microbleeds: 80% [78-82%], infarcts: 66% [63-68%]). Visual rating scores were extracted accurately in both settings, with slight gains from similarity-based few-shot prompting (balanced accuracy: 95% [92–97%] for Fazekas score, 92% [79–100%] for MTA, and 89% [85–93%] for GCA). Performance remained lower on location-specific variables (e.g., 69% for occipital GCA with similarity-based selection).
**Conclusion:** LLaMA 3.1 accurately extracted 24 clinical variables from neuroradiology reports. Few-shot prompting significantly improved performance, especially with similarity-based selection.
**Limitations:** The limitations of the study are that it involves a single LLM, one imaging modality, and reports from one center written in Dutch.
**Funding for this study:** This study was co-funded by an Erasmus MC fellowship 2022. The study is part of TAP-dementia (www.tap-dementia.nl), receiving funding from ZonMw (#10510032120003) in the context of Onderzoeksprogramma Dementie, part of the Dutch National Dementia Strategy. This work was co-funded by Scan2go, a TKI-LSH funded public-private partnership (LSHM22046-H036). This work was co-funded by the European Union under Grant Agreement number 1011100633. Views and opinions expressed are however those of the author(s) only and do not necessarily reflect those of the European Union. Neither the European Union nor the granting authority can be held responsible for them. This work used the Dutch national supercomputer Snellius with the support of the SURF Cooperative and a small compute grant from NWO using grant number EINF-11268.
**Has your study been approved by an ethics committee?** Yes
**Ethics committee - additional information:** The study was approved by the institutional Medical Ethics Committee with a waiver of informed consent (METC-2023-0569).
**Author Disclosures:**
Stefan Klein: Nothing to disclose
Antoine Manenti: Nothing to disclose
Amos Pomp: Nothing to disclose
Francesco Mattace-Raso: Nothing to disclose
Meike W. Vernooij: Nothing to disclose
Harro Seelaar: Nothing to disclose
Esther Bron: Nothing to disclose
Kaouther Mouheb: Nothing to disclose
Frank Wolters: Nothing to disclose

**Automated image quality assessment in shoulder MR arthrography using large language model-generated Python workflows**
*A. Petkoglou*[1], A. Vegter[1], T. Kwee[2], H. Stallmann[1]; [1]Stadskanaal/NL, [2]Groningen/NL

**Purpose:** To compare automated image sharpness measurements with manual signal and contrast to noise radio's (SNR, CNR) assessments and radiologist Likert scores in shoulder MR arthrography using saline versus gadolinium contrast.
**Methods or Background:** 40 patients aged 13-20 years underwent shoulder MRA between 2019-2024 on a 1.5T scanner. 20 received gadolinium (T1-weighted sequences), 20 received saline (T2-weighted sequences). Claude and Google Gemini generated Python code in Colab to convert DICOM to JPEG and perform automated sharpness measurements using variance of the Laplacian in 20 randomly placed 100×100 pixel squares centered around the joint. Manual measurements included normalized SNR/CNR from circular ROIs (1-2mm labrum, 2-5mm contrast, 10-12mm background air). Two musculoskeletal radiologists independently rated image quality using 5-point Likert scales, blinded to contrast type. Statistical analysis included Mann-Whitney U tests, Spearman correlations with FDR correction, and bootstrapped non-inferiority testing ($\delta$=0.15).
**Results or Findings:** Automated sharpness showed plane-dependent differences: gadolinium sharper in axial view (whole image p=0.0006, random crops p=0.0056), saline sharper in coronal view (random crops p=0.0411). Pooled analysis showed no significant sharpness differences (p=0.1371, p=0.7765). Inter-rater agreement ranged from ICC=0.27-0.41 (single rater) to ICC=0.52-0.66 (average raters). Only supraspinatus tendon showed preference for saline by one radiologist (p=0.024). Diagnostic certainty correlated positively with normalized CNR across multiple comparisons (FDR-corrected p<0.05). No significant differences in normalized SNR (p=0.870) or CNR (p=0.271) between contrast agents. Non-inferiority of saline confirmed via bootstrapping (95% CI within ±0.15 margin).
**Conclusion:** AI-generated automated workflows successfully quantified image quality metrics comparable to manual assessment. This approach enables hypothesis testing, upscaling to large datasets, and implementation in quality control and equipment procurement decisions with minimal programming expertise required.
**Limitations:** Single-center study with adolescent population may limit generalizability. Subjective assessments prone to inter-reader variability.
**Funding for this study:** We have applied for a grant from the Stichting De Cock -Hadders (research foundation).
**Has your study been approved by an ethics committee?** Yes
**Ethics committee - additional information:** Ethics approval obtained from Scientific Committee of Treant Zorggroep for this retrospective study.
**Author Disclosures:**
Alie Vegter: Nothing to disclose
Hein Stallmann: Nothing to disclose
Thomas Kwee: Nothing to disclose
Apostolos Petkoglou: Nothing to disclose

**Agentic AI framework for structured reporting (CAD-RADS) and grounding from unstructured CCTA reports**
*A. G. D'Sa*[1], N. Saini[1], A. Vazquez[2], S. Batman[3], G. Urrutia[3]; [1]Bengaluru/IN, [2]Frankfurt/DE, [3]Durham, NC/US
*(sbatman@concertai.com)*

**Purpose:** Clinical radiology priors provide detailed patient history and imaging findings but remain unstructured (CCTA reports) and time-consuming to review. Structured reporting (CAD-RADS) standardizes and condenses reports for clarity and consistency, yet converting unstructured reports is laborious. Advances in Natural Language Processing (NLP) and Large Language Models (LLMs) enables transforming unstructured text into structured reports. However, LLMs can produce unreliable outputs (hallucinations), so detecting errors and linking structured content to the original report is crucial for accuracy and traceability.

**Methods or Background:** The procedure has two phases: (i) structured reporting using multiple agents, and (ii) grounding and hallucination detection (Figure 1). The document is processed by a "Study Understanding Agent" to identify the study type (e.g., plaque analysis). The "Fields Generating Agent" is invoked three times to compile a comprehensive list of fields. Next, the "Value Extraction Agent" extracts corresponding field values from the report. Empty fields are filtered out, followed by "Validation Agent" that detects incorrectly filled or unnecessary fields. On phase two, "Grounding Agent" traces values back to their sources, adding redundancy for validation. This modular approach ensures comprehensive, accurate, and validated structured reporting.
**Results or Findings:** Evaluated on 50 coronary CT angiography (CCTA) reports, our structured reporting framework extracted over 85% of required fields with 96% accuracy for filled values. The grounding agent achieved 88% accuracy.
**Conclusion:** Structured reporting accelerates clinical studies by highlighting essential fields clearly and efficiently. LLMs enable automatic generation of structured reports with high precision. Additionally, LLMs can be leveraged for traceability and validation of generated content, ensuring reliable and accurate reporting.
**Limitations:** Currently, evaluation is limited to CCTA reports. In future we can extend to other report types (Li-RADS, Pi-RADS) and enhance grounding accuracy using traditional NLP techniques alongside LLMs.
**Funding for this study:** ConcertAI
**Has your study been approved by an ethics committee?** Not applicable
**Author Disclosures:**
Gene Urrutia: Employee: ConcertAI
Sinan Batman: Employee: ConcertAI
Nandini Saini: Employee: ConcertAI
Ashwin Geet D'Sa: Employee: ConcertAI
Aldo Vazquez: Employee: ConcertAI

**Initial Insights into an Institutional Secure Large Language model for MRI Examination Requests**
J. T. P. D. Hallinan, N. W. Leow, *Y. X. Low*, A. Lee, W. Ong, D. Z. M. Chan, D. D-L. Loh, A. Makmur, Y. Ting; Singapore/SG

**Purpose:** To compare clinician MRI examination requests (MERs) with institutional secure large language model (sLLM)-augmented MERs for information quality and to evaluate protocoling accuracy of the sLLM versus board-certified radiologists across body, musculoskeletal, and neuroradiology MRI.
**Methods or Background:** Incomplete clinical details on MERs can lead to sub-optimal protocol selection. An institutional sLLM with access to the electronic medical record (EMR) may improve request completeness and protocol accuracy across multiple MRI subspecialties. This retrospective study included 608 consecutive MRI examinations comprising 528 patients performed between September 2023 and July 2024. A privately hosted Anthropic Claude 3.5 model augmented each MER with EMR data and, via rule-based parsing, recommended region/coverage and contrast use. Two experienced radiologists established a consensus reference standard. Two board-certified general radiologists and the sLLM were compared with this standard. Clinical-information quality was graded using the Reason-for-Exam Imaging Reporting and Data System (RI-RADS). Inter-rater reliability was quantified with Gwet's AC1. Paired accuracies were compared with McNemar testing to determine if there was a statistically significant difference.
**Results or Findings:** Limited or deficient clinical information fell to 0–0.7% with sLLM augmentation versus 5.2–20.4% for clinician MERs. Overall protocol accuracy was 93.1% for the sLLM, 91.4% for Rad 3, and 92.1% for Rad 4. Region/coverage accuracy was similar (sLLM 95.2%, Rad 3 96.2%, Rad 4 94.2%). Contrast decisions were more accurate using the sLLM at 94.4% versus Rad 3 at 92.1%.
**Conclusion:** Across subspecialty MRI, sLLM-augmented examination requests had improved clinical context and contrast selection while matching general radiologists for region/coverage. Integrating sLLMs into vetting workflows may reduce manual workload and standardize protocoling.
**Limitations:** This was a single-centre, retrospective evaluation. Whether similar gains would be realised in institutions that use different order-entry systems is a future area of research.
**Funding for this study:** Singapore Ministry of Health National Medical Research Council under the NMRC Clinician Innovator Award (CIAINV23jan-0001, MOH-001405).
**Has your study been approved by an ethics committee?** Not applicable
**Author Disclosures:**
Andrew Makmur: Nothing to disclose
Yonghan Ting: Nothing to disclose
James Thomas Patrick Decourcy Hallinan: Nothing to disclose
Naomi Wenxin Leow: Nothing to disclose
Yi Xian Low: Nothing to disclose
Aric Lee: Nothing to disclose
Daniel De-Liang Loh: Nothing to disclose
Ding Zhou Matthew Chan: Nothing to disclose
Wilson Ong: Nothing to disclose

**Large Language Model-Assisted CT Protocol Selection: A Comparative Study of Finetuning vs Zero-Shot Reasoning**
*P. Arnold*, M. Russe, E. Kotter, M. Scholz, M. Jeuck, L. Heine, T. Stein, S. Walter; Freiburg Im Breisgau/DE
*(philipp.arnold@uniklinik-freiburg.de)*

**Purpose:** To evaluate whether large language models (LLMs) can assist radiologists in selecting the optimal CT protocol by name, correct body region, and appropriate use of intravenous contrast, we compared two strategies: supervised fine-tuning on institutional data versus zero-shot reasoning with structured prompting.
**Methods or Background:** CT protocol selection balances diagnostic yield against radiation and contrast risks. We retrospectively analyzed 20,000 CT exams with clinical indications, history, and standardized protocol labels covering 100 distinct protocol names, body region, and contrast use. Fine-tuning: Qwen-2.5 models (3B–32B) were trained to map clinical text to the correct protocol name, body region, and contrast use. Zero-shot reasoning: Qwen-3 models (4B–32B) received structured prompts with the same inputs, with or without prior imaging reports. Benchmarking: Three resident radiologists independently selected protocols for the same test set. Accuracy was assessed for (1) protocol name, (2) body region, and (3) contrast use.
**Results or Findings:** Fine-tuned LLMs achieved 68–77% accuracy for protocol names across 100 labels (Top-3: 84–87%) and 90–96% accuracy for body region and contrast, with the 7B model performing best. Zero-shot models reached 91–93% accuracy for body region and contrast, comparable to residents (87–90% / 89–94%), but lower for protocol names (53–56% vs. 58–62%). Prior imaging reports improved contrast accuracy by 1–4% across all models.
**Conclusion:** LLMs show potential as decision-support tools for CT protocol selection. Fine-tuned models surpassed radiologists in protocol-name matching, while zero-shot models matched human performance on individual components and leveraged prior reports to enhance contrast decisions. To improve protocol selection, inclusion of key clinical details in the provided data is essential.
**Limitations:** Single-institution data and protocol taxonomy may limit generalizability.
**Funding for this study:** German Research Foundation (DFG) - SFB 1597–499552394. Hans A. Krebs Programme (University Clinic Freiburg im Breisgau)
**Has your study been approved by an ethics committee?** Yes
**Ethics committee - additional information:** Ethics Vote register number: FRKS004287
**Author Disclosures:**
Marvin Jeuck: Nothing to disclose
Laura Heine: Nothing to disclose
Maxim Scholz: Nothing to disclose
Maximilian Russe: Nothing to disclose
Sebastian Walter: Nothing to disclose
Philipp Arnold: Nothing to disclose
Thomas Stein: Nothing to disclose
Elmar Kotter: Nothing to disclose

**The Expertise Paradox: Who Benefits from LLM-Assisted Brain MRI Differential Diagnosis?**
S. Schramm[1], B. Le Guellec[2], L. C. Adams[1], K. Bressem[1], J. S. Kirschke[1], D. M. Hedderich[1], B. Wiestler[1], *S. H. Kim*[1]; [1]Munich/DE, [2]Lille/FR
*(shk.heidelberg@gmail.com)*

**Purpose:** To evaluate how reader experience influences the diagnostic benefit from large language model-assisted brain MRI differential diagnosis.
**Methods or Background:** Neuroradiologists (n = 4), radiology residents (n = 4), and neurology/neurosurgery residents (n = 4) were recruited. A dataset of complex brain MRI cases was curated from the local imaging database (n = 40). For each case, readers provided a textual description of the main imaging finding and their top three differential diagnoses ("Unassisted"). Three state-of-the-art large language models (GPT-4.1, Gemini 2.5 Pro, DeepSeek-R1) were prompted to generate top-three differentials based on the clinical case description and reader-specific findings. Readers then revised their differential diagnoses after reviewing GPT-4.1 suggestions ("Assisted"). To statistically evaluate the association between reader experience and diagnostic benefit, a cumulative link mixed model was fitted with change in diagnostic result as ordinal outcome, reader experience as a fixed effect, and random intercepts for rater and case.
**Results or Findings:** LLM-generated differential diagnoses achieved the highest top-3 accuracy when provided with image descriptions from neuroradiologists (top-3: 78.8-83.8%), followed by radiology residents (top-3: 71.8-77.6%), and other neurology/neurosurgery residents (top-3: 62.6-64.5%). In contrast, relative gains in top-3 accuracy from LLM assistance diminished with increasing experience, with +19.2% for neurology/neurosurgery residents (from 43.2% to 62.6%),+14.7% for radiology residents (from 59.6% to 74.4%), and +4.4% for neuroradiologists (from 83.1% to 87.5%). The cumulative link mixed model confirmed a significant negative association between reader experience and diagnostic benefit from LLM assistance (p = 0.005).
**Conclusion:** With increasing reader experience, absolute diagnostic LLM performance with reader-specific input improved, while relative diagnostic gains through LLM assistance paradoxically diminished. Our findings call attention to the gap between isolated LLM performance and actual clinical relevance, emphasizing the need to account for human-AI interaction.
**Limitations:** N/A
**Funding for this study:** None
**Has your study been approved by an ethics committee?** Yes
**Ethics committee - additional information:** Ethics Committee of the Technical University of Munich
**Author Disclosures:**
Bastien Le Guellec: Nothing to disclose
Jan S Kirschke: Nothing to disclose
Su-Hwan Kim: Nothing to disclose
Severin Schramm: Nothing to disclose
Lisa C. Adams: Nothing to disclose
Keno Bressem: Nothing to disclose
Dennis M. Hedderich: Nothing to disclose
Benedikt Wiestler: Nothing to disclose

### Large Language Model-Assisted Simplification of CT Staging Reports for Cancer Patients: A Prospective Quasi-Randomized Trial

*F. Busch*, P. Prucker, K. K. Bressem, J. Peeken, A. W. Marka, S. H. Kim, S. Ziegelmayer, M. R. Makowski, L. C. Adams; Munich/DE
*(felix.busch@tum.de)*

**Purpose:** To evaluate whether large language model (LLM)-assisted simplification of CT staging reports improves cancer patients' cognitive workload, text comprehension, report perception, and reading time.
**Methods or Background:** Prospective, controlled, open-label, quasi-randomized, pre-registered trial of 200 adult cancer patients undergoing routine CT re-staging with alternate 1:1 allocation to receive either the unmodified report or a locally generated LLM-simplified version (Llama-3.3-70B, on-premise via basebox) with mandatory radiologist review. Co-primary outcomes were reading time and three composite scores (cognitive workload, text comprehension, patient perception), each derived from three 7-point Likert items. Secondary outcomes included readability indices, word count, medical-terminology ratings, and independent radiologist assessments of factual errors, omissions, insertions, clinical usefulness, and overall quality. Logistic regression was performed to analyze patient-reported outcomes, adjusting for patient characteristics.
**Results or Findings:** Simplification reduced median reading time from 7 to 2 minutes (adjusted β: -3.86; 95% confidence interval (CI): -5.46, -2.26; P<.001). Patients reported lower cognitive workload (adjusted odds ratio (aOR): 0.18; 0.13, 0.25), higher text comprehension (aOR: 13.28; 9.31, 18.93), and enhanced perception of report usefulness (aOR: 5.46; 3.55, 8.38; all P<.001). Readability was significantly improved across metrics (e.g., Flesch-Kincaid Grade Level from 13.69 ± 1.13 to 8.89 ± 0.93; P<.001). Two radiologists independently identified factual errors in 6% of simplified reports (2 moderate, 4 severe), omissions in 7% (2 minor, 1 moderate, 4 severe), and unsupported insertions in 3% (1 minor, 2 moderate). The majority of simplified reports were rated clinically useful and of good or better quality.
**Conclusion:** LLM simplification substantially improves patient-centered outcomes and readability of CT staging reports while maintaining generally favorable clinical usefulness and quality. However, clinically relevant errors underscore the need for expert radiologist oversight before clinical implementation.
**Limitations:** Single-center, open-label with alternate allocation, self-reported patient outcomes.
**Funding for this study:** None.
**Has your study been approved by an ethics committee?** Yes
**Ethics committee - additional information:** Technical University of Munich (2025-186-S-KK)
**Author Disclosures:**
Philipp Prucker: Nothing to disclose
Keno K. Bressem: Nothing to disclose
Marcus R. Makowski: Nothing to disclose
Lisa C. Adams: Nothing to disclose
Alexander Wolfgang Marka: Nothing to disclose
Sebastian Ziegelmayer: Nothing to disclose
Jan Peeken: Nothing to disclose
Felix Busch: Nothing to disclose
Su Hwan Kim: Nothing to disclose

### Local large language models for MRI protocol selection: A privacy-preserving alternative to cloud AI

Z. Bendella, *T. J. Neumann*, Z. Ganji, R. Clauberg, N. Lehnen, A. Radbruch, M. Wolter, B. D. Wichtmann; Bonn/DE
*(timotheus.neumann@ukbonn.de)*

**Purpose:** Cloud-based AI like ChatGPT has demonstrated high accuracy for MRI protocol selection. However, transmitting patient data to external servers entails privacy risks. This study evaluated whether LLaMA-3.1-8B, a compact open-source model deployable locally, can achieve comparable accuracy while maintaining complete data sovereignty.
**Methods or Background:** This IRB-approved, retrospective study used real-world radiology referral forms (RRFs) and corresponding MRI sequences from our institutional neuroradiology department. For model development, 8,281 consecutive MRI examinations (November 2023–January 2025) were included, with RRFs extracted from the RIS and executed sequences from the PACS, split into training (n = 6,624) and validation (n = 1,657). For testing, 1,001 consecutive RRFs (August 2023–July 2024) covering the full range of neuroradiological MRI protocols were included, with ground truth protocol selections defined by two board-certified neuroradiologists. LLaMA-3.1-8B was fine-tuned using prompt-tuning and LoRA (Low-Rank Adaptation), and compared against the pretrained model and ChatGPT-4. Accuracy was evaluated using BioBERT-based similarity scores between model outputs and expert ground truth.
**Results or Findings:** ChatGPT-4 achieved 99.7% accuracy on the test set. The locally deployed pretrained LLaMA-3.1-8B achieved 93.2% accuracy, improving to 94.5% with prompt-tuning and 95.4% with LoRA fine-tuning.
**Conclusion:** Despite being orders of magnitude smaller than ChatGPT-4, the locally deployed and fine-tuned LLaMA-3.1-8B achieved near-comparable accuracy in MRI protocol selection. This demonstrates the feasibility of privacy-preserving, institutionally controlled AI solutions to support radiologists without external data transfer, combining clinical utility with data sovereignty. These results establish a performance benchmark for local LLM deployment in radiology protocol selection.
**Limitations:** Fine-tuning refinements may further improve accuracy, though radiologist oversight remains essential for diagnostic safety. As this work employed a comparatively modest 8-billion-parameter model, future investigations with larger locally deployable models are expected to yield even higher performance.
**Funding for this study:** No funding was received for this study.
**Has your study been approved by an ethics committee?** Yes
**Ethics committee - additional information:** The retrospective study received approval from the local Ethics Committee for Clinical Trials on Humans and Epidemiological Research with Personal Data, IRB number: 312/23-EP.
**Author Disclosures:**
Timotheus Josef Neumann: Nothing to disclose
Barbara Daria Wichtmann: Speaker: Barbara Daria Wichtmann has received speaker honoraria from Philips Healthcare.
Moritz Wolter: Nothing to disclose
Zeynep Bendella: Nothing to disclose
Zahra Ganji: Nothing to disclose
Ralf Clauberg: Nothing to disclose
Alexander Radbruch: Nothing to disclose
Nils Lehnen: Nothing to disclose

**08:00 - 09:00** **Research Stage 2**

### Research Presentation Session: Genitourinary

### RPS 1207

### Imaging of benign kidney diseases and renal transplantation

**Moderator**
Jean Michel Correas; Paris/FR

**One-stop early noninvasive evaluation of renal allograft rejection and fibrosis: microstructural mapping via time-dependent diffusion MRI**
*C. Lyu*, Z. Liao, Z. Li; Wuhan/CN

**Purpose:** Functional MRI offers promise for noninvasive detection of renal allograft injury, but conventional models lack biological specificity. This study aimed to evaluate the clinical utility of time-dependent diffusion MRI (td-dMRI) for detecting rejection and fibrosis in kidney transplant recipients.
**Methods or Background:** In this prospective observational study at a tertiary center in China, adult renal allograft recipients undergoing indication biopsies between April 2024 to June 2025 were enrolled. Microstructural maps from td-dMRI based on a Bayesian method, DWI, intravoxel incoherent motion, diffusion kurtosis imaging, stretched exponential model, fractional order calculus, and continuous-time random-walk model. The diagnostic performances of these microstructural parameters in differentiating different degrees of renal allograft fibrosis (mild, moderate and severe fibrosis) and rejection (no-rejection, acute and chronic rejection) were evaluated by areas under the receiver operating characteristic curves (AUC).
**Results or Findings:** A total of 100 patients (67 males, 33 females) were analyzed. Among them, 41 had acute rejection and 13 had chronic rejection; 67 had mild fibrosis, 19 moderate, and 14 severe fibrosis. td-dMRI-derived diameter significantly distinguished fibrosis grades, while cellularity identified early fibrotic changes. For rejection, diameter, fin, and fp differentiated acute rejection, and D-CTRW, fin, fp, and diameter distinguished chronic rejection. Combining td-dMRI metrics with clinical variables yielded high AUCs (0·968 for acute, 0·913 for chronic rejection).
**Conclusion:** This study supports td-dMRI as a biologically meaningful, one-stop contrast-free imaging approach for transplant kidney surveillance. It enables noninvasive detection of structural and cellular changes associated with rejection and fibrosis, even when conventional markers are inconclusive. Integrating td-dMRI with serum biomarkers may improve diagnostic accuracy and reduce unnecessary biopsies, promoting personalized immunosuppression and earlier intervention.
**Limitations:** It was a single-center study with a moderate sample size, which may limit generalizability.
**Funding for this study:** the National Natural Science Foundation of China. (No. 82371942 and No. 82071889）
**Has your study been approved by an ethics committee?** Yes
**Ethics committee - additional information:** This prospective study was approved by the ethic committee of Tongji Hospital, Tongji Medical College, Huazhong University of Science and Technology
**Author Disclosures:**
Zhouyan Liao: Nothing to disclose
Zhen Li: Nothing to disclose
Chenxi Lyu: Nothing to disclose

**Utility of Multi-Model Diffusion MRI for Stratifying High-Risk Complications in Renal Transplant Recipients**
*C. Lyu*, Z. Liao, Z. Li; Wuhan/CN

**Purpose:** This study aims to develop a noninvasive comprehensive diagnostic protocol based on diffusion weighted imaging (DWI) and clinical indicators to achieve accurate and efficient identification of high-risk complications (rejection, acute kidney injury, viral infection, moderate to severe fibrosis) after renal transplantation.
**Methods or Background:** This prospective study enrolled 120 renal allograft recipients. Quantitative parameters of DWI, intravoxel incoherent motion (IVIM), diffusion kurtosis imaging (DKI), stretched exponential model (SEM), fractional order calculus (FROC) and continuous-time random walk (CTRW) were measured. Clinical and pathological features were also recorded. Univariate and multivariate logistic regression analyses were performed to identify independent clinical and imaging predictors of high-risk complications group. Receiver operating characteristic (ROC) curves were used to evaluate diagnostic performance. Internal validation of the combined model was performed using stratified 5-fold cross-validation and bootstrap resampling to assess model stability and generalizability.
**Results or Findings:** Finally, 101 patients were included. Diffusion coefficients differed significantly between superior and inferior graft function groups across models. DDC_SEM_cortex and $HCO_3^-$ emerged as independent predictors in multivariate analysis. The AUCs for HCO3-, DDC_SEM_cortex, and their combined model in identifying patients requiring clinical management changes were 0.888, 0.898, 0.941. Internal validation confirmed strong performance (cross-validation AUC 0.938; bootstrap AUC 0.940, 95% CI 0.931–0.943)
**Conclusion:** DDC_SEM_cortex and serum HCO3- levels are promising noninvasive imaging biomarkers for identifying renal allograft recipients with high-risk complications. It provides a powerful noninvasive tool for clinical decision-making and is expected to guide individualized kidney transplant management.
**Limitations:** It was a single-center study with a moderate sample size, which may limit generalizability. MRI was acquired in free-breathing mode, making image quality susceptible to motion artifacts despite the iliac fossa location
**Funding for this study:** This study has received funding by the National Natural Science Foundation of China. (No. 82371942 and 82071889）
**Has your study been approved by an ethics committee?** Yes
**Ethics committee - additional information:** This prospective study was approved by the ethic committee of Tongji Hospital, Tongji Medical College, Huazhong University of Science and Technology
**Author Disclosures:**
Zhouyan Liao: Nothing to disclose
Zhen Li: Nothing to disclose
Chenxi Lyu: Nothing to disclose

Friday

**Multiparametric Renal MRI for Detecting Active Disease, Remission, and Subclinical Injury in Lupus Nephritis: A Prospective Observational Study**
*X. Zhang*, H. Sun, G. Zhang, C. Huang; Beijing/CN
*(zhang_xiaoxiao23@163.com)*

**Purpose:** To evaluate structural and quantitative multiparametric MRI (mpMRI) features across the lupus nephritis (LN) disease continuum—active LN, clinically stable LN, and healthy controls—and to investigate correlations with clinical indices.
**Methods or Background:** In this prospective study, 40 patients with active LN, 40 with stable LN, and 21 matched healthy volunteers underwent renal MRI at 3.0T, including structural sequences, multi-b-value diffusion-weighted imaging (DWI/IVIM/DKI)and T1/T2 mapping. Structural abnormalities were qualitatively assessed, and quantitative parameters were compared across groups. Correlations with proteinuria, eGFR, complement levels, and disease activity scores were analyzed.
**Results or Findings:** Morphological abnormalities were observed in 40% of active LN and 30% of stable LN, versus none in controls. Exudative lesions were significantly more common in active LN than in stable LN (45.0% vs. 7.5%, $p < 0.001$). Quantitatively, active LN showed markedly reduced cortical ADC ($1.74 \pm 0.24 \times 10^{-3}$ mm²/s vs. $2.05 \pm 0.21$ in controls, $p < 0.001$) and elevated parenchymal T1 ($1632 \pm 123$ ms vs. $1408 \pm 95$, $p < 0.001$). Stable LN showed partial normalization of cortical ADC ($p = 0.022$ vs. active LN) and T1 ($p = 0.007$), but parenchymal ADC and medullary ADC remained depressed ($p < 0.01$ vs. controls). Proteinuria correlated negatively with cortical diffusion ($r = –0.33$, $p = 0.038$) while eGFR correlated positively with ADC (r up to 0.48, $p = 0.002$).
**Conclusion:** Multiparametric renal MRI sensitively detects structural and functional changes across active LN, clinical remission, and health. Crucially, it identifies persistent microstructural injury in clinically stable patients, underscoring its potential as a noninvasive tool for monitoring treatment response and guiding long-term management.
**Limitations:** The lack of direct histopathological limits ability to precisely link imaging findings to specific microscopic changes
**Funding for this study:** Gumuyang Zhang received funding from Peking Union Medical College Hospital Talent Cultivation Program (Category D); GrantID UHB11588. Hao Sun received funding from the CAMS Innovation Fund for Medical Sciences(CIFMS); GrantID2024-12M-C&T-C-0041; Hao sun received funding from the CAMs innoyation Fund for Medical sciences(CIFMS): Grant ID2025-12M-C&T-C-001.
**Has your study been approved by an ethics committee?** Yes
**Ethics committee - additional information:** Pecking Union College Hospital Institutional Review Board approval was obtained
**Author Disclosures:**
Gumuyang Zhang: Nothing to disclose
Can Huang: Nothing to disclose
Hao Sun: Nothing to disclose
Xiaoxiao Zhang: Nothing to disclose

**Diagnostic Value of Renal T1 and T2 Mapping in Biopsy-Proven Renal Amyloidosis: Correlation with Clinical Stage and Renal Function**
*E. Şahin*, F. Alpaslan, T. Y. Kılıç, E. İsmailov, M. F. Kazanbaş, T. Banaz, B. Tütüncüoğlu, M. Kadıoğlu, S. Baş Özkök; Istanbul/TR
*(elifnazsahinn12@icloud.com)*

**Purpose:** To evaluate renal T1 and T2 mapping characteristics in patients with biopsy-proven renal amyloidosis and to explore their correlation with clinical severity and renal function indices.
**Methods or Background:** Renal amyloidosis is characterized by extracellular amyloid deposition that leads to progressive organ dysfunction. Non-invasive CMR-based renal mapping techniques may provide insight into disease activity and stage. Twenty-eight patients with biopsy-confirmed renal amyloidosis (mean age 53.2±12.8 years; 43% female) underwent renal T1 and T2 mapping using a 1.5 Tesla scanner. Disease severity was staged (GRADE I–V) according to clinical and histopathologic criteria. Standard renal function tests (urea, creatinine, eGFR) were obtained. Spearman's rank correlation was used to assess associations between renal mapping parameters, disease grade, and renal function. Healthy volunteers (n=20) served as controls.
**Results or Findings:** Median renal T1 values were significantly higher in the amyloidosis group compared with controls (1245 ms, IQR 1150–1410 vs. 1020 ms, IQR 985–1065; $p<0.01$). Similarly, median renal T2 values were elevated (82 ms, IQR 71–91 vs. 65 ms, IQR 61–70; $p<0.01$). A strong positive correlation was observed between disease grade and renal T1 ($\rho=0.62$, $p<0.001$), while renal T2 demonstrated a weaker but significant association ($\rho=0.38$, $p<0.05$). Renal T1 correlated positively with serum creatinine ($\rho=0.45$, $p<0.05$) and urea ($\rho=0.41$, $p<0.05$), and negatively with eGFR ($\rho=-0.52$, $p<0.01$). Renal T2 values showed a similar but less consistent pattern.
**Conclusion:** Renal T1 and T2 mapping values are elevated in renal amyloidosis and correlate with both disease severity and renal function impairment. These parameters may serve as useful non-invasive biomarkers for staging and monitoring renal involvement, complementing histopathology and laboratory measures.
**Limitations:** Relatively small, single-centre sample size.
**Funding for this study:** Not receive any financial support
**Has your study been approved by an ethics committee?** Yes
**Ethics committee - additional information:** This study was approved by the Başakşehir Çam and Sakura City Hospital Ethical Board. Application was submitted and registered
**Author Disclosures:**
Serçin Baş Özkök: Nothing to disclose
Berk Tütüncüoğlu: Nothing to disclose
Fatih Alpaslan: Nothing to disclose
Taha Yiğid Kılıç: Nothing to disclose
Muhammed Faruk Kazanbaş: Nothing to disclose
Eitibar Ismailov: Nothing to disclose
Tuba Banaz: Nothing to disclose
Elifnaz Şahin: Nothing to disclose
Mehmet Kadıoğlu: Nothing to disclose

**MRI-Based Renal Fat Quantification as a Biomarker for Nephroprotective Therapy Monitoring in Metabolic Syndrome**
*M. I. Scheuer*[1], C. S. Böttger[1], C. Liang[2], E. Bechler[1], A. Ljimani[1]; [1]Düsseldorf/DE, [2]Thuebingen/DE
*(ingemarie.scheuer@med.uni-duesseldorf.de)*

**Purpose:** Metabolic syndrome is a collection of multifactorial conditions associated with combined cardiovascular pathologies and renal dysfunction. Novel therapies such as SGLT2 inhibitors show nephroprotective effects, but their clinical use is limited by the absence of noninvasive tools to monitor renal fat and treatment response. This study investigates MRI-based fat quantification as a method for assessing renal lipid accumulation.
**Methods or Background:** A total of 58 patients from a metabolic outpatient clinic underwent 1.5 T MRI scans (Avanto, Siemens Healthineers, Germany). Participants were divided into two groups by BMI (<30 vs. >30). Renal fat fraction (RFF) was quantified alongside visceral (VAT), subcutaneous (SAT), and abdominal muscle (AM) areas at L1–L2, as well as hepatic fat fraction (HFF). Correlations between renal and abdominal fat depots were analyzed to evaluate the potential of RFF as a biomarker.
**Results or Findings:** Results demonstrated significantly higher RFF in participants with BMI >30 compared to BMI <30. No significant correlations were observed between RFF and HFF, VAT, SAT, or AM. These findings suggest that renal fat accumulation occurs independently of other fat depots. MRI-based renal fat quantification emerges as a promising noninvasive biomarker for monitoring renal lipid content in patients with metabolic syndrome. The observed group differences highlight its potential to detect renal fat burden, while the lack of correlation with hepatic or abdominal fat supports its unique role. A second MRI scan is planned after the treatment by new SGLT2 inhibitors within the study.
**Conclusion:** MRI-based renal fat quantification may serve as a valuable biomarker for monitoring the effectiveness of nephroprotective therapies in patients with metabolic syndrome.
**Limitations:** None.
**Funding for this study:** None.
**Has your study been approved by an ethics committee?** Yes
**Ethics committee - additional information:** The study was approved by the Ethics Committee of the Medical Faculty of Heinrich Heine University Düsseldorf (study number 5891R, approval date August 28, 2018)
**Author Disclosures:**
Eric Bechler: Nothing to disclose
Charlotte Sonja Böttger: Nothing to disclose
Alexandra Ljimani: Nothing to disclose
Marie I. Scheuer: Nothing to disclose
Cecilia Liang: Nothing to disclose

**Deep Learning-Based Kidney Volume Estimation in Polycystic Kidney Disease: Are We Ready for Clinical Practice? A Systematic Review and Meta-Analysis**
*E. Almar Munoz*[1], E. Colliander[2], S. Tupper[2], M. Lansner Kielberg[2], M. L. Liu[2], A. Mayr[1], R. Mirón Mombiela[2]; [1]Innsbruck/AT, [2]Herlev/DK
*(enrique.almar@i-med.ac.at)*

**Purpose:** n patients with autosomal dominant kidney disease (ADPKD), total kidney volume (TKV) is the gold standard biomarker for assessing the risk of progression and the need for drug therapy. However, it is a time-consuming process. In this systematic review, we evaluate the current state of deep learning (DL) algorithms for automatic kidney volume segmentation.
**Methods or Background:** All original research, including the search terms ADPKD, diagnostic imaging, DL, and TVK, was identified in PubMed, Embase, and Ovid MEDLINER databases from January 2000 to October 13, 2024. Articles with insufficient information to assess methodological quality were excluded. The quality was assessed using the QUADAS-2 and Checklist for Artificial Intelligence in Medical Imaging (CLAIM) tools. We focused on Dice Similarity Coefficient (DSC), bias differences, and time efficiency as outcomes.
**Results or Findings:** Nineteen studies were included, with an overall good quality; however, the mean adherence to the CLAIM checklist was 64%. The average DSC was 95 ± 2.89% (95% CI: 94.62–94.85) with relatively low bias for TKV in 5,622 ADPKD patients (mean age, 46.1 years; 45% male) and 9,180 scans (79% MRI). The average segmentation time was decreased by 75% compared to the ground truth. Performance differences were evident among imaging modalities, but 3D models did not outperform their 2D counterparts. No statistically significant evidence of small-study effects or publication bias was detected.
**Conclusion:** DL models for TKV in ADPKD patients demonstrated high precision compared to manual segmentation in a large, pooled sample with heterogeneous study designs and methods. Therefore, clinical implementation is feasible.
**Limitations:** Risk of data and model drift over time. High computational and environmental costs. Lack of task-specific evaluation standards (addressable by a tailored PRISMA for AI). Potential automation bias. Workflow/institutional variability. Regulatory and compliance challenges.
**Funding for this study:** No funding was received for this study.
**Has your study been approved by an ethics committee?** Not applicable
**Author Disclosures:**
Marie Louise Liu: Nothing to disclose
Emil Colliander: Nothing to disclose
Sebastian Tupper: Nothing to disclose
Mira Lansner Kielberg: Nothing to disclose
Enrique Almar Munoz: Nothing to disclose
Rebeca Mirón Mombiela: Nothing to disclose
Agnes Mayr: Nothing to disclose

**Renal Transplant Ultrasound: From Normal Assessment to Early and Late Detection of Complications**
*W. M. Fernández Rios*, N. Lopez-Galiacho, E. Sifre Gómez, L. P. Cervera Bravo, S. Descalzo García, F. C. Mejia Galvis, M. N. Vicente de Vera, T. Vicente García, N. Carreres Creus; Alabcete/ES
*(windymary13@gmail.com)*

**Purpose:** To describe the normal ultrasonographic findings of the renal graft and to review the radiological semiology of the most frequent complications through a series of cases from our department, both in the immediate postoperative period and during long-term follow-up.
**Methods or Background:** Renal transplantation is the most common solid-organ replacement procedure and represents the definitive treatment for chronic kidney disease. Advances in surgical technique, immunosuppression, surveillance imaging, and histopathological diagnosis of rejection have significantly improved graft survival. The increasing number of recipients and the use of higher-risk donors make thorough radiological assessment essential. The radiologist plays a pivotal role throughout the process—from baseline evaluation to the assessment of graft dysfunction and the guidance of interventional procedures.
**Results or Findings:** Doppler ultrasound is the first-line imaging modality in the immediate postoperative period, providing a reference examination and enabling early detection of complications. In cases of dysfunction, contrast-enhanced ultrasound or magnetic resonance imaging can provide greater diagnostic specificity. Complications are classified by timing and aetiology: immunological (rejection), vascular, urological, infectious, surgical/iatrogenic, and neoplastic. The heterotopic placement of the graft in the iliac fossa results in a characteristic spectrum of imaging findings.
**Conclusion:** Detailed knowledge of normal anatomy, vascular variations, surgical techniques, and donor and recipient risk factors is essential for accurate diagnosis and timely management. Ultrasound remains the cornerstone in the evaluation of renal transplantation, both in the early and late postoperative periods.
**Limitations:** No limitations
**Funding for this study:** No disclosure
**Has your study been approved by an ethics committee?** Not applicable
**Author Disclosures:**
Nuria Lopez-Galiacho: Nothing to disclose
Ferney Camilo Mejia Galvis: Nothing to disclose
Elena Sifre Gómez: Nothing to disclose
Luisa Pilar Cervera Bravo: Nothing to disclose
Santiago Descalzo García: Nothing to disclose
Teresa Vicente García: Nothing to disclose
Windy Mary Fernández Rios: Nothing to disclose
Mario Navarro Vicente De Vera: Nothing to disclose
Nuria Carreres Creus: Nothing to disclose

**08:00 - 09:00** **Research Stage 5**

### Research Presentation Session: Musculoskeletal

### RPS 1210

### When MR substitutes CT and CT replaces MR

**Moderator**
Gustav Andreisek; Münsterlingen/CH

**Diagnostic Performance and Reader Confidence of a CT-like MRI Sequence for Femoral Head Necrosis**
*M. L. Schnitzer*, F. Herr, N. Hohmann, C. A. Dascalescu, B. Holzapfel, J. Ricke, N. Hesse, J. Arnholdt, P. Reidler; Munich/DE
*(felix.herr@med.uni-muenchen.de)*

**Purpose:** To evaluate a novel CT-like MRI sequence for ARCO classification, reader confidence, and image quality, and compare its diagnostic performance with standard MRI and radiography.
**Methods or Background:** In this retrospective study, 21 patients with suspected femoral head necrosis underwent MRI (T1, T2, CT-like) and radiography. Two experienced readers independently assessed ARCO stage (I–IV), reader confidence, and image quality using a binary scale (1 = high/good, 2 = low/poor). Mean scores were calculated. Interrater reliability was assessed using intraclass correlation coefficients (ICC); intermodality agreement with weighted Cohen's Kappa. Paired comparisons used Wilcoxon signed-rank tests.
**Results or Findings:** CT-like MRI yielded higher ARCO scores ($2.23 \pm 0.78$) than radiography ($1.75 \pm 1.01$, $p = 0.001$), T1 ($2.08 \pm 0.73$, $p = 0.010$), and T2 ($2.08 \pm 0.69$, $p = 0.023$). Reader confidence was superior with CT-like MRI ($1.11 \pm 0.25$) compared to T1 ($1.53 \pm 0.47$), T2 ($1.50 \pm 0.46$), and radiography ($1.43 \pm 0.37$), all $p < 0.01$. Image quality ratings were highest for CT-like ($1.11 \pm 0.30$), outperforming T1 ($1.86 \pm 0.51$) and T2 ($1.75 \pm 0.51$), $p < 0.001$. Agreement in ARCO staging was excellent with T1 ($\kappa = 0.904$) and T2 ($\kappa = 0.869$), and substantial with radiography ($\kappa = 0.709$). Interrater reliability was high for ARCO staging (ICC 0.82–0.93), lower for confidence (ICC < 0.40), and variable for image quality, with CT-like showing the best agreement (ICC = 0.84).
**Conclusion:** This CT-like MRI sequence improves diagnostic confidence and staging accuracy while avoiding ionizing radiation and reducing the need for additional imaging. It supports a more sustainable approach by minimizing exposure and streamlining diagnostics.
**Limitations:** Single-center design and small sample size may limit generalizability. The sequence requires specialized protocols not yet widely available.
**Funding for this study:** No funding was received for this study.
**Has your study been approved by an ethics committee?** Yes
**Ethics committee - additional information:** The study was approved by the local ethics committee of (clinical trial number: DRKS00030822, date of registration: 12 January 2023).
**Author Disclosures:**
Moritz Ludwig Schnitzer: Nothing to disclose
Jörg Arnholdt: Nothing to disclose
Natascha Hohmann: Nothing to disclose
Boris Holzapfel: Nothing to disclose
Felix Herr: Nothing to disclose
Paul Reidler: Nothing to disclose
Nina Hesse: Nothing to disclose
Christian Alexander Dascalescu: Nothing to disclose
Jens Ricke: Nothing to disclose

**Opportunistic comparative analysis of quantitative radiomics between zero-echo-time MRI and CT on normal hip bone during oncologic metastatic screening**
R. F. Valenzuela, *E. Duran-Sierra*, M. Antony, J. Espinoza, C. M. Costelloe, W. Murphy, D. Wells, B. Amini, S. Lo; Houston, TX/US
*(eduran3@mdanderson.org)*

**Purpose:** Zero-Echo-Time (ZTE) and Ultrashort-Echo-Time (UTE) sequences are designed to characterize short-T2 materials. By utilizing ray-sum-rendering and inverse-logarithm-rescaling, these sequences can enhance bone contrast, resembling CT. ZTE publications often rely on perception analysis to validate its use as a CT-succedaneum. We aim to quantitatively compare Ozteo (GE-ZTE) MRI and CT using radiomic analysis, first on normal bone and subsequently on sclerotic and lucent lesions.
**Methods or Background:** Starting July 2025, Ozteo has become part of our institutional routine MSK-pelvic-MRI across nine GE magnets (two-3T and seven-1.5T). An ongoing prospective collection included 26 patients with bone-metastases screening scanned with ZTE+CT within six months. Manual-volume-of-interest (VOI) segmentations were created from healthy right hips, using the left hip in the presence of right hip pathology or hardware. An in-house Python-based sequence-neutral software extracted 107 radiomic features, comprising 14 shape, 18 first-order, and 75 texture features. Statistical analysis compared ZTE MRI-and CT-derived radiomic features using two-tailed non-parametric Wilcoxon signed-rank paired tests.
**Results or Findings:** Gender: 42% male/58% female. Age-average: 50 years (range: 24-76). 22 right and four left hip VOI were segmented. The average time between ZTE-MRI and CT was two months (range: 1-day/6-months). The ZTE-MRI vs. CT statistical analysis revealed that 16-out-of-107 (15%) radiomic features were significantly similar (P>=0.05), including: glszm_largeareaemphasis (P=0.0796), glszm_zonevariance (P=0.0796), and firstorder_maximum (P=0.0559). Maximum differences (9.3%, P=2.98x10-8) included firstorder_robustmeanabsolutedeviation, firstorder_interquartilerange, firstorder_meanabsolutedeviation, firstorder_variance, glszm_graylevelvariance and firstorder_range.
**Conclusion:** Detached from subjective-perceptual evaluations, radiomics analysis from CT versus ZTE-MRI provided objective-quantitative evidence of maximal-similarities (15% of radiomic features) and maximal-differences (9.3%), outlining the true range of features where ZTE may be considered comparable with conventional CT. Based on preliminary data, we hypothesize that the normal bone feature range may differ from sclerotic and lytic bone lesions.
**Limitations:** Small sample size.
**Funding for this study:** The John S. Dunn, Sr. Distinguished Chair in Diagnostic imaging and M.R Evelyn Hudson Foundation Endowed Professorship.
**Has your study been approved by an ethics committee?** Not applicable
**Author Disclosures:**
Sam Lo: Nothing to disclose
Elvis Duran-Sierra: Nothing to disclose
Jossue Espinoza: Nothing to disclose
Behrang Amini: Nothing to disclose
Colleen M. Costelloe: Nothing to disclose
David Wells: Nothing to disclose
Raul Fernando Valenzuela: Nothing to disclose
William Murphy: Nothing to disclose
Mathew Antony: Nothing to disclose

**Acetabular cup version measurement in total hip arthroplasty: Comparing MRI versus CT**
*F. Abel*, R. Sutter, F. Schmaranzer; Zürich/CH
*(frederikabel@gmx.net)*

**Purpose:** The acetabular cup version in patients with total hip arthroplasty (THA) is a key parameter influencing hip stability and functional outcomes. Although CT is the reference standard for assessing cup orientation, MRI has emerged as the first-line cross-sectional imaging modality for evaluating postoperative complications. This study compared MRI- and CT-based measurements of acetabular cup version in terms of agreement, reproducibility, and reliability.
**Methods or Background:** Patients who underwent THA between 2015 and 2025 and received both postoperative CT and MRI were retrospectively reviewed. MRI was performed using optimized metal-artifact reduction sequences. Acetabular cup version was measured on axial high-bandwidth T1-weighted turbo-spin echo sequences and corresponding CT scans by two musculoskeletal radiologists. Inter- and intra-reader, as well as inter-modality agreement, were assessed using intraclass correlation coefficients (ICC). Bland-Altman plots evaluated systematic bias.
**Results or Findings:** Thirty patients were included (median age, 69.5 years; 14 women). Mean acetabular version ranged from 31.20°–31.22° (standard deviation [SD], 9.40–10.30) and from 30.05°–30.21° (SD, 8.90–9.32) for CT and MRI for both readers, respectively. Inter-reader reproducibility was excellent for CT (ICC, 0.96) and good for MRI (ICC, 0.76). Intra-reader reliability was excellent for both modalities (ICC, CT: 0.99; MRI: 0.94). Bland-Altman analysis showed no statistically significant differences between CT and MRI measurements with a slight positive bias for MRI (mean pooled difference, 1.05°; p = 0.06; limits of agreement, –4.66° to 6.76°) and good to excellent inter-modality agreement for both readers (ICC, 0.86–0.90).
**Conclusion:** MRI provides reliable measurements of acetabular cup version in THA, demonstrating good-to-excellent agreement with CT. MRI can be used for estimating cup version within clinically acceptable limits.
**Limitations:** No limitations were identified.
**Funding for this study:** No funding was received for this study.
**Has your study been approved by an ethics committee?** Yes
**Ethics committee - additional information:** The study was approved by the local ethics committee (Cantonal Ethics Committee Zurich, approval number: 2023-00969).
**Author Disclosures:**
Frederik Abel: Nothing to disclose
Florian Schmaranzer: Nothing to disclose
Reto Sutter: Nothing to disclose

**Diagnostic Performance of Dual-Energy CT for Opportunistic Detection of Rotator Cuff Disease: A Retrospective Multireader Study**
*S. Liu*; Beijing/CN

**Purpose:** Multi-material decomposition (MMD), a key application of DECT, has shown potential in musculoskeletal research. This study aimed to compare the diagnostic performance of DECT-based MMD with standard CT and MRI in detecting rotator cuff disease.
**Methods or Background:** This study evaluated patients diagnosed with rotator cuff disease who underwent third-generation dual-source DECT and 3.0T MRI within a 2-week interval between December 2023 and November 2024. Shoulder arthroscopy served as the reference standard. Six readers independently assessed rotator cuff tears and determine the degree of supraspinatus tendon diseases using standard CT, DECT-based MMD and MRI. Area under the curve (AUC), sensitivity, specificity, positive/negative predictive values and accuracy were calculated for the diagnosis of rotator cuff disease. Friedman test was used to analyze the radiologists' diagnostic confidence across the three image types.
**Results or Findings:** In total of 103 patients (mean age: 50.0 ± 15.6 years) underwent shoulder arthroscopy. MMD demonstrated a higher average AUC for diagnosing rotator cuff tears (88% vs. 65%, P < 0.001) and supraspinatus tendon disease (86% vs. 63%, P < 0.001) compared to standard CT. Its diagnostic performance for supraspinatus tendon disease (91% vs. 90%, P = 0.35) and full-thickness tears (95% vs. 93%, P = 0.11) was comparable to that of MRI.
**Conclusion:** DECT-based MMD demonstrated superior diagnostic performance and reliability for detecting rotator cuff diseases compared to standard CT, with accuracy comparable to that of MRI in detecting supraspinatus tendon tears. DECT-based MMD offers a promising approach for the opportunistic detection of rotator cuff diseases.
**Limitations:** First, as a retrospective single-center study, the generalizability of our findings are limited. Second, its ability to identify partial-thickness tears and subtle tendon degeneration—conditions more frequently encountered in clinical practice—remains limited.
**Funding for this study:** This work was supported by National Key Research and Development Program of China (No.2023YFC2410703)
**Has your study been approved by an ethics committee?** Yes
**Ethics committee - additional information:** This retrospective study was approved by the Institutional Review Board of Peking University Third Hospital (Approval Number: M2024188)
**Author Disclosures:**
Suwei Liu: Nothing to disclose

**Dual-Layer Spectral CT with Electron Density in Bone Marrow Edema Diagnosis: A Valid Alternative to MRI?**
*C. Beltramini*[1], F. Piacentino[1], F. Fontana[1], A. Coppola[1], D. Mesiano[1], C. Recaldini[1], A. M. Ierardi[2], G. Carrafiello[2], M. Venturini[1]; [1]Varese/IT, [2]Milan/IT

**Purpose:** Although MRI with fat-suppression sequences is the gold standard for diagnosis of bone marrow edema (BME), Dual-Layer Spectral CT (DL-SCT) with electron density (ED) provides a viable alternative, particularly in situations where an MRI is not accessible. Using MRI as the reference standard, this study analyzed how DL-SCT with ED reconstructions may be a valid alternative in the detection of BME.
**Methods or Background:** This retrospective study included 28 patients with a suspected diagnosis of BME via MRI conducted between March and September 2024. Patients underwent DL-SCT using ED reconstructions obtained through IntelliSpace software v. 12.1. Images were evaluated by two experienced radiologists and one young radiologist in a blinded way, giving a grade from 0 to 3 to classify BME (0 absence; 1 mild; 2 moderate; 3 severe). To reduce the recall bias effect, the order of image evaluations was set differently for each reader. p-Values were considered significant when <0.05. Fleiss' Kappa was used to assess inter-rater reliability: agreement was considered poor for k < 0; slight for k 0.01-0.20; fair for 0.21-0.40; moderate for 0.41-0.60; substantial for 0.61-0.80; and almost perfect for 0.81-1.00.
**Results or Findings:** All the readers detected the presence or absence of BME using DL-SCT. Inter-rater reliability for grade 0 resulted in 1 (p-value < 0.001); for grade 1: 0.21 (p-value < 0.001); for grade 2: 0.197 (p-value < 0.001); and for grade 3: 0.515 (p-value < 0.001).
**Conclusion:** ED reconstructions allowed the identification of BME presence or absence in all analyzed cases, thus suggesting DL-SCT as a potentially effective method for its detection.
**Limitations:** Heterogeneous sample that doesn't consider metabolic disorders different from osteoporosis or malignancy.
**Funding for this study:** None
**Has your study been approved by an ethics committee?** Not applicable
**Author Disclosures:**
Anna Maria Ierardi: Nothing to disclose
Federico Fontana: Nothing to disclose
Cecilia Beltramini: Nothing to disclose
Andrea Coppola: Nothing to disclose
Chiara Recaldini: Nothing to disclose
Filippo Piacentino: Nothing to disclose
Massimo Venturini: Nothing to disclose
Daniele Mesiano: Nothing to disclose
Gianpaolo Carrafiello: Nothing to disclose

**Diagnostic value of Dual-Energy Cone Beam CT in Musculoskeletal trauma: A comparative evaluation against single-energy CBCT and MRI**
*N. Castro*, J. Catala March; Barcelona/ES
*(ncastro@institutsguirado.com)*

**Purpose:** Dual-energy CBCT (DE-CBCT) represents a significant technological evolution in musculoskeletal imaging, offering superior tissue differentiation compared to single-energy CBCT (SE-CBCT) through material decomposition. While MRI remains the gold standard for soft tissue evaluation, DE-CBCT emerges as a promising alternative when MRI is contraindicated or unavailable. This prospective study evaluated the diagnostic performance of DE-CBCT against SE-CBCT for bone structures and MRI for soft tissues, focusing on its clinical application in trauma and joint diseases.
**Methods or Background:** Thirty participants (45.57±17.93 years) with suspected or confirmed pathology and MRI inclusion criteria were enrolled. Each participant underwent DE-CBCT (80kVp; 140kVp), SE-CBCT (120kVp), and MRI (proton density and T1-weighted). Two blinded, MSK-experienced radiologists independently assessed bone detail, soft tissue contrast, and diagnostic confidence using a 5-point Likert scale.
**Results or Findings:** Inter-reader agreement was higher for DE-CBCT than SE-CBCT (k=0.82 vs 0.71). For soft tissues, MRI showed superior agreement over DE-CBCT (k=0.94 vs 0.76). No significant differences in bone diagnostic accuracy emerged between DE-CBCT and SE-CBCT, although DE-CBCT received higher qualitative scores. Differences with MRI were notable for soft tissues (p<0.05), confirming its superior visualisation. However, DE-CBCT affirmed accurate visualisation of articular cartilage and tendons. Although not statistically significant, DE-CBCT provided greater confidence in complex fractures, such as intra-articular wrist and ankle injuries, while reducing the need for additional MRI and speeding emergency decision-making.

**Conclusion:** DE-CBCT is an effective tool for recent trauma, especially when MRI is unavailable. It accurately detects fractures and soft tissue changes, such as bone oedema and joint effusions, offering a comprehensive post-traumatic assessment. Clinically, DE-CBCT should be integrated into emergency departments for rapid triage of complex trauma and prioritised over SE-CBCT for bone oedema or cartilage evaluation.
**Limitations:** Increment of radiation exposure compared to SE-CBCT
**Funding for this study:** None
**Has your study been approved by an ethics committee?** Not applicable
**Author Disclosures:**
Jordi Catala March: Nothing to disclose
Nagore Castro: Nothing to disclose

**Diagnostic Value of Bone Marrow Edema Maps from Photon-Counting CT in Suspected Fractures**
*E. Vilkelyte*, N. F. Kämmerling, E. Tesselaar; Linköping/SE
*(egleivilk@gmail.com)*

**Purpose:** To evaluate the diagnostic utility of bone marrow edema (BME) maps generated using dual source photon-counting CT (PCCT), compared with ultra-high-resolution (UHR) standard reconstructions, in patients with suspected fractures in different .
**Methods or Background:** This study included 33 patients (37 examinations; mean age 59.5 ± 22.7 years, range 13–88) with suspected fractures at various anatomical sites including spine, hip, and wrist, examined on a clinical PCCT system using a dual-source acquisition mode. Two radiologists with 9, and 10 years of experience, and a second-year radiology resident, independently reviewed anonymised datasets in two sessions: first, UHR images only; second, the same cases (in randomized order) supplemented with BME maps, and assessed fracture presence and diagnostic confidence (5-point scale). Image quality and evaluability of BME maps, and inter-rater agreement were analysed.
**Results or Findings:** Agreement on fracture detection improved from moderate without BME maps (κ=0.514, p<0.001) to substantial when BME maps were available (κ=0.711, p<0.001). Overall diagnostic confidence increased significantly with BME maps (median 4→5, p=0.007). In 7% of cases, BME maps led to newly diagnosed fractures, while 4% of suspected fractures were excluded. The change in diagnosed fractures was more frequent for the radiology resident. Interobserver agreement on the presence of an edema signal in the BME maps was low (AC1=0.197, p=0.074), and image quality ratings for the BME maps differed significantly between readers (p<0.001). BME maps were considered non-evaluable due to artifacts in 4/37 (11%) of examinations.
**Conclusion:** BME maps derived from PCCT improve interobserver agreement and diagnostic confidence in fracture assessment. They may aid in detection and exclusion of subtle fractures beyond UHR images alone.
**Limitations:** Limited sample size; heterogeneous patient cohort; absence of independent diagnostic reference standard for fractures;
**Funding for this study:** N.a.
**Has your study been approved by an ethics committee?** Not applicable
**Author Disclosures:**
Nina Fredäng Kämmerling: Nothing to disclose
Erik Tesselaar: Nothing to disclose
Egle Vilkelyte: Nothing to disclose

**Role of z effective maps for vertebral bone marrow edema assessment with dual energy ct: comparison with water calcium maps and magnetic resonance imaging**
*M. Bozzolo*, G. Gallarato, A. Balderi, E. Roberto, S. Amabile, D. Buccicardi; Cuneo/IT
*(dott.marcobozzolo@gmail.com)*

**Purpose:** To explore the role of Z-effective maps as an additional tool, in combination with Water/Calcium maps, for detecting vertebral bone marrow edema (BME) in spinal fractures with dual energy ct (DECT), with MRI as reference standard.
**Methods or Background:** We analysed 261 vertebral bodies from 41 patients with acute spinal trauma who underwent DECT (GE Revolution CT ES 128, Rapid-Switching 80–140 kVp) and MRI. Qualitative analysis compared Z-effective and Water/Calcium maps with MRI, assessing BME presence and extent. Two independent readers evaluated all examinations blinded to MRI results. Quantitative analysis included HU, Z-effective, Water/Calcium, SNR, and spectral curves between edematous and healthy bone.
**Results or Findings:** Z-effective and Water/Calcium maps showed sensitivity 93.8–96.2%, specificity 98.9–99.5%, accuracy 97.3–98.5%, with inter-reader agreement (κ >0.96). Quantitative values were higher in edematous bone: HU (298 vs 53, p<0.001), Z-effective (10.36 vs 8.39, p<0.001), Water/Calcium (1071 vs 1002 mg/cm³, p<0.001), and SNR (14.4 vs 2.3, p<0.001). Z-effective strongly correlated with HU (r=0.92, p<0.001), while correlation with Water/Calcium was moderate (r=0.37, p=0.016), indicating complementary diagnostic value. Spectral curves confirmed higher attenuation in BME than healthy bone (p<0.001). ROC analysis showed excellent diagnostic performance (AUC Z-effective 0.97–0.98; Water/Calcium 0.96–0.97).
**Conclusion:** Z-effective maps show diagnostic accuracy comparable to Water/Calcium maps in detecting vertebral BME and provide complementary information. Their integration strengthens the role of DECT as a reliable tool for bone marrow edema assessment. In selected clinical and emergency settings, DECT may represent a practical alternative to MRI when not available or contraindicated.
**Limitations:** The time interval, although short, between DECT and MRI may have influenced the extent of edema. Relatively small patient sample. Variability of MRI scanners, potentially affecting quality and interpretation.
**Funding for this study:** This study received no external funding.
**Has your study been approved by an ethics committee?** Not applicable
**Author Disclosures:**
Simone Amabile: Nothing to disclose
Gabriele Gallarato: Nothing to disclose
Marco Bozzolo: Nothing to disclose
Alberto Balderi: Nothing to disclose
Emanuele Roberto: Nothing to disclose
Duccio Buccicardi: Nothing to disclose

**Deep learning-based chemical shift-artifact correction of ZTE MRI for enhanced bone assessment of the lumbar spine**
*C. Obermüller*[1], U. Bach[1], F. Zecca[2], F. Heidt[1], M. Lohezic[1], R. Guggenberger[3], E. Burian[1], J. Kroschke[1], F. Ensle[1]; [1]Zürich/CH, [2]L'Aquila/IT, [3]Winterthur/CH
*(carina.obermueller@live.at)*

**Purpose:** To assess the impact of deep learning-based (DL) chemical shift-artifact correction (CSC) on bone assessment in zero echo time (ZTE) MRI of the lumbar spine, compared to non-DL and DL-reconstructions. ZTE is a bone-specific MRI sequence suffering from CS artifacts, particularly in the spine and at 3T.
**Methods or Background:** This retrospective study included 60 clinical patients with ZTE lumbar spine MRI at 3T. Raw k-space data was reconstructed offline with a prototype DLCSC algorithm (specifically designed for CS reduction), prototype DL algorithm without artifact correction and non-DL technique. Quantitative image sharpness was plotted with line profiles over vertebrae L2-4, analysed with repeated-measures ANOVA. Two radiologists assessed qualitative pathology-related criteria (pathology type, conspicuity, impact of artifacts, diagnostic confidence, n = 22) and diagnostic image quality (cortical and trabecular bone delineation, noise, artifacts, n = 38) using a 4-point scale. Ordinal data was analysed using the Friedman test and weighted Cohen's kappa.
**Results or Findings:** For quantitative sharpness, the mean score for DLCSC (0.388) was significantly higher than for non-DL (0.269; post-hoc p = .010) and DL (0.247; post-hoc p = .001). For the qualitative assessment, mean scores consistently improved from non-DL to DL to DLCSC, respectively: Diagnostic Image Quality (p < 0.001): means were 0.95, 1.63, and 2.37. Pathology Conspicuity (p < 0.001): means were 1.27, 1.82, and 2.18. Diagnostic Confidence (p < 0.001): means were 1.86, 2.32, and 2.59. Image Noise (p < 0.001): means were 1.18, 1.76, and 2.55. Inter-reader agreement for the qualitative assessment was moderate (k=0.45-0.6).
**Conclusion:** The DLCSC technique significantly enhanced bone assessment of the lumbar spine, compared to DL and non-DL reconstructions, including the evaluation of pathologies. This advancement extends the utility of ZTE for comprehensive spine imaging.
**Limitations:** Modest sample size
**Funding for this study:** No funding was received for this study (Basec-Nr 2021-02408).
**Has your study been approved by an ethics committee?** Yes
**Ethics committee - additional information:** This study received Ethics Committee approval.
**Author Disclosures:**
Maelene Lohezic: Nothing to disclose
Franziska Heidt: Nothing to disclose
Fabio Zecca: Nothing to disclose
Falko Ensle: Nothing to disclose
Roman Guggenberger: Nothing to disclose
Ulf Bach: Nothing to disclose
Jonas Kroschke: Nothing to disclose
Carina Obermüller: Nothing to disclose
Egon Burian: Nothing to disclose

08:00 - 09:00 Research Stage 3

### Research Presentation Session: Physics in Medical Imaging Hot Topic with Keynote Lecture

### RPS 1213

### Hot Topic: error reporting

**Moderator**
John Damilakis; Iraklion/GR
*(damilaki@med.uoc.gr)*

**Keynote Lecture**
Donald P. Frush; Durham/US
*(donald.frush@duke.edu)*

**Radiation safety deviation reporting of X-ray practices in Finland: analysis of the last 10 years**

E. Hallinen, *T. Hietamies*, I. P. Kuurne, A. Lajunen, V. Ruonala, J. Suutari, P. Tenkanen-Rautakoski; Vantaa/FI
*(tommi.hietamies@stuk.fi)*

**Purpose:** This study aims to describe the characteristics, frequency, and outcomes of reported radiation safety deviations in healthcare X-ray practices in Finland, and to highlight their role in supporting continuous safety improvement.
**Methods or Background:** In Finland, radiation safety deviations in healthcare X-ray practices must be reported to the Radiation and Nuclear Safety Authority (STUK). Deviations are classified into significant cases, which must be reported immediately, and less significant cases, which are submitted annually. Since 2015, approximately 270 significant and 12,650 less significant deviations have been reported. The reporting system is regarded as an indicator of a strong safety culture. Deviation reports submitted to STUK between 2015 and 2024 were analyzed. Reports were classified into predefined categories. Descriptive analyses included frequency distributions, trends over time, and typical causes. From 2025 onwards, reporting requirements will also include preventive measures taken for each deviation type.
**Results or Findings:** No severe radiation safety incidents have been reported since the implementation of systematic reporting. Patient exposures resulting from deviations remained below 100 mSv, occupational exposures below 1 mSv, and unintended foetal exposures below 40 mGy. The most common deviation was failed examinations due to incident, requiring repetition, typically caused by technical problems or human error. Analysis of reports and anonymized case studies have been used as educational tools in radiation safety training.
**Conclusion:** The systematic reporting of radiation safety deviations has proven effective in monitoring and improving safety culture in Finnish healthcare X-ray practices. The introduction of preventive action reporting from 2025 is expected to further strengthen proactive safety management.
**Limitations:** Not all radiation safety deviations are reported to STUK.
**Funding for this study:** This study is a by-product of statutory regulatory oversight, conducted and funded by STUK.
**Has your study been approved by an ethics committee?** Not applicable
**Author Disclosures:**
Iida Pauliina Kuurne: Nothing to disclose
Elina Hallinen: Nothing to disclose
Petra Tenkanen-Rautakoski: Nothing to disclose
Atte Lajunen: Nothing to disclose
Verneri Ruonala: Nothing to disclose
Tommi Hietamies: Nothing to disclose
Juha Suutari: Nothing to disclose

**Patterns and Safety Implications of Unintended Patient Exposures in Imaging and Radiotherapy Across Multiple Countries**

*C. Paraskevopoulou*[1], A. Papachristodoulou[1], C. D. Filho[2], D. Baltazar[3], C. Colmo[4], A. Roncacci[1]; [1]Hague/NL, [2]Barcelona/ES, [3]Amadora/PT, [4]Padua/IT
*(chryssa.paraskevopoulou@affidea.com)*

**Purpose:** Unintended patient exposures in ionizing radiation procedures, including diagnostic imaging (CT, X-ray, PET-CT, Mammography, SPECT-CT) and therapeutic applications (Radiotherapy, Brachytherapy), pose risks to safety, diagnostic accuracy, and treatment precision. This study analyzed unintended exposure incidents across multiple countries to identify patterns, underlying causes, and opportunities for safety improvement.
**Methods or Background:** Incident data from a centralized multi-national reporting system were analyzed over a three-year period. Incidents were categorized as repeated examination, wrong patient, wrong examination, wrong site, and wrong side. Severity was classified as no physical harm, minor, medium, serious, or severe. Rates were calculated relative to 8.18 million ionizing radiation procedures performed in 376 centers across 15 countries.
**Results or Findings:** Across 8.18 million ionizing radiation procedures, 369 unintended patient exposures were identified (0.0011%). Repeated examinations and wrong examination events together accounted for over half of all incidents, reflecting common issues such as motion artifacts, technical failures, or request-to-procedure mismatches. Wrong patient exposures were also frequent, representing nearly one quarter of cases, while wrong site and wrong side events were less common but clinically significant (Fig.1). Most events were minor or medium in severity, yet a consistent presence of serious cases underscores the importance of systematic monitoring and targeted preventive measures.
**Conclusion:** Although unintended patient exposures represent a very small fraction of ionizing radiation procedures, their implications for safety and quality of care are significant. The high share of repeated and wrong examination events highlights the need for workflow optimization, request-to-procedure verification, improved patient communication, and enhanced personel training. Preventive strategies should also address wrong patient, wrong site, and wrong side incidents. Continuous monitoring are essential to guide systematic improvements and targeted preventive measures, supporting a stronger safety culture across imaging and radiotherapy services.
**Limitations:** N/A
**Funding for this study:** N/A
**Has your study been approved by an ethics committee?** Not applicable
**Author Disclosures:**
Alessandro Roncacci: Nothing to disclose
Athanasia Papachristodoulou: Nothing to disclose
Cristian Colmo: Nothing to disclose
Chrysa Paraskevopoulou: Nothing to disclose
Clodoaldo Domingos Filho: Nothing to disclose
David Baltazar: Nothing to disclose

**Projected Impact of The U.S. Quality Measure, Excessive Radiation Dose or Inadequate Image Quality for Diagnostic CT on Radiation Dose and Projected Cancer Risks**

*R. Smith-Bindman*[1], G. Frija[2]; [1]San Francisco, CA/US, [2]Paris/FR
*(rebecca.smith-bindman@ucsf.edu)*

**Purpose:** Excessive radiation doses in CT exams are a patient safety concern. Provider-based financial incentives in the U.S.have successfully improved patient safety. UCSF, with funding from the Centers for Medicare & Medicaid Services, developed a quality measure for CT to reduce unnecessary radiation doses while maintaining diagnostic value. During the presentation, speakers will detail the measure's methodology, discuss its impact on projected cancers, and explore its potential application in Europe.
**Methods or Background:** The measure automates CT radiation/image noise calculations using standardized data based on scan indications. Eligible adult CT exams are assigned to categories with thresholds for allowable radiation doses and noise based on ICD-10-CM and CPT codes. Radiation dose is adjusted for patient size, and exams are flagged as "out-of-range" if they exceed thresholds supported by radiologists' estimates of diagnostic value. Testing across 7 health systems, 16 hospitals, and 606 clinicians included 48,500 CT exams, with 33% flagged as out-of-range. Data from an international CT registry showed approximately 28% of European exams were out-of-range.
**Results or Findings:** If dose thresholds had been applied to the 90 million CT exams conducted among 59 million adults in the U.S. in 2023, the projected number of future cancers would decrease from 93,000 to 69,000—a 26% reduction. Abdomen and pelvis CT contributed the highest cancer burden, with 31,200 projected cancers, which could be reduced by 9,556 if thresholds were implemented. Chest CT followed, with 21,500 projected cancers, 8,376 of which could be prevented. These two CT categories represent key targets for optimizing and standardizing radiation dose protocols.
**Conclusion:** The measure is being adopted across U.S. hospital and physician payment programs, with over 3,500 hospitals working towards reporting. Financial incentives will drive improvements in patient safety and care quality.
**Limitations:** None
**Funding for this study:** The quality measure was funded by the U.S. Centers for Medicare and Medicaid Services, and the Patient Centered Outcomes Research Institute
**Has your study been approved by an ethics committee?** Not applicable

**Author Disclosures:**
Guy Frija: Nothing to disclose
Rebecca Smith-Bindman: Founder: Smith-Bindman is a co-founder of Alara Imaging, Inc., a company focused on improving the clinical and operational aspects of health systems, including collecting and reporting radiation dose and image quality associated with computed tomography as part of payer-led quality programs. Alara Imaging played no role in any aspect of the paper and the work does not overlap with Alara's commercial activities.

**Organ-specific radiation doses after mispositioning or scan direction variation: Impact of single versus dual scanogram across four different CT-scanners**

*L. D'Hondt*[1], C. Haentjens[2], A. Snoeckx[3], K. Bacher[1]; [1]Ghent/BE, [2]Leuven/BE, [3]Antwerp/BE

**Purpose:** With the considerable number of low-dose CT examinations for lung cancer screening, participant mispositioning or scan direction variations are expected. These variations affect automatic tube current modulation (ATCM), but dose implications across CT-models remain unknown. Moreover, current screening guidelines lack scanogram specifications. This study simulated organ-specific doses when protocol deviations were preceded by either single or dual scanogram.
**Methods or Background:** ATCM-profiles were established using the Alderson Rando phantom on four CT-scanners (GE Revolution, Siemens SOMATOM Definition Flash, Canon Aquilion ONE, Philips Spectral CT 7500). Protocol variations included vertical and lateral mispositioning (2cm increments) and scan direction changes (craniocaudal, caudocranial), each preceded by either a single posteroanterior (PA) or dual (PA+Lateral) scanogram. Each variation was simulated with the ImpactMc Monte Carlo software for 32 patient-specific voxelmodels and organ doses were calculated (lungs, heart, thyroid, liver, breasts).
**Results or Findings:** GE and Siemens scanners showed considerable linear increase in organ doses (respectively 18-50% and 5-25%) with vertical/downward mispositioning using a single PA scanogram. This was eliminated by adding a lateral scanogram. Canon and Philips scanners generally displayed stable ATCM-profiles and dose levels after vertical mispositioning, regardless of scanogram type. Solely the Canon scanner showed substantial average dose increases of 37.5% and 34% after respectively a single and dual scanogram across lateral mispositioning. The ATCM-behaviour upon changing to the craniocaudal scan direction is highly model dependent. However, combined with the dual scanogram, dose deviations are minimised for all scanners except for Siemens, where no differences are seen.
**Conclusion:** An additional lateral scanogram can mitigate considerable organ dose increases related to scan protocol deviations. Since ATCM-behaviour varies across CT-scanner models, scanner-specific evaluation and optimisation should involve medical physics expertise.
**Limitations:** The limitations of the study are the use of a phantom.
**Funding for this study:** Funding was provided by the FWO "Kom op tegen Kanker"-project for lung cancer screening research in Belgium. (Project number: G0B1922N).
**Has your study been approved by an ethics committee?** Not applicable
**Author Disclosures:**
Klaus Bacher: Nothing to disclose
Louise D'Hondt: Nothing to disclose
Annemiek Snoeckx: Nothing to disclose
Claudia Haentjens: Nothing to disclose

**Harmonizing Dose Monitoring and QA Reporting in Dental Cone-beam Computed Tomography**

*S. Inkinen*, J. H. J. Ketola, A-M. Vitikainen, M. Kortesniemi; Helsinki/FI
*(satu.inkinen@hus.fi)*

**Purpose:** We report a case emphasizing the need for harmonized external quality assurance (QA) in dental imaging, specifically regarding dose area product (DAP) monitoring. Our existing QA procedure was modified with an electronic form that radiographers complete during device QA. This form also includes externally verified DAP monitoring values, since, our dose monitoring platform cannot currently track DAP for dental cone-beam computed tomography (CBCT) units.
**Methods or Background:** Dental CBCT devices follow manufacturer-specific QA protocols using custom phantoms. These protocols often omit DAP monitoring, and dose monitoring systems may not receive input from dental devices via Radiation Dose Structured Reports, or only report CTDI instead of DAP that is the dose unit used for diagnostic reference levels in Finland. QA is required at least twice a year or following service. We had a case that after a service, a dental unit's software was reverted to factory settings (exposure settings altered) via service engineer without notifying users, leading to consistently elevated patient doses. This went unnoticed due to the lack of DAP data integration to our dose monitoring systems.
**Results or Findings:** We developed a multi-modality QA system using open-source libraries and a web-based interface at HUS Diagnostic Center. The system now includes dental device QA via electronic forms and monthly DAP value checks for the most common protocol (teeth). The updated system streamlines QA, eliminates parallel spreadsheet logging, and improves data consistency. It also addresses the previously reported adverse event by incorporating DAP monitoring.
**Conclusion:** The revised QA process is scalable across dental imaging units. The web-based form harmonizes documentation and enables dose monitoring, supporting safer and more transparent imaging practices. Manufacturers should ensure DAP values are included in baseline system checks.
**Limitations:** No limitations were identified.
**Funding for this study:** None.
**Has your study been approved by an ethics committee?** Not applicable
**Author Disclosures:**
Mika Kortesniemi: Nothing to disclose
Satu Inkinen: Nothing to disclose
Anne-Mari Vitikainen: Nothing to disclose
Juuso Heikki Jalmari Ketola: Nothing to disclose

**Errors in hybrid nuclear medicine: overview, challenges, and prevention**

*K. Skovorodko*, D. Serėnaitė-Pečiulė, V. Statkus, R. Komiagienė, L. Krynke, B. Griciene; Vilnius/LT
*(kirill.skov@gmail.com)*

**Purpose:** Hybrid nuclear medicine methods such as PET/CT, SPECT/CT and PET/MRI have significantly improved diagnostic accuracy by combining functional and anatomical data. Despite the clinical benefits of these methods, the complexity nuclear medicine procedures and these modalities entails a wide range of potential errors, ranging from technical to human factors. The aim of this study was to analyse the reported errors in Lithuanian hospitals providing nuclear medicine services, identify the main problems associated with these errors and discuss possible ways to prevent them.
**Methods or Background:** An assessment of official error (unintended events and accidents) registration data for the period 2020–2025 in Lithuanian hospitals providing nuclear medicine services was conducted. An oral survey of nuclear medicine department personnel was also conducted to assess potential weaknesses that could lead to errors and identify possible measures to prevent them.
**Results or Findings:** Prevention strategies target both to quality assurance system and human performance should be implemented. Key measures include compressive quality assurance of the equipment, standardized training and practice, and structured reporting to reduce errors. AI and advanced software can automate checks, limit misregistration, and support interpretation. A safety-focused culture with error reporting and regular clinical audits ensures early detection and ongoing improvement.
**Conclusion:** In conclusion, errors in hybrid nuclear medicine are due to many factors, reflecting both technological complexity and human limitations. Some of the potential errors could be minimized by using artificial intelligence solutions in nuclear medicine. By addressing these challenges through training, protocol standardization, and technological innovation, the field can improve diagnostic reliability while ensuring patient safety and optimal clinical outcomes. Errors in hybrid nuclear medicine not only compromise diagnostic reliability but can also lead to inappropriate clinical decisions and increased radiation exposure.
**Limitations:** Not applicable
**Funding for this study:** No funding was provided for this study.
**Has your study been approved by an ethics committee?** Not applicable
**Author Disclosures:**
Renata Komiagienė: Nothing to disclose
Dovilė Serėnaitė-Pečiulė: Nothing to disclose
Kirill Skovorodko: Nothing to disclose
Vaidas Statkus: Nothing to disclose
Birutė Griciene: Nothing to disclose
Leonid Krynke: Nothing to disclose

**Replacing Error-Prone Spreadsheets with a Free and Open-Source QC Management System: A QATrack+ Pilot Study Using the Pylinac ACR MRI Large Phantom Module**

*C. Attard*, D. J. M. Scicluna, K. Schembri, N. Vella, E. Pace, C. J. Caruana; Msida/MT
*(clarissa.attard.21@um.edu.mt)*

**Purpose:** Medical Physics Quality Control (MPQC) often relies on improvised, locally-developed, spreadsheet-based, software tools used to manually collect, analyse, and present results. This may lead to undetected inconsistencies and, data-entry and formula errors. A more reproducible and controlled QC process is essential. This work reviewed spreadsheet-based error in current practice and explored QATrack+ as an alternative robust centrally managed solution.

**Methods or Background:** A literature review was conducted using Google Scholar with the search string "error AND spreadsheet AND medical-physics" (2015–present), which retrieved n = 1,780 papers. QATrack+, a free-open-source-software (FOSS) based alternative to spreadsheets was pilot-tested on annual MRI MPQC based on ACR guidelines.
**Results or Findings:** No previous studies were identified that investigated, classified or quantified errors emerging from the use of spreadsheet software in MPQC, despite hundreds of 'horror-stories' documented by the European Spreadsheet Risks Interest Group. The fact that studies have shown that the vast majority of MPQC rely on spreadsheets which are not compliant with the Flexible, Appropriate, Structured, Transparent (FAST) Standard for spreadsheet document control, indicates that errors are likely to be present which are undetected. QATrack+ supports the following main data-integration routes: external analysis with manual or automated API data entry and internal analysis with either non-version-controlled or version-controlled custom Python code. The latter preferred method was used in this study.
**Conclusion:** A centrally managed, controlled system such as QATrack+ is essential particularly considering that software is legally a medical device under EU regulations. The QATrack+ based MRI pilot programme developed demonstrates that such tools are within reach of all medical physics departments.
**Limitations:** N/A
**Funding for this study:** N/A
**Has your study been approved by an ethics committee?** Not applicable
**Author Disclosures:**
Carmel J. Caruana: Nothing to disclose
Nolan Vella: Nothing to disclose
Eric Pace: Nothing to disclose
Clarissa Attard: Nothing to disclose
Keith Schembri: Nothing to disclose
David Joseph Matthew Scicluna: Nothing to disclose

**Reporting of MRI Incidents and Near-misses at a Multi-site Imaging Centre**
*L-E. Lahtela*, T. Mäkelä, T. Kaasalainen, M. Timonen; Helsinki/FI

**Purpose:** MRI safety relies on comprehensive staff training, well-defined duties, thorough risk awareness, access control, strict screening protocols, emergency preparedness, and management of safety incidents and concerns. Fostering an ongoing safety culture is one of the most influential strategies for sustaining and enhancing safety practices. Operating within a multi-site and multi-departmental environment introduces additional factors that must be addressed to ensure effective communication and coordination. Over the years, we have improved our incident reporting and handling protocols.
**Methods or Background:** Historically, MRI safety practices in Finland have largely been determined by individual imaging facilities and local established protocols. This is in contrast with ionising radiation modalities, where nationwide regulation has long promoted best clinical practices, safety monitoring, and communication.
**Results or Findings:** We created an online tool to report MRI incidents and near misses. Typical reported incidents include small metal objects in the scanning room and uncertainty regarding the safety of undisclosed implants or other foreign bodies. We have also identified the lack of MRI safety training of visiting non-MRI personnel as a recurring and significant risk factor. The main benefit of the tool has been lowering the barrier to report incidents. All findings are communicated in regular meetings and incorporated into staff training sessions. Significant accidents reported by other institutions worldwide are also reviewed and discussed.
**Conclusion:** If the safety culture does not encourage constructive feedback and open discussion, there is a risk of underreporting and an increased likelihood of preventable accidents. MRI safety can be enhanced by promoting the reporting, management, and documentation of all incidents, near-misses, and potential safety issues. The reporting system should be simple and user-friendly to encourage participation and effectiveness, and push towards continuous improvement of MRI safety.
**Limitations:** No limitations were identified.
**Funding for this study:** No funding was received for this study.
**Has your study been approved by an ethics committee?** Not applicable
**Author Disclosures:**
Touko Kaasalainen: Nothing to disclose
Laura-Elina Lahtela: Nothing to disclose
Teemu Mäkelä: Nothing to disclose
Marjut Timonen: Nothing to disclose

**08:00 - 09:00 Research Stage 6**

### Research Presentation Session: Professional Challenges: Shaping our Professional Future

### RPS 1230
### Shaping the future of radiology education

**Moderator**
Magali Hovsepian; Buenos Aires/AR
*(magalihovsepian@hotmail.com)*

**From error to excellence: seven principles for highly effective radiology learning**
*R. Magennis*[1], E. K. Roditi[2]; [1]Manchester/UK, [2]Glasgow/UK
*(racmag2@gmail.com)*

**Purpose:** Errors and discrepancies are an unavoidable part of radiology. The Royal College of Radiologists recommends Radiology Events and Learning Meetings (REALM) as a safe forum for discussing them. Beyond reviewing individual cases, these meetings provide powerful insights into the attitudes and behaviours that help radiologists thrive.
**Methods or Background:** Drawing on a year of REALM discussions and informal participant feedback, we identified seven principles that consistently supported positive learning and professional growth. Own the Learning – contribute, seek feedback, and embrace radiology event and learning (REAL) as a tool for growth. Start with Patient Safety – keep patient outcomes at the centre of reflection. Collaborate to Elevate – value input from colleagues across the team. Listen Before You Lead – understand others' reasoning when offering feedback. Reflect, Don't React – approach discrepancies with curiosity rather than defensiveness. Spot the Good – celebrate strong practice as well as errors. Keep Refining – translate learning into ongoing personal and departmental improvement.
**Results or Findings:** By embedding these habits, REALM creates a culture of openness and continuous improvement. This strengthens individual confidence, enhances team collaboration, and, most importantly, improves patient safety.
**Conclusion:** These 7 simple principles help shift REALM from a retrospective review of errors into a forward-looking, supportive process that normalises error, reduces shame and seeks balance with positive cases, further boosting morale. These encourage radiologists to learn together, improve systems, and recognise good practice as well as pitfalls.
**Limitations:** There are no limitations
**Funding for this study:** No funding received
**Has your study been approved by an ethics committee?** Not applicable
**Author Disclosures:**
Rachel Magennis: Nothing to disclose
Emma Kaija Roditi: Nothing to disclose

**Creating a safe space for learning: resident lead radiology events and learning meeting using a traffic-light addendum system**
*R. Magennis*, N. Edi-Osagie, M. M. Kiandee; Manchester/UK
*(racmag2@gmail.com)*

**Purpose:** Structured learning from diagnostic discrepancies is essential in radiology training, particularly in the high-stakes out-of-hours setting. This project describes the implementation of a resident-led learning meeting, tailored to on-call practice and aligned with the RCR's Radiology Events and Learning (REAL) framework.
**Methods or Background:** A traffic-light system was introduced to classify consultant-issued addenda to resident reports: • Green: Consultant agrees with the provisional report. • Amber: Minor additional finding, not impacting immediate management. • Red: Significant change or addition requiring urgent communication with the clinical team. Trainees' provisional reports may precede consultant review by up to 16 hours. Cases were reviewed from the amber and red categories for educational value, not just in terms of clinical diagnostic decisions but also for decision making and communication lessons or human factor related learning. The dedicated resident REAL meeting provides a safe, peer-led forum to discuss anonymised amber/red cases without the perceived inhibition of senior presence.

**Results or Findings:** Outcomes have included improved confidence in clinical reasoning, greater insight into diagnostic uncertainty, and a stronger culture of reflection and shared learning. Leadership skills were fostered through rotating facilitators, and participants reported feeling empowered to ask questions and openly analyse their decision-making processes. Unforeseen benefits included early identification of recurrent error patterns and strengthened links between the trainee group and the wider department via feedback loops into the main REAL meeting.
**Conclusion:** This pilot demonstrates that a psychologically safe, trainee-led forum focused on local discrepancies enhances learning from error, supports professional growth, and ultimately improves patient care.
**Limitations:** There were no limitations
**Funding for this study:** No funding received
**Has your study been approved by an ethics committee?** Not applicable
**Author Disclosures:**
Ndidi Edi-Osagie: Nothing to disclose
Rachel Magennis: Nothing to disclose
Miraen M. Kiandee: Nothing to disclose

**Long-term Evaluation of the Objective Structured Clinical Examination (OSCE) in Radiology Resident Training: A Multi-dimensional Assessment from Examiners' and Examinees' Perspectives**
*N. Ding*, X. Gao, H. Sun, L. Song, X. Wang, Y. Chen, D. Zhang, H. Xue, Z. Jin; Beijing/CN
*(476497046@qq.com)*

**Purpose:** This study aimed to evaluate the effectiveness, reliability, and validity of the Objective Structured Clinical Examination (OSCE) in radiology resident training, from the perspectives of both examiners and examinees.
**Methods or Background:** This retrospective observational study analyzed subjective evaluations and objective examination data collected over 6 years (2018–2021, 2023, and 2024). Subjective evaluations were gathered via questionnaires from 198 examiners and 818 examinees to assess the difficulty and satisfaction with the OSCE. Objective data, including examination scores, difficulty indices, and discrimination indices, for each OSCE station were analyzed using correlation analysis and t-tests.
**Results or Findings:** The OSCE demonstrated stable performance over 6 years, with consistent difficulty levels and discrimination ability across all stations. The average scores for individual stations varied; however, the overall final scores remained stable. Strong correlations between the station and final scores indicate good discrimination. Examinees rated the overall difficulty higher than examiners, but the objective indices aligned with examiner assessments. Over 6 years (198 examiners, 818 examinees), OSCE scores stabilized (85.48–88.48), with improved consistency (station range narrowed to 85.51–93.9 by 2024). Difficulty (0.12–0.15) and discrimination indices remained stable (most $p < 0.05$). Examinees rated it harder than examiners ($p < 0.001$).
**Conclusion:** The OSCE is a reliable, valid, and effective assessment tool in radiology. Evaluating the OSCE from both subjective and objective perspectives ensured the robustness and validity of the examination.
**Limitations:** This study had certain limitations. First, the retrospective design inherently limits causal inferences, though our 6-year dataset provides robust observational evidence. Second, while the 3-point Likert scale (difficult/moderate/easy) was chosen to maximize response rates during time-sensitive post-exam evaluations, future studies could adopt a 5-point scale for more nuanced feedback.
**Funding for this study:** This study was supported by the Peking Union Medical College, Graduate Education and Teaching Reform Project in 2024 (grant number: 2024yjsjg006)
**Has your study been approved by an ethics committee?** Yes
**Ethics committee - additional information:** This study was approved by the Institutional Review Board of the conducting institution (No. S-K2067). All participants provided written informed consent prior to inclusion in the study.
**Author Disclosures:**
Yuan Chen: Nothing to disclose
Xinyu Gao: Nothing to disclose
Xuan Wang: Nothing to disclose
Hao Sun: Nothing to disclose
Ning Ding: Nothing to disclose
Lan Song: Nothing to disclose
Daming Zhang: Nothing to disclose
Zhengyu Jin: Nothing to disclose
Huadan Xue: Nothing to disclose

**Effect of interruptions on radiologists' active reporting time**
*J. Huovinen*, S. Väänänen, A-P. Ronkainen, J. Hakumäki, O. M. Lahtinen; Kuopio/FI
*(juho.huovinen@pshyvinvointialue.fi)*

**Purpose:** While previous studies have shown that interruptions are common in radiologists' workflow, they rely mostly on qualitative data. Interruptions can originate from consultations from trainees, co-workers and clinicians, IR-procedures and tele-communications. We aim to quantify how interruptions affect active reporting times.
**Methods or Background:** We analyzed radiological reporting between 2019-2025 at the Kuopio University Hospital, Finland. Our dataset included radiological examination and report metadata, user profiles from the reporting databases, and command-level usage logs from the PACS. Reporting times and interruptions were determined from examination opening and closing events in PACS, supplemented by "report completed" timestamps from the RIS. An interruption was defined as accession of an unrelated examination before completing the original report. We investigated the disruptiveness of interruptions by grouping them into categories (Zero, One, Two, Three-or-more interruptions) and conducted multivariable linear regression analysis.
**Results or Findings:** The median active reporting times of N=42772 MRI, N=92765 CT and N=241936 X-ray examinations were 13.95±18.95 min, 12.75±15.27 min and 2.13±5.28 min, respectively. The median active reporting times for interrupted cases were 16.04±19.24 min (N=9414) for MRI, 14.87±16.15 min (N=16065) for CT and 2.94±7.61 min (N=36334) for X-ray. The median duration of the interruption was 3.74±53.55 min for MRI, 3.14±47.30 min for CT and 1.38±50.10 min for X-ray. After adjusting the examination type, one interruption was associated with a 26% increase in active reporting time ($p<0.00$, $R2=0.52$), two interruptions increased the active reporting time 44% and three or more interruptions increased the active reporting time 51%.
**Conclusion:** Interruptions have a significant impact on radiologists' active reporting time.
**Limitations:** Interruption types, subgroups and their effect on report quality will be analyzed in future studies.
**Funding for this study:** This study was financially supported (or partly supported) by the State Research Funding for university-level health research, Kuopio University Hospital, Wellbeing Service County of North Savo, Project Number 5063601
**Has your study been approved by an ethics committee?** Not applicable
**Author Disclosures:**
Ari-Petteri Ronkainen: Nothing to disclose
Juho Huovinen: Nothing to disclose
Juhana Hakumäki: Nothing to disclose
Olli Matias Lahtinen: Nothing to disclose
Sami Väänänen: Nothing to disclose

**Research motivation and success factors in radiology – results of a nationwide survey**
*F. Wegner*[1], H. Heinrichs[2], K. Stahlmann[3], F. Kiessling[2], T. Bäuerle[4], P. Bannas[3]; [1]Lübeck/DE, [2]Aachen/DE, [3]Hamburg/DE, [4]Mainz/DE
*(franz.wegner@uksh.de)*

**Purpose:** Research activities are very important for the advancement of medical disciplines. In the context of increasing workload and persistent staff shortages, both the time and willingness to engage in research are diminishing. A comprehensive understanding of determinants of scientific success is therefore essential to enable its systematic and targeted promotion. The goal of this study was to explore motivation and identify key success factors for research in radiology.
**Methods or Background:** A nationwide online survey (54 items, duration: 3.5 months) was conducted in Germany. The questionnaire assessed demographic, intrinsic and extrinsic success characteristics, as well as personal and organizational determinants of success, based on an available career success model. The results were reported descriptively, and associations between success factors and success characteristics were evaluated by using linear, binary-logistic, and multinomial regression models.
**Results or Findings:** A total of 176 individuals participated (164 with academic activity, 10 without). The majority (80%, 139/174) were employed at university hospitals, and 32% held the academic title of Privatdozent (assistant professor) or professor (56/173). The primary motivation for research was intrinsic scientific interest (55%, 89/163), followed by career advancement opportunities (25%, 41/163). Key predictors of intrinsic success included: a) support from department management ($\beta=0.26$, $p<0.001$), b) good work-life balance ($\beta=0.37$, $p<0.001$), and c) sustained engagement in research beyond career milestones ($\beta=0.16$, $p=0.016$). Extrinsic success was significantly associated with mentoring, protected research time, and active involvement in professional societies.

**Conclusion:** Radiology researchers in Germany are predominantly driven by intrinsic motivation. Established determinants of both intrinsic and extrinsic scientific success were confirmed in this study. Awareness of these factors enables targeted support strategies, potentially enhancing scientific achievement in radiology.
**Limitations:** The survey was available only in German.
**Funding for this study:** Federal Ministry of Education and Research; Bundesministerium für Bildung und Forschung 01GP1910A
**Has your study been approved by an ethics committee?** Not applicable
**Author Disclosures:**
Tobias Bäuerle: Nothing to disclose
Franz Wegner: Nothing to disclose
Fabian Kiessling: Nothing to disclose
Katharina Stahlmann: Nothing to disclose
Helen Heinrichs: Nothing to disclose
Peter Bannas: Nothing to disclose

**Radiologists' Quality of Life and Occupational Stress in India: A Cross-Sectional Study**
*F. Abubacker Sulaiman*; Chennai/IN
*(fasulaiman@gmail.com)*

**Purpose:** To evaluate the quality of life, occupational stress, and burnout prevalence among radiologists in India, and to identify key professional and interpersonal factors contributing to stress in contemporary radiology practice.
**Methods or Background:** A cross-sectional, questionnaire-based study was conducted among 210 practicing radiologists across India between January and June 2025. Data were collected using the WHOQOL-BREF and Perceived Stress Scale (PSS-10) along with structured questions on workload, reporting pressure, interpersonal expectations, and lifestyle habits. Responses were anonymized and analyzed using descriptive statistics and multivariate regression to determine predictors of high stress and reduced life quality.
**Results or Findings:** The mean age of participants was 38.4 ± 8.7 years, with a male-to-female ratio of 1.6:1. The average weekly workload was 58 ± 12 hours, and 68% reported frequent emergency or night duties. Turnaround time (TAT) pressure was identified as a major stressor by 74% of respondents, with additional strain arising from referring clinicians demanding immediate appointments and same-day reports (69%), and patients expecting instant results (63%). Overall, 54% exhibited moderate-to-high stress scores, while 39% met criteria for burnout. Long working hours, administrative load, and unrealistic reporting timelines were significantly associated with stress ($p < 0.01$). Radiologists engaged in teaching or research reported comparatively better psychological well-being.
**Conclusion:** Indian radiologists face significant occupational stress driven by high workload, rapid report turnaround expectations, and external pressure from referring doctors and patients. Institutional measures promoting realistic scheduling, adequate staffing, and wellness initiatives are essential to preserve mental health and professional satisfaction.
**Limitations:** Absence of longitudinal follow-up limit generalizability.
**Funding for this study:** No external funding was received for this study.
**Has your study been approved by an ethics committee?** Yes
**Ethics committee - additional information:** Institutional ethical committee approval was obtained.
**Author Disclosures:**
Farook Abubacker Sulaiman: Nothing to disclose

**09:30 - 11:00** **Research Stage 1**

### Research Presentation Session: Abdominal and Gastrointestinal

### RPS 1301
### AI in liver and pancreatic oncology

**Moderator**
Christoforos Stoupis; Forch/CH

**Moderator's introduction**
Christoforos Stoupis; Forch/CH
*(c.stoupis@spitalmaennedorf.ch)*

**A Multimodal Deep Learning Model for Prediction of Early Progression in Patients with Advanced Hepatocellular carcinoma Treated with Atezolizumab-Bevacizumab**
*G. Crescimanno*, R. Cannella, C. Celsa, S. Contino, L. Cruciata, G. Cirrincione, R. Pirrone, G. Brancatelli, C. Cammà; Palermo/IT
*(gaiacrescimanno@gmail.com)*

**Purpose:** Atezolizumab-Bevacizumab is recommended as first-line treatment for advanced/unresectable hepatocellular carcinoma (HCC). However, validated clinical or radiological systems able to predict early treatment response or identify non-responsive patients at risk of early therapeutic failure are currently lacking. This study developed a multimodal AI model to predict 6-month progression-free survival (PFS).
**Methods or Background:** 51 patients (mean age 78.2±9.2 years, 78.4% male) with advanced/unresectable HCC treated with Atezolizumab-Bevacizumab as first-line systemic treatment between 2021 and 2024 were retrospectively included at a single tertiary referral centre. We designed a multimodal neural architecture using late fusion strategy: (1) convolutional neural network (CNN) for feature extraction from pre-treatment contrast-enhanced CT images using the arterial phase, and (2) multilayer perceptron (MLP) for conventional clinical-laboratory data. Feature vectors were concatenated and fed into a final classifier. Primary outcome was 6-month PFS. Performance metrics included accuracy, precision, recall, F1-score, and area under the curve (AUC).
**Results or Findings:** During median follow-up of 14.5 months, median overall survival was 23.6 months (95%CI:15.1-38.0) and median PFS was 14 months (95%CI:9.9-23.6), with 19.6% of patients experiencing radiological progression or death within 6 months. There were no Traditional statistical analysis failed to identify significant predictors of 6-month PFS by using conventional statistical analysis. The multimodal AI model demonstrated excellent performance for 6-month PFS prediction, with accuracy of 96.25%, precision 97.82%, recall 70.66%, F1-score 82.05%, and AUC 0.95.
**Conclusion:** Multimodal AI framework can successfully address the critical gap in early prediction of treatment failure for HCC patients on Atezolizumab-Bevacizumab. The high precision could make it a reliable clinical decision-support tool for identifying patients at risk of early therapeutic failure, improving personalized treatment.
**Limitations:** Single-center study with a small number of patients.
**Funding for this study:** No
**Has your study been approved by an ethics committee?** Yes
**Ethics committee - additional information:** The study has been approved.
**Author Disclosures:**
Gaia Crescimanno: Nothing to disclose
Ciro Celsa: Nothing to disclose
Roberto Pirrone: Nothing to disclose
Giansalvo Cirrincione: Nothing to disclose
Roberto Cannella: Nothing to disclose
Giuseppe Brancatelli: Nothing to disclose
Calogero Cammà: Nothing to disclose
Salvatore Contino: Nothing to disclose
Luca Cruciata: Nothing to disclose

**Ensemble Deep Learning Models on Multi-Sequence MRI for Enhanced Prediction of Microvascular Invasion in Hepatocellular Carcinoma**
*Y. Pan*; Fuzhou/CN
*(936992526@qq.com)*

**Purpose:** To evaluate and compare three ensemble strategies—soft voting, hard voting, and stacking—in deep learning models integrating multi-sequence MRI data for predicting microvascular invasion (MVI) in hepatocellular carcinoma (HCC).
**Methods or Background:** Retrospective study included 299 postoperatively pathologically confirmed HCC patients from two centers. Six MRI sequences (T2WI, T1WI, arterial/portal venous/delayed/hepatobiliary phases) were manually annotated with bounding boxes to fully cover tumors. Six 3D ResNet-18 single-sequence models were built, with 5-fold cross-validation to ensure training robustness. The three ensemble strategies integrated model output probabilities; stacking used a support vector machine (SVM) as the meta-model for further training. Performance was assessed via receiver operating characteristic (ROC) curves.
**Results or Findings:** In the validation set, five-fold cross-validation showed the following average AUCs for single-sequence models: T2WI (0.685), T1WI (0.690), AP (0.689), PVP (0.712), DP (0.694), and HBP (0.666). For fusion strategies: soft voting aggregated MVI risk scores to reach AUC 0.775 and accuracy 0.721; hard voting yielded AUC 0.733 and accuracy 0.738. Among the three, stacking performed best (AUC 0.791, accuracy 0.740), outperforming the other two in integrating multi-sequence MRI for more accurate MVI prediction.
**Conclusion:** Multi-sequence MRI models consistently outperform single-sequence counterparts in HCC MVI prediction, as they integrate complementary info (e.g., T2WI's soft-tissue contrast, enhanced phases' vascular patterns) single-sequence models miss. Among ensembles, stacking excels by using an SVM meta-model to refine cross-sequence feature fusion—avoiding voting methods' flaws—and delivers the highest performance. This supports more reliable MVI stratification, aiding clinicians in optimizing surgical plans and postoperative follow-up for precise HCC management.
**Limitations:** The sample size needs expansion, and further multi-center studies are required to improve the generalizability of the findings.
**Funding for this study:** None
**Has your study been approved by an ethics committee?** Yes
**Ethics committee - additional information:** The data involved in this study has been approved by the Ethics Committee of the First Affiliated Hospital of Fujian Medical University
**Author Disclosures:**
Yifan Pan: Nothing to disclose

**Interpretable Hepatocellular Carcinoma Risk Stratification Model in Cirrhotic Patients: Integrating MRI-based Radiomics Deep Learning and Body Composition**
*Y. Qin*, D. Xu, X. Zhou, S-T. Feng; Guangzhou/CN
*(qinyj27@mail2.sysu.edu.cn)*

**Purpose:** Purpose: Hepatocellular carcinoma (HCC) poses a significant global health challenge, underscoring the critical importance of accurate risk stratification in cirrhosis, which currently remains limited. This study aims to develop and validate an interpretable risk stratification prediction (called IRSP) model that integrates MRI-based radiomics, deep learning, body composition, and clinical features to enhance early HCC prediction in cirrhotic patients.
**Methods or Background:** Methods: This analysis included 479 cirrhotic patients from three primary cohorts who had undergone gadoxetic acid-enhanced MRI between January 2015 and December 2020. Radiomic and deep learning features were extracted from liver regions of interest at multi-MRI sequences (unenhanced, late arterial, and hepatobiliary phase). Unenhanced MRI-quantified body composition was measured. Using features mentioned above, the IRSP model was developed in the discovery cohort (n = 302), and then validated in an internal validation cohort (n = 73), and an external validation cohort from 2 external centers (n = 104).
**Results or Findings:** Results: The IRSP model effectively predicted short-term HCC development in cirrhotic patients with an area under the curve (AUC) of 0.924 (95% confidence interval 0.876-0.9721) in the discovery cohort, 0.895 (0.835-0.955) in the internal validation cohort, and 0.915 (0.882-0.948) in the external validation cohort. By applying optimal thresholds of 0.31 and 0.67, the high-risk (n = 121, 16.0%) and medium-risk (n = 233, 30.7%) groups, which covered 92.6% (88/95) of the patients who developed HCC, had significantly higher rates of HCC occurrence compared to the low-risk group (n = 404, 53.3%) (17.3% vs 4.8% vs 0.31%, P < 0.001).
**Conclusion:** Conclusion: The novel IRSP model provides reliable estimates of HCC development for cirrhotic patients and may have the potential to improve the precision in clinical decision-making and early initiation of HCC treatments.
**Limitations:** Not applicable.
**Funding for this study:** This work was funded by the National Natural Science Foundation of China (82471948, 82271958) and the Natural Science Foundation of Guangdong Province (2024A1515012149).
**Has your study been approved by an ethics committee?** Yes
**Ethics committee - additional information:** Yes, this study has been reviewed and approved by the Ethics Committee of the First Affiliated Hospital of Sun Yat-Sen University , with approval number [2021]124. All research procedures strictly adhere to relevant ethical guidelines and regulations.
**Author Disclosures:**
Shi-Ting Feng: Nothing to disclose
Danyang Xu: Nothing to disclose
Yanjin Qin: Nothing to disclose
Xiaoqi Zhou: Nothing to disclose

**A CT radiomic-integrated model to predict the risk of Hepatocellular Carcinoma in Cirrhotic patients**
*S. Schirò*[1], L. Leo[1], G. Besutti[2], C. Marrocchio[1], M. V. Bazzocchi[1], D. Stefanelli[1], E. Gjataj[2], G. Missale[1], N. Sverzellati[1]; [1]Parma/IT, [2]Modena/IT
*(silvia.schiro@unipr.it)*

**Purpose:** To develop and externally validate a clinico-radiomic model for predicting the risk of hepatocellular carcinoma (HCC) progress in cirrhotic patients employing non-contrast-enhanced CT scans
**Methods or Background:** In this retrospective bicentric study 141 cirrhotic patients were included and divided into a discovery cohort (n=98) and an independent test cohort (n=43). All subjects underwent at least one non-contrast abdominal CT prior to HCC onset (HCC cohort) or during follow-up (non-HCC cohort). The whole liver parenchyma was manually segmented from non-contrast CTs. Comprehensively 851 radiomic features were extracted and filtered for reproducibility and redundancy. A radiomics-only model (RAD) and a clinico-radiomic model (INT) integrating radiomics with clinical variables (alpha-fetoprotein, FIB-4 index, Child-Pugh score) were trained on the discovery cohort and validated on the test set.
**Results or Findings:** The RAD model, made of three selected features and a decision tree classifier, achieved an AUC of 0.696 in the external test set, with 0.905 sensitivity and 0.500 specificity. The INT model, incorporating RAD-score and clinical parameters via stochastic gradient descent, improved specificity to 0.681 while maintaining good sensitivity (0.809), yielding an AUC of 0.703. Decision curve analysis showed a higher net clinical benefit of both models compared to default strategies across a range of decision thresholds.
**Conclusion:** Radiomic features extracted from whole liver on non-contrast CT, especially when integrated with routine clinical data, can stratify the risk of HCC progress in cirrhotic patients
**Limitations:** Firstly, the retrospective study design may have introduced bias on subjects' selection. Secondly, the size of the study population was small, although this was partly compensated by using an external validation cohort to test model performance. Further validation on larger and prospective cohorts is required to address these limitations and confirm the clinical utility of our model.
**Funding for this study:** No funding was received for this study
**Has your study been approved by an ethics committee?** Yes
**Ethics committee - additional information:** This retrospective bicentric study has been approved by the ethics committee (protocol number 930/2022/OSS/UNIPR)
**Author Disclosures:**
Everest Gjataj: Nothing to disclose
Ludovica Leo: Nothing to disclose
Daniela Stefanelli: Nothing to disclose
Nicola Sverzellati: Nothing to disclose
Gabriele Missale: Nothing to disclose
Cristina Marrocchio: Nothing to disclose
Maria Vittoria Bazzocchi: Nothing to disclose
Giulia Besutti: Nothing to disclose
Silvia Schirò: Nothing to disclose

**Deep learning–accelerated Dixon MRI enables rapid and reliable liver fat quantification**
*S. Rau*[1], A. Fink[1], R. Strecker[2], D. Nickel[2], L. Michel[1], D. I. Klemm[1], F. Bamberg[1], J. Weiß[1], M. Russe[1]; [1]Freiburg Im Breisgau/DE, [2]Erlangen/DE
*(stephan.rau@uniklinik-freiburg.de)*

**Purpose:** To assess the accuracy and clinical feasibility of deep learning–accelerated T1-weighted VIBE Dixon sequences for whole-liver fat-signal fraction (FSF) quantification compared with standard protocols.
**Methods or Background:** In this prospective single-centre study, 60 patients (mean age 64 years, 55% female) underwent abdominal MRI at 1.5 T, including a standard VIBE Dixon sequence and two accelerated protocols ("fast" and "ultra-fast") reconstructed with deep learning. Acquisition times were 15, 10 and 6 seconds, respectively. Whole-liver FSF was calculated using a validated automated convolutional neural network–based segmentation. Agreement between accelerated and standard sequences was evaluated using mean absolute error (MAE) and Spearman's correlation.

**Results or Findings:** Liver volumes obtained from accelerated protocols showed excellent correlation with the standard sequence (ρ = 0.975–0.988, p < 0.001). Median liver fat fractions were 2.25% (standard), 2.61% (fast), and 2.35% (ultra-fast). The MAE from the standard was 0.57% for fast and 0.52% for ultra-fast. Correlations for FSF remained high (ρ = 0.923–0.936, p < 0.001), with no systematic bias across protocols.
**Conclusion:** Deep learning–accelerated Dixon MRI allows reliable and fully automated liver fat quantification with substantial reduction of breath-hold duration, supporting its use as an interchangeable alternative to standard protocols.
**Limitations:** The limitations of the study are its single-vendor setting and use of a two-point Dixon reference rather than a multi-echo or histological gold standard.
**Funding for this study:** No funding was received for this study.
**Has your study been approved by an ethics committee?** Yes
**Ethics committee - additional information:** Ethics Committee of the University Medical Center Freiburg, case number 22-1185
**Author Disclosures:**
Ralph Strecker: Employee: Siemens Healthineers AG
Jakob Weiß: Nothing to disclose
Lea Michel: Nothing to disclose
David Immanuel Klemm: Nothing to disclose
Anna Fink: Nothing to disclose
Maximilian Russe: Nothing to disclose
Fabian Bamberg: Nothing to disclose
Dominik Nickel: Employee: Siemens Healthineers AG
Stephan Rau: Nothing to disclose

**Radiomics of Hepatocellular Carcinoma: Identifying Predictors of Microvascular Invasion Using Multi-Phase CT**
F. Spoto, N. Cardobi, *C. Vitale*, B. Mascarin, L. Ordofendi, F. Apolloni, R. De Robertis Lombardi, M. D'Onofrio; Verona/IT
*(vitalecate@gmail.com)*

**Purpose:** To explore radiomic texture features from multi-phase contrast-enhanced CT as potential predictors of microvascular invasion (MVI) in hepatocellular carcinoma (HCC).
**Methods or Background:** This exploratory single-center study retrospectively analyzed 49 patients (54 HCC lesions) who underwent liver resection between 2018-2022. Radiomic analysis extracted 642 features across arterial, venous, and delayed phases using original and 5mm-expanded tumor margins.
**Results or Findings:** The 20-50mm lesion subgroup (n=37) provided the most reliable results, with arterial phase texture homogeneity features achieving AUC 0.772. Features from lesions <20mm (n=14, 4 MVI+) showed clear evidence of overfitting and were excluded from primary analyses. Delayed phase features showed preliminary associations (AUC 0.8) in a small LR-3/4 subset (n=20).
**Conclusion:** Multi-phase CT radiomic analysis shows potential for MVI prediction in intermediate-sized HCC lesions, though external validation in larger cohorts is essential before clinical application.
**Limitations:** This hypothesis-generating study has significant limitations including small sample size, single-center design, and lack of correction for multiple comparisons.
**Funding for this study:** None
**Has your study been approved by an ethics committee?** Not applicable
**Author Disclosures:**
Riccardo De Robertis Lombardi: Nothing to disclose
Nicolò Cardobi: Nothing to disclose
Caterina Vitale: Nothing to disclose
Federica Apolloni: Nothing to disclose
Mirko D'Onofrio: Nothing to disclose
Beatrice Mascarin: Nothing to disclose
Flavio Spoto: Nothing to disclose
Luca Ordofendi: Nothing to disclose

**Reducing Breath-Hold Time in Liver MRI: Clinical Performance of Deep Learning-Accelerated Post-Contrast T1 VIBE**
*S. Rau*[1], A. Fink[1], V. Sacalean[1], K. Kästingschäfer[1], R. Strecker[2], D. Nickel[2], F. Bamberg[1], J. Weiß[1], M. Russe[1]; [1]Freiburg Im Breisgau/DE, [2]Erlangen/DE
*(stephan.rau@uniklinik-freiburg.de)*

**Purpose:** To assess whether deep learning-accelerated post-contrast liver MRI can shorten breath-holds while maintaining diagnostic image quality.
**Methods or Background:** In this prospective study, ninety-nine patients (mean age 61.0 ± 15.4 years; 49.5% female) underwent three T1-weighted two-point Dixon gradient-echo sequences on a 1.5 T system: a standard protocol (18 seconds) and two deep learning-accelerated protocols (10 and 6 seconds). Three blinded radiologists rated overall image quality, motion artefacts, other artefacts, anatomical differentiability, and lesion conspicuity on five-point Likert scales. Per-patient consensus was the median across readers. Global differences were tested with the Friedman test followed by Holm-adjusted Wilcoxon signed-rank tests. Non-inferiority of diagnostic acceptability (Likert score at least 3) for accelerated versus standard sequences was tested with a non-inferiority margin of minus five percentage points.
**Results or Findings:** The standard sequence yielded higher ratings for anatomical differentiability (median 5 vs 4 and 4) and lesion conspicuity (5 vs 4 and 4; both p<0.001) and slightly higher overall image quality (4 vs 4 and 4; p<0.001). Motion-artefact ratings did not differ across sequences. Diagnostic acceptability met non-inferiority for both accelerated sequences across all items.
**Conclusion:** Deep learning reconstruction enabled substantial acceleration of post-contrast liver MRI, reducing breath-holds by 44% (10 seconds) and 67% (6 seconds) without loss of diagnostic acceptability. Faster, motion-robust acquisitions may benefit patients with limited breath-hold capacity.
**Limitations:** Single-centre study with minor image-quality trade-offs relative to the standard sequence.
**Funding for this study:** No funding was received for this study.
**Has your study been approved by an ethics committee?** Yes
**Ethics committee - additional information:** Ethics commitee of the University Medical Center Freiburg; Nr. 22-1185.
**Author Disclosures:**
Ralph Strecker: Employee: Siemens Healthineers
Jakob Weiß: Nothing to disclose
Kai Kästingschäfer: Nothing to disclose
Anna Fink: Nothing to disclose
Maximilian Russe: Nothing to disclose
Fabian Bamberg: Nothing to disclose
Vlad Sacalean: Nothing to disclose
Dominik Nickel: Employee: Siemens Healthineers
Stephan Rau: Nothing to disclose

**Novel methods using dynamic-gadoxetate enhanced MRI to identify inhibitors of drug metabolism by the liver**
*S. Jenkins*, O. Spear, E. Checkley, S. Sourbron, B. Rea; Sheffield/UK
*(s.jenkins12@nhs.net)*

**Purpose:** To demonstrate that dynamic gadoxetate enhanced MRI (DGE-MRI) can distinguish inhibitors from non-inhibitors of liver transporter function by measuring the effect of ciclosporin (known inhibitor) and metformin (no evidence of inhibition) on hepatocellular gadoxetate uptake and excretion.
**Methods or Background:** The identification of drugs at risk of drug-drug interactions (DDI) early in the drug development life cycle is key to avoid late stage drug development failures. DGE-MRI can potentially detect liver-mediated DDI's by assessing drug effects on liver transporter function, but it is currently unclear whether it is sufficiently sensitive to distinguish levels of inhibition. This prospective study recruited 12 healthy volunteers, split evenly between ciclosporin and metformin groups. Each participant underwent two visits and at each visit underwent two contrast-enhanced (1/4 dose of gadoxetate) MRI liver scans one-hour apart. The second scan ensured biliary excretion could be accurately evaluated. At Visit B, a one-off clinical dose of metformin or ciclosporin was administered prior to imaging. Liver uptake and excretion rates were derived by MRI signal modelling.
**Results or Findings:** Ciclopsorin reduced hepatocellular uptake by 67% (p<0.001, 95%CI 61-72) and biliary excretion rate by 50% (p=0.027), although more variable (95%CI 21-80). Metformin did not affect hepatocellular uptake rate (average -7.3%, p=0.25, 95%CI -2.3-17) and or biliary excretion of gadoxetate (average 11%, p=0.35, 95%CI -8.2-31).
**Conclusion:** DGE can distinguish weak from strong inhibition of uptake and excretion. In future, the method may be of use in drug safety assessment to help predict DDI risk.
**Limitations:** Further assessment is required to develop DGE-MRI as a biomarker, including administration of drugs specific to individual transporters and testing in patients with impaired liver function.
**Funding for this study:** The research leading to these results received funding from the Innovative Medicines Initiatives 2 Joint Undertaking under grant agreement No 116106. This Joint Undertaking receives support from the European Union's Horizon 2020 research and innovation programme and EFPIA.
**Has your study been approved by an ethics committee?** Yes
**Ethics committee - additional information:** HRA and Health and Care Research Wales (HRCW) Approval.
**Author Disclosures:**
Sam Jenkins: Nothing to disclose
Benjamin Rea: Nothing to disclose
Steven Sourbron: Nothing to disclose
Elliot Checkley: Nothing to disclose
Oliver Spear: Nothing to disclose

**Next generation spectral AI-based reconstruction for abdominal spectral detector dual energy CT: superior quality for liver imaging**
*L. Fortmann*[1], J. Lueckel[1], L. Hieronymi[1], S. Skornitzke[2], D. Maintz[1], N. Große Hokamp[1]; [1]Cologne/DE, [2]Hamburg/DE
*(lukas.fortmann@uk-koeln.de)*

**Purpose:** Spectral detector DECT (sdDECT) offers advantages in liver imaging, like virtual monoenergetic images. A new prototype deep learning–based spectral reconstruction algorithm (SAI, Philips) was evaluated for conventional reconstructions (SAI) and virtual monoenergetic images (SAI-VMI) to optimize reconstruction settings for abdominal image quality compared to existing fully-iterative reconstruction (FI-R) and hybrid-iterative reconstruction (HI-R/HI-VMI).
**Methods or Background:** For 20 patients undergoing abdominal sdDECT, conventional images were reconstructed with FI-R, HI-R, and five SAI settings: SAI-Sharper, SAI-Sharp, SAI-Standard, SAI-Smooth, and SAI-Smother. For 55keV-VMI, we compared HI-VMI with the five SAI-VMI settings. Quantitative analysis with eight liver ROIs included signal-to-noise (SNR) and contrast-to-noise ratio (CNR). Image quality was assessed by two radiologists using a two-alternative forced-choice design.
**Results or Findings:** For conventional images, mean attenuation was comparable between FI-R, HI-R, and SAI (103.31HU±18.93HU;p>.05). Noise was lowest for FI-R and SAI-Smoother (4.12HU±0.85HU and 5.05HU±1.11HU;p≤.05 vs. remaining; HI-R:15.08HU±3.91HU). FI-R achieved highest SNR (25.72±6.64;p≤.05) and CNR (51.91±7.59;p≤.05) followed by SAI-Smoother (SNR: 21.13±5.79, CNR: 43.82±8.19; both p≤.05 vs. remaining; HI-R SNR:7.23±2.33 and CNR:15.29±3.31). Regarding subjective quality, radiologists showed a significant preference for SAI-Smoother (81.67%±9.60%) and SAI-Smooth (75.83%±7.60%) compared to FI-R (65.00%±13.13%), HI-R (19.58%±8.67%), and other reconstructions (p≤.05). For 55keV-VMI, there was no significant difference in mean attenuation between HI-VMI and SAI-VMI (146.94HU±28.14HU). Noise was significantly lower with SAI-VMI-Smoother (4.97HU±2.68HU, HI-VMI:12.68HU±3.27HU;p≤.05), resulting in significantly higher SNR (30.40±7.04, HI-VMI:12.10±3.88;p≤.05) and CNR (55.16±10.43, HI-VMI:23.35±4.92;p≤.05). Radiologists significantly preferred SAI-VMI-Smooth (81.50%±7.45%) and SAI-VMI-Smoother (81.00%±15.53%) compared to HI-VMI (38.50%±7.45%) and other reconstructions (p≤.05).
**Conclusion:** For conventional sdDECT, novel SAI-Smooth and SAI-Smoother are preferred by radiologists, despite having lower quantitative SNR and CNR than FI-R. Additionally, SAI-VMI-Smooth and SAI-VMI-Smoother also yield better quantitative and qualitative results than HI-VMI.
**Limitations:** The study is limited by its retrospective design.
**Funding for this study:** This work was funded by Philips Healthcare. The funding source had no involvement in study design, collection or interpretation of data.
**Has your study been approved by an ethics committee?** Yes
**Ethics committee - additional information:** IRB-approved
**Author Disclosures:**
Nils Große Hokamp: Nothing to disclose
Stephan Skornitzke: Employee: Philips Market DACH GmbH
David Maintz: Nothing to disclose
Lina Hieronymi: Nothing to disclose
Lukas Fortmann: Nothing to disclose
Julia Lueckel: Nothing to disclose

**Deep Learning-based segmentation of pancreatic neuroendocrine neoplasms: development and validation of a hierarchical model**
A. Belardo, M. Fois, E. Guccinelli, *F. Prato*, L. Tonelli, D. Palumbo, M. G. Ubeira-Gabellini, C. Fiorino, F. De Cobelli; Milan/IT

**Purpose:** This study aims to train and validate a neural network for pancreatic neuroendocrine neoplasms (PanNENs) segmentation. The enrolled patients belong to a retrospective cohort used in a previous study where baseline radiomic analyses were performed [https://doi.org/10.1007/s00330-022-09351-9].
**Methods or Background:** The training dataset consisted of 107 patients' CTs of patients who underwent surgery for PanNENs between January 2015 and December 2021. For each patient, the previously segmented ROI was transferred onto the arterial phase images. The volume's median value was 5.71cc (IQR=[1.63, 17.55]cc). First, the segmentation of the pancreas was performed using TotalSegmentator. All propagated segmentations were reviewed and verified by an experienced radiologist. Model training was then performed using a nnUNet region class order strategy, in which the network learns to segment anatomical structures following a hierarchical order to improve consistency across labels, restricting the search to the segmented pancreas. The test phase was conducted on 30 additional PanNEN patients who underwent surgery between February 2017 and March 2025. The volume's median value on this cohort was 5.29cc (IQR=[3.87, 8.56]cc).
**Results or Findings:** For 10 out of 107 patients, no prediction was obtained due to failure of the model in finding reasonable contours. For the remaining 97 patients, the model achieved an average Dice value of 0.85 (IQR=[0.84, 0.92]). On the test population, the average Dice score was 0.60 (IQR=[0.54, 0.78]). Given the prevalently small volumes, results indicate good segmentation accuracy, although the significantly worse performance in the test cohort needs further investigation.
**Conclusion:** AI-based segmentation of PanNEN is feasible with performances consistent with inter-observer variability of manual segmentation [https://doi.org/10.1016/j.ejmp.2018.12.005].
**Limitations:** In cases of unclear contours (about 10% of cases), position at the pancreas borders or poor enhancement, the model fails in recognizing PanNEN presence or position.
**Funding for this study:** Not available now.
**Has your study been approved by an ethics committee?** Not applicable
**Author Disclosures:**
Francesco De Cobelli: Nothing to disclose
Claudio Fiorino: Nothing to disclose
Marco Fois: Nothing to disclose
Diego Palumbo: Nothing to disclose
Alfonso Belardo: Nothing to disclose
Maria Giulia Ubeira-Gabellini: Nothing to disclose
Francesco Prato: Nothing to disclose
Edoardo Guccinelli: Nothing to disclose
Lisa Tonelli: Nothing to disclose

**09:30 - 11:00** **Research Stage 3**

### Research Presentation Session: Breast

### RPS 1302
### The state of the art in breast MRI

**Moderator**
Ritse Mann; Nijmegen/NL
*(ritse.mann@radboudumc.nl)*
**Author Disclosures:**
Ritse Mann: Grant Recipient: Siemens Bayer screenpoint bd

**T2 Hyperintensity in Breast MRI: a Useful Marker in Lesion Characterization?**
*E. Rubino*[1], C. M. L. Trombadori[1], C. Boldrini[1], A. Marra[1], A. D'Angelo[1], P. Belli[1], P. Clauser[2]; [1]Rome/IT, [2]Vienna/AT
*(eliana.rubino@gmail.com)*

**Purpose:** In breast magnetic resonance imaging (MRI), T2-weighted (T2w) hyperintensity is typically observed in benign lesions but can also occur in some malignant tumors. The aim of our study was to retrospectively evaluate the association between T2 hyperintensity and histopathological and molecular features of breast lesions.
**Methods or Background:** We retrospectively analyzed 1387 breast MRIs performed between 2022 and 2024. For each lesion, a breast radiologist assessed signal intensity on T2w images using non-fat suppressed T2-TSE sequences, enhancement pattern, histology, molecular subtype (Luminal A, Luminal B, HER2+, triple-negative), and Ki67 proliferation index. Associations among these variables were analyzed using Pearson's chi-squared test, Fisher's exact test, Mann-Whitney U test, and Spearman's correlation.
**Results or Findings:** Out of 1387 patients, 779 lesions were hyperintense on T2w images. No statistically significant association was found between T2 hyperintensity and lesion benignity or malignancy, with similar proportions of hyperintensity in both groups. Analysis of malignant histological subtypes revealed that tumors with an in situ component (DCIS and DCIS + IDC) were more frequent among non-hyperintense lesions, while rare tumor subtypes - such as mucinous, papillary, and metaplastic carcinomas - were significantly more frequent among T2-hyperintense lesions (p<0.001). Molecular subtype analysis showed that Luminal A tumors were underrepresented among T2-hyperintense lesions, whereas Luminal B and triple-negative tumors were more common (p<0.05). Spearman's correlation indicated that higher T2 signal intensity was associated with higher Ki67 levels (p<0.001).
**Conclusion:** In our cohort, T2 hyperintensity showed significant associations with rare tumor subtypes (mucinous, papillary, and metaplastic carcinomas), Luminal B and triple-negative molecular subtypes, and high proliferative activity. These preliminary findings suggest that T2w signal characteristics may contribute to refining lesion interpretation in breast MRI and highlight their potential role in lesion characterization.

**Limitations:** Retrospective single-study center, single reader
**Funding for this study:** No
**Has your study been approved by an ethics committee?** Yes
**Ethics committee - additional information:** Ethics Committee Territorial Lazio Area 3, protocol no. 00000883/24, ID 6699, non-profit study.
**Author Disclosures:**
Cristiana Boldrini: Nothing to disclose
Eliana Rubino: Nothing to disclose
Angelica Marra: Nothing to disclose
Charlotte Marguerite Lucille Trombadori: Nothing to disclose
Paola Clauser: Nothing to disclose
Paolo Belli: Nothing to disclose
Anna D'Angelo: Nothing to disclose

**MRI-based differentiation of breast lesions using high b-value DWI: role of quantitative and qualitative ADC assessment**
*A. Angelino*[1], F. Ferrara[1], V. Longo[1], E. Farina[1], A. Carcagnì[1], C. M. L. Trombadori[1], P. Belli[1], A. D'Angelo[1], P. Clauser[2]; [1]Rome/IT, [2]Vienna/AT
*(alessiaangelino18@gmail.com)*

**Purpose:** To explore the role of high b-value 1400 DWI in breast lesion characterization comparing a quantitative assessment of the apparent diffusion coefficient (ADC) map with a qualitative visual analysis.
**Methods or Background:** This retrospective, IRB-approved, single-center study included women with at least one breast lesion classified as BI-RADS 2–6 at consecutive MRIs performed from January 2022 to February 2024. The reference standard was histology from image-guided biopsy or ≥2 years follow-up for non-suspicious lesions. MRI was performed on 1.5T scanners according to international guidelines, including DWI with b=0,1400 s/mm². ADC maps were generated using mono-exponential fitting. One of six breast radiologists (≥3 years' experience), blinded to clinical data, evaluated lesions quantitatively (ADC measured with a ROI in the darkest area) and qualitatively (5-point visual scale: hyperintense/non-suspicious to markedly hypointense/highly suspicious). ROC analysis determined sensitivity and specificity; the highest Youden index on the ROC curve defined the optimal ADC threshold.
**Results or Findings:** A total of 773 lesions in 760 women (mean age 51.2 years) were analyzed: 597 malignant (77.2%) and 176 benign (22.8%). Malignant lesions had lower ADC values ($0.85\times10^{-3}$ mm²/s, SD 0.21) than benign lesions ($1.23\times10^{-3}$ mm²/s, SD 0.34; p<0.001). Quantitative analysis achieved an AUC of 0.81 (95% CI: 0.78–0.87). ADC threshold for distinguishing malignant from benign lesions was of $1.03\times10^{-3}$ mm²/s (85% sensitivity, 72% specificity). Visual assessment showed an AUC of 0.73 (95% CI: 0.68–0.78), with high sensitivity (89%) but low specificity (49%).
**Conclusion:** Quantitative and qualitative ADC map assessment effectively differentiated benign from malignant breast lesions but remained less accurate than CE-MRI (p=0.001 and p=0.027). Visual ADC analysis achieved good sensitivity yet low specificity.
**Limitations:** Retrospective, single-center study; high cancer prevalence, inter-reader variability, subjective scoring.
**Funding for this study:** No funding was received for this study.
**Has your study been approved by an ethics committee?** Yes
**Ethics committee - additional information:** This study was approved by the local ethics committee (ID 6699).
**Author Disclosures:**
Elisa Farina: Nothing to disclose
Francesca Ferrara: Nothing to disclose
Valentina Longo: Nothing to disclose
Antonella Carcagnì: Nothing to disclose
Alessia Angelino: Nothing to disclose
Charlotte Marguerite Lucille Trombadori: Nothing to disclose
Paola Clauser: Nothing to disclose
Paolo Belli: Nothing to disclose
Anna D'Angelo: Nothing to disclose

**Phase-Dependent Transformation in NME: From Clumped to Clustered Ring on Breast MRI**
*E. N. Korkmaz*, H. Aydin, M. Kamışlık; Ankara/TR
*(edanurdoger3@gmail.com)*

**Purpose:** In breast MRI of non-mass enhancement (NME), BI-RADS internal enhancement patterns—clumped and clustered ring (CRE)—have not been reported to transform across dynamic phases. We observed that lesions appearing clumped on early dynamics often demonstrated CRE on later or high-resolution images, suggesting that limited spatial resolution may obscure early CRE. This study evaluated the malignancy association of this phase-dependent transformation and its added diagnostic value combined with ADC.
**Methods or Background:** This single-centre retrospective study (2022–2025) included 80 patients with histopathology-proven NME. MRI was performed at 1.5 T with a dedicated breast coil. Early and late post-contrast DCE-MRI, a high-resolution sagittal sequence, and diffusion-weighted imaging were obtained. Enhancement patterns were assessed by two blinded radiologists. Statistical analysis included $\chi^2$/Fisher's tests, Mann–Whitney U for age, ROC analysis, and logistic regression.
**Results or Findings:** Among 80 lesions, 47 were benign and 33 malignant. Malignant cases were significantly older (p = 0.015). Late-phase and high-resolution images showed a stronger association of CRE with malignancy. Phase-dependent transformation occurred in 43.8% of lesions and was associated with increased malignancy risk. Washout kinetic curves were more frequent in malignant lesions. ADC showed moderate diagnostic performance, and logistic regression identified CRE and wider distribution as independent predictors.
**Conclusion:** Phase-dependent transformation from clumped to CRE is a frequent and diagnostically valuable feature of NME. Incorporating late-phase, high-resolution imaging and diffusion assessment into breast MRI protocols may improve specificity and guide biopsy decisions.
**Limitations:** This study has several limitations. Its retrospective, single-centre design may limit generalisability. Not all benign lesions had histological confirmation or complete follow-up, possibly underestimating malignancy. Interobserver variability remains a factor despite consensus reading, and ultrafast MRI was not directly evaluated.
**Funding for this study:** This study received no specific grant from any funding agency in the public, commercial, or not-for-profit sectors
**Has your study been approved by an ethics committee?** Yes
**Ethics committee - additional information:** The study was approved by the Institutional Review Board of Gülhane Training and Research Hospital, approval number: 2025/126
**Author Disclosures:**
Eda Nur Korkmaz: Author: The author declares no conflicts of interest related to this study. Author: The author participated solely as a contributor to the research and manuscript preparation. Author: The author has no financial or personal relationships that could have influenced the work.
Hale Aydin: Author: The author participated solely as a contributor to the research and manuscript preparation. Author: The author declares no conflicts of interest in relation to this work. Author: The author has no relevant financial ties or personal relationships to disclose.
Melike Kamışlık: Author: The author reports no conflicts of interest associated with this study. The author has no financial, commercial, or institutional relationships to declare.

**Evaluating Tumor-Infiltrating Lymphocytes in Breast Cancer: The importance of Clinicopathological and MRI Features**
*J. Zhou*, M. Du, M. Wang; Wenzhou/CN
*(zhoujiejie@wmu.edu.cn)*

**Purpose:** Tumor-infiltration lymphocytes (TILs) is a key prognostic factor for breast cancer. It is a crucial component of the tumor microenvironment associated with the metabolism of tumor cells and the local immune response. The study aimed to evaluate the clinicopathological parameters associated with TILs, and MRI features corresponding to TILs expression.
**Methods or Background:** A total of 785 patients were retrospectively investigated in this study, with 676 as training dataset and 109 as testing dataset. The percentage of TILs in the stroma adjacent to the tumor cells was assessed on H&E-stained slides, and they were separated into low (<10%) and high (≥10%) groups. MRI features were reviewed by two radiologists by consensus using BI-RADS lexicon descriptors. Clinicopathological and MRI features were compared between low and high TILs groups, and parameters with P<0.05 were used to build logistic regression model. The analysis of disease-free survival (DFS) was performed to assess the model's performance.
**Results or Findings:** Of 676 patients, 43 (6.4%) underwent recurrence with the DFS 54.1±17.4 (mean±SD) months, compared to non-recurrence group with DFS 29.0±20.4 months. Histological grade, ER, PR, HER2 over-expression, low-HER2, Ki-67≥20%, molecular subtype and the presence of peri-tumor edema showed significant difference between low and high TILs groups. Nomogram was build with above parameters and the AUC was 0.727 in training and 0.710 in testing dataset. Kaplan-Meier survival analysis showed patients with high TILs level had better DFS than who with low TILs, but without showing significant difference (P=0.052, HR=1.80).
**Conclusion:** Certain important pathological parameters and edema presence on MRI are associated with high TILs expression, which may help identify patients who may be candidate for immunotherapy and have better response to neoadjuvant therapy and better survival.
**Limitations:** The cutoff of TILs level hasn't been standardized.
**Funding for this study:** This study was supported by National Natural Science Foundation of China (No. 82572172).
**Has your study been approved by an ethics committee?** Yes

**Ethics committee - additional information:** The study has been approved by the ethics committee in the hospital, and approved document is YS2025-423.
**Author Disclosures:**
Meimei Du: Nothing to disclose
Jiejie Zhou: Nothing to disclose
Meihao Wang: Nothing to disclose

**Increased Breast Density-Breast Cancer Relationship: Volumetric Assessment with MRI**
*S. Kılıçarslan Özdemir*, G. Sezgin, M. F. Tumer, M. Yağtu, K. A. Sinci, B. Gençer; Izmir/TR
*(symklcrslnn@gmail.com)*

**Purpose:** Although many studies have reported an association between breast density and breast cancer, this relationship remains debated. Dense parenchyma is typically defined by visual mammographic assessment. This study aimed to quantitatively evaluate the relationship between breast density and breast cancer using three-dimensional volumetric MR images.
**Methods or Background:** This retrospective study reviewed MR exams from January to December 2022. Of 411 examinations, cases with bilateral malignancy (n=32), breast implants (n=59), prior contralateral mammoplasty (n=74), asymmetric parenchyma (n=3), or pure DCIS (n=2) were excluded. The final cohort included 121 patients with unilateral breast cancer and 121 controls without malignancy; none in the control Group developed cancer during two-year follow-up. MR scans were obtained using a 3T Lumina system with an 18-channel breast coil.Breast density was measured semi-automatically on non–fat-suppressed 3D T1-weighted images. The thoracic region was cropped along the pectoral muscle, and total breast and fat volumes segmented using a bright fat thresholding technique. Parenchymal volume was calculated by subtraction. Two observers performed the measurements. Based on BI-RADS 4th edition, densities ≥50% were classified as “dense.”
**Results or Findings:** Among malignant cases, 83 were invasive ductal, 28 invasive lobular, and 10 other types. The mean age was 52.8 years in the malignant group and 45.2 in controls. Dense parenchyma was found in 15.7% of malignant and 3.3% of controls. Breast density was significantly higher in the malignant group (p=0.000). Logistic regression indicated malignancy risk increased with fat content and age.
**Conclusion:** Quantitative MR analysis confirmed a strong association between increased breast density and breast cancer. Three-dimensional MR provides reliable volumetric assessment, though semi-automatic segmentation is time-consuming.
**Limitations:** Single-center design, limited sample size, and interobserver variability. Future multicenter studies using automated deep learning and radiomics methods may improve reproducibility.
**Funding for this study:** None
**Has your study been approved by an ethics committee?** Yes
**Ethics committee - additional information:** January 2024 / ID:647
**Author Disclosures:**
Mehmet Fatih Tumer: Nothing to disclose
Kazim Ayberk Sinci: Nothing to disclose
Seyma Kılıçarslan Özdemir: Nothing to disclose
Burak Gençer: Nothing to disclose
Gulten Sezgin: Nothing to disclose
Mehmet Yağtu: Nothing to disclose

**Role of the Kaiser Score in breast MRI to reduce unnecessary second-look ultrasounds and biopsies in patients undergoing neoadjuvant chemotherapy: a two-year retrospective study**
*E. Tallamona*[1], P. Francesco[2]; [1]Gela/IT, [2]Misterbianco/IT
*(elianatallamona@gmail.com)*

**Purpose:** To evaluate the role of the Kaiser Score (KS) applied to pre-treatment breast MRI in reducing unnecessary second-look ultrasound examinations and biopsies in patients scheduled for neoadjuvant chemotherapy (NACT).
**Methods or Background:** This retrospective single-center study included consecutive patients with biopsy-proven breast cancer evaluated for NACT between January 2023 and December 2024. All patients underwent baseline breast MRI. For each MRI-detected lesion, the KS was calculated based on morphology, dynamic enhancement, and additional diagnostic features. Lesions with KS ≥5 were considered suspicious, while those with KS ≤4 were considered likely benign. Second-look ultrasound was performed for lesions without sonographic correlates or not already biopsied, and biopsies were conducted according to institutional protocols. Final histopathological results were used as the reference standard. The primary endpoints were the number of second-look ultrasounds and biopsies that could be avoided by applying the KS. Diagnostic performance metrics, including sensitivity, specificity, positive predictive value (PPV), and negative predictive value (NPV), were calculated.
**Results or Findings:** We included in the study 56 patients. Only the "second-look" lesions evaluated as suspicious and biopsied by a team of at least 5 year experienced breast radiologists were included in the study. Histological results were collected and classified according the Easton Ellis classification from B1 to B5 and then compared to the given imaging score. By adopting a KS threshold of 5 both second-look ultrasounds and biopsies could have been safely avoided with a low grade of missing "B3" and malignant lesion.
**Conclusion:** The KS is a reliable tool to stratify MRI-detected lesions in patients undergoing NACT. Its systematic use before second-look ultrasound could substantially reduce unnecessary imaging and biopsies, streamlining pre-treatment workup and minimizing patient burden.
**Limitations:** This study is limited by its retrospective, single-center design,
**Funding for this study:** No funding needed
**Has your study been approved by an ethics committee?** Not applicable
**Author Disclosures:**
Pane Francesco: Nothing to disclose
Eliana Tallamona: Nothing to disclose

**An interpretable model for differentiating early-stage breast cancer and benign lesions, automatically generating structured reports**
J. Qu, Y. Liu, *S. Lui*; Cheng Du/CN

**Purpose:** To develop and evaluate an interpretable model for distinguishing early breast cancer (BC) from benign lesions and generating structured reports.
**Methods or Background:** Preoperative MRI scans and corresponding radiological reports from patients with breast lesions were retrospectively collected across five institutions. Lesion descriptors extracted from MRI reports were translated into standardized BI-RADS lexicon terms (hereafter termed “concepts”) by radiologists. These concepts, alongside multiparametric MRI sequences, were input into a Concept Bottleneck Model (CBM) designed to differentiate BC from benign lesions and to generate structured reports. Classification performance of the CBM was compared against a black-box model. CBM-derived concept accuracy was also assessed. Additionally, a two-phase multi-reader study was conducted to evaluate the clinical utility of the CBM.
**Results or Findings:** A total of 1,695 pathology-confirmed breast lesions (857 malignant and 838 benign) from 1,634 patients were included. In the test set, the CBM achieved an AUC of 0.92 (95%CI 0.90-0.93), comparable to the benchmark black-box model (AUC: 0.93 [95%CI 0.92-0.94]). The accuracy of CBM-generated concepts ranged from 0.62 to 1.00. In the multi-reader study, the CBM yielded an accuracy of 0.89, performing on par with one radiologist and outperforming the other seven (all $P < 0.05$). With CBM assistance, radiologists correctly downgraded 22.1% of the lesions to benign. Diagnostic accuracy improved for three radiologists, increasing from 0.71-0.72 to 0.82-0.91 (all $P < 0.05$). Furthermore, CBM support enhanced inter-reader agreement in both concept recognition and BI-RADS classification, with Gwet’s AC1 increasing from 0.27-1.00 to 0.55-1.00.
**Conclusion:** The CBM enhances intrinsic interpretability through image-concept alignment, effectively discriminates early BC from benign lesions, potentially reduces unnecessary benign breast lesion biopsies, and improves reporting consistency among radiologists.
**Limitations:** The limited size of the dataset warrants further validation in larger, preferably prospective cohorts.
**Funding for this study:** This study was supported by (1) the National Natural Science Foundation of China (Project Nos. 82441007, 82120108014); (2) 1.3.5 project for disciplines of excellence, West China Hospital, Sichuan University (Project No. ZYGD23003); (3) 1.3.5 projects for Artificial Intelligence (Project No. ZYAI24010), West China Hospital, Sichuan University; (4) Chengdu Science and Technology Office, major technology application demonstration project (Project No.2022-GH03-00017-HZ); and (5) the Postdoctor Research Fund of West China Hospital, Sichuan University (2025HXBH040).
**Has your study been approved by an ethics committee?** Yes
**Ethics committee - additional information:** This retrospective study was approved by the Ethics Committee on Biomedical Research, West China Hospital of Sichuan University, Chengdu, Chinal (approval No. 2025-949).
**Author Disclosures:**
Yang Liu: Nothing to disclose
Su Lui: Nothing to disclose
Jiao Qu: Nothing to disclose

**Automatic Segmentation of Breast Ductal Carcinoma in Situ on MRI with Active learning based Deep-learning: A Multicenter Study**
*S. Ham*[1], B. K. Seo[1], S. Wang[2], J. Y. Lee[1], M. S. Bae[1], K. R. Cho[3], O. Woo[3], S. E. Song[3], S-Y. Kim[3]; [1]Ansan/KR, [2]Cambridge/UK, [3]Seoul/KR

**Purpose:** Ductal carcinoma in situ (DCIS) often presents on MRI as nonmass enhancement with variable enhancement than invasive cancer. We aimed to develop and validate a deep learning–based automatic segmentation algorithm for biopsy-proven DCIS on preoperative MRI, achieving robust performance across lesion types, background parenchymal enhancement (BPE), and invasive cancer upgrade.

**Methods or Background:** This retrospective multicenter study included 842 DCIS lesions (Institution A, 342; Institution B, 500) collected over 11 years from nine 3T scanners (five vendors). SAM 2.1-L was initially trained on 292 cases from Institution A, with 50 held out for testing. Active learning was applied to Institution B in three iterations (250, 150, and 50 cases), using expert corrections for retraining. Final evaluation was performed on an independent test set of 100 lesions (50 per institution). Subgroup analyses included lesion type, size, BPE, and invasive upgrade. Segmentation performance was assessed with the dice similarity coefficient (DSC) and compared with training from scratch. Annotation times were compared between manual and active learning–assisted labeling.
**Results or Findings:** Overall DSC improved from 0.68 in the initial model to 0.73 after multiple iterations (p<0.05). DSC did not differ by lesion type (mass, 0.72 vs. nonmass enhancement, 0.73) or invasive upgrade (pure DCIS, 0.77 vs. invasive upgrade, 0.71; both p>0.05), but was significantly higher in tumors >30 mm and with lower BPE (minimal or mild) (all p<0.05). Annotation time decreased from 453 to 35 seconds per lesion (p<0.05).
**Conclusion:** Active learning with SAM 2.1-L enabled robust DCIS segmentation on MRI regardless of lesion type or invasive upgrade, while dramatically reducing annotation time. Gains in accuracy and efficiency support its potential for clinical integration in breast MRI analysis.
**Limitations:** External multicenter datasets is needed to confirm generalizability.
**Funding for this study:** This research was supported by the National Research Foundation of Korea (NRF) grant funded by the Korea government (RS-2024-00347290).
**Has your study been approved by an ethics committee?** Yes
**Ethics committee - additional information:** Approved by the Institutional Review Board (IRB) of Korea University Ansan Hospital.
**Author Disclosures:**
Min Sun Bae: Nothing to disclose
Okhee Woo: Nothing to disclose
Soo-Yeon Kim: Nothing to disclose
Bo Kyoung Seo: Nothing to disclose
Sungwon Ham: Nothing to disclose
Sung Eun Song: Nothing to disclose
Ji Young Lee: Nothing to disclose
Shuncong Wang: Nothing to disclose
Kyu Ran Cho: Nothing to disclose

**Simultaneous Multiparametric 18F-Fluorodeoxyglucose (18F-FDG)-PET/MR-imaging Biomarkers for Overall Survival Prediction in Breast Cancer: Conventional Parameters Remain Strongest**
*T. Spiegel*[1], V. Romeo[2], D. A. Resch[1], O. Lafcı[1], S. Rasul[1], A. Stiglbauer-Tscholakoff[1], P. Clauser[1], K. Pinker-Domenig[3], T. H. Helbich[1]; [1]Vienna/AT, [2]Naples/IT, [3]New York, NY/US
*(thomas.spiegel@meduniwien.ac.at)*

**Purpose:** To evaluate whether multiparametric 18F-Fluorodeoxyglucose (18F-FDG)-PET/MR-imaging biomarkers provide prognostic value for predicting overall survival (OS) in breast cancer (BC) patients.
**Methods or Background:** This retrospective analysis of a prospectively performed single-center study includes 202 BC patients (median age 52 years, range 25-82 years), who underwent simultaneous 18F-FDG-PET/MRI of the breast and whole body using a Biograph mMR system (Siemens, Germany) for staging purposes. Follow-up was available for up to 103 months (median 69 months). Imaging parameters of the index lesion included tumor size, SUVmax, ADC and perfusion parameters (Plasma Flow, Volume Distribution, Mean Transit Time). Histopathological and clinical variables included lymph node (LN) status, presence of distant metastases, and molecular subtype. Kaplan-Meier survival curves with log-rank tests, univariate and multivariate Cox regression analyses using hierarchical modeling to assess incremental prognostic value, were performed; p<0.05 was considered significant.
**Results or Findings:** During follow-up, 28 (13.9%) patients passed away. Tumor size predicted poorer OS (HR 1.224 (95% CI 1.044-1.435), p=0.013). Presence of distant metastases was strongly predictive for OS (HR 3.609 (95%CI 1.632-7.982), p=0.002). OS differed significantly among molecular subtypes (log rank p<0.001). LN status showed only a trend (HR 2.330 (95%CI 0.990-5.482), p=0.053). SUVmax, ADC, and perfusion parameters were not significant in predicting OS. Adding distant metastasis to tumor size improved the model (p=0.013). Further inclusion of LN information (p=0.191), molecular subtype (p=0.084) and imaging parameters (p>0.225) contributed minimally.
**Conclusion:** Among several histopathologic and clinical variables tumor size and distant metastases remain the strongest predictors for OS in BC patients. Several 18F-FDG-PET/MR-imaging biomarkers did not improve prognostic models significantly. This indicates that FDG-PET/MR-imaging parameters do not yet surpass conventional assessments of tumor size and metastases in predicting OS.
**Limitations:** Single-center design, limited number of events, and heterogeneous follow-up duration.
**Funding for this study:** This study is supported by the WWTF (Vienna Science and Technology Fund), grant number LS20-065.
**Has your study been approved by an ethics committee?** Yes
**Ethics committee - additional information:** EK Nr. 510/2009
**Author Disclosures:**
Daphne Ariane Resch: Nothing to disclose
Thomas Spiegel: Nothing to disclose
Katja Pinker-Domenig: Nothing to disclose
Valeria Romeo: Nothing to disclose
Thomas H. Helbich: Nothing to disclose
Alexander Stiglbauer-Tscholakoff: Nothing to disclose
Oğuz Lafcı: Nothing to disclose
Sazan Rasul: Nothing to disclose
Paola Clauser: Nothing to disclose

**T-Stage Accuracy of Invasive Breast Carcinoma (IBC) with CEM and MRI**
*G. Chiffi*[1], Ö. Lafci[2], A. Santonocito[3], N. Pirringer-Pötsch[2], A. Stiglbauer-Tscholakoff[2], M. Costantini[1], T. H. Helbich[2], P. A. Baltzer[2], P. Clauser[2]; [1]Rome/IT, [2]Vienna/AT, [3]Turin/IT
*(chiffi.greta@gmail.com)*

**Purpose:** To evaluate the accuracy of tumor size and T-stage estimation in IBC by Contrast-Enhanced Mammography (CEM) and Magnetic Resonance Imaging (MRI), compared with pathology.
**Methods or Background:** This retrospective single-centre study included 100 patients (mean age 57 years) with biopsy-proven IBC who underwent pre-surgical CEM and MRI, each showing at least a component of mass enhancement. Two radiologists independently measured the maximum invasive mass component. CEM lesions were measured on low-energy and recombined images, MRI measurements on early post-contrast dynamic series (MRI-dyn) and Maximum Intensity Projection reconstructions (MRI-MIP). Pathology was the reference standard. Agreement was evaluated with intraclass correlation coefficients (ICC), Bland–Altman analysis using a ±2.5 mm margin. Impact on T-stage classification was assessed.
**Results or Findings:** A total of 112 invasive lesions were analysed (mean pathological size 13.8 mm). All modalities showed good correlation with pathology (ICC 0.825–0.876, p < 0.001). Mean lesion sizes were close to pathology, but case-wise agreement varied. Bland–Altman analysis showed for CEM mean differences ranged from –0.9 to +0.2 mm across reader, with limits of agreement (LOA) –11.4 to +9.6 mm; for MRI-dyn, –1.1 to 0.0 mm, LOA –11.5 to +9.3 mm. MRI-MIP showed differences –1.6 to +0.5 mm with wider LOA (–12.2 to +11.2 mm). Discrepancies increased with tumor size, trending toward underestimation. T-stage accuracy was highest with MRI-dyn (84%), followed by CEM (80%), while MRI-MIP consistently overstaged. Small but consistent inter-reader shifts were observed.
**Conclusion:** CEM and MRI-dyn showed good correlation with pathology, but accuracy declined for larger tumors. For preoperative size and T-stage assessment, MRI-dyn should be preferred, CEM as an alternative. Standardized measurement rules may reduce inter-reader variability.
**Limitations:** Single-centre retrospective design, modest sample size, exclusion of non-mass enhancement, which-though likely irrelevant for T stage-may influence surgical planning.
**Funding for this study:** Not applicable
**Has your study been approved by an ethics committee?** Yes
**Ethics committee - additional information:** This study was approved by the Institutional Review Board (IRB), which ruled that written informed consent is unnecessary. Data were collected within a prospective study comparing the diagnostic value of CEM to MRI in a problem-solving setting (Ethics Review Board number 2282/2019).
**Author Disclosures:**
Pascal A.T. Baltzer: Nothing to disclose
Alexander Stiglbauer-Tscholakoff: Nothing to disclose
Thomas H. Helbich: Nothing to disclose
Melania Costantini: Nothing to disclose
Nina Pirringer-Pötsch: Nothing to disclose
Greta Chiffi: Nothing to disclose
Ambra Santonocito: Nothing to disclose
Öguz Lafci: Nothing to disclose
Paola Clauser: Nothing to disclose

**Preoperative Detection of Pure Ductal Carcinoma In Situ (pDCIS) of the Breast: A Two-Center Comparison Between CEM and MRI**
*J. Azcona Sáenz*[1], J. Molero-Calafell[1], U. Lalji[2], T. Wegman[2], P. Santiago Díaz[1], J. M. Maiques Llacer[1], R. Alcantara Souza[1], M. d. M. Vernet Tomás[1], T. Van Nijnatten[2]; [1]Barcelona/ES, [2]Maastricht/NL

**Purpose:** To compare contrast-enhanced mammography (CEM) and MRI in the preoperative diagnosis of pure ductal carcinoma in situ (pDCIS), and to evaluate their performance in detecting additional lesions (ALs).

**Methods or Background:** In this international two-center retrospective study, 46 pDCIS lesions at Hospital del Mar and 18 lesions at Maastricht UMC+, all of whom underwent preoperative CEM and MRI, were reviewed. The sensitivity, morphology, and extent of pDCIS on each modality, as well as their ability to detect ALs, were assessed.
**Results or Findings:** CEM and MRI combined with low-energy images (LEI) showed the highest sensitivity for pDCIS detection at both centers (100.0%), followed by MRI (97.5% at Hospital del Mar; 93.3% at Maastricht UMC+), recombined images (RCI) (90.0% and 86.7%), and LEI (80.0% and 86.7%) of CEM. Calcifications were the most frequent LEI descriptor (~60%). On RCI and MRI, pDCIS predominantly presented as non-mass enhancement (60.0–75.0). LEI tended to underestimate tumor size, particularly at Maastricht UMC+, whereas MRI provided the most accurate estimations, closely followed by RCI. MRI detected more ALs than CEM; however, most corresponded to false positives (Hospital del Mar: 21 vs. 12 [MRI vs. CEM]; Maastricht UMC+: 15 vs. 11). CEM failed to identify one additional malignant lesion.
**Conclusion:** CEM and MRI + LEI showed slightly higher sensitivity than MRI for detecting pDCIS. MRI provided the most accurate preoperative size assessment, closely followed by RCI, with both modalities overestimating size. MRI was superior to CEM in detecting additional malignant lesions.
**Limitations:** -Small sample size in both cohorts. -Retrospective design. -Lesion size assessment may have been biased in cases where VAB were performed. -Diagnostic specificity and disease prevalence could not be evaluated. -Not all patients underwent bilateral mastectomies. Therefore, assessment of true false-negative rates was precluded.
**Funding for this study:** No
**Has your study been approved by an ethics committee?** Yes
**Ethics committee - additional information:** This retrospective study was approved by the Institutional Ethics Committees of both centers, with a waiver of informed consent. Maastricht UMC+: 2025-0114 Hospital del Mar: 2025/11910
**Author Disclosures:**
Thiemo Van Nijnatten: Nothing to disclose
Rodrigo Alcantara Souza: Nothing to disclose
Thomas Wegman: Nothing to disclose
Javier Azcona Sáenz: Nothing to disclose
Pablo Santiago Díaz: Nothing to disclose
Javier Molero-Calafell: Nothing to disclose
Ulrich Lalji: Nothing to disclose
María Del Mar Vernet Tomás: Nothing to disclose
Jose María Maiques Llacer: Nothing to disclose

**09:30 - 11:00** | **Research Stage 2**

### Research Presentation Session: Chest

### RPS 1304

### Advances in MRI for pulmonary imaging

**Moderator**
James F Meaney; Dublin/IE
*(jmeaney@meaneys.com)*

### The potential role of MRI in predicting worsening of CTD-ILD

*N. D. I. Colomba*, P. Bica, F. Corrao, C. Zichichi, M. Molino; Trapani/IT
*(nicola.colomb@gmail.com)*

**Purpose:** Interstitial lung disease (ILD) is a common complication in patients with connective tissue disease (CTD). The ILD is generally quantified with HRCT. The purpose of the study is to identify a qualitative evaluation of the CTD-ILD and eventually a biomarker of worsening with the MRI.
**Methods or Background:** We optimized an MRI thorax protocol with a T2W fat sat breath-gated sequences which enhance the fluids. The study started in January 2025 and we selected so far twelve patients with mild to advanced stage CTD-ILD. Only patient with serological or sistemic simphtoms worsening has been enrolled. All patients took a first MRI scan followed by a second scan six months later.
**Results or Findings:** We found a similar pattern in almost all patient with hyperintensity in the subpleural regions of the inferior lobes usually in the context of honey combing areas. Since only water has a high signal in the sequence we used those alterations found in our patiens are very likely to be oedema. We found the amount of oedema was not strictly related to the stage of the ILD. In our group six patients with moderate to severe oedema alterations were basically stable at the six months follow-up. Three patients had a slight remission of the oedema while tree had a subtle worsening. Among the three of the last group two had a real worsening of the stage of the ILD in the following HRCT scans.
**Conclusion:** Our preliminary results suggest that the presence and the evolution of oedema in the MRI scan could relate the potential likelihood of worsening of ILD. We definitely need more patients and a longer follow-up. The study is still in progress.
**Limitations:** No limitations were identified.
**Funding for this study:** No funding was provided for this study.
**Has your study been approved by an ethics committee?** Not applicable
**Author Disclosures:**
Barone Molino Massimo: Nothing to disclose
Pietro Bica: Nothing to disclose
Nicolò Davide Igor Colomba: Nothing to disclose
Fabio Corrao: Nothing to disclose
Clara Zichichi: Nothing to disclose

### Magnetic resonance imaging detects dilated bronchial arteries in patients with primary ciliary dyskinesia

*D. Cetiner*[1], P. Leutz-Schmidt[1], M. Stahl[2], O. Sommerburg[1], C. P. Heußel[1], H-U. Kauczor[1], M. A. Mall[2], M. O. Wielpütz[3], L. Wucherpfennig[1]; [1]Heidelberg/DE, [2]Berlin/DE, [3]Greifswald/DE

**Purpose:** Bronchial arterial dilatation (BAD) is known to be associated with pulmonary hemorrhage and is highly prevalent in patients with chronic muco-obstructive lung diseases such as cystic fibrosis. However, studies on the prevalence of BAD in patients with primary ciliary dyskinesia (PCD) and their association with lung disease severity are lacking. Thus, the aim of our study is to evaluate BAD onset and its association with disease severity in patients from two different centers with PCD by magnetic resonance imaging (MRI).
**Methods or Background:** 68 patients with PCD (mean age at baseline 17.3±14.4 yr) performed annual standardized MRI including dynamic contrast-enhanced (DCE) MRI or angiography, contributing to a total of 155 examinations. The presence of BAD was assessed visually by two readers in consensus. Disease severity was assessed using the validated chest MRI score and spirometry (percent predicted forced expiratory volume in 1s [ppFEV1]).
**Results or Findings:** At baseline, at least one BAD was present in 30 patients (44.1%), with most patients having one or two BAD (53.3% and 26.6%, respectively). The number of BAD correlated strongly with patient age ($r=0.641$, $p<0.001$). The mean MRI global score was significantly higher in patients with BAD at baseline compared to patients without BAD (26.3±6.7 vs. 19±8.1, $p=0.002$), while ppFEV1 did not show differences (76.2±20.8% vs. 73.1±18.5%, $p=0.57$). During follow-up, four patients developed a BAD for the first time (5.8%) and three patients (4.4%) developed an additional BAD. BAD development was neither associated with changes in the MRI score, nor in ppFEV1 ($p>0.99$ and $p=0.87$).
**Conclusion:** Our study demonstrates a high prevalence of BAD in children and adults with PCD and an association between prevalence of BAD markers of greater disease severity.
**Limitations:** Not all patients received angiography additional to DCE-MRI.
**Funding for this study:** This study was supported by grants from the German Federal Ministry of Education and Research (BMBF) (82DZL004A1, 82DZL009C1 and 01GL2401A) and the German Research Foundation (CRC 1449 – project 431232613). MS is participant of the BIH-Charité Clinician Scientist Program funded by the Charité – Universitätsmedizin Berlin and the BIH. LW is participant of the Career Development Programm funded by the University of Heidelberg. Funders had no involvement in the collection, analysis and interpretation of data, in the writing of the report and in the decision to submit the article for publication.
**Has your study been approved by an ethics committee?** Yes
**Ethics committee - additional information:** Ethic commitee approval number: S-509/2019, S-184/2025, EA2/003/21 University Hospital Heidelberg
**Author Disclosures:**
Olaf Sommerburg: Speaker: Vertex Pharmaceuticals (unrelated to the project)
Mirjam Stahl: Other: FGM (chairwomen, unrelated to this project) Other: GPP (president elect, unrelated to this project) Advisory Board: Vertex Pharmaceuticals (unrelated to this project) Grant Recipient: Vertex Pharmaceuticals (unrelated to the project) Other: ERS (group secretary CF, unrelated to this project).
Mark O. Wielpütz: Board Member: ESTI (not related to this project) Speaker: Boehringer Ingelheim (unrelated to this project) Grant Recipient: Vertex Pharmaceuticals (unrelated to this project) Grant Recipient: Boehringer Ingelheim (unrelated to the project) Speaker: Vertex Pharmaceuticals (unrelated to this project) Consultant: Vertex Pharmaceuticals (unrelated to this project) Advisory Board: Boehringer Ingelheim (unrelated to this project) Board Member: IWPFI (unrelated to this project) Research/Grant Support: German Federal Ministry of Education and Research.
Lena Wucherpfennig: Consultant: Antaros Medical (unrelated to the project) Consultant: Boehringer Ingelheim (unrelated to the project) Speaker: Vertex Pharmaceuticals (unrelated to the project).

Hans-Ulrich Kauczor: Speaker: Siemens (unrelated to this project) Research/Grant Support: Siemens (unrelated to this project).
Defne Cetiner: Nothing to disclose
Marcus A. Mall: Research/Grant Support: DFG German Research Foundation Other: Fellow of the European Respiratory Society (FERS) (unrelated to this project) Grant Recipient: Boehringer Ingelheim (unrelated to the project) Grant Recipient: Enterprise Therapeutics (unrelated to this project) Consultant: Boehringer Ingelheim (unrelated to this project) Research/Grant Support: BMBF German Federal Ministry for Education and Research Grant Recipient: Vertex Pharmaceuticals (unrelated to this project) Consultant: Kither Biotech (unrelated to this project) Grant Recipient: German Innovation Fund (unrelated to this project).
Claus Peter Heußel: Nothing to disclose
Patricia Leutz-Schmidt: Nothing to disclose

**Multimodality imaging insights in atypical thoracic hydatidosis**
*M. Kumar*[1], K. K. Ratre[2], A. Rai[2], A. Agrawal[2], V. Kumar[2]; [1]Raipur, Chhatisgarh/IN, [2]Raipur/IN
*(manish2k4@gmail.com)*

**Purpose:** To identify characteristic imaging features of atypical thoracic hydatid disease on multimodality imaging To emphasize the importance of imaging in guiding clinical management and surgical planning in complicated hydatid disease.
**Methods or Background:** 6 patients with confirmed thoracic hydatid disease were retrospectively evaluated using CXR, ultrasound, CECT and MRI. Findings were correlated with clinical and surgical outcomes.
**Results or Findings:** 1.Extrapleural hydatid CXR demonstrated homogeneous radiodense lesion in the left upper zone which on CT appeared solid non-enhancing. MRI revealed a multilocular cystic lesion with internal T2 hypointense membranes located outside the pleural cavity with cervical extension and compressing the left brachial plexus. 2.Endobronchial and pleural rupture of pulmonary hydatid cyst with air and fluid in pleural space. 3.Pleural hydatidosis secondary to ruptured pulmonary hydatid CECT revealed multiple cystic lesions in the lung parenchyma bilaterally with left sided effusion & one of them showing rupture into the left pleural space. Multilocular lesion was seen in the left pleural cavity with pleural thickening, internal membranes and hydatid sand. 4.Primary pleural hydatid Chest CT reveals presence of moderate sized pleural effusion and a large lesion inseparable from the collapsed right lower lobe. Ultrasound showed a multilocular cystic lesion outside the lung. 5.Calcification within pulmonary hydatid cysts. CECT revealed solitary/ multiple lesions with calcification in the wall or membranes.
**Conclusion:** Pleural hydatid cysts may be primary or secondary to rupture of pulmonary cysts. Ultrasound is superior to CT in characterisation of pleural lesions/disease. Extrapleural hydatid disease can mimic chest wall or pleural tumors. Calcification represents chronicity. MRI is reserved for unusual cases like extrapleural or mediastinal lesions. Identifying pleural rupture is paramount as it needs emergency chest tube placement or planned surgical decortication in refractory cases.
**Limitations:** Less number of cases
**Funding for this study:** None recived.
**Has your study been approved by an ethics committee?** Not applicable
**Author Disclosures:**
Anshul Agrawal: Nothing to disclose
Manish Kumar: Nothing to disclose
Kamlesh Kumar Ratre: Nothing to disclose
Virendra Kumar: Nothing to disclose
Ankush Rai: Nothing to disclose

**Diagnostic performance of single breath-hold lung MRI scan with AI-powered compressed sensing for Nodule Detection in comparison toPhoton Counting Detector-CT**
*G. Piccinni*, A. Palmisano, D. Serra, E. Bruno, A. Esposito; Milano/IT
*(giuliapiccinni95@gmail.com)*

**Purpose:** Evaluate the performance of a single breath-hold accelerated 3D-T1-FFE 3T MRI sequence for the screening of lung nodules using Photon-Counting Detector CT as the reference standard.
**Methods or Background:** This single-center prospective study enrolled 97 healthy adults who underwent Photon-Counting Detector CT scans after pulmonary nodules were detected by 3T whole-body magnetic resonance imaging (3D-T1-FFE with AI and breath-hold) between June 2024 and June 2025. All nodules were evaluated in terms of size and composition and sensitivity of MRI in comparision to PCD-CT in nodule detection capability was calculated. Intermodality agreement was assessed with the Prevalence-Adjusted Bias-Adjusted Kappa and Lin's Concordance Correlation Coefficient.
**Results or Findings:** Using CT as the reference standard, MRI achieved a specificity of 97.0% (95% CI: 84.7–99.5) and a positive predictive value of 98.7% (95% CI: 93.1–99.8), indicating that nearly all nodules detected by MRI were true positives. Stratifying lesions by size (<4 mm vs. ≥4 mm), MRI showed a sensitivity of 60.8% (95% CI: 49.7–70.8), specificity of 89.7% (95% CI: 76.4–95.9), PPV of 92.3% (95% CI: 81.8–97.0), NPV of 53.0% (95% CI: 41.2–64.6), and an overall accuracy of 70.3% (95% CI: 61.6–77.8) compared with CT . Stratifying by composition, MRI demonstrated excellent ability to identify solid nodules with a sensitivity of 86.3% (95% CI: 76.6–92.4), specificity of 100% (95% CI: 92.1–100), PPV of 100% (95% CI: 94.3–100), NPV of 81.8% (95% CI: 69.7–89.8), and an accuracy of 91.5% (95% CI: 85.1–95.3), indicating that MRI has an excellent accuracy for detection of solid nodule, whereas false negatives occur in calcific lesions.
**Conclusion:** A single breath-hold accelerated 3D-T1-FFE MRI scan is a fast valuable tool for solid lung nodule screening with an excellent agreement with CT.
**Limitations:** Small patient sample.
**Funding for this study:** No.
**Has your study been approved by an ethics committee?** Yes
**Ethics committee - additional information:** The study was approved by the local Ethic Commitee (AIMOMICS-BANK CET 233-2024), and written informed consent was obtained.
**Author Disclosures:**
Davide Serra: Nothing to disclose
Antonio Esposito: Nothing to disclose
Elisa Bruno: Nothing to disclose
Giulia Piccinni: Nothing to disclose
Anna Palmisano: Nothing to disclose

**Impact of Zonally Magnified Oblique Multi-slice Technique (Zoom DWI) Versus Conventional DWI on Image Quality and ADC Measurements in Mediastinal Lesions**
*Y. Ozawa*, T. Ueda, M. Nomura, T. Yoshikawa, D. Takenaka, Y. Ohno; Toyoake/JP

**Purpose:** Zonally magnified oblique multi-slice DWI (Zoom DWI) enables a smaller field of view than conventional DWI (cDWI), reducing aliasing and echo planar imaging distortion. However, Zoom DWI's impact on image quality and apparent diffusion coefficient (ADC) measurements in mediastinal lesions remains unexplored. This study compared Zoom DWI with cDWI in terms of image quality and ADC assessment.
**Methods or Background:** Thirty patients with mediastinal lesions underwent STIR, Zoom DWI, and cDWI on a 3T MR system. Tumor long- and short-axis diameters (LA and SA) were measured on STIR and each DWI for distortion evaluation. ADC values and signal-to-noise ratios (SNR) of tumor and muscle were obtained from ROI analyses. Two radiologists assessed image quality, artifacts, and diagnostic confidence. Spearman's correlation and Bland-Altman analysis evaluated diameter agreement, while paired t-tests or Wilcoxon tests compared quantitative and qualitative indices.
**Results or Findings:** On correlation between STIR and each DWI, Zoom DWI (LA and SA: r=0.99, p<0.0001) showed significant and better correlations than cDWI (LA: r=0.98, p<0.0001; SA: r=0.97, p<0.0001). The limits of agreement (mean, SD) of Zoom DWI (LA: -0.8, 3.4 mm, SA: -0.3, 1.6 mm) were smaller than those of cDWI (LA: -4.1, 4.4 mm, SA: -2.6, 2.2 mm). ADC values of tumor on Zoom DWI was significantly higher than that of cDWI (p< 0.01). Zoom DWI showed significantly better qualitative image quality indices than cDWI (p<0.01).
**Conclusion:** Zoom DWI has the potential to reduce image distortion and enhance image quality, as well as influence tumor ADC measurements, compared with conventional DWI.
**Limitations:** First, relatively small number of the cases. Second, explore of further clinical Utility for diagnosis of mediastinal lesions is needed.
**Funding for this study:** No funding was provided for this study.
**Has your study been approved by an ethics committee?** Yes
**Ethics committee - additional information:** Institutional Review Board HM24-283
**Author Disclosures:**
Yoshiyuki Ozawa: Nothing to disclose
Masahiko Nomura: Nothing to disclose
Takahiro Ueda: Nothing to disclose
Daisuke Takenaka: Nothing to disclose
Takeshi Yoshikawa: Nothing to disclose
Yoshiharu Ohno: Nothing to disclose

**Early Prediction of Progression-Free Survival in Advanced NSCLC Using Spectral CT Histogram Features**
*W. Yang*, Q. Feng, X. Chen, X. Xin; Nanjing/CN
*(15805159169@163.com)*

**Purpose:** This study aimed to evaluate the predictive value of spectral CT-derived histogram features for progression-free survival (PFS) in patients with advanced NSCLC undergoing immunotherapy.
**Methods or Background:** This retrospective study included 43 patients who underwent baseline spectral contrast-enhanced CT prior to immunotherapy and a first follow-up scan at three months, with progression-free survival (PFS) recorded. Spectral image series-including conventional images, 40/70 keV virtual monoenergetic images (VMI), iodine density, effective atomic number, electron density, and water/iodine-based maps-were reconstructed in arterial and venous phases. Lesions were segmented on VMI-40 keV images for extraction of histogram features. Significant predictors were selected using univariate and variance inflation factor analyses, followed by Cox proportional hazards regression. Patients were stratified into high- and low-risk groups based on Cox-derived risk scores (median cutoff). Kaplan–Meier (KM) analysis compared PFS between groups, and model performance was evaluated by concordance index (C-index).
**Results or Findings:** At follow-up, 12 patients (26.1%) remained progression-free, while 34 (73.9%) progressed (PFS range: 1.7–25 months, mean 9.05 ± 5.61). Three baseline features yielded a Cox model with a C-index of 0.72. Ten follow-up features achieved the same C-index. KM analysis showed significant PFS differences between high- and low-risk groups (log-rank p < 0.01). Median 12-month survival was markedly shorter in high-risk groups compared with low-risk groups.
**Conclusion:** Histogram features derived from spectral CT at baseline or early follow-up are significant predictors of immunotherapy response and PFS in advanced NSCLC, offering potential for early risk stratification.
**Limitations:** Sample size is small.
**Funding for this study:** Not applicable.
**Has your study been approved by an ethics committee?** Yes
**Ethics committee - additional information:** This study was approved by the Institutional Ethics Committee. The requirement for informed consent was waived due to the retrospective nature of the analysis.
**Author Disclosures:**
Xingbiao Chen: Nothing to disclose
Wen Yang: Nothing to disclose
Qianqian Feng: Nothing to disclose
Xiaoyan Xin: Nothing to disclose

**Compared to Perfusion Assessment Capabilities among ECG- and PPG-Gated PREFUL and Dynamic CE-Perfusion MRIs**
Y. Ozawa[1], T. Ueda[1], M. Nomura[1], T. Yoshikawa[1], D. Takenaka[1], M. Endo[2], *Y. Ohno*[1]; [1]Toyoake/JP, [2]Shizuoka/JP
*(yohno@fujita-hu.ac.jp)*

**Purpose:** To directly compare the quantitative capabilities of regional perfusion and pulmonary functional change assessment among electrocardiogram (ECG-) and photoplethysmography (PPG-) phase-resolved functional lung (PREFUL) MRIs and dynamic contrast-enhanced (CE-) perfusion MRI in thoracic oncologic patients.
**Methods or Background:** 17 thoracic oncologic patients prospectively underwent ECG- and PPG-gated PREFUL MRI, dynamic CE-perfusion MRI and pulmonary function test. Then, quantitatively assessed ECG- and PPG-gated perfusion-weighted (PW-) MRI and perfusion map from dynamic CE-perfusion MRI were generated. On each method, regional perfusions were determined by ROI measurements, and overall perfusion was determined as average value of ROI measurements. Then, Pearson's correlations were performed to determine relationship among all regional perfusions. On comparison of regional perfusion among ECG- and PPG-gated PW-MRI and quantitative perfusion maps, Tukey's HSD test was performed. To assess pulmonary functional loss on each MRI method, each overall perfusion was correlated with %VC and %FEV1 by Pearson's correlation.
**Results or Findings:** Correlation of regional perfusion between ECG- and PPG-gated PW-MRIs was determined as significant and good (r=0.79, p<0.0001). However, correlations between ECG- or PPG-gated PW-MRIs and quantitative perfusion map were assessed as significant and fair (ECG: r=0.4, p<0.0001; PPG: r=0.36, p<0.0001). ECG- and PPG-gated PW-MRI demonstrated significantly higher perfusion than quantitative perfusion map (p<0.0001). Moreover, ECG-and PPG-gated PW-MRIs and quantitative perfusion map had significant and moderate correlations with them (%VC: 0.59<r<0.64, p<0.05; %FEV1: 0.51<r<0.7, p<0.05).
**Conclusion:** When compared to others, PPG-gated PREFUL MRI had almost similar potential for quantitatively assessed regional perfusion and pulmonary functional change assessments in thoracic oncologic patients.
**Limitations:** Limited study population
**Funding for this study:** This study was financially and technically supported by Canon Medical Systems. This study was also financially supported by Grants-in-Aid for Scientific Research from the Japanese Ministry of Education, Culture, Sports, Science and Technology.
**Has your study been approved by an ethics committee?** Yes
**Ethics committee - additional information:** This study was approved by the institutional review board of Fujita Health University Hospital (HM24-303).
**Author Disclosures:**
Yoshiyuki Ozawa: Research/Grant Support: LPIXEL Inc. Grant Recipient: Smoking Research Foundation Research/Grant Support: Corelinesoft Co., Ltd.
Masahiko Nomura: Research/Grant Support: Canon Medical Systems Corporation Research/Grant Support: Corelinesoft. Co., Ltd.
Takahiro Ueda: Research/Grant Support: Corelinesoft. Co., Ltd. Grant Recipient: Grant-in-Aid for Scientific Research from the Japanese Ministry of Education, Culture, Sports, Science and Technology
Daisuke Takenaka: Research/Grant Support: Corelinesoft. Co., Ltd. Research/Grant Support: LPIXEL. Inc.
Takeshi Yoshikawa: Nothing to disclose
Yoshiharu Ohno: Grant Recipient: Smoking Research Foundation Grant Recipient: LPIXEL Inc. Grant Recipient: Corelinesoft Co., Ltd. Grant Recipient: Canon Medical Systems Corporation, Grant Recipient: Grants-in-Aid for Scientific Research from the Japanese Ministry of Education, Culture, Sports, Science and Technology.
Masahiro Endo: Nothing to disclose

**09:30 - 11:00** **Research Stage 4**

### Research Presentation Session: Artificial Intelligence and Imaging Informatics

### RPS 1305

### The radiomics reality check: building robust biomarkers for thoracic and abdominal disease

**Moderator**
Oğuz Lafcı; Vienna/AT
*(oguz-oguzz@hotmail.com)*

**Robustness of Radiomics in Dual-Layer Spectral CT: A Phantom Study on the Impact of Acquisition and Reconstruction Parameters**
*J. Jiang*[1], L. Shi[1], M. Lin[1], C. Huang[1], Y. Wang[2], T. OuYang[1], Q. Zhou[1], J. Hu[1], Y. Zhou[1]; [1]Hangzhou/CN, [2]Shanghai/CN
*(13093857028@163.com)*

**Purpose:** To assess the impact of acquisition and reconstruction factors on the robustness of radiomics within Dual-Layer Spectral CT.
**Methods or Background:** A chest phantom consisting of 12 pulmonary nodules was scanned with different acquisition and reconstruction factors, including tube voltage (120 kV vs 100 kV), tube current (10–90mAs vs 100mAs), slice thickness(0.67 mm, 3 mm, 5 mm vs 1 mm), iterative reconstruction levels(idose0–idose7 vs idose4), reconstruction kernels(smooth [A], standard [B], sharp [C], lung [E], y-Sharp [YA] vs y-Detail [YB]), collimation, (16 × 0.625, 32 × 0.625, 64 × 0.625 vs 128 × 0.625) and pitch(0.66, 1.473 vs 1). A total of 31 different scanning sets of 40–100keV virtual monochromatic images and conventional images were reconstructed. Regions of interest were segmented using a semi-automated approach, and 108 radiomics features were extracted. Reproducibility was quantified by the intraclass correlation coefficient (ICC)and concordance correlation coefficient (CCC), while variability was measured using coefficient of variation (CV) and quartile coefficient of dispersion (QCD).
**Results or Findings:** Across all virtual monochromatic and conventional images, the percentages of features were high for both ICC > 0.90 (median: 77.78%; interquartile range [IQR]: 72.22%-80.56%) and CCC > 0.90 (median: 75.93%; IQR: 69.44%-78.70%) when pitch, collimation, tube voltage, or iterative reconstruction level were modified, as well as under higher tube current (≥70 mAs) conditions. Reproducibility was low (median: 30.56%.; IQR: 24.77%-39.12% for ICC; median: 27.78%; IQR: 23.15%-36.57% for CCC) under conditions of altered reconstruction kernel or slice thickness. The inter-protocol variability suggested that 30.56% and 51.85% of features had a CV < 10% and QCD < 10%, respectively.

**Conclusion:** Dual-Layer Spectral CT radiomics was robust to tube voltage, collimation, pitch, iterative reconstruction level, and tube current, but remained sensitive to slice thickness and reconstruction kernel.
**Limitations:** Phantom study.
**Funding for this study:** None
**Has your study been approved by an ethics committee?** Not applicable
**Author Disclosures:**
Jiajie Hu: Nothing to disclose
Tao Ouyang: Nothing to disclose
Chao Huang: Nothing to disclose
Yahong Zhou: Nothing to disclose
Yu Wang: Nothing to disclose
Jinyi Jiang: Nothing to disclose
Qian Zhou: Nothing to disclose
Lei Shi: Nothing to disclose
Manpeng Lin: Nothing to disclose

**Radiomics Feature Reproducibility Across Virtual Monoenergetic and VNC Reconstructions Using Native Phase as Reference**
*A. Ustalov*, S. A. Shmeleva, E. V. Kondratyev, S. Tamaeva, V. Aznaurov, D. Bogomolov, V. Gurina, V. Shirokov, I. Gruzdev; Moscow/RU
*(andreiustalov@gmail.com)*

**Purpose:** To evaluate the reproducibility of radiomics features extracted from virtual monoenergetic (MonoE) and virtual non-contrast (VNC) reconstructions of the liver parenchyma compared with native phase imaging, using a standardized 3D region of interest (ROI).
**Methods or Background:** Fifty patients who underwent abdominal CT were included. A spherical 3D ROI (15 mm diameter) was placed in a homogeneous, vessel-free area of the liver parenchyma. Segmentation was initially performed on one phase and propagated across other reconstructions. Radiomics features were extracted from: native phase (reference standard), VNC from arterial phase, VNC from portal venous phase, MonoE at 200 keV (portal). All features were z-score normalized prior to analysis. Reproducibility was assessed using intra-class correlation coefficient (ICC 2,1), concordance correlation coefficient (CCC), within-subject coefficient of variation (wCV), mean absolute percentage error (MAPE), and paired t-tests with Benjamini–Hochberg FDR correction. A composite instability score was used to identify the least reproducible features.
**Results or Findings:** VNC-portal demonstrated the highest agreement with native phase, with mean CCC >0.90 for shape and first-order features and the largest proportion of “core-like” reproducible features (≈45%). VNC-arterial showed slightly lower reproducibility, particularly for texture classes (GLCM, GLRLM). MonoE200 exhibited significantly reduced reproducibility, with numerous features showing CCC <0.70, MAPE >25%, and systematic bias (q<0.05). The least reproducible features were predominantly wavelet- and LoG-based texture metrics from MonoE200. Shape and first-order features remained robust across all reconstructions.
**Conclusion:** VNC-portal reconstructions provide radiomics features most comparable to native images and may serve as a reliable surrogate when native acquisitions are unavailable. MonoE200 reconstructions introduce substantial variability, particularly in high-frequency texture features, and should be used with caution in radiomics studies.
**Limitations:** Retrospective single-center design, limited sample size
**Funding for this study:** No external funding.
**Has your study been approved by an ethics committee?** Yes
**Ethics committee - additional information:** Approved by the local ethics committee, Protocol № IH-2025/03, 15 June 2025.
**Author Disclosures:**
Evgeny V. Kondratyev: Nothing to disclose
Sofiia Antonovna Shmeleva: Nothing to disclose
Andrey Ustalov: Nothing to disclose
Vladimir Aznaurov: Nothing to disclose
Vera Gurina: Nothing to disclose
Ivan Gruzdev: Nothing to disclose
Danila Bogomolov: Nothing to disclose
Safi Tamaeva: Nothing to disclose
Vadim Shirokov: Nothing to disclose

**Augmented Intelligence for Crohn's Disease: Boosting Prognostic Accuracy by Integrating CT Radiomics with LLM-Processed Electronic Health Records Data**
*Z. Li*[1], Y. Yi[2], S. Li[1], Y. Wang[1], J. Lin[1], S-T. Feng[1], X. Li[2]; [1]Guangzhou/CN, [2]Macau/CD
*(39208300@qq.com)*

**Purpose:** Accurate early prediction of adverse outcomes in Crohn's disease (CD) is paramount for personalized therapy. Current approaches often operate in silos: quantitative CT radiomics models or qualitative clinical narrative assessments. We hypothesize that integrating structured imaging biomarkers with unstructured, context-rich data from Electronic Health Records (EHRs) via Large Language Models (LLMs) will create a superior, holistic predictive framework.
**Methods or Background:** In this multicenter study, we enrolled 212 CD patients from six hospitals. We developed a multimodal fusion model that synergistically combines: 1) A CT Radiomics Model (VAT-RM), extracting 850 features (texture, first-order, wavelet) from baseline CT using pyradiomics and employing a Support Vector Machine (SVM) classifier; and 2) An LLM-based Clinical Narrative Analyzer, processing unstructured EHR data (symptom history, medication usage, endoscopic reports) using Gemini-2.5 to generate quantitative clinical embeddings. The outputs of both models were integrated using a logistic regression meta-learner to generate the final prognostic prediction.
**Results or Findings:** The integrated multimodal model demonstrated exceptional performance, achieving an AUC of 0.920 (95% CI 0.871–0.956) in the external test cohort. This significantly outperformed both the standalone VAT-RM (AUC=0.882, P=0.039) and the best-performing standalone LLM (Gemini-2.5 AUC=0.791, P<0.001). The fusion model also showed superior calibration and reclassification metrics, indicating its enhanced clinical utility. The LLM component provided unique value in interpreting complex clinical narratives, such as subtle symptom progression and medication adherence patterns, complementing the robust imaging biomarkers.
**Conclusion:** Fusing CT radiomics with LLM-derived clinical insights creates a powerful synergistic model that surpasses either modality alone. This integrated approach offers a comprehensive and superior strategy for risk stratification in CD, paving the way for more precise and data-driven clinical decision support.
**Limitations:** Not applicable.
**Funding for this study:** None.
**Has your study been approved by an ethics committee?** Yes
**Ethics committee - additional information:** This study was approved by the Institutional Ethics Review Committee at the First Affiliated Hospital of Sun Yat-sen University (ethics number [2024]668).
**Author Disclosures:**
Shi-Ting Feng: Nothing to disclose
Shaochun Li: Nothing to disclose
Yunqi Yi: Nothing to disclose
Xuehua Li: Nothing to disclose
Yangdi Wang: Nothing to disclose
Zhoulei Li: Nothing to disclose
Jinjiang Lin: Nothing to disclose

**Robustness of radiomics features between poly-energetic images and virtual mono-energetic images: synchronizing kiloelectron-volt level for better radiomics reproducibility**
*J. Zhong*, Y. Song, Z. Xu, H. Zhang, W. Yao; Shanghai/CN
*(wal_zjy@163.com)*

**Purpose:** To find the kiloelectron-volt (keV) level of virtual mono-energetic images (VMIs) matching to the poly-energetic images (PEIs) that provide better radiomics reproducibility for subsequent clinical analysis.
**Methods or Background:** A phantom of twenty diverse texture materials was scanned using single-source mode at tube voltages of 70, 80, 90, 100, 110, 120, 130, 140, and 150 kVp, and dual-source mode at tube voltage combinations of 70/150Sn, 80/140, 80/150Sn, 90/150Sn, and 100/150Sn kVp, respectively, all with a radiation dose of 5 mGy. Nine sets of PEIs were reconstructed as reference. Thirty-one sets of VMIs at 40 to 190 keV with a stepwise of 5 keV were reconstructed for five dual-source scans, resulting 155 sets of VMIs. Ninety-three radiomics features were extracted from each material per PyRadiomics. The reproducibility of features between PEIs and VMIs was evaluated using intraclass correlation coefficient (ICC) and concordance correlation coefficient (CCC).
**Results or Findings:** According to ICC and CCC values, the keV levels of VMIs for highest radiomics reproducibility were 55, 60, 65, 65, 65, 70, 75, 75, and 75 keV for PEIs at 70, 80, 90, 100, 110, 120, 130, 140, and 150 kVp, respectively. The radiomics features showed highest percentage of 82.2% and 79.6% with ICC>0.90 and CCC>0.90 at 70 keV VMI for 120 kVp PEIs.
**Conclusion:** The ideal keV levels of VMIs that provide appropriate radiomics reproducibility increased with the tube voltages for PEIs. Synchronizing keV levels can provide better radiomics reproducibility between VMIs and PEIs. The keV levels are important for generalizability radiomics models across conventional and spectrum CT platforms.
**Limitations:** The limitations of the study are: (1) phantom study; (2) the exact best keV level VMI for PEI at each kVp not confirmed; (3) impact on diagnostic performance not investigated.
**Funding for this study:** Funding was provided by National Natural Science Foundation of China (82302183, 82471935, 82271934), Research Found of Health Commission of Shanghai Municipality (20244Y0214), Research Found of Health Commission of Changing District, Shanghai Municipality (2023QN01), Laboratory Open Fund of Key Technology and Materials in Minimally Invasive Spine Surgery (2024JZWC-ZDA04, 2024JZWC-YBA07), and Research Fund of Tongren Hospital, Shanghai Jiao Tong University School of Medicine (TRKYRC-XX202204, TRYJ2021JC06).
**Has your study been approved by an ethics committee?** Not applicable

**Machine Learning Survival Prediction in Esophageal Cancer Using Radiomics and Body Composition from Pretreatment and Follow-Up T12-Level CT**

*M. Liu*, S-C. Lin, C-H. Liao, W-H. Chen, Y-J. Liu; Taichung/TW
*(penguin2046@gmail.com)*

**Purpose:** This study aimed to develop prognostic models for esophageal cancer by integrating body composition indices and radiomic features of skeletal muscle and adipose tissue at the T12 level from both pretreatment and follow-up CT scans, along with clinical and demographic data.
**Methods or Background:** This retrospective study included 212 esophageal cancer patients who underwent concurrent chemoradiotherapy, with both pretreatment and follow-up chest CT scans available. Body composition analysis (BOA) and radiomic features were extracted from skeletal muscle and adipose tissue at the T12 level using automated tools. Four feature subsets (no-radiomics, pretreatment only, follow-up only, and combined inputs) were developed using logistic regression with LASSO for feature selection, followed by Cox regression. Prognostic models—including nomogram, support vector classifier, logistic regression, and extra trees classifier—were constructed to predict 1-, 2-, and 3-year overall survival.
**Results or Findings:** The model integrating both BOA and radiomics from pretreatment and follow-up CT, combined with clinical data, achieved the highest AUC (0.91), sensitivity (0.81), and specificity (0.88) using the logistic regression model. The most predictive features included both clinical variables, body composition indices, and radiomic features, particularly from follow-up VAT. Follow-up imaging contributed significantly to model performance, reinforcing its value in treatment response evaluation.
**Conclusion:** This is the first study to demonstrate that BOA indices and their corresponding radiomics at the T12-level from both pretreatment and follow-up CT scans—combined with clinical data—can provide accurate prognostic information for esophageal cancer. This approach offers a practical alternative when L3-level imaging is unavailable and supports the clinical integration of automated T12-based imaging biomarkers. The integration of these imaging features with clinical parameters enhances the prediction of survival outcomes.
**Limitations:** Conducted retrospectively at a single center, with relatively small and heterogeneous patient cohort.
**Funding for this study:** None
**Has your study been approved by an ethics committee?** Yes
**Ethics committee - additional information:** This retrospective study was approved by the IRB of Taichung Veterans General Hospital.
**Author Disclosures:**
Mingcheng Liu: Nothing to disclose
Shao-Chieh Lin: Nothing to disclose
Chun-Han Liao: Nothing to disclose
Wen-Hsien Chen: Nothing to disclose
Yi-Jui Liu: Nothing to disclose

**AI-Assisted CT Diagnosis of Appendicits: Synergistic Gains Across Radiologist Experience Levels**

*S. Reischl*, J. D. B. Brandt, F. Reischl, S. Ziegelmayer, A. Sauter, F. Lohöfer, M. R. Makowski, D. Rueckert, R. Braren; Munich/DE

**Purpose:** To evaluate the diagnostic performance of a deep learning system for appendicitis in CT and its synergistic effect when used as decision support across radiologists with varying experience levels.
**Methods or Background:** We developed AIppendix, an end-to-end deep learning pipeline combining a 3D Retina-UNet for detection and a 3D ResNet18 for classification of appendicitis and co-pathologies. Training was performed on 580 annotated CT scans with 116 cases reserved for validation and 162 independent cases for external testing. The system was assessed in a reader study including 12 participants: medical students (n=4), residents with >3 years of experience (n=3), and board-certified radiologists (n=5, including 2 gastrointestinal subspecialists). Readers classified if appendicitis was present on abdominal CT scans of patients with abdominal discomfort first unaided, then with AI support.
**Results or Findings:** The AI system achieved an accuracy 0.93 (sensitivity 0.96, specificity 0.90) on internal data and 0.88/0.87 AUC on external testing, matching senior radiologists and surpassing residents in specificity. Without AI support, accuracy increased with experience (students 0.72, residents 0.91, senior radiologists 0.93). With AI assistance, performance improved in all groups. Students benefited most (accuracy 0.72→0.87; sensitivity 0.78→0.87; specificity 0.67→0.88). Residents improved from 0.91→0.97, mainly through specificity gains (0.83→0.96), while senior radiologists showed only marginal benefit (0.93→0.95). The AI thus compensated for inexperience, reduced false positives among residents, and acted as a safeguard for experts.
**Conclusion:** The model achieves radiologist-level performance in diagnosing appendicitis and demonstrates the strongest impact when combined with less experienced readers, harmonizing diagnostic quality across experience levels.
**Limitations:** Retrospective single-center study with moderate dataset size; end-to-end performance remains limited by detection accuracy.
**Funding for this study:** None
**Has your study been approved by an ethics committee?** Yes
**Ethics committee - additional information:** The study was approved by the ethical review board of the Technical University of Munich. Informed consent was waived according to the regulations of the university for retrospective analyses.
**Author Disclosures:**
Andreas Sauter: Nothing to disclose
Stefan Reischl: Nothing to disclose
Daniel Rueckert: Nothing to disclose
Johannes David Benedict Brandt: Nothing to disclose
Rickmer Braren: Nothing to disclose
Marcus R. Makowski: Nothing to disclose
Fabian Reischl: Nothing to disclose
Sebastian Ziegelmayer: Nothing to disclose
Fabian Lohöfer: Nothing to disclose

**CT-based radiomics for HCC recurrence prediction after liver transplantation: which choices really matter? Insights on segmentation margins, bin width, and contrast phase**

*V. Piva*, G. Zorzi, F. Rizzetto, G. Bruschi, C. De Mattia, A. Vanzulli, P. E. Colombo; Milano/IT
*(virginia.piva@unimi.it)*

**Purpose:** Aim of this study was to evaluate the impact of CT contrast phase, segmentation margins and radiomic features extraction bin width on the performance of a machine learning model for predicting post-transplant HCC recurrence.
**Methods or Background:** We retrospectively included 54 histologically confirmed HCC patients who underwent pre-transplant liver CT imaging between 2010 and 2019 at a single institution, yielding a total of 116 lesions. A single radiologist segmented the lesions with four different peritumoral expansions (PT 0, 5,10 and 15 mm) on arterial, venous and delayed phases. Radiomic features were extracted using PyRadiomics with varying bin widths (3,5,10,20,25) and isotropic resampling. Principal Component Analysis (PCA) was used to select relevant features for each combination. A multilayer perceptron (MLP) model was trained with stratified 10-fold cross-validation and hyperparameters optimized via GridSearchCV. Model performance was evaluated across different combinations of segmentation margin, contrast phase, and bin width to compare their effect.
**Results or Findings:** The MLP achieved its best performance on the arterial phase with no peritumoral expansion and a medium bin width of 10 (AUC 0.82, accuracy 0.80, specificity 0.87). With PT0–PT5, the arterial phase outperformed the delayed phase, achieving higher values for AUC (0.72–0.82 vs. 0.62–0.75), accuracy (0.78–0.80 vs. 0.77–0.79), and comparable specificity (0.87–0.88 vs. 0.88–0.89). Larger expansions (PT10–PT15) decreased performance across phases. The portal phase did not achieve predictive value (AUC<0.60). Model performance also declined with very small or very large bin widths (AUC<0.78).
**Conclusion:** CT-based radiomics can support prediction of post-transplant HCC recurrence; however, model performance is highly dependent on contrast phase, segmentation strategy, and feature extraction settings. Careful optimization of these factors is essential to achieve reliable predictions.
**Limitations:** The small patient cohort and its retrospective nature.
**Funding for this study:** None
**Has your study been approved by an ethics committee?** Yes
**Ethics committee - additional information:** Institutional Review Board approved the retrospective data collection in anonymous form.
**Author Disclosures:**
Francesco Rizzetto: Nothing to disclose
Giulia Bruschi: Nothing to disclose
Giulia Zorzi: Nothing to disclose
Angelo Vanzulli: Nothing to disclose
Virginia Piva: Nothing to disclose
Paola Enrica Colombo: Nothing to disclose
Cristina De Mattia: Nothing to disclose

**The impact of deep learning image reconstruction on image quality and diagnostic confidence of liver tumors in 40 kev virtual monochromatic imaging**

*C. Huang*, C. He; Guiyang/CN
*(huangcaijun1985@163.com)*

**Purpose:** To investigate the effect of deep learning image reconstruction (DLIR) on the synchronous visualization of liver tumors in 40 keV virtual monochromatic images (VMIs).
**Methods or Background:** This prospective study enrolled 50 patients who underwent abdominal contrast-enhanced dual energy CT (DECT) imaging, 40 keV VMIs were obtained during the venous phase. Images were reconstructed with different algorithms: filtered back projection (FBP), adaptive statistical iterative reconstruction V (ASIR-V 60%), DLIR-M, and DLIR-H. Measurements included liver SD, lesion-background contrast-to-noise ratio (CNR). Two radiologists independently scored image quality, lesion conspicuity and diagnostic confidence using a 5-point Likert scale. Lesions were categorized as hypervascular or hypovascular based on blood supply for subgroup analysis. Noise equivalent dose (NED) and image quality index (IQF) were calculated considering image noise and radiation dose.
**Results or Findings:** The smallest SDs of liver was observed in the DLIR-H group, there was no significant difference between ASIR-V 60% and DLIR-M, though both were lower than FBP. For liver lesion CNR, DLIR-H significantly outperformed ASIR-V 60% and FBP, while no significant difference was found between DLIR-M and ASIR-V 60%. In lesion conspicuity and diagnostic confidence assessments—for both hypovascular and hypervascular tumors—DLIR-H yielded superior scores compared to ASIR-V 60%. The NED was lowest in the DLIR-H group, comparable between ASIR-V 60% and DLIR-M, both lower than FBP; conversely, the IQF showed an opposite trend.
**Conclusion:** 40 keV VMIs with DLIR-H markedly enhances the visualization and diagnostic confidence of liver tumors, demonstrating the highest dose-to-image quality efficiency.
**Limitations:** First, single-center study, the distribution of lesion types is uneven, and it is difficult to avoid bias. Second, the number of cases is small, and diagnostic control studies cannot be conducted. Third, no images of other energies are explored.
**Funding for this study:** No funding was received for this study.
**Has your study been approved by an ethics committee?** Yes
**Ethics committee - additional information:** The study was approved by Guiqian International Hospital Institutional Ethics Committee (NO. 20250001)
**Author Disclosures:**
Chunyan He: Nothing to disclose
Caijun Huang: Nothing to disclose

**CT-based Radiomics in Colorectal Liver Metastases: Redefining Pre-operative Survival Prediction**

*A. Ammirabile*[1], G. Matteucci[1], F. Fiz[2], E. Lanza[1], L. Cavinato[1], A. Laghi[1], G. Torzilli[1], F. Ieva[1], L. Viganò[1]; [1]Milan/IT, [2]Genoa/IT
*(ang.ammirabile@gmail.com)*

**Purpose:** Surgery with perioperative chemotherapy is potentially curative for colorectal liver metastases (CRLM). Selection of candidates should rely on survival prediction; however, available prognostic factors have limited reliability, and most biomarkers are assessed on surgical specimens. This study evaluated preoperative CT-based radiomics for overall survival (OS) prediction, focusing on the impact of CT–surgery interval and peritumoral tissue analysis and comparison with clinical scores.
**Methods or Background:** All consecutive patients undergoing CRLM resection (2010-2020) with contrast-enhanced CT performed ≤60 days before surgery and at least one CRLM ≥10 mm were considered. Manual tumor segmentation (Tumor-VOI) and automatic 5-mm peritumoral expansion (Margin-VOI) were performed on portal phase images. From each VOI, 110 IBSI-compliant radiomic features were extracted. Three prediction models were developed: Clinical, Clinical+Tumor-radiomics, Clinical+Tumor/Margin-radiomics. Features selection used Boruta algorithm, followed by Random Forest classification with 10-fold cross-validation. Models were evaluated in the whole cohort and in patients with CT-surgery interval ≤30 days. Inter-tumour heterogeneity was assessed with Tree-Edit Distance and Hierarchical Clustering to stratify patients, and resulting clusters were integrated into survival models.
**Results or Findings:** 306 patients were included (mean age 63 years; 187 men). Five-year survival was 40.9% (mean follow-up 34 months). At validation, the clinical model achieved C-index=0.629. Radiomics provided modest improvement in the entire cohort, with greater impact in the 212 patients with a CT-surgery interval ≤30 days: the Clinical+Tumor-radiomics model reached C-index=0.691, increasing to 0.717 with Margin-VOI features. Clinical–radiomic models outperformed established scores (Fong, GAME, m-CS; C-indices=0.553–0.613). Inter-tumor heterogeneity did not improve prediction.
**Conclusion:** Radiomic features of CRLM and peritumoral tissue improve preoperative survival prediction beyond clinical scores, with better performance at shorter CT-surgery intervals. Combined models may refine CRLM treatment strategies, acting as a preoperative filter.
**Limitations:** Retrospective single-center design; Manual segmentation; OS-only analysis.
**Funding for this study:** AIRC grant #2019−23822
**Has your study been approved by an ethics committee?** Yes
**Ethics committee - additional information:** The study was performed according to the declaration of Helsinki and its later amendments. The local review board approved the study protocol (83/20). Because of the retrospective nature of the study, the need for informed consent was waived.
**Author Disclosures:**
Gilda Matteucci: Nothing to disclose
Angela Ammirabile: Nothing to disclose
Guido Torzilli: Nothing to disclose
Ezio Lanza: Nothing to disclose
Francesco Fiz: Nothing to disclose
Andrea Laghi: Nothing to disclose
Lara Cavinato: Nothing to disclose
Luca Viganò: Nothing to disclose
Francesca Ieva: Nothing to disclose

**Multiple Instance Learning with Radiomics Features Enables Peritoneal Carcinomatosis Detection from CT Images: preliminary results**

*K. Vrettos*[1], B. Huang[2], I. Moberg[2], I. Rouvelas[2], M. Klontzas[1], A. Tzortzakakis[2]; [1]Heraklion/GR, [2]Stockholm/SE

**Purpose:** Peritoneal carcinomatosis (PC) correlates with advanced cancer and its identification can significantly impact treatment planning and patient outcomes. Current detection methods such as CT and circulating protein biomarkers have limitations in terms of accuracy and efficiency. Radiomics, an AI-driven approach that extracts quantitative features from medical images, holds promise for improving PC detection. The aim of this work is to develop an open-access radiomics model that assists radiologists in identifying PC from CT scans, while maintaining human accountability and ethical oversight, through a human-in-the-loop approach.
**Methods or Background:** The model architecture is an attention-based Multiple Instance Learning(MIL) network, which utilizes an instance feature extractor, an attention mechanism and a final classifier operating on aggregated features. The dataset consists of 141 patients with gastric tumors who underwent laparoscopy and biopsy to detect PC. Radiomics features were extracted from CT images for 12 biopsy spots(the tumor was not included) corresponding to the conventional laparoscopic based Peritoneal Cancer index score . Boruta feature selection and interpretability analysis were performed . The model's performance was evaluated using AUC,Accuracy,F1-score,Sensitivity and Specificity on a held-out test set. CLAIM guidelines were followed.
**Results or Findings:** The radiomics model achieves an AUC of 0.7,Accuracy of 0.81 and Specificity of 0.94. The model's performance was non-inferior to expert radiologists' predictions of PC from CT scans. The human-in-the-loop approach yielded comparable results with an increased sensitivity of 0.8, when radiologist performance was combined with model performance. Analysis of existing literature, revealed that the proposed model outperforms current PC detection approaches, including CA125 blood test.
**Conclusion:** This study highlights the potential of radiomics-based models to assist radiologists in detecting PC on CT scans. It further shows that radiologist-AI collaboration can improve detection performance while ensuring transparency.
**Limitations:** Dataset size
**Funding for this study:** Funding from the Cancer Research Funds of Radiumhemmet
**Has your study been approved by an ethics committee?** Yes
**Ethics committee - additional information:** EPM diarienummer: Dnr 2018/970-31/1
**Author Disclosures:**
Michail Klontzas: Nothing to disclose
Antonios Tzortzakakis: Nothing to disclose
Konstantinos Vrettos: Nothing to disclose
Ivan Moberg: Nothing to disclose
Ioannis Rouvelas: Nothing to disclose
Biying Huang: Nothing to disclose

**Effectiveness of Deep-Learning Based Denoising on the Image Quality and Diagnostic Performance of Low-Dose Abdominal CT for Acute Appendicitis**

*C. Shin*, Y. H. Lee; Iksan/KR
*(chung3684@naver.com)*

**Purpose:** The aim of this study is to evaluate the effectiveness of deep-learning based denoising images using low-dose abdominal CT in terms of image quality and diagnostic performance for evaluating acute appendicitis.
**Methods or Background:** We retrospectively analysed 53 patients underwent low-dose abdominal CT for suspected acute appendicitis. Images were reconstructed using filtered-back-projection (FBP) and iterative reconstruction (IR), then processed using deep-learning-based denoising software (ClariCT.AI, Claripi), resulting in four image sets per patient. For quantitative analysis, image noise and signal-to-noise ratio were measured. Two radiologists independently scored for qualitative analysis (noise, sharpness, artifacts, overall) on a 4-point Likert scale and assessed the presence of acute appendicitis. Statistical analyses included repeated measures ANOVA, Friedman test, Wilcoxon signed-rank test, and McNemar's test.
**Results or Findings:** Four image sets demonstrated significant differences in both noise and SNR (p<0.001). The noise progressively decreased in the order of FBP(25.53±1.98), IR(17.07±1.27), denoised FBP(11.45±0.86), and denoised IR(8.36±0.63). SNR was highest in denoised IR (23.11±2.84), followed by denoised FBP(16.92±2.04), IR(11.37±1.36), and FBP(7.65±0.93). Denoised FBP achieved higher overall quality than FBP, IR, and denoised IR in both readers (p<0.01), providing superior sharpness and fewer artifacts, while denoised IR images achieved lower image noise. All image sets showed 100% sensitivity for acute appendicitis. In reader 1, denoised images showed higher specificity (95.35% each) than undenoised FBP and IR(90.70% each). In reader 2, FBP showed lower specificity (88.38%) than other image sets (95.35% each). No statistically significant difference was observed for specificity in either readers.
**Conclusion:** Deep-learning-based denoising markedly improves noise reduction in low-dose abdominal CT. Reconstructed images with denoising provided superior image quality and higher diagnostic accuracy than undenoised FBP or IR images for evaluating acute appendicitis.
**Limitations:** Quantitative assessment was performed in regions unrelated to the appendix.
**Funding for this study:** This study received no specific funding.
**Has your study been approved by an ethics committee?** Yes
**Ethics committee - additional information:** The study was approved with a waiver of informed consent by the institutional review board of Wonkwang University Hospital (IRB No.2023-12-031)
**Author Disclosures:**
Young Hwan Lee: Nothing to disclose
Chunghwan Shin: Author: First author and presenting author

**Deep-learning-based liver age estimation predicts major health outcomes**

*R. T. Schirrmeister*, M. Jung, M. Reisert, F. Bamberg, J. Weiß; Freiburg Im Breisgau/DE
*(robin.schirrmeister@uniklinik-freiburg.de)*

**Purpose:** Various studies have shown that AI can estimate biological age from medical imaging and predict outcomes beyond traditional risk factors. In this study we developed a deep learning model (MR-LiverAge) that estimates liver age from MR imaging and investigated its association with different health outcomes.
**Methods or Background:** MR-LiverAge was developed in a two-step approach using 30025 subjects from the German National Cohort (NAKO): Model 1) segmentation of the liver from T1-weighted abdominal MRI; Model 2) takes the segmented pancreas mask of Model 1 as the only input and outputs a liver age estimate in years.Independent validation was performed in 40151 subjects of the UK Biobank (UKB). To account for potential confounders such as BMI, sex or liver fat, generalized additive models were fitted to predict the AI-estimated MR-LiverAge from those confounders. The remaining difference between MR-LiverAge and confounder-estimated age was used to group individuals into decelerated, normal and accelerated aging groups. Cox proportional hazards regression assessed the association between age groups and incident diabetes, liver disease, cardiovascular events and mortality.
**Results or Findings:** MR-LiverAge had a Pearson-R correlation of 80.4% in the NAKO and 50.9% in the UKB with chronological age. Individuals with accelerated MR-LiverAge in the UKB had a higher risk of incident diabetes (HR=1.66, 95% CI=1.37–2.01, p < 0.001), liver disease (HR=1.52, 95% CI=1.19–1.96, p=0.001), cardiovascular events (HR=1.38, 95% CI=1.1–1.73, p=0.005) and mortality risk (HR=1.45, 95% CI=1.16–1.81, p=0001) independent of traditional risk factors.
**Conclusion:** MR-LiverAge has potential as a novel opportunistic imaging biomarker to estimate risk of major health outcomes.
**Limitations:** Results have only been obtained from MRI on the UKB dataset and would need to be further validated on other datasets and other modalities.
**Funding for this study:** We acknowledge support by the Deutsche Forschungsgemeinschaft (DFG, German Research Foundation), grant number 525002713.
**Has your study been approved by an ethics committee?** Yes
**Ethics committee - additional information:** Study has been approved by the local IRB.
**Author Disclosures:**
Marco Reisert: Nothing to disclose
Jakob Weiß: Nothing to disclose
Matthias Jung: Nothing to disclose
Robin Tibor Schirrmeister: Nothing to disclose
Fabian Bamberg: Nothing to disclose

**09:30 - 11:00** **Research Stage 5**

### Research Presentation Session: Neuro

### RPS 1311

### Imaging the psychiatric brain: new frontiers and insights

**Moderator**
Jean-Pierre Pruvo; Lille/FR
*(jean-pierre.pruvo@chru-lille.fr)*

**Transdiagnostic compression of connectome gradient in major depression, bipolar disorder, and schizophrenia: A large-scale multicenter resting-state fMRI study**

*Q. Guo*, H. Yang; Hangzhou/CN

**Purpose:** To examine connectome gradient dysfunction associated with major depressive disorder (MDD), bipolar disorder (BD), and schizophrenia (SCZ), and to identify transdiagnostic variation patterns.
**Methods or Background:** Using a large cohort of resting-state fMRI data from 3266 participants recruited at 23 sites, including 1275 healthy controls (HC), 1531 MDD, 187 BD and 273 SCZ patients, we applied diffusion map embedding to estimate connectome gradient. Statistical analyses were conducted on range, variance, and gradient values of principal gradient, and correlations were examined with clinical scales.
**Results or Findings:** The principal gradient extends from primary visual (VIS) and somatosensory/motor networks (SMN) to default mode network (DMN). MDD showed less variance than HC. BD and SCZ exhibited narrower range and less variance than HC and MDD. Regionally, gradient values in DMN regions exhibited a pattern of SCZ < BD < MDD < HC. MDD, BD, and SCZ groups had lower gradient scores in VIS regions and higher in SMN compared to HC, indicating opposite changes within primary systems. The mean gradient value in SMN regions of MDD showed significant positive correlations with anxious mood and tension scores (FDR-corrected). MDD and SCZ in VIS exhibited positive correlation trends with scores on several items of their respective scales (p < 0.05 before FDR correction).
**Conclusion:** This study demonstrated that principal gradient compression was both shared and spectrally distributed across MDD, BD, and SCZ. The non-uniform compression was characterized by heterogeneous alterations within primary systems. These findings not only provide a unified neurobiological account for sensory-cognitive integration deficits in these disorders but also propose a new perspective: mental disorder patients may maintain a relatively stable baseline of sensory reality through VIS's protective compensation.
**Limitations:** The sample sizes for BD and SCZ groups are relatively small.
**Funding for this study:** No funding was received for this study.
**Has your study been approved by an ethics committee?** Yes
**Ethics committee - additional information:** The study was approved by the ethical committee of the First Affiliated Hospital of Zhejiang University School of Medicine (Approval No. IIT20220463B-R1).
**Author Disclosures:**
Qinger Guo: Nothing to disclose
Hong Yang: Nothing to disclose

**Brain connectivity during listening to different styles of classical music: a prospective fMRI study**

*C. Dalmasso*, F. Andersson, I. Maldonado, M. A. Siragusa, V. Lefevre, C. Destrieux, W. El-Hage, T. Desmidt, J-P. Cottier; Tours/FR
*(clement.dalmasso@gmail.com)*

**Purpose:** While numerous studies have examined the effects of musical styles on pleasure and fear, few have investigated the overall brain activity underlying these emotional responses, particularly across different styles and periods of classical music. The present study aimed to use fMRI to explore functional connectivity during listening to four excerpts from different periods of Classical music, and to identify musical parameters associated with the engagement of clinically relevant networks.
**Methods or Background:** This prospective, single-centre study included 25 healthy subjects with musician status and psychometric assessment using validated scales. A 3T MRI scanner acquired BOLD fMRI data during a task involving watching emotionally neutral images and listening to four 1-minute excerpts presented in random order (Rossini Guillaume Tell, Minkus La Bayadère, Satie Gymnopédie No. 1, Stravinsky The Rite of Spring). Data were processed using the Conn Toolbox and MatLab®, highlighting significant co-activations for each stimulus (atlas MNI AAL3).
**Results or Findings:** Each contrast (opposing stimuli) revealed significant co-activations (t-tests, $p < 0.05$) between distinct brain areas, recruiting different network profiles: audio–visuo–fronto-linguistic coupling with orbitofrontal involvement (valence/reward) for Rossini; limbic–cerebellar and occipital circuits (contextual encoding, motor imagery) for Minkus; salience/interoception pole (insula) and thalamic-striatal-cerebellar loops compatible with calm attentional state for Satie; orbitofrontal axis–temporal poles (structural complexity, semantic combinatorics, emotional coloring) for Stravinsky. Slow tempi and regular meters favored insula–thalamus/limbic and cerebellar networks, whereas fast tempi and/or irregular meters engaged orbitofrontal and temporal poles.
**Conclusion:** Our results highlight a diversity of cerebral co-activation during listening to different classical styles. Tempo and metrical regularity appeared as modulators of network architecture. This network-centered mapping may pave the way for personalized “musical prescriptions” in music therapy (interoceptive calming, language/rhythm training, cognitive flexibility...).
**Limitations:** Not applicable
**Funding for this study:** Kahler Communication France
**Has your study been approved by an ethics committee?** Yes
**Ethics committee - additional information:** The study protocol was approved by the human ethical committee of Toulouse (France).
**Author Disclosures:**
Marta Andrea Siragusa: Nothing to disclose
Jean-Philippe Cottier: Nothing to disclose
Wissam El-Hage: Nothing to disclose
Valentin Lefevre: Nothing to disclose
Clément Dalmasso: Nothing to disclose
Igor Maldonado: Nothing to disclose
Thomas Desmidt: Nothing to disclose
Christophe Destrieux: Nothing to disclose
Frederic Andersson: Nothing to disclose

**Hypoxic–Ischaemic Brain Alterations and Their Association with Neurodevelopmental Disorders: Focus on Autism Spectrum Disorder and Speech Delay**

*N. Dichko*; Kyiv/UA
*(ekskluziv.da@gmail.com)*

**Purpose:** Hypoxic–ischaemic (HI) events during early brain development can lead to structural and functional alterations that may predispose children to neurodevelopmental disorders (NDDs).
**Methods or Background:** Perinatal and early childhood HI events can disrupt the maturation of neural networks. Increasing evidence suggests that such early-life brain insults may contribute to the development of neurodevelopmental disorders (NDDs). Despite emerging interest in the link between HI injury and NDDs, there is still limited large-scale clinical research exploring this association across diverse pediatric age groups. This study aims to fill this gap by evaluating structural brain alterations following HI injury and examining their potential association with subsequent diagnoses of ASD and/or speech delay in a well-characterized pediatric cohort. This retrospective observational study included 331 pediatric patients, inclusion criteria required evidence of hypoxic–ischaemic brain injury confirmed through neuroimaging (MRI) and availability of clinical neurodevelopmental assessments. Patients with genetic syndromes or major congenital brain malformations unrelated to HI injury were excluded.
**Results or Findings:** Hypoxic–ischaemic brain alterations were predominantly observed in the periventricular white matter (63%), basal ganglia and thalami (41%), temporal lobes (38%), and frontal lobes (26%). Diffuse white matter abnormalities and cortical atrophy were more common in patients diagnosed with ASD or combined ASD and speech delay. • Periventricular leukomalacia (PVL), basal ganglia and thalamic involvement, temporal lobe abnormalities and diffuse cortical atrophy showed a strong correlation of combined ASD and speech delay.
**Conclusion:** Our findings support the growing evidence that early-life HI injury disrupts critical neural pathways involved in language acquisition, social communication, and cognitive functioning. Early neuroimaging, particularly MRI, plays a vital role in identifying at-risk children and may provide predictive value for long-term neurodevelopmental outcomes.
**Limitations:** Retrospective design, heterogeneity of age and timing of injury, imaging modalities and quality.
**Funding for this study:** This study did not receive any specific grant from funding agencies in the public, commercial, or not-for-profit sectors.Future research expansion and longitudinal follow-up will require external funding support.
**Has your study been approved by an ethics committee?** Not applicable
**Author Disclosures:**
Nataly Dichko: Nothing to disclose

**Assessing Glymphatic System Alterations in Autism Spectrum Disorder using DTI-ALPS index**

*S. Reddy Kankara*, D. Jayanna, S. R. Kankara, U. Nayak, A. Josephine, V. M. Tellis; Bangalore/IN

**Purpose:** To assess glymphatic system alterations in children with autism spectrum disorder (ASD) using diffusion tensor imaging along the perivascular space (DTI-ALPS) and to compare findings with age- and sex-matched healthy controls.
**Methods or Background:** The glymphatic system facilitates metabolic waste clearance via perivascular pathways, and dysfunction may contribute to neurodevelopmental disorders. In this retrospective case–control study, 30 children with DSM-5–confirmed ASD (mean age 7.4 ± 2.0 years; 22 males) and 30 matched controls (7.1 ± 2.3 years; 21 males) were included. Eligibility required age 3–12 years and diagnostic-quality 3T MRI with DTI; exclusions were prior CNS infection/trauma, structural brain abnormalities, or motion-degraded scans. ALPS indices were derived at the lateral ventricular body using standardized ROIs in projection and association fibers, placed independently by two blinded raters. Group comparisons were performed with independent-samples t-tests, and age–ALPS relationships were assessed with Pearson correlation.
**Results or Findings:** Children with ASD showed significantly lower ALPS indices than controls (Left: 1.10 ± 0.13 vs 1.32 ± 0.16, $p < 0.001$; Right: 1.12 ± 0.14 vs 1.30 ± 0.15, $p = 0.002$), remaining significant after bilateral correction. Age correlated positively with ALPS in both ASD ($r = 0.38$, $p = 0.04$) and controls ($r = 0.31$, $p = 0.04$), indicating maturational improvement; however, values in ASD remained consistently lower across the age spectrum.
**Conclusion:** Pediatric ASD is associated with reduced DTI-ALPS indices, consistent with impaired glymphatic function. Despite age-related gains, glymphatic activity remains attenuated in ASD. DTI-ALPS provides a feasible, non-contrast imaging biomarker of neurofluid dynamics, with potential relevance for studying neurodevelopmental disorders.
**Limitations:** The study is limited by its retrospective, single-center nature, small cohort, and reliance on manual ROI selection. Future work could include longitudinal evaluations and explore associations with clinical severity.
**Funding for this study:** Not applicable
**Has your study been approved by an ethics committee?** Yes
**Ethics committee - additional information:** The study was approved by the Institutional Ethics Committee
**Author Disclosures:**
Shreyas Reddy Kankara: Nothing to disclose
Dhanush Jayanna: Nothing to disclose
Shravan Reddy Kankara: Nothing to disclose
Unnathi Nayak: Nothing to disclose
Arpitha Josephine: Nothing to disclose
Vilas Melrick Tellis: Nothing to disclose

**Cortical Excitability and Molecular Profiling Reveal Distinct Neurobiological Subtypes of Schizophrenia**

*S. Guo*, Q. Gong, Y. Zhao, S. Lui, L. Li, R. Xie, X. Zhang; Chengdu/CN
*(guosixuan479@163.com)*

**Purpose:** Dysregulation of excitation–inhibition balance in specific neural circuits is believed to underlie the diverse positive and negative symptoms of schizophrenia (SCZ). This study aimed to identify neurobiological subtypes of drug-naïve first-episode SCZ (FES) using cortical excitability (CE) mapping to advance precision-medicine approaches for SCZ.

**Methods or Background:** Resting-state fMRI data from 77 drug-naïve FES patients and 76 matched healthy controls were analyzed using a novel CE-mapping approach. After deriving individual CE profiles, support vector machines (SVM) assessed diagnostic accuracy, and hierarchical clustering delineated neurobiological subtypes. Twelve-month follow-up data evaluated treatment-related changes. Molecular analyses integrated transcriptomic data via partial least squares regression with functional enrichment and JuSpace-based neuroreceptor mapping to identify subtype-specific molecular correlates.
**Results or Findings:** FES patients exhibited significantly reduced CE compared with controls, predominantly in the bilateral frontal lobes, sensorimotor cortex, and right cuneus. SVM achieved 86.7% diagnostic accuracy. Hierarchical clustering identified two subtypes: FES1, showing widespread CE reductions with more severe depressive, negative, and cognitive symptoms; and FES2, showing milder alterations. Longitudinal analysis revealed partial CE recovery in FES1 but persistent reductions in FES2. Molecular profiling suggested that FES1 was characterized by synaptic dysfunction and neurodevelopmental disruption, whereas FES2 reflected multisystem impairments accompanied by compensatory processes.
**Conclusion:** This study delineates two neurobiological subtypes of FES with distinct CE profiles and molecular underpinnings. CE-based subtyping offers a promising framework for linking clinical heterogeneity with underlying neurobiology and may inform precision treatment strategies in SCZ.
**Limitations:** The modest sample size and one-year follow-up may limit generalizability, and clinical assessments were restricted, constraining exploration of CE–FES heterogeneity. Transcriptomic analyses were confined to the left hemisphere, precluding bilateral evaluation of gene–CE associations.
**Funding for this study:** This study was supported by grants from the National Key R&D Program of China (2022YFC2009900), National Natural Science Foundation of China (82027808), and National Natural Science Foundation of China (82302167).
**Has your study been approved by an ethics committee?** Yes
**Ethics committee - additional information:** The study was approved by the Research Ethics Committee of West China Hospital of Sichuan University (Approval No. 2022-886).
**Author Disclosures:**
Xinlan Zhang: Nothing to disclose
Yu Zhao: Nothing to disclose
Sixuan Guo: Nothing to disclose
Luying Li: Nothing to disclose
Qiyong Gong: Nothing to disclose
Ruoxi Xie: Nothing to disclose
Su Lui: Nothing to disclose

**Connectivity Patterns across Bipolar Disorder Stages: a Tractography-based Graph Analysis**
S. Capelli[1], A. Arrigoni[1], S. Saluzzi[2], P. Patani[2], S. Martinelli[2], A. Caroli[1], A. Di Giorgio[2], *S. Gerevini*[2]; [1]Ranica/IT, [2]Bergamo/IT
*(sgerevini@asst-pg23.it)*

**Purpose:** Diffusion-weighted MRI (DW-MRI) tractography enables non-invasive mapping of brain connectivity and may reveal biomarkers of structural network alterations in psychiatric disorders. This study investigated connectome changes across clinical stages of Bipolar Disorder (BD), including individuals at familial risk, using advanced DW-MRI processing and graph analysis.
**Methods or Background:** Forty-five participants were recruited from two Italian centers and classified according to the Kupka–Hillegers staging model: Stage 0–1 (n = 13, familial risk and subthreshold symptoms), Stage 2 (n = 8, first hypo/manic episode), Stage 3 (n = 13, recurrent episodes), and Stage 4 (n = 11, chronic non-remissive course). DW-MRI data were acquired using a 3T scanner and processed with a custom pipeline integrating single-shell 3-tissue constrained spherical deconvolution (SS3T-CSD), anatomically constrained probabilistic tractography (iFOD2-ACT), and graph-based connectome analysis. Tractograms were optimized with the COMMIT2 method, and connectivity metrics were derived using Python's bctpy package. T1-weighted imaging was coregistered to the DW-MRI scan using ANT and parcellated with FreeSurfer. Group differences were assessed with Kruskal-Wallis tests and Wilcoxon pairwise comparisons ($p < 0.05$), with Bonferroni correction applied.
**Results or Findings:** Mean nodal strength showed a significant group effect ($p = 0.024$), with post hoc analysis indicating reduced strength in Stage 4 compared to Stage 2 ($p = 0.002$) and a trend compared to Stage 3 ($p = 0.072$). Among node-wise metrics, the betweenness of the left posterior cingulate cortex (PCC) had the most significant effect ($p < 0.001$), which remained significant after Bonferroni correction.
**Conclusion:** Findings indicate progressive disruption of structural connectivity with advancing BD stage, particularly affecting PCC centrality. DW-MRI tractography and connectome metrics hold promise as biomarkers for disease progression and stratification in BD.
**Limitations:** Single-center image acquisition, modest sample size, and lack of external validation.
**Funding for this study:** NextGeneration EU - PNRR: M6/C2_CALL 2022
**Has your study been approved by an ethics committee?** Yes
**Ethics committee - additional information:** Protocol title: BipOlAR Disorder Integrative stagiNG: incorporating the role of biomarkers into Progression AcrosS Stages (BOARDING-PASS)
**Author Disclosures:**
Serena Capelli: Nothing to disclose
Stefano Martinelli: Nothing to disclose
Alberto Arrigoni: Nothing to disclose
Salvatore Saluzzi: Nothing to disclose
Annabella Di Giorgio: Nothing to disclose
Anna Caroli: Nothing to disclose
Paolo Patani: Nothing to disclose
Simonetta Gerevini: Nothing to disclose

**Integrative brain neural activity and molecular analyses of interaction between chronic nicotine addiction and alcohol dependence**
L. Ma, M. Zhang, *B. Mei*, Y. Wei, S. Han, Y. Zhang; Zhengzhou/CN
*(15539680362@163.com)*

**Purpose:** Previous studies have demonstrated that nicotine addiction and alcohol dependence are frequently comorbid. However, the underlying neurobiological mechanisms remain unclear. Therefore, we will investigate the impacts of two factors on spontaneous neural activity and specific neurotransmitter system.
**Methods or Background:** This study used a two-way factorial design, including the following four groups: 1) Alcohol dependence smokers (n = 32); 2) Non-alcohol dependence smokers (n = 30); 3) Alcohol dependence non-smokers (n = 21); 4) Healthy control (n = 27). Functional magnetic resonance imaging (fMRI) was used to compare the differences in fractional amplitude of low-frequency fluctuation (fALFF) among the four groups to detect the interaction effect of nicotine addiction and alcohol dependence on spontaneous neural activity. Furthermore, correlations between fALFF values and PET- and SPECT-driven maps to examine specific neurotransmitter system alternations underlying two factors.
**Results or Findings:** Our study revealed a significant antagonistic interaction between two factors in the right cerebellum. The main effect of nicotine addiction is in the bilateral occipital lobes. The main effects of alcohol dependence are in the bilateral precentral gyrus. Cross-modal correlations revealed alternations in spontaneous neural activity in interactive brain regions associated with the serotonin system (5-HT1b).
**Conclusion:** This study identified an antagonistic interaction of the cerebellum between nicotine addiction and alcohol dependence. Besides, our study further correlates the serotonin system with the intrinsic neural activity associated with this comorbidity. This may be a potential neurobiological mechanism of smoking and alcohol, offering new directions for the treatment of this comorbidity.
**Limitations:** The Juspace toolbox achieves indirect coupling of functional imaging with neurotransmitter systems. However, these neurotransmitters are limited in types and information. Future studies should use PET and MRI to provide more direct evidence.
**Funding for this study:** This study was supported by the Natural Science Foundation of China [grant numbers 81601467, 81871327, and 62106229] and the Funding for Scientific Research and Innovation Team of The First Affiliated Hospital of Zhengzhou University [grant numbers QNCXTD2023007].
**Has your study been approved by an ethics committee?** Yes
**Ethics committee - additional information:** The First Affiliated Hospital of Zhengzhou University
**Author Disclosures:**
Yarui Wei: Nothing to disclose
Yong Zhang: Nothing to disclose
Mengzhe Zhang: Nothing to disclose
Longyao Ma: Nothing to disclose
Bohui Mei: Nothing to disclose
Shaoqiang Han: Nothing to disclose

**Artificial intelligence–assisted voxel-based morphometry for imaging biomarkers of alcohol-related brain atrophy**
*S. S. Bhoyar*, P. Shah, P. Naik, A. G. Sasane; Pune/IN
*(bshreyashs@gmail.com)*

**Purpose:** Alcohol dependence is associated with selective brain atrophy, yet conventional magnetic resonance imaging remains largely qualitative and observer-dependent. Artificial intelligence–assisted voxel-based morphometry enables automated, reproducible volumetric quantification. This study evaluated its ability to objectively identify region-specific atrophy in alcohol dependence and explored its potential role as a clinically translatable imaging biomarker.
**Methods or Background:** Sixty consecutive adult patients diagnosed with alcohol dependence syndrome (ICD-10 criteria) were prospectively enrolled over 18 months. Magnetic resonance imaging was performed on a 1.5T

system using a three-dimensional T1-weighted spoiled gradient recalled acquisition sequence. Automated segmentation and volumetric analysis were conducted using a validated artificial intelligence–based morphometry pipeline, normalised to intracranial volume. Quantitative volumes of cortical and subcortical regions were compared with established normative references.
**Results or Findings:** Frontal lobe atrophy was universal (100 per cent), with high prevalence also observed in the temporal lobes (93 per cent), cerebellum (90 per cent), basal ganglia (putamen 90 per cent, caudate 88 per cent), and hippocampus (81.7 per cent). The amygdala (68 per cent) and thalamus (65 per cent) were variably affected, while the occipital lobes (40 per cent) and brainstem (23 per cent) were relatively preserved. This pattern highlights selective vulnerability of fronto-limbic and cerebellar circuits, correlating with executive dysfunction, impaired memory, and motor incoordination. The artificial intelligence–assisted pipeline consistently generated robust, reproducible volumetric outputs with reduced observer bias and faster processing compared with conventional approaches.
**Conclusion:** Artificial intelligence–assisted voxel-based morphometry provides objective, reproducible imaging biomarkers of alcohol-related brain atrophy. It paves the way for early diagnosis, personalised risk stratification, and treatment monitoring, positioning artificial intelligence as a transformative tool in neuropsychiatric care.
**Limitations:** Absence of a matched control group and lack of direct neuropsychological correlation.
**Funding for this study:** No funding was received for this study.
**Has your study been approved by an ethics committee?** Yes
**Ethics committee - additional information:** Approved by the Institutional Ethics Committee of SKNMC,Pune, approval number [SKNMC/2023/103]
**Author Disclosures:**
Shreyash Subhash Bhoyar: Nothing to disclose
Amol Gorakhnath Sasane: Nothing to disclose
Prashant Naik: Nothing to disclose
Pooja Shah: Nothing to disclose

**How sleep disorder affect glymphatic circulation and network efficiency in internet gaming disorder: Insights from a DTI-ALPS**
L. Ma, M. Zhang, *B. Mei*, Y. Wei, S. Han, Y. Zhang; Zhengzhou/CN
*(15539680362@163.com)*

**Purpose:** Sleep disorders can impair the glymphatic system and may exacerbate cognitive deficits in internet gaming disorder (IGD). Therefore, this study aimed to investigate whether the glymphatic system and the human brain network are impaired in IGD patients with sleep disorder (IGDSD) compared to IGD patients without sleep disorders (IGDNSD), which is crucial for elucidating the neuropathological mechanisms of IGD.
**Methods or Background:** This study recruited 36 IGDSD, 30 IGDNSD, and 30 healthy controls, who performed magnetic resonance imaging (MRI) scanning and clinical symptom assessment. The glymphatic function and network efficiency were calculated based on diffusion images along the perivascular space (DTI-ALPS) index and resting-state functional MRI, respectively. Differences between groups, neural/clinical correlations, mediation analyses, and diagnostic performance were further explored.
**Results or Findings:** Compared to IGDNSD, IGDSD had a significantly lower ALPS index. The correlation analysis showed that the ALPS index in IGDSD was significantly correlated with the local brain efficiency and the sleep quality. The mediation analysis showed that sleep quality partially mediated the effect of local brain efficiency on glymphatic system function. In addition, the area under the ROC curve of ALPS in distinguishing IGDSD from IGDNSD was 0.93, with a cut-off value of 1.32.
**Conclusion:** These results indicated the severely impaired glymphatic function and network efficiency in IGDSD. And this study also adds important evidence that the brain network efficiency disrupts the glymphatic system function by affecting sleep quality, which may reveal the neuropathological mechanisms of IGDSD.
**Limitations:** The DTI-ALPS method is a deductive measure of glymphatic system function. The ALPS index does not comprehensively reflect the clearance function of the glymphatic system.
**Funding for this study:** This study was supported by the Natural Science Foundation of China [grant numbers 81601467, 81871327, and 62106229] and the Funding for Scientific Research and Innovation Team of The First Affiliated Hospital of Zhengzhou University [grant numbers QNCXTD2023007].
**Has your study been approved by an ethics committee?** Yes
**Ethics committee - additional information:** The First Affiliated Hospital of Zhengzhou University
**Author Disclosures:**
Yarui Wei: Nothing to disclose
Yong Zhang: Nothing to disclose
Mengzhe Zhang: Nothing to disclose
Longyao Ma: Nothing to disclose
Bohui Mei: Nothing to disclose
Shaoqiang Han: Nothing to disclose

**12:30 - 13:30 Research Stage 6**

### Research Presentation Session: Breast Hot Topic with Keynote Lecture

### RPS 1402

### Hot Topic: personalised imaging

**Moderator**
Fiona J. Gilbert; Cambridge/UK
*(fjg28@cam.ac.uk)*

**Keynote Lecture**
Magali Hovsepian; Buenos Aires/AR
*(magalihovsepian@hotmail.com)*

**Breast density and the value of integrated imaging: evidence from the P.I.N.K. study**
*L. A. Incardona*[1], S. Molinaro[2], S. Pieroni[2], B. Di Nubila[3], G. M. Giuseppetti[4], P. Belli[5], E. Montrucchio[1], M. Franchini[2]; [1]La Spezia/IT, [2]Pisa/IT, [3]Milan/IT, [4]Ancona/IT, [5]Rome/IT
*(incardonaludovica@gmail.com)*

**Purpose:** Breast density is a major limitation of mammographic screening, reducing sensitivity and delaying cancer diagnosis. The P.I.N.K. Study was designed to evaluate the contribution of multimodality imaging in breast cancer diagnosis. This sub-analysis quantified mammography (MX) performance according to density and assessed the additional detection (AD) provided by ultrasound (US), digital breast tomosynthesis (DBT), and magnetic resonance imaging (MRI).
**Methods or Background:** The P.I.N.K. database included 29,360 women who underwent 60,270 integrated diagnostic exams between 2017 and 2025. A total of 1,246 breast cancers were surgically confirmed. MX sensitivity and the AD of US, DBT, and MRI were calculated across density categories (A–D).
**Results or Findings:** Of 1,246 cancers, 422 occurred in fatty breasts (A–B) and 824 in dense breasts (C–D). In A–B breasts, MX detected 389 of 422 cancers (92.2%), with 33 additional cancers (7.8%) identified by other modalities. In C–D breasts, MX detected 575 of 824 cancers (69.8%), while integrated imaging identified 249 additional cancers (30.2%), increasing detection by up to 25%. The benefit was greatest in younger women and in those with extremely dense tissue.
**Conclusion:** Integrated imaging adds little in fatty breasts but significantly improves detection in dense breasts. On a large multicentre scale, P.I.N.K. demonstrates that tailoring imaging strategies to breast density enhances early diagnosis while avoiding unnecessary procedures in low-density women, supporting more effective and resource-efficient screening pathways.
**Limitations:** This was a retrospective analysis of clinical data.
**Funding for this study:** No funding was received for this study.
**Has your study been approved by an ethics committee?** Not applicable
**Author Disclosures:**
Sabrina Molinaro: Nothing to disclose
Edgardo Montrucchio: Nothing to disclose
Stefania Pieroni: Nothing to disclose
Michela Franchini: Nothing to disclose
Ludovica Anna Incardona: Nothing to disclose
Gian Marco Giuseppetti: Nothing to disclose
Brunella Di Nubila: Nothing to disclose
Paolo Belli: Nothing to disclose

**Correlation between Background Parenchymal Enhancement in Contrast Enhanced Mammography and breast cancer risk: towards personalised screening**
*E. D'Ascoli*, C. Depretto, G. Rossini, C. De Berardinis, G. Irmici, G. Della Pepa, L. Corradini, G. P. Scaperrotta, F. Sardanelli; Milan/IT

**Purpose:** To evaluate the correlation between background parenchymal enhancement (BPE) on contrast-enhanced mammography (CEM) and breast cancer (BC) risk, in order to identify a useful indicator for risk stratification and the adoption of personalised screening strategies.
**Methods or Background:** We retrospectivly evaluated CEMs performed at our Institution between 2021 and 2022, including patients diagnosed with BC and patients who underwent CEM for problem solving, with at least 2-year negative follow-up. For each examination, breast density (BD) and BPE (none/minimal = 1, mild = 2, moderate = 3, severe = 4) were assessed according to the BI-

RADS lexicon by two independent readers with at least 3 years of CEM experience. For BC cases, BD and BPE were assessed by evaluating the contralateral breast. For each BD class, BPE scores were compared between patients with BC and healthy controls in order to assess the association between BPE and increased cancer risk.
**Results or Findings:** We performed a preliminary analysis on 179 patients. For each BD category, 25 patients with BC and 25 healthy controls were included, with the exception of category A, for which only 4 healthy subjects were identified. In all BD categories, the average BPE was higher in patients with BC than in controls. Specifically, in category A, the average BPE was 1.36 in cancer patients and 1.25 in healthy subjects (increase of 8.8%); in category B, 1.68 vs 1.56 (+7.7%); in category C, 2.20 vs. 1.64 (+34.1%); in category D, 2.92 vs. 2.32 (+25.9%).
**Conclusion:** The analysis showed an association between high BPE and BC, regardless of density; BPE could therefore be integrated into risk assessment models and represent a useful indicator for population stratification, promoting personalised screening.
**Limitations:** Retrospective monocentric study with limited number of patients.
**Funding for this study:** None
**Has your study been approved by an ethics committee?** Yes
**Ethics committee - additional information:** This study was approved by the Independent Ethics Committee at the Fondazione IRCCS Istituto Nazionale dei Tumori, Milano.
**Author Disclosures:**
Gianmarco Della Pepa: Nothing to disclose
Claudia De Berardinis: Nothing to disclose
Elisa D'Ascoli: Nothing to disclose
Francesco Sardanelli: Nothing to disclose
Gabriele Rossini: Nothing to disclose
Gianfranco Paride Scaperrotta: Nothing to disclose
Catherine Depretto: Nothing to disclose
Lisa Corradini: Nothing to disclose
Giovanni Irmici: Nothing to disclose

**Deep Learning with Transfer Learning on Digital Breast Tomosynthesis: A Radiomics-Based Model for Predicting Breast Cancer Risk**

*G. Barcaroli*, F. Galati, R. Maroncelli, C. De Nardo, V. Rizzo, G. Moffa, F. Pediconi; Rome/IT
*(gloria.barcaroli@uniroma1.it)*

**Purpose:** Digital breast tomosynthesis (DBT) is a valuable imaging modality for breast cancer detection; however, its interpretation remains time-consuming and subject to inter- reader variability. This study aimed to develop and evaluate two deep learning (DL) models based on transfer learning for the binary classification of breast lesions (benign vs.malignant) using DBT images to support clinical decision-making and risk stratification.
**Methods or Background:** In this retrospective monocentric study, 184 patients with histologically or clinically confirmed benign (107 cases, 41.8%) or malignant (77 cases, 58.2%) breast lesions were included. Each case underwent DBT with a single lesion manually segmented for radiomic analysis. Two convolutional neural network (CNN) architectures—ResNet50 and DenseNet201—were trained using transfer learning from ImageNet weights. A 10-fold cross-validation strategy with ensemble voting was applied. Model performance was evaluated through ROC–AUC, accuracy, sensitivity, specificity, PPV, and NPV.
**Results or Findings:** The ResNet50 model outperformed DenseNet201 across most metrics. On the internal testing set, ResNet50 achieved a ROC–AUC of 63%, accuracy of 60%, sensitivity of 39%, and speci- ficity of 75%. The DenseNet201 model yielded a lower ROC–AUC of 55%, accuracy of 55%, and sensitivity of 24%. Both models demonstrated relatively high specificity, indicating potential utility in ruling out malignancy, though sensitivity remained suboptimal.
**Conclusion:** This study demonstrates the feasibility of using transfer learning-based DL models for lesion classification on DBT. While the overall performance was moderate, the results highlight both the potential and current limitations of AI in breast imaging. Further studies and approaches are warranted to enhance model robustness and clinical applicability.
**Limitations:** Retrospective and monocentric study, small cohort
**Funding for this study:** None
**Has your study been approved by an ethics committee?** Not applicable
**Author Disclosures:**
Roberto Maroncelli: Nothing to disclose
Chiara De Nardo: Nothing to disclose
Giuliana Moffa: Nothing to disclose
Veronica Rizzo: Nothing to disclose
Gloria Barcaroli: Nothing to disclose
Francesca Galati: Nothing to disclose
Federica Pediconi: Nothing to disclose

**AI-assisted double reading in mammography screening: exam risk score patterns and early cancer risk prediction**

*C. M. Weiss*, E. Di Gaetano, E. Cattarin, R. Cerniato, I. Vinci, G. Soppelsa, G. Morana; Treviso/IT
*(claudiamaria.weiss@aulss2.veneto.it)*

**Purpose:** Recent studies show that AI-algorithms for breast cancer (BC) diagnosis have potential applications in risk-assessment, specifically in utilizing exam risk scores (EXRS) to predict the likelihood of developing the disease. We examined whether ExRS on baseline negative screening mammograms (SM) could predict BC detected in the subsequent round in an AI-supported-screening (AISS) using human-double-reading, and evaluated if these results were consistent across different BI-RADS breast-density categories (BD).
**Methods or Background:** A retrospective analysis was conducted on 135,372 SMs from two consecutive AISS rounds, examining 67,686 women between November 2021 and July 2025, with an average interval of 777 days between rounds. AI assigned an ExRS (0–100) to each SM. ExRS values were compared between women who developed BC (451/67,686) and those who did not (67,235/67,686), with subgroup analyses by BD. Statistical tests included chi-square or z-tests, t-test or Mann–Whitney U-test, and McNemar's test.
**Results or Findings:** Among 451 BC cases, mean ExRS rose from 15.4 at baseline to 73.9 in the subsequent round (median 6.8vs83.0;p<0.001). Women negative at both rounds (n=67,235) had little change: means were 6.7 and 6.4 (medians 2.1 and 2.3;p<0.001). ExRS was significantly higher in BC cases than negatives at both baseline (15.4vs6.7;p<0.001) and subsequent (73.9vs6.4;p<0.001). Baseline ExRS were higher in dense (C–D) than non-dense (A–B) breasts. For BC cases, mean ExRS increased from 13.8 to 73.7 (A–B) and 17.6 to 74.1 (C–D). In negatives, ExRS stayed low: 6.0 to 5.5 (A–B) and 7.6 to 7.9 (C–D).
**Conclusion:** The AI-derived ExRS is able to differentiate between women with varying levels of risk for developing BC at baseline, and this ability was consistent across all BD categories. The results show that ExRS can be also used for risk-based stratification in screening
**Limitations:** No limitations
**Funding for this study:** No fundings
**Has your study been approved by an ethics committee?** Not applicable
**Author Disclosures:**
Giorgia Soppelsa: Nothing to disclose
Eleonora Di Gaetano: Nothing to disclose
Elisa Cattarin: Nothing to disclose
Giovanni Morana: Nothing to disclose
Ivana Vinci: Nothing to disclose
Claudia Maria Weiss: Nothing to disclose
Roberta Cerniato: Nothing to disclose

**External Validation of Four Breast Cancer Risk Models With and Without Breast Density in a prospective Dutch Screening Cohort**

*J. Peters*[1], D. Van Der Waal[1], M. Schmidt[2], C. Van Gils[3], M. Broeders[1]; [1]Nijmegen/NL, [2]Amsterdam/NL, [3]Utrecht/NL
*(jim.peters@radboudumc.nl)*

**Purpose:** Tailoring breast cancer screening to individual risk can improve its harm–benefit ratio versus a 'one-size-fits-all' approach. We externally validated four widely used risk models—Gail, BCSC, BOADICEA, and IBIS—in a large Dutch screening cohort. Recent model updates include breast density using different approaches (categorical or continuous), so we evaluated performance with and without density.
**Methods or Background:** The PRISMA study is a prospective cohort (2014–2019) embedded in the Dutch biennial breast cancer screening program, which invites women aged 50-75 years. Breast cancer risk was predicted for 38,767 participants using four models (Gail, BCSC, BOADICEA, IBIS) based on questionnaire data (personal, lifestyle, hormonal, family history) and breast density. Gail does not include density; BCSC uses density categories; BOADICEA and IBIS allow categories, a continuous measure, or no density. Volumetric percent density and Volpara Density Grades (Volpara version 1.5.0) were measured on raw mammograms. Breast cancers were ascertained via linkage with the Netherlands Cancer Registry until October 2023. Model performance for 5-year risk was evaluated using the concordance index (C-index), observed–expected (O/E) ratio, and calibration slope.
**Results or Findings:** During a median 4.3 years follow-up, 609 breast cancers occurred. Discrimination was poor for Gail (C-index 0.56) and modest for BCSC, BOADICEA and IBIS (C-indices 0.60-0.61). Calibration was good for BCSC (O/E 1.03, slope 0.81) and BOADICEA (O/E 0.96, slope 0.78), but IBIS (O/E 0.71, slope 0.66) and Gail (O/E 0.79, slope 0.59) overpredicted risk. Adding continuous breast density improved discrimination (ΔC-index +0.02–0.04) and calibration most.
**Conclusion:** Traditional breast cancer risk models show at most moderate performance; despite small improvements from continuous breast density, overall accuracy remains limited for personalized screening.

**Limitations:** The limitations of the study are incomplete family history and genetic data, which may particularly underestimate BOADICEA and IBIS performance.
**Funding for this study:** Dutch Cancer Society (KWF7626) and Dutch Research Society (ZonMw 200500004)
**Has your study been approved by an ethics committee?** Yes
**Ethics committee - additional information:** CMO Arnhem-Nijmegen reference no. 2014/177
**Author Disclosures:**
Jim Peters: Nothing to disclose
Carla Van Gils: Nothing to disclose
Mireille Broeders: Nothing to disclose
Danielle Van Der Waal: Nothing to disclose
Marjanka Schmidt: Nothing to disclose

**Epidemiological and deep learning breast cancer risk models compared for an increased-risk population**
*M. Keupers*, W. Sarkol, S. Nijssen, Y-K. Wang, L. Cockmartin, H. Bosmans, C. Van Ongeval; Leuven/BE

**Purpose:** For women with clinically elevated breast cancer risk due to personal risk factors, family history or breast density, screening guidelines remain ambiguous. Epidemiological risk-models like CanRisk and IBIS, are currently used to guide supplemental screening. Recent studies demonstrated that deep learning models might be superior. This study compares risk assessment of two epidemiological models with two deep learning models.
**Methods or Background:** In this single-center cohort 401 women with clinically elevated breast cancer risk were included (2014) with ten-year follow-up info. CanRisk v2.4.2. and IBIS v8.0b assessed lifetime and short-term risk based on patient files. Deep learning models i.e. MIRAI and Transpara were used to predict short-term risk. Model performance was evaluated using receiver operating characteristic (ROC) analysis; areas under the curve (AUCs) were calculated and compared using paired DeLong's test.
**Results or Findings:** Of the 401 women (mean age=49 ±7) 25 developed breast cancer after a ten-year period; 11 cancers were detected after five years. The AUCs for risk assessment within five and ten years for CanRisk were 0.58 (95% confidence interval (CI) [0.37; 0.79]) and 0.61 (95% CI [0.49; 0.73]) resp, and for IBIS lifetime risk it was 0.61 (95% CI [0.49; 0.72]). Deep learning models were evaluated to predict breast cancer risk after five years. Transpara (0.73 (95% CI [0.52; 0.95]) showed superior performance over Mirai (0.57 (95% CI [0.38; 0.77]) (p=0.042).
**Conclusion:** In this single-center cohort with elevated breast cancer risk, one deep learning model outperformed the epidemiological models for short term risk prediction. Generalizability of deep learning models for increased-risk populations should be tested. Further evaluation in larger cohorts is needed.
**Limitations:** Number of patients in this single-center, increased-risk cohort. Still some missing data despite thorough review of electronic patient files.
**Funding for this study:** No funding was received for this study.
**Has your study been approved by an ethics committee?** Yes
**Ethics committee - additional information:** The study was approved by the Ethical Committee of the Catholic University Leuven, Belgium (MP029292 and S70006).
**Author Disclosures:**
Yao-Kuan Wang: Nothing to disclose
Hilde Bosmans: Nothing to disclose
Chantal Van Ongeval: Nothing to disclose
Machteld Keupers: Nothing to disclose
Willem Sarkol: Nothing to disclose
Lesley Cockmartin: Nothing to disclose
Sam Nijssen: Nothing to disclose

**12:30 - 13:30 Research Stage 3**

### Research Presentation Session: Cardiac

### RPS 1403

### Quantitative cardiac imaging and standardisation studies

**Moderator**
Nicola Galea; Rome/IT
*(nicola.galea@uniroma1.it)*

**Impact of scan parameters on the reliability of automatic coronary artery calcium quantification and density measurement in low-dose chest CT**
*W-S. Yoo*[1], S. Hong[1], Y. J. Suh[1], C. Kim[2]; [1]Seoul/KR, [2]Ansan/KR
*(wsyoo0718@yuhs.ac)*

**Purpose:** To evaluate the reliability of AI-based automatic coronary artery calcium (CAC) quantification and density measurement on non-ECG-gated low-dose chest CT (LDCT) compared with manual measurements on calcium scoring CT (CSCT), and to investigate the influence of scan parameters.
**Methods or Background:** This retrospective study included 417 patients from four academic hospitals who underwent both CSCT and LDCT within six months, with CAC scores >0. CT scans were obtained on scanners from four different vendors. CSCT was reconstructed at 3-mm with standard kernel; LDCT was reconstructed in 1-mm and 3-mm pairs with various kernels (identical within each pair). Patients were grouped as high- and low-noise by LDCT noise level. Agatston score, peak density attenuation, and density score (Agatston score divided by total calcium area) were measured manually on CSCT by cardiac radiologists and obtained automatically on LDCT using commercial software. Reliability of each variable between manual CSCT and automatic LDCT (LDCTauto) was evaluated using the concordance correlation coefficient with 95% CIs.
**Results or Findings:** In the low-noise group (n=337), 1-mm LDCTauto demonstrated higher reliability than 3-mm LDCTauto across all variables: Agatston score (0.943 [0.932–0.952] vs. 0.922 [0.908–0.934]), peak density attenuation (0.812 [0.773–0.846] vs. 0.710 [0.664–0.750]), and density score (0.565 [0.496–0.627] vs. 0.556 [0.490–0.615]). However, in the high-noise group (n=80), 1-mm LDCTauto showed lower reliability than 3-mm LDCTauto for Agatston score (0.710 [0.604–0.791] vs. 0.838 [0.763–0.890]) and peak density attenuation (0.157 [0.087–0.226] vs. 0.690 [0.567–0.782]), but slightly higher reliability for density score (0.442 [0.270–0.587] vs. 0.411 [0.219–0.572]).
**Conclusion:** The reliability of automatic CAC measurements on LDCT is influenced by slice thickness and noise level. With lower-noise kernels, 1-mm LDCT may provide more reliable automatic CAC quantification and density measurement than 3-mm LDCT.
**Limitations:** Further validation using different software is required.
**Funding for this study:** None.
**Has your study been approved by an ethics committee?** Yes
**Ethics committee - additional information:** The Institutional Review Boards of the participating hospitals approved this study and waived the requirement for informed consent.
**Author Disclosures:**
Sehyun Hong: Nothing to disclose
Young Joo Suh: Nothing to disclose
Cherry Kim: Nothing to disclose
Won-Seok Yoo: Nothing to disclose

**Spectral Photon-counting CT at reduced tube potential enables high quality coronary imaging at ultra-low-radiation dose: A comparative study to standard dose protocols**
*S. Barus*[1], M. T. Hagar[2], M. Soschynski[1], F. Bamberg[1], C. L. Schlett[1], T. Krauß[1], C. Schuppert[1]; [1]Freiburg im Breisgau/DE, [2]Charleston, SC/US
*(sinan.barus@gmail.com)*

**Purpose:** Photon-counting detector coronary CT angiography (PCD-CCTA) is typically performed at 120/140 kVp (standard dose, SD) to enable full spectral applications, including material decomposition and virtual monoenergetic imaging (VMI). Low-dose (LD) acquisitions at 90/70 kVp are limited to VMI but may suffice to rule out coronary artery disease.

**Methods or Background:** In this single-center post-hoc study, consecutive low-risk-profile patients (age <60 years, BMI <30, Agatston score <200) underwent clinically indicated CCTA using a dual-source PCD-CT system. Scans were randomly acquired in either full (SD) or reduced spectral mode (LD), using high-pitch or prospective sequence acquisition. Images were reconstructed as 55 keV VMIs using a vascular kernel (Bv48, 0.4 mm). Quantitative image quality included signal-to-noise ratio (SNR), contrast-to-noise ratio (CNR) averaged across the ascending aorta, proximal and distal coronaries; CNR of ≥10.0 was defined as diagnostic. Radiation dose was evaluated using the dose-length product (DLP), and multivariate analysis was used to assess indicators of radiation dose.
**Results or Findings:** A total of 192 patients were included (115 LD, 48.1 ± 8.3 years; 77 SD, 53.0 ± 7.1 years). LD reduced radiation dose by 52.6% (91 [58–179] vs. 192 [137–475] mGy·cm, p<0.01), with slightly lower SNR (15.5 [13.1–17.6] vs. 19.2 [15.5–23.3], p<0.001) and CNR (13.0 [11.1–14.9] vs. 16.2 [13.2–20.6], p<0.001), although both exceeded diagnostic thresholds. In multivariate analysis, LD and high-pitch acquisition predicted lower dose (β = -138.8 and -215.6, p<0.001), while higher BMI and male sex increased dose (β = 7.8 and 47.2, p=0.016 and 0.018).
**Conclusion:** PCD-CCTA acquired at low tube potentials enables substantial radiation dose reduction while maintaining diagnostic image quality. Our results indicate that in low-risk patients, patient-protective measures should be favored over full spectral capacity.
**Limitations:** Post-hoc, single-center study.
**Funding for this study:** No funding was provided for this study.
**Has your study been approved by an ethics committee?** Yes
**Ethics committee - additional information:** Ethics Committee of the Medical Center – University of Freiburg (No. 21-1469, approved on September 21, 2021).
**Author Disclosures:**
Martin Soschynski: Nothing to disclose
Tobias Krauß: Nothing to disclose
Muhammad Taha Hagar: Nothing to disclose
Christopher L. Schlett: Nothing to disclose
Fabian Bamberg: Nothing to disclose
Christopher Schuppert: Nothing to disclose
Sinan Barus: Nothing to disclose

**Harmonizing T1 Mapping Across Sequences and Diseases: Insights from a Real-World Dual-Sequence CMR Study**
*H. Zhang*, M. Lu; Peking/CN
*(zhy_dreamer@163.com)*

**Purpose:** The clinical translation of cardiovascular magnetic resonance (CMR) T1 mapping is limited by inconsistent acquisition protocols and reference ranges. We aims to establish the ranges of native T1 and extracellular volume (ECV) in healthy Chinese adults and across various cardiovascular diseases, and to evaluate their diagnostic performance.
**Methods or Background:** This prospective study included 1,237 human subjects with 15 cardiovascular conditions and 12 swine for histopathologic validation. T1 mapping were acquired using Modified Look-Locker Inversion Recovery (MOLLI) and Shortened MOLLI (ShMOLLI) sequences on a 3 Tesla Siemens scanner. Histological collagen volume fraction (CVF) in swine myocardium was quantified and correlated with T1 mapping. Global native T1 and ECV were compared between myocardial disease groups and healthy controls. The differences between MOLLI and ShMOLLI measurements were analyzed using Bland-Altman analysis.
**Results or Findings:** Histological validation in swine confirmed strong correlations between T1 mapping parameters and CVF. In clinic, the references ranges derived from healthy controls were 1179±29 ms (MOLLI) and 1147±33 ms (ShMOLLI) for native T1, and 25.1±2.1% (MOLLI) and 25.5±1.9% (ShMOLLI) for ECV. Gender stratification revealed higher native T1 in females compared to males within healthy population. MOLLI yielded systematically higher native T1 values compared to ShMOLLI across the entire cohort and in all conditions. ECV showed inter-sequence consistency. Across the spectrum of cardiovascular diseases, significant deviations in native T1 and ECV were observed. T1 mapping had exceptional diagnostic performance for cardiac amyloidosis and Fabry disease, and strong diagnostic capabilities for most primary cardiomyopathies, ischemic heart disease, and myocarditis.
**Conclusion:** This large-scale study established comprehensive reference ranges for native T1 and ECV using MOLLI and ShMOLLI at 3 Tesla, highlighting their diagnostic utility across diverse cardiovascular conditions.
**Limitations:** Patients were enrolled based on a single primary cardiovascular diagnosis
**Funding for this study:** National Natural Science Foundation of China (grant no. 82471973)
**Has your study been approved by an ethics committee?** Yes
**Ethics committee - additional information:** The pre-clinical study was approved by Ethics Committee for Animal Study at Fuwai hospital and the Care of Experimental Animals Committee of the Chinese Academy of Medical Sciences and Peking Union Medical College (0102-1-30-ZX(X)22). The clinical study was approved by the local Institutional Ethics Committee. All human participants gave written informed consent.
**Author Disclosures:**
Minjie Lu: Nothing to disclose
Huaying Zhang: Nothing to disclose

**Non-invasive estimation of blood oxygen saturation with Standard T1/T2 CMR mapping: enhanced accuracy through flow-based adjustment**
A. Perazzolo[1], T. C. Chao[2], D. Curione[1], M. Rebonato[1], M. Pilari[1], G. Butera[1], L. Natale[1], T. Leiner[2], *A. Secinaro*[1]; [1]Rome/IT, [2]Rochester, MN/US
*(aurelio.secinaro@opbg.net)*

**Purpose:** Blood oxygen saturation is a key physiological marker of cardiopulmonary function. Differences in magnetic susceptibility of oxygenated versus deoxygenated hemoglobin affect MRI relaxation times. Models such as the Luz–Meiboom (LM) equation link T2 to oxygen saturation, while synthetic hematocrit (HCT) can be inferred from native T1. Nonetheless, flow-related artifacts remain a limitation to quantification. We evaluated the feasibility of non-invasive CMR-based oxygen saturation assessment, validated against simultaneous sampling during MRI-guided catheterization in pediatric patients, and tested a physiological flow-based correction.
**Methods or Background:** Synthetic HCT was calculated from native T1 (MOLLI), and T2 maps were obtained from a T2-prep bSSFP sequence (echo times 0 and 55 ms). These inputs were applied to the LM model to generate saturation maps. Right ventricular blood-pool ROIs provided image-based values, compared with catheter samples from pulmonary arteries under matched conditions. Additional morpho-functional indices (ventricular volumes, ejection fraction, main pulmonary artery [MPA] flow) were extracted to assess their influences on image-based $SbO_2$. A linear correction model was derived associating peak velocity of MPA (VmaxMPA) and $\Delta SbO_2$ (difference between catheter and image-based measurement) verified by leave-one-out cross validation.
**Results or Findings:** Thirteen pediatric patients were retrospectively included (9 restrictive cardiomyopathies, 1 dilated cardiomyopathy, 1 Uhl's anomaly, 1 congenitally-corrected transposition, 1 idiopathic right atrial dilatation). MPA peak velocity (Vmax) correlated with $\Delta SbO_2$ ($R^2$=0.93). Leave-one-out cross validation demonstrated that applying VmaxMPA correction reduced root mean square error from 15.92% to 2.77%, mitigating flow-related artifacts.
**Conclusion:** Non-invasive blood oxygen saturation estimation from standard T1/T2 mapping is feasible when synthetic HCT and the LM equation are combined with a physiological correction for MPA flow. Validation against invasive sampling supports the robustness and clinical applicability of this approach for artifact-resistant quantification.
**Limitations:** Small sample size and absent external validation.
**Funding for this study:** Italian Ministry of Health, Current Research funds.
**Has your study been approved by an ethics committee?** Yes
**Ethics committee - additional information:** The study was approved by the Bambino Gesù Children's Hospital ethics committee.
**Author Disclosures:**
Gianfranco Butera: Nothing to disclose
Aurelio Secinaro: Nothing to disclose
Mara Pilari: Nothing to disclose
Davide Curione: Nothing to disclose
Tim Leiner: Nothing to disclose
Luigi Natale: Nothing to disclose
Alessio Perazzolo: Nothing to disclose
Micol Rebonato: Nothing to disclose
Tzu Cheng Chao: Nothing to disclose

**Retrospective Evaluation of Short Protocols in Cardiac MRI: Role of Mapping and Left Ventricular Function as Decision Making Parameters for Possible Intravenous Contrast Administration**
*E. Cantalamessa*[1], F. Fiocchi[1], G. Battinelli[1], R. Cuoghi Costantini[1], G. Ligabue[2]; [1]Modena/IT, [2]Sassuolo/IT

**Purpose:** The use of shortened non-contrast Cardiac Magnetic Resonance (CMR) protocols may reduce examination time and still maintain diagnostic accuracy. This study aimed to assess the effectiveness of such an approach for detecting left ventricular myocardial disease, using mapping and ventricular function as decision-making criteria.
**Methods or Background:** This single-center retrospective study included 337 patients referred for CMR with heterogeneous clinical indications. Each examination was blindly evaluated using a short protocol including cine sequences, edema-sensitive sequences and T1/T2 mapping. For each one of them, functional parameters (LVEDV, LVEDV/BSA, LVEF, LVSV) and mapping values (T1 MAX, T2 MAX) were collected. T1 MAX and T2 MAX were defined as the highest mean values assigned to a myocardial segment in native T1 and T2 mapping, respectively. The diagnosis obtained with the standard contrast-enhanced protocol served as the reference standard. ROC analysis was used to assess the performance of individual parameters and combined models (logistic regression, decision tree) in predicting LGE positivity.
**Results or Findings:** T1 Max demonstrated an AUC of 0.716 (sensitivity 67.2%; specificity 66.8%), which improved with the addition of LVEDV/BSA (AUC 0.721). The decision tree achieved an AUC of 0.751. A T1 Max cut-off >1113 ms identified a high-risk subgroup (positivity 70%), whereas T1 Max <1025 ms defined a low-risk subgroup (positivity 13%). In intermediate cases, adding LVEDV/BSA >93 ml/m² improved discriminatory capacity.
**Conclusion:** In a heterogeneous population, a short non-contrast CMR protocol based on T1 mapping thresholds and left ventricular function may identify patients at higher risk of myocardial injury. Implementing a shortened protocol could significantly reduce acquisition times, supporting more efficient and personalized diagnostic pathways in everyday clinical practice.
**Limitations:** Limitations include retrospective single-center nature of the study, heterogeneous patient population, and the need for experienced radiologist presence.
**Funding for this study:** No funding was received for this study.
**Has your study been approved by an ethics committee?** Not applicable
**Ethics committee - additional information:** The study is retrospective
**Author Disclosures:**
Federica Fiocchi: Nothing to disclose
Eleonora Cantalamessa: Nothing to disclose
Riccardo Cuoghi Costantini: Nothing to disclose
Guido Ligabue: Nothing to disclose
Giovanni Battinelli: Nothing to disclose

**Prognostic value of CMR parametric mapping in cardiac amyloidosis: An updated systematic review and meta--analysis**
*S. Forouzannia*[1], S. R. Rafiei Alavi[1], S. M. Forouzannia[2], M. Umair[3], G. Francese[4]; [1]Tehran/IR, [2]San Francisco, CA/US, [3]New York, NY/US, [4]Genoa/IT

**Purpose:** Cardiac amyloidosis (CA) is the leading cause of mortality in systemic amyloidosis, highlighting the need for accurate risk assessment to guide patient management. While the diagnostic value of cardiac MR (CMR) parametric mapping is well established, its prognostic utility remains inconsistent across studies. To perform a systematic review and meta-analysis to evaluate the prognostic value of CMR parametric mapping in patients with CA.
**Methods or Background:** An extensive search was conducted in Medline, Scopus, Embase and Web of Science databases. Eligible studies were observational studies that reported HRs for predicting adverse outcomes in patients with CA using CMR parametric mapping.
**Results or Findings:** 22 studies with 3398 patients were included in this systematic review. Higher extracellular volume (ECV) values were associated with increased mortality, both as a dichotomous (HR: 2.90; 95% CI: 1.68 to 5.01) and continuous variable (HR for 1% increase: 1.08; 95% CI: 1.06 to 1.10; HR for 3% increase: 1.17; 95% CI: 1.11 to 1.22 and HR for 10% increase: 2.11; 95% CI: 1.70 to 2.62). Higher native T1 mapping values were associated with mortality as a dichotomous variable (HR: 1.33; 95% CI: 0.79 to 2.24). Native T2 mapping showed inconsistent associations with prognosis across studies.
**Conclusion:** Higher ECV and native T1 values are associated with worse prognosis in CA, supporting their role in risk stratification. Further studies with standardised CMR protocols are needed to enhance the prognostic utility of parametric mapping in clinical practice.
**Limitations:** The heterogeneity in ECV and T1 cut-off values across studies presents a challenge to standardisation, thereby limiting direct comparisons. Another limitation is the insufficient data available on native T2 mapping due to the limited number of studies investigating its values in the setting of CA.
**Funding for this study:** No funding was received for this study.
**Has your study been approved by an ethics committee?** Not applicable
**Author Disclosures:**
Seyed Mohammad Forouzannia: Nothing to disclose
Seyedeh Romina Rafiei Alavi: Nothing to disclose
Giulia Francese: Nothing to disclose
Seyedali Forouzannia: Nothing to disclose
Muhammad Umair: Nothing to disclose

**Myocardial Extracellular Volume difference according to sex, fibrosis and left ventricular hypertrophy in Fabry Disease: A Retrospective Cardiac MRI Study**
*C. Filliol*[1], C. De Bourguignon[2], A. Ghaouar[1], M. Bourmaaz[1], A. Braillon[1], A. Deliniere[1], A. Jobbe-Duval[1], A. Fouilhoux[2], S. A. Si-Mohamed[1]; [1]Bron/FR, [2]Lyon/FR

**Purpose:** To analyze differences in myocardial extracellular volume (ECV) according to sex, left ventricular hypertrophy (LVH) and fibrosis in Fabry disease.
**Methods or Background:** Between February 2017 and August 2023, 64 patients with Fabry disease (36 women [56%], mean age 45 ± 15 years) from our reference center who underwent baseline cardiac magnetic resonance imaging (CMR) on a 1.5 Tesla MRI system (Philips XXX) were retrospectively included. ECV was calculated using the hematocrit level obtained on the same day after semi-automatic segmentation on short-axis T1 mapping images, using a clinical workstation (ISPortal, CM Suite, Philips). The presence of fibrosis and left ventricular hypertrophy (LVH) was assessed in consensus by 2 experienced observers. Following data were registered: age, sex, alpha-galactosidase A levels, troponin levels, left ventricular ejection fraction (LVEF). Mann-Whitney and t- test were used according to their normality.
**Results or Findings:** Age, troponine and LVEF were not significantly different according to sex, Alpha-galactosidase level was significantly lower in men (0.46±0.15 vs 3.07±1.8, p<0.001). LVH status was significantly higher in men (11(44%) vs 5(12.8%), p<0.001) and fibrosis status was significantly higher in men (9(36%) vs 11(28.2%), p<0.001). 13(20%) patients presented both LVH and fibrosis (4 women, 9 men). 46(72%) patients were indemn of LVH and fibrosis (29 women, 17 men). In men, global ECV was significantly higher in presence of LVH (27.7%±3.9 vs 24.4%±2.4, p=0.02), as well as in presence of fibrosis (28.2%±4.2 vs 24.6%±2.3, p=0.04). No statistical difference was found in women.
**Conclusion:** Myocardial ECV is significantly elevated in men with LVH and fibrosis, highlighting sex-specific differences in cardiac tissue alterations. These findings suggest that assessing ECV, particularly in male patients, may offer valuable insights into myocardial involvement and disease progression in Fabry disease
**Limitations:** Retrospective and monocentric study.
**Funding for this study:** None
**Has your study been approved by an ethics committee?** Yes
**Ethics committee - additional information:** HCL ethical commitee N°23257
**Author Disclosures:**
Salim Aymeric Si-Mohamed: Nothing to disclose
Clément Filliol: Nothing to disclose
Asma Ghaouar: Nothing to disclose
Alain Fouilhoux: Nothing to disclose
Meriem Bourmaaz: Nothing to disclose
Alexandra Braillon: Nothing to disclose
Charles De Bourguignon: Nothing to disclose
Antoine Jobbe-Duval: Nothing to disclose
Antoine Deliniere: Nothing to disclose

**Automatic volumetric evaluation of extracellular volume from CT: prognostic value in pre-TAVI risk stratification**
*A. Colombo*, C. Gnasso, D. Vignale, M. Liberotti, A. Palmisano, A. Esposito; Milan/IT
*(colombo.alberto@unisr.it)*

**Purpose:** To develop an automatic software for myocardial extracellular volume (ECV) analysis and to evaluate its association with a composite outcome of death and heart failure at 12 months after transcatheter aortic valve implantation (TAVI) in patients with aortic stenosis (AS).
**Methods or Background:** In this retrospective single-centre study, we considered patients who underwent pre-TAVI CT (10/2020-12/2023) and employed a fully automated pipeline for volumetric quantification of ECV and fibrotic burden (FB), a.k.a. percentage of left ventricular voxels with ECV values above a set threshold. The pipeline consisted of four steps: 1-segmentation of cardiac structures and 2-their post-processing, 3-co-registration of late post-contrast and pre-contrast scans, 4-actual ECV quantification. Agreement between automated (ECVauto) and manual (ECVmanu) measurements by two radiologists was assessed with Bland-Altman analysis, while prognostic value was evaluated using Kaplan-Meier curves with log-rank cutoffs and multivariable Cox models adjusted for clinical and echocardiographic variables. Analyses were performed in Python (v3.9.13), with p=0.05 significance.
**Results or Findings:** Among the 438 included patients (median age 82 years, F221/M217), median EuroSCORE was 5.2, mean gradient 44 mmHg, and ejection fraction 59%. The 12 months composite outcome occurred in 74 patients (16.9%), including 57 deaths (10.7%). The pipeline processed all CTs in ~4.5min/patient. Bias and limits of agreement between ECVauto and ECVmanu were 0.82% (-11.9%/13.6%). ECVauto and ECVmanu were higher in patients with events (32.5vs29.3%, p=0.001; 30.1vs28.0%, p=0.020,

Friday respectively). Elevated ECVauto independently predicted worse outcomes (cutoff 32.9%, p<0.001; HR 1.04 per 1%, p=0.028), as did high FB (cutoff 48.5%, p<0.001; HR 1.02, p=0.031).
**Conclusion:** The pipeline enabled rapid, automated ECV mapping, independently predicting 12-month outcomes. Results further support CT-based fibrosis assessment.
**Limitations:** Limitations include retrospective, single-center design, improving the segmentation step to exclude artifacts, and possible inclusion of undiagnosed amyloidosis.
**Funding for this study:** This study was partially granted by: European Union - Next Generation EU, Mission 4 Component 1, CUP D53D23021100001", Bando PRIN PNRR 2022 (P2022JBKN2), Italian Ministry of University and Research (MUR).
**Has your study been approved by an ethics committee?** Yes
**Ethics committee - additional information:** This monocentric observational study was approved by the institutional review board (CT-based myocardial characterization study: CTMyoC 112/INT/2019 ). The study is a retrospective analysis of prospectively collected data and was conducted according to the Declaration of Helsinki. Written informed consent was obtained for all participants.
**Author Disclosures:**
Davide Vignale: Nothing to disclose
Antonio Esposito: Nothing to disclose
Marta Liberotti: Nothing to disclose
Anna Palmisano: Nothing to disclose
Alberto Colombo: Nothing to disclose
Chiara Gnasso: Nothing to disclose

**Synergistic role of CT-derived Extracellular Volume Fraction (ECV) and Flow-Gradient Patterns in Risk Stratification of Patients with Severe Aortic Stenosis Undergoing TAVI**
*C. Gnasso*, D. Vignale, A. Palmisano, D. Serra, D. Margonato, A. Esposito; Milan/IT
*(gnasso.chiara@hsr.it)*

**Purpose:** Previous studies have demonstrated that CT for transcatheter aortic valve implantation (TAVI) planning can quantify interstitial fibrosis as extracellular volume fraction (ECV), associated with a worse prognosis. Also the echocardiographic flow-gradient pattern (high-gradient [HG]; paradoxical low-flow [LF] low-gradient [LG], and classical LFLG AS) is associated with prognosis, with classical LFLG having the worst. Thus, we aimed to investigate the incremental prognostic value of ECV over flow-gradient pattern.
**Methods or Background:** Single-center prospective observational study. Consecutive patients undergoing TAVI (October 2020-November 2023) were enrolled and categorized as HG, paradoxical or classical LFLG AS. ECV was calculated from a 5-minute delayed scan. After a follow-up of at least 1 year, a composite endpoint of death or heart failure hospitalization was collected.
**Results or Findings:** In the final population of 460 patients (82 [77-85] years), 83% had HG AS, 8.0% paradoxical, and 8.9% classical LFLG AS. After a median follow-up of 468 days, patients with classical LFLG had a higher rate of events (39.0%) than paradoxical LFLG (29.7%), and HG (15.6%) (p=0.004) and a higher ECV (32.0% [27.3-34.9] vs 28.0% [25.2-31.2] vs 28.0% [26.2-33.0], respectively; p=0.001). On Kaplan-Meier analysis, the combination of flow-gradient pattern and ECV distinguished distinct risk profiles: patients with classical LG-LF AS and a high ECV had the worst prognosis (p<0.001). The improved risk stratification capability of ECV was confirmed with the likelihood ratio test (p=0.019) and the net reclassification index (0.3).
**Conclusion:** ECV derived from pre-procedural TAVI planning has an incremental prognostic value over echocardiographic flow-gradient pattern.
**Limitations:** Relatively small sample size; ECV assessed with a single manual ROI with a single-energy CT method; absence of a standard of reference to detect cardiac amyloidosis (to reduce this bias, we excluded patients with an ECV >40%).
**Funding for this study:** This study was partially granted by: European Union - Next Generation EU, Mission 4 Component 1, CUP D53D23021100001", Bando PRIN PNRR 2022 (P2022JBKN2), Italian Ministry of University and Research (MUR).
**Has your study been approved by an ethics committee?** Yes
**Ethics committee - additional information:** The study was approved by the local Ethical Committee (CT-based myocardial characterization study: CTMyoC 112/INT/2019 and amendments) and conducted according to the Declaration of Helsinki.
**Author Disclosures:**
Davide Margonato: Nothing to disclose
Davide Vignale: Nothing to disclose
Davide Serra: Nothing to disclose
Antonio Esposito: Nothing to disclose
Anna Palmisano: Nothing to disclose
Chiara Gnasso: Speaker: NA

**12:30 - 13:30 Research Stage 4**

### Research Presentation Session: Head and Neck Hot Topic with Keynote Lecture

### RPS 1408

### Hot Topic: hybrid solutions for salivary gland imaging

**Moderator**
Roberto Maroldi; Concesio/IT
*(roberto.maroldi@unibs.it)*

**Keynote Lecture**
Osamu Sakai; Boston/US
*(osakai@mgh.harvard.edu)*

**Dynamic MR Sialography with Lemon Juice Stimulation: A Non-Invasive Functional Imaging Biomarker for Sjögren's Syndrome**
*A. A. Basheer Ahmed*[1], F. Abubacker Sulaiman[2], R. Praveenkumar[2], D. Velan[2], J. Lydia[2], P. K. Anbazhagan[2]; [1]Melmaruvathur/IN, [2]Chennai/IN
*(dr.ashrafbasheer@gmail.com)*

**Purpose:** To evaluate the diagnostic potential of dynamic MR sialography following lemon juice stimulation as a non-invasive, functional biomarker for detecting early salivary gland involvement in Sjögren's syndrome. The objective was to assess both morphological and secretory changes of major salivary glands and to correlate these imaging findings with serological and clinical parameters.
**Methods or Background:** A prospective observational study was conducted on 40 participants—20 with clinically confirmed Sjögren's syndrome (based on ACR/EULAR criteria) and 20 healthy controls. Imaging was performed using 3T MRI with high-resolution, heavily T2-weighted 3D MR sialography sequences (SPACE/DRIVE technique). Each subject received 5 mL of freshly squeezed lemon juice orally to stimulate salivary flow. Sequential scans were acquired at baseline, 3 minutes, and 10 minutes post-stimulation. Quantitative evaluation included ductal visualization grading, glandular parenchymal signal intensity, and dynamic excretory flow assessment. All results were correlated with Schirmer's test, unstimulated salivary flow rate, and anti-Ro/La antibody positivity.
**Results or Findings:** Healthy subjects showed a prompt increase in ductal visualization and salivary excretion following stimulation, while Sjögren's patients exhibited reduced or delayed flow, ductal beading, and glandular atrophy. Combined dynamic and structural parameters achieved superior diagnostic accuracy (AUC = 0.93) compared to conventional morphological imaging alone. The technique demonstrated high interobserver reproducibility and excellent gland-to-duct contrast resolution.
**Conclusion:** Lemon juice–stimulated MR sialography represents a promising, radiation-free, and functionally dynamic imaging modality for early detection and grading of Sjögren's syndrome, offering a potential alternative to invasive diagnostic techniques such as parotid sialography or labial gland biopsy.
**Limitations:** The study was limited by a relatively small cohort size, absence of histopathological validation in all cases, and interindividual variability in lemon-induced stimulation response. Larger multicenter studies with standardized stimulation protocols are warranted to establish reproducibility and optimize diagnostic thresholds.
**Funding for this study:** Not Applicable
**Has your study been approved by an ethics committee?** Not applicable
**Author Disclosures:**
Rathinamoorthy Praveenkumar: Nothing to disclose
Pradeep Kumar Anbazhagan: Nothing to disclose
Johnbosco Lydia: Nothing to disclose
Dhanush Velan: Nothing to disclose
Farook Abubacker Sulaiman: Nothing to disclose
Ashraf Ahmed Basheer Ahmed: Nothing to disclose

**Radiological features of recurrent adenoid cystic carcinoma (ACC) of the head and neck following carbon ion radiotherapy**

*L. Levante*[1], C. Fichera[2], G. Fontana[1], V. Dolcetti[1], J. Fragomene[1], B. Vischioni[1], E. Orlandi[1], L. Preda[1], S. Imparato[1]; [1]Pavia/IT, [2]Lodi/IT

**Purpose:** To identify early radiological features of recurrent adenoid cystic carcinoma (ACC) of the head and neck after carbon ion radiotherapy (CIRT).
**Methods or Background:** Radiological features (shape, margins, mass effect, contrast enhancement, DWI, mean ADC on ROI, necrosis, side relative to treated area, location relative to initial tumor volume, growth trend, T2 and T1 signal) were retrospectively analyzed in 51 patients with recurrent ACC treated with CIRT between 2013 and 2022 at CNAO. The same variables were assessed in 51 patients without recurrence (control group). In recurrent cases, imaging was evaluated at three time points (t.p.): 6 months after treatment (t.p.0), 6 months before recurrence (t.p.1), and at recurrence (t.p.2). For controls, t.p.2 corresponded to the mean recurrence time of the other group (34 months). ΔADC values across time points were also calculated in both groups.
**Results or Findings:** Compared with controls, recurrent ACC exhibited significantly more frequent features ($p<0.001$), including rounded or lobulated shape, mass effect, homogeneous enhancement, mean ADC of 1098 $mm^2/s$, ΔADC of –460 $mm^2/s$, progressive growth, peripheral localization, and intermediate T2 signal. At t.p.1, these characteristics were already comparable to those observed at t.p.2, except for mean ADC, which showed a progressive reduction, and ΔADC, which had already increased compared with t.p.0 (1306 and –250 $mm^2/s$, respectively). A reproducible radiological pattern of recurrent ACC after CIRT was identified, with some features being objectively quantifiable, particularly ΔADC, a parameter scarcely investigated to date.
**Conclusion:** This preliminary study outlines the main radiological features of ACC recurrence after CIRT and provides a basis for developing response criteria related to salivary gland tumors of the head and neck treated with particle therapy.
**Limitations:** This is a single-center study
**Funding for this study:** None
**Has your study been approved by an ethics committee?** Yes
**Ethics committee - additional information:** Approved by local ethical committee of Pavia (PV)
**Author Disclosures:**
Jacopo Fragomene: Nothing to disclose
Barbara Vischioni: Nothing to disclose
Ester Orlandi: Nothing to disclose
Giulia Fontana: Nothing to disclose
Sara Imparato: Nothing to disclose
Vincenzo Dolcetti: Nothing to disclose
Lorenzo Preda: Nothing to disclose
Cristina Fichera: Nothing to disclose
Lorena Levante: Nothing to disclose

**Diagnostic Value of Shear Wave Elastography and Multiparametric MRI in The Diagnosis of Parotid Tumors**

*C. Zito*, R. Cannella, V. Pampalone, F. A. Cordaro, F. Pandolfo, A. Lo Casto; Palermo/IT
*(chiarazito153@gmail.com)*

**Purpose:** This study aims to evaluate the diagnostic accuracy of shear wave elastography (SWE) and multiparametric MRI in differentiating benign from malignant parotid gland lesions, based on quantitative assessment of tissue stiffness and perfusion parameters.
**Methods or Background:** From October 2022 to September 2025, 47 patients (24 females, 23 males; age range 15–85 years) presenting with 53 parotid lesions (size range 8–60 mm) were prospectively evaluated at our Department of Radiological Sciences. Each patient underwent ultrasound with SWE using a 5–14 MHz linear probe. Regions of interest (ROI) were sampled multiple times per lesion (10 measurements each). Additionally, all patients underwent 3-Tesla MRI with dynamic contrast-enhanced sequences. MRI curve patterns were classified based on parameters including peak signal intensity, time to peak (T-peak), wash-in, and wash-out rates. Surgical excision and histopathological analysis were performed for definitive diagnosis.
**Results or Findings:** A total of 35 parotid lesions underwent histopathological analysis, of which 26 were benign (including 10 cystadenolymphomas and 9 pleomorphic adenomas) and 9 were malignant. Malignant lesions were significantly larger than benign ones (median size: 31 mm vs. 20 mm; $p = 0.013$). The median SWE values were 40.6 kPa in malignant lesions and 37.5 kPa in benign lesions, with no significant difference ($p=0.437$). On dynamic contrast-enhanced MRI, the brevity of enhancement showed a tendency toward statistical significance (median values: 88.3 in malignant vs. 49.2 in benign lesions; $p = 0.078$).
**Conclusion:** Parotid tumors demonstrated overlapping stiffness values on SWE. However, dynamic contrast-enhanced MRI revealed a trend toward significant differences, suggesting potential value in differentiating malignant from benign lesions.
**Limitations:** Single-center study. Small number of included patients.
**Funding for this study:** No funding
**Has your study been approved by an ethics committee?** Not applicable
**Author Disclosures:**
Francesca Pandolfo: Nothing to disclose
Filippo Angelo Cordaro: Nothing to disclose
Antonio Lo Casto: Nothing to disclose
Chiara Zito: Nothing to disclose
Roberto Cannella: Nothing to disclose
Vito Pampalone: Nothing to disclose

**Quantitative and Histogram-Based MRI Metrics of Salivary Glands in Sjögren's Syndrome**

*Z. N. Doghramachi*, T. Cankurtaran, M. Aliyev, T. Yıldırım, A. Isik, G. Kahraman, C. S. Oygur, D. Kalemci, O. Kayık, A. Beheshtırooy; Ankara/TR
*(zeynepdayican@gmail.com)*

**Purpose:** To investigate structural and diffusion-related heterogeneity in salivary glands of Sjögren patients using quantitative MRI and histogram-derived parameters.
**Methods or Background:** Fifty-six participants (26 with Sjögren's syndrome, 30 controls; mean age 47 years) underwent MRI of parotid and submandibular glands. Volumetric measurements, inter-rater agreement for Kojima and Makula grading, and quantitative metrics (T1 mapping, ADC, fat fraction, R2*) were assessed. Histogram analyses were performed with dedicated software and included mean, standard deviation, interquartile range (IQR), robust mean absolute deviation (rMAD), root mean square (RMS), entropy, skewness, and kurtosis. Group comparisons were performed.
**Results or Findings:** Salivary gland volumes were reduced in Sjögren patients ($p<0.05$). Inter-rater reliability was high for Kojima grading and moderate for Makula. In parotid glands, mean T1 values tended to be higher without significance ($p=0.085$), while entropy, IQR, rMAD, and SD were significantly elevated (all $p<0.05$); skewness and kurtosis showed no differences. Parotid glands ADC metrics (mean, RMS, rMAD, SD) were also higher in Sjögren (all $p<0.05$), whereas submandibular glands showed no significant differences. Fat fraction and R2* values were comparable between groups.
**Conclusion:** Sjögren's syndrome is associated with increased parotid gland heterogeneity in both T1 and ADC histogram metrics, while fat and R2* values remain unchanged. Histogram-derived MRI metrics may provide non-invasive biomarkers for salivary gland involvement.
**Limitations:** Single-center cohort with a moderate sample size; voxel-based or whole-gland segmentation was not performed.
**Funding for this study:** Funding was provided by Siemens Healthineers, Erlangen, Germany.
**Has your study been approved by an ethics committee?** Yes
**Ethics committee - additional information:** The study was approved by Baskent University Institutional Review Board and Ethics Committee. (reference number KA22/180).
**Author Disclosures:**
Atakan Isik: Investigator: none
Cagdas Sahap Oygur: Investigator: none
Zeynep Nazli Doghramachi: Author: Author Author: Author
Tülin Yıldırım: Investigator: none
Mahammad Aliyev: Investigator: none
Gökhan Kahraman: Investigator: none
Tijen Cankurtaran: Investigator: none
Arman Beheshtırooy: Investigator: none
Onurhan Kayık: Investigator: none
Deniz Kalemci: Investigator: none

**Magnetic Resonance Radiomics Analysis for Differentiating Parotid Neoplasms: A Two-Center Study**

*B. Wen*[1], Z. Zhang[1], Y. Zhang[1], D. Zheng[2], H. Cui[3]; [1]Zhengzhou/CN, [2]Beijing/CN, [3]Hangzhou/CN
*(weiwei.deng@139.com)*

**Purpose:** To establish and validate MR-based radiomics models for differentiating benign from malignant parotid tumors (BT vs MT), and pleomorphic adenomas (PA) from Warthin tumors (WT).
**Methods or Background:** This two-center study included 200 patients (BT=133 [PA=87, WT=46], MT=67). Radiomics features were extracted from T1WI, fs-T2WI, and fs-CE-T1WI, reduced with Pearson correlation, and used to build machine learning models. A clinical model was developed from demographics and MR features. Fused models combining radiomics and clinical variables were evaluated by ROC and decision curve analysis.

**Results or Findings:** Radiomics and fused models showed robust diagnostic performance. For BT vs MT, AUCs reached 88.3% (validation) and 86.5% (external test). For PA vs WT, AUCs reached 98.7% and 91.4%, respectively. Clinical models showed lower accuracy. Fused models provided greater clinical benefit than radiomics or clinical models alone.
**Conclusion:** Fused models integrating radiomics and clinical features achieved high accuracy in differentiating BT from MT and PA from WT, and may support preoperative decision-making.
**Limitations:** Limited external validation; larger multicenter studies are warranted.
**Funding for this study:** None
**Has your study been approved by an ethics committee?** Not applicable
**Author Disclosures:**
Baohong Wen: Nothing to disclose
Yong Zhang: Nothing to disclose
Heng Cui: Nothing to disclose
Dandan Zheng: Employee: An employee of Philips Healthcare
Weiwei Deng: Employee: An employee of Philips Healthcare
Zanxia Zhang: Nothing to disclose

**12:30 - 13:30 Research Stage 2**

### Research Presentation Session: Musculoskeletal

### RPS 1410

### Detecting damage: sports and trauma imaging

**Moderator**
Diana Afonso; Lisbon/PT
*(p.diana.a@gmail.com)*

**MRI findings affecting the time to return to play (RTP) and re-injury rate with the potential role of artificial intelligence (AI) in most common thigh muscle injuries in professional football players**
M. Shoura[1], *S. M. Elhennawy*[2], Y. A. A. E. F. Hassan[2]; [1]Boston, MA/US, [2]Cairo/EG
*(dr-sherif-elhanawy@live.com)*

**Purpose:** Evaluate predictive value of MRI-based BAMIC & MLG-R classification systems for RTP time and re-injury risk with thigh muscle injuries in professional football players with the potential enhancement of AI integration.
**Methods or Background:** Retrospective study on 48 professional football players with recent thigh muscle injuries in biceps femoris, adductor longus and rectus femoris muscles between 2018 and 2024. Two professional MSK radiologists independently classified injuries.. Statistical analysis included Pearson correlation, ROC analysis, inter-rater reliability (κ), and others. AI model was trained to predict time to RTP and re-injury rate
**Results or Findings:** BAMIC showed strong correlation with RTP time (r = 0.72) and good predictive accuracy (AUC = 0.81,). MLG-R also demonstrated high correlation (r = 0.66) with AUC = 0.77. Anatomical location significantly impacted outcomes: biceps femoris distal myotendinous T-junction injuries showed longest RTP time and highest re-injury rate (37.5%). Length of injury and intermuscular fluid also showed prolonged RTP. Combined classification model improved correlation to r = 0.76 with AUC = 0.84. AI based model achieved cross-validation accuracy of 79.2% with improved correlation (r = 0.79, AUC = 0.87).
**Conclusion:** BAMIC and MLG-R classification systems demonstrate good-to-strong predictive value for RTP timing in professional football thigh muscle injuries. Specific anatomical injury locations significantly influence recovery outcomes. Other MRI parameters including cranio-caudal length and intermuscular fluid volume provide additional prognostic value. AI-enhanced models incorporating anatomical and quantitative factors achieve superior predictive accuracy
**Limitations:** Retrospective design with inherent selection bias. Limited sample size (n=48) may affect statistical power and generalizability. Study limited to Egyptian and Gulf region professional football players, potentially affecting generalizability to other sports. Machine learning model requires external validation in larger independent cohorts. Rehabilitation protocols varied between clubs, introducing potential confounding variables
**Funding for this study:** Saudi German Hospital and Paxera-health
**Has your study been approved by an ethics committee?** Not applicable
**Author Disclosures:**
Mohamed Shoura: Nothing to disclose
Sherif Mohyeldin Elhennawy: Nothing to disclose
Yasser Ahmed Abd El Fattah Hassan: Nothing to disclose

**MRI-based predictive modelling for estimating anterior cruciate ligament injury timing**
*A. Piacentini*, K. Abdelmaguid, S. Gitto, S. Fusco, C. Messina, F. Ambrogi, A. Vanzulli, L. M. Sconfienza, D. Albano; Milan/IT

**Purpose:** Accurate estimation of anterior cruciate ligament (ACL) tear timing is essential for clinical and medico-legal purposes. We aimed to identify MRI parameters linked to lesion chronological age and develop a logistic-regression model to distinguish acute (≤12 weeks) from chronic (>12 weeks) injuries.
**Methods or Background:** This retrospective study included 426 patients with clinically and MRI-confirmed ACL tears (2014–2024). MRI (1.5/3 T) assessed 11 direct and indirect signs including bone oedema, effusion, tear morphology, ACL atrophy, ACL thinning, "wavy" morphology, anterior tibial translation, angulated posterior cruciate ligament and uncovered lateral meniscus. Firth-penalized logistic regression predicted injury timing; discrimination was assessed by AUC with bootstrap validation (n=200) and a simplified rule-based score was developed for clinical use.
**Results or Findings:** Among the 426 patients (mean age 35±14 years; 71% male), 261 had acute tears, 73 sub-acute, and 92 chronic. The regression model achieved excellent discrimination between recent and chronic tears with AUC 0.97 (bias-corrected 0.96); at the optimal Youden-index threshold (0.56) it yielded 96% sensitivity, 96% specificity, 96% accuracy, and PPV 0.99. Key discriminators of chronicity were ACL atrophy, thinning, and inhomogeneous thickening. The simplified algorithm–requiring either all major (atrophic ACL, thinned ACL, inhomogeneous thickening of the ACL) or ≥5 minor criteria (bone edema, effusion, uncovered lateral meniscus, full thickness tear with fibers edema, absent wavy ligament, absent anterior tibial translation)–retained high performance (sensitivity 94–96%, specificity 95–96%), closely mirroring the full model.
**Conclusion:** MRI-based multifeatured analysis accurately distinguishes acute from chronic ACL tears. The simplified rule-based model retains high diagnostic performance and it is practical for routine imaging and medico-legal timing of ACL injuries.
**Limitations:** Single-center retrospective design; exclusion of clinical variables (age, mechanism of injury) may limit generalizability; the model yields probability estimates rather than a definitive diagnosis.
**Funding for this study:** No funding received.
**Has your study been approved by an ethics committee?** Yes
**Ethics committee - additional information:** Comitato Etico Territoriale Lombardia 1 (RETRO-RAD, CE 61/INT/2017, 12 March 2025).
**Author Disclosures:**
Alessio Piacentini: Nothing to disclose
Stefano Fusco: Nothing to disclose
Angelo Vanzulli: Nothing to disclose
Carmelo Messina: Nothing to disclose
Salvatore Gitto: Nothing to disclose
Luca Maria Sconfienza: Nothing to disclose
Kholoud Abdelmaguid: Nothing to disclose
Federico Ambrogi: Nothing to disclose
Domenico Albano: Nothing to disclose

**Evaluating the Clinical Impact of Radiologist Reporting in AI-Positive Skeletal Radiographs: A Retrospective Single-Site Quality Study**
*R. Sivanandan*[1], L. Tveiten[2], A. J. Fagerlund[3], K. Malm-Nicolaisen[3], B. A. Graff[2]; [1]Asker/NO, [2]Drammen/NO, [3]Oslo/NO
*(sivram@vestreviken.no)*

**Purpose:** To evaluate the therapeutic impact and clinical relevance of radiologist reporting on AI-positive fracture detections in trauma patients, within a workflow where orthopedists often initiate treatment prior to radiologist review.
**Methods or Background:** This retrospective, single-center study was conducted over two months following the implementation of an AI algorithm for fracture detection in trauma care. Of 2,394 consecutive skeletal radiographs reviewed, 1,127 (47%) AI-positive cases were included and detailed analysis on fracture cases performed. Data collected include patient demographics, fracure type, AI-report, radiologist's-report, musculoskeletal-radiologists (MSKr) report and orthopedist's-report . Discordant cases between AI alone and AI+radiologist interpretations were analyzed for therapeutic impact with orthopedists and MSKr as ground truth.
**Results or Findings:** In AI-positive cases, concordance between AI and radiologist reports for fractures was high (97%) and discordance was noticed in 3% (20 cases). Of the 20 discordant cases, the MSK radiologists agreed with AI in 3 cases (15%) and disagreed in 17 (85%). The orthopedists with AI in 6

cases (30%), disagreed in 12 (60%), and was uncertain in 2 cases (10%). Ground truth was defined by the orthopedist's assessment, with the MSKr used to resolve the 2 uncertain cases. False-positive AI results led to 1 over-treatment, 1 under-treatment while others received adequate necessary treatment (Fig-4).
**Conclusion:** In an AI-supported workflow for fracture detection, therapeutic decisions were primarily driven by orthopedists integrating AI with their clinical judgment. Our findings suggest that adding radiologist input to AI may not significantly improve accuracy in discordant cases, whereas an AI+orthopedits approach appears more clinically relevant and efficient—allowing radiologists to focus on complex, high-impact tasks.
**Limitations:** This is a single-site retrospective study and may be feasible when orthopedists assess the radiographs with AI as part of routine clinical care.
**Funding for this study:** No funding
**Has your study been approved by an ethics committee?** Yes
**Ethics committee - additional information:** ID (24/05320) by the Data Protection Officer of the Vestre Viken Hospital Trust
**Author Disclosures:**
Kristian Malm-Nicolaisen: Nothing to disclose
Asbjørn Johansen Fagerlund: Nothing to disclose
Line Tveiten: Nothing to disclose
Ramprabananth Sivanandan: Nothing to disclose
Bjørn Anton Graff: Nothing to disclose

### Ankle Injuries with Deltoid Complex Involvement: MRI findings and Association with Fascial Sleeve and Retinaculum Tears

*S. Fusco*[1], L. Cerezal Pesquera[2], A. M. Perez[3], F. Idoate[4], L. M. Sconfienza[1], E. Llopis San Juan[5]; [1]Milan/IT, [2]Santander/ES, [3]Terrassa/ES, [4]Pamplona/ES, [5]Alzira/ES
*(stefano.fusco@unimi.it)*

**Purpose:** To describe MRI findings associated with ankle injuries involving the deltoid complex, with focus on fascial sleeve avulsion of the medial malleolus and periosteal stripping of the flexor retinaculum, and to assess their relationship with injury timing.
**Methods or Background:** Two musculoskeletal radiologists retrospectively evaluated 165 ankle MRIs with deltoid complex injury. Each study was reviewed for: superficial and deep deltoid tears, fascial sleeve avulsion, flexor retinaculum stripping, lateral ligaments and syndesmotic tears, osteochondral lesions(OCLs), soft tissue impingement, pathology of the posterior tibial tendon(PTT), and peroneal tendons. Injuries were classified as acute, chronic, or acute-on-chronic. Descriptive statistics and chi-square tests were used.
**Results or Findings:** Deep deltoid tears were present in 160/165 cases. Superficial deltoid proximal tear was seen in 84/165(50.9%). Fascial sleeve avulsion occurred in 74/165(44.8%), and flexor retinaculum stripping in 66/165(40.0%); both coexisted as “complete fascial sleeve” in 55/165(33.3%). These lesions were associated with superficial deltoid proximal tears (fascial sleeve in 88.1% vs 0%; retinaculum stripping in 66.7% vs 12.3%; complete fascial sleeve in 65.5% vs 0%; $p<0.0001$). The prevalence of complete fascial sleeve increased with chronicity, being less common in acute (25.4%) and more frequent in chronic (45.2%) and acute-on-chronic injuries (64.1%) ($p<0.001$). Isolated deltoid injuries were rare: >95% also had lateral ligament tears. Additional findings included syndesmotic injuries (9%), OCLs (15%), soft tissue impingement (24%), PTT abnormalities (27%), and peroneal pathology (28%).
**Conclusion:** Deltoid complex injuries are rarely isolated and usually coexist with lateral ligament tears. Fascial sleeve avulsion and retinacular stripping—especially in combination—are closely associated with proximal superficial deltoid tears, likely functioning as a biomechanical unit. These findings are more common in chronic injuries, reflecting their link to chronic lateral ankle instability.
**Limitations:** Retrospective; lack of surgical correlation; lack of long-term outcomes
**Funding for this study:** None
**Has your study been approved by an ethics committee?** Not applicable
**Author Disclosures:**
Eva Llopis San Juan: Nothing to disclose
Luis Cerezal Pesquera: Nothing to disclose
Fernando Idoate: Nothing to disclose
Stefano Fusco: Nothing to disclose
Luca Maria Sconfienza: Nothing to disclose
Alexeys Marcel Perez: Nothing to disclose

### Virtual arthroscopic imaging: validation of a low-field rotating MRI harnessing the magic angle effect in the investigation of suspected anterior cruciate ligament rupture

*D. Amiras*, K. Chappell, C. Tsitsifylla, H. Lanz, Y. Joshi, J. Mcginley, C. Gupte, M. Ristic; London/UK
*(dimitri.amiras@nhs.net)*

**Purpose:** A novel rotating low-field MRI can harness the magic angle effect to identify collagen fibre alignment and orientation in vivo. Typically, imaging collagen fibres relies on negative contrast, caused by adjacent water or haemorrhage, to depict injuries, as collagen produces inherently low signal on most MR techniques. In this study, we utilise magic angle directional imaging (MADI) to image the anterior cruciate ligament (ACL) in patients being investigated for ACL tears and correlate the findings with surgical outcomes.
**Methods or Background:** A novel 0.15T rotating transverse magnet allowed reorientation of the main B0 field. Seven to eight 3D T1 FLASH isotropic volumes at different B0 orientations, relative to the projected orientation of the ACL, were acquired. In post-processing, co-registration of these volumes was performed, and collagen fibre vectors were calculated for regions of interest corresponding to the ACL. These vectors were then visualised as tractograms superimposed on the previously acquired volume. Conventional MRI and magic angle MR images (MAMRI) were compared to arthroscopic findings.
**Results or Findings:** Five cases from four patients were compared with arthroscopic findings. Three cases had complete ACL tears, whereas one case had a 'cyclops' lesion post-ACL reconstruction, and the same patient had an old partial ACL tear on their contralateral knee. MAMRI performed at least as well as conventional MRI and, in one case, correctly predicted a complete tear when conventional MRI suggested a partial-thickness tear.
**Conclusion:** This is the first report demonstrating the clinical applicability of low-field magic angle MRI in the assessment of ACL tears in an injured population. Whilst only five cases had surgical correlation, the images obtained were promising and revealed detail not previously visible on conventional MRI.
**Limitations:** Limited sample size; Arthroscopic subjectivity; Limited data on MAMRI interpretation
**Funding for this study:** NIHR i4i Grant II-LA-1111-20005 Wellcome Trust Innovator Award
**Has your study been approved by an ethics committee?** Yes
**Ethics committee - additional information:** REC reference: 24/LO/0531
**Author Disclosures:**
John Mcginley: Nothing to disclose
Dimitri Amiras: Shareholder: MedicaliSight Ltd
Yash Joshi: Nothing to disclose
Karyn Chappell: Nothing to disclose
Mihailo Ristic: Nothing to disclose
Chinmay Gupte: Nothing to disclose
Harry Lanz: Nothing to disclose
Chrysoula Tsitsifylla: Nothing to disclose

### Frequent musculoskeletal MRI findings in pre-signing medical assessments of professional soccer players

*E. Höhne*, S. Mahmoudi, T. Vogl, K. Eichler, C. Booz, I. Yel; Frankfurt/DE

**Purpose:** Prior to player transfers professional soccer clubs conduct comprehensive musculoskeletal assessments in healthy athletes to evaluate their current condition and injury risk. Magnetic resonance imaging (MRI) plays a central role in this process by detecting musculoskeletal abnormalities even in the absence of symptoms. This study presents common MRI findings in a cohort of professional soccer players and aims to improve understanding of the physical condition of elite athletes.
**Methods or Background:** In this retrospective analysis, 3T MRI examinations performed between August 2019 and March 2025 during pre-signing assessments were reviewed. Clinical data were extracted from medical records and supplemented with functional information. Non-age-typical structural alterations were systematically categorized, and prevalence patterns were analyzed by playing position.
**Results or Findings:** Fifty professional players (mean age 25.4 ± 4.7 years) were included. Secondary cleft signs (42%) and lumbar disc bulging (40%) were the most frequent abnormalities. Additional common findings comprised knee chondropathy (34%), acetabular labral degeneration (26%), femoroacetabular impingement (22%), and various soft tissue or bone-related changes. While most abnormalities were evenly distributed across positions, the prevalence of secondary clefts differed significantly ($\chi^2 = 8.07$, $p = 0.045$), with strikers showing the highest proportion (69%).
**Conclusion:** MRI screening in professional soccer players most frequently revealed structural musculoskeletal abnormalities, many of which were also present in asymptomatic individuals. This underscores the ability of MRI to detect subclinical alterations that may later develop into clinically relevant conditions. Overall, the findings support routine use of MRI in pre-signing assessments to guide individualized prevention, rehabilitation, and long-term player management.

**Limitations:** The relatively small sample size, absence of a control group, and uneven distribution of playing positions and limb dominance may limit the generalizability of these findings.
**Funding for this study:** Not applicable.
**Has your study been approved by an ethics committee?** Yes
**Ethics committee - additional information:** Ethical approval was obtained from the local ethics committee of the university hospital Frankfurt (2024-2129) and informed consent was obtained.
**Author Disclosures:**
Christian Booz: Nothing to disclose
Thomas Vogl: Nothing to disclose
Scherwin Mahmoudi: Nothing to disclose
Ibrahim Yel: Nothing to disclose
Katrin Eichler: Nothing to disclose
Elena Höhne: Nothing to disclose

**Exercise-related signal abnormalities (ERSA) in Lower Limb MRI of Athletes: Prevalence, Patterns, and Symptom Correlation**
*S. Fusco*[1], F. Idoate[2], L. Cerezal Pesquera[3], A. M. Perez[4], L. M. Sconfienza[1], E. Llopis San Juan[5]; [1]Milan/IT, [2]Pamplona/ES, [3]Santander/ES, [4]Terrassa/ES, [5]Alzira/ES
*(stefano.fusco@unimi.it)*

**Purpose:** To describe the prevalence, distribution, and clinical associations of exercise-related signal abnormalities (ERSA) in lower limb MRI of athletes, and to assess their relationship with symptoms and concomitant muscle injuries.
**Methods or Background:** One musculoskeletal radiologist reviewed lower limb MRI of professional and non-professional athletes performed for suspected muscle injury or follow-up, to identify ERSA lesions (ovoid peritendinous and/or ring-shaped perifascial edema). For each MRI, the following parameters were assessed: ERSA type (A/B/C), muscle(s) involved, longitudinal distribution, laterality, relationship to the clinically targeted compartment (site of suspected/ongoing injury). Correlation with reported pain and with acute, subacute, or chronic lesions/scar was assessed. Descriptive statistics and Fisher's exact/Chi-square tests were used.
**Results or Findings:** ERSA lesions were found in 30 athletes (mean age 26.7 years; 27 male; 21 soccer players), involving a single muscle in 19/30 (63.3%), two in 8/30 (26.7%), and ≥3 in 3/30 (10.0%). Subtypes included type B in 15(50.0%), type C in 13(43.3%), and type A in 2(6.7%). The rectus femoris was most frequently affected(n=12), followed by soleus, vastus lateralis, and adductor major (n=4). Pain in the ERSA compartment was present in 20/30(66.7%). In 12, pain was explained by concomitant acute/subacute injuries, while in 8 it occurred with isolated ERSA. Conversely, 10/30 showed ERSA in non-target compartments without pain. No significant association was found between pain and ERSA subtype(p=0.099).
**Conclusion:** ERSA are a relatively frequent MRI finding in athletes investigated for suspected muscle injury. Our data suggest a mixed picture: ERSA may explain pain in the absence of associated muscle injuries but are also frequently found in asymptomatic compartments. Prospective studies are needed to clarify their clinical relevance or determine whether they represent overload-related changes.
**Limitations:** Retrospective design; relatively small sample size; lack of prospective symptom monitoring
**Funding for this study:** None
**Has your study been approved by an ethics committee?** Not applicable
**Author Disclosures:**
Eva Llopis San Juan: Nothing to disclose
Luis Cerezal Pesquera: Nothing to disclose
Fernando Idoate: Nothing to disclose
Stefano Fusco: Nothing to disclose
Luca Maria Sconfienza: Nothing to disclose
Alexeys Marcel Perez: Nothing to disclose

**Comparative Analysis of Deep Learning-Based Reconstruction and Hybrid Iterative Reconstruction in Ultra-Low-Dose Ankle and Foot CT Imaging**
*C. Otgonbaatar*[1], S. Cha[2], S. H. Jeon[2], H. Shim[1], S. M. Ko[2], H. Kim[2]; [1]Seoul/KR, [2]Wonju-si/KR
*(chukarad@gmail.com)*

**Purpose:** This study aimed to evaluate the quantitative and qualitative image analysis of ultra-low-dose CT with deep learning image reconstruction (DLR) compared with hybrid iterative reconstruction (IR) in patients with ankle and foot fractures.
**Methods or Background:** This retrospective study included 32 patients who underwent ultra-low-dose CT of the ankle and foot (effective dose ~0.86 μSv). Images were reconstructed with DLR and hybrid IR. Objective analysis included image noise, SNR, CNR, and sharpness (assessed with a no-reference perceptual sharpness metric). Subjective quality was rated by two board-certified radiologists on a 5-point scale.
**Results or Findings:** DLR demonstrated clear advantages over hybrid IR, with substantially lower image noise (47.33 ± 6.60 HU vs. 87.65 ± 12.48 HU) and significantly higher SNR (31.42 ± 5.81 vs. 19.15 ± 4.48) and CNR (50.59 ± 7.62 vs. 24.08 ± 3.81) (all p < 0.001). Image sharpness was also enhanced (0.76 ± 0.09 for DLR vs. 0.59 ± 0.09 for hybrid IR, p < 0.001). Subjective evaluations confirmed the superiority of DLR, with improved ratings for overall image quality, trabecular architecture, cortical bone delineation, and fracture depiction (all p = 0.001).
**Conclusion:** Ultra-low-dose CT reconstructed with DLR improves image quality while maintaining minimal radiation exposure in the assessment of ankle and foot fractures.
**Limitations:** Not applicable
**Funding for this study:** None
**Has your study been approved by an ethics committee?** Yes
**Ethics committee - additional information:** This study was approved by the Institutional Review Board
**Author Disclosures:**
Sung Min Ko: Nothing to disclose
Sungjin Cha: Nothing to disclose
Hyunjung Kim: Nothing to disclose
Sang Hyeon Jeon: Nothing to disclose
Hackjoon Shim: Nothing to disclose
Chuluunbaatar Otgonbaatar: Nothing to disclose

**ChatGPT as a diagnostic support tool in the preoperative MRI evaluation of ACL tears: a semi-quantitative and surgical validation study**
*A. Şükün*[1], C. Özenbaş[2], B. Sargın[1], K. Gökkuş[1], M. Ş. Sahin[1]; [1]Antalya/TR, [2]Izmir/TR
*(abdullah.sukun@gmail.com)*

**Purpose:** To assess the diagnostic accuracy of a semi-quantitative signal scoring system for anterior cruciate ligament (ACL) tears on preoperative 1.5T knee MRI, and to compare the performance of conventional radiology reports with ChatGPT-based scoring, using arthroscopic findings as the reference standard.
**Methods or Background:** This retrospective single-center study received institutional review board approval (KA25/133, March 26, 2025). Between 2012 and 2024, 185 patients undergoing arthroscopic ACL surgery were screened. Exclusion criteria were the absence of preoperative MRI, poor image quality, or missing PACS data. After applying the criteria, 100 patients with surgically confirmed ACL rupture were included. Sagittal T1- and PD-weighted sequences were anonymized and exported in JPEG format (300 dpi, 1350×1200 pixels). Three PD and three T1 sagittal slices best depicting the ACL were selected. Semi-quantitative scoring was applied exclusively to PD images by freehand ROI placement, classifying ACLs as intact (<2), partial (2–3.5), or complete (>3.5) on a four-point scale. T1 images were used for anatomical correlation. Scoring was performed independently by ChatGPT, with surgery as the gold standard.
**Results or Findings:** A total of 100 patients (86 males [86%], 14 females [14%]; mean age, 31.99 ± 9.25 years) underwent preoperative knee MRI and arthroscopic ACL surgery. All had surgically confirmed complete ACL tears. ChatGPT achieved 64% diagnostic accuracy, compared to 45% for radiology reports. The semi-quantitative scoring system with ChatGPT improved accuracy by ~20% versus conventional reporting.
**Conclusion:** ChatGPT-assisted semi-quantitative scoring significantly enhanced the accuracy of preoperative MRI in diagnosing ACL tears compared to routine radiology reporting, suggesting its potential as a supportive decision-making tool in clinical practice.
**Limitations:** The retrospective design, single-center cohort, and restriction to surgically confirmed complete tears limit generalizability. External validation with larger, multi-center, and prospective datasets is warranted.
**Funding for this study:** No
**Has your study been approved by an ethics committee?** Yes
**Ethics committee - additional information:** KA25/133, March 26, 2025
**Author Disclosures:**
Barış Sargın: Nothing to disclose
Cemre Özenbaş: Nothing to disclose
Kemal Gökkuş: Nothing to disclose
Mehmet Şükrü Sahin: Nothing to disclose
Abdullah Şükün: Nothing to disclose

**12:30 - 13:30** **Research Stage 5**

### Research Presentation Session: Vascular Hot Topic with Keynote Lecture

### RPS 1415

### Hot Topic: photon counting CT

**Moderator**
Guillaume Fahrni; Lausanne/CH
*(guillaume.fahrni@chuv.ch)*

**Keynote Lecture**
Hirofumi Kuno; Kashiwa/JP
*(hkuno@east.ncc.go.jp)*

**Ultra-High Resolution Photon-Counting CTA for Follow-up Assessment of Intracranial Aneurysms after Flow Diverter: Initial Experience**
R. Li[1], *N. He*[1], Y. Cui[2], Z. Xu[1], H. Jiang[3], F. Yan[1], S. Haller[4]; [1]Shanghai/CN, [2]Nanjing/CN, [3]Shagnhai/CN, [4]Geneve/CH

**Purpose:** To evaluate the preliminary diagnostic performance and utility of UHR-PCCTA and standard resolution (SR) PCCTA compared to immediate post-deployment DSA following FD treatment, with emphasis on comprehensive characterization of post-FD follow-up.
**Methods or Background:** This prospective study consecutively enrolled 47 participants treated with FD and subsequently underwent PCCTA with UHR and SR reconstructions between August 2023 and August 2024. Two readers independently analyzed images blindly, including aneurysm size, occlusion status, neck coverage, wall apposition, stent deformation, branch vessel with Likert-scale (1-5). The inter-rater reliability for both UHR and SR-PCCTA was assessed using the intraclass correlation coefficient (ICC) and weighted Cohen's kappa. The diagnostic performance of PCCTA within 1 month after FD was compared with that of immediate post-deployment DSA using a confusion matrix.
**Results or Findings:** The study included 47 participants with 57 intracranial aneurysms treated with 51 FD stents. UHR-PCCTA exhibited higher diagnostic confidence (median=5, IQRs=5-5) compared to SR-PCCTA (median=4, IQRs=4-5, P<0.05). UHR-PCCTA demonstrated a trend toward higher inter-rater agreement (κ range:0.814-1.0; ICC range:0.902-0.960) relative to SR-PCCTA (κ range:0.668-1.0; ICC range:0.873-0.966) among both aneurysm size and stent morphology. Compared with immediate post-deployment DSA, UHR-PCCTA showed excellent diagnostic accuracy for aneurysm neck coverage (93.3%), wall apposition (100.0%), stent deformation (93.8%), and branch vessel inclusion (93.3%), along with very-good accuracy for aneurysm occlusion (88.2%).
**Conclusion:** UHR-PCCTA demonstrates high reliability and diagnostic accuracy in the assessment of intracranial aneurysms following FD treatment. Its comprehensive imaging evaluation capability has the potential to inform clinical decision-making.
**Limitations:** (1) relatively small sample size owing to the preliminary clinical nature; (2) potential changes during the ≤1-month interval between PCCTA and DSA, may affect accuracy assessments; (3) Whether the sub-stent hypodensity detected by UHR-PCCTA suggested neointima requires histopathological confirmation.
**Funding for this study:** No
**Has your study been approved by an ethics committee?** Yes
**Ethics committee - additional information:** This prospective single-center study was approved by the local review board (KY2023-186) and written informed consent was obtained from all participants.
**Author Disclosures:**
Naying He: Nothing to disclose
Ying Cui: Nothing to disclose
Zhihan Xu: Nothing to disclose
Fuhua Yan: Nothing to disclose
Hong Jiang: Nothing to disclose
Ruixi Li: Nothing to disclose
Sven Haller: Nothing to disclose

**Quality improvements in ultra-high resolution photon counting detector CT-angiography of the cerebral vasculature with image acquisition at lower kVp and application of sharper reconstruction kernels**
F. M. E. Pinckaers, T. Flohr, B. A. J. M. Wagemans, I. Huijberts, J. E. Wildberger, *A. Postma*; Maastricht/NL

**Purpose:** To assess quality improvements in ultra-high resolution (UHR) photon counting detector (PCD) CT angiography (CTA) of the cerebral vasculature when moving to image acquisition at lower kVp and image reconstruction with sharper kernels.
**Methods or Background:** Over a period of 25 months, all cerebral CTA imaging on PCD-CT was evaluated. Records were excluded in case of protocol deviations, severe motion artifacts or cerebral circulatory arrest. Using UHR resolution (0.2 mm), three protocols were evaluated: (1) 140 kVp/medium sharp kernel (Hv40); (2) 90 kVp/Hv40; (3) 90 kVp/very sharp kernel (Hv72). Vessel attenuation, signal-to-noise ratios (SNR) and contrast-to-noise ratios (CNR) were derived at four locations in the anterior circulation. Vessel sharpness was quantified using the edge rise distance and edge rise slope. Subjective assessments of image noise, vessel attenuation and vessel sharpness were performed by two experienced readers on a 5-point Likert scale.
**Results or Findings:** Out of 154 consecutive records, 141 (92%) were included. Vessel attenuation, SNR and CNR improved with image acquisition at 90 kVp compared to 140 kVp in the protocols using a medium sharp reconstruction kernel. SNR and CNR decreased when applying (very) sharp reconstruction kernels at 90 kVp due to an increase in noise. However, while an evident decrease in SNR and CNR was observed in smaller calibre vessels when using a medium sharp kernel at 90 kVp, these parameters remained relatively stable when using (very) sharp kernels. Vessel sharpness was markedly improved in the 90 kVp/(very) sharp kernel protocol. Subjective assessments of image quality also favoured the 90 kVp/(very) sharp kernel protocol.
**Conclusion:** Image quality of UHR PCD-CTA of the cerebral vasculature is improved with image acquisition at 90 kVp and image reconstruction with (very) sharp kernels.
**Limitations:** Single-centre, retrospective study.
**Funding for this study:** None.
**Has your study been approved by an ethics committee?** Yes
**Ethics committee - additional information:** The medical ethical committee of the Maastricht University Medical Centre waived the need for individual patient consent.
**Author Disclosures:**
Bart A J M Wagemans: Nothing to disclose
Joachim E. Wildberger: Nothing to disclose
Thomas Flohr: Nothing to disclose
Linda Jacobi-Postma: Nothing to disclose
Florentina Maria Egidius Pinckaers: Nothing to disclose
Ilse Huijberts: Nothing to disclose

**Differentiation of Carotid Atherosclerotic Plaque Components Using Dual-Layer Spectral CTA**
F. Xu, *Y. Wang*, D. Mu; Shanghai/CN
*(yu.wang@philips.com)*

**Purpose:** Carotid plaque composition is a major determinant of plaque vulnerability and thromboembolic stroke. This study investigated the performance of dual-layer spectral CT angiography (DLCTA) parameters to characterize carotid atherosclerotic plaques components, with high-resolution MRI (HR-MRI) or histopathology of postoperative specimens as reference standard.
**Methods or Background:** Fifteen patients with moderate-to-severe carotid bifurcation stenosis were prospectively enrolled. All patients underwent both DLCTA and HR-MRI, with findings correlated with histopathology in surgical cases. Plaque components including intraplaque hemorrhage (IPH), lipid-rich necrotic core (LRNC), fibrous tissue, and calcifications were identified on MRI or histopathology, and mapped to CTA using slice-to-slice coregistration. Quantitative parameters recorded included conventional CT (con-CT) values, virtual monoenergetic images (VMI, 40-140keV, 10-keV intervals), effective atomic number (Z-eff), and electron density (ED) . Diagnostic performance was evaluated by ROC analysis.
**Results or Findings:** A total of 42 slices and 164 ROIs were analyzed. Calcifications were clearly distinguished by conventional CT values, while non-calcified components (LRNC, IPH, and fibrous tissue) showed significant differences on VMI40keV and Z-eff (LRNC: 25.5 ± 16.1 HU, 7.19 ± 0.08; IPH: 122.3 ± 40.5 HU, 7.66 ± 0.43; fibrous tissue: 66.4 ± 20.8 HU, 7.40 ± 0.09; all p < 0.001). VMI40keV and Z-eff demonstrated the highest accuracy for differentiating LRNC from other non-calcified components (AUC 0.99, 95% CI 0.97–1.00 and 0.96, 0.83–1.00, respectively). For distinguishing IPH from fibrous tissue, AUC was 0.82 for VMI40keV.

**Conclusion:** DLCTA, particularly VMI40keV and Z-eff, enables noninvasive differentiation of carotid plaque components and may offer a practical alternative to contrast-enhanced MRI for identifying vulnerable carotid plaques, with potential implications for stroke risk stratification
**Limitations:** Study limited by small sample size and limited histopathologic confirmation.
**Funding for this study:** Not applicable.
**Has your study been approved by an ethics committee?** Not applicable
**Author Disclosures:**
Yu Wang: Employee: Philips healthcare company
Feijia Xu: Nothing to disclose
Dan Mu: Nothing to disclose

**Ultra–High-Resolution Photon-Counting CTA With Black Blood Technique for Carotid Plaque Characterization**
*Z. Li*[1], X. Fan[1], M. Lyu[2], F. Feng[1]; [1]Beijing/CN, [2]Shanghai/CN

**Purpose:** To evaluate the feasibility and performance of photon-counting CT (PCCT) with black-blood technique for carotid plaque characterization, by comparing with vessel wall MRI (VW-MRI) and biphasic CT angiography (CTA).
**Methods or Background:** From January 2024 to May 2025, a total of twenty-five patients scheduled for carotid revascularization underwent CTA with arterial and delayed-phase using PCCT and eight patients voluntarily received vessel wall MRI (VW-MRI) within 2 weeks. Black-blood CT (BBCT) images was generated for each CTA phases. Totally 50 bilateral vessels were included for qualitative analysis between BBCT and biphasic CTA. And thirteen plaques were evaluated for quantitative analysis and plaque type classification. Qualitative scores (including calcified/non-calcified plaque visibility, diagnostic confidence, overall image quality) were independently assessed by two radiologists blindly using a 5-point scale between biphasic CTA and BBCT. Quantitative parameters (stenosis rate, plaque area, calcium area, non-calcium area) were compared between BBCT and VW-MRI using intraclass correlation coefficients (ICCs). Carotid Plaque-RADS evaluation was further applied in BBCT, biphasic CTA and VW-MRI.
**Results or Findings:** Compared with arterial and delayed images, BBCT achieved significantly higher scores in non-calcified plaque visibility, diagnostic confidence, and overall image quality (all $p < 0.001$). BBCT showed excellent agreement with VW-MRI, especially for non-calcified area (ICC = 0.96). Confusion matrix analysis illustrated that BBCT identified more high-risk plaques compared to biphasic CTA (such as intraplaque hemorrhage), and it exhibited strong concordance with VW-MRI.
**Conclusion:** BBCT enabled accurate carotid plaque quantification with excellent agreement to VW-MRI and outperforms biphasic CTA in image quality, diagnostic confidence, and high-risk plaque detection, suggesting its potential utility in stroke risk stratification when combined with Carotid Plaque-RADS.
**Limitations:** The number of carotid plaques with matched VW-MRI is small due to only single-center inclusion.
**Funding for this study:** None.
**Has your study been approved by an ethics committee?** Yes
**Ethics committee - additional information:** The study was conducted in accordance with the Declaration of Helsinki and its subsequent amendments. This study was approved by the Medical Ethics Committee of the Peking Union Medical College Hospital (No.I-24PJ0346). Written consent was obtained from each subject.
**Author Disclosures:**
Zhonghui Li: Nothing to disclose
Mengzhe Lyu: Employee: Engineer
Xiaoyuan Fan: Nothing to disclose
Feng Feng: Nothing to disclose

**Normalization Strategies for Perfused Blood Volume Quantification in Photon-Counting CT of the Lung**
*A. M. Hunkemöller*, K. Höffler, K. Katsirntaki, F. Wacker, B. Wiegmann, H-O. Shin; Hannover/DE

**Purpose:** Photon-counting CT (PCCT) inherently provides spectral data, enabling reconstruction of virtual non-contrast (VNC) and perfused blood volume (PBV) images. However, quantitative PBV assessment remains challenging due to variability in lung inflation and iodine concentration. This study systematically evaluated normalization strategies to improve PBV reproducibility across different inflation states, contrast concentrations, and acquisition conditions in an ex-vivo lung model.
**Methods or Background:** Five explanted porcine lungs were perfused and ventilated on the Organ Care System (OCS™) Lung platform under three standardized inflation states (inspiration, mid-inspiration, expiration) and two contrast concentrations. PCCT datasets were reconstructed into VNC and PBV images. After manual segmentation, three normalization approaches were applied: (1) normalization to an intravascular reference region (PBV_rel), (2) further normalization to total lung volume (PBV_lung), and (3) adjustment for lung tissue fraction derived from VNC attenuation (PBV_VNC). The dependence of PBV on inflation state was analyzed using repeated-measures ANOVA.
**Results or Findings:** Unnormalized PBV demonstrated significant dependence on lung inflation ($p = 0.0143$; SD = 5.00 HU). Normalization to an intravascular reference (PBV_rel) effectively mitigated this effect ($p = 0.9388$; SD = 0.13). PBV_lung retained minor residual variation ($p = 0.0292$; SD = 0.11), while PBV_VNC yielded the most consistent results across inflation states ($p = 0.0825$; SD = 0.08), indicating superior robustness and reduced variability.
**Conclusion:** PBV quantification in PCCT is strongly affected by lung inflation and iodine concentration. Normalization substantially enhances measurement stability, with VNC-based tissue fraction adjustment (PBV_VNC) providing the most reliable and inflation-independent standardization for quantitative pulmonary perfusion assessment.
**Limitations:** This ex-vivo study employed a small sample size and controlled ventilation conditions, which may limit direct translation to in-vivo applications.
**Funding for this study:** No funding.
**Has your study been approved by an ethics committee?** Not applicable
**Author Disclosures:**
Klaus Höffler: Nothing to disclose
Bettina Wiegmann: Nothing to disclose
Katherina Katsirntaki: Nothing to disclose
Anna M. Hunkemöller: Nothing to disclose
Frank Wacker: Nothing to disclose
Hoen-Oh Shin: Nothing to disclose

**Added Value of Extremity-Specific Isotropic Voxel Spacing in Below-Knee Spectral Ultrahigh-Resolution Photon-Counting CT**
*M. T. Hagar*[1], S. Hyska[2], M. Vecsey-Nagy[3], J. I. Griggers[1], J. Osoria-Velasquez[4], T. S. Emrich[5], A. Varga-Szemes[4]; [1]Freiburg Im Breisgau/DE, [2]Munich/DE, [3]Budapest/HU, [4]Charleston, SC/US, [5]Mainz/DE

**Purpose:** Below-knee CT angiography (CTA) is limited by artifacts from calcified plaques in peripheral artery disease (PAD). Combined spectral ultrahigh-resolution (UHR) photon-counting CT (PCD-CT) may improve performance, but optimal conditions remain unclear.
**Methods or Background:** In this IRB-approved post-hoc study, consecutive patients with PAD undergoing clinically indicated PCD-CTA were included. Scans were performed on a dual-source PCD system in combined spectral and UHR mode (collimation 120 × 0.2 mm, 120 kVp). Axial images were reconstructed as down-sampled (DS, 0.8 mm, Bv40), virtual monoenergetic images (VMIs, 45–90 keV, 0.4 mm, Bv60), iodine maps (IM, 0.4 mm, Bv60), and UHR (0.2 mm, Bv60). Additional extremity-specific reconstructions with isotropic voxel spacing were generated (FOV 205 × 205 mm; UHRfocused: 0.2 mm, spectral: 0.4 mm). For small-caliber vessels (2.0 mm) with calcified plaques, perpendicular line profiles were analyzed to derive lumen and plaque widths using full-width-at-half-maximum (FWHM_lumen and FWHM_plaque). Digital subtraction angiography (DSA) served as reference, and diagnostic performance was assessed.
**Results or Findings:** A total of 59 patients (mean age 64.6 ± 13.5 years; 40 men, 68%) with 111 lower extremities were analyzed. Both UHR_focused and iodine map (IM_focused) reconstructions offered superior lumen visualization (FWHM_lumen: UHR 1.68 ± 0.76; IM 1.70 ± 0.76) and minimized blooming (FWHMplaque: UHR 1.01 ± 0.28; IM 0.98 ± 0.27; all $p<0.01$). In contrast, DS reconstructions were limited by blooming artifacts (FWHMlumen: 0.60 ± 0.78; FWHMplaque: 2.11 ± 0.60). UHR_focused and IM_focsued achieved highest sensitivity (93% (95% CI: 77–99%)), with UHRfocused additionally reaching the highest per-segment accuracy of 94% (95% CI: 83–99%, n=50).
**Conclusion:** UHR PCD-CT with per-extremity reconstruction using isotropic voxel spacing enhances image quality, improves lumen and plaque delineation, and yields highest diagnostic accuracy in below-knee CTA.
**Limitations:** The limited sample size requires confirmatory research.
**Funding for this study:** No
**Has your study been approved by an ethics committee?** Yes
**Ethics committee - additional information:** ID: Pro99133632, approval date: 4/25/2025
**Author Disclosures:**
Sardi Hyska: Nothing to disclose
Muhammad Taha Hagar: Speaker: Siemens Healthineers
Milán Vecsey-Nagy: Nothing to disclose
James Ira Griggers: Nothing to disclose
Tilman Stephan Emrich: Nothing to disclose
Jose Osoria-Velasquez: Nothing to disclose
Akos Varga-Szemes: Nothing to disclose

12:30 - 13:30 **Research Stage 1**

### Research Presentation Session: Emergency Imaging Hot Topic with Keynote Lecture

### RPS 1417

### Hot Topic: AI in emergency imaging

**Moderator**
Sujit Vaidya; London/UK

**Keynote Lecture**
Michael N. Patlas; Burlington/CA
*(patlas69@yahoo.com)*

**AI Triage in Emergency Radiology: Enhancing Detection or Adding Noise?**
T. Clement[1], L. Mabit[1], G. Davy[1], G. D'Assignies[2], R. Guillevin[1], *G. Herpe*[1]; [1]Poitiers/FR, [2]Nantes/FR
*(herpeguillaume@gmail.com)*

**Purpose:** AI-driven triage tools are increasingly used in emergency radiology to help detect and prioritize urgent conditions such as brain injuries, pneumothorax, and incidental pulmonary embolism. This study assesses the real-world impact of deploying multiple AI alerting systems.
**Methods or Background:** This retrospective multi-centric study was conducted over two months in three ER department. AI algorithms were applied to detect intracranial hemorrhage on CT, pneumothorax on chest X-ray, and incidental pulmonary embolism on contrast-enhanced CT across 2,336 CT and 119 Chest X-rays. Discrepancies between AI outputs and radiology reports were first reviewed by a radiologist and then resolved by an emergency radiologist. Performance was evaluated using diagnostic metrics.
**Results or Findings:** ICH detection : Among 682 head CT scans, AI flagged 133 positives(19.5%,133/682), including 46 false positives (6.7%,46/682) and 11 false negatives(1.6%,11/682). AI detected 2 additional true positives(1.5%,2/133) missed by radiologists. Pneumothorax Detection: Out of 119 chest X-rays, AI identified 3 positive cases(2.5%,3/119), including 1 missed by the radiologist (0.8%,1/119), with no false positives. Incidental Pulmonary Embolism Detection : In 1654 contrast-enhanced CT scans, AI flagged 70 positives(4.2%,70/1654), with 22 true positives(31.4%,22/70) and 9 missed by radiologists(41%,9/22). AI increased the detected prevalence from 0.8%(14/1654) to 1.3%(22/1654), but the false-positive rate was 68.6%(48/70). Overall, at the cost of about 1.5 false alerts per day, AI helped uncover one life-threatening condition every five days.
**Conclusion:** AI triage tools in emergency radiology enhance detection of missed critical findings and remain valuable if false positives are carefully managed to avoid alert fatigue.
**Limitations:** This retrospective, short-duration study limits generalizability, especially given the small sample size for some modalities. The reference standard relied solely on radiologist re-reads without follow-up confirmation. Finally, AI real impact on workflow, alert fatigue, and patient outcomes was not assessed.
**Funding for this study:** No funding for this study
**Has your study been approved by an ethics committee?** Not applicable
**Author Disclosures:**
Guillaume Herpe: Consultant: Incepto medical
Léo Mabit: Nothing to disclose
Gaspard D'Assignies: Founder: Incepto Medical
Rémy Guillevin: Nothing to disclose
Guillaume Davy: Nothing to disclose
Thomas Clement: Nothing to disclose

**A Machine Learning Model for Predicting Abnormal Head CT Findings in Non-Traumatic Pediatric Emergency Patients: A Multi-center Derivation and Validation Study**
*J. Wu*[1], Y. Li[2], P. Zhang[1], H. Huang[1], J. Hu[1]; [1]Fuzhou/CN, [2]Zhangzhou/CN
*(249961548@qq.com)*

**Purpose:** To identify clinical predictors and develop a prediction model to identify non-traumatic pediatric patients at risk of abnormal head CT findings in emergency department(ED).
**Methods or Background:** Emergency pediatric patients (≤14 years old) were identified from four tertiary general hospitals. Age; Gender; Medical history; Fever;Crying; Seizure; Headache; Dizziness; Syncope; Vomiting; Abnormal Physical examination; Impaired Consciousness, etc., were used as candidate clinical factors. Multivariate logistic regression analysis were used to identify the independent clinical predictors of abnormal head CT findings. Datasets from two hospitals were used for model training and the other two for external validation. We developed models using logistic regression and machine learning (KNN, NNet, Random Forest, XGBoost, Naive Bayes).The best-performing model was identified, assessed with Decision Curve Analysis (DCA), and its feature contributions were explained via SHAP values.
**Results or Findings:** 127 of 2,272 ( 5.6%) cases with abnormal head CT findings. Younger age (OR=0.89), medical history (OR=40.43), non fever(OR=0.35), crying(OR=11.13), vomiting (OR=4.75), headache(OR=2.18), physical examination abnormalities (OR=23.17), and consciousness disorders (OR=21.69) were clinical independent predictors. XGBoost was the top-performing model, with validation AUCs of 0.84 (Test1) and 0.88 (Test2). It showed a balanced sensitivity and specificity(Test1: 67.9% sensitivity, 91.0% specificity; Test2: 81.2% sensitivity, 81.6% specificity) at the Youden threshold and provided the highest net benefit on DCA in the clinically relevant range (0.20-0.30), reducing interventions by 57.8–90.6 per 100 patients. SHAP analysis showed impaired consciousness, age, fever, abnormal physical examination, and seizure were the top predictors of abnormal head CT findings.
**Conclusion:** The validated XGBoost model enables more selective uses of head CT for emergency pediatric patients, potentially reducing unnecessary scans without a loss in sensitivity.
**Limitations:** The relatively small number of abnormal CTs may affect the stability of the estimates for some predictor variables.
**Funding for this study:** Joint Funds for the innovation of science and Technology, Fujian province(Grant number : 2024Y96020147)
**Has your study been approved by an ethics committee?** Yes
**Ethics committee - additional information:** Ethical approval of this study was obtained from the Ethics Committee of these four hospitals（Fujian Provincial Hospital, The First Affiliated Hospital of Fujian Medical University, Fujian Medical University Affiliated Zhangzhou Hospital and the 900th Hospital of the PLA Joint Logistic Support Force ）
**Author Disclosures:**
Jianping Hu: Nothing to disclose
Jianman Wu: Nothing to disclose
Yimin Li: Nothing to disclose
Pan Zhang: Nothing to disclose
Hongbiao Huang: Nothing to disclose

**Real-World Evaluation of an AI-Powered Intracranial Hemorrhage Detection System in Emergency Head CT: A Clinical Validation Study**
*E. E. Aktufan*; Antalya/TR
*(dreminesraktufan@gmail.com)*

**Purpose:** To evaluate the real-world performance of an AI-powered intracranial hemorrhage (ICH) detection system in emergency head CT. After the session, participants will understand the diagnostic strengths and limitations of AI, recognize common causes of false results, and appreciate its implications for clinical workflows.
**Methods or Background:** Deep learning algorithms show high accuracy in ICH detection, but real-world validations are limited. This retrospective study included all emergency non-contrast head CTs performed over three months at a tertiary hospital. Cases with motion and metallic artifacts were retained to reflect real practice. The ground truth was defined by board-certified radiologist reports, which were retrospectively compared with AI outputs (hStroke V1, Hevi AI, Istanbul). The model employs a CNN-RNN architecture with attention for ICH subtype classification.
**Results or Findings:** In 1,421 CTs from 1,379 patients (mean age: 50.6 years; 57% male), 73 were hemorrhage-positive (5.8%). The AI achieved 97.5% accuracy, 95.9% sensitivity, 97.6% specificity, and 68.6% precision. Common subtypes were IPH (n=35), SAH (n=37), and SDH (n=39). Multiple subtypes occurred in 37 cases, most often IPH+SAH+SDH. Three hemorrhages were missed, while 24 false positives mainly arose from artifacts, masses, and calcifications.
**Conclusion:** The AI tool demonstrated high accuracy and sensitivity for ICH detection in a real-world emergency setting. False positives remain a concern, emphasizing radiologist oversight and improved artifact handling.
**Limitations:** This single-center retrospective study relied on board-certified radiologist reports as ground truth, without interobserver analysis. Subtype-specific stratification was limited by sample size. Only axial CTs were analyzed, excluding multiplanar reconstructions that may improve detection in anatomically complex regions.
**Funding for this study:** This research received no specific grant from any funding agency in the public, commercial, or not-for-profit sectors.
**Has your study been approved by an ethics committee?** Yes

**Ethics committee - additional information:** The study protocol was approved by the institutional ethics committee (Protocol No: 2024-122, Decision No: 11/28; Approval Date: August 8, 2024). Informed consent requirements were waived due to the retrospective nature of the study and the anonymization of all datasets
**Author Disclosures:**
Emine Esra Aktufan: Nothing to disclose

**AI Assistance Improves Radiology Resident Reader Performance in CT Diagnosis of Intracranial Hemorrhage**
*P. Reschke*, K. Eichler, T. Vogl, C. Booz; Frankfurt/DE
*(philipp.reschke@outlook.de)*

**Purpose:** Accurate detection of intracranial hemorrhage (ICH) on non-contrast CT is critical in emergency settings, where missed diagnoses may delay treatment and worsen outcomes. While artificial intelligence (AI) models demonstrate high standalone performance, their additive value as a second reader for radiology residents is not well established.
**Methods or Background:** This retrospective study included 1,337 non-contrast head CT scans from 2015–2019 (670 ICH-positive, 667 ICH-negative). A previously validated AI model was used for ICH detection. Two radiology residents reviewed all scans in consensus, first without and later with AI support after a 30-day washout. Ground truth was established by expert consensus. Diagnostic performance metrics were calculated.
**Results or Findings:** AI assistance significantly improved radiology residents' diagnostic performance. Sensitivity increased from 0.85 to 0.94 and specificity from 0.87 to 0.97 (both $p < 0.01$). ROC-AUC rose from 0.86 to 0.95, and PR-AUC from 0.83 to 0.95 ($p < 0.0001$). The number of false negatives dropped from 101 to 41 with AI support. The greatest benefit was observed in subdural hematomas (SDH), where misses declined from 32 to 9 (20.3% to 5.7%; $p < 0.001$), corresponding to a 72% relative risk reduction. Misses also decreased for intraparenchymal hemorrhages (IPH: 37 to 20; RRR 46%) and subarachnoid hemorrhages (SAH: 30 to 11; RRR 63%). AI support reduced common error sources: small hemorrhage volume (48 to 21), atypical locations (30 to 12), and image-degrading artifacts (23 to 8). False positives fell from 87 to 21.
**Conclusion:** By reducing diagnostic errors and supporting learning, AI serves as a valuable second reader for radiology residents—enhancing both patient safety and resident training in ICH detection.
**Limitations:** This study did not evaluate radiologists' trust in or interaction with the system.
**Funding for this study:** None
**Has your study been approved by an ethics committee?** Yes
**Ethics committee - additional information:** Ethical approval was granted by the institutional review board of the University of Frankfurt and written informed consent was waived due to the retrospective nature of the study (approval number: 19-236).
**Author Disclosures:**
Christian Booz: Nothing to disclose
Thomas Vogl: Nothing to disclose
Philipp Reschke: Nothing to disclose
Katrin Eichler: Nothing to disclose

**Artificial Intelligence Software versus Expert Radiologists in Traumatic Ankle and Foot Fracture Detection**
D. Ferreira Branco, *P. Botti*, A. Platon, P-A. A. Poletti, S. Boudabbous; Geneva/CH
*(paul.botti@hug.ch)*

**Purpose:** To evaluate a commercial AI fracture detection tool on foot/ankle radiographs, with emphasis on midfoot fractures (Chopart/Lisfranc), compared with board-certified musculoskeletal radiologists using a composite reference standard (CBCT and/or clinical follow-up).
**Methods or Background:** This retrospective single-center study included all emergency radiographs for adult patients with foot/ankle trauma over six months. Radiographs were first interpreted in routine workflow by radiologists, then independently (stand alone) by the AI tool (BoneView™, Gleamer), each blinded to the other. Descriptive analyses summarized fracture prevalence and distribution (overall, Chopart, Lisfranc). Diagnostic performance of AI and radiologists was assessed, with inter-reader agreement (Cohen's κ). Paired comparisons used McNemar's test.
**Results or Findings:** In total, 701 studies were analyzed; 319 fractures (45.5%) were found: 24 Chopart (7.6%), 22 Lisfranc (6.8%), and 273 (85.6%) other bony structures. AI achieved overall sensitivity 74.3%, specificity 83.0%, accuracy 79.0%. Radiologists achieved sensitivity 84.0%, specificity 95.5%, accuracy 90.3% (p=0.145); κ=0.65. For Chopart fractures, AI sensitivity was 62.1%, specificity 99.6%, accuracy 98.0%; radiologists achieved sensitivity 82.8%, specificity 99.7%, accuracy 99.0% (p=0.180); κ=0.80. For Lisfranc fractures, AI sensitivity was 65.4%, specificity 99.9%, accuracy 98.6%; radiologists achieved sensitivity 80.8%, specificity 100.0%, accuracy 99.3% (p=0.453); κ=0.82
**Conclusion:** AI and radiologists achieved comparable overall performance for detecting foot/ankle fractures on radiographs, with substantial agreement. Radiologists showed a better performance in detecting Lisfranc/Chopart fractures than AI. Our findings support the AI use as complementary help for radiologists in a busy workflow, with targeted attention to midfoot injuries where detection remains challenging.
**Limitations:** Given the low prevalence of Chopart and Lisfranc fractures, the study period was relatively short.
**Funding for this study:** No funding was received for this study.
**Has your study been approved by an ethics committee?** Yes
**Ethics committee - additional information:** Ethical Board approval (CER 2020-02812)
**Author Disclosures:**
Paul Botti: Nothing to disclose
Alexandra Platon: Nothing to disclose
Sana Boudabbous: Nothing to disclose
Pierre-Alexandre Aloïs Poletti: Nothing to disclose
David Ferreira Branco: Nothing to disclose

**Making the small things easy: Hand unfolding in polytrauma whole-body photon-counting CT**
*H-L. Kaatsch*[1], D. Dillinger[1], C. Bauer[1], D. Friedmann[2], B. Schmidt[2], S. Waldeck[1], D. Overhoff[1]; [1]Koblenz/DE, [2]Forchheim/DE

**Purpose:** This study aims to assess a prototype hand unfolding reformation algorithm in polytrauma whole-body photon-counting CT (WB-PCCT) regarding time saving and image quality compared to a conventional bone reconstruction algorithm.
**Methods or Background:** We retrospectively analyzed 20 polytraumatized patients with bone injuries of the hand, who underwent polytrauma WB-PCCT, and reconstructed a total of 68 hands (matching 34 unfolded and 34 conventional (Br60 kernel) reconstructions). The hand unfolding reformation algorithm uses AI to automatically locate the hands in the WB-PCCT. After a deep learning-based detection of the course of the finger bones, the algorithm maps the original image to a predefined template using a deformation algorithm. This effectively unfolds each hand and enables visualization in the standard anatomical position. Quantitative image analysis was performed based on the calculation of the contrast-to-noise ratio (CNR). Two readers randomly assessed hand reconstructions with regard to reading time, subjective image quality (overall image quality, image noise perception, fracture delineation, fracture/joint dislocation) and diagnostic confidence using a 5-point Likert scale.
**Results or Findings:** Unfolded hand reformations significantly reduced ($p<0.001$) reading time for sufficient evaluation compared to conventional hand reconstructions by 57% (mean: 59 seconds versus 138 seconds). The novel reformation algorithm significantly improved CNR (28.18 versus 20.93; $p <0.001$). Subjective evaluation revealed comparably higher ratings for overall image quality, image noise perception, fracture and joint dislocation, and diagnostic confidence for unfolded hand reformations with equal ratings for fracture delineation.
**Conclusion:** The novel hand unfolding reformation algorithm for polytrauma WB-PCCT applied in this study led to 57% reduction in time required for hand assessment in time-critical polytrauma setting and improves both objective as well as subjective image quality compared to conventional bone reconstruction.
**Limitations:** Small sample size (n=20). Retrospective design.
**Funding for this study:** No funding.
**Has your study been approved by an ethics committee?** Yes
**Ethics committee - additional information:** The present study was approved by the Local Ethics Committee of the Chamber of Physicians Rhineland-Palatinate in Mainz, Germany, and conducted in accordance with the Declaration of Helsinki.
**Author Disclosures:**
Bernhard Schmidt: Employee: Siemens Healthineers AG
Daniel Overhoff: Nothing to disclose
Hanns-Leonhard Kaatsch: Nothing to disclose
Christian Bauer: Nothing to disclose
Stephan Waldeck: Nothing to disclose
Dominik Friedmann: Employee: Siemens Healthineers AG
Daniel Dillinger: Nothing to disclose

**Artificial Intelligence (AI) may not increase the sensitivity of chest radiographs in the emergency setting**
*E. C. Stamoulaki*, E. Detorakis, N. Christodoulides, D. Grigoropoulou, M. Klontzas, M. Raissaki; Heraklion/GR
*(xarastam.98@gmail.com)*

**Purpose:** To assess performance of chest radiography without and with AI assistance compared to CT for abnormality detection in the emergency setting.
**Methods or Background:** 150 patients (males 63,33%), aged 17-92 years (mean 72,5) underwent frontal PA or AP and lateral radiographs and CT within 10 days (mean 1 day). A junior radiologist assessed radiographs for lung, mediastinal and pleural/rib abnormalities, initially without AI assistance (reading 1), and following a three-week weaning interval with AI assistance (reading 2). CT findings were subsequently recorded by another junior and a senior radiologist, formulating ground truth. Lesions were marked as 0=not present, 1=present. Statistical analysis was performed using R programming language (v4.2.2 in RStudio for MacOS). Agreement between CT and each reading was assessed with weighted kappa statistics. Confusion matrices were used to calculate sensitivity, specificity, balanced accuracy, positive and negative predictive value. P<0.05 was statistically significant.
**Results or Findings:** Agreement between radiography and CT for presence of abnormalities ranged from slight to moderate (weighted k-values 0.18-0.67). The addition of AI did not increase the sensitivity of radiographs for any of the assessed abnormalities. The weighted kappa ranged from slight for mediastinal masses (k=0.17 without vs 0.17 with AI) to substantial for pleural effusion (k=0.67 without vs 0.69 with AI) (P<0.001). Both readings showed excellent specificity (range 87.2%-100%). Both readings had similar sensitivity which was low for lung and mediastinal masses, nodules and rib fractures (13.3%-33.9%), moderate for consolidation (46.7% without AI vs 54.8% with AI) and high for pleural effusion (80% for both readings).
**Conclusion:** In our small cohort, the addition of AI did not result in a statistically significant improvement of agreement between radiographic findings and CT.
**Limitations:** Small sample size.
**Funding for this study:** No funding was provided for this study.
**Has your study been approved by an ethics committee?** Yes
**Ethics committee - additional information:** Ethics committee of University General Hospital of Heraklion
**Author Disclosures:**
Michail Klontzas: Nothing to disclose
Dimitra Grigoropoulou: Nothing to disclose
Eleftheria Chara Stamoulaki: Nothing to disclose
Efstathios Detorakis: Nothing to disclose
Nikolaos Christodoulides: Nothing to disclose
Maria Raissaki: Nothing to disclose

**Can AI train radiologists to better assess chest radiographs in the emergency setting?**
*D. Grigoropoulou*, E. C. Stamoulaki, E. Detorakis, N. Christodoulides, M. Klontzas, M. Raissaki; Herakleion/GR

**Purpose:** To investigate the educational effect of artificial intelligence on junior radiologists who report chest radiographs.
**Methods or Background:** Two sets of 75 patients who presented in the emergency department underwent PA or AP chest radiography and lateral chest radiography on the same day and chest CT within 0-10 days. The first set were assessed by one junior resident radiologist (reader) for lung, mediastinal and pleural/rib abnormalities separately without and with AI assistance. The second set was subsequently assessed by the same reader, blinded to CT-findings, which were recorded by two additional radiologists in consensus. Lesions were marked as 0=absent, 1=potentially/definitely present. Statistical analysis was performed using R programming language (v4.2.2 in RStudio for MacOS). Agreement between CT and each reading was assessed with weighted kappa statistics. Confusion matrices were used to calculate sensitivity, specificity, balanced accuracy, positive and negative predictive value, which were compared between two sets. P<0.05 was statistically significant.
**Results or Findings:** Assessment of 1st set of radiographs without AI yielded a low to moderate agreement with CT, with a weighted kappa ranging between 0.108 (detection of mediastinal masses) and 0.529 (detection of pleural effusion). This agreement did not change with the assistance of AI, except for pleural effusion detection where agreement increased from 0.529 to 0.689. Assessment of the 2nd set without AI exhibited increased agreement with CT for nodules (0.131 before vs 0.478 after), lung (0.251 before vs 0.681 after) and mediastinal masses (0.108 before vs 0.520 after). Regarding sensitivity of radiographs for nodules, lung and mediastinal masses, it increased by 24-33%. Specificity was similar before and after the use of AI.
**Conclusion:** The addition of AI in chest radiography may increase sensitivity and may accelerate education of junior radiologists.
**Limitations:** Small sample size.
**Funding for this study:** No funding was provided for this study.
**Has your study been approved by an ethics committee?** Yes
**Ethics committee - additional information:** Ethics committee of University General Hospital of Heraklion
**Author Disclosures:**
Michail Klontzas: Nothing to disclose
Dimitra Grigoropoulou: Nothing to disclose
Eleftheria Chara Stamoulaki: Nothing to disclose
Efstathios Detorakis: Nothing to disclose
Nikolaos Christodoulides: Nothing to disclose
Maria Raissaki: Nothing to disclose

**14:00 - 15:30 Research Stage 3**

### Research Presentation Session: Cardiac

### RPS 1503

### AI and deep learning in cardiac imaging: artificial intelligence, motion correction and photon-counting optimisation

**Moderator**
Hildo J. Lamb; Leiden/NL
*(h.j.lamb@lumc.nl)*
**Author Disclosures:**
Hildo J. Lamb: Consultant: Royal Philips

**Deep Learning–Enhanced Post-Hoc Denoising Enables High-Quality Low-Dose Coronary CT Angiography in Obese Patients**
J. Osoria-Velasquez[1], *S. Hyska*[1], N. Fink[2], M. Vecsey-Nagy[3], T. S. Emrich[4], A. Varga-Szemes[1], M. T. Hagar[1]; [1]Charleston, SC/US, [2]Munich/DE, [3]Budapest/HU, [4]Mainz/DE

**Purpose:** To assess the incremental benefit of post-hoc convolutional neural network (CNN)–based denoising for coronary CT angiography (CCTA) performed at low radiation doses, compared with conventional iterative reconstruction.
**Methods or Background:** Consecutive patients who underwent clinically-indicated CCTA on a third-generation dual-source CT system were included. All examinations used a fast-pitch acquisition and a fully individualized contrast protocol comprising 56 tailored combinations of contrast volume and injection rate. Axial datasets were reconstructed with a vascular kernel (Bv40) using iterative reconstruction (Admire 3), with and without additional CNN-based denoising. Two blinded readers assessed signal-to-noise ratio (SNR), contrast-to-noise ratio (CNR), and image noise across proximal and distal coronary segments. Subjective image quality and sharpness were rated on a 4-point scale (4 = excellent). Linear mixed-effects models were used to analyze the impact of body mass index (BMI) on denoising performance.
**Results or Findings:** Fifty-six patients (51.7±12.8 years old; 57% women) were included, 35 (62.5%) were obese. The mean dose-length product was 99±86 mGy·cm. Compared with iterative reconstruction, CNN-denoising decreased image noise (21 ± 5 HU vs 35 ± 8 HU, p<0.001) and enhanced SNR (22 ±6 vs 12 ±3, p<0.001) and CNR (19 ±5 vs 11 ±3, p<0.001), without altering attenuation values (p>0.1). Subjective image quality improved (median 3 [3–4] vs 3 [2–3], p<0.001), while sharpness remained unchanged. Poor-quality segments decreased from 14.7% to 9.0%, and good-to-excellent quality rose from 52.6% to 75.0%. Noise reduction (−10.3 HU, p=0.007) and gains in SNR (+ 8.0, p=0.008) and CNR ( + 6.9, p=0.011) were consistent across BMI categories (p≥0.29).
**Conclusion:** Post-hoc CNN-based denoising enables high-quality, low-dose CCTA -even in obese patients- by markedly reducing image noise and improving SNR/CNR while preserving attenuation accuracy and vessel sharpness.
**Limitations:** The used denoising algorithm is proprietary.
**Funding for this study:** No funding was received for this study.
**Has your study been approved by an ethics committee?** Yes

**Ethics committee - additional information:** This retrospective study was IRB-approved.
**Author Disclosures:**
Muhammad Taha Hagar: Speaker: Siemens Healthineers
Sardi Hyska: Nothing to disclose
Milán Vecsey-Nagy: Nothing to disclose
Tilman Stephan Emrich: Speaker: Siemens Healthineers Research/Grant Support: Siemens Healthineers
Nicola Fink: Nothing to disclose
Jose Osoria-Velasquez: Nothing to disclose
Akos Varga-Szemes: Speaker: Siemens Healthineers Research/Grant Support: Siemens Healthineers Shareholder: Elucid Bioimaging

**Super-resolution deep learning reconstruction for coronary CT angiography：improved coronary stenosis assessment and CAD-RADS reclassification**
*L. Zou*; Beijing/CN
*(1026105497@qq.com)*

**Purpose:** A novel super-resolution deep learning reconstruction (SR-DLR) technique has been developed for coronary CT angiography (CCTA). The purpose of this study was to compare SR-DLR against conventional hybrid iterative reconstruction (HIR) in coronary stenosis assessment, using invasive coronary angiography (ICA) as a reference, and explore the possible impact on patient-level CAD-RADS classification.
**Methods or Background:** From September 2023 to November 2024, patients who underwent clinically indicated CCTA and ICA within 2-month interval were prospectively enrolled from 10 hospitals across China. CCTA images were reconstructed with both HIR and SR-DLR and percentage diameter stenosis of calcified, noncalcified, and mixed plaques were quantified and bias was determined from the ICA-derived quantitative coronary angiography measurements. Changes in patient-level CAD-RADS categories across the reconstructions were also assessed.
**Results or Findings:** 204 patients (mean age, 64.3 years ± 9.1 [SD]; 137 male patients) were included and 605 plaques (175 calcified, 140 noncalcified, and 290 mixed) were identified. Higher agreement with ICA was obtained with SR-DLR as compared to HIR across the plaque type(mean bias:11% vs. 17% for calcified plaques, 0% vs. -4% for noncalcified plaques, and 4% vs. 5% for mixed plaques). While 163 patients remained constant, 25 patients were assigned a lower CAD-RADS category using SR-DLR than assigned using HIR, whereas 16 patients were assigned a higher category.
**Conclusion:** SR-DLR outperformed HIR for coronary stenosis assessment and led to 20% patient-level CAD-RADS reclassification, potentially enhancing the role of CCTA in the diagnosis and patient management of CAD.
**Limitations:** Due to major differences in the image characteristics, observers may have been able to visually distinguish between the reconstruction methods. And further dedicated studies are warranted to investigate the possible impact of CAD-RADS reclassifications enabled by SR-DLR on downstream management and patient outcomes.
**Funding for this study:** This study was supported by the Beijing Natural Science Foundation [Grant No. Z210013, 2021], the National Science Fund for Distinguished Young Scholars [Grant No. 82325026, 2024], the CAMS Innovation Fund for Medical Science[2023-I2M-C&T-A-004], the Non-profit Central Research Institute Fund of Chinese Academy of Medical Sciences[2024-RC320-03] and the National High Level Hospital Clinical Research Funding [2022-PUMCH B-027, 2022-PUMCH B-068]
**Has your study been approved by an ethics committee?** Yes
**Ethics committee - additional information:** Peking union medical college hospital
**Author Disclosures:**
Limiao Zou: Nothing to disclose

**Development of a LASSO-Cox Model for Predicting In-Stent Restenosis After Percutaneous Coronary Intervention**
*J. Ma*, M. Jing, H. Zhang, X. Ming; Lanzhou/CN
*(17339767506@163.com)*

**Purpose:** To construct and validate a predictive model for in-stent restenosis (ISR) following percutaneous coronary intervention (PCI) .
**Methods or Background:** This retrospective study enrolled patients who underwent coronary computed tomography angiography (CCTA) within one week prior to PCI and follow-up CCTA or invasive coronary angiography (ICA) post-PCI. Fat attenuation index (FAI) was measured at the target lesion(FAIp), both proximally and distally(FAIpp). CT-derived fractional flow reserve (CT-FFR) at the lesion and the translesional gradient ($\Delta$CT-FFR) were computed. The LASSO was used to screen for factors influencing ISR occurrence. These factors were incorporated into a multivariate Cox regression analysis to construct a nomogram model.
**Results or Findings:** CCTA-derived FAIp (HR=1.090, 95% CI 1.051–1.131, P < 0.001) and $\Delta$CT-FFR (HR=5.335, 95% CI: 1.085-26.229, P < 0.001) were independent risk factors for ISR. The constructed nomogram model achieved a C-index of 0.869 (95% CI: 0.797-0.941), with good calibration curve fit. The clinical decision curve demonstrated high clinical utility of the model.
**Conclusion:** The integrated model combining FAIp and $\Delta$CT-FFR based on CCTA enables early prediction of ISR after PCI.
**Limitations:** First, as a single-center retrospective study, it is potentially subject to selection bias. The extended follow-up period also contributed to a relatively small sample size. Despite this, the study yielded favorable outcomes. Future investigations will incorporate larger cohorts to enhance model stability and include prospective validation. Second, the use of a fixed tube voltage may limit generalizability; future work should explore other voltage levels. It is noteworthy, however, that consistent scanning equipment and parameters eliminated their potential confounding effects on FAI measurements. Finally, semi-automated quantification of FAI and CT-FFR (using Shukun software) may introduce some bias, although all results were reviewed and corrected by physicians, thereby ensuring a reasonable level of reliability.
**Funding for this study:** This work was supported by the Gansu Provincial Health Industry Research Program for Outstanding and Key Young Talents [Grant No. GSWSQN2023-04], the "Cuiying Graduate Supervisor" Mentorship Program of the Second Hospital of Lanzhou University [Grant No. CYDSPY202003], and the Natural Science Foundation of Gansu Province [Grant No. 23JRRA0997].
**Has your study been approved by an ethics committee?** Yes
**Ethics committee - additional information:** This study complied with the Declaration of Helsinki and was approved by the Institutional Ethics Committee (Approval No. 2025A-703).
**Author Disclosures:**
Xiaojing Ming: Other: Collection and assembly of data
Jinxia Ma: Author: Conception and design Speaker: Manuscript writing
Hongyu Zhang: Other: Provision of study materials or patients
Mengyuan Jing: Other: Provision of study materials or patients Other: Provision of study materials or patients Investigator: none

**Impact of DLR-based whole heart Motion Correction on inter and intra reader reproducibility of pre-TAVR CT measurements**
G. Coutat[1], F. Tatsugami[2], D. Touitou-Gottenberg[1], W. Fukumoto[2], T. Higaki[2], A. Taniguchi[2], K. Haioun[2], Y. Nakamura[2], *M. Ohana*[1]; [1]Strasbourg/FR, [2]Hiroshima/JP
*(mickael.ohana@chru-strasbourg.fr)*

**Purpose:** Reproducibility of aortic annulus sizing and aortic valve opening area planimetry on pre-TAVR cardiac CT is crucial for procedural planning. Whether a DLR-based whole heart Motion Correction algorithm (MC-DLR) combined with Super Resolution Deep Learning Reconstruction (SR-DLR) could affect inter and intra reader reproducibility of these measurements, particularly in heavily calcified aortic cusps, remains unknown. This study aimed to compare inter and intra reader reproducibility of aortic annulus and aortic valve opening planimetry measurements on CT reconstructions without and with MC-DLR.
**Methods or Background:** 60 consecutive pre-TAVR CT scans stratified by heart rate (30 with HR<75bpm, 30 with HR>75) were retrospectively selected from 2 tertiary centers. Systolic phase was reconstructed with SR-DLR (1024 matrix size) without and with MC-DLR. 4 radiologists independently and randomly reviewed all 120 datasets to assess qualitative image quality, aortic annulus area and aortic valve planimetry. 2 readers repeated all measurements following a 4 weeks delay. Statistical analysis was performed using Bland-Altman plots and intraclass correlation coefficient (ICC).
**Results or Findings:** Image quality was higher with MC-DLR (mean 2.82/3 vs 2.67, p=0.07) Inter-reader agreement for aortic annulus area was excellent and similar without (ICC 0.81, 95% CI 0.78-0.83) and with MC-DLR (ICC 0.80, 95% CI 0.77-0.82). Inter-reader agreement for aortic valve planimetry was higher with MC-DLR (ICC 0.90, 95% CI 0.86-0.92) than without (ICC 0.83, 95% CI 0.80-0.85). Intra-reader agreement for both measurements was marginally improved with MC-DLR.
**Conclusion:** DLR-based whole heart MC may improve the reproducibility of aortic valve area planimetry in systolic pre-TAVR CT.
**Limitations:** Potential clinical implications of MC-DLR on device selection were not analyzed in this study.
**Funding for this study:** No funding was received for this study.
**Has your study been approved by an ethics committee?** Yes
**Ethics committee - additional information:** Approved by the ethics committee of Strasbourg University Hospital

**Author Disclosures:**
Diane Touitou-Gottenberg: Nothing to disclose
Guillaume Coutat: Nothing to disclose
Wataru Fukumoto: Nothing to disclose
Karim Haioun: Employee: Canon Medical Systems Japan
Fuminari Tatsugami: Nothing to disclose
Toru Higaki: Nothing to disclose
Yuko Nakamura: Nothing to disclose
Akira Taniguchi: Nothing to disclose
Mickaël Ohana: Speaker: Canon Medical Systems Europe

**Assessment of coronary artery stenosis using virtual non-calcification in photon-counting detector CT**

*H. Shih*[1], M-C. Liu[1], S-W. Chan[1], W-H. Chen[1], J-H. Chen[2]; [1]Taichung/TW, [2]Kaohsiung/TW
*(timeandust@gmail.com)*

**Purpose:** Blooming artifacts from calcified plaques may lead to overestimation of coronary arteries stenosis (OCAS) using virtual monochromatic imaging (VMI). The aim of this study was to test the ability of virtual non-calcification (VNC) of photon-counting detector CT (PCD-CT) to reduce OCAS in VMI.
**Methods or Background:** Retrospective analysis of subjects underwent coronary CT angiography from 2025 Jan. to Mar. was performed. Images acquired by PCD-CT were reconstructed by VMI and VNC. Percentage diameter stenosis (PDS) was compared between the two algorithms. Subgroup analyses by stenosis extent, CAD-RADS and calcium score were also performed.
**Results or Findings:** 344 subjects were initially included. 180 subjects were excluded, 159 due to zero calcium scores, and 21 with stent history. Totally, 164 subjects with 562 plaques were studied. VNC failed to remove calcium in 124 plaques (22.1%). Most of these plaques had density < 1000 HU (average density 887 HU). In 27 dense plaques, VNC erroneously removed intravascular contrast medium. PDS significantly decreased in VNC compared to VMI (VMI: 18.9 ± 16.7; VNC: 8.7 ± 10.5. P<0.01). VNC also showed significant decrease of PDS in three subgroup analyses (all Ps<0.01). For example, subjects with PDS> 50 in VMI decreased from 58.7 ±8.3 in VMI to 26.1 ±13.2 in VNC. Further, VMI may show false positive findings. A 67-year-old male subject showed moderate to severe stenosis (>50%) in VMI. While invasive angiography and VNC images showed no significant stenosis. Overall, subjects with more severe calcified plagues showed more prominent overestimation of stenosis in VMI images.
**Conclusion:** Our preliminary results showed the benefit of VNC to reduce the overestimation of stenosis evaluated by VMI. VNC may potentially reduce the unnecessary invasive coronary angiography.
**Limitations:** Our study lacked invasive angiography for most of the cases.
**Funding for this study:** No funding was provided for this study.
**Has your study been approved by an ethics committee?** Not applicable
**Author Disclosures:**
Ming-Cheng Liu: Nothing to disclose
Hao Shih: Nothing to disclose
Si-Wa Chan: Nothing to disclose
Jeon-Hor Chen: Nothing to disclose
Wen-Hsien Chen: Nothing to disclose

**Feature-tracking CT assessment of LV global longitudinal strain in TAVI candidates: prospective comparison with echocardiography**

*M. J. S. Irding*; Jönköping/SE
*(irding.markus@gmail.com)*

**Purpose:** In patients referred for TAVI prospectively evaluate the determination of left ventricular ejection fraction (LVEF) and global longitudinal strain (GLS) measured by feature-tracking multi-detector CT (MDCT) versus transthoracic echocardiography (TTE).
**Methods or Background:** 77 consecutive TAVI candidates (mean age 80 ± 7 years; 54% women) underwent clinical TTE and multiphase MDCT. LVEF and GLS were analysed using commercially available software.
**Results or Findings:** Mean GLS −13.9 ± 4.2% (TTE) and −11.2 ± 4.1% (MDCT) where MDCT underestimated GLS versus TTE (mean bias 2.7 percentage points; p < 0.001). Moderate correlations were observed for LVEF (r = 0.603, p < 0.001) and GLS (r = 0.452, p < 0.001); correlations improved in a subgroup excluding 12 examinations with compromised image quality (LVEF r = 0.688; GLS r = 0.622). Reproducibility of MDCT measurements was high (intra-reader ICC for LVEF 0.90 and GLS 1.00; inter-reader ICC for LVEF 0.67 and GLS 0.96).
**Conclusion:** Our findings indicate that cardiac MDCT is a feasible and repeatable method for assessing LVEF and GLS in TAVI candidates. However, MDCT shows a tendency to underestimate GLS compared to TTE, warranting the establishment of modality-specific threshold values. Stricter quality criteria yield a higher agreement, suggesting that diagnostic modality selection should consider patient-specific factors. Future research should explore the integration of MDCT-derived GLS in clinical practice, particularly in cases where TTE quality is compromised.
**Limitations:** The main limitations include the variable time interval between echocardiography and MDCT and the relatively small sample size for reproducibility testing. Despite these, the study's prospective design and low intra- and inter-reader variability support the reliability and clinical relevance of our findings.
**Funding for this study:** Funding for this study was generously provided by Futurum, The Academy for Healthcare Region Jönköping County, and FORSS, the Medical Research Council of Southeast Sweden.
**Has your study been approved by an ethics committee?** Yes
**Ethics committee - additional information:** It has been granted authorization from the Swedish ethical review authority.
**Author Disclosures:**
Markus Jean Staffan Irding: Nothing to disclose

**High-Z Contrast Media for Coronary Photon Counting Detector CT Angiography: Improved Quantification of Calcified Stenoses**

*T. T. Demmert*[1], K. Klambauer[1], B. Schmidt[2], V. Mergen[1], L. J. Moser[1], T. Allmendinger[2], T. Flohr[2], M. Eberhard[1], H. Alkadhi[1]; [1]Zürich/CH, [2]Forchheim/DE
*(tristan.demmert@usz.ch)*

**Purpose:** Blooming artifacts from calcified plaques obscure the vessel lumen and cause stenosis overestimation. Spectral coronary angiography with photon-counting CT (PCD-CT) provides virtual monoenergetic images (VMI). While higher VMI energies reduce blooming, iodine contrast is diminished. This study evaluated whether contrast agents with higher atomic numbers (high-Z) preserve vascular contrast at high energies and improve stenosis quantification.
**Methods or Background:** A phantom with 4 mm and 6 mm rods mimicking vessels containing eccentric calcified plaques (25%, 50%, 75% stenoses) was scanned with a dual-source PCD-CT. Five contrast agents (Iodine, Tungsten, Holmium, Hafnium, Bismuth) were tested. VMI were reconstructed from 40–190 keV in 1 keV increments. Vessel attenuation, contrast-to-noise ratio (CNR), and stenoses were measured. Image quality was qualitatively assessed.
**Results or Findings:** Iodine attenuation was high at low energies but dropped below 250 HU at >100 keV. Tungsten, Holmium, Hafnium, and Bismuth maintained >250 HU across the range. Iodine CNR was high at low but decreased at high energies, similar to Holmium and Bismuth. Tungsten and Hafnium showed lower CNR at low energies but stable at high keV; Tungsten rose to ~40. Stenoses were overestimated at low energies (24–32.5% at 40 keV) but decreased at high energies (0–13.5% at 190 keV). At 190 keV, Tungsten, Hafnium, and Bismuth showed ≤2.5% overestimation versus Iodine (10–13.5%). Image quality varied: very high-Z agents achieved highest scores, iodine peaked at 55–70 keV but performed worst overall at high energies.
**Conclusion:** Compared with iodine, very high-Z agents enable superior lumen definition and more accurate stenosis assessment at high VMI energies, minimizing calcium blooming.
**Limitations:** We used a static coronary phantom with uniform vessel diameters and calcified stenoses, not reflecting in vivo complexity. Patient factors including motion, tortuosity, and heterogeneous plaque morphology were not considered.
**Funding for this study:** There was no funding.
**Has your study been approved by an ethics committee?** Not applicable
**Author Disclosures:**
Thomas Flohr: Nothing to disclose
Tristan Thorben Demmert: Nothing to disclose
Bernhard Schmidt: Nothing to disclose
Victor Mergen: Nothing to disclose
Matthias Eberhard: Nothing to disclose
Lukas Jakob Moser: Nothing to disclose
Thomas Allmendinger: Nothing to disclose
Konstantin Klambauer: Nothing to disclose
Hatem Alkadhi: Nothing to disclose

**A practical rule-of-thumb to adapt the contrast media dose in photon-counting detector CT: The 10-to-5-rule**

*C. R. Jeukens*, B. Martens, J. Vandewall, S. Jasper, G. M. Schrijnemaekers, J. E. Wildberger, T. Flohr; Maastricht/NL
*(cecile.jeukens@mumc.nl)*

**Purpose:** It is recognized practice to reduce the contrast media (CM) dose in CT as much as clinically feasible. For conventional CT a practical 10-to-10-rule indicates that a 10% lower CM dose can be used for each 10 kV reduction in tube voltage, while maintaining constant iodine enhancement. This study aims to develop a practical rule how to reduce CM dose in photon-counting detector CT (PCD-CT) when lowering the energy of the reconstructed virtual mono-energetic images (VMI) for parenchymal CT and CTA.

**Methods or Background:** Spectral abdominal and chest CT phantoms, containing a range of iodine concentrations and ICRU muscle tissue, were scanned using a portal venous phase (PVP) abdominal and a high-pitch CTA protocol on a dual-source PCD-CT. Two fat equivalent rings were used to mimic different patient sizes. Iodine Contrast-to-Noise Ratio (CNR) was measured in VMIs at energies from 40 to 60 keV in 5 keV steps. In 15 abdominal and 15 CTA patient scans (body-mass-index 17-37 kg/m2), the CNR at different VMI energies was retrospectively determined.
**Results or Findings:** Each 5 keV reduction in the VMI energy range 60-40 keV maintained a similar CNR when reducing the iodine concentration by 11.7-14.4% for PVP scans and 11.8-14.5% for CTAs. Patient scan analysis showed that each 5 keV reduction resulted in a mean 11.4% and 13.7% increase in CNR for PVP and CTA scans. This can be translated to a corresponding reduction in CM dose at constant CNR. A robust, simple 10-to-5-rule was derived: for each 5 keV reduction of VMI energy, the CNR can be maintained with about 10% less CM dose.
**Conclusion:** Based on this phantom and retrospective proof-of-principle patient study, a practical 10-to-5-rule was derived for contrast-enhanced PCD-CT.
**Limitations:** Validation in larger patient study needed.
**Funding for this study:** N/A
**Has your study been approved by an ethics committee?** Yes
**Ethics committee - additional information:** The proof-of-principle patient study received a waiver of written informed consent from the local ethical committee and institutional review board (METC 2024-0471), due to its retrospective nature.
**Author Disclosures:**
Thomas Flohr: Employee: employee at Siemens Healthineers
Joachim E. Wildberger: Grant Recipient: reports institutional research grants from Abbott, Anaconda Biomed, Asklepios, Bayer, Becton & Dickinson Medical, Bentley, Boston, Brainlab, GE Healthcare, Gleamer, Hologic, Inari Medical, Johnson & Johnson, LCRB, Medtronic, Merit Medical Systems, Microvention, Nico-Lab, Nova Techs, Oldelft Benelux, Ontario Association of Radiologists, Penumbra, Philips, Screenpoint Medical, Siemens, Stryker, Tajpan Sro, all outside the submitted work. Speaker: Bayer and Siemens, all outside the submitted work.
Bibi Martens: Speaker: receives personal fees (speakers bureau) from Bayer outside the submitted work
Janneke Vandewall: Nothing to disclose
Cécile Rlpn Jeukens: Nothing to disclose
Sören Jasper: Employee: employee at Siemens Healthineers
Guy M.M. Schrijnemaekers: Nothing to disclose

### Breaking the heart rate barrier: coronary CT angiography derived from stress dynamic myocardial perfusion on dual wide-coverage CT in patients with heart rate >90 bpm

K. Hou, L. Peng, J. Shen, *S. Xu*, M. Zeng; Shanghai/CN
*(shijie.xu@united-imaging.com)*

**Purpose:** To report the initial experience and evaluate the clinical feasibility of acquiring stress perfusion-derived coronary CT angiography (CCTA) on a newly introduced dual wide-coverage CT, in patients with heart rate >90 bpm.
**Methods or Background:** This retrospective study included 35 patients with heart rate >90 bpm who underwent both stress dynamic myocardial CT perfusion (CTP) and CCTA within one examination on a dual wide-coverage CT. CTP was followed by a routine rest CCTA within a 10-minute interval, using two separate contrast medium injections. Perfusion-derived CCTA was obtained by manually selecting the peak arterial phase from the dynamic perfusion dataset. Two radiologists jointly evaluated the presence of ≥50% stenosis on both perfusion-derived CCTA and routine CCTA. The diagnostic performance was compared between the two CCTA datasets on a per-segment and per-vessel basis, using invasive coronary angiography as the reference standard. The overall image quality was evaluated using a 5-point scale (1=poor, 5=excellent).
**Results or Findings:** The mean heart rates during CTP and routine CCTA acquisitions were 102.3 ± 10.3 bpm and 78.9 ± 7.8 bpm, respectively. There was no significant difference in sensitivity, specificity, and accuracy between two CCTA datasets for diagnosing ≥50% stenosis in both per-segment (perfusion-derived CCTA: 92.0%, 96.4% and 95.7% vs. routine CCTA: 93.3%, 97.0% and 96.5%; all p>0.05) and per-vessel analyses (perfusion-derived CCTA: 96.1%, 95.5% and 95.7% vs. routine CCTA: 98.0%, 95.5% and 96.4%; all p>0.05). No significant difference was found in overall image quality between two CCTA datasets (perfusion-derived CCTA: 4.5±0.5 vs. routine CCTA: 4.6±0.5, p=0.508).
**Conclusion:** It is feasible to obtain stress perfusion-derived CCTA in patients with heart rate >90 bpm using dual wide-coverage CT, where the diagnostic performance of perfusion-derived CCTA was comparable to that of routine rest CCTA.
**Limitations:** N/A
**Funding for this study:** N/A
**Has your study been approved by an ethics committee?** Yes
**Ethics committee - additional information:** This study was approved by the local IRB
**Author Disclosures:**
Mengsu Zeng: Nothing to disclose
Junjian Shen: Nothing to disclose
Kai Hou: Nothing to disclose
Shijie Xu: Nothing to disclose
Liying Peng: Nothing to disclose

### CT-based epicardial adipose tissue change as a predictor of obstructive coronary artery disease progression

*W. Zhang*, Q. Zhou, X. Xu; Hangzhou, China/CN
*(3190105675@zju.edu.cn)*

**Purpose:** To evaluate the predictive value of CT-based longitudinal changes in EAT for obstructive CAD progression.
**Methods or Background:** We included 583 suspected CAD patients underwent coronary computed tomography angiography (CCTA) between 01.06.2010 and 30.06.2017, and received follow-up CCTA at an interval of ≥ two years . 107 patients progressed to obstructive CAD. EAT and coronary artery calcium score (CACS) were measured on non-contrast CCTA. Annualized EAT volume change was calculated as the difference between two scans divided by the follow-up duration. Obstructive CAD progression was defined as new-onset ≥50% stenosis in initially non-obstructive patients. The association of EAT with obstructive CAD progression was evaluated using univariate and multivariate logistic regression.
**Results or Findings:** Annualized EAT volume change was 3.47 $cm^3$ (IQR, 0.33 to 6.73 $cm^3$) in the obstructive progression group and 2.07 $cm^3$ (IQR,–1.16 to 4.97 $cm^3$) in the no obstructive progression group (P = 0.006). In univariate analysis, only age, CACS, baseline largest luminal diameter stenosis, and annualized EAT volume change were significantly associated with obstructive CAD progression. In multivariate analysis, after including variables significant in univariate analysis, annualized EAT volume change remained an independent predictor of obstructive CAD progression (adjusted OR 1.06, P = 0.007).
**Conclusion:** The annualized EAT volume change is an independent predictor of obstructive CAD progression, suggesting that dynamic EAT monitoring on routine CT scans may improve risk stratification for obstructive CAD.
**Limitations:** First, this was a retrospective study and used single-center data. Although patients were included consecutively, selection bias could not be avoided. Second, there were differences in the scan interval, with a median of 3.33 years (IQR, 2.67 to 4.33 years). Therefore, the annualized changes may to some extent adjust the potential differences induced by the various follow-up intervals.
**Funding for this study:** No funding was received for this study.
**Has your study been approved by an ethics committee?** Yes
**Ethics committee - additional information:** The study was conducted in accordance with the Declaration of Helsinki and approved by the Medical Ethics Committee of the Second Affiliated Hospital, Zhejiang University School of Medicine (No. 2024-0566). In our study, an informed consent waiver was obtained from the Clinical Research Ethics Committee.
**Author Disclosures:**
Wenzhuo Zhang: Nothing to disclose
Xiaojun Xu: Nothing to disclose
Qijing Zhou: Nothing to disclose

### A Scanner-Integrated Decision Support Solution for Optimized Protocol Selection in Coronary Angiography with Photon-Counting CT

*C. Schuppert*[1], S. Barus[1], M. Soschynski[1], T. Allmendinger[2], F. Bamberg[1], T. Krauß[1], C. L. Schlett[1]; [1]Freiburg/DE, [2]Forchheim/DE

**Purpose:** Photon-counting CT (PCCT) introduces novel scan modes that assist coronary CT angiography (CCTA) but increase protocol complexity. We developed and evaluated a scanner-integrated decision support solution to optimize protocol selection.
**Methods or Background:** In this prospective, two-center study, adult patients referred for CCTA were scanned on identical dual-source PCCT systems (NAEOTOM Alpha, Siemens Healthineers) with standard preparation. One site used the decision support solution (myExamCompanion, Siemens Healthineers), whereas the control site relied on conventional operator-driven protocol selection. Patient flow was not controlled. The decision support solution considered pre- and intra-scan input parameters reflecting coronary artery disease risk and cardiac dynamics (age, BMI, stents, Agatston score, heart rate, heart rate variability). Guided by the principle of "minimized exposure, full diagnostic performance", it automatically selected from Quantum (70/90 kVp), Quantum Plus (120/140 kVp), Ultra High-Resolution (UHR), and Spectral UHR acquisition modes, as well as ECG-gating strategies (high-pitch spiral, prospective sequence, retrospective spiral). Evaluation focused on radiation dose, diagnostic confidence, and image quality, as measured by coronary signal-to-noise ratio (SNR) in a low-risk subset.

**Results or Findings:** A total of 1,304 patients underwent CCTA, with 727 (56%) scanned using the decision support solution. Baseline patient characteristics were comparable between groups. Use of the decision support solution was associated with lower dose length product (median 269 vs. 370 mGycm), mainly through greater Quantum mode utilization (34% vs. 11%). SNR was slightly lower in Quantum vs. Quantum Plus mode (median 15.3 vs. 17.7, p=0.07). The incidence of CAD-RADS≥3 was 21% vs. 29% with vs. without the solution, while CAD-RADS N was <1% in both groups.
**Conclusion:** The decision support solution optimizes PCCT protocol selection for CCTA, preserving image quality and interpretability while lowering radiation dose.
**Limitations:** Single-vendor, two-center study with site-based, non-randomized allocation.
**Funding for this study:** No funding was provided for this study.
**Has your study been approved by an ethics committee?** Yes
**Ethics committee - additional information:** Ethics Committee of the Medical Center – University of Freiburg (No. 21-1469, approved on September 21, 2021, and amendments).
**Author Disclosures:**
Martin Soschynski: Nothing to disclose
Tobias Krauß: Nothing to disclose
Christopher L. Schlett: Speaker: Bayer Healthcare Speaker: Siemens Healthineers
Thomas Allmendinger: Employee: Siemens Healthineers
Fabian Bamberg: Speaker: Bayer Healthcare Grant Recipient: Siemens Healthineers Speaker: Siemens Healthineers
Christopher Schuppert: Nothing to disclose
Sinan Barus: Nothing to disclose

**Coronary stent assessment: a comparison between photon counting CT and energy integrating detector CT**
*G. Gambato*, D. Vignale, A. Palmisano, A. Esposito; Milano/IT
*(gambato.gioele@hsr.it)*

**Purpose:** To compare photon-counting CT (PCCT) and conventional energy-integrating detector CT (EID-CT) in the in-vivo evaluation of coronary artery stents.
**Methods or Background:** This retrospective study was conducted in patients with coronary stents undergoing cardiac PCCT between May 2024 and September 2025 (NAEOTOM Alpha, Siemens; slice thickness 0.4 mm; kernels: Bv56, n=26; Br56, n=3; Qr56, n=1; Bv48, n=2; Br40, n=1), who had undergone a prior examination with EID-CT (SOMATOM Definition FLASH, Siemens; 120 keV, I36f kernel). Stent models were known. Images were evaluated according to qualitative parameters (Overall Image Quality, Sharpness, Noise, Blooming, and Diagnostic Confidence) by a single rater with three years of experience in cardiovascular radiology, using a five-point Likert scale. Blooming was quantified by comparing nominal stent diameter (median 3.00[2.75,3.50] mm) with measured stent diameters and by assessing intra- and extra-stent attenuation. The Wilcoxon signed-rank test was used for comparisons.
**Results or Findings:** Sixty stents in 33 patients (M:F 32:1, 80.5 [IQR 72.5,84.0] years) were analyzed. PCCT yielded higher scores than EID-CT across all qualitative parameters (p<0.001). Median improvements were +1 [IQR 0–2] for image quality, +1 [IQR 0–2] for sharpness, +1 [IQR 0–2] for noise, +2 [IQR 1–2] for blooming, and +1[IQR 0–1] for diagnostic confidence. All quantitative comparisons were significant (p<0.001). PCCT measurements were closer to the nominal stent diameter (median difference -0.77[IQR -1.10,-0.54]mm) than EID-CT (-1.05[IQR -1.40,-0.75]mm, and PCCT yielded larger in-stent luminal diameters than EID-CT (median difference +0.30[IQR 0.00,0.50]mm). Moreover, PCCT showed less attenuation difference between intra- and extra-stent measurements compared to EID-CT (median 139 [IQR 37–213] vs 236 [IQR 158–312] HU), both indicating a reduction of blooming artifacts.
**Conclusion:** Compared to EID-CT, PCCT improves the in-vivo evaluation of coronary stents by reducing blooming.
**Limitations:** Small sample.
**Funding for this study:** None.
**Has your study been approved by an ethics committee?** Yes
**Ethics committee - additional information:** Ospedale San Raffaele (CTMyoC)
**Author Disclosures:**
Davide Vignale: Nothing to disclose
Antonio Esposito: Nothing to disclose
Anna Palmisano: Nothing to disclose
Gioele Gambato: Nothing to disclose

**14:00 - 15:30** **Research Stage 1**

### Research Presentation Session: Artificial Intelligence and Imaging Informatics

### RPS 1505

### Mining the image: AI-driven diagnosis and prognosis in abdominal imaging

**Moderator**
Ludovica Lofino; Milan/IT
*(ludovica.lofino@humanitas.it)*

**Automated CT Body Composition Analysis for Perioperative Risk Stratification**
*J. Kohnke*, K. A. Borys, C. Bojahr, Y. Wen, S. Warmer, C. S. Schmidt, F. Nensa, R. Hosch; Essen/DE

**Purpose:** To investigate whether body composition parameters derived from CT scans are associated with 90-day survival in patients undergoing surgery within 14 days before or after imaging.
**Methods or Background:** Body composition has emerged as a potential predictor of perioperative risk. Sarcopenia, myosteatosis, and cachexia may indicate vulnerability to poor postoperative outcomes. Thorax and Abdominal CT scans of 1575 cancer patients (844 female, 731 male) who underwent surgery within ±14 days of imaging were retrospectively analyzed, and an automated in-house body composition analysis was performed. Two groups were classified based on 90-day survival (1333 survived, 242 deceased). Differences in sarcopenia, myosteatosis, and cachexia (abdomen and thorax) between survivors and non-survivors were assessed using the Mann–Whitney U test. Correlations between body composition parameters and survival were examined using Spearman's rank correlation.
**Results or Findings:** Mann–Whitney U test revealed significant differences between 90-day survivors and non-survivors for sarcopenia (abdomen: p<0.001; thorax: p<0.001) and myosteatosis (abdomen: p<0.001; thorax: p<0.001), but not for cachexia (abdomen: p=0.203; thorax: p=0.072). Spearman correlation analysis showed positive correlations of sarcopenia with mortality (abdomen: r=0.283, p<0.001; thorax: r=0.295, p<0.001), negative correlations of myosteatosis with mortality (abdomen: ρ=-r.331, p<0.001; thorax: r=-0.273, p<0.001), and no significant correlations for cachexia (abdomen: r=0.009, p=0.728; thorax: r=0.025, p=0.314).
**Conclusion:** Sarcopenia and myosteatosis are significantly associated with 90-day postoperative survival, suggesting that automated CT-based body composition analysis provides valuable prognostic information to aid perioperative risk stratification.
**Limitations:** This is a single-center study. The inclusion was limited to cancer patients, and potential confounding factors were not fully accounted for.
**Funding for this study:** No funding was provided for this study.
**Has your study been approved by an ethics committee?** Yes
**Ethics committee - additional information:** Informed consent was waived by the ethics committee due to the retrospective setting.
**Author Disclosures:**
Christian Bojahr: Nothing to disclose
Sina Warmer: Nothing to disclose
Katarzyna Anna Borys: Nothing to disclose
Judith Kohnke: Nothing to disclose
Yutong Wen: Nothing to disclose
Cynthia Sabrina Schmidt: Nothing to disclose
René Hosch: Nothing to disclose
Felix Nensa: Nothing to disclose

**LymphoGenAI: A regulatory-compliant, interoperable AI platform for multimodal lymph node staging in oncology**
*R. E. Pacios Blanco*, J. Vega, C. Martínez Cabezali, L. A. Herrera Galvez; Madrid/ES
*(eduardo.pacios@gmail.com)*

**Purpose:** Lymph node status is a critical determinant of prognosis and treatment planning in oncology. Current diagnostic pathways rely on invasive procedures and fragmented data interpretation, creating variability and delays. LymphoGenAI addresses this gap by developing a multimodal, generative AI-based decision support system (AI-DSS) for predicting lymph node involvement in breast and head & neck cancers, fully aligned with European digital health and AI regulatory frameworks.

**Methods or Background:** The consortium platform integrates radiological imaging (CT, MRI, PET), digital pathology, structured clinical data and unstructured reports into a harmonized, GDPR-compliant dataset across multiple European hospitals. Built on interoperability standards (DICOM, HL7 FHIR, SNOMED), the system employs advanced vision–language architectures and radiomics pipelines to deliver explainable predictions. Development follows EU Medical Device Regulation (MDR 2017/745) and incorporates transparency, bias monitoring and human oversight in line with the EU AI Act. Clinical validation will be conducted through retrospective and prospective multicenter studies.
**Results or Findings:** The project is expected to deliver a CE-mark-ready AI-DSS that enables accurate, non-invasive lymph node staging, reducing the need for unnecessary biopsies and surgical procedures. By integrating multimodal data and explainable outputs, the system will improve diagnostic consistency across institutions and accelerate time-to-treatment. Its modular, interoperable architecture will allow seamless integration into diverse hospital IT environments and ensure compliance with the European Health Data Space framework. Additionally, it will provide a long-term resource for research in oncology AI.
**Conclusion:** By embedding regulatory compliance, interoperability and explainability from inception, LymphoGenAI sets a benchmark for trustworthy AI in oncology. Its modular design supports scalability to other cancer types, reinforcing Europe's leadership in ethical, human-centric AI for health.
**Limitations:** Compliance with evolving regulatory frameworks such as GDPR, MDR and the EU AI Act introduces complexity and may delay deployment.
**Funding for this study:** Presented to the Horizon Europe Programme – HLTH-2025-01-CARE-01, GenAI4EU.
**Has your study been approved by an ethics committee?** Not applicable
**Author Disclosures:**
Ruben Eduardo Pacios Blanco: Nothing to disclose
Lydia Alba Herrera Galvez: Nothing to disclose
Celia Martínez Cabezali: Nothing to disclose
Juan Vega: Nothing to disclose

### Reproducibility of Manual, Semi-automated, and Fully Automated L3 CT Body-Composition Metrics in Metastatic Renal Cell Carcinoma

*F. Farioli*, L. Casarini, F. Fiocchi, M. Dominici, R. Sabbatini, G. Ligabue, P. Torricelli, C. Baldessari; Modena/IT
*(farioli.francesco.96@gmail.com)*

**Purpose:** To compare the reproducibility of three CT-based body-composition workflows—manual HU thresholding, semi-automated assisted thresholding, and fully automated open-source segmentation—for skeletal muscle and adipose compartments at L3 in patients with metastatic renal cell carcinoma (mRCC).
**Methods or Background:** We retrospectively included 68 patients with mRCC who underwent non-contrast abdominal CT at diagnosis. At the L3 level, cross-sectional area and mean attenuation (Hounsfield units, HU) were obtained for skeletal muscle (MT), subcutaneous adipose tissue (SAT), visceral adipose tissue (VAT), and intramuscular adipose tissue (IMAT). The manual reference applied fixed HU thresholds: adipose −190 to −30 HU; muscle −29 to +150 HU. Semi-automated software generated threshold suggestions with manual slice selection and contour adjustment (areas only). A fully automated open-source pipeline yielded areas and densities for all compartments. Agreement was assessed using two-way mixed-effects ICC(3,1) with 95% confidence intervals; density ICCs were computed only for manual vs automated methods (the semi-automated workflow lacked densities).
**Results or Findings:** Area agreement was excellent for SAT (ICC 0.944; 95% CI 0.918–0.963) and VAT (0.945; 0.920–0.964), and good for muscle (0.847; 0.783–0.897). For densities, reproducibility was moderate for muscle (0.574; 0.391–0.714) and SAT (0.811; 0.710–0.879), but poor for VAT (0.328; 0.099–0.524). IMAT showed low agreement for area (0.319; 0.089–0.517) and density (0.103; −0.138–0.331).
**Conclusion:** Across methods, SAT area, VAT area, and muscle area show high reproducibility, supporting interchangeability in research pipelines and potential clinical use. Density metrics are less robust—particularly for VAT and IMAT—and may require manual verification or method-specific calibration (including explicit threshold specifications) in semi-automated and fully automated pipelines. IMAT remains challenging for automated quantification and warrants algorithmic refinement.
**Limitations:** Single-center retrospective design; non-contrast CT; single-slice L3; semi-automated workflow lacked densities; modest sample; variability in slice selection and software parameters; no outcomes correlation.
**Funding for this study:** None
**Has your study been approved by an ethics committee?** Yes
**Ethics committee - additional information:** SIRER ID 7486 - Studio CO-CO-RE
**Author Disclosures:**
Cinzia Baldessari: Nothing to disclose
Pietro Torricelli: Nothing to disclose
Federica Fiocchi: Nothing to disclose
Roberto Sabbatini: Nothing to disclose
Guido Ligabue: Nothing to disclose
Lorenzo Casarini: Nothing to disclose
Francesco Farioli: Nothing to disclose
Massimo Dominici: Nothing to disclose

### Computed tomography-based Delta radiomics model for predicting response to antiangiogenic therapy in colorectal liver metastases

*L. Yuan*, J. Zhou; Lanzhou/CN
*(yuanl075@163.com)*

**Purpose:** To develop a delta radiomics model based on computed tomography (CT) for predicting the efficacy of bevacizumab in patients with CRLM.
**Methods or Background:** This multicenter retrospective study included 90 patients with CRLM and 255 liver metastases treated with bevacizumab. Center 1 was allocated to training and internal validation, and centers 2 and 3 to external validation. The initial texture features of liver metastases were extracted from pre- and post-treatment CT images, and temporal texture features (Ratio, Delta, and Delta ABS) were calculated. Based on statistically significant clinical and texture features, eight models were constructed using a logistic regression classifier. The performance of eight models was comprehensively evaluated according to the predictive efficacy of therapeutic response to liver metastases and patient efficacy.
**Results or Findings:** Among the eight models, the Ratio, Delta, and COMB models demonstrated the highest predictive performance for therapeutic response to liver metastases and patient efficacy. In the training cohort, AUC values for predicting liver metastasis treatment response and patient efficacy ranged from 0.858 to 0.956 and 0.806 to 0.963, respectively. In the internal validation cohort, the ranges were 0.891–0.899 and 1.000. In the external validation cohort, they were 0.833–0.891 and 0.858–0.896.
**Conclusion:** The Ratio, Delta, and COMB models based on clinical and pre- and post-treatment CT texture feature for predicting the efficacy of bevacizumab therapy in patients with CRLM.
**Limitations:** First, although designed as a multicenter retrospective analysis, the sample size remained relatively modest. Future investigations should expand cohort sizes to enhance the prediction performance. Second, manual segmentation of liver metastasis introduces potential variability; implementing semi-automated or fully automated segmentation methods would improve efficiency and reproducibility.
**Funding for this study:** This work was supported by grants of the National Natural Science Foundation of China (No. 82371914) and the Cuiying Scientific and Technological Innovation Program of Lanzhou University Second Hospital (CY2021-ZD-01).
**Has your study been approved by an ethics committee?** Yes
**Ethics committee - additional information:** The study was approved by the three institutional ethical review board (2023A-379, 2022-254, and P-SL-202219).
**Author Disclosures:**
Junlin Zhou: Nothing to disclose
Long Yuan: Nothing to disclose

### Multi-task Deep Learning Model to Predict Grade and Lymph Node Metastasis in non-functional pancreatic neuroendocrine tumors (NF-PNETs)

*W. Tang*; Shanghai/CN

**Purpose:** To develop and validate a multi-modality deep learning pipeline that first achieves accurate automated lesion segmentation, then integrates imaging features and clinical parameters to predict tumor grade and LNM status in NF-PNETs.
**Methods or Background:** Accurate preoperative assessment of tumor grade and lymph node metastasis (LNM) in NF-PNETs is critical for optimizing surgical strategies and patient outcomes.This multicenter retrospective prognostic study analyzed 931 patients with pathologically confirmed NF-PNETs who underwent preoperative CE-CT between October 2010 and December 2023. Data were collected from 3 tertiary medical centers and divided into internal training (n=425), testing (n=141), and validation (n=174) cohorts, plus two external cohorts (Beijing, n=84; Guangzhou, n=107). Main Outcomes and Measures Primary outcomes were under the receiver operating characteristic curve (AUC) for tumor grade and lymph node metastasis (LNM) predictions. The Dice coefficient quantified segmentation accuracy. Predictive performance was compared against established clinical guidelines and single-modality models.

**Results or Findings:** In this multi-center retrospective study of 931 NF-PNET cases (740 internal; 191 external), the deep learning pipeline achieved superior automated segmentation (Dice coefficient, 0.74) and, building upon this foundation, delivered robustness accross internal and external cohorts on Grading and LNM prediction. The areas under the receiver operating characteristic curve ranged from 0.809 in internal sets to 0.820 and 0.779 in two external sets for grade classification task. For LNM prediction, model ranged from 0.892 in internal sets to 0.710 and 0.872 in two external sets.
**Conclusion:** This study demonstrates that a CE-CT-based deep learning pipeline provides robust, generalizable performance for noninvasive prediction of NF-PNET grade and nodal involvement. This imaging biomarker could enable personalized surgical planning by identifying patients suitable for organ-preserving procedures versus those requiring extended lymph adenectomy, potentially improving surgical outcomes while reducing unnecessary morbidity.
**Limitations:** No
**Funding for this study:** No
**Has your study been approved by an ethics committee?** Yes
**Ethics committee - additional information:** Fudan University Shanghai Cancer Center
**Author Disclosures:**
Wei Tang: Nothing to disclose

**Age- and Sex-Specific Trajectories of Muscle and Fat Depots: A CT Study of 25,092 Adults**
*P. Reschke*, K. Eichler, T. Vogl, L. D. Grünewald; Frankfurt/DE

**Purpose:** Age- and sex-related changes in muscle and adipose tissue are key determinants of frailty, sarcopenia, and metabolic disease, but large-scale CT-based reference data are scarce.
**Methods or Background:** We retrospectively analyzed CT scans of 25,092 adults (8,949 females, 16,143 males; 18–100 years). Automated segmentation quantified skeletal muscle percentage, volumes of major muscle groups, intramuscular fat (IMF), muscle attenuation, and adipose depots (total [TAT], subcutaneous [SAT], visceral [VAT], epicardial [EAT], paracardial [PAT]) at thoracic and abdominal levels. Nonlinear regression characterized age- and sex-specific trajectories.
**Results or Findings:** Skeletal muscle percentage was higher in males than females (thorax: 31.3% vs. 25.9%; abdomen: 30.1% vs. 26.1%; $p < 0.001$) and declined faster in the thorax. Annual losses were −0.49%/year (thorax) and −0.31%/year (abdomen) in males versus −0.27%/year and −0.13%/year in females ($p < 0.001$). Decline peaked earlier and steeper in males (24–38 years) than in females (18–46 years). Muscle volumes were consistently larger in males, including iliopsoas (669 vs. 412 mL), back muscles (978 vs. 676 mL), and gluteus maximus (1,086 vs. 832 mL). IMF was higher in females ($p < 0.001$) and increased with age. Muscle attenuation declined significantly, with quality best preserved in the iliopsoas and poorest in autochthonous muscles. Females had significantly higher SAT and TAT ($p < 0.001$), while males had significantly higher VAT, EAT, and PAT ($p < 0.001$). VAT and especially PAT showed the steepest relative increases with age.
**Conclusion:** This large-scale CT study defines normative age- and sex-specific trajectories of muscle quantity, quality, and fat depots, supporting opportunistic screening, sarcopenia phenotyping, and individualized risk stratification.
**Limitations:** Lack of lifestyle and ethnicity information.
**Funding for this study:** None
**Has your study been approved by an ethics committee?** Yes
**Ethics committee - additional information:** This study was approved by the ethics committee.
**Author Disclosures:**
Thomas Vogl: Nothing to disclose
Philipp Reschke: Nothing to disclose
Leon David Grünewald: Nothing to disclose
Katrin Eichler: Nothing to disclose

**Deep learning-based pancreatic age estimation from MRI predicts incident diabetes beyond known risk factors in the general population**
*M. Jung*, R. T. Schirrmeister, M. Reisert, S. Rospleszcz, Z. Berkarda, C. L. Schlett, F. Bamberg, J. Weiß; Freiburg Im Breisgau/DE
*(matthias.jung@uniklinik-freiburg.de)*

**Purpose:** Individuals and organs age at different rates. Chronological age poorly reflects this variability, whereas organ-specific biological age measures could improve risk assessment. We propose a deep learning (DL) framework (MRI-PancAge) for estimating pancreas age from MRI and investigate its value for predicting incident diabetes.
**Methods or Background:** MRI-PancAge was developed using data from 30,389 individuals (20-75 years) from the German National Cohort. A pancreas segmentation model on abdominal MRI was followed by a second model that takes the 3D-pancreas-segmentation-mask as input and outputs MRI-PancAge in years. MRI-PancAge was converted to a z-score, where z-score>0 refers to older and z-score<0 to younger MRI-PancAge compared to chronological age. Validation was performed in 33,559 UK Biobank (UKB) participants free of diabetes at imaging. Primary outcome was incident diabetes. Associations between MRI-PancAge categories (younger:z<-1; reference:-1–1; older:z>1) and incident diabetes were tested using Cox regression adjusted for age, sex, BMI, race, pancreas volume and fat fraction, hypertension, alcohol consumption, and smoking status. Incremental predictive value of MRI-PancAge was assessed with Harrell’s C-index.
**Results or Findings:** Among 33,559 UKB participants (64.8±7.7 years, 50.9% female; 1.9% incident diabetes; median follow-up 4.7 years), diabetes incidence was higher in older and lower in younger MRI-PancAge (log-rank p<0.001). Multivariable Cox regression revealed an independent association between older MRI-PancAge and incident diabetes (aHR: 1.35, 95%CI[1.10-1.65], p=0.005) after adjustment for all covariates. Adding MRI-PancAge to a baseline model with traditional risk factors showed a small but significantly improved discrimination for incident diabetes (C-index 0.780 to 0.781, p=0.009).
**Conclusion:** DL can estimate pancreas age from MRI and predict incident diabetes in the general population beyond chronological age, pancreas volume and fat fraction, and cardiometabolic risk factors.
**Limitations:** Results may not be generalizable to non-Whites or people outside the United Kingdom.
**Funding for this study:** None
**Has your study been approved by an ethics committee?** Yes
**Ethics committee - additional information:** Informed consent was obtained from all participants in the UK Biobank and the German National Cohort study. In addition, we received local IRB approval (IRB of the University of Freiburg: 23-1316-S1-retro and 24-1099-S1-retro).
**Author Disclosures:**
Christopher L. Schlett: Nothing to disclose
Marco Reisert: Nothing to disclose
Zeynep Berkarda: Nothing to disclose
Jakob Weiß: Nothing to disclose
Matthias Jung: Nothing to disclose
Robin Tibor Schirrmeister: Nothing to disclose
Susanne Rospleszcz: Nothing to disclose
Fabian Bamberg: Nothing to disclose

**Volumetric vs. Slice-Based CT Body Composition Analysis: Systematic Discordance and Prognostic Impact**
*K. A. Borys*, K. Arzideh, J. Kohnke, Y. Wen, C. Bojahr, L. Umutlu, J. Haubold, F. Nensa, R. Hosch; Essen/DE
*(katarzyna.borys@uk-essen.de)*

**Purpose:** To investigate sex-specific and ECOG status-stratified differences in CT-derived body composition analysis (BCA) and to assess concordance between volumetric and slice-based BCA measures.
**Methods or Background:** Baseline whole-body CTs from 23,685 cancer patients (44% female) were automatically segmented to quantify skeletal muscle and adipose compartments both volumetrically (thoracic and abdominal regions) and slice-based (mid-slice at L3 and T4 levels). Prognostic value for overall survival was evaluated using Kaplan-Meier and Accelerated Failure Time models, stratified by sex, ECOG performance status, and metastatic status. Concordance between volumetric and slice-based BCA measures was assessed with Cohen’s kappa.
**Results or Findings:** Volumetric muscle index (MI) provided substantially greater prognostic separation than slice-based measures, with a median survival difference of 94 months between lowest and higher tertiles in females (47 vs. 141 months) and 69 months in males (27 vs. 96), compared with ≤56 months for slice-based muscle area (MA). Subcutaneous adipose tissue also showed stronger volumetric effects, while visceral fat offered limited prognostic value. Increasing physical impairment (ECOG) was associated with muscle and fat loss. Accelerated Failure Time models confirmed consistently higher time ratios (TR) for volumetric vs. slice-based measures (abdominal MI TR=1.89, P<0.001 vs. abdominal MA TR=1.41, P<0.001 in males). Concordance analyses revealed substantial disagreement (~35%) between volumetric and slice-based muscle classifications of high- and low-risk patients, indicating systematic discordance.
**Conclusion:** Volumetric CT analysis provides superior prognostic value compared to slice-based markers, particularly across ECOG strata, highlighting its potential for individualized risk stratification.
**Limitations:** Despite adjusting for key clinical factors, unmeasured confounders such as treatment regimens, comorbidities, and nutritional habits were not assessed. Moreover, the results require external validation.
**Funding for this study:** None
**Has your study been approved by an ethics committee?** Yes
**Ethics committee - additional information:** The approval for this retrospective study was obtained by the Ethics Committee of the University Hospital Essen (approval number 21-10204-BO). Due to the study's retrospective nature, the requirement of written informed consent was waived by the Ethics Committee. All data were fully anonymized.

**Author Disclosures:**
Christian Bojahr: Nothing to disclose
Katarzyna Anna Borys: Nothing to disclose
Johannes Haubold: Nothing to disclose
Judith Kohnke: Nothing to disclose
Yutong Wen: Nothing to disclose
Lale Umutlu: Nothing to disclose
Kamyar Arzideh: Nothing to disclose
René Hosch: Nothing to disclose
Felix Nensa: Nothing to disclose

**Algorithmic Fairness in Radiology: A Practical Deep Dive into AI Challenges**
*B. Mohajer*[1], A. Zain[2], H. Zhang[2], Z. Hu[3], R. Ball[4], J. Gichoya[5], A. E. Flanders[1], M. Ghassemi[2], E. Colak[3]; [1]Philadelphia, PA/US, [2]Boston, MA/US, [3]Toronto, ON/CA, [4]Bar Harbor, ME/US, [5]Atlanta, GA/US
*(mohajer.bahram@gmail.com)*

**Purpose:** Machine learning (ML) models have demonstrated expert-level performance in radiology; however, concerns about fairness persist, potentially reinforcing healthcare inequities. AI competitions have advanced the field by providing the most diverse publicly available datasets and hosting AI challenges. However, even in these controlled settings, concerns remain about the fairness of ML models. This study assessed fairness in top-performing ML models from the Radiological Society of North America (RSNA) Cervical Spine Fracture and Abdominal Trauma Detection AI Challenges, focusing on demographic performance differences.
**Methods or Background:** Predictions from the 9 top-performing models were evaluated using private test sets stratified by age groups, sex, and geographical location. Performance metrics–including false positive rate (FPR), false negative rate (FNR), area under the receiver operating characteristic curve (AUC), and expected calibration error (ECE)–were compared across subgroups.
**Results or Findings:** The study included 788 participants from the Cervical Spine (64% male, mean-age 54.8 years) and 709 participants from the Abdominal Trauma (69% male, mean age 48.7 years) challenges. No significant AUC or FNR differences were observed across subgroups or between sexes. However, age- and region-specific FPR disparities emerged. For cervical spine fractures, older adults (≥61 years) had higher FPRs (9.7% vs. 2.6%, $p<0.05$). In abdominal trauma detection, older adults also showed elevated FPRs (11.6%, $p=0.003$). Geographic variation was notable–Asian patients had higher FPRs (28.0%), while Oceanian patients had lower rates (5.6%, $p<0.05$).
**Conclusion:** Despite being trained on the most diverse datasets available, subgroup-specific differences in FPR–particularly across age groups–persisted. These findings highlight that even diverse training data may not entirely eliminate disparities. Continued efforts to improve demographic representation and integrate fairness-aware approaches into ML development are essential.
**Limitations:** Limited subgroup sample sizes—especially from Africa and South America—may affect robustness of fairness estimates and limit generalizability.
**Funding for this study:** This study received no direct funding. In preparation of the dataset and AI challenges, funding was recieved from Radiological Society of North America (RSNA).
**Has your study been approved by an ethics committee?** Not applicable
**Ethics committee - additional information:** Approved by
**Author Disclosures:**
Robyn Ball: Employee: The Jackson Laboratory
Haoran Zhang: Nothing to disclose
Ahmed Zain: Nothing to disclose
Judy Gichoya: Nothing to disclose
Marzyeh Ghassemi: Nothing to disclose
Errol Colak: Nothing to disclose
Adam E. Flanders: Nothing to disclose
Zixuan Hu: Nothing to disclose
Bahram Mohajer: Nothing to disclose

**Preoperative Radiomics of Fat Stranding Enhancing the 2-Year Recurrence Prediction in Colorectal Cancer Patients**
*J. Z. Urbaniec-Stompor*[1], B. K. Budai[2], C. Zerbato[3], V. Damerell[2], S. Hardikar[4], C. Kahlert[2], C. Ulrich[4], H-U. Kauczor[2], B. Gigic[2]; [1]Olsztyn/PL, [2]Heidelberg/DE, [3]Padua/IT, [4]Utah, UT/US
*(j.urbaniec.stompor@gmail.com)*

**Purpose:** Preoperative computed tomography (CT) in colorectal cancer (CRC) is used for TNM staging at the initial diagnosis. Our study aimed to identify CT-based radiomics features of the tumor and peritumoral fat tissue that could be independent predictors of recurrence.
**Methods or Background:** This single-center analysis of a prospective cohort included 273 patients with venous phase preoperative CT scans from the Heidelberg site of the ColoCare Study recruited between 2010 and 2024. Single-slice tumor segmentation was performed, followed by the segmentation of peritumoral fat and radiomics feature extraction. Patients were divided into training (n=148) and test (n=126) datasets. Clinical and combined models were built to predict 2-year recurrence. Logistic regression was used for radiomics feature selection and model building. Receiver operating characteristic curve (ROC) analysis with area under the curve (AUC), likelihood ratio tests (LRT), Net Reclassification Index (NRI), and Brier scores were used for evaluation.
**Results or Findings:** This study evaluated 274 patients (96 female and 178 males, aged 62.64 ±11.78). TNM classification and T-stage were used as proxy labels guiding radiomics feature selection. The “GLCM-Autocorrelation” texture feature of peritumoral fat was a significant independent predictor (OR: 0.23 [0.07 - 0.88, $p=0.031$) of 2-year recurrence in the adjusted regression model. The combined model, including tumor and peritumoral fat radiomics features, outperformed the clinical models with an AUC, accuracy, and specificity of 0.912, 81.7%, and 80.8%, respectively, at a sensitivity of 85.2%. NRI of 0.111 and Brier score 0.074 vs. 0.119 ($p < 0.001$) also confirmed superiority
**Conclusion:** Radiomics features of peritumoral fat tissue significantly improve the prediction of 2-year disease recurrence when combined with conventional clinical and pathological factors.
**Limitations:** Single-center study design.
**Funding for this study:** This study was supported by National Institutes of Health (NIH)/National Cancer Institute (NCI) grants (U01206110, R01 CA189184, R01 CA207371, R01 CA211705, T32 HG008962, KL2TR002539, K07 CA222060, R03AG067994), German Federal Ministry of Education and Research (BMBF) project PerMiCCion (01KD2101D), and Stiftung LebensBlicke. B.K.B. was supported by the Medical Data Scientist Program of the Medical Faculty of Heidelberg University.
**Has your study been approved by an ethics committee?** Yes
**Ethics committee - additional information:** Institutional Ethics Board (approval number: S-134/2016).
**Author Disclosures:**
Sheetal Hardikar: Nothing to disclose
Joanna Zofia Urbaniec-Stompor: Nothing to disclose
Biljana Gigic: Nothing to disclose
Hans-Ulrich Kauczor: Nothing to disclose
Cornelia Ulrich: Nothing to disclose
Christoph Kahlert: Nothing to disclose
Victoria Damerell: Nothing to disclose
Chiara Zerbato: Nothing to disclose
Bettina Katalin Budai: Nothing to disclose

**Validation of AI-Assisted RECIST Lesion Measurements in Follow-Up CT: A Multi-Center Reader Study**
M. J. J. De Grauw[1], R. Weber[1], M. Westphal[2], T. Lossau[2], J. H. Moltz[2], B. Van Ginneken[1], M. Prokop[1], E. J. Smit[1], *A. Hering*[1]; [1]Nijmegen/NL, [2]Bremen/DE
*(alessa.hering@radboudumc.nl)*

**Purpose:** To evaluate the effect of AI assistance on reading time, inter-reader variability, and RECIST 1.1 response in follow-up CT.
**Methods or Background:** In a retrospective, multi-center reader study, 23 readers (15 radiologists, 8 residents) evaluated follow-up chest–abdomen–pelvis CT of 212 oncology patients with in total 539 lesions under three conditions: unassisted, AI-assisted, and expert-assisted (using a prior radiologist’s measurement as a strong reference condition). To prevent bias, readers were informed that all support originated from AI. Reading time and inter-observer measurement variability were assessed, and outcomes were analyzed with a Bayesian generalized linear mixed model.
**Results or Findings:** AI assistance significantly reduced reading time per patient compared to unassisted reading (–35.96 s; 95% CI: –52.95, –22.07). At the lesion level, inter-reader variability relative to the consensus standard increased slightly with AI assistance (1.32 mm; 95% CI: 0.83, 1.91). In contrast, at the clinically relevant patient level, variability decreased substantially: differences in the Sum of Longest Diameters (SLD) of more than 20% between radiologists were observed in 43.4% of unassisted cases, 28.3% of AI-assisted cases, and 17.4% of expert-assisted cases. These effects were consistent across experience levels.
**Conclusion:** AI assistance reduces patient-level inter-observer variability in RECIST measurements, leading to fewer discrepancies in predicted clinical response, despite a slight increase in lesion-level variability. Combined with the significantly reduced reading time, AI systems have the potential to standardize follow-up assessments, minimize discordant treatment decisions, and increase efficiency in clinical practice. The expert-assisted condition illustrates that even higher consistency is achievable, highlighting both the progress made and potential for further refinement of AI tools.

**Limitations:** The use of national-level data may limit the generalizability of these findings to international populations. Further evaluation across imaging vendors and more diverse patient cohorts is warranted.
**Funding for this study:** This research is funded by the Dutch Research Council (NWO) under grant number Veni-21121 for Applied and Engineering Sciences (Spotting the Differences: AI-based change detection in medical images).
**Has your study been approved by an ethics committee?** Not applicable
**Author Disclosures:**
Mathias Prokop: Speaker: Canon Advisory Board: https://plain-medical.com/ Research/Grant Support: Canon Research/Grant Support: Bracco Speaker: Bracco
Max Jacobus Johannes De Grauw: Nothing to disclose
Tanja Lossau: Nothing to disclose
Alessa Hering: Nothing to disclose
Bram Van Ginneken: CEO: https://plain-medical.com/
Rianne Weber: Nothing to disclose
Max Westphal: Nothing to disclose
Ewoud J. Smit: Nothing to disclose
Jan Hendrik Moltz: Nothing to disclose

**Dual-Habitat Multimodal Imaging with 3D Deep Learning for Predicting Lung Adenocarcinoma Invasiveness: A Radiogenomic Analysis**
*X. Han*[1], L. Xiao[2], C. Zhang[1], C. Han[1]; [1]Xian City/CN, [2]Shanghai/CN
*(1261700592@qq.com)*

**Purpose:** To develop a novel deep learning framework integrating CT-defined anatomical and PET/CT-defined functional habitats to predict lung adenocarcinoma invasiveness and explore radiogenomic correlations.
**Methods or Background:** This retrospective study included 292 patients with separate preoperative diagnostic CT and 18F-FDG PET/CT scans, which were spatially aligned using deep learning-based deformable image registration. A dual-habitat analysis was then performed: tumors were partitioned into distinct anatomical habitats via two-stage clustering on CT data, and into functional habitats using PET/CT data. A multi-stream 3D residual network with a multi-stage intermediate fusion architecture was developed to integrate all imaging and resulting habitat maps. The model was trained to predict invasive (IAC) vs. minimally invasive (MIA) status, with performance evaluated by AUC and external validation on the TCIA TCGA-LUAD cohort.
**Results or Findings:** The analysis identified four distinct anatomical habitats (e.g., solid-core, ground-glass) and three functional habitats (e.g., metabolically active, necrotic). The dual-habitat deep learning model achieved significantly superior performance (Internal Validation AUC: 0.92) compared to single-habitat (AUC: 0.90) and whole-tumor models (AUC: 0.88). External validation on 69 TCIA cases yielded a robust AUC of 0.85, demonstrating strong generalizability. Explainable AI (XAI) maps visually confirmed the model's focus on the critical interface between high-density anatomical and metabolically active functional habitats. Furthermore, the volume of the metabolically active habitat correlated significantly with PD-L1 expression ($p<0.05$), establishing a key radiogenomic link.
**Conclusion:** Integrating anatomical and functional tumor habitats via a multi-stream deep learning model provides a comprehensive characterization of tumor heterogeneity. This dual-habitat approach significantly improves non-invasive prediction of lung adenocarcinoma invasiveness, offering a promising pathway toward a virtual "imaging biopsy".
**Limitations:** The limited sample size and single data source at the data level may affect the model's generalization ability and the promotion of research results.
**Funding for this study:** Not applicable
**Has your study been approved by an ethics committee?** Not applicable
**Author Disclosures:**
Lihao Xiao: Nothing to disclose
Chenchen Han: Nothing to disclose
Chen Zhang: Nothing to disclose
Xiaoyan Han: Nothing to disclose

**14:00 - 15:30 Research Stage 4**

### Research Presentation Session: Interventional Radiology

### RPS 1509

### The many faces of interventional radiology: a journey through innovation and impact

**Moderator**
Fernando Gomez Muñoz; València/ES
*(fernan.m.gomez@gmail.com)*
**Author Disclosures:**
Fernando Gomez Muñoz: Advisory Board: iVascular, Consultant: Boston Sci, iVascular, Bayer, Research Grant/Support: Boston Sci, iVascular, Speaker: Boston Sci, iVascular, Terumo

**Gaps in IVC filter retrieval: Benchmarking outcomes through a global multi-centre analysis**
B. Miles, *A. Sharma*, W. Oza, T. Elwell, J. Moss; Dallas/US
*(akashvikash11@gmail.com)*

**Purpose:** Despite multiple FDA safety communications (2010, 2014) and multidisciplinary guideline statements (CHEST 2016; SIR 2020) recommending timely retrieval of inferior vena cava (IVC) filters, retrieval rates remain low. This represents not a knowledge gap but an implementation failure. We leveraged a global, multi-institutional dataset to quantify morbidity and mortality associated with indwelling filters compared to retrieval, entirely in the post-guideline era.
**Methods or Background:** We queried the TriNetX Global Collaborative Network, encompassing more than 150 health systems and 150 million patient records to identify adults undergoing IVC filter insertion (CPT 37191) between 2018–2025. Patients were stratified into (1) Filter Remains (no retrieval) and (2) Filter Removed (retrieval CPT 37193 within one day to six months). Propensity score matching balanced demographic covariates. Outcomes included severe sepsis, haemorrhage, cerebral infarction, and all-cause mortality. Risk ratios (RR), risk differences, and numbers needed to treat (NNT) were calculated.
**Results or Findings:** A total of 58,549 patients across 155 health systems were analyzed (46,173 Filter Remains; 12,376 FilterRemoved). Despite clear guidelines, only 21% underwent retrieval. Retained filters were associated with significantly worse outcomes: Severe sepsis: 10.8% vs 2.5% (RR 4.3; NNT=12); Haemorrhage: 2.6% vs 1.1% (RR 2.3; NNT=69); Cerebral infarction: 10.0% vs 4.9% (RR 2.0; NNT=20); Mortality: 30.2% vs 4.2% (RR 7.2; NNT=4).
**Conclusion:** In a cohort spanning 2018–2025, only one in five filters was retrieved and retained filters were associated with sharply higher rates of infection, stroke, haemorrhage, and death. Absolute differences translate to one death prevented for every 4 filters retrieved. These findings demonstrate a persistent guidelines-to-practice gap and highlight the need for structured retrieval pathways, automated alerts, and institutional accountability to align practice with safety standards.
**Limitations:** Reasons for leaving IVC filters in place were not available.
**Funding for this study:** None
**Has your study been approved by an ethics committee?** Not applicable
**Author Disclosures:**
Jason Moss: Nothing to disclose
Willa Oza: Nothing to disclose
Brittany Miles: Nothing to disclose
Trevor Elwell: Nothing to disclose
Akash Sharma: Nothing to disclose

**Catheter-directed therapy vs anticoagulation alone for intermediate-risk PE: A propensity-matched global retrospective cohort study**
B. Miles, *S. Patel*, T. Elwell, W. Oza, J. Moss; Dallas/US
*(p.shivampatel@gmail.com)*

**Purpose:** Management of intermediate-risk pulmonary embolism (PE), characterized by right ventricular (RV) strain without hemodynamic compromise, is complicated by a lack of comparative outcome data and variability in clinical guidelines. This study was conducted as a real-world comparative analysis using TriNetX, a federated EHR network with over 100 million patients across U.S. health systems. We evaluated the mortality and bleeding outcomes of CDT versus anticoagulation alone in intermediate-risk PE at one year.
**Methods or Background:** This retrospective cohort study used data from the TriNetX Global Collaborative Network. Adult patients with acute PE (ICD-10-CM I26.9) and evidence of RV strain (troponin ≥ 0.04 ng/mL or BNP ≥ 125 pg/mL) were included. Two cohorts were defined: (1) the CDT cohort, identified via CPT codes for mechanical thrombectomy (37184–37186), thrombolytic infusion (37211–37214), and/or alteplase administration (J2997); and (2) the anticoagulation-only group, defined by heparin or DOAC use within 24 hours of admission without CDT procedure codes. Following propensity score matching, 5,523 patients were included in each group. Primary outcomes included mortality and major bleeding (defined using ICD-10 codes I97.4, K92.2, and D62) within one year of treatment.
**Results or Findings:** One-year mortality was significantly lower in the CDT group (8.88% versus 10.57%), with an adjusted risk ratio (aRR) of 0.84 (95% CI: 0.75–0.90, p < 0.0001). Major bleeding occurred in 8.88% of CDT patients versus 11.25% in the anticoagulation cohort, with an aRR of 0.84 (95% CI: 0.76–0.94, p < 0.0001), indicating that CDT was not associated with increased bleeding risk.
**Conclusion:** CDT is associated with a mortality advantage without increased bleeding. CDT may be an effective and safer option than systemic anticoagulation for treatment of these patients.
**Limitations:** This study was retrospective. Prospective studies will be needed to confirm.
**Funding for this study:** None
**Has your study been approved by an ethics committee?** Not applicable
**Author Disclosures:**
Jason Moss: Nothing to disclose
Willa Oza: Nothing to disclose
Brittany Miles: Nothing to disclose
Trevor Elwell: Nothing to disclose
Shivam Patel: Nothing to disclose

**Risks and Outcomes of Vessel Perforations in Mechanical Thrombectomy: A retrospective multicenter study**
*M. M. Taepper*[1], A. Dmytriw[2], N. Ntoulias[1], A. Brehm[1], M-N. Psychogios[1], V. Schulze-Zachau[1]; [1]Basel/CH, [2]Oxford/UK

**Purpose:** Vessel perforation is a rare but severe complication of mechanical thrombectomy for acute ischemic stroke. We aimed to identify the procedural steps at which perforations most frequently occur and to compare bleeding duration, functional outcome, and survival across these steps.
**Methods or Background:** We conducted a retrospective cohort study of patients with vessel perforation during thrombectomy between January 2015 and September 2022 across 32 centers. Outcomes included bleeding duration, defined as angiographic extravasation; 90-day functional outcome, assessed by the modified Rankin Scale (mRS); and overall survival, evaluated by Kaplan–Meier analysis. Outcomes were stratified by the procedural step of perforation.
**Results or Findings:** Among 447 patients (mean age 73 years, 59% female), perforations occurred during retraction of a stent retriever or aspiration catheter in 45% (n=201), probing beyond the occlusion in 26% (n=117), access to the occlusion in 13% (n=58), device deployment in 9% (n=42), percutaneous transluminal angioplasty (PTA) or stent placement in 5% (n=24), and contrast injection or thrombolysis in 1% (n=5). Perforations during PTA/stent placement were associated with longer bleeding duration (1.82 [1.14 – 2.90 95% CI], p=0.01) and worse 90-day mRS (1.10 [0.33-1.80 95%CI], p=0.01) compared with all other steps. Survival also differed significantly by perforation step (p=0.01), with the lowest survival following PTA/stent-related perforations.
**Conclusion:** Vessel perforations most often occurred during device retraction and probing beyond the occlusion. Although less frequent, perforations during PTA or stent placement carried the poorest prognosis, likely due to prolonged bleeding.
**Limitations:** Retrospective design and incomplete anticoagulation data.
**Funding for this study:** None.
**Has your study been approved by an ethics committee?** Yes
**Ethics committee - additional information:** Ethical commission approval and patient consent were not required according to current local legislation as all data were anonymized before analysis and the project involved assessing safety and quality of routine patient management in the participating institutions.
**Author Disclosures:**
Adam Dmytriw: Nothing to disclose
Marios-Nikos Psychogios: Nothing to disclose
Nikolaos Ntoulias: Nothing to disclose
Alex Brehm: Nothing to disclose
Victor Schulze-Zachau: Nothing to disclose
Maria Marta Taepper: Nothing to disclose

**Does Drug-coated Balloon Angioplasty for all Significant Dialysis Access Arteriovenous Fistula Stenoses Improve Circuit Patency? Preliminary Analysis of a Randomized Controlled Trial**
*B. S. Tan*, A. J. W. Tan, S. Arjunana, W. Lee, Z. Tan, A. Patel, K. H. Tay, T. H. Heng, K. D. Zhuang; Singapore/SG
*(tan.bien.soo@sgh.com.sg)*

**Purpose:** To compare the efficacy of drug-coated balloon angioplasty (DCBA) (Ranger, Boston Scientific; Marlborough, Massachusetts, USA) to conventional balloon angioplasty (CBA) of all stenoses in the peripheral circuit of dysfunctional dialysis arteriovenous fistula (AVF). We present the preliminary analysis of a randomized controlled trial (RCT) comparing DCBA against CBA of our first 73 patients.
**Methods or Background:** This prospective single-center RCT recruited participants with stenoses in the peripheral circuit of failing mature AVF. Participants with central vein stenoses or AVF thrombosis were excluded. After successful angioplasty of all AVF stenoses in the peripheral circuit (lesion preparation achieving < 30% residual stenosis), 94 participants (72 male, 76.6%; mean age 66.8 ± 9.5 years) were randomized to DCBA or CBA in a 1:1 ratio, stratified by the number of AVF stenoses (single versus multiple). The primary outcome measure was clinically driven 6-month access circuit primary patency (ACPP). Participant follow-up for patency was up to 12 months post recruitment.
**Results or Findings:** The outcomes of 73 study participants (DCBA: 35, CBA: 38) (59 males, mean age: 66.2 years old) who had completed a minimum of six months follow-up were analyzed. The types of AVFs were radiocephalic (64.4 %), brachiocephalic (24.7%) and brachiobasilic (11%). In 43 patients, a single lesion was treated, while two or more lesions were treated in 30 patients. ACPP at six months was not significantly different between both groups (DCBA: 79.4%, CBA: 77.8%, p= 0.82).
**Conclusion:** In this initial report of our first 73 patients, the treatment of all peripheral AVF stenoses with DCBA did not yield a statistically significant difference in ACPP at six months when compared to CBA. Further analysis of the full cohort with longer follow-up may yield more definitive results.
**Limitations:** Preliminary results
**Funding for this study:** Boston Scientific Foundation.
**Has your study been approved by an ethics committee?** Yes
**Ethics committee - additional information:** Approved
**Author Disclosures:**
Kun Da Zhuang: Nothing to disclose
Tseng Hui Heng: Nothing to disclose
Zehao Tan: Nothing to disclose
Weiyong Lee: Nothing to disclose
Kiang Hiong Tay: Nothing to disclose
Bien Soo Tan: Nothing to disclose
Ankur Patel: Nothing to disclose
Sarupraba Arjunana: Nothing to disclose
Anne Jing Wen Tan: Nothing to disclose

**Evaluating the Safety of Selective Internal Radiation Therapy for Primary and Secondary Liver Malignancies: A Study of 4,432 Patients**
*A-I. Nica*[1], T. J. Vogl[1], F. Wacker[2], R. Klöckner[3], C. Booz[1], L. S. Alizadeh[1]; [1]Frankfurt/DE, [2]Hannover/DE, [3]Lübeck/DE

**Purpose:** To evaluate the safety profile of Selective Internal Radiation Therapy (SIRT) in the treatment of primary and secondary liver malignancies, with a specific focus on the incidence and management of periprocedural complications.
**Methods or Background:** We conducted a retrospective analysis of data from the national quality assurance registry maintained by the German Society of Minimally Invasive Therapies and Interventional Radiology (DeGIR), covering the period from 2018 to 2023. The study aimed to identify adult patients (aged >18 years) who underwent SIRT for liver tumors. We evaluated the incidence, types, and management of complications, classifying them as either 'procedure-related complications' (PRCs), occurring during or within 24 hours of the intervention, or 'post-procedure complications' (PPCs), defined as those arising more than 24 hours after the procedure.
**Results or Findings:** The study included 4432 patients (1563 females, 35.3%; 2869 males, 64.7%) with a mean age of 67.4 ± 11.4 years. Most SIRT-related complications were PRCs (36 cases, 0.8%), mainly due to drug side effects (5, 0.1%) and arterial bleeding (3, 0.1%). Nearly half (45.7%) were Category 2/B, requiring prolonged monitoring. One death (Category 6/F, 2.9%) occurred within 24 hours post-intervention. In 50% of cases (18), complications were managed by the interventional radiologist, 27.8% (10) by other specialties, 16.7% (6) required no treatment, and 5.6% (2) were unspecified. PPCs occurred in 4 patients (0.09%), for a total of 40 patients (0.9%) with periprocedural complications.
**Conclusion:** Our findings suggest that SIRT is a safe procedure for patients with liver tumors, as it demonstrates a low incidence of complications, with many being effectively managed within the same medical specialty.
**Limitations:** The main limitation of this study is its retrospective design.

**Funding for this study:** This study received no funding.
**Has your study been approved by an ethics committee?** Yes
**Ethics committee - additional information:** This study received approval from the Ethics Committee.
**Author Disclosures:**
Christian Booz: Speaker: I have received speaking fees from Siemens Healthineers.
Roman Klöckner: Nothing to disclose
Andreea-Ioana Nica: Nothing to disclose
Thomas J. Vogl: Nothing to disclose
Leona Soraja Alizadeh: Nothing to disclose
Frank Wacker: Nothing to disclose

**Magnetic Resonance-guided Focused Ultrasound Surgery (MRgFUS) for ablation of osteoid osteoma: results from 10 years of experience**

*M. Adinolfi Falcone*, L. Rinvenuto, F. Arrigoni, L. Zugaro, A. Barile, E. Di Cesare; L'Aquila/IT
*(marica.adi@outlook.it)*

**Purpose:** Osteoid Osteoma (OO) is a benign but extremely painful bone lesion. Magnetic Resonance-guided Focused Ultrasound Surgery (MRgFUS) is an ablative technique in Interventional Radiology that combines high-intensity focused ultrasound (HIFU) with MR imaging. The aim of this study was to evaluate the long-term outcomes and effectiveness of MRgFUS in the treatment of OO compared with other therapeutic options.
**Methods or Background:** A retrospective analysis was performed on all MRgFUS procedures for OO conducted at our center over a 13-year period. 67 patients treated were included, all meeting the same inclusion criteria: radiological findings of OO, symptomatic lesions whit VAS > 7, and an adequate acoustic window for ultrasound beam penetration. Demographic data, morphological features, imaging findings and clinical outcomes wcre collected. All variables were statistically analyzed, and results were evaluated in terms of pain relief and nidus characteristics. Standard follow-up included clinical ooutcomes assessment at 7-10 days, 6 months, 1 year, 2 years, and 4 years; MRI at 6 and 12 months; and CT scan at 12 months.
**Results or Findings:** The procedure was successful in 61 out of 67 patients, with an overall success rate of 91%. All responders showed MRI resolution of bone edema and reduction of perilesional reactive changes. No complications were recorded. Treatment failure was significantly correlated with thick cortical bone overlying the nidus (p=0.014) and with a short nidus-to-ultrasound source distance (p= 0.002).
**Conclusion:** MRgFUS is a safe, minimally invasive, and effective therapy for OO, associated with absence of complications, rapid recovery, and a low recurrence rate. This study represent the largest reported single-center series with the longest follow-up (13 years), confirming the excellent safety profile and long-term efficacy of MRgFUS in the treatment of Osteoid Osteoma.
**Limitations:** No limitations were identified.
**Funding for this study:** No funding was received for this study.
**Has your study been approved by an ethics committee?** Not applicable
**Author Disclosures:**
Francesco Arrigoni: Nothing to disclose
Luigi Rinvenuto: Nothing to disclose
Ernesto Di Cesare: Nothing to disclose
Marica Adinolfi Falcone: Nothing to disclose
Antonio Barile: Nothing to disclose
Luigi Zugaro: Nothing to disclose

**Pectoral Nerve Block for Microwave Ablation of breast benign lesions: The Initial Clinical Experience**

*J. LI*, M. Lu; Chengdu/CN
*(18010620786@163.com)*

**Purpose:** To investigate the effect of pectoral nerve block on pain management in microwave ablation (MWA) for multiple benign breast nodules.
**Methods or Background:** A total of 94 patients undergoing MWA for benign breast nodules were divided into three groups based on the analgesic method used: local anesthesia group (Group A, n = 31), pectoral nerve block group (Group B, n = 30), and local anesthesia combined with pectoral nerve block group (Group C, n = 33). Pain levels were recorded using the Numerical Rating Scale (NRS) during the procedure and at 2, 6, 12, 24, and 48 hours postoperatively. Postoperative analgesic requirements and complications were also documented.
**Results or Findings:** The consumption of lidocaine and ropivacaine during the procedure differed significantly among the groups (p < 0.05), with Group B showing the highest usage and Group C the lowest. Group C exhibited lower intraoperative and postoperative NRS scores compared to Groups A and B (P < 0.05), while no significant difference was observed between Groups A and B (P > 0.05). At 2 and 6 hours postoperatively, Group A had lower NRS scores than Group B (P < 0.05), but no significant differences were noted at 12, 24, and 48 hours (P > 0.05). The postoperative analgesic requirement was significantly lower in Groups B and C compared to Group A (P < 0.05). No serious complications occurred after the procedure.
**Conclusion:** The combination of local anesthesia and pectoral nerve block effectively reduces pain during and after MWA for multiple benign breast nodules and decreases the need for postoperative analgesia.
**Limitations:** No
**Funding for this study:** This study is funding by Sichuan Provincial Science and Technology Support Program, 2024YFHZ0140
**Has your study been approved by an ethics committee?** Yes
**Ethics committee - additional information:** This study was approved by the Institutional Review Board and Ethics Committee of Sichuan Cancer Hospital. All patients provided signed informed consent before the examination. All methods were performed in accordance with the relevant guidelines and regulations.
**Author Disclosures:**
Juan Li: Nothing to disclose
Man Lu: Nothing to disclose

**Bleomycin electrosclerotherapy (BEST) for extracranial slow-flow malformations treatment: a single-center experience**

*F. Lazzarotto*, G. Falcone, A. L. Annese, D. A. Campanacci, F. Fanelli; Florence/IT
*(federica.lazzarotto@unifi.it)*

**Purpose:** BEST (Bleomycin Electrosclerotherapy) has been proposed as a novel approach for treating venous and linfatic malformations (VMs). This study reports a single-center initial experience in treating extracranial symptomatic slow-flow malformations.
**Methods or Background:** A retrospective observational study was conducted from March 2024 to Sep 2025, including 24 patients (28 lesions) with symptomatic VMs. Most had undergone previous unsuccessful treatments. Electrosclerotherapy was performed using reversible electroporation combined with bleomycin. Clinical records, magnetic resonance imaging (MRI) data, previous treatment history, intervention details, complications, and clinical outcomes were analyzed. A postoperative questionnaire assessed both functional and aesthetic effects.
**Results or Findings:** Of the 24 patients (mean age 23,8 ± 8,4 years; 8 females), 7 had undergone an average of 2.5 previous invasive treatments. A total of 34 electrosclerotherapy sessions were performed on 28 lesions. The median dose of bleomycin administered was 2,6 mg. Clinical and MRI follow-up schedules were established. The median MRI-derived lesion volume decreased in 73% of the patients . After a median follow-up of 6 months (range 3-9 mos), 14 patients reported complete symptom resolution, while 8 showed significant improvement, 2 no improvement. 6 minor complications occurred, including transient skin discoloration and swelling.
**Conclusion:** BEST is a safe and effective therapeutic option for LM and VMs, including those resistant to prior treatments.
**Limitations:** Despite promising results, the small sample size and limited FUP warrant further investigation to confirm these findings.
**Funding for this study:** No funding received for this study
**Has your study been approved by an ethics committee?** Not applicable
**Author Disclosures:**
Fabrizio Fanelli: Nothing to disclose
Domenico Andrea Campanacci: Nothing to disclose
Antonio Luca Annese: Nothing to disclose
Gianmarco Falcone: Nothing to disclose
Federica Lazzarotto: Nothing to disclose

**Microwave thermal ablation in cirrhotic patients: comparison of actual ablation volumes to expected ablation volume following manufacturer charts**

K. D. Martins De Mattos[1], *R. Muglia*[2], F. S. Carbone[2], M. Bertuletti[2], L. Dulcetta[2], P. Marra[2], S. Sironi[1]; [1]Milan/IT, [2]Bergamo/IT

**Purpose:** Microwave thermal ablation (MWTA) is an established therapy for HCC in cirrhotic patients, with a low rate of local recurrence. However, in some cases, despite optimal antenna placement and strict adherence to the ablation volume charts, residual disease may persist after treatment. We aimed to evaluate the immediate ablation volume after MWTA in cirrhotic livers, and compare it with the volumes estimated by the manufacturer chart.
**Methods or Background:** This single-center retrospective study was conducted at a tertiary referral center, including cirrhotic patients BCLC 0/A, treated for HCC by MWTA (Emprint™HP, Medtronic, USA) between November 1, 2022, and August 31, 2025. HCCs were categorized by size (small <25mm; large 25<x<30mm) and anatomical location (subcapsular: ≤1cm from the liver capsule; perivascular: ≤1cm from a hepatic vein or intrahepatic portal branch).

All MWTAs were performed under CT-hepatic arteriography guidance; we calculated immediate post-ablation necrotic volumes, and compared them to expected ablation volumes, following the manufacturer chart. Volumes were calculated via multiplanar reconstruction (MPR) using the ellipsoid formula (D1×D2×D3×π/6).
**Results or Findings:** We analyzed 43 HCCs in 29 patients, treated at 100W/150W (36:7). Twelve nodules were perivascular (11 small - 1 large), 31 subcapsular (22 small - 9 large). No nodules were far from liver capsule or veins. The mean achieved volume for perivascular nodules was 12.37cm³, 42.4% lower than the expected 21.48cm³ (p<0.001). For subcapsular nodules the mean achieved volume was 15.25cm³, 36.3% lower than the expected 23.96cm³ (p<0.001). This significant underestimation corresponded to a Bland-Altman systemic bias of -9.04 cm³. Only 2/43 outlying cases (subcapsular nodules) were identified.
**Conclusion:** Our findings suggest a systematic underestimation of achieved ablation volumes compared to expected volumes in cirrhotic livers. Increasing ablation volumes compared to manufacturer chart might improve MWTA efficacy.
**Limitations:** Retrospective-design
**Funding for this study:** No funding was provided for this study
**Has your study been approved by an ethics committee?** Not applicable
**Author Disclosures:**
Martina Bertuletti: Employee: Interventional radiology dept.
Paolo Marra: Employee: Interventional radiology dept.
Francesco Saverio Carbone: Employee: Interventional radiology dept.
Kessy Djonis Martins De Mattos: Other: Interventional radiology resident
Riccardo Muglia: Employee: Interventional radiology dept.
Sandro Sironi: Other: Full Professor at the University of Milano-Bicocca and Head of the Department of Radiology at Papa Giovanni XXIII Hospital
Ludovico Dulcetta: Employee: Interventional radiology dept.

**Use of resorbable gelatin microspheres in genicular artery embolization for treatment-resistant knee pain – Technique, Safety, and Results**
*A. Taheri Amin*[1], L. M. Wilms[2], K. Jannusch[2], F. Ziayee[2], P. Minko[2]; [1]Berlin/DE, [2]Düsseldorf/DE

**Purpose:** Assess the safety, procedural aspects, and clinical outcomes of genicular artery embolization (GAE) with resorbable gelatin microspheres (RGM) in patients suffering from knee osteoarthritis (OA) or ongoing pain following total knee replacement (TKR)
**Methods or Background:** 45 patients were prospectively observed, 35 with knee OA (Kellgren–Lawrence grades 1–4) and 10 with post–TKR pain. GAE was performed using100–300 µm RGM, with embolic volume and treated vessels recorded. Clinical outcomes were measured using KOOS and NRS at baseline, 6 weeks, 3 and 6 months. The HADS questionnaire was used to screen for psychological comorbidities. Adverse events were monitored.
**Results or Findings:** At 6 months, the mean NRS decreased by 35% and the KOOS pain subscale improved by 55% (p < 0.001), with no significant differences observed between OA grades and post-TKR . A median of 3 vessels (range 2–6) were embolized, with an average embolic volume of 6.5 mL (range 2.1–18.0 mL) per procedure. Patients with HADS scores > 8 demonstrated smaller improvements in both KOOS and NRS, with up to 2.2 points less improvement per unit increase in HADS (p < 0.05). Total embolic volume was significantly higher in advanced OA and post-TKR cases, rising by an average of 2.7 mL with each OA grade (p < 0.0001). No major complications occurred.
**Conclusion:** GAE with RGM at doses exceeding 2 mL is safe and provides short-term clinical benefit in all OA grades as well as in post-TKR pain. Patients with advanced OA or post-TKR tend to require higher embolic volumes. Elevated HADS scores are linked to reduced clinical response.
**Limitations:** This study is limited by its small sample size, short-term follow-up, lack of a control group, and reliance on patient-reported outcomes without systematic imaging.
**Funding for this study:** No funding was recieved
**Has your study been approved by an ethics committee?** Yes
**Ethics committee - additional information:** All procedures performed were in accordance with the ethical standards of the institutional research committee Board (Study number: 2021-1777_5) and with the principles of the 1964 Declaration of Helsinki and its later amendments.
**Author Disclosures:**
Farid Ziayee: Nothing to disclose
Peter Minko: Nothing to disclose
Arian Taheri Amin: Nothing to disclose
Lena Marie Wilms: Nothing to disclose
Kai Jannusch: Nothing to disclose

**Connecting the Dots: The impact of Osteoarthritis, Synovitis and Evoked Pain on Embolization Endpoints in Genicular Artery Embolization**
*A. Taheri Amin*[1], E. Kemmer[2], A-J. Hübner[2], K. Jannusch[2], D. Weiß[2], L. M. Wilms[2], F. Ziayee[2], P. Minko[2]; [1]Berlin/DE, [2]Duesseldorf/DE
*(arian.taheri-amin@charite.de)*

**Purpose:** To evaluate the impact of osteoarthritis (OA), synovitis and evoked pain on embolic volume in genicular artery embolization (GAE).
**Methods or Background:** Radiographs, MRI and DSA images of patients undergoing GAE were retrospectively reviewed. OA was graded (Kellgren-Lawrence) and categorized by predominant location (medial/lateral). Synovitis was graded on unenhanced MRIs using the MRI Osteoarthritis Knee Score. Evoked pain during superselective angiography and embolic volume were systematically recorded. Embolic volume was correlated with OA grade, predominant OA location, synovitis grade and evoked pain. Clinical outcome was assessed using the numeric rating scale at six weeks and months.
**Results or Findings:** A total of 1,739 vessels were embolized in 450 patients (median: 4 ± 1 vessels per session). Pre-interventional radiographs were available in all patients, with additional MRI scans in 214 patients. Embolic volume correlated moderately with OA-grade (Spearman's ρ=0,51; p<0.001) and weakly with synovitis grade (ρ=0,20; p<0.01). In patients with predominantly medial OA, embolic volume applied in the medial compartment was significantly higher than in the lateral compartment (2.7±1.5 vs 1.5±1.2 mL; p<0.001), and vice versa for patients with predominantly lateral OA (2.2±1.1 vs 1.6±1.0 mL; p<0.001). Embolic volume was significantly higher in vessels with evoked pain than without (1.4±0.9 vs 0.8±0.3 mL; p<0.001). Pain scores improved significantly at all timepoints (p<0.001).
**Conclusion:** OA grade, predominant OA location, synovitis grade, and evoked pain influence embolic volume in GAE, illustrating that—similar to the multifactorial genesis of OA—embolization endpoints are likewise shaped by multiple variables.
**Limitations:** ChatGPT: The study is limited by its retrospective design, reliance on NRS without long-term follow-up, and MRI data available in only half of patients.
**Funding for this study:** This work was supported by the local Research Committee of the Medical Faculty of Heinrich-Heine-University Düsseldorf (grant number: 9772841).
**Has your study been approved by an ethics committee?** Yes
**Ethics committee - additional information:** All procedures performed in studies involving human participants were in accordance with the ethical standards of the institutional research committee (Study number: 2021-1777_5) and with the 1964 Helsinki declaration and its later amendments or comparable ethical standards
**Author Disclosures:**
Eva Kemmer: Nothing to disclose
Farid Ziayee: Nothing to disclose
Peter Minko: Nothing to disclose
Arian Taheri Amin: Nothing to disclose
Daniel Weiß: Nothing to disclose
Lena Marie Wilms: Nothing to disclose
Ann-Joel Hübner: Nothing to disclose
Kai Jannusch: Nothing to disclose

**14:00 - 15:30** **Research Stage 6**

### Research Presentation Session: Neuro

### RPS 1511

### Modern approaches to dementia evaluation

**Moderator**
Max Scheffler; Geneva/CH

### Mapping Cognitive Signatures through Simultaneous EEG–fMRI Coupling: Insights from MoCA and MMSE across Alzheimer's Disease and Healthy Ageing

*H. Zhang*[1], S. Laws[2], L. XIE[3], S. Ma[3]; [1]DaZhou/CN, [2]Joondalup, WA/AU, [3]Shantou, Guangdong/CN
*(hengzhangchat@gmail.com)*

**Purpose:** To test whether resting-state EEG–fMRI coupling strength ($R^2$) indexes cognitive performance across AD, MCI, and healthy ageing, and to identify stage- and domain-specific brain–cognition signatures via multimodal imaging.
**Methods or Background:** Simultaneous resting-state EEG–fMRI from 99 participants (AD=14, MCI=45, HC=40) was analyzed. EEG features were convolved with a canonical hemodynamic response function and regressed against voxel-wise fMRI via general linear models. Regional $R^2$ maps were derived and correlated with MoCA and MMSE within groups.
**Results or Findings:** In AD, MoCA correlated negatively with $R^2$ in the supplementary motor area (right; r=−0.65, p=.012), inferior temporal gyrus (left; r=−0.72, p=.004), and occipital superior gyrus (right; r=−0.71, p=.005), indicating lower cognition with higher coupling in sensorimotor/visual cortices. In MCI, MoCA showed negative correlations in thalamic regions (pulvinar anterior, left; r=−0.37, p=.013) and the parahippocampal gyrus (r=−0.32, p=.028). For MMSE, AD exhibited positive $R^2$ associations in cerebellar vermis 8 (r=+0.76, p=.002) and cerebellum lobule 10 (right; r=+0.60, p=.022), whereas MCI and HC showed widespread negative $R^2$–MMSE relationships, notably in superior temporal gyrus (right; r=−0.57, p<.001), Rolandic operculum (r=−0.39, p=.012), and thalamic anterior ventral nucleus (r=−0.38, p=.018). Overall patterns differed by group and cognitive domain, underscoring stage specificity.
**Conclusion:** EEG–fMRI coupling strength ($R^2$) delineates discrete, clinically meaningful neural signatures across the dementia continuum. Multidomain cognitive testing captures complementary vulnerability profiles, underscoring the value of coupling metrics for individualized diagnosis and monitoring. Coupling-based biomarkers show promise for early detection, risk stratification, and precision intervention, with clear potential for integration into multimodal clinical workflows.
**Limitations:** Single-center, cross-sectional cohort (n=99) with class imbalance (AD=14) and no external replication limits generalizability and causal inference; reliance on a canonical HRF and atlas parcellation may bias regional estimates.
**Funding for this study:** None
**Has your study been approved by an ethics committee?** Yes
**Ethics committee - additional information:** The study protocol was approved by the Institutional Ethics Committee of the First Affiliated Hospital of Shantou University Medical College (Approval No.: SDFY-EC-SOP-044-B-2022-188).
**Author Disclosures:**
Heng Zhang: Nothing to disclose
Lei Xie: Nothing to disclose
Simon Laws: Nothing to disclose
Shuhua Ma: Nothing to disclose

### DTI-derived radial diffusivity in the fornix as a potential early biomarker for preclinical Alzheimer's disease

*F. Sodaei*[1], M. Noroozian[2], T. M. Sheldrick-Michel[1], H. Salighe Rad[2]; [1]Odense/DK, [2]Tehran/IR
*(fsodaei@health.sdu.dk)*

**Purpose:** Alzheimer's disease (AD) is the most prevalent neurodegenerative disorder, with amnestic mild cognitive impairment (aMCI) representing an early transitional stage. Subtle white matter changes may remain undetected using standard magnetic resonance imaging (MRI). Limbic tracts, crucial for memory, can be examined with diffusion tensor imaging (DTI) to detect microstructural alterations. This study investigates whether DTI-derived measures of limbic fiber bundles can act as sensitive early biomarkers for differentiating aMCI and mild AD from cognitively healthy controls and evaluates their diagnostic potential.
**Methods or Background:** This study involved 46 participants divided into three groups: 17 with AD, 17 with amnestic aMCI, and 12 cognitively normal controls. All participants completed neuropsychological assessments and MRI, including 3D T1-weighted and DTI sequences. Diffusion data were analyzed using ExploreDTI 4.8.6 with atlas-based parcellation of limbic white matter. Fractional anisotropy (FA), mean diffusivity (MD), axial diffusivity (AxD), and radial diffusivity (RD) were extracted. Group differences were assessed with one-way ANOVA, correlations with cognition were examined, and diagnostic performance was evaluated using accuracy, sensitivity, specificity, and area under the curve (AUC).
**Results or Findings:** Region-of-interest analyses showed widespread group differences, including changes in DTI metrics within the cingulum bundle, higher MD in the uncinate fasciculus, and increased RD in the left fornix, all correlating with cognitive performance. Cross-validated diagnostic assessment of left fornix RD demonstrated strong performance (accuracy 83%, sensitivity 85%, specificity 80%, AUC 0.87), indicating early limbic white matter microstructural degeneration, with the fornix exhibiting particularly marked alterations.
**Conclusion:** DTI-derived radial diffusivity in the fornix appears to be a sensitive and non-invasive biomarker for early detection of Alzheimer's disease, which may facilitate the identification of at-risk individuals.
**Limitations:** The limitations of the study are the relatively small sample size and cross-sectional design.
**Funding for this study:** Funding was provided by Tehran University of Medical Sciences.
**Has your study been approved by an ethics committee?** Yes
**Ethics committee - additional information:** The study was approved by the ethics committee of Tehran University of Medical Sciences, Iran.
**Author Disclosures:**
Maryam Noroozian: Nothing to disclose
Hamidreza Salighe Rad: Nothing to disclose
Tanja Maria Sheldrick-Michel: Nothing to disclose
Forough Sodaei: Nothing to disclose

### Role of Arterial-spin labelling Magnetic Resonance Imaging in differentiation of Alzheimer's disease (AD) from frontotemporal dementia (FTD)

*J. Logre*, P. Narang, K. Bansal, A. Ranga; New Delhi/IN
*(logrejaya@gmail.com)*

**Purpose:** To assess the role of arterial spin labeling (ASL-MRI) in detection of perfusion changes and patterns that distinguish Alzheimer's Disease (AD) from Frontotemporal Dementia (FTD).
**Methods or Background:** Fifteen patients each with AD, FTD and healthy controls underwent 3D-Pseudocontinous ASL-MRI in addition to routine structural imaging on 3T MRI. Assessment of regional atrophy was done using visual rating scales with quantification of mean cerebral blood flow (CBF) in various regions of brain.
**Results or Findings:** Regional patterns of atrophy were seen as asymmetrical bifrontal and temporal atrophy in FTD whereas, AD showed parietal and temporal predominance, with further progressive involvement of occipital as well as frontal lobes. Significant hypoperfusion was seen in the areas corresponding to regional atrophy, predominantly in anterior cingulate gyrus in FTD which helped in differentiation of AD from controls with specificity of 80% and sensitivity of 86.6 at a cut-off mean CBF value of 31.91 ml/100gm/min in the anterior cingulate gyrus. There was significant hypoperfusion in posterior cingulate gyrus seen in AD which differentiated it from controls satisfactorily, with a specificity and sensitivity of 73.3% and 86.6% respectively, at a cut-off mean CBF value of 38.29 ml/100gm/min.
**Conclusion:** The use of functional neuroimaging techniques like ASL as an adjunct to conventional MRI can provide unique insights into the changes seen in neurodegenerative diseases as disease- specific patterns of differences in perfusion without radiation exposure, contrast administration or significant increase in scan time.
**Limitations:** The cross-sectional design and duration of study didn't allow establishment of a temporal relationship, hence, limiting our ability to draw inferences.The sample may not be a true representation of the disease population. Since, histopathological examination couldn't be done due to humanitarian reasons, there was a lack of confirmation of the diagnosis.
**Funding for this study:** No funding.
**Has your study been approved by an ethics committee?** Yes
**Ethics committee - additional information:** Institutional Ethics Committee, Maulana Azad Medical College, New Delhi, India-110002
**Author Disclosures:**
Kalpana Bansal: Nothing to disclose
Jaya Logre: Nothing to disclose
Poonam Narang: Nothing to disclose
Anju Ranga: Nothing to disclose

**Multimodal advanced MRI evaluation in patients with Alzheimer's disease and mild cognitive impairment undergoing transcranial pulse stimulation (TPS)**

*F. Bruno*, G. Saporito, G. Saltarelli, A. Innocenzi, G. Di Cerbo, C. De Felici, E. Di Cesare, F. Pistoia, A. Splendiani; L'Aquila/IT
*(federico.bruno.1988@gmail.com)*

**Purpose:** To assess structural and functional brain changes on advanced MRI in patients with Alzheimer's disease (AD) and mild cognitive impairment (MCI) undergoing transcranial pulse stimulation (TPS).
**Methods or Background:** Seventeen consecutive patients with AD/MCI scheduled for TPS underwent 3T MRI at baseline and 6 months. The protocol included structural MRI with volumetry, diffusion tensor imaging (DTI) with whole-brain and hippocampal/cingulate reconstructions, single-voxel spectroscopy of the cingulate cortex, and arterial spin labeling (ASL) perfusion. Brain volumes were segmented with dedicated software; DTI was processed in DSI-Studio; ASL was analyzed in MRI Cloud Brain Mapping to derive absolute and relative cerebral blood flow (CBF).
**Results or Findings:** Spectroscopy showed no significant post-treatment change in CH ratio (mean $\Delta = +0.09$; $p = 0.076$) or NAA ratio, with a non-significant trend toward higher CH ratio. In the hippocampus, fractional anisotropy (FA) and mean diffusivity (MD) decreased after TPS (FA $\Delta = -0.093$; $p = 0.021$; MD $\Delta = -0.255$; $p = 0.039$), while quantitative anisotropy was unchanged. ASL revealed widespread CBF increases, with significant cortical rises bilaterally and in the right frontal lobe ($p = 0.03$), left parietal lobe ($p = 0.05$), right parietal lobe ($p = 0.01$), and right temporal lobe ($p = 0.04$). Among subcortical regions, CBF increased in the right thalamus ($p = 0.05$) and left cingulate gyrus ($p = 0.05$). No significant changes were observed in white matter, occipital and insular cortices, basal ganglia, limbic system, or cerebellum.
**Conclusion:** Advanced MRI sequences, combining volumetry, DTI, spectroscopy, and ASL, can detect subtle structural and perfusion modifications after TPS in AD and MCI.
**Limitations:** These exploratory findings support the feasibility of MRI for monitoring TPS-related effects; larger controlled studies are warranted to confirm clinical significance and durability.
**Funding for this study:** None
**Has your study been approved by an ethics committee?** Yes
**Ethics committee - additional information:** Local IRB
**Author Disclosures:**
Alessandra Splendiani: Nothing to disclose
Gaspare Saltarelli: Nothing to disclose
Antonio Innocenzi: Nothing to disclose
Ernesto Di Cesare: Nothing to disclose
Francesca Pistoia: Nothing to disclose
Giovanni Di Cerbo: Nothing to disclose
Claudia De Felici: Nothing to disclose
Federico Bruno: Nothing to disclose
Gennaro Saporito: Nothing to disclose

**Correlation of Volumetric Changes and Functional Connectivity using rs-fMRI in MCI Patients**

*W. N. Machaj*, P. Podgórski, J. Maciaszek, D. Szcześniak, J. Rymaszewska, P. Piotrowski, A. Zimny; Wrocław/PL
*(wnmachaj@gmail.com)*

**Purpose:** Mild cognitive impairment (MCI) is considered a transitional state between normal aging and dementia, characterized by subjective complaints, measurable cognitive decline, and preserved daily functioning. The aim of this study was to investigate alterations in resting-state functional connectivity and brain structure in individuals with MCI, with particular emphasis on the medial temporal lobe and its interactions with other brain regions.
**Methods or Background:** We included 27 patients with MCI and 25 age-matched healthy controls. 3D T1 volumetric imaging followed by resting-state fMRI were performed on a 3T Philips Ingenia scanner equipped with a 32-channel head and neck coil. Seed-to-voxel functional connectivity analyses were performed using the bilateral hippocampi and anterior/posterior parahippocampal gyri as seed regions. Volumetric analysis was conducted with the Desikan-Killiany and Destrieux atlases to assess cortical thickness.
**Results or Findings:** MCI patients demonstrated significantly increased functional connectivity between bilateral medial temporal lobe regions and the right thalamus compared to controls. Volumetric analysis revealed reduced cortical thickness in temporal, frontal, orbitofrontal, limbic, parietal, sensorimotor, and occipito-temporal regions.
**Conclusion:** Increased medial temporal-thalamic connectivity together with widespread cortical atrophy indicates early neurodegenerative changes in MCI. These alterations may represent compensatory mechanisms in response to cognitive decline and highlight the value of combining functional and structural MRI for early detection of cognitive impairment.
**Limitations:** The main limitations of the study are its cross-sectional design and the small sample size.
**Funding for this study:** This study was supported by Wroclaw Medical University grants SUBZ.C270.24.078 and SUBK.C230.23.065.
**Has your study been approved by an ethics committee?** Yes
**Ethics committee - additional information:** This study was conducted under the guidance and approval of the Bioethical Committee at Wroclaw Medical University (KB-400/2018/2506), date of approval 25 June 2018.
**Author Disclosures:**
Patryk Piotrowski: Nothing to disclose
Julian Maciaszek: Nothing to disclose
Anna Zimny: Nothing to disclose
Weronika Natalia Machaj: Nothing to disclose
Przemysław Podgórski: Nothing to disclose
Joanna Rymaszewska: Nothing to disclose
Dorota Szcześniak: Nothing to disclose

**Examining the independent and additive effect of arterial stiffness and cerebral blood flow on total hippocampal and hippocampal subfield volumes**

*M. T. Horan*, J. F. Meaney, R-A. Kenny, C. DeLooze; Dublin/IE
*(horanmi@tcd.ie)*

**Purpose:** There is a critical link between vascular disease and the progression to dementia. The hippocampus has been implicated in the development of memory decline and whole hippocampal atrophy has been identified consistently in patients with cognitive decline.
**Methods or Background:** We investigate the independent and moderating effects of increased arterial stiffness (AS) and reduced cerebral blood flow (CBF) on total hippocampal volume (HV) in a large MRI sample of community-dwelling older adults (n=395) from a nationally representative population-based study, the Irish Longitudinal Study on Ageing (TILDA). We also examine if these effects are specific to certain hippocampal subfields which are known to be selectively vulnerable to ischemia. Arterial spin labelling MRI was used for CBF quantification. Automated Segmentation of the Hippocampal was performed using FreeSurfer v6.0.
**Results or Findings:** This four-year follow up longitudinal study demonstrated that (i) prolonged elevated AS (at baseline and at four-year follow-up), (ii) the interaction between higher AS at baseline and lower CBF at follow-up 3 and (iii) the interaction between prolonged elevated AS (at baseline and four-year follow-up) and reduced CBF at follow-up were associated with smaller hippocampal volumes. We propose that the subsequent reduction in cerebral blood flow observed with elevated arterial stiffness may be the missing link in the pathway linking arterial stiffness to hippocampal atrophy.
**Conclusion:** Our study shows that increased AS and reduced CBF are not independently associated with whole hippocampal or subfield volumes. However, when combined, increased arterial stiffness for a longer duration in combination with a reduction in cerebral blood flow is associated with lower hippocampal volumes. These effects were equally exerted across all hippocampal subfields tested in this study.
**Limitations:** There are reported limitations to automated segmentation tools in the literature.
**Funding for this study:** Funding for The Irish Longitudinal Study on Ageing (TILDA) is provided by the Irish Government, the Health Research Board (HRB), The Atlantic Philanthropies, and the Irish Life PLC. CDL is supported with funding from the Health Research Board (HRB) of Ireland under an Emerging Investigator Award (EIA-2017-012).
**Has your study been approved by an ethics committee?** Yes
**Ethics committee - additional information:** This study was approved by the Trinity College Faculty of Health Sciences Research Ethics Committee, Dublin, Ireland. Protocols conformed with the Declaration of Helsinki. Signed informed consent was obtained from all respondents prior to participation. Additional ethics approval was received for the magnetic resonance imaging (MRI) sub-study from the St James's Hospital/Adelaide and Meath Hospital, Inc. National Children's Hospital, Tallaght (SJH/AMNCH) Research Ethic Committee, Dublin, Ireland. Those attending for MRI also completed an additional MRI-specific consent form.
**Author Disclosures:**
Rose-Anne Kenny: Nothing to disclose
Celine Delooze: Nothing to disclose
Michelle Therese Horan: Nothing to disclose
James F. Meaney: Nothing to disclose

**Field Strength and Sequence Effects on Cerebral Microbleed Detection in Alzheimer's Disease: Relevance for Anti-Amyloid Antibody Therapy Eligibility**

T. Schömig, J. Kottlors, M. Schlamann, *M. Schönfeld*; Köln/DE
*(michael.schoenfeld@gmx.de)*

**Purpose:** Cerebral microbleeds (CMBs) are a safety concern in patients considered for anti-amyloid antibody therapy in Alzheimer's disease. More than four CMBs constitute an exclusion criterion for Lecanemab or Donanemab.

Detection rates vary with MRI field strength and sequence type. This study evaluated the impact of these parameters on CMB detection and their relevance for treatment eligibility in a clinical cohort.
**Methods or Background:** We retrospectively analyzed 284 of 1575 patients from two memory clinics classified within the Alzheimer's continuum, defined by a pathological cerebrospinal fluid amyloid-β 1-42/1-40 ratio and/or a positive amyloid-PET scan (visual assessment by standardized criteria). CMBs were quantified when a hemorrhage-sensitive MRI sequence was available. Patients were grouped by field strength (1.5T vs. 3T) and sequence type (T2* vs. SWI). Detection rates, CMB counts, and the proportion exceeding exclusion thresholds (>4 CMBs) were compared using Mann–Whitney U and Chi² tests.
**Results or Findings:** Of 284 eligible patients, 263 (mean age 71.3 years, 53 % female) were screened. A total of 162 underwent 1.5T MRI (all T2*) and 101 underwent 3T MRI (66 T2*, 35 SWI). CMBs were detected significantly more often at 3T than at 1.5T (43.6 % vs. 23.5 %, p<0.001), with higher mean counts (1.86 ± 4.7 vs. 1.12 ± 3.9, p=0.004). Within the 3T subgroup, SWI showed a non-significant trend toward higher detection than T2* (54.3 % vs. 37.9 %, p=0.057). Importantly, the proportion with >4 CMBs—rendering them ineligible—was significantly higher at 3T than at 1.5T (10.9 % vs. 4.3 %, p = 0.040).
**Conclusion:** MRI field strength and sequence type substantially affect CMB detection and may directly alter eligibility for anti-amyloid antibody therapy. Standardization of imaging protocols is required to ensure consistent patient selection and reliable risk assessment.
**Limitations:** None
**Funding for this study:** None
**Has your study been approved by an ethics committee?** Not applicable
**Author Disclosures:**
Michael Schönfeld: Nothing to disclose
Jonathan Kottlors: Nothing to disclose
Marc Schlamann: Nothing to disclose
Thomas Schömig: Nothing to disclose

**Neurodegeneration contributes more to cognitive impairment than microvascular injury in middle-aged and young-old patients with type 2 diabetes combined with NAFLD or high BMI**
*X. Li*, B. Zhang; Nanjing/CN
*(lixin_med@smail.nju.edu.cn)*

**Purpose:** Neurodegeneration and microvascular injury were associated with cognitive decline in type 2 diabetes (T2D). Overweight/obesity or non-alcoholic fatty liver disease (NAFLD) were also related to brain damage. We aimed to investigate the gray and white matter changes in T2D patients with NAFLD or high body mass index (BMI).
**Methods or Background:** The 451 patients with T2D and 65 normal controls were enrolled. The patients were further divided into four subgroups based on the presence of NAFLD or high BMI: 156 with both NAFLD and high BMI, 66 with NAFLD, 76 with high BMI, and 153 with neither NAFLD nor high BMI. All participants underwent magnetic resonance, clinical assessments, and cognitive tests. Brain gray volume and cortical thickness represent neurodegeneration, and peak width of skeletonized mean diffusivity (PSMD) and white matter hyperintensity (WMH) reflect microvascular injury. Partial correlation, canonical correlation, and mediation effect were employed to assess relationship between metabolic measures, imaging markers and cognitive performance.
**Results or Findings:** 1) The patients with T2D exhibited widespread brain atrophy involving global gray matter, subcortical structures, and hippocampal subfields. Both NAFLD and high BMI were independently associated with neurodegeneration, with no significant interaction effect; 2) No significant differences were found in PSMD or WMH volume between the T2D and normal controls groups, and neither NAFLD nor high BMI had significant impacts on white matter injury; 3) The T2D group showed multiple damage in cognitive domains, and both NAFLD and high BMI independently effected executive function; 4) Mediation analysis indicated that neurodegeneration mediated the association between metabolic dysregulation and cognitive impairment, while white matter injury did not.
**Conclusion:** The results revealed neurodegeneration contributes more to cognitive impairment than cerebral small vessel injury in middle-aged and young-old patients with T2D.
**Limitations:** Not applicable
**Funding for this study:** Not applicable
**Has your study been approved by an ethics committee?** Yes
**Ethics committee - additional information:** No
**Author Disclosures:**
Bing Zhang: Nothing to disclose
Xin Li: Nothing to disclose

**Diagnostic Performance of Qualitative and Quantitative PET/MRI Biomarkers: Distinct Patterns of Atrophy and Hypometabolism in Dementia**
*S. Petrović*[1], M. Nguyen[2], N. Villain[2], F-X. Lejeune[2], A. Kas[2], M-O. Habert[2], N. Pyatigorskaya[2]; [1]Novi Sad/RS, [2]Paris/FR
*(petrovicsonia9@gmail.com)*

**Purpose:** The aim of this study is to identify distinct patterns of atrophy and hypometabolism for differential diagnosis of dementia and compare qualitative and quantitative biomarker classification performance.
**Methods or Background:** The study included 274 patients from Pitié-Salpêtrière Memory Clinic who underwent PET/MRI scanning with the diagnosis of Alzheimer's disease (AD), frontotemporal dementia (FTD), Lewy Body dementia (LBD). Qualitative and quantitative region of interest (ROI) analysis of T1W and FDG-PET images was performed. ROI were chosen according everyday clinical practice and functional characteristics. Statistical Data Integration Analysis for Biomarker Discovery using the Latent components was performed.
**Results or Findings:** Quantitative metabolism showed good separation of FTD from other groups, mainly involving the temporal pole and caudate, while visual metabolism distinguished FTD from LBD, with hypometabolism in the prefrontal and insular regions versus parietal regions, respectively. AD was separated from the other groups by hypometabolism in the mesial temporal region in both visual and quantitative metabolism, and by posterior cingulate hypometabolism in the quantitative block. The AUC was higher in metabolism blocks (0.778–0.954) compared to volume blocks (0.691–0.864), with the best performance from visual metabolism (LBD vs. others 0.954; FTD vs. others 0.938; AD vs. others 0.81). Qualitative volume contributed mainly to FTD separation, involving temporal pole, prefrontal, and anterior cingulate, while quantitative volume showed no clear separation. No significant AUC differences were found between qualitative and quantitative approaches for both metabolism and atrophy (p>0.05).
**Conclusion:** FTD demonstrated a characteristic pattern across both modalities and analytic approaches, allowing easier differentiation from other dementias. FDG-PET added sensitivity to functional changes, while MRI highlighted complementary structural alterations. Visual and quantitative analyses showed comparable diagnostic performance, suggesting no significant added value of implementing quantitative software in clinical practice.
**Limitations:** No limitations were identified.
**Funding for this study:** No funding was received for this study.
**Has your study been approved by an ethics committee?** Yes
**Ethics committee - additional information:** The study was approved by national ethical committee.
**Author Disclosures:**
Nadya Pyatigorskaya: Nothing to disclose
Aurelie Kas: Nothing to disclose
Sonja Petrović: Nothing to disclose
Nicolas Villain: Nothing to disclose
François-Xavier Lejeune: Nothing to disclose
Mathilde Nguyen: Nothing to disclose
Marie-Odile Habert: Nothing to disclose

**MCI-specific white matter-cognition interactions in dementia transition characterization using OpenMAP-T1 analysis**
*M. Kawaguchi*, D. Yoshimaru, Y. Nakamura, K. Touma, K. Saito; Nishishinnjyuku,Tokyo/JP
*(masukawa0311@gmail.com)*

**Purpose:** While MCI is traditionally viewed as an intermediate stage to AD, the dynamic nature of brain-cognition relationships during this transition remains unclear. This study investigated disease-specific white matter-cognition interaction patterns and their correlations using automated OpenMAP-T1 analysis to characterize the pathological continuum of cognitive decline.
**Methods or Background:** We analyzed 74 participants: 10 healthy controls (68.3±9.1 years), 16 MCI (75.4±4.0 years), and 48 AD patients (78.8±7.5 years). White matter regions were automatically segmented from 3D T1-weighted images using OpenMAP-T1. Multiple regression analyses with/without disease-by-cognition interaction terms were performed for MMSE and MoCA-J, adjusting for TIV, age, and sex. Spearman correlations between white matter volumes and cognitive scores were calculated for interaction-significant regions in the healthy-MCI combined group.
**Results or Findings:** Disease-by-cognition interactions were exclusively observed in MCI but completely absent in AD and controls. MoCA-J demonstrated superior sensitivity, detecting interactions in five regions (SS_R, IFO_L, IFO_R, SS_L, SLF_R; all p<0.05) versus one for MMSE (SS_L, p=0.024). Correlation analysis in MCI revealed that interaction-significant regions maintained strong positive correlations with MoCA-J (SS_L: ρ=0.767, p=0.001; IFO_R: ρ=0.725, p=0.002; SS_R: ρ=0.702, p=0.004; SLF_R: ρ=0.632, p=0.011; IFO_L: ρ=0.622, p=0.013), indicating preserved brain-cognition relationships in MCI. In baseline regression, cognition-related significant regions progressively decreased from controls (MMSE: 17, MoCA-J:

18) through MCI (MMSE: 13, MoCA-J: 12) to AD (MMSE: 4, MoCA-J: 9), demonstrating stage-dependent network disruption.
**Conclusion:** Our findings reveal MCI as a unique dynamic state with active brain-cognition remodeling and preserved correlations, fundamentally distinct from AD's terminal state with disrupted relationships. This distinction indicates MCI represents a critical therapeutic window. MoCA-J emerged as the optimal detection tool, providing important implications for early diagnosis and intervention strategies.
**Limitations:** Cross-sectional design limits assessment of longitudinal changes. Small sample size, particularly in controls (n=10).
**Funding for this study:** None
**Has your study been approved by an ethics committee?** Yes
**Ethics committee - additional information:** Approved by Tokyo Medical University Hospital IRB (T2022-0215).
**Author Disclosures:**
Kazuhiro Saito: Nothing to disclose
Kaori Touma: Nothing to disclose
Daisuke Yoshimaru: Nothing to disclose
Masumi Kawaguchi: Nothing to disclose
Yui Nakamura: Nothing to disclose

**Correlation between visual scales of brain atrophy and volumetric analysis using artificial intelligence software in patients with cognitive impairment and Brain MRI**
*M. B. Nallino*, P. Acevedo, L. Gangui Araoz, M. I. Cañizares, L. Zamer, A. A. Ojeda; Rosario/AR
*(belunallino@hotmail.com)*

**Purpose:** In patients with cognitive impairment (CI), identifying atrophy patterns and assessing white matter integrity is crucial for accurate diagnosis and treatment. This study aims to correlate visual scales of atrophy, including Medial Temporal Atrophy (MTA), Global Cortical Atrophy (GCA), KOEDAM score, Evans Index and evaluation of leukoaraiosis (Fazekas scale) in brain magnetic resonance imaging (bMRI), with automated volumetric analyses using artificial intelligence software.
**Methods or Background:** The retrospective study involved 104 patients (49 women and 55 men) with CI who underwent bMRI at our institution between January 2023 and August 2023. Visual assessments were performed using scales (MTA), (GCA), (KOEDAM), Evans Index, and Fazekas scale by a neuroradiologist with over 15 years of experience. Additionally, automated brain volumetric analyses were conducted using the Entelai artificial intelligence software. Spearman correlation coefficients were computed to assess the relationship between brain volumes and the corresponding visual atrophy scales.
**Results or Findings:** Results revealed a moderate inverse correlation between global brain volume and GCA scores (-0.47), particularly pronounced in patients over 70 years old. Grey matter volume showed a strong inverse correlation with GCA (-0.62). Both right and left hippocampal volumes had significant inverse correlations with MTA (-0.568 and -0.577, respectively). Furthermore, the Evans Index exhibited a strong positive relationship with ventricular volume (0.789), especially in older patients.
**Conclusion:** The results show a consistent association between visual measures of cerebral atrophy and quantitative volumes obtained through software. As scores on the visual scales (GCA, MTA, Koedam, Evans) increase, a reduction in brain volumes or an increase in ventricular volume is observed, with a more significant impact on the older age groups. This supports the validity of the scales used and suggests that automatic quantification could be a useful supplement.
**Limitations:** Patient number
**Funding for this study:** No
**Has your study been approved by an ethics committee?** Not applicable
**Author Disclosures:**
Maria Ines Cañizares: Nothing to disclose
Adriana Alicia Ojeda: Nothing to disclose
Paola Acevedo: Nothing to disclose
Lucia Gangui Araoz: Nothing to disclose
Lisbeth Zamer: Nothing to disclose
María Belen Nallino: Nothing to disclose

**14:00 - 15:30 Research Stage 2**

### Research Presentation Session: Oncologic Imaging

### RPS 1516

### Advancements in hepatocellular carcinoma (HCC) imaging

**Moderator**
Max Seidensticker; Gräfelfing/DE
*(max.seidensticker@med.uni-muenchen.de)*
**Author Disclosures:**
Max Seidensticker: Advisory Board: Siemens, Bayer, SIRTEX, Grant Recipient: SIRTEX, Astra Zeneca, Boston Scientific

**Role of PMFI-CEUS in the differential diagnosis between ICC and HCC**
*G. A. Risoleo*, M. Ferrari, G. Tripodi, A. Teti, G. Casuscelli, D. Ierace, R. Maccarone, M. Lico, F. Loria; Vibo Valentia/IT
*(risoleog@gmail.com)*

**Purpose:** CEUS is used in the differential diagnosis between intrahepatic cholangiocarcinoma (ICC) and hepatocellular carcinoma (HCC). ICC typically shows earlier arterial enhancement and faster wash-out than HCC. Parametric Micro-Flow Imaging (PMFI) provides further information providing a color-coded map of vascularization and allow to make a differential diagnosis. The purpose of this study was to evaluate the diagnostic value of PMFI-CEUS in the differential diagnosis between ICC and HCC.
**Methods or Background:** We retrospectively reviewed CEUS examinations, recorded on digital data, of 52 patients with histologically proven ICC, after CEUS the lesions were analyzed using PMFI which produces a color-coded map of vascularization. During post-processing, the PMFI software generated light-blue for the wash-in phase and red-yellow for the wash-out phase, providing a detailed view of microbubble dynamics within the lesions. For each lesion we assessed: wash-in and wash-out timing; PMFI color pattern, and time–intensity curve (TIC) parameters, including arrival time and time-to-peak. These findings were compared with a control group of 160 patients affected by HCC.
**Results or Findings:** ICC showed earlier wash-in (11–22 s) compared with control group of HCC (20–30 s,). Wash-out occurred between 22–35 s in ICC, while in HCC started after 50–60s. On PMFI-CEUS, ICC were predominantly blue-light color coded, while HCC were more red-color coded. TIC confirmed shorter arrival time and time-to-peak in ICC. These findings showed sensitivity 100%, specificity 100% and accuracy 100% to differentiate ICC from HCC.
**Conclusion:** PMFI-CEUS provides reproducible parameters for differentiating ICC from HCC. Early wash-in with blue-light color coded map support ICC, while delayed wash-in with more red-coded PMFI indicates HCC, improving diagnostic accuracy and clinical decision-making in primary liver tumors.
**Limitations:** The sample is relative small. A multicentric confirmation is needed.
**Funding for this study:** No Funding.
**Has your study been approved by an ethics committee?** Yes
**Ethics committee - additional information:** ASP Vibo Valentia
**Author Disclosures:**
Debora Ierace: Nothing to disclose
Gianluca Tripodi: Nothing to disclose
Marcello Ferrari: Nothing to disclose
Giovanni Antonio Risoleo: Nothing to disclose
Francesco Loria: Nothing to disclose
Angela Teti: Nothing to disclose
Rosario Maccarone: Nothing to disclose
Matilde Lico: Nothing to disclose
Giuseppe Casuscelli: Nothing to disclose

**Preliminary application of time-dependent diffusion MRI based on 5.0T ultra-high field in the diagnosis and pathological grading of HCC**
*S-P. P. Li*[1], D. Yin[1], X. Song[2], Z. Feng[2], Y. Liu[1], W. Wei[1], L. Liu[1]; [1]Hefei/CN, [2]Shanghai/CN
*(lsp_318@139.com)*

**Purpose:** To investigate the feasibility of microstructure analysis using 5.0T ultra-high-field MRI based on time-dependent diffusion imaging(td-dMRI) in differentiating benign and malignant liver lesions and assessing the pathological grading of HCC

**Methods or Background:** 51 patients with clinically confirmed focal liver lesions were enrolled. Td-dMRI were acquired using a 5.0T scanner with pulsed and oscillating gradient spin-echo MRI sequences at equivalent diffusion times ranging from 4.0 to 44 msec. The imaging microstructural parameters using limited spectrally edited diffusion (IMPULSED)–based parameters derived from td-dMRI including cell diameter(d), intracellular volume fraction(vin), cellularity, and extracellular diffusivity(Dex). Differences in these parameters between malignant and benign lesions were compared.
**Results or Findings:** 1. 14 patients had benign lesions and 37 had malignant lesions (including 23 HCC, 5 ICC, and 9 metastases). Benign lesions exhibited lower vin and cellularity but larger cell diameter, with statistically significant differences, and the diagnostic efficacy of cellularity is the highest(AUC=0.714). Dex was higher in benign lesions but not statistically significant. 2. Among 23 HCC cases, 18 were pathologically confirmed, including 7 low-grade (I-II) and 9 high-grade (III-IV) HCC. The high-grade HCC group showed higher vin and cellularity but smaller cell diameter and lower Dex. Statistically significant differences were observed in cellularity and vin between the two groups. 3. When patients were divided into a normal liver group and a hepatitis B virus (HBV)-infected or cirrhotic group, the HBV/cirrhotic group exhibited increased hepatic cellularity and vin, with reduced cell diameter and Dex.
**Conclusion:** Time-dependent diffusion MRI-based microstructural characterization is a feasible and effective method for differentiating benign and malignant liver lesions and assessing HCC pathological grades, with structural correlations to histopathology.
**Limitations:** Its potential value in evaluating post-hepatitis B cirrhosis warrants further investigation.
**Funding for this study:** No
**Has your study been approved by an ethics committee?** Yes
**Ethics committee - additional information:** Approval number：2022KY-267
**Author Disclosures:**
Wei Wei: Author: co-author
Xiaopeng Song: Author: co-author
Shao-Peng Peng Li: Author: presenter/speaker
Yin Liu: Author: co-author
Dawei Yin: Author: co-author
Lianxin Liu: Author: co-author
Zhichao Feng: Author: co-author

**Prognostic modeling using early CT features after TACE in HCC – comparison with established imaging response systems**
*C. B. Fink*, F. Ziayee, J. Böven, K. Jannusch, G. Antoch, P. Minko, E. Tietz; Düsseldorf/DE

**Purpose:** To assess the prognostic utility of early contrast-enhanced CT after drug-eluting bead transarterial chemoembolization (DEB-TACE) in hepatocellular carcinoma (HCC), focusing on validation of CT as a diagnostic tool and development of a simplified prognostic imaging model compared to established response criteria.
**Methods or Background:** We retrospectively analyzed 80 HCC patients who underwent DEB-TACE between 2014–2021. Multiphasic CT was performed before and within 7 days after treatment. Two radiologists evaluated morphologic features including arterial phase hyperenhancement (APHE), non-peripheral washout, and capsule enhancement. Regression was defined as disappearance of each feature. Tumor shrinkage was quantified. A Cumulative Regression Score (CRS, 0–3) was derived, and a CT-based Early Response Model (CERM) combining APHE regression and tumor shrinkage was developed. The primary endpoint was progression-free survival (PFS). Survival analysis used Kaplan–Meier and Cox regression; model performance was compared with mRECIST and LI-RADS TR.
**Results or Findings:** APHE regression was observed in 60.6% of patients and independently predicted longer PFS (HR = 0.49, p = 0.01). was also independently prognostic (HR 0.60; p = 0.03). Washout regression was significant only in univariate analysis; capsule regression was not prognostic. Higher CRS values correlated with stepwise PFS improvement (HR per regressed feature 0.74, p = 0.03). CERM achieved the best prognostic performance (C-index = 0.65), surpassing mRECIST and LI-RADS TR. Internal validation confirmed minimal overfitting and stable calibration.
**Conclusion:** Early CECT after DEB-TACE is feasible and provides reproducible biomarkers for early PFS prediction. The proposed CERM enables individualized risk stratification within one week, without additional laboratory or functional imaging.
**Limitations:** Retrospective single-center design; external validation is needed.
**Funding for this study:** None
**Has your study been approved by an ethics committee?** Yes
**Ethics committee - additional information:** The institutional ethics committee approved the study (ref. 2023-2630) and waived informed consent.
**Author Disclosures:**
Farid Ziayee: Nothing to disclose
Peter Minko: Nothing to disclose
Eric Tietz: Nothing to disclose
Gerald Antoch: Nothing to disclose
Christian Bijan Fink: Nothing to disclose
Judith Böven: Nothing to disclose
Kai Jannusch: Nothing to disclose

**Associations between the SMARS score derived from CT and MRI with histopathological features in HCC**
*D. Romeo*[1], T. Denecke[2], H-J. Meyer[2]; [1]Palermo/IT, [2]Leipzig/DE
*(daniele.romeo.de@gmail.com)*

**Purpose:** Complex associations exist between imaging phenotype and underlying histopathology for hepatocellular carcinoma (HCC). SMARS score discriminates proliferative and non-proliferative HCC in a non-invasive way, associated with treatment outcomes. Systematic validation is needed and it is unclear whether associations between this score with histopathology features exist. This study elucidates correlations between the CT and MRI-defined SMARS score with immunohistochemistry of the pathological specimens in a curatively-treated HCC cohort.
**Methods or Background:** 44 patients (mean age: 59.6±10.7 years) with histologically confirmed HCC after curative surgical resection were included. Contrast-enhanced MRI and CT were performed before surgery and the SMARS score was calculated. Samples were analyzed for programmed death ligand 1 (PD-L1), Glypican-3, CD3-tumour infiltrating lymphocyte, CD68+ cells, CD34+ microvessel density (MVD).
**Results or Findings:** The median MRI-derived SMARS score was 1.4 (IQR: -0.32; 2.18) and the CT-derived was − 0.32 (IQR: -1.08; 0.56). According to the proposed threshold, 29 tumours were categorized as proliferative (82.9%) and six as nonproliferative HCC (17.1%) accordingly to the MRI-derived SMARS score. According to the CT-derived SMARS score 24 tumours were categorized as proliferative (61.5%) and 15 as nonproliferative HCC (38.5%). A moderate association was shown between the MRI-derived SMARS score with the Glypican-3 expression (r = 0.37, p = 0.03). CT-derived SMARS score showed correlations with two PD-L1 parameters (PD-L1 tumour positive score r=-0.37, p = 0.02; PD-L1 combined positive score r=-0.35, p = 0.03). No other association with the remaining parameters was detected.
**Conclusion:** SMARS score is a promising imaging score associated with Glypican-3 and PD-L1 expression in curatively-treated HCC patients. Differences between the CT and MRI-defined score need to be investigated in further trials on larger cohorts.
**Limitations:** Retrospective single-center design, small patient sample, different time periods between imaging and histopathology.
**Funding for this study:** None
**Has your study been approved by an ethics committee?** Yes
**Ethics committee - additional information:** This retrospective study was approved by the institutional ethic committee (University of Leipzig, approval number: 159/25-ek ) and informed consent was waived.
**Author Disclosures:**
Timm Denecke: Nothing to disclose
Daniele Romeo: Nothing to disclose
Hans-Jonas Meyer: Nothing to disclose

**The impact of extrahepatic metastases in patients with hepatocellular carcinoma: A longitudinal analysis**
*A. Mähringer-Kunz*[1], L. Müller[1], M-T. Rüßmann[1], I. Schmidtmann[1], M. Moos[1], D. Pinto Dos Santos[1], T. Bäuerle[1], A. Weinmann[1], R. Klöckner[2]; [1]Mainz/DE, [2]Lübeck/DE

**Purpose:** Extrahepatic metastatic disease (EMD) in patients with hepatocellular carcinoma (HCC) is classified as advanced stage by BCLC. Evidence on prognostic impact of EMD is limited. We investigated the prognostic relevance of EMD — with a special focus on anatomical sites — using a longitudinal approach.
**Methods or Background:** Patients with HCC treated between 01/2007-12/2021 were included. All cross-sectional imaging was re-evaluated by a radiologist to determine the timing and anatomical sites of EMD. Overall survival (OS) was analysed using a multivariable Cox model with site-specific metastases as time-dependent covariates, adjusting for established risk factors.
**Results or Findings:** Of 1,563 patients included, 429 (27.4%) had EMD: 190 (12.2%) at diagnosis (synchronous) and 239 (15.3%) during follow-up (metachronous). Median OS was 5.6 months with synchronous EMD vs 20.0 months in those without EMD or with metachronous EMD (p<0.001). The distribution of metastatic sites and corresponding hazard ratios (HR, 95% CI) was: lung (n=183; HR=1.62 (CI=1.32-2.00)), regional lymph nodes (n=168; HR=1.55 (CI=1.27-1.90)), bone (n=102; HR=1.37 (CI=0.84-2.25)), peritoneum (n=101; HR=1.78 (CI=1.40-2.27)), adrenal glands (n=79; HR=1.42 (CI=1.02-

1.99)), distant lymph nodes (n=53; HR=2.38 (CI=1.74-3.27)), and soft tissue (n=43; HR=1.79 (CI=1.23-2.60)). Metastases at the sites lung, lymph nodes, peritoneum, adrenal glands, and soft tissue were independent predictors of survival. Each additional site increased the HR by 1.64 (95% CI: 1.54-1.74).
**Conclusion:** Importantly, the presence of any metastasis is associated with adverse outcomes and an unfavorable hazard ratio. However, bone involvement appears comparatively less detrimental than other forms of extrahepatic spread, whereas distant lymph node metastases are particularly unfavorable. Moreover, prognosis deteriorates progressively with the number of metastatic sites, with each additional site conferring a substantial increase in risk. Thus, regular re-staging with detailed imaging evaluation is crucial.
**Limitations:** Limitations include the retrospective, single-center design.
**Funding for this study:** No funding was received for this study.
**Has your study been approved by an ethics committee?** Not applicable
**Ethics committee - additional information:** Approval was not required due to the retrospective nature of the study
**Author Disclosures:**
Tobias Bäuerle: Nothing to disclose
Roman Klöckner: Nothing to disclose
Marie-Theres Rüßmann: Nothing to disclose
Maximilian Moos: Nothing to disclose
Aline Mähringer-Kunz: Nothing to disclose
Irene Schmidtmann: Nothing to disclose
Lukas Müller: Nothing to disclose
Daniel Pinto Dos Santos: Nothing to disclose
Arndt Weinmann: Nothing to disclose

**Beyond size and morphology: multiparametric MRI for functional characterization and therapy monitoring in hepatocellular carcinoma**
*E. Hoffmann*, B. Noto, F. Rennebaum, W. Roll, M. Köhler, M. Masthoff, P. Schindler; Münster/DE
*(emily.hoffmann@ukmuenster.de)*

**Purpose:** The tumor microenvironment (TME) plays a crucial role in the progression and treatment response of hepatocellular carcinoma (HCC). This proof-of-concept study examined whether multiparametric magnetic resonance imaging (mpMRI) could noninvasively characterize the TME and detect early, therapy-induced alterations following transarterial chemoembolization (TACE).
**Methods or Background:** 18 patients with HCC underwent comprehensive mpMRI at 3T, including diffusion-weighted imaging (DWI), T1 and T2* mapping, as well as dynamic contrast-enhanced (DCE) MRI. 8 patients underwent scans before and after TACE. Quantitative imaging biomarkers were derived to assess perfusion, endothelial permeability, cellular microstructure, hemorrhage and edema in tumorous and nontumorous liver tissue.
**Results or Findings:** Preliminary quantitative analysis revealed significant differences in TME characteristics between HCC lesions and non-tumor liver tissue. HCC lesions showed disrupted microvascular architecture with increased permeability and altered perfusion [e.g., mean Ktrans ($10^{-3}$/min) HCC: 796 ± 330, non-tumor: 227 ± 165, p = 0.0036]. These vascular alterations were paralleled by restricted diffusivity [mean ADC ($10^{-3}$ $mm^2$/s) HCC: 1.30 ± 0.29, non-tumor: 2.23 ± 0.20, p < 0.0001] and prolonged T2* relaxation times [mean T2* (ms) HCC: 39 ± 4, non-tumor: 18 ± 5, p = 0.004], indicating intratumoral hemorrhage, edema and microstructural disorganization. Early post-TACE scans demonstrated that mpMRI can detect therapy-induced remodeling of the TME before changes in size or morphology become apparent.
**Conclusion:** MpMRI allows for noninvasive characterization of the TME of HCC and demonstrates sensitivity to early treatment-related changes. These findings underscore the potential of mpMRI-derived biomarkers to support personalized therapeutic decisions and improve response evaluation in targeted oncologic therapies.
**Limitations:** These results are limited by the small preliminary sample size. A prospective clinical trial has recently begun to validate these findings and establish robust imaging biomarkers for MRI-based characterization of the TME of HCC.
**Funding for this study:** None
**Has your study been approved by an ethics committee?** Yes
**Ethics committee - additional information:** 2025-207-f-S
**Author Disclosures:**
Florian Rennebaum: Nothing to disclose
Wolfgang Roll: Nothing to disclose
Philipp Schindler: Nothing to disclose
Michael Köhler: Nothing to disclose
Emily Hoffmann: Nothing to disclose
Benjamin Noto: Nothing to disclose
Max Masthoff: Nothing to disclose

**Prediction of microvascular invasion in hepatocellular carcinoma using multiparameter spectral CT and clinical radiological features**
*S. C. Singh*[1], C. Xie[2], Z. Song[2], Y. Liao[2]; [1]Lalitpur,Kathmandu/NP, [2]Guangzhou/CN
*(subhash2121989@gmail.com)*

**Purpose:** This study aims to evaluate the performance of dual-layer spectral CT (DLCT) multi-parameter imaging combined with clinical-radiological features in predicting microvascular invasion (MVI) in hepatocellular carcinoma (HCC), and to develop a logistic regression-based predictive model for clinical decision support.
**Methods or Background:** This study included 67 patients with pathologically confirmed HCC who underwent preoperative multiphase DLCT between Oct 2022 and Oct 2024. Clinical variables, radiological features and DLCT parameters (electron density, iodine density, MONO-E 40/70/100 keV, Zeff) were collected, and normalized iodine concentration was calculated. Univariate tests compared patients with and without MVI. Variables with clinical relevance and low collinearity (VIF < 5, r < 0.7) entered multivariable logistic regression to build four models (DLCT, clinical, radiological and combined). Model performance was assessed using AUC, sensitivity, specificity, accuracy, predictive values, DeLong's test, decision curve analysis, and a nomogram.
**Results or Findings:** Among 67 patients, 21 (31.3%) had MVI. Univariate analysis identified PIVKA-II, tumor size, non-smooth margin, and TTPVI as significant factors, while multivariate analysis retained only TTPVI. Selected DLCT parameters were included for modeling despite non-significance. The combined model demonstrated the highest predictive performance (AUC = 0.880) with balanced diagnostic accuracy (accuracy 0.82, sensitivity 0.76, specificity 0.85). It significantly outperformed the individual models (all p < 0.05), and decision curve analysis indicated clinical benefit. The nomogram provided a straightforward tool for individualized risk estimation.
**Conclusion:** Combining DLCT multi-parameters with clinical-radiomics features significantly improves preoperative prediction of MVI in HCC, supporting individualized treatment decisions.
**Limitations:** Single-center study with a small cohort; external validation and comparison with advanced machine learning models are needed.
**Funding for this study:** Not applicable
**Has your study been approved by an ethics committee?** Not applicable
**Author Disclosures:**
Chuanmiao Xie: Nothing to disclose
Yuting Liao: Nothing to disclose
Zhixuan Song: Nothing to disclose
Subhash Chandra Singh: Nothing to disclose

**Photon-Counting CT for Interventional Oncology: Improving Vascular Mapping, Tumor Detection, and Personalized Treatment Strategies**
*P. Chang*[1], W. Chiou[2]; [1]Taichung/TW, [2]Taithung/TW
*(bibichang1023@gmail.com)*

**Purpose:** To illustrate the clinical applications of photon-counting CT (PCCT) in abdominal interventional radiology (IR) through a series of representative cases, with emphasis on hepatocellular carcinoma (HCC), metastases, and rare hepatic neoplasms.
**Methods or Background:** We collected abdominal PCCT cases performed at a tertiary referral center between April–August 2025. The cases covered three main clinical scenarios: (1) vascular mapping for TACE and Y-90 treatment; (2) detection of recurrence and follow-up after locoregional therapy or immunotherapy; and (3) evaluation of rare hepatic neoplasms. Each case was analyzed for the incremental value of PCCT compared with conventional energy-integrating detector CT. Specific techniques assessed included virtual monoenergetic imaging (VMI), iodine mapping, virtual non-contrast (VNC), and vessel reconstruction.
**Results or Findings:** Across the case collection, PCCT consistently provided higher lesion conspicuity, especially at 40–50 keV VMI, allowing better visualization of arterial-phase hyperenhancement and washout in HCC and metastases. Vascular mapping was improved, with clearer identification of segmental/subsegmental feeders, anatomical variants, and arterioportal shunts—crucial for TACE and Y-90 planning. Dose- and contrast-saving protocols achieved reliable imaging with contrast volumes as low as 45 ml. Surveillance cases demonstrated PCCT's ability to differentiate recurrent HCC from pseudoprogression or ablation changes. Rare tumors such as mucinous cystic neoplasm and IPMN were also better characterized.
**Conclusion:** PCCT is a promising next-generation imaging tool for safe and personalized interventional strategies in liver cancer and abdominal disease.
**Limitations:** Single-center retrospective review. Limited case numbers, especially for rare hepatic neoplasms. Lack of quantitative outcome correlation with treatment efficacy.
**Funding for this study:** None
**Has your study been approved by an ethics committee?** Not applicable
**Author Disclosures:**
Piyi Chang: Nothing to disclose
Weiru Chiou: Nothing to disclose

**Predictive Value of IVIM-Based Habitat Radiomics for Pathological Grading of Hepatocellular Carcinoma**
*J. Pan*[1], J. Dai[2], Q. Wang[1], L. Gao[1]; [1]Shijiazhuang/CN, [2]Shanghai/CN

**Purpose:** To explore the predictive value of habitat radiomics based on intravoxel incoherent motion (IVIM) for the pathological grading of hepatocellular carcinoma (HCC).
**Methods or Background:** Clinical and imaging data were retrospectively collected from 83 patients with pathologically confirmed and Edmondson-Steiner graded HCC (96 lesions in total) between May 2018 and June 2025. HCC was categorized as low-grade ( Ⅰ-Ⅱ ) or high-grade (Ⅲ-Ⅳ). Lesions were randomly split into a training set (n=67) and validation set (n=29) at a 7:3 ratio. All patients underwent preoperative IVIM scans on a 3.0 T MRI system. Based on the k-means clustering results of IVIM parametric maps, the tumor region of interest (ROI) was segmented into multiple subregions, from which radiomic features were subsequently extracted. Univariate/multivariate analyses screened clinical features to construct a logistic regression (LR) clinical model. For habitat features, Spearbvtman correlation and LASSO selected variables used to build multiple classifiers (LR, RF, SVM, k-NN, Bayesian), with the optimal model determined by 5-fold cross-validation. Model performance (AUC, calibration, decision curves) and comparisons (Delong test) were evaluated.
**Results or Findings:** Univariate and multivariate analyses identified age as an independent predictor for HCC pathological grading. The tumor region was partitioned into four subregions using k-means clustering. After radiomic feature screening, 10 features were retained. Among habitat-based models, the RF model performed best (training set AUC: 0.926, 95% CI: 0.851–0.980; validation set AUC: 0.863, 95% CI: 0.712–0.971). The combined model showed the highest diagnostic performance (training set AUC: 0.950, 95% CI: 0.894–0.990; validation set AUC: 0.926, 95% CI: 0.810–1.000).
**Conclusion:** IVIM-based habitat radiomics has favorable predictive value for HCC pathological grading. The combined model further improves performance, providing a novel methodological reference for preoperative non-invasive HCC grading.
**Limitations:** Not applicable.
**Funding for this study:** No funding was provided for this study.
**Has your study been approved by an ethics committee?** Yes
**Ethics committee - additional information:** The ethics committee notification can be found under the number 2021KY121
**Author Disclosures:**
Jiahuan Dai: Nothing to disclose
Lei Gao: Nothing to disclose
Jiangyang Pan: Nothing to disclose
Qi Wang: Nothing to disclose

**Quantitative analysis of spectral CT multi-parameter imaging for evaluating residual tumor activity after transarterial chemoembolization in hepatocellular carcinoma**
C. Liu[1], W. Liang[1], J. Meng[1], *X. Liu*[2], D. Liu[1]; [1]Wuzhou/CN, [2]Guangzhou/CN
*(xiaomin.liu@philips.com)*

**Purpose:** To investigate the diagnostic value of spectral CT multi-parameter imaging in detecting residual tumor activity in hepatocellular carcinoma (HCC) lesions after transarterial chemoembolization (TACE).
**Methods or Background:** Thirty-nine HCC patients with 43 lesions (26 with lipiodol deposition, 17 with residual tumor) who underwent TACE were retrospectively analyzed. Pathology or digital subtraction angiography (DSA) served as the reference standard. All patients received spectral CT plain and four-phase enhanced scans. Quantitative parameters, including conventional CT, virtual mono-energetic images (VMIs at 40/70 keV), iodine concentration (IC), normalized iodine concentration (NIC), effective atomic number (Zeff), spectral slope (λHU), electron density (ED), arterial enhancement fraction (AEF), and extracellular volume fraction (ECV) were measured in the early arterial (EAP), late arterial (LAP), venous (VP), and parenchymal (PP) phases. ROC analysis was performed to assess diagnostic performance.
**Results or Findings:** Significant differences between lipiodol deposition and residual tumor were observed in Zeff, λHU, IC, and NIC in EAP; Zeff, VMI 40 keV, λHU, IC, and NIC in LAP; and ED in PP (all $P < 0.05$). AEF(EAP/VP), AEF-(LAP/VP), AEF-(EAP/PP), AEF-(LAP/PP), and ECV showed no significant differences. The best single-parameter performance was obtained with Zeff and λHU in LAP (cut-off values: 8.24 and 3.44; AUC = 0.725 and 0.724). The combined multi-parameter model achieved excellent diagnostic accuracy (AUC = 0.953, 95% CI: 0.896–1.00, $P < 0.001$), with 100% sensitivity and 76% specificity.
**Conclusion:** Spectral CT multi-parameter imaging enables comprehensive and quantitative evaluation of HCC lesions after TACE, overcoming the limitations of conventional imaging obscured by lipiodol deposition. This approach may facilitate early detection of tumor recurrence and guide personalized treatment strategies.
**Limitations:** None
**Funding for this study:** None
**Has your study been approved by an ethics committee?** Yes
**Ethics committee - additional information:** None
**Author Disclosures:**
Xiaomin Liu: Nothing to disclose
Cuiyu Liu: Nothing to disclose
Dezhang Liu: Nothing to disclose
Jinjin Meng: Nothing to disclose
Weizhi Liang: Nothing to disclose

**16:00 - 17:30 Research Stage 1**

### Research Presentation Session: Abdominal and Gastrointestinal

### RPS 1601
### Updates on pancreatic cysts and gland dysfunction

**Moderator**
John J. Hermans; Nijmegen/NL
*(john.hermans@radboudumc.nl)*

**Long-term outcomes of branch-duct IPMN ≤2 cm: a 10-year MRI follow-up study**
A. Saccomanno[1], *D. Squecco*[2], G. Morana[3]; [1]Varese/IT, [2]Modena/IT, [3]Treviso/IT
*(denidella46@gmail.com)*

**Purpose:** To assess the risk of progression and clinical outcomes in patients with Branch-Duct Intraductal Papillary Mucinous Neoplasms (BD-IPMN) ≤2 cm followed by magnetic resonance imaging (MRI) for at least 10 years, stratified by initial cyst size (1–10 mm vs 11–20 mm).
**Methods or Background:** We performed a retrospective observational study at Ca' Foncello Hospital, Treviso. A total of 174 patients with BD-IPMN ≤2 cm diagnosed between 2007 and 2025 were included, all undergoing MRI follow-up for ≥10 years. Outcomes were time to progression, defined as appearance of worrisome features (WF) or high-risk stigmata (HRS), time to surgery, and overall survival (OS). Kaplan–Meier survival analysis and multivariate Cox regression were used to identify independent predictors.
**Results or Findings:** Median follow-up was 12 years. New WF/HRS occurred in fewer than 15% of patients, with cumulative progression rates of 10.8% at 5 years and 12.6% at 10 years. Stratified by size, 10-year progression was 8.5% in cysts 1–10 mm and 21.4% in cysts 11–20 mm (p=0.028). Surgery was required in 11 patients (6.3%), yielding one high-grade dysplasia and two pancreatic ductal adenocarcinomas. Ten-year OS exceeded 87% in both groups. Multivariate analysis confirmed age at diagnosis (HR 1.22, p<0.001) and active smoking (HR 4.69, p=0.042) as independent predictors.
**Conclusion:** BD-IPMN ≤2 cm show indolent long-term behavior, with most patients free from WF/HRS even after 10 years. Lesions 11–20 mm have a slightly higher risk of progression, but overall rates remain low.
**Limitations:** This is a retrospective, single-center study; not all follow-up MRIs were performed with contrast media; the number of surgical and oncological events was limited.
**Funding for this study:** No external funding was received for this study.
**Has your study been approved by an ethics committee?** Not applicable
**Author Disclosures:**
Giovanni Morana: Nothing to disclose
Denise Squecco: Nothing to disclose
Angiola Saccomanno: Nothing to disclose

**Comparison of single- vs dual-source photon-counting detector CT scanners: phantom and clinical assessment of radiation dose and image quality of abdominal scans**
*I. Dudás*, Z. Somogyiné Nagy, A. Kubovje, A. I. Vigh, T. Marjai, P. Maurovich-Horvat, B. K. Budai; Budapest/HU

**Purpose:** This study aimed to compare image quality and radiation dose between single-source and dual-source photon-counting detector CT (PCD-CT) for pancreatic cystic lesion (PCL) follow-up imaging.
**Methods or Background:** We retrospectively identified 20 patients with pancreatic cystic lesions scanned with single-source PCD-CT between 2024 October and 2024 December who had previous dual-source PCD-CT scans at our institution with the same abdominal imaging protocol as part of their followup. In addition, anthropomorphic phantoms were scanned on both systems. Virtual monoenergetic images (VMI) at 40 and 70 keV were reconstructed. Circular regions of interest were placed in the aorta, pancreas parenchyma, liver parenchyma, cyst fluid, subcutaneous fat, and iliopsoas muscles. Signal-to-noise ratio (SNR) and contrast-to-noise ratio (CNR) were calculated. Pancreatic duct depiction, PCL delineation, image noise, and overall quality were assessed using a 5-point modified Likert scale.
**Results or Findings:** The radiation dose was not significantly different between the scanners in either the unenhanced, pancreatic, or venous phases. At 70 keV, postcontrast CNR did not differ between scanners, whereas at 40 keV, single-source PCD-CT demonstrated significantly higher CNR in the aorta (p=0.014), pancreas (p=0.002), liver (p<0.001), and iliopsoas (p=0.017) for the venous phase. SNR was significantly higher for single-source in multiple tissues at both 40 and 70 keV, most prominently in the venous phase. Subjective assessments favored single-source PCD-CT for image quality, though phantom-based noise power spectrum analysis showed no significant differences.
**Conclusion:** Single-source PCD-CT provides improved SNR, CNR, and subjective image quality compared with dual-source PCD-CT for pancreatic cystic lesion imaging, without increased radiation dose. However, distinction cannot be made whether these improvements originate from hardware or software differences.
**Limitations:** This was a single-center study with a retrospective study design.
**Funding for this study:** No funding was received for this study.
**Has your study been approved by an ethics committee?** Yes
**Ethics committee - additional information:** The study was approved by the institutional ethics committee.
**Author Disclosures:**
Anikó Kubovje: Nothing to disclose
Pál Maurovich-Horvat: Nothing to disclose
Ibolyka Dudás: Nothing to disclose
András István Vigh: Nothing to disclose
Tamás Marjai: Nothing to disclose
Zsófia Somogyiné Nagy: Nothing to disclose
Bettina Katalin Budai: Nothing to disclose

**IPMNs: Imaging Features, Growth, and Outcomes in a Large Cohort**
*B. Abdolalizadeh*, N. Madrid Scheller, S. Jawad, T. Skårup Kristensen, C. Palnæs Hansen, C. Ewertsen; Copenhagen/DK
*(bahareabdolalizade@gmail.com)*

**Purpose:** Intraductal papillary mucinous neoplasms (IPMNs) are common pancreatic cystic lesions with variable malignant potential, making management and surveillance challenging. This study aimed to evaluate imaging characteristics, demographic factors, and clinical outcomes of patients with IPMNs referred to multidisciplinary team conferences at a tertiary reference center between 2019 and 2023.
**Methods or Background:** We included all patients referred to the weekly IPMN MDT conference from January 1, 2019, to December 31, 2023. Exclusion criteria were cystic lesions other than IPMN, uncertain IPMN diagnosis, patients with only a single CT scan available, and cysts <5 mm. Demographic data, imaging features including baseline and newly developed worrisome features (WFs), and histological outcomes of surgically resected patients were collected from electronic health records.
**Results or Findings:** A total of 1082 patients were included (mean age 69.8 years, 57.1% female). Branch-duct IPMN was the predominant subtype (95.3%). Worrisome features (WFs) were present in 207 patients (19.1%) at baseline, and an additional 47 patients (4.1%) developed new WFs during follow-up. Rapid cyst growth was observed in 6.8% according to Fukuoka criteria and in 10.3% according to the updated Kyoto 2024 criteria. Surgical resection was performed in 62 patients (5.7%), with malignant transformation or high-grade dysplasia confirmed in 31 cases (2.9%).
**Conclusion:** Malignant transformation was infrequent. The presence of worrisome features, including rapid cyst growth did not predict malignancy. These findings support a more individualised and potentially less intensive surveillance strategy.
**Limitations:** The limitations of the study are its single-centre , potential selection bias, and interobserver variability in WF assessment.
**Funding for this study:** No funding was provided for this study.
**Has your study been approved by an ethics committee?** Yes
**Ethics committee - additional information:** legal department at Rigshospitalet (ref. 23052217) and the Danish Data Protection Agency (RH - 2015-07, nr. 03616)
**Author Disclosures:**
Samir Jawad: Nothing to disclose
Thomas Skårup Kristensen: Nothing to disclose
Bahareh Abdolalizadeh: Nothing to disclose
Carsten Palnæs Hansen: Nothing to disclose
Caroline Ewertsen: Nothing to disclose
Nikolai Madrid Scheller: Nothing to disclose

**Surveillance of Branch-Duct IPMNs: Comparing Abbreviated and Comprehensive MRI Protocols in Terms of Accuracy, Reproducibility, and Resource Utilization**
*C. Reca*, R. Levi, C. Bonifacio, A. Laghi; Milano/IT
*(reca.christian20@gmail.com)*

**Purpose:** To compare the diagnostic value of Abbreviated MRI protocols (APs)(with/without DWI) versus a comprehensive protocol(CP) for Branch-Duct Intraductal Papillary Mucinous Neoplasms(BD-IPMN) surveillance, evaluating inter-reader agreement, cost-effectiveness and time efficiency.
**Methods or Background:** This single-center retrospective study included patients with BD-IPMN undergoing baseline and follow-up MRI between January2018 and April2024. CP included axial and coronal T2WI, axial fat-saturated T1-weighted images before and after contrast injection (T1WI), 2D and 3D MR MRCP and DWI images.The two APs were obtained from CP follow-up examinations: AP with DWI consisted of axial and coronal T2WI, 2D-3D MRCP, axial fat-saturated T1WI images and DWI, AP without DWI comprised only axial and coronal T2WI and 2D-3D MRCP. A senior radiologist(>20 years of experience in pancreatic imaging) and a junior radiologist(4th-year-radiology resident) independently and blinded analyzed each protocol using Kyoto criteria and recommended management. Inter-reader and inter-protocol agreement were analyzed using Cohen's and Fleiss' Kappa. Acquisition times and healthcare costs were compared using national reimbursement rates(p<0.05 statistical significant).
**Results or Findings:** Overall, 40 patients(25 women and 15 men) with a median age of 67 years(range 48-87) were included. No statistically significant differences were observed among the 3protocols in identifying worrisome features, including high-risk stigmata, cyst size, main pancreatic duct dilation, and mural nodules.The inter-reader agreement was almost perfect(Fleiss' Kappa=0.82). Substantial inter-reader agreement(Cohen's Kappa>0.6) was observed for all protocols, with almost perfect agreement(Cohen's Kappa>0.8) in some instances. APs demonstrated up to a 48%reduction in acquisition time and a 27.4%cost reduction compared to CP. The addition of DWI did not significantly improve diagnostic accuracy.
**Conclusion:** APs, particularly without DWI, are reliable and cost-effective alternatives to CP for BD-IPMN follow-up, maintaining comparable diagnostic performance. Substantial inter-reader agreement highlights the reproducibility of APs, even among less experienced radiologists.
**Limitations:** Retrospective
**Funding for this study:** n/a
**Has your study been approved by an ethics committee?** Not applicable
**Ethics committee - additional information:** n/a
**Author Disclosures:**
Cristiana Bonifacio: Nothing to disclose
Andrea Laghi: Nothing to disclose
Riccardo Levi: Nothing to disclose
Christian Reca: Nothing to disclose

**Radiomic MRI model for predicting the development of worrisome features in branch duct-intraductal papillary mucinous neoplasms (BD-IPMNs)**
S. Boccioli, *S. Bogani*, C. Badii, G. Danti, S. Paolucci, V. Miele; Florence (FI)/IT
*(silviabogani96@gmail.com)*

**Purpose:** The aim of the study was to develop a radiomic MRI model able to predict the future development of worrisome features (WF) in branch duct-intraductal papillary mucinous neoplasms (BD-IPMNs). By analyzing quantitative imaging features from MRI scans, we try to identify biomarkers that could be used as early indicators of disease progression, helping personalized management and reducing unnecessary interventions.
**Methods or Background:** We performed a single-center retrospective study including patients with BD-IPMNs who had at least two contrast-enhanced MRI exams between January 2011 and March 2025. Patients were divided into two groups depending on whether they developed WF or HRS during follow-up. Lesions were manually segmented on T2-weighted images and 107 radiomic features were extracted using PyRadiomics. Feature selection and model building were done using LASSO logistic regression with weighting to account for class imbalance.
**Results or Findings:** A total of 194 patients were included. After a mean follow-up of 4 years and 5 months, 143 patients did not develop WF/HRS (Group 0) while 51 did (Group 1). Out of 107 radiomic features, 52 were initially associated with progression and 9 shape-based features remained significant after correction. The predictive model achieved an AUC of 0.70 (95% CI 0.62–0.79) and a precision-recall AUC of 0.49, showing moderate predictive ability.
**Conclusion:** Radiomic MRI models may help identify BD-IPMN patients at higher risk of developing WF or HRS. By providing quantitative information beyond conventional imaging, it could support earlier and more personalized clinical decisions. Further studies are needed to confirm and refine these findings.

**Limitations:** This study was retrospective, conducted at a single center and included a relatively small sample, which may limit generalizability. MRI scans from different vendors could introduce variability. Moreover the biological significance of radiomic features remains unclear.
**Funding for this study:** No
**Has your study been approved by an ethics committee?** Yes
**Ethics committee - additional information:** The study was approved by the local Ethical Committee of Careggi University Hospital (July 13th, 2021; protocol number: 20256_oss) and conducted in accordance with the Declaration of Helsinki and its later amendments.
**Author Disclosures:**
Vittorio Miele: Nothing to disclose
Ginevra Danti: Nothing to disclose
Chiara Badii: Nothing to disclose
Sebastiano Paolucci: Nothing to disclose
Sofia Boccioli: Nothing to disclose
Silvia Bogani: Nothing to disclose

**Follow-up of side-branch intraductal pancreatic mucinous neoplasms – radiological and clinical outcomes of patients with lesions of different sizes**
A. Chernomorets, D. Ben-Ami Shor, R. Tzadok, H. Yashar, S. Lazar, Y. Katz, *R. Kessner*; Tel Aviv/IL
*(rikikes@gmail.com)*

**Purpose:** -To evaluate the growth of Side-Branch Intraductal Pancreatic Mucinous Neoplasm (SB-IPMN) lesions during a long follow-up period. -To compare between SB-IPMN lesions of different sizes.
**Methods or Background:** The final reports of all the patients that underwent MRI-MRCP at our institution between the years 2011-2021 were reviewed for the diagnosis of SB-IPMN. 483 adult patients were diagnosed with SB-IPMN, however 115 patients were excluded due to: misclassification at diagnosis, clinical or radiological follow-up of less than 12 months, non-detectable lesion on revision or pancreatic malignancy at diagnosis. We evaluated the growth of the largest cystic lesion during follow-up, according to the first and last examinations of the patients. The medical records of the patients were reviewed for demographic data, history of hepatobiliary diseases, and history of non-pancreatic malignancy. Finally, we compared between 3 size groups of SB-IPMN: < 1 cm at diagnosis (smallest), 1-1.9 cm (intermediate) and >= 2 cm (largest).
**Results or Findings:** Our final study population included 368 patients, 254 females and 114 males. The mean radiological follow-up was 54.3 months and the mean clinical follow-up was 67.4 months. The mean absolute growth of the largest cystic lesion during follow-up was 1.3 mm, for the entire population. Only two patients (0.5%) developed pancreatic malignancy, one from the smallest lesions group and one from the largest lesions group. The comparison between the size groups revealed no differences regarding the mean follow-up length, growth during follow-up and development of malignancy. The mean growth during follow-up was 1.4 mm in the smallest and intermediate lesions groups and 0.4 mm in the largest lesions group.
**Conclusion:** Our results suggest that the follow-up guidelines for SB-IPMN should be less strict than for other types of IPMN.
**Limitations:** The study is retrospective.
**Funding for this study:** None.
**Has your study been approved by an ethics committee?** Yes
**Ethics committee - additional information:** Tel Aviv Sourasky Medical Center Institutional Review Board approval (2021-2025)
**Author Disclosures:**
Yuval Katz: Nothing to disclose
Rivka Kessner: Nothing to disclose
Roie Tzadok: Nothing to disclose
Hila Yashar: Nothing to disclose
Sapir Lazar: Nothing to disclose
Dana Ben-Ami Shor: Nothing to disclose
Arthur Chernomorets: Nothing to disclose

**Evaluation of Virtual Non-Contrast Reconstructions from Dual-Layer Spectral CT in Abdominal Imaging**
*A. Del Gaudio*, D. De Santis, B. Masci, B. Catalano, M. Zerunian, G. Tremamunno, T. Polidori, D. Caruso, M. Francone; Rome/IT
*(anton.delgaudio@gmail.com)*

**Purpose:** To evaluate the feasibility of replacing true non-contrast (TNC) images with virtual non-contrast (VNC) reconstructions generated with dual-layer spectral CT (DLCT).
**Methods or Background:** From December 2024 to February 2025, consecutive inpatients who underwent contrast-enhanced abdominal-CT using DLCT scanner (Spectral CT 7500, Philips Healthcare) were retrospectively enrolled. Each examination included a TNC acquisition, followed by arterial and portal venous phases. VNC images were reconstructed from the arterial (VNCa) and portal venous (VNCp) datasets using vendor-provided spectral decomposition algorithms. An independent radiologist measured attenuation values (HU) on TNC, VNCa, and VNCp images for the liver, spleen, pancreas, kidneys, abdominal aorta, portal vein, and hypodense liver and kidney lesions. Signal-to-noise ratio (SNR) and contrast-to-noise ratio(CNR) were calculated for parenchymal organs and vessels across all image types. Lesion size was assessed on TNC, VNCa,and VNCp images.
**Results or Findings:** One hundred patients (mean age: 67.1 ± 11.4 years; 52 males)with 160 hypodense abdominal lesions were analyzed. VNCa and VNCp reconstructions showed lower attenuation than TNC in the liver, spleen, and pancreas(all P < .001).No significant HU differences were observed in the portal vein and kidneys(all P > .540). Aortic HU was higher in VNCa but lower in VNCp compared with TNC(P < .001).Mean HU of hypodense lesions was significantly higher in TNC compared with both VNCa and VNCp(P < .001). SNR was consistently higher in VNCa and VNCp compared to TNC(P < .001). CNR was significantly higher in VNCa compared with TNC for the aorta and spleen, and higher in VNCa than VNCp for the aorta. No other significant differences were observed.
**Conclusion:** VNC images from DLCT underestimated attenuation relative to TNC but provided comparable or higher objective image quality and preserved lesion size measurement.
**Limitations:** Retrospective single-center study, single-vendor reconstruction
**Funding for this study:** N/A
**Has your study been approved by an ethics committee?** Not applicable
**Author Disclosures:**
Damiano Caruso: Nothing to disclose
Benedetta Masci: Nothing to disclose
Bianca Catalano: Nothing to disclose
Marta Zerunian: Nothing to disclose
Giuseppe Tremamunno: Nothing to disclose
Domenico De Santis: Nothing to disclose
Tiziano Polidori: Nothing to disclose
Antonella Del Gaudio: Nothing to disclose
Marco Francone: Nothing to disclose

**Cystic neuroendocrine tumors of the pancreas: imaging features**
*F. Omboni*[1], M. Ronot[2], C. R. A. Corallo[3], C. Triantopoulou[4], N. Kartalis[5], M. Zins[2], G. Zamboni[1]; [1]Verona/IT, [2]Paris/FR, [3]Leeds/UK, [4]Athens/GR, [5]Stockholm/SE
*(fede.omb@gmail.com)*

**Purpose:** To describe a series of cystic panNETs to determine specific imaging features in a large cohort of patients
**Methods or Background:** This is a retrospective multicenter observational study (5 centers in 4 countries) which included cystic panNETs diagnosed with FNA/pathology or by 68GaDOTATOC-PET-CT. Cross-sectional imaging was reviewed for each patient (145 MRI, 11 CT with contrast, 1 non-contrast CT).
**Results or Findings:** 157 patients (85 women, 72 men, mean age 57,5 yrs) with 166 cystic panNETs were included; 9 had multiple lesions. Diagnosis was made by FNA/pathology (143 lesions) or by positivity at DOTATOC-PET-CT (23 lesions). Nine lesions were functioning. ki-67 ranged from 1% to 19% (G1:103; G2: 21; G3: 0). Ki-67% was not available/indeterminate for 42 lesions. Lesions were predominantly located in the body-tail (125) with a mean diameter of 24mm (5-90mm) and a mean wall thickness of 1.9mm. Most patients (130/157) had a solitary lesion; 36 panNETs were associated with other pancreatic lesions (18 IPMN, 14 solid/cystic panNETs, 4 nonspecific cysts). 142 lesions had round margins. On MRI, cyst fluid was predominantly hyperintense on T2-weighted sequences (139/166 lesions) and hypointense on T1-sequences (152/166 lesions). Five lesions contained enhancing mural nodules. MPD was dilated in 13 patients, in 12/13 caused by the cystic lesion. Contrast-enhancement was present in 162 lesions, in 96/162 constant in all phases; 4 lesions showed no enhancement. 15 lesions had wall calcifications. No lesion showed macroscopic nodal or metastatic spread at baseline.
**Conclusion:** In our retrospective multicenter series, we analyzed radiologic features of cystic panNETs. The lack of extra-pancreatic spread at diagnosis, even in large tumors with high Ki67, raises the possibility of an indolent course. Long-term follow-up studies are needed to confirm this hypothesis.
**Limitations:** Retrospective study
**Funding for this study:** None
**Has your study been approved by an ethics committee?** Not applicable
**Author Disclosures:**
Marc Zins: Nothing to disclose
Giulia Zamboni: Nothing to disclose
Nikolaos Kartalis: Nothing to disclose
Maxime Ronot: Nothing to disclose
Charikleia Triantopoulou: Nothing to disclose
Carmelo Ranieri Alessandro Corallo: Nothing to disclose
Federica Omboni: Nothing to disclose

**Natural evolution of cystic neuroendocrine tumors of the pancreas**
*F. Omboni*[1], M. Ronot[2], C. R. A. Corallo[3], C. Triantopoulou[4], N. Kartalis[5], M. Zins[2], G. Zamboni[1]; [1]Verona/IT, [2]Paris/FR, [3]Leeds/UK, [4]Athens/GR, [5]Stockholm/SE
*(fede.omb@gmail.com)*

**Purpose:** To evaluate a population of resected or followed-up cystic panNETs to determine natural evolution.
**Methods or Background:** This retrospective multicentric study included 121 patients (69 women, 52 men, mean age 60 yrs) with 126 cystic panNETs diagnosed by FNA/histology (115 lesions) or 68Ga-DOTATOC-PET-CT (11 lesions). Ki-67 was available for 90 lesions (range 1%-15% (79 G1; 11 G2). For each patient, all available imaging was assessed, at least baseline and most recent scans (119 MRI; 7 CT). Patients were divided in two subgroups: resected versus followed-up.
**Results or Findings:** At baseline, no lesion showed macroscopic nodal or metastatic spread. Resected group (55 lesions): mean diameter 29,9 mm (17 pT1, 26 pT2, 12 pT3). Thirty-three were resected after ≥ 6 months follow-up (mean interval 29 months). Three tumors >40 mm (44, 52, 70 mm), showed peripancreatic nodal involvement (all T3N1M0; 2G1 + 1G2). No lesion < 40 mm have nodal spread. After a median 31,7 months follow-up, no recurrence was observed in 49/55 (6 lost to follow-up). Follow-up group (71 lesions): fifty-nine were followed for >6 months (mean 46,9 months). Mean diameter was 17,5 mm; among the 15 lesions >20 mm, 10 remained stable. Eleven progressed radiologically after a mean of 65.2 months. Detailed data were available for 8/11 growth was limited (<50% of initial diameter). None developed nodal or metastatic spread.
**Conclusion:** In this series, cystic G1-G2 cystic panNETs <40 mm showed an indolent course, with no nodal or metastatic spread during follow-up. In such cases, surveillance may represent a safe alternative to upfront resection.
**Limitations:** Retrospective study
**Funding for this study:** None
**Has your study been approved by an ethics committee?** Not applicable
**Author Disclosures:**
Marc Zins: Nothing to disclose
Giulia Zamboni: Nothing to disclose
Nikolaos Kartalis: Nothing to disclose
Maxime Ronot: Nothing to disclose
Charikleia Triantopoulou: Nothing to disclose
Carmelo Ranieri Alessandro Corallo: Nothing to disclose
Federica Omboni: Nothing to disclose

**Radiomics-Based CT Analysis for Diagnosis and Staging of Chronic Pancreatitis**
*S. Nalliah*[1], S. N. F. Hostrup[1], E. B. Mark[1], M. H. Liedenbaum[2], T. Engjom[2], I. H. S. Haldorsen[2], A. M. Drewes[1], S. S. Olesen[1], J. B. Frøkjær[1]; [1]Aalborg/DK, [2]Bergen/NO
*(surenth.nalliah@gmail.com)*

**Purpose:** To evaluate whether a radiomics-based AI model can (1) classify patients with chronic pancreatitis (CP), (2) identify CP-related complications, and (3) provide quantitative imaging biomarkers of disease severity on routine CT.
**Methods or Background:** CP is a progressive inflammatory disease associated with pain, diabetes, and exocrine pancreatic insufficiency (EPI). Conventional imaging typically detects advanced disease, limiting early diagnosis and accurate staging. Radiomics can reveal subtle imaging alterations beyond visual assessment, offering an opportunity for earlier detection and monitoring. We evaluated whether CT-based radiomics could classify CP, detect complications, and provide biomarkers of disease severity. The study included 468 participants: a training cohort of 359 from Aalborg (201 CP, 148 controls) and a test cohort of 109 (68 external CP patients from Bergen, 41 Aalborg controls). CT scans underwent automated pancreatic segmentation, followed by radiomics feature extraction (PyRadiomics) and feature selection using LASSO regression. AI models were trained to classify CP and complications (EPI, diabetes, pain). Performance was evaluated on the test cohort, and model probability scores were correlated with fecal elastase levels and disease severity.
**Results or Findings:** Forty-six radiomics features were associated with CP and its complications, including markers related to pancreatic volume, calcifications, and ductal dilatation. The CP vs. healthy classification model achieved excellent performance (AUC = 0.97). For complications, AUCs were 0.80 for EPI, 0.63 for diabetes, and 0.59 for pain. Model probability scores correlated with fecal elastase levels ($p < 0.001$) and increased with disease severity ($p = 0.004$).
**Conclusion:** Radiomics-based CT analysis allows accurate CP classification and provides quantitative markers of disease severity and complications. These results support its potential as a non-invasive tool for diagnosis, staging, and longitudinal monitoring of CP
**Limitations:** Retrospective design, and no prospective clinical validation limit this study.
**Funding for this study:** The study was funded from the North Denmark Region Health Innovation Fund (journal number: 2024-0015).
**Has your study been approved by an ethics committee?** Yes
**Ethics committee - additional information:** This retrospective, multi-center study was approved by the Danish National Committee on Health Research Ethics (Ref: 2308560) and The Regional Ethical Committee in Western Norway (Ref: 2019/1037), with data-sharing agreements between Aalborg University Hospital and Haukeland University Hospital approved by the Scandinavian Baltic Pancreas Club (SBPC) steering committee.
**Author Disclosures:**
Marjolein Henrieke Liedenbaum: Nothing to disclose
Ingfrid H. Salvesen Haldorsen: Nothing to disclose
Jens Brøndum Frøkjær: Nothing to disclose
Esben Bolvig Mark: Nothing to disclose
Søren Nicolai Frederiksen Hostrup: Nothing to disclose
Søren Schou Olesen: Nothing to disclose
Trond Engjom: Nothing to disclose
Surenth Nalliah: Nothing to disclose
Asbjørn Mohr Drewes: Nothing to disclose

**CT-guided percutaneous biopsies of suspect pancreatic lesions: Accuracy and safety**
*T. J. Vogl*, H. Adwan, I. Al Haj Ibrahem; Frankfurt/DE

**Purpose:** To retrospectively analyze the accuracy and safety of CT-guided percutaneous core-needle biopsies of pancreatic lesions suspected to be malignant based on MRI or CT scans.
**Methods or Background:** This study evaluated CT-guided percutaneous biopsies of suspicious pancreatic lesions performed at our university hospital. Biopsy was performed using a 17G coaxial needle and an 18G core biopsy needle. Data on patient characteristics, lesions, procedures, and histologic results were recorded.
**Results or Findings:** In total, 90 patients (58.9% males, mean: 65±12.2 years) underwent CT-guided percutaneous biopsies. The lesions had a mean size of 36.8±12.2 mm and were predominantly located in the pancreatic head 61.1% (55/90). Technical success was achieved in all biopsies 100% (90/90). 96.7% of the procedures (87/90) were performed using direct access routes, while 3.3% (3/90) required indirect transhepatic or transgastric approaches. Among the biopsies, 65.6% (59/90) confirmed malignancy, with adenocarcinoma as the most common malignant subtype representing 55.6% (50/90) of all cases. The rate of non-malignant findings was 26.7% (24/90) including chronic pancreatitis at a rate of 5.6% (5/90) as well as pancreatic pseudocysts and pancreatic cystadenoma each at a rate of 2.2% (2/90). Seven cases were identified as false negative, but malignancy was later confirmed after re-biopsy or surgery. The diagnostic accuracy was 92.2% (83/90). The rate of major complications was 1.1% (1/90), of minor complications 2.2% (2/90).
**Conclusion:** This study shows that CT-guided pancreatic biopsy is a safe procedure with high diagnostic accuracy.
**Limitations:** Retrospective study design
**Funding for this study:** No funding
**Has your study been approved by an ethics committee?** Yes
**Ethics committee - additional information:** Approval of the ethics committee of the Johann-Wolfgang-Goethe University Frankfurt
**Author Disclosures:**
Ibrahem Al Haj Ibrahem: Nothing to disclose
Thomas J. Vogl: Nothing to disclose
Hamzah Adwan: Nothing to disclose

**Multiparametric MRI for Differentiating Late-Onset Autoimmune Diabetes and Type 2 Diabetes Mellitus: A Prospective Study**
*S. Seyrek*, A. Avcioglu, S. S. Taflan, B. Tozlu, Ö. B. Kuş, M. G. Kartal, B. Gultekin, A. Sadic, A. K. Uzum; Istanbul/TR
*(sinan.seyrek@istanbul.edu.tr)*

**Purpose:** Late-onset autoimmune diabetes mellitus (LADA) and type 2 diabetes mellitus (DM) are are often misdiagnosed (10–20%) but differ in treatment and prognosis. In this study, multiparametric MRI techniques capable of reliably distinguishing between these two entities within the clinical gray zone were compared.
**Methods or Background:** This prospective single-center study enrolled 104 outpatients (51 LADA, 53 type 2 DM) during July 2024–August 2025. Participants underwent non-contrast abdominal MRI. Pancreatic volume (PV, $cm^3$), proton density fat fraction (PDFF, %), T1 relaxation time (ms), and T2* relaxation time (ms) were measured. In addition, C-peptide levels and fecal elastase were assessed to evaluate endocrine and exocrine pancreatic function.
**Results or Findings:** Compared with type 2 DM, patients with LADA demonstrated significantly lower PV (35.7 vs. 50.1 $cm^3$, $p = 0.0002$), with consistent reductions across the head ($p < 0.0001$) and corpus ($p = 0.0069$). PDFF values were significantly lower in LADA patients both in the whole pancreas (2.3 vs. 5.6%, p = 0.0005) and pancreatic head (p = 0.012). C-peptide was reduced in LADA (0.83 vs. 1.62 ng/ml, p < 0.0001), whereas fecal elastase showed no difference (p = 0.71). Anthropometric measures, including body mass index (26.1 vs. 29.6 kg/m², p = 0.0007) was also significantly lower in LADA patients. Regarding relaxation times, no significant differences were observed for pancreatic T1 (p = 0.28) or T2* (p = 0.028). Additional analyses revealed smaller psoas muscle area (2.98 vs. 4.97 cm², p < 0.0001) and reduced vertebral bone marrow fat fraction (42.0 vs. 49.0%, p = 0.003) in LADA.
**Conclusion:** Multiparametric MRI, particularly pancreatic volume and PDFF, provides reliable markers to distinguish LADA from type 2 DM within the clinical gray zone.
**Limitations:** Patient compliance
**Funding for this study:** This study was supported by the Scientific Research Projects Coordination Unit of Istanbul University (Project No: TTU-2024-40922)
**Has your study been approved by an ethics committee?** Yes
**Ethics committee - additional information:** The study was approved by the Ethics Committee of Istanbul University/Istanbul Faculty of Medicine (Approval No:2023/2373, Date: 29/12/2023). All participants provided informed consent
**Author Disclosures:**
Sinan Seyrek: Nothing to disclose
Özlem Buruc Kuş: Nothing to disclose
Burak Gultekin: Nothing to disclose
Abdurrahman Avcioglu: Nothing to disclose
Merve Gulbiz Kartal: Nothing to disclose
Abdurrahman Sadic: Nothing to disclose
Belis Tozlu: Nothing to disclose
Sıtkı Safa Taflan: Nothing to disclose
Ayse Kubat Uzum: Nothing to disclose

### Prediction of postoperative complications severity after pancreatoduodenectomy using clinical, volumetric, and radiomic analyses of abdominal aortic calcification

*T. Ota*[1], A. Busse-Coté[2], M. Hori[1], R. Sartoris[2], A. Vanzulli[2], V. Vilgrain[2], N. Tomiyama[1], M. Ronot[2]; [1]Suita/JP, [2]Paris/FR
*(t-ota@radiol.med.osaka-u.ac.jp)*

**Purpose:** Abdominal aortic calcification (AAC) has been linked to complications after pancreatoduodenectomy (PD), but the value of volumetric calcification score (VCS) and radiomic features (RFs) remains unclear. We aimed to evaluate whether volumetric AAC metrics and RFs predict postoperative complication severity and to develop integrated prediction models (PMs).
**Methods or Background:** This retrospective study included 380 patients undergoing PD. Clinical variables was summarized as propensity score (PS). AAC was segmented on non-contrast CT to calculate ACV volume (ACV), VCS, and 107 RFs. PMs combining PS, VCS, and selected RF were built using multivariate logistic regression, support vector machine (SVM), and neural network (NN), and PMs were validated by two radiologists. Performance was assessed using area under the receiver operating characteristic curve (AUC) and accuracy.
**Results or Findings:** Of 380 patients, 177 with measurable AAC were included for radiomic analyses, and randomly assigned to training (n=141) and validation (n=36) cohorts. PS was significantly higher in patients with major versus minor complications (p < 0.0001). Neither ACV nor VCS differed significantly between groups. Among RFs, "busyness" showed the strongest discriminatory potential (p = 0.078). Three PMs incorporating PS, VCS, and busyness were constructed. In training, NN-based PM2 (PS + busyness) achieved the best performance (AUC 0.94; accuracy 86.5%). In validation, PM2 with SVM and NN maintained robust accuracy (~80%) and AUCs of 0.86–0.88.
**Conclusion:** Volumetric AAC measures alone were insufficient predictors of complication severity. Combining clinical data with radiomic texture “busyness” in non-linear models improved accuracy, offering a promising preoperative tool for PD risk stratification.
**Limitations:** Single-centre, retrospective design.
**Funding for this study:** N/A
**Has your study been approved by an ethics committee?** Yes
**Ethics committee - additional information:** Institutional Review Board of Hôpital Beaujon, AP-HP
**Author Disclosures:**
Noriyuki Tomiyama: Nothing to disclose
Riccardo Sartoris: Nothing to disclose
Maxime Ronot: Nothing to disclose
Toru Honda: Nothing to disclose
Andrea Vanzulli: Nothing to disclose
Masatoshi Hori: Nothing to disclose
Valérie Vilgrain: Nothing to disclose
Andreas Busse-Coté: Nothing to disclose
Takashi Ota: Nothing to disclose

**16:00 - 17:30** **Research Stage 2**

### Research Presentation Session: Chest

### RPS 1604

### Artificial Intelligence in pulmonary imaging

**Moderator**
Gordon Cowell; Glasgow/UK
*(gordon.cowell@ggc.scot.nhs.uk)*

### Effect of an integrated CT-based lung cancer detection and diagnosis AI tool on radiologists evaluating lung nodules: A multi-reader multi-case study

M. Santos[1], C. Santos[1], J. H. R. Cairns[2], M. Darby[2], A. Johnstone[2], C. Arteta[1], *A. Scarsbrook*[2]; [1]Oxford/UK, [2]Leeds/UK
*(a.scarsbrook@nhs.net)*

**Purpose:** Integration of AI-based computer-aided detection and diagnosis tools into lung cancer screening has the potential to improve and standardize CT reporting, streamline follow-up recommendations, reduce diagnostic errors, and increase efficiency. This multi-reader, multi-case (MRMC) study evaluated the impact of a new AI tool, assessing influence on risk stratification of pulmonary nodules by radiologists.
**Methods or Background:** A fully crossed MRMC design involved twelve radiologists, with varying experience and sub-speciality expertise, retrospectively reviewing 240 screening and non-screening thoracic CTs (95 lung cancers), with and without AI support. AI assistance consisted of automated localisation, measurement, and characterisation of detected lung nodules, including a per-nodule lung cancer risk score. A 30-day washout period separated the two reads of any given case. Sequencing was randomised with AI-assistance occurring either during the first or second read. Percentage likelihood of malignancy was estimated by the reader or AI tool. Performance of AI-assisted versus unassisted read against ground truth was compared using area under the curve (AUC) analysis, averaged across readers. Statistical significance of mean AUC difference was performed using Dorfman-Berbaum-Metz methodology.
**Results or Findings:** Mean effect size between assisted and unassisted reads was 3.92%, 95% confidence interval (CI) [2.00, 5.85] (p < 0.001). When stratified by reader subspeciality, mean effect size for cardiothoracic radiologists (n=7) was lower (2.54%, [0.75, 4.34], p=0.009) compared to other subspecialties (5.86%, [3.52, 8.2], p<0.001). Similarly, when comparing experienced (n=4) versus less experienced participants, mean effect size was lower 2.62%, [-0.16, 5.4], p=0.06 and 4.58%, [2.25, 6.9], p<0.001, respectively.
**Conclusion:** The study illustrates the potential utility of an integrated detection and diagnosis AI tool to support lung cancer screening CT reporting, with higher impact for less experienced and non-specialist radiologists.
**Limitations:** Provisional evaluation with 12 participants in the MRMC study.
**Funding for this study:** The study was jointly funded by the National Institute for Health and Care Research (NIHR) and the Office for Life Sciences (OLS) under project ID NIHR207547.
**Has your study been approved by an ethics committee?** Not applicable
**Author Disclosures:**
Carlos Arteta: Employee: Optellum Ltd
James Henry Robert Cairns: Nothing to disclose
Mauro Santos: Employee: Optellum Ltd
Annette Johnstone: Nothing to disclose
Andrew Scarsbrook: Research/Grant Support: National Institute of Health Research
Catarina Santos: Employee: Optellum Ltd
Michael Darby: Nothing to disclose

### Computational modeling of pulmonary hemodynamics in the context of chronic thromboembolic pulmonary hypertension

*S. Benchara*[1], A. Marchi[2], Q. Herszkowicz[2], A. Decoene[3], S. Jan[2], D. Rodriguez[2], O. Meyrignac[1]; [1]Kremlin-Bicetre/FR, [2]Orsay/FR, [3]Bordeaux/FR

**Purpose:** Pulmonary hypertension (PH) is a chronic, progressive disease defined by a mean pulmonary arterial pressure (mPAP) > 20 mmHg at rest. Right heart catheterization (RHC), the diagnostic gold standard, is invasive, costly, and requires specialized expertise. This study aimed to evaluate a fully open-source, non-invasive computational fluid dynamics (CFD) pipeline for estimating hemodynamic biomarkers in chronic thromboembolic pulmonary hypertension (CTEPH).

**Methods or Background:** A fully open-source workflow, from CT imaging to biomarker extraction, was developed with optimized modeling and simulation steps. The model incorporated flow and resistance data from RHC, assumed rigid vessel walls, and used simplified distal vasculature. Biomarkers included simulated mPAP, wall shear stress (WSS), time-averaged WSS (TAWSS), relative residence time (RRT), oscillatory shear index (OSI), turbulence, and stagnation indices. This workflow was applied to 45 confirmed CTEPH patients (15 with post-treatment follow-up) and 10 healthy controls.
**Results or Findings:** Numerical simulation results were consistent with clinical measurements (simulated vs. measured mPAP: 33.2 ± 17.5 vs. 32.7 ± 12.5 mmHg; r = 0.913, p < 0.0001). The model accurately differentiated patients from controls and effectively captured treatment effects. For example, compared with controls, patients had lower TAWSS (0.81 ± 0.37 vs. 2.47 ± 1.14 $g \cdot mm^{-1} \cdot s^{-2}$, p < 0.0001) and higher RRT (2.55 ± 1.30 vs. 0.80 ± 0.30 $mm \cdot s^2 \cdot g^{-1}$, p < 0.0001). Post-treatment, all biomarkers tended toward normalization (0.70 ± 0.25 to 1.37 ± 0.73 $g \cdot mm^{-1} \cdot s^{-2}$, p < 0.0001 for TAWSS and 3.12 ± 1.73 to 1.48 ± 0.63 $mm \cdot s^2 \cdot g^{-1}$, p < 0.0001 for RRT, for instance).
**Conclusion:** This validated CFD model support personalized PH management by providing robust non-invasive biomarkers, differentiates patients from controls, and detects therapeutic response.
**Limitations:** Model assumptions include rigid vessel walls and simplified distal vasculature, which may affect local flow accuracy.
**Funding for this study:** This research was supported by Siemens Healthineers.
**Has your study been approved by an ethics committee?** Yes
**Ethics committee - additional information:** CNIL approval
**Author Disclosures:**
Astrid Decoene: Nothing to disclose
Quentin Herszkowicz: Nothing to disclose
Antoine Marchi: Nothing to disclose
Olivier Meyrignac: Nothing to disclose
Sebatien Jan: Nothing to disclose
Sara Benchara: Nothing to disclose
Dima Rodriguez: Nothing to disclose

### Impact of using artificial intelligence-based lung nodule evaluation assistant tool for reading low-dose chest CTs : A randomized controlled trial

*E. J. Hwang*, J. M. Goo; Seoul/KR
*(ken921004@hotmail.com)*

**Purpose:** We aimed to evaluate the impact of using an artificial intelligence (AI)-based lung nodule evaluation assistant tool for reading low-dose chest CT (LDCT) on the reading time and lung nodule detection rate.
**Methods or Background:** Consecutive individuals undergoing LDCTs for health check-ups in a single institution were enrolled and randomized into an intervention group and a control group in a 1:1 ratio. For the intervention group, an AI tool for automated lung nodule detection and measurement was integrated into the picture archiving and communication system (PACS) for reading LDCTs. All LDCTs were read by thoracic radiologists, using a structured report, including information regarding the presence, number, consistency, location, and size of pulmonary nodules with a diameter of ≥4 mm. The primary endpoint of the trial was the reading time (time interval between opening the image and completing the structured report), while secondary endpoints included the frequency of detecting lung nodules, the number of detected nodules per exam, and the frequency of recommendations of follow-up LDCT for detected nodules.
**Results or Findings:** We enrolled 901 individuals (male-to-female ratio, 507:394; mean age 62 years; intervention-to-control group ratio, 456:445). The reading time of LDCT did not differ significantly between the two groups (intervention group, 179 seconds; control group, 168 seconds; P=.258). The frequency of detecting nodules (34.6% vs. 25.8%; P=.004), the number of detected nodules (0.4 vs. 0.6 per exam; P=.005), and the frequency of recommendations for follow-up LDCT (13.4% vs. 9.2%; P=.049) were significantly higher in the intervention group.
**Conclusion:** Using a PACS-integrated AI-based lung nodule evaluation assistant tool for reading LDCTs led to increased detection of pulmonary nodules requiring follow-up, without a significant change in the reading time.
**Limitations:** The reproducibility of our result in different populations remains unclear.
**Funding for this study:** This study was supported by Coreline Soft.
**Has your study been approved by an ethics committee?** Yes
**Ethics committee - additional information:** Seoul National University Hospital Institutional Review Board
**Author Disclosures:**
Jin Mo Goo: Grant Recipient: Coreline Soft
Eui Jin Hwang: Grant Recipient: Coreline Soft

### Dual-Source Photon-Counting CT for Thoracic Arteries: Enhancing Image Quality and Diagnostic Assessability with Low Energy Virtual Monoenergetic Imaging

A-I. Nica[1], C. Booz[1], T. J. Vogl[1], G. M. Bucolo[1], T. D'Angelo[2], *H-L. Kaatsch*[3], S. Waldeck[3], D. Overhoff[3]; [1]Frankfurt/DE, [2]Messina/IT, [3]Koblenz/DE

**Purpose:** This study aimed to investigate the impact of low energy virtual monoenergetic imaging (VMI) on quantitative and qualitative image characteristics, as well as its effect on the diagnostic assessability of thoracic arteries in photon-counting computed tomography angiography (CTA).
**Methods or Background:** We retrospectively evaluated 125 patients who underwent dual-source photon-counting CTA scans of the thoracic arteries. We reconstructed standard CT images at 120 kV and VMI series at 15 keV intervals, ranging from 40 to 100 keV, and conducted quantitative and qualitative image analyses. For quantitative analysis, we assessed vascular CT numbers, signal-to-noise ratio (SNR), and contrast-to-noise ratio (CNR). For qualitative evaluation, three board-certified radiologists independently rated image quality, vascular contrast, and diagnostic assessability of the thoracic arteries using a five-point scale.
**Results or Findings:** Quantitative image analysis revealed that 40 keV VMI reconstructions exhibited the highest mean attenuation (HU: 1205 ± 286), SNR (30.4 ± 9.17), and CNR (29.22 ± 9.13), followed by 55 keV series (HU: 679 ± 161, SNR: 24.31 ± 7.57, CNR: 22.54 ± 7.36), significantly improved compared to higher keV levels and the standard 120 kV CT series (p < 0.001). Qualitative analysis showed the highest rating scores for 55 keV reconstructions, significantly higher than those of VMI series at higher energy levels and the standard 120 kV series (p < 0.001).
**Conclusion:** VMI reconstructions at low energy levels (40-55 keV) significantly enhance vascular attenuation, SNR, and CNR, offering superior image quality and diagnostic assessability for thoracic arteries compared to standard CT series in photon-counting CTA.
**Limitations:** Limitations of the study include its retrospective nature.
**Funding for this study:** No funding.
**Has your study been approved by an ethics committee?** Yes
**Ethics committee - additional information:** This study received approval from the Ethics Committee.

### Deep-learning analysis on HRCT for predicting progression and mortality in systemic sclerosis related interstitial lung disease

*I. Friscia*, G. Cicchetti, L. Calandriello, F. Scrocca, L. Cerquiglini, E. De Lorenzis, S. L. Bosello, L. Natale, A. R. Larici; Rome/IT
*(igna.friscia@gmail.com)*

**Purpose:** Interstitial lung disease (ILD) is a major complication in systemic sclerosis (SSc) patients. Artificial intelligence (AI) application on high-resolution Computed Tomography (HRCT) has emerged as a tool that may ensure an objective assessment of ILD. The aim of this study is to correlate measures extracted from HRCT images by a deep-learning based software to assess ILD progression and disease-related mortality in SSc patients.
**Methods or Background:** HRCT scans from a cohort of consecutive SSc-ILD patients at baseline and after 24±3 months were analyzed using AVIEW software (Coreline Soft, South Korea). Quantitative analyses included lung volume, texture, airway, and vascular measurements. Baseline metrics were assessed for their association with ILD progression, defined by criteria based on the INBUILD trial. Changes in AI-derived measurements between two consecutive HRCT scans over the 24-month follow-up were analyzed for their association with SSc-related mortality during the subsequent 36 months.
**Results or Findings:** A total of 146 HRCT scans from 73 SSc-ILD patients were assessed (mean age 58.4±14.3 years). Thirty-one patients (42.4%) experienced ILD progression over 24 months, which was predicted at baseline by higher percentages of ground glass opacities (GGO) (p=0.05) and reticulation (p=0.05), higher subpleural vessel volumes (p=0.017), and a tendency toward larger distal airways (p=0.066). Serial evaluations demonstrated that progression was associated with a reduction in the percentage of normal lung (p=0.044), and absolute volumes (p=0.009). Patients in the upper quartile for changes in reticular score and airway volume exhibited a higher mortality risk, independently from INBUILD progression (reticular score: OR 3.30, 95%CI 1.03–10.61, p=0.045; airway volume: OR 3.37, 95%CI 1.08–10.51, p=0.036).
**Conclusion:** Deep learning-based assessment in SSc-ILD identified distinct modifications in lung anatomical components with significant prognostic implications, potentially enabling a precise patient evaluation and stratification.
**Limitations:** Single center population
**Funding for this study:** None
**Has your study been approved by an ethics committee?** Yes
**Ethics committee - additional information:** The study protocol was approved by the local institutional Committee on Research Ethics

**Author Disclosures:**
Silvia Laura Bosello: Nothing to disclose
Francesco Scrocca: Nothing to disclose
Ignazio Friscia: Nothing to disclose
Enrico De Lorenzis: Nothing to disclose
Anna Rita Larici: Nothing to disclose
Luigi Natale: Nothing to disclose
Giuseppe Cicchetti: Nothing to disclose
Lucia Cerquiglini: Nothing to disclose
Lucio Calandriello: Nothing to disclose

**Artificial Intelligence for Detecting Interstitial Lung Disease in Radiation Therapy Planning CTs**
*S. Kandel*, A. Hope, P. Rogalla; Toronto, ON/CA
*(sonja.kandel@uhn.ca)*

**Purpose:** Interstitial lung disease (ILD) is a significant risk factor for radiation pneumonitis in patients receiving pulmonary stereotactic body radiotherapy (SBRT) for cancer. Pre-treatment CT used for planning offer an opportunity to identify patients with ILD prior to therapy. However, manual CT review by thoracic radiologist isn't always available and labor intensive. We developed and clinically implemented a deep learning tool to automatically detect ILD on planning CTs to support risk stratification and workflow efficiency.
**Methods or Background:** A three-dimensional convolutional neural network (3D VGG16) was trained using 4,393 diagnostic CT scans, including 1,366 ILD cases, normalized for slice thickness and reconstruction algorithm. The algorithm was validated on a cohort of 537 patients treated with SBRT, where ILD prevalence and pneumonitis risk were known. A prospective "silent mode" evaluation was performed on 111 patients prior to live deployment. Two operating thresholds were defined: a low-risk threshold (higher sensitivity) and a high-risk threshold (higher specificity). Clinical integration occurred with automated email notifications to radiation oncologists.
**Results or Findings:** In the validation cohort, ILD was associated with higher rates of pneumonitis (G2+: 20.5% vs. 5.8%; G3: 10.3% vs. 1%; G5: 2/3 patients had ILD features; all p<0.01). In the silent mode phase, the low-risk threshold flagged 52% of patients (missed 1 ILD case), while the high-risk threshold flagged 14% (missed 1 ILD case). Since clinical implementation, 99 patients have been reviewed, with 16 flagged for ILD. Two previously unrecognized ILD cases were detected through the AI tool, leading to changes in patient management.
**Conclusion:** An AI-based screening tool for ILD can be integrated into radiation therapy planning workflows, identifying high-risk patients and improving clinical decision-making without delaying treatment. Ongoing work involves expansion to multi-center clinical trials.
**Limitations:** Single center study.
**Funding for this study:** None.
**Has your study been approved by an ethics committee?** Yes
**Ethics committee - additional information:** REB approval was obtained.
**Author Disclosures:**
Andrew Hope: Nothing to disclose
Sonja Kandel: Nothing to disclose
Patrik Rogalla: Nothing to disclose

**Personalized Medicine in COPD: AI-based Chest CT Analysis Uncovers Body Composition's Effect on the Bone–Vascular Axis of Osteoporosis**
*B. K. Budai*, A. Wagner, O. Havlicek, P. Konietzke, F. Trudzinski, J. Biederer, C. P. Heußel, H-U. Kauczor, V. Palm; Heidelberg/DE

**Purpose:** This study aimed to investigate the association between vertebral bone density (T12 BMD) and total thoracic vascular calcification (TTVC) volume, with a special focus on the effect of body composition. Moreover, we aimed to determine whether body composition indices modify the bone-vascular axis in patients with chronic obstructive pulmonary disease (COPD).
**Methods or Background:** In this prospective multicentric study on 539 COPD patients (COSYCONET Study), chest CT scans were investigated with AI-based tools for T12 BMD, TTVC, and volumetric body composition analysis. Adjusted regression models were constructed to assess the impact of conventional body phenotypes (normal, sarcopenic, non-sarcopenic obesity, and sarcopenic obesity). Stepwise interaction model building included T12 BMD, the intermuscular adipose tissue (IMAT), their interaction, adding BMI, clinical and metabolic covariates, lung function, physical performance, and age.
**Results or Findings:** A consistent inverse association was observed between T12 BMD and TTVC in all phenotypes, reaching significance in normal nutritional status ($\beta = -0.38$, $p < 0.01$), sarcopenia ($\beta = -0.36$, $p < 0.01$), and non-sarcopenic obesity ($\beta = -0.24$, $p < 0.01$). However, the interaction model for TTVC with T12 BMD could not confirm the conventional phenotype's significant effect. In a fully adjusted linear regression model, IMAT was identified as an independent predictor of TTVC. Interaction models confirmed age and pack-years as the strongest risk factors of calcification; moreover, IMAT consistently remained a significant independent predictor even in the fully adjusted model ($\beta$=0.15, 95% CI 0.015–0.28, p=0.029), while the interaction between T12 BMD and IMAT lost significance only once age was included.
**Conclusion:** Thoracic IMAT is independently associated with vascular calcification in COPD, although the modifying effect of IMAT on the bone-vascular axis suggests an age-dependent interaction.
**Limitations:** The absence of a non-COPD control group.
**Funding for this study:** This study was conducted within the framework of COSYCONET and further partially funded through a collaboration with PERMED-COPD (No: 01EK2203A and 01EK2203B). B.K.B. was supported by the Medical Data Scientist Program of the Medical Faculty of Heidelberg University.
**Has your study been approved by an ethics committee?** Yes
**Ethics committee - additional information:** The COSYCONET study was approved by the Ethical Committee of Philipps University Marburg (reference no. AZ 2010-28), as well as the ethics committees of each center.
**Author Disclosures:**
Andreas Wagner: Nothing to disclose
Ondrej Havlicek: Nothing to disclose
Hans-Ulrich Kauczor: Nothing to disclose
Franziska Trudzinski: Nothing to disclose
Phillip Konietzke: Nothing to disclose
Viktoria Palm: Nothing to disclose
Claus Peter Heußel: Nothing to disclose
Jürgen Biederer: Nothing to disclose
Bettina Katalin Budai: Nothing to disclose

**Impact of AI-assisted reading on detection of progressive pulmonary fibrosis (PPF) on serial CT**
*L. Sun*[1], G. Bailey[1], B. Rawal[1], C. Marrocchio[2], P. M. George[1], S. Gerry[3], S. R. Desai[1], A. Devaraj[1]; [1]London/UK, [2]Parma/IT, [3]Oxford/UK
*(logan.sun@doctors.org.uk)*

**Purpose:** To evaluate the impact of AI-assisted reading using quantitative CT (qCT) on the performance of thoracic radiologists in identifying clinically significant PPF on serial CT, in patients with marginal forced vital capacity (FVC) decline.
**Methods or Background:** 102 patients (median age, 60 years [range 34–82]; M=40) with non-IPF fibrotic interstitial lung disease (ILD) with serial CTs >6 months apart and contemporaneous FVC decline of 5.0-9.9% were retrospectively evaluated. Five thoracic radiologists, blinded to clinical data, independently reviewed serial CTs side-by-side, categorising cases as either stable disease (SD) or progressive disease (PD), based on visually estimated changes in ILD extent. Quantitative CT biomarkers of ILD severity were also generated using commercially available AI software (e-Lung, Brainomix). Cases initially categorised as SD, but with a qCT biomarker increase of ≥1.5% on serial CT were re-reviewed in conjunction with software generated fibrosis segmentation overlays, and radiologists then either retained categorisation as SD or changed to PD. Radiologists' performance with and without AI support was analysed using a Cox proportional hazards model based on progression-free survival (defined as time to death, lung transplantation or 10% FVC decline).
**Results or Findings:** During a median follow-up of 1085 days (IQR 522–1694), 44 patients died and 3 had lung transplants. Without AI, hazard ratios (HR) for visually-identified PD were 1.83-2.33 (p=0.001–0.017) for all readers. QCT identified 22 to 40 cases per reader for re-evaluation, leading to readers changing PPF categorisation in 45%-94% (n=10-29) of these cases. With AI-assisted reading, HRs increased for all radiologists to 2.34–3.25 (p<0.001–0.002).
**Conclusion:** In patients with non-IPF fibrotic ILD and marginal FVC decline, AI-based qCT decision support improves reader performance in identifying clinically significant PPF.
**Limitations:** Retrospective, single-centre study
**Funding for this study:** Nil sought.
**Has your study been approved by an ethics committee?** Yes
**Ethics committee - additional information:** Approval by Health Research Authority England
**Author Disclosures:**
Bhavin Rawal: Nothing to disclose
Stephen Gerry: Nothing to disclose
Anand Devaraj: Consultant: Brainomix Speaker: Boehringer Ingelheim
Peter M. George: Shareholder: Brainomix
Logan Sun: Nothing to disclose
Cristina Marrocchio: Nothing to disclose
Sujal R. Desai: Nothing to disclose
Georgina Bailey: Nothing to disclose

**Ultra-low-dose CT lung cancer screening with AI-integrated thin-slice reconstruction: biopsy validation and comparison with radiologist and machine learning"**

*R. J. Prabagaran*[1], K. Venugopal[1], N. D. Kanase[2], M. P. Ghate[2], P. Moorthy[1]; [1]Chennai/IN, [2]Mumbai/IN
*(drsamreefath@gmail.com)*

**Purpose:** To evaluate AI-based ultra-low-dose CT (Delta, 0.5 mm) for lung cancer screening in smokers and non-smokers, validated against histopathology, and compared with a radiologist and machine learning (ML) classifier.
**Methods or Background:** 179 patients undergoing ultra-low-dose CT chest, all suspicious nodules were biopsied. Histopathology was the reference. AI, radiologist, and ML provided binary malignant/benign predictions. Diagnostic accuracy was assessed per-patient (positive if any malignant lesion) and per-lesion. Sensitivity, specificity, PPV, NPV, and accuracy were calculated with 95% confidence intervals. Subgroup analyses were performed in smokers and non-smokers. False positives were reviewed by histopathology. McNemar's test compared paired methods.
**Results or Findings:** Ninety-seven of 179 patients (54.2%) had malignancy. Per-patient accuracy was 81.6% for AI, 88.8% for radiologist, and 87.2% for ML. Sensitivity/specificity were: AI 80.8%/82.9%, radiologist 88.6%/89.0%, ML 87.6%/86.6%. In smokers, radiologist sensitivity/specificity were 86.3%/89.5%; in non-smokers 91.3%/88.6%. False positives were mainly benign fibrosis and granuloma. McNemar's test showed no significant difference between methods.
**Conclusion:** AI applied to ultra-low-dose CT demonstrates clinically useful accuracy for lung cancer screening across smokers and non-smokers, but radiologist and ML interpretation remain superior. Novelty lies in combining AI-integrated ultra-thin reconstruction with systematic biopsy validation, highlighting benign mimics as the main limitation. AI holds promise as an adjunct in screening workflows
**Limitations:** Single-center cohort: Conducted at one institution, which may limit generalizability to broader screening populationsNo longitudinal follow-up: Interval cancers and long-term outcomes were not assessed; thus, the study reflects diagnostic performance at baseline onlyHistopathology spectrum: Benign conditions such as fibrosis, granuloma, and infection were frequent; while biopsy was the reference, some benign diagnoses may evolve over time.
**Funding for this study:** This research received no external funding and was conducted as part of institutional academic activity.No specific grant from funding agencies in the public, commercial, or not-for-profit sectors was received for this study."
**Has your study been approved by an ethics committee?** Yes
**Ethics committee - additional information:** The study was approved by the Institutional Ethics Committee and written informed consent was obtained from all patients. This prospective study was conducted in accordance with the Declaration of Helsinki and approved by the Institutional Review Board
**Author Disclosures:**
Prashant Moorthy: Nothing to disclose
Madhura Pushkar Ghate: Nothing to disclose
Reefath Jabaraj Prabagaran: Nothing to disclose
Krishnaveni Venugopal: Nothing to disclose
Niranjan Dhanaji Kanase: Nothing to disclose

**Development and validation of an explainable machine learning-based model for predicting the interval growth of pulmonary subsolid nodules: a retrospective multicenter cohort study**

*Z. Zhedong*; Beijing, China/CN
*(zhangzdmed@zju.edu.cn)*

**Purpose:** This multicenter study aimed to develop and validate a machine learning model for predicting the growth of pulmonary subsolid nodules (SSN) at different time intervals using CT radiomics. The model is intended to guide personalized follow-up strategies in clinical practice.
**Methods or Background:** We retrospectively analyzed data from 642 patients with 717 SSNs, collected from three medical centers, who underwent long-term follow-up. Patients were classified into growth and non-growth groups based on SSN growth within 2 or 5 years and were randomly divided into training and internal testing sets. Predictive models were developed using optimal ML algorithms for clinical, radiomics, and clinical-radiomics fusion models to assess SSN growth risk. An independent external test set, including 95 patients with 105 SSNs from a health examination center, was used for validation. Model performance was assessed using the AUC. The SHAP method was used to clarify model rationale.
**Results or Findings:** XGBoost and LightGBM showed the highest discriminative ability among eight ML models. For 2-year growth prediction, AUCs were 0.823, 0.889, and 0.911 (internal set), and 0.712, 0.734, and 0.734 (external set). For 5-year growth, AUCs were 0.796, 0.838, and 0.849 (internal set), and 0.672, 0.773, and 0.776 (external set). These insights were integrated into a clinical management framework, enhancing clinical utility.
**Conclusion:** Our interpretable ML model, based on multicenter longitudinal data, accurately predicts SSN changes over 2 years and offers guidance for 5-year follow-up.
**Limitations:** As a multicenter retrospective study, site-to-site variation in follow-up likely introduced selection and temporal bias and precluded robust subgroup analyses. The exclusively Asian cohort limits generalizability to other ethnicities, underscoring the need for international external validation. Moreover, not all enlarging nodules were pathologically confirmed, indicating that surgical thresholds after progression require further study.
**Funding for this study:** Not applicable
**Has your study been approved by an ethics committee?** Yes
**Ethics committee - additional information:** This study received approval from the Institutional Review Boards (IRBs) of three tertiary comprehensive medical centers (No. 2022PHB031-001, No. 2022-0601-01, and No. 2022002).
**Author Disclosures:**
Zhang Zhedong: Nothing to disclose

**Ultralow-dose 60kVp Chest CT with the Artificial Intelligence Iterative Reconstruction for Diagnosing Lobar Pneumonia in Children**

*L. Bai*[1], S. Xu[2], A. Li[1], J. Yang[1]; [1]Xi'an/CN, [2]Shanghai/CN

**Purpose:** Repeated chest CT is crucial for monitoring children with severe lobar pneumonia, but radiation exposure is a major concern. This study aimed to evaluate the diagnostic feasibility of the ultralow-dose CT at 60kVp with artificial intelligence iterative reconstruction (AIIR) for pediatric pneumonia, compared to routine-dose CT.
**Methods or Background:** Thirty-three pediatric patients with severe lobar pneumonia (20 boys; mean age, 7.7±3.5years) undergoing follow-up CT within one-week of baseline routine-dose imaging were prospectively enrolled. Routine-dose protocols were age-specific: 100kVp, reference 70mAs (≤5 years); 120kVp, reference 30mAs (6–18 years), and images were reconstructed with hybrid iterative reconstruction (HIR). Follow-up scans used 60kVp, reference 70mAs, and were reconstructed with AIIR. Image noise was defined as the standard deviation of the CT number in chest-wall fat. Signal-to-noise ratio (SNR) and contrast-to-noise ratio (CNR) were quantified at aortic root. Two blinded radiologists scored anatomical visualization and diagnostic confidence by consensus using 5-point Likert scale (1=poor, 5=excellent). The paired-t test and Wilcoxon signed-rank test were used. The performance of 60kVp-images for demonstrating pneumonia-related imaging findings was evaluated, with routine-dose CT as the reference standard.
**Results or Findings:** The effective radiation doses were 0.17±0.03mSv at 60kVp and 1.14±0.37mSv at routine dose, respectively ($p<0.05$). Compared to routine-dose images, 60kVp-AIIR images demonstrated significantly lower noise, higher SNR and CNR (all $p<0.05$). Subjective anatomical visualization and diagnostic confidence scores were comparable between 60kVp and routine-dose scans (4.00±0.74 vs. 4.42±0.67, $p=0.13$; 4.25±0.62 vs. 4.50±0.67, $p=0.51$). Sensitivity, specificity, negative predictive value, positive predictive value, and accuracy of 60kVp-scans were 95.45%, 100%, 91.67%, 100%, and 96.30%.
**Conclusion:** The 60kVp ultralow-dose chest CT with AIIR provides diagnostic image quality comparable to routine-dose CT while reducing radiation exposure by approximately 85%, demonstrating high potential for safely monitoring pediatric lobar pneumonia.
**Limitations:** Not applicable.
**Funding for this study:** No funding was received by this study.
**Has your study been approved by an ethics committee?** Yes
**Ethics committee - additional information:** Institutional Review Board approval was obtained.
**Author Disclosures:**
Shijie Xu: Nothing to disclose
Ao Li: Nothing to disclose
Lu Bai: Nothing to disclose
Jian Yang: Nothing to disclose

**Quantitative emphysema thresholds for defining combined pulmonary fibrosis and emphysema in idiopathic pulmonary fibrosis relevant to disease progression monitoring**

*J. Choi*, J. Choe, H. J. Hwang, S. M. Lee, E. Chae, J. B. Seo; Seoul/KR

**Purpose:** In idiopathic pulmonary fibrosis (IPF), coexisting emphysema may attenuate declines in forced vital capacity (FVC), masking progression. Visual thresholds have been used to define combined pulmonary fibrosis and emphysema (CPFE), but no quantitative CT (QCT)–based thresholds have been clinically validated. We aimed to identify a QCT-defined emphysema threshold on HRCT relevant to monitoring disease progression.
**Methods or Background:** We retrospectively analyzed IPF patients with baseline and 1-year pulmonary function tests. Emphysema and fibrosis extent were quantified using deep learning–based texture analysis. Prognostic value of 1-year decline in FVC (≥5%) and diffusing capacity of carbon monoxide (DLco,≥10%) for survival was assessed across QCT emphysema thresholds using multivariable Cox models. Longitudinal trajectories were modeled with linear mixed-effects analysis. In a subgroup with follow-up HRCT, prognostic association of QCT-fibrosis progression (≥4.52% increase in fibrosis, predefined DLco-anchored threshold) was evaluated in relation to emphysema burden.
**Results or Findings:** Among 944 patients (mean age, 66.6 ± 7.9 years; 80.6% male), mean QCT emphysema was 1.75±4.29% and fibrosis extent 11.7±9.95%. FVC decline predicted mortality across subgroups based on different emphysema thresholds but showed no significant association in patients with >10% emphysema (HR, 1.54[95%CI: 0.50–4.69]; P=0.45). DLco decline remained robustly associated with mortality regardless of emphysema extent (all, P<0.001). QCT-fibrosis progression also significantly associated with survival across emphysema strata. FVC trajectories differed by the 10% QCT-emphysema threshold (P<0.001), with stability in >10% versus decline in ≤10%. DLco declined in both groups without slope difference (P=0.36).
**Conclusion:** A QCT-emphysema threshold of ≥10% defines a CPFE-IPF phenotype in which FVC is less sensitive for monitoring progression. DLco decline and QCT-fibrosis extent change may serve as preferred surrogate markers.
**Limitations:** Retrospective, single-center study; small sample size for HRCT follow-up subgroup analysis.
**Funding for this study:** This work was supported by the National Research Foundation of Korea (NRF) grant funded by the Korea government (MSIT) (No. RS-2025-16067456).
**Has your study been approved by an ethics committee?** Yes
**Ethics committee - additional information:** This retrospective study was approved by our institutional review board, and the need for written informed consent was waived.
**Author Disclosures:**
Eunjin Chae: Nothing to disclose
Jaeyeon Choi: Nothing to disclose
Joon Beom Seo: Shareholder: Coreline Soft Co., Ltd.
Sang Min Lee: Nothing to disclose
Hye Jeon Hwang: Nothing to disclose
Jooae Choe: Nothing to disclose

**Optimizing 0.55T MRI Lung Visualization Through High-Resolution Respiratory Motion-Resolved 4D UTE: Performance Assessment Against Standard Techniques**
D. C. Rotzinger, *G. Fahrni*, A. Mackowiak, S. Rapacchi, J-B. Ledoux, M. Stuber, C. W. W. Roy, C. Pozzessere; Lausanne/CH
*(guillaume.fahrni@chuv.ch)*

**Purpose:** To assess the performance of free-running 4D (respiratory motion-resolved) ultrashort-echo-time (UTE) sequences at 1.25mm3 and 0.98mm3 resolution on low-field MRI, compared with commercially available 3DT1VIBE (1.25mm3) and 2DT2HASTE (1.56mm2×6mm) for contrast-free lung imaging.
**Methods or Background:** We scanned fourteen volunteers on low-field MRI (0.55T). Three experienced radiologists independently rated image quality/artifacts and vessels/airways conspicuity (4-point Likert scale). We calculated median/IQR ratings and assessed differences of pooled and individual ratings via Kruskal-Wallis with Dunn's post-hoc tests. Inter-rater agreement was evaluated with weighted Cohen's Kappa and interpreted according to Landis & Koch (1977). Quantitative analysis included pulmonary artery-to-parenchyma contrast ratio (CR).
**Results or Findings:** UTE1.25 vs. UTE0.98's pooled qualitative ratings were not significantly different (p=0.757). However, UTEs ratings (average median±SD: 3.25±0.5) outperformed other sequences; 3DT1VIBE ranked third (3.0±0) and 2DT2HASTE last (2.25±0.5), all p.adj<0.001. When looking at individual rating categories, UTEs excelled especially in vessel (median[IQR]: 4[1]) and airways conspicuity (3[1]), outperforming 2DT2HASTE (2[1.75] and 2[1], p.adj<0.001) and 3DT1VIBE (3[1] and 3[1], respectively, p.adj<0.01). UTEs had milder artifacts than 3DT1VIBE, p.adj<0.05, but were not significantly better than 2DT2HASTE (p.ajd>0.19). Inter-rater agreements were as follows (averages): image quality 0.32 (fair), artifacts 0.15 (slight), vessels 0.50 (moderate), airways 0.64 (substantial). In quantitative analysis, 3DT1VIBE outperformed UTE0.98 (CR 4.4±1.6 vs. 1.92±0.5, p.adj<0.01) and 2DT2HASTE (CR 0.64±0.2, p.adj<0.001), but was not significantly superior to UTE1.25 (CR 2.89±1.1, p.adj=0.09), favoring UTE1.25 over UTE0.98 due to its higher contrast ratio.
**Conclusion:** UTE1.25 balanced qualitative superiority and quantitative reliability for low-field 0.55T lung MRI, offering superior depiction of vessels and airways compared to 3DT1VIBE and 2DT2HASTE and less artifacts.
**Limitations:** Pilot study with limited clinical cohort size; no clinical endpoint
**Funding for this study:** This work was funded by a CHUV Radiology Seed Grant
**Has your study been approved by an ethics committee?** Yes
**Ethics committee - additional information:** CER-VD, Vaud's Canton indepedant Ethics Committee.
**Author Disclosures:**
Adèle Mackowiak: Nothing to disclose
Chiara Pozzessere: Nothing to disclose
Jean-Baptiste Ledoux: Nothing to disclose
David Christian Rotzinger: Nothing to disclose
Christopher W. W Roy: Nothing to disclose
Stanislas Rapacchi: Nothing to disclose
Guillaume Fahrni: Nothing to disclose
Matthias Stuber: Nothing to disclose

**16:00 - 17:30** **Research Stage 6**

### Research Presentation Session: Artificial Intelligence and Imaging Informatics Hot Topic with Keynote Lecture

### RPS 1605
### Hot Topic: generative AI in radiology

**Moderator**
Keno K. Bressem; Berlin/DE
**Author Disclosures:**
Keno K. Bressem: Advisory Board: Advisor for the EU IHI Project IMAGIO (101112053), Grant Recipient: Grant from the European Union (101079894), Bayern Innovativ, German Federal Ministry of Research, Technology and Space, Else Kröner-Fresenius-Foundation, Max Kade Foundation and Wilhelm-Sander Foundation.

**Keynote Lecture**
Ramprabananth Sivanandan; Asker/NO
*(sivram@vestreviken.no)*

**Development and Initial Evaluation of a Generative AI Tool for Personalized Automatic Breast Imaging Reporting**
*G. Della Pepa*, G. Irmici, V. Molinari, C. De Berardinis, E. D'Ascoli, L. Corradini, G. Rossini, C. Depretto, G. P. Scaperrotta; Milan/IT
*(gianmarco.dellapepa@gmail.com)*

**Purpose:** To design and evaluate a generative artificial intelligence (AI) tool that automatically produces personalized and structured breast imaging reports (mammography, ultrasound, and interventional procedures) from minimal user input.
**Methods or Background:** A prototype based on a large language model (LLM) was developed to learn each radiologist's unique reporting style. For each user, 50 anonymized previous breast imaging reports were combined and analyzed to generate an automatically created JSON (JavaScript Object Notation) structure encoding linguistic patterns, recurrent terminology, and section hierarchy. A second, handcrafted JSON file defined the functional core of the LLM, including task logic, prompts, and behavioral instructions. The system combines both components to generate full reports from brief notes summarizing the current exam, optionally integrating previous reports for automated comparison. Internal testing was conducted comparing reporting time and usability across manual typing, voice dictation, and the AI-assisted workflow.
**Results or Findings:** The AI tool, tested by five breast radiologists, generated complete, correct, and style-consistent reports within a few seconds from brief inputs. Reporting time was significantly reduced compared with both manual typing and voice dictation. Radiologists reported smoother workflow and lower cognitive load after the initial setup. Mean satisfaction scores were 9.2/10 for usability and 9.0/10 for accuracy. Generated reports required only minimal editing before validation.
**Conclusion:** The developed tool demonstrates that personalized, fast, and consistent breast imaging reporting can be achieved through generative AI, improving workflow efficiency and ensuring high reporting accuracy.
**Limitations:** Preliminary single-center testing. A refined model with expanded parameters and larger datasets is under development to improve precision and scalability
**Funding for this study:** None

**Has your study been approved by an ethics committee?** Not applicable
**Author Disclosures:**
Gianmarco Della Pepa: Nothing to disclose
Elisa D'Ascoli: Nothing to disclose
Valentina Molinari: Nothing to disclose
Gabriele Rossini: Nothing to disclose
Gianfranco Paride Scaperrotta: Nothing to disclose
Catherine Depretto: Nothing to disclose
Lisa Corradini: Nothing to disclose
Claudia De Berardinis: Nothing to disclose
Giovanni Irmici: Nothing to disclose

**Deep-Learning augmented contrast enhancement improves image quality of CTPE: A multi-scanner study**
M. Meyer, *S. Steinmetz*, A. Othman, D. Graafen; Mainz/DE

**Purpose:** Pulmonary embolism (PE) is a potentially life-threatening event requiring accurate imaging and diagnostics. CT pulmonary angiography (CTPA) is widely established as imaging standard for PE detection but can face difficulties in poorly contrasted images or in the detection of small, peripheral PEs. This study aims to evaluate the potential benefits of Deep Learning-based iodine contrast enhancement and denoising in CTPA (DLe-CTPA) compared to conventional CTPA (c-CTPA).
**Methods or Background:** This retrospective, multi-center study analyzed data from 224 patients who underwent CTPA for suspected pulmonary embolism (PE). Imaging data were collected from eight different CT scanners, and a vendor-agnostic Deep Learning algorithm was utilized to enhance iodine contrast. For the quantitative analysis, Signal intensity (SI), Signal-to-Noise Ratio (SNR) and Contrast-to-Noise Ratio (CNR) where obtained at eleven distinct pulmonary artery segments using a Matlab tool. For the qualitative analysis, two radiologists independently evaluated both data sets and performed qualitative assessments using a 4-point Likert scale to evaluate various parameters, including image quality, contrast, image noise and diagnostic confidence.
**Results or Findings:** DLe-CTPA significantly enhanced SI, SNR, and CNR across all pulmonary artery sites and scanners compared to c-CTPA (SI, CNR: $p<0.001$; SNR: $p<0.03$). Qualitative assessment showed improvements for overall image quality, contrast, image noise and diagnostic confidence for DLe-CTPA (all $p<0.001$).
**Conclusion:** The application of DLe-CTPA significantly improves SI, SNR and CNR for all pulmonary artery segments and CT scanners, as well as the qualitative measures overall image quality, contrast, image noise and diagnostic confidence when compared to c-CTPA.
**Limitations:** We are limited by retrospective study design.
**Funding for this study:** No.
**Has your study been approved by an ethics committee?** Yes
**Ethics committee - additional information:** This study was approved by the local ethic commission.
**Author Disclosures:**
Dirk Graafen: Nothing to disclose
Merle Meyer: Nothing to disclose
Sebastian Steinmetz: Nothing to disclose
Ahmed Othman: Nothing to disclose

**Synthetic post-contrast MRI in brain metastases using generative AI: A GAN framework for contrast enhancement**
*M. Solak*, M. Tören, B. Asan, E. Kaba, M. Beyazal, F. Beyazal Çeliekr; Rize/TR
*(mervesolak2555@gmail.com)*

**Purpose:** Contrast-enhanced MRI (CE-MRI) is essential for diagnosing and monitoring brain metastases (BM). However, potential risks of gadolinium-based contrast agents (GBCA) call for alternative methods. This study aimed to generate synthetic CE-T1 and CE-FLAIR from non-contrast MRI in BM patients using Generative adversarial networks (GAN)-based models.
**Methods or Background:** This retrospective study utilized brain MRI scans (3.0T GE Discovery MR750w) acquired between January 2023 and April 2025 from 183 patients with brain metastases (T1: 83, T2: 100). The dataset included 16,250 T1-related and 2,810 T2-related images (T1, CE-T1, T2, and CE-FLAIR sequences). For CE-MRI synthesis, four GAN models (Pix2PixHD, CycleGAN, C-CycleGAN, and CGAN) were applied using sample boundary maps instead of semantic labelling. Data were divided into training (70%), validation (20%), and testing (10%) sets. Generator realism was assessed via G_GAN loss, and visual quality via VGG-based perceptual loss. Model outputs were quantitatively evaluated using MSE, SSIM, PSNR, and RMSE. Qualitative evaluation was performed through a visual Turing test by experts and repeated after one month.
**Results or Findings:** G_GAN losses generally decreased, and T1 and T2 VGG losses showed strong correlation (r = 0.90). Pix2PixHD achieved thr best performance (T1: SSIM 0.80, PSNR 29.2 dB; T2: SSIM 0.90, PSNR 27.3 dB), while CGAN performed worst (T1: SSIM 0.30, PSNR 19.1 dB; T2: SSIM 0.20, PSNR 18.5 dB). Qualitative evaluation showed 61.4% accuracy with moderate inter-observer agreement; repeat testing showed 60% accuracy.
**Conclusion:** GAN-based models can generate CE-T1 and CE-FLAIR images from non-contrast MRI, achieving strong quantitative metrics and moderate diagnostic accuracy, supporting their potential to reduce exposure GBCA in neuro-oncologic imaging.
**Limitations:** Not applicable.
**Funding for this study:** No funding was provided for this study.
**Has your study been approved by an ethics committee?** Yes
**Ethics committee - additional information:** The ethics committee notification can be found under the number Decision No: 2025/280.
**Author Disclosures:**
Mehmet Beyazal: Nothing to disclose
Fatma Beyazal Çeliekr: Nothing to disclose
Murat Tören: Nothing to disclose
Berkutay Asan: Nothing to disclose
Esat Kaba: Nothing to disclose
Merve Solak: Nothing to disclose

**Overcoming Scanner Variability: MRF-Powered Synthesis of Multiparametric MRI for Glioblastoma**
*Y. Ni*, C. Liu, W. Li, H. F. V. Lee, A. E. Helali, Y-L. Wong, G. Ren, J. Cai, T. Li; Hong Kong/HK
*(yiminyle@gmail.com)*

**Purpose:** Multiparametric MRI (mpMRI) is essential for glioblastoma (GBM) management but is protracted and costly. Deep learning synthesis from conventional MRI suffers from poor generalizability across scanners. We propose a novel solution: a quantitative synthesis network (QS-Net) that generates diagnostic-quality mpMRI contrasts directly from quantitative Magnetic Resonance Fingerprinting (MRF) maps, inherently bypassing scanner-specific contrast variability.
**Methods or Background:** A generative adversarial network with deep-supervised residual blocks was developed to synthesize standard mpMRI (T1w, T2w, FLAIR, SWI) from core MRF-derived T1/T2 maps. The model was trained on 32 healthy volunteer scans and fine-tuned on 9 GBM patient scans, with hold-out testing on 9 independent GBM patients. Performance was quantitatively (MAE, SSIM, PSNR) and qualitatively compared against Res-Unet, cGAN, and Swin-Transformer models. A critical generalizability test evaluated all architectures when trained on MRF maps versus conventional MRI inputs.
**Results or Findings:** QS-Net significantly outperformed all state-of-the-art models, achieving superior quantitative metrics across all synthesized contrasts: T1w, T2w, FLAIR, and SWI (e.g., MAE: 1.01-1.45e-02; SSIM: 0.926-0.939; PSNR: 27.56-29.69) and qualitatively reproducing critical tumor boundaries and internal texture. Crucially, models trained on quantitative MRF inputs demonstrated consistently superior generalizability ($p < 0.005$) across all architectures compared to those trained on conventional qualitative MRI.
**Conclusion:** QS-Net establishes a new paradigm for rapid, high-fidelity mpMRI synthesis by leveraging quantitative MRF as a scanner-agnostic source. This directly addresses the critical limitation of generalizability in prior methods, with immediate clinical potential to streamline GBM imaging protocols, reduce acquisition time, and standardize diagnostics across healthcare institutions.
**Limitations:** The study's cohort size, while sufficient for a proof-of-concept, is limited. Further validation on a larger, multi-scanner cohort is necessary to confirm robust clinical deployment.
**Funding for this study:** This study has been supported by: (1) The National Natural Science Foundation of China Young Scientist Fund (NSFC-YSF 82202941) from China; (2) The Innovation and Technology Support Program (ITS/049/22FP) from the Hong Kong Special Administrative Region (HKSAR), China; (3) The General Research Fund (GRF 15104822, GRF 15102219) from HKSAR, China; (4) The Health and Medical Research Fund (HMRF 10211606, HMRF 06173276) from HKSAR, China.
**Has your study been approved by an ethics committee?** Not applicable
**Author Disclosures:**
Ho Fun Victor Lee: Nothing to disclose
Ge Ren: Nothing to disclose
Tian Li: Nothing to disclose
Aya El Helali: Nothing to disclose
Yat-Lam Wong: Nothing to disclose
Jing Cai: Nothing to disclose
Wen Li: Nothing to disclose
Yimin Ni: Nothing to disclose
Chenyang Liu: Nothing to disclose

**Deep Learning Amplifies the Benefits of High Relaxivity in Brain MRI: A Quantitative Assessment of a Contrast Boosting Algorithm Using Gadopiclenol**

*S. Pasumarthi Venkata*[1], S. Colombo Serra[2], J. Vymazal[3], A. Shankaranarayanan[1]; [1]Menlo Park, CA/US, [2]Colleretto Giacosa/IT, [3]Prague/CZ
*(sonia.colombo@bracco.com)*

**Purpose:** Higher-relaxivity gadolinium-based contrast agents (GBCAs) like Gadopiclenol have recently been introduced which are being used at lower dose levels to achieve comparable lesion visualization. In this work, we show that a deep learning (DL) based contrast boosting algorithm further amplifies the benefits of such higher-relaxivity GBCAs, by improving lesion visualization and image quality without increasing the dosage.
**Methods or Background:** T1w pre-contrast and standard-contrast (SC) images from 20 patients (2D and 3D scans) were obtained for this study. The patients were injected with 0.05 mmol/kg of Gadopiclenol. The pre-contrast and SC images were used to generate contrast boosted (CB) images using an FDA-cleared DL algorithm that boosts the contrast signals present in the SC images. Rectangular regions-of-interest (ROIs) were drawn on all enhancing lesions and on the healthy parenchymal tissues. These ROIs were used to compute the contrast-to-noise ratio (CNR), lesion-to-brain ratio (LBR) and contrast enhancement percentage (CEP).
**Results or Findings:** The mean CNR, LBR and CEP of CB images (10.38±1.56, 5.12±1.03 and 1.96±0.35) were greater than that of SC images (2.52±0.85, 2.55±1.85 and 0.99±0.28). The percentage increase of CNR, LBR and CEP from SC to CB images are 314.19%, 118.12% and 335.18% respectively. We separately calculated the percent increase of CNR, LBR and CEP for patients injected with Gadobenate dimeglumine (at 0.1 mmol/kg) and found it to be 205.97%, 60.50% and 83.19%.
**Conclusion:** Quantitative analysis has shown that the CB algorithm has improved the benefits of Gadopiclenol. From the percent increase in CEP it can be seen that the CB images are equivalent to a double dose injection. Comparative analysis with normal GBCAs has revealed that the CB algorithm performance is better in higher relaxivity GBCAs.
**Limitations:** The study is limited to a small sample size.
**Funding for this study:** n/a
**Has your study been approved by an ethics committee?** Yes
**Ethics committee - additional information:** Internal IRB
**Author Disclosures:**
Srivathsa Pasumarthi Venkata: Employee: Subtle Medical Inc
Sonia Colombo Serra: Employee: Bracco Imaging SpA
Josef Vymazal: Investigator: Na Homolce Hospital
Ajit Shankaranarayanan: Employee: Subtle Medical Inc

**Favorable Contrast MRI Synthesis From Non-Contrast Using a 22 fps Diffusion Model**

D. Wang[1], S. Pasumarthi Venkata[1], *T. C. Arnold*[1], A. Shankaranarayanan[1], G. Zaharchuk[2]; [1]Menlo Park/US, [2]Stanford/US

**Purpose:** Gadolinium-based contrast agents (GBCAs) are widely used in brain MRI to enhance lesion visibility for diagnosing and monitoring brain tumors. However, due to safety concerns such as long-term gadolinium retention in tissues, there is growing interest in dose-reduction strategies. In this study, we investigate the feasibility of a fast diffusion model to synthesize contrast-enhanced images from pre-contrast scans.
**Methods or Background:** The proposed method integrates a conditional denoising diffusion probabilistic model with adversarial learning to achieve high-fidelity medical image synthesis. The model maps source domain images to conditioning features to guide the diffusion model during generation. During inference, the model runs on latent features, thus enabling high throughput at 22 fps. The studied dataset includes 126 clinical cases (113 for training and 13 for testing) acquired using a Philips Insignia 3T scanner with Gadoterate meglumine contrast agent. The cohort consists of 55 female and 71 male patients, with a mean age of 48 ± 16 years. All pre-contrast and SOC-Gad images were mean-normalized, skull-stripped, and affine co-registered using the pre-contrast scan as reference.
**Results or Findings:** The Syn-Gad images showed strong similarity to SOC-Gad images, with a mean PSNR of 34.13, SSIM of 0.8906, RMSE of 0.0202, and CNR of 0.0523, further supporting the fidelity of the synthesized images. Qualitative analysis shows that Synth-Gad images preserve enhancement patterns comparable to those in SOC-Gad, particularly around enhancing tissue regions.
**Conclusion:** The proposed diffusion-based synthesis model demonstrates strong potential for contrast dose reduction in brain MRI. This approach offers a promising path toward reducing patient and environmental exposure to gadolinium-based contrast agents in routine brain MRI exams.
**Limitations:** The work still requires clinical validation in larger, pathology-rich cohorts.
**Funding for this study:** None
**Has your study been approved by an ethics committee?** Not applicable
**Author Disclosures:**
Srivathsa Pasumarthi Venkata: Nothing to disclose
Greg Zaharchuk: Nothing to disclose
Thomas Campbell Arnold: Employee: Subtle Medical Inc.
Ajit Shankaranarayanan: Nothing to disclose
Dayang Wang: Nothing to disclose

**Towards Deterministic Synthesis of Lesion Progression: Mask-Guided Brownian Bridge Diffusion for Multiple Sclerosis Imaging**

P. Mathur, *B. S. Kelly*, R. P. Killeen, A. Lawlor; Dublin/IE
*(brendanskelly@me.com)*

**Purpose:** To investigate the determinism and consistency of diffusion-based generative models for synthesising longitudinal magnetic resonance imaging (MRI) progression in Multiple Sclerosis (MS), comparing a state-of-the-art diffusion model, Brownian Bridge Diffusion (BBDM), and its Mask-Guided extension (MGBBDM).
**Methods or Background:** Baseline and follow-up brain MRI slices from 110 patients were used for training, 30 for validation, and 30 for testing, yielding approximately 6,000 paired slices for model training. Three diffusion-based generative models were trained to simulate follow-up images conditioned on baseline scans and corresponding lesion masks (except BBDM). For each model, five synthetic samples were generated per input to assess pixel-wise variability and reproducibility. Consistency was quantified using the coefficient of variation (CV) across Peak Signal-to-Noise Ratio (PSNR), Structural Similarity Index (SSIM), and L1 distance metrics.
**Results or Findings:** The MGBBDM demonstrated the lowest inter-sample variability, achieving PSNR CV = 9.41% and SSIM CV = 8.89% (both Excellent), with moderate L1 CV = 20.64%. In contrast, the Improved Diffusion and standard BBDM exhibited higher variability (PSNR CV ≈ 12–13%, L1 CV > 30%). The BBDM without mask guidance showed no consistent lesion progression, producing follow-up images resembling the baseline. These findings indicate that mask guidance is essential for achieving deterministic and clinically meaningful lesion evolution.
**Conclusion:** Mask-Guided Brownian Bridge Diffusion offers superior reproducibility and anatomical stability compared to standard diffusion models. By enforcing lesion-specific constraints during synthesis, it produces consistent and clinically interpretable MS progression—an essential step toward reliable generative modelling in longitudinal neuroimaging. Deterministic synthesis supports the creation of reproducible imaging datasets, enabling clinically trustworthy simulation and validation for Multiple Sclerosis tracking.
**Limitations:** The study is limited to 2D modelling with a moderate dataset. Future work will extend to 3D evolution and clinical validation.
**Funding for this study:** This research was supported by Science Foundation Ireland (SFI) under Grant Number SFI/12/RC/2289_P2 and the Irish Centre for High-End Computing (ICHEC)
**Has your study been approved by an ethics committee?** Not applicable
**Author Disclosures:**
Aonghus Lawlor: Nothing to disclose
Ronan P. Killeen: Nothing to disclose
Brendan S. Kelly: Nothing to disclose
Prateek Mathur: Nothing to disclose

**Cross-modality synthesis: conditional diffusion-based generation of MRI from FDG-PET scans**

*N. Benchekroun*, G. Webber, M. Alshammari, A. Hammers; London/UK
*(k23010264@kcl.ac.uk)*

**Purpose:** Combined MRI and PET imaging enables fusion of anatomical and metabolic information in neuroimaging, but simultaneous acquisition is limited by the availability of hybrid PET-MR scanners, cost, and patient burden. Shortage of paired PET-MRI datasets further constrains research requiring both modalities. This study aims to evaluate generative AI methods for synthesising MRI brain images from FDG-PET scans.
**Methods or Background:** A Denoising Diffusion Probabilistic Model (DDPM) with U-Net architecture was developed for cross-modality synthesis from FDG-PET to MRI. The model was trained on over 1,400 axial slices extracted from 149 paired PET-MRI volumes and evaluated on 62 independent test subjects. Two approaches were systematically compared: a 2D method processing slices independently, and a 2.5D method incorporating adjacent slice context to enhance spatial consistency and inter-slice coherence. Quantitative performance was assessed using structural similarity index measure (SSIM) and Pearson correlation coefficients, while visual assessment evaluated anatomical structure preservation and artefact patterns.
**Results or Findings:** Both approaches achieved reasonable synthesis quality with modest improvements for the 2.5D method. Quantitative evaluation demonstrated 3-10% improvements across metrics for the 2.5D approach, with SSIM increasing from 0.626±0.064 to 0.651±0.062 and correlation improving from 0.798±0.043 to 0.822±0.049. Visual assessment revealed that while both approaches adequately preserved anatomical structures, the 2.5D method

Friday produced more systematic error patterns concentrated at tissue boundaries, with more randomly scattered artefacts for the 2D approach.
**Conclusion:** Diffusion-based cross-modality synthesis can generate structurally coherent MRI images from FDG-PET scans, with the 2.5D context-aware approach providing modest but consistent improvements in synthesis quality. These methods demonstrate potential for supplementing missing MRI data in research and clinical applications.
**Limitations:** While results are promising, SSIM is still limited, and clinical validation outstanding.
**Funding for this study:** The School of Biomedical Engineering and Imaging Sciences is supported by the Wellcome EPSRC Centre for Medical Engineering at King's College London (WT 203148/Z/16/Z) and the Department of Health via the National Institute for Health Research (NIHR) comprehensive Biomedical Research Centre award to Guy's & St Thomas' NHS Foundation Trust in partnership with King's College London and King's College Hospital NHS Foundation Trust. MA is supported by the Saudi Arabia Cultural Bureau in London under the Saudi scholarship program. NB was supported by a CDT summer studentship.
**Has your study been approved by an ethics committee?** Not applicable
**Author Disclosures:**
Alexander Hammers: Nothing to disclose
George Webber: Nothing to disclose
Noor Benchekroun: Nothing to disclose
Maha Alshammari: Nothing to disclose

**Deep learning-based virtual dynamic contrast enhanced image generation for prostate**

*J. Pfann Hossbach*, H. Schreiter, L. Brock, T-T. Nguyen, S. Heidarikahkesh, A. George, R. Janka, M. Uder, S. Bickelhaupt; Erlangen/DE
*(julian.hossbach@uk-erlangen.de)*

**Purpose:** Avoiding dynamic contrast enhanced (DCE) prostate MRI acquisition can accelerate clinical workflows, increasing the use of prostate MRI. We aim to generate virtual DCE (vDCE) images from multiparametric non-contrast-enhanced sequences using artificial intelligence as potential substitute.
**Methods or Background:** This IRB-approved retrospective study included n=2092 patients who underwent clinical prostate examinations with T1w-DCE at 3T scanners (Siemens Healthineers MAGNETOM Skyra/Vida). T1w, T2w, and DWI (b-values: 50, 800, 1500 s/mm²) acquisitions were used to train a GAN network; a 2.5D U-Net with 2 discriminators (full/half resolution). Data were resampled to a mutual FOV/resolution, sequence-wise normalized, and split into train=1450, validation=419, and test=213 subjects (⌀25 slices). To standardize and reduce temporal resolution, DCE images at 15, 30, 45 and 60s after acquisition start were selected and registered with a separately trained VoxelMorph-Network forming the targets. The training for 100 epochs minimized the combined adversarial (binary cross entropy), perceptual, L1 and SSIM loss between predicted and target slices using the non-DCE images with their ±1 neighboring slices as input.
**Results or Findings:** The generated test data achieved a MS-SSIM of 0.9649, 0.9298, 0.8762 and 0.853, SSIM of 0.9251, 0.8516, 0.7801 and 0.7407 and NRMSE of 0.0318, 0.0484, 0.0657 and 0.0713, respectively, outperforming state-of-the-art single timepoint predictions. Radiologist classified the overall image quality of n=200 targets/predictions into real/generated, yielding near-equal counts: real (n=140 real, n=138 generated) and generated (n=60 real, n=62 generated).
**Conclusion:** Multi-timepoint vDCE image generation was technically feasible and indistinguishable from real images for the reader. Further work is necessary to improve the method and to assess its potential for prostate MRI in clinical practice.
**Limitations:** A lesion enhancement comparison and diagnostic value evaluation was not conducted in this retrospective single-centre study. Furthermore, truly dynamic image generation was not addressed.
**Funding for this study:** This research was funded by the Bavarian State Ministry of Economic Affairs, Regional Development and Energy.
**Has your study been approved by an ethics committee?** Yes
**Ethics committee - additional information:** Study was approved by ethics Committee of FAU with waived infromed consent
**Author Disclosures:**
Sebastian Bickelhaupt: Nothing to disclose
Tri-Thien Nguyen: Nothing to disclose
Aju George: Nothing to disclose
Julian Pfann Hossbach: Patent Holder: Siemens Healthineers
Michael Uder: Nothing to disclose
Hannes Schreiter: Nothing to disclose
Luise Brock: Nothing to disclose
Shirin Heidarikahkesh: Nothing to disclose
Rolf Janka: Nothing to disclose

**Invisible Biases in AI-Generated Medical Images: Implications for Diagnostic Algorithm Equity**

*K. Nechypurenko*, T. Nechypurenko; Kyiv/UA

**Purpose:** To synthesize evidence on hidden feature propagation and demographic bias in generative AI models for synthetic medical image generation, evaluating implications for diagnostic algorithm performance and equity across patient populations.
**Methods or Background:** Narrative synthesis of peer-reviewed literature (2024-2025) examining generative AI in radiology. We analyzed 8 key publications including: one systematic survey of 103 diffusion model studies, three foundation model validation studies with datasets exceeding 20 million images (SA-Med2D-20M, TotalSegmentator, UK Biobank), two multi-institutional bias assessments, and two radiologist evaluation studies. We extracted data on diagnostic performance metrics, demographic bias patterns, and validation methodologies across CT, MRI, chest radiography, and histopathology.
**Results or Findings:** Synthetic image quality varied by modality. Pathologists could not distinguish synthetic histopathology images while radiologists identified 96.6% of synthetic radiological images as non-authentic. Algorithms trained with combined synthetic-authentic data showed improvements: isocitrate dehydrogenase mutation prediction area under curve 0.75, glioma volumetry concordance 0.782, lesion detection (sensitivity improvements up to 25% for specific tasks). However, foundation models exhibited persistent demographic disparities: decreased accuracy for female patients (normal chest radiographs) and patients of African ancestry (pleural effusion), despite massive training datasets. Algorithms extracted patient race through hidden features imperceptible to radiologists. Demographic-balanced fine-tuning provided only partial mitigation.
**Conclusion:** Synthetic medical images enhance diagnostic algorithm performance for rare pathologies and data-scarce scenarios but systematically propagate demographic biases through hidden features undetectable by visual assessment. Clinical deployment requires demographic-stratified validation protocols and continuous performance monitoring across patient populations to ensure equitable diagnostic accuracy.
**Limitations:** Secondary literature synthesis with heterogeneous validation methodologies across studies. Limited long-term clinical outcome data. Geographic and demographic representation varied across included datasets.
**Funding for this study:** No funding was received for this study
**Has your study been approved by an ethics committee?** Not applicable
**Author Disclosures:**
Tetyana Nechypurenko: Nothing to disclose
Kateryna Nechypurenko: Nothing to disclose

**16:00 - 17:30** **Research Stage 3**

### Research Presentation Session: Genitourinary

### RPS 1607

### Imaging of renal lesions

**Moderator**
Iztok Caglič; London/UK
*(iztokcaglic@gmail.com)*

**Non-Contrast MRI Versus Contrast-Enhanced CT for Active Surveillance of Small Solid Renal Masses: An International Multi-Observer Agreement Study**

B. Stensby Breen[1], H. Søgaard Christensen[2], J. Brøndum Frøkjær[2], O. Graumann[3], M. Kleivane[1], A. Negard[1], I. Mjåland Salte[1], *J. Borgbjerg*[1]; [1]Oslo/NO, [2]Aalborg/DK, [3]Aarhus/DK
*(jb@castlemountain.dk)*

**Purpose:** To assess the agreement of maximum tumor diameter and tumor nearness between non-contrast MRI and contrast-enhanced CT for SRM surveillance, and to compare diagnostic confidence in tumor delineation across modalities.
**Methods or Background:** Active surveillance (AS) of small renal masses (SRMs) typically relies on contrast-enhanced CT for serial size measurement. Non-contrast MRI protocols may provide an alternative. We retrospectively identified 50 patients (mean age, 62.9 years; 44% women) who underwent both contrast-enhanced CT and axial T2-weighted MRI within 3 months as part of AS protocol. Nine radiologists from five hospitals independently assessed maximum tumor diameter (axial plane) and tumor nearness to the collecting system (TN, three-tier scale) and subjectively graded their diagnostic confidence in delineating the tumor contour (DC, 5-point scale) in 50 CT and MRI scans across four randomized reading sessions. Observer agreement for diameter assessment was quantified using the limits of agreement with the mean (LOAM), whereas TN and DC was analyzed using Gwet's AC2.
**Results or Findings:** Mean tumor diameters were 19.9 mm for CT and 19.5 mm for MRI (mean difference, 0.4 mm; p=0.051). Reproducibility LOAMs were ±2.9 [2.7-3.2] mm (CT) and ±3.1 [2.9-3.4] mm (MRI), with overlapping confidence intervals. TN ratings and agreement were similar for CT and MRI (p=0.61; AC2=0.67 each), whereas DC ratings were higher for CT (p<0.001).
**Conclusion:** Non-contrast axial T2-weighted MRI demonstrated agreement comparable to contrast-enhanced CT for tumor diameter and tumor nearness, while diagnostic confidence favored CT without affecting agreement.
**Limitations:** A variety of MRI systems with a span in scan parameters. Retrospective study design with patients from a single center. Renal mass sample size is relatively small, albeit comparable to previous studies. Maximum axial tumor diameter was evaluated to remain consistent with clinical standards and previous research.
**Funding for this study:** Akershus University Hospital
**Has your study been approved by an ethics committee?** Yes
**Ethics committee - additional information:** The study was approved by the Norwegian Regional Committees for Medical and Health Research Ethics (reference 285666) and by the institutional data protection officer with a waiver for informed patient consent.
**Author Disclosures:**
Jens Brøndum Frøkjær: Nothing to disclose
Ole Graumann: Nothing to disclose
Bendik Stensby Breen: Nothing to disclose
Mats Kleivane: Nothing to disclose
Anne Negard: Nothing to disclose
Heidi Søgaard Christensen: Nothing to disclose
Jens Borgbjerg: Nothing to disclose
Ivar Mjåland Salte: Nothing to disclose

**CCLS and beyond: are there any additional MR features that can help characterizing Small Renal Masses?**

*G. Imperiale*, G. Brembilla, C. Rodolfi, F. Cei, A. Larcher, G. Rosiello, U. Capitanio, F. De Cobelli; Milan/IT
*(imperiale.giulio@hsr.it)*

**Purpose:** Evaluate the frequency of Clear Cell Likelihood Score (CCLS) features of Small Renal Masses (SRMs) on multi-parametric Magnetic Resonance (mpMRI) and investigate additional imaging characteristics to improve diagnostic accuracy and better define Renal Cell Carcinoma (RCC).
**Methods or Background:** Preoperative multiparametric mpMRI of patients with SRMs subject to surgery were retrospectively evaluated by two radiologists with intermediate experience blinded to the histological diagnosis (gold standard). CCLS features evaluated were: T2 signal intensity, microscopic fat, cortex vascularity, segmental enhancement inversion (SEI), arterial-delayed enhancement ratio (ADER >1.5); additionally, T2-heterogeneity, peritumoral-pseudocapsule and heterogeneity were assessed.
**Results or Findings:** 80 patients with 33 clear cell RCCs (41.25%), 25 papillary RCCs (31.25%), 9 oncocytomas (11.25%), 9 chromophobe RCCs (11.25%) and 4 fat-poor angiomyolipomas (5%) were evaluated. Clear cell RCCs (ccRCC) showed T2-hyperintensity (75,76%), heterogeneous signal (81,82%), pseudocapsule (72,73%), microscopic fat (54,55%) and marked hypervascular enhancement (96,97%), with heterogeneous pattern (81,82%). Papillary RCCs (pRCC) were predominantly T2-hypointense (64%), with lower frequency of pseudocapsule (52%), reduced vascularity (64% hypovascular) and rare heterogeneous pattern of enhancement (24%).
**Conclusion:** MRI characterization of the major renal tumor histotypes confirms established features from the CCLS, such as hypervascularity and T2-hyperintensity in ccRCC and hypovascularity and T2-hypointensity in pRCC. It also identified T2-signal intensity and enhancement heterogeneity and the presence of a pseudocapsule as discriminators. These findings may aid radiologists in refining non-invasive differentiation among renal tumor subtypes and may be added to the CCLS in order to increase specificity.
**Limitations:** These include the retrospective single center design. Confirmation of features identified vs gold standard in this preliminary evaluation should be confirmed on larger cohorts.
**Funding for this study:** This study received no external funding.
**Has your study been approved by an ethics committee?** Yes
**Ethics committee - additional information:** The study was approved by the Ethics Committee.
**Author Disclosures:**
Chiara Rodolfi: Nothing to disclose
Francesco De Cobelli: Nothing to disclose
Giulio Imperiale: Nothing to disclose
Alessandro Larcher: Nothing to disclose
Francesco Cei: Nothing to disclose
Giuseppe Rosiello: Nothing to disclose
Giorgio Brembilla: Nothing to disclose
Umberto Capitanio: Nothing to disclose

**Clear Cell Likelihood Score: A Categorizing and Grading Tool for Small Renal Masses**

*O. M. S. M. Sherif*, A. Mousa, M. Zaky, A. Abdelhamid, M. E. Abou El-Ghar, T. A. El-Diasty; Mansoura/EG
*(drosoliman@yahoo.com)*

**Purpose:** We aimed prospectively to assess effectiveness of Clear Cell Likelihood Score in small renal masses and its role in predicting tumor grade.
**Methods or Background:** The detection of small renal masses has increased due to recent widespread of cross-sectional imaging . Clear cell renal cell carcinoma (ccRCC) is the most common subtype with rapid progression and metastasis. We utilized MRI Clear Cell Likelihood Score to investigate its ability to distinguish and grade different subtypes.
**Results or Findings:** In total, 103 patients with small solid renal masses of stage T1a (≤4 cm) were identified. Mean tumor size was 3.4 ± 0.6 cm. According to our study results, the clear cell Likelihood Score (ccLS) had sensitivity of 75.6 %, specificity of 93.5%, PPV of 88.6 %, NPV of 85.3 % and accuracy of 86.4 % in diagnosing ccRCC using a ccLS threshold of 4 and 5. As regard the assessment of ccLS threshold of 1 or 2 in excluding ccRCC pathological subtype, our study found that out of 29 patients with ccLS 1 or 2, there was only 1 ccRCC case with false result (3% false positive). It was also noted that there is significant relation between Arterial-to-delayed-enhancement-ratio (ADER) value and the grade of the ccRCC. The median interquartile range (IQR) of ADER parameter was statistically significant higher in grade II compared to grade I (Median was 1.6 and 0.9 respectively) and much higher in grade III compared to grades I and II (Median was 2.9) with P value < 0.001.
**Conclusion:** This ccLS showed promising efficacy in prediction and exclusion of ccRCC subtype. Moreover, it aids in predicting the ccRCC grade.
**Limitations:** Few cases of fat poor angiomyolipoma was interpreted as a high risk for being clear cell carcinoma.
**Funding for this study:** Self funding.
**Has your study been approved by an ethics committee?** Yes
**Ethics committee - additional information:** Mansoura University Research Ethics Committee
**Author Disclosures:**
Tarek A. El-Diasty: Nothing to disclose
Abdalla Abdelhamid: Author: Supervisor
Osama Mohamed Soliman Mahmoud Sherif: Author: Author and Investigator
Mohamed Ebrahem Abou El-Ghar: Nothing to disclose
Amany Mousa: Author: Main supervisor
Mona Zaky: Author: Supervisor

**IRM K01: DIAGNOSTIC VALUE OF MULTIPARAMETRIC MR IMAGING OF SMALL SOLID RENAL TUMORS – A Prospective Multicenter Study**

*E. Jambon*, A. Crombé, G. Margue, N. Grenier, F. H. Cornelis, J-C. Bernhard; Bordeaux/FR

**Purpose:** Purpose: Small renal solid tumors (SRST) are increasingly being detected incidentally, posing diagnostic challenges. Up to 20% of SRST result in non-contributive biopsies. The aim was to assess the diagnostic accuracy of multiparametric MRI (mpMRI) in differentiating malignant from benign SRST in patients with suspected renal malignancy without no evident signs of malignancy.
**Methods or Background:** Methods or Background: This is a prospective multicentric French study. A cohort of 387 patients in 18 centers with non-hereditary, SRST between 1.5 and 4 cm in diameter was enrolled between November 2018 and May 2022. mpMRI protocols included T1w, T2w, diffusion-weighted imaging, and dynamic contrast-enhanced sequences. Two radiologists performed blinded readings with a centralized review in case of discordance. The primary endpoint is the negative predictive value (NPV) of a dichotomized Likert scale score, targeting a 98% NPV.

**Results or Findings:** Results or Findings: Among 287 patients analyzed for the primary endpoint, mpMRI achieved high sensitivity (97.4%) and PPV (83.2%) in detecting malignant tumors, but specificity (9.8%) and NPV (45.5%) remained low, limiting its ability to rule out malignancy. Likert scores varied significantly across histological subtypes, with oncocytomas and chromophobe RCCs often misclassified as malignant. In clear cell RCC (n = 121), higher Fuhrman grades were significantly associated with extensive diffusion restriction (p < 0.001) and T2 hypointensity (p = 0.001). The median Likert score increased with tumor grade (p = 0.004), suggesting that certain MRI features may reflect histological aggressiveness in this subtype.
**Conclusion:** Conclusion: MpMRI remains limited in its ability to distinguish benign from malignant tumors based on the predefined criteria used in this study. However, it may offer a noninvasive means of assessing tumor grade in clear cell renal cell carcinoma.
**Limitations:** Predefined criteria
**Funding for this study:** National funding (PHRC)
**Has your study been approved by an ethics committee?** Yes
**Ethics committee - additional information:** The trial protocol (ClinicalTrials.gov identifier: NCT03470285) was approved by the CPP Sud-Méditerranée 5 (Committee for the Protection of Persons – French equivalent of an Ethical Committee) and the authorized by the authorisation from ANSM (the French National Agency for the Safety of Health Products).
**Author Disclosures:**
Jean-Christophe Bernhard: Nothing to disclose
Gaelle Margue: Nothing to disclose
François H Cornelis: Nothing to disclose
Eva Jambon: Nothing to disclose
Amandine Crombé: Nothing to disclose
Nicolas Grenier: Nothing to disclose

**Analysis of clinicopathologic features and imaging findings of TFE3-rearranged renal cell carcinoma Abstract**
*J. Liu*, J. Zhao; Lanzhou/CN
*(1834049392@qq.com)*

**Purpose:** To examine the imaging features and clinicopathologic features of TFE3-rearranged renal cell carcinoma and highlight key imaging findings for clinical decision-making.
**Methods or Background:** The imaging and clinicopathological data of 49 pathologically confirmed cases of TFE3-rearranged renal cell carcinoma from multiple institutions between January 2018 and December 2024 were retrospectively analyzed.
**Results or Findings:** Of the 49 patients, 17 were male and 32 were female; aged 44 ± 15 years (range: 10-82 years) , 22 were asymptomatic and 27 were symptomatic. Tumor size averaged 7.0 (range: 2.0–14.9) cm. Imaging showed 28 round-like and 21 lobulated lesions; 18 had distinct margins, while 31 had indistinct margins. A pseudocapsule was present in 36 cases, 24 of which were incomplete. Additionally, 42 tumors showed uneven density with necrosis or cystic degeneration; there were 25 cases of hemorrhage, 27 cases of irregular calcification, Peritumoral vessels were noted in 23 cases. Imaging modalities included CT (44 cases: 41 with both plain and enhanced scans, 3 with plain only), MRI (13 cases with plain and enhanced scans), and ultrasonography (15 cases, including 5 with contrast enhancement). Treatment included partial nephrectomy (21 cases), total nephrectomy (22 cases) , and biopsy (6 cases).With a median follow-up of 30 months (range: 1–72 months) until December 2024, 6 patients experienced tumor recurrence, 14 developed metastasis, and 7 died, while 5 patients were lost to follow-up.
**Conclusion:** TFE3-rearranged renal cell carcinoma is a rare renal tumor with distinct imaging and clinical features that can inform preoperative diagnosis, treatment, and prognosis.
**Limitations:** This is a retrospective single-center study whose results have not been validated in an independent cohort.
**Funding for this study:** No.
**Has your study been approved by an ethics committee?** Not applicable
**Author Disclosures:**
Jianqiang Liu: Nothing to disclose
Jianhong Zhao: Nothing to disclose

**Differentiating Fat-Poor Angiomyolipoma from Chromophobe Renal Cell Carcinoma Using Preoperative Non-Contrast CT Histogram Analysis**
*J. Wei*, X. Zhang, J. Liu, J. Zhou; Lanzhou/CN
*(weijinyan501@163.com)*

**Purpose:** To investigate the value of clinical-radiologic features and whole-tumor histogram analysis derived from non-contrast CT in differentiating fat-poor angiomyolipoma (fp-AML) from chromophobe renal cell carcinoma (ChRCC).
**Methods or Background:** This retrospective study enrolled 89 patients with pathologically confirmed fp-AML (n=36) or ChRCC (n=53). Clinical, imaging, and pathological data were collected. Demographic data and conventional CT features (sex, age, symptoms, location, shape, margin, attenuation on non-contrast CT, tumor volume) were recorded. Using FireVoxel software, volumetric regions of interest were drawn on axial non-contrast CT images to extract histogram parameters (minimum, maximum, mean, standard deviation, variance, coefficient of variation, skewness, kurtosis, entropy, and percentiles). Statistical analyses included the Chi-square test, Mann-Whitney U test, and independent samples t-test. Multivariable logistic regression was used to identify independent predictors for differentiation and to construct clinical-radiologic and histogram models. Receiver operating characteristic (ROC) curve analysis was performed to evaluate diagnostic performance.
**Results or Findings:** Multivariable analysis identified non-contrast CT attenuation (OR=1.178, 95% CI: 1.087-1.277, P<0.001), tumor volume (OR=0.952, 95% CI: 0.928-0.976, P<0.001), mean attenuation (OR=0.764, 95% CI: 0.589-0.946, P=0.015), skewness (OR=0.135, 95% CI: 0.046-0.396, P<0.001), and the Perc.75 (OR=1.522, 95% CI: 1.169-1.983, P=0.002) as independent predictors for differentiating fp-AML from ChRCC. Both models demonstrated good performance. The clinical-radiologic model achieved an AUC of 0.905 (95% CI: 0.843-0.967), with a sensitivity of 77.8% and specificity of 90.6%. The histogram model achieved an AUC of 0.964 (95% CI: 0.932-0.996), with a sensitivity of 88.9% and specificity of 75.5%.
**Conclusion:** Clinical-radiologic features and whole-tumor histogram analysis based on non-contrast CT can help noninvasively differentiate fp-AML from ChRCC preoperatively, providing valuable guidance for clinical management.
**Limitations:** This study is limited by its retrospective, single-center design, and the findings have not been validated in an independent cohort.
**Funding for this study:** Natural Science Foundation of Gansu Province (22JR11RA060)
**Has your study been approved by an ethics committee?** Yes
**Ethics committee - additional information:** This study was approved by the Medical Ethics Committee of the Second Hospital of Lanzhou University (approval number: 2025A-585) and informed consent was waived.
**Author Disclosures:**
Junlin Zhou: Nothing to disclose
Xueling Zhang: Nothing to disclose
Jianqiang Liu: Nothing to disclose
Jinyan Wei: Nothing to disclose

**Preoperative Prediction of Renal Cell Carcinoma Risk Stratification Using Multiphase CT Features and R.E.N.A.L. Score**
*J. Wei*, X. Zhang, J. Liu, J. Zhou; Lanzhou/CN
*(weijinyan501@163.com)*

**Purpose:** To investigate the association of multiphase computed tomography(CT) features, and R.E.N.A.L. score with Leibovich risk stratification in localized renal cell carcinoma (RCC).
**Methods or Background:** This retrospective study analyzed 265 pathologically confirmed RCC patients. Clinical data, CT semantic features, and R.E.N.A.L. scores were collected. Patients were classified into low-risk (n=124) and intermediate-high-risk (n=141) groups per Leibovich criteria. Clinical characteristics included symptoms, albumin level, neutrophil-to-lymphocyte ratio (NLR), systemic immune-inflammation index (SII), systemic inflammation response index (SIRI), and tumor extracellular volume fraction (ECV). CT semantic features included maximum tumor diameter, location, shape, margin, cystic change/necrosis, calcification, enhancement homogeneity, perinephric neovascularity, and R.E.N.A.L. score. Statistical analyses involved $\chi^2$ tests, t-tests, Mann-Whitney U tests. Univariate and multivariate logistic regression analyses were employed to identify independent predictive factors for Leibovich risk stratification.
**Results or Findings:** Patients in the intermediate-high-risk group were more likely to have clinical symptoms, higher SII, and lower albumin levels compared to the low-risk group (P < 0.05). Tumor shape, margin, cystic change/necrosis, calcification, enhancement homogeneity, and perinephric neovascularity significantly differed between the two groups (P < 0.05). The maximum tumor diameter was larger in the intermediate-high-risk group, while the parenchymal phase enhancement ratio was lower (P < 0.05). Perinephric neovascularity was more frequent in the intermediate-high-risk group (P < 0.05). The R.E.N.A.L. score significantly differed between groups, R.E.N.A.L. score complexity was significantly higher in the intermediate-high-risk group (P<0.05). Multivariate analysis identified cystic change/necrosis, perinephric neovascularity, and R.E.N.A.L. score as independent predictors of intermediate-high Leibovich risk stratification in RCC.
**Conclusion:** Tumor cystic/necrotic change, perinephric neovascularity, and R.E.N.A.L. score are independent preoperative biomarkers for predicting intermediate-high Leibovich risk in RCC.
**Limitations:** This study is limited by its retrospective, single-center design and by the lack of xternal validation
**Funding for this study:** Natural Science Foundation of Gansu Province (22JR11RA060)
**Has your study been approved by an ethics committee?** Yes

**Ethics committee - additional information:** This study was approved by the Medical Ethics Committee of the Second Hospital of Lanzhou University (approval number: 2025A-585) and informed consent was waived.
**Author Disclosures:**
Junlin Zhou: Nothing to disclose
Xueling Zhang: Nothing to disclose
Jianqiang Liu: Nothing to disclose
Jinyan Wei: Nothing to disclose

### Is the Clear Cell Likelihood Score Applicable Across MRI and CT? Evidence from a Single-Center Study

*G. Imperiale*, G. Brembilla, C. Rodolfi, F. Cei, A. Larcher, G. Rosiello, U. Capitanio, F. De Cobelli; Milan/IT
*(imperiale.giulio@hsr.it)*

**Purpose:** Systematically evaluate the concordance of enhancement patterns between MRI and CT in small renal masses. This comparison aims to support the feasibility and broader application of the Clear Cell Likelihood Score (CCLS) in clinical contexts where contrast-enhanced MRI may be impractical, contraindicated or where optimization of healthcare resources is desirable.
**Methods or Background:** Patients with both preoperative CT and MRI were included, with histopathology serving as the reference standard. Two radiologists with intermediate experience, blinded to histology, independently assessed the enhancement patterns on MRI and CT; evaluated parameters included vascularity (hyper-, iso-, hypovascular), presence of segmental enhancement inversion (SEI) and arterial-delayed enhancement ratio (ADER >1.5). Lastly, CCLS was assessed both on MRI alone and on CT-derived enhancement pattern combined with MRI features.
**Results or Findings:** The cohort consisted of 47 patients, with 20 clear cell RCCs (42.6%), 12 papillary RCCs (25.5%), 9 oncocytomas (19.1%), 4 chromophobe RCCs (8.5%) and 2 fat-poor angiomyolipomas (4.3%). Enhancement type was concordant in 41 cases (87.2%). Concordance for SEI was observed in 42 cases (89%) and for ADER in 44 cases (94%). Overall, the CCLS was consistent in 85% of the cohort between MRI only and CT-MRI combined.
**Conclusion:** MRI and CT showed a high level of concordance in the characterization of enhancement patterns in small renal masses, supporting the potential role of CT as a complementary tool when MRI with contrast is not feasible. Importantly, as CT is usually performed before MRI in clinical practice, sparing contrast in MRI could be reasonable, with CT-MRI combined CCLS representing a viable option.
**Limitations:** This study is limited by its single-center design, small cohort and histotype imbalance favoring clear cell and papillary RCCs. Findings should be considered preliminary, with validation in larger, multicenter populations warranted.
**Funding for this study:** This study received no external funding.
**Has your study been approved by an ethics committee?** Yes
**Ethics committee - additional information:** The study was approved by the Ethics Committee.
**Author Disclosures:**
Chiara Rodolfi: Nothing to disclose
Francesco De Cobelli: Nothing to disclose
Giulio Imperiale: Nothing to disclose
Alessandro Larcher: Nothing to disclose
Francesco Cei: Nothing to disclose
Giuseppe Rosiello: Nothing to disclose
Giorgio Brembilla: Nothing to disclose
Umberto Capitanio: Nothing to disclose

### Automated Radiomics-Based Machine Learning for Differentiating Clear Cell from Non-Clear Cell Renal Cell Carcinoma Using Multi-Parametric MRI

M. Liu, S-C. Lin, Y-T. Lin, W-H. Chen, Y-J. Liu, *C-H. Liao*; Taichung/TW
*(springletter@hotmail.com)*

**Purpose:** This study aimed to develop an automated radiomics-based machine learning framework using multiple MRI weightings to distinguish clear cell renal cell carcinoma (ccRCC) from non-clear cell renal cell carcinoma (ncRCC). Accurate preoperative identification of RCC subtypes is critical for selecting optimal surgical strategies and guiding targeted therapies, especially for patients unable to undergo contrast-enhanced imaging.
**Methods or Background:** RCC accounts for 85% of malignant kidney tumors, with ccRCC comprising ~75% of cases. MRI provides diverse weighted sequences (T2WI, T1WI, CE-T1WI, In/Out phase, DWI/ADC) useful for RCC differentiation. Radiomics enables extraction of thousands of quantitative features from tumor ROIs, which can be processed with machine learning. This study employed AutoML via TPOT to identify optimal feature sets and classifiers. MRI data from 76 patients (49 ccRCC, 27 ncRCC) between 2014–2023 were analyzed. Tumor ROIs were segmented in 2D, 3D, and combined formats; features were extracted and reduced through feature selection, followed by AutoML training with 10-fold cross-validation.
**Results or Findings:** Among classifiers, LinearSVC and SGD achieved the best performance. In 3D analyses, AUCs reached 0.81–0.86 across image sets. Importantly, models trained without contrast-enhanced images (C−) still attained robust accuracy (AUC up to 0.81–0.86 when combining 2D and 3D data). This demonstrates that non-contrast MRI radiomics can yield reliable subtype classification, potentially avoiding gadolinium-related risks.
**Conclusion:** The AutoML-based radiomics approach successfully differentiated ccRCC from ncRCC using multiparametric MRI, achieving high performance even without CE-T1WI. This method offers a clinically valuable tool for preoperative planning, particularly in patients with renal dysfunction or contrast contraindications.
**Limitations:** The study's retrospective design and modest cohort size, especially the smaller ncRCC subgroup, may affect generalizability. Larger multicenter datasets and prospective validation are needed to confirm robustness and clinical applicability.
**Funding for this study:** There is no funding for this study
**Has your study been approved by an ethics committee?** Yes
**Ethics committee - additional information:** Institutional Review Board (IRB) of Taichung Veterans General Hospital (TCVGH)
**Author Disclosures:**
Mingcheng Liu: Nothing to disclose
Shao-Chieh Lin: Nothing to disclose
Chun-Han Liao: Nothing to disclose
Wen-Hsien Chen: Nothing to disclose
Yen-Ting Lin: Nothing to disclose
Yi-Jui Liu: Nothing to disclose

### Diagnostic Accuracy of Contrast-Enhanced Ultrasound in Differentiating Renal Cell Carcinoma from Angiomyolipoma in Small Hyperechoic Renal Masses (≤3 cm): A Retrospective Study

*N. Giordano*[1], F. Urraro[2], V. Patanè[1], M. Piscopo[1], A. Russo[1], S. Cappabianca[1], A. Reginelli[1]; [1]Naples/IT, [2]Roma/IT
*(nicolettagiord19@gmail.com)*

**Purpose:** To assess the diagnostic accuracy of contrast-enhanced ultrasound (CEUS) in differentiating renal cell carcinoma (RCC) from angiomyolipoma (AML) in small (≤3 cm), hyperechoic renal masses and to identify predictive enhancement patterns in routine clinical practice.
**Methods or Background:** This retrospective single-center study included 104 patients with incidentally detected hyperechoic renal lesions ≤3 cm examined by CEUS between December 2021 and July 2024. Two blinded radiologists independently assessed wash-in/wash-out dynamics, peak intensity, homogeneity, and perilesional rim-like enhancement. Histopathology was the reference standard when available; lesions stable for ≥18 months on follow-up were considered benign. Diagnostic metrics were calculated, and multivariate logistic regression identified independent predictors. Interobserver agreement was assessed by intraclass correlation coefficient (ICC)
**Results or Findings:** Of 104 lesions, 80 were managed as AMLs, with 4 biopsied during follow-up (2 papillary RCCs, 2 AMLs). Twenty-eight lesions underwent biopsy, confirming 26 RCCs (53% papillary, 32% chromophobe, 15% clear cell) and 2 atypical AMLs. RCCs typically demonstrated heterogeneous hyperenhancement with rapid wash-out and perilesional rim-like enhancement, whereas AMLs showed iso- or homogeneous enhancement with delayed wash-out. Rapid wash-out achieved sensitivity of 87% and specificity of 94% (AUC 0.91), while perilesional rim-like enhancement reached specificity of 98% and PPV of 92%. Combined CEUS features yielded an AUC of 0.93. Interobserver agreement was good (ICC 0.75–0.9).
**Conclusion:** CEUS provides excellent diagnostic accuracy in distinguishing RCC from AML in small hyperechoic renal masses. Rapid wash-out and perilesional rim-like enhancement are independent predictors of malignancy, supporting CEUS as a reliable, radiation-free tool to guide clinical decision-making
**Limitations:** This was a retrospective, single-center study with limited sample size. Histological confirmation was not available for all AMLs, relying on imaging stability as a surrogate of benignity. Larger prospective multicenter studies are required to validate these findings.
**Funding for this study:** No external funding was received for this study

**Has your study been approved by an ethics committee?** Yes
**Ethics committee - additional information:** Approved by the local Ethics Committee of the University Hospital 'Luigi Vanvitelli', Naples, Italy (Protocol 158/i/2022)
**Author Disclosures:**
Anna Russo: Author: The authors declare no conflicts of interest and no role of commercial entities in the dissemination of study results
Nicoletta Giordano: Author: The authors declare no conflicts of interest and no role of commercial entities in the dissemination of study results
Salvatore Cappabianca: Author: The authors declare no conflicts of interest and no role of commercial entities in the dissemination of study results
Fabrizio Urraro: Author: The authors declare no conflicts of interest and no role of commercial entities in the dissemination of study results
Marco Piscopo: Author: The authors declare no conflicts of interest and no role of commercial entities in the dissemination of study results
Vittorio Patanè: Author: The authors declare no conflicts of interest and no role of commercial entities in the dissemination of study results
Alfonso Reginelli: Author: The authors declare no conflicts of interest and no role of commercial entities in the dissemination of study results

**Peritumoral Radiomics Improves Prediction of Occult pT3a in Clear Cell Renal Cell Carcinoma: A Multicenter Study**
*S. Li*, Z. Li; Wuhan/CN
*(15172541672@163.com)*

**Purpose:** Occult pathological T3a (pT3a) upstaging in cT1b–T2a clear cell renal cell carcinoma (ccRCC) correlated with poor prognosis and necessitates modifications in surgical planning. However, predicting it preoperatively remains challenging. Conventional imaging emphasizes intratumoral features, yet early invasion frequently manifests as subtle alterations in peritumoral tissue. This study aimed to evaluate the diagnostic value of peritumoral tissue and to test whether combining peritumoral and intratumoral radiomics improves prediction of occult pT3a.
**Methods or Background:** This multicenter retrospective analysis included 1240 patients initially staged as cT1b–T2a ccRCC. Tumor and peritumoral regions were segmented using nnU-Net, and 107 radiomic features were extracted. Logistic regression, support vector machine, decision tree, and random forest classifiers were trained using tumor-only, peritumor-only, and combined features. Model performance was assessed with AUC, sensitivity, specificity, and accuracy across internal and external cohorts, with DeLong tests for AUC comparisons. SHAP analysis was used to evaluate feature contributions and tumor–peritumor interactions.
**Results or Findings:** In the internal validation cohort, the Radiomics_Combined model achieved an AUC of 0.800, significant outperforming the Radiomics_peritumor model (AUC 0.709, $p = 0.020$) and showing a nonsignificant improvement over the Radiomics_tumor model (AUC 0.787, $p = 0.798$). Similar trends were observed in external validation cohorts. SHAP analysis identified 30 peritumoral features among the top 40 predictors, with peritumor_shape_Maximum3DDiameter ranked highest. SHAP dependence plots further demonstrated nonlinear tumor–peritumor interactions, indicating that the model captures cross-regional effects reflecting subtle morphologic changes associated with local invasion.
**Conclusion:** Peritumoral tissue contributes distinct and complementary signatures of early invasion. Integrating tumor and peritumoral features enables accurate, interpretable prediction of occult pT3a upstaging in cT1b–T2a ccRCC and may improve surgical dicision-making.
**Limitations:** Retrospective design.
**Funding for this study:** This study was supported by grants from the National Natural Science Foundation of China (No. 82371942).
**Has your study been approved by an ethics committee?** Yes
**Ethics committee - additional information:** The ethical approvals for this multi-center retrospective study were provided by the Institutional Review Board of Tongji Hospital, Tongji Medical College, Huazhong University of Science and Technology; the Institutional Review Board of the First Affiliated Hospital of Nanjing Medical University; and the Institutional Review Board of Xiangyang Central Hospital.
**Author Disclosures:**
Zhen Li: Nothing to disclose
Shichao Li: Nothing to disclose

**16:00 - 17:30 Research Stage 4**

### Research Presentation Session: Neuro

### RPS 1611

### Neuroimaging: innovation in techniques and contrast agents

**Moderator**
Nicoletta Emanuela Anzalone; Milan/IT

**First prospective evaluation of post-contrast ultra-low field brain MRI across diverse neurological pathologies**
*B. D. Wichtmann*[1], K. Teichmann[1], N. Lehnen[1], T. Tonguc[1], F. Padormo[2], A. Rastogi[1], Z. Bendella[1], A. Radbruch[1], J. Faber[1]; [1]Bonn/DE, [2]Guilford, CT/US
*(barbara.wichtmann@ukbonn.de)*

**Purpose:** Portable ultra-low-field-(pULF)-MRI enables bedside neuroimaging in resource limited settings. However, lower signal and relaxivity differences at 64mT limit contrast enhancement (CE). This prospective study provides first evaluation of CE-pULF-MRI in a heterogeneous cohort compared to high-field-(HF)-MRI for lesion detection and diagnostic confidence.
**Methods or Background:** This IRB-approved prospective study included 39 patients (58.1±15.7years; 20♀) undergoing clinically indicated CE-HF-MRI at 1.5/3T for oncologic, demyelinating, inflammatory, vascular, postoperative, or degenerative conditions between 04-05/2025. Patients received native pULF-MRI (SWOOP,Hyperfine,Guilford,CT) with axial T1w standard (std) and synthetic echo train length (sETL) sequences, FLAIR, T2w, and DWI. HF-MRI followed clinical protocols with pre-/post-contrast T1w-imaging after IV-administration of 1.0mmol/mL gadobutrol (Gadovist). CE-pULF-MRI was then repeated using the same axial T1w std and sETL sequences. 4 blinded (neuro-)radiologists independently evaluated all T1w scans from both field strengths for post-contrast hyperintense lesions, rating diagnostic confidence per lesion on a 5-point Likert scale (1=very uncertain; 5=very certain).
**Results or Findings:** 29/39 patients had ≥ 1 lesion on CE-HF-MRI. Up to 100 lesions per rater were marked on HF-MRI. On pULF-MRI 23% of lesions were missed, thereof 59% <6 mm, 89% <8 mm. The remaining 11% were located infratentorial, near the venous sinus, subcallosal at the frontal sinus, or outside the pULF-MRI scan volume. One lesion missed on pULF-MRI showed minimal CE, detected on HF-MRI by only two readers with low confidence (2). Diagnostic confidence was slightly higher for pULF T1w std than sETL. In pULF T1w std, ≥2 readers marked 3 false positives; in sETL, 4 false positives, all with low confidence.
**Conclusion:** CE pULF MRI enables detection of enhancing brain lesions ≥6–8 mm and is feasible for bedside imaging. T1w std outperforms sETL.
**Limitations:** Limitations exist for smaller lesions and those in anatomically challenging locations.
**Funding for this study:** None.
**Has your study been approved by an ethics committee?** Yes
**Ethics committee - additional information:** The study was conducted under the ethical approval "Gehirnentwicklung von Früh- und Neugeborenen während des Aufenthaltes auf der neonatologischen Intensivstation" granted by the local institutional review board (Ethics Committee of the University Hospital Bonn). All participants provided written informed consent.
**Author Disclosures:**
Tolga Tonguc: Nothing to disclose
Barbara Daria Wichtmann: Speaker: I have given scientific presentations for Philips GmbH on unrelated topics for which I received monetary compensation.
Jennifer Faber: Nothing to disclose
Kennet Teichmann: Nothing to disclose
Zeynep Bendella: Nothing to disclose
Alexander Radbruch: Nothing to disclose
Aditya Rastogi: Nothing to disclose
Francesco Padormo: Employee: At the time of writing, Senior Clinical Scientist at Hyperfine.
Nils Lehnen: Nothing to disclose

**Pathology-Aware Targeted T1 synthesis from T2 and FLAIR images using Multi-Contrast Multi-Scale Vision Transformers**
S. Pasumarthi Venkata, *T. Campbell Arnold*, A. Shankaranarayanan; Menlo Park, CA/US

**Purpose:** Multi-contrast brain MRI images offer complimentary information that aids in differential diagnosis and treatment. The acquisition of high-quality multi-contrast images is time consuming and is often susceptible to artifacts. In this work, we propose the synthesis of T1w images from T2 and FLAIR images using a multi-contrast multi-scale vision transformer (MMT). T1w images are usually acquired at the start of any brain protocol and provide a structural baseline. Other contrasts like T2, FLAIR and T1C provide additional pathological information. It was found that the basic structure in T1 can be faithfully reconstructed from T2 and FLAIR images thus cutting down on the acquisition time (~4 mins for 3D; ~1.5 mins for 2D). We quantitatively evaluate the synthesized and acquired T1w images using PSNR, SSIM and RMSE.
**Methods or Background:** MMT was pre-trained on all subsets of T1,T1C,T2,FLAIR as input and the missing sequence(s) as the output. This model was further fine-tuned by fixing T2&FLAIR as inputs, and T1 as output. Reconstruction L1-loss was weighted on the lesion maps generated from T1C images using a pre-trained lesion segmentation algorithm. Multi-contrast (T1w,T2&FLAIR) brain MRI images from 45 patients were quantitatively evaluated using PSNR, SSIM and RMSE computed between real and synthesized T1.
**Results or Findings:** The average PSNR, SSIM and RMSE over the 45 patient studies are 33.53±1.75, 0.91±0.13 and 0.18±0.02 respectively. The synthesized T1w images had better overall image quality with less artifacts.
**Conclusion:** In this study, we have shown the quantitative performance of synthesizing T1w images from T2 and FLAIR to reduce acquisition time. The synthesized T1w images provide similar structural information as that of the acquired T1w images.
**Limitations:** Detailed qualitative assessment is missing. Future studies will evaluate different downstream tasks using the synthesized T1 image.
**Funding for this study:** n/a
**Has your study been approved by an ethics committee?** Yes
**Ethics committee - additional information:** Internal IRB
**Author Disclosures:**
Srivathsa Pasumarthi Venkata: Employee: Subtle Medical Inc
Thomas Campbell Arnold: Employee: Subtle Medical Inc
Ajit Shankaranarayanan: Employee: Subtle Medical Inc

**Gadoquatrane Demonstrates Similar Pharmacokinetics and Imaging Compared to Other Macrocyclic Extracellular GBCAs in Phase 2 and 3 CNS CE-MRI Studies**

*B. P. Liu*[1], C. Deuschl[2], K. Kudo[3], A. Liu[4], G. Sutter[5], B. M. Hofmann[5], P. Palkowitsch[5]; [1]Chicago, IL/US, [2]Essen/DE, [3]Sapporo/JP, [4]Whippany, NJ/US, [5]Berlin/DE
*(bliu@nm.org)*

**Purpose:** Compared to macrocyclic gadolinium (Gd)-based contrast agents (GBCA) used at 0.1 mmol Gd/kg body weight (bw), gadoquatrane's tetrameric structure enables high relaxivity, allowing for a reduction of 90% molecule and 60% Gd dose. This analysis of Phase (ph) 2 and 3 data explores the potential effect of the gadoquatrane structure and larger molecular size on imaging and pharmacokinetics (PK) vs. gadoterate meglumine, gadoteridol and gadobutrol.
**Methods or Background:** Two controlled clinical trial studies were conducted in patients with known or suspected CNS pathologies. Patients underwent two CE-MRIs, one with gadoquatrane (0.01 mmol/kg body weight (bw), equal to 0.04 mmol Gd/kg bw) and one with comparator GBCA (0.1 mmol Gd/kg bw). In ph3, MRIs followed clinical practice; in ph2 scans were taken at 5-, 10- and 15-min post injection. Images were evaluated in a blinded central read using 3 visualization parameters: contrast enhancement, delineation, morphology. Signal intensity was also assessed quantitatively in ph2. PK were evaluated via plasma samples taken at predefined time windows.
**Results or Findings:** The integrated analysis included 362 adult CNS patients, with 326 evaluable for efficacy, and 338 for pharmacokinetics. Gadoquatrane MRI was non-inferior to the comparator MRI for all visualization parameters. Quantitative enhancement was similar for gadoquatrane and gadobutrol. Dose normalized plasma concentration-time curves showed overlapping concentrations up to 24 hours post dose, indicating dose-proportionality and essentially the same PK behavior over time for all GBCAs.
**Conclusion:** Gadoquatrane at a 60% reduced Gd dose is non-inferior to comparator GBCAs for CNS CE-MRI. For later imaging time points, qualitative and quantitative assessments were similar to comparator GBCAs. There is no relevant impact of the larger molecular size on imaging properties or PK behavior.
**Limitations:** The integrated analysis includes patients with CNS pathologies only.
**Funding for this study:** Both studies referenced were sponsored by Bayer AG.
**Has your study been approved by an ethics committee?** Yes
**Ethics committee - additional information:** The studies were approved by Institutional Review Boards at all participating investigation sites of these multicenter trials.
**Author Disclosures:**
Alex Liu: Employee: Bayer AG
Benjamin P Liu: Consultant: Bayer AG Advisory Board: Bayer AG
Cornelius Deuschl: Advisory Board: Bayer AG
Petra Palkowitsch: Employee: Bayer AG
Kohsuke Kudo: Advisory Board: Bayer AG
Gabriele Sutter: Employee: Bayer AG
Birte Maria Hofmann: Employee: Bayer AG

**Population PK Modeling of Gadoquatrane: Supporting Regulatory Submission for Use in Patients of All Ages**

*G. Sutter*[1], E. Vendel[2], P. Vis[2], J. Zisowsky[1], M. Feldmueller[1], B. M. Hofmann[1]; [1]Berlin/DE, [2]Leiden/NL
*(gabriele.sutter@bayer.com)*

**Purpose:** An integrated population-pharmacokinetic (popPK) modeling approach was chosen to thoroughly evaluate the pharmacokinetics (PK) of the extracellular macrocyclic gadolinium-based contrast agent (mGBCA) gadoquatrane, in adults and children, utilizing data from all Phase 1-3 clinical trials, including a pediatric study, to support regulatory submission for the use in patients of all ages with CNS or body pathologies.
**Methods or Background:** Data from eight clinical Phase 1-3 studies, covering doses of 0.0025 – 0.05 mmol/kg body weight (bw) gadoquatrane (corresponding to 0.01 – 0.2 mmol Gd/kg bw), and a Phase 1/3 study in pediatric patients aged 0-<18 years (0.04 mmol Gd/kg bw) were analyzed. Non-linear mixed-effects methods were applied for popPK model development including covariate assessment. Analysis started with data from 871 adults (4054 Gd plasma concentrations), forming the basis for the pediatric analysis by applying and adjusting the adult model to pediatric data (92 children with 276 Gd plasma concentrations). Exposure parameters were compared across populations (age, gender, race/ethnicity, renal function, health status) and with other mGBCAs.
**Results or Findings:** The adult model was a linear three-compartment model with allometric scaling based on lean body mass (LBM) and estimated glomerular filtration rate as covariate on clearance (CL). The pediatric data allowed only for identification of two compartments, the resulting model included LBM-based allometric scaling and a maturation factor on CL based on postmenstrual age. Gadoquatrane demonstrated consistent PK behavior across populations, closely matching the patterns observed with other mGBCAs.
**Conclusion:** This integrated popPK modeling approach enabled a thorough and efficient characterization of gadoquatrane's PK in both adults and children, supporting regulatory submission across all indications and ages.
**Limitations:** Limitations included limited representation of some groups (Patients aged <6 months, Black participants, individuals with severe renal impairment).
**Funding for this study:** All studies referenced were sponsored by Bayer AG.
**Has your study been approved by an ethics committee?** Not applicable
**Author Disclosures:**
Jochen Zisowsky: Employee: Bayer AG
Maximilian Feldmueller: Employee: Bayer AG
Peter Vis: Consultant: Bayer AG
Gabriele Sutter: Employee: Bayer AG
Esmée Vendel: Consultant: Bayer AG
Birte Maria Hofmann: Employee: Bayer AG

**Experience with gadopiclenol in the detection of brain metastases: Single and double doses, comparison of standard and delayed imaging**

*A. Rulseh*, Z. Ryznarová, R. Liščák, J. Vymazal; Prague/CZ

**Purpose:** To demonstrate the role of gadopiclenol in MR imaging of brain metastases at standard (0.05 mmol/kg) and double doses (0.1 mmol/kg), including delayed examination (~15 minutes post-contrast).
**Methods or Background:** One hundred sixty-two subjects with known brain metastases underwent MRI prior to radiosurgery (75 female, mean age 63.67 years ± 12.7 SD). A standard T1W 3D SPACE sequence was acquired post-contrast in all subjects (5 subjects at 1.5T, 157 at 3T), followed by a delayed acquisition. In roughly half of subjects (n=83), the delayed acquisition was preceded by additional contrast administration (cumulative double dose). After randomization, 3 blinded readers evaluated each subject in 2 sessions, minimally 4 weeks apart. A one-sided paired t-test was used to compare the number of lesions detected in the 2 groups: single dose (early versus delayed), and double dose (early versus delayed [double]).
**Results or Findings:** Agreement between all blinded readers was used for evaluation. The delayed single dose exam revealed 288 metastases compared to 279 metastases on the early exam. Delayed double-dose exam revealed 258 metastases compared to 221 metastases on the early single dose exam. In subjects that received a double dose, the number of lesions detected on the

Friday early versus delayed (double) dose exams differed significantly (p=0.002), while in subjects that received only a single dose no significant difference in the number of lesions detected on early versus delayed exams was detected (p=0.24).
**Conclusion:** Significantly more metastases were detected on delayed double dose imaging, while no difference was detected on early versus delayed single dose acquisitions. Our results suggest that a delayed, double dose gadopiclenol acquisition may improve the detection of metastases.
**Limitations:** The study was performed at a single institution. Only the SPACE sequence was used for blinded reading.
**Funding for this study:** Ministry of Health CZ, IG244302.
**Has your study been approved by an ethics committee?** Yes
**Ethics committee - additional information:** Ethics committee approval, na homolce hospital ethics committee
**Author Disclosures:**
Roman Liščák: Nothing to disclose
Josef Vymazal: Consultant: Bracco
Aaron Rulseh: Nothing to disclose
Zuzana Ryznarová: Nothing to disclose

**Performance of gadopiclenol in dynamic susceptibility contrast magnetic resonance imaging (DSC-MRI) perfusion of brain gliomas**
*S. Gerevini*[1], G. Hutóczki[2], A. G. Bagó[3], A. Pichiecchio[4], S. Gaudino[5]; [1]Cremona/IT, [2]Debrecen/HU, [3]Gdansk/PL, [4]Pavia/IT, [5]Rome/IT
*(sgerevini@asst-pg23.it)*

**Purpose:** To demonstrate the non-inferiority of DSC-MRI perfusion using gadopiclenol at 0.05 mmol/kg compared to gadoterate meglumine at 0.1 mmol/kg in terms of diagnostic quality of Cerebral Blood Volume (CBV) perfusion map.
**Methods or Background:** In this multicentre randomized controlled trial, 138 adult patients with primary glial tumour scheduled for a follow-up DSC-MRI were randomized to receive either gadopiclenol or gadoterate meglumine. The primary outcome was the CBV map diagnostic quality assessed by two independent blinded readers, using a 4-point scale (poor, fair, good, excellent). Secondary outcomes included glioma grade differentiation by relative CBV (rCBV) quantification, and safety. Non-inferiority was defined as proportion of patients with excellent or good diagnostic quality images for gadopiclenol of no more than 12% below that for gadoterate meglumine.
**Results or Findings:** Per-protocol analysis included 60 patients with gadopiclenol and 64 with gadoterate meglumine. The proportion of patients with CBV map images assessed as “excellent or good” was 96.7% with gadopiclenol and 98.4% with gadoterate meglumine, respectively, with a difference of -1.8% [two-sided 95% CI: - 8.9% to 5.3%], demonstrating the non-inferiority. Overall, rCBV values were significantly different between high-grade and low-grade gliomas with both contrast agents, with a median of 3.11 vs 1.51 (p =0.003) for gadopiclenol and 2.47 vs 1.41 (p <0.001) for gadoterate meglumine. Three patients receiving gadopiclenol and two receiving gadoterate meglumine experienced non-serious adverse events of mild intensity, none related to the contrast agent.
**Conclusion:** DSC-MRI perfusion using gadopiclenol at 0.05 mmol/kg was non-inferior to gadoterate meglumine at 0.1 mmol/kg on diagnostic quality of CBV perfusion map and both enabled brain glioma grade differentiation. This study additionally confirmed the safety profile of gadopiclenol.
**Limitations:** No limitations were identified
**Funding for this study:** This study was funded by Guerbet.
**Has your study been approved by an ethics committee?** Yes
**Ethics committee - additional information:** Approval number for the centers in Hungary : BM/3737-0/2023-EKL Approval number for the centers in Italy : 2023-3.11/17 Approval number for the centers in Poland: KE-0254/60/03/2023
**Author Disclosures:**
Simona Gaudino: Nothing to disclose
Gábor Hutóczki: Nothing to disclose
Anna Pichiecchio: Nothing to disclose
Attila G. Bagó: Nothing to disclose
Simonetta Gerevini: Nothing to disclose

**Rationales for non-standard GBCA dosing – Low? - High? - When? & Why? A literature-based study**
*J. S. Endrikat*[1], I. Siddiqui[2], H. Khater[1], M. Blankenburg[1]; [1]Berlin/DE, [2]Reading/UK
*(jan.endrikat@bayer.com)*

**Purpose:** To systematically explore rationales for non-standard dosing of GBCAs and discuss the potential future impact of high-relaxivity contrast agents.
**Methods or Background:** A systematic literature review was conducted using Embase, covering 1991 to 2024. Publications were categorized by indication, GBCA dose, study design, and rationale for non-standard dosing. The dose of 0.1 mmol Gd/kg body weight was defined as the 'standard' reference for comparison.
**Results or Findings:** Eighty-four publications comparing non-standard dosing regimen with the standard dose were included: 43 high-dose and 55 low-dose studies. The rationales for high-dose administration were to achieve better contrast (25/43; 58%) and improve lesion detection (15/43; 35%). High-dose studies were primarily performed in CNS until 2006. Rationales for using low-dose administration were related to 1) NSF (28/55; 51%); 2) Gd exposure (22/55; 40%); 3) cost (22/55; 40%); 4) unspecified safety (18/55; 33%); 5) Gd retention/presence (16/55; 29%); and 6) environment (6/55; 11%). From 1991 to 2006, cost was the sole rationale for lower dose administration. From 2008, NSF was noted; from 2017 onwards, Gd retention/presence, and most recently, environmental impact emerged as important rationales. Forty-six of 55 studies (84%) investigating low-dose regimen reported comparable outcomes, 7 studies (13%) inferior outcomes. However, 35 of 46 low-dose studies reporting comparable outcomes modified also other parameters or used a study design potentially impacting study strength.
**Conclusion:** For over 34 years, there has been a consistent demand to lower GBCA doses, with an increasing number of rationales over time. The next generation of high-relaxivity, low-dose mGBCAs shows promise for reducing Gd dose while maintaining high image quality, potentially defining a new standard dose.
**Limitations:** The impact of advances in scanner technology and software was beyond the scope of this review.
**Funding for this study:** Funding was provided by Bayer AG.
**Has your study been approved by an ethics committee?** Not applicable
**Author Disclosures:**
Jan Siegfried Endrikat: Author: Bayer AG
Hassan Khater: Author: Bayer AG
Imran Siddiqui: Author: Bayer AG
Michael Blankenburg: Author: Bayer AG

**New horizons in brain tumor surgery: feasibility and diagnostic value of DSC and ASL perfusion in intraoperative MRI**
*M. R. López De La Torre Carretero*, D. A. Zambrano, C. Mbongo, A. M. Delgado Brito, C. D. Solano, M. A. Fernández Seara, M. Calvo Imirizaldu; Pamplona/ES
*(mltorrec@unav.es)*

**Purpose:** The main aim of this study was to assess the diagnostic value of perfusion imaging during intraoperative MRI (iMRI) for detecting residual lesions. We evaluated the image quality of intraoperative dynamic susceptibility contrast (DSC) and arterial spin labeling (ASL) sequences in a cohort of patients undergoing brain surgery, and then compared their ability to identify residual lesions with that of conventional techniques.
**Methods or Background:** Twenty-seven patients (13 men; mean age 60±14) undergoing intraoperative MRI during brain tumor resection were prospectively imaged with structural MRI, DSC, and ASL. Two independent readers rated image quality (1–4; 1–2 non-diagnostic, 3–4 diagnostic) and scored tumour remnants on structural MRI, DSC-CBV, and ASL-CBF as present, absent, or non-evaluable (artifacts); only perfusion studies rated diagnostic were included for the remnant analysis, and interobserver and intermodality agreement were assessed with Cohen’s kappa.
**Results or Findings:** Consensus diagnostic quality was slightly higher for DSC (25/27 cases) compared to ASL (23/26). Interobserver agreement was excellent for DSC (κ=1.00) and substantial for ASL (κ=0.75). Concordance between DSC and ASL in evaluable cases was moderate (κ=0.26). When compared with structural MRI, perfusion-based assessment of residual tumor demonstrated substantial agreement for both DSC (κ=0.89) and ASL (κ=0.69). The performance of the two techniques was not interchangeable: DSC was generally more resilient to image degradation, whereas ASL enabled interpretation in one case where DSC failed.
**Conclusion:** Intraoperative perfusion MRI is feasible. DSC yields slightly better image quality and structural agreement, but ASL proves to be useful when contrast is contraindicated, not needed (i.e. non-enhancing tumors) or DSC is compromised, providing complementary value for the detection of tumor remnants during brain surgery. Their complementary roles support combined use to enhance intraoperative confidence.
**Limitations:** Tumour heterogeneity Qualitative analysis No histopathological gold-standard
**Funding for this study:** No funding was provided for this study
**Has your study been approved by an ethics committee?** Not applicable
**Author Disclosures:**
Carmen Mbongo: Nothing to disclose
María A. Fernández Seara: Nothing to disclose
Manuel Rafael López De La Torre Carretero: Nothing to disclose
Adolfo Manuel Delgado Brito: Nothing to disclose
Marta Calvo Imirizaldu: Nothing to disclose
Daniel Alfonso Zambrano: Nothing to disclose
Carlos Delgado Solano: Nothing to disclose

**Magnetic resonance neurography for evaluating diabetic peripheral neuropathy in type 1 diabetes**

*S. S. Croosu*, J. Røikjer, S. M. Tahami, T. M. Hansen, J. Brøndum Frøkjær, N. Ejskjaer, C. D. Mørch; Aalborg/DK
*(suganthiya.s@rn.dk)*

**Purpose:** Diabetic peripheral neuropathy (DPN) is a frequent complication of type 1 diabetes mellitus (T1DM), causing sensory loss and pain, but reliable imaging biomarkers of nerve damage are lacking. This study investigated whether tibial and sciatic nerve integrity is affected by T1DM with painful or painless DPN using diffusion tensor imaging magnetic resonance neurography (DTI-MRN), and whether these measurements associate with clinical outcomes.
**Methods or Background:** Fifty-eight participants with T1DM were divided into painful DPN(n=20), painless DPN(n=19), and no DPN(n=19), along with 20 healthy controls (HC). DTI-MRN was performed at 10% and 40% above the patella to evaluate the tibial and sciatic nerves, respectively. Fractional anisotropy (FA) and apparent diffusion coefficient (ADC) were measured. Correlations with age, diabetes duration, HbA1c, nerve conduction, and pain scores were assessed.
**Results or Findings:** Higher FA indicates better fibre integrity and coherence, while lower ADC reflects more restricted diffusion, consistent with intact nerve fibres and myelin. Mean tibial nerve FA was lower in painful DPN(0.25±0.08) and painless DPN(0.24±0.05) versus T1DM without DPN(0.33±0.06) and HC(0.32±0.07) (all p<0.002). Tibial ADC was higher in painful DPN(912.2±338.2 $\times10^{-6}$ mm²/s) compared with T1DM without DPN(622.0±152.6 $\times10^{-6}$ mm²/s) and HC(673.6±185.4 $\times10^{-6}$ mm²/s) (all p<0.007). No differences in the sciatic nerve were detected (all p>0.05). Lower FA and higher ADC of the tibial nerve were associated with poorer sural amplitude and velocity, impaired warm/cold detection, and greater pain intensity (all p<0.03).
**Conclusion:** DTI-MRN detects tibial nerve alterations in T1DM with DPN. FA reduction occurred in both painful and painless DPN, while ADC increase was specific to painful DPN. Tibial FA and ADC correlated with nerve dysfunction and pain, suggesting potential as biomarkers of neuropathy severity.
**Limitations:** Sample size and cross-sectional design limit causal interpretation. Longitudinal studies are needed.
**Funding for this study:** This work was partly supported by Augustinus Fonden, København, Denmark (grant 19-1302). The funding source did not influence the study.
**Has your study been approved by an ethics committee?** Yes
**Ethics committee - additional information:** The North Denmark Region Committee on Health Research Ethics granted the ethics approval (N-20190003)
**Author Disclosures:**
Carsten Dahl Mørch: Nothing to disclose
Jens Brøndum Frøkjær: Nothing to disclose
Seyed Mohammad Tahami: Nothing to disclose
Johan Røikjer: Nothing to disclose
Niels Ejskjaer: Nothing to disclose
Suganthiya S. Croosu: Nothing to disclose
Tine Maria Hansen: Nothing to disclose

**Dose management of head CT scans in 2025: the importance of the human factor in the age of artificial intelligence**

*E. Armando*[1], M. Porzio[2], G. Salmè[3], E. Rachetta[1], F. Groppo Marchisio[1]; [1]Caraglio/IT, [2]Borgo San Dalmazzo/IT, [3]Collegno/IT
*(enrico.armando@gmail.com)*

**Purpose:** To evaluate brain CT scan doses in a radiology department with different CT scanners, with different algorythms, from filtered back projection to deep learning reconstructions and with or without camera based patient postioning.
**Methods or Background:** Dose data of all the brain CT scan examinations obtained by seven different scanners between January and September 2025 were included in the analysis, using the dose tracking software (Gray detector, EL.Co, Cairo Montenotte, Italy). DLP and CTDI data were analysed using SAS Studio on demand for academics using the Kruskall Wallis test. Children examinations were excluded
**Results or Findings:** 5064 head CT scans were included in the analysis. There were no statistical differences in CTDI and DLP among the scanners despite differences in technology and protocols. The most recent CT scanner with deep learning reconstruction and a camera for automated positioning of the patient had a 15% lower median DLP than other scanners. This improvement was not immediate but it was obtained after protocol revision, reducing the minimum mA in the protocol setting oif the current modulation.
**Conclusion:** Brain CT scan is the most frequent examination in most radiology departments. However it is the examination with the lowest reduction of CTDI and DLP despite the introduction of iterative and deep learning reconstruction algorythms, as low noise images are needed. New scanners with deep learning based algorythms do not necessarily lead to dose reduction unless protocols are carefully revised to exploit these improvements
**Limitations:** This is a retrospective study and is the basis for further research
**Funding for this study:** None
**Has your study been approved by an ethics committee?** Not applicable
**Author Disclosures:**
Giulio Salmè: Nothing to disclose
Massimiliano Porzio: Nothing to disclose
Enrico Armando: Nothing to disclose
Eleonora Rachetta: Nothing to disclose
Federica Groppo Marchisio: Nothing to disclose

**16:00 - 17:30** **Research Stage 5**

### Research Presentation Session: Oncologic Imaging

### RPS 1616

### Recent developments in colorectal cancer imaging

**Moderator**
Matthias Frölich; Mannheim/DE

**Prognostic impact of lateral nodes size in patients with locally advanced rectal cancer treated with neoadjuvant CRT**

*P. Varotto*, G. Sussan, F. Scannapieco, G. Spolverato, E. Quaia, F. Crimì; Vicenza/IT

**Purpose:** The evaluation of pelvic lymph nodes in rectal cancer has traditionally emphasized mesorectal nodes, whereas the role of lateral pelvic lymph nodes (LLNs: obturator, internal and external iliac) remains less clearly defined. Their prognostic significance, particularly after neoadjuvant chemoradiotherapy (CRT), is debated. International guidelines diverge: some Western centers favor a conservative, rectum-sparing approach, while Japanese guidelines support more radical strategies. Assessment of LLN size, especially short-axis diameter, may provide valuable prognostic and therapeutic insights.
**Methods or Background:** A cohort of 138 patients with locally advanced rectal cancer was retrospectively analyzed (mean age 66 years, range 27–87; 44% female). All patients underwent baseline and restaging pelvic MRI after pCRT. Among the cohort, 96 underwent total mesorectal excision (TME) or Abdomino-Perineal Resection (APR) and 42 were managed with rectum-sparing strategies (watch and wait and transanal local excision). LLNs were evaluated in baseline MRI and considered positive when the short-axis diameter was ≥7 mm.
**Results or Findings:** At baseline, LLNs were detected in 50% of patients. One patient was lost at follow up. Among 137 patients, the median OS was 58 months (IQR 4-96 months). No patient developed metastases in LLNs. The cohorts of patients with short axis of LLNs ≥7 mm and LLNs< 7 mm showed no significant differences in Overall Survival (OS) p=0.2 and Distant Metastases Free Survival (DMFS) p=0.22.
**Conclusion:** This study demonstrates that LLN size is not associated with OS and DMFS in locally advanced rectal cancer treated with CRT. Routine LLN excision, an invasive procedure associated with increased morbidity and functional sequelae, does not appear to provide survival benefit. Our findings support conservative management.
**Limitations:** This was a single-center, retrospective study with a relatively small sample and variable follow-up (4–96 months).
**Funding for this study:** None
**Has your study been approved by an ethics committee?** Yes
**Ethics committee - additional information:** Protocol number 3554/AO/15
**Author Disclosures:**
Giovanni Sussan: Nothing to disclose
Federico Scannapieco: Nothing to disclose
Filippo Crimì: Nothing to disclose
Pietro Varotto: Nothing to disclose
Emilio Quaia: Nothing to disclose
Gaya Spolverato: Nothing to disclose

**Preoperative Prognostic Value of Gadoxetic Acid–Enhanced MRI in Patients with Colorectal Cancer Liver Metastases after Chemotherapy**

*P. Eckwolf*, S. Pötter-Lang, A. Ba-Ssalamah, C. Kuntner,
I. Pashkunova-Martic, U. I. Attenberger, N. Bastati-Huber; Wien/AT

**Purpose:** To evaluate the prognostic value of gadoxetic acid–enhanced MRI (gaMRI) for predicting treatment response (TR) and overall survival (OS) in patients with colorectal cancer liver metastases (CRCLM).
**Methods or Background:** In this retrospective study, 79 patients (mean age, 61.8 ± 9.7 years) with 124 CRCLM underwent gaMRI after chemotherapy and before hepatic resection. Hepatobiliary phase (HBP) enhancement was qualitatively assessed by two independent readers and categorized as homogeneous hypointensity (favorable) or heterogeneous hypointensity (unfavorable). Apparent diffusion coefficient (ADC) values were measured. The primary outcome was residual vital tumor (RVT). Correlations of HBP pattern, TR, and ADC with RVT were analyzed, and OS was assessed using the log-rank test.
**Results or Findings:** Interobserver agreement was substantial (κ = 0.80). Median RVT was 37.8% (range, 0–95%) and was significantly associated with HBP pattern (p < 0.001) and ADC (p = 0.021). Heterogeneous hypointensity correlated with higher RVT (r = 0.510, p < 0.001), unfavorable regression categories (r = 0.501, p < 0.001), lower ADC (r = –0.207, p < 0.05), and higher vitality (r = 0.264, p < 0.05). Conversely, homogeneous hypointensity strongly predicted major pathological response (≤10% RVT, r = 0.547, p < 0.001) and, to a lesser degree, response ≤50% RVT (r = 0.309, p < 0.001). OS analysis showed a trend toward improved survival in patients with homogeneous hypointense lesions, although not statistically significant (p = 0.066).
**Conclusion:** Homogeneous hypointensity on HBP after gaMRI is a favorable biomarker, predicting major pathological response and higher ADC values, while heterogeneous hypointensity indicates poor response, greater residual vitality, and restricted diffusion.
**Limitations:** The retrospective, single-center design and modest sample size limit generalizability. OS analysis did not reach statistical significance.
**Funding for this study:** No funding was provided for this study.
**Has your study been approved by an ethics committee?** Yes
**Ethics committee - additional information:** Ethic committee number: 2064/2015
**Author Disclosures:**
Claudia Kuntner: Nothing to disclose
Sarah Pötter-Lang: Speaker: Bayer
Ahmed Ba-Ssalamah: Consultant: Bayer Speaker: Bayer, Siemens, Sanofi
Philip Eckwolf: Nothing to disclose
Ulrike I. Attenberger: Consultant: Bureau, Bayer Speaker: Siemens Healthineers
Nina Bastati-Huber: Nothing to disclose
Irena Pashkunova-Martic: Nothing to disclose

**Body composition as a predictor of cancer specific death in colon cancer: an AI-based volumetric analysis**

*D. Capece*, M. Polici, B. Masci, L. Nardoni, S. Nardacci, F. Pacelli,
M. Zerunian, D. Caruso, M. Francone; Rome/IT
*(daniela.capece@uniroma1.it)*

**Purpose:** To investigate body composition as a predictive biomarker of cancer specific death in patients with non-metastatic colon cancer.
**Methods or Background:** Patients with colon cancer (stage II-III) treated with up-front surgery, with availability of baseline CT, clinical, and histological data, survival data were retrospectively enrolled. Patients with stage IV disease or those with CT unavailability were excluded. Body composition parameters were derived from baseline abdominal CT using AI-based automatic segmentation software. Up to 76 parameters regarding adipose visceral fat (AVF), subcutaneous fat (SF), bone density, liver density and fat-fraction were automatically extracted from both whole segmentation volume and multi-slices region (last rib-iliac crest). According to the Colon Cancer specific death (CC-specific death), the population was divided into Group 1 (CC-specific death) and Group 2 (non CC-specific death). Body composition features were compared between the two groups. Predictive model and survival analysis were performed with ROC curves, Cox regression and the Kaplan-Meier method. P<0.05 was considered significant.
**Results or Findings:** 293 patients were included in the study, 101/293 (34.5%) with CC-specific deaths. Mean HU of AVF (Group1 vs Group2, -77 HU vs -82 HU) and SF (Group1 vs Group2, -87 HU vs -90 HU) resulted directly correlated with CC-specific death (P=0.004 and HR=1.03, P=0.002 and HR=1.04, respectively) for the multislice analysis. Mean HU of AVF resulted in direct correlation with CC- CC-specific deaths, also for the volumetric analysis (P=0.04 and HR=1.02). In the Kaplan-Meier analysis, the AVF and SF for the multislice analysis resulted in statistically significant differences (P=0.033 and <0.001, Chi-Square=4.56 and 11.7, respectively).
**Conclusion:** In conclusion, our study demonstrated that the body composition metrics of visceral and subcutaneous fat was significantly associated with cancer-specific death in non-metastatic colon cancer patients.
**Limitations:** Retrospective study, lack of validation cohort
**Funding for this study:** Funded by AIRC IG 2020–ID 24974
**Has your study been approved by an ethics committee?** Yes
**Ethics committee - additional information:** This retrospective analysis was conducted within the framework of a 5-year multicenter trial, approved by the ethical committee of Sant'Andrea University Hospital (ref. nr. CE 6597/2021), registered on ClinicalTrials.gov (NCT06108310).
**Author Disclosures:**
Damiano Caruso: Nothing to disclose
Fiammetta Pacelli: Nothing to disclose
Benedetta Masci: Nothing to disclose
Marta Zerunian: Nothing to disclose
Lapo Nardoni: Nothing to disclose
Daniela Capece: Nothing to disclose
Michela Polici: Nothing to disclose
Marco Francone: Nothing to disclose
Stefano Nardacci: Nothing to disclose

**CT-derived visceral adiposity and sarcopaenia demonstrate stronger correlation with epigenetic age compared to age by birth in early onset colorectal cancer (EOCRC) treated with curative intent**

*A. Daly*; Craughwell/IE
*(ainedaly09@gmail.com)*

**Purpose:** EOCRC is increasing globally and in Ireland. One hypothesis is accelerated aging (AA). Epigenetic Age (EpiAge) is calculated using a blood-based algorithm and correlates strongly with age in whole body tissue/cell testing and is considered a more accurate predictor of DNA methylation than age by birth. This study aimed to examine the strength of relationship between dysmetabolic body composition with EpiAge and age by birth to better understand why it is prognostic of worse outcomes in EOCRC. <b>EOCRC patients diagnosed in the MidWest of Ireland from 2015-2024 (inclusive) and treated with curative intent (Stage I-III) were included. EpiAge was calculated using a validated blood-based algorithm. CT-derived body composition was measured on staging CTAP. Visceral adipose area was quantified on a single CT slice 6 cm above L4–L5, and skeletal muscle area at L3. Pearson and Spearman correlation coefficients were used depending on data distribution, significance was observed <0.05.
**Results or Findings:** Ninety one patients were suitable for inclusion (N=49 colon, n=42 rectal). 41% (n=37) had high visceral adiposity and 35% (n=32) sarcopaenia. EpiAge was older than age by birth in 77% (n=70) EOCRC and 83% (n=35) rectal cancers. Visceral adiposity correlated with EpiAge stronger than age by birth in EOCRC (r=0.69, p=0.005; r=0.25, p=0.1). This difference was more significant in colon cancer (r=0.79, p=<0.001; r=0.35, p=0.55) than rectal cancer. Sarcopenia correlated with EpiAge stronger than age by birth also (r=0.94, p=<0.01; r=0.48, p=0.65). EpiAge was significantly associated with disease specific mortality unlike age by birth [OR 6.6 (95%CI 0.84-0.95), p=0.028].
**Conclusion:** EpiAge correlates strongly with dysmetabolic body composition in EOCRC further strengthening the aetiological relationship between accelerated aging and EOCRC.
**Limitations:** Interpretation is limited by the retrospective design and sample size, but the findings highlight important associations warranting further study.
**Funding for this study:** No funding received for this study.
**Has your study been approved by an ethics committee?** Yes
**Ethics committee - additional information:** University Hospital Limerick Research Ethics Committee
**Author Disclosures:**
Aine Daly: Nothing to disclose

**Delta total tumour volume and radiomics as predictive biomarkers in MSI-H metastatic colorectal cancer treated with immunotherapy**

*J. Dupont*[1], S. Journo[1], R. Barbe[1], P-H. Cournède[2], Y. Menu[1],
C. S. Balleyguier[1], A. Hollebecque[1], T. André[3], N. Lassau[1]; [1]Villejuif/FR,
[2]Gif-Sur-Yvettes/FR, [3]Paris/FR
*(jules.dupont@gustaveroussy.fr)*

**Purpose:** To evaluate whether variations (delta-) in total tumour volume (TTV) and radiomic features can early predict Overall Survival (OS) in rare cohort of patients with metastatic colorectal cancer (mCRC) with high microsatellite instability (MSI-H) undergoing immunotherapy.
**Methods or Background:** One hundred forty-five patients diagnosed with MSI-H mCRC (mean age, 56 ± 15 years; 89 men) were retrospectively included from two centers. Radiologists manually annotated all tumours on chest-abdomen-pelvis computed-tomography at baseline (BL) and first evaluation (E1) scans. The median time between BL and E1 was 50 days (interquartile range: 45 - 64 days). Tumour volume and radiomics were extracted for each lesion to predict OS. Every scan was resampled to isotropic voxel spacing of 1×1×1 mm3 to reduce variability. The delta-TTV and delta-radiomics were defined as their relative between BL and E1. The ability to stratify patients into long- and short-term survival groups was assessed using the Restricted Mean Survival Time (RMST) ratio, AUC at 3 years, and C-index. Generalization was assessed through 75 train/test cohort splits.
**Results or Findings:** A total of 2079 lesions at BL and 2112 at E1 were annotated. Delta-TTV outperformed TTV at BL and E1 across all metrics (p<0.001). Specifically, delta-TTV achieved an RMST ratio of 2.215 (95% CI: [2.106-2.324]), an AUC of 0.854 [0.840-0.868], and a C-index of 0.830 [0.819-0.841], with a cut-off of 17.31% relative increase in TTV. Combining delta-radiomics with delta-TTV enhanced performance, yielding an RMST ratio of 2.450 (2.248-2.652, p=0.019), an AUC of 0.875 (0.862-0.888, p<0.001), and a C-index of 0.839 (0.819-0.841, p<0.001).
**Conclusion:** Delta-TTV is a strong predictor of survival in MSI-H mCRC patients. Adding radiomic features enhances this biomarker's efficiency for early survival prediction.
**Limitations:** External validation is recommended for this two-center retrospective study.
**Funding for this study:** No fundings to disclose
**Has your study been approved by an ethics committee?** Yes
**Ethics committee - additional information:** Ethics committee N°2020-CER 2020-6
**Author Disclosures:**
Paul-Henry Cournède: Nothing to disclose
Thierry André: Nothing to disclose
Nathalie Lassau: Nothing to disclose
Corinne S. Balleyguier: Nothing to disclose
Antoine Hollebecque: Nothing to disclose
Rémy Barbe: Nothing to disclose
Jules Dupont: Nothing to disclose
Yves Menu: Nothing to disclose
Segolene Journo: Nothing to disclose

### The Efficacy of Interventional Transarterial Treatments for Unresectable Colorectal Carcinoma

T. J. Vogl, *A-I. Nica*, C. Booz, I. Yel, C. Wolfram, H. Adwan; Frankfurt/DE

**Purpose:** To evaluate the efficacy of transarterial chemoperfusion (TACP) and transarterial chemoembolization (TACE) as palliative and symptomatic treatments for unresectable colorectal carcinoma (CRC), focusing on local tumor response and survival outcomes.
**Methods or Background:** Between January 2000 and October 2023, 67 consecutive patients diagnosed with unresectable CRC underwent 318 TACP and 80 TACE procedures. Of these, 48 patients were treated with TACP, 14 with TACE, and 5 received a combination of both therapies. We retrospectively assessed local tumor response using the Response Evaluation Criteria in Solid Tumors (RECIST) and calculated overall survival (OS) and progression-free survival (PFS) using the Kaplan-Meier estimator.
**Results or Findings:** No major complications were reported. A total of 49 patients (73%) exhibited stable disease, while 3 patients (5%) achieved a partial response. The rate of progressive disease was 22%. The median OS for the entire cohort was 16.17 months, and the median PFS was 11.25 months. There were no statistically significant differences in OS (p=0.82) or PFS (p=0.37) between patients receiving TACP, TACE, or both treatments.
**Conclusion:** TACP and TACE are minimally invasive procedures that provide a viable treatment option for patients with unresectable CRC who cannot tolerate, refuse, or do not respond to chemotherapy. These treatments can serve as both palliative and symptomatic measures, potentially preventing tumor progression and improving quality of life. However, their benefits in the treatment of CRC warrant further investigation.
**Limitations:** Limitations of the study include its retrospective nature.
**Funding for this study:** No funding.
**Has your study been approved by an ethics committee?** Yes
**Ethics committee - additional information:** Ethics Committee approval was obtained for this study.
**Author Disclosures:**
Christian Booz: Speaker: I have received speaking fees from Siemens Healthineers
Ibrahim Yel: Nothing to disclose
Christian Wolfram: Nothing to disclose
Andreea-Ioana Nica: Nothing to disclose
Thomas J. Vogl: Nothing to disclose
Hamzah Adwan: Nothing to disclose

### Cost-effectiveness analysis of dedicated MR imaging vs. surgical staging in patients with colorectal peritoneal metastases

L. Van der Snee, A. Kramer, N. Kok, A. Aalbers, M. Lopez, V. Retel, *M. Lahaye*; Amsterdam/NL
*(mj.lahaye@gmail.com)*

**Purpose:** In patients with colorectal peritoneal metastases (PM-CRC), cytoreductive surgery with hyperthermic intraperitoneal chemotherapy (CRS-HIPEC) is potentially life-prolonging but carries substantial morbidity. Standard staging with computed tomography (CT) and diagnostic laparoscopy often results in preventable surgical procedures. The randomized multicenter DISCO trial demonstrated that adding diffusion-weighted magnetic resonance imaging (DW-MRI) reduces such procedures. This study evaluated the cost-effectiveness of additional DW-MRI in the Netherlands.
**Methods or Background:** A decision-analytic cohort model combined a decision tree of diagnostic strategies with a Markov model simulating survival. Patients were staged with either standard care (CT ± laparoscopy) or additional DW-MRI. Model inputs were derived from 149 Dutch patients randomized in DISCO (DW-MRI n=74, 49.7%; standard care n=75, 50.3%). Outcomes included short-term metrics (staging laparoscopies, CRS-HIPEC rates, diagnostic costs) and long-term outcomes (quality-adjusted life years [QALYs], total costs). Deterministic and probabilistic sensitivity analyses tested robustness.
**Results or Findings:** DW-MRI markedly reduced staging laparoscopies and lowered diagnostic costs, saving €2087 per patient. Over a lifetime horizon, DW-MRI yielded a gain of 0.162 QALYs and total cost savings of €455 compared with standard care. Both deterministic and probabilistic analyses confirmed cost-effectiveness. National implementation could generate annual diagnostic savings of ~€2.8 million.
**Conclusion:** Adding DW-MRI to the staging work-up for PM-CRC is a cost-effective strategy that reduces unnecessary surgical procedures and patient burden. In the Dutch healthcare setting, DW-MRI implementation would improve patient selection for CRS-HIPEC and result in significant cost savings.
**Limitations:** This analysis was based on Dutch trial data, and results may not fully generalize to healthcare systems with different surgical practices, cost structures, or MRI expertise.
**Funding for this study:** This study was investigator-initiated and sponsored by ZonMw. This study was approved by the Medical Ethics Committee of the Netherlands Cancer Institute (13 September 2019; NL70045.031.19) and registered in the ClinicalTrials.gov registry of the U.S. National Library of Medicine (NCT04231175), and the study protocol was published in BMC Cancer (PMID: 33902498).
**Has your study been approved by an ethics committee?** Yes
**Ethics committee - additional information:** The study was approved by the ethics committee.
**Author Disclosures:**
Lizzel Van Der Snee: Nothing to disclose
Max Lahaye: Nothing to disclose
Astrid Kramer: Nothing to disclose
Marta Lopez: Nothing to disclose
Valesca Retel: Nothing to disclose
Niels Kok: Nothing to disclose
Arend Aalbers: Nothing to disclose

### Correlation of FDG Uptake with Serum Tumor Markers in Gynecologic and Gastrointestinal Malignancies

*F. Abubacker Sulaiman*, S. Zainamb Begum, R. Praveenkumar, M. Nivitha, J. Lydia, A. Raashid Ibrahim; Chennai/IN
*(fasulaiman@gmail.com)*

**Purpose:** To evaluate the correlation between ^18F-FDG uptake parameters on PET/CT and serum tumor markers in patients with gynecologic and gastrointestinal malignancies, and to assess their combined prognostic and diagnostic utility in disease characterization.
**Methods or Background:** A prospective study was conducted on 80 patients—40 with gynecologic (ovarian, endometrial, cervical) and 40 with gastrointestinal (colorectal, gastric, pancreatic) cancers—who underwent baseline ^18F-FDG PET/CT and serum tumor marker analysis (CA-125, CEA, and CA 19-9). Quantitative PET parameters including SUVmax, SUVmean, metabolic tumor volume (MTV), and total lesion glycolysis (TLG) were recorded. Correlations between imaging and biochemical markers were analyzed using Pearson's correlation coefficient and regression modeling. Diagnostic and prognostic significance were assessed against histopathologic grade and clinical stage.

**Results or Findings:** A strong positive correlation was observed between SUVmax and CA-125 in ovarian and endometrial carcinomas ($r = 0.76$, $p < 0.001$), and between SUVmax and CEA in colorectal cancer ($r = 0.71$, $p < 0.001$). Elevated TLG values correlated with advanced FIGO and TNM stages. Combined FDG and tumor marker evaluation improved detection of metastatic disease by 19% and provided better prognostic stratification for recurrence risk. Patients with discordantly high CA-125 or CEA and low FDG uptake demonstrated low-grade or mucinous histology, reflecting reduced glycolytic activity.
**Conclusion:** FDG uptake shows significant correlation with serum tumor markers in gynecologic and GI malignancies, supporting their complementary role in staging, prognosis, and treatment monitoring.
**Limitations:** Single-center study with modest sample size and absence of longitudinal follow-up. Multicentric validation is recommended to standardize combined metabolic–biochemical assessment.
**Funding for this study:** No external funding was received for this study.
**Has your study been approved by an ethics committee?** Yes
**Ethics committee - additional information:** Institutional ethical committee approval was obtained.
**Author Disclosures:**
Rathinamoorthy Praveenkumar: Nothing to disclose
Sulaiman Zainamb Begum: Nothing to disclose
Mohanam Nivitha: Nothing to disclose
Johnbosco Lydia: Nothing to disclose
Farook Abubacker Sulaiman: Nothing to disclose
Abubacker Raashid Ibrahim: Nothing to disclose

### Saturday, March 7

**08:00-09:00** **Research Stage 1**

### Research Presentation Session: Abdominal and Gastrointestinal

### RPS 1701

### Liver tumour detection and characterisation

**Moderator**
Hanyu Jiang; Chengdu/CN
*(hanyu_jiang@foxmail.com)*

### Short MRI Surveillance (SMS) for hepatocellular carcinoma screening: preliminary results of the prospective multicentre SMS-HCC study

*C. Van De Braak*, F. Willemssen, R. De Man, A. Van Der Lugt, D. Bos, R. S. Dwarkasing, SMS Consortium; Rotterdam/NL
*(c.vandebraak@erasmusmc.nl)*

**Purpose:** Current guidelines recommend biannual ultrasound (US) screening for high-risk patients for developing hepatocellular carcinoma (HCC), however it has limited sensitivity for early-stage detection of 47%. Our aim is to validate a Short MRI Surveillance (SMS)-protocol for HCC screening, and compare this to US in a prospective multicentre study. Here, we present our preliminary results.
**Methods or Background:** From November 2023, patients included from the current HCC surveillance programme underwent paired US-MRI screening. The abbreviated MRI (aMRI) was performed on 1.5/3.0-T systems from different vendors, using dedicated 8-16 channel range body coil. The SMS-protocol consisted of T1W in-out phase, T2W with fat saturation, and DWI. One radiologist evaluated both US and aMRI images, while two additional radiologists solely evaluated aMRI images. Statistical analyses included inter-rater reliability- and sensitivity/specificity analysis.
**Results or Findings:** For final analyses, 82 participants were included. The most common indication was hepatitis B (53.7%), and cirrhosis (32.9%). Based on US, a total of 20 lesions were found in 11 patients, whereas aMRI detected 132 lesions in 29 patients. Most findings were benign, except for two lesions that were noted on SMS, but undetected on US, and proved to be ≥ LIRADS-3 on subsequent contrast-enhanced MRI, i.e. the golden standard. There was substantial agreement between the readers (Gwet's AC1: 0.70). There was no difference in sensitivity/specificity between US and SMS for detection of suspicious lesions (0.50/0.99, and 0.50/1.00, respectively).
**Conclusion:** Our preliminary results provide a proof of principle that aMRI can be a potential screening modality for HCC surveillance. At this timepoint, with the low number of events, no difference between the sensitivity/specificity of US and SMS could be established. Ongoing enrolment and follow-up are expected to provide more insight.
**Limitations:** Preliminary findings due to ongoing inclusions
**Funding for this study:** Funding was provided by a grant of the Dutch Cancer Society (KWF, grant number: 2021-2/13803)
**Has your study been approved by an ethics committee?** Yes
**Ethics committee - additional information:** This study was approved by the institutional review board, and written informed consent was obtained from all participants.
**Author Disclosures:**
Aad Van Der Lugt: Nothing to disclose
SMS Consortium: Nothing to disclose
Roy S. Dwarkasing: Nothing to disclose
Rob De Man: Nothing to disclose
François Willemssen: Nothing to disclose
Daniel Bos: Nothing to disclose
Céline Van De Braak: Nothing to disclose

### Abbreviated MRI for Hepatocellular Carcinoma Screening: A meta-analysis and Cost-effectiveness

L. Jia[1], *J. Li*[1], Y. Li[2], S. N. Li[1], J. Lei[1]; [1]Lanzhou/CN, [2]Beijing/CN

**Purpose:** While ultrasound has low sensitivity for hepatocellular carcinoma(HCC) screening in high-risk patients, this is particularly pronounced in the growing population with non-viral liver disease (e.g., MASH/NAFLD). This meta-analysis aimed to evaluate the diagnostic accuracy and cost-effectiveness of abbreviated MRI (AMRI) specifically in this subpopulation, and to compare the performance of different AMRI protocols.
**Methods or Background:** PubMed, Embase, Cochrane Library, and Web of Science databases were searched for studies published from inception to March 2025. A pre-specified subgroup analysis was performed for studies focusing on patients with non-viral etiologies of liver disease. Additionally, we directly compared the diagnostic accuracy of hepatobiliary phase AMRI (HBP-AMRI) versus non-contrast AMRI (NC-AMRI) protocols. qualitatively compared the cost-effectiveness of AMRI protocol with ultrasound in HCC screening.
**Results or Findings:** 17 studies (including 3290 high-risk patients) characterized the accuracy of AMRI for surveillance of HCC. The pooled sensitivity and specificity were 88% (95% CI: 84,91) and 95% (95% CI: 92,96). Six studies directly compared diagnostic accuracy of AMRI and ultrasound monitoring end-to-end, the sensitivity of ultrasound was significantly lower at 39% (95% CI: 23,57) than the sensitivity of AMRI at 82% (95% CI: 0.74,0.88, p < 0.001). Hepatobiliary phase contrast-enhanced AMRI (HBP-AMRI) had higher sensitivity than non-contrast AMRI (NC-AMRI) (91% [95% CI: 87, 95] vs 86% [95% CI: 82, 90], p □0.001),also higher than contrast enhanced AMRI (CE-AMRI) (91% [95% CI: 87, 95] vs 90% [95% CI: 86, 95], p □0.001). Most studies found semi-annual abbreviated MRI cost-saving versus ultrasound.
**Conclusion:** Abbreviated MRI demonstrates high performance in HCC screening for patients with both viral and non-viral liver disease and is cost-effective or cost-saving.
**Limitations:** No
**Funding for this study:** No
**Has your study been approved by an ethics committee?** Not applicable
**Author Disclosures:**
Shu Nan Li: Nothing to disclose
Lulu Jia: Nothing to disclose
Junqiang Lei: Nothing to disclose
Jinkui Li: Speaker: RPS 601, Chronic liver disease and portal hypertension, 2026-03-04 16:30 - 17:30
Yang Li: Nothing to disclose

### A Multicenter Multinational Retrospective Study of the 1-Year Natural History of LI-RADS 3 Observations in Patients with Cirrhosis

*L. Asmundo*[1], N. Mercaldo[2], A. J. Herold[3], F. Vernuccio[4], D. V. Sahani[2], M. Renzulli[5], G. Brancatelli[4], A. Vanzulli[1], O. A. Catalano[2]; [1]Milan/IT, [2]Boston, MA/US, [3]Vienna/AT, [4]Palermo/IT, [5]Bologna/IT

**Purpose:** To assess the 1-year natural history of LI-RADS 3 observations on contrast-enhanced MRI in cirrhotic patients across multiple international centers, and to identify clinical and imaging predictors of progression using multivariable and machine learning models.
**Methods or Background:** This retrospective study included 347 cirrhotic patients with 540 LI-RADS 3 observations from six centers across three countries, each with 12-month MRI follow-up. Observations were reassessed using LI-RADS v2018 criteria. Generalized linear mixed-effects models and machine learning (LASSO, random forest) evaluated predictors of progression. Area under the curve (AUC) analysis assessed the predictive performance of clinical and imaging variables.
**Results or Findings:** Within one year, 27% of LI-RADS 3 observations progressed: 13% to LI-RADS 4 and 14% to LI-RADS 5. Independent predictors of progression included lesion size (OR: 1.12, 95% CI: 1.01–1.24), Child-Pugh Class C (OR: 8.36, 95% CI: 1.01–69.27), and alcohol-related liver disease (OR: 0.24, 95% CI: 0.06–0.94). Enhancing capsule and untreated HCV were significant in univariable analysis. Imaging features improved predictive accuracy, increasing AUC from 0.65 to 0.72 (p = 0.01). A lesion size cut-off of 9.5 mm was associated with increased progression risk.
**Conclusion:** One in four LI-RADS 3 observations progress within one year. Lesion size, liver function, and etiology are key predictors. Integration of imaging features enhances risk stratification and supports more personalized follow-up strategies for indeterminate liver lesions.
**Limitations:** The potential selection bias and variability in imaging protocols across centers. Follow-up was limited to contrast-enhanced MRI within 12 months, excluding some patients. Reliance on imaging rather than histopathology may have caused misclassification. Ancillary features were applied in LI-RADS categorization but not independently analyzed to reduce inter-reader variability.
**Funding for this study:** This research received no external funding.
**Has your study been approved by an ethics committee?** Yes
**Ethics committee - additional information:** Institutional review board approval was obtained and informed consent was waived due to the retrospective design. Patient-identifiable information remained secure and encrypted on site servers.

**Author Disclosures:**
Federica Vernuccio: Nothing to disclose
Dushyant V Sahani: Nothing to disclose
Alexander Johannes Herold: Nothing to disclose
Onofrio Antonio Catalano: Nothing to disclose
Angelo Vanzulli: Nothing to disclose
Matteo Renzulli: Nothing to disclose
Giuseppe Brancatelli: Nothing to disclose
Luigi Asmundo: Nothing to disclose
Nathaniel Mercaldo: Nothing to disclose

**Routes to hepatocellular carcinoma diagnosis: A UK-based multicentre service evaluation**

*A. Aslam*[1], J. Cowen[2], T. Parry[1], E. J. Wigmore[3], N. Abimanue[2], A. Gangi-Burton[4], A. Aravinthan[4], J. Franklin[5]; [1]London/UK, [2]Portsmouth/UK, [3]Manchester/UK, [4]Nottingham/UK, [5]Bournemouth/UK

**Purpose:** Six-monthly surveillance for patients with hepatitis B or cirrhosis aims to detect hepatocellular carcinoma (HCC) at an earlier, and more treatable, stage. This UK-based multi-centre service evaluation described the proportion, demographics, and staging of HCC patients diagnosed through surveillance, symptomatically, or incidentally.
**Methods or Background:** This retrospective service evaluation was conducted by the Radiology Academic Network for Trainees (RADIANT). Consecutive patients with a first diagnosis of HCC from 1st January 2020 across 14 centres were included. Data were collected from electronic patient records and Radiology Information Systems, and presented using descriptive statistics.
**Results or Findings:** A total of 723 patients were included (79% [570/723] male, median 71 years [IQR 64-77]). Only 28% (201/723) were diagnosed with HCC through surveillance, whilst 48% (346/723) were diagnosed through symptomatic presentation and 24% (176/723) incidentally. Although 51 % (369/723) of patients had known cirrhosis pre-diagnosis, only 36% (257/723) had been enrolled in a local surveillance programme. A further 20% of patients (143/723) were confirmed to have cirrhosis at diagnosis. Surveillance-diagnosed patients were younger (median 67 years [IQR 61-74]) compared to symptomatic (median 72 years [IQR 65-79]) or incidental (median 73 years [IQR 65-79]) groups, and also had a greater proportion of BCLC 0/A HCC (55%, 110/201), compared to symptomatic (26%, 91/346) or incidental (35%, 62/176) groups. Patients diagnosed symptomatically had highest proportions of ascites, vascular invasion, metastases, and lowest 3-, 6-, and 12-month survival.
**Conclusion:** In the UK, most patients with HCC are diagnosed outside of surveillance. Surveillance-diagnosed patients are identified at earlier stages of disease, with lower mortality rate, reinforcing the need to improve how we identify high-risk patients, enrol and maintain these patients in surveillance, and deliver surveillance itself.
**Limitations:** Retrospective design.
**Funding for this study:** N/A
**Has your study been approved by an ethics committee?** Not applicable
**Author Disclosures:**
James Franklin: Nothing to disclose
Anmol Gangi-Burton: Nothing to disclose
Edward James Wigmore: Nothing to disclose
Nagushan Abimanue: Nothing to disclose
Thomas Parry: Nothing to disclose
Aloysious Aravinthan: Nothing to disclose
Aiman Aslam: Nothing to disclose
Jake Cowen: Nothing to disclose

**Adherence to ultrasound surveillance for hepatocellular carcinoma: A UK-based multicentre service evaluation**

E. J. Wigmore[1], N. Abimanue[2], T. Parry[3], *A. Aslam*[3], J. Cowen[2], A. Gangi-Burton[4], A. Aravinthan[4], J. Franklin[5]; [1]Manchester/UK, [2]Portsmouth/UK, [3]London/UK, [4]Nottingham/UK, [5]Bournemouth/UK

**Purpose:** Hepatocellular carcinoma (HCC) prognosis is suboptimal, typically due to late-stage diagnosis. Although six-monthly ultrasound surveillance (USS) is recommended for high-risk patients, absence of a centralised national programme results in inconsistent implementation. This United Kingdom (UK) multicentre service evaluation assessed provider adherence and patient compliance with HCC USS.
**Methods or Background:** The Radiology Academic Network for Trainees (RADIANT) retrospectively identified consecutive adult patients receiving ≥1 HCC USS in 2021 using the Radiology Information System of 17 participating centres. Records from 2022 were reviewed to assess adherence to six-monthly USS. Provider adherence was defined by scan requests; patient compliance by attendance. Data were presented using descriptive statistics and risk differences with chi-squared confidence intervals.
**Results or Findings:** A total of 1713 patients (58% [993/1713] male; median age 62 years [IQR 54-70]) were included, with 2285 HCC USS requested, of which 88% (2018/2285) were attended. Provider full (2/2 USS), partial (1/2 USS) and non-adherence was 49% (839/1713), 35% (607/1713) and 16% (267/1713) respectively. Of the 84% (1446) of patients for whom ≥1 USS had been requested, full, partial and non-compliance rates were 83% (1199/1446), 11% (160/1446), and 6% (87/1446) respectively. The absolute risk of none relative to full compliance for patients with alcohol history relative to those without was 3% (95% CI 0, 6) higher. For patients with ≥2 comorbidities relative to those without, the absolute risk of none relative to full compliance was 2% (95% CI -1, 4) lower.
**Conclusion:** UK surveillance delivery is suboptimal, due to deficits in provider adherence and patient compliance. National investment in HCC surveillance infrastructure, including tracking and recall systems, is needed, as well as targeted interventions to promote patient compliance.
**Limitations:** Retrospective. Patients eligible for USS but not scanned in 2021 were not captured.
**Funding for this study:** N/A
**Has your study been approved by an ethics committee?** Not applicable
**Author Disclosures:**
James Franklin: Nothing to disclose
Anmol Gangi-Burton: Nothing to disclose
Edward James Wigmore: Nothing to disclose
Nagushan Abimanue: Nothing to disclose
Thomas Parry: Nothing to disclose
Aloysious Aravinthan: Nothing to disclose
Aiman Aslam: Nothing to disclose
Jake Cowen: Nothing to disclose

**Enhanced CT Characteristics of P53-mutated Hepatocellular Carcinoma**

L. Jia[1], *S. N. Li*[1], Y. Li[2], J. Li[1], J. Lei[1]; [1]Lanzhou/CN, [2]Beijing/CN

**Purpose:** P53-mutated hepatocellular carcinoma (HCC) is a highly aggressive subtype with profound implications for prognosis and treatment planning. However, non-invasive preoperative identification remains challenging. The purpose of this study was to assess the diagnostic utility of enhanced CT imaging characteristics for P53-mutated HCC and to elucidate their imaging characteristics.
**Methods or Background:** Patients with surgically resected HCC in a university hospital between January 2020 and December 2023 were retrospectively evaluated. Two radiologists independently evaluated CT imaging features and assessed inter-reader agreement.The Mann-Whitney test, □2 test, and logistic regression analysis were used to compare the enhanced CT characteristics, clinical features, and pathologic results. Using area under the curve, sensitivity and specificity to assess model performance.
**Results or Findings:** A total of 194 patients (mean age, 56 ± 10 years; 151 men) were included. P53 mutations were significantly associated with several aggressive pathological features (p-values ranging from <0.001 to 0.016). On enhanced CT, the presence of intratumoral arteries (p < 0.001) and an arterial phase hypovascular component (p < 0.001) were strong predictors of mutation. Multivariate analysis confirmed these as independent predictors, with high odds ratios of 9.4 (95% CI: 3.3, 26.8) and 10.6 (95% CI: 4.1, 27.6), respectively. The logistic regression model integrating these two features demonstrated excellent diagnostic performance, with an AUC of 0.82 (95% CI: 0.76, 0.89), a sensitivity of 71%, and a specificity of 79%.
**Conclusion:** Our study identifies two specific enhanced CT features—intratumoral arteries and an arterial phase hypovascular component—that are independently associated with P53 mutations in HCC. The model enables noninvasive, preoperative detection of this aggressive HCC subtype to guide risk stratification and personalize treatment.
**Limitations:** None
**Funding for this study:** None
**Has your study been approved by an ethics committee?** Yes
**Ethics committee - additional information:** This study was approved by the institutional review boards of all participating centers, and the requirement for obtaining informed consent from patients was waived.
**Author Disclosures:**
Shu Nan Li: Nothing to disclose
Lulu Jia: Nothing to disclose
Junqiang Lei: Nothing to disclose
Jinkui Li: Nothing to disclose
Yang Li: Nothing to disclose

**Gadoxetic Acid-Enhanced MRI Features Correlated with Transcriptomic Subtypes of Hepatocellular Carcinoma**
*D. Choi*[1], H. Rhee[1], Y. N. Park[1], H. G. Woo[2], T. Chung[1], J.-H. Choi[2], J. E. Yoo[1]; [1]Seoul/KR, [2]Suwon/KR
*(julie715@yuhs.ac)*

**Purpose:** To identify gadoxetic acid-enhanced MRI findings associated with transcriptomic subtypes of hepatocellular carcinoma (HCC).
**Methods or Background:** We retrospectively enrolled treatment-naïve patients with pathologically confirmed HCC who underwent surgery between January 2008 and December 2019. Eligible patients had preoperative gadoxetic acid–enhanced MRI within 3 months of surgery and available RNA sequencing data. In cases with multiple tumors, only the largest lesion was analyzed. Transcriptomic subtypes were classified according to Montironi, Hoshida, and Chiang frameworks. Two radiologists independently evaluated major, ancillary, and LR-M LI-RADS features, as well as prognostic imaging findings on gadoxetic acid–enhanced MRI.
**Results or Findings:** This study included 89 HCCs from 89 patients (median age, 55 years; interquartile range, 49–64 years; 68 men). By Montironi's classification, 36 tumors (40%) were inflamed and 53 (60%) non-inflamed. Hoshida's system identified S1 (n=26, 29%), S2 (n=16, 18%), S3 (n=38, 43%), and unclassified (n=9, 10%) tumors. Chiang's framework grouped them into CTNNB1 (n=17, 19%), proliferation (n=32, 36%), interferon (n=8, 9%), polysomy 7 (n=15, 17%), unannotated (n=8, 9%), and unclassified (n=9, 10%). In the proliferation subtype, marked HBP hypointensity was positively correlated (OR 4.214, P=0.005). For Hoshida S1, peritumoral HBP hypointensity (OR 5.715, P=0.028) and transitional-phase targetoid appearance (OR 18.118, P=0.037) were positive correlates, while capsule presence was negative (OR 0.276, P=0.037). In CTNNB1, marked HBP hypointensity was negatively correlated (OR 0.137, P =0.001) and intratumoral fat was positive (OR 3.980, P=0.040). For the inflamed subtype, capsule presence (OR 0.243, P=0.007) and substantial necrosis (OR 0.062, P=0.019) were negative correlates, with no other significant associations.
**Conclusion:** Ancillary, LR-M features, and prognostic imaging features on gadoxetic acid-enhanced MRI were significantly associated with established transcriptomic subtypes of HCC.
**Limitations:** Limitations include the single-center, retrospective design, lack of validation, and small sample size.
**Funding for this study:** No funding was provided for this study.
**Has your study been approved by an ethics committee?** Yes
**Ethics committee - additional information:** This study was approved by the Institutional Review Board of Severance Hospital.
**Author Disclosures:**
Ji-Hye Choi: Nothing to disclose
Daeun Choi: Nothing to disclose
Young Nyun Park: Nothing to disclose
Hyun Goo Woo: Nothing to disclose
Jeong Eun Yoo: Nothing to disclose
Hyungjin Rhee: Nothing to disclose
Taek Chung: Nothing to disclose

**08:00 - 09:00** **Research Stage 6**

### Research Presentation Session: Breast

### RPS 1702

### Predicting response and recurrence in breast cancer

**Moderator**
Nuala Healy; Dublin/IE
*(nualahealy@rcsi.com)*

**Early Monitoring of NAC in Breast Cancer Using CEUS: A Systematic Review and Meta-Analysis of Predictive Accuracy for pCR**
*F. Ramos Barra*[1], V. A. Ohannesian[2], P. Q. Monteiro[3], M. S. Dias[4]; [1]Brasilia/BR, [2]São Paulo/BR, [3]Salvador/BR, [4]Goiania/BR
*(filipebarra@gmail.com)*

**Purpose:** This systematic review and meta-analysis aimed to evaluate the diagnostic accuracy of early, mid-treatment Contrast-Enhanced Ultrasound (CEUS) for predicting pathological Complete Response (pCR) in patients with primary, non-metastatic breast cancer undergoing Neoadjuvant Chemotherapy (NAC).
**Methods or Background:** Following PRISMA guidelines, we systematically searched PubMed, Embase, and Web of Science for studies assessing the predictive accuracy of CEUS after one or more cycles of NAC. The reference standard for pCR varied across studies (absence of invasive cancer or Miller-Payne Grade ≥4). A potential source of heterogeneity was addressed by selecting the best performing CEUS parameter from each study for analysis.
**Results or Findings:** Seven eligible studies, comprising 979 patients, were included in the bivariate meta-analysis. The overall diagnostic accuracy of early CEUS for predicting pCR was high, demonstrated by an Area Under the Summary Receiver Operating Characteristic Curve (AUROC) of 0.90 (95% CI: 0.87–0.93) and a Diagnostic Odds Ratio (DOR) of 25 (95% CI: 13, 48). The pooled sensitivity and specificity were 0.83 (95% CI: 0.74, 0.89) and 0.84 (95% CI: 0.76, 0.90), respectively, with low heterogeneity (I2=8.4%). Subgroup analysis indicated that assessment after 2nd or 3rd cycles offered slightly higher sensitivity (0.86; 95% CI: 0.76–0.96 versus 0.80; 95% CI: 0.68-0.92) than assessment at the end of the 1st cycle.
**Conclusion:** Early monitoring of NAC response in breast cancer using CEUS demonstrates high diagnostic accuracy for predicting pCR. This non-invasive tool is valuable for guiding mid-treatment decisions and tailoring subsequent therapeutic strategies for patients.
**Limitations:** Limitations included the variability in the pathological complete response definition used across studies (absence of invasive cancer or Miller-Payne Grade ≥4) and the methodological choice to utilize the best-performing CEUS parameter from each study to synthesize the findings.
**Funding for this study:** None
**Has your study been approved by an ethics committee?** Not applicable
**Author Disclosures:**
Filipe Ramos Barra: Nothing to disclose
Philippe Quadros Monteiro: Nothing to disclose
Victor Arthur Ohannesian: Nothing to disclose
Moises Souza Dias: Nothing to disclose

**The Role of Pre-treatment Contrast-Enhanced Mammography Findings in Predicting Response to Neoadjuvant Chemotherapy in Breast Cancer**
*Ö. Ezmeci*, S. Kul; Trabzon/TR
*(ozlemezmeci@gmail.com)*

**Purpose:** Pathological complete response (pCR) after neoadjuvant chemotherapy (NAC) has gained importance as a favorable prognostic indicator in breast cancer (BC). Pretreatment imaging biomarkers may predict tumors likely to achieve pCR and support personalized NAC planning. This study investigated the role of pretreatment contrast-enhanced mammography (CEM) findings in predicting the NAC response in BC.
**Methods or Background:** This retrospective, ethics-approved single-center study (2021–2024) included BC patients treated with NAC who had pretreatment CEM and surgical pathology. Patients who did not complete NAC, did not undergo surgery or lacked histopathological results were excluded. Pre-treatment CEM images were evaluated for breast density, background enhancement (BPE), lesion size, shape, margin, internal enhancement, conspicuity, and presence of calcifications using BI-RADS lexicon. Tumor contrast-to-noise ratio (CNR) was calculated from early-phase recombined images using the formula: (mean tumor SI – mean background SI) / standard deviation of background SI). Tumor enhancement kinetics were assessed by visually comparing early and delayed phase images. Imaging parameters were correlated with response status. Chi-square and Mann–Whitney U tests were used (p<0.05).
**Results or Findings:** In 15 (26%) of the 58 cases pCR was achieved. The relationship between pre-treatment CEM parameters and the presence of pCR was documented in Table 1. Tumor visibility and CNR values were significantly higher in patients with pCR (p<0.049) (Fig 1). pCR rates were 0%, 24%, and 43% in tumors with type 1, 2, and 3 enhancement kinetics, respectively (p=0.025) (Fig 2). Other CEM parameters showed no correlation with pCR.
**Conclusion:** This study suggests that CEM-derived tumor enhancement intensity and kinetics may serve as markers in predicting NAC response. Previous radiomics-based studies also support these findings (2-4).
**Limitations:** Small cohort and lack of inter-reader analysis.
**Funding for this study:** No funding was received.
**Has your study been approved by an ethics committee?** Yes
**Ethics committee - additional information:** Approved by the Ethics Committee of Karadeniz Technical University, Faculty of Medicine (approval no: 2024/280, year 2025)
**Author Disclosures:**
Özlem Ezmeci: Nothing to disclose
Sibel Kul: Nothing to disclose

**Refining CEM Protocols to Predict pCR in Breast Cancer: Early, Delayed, or Both?**

*C. Bellini*[1], G. Bicchierai[1], F. Amato[2], C. Maiello[1], F. Di Naro[1], D. De Benedetto[1], S. Vidali[1], V. Miele[1], J. Nori[1]; [1]Florence/IT, [2]Agrigento/IT
*(1chiarabellini@gmail.com)*

**Purpose:** To evaluate which contrast-enhanced mammography (CEM) acquisition protocol—early, delayed, or combined—is most accurate in assessing pathological complete response (pCR) after neoadjuvant chemotherapy (NAC) in breast cancer.
**Methods or Background:** This retrospective study included 202 women who underwent CEM after NAC and before surgery, with both early and delayed acquisitions. Pathological tumor status (ypT) at surgery was the gold standard, with pCR defined as ypT0 or ypTis. Two breast radiologists reviewed images in consensus. Early, delayed, and combined image sets were assessed in randomized sessions. Radiologic response was categorized per RECIST criteria and then dichotomized as complete response vs residual disease. Sensitivity, specificity, predictive values, accuracy, and ROC-AUCs were calculated and compared.
**Results or Findings:** Sensitivity was higher for combined (86.6%) and delayed (85.7%) acquisitions than for early images (68.1%), whereas specificity was superior in early acquisitions (63.9%). Accuracy was 66.3% for early, 71.3% for delayed, and 69.8% for combined CEM. NPV improved from early (58.2%) to delayed (71.2%) and combined acquisitions (70.4%). ROC curve comparisons showed no statistically significant differences between protocols. Delayed acquisitions reclassified 57.9% of early false negatives as true positives and corrected 13.3% of early false positives but converted 28.3% of early true negatives into false positives. A relevant proportion of false positives in both protocols corresponded to residual in situ carcinoma at histology.
**Conclusion:** Delayed acquisitions improve sensitivity and reduce false negatives in NAC response assessment with CEM, although at the cost of decreased specificity due to overestimation of residual disease, particularly in cases with in situ carcinoma. The combined protocol enhances overall performance but does not significantly differ from early or delayed acquisitions alone.
**Limitations:** Limitations include the retrospective, single-center design, consensus readings without interobserver analysis, absence of comparison with MRI.
**Funding for this study:** No funding
**Has your study been approved by an ethics committee?** Yes
**Ethics committee - additional information:** Comitato Etico Area Vasta Centro 16.251
**Author Disclosures:**
Vittorio Miele: Nothing to disclose
Chiara Maiello: Nothing to disclose
Giulia Bicchierai: Nothing to disclose
Federica Di Naro: Nothing to disclose
Diego De Benedetto: Nothing to disclose
Jacopo Nori: Speaker: Hologic, Giotto
Sofia Vidali: Nothing to disclose
Francesco Amato: Nothing to disclose
Chiara Bellini: Speaker: Hologic

**Diagnostic Accuracy of Contrast-Enhanced Mammography in Evaluating Breast Cancer Response to Neoadjuvant Therapy: A Systematic Review and Meta-Analysis**

G. Barcaroli, F. Galati, F. Shakki Katouli, *R. Maroncelli*, F. Cicciarelli, M. Pasculli, F. Pediconi, C. Catalano; Rome/IT
*(roberto199426@gmail.com)*

**Purpose:** Neoadjuvant therapy (NAT) in breast cancer enables tumor downstaging and response evaluation. Although magnetic resonance imaging (MRI) is the gold standard, its limitations have prompted growing interest in alternatives such as contrast-enhanced mammography (CEM).
**Methods or Background:** This review was conducted according to PRISMA guidelines. We evaluated CEM accuracy in predicting pathological complete response after NAT, using surgery as reference standard. A comprehensive search was conducted in PubMed/Medline, Embase, Cochrane, and Web of Science. Two reviewers screened studies, extracted data, and evaluated risk of bias with QUADAS-2 tool. A bivariate random-effects model estimated diagnostic odds ratio (DOR), sensitivity, specificity, and likelihood ratios. Summary receiver operating characteristic (sROC) curves were generated, and heterogeneity was quantified using Higgins'$I^2$. Publication bias was assessed via Deek's funnel plot.
**Results or Findings:** 13 studies, including 582 patients, met inclusion criteria. The pooled analysis showed a DOR of 8.60 (95%,CI:3.29–22.52) with substantial heterogeneity ($I^2$=70.06%). Pooled sensitivity and specificity were 72% (95%,CI:63%–79%) and 83% (95%,CI:68%–92%), respectively, with an sROC AUC of 0.82 (95%,CI:0.78–0.85). Excluding the study contributing most to heterogeneity improved the DOR to 9.76 (95%,CI:6.00–15.89) and reduced $I^2$ to 0.00%.
**Conclusion:** CEM demonstrates good diagnostic accuracy for assessing NAT response, performing comparably to MRI.
**Limitations:** Retrospective study, limited number of studies included
**Funding for this study:** None
**Has your study been approved by an ethics committee?** Not applicable
**Author Disclosures:**
Roberto Maroncelli: Nothing to disclose
Gloria Barcaroli: Nothing to disclose
Francesca Galati: Nothing to disclose
Marcella Pasculli: Nothing to disclose
Federica Cicciarelli: Nothing to disclose
Fatemeh Shakki Katouli: Nothing to disclose
Federica Pediconi: Nothing to disclose
Carlo Catalano: Nothing to disclose

**MRI-Based Evaluation for Predicting Pathological Response and Monitoring Reactive Angioma Evolution in Triple-Negative Breast Cancer Undergoing Neoadjuvant Chemoimmunotherapy**

*L. Lin*, J. Zheng, C. You, Y. Gu; Shanghai/CN
*(linly_1995@163.com)*

**Purpose:** Neoadjuvant chemotherapy combined with anti-programmed cell death protein-1 (PD-1) immunomodulators (NACI) has been proven to improve outcomes in triple-negative breast cancer (TNBC). However, the role of MRI in predicting therapeutic response remains underexplored. This study aims to investigate the utility of MRI for early response prediction and longitudinal assessment of reactive angiomatous changes in TNBC patients undergoing NACI therapy.
**Methods or Background:** This secondary analysis pooled data from the CamRelief and NeoTENNIS RCTs, among others, investigating NACI at our center (2020-2023). We analyzed baseline clinicopathological characteristics, MRI features (including BI-RADS descriptors and whole-tumor ADC histogram metrics), and response outcomes. Independent predictors of pathological complete response (pCR) were identified via univariate and multivariate logistic regression to develop a prognostic model. Longitudinal evolution of reactive angiomas during NACI was assessed with non-parametric and chi-square tests.
**Results or Findings:** A total of 61 TNBC patients undergoing NACI were enrolled. Univariate analysis identified four independent predictors of pCR. While elevated Ki-67 index ($p<0.001$) and CD8-T cell infiltration ($p=0.004$) reflected baseline pathological predictors, type III kinetic curves ($p=0.004$) and reduced median_ADC value ($p=0.021$) of MRI provided unique insights with pCR. Moreover, the combined multi-dimensional model achieved a high prediction accuracy, got AUC of 0.89. Serial MRI monitoring identified transient reactive changes characterized by multiple small masses emerging (mean 8.82±0.67mm) during NACI unrelated to response, peaking at post-cycle-2 MRI, followed by progressive regression. One was histopathologically confirmed as benign reactive angiomatous remodeling.
**Conclusion:** MRI played a dual role in managing NACI for TNBC. An integrative model of MRI biomarkers and clinicopathology superiorly predicted pCR, whereas reactive angiomatous remodeling was transient.
**Limitations:** Small sample size and and paucity of quantitative features on MRI.
**Funding for this study:** Special Research Fund for Clinical Studies of Innovative Drugs after Market Launch (WKZX2024CX103301)
**Has your study been approved by an ethics committee?** Yes
**Ethics committee - additional information:** The study was approved by the Ethics Committee of the Institutional Review Board (IRB) of our institution. All patients provided written informed consent before enrollment.
**Author Disclosures:**
Yajia Gu: Nothing to disclose
Jinlong Zheng: Nothing to disclose
Chao You: Nothing to disclose
Luyi Lin: Nothing to disclose

**Predicting pathologic complete response before neoadjuvant therapy: pre-treatment breast MRI-derived radiomics with radiologic-pathologic integration**

*C. Yanardağ*, M. G. Akpınar, G. Durhan, F. Demirkazık; Ankara/TR
*(cennetyanardagmd@gmail.com)*

**Purpose:** This study evaluates whether pre-treatment breast Magnetic Resonance Imaging (MRI)-derived radiomic features, alone and combined with radiologic and pathologic variables, can predict pathologic complete response (pCR) before neoadjuvant therapy (NAT) in breast cancer.
**Methods or Background:** We retrospectively included 154 patients who underwent pre-treatment breast MRI, received NAT, and subsequently had surgery at our center (2016-2024). Patients were split 70/30 into training

(n=109) and test (n=45) sets. Pathologic variables (estrogen receptor, progesterone receptor, HER2 status, and histologic grade) were obtained from pre-treatment biopsies. Radiologic variables included tumor size, distribution, shape, and axillary lymph-node status. Variables showed balanced distributions between training and test sets (p>0.05). Tumors were manually volumetrically segmented on early post-contrast fat-suppressed T1-weighted images with syngo.via. We extracted 1,691 radiomic features, retained 652 after applying an intraclass correlation coefficient threshold of 0.90 (one reader, repeated segmentations, 38-case subset). Radiomic features underwent elastic-net selection in the training set with cross-validation, yielding nine features. Pure radiomic, radiologic, and pathologic, as well as combined (radiomics-radiologic, radiomics-pathologic, and radiomics-radiologic-pathologic) models were developed using a Support Vector Machine (SVM). The area under the curve (AUC), accuracy, sensitivity, and specificity were calculated for both sets.
**Results or Findings:** A total of 42.9% had pCR. Test-set AUCs: radiomics 0.563; radiologic 0.558; pathologic 0.704; radiomics+radiologic 0.557; radiomics+pathologic 0.662; radiomics-radiologic-pathologic 0.607. The pathologic model had the highest AUC. Specificities: radiomics 0.846; radiologic 0.807; pathologic 0.692; radiomics+radiologic 0.846; radiomics+pathologic 0.539; radiomics-radiologic-pathologic 0.654. Radiomics-only and radiomics+radiologic models showed higher specificity.
**Conclusion:** Radiomics alone offered modest discrimination, but adding pathologic variables improved performance. Radiomic models have higher specificity, useful for ruling out pCR. Combining radiologic and pathologic findings before treatment can help with risk stratification and customizing therapy prior to NAT.
**Limitations:** A modest sample size, retrospective, single-center design.
**Funding for this study:** No funding was received for this study.
**Has your study been approved by an ethics committee?** Yes
**Ethics committee - additional information:** This study received approval from the Hacettepe University Health Sciences Research Ethics Committee (decision no. SBA 24/492) and was conducted in accordance with the Declaration of Helsinki.
**Author Disclosures:**
Gamze Durhan: Nothing to disclose
Figen Demirkazık: Nothing to disclose
Cennet Yanardağ: Nothing to disclose
Meltem Gülsün Akpınar: Nothing to disclose

**Tumor to breast ratio as radiological tool to predict breast-conserving surgery vs mastectomy in patients with pathologic partial response after neoadjuvant systemic treatment: a retrospective study**
*E. Tallamona*[1], R. Gioco[2], C. Giuseppe[2], P. Francesco[2]; [1]Gela/IT, [2]Misterbianco/IT
*(elianatallamona@gmail.com)*

**Purpose:** This study aims to identify radiological features along with clinical and pathological predictors that influence the choice of mastectomy in non-metastatic breast cancer patients who demonstrate a pathological partial response (pPR) after neoadjuvant systemic therapy (NACT), despite presenting a favorable tumor-to-breast ratio that could allow breast-conserving surgery (BCS). The goal is to better understand factors that lead to over-treatment and to prove how radiologists could help to reach a more evidence-based surgical approach.
**Methods or Background:** This retrospective single-center study included 165 female patients treated with NACT followed by surgery between 2021 and 2023 at Humanitas Istituto Clinico Catanese in Catania. Only patients with residual cancer burden class I or II were included. Patients with RCB III, metastatic disease, BRCA mutations, or male gender were excluded. For a subgroup of 26 patients, a tumor-to-breast volume ratio was calculated using mammographic measurements before and after NACT to determine suitability for BCS. Statistical analysis was performed to identify correlations between radiological measurement, clinical characteristics and surgical choice (BCS vs mastectomy).
**Results or Findings:** Among 165 patients with partial pathological response (pPR), 43% underwent BCS and 57% underwent mastectomy. Mastectomy was more frequently performed in younger and premenopausal patients, those with multifocal or larger tumors, and higher clinical staging at diagnosis. In the subgroup with a post-NACT favorable tumor-to-breast ratio, 38.5% still underwent mastectomy.
**Conclusion:** Despite partial tumor response and anatomical feasibility, many patients undergo mastectomy due to factors beyond tumor biology. A multidisciplinary, patient-centered approach, integrating predictive tools like tumor-to-breast ratio analysis and shared decision-making, may promote a more conservative and personalized surgical strategy leading to reduce unnecessary mastectomies and enhance patient outcomes and satisfaction
**Limitations:** Main limitation include the single-center design and limited sample size
**Funding for this study:** No funding was required for this study
**Has your study been approved by an ethics committee?** Not applicable
**Author Disclosures:**
Pane Francesco: Nothing to disclose
Catanuto Giuseppe: Nothing to disclose
Eliana Tallamona: Nothing to disclose
Rossella Gioco: Nothing to disclose

**Digital twins for breast cancer surgery**
*R. Timóteo*, A. Laborde, Y. Forghani, N. Loução, A. Cardoso, M. Correia, J. Santinha, T. Marques, P. Gouveia; Lisboa/PT
*(rafaela.timoteo@research.fchampalimaud.org)*

**Purpose:** Breast cancer (BC) is the second most common cancer worldwide, and breast conservative surgery (BCS) is its primary treatment. Accurate tumor localization is essential to achieve negative margins and reduce recurrence, yet current methods are invasive and often imprecise, leading to re-excision. Digital twins (DTs) are emerging in healthcare as dynamic virtual replicas of patients, integrating both anatomy and physiology, and serving as a powerful tool for treatment, decision-making, and outcome prediction. By providing a non-invasive and precise approach to tumor localization, DTs may enhance surgical accuracy, reduce re-excision rates, and ultimately improve patient outcomes.
**Methods or Background:** Four breast cancer patients proposed for BCS were selected between January and September 2025. Patient-specific DTs were built from annotated supine breast MRI and automatically aligned to the patient in real-time with a 3D surface scanning system in the operating room (OR). DTs were visualized through an Augmented Reality (AR) interface that was co-designed with breast surgeons to ensure suitability for the OR environment. Tumor marking with AR was compared to carbon tattoo marking and intraoperative findings.
**Results or Findings:** Results reveal a high degree of tumor localization accuracy in most surgeries, with fast setup time and tumor marking. Usability and workload questionnaires highlighted our system's excellent user experience.
**Conclusion:** Integrating DTs in AR environments represents the next era of breast cancer surgery, offering surgeons a new paradigm for interacting with patient-specific data, enhancing their performance in the OR, and leading to better surgical outcomes.
**Limitations:** Future work should consider a clinical study with a larger patient cohort, accuracy measurement through rate of positive margins, non-rigid registration of DTs with volumetric correction, and biomechanical simulation capabilities.
**Funding for this study:** This research is part of the Health for Portugal (HfP), funded by Agendas Mobilizadoras para a 5 Inovação Empresarial - Plano de Recuperação e Resiliências Português (PRR). A special thanks to the Breast Unit at Champalimaud Clinical Center for their collaboration, and to all of those who participated in this study, to contribute to the evolution of health technologies and consequent patient care.
**Has your study been approved by an ethics committee?** Yes
**Ethics committee - additional information:** This study was approved by the ethics committee of Champalimaud Foundation.
**Author Disclosures:**
André Cardoso: Nothing to disclose
Pedro Gouveia: Nothing to disclose
Yasna Forghani: Nothing to disclose
Mariana Correia: Nothing to disclose
Joao Santinha: Nothing to disclose
Tiago Marques: Nothing to disclose
Nuno Loução: Nothing to disclose
Rafaela Timóteo: Nothing to disclose
Alexandre Laborde: Nothing to disclose

**Local Recurrence After Treatment of Pure Ductal Carcinoma In Situ: A Radiological Retrospective Study**
*A. G. Azzarito*, M. C. Torrione, A. Delli Pizzi, M. F. Savina, L. Di Pietrantonio, A. Di Credico, A. Figorilli, M. Muzi, M. Caulo; Chieti/IT
*(andreagaiaazzarito@gmail.com)*

**Purpose:** To evaluate the local recurrence rate of pure ductal carcinoma in situ (DCIS) after surgery, with particular focus on the impact of adjuvant radiotherapy and clinicopathological factors, in order to explore the potential for more personalized management strategies.
**Methods or Background:** We retrospectively analyzed 403 patients with histologically confirmed pure DCIS treated with breast-conserving surgery or mastectomy between 2014 and 2023. Imaging and pathological variables were assessed. Recurrence rates were correlated with tumor grade, radiotherapy, and other clinicopathological parameters. Statistical analyses included Kaplan–Meier survival estimates and multivariate Cox regression.
**Results or Findings:** Among 417 lesions, 21 ipsilateral recurrences (5%) were observed, of which 57% were non-invasive and 38% invasive. Recurrences occurred predominantly in patients not treated with adjuvant radiotherapy

(52%) and in those with breast-conserving surgery. All recurrent tumors were estrogen receptor–positive, yet none received endocrine therapy. Tumor grade and size were not significantly associated with recurrence. No distant metastases or disease-related deaths were recorded.
**Conclusion:** Recurrence after treatment for pure DCIS is relatively uncommon and often non-invasive. Traditional prognostic markers such as tumor grade and size did not predict recurrence. These findings support a more individualized approach to treatment, where selected patients may avoid overtreatment. Further studies incorporating molecular profiling are warranted to refine risk stratification.
**Limitations:** This study is limited by its retrospective design and by the relatively short follow-up in some patients, which may not capture late recurrences. Moreover, the absence of molecular or genomic profiling restricts the ability to refine risk stratification.
**Funding for this study:** None
**Has your study been approved by an ethics committee?** Not applicable
**Author Disclosures:**
Andrea Gaia Azzarito: Nothing to disclose
Andrea Delli Pizzi: Nothing to disclose
Maria Francesca Savina: Nothing to disclose
Laura Di Pietrantonio: Nothing to disclose
Maria Concetta Torrione: Nothing to disclose
Andrea Figorilli: Nothing to disclose
Massimo Caulo: Nothing to disclose
Angela Di Credico: Nothing to disclose
Marzia Muzi: Nothing to disclose

**08:00 - 09:00** **Research Stage 2**

### Research Presentation Session: Chest

### RPS 1704
### Advanced chest imaging

**Moderator**
Jonas Kroschke; Zurich/CH
*(jonas.kroschke@usz.ch)*

### Navigating the CT Wave: Multicentric Chest Imaging Trends Before, During, and After COVID-19 pandemic
*T. Viana Miranda Lima*, N. Saltybaeva, T. Gassenmaier, L. Ebner, J. E. Roos; Lucerne/CH
*(thiago.lima@luks.ch)*

**Purpose:** The COVID-19 pandemic has significantly impacted healthcare practices worldwide, including the use of CT in chest examinations. This study is aimed to analyse the trends in CT chest examinations before, during, and after the COVID-19 pandemic.
**Methods or Background:** Data was retrospectively collected from 10 public hospitals over a period pre-pandemic years (2019 until February 2020), through the pandemic (March 2020- April 2023) (as defined by the World Health Organisation) and post-pandemic (May 2023- December 2024). Patient exposure information from more than 240 000 CT examinations was collected and analysed using a commercial dose management system. Statistical analysis was performed with descriptive and inferential statistics employed to compare the number of CT chest examinations and patient radiation exposure across different periods.
**Results or Findings:** The results indicate a increase in CT chest examinations during the pandemic, which reached a plateau and remained stable post-pandemic (p-value < 0.001). Importantly, the average radiation exposure per patient has decreased with the evolution of technology. There was also a shift between protocols used during the pandemic with the move towards more dedicated procedures.
**Conclusion:** These findings highlight the sustained demand for CT chest examinations and the effective management of patient radiation exposure during and after the pandemic. The study underscores those possible practices obtained during the pandemic became the normal after the end of the pandemic.
**Limitations:** This study has few limitations: firstly, the data are derived from DICOM data, which, while ensuring reproducibility and objectivity, may not capture all clinical nuances for example a patient being imaged on the wrong protocol. Secondly, the analysis of radiation exposure was conducted primarily to demonstrate overall trends in dose levels rather than to thoroughly investigate or attribute the main factors underlying the observed changes.
**Funding for this study:** None
**Has your study been approved by an ethics committee?** Yes
**Ethics committee - additional information:** Ethics was waived for this project by the North- and Central Switzerland Ethics Commission (Req-2025-00111), and all patient data was anonymised.
**Author Disclosures:**
Tobias Gassenmaier: Nothing to disclose
Justus Erasmus Roos: Nothing to disclose
Natalia Saltybaeva: Nothing to disclose
Thiago Viana Miranda Lima: Nothing to disclose
Lukas Ebner: Nothing to disclose

### Deep Learning-based analysis of HRCT images for outcome prediction in pulmonary fibrosis: the added value over clinico-functional assessment in a prospective longitudinal study
*F. Villone*, L. Calandriello, G. Cicchetti, R. D'Abronzo, F. Scrocca, F. Del Prete, G. Sgalla, L. Natale, A. R. Larici; Roma/IT
*(federicovillone@libero.it)*

**Purpose:** Prognostic assessment in interstitial lung disease (ILD) is challenging, due to unpredictable behaviour and scarcity of validated progression markers. Clinico-functional progression criteria are limited by subjectivity and variability, while radiological progression relies on visual assessment, affected by inter-observer variability. Artificial intelligence(AI) on high-resolution Computed Tomography(HRCT) has emerged as a reliable tool for ILD assessment. This study evaluated deep-learning HRCT analysis with commercially available software in assessing progression and mortality in a real-life prospective ILD patients cohort.
**Methods or Background:** HRCT scans from ILD patients (idiopathic pulmonary fibrosis [IPF] and non-IPF ILDs) at baseline and after 12±4 months were analyzed using AVIEW software (Coreline Soft). Correlation among baseline AI variables and baseline forced vital capacity (FVC) categories(≥80%; 79-50%; ≤49%) and between AI changes and FVC decline categories(≥10%; 5-9%; ≤4%) were assessed. Multivariate logistic regression tested correlation of AI variables with all-cause mortality and functional progression. Integration of quantitative data into clinico-functional models to predict mortality was evaluated with C-index.
**Results or Findings:** 148 HRCT scans from 74 ILD patients were assessed (mean age 73.3±7.8 yrs; male 61.3%;IPF 68.8%). Thirty-nine patients(52.7%) progressed and twenty-four(32.4%) died. At baseline, most AI variables inversely correlate with the FVC categories; changes in the quantitative measurements correlates with the categorial FVC decline, showing responsiveness. At multivariate analysis, 19 variables at baseline were independently predictive of mortality; at follow-up, changes in 5 variables predicted mortality, with the best predictor being Δ% of consolidation(HR 4.01, 95%CI 1.69-10.13, p=0.003). Incorporating AI variables into clinico-functional models improved mortality prediction at baseline (C-index 0.707 vs 0.57; p=0.012) and follow-up(0.866 vs 0.763; p=0.005).
**Conclusion:** Deep-learning-based HRCT measurements and longitudinal changes predict progression and mortality in fibrotic ILD. Composite AI clinico-functional models may allow more precise prognostic assessment .
**Limitations:** Single-center study
**Funding for this study:** None
**Has your study been approved by an ethics committee?** Yes
**Ethics committee - additional information:** The study protocol was approved by the local institutional commmittee on research ethics.
**Author Disclosures:**
Giacomo Sgalla: Nothing to disclose
Francesco Scrocca: Nothing to disclose
Anna Rita Larici: Nothing to disclose
Federico Villone: Nothing to disclose
Luigi Natale: Nothing to disclose
Giuseppe Cicchetti: Nothing to disclose
Lucio Calandriello: Nothing to disclose
Federica Del Prete: Nothing to disclose
Rosa D'Abronzo: Nothing to disclose

### Microradiological Characterization and Novel Growth Pattern Insights of Ultra-Early-Stage Lung Squamous Cell Carcinoma
*H. Zhang*[1], L. Xu[2]; [1]Joondalup/CN, [2]Dazhou/CN
*(hengzhangchat@gmail.com)*

**Purpose:** To delineate the microradiological features of ultra-early-stage lung squamous cell carcinoma (UESLSCC) and investigate hypotheses on growth dynamics, vascular supply, and early morphological evolution between central- and peripheral-type lesions.
**Methods or Background:** A retrospective study included 38 patients with pathologically confirmed UESLSCC identified via opportunistic screening or health examinations. Pre-diagnostic thin-section CT was reviewed, with microradiological post-processing (MPR, CPR, MIP, VB) to enhance visualization. Lesions were classified by anatomical location (central- vs peripheral-type). Recognition intervals, morphological subtypes, microradiological signs (tail sign, microvascular convergence), and relationships with bronchovascular structures were evaluated by experienced thoracic radiologists. The study was approved by the institutional ethics committee, and informed consent was waived due to its retrospective design.
**Results or Findings:** Central-type lesions had significantly shorter recognition intervals than peripheral-type (P < 0.05), plausibly due to earlier respiratory symptoms, earlier bronchoscopy, and/or larger lesion volumes. We hypothesize that central lesions—supported by richer bronchial arterial flow and larger airway caliber—proliferate and present earlier, whereas peripheral lesions—reliant on pulmonary circulation in narrower distal airways—grow more slowly. Morphologically, wall-type and luminal-type patterns predominated in central lesions, while non-specific alveolar-type and pleural-type patterns were more common peripherally, suggesting an alveolar origin. Peripheral lesions frequently arose near bronchial bifurcations and showed tail signs, indicating bidirectional growth along adjacent bronchioles. Microradiological post-processing significantly improved detection and characterization of ultra-early lesions, especially subtle bronchovascular relationships.
**Conclusion:** Ultra-early–stage lung squamous cell carcinoma predominantly originates from the bronchial wall. Microradiological imaging is pivotal for detecting and characterizing these lesions, revealing distinct vascular supply patterns and growth dynamics between central and peripheral types. These findings propose new pathophysiological hypotheses and provide a foundation for early screening, near-pathological preoperative diagnosis, and tailored management of UESLSCC.
**Limitations:** Single-center, retrospective design, a small opportunistically screened cohort.
**Funding for this study:** None
**Has your study been approved by an ethics committee?** Not applicable
**Author Disclosures:**
Heng Zhang: Nothing to disclose
Lin Xu: Nothing to disclose

**High-Pitch Low-Dose Chest CT with Calcium-Aware Reconstruction for Coronary Artery Calcium Scoring: Accuracy and Dose Reduction**
*H. Zhang*, Y. Meng; Xuzhou/CN

**Purpose:** To evaluate the accuracy, radiation dose, and clinical feasibility of a high-pitch low-dose chest CT (Sa36LDCT) protocol with calcium-aware reconstruction for coronary artery calcium scoring (CACS).
**Methods or Background:** This prospective study included 90 patients underwent both standard CAC scans and Sa36LDCT. CACS was quantified as Agatston score, calcium volume, and equivalent mass. Risk stratification was assessed using Agatston categories. Agreement between protocols was evaluated using ICC, Bland–Altman analysis, and weighted kappa. Subgroup analyses were performed according to heart rate (≤75 vs >75 bpm). Radiation dose and subjective image quality were compared.
**Results or Findings:** CAC metrics showed no significant difference between protocols, with excellent agreement (ICC=0.983-0.996). Subgroup analysis revealed that heart rate didn't significantly influence CAC quantification or risk classification with Agatston score ICC values of 0.98 for ≤75 bpm and 0.99 for >75 bpm, demonstrating accuracy in both subgroups. Radiation dose reduction was consistently observed across both subgroups, with Sa36LDCT reducing effective radiation dose by 62% (0.74 vs 0.28 mSv, P<0.001). Risk stratification showed near-perfect consistency across heart rate subgroups.
**Conclusion:** High-pitch Sa36LDCT provides accurate CACS equivalent to standard CACS with radiation dose reduction and preserved image quality. The protocol's robustness across heart rate subgroups supports its clinical use as a safe and practical one-stop approach for combined pulmonary and cardiovascular screening.
**Limitations:** First, it was conducted at a single center with a relatively limited sample size. Second, calcium-aware Sa36 reconstruction kernel is manufacturer-specific, and our results may not be directly applicable to scanners from other vendors or reconstruction algorithms with different technical implementations. Third, we did not specifically investigate the influence of BMI on calcium quantification.
**Funding for this study:** We acknowledge financial support from the Jiangsu Traditional Chinese Medicine Science and Technology Development Plan Project (MS2021100), and the Key Research and Development Program of Xuzhou Science and Technology Bureau (KC20159).
**Has your study been approved by an ethics committee?** Yes
**Ethics committee - additional information:** This prospective study was approved by the Institutional Review Board of our institution (XYFY2025-KL037)

**Regional ventilation imaging in normal and bronchoconstricted in vivo rabbit lungs using spectral Xe-enhanced CT**
*E. Verelst*[1], S. Bayat[2], S. Verbanck[1], G. Van Gompel[1], J. De Mey[1], N. Buls[1]; [1]Brussels/BE, [2]Grenoble/FR
*(emma.verelst@vub.be)*

**Purpose:** To investigate regional ventilation in healthy and pathological in vivo rabbit lungs using Xenon (Xe)-enhanced spectral CT-based ventilation maps.
**Methods or Background:** Six anesthetized rabbits [2.6 ± 0.2 (SD) kg] were mechanically ventilated. Dynamic multi-breath imaging was performed during Xe washout using a clinical DECT (Revolution, GE Healthcare) before and after the administration 40 mg/mL Methacholine (MCh) to induce bronchoconstriction (post-MCh). Xe concentration (Xeconc) was monitored using a mass spectrometer (LR6000, Logan Research). The dynamic DECT data was decomposed into Xe densities images. Ventilation maps were generated depicting specific ventilation (sV), quantified as the time constant (min-1) of a fitted mono-exponential model, following image registration. Agreement between global sV derived from ventilation maps (sV_image) and mass spectrometry data (sV_ms) was assessed. Ventilation defect fraction (VDF) was computed as voxels with sV below 25% of the mean and compared between baseline and post-MCh. Regional ventilation was evaluated by comparing the mean sV within the VD-area (sV_VDF) compared to the non-VD area (sV_non-VDF).
**Results or Findings:** The ventilation maps revealed differences in ventilation distribution between baseline and post-MCh conditions. VDF increased from 22.6% ± 10.6% at baseline, to 37.2% ± 9.8% post-MCh, p = 0.003. At baseline and post-MCh, sV_VDF (6.6 ± 9.92 min-1, 5.27 ± 8.38 min-1) was reduced compared to sV_non-VDF (0.29 ± 0.09 min-1, 0.22 ± 0.03 min-1), p < 0.001. Lastly, a strong agreement was found between global sV_image and sV_ms at baseline (3.0 min-1 ± [-0.77 min-1, 6.7 min-1]) and post-MCh (3.8 min-1 ± [-1.15 min-1, 8.8 min-1]).
**Conclusion:** This study shows the potential of dynamic Xe-enhanced spectral CT-imaging for quantitative studies of ventilation defects, supporting its utility for regional assessments of pulmonary function.
**Limitations:** This study was an in vivo animal study.
**Funding for this study:** Flemish Research Foundation (FWO) personal grant, number: 1SH1Z24N.
**Has your study been approved by an ethics committee?** Yes
**Ethics committee - additional information:** Ethical approval obtained by the Ethical committee on Animal T esting of the Vrije Universiteit Brussel (18-272-15).
**Author Disclosures:**
Johan De Mey: Nothing to disclose
Sylvia Verbanck: Nothing to disclose
Emma Verelst: Nothing to disclose
Gert Van Gompel: Nothing to disclose
Nico Buls: Nothing to disclose
Sam Bayat: Nothing to disclose

**Quantitative CT of Lung Parenchyma and Airways in Primary Ciliary Dyskinesia**
*O. Weinheimer*[1], S. Riepenhausen[2], R. Tenardi-Wenge[2], M. A. Mall[3], H-U. Kauczor[1], H. Omran[2], M. O. Wielpütz[4], J. Raidt[2]; [1]Heidelberg/DE, [2]Muenster/DE, [3]Berlin/DE, [4]Greifswald/DE
*(weinheimer@uni-heidelberg.de)*

**Purpose:** Primary Ciliary Dyskinesia (PCD) is a rare genetic disorder where tiny hair-like structures called cilia don't work properly. Various quantitative CT (QCT) techniques have been proven to deliver useful and objective biomarkers describing lung parenchyma and airways. This study explores which QCT biomarkers best characterize lung and airway abnormalities in PCD.
**Methods or Background:** A total of 148 inspiratory CT scans from 106 genetically confirmed PCD subjects (55 female, mean age 29.2 ± 17.7 years), collected from multiple medical centers, were automatically analyzed using YACTA software. 59 CT scans from 35 individuals with situs inversus were mirrored to enable standard YACTA evaluation. Quantitative CT parameters of the airways and lung parenchyma were assessed on a six-lobe basis.
**Results or Findings:** Mirroring the CT scans allowed for the inclusion of all situs inversus subjects in the analysis. Airway wall percentage (AWP) was significantly higher in the lower lobes compared to the upper lobes (60.54% vs 55.89%, p<2.2e-16). Similarly, the bronchiectasis index (BEI) was elevated in the lower lobes (1.64 vs 0.45, p<2.2e-16). Mean lung density (MLD) and the 75th percentile of the lung histogram were also higher in the lower lobes (–729.39 HU vs –763.45 HU, p=1.95e-11 and –854.42 HU vs –892.74 HU, p=3.79e-10). The values in the right middle lobe and lingula were comparable to those in the lower lobes. AWP, BEI and MLD showed a moderate correlation with subject age (R=-0.57, p=8.91e-14 and R=0.27, p<0.001 and R=-0.47, p=2.85e-09).

Saturday

**Conclusion:** QCT showed significant changes in airway and lung parenchyma paramaters predominantly in the lower lobes, differing from the typical pattern seen in cystic fibrosis, where changes are distributed across all lobes. QCT may be a valuable tool for PCD research.
**Limitations:** Retrospective study, no uniform CT protocol.
**Funding for this study:** DFG OM6/7, OM6/8, OM6/10, OM6/14, OM6/16, CRU 326 (subprojects OM6/11 (H. Omran), RA3522/1 (J. Raidt)) IZKF Muenster (Om2/010/20; OM2/014/24), BMFTR - project ReproTrackMS (grant 01GR2303). Part of the authors are Healthcare Professionals in the European Reference Network ERN LUNG.
**Has your study been approved by an ethics committee?** Yes
**Ethics committee - additional information:** The study was approved by the Ethics Committee of the Medical Association of Westphalia-Lippe and the local ethics committee of the University of Münster (Münster, Germany; AZ 2011-270-f-S).
**Author Disclosures:**
Mark O. Wielpütz: Nothing to disclose
Oliver Weinheimer: Nothing to disclose
Hans-Ulrich Kauczor: Nothing to disclose
Marcus A Mall: Nothing to disclose
Heymut Omran: Nothing to disclose
Johanna Raidt: Nothing to disclose
Sarah Riepenhausen: Nothing to disclose
Retno Tenardi-Wenge: Nothing to disclose

**Computed Tomography Pulmonary Angiography biomarkers of peripheral vascular disease in patients treated for chronic thromboembolic pulmonary hypertension: prognostic evaluation**
*E. Muscogiuri*, F. De Keyzer, W. De Wever, L. Hardy, G. Aerts, R. Quarck, M. Delcroix, T. Verbelen, L. Godinas; Leuven/BE
*(emanuele.muscogiuri@uzleuven.be)*

**Purpose:** Chronic Thromboembolic Pulmonary Hypertension (CTEPH) is a rare form of pulmonary hypertension, with pulmonary endarterectomy (PEA) being the treatment for suitable candidates. This study investigates the prognostic value of quantitative imaging biomarkers from Computed Tomography Pulmonary Angiography (CTPA) in patients undergoing PEA.
**Methods or Background:** We retrospectively enrolled CTEPH patients who underwent CTPA within 6 months pre-PEA, and right heart catheterization (RHC) pre- and post-PEA. CTPA scans were analyzed with a dedicated software (Aview, Coreline Soft, Inc) to extract BV5 and BV10 (representing total blood volume of pulmonary vessels with cross-sectional areas <5mm2 and <10mm2, respectively), also indexing them on total blood (TBV) and lung volume (respectively BV5/TBV, BV10/TBV; ρBV5, ρBV10). Significant associations ($p<0.05$) with RHC parameters, including their percentage change (Δ%), were evaluated using Spearman's rank correlation.
**Results or Findings:** Thirty-three patients (16 men, mean age 60.2 years) were included. Pre-operatively, BV5/TBV and BV10 negatively correlated with cardiac output (CO) and cardiac index (CI). Post-operative analysis revealed a negative correlation between BV5/TBV and the RHC parameters mean pulmonary artery pressure (mPAP), right atrial pressure (RAP), and pulmonary artery wedge pressure (PAWP), with r=-0.39, -0.43, -0.55 respectively. A negative correlation was also observed between ρBV5 and both post-operative pulmonary vascular resistance (PVR) and percentage change (Δ%) in PVR (r=-0.38 for both), highlighting the association between increased peripheral involvement and a reduced improvement of vascular resistance.
**Conclusion:** CTPA-derived quantitative biomarkers show that a lesser involvement of the peripheral vasculature results in a better post-PEA outcome. The extent of peripheral disease, quantified with CTPA, correlates with greater reductions in pulmonary pressures and vascular resistances, and improved cardiac function post-PEA, supporting the prognostic value of these quantitative imaging metrics.
**Limitations:** Small study population with possible selection bias.
**Funding for this study:** None
**Has your study been approved by an ethics committee?** Yes
**Ethics committee - additional information:** The study has been approved by the institutional IRB (S70797).
**Author Disclosures:**
Tom Verbelen: Nothing to disclose
Laurent Godinas: Nothing to disclose
Frederik De Keyzer: Nothing to disclose
Gitte Aerts: Nothing to disclose
Laura Hardy: Nothing to disclose
Emanuele Muscogiuri: Nothing to disclose
Walter De Wever: Nothing to disclose
Rozenn Quarck: Nothing to disclose
Marion Delcroix: Nothing to disclose

**Deep learning motion reduction algorithm: Its efficacy of improving image clarity in CT pulmonary angiogram**
*K. Z. Lim*, E. Yang, B. Jong, K. K-P. Lau; Melbourne/AU
*(teddylimkz@outlook.com)*

**Purpose:** Deep-learning motion correction algorithm (DLMCA) (CLEAR MOTION, Canon Medical Systems) works by creating a 4D motion vector-map from raw CT data to compensate for coronary artery movement during reconstruction and corrects motion artifacts without increasing radiation dose. Reducing motion/pulsation artifacts in CT pulmonary angiography (CTPA) is essential in evaluating pulmonary arteries (PA) and the adjacent ascending aorta (Ao) and aortic root (Ar). We aimed to investigate the efficacy of DLMCA in CTPA in image quality (IQ) improvement.
**Methods or Background:** All adult consecutive CTPAs over 3-month period in a specialist cardiac-lung hospital were included. Images of each CTPA were reconstructed with standard deep-learning image-reconstruction (SDLIM) and DLMCA. Two radiologists independently and randomly assessed the image quality using Likert 5-point scale of PA, Ao and Ar. Interobserver agreement with Kappa score was determined. Objective IQ using signal-to-noise (SNR) and contrast-to-noise ratio (CNR) were calculated. Quantitatively, the degrees of motion artifact (the width of double-shadow) at PA and Ao were assessed. Statistical analysis with paired t-test and Wilcoxon signed-rank test was undertaken (p-value <0.05 as significant).
**Results or Findings:** 100 CTPAs (44% male, mean age of 65.5 years) were included. There was substantial to excellent interobserver agreement with kappa-score of 0.73 for SDLIM and 0.81 for DLMCA. Significant improvement in subjective image clarity with all three structures and significant reduction of double shadow sign of ascending aorta (mean 1.9mm difference, $p<0.0001$) and pulmonary trunk (mean 1.2mm difference, $p<0.0001$) were achieved. However, mean SNR and CNR for both aorta and pulmonary trunk were reduced with DLMCA ($p<0.0001$).
**Conclusion:** DLMCA has benefit of reducing motion artifact of PA, Ao and Ar in CTPA, and therefore, improving image clarity, despite some degradation of SNR and CNR.
**Limitations:** Single centre retrospective study.
**Funding for this study:** None.
**Has your study been approved by an ethics committee?** Not applicable
**Author Disclosures:**
Eric Yang: Nothing to disclose
Kai Zheong Lim: Nothing to disclose
Kenneth Kwok-Pan Lau: Nothing to disclose
Beven Jong: Nothing to disclose

**08:00 - 09:00** **Research Stage 3**

### Research Presentation Session: Musculoskeletal

### RPS 1710

### Advanced CT imaging: PCCT, 4D CT, and metal artefacts

**Moderator**
Reto Sutter; Zurich/CH
**Author Disclosures:**
Reto Sutter: Other: Balgrist University Hospital has an academic research collaboration with Siemens Healthineers and Bayer.

**Assessment of Trabecular Microstructure using Photon-Counting CT: Influence of Dose, Spatial Resolution and Reconstruction Algorithm**
*J. A. Pena*[1], F. Thomsen[2], T. Damm[1], M. Frölich[3], S. O. Schönberg[3], C-C. Glüer[1], J-B. Hövener[1], M. Kachelrieß[4], S. Sawall[4]; [1]Kiel/DE, [2]Minden/DE, [3]Mannheim/DE, [4]Heidelberg/DE
*(jaime.pena@rad.uni-kiel.de)*

**Purpose:** To evaluate the feasibility and accuracy of photon-counting computed tomography (PCCT) for assessing trabecular microstructure in human vertebrae at clinically realistic dose levels, while investigating the effects of radiation dose, spatial resolution, and reconstruction method.

**Methods or Background:** Seven excised human vertebrae were imaged with a PCCT system (Naeotom Alpha) in ultra-high-resolution mode. To reproduce clinically relevant intersection lengths, the vertebrae were placed inside a semi-anthropomorphic thorax phantom and scanned at 120 kV and dose levels between 6-20 mGy (CTDI32cm). Additional scans were performed without the phantom for high-dose reference measurements. Data were reconstructed with filtered backprojection (FBP) and quantum iterative reconstruction (QIR3) using three kernels of increasing resolution (Br56, Br76, Br89) and 0.2 mm slice thickness. Bone mineral density (BMD), bone volume fraction (BV/TV), trabecular separation (Tb.Sp), and trabecular thickness (Tb.Th) were quantified within standardized volumes of interest. Agreement with high-dose reference scans was evaluated using Wilcoxon signed-rank tests, Bland-Altman analysis, and Lin's concordance correlation coefficient (rccc).
**Results or Findings:** BMD and BV/TV were comparable between FBP and QIR3 with no significant differences. Sharper kernels increased noise, particularly for FBP, which compromised microstructural accuracy. Br56 resolution was insufficient to resolve trabecular microstructure. Bland-Altman and rccc analyses showed Br76 with QIR3 achieving the best agreement to the high-dose reference. For Br76, rccc ranged between 0.66-0.94 for Tb.Sp across dose levels, compared with 0.05-0.58 for Br89. Similar trends were observed for other parameters.
**Conclusion:** PCCT enables reliable estimation of vertebral trabecular microstructure at diagnostic dose levels and clinically relevant intersection lengths. Br76 with QIR3 was the most robust setting across dose levels and offered a good balance between noise suppression and microstructural fidelity.
**Limitations:** Small sample size of cadaveric vertebrae.
**Funding for this study:** This study did not receive any funding.
**Has your study been approved by an ethics committee?** Not applicable
**Author Disclosures:**
Timo Damm: Nothing to disclose
Claus-Christian Glüer: Nothing to disclose
Stefan Oswald Schönberg: Nothing to disclose
Jan-Bernd Hövener: Nothing to disclose
Matthias Frölich: Nothing to disclose
Jaime Andres Pena: Nothing to disclose
Felix Thomsen: Nothing to disclose
Marc Kachelrieß: Nothing to disclose
Stefan Sawall: Nothing to disclose

### Gantry-based Cone-beam CT of the thoracolumbar spine: A phantom comparison with Photon-Counting CT and Energy-Integrating CT

U. Bach, *C. Obermüller*, D. Cester, F. Ensle; Zürich/CH
*(carina.obermueller@live.at)*

**Purpose:** This study aimed to evaluate the performance of a novel gantry-based, multi-scan CBCT system for spinal imaging with complete anatomic coverage and compare it to energy integrating (EI)CT and photon counting (PC)CT using dose-matched protocols.
**Methods or Background:** An anthropomorphic torso phantom was used to simulate human anatomy. Gantry-based CBCT scans of the thoracolumbar spine were performed using different presets (low-dose, enhanced, best quality), while EICT and PCCT scans followed dose-matched clinical protocols. Qualitative image analysis was assessed by three blinded readers using a 4-point Likert scale, and quantitative analysis was conducted using global noise level (GNL) measurements.
**Results or Findings:** CBCT achieved diagnostic-quality imaging for the thoracic and lumbar spine, particularly with "best" and "enhanced" presets. Subjective image quality was highest for PCCT, followed by EICT and CBCT. CBCT demonstrated lower GNL than EICT, nearing PCCT levels. However, high radiation doses (5 mGy) were required for CBCT imaging of the upper thoracic spine (Th1–Th6) due to anatomical complexity, while low doses (0,5 mGy) sufficed for the lower thoracolumbar spine (Th7–S1).
**Conclusion:** Gantry-based CBCT generated diagnostic-quality images of large spinal regions at relatively low radiation doses, although the upper thoracic spine (above Th6) required higher doses. The overall subjective image quality remained below EICT and PCCT.
**Limitations:** Limitations of this study include its reliance on a single-sized phantom, which does not fully capture the complexity and variability of human anatomy and physiology. Although the phantom imitates the diverse tissue composition of the human body, it lacks pathological conditions such as fractures, and the dynamic factors present in clinical settings, including motion artifacts.
**Funding for this study:** None
**Has your study been approved by an ethics committee?** Not applicable
**Author Disclosures:**
Davide Cester: Nothing to disclose
Falko Ensle: Nothing to disclose
Ulf Bach: Nothing to disclose
Carina Obermüller: Nothing to disclose

### Optimising CT metal artefact reduction: quantitative evaluation of acquisition and reconstruction strategies across nine orthopaedic alloys

*M. A. Spurek*, J. P. Janssen, S. Skornitzke, N. Große Hokamp; Cologne/DE

**Purpose:** CT evaluation in patients with orthopaedic implants is often complicated by metal artefacts. Although methods such as iterative reconstruction and orthopaedic metal artefact reduction (O-MAR) have shown promising results, evidence on interaction effects with alloy density, acquisition parameters, and with each other, remains limited. This study aimed to systematically assess both main and interaction effects on metal artefact reduction.
**Methods or Background:** Cylindrical rods of nine implant alloys were scanned using a CT7500 and IQon (Philips) at 100/120/140 kVp and 2/5/10 CTDIvol. Conventional and iterative model reconstructions (IMR) were generated with and without O-MAR. Artefacts were assessed across 20 cross-sections using an objective quantitative approach based on Fourier analysis. Mixed-effects regression was used to quantify the independent and combined effects of physical alloy density, kVp, CTDIvol, reconstruction method, and O-MAR on metal artefacts.
**Results or Findings:** Artefact levels increased with higher alloy density. O-MAR, IMR, higher kVp, and higher CTDIvol substantially reduced artefacts, with O-MAR exerting the strongest and IMR the second-strongest effect. The artefact-increasing effect of alloy density was particularly reduced by O-MAR and higher kVp, while the interaction effects with both IMR and CTDIvol were small. Combining O-MAR and IMR with higher kVp yielded a small additional artefact reduction, whereas the artefact-reducing effect of higher CTDIvol was diminished by the combined use of O-MAR and IMR.
**Conclusion:** On standard scanners, O-MAR is the most, and IMR the second-most, effective tool for reducing metal artefacts, and both should be routinely applied. Higher kVp provides additional benefit, particularly in dense alloys, whereas increasing CTDIvol yields little extra improvement and should not be prioritised considering patient radiation exposure. These findings provide practical guidance for optimising CT protocols in patients with orthopaedic implants.
**Limitations:** Results should be verified in vivo.
**Funding for this study:** No funding was received for this study.
**Has your study been approved by an ethics committee?** Not applicable
**Author Disclosures:**
Nils Große Hokamp: Speaker: Philips Healthcare Research/Grant Support: Philips Healthcare
Marvin A. Spurek: Nothing to disclose
Jan Paul Janssen: Nothing to disclose
Stephan Skornitzke: Employee: Philips Healthcare

### A preliminary study of automated segmentation and quantification of human knee trabecular bone in vivo using photon-counting CT

*K. Wen*[1], Y. Zhu[1], G. Fan[1], B. Zhang[1], Y. Ling[2], X. Liang[2], B. Li[1], H. Li[1], K. Zhang[1]; [1]Shenyang/CN, [2]Shanghai/CN
*(wenkcdr@163.com)*

**Purpose:** To explore the feasibility of evaluating knee trabecular microstructure in vivo using photon-counting CT (PCCT) with automated segmentation and quantitative analysis.
**Methods or Background:** Changes of knee trabecular microstructure are associated with the onset and progression of bone diseases such as osteoporosis and osteoarthritis. High-resolution peripheral quantitative CT (HR-pQCT) has been used to quantify trabecular microstructure in vivo, but is limited by a relatively small field of view. PCCT enables superior spatial resolution and dose efficiency for whole-body imaging. Seven volunteers (age range 30-69 years) were recruited in this preliminary study for PCCT scans (P10, Neusoft Medical Systems; 104µm @2%MTF and 120 kV) of both knees. Images were reconstructed with a 1024 matrix and slice thickness of 274 µm. Subchondral trabecular bone of femoral and tibial condyle was automatically segmented using a ResUNet-based framework. Trabecular parameters, including bone volume fraction (BV/TV), trabecular thickness (Tb.Th), trabecular separation (Tb.Sp), trabecular number (Tb.N), and synthetic images (SMI), were computed via the NeuSpace platform (Neusoft Medical Systems) and compared with values previously reported in cadaveric PCCT studies.
**Results or Findings:** The automated pipeline successfully segmented and quantified in vivo subchondral trabecular microstructure. Compared to cadaveric literature of knee PCCT, our in vivo cohort exhibited a 65% higher bone volume fraction (BV/TV: 48.90±8.74 % vs. 29.61±11.06 %), 74% thicker trabeculae (Tb.Th: 0.75±0.08 mm vs. 0.43±0.09 mm), and more plate-like structure (SMI: 0.68±0.26 vs. 1.64±0.74).
**Conclusion:** This study provides preliminary subchondral trabecular evidence in vivo using PCCT, and demonstrates the feasibility of automated segmentation and quantitative analysis with NeuSpace, suggesting potential applications for osteoporosis and osteoarthritis assessment.
**Limitations:** This is a preliminary study with a small sample size. Larger-scale studies are needed to confirm the clinical value.
**Funding for this study:** No funding was received for this study.

**Has your study been approved by an ethics committee?** Yes
**Ethics committee - additional information:** This study was approved by the Institutional Ethical Committee (2025QL004).
**Author Disclosures:**
Kaicheng Wen: Nothing to disclose
Bingsheng Li: Employee: Neusoft Medical Systems
Yuting Ling: Employee: Neusoft Medical Systems
Guoguang Fan: Nothing to disclose
Xiaoyun Liang: Employee: Neusoft Medical Systems
Kai Zhang: Employee: Neusoft Medical Systems
Yue Zhu: Nothing to disclose
Hechen Li: Employee: Neusoft Medical Systems
Bin Zhang: Employee: Neusoft Medical Systems

**Impact of motion artifacts on registration accuracy in dynamic CT images of the wrist joint: a multivendor phantom study**
*H. Vries*[1], B. van der Heijden[1], S. Hummelink[1], J. G. G. Dobbe[2], I. Sechopoulos[1], G. J. Streekstra[2]; [1]Nijmegen/NL, [2]Amsterdam/NL
*(hanne.vries@radboudumc.nl)*

**Purpose:** To assess how motion artifacts influence registration accuracy across acquisition and reconstruction protocols of different CT systems in 4D CT images of a rotating wrist phantom.
**Methods or Background:** A rotating wrist phantom with three 3D-printed bones (scaphoid, lunate, capitate) was scanned on four CT systems (two vendors; single- and dual-source). One static 3D scan and multiple axial 4D scans were acquired at different phantom rotation speeds, each lasting 10s. Single-source covered 0.5–3 cycles, and dual-source 1–6 cycles (one cycle represents radial–ulnar–radial; 0.5 cycle represents radial–ulnar). Dose dependence was evaluated on two systems (80 kV/40 mA and 120 kV/100 mA). Images were reconstructed in full and partial modes, segmented, and registered using point-to-image registration. Registration accuracy was calculated as translation and rotation errors of the scaphoid and capitate relative to the lunate, referenced to the static scan, and reported as median with interquartile range per wrist cycle.
**Results or Findings:** Registration accuracy was unaffected by dose. For one cycle, the errors of the best single-source system were 0.29(0.21–0.36) mm and 1.37(0.82–2.18) degrees for full and 0.21(0.16–0.25) mm and 0.70(0.52–0.95) degrees for partial reconstructions for the capitate; the dual-source system showed the highest accuracy (errors of 0.14(0.12 – 0.17) mm and 0.48(0.39 – 0.62) degrees). For all systems with partial reconstructions, the translation errors remained below the voxel size (0.28mm) for ≤one cycle per 10s.
**Conclusion:** Dynamic 4DCT of the wrist has shown promise, but the impact of motion artifacts on bone-to-bone measurements needs to be understood. Partial reconstructions kept registration errors below the pixel size for wrist rotations up to one cycle per 10s, while dual-source scans reached similar accuracy at higher speeds.
**Limitations:** Limited to a phantom with 3D-printed bones and not yet validated in clinical setting.
**Funding for this study:** Funding was provided by NWO
**Has your study been approved by an ethics committee?** Not applicable
**Author Disclosures:**
Hanne Vries: Nothing to disclose
Johannes G. G. Dobbe: Nothing to disclose
Ioannis Sechopoulos: Speaker: Siemens Healthcare New boost Research/Grant Support: Siemens Healthcare, Canon Medical, ScreenPoint Medical, Sectra Benelux, Volpara Healthcare, Lunit, iCAD.
Brigitte Van Der Heijden: Nothing to disclose
Geert J. Streekstra: Nothing to disclose
Stefan Hummelink: Nothing to disclose

**08:00 - 09:00** **Research Stage 4**

### Research Presentation Session: Neuro

### RPS 1711

### Parkinson's disease and amyotrophic lateral sclerosis (ALS): challenges and innovations

**Moderator**
Zoran Rumboldt; Rovinj-Rovigno/HR
*(puz3@yahoo.com)*

**Diagnostic Performance of Standard versus SWI- and Neuromelanin-Sensitive Imaging-Reconstructed Susceptibility Map–Weighted Imaging in Early Parkinson's Disease**
*S. Lee*, J. Youn, E. Y. Kim, B. Sohn; Seoul/KR

**Purpose:** Susceptibility map-weighted imaging (SMwI) provides a reliable marker for the loss of nigral hyperintensity in Parkinson's disease (PD), yet requires a dedicated high-resolution oblique-coronal acquisition. To improve feasibility and broader applicability, we investigated whether SMwI reconstructed from existing sequences—susceptibility-weighted imaging (SWI) and neuromelanin-sensitive imaging (NM)—could approximate the diagnostic performance of standard SMwI in differentiating early PD from disease controls (DC). Diagnostic performance was further evaluated with reference to 18F-FP-CIT PET as an independent standard.
**Methods or Background:** We retrospectively reviewed 187 early PD and 43 DC subjects imaged at Samsung Medical Center between 2021 and 2022. Source data for SMwI, SWI, and NM were acquired perpendicular to the midbrain, along the anterior commissure–posterior commissure plane, and perpendicular to the fourth ventricle, respectively, with resolutions of $0.5 \times 0.5 \times 1$ $mm^3$, $0.5 \times 0.5 \times 2$ $mm^3$, and $0.7 \times 0.7 \times 1.2$ $mm^3$. Standard SMwI, SWI-driven SMwI, and NM-driven SMwI were reconstructed in MATLAB. Two blinded neuroradiologists evaluated nigral hyperintensity, and diagnostic accuracy was assessed relative to both clinical diagnosis and PET.
**Results or Findings:** Standard SMwI demonstrated the highest accuracy against clinical diagnosis, significantly outperforming reconstructed alternatives, with the largest disparity observed for NM-driven SMwI. In both lesion- and patient-level analyses referenced to PET, standard SMwI consistently exceeded NM-driven SMwI, whereas its performance was not statistically distinguishable from SWI-driven reconstructions. These differences appear attributable to variations in spatial resolution, slice thickness, and acquisition geometry.
**Conclusion:** Standard SMwI outperformed reconstructed methods against clinical diagnosis, but SWI-driven SMwI showed comparable accuracy to the standard with PET, indicating its potential as a practical alternative when standard acquisition is not feasible.
**Limitations:** Because the compared methods varied simultaneously in in-plane resolution, slice thickness, and acquisition plane, the independent contribution of each factor could not be isolated.
**Funding for this study:** No funding was received for this study.
**Has your study been approved by an ethics committee?** Yes
**Ethics committee - additional information:** This study was approved by the Institutional Review Board of Samsung Medical Center.
**Author Disclosures:**
Beomseok Sohn: Nothing to disclose
Jinyoung Youn: Nothing to disclose
Seon Lee: Nothing to disclose
Eung Yeop Kim: Nothing to disclose

**Substantia Nigra and Neuromelanin Volume Changes in Association with Motor Symptoms in Parkinson's Disease Using Semi-Automated Neuromelanin Quantification**
*A. Silovs*, N. Zdanovskis, G. K. Skuburs, S. Umbrasko, A. Sevcenko, J. Mednieks, A. Platkājis, J. Skilters, S. Bartusevica; Riga/LV
*(arturshilov@gmail.com)*

**Purpose:** Parkinson's disease (PD) is characterised by progressive dopaminergic neuronal loss in the substantia nigra pars compacta. Neuromelanin-sensitive MRI (NM-MRI) allows in vivo assessment of this degeneration. We aimed to evaluate substantia nigra neuromelanin volume changes in PD patients and their association with motor symptoms using semi-automated volumetric analysis.

**Methods or Background:** This prospective case-control study enrolled 23 PD patients, mean age 64.9 ± 12.3 years; 23 controls, mean age 50.8 ± 11.1 years. High-resolution 3T NM-MRI was acquired using a T1-weighted spin-echo sequence. Semi-automated segmentation (Mango v3.5.1) with intracranial volume correction (FreeSurfer 7.3) was performed. Clinical severity was assessed with the Unified Parkinson's Disease Rating Scale (UPDRS) and Hoehn & Yahr (H&Y) staging.
**Results or Findings:** Corrected substantia nigra volume was significantly reduced in PD patients (0.0276 ± 0.0058 $mm^3$) compared with controls (0.0337 ± 0.0102 $mm^3$), representing an 18% reduction (p = 0.014, t-test; p = 0.039, Mann–Whitney U). ROC analysis demonstrated moderate diagnostic performance (AUC = 0.700; sensitivity = 68.4%; specificity = 74.1%). Progressive reduction was observed across disease stages: H&Y stage 1 (0.0285 ± 0.0054 $mm^3$) and stage 2 (0.0279 ± 0.0064 $mm^3$), both significantly lower than controls ($p < 0.05$). No significant correlation was found between corrected volumes and UPDRS-III motor scores. Semi-automated segmentation showed excellent agreement with manual measurements (ICC = 0.945).
**Conclusion:** Semi-automated NM-MRI reliably detects substantia nigra volume loss in early-stage PD with moderate diagnostic accuracy. While not strongly correlated with UPDRS-III, corrected NM volume reflects disease staging and may serve as a supportive biomarker for PD diagnosis and monitoring.
**Limitations:** The main limitation is age mismatch between groups (mean difference 14 years), representing a potential confounder. Additional limitations include modest sample size and cross-sectional design.
**Funding for this study:** This research was funded by the Latvian Council of Science, project No. lzp-2022/1-0100
**Has your study been approved by an ethics committee?** Yes
**Ethics committee - additional information:** The study was conducted in accordance with the Declaration of Helsinki, and approved by the Institutional Ethics Committee of Paul Stradins University Hospital protocol code Nr 240423 - 211 24.04.2023
**Author Disclosures:**
Ardis Platkājis: Nothing to disclose
Gvido Karlis Skuburs: Nothing to disclose
Arturs Silovs: Nothing to disclose
Janis Mednieks: Nothing to disclose
Solvita Umbrasko: Nothing to disclose
Aleksejs Sevcenko: Nothing to disclose
Nauris Zdanovskis: Nothing to disclose
Santa Bartusevica: Nothing to disclose
Jurgis Skilters: Nothing to disclose

### Resting-State brain connectivity in Parkinson's disease: a randomized controlled trial following the Quadrato Motor Training

*F. De Rosa*[1], R. Franciosi[2], D. Vo[1], G. Greco[1], M. Fusina[1], C. C. Quattrocchi[1]; [1]Trento/IT, [2]Rovereto/IT

**Purpose:** To evaluate the effects of Quadrato Motor Training (QMT) on resting-state FC in PD patients.
**Methods or Background:** Parkinson's disease (PD) is characterized by motor and cognitive impairments, associated with alterations in functional connectivity (FC) across brain networks. Motor interventions with cognitive engagement may foster neuroplasticity and compensatory mechanisms.
**Results or Findings:** Out of 50 randomized PD patients, 48 completed the trial (QMT n=23, control n=25). Independent component analysis identified 11 major resting-state networks (RSNs). Control group showed significant FC decrease (p<0.01 FDR-corrected) across multiple RSNs including default mode (Tmax=5.03, L frontal pole), dorsal attention (Tmax=5.03, R occipital cortex), executive control (Tmax=3.72, paracingulate gyrus), sensorimotor (Tmax=4.80, precuneus; Tmax=4.38, R postcentral gyrus) and cerebellar networks (Tmax=4.89, L Crus II). QMT group exhibited increased FC in the right frontoparietal network (T=3.99, R angular gyrus) and in the sensorimotor II network (T=3.62, R postcentral gyrus), with no significant reductions in any RSN. Between-group comparisons (ΔFC): QMT preserved connectivity in the left postcentral gyrus (T=4.41), supplementary motor area (T=3.72), and cerebellar Crus II (T=3.83), while controls showed significant decline.
**Conclusion:** By combining motor and cognitive stimulation,QMT may prevent FC decline and enhance connectivity within key motor and cognitive networks in PD. Brain functional connectivity measures are suitable to measure the effect of rehabilitation strategies in PD.
**Limitations:** The study was limited to 4 weeks; pre- and post-intervention assessments of motor and cognitive functions (beyond fMRI) were not included; control groups: only a sham motor control was used, without an active cognitive control group.
**Funding for this study:** Brain functional connectivity (FC) emerged as a sensitive marker to capture the neural effects of rehabilitation strategies in Parkinson's disease (PD). Resting-state fMRI revealed that controls (sham exercise) exhibited a widespread decline in FC across default mode, executive, dorsal attention, sensorimotor, and cerebellar networks, confirming the progressive disconnection typical of PD. QMT group: showed preserved or increased FC, with significant gains in the right frontoparietal and sensorimotor networks. Between-group comparison: highlighted that QMT protected connectivity in regions critical for motor control and compensation, including the supplementary motor area and cerebellar Crus II, which were selectively impaired in controls. These findings demonstrate that resting-state FC is not only suitable but highly informative to assess the impact of motor-cognitive rehabilitation in PD. The ability of QMT to enhance or stabilize FC in key brain networks supports its potential role as a neuroplastic and neuroprotective intervention, measurable with functional imaging biomarkers.
**Has your study been approved by an ethics committee?** Not applicable
**Author Disclosures:**
Flora De Rosa: Nothing to disclose
Rossana Franciosi: Nothing to disclose
Giuseppe Greco: Nothing to disclose
Daniele Vo: Nothing to disclose
Carlo Cosimo Quattrocchi: Nothing to disclose
Marianna Fusina: Nothing to disclose

### Five-Year MRI Visibility of MRgFUS Thalamotomy in Essential Tremor and Parkinson's Disease

*G. Di Cerbo*, G. Saltarelli, A. Innocenzi, C. De Felici, S. Di Terlizzi, A. Catalucci, F. Bruno, E. Di Cesare, A. Splendiani; L'Aquila/IT
*(giovannidicerbo96@gmail.com)*

**Purpose:** To evaluate long-term clinical and radiological outcomes of unilateral MR-guided focused ultrasound (MRgFUS) Vim thalamotomy for essential tremor over five years, assessing durability of tremor control and lesion evolution on MRI.
**Methods or Background:** Retrospective single-centre cohort of 28 right-handed, medication-refractory essential tremor patients who underwent unilateral (left) Vim MRgFUS thalamotomy between February 2018 and December 2019 (San Salvatore Hospital, L'Aquila). Clinical evaluations used the Fahn–Tolosa–Marin Tremor Rating Scale (FTM-TRS) at baseline, 24 h, 1 month, 1, 2, and 5 years. Brain MRI (T1, T2, FLAIR, DWI, SWI, DTI) was acquired at the same time points. Lesion presence and volume were assessed on all sequences, and whole-brain volumetry was performed with dedicated software. DTI tractography reconstructed the dentato-rubro-thalamic tract (DRTT) in the treated hemisphere using ROIs in the dentate nucleus, red nucleus, and Vim.
**Results or Findings:** Tremor severity improved and remained stable (FTM-TRS 5.1 ± 0.79 baseline vs 1.3 ± 0.61 at 5 years; −74.5%). Recurrence occurred in 2 patients. No adverse events at 5-year follow-up. MRI showed progressive lesion shrinkage. At 5 years, lesions were visible in 56% on fluid-sensitive sequences, 73% on T1-weighted, and 100% on SWI. No lesion was visible on DWI after 6 months. Only the treated thalamus showed significant long-term volume reduction (7.36 ± 0.79 mL at 1 year vs 7.15 ± 0.71 mL at 5 years; p = 0.001).
**Conclusion:** MRgFUS thalamotomy provides sustained tremor control with a favorable safety profile and durable imaging signatures of thalamic ablation.
**Limitations:** The main limitation is the small sample size, which may reduce statistical power.
**Funding for this study:** This research did not receive any specific grant from funding agencies in the public, commercial, or not-for-profit sectors.
**Has your study been approved by an ethics committee?** Not applicable
**Author Disclosures:**
Alessandra Splendiani: Nothing to disclose
Gaspare Saltarelli: Nothing to disclose
Antonio Innocenzi: Nothing to disclose
Ernesto Di Cesare: Nothing to disclose
Sandro Di Terlizzi: Nothing to disclose
Giovanni Di Cerbo: Nothing to disclose
Claudia De Felici: Nothing to disclose
Alessia Catalucci: Nothing to disclose
Federico Bruno: Nothing to disclose

### Neuromelanin-sensitive MRI for locus coeruleus imaging in parkinson's disease and multiple system atrophy

*Y. Yang*, H. Lyu, Y. Lu; Shanghai/CN
*(moutonlimpide@outlook.com)*

**Purpose:** The locus coeruleus (LC), brain's principal noradrenergic nucleus, contributes to cognitive and non-motor symptoms in Parkinson's disease (PD), yet its role in multiple system atrophy (MSA) and its subtypes—cerebellar (MSA-C) and parkinsonian (MSA-P)—remains unclear. This study quantified LC relative contrast ratios (rCR) on neuromelanin-sensitive MRI (NM-MRI), compared LC integrity among PD, MSA subtypes, and healthy controls (HC), and examined associations with motor, autonomic, cognitive, and sleep functions.

**Methods or Background:** A total of 147 participants (42 PD, 82 MSA, 23 HC) underwent 2D NM-MRI. LC signal intensity was normalized to pontine white matter (rCR-LC-Pons). Group differences were assessed using Kruskal–Wallis with Bonferroni correction; clinical associations were examined via Spearman correlations and age-, sex-, and education-adjusted GLMs. Receiver operating characteristic (ROC) analyses evaluated the diagnostic performance of rCR-LC-Pons across groups.
**Results or Findings:** rCR-LC-Pons showed strong group differences ($p < 0.001$), indicating a progressive LC signal decline from HC to PD to MSA and MSA subtypes. rCR-LC-Pons correlated negatively with disease duration in both PD and MSA, reflecting ongoing neuro degeneration. Adjusted models showed rCR-LC-Pons 5.44 units lower in MSA than PD at matched duration, independent of age or sex. Diagnostic accuracy was high for distinguishing MSA from HC (AUC = 0.93) and PD from HC (AUC = 0.82). In PD, LC integrity predicted cognitive performance (MoCA: $\beta = 0.46$, $p < 0.01$; $R^2 \approx 0.20$), supporting its value as a marker of cognitive vulnerability and disease stratification.
**Conclusion:** NM-MRI reliably detects LC degeneration across PD and MSA spectra. Pons-referenced normalization provides superior discrimination, and LC integrity partly explains cognitive variance in PD, supporting its utility as a clinical imaging biomarker.
**Limitations:** Cross-sectional design and modest model fits limit causal inference; longitudinal and multimodal validation is needed.
**Funding for this study:** This work was supported by Shanghai Jiao Tong University Trans-med Awards Research (YG2023LC02), Shanghai Science and Technology Commission of China (SHDC2022CRD017)
**Has your study been approved by an ethics committee?** Yes
**Ethics committee - additional information:** Our study has been approved by the ethics committee of Shanghai Jiao Tong University Medical School Affiliated Ruijin Hospital
**Author Disclosures:**
Yong Lu: Nothing to disclose
Yiqing Yang: Nothing to disclose
Haiying Lyu: Nothing to disclose

**Cortical susceptibility mapping in Parkinson's Disease reveals spatial iron accumulation and its molecular basis**
*J. Qin*, M. Zhang; Hangzhou/CN
*(12118270@zju.edu.cn)*

**Purpose:** Parkinson's disease (PD) is increasingly recognized to involve cortical regions. Quantitative susceptibility mapping (QSM), a marker of iron accumulation, may sensitively capture such changes. We sought to characterize whole-brain susceptibility alterations in PD and evaluate their structural and molecular correlates.
**Methods or Background:** We studied 355 PD patients and 206 healthy controls with 3T MRI, including QSM and T1-weighted imaging. Cortical QSM values were extracted using Desikan–Killiany parcellation with multiscale vessel filter to minimize venous contamination, while cortical thickness and subcortical volumes were derived from FreeSurfer. Group comparisons used general linear models adjusting for age, sex, and intracranial volume. Spatial correlations were tested against ENIGMA PD cortical atrophy maps (3092 PD patients and 1262 healthy controls), intrinsic functional networks, cytoarchitectonic classes, and neurotransmitter receptor distributions, with spin-test spatial null models. Regional gene expression from the Allen Human Brain Atlas was analyzed using partial least squares (PLS), followed by gene set enrichment analysis to identify underlying biological pathways.
**Results or Findings:** QSM revealed widespread cortical and subcortical susceptibility increases, already evident in early-stage PD, while morphometric measures showed limited changes. Cortical QSM abnormalities show strong spatially overlapped with ENIGMA PD atrophy patterns, and were centered in frontoparietal and association cortices. This susceptibility abnormalities pattern correlated with serotonin (5-HT6, 5-HT2A), dopamine transporters, and cholinergic systems. PLS analysis revealed gene expression profiles associated with cortical QSM alterations, highlighting pathways related to mitochondrial dysfunction, impaired synaptic signaling, and immune–glial processes.
**Conclusion:** QSM detects early cortical iron-related abnormalities in PD, aligning with large-scale atrophy patterns and linking to neurotransmitter and molecular pathways. These findings support cortical susceptibility as a potential biomarker and implicate neuronal vulnerability in disease mechanisms.
**Limitations:** Longitudinal imaging and multi-cohort replication will be needed to validate and extend these findings.
**Funding for this study:** This work was supported by the National Natural Science Foundation of China (Grant Nos. 82271935, 82171888, 82202091, 82001767 and 82302132), the China Postdoctoral Science Foundation (Grant Nos. 2023M733085), and the 13th Five-year Plan for National Key Research and Development Program of China (Grant No. 2016YFC1306600).
**Has your study been approved by an ethics committee?** Yes
**Ethics committee - additional information:** This research was conducted in accordance with the ethical standards of the Declaration of Helsinki and was approved by the Ethics Committee of The Second Affiliated Hospital of Zhejiang University School of Medicine.
**Author Disclosures:**
Minming Zhang: Nothing to disclose
Jianmei Qin: Nothing to disclose

**Microstructure and gene expression influence gyrification in Amyotrophic Lateral Sclerosis**
*X. Yi*[1], B. T. Chen[2]; [1]Chongqing/CN, [2]Duarte, CA/US
*(doctoryixiaoping@126.com)*

**Purpose:** Beyond well-established gray and white matter pathology, alterations in cortical gyrification have recently been observed in Amyotrophic lateral sclerosis (ALS) , yet their clinical relevance and molecular underpinnings remain to be understood. We aimed to perform an in-depth investigation of the cortical gyrification changes and their microstructural and transcriptional correlates in symptomatic patients with ALS.
**Methods or Background:** Here, we investigated this premise by examining its microstructural and transcriptional basis in 60 patients with ALS (median age = 55, range = 25-72 years) and 60 matched controls (median age = 56, range = 27-72 years) using structural and diffusion MRI.
**Results or Findings:** Patients exhibited a significant reduction in local gyrification index (LGI) within bilateral precentral and postcentral gyri, left middle frontal gyrus, and left superior parietal lobule. This was accompanied by reduced fractional anisotropy (FA) in the white matter tracts, primarily involving the corticospinal tract and corpus callosum. Higher LGI and FA values were associated with better motor function as measured by the ALS Functional Rating Scale-Revised, and LGI also showed positive associations with global cognitive status. A mediation analysis indicated that FA partially accounted for the relationship between LGI and functional disability, suggesting that disrupted white matter pathways contribute to the clinical impact of gyrification changes. Regions of reduced LGI showed spatial convergence with cortical expression of ALS-related genes such as TARDBP and C9orf72, enriched for biological processes related to protein aggregation, axon guidance, and synaptic signaling.
**Conclusion:** These findings suggest that cortical gyrification abnormalities in ALS are closely linked to white matter degeneration, functional impairment, and genetic vulnerability, thereby offering an integrative window into the multiscale pathology of ALS.
**Limitations:** The sample size was relatively small, and this was a cross-sectional study.
**Funding for this study:** None
**Has your study been approved by an ethics committee?** Yes
**Ethics committee - additional information:** The study was approved by the Ethics Committee and the Expert Committee of Xiangya Hospital, Central South University , and written informed consent was obtained from all participants.
**Author Disclosures:**
Bihong T. Chen: Nothing to disclose
Xiaoping Yi: Nothing to disclose

**08:00 - 09:00** **Room G1**

### Research Presentation Session: Radiographers

### RPS 1714
### Advances in women's health imaging

**Moderators**
Rahel Kubik-Huch; Baden/CH
*(kubik@hispeed.ch)*
Lyanne Molenaar; Amsterdam/NL
*(l.molenaar@nki.nl)*

### Understanding Mammography Care for People with Dementia-Related Cognitive Disorders

*S. Frei*, M. Champendal, R. Freund, C. S. D. Reis; Lausanne/CH
*(w4women@outlook.com)*

**Purpose:** The care of individuals with cognitive impairment related to dementia (CI) in the context of mammography remains insufficiently documented, despite its potential impact on examination quality. This study aimed to identify facilitators and barriers influencing the performance of mammography examinations in this context.
**Methods or Background:** A cross-sectional design was applied using a questionnaire developed in French, translated into German, and disseminated via professional radiography bodies. The survey covered four domains: sociodemographic characteristics, training, professional practices, structural environment. Data were analysed using descriptive and differential statistics (test exact de Fisher), supplemented by qualitative thematic analysis for open-ended questions. As no sensitive data were collected, ethics approval was not required.
**Results or Findings:** A total of 243 radiographers (233 females,10 males) responded. Only 2.8%(n=7) reported having received specific training on dementia-related care, while 82.3%(n=200) expressed a need for such preparation. Formal procedures were rare (1.6%,n=4), with 17.6%(n=43) of participants indicating adaptations as allocation of extra time to perform the examination. Implementation of facilitators varied according to experience and education but the presence of caregivers was highlighted. Structural limitations were also evident: over half of respondents (51.8%,n=126) reported working in departments with inadequate floors, raising concerns about accessibility and mobility of this population. Statistical significant differences ($p<0.05$) were identified according to the type of institution.
**Conclusion:** Findings demonstrate that recommended strategies to adapt mammography examinations for people with CI are inconsistently applied within practice. The absence of training, protocols, and supportive environments represents a barrier to provide adequate care. Targeted professional development, explicit procedural guidelines, and organisational adjustments are required to optimise patient management and ensure examination quality.
**Limitations:** Conducted in several languages
**Funding for this study:** Not applicable
**Has your study been approved by an ethics committee?** Not applicable
**Author Disclosures:**
Claudia Sa Dos Reis: Nothing to disclose
Romain Freund: Nothing to disclose
Stephanie Frei: Nothing to disclose
Mélanie Champendal: Nothing to disclose

### Assessing image acquisition in Contrast-enhanced mammography: dosimetric, temporal and qualitative evaluation

F. E. El Archa, *E. Di Paola*, V. Magni, S. Marziali, M. Zanardo, F. Sardanelli; Milan/IT
*(eleonora.dipaola@grupposandonato.it)*

**Purpose:** Since technical variability in contrast-enhanced mammography (CEM) may compromise diagnostic performance, the aim of this study was to retrospectively evaluate adherence to acquisition protocols and image quality.
**Methods or Background:** A retrospective analysis was conducted on 122 CEM examinations performed at the Radiology department of IRCCS Policlinico San Donato between January 2019 and August 2020, using a dedicated digital mammography system (Senographe Pristina, GE Healthcare). Three domains were assessed: dosimetric parameters (kVp, mAs, compressed breast thickness); temporal data (acquisition times, overall examination duration); and qualitative criteria derived from the PGMI system, including pectoral muscle visualisation, nipple profile alignment, posterior nipple line (PNL) consistency, inclusion of the inframammary fold, pectoral muscle shape, and the presence of artefacts or excluded tissue. Descriptive statistics were applied.
**Results or Findings:** Median kVp was 34 (IQR 34–34), median mAs 40.6 (IQR 32.3–49.1), and mean compressed breast thickness 5.5 ± 1.3 cm. The recommended 10-minute post-contrast window was consistently respected, with a mean examination duration of 1:22 minutes (maximum 4:13). Qualitative analysis showed frequent deviations from positioning standards. The pectoral muscle was absent in 106/122 (87%) CC-right views and in 103/122 (84%) CC-left views. Nipple profile was inadequate in 22/122 (18%) CC views and 43/122 (35%) MLO views. PNL discrepancies >1 cm occurred in 81% patients. The inframammary fold was included in 101/122 (83%) MLO-right views and in 99/122 (81%) MLO-left views, but perfectly visualised in only 3/122 (2%) and 5/122 (4%) cases, respectively. Exclusion of glandular tissue occurred in 115/122 (94%) cases.
**Conclusion:** CEM examinations demonstrated marked heterogeneity in image acquisition, with frequent failure to meet positioning criteria. These findings highlight the urgent need for standardised acquisition protocols and quality assurance frameworks to optimise clinical implementation of CEM.
**Limitations:** Single-centre retrospective study.
**Funding for this study:** No funding was provided for this study.
**Has your study been approved by an ethics committee?** Not applicable
**Author Disclosures:**
Sara Marziali: Nothing to disclose
Francesco Sardanelli: Nothing to disclose
Fatima Ezzahrae El Archa: Nothing to disclose
Eleonora Di Paola: Nothing to disclose
Moreno Zanardo: Nothing to disclose
Veronica Magni: Nothing to disclose

### Breast screening attendance amongst culturally and linguistically diverse women in Ireland

C. Power, M. F. Mcentee, *C. Rainey*, J. ONeill, L. McLaughlin, P. C. Murphy, A. England; Cork/IE
*(clare.rainey@ucc.ie)*

**Purpose:** To identify the gap in breast screening services in Ireland for this population of women, promoting inclusiveness in breast cancer services.
**Methods or Background:** Cultural and linguistic diversity is increasing worldwide with a significant increase in migration. Access of breast cancer services for cultural and linguistically diverse women in Ireland may impose challenges, due to many factors including lack of education, language, social and cultural factors. Breast cancer is one of the most common cancers diagnosed in women worldwide, however screening rates vary amongst populations with cultural and linguistically diverse women having lower screening rates when compared with the general population of women. Using a qualitative approach, this study aims to explore the personal perspectives of cultural and linguistically diverse women in accessing breast cancer services in Ireland, through 1:1 face-to-face online interviews.
**Results or Findings:** Five themes and two subthemes emerged throughout discussion which included, barriers and facilitators to accessing care, reflection on previous experience in home country, knowledge and awareness of breast cancer/breast health, knowledge and awareness of screening/health care provision in Ireland for breast cancer and breast health, and recommendations/ healthcare strategies to improve services for the culturally and linguistically diverse population.
**Conclusion:** Culturally and linguistically diverse women's access to breast cancer screening and services in Ireland is influenced by a variety of factors, some favourable and others unfavourable. Raising education and awareness for culturally and linguistically diverse women around breast health and breast cancer services may lead to increased screening rates and improved outcomes for these women.
**Limitations:** This study included only those who were proficient in English, thus might not be representative of all seeking asylum.
**Funding for this study:** None
**Has your study been approved by an ethics committee?** Yes
**Ethics committee - additional information:** University College Cork (UCC) Medical School Social Research Ethics Committee (SREC) on 7th of November 2024.
**Author Disclosures:**
John O'Neill: Nothing to disclose
Mark F. Mcentee: Board Member: EFRS
Clare Rainey: Nothing to disclose
Laura Mclaughlin: Nothing to disclose
Andrew England: Nothing to disclose
Peter C. Murphy: Nothing to disclose
Courtney Power: Nothing to disclose

**AI in action: a European radiography survey on Endometriosis diagnostic protocols**

*M. Haber*, M. Montebello, K. Borg Grima; Msida/MT
*(martinahaber90@gmail.com)*

**Purpose:** Endometriosis is a chronic, gynaecological condition that affects approximately 10% of females worldwide, with an average diagnostic delay of 6-12 years. While, AI shows promise in aiding diagnosis, a gap exists regarding its practical applications and the radiography professionals' perceptions on its use. The study aims to compare imaging protocols for endometriosis used within Europe in MRI and gynaecological ultrasound. It will assess radiographers' knowledge and awareness of AI-based diagnostic tools.
**Methods or Background:** This study comprises a phase of an ongoing PhD study. An online survey will be distributed through the European Federation of Radiographer Societies (EFRS). The survey is targeted at radiographers working in MRI or gynaecological ultrasound. Its objective is to gather insights into the current medical imaging procedures and protocols used in diagnosing Endometriosis, while collecting information on the awareness and use of AI-based tools in clinical practice.
**Results or Findings:** A systematic review of 116 articles conducted in February 2025 highlighted a need for further research on the use of AI in the diagnosis of Endometriosis. The results of this survey will reveal region-specific practices, variations in imaging protocols, and the perceived value for the adoption of AI-based diagnostic tools within the radiography community.
**Conclusion:** The findings from this survey will provide an overview of radiography practices and the perceived utility of AI in Endometriosis diagnosis. The results could inform the standardisation of imaging protocols used in MRI and gynaecological ultrasound to diagnose this pathology. Data collected can provide information to professional bodies and educational institutions on the integration of AI-based tools.
**Limitations:** Limitations of the study relate to the use of an online survey which may limit responses while introducing a response bias, since self-reported data reflects participants' perceptions and practices.
**Funding for this study:** No funding was received for this study.
**Has your study been approved by an ethics committee?** Yes
**Ethics committee - additional information:** The study was approved by the University of Malta Research Ethics committee (reference number: FHS-2025-00146).
**Author Disclosures:**
Karen Borg Grima: Nothing to disclose
Matthew Montebello: Nothing to disclose
Martina Haber: Nothing to disclose

**Evaluation of Postpartum Abdominal Diastasis in Vaginal Births vs. Cesarean Sections Using Ultrasound**

*M. M. Silva*[1], A. F. C. L. Abrantes[2], L. P. V. Ribeiro[3], S. I. Rodrigues[1], R. P. P. Almeida[4], R. Van Hauwaert[4], A. Onofre de Carvalho[1], R. Lopes[5], S. Tavares[5]; [1]Faro/PT, [2]Évora/PT, [3]Lagoa/PT, [4]São Brás de Alportel/PT, [5]Lisboa/PT
*(magdamsilva17@gmail.com)*

**Purpose:** This study aims to assess the prevalence of rectus abdominis diastasis in nulliparous postpartum women, identifying associated factors and differences between vaginal delivery and cesarean section, reinforcing the role of ultrasound as a diagnostic and monitoring method.
**Methods or Background:** A quantitative descriptive correlational design, was applied to a convenience sample of 50 women (25 vaginal, 25 cesarean), aged 18–40 years, with 37–42 weeks of gestation. Diastasis was assessed by ultrasound at three standardized points: ½ supraumbilical (>1.5 cm), ¼ supraumbilical (>2.6 cm), and ½ infraumbilical (>1.8 cm). Total diastasis was defined when all three points were positive, and classified as severe if any exceeded 2.5 cm.
**Results or Findings:** No statistically significant associations were observed between the type of delivery or the percentile of the newborn and abdominal diastasis, although a moderate negative correlation was observed between higher percentiles and diastasis in the supraumbilical region in eutocic deliveries (rs=-0.45, p=0.02). Pre-delivery body mass index (BMI) was found to be a significant variable, showing a moderate negative correlation with supraumbilical diastasis (rs=-0.390, p=0.01), especially in eutocic deliveries. Pre-pregnancy physical activity did not show significant influence. Diastasis prevalence was highest in supra- and infraumbilical regions, with lower incidence at the umbilicus.
**Conclusion:** Postpartum abdominal diastasis was common, especially supra- and infraumbilically. Pre-pregnancy BMI emerged as the main factor influencing its extent, while delivery type, neonatal percentile, and exercise showed limited impact. These findings highlight theimportance of standardized ultrasound protocols for early detection, follow-up, and guidance of preventive and therapeutic strategies in abdominal wall recovery after childbirth.
**Limitations:** The sample size.
**Funding for this study:** I did not receive any funding for this research.
**Has your study been approved by an ethics committee?** Yes
**Ethics committee - additional information:** In accordance with ethical standards, this study obtained ensured privacy, and received approval from the institutional ethics committee (CEUAlg Pn° 90 /2024).
**Author Disclosures:**
Roxane Van Hauwaert: Nothing to disclose
António Fernando Caldeira Lagem Abrantes: Nothing to disclose
Rui Pedro Pereira Almeida: Nothing to disclose
Sónia Isabel Rodrigues: Nothing to disclose
Magda Miranda Silva: Nothing to disclose
Sérgio Tavares: Nothing to disclose
Ana Onofre De Carvalho: Nothing to disclose
Revelino Lopes: Nothing to disclose
Luís Pedro Vieira Ribeiro: Nothing to disclose

**Improving Early Pregnancy Outcomes: Predictive Tools for Threatened Miscarriage**

*L. Sammut*; Msida/MT
*(lara.sammut@um.edu.mt)*

**Purpose:** To investigate the prevalence, risk factors, and predictive markers of threatened miscarriage (TM) through a structured three-part research design in a national state hospital.
**Methods or Background:** The study comprised three components: - A retrospective cohort of 711 women presenting with first-trimester bleeding. - A scoping review of 128 studies evaluating ultrasound and biochemical predictors. - A prospective case-control study of 118 TM cases and 59 controls, using ultrasound, biochemical, clinical, and demographic data, analysed through logistic regression and random forest modelling.
**Results or Findings:** - Retrospective cohort: 33.9% of TM cases progressed to live births beyond 22 weeks. Advanced maternal age (≥35 years) increased miscarriage risk. Neonates following TM had lower birthweights (female: 3008g vs 3239g; male: 3085g vs 3346g, P < 0.001) and shorter gestational ages (female: 38.1 vs 39.0 weeks; male: 38.0 vs 38.6 weeks, P < 0.05). - Scoping review: Intrauterine haematoma, yolk sac abnormalities, and fetal heart rate deviations emerged as strong ultrasound predictors. Combined with biochemical markers (e.g., beta-hCG), predictive value improved, but heterogeneity limited generalisability. - Prospective case-control: Key predictors included progesterone, trophoblast thickness, mean gestational sac diameter, cervical length, sFlt-1:PlGF ratio, and maternal age. Logistic regression and random forest models performed strongly (AUC 0.85 and 0.968; accuracies 82.7% and 93.1%).
**Conclusion:** TM significantly affects perinatal outcomes. Integrated ultrasound, biochemical, and clinical markers can reliably predict miscarriage risk. These findings support the development of structured early pregnancy pathways to improve care delivery.
**Limitations:** Single-centre recruitment, and limited sample sizes for some analyses may affect generalisability.
**Funding for this study:** None
**Has your study been approved by an ethics committee?** Yes
**Ethics committee - additional information:** Ethical approval was obtained from the hospital's Data Protection Officer (DPO) and Chief Executive Officer (CEO) and the University of Malta Research Ethics Committee (UREC).
**Author Disclosures:**
Lara Sammut: Nothing to disclose

**Radiofrequency Echographic Multispectrometry (REMS) as a Tool for Osteoporosis Screening in Primary Care**

*R. Santos*, A. I. Saraiva Vieira; Coimbra/PT
*(rutemartinssantos@gmail.com)*

**Purpose:** Radiofrequency echographic multispectrometry (REMS) has emerged as a promising, non-ionising, portable, and accessible alternative for osteoporosis diagnosis, with potential for use in primary healthcare. This study aimed to evaluate the effectiveness of REMS in detecting osteoporosis in this setting.
**Methods or Background:** Bone mineral density (BMD) was assessed in 86 participants through 172 REMS scans of the lumbar spine and femur, conducted in two Portuguese primary healthcare units (Guarda region).
**Results or Findings:** In the lumbar spine, 51.2% of participants were classified with osteopenia and 31.4% with osteoporosis. In the femur, 43.0% had osteopenia and 34.9% osteoporosis. These findings reveal a high prevalence of bone fragility. REMS-derived BMD showed good concordance with clinical diagnosis, supporting its reliability for early detection.
**Conclusion:** REMS-based bone densitometry, performed by radiographers in primary healthcare, represents a feasible and innovative alternative for the early and effective detection of osteoporosis and osteopenia.

**Limitations:** This study is limited by its small, exclusively Caucasian sample, which may reduce the generalisability of the results. The use of self-reported questionnaires for fracture history and lifestyle factors introduces potential recall bias. Moreover, the absence of DXA comparison and clinical confirmation of osteoporosis/osteopenia constrains the robustness of the findings. Finally, the lack of frailty and body composition analysis restricted a more comprehensive assessment of fracture risk.
**Funding for this study:** N/A
**Has your study been approved by an ethics committee?** Yes
**Ethics committee - additional information:** The study was approved bythe Board of Directors and the Ethics Committee of the Unidade Local de Saúde da Guarda(No. UI-NS-240033).
**Author Disclosures:**
Ana Isabel Saraiva Vieira: Nothing to disclose
Rute Santos: Nothing to disclose

**Hand bone densitometry: a more sensitive standard for the early bone damage in rheumatoid arthritis**
*M. A. Deshmukh*, P. C. P. Joshi, J. D' Souza; Pune/IN
*(meghanaadeshmukh@gmail.com)*

**Purpose:** To determine the role of dual-energy X ray absorptiometry (DEXA) compared with plain radiography in early rheumatoid arthritis of hand who have active disease.
**Methods or Background:** This study included 69 patients with suspicion of rheumatoid arthritis underwent DEXA scans of both hands, performed according to a standardised procedure for each site. For whole hand DEXA, all hand bones distal from the wrist joint were included in the measurement, the hand is put flat on a table and fingers extended. Plain radiographs were also performed of both hands and the joint damage score was calculated by van der Heijde modification of the Sharp method. Final diagnosis was made on RA Factor test. Diagnostic accuracy was evaluated via sensitivity and specificity.
**Results or Findings:** In this cross-sectional study of 69 patients with suspected early rheumatoid arthritis of the hand dual-energy X-ray absorptiometry (DEXA) and plain radiography were used to assess BMD loss. DEXA identified BMD loss in 49 out of 69 patients (71.0%). Plain radiography detected BMD loss in only 12 patients (17.4%). All patients who were positive on X-ray were also detected as positive by DEXA, indicating 100% agreement in radiographically confirmed cases. Additionally, DEXA identified 37 patients with BMD loss not detected by X-ray, who came out to be positive for rheumatoid arthritis.
**Conclusion:** This study underscores the superior sensitivity of hand DEXA over conventional hand radiographic joint damage scoring in detecting early bone damage in patients with rheumatoid arthritis. Traditional radiography, it often fails to identify subtle, preclinical changes in bone mineral density that precede visible erosions. The enhanced sensitivity of hand DEXA not only facilitates timely diagnosis but also enables more accurate monitoring of disease activity and treatment response.
**Limitations:** Loss to followup.
**Funding for this study:** No funding.
**Has your study been approved by an ethics committee?** Not applicable
**Author Disclosures:**
John D' Souza: Nothing to disclose
Priscilla Col Priscilla Joshi: Nothing to disclose
Meghana Avinash Deshmukh: Nothing to disclose

**Fetal MRI: Challenges and Strategies for Optimal Patient Management**
*A. Collaku*[1], E. Dybeli[2], Z. Mulla[1], F. Goga[1]; [1]Tirana/AL, [2]Elbasan/AL
*(alket_collaku@yahoo.com)*

**Purpose:** To discuss the main challenges of fetal MRI and propose practical approaches for handling different patients, with focus on maternal comfort, technical optimization, and safety.
**Methods or Background:** Technical and patient-related factors influencing fetal MRI were reviewed. Special attention was given to motion artifacts, sequence selection, maternal positioning, and safety considerations. Strategies were evaluated across different gestational ages and maternal conditions, including obesity, claustrophobia, and multiple gestations.
**Results or Findings:** Fetal MRI provides high diagnostic value when ultrasound findings are inconclusive, especially in central nervous system, thoracic, and abdominal evaluation. However, challenges include fetal/maternal motion, maternal discomfort, and safety limitations. Optimal results are achieved between 20–28 weeks of gestation, when fetal size and mobility allow better image quality. Later stages require protocol adaptation with ultrafast sequences and repeated acquisitions. Maternal positioning with left lateral tilt reduces vena cava compression and improves tolerance. Wider bore scanners and effective patient communication are essential for obese and anxious patients. Gadolinium should be avoided, while SAR and acoustic noise must be carefully monitored.
**Conclusion:** Fetal MRI is a safe and effective diagnostic tool when protocols are tailored to gestational age and maternal condition. Success relies on balancing technical parameters with patient-centered care, ensuring both image quality and maternal-fetal safety.
**Limitations:** No
**Funding for this study:** No
**Has your study been approved by an ethics committee?** Not applicable
**Author Disclosures:**
Flogerta Goga: Nothing to disclose
Zane Mulla: Nothing to disclose
Elona Dybeli: Nothing to disclose
Alket Collaku: Nothing to disclose

**08:00 - 09:00** **Research Stage 5**

### Research Presentation Session: Oncologic Imaging

### RPS 1716

### Innovations in pancreatic cancer imaging

**Moderator**
Andrea Vanzulli; Tradate/IT
*(andrea.vanzulli@istitutotumori.mi.it)*

**A combined diagnostic model to evaluate the outcome of neoadjuvant chemotherapy for pancreatic ductal adenocarcinoma**
E. V. Kondratyev, *A. Zharikova*, I. Gruzdev, A. Ustalov, S. A. Shmeleva, V. Egorov, E. P. Yasakova, P. V. Markov, D. V. Kalinin; Moscow/RU
*(zha-vit@yandex.ru)*

**Purpose:** To develop and compare diagnostic models, including a combined model, so as to predict the patologic responce to neoadjuvant chemotherapy (NAC) for pancreatic ductal adenocarcinoma (PDAC).
**Methods or Background:** 59 patients with histologically confirmed PDAC and preoperative computed tomography (CT) were included in the study. Patients were divided into two groups depending on the grade of histological response of the tumour based on Tumour Regression Score (TRS) criteria. The first group had a favourable response (TRS 0, 1 ,2), the second - unfavourable response (TRS 3). A radiologist with 6 years of experience, segmented the region of interest (lesion) for radiomics structure analysis in the arterial and venous CT phases before and after NAC. The extracted texture features were divided into 3 groups (pre-NAC, post-NAC, combined model) and analysed using machine learning techniques.
**Results or Findings:** The AdaBoost ensemble model (pre-NAC) - ROC AUC (0,831), PR-AUC (0,874) и F1 Score (80%), accuracy (77%), precision (88%), specificity (85,7%) and the Optimized Random Forest (post-NAC) - ROC AUC (0,870), PR-AUC (0,941), F1 Score (85,7%), accuracy (83,3%), precision (90%), recall/sensitivity (81,8%) are the best models for recognising tumours with an unfavourable response, if the high accuracy is priority. Gradient Boosting is the best fitting model both pre- and post-NAC, when focusing on ROC AUC(0.896) and PR-AUC (0.95). Comparing the results of the pre- and post-NAC models, the latter were more efficient.
**Conclusion:** Machine learning models, specifically Optimized Random Forest and Gradient Boosting, trained on texture features from post-NAC CT scans demonstrated high accuracy in detecting non-responders with an unfavorable prognosis.
**Limitations:** A relatively small sample size and the absence of an external validation group, which complicates the wider application of our model.
**Funding for this study:** It was not required.

**Has your study been approved by an ethics committee?** Yes
**Ethics committee - additional information:** Extract from the minutes № 003-2025 of the meeting of the Scientific Research Ethics Committee of the Federal State Budgetary Institution "A.V. Vishnevsky National Medical Research Center of Surgery" of the Ministry of Health of the Russian Federation dated March 21, 2025.
**Author Disclosures:**
Alexandra Zharikova: Nothing to disclose
Evgeny V Kondratyev: Nothing to disclose
Sofiia Antonovna Shmeleva: Nothing to disclose
Andrey Ustalov: Nothing to disclose
Elena Petrovna Yasakova: Nothing to disclose
Ivan Gruzdev: Nothing to disclose
Vyacheslav Egorov: Nothing to disclose
Dmitry V. Kalinin: Nothing to disclose
Pavel V. Markov: Nothing to disclose

**Dual-energy CT extracellular volume fraction to predict tumor collagen ratio and to assess response to neoadjuvant chemotherapy in pancreatic ductal adenocarcinoma**
*S. Ming*, W. Wei; Hefei/CN

**Purpose:** To investigate the value of dual-energy computed tomography (DECT)-derived extracellular volume fraction (ECV) for predicting tumor collagen ratio and assessing the response to neoadjuvant chemotherapy (NAC) in pancreatic ductal adenocarcinoma (PDAC).
**Methods or Background:** This retrospective study enrolled 176 patients with pathologically confirmed PDAC (64 resected, 112 unresectable) who underwent DECT. The ECV fraction based on iodine concentration (ECV_IC) was calculated. Histological collagen ratio was measured from surgical specimens using Masson's trichrome staining and digital image analysis. For unresectable patients, chemotherapy response was evaluated according to RECIST 1.1. Univariate and multivariate analyses were performed to identify predictors of collagen ratio and treatment response. Receiver operating characteristic (ROC) curves were used to evaluate predictive performance.
**Results or Findings:** ECV_IC showed a strong positive correlation with the histological collagen ratio (r = 0.618, p < 0.001) and was an independent predictor of high collagen ratio (HR = 1.420, p = 0.009). In the unresectable cohort, ECV_IC was also an independent predictor of poor response to NAT (HR = 1.259, p = 0.023). ROC analysis demonstrated that ECV_IC could discriminate between responders and non-responders with area under the curve (AUC) of 0.83 (sensitivity 81.0%, specificity 70.4%) at a cutoff value of 31.8%, significantly outperforming venous-phase iodine concentration (IC_VP; AUC = 0.73, P = 0.026).
**Conclusion:** DECT-derived ECV_IC is a promising non-invasive biomarker for predicting tumor collagen ratio and assessing response to NAC in PDAC, with potential to guide personalized treatment strategies.
**Limitations:** First, its retrospective and single-center nature introduces potential selection bias. Second, the chemotherapy regimens were not completely standardized, which might have influenced the response assessment. Third, the evaluation of treatment response was based on RECIST 1.1 criteria rather than pathological confirmation, which remains the gold standard.
**Funding for this study:** This study warted by the following projects:the National Natural Science Foundation of China (NSFC, No.82271991);Joint Fund for Medical Artificial Intelligence of USTC (No.MAI2023C006);Level A Funding Project for Reserve Candidates of Academic and Technical Leaders in Anhui Province (No.2022H279).
**Has your study been approved by an ethics committee?** Yes
**Ethics committee - additional information:** This retrospective study was approved by the ethics committee of First Affiliated Hospital of University of Science and Technology of China (Anhui Provincial Hospital), Approval No: 2024-RE-438.
**Author Disclosures:**
Wei Wei: Nothing to disclose
Shuai Ming: Nothing to disclose

**Imaging Pancreatic Cancer with Photon-Counting CT: image quality, dose reduction and clinical impact**
*L. Lofino*, A. Ammirabile, R. Levi, C. Bonifacio, A. Laghi; Rozzano/IT
*(ludovica.lofino@humanitas.it)*

**Purpose:** To compare quantitative and qualitative image quality parameters and radiation dose between photon-counting CT (PCCT) and energy-integrating detector CT (EIDCT) for the detection of pancreatic cancer.
**Methods or Background:** In this IRB-approved prospective study, 32 patients pancreatic cancer underwent multiphase CT (16 PCCT, 16 EIDCT). Patients were matched by age and BMI. Arterial and 5-minute delayed venous phase imaging were retrieved and analyzed. Contrast-to-noise ratio (CNR) was calculated as follows: (pancreatic parenchyma HU - pancreatic cancer HU) / noise, where noise was considered as the standard deviation of subcutaneous fat attenuation. Three radiologists with 7, 10 and 22 years of experience independently evaluated tumor conspicuity and overall image quality on a 5-point scale. Dose-length product (DLP) was retrieved for all examinations. Statistical analysis included t-tests, ANOVA and inter-reader agreement.
**Results or Findings:** PCCT demonstrated significantly higher tumor CNR than EIDCT in both arterial (5.3 ± 0.8 vs 4.0 ± 1.1, P<0.01) and delayed venous phases (4.9 ± 1.0 vs 2.3 ± 0.6, P<0.01). Image quality scores were consistently higher with PCCT for overall image quality (arterial: 4.5 ± 0.4 vs 3.3 ± 1.2; venous: 4.2 ± 0.8 vs 3.0 ± 1.1) and tumor conspicuity (all P<0.03). DLP was significantly lower for PCCT compared with EIDCT (1,050 ± 120 vs 2,350 ± 750 mGy·cm, P<0.01). Inter-reader agreement was strong both for PCCT and for EIDCT. Of note, in 2 patients imaged with both modalities, PCCT showed a clearer tumor delination which allowed for downstaging.
**Conclusion:** In our population, PCCT has shown higher objective and subjective image quality with reduced radiation dose compared to EIDCT. Enhanced delineation of pancreatic cancer and surrounding structures may improve staging and increase the cohort of potentially operable patients.
**Limitations:** Small sample size, single center.
**Funding for this study:** This study was funded by ANTHEM foundation.
**Has your study been approved by an ethics committee?** Yes
**Ethics committee - additional information:** Ethics committee reviewed and approved the study protocol.
**Author Disclosures:**
Ludovica Lofino: Nothing to disclose
Angela Ammirabile: Nothing to disclose
Cristiana Bonifacio: Nothing to disclose
Andrea Laghi: Nothing to disclose
Riccardo Levi: Nothing to disclose

**From Scan to Surgery: Comparison of CT-based vascular status with intraoperative findings in pancreatic cancer**
*N. G. Nacul Mora*, A. Andreou, B. Strücker, S. Katou, H. Morgül, F. Becker, A. Pascher, M. Köhler, G. H. Pöhler; Muenster/DE
*(galanaculm@gmail.com)*

**Purpose:** To compare NCCN radiological vessel contact grading and binary surgical infiltration assessment in pancreatic cancer with surgical and histological finding as reference.
**Methods or Background:** Single-center, retrospective cohort study including 103 patients (mean age 68 years, male 61%) with CT-based diagnosis of pancreatic carcinoma (2012 - 2023) undergoing primary resection. CT-tumor-vessel contact was blindly classified following NCCN criteria by two abdominal radiologists. By discrepancies, the statement of the radiologist with longer experience was chosen. Radiological infiltration was defined as any-contact / no-contact and compared to surgical and histological findings using McNemar-test, Spearman-correlation, sensitivity (how well CT detects actual infiltration) and specificity (how well CT rules out infiltration when there's none). Subgroup analysis of CT interval to operation ≤4 weeks (n=83) vs. >4 weeks (n=20) was compared using ROC-Analysis.
**Results or Findings:** Vessel infiltration was radiologically assessed higher (radiological 51%, surgical 39%, p= 0.041): arterial infiltration was comparable (radiological 7%, surgical 5%, p= 0.625), but venous infiltration was radiologically higher (radiological 44%, surgical 34%, p= 0.019), with 83.5% overall agreement but systematic radiological overestimation of venous involvement. Radiological artery infiltration sensitivity was 99% and specificity 98%, venous infiltration sensitivity was 88% and specificity 81%. CT ≤4 weeks subgroup showed enhanced correlations (arterial AUC: 0.731, venous AUC: 0.888). 69% of patients had R0 resection status, 28% R1 status. The histological finding of R0 patients did not show arterial invasion. There was a significant difference in venous infiltration between surgical and histological invasion (p < 0.001).
**Conclusion:** The interdisciplinary study reveals the complexity of vascular status in NCCN-based grading, resulting in radiological overestimation of venous infiltration, which may lead to over-recommendation for neoadjuvant therapy in interdisciplinary tumor boards. A CT-surgery interval ≤4 weeks should be prioritized.
**Limitations:** Small cohort, single-centre Study
**Funding for this study:** No funding.
**Has your study been approved by an ethics committee?** Yes
**Ethics committee - additional information:** Ethik Komission Westfalen Lippe (2025-506-f-S)

**Author Disclosures:**
Andreas Pascher: Nothing to disclose
Felix Becker: Nothing to disclose
Shadi Katou: Nothing to disclose
Michael Köhler: Nothing to disclose
Andreas Andreou: Nothing to disclose
Haluk Morgül: Nothing to disclose
Gesa Helen Pöhler: Nothing to disclose
Benjamin Strücker: Nothing to disclose
Nabila Gala Nacul Mora: Nothing to disclose

**The role of FDG-PET based systemic surveillance in re-staging of borderline resectable pancreatic cancer after neo-adjuvant treatment: a multicenter study**

S. Han, *D. H. Lee*; Seoul/KR
*(dhlee.rad@gmail.com)*

**Purpose:** To retrospectively evaluate the clinical significance of PET based systemic surveillance for distant metastasis in borderline resectable pancreatic cancer (BRPC) patients who are undergoing FOLFIRINOX based neoadjuvant chemotherapy (NAC).
**Methods or Background:** 161 patients who underwent FOLFIRINOX based NAC for BRPC from two institutes during January 2013 to December 2019 were retrospectively reviewed. All of the patients underwent an initial PET scan to confirm local disease. Image review including pre- and post-NAC images was done by 2 radiologists in consensus based on the NCCN 2020 guideline. Clinical information were obtained from patients records. Risk factor analysis for metastasis development was performed.
**Results or Findings:** Among the 161 patients who underwent NAC, 22.4% (36/161) converted to palliative setting due to local progression (14.3%, 23/161) or distant metastasis (8.1%, 13/161). The remaining 84.6% (125/161) proceeded to preoperative evaluation with (n=70) or without (n=55) PET evaluation. Among the patients with preoperative PET scan, distant metastasis was detected in 7.1% (5/70) of the patients. Among them, one liver metastasis (20%, 1/5) in one patient was only found on PET scan. Overall, 11.8% (19/161) patients developed metastasis during NAC. Among them, 2 cases of metastasis were only detected in the PET scan (2/19, 10.5%). Regarding the risk factors, the increment of CA 19-9 level during treatment (OR 4.43 [1.62-12.05], p=0.004) and present of major vein invasion before NAC (OR 5.70 [1.27-25.62], p=0.02) was associated with development of metastasis during NAC.
**Conclusion:** Considerable proportion of patients undergoing NAC for BRPC develop distant metastasis during treatment, especially for patients with increased CA 19-9 during the NAC or patients with initial major vein invasion. Systemic PET can aid in detection before undergoing curative resection.
**Limitations:** This is a retrospective study with heterogenous patient group.
**Funding for this study:** N/A
**Has your study been approved by an ethics committee?** Yes
**Ethics committee - additional information:** Approved by the IRB of the appropriate institutions.
**Author Disclosures:**
Seungchul Han: Nothing to disclose
Dong Ho Lee: Nothing to disclose

**Baseline tumor stiffness measurement by MR elastography predicts survival in pancreatic ductal adenocarcinoma**

*S. Poli*[1], A. Wenning[2], P. Lombardo[2], V. C. Obermann[2], A. T. Huber[1]; [1]Lucerne/CH, [2]Bern/CH
*(simone.poli@luks.ch)*

**Purpose:** Investigate the association between baseline tumor stiffness measured by magnetic resonance elastography (MRE) and survival in pancreatic ductal adenocarcinoma (PDAC). We hypothesized that patients with an MRE “ring sign” (high stiffness in the periphery but low in the center, indicating necrosis) or homogeneously high-stiffness have worse survival than those with homogeneous lower stiffness.
**Methods or Background:** 55 patients with newly diagnosed PDAC (Jan-21 to April-23, ethics approval Bern, registry-ID:NCT03469726) underwent baseline fasting (≥6 hours) multiparametric-MRI and 3D-MRE (3T Magnetom Prisma, Siemens) for cancer staging and stiffness assessment, and exclusion of liver metastasis. 3D-MRE was performed with a pneumatic driver (Resoundant, 40Hz, 40A), and a spin-echo 3D-MRE sequence (10 slices, 3.5 mm, end-expiration), providing pancreatic coverage. Kaplan-Meier curves and Cox-proportional hazard models were used to compare mortality.
**Results or Findings:** During follow-up, unresectable PDAC showed poorest survival, confirming its adverse prognosis (0% 3-years survival). Patients with homogeneous stiffness <13.3 kPa demonstrated significantly better outcomes (39% 3-year survival). In contrast, high tumor stiffness (hazard ratio, HR[95%CI] = 2.3[1.2,4.3]; p=<.05) and the presence of the MRE ring sign (HR[95%CI] = 2.1 [1.1,4.0]; p=<.05) were independently associated with increased 3-year mortality (26% and 0% survival, respectively). Resected patients with a ring sign had a prognosis comparable to that of patients with unresectable disease. As tumor necrosis has been histologically linked to aggressive PDAC, our findings support MRE-derived stiffness as a promising, noninvasive survival imaging biomarker.
**Conclusion:** A single baseline measurement of tumor stiffness by MRE is predictive of patient survival in newly diagnosed PDAC. MRE may serve as a valuable imaging biomarker for risk stratification in PDAC and could be incorporated into standard clinical MRI.
**Limitations:** The limitation of the study is the relatively small, single-center patient cohort, which may limit generalizability.
**Funding for this study:** SNF-project:REPORT-IT(#10003604)
**Has your study been approved by an ethics committee?** Yes
**Ethics committee - additional information:** This was a local, single-center sub-study of the DIA-PANC multi-center study (registry ID NCT03469726). The registry was approved by the Bern cantonal ethics committee, and the study was carried out in accordance with the principles of the Declaration of Helsinki.
**Author Disclosures:**
Verena Carola Obermann: Nothing to disclose
Simone Poli: Nothing to disclose
Paolo Lombardo: Nothing to disclose
Anna Wenning: Nothing to disclose
Adrian Thomas Huber: Nothing to disclose

**Multi-frequency magnetic resonance elastography for predicting pancreatic cancer aggressiveness and patient survival**

*Q. Wang*, L. Zhu, J. Liu, M. Dai; Beijing/CN

**Purpose:** To compare the stiffness and fluidity in the central part and peripheral part of pancreatic cancer using multi-frequency magnetic resonance elastography (MF-MRE), to correlate the MF-MRE parameters with clinicopathological factors that indicates tumor aggressiveness, and to identify risk factors for patient survival.
**Methods or Background:** MF-MRE was performed in 97 pancreatic cancer patients before treatment. High-resolution shear wave speed (SWS) and loss angle (φ) maps were generated, representing tissue stiffness and fluidity. SWS and φ were measured in the central part, the peripheral part and covering the entire pancreatic lesion, respectively. Pearson’s and Spearman’s correlation analysis were performed to evaluate the relationship between MRE parameters and tumor stage; grade; vascular and perineural invasion, regional lymphadenopathy and distant metastasis. Kaplan-Meier and Cox proportional hazards models were used to identify prognostic factors in patients with and without R0 resection (n=48 and 30, respectively).
**Results or Findings:** Fluidity in the peripheral part of the tumor was higher compared to the central part (1.23±0.25 vs. 0.96±0.20 rad, p＜0.001), whereas stiffness of the tumor didn’t show regional difference (p=0.064). In all patients, tumors with higher stiffness had more frequent regional lymphadenopathy (p=0.038), and tumors with higher fluidity in the peripheral part had more frequent vascular invasion, regional lymphadenopathy and distant metastasis (p=0.037, 0.007, and 0.027, respectively). Fluidity in the peripheral part of the tumor was positively correlated with tumor stage (rho=0.26; p=0.010). For patients with R0 resection, patients with higher fluidity in the peripheral part of the tumor (φ＞1.17 rad) had shorter disease-free survival (12.1 vs. 19.4 months, p=0.022).
**Conclusion:** MF-MRE may help to predict pancreatic cancer aggressiveness and patient survival.
**Limitations:** Not applicable.
**Funding for this study:** Funding was provided by National Natural Science Foundation of China (grant number: 82371950).
**Has your study been approved by an ethics committee?** Yes
**Ethics committee - additional information:** The study was approved by Peking Union Medical College Hospital Institutional Review Board (I-24PJ2450).
**Author Disclosures:**
Qi Wang: Nothing to disclose
Liang Zhu: Nothing to disclose
Jingyi Liu: Nothing to disclose
Menghua Dai: Nothing to disclose

**09:30 - 11:00** **Research Stage 1**

### Research Presentation Session: Abdominal and Gastrointestinal

### RPS 1801

### Pancreas multi-modality imaging innovation

**Moderator**
Ahmed Ba-Ssalamah; Vienna/AT
*(ahmed.ba-ssalamah@meduniwien.ac.at)*
**Author Disclosures:**
Ahmed Ba-Ssalamah: Consultant: Bayer, Speaker: Bayer, Siemens

**Combined Pancreatic Attenuation and Pancreas-to-Spleen Ratio on Non-Contrast CT Improve Diabetes Risk Stratification over Single Biomarkers: A Retrospective Analysis**
*C. Booz*, A-I. Nica, I. Yel, V. Koch, L. D. Grünewald, P. Reschke, J. Gotta, K. Eichler, T. J. Vogl; Frankfurt/DE
*(boozchristian@gmail.com)*

**Purpose:** Non-invasive imaging biomarkers derived from routine CT may assist in the early identification of individuals at risk for diabetes mellitus (DM). While both decreased pancreatic attenuation and a reduced pancreas-to-spleen attenuation ratio (PSR) have been associated with dysglycemia, it remains unclear whether a combination of both parameters yields additional diagnostic benefit. This study aimed to evaluate the added value of combining pancreatic attenuation and PSR for risk stratification of DM.
**Methods or Background:** We retrospectively analyzed non-contrast abdominal CT scans from 5624 patients (51% male; mean age 52 ± 10 years). Patients were categorized into normoglycemic (n=2900), prediabetic (n=1569), and diabetic (n=1155) groups based on clinical and laboratory data. Mean pancreatic attenuation (HU) was measured in head, body, and tail regions and compared to splenic attenuation to calculate PSR. ROC curve analyses and multivariate logistic regression were used to evaluate diagnostic performance for identifying diabetic status using individual and combined CT parameters.
**Results or Findings:** Pancreatic attenuation decreased progressively from normoglycemic (43 HU) to prediabetic (36 HU) to diabetic patients (32 HU) (all $p < 0.001$). PSR showed stronger correlation with HbA1c ($r = -0.90$) than pancreatic HU alone ($r = -0.76$). The AUC for PSR alone was 0.88, outperforming pancreatic HU (AUC: 0.79). However, combining both parameters slightly improved diagnostic accuracy (AUC: 0.92; $p = 0.01$ vs. PSR alone). In multivariate models, the combined approach remained an independent predictor of diabetes, offering both improved sensitivity and specificity.
**Conclusion:** Although PSR is a superior single CT-derived biomarker for assessing diabetes risk, combination of PSR and pancreatic attenuation provides a small but statistically significant improvement in diagnostic performance. This supports usage of combined attenuation-based metrics for more precise opportunistic screening in routine CT.
**Limitations:** Single-center study; retrospective study design
**Funding for this study:** No funding
**Has your study been approved by an ethics committee?** Yes
**Ethics committee - additional information:** Ethics committee approval number 2023-1216
**Author Disclosures:**
Christian Booz: Speaker: Bracco Speaker: Siemens Healthineers
Vitali Koch: Nothing to disclose
Ibrahim Yel: Nothing to disclose
Philipp Reschke: Nothing to disclose
Andreea-Ioana Nica: Nothing to disclose
Thomas J. Vogl: Nothing to disclose
Leon David Grünewald: Nothing to disclose
Katrin Eichler: Nothing to disclose
Jennifer Gotta: Nothing to disclose

**Alterations of the pancreas in type 1 diabetes - from prior to diagnosis to long-standing disease**
*N. Missima*, H. Hill, C-S. Aioanei, P. Liss, D. Espes; Uppsala/SE

**Purpose:** In type 1 diabetes (T1D), the loss of insulin-producing beta-cells is the hallmark pathophysiological alteration. However, volumetric and functional abnormalities of the exocrine pancreas are also observed. These changes may result from the loss of insulin's anabolic effects or reflect an underexplored aspect of T1D. Imaging techniques have enabled a better characterization of pancreatic morphology throughout T1D progression. This study examines exocrine pancreatic alterations at various stages of T1D using CT scans, including assessments conducted prior to diagnosis.
**Methods or Background:** The study utilized retrospective abdominal CT scans and clinical data collected from Uppsala University Hospital, including 150 T1D subjects, with 15 examined before diagnosis, and 61 age- and gender-matched non-diabetic controls. Volume segmentation and 3D reconstruction assessed the exocrine pancreas, and pancreas volume index (PVI) calculations were standardized using body weight, BMI, and body surface area (BSA). Descriptive and laboratory data were obtained from electronic medical records.
**Results or Findings:** Pancreas volume was significantly reduced in T1D patients. The reduction was more pronounced in patients diagnosed before the age of 20. No significant volume difference was noted in patients before their T1D diagnosis compared to controls, however, a reduction was observed post-diagnosis. Pancreas volume correlated negatively with disease duration and HbA1c levels and correlated positively with body surface area and plasma amylase levels.
**Conclusion:** Pancreas volume reduction is a consistent feature in T1D, correlating with both disease duration and markers of metabolic control. These findings support the potential of using imaging techniques as a non-invasive method for monitoring T1D progression.
**Limitations:** Lack of standardization, absence of relevant laboratory measurements (e.g. amylase, C-peptide) in the original clinical evaluations. Variability in imaging protocols. Many T1D patients and control subjects with native CT scans were excluded due to segmentation challenges.
**Funding for this study:** Research grants from SciLife Lab, Barndiabetesfonden, Diabetesfonden, Familjen Ernfors fond, EXODIAB and Göran Gustafssons Stiftelse.
**Has your study been approved by an ethics committee?** Yes
**Ethics committee - additional information:** The presented study is based on retrospective data collected at Uppsala University Hospital, Sweden. The study has been approved by the Swedish Ethical Review Authority (Dnr 2018/391).
**Author Disclosures:**
Daniel Espes: Nothing to disclose
Casian-Simon Aioanei: Nothing to disclose
Per Liss: Nothing to disclose
Henrik Hill: Nothing to disclose
Nathalia Missima: Nothing to disclose

**Diffusion-Weighted Imaging of the Healthy Pancreas: Association Between Subcutaneous Adipose Tissue Thickness and Pancreatic ADC Values**
S. Kaya, H. Atasoy, *M. A. Arıkan*, A. H. H. Yardımcı, M. Karagülle, C. C. Arıkan; Istanbul/TR
*(m.aliarikan@hotmail.com)*

**Purpose:** To evaluate the relationship between abdominal subcutaneous adipose tissue thickness and pancreatic apparent diffusion coefficient (ADC) values in healthy individuals, using diffusion-weighted imaging (DWI).
**Methods or Background:** A total of 100 abdominal MRI scans (69 females, 31 males; mean age 42.8 ± 12.0 years) performed on a 1.5T scanner were retrospectively reviewed. ADC values were measured in the head-neck, body, and tail of the pancreas with free-hand regions of interest encompassing the entire visible segment. Subcutaneous adipose tissue thickness was measured at the umbilical level. Two radiologists independently performed measurements, with interobserver agreement assessed by intraclass correlation coefficients (ICC). Correlations between ADC values, adipose thickness, age, and gender were analyzed using Pearson correlation and Mann–Whitney U tests.
**Results or Findings:** No significant differences in ADC values were observed among pancreatic segments or between genders, and no correlation with age was found ($p > 0.05$ for all). Subcutaneous adipose tissue thickness showed a significant inverse correlation with ADC values across all pancreatic segments (head-neck $r = -0.296$, $p = 0.003$; body $r = -0.302$, $p = 0.002$; tail $r = -0.310$, $p = 0.002$). Interobserver agreement was excellent (ICC range: 0.810–0.862).
**Conclusion:** Pancreatic ADC values appear stable across age and gender but decrease significantly with increasing subcutaneous adipose tissue thickness. This novel finding identifies adipose thickness as a potential confounding factor in pancreatic DWI interpretation and may improve the reliability of pancreatic imaging in clinical and research settings.

**Limitations:** This study has no limitations.
**Funding for this study:** No funding was received for this study.
**Has your study been approved by an ethics committee?** Not applicable
**Author Disclosures:**
Serhat Kaya: Nothing to disclose
Aytül Hande Hande Yardımcı: Nothing to disclose
Halit Atasoy: Nothing to disclose
Ceyda Ceren Arıkan: Nothing to disclose
Mehmet Karagülle: Nothing to disclose
Mehmet Ali Arıkan: Nothing to disclose

**CT-Based Pancreatic Biomarkers for Risk Stratification of Diabetes Mellitus: A Retrospective Analysis in A Huge Patient Cohort**
*C. Booz*, A-I. Nica, I. Yel, V. Koch, L. D. Grünewald, P. Reschke, J. Gotta, K. Eichler, T. J. Vogl; Frankfurt/DE
*(boozchristian@gmail.com)*

**Purpose:** Non-invasive imaging biomarkers may aid in early detection of individuals at risk for diabetes mellitus (DM). While decreased pancreatic attenuation on non-contrast CT has been associated with metabolic disorders, recent evidence suggests that the pancreas-to-spleen attenuation ratio (PSR) may offer improved diagnostic performance by minimizing inter-individual and technical variability. This study aimed to compare the diagnostic value of pancreatic attenuation versus PSR in assessing diabetes risk.
**Methods or Background:** In this retrospective study, non-contrast abdominal CT scans from 5634 patients (52% male, mean age 53 ± 8 years) were analyzed. Patients were categorized into normoglycemic (n=2902), prediabetic (n=1572), and diabetic groups (n=1160) based on clinical and laboratory data. Mean pancreatic attenuation (HU) was measured at three anatomical regions and compared with splenic attenuation to derive the PSR. Diagnostic performance was evaluated using ROC curve analysis and multivariate logistic regression.
**Results or Findings:** Mean pancreatic attenuation was significantly lower in diabetic patients (HU:31) compared to prediabetic (HU:37) and normoglycemic controls (HU:44) (all comparisons, $p < 0.001$). PSR showed a stronger inverse correlation with HbA1c ($r = -0.89$ vs $-0.75$) and yielded a higher diagnostic accuracy for identifying diabetes (AUC: 0.88) compared to pancreatic attenuation alone (AUC: 0.79) ($p<.001$). Optimal cut-off values for pancreatic attenuation and PSR were 35 HU and 0.85, respectively. In multivariate analysis, the PSR remained an independent predictor of diabetic status, outperforming absolute pancreatic HU values.
**Conclusion:** PSR is a more robust and reliable CT-based biomarker for assessing diabetes risk than pancreatic attenuation alone. Its relative nature reduces scanner- and patient-related variability, making it a preferable parameter for opportunistic screening in routine CT.
**Limitations:** Single-center study; retrospective study design
**Funding for this study:** No funding
**Has your study been approved by an ethics committee?** Yes
**Ethics committee - additional information:** Ethics committee approval number 2023-1216
**Author Disclosures:**
Christian Booz: Speaker: Bracco Speaker: Siemens Healthineers
Vitali Koch: Nothing to disclose
Ibrahim Yel: Nothing to disclose
Philipp Reschke: Nothing to disclose
Andreea-Ioana Nica: Nothing to disclose
Thomas J. Vogl: Nothing to disclose
Leon David Grünewald: Nothing to disclose
Katrin Eichler: Nothing to disclose
Jennifer Gotta: Nothing to disclose

**Topographical classification of postoperative pancreatic fistula: a radiological proposal for a novel, prospective classification system**
*B. De Franciscis*, D. Palumbo, A. Campisi, D. Tamburrino, S. Partelli, S. Crippa, M. Falconi, F. De Cobelli; Milano/IT
*(defranciscis.benedet@hsr.it)*

**Purpose:** Postoperative pancreatic fistula (POPF) remains the major cause of morbidity/mortality after pancreaticoduodenectomy (PD). The 2016 revision of the International Study Group for Pancreatic Surgery defines only grade B and grade C POPF as clinically relevant: grade B requires non surgical interventions, whereas grade C involves organ failure, reoperation or death. However, such classification is burdened by significant limitations, the main one being its retrospective nature. Radiological tools able to early stratify POPF severity and, more generally, morbidity burden after PD remain limited.
**Methods or Background:** We retrospectively analyzed 809 patients who underwent PD (01/2015–12/2023) and subsequent postoperative CT. 309 patients suffered from clinically relevant (CR)-POPF. Radiological variables (anastomotic defect/dehiscence, fluid collections, perianastomotic air bubbles, HU values of pancreatic remnant) were considered. A scoring system was derived from significant radiologic features. Model performance was assessed using area under the ROC curve (AUC).
**Results or Findings:** Among 309 CR-POPF patients, 259 were grade B and 50 grade C. Mortality was 44 % in grade C versus 0.4 % in grade B. Univariate analysis revealed that the presence of i) posterior anastomotic defect [2 points], ii) intra-abdominal fluid collections [1 point1], iii) perianastomotic air [2 points] and iv) lower pancreatic attenuation on CT [2 points] was associated with grade C POPF ($p < 0.05$). A postoperative CT score achieved AUC 0.74; a cut-off ≥ 4 provided 84 % sensitivity and 73 % specificity. Also, the score strongly predicted morbidity (in terms of Clavien-Dindo grading) and mortality (AUC: 0.68).
**Conclusion:** Several early CT findings differentiate grade C from grade B POPF. A simple CT-based score offers good discrimination. Prospective validation is necessary to establish these tools for early risk stratification and timely intervention.
**Limitations:** Retrospective Single Center
**Funding for this study:** None
**Has your study been approved by an ethics committee?** Yes
**Ethics committee - additional information:** The number of Ethics committee's approvment is 06/INT/2022
**Author Disclosures:**
Domenico Tamburrino: Nothing to disclose
Francesco De Cobelli: Nothing to disclose
Diego Palumbo: Nothing to disclose
Massimo Falconi: Nothing to disclose
Antonino Campisi: Nothing to disclose
Stefano Partelli: Nothing to disclose
Stefano Crippa: Nothing to disclose
Benedetta De Franciscis: Nothing to disclose

**Iodine concentration in photon counting computed tomography: correlation with tumor grade and Ki-67 in pancreatic NEN**
*M-J. Sähn*, S. Waltermann, J. Ottemöller, R. I. Danebrock, J. H. Niehoff, B. Gerdes, J. Borggrefe, N. Begum, A. S. Surov; Minden/DE
*(marwin.saehn@web.de)*

**Purpose:** Iodine concentration (IC) derived from dual energy computed tomography was recently explored as a potential biomarker for pancreatic NEN grade and Ki-67. With methodologies exhibiting significant variability, outcomes in recent publicatiobns were ambigous - ranging from, at best, weak correlation to strong predictive performance in complex multivariate analyses. This study revisits the issue and aims to provide evidence for tumor characterisation using PCCT derived iodine concentration in pancreatic NEN.
**Methods or Background:** IC in neuroendocrine pancreatic primaries were analyzed in venous phase pre treatment regarding correlation with histopathological tumor grade and Ki-67. Pancreatic NEN IC was normalized vs. aortic IC (NIC), as well as relative to unaffected pancreatic tissue (RIC). Correlations were analyzed using Spearman correlation, mean concentrations were analyzed using Mann-Whitney-U-test.
**Results or Findings:** Iodine concentration in P-NEN compared to normal pancreatic tissue (RIC) exhibited a strong correlation with tumor grade ($\rho = 0.54$, $p = 0{,}021$) and Ki-67 ($\rho = 0.54$, $p = 0.021$). Mean RIC were higher in high grade tumors ($p = 0.028$). NIC exhibited weak, non statistically significant correlation with tumor grade ($\rho = 0.328$, $p = 0.183$) and Ki-67 ($\rho = 0.302$, $p = 0.224$). Mean NIC did not diverge significantly in low grade vs. high grade pancreatic NET.
**Conclusion:** PCCT derived IC and especially RIC is a promising biomarker for tumor grade and Ki-67 prediction with strong, statistically significant correlations in untreated pancreatic NEN. The method is non-invasive, requires little to no additional resources and may support early, valid therapeutic decisions.
**Limitations:** Retrospective analysis
**Funding for this study:** None
**Has your study been approved by an ethics committee?** Yes
**Ethics committee - additional information:** This retrospective study was approved by the Ethics Committee of the Faculty of Medicine, Ruhr-University Bochum (2021–827).
**Author Disclosures:**
Alexey Surov: Nothing to disclose
Nehara Begum: Nothing to disclose
Simon Waltermann: Nothing to disclose
Julius Henning Niehoff: Nothing to disclose
Marwin-Jonathan Sähn: Nothing to disclose
Jonas Ottemöller: Nothing to disclose
Raihanatou Ina Danebrock: Nothing to disclose
Jan Borggrefe: Nothing to disclose
Berthold Gerdes: Nothing to disclose

**Automated Bone Mineral Density from Routine Staging CT as a Predictor of Progression-Free Survival in Resected Pancreatic Cancer under Adjuvant Chemotherapy**

*L. Endrös*[1], J. H. W. Bodden[2], F. Jungmann[2], R. L. Walter[2], M. Eiber[2], J. S. Kirschke[2], R. Braren[1], L. Steinhelfer[2]; [1]Hamburg/DE, [2]Munich/DE
*(lukas.endroes@t-online.de)*

**Purpose:** To evaluate whether automated, opportunistic analysis of volumetric bone mineral density (BMD) from routine staging CT predicts progression-free survival (PFS) in resected pancreatic ductal adenocarcinoma (PDAC) under adjuvant chemotherapy, and to compare its prognostic value with CA 19-9, as established biomarkers such as CA 19-9 have known limitations.
**Methods or Background:** We retrospectively analyzed 112 patients with resected PDAC who received gemcitabine-based or FOLFIRINOX chemotherapy. Vertebral BMD was quantified preoperatively and after completion of adjuvant therapy using SpineQ (BoneScreen), a CE-certified AI tool providing absolute volumetric BMD values (mg/cm³). Associations with PFS were tested using univariate and multivariate Cox regression models.
**Results or Findings:** During adjuvant chemotherapy, mean BMD declined from 97.6 ± 42.5 to 73.7 ± 34.1 mg/cm³. In univariate Cox regression, relative BMD change, follow-up BMD, and baseline CA 19-9 were significantly associated with PFS, whereas CA 19-9 change was not. In multivariate analysis, only relative BMD change remained predictive (HR 0.947, $p < 0.0001$). Each 1% decline in BMD corresponded to an approximately 5% higher risk of progression. Follow-up BMD and baseline CA 19-9 lost significance in the adjusted model.
**Conclusion:** Automated CT-based volumetric BMD analysis provides robust, independent prognostic information in resected PDAC and outperforms CA 19-9 for predicting PFS. Opportunistic skeletal assessment from routine staging CT enables longitudinal monitoring and individualized risk stratification without additional imaging or patient burden.
**Limitations:** This was a retrospective single-center study with a moderate sample size. Validation in larger, prospective, multicenter cohorts is required before clinical implementation.
**Funding for this study:** No funding was provided for this study.
**Has your study been approved by an ethics committee?** Yes
**Ethics committee - additional information:** This study was approved by the TUM Ethics Committee (reference number 2024-531-CD).
**Author Disclosures:**
Jan S Kirschke: Nothing to disclose
Robert Leon Walter: Nothing to disclose
Rickmer Braren: Nothing to disclose
Matthias Eiber: Nothing to disclose
Lukas Endrös: Nothing to disclose
Friederike Jungmann: Nothing to disclose
Lisa Steinhelfer: Nothing to disclose
Jannis Hubert Wilhelm Bodden: Nothing to disclose

**Less Dose, More Signal: Clinical Evaluation of Photon-Counting Detector CT in Pancreatic Patients**

A. I. Vigh, S. Nettesheim, T. Ojejinmi, M. Benke, Á. Szücs, A. Szijártó, P. Maurovich-Horvat, *B. K. Budai*, I. Dudás; Budapest/HU

**Purpose:** To compare radiation dose and image quality between photon-counting detector CT (PCD-CT) and energy-integrating detector CT (EID-CT) in patients with pancreatic disease who underwent abdominopelvic venous phase imaging.
**Methods or Background:** We retrospectively identified patients scanned with both PCD-CT and EID-CT between February 2022 and August 2023. Only patients without significant body-size change (L3 axial slice area difference <15%) were included, yielding 58 matched pairs. Radiation dose was compared using CTDIvol. Agreement in body size between scans was assessed using concordance correlation coefficients (CCC) and Bland–Altman analysis. Linear regression models tested the interaction between modality and body size. In a subset of 32 patients with identical venous phase protocols (3.0 mm slice thickness), signal-to-noise ratio (SNR) and contrast-to-noise ratio (CNR, muscle–fat) were measured at the L3 level.
**Results or Findings:** The mean time interval between scans was 116 ± 99 days, with a body-size difference of 4.3 ± 3.3 cm². Body size agreement was excellent (CCC = 0.968, LoA: –10.0 to 11.3 cm²). CTDIvol was significantly lower for PCD-CT than EID-CT (paired $p < 0.0001$), independent of body size (interaction $p = 0.291$). In protocol-matched cases, muscle SNR was significantly higher for PCD-CT ($p < 0.0001$), while muscle–fat CNR was not significantly different ($p = 0.875$).
**Conclusion:** PCD-CT provides substantial radiation dose reduction compared with EID-CT without compromising body-size comparability. In addition, PCD-CT improves muscle SNR, although CNR between muscle and fat remains unchanged. These results support the potential of PCD-CT to enhance dose efficiency and image quality in abdominopelvic imaging of pancreatic disease.
**Limitations:** This was a single-center study with a retrospective study design. Only venous phase 3.0 slice thickness scans were investigated.
**Funding for this study:** No funding was received for this study.
**Has your study been approved by an ethics committee?** Yes
**Ethics committee - additional information:** The study was approved by the institutional ethics committee.
**Author Disclosures:**
Attila Szijártó: Nothing to disclose
Pál Maurovich-Horvat: Nothing to disclose
Ákos Szücs: Nothing to disclose
Ibolyka Dudás: Nothing to disclose
Toluwalase Ojejinmi: Nothing to disclose
András István Vigh: Nothing to disclose
Sophie Nettesheim: Nothing to disclose
Márton Benke: Nothing to disclose
Bettina Katalin Budai: Nothing to disclose

**An Explainable AI Workflow Integrating Automated Volumetric Body Composition Analysis for Predicting Pathological Grading of Gastroenteropancreatic Neuroendocrine Neoplasms: A Multicenter Cohort Study**

*C. Lyu*, W. Qu, J. Li, Z. Li; Wuhan/CN

**Purpose:** To develop a non-invasive predictive model by integrating auto-segmented abdominal CT-based volumetric body composition with machine learning to preoperatively distinguish low-grade from high-grade Gastroenteropancreatic Neuroendocrine Neoplasms(GEP-NENs).
**Methods or Background:** This multicenter analysis enrolled 633 GEP-NENs patients from three institutions. Patients were divided into: Training set (n=403) and internal validation (n=174) (7:3 ratio from Hospital 1); test set (n=56 from 2 other hospitals). An nnUNetv2-based automatic segmentation algorithm of abdominal fat tissue and skeletal muscle on arterial-phase CT was applied. Visceral fat index, subcutaneous fat index, intermuscular fat index and skeletal muscle index were calculated. Features with a p-value < 0.05 were selected using univariate logistic regression and included in the prediction model built with extreme gradient boosting algorithm. Receiver operating characteristic curves and decision curve analysis were performed to evaluate the utility of the model. SHapley Additive explanation was conducted to enhance the model interpretability and visualization.
**Results or Findings:** The automatic segmentation achieved a Dice coefficient of 0.90. For pathological grading, a model built with body composition parameters achieved an AUC of 0.863 in the training set, 0.750 in the validation set, and 0.717 in the test set. SHAP analysis revealed that the relative intermuscular adipose tissue(rIMAT) contributed most among the body composition parameters to the model decision-making. And rIMAT levels were higher in P53-mutant and CK19-positive cases compared to negative cases.
**Conclusion:** Auto-segmented abdominal body composition combined with machine learning-based model could provide an assisted, non-invasive tool for prediction of pathological grade in GEP-NENs.
**Limitations:** First,changes in CT scanning protocols are inevitable. Second, retrospective and observational designs may be prone to have selection bias.
**Funding for this study:** National Natural Science Foundation of China (NSFC) [grants number 82202127, 82071889 and 62131009].
**Has your study been approved by an ethics committee?** Yes
**Ethics committee - additional information:** The study received approval from the institutional review board of our institution (TJ-IRB202411010) on 4 November 2024. We have registered it on the https://www.clinicaltrials.gov/ and the Unique Identifying Number (UIN) is NCT06983106.
**Author Disclosures:**
Weinuo Qu: Nothing to disclose
Zhen Li: Nothing to disclose
Chenxi Lyu: Nothing to disclose
Jiali Li: Nothing to disclose

**Patient-Derived Predictors of Peak Pancreatic Enhancement in CT Perfusion**

B. Kataria, I. Khudair, W. C. Bartholomä, P. Sandström, B. Björnsson, *M. Woisetschlager*; Linköping/SE

**Purpose:** For accurate diagnosis, optimal contrast timing is crucial in pancreatic CT imaging. To standardize scan timing, fixed protocols or bolus-tracking techniques are used. However, these methods often fail to account for individual physiological differences, potentially compromising image quality. The purpose of this study was to identify patient-specific parameters in order to individualize scan timing.
**Methods or Background:** Data from 16 patients who underwent a CT perfusion and a 4-phase abdominal examination at the same time point were used to evaluate the relationship between patient-specific parameters and contrast enhancement timing across three specific time intervals: t1: Time to aortic threshold >100 HU t2: Time from aortic threshold to maximum pancreatic enhancement t3: Total time from injection start to peak pancreatic enhancement Correlation analyses were performed between the three time intervals and variables including height, cardiac volume, lean body mass (LBM), and fat-related metrics.
**Results or Findings:** At time t1, significant correlations were observed for height (r = 0.550, p = 0.027) and cardiac volume (r = 0.595, p = 0.015); and at time t3 for LBM (r = 0.501, p = 0.048) and cardiac volume (r = 0.528, p = 0.036). No significant associations were observed at time t2. Strong inter-parameter relationships were noted between height and cardiac volume (r = 0.758,p=0.0007), and between cardiac volume and LBM (r = 0.812, p=0.0001).
**Conclusion:** Cardiac volume and LBM are easily accessible promising predictors of contrast timing in abdominal CT and may enable individualized timing, thus improving diagnostic accuracy in patients with diverse body compositions. Further research is warranted to validate these findings and to support clinical implementation.
**Limitations:** Small sample size
**Funding for this study:** LFoU from Region Östergötland, Sweden
**Has your study been approved by an ethics committee?** Yes
**Ethics committee - additional information:** Ref. no. 2016/43-31
**Author Disclosures:**
Bharti Kataria: Nothing to disclose
Wolf Claus Bartholomä: Nothing to disclose
Per Sandström: Nothing to disclose
Mischa Woisetschlager: Nothing to disclose
Ingrid Khudair: Nothing to disclose
Bergthor Björnsson: Nothing to disclose

**09:30 - 11:00** **Research Stage 4**

### Research Presentation Session: Artificial Intelligence and Imaging Informatics

### RPS 1805

### Beyond text mining: large language models as diagnostic and prognostic tools in radiology

**Moderator**
Marco Parillo; Rovereto/IT
*(marco.parillo@apss.tn.it)*

### When AI Joins the Table: Evaluating Large Language Model Performance in Sarcoma Tumor Board Decisions

*R. Dehdab*, F. K. E. Mankertz, N. Maalouf, S. Afat, C. Deinzer; Tübingen/DE
*(reza.dehdab@med.uni-tuebingen.de)*

**Purpose:** Multidisciplinary tumor boards (MDTs) are critical for the personalized management of soft tissue sarcomas (STS), but they are limited by time, cost, and resource demands. With recent advances in large language models (LLMs) like ChatGPT, there is growing interest in evaluating their potential role in augmenting MDT workflows. This study aimed to assess the clinical performance of ChatGPT-4o in real-world STS cases using predefined evaluation criteria, comparing its treatment suggestions with expert MDT decisions.
**Methods or Background:** We retrospectively analyzed 152 sarcoma cases presented to a single-center MDT between July 2023 and April 2024; 13 cases were used for prompt development and excluded. ChatGPT-4o generated guideline-based treatment suggestions from anonymized tumor board registration letters. Two blinded experts independently scored outputs using a five-domain framework: diagnostics, therapeutics, sequencing, chemotherapy, and contextualization. Scores were normalized to 1.0. Descriptive statistics and non-parametric ANOVA with post hoc tests assessed performance, including subgroup analysis by sarcoma subtype.
**Results or Findings:** The final cohort included 138 sarcoma cases (median age: 66; 51% male). The most common subtypes were leiomyosarcoma (n=22), dedifferentiated liposarcoma (n=16), and myxofibrosarcoma (n=15). The median normalized score was 0.857 (IQR: 0.75–1.0), significantly below the maximum achievable score (p < 0.05). Clinical contextualization scored highest (p < 0.05 vs. other criteria). No significant performance differences were observed across sarcoma subtypes (p = 0.138).
**Conclusion:** ChatGPT-4o showed high but imperfect concordance with sarcoma tumor board decisions, performing best in individualized reasoning. While overall appropriate, gaps in sequencing and chemotherapy selection highlight the need for further refinement before clinical use.
**Limitations:** Limitations include prompt development on a limited internal case set, reliance on internal expert consensus without external validation, and the absence of testing across different model temperature settings.
**Funding for this study:** No funding was received for the conduct of this study.
**Has your study been approved by an ethics committee?** Yes
**Ethics committee - additional information:** Ethical approval was granted by the Institutional Review Board 418/2024BO2
**Author Disclosures:**
Saif Afat: Nothing to disclose
Reza Dehdab: Nothing to disclose
Fiona Karola Elisabeth Mankertz: Nothing to disclose
Nour Maalouf: Nothing to disclose
Christoph Deinzer: Nothing to disclose

### LLMs Software Performance Evaluation for Prostate mpMRI Reports Interpretation

*B. Masci*[1], L. Nardoni[1], M. Polici[1], M. Zerunian[1], G. Argento[1], D. Caruso[1], M. Francone[1], A. Laghi[2]; [1]Rome/IT, [2]Milan/IT
*(benedetta.masci@uniroma1.it)*

**Purpose:** To evaluate a large language model(LLM)-based software for simplifying prostate multiparametric MRI(mpMRI) reports, focusing on clinical accuracy and communication clarity.Special attention was given to correct identification of key diagnostic elements, including PIRADS category and appropriate diagnostic work-up.
**Methods or Background:** This prospective single-center study analyzed 40 prostate mpMRI reports(January–May 2025) using a custom LLM-based software designed to process clinical data quickly and improve patient understanding.Reports were created by multiple board-certified radiologists to reduce bias and then simplified by the LLM tool. Two radiologists(Reader1,R1;Reader2,R2) independently evaluated each AI-generated report in six domains:clarity,completeness,capacity of identifying patient-relevant information,accuracy,communicative safety and overall satisfaction,with a 5-point Likert scale.Interpretative errors and correct identification of PIRADS-based diagnostic pathway were also assessed.Statistical analysis included Wilcoxon signed-rank test and inter-reader agreement was assessed.
**Results or Findings:** Both radiologists evaluated all reports across the domains. Language clarity of LLM-generated reports was moderate (R1: 3.35±0.98; R2: 3.08±0.80;p<0.05).Completeness showed greater variability (R1: 3.85±1.14; R2: 3.58±0.87;p<0.05). Capacity of identifying patient-relevant information was rated 3.25±0.81 (R1) and 3.13 ± 0.65 (R2;p=0.1970). Clinical accuracy scored 3.73±1.09 (R1) vs 3.45±0.82 (R2;p<0.05).Communicative safety was 3.58±0.98 for R1 and 3.23±0.83 for R2 (p<0.05); finally,overall satisfaction was moderate for both readers, with a mean score of 3.43±0.93 for R1 and 3.08±0.76 for R2, (p<0.05).Inter-reader agreement was moderate-to-good(ICC 0.65–0.78).Errors occurred in 35% of reports, mostly minor; 77.5% preserved correct PI-RADS-based diagnostic pathways.These findings highlight the potential to enhance patient communication but support maintaining radiologist oversight.
**Conclusion:** A tailored LLM-based software showed encouraging performances in simplifying prostate mpMRI reports, with moderate inter-reader agreement. This tool may improve communication and patient empowerment in complex diagnostic settings such as prostate mpMRI, helping reduce communication gaps in imaging workflows.
**Limitations:** Single software evaluation; no patients' assessments of the AI generated reports
**Funding for this study:** No funding was received for this study
**Has your study been approved by an ethics committee?** Yes
**Ethics committee - additional information:** Ethics committee approved
**Author Disclosures:**
Damiano Caruso: Nothing to disclose
Benedetta Masci: Nothing to disclose
Marta Zerunian: Nothing to disclose
Lapo Nardoni: Nothing to disclose
Andrea Laghi: Nothing to disclose
Michela Polici: Nothing to disclose
Marco Francone: Nothing to disclose
Giuseppe Argento: Nothing to disclose

**Democratizing Radiomics: Comparing Expert, "Vibe Coding" (LLM-Generated), and Ai-Assisted Pipelines For Clinical Prediction**

*P. P. Azzaro*, G. Avesani, R. Chianura, M. Dolciami, B. Gui, E. Sala; Rome/IT
*(pp.azzaro@gmail.com)*

**Purpose:** Evaluate the performance and usability of three approaches for building radiomics machine-learning (ML) pipelines: an expert-crafted pipeline, a semi-automated AutoML tool (CLIMB), and a pipeline generated by a large language model (ChatGPT-o3).
**Methods or Background:** Radiomic and clinical data from 94 patients with ovarian cancer (1,702 features from the primary mass and metastatic sites) were used to predict BRCA status. The expert pipeline, implemented in Python with PyRadiomics and scikit-learn, employed SHAP-based feature selection and grid-search hyperparameter tuning. The LLM pipeline was created via iterative prompting of ChatGPT-o3 by a radiologist with good radiomics/ML knowledge but limited coding experience. CLIMB was attempted but could not execute because the feature matrix exceeded token capacity. Both working pipelines evaluated four classifiers—Random Forest, Support Vector Machine, Logistic Regression, and XGBoost—within a SMOTE-based workflow to address class imbalance. Five-fold cross-validation generated fold-wise accuracy and precision.
**Results or Findings:** Mean accuracy across classifiers was 0.76 ± 0.03 for the expert pipeline and 0.73 ± 0.04 for the ChatGPT pipeline; the difference was not statistically significant (p = 0.27). The best individual model in both pipelines was XGBoost (expert 0.78; ChatGPT 0.75). CLIMB yielded no executable model due to input-size limitations. Development effort differed: the expert pipeline required about four programmer-hours and ~220 lines of bespoke code, whereas the ChatGPT pipeline required roughly ten hours of dialog-driven corrections but only ~110 lines. The expert code offered maximal transparency and tunability; the ChatGPT code was functional yet less flexible and relied on the prompt history for reproducibility.
**Conclusion:** LLM assistance enabled a non-programmer to build a radiomics pipeline with accuracy comparable to an expert benchmark.
**Limitations:** Single-dataset design, non-deterministic LLM behavior, assessment of only one LLM and one AutoML tool, and no prospective clinical evaluation.
**Funding for this study:** None
**Has your study been approved by an ethics committee?** Yes
**Ethics committee - additional information:** The study was approved by the ethics committee under code 3311
**Author Disclosures:**
Giacomo Avesani: Consultant: Lucida Medica
Roberta Chianura: Nothing to disclose
Pietro Paolo Azzaro: Nothing to disclose
Benedetta Gui: Nothing to disclose
Evis Sala: Nothing to disclose
Miriam Dolciami: Nothing to disclose

**A major potential of LLMs: determining skeletal biological age in breast cancer to predict vertebral compression fractures**

*C. Wan*, Z. Zhang, L. Kong, J. Hao, L. Fajin; Chongqing/CN
*(chengxinwan@hospital.cqmu.edu.cn)*

**Purpose:** To determine whether an unsupervised large language model (LLM)–inferred skeletal biological age gap (BAG: LLM bone age minus chronological age) from routine CT–based reports predicts incident vertebral compression fractures (VCF) in women with breast cancer and improves risk stratification beyond conventional metrics.
**Methods or Background:** We retrospectively included 528 consecutive, newly diagnosed, surgically treated breast cancer patients (2018–2024; 46–76 years). Baseline assessments comprised thoracolumbar CT (T12–L2), quantitative CT volumetric BMD, dual-energy X-ray absorptiometry T-scores, bone-metabolism labs and lifestyle data; patients with baseline VCF or bone metastasis were excluded. All variables were transcribed into a structured Chinese radiology/clinic report. A domain-adapted LLM performed prompt-based, label-free inference of skeletal bone age from the textual report (including CT-derived metrics); BAG (years) was computed. The primary endpoint was first low-energy VCF within 3 years. Cox and Fine–Gray models estimated hazard ratios (HR) per 1-year BAG, adjusting for age, body mass index and Hounsfield units. Discrimination was compared against Hounsfield units, DXA and FRAX using C-index, time-dependent AUC and net reclassification improvement (NRI).
**Results or Findings:** Fifty-four VCFs occurred (3-year cumulative incidence 10.2%). Each 1-year increase in BAG was associated with 6% higher VCF risk (HR 1.06; 95% confidence interval 1.02–1.11; P=0.002). Adding BAG to a base model (age, body mass index, Hounsfield units) improved 3-year C-index from 0.66 to 0.73 (Δ0.07; P<0.001), increased time-AUC by 0.06 and yielded an NRI of 0.15. Results were consistent in competing-risk analysis, bootstrap validation and endocrine-therapy strata.
**Conclusion:** Unsupervised LLM-inferred skeletal BAG from routine CT reports independently predicts VCF in breast cancer and meaningfully enhances discrimination beyond density-based metrics, supporting targeted post-operative fracture prevention.
**Limitations:** Single-center, retrospective design; external validation pending; potential residual confounding.
**Funding for this study:** The First Affiliated Hospital of Chongqing Medical University 2025 Science and Technology Innovation Project
**Has your study been approved by an ethics committee?** Yes
**Ethics committee - additional information:** Informed consent was waived after approval by the Medical Research Ethics Review Committee of the First Affiliated Hospital of Chongqing Medical University (No. 276-02).
**Author Disclosures:**
Lv Fajin: Nothing to disclose
Zhiwei Zhang: Nothing to disclose
Lingquan Kong: Nothing to disclose
Jie Hao: Nothing to disclose
Chengxin Wan: Nothing to disclose

**Multidisciplinary Management of B3 Breast Lesions: A Comparative Performance Analysis of General and Custom-Trained LLM Models**

*G. Della Pepa*, G. Irmici, M. Cao, C. De Berardinis, E. D'Ascoli, L. Corradini, G. Rossini, C. Depretto, G. P. Scaperrotta; Milan/IT

**Purpose:** To retrospectively compare GPT-4o, a general-purpose large language model (LLM), and a custom-trained GPT adapted to breast imaging practice, in supporting clinical decision-making for B3 breast lesions. The goal was to assess concordance with multidisciplinary team (MDT) decisions and evaluate clinical utility.
**Methods or Background:** Clinical, imaging, and histopathological data of consecutive biopsy-confirmed B3 breast lesions discussed at the institutional MDT between February and July 2024 were anonymized and standardized for text-only input. Two LLMs were tested: GPT-4o and a custom GPT trained using retrospective institutional cases, internal MDT protocols, and international guidelines and consensus. Each case was submitted to both models using a two-step prompt: generation of management options, followed by a single best recommendation with rationale. Three breast radiologists reviewed outputs and rated option accuracy and recommendation appropriateness (1–5 scale). Concordance with MDT decision was recorded.
**Results or Findings:** Forty-nine cases were included. Both models generated accurate management options (mean scores: GPT-4o 4.6/5; custom GPT 4.9/5). Appropriateness of the final recommendation was lower for GPT-4o (3.8/5) compared to the custom GPT (4.5/5). Concordance with MDT decisions was 65.3% (32/49) for GPT-4o and 83.7% (41/49) for the custom GPT. Weighted Kappa values were 0.41 and 0.68, respectively. McNemar's test confirmed a significant difference in concordance (p=0.03); Wilcoxon signed-rank test confirmed the difference in appropriateness (p=0.002). Inter-reader agreement was substantial (Fleiss' Kappa 0.72).
**Conclusion:** Both models showed high accuracy in understanding complex cases. While GPT-4o aligned only moderately with MDT decisions, the custom GPT demonstrated improved concordance and appropriateness. Carefully trained LLMs may provide valuable decision support in challenging scenarios, particularly when MDT expertise is not readily available.
**Limitations:** Single-institution pilot with a small sample size. Broader validation and training on larger, more diverse datasets are needed.
**Funding for this study:** None
**Has your study been approved by an ethics committee?** Yes
**Ethics committee - additional information:** Approved by: Comitato Etico Territoriale Lombardia 4
**Author Disclosures:**
Gianmarco Della Pepa: Nothing to disclose
Menmeng Cao: Nothing to disclose
Elisa D'Ascoli: Nothing to disclose
Gabriele Rossini: Nothing to disclose
Gianfranco Paride Scaperrotta: Nothing to disclose
Catherine Depretto: Nothing to disclose
Lisa Corradini: Nothing to disclose
Claudia De Berardinis: Nothing to disclose
Giovanni Irmici: Nothing to disclose

**Potential value of the commercialised large language models for cancer staging based on head and neck MRI reports**

*Q. H. Ai*, H. H. Leung, H. M. Kwok, K. F. Hung, L. M. Wong, T. Y. So, A. D. King, K. T. Bae; Hong Kong/HK
*(aqy0621@gmail.com)*

**Purpose:** MRI is routinely used for staging head and neck cancer (HNC), which is a vital step for disease management. However, T and N category criteria for HNC staging are complex and require specialized expertise, and so in many institutions only descriptive MRI reports without formal staging are available. This study aimed to evaluate the potential of commercialized large language models (LLMs) in staging HNC based on the descriptive MRI reports for oral cavity cancer (OCC) by comparing the accuracies of LLMs for T and N categorization and overall stage with that of human experts.
**Methods or Background:** Descriptive contents in 70 eligible MRI reports were retrospectively input to three commercialised LLMs (ChatGPT5.0, ChatGPT4.0 and DeepSeekV3), respectively. The T- and N- categorisation, and overall stage were extracted from the outputs for further analysis. Accuracies of the LLMs for HNC staging were assessed by the gold standard (confirmed by other two senior head and neck radiologists), and compared using McNamar test.
**Results or Findings:** The LLMs staged all cases based on the 8th edition of the AJCC cancer staging manual. The ChatGPT5.0, ChatGPT4.0 and DeepseekV3 achieved an accuracy of 74.3%, 72.8%, and 57.1%, respectively for T-categorisation; 85.7%, 82.8%, and 61.4%, respectively for N-categorisation; and 75.7%, 72.8%, and 52.8%, respectively for overall stage. Compared with DeepseekV3, ChatGPT5.0 and ChatGPT4.0 showed higher accuracies for T- and N-categorisation, and overall stage (all $p<0.05$). No differences in accuracies for T- and N- categorization and overall stage between ChatGPT5.0 and ChatGPT4.0 (all $p>0.05$).
**Conclusion:** Results suggested that the current commercialised LLMs may not be able to assist HNC staging based on descriptive MRI reports for OCC.
**Limitations:** Small sample size; Only tested the LLMs using OCC MRI reports
**Funding for this study:** Not applicable
**Has your study been approved by an ethics committee?** Yes
**Ethics committee - additional information:** Joint CUHK-NTEC (Ref. 2025-567
**Author Disclosures:**
Tiffany Y. So: Nothing to disclose
Kyongtae Ty Bae: Nothing to disclose
Kuo Feng Hung: Nothing to disclose
Lun Matthew Wong: Nothing to disclose
Ann Dorothy King: Nothing to disclose
Hoi Ming Kwok: Nothing to disclose
Hanson Hs Leung: Nothing to disclose
Qiyong Hemis Ai: Nothing to disclose

**Turning Reports into Labels: LLM-Driven Extraction of Tumor Progression in Lung Cancer Patients using Radiological Reports**
*S. Warmer*, Y. Wen, C. Bojahr, L. Umutlu, J. Haubold, J. Kohnke, K. A. Borys, F. Nensa, R. Hosch; Essen/DE

**Purpose:** Radiology reports routinely describe tumor response, yet this information remains embedded in unstructured text, limiting its clinical structured availability. Therefore, this study investigates whether a large language model (LLM) can accurately classify tumor progression, regression, or stability from free-text radiological reports using a zero-shot approach.
**Methods or Background:** 223 radiology reports were randomly selected from a retrospective cohort of 100 lung cancer patients (female=41, 65±9.72 years, NSCLC=96, SCLC=4) who underwent CT or PET/CT imaging between 2003 and 2021. The dataset consisted of thoracic CT scans (44%), PET/CT whole-body scans (34%), and abdominal CT scans (22%). Clinical experts independently annotated reports to indicate whether they showed progression, regression, or stability. A general-purpose, open-source LLM (Qwen3-235B) was prompted in a zero-shot setting to classify each report. Performance was evaluated for two tasks: binary classification (progression vs. no progression) and multiclass classification (progression, regression, stability, non-classifiable). Performance was measured using accuracy and F1 score.
**Results or Findings:** In the binary classification task, the model achieved 80% accuracy with an F1 score of 0.71 and strong agreement with expert labels. In the multiclass setting, accuracy was 0.74, with per-class F1 scores of 0.74 for progression, 0.9 for regression, and 0.71 for stability. The model achieved 70% accuracy on non-classifiable cases. These results were achieved without fine-tuning, relying solely on zero-shot prompting, which underscores the potential of open-source LLMs for clinical information extraction.
**Conclusion:** This study demonstrates the potential of zero-shot LLMs to extract structured tumor responses directly from free-text radiology reports. Such models offer a scalable, training-free solution to support longitudinal therapy monitoring and improve access to critical clinical information written in radiological reports.
**Limitations:** The limitations of the study are the need for further evaluations of different llms and prompting strategies.
**Funding for this study:** No funding was provided for this study.
**Has your study been approved by an ethics committee?** Yes
**Ethics committee - additional information:** The study was approved by the ethics commitee of the University Hospital Essen.
**Author Disclosures:**
Christian Bojahr: Nothing to disclose
Sina Warmer: Nothing to disclose
Katarzyna Anna Borys: Nothing to disclose
Johannes Haubold: Nothing to disclose
Judith Kohnke: Nothing to disclose
Yutong Wen: Nothing to disclose
Lale Umutlu: Nothing to disclose
René Hosch: Nothing to disclose
Felix Nensa: Nothing to disclose

**Large Language Models in Medical Data Structuring: A Case Study on Neuro-Oncological Cohorts**
*R. Hahnfeldt*, M. Schönfeld, T. Schömig, J. P. Janssen, S. Lennartz, D. Maintz, M. Schlamann, K. R. Laukamp, J. Kottlors; Cologne/DE

**Purpose:** Primary brain tumors form a heterogeneous group of neoplasm. Standardized follow-up protocols lead to growing institutional MRI datasets that reflect local epidemiology and offer a basis for clinical research. Large language models (LLMs) offer a novel approach by enabling automated structuring and real-time summarization of clinical cohorts. This study evaluated LLM use for automated analysis of a neuro-oncological database, including estimation of entity distributions, follow-up frequencies, and progression patterns.
**Methods or Background:** A total of 248 patients with intracranial neoplasms treated between 2014 and 2023 at a neuro-oncological center were included, with 165 glioblastoma patients. An anonymized institutional database was processed using Claude Sonnet 3.5 to autonomously generate descriptive statistics and cohort-specific estimates of follow-up intervals and progression rates. Model outputs were cross-validated using conventional statistical techniques.
**Results or Findings:** Patients underwent an average of 11 MRI examinations during their disease course. In glioblastoma cases, the shortest intervals between imaging and clinical progression were observed (mean: 348 days for imaging; 533 days for clinical symptoms). The LLM successfully extracted these patterns and predicted progression timelines that plausible matched manual statistical analysis results.
**Conclusion:** LLM-based cohort analysis enables reliable, automated extraction of key metrics from routine clinical data. The high concordance with classical statistics underscores the potential of these models to support longitudinal data analysis, resource planning, and individualized follow-up strategies in neuro-oncology.
**Limitations:** Potential bias due to incomplete or inconsistent documentation in the past The use of Claude Sonnet 3.5 for statistical analyses is not yet established and validated.
**Funding for this study:** This study was conducted with project funding from the Else Kröner-Fresenius-Stiftung (Grant-Number: 2023_EKEA.77)
**Has your study been approved by an ethics committee?** Not applicable
**Author Disclosures:**
Simon Lennartz: Nothing to disclose
Robert Hahnfeldt: Nothing to disclose
Kai Roman Laukamp: Nothing to disclose
David Maintz: Nothing to disclose
Michael Schönfeld: Nothing to disclose
Jonathan Kottlors: Nothing to disclose
Jan Paul Janssen: Nothing to disclose
Marc Schlamann: Nothing to disclose
Thomas Schömig: Nothing to disclose

**Symptom-Only Localization of Brainstem Ischemia: LLM vs Neurologists in 109 DWI-Positive Cases**
*N. Beste*[1], T. Dratsch[1], J. Kottlors[1], P. Floßdorf[1], A-M. Konitsioti[1], L. Volz[1], D. Pinto Dos Santos[2], M. Schönfeld[1]; [1]Köln/DE, [2]Mainz/DE
*(nedim25@icloud.com)*

**Purpose:** To evaluate the diagnostic accuracy of large language models (LLMs) in localizing brainstem ischemic lesions based solely on neurological symptoms, compared with experienced neurologists.
**Methods or Background:** We retrospectively included 109 patients with diffusion-weighted imaging (DWI)-confirmed acute brainstem ischemia. Clinical symptoms were provided to three neurologists and five LLMs (GPT-5, GPT-4, GPT-4.1, GPT-4o, o3, o3 pro), which were tasked to predict lesion site (midbrain, pons, medulla) and laterality (left/right). Accuracy, Cohen’s κ, region-specific performance, and correlations with symptom count were analyzed.

**Results or Findings:** GPT-4 and GPT-4o achieved the highest overall accuracy (56.0%), outperforming GPT-5 (48.6%), GPT-4.1 (41.3%), GPT-o3 (34.9%), GPT-o3 pro (10.1%), and all neurologists (32.1–36.7%). Cohen's κ was highest for GPT-4o (κ = 0.29). LLMs performed best in pontine strokes (GPT-4: 74.0%, GPT-4o: 68.8%), while performance in midbrain and medulla lesions was substantially lower. A weak but significant correlation between number of symptoms and prediction accuracy was found for GPT-4 ($r = 0.28$, $p < 0.01$), GPT-5 ($r = 0.26$, $p < 0.01$), and one neurologist ($r = 0.29$, $p < 0.01$).
**Conclusion:** GPT-4 and GPT-4o outperformed neurologists in localizing brainstem lesions based on clinical symptoms alone, while GPT-5 also exceeded human performance but remained less accurate than GPT-4/4o. Accuracy was modest overall, especially outside pontine strokes.
**Limitations:** Retrospective design, small cohort size, absence of multimodal input, high percentage of pontine strokes and lack of external validation limit generalizability. Prospective studies with integrated imaging and reasoning-augmented models are needed.
**Funding for this study:** 1. GPT-4 and GPT-4o reached the highest overall accuracy (56.0%), surpassing GPT-5 (48.6%), other LLMs (41.3–10.1%), and all neurologists (32.1–36.7%) in localizing brainstem lesions based on clinical symptoms alone . 2. Agreement with imaging (Cohen's κ) was highest for GPT-4o (κ = 0.29). 3. Performance was best in pontine strokes (GPT-4: 74.0%, GPT-4o: 68.8%), but substantially lower in midbrain and medulla lesions. 4. Weak yet significant correlations between number of symptoms and accuracy were found for GPT-4 (r = 0.28), GPT-5 (r = 0.26), and one neurologist (r = 0.29).
**Has your study been approved by an ethics committee?** Not applicable
**Author Disclosures:**
Pia Floßdorf: Nothing to disclose
Thomas Dratsch: Nothing to disclose
Nedim Beste: Nothing to disclose
Michael Schönfeld: Nothing to disclose
Jonathan Kottlors: Nothing to disclose
Agni-Maria Konitsioti: Nothing to disclose
Lukas Volz: Nothing to disclose
Daniel Pinto Dos Santos: Nothing to disclose

**ChexFract: Fracture-Focused Vision–Language Models for Chest Radiography**
N. Nechaev, E. Przhezdzetskaya, D. Umerenkov, V. Gombolevskiy, *E. Petrash*, D. Dylov; Moscow/RU
*(cherkun.ekaterina@gmail.com)*

**Purpose:** State-of-the-art vision–language models (VLMs) often miss or underspecify fractures—rare yet clinically critical findings on chest radiographs. We aimed to (i) build a fracture-focused dataset for report generation and (ii) train specialized VLMs that produce precise, structured fracture descriptions, improving clinical utility over general-purpose systems.
**Methods or Background:** We curated ChexFract, a public set of 18,710 CXR–text pairs with standardized, template-based fracture mentions. Fracture sentences were extracted from original reports and normalized to a schema capturing presence, location, side, stage (acute/healed/other), and implants. As the language backbone we used Phi-3.5 Vision Instruct. We compared two domain visual encoders—Rad-DINO (MAIRA-2) and CheXagent—by training lightweight projection heads and fine-tuning end-to-end for free-text generation. To score model outputs, we parsed generated text to the same schema and computed standard classification metrics against ground truth.
**Results or Findings:** Fracture-specialized VLMs consistently outperformed general baselines. With the MAIRA-2 encoder, end-to-end fine-tuning achieved ROC-AUC 0.715 and F1 0.629; with the CheXagent encoder, fine-tuned models reached ROC-AUC 0.697 and F1 0.591. General baselines were lower (e.g., MAIRA-2 baseline ROC-AUC 0.518, F1 0.085; CheXagent baseline ROC-AUC 0.604, F1 0.376). Stratified analyses by fracture type and anatomical site (ribs, clavicle, shoulder, spine, sternum, scapula, sternal wires/other) showed complementary strengths across encoders and informed selection for trauma-focused reporting workflows.
**Conclusion:** ChexFract enables accurate, structured fracture reporting directly from chest radiographs by focusing learning on clinically salient trauma findings. Specialized VLMs trained on ChexFract markedly improve detection and description quality versus general models, with best configurations achieving ROC-AUC ≈0.71 and F1 ≈0.63.
**Limitations:** Relabeling used an LLM-assisted protocol and may inherit parsing/bias artifacts; external, multi-center validation is pending. Prospective workflow impact, safety, and hallucination controls require clinical studies.
**Funding for this study:** No external funding was received; the work was conducted as part of the authors' institutional duties.
**Has your study been approved by an ethics committee?** Not applicable
**Author Disclosures:**
Victor Gombolevskiy: Nothing to disclose
Dmitry Dylov: Nothing to disclose
Evgenia Przhezdzetskaya: Nothing to disclose
Nikolay Nechaev: Nothing to disclose
Dmitry Umerenkov: Nothing to disclose
Ekaterina Petrash: Nothing to disclose

**09:30 - 11:00** **Research Stage 6**

### Research Presentation Session: Musculoskeletal

### RPS 1810

### Radiologic insights into skeletal ageing: osteoporosis and frailty

**Moderator**
Christian Krestan; Wien/AT
*(christian.krestan@gesundheitsverbund.at)*

**Ultrasound Assessment of Therapeutic Water-based Exercise in a Frail Population - A Randomized Controlled Trial**
J. S. Cárdenas Herran[1], J. Vucetic[1], M. Inglés[1], C. Borras[1], *R. Mirón Mombiela*[2]; [1]Valencia/ES, [2]Herlev/DK
*(mirona@ufm.edu)*

**Purpose:** Frailty, an age-related functional capacity loss, constitutes a significant health concern. High-impact exercises are effective in improving frailty, while the effect of therapeutic water-based exercise remains poorly studied. We aimed to investigate the impact of a therapeutic water-based exercise program on ultrasound parameters related to frailty and functional capacity in older adults.
**Methods or Background:** This was a randomized controlled trial set in a tertiary-care hospital and a private swimming pool. Thirty-five older adults, median age 72 (+/-10), were divided into two groups: i) therapeutic water-based exercise group (WEG) (n=19) and ii) control group (CG) (n=16). WEG underwent a twelve-week water-based exercise intervention supervised by a physiotherapist. CG did not receive any intervention. Frailty status, quantitative muscle ultrasound parameters, and functional outcomes were assessed before and after the intervention. Possible differences between quantitative variables were determined using a two-way ANOVA model with interaction. Minimum detectable change (MDC) between the quantitative variables was also determined.
**Results or Findings:** Frailty status, sex, physical function, and comorbidities were homogeneously distributed. There was a statistically significant effect on the ultrasound variables: Echo-Intensity ($p = 0.013$), Muscle-Thickness ($p = 0.011$), and Subcutaneous-Fat-Thickness ($p = 0.010$) with respect to treatment and time. We also observed differences in the values of Echo-Intensity in the treatment group after the intervention exceeded the MDC values, thus demonstrating a change that can be attributed to the intervention.
**Conclusion:** A therapeutic water-based exercise program improves parameters related to frailty and physical condition in older adults, likely by enhancing muscle quality or Echo-Intensity as measured by ultrasound. This suggests that ultrasound can be used as a tool to measure the effect of exercise-based treatments in frail older adults.
**Limitations:** The study was not double-blind, and the sample size was small.
**Funding for this study:** Not Applicable.
**Has your study been approved by an ethics committee?** Yes
**Ethics committee - additional information:** The protocol was approved by the Research Ethics Committee of the University of Valencia (num. H1520499833822) and by the Research Ethics Committee of the Consorcio Hospital General Universitario de Valencia. The study was also prospectively registered at www.clinicaltrials.gov as SWIMFRAIL (NCT03955302).
**Author Disclosures:**
Marta Inglés: Nothing to disclose
Rebeca Mirón Mombiela: Nothing to disclose
Consuelo Borras: Nothing to disclose
Jenny Soraya Cárdenas Herran: Nothing to disclose
Jelena Vucetic: Nothing to disclose

**Association between spinal deformity and pulmonary function in patients with interstitial lung disease**

L. Pinali, A. Fassio, *A. Ventura*, L. Carobene, C. Micheletto, D. Gatti, M. Rossini, G. Puppini, S. Baltieri; Verona/IT
*(angela.ventura@aovr.veneto.it)*

**Purpose:** Vertebral fractures and spinal deformities are common in patients with interstitial lung disease (ILD). We aimed to explore impact of vertebral damage on pulmonary restriction evaluating the association between the Spinal Deformity Index (SDI) and lung volumes in patients with ILD.
**Methods or Background:** This retrospective study included 200 patients with ILD: 76 idiopathic pulmonary fibrosis (IPF), 65 systemic sclerosis-associated ILD (SSc-ILD), 31 idiopathic inflammatory myopathy-associated ILD (IIM-ILD) and 28 other ILDs. SDI was assessed on multiplanar reconstructions of CT thoracic examinations. Pulmonary function tests included percent predicted forced vital capacity (ppFVC), total lung capacity (ppTLC), absolute FVC and TLC volumes. Multivariable linear regression models were used to evaluate the association between SDI and lung function parameters, adjusted for age, sex, ILD subtype and presence of diffuse idiopathic skeletal hyperostosis or osteoarthritis (DISH/OA).
**Results or Findings:** In adjusted models, higher SDI was significantly associated with lower ppFVC (2.9% per SDI unit, p<0.01) and ppTLC (2.7%, p<0.01). These associations persisted across ILD subtypes. SDI was inversely associated with absolute lung volumes: each unit increase in SDI corresponded to a 95.64 ml reduction in FVC (P = 0.02) and 199.46 ml in TLC (p<0.05 for both). No significant relationship was observed between SDI and ppDLCO.
**Conclusion:** Spinal deformity, as quantified by SDI, is independently associated with reduced lung volumes in ILD patients, irrespective of ILD subtype. Spinal integrity assessment may be a valuable addition to the interpretation of restrictive ventilatory defects, especially in elderly and osteoporotic populations.
**Limitations:** No limitations were identified.
**Funding for this study:** No funding was received for this study.
**Has your study been approved by an ethics committee?** Not applicable
**Author Disclosures:**
Angela Ventura: Nothing to disclose
Giovanni Puppini: Nothing to disclose
Lucia Pinali: Nothing to disclose
Susanna Baltieri: Nothing to disclose
Claudio Micheletto: Nothing to disclose
Loredana Carobene: Nothing to disclose
Angelo Fassio: Nothing to disclose
Davide Gatti: Nothing to disclose
Maurizio Rossini: Nothing to disclose

**Intravenous Contrast Effects on Opportunistic Osteoporosis Assessment: Impact on Absolute Values, T-Scores, and Classifications in Single- and Dual-Energy CT**

*S. Bernatz*, J. Gotta, V. Koch, S. Mahmoudi, R. Hammerstingl, T. Vogl, L. D. Grünewald; Frankfurt/DE
*(s.bernatz@web.de)*

**Purpose:** This study evaluates the effect of contrast agents on Hounsfield measurements, T-scores, and Z-scores, assessing their impact on diagnostic accuracy to reduce misclassification and optimize CT-based BMD assessment.
**Methods or Background:** A retrospective analysis of 597 patients (median age:66 years, 157 females, 440 males) was performed using dual-energy CT (DECT) scans of the abdomen and chest. All patients underwent non-contrast, arterial, and venous phase CT. Automated segmentation (nnU-Net) delineated L1 and L1-L4 trabecular bone, validated by two radiologists. T-scores were calculated according to DEXA-equivalent guidelines.
**Results or Findings:** Based on non-contrast CT, 35% were diagnosed with osteoporosis, 46% with osteopenia, and 18% had normal bone status. Median T-score was -2.0 (L1) and -2.1 (L1-L4)(p<0.001). Contrast agents significantly altered BMD values, with median changes of 22.9% (arterial) and 20.1%(venous). The most pronounced changes occurred in patients under 50 years (+99% at L1, p<0.001). In older females, 21% were misclassified as osteopenic instead of osteoporotic (p<0.001).
**Conclusion:** Contrast agents significantly affect BMD measurements, leading to diagnostic misclassification. This effect should be considered when using CT for osteoporosis diagnosis and treatment planning.
**Limitations:** It was conducted at a single center, which may limit generalizability across different scanners. Slice thickness was not assessed, despite its known influence on body composition parameters. Bone density was evaluated using T-scores derived from routine CT rather than quantitative CT, while pragmatic and clinically familiar, this approach may lack the precision of QCT. The cohort was imbalanced, with a predominance of male patients and relatively few individuals under 50 years, reflecting the underlying clinical population but restricting subgroup analyses. Finally, only cases with complete agreement between two readers were included; although this ensured high-quality ground truth, a consensus process or third reviewer would have been more robust.
**Funding for this study:** No funding
**Has your study been approved by an ethics committee?** Yes
**Ethics committee - additional information:** Ethics commitee of the University Hospital Frankfurt
**Author Disclosures:**
Thomas Vogl: Nothing to disclose
Vitali Koch: Nothing to disclose
Scherwin Mahmoudi: Nothing to disclose
Renate Hammerstingl: Nothing to disclose
Leon David Grünewald: Nothing to disclose
Jennifer Gotta: Nothing to disclose
Simon Bernatz: Nothing to disclose

**Bone health assessment in patients with prostate cancer: the role of REMS technology**

*M. Montatore*[1], S. Casciaro[2], F. R. Contaldo[2], F. Conversano[2], F. A. Lombardi[2], M. Muratore[2], P. Pisani[2], C. Stomaci[2], G. Guglielmi[1]; [1]Foggia/IT, [2]Lecce/IT
*(manuela.montatore@unifg.it)*

**Purpose:** Prostate cancer (PCa) represents one of the most prevalent malignancies among men, and its management often leads to significant complications, particularly concerning bone health. The interaction between prostate cancer and osteoporosis is multifaceted, involving both the disease itself and the therapies employed in its treatment (such as Androgen Deprivation Therapy (ADT)), which accelerates bone loss and increases fracture risk. Given the intricate relationship between PCa, its treatments, and bone health, proactive monitoring and management of osteoporosis in these patients are essential. This study aims to evaluate the impact of PCa and ADT on bone mineral density (BMD) using the non-ionizing REMS (Radiofrequency Echographic Multi-Spectrometry), the ultrasound-based method for diagnosing osteoporosis and predicting fracture risk on spine and femur
**Methods or Background:** A cohort of 51 Caucasian men with PCa and a healthy control group matchedfor gender, ethnicity, age, and body mass index (BMI), underwent REMS scans on the femoral neck (FN). A t-test to evaluate the differences in the measured BMD values between the two groups was conducted.
**Results or Findings:** In subjects with PCa and healthy controls, the mean ± standard deviation for age, weight, height, and BMI were73.5±7.3years, 25.3 ± 2.9 kg/m2) and (73.4 ± 7.2 years, 25.3 ± 3.1 kg/m², respectively. Patients with PCa had a significantly reduced BMD, T- and Z-score at the femoral neck compared to the healthy control group, table 1. BMD percentage difference: -3.4% g/cm2
**Conclusion:** The obtained significant decrease in FN BMD in PCa patients, as measured by REMS, underlines the disease's negative impact on bone health. The accuracy and precision of REMS allows it to early detect a bone damage for better management of PCa patients, especially those at higher risk due to ADT.
**Limitations:** No limitation were identified
**Funding for this study:** No funding was received for this study
**Has your study been approved by an ethics committee?** Not applicable
**Author Disclosures:**
Chiara Stomaci: Nothing to disclose
Maurizio Muratore: Nothing to disclose
Francesco Conversano: Nothing to disclose
Giuseppe Guglielmi: Nothing to disclose
Sergio Casciaro: Nothing to disclose
Fiorella Anna Lombardi: Nothing to disclose
Manuela Montatore: Nothing to disclose
Fabiola Rosa Contaldo: Nothing to disclose
Paola Pisani: Nothing to disclose

**Machine learning for the prediction of fragility fractures by bone and body composition parameters: the OsteoLaus 10 years populational cohort**

*C. Vendrami*, G. Gatineau, E. Shevroja, E. Gonzalez Rodriguez, O. Lamy, D. Hans; Lausanne/CH
*(colin.vendrami@chuv.ch)*

**Purpose:** Recent reviews have highlighted the potential value of machine learning (ML) in improving fragility fracture prediction. We aim to analyse the prediction of fragility fractures using regional and total body dual X-ray absorptiometry (DXA) assessments using a ML pipeline.
**Methods or Background:** A total of 1475 Swiss postmenopausal women (mean age 63.3±7.1 years, body mass index 25.4±4.3kg/m2) were followed for 10 years between 2010 and 2022. Parameters of bone health (from regional hip and spine DXA: 34 variables) and body composition (total body DXA: 65 variables) were assessed by DXA scans at baseline. Vertebral fractures were screened with lateral DXA during follow-up. Other risk factors (15 variables) and fragility fractures were collected from questionnaires at baseline and during follow up, respectively. All datasets were split for training (85%) and testing (15%) with a balanced fragility fractures proportion. Eight ML models were trained with hyperparameters tunning through 5-fold cross-validation to maximize the area under the curve (AUC).
**Results or Findings:** Using different combination of variables in the final complete case analysis, a total of 590 to 957 participants with 115-221 fragility fractures were included. The combination of all 114 variables in the same dataset (n=590) achieved the best AUC in both the training set (1.00-0.70) and test set (0.79-0.64). Logistic regression demonstrated the best balance in performance metrics: AUC 0.78, specificity 0.82, and sensitivity 0.71. ML models tend toward higher sensitivity and lower specificity.
**Conclusion:** These findings emphasize the combination of DXA-derived bone and body composition parameters for fragility fracture prediction. Logistic regression produced the most promising results. ML models remain at interest for further analysis in combination with image based analysis.
**Limitations:** Further studies including comparisons with FRAX®, larger sample sizes and external validation are needed.
**Funding for this study:** This study was funded by the Swiss National Science Foundation (SNSF 32473B_156978 and 320030_188886).
**Has your study been approved by an ethics committee?** Yes
**Ethics committee - additional information:** The OsteoLaus study received approval from the Institutional Ethics Committee of the University of Lausanne, and all participants signed informed consent (reference 215/09).
**Author Disclosures:**
Guillaume Gatineau: Nothing to disclose
Colin Vendrami: Nothing to disclose
Didier Hans: Nothing to disclose
Olivier Lamy: Nothing to disclose
Enisa Shevroja: Nothing to disclose
Elena Gonzalez Rodriguez: Nothing to disclose

**Prospective assessment of bone mineral density in spectral localizer radiographs from photon counting detector CT: A prospective in-vivo study**
*L. J. Moser*[1], K. Klambauer[1], V. Mergen[1], M. Eberhard[1], T. Nowak[2], B. Schmidt[2], T. Flohr[2], O. Distler[3], H. Alkadhi[1]; [1]Zürich/CH, [2]Forchheim/DE, [3]Zurich/CH

**Purpose:** To determine in a prospective patient study the accuracy of areal bone mineral density (aBMD) measurements with spectral localizer radiographs obtained with a clinical photon-counting detector (PCD)-CT scanner in comparison with dual-energy x-ray absorptiometry (DXA).
**Methods or Background:** In this IRB-approved, prospective study, 41 patients (15 females, 26 males; mean age 61.3 years, age range 35 - 78 years) underwent PCD-CT of the abdomen with a spectral localizer radiograph (tube voltage 140 kVp, tube current 30 mA) and DXA within a median of 45 days. Areal bone mineral density (aBMD) values were derived for lumbar vertebrae L1 - L4 from both methods and were compared with linear regression, Pearson correlation, intraclass correlation coefficients (ICCs), and Bland-Altman plots. T-scores were calculated on a patient level and were compared between methods.
**Results or Findings:** DXA and spectral localizer radiographs showed strong correlation in aBMD measurements (R = 0.97, p < 0.001) and patient level T-scores (R = 0.99, p < 0.001). There was a strong agreement between aBMD from both methods (ICC, 0.96 (95% CI [0.94, 0.97])). Bland-Altman analysis revealed a very small mean difference in aBMD between methods (mean absolute error 0.019 g/cm2) with narrow limits of agreement (-0.083 g/cm2 to 0.121 g/cm2). Similarly, there were small differences in regard to the T-score (mean absolute error 0.156) with narrow limits of agreement (-0.422 to 0.734) between methods. ICCs indicated an excellent agreement between T-scores from DXA and spectral localizer radiographs (ICC, 0.98 (95% CI [0.95, 0.99])).
**Conclusion:** Our prospective patient study indicates that spectral localizer radiographs obtained with a clinical PCD-CT system enable accurate quantification of the lumbar bone areal mineral density.
**Limitations:** The limitations of this study are as follows: Small sample size. Manual segmentation process. Prototype software.
**Funding for this study:** No funding was received for this study.
**Has your study been approved by an ethics committee?** Yes
**Ethics committee - additional information:** Approval by the local EC
**Author Disclosures:**
Thomas Flohr: Employee: Siemens Healthineers AG
Victor Mergen: Nothing to disclose
Matthias Eberhard: Nothing to disclose
Bernhard Schmidt: Employee: Siemens Healthineers AG
Oliver Distler: Nothing to disclose
Lukas Jakob Moser: Nothing to disclose
Konstantin Klambauer: Nothing to disclose
Tristan Nowak: Employee: Siemens Healthineers AG
Hatem Alkadhi: Nothing to disclose

**Longitudinal Changes of Muscle and Bone Marrow Fat Fraction over 5 years Postpartum in Premenopausal Women with Prior Gestational Diabetes**
*Y. Stohldreier*, U. Ferrari, S. Schläger, O. Dietrich, H. Hermann, E. Pappa, J. Seißler, A. Gersing, N. Hesse; Munich/DE
*(yannick.stohldreier@med.uni-muenchen.de)*

**Purpose:** This study investigates the longitudinal associations between transient insulin resistance during gestational diabetes mellitus (GDM), a prediabetic state, and the composition of vertebral bone marrow (VBM) and paraspinal muscles assessed using quantitative magnetic resonance imaging (MRI).
**Methods or Background:** We included 78 premenopausal women, 46 with a history of GDM (GDM group) and 32 healthy controls (HC) after normoglycemic pregnancy. At the 5-year-follow-up the glucose metabolism remained pathologic (pGM) in 10 women, based on oral glucose tolerance testing. 3T MRI including quantitative spine imaging was acquired at 1 year and 5 years postpartum, from which proton density fat fraction (PDFF) maps were calculated and autochthone muscles (AM), psoas, and VBM of the thoracic (T9-12) and lumbar (L1-L4) spine were assessed.
**Results or Findings:** At 1-year-follow-up, all women with prior GDM showed a significantly higher PDFF in the VBM (p=0.03) and AM (p=0.04) compared with controls, suggesting increased fat content and reduced quality. At 5-year-follow-up, no significant group differences were observed, indicating a potential reversal of the previous composition alterations. In contrast, after 5 years, the pGM group exhibited higher PDFF values in the PM and AM (both p=0.01), but not in VBM at 5-year-follow-up (p>0.05). Elevated muscle PDFF was associated with significantly increased odds of pGM at 5-year-follow-up after adjusting for age (PDFF AM OR 1.26 [95% CI 1.05, 1.56], p=0.02; PDFF psoas OR 1.49 [95% CI 1.07, 2.24], p=0.03).
**Conclusion:** Vertebral bone marrow fat fraction was elevated 1 year after GDM and normalized after 5 years postpartum, while persistent impairment of glucose metabolism was associated with increased PDFF, suggesting reduced skeletal muscle quality 5 years postpartum. Therefore, muscle PDFF may be a useful biomarker for monitoring muscle health in patients with metabolic diseases.
**Limitations:** Retrospective monocentric study.
**Funding for this study:** This work was funded by the Munich Clinician Scientist Program (MCSP) of the University of Munich (LMU; grant number ACS-10), LMU Klinikum, the German Center for Diabetes Research (DZD), and the Helmholtz Zentrum München
**Has your study been approved by an ethics committee?** Yes
**Ethics committee - additional information:** The study was approved by the local institutional review board (Ethics Commission of the Medical Faculty, Ludwig-Maximilians Universität München) and all study participants provided written informed consent prior to their participation in the study, which was conducted in accordance with the declaration of Helsinki.
**Author Disclosures:**
Olaf Dietrich: Nothing to disclose
Jochen Seißler: Nothing to disclose
Sarah Schläger: Nothing to disclose
Yannick Stohldreier: Nothing to disclose
Alexandra Sophia Gersing: Nothing to disclose
Nina Hesse: Nothing to disclose
Hana Hermann: Nothing to disclose
Eleni Pappa: Nothing to disclose
Uta Ferrari: Nothing to disclose

**Assessment of bone microarchitecture and its correlation with trabecular fracture predictor using ImageView**
*N. Castro*, J. Catala March; Barcelona/ES
*(ncastro@institutsguirado.com)*

**Purpose:** This preliminary study evaluates ImgView, a novel software for high-resolution CT (HR-CT) analysis, in quantifying bone microarchitecture and predicting trabecular fracture risk. The relationship between microarchitectural parameters and predicted fracture risk is assessed in skeletal regions affected by morphological alterations or metabolic bone disease.
**Methods or Background:** Ten healthy subjects underwent 5G Cone Beam CT (CBCT) imaging. Scans were analysed using ImgView (RAR SRL, Italy) and ImageJ software to obtain bone volume fraction (BV/TV), trabecular thickness (Tb.Th), and trabecular spacing (Tb.Sp). ImgView automatically segments cortical and trabecular bone within a 9.5 mm distal radial ROI, computing densitometric and morphometric indices including bone mineral density, cortical thickness and porosity, and detailed trabecular parameters. A subtool predicts fracture risk by applying a uniform 1000N force to the reconstructed bone structure. Statistical analysis used paired t-tests, Bland–Altman plots, and linear regression to evaluate bone parameters against fracture prediction.
**Results or Findings:** No significant differences were found for BV/TV between methods (p > 0.05). In contrast, minor differences were identified for Tb.Th (p=0.034) and Tb.Sp (p=0=0.028), Bland-Altman analysis confirmed these remained within acceptable clinical limits. Regression analysis demonstrated strong, significant correlations: BV/TV with fracture prediction (p=0.002; $R^2$=0.764), and Tb.Sp with fracture prediction (p=0.001; $R^2$=0.787). Tb.Th showed a weaker, marginally significant association (p=0.05; $R^2$=0.444).
**Conclusion:** This study confirms that favourable bone microarchitecture, with greater bone volume and thickness, correlates with lower fracture risk, while increased intertrabecular spacing indicates higher risk. Despite data variability, this study validates ImgView's indicators for fracture risk assessment. ImgView offers automated metrics to enhance diagnostic accuracy by detecting subtle trabecular changes in suspected osteoporosis, enabling radiologists to target high-risk fracture areas. This tool represents a significant advancement in objective bone health assessment, promising improved patient outcomes.
**Limitations:** Sample size.
**Funding for this study:** None
**Has your study been approved by an ethics committee?** Not applicable
**Author Disclosures:**
Jordi Catala March: Nothing to disclose
Nagore Castro: Nothing to disclose

### Opportunistic screening of lumbar vertebrae in CT using TotalSegmentator to prevent osteoporotic vertebral fractures

*M. Seng*, J. Wasserthal, M. Bach, D. Harder, M. Obmann, C. Meier, E. M. Merkle, H-C. Breit, M. Segeroth; Basel/CH

**Purpose:** Osteoporotic vertebral fractures impair quality of life but are preventable. Routine CT examinations offer an opportunity for early risk stratification. This study assessed quantitative metrics of vertebral and paraspinal tissue extracted by TotalSegmentator to identify predictors of osteoporotic vertebral fractures.
**Methods or Background:** 3299 lumbar spine CT datasets were retrospectively identified in our PACS (2020-2025). 1469 were excluded using large language models (contrast agent, bone metastasis, severe artefacts, hematological diseases, high impact trauma) and 370 due to incompatible imaging parameters (image type, bone kernel, slice thickness). Automated segmentation of vertebrae and paraspinal tissue was performed using TotalSegmentator. Quantitative measures included mean attenuation values and volumes. Vertebral heights of L1 to L4 were assessed on three coronal slices, with fractures defined by a height reduction of >20%. Associations were assessed with logistic regression and ROC analyses.
**Results or Findings:** Automated vertebral segmentation was successful in 1185 of 1460 cases. The mean attenuation of the non-fractured lumbar vertebrae was lower in patients with an osteoporotic fracture (85.08 (CI: 81.25 - 89.17) n=612) compared to patients without any fracture (125.75 (CI: 120.50 - 131.13) n=573, p < 0.001). Attenuation alone showed an AUC of 0.70 (95% CI: 0.67–0.73) with an optimal cut-off at 90 Hounsfield units (sensitivity 0.71, specificity 0.59). Volume and attenuation of the iliopsoas and autochthon muscles differed significantly between groups (p < 0.001), whereas subcutaneous and visceral fat did not. A combined logistic model (AUC 0.77 (95% CI: 0.71–0.82)) rendered the attenuation of the non-fractured vertebrae as most relevant followed by the attenuation of L1, L3, L2 and L4.
**Conclusion:** Vertebral attenuation, below 90 HU, is a robust predictor of osteoporotic fractures. Opportunistic CT-based screening could support early identification and timely intervention in patients at risk.
**Limitations:** None.
**Funding for this study:** None.
**Has your study been approved by an ethics committee?** Yes
**Ethics committee - additional information:** The requirement for informed consent was waived as this was a retrospective study.
**Author Disclosures:**
Markus Obmann: Nothing to disclose
Jacob Wasserthal: Nothing to disclose
Michael Bach: Nothing to disclose
Hanns-Christian Breit: Nothing to disclose
Dorothee Harder: Nothing to disclose
Magdalena Seng: Nothing to disclose
Martin Segeroth: Nothing to disclose
Christian Meier: Nothing to disclose
Elmar M. Merkle: Nothing to disclose

### Novel and Compact Digital Tomosynthesis DXA Device for Bone Mineral Density

J. Hu[1], J. Chen[1], S. Wells[2], V. Soloviev[2], S. Coulson[2], S. Phillips[2], J. Evans[1], A. Forbes-Brown[1], *K. Knapp*[1]; [1]Exeter/UK, [2]Oxford/UK
*(k.m.knapp@exeter.ac.uk)*

**Purpose:** Bone mineral density (BMD) measurement is central to the diagnosis and management of osteoporosis [1]. Dual-energy X-ray absorptiometry (DXA) remains the most widely used clinical tool, but is limited by large, immobile scanners restricted to specialist centres, reducing accessibility [2] and two-dimensional (2D), low-resolution measurements of areal BMD (aBMD) that do not capture the true volumetric density of local bone variation [3].
**Methods or Background:** We present early feasibility results from an ongoing collaboration with Adaptix Ltd. to develop a digital tomosynthesis–based DXA (DT-DXA) system. It employs a compact x-ray source that can sequentially fire X-rays from an array of positions with a short source–detector distance, enabling portable deployment and three-dimensional tomographic capability with areal or volumetric BMD (vBMD) as an add-on to extremity orthopaedic imaging. DT-DXA can use synthetic 2D to calculate aBMD with the potential advantage of being able to exclude slices containing only soft tissue from the calculation. 3D-printed trabecular bone phantoms [4] and 30 surgically replaced human femoral heads, representing a broad spectrum of densities and microarchitectures, were used to compare DT-DXA performance against iDXA (GELunar) and microCT and to evaluate its accuracy and robustness across clinically relevant bone conditions.
**Results or Findings:** DT-DXA aBMD and vBMD results were benchmarked against GE iDXA. Phantom and femoral head studies showed close agreement.
**Conclusion:** DT-DXA offers BMD measurement in a compact, mobile platform. These results highlight its potential for offering point-of-care access to bone density assessment, with the potential to expedite diagnosis and treatment of osteoporosis.
**Limitations:** In vivo patient imaging has not yet been performed. The radiation dose of DT-DXA was higher than the iDXA, but far lower than medical CT, with scope for further optimisation.
**Funding for this study:** UK Research and Innovation
**Has your study been approved by an ethics committee?** Yes
**Ethics committee - additional information:** Exeter Clinical Research Facility
**Author Disclosures:**
Sian Phillips: Nothing to disclose
Karen Knapp: Nothing to disclose
Junning Chen: Nothing to disclose
Andrew Forbes-Brown: Nothing to disclose
Steve Technology Wells: Nothing to disclose
Jingrui Hu: Nothing to disclose
Vadim Soloviev: Nothing to disclose
Jonathan Evans: Nothing to disclose
Sophie Coulson: Nothing to disclose

### Reproducibility of DXA BMD measurements: effect of soft-tissue thickness and acquisition mode

*F. Cicchetti*, G. De Padova, L. Macrì, M. Costa, K. Caringal, D. Albano, A. Zagarella, C. Messina; Milan/IT

**Purpose:** To evaluate how acquisition mode and simulated soft-tissue thickness affect the stability of dual-energy X-ray absorptiometry (DXA) measurements.
**Methods or Background:** DXA scans were performed with Stratos DR densitometer (DMS, France), software version 5.3.3.3. Three acquisition modes (Fast, Normal, Precision) were tested under four simulated adipose tissue thicknesses (0, 1, 3, 6 cm, applied with pork rind). Each condition had 25 repeated acquisitions, totalling 300 measurements. Data were analyzed with two-way ANOVA and Tukey's HSD test (p < 0.05).
**Results or Findings:** ANOVA demonstrated a highly significant effect of tissue thickness (F = 27.0, p < 0.0001), while acquisition mode alone was not significant (F = 0.72, p = 0.49). A significant thickness × mode interaction was observed (F = 3.26, p = 0.004). Post-hoc analysis revealed that at the same thickness no differences were observed between Fast, Normal, and Precision modes. Within modes, Normal and Precision showed significant changes between 0 cm and 1 cm (p ≤ 0.04), and between 1 cm and thicker layers (p ≈ 0.001–0.002). The Fast mode showed significance only between 0 cm and 1 cm (p = 0.0002). Although the 0 cm vs 6 cm comparison was not statistically significant (p = 0.53), reproducibility indices indicated a reduction in measurement precision from ~98.5% at 0 cm to ~94% at 6 cm, suggesting a relevant downward trend.
**Conclusion:** Simulated soft tissue thickness is the main determinant of DXA measurement variability. Although the three acquisition modes provide comparable results under the same thickness, Normal and Precision modes are more sensitive to minimal increments in tissue layers, whereas the Fast mode appears less sensitive but more variable.
**Limitations:** The limitations of the study are the phantom design and use of simulated soft tissue.
**Funding for this study:** No funding was received for this study.
**Has your study been approved by an ethics committee?** Not applicable

**Author Disclosures:**
Giuseppe De Padova: Nothing to disclose
Marco Costa: Nothing to disclose
Laura Macrì: Nothing to disclose
Francesco Cicchetti: Nothing to disclose
Carmelo Messina: Nothing to disclose
Andrea Zagarella: Nothing to disclose
Domenico Albano: Nothing to disclose
Krismalyn Caringal: Nothing to disclose

**HR-pQCT versus DXA for longitudinal monitoring of patients with osteogenesis imperfecta: results at 1-year follow-up**

*S. Gazzotti*[1], G. Fraterrigo[1], E. Schileo[1], R. Sassi[1], M. P. Aparisi Gomez[2], A. Moroni[1], L. Sangiorgi[1], F. Taddei[1], A. Bazzocchi[1]; [1]Bologna/IT, [2]Auckland/NZ

**Purpose:** Osteogenesis imperfecta (OI) is a rare genetic disease characterised by frequent fractures. Affected patients undergo periodic dual-energy X-ray absorptiometry (DXA), but this has limitations. High-resolution peripheral quantitative computed tomography (HR-pQCT) is an alternative low-dose technique providing information on volumetric density and microstructure, but there are scarce data on its use for follow-up. The aim of this work is to compare the performance of DXA and HR-pQCT in the longitudinal monitoring of OI.
**Methods or Background:** This preliminary analysis includes 19 adult patients (median age: 35.5 years) with OI type I who underwent DXA scans at lumbar spine, hip, total body, and forearm and HR-pQCT at distal radius and tibia at baseline and at 1-year follow-up.
**Results or Findings:** Median Z-scores at the femoral neck and lumbar spine were -1.3 and -1.9 at baseline versus -1.4 and -1.9 at 1-year, respectively. No significant changes in DXA-areal bone mineral density (aBMD) could be detected in 1-year at central sites. HR-pQCT parameters overall showed minimal variations (median change: +1.5% to -6.4% at radius; +1.75% to -0.4% at tibia). DXA-aBMD at ultradistal radius was correlated with total volumetric BMD by HR-pQCT at radius both at baseline (Spearman’s rho = 0.70; $p < 0.05$) and at 1-year (Spearman’s rho = 0.88; $p < 0.001$). However, when the respective longitudinal changes were directly compared, the results were not statistically significant.
**Conclusion:** While data indicate some site-specific agreement between DXA and HR-pQCT, the two modalities are likely to play a differential role in the longitudinal monitoring of OI. A 1-year time frame may be too short to observe significant changes in measured parameters, which could have important implications for selecting the most appropriate interval for follow-up in clinical practice.
**Limitations:** Ongoing study, small sample size.
**Funding for this study:** No funding was received for this study.
**Has your study been approved by an ethics committee?** Yes
**Ethics committee - additional information:** CE-AVEC 890/2022/Sper/IOR
**Author Disclosures:**
Luca Sangiorgi: Nothing to disclose
Alberto Bazzocchi: Nothing to disclose
Giulia Fraterrigo: Nothing to disclose
Enrico Schileo: Nothing to disclose
Alice Moroni: Nothing to disclose
Fulvia Taddei: Nothing to disclose
Maria Pilar Aparisi Gomez: Nothing to disclose
Rebecca Sassi: Nothing to disclose
Silvia Gazzotti: Nothing to disclose

**09:30 - 11:00 Research Stage 5**

### Research Presentation Session: Neuro

### RPS 1811

### Non-ischemic vascular pathology of the brain

**Moderator**
Alexandre Krainik; Grenoble/FR
**Author Disclosures:**
Alexandre Krainik: Advisory Board: Geadaisics, Lilly, Consultant: Olea, Investigator: Bayer, Share Holder: Geaodaisics, Gleamer, Speaker: GE Healthcare, Other: Philips, Guerbet, Bracco

**Real-World Diagnostic Accuracy of 4D-CT Angiography for the Detection and Localization of Cerebrovascular Arteriovenous Shunts: a 15-year Cohort Study**

*F. J. A. Meijer*, O. Van Hulst, S. Pegge, H. D. Boogaarts; Nijmegen/NL
*(anton.meijer@radboudumc.nl)*

**Purpose:** The aim of this study was to evaluate the indications for four-dimensional computed tomography angiography (4D-CTA), and to assess its diagnostic accuracy for detecting and localizing cerebrovascular arteriovenous shunting, in a real-world clinical setting.
**Methods or Background:** A single-center retrospective cohort study was performed, where all patients who underwent 4D-CTA in the period January 2010 and January 2025 were retrieved. Demographic and clinical parameters including age, sex, and presenting symptoms were obtained from the medical records. The indications and results of angiographic studies (shunt presence, type, location, and classification) were retrieved from the study reports. Diagnostic accuracy of 4D-CTA for the detection of AV-shunting was evaluated in a subgroup of patients who underwent digital subtraction angiography (DSA) within 6 months of 4D-CTA, with unchanged clinical status.
**Results or Findings:** Among 366 patients, the most common indications for 4D-CTA were pulsatile tinnitus (37%) and the diagnostic work-up of cerebral hemorrhage (19%). In the subgroup of patient who also underwent DSA (n=71), 4D-CTA demonstrated a sensitivity of 96.2%, positive predictive value of 98.0%, specificity of 94.7%, and negative predictive value of 90.0% for the detection of AV-shunting. 4D-CTA demonstrated high accuracy (98%) in distinguishing between arteriovenous malformations (AVM) and dural arteriovenous fistulas (AVF).
**Conclusion:** 4D-CTA exhibits high sensitivity and specificity for the detection of cerebrovascular arteriovenous shunting, and high accuracy to differentiate between AVM or AVF, supporting its potential role as a first-line imaging modality prior to invasive DSA.
**Limitations:** The lack of confirmatory DSA following negative 4D-CTA examinations may have introduced a selection bias, as a majority of patients with positive findings on 4D-CTA underwent subsequent DSA. The generalizability of our findings may be constrained to tertiary neurovascular centers, where referred populations often differ from broader clinical cohorts.
**Funding for this study:** None
**Has your study been approved by an ethics committee?** Not applicable
**Author Disclosures:**
Sjoert Pegge: Nothing to disclose
Frederick Jan Anton Meijer: Nothing to disclose
Hieronymus D. Boogaarts: Nothing to disclose
Owen Van Hulst: Nothing to disclose

**Automated Assessment Pipeline for Aneurysm Rupture Risk: Automatic Segmentation, Classification, and External Testing**

*F. Yao*, D. Zhu, Y. Yang; Wenzhou/CN

**Purpose:** Current research on intracerebral aneurysms (IAs) rupture risk assessment mainly focuses on deep learning and radiomics models, both of which rely on accurate segmentation of aneurysm regions. This study aims to create a robust, fully automated segmentation network for multi-center use and compare deep learning and radiomics models for rupture prediction.
**Methods or Background:** The study utilized data from the MIRACLE cohort, applying the DGIS method for automatic aneurysm segmentation. Five machine learning (ML) and deep learning models were developed for rupture prediction. The models were trained on 593 aneurysms and validated on 75 aneurysms from four hospitals. Model performance was evaluated using AUC, accuracy, sensitivity, specificity, and F1 score, along with SHAP analysis for feature importance and Grad-CAM for interpretability.

**Results or Findings:** The DGIS segmentation method achieved high accuracy, with Dice coefficients of 0.98 in the source domain and 0.75 in the target domain. The DRE deep learning model outperformed other models, achieving an AUC of 0.995 in training and 0.851 in external testing, surpassing radiomics AdaBoost (AUC, 0.983 and 0.634) and SVM (AUC, 0.712 and 0.656). SHAP analysis revealed key rupture risk features, and Grad-CAM highlighted the model's focus on the aneurysm region.
**Conclusion:** This study presents an automated, generalizable approach for IA rupture risk analysis, offering an efficient and clinically applicable method for aneurysm detection and rupture prediction across multi-center datasets.
**Limitations:** First, the study primarily focused on a single type of aneurysm (middle cerebral artery aneurysms), which may limit the generalizability of the findings to other types of aneurysms. Even though the attention regions of the model can be visualized, fully understanding the internal workings of the model is still challenging.
**Funding for this study:** This study has received funding by the Key Laboratory of Novel Nuclide Technologies on Precision Diagnosis and Treatment & clinical Transformation of Wenzhou (Grant No. 2023HZSY0012), the Discipline Cluster of Oncology, Wenzhou Medical University, China (Grant No. z1-2023008), the Summit Advancement Disciplines of Zhejiang Province (Wenzhou Medical University – Pharmaceutics).
**Has your study been approved by an ethics committee?** Not applicable
**Author Disclosures:**
Dongqin Zhu: Nothing to disclose
Fei Yao: Nothing to disclose
Yunjun Yang: Nothing to disclose

**Deep learning assistance improves clinician performance in detecting cerebral aneurysms on MRA**
*G. Jung*, J. Y. Lee, H. Kim, S-M. Gho, Y. Yim, J-B. Lee, T-K. Nam, C-Y. Park, K-Y. Park; Seoul/KR
*(jungkc123@naver.com)*

**Purpose:** Reliable detection of intracranial aneurysms on MRA requires substantial expertise, yet diagnostic variability among physicians remains a challenge. As clinical use of MRA expands, there is a growing demand for consistent and accurate aneurysm detection. This study aimed to assess interobserver variability in detection performance across physicians with different levels of experience and to explore the potential role of artificial intelligence as a decision-support tool.
**Methods or Background:** TOF-MRA studies with and without intracranial aneurysms were retrospectively collected. An inexperienced physician and a neurology resident with one year of training independently reviewed the images and marked suspected aneurysms. Their findings were compared with reference annotations provided by two board-certified neuroradiologists. After a washout period, the same readers reinterpreted the MRAs with the assistance of deep learning–based software. Diagnostic accuracy and interpretation time were compared between readings with and without AI support.
**Results or Findings:** A total of 642 patients were included, among whom 321 patients had 398 aneurysms (up to five per patient). Without AI assistance, the inexperienced physician required an average of 85 seconds per case, achieving a sensitivity of 63.6% (253/398) and an accuracy of 74.5%. The neurology resident required 33 seconds per case, with a sensitivity of 60.0% and an accuracy of 66.8%. With AI support, the inexperienced physician improved to 42 seconds per case, with a sensitivity of 83.4% and an accuracy of 80.2%. The resident improved to 29 seconds per case, with a sensitivity of 85.9% and an accuracy of 86.1%.
**Conclusion:** Deep learning–based AI assistance improved sensitivity and accuracy of intracranial aneurysm detection on MRA while reducing interpretation time.
**Limitations:** As the study is ongoing, the findings represent a preliminary analysis of the available data.
**Funding for this study:** This work was supported by 'Supporting Project to Clinical evaluation Domestic Medical Devices in Hospitals' funded by 'Ministry of Health and Welfare (MOHW)' and 'Korea Health Industry Development Institute (KHIDI)'
**Has your study been approved by an ethics committee?** Yes
**Ethics committee - additional information:** Approved by the Chung-Ang University Hospital Institutional Review Board. (IRB No. 2504-007-19570)
**Author Disclosures:**
Taek-Kyun Nam: Nothing to disclose
Gucheol Jung: Nothing to disclose
Chan-Young Park: Nothing to disclose
Jung-Bin Lee: Nothing to disclose
Hanna Kim: Nothing to disclose
Ju Young Lee: Nothing to disclose
Kwang-Yeol Park: Nothing to disclose
Sung-Min Gho: Nothing to disclose
Younghee Yim: Nothing to disclose

**Clinical verification of deep learning enabled CTA-based intracranial aneurysm auxiliary diagnostic systems: dual-center clinical trial**
*D. Wang*, S. Wang, C. Xia; Beijing/CN
*(wdwwang123@126.com)*

**Purpose:** Mounting evidence indicates that early treatment of the aneurysm and aggressive prevention and management of complications can both improve patients' clinical outcomes. This study intends to verity the effectiveness of a CT-angiography-based auxiliary triage and evaluation system for intracranial aneurysms via a clinical trial.
**Methods or Background:** A dual-center, retrospective, single-arm target-value design was used to evaluate the triage performance of a deep learning-based intelligent system (InferRead CTA Stroke. A total of 485 subjects were enrolled from two centers. Of these, 481 subjects completed the trial: 321 from Center 1 and 160 from Center 2. Reference standard was established by three experienced cerebrovascular imaging experts who were independent of the AI system. Primary endpoints are aneurysm triage sensitivity and specificity both over 80%. Secondary endpoint is saved time by the AI system compared to the hospital's routine imaging-report turnaround time.
**Results or Findings:** The system achieved a triage sensitivity of 88.14 % (95 % CI: 82.44 %–92.50 %) and specificity of 88.16 % (95 % CI: 83.98 %–91.57 %). Because the lower bound of the confidence interval exceeded the pre-specified targets of 80% (sensitivity, $p = 0.0068 < 0.01$; specificity, $p = 0.0004 < 0.001$), the system can be considered effective in identifying positive cases and ruling out negative cases. Median software reading time was 843s, which was 3 545s faster than the hospital's routine reporting time ($p < 0.0001$). In terms of mean time consumption, the system increased diagnostic efficiency by 94%. Thus, the software is expected to significantly improve reading efficiency.
**Conclusion:** The validated AI auxiliary diagnostic system is effective in identifying aneurysm cases in a more efficient way, exhibiting great potential to improve the clinical workflow for early detection of intracranial aneurysm.
**Limitations:** N/A
**Funding for this study:** N/A
**Has your study been approved by an ethics committee?** Yes
**Ethics committee - additional information:** Ethics Committee of Zhongnan Hospital of Wuhan University and Affiliated Zhongshan Hospital of Dalian University
**Author Disclosures:**
Dawei Wang: Employee: Infervision
Chen Xia: Nothing to disclose
Shaokang Wang: Employee: Infervision

**Preoperative high-resolution vessel wall MRI of unruptured intracranial aneurysms: correlation of bleb enhancement with wall inflammation and instability**
*A. Brancasi*, D. Parisi, A. Perna, G. Marziali, R. Di Bonaventura, M. Galeazzi, R. Russo, A. Albanese, S. Gaudino; Rome/IT
*(alessandrobrancasi@gmail.com)*

**Purpose:** This study investigates the diagnostic value of high-resolution preoperative MRI for unruptured intracranial aneurysms and the association between wall bleb enhancement and aneurysm instability.
**Methods or Background:** Intracranial aneurysm rupture is life-threatening, yet non-invasive risk assessment remains difficult. Blebs—focal protrusions of the aneurysmal sac—are considered markers of instability, though their predictive value is inconsistent. High-resolution vessel wall imaging (VWI) with post-contrast black-blood sequences suppresses intraluminal flow and depicts wall thickening and enhancement as indicators of inflammation and remodeling. Although enhancement has been linked to unstable phenotypes, its association with blebs remains uncertain. We conducted a prospective single-center study of 30 patients scheduled for surgery for unruptured aneurysms. All patients underwent preoperative 3T MRI (GE, 48-channel coil) with Gadovist, including 3D TOF and pre- and post-contrast black-blood sequences. Imaging findings were compared with intraoperative appearance, and wall tissue was collected for histopathology in nine cases.
**Results or Findings:** Of 30 aneurysms, 22 (73.3%) had at least one bleb confirmed by imaging and surgery. Contrast enhancement was significantly associated with blebs (73% vs. 25%; $P = 0.0492$). Blebs were classified as red, yellow, or white. Enhancement was most frequent in white blebs (77%), followed by red (63%) and yellow (60%), with no link between morphology and inflammation. In all histological samples, wall enhancement matched inflammatory infiltration.
**Conclusion:** High-resolution VWI with black-blood sequences is a valuable tool for preoperative aneurysm assessment. Bleb wall enhancement correlates with histological inflammation, supporting its role as a non-invasive biomarker of wall instability and a tool for rupture risk stratification.
**Limitations:** No limitations were identified.
**Funding for this study:** No funding was received for this study.
**Has your study been approved by an ethics committee?** Not applicable

Saturday

**Author Disclosures:**
Rosellina Russo: Nothing to disclose
Marco Galeazzi: Nothing to disclose
Giammaria Marziali: Nothing to disclose
Simona Gaudino: Nothing to disclose
Alessandro Perna: Nothing to disclose
Alessio Albanese: Nothing to disclose
Davide Parisi: Nothing to disclose
Rina Di Bonaventura: Nothing to disclose
Alessandro Brancasi: Other: Ethics committee approval has been requested and is currently pending.

**Quantitative angiographic parameters associated with symptomatic radiation induced changes in supratentorial brain arteriovenous malformations after radiosurgery**

*W. L. Kao*[1], Y-S. Hu[2], C-J. Lin[1], J-K. Loo[2], H-C. Yang[1], C-C. Lee[1], H-J. Chiou[1], F-C. Chang[1], K-D. Liu[1]; [1]Taipei/TW, [2]New Taipei/TW

**Purpose:** Radiation-induced changes (RICs) may cause neurological deficits in patients with brain arteriovenous malformations (BAVMs) after radiosurgery. The present study investigated quantitative angiographic parameters contributing to symptomatic RICs.
**Methods or Background:** A total of 131 patients with supratentorial BAVMs who had not received prior treatment and underwent radiosurgery between 2011 and 2020 were included. Patients completed ≥ 24 months of MRI and clinical follow-up. MRIs and angiograms taken before radiosurgery were analyzed for morphological characteristics and quantitative angiographic parameters. Symptomatic RICs were defined as neurological symptoms attributed to RICs. The vein-artery (VA) ratio was defined as the sum of all draining vein diameters divided by the sum of all supplying artery diameters. The modified cerebral circulation time (mCCT) was defined as the interval between the bolus arrival time of the ipsilateral cavernous internal carotid artery and the parietal vein. Logistic regression models were used to evaluate associations between these parameters and symptomatic RICs.
**Results or Findings:** Symptomatic RICs developed in 27 (20.6%) of 131 patients. Nine patients with symptomatic RICs were hospitalized. Multivariable analysis revealed that a lower VA ratio and shorter mCCT were independently associated with symptomatic RICs. Furthermore, the quantitative angiographic model exhibited a higher performance in association with symptomatic RICs than the angioarchitectural model did.
**Conclusion:** A lower VA ratio and shorter mCCT were quantitative angiographic parameters of venous outflow impairment and high blood flow of BAVMs, respectively. These parameters may quantify the hemodynamic effect that contributes to symptomatic RICs development in patients with BAVMs after radiosurgery.
**Limitations:** Our single-center, retrospective study may have overestimated symptomatic RIC rates due to follow-up bias and excluded certain BAVM cases, limiting generalizability to other patient groups and treatments.
**Funding for this study:** Funding was provided by Taiwan’s Ministry of Science and Technology (grant number: MOST 112-2314-B-A49-064- to C.J.L. and MOST 113-2314-B-A49-051- to C.J.L.) and Melissa Lee Cancer Foundation.
**Has your study been approved by an ethics committee?** Yes
**Ethics committee - additional information:** The study was approved by the Institutional Review Board (3) of Taipei Veterans General Hospital and supervised by the Institutional Review Board (3) of Taipei Veterans General Hospital. After the review by Human Research Protection Center, the implementation of the study was approved.
**Author Disclosures:**
Kang-Du Liu: Nothing to disclose
Jing-Kai Loo: Nothing to disclose
Cheng-Chia Lee: Nothing to disclose
Feng-Chi Chang: Nothing to disclose
Hong-Jen Chiou: Nothing to disclose
Huai-Che Yang: Nothing to disclose
Chung-Jung Lin: Nothing to disclose
Yong-Sin Hu: Nothing to disclose
Wei Lun Kao: Nothing to disclose

**Evaluation of cerebral perfusion patterns in patients with chronic internal carotid artery occlusion for EC-IC bypass surgery selection**

*T. Nemirovskaya*[1], A. Nemirovskiy[2]; [1]Kazan, Moscow/RU, [2]Kazan/RU
*(tanya.nemirovsky@gmail.com)*

**Purpose:** Extracranial-intracranial bypass (ECIC) surgery may be useful for internal carotid artery (ICA) chronic occlusion. Most cases are secondary to atherosclerosis, however other mechanisms exist, e.g. post-radiotherapy damage, vasculopathy, developmental anomalies. We use CT-perfusion (CTP) as key modality for revascularization candidates selection. Purpose of study was assessment of perfusion patterns in unilateral, bilateral atherosclerotic and moya-moya disease/syndrome ICA occlusion.
**Methods or Background:** Retrospective assessment of pre-operation CTP-maps of 271 patients, later underwent ECIC in our Neurosurgery department, was performed. All underwent CTP according to standard institutional protocol. Perfusion maps: cerebral blood flow (CBF), cerebral blood volume (CBV), mean transit time (MTT). Six regions of interest were manually outlined bilaterally in main supply areas. Mean hemispheric values and mean ICA-area values were calcualted with interhemispheric ratios assessment.
**Results or Findings:** A total 271 patients with chronic unilateral (234) and bilateral (27) ICA occlusion and moya-moya disease (10) were selected for ECIC surgery. Candidates criteria were ipsilateral MTT elevation, CBF reduction, increased CBV. Unilateral occlusion group CBF was 34.785 ml/100g/min, interhemispheric asymmetry 76.59% , ICA areas asymmetry 65.159% . No significant difference of interhemispheric CBF in bilateral occlusion group, 37.612 and 35.933ml/100 g/min and symmetry 104.67%, were observed, however anterior-posterior asymmetry was detected. Moya-moya group demonstrated highest CBF, 49.251 and 57.913ml/100 g/min, with asymmetry 81,806%. P-value <0.0001. The highest MTT values were observed in unilateral occlusion group (P = 0.05). CBV values were high and not significantly differed. We established perfusion patterns for each group with highest interhemispheric asymmetry in unilateral occlusion, anterior-posterior pattern in bilateral occlusion and asymmetric increased values in moya-moya group.
**Conclusion:** Specific CTP patterns depending on different aetiology of chronic ICA occlusion should be considered surgical treatment candidates evaluation.
**Limitations:** Retrospective assessment of CT perfusion maps
**Funding for this study:** No
**Has your study been approved by an ethics committee?** Not applicable
**Author Disclosures:**
Alexander Nemirovskiy: Nothing to disclose
Tatiana Nemirovskaya: Nothing to disclose

**Berlin EMMA Score (BE-EMMA): Branch-enhanced grading of middle meningeal artery embolization in chronic subdural hematoma**

*L. Fetscher*[1], E. Siebert[1], C. Csapo-Schmidt[1], N. Mahmoudi[1], O. Reihani[1], D. Kupka[2], M. P. Wattjes[1], J. Nawabi[1]; [1]Berlin/DE, [2]Falun/SE
*(leonard.fetscher@icloud.com)*

**Purpose:** Endovascular treatment of chronic subdural hematoma (cSDH) by middle meningeal artery embolization (MMAE) is rapidly expanding, yet treatment success remains difficult to predict. Existing grading tools on angiographic imaging neglect branch anatomy, dominance, and collateral supply. We therefore developed the BE-EMMA Score, a branch enhanced, angiographic grading system, intended to improve reliability and enable prognostic correlation.
**Methods or Background:** We retrospectively reviewed 50 MMAEs in 38 patients with cSDH (Charité, 2023-2024). Initial inter-rater testing of a preliminary version of the BE-EMMA Score (capturing branch dominance, occlusion, and collaterals) in 19 cases was followed by refinement in consensus with four interventional (neuro-)radiologists. The refined scoring system integrates pre-, intra- and post-embolization parameters: (i) branch-specific occlusion grade (0-3; grade 4 = proximal stasis without distal penetration, liquid agents only), (ii) branch dominance (F/f = frontal, P/p = posterior), (iii) aplasia or surgical interruption, (iv) main trunk diameter (D/d for ≥/<1.5 mm), and (v) collateral status (C+ = relevant supply, C− = hazardous collaterals prohibiting embolization). Evaluation of inter-rater reliability (four inter-raters) and correlation with treatment outcome is ongoing in a final dataset of 50 MMAE-cases.
**Results or Findings:** Using the preliminary version, near-complete occlusion (grade ≥2) was achieved in 84% of divisions; full bilateral occlusion in 53%; relevant collaterals (C+) in ~20%. Inter-rater agreement was strong (κ = 0.84 overall; occlusion κ = 0.88; collaterals κ = 0.85).
**Conclusion:** The BE-EMMA Score provides an accurate, imaging based, reproductible, and anatomically detailed evaluation of MMAE. By integrating multiple anatomical and procedural dimensions, it extends beyond descriptive reporting toward a standardized framework that may facilitate future correlation with treatment success and clinical outcome.
**Limitations:** Broader validation is needed to confirm prognostic value and clarify potential use in clinical decision-making.
**Funding for this study:** Future prospective research including validation of the BE-EMMA Score is funded by the ESMINT Pilot Research Grant. Development of the score itself was not funded.
**Has your study been approved by an ethics committee?** Yes
**Ethics committee - additional information:** Ethics approval for the retrospective study of MMAE cases (2022–2024) was granted by the institutional review board of Charité – Universitätsmedizin Berlin (EA4/181/24).

**Author Disclosures:**
Mike P. Wattjes: Nothing to disclose
Nima Mahmoudi: Nothing to disclose
Leonard Fetscher: Nothing to disclose
Eberhard Siebert: Nothing to disclose
Daniel Kupka: Nothing to disclose
Christoph Csapo-Schmidt: Nothing to disclose
Jawed Nawabi: Nothing to disclose
Omid Reihani: Nothing to disclose

**Birth-Related Subdural Hemorrhage in Asymptomatic Newborns: Magnetic Resonance Imaging Prevalence and Evolution of Intracranial and Intraspinal Localization**

D. Turilli, L. Piscopo, A. Dessì, C. Pinna, *L. M. Fattacciu*, E. Solinas, I. Conti, S. Masala, M. Scaglione; Sassari/IT
*(lialafattacciu@gmail.com)*

**Purpose:** Neonatal birth-related intracranial subdural hemorrhages (SDHs) represent a form of bleeding inside the skull that occurs in newborns. This condition includes the extravasation of blood both in the encephalic parenchyma and in the extra-axial spaces. Recent studies have shown that SDH and particularly post-traumatic birth-related hemorrhages represent a frequent occurrence, but they are often asymptomatic. The gold standard for the diagnosis and follow-up of patients with SDH is multiparametric Magnetic Resonance Imaging. The aim of this study is to describe our experience by reporting several cases of SDH with different distribution and Central Nervous System involvement by the MRI of this pathology in infants up to 30 days of age.
**Methods or Background:** We analyzed the age and sex of the patients included in this study, the localization of SDH in different CNS areas, and their frequency using distribution plots and pie charts.
**Results or Findings:** About the analysis of the SDH locations in the 32 patients, the most common location was the cerebellum (31/32, 96.9%), followed by parietal and occipital lobes (19/32, 59.4%; 18/32, 56.2%, respectively), falx cerebri (11/32, 34.4%), tentorium cerebelli (10/32, 31.2%), temporal lobes (6/32, 18.7%), and finally cervical and dorsal spine in the same patients (4/32, 12.5%). According to SDH locations, the patients were divided into supratentorial, infratentorial, both, and Spinal Canal.
**Conclusion:** Our study confirmed the literature data regarding the neonatal birth-related SDH high frequency, but also allowed us to focus our attention on the rarest spinal SDH localizations with the same benign evolution.
**Limitations:** The small sample object of the study.
**Funding for this study:** This research received no external funding.
**Has your study been approved by an ethics committee?** Yes
**Ethics committee - additional information:** The local Institutional review board approved the study. The Ethical Committee’s approval was obtained (2022030) (28 December 2023).
**Author Disclosures:**
Davide Turilli: Nothing to disclose
Liala Mirella Fattacciu: Nothing to disclose
Ilaria Conti: Nothing to disclose
Alberto Dessì: Nothing to disclose
Mariano Scaglione: Nothing to disclose
Salvatore Masala: Nothing to disclose
Claudia Pinna: Nothing to disclose
Leandra Piscopo: Nothing to disclose
Emma Solinas: Nothing to disclose

**Radiological Management Of Reversible Cerebral Vasoconstriction Syndrome (RCVS) In France: A Multicenter Survey**

*V. L'Allinec*; Angers/FR
*(vincent.lallinec@gmail.com)*

**Purpose:** Reversible Cerebral Vasoconstriction Syndrome (RCVS) is characterized by thunderclap headaches and reversible segmental cerebral arterial narrowing. In the absence of guidelines, radiological management remains heterogeneous. This study aimed to evaluate diagnostic and follow-up imaging practices across French hospitals.
**Methods or Background:** A multicenter cross-sectional survey was conducted between February and May 2024 in 25 French neuroradiology centers. A self-administered online questionnaire explored initial imaging strategies, CT and MRI protocols, confirmatory and long-term follow-up imaging, and the role of invasive techniques. Responses were analyzed descriptively.
**Results or Findings:** Twenty centers (80%) responded, mostly university hospitals. CT was the preferred first-line modality (85%), mainly to exclude aneurysmal subarachnoid hemorrhage. MRI was used in all centers at some stage, with a common protocol including DWI, FLAIR, SWI, and TOF-MRA. Practices diverged beyond this: CTA coverage varied (head and neck vs intracranial only), advanced MRI sequences such as vessel wall imaging (40%) and ASL (15%) were inconsistently used. After positive initial imaging, 40% of centers did not perform confirmatory exams, while 75% repeated imaging after negative but clinically suspicious cases. Long-term follow-up was performed by 90% of centers, most often at 3 months, to confirm reversibility. No center reported invasive endovascular treatments.
**Conclusion:** This first national survey highlights consensus on initial non-invasive imaging but substantial heterogeneity in subsequent diagnostic and follow-up pathways. Such variability reflects the low incidence of RCVS and the absence of formal guidelines. These findings emphasize the need for national recommendations and collaborative prospective studies.
**Limitations:** Declared practices may differ from real-world management. The predominance of university hospitals may limit generalizability, and descriptive design precludes outcome comparisons. Rapidly evolving imaging technologies could also impact practices
**Funding for this study:** Investigator-initiated study with no external funding.
**Has your study been approved by an ethics committee?** Not applicable
**Author Disclosures:**
Vincent L'Allinec: Nothing to disclose

**Persistent Dizziness After EDAS Surgery in Adult Moyamoya Disease Patients: Identifying Risk Factors**

*B. Li*[1], Y. Liu[1], X. Du[1], Z. Hong-Tao[1], R. Xie[1], M. Lu[2], X. Zhao[1], J. Cai[1]; [1]Beijing/CN, [2]Tianjin/CN
*(lbb826@126.com)*

**Purpose:** This study aims to examine the determinants associated with persistent dizziness (RD) in adult patients diagnosed with Moyamoya disease (MMD) following successful Encephalo-Duro-Arterial-Synangiosis (EDAS) surgery.
**Methods or Background:** In patients with dizziness as the initial symptom of MMD, post-surgery dizziness improvement is noted, but mechanisms remain unclear. This study included 78 adults with MMD who initially experienced dizziness. We recorded patient characteristics, comorbidities, and DHI scores. CVR was assessed pre- and post-EDAS surgery with breath-modulated BOLD-fMRI. DSC-MRI measured TTP, MTT, CBV, and CBF in middle cerebral artery (MCA) regions. White matter was evaluated with LA-score MRI. Patients were categorized into RD and non-RD groups based on dizziness within 3 months post-surgery. We then assessed the impact of CVR, perfusion, and white matter on RD in MMD patients.
**Results or Findings:** In the RD group, pre- and post-surgical CVR was markedly lower than in the non-RD group, accompanied by elevated DHI and LA scores, with statistically significant differences ($P < 0.05$). Decreased CVR in MCA territory, along with increased DHI and LA scores, were identified as risk factors for early RD post-indirect surgery in adults with MMD. Multivariate logistic regression confirmed CVR, DHI, and LA scores as independent RD risk factors. ROC analysis revealed AUCs of 0.705 for CVR, 0.720 for LA score, and 0.782 for DHI score. Combined, these indicators achieved an AUC of 0.821, indicating high diagnostic accuracy (sensitivity 68.93%, specificity 89.54%).
**Conclusion:** Reduced CVR in MCA region, along with increased DHI and LA scores, predict short-term residual dizziness in adult MMD patients post-EDAS surgery. The combined assessment of CVR, DHI, and LA scores provides superior predictive value for RD.
**Limitations:** The study was conducted at a single medical center, which may limit the generalizability.
**Funding for this study:** None
**Has your study been approved by an ethics committee?** Yes
**Ethics committee - additional information:** the fifth medical center of Chinese PLA general hospital; Institutional Review Board (IRB number: KY-2022-4-29-1)
**Author Disclosures:**
Yuan Liu: Nothing to disclose
Xiangrong Du: Nothing to disclose
Zhang Hong-Tao: Nothing to disclose
Mingming Lu: Nothing to disclose
Rongchao Xie: Nothing to disclose
Xihai Zhao: Nothing to disclose
Jianming Cai: Nothing to disclose
Baobao Li: Nothing to disclose

09:30 - 11:00 **Research Stage 3**

### Research Presentation Session: Vascular

### RPS 1815

### Predictive power of vascular imaging

**Moderator**
Andrej Klepanec; Bratislava/SK
*(andrej.klepanec@fmed.uniba.sk)*

**Glucometabolic Dysregulation Drives White Matter Hyperintensity Progression in Cerebral Small Vessel Disease: Longitudinal Evidence from the UK Biobank and Mendelian Randomization Analysis**
*X. Han*, X. Yu, Y. Zhou; Shanghai/CN

**Purpose:** To develop a prediction model for WMH progression and to investigate whether glucometabolic dysregulation causally influences WMH progression via microstructural damage.
**Methods or Background:** The study analyzed data from UK Biobank participants of European descent with serial brain MRI scans. Participants took part in both imaging visit and with a diagnosis of CSVD were included in the study. For machine learning, a total of 8 key features were identified from Akaike information criterion, including age, body mass index, cystatin C, glucose, fractional anisotropy, mean diffusivity, intracellular volume fraction, and isotropic volume fraction. For Mendelian Randomization, 4 glucose indexes including fasting plasma glucose, 2-hour plasma glucose after an oral glucose tolerance test, HbA1c, fasting insulin, and 75 diffusion MRI (dMRI)-derived microstructural metrics were applied. Machine learning models were constructed to predict WMH progression, while structural equation modeling tested mediation pathways. Bidirectional two-sample Mendelian Randomization was employed to establish causal relationships between glucose metabolism indices and white matter microstructure using genome-wide association study data.
**Results or Findings:** Of 1616 participants included, 902 had WMH increase and 714 had WMH. Seven algorithms were employed to develop WMH prediction models, with logistic regression and support vector machine (SVM) demonstrating optimal performance. Structural equation modeling (SEM) revealed that glucose partially mediates WMH progression through ISOVF. Mendelian randomization (MR) analyses indicated that genetic susceptibility to hemoglobin A1c (HbA1c) significantly altered free water content in the left cerebral peduncle, right hippocampal gyrus, left anterior thalamic radiation, and left corticospinal tract.
**Conclusion:** Glucometabolic dysregulation contributed to WMH progression via microstructural damage.
**Limitations:** our study population was predominantly of European cohort from UK Biobank, which may limit the generalizability of the findings to other ethnic groups.
**Funding for this study:** This work was supported by National Natural Science Foundation of China (82171885), Shanghai Natural Science Foundation (25ZR1401225), Eastern Talent Plan Leading Project (LJ2023127), the Shanghai Science and Technology Committee Project, Explorer Project Funding (24TS1414800), the Leading Talent Program of Shanghai Municipal Health Commission (2022LJ023), Shanghai Engineering Research Center of Peri-operative Organ Support and Function Preservation (20DZ2254200), Renji Hospital Project (RJTJ25-QN-064, RJTJ23-RC-013, RJTJ25-MS-014, RJKY24-004).
**Has your study been approved by an ethics committee?** Yes
**Ethics committee - additional information:** Participant data were obtained from the UK Biobank cohort (Approved Application No. 117280). Ethical approval for the UK Biobank Study was granted by the National Information Governance Board for Health and Social Care and the NHS North West Multicentre Research Ethics Committee.
**Author Disclosures:**
Xu Han: Nothing to disclose
Yan Zhou: Nothing to disclose
Xiao Yu: Nothing to disclose

**Carotid Plaque Dynamic Contrast-enhanced MRI Normalised Signal Intensity as a Simple Surrogate for Neovascularisation: A Reproducible Alternative to Kinetic Modelling of Gadolinium Contrast Agents**
*T. R. Readford*[1], G. Martínez Rodríguez[2], S. Patel[1], M. Ugander[3], P. Kench[1], N. Giannotti[1]; [1]Sydney/AU, [2]Santiago/CL, [3]Solna/SE
*(nicola.giannotti@sydney.edu.au)*

**Purpose:** Dynamic contrast-enhanced MRI (DCE-MRI) can non-invasively characterise carotid atherosclerotic plaque vulnerability by quantifying measures related to arterial wall neovascularisation and endothelial permeability, which are linked to stroke risk. However, quantitative perfusion metrics such as Ktrans and Kep require complex kinetic modelling and generate disagreement regarding optimal methodology, hindering clinical interpretation. The purpose of this study was to assess the reproducibility of a simple method for carotid plaque DCE quantification using signal intensity in the vessel wall normalised to skeletal muscle signal intensity, as an accessible surrogate marker of neovascularisation.
**Methods or Background:** This was a sub-study of the CAPRI trial, which had a neutral outcome for the effect of colchicine versus placebo on carotid atherosclerotic plaque volume after six months. All participants underwent T1-weighted black-blood DCE-MRI of the carotid arteries at baseline and six months, with images acquired every 10 seconds over 210 seconds following intravenous administration of 0.1 mmol/kg gadoteric acid. Plaque core and remote unaffected vessel wall were manually delineated in the same slice. Signal intensities were normalized to skeletal muscle signal intensity from the same slice and timepoint.
**Results or Findings:** Among included patients (n=28, median [interquartile range] age 72 [64–74] years, 36% female), normalised peak signal intensity of the plaque core was greater than the remote vessel wall at both baseline (3.5 [2.3–4.1] vs 2.1 [1.7–2.5], p<0.001) and six months (3.2 [2.5–4.4] vs 2.0 [1.7–2.5], p<0.001; but compared to baseline: p≥0.81 for both, mean±SD differences 0.7±0.7 and 0.6±0.4, respectively).
**Conclusion:** DCE-MRI normalised peak intensity was greater for plaque core compared to remote vessel wall, and both measurements are reproducible over six months. This simplified quantification approach may facilitate future assessment of neovascularisation and plaque phenotypes in clinical practice.
**Limitations:** Lack of
**Funding for this study:** No funding was received for this study.
**Has your study been approved by an ethics committee?** Yes
**Ethics committee - additional information:** This study was approved by the local human subject research ethics committee and all subjects provided written informed consent.
**Author Disclosures:**
Peter Kench: Nothing to disclose
Martin Ugander: Nothing to disclose
Nicola Giannotti: Nothing to disclose
Thomas Ramsay Readford: Nothing to disclose
Sanjay Patel: Nothing to disclose
Gonzalo Martínez Rodríguez: Nothing to disclose

**Improving Aortic Calcium Assessment in Contrast-Enhanced CT: Exploring HU Thresholds for Reliable Cardiovascular Scoring**
*P. Reschke*, K. Eichler, T. Vogl, L. D. Grünewald; Frankfurt/DE

**Purpose:** Cardiovascular disease is the leading cause of death in the Western world, with vascular calcification serving as a marker of advanced pathology. The Agatston score is only applicable in non-contrast CT scans. However, no standardized method exists for quantifying aortic calcification on contrast-enhanced CT scans.
**Methods or Background:** This retrospective study included 1125 patients (344 women, 781 men) who underwent triphasic CT angiography. Patients with metallic stents, prosthetic grafts, or poor image quality were excluded. Fixed (100–900 HU) and dynamic thresholds were applied to arterial and venous phases for aortic calcium quantification and compared with non-contrast Agatston scores.
**Results or Findings:** In the venous phase, the 300 HU threshold demonstrated the strongest correlation with the Agatston score (thoracic aorta Pearson's r = 0.81,; abdominal aorta r = 0.83; all p < 0.001) and the highest predictive accuracy ($R^2$ = 0.66 and 0.68). In the arterial phase, higher thresholds were required, with optimal performance at 900 HU for the thoracic aorta (r = 0.78, $R^2$ = 0.51, p < 0.001) and 800 HU for the abdominal aorta (r = 0.80, $R^2$ = 0.45, p < 0.001). Dynamic thresholding performed significantly worse in both phases, with $R^2$ values of 0.56 (venous) and 0.07 (arterial) for thoracic calcifications and 0.62 (venous) and 0.09 (arterial) for abdominal calcifications (all p < 0.05).
**Conclusion:** A fixed threshold of 300 HU in the venous phase enables accurate aortic calcium quantification on contrast-enhanced CT, closely aligning with Agatston scoring and potentially allowing broader clinical application without the need for additional non-contrast imaging.

**Limitations:** The fixed HU thresholds used may not be universally applicable across different CT protocols, as variations in the contrast agent volume, flow rate, and contrast concentration can influence HU measurements.
**Funding for this study:** None
**Has your study been approved by an ethics committee?** Yes
**Ethics committee - additional information:** The study was approved by the ethics committee in Frankfurt.
**Author Disclosures:**
Thomas Vogl: Nothing to disclose
Philipp Reschke: Nothing to disclose
Leon David Grünewald: Nothing to disclose
Katrin Eichler: Nothing to disclose

**Periaortic Adipose Tissue Attenuation on Preprocedural CT Represents a Novel Predictor of Mortality in Patients Undergoing TAVR**
*J. M. Brendel*, I. Hadzic, T. Mayrhofer, L. H. Cooke, E. Yucel, N. K. Patel, V. Raghu, B. Ghoshhajra, B. Foldyna; Boston, MA/US
*(jan.brendel@med.uni-tuebingen.de)*

**Purpose:** To determine whether periaortic adipose tissue (PAAT) attenuation predicts long-term mortality in transcatheter aortic valve replacement (TAVR) patients.
**Methods or Background:** Higher PAAT attenuation has been linked to periaortic inflammation and can be measured on routine preprocedural CT. Its prognostic value in patients undergoing TAVR remains unclear. In this retrospective multicenter study, we analyzed preprocedural CT scans from consecutive TAVR patients between 2014 and 2023. PAAT was segmented using a dedicated deep learning tool (TotalSegmentator) that delineates the entire aorta, followed by logical operations creating a 10-mm radial cylinder around the aortic wall with attenuation thresholds of (–190 to –30 HU). Associations with all-cause mortality were assessed using Cox regression adjusted for CT technical parameters (tube voltage, signal-to-noise ratio, slice thickness) and the Society of Thoracic Surgeons (STS) risk score.
**Results or Findings:** Among 928 patients from four tertiary medical centers (mean age 81±8 years; 58% male), 222 (23.9%) died during a median follow-up of 22 (14–36) months. Mean PAAT attenuation was -77.3±7.2 HU and was higher in women (+2.1±0.5 HU), nonobese patients (+4.7±0.5 HU), and those with moderate or high (≥4%) STS risk score (+1.7±0.5 HU), all p<0.001. Each 10 HU increase in PAAT attenuation was associated with an 82% higher risk of death independent of STS risk (aHR 1.82: 95%CI: 1.36–2.43; p<0.001). Patients with PAAT attenuation above -77 HU had nearly a two-fold greater mortality risk (aHR 1.97; 95%CI: 1.36–2.85, p<0.001).
**Conclusion:** High PAAT attenuation represents a novel opportunistic CT marker that predicts mortality in TAVR patients, and may inform long-term risk stratification and management.
**Limitations:** No histopathologic validation of PAAT attenuation was available, limiting mechanistic interpretation.
**Funding for this study:** N/A
**Has your study been approved by an ethics committee?** Yes
**Ethics committee - additional information:** The institutional review board approved the study protocol, with a waiver for written informed consent.
**Author Disclosures:**
Brian Ghoshhajra: Nothing to disclose
Jan Michael Brendel: Grant Recipient: Deutsche Forschungsgemeinschaft (DFG, German Research Foundation) #540505270
Ibrahim Hadzic: Nothing to disclose
Evin Yucel: Nothing to disclose
Thomas Mayrhofer: Nothing to disclose
Borek Foldyna: Grant Recipient: NIH/NHLBI, AstraZeneca, MedImmune, Cleerly, and MedTrace
Nilay K Patel: Nothing to disclose
Vineet Raghu: Shareholder: NVIDIA, Alphabet, Apple, Meta, and ASML Grant Recipient: NIH/NHLBI, American Heart Association, Norn Group, and the National Academy of Medicine
Lauren Hannah Cooke: Nothing to disclose

**CT-Based Radiomic Assessment of Periaortic Adipose Tissue in Takayasu Arteritis and Atherosclerosis: A Multi-Segmental Analysis**
*M. Kadıoğlu*, S. Ozkök; Istanbul/TR
*(mehmetkol88@gmail.com)*

**Purpose:** To evaluate microstructural differences in periaortic adipose tissue among patients with Takayasu arteritis, atherosclerosis, and healthy controls using CT-based radiomics, and to investigate the potential of radiomic parameters as imaging biomarkers for subclinical inflammation
**Methods or Background:** A total of 66 subjects were included: 26 patients with Takayasu arteritis, 20 with atherosclerotic vascular disease, and 20 healthy controls. For the Takayasu group, pre-treatment CT angiograms obtained at the time of diagnosis were analyzed. All scans were performed using Philips 128-slice CT system (100–120 kVp, 1 mm slice thickness, B kernel). The aorta was divided into ascending, aortic arch, descending, and abdominal segments. Periaortic adipose tissue was semi-automatically segmented in 3D Slicer v5.8.1, and over 300 radiomic features were extracted using PyRadiomics, following standardization guidelines proposed by Koçak B et al. Group comparisons were conducted using ANOVA and Kruskal–Wallis tests with FDR correction (p < 0.05 considered significant).
**Results or Findings:** A total of 284 segments were analyzed. Statistically significant radiomic differences were detected in the ascending (n = 91), aortic arch (n = 178), descending (n = 199), and abdominal aorta (n = 189) segments. Key discriminative parameters included Entropy, Joint Energy, Busyness, and the 90th percentile intensity, all showing p < 0.001–0.01. Compared with atherosclerotic patients, Takayasu arteritis demonstrated markedly higher heterogeneity and entropy metrics. Notably, even CT segments without visible wall thickening or enhancement revealed microstructural alterations in Takayasu patients.
**Conclusion:** CT-based radiomics can identify inflammation-related textural alterations in periaortic adipose tissue, even in morphologically normal aortic segments. Compared with atherosclerosis, Takayasu arteritis exhibits significantly higher heterogeneity parameters, suggesting that perivascular fat radiomics may serve as a non-invasive biomarker of early vascular inflammation and tissue remodeling.
**Limitations:** Single-center study, limited sample size; lack of histopathologic correlation.
**Funding for this study:** No financial support was received.
**Has your study been approved by an ethics committee?** Not applicable
**Author Disclosures:**
Serçin Ozkök: Nothing to disclose
Mehmet Kadıoğlu: Nothing to disclose

**Impact of the Mesenteric Calcium Score on Mortality and Morbidity in Acute Occlusive Arterial Mesenteric Ischaemia**
P. Jessua[1], R. Sartoris[1], M. Ronot[1], *L. Garzelli*[2]; [1]Clichy/FR, [2]Paris/FR
*(lorenzo.garzelli@aphp.fr)*

**Purpose:** To assess whether the mesenteric calcium score (MCS) predicts mortality or morbidity in acute occlusive arterial mesenteric ischaemia (AOAMI), of embolic or atherothrombotic origin.
**Methods or Background:** Retrospective cohort study of patients with AOAMI admitted to our intestinal stroke unit. Clinical, biological, imaging, treatment and outcomes data were analysed. MCS of the superior mesenteric artery was calculated using the Agatston method and compared between survivors and non-survivors at 30 days. The primary endpoint was 30-day mortality; the secondary endpoint was a composite of 30-day mortality and morbidity (short bowel syndrome, permanent stoma or home parenteral nutrition). Inter- and intra-observer reproducibility was assessed with intraclass correlation coefficients (ICC).
**Results or Findings:** Among 506 patients screened, 179 were included (77 women, mean age 69.5 years). Thirty-day mortality was 13% with a median overall survival of 101 months. Morbidity was significant: short bowel syndrome (27%), permanent stoma (17%), and long-term parenteral nutrition (26%), with a composite morbidity score ≥1 in 37%. Median MCS was 27, differing by etiology (embolic: 0, atherothrombotic: 375). However, no significant association was foun between MCS and 30-day mortality (p=0.34) or composite mortality-morbidity (p=0.40). Subgroup analyses by etiology confirmed these findings (embolic p=0.5 and 0.34, atherothrombotic p=0.08 and 0.63). Reproducibility of MCS were excellent (inter-observer ICC 0.94, intra-observer ICC 0.98).
**Conclusion:** The superior mesenteric artery calcium score does not predict short-term mortality or morbidity in AOAMI, including in atherothrombotic patients.
**Limitations:** Retrospective design, potential missing data, and limited power given to the relatively low mortality rate.
**Funding for this study:** None
**Has your study been approved by an ethics committee?** Not applicable
**Author Disclosures:**
Riccardo Sartoris: Nothing to disclose
Maxime Ronot: Nothing to disclose
Paul Jessua: Nothing to disclose
Lorenzo Garzelli: Nothing to disclose

**Identifying predictors for limb loss and death in patients with diabetic foot disease by combining patient metadata and MRI based imaging findings**

*M. Ahmad*, J. Shalhoub, D. Amiras, A. G. Rockall, A. Davies; London/UK
*(manalahmad.ma@gmail.com)*

**Purpose:** Diabetic foot disease (DFD) is a complex disease and is associated with lower limb amputation. Magnetic resonance imaging (MRI) is commonly used in patients with DFD. Early detection of surrogate markers on MRI may help to predict the risk of limb loss. Our aim was to explore the use of deep learning models to identify predictors on MRI for limb loss in patients with DFD.
**Methods or Background:** Sarcopenia severity grading and the pseudo fat fraction values calculated from 824 T1-weighted coronal MRI scans of the foot across 427 patients were combined with patient metadata to identify salient predictors for limb loss, major amputation and death and all amputation and death as composite outcomes. Least Absolute Shrinkage and Selection Operator (LASSO), Ridge Regression and Random Forest (RaF) models were used. Further analysis was also undertaken using Cox proportional hazard.
**Results or Findings:** The converging variables which appeared in all three models were Haemoglobin, HbA1c and renal status. The overlapping variables across ridge regression and RaF for all amputations were C-reactive protein (CRP), age, albumin and the pseudo fat fraction. Further analysis with a cox proportional hazard model found CRP was a strong predictor (Hazard Ratio 1.004 [p-value = 0.02]). Former history of transient ischaemic attacks was identified as a predictor for all amputation/death on LASSO and ridge regression. Pseudo fat fraction, weight and HbA1c were highlighted as features of importance for composite outcomes (major lower limb amputation/death and all amputation/death) on random forest.
**Conclusion:** There may be merit in combining MRI based image findings with patient metadata to stratify risk.
**Limitations:** Further internal and external validation is required with a more heterogenous cohort of patients.
**Funding for this study:** Imperial College Healthcare NHS Trust radiology pump priming fund
**Has your study been approved by an ethics committee?** Yes
**Ethics committee - additional information:** Ethics approval from the Health Research Authority UK
**Author Disclosures:**
Dimitri Amiras: Nothing to disclose
Alun Davies: Nothing to disclose
Manal Ahmad: Nothing to disclose
Joseph Shalhoub: Nothing to disclose
Andrea Grace Rockall: Nothing to disclose

**Using deep learning models (AI) on diabetic foot disease related MRI reports of the foot to identify salient predictors for limb loss**

*M. Ahmad*, K. C. Soh, J. Shalhoub, D. Amiras, A. Davies, A. G. Rockall; London/UK
*(manalahmad.ma@gmail.com)*

**Purpose:** Diabetic foot disease (DFD) is a complex disease and is associated with lower limb amputation. Magnetic resonance imaging (MRI) is commonly used in patients with DFD.
**Methods or Background:** 419 MRI reports were reviewed to identify salient predictors for no amputation versus major versus minor limb loss from a list of features including collections, ulcer, osteomyelitis, diabetic myopathy/sarcopenia and Charcot foot. Random Forest, Extreme Gradient Boosting and a Multilayer Perceptron model was applied. Further composite analysis was undertaken to compare no amputation versus major lower limb amputation, no amputation versus all amputation and death and all amputation versus death.
**Results or Findings:** Extreme gradient boosting comparing the no amputation versus minor amputation versus major amputation group using extreme gradient boosting was the only model of statistical significance. The model had a 63.4% accuracy [95% CI 0.52-0.7; p-value <0.001]. The ROC-AUC values were 0.724 (no amputation group), 0.779 (minor amputation group) and 0.439 (major amputation group). Features highlighted as potential predictors included absence of osteomyelitis and presence of Charcot as variables which influenced the outcome.
**Conclusion:** MRI reports may provide useful information in highlighting features which could potentially predict the risk of adverse outcomes.
**Limitations:** Variability in reporting can influence the outcome. Uniformity in reporting may allow this to be explored further and to create a standardised prediction model.
**Funding for this study:** Imperial College Healthcare NHS Trust radiology pump priming fund.
**Has your study been approved by an ethics committee?** Yes
**Ethics committee - additional information:** Ethics approval received from Health Research Authority UK
**Author Disclosures:**
Dimitri Amiras: Nothing to disclose
Alun Davies: Nothing to disclose
Karen Chien Soh: Nothing to disclose
Manal Ahmad: Nothing to disclose
Joseph Shalhoub: Nothing to disclose
Andrea Grace Rockall: Nothing to disclose

**Identifying High-Risk Patients for Endovascular Failure with UTE-MRI: Evidence from an NIH-Funded Study**

*J. Csőre*[1], A. Crichton[2], E. Pomozi[1], J. Lamichhane[2], A. B. Lumsden[2], T. L. Roy[2]; [1]Budapest/HU, [2]Houston, TX/US

**Purpose:** Endovascular treatment failure in chronic limb-threatening ischemia (CLTI) is not fully understood, and patient selection is crucial for optimal results. Ultrashort Echo Time (UTE) MRI is a non-contrast method shown ex vivo to characterize plaque composition. This study assessed whether MRI-defined plaque morphology influences procedural difficulty and immediate technical failure (ITF) in peripheral vascular interventions (PVI).
**Methods or Background:** Patients with CLTI undergoing PVI at a tertiary vascular center were enrolled and underwent 3T UTE-MRI. Lesions were classified as predominantly soft (>50% thrombus, cholesterol/lipid plaque) or hard (calcific/collagenous plaque). Operators were blinded to MRI findings and used standard pre-procedural imaging. Primary outcome was ITF; secondary outcome was lesion crossing time.
**Results or Findings:** A total of 43 patients (86 lesions; mean age 67.5 years, 40% female) were included; 14 lesions were excluded (7 diagnostic DSA only, 7 non-diagnostic MRI). Of the 72 evaluable lesions, 66.7% were scored as soft and 33.3% were hard. ITF occurred in 15.3% of lesions, most commonly due to crossing failure (72.7%). Hard lesions had a markedly higher ITF rate compared with soft (41.7% vs 2.1%, p<0.001, $Chi^2$=19.3). In a subgroup analysis among >75% stenosis/occlusions, ITF was 58.8% in hard vs 4.3% in soft lesions (p<0.001, $Chi^2$=14.5). Median crossing time was significantly prolonged in hard lesions (535s vs 38s, p=0.024).
**Conclusion:** UTE-MRI enables in vivo characterization of vessel wall composition and predicts procedural complexity and ITF in PVI. Despite its potential, assessment of plaque morphology remains underused in CLTI care. Incorporating UTE-MRI into clinical workflows may refine patient selection, guide interventional strategy, and improve outcomes.
**Limitations:** Single-center study, sample size
**Funding for this study:** Jerold B. Katz Academy of Translational Science under project number 15790002 (recipient's name: Trisha Roy), the American Heart Association Transformational Award under project ID 17590004 (recipient's name: Trisha Roy), and the National Institutes of Health Research Project grant (R01) under award number R01HL174587 (recipient's name: Trisha Roy).
**Has your study been approved by an ethics committee?** Yes
**Ethics committee - additional information:** Study ID 15790002
**Author Disclosures:**
Alexander Crichton: Nothing to disclose
Enikő Pomozi: Nothing to disclose
Janak Lamichhane: Nothing to disclose
Judit Csőre: Nothing to disclose
Alan B. Lumsden: Nothing to disclose
Trisha L. Roy: Research/Grant Support: Baylis Medical Technologies, Boston Scientific, Light Matter Interaction Owner: Magellan Biomedical Inc.

09:30 - 11:00 **Research Stage 2**

### Research Presentation Session: Oncologic Imaging

### RPS 1816

### Modern strategies in thoracic oncologic imaging

**Moderator**
Giuseppe Cicchetti; Rome/IT
*(cicchetti.giuseppe88@gmail.com)*

### CT-Derived Cardiovascular and Metabolic Biomarkers for Risk Stratification in Locally Advanced Lung Cancer Undergoing Combined-Modality Therapy

*A. Cicchetti*[1], P. Vallerio[1], A. Catalano[1], C. Sangalli[1], L. Marrazzo[2], R. Tummineri[1], A. Botti[3], F. Dionisi[4], E. Gioscio[1]; [1]Milan/IT, [2]Firenze/IT, [3]Reggio Emilia/IT, [4]Rome/IT
*(alessandro.cicchetti@istitutotumori.mi.it)*

**Purpose:** To assess whether baseline CT-derived cardiovascular and metabolic biomarkers can stratify intermediate-risk patients with locally advanced non-small cell lung cancer (LA-NSCLC) treated with chemoradiotherapy+immunotherapy, thereby supporting personalized follow-up strategies and identifying candidates for advanced cardiological imaging.
**Methods or Background:** Baseline CT-scans from 305 LA-NSCLC patients enrolled in four Italian centers were retrospectively analyzed. Two-year overall survival (OS2Y) was recorded. Image-derived features included the Agatston score, body composition metrics, aorta diameters, and percent Emphysema. Gross tumor volume (GTV) was contoured by experts and added to the analysis together with chemo/immune information. Automatic segmentation and feature extraction was performed using open-source tools and locally developed scripts. A Random Forest classifier was trained following feature selection; SHAP values were used for feature importance interpretation, and UMAP+HDBSCAN clustering was applied to identify patient subgroups.
**Results or Findings:** OS2Y was 62%. Patients receiving chemoradiotherapy (66%) combined with immunotherapy were 34%. Four distinct survival clusters were identified based on selected features(GTV, Visceral Fat, Hepatic Fat, Agatston score, and Emphysema): Cluster1 (n=69) – OS2Y 83.6%: characterized by small tumor volumes and absence of imaging risk factors above the median. Cluster2 (n=24) – OS2Y 62.5%: tumor volumes comparable to Cluster 1, but high torso fat, very-high Agatston score and hepatic fat. Cluster3 (n=201) – OS2Y 59.7%: features around median values, representing the average population group. Cluster4 (n=11) – OS2Y 11.1%: characterized by large GTV volumes and moderate-to-high emphysema.
**Conclusion:** This unsupervised clustering approach demonstrated the ability to identify patient subgroups with shared clinical characteristics but distinct risk profiles. Importantly, it highlighted a significant survival impact (cl1-cl2=21.1%) for patients with baseline cardiac calcifications, visceral and hepatic fat, underlining their potential role as prognostic imaging biomarkers to guide follow-up strategies.
**Limitations:** Lack of systematic cardiological test, retrospective analysis
**Funding for this study:** The study LOCATION MATTERS was funded by AIRC MFAG 27480
**Has your study been approved by an ethics committee?** Yes
**Ethics committee - additional information:** Num INT 36/23
**Author Disclosures:**
Eliana Gioscio: Nothing to disclose
Claudia Sangalli: Nothing to disclose
Paola Vallerio: Nothing to disclose
Andrea Botti: Nothing to disclose
Livia Marrazzo: Nothing to disclose
Alessandro Cicchetti: Nothing to disclose
Alessandra Catalano: Nothing to disclose
Roberta Tummineri: Nothing to disclose
Francesco Dionisi: Nothing to disclose

### Node-RADS v1.0 on chest CT for lung cancer lymph node staging: a multi-reader agreement study

*F. Sparascio*[1], E. Versienti[1], L. Cereser[1], T. Nadarević[2], C. Ciancimino[1], A. P. Pace[1], G. Como[1], R. Girometti[1], C. Zuiani[1]; [1]Udine/IT, [2]Rijeka/HR
*(sparascio@icloud.com)*

**Purpose:** To assess intra- and inter-reader agreement for Node-RADS v1.0 in mediastinal lymph node evaluation on chest CT in stage I–III non-small cell lung cancer (NSCLC) and determine its diagnostic performance.
**Methods or Background:** This retrospective, single-center study included 46 patients (38 adenocarcinomas, 8 squamous cell carcinomas) with 158 pathologically confirmed mediastinal lymph nodes (22 malignant, 136 benign). A contrast- enhanced chest CT scan was available for all patients. Four radiologists (two experts, two juniors) independently assigned Node-RADS scores and descriptors (“size” and “configuration”) in two sessions, three weeks apart. Intra- and inter-reader agreement were assessed using Gwet’s AC2. Diagnostic performance was assessed by ROC analysis; sensitivity, specificity, and predictive values were calculated at a Node-RADS score ≥3 threshold.
**Results or Findings:** Inter-reader agreement for Node-RADS scores was almost perfect for experts (Gwet’s AC2 = 0.97; 95% CI: 0.96–0.99) and juniors (Gwet’s AC2 = 0.95; 95% CI: 0.93–0.97). Intra-reader agreement Gwet’s AC2 values ranged from 0.95–0.99. Descriptor agreement was similarly high (Gwet’s AC2 ≥ 0.85). ROC AUCs ranged from 0.71–0.76 for experts and 0.68–0.84 for juniors. At the ≥3 threshold, specificity and negative predictive value were consistently ≥90%, while sensitivity remained limited (<64%) for all readers.
**Conclusion:** Node-RADS v1.0 shows excellent reproducibility across radiologists with different expertise for mediastinal lymph node assessment on CT in stage I–III NSCLC. Its high specificity and negative predictive value suggest a supportive role in excluding malignancy, although limited sensitivity warrants cautious interpretation and complementary diagnostic assessment.
**Limitations:** Single-center and retrospective study.
**Funding for this study:** No funding was received for this study.
**Has your study been approved by an ethics committee?** Yes
**Ethics committee - additional information:** This retrospective study has been approved by the Institutional Review Board (IRB) of the Department of Medicine, University of Udine. Study protocol code: 019/2025. Approval date: 15/01/2025.
**Author Disclosures:**
Federica Sparascio: Nothing to disclose
Eugenia Versienti: Nothing to disclose
Consuelo Ciancimino: Nothing to disclose
Chiara Zuiani: Nothing to disclose
Rossano Girometti: Nothing to disclose
Tin Nadarević: Nothing to disclose
Lorenzo Cereser: Nothing to disclose
Antonia Pia Pace: Nothing to disclose
Giuseppe Como: Nothing to disclose

### Spectral CT Histogram Features for Early Prediction of Immunotherapy Response in Advanced Lung Cancer

*W. Yang*, Q. Feng, X. Chen, X. Xin; Nanjing/CN
*(15805159169@163.com)*

**Purpose:** This study evaluated the potential of spectral image-based histogram features for early assessment of immunotherapy response in advanced lung cancer.
**Methods or Background:** Thirty-five patients who underwent baseline and follow-up spectral contrast-enhanced CT scans before and during immunotherapy were retrospectively analyzed. Treatment response at the 4th follow-up was determined using RECIST 1.1 and categorized as response (CR, PR) or non-response (SD, PD). Spectral image series-including conventional images, 40/70 keV virtual monoenergetic images (VMI), iodine density, effective atomic number, electron density, and water/iodine-based maps-were reconstructed in arterial and venous phases. VMI-40 keV images were used for 3D semi-automatic lesion segmentation, and first-order histogram features were extracted. Features were standardized with Z-scores, and significant predictors were identified by Mann-Whitney U-test. Logistic regression models were built, and discriminatory ability was evaluated with ROC AUC; AUC differences were compared with the DeLong test.
**Results or Findings:** Based on RECIST 1.1, 2 patients achieved CR, 23 PR, 9 SD, and 1 PD. From baseline spectral data, three histogram features distinguished response from non-response with an AUC of 0.796. When combining baseline and first follow-up data, three features achieved an improved AUC of 0.852. No predictive features were identified from conventional images.
**Conclusion:** Histogram features derived from spectral CT, particularly when incorporating both baseline and early follow-up data, show promise for early prediction of immunotherapy response in advanced lung cancer patients.

**Limitations:** Sample size is small.
**Funding for this study:** Not applicable
**Has your study been approved by an ethics committee?** Yes
**Ethics committee - additional information:** This study was approved by the Institutional Ethics Committee. The requirement for informed consent was waived due to the retrospective nature of the analysis.
**Author Disclosures:**
Xingbiao Chen: Nothing to disclose
Wen Yang: Nothing to disclose
Qianqian Feng: Nothing to disclose
Xiaoyan Xin: Nothing to disclose

**Preliminary findings of the lung cancer screening campaign at a single centre in 796 individuals**
H. W. Phillips[1], *P. Marra*[1], A. Smedile[2], M. Bertuletti[2], C. Gargiulo[1], K. D. Martins De Mattos[1], P. A. Bonaffini[2], G. Muscogiuri[2], S. Sironi[1]; [1]Milan/IT, [2]Bergamo/IT
*(pmarra@asst-pg23.it)*

**Purpose:** Lung cancer screening of at-risk individuals remains under investigation, with heterogeneous results being expected due to local environmental and epidemiological factors. We present the preliminary experience of the experimental low-dose chest CT screening campaign at a single Italian centre.
**Methods or Background:** Eligibility criteria include age (55-75) and positive smoking-history (≥30 pack years, active or cessation within the last 15 years) with follow-up imaging at variable intervals based on Lung-RADS 1.1 (2019) risk stratification. Suspect nodules underwent further evaluation following specialist review, often 18FDG-PET, biopsy and/or surgical excision. A review of all lung and collateral oncological findings in the study population was performed.
**Results or Findings:** From 11/02/23, 796 individuals have been imaged, with 593 having a second scan by 30/06/25. 16 diagnoses of NSCLC (2% detection rate) have been made, all categorised as LR4 (sensitivity 100%, specificity 89.5%, PPV 16.3%, NPV 100%), 15/16 at initial CT. All underwent 18FDG-PET (seven positive, nine nonspecific), 12 were biopsied (nine CT-guided, three by EBUS-TBNA) whilst four proceeded directly to surgery. Initial CT also identified 56 others with LR4 nodules, 19 of whom were evaluated by 18FDG-PET. Four had EBUS-TBNA biopsies whilst three were directly excised. Follow-up scans showed evolution to LR4 in 26 others, resulting in six 18FDG-PET scans, one EBUS-TBNA biopsy and one excision. During the project, 17 participants were diagnosed with extra-pulmonary malignancies, seven as a result of their screening CT: two thyroid, two breast, one tonsillar, one lymphoma and one thymoma.
**Conclusion:** Lung cancer screening at a national level and its potential to provide earlier detection seems valuable, though false positives and resultant investigations deserve consideration.
**Limitations:** Limitations include provisional data use, cohort factors with strict eligibility criteria, compliance and imaging interpretation, particularly of slow growing malignancies.
**Funding for this study:** None.
**Has your study been approved by an ethics committee?** Yes
**Ethics committee - additional information:** Data obtained from a national multicentre prospective study.
**Author Disclosures:**
Martina Bertuletti: Nothing to disclose
Paolo Marra: Nothing to disclose
Pietro Andrea Bonaffini: Nothing to disclose
Kessy Djonis Martins De Mattos: Nothing to disclose
Carlotta Gargiulo: Nothing to disclose
Antonella Smedile: Nothing to disclose
Harry William Phillips: Nothing to disclose
Giuseppe Muscogiuri: Nothing to disclose
Sandro Sironi: Nothing to disclose

**Machine learning-based quantitative prediction of spread through air spaces in primary lung adenocarcinoma using intratumoural heterogeneity scores**
Y. Li[1], X. Yang[1], P. Wang[2], *W. Meng*[1]; [1]Harbin/CN, [2]Beijing/CN

**Purpose:** This study aimed to quantify intratumoural heterogeneity (ITH) to preoperatively predict the spread through air spaces (STAS) status of lung adenocarcinoma (LUAD) and further explore the potential biological basis underlying the prediction model.
**Methods or Background:** STAS is an aggressive pattern of primary LUAD that affects both prognosis and treatment strategies for patients. In our study, conventional radiomics features and habitat features were extracted from intratumoural and peritumoural regions on preoperative CT images. A new index, the ITH score, was developed to quantify ITH. Clinical-radiologic characteristics associated with STAS were identified by multivariable logistic regression analyses. Additionally, intratumoural-peritumoural habitat features, ITH score, and clinical-radiologic characteristics were integrated into a combined model by various machine learning algorithms. Finally, 24 patients with RNA sequencing data were utilised for gene expression analysis.
**Results or Findings:** A total of 1268 patients (median age, 60 years; IQR, 53.8–66.0 years; 850 female) were divided into the training set (n=943), validation set (n=236), and external test set (n=89). Using the Light Gradient Boosting Machine classifier, the combined model demonstrated the highest predictive performance for STAS, achieving an AUC value of 0.97 in the training, 0.98 in the validation, and 0.91 in the external test set. Differentially expressed genes in high probability group were associated with monocarboxylic acid transport and metabolism.
**Conclusion:** The combined model demonstrated superior performance in predicting STAS in primary LUAD.
**Limitations:** First, its retrospective design may introduce bias, and further prospective studies are needed to validate the model's accuracy. Second, manual tumor delineation by different radiologists could affect the consistency of radiomic features, so future research should prioritize automated segmentation methods. Third, the limited RNA-seq sample size may weaken biological validation, and larger, more diverse cohorts are needed in future studies.
**Funding for this study:** This study was supported by the Scientific and Technological Innovation 2030-New Generation Artificial Intelligence Project of the National Key Research and Development Program of China, the National Natural Science Foundation of China, and the Climbing Fund of the National Cancer Center.
**Has your study been approved by an ethics committee?** Yes
**Ethics committee - additional information:** Ky2024-104
**Author Disclosures:**
Xiaolong Yang: Nothing to disclose
Yingjie Li: Nothing to disclose
Wei Meng: Nothing to disclose
Ping Wang: Nothing to disclose

**Comparative Analysis of Modern 18F-FDG PET/CT Imaging and Histopathology for Lymph Node Staging in Lung Cancer Patients**
*P. Eckwolf*, D. Kifjak, H. Prosch, L. Beer; Wien/AT

**Purpose:** To assess whether quantitative 18F-fluoro-2-deoxy-D-glucose (FDG) positron emission tomography/computed tomography (PET/CT) parameters improve preoperative thoracic lymph node (LN) staging accuracy in non-small-cell lung cancer (NSCLC).
**Methods or Background:** Thirty-eight treatment-naïve patients (mean age 67 ± 9 years) with confirmed NSCLC (29 adenocarcinoma [76.3%], 8 squamous cell carcinoma [21.1%], 1 large cell carcinoma [2.6%]) underwent pre-interventional long field-of-view 18F-FDG PET/CT. A total of 149 thoracic LN were sampled intraoperatively, by transbronchial needle aspiration (TBNA), or both. PET metrics included maximum, peak, and mean standardized uptake value (SUVmax, SUVpeak, SUVmean), metabolic tumor volume (MTV), and total lesion glycolysis (TLG).
**Results or Findings:** Twelve patients (32%) had metastatic thoracic LN (19/149, 12.8%). Metastatic LN were significantly larger (P=0.013) and more metabolically active (P<0.001) than non-metastatic nodes. ROC analysis showed the best diagnostic performance for SUVmax and SUVpeak (AUC 0.86 and 0.862). Using a sensitivity-optimized SUVmax cutoff of 2.82 yielded a negative predictive value (NPV) of 98%, moderate positive predictive value (PPV, 38%), and overall accuracy of 80%. False positives included reactive LN with SUVmax up to 12, limiting PPV despite high NPV.
**Conclusion:** Quantitative 18F-FDG PET/CT, particularly SUVmax and SUVpeak, enables highly reliable exclusion of thoracic LN metastasis in NSCLC, with excellent NPV for nodes showing low FDG uptake. However, positive findings remain nonspecific and require histopathological confirmation.
**Limitations:** Retrospective single-center design and modest sample size.
**Funding for this study:** The financial support by the Austrian Federal Ministry for Digital and Economic Affairs, the National Foundation for Research, Technology and Development and the Christian Doppler Research Association is gratefully acknowledged.
**Has your study been approved by an ethics committee?** Yes
**Ethics committee - additional information:** Ethic committee number: 1187/2025
**Author Disclosures:**
Helmut Prosch: Nothing to disclose
Lucian Beer: Nothing to disclose
Philip Eckwolf: Nothing to disclose
Daria Kifjak: Nothing to disclose

**CT-guided percutaneous cryoablation of lung tumors: A retrospective single-center cohort**
*E. Suderland*, G. J. Schmid; Neuss/DE

**Purpose:** To evaluate feasibility, safety, and local tumour control after CT-guided percutaneous cryoablation of primary and metastatic lung cancer.
**Methods or Background:** This retrospective single-center study included 29 patients (11 men, 18 women; mean age 67.8 years) with 33 pulmonary lesions ≤3 cm treated between 2012 and 2025. Mean lesion size was 17.3 mm (range 7-30 mm). Eight lesions represented primary lung cancer (1 SCLC, 1 SCC, 6 Adenocarcinomas), and 25 metastases (14 colorectal, 4 breast, 3 uterine, 4 others). Cryoablation was performed under general anaesthesia with CT-guidance. The cryoablation protocol usually consisted of 3 freezing cycles of 3/7/10 minutes, followed by passive and active thawing. Depending on tumor size and location 1-3 cryoprobes were used. Chest tubes were placed during or after treatment in cases of pneumothorax. Treatment decisions were approved by a multidisciplinary tumour board and oncologic consultation.
**Results or Findings:** Initial technical success was 100%. No major adverse events occured. Minor complications included 18 peri-interventional pneumothoraces and 6 post-interventional pneumothoraces; in total, 18 chest tubes were placed. Additional minor events were perifocal bleeding, post-procedural haemoptysis and pneumonia. Mean hospital stay was 4.2 days (SD 2.1). After a mean follow-up of 14.7 months (SD 13.6), 21 lesions showed local tumor control, 6 showed progression and for 6 lesions follow-up was not available.
**Conclusion:** CT-guided cryoablation is safe and effective for selected primary and secondary lung tumours, achieving high technical success with low complication rates and good intermediate local tumor control.
**Limitations:** The study is limited by its retrospective, single-centre design and the relatively small patient cohort. In 6 cases follow-up was missing to fully assess local tumour control.
**Funding for this study:** No funding was received for this study.
**Has your study been approved by an ethics committee?** Not applicable
**Author Disclosures:**
Elena Suderland: Nothing to disclose
Gebhard Josef Schmid: Nothing to disclose

**12:30 - 13:30** **Research Stage 4**

### Research Presentation Session: Abdominal and Gastrointestinal

### RPS 1901
### Exploring fatty liver disease

**Moderator**
Ilkay Sedakat Idilman; Ankara/TR

**Moderator's introduction**
Ilkay Sedakat Idilman; Ankara/TR
*(ipolater@yahoo.com)*

**New horizons in liver steatosis imaging - multiparametric ultrasound - preliminary study**
*M. Zbroja-Putowska*, M. A. Kuczyńska, A. Brodzisz, P. Krawiec, M. M. Wozniak; Lublin/PL
*(m.zbroja8888@gmail.com)*

**Purpose:** The aim of the study is to evaluate the use of multiparametric liver ultrasound in the diagnosis and monitoring of liver steatosis and fibrosis.
**Methods or Background:** Forty-five pediatric patients (20 W, 25 M) with increased liver laboratory parameters (AST, ALT) were included in the study. They were subjected to multiparametric liver ultrasound with evaluation of SWE (Emedian [kPa]), attenuation (ATI [dB/cm/MHz]) and dispersion (SWD [(m/s)/kHz]). All ultrasound examinations were performed with a Canon Aplio i800, with a convex i8CX1 probe from intercostal access; the sampling gate was positioned 1-2 cm from the liver pouch. Min. 5 measurements for all parameters, determining the median and the IQR coefficient for the median.
**Results or Findings:** 40 out of 45 pediatric patients examined turned out to have liver steatosis (mostly mild and moderate). In 32 of them the level of steatosis was proportional to the level of obesity. 8 patients didn't have obesity but the liver steatosis turned out to be mild or moderate, 1 of them had fibrosis (F1/F2). These patients were taking steroid medications. After one year, 8 patients had follow up examination. In 6 patients liver steatosis and fibrosis levels have decreased, in 2 patients steatosis maintainted on the same level – these patients didn't follow the recommendations.
**Conclusion:** Liver parenchymal steatosis remains asymptomatic for a long time, posing a serious clinical problem. Multiparametric liver assessment provides a rapid, accessible means of reliably evaluating not only the degree of parenchymal steatosis, but also the risk of progression or transformation to cirrhosis. Thus, mpUSG now has the potential to become the most important non-invasive diagnostic tool in the screening assessment and monitoring of patients with metabolic syndromes as well as those undergoing hepatotoxic treatment.
**Limitations:** Small group
**Funding for this study:** n/a
**Has your study been approved by an ethics committee?** Not applicable
**Author Disclosures:**
Monika Zbroja-Putowska: Nothing to disclose
Agnieszka Brodzisz: Nothing to disclose
Magdalena Maria Wozniak: Nothing to disclose
Maryla Anna Kuczyńska: Nothing to disclose
Paulina Krawiec: Nothing to disclose

**Hepatic steatosis and fibrosis assessment in MASLD using UDFF and pSWE in routine clinical practice: a retrospective analysis of multi-centre real-world data**
X. Hong, B. Sadegi, L. Alvarez, A. Klunk, C. Kruse, E. Sober, H. Simon, *S. Raj*; Scottsdale, AZ/US
*(sean.raj@gmail.com)*

**Purpose:** Metabolic dysfunction-associated steatotic liver disease (MASLD) can progress from fat accumulation to fibrosis, cirrhosis, or hepatocellular carcinoma. We evaluated the clinical utility of Ultrasound Derived Fat Fraction (UDFF) combined with Point Shear Wave Elastography (pSWE) for assessment of steatosis and fibrosis in routine clinical practice.
**Methods or Background:** A cohort of 220 patients who underwent abdominal ultrasound at SimonMed Imaging between January and April of 2025 was selected. UDFF with pSWE was integrated into the routine abdominal exam. Measurements were categorized per clinical indications (abnormal labs, abdominal complaints, MASLD, non-liver disease, and other liver disease) and BMI. Steatosis severity was assessed using UDFF: ≤5% (Normal), (5,10] (Minimal to Mild), (10,15] (Mild to Moderate), (15, 20] (Moderate to Severe), and >20% (Severe). The SRU "rule of four" was used for fibrosis assessment using pSWE: ≤5kPa (Normal), (5,9) (Rule-out cACLD), [9,13] (Suggestive of cACLD), (13, 17] (Rule-in cACLD), (17,21] (Suggestive of CSPH), and >21 (CSPH).
**Results or Findings:** Across 9 cities in Arizona (7; n=167) and Nevada (2; n=53), median UDFF was 8% (5-14%) and median liver stiffness measurement was 3.56 kPa (2.88-4.65kPa). UDFF varied across the five clinical indications ($p<0.01$) with the MASLD cohort (70/220) having the highest median UDFF of 10% (6-16%). The pSWE measurements did not vary across the clinical indications, BMI and the steatosis grades; 177/220 were normal, 35/220 ruled-out cACLD and 8/220 were suggestive of cACLD. Higher BMI was associated with greater steatosis severity ($p<0.001$).
**Conclusion:** Our results highlight the utility of UDFF and pSWE in assessing steatosis and fibrosis in patients with MASLD in routine practice. These results are of interest in MASLD management for risk stratification to treatment monitoring.
**Limitations:** N/A
**Funding for this study:** N/A
**Has your study been approved by an ethics committee?** Yes
**Ethics committee - additional information:** Waiver of IRB review
**Author Disclosures:**
Xiaowei Hong: Nothing to disclose
Lexy Alvarez: Nothing to disclose
Howard Simon: Nothing to disclose
Cody Kruse: Nothing to disclose
Erik Sober: Nothing to disclose
Sean Raj: Nothing to disclose
Amanda Klunk: Nothing to disclose
Barry Sadegi: Nothing to disclose

**Diagnostic Accuracy of Quantitative Ultrasound Derived Metrics with Histological steatosis grade as Reference Standard in patients with mixed aetiology Chronic Liver Disease**
*N. Seneviratne*, C. Fang, D. Quinlan, P. S. Sidhu; London/UK
*(nuran.seneviratne@nhs.net)*

**Purpose:** Quantitative ultrasound (QUS) techniques—including tissue attenuation imaging (TAI), tissue scatter-distribution imaging (TSI), and ultrasound derived fat fraction (USFF)—are emerging non-invasive tools for quantifying hepatic steatosis. We assessed their diagnostic accuracy against histological steatosis grade and compared them with the Controlled Attenuation Parameter (CAP).
**Methods or Background:** One hundred and twenty patients with chronic liver disease were prospectively recruited for contemporaneous liver biopsy and QUS imaging. Reference was histological steatosis grade (S0-3) scored by consensus by liver pathologists. Diagnostic accuracy of USFF, TSI, TAI and CAP was evaluated using ROC analysis and Youden optimal cut-offs. Multivariable ordinal logistic regression examined independent predictors of steatosis grade.
**Results or Findings:** The final cohort comprised 115 patients (57 men, 58 women; median age 45 years; median BMI 27.8 kg/m2). Histological steatosis grades were S0=65, S1=28, S2=14, S3=8. USFF, TSI and TAI correlated strongly with histological grade (ρ=0.81, 0.76 and 0.75, all P<0.001), while BMI showed a moderate correlation (ρ=0.56, P<0.001). Age and gender were not significant. USFF achieved excellent AUCs of 0.95 for S≥1 and 0.94 for S≥2, with optimal cut-offs of 8.8% and 15.4%. At these thresholds, sensitivity/specificity were 88%/89% (S≥1) and 91%/85% (S≥2). TAI and TSI showed similar accuracy (AUCs 0.89–0.94) with cut-offs of 0.83 and 89.2 (S≥1), 0.91 and 93.1 (S≥2). In a subset (N=61), CAP showed comparable accuracy (AUCs 0.93, 0.94). On multivariate analysis, USFF (OR 18.0, P<0.001), TSI (OR 12.8, P<0.001) and TAI (OR 8.4, p<0.001) remained independent predictors; BMI was also significant in the USFF model (OR 1.94, P=0.038).
**Conclusion:** QUS-derived USFF, TSI and TAI provide excellent diagnostic accuracy for histological steatosis, performing at least as well as CAP.
**Limitations:** Possibility of sampling error at liver biopsy. Relatively small high steatosis grade patients.
**Funding for this study:** Samsung Medison
**Has your study been approved by an ethics committee?** Yes
**Ethics committee - additional information:** Research Ethics [REC] reference: 22/NW/0401, IRAS Project ID: 212836
**Author Disclosures:**
Nuran Seneviratne: Nothing to disclose
Dan Quinlan: Nothing to disclose
Cheng Fang: Nothing to disclose
Paul S. Sidhu: Equipment Support Recipient: Siemens, Phillips, GE Healthcare Consultant: Itreas, Bracco Research/Grant Support: Samsung Speaker: Itreas, Samsung, Philips, Siemens, Mindray Grant Recipient: Siemens, Phillips, GE Healthcare.

**Impact of Ultrasound-Assessed Diffuse Hepatic Steatosis on Liver-Related Events Risk in Patients with Chronic Hepatitis B**
*M. Xiao*, Y. Wu, N. Wang, X. Wang, Y. Chong, J. Ren, L. Wu; Guangzhou/CN
*(xmingyue56@126.com)*

**Purpose:** To assess the impact of ultrasonographically assessed diffuse hepatic steatosis and metabolic factors on the risk of liver-related events (LREs) in chronic hepatitis B (CHB) patients.
**Methods or Background:** We conducted a retrospective cohort study of patients with CHB who attended regular follow-up visits at our institution from January 2009 to December 2022. Diffuse hepatic steatosis was evaluated using ultrasonography. The primary endpoint was the development of LREs.Kaplan-Meier curves were generated to compare LRE-free survival between groups. Propensity score matching (PSM) was performed in a 1:1 ratio to minimize confounding effects. Univariate and multivariate Cox regression models were used to identify independent predictors associated with LREs.
**Results or Findings:** A total of 1808 patients with CHB were included, with a median follow-up of 104 months. LREs occurred in 134 patients (7.4%), among which hepatocellular carcinoma (HCC) accounted for 74.6% (n=100), decompensated cirrhosis for 21.0% (n=28), liver transplantation for 2.2% (n=3), and liver failure-related death for 2.2% (n=3). The group with diffuse hepatic steatosis exhibited a significantly lower risk of LREs compared to those without steatosis (HR: 0.398, 95% CI: 0.232-0.682). This protective association remained significant after PSM (HR: 0.484, 95% CI: 0.250-0.940). Multivariate analysis identified type 2 diabetes (HR: 1.69) and cirrhosis (HR: 5.15) as independent risk factors for LREs, while female sex (HR: 0.56) and higher albumin levels (HR: 0.89) were associated with a reduced risk.
**Conclusion:** Diffuse hepatic steatosis is not a primary driver of LREs in patients with CHB, and its potential protective effect should be interpreted within an individualized clinical context. Beyond antiviral therapy, proactive management of metabolic factors—such as glycemic and blood pressure control—can effectively reduce the risk of LREs.
**Limitations:** This study lacks dynamic assessment of hepatic steatosis.
**Funding for this study:** Guangzhou Science and Technology Plan Project under Grant No: 2023A03J0728.
**Has your study been approved by an ethics committee?** Yes
**Ethics committee - additional information:** Third Affiliated Hospital of Sun Yat-sen University (Approval No. [2021]02-387-01).
**Author Disclosures:**
Jie Ren: Nothing to disclose
Nana Wang: Nothing to disclose
Xiaomei Wang: Nothing to disclose
Yuankai Wu: Nothing to disclose
Mingyue Xiao: Nothing to disclose
Yutian Chong: Nothing to disclose
Lili Wu: Nothing to disclose

**Correlation of fat quantification on UGAP Ultrasound with MR Spectroscopy and mDIXON Imaging in the evaluation of non-alcoholic fatty liver disease: A Pilot Study**
P. C. P. Joshi, A. K. K. O. Dr, I. S. Shah, *A. Borse*; Pune/IN
*(aishwaryaborse1011@gmail.com)*

**Purpose:** To correlate fat quantification on ultrasound-guided attenuation parameter with MR Spectroscopy and mDIXON fat fractions in patients with non-alcoholic fatty liver disease (NAFLD).
**Methods or Background:** This prospective study was conducted at a tertiary care hospital in western india after obtaining ethical clearance from the institute. Thirty patients with diffuse hepatic steatosis on ultrasound / CT were included in this study. Patients with chronic alcoholism, chronic viral hepatitis and hepatocellular carcinoma were excluded. Each participant underwent UGAP ultrasound and 3T MRI (Philips) with body coil. Hepatic fat fraction was calculated using the lipid-to-water peak ratio in MR spectroscopy and fat fraction value in mDIXON imaging. UGAP parameters including attenuation coefficient were also recorded.
**Results or Findings:** Pearson correlation was used to assess the association between UGAP grading, MR Spectroscopy and mDIXON fat fraction. Correlation is significant at the 0.01 level. UGAP showed a strong positive correlation with both MR Spectroscopy and mDIXON results, with a correlation coefficient of 0.869 and 0.897 respectively. Notably, the mDIXON method demonstrated a higher level of agreement with MR Spectroscopy, with a correlation coefficient of 0.892.
**Conclusion:** UGAP shows high degree of correlation with MR spectroscopy and mDIXON imaging as a non-invasive method for the evaluation of non-alcoholic fatty liver disease. This study highlights that UGAP can be used in isolation, potentially providing a cost-effective and accessible alternative technique to MRI-based techniques in the assessment and monitoring of hepatic steatosis.
**Limitations:** Histological confirmation was not included in this study. Since the MR Spectroscopy fat fraction method is advised for measuring hepatic steatosis in a clinical context, it was utilized as a reference instead. Only Indian patients with NAFLD were included. Only GE scanners are compatible with the UGAP used in this study.
**Funding for this study:** No
**Has your study been approved by an ethics committee?** Yes
**Ethics committee - additional information:** Bharati Vidyapeeth Medical College,PUNE-411043 Institutional Ethics Committe (DHR Reg. No:EC/NEW/INST/2022/MH/0150)
**Author Disclosures:**
Aishwarya Borse: Nothing to disclose
Priscilla Col Priscilla Joshi: Nothing to disclose
Aung Ko Ko Oo Dr: Nothing to disclose
Isha Sandip Shah: Nothing to disclose

**Hepatic fat quantification via three-material decomposition in dual-layer CT: Reproducibility and interobserver agreement**
*T. Schömig*[1], S. Skornitzke[2], L. Hieronymi[1], D. Maintz[1], N. Große Hokamp[1]; [1]Cologne/DE, [2]Hamburg/DE
*(thomas.schoemig@uk-koeln.de)*

**Purpose:** This study investigates reproducibility and interobserver agreement of a previously published three-material-decomposition (3MD) algorithm for hepatic fat quantification using dual-layer dual-energy CT (dlDECT). Prior research demonstrated good correlation between dlDECT and MR fat quantification. As a next step toward clinical integration, we aim to evaluate consistency and reliability of this dlDECT-based approach.
**Methods or Background:** In this retrospective study, we evaluated contrast-enhanced abdominal dual-layer CT scans (IQon, Philips Healthcare) of 58 patients obtained twice within 60 days (mean interval: 34.3± 20.2 days). Patients with severe changes in clinical status, such as initiation of chemotherapy, or findings affecting liver perfusion, were excluded. Fat fraction maps were generated using the published 3MD-algorithm for native, arterial, venous and delayed phases. Two radiologists independently placed five circular ROIs per scan, four in the right and one in the left hepatic lobe, avoiding vessels. Reproducibility between scans and interobserver agreement were assessed using Pearson's correlation, Bland-Altman analysis, repeated measures ANOVA, and intraclass-coefficients (ICC).

**Results or Findings:** The mean hepatic fat fraction across all patients and contrast phases was 4.91%±5.91, no significant difference was found between phases (p=0.07). Averaging across phases and readers, measurements at the two time points strongly correlated (4.87%±6.04 vs. 4.94%±6.13, $R^2$=0.78) with a low mean difference (0.34±3.09 percentage points, not significant, p=0.61), indicating good reproducibility. Interobserver agreement was excellent (ICC=0.92), with a mean difference of 0.62±1.3 percentage points, consistent across all phases.
**Conclusion:** The novel 3MD-algorithm enabled through dlDECT provides reproducible and consistent hepatic fat quantification with excellent interobserver agreement, supporting its potential role in clinical practice as a reliable alternative to MRI or ultrasound.
**Limitations:** The limitations of the study are the single-center, retrospective design, potential reader dependency of ROI-placement and limited sample size.
**Funding for this study:** This Work was funded by Philips Healthcare. The funding source had no involvement in study design, collection or interpretation of data.
**Has your study been approved by an ethics committee?** Not applicable
**Author Disclosures:**
Nils Große Hokamp: Nothing to disclose
David Maintz: Nothing to disclose
Lina Hieronymi: Nothing to disclose
Stephan Skornitzke: Employee: Philips GmbH DACH
Thomas Schömig: Nothing to disclose

**12:30 - 13:30** **Research Stage 3**

### Research Presentation Session: Artificial Intelligence and Imaging Informatics

### RPS 1905

### Building the AI breast imaging service: from deployment to clinical practice

**Moderator**
Joshua Rothwell; Cambridge/UK
*(jr947@cam.ac.uk)*
**Author Disclosures:**
Joshua Rothwell: Research Grant/Support: Merantix, ScreenPoint Medical, Lunit, iCAD, Google, Therapixel, and Volpara

### Improving time to breast cancer detection: AI identification of screening-detected cancers on prior-year digital breast tomosynthesis

*M. Bahl*, H. Kim, K-S. Kim, S. Do, L. Lamb; Boston, MA/US
*(mbahl1@mgh.harvard.edu)*

**Purpose:** To evaluate whether a commercial AI-based computer-assisted detection/diagnosis (CADe/x) algorithm for digital breast tomosynthesis (DBT) could have identified screening-detected breast cancers on the prior year's DBT examination, potentially enabling earlier diagnosis.
**Methods or Background:** This retrospective study included consecutive women with screening-detected breast cancers on DBT from 2016 to 2019 who also had a prior screening DBT within 18 months interpreted as negative. Both the index examination (the screening examination on which cancer was detected by the radiologist) and the prior examination were analyzed using a commercial CADe/x algorithm (Genius AI® Detection 2.0; Hologic, Inc.). AI scores ranged from 0-100, with values ≥22 considered positive per vendor recommendation. A breast imaging radiologist reviewed all AI-positive cases to determine whether AI marks corresponded to the site of the subsequently diagnosed cancer.
**Results or Findings:** Four hundred women (mean age 64±10) met inclusion criteria. On the index mammogram, AI detected and correctly localized 88.8% (355/400) of cancers. Among these 355 AI-detected cases, 44.5% (158/355) were also identified and correctly localized by AI on the prior-year exam (radiologist-negative). Thus, 39.5% (158/400) of patients in the cohort may have benefited from earlier cancer detection. Of the 158 cancers flagged by AI on the prior exam, 79.7% (126/158) were invasive. Among these invasive cancers, 73.8% (93/126) were grade 2-3, and 9.5% (12/126) were node-positive at diagnosis.
**Conclusion:** A commercial AI algorithm retrospectively identified and localized nearly 40% of screening-detected breast cancers on prior DBT - approximately one year before radiologic diagnosis - suggesting a potential role for AI in facilitating earlier detection, even outside classic false-negative or interval cancer categories.
**Limitations:** The limitations of the study are single-center, retrospective design and use of one vendor/algorithm with a vendor-defined threshold, which may limit generalizability.
**Funding for this study:** Funding was provided by Hologic, Inc. The authors, none of whom are employees of Hologic, maintained full control over the data and the submitted information.
**Has your study been approved by an ethics committee?** Yes
**Ethics committee - additional information:** This retrospective Health Insurance Portability and Accountability Act (HIPAA)-compliant study was granted an exemption from the requirement for written informed consent by the institutional review board at the Massachusetts General Hospital (Protocol #: 2023P003130).
**Author Disclosures:**
Kyung-Su Kim: Nothing to disclose
Leslie Lamb: Nothing to disclose
Manisha Bahl: Consultant: Hologic, Inc. Research/Grant Support: Hologic, Inc.
Synho Do: Nothing to disclose
Hyunji Kim: Nothing to disclose

### Comparative Study of Radiologists' Diagnostic Performance With and Without Artificial Intelligence Support for Breast Lesion Detection in 2D Mammography

*L. Lassalle*[1], J. Ventre[2], V. Marty[2], L. Clovis[2], N. Nitche[2], J. Hadchiti[3], N-E. Regnard[1], A-L. Hermann[4], E. Kotter[5]; [1]Lieusaint/FR, [2]Paris/FR, [3]Villejuif/FR, [4]Lyon/FR, [5]Freiburg Im Breisgau/DE

**Purpose:** Mammography is a key domain where AI may improve early breast cancer detection. The study compared the diagnostic performance of radiologists with and without support from a new AI system (BreastView, Gleamer) in detecting breast lesions.
**Methods or Background:** We retrospectively collected mammographies from three imaging centers across France (2018-2023), sourced from three manufacturers (Hologic, Siemens, GE). Eligible patients were women over 18 who underwent 2D mammography with CC and MLO views and had either biopsy or 18-month follow-up. Poor-quality exams were excluded. Ground truth was determined by an experienced breast radiologist who had access to the entire patient file, including anterior and posterior mammographies and, when available, digital breast tomosynthesis, MRI, ultrasound, clinical reports, and biopsy reports for cancer cases. Nine radiologists participated: five "non-subspecialists" (250 and 750 mammographies/year), and four "subspecialists" (>1000 mammographies/year). They annotated all visible lesions and assigned malignancy scores from 0 to 100. Each completed two reading sessions, unaided and with AI support, separated by a 12-month washout.
**Results or Findings:** The dataset included 319 patients (age: 58 ± 13 years): 159 with a malignant biopsy-proven lesion, 39 with only benign lesions confirmed by biopsy or 18-month follow-up, and 121 with no lesion confirmed by 18-month follow-up. The stand-alone AI achieved an AUC of 0.911 [0.881–0.942] for malignant lesion detection, outperforming the mean radiologist AUC of 0.801 [0.769–0.832]. With AI assistance, radiologists significantly improved their AUC (0.880 [0.865–0.896]), sensitivity (+18.7 points, p<.001), and specificity (+2.6 points, p=.042). Gains were lower for subspecialists but still significant.
**Conclusion:** The AI system showed robust performance, enhancing radiologists' diagnostic accuracy and supporting its potential as a clinical decision-support tool.
**Limitations:** The study was retrospective, with an enriched cancer dataset, and readers had no access to clinical information.
**Funding for this study:** Gleamer
**Has your study been approved by an ethics committee?** Not applicable
**Author Disclosures:**
Nor-Eddine Regnard: Shareholder: Gleamer
Vincent Marty: Employee: Gleamer
Lauryane Clovis: Employee: Gleamer
Louis Lassalle: Shareholder: Gleamer Employee: Gleamer
Jeanne Ventre: Employee: Gleamer
Joya Hadchiti: Nothing to disclose
Nicolas Nitche: Employee: Gleamer
Elmar Kotter: Nothing to disclose
Anne-Laure Hermann: Nothing to disclose

**Should AI results be disclosed in mammography reports? A randomized survey study of patient responses to concordant and discordant interpretations**

*G. Irmici*, F. Pesapane, C. Depretto, L. Nicosia, G. Della Pepa, A. Rotili, S. Santicchia, G. P. Scaperrotta, E. Cassano; Milan/IT
*(giovanni.irmici@icloud.com)*

**Purpose:** To assess how disclosing artificial intelligence (AI) results, particularly discordant findings, affects patient trust, anxiety, follow-up intentions, and attitudes toward AI in mammography. The study also evaluated whether a brief explanatory note mitigates adverse reactions.
**Methods or Background:** A cross-sectional randomized experimental survey was conducted among 600 women (mean age 55.4 ± 6.8 years) undergoing mammography in two academic breast imaging centers in Milan, Italy, between January 2023 and June 2024. Participants were randomized into four hypothetical BI-RADS 1 scenarios: Radiologist Only (control), AI No-Flag (AI concordant with radiologist), AI Flagged (AI discordant false-positive), and AI Flagged + Explanation (discordant AI with contextual information). Outcomes included trust (0–100 scale), worry, second-opinion intent, legal action intent, and AI approval. Statistical analyses involved ANOVA, chi-square tests, and logistic regression with Bonferroni correction.
**Results or Findings:** Discordant AI disclosure significantly reduced trust in the radiologist (73.0 vs. 90.1; p<0.001), increased anxiety (58.0% vs. 16.0%; OR=15.4), second-opinion intent (50.0% vs. 8.7%; OR=10.2), and legal action consideration (60.7% vs. 38.7%; OR=2.49). Adding explanatory context significantly alleviated these effects (e.g., anxiety: 25.3%; OR=0.26). AI approval remained high (>85%) across AI scenarios.
**Conclusion:** Disclosing discordant AI results negatively impacts patient trust and anxiety, prompting increased intentions for second opinions and legal actions. Providing explanatory context mitigates these adverse effects, supporting transparent but contextually informed disclosure as essential for patient communication strategies in AI-integrated mammography.
**Limitations:** Limitations of our study include the hypothetical scenario nature. The explanation tested in this study focused on FP rates; future work should explore the effects of alternative or additional types of contextual information. Additionally, we measured stated intentions rather than observed behaviours.
**Funding for this study:** No funding was received for this study.
**Has your study been approved by an ethics committee?** Yes
**Ethics committee - additional information:** Institutional Review Board approval was obtained.
**Author Disclosures:**
Anna Rotili: Nothing to disclose
Gianmarco Della Pepa: Nothing to disclose
Luca Nicosia: Nothing to disclose
Filippo Pesapane: Nothing to disclose
Gianfranco Paride Scaperrotta: Nothing to disclose
Enrico Cassano: Nothing to disclose
Catherine Depretto: Nothing to disclose
Sonia Santicchia: Nothing to disclose
Giovanni Irmici: Nothing to disclose

**AI performance on an interval cancer mammography dataset and its role in audit triage**

A. Taib[1], *K. Wells*[1], I. Darker[1], J. James[1], N. Sharma[2], Y. Chen[1]; [1]Nottingham/UK, [2]Leeds/UK
*(keenandwells@gmail.com)*

**Purpose:** Interval cancers (IC, cancers arising between screening rounds) are an important performance indicator in breast cancer screening. The NHSBSP requires a minimum of two readers to classify the previous screening mammograms into one of three categories: (1) Satisfactory, (2) Satisfactory with learning points and (3) Unsatisfactory. We assessed whether artificial intelligence (AI) could be applied retrospectively to reliably classify IC to help standardise this process.
**Methods or Background:** IC cases were collected from January 2015 to December 2024 from two large UK screening centres. An AI model (Lunit) analysed all cases and provided a continuous malignancy probability score (0-100) for each breast. A ROC analysis was performed to determine the ability of AI to distinguish Category 1 cases separately from 2 and 3 using malignancy scores and thresholding, where cases with scores less than the threshold were classified as Category 1, and those over classified as 2 and 3.
**Results or Findings:** 409 IC cases were included which were previously classified by readers at each centre (79% Category 1, 20% Category 2 and 1% Category 3). At a threshold of 0.5, AI classified 65 cases as Category 1 (63 correct, 2 misclassified Category 2 cases). At a threshold of 10, AI classified 229 cases as Category 1 (206 correct, 23 misclassified Category 2 cases). No Category 3 cases were misclassified as Category 1 by either threshold.
**Conclusion:** AI shows promise as a triage tool to improve the IC audit process by reliably classifying Category 1 cases, which make up most interval cancer, while still correctly identifying most category 2 & 3 cases for human review.
**Limitations:** Predominantly Category 1 cases, with limited Category 3. Limited number of centres may restrict generalisability.
**Funding for this study:** Funding was provided by Lunit Inc.
**Has your study been approved by an ethics committee?** Yes
**Ethics committee - additional information:** The study was approved by the local research and ethics committee of St James's University Hospital, Leeds, England.
**Author Disclosures:**
Nisha Sharma: Nothing to disclose
Keenan Wells: Nothing to disclose
Adnan Taib: Nothing to disclose
Iain Darker: Nothing to disclose
Jonathan James: Nothing to disclose
Yan Chen: Grant Recipient: Study funded by Lunit Inc.

**Beyond Screening: AI Performance in Mammographic Breast Cancer Recurrence Detection**

*Z. Aghdam*, X. Wang, A. Portaluri, J. Kroes, J. Teuwen, K. Lipman, R. Mann; Amsterdam/NL
*(z.aghdam@nki.nl)*

**Purpose:** To evaluate the diagnostic performance of a commercial mammography-based AI system for recurrence detection in the post-operative breast, addressing a critical evidence gap.
**Methods or Background:** This retrospective single-center diagnostic accuracy study included patients after breast-conserving surgery undergoing digital mammography for follow-up between 2004 and 2022. Recurrence was defined as ipsilateral malignancy irrespective of tumor biology. Reference standard was pathology. The most recent mammogram ≤3months before recurrence was analysed. Mahalanobis-distance matching was used to select one mammogram per control(≥24months negative follow-up), aligning the age and time from the primary tumor between cohorts. 4,235 exams(384 recurrence-3,851 controls)were included. BI-RADS categories were extracted from reports; sensitivity and specificity calculated at BI-RADS≥3. For each exam, AI(Transpara version-2.1.0)yielded highest region score at breast level(0–100) and a risk category(low-intermediate-elevated). AI sensitivity was assessed at radiologists’ specificity. A combined AI+BI-RADS score was derived via logistic regression. Risk category changes from preceding year exams were evaluated using chi-square with Bonferroni-adjusted pairwise comparisons(p<0.05).
**Results or Findings:** Radiologists’ sensitivity, specificity were 0.75[0.68-0.81] and 0.98[0.98-0.99], respectively. AI sensitivity at matched specificity was 0.28[0.21-0.34] and at Youden-derived threshold was 0.77[0.71-0.84]. AUC of AI was 0.88[0.85-0.90]. Combined AI+BI-RADS had a sensitivity of 0.77[0.70-0.83] while improving AUC to 0.95[0.93-0.97](vs AI alone,p<0.001). AI risk category transitions over one year differed significantly between cases and controls(132 recurrences vs 2985 controls),($\chi^2$=336.0, p<0.001). Recurrences were more likely to transition to a higher risk category(76.5% vs 14.8%,RR=5.17[4.55–5.87]), and less likely to remain in the same category(22% vs 65.4%,RR=0.34[0.24–0.46]) or have decreased risk(1.5% vs 19.8%,RR=0.08[0.02–0.30]).
**Conclusion:** Sensitivity of stand-alone AI for recurrence detection was lower than for radiologists at the same specificity. Increase in risk category was, however, associated with local recurrence.
**Limitations:** Single-center retrospective design, use of varied mammography systems, assessment of only one AI model
**Funding for this study:** Health Holland
**Has your study been approved by an ethics committee?** Yes
**Ethics committee - additional information:** IRBd21-060
**Author Disclosures:**
Jaap Kroes: Employee: Screen Point Medical
Kevin Lipman: Other: public–private research funding with ScreenPoint Medical. Co-development with kaiko.ai (unpaid)
Jonas Teuwen: Other: shareholder of Ellogon.ai; has a patent pending on Cone Beam Computed Tomography reconstruction
Xin Wang: Nothing to disclose
Antonio Portaluri: Nothing to disclose
Zahra Aghdam: Nothing to disclose
Ritse Mann: Other: has grants or grants pending with European Research Council, Horizon Europe, The Netherlands Research Council, The Dutch Cancer Society, Health Holland, Siemens Healthineers, Bayer Healthcare, Beckton & Dickinson, Screenpoint Medical, Lunit, PA Imaging, and Koning; royalties or licenses with Elsevier; consulting fees with Screenpoint Medical, Beckton & Dickinson, Siemens Healthineers, and Bayer Healthcare; and participation on a Data Safety Monitoring Board or Advisory Board with the SMALL study

**Comparing the Performance of Top Ranked AI Models from the RSNA 2023 Screening Mammography Breast Cancer Detection AI Challenge to Commercial AI Models**

G. J. W. Partridge[1], H. Jupp[1], T. Zhang[2], X. Wang[2], R. Mann[2], L. Moy[3], *Y. Chen*[1]; [1]Nottingham/UK, [2]Nijmegen/NL, [3]New York, NY/US
*(yan.chen@nottingham.ac.uk)*

**Purpose:** In 2023, the RSNA hosted a Screening Mammography Breast Cancer Detection Artificial Intelligence (AI) Challenge, where participants were invited to develop AI models for cancer detection in mammograms. Here we assess the performance of the Top 7 ranked models compared to 4 commercially available AI products, using a multi-national dataset.
**Methods or Background:** A large multi-national evaluation dataset was sourced from the USA, UK, Australia and Brazil as part of the 2023 RSNA Challenge, consisting of 20,365 cases. Cases consisted of 2-view 2D mammography screening cases, where cancers were pathology proven and non-cancer cases had at least 1-year of normal follow-up. The Top 7 ranked RSNA challenge AI models (as per pF1 score in the RSNA Challenge https://www.kaggle.com/competitions/rsna-breast-cancer-detection/leaderboard), and 4 commercially available AI products (Lunit, Transpara, Therapixel, iCAD) will analyse all cases, and the performance of all AI models will be compared.
**Results or Findings:** In the current dataset including 4,811 cases (USA and Australia), equating to 9,622 single breast exams, 193 (2.0%) had biopsy proven cancer, and 9,429 were non-cancer (98.0%). The Top 7 Challenge algorithms achieved AUCs between 0.903 and 0.947, and the commercial AI product achieved an AUC of 0.933. The Top ranked Challenge model AUC was not different to the commercial AI AUC (Delong's method: P = .18).
**Conclusion:** The Top 7 ranked challenge algorithms performed very well compared to the commercial product on the current dataset sourced from the USA and Australia. Inclusion of data from the UK and Brazil will enable an analysis of AI generalisability and robustness across different populations.
**Limitations:** Relatively small size of evaluation test-set; low cancer prevalence (but screening setting).
**Funding for this study:** N/A
**Has your study been approved by an ethics committee?** Not applicable
**Author Disclosures:**
Harry Jupp: Nothing to disclose
Linda Moy: Nothing to disclose
George J W Partridge: Nothing to disclose
Xin Wang: Nothing to disclose
Tianyu Zhang: Nothing to disclose
Yan Chen: Nothing to disclose
Ritse Mann: Nothing to disclose

**Large-scale AI implementation in Radiology: technical, operational, and behavioral adoption patterns across a 20-center Swiss Imaging Network**

S. Morozov, N. Heracleous, *B. Rizk*, C. Thouly, B. Dufour, O. Novarina; Sion/CH
*(benoit.rizk@groupe3r.ch)*

**Purpose:** To evaluate technical efficiency and radiologist adoption of AI tools across musculoskeletal, chest, breast, and neurologic imaging in Switzerland's largest radiology network, establishing benchmarks for processing, workflow benefit, and user acceptance.
**Methods or Background:** A retrospective analysis included 397,694 radiological studies processed by AI at 20 Swiss centers between January 2022 and June 2025. Survey data (53/58 radiologists, 91.4% response) captured usage frequency, trust, satisfaction (Net Promoter Score), and desired improvements. Time metrics included AI processing latency, report turnaround with vs. without AI, and the proportion of results available at reporting. AI implementation covered multiple modalities and anatomies.
**Results or Findings:** Musculoskeletal AI accounted for 67.8% of use (trauma radiography 37%). Breast, chest, and brain AI demonstrated 76% specialty adoption. Frequent AI use was reported by 66.1% of radiologists, primarily mid-career. Perceived benefits were error protection (71.7%) and time savings (56.6%). Key requests: faster processing (48%) and automated report integration (34%). NPS varied: +86.7 for bone age, +65.2 orthopedic radiography, −55.3 spine MRI, and +38.5 for chest CT (78% use). Technical bottlenecks were data transfer for radiography/mammography (2.1–3.5min) and AI computation for brain/spine MRI (8.2min). Only 21.6% of AI results were ready before reporting; 5% were missing at validation. AI reduced report times by 12–57% in targeted modalities (knee MRI 16→14min, trauma radiography 7→3min, bone age 3.5→1.6min).
**Conclusion:** AI tools produced measurable workflow gains (12–57% turnaround reduction) and 90%+ adoption, but improvements in speed, integration, and interoperability are required. Addressing technical and workflow barriers and focusing on user feedback will be critical for effective specialty adaptation.
**Limitations:** AI evaluation focused on technical and operational metrics, with indirect assessment of clinical outcomes. Results reflect the experience of a mature, multi-site Swiss network, which may limit generalizability to other settings.
**Funding for this study:** Self-funded
**Has your study been approved by an ethics committee?** Not applicable
**Author Disclosures:**
Cyril Thouly: Shareholder: SMA, 3R Swiss Imaging Network
Sergey Morozov: Research/Grant Support: Lunit Board Member: Vital Radiology Services Research/Grant Support: GE Healthcare Founder: Medlogic Research/Grant Support: Guerbet
Benoît Dufour: Shareholder: SMA – Holding of 3R Swiss Imaging Network
Natalie Heracleous: Nothing to disclose
Benoît Rizk: Shareholder: SwissMed Holding Shareholder: Holding G
Octave Novarina: Nothing to disclose

**Efficient external validation of AI models using nested case–control designs: Application to breast cancer risk prediction**

*J. Peters*[1], D. Sergeev[2], C. Jacobs[1], D. Van Der Waal[1], M. Broeders[1]; [1]Nijmegen/NL, [2]Heidelberg/DE
*(jim.peters@radboudumc.nl)*

**Purpose:** External validation of AI models in large cohorts is critical but computationally and resource-intensive, especially when multiple models are compared or evaluations repeated for quality control. Nested case-control (NCC) designs could enable faster and more sustainable validation by reducing data requirements, but it is unclear whether they yield unbiased estimates across all recommended performance metrics. We investigated whether an NCC design with weighted estimators can accurately evaluate discrimination and calibration of breast cancer risk prediction models.
**Methods or Background:** We used data from the PRISMA study, a Dutch population-based breast cancer screening cohort including 38,742 women with questionnaire data and mammograms. Two prediction models for 5-year breast cancer risk were evaluated: the Tyrer–Cuzick model and Mirai, a mammography-based AI model. For the NCC design, all cases were matched to four controls. Weighted estimators were applied for the concordance index (C-index), time-dependent AUC (tAUC), observed/expected ratio (O/E), and calibration slope. To assess sampling variability, NCC samples were drawn 100 times. Averaged estimates of performance metrics were compared with the full cohort.
**Results or Findings:** There were 571 breast cancer cases after a median follow-up of 4.3 years. Average performance of Tyrer-Cuzick in 100 NCC samples (all cases, 2,284 controls) was: C-index 0.598 (SD 0.007), tAUC 0.587 (0.010), O/E ratio 0.701 (0.010), slope 0.636 (0.041). In the full cohort, results were: C-index 0.585 (95% CI 0.558–0.613), tAUC 0.574 (0.536–0.607), O/E ratio 0.693 (0.625–0.775), slope 0.563 (0.399–0.726).
**Conclusion:** Using only 8% of the data, the NCC design produced performance estimates closely matching full-cohort results. This approach may enable faster, more resource-efficient external validation, particularly useful for comparing AI models or repeated quality control evaluations.
**Limitations:** Current results are based on one model; analyses with Mirai will be available at ECR 2026.
**Funding for this study:** Dutch Cancer Society (KWF7626) and The Netherlands Organisation for Health Research and Development (ZonMw 200500004)
**Has your study been approved by an ethics committee?** Yes
**Ethics committee - additional information:** CMO Arnhem-Nijmegen reference no. 2014/177
**Author Disclosures:**
Dmitry Sergeev: Nothing to disclose
Jim Peters: Nothing to disclose
Mireille Broeders: Nothing to disclose
Danielle Van Der Waal: Nothing to disclose
Colin Jacobs: Nothing to disclose

**12:30 - 13:30** **Research Stage 2**

### Research Presentation Session: Hybrid, Molecular and Translational Imaging

### RPS 1906

### Moving molecular imaging forward: experimental and beyond oncology

**Moderator**
Mike Sathekge; Pretoria/ZA
*(mike.sathekge@up.ac.za)*

**Multimodal Imaging for Response Assessment to Combined anti-PD-L1/anti-CTLA-4 Immunotherapy in a Murine Melanoma Model: A Comparative Evaluation of [18F]FDG-PET/CT, MRI and CEUS**
*M. J. Antons*, F. Herr, M. Heimer, S. Lindner, M. Brendel, D-A. Clevert, J. Ricke, R. Werner, C. C. Cyran; Munich/DE
*(melissa.antons@med.uni-muenchen.de)*

**Purpose:** While immune checkpoint inhibitors have demonstrated considerable efficacy in malignant melanoma, interindividual variability necessitates robust imaging biomarkers for response evaluation. This study compared the diagnostic performance of [18F]FDG-PET/CT, multiparametric MRI and contrast-enhanced ultrasound (CEUS) in monitoring the effects of a combined anti-PD-L1/anti-CTLA-4-immunotherapy in a murine melanoma model with immunohistochemical validation.
**Methods or Background:** C57BL/6 mice (female, n=88) were inoculated subcutaneously with B16-F10 melanoma cells into the left abdominal flank and randomized into therapy and control groups. The therapy group received intraperitoneal injections of anti-PD-L1- and anti-CTLA-4-antibodies, while the control group received sham treatment. Imaging assessments at baseline (day 7 post inculation), follow-up 1 (FU-1; day 13) and follow-up 2 (FU-2; day 19) included [18F]FDG-PET/CT (n=40), multiparametric MRI (n=28) and CEUS (n=20). Tumor allografts were harvested for ex vivo immunohistochemical validation (CD8, Ki-67, TUNEL, CD31).
**Results or Findings:** [18F]FDG-PET/CT revealed significantly lower MTV and SUVmax in the therapy group (FU-1: MTV: p=0.004; FU-2: MTV: p=0.008, SUVmax: p=0.0003). MRI demonstrated significantly lower ADC in the therapy group at follow-up (FU-1: p = 0.002). CEUS showed significant reduction in tumor perfusion (WiAUC, p=0.0152) and a significantly lower number of VEGFR2-targeted microbubbles in the therapy group (SI8min: p=0.003; SI10min: p=0.0019). Immunohistochemistry confirmed significantly higher apoptosis rates (FU-1: p=0.012; FU-2: p=0.001), more CD8-positive T-cells (FU-2: p=0.003), lower tumor cell proliferation (FU-1: p=0.012; FU-2: p=0.012) and lower microvascular density (FU-1: CD31: p<0.001) in the therapy group.
**Conclusion:** This study highlights the complementary strengths of PET/CT, MRI, and CEUS in assessing immunotherapy response in malignant melanoma. [18F]FDG-PET/CT provided early metabolic response parameters, MRI parameters reflected cellular and microstructural tumor alterations and CEUS detected vascular remodelling.
**Limitations:** The observation period was relatively short and no clinical endpoints such as overall survival of the animals were determined.
**Funding for this study:** This work was supported by a research grant from Bracco Imaging SA, Geneva, Switzerland, which provided the BR55 free of charge.
**Has your study been approved by an ethics committee?** Not applicable
**Author Disclosures:**
Rudolf Werner: Nothing to disclose
Dirk-André Clevert: Nothing to disclose
Clemens C. Cyran: Grant Recipient: Bracco Imaging SA, Geneva, Switzerland
Simon Lindner: Nothing to disclose
Maurice Heimer: Nothing to disclose
Felix Herr: Grant Recipient: Bracco Imaging SA, Geneva, Switzerland
Melissa J. Antons: Nothing to disclose
Matthias Brendel: Nothing to disclose
Jens Ricke: Nothing to disclose

**Impact of Ischemia Duration on MRI-Derived Quantitative Perfusion Parameters in a Mouse Kidney Transplant Model**
*F. Herr*, S. Kloiber-Langhorst, M. M. Li, O. Dietrich, J. Ricke, C. C. Cyran, J. Andrassy; Munich/DE
*(felix.herr@med.uni-muenchen.de)*

**Purpose:** To assess the effect of cold ischemia duration on MRI-derived perfusion parameters in kidney transplants of mice after long-term post-transplant survival.
**Methods or Background:** Fifteen C57BL/6 mice underwent kidney transplantation and were divided into two groups based on cold ischemia time: 30 minutes (n = 6) and 16 hours (n = 9). Dynamic contrast-enhanced MRI was performed at a mean of 268 ± 29.9 days post-transplantation. Perfusion parameters were calculated using the Patlak model, yielding the plasma volume fraction (vp), representing blood volume/perfusion, and the volume transfer constant (Ktrans), reflecting vascular permeability. Intergroup comparisons were performed using the Mann–Whitney U-test.
**Results or Findings:** Significant differences were observed in the Ktrans parameter of transplanted kidneys between the ischemia groups. The median Ktrans was significantly elevated in the 16 h group (2.82 ± 0.47 mL/100 mL/min) versus the 1 h group (1.16 ± 0.53 mL/100 mL/min; p = 0.008). In contrast, vp did not differ significantly between the groups. Median vp was 20.56 ± 7.02 mL/100 mL (16 h) versus 30.69 ± 8.11 mL/100 mL (1 h; p = 0.151). No significant differences were observed between the transplanted kidney and the contralateral native kidneys for any of the Patlak-derived parameters.
**Conclusion:** Prolonged cold ischemia of 16 hours leads to significantly increased Ktrans values in murine kidney transplants, indicating impaired microvascular integrity after long-term engraftment. These finding suggest that cold ischemia duration may be a critical factor influencing graft microcirculation even in the chronic post-transplant phase.
**Limitations:** The study includes a relatively small sample size, which limits statistical power. Additionally, potential interindividual anatomical variations in the small mouse kidneys could influence quantification; however, we largely counteracted this through standardized ROI placements and modeling.
**Funding for this study:** No funding was received for this study.
**Has your study been approved by an ethics committee?** Yes
**Ethics committee - additional information:** The study was approved by the government of Upper Bavaria (ROB-55.2-2532.Vet_02-20-186) and carried out in accordance to the EU Directive 2010/63 for the protection of animals used for scientific purposes and was reported in compliance to ARRIVE (Animal Research: Reporting of In Vivo Experiments) guidelines.
**Author Disclosures:**
Olaf Dietrich: Nothing to disclose
Clemens C. Cyran: Nothing to disclose
Ming Ming Li: Nothing to disclose
Felix Herr: Nothing to disclose
Joachim Andrassy: Nothing to disclose
Sandra Kloiber-Langhorst: Nothing to disclose
Jens Ricke: Nothing to disclose

**Drug-Loaded Mesoporous Nanozyme for In Vivo Imaging and Lipid Reprogramming-Enhanced Synergistic Therapy of Pancreatic Tumors in Mice**
*J. Dai*, X-G. Peng; Nanjing/CN

**Purpose:** Pancreatic tumor therapy is severely hindered by chemotherapy resistance. Oxaliplatin (OXA) efficacy is limited by dual resistance mechanisms: glutathione (GSH)-mediated detoxification and lipid metabolic reprogramming. Although the metabolic hormone Irisin represents a potential strategy to modulate lipid metabolism, its systemic delivery raises concerns about potentially exacerbating cancer-associated cachexia. Furthermore, non-invasive methods to dynamically monitor metabolic changes and treatment response are critically needed.
**Methods or Background:** The platinum-palladium-rhodium-iron quaternary mesoporous nanozyme co-loaded with OXA and Irisin (PtPdRhFe@OXA/Irisin) was developed for the targeted co-delivery to pancreatic tumors in mice. This theranostic platform was assessed via a multi-modal imaging strategy: 1) Near-infrared fluorescence (NIRF) imaging for real-time tracking of tumor accumulation; 2) Serial MRI at weekly intervals to quantitatively monitor tumor volume changes and assess lipid content, providing a non-invasive measure of metabolic response.
**Results or Findings:** The nanosystem demonstrated effective CD44-targeted delivery to tumors, as confirmed by NIRF signals exclusively at the tumor site. Weekly MRI scans revealed that mice treated with PtPdRhFe@OXA/Irisin exhibited a significant suppression in tumor growth compared to control groups. MRI-based assessment indicated a notable decrease in intratumoral lipid content, suggesting Irisin affects pancreatic tumor lipid metabolic reprogramming in mice. This metabolic shift sensitized tumors to OXA, whose effect was further enhanced by the nanozyme's dual enzyme-mimetic activities, scavenging GSH and elevating ROS.

**Conclusion:** The multifunctional nanosystem PtPdRhFe@OXA/Irisin exerts potent anti-pancreatic tumor effects by targeting lipid metabolic reprogramming in synergy with GSH depletion and oxidative stress induction, while enabling non-invasive monitoring via multi-modal imaging.
**Limitations:** The precise molecular pathways by which Irisin reprograms lipid metabolism require further elucidation.
**Funding for this study:** National Natural Science Foundation of China (82272064)
**Has your study been approved by an ethics committee?** Yes
**Ethics committee - additional information:** The Institutional Animal Care and Use Committee of the Medical School of Southeast University (Approve ID: 20240226015)
**Author Disclosures:**
Jingyue Dai: Nothing to disclose
Xin-Gui Peng: Nothing to disclose

**Whole-Body 18F-FDG Parametric PET/CT in people with Long COVID: The ERASE trial protocol**
H. Abdul Razak[1], W. D. Strain[1], P. Rogers[1], M. Faghy[2], S. Kranen[1], K. Mokbel[1], M. Scott-Cleasby[1], J. Hoare[1], *K. Knapp*[1]; [1]Exeter/UK, [2]Derby/UK
*(k.m.knapp@exeter.ac.uk)*

**Purpose:** Long-COVID is a long-term sequela arising from an acute infection with SARS-CoV-2. To date there is no known cure. The ERASE feasibility trial will evaluate the efficacy of Remdesivir for the treatment of Long COVID with a wide range of outcome measures. This abstract outlines the nested parametric PET/CT study and the novel protocol utilised.
**Methods or Background:** Whole body (WB) 18F-FDG PET/CT scans were performed in 20 participants at one centre on day 11 for the baseline scan and day 55 for the follow-up scan with a maximum dose constraint of 31.2mSv for both scans, but a typical dose of 22mSv. Dynamic scans were acquired using the Siemens Motionflow acquisition on a Siemens Biograph Vision 600 scanner (Siemens, Germany) with an acquisition time of 75 to 90 minutes depending on the height of the participant. Patient public involvement and engagement was undertaken throughout the study design.
**Results or Findings:** The long scan duration was generally well tolerated by the participants. The image quality was diagnostic and both SUV and Ki were calculated for major organs and bone marrow, the latter using the automated Patlak algorithm in Siemens Syngo Via software (Siemens Healthineers, Germany).
**Conclusion:** In conclusion, this protocol demonstrates a novel use of parametric PET/CT 18F-FDG WB scans for the assessment of Long COVID and response to Remdesivir. The opportunity to quantify metabolic changes in a disease which has a range of presentations provides the potential to identify drug efficacy in different organs, which could provide useful insights for the future in both diagnosing and treating this disease.
**Limitations:** This study was performed at a single centre on a single scanner and further quantitative analysis of image quality and baseline to follow-up changes is required.
**Funding for this study:** This study was funded by Gilead Sciences
**Has your study been approved by an ethics committee?** Yes
**Ethics committee - additional information:** IRAS number: 1007101
**Author Disclosures:**
Patrick Rogers: Research/Grant Support: Gilead grant
Karen Knapp: Research/Grant Support: Research funding from UKRI. Adaptix, ESA, NIHR. Investigator: Co-Investigator on Gilead project Speaker: ECR
William David Strain: Nothing to disclose
Kinan Mokbel: Nothing to disclose
Mark Faghy: Research/Grant Support: Gilead project PI
Jackie Hoare: Nothing to disclose
Michelle Scott-Cleasby: Nothing to disclose
Sascha Kranen: Nothing to disclose
Hairil Abdul Razak: Research/Grant Support: Gilead grant

**12:30 - 13:30** **Research Stage 1**

### Research Presentation Session: Genitourinary Hot Topic with Keynote Lecture

### RPS 1907

### Hot Topic: AI-assisted prostate cancer diagnosis

**Moderator**
Anwar R. Padhani; Northwood/UK
*(anwar.padhani@btinternet.com)*

**Keynote Lecture**
Binit Sureka; Jodhpur/IN
*(binitsurekapgi@gmail.com)*

**Improving biopsy decision-making in prostate cancer using AI-assisted biparametric MRI**
*S. Bottazzi*, A. Iacono, I. Isufi, L. D'Erme, S. Persiani, G. Avesani, L. Russo, E. Sala, B. Gui; Rome/IT

**Purpose:** To evaluate the biopsy benefit-to-harm ratio of biparametric MRI (bpMRI) alone versus AI-assisted bpMRI in a decision-support setting.
**Methods or Background:** This retrospective study included patients undergoing pre-biopsy MRI between 2021 and 2024. The MRIs were analysed in two sequential phases with a 45-day washout period. Initially, five readers (2 experts and 3 basics according to ESUR-ESUI) independently assigned PI-RADS on bpMRI. After, AI-generated reports were available, and readers reassessed bpMRI with AI decision support. Benefit-to-harm metrics were biopsy selectivity (GG≥2/GG1) and biopsy efficiency (GG≥2/[GG1+benign]). They were calculated across two different biopsy thresholds: PI-RADS ≥3 and a combined threshold of PI-RADS ≥4 or PI-RADS 3 with PSA density (PSAd) ≥0.15 ng/ml². Metrics were computed per reader and summarised as overall and subgroups (experts and basics readers) means (±SD).
**Results or Findings:** AI assistance improved benefit-to-harm metrics overall. At PI-RADS ≥3, biopsy selectivity increased from 3.889±0.884 to 5.078±1.602 and efficiency from 1.688±0.404 to 1.992±0.053. At the composite threshold (PI-RADS ≥4 or PI-RADS 3 with PSAd ≥0.15 ng/ml²), selectivity increased from 4.317±0.735 to 5.400±1.188 and efficiency from 2.166±0.699 to 2.363±0.250. Basic readers showed the greatest benefit: at PI-RADS ≥3, efficiency reached 2.000 and NNB 0.50; at the composite threshold, efficiency reached 2.260, with higher selectivity at both thresholds. Among experts, selectivity improved at PI-RADS ≥3 (from 4.688 to 5.378) and at the composite threshold (from 4.989 to 5.833), while efficiency remained stable.
**Conclusion:** Across the evaluated thresholds, the composite criterion—combining PI-RADS with PSAd—provided the highest biopsy selectivity and efficiency. With AI decision support, basic readers achieved performance comparable to experts, while expert metrics remained stable (efficiency) or slightly improved (selectivity). These findings support AI-assisted bpMRI interpretation to improve biopsy decision-making.
**Limitations:** Retrospective study with limited number of readers
**Funding for this study:** Na
**Has your study been approved by an ethics committee?** Yes
**Ethics committee - additional information:** Study ID7680
**Author Disclosures:**
Giacomo Avesani: Consultant: Lucida Medical
Ina Isufi: Nothing to disclose
Benedetta Gui: Nothing to disclose
Alessandra Iacono: Nothing to disclose
Silvia Bottazzi: Nothing to disclose
Evis Sala: Shareholder: Lucida Medical Speaker: GE Healthcare Speaker: Bayer
Salvatore Persiani: Nothing to disclose
Luca D'Erme: Nothing to disclose
Luca Russo: Advisory Board: Lucida Medical Speaker: GE Healthcare

**Artificial intelligence or radiologist interpretation for prostate cancer diagnosis**

*A. Ng*, A. Asif, A. Shah, A. Dudko, R. Kumar, P. Rajwa, F. Giganti, D. Pendse, V. Kasivisvanathan; London/UK

**Purpose:** With the global incidence of prostate cancer predicted to double in the next 20 years, alongside the potential adoption of biparametric MRI and national MRI screening programmes, demand for prostate MRI is set to rise substantially. Interpretation, however, has a steep learning curve, with optimal performance achieved by expert genitourinary radiologists. With a rising demand for medical imaging and a projected 40% radiologist shortfall by 2027, a prompt international solution is warranted. PARADIGM aims to evaluate whether artificial intelligence (AI) is non-inferior to radiologists in detecting clinically significant prostate cancer (Gleason grade group ≥ 2).
**Methods or Background:** PARADIGM is an international, prospective, multicentre, non-inferiority, within-patient, level-1 evidence diagnostic study. 500 men will be recruited over 18 months. Men will undergo standard of care MRI with either 1.5 or 3.0 T with at least a pelvic phased array coil. The radiologist and a primary AI algorithm will report the MRI blinded to each other. The radiologist will then be unblinded and produce a merged report, with the ability to overrule AI findings for safety. Suspicious lesions identified by either AI or radiologist will undergo targeted biopsies, with optional perilesional and/or systematic biopsies. The primary outcome is the proportion of men with clinically significant cancer. Planned secondary outcomes include the proportion of men with clinically insignificant cancer (Gleason grade group 1), and test performance characteristics of AI and radiologists.
**Results or Findings:** 45 centres across 14 countries expressing interest are undergoing pre-trial MRI quality control. PARADIGM will open to recruitment in Q1 2026.
**Conclusion:** PARADIGM will provide the first prospective, level-1 evidence on the diagnostic performance of AI in the detection of clinically significant prostate cancer on MRI.
**Limitations:** PARADIGM will not investigate workload reductions. Approvals are in progress.
**Funding for this study:** The PARADIGM trial is supported by The John Black Charitable Foundation and the European Association of Urology Research Foundation.
**Has your study been approved by an ethics committee?** Yes
**Ethics committee - additional information:** The PARADIGM trial has not yet been approved by an ethics committee. Regulatory approvals are currently underway and results of ethics committee approvals will be shown during the presentation if received by date of presentation.
**Author Disclosures:**
Aqua Asif: Nothing to disclose
Alexander Ng: Nothing to disclose
Pawel Rajwa: Nothing to disclose
Alexander Dudko: Nothing to disclose
Douglas Pendse: Nothing to disclose
Aishwarya Shah: Nothing to disclose
Veeru Kasivisvanathan: Nothing to disclose
Francesco Giganti: Nothing to disclose
Ranya Kumar: Nothing to disclose

**AI-accelerated versus conventional diffusion-weighted imaging for prostate MRI: A head-to-head comparison of quality and quantitative metrics**

*V. Sacalean*[1], O. Gebler[1], W. Liu[2], R. Strecker[2], E. Weiland[2], F. Bamberg[1], J. Weiß[1], M. Russe[1], H. Engel[1]; [1]Freiburg Im Breisgau/DE, [2]Erlangen/DE
*(vlad.sacalean@uniklinik-freiburg.de)*

**Purpose:** Diffusion-weighted-imaging (DWI) is central to prostate MRI but prolongs examinations. We compared an AI-accelerated reduced-field-of-view readout-segmented EPI diffusion sequence (AI-DWI) with a conventional full-field readout-segmented sequence (c-DWI), hypothesising shorter acquisition with non-inferior perceived diagnostic quality and characterising effects on diffusion metrics.
**Methods or Background:** This prospective, single-center study of diagnostic accuracy enrolled 62 consecutive men with elevated PSA-levels between March and May 2025. The AI-DWI sequence was compared against the standard c-DWI sequence for each patient. Three radiologists independently scored overall image quality, anatomic differentiability, lesion conspicuity and artefacts on five-point Likert scales. Quantitative analysis involved comparing mean Apparent Diffusion Coefficient (ADC) and seven additional texture features (maximum, minimum, standard-deviation, coefficient of variation, entropy, skewness and kurtosis) using a five-millimetre region of interest in each index lesion. Wilcoxon-signed-rank tests assessed ordinal scores, and paired t-tests were used for quantitative metrics.
**Results or Findings:** The AI-DWI sequence demonstrated a significantly shorter acquisition time compared to c-DWI (3 min 59 s vs. 4 min 21 s; $p < 0.01$). There was no significant difference in subjective scores for overall image quality, lesion conspicuity, artefacts, or anatomic differentiability ($p > 0.05$ for all). AI-DWI yielded significantly lower mean ADC values (975.92 ± 174.57 vs. 1013.21 ± 189.34; adj. $p < 0.01$) and maximum ADC values (adj. $p < 0.01$). No significant differences were found for standard deviation, coefficient of variation, entropy, kurtosis, minimum, or skewness (adj. $p > 0.05$).
**Conclusion:** The AI-DWI sequence allows for a meaningful reduction of acquisition time while preserving excellent subjective image quality compared to the c-DWI. Quantitatively, it yields lower mean and maximum ADC values, while showing no significant differences in the rest of the quantiative metrics.
**Limitations:** Single-centre design. Lack of histopathological correlation.
**Funding for this study:** The research MRI sequence was provided under an unrestricted collaboration agreement between Siemens Healthineers and the Department of Diagnostic and Interventional Radiology, Medical Center – University of Freiburg. Siemens Healthineers AG provided technical support; study conception and design, as well as data analysis and interpretation, were conducted independently.
**Has your study been approved by an ethics committee?** Yes
**Ethics committee - additional information:** The study protocol was approved by the Ethics Committee of the Albert-Ludwigs-Universität Freiburg (Approval #22-1185). All participants provided written informed consent prior to inclusion. This prospective study consecutively enrolled 64 men referred for multiparametric prostate MRI between March and May 2025; two were excluded due to incomplete or severely degraded studies, yielding a final cohort of 62 participants.
**Author Disclosures:**
Elisabeth Weiland: Employee: Research & Clinical Translation, Magnetic Resonance, Siemens Healthineers AG, Erlangen, Germany
Ralph Strecker: Employee: EMEA Scientific Partnerships, Siemens Healthineers AG, Forchheim, Germany
Jakob Weiß: Nothing to disclose
Maximilian Russe: Nothing to disclose
Oliver Gebler: Nothing to disclose
Fabian Bamberg: Nothing to disclose
Wei Liu: Employee: Research & Clinical Translation, Magnetic Resonance, Siemens Healthineers AG, Erlangen, Germany
Vlad Sacalean: Nothing to disclose
Hannes Engel: Nothing to disclose

**In Prostate Cancer Diagnosis, Deep Learning Model's Performance Declines as MRI Quality Reduces, while Radiologists' Performance Remains the Same**

*E. H. P. Pooch*, G. Agrotis, S. Ursprung, A. Dehghanpour, R. Beets-Tan, T. Janssen, I. G. Schoots; Amsterdam/NL
*(e.pais.pooch@nki.nl)*

**Purpose:** To assess the impact of biparametric MRI (bpMRI) scan quality, as determined by PI-QUAL scores, on the diagnostic performance of a deep learning (DL) model and radiologists in correctly detecting Grade Group (GG) ≥2 prostate cancer in prostate cancer suspected men.
**Methods or Background:** A nnU-Net GG≧2 cancer segmentation model used 1500 bpMRI scans for training, 1000 for testing (PI-CAI cohort), and 573 scans for external validation (PROMIS cohort). The external cohort analysis included MRI assessment by a radiologist (R1) using PI-RADS v2.1 and the original PROMIS study Likert scores as the second assessment (R2). Two readers (QR1, QR2) assessed the image quality of the PROMIS MRI scans and determined a consensus Prostate Imaging Quality (PI-QUAL) v2 score. The reference standard was GG≧2 cancer, confirmed by transperineal saturation biopsy. The model's and radiologists' diagnostic performance (AUCs) were compared. Bootstrap testing (1000 iterations) was used to calculate 95% Confidence Intervals (CI) and determine the statistical significance of the performance differences between quality subgroups.
**Results or Findings:** On the external dataset, readers' performance achieved AUC[R1] 0.85 [0.82-0.88] and AUC[R2] 0.76 [0.73-0.79], respectively. The DL model's performance on reduced-quality scans (n=141) declined (AUC[DL-PIQUAL1] 0.63 (0.53-0.71), whereas on high-quality scans (n=432), performance increased (AUC[DL-PIQUAL2-3] 0.71 [0.66-0.75] ($p<0.05$)). In contrast, the readers' performance remained consistent across scan quality (AUC[R1-PIQUAL1] 0.88 [0.82-0.93] and AUC[R1-PIQUAL2-3] 0.91 [0.88-0.93] ($p=0.35$); AUC[R2-PIQUAL1] 0.79 [0.71-0.86] and AUC[R2-PIQUAL2-3] 0.80 [0.76-0.84] ($p=0.49$).
**Conclusion:** The diagnostic performance of the AI model declined significantly on reduced-quality MRI scans as determined by PI-QUAL v2 scoring, whereas radiologists maintained consistent performance regardless of scan quality.
**Limitations:** Single external cohort, single AI model, subjective quality evaluation
**Funding for this study:** No

**Has your study been approved by an ethics committee?** Not applicable
**Author Disclosures:**
Georgios Agrotis: Nothing to disclose
Ailin Dehghanpour: Nothing to disclose
Regina G.H. Beets-Tan: Nothing to disclose
Eduardo H. P. Pooch: Nothing to disclose
Tomas Janssen: Nothing to disclose
Ivo Gerardus Schoots: Nothing to disclose
Stephan Ursprung: Nothing to disclose

**AI in the Hot Seat: Can a Commercial AI tool Triage Prostate MR using a dedicated 3-point scale?**
*F. Giganti*; London/UK

**Purpose:** To evaluate a commercial AI algorithm's ability to triage prostate MRI into actionable risk categories for clinically significant prostate cancer (csPCa), benchmarked against radiologist-assigned PI-RADS scores.
**Methods or Background:** Pathology reports from targeted biopsies of 39 consecutive biopsy-naive men with MRI studies at Rolling Oaks Radiology (CA, USA) were used as ground truth. Radiologists assigned PI-RADS scores in their original reports. Imaging was analysed by the DeepHealth algorithm, which automatically segmented the prostate and lesions using a 3-point scale: Low (unlikely csPCa, avoid biopsy); Medium (likely csPCa, consider biopsy); High (likely csPCa, biopsy recommended). CsPCa was defined as grade group ≥2. AI predictions were dichotomised using High alone or High+Medium as positive. PI-RADS thresholds of ≥3, ≥4, and 5 were compared. Patient-level analysis used the highest scoring lesion.
**Results or Findings:** Twenty patients (51%) had csPCa. Studies included four scanner manufacturers (GE, Siemens, Philips, Hitachi) with 3T, open, and hybrid PET/MR systems. For ≥Medium risk, the AI sensitivity was 89% (69–97%) and specificity was 45% (26–66%). PI-RADS ≥3 and ≥4 yielded 100% (83–100%) and 95% (75–99%) sensitivity, with specificities of 25% (11–47%) and 50% (30–70%). The PPV for ≥Medium risk was 61% (42–76%), comparable to that of PI-RADS ≥3 (56%, 39–71%) and ≥4 (64%, 46–79%). High risk achieved 47% (27–68%) sensitivity and 100% (84–100%) specificity, versus PI-RADS 5 with 42% (23-64%) sensitivity and 95% (76-99%) specificity. AI's High risk had 100% PPV (70–100%) with 67% NPV (49–81%).
**Conclusion:** This pilot study demonstrates the feasibility of AI-based prostate MRI stratification using a 3-point scale, with classifications aligned with expert PI-RADS scores. Further validation is ongoing.
**Limitations:** Small sample size and a case-level analysis only.
**Funding for this study:** None
**Has your study been approved by an ethics committee?** Not applicable
**Author Disclosures:**
Francesco Giganti: Consultant: DeepHealth Consultant: SpectraCure Consultant: Procept Speaker: Bayer Speaker: Siemens

**Artificial intelligence-assisted reading of non-contrast prostate MRI: Application and concordance with expert interpretation in a screening population within the PROSA Trial**
*E. Messina*, A. Borrelli, L. Laschena, A. Dehghanpour, M. Pecoraro, V. Panebianco; Rome/IT
*(emanuele.messina@uniroma1.it)*

**Purpose:** Bi-parametric MRI (bpMRI), a non-contrast imaging approach, has been explored as potential method for screening clinically significant prostate cancer (csPCa). At the same time, artificial intelligence (AI) is increasingly recognized as potential supportive tool. This study aimed to assess the performance of an AI-based software for csPCa screening with bpMRI, focusing on its value in assisting less-experienced readers.
**Methods or Background:** Retrospective analysis of the PROSA trial, a prospective, randomized, single-center study that enrolled 759 men eligible for csPCa screening. BpMRI scans were obtained following PI-RADS v2.1 guidelines and independently reviewed by an expert radiologist, a less-experienced reader, AI-software, and the less-experienced reader assisted by AI. Diagnostic accuracy was evaluated through ROC curve analysis and inter-reader agreement (Cohen's kappa), with the expert's assessment serving as reference standard.
**Results or Findings:** Out of 499 bpMRI scans, the less-experienced reader supported by AI achieved the best diagnostic performance (sensitivity 76.5%, specificity 97.2%, accuracy 95.8%, AUC 0.868), outperforming both AI-alone (sensitivity 58.8%, specificity 96.6%, accuracy 94.0%, AUC 0.777) and the unaided less-experienced reader (sensitivity 67.6%, specificity 95.1%, accuracy 93.2%, AUC 0.814). AI support also enhanced inter-reader agreement (κ=0.84), reducing the number of PI-RADS 3 cases (77→53), and increased exact concordance with the expert from 32.5% to 54.5%, while lowering diagnostic discordance.
**Conclusion:** AI has the potential to assist less-experienced radiologists and improve the consistency of bpMRI readings, especially considering equivocal cases. In addition, its integration into radiology workflows may reduce reporting workload and facilitate prioritization of suspicious findings, providing important benefits in large-scale screening programs.
**Limitations:** Reference standard: expert reader's assessment (not histopathology), since only MRI-positive cases undergo biopsy; histology for all would be unfeasible in screening. AI-software trained mostly on older, clinically suspected patients, not younger screening population.
**Funding for this study:** No
**Has your study been approved by an ethics committee?** Yes
**Ethics committee - additional information:** Local EC
**Author Disclosures:**
Valeria Panebianco: Nothing to disclose
Antonella Borrelli: Nothing to disclose
Emanuele Messina: Nothing to disclose
Ailin Dehghanpour: Nothing to disclose
Martina Pecoraro: Nothing to disclose
Ludovica Laschena: Nothing to disclose

**AI-supported prostate MRI lesion classification: impact at different reader experience levels**
*H. Bayraktaroglu*, M. Heimer, P. Lohse, J. F. Kuhnle, R. Lorbeer, C. Stief, J. Ricke, C. C. Cyran, P. M. Kazmierczak; Munich/DE

**Purpose:** To investigate the impact of a commercially available AI tool for prostate lesion classification according to PI-RADS v2.1 on the diagnostic accuracy of radiologists at different experience levels.
**Methods or Background:** In this IRB-approved retrospective single-center study, prostate MRI datasets of 477 patients (470 mpMRI, 7 bpMRI; median age 68 years) acquired at 3 Tesla between 09/22 and 11/24 were analysed. Three blinded radiologists with different experience levels (low, intermediate, high) independently assessed the datasets in a two-step approach: first without AI support, then after reviewing AI results with the option to change the initial PI-RADS score. Scoring differences were tested using the McNemar-Bowker test and agreement with the reference standard (board-certified radiologist, >12 years of experience in abdominal imaging) was quantified by weighted Cohen's k.
**Results or Findings:** Across all readers, AI support led to redistribution of PI-RADS scores, most pronounced in the least experienced reader (p=0.002), including upscoring of PI-RADS 2 lesions: ten to PI-RADS 3, five to PI-RADS 4, six to PI-RADS 5 [21/194, 10.8%]. In 7/11 of the upscored cases, TRUS-guided transperineal fusion biopsy confirmed csPCa (ISUP ≥2). AI improved agreement with the reference standard in all readers, reaching statistical significance in the low experience reader (Cohen's k from 0.54 [0.46-0.61] to 0.59 [0.52-0.66], p=0.009), whereas changes for the other readers did not prove statistically significant (p=0.143 and p=0.731). Overall, agreement of the AI results with the reference standard remained moderate (k=0.59 [0.52-0.66], p<0.001).
**Conclusion:** AI support significantly improved diagnostic accuracy of low, but not of intermediately or highly experienced readers. However, in view of the moderate agreement of the AI results with the reference standard, supervision by an experienced uroradiologist remains mandatory.
**Limitations:** No limitations were identified.
**Funding for this study:** No funding was received for this study.
**Has your study been approved by an ethics committee?** Yes
**Ethics committee - additional information:** The study was approved by the local institutional review board (project nr.: 24-0945).
**Author Disclosures:**
Philipp M. Kazmierczak: Nothing to disclose
Roberto Lorbeer: Nothing to disclose
Philipp Lohse: Nothing to disclose
Hüdanur Bayraktaroglu: Nothing to disclose
Clemens C. Cyran: Nothing to disclose
Maurice Heimer: Nothing to disclose
J. Felix Kuhnle: Nothing to disclose
Christian Stief: Nothing to disclose
Jens Ricke: Nothing to disclose

**Evaluation of an AI algorithm's performance in MRI follow-up of patients undergoing active surveillance for prostate cancer**
J. Spilleboudt[1], C. Ruppli[2], G. Herpe[3], D. Bouda[4], *G. D'Assignies*[2], L. Beuzit[1]; [1]Rennes/FR, [2]Paris/FR, [3]Poitiers/FR, [4]Nantes/FR

**Purpose:** The PRECISE scoring system is a recently developed tool designed to standardize MRI follow-up in patients undergoing active surveillance (AS) for prostate cancer. This study aimed to evaluate the performance of a deep-learning–based artificial intelligence (AI) software prototype in assessing the likelihood of radiological tumor progression on serial MRI scans using the PRECISE criteria. The performances of the AI prototype and a junior radiologist were compared to those of an expert radiologist, who served as the ground truth.

**Methods or Background:** A total of 96 patients undergoing active surveillance were included, each with two available MRI scans. For each patient, prostate lesions were detected, measured and classified according to the Pi-RADS 2.1 guidelines by a junior radiologist, an expert radiologist, and the AI algorithm. The PRECISE score was independently assessed by each radiologist, while the AI algorithm inferred the score based on predefined calculation rules. Balanced accuracy was calculated using a threshold of PRECISE score 3 (i.e., scores ≤3 vs >3), comparing the predictions of the junior radiologist and the AI software to those of the expert radiologist.
**Results or Findings:** The difference between AI protype and junior radiologist PRECISE scores inferences was not statistically significant. AI prototype inferred PRECISE scores with non significant lower accuracy than the junior radiologist. Using a threshold at PRECISE 3, the balanced accuracy was 0.67 for the junior reader and 0.62 for the AI (p-value 0.44).
**Conclusion:** The AI prototype inferred PRECISE scores with lower accuracy than a junior radiologist, though the difference was not statistically significant. However, AI prototype demonstrated superior performance in lesion detection and segmentation.
**Limitations:** The ground truth is assessed by a single expert radiologist. Our study is also limited by its retrospective design and single-center setting.
**Funding for this study:** No funding
**Has your study been approved by an ethics committee?** Not applicable
**Author Disclosures:**
Damien Bouda: Nothing to disclose
Guillaume Herpe: Consultant: Incepto medical
Juliette Spilleboudt: Nothing to disclose
Camille Ruppli: Employee: Incepto medical
Gaspard D'Assignies: Founder: Incepto medical
Luc Beuzit: Nothing to disclose

**12:30 - 13:30 Research Stage 5**

### Research Presentation Session: Interventional Radiology

### RPS 1909

### Portal pathways: precision interventions in hepatic vascular care

**Moderator**
Charlotte Ebeling Barbier; Uppsala/SE

**Portal hemodynamic response to tips assessed by 4D flow MRI: Association with procedural strategies and prediction of postoperative outcomes in a prospective cohort study**
*S. Wan*, J. He, X. Luo, B. Song; Chengdu/CN
*(604775856@qq.com)*

**Purpose:** Transjugular intrahepatic portosystemic shunt (TIPS) is an essential intervention for portal hypertension but significantly risks postoperative complications such as hepatic encephalopathy (HE). This study aimed to investigate the prognostic value of portal hemodynamics assessed by four-dimensional (4D) flow MRI in predicting post-TIPS outcomes and to identify associations with procedural strategies
**Methods or Background:** This prospective single-center study included 20 patients who underwent TIPS between September 2023 and November 2024 and completed both pre- and post-procedural 4D flow MRI for quantitative hemodynamic assessment. Parameters measured included forward volume(FV), total volume(TV), maximum flow(MF), backward volume(BV), peak velocity(PV), pressure difference(PD), wall shear stress(WSS), and regurgitation fraction(RF) across the portal venous system and TIPS-stent. Differences by procedural strategies were evaluated, and correlations with portal pressure gradient(PPG) were analyzed. Cox regression assessed associations with postoperative outcomes.
**Results or Findings:** Overall, FV, TV, MF, PV, WSS, and PD increased significantly across the portal venous system after TIPS (all P<.001). Patients developing HE (n=4) showed greater FV (P=.02) and MF (P=.007) changes at the proximal main portal vein. VIATORR Controlled Expansion stents had significantly higher mid-stent PD than conventional stents(P<.05). Mid-stent BV correlated positively with post-TIPS PPG (r=0.498, P=.04), whereas higher distal-stent BV was associated with reduced risk of HE or variceal rebleeding(HR=0.48; 95% CI, 0.26-0.90; P=.02).
**Conclusion:** TIPS significantly improved portal hemodynamics as measured by 4D flow MRI. Backward flow volume emerged as a potential predictor of adverse outcomes, and distinct procedural strategies were associated with specific flow patterns, offering insights for surgical planning and risk stratification.
**Limitations:** The sample size for this exploratory study was relatively small
**Funding for this study:** N/A
**Has your study been approved by an ethics committee?** Yes
**Ethics committee - additional information:** West China Hospital of Sichuan University
**Author Disclosures:**
Jiajia He: Nothing to disclose
Bin Song: Nothing to disclose
Shang Wan: Nothing to disclose
Xuefeng Luo: Nothing to disclose

**Increasing experience with portal vein recanalization in pediatric patients with non-cirrhotic extrahepatic portal vein obstruction: technique and outcomes**
*P. Marra*, K. D. Martins De Mattos, R. Muglia, F. S. Carbone, L. Dulcetta, M. Bertuletti, M. Cheli, L. D'Antiga, S. Sironi; Bergamo/IT
*(pmarra@asst-pg23.it)*

**Purpose:** Portal vein recanalization (PVR) without TIPS is being investigated to treat non-cirrhotic portal hypertension resulting from extrahepatic portal vein obstruction (EHPVO). We describe advanced techniques and outcomes of PVR attempted in a cohort of pediatric patients and young adults.
**Methods or Background:** Consecutive patients suffering from non-cirrhotic portal hypertension due to perinatally-acquired EHPVO were prospectively enrolled since 2021. Wedge hepatic venography and percutaneous portal access (transhepatic and/or transplenic), were performed upon multidisciplinary discussion to attempt PVR. Clinical and procedural data, technical and clinical success, complications and follow-up data were recorded. Technical success was considered at least the partial revascularization of the native portal system.
**Results or Findings:** Twenty-three patients (16 males; median age 10 years) with severe portal hypertension due to EHPVO underwent 33 percutaneous transhepatic (n=2), transplenic (n=24) or simultaneous transhepatic/transplenic (n=7) for PVR. All but two were judged not eligible for Meso-Rex bypass while it failed 2 patients. Technically successful recanalization was achieved in 18/23 patients (78%), by means of advanced techniques in 4 cases, respectively 2 gun-sight sharp recanalization and 2 collateral vessel angioplasty/stenting. Severe arterial bleeding complications occurred in 2 patients, managed by transarterial embolization. After successful angioplasty, 17/18 patients required primary or secondary stenting to obtain sustained patency. Revisions due to thrombosis/stenosis were necessary in 8/18 patients. After a median follow-up of 12 months, portal vein patency was demonstrated in all but one patients who achieved successful PVR, with clinical and laboratory improvement of portal hypertension.
**Conclusion:** With increasing experience, more than 70% of patients with non-cirrhotic portal hypertension due to EHPVO can restore the portal flow by endovascular treatment, even when Meso-Rex is unfeasible. Technical challenges still remain but clinical outcomes seem promising.
**Limitations:** Retrospective cohort. Limited reproducibility due to technical challenges.
**Funding for this study:** None
**Has your study been approved by an ethics committee?** Yes
**Ethics committee - additional information:** Authorization code: Primo-01
**Author Disclosures:**
Martina Bertuletti: Nothing to disclose
Paolo Marra: Nothing to disclose
Francesco Saverio Carbone: Nothing to disclose
Kessy Djonis Martins De Mattos: Nothing to disclose
Riccardo Muglia: Nothing to disclose
Lorenzo D'Antiga: Nothing to disclose
Maurizio Cheli: Nothing to disclose
Sandro Sironi: Nothing to disclose
Ludovico Dulcetta: Nothing to disclose

**Impact of Right Hepatic Artery Caliber on Future Liver Remnant Hypertrophy After Liver Venous Deprivation**
*D. Santangelo*, R. Vitali, A. Campisi, F. Cipriani, D. Palumbo, C. Canevari, F. Ratti, A. Chiti, F. De Cobelli; Milan/IT
*(santangelo.domenico@hsr.it)*

**Purpose:** To evaluate the influence of right hepatic artery (RHA) caliber on liver regeneration in patients with primary or secondary liver malignancies undergoing liver venous deprivation (LVD), and to assess the impact of the hepatic arterial buffer response (HABR) on post-LVD regeneration.

**Methods or Background:** Fifty-nine patients (June 2019–September 2024) who underwent LVD were retrospectively analyzed. Right hepatic artery (RHA) caliber was measured on CT scans obtained both before LVD and within 30 days after the procedure. All patients underwent FLR volumetry on CT at three timepoints; pre-LVD (baseline), 5–15 days post-LVD (timepoint 1), and 15–30 days post-LVD (timepoint 2). FLR function was assessed with 99mTc-mebrofenin hepatobiliary scintigraphy pre-LVD and within 30 days post-LVD. Correlation analyses and linear regression were performed.
**Results or Findings:** A significant correlation between total liver volume and right hepatic artery caliber at baseline was found; therefore, all measured RHA caliber were standardized to the total liver volume, obtaining the new variable "s-caliber". At baseline, RHA s-caliber showed an inverse correlation with post-procedural volumetric FLR volume ($p<0.001$), degree of hypertrophy ($p=0.008$), and kinetic growth rate ($p=0.014$). No significant associations were observed between s-caliber and functional hypertrophy metrics. The RHA caliber change after LVD didn't correlate with any volumetric/functional hypertrophy parameters.
**Conclusion:** This is the first report demonstrating the potential impact of the RHA on volumetric FLR hypertrophy after LVD. Larger arterial calibers may attenuate hypertrophy, likely by sustaining arterial inflow to embolized segments.
**Limitations:** Retrospective. Small sample size.
**Funding for this study:** None.
**Has your study been approved by an ethics committee?** Yes
**Ethics committee - additional information:** Leatum 64/INT/2021
**Author Disclosures:**
Francesco De Cobelli: Nothing to disclose
Diego Palumbo: Nothing to disclose
Arturo Chiti: Nothing to disclose
Roberta Vitali: Nothing to disclose
Federica Cipriani: Nothing to disclose
Carla Canevari: Nothing to disclose
Antonino Campisi: Nothing to disclose
Francesca Ratti: Nothing to disclose
Domenico Santangelo: Nothing to disclose

**Retrograde transvenous obliteration (RTO) versus endoscopic ultrasound (EUS)-guided therapies in fundal varices- A comparative analysis**
*R. K. Patel*, T. P. Tripathy, M. Panigrahi, H. Nayak; Bhubaneswar/IN
*(ranjanair1@gmail.com)*

**Purpose:** To compare the outcome of RTO and EUS-guided therapies in the management of fundal varices.
**Methods or Background:** We retrospectively analyzed the data of patients with fundal varices undergoing EUS-guided intervention or RTO, and both groups were compared after propensity matching. The study's primary outcome was the incidence of variceal bleeding within 1 year. The secondary outcomes included procedure-related adverse events (AEs), variceal obliteration, reintervention, and mortality within 1 year.
**Results or Findings:** 167 patients (EUS-guided intervention: 108, RTO: 59) were included in the analysis, of which 59 patients were included in each group after propensity matching. The incidence of variceal obliteration at 4 weeks was comparable between groups (83.1% vs. 91.5%, p = 0.167). The incidence of variceal bleeding (15.3% vs. 13.6%, p = 0.793) within 1 year was also comparable between the EUS and RTO groups. Nevertheless, the need for reintervention for GVs was higher in the EUS group (28.8% vs. 5.1%, p = 0.001), and the need for reintervention for esophageal varices (EVs) was higher in the RTO group (16.9% vs. 1.7%, p = 0.008). Procedure-related adverse events (AEs), primarily new onset or worsening of ascites, were higher in the RTO group. None of the AEs were life-threatening.
**Conclusion:** RTO provides more complete fundal variceal obliteration, requiring a significantly lower number of reintervention than EUS-guided therapies. Thus, RTO may be considered as a more definite therapy for fundal variceal obliteration
**Limitations:** Retropsective analaysis Operator bias
**Funding for this study:** None
**Has your study been approved by an ethics committee?** Yes
**Ethics committee - additional information:** IEC, AIIMS-Bhubaneswar IEC number: T/IM-NF/Radiodi/25-26/48
**Author Disclosures:**
Ranjan Kumar Patel: Nothing to disclose
Manas Panigrahi: Nothing to disclose
Hemanta Nayak: Nothing to disclose
Tara Prasad Tripathy: Nothing to disclose

**From surgical to endovascular approach in the management of pediatric congenital portosystemic shunts**
*P. Marra*, K. D. Martins De Mattos, G. De Petri, V. Casotti, R. Muglia, F. S. Carbone, M. Bertuletti, L. Dulcetta, S. Sironi; Bergamo/IT
*(pmarra@asst-pg23.it)*

**Purpose:** Congenital portosystemic shunts (CPSS) are rare vascular malformations potentially leading to liver transplantation if untreated. We evaluated whether timely, multidisciplinary use of interventional radiology (IR) for diagnostic and therapeutic management reduces complications and the need for liver transplantation in a retrospective cohort.
**Methods or Background:** In this single-center cohort of pediatric CPSS, all patients underwent multidisciplinary workup. Due to substantial improvement in the management protocol over time, patients were divided into two groups according to treatment period: Early (treatment before 2019) and Late (treatment in 2019 or later). In the Late cohort IR procedures included diagnostic retrograde portal venography with balloon occlusion test and endovascular shunt closure, when technically feasible; alternatively, surgical shunt closure was performed. In the Early cohort, endovascular and surgical closure were not routinely performed. We compared time from diagnosis to treatment, frequency of liver transplantation, and the number of CPSS-related complications between groups.
**Results or Findings:** Twenty-four patients (male n = 9) were analyzed, 11 in the Early group and 13 in the Late group. IR procedures were undertaken in 4/11 (36%) Early versus 8/13 (62%) Late patients. Liver transplantation was performed in 9/11 (82%) Early patients and in 1/13 (8%) Late patients. Surgical shunt ligation was performed in 3/24 (13%) overall. Mean age at diagnosis was similar between cohorts (~5 years). Median time from diagnosis to treatment decreased from 4 years in the Early cohort to 1 year in the Late cohort. Patients who underwent IR management had fewer documented CPSS-related complications.
**Conclusion:** Incorporation of IR into a multidisciplinary care pathway was associated with shorter diagnostic-to-treatment intervals, increased utilization of minimally invasive management, and a substantially lower transplant rate in children with CPSS.
**Limitations:** Retrospective cohort with management shift over a long time.
**Funding for this study:** None
**Has your study been approved by an ethics committee?** Yes
**Ethics committee - additional information:** Multicenter registry IRCPSS
**Author Disclosures:**
Martina Bertuletti: Nothing to disclose
Giacomo De Petri: Nothing to disclose
Paolo Marra: Nothing to disclose
Francesco Saverio Carbone: Nothing to disclose
Kessy Djonis Martins De Mattos: Nothing to disclose
Valeria Casotti: Nothing to disclose
Riccardo Muglia: Nothing to disclose
Sandro Sironi: Nothing to disclose
Ludovico Dulcetta: Nothing to disclose

**Percutaneous recanalization of chronic total occlusion of the portal vein: technical aspects and outcomes**
*L. Dulcetta*, P. Marra, R. Muglia, F. S. Carbone, M. Bertuletti, S. Sironi; Bergamo/IT
*(l.dulcetta@campus.unimib.it)*

**Purpose:** Chronic total occlusion (CTO) of the portal vein is a major cause of portal hypertension, which may lead to life-threatening complications often managed by interventional radiology (IR). The aim of this study was to evaluate the feasibility, safety and clinical outcomes of percutaneous revascularization therapy for CTO of the portal vein in pediatric and adult patients.
**Methods or Background:** From January 2020 to June 2025, consecutive patients with severe portal hypertension due to portal vein CTO undergoing percutaneous recanalization were retrospectively reviewed. Technical success was defined as restoration of portal vein patency at angiography; clinical success was defined as improvement of clinical and laboratory signs of portal hypertension and control of variceal bleeding.
**Results or Findings:** Twenty-two patients (median age 27 years; range 6–60; 14 males; 5 children) underwent 32 procedures. Eleven (50%) were liver transplant recipients. Cavernous transformation was present in 21/22, with splenomesenteric confluence involvement in 10 patients. Technical success was achieved in 18/22 patients (82%), 11 of whom underwent portal revascularization through extrahepatic stent placement; in 6 cases, a TIPS was performed to achieve sustained portal vein patency. Embolization of varices and/or cavernoma was performed in 17 patients. Three complications (two splenic artery perforation, one hemoperitoneum) were managed conservatively. Clinical success was achieved in all 18 technically successful cases, with a median follow-up of 32 months (IQR 3–41).

**Conclusion:** Percutaneous revascularization is feasible and effective in managing portal vein CTO, with no significant technical contraindications to attempting the procedure. Restored portal flow physiology alone is possible in most patients; TIPS is not essential to achieve portal vein patency, but it may be useful in a few selected cases to maintain long-term patency and control portal hypertension.
**Limitations:** Retrospective nature and the small sample size.
**Funding for this study:** No funding was received for this study.
**Has your study been approved by an ethics committee?** Yes
**Ethics committee - additional information:** The Ethical Committee of Bergamo authorized this retrospective study (Portal01; N.92/21) that was conducted in respect of the ethical standards laid down in the 1964 Declaration of Helsinki.
**Author Disclosures:**
Martina Bertuletti: Nothing to disclose
Paolo Marra: Nothing to disclose
Francesco Saverio Carbone: Nothing to disclose
Riccardo Muglia: Nothing to disclose
Sandro Sironi: Nothing to disclose
Ludovico Dulcetta: Nothing to disclose

**Antegrade Transvenous Obliteration for Refractory Esophageal Variceal Hemorrhage in Patients Unsuitable for TIPS: A Multicenter Retrospective Study**
S. H. Lee, D. Shim, S. Baek, D. Kim, *J. Yoo*; Incheon/KR

**Purpose:** Esophageal variceal bleeding (EVB) refractory to endoscopic treatment in some patients with advanced cirrhosis, leaving few options other than transjugular intrahepatic portosystemic shunt (TIPS). However, patients with encephalopathy or heart failure are often ineligible for TIPS, alternatively opt for antegrade transvenous obliteration (ATO). This study aimed to assess the safety and efficacy of ATO compared to TIPS for refractory EVB.
**Methods or Background:** This study retrospectively reviewed medical records from clinical data warehouse the Catholic University of Korea which encompass eight tertiary hospitals between 2012 and 2024. Although TIPS was initially considered, ATO was indicated for patients who were ineligible for TIPS. ATO were implemented in 20 patients (age=62±10.2 years, men=13), while TIPS were created in 35 patients (age=58±12.6 years, men=26) for refractory EVB. The primary outcome was the difference in the 30-month overall survival (OS) rate between two groups, a noninferiority comparison to exclude a difference of >30 percentage point. The secondary outcomes were comparison of postprocedural adverse events and bleeding-free survival (BFS) between the ATO and TIPS groups.
**Results or Findings:** At a median 31-month follow-up, 30-month BFS and OS for ATO were 23.2% (95% CI, 7.8–69.7%) and 50.9% (95% CI, 32.0–81.0%); for TIPS, 63.8% (95% CI, 45.4–89.7%) and 52.2% (95% CI, 34.1–79.8%). The absolute OS difference was 1.3% (95% CI, –31.1–33.7%). One-week mortality or liver transplant occurred in 5 TIPS patients (14.3%) but none with ATO. Kaplan-Meier/log-rank analysis showed no significant differences in BFS (p=0.07) or OS (p=0.41).
**Conclusion:** Although the outcomes fall short for the specified noninferiority, the ATO might be an alternative for those who are ineligible for TIPS in treatment of refractory EVB.
**Limitations:** This study is limited by its retrospective design and sample size.
**Funding for this study:** No funding was received for this study.
**Has your study been approved by an ethics committee?** Not applicable
**Author Disclosures:**
Seung Hyun Lee: Nothing to disclose
Doyoung Kim: Nothing to disclose
Dongjae Shim: Nothing to disclose
Jimin Yoo: Nothing to disclose
Seunghwan Baek: Nothing to disclose

**Non-invasive estimation of hepatic venous pressure gradient in patients undergoing TIPS: A predictive approach**
*F. Schön*, P. Hahlbohm, T. Helmberger, S. F. U. Blum, M. Berning, S. Löck, R-T. Hoffmann, J-P. Kühn; Dresden/DE

**Purpose:** To develop non-invasive models for estimating the hepatic venous pressure gradient (HVPG) using computed tomography (CT) in patients receiving transjugular intrahepatic portosystemic shunt (TIPS).
**Methods or Background:** Patients with therapy-refractory ascites who underwent TIPS between 2017 and 2024 were retrospectively enrolled. Baseline characteristics were collected, and pre-interventional CT scans were analyzed to extract quantitative parameters (e.g., hepatic vessel diameters, sarcopenia scores). The HVPG was measured during the TIPS procedure prior to shunt placement. Univariate and multivariate linear and logistic regression analyses using backward elimination were performed to (1) predict absolute HVPG values, and (2) distinguish between patients with HVPG ≤20 mmHg and those with HVPG >20 mmHg. Model performances were assessed by area under the receiver operating characteristic curve (AUC), $R^2$, and mean absolute error (MAE).
**Results or Findings:** A total of 129 patients (87 men; 61.0+/-10.1 years) were enrolled with an intra-procedural HVPG value prior to shunt placement of 20.21+/-3.61 mmHg. In multivariate linear regression, the superior mesenteric vein diameter (ß = 0.431; p = 0.012) and MELD-Na Score (ß = 0.152; p = 0.031) performed best to estimate the absolute HVPG ($R^2$ = 0.134; MAE = 2.53+/-2.10 mmHg). Multivariate logistic regression identified the superior mesenteric vein diameter (OR = 1.186; 95%-CI: 1.002-1.405; p = 0.048) and ALAT (OR = 24.218; 95%-CI: 2.395-244.913; p = 0.007) as independent predictors for HVPG ≤ 20 vs. >20 mmHg (AUC = 0.729; 95%-CI: 0.638-0.820). Sarcopenia scores only revealed significant predictive value in univariate analysis.
**Conclusion:** Non-invasive prediction models may estimate the HVPG in patients undergoing TIPS for therapy-refractory ascites. These models could help identify patients at earlier disease stages with less severe portal hypertension, who might benefit from TIPS placement.
**Limitations:** Retrospective study in a single-center setting. Small sample size.
**Funding for this study:** None.
**Has your study been approved by an ethics committee?** Yes
**Ethics committee - additional information:** Approved by the local ethics committee of the Technical University Dresden (BO-EK-501122023_2).
**Author Disclosures:**
Ralf-Thorsten Hoffmann: Nothing to disclose
Marco Berning: Nothing to disclose
Patricia Hahlbohm: Nothing to disclose
Jens-Peter Kühn: Nothing to disclose
Felix Schön: Nothing to disclose
Sophia Freya Ulrike Blum: Nothing to disclose
Thomas Helmberger: Nothing to disclose
Steffen Löck: Nothing to disclose

**12:30 - 13:30** **Research Stage 6**

### Research Presentation Session: Paediatric Hot Topic with Keynote Lecture

### RPS 1912
### Hot Topic: AI in paediatric radiology

**Moderator**
Katharine Halliday; Wysall/UK

**Keynote Lecture**
Andrea Vanzulli; Tradate/IT
*(andrea.vanzulli@istitutotumori.mi.it)*

**AI-Enhanced Placental Radiomics on Diffusion and T2 MRI for Early Prediction of Preeclampsia and Fetal Growth Restriction**
*K. Saravanan*[1], F. Abubacker Sulaiman[2], R. Praveenkumar[2], J. Lydia[2], D. Velan[2]; [1]Melmaruvathur/IN, [2]Chennai/IN
*(dr.saravana.k@gmail.com)*

**Purpose:** To develop and validate an artificial intelligence (AI)–based radiomics model integrating diffusion-weighted imaging (DWI) and T2-weighted MRI features of the placenta for early prediction of preeclampsia (PE) and fetal growth restriction (FGR). The objective is to identify microstructural and textural biomarkers preceding clinical manifestation, enabling proactive obstetric management and improved perinatal outcomes.
**Methods or Background:** This prospective study included 80 pregnant women (20–32 weeks gestation) who underwent placental MRI on a 3-Tesla scanner. DWI (b-values 0, 800 s/mm²) and high-resolution T2 sequences were analyzed. Placental volumes were segmented semi-automatically, and radiomic features (first-order, texture, and shape) were extracted. Machine learning models—including Random Forest and Gradient Boosting—were trained to classify risk for PE/FGR, with cross-validation and feature selection via LASSO regression. Clinical parameters (blood pressure, uterine artery Doppler indices) were integrated into a multimodal model for comparison.
**Results or Findings:** The AI-radiomics model demonstrated an AUC of 0.93 for predicting PE and 0.91 for FGR, outperforming conventional Doppler parameters (AUC 0.76). Key discriminative features included gray-level co-occurrence entropy and ADC histogram kurtosis, reflecting placental microstructural heterogeneity. Combined clinical-radiomics integration improved specificity and early detection (mean 5 weeks before clinical onset).
**Conclusion:** AI-enhanced placental radiomics from DWI and T2 MRI enables noninvasive early prediction of preeclampsia and FGR with high accuracy. This approach holds promise for precision obstetric imaging, facilitating timely intervention and reducing perinatal morbidity.
**Limitations:** Single-center design, limited sample size, and variability in placental segmentation may restrict generalizability. Lack of histopathological correlation and absence of external validation cohorts warrant further multicentric studies for clinical translation.
**Funding for this study:** Not Applicable
**Has your study been approved by an ethics committee?** Not applicable
**Author Disclosures:**
Rathinamoorthy Praveenkumar: Nothing to disclose
K. Saravanan: Nothing to disclose
Johnbosco Lydia: Nothing to disclose
Dhanush Velan: Nothing to disclose
Farook Abubacker Sulaiman: Nothing to disclose

**Addressing data scarcity in paediatric head and neck CT: Cross-age training enables reliable automated lymph node segmentation**
B. Wichtlhuber[1], E. Frodl[1], M. Sayed[1], T. Persigehl[2], M. Neitzel[1], J. Dietz[1], M. Eicke[3], D. M. Renz[3], *A. M. Bucher*[1]; [1]Frankfurt/DE, [2]Koeln/DE, [3]Hannover/DE
*(bucher@med.uni-frankfurt.de)*

**Purpose:** Paediatric lymph node segmentation faces critical data scarcity challenges. We hypothesised that incorporating adult CT data into deep learning training would overcome this limitation while maintaining clinical relevance for paediatric oncology applications, particularly for detecting pathologically enlarged nodes requiring follow-up.
**Methods or Background:** We analysed 418 head/neck CTs from University Hospital Frankfurt: 146 paediatric (<18 years) and 272 adult cases. A 3D full-resolution nnU-Net underwent five-fold cross-validation training (250 epochs) using three strategies: paediatric-only, adult-only, and combined training. Approximately 20% of cases were reserved for independent testing. Evaluation metrics included the Dice coefficient, Intersection over Union (IoU). Clinical relevance was assessed through volumetric coverage analysis, with particular focus on nodes >10mm short-axis diameter—the threshold for pathological enlargement requiring clinical action. Values are presented as median and standard deviation.
**Results or Findings:** Cross-age training outperformed paediatric-only approaches. The combined model achieved Dice=0.714 ±0.151 and IoU=0.575 ±0.176 on paediatric test data versus Dice=0.695 ±0.155 and IoU=0.553 ±0.179 for paediatric-only training, showing clear improvement. Interestingly, this benefit was unidirectional—paediatric patients gained from mixed training, whereas adult test performance remained unchanged (combined model Dice=0.643 ±0.125 vs. adult-only Dice=0.647 ±0.113). Clinical utility analysis showed strong performance: 88.97% of enlarged nodes (>10mm) achieved ≥10% volumetric coverage, ensuring reliable oncological detection. Moreover, 69.08% of all lymph nodes reached ≥50% coverage, supporting accurate volumetric measurements. These metrics translate into improved workflow efficiency, reducing missed findings while preserving precision for follow-up.
**Conclusion:** Cross-age training addresses the key challenge of paediatric data scarcity in medical imaging AI, improving technical metrics and enabling clinically meaningful detection of pathological lymphadenopathy. This approach supports robust AI use in paediatric radiology with limited annotated data, potentially accelerating adoption in oncology workflows.
**Limitations:** A limitation of the study is its single-center validation.
**Funding for this study:** Funding was provided by the German Federal Ministry of Education and Research through the RACOON project (reference number 01KX2021).
**Has your study been approved by an ethics committee?** Yes
**Ethics committee - additional information:** Ethics approval was obtained by University Medicine Frankfurt (Reference 2023-1459)
**Author Disclosures:**
Eric Frodl: Nothing to disclose
Julia Dietz: Nothing to disclose
Marcel Eicke: Nothing to disclose
Diane Miriam Renz: Nothing to disclose
Matthias Neitzel: Nothing to disclose
Mattin Sayed: Nothing to disclose
Benedikt Wichtlhuber: Nothing to disclose
Andreas Michael Bucher: Nothing to disclose
Thorsten Persigehl: Nothing to disclose

**The Anatomical Advantage: CT Radiomics Outperform PET Radiomics and Clinical–PET Models in Pediatric High-Risk Hodgkin Lymphoma**
*L. Ibrahim*, A. Ilivitzki, M. Freiman; Haifa/IL
*(lamaibraheem7@gmail.com)*

**Purpose:** To investigate whether radiomic features from post–first-cycle PET and CT scans, alone or combined with clinical variables, improve the prediction of event-free survival (EFS) in pediatric high-risk Hodgkin lymphoma (HL, stage IIIB/IVB), compared with a baseline model incorporating clinical variables and radiological PET assessment.
**Methods or Background:** Outcome prediction in pediatric HL traditionally relies on risk-adapted protocols combining clinical factors with interim FDG PET/CT, mainly focused on the isotopic PET component. Prior evidence suggests that CT provides additional prognostic information, underscoring the importance of anatomical features. In this study, post–first-cycle PET/CT scans from 137 patients enrolled in the Children's Oncology Group AHOD0831 trial (NCT01026220) were analyzed. Up to five lesions per patient were segmented; PET images were SUV-standardized, and radiomic features from PET and CT were extracted and averaged using tumor volume weighting. Clinical variables included demographics, histology, stage, bulky disease, and radiological PET response. Machine learning models were trained with stratified 5-fold cross-validation, and performance metrics were averaged across folds
**Results or Findings:** EFS events occurred in 27 patients (19.7%). CT radiomics outperformed the baseline clinical + PET model (accuracy: 0.78 ± 0.10; balanced accuracy: 0.71 ± 0.09 vs. 0.70 ± 0.11; 0.66 ± 0.04) and exceeded PET radiomics (accuracy: 0.77 ± 0.07; balanced accuracy: 0.66 ± 0.07). The strongest predictive performance was achieved when CT radiomics were combined with clinical variables.
**Conclusion:** Radiomics from CT improved prognostic prediction beyond the standard clinical + PET model and outperformed PET radiomics, reinforcing the anatomical contribution to risk stratification. CT radiomics represent a promising complementary tool for early prognostic assessment in pediatric high-risk HL.
**Limitations:** This study was based on internal cross-validation within a single trial cohort (AHOD0831), without external or temporal validation, which may limit generalizability.
**Funding for this study:** None
**Has your study been approved by an ethics committee?** Not applicable
**Author Disclosures:**
Moti Freiman: Nothing to disclose
Anat Ilivitzki: Nothing to disclose
Lama Ibrahim: Nothing to disclose

**Deep Learning Reconstruction in Pediatric Chest CT: A Radiation-Sparing Technique with Enhanced Image Quality**
*I. Bianco*, D. Ippolito, C. Maino, C. R. G. L. O. M. Talei Franzesi, P. N. Franco, D. G. Gandola, R. Corso; Monza/IT
*(ilaria.bianco.ib@gmail.com)*

**Purpose:** To compare image quality and radiation dose between deep learning reconstruction (DLR) and hybrid iterative reconstruction (HIR) algorithms in unenhanced chest CT scans of pediatric patients.
**Methods or Background:** We retrospectively reviewed 142 unenhanced, single-phase chest CT scans performed for routine diagnostic purpose. 71 pediatric patients were examined using a 128-slice MDCT scanner (100kV) with a DLR algorithm (Precise Image), while the control group of 71 patients underwent scanning with a 256-slice MDCT scanner (100kV) using HIR algorithm (iDose4). Subjective image quality was assessed using a 5-point Likert scale, and objective quality was evaluated by measuring Hounsfield Units (HU) and Standard Deviations (SD) in lung parenchyma, tracheal lumen, air, aorta and muscle. Radiation dose metrics (CTDIvol and DLP) were recorded for both groups, and the estimated effective dose (EED) was calculated using age-specific k-factors.
**Results or Findings:** A total of 142 pediatric patients were included (median age:10 years).Inter-reader agreement for Likert scale image quality assessment was moderate (κ=0.432) with significantly higher subjective image quality scores for DLR group compared to HIR group (p=0.03).Quantitatively, air and muscle HU differed significantly between the two scanners (air, p=0.036; muscle, p<0.001). Image noise, assessed by SD was significantly lower in DLR group across the lung parenchyma (p=0.007), tracheal lumen (p=0.002) and muscle (p<0.001). Radiation dose metrics were significantly reduced in DLR group compared to control group (mean DLP: 49.6 vs 188.9 mGy·cm;CTDIvol: 1.6 vs 5.6 mGy;EED:0.8 vs 3.2 mSv;all p<0.001).Age-based sub-analysis using quartiles (0–5,6–12,>13 years) confirmed a consistent threefold reduction in DLP and CTDIvol, and a fourfold reduction in EED across all age groups (all p<0.001).
**Conclusion:** DLR in unenhanced chest CT allows significant radiation dose reduction in pediatric population while delivering superior image quality compared to HIR algorithms.

**Limitations:** Monocentric study
**Funding for this study:** None
**Has your study been approved by an ethics committee?** Not applicable
**Author Disclosures:**
Davide Giacomo Gandola: Nothing to disclose
Cesare Maino: Nothing to disclose
Cammillo Roberto Giovanni Leopoldo Oreste Massimiliano Talei Franzesi: Nothing to disclose
Ilaria Bianco: Nothing to disclose
Rocco Corso: Nothing to disclose
Paolo Niccolò Franco: Nothing to disclose
Davide Ippolito: Nothing to disclose

**14:00 - 15:30** **Research Stage 2**

### Research Presentation Session: Abdominal and Gastrointestinal

### RPS 2001
### Upper GI imaging

**Moderator**
Milica Mitrovic; Belgrade/RS
*(dr_milica@yahoo.com)*

**Diagnostic value of spectral CT–derived extracellular volume fraction for gastric cancer: preoperative staging, histologic differentiation, and peritumoral invasion**
J. Liu[1], B. Fan[1], Y. Liao[2], X. Li[1], *W. Deng*[2]; [1]Nanchang/CN, [2]Guangzhou/CN

**Purpose:** Extracellular volume fraction (ECV) derived from spectral CT has been proposed as a potential imaging biomarker reflecting tumor stromal characteristics. This study aim to investigate the diagnostic value of spectral CT-derived ECV for preoperative gastric cancer staging, histological differentiation and peritumoral invasion.
**Methods or Background:** Eighty-six patients with surgically resected and pathologically confirmed gastric cancer were prospectively enrolled. Pathological results served as the reference standard, and patients were grouped according to: T stage (T1–2, n=30 vs T3–4, n=56), N stage (negative, n=31 vs positive, n=55), perineural and vascular invasion (negative, n=35 vs positive, n=51), and histologic differentiation (low differentiation, n=49 vs moderate–high differentiation, n=37). Delayed-phase images were acquired 180 seconds after contrast injection. Regions of interest (ROIs) were manually delineated and ECV values calculated based on iodine concentration. Receiver operating characteristic (ROC) analysis was performed to evaluate the diagnostic efficacy of ECV in different pathological groups.
**Results or Findings:** ECV showed limited diagnostic performance for discriminating histologic differentiation (AUC=0.602, 95%CI:0.461-0.740 ), but demonstrated good diagnostic value for predicting T stage (AUC=0.852, 95%CI: 0.747-0.946), lymph node metastasis (AUC=0.827, 95%CI: 0.727-0.927) and perineural/vascular invasion (AUC=0.821, 95%CI: 0.726-0.916). These findings suggest that ECV predominantly reflects tumor stromal features, with advantages in assessing tumor- and peritumoral infiltration in gastric cancer.
**Conclusion:** Delayed-phase ECV derived from spectral CT may serve as a non-invasive imaging biomarker for gastric cancer aggressiveness, particularly for tumor infiltration, lymph node metastasis, and perineural/vascular invasion, while its value for assessing histologic differentiation is limited.
**Limitations:** This single-center retrospective study with a limited sample size did not include subgroup analyses, which may affect the stability and generalizability of the results.
**Funding for this study:** None
**Has your study been approved by an ethics committee?** Yes
**Ethics committee - additional information:** Jiangxi Provincial People's Hospital Ethics Committee
**Author Disclosures:**
Bing Fan: Nothing to disclose
Weiwei Deng: Nothing to disclose
Yuting Liao: Nothing to disclose
Jiaqi Liu: Nothing to disclose
Xiaofen Li: Nothing to disclose

**Impact of baseline CT-assessed sarcopenia on survival in patients with locally advanced gastric cancer undergoing neoadjuvant chemotherapy and surgery**
*I. Cecchi*, D. Cozzi, G. Angeluzzi, E. Buttitta, D. Rossini, D. Lavacchi, L. Antonuzzo, V. Miele; Firenze/IT
*(ilaria.cecchi@unifi.it)*

**Purpose:** The aim of this study is to investigate if CT assessment of baseline sarcopenia could be considered as a prognostic factor for overall survival (OS) and relapse-free survival (RFS) in a population of 133 patients with locally advanced gastric cancer recruited from Careggi Hospital, Florence.
**Methods or Background:** We included 133 patients with a diagnosis of locally advanced gastric cancer who underwent the same neoadjuvant chemotherapy followed by surgery. Skeletal muscle cross-sectional area at the third lumbar vertebra (L3) was measured using 3D Slicer software on non-contrast-enhanced CT scan performed before neoadjuvant therapy. Different cut-off points for men and women were used to classify individuals as sarcopenic or non-sarcopenic.
**Results or Findings:** Sarcopenia was identified in 26.3% of patients. Median OS was numerically different between sarcopenic and non-sarcopenic patients (59.0 vs 67.3 months); in the same way, RFS was numerically lower in sarcopenic subjects compared with non-sarcopenic ones (38.0 vs 51.0 months). Nevertheless, no statistically significant differences in survival outcomes were observed between the two groups.
**Conclusion:** Baseline sarcopenia, as assessed by CT, was not associated with a significant difference in OS or RFS among patients with locally advanced gastric cancer undergoing neoadjuvant chemotherapy and surgery.
**Limitations:** The limitations of the study are the small and heterogeneous population. Moreover, sarcopenia was examined only at baseline. Future studies should expand the patient cohort, assessing sarcopenia at the end of neoadjuvant therapy to evaluate longitudinal changes and considering a multivariate analysis including socio-demographic and clinical variables.
**Funding for this study:** No funding was received for this study.
**Has your study been approved by an ethics committee?** Not applicable
**Author Disclosures:**
Vittorio Miele: Nothing to disclose
Daniele Rossini: Nothing to disclose
Daniele Lavacchi: Nothing to disclose
Gabriele Angeluzzi: Nothing to disclose
Ilaria Cecchi: Nothing to disclose
Eleonora Buttitta: Nothing to disclose
Lorenzo Antonuzzo: Nothing to disclose
Diletta Cozzi: Nothing to disclose

**VIGA: A virtual gastric analysis tool for MRI-based assessment of stomach structure and function**
D. Liao[1], *E. B. Mark*[1], M. B. Poulsen[1], L. Davidsen[1], V. Rosca[2], V. Drug[2], C. Brock[1], J. B. Frøkjær[1], A. M. Drewes[1]; [1]Aalborg/DK, [2]Iasi/RO
*(e.mark@rn.dk)*

**Purpose:** Traditional techniques for evaluating gastric physiology, such as scintigraphy and gastric manometry, face noteworthy limitations, especially in capturing the dynamic behaviour of the stomach wall during phases of filling and emptying. To overcome these challenges, we introduce VIGA (VIrtual GAster), a novel integrated analytical platform that leverages Magnetic Resonance Imaging (MRI) to quantify stomach geometry and motility.
**Methods or Background:** VIGA and its standardized analytical pipeline were developed and applied to static and dynamic MRI datasets from 12 healthy participants following the consumption of a 600 ml liquid test meal (300 kcal). This enabled comprehensive assessment of stomach morphology and wall dynamics. Morphological analysis involved measuring the volume and surface geometry of the fundus, corpus, and antrum. Motility was characterized through a detailed motility map, from which contraction frequency, amplitude, and propagation speed were extracted.
**Results or Findings:** The mean volume of the entire stomach was 785.7 ± 220 ml after the test meal, where the gastric liquid volume was 464.1 ± 59 ml. The mean contraction frequency was 2.83 ± 0.06 cycles per minute. The analysis identified distinct regional differences in contraction patterns, with the distal stomach showing significantly greater contraction occlusion (15.8±4.0%) compared to the proximal region (8.9±4.0%, p<0.001). Furthermore, contraction propagation speed tended to be higher in the distal stomach (4.87±1.7 mm/s) than in the proximal (4.01±0.9 mm/s, p=0.07). Surface geometry also varied across compartments, with the fundus exhibiting the highest inverse mean curvature, an indicator of the ratio between wall tension and intragastric pressure (p=0.003).
**Conclusion:** VIGA offers a robust, non-invasive imaging-based framework for evaluating gastric function, with promising implications for enhancing clinical assessment of gastric disorders.
**Limitations:** The subjects were investigated in supine position, negating the effects of gravity in the gastric content.

**Funding for this study:** This study was supported by the North Denmark Region's Health Innovation Fund.
**Has your study been approved by an ethics committee?** Yes
**Ethics committee - additional information:** The study was approved by the regional ethics committee (N-20220059).
**Author Disclosures:**
Jens Brøndum Frøkjær: Nothing to disclose
Line Davidsen: Nothing to disclose
Esben Bolvig Mark: Nothing to disclose
Vasile Drug: Nothing to disclose
Christina Brock: Nothing to disclose
Vadim Rosca: Nothing to disclose
Maria Bitsch Poulsen: Nothing to disclose
Donghua Liao: Nothing to disclose
Asbjørn Mohr Drewes: Nothing to disclose

**Reproducibility of CT Classification for the esophageal and gastric lesions in patients with caustic Ingestions: A Retrospective Analysis in a Specialized Treatment Center**
*G. Rinaldi*, M. C. Ambrosetti, M. Bariani, E. Strazimiri, G. Zamboni, S. Giacopuzzi, J. Weindelmayer, M. Sacco, G. Puppini; Verona/IT
*(gabriele.rinaldi26@gmail.com)*

**Purpose:** Although in the case of caustic ingestion treatment is usually based on endoscopic evaluation (Zargar classification), CT could provide an accurate assessment and potentially reduce overtreatment. Our aim was to assess reproducibility and effectiveness of a CT-based classification of caustic injuries in a specialized center, for predicting need for emergency intervention and long-term complications.
**Methods or Background:** five radiologists and six surgeons blinded to patient outcomes independently reviewed CT of 36 patients admitted to the Emergency Department between 2014-2025 for caustic ingestion and who underwent endoscopy, applying the 3-grade score by Chirica: grade I (regular), grade IIA (thickened, hypointense and vascularized mucosa, wall/perivisceral fat tissue edema), grade IIB (necrotic mucosa with slight external wall enhancement), grade III (transmural necrosis/lack of wall enhancement) for both esophagus and stomach. Kendall's Kappa inter-operator concordance and CT sensitivity and specificity for each physician were calculated for emergency treatment (grade III) and stenosis risk (grades IIa-IIb), comparing these results to the Zargar classification.
**Results or Findings:** for emergency treatment (Zargar III), mean sensitivity was 37.1% for esophagus evaluation, 30.2% for stomach, and 34.2% for both, with the best performance reached by the radiologist for esophageal evaluation (SE38.4%). For stenosis prediction (Zargar IIA-IIB), the mean sensitivity was 69% for esophagus evaluation, 59.7% for stomach, and 68% for both, with the best performance reached by both radiologist and surgeon for stomach evaluation (SE100%). Interobserver concordance was higher for radiologists than for surgeons (k=0.867-0.895 vs k=0.198-0.573).
**Conclusion:** CT classification, compared to endoscopic evaluation, is not sensitive enough for predicting the need for emergency surgery. Sensitivity is higher for risk of stenosis. While CT offers a promising alternative by potentially reducing overtreatment and predicting long-term complications, the interobserver variability highlights the need for standardization.
**Limitations:** Retrospective study
**Funding for this study:** None
**Has your study been approved by an ethics committee?** Not applicable
**Author Disclosures:**
Giulia Zamboni: Nothing to disclose
Gabriele Rinaldi: Nothing to disclose
Matilde Bariani: Nothing to disclose
Maria Chiara Ambrosetti: Nothing to disclose
Michele Sacco: Nothing to disclose
Giovanni Puppini: Nothing to disclose
Endrit Strazimiri: Nothing to disclose
Jacopo Weindelmayer: Nothing to disclose
Simone Giacopuzzi: Nothing to disclose

**CT-based topographical classification of anastomotic leakage after Ivor Lewis esophagectomy: prognostic implications and pathophysiology insights**
*G. Veneziano*, D. Palumbo, S. Battaglia, C. Sarzo, U. Elmore, R. Rosati, F. De Cobelli, F. Puccetti, A. Cossu; Milano/IT
*(veneziano.gaia@hsr.it)*

**Purpose:** Anastomotic leakage (AL) remains the most feared complication after Ivor Lewis esophagectomy, with significant morbidity/mortality and difficult, non-standardized, management. CT-based topographical characterization of AL may provide crucial insights into its pathogenesis, clinical course, and prognosis, potentially guiding risk stratification and therapeutic decisions.
**Methods or Background:** This is a single-center retrospective analysis of 85 consecutive patients who underwent Ivor Lewis esophagectomy (01/2015-12/2024) and performed contrast enhanced CT scan because of AL development. ALs were classified into four topographical subtypes based on CT findings: left-anterior, right-anterior, left-posterior, right-posterior. Radiological features (perianastomotic collections, air within collections, pleural effusion, pneumothorax, pneumomediastinum) and clinical outcomes were analyzed using bivariate and multivariate regression models; the course of right gastroepiploic arteries was also noted.
**Results or Findings:** The most common subtype of AL was found to be the left-anterior one (40%), followed by right-anterior (20%), left-posterior (14%), and right-posterior (11%). Most ALs manifested within eight postoperative day (72%). Left-anterior leaks demonstrated strongest association with perianastomotic collections containing air (p<0.001) and significantly correlated with respiratory and circulatory complications. Multivariate analysis revealed left-anterior leaks as independent predictors of severe outcomes (Clavien-Dindo 4b-5; p<0.001), with highest mortality rates predominantly from respiratory failure. Notably, the left-anterior position corresponds to the region opposite to the right gastroepiploic artery vascular territory, potentially representing an area of relative hypoperfusion.
**Conclusion:** CT-based topographical classification of AL after Ivor Lewis esophagectomy provides valuable prognostic stratification. The predominance and severity of left-anterior leaks may reflect underlying vascular vulnerability mechanisms that warrant further investigation. These findings underscore the need for targeted preventive strategies, including enhanced intraoperative vascular assessment and potentially modified surgical techniques focusing on this high-risk anatomical region
**Limitations:** Small sample of patients in one high-volume center and need for standardization of indications.
**Funding for this study:** None
**Has your study been approved by an ethics committee?** Yes
**Ethics committee - additional information:** San Raffaele Scientific Institute Ethics Committee
**Author Disclosures:**
Francesco Puccetti: Nothing to disclose
Francesco De Cobelli: Nothing to disclose
Diego Palumbo: Nothing to disclose
Andrea Cossu: Nothing to disclose
Silvia Battaglia: Nothing to disclose
Gaia Veneziano: Nothing to disclose
Carlotta Sarzo: Nothing to disclose
Riccardo Rosati: Nothing to disclose
Ugo Elmore: Nothing to disclose

**Radiologic Assessment of Esophagogastric Junction Outflow Obstruction: Diagnostic Accuracy of Timed Barium Esophagogram**
*V. Patanè*, P. Senneca, M. C. Brunese, G. Sarnelli, R. Grassi, S. Cappabianca, A. Reginelli; Naples/IT
*(patavittorio@gmail.com)*

**Purpose:** Esophagogastric junction outflow obstruction (EGJOO) according to Chicago Classification v4.0 requires manometric, clinical, and supportive evidence of impaired bolus transit. Timed barium esophagogram (TBE) is a widely available imaging test providing objective evidence of functional relevance. This study evaluated the diagnostic performance and reproducibility of standardized TBE in suspected EGJOO.
**Methods or Background:** In this prospective single-center study, 124 consecutive patients underwent standardized TBE for esophageal emptying assessment. Examinations were performed and interpreted blinded to symptoms and high-resolution manometry (HRM). Barium column height and width were measured at 1, 2, and 5 minutes after ingestion of low-density barium sulfate. HRM, performed according to CCv4.0, served as the reference. Interobserver agreement between a senior radiologist and a junior resident was assessed.
**Results or Findings:** TBE was positive in 20 patients (16%), all with functionally relevant EGJOO (FR-EGJOO) on HRM. The mean barium column height at 1 minute was 122.9 ± 65.4 mm, decreasing to 89.0 ± 54.6 mm at 2 minutes. Residual barium at 5 minutes occurred in eight patients, all with the largest 1-minute dimensions. Compared with HRM, TBE showed specificity and positive predictive value of 100%, sensitivity of 31%, and negative predictive value of 58%. Interobserver agreement was excellent.
**Conclusion:** Standardized TBE is a highly specific and reproducible tool for confirming the functional relevance of EGJOO diagnosed by CCv4.0. Although sensitivity is modest, a positive TBE strongly correlates with manometric findings and helps differentiate EGJOO from achalasia. Integration into the diagnostic pathway supports accurate patient stratification and may avoid unnecessary treatment.
**Limitations:** Being a single-center study with relatively few positive cases may limit generalizability. Moreover, suboptimal sensitivity could underestimate EGJOO in milder presentations. Larger multicenter cohorts are required to validate findings and refine cutoff values.

**Funding for this study:** This research received no external funding
**Has your study been approved by an ethics committee?** Yes
**Ethics committee - additional information:** The study was conducted in accordance with the Declaration of Helsinki (as revised in 2013). The study's protocol was approved by the local ethics committee at the University Hospital of Campania "L. Vanvitelli" and AORN "Ospedale dei Colli".
**Author Disclosures:**
Giovanni Sarnelli: Nothing to disclose
Salvatore Cappabianca: Nothing to disclose
Roberto Grassi: Nothing to disclose
Vittorio Patanè: Nothing to disclose
Alfonso Reginelli: Nothing to disclose
Piera Senneca: Nothing to disclose
Maria Chiara Brunese: Nothing to disclose

**Imaging in Gastric Cancer Staging: The Emerging Role of Magnetic Resonance and Advanced Techniques**
*G. Balestrucci*[1], M. Brunese[1], V. Patanè[2], N. Giordano[2], M. Alessandrella[2], S. Cappabianca[2], A. Reginelli[2]; [1]Campobasso/IT, [2]Naples/IT
*(bale21995@gmail.com)*

**Purpose:** To summarize the current role of imaging modalities in gastric cancer staging and highlight the emerging potential of magnetic resonance imaging (MRI) with advanced functional sequences and artificial intelligence (AI) applications.
**Methods or Background:** A systematic literature search was conducted in PubMed using predefined search strings. We included systematic reviews and meta-analyses published from January 2020 to December 2024 focusing on imaging techniques for gastric cancer staging. About 30 studies met the inclusion criteria, covering CT, PET/CT, endoscopic ultrasound, MRI, and AI-based approaches.
**Results or Findings:** Computed tomography (CT) remains the reference technique for baseline staging, although it has limited sensitivity in assessing tumor wall invasion (T-staging) and in detecting peritoneal metastases. PET/CT improves distant staging accuracy; innovative tracers such as Ga-FAPI have demonstrated significantly higher sensitivity than FDG-PET, particularly for peritoneal and nodal metastases. EUS remains the most accurate method for local T-staging and nodal sampling, but its performance is strongly operator-dependent. Magnetic resonance imaging is emerging as a promising complementary tool, with DWI enabling improved differentiation between malignant and non-malignant tissues and perfusion MRI providing insights into tumor vascularity and microenvironment. Several studies reported superior accuracy of MRI compared to CT for local staging and prediction of treatment response. AI and radiomics approaches further expand diagnostic potential, showing promise in predicting tumor aggressiveness and therapy response.
**Conclusion:** MRI, especially when combined with functional sequences and AI, represents an emerging complementary modality for gastric cancer staging. It has the potential to improve diagnostic accuracy, refine patient stratification, and support personalized surgical and oncological management.
**Limitations:** Evidence remains limited, with heterogeneous imaging protocols and few prospective multicenter studies. Larger standardized investigations are warranted to validate the clinical role of MRI and AI in gastric cancer staging.
**Funding for this study:** None
**Has your study been approved by an ethics committee?** Not applicable
**Author Disclosures:**
Mariachiara Brunese: Nothing to disclose
Marina Alessandrella: Nothing to disclose
Giovanni Balestrucci: Nothing to disclose
Salvatore Cappabianca: Nothing to disclose
Vittorio Patanè: Nothing to disclose
Alfonso Reginelli: Nothing to disclose
Nicoletta Giordano: Nothing to disclose

**14:00 - 15:30 Research Stage 4**

### Research Presentation Session: Cardiac

### RPS 2003

### Flow, perfusion and functional assessment in cardiac imaging

**Moderator**
Sara Boccalini; Rouen/FR
*(sara.boccalini@yahoo.com)*
**Author Disclosures:**
Sara Boccalini: Speaker: Philips Healthcare, Bayer

**The influence of a myocardium-specific reconstruction filter on myocardial texture analysis in static spectral cardiac CT perfusion**
*L. R. Koetzier*[1], N. R. van der Werf[2], P. M. Tetteroo[1], B. K. Velthuis[1], D. Suchá[1]; [1]Utrecht/NL, [2]Best/NL
*(l.r.koetzier@umcutrecht.nl)*

**Purpose:** To evaluate the effect of a myocardium-specific reconstruction filter on myocardial texture analysis in static spectral cardiac CT perfusion.
**Methods or Background:** In this retrospective single-center study, 108 patients underwent prospective ECG-triggered coronary CT angiography (CCTA) with spectral dual-layer CT to assess coronary artery disease. Images were reconstructed using two filters (coronary-optimized CB, myocardium-specific MCA), and conventional 120 kVp and iodine density images at 3-mm slice thickness. The left ventricular myocardium was automatically segmented, with intensities normalized and quantized to 64 gray-levels. Texture analysis included six histogram-based features (intensity distribution, ignores spatial context) and three gray-level co-occurrence matrix features (GLCM; captures relationships between neighboring voxels). MCA was compared against CB on conventional and iodine density images using paired Wilcoxon signed-rank tests with Benjamin-Hochberg correction ($\alpha$=0.05) and reported as median difference divided by the interquartile range per feature across patients.
**Results or Findings:** Eighteen patients were excluded with insufficient quality CCTA. Ninety-two patients were analyzed. On conventional images, the MCA filter significantly altered all texture features compared to CB. The largest effects were seen for entropy (−2.71), contrast (−2.12), and homogeneity (+4.10), whereas mean intensity (+0.02) and skewness (+0.43) showed only small effects. On iodine density images, the impact of the MCA filter was more pronounced. Strongest effects were observed for entropy (−5.99), variance (−3.83), kurtosis (+4.74), and homogeneity (+6.07). In contrast, mean intensity (−0.03) and correlation (−0.73) were minimally affected.
**Conclusion:** The MCA filter significantly increased myocardial homogeneity on conventional and iodine density images. Although MCA reduces noise and improves homogeneity, it simultaneously changes texture. Standardized reconstruction choices are therefore necessary when applying texture analysis for myocardial characterization in cardiac CT.
**Limitations:** This retrospective single-center study did not assess the impact of reconstruction protocols on myocardial disease detection.
**Funding for this study:** N/A
**Has your study been approved by an ethics committee?** Not applicable
**Author Disclosures:**
Niels R. Van Der Werf: Employee: Philips Healthcare
Dominika Suchá: Nothing to disclose
Philip Martin Tetteroo: Nothing to disclose
Lennart Roelof Koetzier: Nothing to disclose
Birgitta K. Velthuis: Nothing to disclose

**Reproducibility of 4D flow MRI derived diastolic function testing by mitral and pulmonary venous flow indices**
*T. in de Braekt*[1], P. R. Roos[2], H. van den Bosch[1], H. J. Lamb[2], J. Westenberg[2]; [1]Eindhoven/NL, [2]Leiden/NL

**Purpose:** To investigate scan-rescan reproducibility of mitral valve (MV) and pulmonary vein (PV) flow velocities using 4D flow MRI in healthy volunteers for diastolic function assessment.
**Methods or Background:** Twenty-one volunteers (mean age 27 ± 3 years) underwent whole-heart 4D flow MRI at 3T twice with a 10-minute interval, allowing repositioning. One group (n=11) was scanned during free-breathing without respiratory motion compression, while the other (n=10) used weighted-gating respiratory motion compensation, with an additional 4D flow acquisition without respiratory motion compensation. MV parameters included E wave, A

wave, E/A ratio, annular e' velocity, and E/e' ratio. PV velocities included S wave, D wave, S/D ratio, and AR wave. Parameters were derived from flow velocity-time curves and compared between acquisitions using Pearson's correlation coefficient (ρ). Due to spatial resolution limitations and anatomical variation, PV flow was measured in the same PV (preferably right inferior PV) per volunteer. Subanalysis in the second group compared the effect of respiratory weighted-gating versus free-breathing on flow measurements. Intra- and interobserver agreement was assessed with intraclass correlation coefficient (ICC).
**Results or Findings:** Scan-rescan comparison showed moderate-to-good agreement for MV parameters (ρ=0.51–0.74), except e' velocity and E/e' ratio (ρ=-0.13 and -0.14, respectively). PV flow velocities showed strong reproducibility (ρ=0.88–0.92), except AR wave (ρ=0.47). Subanalysis showed moderate-to-strong agreement for MV parameters (ρ=0.69–0.92), and good-to-excellent agreement for PV parameters (ρ=0.82–0.99). Intraobserver agreement was moderate-to-excellent for MV and PV parameters (ICC=0.62–0.97), except S/D ratio (ICC=0.48). Interobserver agreement was moderate-to-excellent for MV parameters (ICC=0.59–0.94), and poor for PV parameters (ICC=-0.12–0.33).
**Conclusion:** For most diastolic flow parameters, 4D flow MRI demonstrated moderate-to-excellent reproducibility, with or without respiratory motion compensation, supporting its potential for diastolic function assessment.
**Limitations:** Data was not compared to gold standard echocardiography.
**Funding for this study:** Funding for this study was part of an unrestricted grant (number 1261584) provided by the Dutch Society for Radiology (NVvR).
**Has your study been approved by an ethics committee?** Not applicable
**Author Disclosures:**
Paul Ruben Roos: Nothing to disclose
Jos Westenberg: Nothing to disclose
Thomas In De Braekt: Nothing to disclose
Hildo J. Lamb: Nothing to disclose
Harrie Van Den Bosch: Nothing to disclose

**Myocardial Stress CMR Perfusion to Differentiate between Athlete's Heart and Ischemic and Nonischemic Cardiomyopathies: The MYO-SPORT Study**
*C. Mantini*, C. Di Barbora, D. Costantini, S. Paolini, F. Ricci; Chieti/IT
*(cesare.mantini@gmail.com)*

**Purpose:** To assess the incremental value of a stress CMR perfusion study in the diagnostic workup of master athletes with suspected cardiovascular pathology
**Methods or Background:** Between 2020–2024, we enrolled master athletes referred for stress CMR following abnormal sports pre-participation screening.All underwent vasodilator stress CMR with visual and semi-quantitative myocardial perfusion analysis.A multidisciplinary team adjudicated diagnoses as either normal heart (including exercise-induced cardiac remodeling) or abnormal heart (ischemic disease, non-ischemic left ventricular scar, or cardiomyopathy).Diagnostic performance was assessed using the area under the receiver operating characteristic curve (AUC) and global chi-squared statistics across sequential models:baseline function/volumes,baseline+LGE (model 1),model 1+visual perfusion (model 2),model 2+myocardial perfusion reserve index (MPRI) (model 3)
**Results or Findings:** We enrolled 68 recreational master athletes (57±11 yo, 82% male),with 18(26%) training more than 8 hours per week.Referral reasons included chest pain(47%),premature ventricular contractions (18%),abnormal ECG (16%),abnormal exercise stress testing (11%),and abnormal resting echocardiography (8%).Vasodilator stress CMR was performed using regadenoson(57%),adenosine(42%), or dipyridamole(1%).The final adjudicated diagnosis classified 26 (38%) cases as having a normal heart, while42(62%) were diagnosed with heart disease,including ischemic heart disease(n=24),nonischemic left ventricular scar(n=10), and cardiomyopathy(n=8).LGE was present in 53% of cases,and stress-induced myocardial perfusion defects were visually identified in 17%.Myocardial perfusion reserve index was lower in abnormal hearts compared to normal hearts(1.6±0.3vs1.3±0.3;p<0.001), yielding alone an AUC of 0.76 with best cut-off of 1.29.The diagnostic yield improved with sequential imaging integration:baseline assessment had limited discrimination (AUC 0.67),+LGE(AUC 0.87),+visual stress perfusion(AUC 0.93),+MPRI(AUC 0.97). The global chi-squared statistic increased stepwise from 7.4 to 49.5 across models (p < 0.0001 for all comparisons).At a median follow-up of 31 months,no deaths occurred
**Conclusion:** Stress CMR perfusion provides significant incremental value in distinguishing physiological remodeling from ischemic and nonischemic disease in master athletes with abnormal pre-participation screening
**Limitations:** Small sample size
**Funding for this study:** No Founding
**Has your study been approved by an ethics committee?** Not applicable
**Author Disclosures:**
Cesare Mantini: Nothing to disclose
Daniele Costantini: Nothing to disclose
Claudia Di Barbora: Nothing to disclose
Kristian Galanti: Nothing to disclose
Massimo Caulo: Nothing to disclose
Fabrizio Ricci: Nothing to disclose
Giandomenico Bisaccia: Nothing to disclose

**Dynamic stress CT perfusion for ischemia detection in non–infarct-related intermediate coronary stenoses: comparison with instantaneous wave free ratio**
Z. Magomedova, *V. Meshkov*, E. Pershina, M. Serova, E. Ploschenkov; Moscow/RU
*(ryloosha@gmail.com)*

**Purpose:** Dynamic stress CT myocardial perfusion imaging (CT MPI) combines anatomic and physiologic coronary assessment in a single test. Its performance in patients with prior myocardial infarction (MI) and residual, non–infarct related artery (non IRA) stenoses is not well defined. We evaluated CT MPI against invasive instantaneous wave free ratio (iFR) in post MI patients with visually intermediate non IRA stenoses.
**Methods or Background:** 61 consecutive post PCI MI patients with at least one 50–90% non IRA stenosis underwent adenosine (160 $\mu g \cdot kg^{-1} \cdot min^{-1}$) stress and rest dynamic CT MPI (16 cm coverage; Revolution CT, GE Healthcare) with 10 minute delayed iodine enhancement for scar detection, followed by invasive angiography with selective iFR in 50–90% lesions. Hemodynamically significant stenosis was defined as iFR <0.90. Voxel based quantitative myocardial blood flow (MBF) maps were generated; MBF <100 $mL \cdot min^{-1} \cdot 100\ mL^{-1}$ was considered abnormal. Diagnostic performance was calculated per vessel using iFR as reference and per patient.
**Results or Findings:** Hemodynamically significant stenoses by iFR were present in 21/61 patients (34%). Delayed enhancement identified postischemic scar in 48/61 (79%). Against iFR, CT MPI yielded per vessel sensitivity 99%, specificity 97%, and accuracy 98%; per patient - 97%, 90%, 94%. Hemodynamically significant stenoses by iFR were present in 21/61 patients (34%). Delayed enhancement identified postischemic scar in 48/61 (79%). CT MPI yielded per vessel sensitivity 99%, specificity 97%, and accuracy 98%; per patient sensitivity 97%, specificity 90%, and accuracy 94%. Three patients (5%) with iFR ≥0.90 showed regional hypoperfusion with reduced MBF, consistent with microvascular dysfunction.
**Conclusion:** Whole heart dynamic stress CT MPI demonstrates excellent vessel level sensitivity and specificity and high patient level accuracy for detecting functionally significant non IRA stenoses in post MI patients when benchmarked to iFR.
**Limitations:** Single centre study
**Funding for this study:** No funding
**Has your study been approved by an ethics committee?** Yes
**Ethics committee - additional information:** Institutional board
**Author Disclosures:**
Zaynab Magomedova: Nothing to disclose
Ekaterina Pershina: Nothing to disclose
Evgenii Ploschenkov: Nothing to disclose
Maria Serova: Nothing to disclose
Vladimir Meshkov: Nothing to disclose

**Development and validation of a computed tomography myocardial perfusion imaging radiomic model for major adverse cardiovascular events prediction: a multicenter study**
*Z. Zhong*, J. Zhang; Shanghai/CN
*(gluacid@163.com)*

**Purpose:** This study aimed to develop a combined model, including clinical risk factors, coronary atherosclerotic characteristics, and radiomic features derived from CT myocardial perfusion imaging (CT-MPI), to predict major adverse cardiovascular events (MACEs).
**Methods or Background:** Accurate prediction of MACEs is crucial for risk stratification in patients with suspected coronary artery disease. CT-MPI provides various parameters, which may help comprehensively characterize perfusion features. 712 patients who underwent coronary CT angiography (CCTA) and CT-MPI from seven hospitals were enrolled. Radiomic analysis was performed on eight perfusion parameter maps. Three prediction models were established accordingly: Model 1 (clinical risk factors and coronary atherosclerotic characteristics), Model 2 (incorporating myocardial blood flow values upon Model 1), and Model 3 (integrating radiomic scores upon Model 2).

**Results or Findings:** The C-indices for Model 3 in the training, internal validation, and external validation sets were 0.901 (95% confidence interval [CI]: 0.856–0.947), 0.841 (95% CI: 0.783–0.899), and 0.832 (95% CI: 0.761–0.903), respectively, demonstrating significant improvements over Model 1 and Model 2 (all $p < 0.05$). In the external validation set, Model 3 had the largest time-dependent areas under the curve (AUC) values for 1-, 3-, and 5-year MACEs prediction (0.878 [95% CI: 0.775-0.981], 0.851 [95% CI: 0.756-0.946], and 0.747 [95% CI: 0.603-0.891]), compared to Model 1 and Model 2.
**Conclusion:** The radiomic features from multiparametric CT-MPI maps simultaneously captured perfusion features associated with MACEs at both macrovascular and microvascular levels. The combined model exhibited superior MACEs prognostic performance compared with conventional models while maintaining high interpretability.
**Limitations:** Studies involving patients at lower pretest probability of obstructive CAD and multivendor CT-MPI data are needed to confirm the utility and reproducibility of the radiomic model for MACEs prediction in broader populations and different scanner platforms or protocols.
**Funding for this study:** This study is supported by National Natural Science Foundation of China (Grant No.: 82471982, 82271990), National Key Research and Development Program of China (Grant No.: 2021YFF0501402), Shanghai Municipal Science and Technology Commission Discipline Leader Project (Grant No.: BJKJ2024052), Shanghai Health Commission Discipline Leader Project (Grant No.: 2022XD031) and the Key Project of Shanghai Municipal Education Commission (Grant No.: 2024AIZD017).
**Has your study been approved by an ethics committee?** Yes
**Ethics committee - additional information:** The ethics committee of Shanghai General Hospital
**Author Disclosures:**
Zhiqi Zhong: Nothing to disclose
Jiayin Zhang: Nothing to disclose

**Feasibility of stress myocardial perfusion imaging with photon-counting CT: Initial validation against reference modalities**
*B. Longere*[1], H. Caseneuve[1], C. V. Gkizas[1], A. Rodriguez Musso[1], C. Croisille[2], M. Haidar[1], F. A. Pontana[1]; [1]Lille/FR, [2]Bordeaux/FR
*(benjamin.longere@chu-lille.fr)*

**Purpose:** To evaluate the feasibility and diagnostic performance of stress myocardial perfusion imaging (MPI) using a first-generation dual-source photon-counting CT (PCCT),compared to reference standards.
**Methods or Background:** Consecutive patients referred for coronary CT angiography (CCTA) with concomitant stress MPI using PCCT scanner and at least one functional reference test within one month were included. Static PCCT-MPI with regadenoson was acquired 12s after intravenous injection of 40mL of iodine contrast medium and paired with myocardial delayed enhancement imaging. Reference tests for ischemia included stress cardiac magnetic resonance imaging, stress echocardiography, single-photon emission computed tomography, or invasive coronary angiography. Diagnostic accuracy was evaluated using ROC curve analysis and Youden index-derived thresholds.
**Results or Findings:** Forty-one patients (28 males, median age=62 years) were included; 35 (85.4%) had obstructive coronary artery disease (CAD) on CCTA. PCCT-MPI identified myocardial hypoperfusion in 18 patients (43.9%) with a median dose-length product of 97mGy.cm (Q1, 76.8.0; Q3, 155.8mGy.cm). Visual assessment yielded 100% sensitivity (95%CI: 81.5–100.0%), 95.7% specificity (95%CI: 78.1–99.9%), PPV 94.7%, and NPV 100%, with an AUC of 0.98. An optimal defect-to-remote iodine ratio cutoff of 0.79 showed excellent accuracy (97.6%, AUC=0.995). Integration of PCCT-MPI with CCTA could have obviated additional functional testing or invasive angiography in up to 50% of patients with obstructive CAD.
**Conclusion:** Stress static PCCT-MPI is feasible and demonstrates excellent diagnostic accuracy for detecting myocardial ischemia. Defect-to-remote iodine ratio from spectral imaging further enhances performance, establishing PCCT as a comprehensive imaging modality that unifies anatomic and functional coronary assessment at a reasonable radiation dose, within a single examination.
**Limitations:** Retrospective, single-center study Modest sample Heterogeneity of reference standards
**Funding for this study:** None
**Has your study been approved by an ethics committee?** Yes
**Ethics committee - additional information:** IRB number: CMR-2408-417
**Author Disclosures:**
Benjamin Longere: Nothing to disclose
Christos Vasileiou Gkizas: Nothing to disclose
Mehdi Haidar: Nothing to disclose
Cedric Croisille: Nothing to disclose
Hippolyte Caseneuve: Nothing to disclose
Aimée Rodriguez Musso: Nothing to disclose
François Ascagne Pontana: Nothing to disclose

**Stress myocardial perfusion CT after arterial Switch for Transposition of the Great Arteries**
*C. V. Gkizas*[1], B. Longere[1], A. Rodriguez Musso[1], C. Croisille[2], O. Domanski[1], F. Godart[1], F. A. Pontana[1]; [1]Lille/FR, [2]Bordeaux/FR

**Purpose:** Coronary anomalies and neo-aortic root dilatation are recognized as late complications after the arterial switch operation for transposition of the great arteries (TGA), although most patients remain asymptomatic for many years. The purpose of this study was to assess the feasibility and diagnostic performance of CT stress myocardial perfusion imaging (CT-MPI) compared with reference standards in patients with repaired TGA.
**Methods or Background:** Consecutive patients referred for clinically indicated coronary CT angiography (CCTA) underwent CT-MPI—either static with a photon-counting detector CT (PCCT) or dynamic with a third-generation dual-energy CT (DECT)— with regadenoson administration followed by myocardial late iodine enhancement. Within 1 month, each patient underwent ≥1 reference test (stress CMR, stress echocardiography, SPECT, or invasive coronary angiography). Visual analysis thresholds were derived from the Youden index. Radiation dose metrics were compared between scanners.
**Results or Findings:** Thirty patients were included (25 males; median age,= 30 years). Median neo-aortic diameter was 42 mm. CT-MPI detected hypoperfusion in 6/30 patients (20%), all of whom had obstructive coronary disease on CCTA. Diagnostic performance versus reference standards was excellent: sensitivity 85.7% (95% CI, 48.7–97.4), specificity 100% (95% CI, 85.7–99.9), positive predictive value 100%, and negative predictive value 95.8%. Integrating CT-MPI with CCTA could have obviated additional functional testing or invasive angiography in 23/30 patients (77%). Radiation dose was significantly lower with PCCT-MPI than with DECT-MPI (DLP −39%, CTDI −47%; $p < 0.05$).
**Conclusion:** CT stress perfusion is feasible and demonstrates excellent diagnostic performance in patients with repaired TGA and may represent a valuable tool for clinical decision-making during long-term follow-up.
**Limitations:** Single-center retrospective study Modest sample Heterogeneity of reference modalities
**Funding for this study:** None
**Has your study been approved by an ethics committee?** Not applicable
**Author Disclosures:**
Olivia Domanski: Nothing to disclose
Francois Godart: Nothing to disclose
Benjamin Longere: Nothing to disclose
Christos Vasileiou Gkizas: Nothing to disclose
Cedric Croisille: Nothing to disclose
Aimée Rodriguez Musso: Nothing to disclose
François Ascagne Pontana: Nothing to disclose

**Automated liver T1-time assessment in cardiac MRI: Associations with cardiac structure and function in the NAKO study**
*J. Jahn*, H. Thomas, M-N. Von Itter, M. Reisert, S. Rospleszcz, C. Schuppert, C. L. Schlett; Freiburg Im Breisgau/DE
*(johannes.jahn94@gmx.de)*

**Purpose:** Patients with cardiovascular disease often exhibit concomitant liver changes, yet their systematic evaluation in cardiac MRI remains limited. We developed and validated a deep learning approach for automated liver segmentation in cardiac T1 maps to investigate associations between liver T1 times and cardiac function within the German National Cohort (NAKO) study.
**Methods or Background:** A patchwork-based convolutional neural network (CNN) was trained on 323 manually annotated cardiac short-axis T1 maps spanning the mid of the left ventricle [LV] and independently tested on 68 cases. Segmentation performance was assessed using the Dice similarity coefficient and Pearson correlation with manual liver T1 measurements. Associations between automated liver T1 times and cardiac parameters (biventricular volumes, ejection fractions, stroke volumes, and LV mass) were analyzed using linear regression adjusted for age, sex, body mass index, liver fat, and liver iron. Restricted cubic spline (RCS) models tested for nonlinearity.
**Results or Findings:** The CNN achieved a Dice coefficient of 0.92 and excellent correlation with manual liver T1 segmentations ($r=0.95$). Among 26,662 participants (47.8±12.3 years, 46.8% female), median liver T1 values were higher in women (841±77 ms) than in men (787±97 ms, $p<0.001$). Higher liver T1 was strongly associated with smaller biventricular volumes, stroke volumes, LV mass, and lower ejection fractions (all $p<0.01$). For example, each 100 ms increase in liver T1 was associated with lower indexed LV end-diastolic volume ($\beta=-1.41$, $p<0.001$) and LV ejection fraction ($\beta=-0.17$, $p<0.01$). RCS analyses confirmed significant nonlinear relationships for most outcomes.
**Conclusion:** We present a robust deep learning approach for automated liver segmentation in cardiac T1 mapping. Liver T1 times are independently and nonlinearly associated with cardiac structure and function in a large cohort and may provide novel insights into cardio-hepatic interactions.
**Limitations:** No histological confirmation was performed.

**Funding for this study:** The NAKO is funded by the Federal Ministry of Research, Technology and Space (BMFTR) [project funding reference numbers: 01ER1301A/B/C, 01ER1511D, 01ER1801A/B/C/D and 01ER2301A/B/C], federal states of Germany and the Helmholtz Association, the participating universities and the institutes of the Leibniz Association.
**Has your study been approved by an ethics committee?** Yes
**Ethics committee - additional information:** The study received institutional review board approval and written informed consent was obtained from all participants.
**Author Disclosures:**
Christopher L. Schlett: Nothing to disclose
Marco Reisert: Nothing to disclose
Susanne Rospleszcz: Nothing to disclose
Marc-Nicolas Von Itter: Nothing to disclose
Christopher Schuppert: Nothing to disclose
Johannes Jahn: Nothing to disclose
Helene Thomas: Nothing to disclose

**Diagnostic value of mitral annular plane systolic excursion (MAPSE) in cardiac sarcoidosis: comparison with myocardial late gadolinium enhancement and ventricular function**
*E. İsmailov*, T. Y. Kılıç, Y. B. Uluman, K. B. Bekaroğlu, F. Köse, B. Tütüncüoğlu, T. Banaz, M. Kadıoğlu, S. Ozkök; Istanbul/TR
*(eitibar222@gmail.com)*

**Purpose:** To investigate the relationship between MAPSE values and myocardial fibrosis in patients with cardiac sarcoidosis, and to compare MAPSE with conventional CMR-derived cardiac function parameters. Cardiac involvement in sarcoidosis is associated with poor prognosis, and because early functional impairment may precede detectable fibrosis on CMR, MAPSE, as a sensitive marker of longitudinal function, could offer valuable diagnostic insight at an earlier stage.
**Methods or Background:** 47 patients with histologically confirmed systemic sarcoidosis underwent 1.5 Tesla CMR with CİNE, T1/T2 mapping, and LGE were retrospectively analyzed. MAPSE was measured at the medial and lateral mitral annulus in the 4-chamber CİNE view. Ventricular function, and LGE extent/localization were also measured. ECG/Holter were used to assessed arrhythmia. Correlation analyses were performed using Spearman’s rank test, and the predictive performance of MAPSE was assessed using ROC curve analysis.
**Results or Findings:** A total of 47 patients with systemic sarcoidosis (mean age 50.6±12.7 years, 45% male) were analyzed. MAPSE was significantly reduced compared with healthy controls (7.25 vs. 11.60 mm, p<0.001), indicating impaired longitudinal function. A significant inverse correlation was found between MAPSE and myocardial fibrosis assessed by LGE percentage (r=–0.43, p=0.003). Patients with arrhythmia had significantly lower MAPSE values than those without (p=0.026), and LGE percentage correlated positively with arrhythmia presence (r=0.37, p=0.012). ROC analysis demonstrated fair diagnostic accuracy of MAPSE for arrhythmia (AUC=0.70, p=0.01). An optimal cutoff ≤ 6 mm yielded high specificity (93%) but limited sensitivity (42%).
**Conclusion:** These findings suggest MAPSE as a practical marker of fibrosis and arrhythmic risk. MAPSE is significantly reduced in sarcoidosis patients, correlates inversely with myocardial fibrosis and arrhythmia, and may serve as a simple CMR-derived marker of subclinical involvement and arrhythmic risk even in the absence of overt systolic dysfunction.
**Limitations:** Single-center design
**Funding for this study:** Not receive any financial support
**Has your study been approved by an ethics committee?** Yes
**Ethics committee - additional information:** Ethical Board: Başakşehir Cam and Sakura City Hospital EthİcaI Board (Date: 26.06.2024 ; No: E-96317027-514.10-25144424)
**Author Disclosures:**
Serçin Ozkök: Nothing to disclose
Berk Tütüncüoğlu: Nothing to disclose
Taha Yiğid Kılıç: Nothing to disclose
Fadime Köse: Nothing to disclose
Kemal Burak Bekaroğlu: Nothing to disclose
Eitibar Ismailov: Nothing to disclose
Yusuf Bahtiyar Uluman: Nothing to disclose
Tuba Banaz: Nothing to disclose
Mehmet Kadıoğlu: Nothing to disclose

**A fasting-mimicking program improves cardiac diastolic function in patients with Type 2 Diabetes Mellitus: a comprehensive MRI study**
*R. Chen*, J. Westenberg, E. van den Burg, M. Schoonakker, S. Cessie, H. Pijl, H. J. Lamb; Leiden/NL
*(r.chen@lumc.nl)*

**Purpose:** Fasting-mimicking diet (FMD) has shown positive effects in reducing risk factors for diabetes, cardiovascular disease, and aging. However, it is unclear whether FMD improves cardiovascular function. We evaluated the effects of periodic use of a five-day FMD each month on left ventricular (LV) geometry and function in patients with type 2 diabetes mellitus (T2DM).
**Methods or Background:** The study involved 100 patients (aged 63±8 years) with T2DM treated in primary care. We compared patients who received the FMD for five consecutive days each month as adjunct to usual care with those who received usual care alone as controls over twelve months. Magnetic resonance imaging(MRI) data was acquired on a 3.0-T MRI scanner at baseline, six months, and twelve months. LV geometry and systolic function were assessed from cine short-axis images, while diastolic function was analyzed using trans mitral flow curves from 4D flow MRI.
**Results or Findings:** Intention-to-treatment analysis, using linear mixed model, demonstrated FMD cycles had a significant effect on LV mass (from 116.6±23.6 to 115.4±25.5g for FMD vs. 109.6±27.1 to 112.7±27.9g for controls, p=0.022) at six months. The early-to-late peak filling ratio (E/A) increased at twelve months, from 0.87±0.25 to 1.03±0.33 for FMD, versus 0.89±0.24 to 0.88±0.26 for controls, p=0.006). Additionally, the mitral annulus through-plane velocity at early peak filling (e’) also increased at twelve months, from 5.8±2.1 to 6.0±1.8 cm/s for FMD versus a decline from 6.4±1.7 to 5.5±1.7 cm/s for controls, indicating improved diastolic function.
**Conclusion:** Fasting mimicking diet cycles over 12 months have a beneficial effect on LV mass, and improve LV diastolic function in T2DM patients.
**Limitations:** This study lasted twelve months, which limits the ability to evaluate long-term effects of treatment on cardiovascular health.
**Funding for this study:** Health~Holland Top Sector Life Sciences & Health the Dutch Diabetes Foundation and L-Nutra
**Has your study been approved by an ethics committee?** Yes
**Ethics committee - additional information:** The Medical Research Ethics Committee of Leiden University Medical Centre approved the protocol and amendments.
**Author Disclosures:**
Elske Van Den Burg: Nothing to disclose
Ruixin Chen: Nothing to disclose
Marjolein Schoonakker: Nothing to disclose
Jos Westenberg: Nothing to disclose
Saskia Cessie: Nothing to disclose
Hildo J. Lamb: Consultant: Philips
Hanno Pijl: Nothing to disclose

**Impact of left ventricular dilatation on left ventricular function and deformation in diabetes mellitus with reduced ejection fraction: a CMR feature tracking study**
*W-R. Li*, R. Shi, J-K. Li, H. Xu, Y. Li, Z-G. Yang; Chengdu/CN
*(liwenrong2023@163.com)*

**Purpose:** Left ventricular (LV) dilatation has been found to be associated with poor prognosis in patients with reduced left ventricular ejection fraction (LVEF). However, the relationship between LV dilatation and LV myocardial dysfunction in patients with diabetes mellitus (DM) and reduced LVEF unclear.
**Methods or Background:** Patients who underwent CMR scanning in our hospital for clinically diagnosed DM between 2017 and 2024. According to LVEF and LV dilatation status, these patients were divided: DM with preserved LVEF (DMpEF) group, DM with reduced LVEF and no LV dilatation (DMrEF-NLVD) group, and DM with dilated cardiomyopathy (DMrEF-DCM) group. LV global radial strain (LVGRS), LV global circumferential strain (LVGCS), and LV global longitudinal strain (LVGLS) were evaluated and compared among the three groups. Additionally, multiple linear regression analysis was performed to assess the independent effect of LV dilatation on LV strains in DM patients with reduced LVEF.
**Results or Findings:** 122 DMpEF group, 51 DMrEF-NLVD group, and 92 DMrEF-DCM group were included. Significant differences were observed in LV strain parameters among the three groups. LV global strains progressively declined from the DMpEF group to the DMrEF-NLVD group and further to the DMrEF-DCM group (all p < 0.001). In DM patients with reduced LVEF, multivariable linear regression analysis revealed that LV dilatation was independently associated with reduced LVGCS (β = 0.176, p = 0.009).
**Conclusion:** In DM patients with reduced LVEF, LV dilatation has an additive deleterious effect on LV dysfunction. LV dilatation was found to be associated with impaired LVGCS. These findings support that early identification and initiation of treatment for LV dilatation in these patients may improve risk stratification in this high-risk population.

**Limitations:** The retrospective study design limits the ability to evaluate temporal changes in cardiac structure and function.
**Funding for this study:** No funding was received for this study.
**Has your study been approved by an ethics committee?** Not applicable
**Author Disclosures:**
Yuan Li: Nothing to disclose
Rui Shi: Nothing to disclose
Huayan Xu: Nothing to disclose
Jia-Ke Li: Nothing to disclose
Wen-Rong Li: Nothing to disclose
Zhi-Gang Yang: Nothing to disclose

**Cardiac CT vs Cardiac MR for Left Ventricular Function and Myocardial Strain Evaluation: Preliminary Results from the CT-STEMI Study**
*D. Verna*[1], G. Argento[1], T. D'Angelo[2], M. Olivieri[3], L. Blasi[1], M. Moretti[1], R. Aroasio[1], R. Faletti[4], M. Gatti[1]; [1]Turin/IT, [2]Messina/IT, [3]Chieti/IT, [4]Candiolo/IT
*(daniele.verna96@gmail.com)*

**Purpose:** To evaluate the concordance between cardiac computed tomography (CCT) and cardiac magnetic resonance (CMR) in the assessment of left ventricular (LV) function and global myocardial strain (GLS, GCS, GRS) in post-STEMI patients.
**Methods or Background:** Forty-eight patients (mean age 62 ± 11 years, 87.5% male) were prospectively enrolled in the multicenter CT-STEMI study (NCT05941585). Patients were randomized to undergo either CCT or CMR within 6.8 ± 1.8 days after STEMI, followed by the complementary exam within 5.3 ± 2.8 days. LV volumes and function (LVEDV, LVESV, LVEF, LV mass) and global strain indices (GLS, GCS, GRS) were derived using CVI42 software (Circle Cardiovascular Imaging).
**Results or Findings:** CCT demonstrated excellent concordance with CMR for LV function: LVEF (45 ± 10.9% vs. 46 ± 11.0%, p = 0.40), LVEDV (167 ± 38 ml vs. 171 ± 38 ml, p = 0.16), and LVESV (93 ± 33 ml vs. 94 ± 32 ml, p = 0.58). Strain analysis showed good concordance, with strong correlations for GLS (r = 0.74; bias −0.022) and GRS (r = 0.82; bias 0.014), and moderate concordance for GCS (r = 0.65; bias −0.001).
**Conclusion:** CCT provides high concordance with CMR in the evaluation of LV volumes and function, and good concordance for global myocardial strain, particularly GLS and GRS. These results support CCT as a valid alternative to CMR for comprehensive functional assessment of left ventricular performance.
**Limitations:** Despite its prospective, multicenter design, this preliminary analysis is limited by small sample size and predominant use of a single scanner. Planned expansion to 200 patients, inter-scanner validation, assessment of observer variability, and correlation with prognostic endpoints will strengthen the clinical applicability of these findings.
**Funding for this study:** This research was supported by a grant from the Italian Ministry of Health under the “Ricerca Finalizzata 2021 – Giovani Ricercatori” program, project number GR-2021-12372092. The funding was allocated to the project titled “Cardiac Computed Tomography for Comprehensive Risk Stratification of Arrhythmic, Atherothrombotic, and Heart Failure Events Following Reperfused ST-Segment Elevation Myocardial Infarction”. The funder had no role in the design, data collection, analysis, or interpretation of this study
**Has your study been approved by an ethics committee?** Yes
**Ethics committee - additional information:** No additional information
**Author Disclosures:**
Giacomo Argento: Nothing to disclose
Marco Gatti: Nothing to disclose
Ludovica Blasi: Nothing to disclose
Riccardo Faletti: Nothing to disclose
Marta Moretti: Nothing to disclose
Tommaso D'Angelo: Nothing to disclose
Marzia Olivieri: Nothing to disclose
Daniele Verna: Nothing to disclose
Roberta Aroasio: Nothing to disclose

**14:00 - 15:30** **Research Stage 3**

### Research Presentation Session: Artificial Intelligence and Imaging Informatics

### RPS 2005

### How large language models are transforming radiological reporting

**Moderator**
Martin Segeroth; Basel/CH
*(martin.segeroth@gmail.com)*

**Multicenter Clinical Trial on the Application of a Self-Developed AI-Assisted Detection Software for Intracranial Aneurysms on MRA Images**
Y. Bao, *X. Cao*, Q. Zhao, X. Zhao, B. Huang, Y. Luo, Z. Zheng, W. Liu, D. Geng; Shanghai/CN
*(13262566515@163.com)*

**Purpose:** Our team has independently developed AIneurysm, a computer-aided detection software for TOF-MRA, which has obtained the Class III Medical Device Registration Certificate in China. By accurately segmenting the cerebral arteries, it assists physicians in detecting aneurysm lesions. The multicenter clinical trial was subsequently launched to rigorously validate its diagnostic performance.
**Methods or Background:** This study is a prospective, multicenter, fully-crossed multi-reader multi-case trial of the AIneurysm, conducted from December 2024 to November 2027. Five medical centers are prospectively gathering cranial MRA data, targeting enrollment of 1,050 cases. Each center assigned two junior radiologists as the participating readers. They conducted two rounds of full-slice reading for all images in their respective centers: a physician-independent group (control) and an AI-assisted group (experimental). The presence or absence of aneurysms, as well as the location of the lesions and the maximum diameter of the aneurysms, were recorded. The consensus reference standard was established by three senior radiologists. The Dorfman-Berbaum-Metz-Hillis analysis was used to compare the area under the alternative free-response receiver operating characteristic (AFROC) curves, sensitivity, and specificity between the two groups.
**Results or Findings:** A total of 484 cases with 258 lesions have been enrolled until now. The difference in AFROC AUC between the experimental and control groups was 0.0409 (P=0.001, 95%CI:0.020–0.062), indicating superiority of the experimental group. The experimental arm outperformed the control arm with an AFROC AUC gain of 0.0409 (P=0.001, 95%CI:0.020–0.062). Lesion-level sensitivity improved by 0.070 (P=0.010, 95%CI:0.036–0.104), surpassing the predefined superiority threshold, while case-level specificity rose by 0.012 (F=0.457, P=0.010, 95%CI:0.016–0.040), satisfying the non-inferiority criterion.
**Conclusion:** The interim results show that the AI-assisted detection software AIneurysm for intracranial aneurysms on MRA images demonstrated superior diagnostic performance compared to independent reading by junior physicians.
**Limitations:** Omitted.
**Funding for this study:** Science and Technology Commission of Shanghai Municipality (24SF1904200).
**Has your study been approved by an ethics committee?** Yes
**Ethics committee - additional information:** Ethics Committees of the five medical centers: Huashan Hospital Institutional Review Board, Ethics Committee of Longhua Hospital affiliated to Shanghai University of Traditional Chinese Medicine, Medical Ethics Committee of Shanghai Pudong New Area Gongli Hospital, Medical Ethics Committee of Shanghai Fifth People's Hospital Affiliated to Fudan University, and Medical Ethics Committee of Shanghai Fourth People's Hospital.
**Author Disclosures:**
Qiufeng Zhao: Nothing to disclose
Yifang Bao: Nothing to disclose
Daoying Geng: Nothing to disclose
Zhiji Zheng: Author: Fuying (Shanghai) Medical Technology Co., Ltd.
Yu Luo: Nothing to disclose
Xiaohu Zhao: Nothing to disclose
Wanchen Liu: Nothing to disclose
Xin Cao: Nothing to disclose
Bingcang Huang: Nothing to disclose

**Report-Driven Segmentation: Zero-Shot LLM Extraction of SUVmax and Lesion Location from PET/CT for automatic tumor segmentation**
S. Warmer, L. Umutlu, J. Haubold, Y. Wen, C. S. Schmidt, *C. Bojahr*, K. A. Borys, F. Nensa, R. Hosch; Essen/DE

**Purpose:** Standardized uptake value (SUV) metrics, particularly SUVmax, quantify PET/CT radiotracer uptake, which is relevant for tumor identification and detailing. However, this condensed critical information is often hidden in radiological reports. We evaluate a large language model (LLM) for extracting SUVmax values with corresponding anatomical sites and examine its impact on initiating automated tumor segmentation.
**Methods or Background:** We selected PET/CT reports from 100 patients (female=38, 66±9.93 years, NSCLC=97, SCLC=3) diagnosed with lung cancer between 2006 and 2020, each performed within ±30 days of initial diagnosis. The reports were analyzed using a 70B LLaMA 3.3 instruct model with zero-shot prompting for extracting SUVmax values including its corresponding body region. Radiological experts evaluated the extracted data using a questionnaire to assess whether the SUVmax values and locations were correctly extracted. In a subsequent use case, the extracted SUVmax values and location were used to define a seed coordinate for initializing automatic tumor segmentation using the Body and Organ Analysis (BOA) and the nnInteractive. A radiologist then evaluated the resulting segmentation masks case by case.
**Results or Findings:** The LLM accurately extracted SUVmax values and their corresponding locations in 97% of cases, demonstrating high consistency across report styles and time periods. The automatically generated segmentation masks based on the extracted coordinates and values were clinically usable without modification in 70% of the cases.
**Conclusion:** This study demonstrates that LLMs accurately extract SUVmax and anatomical context from unstructured PET/CT reports. The structured output enabled automated tumor segmentation, underscoring the potential of LLMs as integral components in clinical segmentation pipelines.
**Limitations:** The limitations of the study are the small sample size, single tumor type focus, an imbalance between NSCLC and SCLC cases, and the use of one LLM with limited prompt strategies.
**Funding for this study:** No funding was provided for this study.
**Has your study been approved by an ethics committee?** Yes
**Ethics committee - additional information:** This study was performed in adherence to all guidelines defined by the approving institutional review board of the investigating hospital. The Institutional Review Board waived written informed consent due to the retrospective nature of the study. Complete anonymization of all data was performed before inclusion in the study.
**Author Disclosures:**
Christian Bojahr: Nothing to disclose
Sina Warmer: Nothing to disclose
Katarzyna Anna Borys: Nothing to disclose
Johannes Haubold: Nothing to disclose
Yutong Wen: Nothing to disclose
Lale Umutlu: Nothing to disclose
Cynthia Sabrina Schmidt: Nothing to disclose
René Hosch: Nothing to disclose
Felix Nensa: Nothing to disclose

**Large language models-based simplification of Breast Imaging Reports: A prospective multicentric study**
V. Magni[1], *M. Pavan*[1], A. Cozzi[2], A. Liguori[1], F. Pesapane[1], S. Carriero[1], G. Carrafiello[1]; [1]Milan/IT, [2]Lugano/CH
*(matilde.pavan@unimi.it)*

**Purpose:** To evaluate patients' perception of breast imaging reports simplified by ChatGPT-4 compared to radiologist-written reports, focusing on simplicity, comprehensibility, and empathy, and to assess the role of educational level in shaping preferences.
**Methods or Background:** This prospective multicenter study included 10 anonymized mammography and ultrasound reports (BI-RADS 1–5), each simplified by ChatGPT-4 with a 50-word limit. Report pairs (original vs. AI-simplified) were assessed by 300 patients (2965 responses) and 20 physicians using a Likert-scale questionnaire on simplicity, comprehensibility, and empathy. Preferences and demographic data were collected, and logistic regression analyzed factors influencing choices.
**Results or Findings:** AI-generated reports were preferred in 63.3% of responses. They scored higher for simplicity (69.7% levels 4–5), comprehensibility (67.8% levels 4–5), and empathy (predominantly levels 3–4). Higher scores in all three domains significantly increased the likelihood of AI preference. Participants with advanced education (Bachelor's/Master's degrees) showed a stronger inclination toward AI-simplified reports. Physicians confirmed the clinical accuracy and safety of AI outputs.
**Conclusion:** ChatGPT-4 can generate simplified breast imaging reports that patients perceive as clearer, more comprehensible, and more empathetic than traditional versions. This approach may enhance patient understanding and engagement, while maintaining accuracy. Broader validation in different languages, clinical contexts, and AI platforms is warranted.
**Limitations:** Only 10 report pairs were tested, all in Italian, and only ChatGPT-4 was evaluated. More complex BI-RADS categories remain challenging for AI simplification. These factors may limit generalizability.
**Funding for this study:** This study received no external funding. Institutional resources from participating centers supported the project.
**Has your study been approved by an ethics committee?** Not applicable
**Author Disclosures:**
Andrea Cozzi: Nothing to disclose
Alessandro Liguori: Nothing to disclose
Serena Carriero: Nothing to disclose
Filippo Pesapane: Nothing to disclose
Veronica Magni: Nothing to disclose
Matilde Pavan: Nothing to disclose
Gianpaolo Carrafiello: Nothing to disclose

**RAG across scales: A multi-backbone comparison of guideline-grounded LLM-agent sequential decision-making for ED acute abdominal pain**
*R. Andre*, H. E. Huisman; Nijmegen/NL
*(romain.andre@radboudumc.nl)*

**Purpose:** Assess how guideline-grounded retrieval-augmented generation (RAG) improves diagnostic performance and sequential imaging/laboratory request-behavior of LLM-agents across backbone sizes (1B-70B) and domain-specific trainings (general/biomedicine) for emergency-department acute abdominal pain pathologies.
**Methods or Background:** Using the MIMIC-IV-Ext Clinical-Decision-Making dataset (2,400 ED pathways: appendicitis, cholecystitis, diverticulitis, pancreatitis, sharing acute abdominal pain as initial symptom), we compared seven instruction-tuned backbones: Llama-3.2-1B, Mistral-7B-v0.3, Gemma-2-9B, Llama-3.1-8B-UltraMedical, Qwen3-30B, Llama-3.1-70B, Llama-3.1-70B-UltraMedical, spanning both generalist and biomedical-fine-tuned models. LLM-Agents iteratively requested physical-examination, laboratory tests, or imaging (modality and region), received the corresponding reports, then autonomously finalized once judged sufficient evidence is retrieved, issuing a diagnosis and care plan without assistance. With RAG, guidelines snippets were retrieved from a maintainable, disease-scoped knowledge-base at each thinking step and appended to the working context before each action, grounding the iterative process in citable, expert-authored sources.
**Results or Findings:** RAG improved average diagnostic accuracy across every backbone. Relative gains were most notable for smaller models (1-9B: from 46.5% to 55.1%), larger models (30-70B) also improved (67.4% to 72.8%). RAG reduced requests for non-existent tools (i.e. hallucinations), while increasing alignment of imaging orders with clinician trajectories and guideline indications, and maintained disciplined laboratory selection. RAG-equipped agents gathered more evidence before finalization and specified imaging parameters (modality/region) more consistently. Overall, RAG enhanced transparency by surfacing citable guidance throughout the decision chain.
**Conclusion:** Across seven backbones from 1B to 70B, including both generalist and biomedical-tuned models, guideline-grounded RAG consistently improves diagnostic accuracy and imaging decision behavior, supporting safer, more auditable LLM assistance for ED acute abdominal pain.
**Limitations:** Work limited to the ER domain, focusing only on four pathologies from a single-centre, English-language dataset. Only open-weight models were explored to respect MIMIC-IV's data use agreement. Prospective, multi-institutional validation and broader symptom coverage are needed.
**Funding for this study:** This study is part of the HealthyAI project with number KICH3.LTP.20.006 of the research programme KIC which is (partly) financed by the Dutch Research Council (NWO) and with co-funding by Siemens Healthineers.
**Has your study been approved by an ethics committee?** Not applicable
**Author Disclosures:**
Henkjan En Huisman: Nothing to disclose
Romain Andre: Nothing to disclose

**MumbleMED: Introducing a Framework for Fine-Tuning Medical Speech Transcription in Radiology Utilising Large Language and Text-to-Speech Models**
*S. Warmer*, A. Idrissi-Yaghir, K. A. Borys, J. Haubold, C. S. Schmidt, Y. Wen, K. Arzideh, F. Nensa, R. Hosch; Essen/DE

**Purpose:** General-purpose speech recognition models, such as OpenAI's Whisper, struggle with the complex terminology and structure of medical language, which limits their use in radiology. We present MumbleMED, a domain-adapted open-source speech-to-text model fine-tuned using a pipeline that combines large language models (LLMs) and text-to-speech (TTS) to generate high-quality medical training data.
**Methods or Background:** Synthetic medical German texts were created by random sampled structured concepts from ICD, SNOMED CT, and RadLex using Qwen3-235B. These texts were converted to audio using a TTS engine featuring 17 distinct speakers (female=47%) from our institution. The resulting dataset of 13689 samples (4530 minutes of total audio time) was used to fine-tune OpenAI's Whisper model (V2-large), resulting in MumbleMED, a medical variant in German language. Model performance was evaluated on a test set of 450 synthetic samples and 97 real radiology report dictations using Word Error Rate (WER), Character Error Rate (CER), and compared to the unmodified Whisper baseline.
**Results or Findings:** MumbleMED achieved a WER of 3.73% and CER of 1.97% on the synthetic test set, outperforming the baseline Whisper model (WER=20.33%, CER=11.65%). On real radiology reports, MumbleMED (WER=39.78%, CER=17.92%) also outperformed Whisper (WER=70.65%, CER=46.88%), showing strong recognition of medical domain-specific vocabulary and typical terminology of German radiology reports.
**Conclusion:** MumbleMED shows that LLM- and TTS-based synthetic data can effectively fine-tune speech-to-text models for clinical use. The approach enables more accurate and reliable transcription of radiological dictations, reducing the need for manual correction and supporting faster, streamlined reporting workflows. In addition, this pipeline can be used to fine-tune the model for any language in the medical domain.
**Limitations:** The limitations of the study are the synthetic training data and limited speaker variations, which may not fully capture spontaneous or accented speech patterns.
**Funding for this study:** No funding was provided for this study.
**Has your study been approved by an ethics committee?** Yes
**Ethics committee - additional information:** This study was performed in adherence to all guidelines defined by the approving institutional review board of the investigating hospital. The Institutional Review Board waived written informed consent due to the retrospective nature of the study. Complete anonymization of all data was performed before inclusion in the study.
**Author Disclosures:**
Sina Warmer: Nothing to disclose
Katarzyna Anna Borys: Nothing to disclose
Johannes Haubold: Nothing to disclose
Yutong Wen: Nothing to disclose
Kamyar Arzideh: Nothing to disclose
Cynthia Sabrina Schmidt: Nothing to disclose
René Hosch: Nothing to disclose
Ahmad Idrissi-Yaghir: Nothing to disclose
Felix Nensa: Nothing to disclose

### Evaluating Large Language Models for FHIR-Compatible Structured Reporting from Kidney Stone CT Reports

*P. Arnold*, E. Kotter, J. Jahn; Freiburg Im Breisgau/DE
*(philipp.arnold@uniklinik-freiburg.de)*

**Purpose:** To assess whether large language models (LLMs) can convert free-text kidney stone CT reports into standardized HL7 FHIR Questionnaire format and to compare performance across input styles and model sizes.
**Methods or Background:** We collected 99 German abdominal CT reports (50 free-text, 49 semi-structured with section headings). A kidney stone FHIR Questionnaire with 33 key fields was derived from a published consensus template. Three locally hosted Qwen models (8B, 14B, 32B parameters) were prompted field by field to generate FHIR QuestionnaireResponses, which were compared with a radiologist-annotated ground truth. Metrics included ground-truth completeness (proportion of fields present in the source report), AI completeness (proportion of ground-truth fields correctly retrieved by the model), and per-field accuracy (exact or semantically equivalent match across all fields).
**Results or Findings:** Semi-structured source reports contained more of the expected information (ground-truth completeness 77% vs. 65% for free text). Across all models, both accuracy and AI completeness were higher for semi-structured inputs. Qwen-32B achieved 93% per-field accuracy and 97% AI completeness on semi-structured reports (vs. 82% and 92% on free text). The 14B model reached 91% accuracy and 95% AI completeness (vs. 83%/94%), while the 8B model achieved 83%/95% (vs. 69%/87%).
**Conclusion:** LLMs can automatically generate FHIR-compliant structured kidney stone CT reports from textual input with high accuracy. Semi-structured reports yield higher accuracy and completeness. The approach supports prospective workflows, where radiologists dictate freely while an LLM drafts a structured report for rapid review, as well as retrospective extraction of structured data from existing reports.
**Limitations:** Single-center study focused on one exam type; performance on more heterogeneous imaging reports remains to be validated.
**Funding for this study:** German Research Foundation (DFG) - SFB 1597–499552394. Hans A. Krebs Programme (University Clinic Freiburg im Breisgau)
**Has your study been approved by an ethics committee?** Yes
**Ethics committee - additional information:** Ethics Vote register number: FRKS004287
**Author Disclosures:**
Philipp Arnold: Nothing to disclose
Johannes Jahn: Nothing to disclose
Elmar Kotter: Nothing to disclose

### Evaluating the Accuracy of Privacy-Preserving Large Language Models in Calculating the Spinal Instability Neoplastic Score (SINS)

*L. Y. T. Chan*, D. Z. M. Chan, Y. L. Tan, Q. V. Yap, W. Ong, A. Lee, J. H. Tan, N. Kumar, J. T. P. D. Hallinan; Singapore/SG

**Purpose:** In diagnostic radiology, LLMs can assist in the computation of the Spine Instability Neoplastic Score (SINS), which is a critical tool for assessing spinal metastases. However, the accuracy of LLMs in calculating the SINS based on radiological reports remains under-explored. This study evaluates the accuracy of two institutional privacy-preserving LLMs - Claude 3.5 and Llama 3.1 - in computing the SINS from radiology reports and electronic medical records.
**Methods or Background:** A retrospective analysis was conducted on 124 radiology reports from patients with spinal metastases. Three expert readers established a reference standard for the SINS calculation. Two orthopaedic surgery residents and two LLMs (Claude 3.5 and Llama 3.1) independently calculated the SINS. The intraclass correlation coefficient (ICC) was used to measure the inter-rater agreement for the total SINS, while Gwet's Kappa was used to measure the inter-rater agreement for the individual SINS components.
**Results or Findings:** Both LLMs and clinicians demonstrated almost perfect agreement with the reference standard for the total SINS. Between the two LLMs, Claude 3.5 (ICC = 0.984) outperformed Llama 3.1 (ICC = 0.829). Claude 3.5 was also comparable to the clinician readers with ICCs of 0.926 and 0.986, exhibiting a near-perfect agreement across all individual SINS components [0.919–0.990].
**Conclusion:** Claude 3.5 demonstrated high accuracy in calculating the SINS and may serve as a valuable adjunct in clinical workflows, potentially reducing clinician workload while maintaining diagnostic reliability. However, variations in LLM performance highlight the need for further validation and optimisation before clinical integration.
**Limitations:** The training of LLMs was performed with only one prompt strategy, and performance may vary with alternative prompt methods. The evaluation of LLMs were assessed at a single time point using a single institution dataset, limiting conclusions about reproducibility and generalisability.
**Funding for this study:** Direct Funding from MOH/NMRC. This research is supported by the Singapore Ministry of Health National Medical Research Council under the NMRCClinician Innovator Award (CIA). Grant Title: Deep learning pipeline for augmented reporting of MRI whole spine (CIAINV23jan-0001, MOH-001405)
**Has your study been approved by an ethics committee?** Not applicable
**Ethics committee - additional information:** Ethical review and approval were waived for this study as it was granted a Domain-Specific Review Board waiver owing to minimal risk
**Author Disclosures:**
Qai Ven Yap: Nothing to disclose
Yi Liang Tan: Nothing to disclose
Naresh Kumar: Nothing to disclose
James Thomas Patrick Decourcy Hallinan: Grant Recipient: This research was funded as follows: Direct Funding from MOH/NMRC. This research is supported by the Singapore Ministry of Health National Medical Research Council under the NMRC Clinician Innovator Award (CIA). Grant Title: Deep learning pipeline for augmented reporting of MRI whole spine (CIAINV23jan-0001, MOH-001405).
Li Yi Tammy Chan: Nothing to disclose
Aric Lee: Nothing to disclose
Jiong Hao Tan: Nothing to disclose
Ding Zhou Matthew Chan: Nothing to disclose
Wilson Ong: Nothing to disclose

### Empowering radiology training with AI: Large Language Models in resident error detection and feedback

*A. Kyling*, M. Salinas, G. Briceño, C. Pizarro, P. F. Guzman, D. Ladrón de Guevara; Santiago/CL
*(akylingd@gmail.com)*

**Purpose:** The implementation of artificial intelligence in healthcare is advancing globally. Automated analysis of errors in radiology reports could provide objective and personalized feedback, supporting staff radiologists' teaching, optimizing resident training, and improving radiological care quality.

**Methods or Background:** This observational, retrospective study analyzed 213 paired radiology reports (CT, MRI, US) from first- and second-year residents, validated by staff radiologists. Reports were anonymized and processed by a LLM (Gemini-3-Pro) to classify errors (structural, semantic, diagnostic) and measure textual concordance via cosine-similarity embeddings. A random subset of 40 report pairs (18%) underwent independent senior radiologist validation.
**Results or Findings:** 213 report pairs plus 40 controls were analyzed. Median cosine similarity was 0.99 for error-free reports and 0.91 for those with ≥5 diagnostic errors; 0.90 served as discriminant threshold (specificity 100%, sensitivity 13.1%). Spearman correlation between similarity and diagnostic error count was ρ=–0.53 (p <0.001). Controls (deliberately erroneous or incoherent) consistently scored <0.85. Diagnostic errors averaged 2.10±1.43 per report (85.5% had ≥1 error), with omission/false negatives comprising 67.8%. Triaxial-qualitative classification showed poor reliability for error detection (Kappa 0.04 at threshold 0.90), but acceptable reliability for ruling out error-free reports (Kappa 0.36 at threshold 0.99). Manual validation achieved 100% concordance (40/40).
**Conclusion:** The LLM reliably identified discrepancies and graded severity (ρ=–0.53). While not robust for error detection, it showed acceptable reliability in validating error-free reports, suggesting its potential as an educational support tool, particularly for targeting omission errors which comprised the majority of diagnostic discrepancies.
**Limitations:** Single-center, limited sample size, risk of hallucinations, and reliance on staff reports as reference standard constrain generalizability, highlighting the need for larger, multicenter, validation studies.
**Funding for this study:** This research received no external funding and was conducted without dedicated financial support.
**Has your study been approved by an ethics committee?** Yes
**Ethics committee - additional information:** Approved by the Ethics Committee of Hospital San Juan de Dios, Santiago, Chile.
**Author Disclosures:**
Marcelo Salinas: Nothing to disclose
David Ladrón De Guevara: Nothing to disclose
Gonzalo Briceño: Nothing to disclose
Alberto Kyling: Nothing to disclose
Cristobal Pizarro: Nothing to disclose
Patricia Falcon Guzman: Nothing to disclose

### Beyond Evans Index: CT Ventricular Nomograms with 12 Sub-compartments from an LLM-Curated Cohort with nnU-Net Segmentation

*N. Vishwanathan*, S. Griot, J. Wasserthal, S. Yang, M. Segeroth, J. M. Lieb, M. Bach, M-N. Psychogios, M. A. Mutke; Basel/CH
*(nathan.vishwanathan@usb.ch)*

**Purpose:** To build age- and sex-specific CT ventricular volume nomograms (12 subcompartments) and show how they can be used in everyday reporting.
**Methods or Background:** Single-centre retrospective study, 2019–2024. A locally run, German-tuned large language model screened head-CT reports and brief clinical summaries to exclude stroke, mass lesions, relevant white-matter disease, dementia/MCI, or cognitive decline. The final “normal” cohort was 3,086 examinations from 2,964 adults (14–98 years). Ventricular volumes were segmented into 12 subcompartments with an nnU-Net model. We report decade- and sex-stratified values, model performance with 95% CIs, false-discovery correction for multiple tests, and effect sizes.
**Results or Findings:** LLM labelling accuracy across diagnostic categories was 0.966–0.992; for enlarged ventricles 0.992 (95% CI 0.978–0.997). Segmentation achieved median Dice 0.918 (95% CI 0.910–0.923). Total ventricular volume rose with age, with a clear step from 50–59 to 60–69: +33.9% in females and +49.4% in males. Most subcompartments showed moderate to strong age correlations (r=0.44–0.68), while the fourth ventricle changed little (r=0.02). Male volumes were 8–19% higher than female volumes after correction. We provide percentile nomograms (5th–95th) with 95% CIs and decade means/SDs for each subcompartment and sex.
**Conclusion:** A simple, on-premises LLM + nnU-Net workflow can curate large CT datasets and produce reliable ventricular nomograms. These charts make reporting more objective: a scan can be flagged when a volume is at or above the 95th percentile for the patient’s age and sex, supporting earlier recognition of disproportionate dilatation and focused clinical follow-up.
**Limitations:** Single-centre, retrospective Swiss cohort, limited generalisability; no external validation. Cross-sectional nomograms; extreme ages (≥90 y) have wide CIs; no longitudinal/test–retest data. nnU-Net performance validated internally only; no multi-centre benchmark.
**Funding for this study:** This study received no external funding.
**Has your study been approved by an ethics committee?** Yes
**Ethics committee - additional information:** Ethics waiver was granted.
**Author Disclosures:**
Jacob Wasserthal: Author: Parts of this work were presented as an EPOS poster at ECR 2025 (C-28177); the current submission expands the previous work.
Michael Bach: Author: Parts of this work were presented as an EPOS poster at ECR 2025 (C-28177); the current submission expands the previous work.
Matthias Anthony Mutke: Author: Parts of this work were presented as an EPOS poster at ECR 2025 (C-28177); the current submission expands the previous work. Author: Parts of this work were presented as an EPOS poster at ECR 2025 (C-28177); the current submission expands the previous work.
Marios-Nikos Psychogios: Author: Parts of this work were presented as an EPOS poster at ECR 2025 (C-28177); the current submission expands the previous work. Author: Parts of this work were presented as an EPOS poster at ECR 2025 (C-28177); the current submission expands the previous work.
Nathan Vishwanathan: Author: Parts of this work were presented as an EPOS poster at ECR 2025 (C-28177); the current submission expands the previous work.
Martin Segeroth: Author: Parts of this work were presented as an EPOS poster at ECR 2025 (C-28177); the current submission expands the previous work.
Stephanie Griot: Author: Parts of this work were presented as an EPOS poster at ECR 2025 (C-28177); the current submission expands the previous work.
Johanna Maria Lieb: Author: Parts of this work were presented as an EPOS poster at ECR 2025 (C-28177); the current submission expands the previous work.
Shan Yang: Author: Parts of this work were presented as an EPOS poster at ECR 2025 (C-28177); the current submission expands the previous work.

### Towards speech assistants in reporting workflows: interoperability challenges of structured reporting, speech recognition and large language models

*B. Kämpgen*[1], G. Arnhold[2], J. Stöckmann[3], I. Schmittel[1], F. Jungmann[2], D. Feiler[3], D. Pinto dos Santos[2], P. Mildenberger[2], T. Jorg[2]; [1]Würzburg/DE, [2]Mainz/DE, [3]Munich/DE
*(benedikt.kaempgen@empolis.com)*

**Purpose:** Speech-based dialogue systems for structured reporting (SR) promise to improve both reporting quality and efficiency (Jorg et al., 2023, https://doi.org/10.1186/s13244-023-01392-y). For studies involving multiple templates, however, the system must be seamlessly integrated into radiologists’ workflows. This reveals interoperability challenges between structured reporting, speech recognition and large language model components.
**Methods or Background:** We designed an architecture with defined interfaces connecting an open-source structured reporting tool (Dos Santos et al., 2017, https://doi.org/10.1007/s00330-016-4344-0), a commercial speech recognition system (DFC-SYSTEMS), and a commercial large language model (Empolis). The reporting tool is launched from the RIS, where an appropriate SR template (e.g., for urolithiasis) is automatically selected based on the examination. A "speech assistant" button opens a chat window, allowing the user to answer template-guided questions via microphone. At any point, particularly when the system has no further questions, the user may accept or reject the prefilled SR template. This workflow minimises look-away interruptions and maximises efficiency gains in structured reporting.
**Results or Findings:** The application captures audio from the microphone, performs server-based speech-to-text conversion, and forwards the text to a server for text-to-structure processing. Integration across components is achieved via a unified JSON data model, which stores the iteratively completed template and message context/history. Functionality and integration tests demonstrated full vocabulary mapping across the template, speech recognition, and language model components.
**Conclusion:** Speech-based structured reporting, supported by large language models, is approaching clinical deployment. Our results offer valuable insights for initiatives addressing workflow integration and interoperability challenges.
**Limitations:** The limitations of the study are that it only included automatic functionality and integration testing based on fictional and anonymised cases (n > 50) but no clinical assessment of efficiency gains. A clinical study with speech assistant reporting is still in progress.
**Funding for this study:** Funding was provided by the Bundesministerium für Bildung und Forschung (BMBF), 2022-2025, grant agreement number: 16SV9045, project KIPA.
**Has your study been approved by an ethics committee?** Not applicable
**Author Disclosures:**
Gordon Arnhold: Nothing to disclose
Benedikt Kämpgen: Employee: Empolis Information Management GmbH
Jan Stöckmann: Employee: DFC-SYSTEMS GmbH
Tobias Jorg: Nothing to disclose
Florian Jungmann: Nothing to disclose
Dennis Feiler: CEO: DFC-SYSTEMS GmbH
Peter Mildenberger: Nothing to disclose
Daniel Pinto Dos Santos: Nothing to disclose
Isabel Schmittel: Employee: Empolis Information Management GmbH

**Benchmarking Large Language Models for Follow-up Recommendations After Abdominal Ultrasound**

*V. Vingiani*, R. Valletta, N. Cortellini, B. Proner, V. Corato, L. Hoxha, F. Ferro, M. Bonatti; Bolzano/IT
*(vincenzovingiani@gmail.com)*

**Purpose:** To evaluate whether large language models (LLMs) provide consistent, guideline-concordant follow-up recommendations after abdominal ultrasound (US) and to benchmark multiple systems.
**Methods or Background:** We assembled 200 simulated abdominal US cases covering liver, gallbladder/biliary tree, pancreas, spleen, kidneys, and retroperitoneum. Two expert abdominal radiologists defined the ground-truth management (no imaging follow-up, only US follow-up, or additional diagnostic work-up), including modality and timing when further imaging was indicated. Thirty cases refined prompts with GPT-4o; 170 were held out for evaluation. Seven LLMs (GPT-5, GPT-4o, GPT-4o mini, Gemini 2.5 Flash, Gemini 2.5 Pro, Claude 4 Sonnet, and DeepSeek-V3) were tested in zero-shot mode through Firefox with cache cleared and sessions restarted before each query. Stability was assessed across five independent runs. Agreement with the consensus was quantified using Cohen's κ (unweighted and weighted) and F1 scores.
**Results or Findings:** GPT-5 achieved the highest accuracy for the three management categories (no imaging follow-up, only US follow-up, or additional diagnostic work-up), 0.988; the lowest was DeepSeek-V3 with 0.829. Weighted κ ranged from 0.755 (DeepSeek-V3) to 0.964 (GPT-5). F1 scores mirrored this pattern, with GPT-5 at 0.988, followed by Gemini 2.5 Pro (0.954) and Gemini 2.5 Flash (0.948). Management-decision stability across five runs was high for all models (0.928–0.968). When further imaging was required, correct selection of the second-level modality and timing was highest for GPT-5 (accuracy 0.779) and lowest for Gemini 2.5 Flash (0.609).
**Conclusion:** LLMs can translate abdominal US reports into actionable, guideline-aligned follow-up recommendations. GPT-5 performed best overall, supporting the role of LLMs as adjunctive decision support to standardise post-ultrasound imaging decisions across healthcare settings.
**Limitations:** Synthetic, text-only cases may limit generalizability; zero-shot, browser-based testing may reduce performance in optimised deployments; evolving model versions may affect reproducibility and external validity.
**Funding for this study:** None
**Has your study been approved by an ethics committee?** Not applicable
**Author Disclosures:**
Matteo Bonatti: Nothing to disclose
Valentina Corato: Nothing to disclose
Laurjan Hoxha: Nothing to disclose
Bernardo Proner: Nothing to disclose
Riccardo Valletta: Nothing to disclose
Nino Cortellini: Nothing to disclose
Federica Ferro: Nothing to disclose
Vincenzo Vingiani: Nothing to disclose

**Feasibilty of using a Large Language Model for automated extraction of clinically relevant findings from whole-body MRI reports**

*L. Di Palma*, M. Alì, A. Lad, G. D'Anna, F. Darvizeh, I. Castiglioni, D. Fazzini; Milan/IT
*(luca.dipalma@cdi.it)*

**Purpose:** To evaluate the feasibility and accuracy of using a large language model (LLM) to extract and structure clinically relevant information from free-text whole-body MRI (WB-MRI) reports.
**Methods or Background:** This study included 327 WB-MRI reports from a preventive health screening program. Reports were processed with the DeepSeek-R1-Llama3.3 LLM to extract findings classified according to the ONCO-RADS system, including their anatomical locations. Only ONCO-RADS ≥3 findings were analyzed, as they represent suspicious or actionable abnormalities; ONCO-RADS 1–2 were excluded. LLM outputs were compared with original reports, independently reviewed and annotated by three subspecialist radiologists (neuroradiology, musculoskeletal, body imaging; >5 years' experience). Radiologists validated whether extracted ONCO-RADS scores and locations matched the reports. Discrepancies were categorized as: (1) missing findings, (2) localization errors (minor: ovary vs uterus; pleura vs lung; major: different organ), and (3) false positives.
**Results or Findings:** Out of 4,902 total findings, radiologists identified 237 as ONCO-RADS ≥3. Among these, 232 (97.9%) were categorized as ONCO-RADS 3, 3 (1.3%) as ONCO-RADS 4, and 2 (0.8%) as ONCO-RADS 5. The LLM accurately extracted 207 of these cases (87.3%) with full agreement in both classification and location. There were 30 discrepancies (12.7%), comprising 17 missed findings (7.2%) and 13 localization errors (5.5%). Of the localization errors, 11 were minor, while 2 were considered major. Additionally, LLM reported 16 false positives.
**Conclusion:** This study shows that LLMs can accurately extract clinically relevant findings from free-text WB-MRI reports, with high concordance and minimal clinically significant errors. This suggests strong potential for LLMs in supporting report structuring in radiology.
**Limitations:** Use of a single LLM for ONCO-RADS extraction may limit generalizability.
**Funding for this study:** C.D.I. Ricerca Innovazione e Sviluppo
**Has your study been approved by an ethics committee?** Yes
**Ethics committee - additional information:** Ethical committee was requested (study ID: 6309)
**Author Disclosures:**
Gennaro D'Anna: Nothing to disclose
Isabella Castiglioni: Nothing to disclose
Akash Lad: Nothing to disclose
Deborah Fazzini: Nothing to disclose
Marco Alì: Consultant: Bracco
Luca Di Palma: Nothing to disclose
Fatemeh Darvizeh: Nothing to disclose

**The utility of "thinking" in CAD-RADS scoring of an open-source hybrid-reasoning large language model**

V. Sandfort[1], D. Vigneault[1], *L. R. Koetzier*[1], M. J. Willemink[2], J. Wu[2], R. Hallett[1], K. Nieman[1], D. Fleischmann[1], D. Mastrodicasa[3]; [1]Stanford, CA/US, [2]Palo Alto, CA/US, [3]Seattle, WA/US
*(l.r.koetzier@umcutrecht.nl)*

**Purpose:** Large language models (LLMs) are developed to answer questions or follow instructions. While earlier generation LLMs gave quick, general responses, they could not perform multi-step reasoning. Reinforcement-learning has enabled "reasoning" models, such as Qwen3-235B, which operate in both "thinking" and "non-thinking" modes. Although LLMs have been evaluated for extracting information from radiology reports, the effect of "thinking" on this task remains unclear. We evaluated the effect of "thinking" in Qwen3-235B on the performance of determining CAD-RADS scores from cardiac CT-reports.
**Methods or Background:** We retrospectively included 500 de-identified cardiac CT-reports from four hospitals across three USA regions using an online platform (Segmed). CAD-RADS categories were determined in consensus by three cardiovascular imaging experts. Qwen3-235B was run in fp8-quantization via API (together.ai) in both "thinking" and "non-thinking" mode. Thinking was measured in thinking-characters between thinking-tags, divided into quintiles (Q1=least thinking; Q5=most thinking). Model performance was assessed using unweighted Cohen's kappa.
**Results or Findings:** Model performance in "thinking" mode was numerically higher than "non-thinking" mode (0.791 [0.751-0.835] vs 0.732 [0.686-0.777], respectively). In "thinking" mode, best performance was seen in Q1 (kappa=0.893) and declined with more thinking (Q5-kappa=0.452). In "non-thinking" mode, performance was highest in Q1 (kappa=0.944) and Q2 (kappa=0.901), but lowest in Q5 (kappa=0.365).
**Conclusion:** An open-source hybrid-reasoning LLM accurately determined CAD-RADS scores from cardiac CT-reports. Very long "thinking" (Q5) was associated with poor performance, suggesting it may serve as a model-confidence indicator. "Non-thinking" worked better for easy cases, while "thinking" was advantageous in difficult cases.
**Limitations:** Qwen3-235B is not intended for medical use, and our study only evaluated performance.
**Funding for this study:** None
**Has your study been approved by an ethics committee?** Not applicable
**Author Disclosures:**
Dominik Fleischmann: Shareholder: Segmed, Inc.
Koen Nieman: Nothing to disclose
Domenico Mastrodicasa: Shareholder: Segmed, Inc.
Davis Vigneault: Nothing to disclose
Veit Sandfort: Nothing to disclose
Lennart Roelof Koetzier: Nothing to disclose
Martin J. Willemink: Founder: Segmed, Inc.
Jie Wu: Founder: Segmed, Inc.
Richard Hallett: Nothing to disclose

14:00 - 15:30 Research Stage 1

### Research Presentation Session: Neuro Hot Topic with Keynote Lecture

### RPS 2011

### Hot Topic: AI-assisted neuroimaging and image analysis

**Moderator**
Antonella Castellano; Milan/IT
*(castellano.antonella@hsr.it)*

**Keynote Lecture**
Ji-Hoon Kim; Seoul/KR
*(jihnkim@gmail.com)*

### Artificial Intelligence in Target Definition for Brain Metastases: Opportunities and Challenges

*N. Snoeijink*, J. D. J. Slotman, S. Kamerbeek, I. M. Nijholt, M. F. Boomsma, E. Wiegman; Zwolle/NL
*(j.a.n.snoeijink@isala.nl)*

**Purpose:** To assess inter-observer variability of expert-based radiation target definition with and without AI assistance in gamma knife radiosurgery for brain metastases on MRI scans.
**Methods or Background:** A deep learning-based model was developed for automatic detection and delineation of brain metastases using contrast-enhanced T1-weighted (CE-T1w) and black blood (BB) MR sequences. Data from 224 consecutively treated patients were randomly split into training (n=162) and testing (n=64) sets. The AI model achieved an overall F1-score of 0.93, sensitivity of 0.88, PPV of 0.98, and a DICE score of 0.82. Subsequently, it was tested on an independent dataset consisting of 15 patients, with in total 93 brain metastases, classified as S (< 0.1 cc), M (≥0.1 cc & <1 cc), or L (≥1 cc). Four observers evaluated each case: two radiotherapists independently, AI model alone, and combination of AI with a radiotherapist able to refine results manually. Inter-observer variability was quantified using the DICE-score using clinical delineation by one of the radiotherapists as reference.
**Results or Findings:** Inter-observer variability in DICE-scores was observed among radiotherapists (0.88) and between radiotherapist and the AI model (0.73), particularly for smaller metastases (<0.1 cc). Adjustments made by the radiotherapist to the AI-generated delineations increased the DICE-score to 0.84. The largest improvement was seen for small lesions (S: 0.54 --> 0.76, M: 0.81 --> 0.86, L: 0.87 --> 0.88).
**Conclusion:** The AI model is trained on delineations from multiple radiotherapists, who do not always agree with each other. This inter-expert variability may limit AI’s ability to achieve a maximal DICE, potentially challenging effective implementation of AI in clinical practice.
**Limitations:** Small sample size
**Funding for this study:** Innovation and Science fund Isala, The Netherlands
**Has your study been approved by an ethics committee?** Not applicable
**Author Disclosures:**
Erwin Wiegman: Nothing to disclose
Jorik Derk Jan Slotman: Nothing to disclose
Nadine Snoeijink: Nothing to disclose
Martijn Franklin Boomsma: Nothing to disclose
Sander Kamerbeek: Nothing to disclose
Ingrid M. Nijholt: Nothing to disclose

### Predicting Meningioma Recurrence/Progression Based on Multiparametric MRI Intratumoral and Peritumoral Radiomics Models : A Multicenter Study

*T. Han*, J. Zhou; Lanzhou/CN
*(1817120696@qq.com)*

**Purpose:** This study aimed to evaluate the value of intratumoral and peritumoral radiomics models based on multiparametric MRI in predicting meningiomas recurrence/progression (R/P).
**Methods or Background:** A total of 623 patients from Hospital A were divided into a training set (n=294) and a test set (n=127); 202 patients from Hospital B comprised the external validation set. A Clinical-Radiological (C-R) model was developed using significant clinical/MRI features. Radiomics features were extracted from intratumoral (Intra) and peritumoral regions at 5 mm (Peri-5) and 10 mm (Peri-10). Models were constructed using Lasso and evaluated via ROC, calibration curves, and decision curve analysis (DCA). Interpretability was assessed with SHAP (SHapley Additive exPlanations) plots.
**Results or Findings:** The training set AUCs for C-R, Intra, Peri-5, and Peri-10 models were 0.820, 0.880, 0.860, and 0.840, respectively. Test set and external validation AUCs ranged from 0.820–0.910 and 0.620–0.750. The results of the DeLong test indicated that, in external validation set, the predictive performance of the Intra model was significantly superior to that of the C-R, Peri-5 mm, and Peri-10 mm models (P=0.046, P=0.024, P=0.035).
**Conclusion:** The Intra, Peri-5 mm, and Peri-10 mm radiomics models and C-R model, demonstrated good predictive performance in predicting meningioma R/P, providing a theoretical reference for formulating personalized treatment plans for meningioma patients.
**Limitations:** Firstly, it is a retrospective study. Secondly, this study only analyzed the tumor and peritumoral regions, while the predictive value of the peritumoral edema region remains to be further explored. Lastly, manual segmentation methods were used to delineate the VOI of the tumor in this study. In future studies, we aim to explore the automatic segmentation method and larger prospective datasets to improve enhance and validate the model's performance and robustness.
**Funding for this study:** This study was supported by grants of Natural Science Foundation of China (82371914).
**Has your study been approved by an ethics committee?** Yes
**Ethics committee - additional information:** This retrospective study received approval from the Ethics Review Committees of the Hospital A (2023A-169) and Hospital B (2024262K), with the need for informed consent waived.
**Author Disclosures:**
Tao Han: Nothing to disclose
Junlin Zhou: Nothing to disclose

### From MRI to MoCA - Machine Learning Models for Cognitive Impairment Prediction

*N. Zdanovskis*, K. Šneidere, K. Kalva, Z. A. Litauniece, A. Usacka, Z. Freibergs, A. Platkajis, A. Stepens; Riga/LV

**Purpose:** To assess whether machine learning algorithms can predict Montreal Cognitive Assessment (MoCA) scores from MRI morphometry in patients with cognitive impairment.
**Methods or Background:** Eighty subjects were included, with 70 used for training and 10 for testing. From structural MRI, 101 morphometric features were extracted, including cortical thickness (68 regions), subcortical volumes (20 bilateral structures), corpus callosum subdivisions (5), and global volumetric measures (8). Six supervised regression models were evaluated: Linear Regression, Random Forest, Gradient Boosting, AdaBoost, Support Vector Machine (SVM), and k-Nearest Neighbors (kNN). Performance was assessed using mean squared error (MSE), mean absolute error (MAE), and $R^2$.
**Results or Findings:** Random Forest achieved the best accuracy (MSE = 32.7, MAE = 4.55, $R^2$ = 0.655), followed by Gradient Boosting (MSE = 47.8, MAE = 5.91, $R^2$ = 0.496) and AdaBoost (MSE = 58.4, MAE = 6.80, $R^2$ = 0.384). Linear Regression and kNN showed weak predictive value ($R^2$ = 0.001 and 0.10), while SVM performed poorly ($R^2$ = –0.145). Neural Networks failed to converge ($R^2$ = –3.14). At the subject level, Random Forest predictions were closest to actual scores; for example, a patient with MoCA = 24 was predicted as 25, while another with MoCA = 7 was predicted as 13, and one with MoCA = 30 as 24.
**Conclusion:** Machine learning models demonstrated the ability to approximate MoCA scores from MRI-derived morphometric features with clinically meaningful accuracy. These findings suggest that in future ML-based approaches could be applied in clinically relevant scenarios, such as supporting early detection of cognitive impairment and stratifying patients for further testing.
**Limitations:** Single-center retrospective study with a modest sample size. External validation is required to confirm generalizability. MoCA, while widely used, may not capture the full spectrum of cognitive domains.
**Funding for this study:** Modifiable bio and life-style markers in predicting cognitive decline (MOBILE-COG) No: RSU-PAG-2024/1-0014 is financed by the investment of the European Union Recovery and Resilience Facility and the state budget within the project "RSU internal and RSU with LASE external consolidation" No. 5.2.1.1.i.0/2/24/I/CFLA/055.
**Has your study been approved by an ethics committee?** Yes

**Ethics committee - additional information:** Approval was obtained from the institutional ethics committee, and all participants provided informed consent.
**Author Disclosures:**
Agnese Usacka: Nothing to disclose
Zane Anna Litauniece: Nothing to disclose
Ardis Platkajis: Nothing to disclose
Zigmunds Freibergs: Nothing to disclose
Nauris Zdanovskis: Nothing to disclose
Kalvis Kalva: Nothing to disclose
Aināars Stepens: Nothing to disclose
Kristīne Šneidere: Nothing to disclose

**Influence of deep learning image reconstruction and adaptive statistical iterative reconstruction-V on automated ASPECTS evaluation**
*E. Akl*[1], E. Beller[1], D. Cantré[1], F. G. Meinel[1], M-A. Weber[1], S. Langner[2], W. Hermann[1], M. Lütgens[1], A-C. Klemenz[1]; [1]Rostock/DE, [2]Greifswald/DE
*(aklestelle@gmail.com)*

**Purpose:** The Alberta Stroke Program Early CT Score (ASPECTS) and advances in CT reconstruction, such as Adaptive Statistical Iterative Reconstruction-V (ASIR-V), and Deep Learning Image Reconstruction (DLIR) play an important role in the neurodiagnostic workflow. This study examines the effect of these reconstruction techniques on automated ASPECTS.
**Methods or Background:** In this retrospective study, 173 patients (mean age 77 ± 12 years, 39% female) with suspected middle cerebral artery infarction underwent non-contrast CT, reconstructed with FBP, ASIR-V (at 30% and 60%), and DLIR (low, medium, and high). Automated ASPECTS were analyzed, with FBP as the reference standard.
**Results or Findings:** Compared to FBP, ASIR and DLIR reconstructions resulted in a mild overall underestimation of automated ASPECTS, with the least pronounced underestimation for ASIR-V 30% and DLIR-L, and a more marked underestimation for ASIR-V 60% and DLIR-M/H. Most re-classifications involved shifts of ASPECTS from moderate (5–7) to high (8–10), with DLIR-H showing the greatest effect. DLIR more frequently up-classified patients from ≤5 to ≥6, whereas down-classifications were rare. Regionally, the insular ribbon was most underestimated and M3 most overestimated, with DLIR-H exhibiting the largest total regional discrepancies. Agreement with expert consensus was highest for DLIR-M, followed by ASIR-V, while FBP and DLIR-H showed lower concordance.
**Conclusion:** Both ASIR-V and DLIR showed generally minor underestimation of ASPECTS compared to FBP. Occasional overestimation, however, resulted more often in reclassifications of the ASPECTS score, which affected patient eligibility for endovascular therapy (ASPECTS ≥ 6 vs. ASPECTS ≤ 5). DLIR-M was most accurate compared with expert consensus. Careful selection, optimization, and standardization of reconstruction parameters are essential for consistent and reliable stroke imaging.
**Limitations:** No limitations were identified.
**Funding for this study:** No funding was received for this study.
**Has your study been approved by an ethics committee?** Yes
**Ethics committee - additional information:** Not applicable - retrospective study.
**Author Disclosures:**
Sönke Langner: Nothing to disclose
Matthias Lütgens: Nothing to disclose
Ann-Christin Klemenz: Nothing to disclose
Felix G. Meinel: Nothing to disclose
Wiebke Hermann: Nothing to disclose
Marc-André Weber: Nothing to disclose
Daniel Cantré: Nothing to disclose
Estelle Akl: Nothing to disclose
Ebba Beller: Nothing to disclose

**MRI Radiomics-Based Machine Learning Model for Stroke Severity Combining Carotid Plaque and White Matter Hyperintensity**
*Z. Cui*, J. Zhang; Shanghai/CN
*(510349687@qq.com)*

**Purpose:** To develop and validate a reliable machine learning (ML) model combining carotid plaque and white matter hyperintensity (WMH) radiomics features with radiological characteristics to assess acute ischemic stroke (AIS) severity.
**Methods or Background:** Retrospective data were collected from patients with symptomatic carotid artery stenosis (CAS) and AIS between January 2017 and December 2023, and a prospective cohort from October 2023 to October 2024. Based on admission NIHSS scores, patients were categorized into NIHSS score ≤ 1 and > 1 groups. All patients underwent high-resolution vessel wall MRI and brain MRI. Conventional imaging features of carotid plaques and WMH were used to build a conventional imaging model. Radiomics features were extracted from carotid plaque (T1WI and contrast-enhanced T1WI) and WMH (FLAIR), and multiple ML algorithms were applied to build radiomics models. A hybrid model was subsequently developed by combining radiomics and conventional imaging features. Model performance was evaluated in the test set and prospective validation cohort.
**Results or Findings:** Three cohorts were included: retrospective training (140 patients), testing (59 patients), and prospective validation (71 patients). The conventional imaging model achieved areas under the curve (AUC) of 0.94, 0.88, and 0.75 in the training, test, and validation cohorts, respectively. The radiomics model achieved corresponding AUCs of 0.87, 0.79, and 0.73. The hybrid model, which incorporated 9 conventional imaging features and 23 radiomics features (16 plaque-derived and 7 WMH-derived), yielded superior performance, with AUCs of 0.97, 0.96, and 0.87. Decision curve analysis demonstrated that the hybrid model provided greater net clinical benefit.
**Conclusion:** The ML-based hybrid model enables accurate, non-invasive AIS severity assessment and demonstrates strong clinical potential.
**Limitations:** First, the retrospective design may have introduced selection bias. Second, the lack of multicenter validation and the modest sample size remain limitations.
**Funding for this study:** This work was supported by the National Key R&D Program of China (Grant no. 2022YFF0708700), Shanghai Science and Technology Program (Grant no. 22S31905300, 22YF1405000), the National Natural Science Foundation of China Youth Program (Grant no. 82402393).
**Has your study been approved by an ethics committee?** Yes
**Ethics committee - additional information:** The study protocol was approved by the Institutional Review Board of our hospital, and written informed consent was obtained from all participants.
**Author Disclosures:**
Jun Zhang: Nothing to disclose
Zhimeng Cui: Nothing to disclose

**Multimodal Integration of Radiomics, Clinical, and Immune Features Improves Survival Prediction in Glioblastoma**
*Y. Huang*[1], J. Chu[1], L. Zhao[2], R. Xu[2], W. Zhou[3], H. Wei[3]; [1]Guangzhou/CN, [2]Beijing/CN, [3]Shanghai/CN
*(huangyq285@mail.sysu.edu.cn)*

**Purpose:** Accurate preoperative prognosis remains challenging in glioblastoma (GBM). This study developed a multimodal nomogram integrating radiomic, clinical, and immunological features to predict overall survival (OS).
**Methods or Background:** We retrospectively analyzed 298 training and 123 validation GBM patients. A total of 6,792 radiomic features were extracted from tumor core and peritumoral edema regions on T1CE, T2WI, and T2-FLAIR MRI. Clinical variables (cortical, ependymal, and contralateral invasion) and immune markers (PD-1, CD68+, CD86+, CD163+, HIF-1α+, etc.) were evaluated. Tumor and peritumoral radiomic scores were constructed via LASSO-Cox regression. A Cox model integrated these scores with clinical and immune scores to predict OS.
**Results or Findings:** Tumor-core radiomic scores: 2 wavelet-based features were selected via LASSO-Cox regression to construct the tumor core score (HR=3.02, 95%CI 1.38–6.64, p=0.006). Peritumoral radiomic scores: 5 skewness-based features were identified for the peritumoral score (HR=3.18, p=0.049). Clinical score: Multivariate Cox analysis confirmed cortical invasion, ependymal invasion, and contralateral invasion as independent prognostic factors (all p<0.05). The clinical score stratified patients into high- and low-risk groups with distinct survival trajectories (p<0.05). Immune stratification: Based on densities of nine immune markers (PD1+, CD68+, etc.), patients were classified into immune-cold, -intermediate, and -hot subgroups. Survival analysis revealed significant OS differences among subgroups (p<0.05). Multimodal integration: The integrated nomogram combining Tumor-core radiomic scores, peritumoral radiomic scores, clinical scores, and immune scores demonstrated superior predictive accuracy compared to unimodal models.It achieved an AUC of 0.86. High-risk patients had significantly shorter OS (p<0.05).
**Conclusion:** The nomogram synergizes radiological, pathological, and immunological data to refine GBM prognostication. Peritumoral skewness features and specific invasion patterns signal aggressive biology, while immune-hot phenotypes correlate with poorer outcomes. This framework aids personalized therapy decisions.
**Limitations:** This study was a single-center design.
**Funding for this study:** No.
**Has your study been approved by an ethics committee?** Not applicable
**Author Disclosures:**
Hongjiang Wei: Nothing to disclose
Rulin Xu: Nothing to disclose
Wenyu Zhou: Nothing to disclose
Jianping Chu: Nothing to disclose
Yingqian Huang: Nothing to disclose
Longfei Zhao: Nothing to disclose

**Hospital-integrated deep learning system for real-time glioma analysis with advanced diffusion MRI**

*M. Nemy*, E. Stoklasa, D. Bojko, V. Sedlák, A. Kavková, L. Polášková, I. Jacečková, M. Majovsky; Prague/CZ
*(milan.nemy@cvut.cz)*

**Purpose:** This study presents a hospital-integrated real-time system for multi-shell diffusion MRI (dMRI) analysis in gliomas, including its architecture, secure PACS-connected workflow, and embedded machine-learning models for molecular prediction and automatic segmentation.
**Methods or Background:** A cohort of 210 patients with histologically confirmed intra-axial gliomas underwent a 3T MRI protocol including high-angular, multi-shell dMRI (134 directions, 7 b-values). Advanced diffusion models (DKI, RSI, VERDICT, NODDI) were derived to characterize microscopic tissue composition. Cellularity maps were used to train classical machine-learning models (XGBoost, random forest) and deep-learning models (CNNs, ResNet50), with data augmentation and transfer learning to address sample size. Automatic tumor and peritumoral segmentation was performed using CNNs and benchmarked against semi-automatic annotations. A secure hospital-integrated pipeline was implemented to enable real-time use: scans are anonymized at acquisition, transferred via encrypted channels to a high-performance cluster, processed under strict time limits, and results returned directly into PACS for radiologist review.
**Results or Findings:** Advanced dMRI models outperformed conventional ADC in glioma characterization. For IDH mutation prediction, AUC improved from 0.82 (ADC) to 0.91 with advanced models, and for glioma grading from 0.88 to 0.94. Deep-learning achieved the best performance with IDH classification (AUC >0.95, sensitivity >0.93). Automatic segmentation achieved mean Dice coefficients of >0.83 for enhancing tumor and >0.78 for peritumoral zones. The hospital-integrated system processed cases end-to-end robustly within clinically acceptable time limits.
**Conclusion:** This work demonstrates a clinically viable hospital-integrated system that combines advanced multi-shell dMRI and machine learning for accurate molecular prediction and automatic glioma segmentation. The architecture enables secure, real-time analysis with seamless PACS integration, provides advanced imaging biomarkers directly to radiologists, and may reduce manual workload in routine practice.
**Limitations:** The limitations of the study are its single-center design and relatively limited sample size.
**Funding for this study:** Funding was provided by the Ministry of Health of the Czech Republic, grant no. NW25J-08-00023, and by Charles University, project GA UK no. 222623.
**Has your study been approved by an ethics committee?** Yes
**Ethics committee - additional information:** This study was approved by the Ethics Committee of the Military University Hospital Prague.
**Author Disclosures:**
David Bojko: Nothing to disclose
Martin Majovsky: Nothing to disclose
Milan Nemy: Nothing to disclose
Vojtěch Sedlák: Nothing to disclose
Anna Kavková: Nothing to disclose
Lenka Polášková: Nothing to disclose
Erik Stoklasa: Nothing to disclose
Ivana Jacečková: Nothing to disclose

**OMT and tensor SVD based deep learning model for segmentation and predicting genetic markers of glioma: a multicenter study**

*Z. Zhu*[1], H. Yang[1], H. Wang[2], Y. Song[1], M. Xu[1], X. Zhang[1], W-W. Lin[2], T. Li[2], B. Zhang[1]; [1]Nanjing/CN, [2]Shanghai/CN
*(161230042@smail.nju.edu.cn)*

**Purpose:** Our study focused on tumor regions segmentation and predicting the World Health Organization (WHO) grade, isocitrate dehydrogenase (IDH) mutation, and 1p/19q codeletion status using deep learning models on preoperative Magnetic Resonance Imaging (MRI).
**Methods or Background:** Glioma is the most common primary malignant brain tumor and preoperative genetic profiling is essential for management of glioma patients. We developed an optimal mass transport (OMT) approach to transform irregular MRI brain images into tensors. In addition, we proposed an algebraic pre-classification (APC) model utilizing multi-mode OMT tensor singular value decomposition (SVD) to estimate pre-classification probabilities. The fully automated deep learning model named OMT-APC was used for multitask classification. Our study incorporated preoperative brain MRI data from 3,565 glioma patients across 16 datasets spanning Asia, Europe, and America. Among these, 2,551 patients from 5 datasets were used for training and internal validation. In comparison, 1,014 patients from 11 datasets, including 242 patients from The Cancer Genome Atlas (TCGA), were used as independent external test.
**Results or Findings:** OMT segmentation model achieved mean lesion-wise Dice scores of 0.880. The OMT-APC model was evaluated on the TCGA dataset, achieving accuracies of 0.855, 0.917 and 0.809, with AUC scores of 0.845, 0.908 and 0.769 for WHO grade, IDH mutation, and 1p/19q codeletion, respectively, which outperformed the four radiologists in all tasks.
**Conclusion:** These results highlighted the effectiveness of our OMT and tensor SVD-based methods in brain tumor genetic profiling, suggesting promising applications for algebraic and geometric methods in medical image analysis.
**Limitations:** Lacking prospective validation cohort.
**Funding for this study:** National Science and Technology Innovation 2030 -- Major program of "Brain Science and Brain-Like Research" (2022ZD0211800), National Natural Science Foundation of China (82271965, 82330059, 12371377)
**Has your study been approved by an ethics committee?** Yes
**Ethics committee - additional information:** Medical Ethical committee of Nanjing Drum Tower Hospital
**Author Disclosures:**
Wen-Wei Lin: Nothing to disclose
Bing Zhang: Nothing to disclose
Yang Song: Nothing to disclose
Huiquan Yang: Nothing to disclose
Zhengyang Zhu: Nothing to disclose
Mengying Xu: Nothing to disclose
Xin Zhang: Nothing to disclose
Han Wang: Nothing to disclose
Tiexiang Li: Nothing to disclose

**14:00 - 15:30** **Room G1**

### Research Presentation Session: Radiographers

### RPS 2014

### Novel techniques and biomarkers for precision care

**Moderators**
Vassilis Georgios Syrgiamiotis; Haidari, Athens/GR
*(syrgiamiotisvasilis@gmail.com)*
Marie Staunton; Galway/IE

**Ultra-low iodine PE-CTA across body-weight groups and PSI adjustments: achieving the ≥250 HU target in real-world practice**

J. T. d. S. d. Castro, *H. d. J. Ferreira*, D. C. Novais da Silva, D. Yamada, S. San Juan Dertkigil, F. Reis; Campinas/BR
*(horjesus@unicamp.br)*

**Purpose:** Computed tomographic pulmonary angiography (PE-CTA) must achieve ≥250 HU for reliable diagnosis; doing so with ultra-low iodine volumes across different body-weight groups is challenging. The aim is to evaluate the impact of weight-stratified, PSI-adapted PE-CTA protocols on arterial enhancement and iodine load.
**Methods or Background:** Single-centre retrospective longitudinal analysis within ANGIO-MONITOR. Consecutive PE-CTA exams were split by protocol change (go-live 01-Jun-2025): pre (fixed volumes; N=339) vs post (personalised 30–50 mL by weight with PSI ceilings; N=129). Primary endpoint: arterial attenuation (HU) in the pulmonary trunk. Secondary endpoints: proportion ≥250 HU, contrast volume (mL; mL/kg), peak PSI, and repeat acquisitions. Statistics: Welch's tests for means; $\chi^2$ for proportions; 95% CIs.
**Results or Findings:** Arterial enhancement increased from 313.5 HU (95% CI 301.3–325.7) to 499.7 HU (95% CI 465.5–533.9); $p=5.38\times10^{-19}$. The proportion ≥250 HU rose from 72.3% (95% CI 67.3–76.8; 245/339) to 95.3% (95% CI 90.2–97.9; 123/129); absolute Δ +23.1 pp, RR 1.32; $p=5.26\times10^{-8}$. Mean contrast volume fell from 60.0 mL (95% CI 58.3–61.8) to 43.8 mL (95% CI 41.5–46.1); −27.0%, $p=1.22\times10^{-24}$. Normalised dose decreased from 0.825 mL/kg (95% CI 0.797–0.853) to 0.577 mL/kg (95% CI 0.547–0.606); −30.1%, $p=2.31\times10^{-28}$. Peak PSI remained within operational safety, with a modest rise (98.9 to 107.8; 95% CIs 95.5–102.3 vs 102.1–113.5; p=0.0089). Repeat-acquisition rates were rare and stable (p=0.45).
**Conclusion:** Weight-stratified, PSI-adapted ultra-low iodine PE-CTA substantially increased enhancement above the 250 HU threshold while reducing iodine by ~27% (mL) and ~30% (mL/kg), without operational penalty. This supports iodine stewardship and safe high-flow practice in routine care, highlighting technologist-led implementation and hospital–industry synergy.

**Limitations:** The study is a single-center retrospective analysis, which may limit the generalizability of the findings.
**Funding for this study:** Not applicable.
**Has your study been approved by an ethics committee?** Yes
**Ethics committee - additional information:** The work has been submitted to the university's Research Ethics Committee (CEP) and is currently under review for evaluation and final opinion.
**Author Disclosures:**
Hortência De Jesus Ferreira: Nothing to disclose
Fabiano Reis: Nothing to disclose
José Thiago De Souza De Castro: Nothing to disclose
Danielle Cristina Novais Da Silva: Nothing to disclose
Sergio San Juan Dertkigil: Nothing to disclose
Daniel Akio Yamada: Nothing to disclose

**Image Quality Evaluation of Standard Knee versus AI-Enhanced Knee MRI Protocols**
*L. Buttex*, L. Gallus, Y. Cottier, C. S. D. Reis, S. S. Ghotra; Lausanne/CH

**Purpose:** To evaluate the application of artificial intelligence(AI) to reconstruct knee MRI imaging compared to standard protocols and identify if there is add-value for clinical practice.
**Methods or Background:** An exploratory study was conducted to evaluate AI-based image reconstruction methods in MRI compared to the standard(STD) knee MRI protocol. T1 Fast Spin Echo(FSE), PD FSE Fat saturation(FS) and T2 FSE in sagittal view, with PD FSE FS axial, and PD FSE FS coronal views were acquired. Protocols were optimised to enhance image quality(IQ) and reduce acquisition time. The final protocol was applied in 10 healthy volunteers using a 3T MRI to acquire the images. Visual assessment was performed by three MRI-experienced observers using the ViewDEX software. Visual Grading Analysis(VGA), Kappa statistics for inter-observer agreement and Visual Grading Characteristics(VGC) were performed to analyse data.
**Results or Findings:** Overall, the VGA scores indicate that the values for AI protocol are generally higher or equivalent to those for STD sequences across nearly all criteria of anatomical reproduction and IQ and 36.9% of time reduction was achieved as well 8min22s versus 13min15s. Kappa statistics indicated a medium to good agreement for AI protocol sequences(0.44-0.71) compared to low to good agreement for STD protocol sequences(0.13-0.71). VGC analysis showed that sequences reconstructed with the AI tool performed statistically better than STD sequences at 3T, with AUCVGC of 0.76 to 0.81(95% CI and p-value≤0.05).
**Conclusion:** This study demonstrates that the use of AI in knee MRI investigations has a tangible impact on the overall IQ, as well as on acquisition time, which was reduced by 36.9%. Further research is required to emphasise the clinical validation of the results with a larger sample size and cases including pathology.
**Limitations:** Small sample of healthy volunteers
**Funding for this study:** Not applicable
**Has your study been approved by an ethics committee?** Yes
**Ethics committee - additional information:** Ethical approval Number: 2024-01679
**Author Disclosures:**
Léo Gallus: Nothing to disclose
Claudia Sa Dos Reis: Nothing to disclose
Yann Cottier: Nothing to disclose
Switinder Singh Ghotra: Nothing to disclose
Lucas Buttex: Nothing to disclose

**Quantifying the carbon footprint of [$^{18}$F]FDG PET/CT imaging through Life Cycle Assessment**
*A. Roletto*[1], A. Savio[2], E. Bertagna[2], N. S. Chiodini[1], G. R. Bonfitto[1], A. Chiti[1], S. Zanoni[2]; [1]Milan/IT, [2]Brescia/IT
*(rolettoandrea@yahoo.it)*

**Purpose:** While the environmental impact of diagnostic imaging is increasingly acknowledged, comprehensive analyses of nuclear medicine remain scarce. This study presents a Life Cycle Assessment (LCA) of routine [$^{18}$F]FDG PET/CT examinations, with the aim of quantifying their Greenhouse Gas (GHG) footprint and identifying opportunities for impact reduction.
**Methods or Background:** A cradle-to-grave LCA was performed in accordance with ISO 14040 standards within a nuclear medicine department. A standard [$^{18}$F]FDG PET/CT protocol was evaluated. Direct measurements were used to determine scanner electricity consumption, whereas heating, ventilation, and air conditioning (HVAC) energy demand was estimated for each relevant area based on data from the hospital trigeneration plant. Radiopharmaceutical production and single-use materials were modeled using SimaPro software.
**Results or Findings:** GHG footprint of a single PET/CT examination was estimated at 7.6 kg $CO_2$ eq. HVAC systems were the largest contributor (47% of $CO_2$ eq, 3.6 kg $CO_2$ eq.), encompassing the scan room, control room, injection room, and cyclotron facility. Radiopharmaceutical production represented 36% of the footprint, while direct scanner electricity accounted for only 5.4%. Single-use disposables were notable contributors to toxicity-related impacts and waste generation (2.1 kg $CO_2$ eq., 28%). Sensitivity scenarios suggested that ongoing decarbonization of the electricity grid could lower PET/CT-related carbon emission by up to 50%.
**Conclusion:** The environmental impact of [$^{18}$F]FDG PET/CT is predominantly driven by HVAC energy demand and radiopharmaceutical production. Mitigation strategies, such as enhancing HVAC efficiency, increasing reliance on renewable energy, and replacing single-use items with reusable or lower-impact alternatives, could substantially reduce the carbon emission of nuclear medicine services.
**Limitations:** This analysis was conducted in a single-center setting with one representative PET/CT protocol, which may limit generalizability of the results.
**Funding for this study:** n/a
**Has your study been approved by an ethics committee?** Not applicable
**Author Disclosures:**
Anna Savio: Nothing to disclose
Enrico Bertagna: Nothing to disclose
Andrea Roletto: Nothing to disclose
Arturo Chiti: Nothing to disclose
Giuseppe Roberto Bonfitto: Nothing to disclose
Simone Zanoni: Nothing to disclose
Nicolò Sean Chiodini: Nothing to disclose

**Predicting radioiodine-refractory thyroid cancer through the use of nuclear medicine and radiogenomic biomarker analysis: The RADAR study**
*K. Borg Grima*, J. L. Portelli, J. Borg, A. Rasalam Iris; Msida/MT
*(karen.borg-grima@um.edu.mt)*

**Purpose:** Differentiated thyroid cancer (DTC) is primarily treated with surgery and radioactive iodine (RAI) ablation, achieving excellent outcomes in most patients. However, 5–10% of metastatic cases develop RAI-refractory disease (RAI-R), representing 60–70% of those with progressive disease. RAI-R patients face poor prognoses, with an average survival of 3–5 years. At present, clinicians lack biomarkers to predict RAI refractoriness, delaying the initiation of targeted therapies. The RADAR project aims to integrate the results of nuclear medicine imaging and molecular profiling into a radiogenomic framework of a unique population sample.
**Methods or Background:** This prospective study involves patients with DTC presenting at the only oncology centre in Malta. Data relating to clinical, biochemical, and histopathological findings, combined with imaging (I-131 SPECT/CT, 18F-FDG PET/CT) and molecular analyses, such as the microRNA profile, are collected longitudinally. Radiogenomic correlations are performed to identify patterns of RAI uptake and FDG avidity, potentially predictive of resistance. Candidate biomarkers will be validated and incorporated into a predictive risk algorithm.
**Results or Findings:** Previous research has shown that high FDG uptake (specificity 87.5%; sensitivity 83.3%) and certain genetic variants in the symporters are strongly associated with early loss of RAI avidity. By project completion, the study aims to achieve Technology Readiness Level 5 by delivering a validated biomarker panel and predictive model capable of stratifying high- versus low-risk patients for RAI-R progression.
**Conclusion:** The RADAR study introduces an innovative radiogenomic precision oncology approach, combining data obtained from nuclear medicine, radioiodine therapy and pathology. Early prediction of RAI-R could enable personalised treatment planning, reduce unintended toxicity and improve overall survival of DTC patients.
**Limitations:** RAI-R cases present a limited number of patients; however, data collection will run for the whole duration of the RADAR project.
**Funding for this study:** Funding for the RADAR project was obtained from the government agency , Xjenza Malta, responsible for promoting and coordinating scientific research in Malta.
**Has your study been approved by an ethics committee?** Yes
**Ethics committee - additional information:** RADAR project team are currently in the process of obtaining ethical permission from the University of Malta Research Ethics Committee (UREC)
**Author Disclosures:**
Karen Borg Grima: Nothing to disclose
Joseph Borg: Nothing to disclose
Anu Rasalam Iris: Nothing to disclose
Jonathan Loui Portelli: Nothing to disclose

**Assessing Image Quality in Adolescent Idiopathic Scoliosis: A Comparative Study of EOS and X-ray Imaging**

M. F. Mcentee, A. England, C. Rainey, A. O'Donovan, S. Coakley, *J. O'Neill*; Cork/IE
*(johnoneill80@ucc.ie)*

**Purpose:** Adolescent Idiopathic Scoliosis (AIS) is a spinal deformity requiring accurate, high-quality imaging for diagnosis and treatment. While Digital Radiography (DR) remains the gold standard, EOS, a low-dose, biplanar imaging system, have emerged as an alternative. This study compares EOS and DR formage quality for whole-spine X-rays.
**Methods or Background:** A quantitative design assessed whole-spine imaging using anthropomorphic phantoms in two size configurations: Standard and Large. AP and lateral images were acquired with EOS and DR. Subjective image quality was assessed via a Visual Grading Analysis (VGA) questionnaire completed by 39 radiology professionals. Objective image quality was evaluated using Signal-to-Noise Ratio (SNR) and Contrast-to-Noise Ratio (CNR) metrics on ImageJ software, with statistical analysis via SPSSv27.
**Results or Findings:** Subjectively, DR scored higher in AP projections (standard: 72%vs.68%, p = 0.015; large:64%vs.60%, p=0.0004), while EOS led in lateral views (standard: 73%vs.69%, p=0.0001; large: 66%vs.60%, p<0.0001).EOS received more "Good" and "Moderate" ratings, but DR had more "Perfect" scores. Both DR and EOS had nearly identical numbers of "Inadequate" ratings. Objectively, DR showed higher SNR and CNR values, but significance was only seen for SNR in the large phantom AP view (DR 46.05 vs. EOS 28.00, p=0.004). Both systems showed reduced image quality with increased phantom size.
**Conclusion:** EOS demonstrated more consistent imaging across sizes and projections. DR showed marginally better SNR/CNR in some conditions. Subjective results favoured DR in AP views, and EOS in the lateral. Both modalities offer comparable image quality. EOS remains a promising option for AIS imaging; however, several key factors must be considered.
**Limitations:** Future research should involve real patient imaging, ideally in a multicenter, prospective design. Including a diverse sample of AIS patients would allow assessment of diagnostic performance, interobserver agreement, and real-world utility.
**Funding for this study:** None
**Has your study been approved by an ethics committee?** Yes
**Ethics committee - additional information:** Ethics was approved by the School of Medicine's Social Ethics committee
**Author Disclosures:**
John O'Neill: Nothing to disclose
Sarah Coakley: Nothing to disclose
Mark F. Mcentee: Nothing to disclose
Alison O'Donovan: Nothing to disclose
Clare Rainey: Nothing to disclose
Andrew England: Nothing to disclose

**From Pixels to Catheters: Correlating CTA with Coronary Angiography**

*A. Collaku*[1], Z. Mulla[1], E. Dybeli[2], F. Goga[1]; [1]Tirana/AL, [2]Elbasan/AL
*(alket_collaku@yahoo.com)*

**Purpose:** Coronary Computed Tomography Angiography (CTA) has emerged as a powerful non-invasive tool for evaluation of coronary artery disease (CAD). this study aimed to corelate CTA findings with Invasive Coronary Angiography (ICA), consider the gold standard, over a 10 year experience.
**Methods or Background:** We retrospectively reviewed cases in which patients underwent both CTA and ICA within a comparable clinical timeframe. Degree of stenosis in major coronary arteries was categorized, and CTA results were compared with ICA findings. Diagnostic accuracy, including sensitivity, specificity, positive predictive value (PPV), and negative predictive value (NPV), was calculated for detection of significant stenosis (≥50%)
**Results or Findings:** CTA demonstrated high sensitivity and NPV in ruling out significant CAD, showing strong concordance with ICA in the majority of cases. The technique was particularly reliable in single- and double-vessel disease, whereas specificity decreased in the presence of extensive calcification or motion artifacts. Despite these limitations, CTA consistently reduced the need for unnecessary invasive procedures.
**Conclusion:** CTA correlates strongly with ICA in the detection of significant CAD and can serve as an effective gatekeeper to invasive testing. In routine clinical practice, CTA offers a safe, accurate, and patient-friendly alternative that complements—but does not fully replace—the diagnostic value of ICA.
**Limitations:** No
**Funding for this study:** No
**Has your study been approved by an ethics committee?** Not applicable
**Author Disclosures:**
Flogerta Goga: Nothing to disclose
Zane Mulla: Nothing to disclose
Elona Dybeli: Nothing to disclose
Alket Collaku: Nothing to disclose

**Performance evaluation of the Hyperscint™ RP-200 scintillation detector for dosimetry in FLASH radiotherapy**

*S. Recordon*, M. Scheidegger, M. Thiébaud, I. Buchillier-Decka, K. Sprengers, V. Grilj; Lausanne/CH
*(sujeeta.recordon@gmail.com)*

**Purpose:** FLASH radiotherapy (FLASH-RT) delivers ultra-high-dose rates (UHDR >40 Gy/s) with the potential to preserve healthy tissue while maintaining tumor reduction efficacy. Accurate dosimetry is essential for clinical implementation. This study evaluates the performance of the Hyperscint™ RP-200 plastic scintillation detector for routine dosimetry in FLASH-RT, in comparison with detectors considered as references.
**Methods or Background:** The experiments were performed with the Oriatron eRT6 linear accelerator (CHUV, Lausanne) delivering 6 MeV pulsed electron beams in conventional (CONV) and FLASH modes. The RP-200 was tested for linearity, pulse dose response (PDR), repetition rate (PRF), and depth dose profiles (DDP). The measurements were compared with those of a AdvancedMarkus ionisation chamber and Gafchromic™ EBT3 films. The data were normalised by beam monitor and analysed by linear regression and dose ratios.
**Results or Findings:** The RP-200 demonstrated excellent linearity in CONV mode ($R^2$=0.9996) with doses consistent with the reference detectors. In FLASH mode, a systematic underestimation occurred with deviations of 9 to 30%, increasing with PRF and DPP. PDD profiles differed from the references, particularly at a source-surface distance of 600 mm. These results confirm the limitations of the RP-200 in UHDR conditions, potentially related to Cherenkov light contamination.
**Conclusion:** Although reliable in conventional dosimetry, the RP-200 requires correction factors for routine dosimetry in FLASH-RT. Newer models such as the RP-100 may offer better UHDR performance. Further research is needed to optimise scintillator dosimetry in clinical FLASH.
**Limitations:** The main study limitation is the definition with certainty of the exact causes leading to differences in doses measured by the scintillator compared to the doses indicated by the films.
**Funding for this study:** Not applicable
**Has your study been approved by an ethics committee?** Not applicable
**Author Disclosures:**
Ina Buchillier-Decka: Nothing to disclose
Marinette Scheidegger: Nothing to disclose
Sujeeta Recordon: Nothing to disclose
Mike Thiébaud: Nothing to disclose
Kevin Sprengers: Nothing to disclose
Veljko Grilj: Nothing to disclose

**Orthopedic Metal Artifact Reduction (OMAR) combined with Dual Energy Computed Tomography (DECT) improves diagnostic image value**

*I. Blom*[1], M. Olsthoorn[2]; [1]De Lier/NL, [2]Rotterdam/NL
*(i.blom@rdgg.nl)*

**Purpose:** Computed Tomography (CT) is frequently used in orthopedic imaging to assess implant failure and complications such as periprosthetic fractures. However, the presence of metal implants often leads to metal artifacts (MA), which can significantly impair diagnostic image quality. Due to the wide variety of implant types and materials, the extent of MA varies considerably. As a result, a combination of artifact reduction techniques is often required to optimize visualization of both the implant and surrounding tissues. To evaluate the effectiveness of metal artifact reduction, multiple kiloelectron volt (keV) reconstructions were performed, with and without Orthopedic Metal Artifact Reduction (OMAR).
**Methods or Background:** Various orthopedic implants (hip, knee, shoulder, and spine) were scanned using a spectral CT scanner (IQon, Philips, The Netherlands) at 140 kV to reduce metal artifacts. Image reconstructions were performed both with and without OMAR, across a range of virtual monochromatic energy levels up to 200 keV. Hounsfield Units (HU) were measured using regions of interest (ROIs), with average (AVG) and standard deviation (SD) values recorded. Two musculoskeletal radiologists independently assessed diagnostic image quality using a 5-point Likert scale.
**Results or Findings:** Application of OMAR reduced SD in bone by approximately 10 HU, while increasing metal HU by 40. Bone AVG HU decreased by roughly 150 with OMAR across most joint revisions. Combining OMAR with high keV levels resulted in a 200 HU (AVG) increase in metal component.
**Conclusion:** Images reconstructed at 130–150 keV with OMAR yielded the highest diagnostic quality scores. However, 130 keV images without OMAR provided the most balanced HU values for both bone and metal, suggesting optimal contrast for diagnostic assessment.
**Limitations:** Case Study
**Funding for this study:** Not Applicable
**Has your study been approved by an ethics committee?** Not applicable

Author Disclosures:
Mariëlle Olsthoorn: Nothing to disclose
Ian Blom: Nothing to disclose

**Optimisation of chest computed tomography dose values considering image quality indicators**
*J. Santos*, F. Caseiro Alves; Coimbra/PT
*(joanasantos@estesc.ipc.pt)*

**Purpose:** For lung diseases the literature recommends Computed Tomography (CT) for diagnostic, follow-up, planning and intervention. This study aims to redefine image quality indicators in chest CT, for pulmonary pathology, analysing CT exposure and image quality values.
**Methods or Background:** Ethical approval was obtained. In phase 1, the most relevant clinical CT indications for pulmonary pathology were identified. Dose values (n=90), described in the CT Dose Index (CTDIvol-mGy) and Dose Length Product (mGy.cm), were analysed. In phase 2, new imaging criteria were defined by 10 radiologists, using Delphi method. Image quality was evaluated objectively [10 Regions of Interest (ROI's)] and subjectively [(2 radiologists - Visual Grading Characteristic (VGC)]. In phase 3, dose values were analysed (n=100). Examinations performed on the same patients were analysed in terms of exposures and image quality.
**Results or Findings:** Microbacterial pathologies (MB), Lung nodules (NO) and Pulmonary fibrosis (FB) were the defined pathologies. The DLP values on phase 1, were 457mGy.cm for MB, 430mGy.cm for NO and 372mGy.cm for FB, without differences in quality criteria. The exposure values on phase 2, were 305mGy.cm for MB, 300mGy.cm for NO, and 372mGy.cm for FB. Analysis of the dose values of the same patients, revealed a variation from 399 to 324mGy.cm for MB, and from 378 to 324mGy.cm for NO. On phase 3 objective image quality identified lower noise values and higher area under the VGC curve (MB and NO).
**Conclusion:** The definition of new image quality criteria, with the application of robust statistical methods, allows a detailed analysis per imaging quality criteria. A reduction in DLP of 33% for MB, 30% for NO was obtains with an image quality improvement.
**Limitations:** The chest pathologies in study are focus on lung parenquima.
**Funding for this study:** No funding.
**Has your study been approved by an ethics committee?** Yes
**Ethics committee - additional information:** CHUC ethical committe approval.
**Author Disclosures:**
Joana Santos: Other: ECR 2026 PPC
Filipe Caseiro Alves: Nothing to disclose

**Reducing the Need for Sedation in Pediatric CT: Experience with Aquilion ONE 640-Slice**
Z. Mulla[1], *A. Collaku*[1], E. Dybeli[2], F. Goga[1]; [1]Tirana/AL, [2]Elbasan/AL
*(alket_collaku@yahoo.com)*

**Purpose:** To evaluate the impact of ultra-fast 640-slice CT (Aquilion ONE) on the reduction of sedation requirements in pediatric patients undergoing emergency and routine imaging.
**Methods or Background:** A retrospective review was performed on pediatric CT examinations (ages 1–12 years) conducted with the Aquilion ONE 640-slice system over a 12-month period. Parameters recorded included scan time, diagnostic image quality, presence of motion artifacts, and the rate of sedation. Protocols were optimized using individualized low-dose settings and iterative reconstruction techniques.
**Results or Findings:** The mean scan time was below 1 second for most brain, chest, and abdominal studies. Diagnostic quality was preserved in 95% of cases, with minimal need for repeat scanning. Only 7% of patients required sedation, compared with published rates of 30–40% with conventional CT. Motion artifacts were significantly reduced due to volumetric coverage in a single rotation.
**Conclusion:** The Aquilion ONE 640-slice CT significantly decreases the need for sedation in pediatric imaging by providing ultra-fast acquisitions with high diagnostic quality at optimized radiation doses. This approach improves patient safety, comfort, and clinical workflow, representing an important advancement in pediatric CT practice.
**Limitations:** No
**Funding for this study:** No
**Has your study been approved by an ethics committee?** Not applicable
**Author Disclosures:**
Flogerta Goga: Nothing to disclose
Zane Mulla: Nothing to disclose
Elona Dybeli: Nothing to disclose
Alket Collaku: Nothing to disclose

**Quantitative Differentiation of Myocardial Tissue Using Photon-Counting CT Cardiac Imaging: A Pilot Study**
*K. Tissir*, P. Spagnolo; Milan/IT

**Purpose:** To quantitatively evaluate Cardiac imaging with Photon-Counting Computed Tomography (PCCT) for differentiating healthy myocardium, scar tissue, and edema using material decomposition maps and objective imaging metrics.
**Methods or Background:** Anonymised images from twenty-five patients with confirmed myocardial disease (acute or chronic infarction, myocarditis-related edema) undergoing PCCT with ECG-synchronised iodinated contrast were analysed. Reconstructions included conventional images, monoenergetic images (40–70 keV), and material decomposition maps (iodine, calcium, soft tissue). Myocardial regions of interest were segmented using Cardiac Mgnetic Resonance (CMR) imaging as reference. For each tissue type (healthy, scar and edema), mean attenuation associated to HU or iodine concentration, standard deviation, Signal-to-Noise Ratio (SNR), and Contrast-to-noise Ratio (CNR) were calculated. Receiver Operating Characteristic (ROC) analysis assessed discrimination performance between healthy and pathological tissue.
**Results or Findings:** Mean attenuation or iodine concentration differed significantly among tissues: healthy myocardium 55 ± 5 HU, scar 80 ± 8 HU, edema 65 ± 6 HU. CNR between scar and healthy tissue was 5.8 ± 1.2, SNR of healthy myocardium 32 ± 4. ROC analysis yielded area under the curve (AUC) of 0.87 for scar versus healthy myocardium and 0.81 for edema versus healthy myocardium. Voxel-wise concordance with CMR segmentation was 86%, indicating high agreement.
**Conclusion:** PCCT enables quantitative differentiation of myocardial tissues through material decomposition maps, providing reproducible SNR and CNR measurements. This pilot study demonstrates the feasibility of using PCCT for objective assessment of scar and edema, potentially complementing CMR in clinical practice.
**Limitations:** The study is limited by the small sample size and single-center design. Material decomposition maps require further validation against larger cohorts, and current results may not generalize to all PCCT scanners or acquisition protocols.
**Funding for this study:** n/a
**Has your study been approved by an ethics committee?** Not applicable
**Author Disclosures:**
Karima Tissir: Nothing to disclose
Pietro Spagnolo: Nothing to disclose

**14:00 - 15:30** **Research Stage 5**

### Research Presentation Session: Oncologic Imaging

### RPS 2016

### The evolving landscape of imaging for neuroendocrine tumours and sarcomas

**Moderator**
Cecília Vieira-Leite; Santiago De Compostela/ES
*(mariaceciliavll@hotmail.com)*

**Accurate Noninvasive Differentiation of Intrapancreatic Ectopic Spleen from Pancreatic NET with SPIO-Enhanced MRI**
P. Summer[1], *P. Sorgo*[1], P. M. Sbeghen[2], A. Y. Yasin[1], S. Pötter-Lang[1], N. Bastati-Huber[1], A. Ba-Ssalamah[1], U. I. Attenberger[1]; [1]Vienna/AT, [2]Verona/IT

**Purpose:** Intrapancreatic ectopic spleen (IPES) is a benign entity that often mimics pancreatic neuroendocrine tumors (NETs) on imaging. Conventional CT, ECCM-MRI, Ga-68 DOTA-NOC PET/CT, and Tc-99m HDRBC SPECT have important diagnostic limitations. Superparamagnetic iron oxide (SPIO)–enhanced MRI may allow reliable noninvasive differentiation. We evaluated the diagnostic performance of multimodal imaging with emphasis on SPIO-MRI.
**Methods or Background:** This retrospective study included 23 patients (10 NET, 13 IPES; mean age 59 ± 14 years) who underwent ECCM-MRI and SPIO-MRI. Subsets also had CECT (n=22), Ga-68 DOTA-NOC PET/CT (n=13), and Tc-99m HDRBC SPECT (n=6). Two blinded abdominal radiologists assessed qualitative and quantitative imaging features and diagnostic confidence. ROC analyses were performed to evaluate modality performance.

**Results or Findings:** NETs were significantly larger than IPES (1.9 vs. 1.3 cm, p<0.05). ECCM-MRI and CECT showed overlapping enhancement patterns with low accuracy (~50%). Ga-68 DOTA-NOC PET/CT demonstrated higher uptake in NETs compared with IPES (SUVmax 82 vs. 36, p=0.06), but with poor discriminatory value. Tc-99m HDRBC SPECT was inconclusive due to limited spatial resolution. In contrast, all IPES lesions showed marked T2/T2* signal loss after SPIO administration, whereas NETs remained unchanged. Both readers achieved 100% accuracy, with excellent interobserver agreement (κ=0.95, ICC=0.91). ROC analysis confirmed SPIO-MRI as the only modality with near-perfect diagnostic performance (AUC 0.79–1.00, p<0.001).
**Conclusion:** SPIO-enhanced MRI enables confident, noninvasive differentiation between IPES and NET, clearly outperforming conventional imaging and nuclear medicine techniques. It should be implemented as the preferred next step in suspected IPES to avoid unnecessary interventions.
**Limitations:** This retrospective, single-center study included a small cohort with uneven availability of comparator imaging modalities, limiting generalizability. The reported 100% accuracy of SPIO-MRI requires prospective validation in larger, multicenter cohorts.
**Funding for this study:** No funding.
**Has your study been approved by an ethics committee?** Yes
**Ethics committee - additional information:** Protocol Number: 1441/2016
**Author Disclosures:**
Paolo Maria Sbeghen: Nothing to disclose
Sarah Pötter-Lang: Speaker: Bayer
Priska Summer: Nothing to disclose
Ahmet Yitik Yasin: Nothing to disclose
Ahmed Ba-Ssalamah: Speaker: Bayer Speaker: Siemens Consultant: Bayer Speaker: Sanofi
Philipp Sorgo: Nothing to disclose
Ulrike I. Attenberger: Speaker: Siemens Healthineers Consultant: Bureau Consultant: Bayer
Nina Bastati-Huber: Nothing to disclose

**Whole-body magnetic resonance imaging (WB-MRI) for cancer screening in adult patients with neurofibromatosis type 1 (NF1)**
L. Carone, *M. G. Di Niso*, S. Kalantari, M. Zacchino, F. Sirchia, L. Preda; Pavia/IT
*(mariagiovanna03@gmail.com)*

**Purpose:** NF1 is a rare inherited genetic syndrome characterized by wide phenotypic variability and a predisposition to tumor development. In recent years, WB-MRI has emerged as a valuable imaging tool for the comprehensive evaluation of multisystem alterations associated with NF1 and for the early detection of incidental findings of potential clinical relevance. This prospective, monocentric observational study aimed to assess the role of WB-MRI in the characterization of clinically relevant radiological findings (ONCO-RADS) in adult NF1 patients, promoting a tailored approach.
**Methods or Background:** Since July 2024, 20 adult patients with NF1 (mean age 36 years, range 18–68) referred to the Medical Genetics Unit of Policlinico San Matteo, Pavia, underwent WB-MRI from head to toe. Each case was discussed by a multidisciplinary team to evaluate disease-related alterations and define appropriate management.
**Results or Findings:** Plexiform neurofibromas (PN), with variable size and location, were identified in 11/20 patients (55%), of which 6 were newly diagnosed. Other extracranial findings included cutaneous and subcutaneous neurofibromas (100%), skeletal alterations (60%), thyroid nodules (20%), splenomegaly (15%), diffuse neurofibromas (15%), and bullous dystrophy (5%). In one patient, an early-stage malignant lung tumor was diagnosed, allowing timely treatment. In another patient with a symptomatic inoperable PN, experimental treatment with Selumetinib was proposed.
**Conclusion:** This case series confirms the established role of WB-MRI as a safe tool for surveillance and multidisciplinary management of NF1-related manifestations, enabling the detection of previously unknown and clinically relevant lesions.
**Limitations:** Small cohort study; limited observation period; only preliminary restults are avalaible..
**Funding for this study:** No
**Has your study been approved by an ethics committee?** Not applicable
**Author Disclosures:**
Silvia Kalantari: Nothing to disclose
Maria Giovanna Di Niso: Nothing to disclose
Luisa Carone: Nothing to disclose
Michela Zacchino: Nothing to disclose
Lorenzo Preda: Nothing to disclose
Fabio Sirchia: Nothing to disclose

**The Role of Molecular Imaging in the Diagnostic Process of Unknown Primary Origin and Suspected NETs**
L. Alonzo[1], R. Cannella[1], R. Laudicella[2], V. Benfante[1], P. Purpura[1], G. Micci[1], M. Galia[1], G. Brancatelli[1], *P. Alongi*[1]; [1]Palermo/IT, [2]Messina/IT
*(alongi.pierpaolo@gmail.com)*

**Purpose:** The diagnosis of Neuroendocrine Tumors (NETs) can be challenging because of small lesion size, indolent growth, and variable metabolic activity, particularly in cases of unknown primary origin (CUP-NETs). Early and accurate identification of the primary lesion is essential for therapeutic decisions, including surgery, peptide receptor radionuclide therapy (PRRT), or somatostatin analogues. This systematic review investigated the diagnostic accuracy and clinical impact of molecular imaging, with particular emphasis on PET/CT with radiolabeled somatostatin analogs, in patients with suspected NETs and CUP-NETs.
**Methods or Background:** A comprehensive search of PubMed and Scopus was performed following PRISMA guidelines. Twenty-one clinical studies published between 2010 and 2024 were included, encompassing 1,857 patients (420 CUP-NETs and 452 suspected NETs). Extracted data included study design, radiotracers, detection rates, and changes in patient management. Methodological quality was assessed using CASP tools.
**Results or Findings:** PET/CT with [68Ga]Ga-DOTA-peptides consistently outperformed conventional [111In]Pentetreotide SPECT. Reported detection rates for primary tumors in CUP-NETs ranged from 38% to 83% with PET/CT, compared with less than 10% with SPECT. In suspected NETs, PET/CT achieved sensitivity up to 95% and specificity above 85%, leading to therapeutic changes in up to one-third of cases. Additional diagnostic yield was provided by dual-tracer protocols combining [68Ga]Ga-DOTATATE with [18F]FDG and by newer tracers such as [18F]DOPA and [18F]-OC, particularly for aggressive variants and rare subtypes (e.g., paragangliomas, insulinomas). Detection of unknown primaries frequently enabled curative surgery or more tailored systemic treatment.
**Conclusion:** [68Ga]Ga-DOTA-peptide PET/CT is a key tool in the diagnostic evaluation of suspected and unknown primary NETs. Beyond its superior sensitivity compared with conventional imaging, it significantly influences clinical decision-making and should be integrated into standard diagnostic algorithms.
**Limitations:** Several retrospective, single-center, and with small sample sizes articles, which may limit generalizability.
**Funding for this study:** None
**Has your study been approved by an ethics committee?** Not applicable
**Author Disclosures:**
Laura Alonzo: Nothing to disclose
Pierpaolo Alongi: Nothing to disclose
Giuseppe Micci: Nothing to disclose
Pierpaolo Purpura: Nothing to disclose
Massimo Galia: Nothing to disclose
Riccardo Laudicella: Nothing to disclose
Roberto Cannella: Nothing to disclose
Giuseppe Brancatelli: Nothing to disclose
Viviana Benfante: Nothing to disclose

**Quantitative magnetic resonance imaging for the differentiation of soft tissue tumors**
*F. Schön*, P. Günther, A. Weidlich, J. Kirchberg, M-L. Kromrey, R-T. Hoffmann, H. Fritzsche, J-P. Kühn; Dresden/DE

**Purpose:** This study investigated the potential of quantitative magnetic resonance imaging (MRI) to differentiate benign from malignant soft tissue tumors.
**Methods or Background:** Between 10/2022 and 01/2025, patients with soft tissue tumors were prospectively enrolled for quantitative MRI prior to histopathological confirmation. All 3.0T MRI examinations included chemical shift-encoded MRI, T1-/T2-mapping, and diffusion-weighted imaging (intravoxel incoherent motion [IVIM]). All MRI datasets were post-processed to generate R2*-, PDFF- (proton density fat fraction), T1-/T2-maps, and Diffusion (IVIM D), Pseudo-Diffusion (IVIM D*), Perfusion-Fraction (IVIM f). Histopathological diagnosis was defined as standard of reference. Mean values of two readers of each map were compared using Mann-Whitney U tests. A logistic regression model was established to predict malignancy.
**Results or Findings:** Fifty-nine patients (30 women; mean age 59.1+/-15.5 years) were examined, including 23 with malignant and 36 with benign tumors. Malignant tumors revealed significantly lower values for R2* (13.5+/-25.9 vs. 42.7+/-29.2; p<0.001), PDFF (29.3+/-38.1 vs. 60.2+/-42.4; p=0.003), IVIM D (1221.5+/-716.6 vs. 683.4+/-810.7; p=0.007), and IVIM D* (26.0+/-48.2 vs. 107.0+/-170.6; p=0.004) compared to benign tumors. In contrast, T1 relaxation times were significantly higher (1843.0+/-1065.4 vs. 1113.6+/-948.3; p=0.031). No significant differences were observed for IVIM f and T2 relaxation times (p>0.05). Univariate logistic regression analyses identified R2*, PDFF, IVIM D, and T1 as predictors for malignancy (p<0.05). Following backward elimination,

R2* remained as independent variable in the multivariate model (p=0.002; odds ratio=0.96), yielding an AUC of 0.800 with a Youden-Index of 0.539.
**Conclusion:** By enabling non-invasive differentiation of soft tissue tumors, quantitative MRI - particularly through the independent predictive value of R2* - may serve as a valuable tool to determine lesion dignity in clinical practice.
**Limitations:** Limited sample size and single-center setting.
**Funding for this study:** This work was supported by the Clinical Scientist Program MeDDrive of the University Medicine Dresden.
**Has your study been approved by an ethics committee?** Yes
**Ethics committee - additional information:** Approved by the local ethics committee of the Technical University Dresden (BO-EK-342072022_2)
**Author Disclosures:**
Ralf-Thorsten Hoffmann: Nothing to disclose
Jens-Peter Kühn: Nothing to disclose
Marie-Luise Kromrey: Nothing to disclose
Felix Schön: Nothing to disclose
Hagen Fritzsche: Nothing to disclose
Johanna Kirchberg: Nothing to disclose
Anne Weidlich: Nothing to disclose
Philipp Günther: Nothing to disclose

**Beyond Nodules: CT Spectrum of Pulmonary Metastases in osteosarcoma**
*A. K. Nawaz*, S. Ahmed, K. Siddique; Lahore/PK
*(dr.arubanawaz@gmail.com)*

**Purpose:** - To illustrate diverse diverse CT appearances of pulmonary metastasis in osteosarcoma. - To correlate imaging appearances with their frequency in clinical cohort. - To highlight the role of CT in staging, surveillance and treatment response assessment.
**Methods or Background:** Lungs are the most common site of metastasis in osteosarcoma. While soft tissue and calcified nodules are well known, atypical patterns such as cavitary, pleural or endobronchial disease are often underappreciated. Pulmonary metastasis in osteosarcoma are not just nodules - they represent a spectrum every radiologist must recognize. For this purpose, we retrospectively reviewed chest CTs of 46 patients with histologically confirmed osteosarcoma and pulmonary metastasis betrween 2023-2025. Imaging appearances were categorized into six groups: soft tissue nodules, calcified nodules, cavitary lesions, pleural involvement, endobronchial disease and mass like lesions.
**Results or Findings:** Soft tissue nodules were frequent finding, observed in 15 patients (32.6%). Calcified metastases were present in 14 (30.4%), pleural involvement in 11 (23.9%) and mass like lesions or consolidations in 5 (10.8%). Cavitary lesions and endobronchial disease were less common, identified in 2 patients each (4.3%).
**Conclusion:** Pulmonary metastases in osteosarcoma demonstrate a wide CT spectrum. Beyond the classic nodule, recognizing calcified, cavitary, pleural and airway patterns is essential for accurate staging, guiding treatment decisions and monitoring disease progression.
**Limitations:** None
**Funding for this study:** None
**Has your study been approved by an ethics committee?** Yes
**Ethics committee - additional information:** This retrospective anonymized review was conducted in accordance with our institutional IRB guidelines. No additional patient consent was required.
**Author Disclosures:**
Aruba Nawaz Khattak: Nothing to disclose
Kashif Siddique: Nothing to disclose
Shahroze Ahmed: Nothing to disclose

**Extra skeletal Ewing Sarcoma: Five-Year Review of 12 Rare Presentations**
*A. K. Nawaz*, M. Nawab, A. Usman, K. Siddique; Lahore, Pakistan/PK
*(dr.arubanawaz@gmail.com)*

**Purpose:** Ewing sarcoma is typically a bone tumor, but extra skeletal forms, though uncommon, can occur in diverse anatomical sites. Early recognition of these rare presentations is essential for accurate diagnosis and management. The objective of this study was to analyze the anatomical distribution and imaging characteristics of 12 histologically proven extra skeletal Ewing sarcoma cases from 2020–2025.
**Methods or Background:** In this retrospective study, we analyzed 208 cases of histologically proven cases of Ewing Sarcoma from January 20202 to January 2025. From these 208 cases, 12 extra skeletal cases were identified after excluding lesions in the upper limb, lower limb, mandible, and thorax. Demographic, anatomical, and imaging data were reviewed.
**Results or Findings:** Extra skeletal disease represented 5.8% of the cohort. Sites included: head & neck (5, 41.7%), pelvis (3, 25%), abdomen/retroperitoneum (2, 16.7%), thyroid (1, 8.3%), and uterus (1, 8.3%). Patients ranged from 14–32 years; male-to-female ratio was 1.3:1. Imaging revealed well-defined soft tissue masses without bone involvement.
**Conclusion:** Although rare, extra skeletal Ewing sarcoma can involve head & neck, pelvis, abdomen, thyroid, and uterus. Recognizing these histologically proven atypical sites is vital for timely diagnosis and guiding therapy. This five-year review highlights their frequency, distribution, and imaging features, emphasizing the need for awareness among radiologists and oncologists.
**Limitations:** None
**Funding for this study:** None
**Has your study been approved by an ethics committee?** Yes
**Ethics committee - additional information:** This retrospective anonymized review was conducted in accordance with our institutional IRB guidelines. No additional patient consent was required.
**Author Disclosures:**
Asma Usman: Nothing to disclose
Aruba Nawaz Khattak: Nothing to disclose
Kashif Siddique: Nothing to disclose
Mehreen Nawab: Nothing to disclose

**Primary hepatic angiosarcoma: Distinct imaging phenotypes mirroring histopathologic growth patterns**
B. J. Kim, M. J. Kim, *J. Y. Kim*; Daegu/KR
*(wldud971114@naver.com)*

**Purpose:** To date, there are no studies examining radiologic findings based on histologic patterns of primary hepatic angiosarcoma. This study aims to elucidate radiologic findings of primary hepatic angiosarcoma according to distinct histologic patterns.
**Methods or Background:** From January 2010 to October 2024, a cohort of 17 patients was included in this study. All patients were pathologically diagnosed with primary hepatic angiosarcoma and underwent CT with or without MRI. Histologic patterns were categorized into mass-forming and non-mass forming groups. The mass-forming type was subdivided into vasoformative and non-vasoformative (epithelioid, spindled) patterns, while the non-mass forming type was divided into sinusoidal and peliotic patterns. Two radiologists independently reviewed the CT and MRI images, categorizing the lesions into diffusely infiltrating and mass-forming types. Additionally, the presence of hypervascular portions and targetoid patterns was analyzed. The correlation between histologic patterns and radiologic findings was assessed using Fisher’s exact test.
**Results or Findings:** The mass-forming pattern was observed in 13 patients (76.5%), while the non-mass-forming pattern was identified in 4 patients (23.5%). There was a significant correlation (P<.05) between the radiologically classified patterns of diffusely infiltrating and mass-forming lesions and their pathological counterparts. However, the pathological classification of vasoformative and non-vasoformative lesions did not show a significant correlation with the presence of a hypervascular portion on imaging.
**Conclusion:** The pathological classification of hepatic angiosarcoma into mass-forming and non-mass-forming patterns closely corresponds to the radiological classification of mass-forming and diffusely infiltrating lesions, indicating that pathological findings are well reflected in imaging characteristics.
**Limitations:** Due to its retrospective design and relatively small number of patients, there may be inherent selection and sampling biases, and in cases with mixed histologic patterns, both histologic classification and radiologic interpretation were based on the predominant component, potentially limiting accuracy.
**Funding for this study:** The histologic pattern of primary hepatic angiosarcoma may be reflected in imaging as diffusely infiltrating or mass-forming appearances.
**Has your study been approved by an ethics committee?** Not applicable
**Author Disclosures:**
Byoung Je Kim: Nothing to disclose
Mi Jeong Kim: Nothing to disclose
Ji Yeong Kim: Nothing to disclose

**Diagnostic performance of Node-RADS in predicting lymph node metastases in abdominal and pelvic malignancies: a systematic review and meta-analysis**
*M. Ben salah Faria*, G. Raia, F. Peier, L. Widmer, H. Thoeny; Fribourg/CH
*(mariem.bsf7@gmail.com)*

**Purpose:** Our systematic review and meta-analysis aim to evaluate the diagnostic performance of Node Reporting and Data System 1.0 (Node-RADS) for identifying lymph node (LN) metastases, using histopathology as a reference standard, across a range of abdominal and pelvic malignancies.
**Methods or Background:** This review was conducted according to the Preferred Reporting Items for Systematic Reviews and Meta-Analyses (PRISMA) guidelines. PubMed, ScienceDirect and Cochrane Library databases were systematically searched until August 2025. We included studies analysing patients diagnosed with an abdominal or pelvic malignancy, who underwent contrast-enhanced CT or MRI for loco-regional work-up, with

LN assessment by Node-RADS, associated with LN histopathological analysis. Study quality was assessed by modified Quality Assessment of Diagnostic Accuracy Studies (QUADAS-2) tool. Diagnostic performance was estimated by the random-effects model. Forest plots of pooled sensitivity and specificity were generated for two different Node-RADS thresholds.
**Results or Findings:** Eighteen retrospective studies were included, comprising 10 using CT and 8 using MRI. These studies covered a variety of malignancies: gynecologic (endometrial, ovarian, cervical), urologic (prostate, bladder, renal), and gastrointestinal cancers (colon, rectal, gastric, cholangiocarcinoma, periampullary). When applying a Node-RADS threshold of ≥3 to define malignant LN, the pooled sensitivity and specificity were 0.80 (95%CI, 0.69-0.87) and 0.79 (95%CI, 0.68-0.87), respectively. For a threshold of ≥4, sensitivity decreased to 0.60 (95%CI, 0.50-0.69), while specificity improved to 0.94 (95%CI, 0.90-0.96). The hierarchical summary receiver operating characteristic (HSROC) analysis showed area under the curves (AUCs) of 0.85 (95%CI, 0.79-0.89) and 0.87 (95%CI, 0.80-0.92) for thresholds ≥3 and ≥4, respectively.
**Conclusion:** Node-RADS score demonstrated reliable diagnostic performance in assessing lymph node malignancy across abdomino-pelvic malignancies, allowing a standardized approach that enhances reproducibility and supports clinical decision-making in oncologic staging.
**Limitations:** Heterogeneity of the included studies, which are of retrospective design.
**Funding for this study:** None
**Has your study been approved by an ethics committee?** Not applicable
**Author Disclosures:**
Giorgio Raia: Nothing to disclose
Mariem Ben Salah Faria: Nothing to disclose
Franziska Peier: Nothing to disclose
Harriet Thoeny: Nothing to disclose
Lucien Widmer: Nothing to disclose

**16:00 - 17:30** **Research Stage 5**

### Research Presentation Session: Breast

### RPS 2102

### Current innovations in contrast enhanced mammography

**Moderator**
Eva M. Fallenberg; München/DE

### Integrating qualitative and quantitative features from contrast-enhanced mammography to predict breast lesion malignancy

*I. Allajbeu*[1], R. Manavaki[1], T. Wegman[2], J. A. Saenz[3], T. Van Nijnatten[2], F. J. Gilbert[1]; [1]Cambridge/UK, [2]Maastricht/NL, [3]Barcelona/ES
*(ia359@cam.ac.uk)*

**Purpose:** Quantitative analysis of enhancement on contrast-enhanced mammography (CEM) has shown promise in distinguishing benign from malignant breast lesions. We evaluated diagnostic models combining low-energy (LE) mammographic features with quantitative enhancement metrics.
**Methods or Background:** Data from 251 CEM examinations (2018-2021) with identifiable, histologically confirmed lesions were retrospectively analysed. Lesion characteristics, including lesion diameter (LD), type (mass, calcification, distortion, asymmetry), background parenchymal enhancement (BPE), breast density (BD), and conspicuity, were assessed on LE and recombined images by three radiologists using BI-RADS criteria. Enhancement metrics were computed from early (CEearly) and late (CElate) views, with percent residual signal difference (%RSD) used to classify enhancement patterns as progressive, plateau, or wash-out. Model construction utilised nested cross-validation (CV) with stratified sampling. In each of ten outer folds, data were stratified into 90% training and 10% testing subsets. Feature selection was optimised within the training data using five-fold inner CV. Minimum redundancy–maximum relevance identified non-redundant, informative predictors for logistic regression. Performance was assessed on the outer test sets and averaged across folds using area-under-the-curve (AUC), accuracy, sensitivity, and specificity.
**Results or Findings:** Of 251 lesions, 155 (61.7%) were malignant and 96 (38.3%) benign. Eleven features (age, LD, type, BI-RADS score, BD, BPE, conspicuity, CEearly, CElate, RSD, enhancement type) were considered for model construction. The most predictive subset included BI-RADS score, age, lesion conspicuity, BD, CELate, and LD. Models with these features outperformed those using BI-RADS score alone, with higher AUC (0.88 vs 0.84), accuracy (83% vs 78%), sensitivity (89% vs 72%), and comparable specificity (85%).
**Conclusion:** Integrating standard BI-RADS descriptors with quantitative enhancement metrics can improve lesion discrimination on CEM versus BI-RADS alone. This combined approach may enhance diagnostic confidence and reduce unnecessary biopsies in clinical practice
**Limitations:** Single-centre study
**Funding for this study:** Cambridge Biomedical Research Centre (BRC) CUH NHS Foundation Trust
**Has your study been approved by an ethics committee?** Not applicable
**Author Disclosures:**
Thiemo Van Nijnatten: Speaker: GE Healthcare
Javier Azcona Saenz: Speaker: Bayer and GE healthcare
Thomas Wegman: Nothing to disclose
Roido Manavaki: Nothing to disclose
Fiona J. Gilbert: Grant Recipient: CRUK, NIHR , Bayer and GE funding for research
Iris Allajbeu: Speaker: GE healthcare Grant Recipient: BRC pump priming funding, Isaac Newton bridging funding, CUH NHS funding for research

### Comparison of Detection Rate, Lesion Visibility and Lesion Conspicuity in Contrast-Enhanced Mammography Using Two Different Doses

*A. Stiglbauer-Tscholakoff*[1], G. Chiffi[2], P. D. Stelzer[1], T. H. Helbich[1], P. Clauser[1], P. A. Baltzer[1]; [1]Wien/AT, [2]Rome/IT
*(alexander.stiglbauer@meduniwien.ac.at)*

**Purpose:** To compare detection rate, lesion visibility and conspicuity in contrast enhanced mammography using two different doses.
**Methods or Background:** A total of 256 lesions were included: in 122 lesions Iomeron®400 was administered with a dose of 1 ml/kg bodyweight at 2 ml/s flow (group 1) and in 134 lesions with 1.5 ml/kg at 2 ml/s (group 2). Two breast radiologists independently evaluated lesion visibility (overall, 1-5) and conspicuity (in comparison to background enhancement, 0-3). Lesions were classified as benign/malignant (biopsy-proven). Distribution of malignant/benign lesions (chi-square test), diagnostic performance, intra-reader comparisons, and inter-reader differences (Student's t-test) were assessed.
**Results or Findings:** There was no significant difference in the distribution of malignant/benign lesions in group1/group2 (P = 0.4627). Almost all malignant lesions were detected by both readers (group 1: 70/71, group 2: 83/84). Mean lesion conspicuity in group 1 was 1.7 (SD 0.9) for Reader 1 and 1.5 (SD 1.1) for Reader 2, in group 2 2.2 (SD 0.8, R1) and 2.1 (SD 1.0, R2). Mean lesion visibility in group 1 was 3.4 (SD 1.5, R1) and 2.9 (SD 1.3, R2), in group 2 3.5 (SD 1.4, R1) and 3.7 (SD 1.3, R2). Intra-reader analyses showed lower conspicuity for R1 and R2 in group 1 (R1&R2, t-test P < 0.0001), equal visibility (R1, t-test P = 0.5816) and lower visibility (R2, t-test P < 0.0001) in this group.
**Conclusion:** Lesion visibility in CEM was higher using 1,5 ml. Lesion conspicuity was reader dependent. The detection rate of malignant lesion was high with both doses, suggesting that a higher dose of contrast agent may improve lesion conspicuity in comparison to background enhancement but a lower dose might be sufficient for lesion detection.
**Limitations:** Single-centre design; selection bias; no clinical outcomes correlation.
**Funding for this study:** Bracco Imaging
**Has your study been approved by an ethics committee?** Yes
**Ethics committee - additional information:** Data were collected within a prospective study comparing the diagnostic value of CEM to CE-MRI in a problem-solving setting (Ethics Review Board number 2282/2019).
**Author Disclosures:**
Pascal A.T. Baltzer: Grant Recipient: Bracco Grant Recipient: Siemens
Alexander Stiglbauer-Tscholakoff: Nothing to disclose
Philipp Dominik Stelzer: Nothing to disclose
Thomas H. Helbich: Grant Recipient: Siemens
Greta Chiffi: Nothing to disclose
Paola Clauser: Grant Recipient: Siemens

**Preoperative contrast-enhanced mammography: the contribution of 7-minute delayed acquisition**
*L. Corradini*, C. Depretto, C. De Berardinis, G. Della Pepa, E. D'Ascoli, G. Irmici, A. Bonanomi, F. Sardanelli, G. P. Scaperrotta; Milan/IT
*(corradinilisa@gmail.com)*

**Purpose:** To evaluate the diagnostic performance of contrast-enhanced mammography (CEM) in preoperative locoregional staging of breast cancer, with a specific focus on the contribution of 7-minute acquisitions, through a retrospective analysis of consecutive cases at a reference oncology center.
**Methods or Background:** We included patients who underwent CEM between January 2018 and January 2024 at the National Cancer Institute (Milan). Two independent readers, each with over five years of experience in CEM, performed blinded assessments without knowledge of clinical history or biopsy results. A BI-RADS score (0–5) was assigned to each breast, first based soley on 2-minute acquisitions and subsequently incorporating information from 7-minute images. The reference standard included histopathological findings (diagnostic or definitive), follow-up, or comparison with prior imaging. Diagnostic performance was compared using McNemar's test.
**Results or Findings:** A total of 286 breasts in 144 patients (including two cases of prior unilateral mastectomy) were evaluated. The additional review of 7-minute acquisitions led to a significant modification of the BI-RADS score and/or other assessments in 12/286 breasts (4.2%), with clinical impact in 2/144 patients (1.39%, 95% CI 0.17−4.93%). The diagnostic performance of 2-minute acquisitions alone was not significantly different from that obtained when including 7-minute acquisitions: sensitivity 94.0% vs. 94.6% (p = 1.00); specificity 97.7% vs. 96.2% (p = 0.48).
**Conclusion:** Delayed 7-minute CEM acquisitions do not provide clinically relevant additional information for preoperative breast cancer assessment. A protocol limited to 2-minute acquisitions reduces exposure to ionizing radiation and simplifies workflow without compromising the diagnostic value of the examination.
**Limitations:** Retrospective, single-center study with a limited number of patients.
**Funding for this study:** This study received no funding.
**Has your study been approved by an ethics committee?** Yes
**Ethics committee - additional information:** The study was approved by Comitato Etico Territoriale Lombardia 4
**Author Disclosures:**
Gianmarco Della Pepa: Nothing to disclose
Elisa D'Ascoli: Nothing to disclose
Francesco Sardanelli: Nothing to disclose
Alice Bonanomi: Nothing to disclose
Gianfranco Paride Scaperrotta: Nothing to disclose
Catherine Depretto: Nothing to disclose
Lisa Corradini: Nothing to disclose
Claudia De Berardinis: Nothing to disclose
Giovanni Irmici: Nothing to disclose

**Diagnostic Accuracy of Contrast-Enhanced Mammography Compared with Breast MRI in Women at Increased Breast Cancer Risk**
*G. Gennaro*[1], E. Baldan[1], P. Belli[2], D. Bernardi[3], E. Bezzon[1], A. Coran[1], V. Iotti[4], I. Polico[1], F. Caumo[1]; [1]Padua/IT, [2]Rome/IT, [3]Rozzano/IT, [4]Reggio Emilia/IT
*(gisella.gennaro@iov.veneto.it)*

**Purpose:** To compare the diagnostic performance of contrast-enhanced mammography (CEM) versus breast MRI in women at increased risk for breast cancer.
**Methods or Background:** In this paired-case study, 462 women underwent both CEM and MRI. Four breast radiologists interpreted CEM, while a separate group of four radiologists evaluated MRI. Multi-reader, multi-case (MRMC) ROC analysis was used to calculate AUC for each modality. Sensitivity, specificity, positive predictive value (PPV), negative predictive value (NPV), and overall accuracy were also estimated. Low-energy CEM (LE-CEM) readings were included as a baseline reference. Statistical significance was set at p<0.05 for all comparisons.
**Results or Findings:** LE-CEM to CEM comparisons confirmed prior evidence, with mean AUC improving from 0.832 (95% CI: 0.809–0.855) to 0.913 (95% CI: 0.897–0.930, p<0.001). For CEM versus MRI, mean AUCs were 0.936 (95% CI: 0.915–0.958) for CEM and 0.933 (95% CI: 0.916–0.949) for MRI (p=0.665). Sensitivity, specificity, PPV, NPV, and accuracy were also similar between CEM and MRI (sensitivity 0.83 vs 0.86, specificity 0.91 vs 0.87, PPV 0.77 vs 0.70, NPV 0.94 vs 0.95, accuracy 0.89 vs 0.86; all p>0.30).
**Conclusion:** CEM demonstrates diagnostic performance equivalent to breast MRI and significantly outperforms mammography (LE-CEM). These results support CEM as a high-quality, more accessible alternative to MRI for risk-adapted screening.
**Limitations:** Retrospective analysis with readers not fully crossed between modalities.
**Funding for this study:** Veneto Region, grant number RSF-2017-00000562
**Has your study been approved by an ethics committee?** Yes
**Ethics committee - additional information:** Institutional Ethics Committee Veneto Institute of Oncology (IOV)— IRCCS, Padua, Italy. Protocol code CE IOV #2017/92
**Author Disclosures:**
Valentina Iotti: Speaker: GE Healthcare
Francesca Caumo: Speaker: Hologic Speaker: GE Healthcare Speaker: BD
Enrica Baldan: Nothing to disclose
Elisabetta Bezzon: Nothing to disclose
Gisella Gennaro: Speaker: Hologic Speaker: GE Healthcare Speaker: BD
Ilaria Polico: Nothing to disclose
Alessandro Coran: Nothing to disclose
Paolo Belli: Nothing to disclose
Daniela Bernardi: Speaker: Siemens

**Contrast-Enhanced Mammography versus Ultrasound in the Assessment of Recall Examinations for Suspicious Findings in Women with a Personal History of Breast Cancer: A Retrospective Comparative Study**
*V. Lamagna*, C. Bellini, G. Bicchierai, D. De Benedetto, F. Di Naro, S. Vidali, F. Pugliese, V. Miele, J. Nori; Florence/IT
*(verdiana.lamagna@unifi.it)*

**Purpose:** To compare the performance of Contrast-Enhanced Mammography (CEM) vs. ultrasound (US) in women with personal history of breast cancer (PHBC) who are recalled for suspicious mammographic findings.
**Methods or Background:** This retrospective single-center study included women with PHBC recalled for suspicious findings on digital mammography between November 2016 and June 2023. All underwent both CEM and targeted US. Two experienced breast radiologists independently reviewed the images in a blinded manner. For each modality, findings were categorized as positive (BIRADS 4 o 5) or negative (BIRADS 1,2 or 3) based on BI-RADS classification. Histopathology served as the reference standard for positive cases, while a negative 24-month follow-up was used for negative cases. Sensitivity, specificity, positive and negative predictive values, and overall accuracy were calculated for each modality.
**Results or Findings:** Among 295 women included, 54 (18.3%) had malignant lesions. CEM demonstrated significantly higher sensitivity than ultrasound for lesion detection (98.2% vs 35.2%, p<0.001) and higher specificity (91.7% vs 72.2%, p<0.01). CEM accurately identified 53 of 54 malignant lesions confirmed on histopathology with a PPV of 72.6% and a NPV of 99.5%, including several that were occult or equivocal on ultrasound that showed a PPV of 22.1% and a NPV of 83.3%. The only missed case was a non-enhancing ductal carcinoma in situ. Overall diagnostic accuracy was higher for CEM than for ultrasound (92.9% vs 65.4%, p<0.001).
**Conclusion:** CEM compared to US is a reliable and efficient first-line imaging technique in in women with a personal history of breast cancer, potentially improving workflow efficiency in breast cancer follow-up imaging.
**Limitations:** This study was limited by its retrospective single-center design.
**Funding for this study:** No funding was received for this study.
**Has your study been approved by an ethics committee?** Not applicable
**Author Disclosures:**
Vittorio Miele: Nothing to disclose
Giulia Bicchierai: Nothing to disclose
Federica Di Naro: Nothing to disclose
Francesca Pugliese: Nothing to disclose
Diego De Benedetto: Nothing to disclose
Jacopo Nori: Nothing to disclose
Sofia Vidali: Nothing to disclose
Chiara Bellini: Nothing to disclose
Verdiana Lamagna: Nothing to disclose

**Kidney function determination in recalled women from screening scheduled for contrast-enhanced mammography – a single center retrospective analysis**
*E. Meltem*[1], R. Wijgers[2], U. Lalji[2], C. R. Jeukens[2], J. E. Wildberger[2], E. C. Nijssen[2], T. Van Nijnatten[2]; [1]Istanbul/TR, [2]Maastricht/NL
*(meltem_emine@hotmail.com)*

**Purpose:** To evaluate the true extent of iodine-based contrast media (ICM)-related risk of kidney function decline in a breast cancer screening population, recalled and scheduled for contrast-enhanced mammography (CEM).
**Methods or Background:** This retrospective study included women recalled from the Dutch National Breast Cancer Screening Program to Maastricht University Medical Centre+ (MUMC+) between January 2013 and January 2022. Recalled patients routinely underwent CEM, while those with contraindications received full-field digital mammography (with or without digital breast tomosynthesis). The most recent estimated glomerular filtration rate (eGFR) before and after imaging was obtained from electronic medical records. Renal safety was assessed by changes in eGFR; a ≥25% decline within 30 days after CEM was considered clinically relevant.

Saturday

**Results or Findings:** A total of 2707 women were recalled, of whom 2437 (90%) underwent CEM. Paired pre- and post-CEM eGFR values were available in 1184 patients, with pre-CEM values obtained a median of 177 days before (IQR 420-50) and post-CEM values 141 days after imaging (IQR 28-423). The median eGFR difference was 0.5 (IQR -5.4 to 6.3). A ≥25% decline in eGFR occurred in 31 patients (2.6%); only one (0.08%) dropped below an eGFR of 30. Most declines reflected shifts from baseline eGFR >60 to the 45–60 range (n=74, 8%), while eGFR remained stable in those with baseline values <45 (n=13). In the subgroup with post-CEM eGFR within 30 days (n=306), ≥25% decline was observed in six patients(2.0%); none dropped below eGFR 45, and changes were again mainly shifts from >60 to 45–60 (n=15, 6%).
**Conclusion:** Clinically relevant renal function decline after CEM was rare and never severe, supporting the notion that kidney safety concerns should not restrain the implementation of CEM in clinical practice.
**Limitations:** Variability in the timing of eGFR measurements.
**Funding for this study:** Institutional grant support from Bayer
**Has your study been approved by an ethics committee?** Yes
**Ethics committee - additional information:** Institutional ethical approval/2023
**Author Disclosures:**
Thiemo Van Nijnatten: Speaker: Hologic Inc. (Speaker honoraria) Consultant: ScreenPoint Medical (Consultancy agreement) Research/Grant Support: Bayer and GE Healthcare (Institutional grant support) Advisory Board: Bayer and GE Healthcare.
Renske Wijgers: Nothing to disclose
Joachim E. Wildberger: Nothing to disclose
Cécile Rlpn Jeukens: Nothing to disclose
Estelle Claire Nijssen: Nothing to disclose
Emine Meltem: Nothing to disclose
Ulrich Lalji: Nothing to disclose

**Lesion conspicuity on contrast-enhanced mammography as a predictor of response to Cryoablation in breast cancer**
F. Pugliese[1], *F. Di Naro*[1], S. E. Baldi Giorgi[1], C. Maiello[2], V. Lamagna[1], D. De Benedetto[1], G. Bicchierai[1], C. Bellini[1], J. Nori[1]; [1]Florence/IT, [2]Casoria (Na)/IT
*(federicanarella@gmail.com)*

**Purpose:** To evaluate the role of lesion conspicuity (LC) at 12 months on contrast-enhanced mammography (CEM) follow-up as a predictor of cryoablation outcome in breast cancer.
**Methods or Background:** We conducted a retrospective analysis of 69 patients (mean age: 86 years; range: 60–94) with unifocal invasive breast cancer who underwent percutaneous cryoablation. CEM imaging was performed at baseline and within 12 months post-ablation. LC was categorized based on the degree of enhancement relative to background as: low, moderate, or high. Tumor response was assessed at 12 months using standard imaging modalities (ultrasound and CEM) according to RECIST 1.1 criteria. Statistical analyses were conducted to evaluate the correlation between changes in post-treatment conspicuity and later tumor response.
**Results or Findings:** At 12 months post-ablation, CEM showed a complete absence of enhancement in 68.1% of lesions. Among the 22/69 (32%) lesions showing enhancement, 6 had low LC, 11 moderate, and 5 high. The agreement between CEM and biopsy results was strong, with 100% sensitivity and positive predictive value for CEM. A substantial concordance (Kappa=0.80) was observed between CEM and RECIST criteria. Among lesions that were minimally conspicuous or non-enhancing early post-treatment, 80.7% had a complete response at 12 months, compared to only 1.8% of lesions that retained enhancement. In tumors ≤2 cm, early post-ablation CEM showing reduced or absent conspicuity was significantly associated with complete response (Kappa=0.74), with a 78.8% complete response rate in this subgroup.
**Conclusion:** A reduction in lesion conspicuity on early post-ablation CEM strongly correlates with a favorable tumor response in breast cancer patients treated with cryoablation. This imaging feature may serve as an early, noninvasive biomarker for treatment evaluation and could help guide clinical decision-making.
**Limitations:** retrospective study small sample size did't compare CEM-MRI
**Funding for this study:** None
**Has your study been approved by an ethics committee?** Yes
**Ethics committee - additional information:** Meyer Hospital Florence
**Author Disclosures:**
Giulia Bicchierai: Nothing to disclose
Federica Di Naro: Nothing to disclose
Francesca Pugliese: Nothing to disclose
Diego De Benedetto: Nothing to disclose
Jacopo Nori: Nothing to disclose
Sofia Elisabetta Baldi Giorgi: Nothing to disclose
Chiara Bellini: Nothing to disclose
Verdiana Lamagna: Nothing to disclose
Chiara Maiello: Nothing to disclose

**A trial-based economic evaluation of contrast-enhanced mammography (CEM) compared to conventional imaging in the work-up of women recalled from the Dutch breast cancer screening program (RACER trial)**
*L. M. Neeter*[1], M. B. I. Lobbes[2], K. M. Duvivier[3], F. Raat[4], M. Smidt[1], J. E. Wildberger[1], B. Essers[1]; [1]Maastricht/NL, [2]Sittard-Geleen/NL, [3]Amsterdam/NL, [4]Roermond/NL

**Purpose:** The randomised controlled clinical RACER trial studied the diagnostic work-up with contrast-enhanced mammography (CEM) compared to conventional imaging as primary tool in women recalled from breast cancer screening. This current trial-based economic evaluation was performed from a hospital perspective.
**Methods or Background:** Cost prices were retrieved from the financial departments of the Maastricht University Medical Center. Health-related quality of life was measured five times over 18 months using the EQ-5D-5L questionnaire. Cost-utility analysis outcome was expressed as costs per quality-adjusted life year (QALY). Multiple imputation was used for missing data, and non-parametric bootstrap analysis was performed to examine uncertainty in the difference in costs and incremental costs per QALY. Post hoc subgroup analysis was performed per BI-RADS recall score.
**Results or Findings:** Work-up with CEM showed lower total costs of €-117 (95% CI €-254; €+22) compared to conventional imaging. Average imaging costs were significantly lower with CEM (mean difference €-130; 95% CI €-153; €-105). Mean QALY for the CEM group was 1.2034 versus 1.2137 for the control group. Bootstrap analysis showed that 95% of all simulated ICERs were in the quadrants that indicate cost-savings, although 25% of the ICERs showed a small gain in QALY and 70% a small QALY loss. The probability of CEM being cost-effective is 53% at a threshold of €10,000 to accept a QALY loss. In the subgroup of BI-RADS 0 recalls, specifically, this probability is 85%.
**Conclusion:** CEM as primary tool in the diagnostic work-up is a cost-effective diagnostic strategy, especially in BI-RADS 0 (low suspicion for breast cancer) recalls.
**Limitations:** The proportion of missing data related to the EQ-5D-5L questionnaire increased over time up to 42% and 34% in the control and CEM group, respectively.
**Funding for this study:** ZonMw Efficiency Studies (Grant: 843001801); GE Healthcare
**Has your study been approved by an ethics committee?** Yes
**Ethics committee - additional information:** Institutional Review Board approval: METC171082/NL62788.068.17
**Author Disclosures:**
Marjolein Smidt: Nothing to disclose
Lidewij M.F.H. Neeter: Nothing to disclose
Marc B I Lobbes: Advisory Board: received honorary fees for presentations and participation of medical advisory boards from GE Healthcare, Hologic Inc., Sirius Medical B.V., Bayer, Guerbet, and Tromp Medical B.V. Research/Grant Support: received research funding from GE Healthcare, Hologic Inc., and Sirius Medical B.V. Speaker: received honorary fees for presentations and participation of medical advisory boards from GE Healthcare, Hologic Inc., Sirius Medical B.V., Bayer, Guerbet, and Tromp Medical B.V.
Joachim E. Wildberger: Other: J.W. received institutional grants from Abbott, Anaconda Biomed, Asklepios, Bayer, Becton & Dickinson medical, Bentley, Boston, Brainlab, GE Healthcare, Gleamer, Hologic, Inari Medical, Johnson & Johnson, Merit Medical Systems, Nico-Lab, Medtronic, Microvention, Nova Techs, Oldelft Benelux, Ontario Associatino of Radiologists, Penumbra, Philips, Siemens, Stryker, Tajpan Sro.
Katya M. Duvivier: Nothing to disclose
Frank Raat: Nothing to disclose
Brigitte Essers: Nothing to disclose

**How Women Experience Contrast-Enhanced Mammography**
*J. Theunissen*[1], Y. Vissers[1], B. Van Grinsven[2], A. Valentijn-Morsing[1], M. B. I. Lobbes[1], L. Bouwman[2]; [1]Sittard-Geleen/NL, [2]Maastricht/NL
*(ja.theunissen@zuyderland.nl)*

**Purpose:** Contrast-enhanced mammography (CEM) has witnessed an increasing implementation in clinical practice and is currently even considered for large-scale screening programs. However, successful adoption of screening CEM is dependent on patient acceptance. This study investigated women's experiences with CEM, focusing on pain and willingness for future CEM examinations.
**Methods or Background:** In this prospective survey at a specialized breast center, women undergoing CEM completed a 10-minute questionnaire. A questionnaire collected data on demographics, medical history, expectations, and patient-reported outcomes. Procedural data such as prior imaging, biopsy during the same visit, and compression force were collected from clinical records.
**Results or Findings:** Currently, 478 women (median age 58 years) completed the questionnaire, with 382 (79.9%) also participating in the national screening program. Median Visual Analogue Scale pain scores were 1 (IQR 0 – 2) for intravenous cannulation and 3 (IQR 1-6) for breast compression. Overall, 475

(99.4%) participants reported willingness to undergo CEM again if indicated, and 458 (95.8%) would undergo CEM screening if it improved cancer detection rates. Twenty-three participants (4.8%) reported pain during compression as a potential obstacle, and 15 participants (3.1%) reported the same for intravenous cannulation. Amongst 25 women who had undergone a prior MRI, 16 (64%) considered CEM more patient-friendly, 7 (28%) rated both modalities equally, and 2 (8%) preferred MRI. Multivariable analysis of factors associated with pain experience and willingness for CEM screening is planned once all data are collected.
**Conclusion:** CEM is well tolerated, with limited discomfort reported during both intravenous cannulation and compression. Nearly all participants expressed willingness to undergo CEM again, both clinically and for screening.
**Limitations:** The single-center questionnaire may limit generalizability. Outcomes relied on self-reported data which introduces possible response bias.
**Funding for this study:** Zuyderland MC
**Has your study been approved by an ethics committee?** Not applicable
**Author Disclosures:**
Anja Valentijn-Morsing: Nothing to disclose
Yvonne Vissers: Nothing to disclose
Marc B. I. Lobbes: Nothing to disclose
Bart Van Grinsven: Nothing to disclose
Jarn Theunissen: Nothing to disclose
Lee Bouwman: Nothing to disclose

**Patient Acceptance and Experience of Contrast-Enhanced Mammography in Breast Cancer follow-up: results from a large prospective cohort**
*G. Vatteroni*[1], R. Levi[1], M. Filippini[2], M. Costa[3], R. M. Trimboli[1], D. Bernardi[1]; [1]Milan/IT, [2]Nuvolera/IT, [3]Catania/IT
*(giulia.vatteroni@gmail.com)*

**Purpose:** To evaluate patient acceptance and experience of contrast-enhanced mammography (CEM) compared to standard digital mammography (DM) in women operated for breast cancer (BC) undergoing follow-up.
**Methods or Background:** This study included women operated for BC who underwent CEM instead of DM for BC surveillance as part of a prospective study (COMBO-TRIAL). A 16-item anonymous questionnaire was administered after the exam, evaluating perceived discomfort, pain, exam duration, reassurance, information received, anxiety, contrast injection, satisfaction and comparative experience with prior DM. Responses used Likert-type scales (1-5). Descriptive and comparative analyses (T-Test or Mann-Whitney U test, Pearson's R, Spearman's R correlation) were conducted (p<0.05 significant).
**Results or Findings:** Among 969 patients, 936 (median-age 58.2 years, IQR 51–66.25) completed the questionnaire. CEM was reported to cause similar discomfort compared to DM by 67.4% of women, while 14.1% experienced greater discomfort, strongly associated with higher pre-exam anxiety(p<0.001) and lower reassurance(p<0.001). Likewise, 77.5% reported similar pain, whereas 7.4% perceived CEM as more painful, also linked to increased anxiety(p<0.001) and reduced reassurance (p<0.001). Examination positioning was considered comparable by 76.6% of patients, and with a similar duration (64.2%). Overall, 92.6% felt reassured and 95% well informed. Elevated anxiety was reported by 21.2%, with only 32.6% reporting high comfort during contrast injection. Pre-exam anxiety was inversely correlated with injection comfort (r =–0.265, p<0.001). Importantly, 90.6% accepted the risk of contrast-related events if CEM enabled earlier BC detection. Patient satisfaction was high (96.1%), and nearly all (99.6%) would recommend CEM, highlighting its perceived diagnostic value and role in early detection.
**Conclusion:** CEM showed excellent acceptance, tolerability and satisfaction compared with DM in BC follow-up. Distress was limited to a small subgroup with elevated pre-exam anxiety, highlighting the radiologist's key role in communication and patient reassurance.
**Limitations:** n/a
**Funding for this study:** n/a
**Has your study been approved by an ethics committee?** Yes
**Ethics committee - additional information:** n/a
**Author Disclosures:**
Rubina Manuela Trimboli: Nothing to disclose
Daniela Bernardi: Nothing to disclose
Martina Costa: Nothing to disclose
Giulia Vatteroni: Nothing to disclose
Marco Filippini: Nothing to disclose
Riccardo Levi: Nothing to disclose

**Assessing the optimal amount of tissue sampling required in contrast-enhanced stereotactic biopsy (CESB)**
M. B. I. Lobbes[1], *J. Theunissen*[1], R. Körver-Steeman[1], C. Frotscher[1], A. Valentijn-Morsing[1], B. De Vries[1], W. L. Mok[2], Y. Vissers[1], L. Bouwman[1]; [1]Sittard-Geleen/NL, [2]Roermond/NL
*(ja.theunissen@zuyderland.nl)*

**Purpose:** Contrast-enhanced mammography (CEM) can reveal lesions not visible on low-energy images, targeted ultrasound, or tomosynthesis. When such lesions are detected, contrast-enhanced stereotactic biopsy (CESB) is required for tissue diagnosis. However, the optimal number of tissue samples needed for a reliable diagnosis remains unclear.
**Methods or Background:** We included patients with lesions visible only on recombined CEM-images, warranting CESB. Using a 9-gauge vacuum-assisted biopsy (VAB) needle, 18 consecutive tissue samples were obtained per protocol. A pathologist evaluated each sample in the order collected to determine the cumulative diagnostic yield per sample. Additional parameters assessed included patient age, procedure duration (excluding IV catheter placement), adverse events, patient-reported discomfort (10-point scale), and the positive predictive value (PPV) for malignancy.
**Results or Findings:** Sixty women (mean age 57.8 years, SD 10.6) underwent CESB. Mean procedure time was 13.5 minutes. One procedure was discontinued due to syncope. Median discomfort score was 3. The PPV for malignancy was 31.7%. The median number of samples needed to establish a diagnosis was five (range: 1–18). Diagnostic yield increased with each additional sample, reaching 98.3% by the 12th biopsy. In one case, all 18 samples were required, as no lesion was identified; follow-up is ongoing.
**Conclusion:** CESB is a well-tolerated and safe technique with minimal discomfort and low complication rates. Our findings suggest that obtaining 12 samples with a 9-gauge VAB needle provides a high diagnostic yield and is sufficient in most cases to reach a reliable diagnosis.
**Limitations:** Relatively small sample size, but study is currently recruiting.
**Funding for this study:** Hologic Inc.
**Has your study been approved by an ethics committee?** Yes
**Ethics committee - additional information:** Ethical committee approval acquired.
**Author Disclosures:**
Anja Valentijn-Morsing: Author: Author
Yvonne Vissers: Author: Author
Marc B. I. Lobbes: Author: Author Research/Grant Support: Hologic Inc. Speaker: Hologic Inc.; Tromp Medical
Wai Lan Mok: Author: Author
Bart De Vries: Author: Author
Caroline Frotscher: Author: Author
Jarn Theunissen: Author: Author
Rachelle Körver Steeman: Author: Author
Lee Bouwman: Author: Author

**RC-only lesions in CEM: false-positive outcomes and upgrade rates from CEM-guided biopsy**
*R. Alcantara Souza*, J. Saenz, M. Pitarch, P. Miranda Martínez, E. Vall Foraster, E. Vila-Trias Jover, N. Arenas, X. Castells; Barcelona/ES
*(ralcantara@psmar.cat)*

**Purpose:** To evaluate the false-positive rate and histopathological spectrum of recombined (RC)-only lesions requiring contrast-enhanced mammography (CEM)-guided biopsy.
**Methods or Background:** Retrospective analysis of consecutive CEM-guided biopsies performed at a tertiary centre between 2019–2025. Demographics, CEM descriptors (mass, non-mass, enhancing asymmetry), and histopathology were extracted from a REDCap database. Pathology was categorised according to the NHSBSP system: B1 normal, B2 benign, B3 lesions of uncertain malignant potential, and B5 malignant. False positives were defined as B1–B3. RC-only lesions were enhancing findings without a low-energy correlate and represented the main indication for CEM-guided biopsy at our centre.
**Results or Findings:** A total of 174 RC-only lesions in 170 patients (median age 59 years, range 22–88) underwent CEM-guided biopsy with pathology. B1 was found in 3 (1.7%), B2 in 73 (42.0%), B3 in 27 (15.5%), and B5 in 71 (40.8%). The false-positive rate (B1–B3) was 59.2%. Malignancy rates were 45.4% in masses, 36.5% in non-mass enhancements, and 33.3% in enhancing asymmetries (OR mass vs non-mass 1.45; 95% CI 0.78–2.69). Benign outcomes included fibroadenomas, UDH, adenosis, and fat necrosis. Among B3 lesions, 18 underwent intervention (14 lumpectomies, 3 mastectomies, 1 vacuum-assisted excision) and 10 upgraded (55.6%). By subtype: ADH 5/6 (83%), papillary 3/5 (60%) of 9 papillary overall, LIN 1/3 (33%), FEA 0/2, phyllodes 0/1, radial scar 0/0, and other atypia 1/1 (100%).

**Conclusion:** False-positive outcomes are frequent among RC-only CEM findings requiring biopsy, spanning benign and B3 lesions. Although many yield non-malignant results, the high upgrade risk of some subtypes supports intervention. Findings are consistent with published CEM series and comparable to MRI-guided biopsy. Despite this false-positive burden, CEM-guided biopsy remains essential for characterisation of RC-only lesions not accessible with conventional guidance.
**Limitations:** Retrospective design, single-centre setting, and small B3 subgroups.
**Funding for this study:** No funding was received for this study.
**Has your study been approved by an ethics committee?** Yes
**Ethics committee - additional information:** Protocol number 2019/8890
**Author Disclosures:**
Rodrigo Alcantara Souza: Speaker: GE Healthcare, BD, Bayer
Javier Saenz: Speaker: GE Healthcare, BD and Bayer
Elisenda Vall Foraster: Nothing to disclose
Paulina Miranda Martínez: Nothing to disclose
Natalia Arenas: Speaker: BD
Elisabet Vila-Trias Jover: Nothing to disclose
Mireia Pitarch: Nothing to disclose
Xavier Castells: Nothing to disclose

**Clinical and imaging Factors Predictive of Malignancy after CEM-Guided Stereotactic Biopsy: a multicenter study**
*A. F. Murano*[1], D. De Benedetto[2], M. Sacco[1], L. R. La Rocca[1], V. Lamagna[2], F. Di Naro[2], M. G. Vigilante[1], J. Nori[2], V. Romeo[1]; [1]Naples/IT, [2]Firenze/IT
*(alfredofabio.murano@gmail.com)*

**Purpose:** Contrast-enhanced mammography (CEM)-guided biopsy allows the histological characterization of “enhancing only” CEM or MRI findings. Its recent introduction made it a valuable alternative the less available and more expensive MRI guided biopsy. However, “contrast-enhanced only” findings may be non-specific, raising the risk of unnecessary biopsies. The study aimed to identify clinical and/or imaging parameters predictive of malignancy in patients undergoing CEM-guided biopsy in a multicentric setting.
**Methods or Background:** We retrospectively included women with suspicious “contrast-enhanced only” lesions visible only on CEM and sampled with CEM-guided biopsy in two different institutions. Clinical data (age, concurrent/previous breast cancer (BC), family history of BC, high-risk factors) and CEM features (size, enhancement type, distribution, pattern, conspicuity, background parenchymal enhancement) were collected. Univariate and logistic regression analyses were performed to identify clinical and/or CEM findings that could independently predict malignancy after CEM-guided biopsy.
**Results or Findings:** A total of 126 women with 126 suspicious CEM “enhancing only” findings, of which 98 benign and 28 malignant, were included. Among the clinical and imaging parameters, patient age, the presence of concurrent breast cancer, and early contrast enhancement were statistically significant at both univariate (p value: < 0.001, 0.004, and 0.015, respectively) and logistic regression analyses, with a combined AUC of 0.798 (95% CI: 0.718–0.864) and accuracy of 81.75% (95% CI: 73.9% - 81%) in predicting malignancy.
**Conclusion:** Patient age, the presence of a concurrent breast cancer, and early enhancement may help selecting patient who could benefit from CEM-guided biopsy. Larger studies are needed to confirm these preliminary results, improve the performance of the model, and develop clinical decision tools.
**Limitations:** Small sample size
**Funding for this study:** None
**Has your study been approved by an ethics committee?** Yes
**Ethics committee - additional information:** The study was approved by the ethics committee Campania 3
**Author Disclosures:**
Mariateresa Sacco: Nothing to disclose
Maria Grazia Vigilante: Nothing to disclose
Federica Di Naro: Nothing to disclose
Ludovica Rita La Rocca: Nothing to disclose
Diego De Benedetto: Nothing to disclose
Valeria Romeo: Nothing to disclose
Jacopo Nori: Nothing to disclose
Verdiana Lamagna: Nothing to disclose
Alfredo Fabio Murano: Nothing to disclose

**16:00 - 17:30 Research Stage 3**

### Research Presentation Session: Cardiac

### RPS 2103

### Cardiac imaging biomarkers for prognosis and clinical outcomes

**Moderator**
Moritz Christian Halfmann; Mainz/DE
**Author Disclosures:**
Moritz Christian Halfmann: Other: Travel Support Siemens Healthineers

**Adipose tissue compartments predict cardiometabolic outcomes in healthy non-obese individuals: an opportunistic screening approach**
*B. Bogner*, M. Jung, M. Reisert, J. Maushagen, S. Rospleszcz, C. L. Schlett, F. Bamberg, J. Weiß; Freiburg Im Breisgau/DE

**Purpose:** To determine whether MRI-derived visceral-to-subcutaneous adipose tissue (VAT/SAT) ratio predicts incident diabetes and major adverse cardiovascular events (MACE) beyond other risk factors in metabolically healthy non-obese (MHN) individuals.
**Methods or Background:** We analyzed UK Biobank participants who were MHN (absence of hyperlipidemia or hypertension, diabetes, and BMI <30 kg/m²). 3D VAT/SAT volumes (dm3) were extracted from Dixon whole-body MRI using a validated deep learning framework. Sex-specific VAT/SAT ratio cutoffs (≥0.15 for females, ≥0.35 for males) were derived from C-index optimization for incident cardiometabolic outcomes. Cox proportional hazards models assessed associations with 1) incident diabetes and 2) MACE, with stepwise adjustment for age, sex, smoking, waist circumference (WC), and BMI. Multivariable nested models with and without VAT/SAT ratio were compared to test for added value beyond other factors. Net reclassification improvement (NRI) compared risk classification by VAT/SAT versus WC.
**Results or Findings:** Among 22,040 participants (mean age 64.6±7.8 years, 53% female), over a median 4.2-year follow-up, 251 diabetes (1.14%) and 297 MACE (1.35%) events occurred. High VAT/SAT ratio was independently associated with diabetes (aHR 1.77, 95% CI 1.34-2.33, p<0.001) and MACE (aHR 1.30, 95% CI 1.02-1.66, p=0.037) after adjustment for age, sex, smoking, BMI, and WC. Adding VAT/SAT to fully adjusted models significantly improved discrimination for diabetes (C-index 0.723 vs. 0.715, p<0.001) and MACE (C-index 0.694 vs. 0.690, p=0.036). VAT/SAT outperformed WC with significant NRI for diabetes (NRI 0.102, p=0.010) and MACE (NRI 0.088, p=0.013).
**Conclusion:** VAT/SAT ratio independently predicts diabetes and MACE in MHN individuals and demonstrates superior risk classification over WC, highlighting the potential of body composition analysis for personalized risk assessment in apparently healthy individuals.
**Limitations:** The generalizability is limited by the UK Biobank's selection bias, the VAT/SAT cut-off derivation, and the focus on MHN individuals.
**Funding for this study:** None.
**Has your study been approved by an ethics committee?** Yes
**Ethics committee - additional information:** University of Freiburg
**Author Disclosures:**
Christopher L. Schlett: Speaker: Siemens Healthineers, Bayer Healthcare
Marco Reisert: Nothing to disclose
Jakob Weiß: Consultant: Onc.AI.
Balazs Bogner: Nothing to disclose
Matthias Jung: Nothing to disclose
Susanne Rospleszcz: Nothing to disclose
Juliane Maushagen: Nothing to disclose
Fabian Bamberg: Grant Recipient: Siemens Healthineers, Bayer Healthcare
Speaker: Siemens Healthineers, Bayer Healthcare

**Prognostic value of CT-defined coronary calcifications in COVID-19: Results of a multicenter study based on the Weston score**
A. M. Bucher[1], *M. Neitzel*[1], C. Ehrengut[2], T. S. Emrich[3], R. Klöckner[4], M. M. Sieren[4], M. A. Fink[5], F. G. Meinel[6], A. S. Surov[7]; [1]Frankfurt/DE, [2]Leipzig/DE, [3]Mainz/DE, [4]Lübeck/DE, [5]Heidelberg/DE, [6]Rostock/DE, [7]Minden/DE
*(ma.neitzel@med.uni-frankfurt.de)*

**Purpose:** To investigate the prognostic value of CT-defined coronary calcifications in COVID-19 patients using the semiquantitative Weston score.
**Methods or Background:** This retrospective multicenter study included 541 PCR-confirmed COVID-19 patients (176 female, mean age 61.2±15.6 years) from 11 German university hospitals. Coronary calcifications were assessed on chest CT using the Weston score (0-12 points), evaluating four coronary segments on a 4-point scale. Primary endpoint was 30-day mortality. Statistical analysis included univariable and multivariable regression.
**Results or Findings:** Overall 30-day mortality was 21.2% (115/541). Mean Weston score was 3.0±3.6, with 23.7% showing no calcifications. Patients with fatal outcomes had significantly higher Weston scores (4.1±4.01 vs. 2.7±3.4, p=0.0007). While presence of calcifications showed association with mortality in univariable analysis (OR 1.68, 95%CI 1.08-2.59, p=0.01), this lost significance in multivariable analysis (p=0.49). However, Weston score as continuous variable remained significant in both univariable (OR 1.10, 95%CI 1.04-1.14, p<0.001) and multivariable analysis (OR 1.06, 95%CI 1.005-1.138, p=0.036) after adjusting for age and COVID-19 CT score.
**Conclusion:** The extent of coronary calcifications, quantified by Weston score, is an independent prognostic factor for 30-day mortality in COVID-19 patients. The degree of calcification provides greater prognostic value than mere presence. These findings support integrating coronary calcification assessment into COVID-19 risk stratification using routine chest CT without dedicated cardiac imaging.
**Limitations:** The limitation of the study is the retrospective study design.
**Funding for this study:** Funding was provided by the German Federal Ministry of Education and Research (BMBF), Project RACOON (01KX2021).
**Has your study been approved by an ethics committee?** Yes
**Ethics committee - additional information:** Multicentric ethics approval was obtained by University Medicine Frankfurt (Reference 20-719jK).
**Author Disclosures:**
Alexey Surov: Nothing to disclose
Roman Klöckner: Nothing to disclose
Matthias Neitzel: Nothing to disclose
Constantin Ehrengut: Nothing to disclose
Matthias Alexander Fink: Nothing to disclose
Felix G. Meinel: Nothing to disclose
Malte Maria Sieren: Nothing to disclose
Tilman Stephan Emrich: Nothing to disclose
Andreas Michael Bucher: Nothing to disclose

**Virtual IVUS on Coronary CT: Mid LAD Intimal Thickening as a Clinical Predictor of Early CAD-RADS Progression**
S. S. Dereli Bulut, *G. Karamustafao*; Istanbul/TR
*(ssanembulut@gmail.com)*

**Purpose:** To evaluate whether virtual intravascular ultrasound (IVUS)-derived intimal thickness, calcium scoring, and positive remodeling on coronary CT can predict progression from CAD-RADS 0 to CAD-RADS 1, and to assess their relationship with cardiovascular risk factors.
**Methods or Background:** In this retrospective pilot study, 91 patients with CAD-RADS 0–1 (CAD-RADS 2.0)who underwent 128-slice CCTA (2021–2025) were analyzed. Intimal thickness was measured on reformatted axial planes using virtual IVUS,defined as (outer diameter–inner diameter)/2. Two radiologists performed independent measurements, with interobserver agreement assessed by ICC. Calcium scoring (Smart Score) and positive remodeling (Plaque ID) were also evaluated, and risk factors (hypertension, diabetes, smoking, LDL) retrieved from medical records.
**Results or Findings:** CAD-RADS 0 (n=45: 44.8 ± 11.6 years, 46.7% male, BMI 26.9 ± 3.7) and CAD-RADS 1 (n=46: 51.6 ± 9.9 years, 52.6% male, BMI 28.4 ± 2.8) differed significantly. Intimal thickness was greater in CAD-RADS 1, especially mid LAD, LCX, and RCA (p<0.001). Calcium scores were higher, particularly LAD (0.0 vs 21.7; p<0.001). Positive remodeling occurred in 93% vs 42% (p<0.001). Intimal thickness correlated with calcium scores, strongest in mid LAD (r=0.71, p<0.001). Mid LAD thickness was the strongest predictor (p=0.01); the combined model (LAD-mid + LDL + HT) yielded AUC=0.96 (98% sensitivity, 89% specificity).
**Conclusion:** IVUS-derived intimal thickening, particularly in the mid LAD, may serve as an early imaging marker of CAD-RADS progression. Combined with calcium scoring and remodeling, IVUS provides value for early risk stratification. These preliminary results are hypothesis-generating and need confirmation in larger prospective cohorts.
**Limitations:** This single-center pilot study had a modest sample size, included only CAD-RADS 0–1 patients without longitudinal follow-up, and may still be subject to measurement variability despite good reproducibility. Other biomarkers such as inflammation or genetics were not assessed.
**Funding for this study:** No funding was received for this study.
**Has your study been approved by an ethics committee?** Yes
**Ethics committee - additional information:** This retrospective study was approved by the Ethics Committee of Umraniye Training and Research Hospital (Approval number: E-54132726-000-282160058)
**Author Disclosures:**
Safiye Sanem Dereli Bulut: Nothing to disclose
Gorkem Karamustafao: Nothing to disclose

**Novel CT Biomarkers for Preoperative Prognostic Stratification in Patients with Severe Aortic Stenosis Undergoing TAVI**
*F. Piccione*[1], D. Vignale[2], A. Colombo[2], A. Palmisano[2], C. Gnasso[2], G. Amaro[2], A. Esposito[2]; [1]Turin/IT, [2]Milan/IT
*(federica.piccione@unito.it)*

**Purpose:** Assessing the prognostic value of myocardial strain and extracellular volume fraction (ECV) measured by pre-procedural CT in patients with severe aortic stenosis (AS) undergoing transcatheter aortic valve implantation (TAVI).
**Methods or Background:** From October 2020 to December 2023 patients were screened using as exclusion criteria aortic regurgitation, peri-procedural complications, incomplete strain/ECV data. Global strain (longitudinal, radial, and circumferential) was quantified using dedicated software (CVI42 v6.3.0). ECV was measured manually and automatically (in-house built software) in the interventricular septum as the ratio of the differential attenuation of myocardium and blood pool before and after contrast administration. Primary endpoint: death or HF hospitalization after a follow-up of 12+ months. Outcomes were analyzed with Kaplan–Meier survival and Cox regression.
**Results or Findings:** In 241 patients (median age 82 (IQR 78–86) years, 53.9% females) primary endpoint occurred in 18.3%. Patients with events had higher ECV (31.6% vs 27.6% manual, p<0.001; 31.7% vs 28.7% automatic, p=0.003), more impaired longitudinal (-13.0% vs -15.3%, p=0.035) and radial strain (18.1% vs 23.2%, p=0.030). Survival curves identified ECV cut-offs of 30.6% (manual) and 28.9% (automatic), both associated with worse event-free survival (p<0.001). In multivariate analysis, ECV remained an independent predictor (HR 2.12 manual; HR 2.45 automatic), while strain lost significance (HR 1). Patients with elevated ECV and impaired longitudinal strain had the highest risk (HR up to 3.04). Classical low-flow low-gradient AS showed the worst remodeling (ECV 33–34%, GLS –9%).
**Conclusion:** Pre-procedural CT-derived ECV represents an independent and robust predictor of adverse prognosis after TAVI. Combined ECV–strain assessment may provide a more comprehensive characterization of myocardial remodeling and improve risk stratification. This approach offers the additional advantages of being automatable, reproducible, and objective. Larger prospective studies are warranted to confirm its clinical applicability.
**Limitations:** Small single-center cohort.
**Funding for this study:** No funding
**Has your study been approved by an ethics committee?** Yes
**Ethics committee - additional information:** Retrospective study, based on prospectively collected data, and was conducted in accordance with the Declaration of Helsinki. Written informed consent was obtained from all participants.
**Author Disclosures:**
Gabriella Amaro: Nothing to disclose
Davide Vignale: Nothing to disclose
Antonio Esposito: Nothing to disclose
Anna Palmisano: Nothing to disclose
Federica Piccione: Nothing to disclose
Alberto Colombo: Nothing to disclose
Chiara Gnasso: Nothing to disclose

**Assessing left ventricular strain across all stages of chronic kidney disease – a cardiac MRI study**
*P. Kamieniarz*, A-S. Plietz, L. V. Urbach, M. Taupitz, L-A. Schaafs; Berlin/DE
*(paul.kamieniarz@charite.de)*

**Purpose:** Chronic kidney disease (CKD) is a global health burden with increasing prevalence. Those effected have an increased risk for cardiovascular disease (CVD) and higher mortality. Myocardial strain derived from cardiac MRI (cMRI) is shown to be an important prognostic marker in a variety of CVD. We aimed to assess left ventricular (LV) strain in patients with CKD and compare them to a healthy control group.
**Methods or Background:** In this single centre, retrospective study we included 288 patients with CKD in all stages of disease (G1-G5) and 74 healthy controls. All patients underwent comprehensive cMRI. Functional analysis was performed using CVI 42 (Circle Vascular Imaging, Calgary, Canada). For strain analysis Segment® (Medviso, Lund, Sweden) was used. Differences between groups were assessed using ANOVA.
**Results or Findings:** LV ejection fraction (59.4±6.4 vs. 47.5±16.1; 51.0±16.0; 46.1±17.4; 48.8±20.8; 49.0±18.2; p<0.001 ), LV global longitudinal strain (-18.5±3.1 vs. -13.9±5.5; 15.3±4.9; -13.7±5.8; -11.8±5.8; -11.5±4.6; -11.3±4.6; p<0.001), LV global circumferential strain (-21.8±3.5; -16.4±7.7; -19.0±6.1; -17.2±6.8; -15.9±7.2; -15.9±6.4; p<0.001) and LV global radial strain (50.3±9.8 vs. 37.4±19.5; 45.7±18.6; 39.8±18.5; 34.8±19.4; 40.0±18.7; 36.2±18.1; p<0.001) were statistically significant impaired in patients with CKD stages G1 – G5 compared to healthy controls.
**Conclusion:** Patients with CKD had impaired LV strain with the biggest differences observed in Groups G1-G3a. In groups G3b – G5 a gradual recovery of LV strain parameters was noticed. With LV strain severely impaired in patients with CKD, LV ejection fraction was only mildly reduced. Influencing factors such as age, sex and volume status need to be analysed in future studies.
**Limitations:** The limitations of this study are the retrospective design, variance in comorbidities and that groups could not be matched for sex and age.
**Funding for this study:** This research was funded by the Deutsche Forschungsgemeinschaft (DFG, German Research Foundation) - Grant No. 372486779 (SFB 1340/2)
**Has your study been approved by an ethics committee?** Yes
**Ethics committee - additional information:** Ethik-Kommission der Charité (EA2/237/21)
**Author Disclosures:**
Anna-Sophie Plietz: Nothing to disclose
Paul Kamieniarz: Nothing to disclose
Matthias Taupitz: Nothing to disclose
Lars-Arne Schaafs: Nothing to disclose
Louisa Victoria Urbach: Nothing to disclose

**CARE-LIVER – Cardiovascular Risk Evaluation by Coronary Tomography in LIVER Transplant Candidates: A Retrospective Study on Diagnostic Accuracy and Early Outcomes**
*R. Aroasio*[1], L. Tampellini[1], F. Minonne[1], S. Tibaldi[1], G. Balladori[1], L. Blasi[1], M. Moretti[1], M. Gatti[1], R. Faletti[2]; [1]Turin/IT, [2]Torino/IT
*(roberta.aroasio@gmail.com)*

**Purpose:** Evaluate the impact of coronary CT angiography (CCTA) on cardiovascular risk stratification and the prevention of MACE in liver transplant (OLT) candidates.
**Methods or Background:** Single-center retrospective study on 140 consecutive OLT candidates enrolled between April 2021 and October 2024, all with ≥ 2 coronary risk factors and undergoing CCTA. Positive exams, when clinically indicated, were further investigated with invasive coronary angiography (ICA) and possible revascularization; negative CCTAs were considered true negatives (TN) if supported by MACE-free clinical follow-up ≥ 6 months. Primary endpoint: MACE within 30 days after OLT.
**Results or Findings:** A total of 140 OLT candidates (78% male) were consecutively enrolled; 55% were > 65 years old and 32% had NASH cirrhosis. CCTA excluded significant stenosis in 95/140 patients (68%) and detected it in 27/140 (19%); 23/27 (85%) underwent ICA, with confirmation and PCI in 14/23 (61%). Using the TN6m definition (100 patients), the 2 × 2 matrix showed 14 true positives, 9 false positives, 0 false negatives, and 100 TN, with sensitivity and NPV 100%, specificity 91%, and PPV 60.9%. Among 91 transplanted patients, no MACE occurred within 30 days (incidence 0%). These patients accumulated 27,097 days (74.2 patient-years) of event-free follow-up; estimated incidence would be 1.1% assuming a single MACE and 5.5% with five events.
**Conclusion:** With 100% sensitivity and NPV at 6 months, CCTA proves to be a highly reliable screening tool to exclude significant coronary artery disease in OLT candidates with cardiovascular risk factors. The absence of MACE within 30 days post-transplant highlights the effectiveness of the CCTA-first strategy in perioperative protection. Prospective multicenter studies are needed for long-term validation.
**Limitations:** Retrospective and single-center design, relatively small sample size, short- to mid-term clinical follow-up.
**Funding for this study:** No funding for this study
**Has your study been approved by an ethics committee?** Yes
**Ethics committee - additional information:** No additional information
**Author Disclosures:**
Francesco Minonne: Nothing to disclose
Marco Gatti: Nothing to disclose
Ludovica Blasi: Nothing to disclose
Giulia Balladori: Nothing to disclose
Lorenzo Tampellini: Nothing to disclose
Riccardo Faletti: Nothing to disclose
Marta Moretti: Nothing to disclose
Stefano Tibaldi: Nothing to disclose
Roberta Aroasio: Nothing to disclose

**Regional Entropy of Epicardial Adipose Tissue on Spectral CT: A Novel Imaging Biomarker for Atrial Fibrillation Recurrence**
Y. Hu[1], *W. Deng*[2], J. Zhang[2]; [1]Yan'cheng, Jiangsu/CN, [2]Shanghai/CN
*(weiwei.deng@139.com)*

**Purpose:** Epicardial adipose tissue (EAT) is an important biomarker in atrial fibrillation (AF). This study evaluated whether EAT histogram parameters from virtual noncontrast and contrast-enhanced dual-layer spectral CT predict AF recurrence after ablation.
**Methods or Background:** In this prospective study, patients undergoing first-time AF ablation (Jan 2021–Jul 2024) underwent preoperative dual-energy cardiac CT. EAT on contrast-enhanced images (-190 to -30 HU) was segmented using a deep learning model. VNC images were reconstructed, and density difference maps were generated. Regional histogram parameters were extracted. Continuous variables were compared using Mann–Whitney U test, and Cox models evaluated predictors of AF recurrence.
**Results or Findings:** A total of 492 patients (mean age, 66 years; 316 men) were enrolled (paroxysmal AF, 54%; persistent AF, 46%). The median follow-up was 22 months. EAT volume was significantly greater in patients with persistent AF compared with those with paroxysmal AF (144 cm³ [IQR, 105–184] vs 126 cm³ [IQR, 100–154]; P < .001). Multiple EAT histogram parameters differed significantly between groups, including Max, Mean, Median, Std, Q1, Q3, Variance, IQR, 90th percentile, MAD, Skewness, Energy, Total Energy, RMS, Entropy, and Uniformity. During follow-up through July 2025, AF recurrence occurred in 73 patients (15%). Among EAT regional histogram parameters, entropy was significantly associated with AF recurrence (hazard ratio [HR], 2.0; 95% CI: 1.2–3.3; P < .01). In multivariable Cox regression, EAT entropy remained an independent predictor after adjustment for age, sex, AF phenotype, and EAT volume (HR, 1.97; 95% CI: 1.2–3.3; P = .013).
**Conclusion:** DLCT-based EAT histogram analysis, especially regional entropy, offers novel biomarkers for AF recurrence, supporting enhanced pre-ablation risk stratification.
**Limitations:** One-center study
**Funding for this study:** This study is supported by The National Key Research and Development Program of China (Grant No.: 2021YFF0501402), National Natural Science Foundation of China (Grant No.: 82471982, 82271990), Shanghai Municipal Science and Technology Commission Discipline Leader Project (Grant No.: BJKJ2024052), Shanghai Health Commission Discipline Leader Project (Grant No.: 2022XD031), and Medical research project of Yancheng Municipal Health Commission (Grant No.: YK2024225).
**Has your study been approved by an ethics committee?** Yes
**Ethics committee - additional information:** The institutional review board of each participating hospital approved this prospective study (2018-014)
**Author Disclosures:**
Jiayin Zhang: Nothing to disclose
Yifan Hu: Nothing to disclose
Weiwei Deng: Nothing to disclose

**Peri-coronary adipose tissue of the left circumflex artery as a potential biomarker for the recurrent atrial fibrillation after catheter ablation**
*E. Zuhri*[1], E. Elen[1], S. J. S. Gardezi[2], D. Kumar[2], G. Marcel[1], C. A. Atmadikoesoemah[1]; [1]Jakarta/ID, [2]Shanghai/CN
*(ervanzuhri@gmail.com)*

**Purpose:** Atrial fibrillation (AF) is one of the most common cardiac arrhythmias and poses a major challenge in the management of cardiovascular disease. Catheter ablation (CA) is an established therapeutic option for patients with drug-resistant AF. However, recurrent AF following CA remains a major clinical challenge. Recent studies suggest that peri-coronary adipose tissue (PCAT), particularly surrounding the left circumflex artery (LCx), may contribute to AF recurrence after CA. This study aims to identify this potential factor for prediction of recurrent AF after CA.
**Methods or Background:** Patients with recurrent AF after CA were retrospectively enrolled. We investigated the predictive value of PCAT and other clinical factors for AF recurrence after CA. PCAT was measured using AI-based tool (uAI Fat Attenuation Index) developed by United Imaging Intelligence. Bivariate and multivariate logistics regression analyses were performed
**Results or Findings:** During 1-year follow-up, 18 of 33 patients experienced AF recurrence. The multivariable analysis revealed that PCAT of the LCx was independently associated with AF recurrence. PCAT of the LCx showed a significant predictive value, with a cut-off value of >−86.5 HU, yielding 78% sensitivity and 67% specificity. The area under the receiver operating characteristic curve (AUC) was 0.720, indicating good discriminative ability. Other factors-including age, sex, obesity, hypertension, diabetes, dyslipidemia, smoking, chronic kidney disease, heart failure, reduced TAPSE, valvular heart disease, coronary artery disease, and use of antiarrhythmic drugs (AADs, Class II/III)-were not significantly associated AF recurrence.
**Conclusion:** PCAT of the LCx was associated with AF recurrence after CA and may have important clinical value in predicting recurrence.
**Limitations:** Larger sample are needed to validate these findings and further refine risk prediction models for AF recurrence.
**Funding for this study:** None
**Has your study been approved by an ethics committee?** Not applicable
**Author Disclosures:**
Syed Jamal Safdar Gardezi: Nothing to disclose
Gamael Marcel: Nothing to disclose
Ervan Zuhri: Nothing to disclose
Dileep Kumar: Nothing to disclose
Elen Elen: Nothing to disclose
Celly Anantaria Atmadikoesoemah: Nothing to disclose

**Incremental prognostic role of fat replacement over late gadolinium enhancement in patients with non-dilated left ventricular cardiomyopathy and preserved systolic function**

*L. Fulceri*, L. Taddei, A. Marcucci, L. Faggioni, D. Cioni, E. Neri, G. Aquaro; Pisa/IT
*(lisafulceri1@gmail.com)*

**Purpose:** To evaluate the prognostic role of FR compared to late gadolinium enhancement (LGE) in subjects with NDLVC, preserved LVEF and frequent premature ventricular complexes (PVC).
**Methods or Background:** Cardiac magnetic resonance was performed in 900 consecutive patients with frequent PVC, preserved LVEF. Patients with diagnostic criteria for other cardiomyopathies, channelopathies and ischemic disease were excluded. A clinical follow-up was performed and major ventricular arrhythmias (MVA), including sudden cardiac death, appropriated ICD intervention, and sustained VT, were considered endpoints.
**Results or Findings:** The final population included 876 patients. Overall non-ischemic LGE was found in 101 (12%) patients and FR in 38 (4%): 87 (10%) presenting LGE without FR, 14 (1,6%) FR without LGE and 24 (3%) LGE with FR. After a median follow-up of 4 (3-6) years, MVA occurred in 43 patients. At Kaplan-Meier survival curve analysis (figure 1) the presence of LGE and/or FR was associated with worse prognosis than their absence. The group of patients with worse prognosis was that with the combined LGE+FR. A similar prognosis was found in patients with LGE alone and in those with FR alone (p=0.19). Patients with LGE+FR had worse prognosis than those with LGE alone (P=0.01). At univariate analysis non-sustained VT (NSVT), syncope, LGE, FR and LGE+FR were associated to MVA. At multivariable logistic regression analysis the combination LGE+FR was an independent predictor of MVA together with history of NSVT and syncope.
**Conclusion:** In NDLVC with preserved LVEF, the combination of FR+LGE was a stronger prognostic marker than the presence of LGE alone. FR and LGE had similar prognostic role when found separately.
**Limitations:** Relatively small population
**Funding for this study:** Istitutional funds of the University of Pisa
**Has your study been approved by an ethics committee?** Not applicable
**Author Disclosures:**
Emanuele Neri: Nothing to disclose
Lisa Fulceri: Nothing to disclose
Alessandro Marcucci: Nothing to disclose
Giovanni Aquaro: Nothing to disclose
Lorenzo Faggioni: Nothing to disclose
Lapo Taddei: Nothing to disclose
Dania Cioni: Nothing to disclose

**Prognostic value of pulmonary transit time assessed by cardiac magnetic resonance in patients with dilated cardiomyopathy**

*W. Zhang*, X. Xu, W. Peng, K. Shi, X. Lyu, C. Xia, Z. Li; Chengdu/CN
*(zhangwendipaper@163.com)*

**Purpose:** To evaluate the prognostic value of pulmonary transit time (PTT) assessed by cardiac magnetic resonance (CMR) in patients with dilated cardiomyopathy (DCM).
**Methods or Background:** Recent studies confirm CMR first-pass perfusion imaging offers a convenient, non-invasive method to measure PTT, a marker of global cardiopulmonary circulation. However, its prognostic value in DCM patients has not been established. This retrospective study enrolled patients with DCM who underwent CMR at our institution between July 2019 and January 2022. PTT was measured as the peak-to-peak time between the contrast time-intensity curves of the right and left ventricles in first-pass perfusion imaging. Cox regression analysis was performed to assess the association between PTT and a composite endpoint comprising all-cause mortality, heart failure rehospitalization, and heart transplantation.
**Results or Findings:** A total of 140 patients (71.4% males, mean age 52±13 years) were included and followed for a median of 4.6 years (interquartile range, 4.1–5.4 years). During this period, 70 patients (50.0%) reached the endpoint. Multivariable linear regression identified PTT as an independent determinant of left ventricular (LV) remodeling and dysfunction, as it correlated with increased LV end-diastolic dimension (β=0.431), reduced LV ejection fraction (β=-0.391), and impaired LV global longitudinal peak strain (β=0.276). Receiver operating characteristic curve analysis, using the maximization of Youden's index, identified an optimal PTT cut-off of 10.7 seconds for endpoint prediction. Kaplan–Meier survival analysis revealed that patients with a PTT greater than 10.7 seconds had a significantly higher risk of experiencing the composite endpoint (log-rank p<0.001). In multivariable Cox regression analysis, PTT was an independent predictor of the endpoint (adjusted hazard ratio: 1.072 per second; 95% confidence interval: 1.006–1.144; p=0.033).
**Conclusion:** PTT derived from CMR independently predicted adverse cardiovascular outcomes in patients with DCM.
**Limitations:** Not applicable
**Funding for this study:** Not applicable
**Has your study been approved by an ethics committee?** Not applicable
**Author Disclosures:**
Chunchao Xia: Nothing to disclose
Zhenlin Li: Nothing to disclose
Wanlin Peng: Nothing to disclose
Wendi Zhang: Nothing to disclose
Ke Shi: Nothing to disclose
Xinyang Lyu: Nothing to disclose
Xu Xu: Nothing to disclose

**16:00 - 17:30** **Research Stage 6**

### Research Presentation Session: Head and Neck

### RPS 2108

### Head and neck cancer imaging: what's new?

**Moderator**
Maartje De Win; Amsterdam/NL
*(m.m.dewin@amsterdamumc.nl)*

**Trajectory Matters: Longitudinal Evolution of NI-RADS Categories and Risk of Recurrence in Head & Neck Cancer**

*A. H. Shah*, A. Chandra, S. Sen, S. Mukhopadhyay, P. Ghosh, A. Chatterjee, A. Gehani, J. Khoda, A. Patra; Kolkata/IN
*(shahakshat1788@gmail.com)*

**Purpose:** NI-RADS has been validated as a single-time point tool for recurrence prediction. Yet in the clinic, patients return repeatedly for follow-up, and radiologists often see categories shift over time. The prognostic meaning of these longitudinal NI-RADS trajectories has never been systematically studied. We evaluated whether stability or change in NI-RADS scores across surveillance scans influences recurrence risk.
**Methods or Background:** We retrospectively studied 628 patients with head and neck squamous cell carcinoma treated between 2012 and 2024 who had ≥3 consecutive surveillance CT or MRI scans. Two head and neck radiologists independently assigned NI-RADS categories. Trajectories were defined as: stable low-risk (repeated NI-RADS 1–2), escalating risk (progression from NI-RADS 2 → 3/4), or stable high-risk (repeated NI-RADS 3–4). Outcomes were verified by histopathology, MDT consensus, or ≥12-month follow-up. Kaplan–Meier survival and hazard ratios (HR) for progression-free survival (PFS) were calculated.
**Results or Findings:** We retrospectively studied 628 patients with head and neck squamous cell carcinoma treated between 2012 and 2024 who had ≥3 consecutive surveillance CT or MRI scans. Two head and neck radiologists independently assigned NI-RADS categories. Trajectories were defined as: stable low-risk (repeated NI-RADS 1–2), escalating risk (progression from NI-RADS 2 → 3/4), or stable high-risk (repeated NI-RADS 3–4). Outcomes were verified by histopathology, MDT consensus, or ≥12-month follow-up. Kaplan–Meier survival and hazard ratios (HR) for progression-free survival (PFS) were calculated.
**Conclusion:** Recurrence risk is not defined by a single scan but by the trajectory of NI-RADS scores over time. Stability reassures, escalation warns, and downward movement inspires hope. Incorporating trajectory analysis into structured reports could help radiologists guide MDT decisions more confidently, aligning imaging with the lived journey of cancer survivors.
**Limitations:** Single-center retrospective study; modality allocation by subsite.
**Funding for this study:** None
**Has your study been approved by an ethics committee?** Not applicable
**Author Disclosures:**
Aditi Chandra: Nothing to disclose
Anisha Gehani: Nothing to disclose
Sumit Mukhopadhyay: Nothing to disclose
Argha Chatterjee: Nothing to disclose
Saugata Sen: Nothing to disclose
Akshat Hitesh Shah: Nothing to disclose
Anurima Patra: Nothing to disclose
Priya Ghosh: Nothing to disclose
Jeevitesh Khoda: Nothing to disclose

**Significance of Cross-Sectional Imaging characteristics of Orbital Metastases**
A. Samad, *S. S. A. Khatian*; Karachi/PK
*(kktshaista@gmail.com)*

**Purpose:** Orbital metastases are not very common but they tend to be signify progression of malignancy in the entire body and may considerably affect the prognosis of the patient. They can have an appearance similar to primary orbital tumors or inflammatory disorders/changes in clinical practice, and radiologic identification is important.
**Methods or Background:** A cross-sectional study was designed as a descriptive study that was carried out at Radiology, JPMC -Karachi. 144- patients who were known to have cross-sectional imaging (CT and/or MRI) and was histologically confirmed to have orbital metastases were included. Data was analyzed based on demographic profile, primary site of tumor, clinical presentation and detailed imaging features such as location, margins, enhancement, necrosis and bone involvement.
**Results or Findings:** Summary N=104 (mean age of patients 55.6 ±11.8 years, 62 females and 42 males).The most common primary malignancy was breast carcinoma (n=46, 44%), lung carcinoma (n=29, 28%), renal cell carcinoma (n=15, 14%), and other primaries (n=14, 14%) such as gastrointestinal and thyroid malignancies. Breast carcinoma metastases were usually poorly defined, infiltrating lesions of soft tissue in fat in the orbit and extraocular muscles, without extensive bone destruction. .Lung carcinoma metastases were commonly well-defined and rapidly growing and massively enlarging the orbit with evidence of necrotic tissue. Renal cell carcinoma metastases presented with intensely enlarging, well-defined, vascular lesions, which are often accompanied with hemorrhage and bone erosion.
**Conclusion:** Orbital metastases though very uncommon and has characteristic imaging appearances that depends on the nature of the underlying primary tumor. The knowledge of these radiological patterns may help radiologists to make nearly accurate diagnosis proposal, facilitate systemic assessment, and prevent unnecessary procedures. It is also especially important in oncology patients with new orbital complaints who have to be identified early.
**Limitations:** Nil
**Funding for this study:** Nil
**Has your study been approved by an ethics committee?** Yes
**Ethics committee - additional information:** Nil
**Author Disclosures:**
Shaista Shoukat Ali Khatian: Nothing to disclose
Abdul Samad: Nothing to disclose

**Comparison of diagnostic accuracy of CT, MRI and US in detecting vascular involvement in patients with head and neck tumours and metastasis: systematic review and meta-analysis**
*U. B. Abdullaeva*[1], B. Pape[2], J. Hirvonen[3]; [1]Tashkent/UZ, [2]Turku, Vaasa/FI, [3]Tampere/FI
*(umidasamira2@gmail.com)*

**Purpose:** To review the diagnostic performance of MRI, CT and US for detecting vascular invasion in head and neck primary tumours or metastases, using histopathology or surgical findings as the reference standard.
**Methods or Background:** This review was registered in Prospero (CRD42024584016). We searched PubMed and Embase for studies in English, published from 01.01.1990 to 31.12.2024, that used CT, MRI or ultrasound to assess vascular involvement in head and neck primary tumours or metastases, confirmed by histology or surgery and provided data to calculate diagnostic accuracy. Outcomes included sensitivity, specificity, PPV, and NPV, with heterogeneity assessed using Cochran's Q test.
**Results or Findings:** In the systematic review, we included 28 studies with 1687 patients (mean age 57.2): 17 retrospective, seven prospective, one mixed, and the rest unspecified. The meta-analysis included 19 studies (1041 patients), while nine studies (646 patients) were analysed qualitatively. Overall diagnostic accuracy was 0.87 (95% CI: 0.81–0.91). Pooled sensitivity, specificity, PPV, and NPV were 0.63 (0.48–0.75), 0.81 (0.73–0.87), 0.60 (0.52–0.68), and 0.90 (0.78–0.96), respectively. US performed best (accuracy 0.95, sensitivity 0.86, specificity 0.93, NPV 0.96, PPV 0.73), MRI showed intermediate results, and CT the lowest. Moderation effect for accuracy was significant ($p = 0.041$), with US outperforming CT ($p = 0.023$). Significant heterogeneity was found for sensitivity ($Q = 57.81$), specificity ($Q = 109.01$), NPV ($Q = 79.42$), and accuracy ($Q = 170.25$) (all $p < 0.0001$).
**Conclusion:** US is the most effective and precise modality, outperforming MRI and CT, though MRI is superior to CT. Overall modest sensitivity suggests the possibility of false negative findings. Significant heterogeneity requires integration of imaging findings with intraoperative validation.
**Limitations:** The study is largely retrospective, with sampling bias, incomplete data, and significant heterogeneity.
**Funding for this study:** No funding was received for this study.
**Has your study been approved by an ethics committee?** Not applicable
**Author Disclosures:**
Bernd Pape: Nothing to disclose
Jussi Hirvonen: Nothing to disclose
Umida Bafoevna Abdullaeva: Nothing to disclose

**MRI in Differentiating HPV-Positive and HPV-Negative Oropharyngeal Squamous Cell Carcinoma**
*E. Gattuso*, F. Passalacqua, F. Bencivinni, A. Lo Casto; Palermo/IT
*(emanuelegattuso96@gmail.com)*

**Purpose:** Human papillomavirus (HPV) status plays a key role in oropharyngeal squamous cell carcinoma (OPSCC). Our study aims to evaluate MRI features of locoregional metastatic lymph nodes and apparent diffusion coefficient (ADC) values of the primary tumor in HPV+ and HPV– OPSCC to identify distinguishing patterns.
**Methods or Background:** MRI scans of 87 patients with suspected OPSCC were retrospectively reviewed. Exclusion criteria were prior treatment, uncertain diagnosis, or non-squamous histology. Twenty-four patients with pre-biopsy MRI and histological confirmation, including p16 immunohistochemistry evaluation, were included. For each case, lymph node morphology and mean ADC values of the primary lesion were assessed.
**Results or Findings:** Among HPV+ patients (14/24), 12/14 (85.7%) had nodal involvement, mainly ipsilateral (11/12; 91.7%). Mean nodal diameter was 2.33 cm (range 1.1–4.4); cystic morphology was seen in 7/12 (58.3%). Jugular vein thrombosis occurred in 3/12 (25%), and extracapsular spread in 1/12 (8.3%). Mean primary ADC was $0.79 \times 10^{-3}$ mm²/s (range 0.6–1.0). In HPV– patients (10/24), 6/10 (60%) had nodal metastases, ipsilateral in 3/6 (50%) and bilateral in 3/6 (50%). Mean diameter was 2.53 cm (range 1.5–4.5), with solid morphology in 5/6 (83.3%). Mean primary ADC was $1.15 \times 10^{-3}$ mm²/s (range 0.7–1.5).
**Conclusion:** HPV+ OPSCC was associated with smaller, predominantly ipsilateral cystic nodes and lower ADC values, while HPV– OPSCC showed larger, bilateral, solid nodes and higher ADC values. MRI thus offers a valuable non-invasive tool for HPV status characterization.
**Limitations:** This was a single-center study with a relatively small sample size, which may limit the generalizability of the findings. Furthermore, no correlation with patient outcome or survival was performed, preventing assessment of the prognostic significance of the imaging features.
**Funding for this study:** This study received no external funding.
**Has your study been approved by an ethics committee?** Not applicable
**Author Disclosures:**
Fabrizio Passalacqua: Nothing to disclose
Emanuele Gattuso: Nothing to disclose
Francesco Bencivinni: Nothing to disclose
Antonio Lo Casto: Nothing to disclose

**Efficacy of Combined Local and Target Therapy in Radioactive Iodine–Refractory Locally Advanced or Metastatic Thyroid Cancer**
*Y-C. Tsai*, W-C. Lin; Kaohsiung/TW

**Purpose:** Patients with radioactive iodine–refractory (RAIR) metastatic or locally advanced thyroid cancer have a poor prognosis and limited treatment options. This study aimed to evaluate the efficacy of targeted therapy alone versus targeted therapy in combination with local treatment in this patient population.
**Methods or Background:** From 2017 to 2024, a total of 52 patients aged ≥18 years with RAIR locally advanced or metastatic thyroid cancer receiving targeted therapy were evaluated. Baseline imaging with computed tomography (CT) was performed prior to initiation of therapy. Disease progression was assessed using serial CT and/or PET/CT (Positron Emission Tomography-Computed Tomography) imaging, based on a combination of Response Evaluation Criteria in Solid Tumors (RECIST) and PET Response Criteria in Solid Tumors (PERCIST). Associations between clinical factors and disease progression, as well as treatment-related adverse events, were also analyzed.
**Results or Findings:** Among the 52 patients, 20 received additional local treatment. This group demonstrated significantly improved progression-free survival (PFS) compared to those receiving targeted therapy alone: median PFS of 35.2 months (95% CI: 22.95–47.42) vs. 18.9 months (95% CI: 11.23–26.74); $p = 0.038$. Multivariate analysis identified combination therapy as an independent factor associated with favorable PFS (HR: 0.327; 95% CI: 0.140–0.763; $p = 0.010$). There was no significant difference in the incidence of adverse events between the two groups. The most common adverse event was proteinuria (21 patients, 40.4%).
**Conclusion:** Local treatment should be actively considered in eligible RAIR locally advanced or metastatic thyroid cancer patients receiving targeted therapy, as it can significantly enhance survival outcomes with acceptable safety.

**Limitations:** This study was limited by its retrospective, single-center design, relatively small sample size, heterogeneous treatment and limited follow-up duration. Further investigation is needed to clarify the long-term outcome.
**Funding for this study:** No funding was received for this study.
**Has your study been approved by an ethics committee?** Yes
**Ethics committee - additional information:** Chang Gung medical foundation institutional review board
**Author Disclosures:**
Yi-Chia Tsai: Nothing to disclose
Wei-Che Lin: Nothing to disclose

**Optimization of staging system for nasopharyngeal carcinoma based on the detailed MRI features and compared with AJCC/UICC staging**
*S. Li*, H. LI, L-Z. Liu; Guang Zhou/CN
*(lisq1@sysucc.org.cn)*

**Purpose:** This study aimed to develop a new clinical staging system integrating detailed MRI features for non-metastatic nasopharyngeal carcinoma, to address the limitations in risk discrimination of the current 9th AJCC system.
**Methods or Background:** This retrospective study included 1,897 patients with non-metastatic NPC from three hospitals. MRI features for primary tumor and metastatic lymph nodes were evaluated. The primary endpoint was 5-year overall survival (OS). T, N and clinical stage were optimized using univariate and multivariate analyses. Survival curves were generated with the kaplan–meier method. The proposed system was compared with the authoritative 8th/9th AJCC/UICC staging systems using risk stratification, C-index, and AIC.
**Results or Findings:** Among T3 patients, those with severe skull base-foramina invasion (HR:1.99, 95% CI:1.22–3.34, P=0.006) were proposed for upstaging to T4, whereas those with only slight skull base-bone invasion (HR:0.54, 95%CI:0.34–0.85, adjusted P=0.009) were recommended for downstaging to T2 (SBFI_T). The number of MRI-positive lymph nodes was independent prognosticator, supporting an N categorization based on nodal count (LNN_N: N0, 0; N1,1-4; N2, 5-9; N3,≥9). The proposed stage, which integrates SBFI_T and LNN_N, grouped T2N0 and T1N0 as stage Ia, and T1–2N1 as stage Ib. The proposed SBFI_T,LNN_N and stage systems outperformed the 8th/9th editions, achieving higher C-indices, lower AIC values, and significant risk stratification for OS across all subgroups (all P < 0.05).
**Conclusion:** The proposed staging system outperforms current mainstream systems. It also emphasizes critical MRI features that require clinical attention and highlights their role in accurate prognostic prediction.
**Limitations:** As only M0 cases were included, the inclusion of metastatic cases is needed in future studies to optimize the M-stage and develop a comprehensive staging framework.
**Funding for this study:** None
**Has your study been approved by an ethics committee?** Yes
**Ethics committee - additional information:** The ethics committee of Sun Yat-sen university cancer center
**Author Disclosures:**
Li-Zhi Liu: Nothing to disclose
Shuqi Li: Nothing to disclose
Haojiang Li: Nothing to disclose

**Cascade-penetrating domino-ferroptosis nano inducer synergizes with sonodynamic therapy for anaplastic thyroid cancer**
*P. Dong*; Changchun/CN
*(dongpeng940102@163.com)*

**Purpose:** Ferroptosis has become a new approach for antitumor treatment. However, the insufficient accumulation and poor penetration of ferroptosis inducers deep in tumors greatly limit their therapeutic effects. Herein, we aim to construct a cascade penetrating metal-polyphenol ultrasonic molecular probe, Fe3+Cur-PFP@IR780-LIP (FCIPL). Under ultrasonic stimulation, the nanoparticles can targeted cascade-penetrate deep into tumors, induce domino-ferroptosis reaction, enhance sonodynamic therapy (SDT), and realize multimodal imaging.
**Methods or Background:** FCIPL were based on the core of FCr consists of ferroptosis inducer curcumin and Fe3+, and co-embedded with PFP in IR780-loaded liposomes. We examined their characterization, basic properties, the safety, uptake capacity, targeting function, cytotoxicity, ferroptosis potentiating SDT effect and mechanism of action, the deep penetration and multimodal imaging capabilities of nanoparticles in vitro and in vivo.
**Results or Findings:** The FCIPL were regular spherical with uniform size, hydrated particle size of 218.6nm, with good dispersion, stability, and encapsulation rates. Ultrasonic excitation and tumor microenvironment can promote the disassembly of FCIPL, releasing Cur and Fe2+, jointly promotes the domino effect of lipid peroxide accumulation. Vivo and vitro experiments confirmed the good tumor targeting, cascade response and deep penetration ability of the FCIPL with good biosafety, and also verified the ferroptosis potentiating SDT efficacy of the nanoprobe and its mechanism of action, which together effectively demonstrated excellent tumor killing effects. In terms of imaging, FCIPL successfully realizing multi-modal imaging integrating ultrasound, photoacoustic, MRI and fluorescence imaging.
**Conclusion:** A cascade penetrating metal-polyphenol ultrasonic molecular probe FCIPL was successfully synthesised. Realized multimodal imaging in diagnosis. In terms of treatment, ferroptosis in deep tumors was achieved through targeted osmosis and cascaded response drug delivery system, enhanced sonodynamic therapy, and provide new ideas for integrating ATC diagnosis and treatment.
**Limitations:** Subsequent clinical transformation.
**Funding for this study:** Technology Department of Jilin Province (No. YDZJ202201ZYTS248)
**Has your study been approved by an ethics committee?** Yes
**Ethics committee - additional information:** Laboratory Animal Center, College of Life Science, Jilin University
**Author Disclosures:**
Peng Dong: Nothing to disclose

**Prognostic Value Optimization of T Staging in Nasopharyngeal Carcinoma Based on MRI Grading of Skull Base Structure Invasion**
*F. Wu*, S. Li, D. Cao, H. Ya, H. LI, B. Chen; Guangzhou/CN
*(wufz@sysucc.org.cn)*

**Purpose:** To explore the prognostic value of the grading of skull base structure (including soft tissue, bone, and skull base foramina) invasion (SBSI) and their potential improvement for the T staging in nasopharyngeal carcinoma (NPC).
**Methods or Background:** This retrospective study enrolled M0 NPC patients from three medical institutions (1320, 432, and 249, respectively). The primary endpoint was 5-year overall survival (OS). Cluster heatmaps and network analysis were used to explore the grading of SBSI. Univariate and multivariate analyses with Kaplan–Meier method are performed to compare survival outcomes.
**Results or Findings:** This study included 2001 patients (median age, 46 years [IQR, 39-55]; 1469 males). Multivariate analyses identified invasion of the jugular foramen or foramen ovale (severe skull base foramina invasion, SSBFI) as an independent prognostic factor (HR: 2.24, 95% CI: 1.42-3.54, p=0.001), which conferred a poor prognosis similar to T4 disease(5-year OS: 69.7% vs 75.6%, p=0.421). Conversely, the prognosis of patients with pterygoid and sphenoid base (minor skull base invasion, MSBI) was superior to other T3 skull base involvement (5-year OS: 91% vs 82.4%, p= 0.003), was comparable to that of T2 disease (5-year OS: 91% vs 86.3%, p= 0.267). A proposed T staging(proposed_T) system was created by upstaging SSBFI to T4 and downstaging MSBI to T2. This proposed_T category outperformed the 8th/9th edition and other proposed T staging systems in risk stratification and prognostic prediction.
**Conclusion:** The grade of SBSI is a critical factor for risk stratification in T3 NPC patients, and its incorporation into T staging leads to a more precise prognostic stratification.
**Limitations:** The study design was a retrospective analysis and did not include a prospective validation cohort.
**Funding for this study:** National Natural Science Foundation of China ( No.82171906 ) Guangdong Basic and Applied Basic Research Foundation (2025A1515011590)
**Has your study been approved by an ethics committee?** Yes
**Ethics committee - additional information:** The ethical approval for this study was granted by the institutional ethics committees of the three hospitals (Approval No: B2019-222-01)
**Author Disclosures:**
Di Cao: Nothing to disclose
Biyun Chen: Nothing to disclose
Shuqi Li: Nothing to disclose
Fengze Wu: Nothing to disclose
Haojiang Li: Nothing to disclose
Hui Ya: Nothing to disclose

**16:00 - 17:30** **Research Stage 2**

### Research Presentation Session: Interventional Radiology

### RPS 2109

### Plug, block, heal: the art and science of embolotherapy

**Moderator**
Federico Collettini; Berlin/DE
*(federico.collettini@charite.de)*
**Author Disclosures:**
Federico Collettini: Advisory Board: Sirtex, Guerbet, Nextbiomedical, Magle Pharmacept, Medtronic, Terumo, Astrazeneca, Consultant: Sirtex, Guerbet, Nextbiomedical, Magle Pharmacept, Medtronic, Terumo, Asahi, Grant Recipient: Sirtex, Guerbet, Nextbiomedical, Magle Pharmacept, Terumo, Siemens, Philips, Research Grant/Support: Sirtex, Guerbet, Nextbiomedical, Magle Pharmacept, Terumo, Siemens, Philips, Speaker: Sirtex, Guerbet, Nextbiomedical, Magle Pharmacept, Medtronic, Terumo, Asahi

**Transarterial Embolization with Ethylene Vinyl Alcohol Copolymer (EVOH) for Unresectable Hepatocellular Carcinoma: Preliminary Results from the TAEVOH Study**
*C. Viola*, F. C. Bodini, M. Graffeo, C. Sallemi; Brescia/IT
*(viola.costanza@gmail.com)*

**Purpose:** Hepatocellular carcinoma (HCC) is the most common primary liver malignancy, and transarterial embolization is the standard treatment for intermediate-stage disease. While conventional transarterial chemoembolization (TACE) is widely used, the added value of chemotherapy over bland embolization remains debated, with concerns about increased toxicity and costs. In large tumors, particle-based embolization may result in incomplete coverage, leaving viable tumor tissue. Ethylene-vinyl-alcohol-copolymer (EVOH) is a non-adhesive liquid embolic agent with controlled, progressive delivery that provides durable and uniform vasculature filling. The TAEVOH study aimed to evaluate the feasibility, safety, and oncological outcomes of EVOH embolization in clinical practice.
**Methods or Background:** This retrospective single-center study included consecutive patients with unresectable HCC treated with EVOH embolization between 2020 and 2025. Eligible patients had BCLC B disease, no portal vein thrombosis or extrahepatic spread, and Child-Pugh A–B liver function. Procedures were performed under conscious sedation with selective or superselective microcatheterization and controlled EVOH injection until complete tumor filling. Primary endpoints were radiologic response (mRECIST) and safety (CIRSE/CTCAE v5.0). Secondary endpoints included AFP reduction and downstaging to curative treatment.
**Results or Findings:** Forty patients (median age 78 years, 56% male, Child-Pugh A in 82% and B in 18%) underwent 52 procedures with 100% technical success. At first imaging assessment, complete response was observed in 8 of 27 evaluable patients (30%). AFP decreased by >50% in evaluable patients. Eight patients (27%) achieved downstaging and underwent curative therapies. Minor adverse events were limited to post-embolization syndrome and transient grade 1–2 hepatic toxicity, without major complications.
**Conclusion:** EVOH embolization is a feasible, safe, and effective treatment for unresectable HCC, with encouraging radiologic response and downstaging rates. Prospective studies are warranted to validate these results.
**Limitations:** Retrospective, single-center design with a limited cohort; long-term follow-up is ongoing.
**Funding for this study:** No external funding was received for the completion of this study
**Has your study been approved by an ethics committee?** Yes
**Ethics committee - additional information:** Approved by the Territorial Ethics Committee Lombardia 6 (Pavia, Italy), session of July 29, 2025 (Protocol No. TAEVOH-1, Prot. 0045956/25)
**Author Disclosures:**
Massimo Graffeo: Nothing to disclose
Costanza Viola: Nothing to disclose
Claudio Sallemi: Nothing to disclose
Flavio Cesare Bodini: Nothing to disclose

**Real-world breakthrough analysis from the Italian Group of Regenerative and Occlusive Worldwide-used techniques of hepatic Hypertrophy (I GROWtoH) registry**
M. Serenari[1], F. Ratti[2], M. Cescon[1], G. Ercolani[3], V. Mazzaferro[2], D. Patrono[4], E. Jovine[1], *C. Mosconi*[1], F. De Cobelli[2]; [1]Bologna/IT, [2]Milan/IT, [3]Forlì/IT, [4]Torino/IT
*(cristina.mosconi@aosp.bo.it)*

**Purpose:** To present multicentre real-world data on safety and efficacy of liver hypertrophy-inducing procedures (HIP) aiming to track trends in these approaches in Italy, and support a collaborative multidisciplinary network to reinforce the fundamental synergy between surgeons and radiologists.
**Methods or Background:** The I GROWtoH registry prospectively collects data on HIPs performed in candidates for major hepatectomy. For this analysis, the retrospective cohort (Jan 2014–Sep 2024) was reviewed. Demographics, indication for surgery, type of hypertrophy technique, imaging-based functional/volumetric assessment and peri-procedural outcomes were analysed.
**Results or Findings:** A total of 464 patients from 17 centres were included: 169 colorectal liver metastases (36.4%), 97 intrahepatic cholangiocarcinoma (20.9%), 91 perihilar cholangiocarcinoma (19.6%), and 77 hepatocellular carcinoma (16.6%). HIPs performed comprised 224 portal vein embolisations (48.3%; 8% including segment 4), 73 portal vein ligations (15.7%), 44 double vein embolisations/liver venous deprivations (9.5%) and 5 radiation lobectomies (1.1%). Complications occurred in only 3 patients after DVE/LVD. Hepatobiliary scintigraphy was performed in 105 patients after HIP (22.6%) while median increase of future liver remnant volume was 43% (IQR 30–66). Overall, 353 patients (76.1%) underwent curative major hepatectomy; 90-day mortality after HIPs was 5.0%.
**Conclusion:** Curative hepatectomy was achieved in over three-quarters of patients, underscoring the role of these procedures in expanding surgical options for complex hepatobiliary malignancies and witnessing the need for a shift from a “surgical-based” management to a multidisciplinary approach (from definition of surgical candidates, to choice of hypertrophy techniques, to management of intra and postoperative challenges). This trend reflects the collective effort of the Italian surgical community to build consensus on key aspects of regenerative liver surgery, laying the groundwork for a stable and synergic collaboration between surgeons and radiologists.
**Limitations:** Retrospective design and heterogeneity of techniques across centres.
**Funding for this study:** None
**Has your study been approved by an ethics committee?** Yes
**Ethics committee - additional information:** CE AVEC 450/2024/Oss/AOUBo
**Author Disclosures:**
Francesco De Cobelli: Nothing to disclose
Damiano Patrono: Nothing to disclose
Matteo Serenari: Nothing to disclose
Vincenzo Mazzaferro: Nothing to disclose
Matteo Cescon: Nothing to disclose
Cristina Mosconi: Nothing to disclose
Giorgio Ercolani: Nothing to disclose
Elio Jovine: Nothing to disclose
Francesca Ratti: Nothing to disclose

**Investigating the effectiveness and safety of embolic agents in bronchial artery embolization- A Systematic Review and Meta-aAnalysis**
*D. Laczko*, B. Nemes; Budapest/HU
*(laczkodavid97@hotmail.com)*

**Purpose:** Bronchial artery embolization effectively treats severe and recurrent hemoptysis and dramatically reduces mortality rates from 80-90% to less than 10%. Despite its success, no previous meta-analysis has focused on comparing the effectiveness and safety of particle and liquid embolic agents. This study aims to address this gap by comparing the effectiveness and safety of these available embolic agents.
**Methods or Background:** : We systematically searched MEDLINE, EMBASE, CENTRAL, Scopus, and Web of Science for relevant studies from inception until November 02, 2023. Eligible studies included cohort studies of patients who were treated with either particle or liquid embolic agents. The technical and clinical success, hemoptysis recurrence rates were meta-analyzed. Risk of bias was assessed using MINORS. Forest plots visualized meta-analytical results. The study was registered with PROSPERO (CRD42023480289).
**Results or Findings:** Of 2,502 studies, 59 met the inclusion criteria, including 4,660 patients. No significant difference between particle and liquid agents was observed in the proportion of immediate clinical success (0.95 vs 0.95, p=0.935). The proportion of 12-month hemoptysis recurrence did not differ between particle and liquid embolic agents (0.14 vs 0.17, p= 0.663). Significantly higher proportions of hemoptysis recurrence were observed between patients with malignant and non-malignant lung disease at all follow-up times (1 month: 0.26 vs 0.05; p<0.001; 3 months: 0.44 vs 0.08; p<0.001; 6 months: 0.56 vs 0.12; p<0.005; 12 months: 0.66 vs 0.11; p<0.001).
**Conclusion:** Our findings suggest that both particle and liquid embolic agents are effective and safe in treating hemoptysis. Patients with malignant lung diseases had shorter hemoptysis-free periods, highlighting that this subpopulation might benefit from a closer follow-up.
**Limitations:** The lack of reporting patient level data prevented us from conducting further analyses based on different etiologies and further exploring the high heterogeneity.
**Funding for this study:** None
**Has your study been approved by an ethics committee?** Not applicable
**Author Disclosures:**
Balázs Nemes: Nothing to disclose
David Laczko: Nothing to disclose

**Efficacy and Safety of TACE in Combination with Targeted Therapy and Immunotherapy for Patients with CNLC stage IIb/IIIa Hepatocellular Carcinoma Abstract**

*Z. Hu*; Hangzhou/CN
*(huzexin111@163.com)*

**Purpose:** To evaluate the efficacy and safety of first-line transcatheter arterial chemoembolization (TACE) combined with targeted therapy and immunotherapy in patients with stage IIb/IIIa hepatocellular carcinoma (HCC).
**Methods or Background:** A total of 198 patients who received first-line TACE combined with targeted immunotherapy or TACE alone from January 2015 to December 2022 were selected. Using propensity score matching, 50 patients were included in both the combination group and the TACE-alone group(Figure 1) . Kaplan-Meier method was used to calculate the median overall survival (mOS) and median progression-free survival (mPFS) with 95% confidence interval (CI). Objective response frate (ORR) and disease control rate (DCR) were assessed based on modified Response Evaluation Criteria in Solid Tumors (mRECIST).
**Results or Findings:** The mOS in the combination group was 30.1 months (95% CI: 21.9-38.3) compared to 14.5 months (95% CI: 11.0-18.0) in the TACE-alone group, showing a statistically significant difference (P < 0.001). (Figure 2.) The mPFS in the combination group was 10.3 months (95% CI: 8.8-11.8) compared to 7.1 months (95% CI: 5.8-8.4) in the TACE-alone group, with a statistically significant difference (P < 0.001). The ORR and DCR in the combination group were 84% and 94% respectively, while in the TACE-alone group, they were 58% and 80% respectively. The differences in ORR and DCR between the two groups were statistically significant (P < 0.05). The incidence of severe adverse events was 24% in the combination group and 16% in the TACE-alone group(P= 0.317).
**Conclusion:** Compared to TACE alone, TACE combined with targeted therapy and immunotherapy provides better efficacy without increasing the incidence of severe adverse events in patients with CNLC stage IIb/IIIa HCC.
**Limitations:** This is a retrospective study with selection bias."
**Funding for this study:** No funding
**Has your study been approved by an ethics committee?** Yes
**Ethics committee - additional information:** the study protocol was approved by the ethical committee of the First Affiliated Hospital of Soochow University
**Author Disclosures:**
Zexin Hu: Nothing to disclose

**Complication Rates and Effectiveness of Renal Angiomyolipoma Embolisation: A Systematic Review and Meta-Analysis**

*M. Duffy*[1], A. Deshwal[1], R. Donnelly[1], M. J. Mullins[2]; [1]Dublin/IE, [2]East Fremantle/AU
*(michaelduffy9595@gmail.com)*

**Purpose:** To assess the rates of success, complications, and re-interventions of selective arterial embolisation of renal angiomyolipomas
**Methods or Background:** A systematic review and meta-analysis was conducted, including studies reporting outcomes of selective arterial embolisation for renal angiomyolipomas. Pooled estimates were calculated for technical success, radiological success, clinical success, tumour shrinkage, re-embolisation rates, surgical re-intervention, and complication rates. Heterogeneity was assessed using $I^2$ statistics. Exploratory regression modelling was performed to assess factors associated with re-intervention.
**Results or Findings:** Thirteen studies comprising of 478 patients and 542 AMLs were included. Technical success – successful delivery and occlusion of the embolic agent – was achieved in 86.2% of cases (95% CI: 80-89.1%; $I^2$ = 36.1%). Radiological success, based on CT-assessed tumour shrinkage, was observed in 94% of cases (95% CI:91.2-97.3%; $I^2$ = 15.6%). Subgroup analysis revealed ethanol-alone embolisation achieved the highest shrinkage (95.8%) and lowest re-intervention rate (3.4%). Pooled re-intervention (re-embolisation or surgery) was required in 19% of cases (95% CI: 10–34%; $I^2$ = 85.9%). Minor complications occurred in 46% of procedures, predominantly post-embolisation syndrome (PES, 43%; 95% CI: 35–53%, $I^2$ = 88.8%). Major complications, such as retroperitoneal bleeding, significant haematuria, abscesses or prolonged hospital admission, were rare (pooled rate: 5.7%; 95% CI: 3.9–8.2%), and the pooled rate for surgical re-intervention was 4% (95% CI: 2-7%) with low heterogeneity ($I^2$ = 9.6%, p = 0.333).
**Conclusion:** Embolisation is a safe primary treatment option for renal angiomyolipomas with high technical and radiologic success, low major complications, and rates of surgical intervention. Embolic agent choice significantly impacted outcomes in this cohort.
**Limitations:** Clinical heterogeneity reflects variable techniques, definitions, follow-up, and patient selection. Standardised prospective studies with uniform embolic protocols and eGFR tracking are needed to optimise embolisation strategies and assess long-term renal outcomes.
**Funding for this study:** None
**Has your study been approved by an ethics committee?** Not applicable
**Author Disclosures:**
Michael Duffy: Nothing to disclose
Matthew James Mullins: Nothing to disclose
Ryan Donnelly: Nothing to disclose
Avinash Deshwal: Nothing to disclose

**Effectiveness and Safety of Uterine Artery Embolization in Managing Postpartum Hemorrhage Associated with Placenta Accreta: A Systematic Review and Meta-Analysis**

*L. Habiro Alves*[1], G. Martin[1], A. C. Servidoni[2], H. Provinciatto[3], G. Franceschini Machado[1], K. Miller[4], B. Taneja[5], A. K. Taneja[6], A. Yousif[7]; [1]São Paulo/BR, [2]New Haven, CT/US, [3]Ribeirao Preto/BR, [4]Sao Paulo/BR, [5]Boston, MA/US, [6]Miami, FL/US, [7]El Paso, TX/US
*(lucashabiro199@gmail.com)*

**Purpose:** Placenta accreta spectrum disorder is a pregnancy complication associated with a substantially increased risk of maternal mortality. With the advancements of interventional radiology, uterine artery embolization has emerged as an effective minimally-invasive alternative to avoid hysterectomy for severe postpartum hemorrhage. The aim of this systematic review and meta-analysis is to evaluate whether uterine artery embolization is an effective and safe method to manage post-partum hemorrhage associated with placenta accreta spectrum.
**Methods or Background:** PubMed, Embase, and Cochrane Central were systematically searched for studies published from inception until September 2025. A random-effects meta-analysis was conducted through RStudio version 4.4.1 to pool prevalences and rates. The Cochrane Collaboration's Risk Of Bias In Non-randomized Studies of Interventions tool was used for quality assessment.
**Results or Findings:** Six studies including 215 participants were analyzed. The pooled clinical success rate of uterine artery embolization was 85.0% (95% CI, 77.7–90.2), while the pooled prevalence of hysterectomy after clinical failure was 7.9% (95% CI, 4.9–12.5). Postoperative complications occurred in 19.0% of cases (95% CI, 6.2–45.5) and the pooled mean estimated blood loss was 1825 mL (95% CI, 1116–2533).
**Conclusion:** Uterine artery embolization is an effective and safe interventional radiology strategy for PPH due to placenta accreta, offering high success rates while preserving uterine function and reducing surgical morbidity.
**Limitations:** First, there are no published randomized controlled trials on management of PHH secondary to PAS, which restricts the number of studies evaluated and overall statistical power. Second, long-term maternal outcomes, such as subsequent fertility and menstrual function, were not consistently reported, limiting the ability to assess the broader safety profile.
**Funding for this study:** None
**Has your study been approved by an ethics committee?** Not applicable
**Author Disclosures:**
Katherine Miller: Nothing to disclose
Guilherme Franceschini Machado: Nothing to disclose
Atul Kumar Taneja: Nothing to disclose
Giovanna Martin: Nothing to disclose
Lucas Habiro Alves: Nothing to disclose
Abdelrahman Yousif: Nothing to disclose
Bruna Taneja: Nothing to disclose
Henrique Provinciatto: Nothing to disclose
Ana Clara Servidoni: Nothing to disclose

**Local intervention with and without embolization in primary and secondary lung tumors: a prospective randomized stuy**

*T. J. Vogl*, B. Fischer, T. Gruber-Rouh, H. Adwan; Frankfurt/DE

**Purpose:** To evaluate unresectable lung metastases of different primary lesions and primary lung cancers treated by transpulmonary chemoperfusion (TPCP) or transpulmonary chemoembolization (TPCE) regarding size and ADC-values.

**Methods or Background:** In this prospective randomized study 60 patients (30 women, 30 men; mean: 64.5y; range: 29-84y) were included who were treated by TPCP (n=30) and TPCE (n=30). Malignant lesions were from primary lung cancers (n=15), colorectal (n=13), breast cancer (n=10), and other primaries (n=22). All patients were treated via a transfemoral venous approach selectively into the segmental pulmonary artery using Cisplatin and MitomycinC. The TPCE patients received additionally EmboCept®S for the embolization. Follow-up was performed using MRT and CT in 3-month intervals post final therapy session.
**Results or Findings:** No major side effects were observed. In the TPCE group, mean value in the change of volume was 0.97±0.54, in the TPCP group 1.43±1.14 (p=0.08). Change of the ADC-value was 1.08±0.33 in TPCE and 1.13±0.50 in TPCP (p=0.7). According to RECIST 1.1, TPCE patients showed partial response (PR) in 4 cases and progressive disease (PD) in 4 cases (PR=14.81%, PD=14.81%). The TPCP group showed 1 case of PR and six cases of PD (PR=3.57%, PD=21.43%). The overall survival time in the TPCE group was 732.70±379.41 days, in the TPCP group 552.82±350.23 days (p=0.09). The progression-free time had a p-value of 0.14 (TPCP: 405.36±324.37 days; TPCE: 554.07±397.49 days)
**Conclusion:** TPCP and TPCE can be safely performed. TPCE resulted in a more stable treatment outcome and a better prognostic trend than TPCP, even though there was no statistically significant difference between TPCP and TPCE concerning overall survival.
**Limitations:** The number of study participants for each group was very limited. The groups were not as homogeneous as a statistical evaluation of small groups requires.
**Funding for this study:** No funding
**Has your study been approved by an ethics committee?** Yes
**Ethics committee - additional information:** By the Ethics Committee of the university hospital
**Author Disclosures:**
Barbara Fischer: Nothing to disclose
Thomas J. Vogl: Nothing to disclose
Tatjana Gruber-Rouh: Nothing to disclose
Hamzah Adwan: Nothing to disclose

### Efficacy comparison between endovascular embolization with coils vs coils plus PVA particles in spontaneous soft tissue haemorrhage in anticoagulated patients – a retrospective study

J. Pereira, *L. A. Ferreira*, B. Oliveiros, P. Donato; Coimbra/PT
*(luisamaralferreira@gmail.com)*

**Purpose:** To compare different embolic agents' efficacy in the treatment of spontaneous soft tissue haemorrhage in anticoagulated patients.
**Methods or Background:** Spontaneous soft tissue haemorrhage is a rare but potentially life-threatening condition, affecting anticoagulated patients. Endovascular embolization has emerged as the preferred treatment when conservative management fails. This retrospective observational study analysed 60 anticoagulated patients treated with transarterial embolization between January 2022 and December 2023. Patients were stratified based on embolic agents used: coils-only versus coils combined with polyvinyl alcohol (PVA) particles. Demographics, clinical presentation, procedural details, and outcomes were collected. Technical and clinical success, post-procedural haemoglobin concentration levels, reintervention rates, and 15-day mortality were assessed.
**Results or Findings:** Technical success was achieved in all cases with no major complications. No statistically significant differences were found between coils and coils plus PVA groups in terms of haemoglobin concentration variation, transfusion or reintervention need, nor mortality. Mortality at 15 days was 28,6%. Lower systolic and diastolic blood pressures, as well as higher international normalized ratio and prothrombin time, were associated with increased mortality. Higher diastolic blood pressure emerged as a strong predictor of survival. Although literature suggests possible benefits of combining coils with PVA, our findings did not demonstrate superior outcomes for the combination therapy. Nonetheless, the study supports the safety and efficacy of transarterial embolization in this clinical setting.
**Conclusion:** Early intervention and haemodynamic stability appear more critical for survival than the choice between coils versus coils plus PVA particles.
**Limitations:** The main limitation is the retrospective observational nature of the study.
**Funding for this study:** This study did not receive any funding.
**Has your study been approved by an ethics committee?** Yes
**Ethics committee - additional information:** This study was submitted and approved by the ethics committee of the institution where it was carried out.
**Author Disclosures:**
Paulo Donato: Nothing to disclose
Luis Amaral Ferreira: Nothing to disclose
Bárbara Oliveiros: Nothing to disclose
João Pereira: Nothing to disclose

### Prophylactic transarterial embolization after endoscopic hemostasis in high-risk patients with non-variceal upper gastrointestinal bleeding - a systematic review

*E. Battiato*, F. Tiralongo, M. D'Urso, G. Vitaliti, D. G. Castiglione, D. Falsaperla, F. Libra, F. Vacirca, A. Basile; Catania/IT
*(eb90@hotmail.com)*

**Purpose:** To provide a systematic review on prophylactic transarterial embolization (pTAE) after endoscopic hemostasis in high-risk non-variceal upper gastrointestinal bleeding (NVUGIB), focusing on selection criteria, technical conduct by arterial territory and embolic material, clinical outcomes, complications.
**Methods or Background:** Systematic PubMed and Scopus search (Jan 2010–Sep 2025) included original pTAE studies after successful endoscopic hemostasis and excluded case reports, abstracts, studies focused on variceal bleeding, empiric embolization for angiography-negative bleeding, and systematic reviews/meta-analyses. Finally, 10 studies were included in this review: two randomized clinical trials, three prospective studies, five retrospective studies.
**Results or Findings:** Randomized evaluation did not show an overall reduction in 30-day rebleeding in intention-to-treat analysis, but suggested benefit in large ulcers (≥15 mm) with lower transfusion needs, and no embolization-related ischemia among treated patients. Kaminskis et al. reported lower rebleeding and fewer surgery with prophylaxis across matched comparators, also in high-risk profile (Rockall ≥ 7) without excess mortality or ischemic events. In a series treating duodenal ulcer bleeding, early rebleeding occurred in 11% of cases. Major complications (4%) including hepatic coil migration, highlighting the need for meticulous technique. Prophylactic TAE showed numerically less rebleeding and 30-day mortality than therapeutic TAE, with comparable complications. Endovascular therapy compared with surgery shows similar mortality rates, higher rebleeding rates, and substantially fewer major complications. TAE versus surgery after failed endoscopy showed similar 30-day mortality but fewer complications with TAE, with a pTAE subset present but not separately analyzed, underscoring endovascular safety while recognizing rebleeding as the key trade-off in this pathway.
**Conclusion:** pTAE is feasible, generally safe, and appears to reduce rebleeding and surgical rescue in well-selected patients. Routine application across high-risk presentations is not supported by randomized evidence or guidelines.
**Limitations:** N/A
**Funding for this study:** N/A
**Has your study been approved by an ethics committee?** Not applicable
**Author Disclosures:**
Daniele Falsaperla: Nothing to disclose
Gioele Vitaliti: Nothing to disclose
Davide Giuseppe Castiglione: Nothing to disclose
Mattia D'Urso: Nothing to disclose
Federica Libra: Nothing to disclose
Enrico Battiato: Author: Presenter
Antonio Basile: Nothing to disclose
Francesco Vacirca: Nothing to disclose
Francesco Tiralongo: Nothing to disclose

### Liver Venous Deprivation for Perihilar Cholangiocarcinoma: Safety, Hypertrophic Efficacy, and Impact on Resectability

*D. Santangelo*, A. Campisi, D. Palumbo, C. Canevari, F. Ratti, F. Cipriani, A. Chiti, M. Catena, F. De Cobelli; Milan/IT
*(santangelo.domenico@hsr.it)*

**Purpose:** To evaluate the safety and efficacy of liver venous deprivation(LVD) in inducing volumetric and functional hypertrophy, and its impact on resectability in patients with perihilar cholangiocarcinoma(pCCA).
**Methods or Background:** We retrospectively analyzed 25 patients with histologically confirmed pCCA who underwent LVD between May 2020 and May 2025. LVD was indicated when volumetric future liver remnant (FLR) was <35% or functional FLR was <2.69%/min/m² on 99mTc-mebrofenin hepatobiliary scintigraphy. FLR volumetry was measured at baseline (pre-LVD), 5–15 days post-LVD (time point 1), and 15–30 days post-LVD (time point 2). FLR function was assessed with 99mTc-mebrofenin scintigraphy at baseline and within 30 days after LVD. Primary endpoints were technical success, safety, and post-LVD resection rate. Secondary endpoints included volumetric and functional hypertrophy metrics (degree of hypertrophy (DH) and kinetic growth rate (KGR)) and surgical outcomes, including post-hepatectomy liver failure (PHLF) per ISGLS criteria.
**Results or Findings:** Baseline median volumetric FLR was 30.0%, and functional FLR was 1.81%/min/m². Biliary drainage was present in 21/25 (84%); median bilirubin was 3.5 mg/dL. Technical success was 100%; one patient (4%) developed a subcapsular hematoma, managed conservatively. At time point 1, median volumetric FLR was 37% (DH 12.7%; KGR 8.1%/week). At time point 2, median volumetric FLR was 41% (DH 16%; KGR 5%/week). In 15 patients with scintigraphy, median post-LVD functional FLR increased to

Saturday

3.08%/min/m². Surgical resection was performed in 19/25 (76%) at median 40 days post-LVD. Major complications (Clavien–Dindo ≥III) in 14/19 (73.7%). Grade C PHLF in 3/19 (15.8%); 90-day mortality was 15.7%.
**Conclusion:** LVD was feasible and safe in pCCA, produced substantial volumetric and functional hypertrophy of the FLR, and increased resectability in this cohort. High postoperative morbidity underscores the importance of careful patient selection and perioperative optimization strategies.
**Limitations:** retrospective. Small sample size.
**Funding for this study:** None
**Has your study been approved by an ethics committee?** Yes
**Ethics committee - additional information:** Leatum 64/INT/2021
**Author Disclosures:**
Francesco De Cobelli: Nothing to disclose
Diego Palumbo: Nothing to disclose
Arturo Chiti: Nothing to disclose
Federica Cipriani: Nothing to disclose
Carla Canevari: Nothing to disclose
Antonino Campisi: Nothing to disclose
Marco Catena: Nothing to disclose
Francesca Ratti: Nothing to disclose
Domenico Santangelo: Nothing to disclose

**16:00 - 17:30 Research Stage 1**

### Research Presentation Session: Musculoskeletal

### RPS 2110

### Imaging musculoskeletal tumours

**Moderator**
Salvatore Gitto; Milan/IT
*(sal.gitto@gmail.com)*

### Accelerated whole-body diffusion-weighted MRI in multiple myeloma patients using deep learning image reconstruction: a retrospective comparison with standard diffusion-weighted imaging

*J. Hofmann*, S. Afat, K. Nikolaou, J. Herrmann; Tübingen/DE

**Purpose:** Whole-body MRI (WB-MRI) is routine but time-intensive examination in patients with multiple myeloma. Prolonged scan duration may decrease image quality through motion artifacts, reduce throughput, and increase patient discomfort. This study aimed to evaluate the impact of deep learning–accelerated diffusion-weighted imaging (DWIDL) in 3T WB-MRI on overall and medullary image quality, sharpness, noise, lesion detectability, and diagnostic confidence.
**Methods or Background:** This retrospective study included 40 patients with multiple myeloma (21 men, 19 women; mean age, 62 ± 11 years; range, 35–82 years) who underwent 3T WB-MRI, including DWI with two b-values (50 and 800 s/mm²) between February 2023 and April 2024. Single-average raw data were processed using a deep learning (DL) image reconstruction algorithm, yielding a simulated acquisition time of 3 minutes 3 seconds for DWIDL compared with 7 minutes 6 seconds for standard DWI (DWIStd) by reducing the number of signal averages. Two experienced radiologists independently evaluated all DWI datasets using a 5-point Likert scale assessing noise level, artifact severity, sharpness, overall image quality, and diagnostic confidence.
**Results or Findings:** There were no significant differences between DWIDL and DWIStd in artifact severity, sharpness, overall image quality, lesion detectability, or diagnostic confidence. However, DWIDL demonstrated significantly lower noise levels and higher contrast compared with standard DWI ($P < .05$).
**Conclusion:** Deep learning–based image reconstruction for WB-DWI at 3T is feasible and enables a significant reduction in acquisition time without compromising image quality. Accelerated acquisition particularly benefits multiple myeloma patients by reducing motion artifacts, improving comfort, and potentially enabling MRI instead of CT for better assessment of bone marrow infiltration.
**Limitations:** This retrospective single-center study with a limited sample size, subjective image quality assessment, and no cost or workflow analysis limits the generalizability and strength of the findings.
**Funding for this study:** None
**Has your study been approved by an ethics committee?** Yes
**Ethics committee - additional information:** University of Tuebingen
**Author Disclosures:**
Judith Herrmann: Nothing to disclose
Konstantin Nikolaou: Nothing to disclose
Saif Afat: Nothing to disclose
Johannes Hofmann: Nothing to disclose

### Assessment of volumetric and modified Choi MRI response in Desmoid Fibromatosis treatment with Sorafenib versus Nirogacestat

*R. F. Valenzuela*, E. Duran-Sierra, M. Antony, J. Espinoza, C. M. Costelloe, J. E. Madewell, W. Murphy, B. Amini, S. Lo; Houston, TX/US
*(raulvp@estigia.cl)*

**Purpose:** Desmoid fibromatosis patients are typically treated with active surveillance and drugs on progression, particularly sorafenib, and most recently, with nirogacestat. This study aimed to determine the signal and volume changes after therapy and over time, comparing both drugs using MRI T2-WI-derived features, including modified Choi (mChoi) and volumetric measurements.
**Methods or Background:** Retrospective study including 17 patients with single-lesion extremity desmoid fibromatosis and standard-of-care MRI, including T2-WI, from March 2021-February 2024. Volumetric tumor segmentations were created on T2-STIR images. Diameter, mChoi, and volume measurements were computed at four time points across the patient's treatment: Pre-treatment (Pre-Rx), post-treatment 1 (Rx1), post-treatment 2 (Rx2), and post-treatment 3 (Rx3). The percentage change in diameter (RECIST), mChoi, and volume across time were computed with respect to Rx1 and compared in nirogacestat vs. sorafenib patients.
**Results or Findings:** Eight patients were treated with nirogacestat and nine patients with sorafenib. Nirogacestat-group: 1) mChoi detected two cases of progression not detected by RECIST or volume, 2) from Pre-Rx to Rx3, the average mChoi decreased by 66% over 20 months (-3.3%/month), and 3) from Pre-Rx to Rx3, the average volume reduced by 49% over 20 months (-2.45%/month). Sorafenib group: 1) RECIST, mChoi, and volume detected two progressions. mChoi detected one response not detected by RECIST or volume, 2) from Pre-Rx to Rx3, the average mChoi decreased by 49% over 34 months (-1.44%/month), and 3) from Pre-Rx to Rx3, the average volume decreased by 4% over 34 months (-0.09%/month).
**Conclusion:** Patients treated with nirogacestat exhibited larger volumetric and mChoi changes, with the fastest response and similar sustained positive treatment effects compared to those treated with sorafenib, indicating the potential of nirogacestat as an effective treatment option for desmoid tumors.
**Limitations:** Small sample size.
**Funding for this study:** The John S. Dunn, Sr. Distinguished Chair in Diagnostic imaging and M.R Evelyn Hudson Foundation Endowed Professorship.
**Has your study been approved by an ethics committee?** Not applicable
**Author Disclosures:**
Sam Lo: Nothing to disclose
Elvis Duran-Sierra: Nothing to disclose
Jossue Espinoza: Nothing to disclose
Behrang Amini: Nothing to disclose
Colleen M. Costelloe: Nothing to disclose
Raul Fernando Valenzuela: Nothing to disclose
William Murphy: Nothing to disclose
Mathew Antony: Nothing to disclose
John Edward Madewell: Nothing to disclose

### Does Extra-Skeletal Chondrosarcomas manifest distinct MRI radiomics features compared to myxoid liposarcoma and pleomorph sarcoma, and does it have common features with skeletal chondrosarcoma?

*R. Perraut-Gattegno*, J. Stadelmann, P-A. A. Poletti, S. Boudabbous; Geneva/CH

**Purpose:** The aim of the study was to assess if some features of radiomics are common to extra-skeletal chondrosarcoma and skeletal chondrosarcoma, and to distinguish this rare entity from the most frequent encountered soft tissue sarcoma (myxoid liposarcoma and pleomorphic sarcoma) in MRI imaging.
**Methods or Background:** Retrospective study on our radiological data base (PACS archive) confronted to data from multidisciplinary sarcoma Concilium, with the evaluation of 150 radiomics features were extracted from volume-of-interest on T1 and T2-weighted spin-echo MRI, from 19 tumor cases (4 ESC, 5 CS, 5 PS and 5 MLS, based on histopathological diagnosis). These radiomics features were then used to train Random Forests to select the best 10 features for the classification of the 4 types of tumors. Then we used a logistic regression to evaluate the best pair of features for dichotomy classification: EOC and OC versus the other types (MLS, PS).

**Results or Findings:** The visual evaluation of the feature scatter plot confirms the separability of the ESC and SC against the other types of tumours. The evaluation of the logistic regression resulted of a sensitivity of 0.93 specificity of 0.92 for an overall accuracy of 0.92 with the following features: GLCM inverse difference moment normalized (on T2 sequences) and GLCM correlation (on T1 sequences). We consider this preliminary study promising for the use of radiomic features in the differentiation of extra-skeletal and skeletal chondrosarcoma from other soft tissue tumors.
**Conclusion:** Despite being developed on few cases, 2 radiomics features accurately distinguishes the chondrosarcoma group (ESC + SC) from the MLS and PS on MRI with an overall accuracy of 0.92. We as well evaluated that the chondrosarcoma group had identical profiles on all the radiomic features tested.
**Limitations:** Small number of cases
**Funding for this study:** None
**Has your study been approved by an ethics committee?** Yes
**Ethics committee - additional information:** Geneva ethics committee.
**Author Disclosures:**
Joël Stadelmann: Nothing to disclose
Sana Boudabbous: Nothing to disclose
Pierre-Alexandre Aloïs Poletti: Nothing to disclose
Roman Perraut-Gattegno: Nothing to disclose

**Assessment of neoadjuvant therapy response in extremity undifferentiated pleomorphic sarcoma using multiparametric MRI: A comparison between radiation vs consecutive chemotherapy and radiation**

R. F. Valenzuela, E. Duran-Sierra, M. Antony, J. Espinoza, C. M. Costelloe, J. E. Madewell, W. Murphy, D. Wells, *B. Amini*; Houston, TX/US
*(bamini@mdanderson.org)*

**Purpose:** Undifferentiated pleomorphic sarcoma (UPS) patients are typically treated with either consecutive chemotherapy and radiotherapy (CT-RT) or RT alone. This study aimed to determine the impact of the treatment strategy on imaging and pathology in UPS response assessment based on multiparametric MRI (mp-MRI).
**Methods or Background:** This retrospective study included 30 extremity UPS patients with preoperative mp-MRI, including contrast-enhanced susceptibility-weighted-imaging (CE-SWI) and perfusion-weighted-imaging with dynamic-contrast-enhancement (PWI/DCE), with surgery performed from February 2021-May 2023. Lesions were visually classified on CE-SWI into one of six morphology patterns. On PWI/DCE, lesions were classified into one of six enhancement patterns. Time-intensity curves (TICs) were classified as types I-V. Patients demonstrating >=90% pathology-assessed-treatment-effect (PATE) on the surgical specimen were labeled as responders (n=15), and those with <90% PATE were labeled as partial/non-responders (n=15).
**Results or Findings:** 19 patients were treated with presurgical consecutive CT-RT, and 11 with presurgical RT. CT-RT group displayed a higher number of responders (53%) vs. RT (45%). Consecutive CT-RT demonstrated a higher average PATE (78%) compared to the RT group (63%). CT-RT group: 80% of responders displayed a CE-SWI complete-ring pattern (P=1.77x10-5), 90% a PWI/DCE capsular-enhancement (P=3.06x10-6), and 100% a TIC II (P=4.54x10-5). A predictive model combining the PWI/DCE TIC II, PWI/DCE capsular-enhancement, and CE-SWI complete-ring yielded the highest classification performance (AUC = 0.98), discriminating responders from partial/non-responders, outperforming RECIST (AUC = 0.49). RT group: 100% of responders displayed a TIC II (P=0.0253). Perfect classification performance was achieved by a model based on the PWI/DCE TIC II (AUC=1.0).
**Conclusion:** Predictive models based on PWI/DCE and CE-SWI features can outperform RECIST, predicting pathology-assessed response. Furthermore, classification models that best separate responders from partial/non-responders can vary not only by tumor type but also by treatment regimen.
**Limitations:** Small sample size.
**Funding for this study:** The John S. Dunn, Sr. Distinguished Chair in Diagnostic imaging and M.R Evelyn Hudson Foundation Endowed Professorship.
**Has your study been approved by an ethics committee?** Not applicable
**Author Disclosures:**
Elvis Duran-Sierra: Nothing to disclose
Jossue Espinoza: Nothing to disclose
Behrang Amini: Nothing to disclose
Colleen M Costelloe: Nothing to disclose
David Wells: Nothing to disclose
Raul Fernando Valenzuela: Nothing to disclose
William Murphy: Nothing to disclose
Mathew Antony: Nothing to disclose
John Edward Madewell: Nothing to disclose

**Radiomic Biomarkers from MRI for Predicting Chemotherapy Response in Synovial Sarcoma: A Retrospective Single-Center Study**

*F. P. Papa*[1], R. Cuocolo[2], F. Molea[1], L. D'Ambrosio[3], G. Grignani[4], T. Robba[1]; [1]Turin/IT, [2]Naples/IT, [3]Orbassano/IT, [4]Candiolo/IT
*(francescop.papa@gmail.com)*

**Purpose:** Synovial sarcoma is an aggressive malignancy that necessitates innovative diagnostic and therapeutic strategies. Predicting chemotherapy response is a pivotal step in the diagnostic-therapeutic pathway, enabling personalized treatment approaches. Radiomics, combined with machine learning, offers a promising avenue to identify imaging biomarkers that predict treatment response.
**Methods or Background:** In this retrospective, monocentric study, 51 patients diagnosed with synovial sarcoma underwent peri-treatment MRI acquisition. T1-weighted sequences were analyzed to extract 1116 radiomic features, which were subjected to feature selection processes. Machine learning models were developed to predict chemotherapy response. Data were intentionally gathered from heterogeneous MRI scanners to ensure the robustness of the models against clinical variability. Performance metrics included area under the curve (AUC) and accuracy assessments on both training and validation sets.
**Results or Findings:** The final ExtraTrees model, trained on 20 selected features, showed promising performance on the training set with an accuracy of 0.81 (±0.34), precision of 0.88 (±0.37), recall of 0.87 (±0.42), F1-score of 0.84 (±0.28), and AUC of 0.89 (±0.35), despite high variability. On the test set (n=17), accuracy was 64.7%, with good sensitivity for non-responders (80%) but lower for responders (58%). Precision was high for responders (0.88) but lower for non-responders (0.44). The test AUC was modest (0.60), while the Precision-Recall Curve (0.72) indicated reasonable precision for the positive class. The Brier score (0.250) suggested moderate model calibration.
**Conclusion:** This study highlights the potential of radiomics to predict chemotherapy response in synovial sarcoma, despite challenges posed by scanner heterogeneity and intra-reader variability. Further research is required to refine these methods and integrate them into routine clinical practice.
**Limitations:** This study is limited by single-reader segmentation, small sample size, and lack of external validation.
**Funding for this study:** No fundings
**Has your study been approved by an ethics committee?** Not applicable
**Author Disclosures:**
Francesca Molea: Nothing to disclose
Francesco Pio Papa: Nothing to disclose
Tiziana Robba: Nothing to disclose
Lorenzo D'Ambrosio: Nothing to disclose
Giovanni Grignani: Nothing to disclose
Renato Cuocolo: Nothing to disclose

**Imaging and Epidemiology of the Follow-up of Massive Knee Prostheses**

*Y. Abdelouahab*, F. Mihoubi Bouvier, J-L. Drape, A. Feydy, P. Anract; Paris/FR

**Purpose:** Massive knee prostheses are increasingly used after tumor resections or complex revisions. While they allow limb preservation, their imaging follow-up remains poorly standardized, and no dedicated imaging study had previously focused on these implants. Objectives : (i) To describe physiological and pathological imaging features of massive knee prostheses and propose a surveillance scheme; (ii) to estimate complication rates, implant survival, and associated prognostic factors.
**Methods or Background:** A retrospective monocentric study was conducted at Cochin Hospital (2010–2022), including 88 patients with distal femoral (n = 71) or proximal tibial (n = 17) prostheses. Over 1,000 imaging examinations were analyzed (radiographs, CT, MRI, EOS, ultrasound). Complications were classified using Henderson’s system, expanded with a type VI neuro-functional category.
**Results or Findings:** Median follow-up was 37 months. Implant survival was 50% at 37 months (mean 58). The complication rate was 73%: infections (30%), aseptic loosening (13.2%), neuro-functional complications (13.2%), and recurrences (15%). Radiography remained central for mechanical issues. Peri-prosthetic ossifications developed around the prosthetic collar: ossification without osteointegration was strongly associated with loosening (61.9% vs 6.9% and 18.2%), while ossification with osteointegration was protective. New calcifications beyond six months, especially in osteosarcoma patients, were highly suggestive of recurrence. Ultrasound proved particularly effective in the popliteal fossa (recurrences, thrombosis, peri-prosthetic changes), while MRI was more useful for soft tissues outside this area.
**Conclusion:** This first dedicated imaging study on massive knee prostheses establishes peri-collar ossification without osteointegration as a prognostic marker. Above all, it highlights the novel role of ultrasound as a non-invasive, accessible, and highly sensitive modality for surveillance—particularly in the popliteal fossa—offering a decisive new tool to improve early detection and long-term outcomes.

**Limitations:** Retrospective, single-center design and heterogeneous imaging protocols may limit generalizability.
**Funding for this study:** This study received no specific funding.
**Has your study been approved by an ethics committee?** Yes
**Ethics committee - additional information:** The Ethical Review Committee for publications of the Cochin university Hospital (CLEP)
**Author Disclosures:**
Antoine Feydy: Nothing to disclose
Fadila Mihoubi Bouvier: Nothing to disclose
Yacine Abdelouahab: Nothing to disclose
Jean-Luc Drape: Nothing to disclose
Philippe Anract: Nothing to disclose

**Imaging-based Metastatic Risk Model for Tibial Adamantinoma**
*P. Spinnato*[1], M. Colangeli[1], G. Bilancia[1], A. Crombé[2], M. Simonetti[1]; [1]Bologna/IT, [2]Talence/FR
*(paolo.spinnato1982@gmail.com)*

**Purpose:** Adamantinoma is a primary malignant bone tumor that is extremely uncommon. The categorization of metastatic risk at diagnosis is difficult since there is currently no histological grading. Imaging may therefore be useful in predicting prognosis. Our goal was to assess baseline imaging characteristics and how they relate to the emergence of metastatic disease.
**Methods or Background:** We retrospectively collected baseline radiological (CT, MRI, and conventional radiography) and clinical (metastatic disease) data from all consecutive patients with a histological diagnosis of adamantinoma between 2006 and 2022 at our single Sarcoma Center. Lodwick-Madewell grading, periosteal reaction, multifocality, soft-tissue extraskeletal component, tumor location, size, main radiological pattern (lytic, sclerotic, mixed), peritumoral edema, and vascular invasion were all examined. At diagnosis or during follow-up, correlations between the radiological characteristics listed above and metastatic disease were evaluated.
**Results or Findings:** Twenty-two patients were included (15 female, 7 male - mean age 28.8±13.3 years, range 7-58 years old). Six out of 22 patients (27.3%) developed distant metastases at diagnosis or during follow-up controls (minimum follow-up 24 months). The following radiological features were significantly correlated with the development of metastases at baseline or during follow-up controls: presence of an extra-skeletal component (p=0.0004), vascular invasion (p=0.0002), and diffuse peritumoral edema (p=0.0004). A risk model including two of the above mentioned imaging features, provided a sensibility of 100% (54.07%-100%), a specificity of 93.75% (69.67%-99.84%), with an accuracy of 95.45% (77.16%-99.88%) in predicting metastatic disease.
**Conclusion:** An accurate evaluation of baseline imaging studies in patients affected by adamantinoma may significantly aid in prognosis prediction and the selection of high metastatic-risk patients. For these patients, strict follow-up controls and more aggressive treatments should be suggested after multidisciplinary discussions in sarcoma centers.
**Limitations:** Retrospective, single-center analysis.
**Funding for this study:** No funding received.
**Has your study been approved by an ethics committee?** Yes
**Ethics committee - additional information:** CE-AVEC code: RX.RARE.SARC (2025)
**Author Disclosures:**
Gabriele Bilancia: Nothing to disclose
Amandine Crombé: Nothing to disclose
Mario Simonetti: Nothing to disclose
Marco Colangeli: Nothing to disclose
Paolo Spinnato: Nothing to disclose

**Quantitative Comparison of Contrast Enhancement Dynamics between Lipid-poor and Lipid-rich Vertebral Hemangiomas on MRI**
*T. B. Rodrigues*, C. Rodrigues, A. M. Gaspar, M. Ramalho; Lisbon/PT

**Purpose:** To quantitatively compare the contrast enhancement dynamics of lipid-poor and lipid-rich vertebral hemangiomas on MRI and assess whether enhancement parameters can reliably distinguish these subtypes.
**Methods or Background:** Lipid-poor vertebral hemangiomas are rare and diagnostically challenging due to their high T2 signal intensity on fat-suppressed MRI sequences, often mimicking more aggressive lesions. A word-search retrospective analysis included 43 vertebral hemangiomas: 21 lipid-poor (12 females, 9 males; mean age 58.6 ± 13.7 years; mean lesion size 15.5 ± 7.4 mm) and 22 lipid-rich (15 females, 7 males; mean age 57.0 ± 12.6 years; mean size 11.5 ± 5.7 mm). Lesions were confirmed via CT attenuation or MRI follow-up stability. Signal intensity was measured using a fixed region of interest across pre-contrast T1-weighted fat-suppressed, arterial, venous, and delayed (interstitial) phases. Percentage enhancement was calculated relative to pre-contrast signal. Statistical comparisons employed Student's t-test, Mann-Whitney U test, and Chi-square test.
**Results or Findings:** Lipid-poor hemangiomas exhibited significantly greater enhancement across all post-contrast phases compared to lipid-rich counterparts. In the arterial phase, lipid-poor lesions showed a mean enhancement of 206.8 ± 131.8%, versus 118.0 ± 82% for lipid-rich lesions ($p < 0.05$). Venous phase enhancement was also higher in lipid-poor hemangiomas (278.1 ± 117.0%) than in lipid-rich ones (166.3 ± 97.7%, $p < 0.05$). In the delayed (interstitial) phase, lipid-poor enhancement (267.0 ± 100.2%) remained markedly superior to lipid-rich enhancement (139.4 ± 90.9%, $p < 0.05$). No significant differences were found in age or sex ($p > 0.05$), though lipid-poor lesions trended toward larger size (p = 0.051).
**Conclusion:** Lipid-poor vertebral hemangiomas demonstrate significantly stronger and more persistent enhancement than lipid-rich lesions, suggesting that quantitative enhancement metrics may serve as a valuable tool for subtype differentiation on MRI.
**Limitations:** Retrospective; Small sample size
**Funding for this study:** None
**Has your study been approved by an ethics committee?** Not applicable
**Author Disclosures:**
Miguel Ramalho: Nothing to disclose
Augusto Marques Gaspar: Nothing to disclose
Carlota Rodrigues: Nothing to disclose
Teresa Barata Rodrigues: Nothing to disclose

**Bipolar Radiofrequency Ablation–Assisted Kyphoplasty and Osteoplasty for Malignant Pathological Fractures: Safety and Clinical Outcomes**
*R. Marcello*[1], A. Ciabattoni[1], M. Cuccarelli[2], G. Marcello[2], S. Vitale[1]; [1]Roma/IT, [2]Rome/IT
*(robermarcello@gmail.com)*

**Purpose:** Pathological fractures due to metastatic bone lesions are associated with significant pain and functional impairment. Minimally invasive treatments such as kyphoplasty and osteoplasty offer symptomatic relief, but the risk of cement leakage remains a major concern. This study evaluates the safety and efficacy of bipolar radiofrequency ablation (RFA) prior to cement injection to improve procedural control and patient outcomes.
**Methods or Background:** We retrospectively reviewed 37 patients with symptomatic malignant pathological fractures who underwent bipolar RFA-assisted kyphoplasty (n=31) or osteoplasty (n=6) between January 2011 and May 2019. Bipolar RFA was performed using the Osteocool RF Ablation System (Medtronic), achieving a constant temperature of 70°C over 7–15 minutes. Cement (PMMA) was then injected into the thermally ablated cavity. Clinical outcomes were assessed via VAS pain scores, and radiological results were evaluated by MR to determine cement distribution and leakage.
**Results or Findings:** All procedures were technically successful. No peri-procedural morbidity or mortality occurred. Post-procedural VAS scores demonstrated a significant decrease in pain for all patients ($p < 0.05$). MR imaging showed no significant venous or cortical cement leakage. The thermally created cavity facilitated controlled cement deposition.
**Conclusion:** Bipolar RFA-assisted cementoplasty appears to be a safe and effective technique for managing malignant pathological fractures. This approach reduces the risk of cement leakage while improving pain control and procedural precision. It may serve as a valuable adjunct in interventional strategies for metastatic bone disease. Prospective studies are needed to confirm these findings.
**Limitations:** Any
**Funding for this study:** Any
**Has your study been approved by an ethics committee?** Not applicable
**Author Disclosures:**
Antonella Ciabattoni: Nothing to disclose
Martina Cuccarelli: Nothing to disclose
Gianmarco Marcello: Nothing to disclose
Roberto Marcello: Nothing to disclose
Stefano Vitale: Nothing to disclose

**Feasibility study of combined diagnosis of multiple functional imaging modalities in the identification of benign and malignant bone tumors**
Y. Li[1], W. Zhang[1], *L. Ma*[1], L. Liangjie[2], Y. Zhang[1]; [1]Zhengzhou/CN, [2]Beijing/CN

**Purpose:** Amide proton transfer weighted imaging (APTWI) has been widely explored for tumor evaluation. This study attempted to evaluate the added value of APTWI to multiple model diffusion imaging for differentiating benign and malignant bone tumors.
**Methods or Background:** This prospective study included 68 patients with pathologically confirmed bone tumors (25 benign and 43 malignant) . All patients underwent MRI examinations including conventional DWI, intravoxel incoherent motion (IVIM), diffusion kurtosis imaging (DKI), and APTWI. The apparent diffusion coefficient (ADC), pure molecular diffusion (D), perfusion fraction (f), pseudo-diffusion (D*), mean kurtosis (MK), and mean diffusivity (MD) were calculated from multiple diffusion models. The magnetization transfer ratio asymmetry at a chemical shift of 3.5 ppm [MTRasym(3.5 ppm)] were derived from APTWI. Mann-Whitney U test or independent samples t-test was used for group comparison between benign and malignant tumors. Logistic regression modeling was used to identify independent predictors and performed combination diagnosis. Receiver operating characteristic (ROC) curve analysis was performed to evaluate the diagnostic performance.
**Results or Findings:** Malignant tumors showed significantly lower ADC, D, and MD, while significantly higher MK and MTRasym(3.5 ppm) values than those of benign tumors. ROC analysis yielded AUC values of 0.838 (ADC), 0.826 (D), 0.768 (MD), 0.820 (MK), and 0.761 [MTRasym(3.5 ppm)], respectively, for differentiation between benign and malignant bone tumors. Multivariate logistic regression revealed that MTRasym(3.5 ppm) and MK were independent factors for prediction of benign and malignant tumors. The combination of MTRasym(3.5 ppm) and MK showed significant improved diagnostic performance with the AUC of 0.901.
**Conclusion:** The integration of APTWI and advanced diffusion models provides additional insights into tumor metabolism and microstructure of bone tumors, thus significantly enhancing the diagnostic efficacy.
**Limitations:** Single-center study, small tumor cohort, and lack of external validation.
**Funding for this study:** We don't have any funding support.
**Has your study been approved by an ethics committee?** Yes
**Ethics committee - additional information:** This retrospective investigation was permitted by the Review Committee of the First Affiliated Hospital of Zhengzhou University (2023-KY-0888-003).
**Author Disclosures:**
Wenhua Zhang: Nothing to disclose
Yong Zhang: Nothing to disclose
Lin Liangjie: Nothing to disclose
Ying Li: Nothing to disclose
Longyao Ma: Nothing to disclose

**Response Patterns During Neoadjuvant Radiation Therapy in Soft Tissue Sarcomas: A Multiparametric MRI Clustering Analysis**
*B. Bogner*, A. Runkel, M. Reisert, T. D. Diallo, P. M. Jungmann, F. Bamberg, M. Jung; Freiburg Im Breisgau/DE

**Purpose:** To identify distinct treatment response patterns in soft tissue sarcomas (STS) during neoadjuvant radiation therapy (NRT) using multiparametric MRI (mpMRI).
**Methods or Background:** In this prospective study, 25 STS patients (intermediate or high grade) underwent 3T mpMRI at baseline, mid-treatment (week 3), and post-NRT (week 5). Quantitative parameters were extracted from T2-weighted, diffusion-weighted imaging (DWI), intravoxel incoherent motion (IVIM), and dynamic contrast-enhanced (DCE) sequences following 3D tumor segmentation. Principal component analysis (PCA) and unsupervised k-means clustering identified response patterns based on percentage changes from baseline to post-treatment. Representative whole-mount histology sections were obtained using spatially co-registered in vivo and ex vivo MRI, with necrosis quantified from three representative slices per tumor. Clusters were compared using Mann-Whitney U tests and validated against histopathological necrosis.
**Results or Findings:** Among 25 patients (mean age 68±14 years, BMI 25.6±5.5 kg/m², 35% female), k-means clustering identified two distinct response patterns. Cluster 1 (n=13) demonstrated variable changes in vascularity/permeability (Ktrans: +32.8±83.5%, Kep: +23.2±27.5%) and perfusion (f: +13.7±35.8%, fD: +10.0±40.1%) with stable cellularity (ADC: +6.2±7.7%, D: +8.4±10.0%). In contrast, cluster 2 (n=12) showed reduced vascularity/permeability (Ktrans: -42.9±30.3%, Kep: -29.8±25.0%, both p<0.01 vs. cluster 1) and perfusion (f: -11.4±21.0%, fD: -24.1±25.0%, both p=0.024) with reduced cellularity (ADC: +31.7±11.6%, D: +33.9±14.0%, both p<0.001). Cluster 2 demonstrated significantly higher post-surgical necrosis (60±30% vs. 30±30%, p=0.021), indicating superior treatment response. Notably, volumetric changes did not differ significantly between clusters (p=0.568), despite distinct functional imaging patterns and different necrosis levels.
**Conclusion:** Unsupervised clustering identified two distinct functional response patterns during NRT in STS, independent of volumetric changes. These patterns corresponded to different levels of histopathological necrosis, suggesting that mpMRI could non-invasively identify treatment responders during neoadjuvant therapy.
**Limitations:** Small sample size.
**Funding for this study:** None.
**Has your study been approved by an ethics committee?** Yes
**Ethics committee - additional information:** University of Freiburg.
**Author Disclosures:**
Marco Reisert: Nothing to disclose
Balazs Bogner: Nothing to disclose
Matthias Jung: Nothing to disclose
Fabian Bamberg: Nothing to disclose
Pia M. Jungmann: Nothing to disclose
Alexander Runkel: Nothing to disclose
Thierno D. Diallo: Nothing to disclose

**16:00 - 17:30 Research Stage 4**

### Research Presentation Session: Neuro

### RPS 2111

### Gliomas: advanced techniques in neuro-oncology imaging

**Moderator**
Pia C. Maly Sundgren; Malmö/SE

**Moderator's introduction**
Pia C. Maly Sundgren; Malmö/SE

**When Structured Meets Traditional: BT-RADS versus RANO 2.0 in Treated Gliomas with Survival and Decision Impact**
*A. H. Shah*, S. Sen, A. Chandra, A. Gehani; Kolkata/IN
*(shahakshat1788@gmail.com)*

**Purpose:** RANO 2.0 refines trial-grade response criteria but remains complex and inconsistently applied in routine practice. The Brain Tumor Reporting and Data System (BT-RADS) offers a structured lexicon for follow-up, yet head-to-head outcome-based validation against RANO 2.0 is lacking. We compared diagnostic accuracy, reproducibility, survival stratification, and downstream management impact of BT-RADS versus RANO 2.0 in treated gliomas.
**Methods or Background:** We retrospectively reviewed 536 patients with treated gliomas (2012–2024; 1,082 follow-up MRIs). Two neuroradiologists independently applied both BT-RADS and RANO 2.0. Truth standards were histopathology, MDT consensus, or ≥12-month follow-up. Endpoints included diagnostic performance, interobserver agreement (κ), Kaplan-Meier progression-free survival (PFS) and overall survival (OS), and concordance with MDT management decisions. Published BT-RADS work confirms feasibility, but survival-based, RANO-anchored validation has not been reported.
**Results or Findings:** BT-RADS achieved higher diagnostic accuracy (88%) than RANO 2.0 (77%), with better reproducibility (κ = 0.80 vs 0.61). Survival separated cleanly by BT-RADS: median PFS 12 months / OS 22 months for BT-RADS 3, versus PFS 6 months / OS 11 months for BT-RADS 4 (log-rank p<0.001). RANO 2.0 categories overlapped substantially, limiting prognostic clarity. BT-RADS also reduced false positives from pseudoprogression, operationalizing RANO's "confirm on repeat scan" rule. Clinical impact was evident: MDT escalation occurred in 78% of BT-RADS 4 and observation in 85% of BT-RADS 2, higher alignment than with RANO 2.0.
**Conclusion:** In real-world follow-up, BT-RADS is not only more reproducible but also more clinically meaningful than RANO 2.0-cleanly stratifying survival and aligning with actual MDT decisions. This is the first survival-stratified, decision-linked validation of BT-RADS versus RANO, supporting structured lexicons in daily glioma care.
**Limitations:** Single-center retrospective design; heterogeneous treatment regimen.
**Funding for this study:** None
**Has your study been approved by an ethics committee?** Not applicable
**Author Disclosures:**
Aditi Chandra: Nothing to disclose
Anisha Gehani: Nothing to disclose
Saugata Sen: Nothing to disclose
Akshat Hitesh Shah: Nothing to disclose

**Multicentric study on the pre-surgical differentiation of IDH-wildtype glioblastoma and IDH-mutant grade 4 astrocytoma using structured MRI visual assessment**
*A. M. Viveros*[1], C. Majós[1], P. Naval-Baudin[1], J. I. García García[1], M. Cos Domingo[1], L. Oleaga[1], M. Smits[2], A. Pons Escoda[1]; [1]Barcelona/ES, [2]Rotterdam/NL
*(mildred.viveros@hotmail.com)*

**Purpose:** This study aims to assess the diagnostic performance of VASARI (Visually Accessible Rembrandt Images) criteria in the pre-surgical differentiation of IDH-mutant-grade-4 astrocytomas(A4-mut) and IDH-wildtype-glioblastomas(Gb-wt), and the potential added-value of incorporating the T2/FLAIRmismatch into the visual evaluation features.
**Methods or Background:** Histomolecularly confirmed A4-mut or Gb-wt(age-balanced) with available pre-surgical-MRI were recruited retrospectively (2016-2022) from three tertiary hospitals in Spain and The-Netherlands. Blinded, independent assessments of VASARI_features and T2/FLAIRmismatch were performed by three-experienced-neuroradiologists (5, 10, and >20years of experience), and a final consensus reading was obtained. Statistical differences(x2-test) and potential discriminatory performance (AUC-ROC) for each VASARI_features were assessed in univariate analysis. Additionally, two multivariable logistic-regression models were developed: VASARI-only and VASARI+T2/FLAIRmismatch. The discriminative performance of the models was assessed by AUC-ROC values (accuracy, sensitivity and specificity were derived from Youden-index). All analyses were validated using 5-fold cross-validation.
**Results or Findings:** A total of 163 patients were included: 43 A4-mut(mean-age 41y.o.,28males) and 120 Gb-wt(mean-age 47y.o.,87males). Univariate diagnostic performance: The highest AUC-ROC values (>0.70) were T2/FLAIRmismatch, proportion of non-enhancing tumor, definition of enhancing margin, and thickness of enhancing margin (AUC=0.72-0.76). 30% of variables showed AUC=0.60-0.70 and 50% of variables showed AUC<0.60. The performance of the multivariable models was as follows: the VASARI-only model (three most discriminative features) achieved an AUC=0.82 (accuracy=0.77); whereas the VASARI+T2/FLAIRmismatch model achieved an AUC=0.87 (accuracy=0.88). Expert neuroradiologists' consensus accuracy was 0.83.
**Conclusion:** A4-mut exhibited higher proportions of non-enhancing-tumor, ill-defined enhancing borders, and thinner enhancing margins as the most prominent radiological differences. The T2/FLAIRmismatch, present in any proportion (in some cases focal and involving <25% of the total MRI abnormality extent), showed almost perfect specificity for A4-mut. An accuracy of 0.88 was achieved by the multivariable model (VASARIs plus the T2/FLAIRmismatch), exceeding the neuroradiologists' consensus.
**Limitations:** None
**Funding for this study:** None
**Has your study been approved by an ethics committee?** Yes
**Ethics committee - additional information:** None
**Author Disclosures:**
Carlos Majós: Nothing to disclose
Juan Ignacio García García: Nothing to disclose
Pablo Naval-Baudin: Nothing to disclose
Albert Pons Escoda: Nothing to disclose
Mónica Cos Domingo: Nothing to disclose
Laura Oleaga Zufiria: Nothing to disclose
Marion Smits: Nothing to disclose
Ady Mildred Viveros: Nothing to disclose

**Diffusion-Weighted MRI Radiomics Model in Predicting IDH Status of Non-Enhancing (Low-Grade-Appearing) Adult Diffuse Gliomas**
Y. Liang, Y. Liu, Z. Chen, *M. Ge*, Y. Wang; Beijing/CN
*(gml20030825@163.com)*

**Purpose:** In non-contrast-enhanced (non-CE) adult diffuse gliomas, a significant proportion (19–44%) exhibit aggressive behavior due to high-grade molecular features like IDH wild-type status. Precise preoperative IDH prediction is crucial for optimizing treatment. The 2021 WHO Classification of CNS Tumours designates IDH status as a core determinant for molecular subtyping and grading. IDH wild-type gliomas, even those with low-grade histological appearance, are classified as high-grade and require intensified treatment. Conventional imaging lacks quantitative characterization, whereas radiomics non-invasively reveals tumor heterogeneity by extracting high-throughput quantitative features. This study aims to develop a multimodal MRI radiomics model for preoperative non-invasive prediction of IDH mutation status to guide individualized therapy.
**Methods or Background:** A retrospective analysis included 151 patients (158 lesions) with pathologically confirmed non-CE adult diffuse gliomas (2016–2023). Based on pathology, patients were stratified into IDH-mutant (44 lesions) and IDH-wild-type (114 lesions) groups and randomly divided into training and validation sets (7:3 ratio). Regions of interest were manually delineated on T2WI, followed by co-registration with ADC and T1CE sequences. A total of 1,132 radiomics features were extracted from T2WI, T1WI, ADC, and T1CE sequences. Feature selection involved ICC (≥0.85), statistical tests, and LASSO regression. Six machine learning models (LR, SVM with RBF/linear kernels, KNN, DT, NB) were evaluated using AUC to assess single-sequence and multi-sequence predictive performance.
**Results or Findings:** Multi-parametric models combining T2WI, T1CE, and ADC outperformed single-sequence models. The SVM (RBF kernel) classifier with multi-parametric features achieved optimal performance (training AUC = 0.969, sensitivity 88.6%, specificity 100%, accuracy 85.4%; validation AUC = 0.922, sensitivity 82.3%, specificity 90.3%, accuracy 87.3%), significantly surpassing single-sequence models.
**Conclusion:** The multimodal MRI radiomics model enables accurate, non-invasive IDH status prediction in non-enhancing gliomas (AUC>0.9), assisting surgical planning and advancing imaging-genomic diagnostics.
**Limitations:** Not applicable
**Funding for this study:** Not applicable
**Has your study been approved by an ethics committee?** Not applicable
**Author Disclosures:**
Zelong Chen: Nothing to disclose
Yanhong Liu: Nothing to disclose
Menglin Ge: Nothing to disclose
Yuhan Liang: Nothing to disclose
Yulin Wang: Nothing to disclose

**Benchmarking Advanced Diffusion MRI Models for Preoperative Glioma Characterization: A Multi-Compartment and Zone-Specific Analysis**
*I. Jacečková*, V. Sedlák, M. Majovsky, A. Kavkova, D. Netuka, T. Belsan, K. Sichova, E. Stoklasa, M. Nemy; Prague/CZ
*(jaceciva@uvn.cz)*

**Purpose:** To benchmark the diagnostic performance of conventional and advanced diffusion MRI models, including ADC, DTI, DKI, SMT, NODDI, and RSI, in predicting histologic grade and IDH mutation status in adult-type diffuse gliomas, and to investigate whether model performance differs between various tumor zones and peritumoral regions.
**Methods or Background:** A cohort of 200 patients with histologically confirmed adult-type gliomas underwent a standardized 3T MRI protocol including multi-shell diffusion imaging. Quantitative parametric maps were reconstructed from six diffusion models (ADC, DTI, DKI, SMT, NODDI, RSI). Tumoral and peritumoral regions were delineated automatically by a convolutional neural network on structural MRI. Zone-specific diffusion parameters were extracted and evaluated for prediction of histologic grade and IDH mutation. ROC analysis and cross-validation were performed to compare model performance, with ADC serving as the clinical benchmark.
**Results or Findings:** In addition to significantly increasing diagnostic performance in predicting grade and IDH, advanced multi-compartment models revealed marked spatial heterogeneity not captured by conventional ADC. IDH-wildtype gliomas exhibited elevated cellularity indices in both enhancing tumor and peritumoral regions, whereas IDH-mutant gliomas showed more confined abnormalities. Higher-grade gliomas displayed pronounced microstructural disruption extending into edema and peritumoral tissue.
**Conclusion:** This study establishes a comparative benchmark of diffusion models for glioma characterization. NODDI, SMT, and RSI outperform conventional ADC and show robust performance across both tumoral and peritumoral compartments. By leveraging automated segmentation and multi-shell diffusion analysis, zone-specific biomarkers can be extracted with minimal human input, supporting precision preoperative assessment and guiding model selection in clinical neuro-oncology practice.
**Limitations:** The main limitations of this study are its single-center nature and still a relatively small sample size, despite being one of the largest advanced diffusion datasets published. Also, less common models (e.g. CHARMED, VERDICT) were not evaluated.
**Funding for this study:** Funding was provided by the Ministry of Health of the Czech Republic, grant no. NW25J-08-00023, and by Charles University, project GA UK no. 222623.
**Has your study been approved by an ethics committee?** Yes
**Ethics committee - additional information:** This study was approved by the Ethics Committee of the Military University Hospital Prague.
**Author Disclosures:**
Martin Majovsky: Nothing to disclose
Milan Nemy: Nothing to disclose
Vojtěch Sedlák: Nothing to disclose
David Netuka: Nothing to disclose
Erik Stoklasa: Nothing to disclose
Ivana Jacečková: Nothing to disclose
Kristyna Sichova: Nothing to disclose
Tomas Belsan: Nothing to disclose
Anna Kavkova: Nothing to disclose

**Radiogenomic Signatures of Histological Heterogeneity in H3 K27-Altered Diffuse Midline Gliomas**
*Q. Zheng*, X. Su, Y. Deng, X. Yang, L. Wang, S. Tang, Y. Jin, Q. Yue; Chengdu, Sichuan Province/CN
*(2325526330@qq.com)*

**Purpose:** Diffuse midline gliomas, H3 K27-altered (DMGs) are classified as WHO grade 4 tumors regardless of histopathological appearance. Recent advances reveal substantial heterogeneity among these patients, with some exhibiting histological features resembling lower-grade (LGG, histological grade 2-3) group and others resembling high-grade (HGG, histological grade 4) group. This research aims to identify distinct radiomics and genomics features in LGG and HGG groups with H3 K27-altered and link radiomic signatures with relevant genomic alterations.

**Methods or Background:** A cohort of 104 H3 K27-altered DMG patients, diagnosed between December 2016 and February 2023, were classified as LGG or HGG by experienced pathologists. Of these, 48 patients comprised the radiomics set from which radiomic features were extracted from preoperative MRI and selected using least absolute shrinkage and selection operator (LASSO) regression. A separate radiogenomics set of 27 patients with both MRI and whole-exome sequencing (WES) data was used to explore associations between radiomic and genomic features. The biological meaning of radiomics-associated key genes was explored using a public glioma dataset from the Chinese Glioma Genome Atlas (CGGA).
**Results or Findings:** Seven radiomic features were finally selected. Among 35 differential mutation genes, original_glszm_LargeAreaHighGrayLevelEmphasis was associated with AATK, GRIN2A, NEFH and TNK2. In CGGA, GSVA analysis revealed enrichment of pathways related to these radiomics-correlated genes' product. Furthermore, GRIN2A, NEFH and TNK2 emerged as independent prognostic factors for overall survival (OS) in glioma.
**Conclusion:** Histological grade-specific radiomic features derived from preoperative MRI in H3 K27-altered DMGs are linked to key prognostic genes, offering novel insights into the pathophysiological mechanisms of these tumors and their imaging correlates.
**Limitations:** This study is limited by its retrospective design and the relatively small sample size. Furthermore, multi-center validation is needed.
**Funding for this study:** The National Natural Science Foundation of China (Grant No. 82271961) ; 1·3·5 projects for Artificial Intelligence (ZYAI24050), West China Hospital, Sichuan University
**Has your study been approved by an ethics committee?** Yes
**Ethics committee - additional information:** The research protocol was approved by the ethics commissions of the West China Hospital Ethics Committee with a waiver of informed consent (number IRB-2023-745)
**Author Disclosures:**
Qianqian Zheng: Nothing to disclose
Xibiao Yang: Nothing to disclose
Yujiao Deng: Nothing to disclose
Shuang Tang: Nothing to disclose
Yuqi Jin: Nothing to disclose
Lang Wang: Nothing to disclose
Xiaorui Su: Nothing to disclose
Qiang Yue: Nothing to disclose

### Contra-lesional hippocampus in low grade glioma patients with no hippocampal infiltration - a study of structural remodeling

*M. R. Stański*, M. Goralewski, J. Watorek, S. Antczak, J. Moskal, K. Katulska; Poznań/PL
*(marcin_stanski@hotmail.com)*

**Purpose:** Low grade gliomas (LGG) are slowly growing tumours which induce neuroplasticity. It was previously shown that the invasion of hippocampus by LGG may induce enlargement of contra-lesional hippocampus, including its gray matter volume (GMV) and volumes of its subfields. Our goal was to assess if similar changes may occur also in patients with LGG which do not invade hippocampus.
**Methods or Background:** This was a retrospective study of 3D T1 MRI scans of 30 LGG patients (17 left-, 13 right-sided) without hippocampal invasion and 26 healthy controls. In each case a neuroradiologist drew a 3D volume of interest delineating the tumor. We used virtual brain grafting (VBG) to replace abnormal tissue with a clipping of healthy brain template. Using synthetic images we analyzed GMV with voxel-based morphometry (VBM) and volume of hippocampal subfields with FreeSurfer 6.0. We focused on regions previously reported in the literature to be enlarged in LGG with hippocampal infiltration.
**Results or Findings:** Contra-lesional hippocampal GMV was significantly larger in left- ($T = 7.78$, $p < 0.05$) and right-sided LGG ($T = 6.39$, $p < 0.05$). A trend towards larger hippocampal-amygdala transitional area (HATA) was seen in both groups, reaching significance in right-sided LGG (Bonferroni corrected $p = 0.04$). In right-sided LGGs, trends for enlargement were also observed in GC-ML-DG head (the granule cell molecular layer of the dentate gyrus head) , CA1 head, and CA3 head.
**Conclusion:** The hippocampus contralateral to LGG may undergo structural remodeling even when the ipsilateral one is not affected by tumour infiltration.
**Limitations:** The study had retrospective design. Our cohort size was small due to epidemiology of LGGs and strict inclusion criteria. Groups were heterogenous regarding tumor volumes due to difficulty in adjusting it with small samples.
**Funding for this study:** The study received no external funding.
**Has your study been approved by an ethics committee?** Yes
**Ethics committee - additional information:** The study was approved by the Bioethics Committee of Poznań University of Medical Sciences
**Author Disclosures:**
Mikołaj Goralewski: Nothing to disclose
Marcin Radosław Stański: Nothing to disclose
Katarzyna Katulska: Nothing to disclose
Jacek Watorek: Nothing to disclose
Sylwia Antczak: Nothing to disclose
Jakub Moskal: Nothing to disclose

### The 2025 BraTS MICCAI lighthouse challenge: glioma segmentation on pre- and post-treatment MRI

*M. Correia De Verdier*[1], R. Saluja[2], L. Gagnon[3], U. Baid[4], M. Astaraki[5], R. Huang[6], S. Bakas[4], E. Calabrese[7], J. D. Rudie[8], BraTS MICCAI Lighthouse Glioma Consortium [1]; [1]Uppsala/SE, [2]New York, NY/US, [3]Québec City, QC/CA, [4]Indianapolis, IN/US, [5]Stockholm/SE, [6]Boston, MA/US, [7]Durham, NC/US, [8]La Jolla, CA/US
*(maria.correiadeverdier@uu.se)*

**Purpose:** The 2025 Brain Tumor Segmentation (BraTS) challenge on pre- and post-treatment glioma aims to create a large public dataset of annotated diffuse glioma MRIs and a benchmarking environment for developing and evaluating deep learning segmentation models to address challenges in treatment planning and disease monitoring.
**Methods or Background:** Eighteen institutions on four continents contributed 4401 MRIs of patients with diffuse gliomas, acquired pre- or post-surgery, radiation, or systemic therapy. MRIs included pre-contrast and contrast-enhanced T1-weighted, T2-weighted, and T2-FLAIR sequences. Data preprocessing and annotation followed established BraTS guidelines. Neuroradiologists approved annotations for four sub-regions: enhancing tissue (ET), surrounding non-enhancing FLAIR hyperintensity (SNFH), non-enhancing tumour core (NETC), and resection cavity (RC). Participants used the dataset to develop and evaluate their segmentation models, predicting ET, RC, tumour core (TC = ET + NETC) and whole tumour (WT = TC + SNFH). Forty-four teams participated in the validation phase and 12 teams in the testing phase. Evaluation was performed using lesion-wise Dice Similarity Coefficient (L-DSC) and Normalized Surface Distance (L-NSD).
**Results or Findings:** The best performing team's L-DSC (mean (median) ± SD) were: ET – 0.81 (0.93) ± 0.26, RC – 0.89 (1.00) ± 0.25, TC – 0.81 (0.94) ± 0.28, WT – 0.88 (0.96) ± 0.18, and L-NSD were: ET – 0.86 (0.96) ± 0.25, RC – 0.89 (1.00) ± 0.25, TC – 0.82 (0.94) ± 0.27 and WT – 0.85 (0.94) ± 0.19.
**Conclusion:** The 2025 BraTS challenge sets a benchmark for deep learning segmentation models using the largest expert-annotated glioma dataset. The winning team achieved excellent performance with high L-DSC and L-NSD. The developed models may aid objective tumour assessment thought the patient's entire clinical course.
**Limitations:** The annotator model does not directly take into account inter-observer variability.
**Funding for this study:** Not applicable.
**Has your study been approved by an ethics committee?** Yes
**Ethics committee - additional information:** Institutional review board approval from each participating institution.
**Author Disclosures:**
Raymond Huang: Consultant: Nuvation Bio, Telix, Servier Advisory Board: Vysioneer
Rachit Saluja: Nothing to disclose
Ujjwal Baid: Nothing to disclose
BraTS MICCAI Lighthouse Glioma Consortium: Nothing to disclose
Spyridon Bakas: Nothing to disclose
Evan Calabrese: Consultant: Servier Pharmaceuticals
Mehdi Astaraki: Nothing to disclose
Jeffrey D. Rudie: Advisory Board: Cortechs.ai and Subtle Medical Shareholder: Cortechs.ai and Subtle Medical Consultant: Cortechs.ai and Subtle Medical
Louis Gagnon: Nothing to disclose
Maria Correia De Verdier: Nothing to disclose

### Molecular versus Histologic Glioblastoma: Tumor Heterogeneity from Multiparametric Physiologic MRI

*M. Choi*[1], Y. Choi[2], J. Lee[1], M. Kim[1], I. Hwang[1], Y. W. Park[1], J. E. Park[1], S. H. Choi[1], K. Choi[1]; [1]Seoul/KR, [2]Pocheon/KR
*(mschoi.rad@gmail.com)*

**Purpose:** The 2021 WHO classification redefines Grade 2/3 IDH-wildtype diffuse astrocytic tumors with specific molecular alterations as molecular glioblastoma (mol-GBM), grade 4. Since mol-GBM lacks the histological hallmarks of conventional glioblastoma (hist-GBM), it is hypothesized to exhibit a distinct pattern of tumor heterogeneity. This study aimed to explore how mol-GBM differs from hist-GBM in tumor heterogeneity using multi-parametric physiologic MRI and provide novel radiologic insights into the characteristic features of this newly defined subtype.

**Methods or Background:** In this multi-institutional retrospective study, imaging data were collected from two tertiary centers: 13 mol-GBMs and 39 hist-GBMs from institution 1 (2007–2024) for development, and 9 mol-GBMs from institution 2 (2020–2024) for external validation. Apparent diffusion coefficient (ADC; cellularity), relative cerebral blood volume (rCBV; vascularity), and volume transfer constant (Ktrans; permeability) were binarized into high/low categories, yielding eight spatial habitat clusters (2×2×2) to visualize tumor heterogeneity. Habitat distributions and histogram features (mean, median, 10th, 90th percentile) were compared between groups. Diagnostic performance was assessed by multivariable logistic regression with ROC analysis.
**Results or Findings:** The tumor volume of high Ktrans clusters was significantly smaller in mol-GBM than hist-GBM (median; 2.5cm³ vs. 14.6cm³, p<0.001), particularly for the most malignant cluster (low ADC, high rCBV, high Ktrans; 0.06cm³ vs. 7.0cm³, p<0.001). Mol-GBM showed lower mean and 90th percentile values of rCBV (p=0.013) and Ktrans (p<0.001), whereas no difference was observed for ADC. Consequently, adding rCBV and Ktrans to ADC significantly improved differentiation, increasing the AUC from 0.70 to 0.89 (p=0.002).
**Conclusion:** Non-invasive characterization of tumor heterogeneity using multi-parametric MRI habitat analysis revealed distinct patterns in mol-GBM versus hist-GBM. The most discriminating parameter was tumor permeability (Ktrans), with mol-GBM showing smaller hyperpermeable habitats. In contrast, cellularity (ADC) was comparable across both subtypes.
**Limitations:** Not applicable.
**Funding for this study:** This work was supported by the National Research Foundation of Korea (NRF) grant funded by the Korea government (MSIT) (No. RS-2023-00251022) (K.S.C.); the Phase III (Postdoctoral fellowship) grant of the SPST (SNU-SNUH Physician Scientist Training) Program (K.S.C.); the SNUH Research Fund (No. 04-2024-0600) (K.S.C.); and the Korea Health Technology R&D Project through the Korea Health Industry Development Institute (KHIDI) grant funded by the Ministry of Health&Welfare (No. RS-2024-00439549) (K.S.C.).
**Has your study been approved by an ethics committee?** Not applicable
**Author Disclosures:**
Minchul Kim: Nothing to disclose
Inpyeong Hwang: Nothing to disclose
Yae Won Park: Nothing to disclose
Minseo Choi: Nothing to disclose
Kyusung Choi: Nothing to disclose
Ji Eun Park: Nothing to disclose
Yunseo Choi: Nothing to disclose
Junhyeok Lee: Nothing to disclose
Seung Hong Choi: Nothing to disclose

### CBV- vs PSR- optimized MR-DSC-Perfusion sequences for presurgical diagnosis of brain tumors: from demytification to synergy

*C. García*[1], I. Martinez-Zalacain[2], P. Naval-Baudin[2], A. Camins Simó[2], A. Jareño Badenos[2], M. Cos Domingo[2], C. Majós[2], A. Pons Escoda[2]; [1]Murcia/ES, [2]Hospitalet de Llobregat/ES
*(clemente292@gmail.com)*

**Purpose:** To compare the diagnostic performance of CBV- and PSR-optimized sequences in the presurgical differentiation of glioblastoma, brain-metastasis, lymphoma, and meningioma, and to assess the additive value of combining both metrics within and across the sequences.
**Methods or Background:** Retrospective single-center cohort: glioblastoma (n=121), metastasis (n=63), meningioma (n=55), lymphoma (n=13). Each patient underwent two consecutive DSC-acquisitions within the same MR-session: 1st- non-preloaded, high flip-angle (PSR-optimized); and 2nd- full-dose preloaded (using prior contrast), intermediate flip-angle (CBV-optimized, standardized, consensus-compliant). From enhancing tumor masks (plus edema masks for glioblastoma vs. metastasis), we extracted rCBV (NAWM-normalized, leakage-corrected) and PSR statistics. For each tumor-pair we identified the best single-acquisition metric, and trained bivariate logistic models to combine CBV and PSR both within and across both protocols.
**Results or Findings:** Single-metric AUCs for the 1st DSC-sequence ranged 0.72 (Gb_vs_Metastasis, nrCBVmin_edema)- 0.91 (Meningioma_vs_Lymphoma, PSRp75), average AUC=0.79. For the 2nd, AUCs ranged 0.72 (Gb_vs_Metastasis, PSRmax_edema)- 0.89 (Meningioma_vs_Lymphoma, nrCBV_p75), average AUC=0.80. Overall, CBV and PSR were the best metrics in 50% of comparisons each. Selecting the best-performing protocol per tumor-pair increased average AUC to 0.82. The bivariate intraprotocol models combining nrCBV+PSR in the 1st DSC AUCs ranged 0.76 (Gb_vs_Metastasis, PSRmean_edema+nrCBVmin_edema)- 0.94 (Meningioma_vs_Lymphoma, PSRp75+nrCBVp75), average AUC=0.85. In the 2nd DSC AUCs ranged 0.76 (Gb_vs_Metastasis, PSRmax_edema+nrCBVmax_edema)- 0.93 (Meningioma_vs_Lymphoma, PSRp75+nrCBVp75), average AUC=0.83 The bivariate cross-protocols models combining nrCBV+PSR AUCs ranged 0.8 (Gb_vs_Metastasis, PSRmin_enhancing_2nd+PSR_mean_edema_1st) to 0.94 (Meningioma_vs_Lymphoma, PSRp75_2nd+nrCBVp75_1st), average AUC=0.87.
**Conclusion:** Both sequences performed similarly in pairwise tumor classification, with CBV and PSR showing no clear predominance in either acquisition. Within each protocol, models combining CBV and PSR outperformed single-metric approaches, with optimal performance achieved when integrating both protocols. Our findings indicate that implementing the dual-DSC protocol in clinical practice could maximize the accuracy of presurgical diagnosis.
**Limitations:** Single-centre, retrospective design.
**Funding for this study:** No funding was received for this study.
**Has your study been approved by an ethics committee?** Yes
**Ethics committee - additional information:** The study was approved by the Research Ethics Committee of Hospital Universitari de Bellvitge.
**Author Disclosures:**
Carlos Majós: Nothing to disclose
Pablo Naval-Baudin: Nothing to disclose
Albert Pons Escoda: Nothing to disclose
Ignacio Martinez-Zalacain: Nothing to disclose
Aleix Jareño Badenos: Nothing to disclose
Mónica Cos Domingo: Nothing to disclose
Angels Camins Simó: Nothing to disclose
Clemente García: Nothing to disclose

### Language lateralization in brain tumor patients estimated by resting-state fMRI vs. task-based fMRI

*S. Suvak*, B. Papazov, E. Schulz, J. Ricke, F. Ringel, S. Stöcklein, V. Stöcklein; Munich/DE

**Purpose:** Accurate determination of language lateralization is critical for surgical planning in glioma patients, particularly when tumors involve the dominant hemisphere. Task-based fMRI is currently used but is limited by availability and patient compliance. Resting-state fMRI (rs-fMRI) may provide a feasible alternative for identifying the language-dominant hemisphere.
**Methods or Background:** 22 glioma patients prospectively underwent structural MRI (3D T1w and FLAIR), task-based fMRI (sentence generation), and rs-fMRI (6 min each). Broca's and Wernicke's areas and tumors were semi-automatically segmented. For task-fMRI, activation volumes were determined, while rs-fMRI assessed volumes of regions functionally connected to bilateral Broca seeds (r = 0.35). Language lateralization was quantified using the lateralization index (LI = [L–R]/[L+R]), with values near ±1 indicating strong lateralization. Task- vs. rs-fMRI-derived LI were compared using paired t-tests and Pearson correlation.
**Results or Findings:** 22 patients (56 ± 18 yrs) were analyzed; two were excluded due to motion artifacts. Hemispheric lateralization was concordant between task-based and rs-fMRI in 19 patients (95.0%). Rs-fMRI identified left-hemispheric dominance in 17 (mean LI = 0.28 ± 0.17) and right-hemispheric dominance in 3 (LI = –0.11 ± 0.15), while task-fMRI showed left dominance in 18 (LI = 0.49 ± 0.22) and right dominance in 2 (LI = –0.42 ± 0.41). A strong positive correlation was observed between methods (r = 0.69, p = 0.0007).
**Conclusion:** Rs-fMRI identified the language-dominant hemisphere in 95% of glioma patients, showing strong concordance with task-based fMRI. As a non-task-dependent, brief, and automatable technique, rs-fMRI may serve as a standardized alternative for preoperative language mapping, facilitating surgical planning and risk assessment.
**Limitations:** This is a single-center study and intraoperative mapping will be needed to further confirm language lateralization.
**Funding for this study:** This work is the result of a research cooperation with Brainlab SE, Munich, Germany. Otherwise, all authors declare no conflict of interest regarding the materials used or the results presented in this study. All authors declare no other relationships or activities that could appear to have influenced the submitted work.
**Has your study been approved by an ethics committee?** Yes
**Ethics committee - additional information:** We performed the study in accordance with the Declaration of Helsinki and the STROBE statement. All patients included in this study provided written informed consent.
**Author Disclosures:**
Stefan Suvak: Nothing to disclose
Boris Papazov: Nothing to disclose
Sophia Stöcklein: Nothing to disclose
Veit Stöcklein: Nothing to disclose
Enrico Schulz: Nothing to disclose
Florian Ringel: Nothing to disclose
Jens Ricke: Nothing to disclose

### Sunday, March 8

**08:00-09:00** **Research Stage 1**

### Research Presentation Session: Breast

### RPS 2202

### Advanced breast imaging and interventions: CT and minimally invasive approaches

**Moderator**
Mirjam Wielema; Lent/NL

**Ultrasound-guided vacuum-assisted excision: a minimally invasive solution for managing imaging-histological discordant breast lesions after core needle biopsy**

*E. D'Ascoli*, C. Depretto, G. Rossini, G. Irmici, C. De Berardinis, G. Della Pepa, A. Bonanomi, L. Corradini, G. P. Scaperrotta; Milan/IT

**Purpose:** To evaluate the role of US-guided Vacuum-assisted excision (VAE) in the management of imaging-histological discordant lesions after core needle biopsy (CNB), avoiding unnecessary surgical excision.
**Methods or Background:** We retrospectively evaluated all patients who underwent US-guided VAE with a 9 G needle at our Institution between January 2015 and August 2023 for imaging-histological discordant lesions after CNB with a 14 G needle. We determined the upgrade rate to malignancy (UR) after VAE and delayed false-negative results after surgery or follow-up. We also assessed the diagnostic performance of the procedure, technical efficacy (TE), technical success (TS), the occurrence of complications and the correlation between upgrade to malignancy and lesion size, BI-RADS category, morphological characteristics, presence of calcifications and history of breast cancer ($\chi^2$ and Mann-Whitney tests).
**Results or Findings:** We collected data from 334 patients. After VAE, 65.3% (N=218) of the excised lesions were classified as benign, 25% (N=85) were B3 lesions and 9.3% (N=31) were malignant (B5). The upgrade rate to malignancy after VAE was 9.3%. The delayed false negative results were 1.3%. Accuracy was 98.2%, SE was 88.6%, SP was 100%, PPV was 100% and NPV was 97.9%. TE of the procedure was 84.4%; TS was 100%. No major complications were observed. BIRADS category, morphology, margins, and orientation of the lesion were significantly associated with upgrade to malignancy ($p<0.001$).
**Conclusion:** VAE reduces the risk of underestimation of malignancy in cases of imaging-histological discordance, thus avoiding the need for surgery and reducing patients' stress, overtreatment, complications and costs, without compromising the quality of patient care.
**Limitations:** Retrospective monocentric study with a limited number of patients.
**Funding for this study:** None
**Has your study been approved by an ethics committee?** Yes
**Ethics committee - additional information:** This study was approved by the Independent Ethics Committee at the Fondazione IRCCS Istituto Nazionale dei Tumori, Milano.
**Author Disclosures:**
Gianmarco Della Pepa: Nothing to disclose
Claudia De Berardinis: Nothing to disclose
Elisa D'Ascoli: Nothing to disclose
Alice Bonanomi: Nothing to disclose
Gabriele Rossini: Nothing to disclose
Gianfranco Paride Scaperrotta: Nothing to disclose
Catherine Depretto: Nothing to disclose
Lisa Corradini: Nothing to disclose
Giovanni Irmici: Nothing to disclose

**MINIVAB as a treatment method for small invasive breast cancers – initial results**

*W. Sanderink*[1], C. Meeuwis[2], S. De Waard[3], A. Zouzos[3], E. J. M. Wolters-Van Der Ben[4], F. Van Vliet - Moret[4], R. F. Lim[5], J. Camps Herrero[6], R. Mann[1]; [1]Nijmegen/NL, [2]Arnhem/NL, [3]Stockholm/SE, [4]Nieuwegein/NL, [5]Bergen Op Zoom/NL, [6]Alzira/ES

**Purpose:** To assess feasibility of complete removal of small breast cancers using Vacuum Assisted Biopsy (VAB) as a therapeutic procedure under ultrasound (US) guidance.
**Methods or Background:** From August 2021 to May 2025, 35 patients with invasive carcinomas diagnosed by core needle biopsy (CNB), measuring ≤15 mm on US and MRI were enrolled in this international, multicenter, prospective study. One patient withdrew after VAB and was excluded from analysis. All lesions were excised under local anesthesia using a 7G VAB system under US guidance. Three weeks later, breast-conserving surgery excised the VAE cavity. Histopathological findings from VAB were compared with subsequent surgical specimens, and the rate of complete or marginally involved tumor excision by VAB was assessed.
**Results or Findings:** 34 patients completed the study (mean age, 63.3 years; 13 Netherlands, 20 Spain, 1 Sweden). The mean MRI lesion size was 10.1 mm (range 4-13.7 mm). VAB histopathology identified 33 invasive carcinomas of no special type and 1 with medullary features. Complete excision by VAB was achieved in 12 of 34 patients (35.3%). An additional 6 patients had marginally involved tumor excisions, meeting Dutch guidelines for successful removal (18/34, 52.9%). 13 patients (38.2%) showed more than focal margin involvement (mean residual tumor size, 9.4 mm; range, 5-13 mm). In 3 patients, residual tumor was present in the surgical specimen, though margin involvement >4 mm was unclear.
**Conclusion:** US-guided VAB achieved complete or marginally adequate excision in a subset of small breast cancers. Residual tumor in several cases indicates that careful patient selection is essential before considering VAB as a standalone treatment.
**Limitations:** The small sample size and the need for further analysis of patient and procedural variables limit the ability to identify factors that may predict successful VAB excision.
**Funding for this study:** The study was an investigator initiated study sponsored by an unrestricted grant from BD.
**Has your study been approved by an ethics committee?** Yes
**Ethics committee - additional information:** METC-Oost Nederland
**Author Disclosures:**
Robert Franciscus Lim: Nothing to disclose
E.J.M. Wolters-Van Der Ben: Nothing to disclose
Stephanie De Waard: Nothing to disclose
Athanasios Zouzos: Nothing to disclose
Julia Camps Herrero: Nothing to disclose
Carla Meeuwis: Nothing to disclose
Fréderique Van Vliet - Moret: Nothing to disclose
Wendelien Sanderink: Nothing to disclose
Ritse Mann: Nothing to disclose

**Non-surgical treatment of breast cancer: A comparison of outcomes between cryoablation with hormonal therapy versus cryoablation alone and hormonal therapy alone in patients not eligible for surgery**

*F. Di Naro*, S. E. Baldi Giorgi, F. Pugliese, D. De Benedetto, G. Migliaro, S. Vidali, C. Bellini, T. Amadori, J. Nori; Florence/IT
*(federicanarella@gmail.com)*

**Purpose:** To evaluate the most effective non-surgical treatment for breast cancer in surgery-ineligible patients, comparing ultrasound-guided Cryoablation combined with hormonal therapy (HT) versus Cryoablation and hormonal therapy alone.
**Methods or Background:** 111 patients (mean age 81.2 years) not-suitable for surgery due to comorbidities or advanced age was enrolled, total of 125 biopsy-confirmed breast cancers. All the lesions were invasive ductal carcinomas (mean size 17.7 mm), hormone-positive and HER2-negative, with no ultrasound-visible lymph-node involvement. Of these, 41 treated with Cryoablation and HT, 36 with Cryoablation and 36 with HT . Locoregional staging was performed with contrast-enhanced-mammography (CEM), followed by CEM and ultrasound follow-up at 12 months post-treatment. Only patients completing follow-up were included. Lesion size was compared at baseline and 12 months after-treatment.
**Results or Findings:** Of the 125 lesions, 98 completed the 12-months follow-up; 39 in the Cryoablation+HT group, 23 in the Cryoablation-only group, and 36 in the HT-only group. Tumor size reduction was significantly different between the groups (P=0.0005), with greatest reduction in the Cryoablation-with-HT group (83.3%, mean reduction 13.6 mm), followed by Cryoablation-only (61.7%, mean reduction 8.2 mm), and HT-only (42.1%, mean reduction 7.4 mm).Tumors with no CEM-enhancement, suggesting no residual-disease, were most frequent in the Cryoablation-with-HT group (69.2%), followed by Cryoablation-only (60.9%),and HT-only (36.1%). Tumors with complete remission (CR, RECIST 1.1) were similar between the Cryoablation-with-HT and Cryoablation-only groups (74.4% and 78.3%, respectively), followed by the HT-only (36.1%). Pairwise comparisons revealed significant differences between Cryoablation-with- HT and HT-only groups for CEM-enhancement, size reduction, and CR, expressing the added value of Cryoablation (p=0.0041, P<0.0001 and P<0.0001,respectively).
**Conclusion:** Cryoablation with hormonal-therapy significantly reduces tumor size and residual disease more effectively than therapy alone, making it a promising option for patients not-eligible for surgery.

**Limitations:** Single-center-retrospective No-long-term follow-up Small-cohort
**Funding for this study:** None
**Has your study been approved by an ethics committee?** Yes
**Ethics committee - additional information:** Meyer hospital florence
**Author Disclosures:**
Federica Di Naro: Nothing to disclose
Francesca Pugliese: Nothing to disclose
Diego De Benedetto: Nothing to disclose
Jacopo Nori: Nothing to disclose
Sofia Vidali: Nothing to disclose
Sofia Elisabetta Baldi Giorgi: Nothing to disclose
Tommaso Amadori: Nothing to disclose
Chiara Bellini: Nothing to disclose
Giuliano Migliaro: Nothing to disclose

**Infection Rate Following Percutaneous Biopsy: A Multicentric Prospective Study**

*M. Pasculli*[1], E. Giannotti[2]; [1]Rome/IT, [2]Cambridge/UK
*(marcella1.pasculli@gmail.com)*

**Purpose:** Our primary aim was to compare infection rates between sterile and aseptic technique. Secondary aims included identifying possible predictors of infection—such as hematoma, diabetes, or immunosuppression—and explore options to reduce environmental impact.
**Methods or Background:** This prospective multicenter study included 15 centers: 6 using sterile technique (sterile gloves, gel, probe cover, gauze) and 9 using aseptic technique (clean but not fully sterile). Consecutive patients undergoing percutaneous breast biopsy were enrolled over two months (≥50 per center). At 1-month follow-up, patients completed a questionnaire on biopsy type, infection (medical assistance, antibiotics), pain, hematoma, comorbidities, and lesion characteristics. Infection rates were compared between sterile vs aseptic groups and CNB vs VAB using Fisher's exact test, with risk ratios and Wilson 95% CIs. Due to rare events, Firth's penalized logistic regression was applied. Infections were defined as cases requiring antibiotics post-biopsy.
**Results or Findings:** We analyzed 1,560 biopsies (CNB 1,249; VAB 311), of which 531 were performed under sterile and 1,029 under aseptic conditions. Overall, 9 infections (0.58%) occurred. Infection rates were 0.94% (5/531) in the sterile group and 0.39% (4/1,029) in the aseptic group (risk ratio 2.42, 95% CI 0.65–8.98; p=0.18). No significant differences were observed. Hematoma occurred in 6.4% of CNB and 15.4% of VAB. No infections occurred among patients with diabetes (n=45) or under immunosuppressive therapy (n=18).
**Conclusion:** Infections after breast biopsy were extremely rare, occurring in less than 1% of procedures. We found no difference between sterile and aseptic technique, or between core biopsy and vacuum-assisted biopsy. The aseptic approach is safe, faster, cheaper, and environmentally friendly. Overall, our results support best practice guidelines that integrate patient safety with sustainability.
**Limitations:** The low number of infections limited statistical power and led to imprecise estimates in stratified analyses.
**Funding for this study:** No funding was required for this study
**Has your study been approved by an ethics committee?** Yes
**Ethics committee - additional information:** Ethical approval was obtained in some centers, while in others it was not required, in accordance with their local institutional policies.
**Author Disclosures:**
Elisabetta Giannotti: Nothing to disclose
Marcella Pasculli: Nothing to disclose

**Feasibility of Prone Spectral CT in Women with locoregionally advanced breast cancer and Dense Breasts**

*R. Duteweert*[1], M. Schyns[1], L. Deden[1], C. Meeuwis[1], M. Dorrius[2]; [1]Arnhem/NL, [2]Groningen/NL
*(rduteweert@rijnstate.nl)*

**Purpose:** In women with breast cancer and dense breasts, additional contrast-enhanced imaging modalities are needed to determine the extent of the disease. This study aimed to evaluate the feasibility of spectral CT for breast cancer detection in these women, compared to MRI , using quantitative spectral analysis and qualitative image assessment.
**Methods or Background:** This single-centre feasibility study prospectively included 12 patients with locoregionally advanced breast cancer and dense breasts (ACR 3–4) between February 2023 and May 2025. All patients underwent breast MRI and 18F-FDG PET/CT as part of routine staging, followed by an additional prone spectral CT on a dual-layer spectral CT scanner. Quantitative analyses included spectral slope (λHU, defined as the attenuation slope between 40 and 70 keV) and iodine concentration in 21 histopathologically confirmed breast lesions (20 malignant and 1 benign), fibroglandular tissue, and pectoral muscle. Three radiologists independently conducted the qualitative image assessment, which comprised evaluation of background enhancement and lesion detection, with outcomes compared to MRI.
**Results or Findings:** Spectral CT allowed quantitative identification of breast cancer in all patients. Lesions showed a mean λHU of 2.8±1.2, compared to −0.4±0.4 in fibroglandular tissue and 0.7±0.7 in pectoral muscle. Mean iodine concentration was 1.4±0.6mg/mL in lesions, higher than in pectoral muscle (0.4±0.3mg/mL) and fibroglandular tissue (0.0±0.1mg/mL). Background enhancement was scored lower on spectral CT than on MRI across all patients (p=0.07). For lesion detection, spectral CT yielded 56 true positives, 7 false negatives, and 3 false positives, distributed across readers, compared with 55, 8, and 0 on MRI, respectively.
**Conclusion:** Spectral CT is feasible for breast cancer detection in women with dense breasts, providing quantitative lesion characterization and reader performance comparable to MRI.
**Limitations:** No pathological proof for all reader-marked lesions, therefore excluded from analysis.
**Funding for this study:** No
**Has your study been approved by an ethics committee?** Yes
**Ethics committee - additional information:** METC Oost Nederland
**Author Disclosures:**
Laura Deden: Nothing to disclose
Monique Dorrius: Nothing to disclose
Maxime Schyns: Nothing to disclose
Carla Meeuwis: Nothing to disclose
Robin Duteweert: Nothing to disclose

**Detectability of breast lesions on conventional and photon-counting computed tomography: an anthropomorphic phantom study**

*S. Joomun*[1], M. Nicoara[1], L. J. Oostveen[2], I. Sechopoulos[2], G. De Bock[1], M. Greuter[1], M. Dorrius[1]; [1]Groningen/NL, [2]Nijmegen/NL

**Purpose:** To evaluate influence of dose, reconstruction kernel, matrix size, and virtual monoenergetic imaging (VMI) level on breast lesion detectability on conventional energy integrated (EID) computed tomography (CT) and photon-counting detector (PCD) CT.
**Methods or Background:** A semi-anthropomorphic breast phantom containing hyperdense masses, calcification clusters, and fibers of different diameters was attached to an anthropomorphic thorax phantom and positioned on a dedicated breast support cushion. Scans were performed in the prone position, with the breast hanging freely, on EID-CT (Somatom Force, Siemens) and PCD-CT (Naeotom Alpha, Siemens). Dose (CTDIvol) and reconstruction kernel were varied on both systems, while matrix size and VMI level were varied on the PCD-CT system. Qualitative analysis was assessed by lesion visibility using a 5-point Likert scale.
**Results or Findings:** 0.40mm calcification cluster was visible on both systems above 21mGy, whereas 0.29mm cluster was only visible on PCD-CT above 29mGy. 1.80mm mass was not detected on EID-CT but was visible on PCD-CT above 29mGy. On EID-CT, 3.18, 4.76, and 6.32mm masses were visible above 7.4, 1.8, and 0.9mGy, respectively, while on PCD-CT, the 3.18mm mass was detectable above 3.7mGy and the 4.76 and 6.32mm masses above 0.9mGy. Fibers of 0.41 and 0.60mm were visible on EID-CT above 7.4 and 1.8mGy, whereas on PCD-CT, fibers of 0.23, 0.41, and 0.60mm were detected above 29, 7.4, and 0.9mGy, respectively. Optimal visibility was achieved at VMI 70 keV. Sharper kernel enhanced visibility of smaller structures. Use of 1024 matrix on PCD-CT improved detection of smaller structures.
**Conclusion:** PCD-CT demonstrated higher detectability of breast lesions compared with EID-CT, particularly for small structures.
**Limitations:** The breast phantom used in this study only contained adipose tissue and no glandular tissue.
**Funding for this study:** Funding was received from KWF PPS and Siemens Healthineers
**Has your study been approved by an ethics committee?** Not applicable
**Author Disclosures:**
Mara Nicoara: Nothing to disclose
Monique Dorrius: Nothing to disclose
Geertruida De Bock: Nothing to disclose
Luuk J. Oostveen: Nothing to disclose
Shivaira Joomun: Nothing to disclose
Ioannis Sechopoulos: Nothing to disclose
Marcel Greuter: Nothing to disclose

**Response assessment with 18F-FDG PET/CT after neoadjuvant chemotherapy in breast cancer patients - A review and meta-analysis enclosing axillary, internal mammary, and periclavicular lymph nodes**
F. V. Amstel[1], M. Lenaerts[1], F. M. Mottaghy[1], J. Simons[2], M. Smidt[1], *T. Van Nijnatten*[1]; [1]Maastricht/NL, [2]Rotterdam/NL
*(thiemovn@gmail.com)*

**Purpose:** The diagnostic accuracy and prognostic relevance of 18F-FDG PET/CT for regional nodal response assessment after neoadjuvant chemo-immunotherapy (NAC) in clinically node-positive (cN+) breast cancer remains unclear. Therefore, this review and meta-analysis aims to determine the diagnostic performance of post-NAC 18F-FDG PET/CT for axillary lymph node (ALN) response assessment and to evaluate the prognostic value of post-NAC 18F-FDG PET/CT for internal mammary lymph node (IMLN), and periclavicular lymph node response assessment.
**Methods or Background:** PubMed, Embase, Cochrane, and Web of Science were searched for studies using post-NAC 18F-FDG PET/CT for nodal response assessment in cN+ patients treated with NAC, up to 17/02/2025. Pooled sensitivity, specificity, positive predictive value (PPV), and negative predictive value (NPV) were calculated. For ALNs, surgical pathology served as reference standard. For IMLNs and periclavicular lymph nodes, locoregional or distant recurrence was used as surrogate reference standard.
**Results or Findings:** Eight studies (574 patients) were included: six (558 patients) on ALNs, two (16 patients) on IMLNs, and one (1 patient) on periclavicular lymph nodes. Regarding ALNs, the overall axillary pathologic complete response rate was 45.2%. The pooled sensitivity, specificity, PPV, and NPV were 52% (95% CI:0.24-0.79), 83% (95% CI:0.72-0.91), 78% (95% CI:0.62-0.89), and 59% (95% CI:0.30-0.82). Regarding IMLNs, the pooled sensitivity, specificity, PPV, and NPV were 40% (95% CI:0.05-0.85), 100% (95% CI:0.72-1.00), 100% (95% CI:0.16-1.00), and 78.6% (95% CI:0.49-0.95). Regarding periclavicular lymph nodes, findings were limited to only one patient.
**Conclusion:** The diagnostic performance of post-NAC 18F-FDG PET/CT to accurately identify ALN response is limited. Regarding post-NAC 18F-FDG PET/CT in assessing IMLN and periclavicular lymph node response, additional evidence is needed to determine prognostic relevance.
**Limitations:** For periclavicular lymph nodes, data pooling was not possible given low number of included studies
**Funding for this study:** Florien J.G. van Amstel received funding for this study by the Dutch Cancer Society (REFINE-trial; project 14055). Melissa Lenaerts received funding from Kankeronderzoeksfonds Limburg. None of the other authors received support from any organization for the submitted work.
**Has your study been approved by an ethics committee?** Not applicable
**Author Disclosures:**
Marjolein Smidt: Nothing to disclose
Thiemo Van Nijnatten: Board Member: Thiemo van Nijnatten reports participation in medical advisory board meetings for Bayer and GE Healthcare and for Screenpoint Medical, not related to the content of this study Speaker: Thiemo van Nijnatten received speaker honoraria from Bayer, GE Healthcare and Hologic Inc, not related to the content of this study Grant Recipient: institutional grant support from Bayer and GE Healthcare, not related to the content of this study
Melissa Lenaerts: Nothing to disclose
Florien Van Amstel: Nothing to disclose
Janine Simons: Nothing to disclose
Felix M. Mottaghy: Nothing to disclose

**Utility of the RCR CT Staging Guidelines for Breast Cancer: A Retrospective Observational Study**
*M. Elghobashy*, P. Fletcher, I. Masroor, F. Peters, S-W. Tan; UK

**Purpose:** This study aims to demonstrate a centre's experience of the utility of the RCR guidelines for CT staging in breast cancer.
**Methods or Background:** In the United Kingdom, around 57,000 new cases of breast cancer are diagnosed annually [1]. Early identification of metastatic disease is crucial for improving survival and morbidity. The Royal College of Radiologists (RCR) recommends use of whole-body CT staging for distant metastatic disease in those presenting with T3/T4 cancer, those with 4 or more abnormal lymph nodes at axillary ultrasound and those with symptoms that raise suspicion of metastasis [2]. Despite clear guidelines, patients often undergo CT imaging without fulfilling the aforementioned criteria, thus leading to increased and potentially unnecessary radiation exposure. This study explores the utility of the RCR guidelines in stratifying patients with and without metastasis.
**Results or Findings:** A retrospective observational study was undertaken with data extracted from a 5-year period. 526 patients were identified who had a breast cancer staging CT. 214 of these studies were excluded from analysis as they represented patients with disease recurrence, known metastatic disease or treatment surveillance. Of the remaining 312 studies, the requests of 187 (60%) did not meet the RCR guidelines. Of these patients, 107 (57%) had no significant findings, 51 (27.3%) had incidental findings requiring further investigations, 25 (13%) had indeterminate findings of possible metastasis (of which only 3 were later proven to be metastasis) and 4 (2%) had CT proven metastasis.
**Conclusion:** Identifying metastatic disease is key in the management of breast cancer, however, the overuse of CT generates further investigations, and may subject patients to radiation without benefit. The RCR guidelines appear useful in identifying patients who are most likely to benefit from whole body staging CT.
**Limitations:** N/A
**Funding for this study:** N/A
**Has your study been approved by an ethics committee?** Not applicable
**Author Disclosures:**
Peter Fletcher: Nothing to disclose
Maiar Elghobashy: Nothing to disclose
Imrana Masroor: Nothing to disclose
Sue-Wei Tan: Nothing to disclose
Francesca Peters: Nothing to disclose

**Total-body staging and surgical planning in breast cancer with photon-counting CT**
*F. Darvizeh*, D. M. G. Bossi, M. Gerli, M. Alì, I. Castiglioni, D. Fazzini; Milan/IT
*(fdarvizeh@gmail.com)*

**Purpose:** To assess photon counting detector computed tomography (PCD-CT) as a single-session modality for locoregional and systemic total-body staging in breast cancer, aiming to reduce reliance on MRI, lower contrast and radiation dose, and improve patient experience and surgical planning.
**Methods or Background:** A PCD-CT protocol was developed including virtual non-contrast, arterial (upper abdomen), portal (thorax/abdomen), and delayed phases (abdomen/brain) with spectral reconstruction. Contrast (Iomeron 400 mg/mL) was given at 0.7 mL/kg via the arm contralateral to the tumour. Seventeen consecutive women (mean age 51; range 30–78) with biopsy-proven breast cancer scheduled for neoadjuvant chemotherapy or mastectomy were retrospectively compared with MRI. In mixed benign/malignant cases, an extra thoracic arterial phase was added. Two radiologists independently reviewed tumour, lymph nodes, and extramammary findings.
**Results or Findings:** PCD-CT enabled accurate detection of the primary tumour and lymphadenopathy, with diagnostic efficacy comparable to MRI. In all cases, findings matched MRI for tumour detection, size, multifocality, lymph node status, and pectoralis muscle involvement. Microcalcifications were seen only on PCD-CT, reflecting superior spatial resolution. Among 17 patients, PCD-CT revealed axillary adenopathy (n=12), internal mammary adenopathy (n=3), pectoralis fascia invasion (n=1), brain metastasis (n=1), and suspicious liver lesions (n=1). Compared with MRI+CT, PCD-CT was faster (10 vs 30 min), required a single contrast injection, and delivered ~35 mSv, ~60% lower than 64-slice CT. Supine positioning was judged more surgically favourable than prone MRI.
**Conclusion:** PCD-CT provides efficient, accurate locoregional and systemic staging in a single session, reducing contrast, radiation, time, and cost compared with MRI+CT.
**Limitations:** Small, single-centre cohort.
**Funding for this study:** None
**Has your study been approved by an ethics committee?** Yes
**Ethics committee - additional information:** Ethical committee was requested (study ID: 6470)
**Author Disclosures:**
Deborah Fazzini: Nothing to disclose
Marco Alì: Nothing to disclose
Daniela Maria Giacinta Bossi: Nothing to disclose
Isabella Castiglioni: Nothing to disclose
Marco Gerli: Nothing to disclose
Fatemeh Darvizeh: Nothing to disclose

**08:00 - 09:00** **Research Stage 2**

### Research Presentation Session: Chest

### RPS 2204

### Updates in thoracic intervention

**Moderator**
Ante Marušić; Samobor/HR

**Pulmonary nodule risk stratification in robotic-assisted bronchoscopy referrals: Can biopsy be avoided?**

*J. Kroschke*, B. J. Kerber, J. Happe, C. Steinack, P. Baumgartner, R. Engeli, S. Ulrich, T. Frauenfelder, T. Gaisl; Zurich/CH
*(jonas.kroschke@usz.ch)*

**Purpose:** Malignancy risk stratification of pulmonary nodules remains a major challenge in lung cancer screening and incidentally detected nodules. Patients referred for robotic-assisted bronchoscopy with integrated cone-beam CT (RAB+CBCT) at a tertiary hospital represent a distinct, high-risk cohort and it is unclear whether existing stratification tools could reduce biopsy needs. This study evaluated the performance of statistical and deep-learning risk models in this setting.
**Methods or Background:** We retrospectively analyzed 130 patients (54.5% male, mean age 68.1±9.6 years) with 176 nodules who underwent RAB+CBCT biopsy for suspected lung cancer. Biopsied nodules were visually matched to pre-procedural CT scans using bronchoscopy reports and intraprocedural CBCT. Clinical data were extracted from medical records. Automated segmentation was performed, and a deep learning algorithm (malignancy similarity index, mSI; RevealDx) was applied to CT-based thumbnails including perifocal parenchyma. Brock and Mayo risk scores were calculated.
**Results or Findings:** Histopathology confirmed lung cancer in 68.8% of nodules. Nodule characteristics were 53.4% solid (62.8% malignant), 21.0% part-solid (56.8% malignant), and 25.6% ground-glass (91.1% malignant). Median nodule size was 12.00mm (IQR 9.00-16.12mm) and volume 670.00mm³ (IQR 290.50-1710.75 mm³). Most nodules (62.5%) were located in upper lobes, without consistent differences in malignancy rate across lobes (e.g. right upper 64.9% vs. right lower 71.4%). ROC analysis demonstrated limited discrimination with Brock (AUC=0.62), Mayo (AUC 0.63), and mSI (AUC=0.50). In patients with prior scans, volumetric growth achieved higher accuracy (AUC=0.75).
**Conclusion:** Our RAB+CBCT cohort shows a distinct risk profile, especially unexpectedly high malignancy rates in ground-glass nodules. Existing risk models, including deep learning, underperform in this population, making biopsy currently unavoidable. Future research should target tailored stratification models to improve decision-making in high-risk cohorts.
**Limitations:** Single-center, single-vendor. Limited patient numbers. Selection bias. AI-model not trained for this application.
**Funding for this study:** No external funding was provided for this study.
**Has your study been approved by an ethics committee?** Yes
**Ethics committee - additional information:** Approval for this study was granted by the local ethics committee (BASEC2025-D0037).
**Author Disclosures:**
Thomas Frauenfelder: Nothing to disclose
Carolin Steinack: Nothing to disclose
Silvia Ulrich: Nothing to disclose
Thomas Gaisl: Nothing to disclose
Jasmin Happe: Nothing to disclose
Bjarne Jonas Kerber: Nothing to disclose
Jonas Kroschke: Nothing to disclose
Reto Engeli: Nothing to disclose
Patrick Baumgartner: Nothing to disclose

**Evaluation of pneumothorax predictionability by deep learning method before transtoracic fine needle aspiration biopsy (ttiiab) in lung masses**

*N. Gasimli*, V. Mammadlı, H. Ayyildiz, S. M. Ertürk, R. Mammadzada; Istanbul/TR
*(nilufergasimli@gmail.com)*

**Purpose:** Our aim in this study is to evaluate the predictability of pneumothorax, the most common complication of PTB, before TTNAB with deep learning algorithms, and to plan patient management.
**Methods or Background:** A total of 403 PTB procedures performed under CT guidance in our institution were included in our retrospective study. In our hospital system, images of patients who underwent TTIAB between 2018 and 2023 were downloaded and the data was processed with deep learning algorithms in anonymized form. Control CT images of the patients taken during the PTB planning phase before biopsy and after PTB were used. In our study, a deep learning model was created using the VGG-19 algorithm to classify DICOM images.
**Results or Findings:** Pneumothorax did not develop in 271 (67%) of the patients who underwent the procedure, while pneumothorax development was observed in 132 cases (33%). 76 of these 132 cases (58%) did not require hospitalization, but 54 cases (42%) required hospitalization. According to the results obtained as a result of the analysis process in our study, the VGG-19 model has 68.7% accuracy and 0.68 AUC values. However, the sensitivity value calculated as 52% as a result of the analysis shows that approximately half of the positive cases are predicted correctly. As a result of the application of the model, 73% of negative cases were classified correctly. This can be attributed to the fact that the chosen method has more effective success on negative cases.
**Conclusion:** These values, which express the general accuracy rate of the model, show that the selected method has an acceptable success in predicting the risk of pneumothorax.
**Limitations:** Our study is single-center and one of the limitations is that our sample is not larger.
**Funding for this study:** None.
**Has your study been approved by an ethics committee?** Yes
**Ethics committee - additional information:** Ethics committe: 09.05.2023
**Author Disclosures:**
Ruslan Mammadzada: Nothing to disclose
Vusal Mammadlı: Nothing to disclose
Hakan Ayyildiz: Nothing to disclose
Nilufar Gasimli: Nothing to disclose
Sükrü Mehmet Ertürk: Nothing to disclose

**Comparison of Fish-hook and Spiral Shaped Wires in CT-guided Pulmonary Nodule Localization: Impact on Complications and Clinical Outcomes**

*X. Zhang*, S. Thom R.G., G. J. De Jonge, M. Van Tuinen, W. Caroline van de, E. Michiel, W. F. A. Den Dunnen, G. De Bock, M. Dorrius; Groningen/NL
*(x.zhang03@umcg.nl)*

**Purpose:** In December 2020, our center transitioned from using a wire with a fish-hook shape to a wire with a spiral shape for CT-guided pulmonary nodule localization. This study compared the effectiveness and safety of both techniques.
**Methods or Background:** We retrospectively reviewed 157 consecutive patients who underwent CT-guided wire localization between November 2017 and December 2024 in University Medical Center Groningen (UMCG), Netherlands. Patient demographics, lesion characteristics, procedural and surgical details, and pathological findings were collected. Procedure-related complications were classified as minor (pneumothorax or hemoptysis without clinical consequences) or major (events requiring intervention, including air embolism). Logistic regression was performed to identify factors associated with localization success and procedure-related events.
**Results or Findings:** Fish-hook shaped wires were used in 79 patients and spiral shaped wires in 78 patients. Technical success was similar between groups (94.9% vs. 96.2%). Minor complications occurred more frequently with fish-hook shape wires (34.2% vs. 21.8%), while major complications were rare and comparable. Pneumothorax rates (minor and major combined) were 31.6% for fish-hook shape wire and 20.5% for spiral shape wires. Pathological representativeness did not differ significantly. In multivariable regression, longer procedure duration and fish-hook shape wire use were independently associated with an increased risk of minor complications.
**Conclusion:** Both wire types demonstrated high and comparable effectiveness and safety for CT-guided pulmonary nodule localization. However, spiral-shaped wires are associated with fewer minor complications, supporting their preferential use in clinical practice.
**Limitations:** This is a single-center study with a relatively small sample size, which may limit the generalizability of our findings. In addition, pain scores are not collected, precluding evaluation of this potentially relevant outcome.
**Funding for this study:** This research did not receive any specific grant from funding agencies in the public, commercial, or not-for-profit sectors.
**Has your study been approved by an ethics committee?** Not applicable
**Author Disclosures:**
Monique Dorrius: Nothing to disclose
Xiaowen Zhang: Nothing to disclose
Geertruida De Bock: Nothing to disclose
Marcel Van Tuinen: Nothing to disclose
Erasmus Michiel: Nothing to disclose
Wilfred F. A. Den Dunnen: Nothing to disclose
Stams Thom R.G.: Nothing to disclose
Gonda J. De Jonge: Nothing to disclose
Wauwer Caroline Van De: Nothing to disclose

**AI Scoring on Chest Radiographs to Guide Biopsy Decisions in Suspected Lung Cancer: Evidence from a Biopsy-Proven Cohort**

E. G. Kahraman, Y. Varol, *A. Selver*, O. Ozdemir, E. Hasbay, Y. Erol; Izmir/TR
*(alper.selver@deu.edu.tr)*

**Purpose:** To develop and evaluate a novel biopsy-indication scoring system based on TorchXRayVision (TxRV), an open-source deep learning model trained on large chest radiograph datasets, aiming to support clinical decision-making in differentiating malignant from benign lung lesions.
**Methods or Background:** Chest radiographs of 300 patients were screened; 285 (206 malignant, 79 benign) were eligible after excluding anterior–posterior views and indeterminate pathology. TxRV outputs for 18 radiological findings were extracted. For biopsy indication, six core features (effusion, pneumonia, nodule, mass, lung lesion, opacity) were used to generate three handcrafted scores (simple sum, weighted, maximum). For malignancy prediction, extended 18-feature models were tested: simple sum, weighted sum, logistic regression, and random forest. Statistical analyses included Mann–Whitney U tests, ROC/AUC, confusion matrices, and feature importance mapping.
**Results or Findings:** Nodule, mass, and lung lesion scores were significantly higher in malignant cases ($p < 0.01$), while opacity showed borderline association ($p = 0.06$). Weighted biopsy scoring yielded the highest discriminatory capacity, with a malignancy prevalence of ~80% above the 75th percentile cut-off. Logistic regression improved interpretability, achieving AUC 0.71 with balanced sensitivity/specificity. Random forest demonstrated superior performance (AUC 0.94, accuracy 90%), but feature importance confirmed that classic oncologic signs (mass, nodule, lesion) remained the strongest predictors. Precision–recall analysis supported these findings, with Random Forest showing F1 = 0.92, PPV 0.94, and NPV 0.85, underscoring robust diagnostic value.
**Conclusion:** TxRV-derived scoring provides an interpretable, reproducible framework to guide biopsy indication and malignancy risk stratification. Weighted scoring improved diagnostic balance, and logistic regression offered stable performance suitable for clinical translation. Such modeling may help reduce unnecessary lung biopsies.
**Limitations:** This was a single-center retrospective study with modest sample size. Random forest results suggest possible overfitting, highlighting the need for external validation in larger, multi-center cohorts.
**Funding for this study:** None
**Has your study been approved by an ethics committee?** Yes
**Ethics committee - additional information:** This study was approved by the Ethics Committee of Izmir City Hospital (Approval Number: 2025/364 ) All procedures were conducted in accordance with the Declaration of Helsinki.
**Author Disclosures:**
Alper Selver: Nothing to disclose
Yeliz Erol: Nothing to disclose
Yelda Varol: Nothing to disclose
Ozlem Ozdemir: Nothing to disclose
Ebru Hasbay: Nothing to disclose
Emir Gokhan Kahraman: Nothing to disclose

**Diagnostic yield, accuracy, and complications following CT guided anterior mediastinal biopsy: a single centre 10-year review**

*R. Ahmed*, P. Beddy, J. Kavanagh, D. Murphy, V. Young, S. Nicholson, J. F. Meaney; Dublin/IE

**Purpose:** Anterior mediastinal masses are uncommon, with an estimated prevalence of 0.4-0.9%. Computed tomography (CT) guided core needle biopsy (CNB) facilitates a histological diagnosis and appropriate management. The mediastinum contains several vital structures which must be considered when deciding a trajectory for biopsy. The aim of this study is to review technique, yield, and complications to describe a safe and effective method for biopsy.
**Methods or Background:** All CT-guided CNB for anterior mediastinal masses (2015 -2025) at a single centre were included. All images were reviewed by two experienced radiologists. Univariable comparisons between the diagnostic yield and non-diagnostic yield groups were performed.
**Results or Findings:** 52 patients were evaluated. 37 (71.15%) were diagnostic and 15 were (28.85%) non-diagnostic. Of the diagnostic samples, 16 (43.24%) were thymic, 14 (37.85%) lymphomas, and 7 (18.92%) other diagnoses. Minor complications included 6 (11.54%) pneumothoraxes, 6 (11.54%) haematomas, 5 (9.62%) pneumomediastinum, 3 (5.77%) pleural effusions, 2 (3.85%) haemothoraxes, and 1 pulmonary haemorrhage (1.92%). Major complications include 1 (1.92%) haemopericardium requiring pericardiocentesis and 1 (1.92%) fatal pulmonary artery puncture. On univariable analysis, a larger anteroposterior dimension ($p < 0.017$), larger mediolateral dimension ($p < 0.041$), and a greater number of biopsy samples taken ($p < 0.002$) were associated with a lower risk of non-diagnostic yield.
**Conclusion:** CNB is effective for confirming the nature of anterior mediastinal masses, but complications can arise, particularly from inadvertent puncture of the many vascular structures within this area. To our knowledge, this is the first reported case of a biopsy related fatality. We highlight a method to avoid vascular puncture to ensure safe outcome.
**Limitations:** Retrospective cohort study. Small sample size.
**Funding for this study:** No funding support was available for this study.
**Has your study been approved by an ethics committee?** Yes
**Ethics committee - additional information:** Ethical approval was gained from the local ethics committee at St James's Hospital
**Author Disclosures:**
Peter Beddy: Nothing to disclose
Siobhan Nicholson: Nothing to disclose
John Kavanagh: Nothing to disclose
Darra Murphy: Nothing to disclose
Vincent Young: Nothing to disclose
Rakesh Ahmed: Nothing to disclose
James F. Meaney: Nothing to disclose

**CT-guided chest biopsies: impact of operator experience on diagnostic yield and complication rate**

*H. D. J. Rueda-Chaparro*[1], A. Al Mutairi[2], F. Cadour[3], S. Kandel[3], P. Rogalla[3]; [1]Bogotá/CO, [2]Riad/SA, [3]Toronto, ON/CA
*(helidrueda@javeriana.edu.co)*

**Purpose:** To assess the effect of operator experience on diagnostic yield and complication rates in computed tomography (CT)-guided percutaneous chest biopsies.
**Methods or Background:** This retrospective single-center cohort study included 1,453 consecutive adult patients who underwent CT-guided chest biopsy between July 2022 and June 2024. Exclusion criteria were incomplete clinical/procedural data, ultrasound-guided biopsies, and operators with less than 20 procedures performed during the study period. Eight operators were stratified into three groups based on procedural volume: Group A (<100/year, n=3), Group B (100–200/year, n=3), and Group C (>200/year, n=2). Outcomes included diagnostic yield, complication rates, and predictors of both, assessed using univariable and multivariable logistic regression.
**Results or Findings:** Adequate tissue for definitive histopathology was obtained in 1,392 cases (96%). Diagnostic yield differed across operator groups: 93% in Group A, 94% in Group B, and 98% in Group C ($P=.002$). Group C remained independently associated with higher diagnostic yield (OR 2.69; 95%CI 1.26–5.73; $P=.011$). Complications occurred in 27% of patients, including pneumothorax (20%), chest tube insertion (2%) and other events (5%). Complication rates were highest in Group A (16%) and lowest in Group B (9%), yet operator experience was not independently associated with complications. Independent predictors of higher complication risk included smaller lesion size (OR 0.98 per mm, 95%CI 0.97–0.99; $P=.005$), use of sedation (OR 1.55; 95%CI 1.09–2.21; $P=.016$), and lesion location in the right upper lobe (OR 1.73; 95%CI 1.03–2.91; $P=.038$).
**Conclusion:** Operator experience was independently associated with higher diagnostic yield in CT-guided chest biopsies, with the most experienced operators achieving superior diagnostic performance. Complication risk related chiefly to lesion size, sedation, and anatomical location.
**Limitations:** Limitations include the retrospective single-center design, reliance on a two-year procedural volume, and possible dilution of operator-specific effects due to trainee supervision.
**Funding for this study:** None
**Has your study been approved by an ethics committee?** Yes
**Ethics committee - additional information:** The Research Ethics Board at University Health Network, Toronto, Canada approved this research.
**Author Disclosures:**
Farah Cadour: Nothing to disclose
Sonja Kandel: Nothing to disclose
Patrik Rogalla: Nothing to disclose
Arwa Al Mutairi: Nothing to disclose
Helí De Jesús Rueda-Chaparro: Nothing to disclose

**Safety of Ultrasound-Guided Pleural Drain Placement in Patients on Dual Antiplatelet Therapy: A Single-Centre Audit**

*D. Saeed*[1], N. D. Adroja[1], E. Obasi[1], C. Ridge[2]; [1]London/UK, [2]London/IE
*(danial.saeed@nhs.net)*

**Purpose:** Pleural drain insertion is frequently required in patients on dual antiplatelet therapy (DAPT) at our tertiary cardiothoracic centre. Departmental policy advises withholding P2Y12 inhibitors for seven days prior to drain placement, however this is often impractical in urgent settings. Evidence on bleeding risk in this group is limited. This audit evaluated complication rates in DAPT patients undergoing ultrasound (US)-guided pleural drain placement.

**Methods or Background:** A retrospective review was conducted of all US thorax and interventional radiology guided US drainage procedures from April 2024 to April 2025. Inclusion criteria were documented DAPT use at the time of procedure. Data collected included demographics, antiplatelet regimen, drug-hold duration, procedural details, operator grade, laboratory results, and complications.
**Results or Findings:** Of 368 drain requests, 33 (9%) were in patients on DAPT (median age 73 years; range 42-81; 64% male). Most received aspirin + clopidogrel (94%), with 70% continuing DAPT without interruption. Drains were predominantly 8 Fr (88%), left-sided (73%), and inserted in the radiology department (73%); 27% were performed in ICU. Operators included consultants (24%), fellows (21%), and registrars (55%). Mean interval from request to insertion was 0.6 days. No major or minor bleeding complications occurred. Two patients received transfusions for unrelated reasons. Referral documentation omitted complete antiplatelet / anticoagulant details in 68% of cases.
**Conclusion:** In this audit, US-guided small-bore pleural drain placement in patients on uninterrupted DAPT was not associated with bleeding complications, supporting British Society of Interventional Radiology guidance that classifies the procedure as low risk. The findings question the necessity of a seven-day P2Y12 inhibitor hold and highlight the need for improved documentation on referral requests.
**Limitations:** Small sample size, retrospective design, incomplete documentation, single-centre setting, and lack of long-term follow-up limit generalisability.
**Funding for this study:** No funding
**Has your study been approved by an ethics committee?** Not applicable
**Author Disclosures:**
Carole Ridge: Nothing to disclose
Danial Saeed: Nothing to disclose
Naimish D. Adroja: Nothing to disclose
Emeka Obasi: Nothing to disclose

**Imaging predictors of diagnostic success in Navigational Bronchoscopy**
*V. Gnananathan*, M. Morgan, S. Lam, H. Kolajian, A. El-Zeki, A. Metwalli, C. Peebles, A. Alzetani, L. Veres; Southampton/UK

**Purpose:** Navigational bronchoscopy (NB) is a recognized and increasingly available technique for tissue diagnosis of central pulmonary lesions unamenable to percutaneous CT guided approach. The use of intra-procedural 3D-CT has helped increase diagnostic yield and recent studies have demonstrated good safety profile with fewer complications compared to CT guided biopsy. However, NB remains resource intensive, requiring general anaesthetic and theatre access. Using CT lesion characteristics to predict features favouring diagnostic sampling will allow patient stratification in MDT, avoiding risks of anaesthetic and diagnostic delay.
**Methods or Background:** This retrospective study included 101 consecutive, eligible patients who underwent NB biopsy at University Hospital Southampton between June 2023 and February 2025. Patients with available histology reports and diagnostic CT imaging preceding NB were included. Diagnostic success was defined as an unequivocal malignant or benign histological diagnosis. Insufficient and equivocal results were classed as non-diagnostic. Pre-NB CT imaging was analysed for lesion characteristics by a team of radiologists.
**Results or Findings:** NB demonstrated good safety profile with 1% (n=1) post-procedure pneumothorax and no post-procedure haemorrhage . 60% (n=60) of procedures yielded diagnostic sample. Greater lesion size showed weak but statistically significant correlation with diagnostic sampling rate (r=0.20, p=0.04). Lesions ≥2cm were significantly more likely to yield diagnostic sample (p=0.035) as were lesions adjacent to more central airways (Weibel airway generation ≤4 ) (p=0.008). Solid vs subsolid lesions, lobar location and presence of a bronchus sign showed no significant correlation to diagnostic sampling.
**Conclusion:** CT lesion size cut-off (2cm) and assessment of proximity to central bronchial tree (airway generation ≤4) are significant predictors of success and will help stratify patients referred for NB and streamline cancer diagnostic pathways.
**Limitations:** Patients with unavailable diagnostic CT imaging were excluded
**Funding for this study:** No funding was obtained for this study.
**Has your study been approved by an ethics committee?** Not applicable
**Author Disclosures:**
Aiman Alzetani: Nothing to disclose
Stefan Lam: Nothing to disclose
Ahmed El-Zeki: Nothing to disclose
Harout Kolajian: Nothing to disclose
Vaishnavi Gnananathan: Nothing to disclose
Lukacs Veres: Nothing to disclose
Matthew Morgan: Nothing to disclose
Amr Metwalli: Nothing to disclose
Charles Peebles: Nothing to disclose

**Percutaneous CT-guided Lung Core Biopsy - Safety and Diagnostic Adequacy in our hospital**
*A. K. Geetha Virupakshappa*; Bedford/UK

**Purpose:** Aim is to evaluate the histological adequacy and safety of the CT guided percutaneous lung core biopsy done in our radiology department
**Methods or Background:** Adequacy and safety parameters: (First cycle) Diagnostic adequacy : 95 % of samples were adequate. Only one sample was inadequate, and this was lesion >2cm, done with an 20G needle and 5 passes obtained. Sensitivity for malignancy 95% for lesions >2 cm False positive rate =0%. Complication rates: Pneumothorax =20% Pneumothorax needing drainage= 15% (3cases) Haemoptysis =0% Death < 0% Adequacy and safety parameters (Second cycle): Diagnostic adequacy : 100 % of samples were adequate. Sensitivity for malignancy 100% for lesions >2 cm False positive rate =0%. Complication rates: Pneumothorax =40% (10cases) Pneumothorax needing drainage= 0% Haemoptysis =16% (4cases) Death < 0%
**Results or Findings:** We have a diagnostic accuracy rate of 95% in first cycle and 100% in second cycle, higher than the standard (90%). We have a pneumothorax rate equal to the standard, 20% in first cycle and 40% in second cycle, but none of them required drainage (0%, standard is 3%). 16% of patients experienced haemoptysis after the procedure (Standard <5%), which were managed conservatively (none of them required hospitalization). No death resulting from the procedure was recorded.
**Conclusion:** Documentation of the procedure (mode of imaging, needle size, core samples, number of passes) 100%. Diagnostic adequacy should be >90%. Sensitivity for malignancy > 85%. False positive rate <1%. complication rates: Pneumothorax <20%, pneumothorax requiring drainages <3%, haemoptysis<5%
**Limitations:** Individual radiologist variation
**Funding for this study:** No funding for the study
**Has your study been approved by an ethics committee?** Not applicable
**Author Disclosures:**
Anil Kumar Geetha Virupakshappa: Nothing to disclose

**08:00 - 09:00** **Research Stage 5**

### Research Presentation Session: Artificial Intelligence and Imaging Informatics

### RPS 2205

### AI beyond diagnosis: education, regulation and implementation

**Moderator**
Suyash Mohan; Philadelphia, PA/US
*(suyash.mohan@pennmedicine.upenn.edu)*
**Author Disclosures:**
Suyash Mohan: Advisory Board: Guerbet, Servier Pharmaceuticals, Author: Brain Imaging Case Review Series 3e, Research Grant/Support: NIH/NCI, NovoCure and Galileo CDS

**When to validate what: a framework for local performance assessment of AI in radiology**
K. G. Van Leeuwen, M. Horton, S. Krijgsman, *S. Romeijn*; Utrecht/NL
*(stephan.romeijn@healthairegister.com)*

**Purpose:** To present a structured framework that guides decisions on local performance assessment steps needed for safe and efficient AI adoption.
**Methods or Background:** Local quality assurance is crucial for the responsible implementation of commercially available AI solutions in radiology. A common belief is that every AI tool must always be extensively validated on local data before use. However, this is often impractical, particularly in non-academic hospitals, where resources are limited. To enable responsible yet feasible adoption, we present a framework that aligns the extent of performance validation with the available clinical evidence and the clinical risk of the application.
**Results or Findings:** The framework starts with an analysis of existing scientific evidence to assess whether sufficient clinical evidence is available on relevant population data. The next step is to consider the clinical risk (low or high). Based on these two factors, different validation types are recommended: • Low-risk with sufficient evidence: acceptance test and post-deployment monitoring may suffice. E.g. bone age prediction. • High-risk with sufficient evidence: acceptance test, pilot (shadow-mode or restricted implementation), and post-deployment monitoring are indicated. E.g. breast cancer screening AI. Low-risk with limited evidence: retrospective analysis, acceptance test, and post-deployment monitoring are advised. E.g. vertebral fracture detection for opportunistic osteoporosis screening. High-risk with limited evidence: retrospective analysis, acceptance test, pilot (shadow-mode or restricted implementation), and post-deployment monitoring are recommended. E.g. stand-alone stroke detection AI.
**Conclusion:** By tailoring local performance assessment to risk and evidence, this framework balances patient safety with feasibility, supporting responsible AI implementation in both academic and non-academic hospitals.
**Limitations:** The scope of this framework is limited to commercially available AI systems used in accordance with their intended use and does not address IT or data flow quality assurance.
**Funding for this study:** No funding was received for this study.
**Has your study been approved by an ethics committee?** Not applicable
**Author Disclosures:**
Stephan Romeijn: Founder: Romion Health
Margaret Horton: Nothing to disclose
Sade Krijgsman: Nothing to disclose
Kicky Gerhilde Van Leeuwen: Founder: Romion Health

**Clinician-Centric Explainable AI: Building Trust in Radiology Diagnosis**
*T. Schnitzler*[1], H. Zaytoun[1], A. P. Gehret[1], O. E. Oğretmen[2], M. Yavuz[2]; [1]Aarau/CH, [2]Istanbul/TR
*(tician.schnitzler@ksa.ch)*

**Purpose:** Artificial intelligence (AI) in radiology continues to face adoption barriers due to opaque decision-making processes that limit clinical trust. Traditional explainable AI (XAI) tools—such as saliency maps or feature importance rankings—rarely align with radiologists' diagnostic reasoning. We propose a Clinician-Centric Explainable AI (CC-XAI) framework to embed clinically meaningful, anatomy- and workflow-rooted explanations directly into radiological decision support.
**Methods or Background:** A conceptual and applied analysis of CC-XAI was performed, contrasting its interpretability with general-purpose XAI. We developed a five-category taxonomy comprising: (1) Visual (anatomy-aware saliency maps), (2) Textual (clinical language generation), (3) Example-Based (retrieval of annotated similar cases), (4) Quantitative (comparative measurement over time), and (5) Rule-Based (integration of clinical decision logic). A modular CC-XAI system was illustrated using a representative lung nodule case, demonstrating integration of lesion localization, volumetric growth analysis, similar case retrieval, and risk-factor-based reasoning into one cohesive explanation output.
**Results or Findings:** The proposed CC-XAI framework translates AI predictions into structured, interpretable outputs aligned with clinical workflows and cognitive heuristics. In our example, a spiculated nodule with growth from 8 mm to 15 mm over one year (estimated doubling time of 1119 days) was explained via anatomy-aware localization, growth kinetics, retrieval of two malignant-confirmed prior cases, and rule-based consideration of smoking, cancer history, and pulmonary fibrosis. Each module contributes to a cohesive final interpretation that improves explainability and trust calibration.
**Conclusion:** Clinician-centric XAI addresses a fundamental barrier to AI adoption in radiology by offering domain-specific, multi-dimensional explanations that mirror radiologists' decision-making processes. Embedding these modules into radiology workflows can facilitate clinically robust, interpretable, and trustworthy AI integration.Its modular design supports integration into clinical workflows for safer, more interpretable, and diagnostically aligned AI usage—especially in high-stakes areas such as lung cancer evaluation.
**Limitations:** No limitations.
**Funding for this study:** No Funding.
**Has your study been approved by an ethics committee?** Not applicable
**Author Disclosures:**
Adrienne Patrizia Gehret: Nothing to disclose
Okan Efe Oğretmen: Nothing to disclose
Tician Schnitzler: Grant Recipient: 2024 RSNA Research Fellow Grant Grant Recipient: Gottfried&Julia Bangerter-Rhyner Foundation Grant Recipient: Swiss Society of Radiology Consultant: bray-Z AG, Zurich
Hasan Zaytoun: Nothing to disclose
Mehmet Yavuz: Nothing to disclose

**Environmental Impact Reduction through Deep Learning Reconstruction in Clinical CT Imaging: A Hospital-Based Assessment**
*P. N. Franco*, C. Maino, C. R. G. L. O. M. Talei Franzesi, D. G. Gandola, I. Bianco, R. Corso, D. Ippolito; Monza/IT
*(francopaoloniccolo@gmail.com)*

**Purpose:** The healthcare sector accounts for approximately 4.4% of global greenhouse gas (GHG) emissions, with diagnostic imaging being one of the most energy-intensive activities. Computed Tomography (CT) is a major contributor due to high electricity demand and associated emissions. This study aims to evaluate the environmental impact of implementing deep learning-based image reconstruction (DLIR) in CT imaging in a large hospital, focusing on electricity, $CO_2$ emissions, and iodinated contrast media (ICM).
**Methods or Background:** Over the course of one year, data were collected from four CT scanners: two newly installed scanners utilizing DLIR technology and two older scanners operating with hybrid and model-based iterative reconstruction algorithms. Energy consumption, $CO_2$ emissions, and ICM usage were monitored and compared between the two groups. DLIR protocols were introduced with reduced tube voltage (from 120 kV to 80–100 kV) and optimized ICM dose. Energy savings were estimated from the quadratic relationship between kV and power. Emission reduction was quantified in $CO_2$-equivalent ($CO_2$e) tons. Water savings were derived from ICM production impact.
**Results or Findings:** Approximately 28,000 CT exams were performed (54% on iterative scanners, 40% on DLIR). Standard CT scanners consumed ~41,000 kWh/year, equating to ~10.25 tons $CO_2$/year per device. DLIR implementation allowed for a 30–55% energy reduction depending on scan type. Total annual savings per CT unit were up to 22,550 kWh and 5.6 tons $CO_2$e. ICM was reduced by 30%, saving ~2,100 liters/year per DLIR scanner and preventing ~81 tons of $CO_2$e.
**Conclusion:** Deep learning reconstruction in CT imaging can significantly reduce energy use, $CO_2$ emissions, and contrast media consumption without compromising diagnostic quality. These results support its implementation as a sustainable innovation in clinical radiology.
**Limitations:** This study was conducted in a single center, limiting generalizability.
**Funding for this study:** None.
**Has your study been approved by an ethics committee?** Not applicable
**Author Disclosures:**
Davide Giacomo Gandola: Nothing to disclose
Cesare Maino: Nothing to disclose
Cammillo Roberto Giovanni Leopoldo Oreste Massimiliano Talei Franzesi: Nothing to disclose
Ilaria Bianco: Nothing to disclose
Rocco Corso: Nothing to disclose
Paolo Niccolò Franco: Nothing to disclose
Davide Ippolito: Nothing to disclose

**Algorithmic Fairness Unfolded: Collaborative Ethnography within a Medical Imaging AI Lab for Lung Cancer Screening**
*M. Vitale*, M. Vegter, C. Jacobs, M. Boenink; Nijmegen/NL
*(michel.vitale@radboudumc.nl)*

**Purpose:** This study reports on collaborative efforts within an AI lab developing solutions for lung cancer screening (LCS) to investigate and negotiate how fairness is framed and operationalized in practice, and to develop actionable recommendations for better algorithmic fairness practices in medical imaging AI. It contributes to calls for more context-sensitive, morally robust, and interdisciplinary approaches to algorithmic fairness.
**Methods or Background:** We draw on a two-year collaboration between engineers and an ethicist in a UMC AI lab, considering fieldnotes and observations from meetings, ethics roundtables, and a six-month project assessing the fairness of AI-based risk models for LCS (Sybil, Venkadesh21, PanCan2b). Leveraging "ethics parallel research" alongside elements of the JustEFAB framework, we reconstruct and critically examine practices, assumptions, and constraints shaping engineers' approach. We trace how fairness was framed, bias assessed, and explore how fairness practices can be improved. Findings are validated in a follow-up roundtable, informing an agenda for more comprehensive, situated, and auditable fairness practices.
**Results or Findings:** We observe tensions between comprehensive theoretical frameworks of algorithmic fairness and narrow approaches prevalent in engineering practices, where fairness is often overlooked or reduced to technical optimization. Practical constraints (data availability, timelines, unclear responsibilities, limited resources and interdisciplinary opportunities) foster statistical approaches with limited connection to clinical needs or broader contextual ethical considerations. Bridging this gap requires reflexivity tools, transparent decision-making, interdisciplinary research, and recognition of fairness as a multifaceted ethical issue.
**Conclusion:** Current fairness assessments insufficiently engage with AI socio-technical and ethical dimensions. Interdisciplinary collaboration can bridge theory and practice, helping developers move beyond technical metrics. Ethics parallel research made these dimensions visible, contextual, and clinically relevant, while highlighting barriers and enablers of better fairness practices.
**Limitations:** Qualitative, single-center study focused on AI for medical imaging only.
**Funding for this study:** This research is funded by a public-private project with funding from the Dutch Science Foundation, the Dutch Ministry of Economic Affairs, and MeVis Medical Solutions (Bremen, Germany).
**Has your study been approved by an ethics committee?** Not applicable

**Author Disclosures:**
Marianne Boenink: Other: Research projects supported by the Dutch Science Foundation (NWO), the Dutch Ministry of Economic Affairs,), the Dutch Organisation for knowledge and innovation in health, healthcare and well-being (ZonMw), Reinier Post Foundation, the Dutch Brain Foundation, Centre for Society & the Life Sciences, Centre for Translational Molecular Medicine; some of which were co-funded by MeVis Medical Solutions (Bremen, Germany), Verily Life Sciences (USA), TMSi (the Netherlands), CSS (the Netherlands).
Michel Vitale: Other: Conducting research funded by a public-private project with funding from the Dutch Science Foundation, the Dutch Ministry of Economic Affairs, and MeVis Medical Solutions (Bremen, Germany).
Colin Jacobs: Other: Research grants and royalties to host institution from MeVis Medical Solutions (Bremen, Germany); payment for lectures from Canon Medical Systems and Johnson & Johnson; collaborator in a public-private research project where Radboud University Medical Center collaborates with Philips Medical Systems (Best, the Netherlands).
Mira Vegter: Nothing to disclose

**The Influence of Artificial Intelligence on Radiologists' Diagnostic Performance in Fatigue**
*M. Khoobi*, D. Truhn, C. K. Kuhl, S. Nebelung, R. Siepmann; Aachen/DE
*(mahta.khoobi@rwth-aachen.de)*

**Purpose:** Night-shift duty can cause fatigue in radiologists, potentially impairing diagnostic performance and compromising patient safety. This study aims to evaluate the impact of night-shift-induced fatigue on radiologists' diagnostic accuracy and whether artificial intelligence (AI) can mitigate the expected decline in performance.
**Methods or Background:** This prospective intra-individual reader study (July 2024–March 2025) involved 10 radiology residents (mean clinical experience: 35 months; three women) interpreting three sets of 33 chest radiographs before and after a first-call on-site night shift. Sessions included two pre-shift readings (without AI) and two post-shift readings (with and without AI). Eye tracking captured fixation duration and saccade count, and fatigue was classified as a ≥5% reduction in the slope of the saccadic main sequence between pre- and post-shift sessions. Diagnostic accuracy (Cohen's $\kappa$ vs expert majority vote), efficiency (mean reporting time per image), eye-tracking metrics, and surveys were analyzed using generalized linear mixed models with Tukey-adjusted post-hoc comparisons.
**Results or Findings:** Six of 10 radiologists were classified as fatigued. In fatigued radiologists, diagnostic accuracy declined significantly post-shift (from $\kappa=0.71\pm0.05$ to $\kappa=0.61\pm0.10$; $P<.001$), accompanied by reductions in fixation duration ($P=.010$) and saccade count ($P=.015$). Non-fatigued radiologists showed a smaller decline in accuracy (from $\kappa=0.72\pm0.03$ to $\kappa=0.65\pm0.03$, $P=.037$). Reporting times remained stable pre- and post-shift ($P\geq.463$). AI assistance modestly increased accuracy in both groups ($\Delta\kappa=0.03$–$0.04$; $P\geq.525$) and reduced reporting times in fatigued ($P=.122$) and non-fatigued radiologists ($P=.031$). User surveys indicated increased mental exhaustion in fatigued radiologists.
**Conclusion:** Night-shift-induced fatigue impaired diagnostic accuracy without slowing reporting speed, thereby raising concerns about patient safety. AI modestly improved performance but failed to restore pre-shift performance. Institutional fatigue-management strategies are essential, with AI serving as a supportive adjunct.
**Limitations:** A small sample size and restriction to chest radiographs limited the study.
**Funding for this study:** The Deutsche Forschungsgemeinschaft supports this research (DFG 701010997, 517243167, 515639690), the German Federal Ministry of Research, Technology, and Space (Transform Liver - 031L0312C, DECIPHER-M, 01KD2420B), and the European Union Research and Innovation Programme (ODELIA - GA 101057091).
**Has your study been approved by an ethics committee?** Yes
**Ethics committee - additional information:** Ethical approval was obtained (Medical Faculty, RWTH Aachen University, Germany, reference number 24/376), and informed consent was waived.
**Author Disclosures:**
Christiane K. Kuhl: Employee: Department of Diagnostic and Interventional Radiology, University Hospital Aachen, Aachen, Germany
Daniel Truhn: Research/Grant Support: Lab for Artificial Intelligence in Medicine, Department of Diagnostic and Interventional Radiology, University Hospital Aachen, Aachen, Germany Employee: Department of Diagnostic and Interventional Radiology, University Hospital Aachen, Aachen, Germany
Robert Siepmann: Employee: Lab for Artificial Intelligence in Medicine, Department of Diagnostic and Interventional Radiology, University Hospital Aachen, Aachen, Germany Employee: Department of Diagnostic and Interventional Radiology, University Hospital Aachen, Aachen, Germany
Mahta Khoobi: Employee: Lab for Artificial Intelligence in Medicine, Department of Diagnostic and Interventional Radiology, University Hospital Aachen. Aachen, Germany Employee: Department of Diagnostic and Interventional Radiology, University Hospital Aachen, Aachen, Germany,
Sven Nebelung: Research/Grant Support: Lab for Artificial Intelligence in Medicine, Department of Diagnostic and Interventional Radiology, University Hospital Aachen. Aachen, Germany Employee: Department of Diagnostic and Interventional Radiology, University Hospital Aachen, Aachen, Germany

**RadGame: an AI-powered platform for radiology education**
*M. Baharoon*[1], J. Jun[1], S. Raissi[1], T. Heintz[2], M. Alabbad[3], A. Alburkani[4], M. Mohammed[4], H. R. Alomaish[4], P. Rajpurkar[1]; [1]Boston, MA/US, [2]Maastricht/NL, [3]Hofuf/SA, [4]Riyadh/SA
*(mohammed_baharoon@hms.harvard.edu)*

**Purpose:** The purpose of this study is to improve radiology education by teaching trainees two essential skills: localizing imaging findings and generating structured clinical reports. Traditional methods often lack interactive feedback and rely on limited datasets, restricting learning opportunities. RadGame addresses these gaps by combining gamification with large-scale datasets and AI-driven feedback to provide scalable, personalized, and engaging training.
**Methods or Background:** We conducted a prospective, multi-institutional user study with 18 medical students who completed both RadGame modules (Localize and Report) under gamified and traditional learning conditions. Participants were evaluated with pre- and post-tests, and performance was measured using radiologist-verified ground truths, the CRIMSON reporting metric, and efficiency metrics such as time per case.
**Results or Findings:** Students using RadGame demonstrated significantly greater gains than those in traditional learning, with a 68% improvement in localization accuracy versus 17% and a 31% improvement in report-writing accuracy versus 4%. Additionally, the gamified group showed increased efficiency, completing cases faster over time while maintaining higher diagnostic accuracy.
**Conclusion:** RadGame demonstrates that AI-powered gamification can significantly enhance radiology training by integrating structured, real-time feedback into localization and report-writing tasks. Compared to traditional passive methods, the platform produced larger improvements in diagnostic accuracy and efficiency among medical trainees. Beyond its educational impact, RadGame also serves as a human-in-the-loop testbed for refining AI evaluation metrics such as CRIMSON, making them more clinically meaningful. These findings highlight the potential for repurposing medical AI resources into scalable, learner-centered educational tools.
**Limitations:** The limitations of the study are due to a relatively small sample size of 18 students, which reduced the statistical power for some comparisons. Additionally, the gamified module required more interaction time than traditional methods, reflecting trade-offs between active engagement and efficiency.
**Funding for this study:** No funding was received for this study.
**Has your study been approved by an ethics committee?** Yes
**Ethics committee - additional information:** This study was reviewed by the Harvard Faculty of Medicine Institutional Review Board (Protocol \#IRB25-0694) and determined to be exempt under 45 CFR 46.104(d)(2)(3).
**Author Disclosures:**
Thibault Heintz: Nothing to disclose
Pranav Rajpurkar: Nothing to disclose
Mohammed Mohammed: Nothing to disclose
Hassan Rayhan Alomaish: Nothing to disclose
Mohammed Baharoon: Nothing to disclose
Mahmoud Alabbad: Nothing to disclose
Ali Alburkani: Nothing to disclose
John Jun: Nothing to disclose
Siavash Raissi: Nothing to disclose

**Economic Evaluations of AI Applications in Radiology: A Systematic Review**
*F. Zanca*[1], L. Gregory[2], F. Lock[2], H. Harvey[2]; [1]Leuven/BE, [2]Haywards Heath/UK
*(federica.zanca@ec.europa.eu)*

**Purpose:** Artificial intelligence (AI) in radiology offers promising applications to enhance clinical and patient outcomes. However, adoption has been slow, partly due to a lack of robust health economic evidence increasingly required for technology integration. While established tools focus on clinical evidence, they often overlook economic impact. This review synthesises literature on economic evaluations of AI in radiology.
**Methods or Background:** A systematic review of health economic evaluations of radiology AI was conducted and reported following PRISMA guidelines and economic review recommendations. MEDLINE and Cochrane Central were searched using a targeted strategy. Eligible studies were screened, with conflicts resolved by consensus review. Data were extracted into economic, patient, and clinical outcome domains, and reporting quality assessed using CHEERS-AI for evaluations using decision-analytic models.

**Results or Findings:** Thirty-one studies were included. Reported outcomes varied widely. Most focused on direct costs, cost-effectiveness, and diagnostic accuracy with limited attention to productivity or workflow metrics. Quality-adjusted life years (QALYs) were the predominant outcome, although alternative measures such as cost per patient screened or per correct diagnosis were also used. Half of the studies applied decision-analytic modelling, predominantly in opportunistic imaging contexts, and most originated from the US and UK. Evidence from European settings was scarce.
**Conclusion:** There is a significant gap between the rapid proliferation of radiology AI tools and the availability of health economic evidence to support their adoption. Economic outcomes and metrics have been largely based on the pharmaceutical best practices. Future studies should broaden outcomes assessed to include productivity, workflow efficiency, and access to care. Cross-disciplinary collaboration and harmonised international guidance are essential to ensure that radiology AI adopted responsibly.
**Limitations:** Many included studies predated CHEERS-AI. We excluded cardiology and invasive imaging studies. All included studies focused on deep learning.
**Funding for this study:** This work was supported by the European Innovation Council and SMEs Executive Agency (EISMEA).
**Has your study been approved by an ethics committee?** Not applicable
**Author Disclosures:**
Lucy Gregory: Nothing to disclose
Hugh Harvey: Nothing to disclose
Felicity Lock: Nothing to disclose
Federica Zanca: Nothing to disclose

**AI Act and AI-based radiology software: Implications for clinical use**
*J. Kohler*[1], J. Jesser[1], A. Seehafer[2], B. Kohler[3], S. Heiland[1], M. Bendszus[1], J. Kernbach[1], M. Schell[1]; [1]Heidelberg/DE, [2]Berlin/DE, [3]Zurich/CH
*(joel.kohler@gmx.de)*

**Purpose:** Hospitals and other healthcare providers increasingly implement AI-based radiology software in their clinical practice. We investigated how the European AI Act (AIA) might affect this usage from 2027 on.
**Methods or Background:** We applied qualitative and quantitative legal interpretation methods to simulate how the AIA could apply to hospitals. To analyze market effects, we evaluated the landscape of commercial clinical radiology AI applications. We used data provided in the EU Impact Assessment to estimate costs of compliance.
**Results or Findings:** The AIA intensely regulates 'high-risk' AI systems (assumed 5-15 % of all AI applications). 98.6 % of current products and most future clinical radiology AI-based software will represent such high-risk AI systems. If a hospital uses an AI software as provided by a third-party company, the hospital will act as a ‘deployer' (low level of legal duties and moderate costs associated) and the company will be a 'provider' (high level of legal duties). However, not only in case of placing its own AI system on the market but also in certain other scenarios, a hospital might itself become a 'provider' - in some cases unexpectedly and unintentionally. This provider role may lead to roughly 30000 EUR compliance costs per application and up to 330000 EUR one-off and 71000 EUR annual costs on top if a quality management system has to be set up.
**Conclusion:** Clinical stakeholders should prepare for potentially severe legal and economic implications of AI usage. The AIA might hamper broad innovation adaption and leveraging clinical benefits for providers of AI systems in radiology. Public authorities should consider mitigating these effects.
**Limitations:** The limitations of the study include legal and economic uncertainties.
**Funding for this study:** No funding was received for this study.
**Has your study been approved by an ethics committee?** Not applicable
**Author Disclosures:**
Jessica Jesser: Nothing to disclose
Astrid Seehafer: Nothing to disclose
Joel Kohler: Nothing to disclose
Sabine Heiland: Nothing to disclose
Martin Bendszus: Nothing to disclose
Benjamin Kohler: Nothing to disclose
Julius Kernbach: Nothing to disclose
Marianne Schell: Nothing to disclose

**PAIR: A Framework for Standardized Reporting of Real-World AI Implementation in Hospitals**
*R. Sivanandan*[1], W. Grootjans[2]; [1]Asker/NO, [2]Leiden/NL
*(sivram@vestreviken.no)*

**Purpose:** Artificial intelligence (AI) is moving rapidly from development to deployment in radiology. While reporting standards such as CLAIM, CONSORT-AI, DECIDE-AI, STARD-AI, and TRIPOD-AI address validation and trials, they overlook the complex realities of implementation in radiology workflows. This lack of structure hinders comparison, reproducibility, across institutions and potentially limits added value of AI solutions.
**Methods or Background:** We developed the Protocol for AI Implementation Reporting (PAIR) to provide a standardized, practice-oriented framework for documenting and guiding AI implementation in hospitals. PAIR integrates and extends existing reporting guidelines, and it is organized around four domains. The first domain, problem and purpose, describes the clinical need, clarifies the value proposition, and supports application selection. The second is the AI system, which details the technical specifications, regulatory status, and intended use. The third domain addresses implementation, including governance, workflow integration, interoperability, user training, and change management. The fourth domain, results and sustainability, encompasses safety monitoring, drift and bias control, equity, cost-effectiveness, adoption, and retirement criteria.
**Results or Findings:** PAIR includes a reporting checklist, manuscript template, and workflow diagram to support transparent documentation and knowledge sharing. Beyond reporting, it also functions as a practical guide for hospitals to structure AI adoption and assess whether an application adds measurable value to radiological practice.
**Conclusion:** PAIR fills an existing gap between reporting standards and enables transparent and reproducible reporting while guiding safe, equitable, and value-driven AI implementation in clinical practice. Its adoption will accelerate the adoption of trustworthy AI in radiology and beyond.
**Limitations:** At present, PAIR remains conceptual and awaits empirical validation
**Funding for this study:** No funding
**Has your study been approved by an ethics committee?** Not applicable
**Author Disclosures:**
Willem Grootjans: Nothing to disclose
Ramprabananth Sivanandan: Nothing to disclose

**08:00 - 09:00** **Research Stage 3**

### Research Presentation Session: Neuro

### RPS 2211

### Photon-counting CT in neuroradiology: from artifact reduction to high-resolution surgical planning

**Moderator**
Linda Jacobi-Postma; Maastricht/NL
*(l.jacobi@mumc.nl)*
**Author Disclosures:**
Linda Jacobi-Postma: Advisory Board: Siemens Healthineers, Research Grant/Support: Siemens Healthineers, Speaker: Siemens Healthineers

**Photon-Counting CT in Spinal Implants: Artifacts Reduction and Image Quality Improvement**
*L. A. Cappellini*[1], G. Ressa[1], M. De robertis[1], G. Savini[1], R. Levi[1], C. Brembilla[2], M. Fornari[2], L. S. Politi[1]; [1]Pieve Emanuele/IT, [2]Rozzano/IT
*(luca.cappellini@humanitas.it)*

**Purpose:** The aim of this study was to qualitatively and quantitatively determine whether Photon-Counting CT (PCCT) with Virtual Monoenergetic Imaging (VMI) improves postoperative assessment of spinal implants by reducing metallic artifacts, enhancing diagnostic confidence, and better evaluating postoperative complications, in comparison with conventional CT.
**Methods or Background:** A total of 16 subjects who underwent PCCT (NAEOTOM Alpha, Siemens) for postoperative evaluation following anterior, posterior, or combined spinal fixation were included. Scans were performed at 120 or 140 kVp, with and without IMAR. Images were evaluated using VMI (60–190 keV). For each examination, 13 ROIs were placed to calculate Artifact Index (AIx), HU values, and Standard Deviation (SD). Subjective evaluation was performed by three radiologists using a Likert scale (1–5) at different energy levels. Where available PCCT was compared to conventional CT.
**Results or Findings:** PCCT proved effective in reducing soft tissue artifacts, with optimal performance between 110 and 140 keV. At energies above 150 keV, loss of contrast were observed impairing tissue differentiation. Images at 0.2 mm with Br76 kernel showed higher SD and AIx but were subjectively superior for evaluating screws, bone contours, and metal-bone interfaces, compared to IMAR and VMI, due to overcorrection bands, shading, and native software limitations. The subjectively selected optimal reconstruction did not always correspond to the image with the lowest AIx. Compared to available conventional CT scans, monoenergetic PCCT images achieved higher Likert scores and lower AIx values.
**Conclusion:** PCCT provides high subjective and objective image quality in patients with spinal metallic implants. For the evaluation of periprosthetic bone changes, ultra-high-resolution reconstructions with bone kernel images are preferred. Virtual Monoenergetic Imaging (VMI) improves soft tissue assessment, particularly in the setting of postoperative complications.
**Limitations:** Sample size, Comparison with conventional CT was limited
**Funding for this study:** ANTHEM Project
**Has your study been approved by an ethics committee?** Yes
**Ethics committee - additional information:** The study was approved by our institutional ethics committee.
**Author Disclosures:**
Mario De Robertis: Nothing to disclose
Gaia Ressa: Nothing to disclose
Letterio Salvatore Politi: Nothing to disclose
Carlo Brembilla: Nothing to disclose
Giovanni Savini: Nothing to disclose
Luca Alessandro Cappellini: Nothing to disclose
Riccardo Levi: Nothing to disclose
Maurizio Fornari: Nothing to disclose

**Impact of Deep Learning-Based Denoising on Image Quality and Diagnostic Confidence in Neurovascular Ultrahigh-Resolution Photon-Counting CT Angiography**
*A. Toth*, M. T. Hagar, M. Silbergleit, J. Y. Cho, M. Vecsey-Nagy, A. Varga-Szemes, M. V. Spampinato; Charleston, SC/US
*(adrienntoth706@gmail.com)*

**Purpose:** Ultra-high-resolution (UHR) photon-counting detector (PCD)-CT angiography with sharp neurovascular kernels (Hv72) and high-level quantum iterative reconstruction (QIR-4) yields excellent intracranial image quality but often suffers from elevated image noise in the lower neck. This study evaluated the added value of a post-hoc convolutional neural network (CNN) denoising algorithm on quantitative image quality metrics in head and neck UHR PCD-CTA.
**Methods or Background:** In this retrospective study, head and neck UHR PCD-CTA datasets were reconstructed using a sharp neurvascular kernel (Hv72) and iterative reconstruction (QIR-4), with and without post-hoc CNN–denoising. Image noise, intraluminal attenuation, signal-to-noise ratio (SNR), and contrast-to-noise ratio (CNR) were measured at the ’lower–neck’ (CCA and vertebral artery segment V1), ’mid–neck’ (carotid bifurcation and V2), ’intracranially’ (ICA and V4), and compared using paired t-test.
**Results or Findings:** 25 consecutive patients were enrolled. CNN-denoising reduced image noise at all vascular levels ($p<0.001$), with the largest absolute reduction in the ’lower-neck’ (–28.6 HU; 43.3%, $p<0.001$). Mean intraluminal attenuation was preserved across all vascular levels with only minimal differences (<3 HU), and signal variability (SD) remained unchanged. SNR and CNR improved significantly throughout the vasculature. In the ’lower-neck’, CNR increased by 79.4% (6.3 ± 5.8 vs 11.3 ± 9.2; $p<0.001$). In the ’mid-neck’, SNR and CNR increased from 35.8 ± 11.3 to 74.8 ± 72.5 ($p<0.001$). ’Intracranially’, SNR rose from 27.8 ± 6.4 to 51.4 ± 11.6 and CNR from 25.1 ± 6.3 to 46.5 ± 11.2 ($p<0.001$).
**Conclusion:** CNN–denoising selectively suppressed image noise without altering vessel attenuation, providing SNR and CNR improvements across all vascular levels. Improvements were most pronounced in the mid neck, while intracranial regions demonstrated stable and reproducible enhancement. While lower neck values improved, variability remained higher due to artifact-prone anatomy.
**Limitations:** No limitations.
**Funding for this study:** No funding was received for this study.
**Has your study been approved by an ethics committee?** Yes
**Ethics committee - additional information:** The study was approved by the Institutional Review Board (Pro00142731).
**Author Disclosures:**
Muhammad Taha Hagar: Nothing to disclose
Milán Vecsey-Nagy: Nothing to disclose
Matthew Silbergleit: Nothing to disclose
Jennifer Yongjoo Cho: Nothing to disclose
Adrienn Toth: Nothing to disclose
Akos Varga-Szemes: Nothing to disclose
M. Vittoria Spampinato: Nothing to disclose

**Put an eye on the brachial plexs: initial experience with Photon Counting CT**
G. D'Anna, *S. Barazzetta*, M. Alì, A. Licata, M. Gerli, F. Darvizeh, L. Di Palma, I. Castiglioni, D. Fazzini; Milan/IT
*(gennaro.danna@gmail.com)*

**Purpose:** To evaluate the possibility to detect brachial plexus on Total Body PCCT. We evaluated post-contrast images looking for the divisions and relations with surrounding structures.
**Methods or Background:** We retrospectively analyzed the first 11 patients scanned at our Institution with photon-counting CT (Siemens Naetom Alpha), comparing similar patients evaluated with our other scanner (Siemens Magnetom Flash). We established as “well-visible” a case with good delineation of roots and trunks, “medium visible” a case with weak delineation of roots and trunks, “poor visible” a case with poor delineation of the structures.
**Results or Findings:** In the reprocessed images at 70 keV, based on the acquisition made with Photon-Counting technology scanner, out of a total of 11 patients the brachial plexus anatomy is clearly visible in 4 patients (36.36%). In 5 patients it is moderately visible (45.45%) while in 2 cases (18.18%) it is not possible to discern the anatomical structures reliably. In images reprocessed at 140 keV brachial plexus anatomy is clearly visible in 3 patients (27.27%). In 3 patients it is moderately visible (27.27%) while in 5 cases (45.45%) it is not possible to discern the anatomical structures reliably. Aat 70 keV (No. 4), only No. 3 is also clearly visible in the reworks at 140 keV. (75%). Cases with average brachial plexus anatomy visible at 70 keV are No. 5, while at 140 keV they turn out to be No. 3 (60%). All the cases evaluated on Somatom Flash showed poor delineation of brachial plexus.
**Conclusion:** PCCT may evaluate anatomical structures of brachial plexus, allowing more precise and effective diagnosis and patient management.
**Limitations:** Small sample size
**Funding for this study:** None
**Has your study been approved by an ethics committee?** Not applicable
**Author Disclosures:**
Gennaro D'Anna: Nothing to disclose
Antonino Licata: Nothing to disclose
Isabella Castiglioni: Nothing to disclose
Stefano Barazzetta: Nothing to disclose
Deborah Fazzini: Nothing to disclose
Marco Alì: Nothing to disclose
Marco Gerli: Nothing to disclose
Luca Di Palma: Nothing to disclose
Fatemeh Darvizeh: Nothing to disclose

**3D Volume Rendering of Ultra-High-Resolution Photon-Counting CTA: A Useful Tool for Neurosurgical Evaluation of Intracranial Aneurysms**
*A. Toth*, S. Al Kasab, K. Kicielinski, M. Silbergleit, J. Y. Cho, M. T. Hagar, M. Vecsey-Nagy, A. Varga-Szemes, M. V. Spampinato; Charleston, SC/US
*(adrienntoth706@gmail.com)*

**Purpose:** Three-dimensional (3D) volume rendering (VR) reconstructions offer comprehensive illustration of anatomy and pathology. We hypothesized that combining the high spatial resolution of ultra-high-resolution (UHR) photon-counting detector (PCD)-CTA with 3D VR post-processing could provide significant benefits for the neurosurgical evaluation and management of intracranial aneurysms.
**Methods or Background:** In this IRB-approved retrospective study, consecutive patients with diagnosed intracranial aneurysms were included. Image acquisition was performed using a clinical PCD-CT scanner in UHR mode. Two neurosurgeons independently evaluated both conventional 2D images and 3D VR reconstructions. A 13-item questionnaire was used to assess image quality, aneurysm characterization, and the clinical usefulness of the 3D VR datasets. Aneurysm neck and dome dimensions were measured on both 2D source images and 3D VR reconstructions to determine inter-reader agreement.
**Results or Findings:** 21 patients with 32 aneurysms were enrolled. Image quality and clinical usefulness of 3D VR images were rated as excellent, with percentage agreement ranging from 58% to 100%, primarily differing between ratings of 4 and 5. Inter-reader reliability for aneurysm size measurements on 3D VR reconstructions was good for dome size (ICC=0.74) and moderate for neck size (ICC=0.55). Bland–Altman analysis showed measurement differences were generally minor, with a few larger discrepancies for bigger aneurysms.
**Conclusion:** UHR CTA-based 3D VR reconstructions provided high subjective image quality, allowing for the precise depiction of aneurysm morphology and reproducible size measurements with good inter-reader agreement. UHR CTA-based 3D VR reconstructions may complement or potentially replace confirmatory invasive angiography in selected cases by providing reliable morphological information, streamlining preoperative workflows, and supporting surgical or endovascular planning.

**Limitations:** Digital subtraction angiography (DSA), MR angiography, or surgical records were not used as reference standard, precluding direct confirmation of aneurysm size and morphology.
**Funding for this study:** No funding was received for this study.
**Has your study been approved by an ethics committee?** Yes
**Ethics committee - additional information:** The study was approved by the Institutional Review Board (Pro00142731).
**Author Disclosures:**
Muhammad Taha Hagar: Nothing to disclose
Milán Vecsey-Nagy: Nothing to disclose
Kimberly Kicielinski: Nothing to disclose
Sami Al Kasab: Nothing to disclose
Matthew Silbergleit: Nothing to disclose
Jennifer Yongjoo Cho: Nothing to disclose
Adrienn Toth: Nothing to disclose
Akos Varga-Szemes: Nothing to disclose
M. Vittoria Spampinato: Nothing to disclose

**Advances in glioblastoma and various grade glioma using Photon-Counting CT**
*I. Conti*, G. De Paula, D. Turilli, L. Piscopo, E. Solinas, L. M. Fattacciu, M. Pedde, M. Scaglione, S. A. Masala; Sassari/IT
*(ilaria0593@gmail.com)*

**Purpose:** This study aims to develop autonomous Generative AI agents capable of integrating entropy maps derived from Photon-Counting Computed Tomography (PCCT), magnetic resonance imaging (MRI), and, when available, histopathological data, to enable personalized diagnostic profiling of brain tumors (specifically glioblastomas and high-grade gliomas) thereby facilitating advanced preoperative planning and intraoperative guidance.
**Methods or Background:** A prospective analysis was conducted on 25 patients affected by glioblastoma or various grade glioma who underwent PCCT brain imaging at our Radiology Department. A dedicated angiographic protocol was applied to assess perilesional vascularization, in addition to generating iodine concentration maps and entropy maps for lesion characterization and perfusion analysis. Imaging datasets underwent radiomic feature extraction and were co-registered with multiparametric MRI, including MR spectroscopy and, when available, with histopathological results.
**Results or Findings:** This study demonstrated that the use of PCCT provides enhanced spatial resolution and allows evaluation of post-treatment tumor activity using entropy and iodine maps, while achieving significant reduction in both radiation dose and intravenous contrast medium volume. integrating traditional AI with advanced radiological imaging significantly enhances the quality of care for patients with glioblastoma and high-grade gliomas. The use of multimodal imaging and AI-driven interpretation enables advanced diagnosis and personalized treatment planning, resulting in increased survival rates, fewer re-interventions, and overall cost-effectiveness.
**Conclusion:** Since clinicians often struggle to integrate complex, heterogeneous, and large-scale data into a comprehensive view of oncologic patient management, the assistance of an autonomous Generative AI agents play a pivotal role to fully leverage all available data.
**Limitations:** No limitations were identified.
**Funding for this study:** No funding was received for this study
**Has your study been approved by an ethics committee?** Not applicable
**Ethics committee - additional information:** The study is educational and is under ethical committee evaluation
**Author Disclosures:**
Davide Turilli: Nothing to disclose
Liala Mirella Fattacciu: Nothing to disclose
Ilaria Conti: Nothing to disclose
Mariano Scaglione: Nothing to disclose
Giovanni De Paula: Nothing to disclose
Salvatore Masala: Nothing to disclose
Leandra Piscopo: Nothing to disclose
Emma Solinas: Nothing to disclose
Milena Pedde: Nothing to disclose

**08:00 - 09:00** **Room G1**

### Research Presentation Session: Radiographers

### RPS 2214

### Radiographers' roles in angiography and interventional procedures

**Moderators**
Irene Nieri; Paris/FR
*(irene.n@hotmail.it)*
Ruediger Schernthaner; Vienna/AT
*(ruediger.schernthaner@gesundheitsverbund.at)*

**Establishing Diagnostic Reference Levels (DRLs) for Neurointerventional Procedures in Malta**
*M. Grech*, F. Zarb, R. Grech, P. Bezzina; Msida/MT
*(marvin.grech@gov.mt)*

**Purpose:** Interventional neuroradiology procedures are vital for managing neurological conditions but expose patients to ionising radiation. The aim of the study was to assess the level of radiation doses during diagnostic and therapeutic Neurointerventional procedures in Malta and to establish and compare local Dose Reference Levels (DRLs) with international standards.
**Methods or Background:** Retrospective data were collected from DoseWatch for the period January 2020 to May 2025, focusing on Air Kerma Area Product (PKA), Reference Air Kerma (RAK), and Fluoroscopy Time (FT) for the following procedures: Cerebral Angiography (CA), Stroke Thrombectomy (ST), Aneurysm Coiling (AC), and AVM/AVF embolization. DRLs were calculated as the 3rd quartile (75th percentile) of the dose distributions.
**Results or Findings:** In Malta, the 3rd quartile values for CA were considerably lower than those reported in the literature, with PKA at 58 Gycm², RAK at 330 mGy, and FT at 9 minutes. ST revealed radiation doses approximately half of those reported in the literature (PKA: 70 Gycm²; RAK: 372 mGy; FT: 23 minutes). For AC, the doses were comparable to international standards (PKA: 182 Gycm²; RAK: 3384 mGy; FT: 45 minutes). Conversely, radiation doses for AVM/AVF embolisation were considerably higher (PKA: 1463 Gycm²; RAK: 7900 mGy; FT: 168 minutes).
**Conclusion:** The findings emphasise the importance of understanding radiation exposure to enhance patient safety. Establishing local DRLs will provide benchmarks for practitioners in Malta, while enabling continuous monitoring and development of optimisation strategies to mitigate risks associated with ionising radiation.
**Limitations:** This was a single center study establishing local DRLs and comparing these to literature findings. The center where study took place is however the only center providing neurological services in Malta.
**Funding for this study:** No funding required
**Has your study been approved by an ethics committee?** Yes
**Ethics committee - additional information:** Ethical approval was sought and obtained from the University of Malta Research and Ethics Committee (UREC): (FHS-2024-00575)
**Author Disclosures:**
Francis Zarb: Nothing to disclose
Paul Bezzina: Nothing to disclose
Reuben Grech: Nothing to disclose
Marvin Grech: Nothing to disclose

**Saving lives and preventing harm in Jersey: Radiographer-Led Nasogastric Tube Position Check Pathway Pilot**
*G. Cain*[1], N. Aslam[1], D. J. M. Tolan[2], G. Roe[2], L. Horder[3], A. Gill[4];
[1]St. Helier, Jersey (Channel Islands)/UK, [2]Leeds/UK, [3]London/UK, [4]Oxford/UK

**Purpose:** To evaluate the safety, timeliness, and accuracy of a radiographer-led nasogastric (NG) tube position check pathway pre- and post-implementation.
**Methods or Background:** A collaborative initiative involving professional bodies, including the Society and College of Radiographers and the Royal College of Radiologists, launched a national radiographer-led NG tube pathway. This empowers radiographers to assess NG tube placement on chest X-rays and take corrective action if tubes are misplaced. It's a response to repeated safety alerts related to the inadvertent use of misplaced NG tubes—classified as 'Never Events' by the NHS. On 30th June 2025, Jersey General Hospital became the first pilot site in the UK and Channel Islands to implement the pathway. A retrospective audit compared the first 12 weeks post-implementation (radiographers performing first-line placement checks) with a pre-implementation period (radiologist-only X-ray reporting). Metrics assessed included time from image acquisition to clinical evaluation, misplacement detection rate, corrective action on misplaced tubes, and concordance between radiographer evaluations and the definitive radiology report. Radiographers completed a national competency-based e-learning programme supported by governance and a Standard Operating Procedure.
**Results or Findings:** Median turnaround time for clinical evaluation was reduced from 242.2 hours (10.1 days) to 12 minutes post-implementation. Radiographers detected 100% of misplaced NG tubes (12/12) and took corrective action on all misplaced tubes identified in the department (6/6). Concordance with the definitive radiology report was 96.8% (60/62).
**Conclusion:** The radiographer-led pathway is safe and effective, significantly reducing delays and minimising the risk of Never Events. The Jersey pilot reinforces evidence that with appropriate training and governance, radiographers can enhance NG tube safety through assessment and action at the point of image acquisition.
**Limitations:** Small sample size; focused on radiographer accuracy rather than patient outcome.
**Funding for this study:** No
**Has your study been approved by an ethics committee?** Not applicable
**Author Disclosures:**
Noreen Aslam: Nothing to disclose
Damian John Michael Tolan: Nothing to disclose
Avneet Gill: Nothing to disclose
Gavin Cain: Nothing to disclose
Gillian Roe: Nothing to disclose
Lucy Horder: Nothing to disclose

**Enhanced Arterial Imaging with Reduced Contrast Load: A Longitudinal Study of Weight-Based CTA Protocol Implementation**
J. T. d. S. d. Castro, *H. d. J. Ferreira*, D. C. Novais da Silva, D. Yamada, S. San Juan Dertkigil, F. Reis; Campinas - Sao Paulo/BR
*(horjesus@unicamp.br)*

**Purpose:** Weight-based personalisation of contrast media in CTA can maintain or increase arterial enhancement while reducing contrast load; longitudinal real-world evidence remains scarce. The aim is to quantify the impact of weight-stratified CTA protocols on arterial enhancement and iodine use in a real-world setting.
**Methods or Background:** Single-centre retrospective longitudinal study within ANGIO-MONITOR. Consecutive angiographic CTAs were analysed across two periods: pre-implementation (fixed protocols; N=1,626) and post-implementation (from 01-Jun-2025; weight-based volumes/flows and PSI ceilings; N=558). Primary endpoint: arterial attenuation (HU) in the target vessel per exam. Secondary endpoints: proportion of “optimal” studies (≥350 HU), contrast volume (mL/exam; mL/kg), and repeat-acquisition rate. Statistical tests: Welch’s test for means, proportion comparisons with 95% CIs; exam-level analyses with temporal stratification.
**Results or Findings:** Arterial enhancement increased from 315.5 HU (95% CI 311.1–319.9) to 484.4 HU (469.8–499.1); $p=7.1\times10^{-79}$. Mean contrast volume decreased from 79.5 mL (78.3–80.7) to 67.9 mL (65.8–69.9); $p=1.1\times10^{-20}$. mL/kg fell from 1.09 (1.07–1.11) to 0.91 (0.88–0.94); $p=5.8\times10^{-25}$. The proportion of optimal studies rose from 30.0% to 76.5% (absolute Δ +46.6 pp; RR 2.55; $p<10^{-16}$). Repeat-acquisition rates were low and similar (1.91% vs 2.15%; p=0.72). Effects were observed across angiographic indications in routine operations.
**Conclusion:** Weight-based CTA protocols, implemented under technologist-led governance and injector/CT synchronisation, substantially increased arterial enhancement while reducing contrast exposure in real-world practice. Findings support institutional scale-up, underpin iodine stewardship and cost savings, and provide a foundation for indication-specific analyses and prospective predictive modelling.
**Limitations:** The study is a single-center retrospective analysis, which may limit the generalizability of the findings.
**Funding for this study:** Not Applicable
**Has your study been approved by an ethics committee?** Yes
**Ethics committee - additional information:** The work has been submitted to the university's Research Ethics Committee (CEP) and is currently under review for evaluation and final opinion.
**Author Disclosures:**
Hortência De Jesus Ferreira: Nothing to disclose
Fabiano Reis: Nothing to disclose
José Thiago De Souza De Castro: Nothing to disclose
Danielle Cristina Novais Da Silva: Nothing to disclose
Sergio San Juan Dertkigil: Nothing to disclose
Daniel Akio Yamada: Nothing to disclose

**Predictive Modeling and Image Quality Metrics for Dose Reduction in CT-Guided Liver Ablation**
*T. Antheunis*[1], M. Hakkert[2], B. Vermolen[1]; [1]Ede/NL, [2]Haarlem/NL
*(tim.antheunis@gmail.com)*

**Purpose:** CT-guided liver ablation is a minimally invasive procedure associated with substantial radiation exposure for both patients and staff. Current protocols are not optimized, resulting in unnecessary dose. This study aimed to achieve dose reduction through protocol optimization, validated by a predictive model correcting for clinical factors.
**Methods or Background:** A single-center interventional study was conducted at Ziekenhuis Gelderse Vallei. Group A (n=76) underwent ablation before protocol adjustment; Group B (n=7) after implementation. A linear regression model was developed from Group A to predict dose-length product (DLP) based on significant clinical factors (patient size, number of lesions). In parallel, a phantom study evaluated the impact of slice thickness, tube current, and reconstruction technique (hybrid iterative reconstruction vs. model based reconstruction) on task-based image quality using the detectability index (d’). The optimized protocol was applied to Group B and validated against model predictions.
**Results or Findings:** Patient size (r=0.76; p<0.001) and number of lesions (p=0.016) were the strongest dose determinants. The model explained 59% of DLP variance (RMSE=532 mGy·cm). Phantom analysis showed model based reconstructions maintained higher d’ at low dose compared to iDose (at 2 mGy: IMR=4.71 vs. iDose=1.66). The new protocol reduced CTDIvol for spiral scans to 2–4 mGy and differentiated between planning and control scans. Group B achieved a 40–70% DLP reduction versus predicted values, with clinically acceptable image quality.
**Conclusion:** Task-based image quality analysis combined with predictive modeling enables substantial radiation dose reduction without compromising diagnostic utility. The integration of IMR, reduced tube current, and adjusted slice thickness was key. This approach provides a robust framework for task-specific CT protocol optimization and is transferable to other interventional procedures.
**Limitations:** The limitations of the study are single centre study and
**Funding for this study:** No funding was received for this study.
**Has your study been approved by an ethics committee?** Not applicable
**Author Disclosures:**
Bart Vermolen: Nothing to disclose
Tim Antheunis: Nothing to disclose
Marcel Hakkert: Nothing to disclose

**Expanding radiographers’ knowledge and skills through Virtual Reality (VR): A bridging role in preoperative assessment. A pilot experience**
*A. Masperi*, G. Di Stefano, C. Gallotta, M. Vertemati; Milan/IT
*(andrea.masperi1992@gmail.com)*

**Purpose:** To evaluate the value of 3D CT reconstructions visualised in VR for preoperative planning in paediatric and adult thoracic surgery, and to assess through a pilot study the role of radiographers as key professionals in the workflow, from image acquisition to VR scenario creation and clinical collaboration.
**Methods or Background:** We conducted a feasibility study including eight patients, five paediatric and three adults, selected in collaboration with the surgical team to represent different anatomical and pathological scenarios. CT datasets were processed into patient-specific 3D models and integrated into immersive VR environments. The workflow included: 1) segmentation with 3D Slicer, 2) rendering optimisation in Blender, 3) VR development in Unity, and 4) deployment on Meta Quest 3. Models were validated with surgeons through a mixed assessment: a seven-item Likert survey (1–5) and open-ended feedback on anatomical accuracy and surgical planning.
**Results or Findings:** In paediatric cases, VR improved vascular and bronchial assessment, supporting thoracoscopic or conservative strategies. In adults, it enhanced anatomical understanding and team communication, with limited impact on surgical choices. All models scored highly (4–5/5) for accuracy and clinical value.
**Conclusion:** Radiographers demonstrated the ability to manage the full workflow from imaging to VR, enhancing data optimisation, 3D modelling, and teamwork to support surgical planning. By streamlining processes and reinforcing clinical decision-making, they establish themselves as key professionals in precision surgery.
**Limitations:** This study involved a small and heterogeneous cohort without a control group and relied on qualitative data. Model accuracy depended on CT quality, while the VR workflow required specific skills that were progressively acquired during the process. Nevertheless, it represents the first Italian experience of VR-based surgical planning led by radiographers.
**Funding for this study:** This study is based on the ongoing agreement for the provision of virtual 3D models between PRINTMED-3D (Integrated Platform for Three-Dimensional Medical Technologies), awarded in 2019 under the “Call Hub Research and Innovation” program by Regione Lombardia, supported within the MUSA (Multilayered Urban Sustainability Action) project funded in

2022 by the National Recovery and Resilience Plan (PNRR), and the Vittore Buzzi Children's Hospital, part of ASST FBF-Sacco.
**Has your study been approved by an ethics committee?** Not applicable
**Author Disclosures:**
Maurizio Vertemati: Nothing to disclose
Gianvincenzo Di Stefano: Nothing to disclose
Cristina Gallotta: Nothing to disclose
Andrea Masperi: Nothing to disclose

**Evaluation of Needle Artefact Reduction Techniques in CT-Guided Biopsy Imaging**
S. Toomey, B. Normoyle, A. England, *C. Rainey*, N. Moore, L. McLaughlin, M. F. Mcentee, M. Maher; Cork/IE
*(clare.rainey@ucc.ie)*

**Purpose:** CT-guided lung biopsy plays an important role in diagnosing malignant lung lesions. However, the presence of biopsy needle containing metal components introduces unwanted streak artefact that can obscure needle tip visualisation. This study aims to evaluate the impact of various protocols and needle size on axial streak artefacts.
**Methods or Background:** Two needles 18G and 20G with and without stylets were imaged in a watermelon using protocols at 70,80,100,120 kV with multiple reconstruction techniques including ASIR, DLIR and MAR. Signal-to-noise ratio (SNR) and contrast-to-noise ratio (CNR) were calculated across multiple ROIs to objectively compare image quality. Line plot profiles were used to calculate the artefact width for each protocol.
**Results or Findings:** The Interventional Protocol produced the highest SNR values for 18G and 20G biopsy needle. In line plot profile analysis, less streak artefact from the biopsy needle was produced in the Interventional Protocol. In the 20G smaller gauge needle, produced less streak artefact with the stylet out compared to the 18G. The higher tube voltages particularly in 100 and 120 kV reduced streak artefact for both biopsy needles.
**Conclusion:** The Interventional Protocol demonstrated consistent metal artefact reduction generated by 18G and 20G biopsy needle. This reduction may lead to a better depiction of the needle tip inside the nodule of interest.
**Limitations:** Study acquisitions were based on imaging a watermelon, watermelons have been used in similar experiments but are not necessarily a valid substitute for humans,.
**Funding for this study:** None
**Has your study been approved by an ethics committee?** Yes
**Ethics committee - additional information:** Medical School Social Research Ethics Committee - University College Cork
**Author Disclosures:**
Mark F. Mcentee: Nothing to disclose
Niamh Moore: Nothing to disclose
Clare Rainey: Nothing to disclose
Siobhan Toomey: Nothing to disclose
Laura Mclaughlin: Nothing to disclose
Brid Normoyle: Nothing to disclose
Andrew England: Nothing to disclose
Michael Maher: Nothing to disclose

**Shaping Radiography Education: Radiographers Designing Modular 3D-Printed Angiographic Simulators**
*A. Masperi*, F. Cavaliere, S. Boniardi, M. Vertemati, F. Pezzotta; Milan/IT
*(andrea.masperi1992@gmail.com)*

**Purpose:** To pioneer a shift in radiographer education by moving beyond traditional lectures toward immersive, practice-based learning through simulation. Within the specialized field of interventional and angiographic radiology, radiography students conceived and built a modular 3D-printed angiographic simulator.
**Methods or Background:** A modular 3D-printed angiographic simulator was developed from angio-CT datasets extending from femoral to supra-aortic arteries. The workflow included: 1) image segmentation with 3D Slicer, 2) mesh refinement in Meshmixer, 3) modeling and support design with Fusion, 4) resin-based additive printing of vascular structures, and 5) post-processing and casting to assemble the simulator. Validation was conducted by radiographers and interventional radiologists using a 5-point Likert scale to assess anatomical fidelity and suitability for practice.
**Results or Findings:** Five radiographers and three interventional radiologists evaluated the 1:1 scale simulator, reporting high anatomical fidelity (median rating ≥4/5) and strong suitability for practicing catheter navigation and angiographic techniques, highlighting its potential as a safe and reproducible training tool.
**Conclusion:** This project established an innovative educational paradigm in interventional radiology through the creation of a modular 3D-printed angiographic simulator, designed by radiographers for radiographers. Beyond enhancing technical competence, critical reasoning, and professional autonomy, the simulator represents a scalable and reproducible model that can transform radiographer education internationally, fostering a new generation of professionals equipped for the evolving challenges of interventional practice.
**Limitations:** This study has some limitations. High production and assembly costs may hinder broader implementation, while radiographers' skills in 3D printing and post-processing are still developing. Moreover, the simulator has not yet undergone structured testing with students, and its educational effectiveness requires further validation.
**Funding for this study:** None
**Has your study been approved by an ethics committee?** Not applicable
**Author Disclosures:**
Maurizio Vertemati: Nothing to disclose
Federico Pezzotta: Nothing to disclose
Samuele Boniardi: Nothing to disclose
Francesco Cavaliere: Nothing to disclose
Andrea Masperi: Nothing to disclose

**Radiation Dose Monitoring in Interventional Cardiology: Patient, Occupational, and Pediatric Perspectives from Latin America**
*M. V. L. Oliveira*[1], J. Ferreira[1], I. Modesto[1], E. Matos[1], D. Andrade[1], L. Santana[1], C. Ubeda[2], M. Navarro[1]; [1]Salvador/BR, [2]Arica/CL
*(marcusradiology@gmail.com)*

**Purpose:** To review and synthesize recent Latin American research strategies on dose monitoring in fluoroscopy-guided and cardiology procedures, highlighting patient, occupational, and quality-assessment perspectives.
**Methods or Background:** A PubMed search was conducted using the terms Diagnostic reference level OR Radiation dose monitoring AND (fluoroscopy OR cardiology OR fluoroscopy guided interventional procedures) AND ("Latin America" OR Brazil OR Argentina OR Chile OR Colombia OR Peru OR Venezuela OR Ecuador OR Uruguay OR Paraguay OR Bolivia) for studies published between 2010and 2025. A thematic synthesis was applied to group studies into patient monitoring, risk assessment, pediatric DRLs, and occupational exposure.
**Results or Findings:** Twenty-two articles were selected. Brazilian institutions reported comprehensive patient dose registries, with one multicenter database covering over 10,000 cardiology procedures and identifying patients reaching substantial radiation dose levels (SRDLs), triggering dermatological follow-up. The MARP (Modelo de Avaliação de Risco Potencial) model compiled more than 200,000 records across 121 institutions, providing structured indicators for quality evaluation. Pediatric studies proposed initial DRLs using age- and weight-based criteria, addressing a critical gap in radiation protection for children. Occupational assessments revealed lens doses averaging 25–47 μSv/procedure, with estimated annual exposures of 6–11 mSv, and frequent extremity overexposures due to hand positioning.
**Conclusion:** Brazil's initiatives illustrate the feasibility of structured dose monitoring and DRL implementation in interventional cardiology. Expanding these models through regional collaboration, standardized protocols, and occupational protection strategies is essential to strengthening radiation safety across Latin America.
**Limitations:** Coverage across Latin America remains limited, and gaps persist in multinational registries, prospective interventions, and long-term patient outcomes.
**Funding for this study:** Not applicable
**Has your study been approved by an ethics committee?** Not applicable
**Author Disclosures:**
Eric Matos: Nothing to disclose
Lara Santana: Nothing to disclose
Marcus Navarro: Nothing to disclose
Carlos Ubeda: Nothing to disclose
Igor Modesto: Nothing to disclose
Daniele Andrade: Nothing to disclose
Marcus Vinicius Linhares Oliveira: Nothing to disclose
Jeovana Ferreira: Nothing to disclose

**Personalized Weight-Tiered Protocols in Angiotomography: The Synergy Between Canon Prime SP and Stellant SmartFlow Injector in Improving Diagnostic Quality**
*J. T. d. S. d. Castro*, D. C. Novais da Silva, H. d. J. Ferreira, D. Yamada, S. San Juan Dertkigil, F. Reis; Campinas/BR
*(jthiago@unicamp.br)*

**Purpose:** Diagnostic quality in angiotomography relies on adequate vascular opacification (arterial Hounsfield Units, HU). Even with advanced technologies, standardized protocols ignoring patient weight often lead to suboptimal contrast. A study at HC-UNICAMP with 1,082 CTAs confirmed significant influence of weight, venous access type/caliber, contrast flow on arterial density. These findings informed personalized, weight-tiered protocols starting June 1, 2025.

**Methods or Background:** Retrospective analysis included 1,082 CTA exams performed with a Canon Prime SP 80-channel CT, Stellant SmartFlow injector (Bayer), and 300 mg/mL iodinated contrast. Variables collected included weight, age, venous access type/caliber, contrast flow, and arterial HU. Exams were quality-classified per ACR criteria: <250 HU (poor), 251–349 HU (average), and ≥350 HU (excellent). Following this analysis, new personalized weight-tiered protocols were implemented from June 2025, adjusting kVp, flow, contrast volume, and dose modulation for specific weight ranges (e.g., PE protocol for weights up to >100kg), with tailored parameters for each.
**Results or Findings:** Preliminary data shows significant improvement in contrast opacification: the percentage of exams >350 HU increased from 30% to 70%. An approximate 15% reduction in effective radiation dose was also observed. These are partial results from over 140 exams performed up to June 25, 2025, indicating that the personalized angiotomography protocols are extremely promising.
**Conclusion:** The implementation of personalized weight-tiered protocols in angiotomography at HC-UNICAMP marks a data-driven shift, optimizing diagnostic quality while reducing radiation dose and contrast volume. The synergy between the Canon Prime SP CT and Stellant SmartFlow injector, coupled with the radiology team's input, was crucial. This project serves as a model for other institutions, with prospective monitoring and full results to be presented at ESR2026.
**Limitations:** Results are being updated according to the new protocol's implementation, with ongoing data collection.
**Funding for this study:** Not applicable.
**Has your study been approved by an ethics committee?** Yes
**Ethics committee - additional information:** The submission to the Ethics Committee/IRB is currently under review
**Author Disclosures:**
Hortência De Jesus Ferreira: Nothing to disclose
Fabiano Reis: Nothing to disclose
José Thiago De Souza De Castro: Nothing to disclose
Danielle Cristina Novais Da Silva: Nothing to disclose
Sergio San Juan Dertkigil: Nothing to disclose
Daniel Akio Yamada: Nothing to disclose

**08:00 - 09:00** **Research Stage 4**

### Research Presentation Session: Vascular

### RPS 2215

### Treatment planning and surveillance in vascular imaging

**Moderator**
Judit Csőre; Budapest/HU

**Preoperative Contrast-Enhanced Magnetic Resonance Angiography for Perforator Mapping in Free Flap Reconstruction after Oncological Head and Neck Surgery: Protocol Optimization and Clinical Feasibility**

*F. De Geer*[1], L. C. ter Beek[1], A. Te Boekhorst[1], B. Plakke[1], L. Karssemakers[1], R. Dirven[1], F. Siepel[2], M. Van Alphen[1], W. Schreuder[1]; [1]Amsterdam/NL, [2]Enschede/NL
*(a.d.geer@nki.nl)*

**Purpose:** Large head and neck defects following oncological resection are often reconstructed using free vascularized tissue flaps such as the anterolateral thigh flap (ALT) and fibula free flap (FFF). These flaps depend on small perforator vessels for perfusion. Preoperative Magnetic Resonance Angiography (MRA) could offer detailed insights into perforator course, caliber, origin and main pedicle length and could aid in successful surgical flap design and elevation. This study presents an optimized MRA protocol for head and neck cancer patients scheduled for oncological resection with ALT or FFF reconstruction.
**Methods or Background:** Using a 3.0-T Philips MRI system (dStream Achieva), T1-weighted 3D gradient echo DIXON sequences are acquired: pre-contrast, arterial, and early venous phases after gadolinium-based contrast injection. Scans are fluoroscopically triggered with centra k-space acquisition initiated two seconds post-trigger. Post-contrast axial slices are analyzed for perforator identification. Vascular structures and surrounding anatomy are segmented and rendered into 3D models. Based on the MRA-based 3D models, a surgical plan is created and translated into the operating room with 3D printed perforator marking guides.
**Results or Findings:** Nineteen patients underwent surgery using this protocol. In all cases, intraoperative perforators matched those visualized on MRA. Seventeen surgeries followed the preoperative plan; two required intraoperative adjustments. The mean System Usability Score (SUS) for the 3D models as evaluated by the operating surgeons was 73.7 (SD 9.0), indicating the clinical utility of this method.
**Conclusion:** Preoperative MRA combined with 3D modeling enables accurate perforator mapping and enhances surgical planning for head and neck reconstruction using lower extremity perforator flaps.
**Limitations:** The current protocol requires gadolinium-based contrast agents, though MRI offers potential for non-contrast-enhanced vascular imaging. Research in our institute is ongoing to optimize such sequences and preliminary results will be presented at ECR.
**Funding for this study:** Not applicable.
**Has your study been approved by an ethics committee?** Yes
**Ethics committee - additional information:** Approved by Institutional Review Board from Netherlands Cancer Institute - Antoni van Leeuwenhoek with registration number IRBdm22-042
**Author Disclosures:**
Willem Schreuder: Nothing to disclose
Luc Karssemakers: Nothing to disclose
Fleur De Geer: Nothing to disclose
Brenda Plakke: Nothing to disclose
Arjan Te Boekhorst: Nothing to disclose
Richard Dirven: Nothing to disclose
Francoise Siepel: Nothing to disclose
Maarten Van Alphen: Nothing to disclose
Leon C. Ter Beek: Nothing to disclose

**Unique aortic remodeling and device-related complications after PETTICOAT endovascular repair**

*N. Kerschbaumer*[1], A. Fereydooni[1], D. Vigneault[1], K. Bäumler[1], G. Mistelbauer[1], D. Mastrodicasa[2], E. George[1], D. Fleischmann[1]; [1]Stanford, CA/US, [2]Seattle, WA/US

**Purpose:** To identify anatomic and device-related factors associated with aortic remodeling and complications following PETTICOAT(Provisional-Extension-To-Induce-Complete-Attachment).
**Methods or Background:** The concept of scaffolding the aortic true lumen (TL) with bare-stent distal to standard TEVAR has become valuable in dissection treatment. However, anatomical and technical factors associated with treatment outcomes have not been established. In this observational, single-center study two cardiovascular radiologists reviewed a retrospective cohort of 64 patients with complicated type-B-dissection or residual descending dissection after type-A-repair, treated with distal bare-stent extension. Available pre-/postoperative and follow-up (median 15 months) CT-angiograms were assessed.
**Results or Findings:** We identified four distinct, PETTICOAT-specific phenomena: First, small contrast jets into the false lumen (FL) through dissection flap fenestrations corresponding to intercostal arteries were observed at several bare-stent levels, typically thrombosing throughout follow-up. Second, in contrast to covered endografts, the aortic TL can fully re-expand, even beyond the diameter of the bare-stent, with gradual complete absorption of the FL thrombus (n=1). Third, we observed new focal contrast outpouchings in the dissection flap separating the TL from the thrombosed FL (proposed term: 'junctional SINE') at the junction between covered and uncovered stents (n=2). These can be explained as intimal erosions caused by protruding bare-stent wires, associated with abrupt caliber changes and 'telescoping' where the bare-stent exits the endograft. Fourth, all cases of bare-stent migration (n=3), were associated with junctional SINEs. Predisposing factors for migration were short device overlaps within angulated aortic segments.
**Conclusion:** Temporary intercostal fenestrations and TL re-expansion beyond device diameter may be part of normal healing. The prognosis of 'junctional SINEs' and their association with aortic angulation and device migration may impact patient selection and device design.
**Limitations:** This study identified unique imaging features, but is underpowered to establish prognostic significance of these observations.
**Funding for this study:** None
**Has your study been approved by an ethics committee?** Yes
**Ethics committee - additional information:** IRB Stanford University, eProtocol #52791
**Author Disclosures:**
Dominik Fleischmann: Nothing to disclose
Kathrin Bäumler: Nothing to disclose
Elizabeth George: Nothing to disclose
Domenico Mastrodicasa: Nothing to disclose
Gabriel Mistelbauer: Nothing to disclose
Davis Vigneault: Nothing to disclose
Nina Kerschbaumer: Nothing to disclose
Arash Fereydooni: Nothing to disclose

**Imaging role in diagnosing and monitoring Takayasu arteritis in pediatric patients**

*S. Santra*, B. P. Lohith Kumar, E. Joseph, A. Irodi, L. Robinson Vimala ; Vellore/IN
*(shreyosisantra@gmail.com)*

**Purpose:** To highlight the significance of radiological imaging in evaluating disease progression and guiding immunomodulation in pediatric patients with Takayasu arteritis. To develop an optimal workflow of radiological techniques to minimise radiation exposure in this vulnerable age group.
**Methods or Background:** A retrospective analysis of fifteen pediatric patients diagnosed with Takayasu arteritis at our institute between 2014 and 2024 was conducted. The study evaluated the role of Computed Tomography (CT) angiography, Magnetic Resonance angiography (MRA), and Positron Emission Tomography-Computed Tomography (PET-CT) in diagnosis and follow-up in pediatric patients with Takayasu arteritis. Radiological findings were compared with clinical presentation and lab parameters- ESR & CRP.
**Results or Findings:** Wall thickening, stenosis, dilation, aneurysm, signs of organ damage, such as a contracted kidney in renal artery stenosis, and collateral vessel formation were the observed imaging features. The majority of patients underwent CT angiography for diagnosis. Dose-limiting techniques such as high pitch, iterative reconstructions, body-adapted protocols, and shielding for thyroid and gonads should be included in the workflow of evaluation of pediatric patients with Takayasu arteritis. The CARDS score can be used to assess the disease severity. On comparing the CARDS score with laboratory parameters –CRP & ESR in follow-up patients, no definitive pattern was found, thus making imaging crucial for disease assessment.
**Conclusion:** Radiological investigations are crucial for evaluating disease activity and progression, as the accuracy of lab parameters for Takayasu arteritis is limited in diagnosis and disease progression. However, due to significant concerns regarding radiation exposure to this age group requiring repeated follow-up examinations, judicious use of radiation and non-radiation-emitting techniques is highly recommended in managing these patients.
**Limitations:** No limitations
**Funding for this study:** No funding
**Has your study been approved by an ethics committee?** Not applicable
**Author Disclosures:**
B. P. Lohith Kumar: Nothing to disclose
Leena Robinson Vimala: Nothing to disclose
Aparna Irodi: Nothing to disclose
Elizabeth Joseph: Nothing to disclose
Shreyosi Santra: Nothing to disclose

**Dual-Energy CT in the Follow-Up of endovascular treated Visceral Aneurysms: Overcoming the Metal Artifact Challenge**

*G. Francavilla*, L. M. Cacioppa, M. Rosati, P. Boscarato, N. Rossini, S. Ielpo, R. Candelari, C. Floridi, A. Giovagnoni; Ancona/IT

**Purpose:** Follow-up of visceral aneurysms treated with metallic coils remains challenging for conventional CT due to metal artifacts that limit evaluation of the aneurysm sac and adjacent vessels. Dual-Energy CT (DECT), through virtual monoenergetic images (VMI) and iodine maps, may improve diagnostic accuracy and reduce the need for confirmatory angiography.
**Methods or Background:** Seventeen patients with true visceral aneurysms (splenic, hepatic, renal, and mesenteric) treated by coil embolization were retrospectively analyzed. All underwent DECT follow-up between 1 and 6 months. High-keV VMI (100–140 keV) were evaluated for artifact reduction and morphological assessment of the aneurysm sac and surrounding tissues, while low-keV VMI (40–60 keV) and iodine maps were used for detection of in/out arterial feeders and residual perfusion. The analysis was performed both qualitatively and quantitatively: qualitative assessment was conducted using a 5-point Likert scale for image quality and diagnostic confidence, while quantitative evaluation included placement of circular regions of interest (ROIs) to measure attenuation values and image noise. Findings were compared with conventional CT reconstructed at 120 kVp and, when available, with angiography as the reference standard.
**Results or Findings:** High-keV VMI significantly reduced coil-related artifacts, improving diagnostic readability ($p < 0.05$). Low-keV VMI increased sensitivity for intravascular contrast, allowing identification of feeders and residual perfusion not visible on conventional CT. Iodine maps confirmed these findings, helping to differentiate true enhancement from artifacts.
**Conclusion:** DECT provides clear advantages over conventional CT in the follow-up of coil-embolized visceral aneurysms. High-keV VMI reduce metallic artifacts, while low-keV VMI and iodine maps enhance detection of feeders and residual flow. DECT may become the reference imaging tool for post-procedural monitoring, improving patient management and reducing the need for angiographic confirmation.
**Limitations:** The limited number of cases
**Funding for this study:** This study received no external funding
**Has your study been approved by an ethics committee?** Not applicable
**Ethics committee - additional information:** Ethical review and approval were waived for this study since all data were anonymized and collected as part of routine clinical follow-up.
**Author Disclosures:**
Marzia Rosati: Nothing to disclose
Chiara Floridi: Nothing to disclose
Sergio Ielpo: Nothing to disclose
Laura Maria Cacioppa: Nothing to disclose
Pietro Boscarato: Nothing to disclose
Roberto Candelari: Nothing to disclose
Giangabriele Francavilla: Nothing to disclose
Andrea Giovagnoni: Nothing to disclose
Nicolo' Rossini: Nothing to disclose

**Analysis of Factors Influencing the Efficacy of Ovarian Vein Embolization for Pelvic Venous Insufficiency and Construction of and Performance Evaluation of a Short-Term Efficacy Prediction Model**

*B. Zheng*; Beijing/CN
*(zhengby125@163.com)*

**Purpose:** The study aims to elucidate the related factors of short-term efficacy of Ovarian Vein Embolization (OVE) in the treatment of pelvic venous insufficiency (PVI), constructs a predictive model for short-term efficacy of OVE, and evaluates its predictive efficacy.
**Methods or Background:** Clinical and ultrasound data were retrospectively collected from female patients with PVI who underwent OVE at Shijitan Hospital. This study was the first to apply the Minimal Clinically Important Difference to define symptomatic improvement in the Pelvic Venous Clinical Severity Score. Symptomatic improvement was the evaluation standard, and factors affecting short-term efficacy were analyzed. The ROC curve assessed predictive efficacy. Internal validation used k-fold cross-validation to evaluate discrimination, calibration, and clinical utility.
**Results or Findings:** (1) The study included 82 patients: 43 in the remission group and 39 in the non-remission group. (2)There were significant differences between the two groups in the duration of abdominal discomfort, the diameters of the left ovarian and parametrial veins, and the presence of internal iliac vein reflux..(3) Logistic regression revealed that longer abdominal discomfort, smaller left ovarian vein diameter, and internal iliac vein reflux were independent risk factors for poor short-term efficacy of OVE. The ROC curve AUC was 0.807, with a best cut-off value of 0.453. Sensitivity and specificity for predicting efficacy were 82.1% and 74.4%. Internal validation showed discrimination (AUC=0.779), a Brier score of 0.176 indicating accuracy, reasonable calibration, and positive net clinical benefit in decision curve analysis.
**Conclusion:** (1) The duration of lower abdominal discomfort, ovarian vein diameter and internal iliac vein reflux are independent predictors of the short-term efficacy of OVE for PVI. (2) The prediction model of short-term efficacy of OVE for PVI in this study has satisfactory validity.
**Limitations:** No external validation
**Funding for this study:** China State Railway Group Co. Ltd (J2023Z604) and Capital's Funds for Health Improvement and Research (2024-2-2085).
**Has your study been approved by an ethics committee?** Yes
**Ethics committee - additional information:** Beijing Shijitan Hospital, Capital Medical University (protocol No. IIT2024-016-003)
**Author Disclosures:**
Binyu Zheng: Nothing to disclose

**PAD-RADS: A Novel Comprehensive Reporting System for Peripheral Artery Disease**

*A-E. Iacobut*[1], P. J. Kettnaker[2], R. W. Huegli[1], D. Bilecen[2]; [1]Bruderholz/CH, [2]Liestal/CH
*(andreea25emilia@gmail.com)*

**Purpose:** To introduce PAD-RADS, a novel, comprehensive Peripheral Artery Disease Reporting and Data System for standardizing computed tomography angiography (CTA) reporting in patients with known peripheral artery disease (PAD).
**Methods or Background:** Despite advances in CTA technology and increasing use for PAD assessment, there is currently no standardized reporting system equivalent to CAD-RADS for coronary CTA. The lack of structured reporting hinders effective communication between interpreting and referring physicians, limits multicenter research, and creates inconsistencies in patient management recommendations. PAD-RADS was developed to address these gaps by categorizing lesion severity, quantifying overall atherosclerotic burden, and incorporating key modifiers relevant for clinical decision making.

**Results or Findings:** PAD-RADS assigns a grade (0–5) based on the most severe arterial lesion per limb, with 0 indicating no stenosis and 5 representing total occlusion. The system is complemented by a plaque burden score (P0–P4) and a set of clinical modifiers (e.g., non-diagnostic segments, stents, grafts, collateralization, uncalcified plaques etc.). Case-based application demonstrates enhanced reproducibility, clarity in identifying culprit and incidental lesions, and improved reporting quality in both outpatient and acute care settings. The proposed system serves as a framework for quality assurance and education.
**Conclusion:** PAD-RADS offers a structured, reproducible method for reporting peripheral artery imaging that has the potential to improve communication, research, and clinical care in PAD. Prospective studies are needed to validate its utility and impact on patient outcomes.
**Limitations:** PAD-RADS may oversimplify complex clinical scenarios and requires careful integration with clinical context. Its applicability in diverse populations, interobserver reliability, and effectiveness across different healthcare settings require further investigation. Adoption will necessitate prospective validation, multidisciplinary education and consensus building.
**Funding for this study:** No funding was received for the present study.
**Has your study been approved by an ethics committee?** Not applicable
**Author Disclosures:**
Deniz Bilecen: Nothing to disclose
Paul Jakob Kettnaker: Nothing to disclose
Rolf W. Huegli: Nothing to disclose
Andreea-Emilia Iacobut: Nothing to disclose

**Radiation protection: Safety measures and knowledge among interventional radiologists – a UK-based analysis of current practices and recommendations for improvement**
*R. Y. Gasiea*[1], A. T. Rogers[2], R. Lakshminarayan[3], M. Hamady[4], B. Huasen[5]; [1]Blackburn/UK, [2]Nottingham/UK, [3]Hull/UK, [4]London/UK, [5]Manchester/UK
*(rayhangasiea@nwsor.co.uk)*

**Purpose:** Minimally invasive procedures using X-rays have been taken over many traditional surgical techniques, less focus has been placed on the complications faced by primary operators compared to achieving better success rates and outcomes for procedures. The major focus has always been on patient safety and patient dose, which is important. However, there is no doubt that radiation exposure also poses risks to staff working in Interventional Radiology (IR). Over the years, protection for staff has improved, although this progress has unfortunately been driven by first-hand complications observed in healthcare providers.
**Methods or Background:** Together with the British Society of Interventional Radiology (BSIR), a survey was conducted to better understand current practice and availability to operators who use ionising radiation for their vascular and non-vascular practice. This was sent out to members over the 2023-2024 period.
**Results or Findings:** 112 respondents received across 22 vascular centres in the UK, including pregnant female members, helped gauge an understanding of current practice. We review and discuss the results obtained from the 30 questions asked.
**Conclusion:** The survey demonstrates some concern and also positive findings in terms of awareness. Clinicians are innovators and problem solvers. With the increased use of ionising radiation in clinical practice, radiation from scatter and strain from garments.
**Limitations:** N/A
**Funding for this study:** N/A
**Has your study been approved by an ethics committee?** Not applicable
**Author Disclosures:**
Bella Huasen: Nothing to disclose
Raghuram Lakshminarayan: Nothing to disclose
Andrew Timothy Rogers: Nothing to disclose
Rayhan Yousef Gasiea: Nothing to disclose
Mohamad Hamady: Nothing to disclose

**09:30 - 11:00 Research Stage 1**

### Research Presentation Session: Artificial Intelligence and Imaging Informatics

### RPS 2305

### How AI is redefining prostate cancer detection, risk stratification and prognosis: from promising research to clinical impact

**Moderator**
Tobias Penzkofer; Berlin/DE
**Author Disclosures:**
Tobias Penzkofer: Research Grant/Support: T.P. receives funding from Berlin Institute of Health (Advanced Clinician Scientist Grant, Platform Grant), Ministry of Education and Research (BMBF, 01KX2021 (RACOON), 01KX2121 („NUM 2.0“, RACOON), 01KX2524 (NUM 3.0), 68GX21001A, 01ZZ2315D), German Research Foundation (DFG, SFB 1340/2), European Union (H2020, CHAIMELEON: 952172, DIGITAL, EUCAIM:101100633, ARTEMIS). T.P. reports research agreements (no personal payments, outside of the submitted work) with AGO Research GmbH, Aravive, Inc., ARCAGY-GINECO, Astellas Pharma Global Development Inc., AstraZeneca AB, AstraZeneca GmbH, Clovis Oncology, EQRx International, Inc., F. Hoffmann-La Roche Ltd, GlaxoSmithKline Research & Development Limited, Grupo Español de Investigación en Cáncer de Ovario (GEICO), ImmunoGen Inc, Incyte Corporation, Karyopharm Therapeutics, Mario Negri Gynecology Oncology Group (MaNGO) (111), Merck KGaA, Merck Sharp & Dohme Corp., NOGGO e.V., Nordic Society of Gynaecological Oncology – Clinical Trial Unit (NSGO-CTU), Novartis Pharma GmbH, NovoCure GmbH, Sutro Biopharma, Inc., TESARO Inc., TORL Biotherapeutics, LLC, Tubulis GmbH, Universitätspoliklinik A. Gemelli, and Verastem Inc, as well as fees for a book translation (Elsevier) and speaking engagements (Bayer Healthcare).

**Evaluating AI assistance in clinically significant prostate cancer diagnosis using MRI**
*Y. Hou*; Nanjing/CN

**Purpose:** Artificial intelligence (AI) is potential as an assistant in support of human in diagnosing clinically significant prostate cancer (csPC) with MRI. We aimed to test the noninferiority and superiority of human-AI collaboration to human stand-alone review in csPC diagnosis with MRI.
**Methods or Background:** This observer study was conducted in four randomized cohorts (n = 1,305) who initially underwent MRI for csPC from two medical centers. A clinically available AI system was implemented as an assistant or not to stand-alone double reading in 21 readers to flag cases for further arbitration review among MRI-screened men with suspicious csPC. The secondary outcome was the clinically insignificant prostate cancer (ciPC) diagnosis.
**Results or Findings:** In four observer groups, except for junior readers in center 1, Human-AI collaboration was equal to human-alone (AUROC, 0.87 vs 0.82; P = .069), regardless of experience, readers with human-AI collaboration were superior to readers with human-alone (AUROC, 0.86 ~ 0.94 vs 0.70 ~ 0.86; all P-values < .001) in patient-level csPC diagnosis. Using PI-RADS ≥ 3 as diagnosing indication, human-AI collaboration achieved higher sensitivity (92.9% [688/740] vs 87.6% [617/704]; Odds ratio [OR], 1.86 [1.30- 2.67], P = .0007), higher specificity (66.2% [408/616] vs 46.7% [257/550]; OR, 2.23 [1.76- 2.83], P < .0001) than human-alone for csPC diagnosis. For ciPC, human-AI collaboration did not increase risk for over cancer detection rate (6.2% [38/616] vs 4.7% [26/550], OR, 1.30 [0.80- 2.21], P = .276), while achieved higher specificity (68.9% [376/546] vs 46.4% [231/498], OR, 2.56 [1.99- 3.29], P < .0.001) than human-alone review.
**Conclusion:** Human-AI collaboration is noninferior and superior to human-alone review regarding improvement of accuracy in csPC diagnosis and not increase risk for ciPC overdiagnosis at MRI.
**Limitations:** Further prospective validation
**Funding for this study:** None
**Has your study been approved by an ethics committee?** Yes
**Ethics committee - additional information:** grant no. 2019-SR-396
**Author Disclosures:**
Ying Hou: Speaker: None

**Automated MRI-based segmentation of the prostate, prostatic zones, periprostatic space, urethra and seminal vesicles using Convolutional Neural Networks trained on expert-annotated data**

*S. Tótin*[1], W. Holmlund[2], A. T. Simkó[2], T. Z. Kincses[1], E. Koós[1], P. Palásti[1], Z. Fejes[1]; [1]Szeged/HU, [2]Umeå/SE

**Purpose:** Accurate delineation of prostate zones and adjacent structures is essential for diagnosis, radiotherapy planning and surgical decision-making in prostate cancer. Manual contouring is time consuming and prone to variability. This study aimed to develop and validate convolutional neural networks (CNNs) for automated segmentation of the prostate zones, urethra, periprostatic neurovascular bundle and seminal vesicles on MRI, using a large expert-annotated dataset.
**Methods or Background:** We used T2-weighted multiparametric MRI scans from 200 PROSTATEx patients. Manual segmentations of the peripheral, central and transitional zones, anterior fibromuscular stroma were performed in 3D Slicer following PI-RADS v2.1 supplemented by the urethra, seminal vesicles and periprostatic bundle. 40 cases were independently annotated by two radiologists to provide inter-reader variability. The CNNs were trained on 160 cases and validated on 40 test cases. Performance was assessed using Dice Similarity Coefficient (DSC), Surface Dice Measurement (SDM) at multiple tolerance levels and Center Line Distance (CLD) for urethral evaluation, using Hero Imaging software.
**Results or Findings:** The CNNs achieved segmentation accuracy comparable to expert readers. For the prostate, the mean DSC value exceeded 0.90, matching inter-reader variability (0.913±0.0027). Urethral delineation achieved CLD values of 3.0 mm, similar to radiologist agreement (3.6 mm). The CNNs extended reliable segmentations to the central, peripheral and transitional zones, anterior fibromuscular stroma, periprostatic bundle and seminal vesicles, reaching agreement levels comparable to experienced radiologists.
**Conclusion:** Our CNN framework enables realiable, automated MRI segmentation of the prostate zones, urethra, periprostatic space and seminal vesicles. By reducing workload it has the potential to support standardized diagnosis, precise radiotherapy with urethral and periprostatic sparing and informed surgical planning.
**Limitations:** Partial external validation (performed on the prostate zones only). Some regions showed high inter-reader variability, which may indicate the need to harmonize image interpretation.
**Funding for this study:** No funding was provided for this study.
**Has your study been approved by an ethics committee?** Not applicable
**Author Disclosures:**
William Holmlund: Nothing to disclose
Attila Tibor Simkó: Owner: Hero Imaging
Tamás Zsigmond Kincses: Nothing to disclose
Zsuzsanna Fejes: Nothing to disclose
Szilvia Tótin: Nothing to disclose
Enikő Koós: Nothing to disclose
Péter Palásti: Nothing to disclose

**Integrating Automated Neurovascular Bundle Segmentation and Radiomics Biomarkers for Diagnostic and Prognostic Modeling in Prostate Cancer**

*G. Urbanos*, A. Jimenez-Pastor, A. Nogue, G. Ribas, F. J. E. Higa, C. Fontenla Martínez, V. Belloch Ripollés, L. Marti-Bonmati, A. Alberich-Bayarri; Valencia/ES
*(gemmaurbanos@quibim.com)*

**Purpose:** Accurate evaluation of the prostatic neurovascular bundles (NVBs) in prostate cancer (PCa) informs staging and treatment but remains difficult to standardize. This study developed an automated pipeline for NVB segmentation, tumor–NVB invasion risk stratification, and radiomics-based prediction of biochemical recurrence (BCR), perineural invasion (PNI), and extraprostatic extension (EPE).
**Methods or Background:** We collected 807 PCa MRI real-world exams from three sources. Apparent Diffusion Coefficient (ADC) maps and prostate/lesion segmentations were obtained with QP-Prostate®. Experts manually annotated NVBs in 470 T2-weighted (T2w) series, used to train a nnU-Net 3D full-resolution model. Data were split into 80% training/validation and 20% testing. In patients with peripheral zone (PZ) lesions (N=65), minimum lesion–NVB distance was computed and categorized as high (<2 mm), intermediate (2–5 mm), or low (>5 mm) risk. The trained model was applied to 280 diagnostic cases with PZ lesions and clinical data. Radiomic features from lesions and NVBs (ADC and T2w), extended with PSA and age, were used to predict PNI (185-, 91+), EPE (131-, 64+), and BCR (35-, 43+). Pipelines included normalization, feature selection, outlier removal, and class balancing. Different classification models were trained with 5-fold cross-validation.
**Results or Findings:** The results were assessed on the test set. NVB segmentation showed a mean surface distance of 1.02 (0.58–1.93) mm and volume difference of 0.41 (0.23–0.76) cc. Tumor–NVB invasion risk classification achieved accuracy of 0.89. Prediction AUCs were 0.73±0.07 for BCR, 0.80±0.05 for PNI, and 0.80±0.05 for EPE. Models combining NVB and lesion radiomics outperformed either region alone.
**Conclusion:** Automated NVB segmentation enables accurate invasion risk classification and improves prediction of BCR, PNI, and EPE, supporting integration into clinical risk stratification.
**Limitations:** Retrospective design, moderate segmentation overlap, and limited BCR prediction performance require external validation.
**Funding for this study:** Funding was provided by the project ProCanAid (PLEC2021-007709)
**Has your study been approved by an ethics committee?** Yes
**Ethics committee - additional information:** CEIM-Hopital Universitario y Politécnico la Fe (ProcanAid) – Nº de registro 2021-471-1.
**Author Disclosures:**
Luis Marti-Bonmati: Nothing to disclose
Vicente Belloch Ripollés: Nothing to disclose
Ana Jimenez-Pastor: Nothing to disclose
Anna Nogue: Nothing to disclose
Francisco Julian Eduardo Higa: Nothing to disclose
Gloria Ribas: Nothing to disclose
Angel Alberich-Bayarri: Nothing to disclose
Claudia Fontenla Martínez: Nothing to disclose
Gemma Urbanos: Nothing to disclose

**DeepSector-PI: Automated PI-RADS-Compliant Lesion-to-Sector Mapping**

*R. J. Jóźwiak*[1], J. Mycka[1], M. Gonet[1], I. Mykhalevych[1], T. Lorenc[1], A. Zacharzewska-Gondek[2], J. Dołowy[2], K. Tupikowski[2]; [1]Warsaw/PL, [2]Wrocław/PL
*(rafal.jozwiak@opi.org.pl)*

**Purpose:** The PI-RADS 2.1 sector map is a standardized 39-sector grid of the prostate that enables unambiguous lesion localization on mpMRI. We aimed to train a DL-based model for automatic prostate sector mapping and to evaluate its performance on real-world data drawn from an expert-curated mpMRI reference dataset.
**Methods or Background:** DeepSector-PI is built on a DenseNet-based classification network. To evaluate its performance, we assembled data from 321 mpMRI cases with identified suspicious lesions that were independently reported by three expert radiologists, who annotated all lesions and completed structured reports (SR) including the PI-RADS 2.1 sector map. In total, 845 lesion–sector pairs were used for training, and 106 pairs were reserved for validation. Model performance was stratified by zonal location (PZ/TZ/both), anatomical level (base/mid/apex), and lesion size quantified by the number of sectors involved.
**Results or Findings:** F1 scores and balanced accuracy were computed across all stratifications. By lesion size, F1 was 0.65 / 0.78 / 0.86 for lesions involving <4, 4–6, and ≥7 sectors, respectively; by zonal location, F1 was 0.77 / 0.73 / 0.67 for lesions confined to PZ, TZ, and both zones, respectively. Balanced accuracy for the same stratifications was 0.90 / 0.87 / 0.91 (size) and 0.92 / 0.91 / 0.84 (zone). We also remapped predictions to a simplified 24-sector scheme, yielding F1 of 0.72 / 0.84 / 0.86 (size) and 0.83 / 0.75 / 0.77 (zone), with corresponding balanced accuracy of 0.91 / 0.90 / 0.90 (size) and 0.94 / 0.92 / 0.86 (zone).
**Conclusion:** DeepSector-PI provides robust classification of lesion-involved prostate sectors, supporting standardized reporting and targeted biopsy planning. DeepSector-PI performance depends on lesion extent and zonal/anatomical level.
**Limitations:** Classification requires sector-labeled data from experts or another AI model.
**Funding for this study:** This work has been funded by the Polish National Centre for Research and Development under the program INFOSTRATEG I, project INFOSTRATEG-I/0036/2021 “AI-augmented radiology - detection, reporting and clinical decision making in prostate cancer diagnosis”.
**Has your study been approved by an ethics committee?** Not applicable
**Author Disclosures:**
Jan Mycka: Nothing to disclose
Ihor Mykhalevych: Nothing to disclose
Krzysztof Tupikowski: Nothing to disclose
Tomasz Lorenc: Nothing to disclose
Rafał Janusz Jóźwiak: Nothing to disclose
Michał Gonet: Nothing to disclose
Anna Zacharzewska-Gondek: Nothing to disclose
Joanna Dołowy: Nothing to disclose

**From Imaging to Outcomes: A PI-RADS–Driven Radiomics and Clinical Machine Learning Model for Detecting Clinically Significant Prostate Cancer**

*D. Samaras*[1], G. Agrotis[2], M. Vakalopoulou[3], M. Vlychou[1], I. Tsougos[1];
[1]Larissa/GR, [2]Amsterdam/NL, [3]Paris/FR
*(dimitris.samaras27@gmail.com)*

**Purpose:** This study aimed to develop and evaluate a machine-learning (ML) framework based on the PI-RADS protocol for detecting clinically significant prostate cancer (csPCa) using multiparametric MRI (mpMRI), simulating radiologists' decision-making.
**Methods or Background:** The publicly available PI-CAI (Prostate Imaging Cancer AI) dataset was employed, comprising 1,500 cases from 1,476 patients across 11 centers using seven MRI scanners. Among these, 1,075 cases were benign or clinically insignificant prostate cancer (cinsPCa), while 425 represented csPCa, defined as Gleason score (GS) ≥ 3+4. Ground truth labels were derived from biopsy results conducted by urologists, radiologists, or trained medical personnel under supervision. The ML framework followed a two-branch architecture: T2-weighted (T2W) images for the transition zone, and diffusion-weighted imaging (DWI) with apparent diffusion coefficient (ADC) maps for the peripheral zone. In addition to a radiomics-only model, a combined radiomics + clinical model was developed incorporating PSA, age, and prostate volume. Feature extraction was performed using Pyradiomics, including shape and texture features from original and filtered images. Feature space dimensionality was progressively reduced through a multi-stage pipeline: low-variance filtering (threshold 0.01), Pearson correlation pruning ($\rho \geq 0.85$), and Wilcoxon rank-sum testing ($p \leq 0.1$), followed by supervised feature selection restricted to training folds. The dataset was split into 80% training/validation and 20% testing, with five-fold cross-validation. Performance metrics included AUC, sensitivity, specificity, accuracy, balanced accuracy, and F1-score.
**Results or Findings:** The combined model (radiomics+clinical) outperformed the radiomics-only model, achieving higher AUC in both training (0.79±0.02 vs. 0.76±0.02) and testing set (0.78 vs. 0.73).
**Conclusion:** Our approach demonstrates strong potential for improving csPCa detection, supporting biopsy decisions, and enhancing patient outcomes.
**Limitations:** The modest external test set and absence of deep learning benchmarks limit generalizability. Validation on larger, multicenter cohorts and integration into clinical workflows are warranted.
**Funding for this study:** This work has been partially supported by project MIS 5154714 of the National Recovery and Resilience Plan Greece 2.0 funded by the European Union under the NextGenerationEU Program.
**Has your study been approved by an ethics committee?** Not applicable
**Author Disclosures:**
Maria Vakalopoulou: Nothing to disclose
Georgios Agrotis: Nothing to disclose
Ioannis Tsougos: Nothing to disclose
Marianna Vlychou: Nothing to disclose
Dimitrios Samaras: Nothing to disclose

**AI-Assisted Prostate Cancer Detection with MRI: A Clinical Routine Simulation Study**

*L. Pinto*[1], G. Di Costanzo[1], C. Riccio[2], A. G. Tucci[1], L. Palumbo[1], R. Cuocolo[3], A. R. R. Padhani[4], M. Imbriaco[2], A. Ponsiglione[2]; [1]Pozzuoli/IT, [2]Napoli/IT, [3]Salerno/IT, [4]Northwood/UK

**Purpose:** Interest in AI-driven detection of clinically significant prostate cancer (PCa) on MRI is growing. We evaluated a commercial AI system as a concurrent decision-support tool, assessing its impact on radiologists of varying expertise.
**Methods or Background:** In our retrospective study, consecutive patients underwent multiparametric MRI (mpMRI) for clinical suspicion of PCa. Scans were reviewed by six readers with different expertise, with and without AI assistance. Intra- and inter-reader agreements and the impacts of AI-assisted on patient-level csPCa scores were assessed. Diagnostic performance metrics at patient level and benefit-to-harm ratios were evaluated.
**Results or Findings:** The study included 100 patients (26% with csPCa). There was no improvement in inter-reader agreement with AI readings (0.584 vs 0.573). Residents were most likely to change PI-RADS scores with AI assistance compared to basic and expert readers (19, 9, and 7 changes, respectively). Overall, there was no significant difference in AUROC between AI-assisted and unassisted readings (0.87 vs 0.86; p = 0.734). At a PI-RADS ≥3 threshold, sensitivity was slightly lower with AI (0.87 vs 0.89), while specificity (0.73), PPV (0.53–0.54), and NPV (0.94–0.95) remained similar. Subgroup analyses showed no significant differences in diagnostic performance. A slight increase in grade selectivity and selective biopsy avoidance was observed among experts and residents, respectively, with AI-assisted readings when applying a PI-RADS cut-off of 3 or PSA density ≥0.15.
**Conclusion:** AI decision support does not significantly improve diagnostic accuracy for csPCa detection across readers of varying expertise, with minor impacts on benefit-to-harm ratios.
**Limitations:** We did not fully account for MRI-negative patients who avoided biopsies, so accuracy metrics should be interpreted relative to the evaluated cohort. Lack of per-lesion analysis.
**Funding for this study:** None
**Has your study been approved by an ethics committee?** Yes
**Ethics committee - additional information:** Local IRB
**Author Disclosures:**
Anna Giacoma Tucci: Nothing to disclose
Giuseppe Di Costanzo: Nothing to disclose
Luigi Palumbo: Nothing to disclose
Ciro Riccio: Nothing to disclose
Lorenzo Pinto: Nothing to disclose
Andrea Ponsiglione: Nothing to disclose
Anwar R. Padhani: Consultant: Lucida Medica, Siemens Healthineers
Massimo Imbriaco: Nothing to disclose
Renato Cuocolo: Nothing to disclose

**Artificial Intelligence as a Gatekeeper in Prostate Cancer Imaging: Can We Avoid Unnecessary Biopsies?**

*K. A. Bolivar Gil*, F. D. Losada Lopez, J. Camean, L. A. Miquelini;
Buenos Aires/AR
*(karen.med.ucv@gmail.com)*

**Purpose:** Detecting suspicious lesions suggestive of clinically significant cancer which require biopsy for diagnosis, remains an imaging challenge. The aim of this study is to evaluate the added value of artificial intelligence in radiology reports, in order to avoid unnecessary biopsies.
**Methods or Background:** This retrospective study analysed a cohort of 141 patients who underwent prostate mpMRI between 2022 and 2025 with the assistance of an AI-based diagnostic tool. All examinations were initially interpreted by an experienced radiologist. Subsequently, patients underwent transperineal cognitive fusion biopsy (MRI–US) targeting between one and four lesions per patient, resulting in a total of 163 lesions. Histopathological evaluation of the biopsy specimens served as the reference standard.
**Results or Findings:** Diagnostic concordance between expert radiologists and the AI program was observed in 85 cases (60.3%); of these, 68 (80.0%) were positive for clinically significant prostate cancer (Gleason score ≥7) and 17 (20.0%) negative. On independent analysis, expert radiologists reported 81/141 positive cases (57.4%) and 60/141 negative cases (42.6%). The AI, when limited to concordant cases, identified 68 positives (80.0%) and 17 negatives (20.0%). AI detected suspicious lesions in 104/141 patients (73.8%). Among the 37 patients without AI-reported lesions (26.2%), histopathology was also negative in 30 (81.1%) and positive for clinically significant prostate cancer in 7 (18.9%). The AI system achieved a sensitivity of 90.7% and specificity of 45.5% for detecting clinically significant prostate cancer, with a PPV of 65.4%, NPV of 81.1%, and overall accuracy of 69.5%. AI improved the NPV to 85% in low to intermediate risk lesions.
**Conclusion:** The AI system demonstrated a high NPV, enhancing triage of low to intermediate risk lesions and potentially helping to avoid unnecessary prostate biopsies.
**Limitations:** Due to the retrospective design, not all AI-reported positive cases underwent biopsy.
**Funding for this study:** No funding was provided for this study.
**Has your study been approved by an ethics committee?** Not applicable
**Author Disclosures:**
Juan Camean: Nothing to disclose
Karen Andreina Bolivar Gil: Nothing to disclose
Luis Ariel Miquelini: Nothing to disclose
Fernando Daniel Losada Lopez: Nothing to disclose

**Adapting Generative Cross-Modality Image Translation Models for Prostate Magnetic Resonance Imaging (MRI) Denoising: A Large-Scale Study on T2-Weighted Imaging**

*R. Moreno Calatayud*, P. Rodríguez Belenguer, J. Gómez-Martínez, J. Aquerreta-Escribano, G. Ribas, A. Galiana-Bordera, L. Cerda Alberich, L. Marti-Bonmati; Valencia/ES

**Purpose:** Image quality is critical in prostate MRI, as noise and artifacts can obscure anatomical structures, reduce diagnostic confidence, and compromise the performance of downstream automated algorithms. This study evaluated translation-inspired generative models for denoising prostate T2-weighted MRI, aiming to improve image quality while preserving anatomical fidelity.
**Methods or Background:** This retrospective study included a cohort of 805 patients (>18 years old), divided into a training set and a validation set of 700 and 105 patients respectively, with a confirmed pathology diagnosis of prostate cancer who underwent T2-weighted MRI volumes at Hospital Universitari i Politècnic La Fe from January-2015 to December-2022. Synthetic low-quality images were generated by adding Gaussian noise, bias field inhomogeneities, blurring, and ghosting, while the original scans were used as high-quality ground truths. Two state-of-the-art generative models were adapted to this framework: Biting et al.'s edge-aware GANs (Ea-GAN) and Kim et al.'s adaptive latent diffusion model (ALDM VQGAN). These models were developed for brain MRI to translate T1-weighted images into T1ce, T2, and FLAIR sequences but were repurposed to denoise prostate T2-weighted MRI.
**Results or Findings:** Quantitative image quality metrics calculated on the model output resulted in a peak signal-to-noise ratio (PSNR), structural similarity index (SSIM) and normalized mean square error (NMSE) of 29.5, 0.86 and 0.04 respectively with the high-quality ground truth image. These values indicate a strong resemblance between the reconstructed and reference images, highlighting the model's ability to reduce noise while preserving underlying anatomical structures.
**Conclusion:** This pilot study showed generative models can improve image quality of prostate T2-weighted MRI, highlighting potential clinical utility in the early diagnosis of prostate cancer when dealing with noisy acquisitions. Further research is required to validate the model in larger cohorts.
**Limitations:** No limitations were identified.
**Funding for this study:** Funding was provided by the Instituto de Salud Carlos III call for Research, Development, and Innovation (R&D&i) Projects related to Personalized Medicine and Advanced Therapies (Transmissions Initiative), co-financed by the European Union-NextGenerationEU /Recovery Plan, transformation and Resilence (RPTR).
**Has your study been approved by an ethics committee?** Yes
**Ethics committee - additional information:** This study was approved by an Ethics Committee with the following reference number 2023-1107-1.
**Author Disclosures:**
Rafael Moreno Calatayud: Nothing to disclose
Luis Marti-Bonmati: Nothing to disclose
Pablo Rodríguez Belenguer: Nothing to disclose
Judith Gómez-Martínez: Nothing to disclose
Javier Aquerreta-Escribano: Nothing to disclose
Gloria Ribas: Nothing to disclose
Leonor Cerda Alberich: Nothing to disclose
Adrian Galiana-Bordera: Nothing to disclose

### Integrating Radiomics and CNN-Based Approaches for Automated PI-RADS 3–5 Classification in Prostate MRI

*S. Fouladi*, F. Darvizeh, R. Di Meo, L. Di Palma, A. Maiocchi, E. Damiani, M. Alì, D. Fazzini, G. Gianini; Milan/IT
*(saman.fouladi@unimi.it)*

**Purpose:** Accurate classification of clinically significant prostate cancer remains challenging, particularly in distinguishing aggressive tumors from indolent ones. Although multiparametric MRI (mpMRI) has enhanced lesion detection, effective categorization using the Prostate Imaging Reporting and Data System (PI-RADS) remains complex. This study aims to develop and evaluate complementary automated approaches for PI-RADS classification, focusing on categories 3, 4, and 5, using ADC, DWI, and T2W sequences.
**Methods or Background:** Three approaches were investigated. First, hand-crafted radiomic features were extracted from manually segmented lesions using the PyRadiomics library. Second, we extended this approach by incorporating fully automated lesion and zonal segmentation to simulate a practical, manual-free pipeline. Third, a custom convolutional neural network (CNN) was trained on ADC images and lesion masks to learn high-level features directly. These features were subsequently used to train multiple machine learning models for multi-class PI-RADS classification.
**Results or Findings:** Features derived from ADC consistently achieved superior performance, with one ensemble model reaching an accuracy of 0.77 and an AUC of 0.83. Combining features from all sequences further improved robustness (accuracy = 0.73, AUC = 0.84). PI-RADS 5 classification proved most reliable (AUC ≥ 0.94), whereas PI-RADS 3 remained the most challenging to distinguish.
**Conclusion:** ADC-derived features are highly effective for PI-RADS classification, and integrating automated radiomic extraction with deep learning enhances robustness and practical applicability. Combining multi-sequence information and learning-based approaches offers a promising pathway for automated risk stratification in prostate cancer.
**Limitations:** The study is limited by the dataset size and the reliance on manually segmented lesions in some approaches, which is time-consuming. Nevertheless, the results are promising, and performance is expected to improve further with larger datasets and fully automated pipelines.
**Funding for this study:** Funding The work was partially supported by the MUSA-Multilayered Urban Sustainability Action project, funded by the European Union-NextGenerationEU, under the Mission 4 Component 2 Investment Line of the National Recovery and Resilience Plan (NRRP) Mission 4 Component 2 Investment Line 1.5: Strengthening of research structures and creation of R&D ”innovation ecosystems”, set up of ”territorial leaders in R&D” (CUP G43C22001370007, Code ECS00000037); Program ”piano sostegno alla ricerca” PSR and the PSR-GSA-Linea 6; Project ReGAInS (code 2023-NAZ-0207/DIP-ECC-DISCO-23), funded by the Italian University and Research Ministry, within the Excellence Departments program 2023-2027 (law 232/2016).
**Has your study been approved by an ethics committee?** Yes
**Ethics committee - additional information:** Approval on September 11, 2024 by CET Lombardia 3 Ethical Committee (Study ID: 5105)
**Author Disclosures:**
Rosario Di Meo: Nothing to disclose
Ernesto Damiani: Nothing to disclose
Deborah Fazzini: Nothing to disclose
Marco Alì: Nothing to disclose
Gabriele Gianini: Nothing to disclose
Saman Fouladi: Nothing to disclose
Alessandro Maiocchi: Nothing to disclose
Luca Di Palma: Nothing to disclose
Fatemeh Darvizeh: Nothing to disclose

### Diagnostic Pitfalls of AI-Assisted Multiparametric MRI in Prostate Cancer Detection: When AI Gets It Wrong

*A. Borrelli*, L. Laschena, M. Pecoraro, S. Lucciola, E. Messina, V. Panebianco; Rome/IT
*(antonella.borrelli@uniroma1.it)*

**Purpose:** To evaluate the diagnostic pitfalls of AI-assisted mpMRI in prostate cancer detection
**Methods or Background:** Artificial Intelligence (AI) applied to mpMRI is increasingly integrated into prostate cancer (PCa) workflows. Its potential in PCa detection has been investigated, but limitations remain underexplored. MRI pitfalls are a major source of false positives. Prostate mpMRI, although accurate, is influenced by anatomical, clinical, and technical factors that can mimic cancer. In this retrospective single-center study, 458 mpMRI scans were reviewed: 150 biopsy-proven PCa, 156 true negatives, and 152 scans previously identified as Pitfalls. After exclusions, 362 cases were processed with AI software.Diagnostic performance was compared with expert radiologist reports and histopathology when available.Errors were classified as false positives and recurring misclassification patterns.
**Results or Findings:** In the overall cohort of 458 patients (150 cancers, 152 pitfalls, 156 negatives), AI demonstrated a sensitivity of 92.0%, but specificity was only 70.5%, leading to an accuracy of 77.5% and an AUC of 0.66. In the restricted cohort of 306 patients (150 cancers and 156 negatives, excluding pitfalls),sensitivity remained stable at 92.0%,while specificity improved to 93.6%,yielding an accuracy of 92.8% and an AUC of 0.93 in line with literature. BPH caused the largest number of AI errors, DWI artifacts and ectopic BPH showed the highest misclassificatio rate resembling novice reader challenges and confirming that AI currently mirrors known interpretative limitations.
**Conclusion:** These results highlight the current limitations of AI tools in prostate imaging, particularly in differentiating cancer from benign mimics, reinforcing the need for expert radiologist oversight in clinical practice.
**Limitations:** Single-center retrospective study. The tested AI software may not represent other platforms. Our Cohort intentionally enriched with pitfalls and challenging cases making it less comparable to everyday practice
**Funding for this study:** None
**Has your study been approved by an ethics committee?** Not applicable
**Author Disclosures:**
Sara Lucciola: Nothing to disclose
Valeria Panebianco: Nothing to disclose
Antonella Borrelli: Nothing to disclose
Emanuele Messina: Nothing to disclose
Martina Pecoraro: Nothing to disclose
Ludovica Laschena: Nothing to disclose

09:30 - 11:00 **Research Stage 2**

### Research Presentation Session: Head and Neck

### RPS 2308

### Stepping forward in paranasal and skull-based imaging

**Moderator**
Gillian Margaret Potter; Hale/UK

**Photon-Counting CT Provides Superior Qualitative Evaluation of Otologic Diseases Compared to Conventional CT: Analysis of 32 Ear Sections**

*G. Ressa*, L. A. Cappellini, R. Levi, G. Savini, S. Aimola, A. Spinello, D. Villari, G. Spriano, L. S. Politi; Milan/IT
*(gaia.ressa@humanitas.it)*

**Purpose:** Compare Photon Counting CT (PCCT) with conventional CT in detecting pathological alterations in fine anatomical ear structures, also evaluating potential radiation dose reduction with PCCT.
**Methods or Background:** In this prospective, single-center study, patients with otolaryngological clinical indications (e.g., hearing loss, otorrhea, tinnitus) underwent PCCT (NAEOTOM Alpha, Siemens Healthineers). PCCT imaging employed an Ultra High Resolution Mode protocol (120 × 0.2 mm collimation), Hr92 kernel, 0.2 mm slice thickness, tube voltage of 120 or 140 kV with Sn filter, pitch 0.55, rotation time 1s, and IQ level 150. Images were reconstructed axially and coronally with 0.2 mm slices bilaterally. All patients had prior conventional CT scans (100–140 kV, 0.4–0.7 mm slice thickness). Three neuroradiologists (3, 4, and >25 years experience) independently reviewed PCCT and conventional CT images for 15 otologic pathologies, scoring likelihood via a 5-point Likert scale after a 3-month washout. Interobserver agreement was assessed with Spearman's correlation, and diagnostic improvement with linear regression. Radiation doses (DLP) were compared using Mann–Whitney U test.
**Results or Findings:** Thirty-two ears sections from 16 patients (mean age 55, 8 women) were evaluated. PCCT demonstrated superior detection of otospongiotic foci (OR 1.56; p=0.006), tegmen tympani erosion (OR 1.79; p=0.004), ossicular erosion (ORs 1.72–2.15; p<0.05), tympanic membrane thickening/retraction (ORs 2.21, 2.72; p=0.001) and facial canal erosion (OR 1.5; p=0.01) compared to conventional CT. PCCT reduced mean DLP by 60% (236 ± 23 vs. 525 ± 277 mGy*cm; p=0.004).
**Conclusion:** PCCT improves detection of otologic pathology while significantly reducing radiation dose compared to conventional CT.
**Limitations:** Small sample size
**Funding for this study:** ANTHEM (AdvaNced Technology for Human centEred Medicine)
**Has your study been approved by an ethics committee?** Yes
**Ethics committee - additional information:** Humanitas Research Hospital : comitato.etico@humanitas.it
**Author Disclosures:**
Gaia Ressa: Nothing to disclose
Letterio Salvatore Politi: Nothing to disclose
Luca Alessandro Cappellini: Nothing to disclose
Domenico Villari: Nothing to disclose
Giovanni Savini: Nothing to disclose
Silvia Aimola: Nothing to disclose
Alessandro Spinello: Nothing to disclose
Riccardo Levi: Nothing to disclose
Giuseppe Spriano: Nothing to disclose

**MRI Predictors of Baseline Vestibular Function in Vestibular Schwannoma**

*S. Neveu*, D. Daskalou, N. Guinand, P. Senn, M. Becker; Geneva/CH
*(sophie.neveu@hcuge.ch)*

**Purpose:** Vestibular dysfunction is frequent in vestibular schwannoma (VS) and has a major impact on quality of life. We aimed to determine whether MRI-derived features are associated with baseline vestibular function as measured by objective testing.
**Methods or Background:** We retrospectively studied untreated, unilateral VS patients evaluated between 2014 and 2021 at a tertiary center. Vestibular function was assessed using bithermal bilateral caloric testing, video head impulse testing (vHIT), and ocular and cervical vestibular evoked myogenic potentials (oVEMP; cVEMP). MRI predictors included tumor volume (small 0–100 mm³, medium 101–1000 mm³, large >1000 mm³), Koos grade, and fundal contact.
**Results or Findings:** A total of 149 patients were included (median age 58 years). Large tumours showed markedly greater caloric weakness compared with small tumours (median 52% vs 10%, p<0.0001). For the lateral canal, vHIT gain was significantly lower in large than in small tumours (0.77 vs 0.98, p<0.0001), with significant reductions also observed in the anterior (p<0.0001) and posterior (p=0.004) canals. Fundal contact was associated with greater caloric weakness (median 39% vs 24%, p=0.003) and predicted reduced anterior (p=0.04) and lateral (p=0.03) canal vHIT gains. In multivariable analysis, tumour volume, Koos grade and fundal contact emerged as independent predictors of caloric weakness and vHIT gain of the lateral canal. Absent oVEMP and cVEMP responses were observed predominantly in large tumours and Koos 4 lesions and were not associated with fundal contact.
**Conclusion:** Tumor volume, Koos grade, and fundal contact are independent MRI predictors of vestibular dysfunction in untreated VS. Recognizing these imaging markers at diagnosis can prompt targeted vestibular testing, inform counselling and treatment planning, and lay the groundwork for predictive models of functional decline.
**Limitations:** Not applicable.
**Funding for this study:** No funding was provided for this study.
**Has your study been approved by an ethics committee?** Yes
**Ethics committee - additional information:** The study was approved by the Regional Research Ethics Committee (CCER, Project ID: 2022-01257) and conducted in accordance with the principles of the Declaration of Helsinki.
**Author Disclosures:**
Dimitrios Daskalou: Nothing to disclose
Minerva Becker: Nothing to disclose
Nils Guinand: Nothing to disclose
Pascal Senn: Nothing to disclose
Sophie Neveu: Nothing to disclose

**Comparison of CT with and without artefact removal algorithms and CBCT in postoperative cochlear implant imaging – a temporal bone study**

*H. Viitanen*[1], J. Hakumäki[1], H. P. Matikka[1], S. M. Kaartinen[1], A-P. Ronkainen[1], J. Koivisto[2], S. Sipari[1], A. Dietz[1], M. Iso-Mustajärvi[1]; [1]Kuopio/FI, [2]Helsinki/FI
*(henri.viitanen@planmeca.com)*

**Purpose:** Artefact removal algorithms (ARA) for pacemakers (PM) and neurostimulators (NS) are available but the use of ARAs haven't been reported in cochlear implant (CI) imaging, where metal artefacts obscure the evaluation of electrode placement. Additionally, cone-beam computed tomography (CBCT) has been shown to be superior in postoperative CI imaging regarding metallic artefacts. The aim of the present study is to evaluate computed tomography (CT) images with and without ARA in comparison with CBCT images to determine the most suitable postoperative imaging modality for clinical use.
**Methods or Background:** Three SlimJ electrodes and three MidScala electrodes (Advanced Bionics) were inserted into six freshly frozen temporal bones (TB), which were scanned with CT (Siemens Somatom) and CBCT (Promax Planmeca) with our institution's clinical protocols with and without ARAs (PM and NS). Images were evaluated by two neuroradiologists and two ENT specialists. Scores (1-5) were given to the overall image quality (OIQ), suitability for trauma evaluation (TE), and for blooming, ring, and streak artifacts.
**Results or Findings:** The CBCT protocols were ranked best in OIQ (means 4.12 and 4.08), TE (means 4.42 and 4.08) and blooming (means 4.04 and 4.21). The difference between CBCT protocols was not statistically significant (p=0.775) in a pairwise comparison. The results were mixed with ring and streak artifacts. CT protocols without ARA had a mean OIQ of 2.92 and 3 and with NC of 1.29 and PM of 2.92. CBCT protocols were better than CT protocols in OIQ, TE and blooming (p<0.001).
**Conclusion:** CBCT was better than CT in postoperative CI imaging of TBs, and ARAs did not provide any benefit. No specific ARA exists for CI imaging and the use of ARAs developed for other regions/implants cannot be recommended.
**Limitations:** Cadaver study, limited sample size.
**Funding for this study:** None.
**Has your study been approved by an ethics committee?** Yes
**Ethics committee - additional information:** Valvira 9202/06.01.03.01/2013

**Author Disclosures:**
Hanna Pauliina Matikka: Nothing to disclose
Sini Sipari: Nothing to disclose
Henri Viitanen: Employee: Planmeca Oy
Juha Koivisto: Employee: Planmeca Oy
Ari-Petteri Ronkainen: Nothing to disclose
Juhana Hakumäki: Nothing to disclose
Matti Iso-Mustajärvi: Nothing to disclose
Siru M. Kaartinen: Nothing to disclose
Aarno Dietz: Nothing to disclose

**Temporal Bone Pneumatization and Its Relationship with Mastoiditis: A CT-Based Case–Control Radiologic Study**

*F. Azizi*; Istanbul/TR
*(azizifateme13@gmail.com)*

**Purpose:** To evaluate temporal bone pneumatization patterns in adult patients with mastoiditis using a sigmoid sinus-based classification system and to investigate the association between limited mastoid pneumatization and susceptibility to mastoid infection, through comparison with a control group.
**Methods or Background:** This retrospective case–control study included 250 adult patients (aged 20–45 years) with radiologically confirmed mastoiditis and 200 age-matched control subjects without mastoiditis who underwent high-resolution computed tomography (HRCT) of the temporal bone for other clinical indications. Pneumatization patterns were assessed on axial CT images and classified into four groups—hypopneumatization, moderate, good, and hyperpneumatization—based on their extent relative to the sigmoid sinus. The distribution of pneumatization types was compared between the mastoiditis and control groups using descriptive and inferential statistics.
**Results or Findings:** In the mastoiditis group, hypopneumatization (58.4%) and moderate pneumatization (39.2%) were predominant, while good (2.4%) and hyperpneumatization (1.2%) patterns were rare. In contrast, the control group exhibited a significantly higher prevalence of good (37.0%) and hyperpneumatization (20.5%) patterns. Chi-square analysis confirmed a statistically significant association between limited pneumatization and mastoiditis ($p < 0.001$).
**Conclusion:** Limited mastoid pneumatization is strongly associated with the presence of mastoiditis and may serve as a radiologic marker of increased disease susceptibility. The sigmoid sinus-based classification system provides a practical and reproducible method for assessing pneumatization and may assist in clinical risk stratification, diagnosis, and surgical planning.
**Limitations:** This retrospective study is limited by potential selection bias and the qualitative assessment of pneumatization rather than volumetric analysis. Clinical outcomes were not evaluated, and the restricted age range (20–45 years) limits generalizability to other populations. Future prospective studies with broader age groups, clinical follow-up, and advanced imaging are needed.
**Funding for this study:** None
**Has your study been approved by an ethics committee?** Yes
**Ethics committee - additional information:** This study was approved by the IMU Faculty of Medicine Ethics Committee (IRB: 2025.05.22–586).
**Author Disclosures:**
Fatemeh Azizi: Nothing to disclose

**Comparative evaluation of neurography sequences for facial nerve visualisation across 3T MR scanners**

*M. M. Moll*, L. Karssemakers, B. Plakke, A. Te Boekhorst, L. C. ter Beek, M. Valstar, M. Van Alphen, L. Braun; Amsterdam/NL
*(man.moll@nki.nl)*

**Purpose:** Facial nerve injury is a concerning complication in parotid tumour surgery, affecting the patient's quality of life. A tool that facilitates preoperative visualisation of the facial nerve and its spatial relationship to parotid tumours may complement intraoperative facial nerve monitoring. Due to advances in high-resolution MR, especially in the field of neurography, the development of such a tool is more feasible. This study aims to evaluate and compare various neurography sequences for facial nerve imaging across different sequences from different MR manufacturers.
**Methods or Background:** We scanned fifteen healthy volunteers to compare four promising 3D sequences described in the literature: Philips NerveVIEW (turbo spin echo sequence with motion sensitised driven equilibrium pulse), Siemens CISS (Constructive Interference in Steady State), Siemens DESS-WE (double-echo steady-state water-excitation), and Siemens SPACE (Sampling Perfection with Application optimised Contrast using different flip angle Evolution). Sequences were compared based on qualitative (radiologist assessment) and quantitative assessments (signal-to-noise ratio and signal-to-contrast ratio).
**Results or Findings:** Preliminary observations indicate that different neurography sequences yield distinct signal characteristics for the intraparotid facial nerve, with hypointense visualisation in CISS and SPACE, and hyperintense visualisation in DESS-WE and NerveVIEW. Complete qualitative and quantitative analyses are currently underway.
**Conclusion:** The neurography sequences exhibit distinct signal characteristics in visualising the intraparotid facial nerve. Ongoing comprehensive qualitative and quantitative analyses will provide further insight into the optimal imaging approach for preoperative visualisation in parotid gland surgery.
**Limitations:** As the scans were performed on volunteers, no contrast was administered.
**Funding for this study:** No funding was provided for this study.
**Has your study been approved by an ethics committee?** Yes
**Ethics committee - additional information:** This study was approved Institutional Review Board of the Netherlands Cancer Institute – Antoni van Leeuwenhoek (IRBd25-044) and written informed consent was obtained from participants.
**Author Disclosures:**
Matthijs Valstar: Nothing to disclose
Luc Karssemakers: Nothing to disclose
Brenda Plakke: Nothing to disclose
Manon M.M. Moll: Nothing to disclose
Arjan Te Boekhorst: Nothing to disclose
Loes Braun: Nothing to disclose
Maarten Van Alphen: Nothing to disclose
Leon C. Ter Beek: Nothing to disclose

**Specific anatomical characteristics of the styloid process as risk factors for internal carotid artery dissections: a systematic review and meta-analysis of controlled trials**

*T. Klail*[1], E. Kalioras[2], M. Von Gernler[2], R. Giger[2], A. K. Exadaktylos[2], M. Müller[2], F. Wagner[3]; [1]Münsterlingen/CH, [2]Bern/CH, [3]Aarau/CH
*(tomklail@gmail.com)*

**Purpose:** Internal carotid artery dissection (ICA-D) is a cause of stroke in adults. The styloid process may be a risk factor for ICA-D due to potential (micro)trauma from its proximity to the ICA. This systematic review with meta-analysis investigates the association between SP characteristics, particularly SP-ICA distance, and ICA-D.
**Methods or Background:** A systematic review was conducted across six databases to identify observational studies comparing ICA-D patients to controls. The primary association of interest was SP-ICA distance and ICA-D. Secondary studied associations were ICA-D with SP length and angulation. Random-effects meta-analysis evaluated associations, with a subgroup analysis of moderate/high-quality studies with standardised mean difference (SMD, Hedges' g) as an effect size.
**Results or Findings:** Six studies were included. Subgroup analysis of moderate/high-quality studies with measurement of the SP-ICA distance ipsilateral to the ICA-D (4 studies) showed a negative SMD -0.29, $p = 0.047$ indicating shorter SP-ICA distance in ICA-D cases. Moderate heterogeneity ($I^2 = 63\%$) existed. A pooled meta-analysis of all five case-control studies (270 ICA-D patients, 377 controls) showed no significant difference in SP-ICA distance (SMD = -0.92, $p = 0.143$) with high degree of heterogeneity. SP length meta-analysis (3 studies) found no significant differences (SMD 0.24, $p = 0.139$) and two studies found no significant association between ICA-D and SP angulation.
**Conclusion:** Shorter SP-ICA distance might be a risk factor for ICA-D. SP-ICA distance seems to be a more reliable parameter than SP length for risk stratification in evaluating potential underlying cause in ICA-D.
**Limitations:** Despite excluding studies with significant methodological differences to address heterogeneity, inconsistencies in SP-ICA distance measurement persist and may obscure a true association. Furthermore, the inclusion of studies utilizing adjusted SP-ICA distances could have influenced the overall results.
**Funding for this study:** The authors declare no financial support for the present study.
**Has your study been approved by an ethics committee?** Not applicable
**Author Disclosures:**
Tomas Klail: Nothing to disclose
Roland Giger: Nothing to disclose
Emmanouil Kalioras: Nothing to disclose
Marc Von Gernler: Nothing to disclose
Franca Wagner: Nothing to disclose
Aristomenis K. Exadaktylos: Nothing to disclose
Martin Müller: Nothing to disclose

**Onset and Development of Chronic Rhinosinusitis from Infancy through Adolescence in Primary Ciliary Dyskinesia Detected by Longitudinal Magnetic Resonance Imaging**

*L. Wucherpfennig*[1], M. Lulay[1], A. Seitz[1], I. Baumann[1], M. Stahl[2], H-U. Kauczor[1], M. A. Mall[2], O. Sommerburg[1], M. O. Wielpütz[3]; [1]Heidelberg/DE, [2]Berlin/DE, [3]Greifswald/DE
*(lena.wucherpfennig@med.uni-heidelberg.de)*

**Purpose:** Previous cross-sectional study employing magnetic resonance imaging (MRI) of paranasal sinuses showed a high extent of chronic rhinosinusitis (CRS) in children with primary ciliary dyskinesia (PCD). However, longitudinal imaging data on CRS onset and progression is lacking. The aim of this study is to longitudinally assess CRS in PCD from infancy through adolescence with MRI.
**Methods or Background:** 22 children with PCD (mean baseline age 7.6±5.7yr, range 0-18yr) underwent a mean of 2.8±1.8 (range 1-9) annual standardised paranasal sinus MRI examinations. MRI were assessed using the previously evaluated CRS-MRI score by two readers.
**Results or Findings:** At infancy (0yr), 83-100% of paranasal sinuses and mastoid cells were opacified. Mucosal swelling was the most prevalent (83-100%) and dominant abnormality (50-100%). At preschool age (1-5yr), the prevalence of opacified sphenoid sinus (83% vs. 100%, p<0.01) as well as mastoid cells increased (p=0.567-0.999 vs. infancy). Mucosal swelling remained the most prevalent (100%) and dominant abnormality (47-100%), while the prevalence and subscore of mucopyoceles increased (81% vs. 13% and 2.0 ± 1.6 vs. 4.1 ± 2.1, respectively; p<0.05). At school age (≥6yr), almost all maxillary, sphenoid and ethmoid sinuses (91-100%), and 87% of mastoid cells were opacified (p=0.122-0.999 vs. preschool age). The prevalence of mucopyoceles, polyps and sinus deformation decreased (60% vs. 81%, 40% vs. 70% and 51% vs. 78%, respectively; p<0.05 vs. preschool age), especially in maxillary sinuses. The CRS-MRI sum score averaged 27.8±4.9 at infancy, tended to increase at preschool age (31.9±5.6, P>0.999) and decreased at school age to 25.6±8.3 (p<0.01).
**Conclusion:** Longitudinal paranasal sinus MRI detects high prevalence and extent of paranasal sinuses abnormalities from infancy. Our data support its role for comprehensive non-invasive monitoring of CRS in PCD.
**Limitations:** The number of patients was only moderate.
**Funding for this study:** This study was supported by grants from the German Federal Ministry of Education and Research (BMBF) (82DZL004A1, 82DZL009B1, 82DZL009C1 and 01GL2401A), and the German Research Foundation (STA 1685/1-1 and CRC 1449 – project 431232613). MS participates in the BIH-Charité Clinician Scientist Program funded by the Charité – Universitätsmedizin Berlin and the BIH. LW is participant of the Career Development Programm funded by the University of Heidelberg. Funders had no involvement in the collection, analysis and interpretation of data, in the writing of the report and in the decision to submit the article for publication.
**Has your study been approved by an ethics committee?** Yes
**Ethics committee - additional information:** Approved by institutional ethics committees (S-509/2019, S-184/2025, EA2/003/21)
**Author Disclosures:**
Olaf Sommerburg: Speaker: Vertex Pharmaceuticals (unrelated to this project).
Mirjam Stahl: Board Member: German Society of Pediatric Pulmonology (Gesellschaft für Pädiatrische Pneumologie, GPP) (unrelated to this project) Board Member: German CF association (Forschungsgemeinschaft Mukoviszidose, FGM) (unrelated to this project) Board Member: European Respiratory Society (ERS) (unrelated to this project) Speaker: Vertex Pharmaceuticals (unrelated to this project).
Mark O. Wielpütz: Speaker: Vertex Pharmaceuticals (unrelated to this project) Speaker: Boehringer Ingelheim (unrelated to this project) Grant Recipient: Boehringer Ingelheim (unrelated to this project) Board Member: European Society of Thoracic Imaging (ESTI) (unrelated to this project) Grant Recipient: Federal Ministry of Education and Research (Bundesministerium für Bildung und Forschung, BMBF) Grant Recipient: Vertex Pharmaceuticals (unrelated to this project) Board Member: International Workshop on Pulmonary Functional Imaging (IWPFI) (unrelated to this project).
Lena Wucherpfennig: Consultant: Boehringer Ingelheim (unrelated to this project) Speaker: Vertex Pharmaceuticals (unrelated to this project) Consultant: Antaros Medical (unrelated to this project).
Angelika Seitz: Nothing to disclose
Hans-Ulrich Kauczor: Consultant: Siemens (unrelated to this project) Speaker: Siemens (unrelated to this project).
Marcus A. Mall: Grant Recipient: German Research Foundation (Deutsche Forschungsgemeinschaft, DFG) Grant Recipient: German Innovation Fund (unrelated to this project) Grant Recipient: Federal Ministry of Education and Research (Bundesministerium für Bildung und Forschung, BMBF) Speaker: Vertex Pharmaceuticals (unrelated to this project) Grant Recipient: Enterprise Therapeutics (unrelated to this project) Grant Recipient: Vertex Pharmaceuticals (unrelated to this project) Grant Recipient: Boehringer Ingelheim (unrelated to this project).
Ingo Baumann: Board Member: Pediatric ENT working group of German ENT Society (unrelated to this project) Board Member: Spanish German ENT Society (unrelated to this project) Speaker: Sanofi (unrelated to this project) Speaker: GlaxoSmithKline (GSK) (unrelated to this project).
Maximilian Lulay: Nothing to disclose

**Radiological Phenotypes in Rhinogenic Contact Point Headache: A Novel Prognostic Classification**

*S. Lavalle*[1], E. Masiello[2], P. Romeo[3], A. Maniaci[1], A. Lo Casto[4]; [1]Enna/IT, [2]Milan/IT, [3]Catania/IT, [4]Palermo/IT
*(slavalle6@gmail.com)*

**Purpose:** Rhinogenic contact point headache (RCPH) is still an unsolved diagnostic problem in routine medical practice. In this prospective study, we sought to create and validate a complete radiologic classification system built on anatomic phenotypes, to standardize the diagnosis and inform treatment.
**Methods or Background:** A prospective observational study of 120 patients with suspected RCPH was performed from January 2023 to June 2025. All the patients received high-resolution CT scans and clinical data were retrieved. Five radiological anatomical features (CP, CI, SD, CB, ME, TH, and AV) were evaluated.
**Results or Findings:** In our cohort (42.7±13.5 years and 58.3% female), CP-I (septum-middle turbinate contact) was the most frequent contact type (37.5%), followed by CP-II (22.5%), CP-III (18.3%), CP-IV (12.5%), and CP-V (9.2%). Moderate contact intensity (CI-2) was observed in 45.8% of cases. Septal deviation was categorised as mild (SD-A) in 28.3%, 1.7%, moderate in 41.7% (SD-B) and severe (SD-C) in 30.0% of patients. Concha bullosa was observed in 54.2% of patients and the bulbous type (CB-B) was most frequent (26.7%). Mucosal oedema at contact point (ME-1) was significantly correlated with higher pain scores (p=0.003). Mild and moderate turbinate hypertrophy (TH-I and TH-II) were found in 10% and 40.8% of subjects, respectively. Paradoxical middle turbinate (AV-P) was the most common anatomical variation (22.5%) followed by Haller cells (18.3%).
**Conclusion:** Based on the radiological phenotype, a standardized diagnostic method can guide targeted therapies. The importance of anatomical variants is crucial for proper management.
**Limitations:** Validation in larger series is warranted. It is could be useful to evaluate the inter-reader agreement on larger shorts
**Funding for this study:** No funding
**Has your study been approved by an ethics committee?** Yes
**Ethics committee - additional information:** approval number N. 00208/2024
**Author Disclosures:**
Antonino Maniaci: Nothing to disclose
Antonio Lo Casto: Nothing to disclose
Placido Romeo: Nothing to disclose
Edoardo Masiello: Nothing to disclose
Salvatore Lavalle: Nothing to disclose

**09:30 - 11:00** **Research Stage 3**

### Research Presentation Session: Musculoskeletal

### RPS 2310
### From inflammation to degeneration

**Moderator**
Iwona Sudol-Szopinska; Warsaw/PL
*(sudolszopinska@gmail.com)*

**Structural imaging findings of costovertebral joints by low dose spine CT-scan in patients suspected of axial spondyloarthritis**

*M. A. Korteweg*, M. Wessels, B. Bartels, M. Gerritsen, W. Van der Heijden, C. Van der Laken; Amsterdam/NL
*(mieskorteweg@gmail.com)*

**Purpose:** Axial spondyloarthritis (axSpA) affects costovertebral joints (CVJs). Radiographs visualize CVJs poorly. Advancements in low-dose (ld)CT facilitate spine imaging with 2-7 fold lower effective dose (ED) than radiography (1.4mSv). This study describes structural CVJ changes in suspected axSpA patients.
**Methods or Background:** Between 1/8/24-30/11/24 consecutive suspected axSpA patients were included. Final clinical diagnosis (axSpA, diffuse

Sunday idiopathic skeletal hyperostosis (DISH), rheumatoid arthritis (RA), osteoarthritis (OA)), was outcome variable. Two radiologists, blinded for diagnosis, evaluated ldCTs. 24 medial (M), 20 lateral (L) CVJs were scored per patient using Kellgren-Lawrence OA-classification; 0-4 (no pathology-complete ankyloses)- as no CVJ-scoring system is known. Joints scored 3-4 were rated as significant pathology; and reported as 1 for final analyses. These were compared per level between axSpA and other diagnosis groups using Student's t-test for independent samples, assuming equal variance, with two-tailed distribution (p-value ≤0.05 = statistically significant). Erosions were assessed visually. Interobserver agreement and ED were determined.
**Results or Findings:** 77 patients (48 female, mean age 45y; range 19-74y) were included with clinical diagnosis: axSpA (n=26); DISH (n=6); RA (n=5), OA (n=40). Structural changes occur at different levels in all groups. Average number of significantly affected CVJs/patient in axSpA, DISH, RA, OA: 8.5; 12; 6; 8.5. Most prevalent is; axSpA at CVJ4L (p=0.05), DISH at CVJ10L (p=0.02). Mean ED; 1.3 mSv (± SD 0.4). 1 patient had erosions (20 CVJs); diagnosed axSpA. Interobserver agreement; kappa 0.86 (95% CI 0.73-1.00).
**Conclusion:** LdCT detects structural CVJ changes at low ED. AxSpA structural abnormalities overlap in amount and number with; DISH, RA, OA. RA shows the least and DISH the most changes- mainly caudally. Erosions are scarce and only in axSpA. Potential differential features in structural imaging findings at affected sites warrant further investigation.
**Limitations:** No major limitations.
**Funding for this study:** No funding
**Has your study been approved by an ethics committee?** Yes
**Ethics committee - additional information:** The METC approved this study.
**Author Disclosures:**
Martijn Gerritsen: Nothing to disclose
Mascha Wessels: Nothing to disclose
Conny Van Der Laken: Research/Grant Support: GlaxoSmithKline GSK Research/Grant Support: Pfizer Consultant: UCB Pharma Research/Grant Support: Abbvie Research/Grant Support: Novartis Consultant: Lily Consultant: Galapagos Consultant: GlaxoSmithKline GSK Research/Grant Support: UCB Pharma.
Bart Bartels: Nothing to disclose
Mies A. Korteweg: Nothing to disclose
Wouter Van Der Heijden: Nothing to disclose

### Novel Source-Separated QSM for Improved Hemosiderin Assessment in Hemophilic Arthropathy

*S. Sedaghat*[1], J. Park[2], E. Fu[2], M. Grözinger[1], H-U. Kauczor[1], H. Jang[2]; [1]Heidelberg/DE, [2]Davis/US

**Purpose:** To evaluate a novel source-separated quantitative susceptibility mapping (QSM) technique integrated with ultrashort echo time (UTE) for accurate detection and quantification of hemosiderin in hemophilic arthropathy (HA), and to compare it with conventional QSM methods.
**Methods or Background:** This prospective, multicenter study included 10 MRI scans at 3T of knee and ankle joints (5 HA patients and 5 healthy controls). A validation phantom containing calcium carbonate (diamagnetic) and iron oxide (paramagnetic) was used. Three susceptibility mapping approaches were compared: (1) the novel multi-step source-separated UTE-QSM, (2) a non-source-separated UTE-QSM, and (3) a conventional non-UTE QSM. Susceptibility values were quantified in the phantom, and in vivo image quality was assessed by two musculoskeletal radiologists using a 5-point Likert scale.
**Results or Findings:** In the phantom, conventional QSM underestimated susceptibility (up to 0.236 ppm), whereas source-separated UTE-QSM yielded distinct diamagnetic (–0.704 to –0.410 ppm) and paramagnetic (0.484 to 0.857 ppm) values, strongly correlating with true concentrations ($R = -0.99 / 0.95$, $p < 0.05$). In vivo, visual Likert-scale ratings confirmed that source-separated UTE-QSM maps provided the highest image quality and diagnostic confidence. They consistently detected hemosiderin deposits in all HA patients, delineating both subtle focal accumulations and extensive joint involvement. These findings were underestimated on conventional QSM and only partially visible on non-source-separated UTE-QSM, particularly in regions with mixed tissue composition. Lesion conspicuity and boundary sharpness were superior on source-separated maps, facilitating reliable tissue differentiation. No false-positive paramagnetic signals were observed in healthy controls, underscoring the technique's specificity.
**Conclusion:** Source-separated UTE-QSM enables superior quantification and visualization of hemosiderin in hemophilic arthropathy compared with conventional approaches, supporting its potential as a valuable tool for diagnosis and longitudinal monitoring in HA.
**Limitations:** Small cohort and specialized post-processing requirements limit immediate clinical implementation.
**Funding for this study:** This research received funding from the National Institutes of Health (NIH) in the US and from the Deutsche Forschungsgemeinschaft (DFG) in Germany.
**Has your study been approved by an ethics committee?** Yes
**Ethics committee - additional information:** University of California San Diego
**Author Disclosures:**
Jinil Park: Nothing to disclose
Sam Sedaghat: Nothing to disclose
Martin Grözinger: Nothing to disclose
Hans-Ulrich Kauczor: Nothing to disclose
Hyungseok Jang: Nothing to disclose
Eddie Fu: Nothing to disclose

### Imaging of polymyalgia rheumatica: A comparison between contrast-enhanced whole-body MRI and (18F)-FDG-PET

*S. Mema*, R-T. Hoffmann, R. Winzer, M. Miederer, I. Platzek; Dresden/DE

**Purpose:** The aim of the present study is to assess the agreement between contrast-enhanced whole-body MRI and FDG-PET in the detection of joint and entheses involvement in patients with polymyalgia rheumatica.
**Methods or Background:** The current retrospective study included 26 (15 women and 11 men, mean age 70.4 y, SD 8.1) with polymyalgia rheumatica who received a contrast-enhanced FDG-PET-MRI in the period between 2013 and 2023. 10 anatomical structures per patient (glenohumeral, acromioclavicular and hip joints, greater trochanters and ischial tuberosities) were evaluated independently by two nuclear medicine physicians and two radiologists. A total of 155 joints and 102 tendon insertions were evaluated. A four-point Likert scale was used to rate the signs of inflammation (0 = no signs of inflammation ....up to 3 = pronounced signs of inflammation). Differing results were resolved in consensus. Weighted kappa (k) was used to quantify the agreement between methods (MRI and FDG-PET) as well as the interrater agreement.
**Results or Findings:** Scores based on FDG-PET and MRI were identical for 90 anatomical sites and differed for 167 sites. The resulting weighted kappa was k = 0.45, indicating moderate agreement according to Landis and Koch´s criteria.
**Conclusion:** Whole-body MRI revealed bilateral symmetric synovitis and enthesitis in the majority of patients. The agreement between MRI and FDG-PET regarding the extent of inflammation of the synovium and entheses was moderate. A substantial number of joints and entheses identified as inflamed on FDG-PET were interpreted as normal on MRI. These findings suggest that whole-body MRI may underestimate inflammatory activity in PMR compared to FDG-PET.
**Limitations:** Limitations include the small patient cohort and the fact that many patients had already initiated treatment at the time of FDG-PET/MRI.
**Funding for this study:** Internal funds
**Has your study been approved by an ethics committee?** Yes
**Ethics committee - additional information:** This study was approved by the ethics committee of Dresden University of Technology (file number BO-EK-307082024)
**Author Disclosures:**
Ralf-Thorsten Hoffmann: Nothing to disclose
Matthias Miederer: Nothing to disclose
Robert Winzer: Nothing to disclose
Ivan Platzek: Nothing to disclose
Suela Mema: Nothing to disclose

### To X-ray or not to X-ray - Preliminary results of a comparative study on first-line imaging in suspected axial Spondyloarthritis (axSpA)

*D. Deppe*, S. T. Ulas, M. Koka, F. N. Proft, V. Rios Rodriguez, M. Protopopov, J. Rademacher, D. Poddubnyy, T. Diekhoff; Berlin/DE
*(dominik.deppe@charite.de)*

**Purpose:** In routine practice, patients with suspected axial Spondyloarthritis (axSpA) usually undergo sacroiliac joint radiography, followed by MRI if negative. However, X-rays (XR) are limited by low sensitivity and specificity. Computed tomography (CT) is gold standard for detecting structural lesions and can now be performed with ultra-low-dose (uld) techniques at radiation exposure comparable to or lower than XR. This study evaluates the diagnostic efficacy of XR-, MRI-, and uld-CT -based pathways in suspected axSpA.
**Methods or Background:** 231 patients with suspected axSpA were randomized into three arms: 1. Standard (XR first, followed by MRI if negative; followed by uld-CT if negative) 2. MRI-first (followed by uld-CT if MRI was negative) 3. CT-first (followed by MRI if CT was negative). Positive imaging was defined by the unambiguous identification of active inflammatory (according to updated ASAS definition) or structural lesions (e.g. extensive erosions), rated in consensus reading by two radiologist specialists in musculoskeletal imaging.
**Results or Findings:** In the standard-imaging Arm (42 patients), XR was positive in 4/42 patients (9.52%). Subsequent MRI was positive in three patients (3/38; 7.89%) and negative in the other 35 patients. All 35 patients who underwent further subsequent CT had negative results. In the MRI-first arm (97 patients), MRI was positive in 21/97 (21.65%) patients. Subsequent CT in 76 patients showed no positive results (0/76). In the CT-first arm (92

Sunday patients), initial CT was positive in 23/92 (25 %). Subsequent MRI, performed in 69 patients, was positive in two patients (2.9%).
**Conclusion:** The standard imaging pathway demonstrated the lowest diagnostic efficacy compared to the MRI-first and CT-first approaches, raising the question of whether the current imaging pathway should be updated.
**Limitations:** This is an ongoing study and clinical final diagnosis as the gold standard is missing.
**Funding for this study:** No funding was provided for this study.
**Has your study been approved by an ethics committee?** Yes
**Ethics committee - additional information:** The study was approved by the local ethics committee (EA1/145/22).
**Author Disclosures:**
Dominik Deppe: Nothing to disclose
Denis Poddubnyy: Nothing to disclose
Torsten Diekhoff: Nothing to disclose
Mikhail Protopopov: Nothing to disclose
Mariam Koka: Nothing to disclose
Fabian N. Proft: Nothing to disclose
Valeria Rios Rodriguez: Nothing to disclose
Sevtap Tugce Ulas: Nothing to disclose
Judith Rademacher: Nothing to disclose

**Predicting total knee arthroplasty in osteoarthritis: comparative performance of radiomics, clinical, and combined models**
*A. Pooyan*[1], E. Alipour[2], M. Nyflot[2], M. Chalian[2]; [1]Philadelphia, PA/US, [2]Seattle, WA/US

**Purpose:** Knee osteoarthritis (OA) is a prevalent disabling disease. Total knee arthroplasty (TKA) treats end-stage OA. Early identification of patients at high risk for TKA may improve understanding of disease progression. This study aimed to find the features associated with TKA and compared the performance of clinical, radiomics, and combined models for predicting TKA in OA patients.
**Methods or Background:** We analyzed 507 knees from the OAI-ZIB dataset with manually segmented baseline MRIs. The outcome was TKA within 9 years in the segmented knee. Radiomics features (n=400) were extracted from femoral bone, femoral cartilage, tibial bone, and tibial cartilage using PyRadiomics. Clinical variables (n=13) included demographics, comorbidities, medication use, and baseline WOMAC scores. Univariate logistic regression with Benjamini-Hochberg correction identified significant features. Three model types: logistic regression, Least Absolute Shrinkage and Selection Operator (LASSO), and Extreme Gradient Boosting (XGBoost), were trained on three feature sets: radiomics only, clinical only, and combined. Performance was assessed by AUC-ROC, AUC-PR, and F1-score with 95% confidence intervals. Feature importance for XGBoost was evaluated using SHAP values.
**Results or Findings:** 45 radiomics features and three clinical variables (WOMAC pain, disability, stiffness) were significantly associated with TKA in univariate analysis. The combined XGBoost model performed best, achieving an AUC-ROC of 0.95 (95% CI: 0.92-0.97), AUC-PR of 0.72 (0.58-0.82), and F1 of 0.56 (0.46-0.64) in cross-validation, and an AUC-ROC of 0.72 (0.57-0.84), AUC-PR of 0.30 (0.16-0.47), and F1 of 0.48 (0.31-0.63) on the test set. Final model included 38 of 413 features, with femoral bone sphericity, WOMAC disability, and WOMAC pain as top predictors.
**Conclusion:** Combining radiomics with clinical features improves TKA prediction in OA, with XGBoost showing the strongest performance.
**Limitations:** Study was limited by using TKA as a surrogate outcome influenced by non-disease factors.
**Funding for this study:** No funding was received for this study.
**Has your study been approved by an ethics committee?** Not applicable
**Author Disclosures:**
Ehsan Alipour: Nothing to disclose
Atefe Pooyan: Nothing to disclose
Majid Chalian: Nothing to disclose
Matthew Nyflot: Nothing to disclose

**Long-Term Impact of Intralesional Bony Overgrowth on Opposing Cartilage Integrity: Five-Year Results Following Cartilage Repair**
*F. R. M. König*, M. W. Raudner, G. Wollner, V. Juras, P. Szomolanyi, V. Vetchy, J. Leitner, V. Schmidbauer, S. Trattnig; Vienna/AT
*(felix.koenig@meduniwien.ac.at)*

**Purpose:** This study aimed to assess the impact of intralesional bony overgrowth (ILBO) after cartilage repair on the integrity of opposing articulating cartilage (OpaC) using T2 mapping and to correlate these findings with clinical outcomes in a five year follow up.
**Methods or Background:** In this multicenter study, MRI examinations were conducted in 45 patients up to 5 years after cartilage repair (Microfracturing [MFX] and Matrix-induced Autologous Chondrocyte Implantation [MACI]). ILBO was classified into <50% and ≥50% of the transplant thickness. T2 values of the opposing articulating cartilage (OpAC) were measured at 3, 12, and 60 months post-surgery in patients with and without ILBO at 60 months, together with clinical assessments using the International Knee Documentation Committee (IKDC) and Knee injury and Osteoarthritis Outcome Score (KOOS).
**Results or Findings:** At 60 months post-surgery, 44.4% of patients had ILBO, associated with significantly higher OpAC T2 values (P = 0.004). A trend toward increased T2 values at 12 months was observed but was not statistically significant (P = 0.06). No significant differences in clinical outcomes were found between patients with or without ILBO, nor between those with higher or lower OpAC T2 values. Notably, even small ILBO (<50%) significantly elevated OpAC T2 ratios.
**Conclusion:** Even small ILBO (<50% or ≥50% of transplant thickness) significantly alters the biophysical MRI properties of OpAC, shown as higher T2 values after 60 months. Although these changes are not yet reflected in clinical scores, they may indicate early cartilage degeneration and could guide future monitoring strategies after cartilage repair.
**Limitations:** ILBO was categorized as <50% or ≥50% overgrowth. While useful, finer 25% intervals could clarify ossification effects on OpAC. Additionally, a 60-month follow-up may be too short to reveal long-term clinical implications.
**Funding for this study:** This study has received funding from FWF Der Wissenschaftsfonds (grant no. KLI917) and the Slovak Research and Development Agency (grant no. APVV-21-0299)
**Has your study been approved by an ethics committee?** Yes
**Ethics committee - additional information:** Approval was obtained from the local ethics committees and federal authorities at each participating site prior to patient enrollment. The study was conducted from May 2013 to June 2023 to compare the outcomes after MFX and MACI. This trial was registered under EudraCT number 2011-005798-22 and ClinicalTrials.gov identifier NCT01656902.
**Author Disclosures:**
Victor Schmidbauer: Nothing to disclose
Pavol Szomolanyi: Nothing to disclose
Vladimir Juras: Nothing to disclose
Felix Ragnar Merlin König: Nothing to disclose
Veronika Vetchy: Nothing to disclose
Gregor Wollner: Nothing to disclose
Siegfried Trattnig: Nothing to disclose
Marcus Wolfgang Raudner: Nothing to disclose
Johannes Leitner: Nothing to disclose

**Impact of type II lunate morphology on hamatolunate joint impingement and wrist osteoarthritis in patients with wrist pain**
*M. Aslan*, T. Pınarbaşılı, I. Erdem Toslak; Antalya/TR
*(drmelisaaslan@gmail.com)*

**Purpose:** The aim of the study is to evaluate hamatolunate facet morphology and association with cartilage lesions using magnetic resonance imaging(MRI) in patients with wrist pain
**Methods or Background:** From January to December 2024 wrist MRI of patients with Type II lunate were retrospectively evaluated.Geometric characteristics of the hamate(tapered/round) and lunate facet(concave/straight) was decided.Maximum diameter of the facet of the lunate and hamate, the angle between the ulnar and radial margins of the hamate were measured from coronal images. Ulnar variance was evaluated from X-Rays. On MRI, triangularfibrocartilage complex lesions were noted and cartilage surface of the hamatolunate facet was subjectively graded as 0;normal, 1;partial thickness, 2; full thickness cartilage defect, 3; full thickness cartilage defects with signal changes in the subchondral bone.Data was statistically analyzed.
**Results or Findings:** We included 137 wrists of 127 patients. The mean coronal sizes of the lunate and hamate facet were 2.99(±0,92) and 3.55(±1.2) mm respectively. The mean angle between margins of the hamate was 43.8°(±9.3). %36.5 of the patients had positive, %36.5 had negative ulnar variance. 46.7% of patients had grade o, 20.4% had grade 1 , 21.9% had grade 2, 10.9 % had grade 3 cartilage lesions. There was not any correlation between the measurements or geometrical analysis of hamate,TFCC lesions or ulnar variance with the cartilage lesions. Logistic regression analysis revealed a trend towards presence of cartilage lesions as the lunate transverse diameter decreases (p=0.083).There was a statistically significant correlation between age and grade 3 lesions (p=0.047)
**Conclusion:** In patients with type II lunate, age is an important contributor factor for grade 3 cartilage lesions, Patients with shorter lunate facet are more likely to have cartilage lesions.
**Limitations:** Small sample size and retrospective nature of the study are the limitations.
**Funding for this study:** No funding was received.
**Has your study been approved by an ethics committee?** Yes
**Ethics committee - additional information:** Antalya Training and Research Hospital Ethics Commitee 2025-80
**Author Disclosures:**
Iclal Erdem Toslak: Nothing to disclose
Tansu Pınarbaşılı: Nothing to disclose
Melisa Aslan: Nothing to disclose

**A new approach to whole body-MRI for rheumatological imaging in psoriatic arthritis using dixon fat fraction and water fraction mapping**

*S. Lavalle*[1], A. Montana[2], F. Aiello[1], G. Privitera[2], R. Foti[2], R. Foti[2], P. Romeo[2]; [1]Enna/IT, [2]Catania/IT
*(slavalle6@gmail.com)*

**Purpose:** Whole-body MRI (wbMRI) is an advanced scan modality which provides high-contrast resolution images of the entire body and it's a valuable tool for diagnosing and monitoring systemic arthritides by virtue of its comprehensive coverage . We aim to investigate how quantification of DIXON fat fraction (FF) mapping and water fraction (WF) mapping on diagnostic accuracy and how it change in relaction to sex and age.
**Methods or Background:** In this prospective imaging study, we enrolled 67 patients with confirmed or suspected rheumatologic disease, with possible axial involvement, who underwent whole-body MRI. We assessed both sacroiliac joints at four sites. Differences in fat fraction and water fraction were observed and correlated. To obtain robust and accurate confidence intervals, we performed all analyses with the nonparametric Mann-Whitney and Kruscal-Wallis tests.
**Results or Findings:** There is a significant correlation between age and FF values, in particular as age increases, FF values increase and older subjects present lower WF values. Sex does not determine significant differences in the mappings. Furthermore, patients with psoriatic arthritis have higher FF values than other rheumatological patients (p value 0.008).
**Conclusion:** These preliminary data suggest that age and the presence of psoriatic arthritis are correlated with elevated FF values. Larger studies may help identify the optimal cutoff for predicting disease activity and progression.
**Limitations:** Small simple size, inter-reader agreement
**Funding for this study:** No funding
**Has your study been approved by an ethics committee?** Not applicable
**Author Disclosures:**
Placido Romeo: Nothing to disclose
Salvatore Lavalle: Nothing to disclose
Roberta Foti: Nothing to disclose
Fabio Aiello: Nothing to disclose
Rosario Foti: Nothing to disclose
Giambattista Privitera: Nothing to disclose
Angelo Montana: Nothing to disclose

**Ultrasound Detection of Synovitis in Immune Checkpoint Inhibitor Therapy: Prevalence and Clinical Correlates**

*A. Shah*, M. H. Mir, N. A. Choh; Srinagar/IN
*(shaaamer@gmail.com)*

**Purpose:** The primary objective was to estimate the prevalence of ultrasound-detected synovitis in patients receiving ICIs(Immune checkpoint inhibitors ). Secondary objectives were to evaluate correlations between ultrasound findings and musculoskeletal symptoms, to assess associations with laboratory biomarkers, and to describe the joint distribution.
**Methods or Background:** In this prospective cohort, 120 patients starting ICI therapy were screened and 110 enrolled. All underwent clinical evaluation, laboratory testing(ESR, CRP, RF, anti-CCP, ANA), and standardized US of wrists, MCP(metacarpals), and knees three months after ICI initiation. Synovitis was defined as gray-scale (GS) ≥1 and/or power Doppler (PD) ≥1 using OMERACT scoring. Prevalence was estimated with 95% CIs(Confidence Intervals), and predictors were assessed using logistic regression.
**Results or Findings:** US-detected synovitis was present in 62/110 patients (56.4%, 95% CI 47.0–65.3%). PD activity was seen in 19%, and effusion in 71%. Wrists were most often affected (52%), followed by MCP joints (38%) and knees (24%). Prevalence did not differ by cancer type or ICI regimen. Synovitis was far more frequent in symptomatic patients (84%) than in asymptomatic patients (28%). Inflammatory markers and autoantibodies showed no significant association. On multivariable analysis, musculoskeletal symptoms were the only independent predictor (OR 3.1, 95% CI 1.4–7.8).
**Conclusion:** This study revealed a high prevalence of US detected synovitis in ICI-treated patients, including many without joint symptoms. Conventional biomarkers were unhelpful, highlighting US as a valuable tool for early detection and monitoring of musculoskeletal irAEs.
**Limitations:** This was a single-centre study with a modest sample size, restricting subgroup analyses by cancer type and ICI regimen. Patients were evaluated only at a three-month time point, so the prognostic significance of subclinical synovitis remains uncertain. Longitudinal follow-up is needed to establish whether subclinical synovitis predicts clinical progression or necessitates treatment modification.
**Funding for this study:** No funding was provided for this study
**Has your study been approved by an ethics committee?** Yes
**Ethics committee - additional information:** The ethics committee notification can be found under the number IEC/SKIMS Protocol # 91/2025
**Author Disclosures:**
Aamir Shah: Nothing to disclose
Naseer Ahmad Choh: Nothing to disclose
Mohammad Hussain Mir: Nothing to disclose

**A Comparison of T2 Relaxometry, ADC Imaging& Diffusion Tensor Imaging in assessment of early onset Articular Cartilage damage of the knee**

*R. B. Venkatasubramanian*[1], R. Sivakumar[1], G. Murugan[1], L. Murali[2], V. Sai[1], R. Ramachandran[1], B. Dev[1], R. Rangasami[1], G. Ilangovan[2]; [1]Chennai/IN, [2]Tirupattur/IN
*(vrajeshwarbalaji@gmail.com)*

**Purpose:** To compare&evaluate T2 Relaxometry, ADC /DWI and Diffusion Tensor Imaging in assessment of early-onset articular cartilage damage of knee in young adults.
**Methods or Background:** Certain routine sequences in MRI knee protocol like T2,GRE can help in assessing cartilage damage once macrostructural damage had already occured. However special sequences like T2 Relaxometry, ADC and DTI/FA are increasingly used in cases with high suspicion of microstructural /impending cartilage damage, especially in athletes and trauma victims. T2 relaxometry is sensitive to collagen integrity and water content. ADC/diffusion weighted sequence can identify early stages of inflammation. Diffusion Tensor Imaging sensitively depicts proteoglycan content and collagen structure in cartilage. 40 adults(18-30 years age) who presented for knee MRI for indications other than trauma/pain and without gross signal changes in T2/PDFS were chosen. Additional primary sequences of T2 Relaxometry, ADC/DWI and DTI centered for cartilage and derivative sequences like T2*, R2*, AvDC and Fractional anisometry were obtained. Qualitative&quantitative assessment of cartilage was done with these sequences to establish the more sensitive/specific sequence which can help to detect cartilage damage earlier than conventional sequences.
**Results or Findings:** T2 relaxometry showed qualitative signal changes in articular cartilage earlier than ADC&DTI, predominantly in tibiofemoral joint surface. Healthy controls had mean T2 values 29.1 +/- 3.2 msec, while early-onset cartilage damage had 35.2+/- 3.3 [p<0.05] in affected areas. ADC values in healthy controls were 1.81 +/- 0.44 x 10−3mm2/s compared to 1.21 +/- 0.89 x 10-3mm2/s.[p<0.05].FA values showed minimal difference even in cartilage damage identified in other 2 sequences.
**Conclusion:** T2 relaxometry is a sensitive sequence for cartilage microstructural damage. T2 relaxometry and ADC mapping can be done routine in athletes and young adults for early detection of cartilage damage.
**Limitations:** Limited sample size can be increased.
**Funding for this study:** Self funded
**Has your study been approved by an ethics committee?** Yes
**Ethics committee - additional information:** PhD Radiology dissertation study
**Author Disclosures:**
Rajalakshmi Sivakumar: Nothing to disclose
Venkata Sai: Nothing to disclose
Bhawna Dev: Nothing to disclose
Rajoo Ramachandran: Nothing to disclose
Lokesh Murali: Nothing to disclose
Rajeshwar Balaji Venkatasubramanian: Nothing to disclose
G. Murugan: Nothing to disclose
Gurubharath Ilangovan: Nothing to disclose
Rajeswaran Rangasami: Nothing to disclose

**Quantitative Elastography of the Achilles Tendon Detects Subclinical Enthesopathy in Psoriatic Arthritis**

V. Burulday, *A. Gündüz Uslu*, S. Yolbaş; Malatya/TR
*(gunduzaysun4@gmail.com)*

**Purpose:** To compare Achilles tendon elasticity, measured by shear wave elastography (SWE), between asymptomatic psoriatic arthritis (PsA) patients and healthy controls. PsA frequently involves entheseal structures, but subclinical changes often escape clinical detection. SWE provides a non-invasive, quantitative method to assess tendon stiffness and may reveal early abnormalities.
**Methods or Background:** This prospective case–control study was conducted at Inonu University (Oct 2024–Feb 2025). Thirty-four PsA patients (≥2 years disease duration, 18–65 years, no Achilles symptoms) and 34 age- and sex-matched healthy volunteers were enrolled. Exclusion criteria included diabetes, rheumatoid arthritis, ankylosing spondylitis, tendinopathy, systemic steroids or biologics, recent ankle trauma or surgery. Ultrasound and SWE were performed with a Mindray Resona I9, L14-3Ws probe. Participants lay prone with feet relaxed. Achilles thickness was measured 3 cm proximal to the calcaneal insertion. SWE was performed longitudinally with a 3 mm ROI; six measurements per tendon were averaged. Values were recorded in m/s and kPa. Only high-quality images (M-STB = 5) were analyzed.

**Results or Findings:** Demographics did not differ between groups. Tendon thickness was similar bilaterally ($p>0.5$). SWE values were significantly higher in PsA patients. Left tendon: 4.89 ± 2.52 m/s vs 3.23 ± 0.41 m/s ($p<0.001$). Right tendon: 4.88 ± 1.94 m/s vs 3.12 ± 0.30 m/s ($p<0.001$). kPa values showed the same pattern, indicating subclinical stiffening in PsA.
**Conclusion:** SWE demonstrated increased Achilles tendon stiffness in asymptomatic PsA patients despite normal thickness. Elastography may serve as a sensitive imaging biomarker for early detection and monitoring of enthesopathy. Larger, multicentre studies are needed.
**Limitations:** Single-centre design, modest sample, no EMG or histopathology, and no treatment-based analysis.
**Funding for this study:** Supported by Inonu University Scientific Research Projects Coordination Unit (BAP). No external funding.
**Has your study been approved by an ethics committee?** Yes
**Ethics committee - additional information:** Approved by Inonu University Clinical Research Ethics Committee (protocol 2024/119). Written informed consent was obtained from all participants.
**Author Disclosures:**
Aysun Gündüz Uslu: Nothing to disclose
Servet Yolbaş: Nothing to disclose
Veysel Burulday: Nothing to disclose

**09:30 - 11:00** **Research Stage 4**

### Research Presentation Session: Paediatric

### RPS 2312

### Future directions of paediatric neuroradiology

**Moderator**
Chiara Carducci; Roma/IT
*(chi.carducci@gmail.com)*

### Melatonin alleviates lactate and promotes neurogenesis via the JAK2/STAT3 pathway in hypoxic-ischemic neonatal rats

X. Wang[1], Y. Wang[1], M. Yu[1], Y. Li[1], *S. Gui*[2], X. Zhang[1], Y. Qin[1], N. Xue[1], Y. Zeng[1]; [1]Weifang/CN, [2]Shanghai/CN
*(shen.gui@philips.com)*

**Purpose:** Multimodal magnetic resonance imaging (MRI) combined with multiple histological techniques was employed to investigate the effects of melatonin (Mel) treatment on endogenous neural stem cells (NSCs) regeneration and metabolic conditions in the hippocampal dentate gyrus, and explore the potential neuroprotective effect via the JAK2/STAT3 pathway in neonatal rats with hypoxic-ischemic brain damage (HIBD).
**Methods or Background:** Cerebral blood flow was dynamically monitored by laser speckle imaging. Multimodal MRI was applied in vivo to assess metabolic alterations in the cerebral microenvironment. Multiple histological techniques were employed to evaluate the effect of endogenous neurogenesis in the dentate gyrus (DG). 28 days post-modeling, the Morris water maze (MWM) was used to assess behavioral and cognitive function.
**Results or Findings:** Laser speckle imaging revealed that cerebral blood flow decreased most significantly at 24 h after HIBD . T2WI demonstrated a marked reduction in infarct volume following Mel treatment, accompanied by decreased abnormal DWI signals and significantly increased rADC values. Both Amide-CEST and NOE-CEST showed reduced abnormal signals in the injured hemisphere after Mel treatment, with LD-Amide and LD-NOE values significantly elevated MRS indicated a reduction in lactate levels in the Mel group. EdU/Nestin and EdU/DCX immunofluorescence staining revealed that Mel promoted the proliferation and migration of endogenous NSCs. The JAK2 inhibitor (WP1066) and agonist (C-A1) were used and results demonstrated that Mel exerts protective effects by inhibiting the JAK2/STAT3 pathway. MWM further confirmed that Mel improved memory function in neonatal rats with HIBD.
**Conclusion:** Multimodal MRI offers a visual basis for monitoring metabolic changes and reveals that Mel treatment can improve cerebral microenvironmental metabolism in HIBD neonatal rats. Moreover, Mel promotes endogenous NSC proliferation and migration by inhibiting the JAK2/STAT3 pathway.
**Limitations:** We recommend conducting further clinical studies.
**Funding for this study:** This work was supported by grants from the National Natural Science Foundation of China (Grant No. 82071888), the Natural Science Foundation of Shandong Province (Grant No. ZR2024MH067), the 2019 Young Creative Talent Induction Program for Higher Education Institutions, Shandong medical health science and technology project (Grant No. 202309041351)
**Has your study been approved by an ethics committee?** Yes
**Ethics committee - additional information:** This study was approved by the Ethics Committee for Animal Experiments of Weifang Medical College (reference number: 2019 SDL 024, China).
**Author Disclosures:**
Xiaoli Wang: Nothing to disclose
Yanyu Zeng: Nothing to disclose
Xiaozu Zhang: Nothing to disclose
Youcheng Qin: Nothing to disclose
Shen Gui: Nothing to disclose
Ning Xue: Nothing to disclose
Yijing Wang: Nothing to disclose
Yan Li: Nothing to disclose
Miao Yu: Nothing to disclose

### Glymphatic system dysfunction in children with MRI-Negative focal epilepsy and relationship to cognitive impairment: A multimodal MRI study

*H. Ran*, T. Zhang, K. Huang; Zunyi/CN
*(haifengran_zmu@163.com)*

**Purpose:** Epilepsy is a common pediatric disease that often leads to cognitive and intellectual impairments, but the relationship between glymphatic system function and cognitive performance in children with focal epilepsy(FE) remains unclear. This study investigated the association between glymphatic system markers and cognitive outcomes in childhood FE.
**Methods or Background:** 80 FE children and 87 healthy controls(HC) were recruited, all subjects underwent multimodal MRI scan and cognitive performance assessment. And the glymphatic markers were acquired based on MRI data, including choroid plexus(CP) volume, cerebrospinal fluid(CSF) volume, diffusivity along perivascular spaces(ALPS), blood-oxygen-level-dependent(BOLD) signals, and CSF(BOLD-CSF) coupling. Comparisons of glymphatic markers were conducted between the FE and HC groups. The correlation analysis was performed to assess the association between glymphatic markers and cognitive function scores and clinical variables.
**Results or Findings:** The ratio of CP, CSF volume to intracranial volume was significantly higher in children with FE compared to HC($p<0.05$). Additionally, the BOLD-CSF coupling in the whole and middle cortical regions and the left hemisphere and global ALPS index were significantly lower in FE children compared to HC($p<0.05$). the ratio of CP volume changes were negatively correlated with PRI and onset age($p<0.05$), the ratio of CSF volume changes were negatively correlated with PRI and FSIQ($p<0.05$), and the BOLD-CSF coupling in the whole and middle cortical regions were negatively correlated with the age($p<0.05$) , while the left hemisphere ALPS index was positively correlated with VCI, PRI, WMI, PSI, FSIQ, respectively($p<0.05$), the global ALPS index was positively correlated with VCI, WMI, PSI, FSIQ, respectively($p<0.05$) and negatively correlated with the disease duration($p<0.05$).
**Conclusion:** These findings identify an association between glymphatic system dysfunction and cognitive impairment in children with FE.
**Limitations:** The cross-sectional design limits the ability to track spatiotemporal changes in glymphatic dysfunction.
**Funding for this study:** This research was supported by the National Natural Science Foundation of China (Grant Nos .82171919) and Intelligent Medical Imaging Engineering Research Center of Guizhou Higher Education Institutions project (Grant No. Qianjiaoji [2023] 038)
**Has your study been approved by an ethics committee?** Yes
**Ethics committee - additional information:** The Ethics Committee of Zunyi Medical University reviewed and granted ethical approval for this study (Ethical Batch Number: lunshen [2021] 1-080).
**Author Disclosures:**
Tijiang Zhang: Nothing to disclose
Kexin Huang: Nothing to disclose
Haifeng Ran: Nothing to disclose

### Glymphatic system dysfunction in children with focal epilepsy evidenced by DKI-ALPS and DTI-ALPS index

*H. Ran*, T. Zhang, K. Huang; Zunyi/CN
*(haifengran_zmu@163.com)*

**Purpose:** The diffusion tensor imaging analysis along the perivascular space(DTI-ALPS) method has been used to evaluate glymphatic system function in patients with epilepsy. However, since the diffusion tensor model may fail to describe the diffusion coefficient of the nerve fibre crossing region accurately. Thus, this study aimed to combined diffusion kurtosis imaging ALPS(DKI-ALPS) and DTI-ALPS methods to evaluate glymphatic system function in children with focal epilepsy(FE).

**Methods or Background:** The study finally included 63 children with FE and 58 healthy controls (HC).All participants underwent MRI scans to acquire DKI and DTI data, and they also received a cognitive assessment on the same day as their MRI scan. We calculated the DTI-ALPS and DKI-ALPS indices for the left brain, the right brain and the whole brain. A two-sample t-test was then used to compare the DTI-ALPS and DKI-ALPS indices of the two groups. We examined the correlation between the DTI-ALPS and DKI-ALPS indices and clinical variables. The statistical threshold was set at $P < 0.05$.
**Results or Findings:** Both groups were matched for age, sex, and education. Compared to HC, FE children showed decreased DTI-ALPS and DKI-ALPS indices. However, both left, right, mean DKI-ALPS index and only mean DTI-ALPS index was significantly decreased($P < 0.05$). Correlation analysis revealed a significant positive correlation between the decreased DTI-ALPS index and the VCI, PRI, WMI, PSI and FSIQ ($P < 0.05$). Regrettably, there was no significant correlation between DKI-ALPS indices and clinical variables.
**Conclusion:** This study demonstrates that abnormal glymphatic function in children with FE and associated with cognitive impairment. DKI-ALPS index showed higher sensitivity than DTI-ALPS in detecting group differences, and DTI-ALPS index may be a potential biomarker for cognitive impairment.
**Limitations:** Due to its cross-sectional design, causal relationships cannot be established.
**Funding for this study:** This study was supported by National Natural Science Foundation of China (grant No. 82171919), Intelligent Medical Imaging Engineering Research Center of Guizhou Higher Education Institutions project (grant No. Qianjiaoji [2023] 038), and Construction of Scientific and Technological Innovation Talent Team of Functional Imaging and Artificial Intelligence Application Research in Guizhou Province (grant No. QianKeHeRenCai CXTD [2025] 047).
**Has your study been approved by an ethics committee?** Yes
**Ethics committee - additional information:** The Ethics Committee of Zunyi Medical University reviewed and granted ethical approval for this study (Ethical Batch Number: lunshen [2021] 1-080).
**Author Disclosures:**
Tijiang Zhang: Nothing to disclose
Kexin Huang: Nothing to disclose
Haifeng Ran: Nothing to disclose

### Impact of intrauterine fetoscopic spina bifida repair on foetal brain edema

*M. Schulze*[1], P. J. Orbán[2], C. Keil[1], S. Köhler[1], R. Axt-Fliedner[2], G. Kasprian[3], I. Bedei[2]; [1]Marburg/DE, [2]Giessen/DE, [3]Vienna/AT
*(maximilian_schulze@gmx.de)*

**Purpose:** Neural tube defects such as myelomeningocele (MMC) and rachischisis are frequently associated with secondary central nervous system alterations including hindbrain herniation, edema, and aqueductal obstruction. Surgical repair aims to reduce these sequelae, but postoperative radiological outcomes remain incompletely characterized.
**Methods or Background:** A retrospective cohort of 31 patients with MMC (n=18) or rachischisis/myelocele (n=13) was analyzed using pre- and postoperative foetal MRI. Parameters included atlanto-occipital membrane bulging, extracerebral space size, brain edema, aqueductal patency, and cavum septum pellucidum (CSP) perforation. Statistical methods comprised descriptive analysis, correlation tests, and McNemar's test for paired pre/post comparisons.
**Results or Findings:** Preoperatively, 71% of patients demonstrated atlanto-occipital bulging, 68% brain edema, and 74% aqueductal stenosis; mean extracerebral space was 3.1 mm. Postoperatively, bulging decreased to 16%, edema to 16% (McNemar $p < 0.001$), and extracerebral space increased to 8.2 mm. However, aqueductal obstruction was observed in 39% of cases postoperatively, and CSP findings remained unchanged or worsened in some patients. A very strong correlation was detected between postoperative hindbrain herniation and postoperative brain edema ($r \approx 0.94$; Chi² $p = 0.0005$).
**Conclusion:** Surgical repair significantly improves edema and hindbrain bulging, with clear enlargement of the extracerebral space. Nevertheless, aqueductal obstruction and CSP changes persist as postoperative risks. The close association between hindbrain herniation and postoperative edema highlights the need for vigilant MRI follow-up and may inform tailored therapeutic strategies.
**Limitations:** Retrospective design. Clinical endpoints such as neurological function or developmental milestones were not evaluated.
**Funding for this study:** There was no funding for this study.
**Has your study been approved by an ethics committee?** Yes
**Ethics committee - additional information:** Ethics committee approval was obtained.
**Author Disclosures:**
Siegmund Köhler: Nothing to disclose
Roland Axt-Fliedner: Nothing to disclose
Philip Julian Orbán: Nothing to disclose
Maximilian Schulze: Nothing to disclose
Corinna Keil: Nothing to disclose
Gregor Kasprian: Nothing to disclose
Ivonne Bedei: Nothing to disclose

### Abnormalities present on MRI in monochorionic twin pregnancies with demise of 1 twin

*P. H. Tang*, W. C. Tan, A. Bhatia, G. Y. H. Ng, J. Y. Kong, A. J. Anand, P. L. Tan, C. L. Ong; Singapore/SG

**Purpose:** Monochorionic twin pregnancies with single intrauterine foetal demise are reported as having highest risk of cerebral injury. Objective is to review our institution's foetal MRI cases in the past decade to determine abnormalities seen in monochorionic twin pregnancies with demise of 1 twin.
**Methods or Background:** Retrospective review of foetal MRI brain performed for monochorionic twin pregnancies for foetal demise / post in utero intervention from 1 Jan 2014 to 31 Dec 2024 was performed. Indication for MRI, gestational age at imaging, type of intervention performed (cord coagulation, ligation of vessel, radiofrequency ablation) were obtained from the radiology report.
**Results or Findings:** There were 54 MRI done for monochorionic twin pregnancies for foetal demise / post in utero intervention from 1 Jan 2014 to 31 Dec 2024. MRI was performed at an average 23.0 weeks of gestation (range from 21.0 to 31.1 weeks.) There were 11 pairs of live twins post intervention with 2 foetuses having mild ventriculomegaly. Only 1 pregnancy being investigated for demise of 1 twin showed demise of both twins, both showing acute brain infarcts. Of the other 42 pregnancies having demise of 1 twin, 36 (85.7%) of the live twins were normal, 5 (11.9%) live twins had brain abnormalities, 1(2.3%) live twin had isolated pericardial effusion. Brain abnormalities were acute infarcts in 2 foetuses, smaller cerebral hemisphere in 1 foetus, thin bilateral cerebral parenchyma in 2 foetuses, possible subependymal heterotopia in 1 foetus, giving cerebral abnormality rate of 13.9% (6/43).
**Conclusion:** Cerebral abnormalities are seen in 13.9% of monochorionic twin pregnancies with demise of 1 twin.
**Limitations:** Postnatal follow up not available at the time of preparation of this abstract.
**Funding for this study:** Nil
**Has your study been approved by an ethics committee?** Not applicable
**Author Disclosures:**
Pih Lin Tan: Nothing to disclose
Wei Ching Tan: Nothing to disclose
Phua Hwee Tang: Nothing to disclose
Chiou Li Ong: Nothing to disclose
Anju Bhatia: Nothing to disclose
Grace Yang Huang Ng: Nothing to disclose
Amudha Jayanthi Anand: Nothing to disclose
Juin Yee Kong: Nothing to disclose

### Morphometric similarity mapping in pediatric migraine without aura: unravelling the role of sex

*A. Guarnera*[1], A. Napolitano[2], F. Dellepiane[2], A. Carboni[2], M. C. Rossi Espagnet[2], G. Moltoni[1], C. Gandolfo[2], D. Longo[2]; [1]Rome/IT, [2]Vatican City/VA
*(guarneraalessia@gmail.com)*

**Purpose:** Pediatric migraine without aura (pmwa) is suspected to cause structural and functional alterations in the brain connectome. MSM (morphometric similarity mapping), which has never been applied to pmwa, is an advanced technique which identifies anomalies of the cerebral cortex and connectivity. The paper aims to: a) identify differences in morphometric similarity networks (MSNs) in pediatric patients affected by mwa compared to controls and in subgroups of patients; and b) correlate these differences in MSNs with patients' demographic and clinical characteristics.
**Methods or Background:** 83 patients (6-18 years), and 81 controls were retrospectively recruited from our Institution's archive if the subjects showed: normal MRI exams and neurological examination, and no comorbidities. 3D T1 MPRAGE and DWI were pre-processed to obtain eleven cortical parameters for the MSN analysis. The global and regional mean morphometric similarities were calculated. We performed separate linear regression models, followed by an ANOVA with an FDR correction and a p-value threshold of 0.05 for all subjects and subgroups of patients in the global and regional models. For significant predictors, a post hoc analysis using the Benjamini-Hochberg method was performed.

**Results or Findings:** MSM identified significant differences in MSNs in patients based on clinical and demographic parameters, particularly biological sex. The cerebral pathways that showed the most significant alterations encompassed the executive function pathway, the nociceptive pathway, and the default mode network ($p<0.001$).
**Conclusion:** Differences in MSNs in the executive function pathway, the nociceptive pathway, and the default mode network demonstrated that migraine shapes the cerebral connectome and favours neuronal plasticity and may lead to chronicity of migraine symptoms. Biological sex is confirmed as a paramount parameter in migraine, supporting the hypothesis of a "sex-specific phenotype".
**Limitations:** The key limitation is the retrospective design of the study.
**Funding for this study:** No funding was received for this study.
**Has your study been approved by an ethics committee?** Not applicable
**Ethics committee - additional information:** The study is retrospective.
**Author Disclosures:**
Carlo Gandolfo: Nothing to disclose
Antonio Napolitano: Nothing to disclose
Alessia Guarnera: Nothing to disclose
Alessia Carboni: Nothing to disclose
Daniela Longo: Nothing to disclose
Giulia Moltoni: Nothing to disclose
Maria Camilla Rossi Espagnet: Nothing to disclose
Francesco Dellepiane: Nothing to disclose

**MRI findings in pediatric idiopathic intracranial hypertension: evaluating the oculomotor nerve cistern as a potential new diagnostic marker**
*B. Arıkan Ergün*, S. Yilmaz, H. Bulut, B. Uçan, S. Seçer, S. Celik, D. Yüksel, Ç. Üner; Ankara/TR
*(bilgesu.arikan@hotmail.com)*

**Purpose:** Idiopathic intracranial hypertension (IIH) is characterized by elevated intracranial pressure without an identifiable cause. Although headache is the most common symptom, prepubertal children often present with ocular motor nerve involvement, differing from older patients. This study aimed to assess the diagnostic value of oculomotor cistern (OMC) enlargement in pediatric IIH, compare it with established MRI findings, and propose it as a potential biomarker.
**Methods or Background:** Brain MRI and contrast-enhanced MR venography of patients under 18 years with clinical signs of increased intracranial pressure were retrospectively reviewed independently by two blinded radiologists. Pituitary morphology, perioptic CSF space, optic nerve tortuosity, posterior scleral flattening, Meckel's cave dimensions, OMC CSF space, and venous sinus narrowing were evaluated. Demographic data, ophthalmologic findings, and CSF pressure measured by lumbar puncture were recorded. Patients with elevated CSF pressure were compared with those with normal pressure.
**Results or Findings:** A total of 76 children with suspected IIH were evaluated, 10 of whom were excluded due to secondary causes. Among the remaining 66, 44 (67%) had elevated and 22 (33%) normal CSF pressure. In addition to established imaging findings—such as increased perioptic CSF, posterior scleral flattening, and optic nerve tortuosity—increased CSF space in the OMC was also statistically significant.
**Conclusion:** Neuroimaging is essential in IIH evaluation. Alongside recognized findings such as perioptic CSF widening, optic nerve tortuosity, posterior globe flattening, sellar changes, and venous sinus stenosis, increased OMC CSF space may serve as a novel imaging marker for diagnosing pediatric IIH.
**Limitations:** The limitations of the study are its retrospective and single-center design, which may limit generalizability, the small sample size, non-homogeneous patient age distribution, and the lack of long-term clinical correlation of oculomotor cistern enlargement with visual outcomes or disease progression.
**Funding for this study:** No funding was received for this study.
**Has your study been approved by an ethics committee?** Yes
**Ethics committee - additional information:** The study was approved by the Ankara Etlik City Hospital Ethics Committee, Ankara, Turkey (no: 4/12)
**Author Disclosures:**
Deniz Yüksel: Nothing to disclose
Selda Celik: Nothing to disclose
Hasan Bulut: Nothing to disclose
Sükriye Yilmaz: Nothing to disclose
Çiğdem Üner: Nothing to disclose
Bilgesu Arıkan Ergün: Nothing to disclose
Sevde Seçer: Nothing to disclose
Berna Uçan: Nothing to disclose

**Transient brain injury in utero imaging among surviving and recipient co-twins in demised, TTTS, or monochorionic pregnancy evaluations in second and third trimester**
S. Babapour, A. Demiray, *J. M. Johnson*; New Haven, CT/US

**Purpose:** There is an increased risk of neurologic damage, 20- 40%, in the surviving twin if one twin dies in utero. This single-institution retrospective study evaluated the effects of co-twin demise and twin-twin transfusion syndrome (TTTS) on fetal brain and vasculature in monochorionic twin pregnancies.
**Methods or Background:** Using fetal brain MRI and ultrasound imaging, 16 defects were identified among 53 in utero twins. The study focused on cases within the first 30 days after a demise during the second and third trimesters. The average gestational age at initial MRI evaluation and based on LMP was 23 weeks and 4 days.
**Results or Findings:** 24 (48%) pregnancies involved a demised twin, most frequently associated with advanced TTTS (stage 3-4) or following radiofrequency ablation. Maternal demographic data were generally unremarkable except for two cases of maternal obesity. The imaging findings of the surviving co-twins, using both ultrasound and MRI, were largely consistent and indicated normal neurodevelopmental growth and cerebral vascular flow. The demise of donor twins occurred within three days post-procedure. In most cases, no evidence of acute brain injury, asymmetric development, or compensatory vascular overflow was detected in the surviving or recipient twins within one month following the co-twin's demise. Notable exceptions included single cases of asymmetric biometry and increased corpus callosum length. Other observed abnormalities among co-existing twins with early-stage TTTS included heart enlargement, ventriculomegaly, and Dandy-Walker malformation, although five cases demonstrated intact brain and vascular flow. There was a high concordance (95.35%) between ultrasound and MRI findings.
**Conclusion:** After advanced TTTS and fetal demise, surviving co-twins generally do not exhibit early brain injury or abnormal vascular compensation within one month; however, comprehensive soft tissue evaluation remains essential.
**Limitations:** A lack of long-term follow-up beyond one month post-demise.
**Funding for this study:** Internal Yale funding
**Has your study been approved by an ethics committee?** Yes
**Ethics committee - additional information:** 2000037822 IRB# informed consent was waived
**Author Disclosures:**
Atalay Demiray: Nothing to disclose
Jason Michael Johnson: Nothing to disclose
Sara Babapour: Nothing to disclose

**Evaluation of fetal brain changes between fetal growth restricted and small-for-gestional age pregnancies using T1 mapping sequence**
*L. Ji*, X. Zhang, M. Zhao; Nanjing/CN
*(jiliangyu943@163.com)*

**Purpose:** Fetal growth restriction (FGR) poses challenges in both the timing and detection of altered brain development, particularly in distinguishing pathological FGR from small for gestational age (SGA) fetuses. This study aimed to investigate differences in T1 relaxation times in the fetal brain between FGR and SGA cases.
**Methods or Background:** STAGE data were collected using two flip angles(15°and 60°, with a TR=600ms) at 1.5T. T1app maps of the fetal brain were generated from the STAGE data. T1 values were measured and compared in the following regions: frontal white matter (FWM), occipital white matter (OWM), centrum semiovale (CSO), thalami (T), cerebellar hemisphere (HC), basal ganglia (BG), and brainstem. T1 values among FGR, SGA, and AGA groups were compared using independent or Welch's t-tests, as appropriate. A p-value $< 0.05$ was considered statistically significant.
**Results or Findings:** Among 50 cases (16 SGA, 13 FGR, 21 AGA), FGR fetuses exhibited significantly higher T1 values in FWM, OWM and CSO compared to the other two groups. FGR showed differences in the BG(1593.21±205.60ms vs 1368.92±233.12ms P=0.006) and brainstem(1315.11±263.57ms vs 1142.69±217.58ms P=0.039) compared to AGA fetuses. The T1 values of AGA fetuses showed negative correlations with gestational weeks in BG (r=-0.660), brainstem (r=-0.603), HC (r=-0.469), FWM (r=-0.646), OWM (r=-0.540) and thalami (r =-0.513).
**Conclusion:** T1 mapping offers a sensitive, non-invasive method for detecting microstructural brain alterations in FGR fetuses, especially in white matter and subcortical regions.
**Limitations:** First, some images were of suboptimal quality due to a small gestational age or polyhydramnios. Second, the study did not assess long-term neurodevelopmental outcomes, so the clinical relevance of the observed differences in T1 values remains uncertain.

**Funding for this study:** Maternal and Child Health Hospital High-level Construction Project(GZL2529)
**Has your study been approved by an ethics committee?** Yes
**Ethics committee - additional information:** It was approved by the institutional ethics committee of The First Affiliated Hospital of Nanjing Medical University.
**Author Disclosures:**
Liangyu Ji: Nothing to disclose
Xuan Zhang: Nothing to disclose
Meng Zhao: Nothing to disclose

**Prenatal Corpus-Callosum Abnormalities: Maternal, Gestational and Brain-Biometric Profiles in a 61-Case MRI Cohort**
S. Babapour, J. Pearce, L. Ment, M. Bahtiyar, K. Kohari, E. Dave, *J. M. Johnson*; New Haven, CT/US

**Purpose:** To characterize maternal and fetal features quantitatively with a standardized panel of brain MRI biometrics among referrals with suspected corpus callosum (CC) anomalies.
**Methods or Background:** In this single-center retrospective evaluation of 61 consecutive fetal MRIs (2013-2025) reported as CC abnormality, morphometric variables were reviewed: bilateral ventricular-atria (VA) diameters, CC length, cerebral and bone biparietal diameters (BPD), vermis antero-posterior (AP) diameter & height, coronal cerebellar diameter and pons AP diameter (table 1). Gestational age (GA) was taken from ultrasound dating or, if absent, last menstrual period.
**Results or Findings:** Mean maternal age at MRI was 31.8 ± 4.7 years (22–42). Median GA was 25 weeks (IQR 22–28). • CC morphology: complete agenesis in 42 (69 %), partial agenesis or hypoplasia in 10 (16 %), dysgenesis in 9 (15 %). • CC length (non-agenesis, n = 32): 29.7 ± 6.1 mm (-1.8 SD vs normative medians). • VA diameters: dilatation ≥ 10 mm in 34/55 measurable cases (62 %). VA_mean inversely correlated with CC length (r = -0.58, p < 0.001). • Posterior fossa: vermis height < 5th percentile in 10/43 (23 %), predominating in complete agenesis (8/10). • Parenchyma–skull discordance: cerebral BPD lagged behind bone BPD by ≥ 5 mm in 28/50 (56 %). No significant association was found between maternal age tier (< 30 vs ≥ 30 yr) and degree of VA dilatation or CC subtype (p > 0.3).
**Conclusion:** In this in utero second and early third trimester MRI series, CC malformations were assessed in mothers of reproductive age. Ventriculomegaly and posterior-fossa under-development were common co-findings and showed quantifiable relationships to CC size. Routine extraction of these biometric signatures may refine prenatal counselling and prognostication in CC anomaly.
**Limitations:** Further multicenter validation and collaboration are required.
**Funding for this study:** Yale internal funding
**Has your study been approved by an ethics committee?** Yes
**Ethics committee - additional information:** IRB-approved
**Author Disclosures:**
Laura Ment: Nothing to disclose
Jason Michael Johnson: Nothing to disclose
Mert Bahtiyar: Nothing to disclose
Sara Babapour: Nothing to disclose
Eesha Dave: Nothing to disclose
Jackson Pearce: Nothing to disclose
Katherine Kohari: Nothing to disclose

**09:30 - 11:00** **Research Stage 5**

### Research Presentation Session: Emergency Imaging

### RPS 2317

### From the abdomen up to the head: new emergency imaging approaches!

**Moderator**
Frank Volker Bensch; Helsinki/FI
*(frank.bensch@hus.fi)*

**Improved Detection of Bowel Ischemia in Emergency CT: Diagnostic Value of Spectral Reconstructions Across DECT and PCCT Platforms**
*F. K. E. Mankertz*, N. Maalouf, J. Berger, J. Herrmann, R. Dehdab, K. Nikolaou, S. Afat; Tübingen/DE

**Purpose:** To assess whether spectral CT reconstructions improve diagnostic accuracy of bowel ischemia detection compared with blended images and stratified by dual energy CT and photon counting CT platforms.
**Methods or Background:** This retrospective single centre study included 378 emergency spectral CT examinations for suspected bowel ischemia between January 2023 and July 2025, with 265 dual energy CT and 113 photon counting CT. Exclusions were absent spectral data, non diagnostic image quality, incomplete bowel coverage, no reference standard within 72 hours, or age <18 years. Two abdominal radiologists with four years subspecialty experience independently reviewed examinations first with blended images and after a four week washout with spectral reconstructions. Readers assigned 1 to 5 suspicion scores per examination and duplicate cases assessed intra reader repeatability. Outcomes were analysed with generalized linear mixed models with random intercepts for reader and case and with DeLong testing.
**Results or Findings:** Bowel ischemia was present in 126/378 examinations (33%). Sensitivity increased from 75% with blended images to 87% with spectral reconstructions (p = 0.008) and specificity from 72% to 86% (p < 0.001). Per reader AUC rose from 0.81 and 0.82 to 0.91 and 0.92, both p < 0.001. Diagnostic confidence improved from 3 to 5 on a 7 point scale. Inter reader agreement rose from kappa 0.56 to 0.71 and intra reader repeatability was high at 0.82. Platform stratified analysis showed significantly greater gains with photon counting CT than with dual energy CT (interaction p = 0.03).
**Conclusion:** Spectral reconstructions improved diagnostic accuracy, confidence and agreement for bowel ischemia detection. Gains were greater with photon counting CT. Integrating spectral reconstructions into emergency CT workflows may improve reliability without added contrast or radiation.
**Limitations:** Single-centre design and reader pool may limit generalisability.
**Funding for this study:** None
**Has your study been approved by an ethics committee?** Not applicable
**Author Disclosures:**
Judith Herrmann: Nothing to disclose
Konstantin Nikolaou: Nothing to disclose
Saif Afat: Nothing to disclose
Josephine Berger: Nothing to disclose
Reza Dehdab: Nothing to disclose
Fiona Karola Elisabeth Mankertz: Nothing to disclose
Nour Maalouf: Nothing to disclose

**Difficult abdominal closure after emergency laparotomy: predictive role of morphometric abdominal indices on CT imaging**
*F. Rizzetto*, R. Bini, P. Chiara, T. Pilia, F. Cammarata, C. B. Monti, S. P. B. Cioffi, D. Albano, A. Vanzulli; Milan/IT
*(francesco.rizzetto@ospedaleniguarda.it)*

**Purpose:** Predicting difficult abdominal closure after emergency laparotomy remains a clinical challenge. This study aimed to assess whether morphometric abdominal indices derived from preoperative CT can predict the need for multiple surgical procedures to achieve definitive closure.
**Methods or Background:** We retrospectively reviewed emergency abdominal CT scans performed between 2010 and 2024 in patients who subsequently underwent open abdominal surgery. Morphometric measurements included maximum latero–lateral diameters of the rib cage, abdomen, and pelvis; maximum anteroposterior and cranio–caudal abdominal diameters; and total abdominal cavity volume. Thickness of the anterolateral abdominal wall muscles, rectus abdominis, and length of the linea semilunaris were also recorded. Demographic data and surgical indication were also collected.

The primary endpoint was difficult abdominal closure, defined as requiring more than one surgical procedure for closure. Variables with p<0.10 at univariate analysis were entered into multivariate logistic regression (significance p<0.05), reporting odds ratios (ORs) and 95% confidence intervals (CIs).
**Results or Findings:** A total of 144 patients (105 males, 72%; mean age 47±21 years) were included. Difficult closure occurred in 35 patients (24%). These patients were more frequently male (86% vs. 69%; p=0.063) and more often underwent surgery for trauma (83% vs. 43%; p<0.001). Among morphometric parameters, only abdominal cavity volume (p=0.002) and the minimum thickness measured among the rectus abdominis muscle bellies (p=0.063) were associated with difficult closure. In multivariate analysis, abdominal cavity volume (OR=1.45; 95%CI: 1.12–1.91; p=0.006) and trauma (OR=5.22; 95%CI: 1.77–18.4; p=0.005) remained independent predictors.
**Conclusion:** Total abdominal cavity volume was the only independent morphometric predictor of difficult closure, together with trauma as the surgical indication. Incorporating this parameter into preoperative assessment may improve risk stratification and guide surgical management in emergency patients.
**Limitations:** Retrospective design and variability in surgical decision-making could limit generalizability.
**Funding for this study:** No funding was received for this study.
**Has your study been approved by an ethics committee?** Yes
**Ethics committee - additional information:** The study protocol was approved by the local Ethical Committee Milano Area 3 (record number: 534-102018)
**Author Disclosures:**
Francesco Rizzetto: Nothing to disclose
Caterina Beatrice Monti: Nothing to disclose
Tiziana Pilia: Nothing to disclose
Stefano P. B. Cioffi: Nothing to disclose
Angelo Vanzulli: Nothing to disclose
Roberto Bini: Nothing to disclose
Pietro Chiara: Nothing to disclose
Domenico Albano: Nothing to disclose
Francesco Cammarata: Nothing to disclose

**Enhancing Adherence to Imaging Guidelines in Acute Pancreatitis: Impact of a Two-Cycle Quality Improvement Audit**
*M. Maki*; Leicester/UK
*(mish12349@gmail.com)*

**Purpose:** The general objective of the audit, through local data utilization, was to support the continued quality enhancement by reassessing the imaging practice of AP across two audit cycles
**Methods or Background:** 619 inpatient patients were retrospectively followed between 2023 and 2025. The first audit cycle involved thirty patients and the second audit cycle involved fifty patients. Intervention activities were comprised of electronic reminding of imaging request and posters in high-traffic zones, and training for clinicians. The imaging conducted within 72 hours of the request was termed to be compliant, and the modality choice or the use of needless premature CT used as additional
**Results or Findings:** The compliance rate was 85 percent and the compliance rates in cycle one and cycle two were elevated by 20.3 percent (66.7 percent) and 20.3 percent respectively. The use of ultrasound increased by 70-90 percent at admission and decreased by 30-10 percent in the unnecessary use of CT
**Conclusion:** The two- cycle audit carried out within the University Hospitals of Leicester (UHL) Trust shows the appropriateness of continuous quality improvement in the alignment of clinical practice with evidence-based suggestions in imaging of acute pancreatitis (AP). It has been discovered that the overall compliance rates regarding the use of the 600 patients 72-hour-long imaging standard were higher than the 66.7% of the first cycle to 96% of the second cycle. Notably, the percentage of the unnecessary premature scan CT minimized (30 to 10), and the percentage of admission ultrasounds rose 70 to 90
**Limitations:** Despite the huge improvements, delays were only in two patients in the second round, both of which had issues related to the patient such as imaging intolerance, but 15% of all the patients in the dataset still had delays.
**Funding for this study:** I do not need funding for this audit, I just need an opportunity to present my audit either orally or by eposter.
**Has your study been approved by an ethics committee?** Not applicable
**Ethics committee - additional information:** I wiill appreciate the
**Author Disclosures:**
Minouche Maki: Other/Author: I am a resident Doctor UHL

**Provision of Emergency Non-Traumatic Abdominal Imaging in the UK: A National Snapshot Audit**
*N. Govindarajah*, J. K. C. Mak, S. Qadri, K. Drinkwater, H. Roach, R. Greenhalgh, G. Retnasingam, H. Addley; London/UK
*(narendranath.govindarajah@stgeorges.nhs.uk)*

**Purpose:** To evaluate the provision of emergency non-traumatic abdominal imaging in the United Kingdom.
**Methods or Background:** Prompt abdominal imaging is central to the diagnosis and management of acute surgical emergencies. The Royal College of Radiologists (RCR) led a national audit to assess the availability, organisation, and reporting practices of emergency non-traumatic abdominal CT imaging across United Kingdom (UK) hospitals. A structured online survey was distributed to radiology departments nationwide. Data were collected from 103 hospitals which included tertiary and district-general (DGH) institutions. Questions addressed service provision, vetting processes, reporting responsibilities and radiologist–clinician communication.
**Results or Findings:** Only 37% of centres recorded a dedicated National Emergency Laparotomy Audit (NELA) Radiology lead at their institutions. All centres (100%) provided both in and out-of-hours CT scanning for acute abdominal emergencies. Only 51% had a standardised flagging system to prioritise patients in-hours, falling to 49% out-of-hours. Acute abdominal imaging pathways were available in 41% of hospitals in-hours and 39% out-of-hours. Vetting of CT requests was primarily undertaken by on-site radiology consultants during in-hours (98%), but responsibility shifted largely to teleradiology providers out-of-hours (74%). On-site consultants almost universally provided the first report in-hours (97%), whereas out-of-hours this was delivered by teleradiology (76%). Documentation of radiologist–clinician communication was inconsistent, with 56% of departments recording formal discussion in-hours and a similar proportion (56%) out-of-hours.
**Conclusion:** This audit demonstrates that although universal CT access exists for emergency non-traumatic abdominal imaging in the UK, significant variation persists in pathways, request vetting, reporting practices, and documentation. This audit has highlighted the reliance on off-site and teleradiology reporting out-of-hours nationally. The findings support the need for clearer national standards and improved consistency in pathways to ensure effective imaging provision for acutely unwell patients.
**Limitations:** Snapshot audit
**Funding for this study:** No funding.
**Has your study been approved by an ethics committee?** Not applicable
**Author Disclosures:**
Ganesh Retnasingam: Nothing to disclose
Naren Govindarajah: Nothing to disclose
Syed Qadri: Nothing to disclose
Karl Drinkwater: Nothing to disclose
Helen Addley: Nothing to disclose
Rebecca Greenhalgh: Nothing to disclose
Huw Roach: Nothing to disclose
Jason Kei Chak Mak: Nothing to disclose

**The use of imaging in patients with a necrotising soft tissue infection**
*L. M. Neeter*, J. Suijker, M. De Lijster, A. Pijpe, A. Meij - de Vries; Beverwijk/NL

**Purpose:** Necrotising soft tissue infections (NSTIs) are infections of the deeper tissues; necrotising fasciitis and Fournier's disease being the most well-known. Due to the rapid progressive destruction of tissue and systemic toxicity, time is of the essence for successful treatment. However, recognition of NSTIs, which predominantly relies on symptom recognition combined with surgical inspection, is challenging and patients are often misdiagnosed. We describe the use of imaging in a Dutch cohort of NSTI patients.
**Methods or Background:** The database of a Dutch multicentre retrospective cohort on 271 NSTI patients from 11 hospitals treated between 2013 and 2017, was accessed for information on the location of NSTI, the imaging performed, and its outcomes.
**Results or Findings:** Imaging was performed in 152 (56.1%) patients. Sixty-three patients underwent ultrasound (41.4%), 28 x-ray (18.4%), 89 CT (58.6%), and 12 MRI (7.9%). In 56 patients, NSTI was considered as potential diagnosis before the imaging examination was performed. In those patients, the ultrasound was suggestive for NSTI in seven patients (70.0%), x-ray in two patients (50.0%), CT in 37 patients (88.1%), and MRI in two patients (66.7%). CT supported the diagnosis of NSTI in the head/neck, arm/thorax, leg, anogenital/gluteal, and abdominal region in two (50.0%), six (100.0%), seven (100.0%), seventeen (94.4%), and eight patients (75.0%), respectively.
**Conclusion:** In this retrospective cohort, imaging was performed in the majority of NSTI patients. CT was most commonly used in case of suspected NSTI and seems able to recognise NSTI in the vast majority of cases. However, the benefits of imaging and potential disadvantages caused by imaging, such as delay in surgical inspection, remains a challenging and debatable trade-off.

**Limitations:** Acquiring diagnostic accuracy was not possible; the cohort consisted solely of NSTI patients.
**Funding for this study:** Dutch Burns Foundation (Grants: 17.109 & 22.111).
**Has your study been approved by an ethics committee?** Yes
**Ethics committee - additional information:** The medical ethics committee of Amsterdam University Medical Centre determined that our study was not subject to the Medical Research Involving Human Subjects Act (WMO).
**Author Disclosures:**
Lidewij M.F.H. Neeter: Nothing to disclose
Manou De Lijster: Nothing to disclose
Jaco Suijker: Nothing to disclose
Anouk Pijpe: Nothing to disclose
Annebeth Meij - De Vries: Nothing to disclose

**Diagnostic Failures in Abdominal Parenchymal Trauma: The Mistake Just Around the Corner**
*M. C. Firetto*, G. Carrafiello; Milan/IT
*(firettosirm@gmail.com)*

**Purpose:** Trauma causes 9% of global deaths (case fatality 4.6%–10.1%), and whole-body CT reduces mortality by 13%. Yet diagnostic failures—seen in up to 12.9% of scans—contribute to 11% of trauma deaths. This study aims to characterize both technical/methodological and interpretative errors in detecting parenchymal injuries of abdominal organs (lacerations, hematomas, infarctions, and associated vascular lesions such as active bleeding, pseudoaneurysms, and arteriovenous fistulae) on emergency CT, and to propose targeted strategies for error reduction.
**Methods or Background:** A structured narrative review of published series on parenchymal injuries was performed. Diagnostic failure rates were analyzed for abdominal parenchymal and associated vascular injuries. Errors were classified as: - Technical/methodological: incomplete multiphase protocols, motion artifacts, beam-hardening, streak artifacts. - Interpretative: false positives (overcalls of congenital clefts, normal variants, artifacts), false negatives (missed pseudoaneurysms, subtle hematomas, delayed bleeds). Representative cases illustrate common diagnostic pitfalls.
**Results or Findings:** Technical errors accounted for ~60% of failures. Monophasic protocols missed: 10%–15% of splenic/hepatic injuries, 5%–20% of vascular lesions, 8%–10% of renal parenchymal injuries, up to 50% of ureteropelvic junction injuries. Artifacts masked both parenchymal and vascular lesions. Pancreatic and adrenal injuries were described qualitatively; quantitative data on missed diagnoses are lacking in the available series. Interpretative errors (~40%) included overcalls due to enhancement heterogeneity and undercalls of vascular injuries and delayed hemorrhage. Dual-/multiphase CT, orthogonal reconstructions, structured reporting, and systematic search patterns improved detection and reduced delays.
**Conclusion:** Optimizing acquisition protocols, interpretative training, structured reporting, and multidisciplinary review can reduce diagnostic errors and improve trauma outcomes.
**Limitations:** The narrative review design and heterogeneity of published protocols limit quantitative synthesis; data on pancreatic and adrenal parenchymal injuries remain insufficient, and the proposed error-reduction strategies lack prospective validation.
**Funding for this study:** No funding for this study
**Has your study been approved by an ethics committee?** Not applicable
**Author Disclosures:**
Maria Cristina Firetto: Nothing to disclose
Gianpaolo Carrafiello: Nothing to disclose

**Reducing ct overuse in minor head injury: a local campaign**
*K. S. Chhabra*, T. Sehgal, D. B. Dahiphale, B. Patel; Aurangabad/IN

**Purpose:** To evaluate the impact of a local awareness and guideline-based campaign on reducing unnecessary CT brain scans in patients with minor head injury at a tertiary care centre in India.
**Methods or Background:** Background CT brain is often overused in cases of minor head injury, exposing patients to unnecessary radiation, increasing costs, and burdening radiology services. International guidelines such as the Canadian CT Head Rule (CCHR) and NICE criteria provide evidence-based thresholds for imaging. However, adherence in busy emergency settings remains inconsistent. Methods or Approach A baseline audit was performed of 120 consecutive patients with Glasgow Coma Scale (GCS) 13–15 presenting with head injury between January and March 2025. The proportion of CT scans meeting CCHR criteria was calculated. A local campaign was then implemented, including educational sessions for emergency physicians, pocket guideline cards, and posters displayed in the emergency department. A re-audit of 100 patients was conducted three months later.
**Results or Findings:** At baseline, 62% of CT scans were performed without meeting guideline criteria. Following the campaign, inappropriate CT utilisation decreased to 32%, reflecting a 30% improvement. No clinically significant injuries were missed during the re-audit period. Emergency physicians reported improved confidence in applying CCHR criteria, and turnaround time in the CT suite improved due to reduced scan load.
**Conclusion:** A simple, low-cost educational campaign significantly reduced inappropriate CT brain scans for minor head injury without compromising patient safety. Sustained adherence to clinical decision rules can improve resource utilisation, reduce radiation exposure, and optimise emergency imaging services.
**Limitations:** Single-centre data.
**Funding for this study:** No funding was received for this work.
**Has your study been approved by an ethics committee?** Not applicable
**Author Disclosures:**
Binitkumar Patel: Nothing to disclose
Devidas Baburao Dahiphale: Nothing to disclose
Karanvir Singh Chhabra: Nothing to disclose
Tarun Sehgal: Nothing to disclose

**Incidental Findings on Head CT: What to Report, What to Ignore**
*E. M. Rajali*; Casablanca/MA
*(rajalimedi1@gmail.com)*

**Purpose:** To guide radiologists through the growing challenge of incidental findings on head CT, providing a practical framework for distinguishing harmless variants from clinically significant lesions.
**Methods or Background:** Head CT is the first-line exam in trauma and emergency neurology. Its systematic use in high-volume, time-sensitive settings frequently reveals incidental abnormalities. While some are benign and can be ignored, others require recognition and follow-up. The core challenge lies in balancing thoroughness with clinical relevance, avoiding both unnecessary investigations and missed diagnoses.
**Results or Findings:** In daily practice, radiologists often struggle with this balance: over-reporting benign variants such as physiologic calcifications, cavum septum pellucidum, or mild sinus changes may create anxiety and lead to unwarranted work-up. On the other hand, subtle but significant findings — such as silent infarcts, meningiomas, or hydrocephalus — may be overlooked, with important clinical consequences. Age-related atrophy further illustrates this dilemma, requiring correlation with patient age and symptoms before deciding on its significance. The key is a structured, context-driven approach supported by clear reporting recommendations.
**Conclusion:** The challenge of incidental findings is not to see “more,” but to know what truly matters. By applying a systematic approach, radiologists can remain consistent, provide reassurance to clinicians, and avoid sending patients into unnecessary diagnostic odysseys, while still capturing findings with real clinical impact.
**Limitations:** May not encompass all potential incidental findings
**Funding for this study:** No funding
**Has your study been approved by an ethics committee?** Not applicable
**Author Disclosures:**
Najwa Touil: Nothing to disclose
Houria Tabakh: Nothing to disclose
Omar Kacimi: Nothing to disclose
Youssef Sakhy: Nothing to disclose
Anas Alami Idrissi: Nothing to disclose
Abdellatif Siwane: Nothing to disclose
El Mehdi Rajali: Nothing to disclose

**CT and MRI predictors of early myelopathy after spinal trauma: a preliminary study**
*I. C. Koc*, K. Aslan, B. Genç; Samsun/TR

**Purpose:** The purpose of this study is to determine the CT parameters that can predict myelopathy risk in spinal trauma patients, to investigate the MRI parameter differences between patients with and without myelopathy and compare the spinal cord changes between these groups during early trauma period (120 days).
**Methods or Background:** 115 patients who suffered spinal trauma, had CT taken within 24 hours and spinal MRI taken within first 48 hours were included in this retrospective single-center study. 76 patients had myelopathy while 39 patients didn’t. Paraspinal muscle area (PMA) and density (PMD), bone density (BD), subcutaneous fat thickness (SFT) at the level of trauma, spinal canal AP diameter measured in CT and spinal cord area, spinal canal area, cord compression ratio measured in MRI were compared between groups. Nine patients without myelopathy and 14 patients with myelopathy had control MRI within 120 days after trauma. Longitudinal comparisons were made between groups.

Sunday

**Results or Findings:** There were no statistically significant differences between groups for PMA, PMD, bone density, SFT. Spinal canal AP diameter below the trauma level (SCAPBTL) showed significant difference (p=0.037). Spinal cord area at trauma level and spinal canal area below the trauma level (SCABTL) showed significant difference (p=0.026; p=0.024). AUC of 0.624 was determined for SCA-BTL through ROC analysis to distinguish myelopathy (75% sensitivity, 56.41% specificity). In longitudinal analysis there were no significant differences for SCA-TL neither within nor between groups (p=0.26; p=0.975; p=0.45).
**Conclusion:** Our study shows only SCAPBTL was meaningful in CT. SCA-BTL can be used to differentiate myelopathy. Spinal cord doesn't show significant change both within and between groups in early trauma period.
**Limitations:** Small sample size. Since data regarding spinal injury impairment scale wasn't available, a clinical correlation couldn't be analyzed.
**Funding for this study:** No funding was received for this study.
**Has your study been approved by an ethics committee?** Yes
**Ethics committee - additional information:** This study was approved by Ondokuz Mayıs University Hospital Ethics Committee (KAEK 2025)
**Author Disclosures:**
Bariş Genç: Nothing to disclose
Kerim Aslan: Nothing to disclose
Irem Ceren Koc: Nothing to disclose

**Acute low back pain of extra-axial non-traumatic origin: clinical and radiological overview from a high-load tertiary emergency department**
*P. A. Bonaffini*[1], E. Piccin[1], C. Preti[2], R. Cosentini[2], C. Valle[1], P. Marra[1], S. Sironi[1]; [1]Monza/IT, [2]Bergamo/IT
*(pa.bonaffini@gmail.com)*

**Purpose:** To report prevalence and etiologies of acute extra-axial non-traumatic low back pain (ALBP, <3 months onset), in patients admitted in a high-load tertiary emergency department (ED) over 2-year (2018-2020). To review radiological features and imaging contribution in ALBP, using discharge or ward admittance diagnosis as reference.
**Methods or Background:** Patients admitted to ED were retrospectively divided according to back pain onset (chronic/acute) and origin (traumatic/non-traumatic, axial/extra-axial): chronic pain, trauma-related and axial causes were excluded. Clinical evaluation during admittance was checked; then, abdominal, genitourinary, gynecological, infectious, rheumatological and cardio-pulmonary etiologies were categorized by final diagnosis at discharge or ward admittance. Then imaging results (plain radiography, US, CT) for each category were reviewed and reported.
**Results or Findings:** Among 46.449 patients, 2024 (23%) were admitted for lumbar pain: in 78.6% chronic-traumatic-musculoskeletal, in 21,4% extra-axial non-traumatic (ALBP). In ALBP final diagnosis was: 212 patients(49,5%) with urinary disease (kidney stones, pyelonephritis, cystitis), 95(22,2%) abdominal (constipation, biliary cholic), 67(15,6%) gynecologic (including pregnancy), 24(5,6%) infectious (influenza, fever), 21(4,9%) cardio-pulmonary (pneumonia, pericarditis), 7(1,6%) rheumatologic (polyarthritis) and 1(0,2%) vascular (dissection). US was the most employed technique (26,9%; 31,6% patients with urinary, 24,2% abdominal, 14,9% gynecological, 28,9% infectious diseases and for aortic dissection). Plain radiography was used to exclude perforation/occlusion (13,4%). CT used was in 9 patients (2,1%) as completion (inconclusive US/plain radiography), including aortic dissection. In 264 patients (61,7%) imaging was not performed: diagnosis was gained through clinical/laboratory data.
**Conclusion:** ALBP is a relevant cause of access to ED. In 20% of cases origin is related to abdominal causes, being urinary diseases the most common. With inconclusive clinical-laboratory evaluation (40%) imaging can aid in confirming pain origin. US is essential for kidney-abdominal evaluation, CT could be reserved to selected cases, sparing unnecessary radiation exposure.
**Limitations:** Retrospective
**Funding for this study:** Not applicable
**Has your study been approved by an ethics committee?** Not applicable
**Author Disclosures:**
Roberto Cosentini: Nothing to disclose
Eleonora Piccin: Nothing to disclose
Clarissa Valle: Nothing to disclose
Paolo Marra: Nothing to disclose
Pietro Andrea Bonaffini: Nothing to disclose
Carlo Preti: Nothing to disclose
Sandro Sironi: Nothing to disclose

**Economic potential of optimizing mobile stroke unit implementation in Germany : A markov model based analysis**
*N. Castioni*, J. S. Rink; Mannheim/DE

**Purpose:** To assess the economic and health system impact of optimized mobile stroke unit (MSU) care in Germany, specifically incorporating a newly developed mobile head CT scanner that delivers high-quality imaging directly in the prehospital setting. This study also highlights, for the first time, the impact of (1) dispatcher sensitivity as a dominant driver of MSU case capture and cost-effectiveness and (2) explicit modeling of endovascular therapy (EVT) workflow acceleration—two aspects not fully captured in prior MSU cost-effectiveness studies.
**Methods or Background:** Expanding on and going beyond prior CEAs, the Markov model included both intravenous thrombolysis (IVT) and, for the first time, EVT care pathways, incorporating time and outcome data from recent clinical registries. Dispatcher sensitivity was systematically varied and analyzed for its effect on case capture, improving on the previous static approach. Model scenarios compared EMS with MSU services for different catchment areas, schedules, and resource-sharing settings, capturing system-level effects of greater dispatch precision and faster EVT.
**Results or Findings:** Improved dispatcher sensitivity yielded a more substantial reduction in incremental cost-effectiveness ratios (ICERs) than adjustments in hardware or staffing costs. Explicit inclusion of EVT workflow improvements led to a higher rate of good outcomes (29.6% mRS 0–2 with MSU vs. 27.5% EMS in EVT-eligible patients), with superior QALYs and costs. While cost-effectiveness was achieved in large catchment areas, scenarios with high dispatcher sensitivity achieved economic viability even at a more modest scale, unlike prior findings.
**Conclusion:** Focusing on dispatcher performance and system-wide EVT optimization enables a broader range of MSU designs to achieve cost-effectiveness, going beyond previous reviews that emphasized only scale. These innovations maximize health and economic benefit.
**Limitations:** Model outcomes rely on estimated EVT gains and dispatch characteristics; real-world validation remains needed.
**Funding for this study:** None
**Has your study been approved by an ethics committee?** Not applicable
**Author Disclosures:**
Noah Castioni: Nothing to disclose
Johann S Rink: Nothing to disclose

**Radiation Exposure from Paediatric Head CT and CT Angiography in Trauma Care**
*D. Rosok*, M. Opitz, D. Bos, Y. L. Thal, J. Haubold, B. Schweiger, M. Forsting, C. Deuschl, S. Zensen; Essen/DE

**Purpose:** Head CT and CT angiography (CTA) of the craniocervical vessels are essential in emergency diagnostics in children, despite their heightened sensitivity to ionising radiation. This study evaluates the radiation exposure associated with head CT and CTA in paediatric patients examined in the trauma resuscitation unit (TRU).
**Methods or Background:** Patients aged 0–15 years undergoing head CT and CTA in the TRU between 04/2020 and 08/2023 were included. Volume-weighted CT dose index (CTDIvol), dose-length product (DLP), and effective dose (ED) were analysed for three age groups according to national diagnostic reference levels (DRL): I (0–<5 years), II (5–<10 years), III (10–<15 years). ED was derived using conversion factors and organ doses were estimated via Monte Carlo simulation.
**Results or Findings:** Of 212 children treated in the TRU, 62.7% (133/212) had CT, and 72.2% (96/133) underwent both head CT and CTA. Median CTDIvol, DLP, and ED (IQR) by age group were: head CT: I: 18.9mGy (17.5–20.1mGy), 282mGycm (253–301mGycm), 3.9mSv (3.6–4.5mSv); II: 23.9mGy (22.3–26.2mGy), 381mGycm (351–413mGycm), 2.7mSv (2.5–2.9mSv); III: 29.4mGy (27.1–32.5mGy), 460mGycm (430–533mGycm), 1.8mSv (1.7–2.1mSv); CTA: I: 1.4mGy (1.4–1.5mGy), 40mGycm (37– 43mGycm), 5.7mSv (5.5–6.2mSv); II: 2.0mGy (1.5–2.1mGy), 68mGycm (50–72mGycm), 5.4mSv (4.0–5.7mSv); III: 2.2mGy (2.2–2.3mGy), 83mGycm (75–88mGycm), 2.8mSv (2.5–3.0mSv). All doses were below national DRLs across age groups.
**Conclusion:** Head CT and CTA in paediatric trauma care can be performed at moderate radiation levels. Continued dose optimisation is vital to further reduce long-term risks such as radiation-induced malignancy.
**Limitations:** The limitations of the study are its single-centre design and use of a specific scan protocol, which may limit the generalisability of our findings. Nevertheless, this setting also allows for a detailed analysis of the protocol and its effects on radiation doses in children.
**Funding for this study:** No funding was received for this study.
**Has your study been approved by an ethics committee?** Yes

**Ethics committee - additional information:** Ethical approval was received by the Ethics committee of the Medical Faculty of the University of Duisburg-Essen, Germany (23-11461-BO).
**Author Disclosures:**
Michael Forsting: Nothing to disclose
Denise Bos: Nothing to disclose
Bernd Schweiger: Nothing to disclose
Cornelius Deuschl: Nothing to disclose
Johannes Haubold: Nothing to disclose
Sebastian Zensen: Nothing to disclose
Yannick Laurent Thal: Nothing to disclose
Daniel Rosok: Nothing to disclose
Marcel Opitz: Nothing to disclose

**11:30 - 12:30** **Research Stage 1**

### Research Presentation Session: Abdominal and Gastrointestinal

### RPS 2401

### Pancreas cancers and high-risk pancreatic lesions

**Moderator**
Maria Antonietta Bali; Brussels/BE
*(mariaantonietta.bali@policlinicogemelli.it)*

**ML radiomics diagnostic model for differential diagnosis of small Solid Pseudopapillary Epithelial Neoplasm of the pancreas**
*A. I. Baeva*, A. Ustalov, S. A. Shmeleva, I. Gruzdev, V. Struchkov, E. V. Kondratyev, A. Pokshubina; Moscow/RU
*(nastya.baeva.2016@mail.ru)*

**Purpose:** The five-year survival rate for patients with solid pseudopapillary epithelial neoplasm (SPEN) after surgery exceeds 98%, whereas pancreatic ductal carcinoma (PDAC) is highly aggressive, characterized by invasive growth and early metastasis. Differential diagnosis of small SPEN and PDAC is challenging. The aim of the present study was to develop and evaluate a ML-radiomic model for distinguishing SPEN from PDAC to improve diagnosis in pancreatic cancer.
**Methods or Background:** A cohort of 97 patients was retrospectively analyzed, including 39 with SPEN under 30 mm in diameter without local invasive growth and 58 with PDAC, all undergoing contrast-enhanced CT scans and histopathological examination of tumor tissues between 2022 and 2025. Tumor segmentation was manually performed on arterial and venous images using a 3D ROI. Radiomic features were extracted with the "Radiomics" extension in 3D-Slicer. Seven machine learning (ML) models were developed based on these features, and their performance was evaluated using ROC AUC to identify the best classifier.
**Results or Findings:** The AutoML_Stacking model achieved the highest ROC-AUC of 0.971, demonstrating excellent discriminatory ability. Similar results were obtained for the LGBM and LR models (ROC-AUC of 0.965). All models had ROC-AUC values between 0.920 and 0.971, indicating robustness of the radiomic features. Sensitivity ranged from 0.972 to 1.0, reflecting good SPEN detection.
**Conclusion:** ML-radiomics diagnostic models for distinguishing SPEN from PDAC were developed. The AutoML_Stacking and LightGBM models exhibit high diagnostic efficiency, highlighting the potential of radiomics for differentiating small SPEN from PDAC, aiding early diagnosis in pancreatic cancer.
**Limitations:** Not applicable.
**Funding for this study:** No funding was provided for this study.
**Has your study been approved by an ethics committee?** Not applicable
**Author Disclosures:**
Anna Pokshubina: Nothing to disclose
Evgeny V Kondratyev: Nothing to disclose
Sofiia Antonovna Shmeleva: Nothing to disclose
Andrey Ustalov: Nothing to disclose
Ivan Gruzdev: Nothing to disclose
Anastasiia Igorevna Baeva: Nothing to disclose
Vladimir Struchkov: Nothing to disclose

**Relationships of Fat Infiltration and R2* with Hepato-pancreatic Diffusion Metrics: A Quantitative MRI Study Using the Spleen as Reference**
*Z. N. Doghramachi*, T. Cankurtaran, D. Kalemci, M. Aliyev, Z. N. Dayican, O. Kayık, B. Yavuz Sarsam, A. Isik; Ankara/TR
*(zeynepdayican@gmail.com)*

**Purpose:** To investigate the independent effects of fat infiltration (Proton Density Fat Fraction, PDFF) and iron overload (R2∗) on quantitative diffusion-weighted imaging (DWI) metrics in the liver and pancreas, using the spleen as a parenchymal reference tissue.
**Methods or Background:** This cross-sectional study included 102 adults (55.8 ± 11.3 years). Abdominal quantitative MRI comprised multi-echo gradient-echo acquisitions for PDFF and R2∗ mapping, and multi-b-value DWI (b=0–2500 s/mm2) for diffusion analysis (monoexponential ADC, logarithmic ADC, and biexponential fast/slow components). Statistical analyses included partial correlation (adjusted for R2∗) and multivariable linear regression (adjusted for age, sex, and BMI) to assess associations between PDFF, R2∗, and diffusion metrics in the liver and pancreas, as well as their ratio to spleen values.
**Results or Findings:** In the liver, Fat Fraction (FF) showed positive associations with the biexponential fast component (r=0.280, p=0.004) and the ratio of fast/slow components (r=0.293, p=0.003). No independent associations were found for monoexponential or logarithmic ADC. In the pancreas, R2∗ was independently and positively related to monoexponential ADC (β=0.380, p<0.001) and negatively to logarithmic ADC (β=−0.293, p=0.003). The association between pancreatic FF and logarithmic ADC lost significance after R2∗ adjustment. Ratio-based analysis (Pancreas/Spleen) further demonstrated an independent effect of pancreatic FF on the biexponential slow ADC component.
**Conclusion:** Hepatopancreatic fat infiltration (PDFF) and R2∗ showed independent, albeit weak, associations with specific DWI metrics. These findings suggest that the effects of both fat and iron need to be considered when interpreting quantitative diffusion measurements in the liver and pancreas.
**Limitations:** This study was limited by the relatively small sample size, the absence of participants with high-grade steatosis, and the lack of voxel by voxel analysis, as all measurements were ROI-based.
**Funding for this study:** Funding was provided by Siemens Healthineers, Erlangen, Germany.
**Has your study been approved by an ethics committee?** Yes
**Ethics committee - additional information:** The study was approved by [Baskent University]
**Author Disclosures:**
Atakan Isik: Advisory Board: Advisory
Busra Yavuz Sarsam: Author: Author
Zeynep Nazli Dayican: Author: Author
Zeynep Nazli Doghramachi: Author: Presentation
Mahammad Aliyev: Author: Author
Tijen Cankurtaran: Author: Author
Onurhan Kayık: Author: Author
Deniz Kalemci: Author: Author

**Refining baseline staging in the era of neoadjuvant chemotherapy for resectable and borderline resectable pancreatic cancer: the role of FDG-PET and EOB-MRI**
*F. Prato*, D. Palumbo, G. Orsi, M. Macchini, C. Carconi, A. Chiti, M. Falconi, M. Reni, F. De Cobelli; Milan/IT

**Purpose:** Neoadjuvant chemotherapy (NAT) is becoming the treatment of choice for pancreatic ductal adenocarcinoma (PDAC). In this scenario, the traditional classification of borderline-resectable and anatomically resectable disease—based mainly on CT—appears progressively outdated, also due to inter-observer variability. Although upfront surgery is rare, accurate baseline staging remains crucial to define prognosis and guide therapy, while avoiding futile surgery in patients with occult systemic disease. Advanced imaging such as FDG-PET and MRI with liver-specific contrast media (EOB-MRI) may provide incremental value over CT.
**Methods or Background:** In this prospective study, patients with a resectable or borderline resectable (according to CT scan), were screened using both FDG-PET and 3T-EOB-MRI before the initiation of NAT.
**Results or Findings:** Of 154 screened patients, 124 were enrolled (upfront resectable = 71, borderline = 53); one patient was later reclassified as locally advanced. Additional imaging excluded 24 patients (18.8%) due to previously undetected metastases, while 5 (3.3%) were excluded for protocol deviations. After a median follow-up of 20 months, 18/154 (11.6%) were considered true positives (“screening failures”), while 117/154 (76%) were true negatives (correctly classified as resectable/borderline). False negatives occurred in 8/154 cases (5.2%) and false positives in 6/154 (3.9%), mostly related to PET overestimation. Retrospective analysis suggested that MRI alone, particularly when interpreted by expert radiologists, could further reduce both false positives and negatives. In upfront resectable patients, predictors of occult metastases (on baseline CT scan) included tumor necrosis (p=0.009), dilated Wirsung duct (p=0.04), and CA19.9 ≥200 U/mL (p<0.001).
**Conclusion:** EOB-MRI proved more effective than FDG-PET in identifying occult metastases, especially in the liver and peritoneum, thereby reducing futile surgeries. These findings support prioritizing EOB-MRI in baseline staging protocols for PDAC, particularly in high-risk patients, to optimize therapeutic strategies and patient selection.
**Limitations:** Monocentric
**Funding for this study:** Patient Associations
**Has your study been approved by an ethics committee?** Yes
**Ethics committee - additional information:** Ethics Committee of Ospedale San Raffaele (Milan, IT) (06/INT/2022)
**Author Disclosures:**
Francesco De Cobelli: Nothing to disclose
Catia Carconi: Nothing to disclose
Diego Palumbo: Nothing to disclose
Arturo Chiti: Nothing to disclose
Massimo Falconi: Nothing to disclose
Giulia Orsi: Nothing to disclose
Marina Macchini: Nothing to disclose
Michele Reni: Nothing to disclose
Francesco Prato: Nothing to disclose

**Contrast-enhanced ultrasound combined with percutaneous ultrasound-guide core needle biopsies in the diagnosis of pancreatic lesions**

*Q. Dai*, M. Lu; Chengdu, China/CN
*(520527966@qq.com)*

**Purpose:** To compare the diagnostic performance of pancreatic lesions using percutaneous ultrasound (US)-guided core needle biopsy (CNB) with and without contrast-enhanced ultrasound (CEUS).
**Methods or Background:** The patients were divided into two groups, US and CEUS group, based on whether CEUS was performed prior to biopsy. Before and after propensity score matching (PSM), the CNB-relevant characteristics of the two groups, including the first puncture success rate, the number of sampling, complication rate, type of complications, and degree of abdominal pain, were compared. The accuracy, sensitivity, specificity, positive predictive value (PPV), negative predictive value (NPV), and area under the curve (AUC) of percutaneous US-guided CNB were compared between the groups.
**Results or Findings:** This study included 277 patients with pancreatic lesions who underwent percutaneous CNB before PSM. After controlling for potential biases using PSM, significant differences were observed in the first puncture success rate and the number of sampling (P<0.05). Importantly, before and after PSM, the CEUS group achieved a higher first-puncture success rate while obtaining a lower number of sampling (P<0.05). Furthermore, the significant difference was observed in the AUC for diagnostic performance between the two groups when compared using DeLong's test (P=0.043).
**Conclusion:** Performing CEUS before percutaneous CNB for pancreatic lesions can help achieve better biopsy results, reduce the number of punctures samples, increase the success rate of biopsies, and avoid the need for repeat biopsies.
**Limitations:** First, the sample size was derived from a single center, and the percutaneous CNB technique for pancreatic lesions requires radiologists with extensive experience in US intervention, which may have affected the generalizability of our findings. Second, as a retrospective study, there remains a potential for selection bias despite conducting a PSM analysis.
**Funding for this study:** No.
**Has your study been approved by an ethics committee?** Yes
**Ethics committee - additional information:** Ethics Committee of Sichuan Cancer Hospital (approval number: SCCHEC-02-2024-127)
**Author Disclosures:**
Quan Dai: Nothing to disclose
Man Lu: Nothing to disclose

**Prospective evaluation of MR elastography for pancreatic cancer characterization and treatment response**

*V. Koch*[1], L. D. Grünewald[1], K. Eichler[1], R. Hammerstingl[1], O. Darwish[2], J. Gotta[1], T. Vogl[1], R. Sinkus[2]; [1]Frankfurt/DE, [2]Paris/FR
*(vitali-koch@gmx.de)*

**Purpose:** The aim of this study was to evaluate magnetic resonance elastography (MRE) using the acoustic (2D) and gravitational transducer (2D/3D) concept for detecting pancreatic cancer and assessing its potential as a marker of chemotherapy response.
**Methods or Background:** In this prospective study, 51 patients with confirmed pancreatic cancer undergoing upper abdominal MRI were enrolled between June 2023 and April 2024. An additional 15 healthy volunteers were included as a reference standard. All participants underwent two examinations at 40 Hz (Aera, 1.5 T, Siemens Healthineers, Germany): first with a commercial MRE system (Resoundant, 2D-MRE, SE-EPI sequence, 11 s breath-hold), and subsequently with the gravitational transducer approach (2D- and 3D-MRE, GRE sequence, TE = 9.2 ms [in-phase], fractional motion encoding at 30 mT/m, 14 s breath-hold). Data were independently analyzed by three experienced readers. To assess potential biases in 2D/3D stiffness reconstruction, analytic plane waves with known wavelength and absorption were superimposed at varying amplitudes and temporal noise levels, and used to derive quality indices for 2D and 3D MRE.
**Results or Findings:** Significant differences in stiffness, shear wave speed, and phase angle were observed between healthy volunteers and pancreatic cancer patients (p < .05). Subtype-specific signatures enabled differentiation between ductal adenocarcinoma and neuroendocrine tumors (p < .05). Stiffness changes tracked chemotherapy response (p < .05), while higher baseline values correlated with longer hospitalization. 2D-MRE systematically overestimated stiffness, whereas proposed 3D-MRE Quality Indices improved reliability.
**Conclusion:** Gravitational MRE enables accurate, noninvasive characterization of pancreatic tissue. Both approaches correlated well with the commercial standard, while 3D-MRE uniquely delivered functional imaging markers, opening new avenues in abdominal imaging.
**Limitations:** Limited study population. Prospective validation in larger patient cohorts needed.
**Funding for this study:** No funding.
**Has your study been approved by an ethics committee?** Yes
**Ethics committee - additional information:** Approved by the institutional ethics committee.
**Author Disclosures:**
Omar Darwish: Nothing to disclose
Thomas Vogl: Nothing to disclose
Vitali Koch: Nothing to disclose
Renate Hammerstingl: Nothing to disclose
Ralph Sinkus: Nothing to disclose
Leon David Grünewald: Nothing to disclose
Katrin Eichler: Nothing to disclose
Jennifer Gotta: Nothing to disclose

**Prognostic Value of the Node Reporting and Data System (Node-RADS) in Patients with Pancreatic Ductal Adenocarcinoma Undergoing Pancreatoduodenectomy**

*Y. Wang*, Y. Li, H. Yu, Z. Huang, B. Song; Chengdu/CN

**Purpose:** To compare the prognostic performance of pathological (pN) and clinical N staging (cN) with Node-RADS for recurrence-free survival (RFS) in patients with pancreatic ductal adenocarcinoma (PDAC) following pancreatoduodenectomy.
**Methods or Background:** This retrospective study included patients with pathologically confirmed PDAC underwent pancreatoduodenectomy between June 2018 and January 2023 and had preoperative contrast-enhanced abdominal CT. Two observers independently assessed lymph nodes ≥0.4 cm in short-axis diameter on venous-phase images using conventional CT criteria and Node-RADS, with a 4-week interval. Node-RADS scores ≥3 were defined as positive. For each patient, total and station-based Node-RADS scores were calculated. Prognostic performance was evaluated using time-dependent ROC curves and Cox regression. Interobserver agreement was assessed with intraclass correlation coefficients (ICC).
**Results or Findings:** 220 patients (median age, 63 years; 138 men) were analyzed. Median RFS was 10 months. The AUCs of pN for 12-, 24-, and 36-month prediction were 0.546, 0.560, and 0.581, while cN yielded 0.541, 0.501, and 0.538. Node-RADS total scores showed AUCs of 0.581, 0.654, and 0.605. The number of positive stations achieved higher performance (0.611, 0.717, 0.695). Station 16a2 scores showed prognostic value (AUCs 0.533, 0.653, 0.631). Node-RADS total score (p=0.0368), positive station number (p=0.00163), and stations 16a2 (p=0.0312) and 17 (p<0.001) as adverse prognostic factors. Interobserver agreement was moderate (ICC = 0.528).
**Conclusion:** Node-RADS outperformed pN and cN in predicting RFS after pancreatoduodenectomy. Positive station number and stations 16a2 and 17 provided additional prognostic value, supporting Node-RADS as a practical tool for preoperative risk stratification in PDAC.
**Limitations:** This retrospective single-center study lacks external validation, which may restrict generalizability. Additionally, CT images obtained over four years may have been affected by inter-scanner and technical variability, potentially influencing Node-RADS assessment.
**Funding for this study:** This study received no external funding.
**Has your study been approved by an ethics committee?** Yes
**Ethics committee - additional information:** West China Hospital, Sichuan University - approval number 2025 − 1152
**Author Disclosures:**
Yu Li: Nothing to disclose
Haopeng Yu: Nothing to disclose
Zixing Huang: Nothing to disclose
Bin Song: Nothing to disclose
Yuqi Wang: Nothing to disclose

**Targeting Inflammation: A Biologically Justified MRI Fat-Fraction-Enhanced Tool for High-Risk IPMN Prediction**

*D. Palumbo*, F. Prato, G. Belfiori, P. Camisa, B. Ferrara, L. Piemonti, M. Falconi, S. Crippa, F. De Cobelli; Milan/IT

**Purpose:** Intraductal papillary mucinous neoplasms (IPMNs) are established precursors of pancreatic ductal adenocarcinoma (PDAC). Current guidelines recommend surgery based on high-risk stigmata (HRS) and worrisome features (WF), yet these criteria lack accuracy and no reliable non-invasive biomarker of malignancy is available. We aimed to identify preoperative radiological biomarkers to distinguish low-grade from high-grade/invasive IPMNs.
**Methods or Background:** Between June 2020 and January 2025, 206 patients undergoing surgical resection for suspected pancreatic cystic neoplasms were prospectively enrolled. Final pathology showed 49 low-grade dysplasias (LGD), 42 high-grade dysplasias (HGD), and 40 invasive carcinomas (IPMC). For the present analysis, IPMN were stratified into low-risk (LGD) and high-risk (HGD + IPMC) groups. Preoperative imaging included CE-MRI with dedicated MRCP incorporating mDixon Quant Fat Fraction sequences and contrast-enhanced CT. Inter-modality agreement between CT and MRI for WF/HRS was evaluated. A composite risk score was constructed by summing WF and HRS, weighted according to hazard ratios reported in the literature. Biological samples (peripheral/portal blood, cyst fluid) were systematically collected intraoperatively and analyzed for inflammatory and metabolic markers.
**Results or Findings:** Pancreatic fat fraction (assessed on non-atrophic parenchima) was significantly higher in high-risk vs low-risk IPMN (15.7% ± 13.8 vs 6.9% ± 5.0, p=0.023), but showed no correlation with molecular variables. Distal pancreatic atrophy showed a trend toward significance (p=0.062). Individual WF/HRS performed poorly, with wide inter-modality variability (K-Cohen 0.02 to 0.87). Conversely, their additive integration into a risk score significantly predicted high-risk IPMN and correlated with inflammatory markers on cyst-fluid samples, particularly TNF-α (p=0.005).
**Conclusion:** Pancreatic fat fraction on MRI emerges as a promising non-invasive biomarker for stratifying IPMN. WF/HRS are inconsistent as isolated criteria, but their cumulative integration into a risk score enhances predictive accuracy and correlates with local inflammatory mediators.
**Limitations:** Monocentric
**Funding for this study:** IRCCS Ospedale San Raffaele
**Has your study been approved by an ethics committee?** Yes
**Ethics committee - additional information:** Ethics Committee of Ospedale San Raffaele (Milan, IT) (06/INT/2022)
**Author Disclosures:**
Francesco De Cobelli: Nothing to disclose
Benedetta Ferrara: Nothing to disclose
Diego Palumbo: Nothing to disclose
Lorenzo Piemonti: Nothing to disclose
Massimo Falconi: Nothing to disclose
Giulio Belfiori: Nothing to disclose
Paolo Camisa: Nothing to disclose
Francesco Prato: Nothing to disclose
Stefano Crippa: Nothing to disclose

**11:30 - 12:30** **Research Stage 2**

### Research Presentation Session: Cardiac

### RPS 2403

### Photon-counting CT and contrast media optimisation

**Moderator**
Matthias Eberhard; Zurich/CH
**Author Disclosures:**
Matthias Eberhard: Speaker: Siemens Healthineers

**Dosimetric study on cardiac photon counting CT**

*P. Levoni*[1], F. Catapano[1], C. Lisi[1], A. Laghi[1], M. Francone[2], L. S. Politi[1]; [1]Milan/IT, [2]Rome/IT
*(pietro.levoni@hunimed.eu)*

**Purpose:** Photon-counting detector CT (PCD-CT) has been proposed to reduce radiation exposure compared with energy-integrating detector CT (EID-CT). Nonetheless, data on patients' radiation dose in cardiac applications are still limited. The aim of the present study is to quantify and analyse radiation dose across three cardiac PCD-CT protocols, and to compare the results with a standard cardiac EID-CT protocol.
**Methods or Background:** A cohort of 268 patients who underwent coronary CT angiography between December 2024 and July 2025 was retrospectively included. Each patient underwent one of four acquisition protocols: three PCD-CT protocols - Spectral; Ultra-High Resolution (UHR); UHR Spectral - and one EID-CT protocol. For each acquisition volume, CT dose index (CTDIvol), dose length product (DLP), and size-specific dose estimate (SSDE), were retrieved, and total DLP was calculated. Statistical analysis included descriptive assessment and Kruskal–Wallis H-tests to compare dose distributions across protocols.
**Results or Findings:** Mean±SD total DLP values were 653±192 mGy·cm for the Spectral protocol, 947±233 mGy·cm for the UHR protocol, 1373±316 mGy·cm for the UHR Spectral protocol, and 390±114 mGy·cm for the EID-CT protocol. Kruskal-Wallis H-tests showed that each pair of distributions is different with statistical relevance. The median value of total DLP for the UHR Spectral protocol (1356 mGy·cm) results greater than the corresponding Italian Dose Reference Level (1200 mGy·cm).
**Conclusion:** Cardiac PCD-CT protocols resulted in higher radiation doses compared with a conventional cardiac EID-CT protocol. Among PCD-CT protocols, UHR Spectral acquisitions frequently exceeded national reference limits. These findings suggest that PCD-CT protocols with default high-dose settings should be reserved for cases where maximal image quality is strictly required.
**Limitations:** The patient cohort undergoing the UHR protocol included a limited number of 20 patients.
**Funding for this study:** Funding was provided by the National Plan for NRRP Complementary Investments (PNC, established with the decree-law 6 May 2021, n. 59, converted by law n. 101 of 2021) in the call for the funding of research initiatives for technologies and innovative trajectories in the health and care sectors (Directorial Decree n. 931 of 06-06-2022) - project n. PNC0000003 - AdvaNced Technologies for Human-centrEd Medicine (project acronym: ANTHEM). This work reflects only the authors' views and opinions, neither the Ministry for University and Research nor the European Commission can be considered responsible for them.
**Has your study been approved by an ethics committee?** Not applicable
**Ethics committee - additional information:** The study is retrospective.
**Author Disclosures:**
Costanza Lisi: Nothing to disclose
Letterio Salvatore Politi: Nothing to disclose
Federica Catapano: Nothing to disclose
Andrea Laghi: Nothing to disclose
Marco Francone: Nothing to disclose
Pietro Levoni: Nothing to disclose

**Coronary Artery Calcium Score calculated on Photon Counting Computer Tomography scans: comparison of different reconstruction algorithms and scan protocols for measurement and risk stratification**

*E. Muscogiuri*, T. Busselot, E. Di Dedda, S. Dymarkowski, H. Bosmans; Leuven/BE
*(emanuele.muscogiuri@uzleuven.be)*

**Purpose:** Compare coronary artery calcium score (CACS) from Photon Counting Computed Tomography (PCCT) images, acquired with contrast-enhanced ECG-gated and non-contrast, non-gated scan protocols, and reconstructed with different algorithms: "Pure Calcium" (PC) and "Virtual non-contrast" (VNC).
**Methods or Background:** Patients undergoing a PCCT scan before Transcatheter Aortic Valve Implantation were included. For each patient, CACS was measured in five datasets with dedicated software: "true" non-contrast ECG-scan (TNC, reference); PC and VNC reconstructions of the ECG-gated (PCGATED, VNCGATED) and non-gated contrast-enhanced scans (PCNON-GATED, VNCNON-GATED). Statistical pairwise comparisons per patient and per vessel (LM+LAD, LCX, RCA) were made using a Friedman test and Dunn's multiple comparison test. Additionally, CACS was classified in risk categories (0; 0-10; 10-100; 100-400; >400) and compared using weighted Cohen's κ.
**Results or Findings:** Sixty-one patients were included (25 males, mean age 78,4 years). The analysis per patient showed that TNC CACS was not significantly different (p<0.05) from PCGATED, while it was significantly different from PCNON-GATED and VNC reconstructions. The analysis per vessel showed that CACS of LM+LAD calculated on PC reconstructions were not significantly different from TNC, while all the CACS of LCX were different from TNC. CACS of RCA calculated on PCGATED images were not significantly different from TNC. Classification comparison per CACS thresholds showed excellent agreement for PCGATED and PC-NONGATED (0.86 and 0.83, respectively) and a moderate to good agreement for VNCGATED and VNC-NONGATED (0.64 and 0.59, respectively).

**Conclusion:** The CACS measurements performed on PC reconstructions (particularly with ECG-gating) proved more reliable than those obtained from VNC reconstructions. However, it has to be noted that significant differences persist in the evaluation of CACS per vessel and in the risk stratification of patients among the different reconstructions.
**Limitations:** Small study population with the same disease.
**Funding for this study:** None
**Has your study been approved by an ethics committee?** Yes
**Ethics committee - additional information:** Study number is S68949, approved on 20 June 2024.
**Author Disclosures:**
Emanuele Di Dedda: Nothing to disclose
Hilde Bosmans: Nothing to disclose
Tim Busselot: Nothing to disclose
Steven Dymarkowski: Nothing to disclose
Emanuele Muscogiuri: Nothing to disclose

**Dual-layer spectral CTA: assessment of coronary vessels using 35mL of high concentration iodinated contrast agent and comparison with standard techniques**
*S. Tamietti*[1], F. Piacentino[2], F. Fontana[3], A. Gnesutta[2], M. Venturini[4], C. Beltramini[5], E. Macchi[2]; [1]Biella, BI/IT, [2]Varese/IT, [3]Vergiate/IT, [4]Segrate/IT, [5]Golasecca/IT
*(silvia.tamietti23@gmail.com)*

**Purpose:** To evaluated the image quality and plaque characterization in coronary CT angiography (CCTA) using dual-layer spectral CT (DL-SCT) with a reduced iodinated contrast bolus (35 mL) compared to the standard dose (70 mL). We focused on the role of virtual monoenergetic imaging (VMI) and Z-effective reconstructions.
**Methods or Background:** A cohort of 40 patients, with low-to-intermediate cardiovascular risk who underwent CCTA with DL-SCT (IQon Spectral CT Philips, Amsterdam, The Netherlands) were retrospectively divided in two groups based on contrast dose (70mL vs. 35mL of iomeron 400mg/mL). Quantitative parameters (contrast attenuation, signal-to-noise ratio [SNR], contrast-to-noise ratio [CNR]) were measured. Qualitative image analysis was performed by two radiologists. Plaque characterization was compared across conventional, VMI, and Z-effective reconstructions.
**Results or Findings:** In the reduced-contrast group, VMI at 50 keV achieved attenuation values comparable to standard acquisitions, while SNR and CNR were significantly higher ($p < 0.05$). Qualitative image scores were similar between groups; however, stenosis evaluation improved in the reduced-dose group due to fewer blooming artifacts in spectral reconstructions. Plaque analysis showed significant reduction in calcified plaque area and stenosis with VMI at 200 keV and Z-effective images, while VMI at 50 keV improved delineation of fibro-lipidic plaques and stenosis assessment ($p < 0.05$).
**Conclusion:** DL-SCT allows a 50% reduction in iodine load without compromising diagnostic quality. Low-energy VMI reconstructions maintain contrast enhancement, while VMI and Z-effective images enhance plaque characterization, supporting more accurate clinical management.
**Limitations:** The limitations of the study are the requirement for additional research on large, multicenter cohorts employing intra-patient protocols, in order to validate and improve future clinical practice.
**Funding for this study:** Not applicable.
**Has your study been approved by an ethics committee?** Not applicable
**Author Disclosures:**
Silvia Tamietti: Nothing to disclose
Federico Fontana: Nothing to disclose
Cecilia Beltramini: Nothing to disclose
Edoardo Macchi: Nothing to disclose
Filippo Piacentino: Nothing to disclose
Massimo Venturini: Nothing to disclose
Aroa Gnesutta: Nothing to disclose

**Contrast Dose Reduction in infants and children with Complex Congenital Heart Disease Using Virtual Monoenergetic Imaging on Dual-Layer Spectral Detector CT**
X. Cao, J. Li, X. Zhong, *X. Liu*; Guangzhou/CN
*(xiaomin.liu@philips.com)*

**Purpose:** To investigate the feasibility of reducing the contrast dose and the optimal keV of virtual monoenergetic images (VMIs) on dual layer spectral detector CT (DLCT) in infants and children with complex congenital heart disease (CHD).
**Methods or Background:** Prospectively enrolled 80 infants and children with complex CHD and underwent preoperative DLCT. Patients were randomly divided into two groups: group A (N=36, standard dose, 1.5~2 ml/kg) and group B (N=44, low dose, 1ml/kg). Conventional image (CI) and VMIs in range of 40-85 keV were reconstructed in group B while only CI was reconstructed in group A. Image quality was evaluated using qualitative scores and objective metrics including CT value, standard deviation (SD), signal-to noise ratio (SNR), and contrast-to-noise ratio (CNR) for the pulmonary artery and ascending aorta.
**Results or Findings:** Group B received 24% less contrast than group A [8.00 (5.00;11.00) ml vs 10.50 (8.00;21.00) ml, $P<0.05$]. VMI 40 keV in group B yielded the highest CT value, SNR, CNR of pulmonary artery and ascending aorta among different VIMs, and significantly outperformed CI in group A ($P<0.05$). Subjective evaluation score on VMI 40-50 keV of group B was significantly higher than CI of group A ($P<0.05$). Diagnostic accuracy for CHD at 40-50 keV in group B was 97.73%, significantly higher than group A (93.40%, $P<0.05$), while no significant difference in sensitivity and specificity.
**Conclusion:** VMI 40-50 keV in DLCT can significantly improve image quality, allowing contrast dose reduction in infants and children with complex CHD without compromising diagnostic performance.
**Limitations:** None
**Funding for this study:** None
**Has your study been approved by an ethics committee?** Yes
**Ethics committee - additional information:** None
**Author Disclosures:**
Xiaomin Liu: Nothing to disclose
Jinglei Li: Nothing to disclose
Ximing Cao: Nothing to disclose
Xiaomei Zhong: Nothing to disclose

**Clinical feasibility and safety of low-iodine-concentration contrast media in low-tube-voltage, low-dose coronary CT angiography for suspected coronary artery disease**
*C. H. Park*, S. Lee, J. W. Kim, J. E. Nam, E-Y. Choi, T. H. Kim; Seoul/KR
*(park_chulhwan@yuhs.ac)*

**Purpose:** Recent advances in low-kVp coronary CT angiography (CCTA) allow reduced iodine use, enabling the use of low-concentration iodine contrast media (ICM) while lowering radiation exposure. This study compared image quality and radiation dose between high- and low-concentration ICM in low-kVp, low-dose CCTA.
**Methods or Background:** 120 patients with suspected coronary artery disease were prospectively randomized to receive either high-concentration (Optiray, 350 mgI/mL) or low-concentration (Visipaque, 270 mgI/mL) contrast media. All CCTAs were performed using a 64-slice CT scanner with tube voltage and contrast volume adjusted to BMI. Quantitative (attenuation, noise, SNR, CNR) and qualitative (4-point scale) image quality were assessed. Acute and delayed adverse effects were monitored. Radiation dose was measured as dose-length product (DLP).
**Results or Findings:** No significant differences were found in demographic data, including heart rate. Attenuation (408.0 ± 67.9 HU vs. 424.5 ± 64.4 HU, $p = 0.174$), image noise, SNR, CNR, and qualitative image quality (mean scores: 3.34 vs. 3.46, $p = 0.075$) were comparable between groups. No acute adverse reactions occurred. Delayed adverse reactions were rare and non-severe, and resolved without sequelae. The radiation dose was not significantly different between the two groups ($p = 0.842$).
**Conclusion:** ICM dose reduction using low-concentration ICM is clinically feasible and provides comparable qualitative image quality in CCTA when using BMI-adapted low-kVp protocols in patients with suspected coronary artery disease.
**Limitations:** In this study, both groups used a low radiation dose protocol of either 100 kVp or 80 kVp. Subjects were randomly assigned to the two groups, resulting in no difference in body mass index. Tube voltage was determined by body mass index. Therefore, the radiation doses for both groups were similar, making it difficult to emphasize
**Funding for this study:** This study was funded by GE Healthcare. The funding sources had no role in the study design, data collection, data analysis, interpretation, or manuscript writing. The investigators maintained full responsibility for the content and the decision to submit the manuscript.
**Has your study been approved by an ethics committee?** Yes
**Ethics committee - additional information:** This prospective study was approved by the Ethics Committee and Institutional Review Board of Gangnam Severance Hospital. All procedures followed established ethical guidelines. Informed consent was obtained from all participants.
**Author Disclosures:**
Ji Won Kim: Nothing to disclose
Tae Hoon Kim: Nothing to disclose
Chul Hwan Park: Nothing to disclose
Eoi-Young Choi: Nothing to disclose
Ji Eun Nam: Nothing to disclose
Seunghyun Lee: Nothing to disclose

**Reducing Contrast Dose In Pulmonary Vein CT Imaging With Spectral Dual-Layer CT**

*P. M. Tetteroo*[1], C. Grinwis[1], L. R. Koetzier[1], D. Suchá[1], E. Langzam[2], N. R. van der Werf[3], B. K. Velthuis[1]; [1]Utrecht/NL, [2]Haifa/IL, [3]Best/NL
*(p.m.tetteroo-2@umcutrecht.nl)*

**Purpose:** To evaluate whether contrast-medium (CM)-dose in a pulmonary vein CT protocol can be safely reduced while maintaining diagnostic image quality using injection protocol adjustments and mono-energetic 55 keV reconstructions (MonoE55) on a dual-layer spectral CT (Philips CT7500).
**Methods or Background:** Sixty-eight patients were scanned with CM-dose concentrations progressively reduced from 100%, to 86%, 72%, 58% (N=17/group) with weight-adjusted (<80/≥80 kg) dual-phase contrast-injection protocols. Baseline contrast was 108/120 mL for <80/≥80 kg. Timing thresholds and injection speeds were modified from 200 HU in the descending aorta with 6/6.7 mL/s (<80/≥80 kg) for both 100%/86% CM-doses to 100 HU in the ascending aorta with 5/5.7 and 4.5/5.5 mL/s for 72%/58% CM-doses to optimize contrast timing. Subjective image quality (SIQ) was scored on MonoE55 and conventional images by one experienced, blinded cardiovascular radiologist using a 5-point Likert-scale for noise, contrast, attenuation, overall, and 3D volume. Objective image quality (OIQ) >200 HU was considered diagnostic and was assessed using 25 mm² regions-of-interest in four pulmonary veins, left atrial appendage, left atrium, and descending aorta. Data were compared to conventional 100% CM-dose images (reference) using Wilcoxon signed-rank or Mann–Whitney U tests for OIQ, and Wilcoxon signed-rank or chi-squared tests for SIQ.
**Results or Findings:** MonoE55 significantly improved SIQ across all CM-doses (p≤.026) compared with the reference, regardless of body weight, except for noise at 86% CM-dose, which was not significantly different (p=.295). MonoE55 significantly increased OIQ across all locations and for all CM-dose groups (p<.001). No MonoE55 attenuation measurements fell <200 HU.
**Conclusion:** CM-dose for pulmonary vein CT protocols can safely be reduced by 42% without compromising SIQ and OIQ when using spectral dual-layer MonoE55 reconstructions in combination with adjusted contrast timing and injection speed.
**Limitations:** Small sample size and single-reader design.
**Funding for this study:** No funding was received for this study.
**Has your study been approved by an ethics committee?** Not applicable
**Author Disclosures:**
Niels R. Van Der Werf: Employee: Philips Healthcare
Dominika Suchá: Nothing to disclose
Philip Martin Tetteroo: Nothing to disclose
Lennart Roelof Koetzier: Nothing to disclose
Eran Langzam: Employee: Philips Healthcare Global
Coraline Grinwis: Nothing to disclose
Birgitta K. Velthuis: Nothing to disclose

**Free-breathing isotropic cardiovascular 3D cine MRI without contrast agent - comparison to clinical standard**

*R. Chen*[1], H. Lu[2], A. Cernicanu[3], J. Westenberg[1], M. Sevenster[4], J. Keupp[4], J. Meineke[4], H. J. Lamb[1]; [1]Leiden/NL, [2]Amsterdam/NL, [3]Eindhoven/NL, [4]Hamburg/DE
*(r.chen@lumc.nl)*

**Purpose:** To develop and evaluate a free-breathing isotropic cardiovascular 3D cine MRI 1.5T framework that can assess cardiovascular anatomy and function without using contrast agent.
**Methods or Background:** A cartesian spiral(CASPR) bSSFP continuous k-space acquisition with 3D sagittal coverage of the whole heart and thoracic aorta was acquired twice, with 2.5mm and 2mm isotropic resolution. GPU-based reconstruction included retrospective binning of k-space profiles with respect to ECG and respiratory self-gating using a 1D feet-head projection. Conventional breath-hold 2D cine and 3D mDixon MR angiography sequences were acquired for comparison. Cardiovascular measurements were compared with Bland-Altman analysis and Intraclass Correlation Coefficient(ICC) for observer agreement(p<0.05).
**Results or Findings:** 24 volunteers were evaluated. Acquisition took 5-7minutes, with reconstruction about 5minutes per sequence. Compared to 2D cine, 3D cine demonstrated accurate measurement of left-ventricle ejection fraction, with a bias of 1.38% and limits of agreement(LoA) from −2.85% to 5.61% at 2.5mm, and a bias of 1.39% with LoA from −1.53% to 4.30% at 2.0mm. Aorta root sizes measured by 2D and 3D cine were comparable, showing a mean difference of 0.28mm2 with 95% LoA from -0.33mm2 to 0.89mm2 at 2.5mm, and a mean difference of 0.25mm2 with 95% LoA from -0.24mm2 to 0.74mm2 at 2.0mm. Other measurements showed strong agreement between 2D and 3D cine, with minimal mean differences and narrow limits of agreement. Intra- and interobserver for all the measurements showed at least moderate agreement, with ICC of 0.75 to 0.998.
**Conclusion:** Free-breathing isotropic cardiovascular 3D cine MRI using cartesian spiral sampling, without contrast agents, enables a simplified and time-efficient assessment of cardiovascular anatomy and function compared to 2D cine at 1.5T.
**Limitations:** There is a lack of studies including patients with arrhythmias and irregular breathing to validate image quality and quantification.
**Funding for this study:** No
**Has your study been approved by an ethics committee?** Yes
**Ethics committee - additional information:** Medical Research Ethics Committee of Leiden University Medical Center
**Author Disclosures:**
Alexandru Cernicanu: Employee: Philips
Jakob Meineke: Employee: Philips
Ruixin Chen: Nothing to disclose
Merlijn Sevenster: Employee: Philips
Jos Westenberg: Nothing to disclose
Hildo J. Lamb: Consultant: Philips
Huangling Lu: Nothing to disclose
Jochen Keupp: Nothing to disclose

**Contrast-free postoperative imaging of the pulmonary artery vasculature: Comparison of Relaxation-Enhanced Angiography without Contrast and Triggering (REACT) and time-resolved contrast-enhanced MRA**

*V. Schmidt*, L. Goertz, J. Tristram, R. A. Terzis, K. Kaya, D. Maintz, C. H. Gietzen, L. Pennig, J. P. Janssen; Köln/DE

**Purpose:** To compare REACT, a flow-independent 3D isotropic non-contrast MRA, with time-resolved contrast-enhanced MRA (4D CE-MRA) for postoperative assessment of the pulmonary arteries and right ventricular outflow tract, with emphasis on different implant types.
**Methods or Background:** In this retrospective single-center study, 53 patients with congenital heart disease underwent both 4D CE-MRA and REACT at 1.5 T. Three radiologists independently scored image quality (IQ), motion, and susceptibility on 5-point Likert scales for MPA, LPA, and RPA; diameters were measured and stenosis/dilatation recorded. Subgroup analyses were performed for stents, conduit/patch/valve (CPV), and no implant.
**Results or Findings:** REACT achieved higher overall IQ than 4D CE-MRA and significantly better motion scores in all vessels (all p < 0.001), while susceptibility scores were comparable (ns). The proportion of fully diagnostic studies (3/3 segments) was similar (REACT 77.4%, 41/53; 4D CE-MRA 83.0%, 44/53; McNemar ns). Diameter measurements showed excellent inter-reader agreement (ICC ≈ 0.89–0.95) and minimal bias between techniques; only the RPA was slightly smaller with REACT (mean difference –0.85 mm, p < 0.001), with Bland-Altman limits around ± 4 mm. In subgroup analyses, stented segments exhibited no IQ advantage of REACT and remained limited by susceptibility; motion scores remained superior with REACT, and diagnostic yield differences were not significant. In CPV and no-implant groups, REACT provided about +1 median IQ and motion score, with high diagnostic rates for both techniques.
**Conclusion:** REACT enables robust, contrast-free postoperative imaging of the pulmonary arteries with superior image quality and reduced motion artifacts compared to 4D CE-MRA, while maintaining highly reproducible diameter measurements. Stent segments remain a shared limitation; neither technique reliably evaluates stent lumen, and alternative modalities should be considered for targeted stent assessment.
**Limitations:** Non
**Funding for this study:** Non
**Has your study been approved by an ethics committee?** Yes
**Ethics committee - additional information:** The local ethics committee approved this study.
**Author Disclosures:**
Lenhard Pennig: Nothing to disclose
Carsten H. Gietzen: Nothing to disclose
David Maintz: Nothing to disclose
Juliana Tristram: Nothing to disclose
Kenan Kaya: Nothing to disclose
Vanessa Schmidt: Nothing to disclose
Robert Angelo Terzis: Nothing to disclose
Jan Paul Janssen: Nothing to disclose
Lukas Goertz: Nothing to disclose

**11:30 - 12:30** **Research Stage 3**

### Research Presentation Session: Genitourinary

### RPS 2407

### Female pelvic cancer imaging: what's new?

**Moderator**
Elisabeth Hedlund; Uppsala/SE
*(elisabeth.hedlund@gmail.com)*

**Immersive imaging for gynecologic oncology: planning beyond slices**
*R. Chianura*, P. P. Azzaro, C. Panico, B. Gui, E. Sala, G. Avesani, A. Fagotti, A. Rosati, D. Arrigo; Rome/IT
*(roberta.chianura01@icatt.it)*

**Purpose:** To assess the usefulness of three-dimensional reconstructions (3DR) and virtual reality (VR) derived from CT/MRI datasets in complex gynecologic malignancies, with a focus on multidisciplinary decision-making and surgical planning.
**Methods or Background:** In this single-center pilot study, 40 patients undergoing highly challenging surgery (e.g., pelvic exenteration; recurrences involving muscle, vessels, or bone; tumors at difficult sites such as the hepatic hilum) were included. CT (n = 24) and multiparametric MRI (n = 16) datasets were segmented and converted into interactive 3D models; VR simulations were generated from the same meshes. Four gynecologic oncology surgeons, assisted by a urogenital radiologist, evaluated each case first with standard imaging and then with 3DR/VR. After review, they completed a 5-point Likert questionnaire (1 = no benefit, 5 = maximum benefit) covering patient counseling, tumor board decisions (surgery vs. alternative therapy) and surgical planning. Pre-processing time was recorded.
**Results or Findings:** Mean usefulness scores increased from 3.0 ± 0.5 with standard imaging to 3.8 ± 0.3 for 3DR ($p < 0.01$) and 4.6 ± 0.5 for VR ($p < 0.001$). Consensus on surgical strategy during multidisciplinary discussions was achieved in 34/40 cases, with VR prompting management-related questions in 14 cases. Surgeons reported improved spatial understanding in 63% of cases and predicted safer resection planes. Median preparation time was 120 minutes (IQR 100–130), representing the main barrier to daily practice.
**Conclusion:** Immersive 3D visualization significantly improves surgical planning and decision-making in complex gynecologic oncology. Streamlining segmentation and workflow processes will be essential for clinical implementation. In future phases, intraoperative augmented reality (AR) will be tested, with early results showing stable hologram registration and promising integration into surgical practice.
**Limitations:** The small sample size and reliance on simulated MDT limit the generalizability of the findings
**Funding for this study:** No funding was received for this study.
**Has your study been approved by an ethics committee?** Yes
**Ethics committee - additional information:** ID prot.7640
**Author Disclosures:**
Giacomo Avesani: Nothing to disclose
Roberta Chianura: Nothing to disclose
Pietro Paolo Azzaro: Nothing to disclose
Benedetta Gui: Nothing to disclose
Evis Sala: Nothing to disclose
Andrea Rosati: Nothing to disclose
Camilla Panico: Nothing to disclose
Anna Fagotti: Nothing to disclose
Davide Arrigo: Nothing to disclose

**Transarterial Chemoperfusion (TACP) and Transarterial Chemoembolization (TACE) in the Treatment of Advanced Pelvic Gynecological Cancer: Tumor Response and Survival**
*T. J. Vogl*, A-I. Nica, L. S. Alizadeh, I. Yel, C. Wolfram, H. Adwan; Frankfurt/DE

**Purpose:** To retrospectively evaluate the efficacy of transarterial chemoperfusion (TACP) and transarterial chemoembolization (TACE) in the treatment of locally recurrent, unresectable gynecologic cancer regarding local tumor response and survival outcome.
**Methods or Background:** Between January 2000 and October 2023, 75 female patients diagnosed with locally recurrent, unresectable gynecologic tumors (cervical cancer, ovarian cancer) underwent 213 TACP and 154 TACE procedures. Among these, 33 underwent TACP, 20 received TACE, and 22 a combination of both therapies. Local tumor response was evaluated using the RECIST criteria and survival rates were determined with the Kaplan-Meier estimator.
**Results or Findings:** 50 patients (66.67%) maintained stable disease until completing therapy, while 10 patients (13.33%) exhibited partial response, 2 patients (2.67%) achieved complete response and 13 (17.33%) experienced progressive disease. Thus, 62 of 75 patients (87%) were classified as therapy responders. Overall, we observed a decrease of 5.53% in tumor size and 7.71% in tumor volume compared to baseline measurements. The median overall survival for all patients was 16.05 months, the median progression-free survival 13.5 months. The patients treated with TACP had the highest therapy response (90.91%) vs 81.92% of patients treated with both therapies and 70% of patients treated with TACE.
**Conclusion:** TACP and TACE are minimally invasive treatment options for patients with recurrent unresectable cervical and ovarian cancer. These therapies are particularly beneficial for patients who do not tolerate, refuse or do not respond to conventional treatment like chemo- or radiotherapy. However, further studies are still necessary
**Limitations:** -Retrospective and single-center study design -Varying follow-up intervals and, in many cases, short follow-up period. -Heterogeneous cohort, encompassing numerous tumor types with varying numbers of patients. -Heterogeneous treatment strategy with transarterial therapies for both palliative and neoadjuvant purposes.
**Funding for this study:** No funding
**Has your study been approved by an ethics committee?** Yes
**Ethics committee - additional information:** Approved by the ethics committee of the University Hospital Frankfurt
**Author Disclosures:**
Ibrahim Yel: Nothing to disclose
Christian Wolfram: Nothing to disclose
Andreea-Ioana Nica: Nothing to disclose
Thomas J. Vogl: Nothing to disclose
Hamzah Adwan: Nothing to disclose
Leona Soraja Alizadeh: Nothing to disclose

**Assessment of adherence to ESUR guidelines in MRI acquisition for uterine cancers and its impact on multidisciplinary tumour boards (MDTs) management decisions: preliminary results**
*G. Imbemba*, S. Bottazzi, V. Celli, E. S. Trogu, F. L. Lia, M. Dolciami, L. D'Erme, E. Sala, B. Gui; Rome/IT
*(gimbemba@gmail.com)*

**Purpose:** To systematically evaluate the adherence of external pelvic MRI studies to ESUR guidelines in patients referred to the uterine MDTs at our tertiary centre, identifying the most frequent protocol deviations, assessing their impact on patient's management.
**Methods or Background:** This retrospective, observational, single-centre study includes patients with cervical cancer (CC) and endometrial cancer (EC) discussed at the uterine MDTs between September 2024 and September 2025, whose external pelvic MRI scans are reviewed by a dedicated GYN radiologist.
**Results or Findings:** Between September 2024 and September 2025, 1820 cases were discussed and 607 required imaging revision. Of these, 277/607 were acquired at our Institution, while 330/607 were not and 110/330 (33%) were deemed non-compliant to ESUR guidelines (84 CC and 26 EC cases). Among CC MRIs, the main causes of non-adherence were absence of axial-oblique T2-WI in 68/84, absence of axial-oblique DWI in 68/84, absence of DWI images in 14/84, slice thickness >4mm in 19/84 cases. Also, contrast media was administered in 72/84 cases (86%). 24/84 patients required repeating MRI. Among those who did not: in 38 cases the necessary information was obtained through PET/CT and in 22 cases MRI was deemed sufficient to exclude locally-advanced CC and proceed directly to surgery. Among EC MRIs, the main non-adherence causes were: absence of axial-oblique DWI in 24/26 (92%), absence of axial-oblique T2-WI in 22/26 (85%), absence of axial-oblique post-contrast images in 25/26 (96%). Five patients required repeat MRI to assess the suitability for fertility-sparing procedures.
**Conclusion:** Adherence to ESUR guidelines for CC and EC MRI varies across institutions, missing recommended sequences and including non-recommended ones. This may reduce diagnostic accuracy, delay treatment initiation, and increase economic and environmental burden due to the unnecessary use of contrast agents.
**Limitations:** Na
**Funding for this study:** None
**Has your study been approved by an ethics committee?** Yes
**Ethics committee - additional information:** The study has been registered to the Ethical committee with the ID: ID8033.

**Author Disclosures:**
Benedetta Gui: Nothing to disclose
Guido Imbemba: Nothing to disclose
Veronica Celli: Nothing to disclose
Francesca Lourdes Lia: Nothing to disclose
Silvia Bottazzi: Nothing to disclose
Evis Sala: Nothing to disclose
Luca D'Erme: Nothing to disclose
Miriam Dolciami: Nothing to disclose
Elena Sofia Trogu: Nothing to disclose

**Radiologic–Pathologic Correlation of Ovarian Masses: Use of Diffusion, DCE and Radiomics**

*F. Abubacker Sulaiman*, R. Praveenkumar, S. Zainamb Begum, M. Nivitha, J. Lydia, A. Raashid Ibrahim; Chennai/IN
*(fasulaiman@gmail.com)*

**Purpose:** To assess the diagnostic performance of diffusion-weighted imaging (DWI), dynamic contrast-enhanced MRI (DCE-MRI), and radiomics features in differentiating benign and malignant ovarian masses, and to correlate imaging biomarkers with histopathological findings.
**Methods or Background:** A prospective study was conducted on 80 patients presenting with ovarian masses who underwent multiparametric MRI prior to surgery or biopsy. MRI protocol included T1- and T2-weighted sequences, DWI (b-values 0, 800 s/mm²), and DCE-MRI with quantitative perfusion analysis. Apparent diffusion coefficient (ADC) values, enhancement curve types, and radiomics features (first-order, texture, and shape-based metrics) were extracted from tumor regions using semi-automated segmentation. Histopathological diagnosis served as the reference standard. Statistical analysis included ROC curve evaluation and logistic regression to identify the best predictive model.
**Results or Findings:** Malignant lesions demonstrated significantly lower mean ADC values ($0.89 \pm 0.17 \times 10^{-3}$ mm²/s) and higher K^trans values on DCE compared to benign lesions ($1.45 \pm 0.24 \times 10^{-3}$ mm²/s, $p < 0.001$). Radiomics parameters such as gray-level entropy and surface irregularity showed strong correlation with tumor grade ($r = 0.71$, $p < 0.001$). The combined model integrating ADC, DCE kinetics, and key radiomics features achieved an AUC of 0.94 for malignancy prediction, outperforming any single parameter. Imaging patterns corresponded well with histopathologic features such as cellular density, necrosis, and stromal vascularity.
**Conclusion:** Multiparametric MRI incorporating diffusion, perfusion, and radiomics analysis enhances non-invasive characterization of ovarian masses and correlates strongly with histopathologic aggressiveness. This integrated approach may improve preoperative risk stratification and treatment planning.
**Limitations:** Single-center design, modest sample size, and absence of external radiomics validation restrict generalizability. Larger multicentric datasets and standardized acquisition protocols are needed for clinical translation.
**Funding for this study:** No external funding was received for this study.
**Has your study been approved by an ethics committee?** Yes
**Ethics committee - additional information:** Institutional ethics commitee approval obtained.
**Author Disclosures:**
Rathinamoorthy Praveenkumar: Nothing to disclose
Sulaiman Zainamb Begum: Nothing to disclose
Mohanam Nivitha: Nothing to disclose
Johnbosco Lydia: Nothing to disclose
Farook Abubacker Sulaiman: Nothing to disclose
Abubacker Raashid Ibrahim: Nothing to disclose

**The added value of ADC in multiparametric MRI for uterine smooth muscle tumor characterization**

*F. Masino*[1], C. Locorotondo[2], A. Torrente[2], M. Montatore[1], M. Balbino[1], V. Buonocore[2], M. Morgillo[2], G. Guglielmi[1], E. Muscogiuri[2]; [1]Foggia/IT, [2]Brindisi/IT
*(federicamasino@gmail.com)*

**Purpose:** To evaluate the added diagnostic value of integrating ADC with conventional MRI features in the differentiation of uterine smooth muscle tumors.
**Methods or Background:** Preoperative distinction between leiomyomas, STUMP, and leiomyosarcomas is critical due to radically different therapeutic implications. While MRI represents the reference imaging technique, morphology alone is insufficient to ensure accuracy. In this retrospective single-center cohort of 27 surgically confirmed cases, both qualitative MRI features (shape, margins, necrosis, T1/T2 signal, contrast enhancement, DWI) and quantitative ADC values were systematically assessed. Logistic regression and ROC analysis tested the incremental value of ADC.
**Results or Findings:** Among 27 patients, 22 had benign lesions and 5 had non-benign tumors. Morphological features such as irregular margins, necrosis, and hemorrhagic T1 foci correlated significantly with malignancy ($p<0.05$), but showed partial overlap with atypical leiomyomas. The ADC alone yielded excellent discrimination (AUC 0.995). Importantly, when ADC was combined with morphological parameters, diagnostic performance improved from an AUC of 0.982 (morphology alone) to 1.000 (integrated model). This integration enhanced risk stratification, supporting accurate identification of leiomyosarcomas while avoiding unnecessary radical surgery in benign cases.
**Conclusion:** A multiparametric MRI approach integrating ADC with morphological features achieves nearly ideal accuracy in differentiating uterine smooth muscle tumors, providing radiologists with a powerful tool to optimize patient management.
**Limitations:** No limitations were identified.
**Funding for this study:** No funding was received for this study.
**Has your study been approved by an ethics committee?** Not applicable
**Author Disclosures:**
Marina Balbino: Nothing to disclose
Giuseppe Guglielmi: Nothing to disclose
Eluisa Muscogiuri: Nothing to disclose
Andrea Torrente: Nothing to disclose
Manuela Montatore: Nothing to disclose
Martina Morgillo: Nothing to disclose
Chiara Locorotondo: Nothing to disclose
Federica Masino: Nothing to disclose
Valeria Buonocore: Nothing to disclose

**11:30 - 12:30** **Research Stage 4**

### Research Presentation Session: Physics in Medical Imaging

### RPS 2413

### Upcoming technologies and deep learning reconstruction across different imaging modalities

**Moderator**
Sabina Strocchi; Varese/IT

**Moderator's introduction**
Sabina Strocchi; Varese/IT
*(sabina.strocchi@asst-settelaghi.it)*

**Advancing Patient-Specific Dosimetry for Synchrotron Phase-Contrast Breast CT: A Monte Carlo Framework for Clinical Translation at the Australian Synchrotron**

*A. Entezam*[1], A. Pakzad[1], A. Maksimenko[1], C. Hall[1], M. John Cameron[1], P. Brennan[2], D. Hausermann[1], T. Gureyev[1], H. M. Quiney[1]; [1]Melbourne/AU, [2]Sydney/AU
*(amir.entezam@unimelb.edu.au)*

**Purpose:** To develop and validate a patient-specific Monte Carlo (MC) dosimetry framework for propagation-based X-ray phase-contrast breast computed tomography (BCT) at the Imaging and Medical Beamline (IMBL) of the Australian Synchrotron, enabling accurate estimation of mean glandular dose (MGD) for clinical translation.
**Methods or Background:** BCT provides 3D imaging without breast compression, improving comfort and enhancing visualization of internal structures—crucial for accurate cancer detection. Propagation-based phase-contrast BCT offers superior soft-tissue contrast at equal resolution and comparable or lower doses than conventional absorption-based BCT. Accurate radiation dosimetry is vital for patient safety and optimal imaging performance. Most existing MC-based MGD studies use homogeneous phantoms, neglecting patient-specific anatomy that affects dose distribution. To overcome this, a voxel-based MC dosimetry framework was developed using EGSnrc to calculate MGD in realistic anthropomorphic breast phantoms derived from synchrotron BCT images, tailored to IMBL beam properties. Corresponding homogeneous phantoms were also generated, and air kerma–to–MGD conversion factors were obtained and compared with heterogeneous phantom results. Simulations were performed for varying breast densities, dimensions, and skin thicknesses across 28–38 keV.

**Results or Findings:** Breast anatomy and beam energy strongly influenced MGD. Higher glandular density and larger breasts increased MGD, while a 2 mm thicker skin layer reduced it by about 10% at equal air kerma. MGDs decreased with rising beam energy, though less markedly in larger breasts. Comparisons between heterogeneous and homogeneous phantoms showed clear differences in conversion coefficients, underscoring the need to include anatomical heterogeneity in dosimetric modelling.
**Conclusion:** The voxel-based MC framework offers an anatomically accurate, patient-specific approach to dosimetry in synchrotron phase-contrast BCT, enabling precise dose estimation and supporting safe, effective clinical implementation at the Australian Synchrotron..
**Limitations:** This study focused on simulations performed using only EGSnrc MC code.
**Funding for this study:** Funding was provided by Australian National Health and Medical Research Council (NHMRC) Synergy Grant (APP2011204).
**Has your study been approved by an ethics committee?** Yes
**Ethics committee - additional information:** Ethical approval was received from the Human Research Ethics Committee of Monash University (project number: CF15/31382015001340).
**Author Disclosures:**
Anton Maksimenko: Nothing to disclose
Daniel Hausermann: Nothing to disclose
Matthew John Cameron: Nothing to disclose
Patrick Brennan: Nothing to disclose
Christopher Hall: Nothing to disclose
Amir Entezam: Nothing to disclose
Harry M. Quiney: Nothing to disclose
Timur Gureyev: Nothing to disclose
Ashkan Pakzad: Nothing to disclose

### 3D-printed anthropomorphic breast phantoms with iodinated resin disks to evaluate the performance of Contrast-Enhanced Mammography

*A. Belarra*[1], M. Castillo[1], I. Hernandez-Giron[2], N. Amallal El Ouahabi[1], A. Tejerina[1], P. Homolka[3], M. Chevalier[1]; [1]Madrid/ES, [2]Dublin/IE, [3]Vienna/AT
*(abelarra@ucm.es)*

**Purpose:** Contrast-enhanced mammography (CEM) is a widespread imaging modality to improve lesion detection. In this study, a 3D-printed anthropomorphic phantom including iodinated resin disks is employed to evaluate CEM performance.
**Methods or Background:** A breast-CT volume segmented into gland/skin and fat and further compressed (thickness: 4.8 cm) was used to generate four 1.2cm-thick slices, which were 3D-printed (BambuLab X1C, 0.2 mm nozzle, 0.14 mm layer height, 100% infill-lines) selecting PLA for glandular tissue/skin and ABS for fat. One of the intermediate slices included five cylindrical recesses (8mm diameter, 0.8mm height) located over different backgrounds. Three sets of four resin iodinated disks (Iomeron400) were SLA-printed with varying concentrations (0.50, 0.75, 1.00 mg/cm²). A fifth disk was PLA-printed as a control. Three CEM phantom images were acquired (Hologic-3Dimensions)for each set of resins+PLA disks. On the images, the difference (S) between MPV of a 7mm-circular-ROI inside each disk and MPV of a 1mm-band-ROI on the background were computed. For each set, the average S was obtained across images (and all disks for resins ones).
**Results or Findings:** The phantom background was successfully removed in CEM images. S values were [average±SD(range)]: [0±2(-2,1) for the PLA disk] and for iodinated disks: [23±5(17,28); 0.50mg/cm2], [30±7(24,39); 0.75mg/cm2] and [44±4(40,49); 1.00mg/cm2]. S value and iodine concentration were linearly dependent (43.1 slope; R2=0.998).
**Conclusion:** In CEM images, both PLA (glandular tissue) and background (parenchyma) were successfully removed, whereas resin-disk signals remained visible. As expected, S values increased with iodine concentration. These results support the use of 3D-printed anthropomorphic phantoms for evaluating the CEM performance under conditions that better mimic the clinical scenario.
**Limitations:** A wider range of concentrations must be evaluated and a better recess-disk fitting achieved to reduce the air artefact on the disk borders.
**Funding for this study:** This work was supported by the Spanish Ministry of Science and Innovation under grants PID2021-123390OB-C22 and PID2021-123390OB-C22.
**Has your study been approved by an ethics committee?** Not applicable
**Author Disclosures:**
Adrián Belarra: Nothing to disclose
Peter Homolka: Nothing to disclose
María Castillo: Nothing to disclose
Irene Hernandez-Giron: Nothing to disclose
Alejandro Tejerina: Nothing to disclose
Margarita Chevalier: Nothing to disclose
Najim Amallal El Ouahabi: Nothing to disclose

### Manufacturing and validation of part-solid pulmonary nodule phantoms with realistic morphology and multiple densities using stereolithography-based 3D-printing

*L. D'Hondt*[1], D. Buytaert[1], P-J. Kellens[1], A. Snoeckx[2], K. Bacher[1]; [1]Gent/BE, [2]Antwerp/BE

**Purpose:** While phantoms support objective CT image quality and dose evaluations, off-the-shelf models insufficiently reflect complex morphologies and radiodensity heterogeneities, thereby limiting clinical translation of results. Part-solid pulmonary nodules exemplify intricate structures inadequately represented by generic models. Adaptation of 3D-printing in research could improve phantoms. This study aimed to 3D-print part-solid nodule phantoms that mimic patient nodules, as validated by a human reader study.
**Methods or Background:** Seven part-solid pulmonary nodules on patient-derived CT series were segmented and solid core and ground glass densities were measured through Hounsfield Unit (HU) thresholding. Four target radiodensity ranges were defined and stereolithography-based 3D-printing parameters were optimised towards 3D-designs of nodules with a solid core (>0HU) and three gradients mimicking ground glass opacity ([0; -300HU[, [-300; -500HU[, [-500; -750HU]). Low-dose CT acquisitions of 3D-prints incorporated in a Lungman phantom were randomised with patient scans. A multicentre, single-blinded reader study, involving seventeen radiologists, assessed whether 3D-printed nodules were distinguishable from real part-solid nodules using $\chi 2$ goodness-of-fit testing.
**Results or Findings:** Final 3D-printed nodule models consisted of the four target radiodensities measured on CT-images of clinical nodules. Overall accuracy of all scored nodules was 53.50%. Importantly, the false negative rate indicates that, across all radiologists, 47.50% of 3D-printed nodules was incorrectly classified as a real patient nodule, while 44.54% of patient nodules were thought to be 3D-prints. There was significant evidence ($\chi 2$= 136.13; p= 1.864e-31; 5% confidence level) against readers reliably distinguishing patient nodules from our 3D-printed phantoms.
**Conclusion:** We were able to manufacture nodule phantom models with morphological and radiological properties that are highly customisable, relatively inexpensive and indistinguishable from real clinical examples. As such, this study demonstrates that adaptation of 3D-printing can enhance clinical relevance of phantom research.
**Limitations:** No limitations were identified.
**Funding for this study:** Funding was provided by the FWO “Kom op tegen Kanker”-project for lung cancer screening research in Belgium. (Project number: G0B1922N).
**Has your study been approved by an ethics committee?** Yes
**Ethics committee - additional information:** The retrospective use of patient CT series was approved by the Antwerp University Hospital Ethics Committee (Approval Number: EC/PM/eva/2024.048).
**Author Disclosures:**
Klaus Bacher: Nothing to disclose
Louise D'Hondt: Nothing to disclose
Annemiek Snoeckx: Nothing to disclose
Dimitri Buytaert: Nothing to disclose
Pieter-Jan Kellens: Nothing to disclose

### Deep Learning-Based Anatomical Segmentation for Beam Hardening Correction in Clinical Dark-Field Chest Radiography

*L. Kaster*[1], M. Lochschmidt[1], A. M. Bauer[1], T. Dorosti[1], S. Demianova[1], T. Koehler[2], D. Pfeiffer[1], F. Pfeiffer[1]; [1]Munich/DE, [2]Hamburg/DE
*(lennard.kaster@tum.de)*

**Purpose:** Dark-field chest radiography has demonstrated superiority over conventional radiography for diagnosing and staging pulmonary diseases by visualising microstructural lung changes. However, rib- and clavicle-induced beam hardening artefacts generate false dark-field signals that impair diagnostic accuracy. This study introduces an AI-based segmentation framework for anatomically adaptive beam hardening correction (BHC) in clinical dark-field radiographs.
**Methods or Background:** Rib and clavicle segmentations were obtained using U-Net models trained on the VinDr-RibCXR dataset and clinical attenuation images. Masks were refined into anterior and posterior rib components and combined with attenuation-contribution weights derived from dual-energy CT material decomposition (aluminium and water as surrogates for bone and soft tissue). Patient-specific correction maps were generated and subtracted from raw dark-field images. The method was evaluated on 174 radiographs (healthy, COPD, COVID-19) using qualitative assessment and quantitative measures of signal homogeneity and diagnostic separability.
**Results or Findings:** Uncorrected dark-field radiographs showed elevated signals at rib and clavicle locations, obscuring the lung parenchyma and reducing interpretability. The proposed segmentation-based BHC suppressed these artefacts, yielding a more homogeneous dark-field signal and improved visualisation of pulmonary structures. Improvements were consistently observed across all cohorts. Quantitatively, intra-pulmonary signal variability decreased (coefficient of variation −16%, p < 0.001), and diagnostic separability improved, with overlap between healthy and diseased lungs reduced by up to 18.8%.
**Conclusion:** AI-segmentation-based BHC enables regionally adaptive correction of bone-induced artefacts in dark-field chest radiography, improving image homogeneity and visual differentiation of pathological changes. This addresses a key limitation of the modality and strengthens its clinical potential for pulmonary disease assessment.
**Limitations:** The limitations of the study are that correction performance depends on segmentation accuracy and on contribution weights derived from limited spectral CT datasets. Patients with atypical anatomy or metallic implants may still exhibit residual artefacts.
**Funding for this study:** Funding was provided by the European Research Council (ERC Synergy Grant SmartX, SyG 101167328), the Free State of Bavaria under the Excellence Strategy of the Federal Government and the Länder, and the Institute of Advanced Study, Technical University of Munich.
**Has your study been approved by an ethics committee?** Yes
**Ethics committee - additional information:** The study was approved by the Ethics Commission of the Medical Faculty, Technical University of Munich (reference number 166/20S and 587/16S) .
**Author Disclosures:**
Sofia Demianova: Nothing to disclose
Thomas Koehler: Employee: Philips Innovative Technologies
Anne M. Bauer: Nothing to disclose
Tina Dorosti: Nothing to disclose
Lennard Kaster: Nothing to disclose
Franz Pfeiffer: Nothing to disclose
Maximilian Lochschmidt: Nothing to disclose
Daniela Pfeiffer: Nothing to disclose

**Characterization of a Deep Learning CT Reconstruction Algorithm and Comparison with Iterative Reconstruction Algorithms**
*R. Villa*, N. Paruccini, E. De Ponti; Monza/IT
*(raffaele.vill@gmail.com)*

**Purpose:** To characterize the image quality performance of a deep learning-based (DL) reconstruction algorithm, Philips Precise Image, and compare it with iterative reconstruction (IR) algorithms (iDose 4 and IMR) under clinically relevant conditions. The goal is to evaluate its potential in optimizing CT protocols.
**Methods or Background:** Two phantoms were used for this study: an anthropomorphic abdomen phantom (PhantomX, Germany) and a Catphan phantom (The Phantom Laboratory, USA). Both phantoms were acquired at five dose levels (1.7-6.8 mGy), with each acquisition repeated five times. Two CT scanners were used: one with Philips iDose 4 and IMR, and another with Philips iDose 4 and Precise Image. Image quality was evaluated in terms of noise, spatial resolution, blur, visual artifacts, and detectability index with a channelized Hotelling observer.
**Results or Findings:** Precise Image significantly improved all image quality parameters compared to iDose 4. Furthermore, noise reduction was up to 70%, spatial resolution improved by up to 20%, and CHO scoring increased by up to 90%. The improvement was less pronounced with IMR; however, Precise Image demonstrated behaviour more similar to filtered back projection (FBP) than IMR, with less dependency of image quality on contrast. This similarity makes it easier to optimize CT protocols. Additionally, Precise Image produced fewer visual artifacts than IMR, resulting in images that were much more similar in appearance to FBP images and reducing the synthetic texture typically associated with iterative reconstruction techniques.
**Conclusion:** The DL reconstruction algorithm demonstrates superior image quality performance compared to conventional iterative reconstruction methods. Its image characteristics, which closely resemble those of FBP, suggest it could be a valuable tool for simplifying and improving CT protocol optimization.
**Limitations:** Phantom size, small size compared to an adult patient.
**Funding for this study:** None
**Has your study been approved by an ethics committee?** Not applicable
**Author Disclosures:**
Raffaele Villa: Nothing to disclose
Nicoletta Paruccini: Nothing to disclose
Elena De Ponti: Nothing to disclose

**Assessment of Low-Contrast Lesion Detectability in CT: Evaluation of Super Resolution Deep-Learning Reconstruction Using 3D-Printed Lifelike Abdominal Phantoms**
D. W. Shin[1], J. Im[2], *L. Liu*[2], B. E. Hoppel[1], T. Hagio[3], A. Dhanantwari[1], K. Boedeker[3], P. Noël[2]; [1]Tustin, CA/US, [2]Philadelphia, PA/US, [3]Ōtawara/JP

**Purpose:** To use 3D-printed anthropomorphic abdominal phantoms to compare trends in low contrast detectability (LCD) across various reconstruction algorithms and doses.
**Methods or Background:** An anthropomorphic abdominal phantom, consisting of two modules, was 3D-printed with PixelPrint, a technique that converts patient CT images and/or synthetic datasets into printer instructions which modulate printed density at sub-resolution scale to mimic realistic tissue densities and textures. The first module consisted of native anatomy, while the second incorporated ten digitally added rods (5 mm diameter, 30 HU contrast) within the same native anatomical background. Scans were acquired using an abdominal CT protocol on a clinical CT scanner (Aquilion ONE INSIGHT, Canon Medical Systems) across four water-equivalent diameters (Dw = 15, 27, 31, and 35 cm) at dose levels from 4-30 mGy. Images were reconstructed with Filtered Back Projection (FBP), Hybrid Iterative Reconstruction (HIR), two Deep Learning Reconstructions (DLR1 and DLR2), and Super Resolution-Deep Learning Reconstruction (SR-DLR). The LCD was determined via a Non-Prewhitening Model Observer with an eye filter. The trend in detectability index (d′) across reconstruction algorithms was compared at various dose levels and phantom sizes.
**Results or Findings:** At the highest dose (30 mGy) and largest size (Dw 35 cm), the d’ were 1.84, 1.56, 1.44, 1.23, and 0.75 for SR-DLR, DLR1, DLR2, HIR, and FBP, respectively. At the lowest dose (4 mGy), the corresponding d’ were 0.95, 0.99, 0.85, 0.89, and 0.30. The SR-DLR generally showed better LCD performance than conventional algorithms across dose levels and phantom sizes.
**Conclusion:** Lifelike 3D-printed abdominal phantoms replicate clinical imaging conditions, demonstrating in this study that SR-DLR improves visualization of subtle lesions—even in larger patients or at reduced doses—thereby supporting greater diagnostic confidence.
**Limitations:** Future reader studies are required for full clinical translation.
**Funding for this study:** Canon Medical Systems USA
**Has your study been approved by an ethics committee?** Not applicable
**Author Disclosures:**
Jessica Im: Nothing to disclose
Peter Noël: Nothing to disclose
Kirsten Boedeker: Employee: Canon Medical Systems Corporation
Tomoe Hagio: Employee: Canon Medical Systems Corporation
Daniel W. Shin: Employee: Canon Medical Systems USA
Leening Liu: Nothing to disclose
Bernice E. Hoppel: Employee: Canon Medical Systems USA
Amar Dhanantwari: Employee: Canon Medical Systems USA

**Contribution of Photon-Counting on Slit-Beam Radiography Image Quality: Cross-Center Benchmarking and Validation**
I. Fitton[1], S. Alkhazzam[2], M. H. Kharita[2], M. Dimailing[1], M. Khalife[1], C. Van Ngoc Ty[1], *V. Tsapaki*[3]; [1]Paris/FR, [2]Doha/QA, [3]Vienna/AT
*(v.tsapaki@iaea.org)*

**Purpose:** Slit-beam radiography is recognized as a low-dose imaging technique particularly suitable for spine, hips, and knees. Additional clinical applications could be expanded with photon-counting technology. The aim of this study was to evaluate the image quality of photon-counting (PCD) compared to pressured Xenon gaseous linear (GLD) detectors, using the ATIA International Atomic Energy Agency (IAEA) methodology.
**Methods or Background:** Seven EOS imaging systems including two models based on PCD and GLD technologies were studied. The IAEA test-object was propped along each detector. A block of 30 × 30 × 10 cm3 PMMA plates was placed at the output of the X-ray tube as an attenuator. Acquisition parameters were fixed : 70kV, 20mA, scanning speed of 6, no modulation. Image acquisitions were repeated five times on each of the detectors. « For-processing » (FPRO) and « For-presentation » (FPRE) image formats were compared when available. Signal-difference-to-noise ratio (SDNR), Normalized Noise Power Spectrum (NNPS), Modulation Transfer Function (MTF), detectability indexes (d') for small lesions with diameters of 0.3mm and 4mm were automatically quantified using PyATIA software. A Wilcoxon-test was used to compare both technologies.
**Results or Findings:** Results showed that task-based detectability metrics (SDNR and d′) were substantially higher on photon-counting. The spatial resolution was higher on PCD compared to GLD at high frequencies in FPRE: 11.21 lp/mm[8.55–12.24] vs. 2.64 lp/mm[2.16–3.44] ($p<0.05$). The noise variance was greatly reduced on PCD and close to zero. The noise level was four times lower on PCD compared to GLD.
**Conclusion:** The IAEA ATIA methodology proved valid for inter-technology comparison, enabling benchmarking between PCD and GLD detectors. PCD demonstrated statistically superior image quality compared to GLD. This paves the way to a range of clinical applications of slit-beam radiography beyond spinal conditions.
**Limitations:** None
**Funding for this study:** None
**Has your study been approved by an ethics committee?** Not applicable

**Author Disclosures:**
Marc Khalife: Nothing to disclose
Virginia Tsapaki: Nothing to disclose
Shady Alkhazzam: Nothing to disclose
Isabelle Fitton: Nothing to disclose
Mohammed Hassan Kharita: Nothing to disclose
Claire Van Ngoc Ty: Nothing to disclose
Maureen Dimailing: Nothing to disclose

**Robust VFA T1 Mapping with Optimal-Control RF Excitation**
*C. Graf*[1], A. Jaffray[1], A. Rund[1], S. Steinerberger[2], A. Rauscher[1]; [1]Vancouver, BC/CA, [2]Seattle, WA/US
*(alexander.rauscher@ubc.ca)*

**Purpose:** The T1 relaxation time of the MRI signal can characterize brain tissue microstructure, e.g. to monitor disease progression and treatment response in conditions such as multiple sclerosis, tumours, or brain trauma. Variable flip angle (VFA) T1-mapping is fast but prone to bias from off-resonance, B1-inhomogeneity, imperfect spoiling, and magnetization transfer (MT). Our aim was to design excitation radiofrequency (RF) pulses with optimal control (OC) that are robust to B0/B1-variations and satisfy controlled-saturation MT (CSMT) to reduce MT-related bias, enabling accurate VFA-T1 without B1-mapping.
**Methods or Background:** Non-selective OC excitation pulses were designed for α=6° and 15° at 3T and 7T. Robustness levels covered B1-scales of 80-120% of nominal and B0-offsets of ±2.4ppm. Pulses were optimized by a semi-smooth quasi-Newton method with exact discrete derivatives; Bloch dynamics were solved via symmetric operator splitting. To enforce CSMT, pulses for both flip angles used identical duration and constant maximum amplitude, yielding matched RF energy. Numerical VFA-T1 simulations compared OC versus block pulses at 3T and 7T, sampling wide B0/B1-ranges. Summary metrics within the target robustness box included min/max/mean T1 and the central 90%-range.
**Results or Findings:** OC pulses produced tighter T1-distributions than block pulses. At 3T, the 90% T1-range for OC was 748-882ms (≤10% from nominal 800ms), versus 540-1129ms (>40%) for block; at 7T, OC yielded 1255-1384ms (<7% from nominal 1300ms) versus 879-1842ms (~40%) for block. OC-T1 remained largely insensitive to B1 within 80-120% of nominal B1, whereas block-pulse T1 increased quadratically with B1.
**Conclusion:** OC-designed, CSMT-compliant excitation pulses substantially reduce B0/B1-related VFA-T1 bias at 3T and 7T while removing the need for B1-mapping. The approach is vendor-agnostic, patient-independent, and requires no specialized hardware, supporting practical deployment for robust quantitative T1-mapping.
**Limitations:** Future work will verify results experimentally.
**Funding for this study:** nA
**Has your study been approved by an ethics committee?** Not applicable
**Author Disclosures:**
Armin Rund: Founder: phaseLab Dynamics Inc.
Alexander Jaffray: Founder: phaseLab Dynamics Inc.
Alexander Rauscher: Founder: phaseLab Dynamics Inc.
Stefan Steinerberger: Nothing to disclose
Christina Graf: CEO: phaseLab Dynamics Inc.

**Towards Non-Invasive Characterization of Thyroid Tissue: Feasibility of Magnetic Resonance Elastography with a Custom Passive Driver**
*V. Atamaniuk*, L. Hanczyk, M. Obrzut; Rzeszów/PL
*(vitaliyacera500@gmail.com)*

**Purpose:** Magnetic resonance elastography (MRE) is an advanced non-invasive imaging technique that quantifies the biomechanical properties of soft tissue. While clinical MRE is mainly applied to the liver, stiffness-related biomarkers may be valuable for other organs, including the thyroid, where tissue stiffening occurs in multiple pathological conditions. However, due to the gland's small size and anatomical location, MRE of the thyroid is challenging without dedicated hardware. This study aimed to assess the feasibility of thyroid MRE using a custom-designed passive driver.
**Methods or Background:** 6 adult volunteers (aged 29–59 years) underwent thyroid MRE. A custom two-branch passive driver was built and positioned on the neck to cover both lobes of the thyroid. Vibrations at 120 Hz were generated by an active driver and transmitted through the passive driver to induce shear waves. Imaging was performed on a 1.5T whole-body MRI scanner using a SE-EPI-based 3D vector MRE sequence. Shear modulus magnitude and its storage and loss components were reconstructed using direct inversion. Wave field quality was assessed with octahedral shear strain SNR (OSS-SNR) and a confidence metric. Regions of interest were manually defined on T2-weighted images by a radiologist.
**Results or Findings:** The custom driver consistently provided excellent shear wave illumination in the thyroid. Mean ± SD OSS-SNR was 4.09±2.11, with confidence 81.39±3.45%. Displacement amplitudes ranged from 6.45 to 149.16 µm (mean ± SD: 51.24±21.45 µm). Mean ± SD stiffness, storage modulus, and loss modulus were 4.11±0.67 kPa, 3.87±0.70 kPa, and 1.22±0.61 kPa, respectively.
**Conclusion:** A dedicated passive driver makes thyroid MRE technically feasible, yielding reliable shear wave propagation and stiffness quantification. These findings support further research into the role of MRE for non-invasive thyroid characterization.
**Limitations:** Small sample size and lack of clinical data.
**Funding for this study:** National Science Centre, Poland (Grant ID: 2024/53/N/ST7/00358) Polish National Agency for Academic Exchange (Grant ID: 25/NAWA-PROM-UR/2019)
**Has your study been approved by an ethics committee?** Yes
**Ethics committee - additional information:** Approved by the Regional Medical Chamber ethics committee (Resolution No 60/2022/B); written informed consent was obtained from all participants.
**Author Disclosures:**
Marzanna Obrzut: Nothing to disclose
Lukasz Hanczyk: Nothing to disclose
Vitaliy Atamaniuk: Nothing to disclose

**11:30 - 12:30** **Research Stage 5**

### Research Presentation Session: Oncologic Imaging

### RPS 2416
### Current advances in lymphoma imaging

**Moderator**
Doris Leithner; New York/US
*(doris.leithner@googlemail.com)*

**Stage-Specific Prognostic Value of Tumor Burden in Lymphoma Patients Treated With CAR T-Cell Therapy**
M. Kassube, P. F. Linden, K. Rejeski, G. Sheikh, R. Werner, M. Subklewe, J. Ricke, W. G. Kunz, *M. Winkelmann*; Munich/DE
*(michael.winkelmann@med.lmu.de)*

**Purpose:** CAR T-cell therapy (CART) has transformed treatment for relapsed or refractory llymphomas. While Ann Arbor staging remains important for risk stratification, tumor burden (TB) may offer additional prognostic insight. The differential impact of TB in early- (I/II) versus advanced-stage (III/IV) disease remains unclear in the context of CART. We assessed the added prognostic value of Lugano-based sum of product diameters (SPD) in stage I/II compared with stage III/IV patients for progression-free (PFS) and overall survival (OS).
**Methods or Background:** This study was conducted on a cohort of 103 patients treated with CART. Tumor burden at baseline was quantified using SPD according tu Lugano Criteria. Patients were stratified by Ann Arbor stage and SPD levels. PFS and OS were analyzed using Kaplan-Meier curves and log-rank tests.
**Results or Findings:** Median age was 67 years; 38% female. Patients with Stage III/IV disease had significantly higher baseline SPD compared to Stage I/II (median SPD: 6,088 mm² vs. 1,504 mm²; p<0.001). Survival analysis demonstrated significantly inferior PFS (p<0.001) and OS (p=0.018) in patients with advanced disease. The subdivision of the early stage patients according to their median SPD of 1,504 mm2 resulted in a significant separation of the two groups. The early stage patients with a low SPD showed a significantly longer OS and a slightly longer PFS than those with a high SPD. Interestingly, stage I/II patients with higher TB showed a similar overall survival curve as the advanced stage patients.
**Conclusion:** Baseline tumor burden, as measured by SPD, had a prognostic value in early-stage lymphoma patients undergoing CART. In this subgroup, higher SPD was associated with inferior OS and slightly inferior PFS. In contrast, SPD did not show a prognostic impact among patients with advanced-stage disease.
**Limitations:** Monocentric, retrospective study.
**Funding for this study:** BZKF, FöFoLe (LMU Munich)
**Has your study been approved by an ethics committee?** Yes

**Ethics committee - additional information:** All medical records and imaging studies underwent review with approval from the LMU Munich Institutional Review Board (LMU Ethics Committee, project number 19-817).
**Author Disclosures:**
Marion Subklewe: Research/Grant Support: Amgen; BMS/Celgene; Gilead; Janssen; Miltenyi Biotec; Novartis; Roche; Seattle Genetics; Takeda Consultant: Aven Cell; CDR-Life; Ichnos Sciences; Incyte Biosciences; Janssen; Miltenyi Biotec; Pfizer Speaker: Amgen; Astra Zeneca; BMS/Celgene; Gilead; GSK
Rudolf Werner: Nothing to disclose
Philipp Franz Linden: Nothing to disclose
Matthias Kassube: Nothing to disclose
Michael Winkelmann: Nothing to disclose
Gabriel Sheikh: Nothing to disclose
Kai Rejeski: Consultant: CSL Baehring Grant Recipient: Kite/Gilead; Novartis
Wolfgang Gerhard Kunz: Nothing to disclose
Jens Ricke: Nothing to disclose

**Second Progression-Free Survival (PFS2) as a Surrogate Endpoint for Overall Survival in Patients Treated with CD19-Targeted CAR T-Cell Therapy**

*P. F. Linden*, M. Kassube, K. Rejeski, G. Sheikh, R. Werner, M. Subklewe, J. Ricke, W. G. Kunz, M. Winkelmann; München/DE

**Purpose:** CD19-directed chimeric antigen receptor T-cell therapy (CART) has improved outcomes in relapsed/refractory (r/r) B-cell lymphomas. However, long-term survival impact is difficult to assess using conventional endpoints such as progression-free survival (PFS1). Second progression-free survival (PFS2) has been proposed as a more comprehensive endpoint, but has not been studied in CART. This study evaluates associations among PFS1, PFS2, and overall survival (OS).
**Methods or Background:** We analyzed patients with r/r lymphomas treated with CART at our center. PFS1 was defined as time to first progression, PFS2 as time to second progression. To avoid overfitting, patients in whom death was the event for both PFS1 and PFS2 were excluded. Associations among PFS1, PFS2, and OS were assessed by linear regression and Spearman correlation. Tumor burden prior to CART was calculated as the sum of product of diameters (SPD). Box plots explored SPD outcome associations. Kaplan–Meier estimates were used for survival endpoints.
**Results or Findings:** Of 140 patients, 51 were excluded (death as event for PFS1: n=13; PFS2: n=38). Median PFS1, PFS2, and OS were 9, 14, and 18 months. PFS2 differed significantly from PFS1 (p=0.044) and was more comparable to OS (p=0.256). PFS2 correlated more strongly with OS (r=0.91) than PFS1 (r=0.77), suggesting superior reflection of outcomes. Patients with shorter PFS1 had higher baseline tumor burden; this association was more pronounced when stratified by median PFS2. Patients with two progressions had shorter OS than those with one, progression-free patients achieved the longest OS.
**Conclusion:** PFS2 shows strong association with OS in CD19 CART recipients, supporting its use as an efficacy endpoint to capture long-term benefit. Incorporating PFS2 into trial design and real-world assessments may improve evaluation of therapeutic benefit and guide treatment sequencing in CART.
**Limitations:** Retrospective, monocentric study
**Funding for this study:** BZKF,FöFoLe
**Has your study been approved by an ethics committee?** Yes
**Ethics committee - additional information:** All medical records and imaging studies underwent review with approval from the LMU Munich Institutional Review Board (LMU Ethics Committee, project number 19-817).
**Author Disclosures:**
Marion Subklewe: Consultant: Aven Cell; CDR-Life; Ichnos Sciences; Incyte Biosciences; Janssen; Miltenyi Biotec; Pfizer, Speaker: Amgen; Astra Zeneca; BMS/Celgene; Gilead; GSK Research/Grant Support: Amgen; BMS/Celgene; Gilead; Janssen; Miltenyi Biotec; Novartis; Roche; Seattle Genetics; Takeda,
Rudolf Werner: Nothing to disclose
Matthias Kassube: Nothing to disclose
Michael Winkelmann: Nothing to disclose
Philipp Franz Linden: Nothing to disclose
Gabriel Sheikh: Nothing to disclose
Kai Rejeski: Grant Recipient: Kite/Gilead; Novartis Consultant: CSL Baehring
Wolfgang Gerhard Kunz: Nothing to disclose
Jens Ricke: Nothing to disclose

**Prognostic relevance of Splenomegaly in CAR T-Cell Therapy for Relapsed Myeloma**

*H-J. Meyer*, V. Sotikova, T. Denecke, M. Merz; Leipzig/DE
*(jonas90.meyer@web.de)*

**Purpose:** B-cell maturation antigen (BCMA)-directed chimeric antigen receptor (CAR) T-cell therapy has shown significant promise for patients with relapsed or refractory multiple myeloma (RRMM). Despite its efficacy, treatment is frequently complicated by adverse events such as cytokine release syndrome and hematologic toxicities, including severe thrombocytopenia. Identifying reliable prognostic markers is essential to improve patient risk stratification, optimizing treatment strategies, and managing complications effectively. While various prognostic markers have been explored, spleen size has not been extensively studied in this context.
**Methods or Background:** This study aims to evaluate spleen size as a prognostic marker in RRMM patients receiving CAR T-cell therapy. Specifically, we examine its association with thrombocytopenia, metabolic tumor volume, soluble BCMA (sBCMA) levels, progression-free survival (PFS), and overall survival (OS). Additionally, we compare spleen size to established prognostic markers, including baseline sBCMA, EASIX, and CAR-HEMATOTOX scores, to determine its predictive value. The association of spleen size, assessed via computed tomography imaging, with clinical outcomes was evaluated.
**Results or Findings:** Splenomegaly (spleen size >340 cm³) was found to be significantly associated with severe and prolonged thrombocytopenia, higher metabolic tumor volumes, and elevated sBCMA levels. In our cohort, splenomegaly emerged as an independent prognostic factor for both PFS and OS, showing stronger associations than other markers such as sBCMA, EASIX, and CAR-HEMATOTOX scores.
**Conclusion:** Spleen size may serve as a promising prognostic marker in CAR T-cell therapy for RRMM patients, providing a simple and readily accessible tool for enhancing risk stratification. This finding could inform monitoring strategies and optimize healthcare resource management for these patients.
**Limitations:** The present study has to address the limitation of its retrospective design. The measurement of the spleen sizes were performed by one reader with potential small reader bias.
**Funding for this study:** None
**Has your study been approved by an ethics committee?** Yes
**Ethics committee - additional information:** University Hospital of Leipzig, 361/22-ek
**Author Disclosures:**
Maximilian Merz: Nothing to disclose
Timm Denecke: Nothing to disclose
Hans-Jonas Meyer: Nothing to disclose
Veronika Sotikova: Nothing to disclose

**Mapping Organ-Specific Depth of Response in Relapsed/Refractory Non-Hodgkin Lymphoma Treated with CD19 CAR T-Cell Therapy**

*M. Kassube*, P. Achhammer, P. F. Linden, K. Rejeski, G. Sheikh, M. Subklewe, J. Ricke, W. G. Kunz, M. Winkelmann; Munich/DE

**Purpose:** CD19-directed CAR T-cell therapy improves outcomes in relapsed/refractory (r/r) B-cell non-Hodgkin lymphoma (NHL). However, relapse remains common, particularly with extranodal disease. Organ-specific involvement may affect prognosis, yet spatial response data are limited. Radiologic assessment enables evaluation at the organ level. We aimed to identify organ systems whose involvement is linked to poor response or early relapse after CAR T-cell therapy.
**Methods or Background:** This retrospective study included 153 patients with r/r B-cell NHL treated with CD19 CAR T-cells. Baseline and follow-up imaging (FDG-PET/CT or contrast-enhanced CT) at day 30 (FU1) and 3 months (FU2) was analyzed. Extranodal lesions were documented by frequency, size, and number per organ system. Depth of response (DoR) was assessed by percentage change in sum of product diameters (SPD) according to Lugano criteria.
**Results or Findings:** Responses were heterogeneous across organ systems. Nodal disease showed stronger reductions compared with extranodal sites (median DoR –89.4% vs. –90.4%). Significant decreases in lesion size occurred from baseline to FU2 in both nodal (p<0.001) and extranodal sites (p<0.001). Among the five most frequently involved extranodal organs, muscle and pleura demonstrated significant reductions (muscle: p=0.020; pleura: p=0.008). Bone and lung showed favorable responses (≈–67% SPD) without reaching statistical significance. In contrast, hepatic lesions responded least, with an average reduction of –29%.
**Conclusion:** Radiologic assessment reveals organ-specific heterogeneity after CD19 CAR T-cell therapy, with lymphoid tissues exhibiting more profound responses than non-lymphoid sites. Specific extranodal involvement, especially of the liver, may limit therapeutic durability and should be validated in future studies.
**Limitations:** Limitations include the single-center design.
**Funding for this study:** This study received no external funding and was conducted with institutional resources only.

**Has your study been approved by an ethics committee?** Yes
**Ethics committee - additional information:** All medical records and imaging studies underwent review with approval from the LMU Munich Institutional Review Board (LMU Ethics Committee, project number 19-817)
**Author Disclosures:**
Marion Subklewe: Speaker: Amgen; Astra Zeneca; BMS/Celgene; Gilead; GSK Research/Grant Support: Amgen; BMS/Celgene; Gilead; Janssen; Miltenyi Biotec; Novartis; Roche; Seattle Genetics; Takeda Consultant: Aven Cell; CDR-Life; Ichnos Sciences; Incyte Biosciences; Janssen; Miltenyi Biotec; Pfizer.
Philipp Achhammer: Nothing to disclose
Philipp Franz Linden: Nothing to disclose
Matthias Kassube: Nothing to disclose
Michael Winkelmann: Nothing to disclose
Gabriel Sheikh: Nothing to disclose
Kai Rejeski: Consultant: CSL Baehring Grant Recipient: Kite/Gilead; Novartis
Wolfgang Gerhard Kunz: Nothing to disclose
Jens Ricke: Nothing to disclose

### Whole-Body MRI Versus 18F-FDG PET/CT for Lesion Detection in Multiple Myeloma and Their Association with Hematologic Findings

B. Baysal, *H. Ardalı Çakır*, M. Gezgin, B. Canitez, I. Erdogan Ozunal, M. Tiglioglu, E. Ozturk, H. S. Uslu; Istanbul/TR
*(hilalardali@gmail.com)*

**Purpose:** This study investigates the diagnostic performance of whole-body magnetic resonance imaging (WB-MRI) with diffusion-weighted imaging (DWI) compared with 18F-FDG PET/CT in detecting lesions in multiple myeloma and examines the relationship of imaging findings with serum parameters and bone marrow involvement.
**Methods or Background:** This retrospective study included newly diagnosed multiple myeloma patients who underwent both WB-MRI and 18F-FDG PET/CT. Demographic, laboratory, and imaging data were collected. Lesion presence in the cranial, cervical, thoracic, lumbar, pelvic, costal–sternal–scapular–clavicular, and extremity regions was compared between modalities using the McNemar test, and the relationship of imaging findings with immunoglobulin subtypes was evaluated.
**Results or Findings:** Twenty eight newly diagnosed multiple myeloma patients (mean age, 66.1 ± 10.9 years; 53.6% female) were evaluated. IgG was the most frequent heavy chain subtype (46.4%), followed by IgA (32.1%), while 21.4% had no heavy chain; kappa light chain predominated (57.1%). MRI identified marrow involvement as diffuse (35.7%), focal (32.1%), micronodular (14.3%), or absent (17.9%). Compared with PET/CT, MRI showed higher sensitivity in cervical ($p = 0.001$), pelvic ($p = 0.031$), and extremity ($p = 0.008$) regions, with a nonsignificant trend in the cranium ($p = 0.063$). No significant differences were observed in thoracic, lumbar, or costal–sternal–scapular–clavicular regions. PET/CT revealed more cervical lesions in IgA patients and more lumbar lesions in IgA and heavy chain–negative groups, while MRI detection did not differ by heavy or light chain subtype.
**Conclusion:** Whole-body MRI with diffusion-weighted imaging demonstrated higher sensitivity than 18F-FDG PET/CT in multiple myeloma, particularly in the cervical spine, pelvis, and long bones. These findings suggest that WB-MRI may serve as a prominent diagnostic tool, while PET/CT provides complementary metabolic insights.
**Limitations:** The main limitations are the small sample size and retrospective design.
**Funding for this study:** No funding was received for this study.
**Has your study been approved by an ethics committee?** Yes
**Ethics committee - additional information:** Currently in the process
**Author Disclosures:**
Erman Ozturk: Nothing to disclose
Merve Gezgin: Nothing to disclose
Isil Erdogan Ozunal: Nothing to disclose
Hatice Sinav Uslu: Nothing to disclose
Hilal Ardalı Çakır: Nothing to disclose
Burak Canitez: Nothing to disclose
Mesut Tiglioglu: Nothing to disclose
Begumhan Baysal: Nothing to disclose

### Whole-Body Diffusion-Weighted MRI for Assessing Therapeutic Response in Lymphoma Patients: An Alternative to PET/CT

*F. Maccioni*, L. Bottino, L. Busato, A. Valenti, M. Rutigliano, C. Catalano; Rome/IT
*(francesca.maccioni@uniroma1.it)*

**Purpose:** This study aims to assess the performance of whole-body DWI MRI (WB-DWI MRI), for staging and monitoring lymphomas. WB-DWI MRI was prospectively compared to PET/CT. The ultimate goal is to propose WB-DWI MRI as a viable alternative or complementary tool to PET/CT, especially in younger patients.
**Methods or Background:** In this single-center study, 71 patients with HL or DLBCL prospectively underwent both ^18F-FDG PET/CT and WB-MRI (within 15 days). Inclusion required histological confirmation, age ≥18, and ≤2 chemotherapy cycles. Exclusion criteria included contraindications to MRI, other neoplasms, or delayed imaging. WB-MRI included T1, T2, and DWI sequences (b-values: 50–800 s/mm²). Qualitative analysis included assessment of 10 different lymphnodal stations and 3 extranodal organans (liver, spleen, bone marrow) and quantitative parameters (SUVmax/ SUVmean vs. DWI/ADC). PET/CT and WB-MRI images were independently evaluated for qualitative and quantitative parameters (SUVmax/ SUVmean; ADCmin/ADCmean). Statistical analyses included concordance (Cohen’s κ), predictive values (PPV/NPV), and correlation tests. Strong inverse correlations were found between SUV and ADC values.
**Results or Findings:** PET/CT and WB-MRI showed excellent agreement (Cohen’s $\kappa = 0.91$; PPV/NPV: 0.96/0.97), including 100% concordance in extranodal sites.
**Conclusion:** Quantitative analysis demonstrated an inverse correlation between ADC and SUV values, reflecting tumor cellularity. Whole-body DWI-MRI, a radiation-free modality, showed high concordance with PET/CT, supporting its integration as a complementary tool for lymphoma management, particularly in younger or radiation-sensitive patients.
**Limitations:** Limited number of patients.
**Funding for this study:** None
**Has your study been approved by an ethics committee?** Yes
**Ethics committee - additional information:** Local ethics committee
**Author Disclosures:**
Alessandra Valenti: Nothing to disclose
Lorenza Bottino: Nothing to disclose
Mariateresa Rutigliano: Nothing to disclose
Carlo Catalano: Nothing to disclose
Ludovica Busato: Nothing to disclose
Francesca Maccioni: Nothing to disclose

### Nomograms based on clinical characteristics and dual-layer spectral detector computed tomography imaging parameters: Potential role in predicting the efficacy of first-line lymphoma treatment

Z. Tan, *S. Gui*, J. Wang; Wuhan/CN
*(shen.gui@philips.com)*

**Purpose:** To develop spectral CT-based nomogram for predicting response and prognosis in lymphoma patients after first-line therapy.
**Methods or Background:** The baseline clinical and spectral CT data of 91 lymphoma patients were retrospectively analyzed. Patients were assessed for therapy response using the Lugano criteria and were categorized into complete response (CR) and non-complete response (non-CR) groups. Spectral CT parameters of each patient's largest lesion, including the arterial phase (AP) and venous phase (VP) were measured. Univariable and multivariable logistic regression were performed to identify independent predictors of response. In addition, the model was visualized using a clinical model, spectral CT model, and nomograms, and its performance was assessed via receiver operating characteristic curves. Overall survival and progression-free survival (PFS) in patients with different parameters were estimated using the Kaplan-Meier method.
**Results or Findings:** In the multivariate analysis, B symptoms, Ki-67, iodine concentration (IC), normalized IC at AP, and arterial enhancement fraction based on IC (AEF-IC) were identified as independent predictor factors for CR. The nomogram incorporating all five independent predictors outperformed spectral CT and the clinical model with the highest AUC of 0.856 (95% CI: 0.767-0.921). AEF-IC, the clinical model, spectral CT model, and nomogram were associated with PFS, and the nomogram had the strongest association with unfavorable PFS, with a hazard ratio of 4.725 (95% CI: 2.275-9.817, $P<0.001$).
**Conclusion:** Spectral CT and the clinical models were useful in predicting efficacy and PFS in lymphoma patients. Combining spectral CT parameters and clinical characteristics in a nomogram improved predictive performance.
**Limitations:** A retrospective study conducted at a single center with a small sample size that used a spectral CT scanner from a specific manufacturer, the results may lack generalizability.
**Funding for this study:** This spectral CT-based nomogram integrating clinical characteristics is a non-invasive and easily obtainable tool for accurately predicting first-line treatment response and prognosis, thereby aiding clinicians in performing personalized treatment planning in lymphoma patients.
**Has your study been approved by an ethics committee?** Not applicable
**Author Disclosures:**
Zhengwu Tan: Nothing to disclose
Shen Gui: Nothing to disclose
Jing Wang: Nothing to disclose
